# A global database of large-scale transverse drainages

**DOI:** 10.1016/j.dib.2018.12.088

**Published:** 2019-01-14

**Authors:** Jacqueline Lee

## Abstract

Transverse drainages, also known as water gaps, are streams which appear to bypass the path of least resistance and flow directly through topographic highlands. Here, a global dataset is presented of over 1500 large-scale transverse drainages (>100 m depth) which are identified and described based on physical characteristics alone, independent of any presumed method of origin.

**Specifications table**

**Table:** 

Subject area	*Earth Science*
More specific subject area	*Transverse drainages*
Type of data	*KMZ files, table, PDF files*
How data was acquired	*Geographical Information System (Global Mapper)*
Data format	*Topographic measurements, geographic descriptions, lithologic description where available*
Experimental factors	*Global Mapper, a Geographic Information System (GIS), was used to collect topographic and hydrologic data on large-scale transverse drainages*
Experimental features	*GIS functions were used to quantify and classify the transverse drainages*
Data source location	*Global (latitude 90°N-90°S, longitude 180°E-180°W)*
Data accessibility	*Google Earth^©^ KMZ files, Attribute Table, and map-view images in PDF files available with this article*
Related research article	*Giant gorges: The challenge of large-scale transverse drainages. Lee, J., in prep.*

**Value of the data**

• Transverse drainages, also known as water gaps, are enigmatic geomorphic features that have been widely studied, but little attention has been paid to their actual physical characterization.
• The dataset presented here is a global survey of over 1500 large-scale transverse drainages, classified solely by physical and topographic attributes.
• Knowledge of the geographic location and physical and hydrologic characteristics of transverse drainages can help in constraining theories for their formation.

## 1 Data

**Fig. 1:**
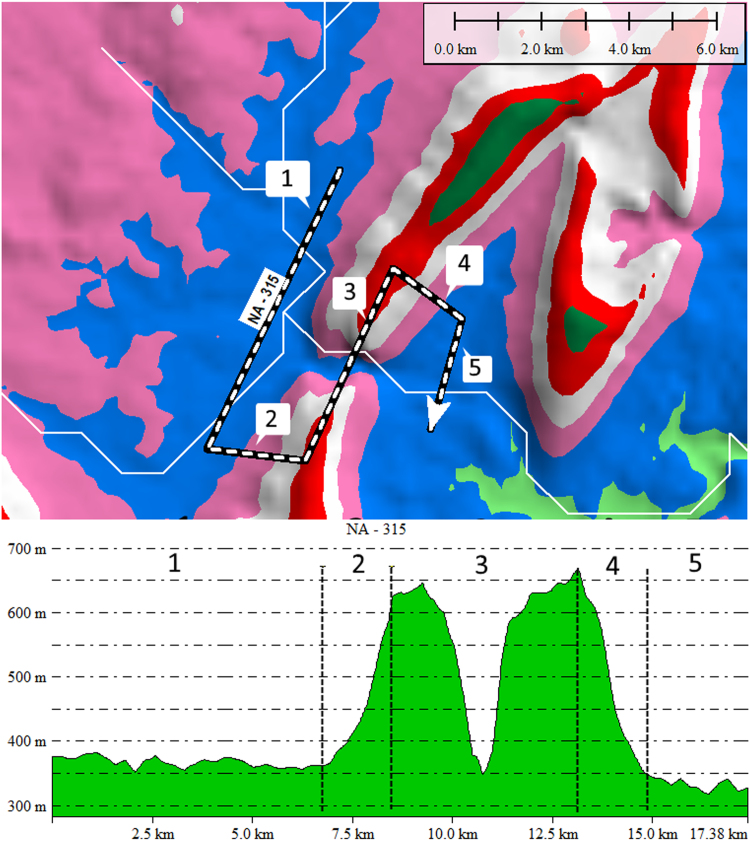
Plan view (top) and profile (bottom) of TD S-line (black and white dashed line) superimposed on ETOPO DEM (2 km spacing of elevation points) with contour areas changing color every 100 m. Segments 1, 3 and 5 are constructed upstream, across and downstream of the topographic high. White lines are HydroSHEDS streams. Profile displays the typical “M” shape of a TD with simple ridge morphology. To emphasize the difference in elevation between upstream and cross-TD segments, TDs in this study were limited to those with an average elevation difference of at least 100 m between Segment 1 and Segment 3.

Transverse drainages, also known as water gaps, are streams which appear to bypass the path of least resistance by flowing directly through topographic highs [1]. The dataset presented here is a global survey of 1549 large-scale transverse drainages (> 100 m depth) which have been identified and classified based upon their physical and topographic characteristics (Table 1 and Supplementary Files). Each transverse drainage (TD) is represented by an S-shaped line joining three parallel traverses of the stream channel, upstream, across and downstream of the topographic high (Fig. 1). Physical and hydrologic data were extracted from the S-lines using Global Mapper (a Geographic Information System program (GIS)), and several digital elevation models (DEMs) of different resolutions. The S-lines were then converted into Google Earth^©^ KMZ files, and an attributes table was created in Excel of the physical and hydrologic characteristics of the transverse drainages (Table 1). Additionally, images of each TD were generated within Global Mapper and combined into PDFs, one file for each continent (excluding Greenland and Antarctica).

## 2 Experimental design, materials and methods

The process of creating the dataset consisted of three parts:

1. Identification
2. Quantification
3. Classification

### 2.1 Identification

Identification of TDs is made possible by the recognition that there are two mappable characteristics that distinguish transversely draining streams from normal stream segments [2], [3]:

1. Transversely draining streams flow from a region of lower elevation into a region of higher elevation and then back into a region of lower elevation
2. Contour lines constrict through the breached topographic high

The constriction of contour lines was visualized through a two-part process:

1. A series of contour areas of contrasting colors was created from the ETOPO DEM (1 arc-minute resolution [4]) at 100 m intervals from 0–3000 m
2. A global-scale vector shapefile of rivers from the HydroSHEDS dataset [5] was superimposed upon the contour areas, and visual inspection used to identify areas of contour constriction through TDs

**Fig. 2:**
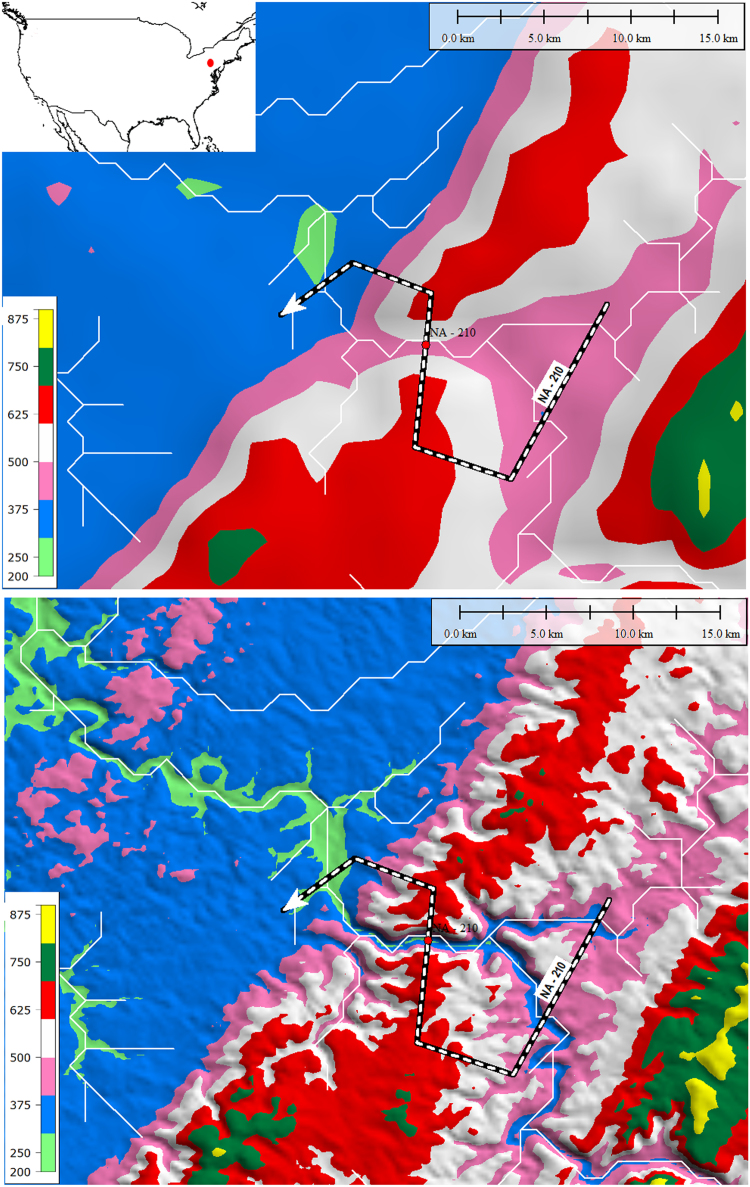
Comparison of the same TD S-line (black and white dashed line) on ETOPO DEM (top, 2 km spacing of elevation points) and higher resolution CGIAR DEM (bottom, 250 m spacing of elevation points). Contour areas change color every 100 m, white lines are HydroSHEDS streams. Note how the pink 500 m contour area constricts through the TD. Inset figure on top shows location of TD, red dot on TD indicates minimum elevation of cross-TD segmen

Once a TD was identified by visual inspection, an S-shaped line was constructed joining three parallel traverses of the stream channel, upstream, across and downstream of the topographic high. The elevation generated by this S-line displays a characteristic M-shaped profile (Fig. 1, Fig. 2). The dataset was re-examined, first in Google Earth^©^ and then in Global Mapper with a higher resolution DEM (SRTM resampled to 250 m [6]), and additional TDs were added as noted. The final TD dataset was then exported as a Google Earth^©^ KMZ file in vector format (Supplementary File).

The final criteria for TD identification included:

1. Visual observation within Global Mapper or Google Earth^©^ of a stream through the TD
2. Observed constriction of 100 m contour areas upon entering the topographic high
3. Continuity of structure observed across the TD
4. Diagnostic S-line and M-shaped profile constructed.

### 2.2 Quantification

Each S-line was split into five segments and the following measurements calculated within Global Mapper using a SRTM DEM (3 arc-second resolution) [7] for TDs between 60° north and 60° south and a GMTED2010 DEM (7.5 arc-second resolution) [8] for TDs outside the SRTM data range:

1. Elevation and X-Y coordinate of the maximum elevation for the segment
2. Elevation and X-Y coordinate of the minimum elevation for the segment
3. Average elevation
4. Average slope
5. Bearing
6. Length

The segment measurements were then used to calculate the following measurements for each TD (Table 1):

1. Difference between the average elevation of Segment 1 and Segment 3. To emphasize the distinction between the lower upstream elevation area and the higher elevation of breached high ground, only TDs with a Segment 1-Segment 3 elevation difference of >=100 m were selected for the dataset. (TDs with differences of >=97 m) were included based upon a +-3m margin of elevation measurement error calculated by accuracy assessments of the DEM [9]
2. Total length of Segments 2 and 4 as a proxy for TD size
3. Average bearing of Segments 2–4 as a proxy for the TD bearing. To normalize the TD compass directions, bearings were measured in gradians, units of angular measure in which the right angle is divided into 100 parts. One gradian equals 1/400 of a circle, so 100 grads = 90 degrees, 200 grads = 180 degrees, etc.
4. As a proxy for the stream gradient through the TD, the difference between the minimum elevations of Segments 1 and 5 divided by the total lengths of Segments 2 and 4 (calculated in meters)
5. TD depth in meters, as calculated by the difference between the maximum and minimum elevation points on Segment 3

### 2.3 Classification

**Fig. 3:**
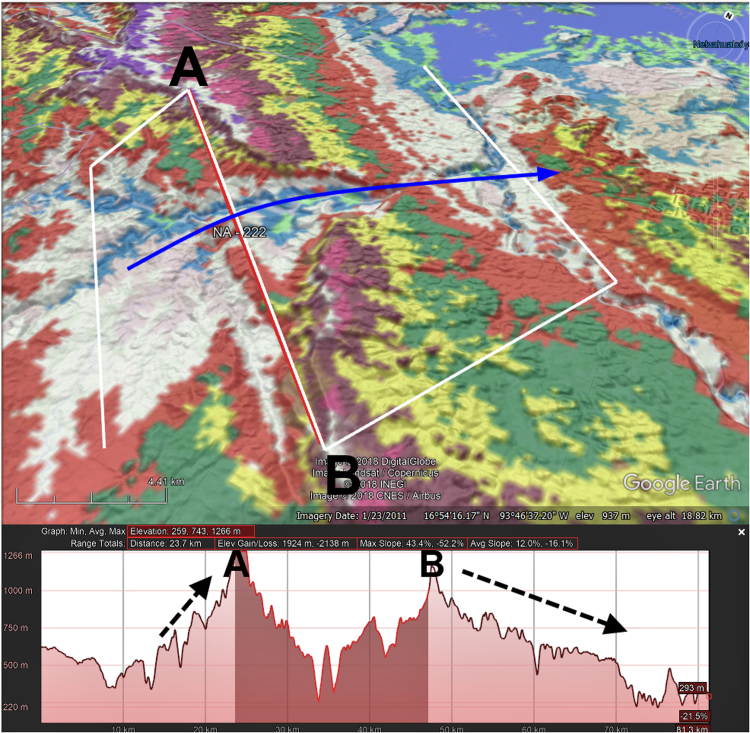
Google Earth^©^ image with 100 m contour overlay draped over topography showing profile of TD with downdip plateau morphology. Blue arrow in top image shows direction of streamflow, darkened area on profile indicates line segment A-B in top image. Note how TD opens in a V toward the upstream direction. Dotted lines on profile shows how downstream slopes of downdip plateau TDs are typically gentler than upstream slopes.

**Fig. 4:**
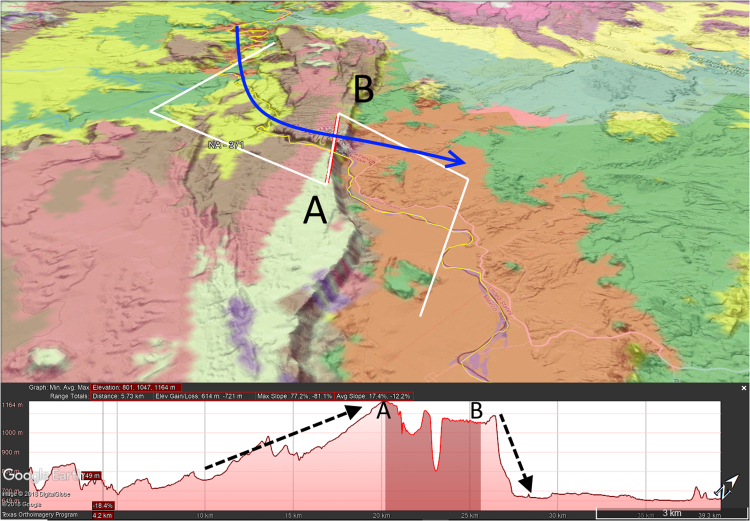
Google Earth^©^ image with 100 m contour overlay draped over topography showing profile of TD with updip plateau morphology. Blue arrow in top image shows direction of streamflow, darkened area on profile indicates line segment A-B in top image. Dotted lines on profile shows how upstream slopes of updip plateau TDs are typically gentler than downstream slopes.
  c. Irregular high ground (Fig. 5)

The following attributes were assigned for each TD using a generalized global geology map created by the Geological Survey of Canada [10], the HydroBASINS hydrologic dataset [11], Natural Earth shapefiles [12], and a set of GIS streamlines from the Elevation Derivatives for National Applications (EDNA) Seamless Three-Dimensional Hydrologic Database [13] (Table 1):

1. Continent
2. Country
3. Lithology
4. Morphology of breached structure:
  a. Ridge (for example, see Fig. 2)
  b. Plateau (updip or downdip if applicable) (Fig. 3, Fig. 4)

**Fig. 5:**
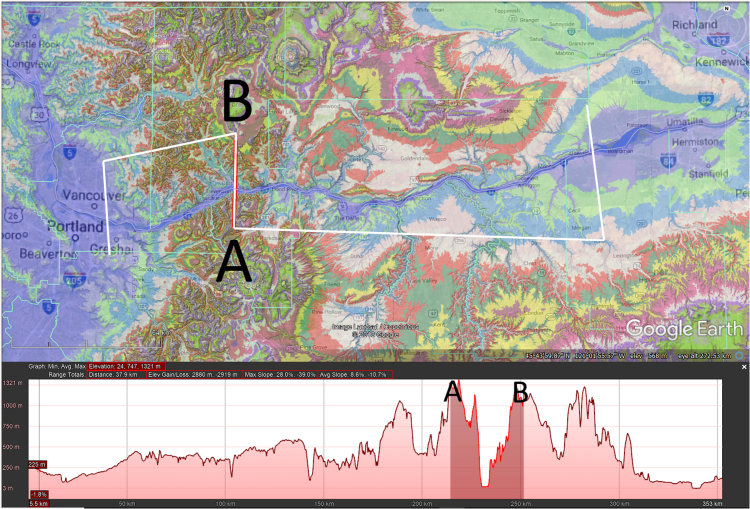
Google Earth^©^ image with 100 m contour overlay draped over topography showing irregular high ground morphology of TD of Columbia River, US, through Cascade Range. Streamflow is from right to left, darkened area on profile indicates line segment A-B in top image.
5. River basin name for exorheic watersheds, taken from the largest river in the HydroBASINS watershed in which the TD was located. If no named river could be located at the exit to the ocean, a significant geographic feature was used instead.
6. Major geographic feature, usually the name of the stream running through the TD. If no stream name could be identified, this attribute was either left blank, or a nearby feature such as the name of a pass or a dam was substituted for a stream name.
7. Classification of TD stream as head stream or trunk stream. If no more than one junction of HydroSHEDS stream segments was located upstream of the TD, it was classified as a head stream (Fig. 6).

**Fig. 6:**
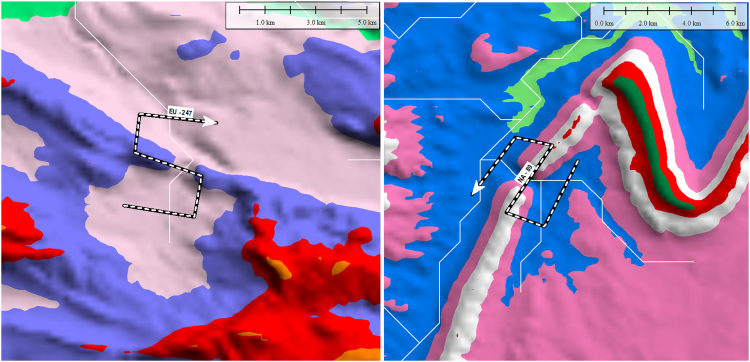
Typical head stream TDs. White lines indicate HydroSHEDS streams. TDs were classified as head streams if no more than one junction of HydroSHEDS streams occurred upstream of the cross-TD segment.
8. Total upstream area. For trunk streams, this attribute was taken directly from HydroBASINS, while for headstreams, the upstream area was calculated using the Generate Watershed process within Global Mapper.
9. Maximum discharge in cubic meters/sec of streams entering TDs from upstream watersheds. TDs were classified as receiving maximum discharge of either >10 cms or <10 cms, based on the size of their upstream watershed. This was derived from an analysis of 263 TDs located within the watersheds of the EDNA dataset which determined that 10 cms was the maximum stream discharge from upstream watersheds of 1000 sq km or less.
10. Classification of TD stream as endorheic (internally draining) or exorheic (externally draining) (from HydroBASINS)

### 2.4 Areas of uncertainty

1. Calculation of TD depth

**Fig. 7:**
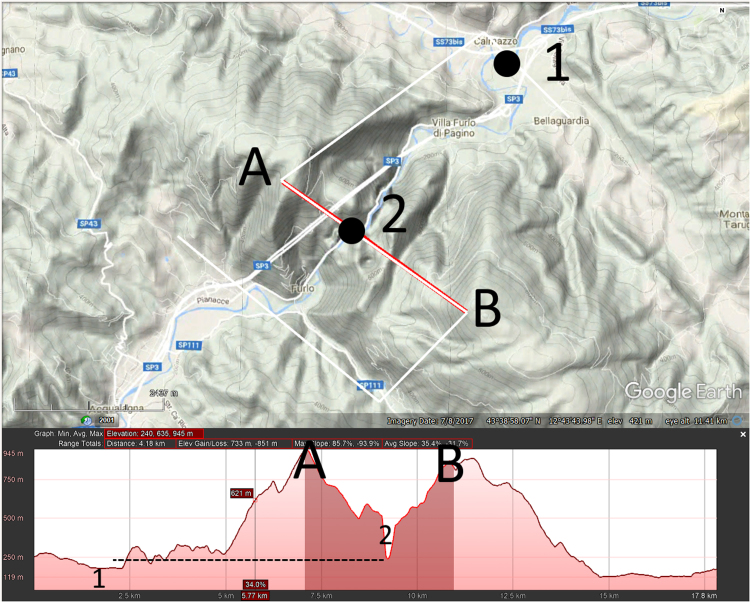
Google Earth© image of TD with false Segment 3 minimum elevation. Cross-TD segment A-B shown by darkened area on profile. Channel width through TD is too narrow to be resolved by spacing of elevation points in Google Earth©, resulting in Point 2 appearing higher than upstream Point 1. This is clearly a false elevation, since Google Earth© shows a stream flowing from Points 1–2.

In 232 TDs, the stream channel was too narrow to be resolved by the elevation spacing of even the highest resolution DEM, resulting in the Segment 3 minimum elevation point being artificially elevated above the Segment 1 minimum elevation (Fig. 7). TDs with inaccurate Segment 3 minimum elevations were confirmed to be true TDs by the identification of actual streams in either Google Earth^©^ or the HydroSHEDS streams dataset. For these TDs, a replacement TD depth was calculated by multiplying the derived TD stream gradient by the Segment 2 length and subtracting the result from the Segment 1 minimum elevation. When this equation was tested by applying it to the 1317 TDs with accurate Segment 3 minimums, the average difference between the calculated TD depth and the true TD depth was 2%, leading to confidence in the accuracy of the replacement TD depth for the TDs with false Segment 3 minimum elevations.

2. Structural continuity across TDs
  Determining structural continuity was at times a subjective process, especially when the breached high ground consisted of irregular topography. Factors used to determine structural continuity included the existence of a well-defined, steep-sided channel within the TD as opposed to a gently sloping saddle, and a similar length, height and width of the breached high across the TD. High resolution geologic maps may be used in further studies to clarify the continuity of the breached high ground.
3. Number of TDs

This dataset is meant to be an illustrative but not an exhaustive sample of the global distribution of large-scale TDs. Researchers can expand the dataset by using the workflow to identify and categorize TDs across all size scales. For example, a study in the Susquehanna River basin identified 653 TDs of >=30 m depth [3].
