# Supplementary material for: A global database of large-scale transverse drainages
Source: Data Brief. 2019 Jan 14;23:103650. doi: 10.1016/j.dib.2018.12.088 (PMC6369416; doi:10.1016/j.dib.2018.12.088)
Supplement: Supplementary file 4 — Supplementary material [file mmc4.pdf]

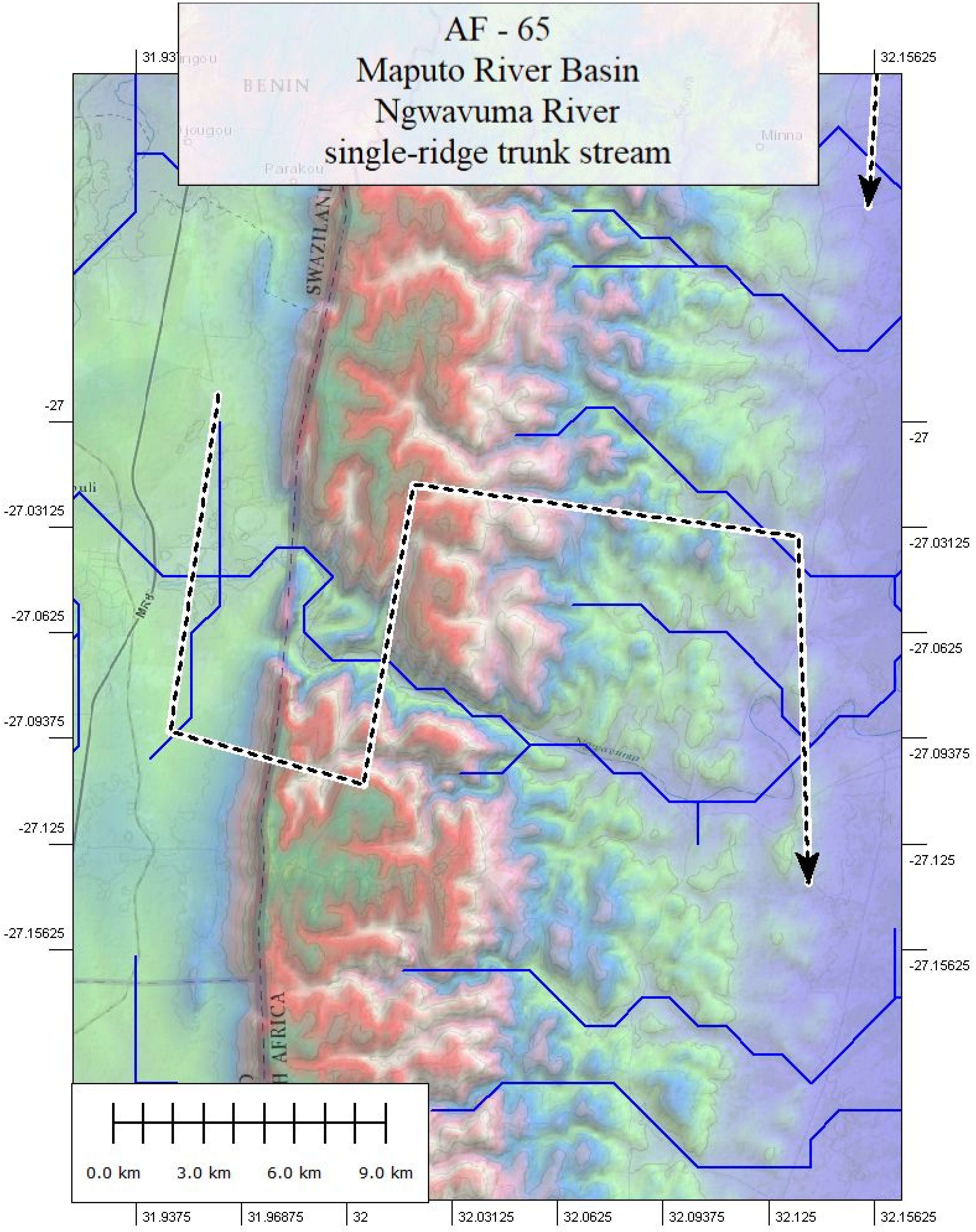
AF - 65
Maputo River Basin
Ngwavuma River
single-ridge trunk stream
BENIN
Parakou
Minna
SWAZILAND
32.15625
-27
-27.03125
-27.0625
-27.09375
-27.125
-27.15625
31.9375
31.96875
32
32.03125
32.0625
32.09375
32.125
32.15625
0.0 km
3.0 km
6.0 km
9.0 km

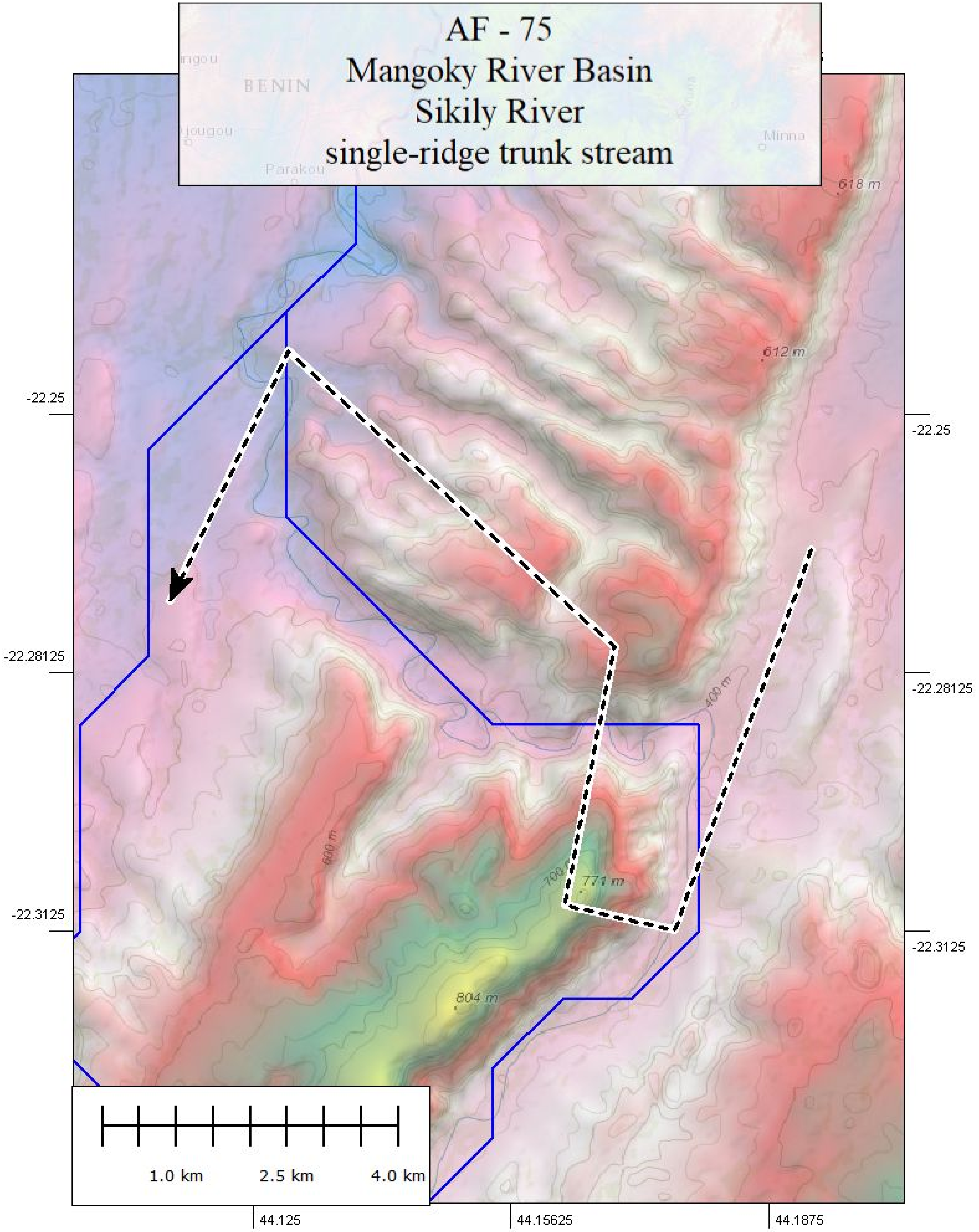

AF - 75
Mangoky River Basin
Sikily River
single-ridge trunk stream
BENIN
Parakou
Minna
618 m
612 m
400 m
600 m
700
771 m
804 m
-22.25
-22.28125
-22.3125
44.125
44.15625
44.1875
1.0 km
2.5 km
4.0 km

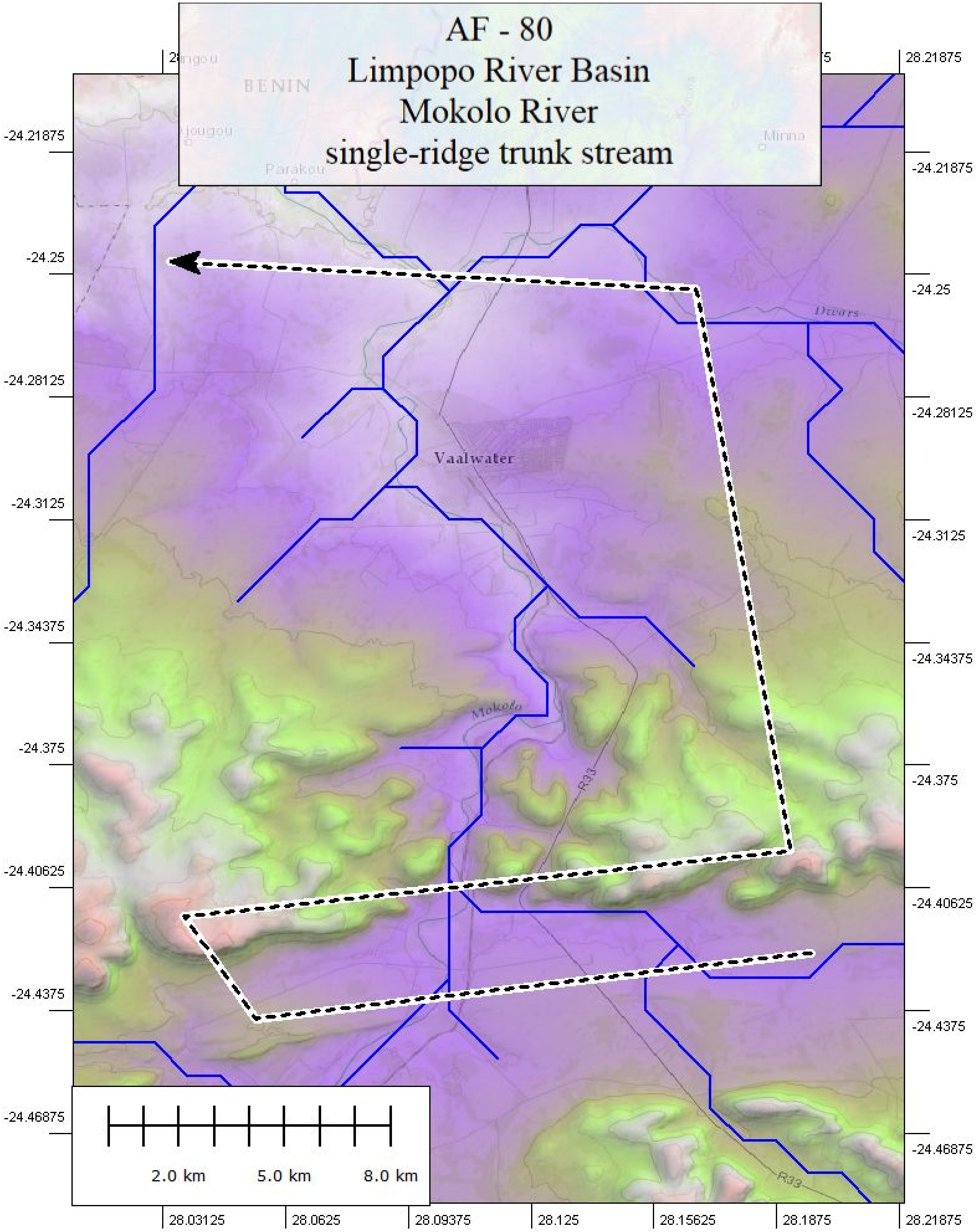
AF - 80
Limpopo River Basin
Mokolo River
single-ridge trunk stream
BENIN
Parakou
Minna
Vaalwater
Mokolo
Dwars
R33
2.0 km
5.0 km
8.0 km
-24.21875
-24.25
-24.28125
-24.3125
-24.34375
-24.375
-24.40625
-24.4375
-24.46875
28.03125
28.0625
28.09375
28.125
28.15625
28.1875
28.21875

# AF - 168
# Maputo River Basin
# Phongolo River
# single-ridge trunk stream

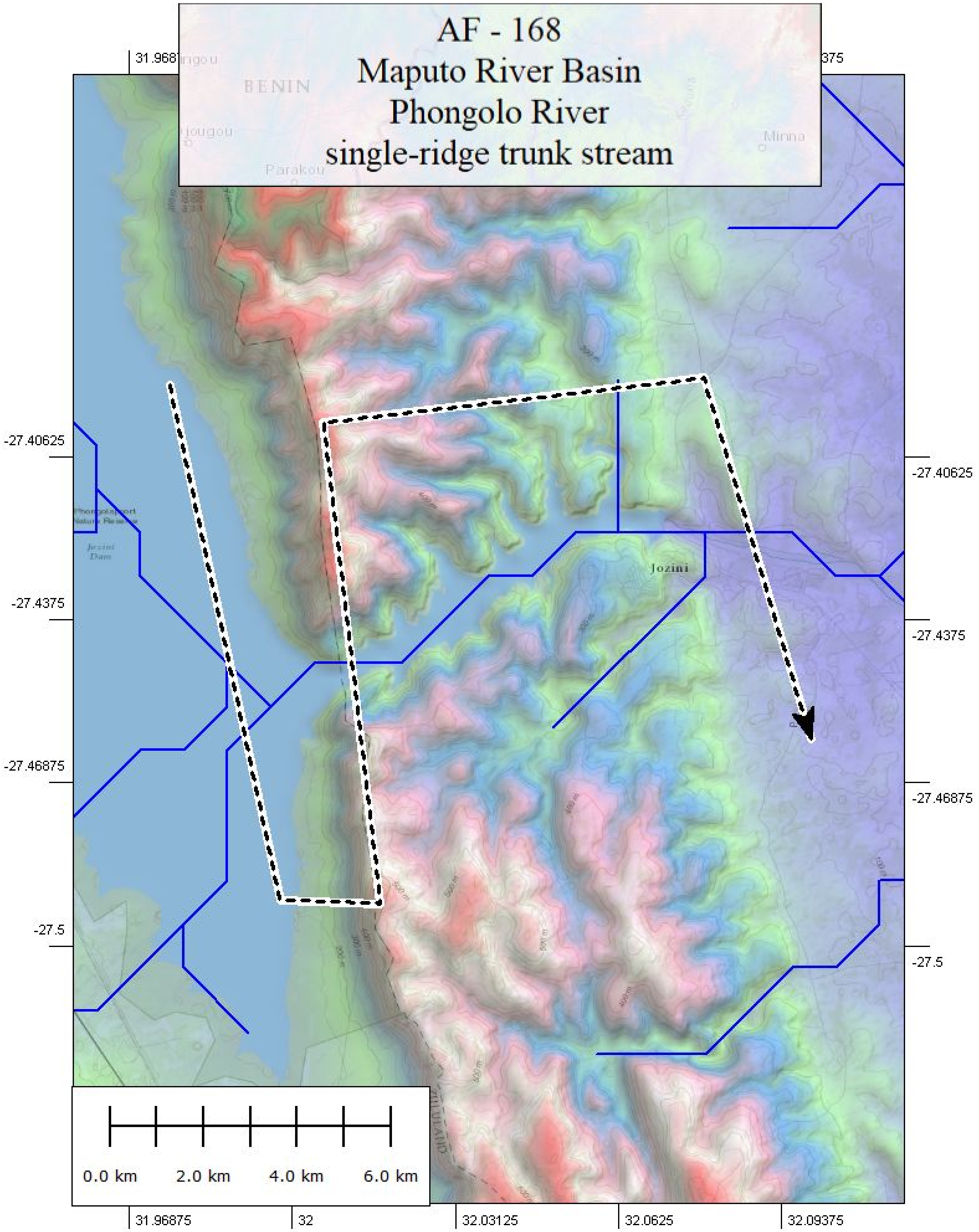

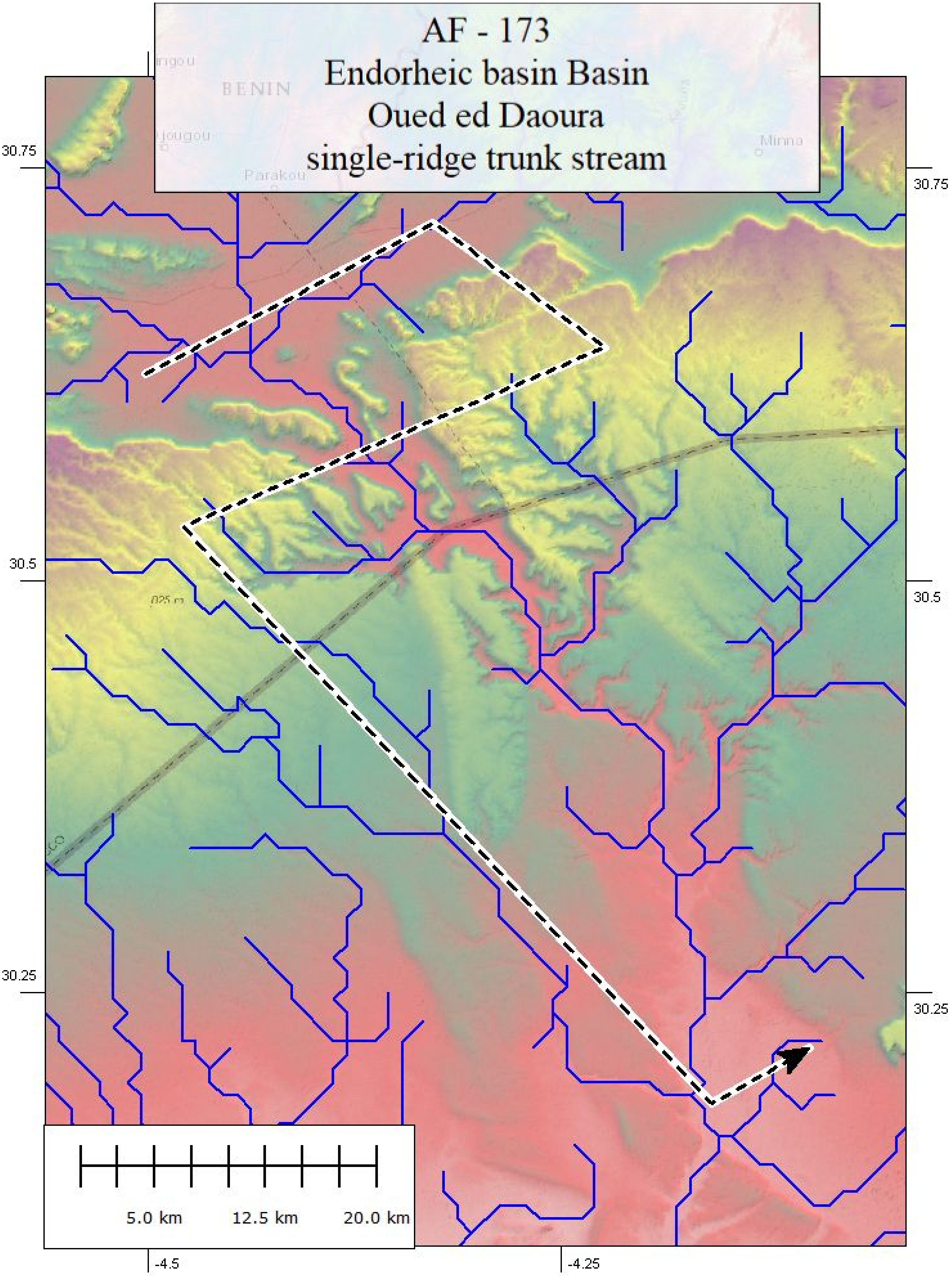
AF - 173
Endorheic basin Basin
Oued ed Daoura
single-ridge trunk stream
30.75
30.5
30.25
-4.5
-4.25
5.0 km
12.5 km
20.0 km

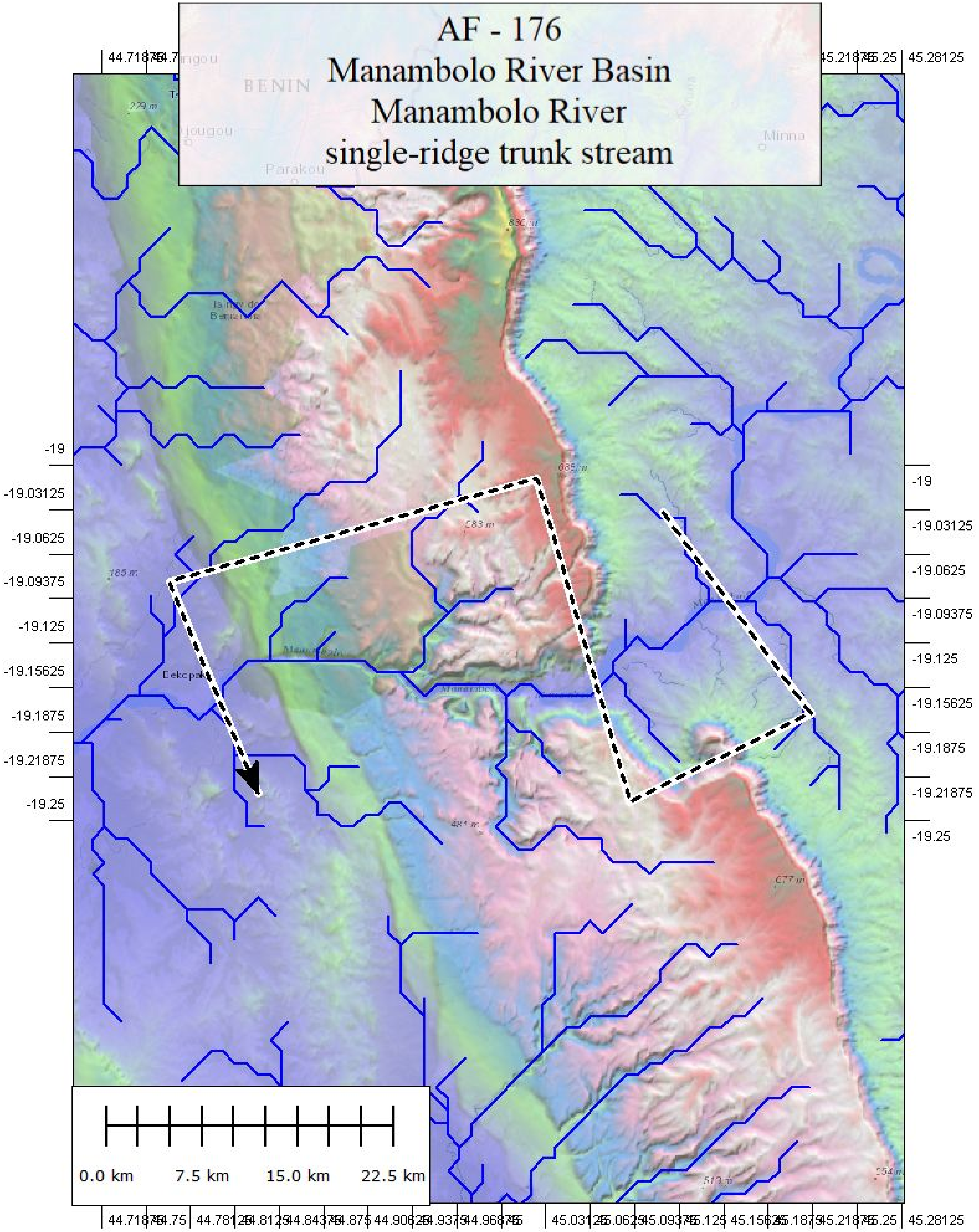

AF - 176
Manambolo River Basin
Manambolo River
single-ridge trunk stream
BENIN
Parakou
Minna
830 m
685 m
583 m
185 m
481 m
677 m
-19
-19.03125
-19.0625
-19.09375
-19.125
-19.15625
-19.1875
-19.21875
-19.25
45.28125
45.03125
0.0 km
7.5 km
15.0 km
22.5 km

# AF - 115
# Sofia River Basin
# Sofia River
# single-ridge trunk stream

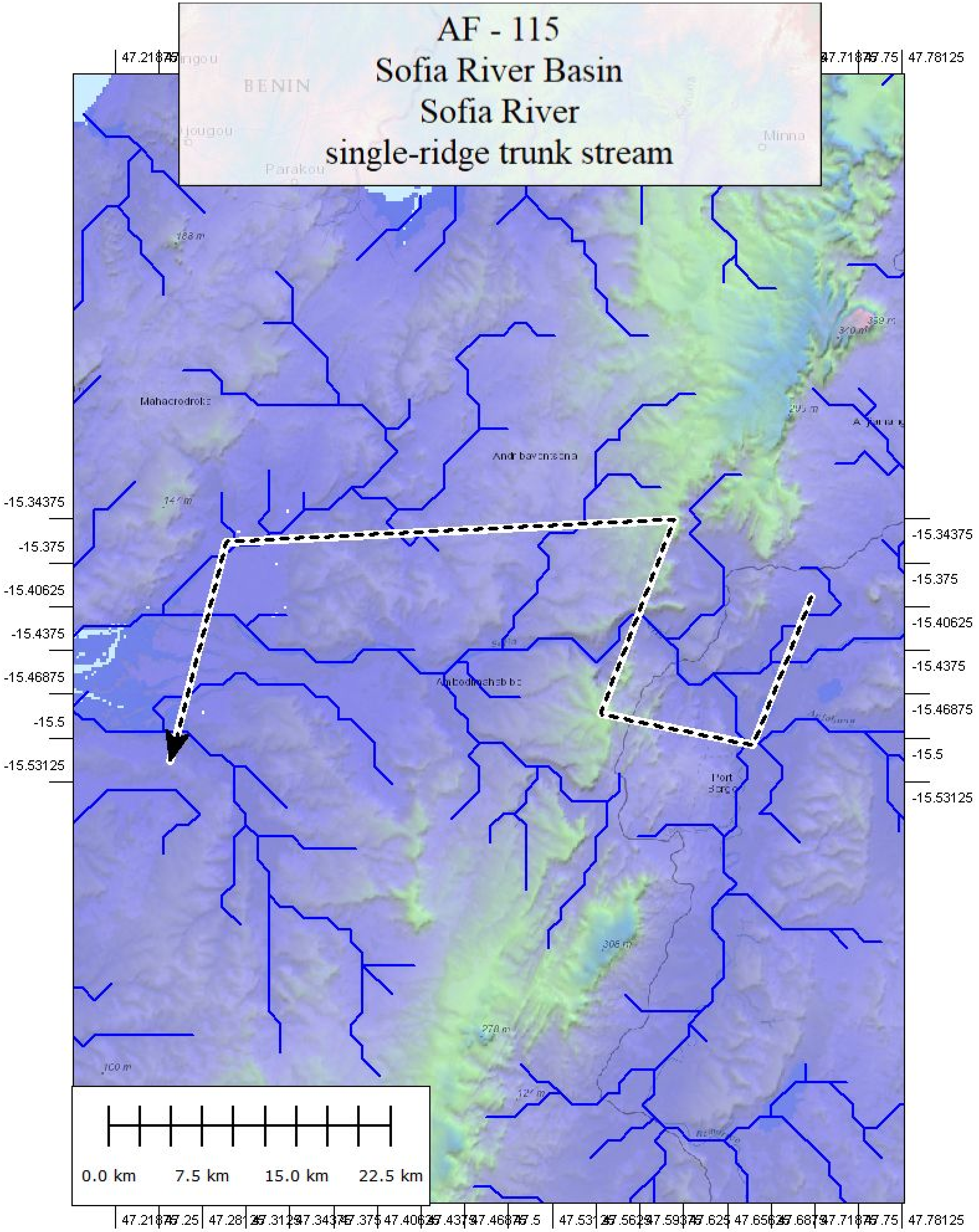

# AF - 186 Mzimkhulu River Basin Mzimkhulwana River single-ridge trunk stream

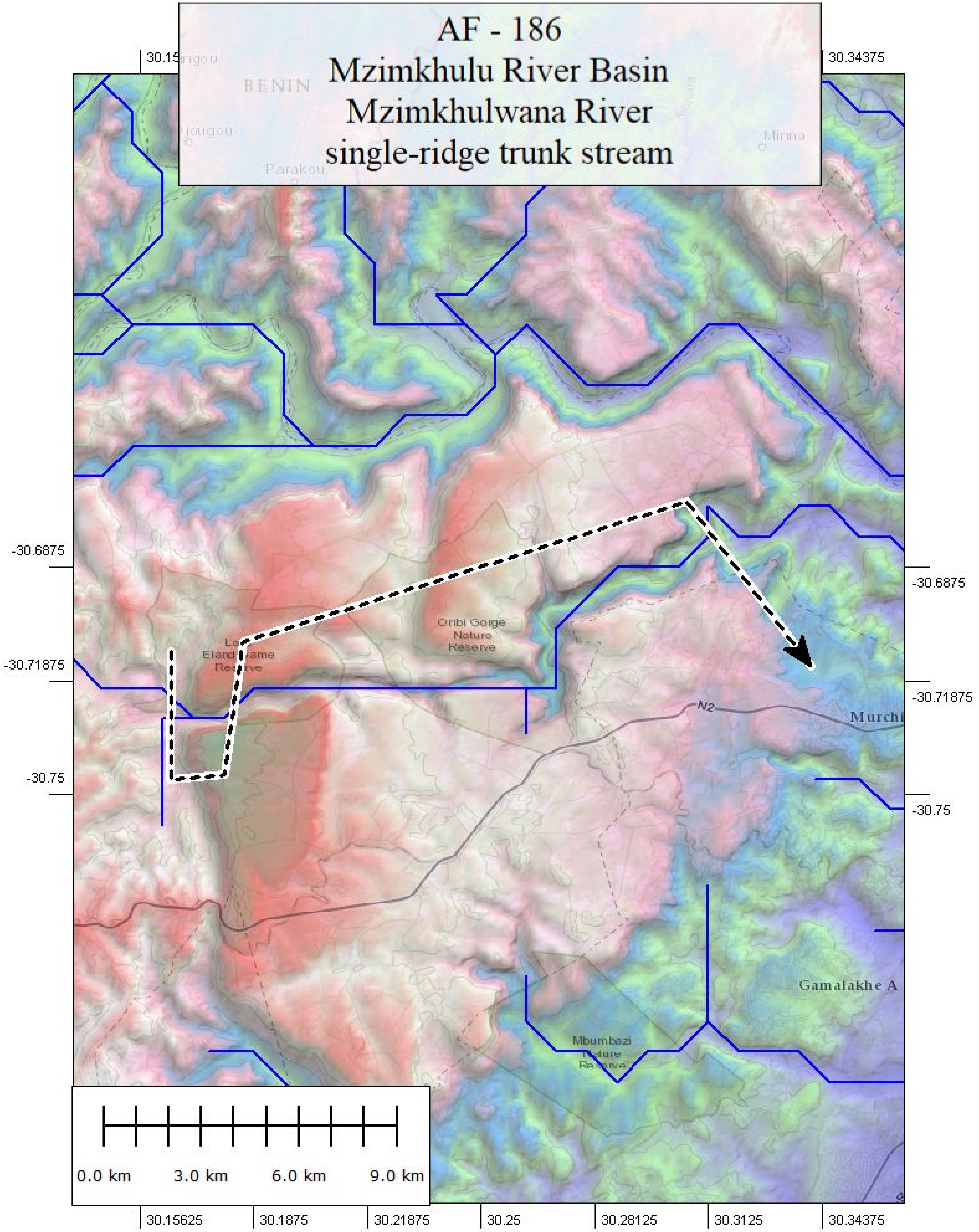

# AF - 68
## Oued Draa Basin
## Oued Draa
## single-ridge trunk stream

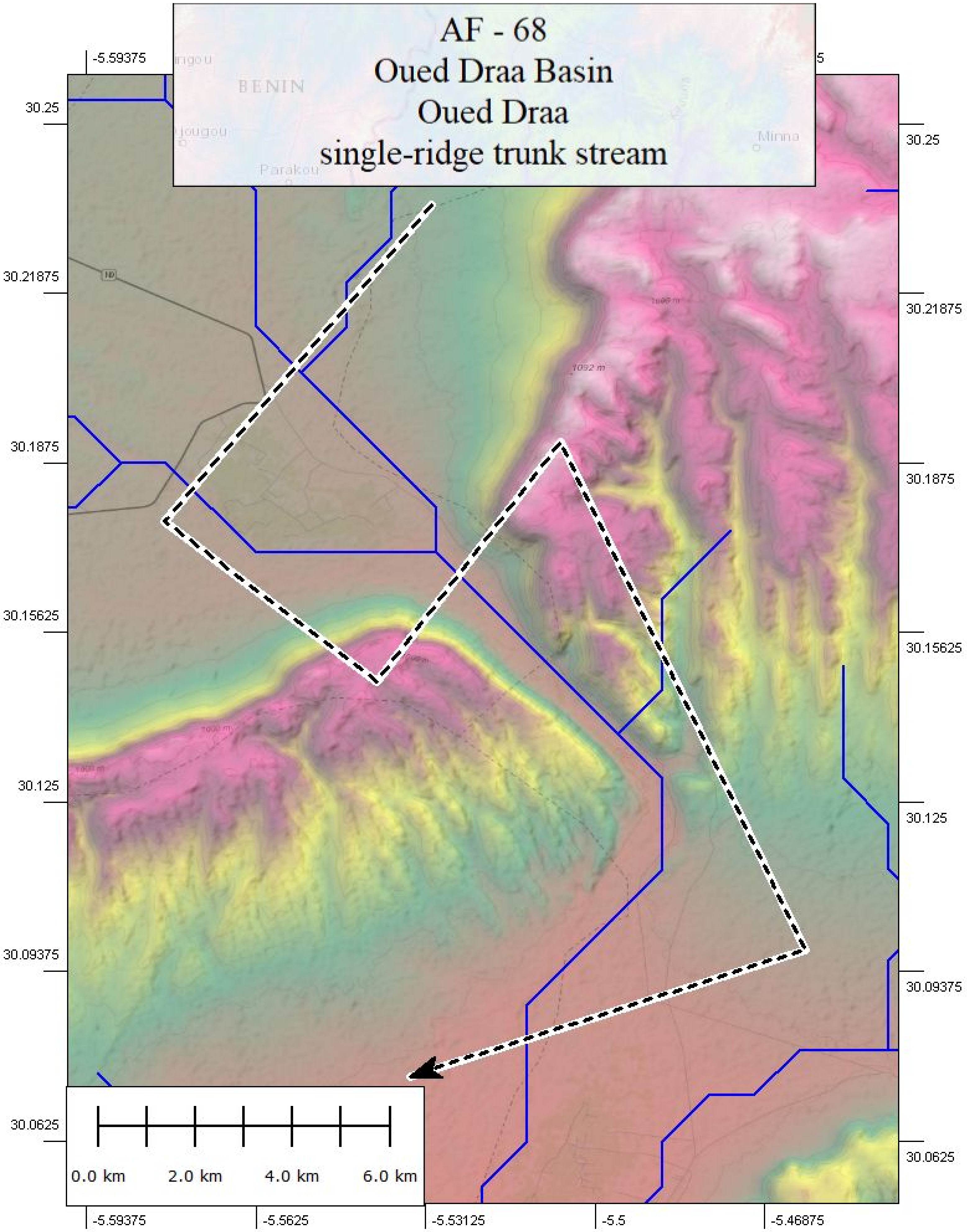

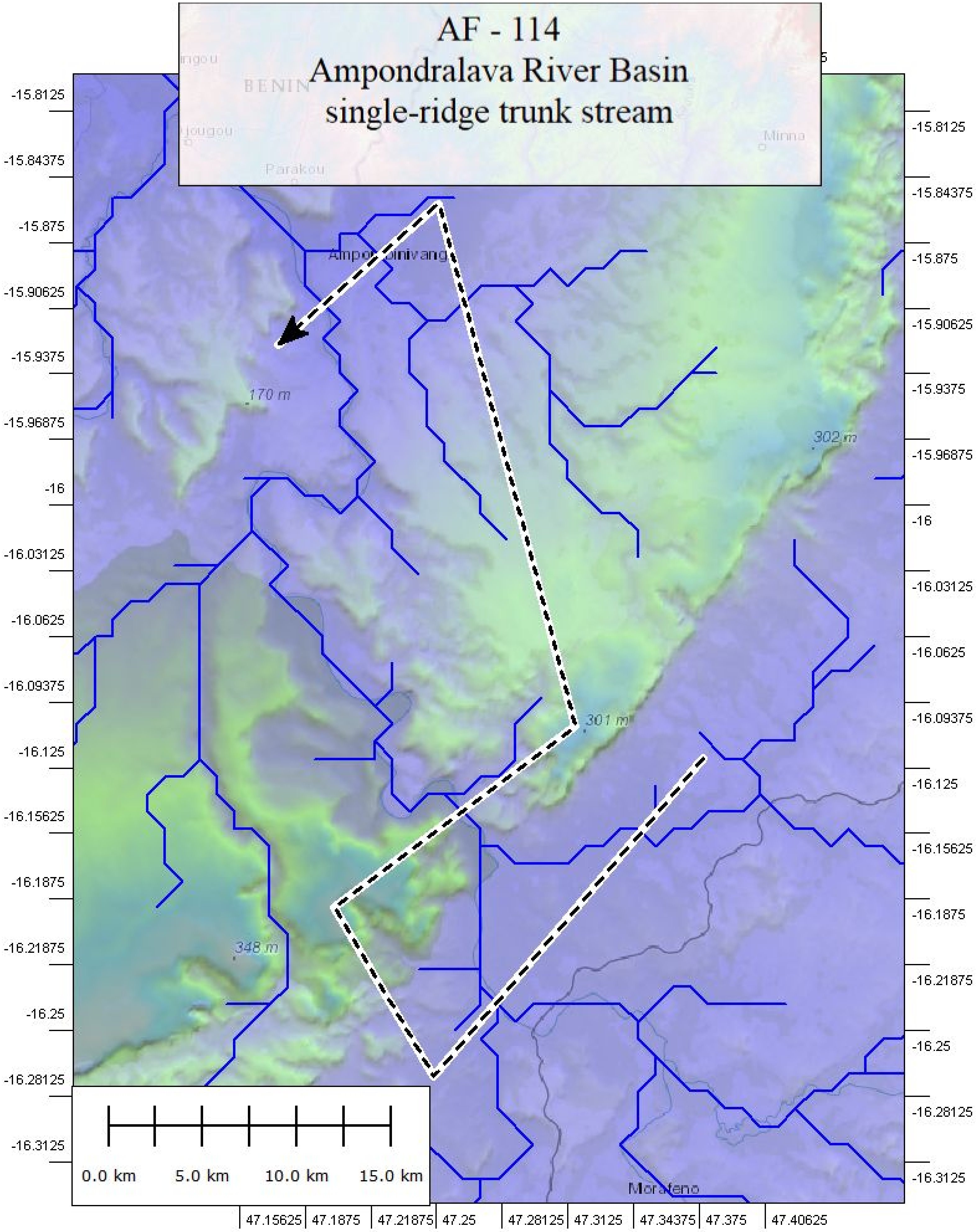
AF - 114
Ampondralava River Basin
single-ridge trunk stream
BENIN
Parakou
Minna
170 m
302 m
301 m
348 m
Morafeno
0.0 km
5.0 km
10.0 km
15.0 km
-15.8125
-15.84375
-15.875
-15.90625
-15.9375
-15.96875
-16
-16.03125
-16.0625
-16.09375
-16.125
-16.15625
-16.1875
-16.21875
-16.25
-16.28125
-16.3125
47.15625
47.1875
47.21875
47.25
47.28125
47.3125
47.34375
47.375
47.40625

# AF - 118
## Endorheic basin Basin
## single-ridge trunk stream

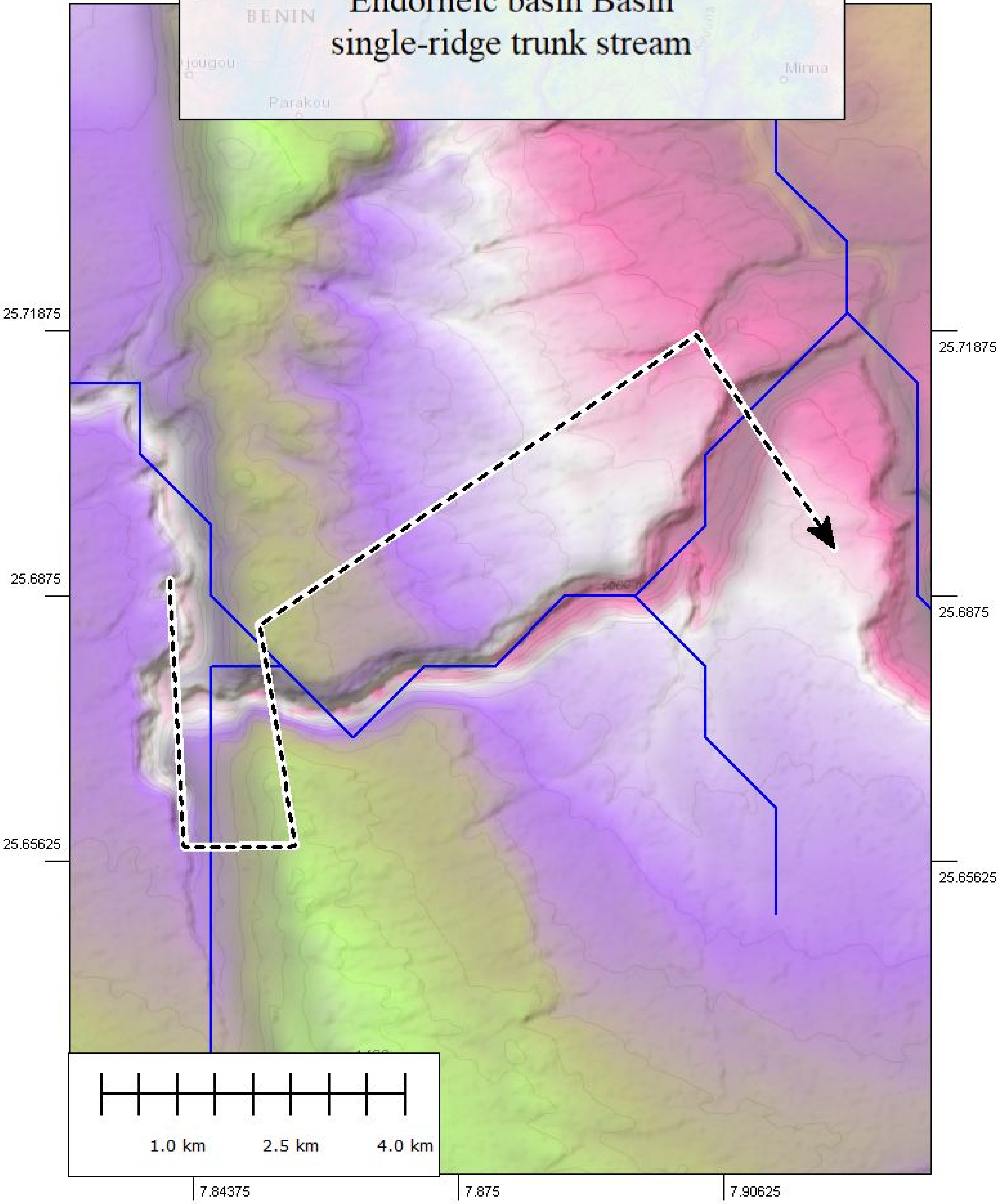

# AF - 157
## Oued Sebou Basin
## single-ridge trunk stream

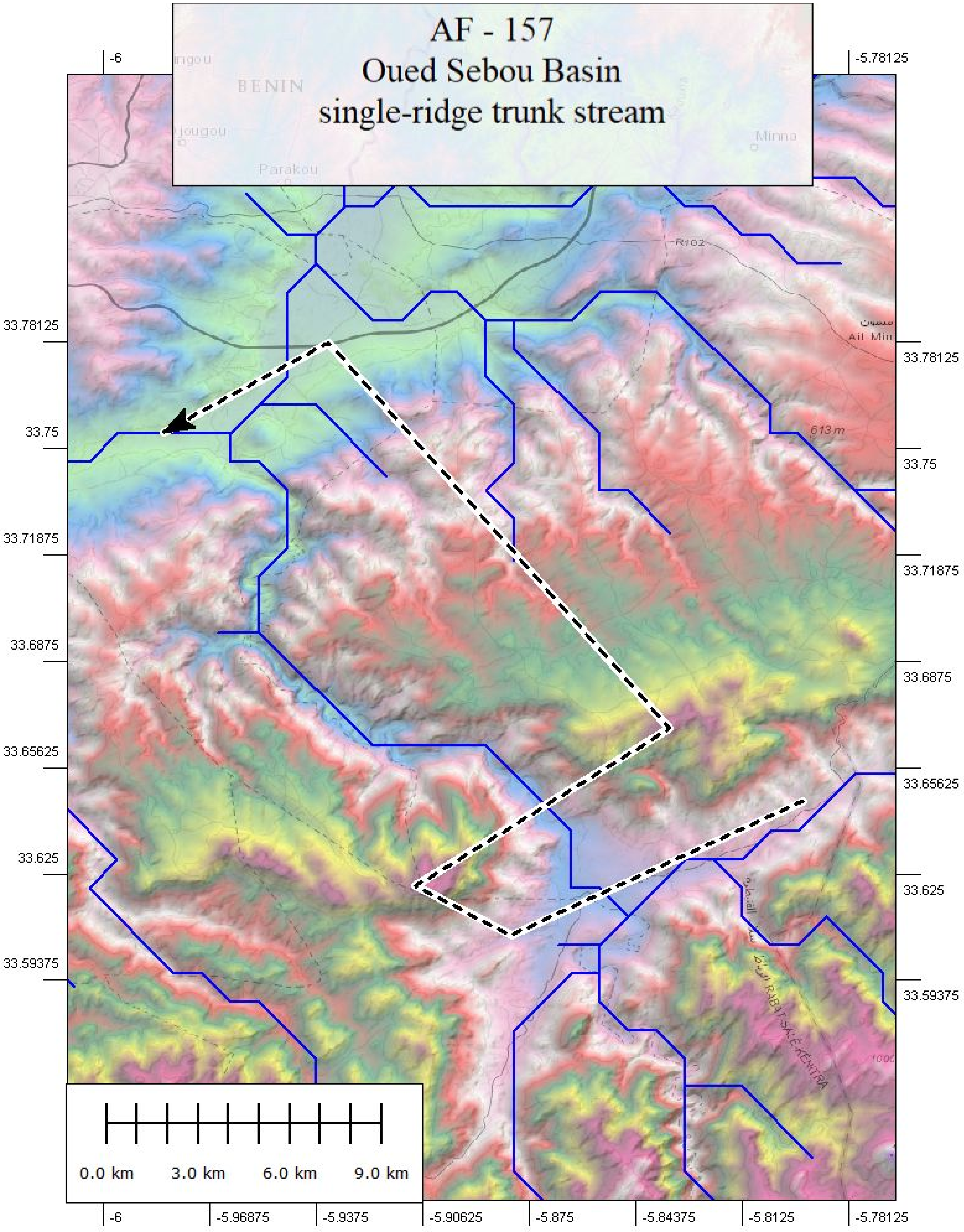

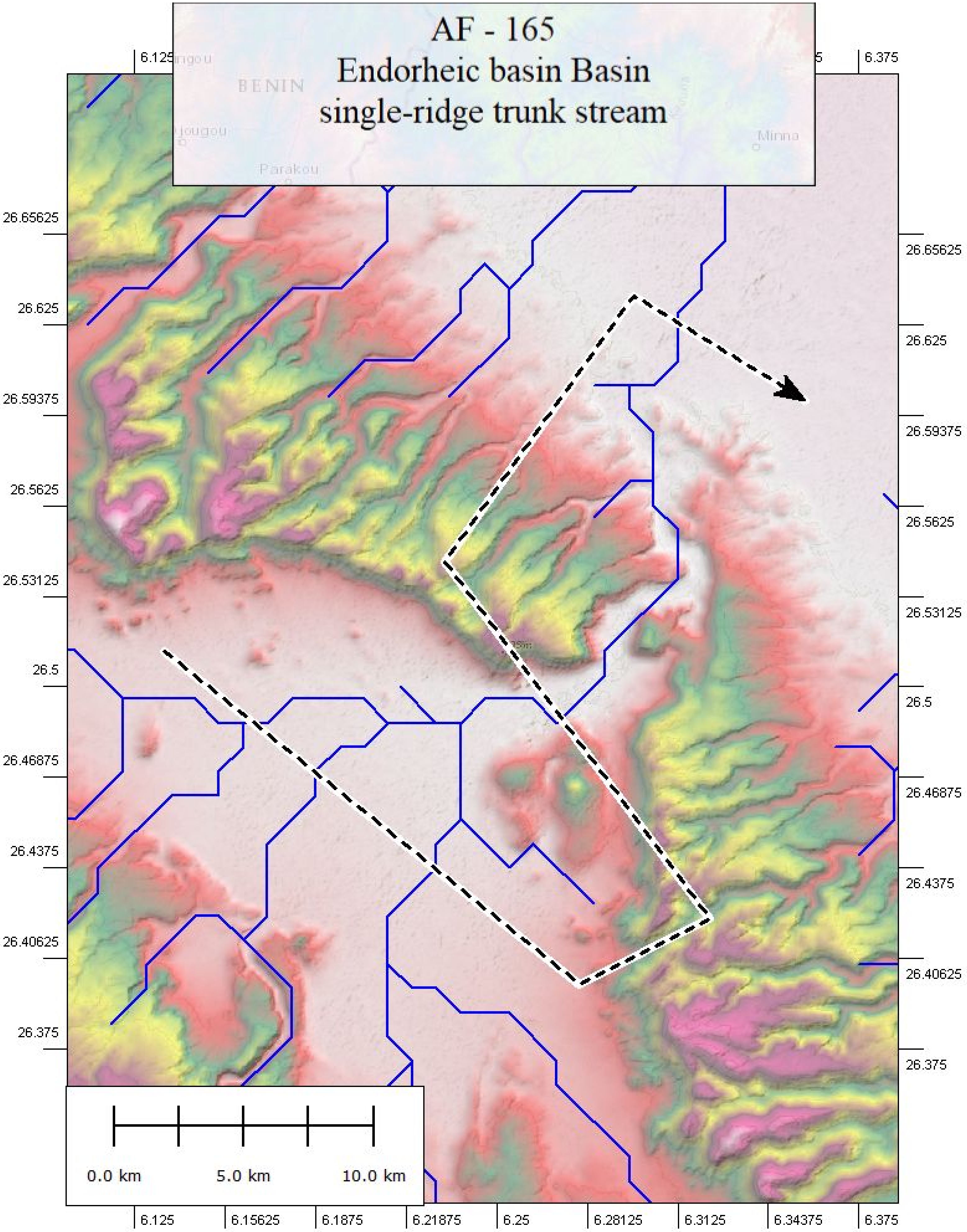
AF - 165
Endorheic basin Basin
single-ridge trunk stream
BENIN
Parakou
Minna
6.125
6.15625
6.1875
6.21875
6.25
6.28125
6.3125
6.34375
6.375
26.65625
26.625
26.59375
26.5625
26.53125
26.5
26.46875
26.4375
26.40625
26.375
0.0 km
5.0 km
10.0 km

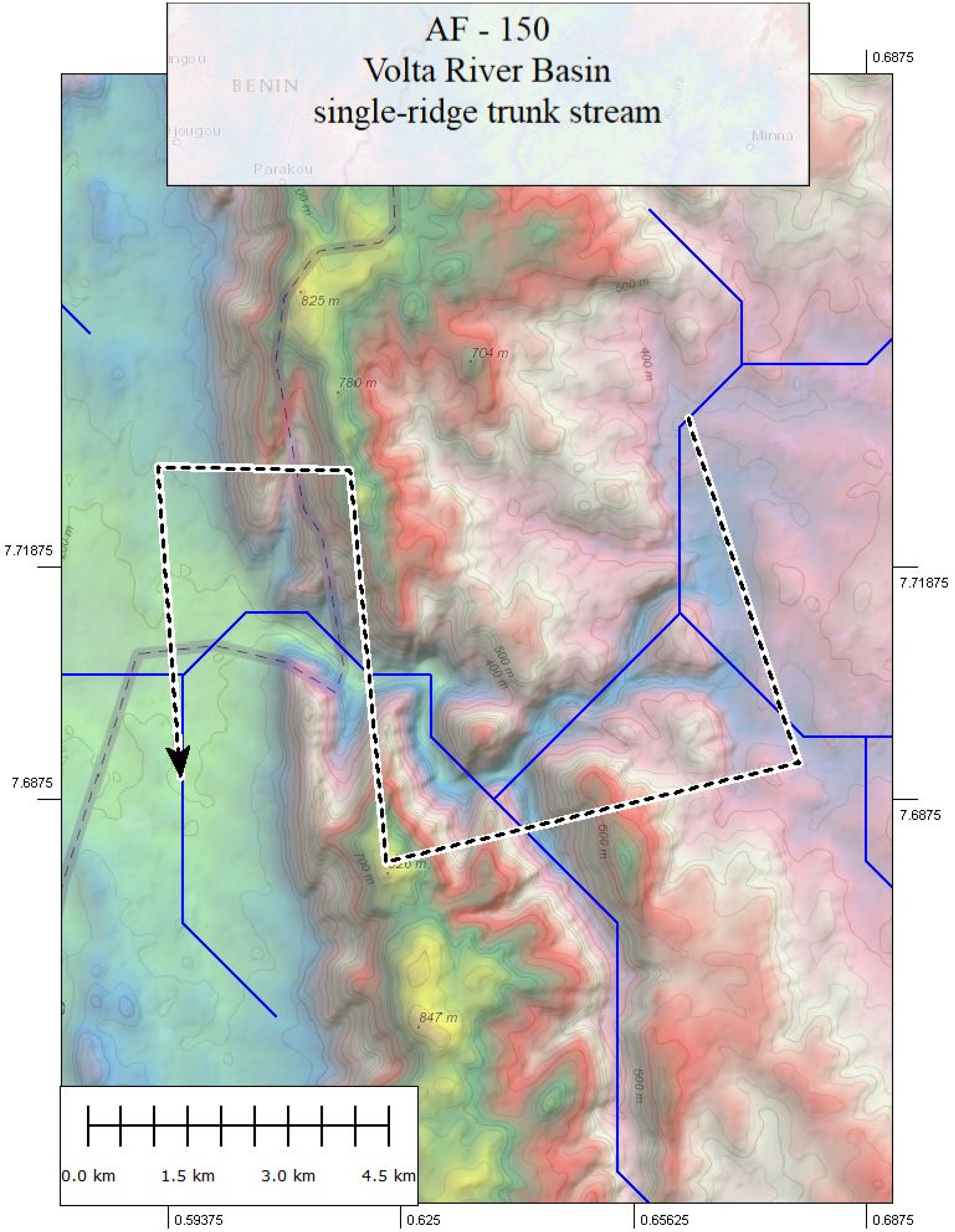

AF - 150
Volta River Basin
single-ridge trunk stream
BENIN
Parakou
Minna
825 m
704 m
780 m
500 m
400 m
600 m
700 m
847 m
0.0 km
1.5 km
3.0 km
4.5 km
7.71875
7.6875
0.59375
0.625
0.65625
0.6875

# AF - 24
## Gourits River Basin
## Doring River
## single-ridge head stream

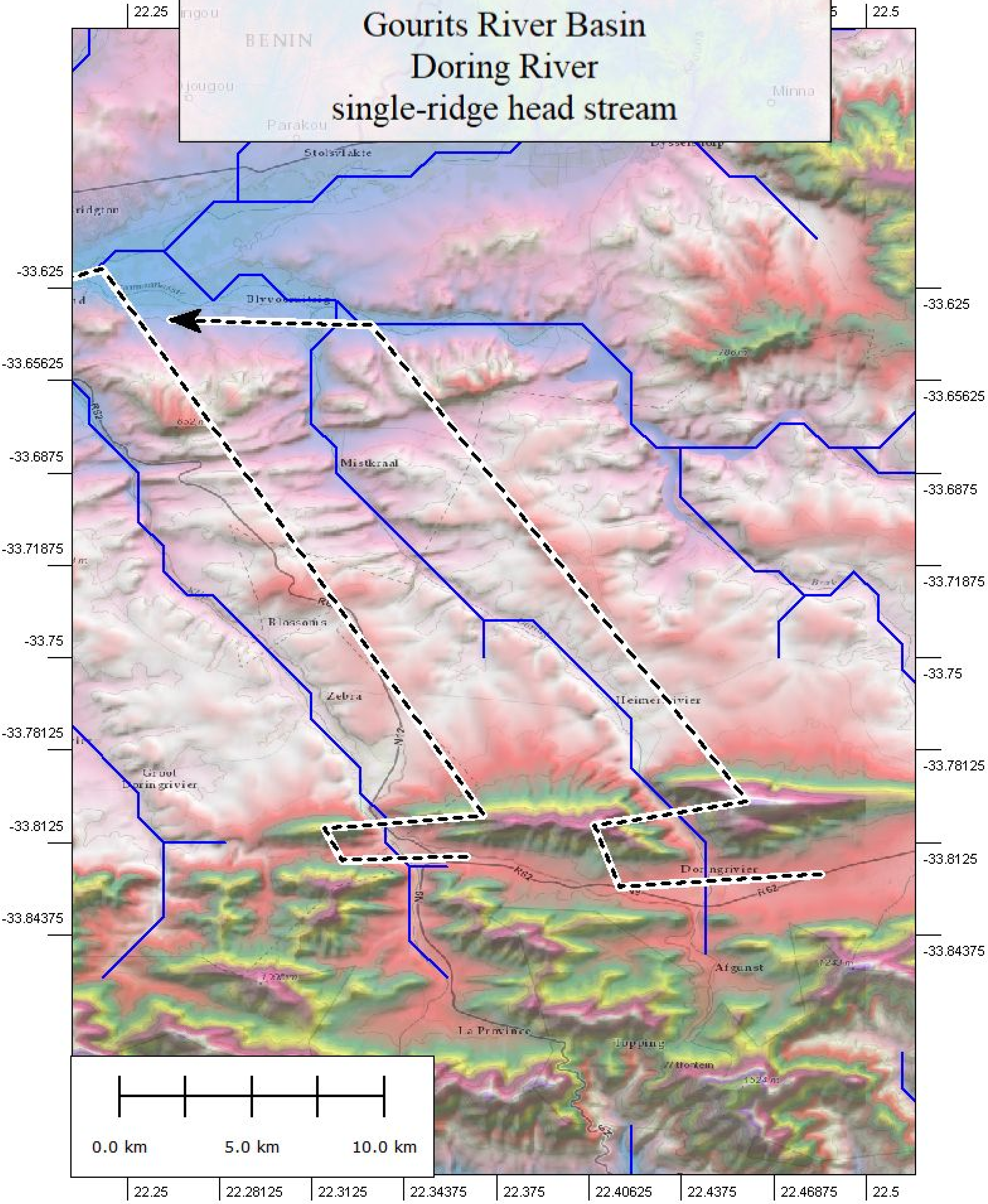

# AF - 55
# Endorheic basin Basin
# Turkwel River
# single-ridge trunk stream

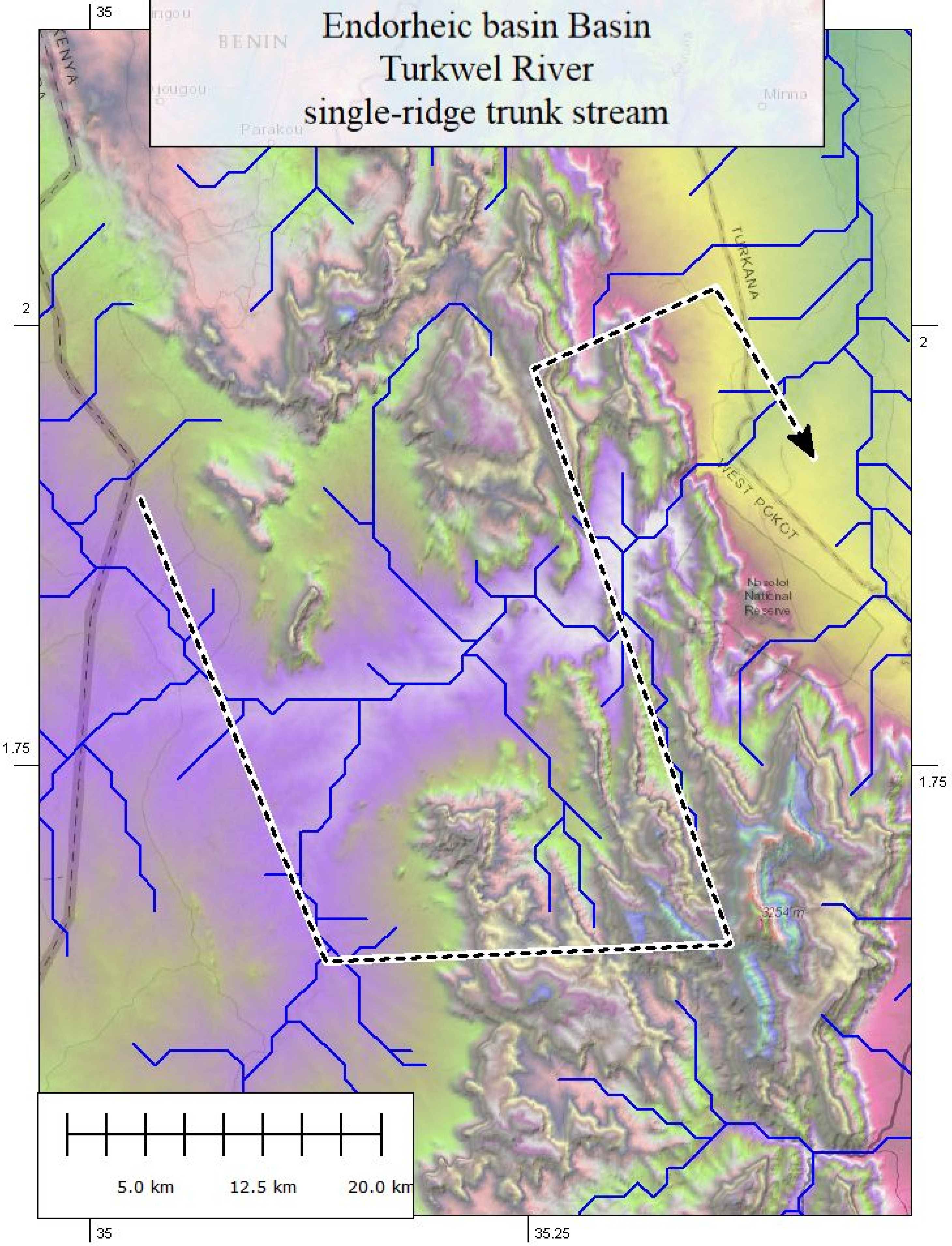

# AF - 57
## Congo River Basin
## Lufira River
## single-ridge trunk stream

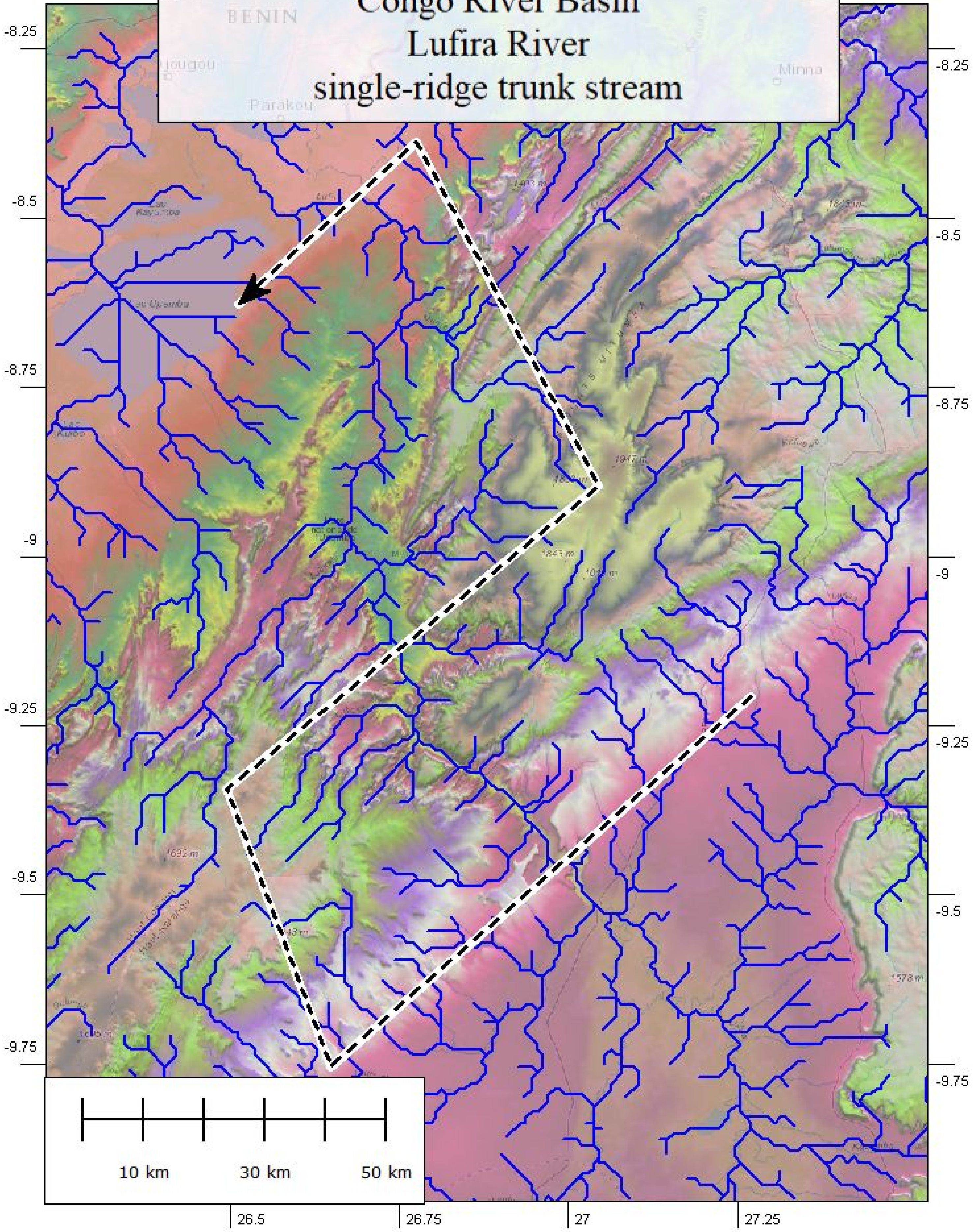

# AF - 58
## Congo River Basin
## Luvua River
## single-ridge trunk stream

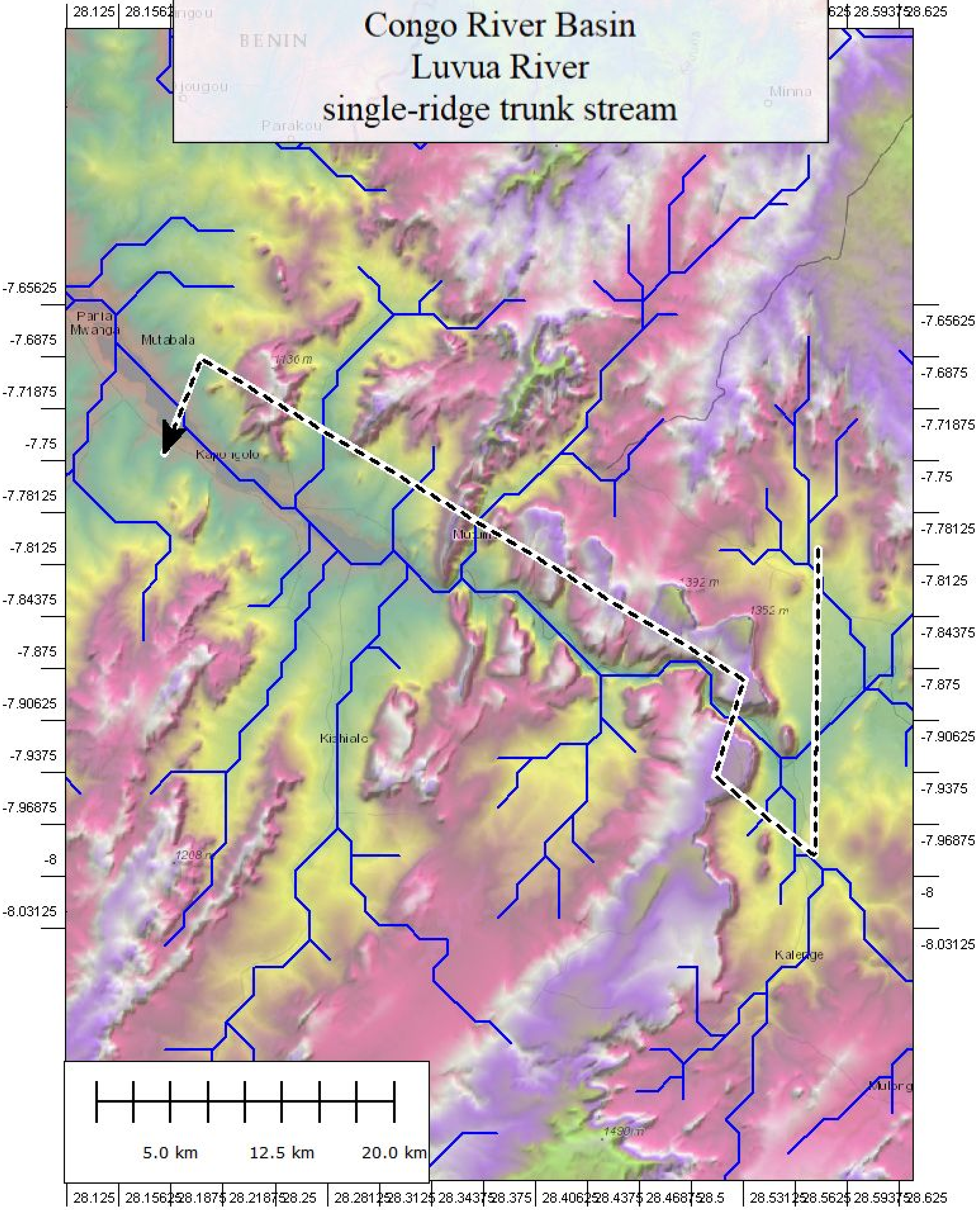

# AF - 61
# Sonderend River Basin
# Hex River
# single-ridge trunk stream

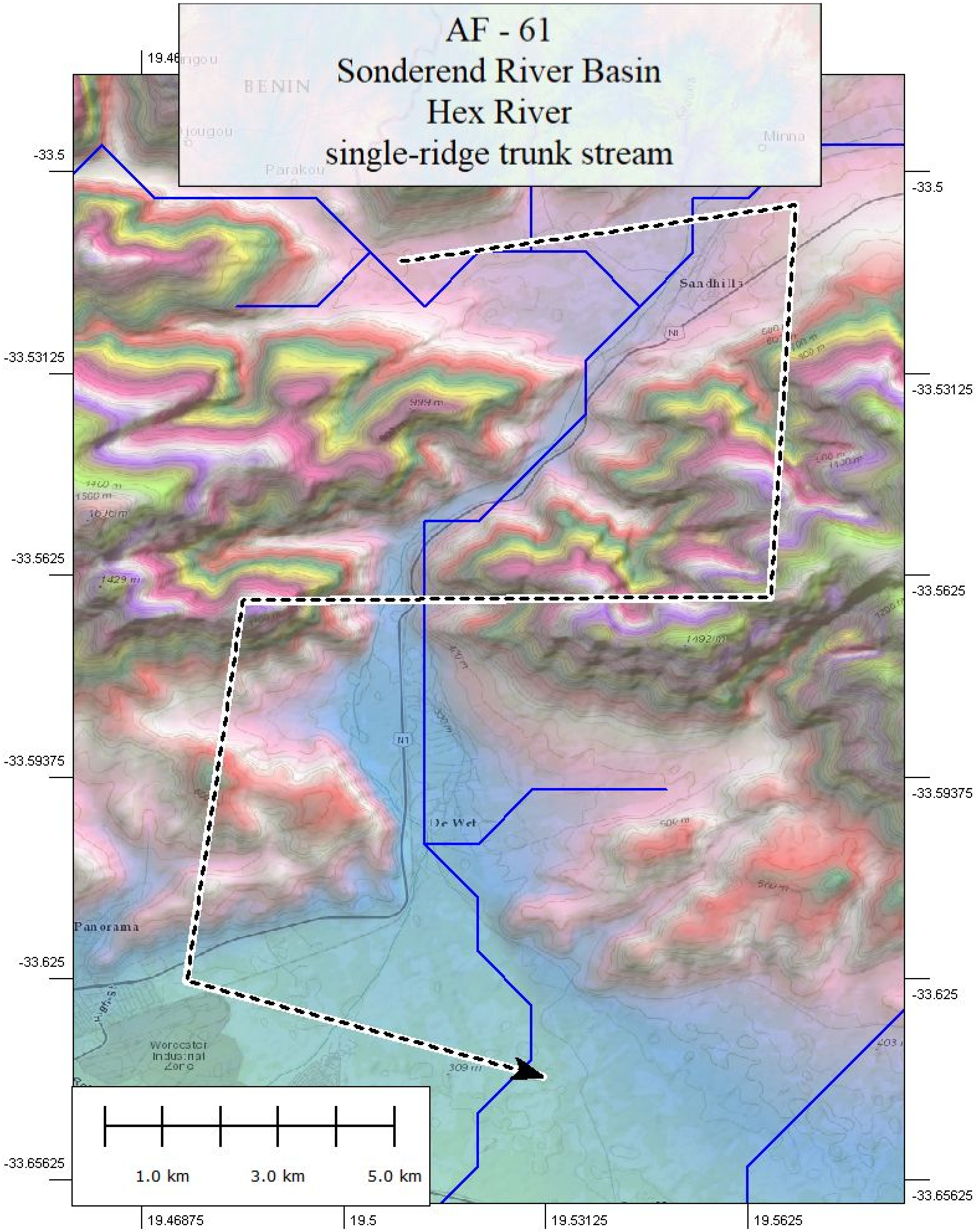

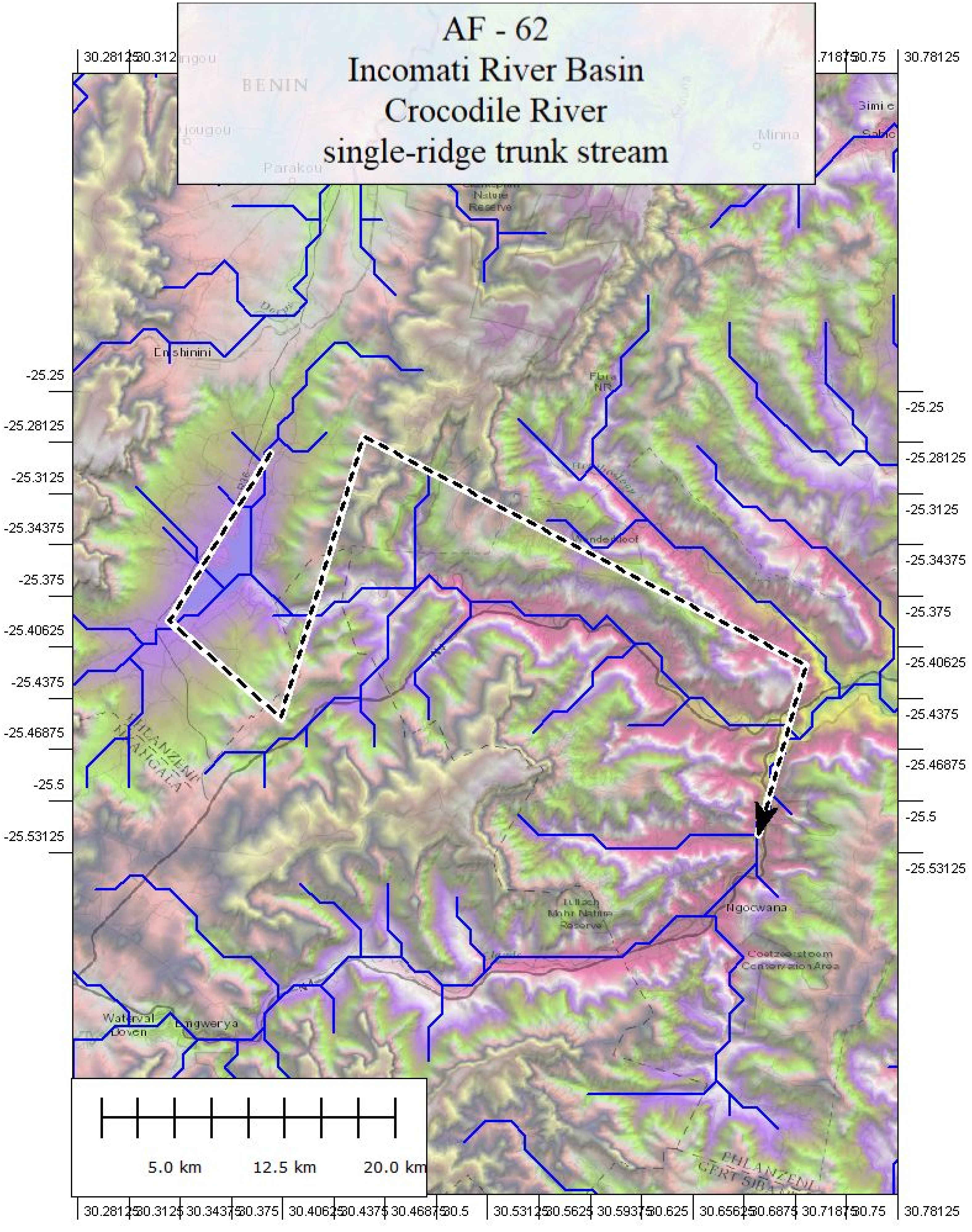
AF - 62
Incomati River Basin
Crocodile River
single-ridge trunk stream
30.28125
30.3125
30.34375
30.375
30.40625
30.4375
30.46875
30.5
30.53125
30.5625
30.59375
30.625
30.65625
30.6875
30.71875
30.75
30.78125
-25.25
-25.28125
-25.3125
-25.34375
-25.375
-25.40625
-25.4375
-25.46875
-25.5
-25.53125
BENIN
Parakou
Minna
Emshinini
Ngocwana
Coetzeestroom Conservation Area
Waterval Boven
Emgwenya
5.0 km
12.5 km
20.0 km

# AF - 70
## Oued Draa Basin
## Oued Draa
## single-ridge trunk stream

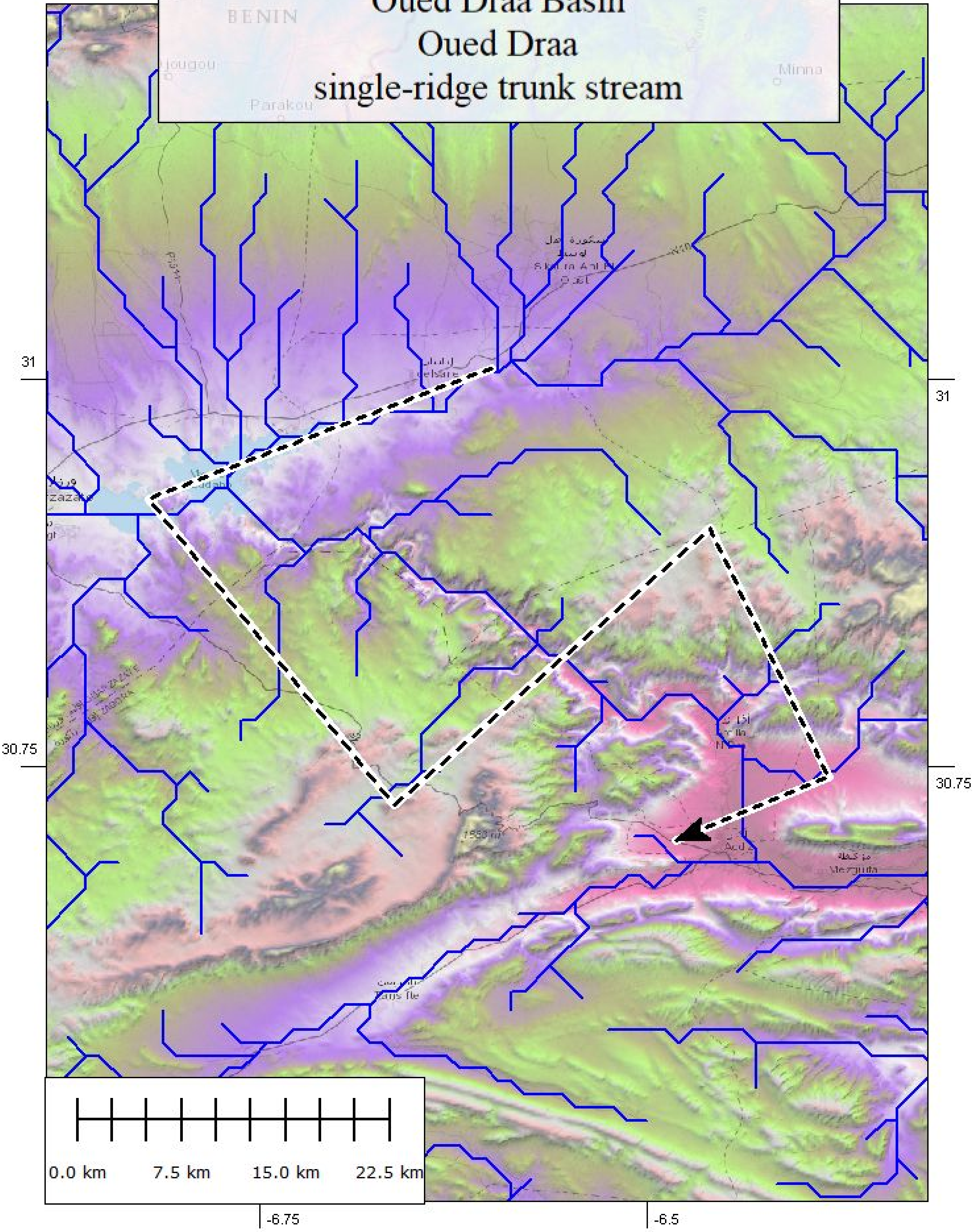

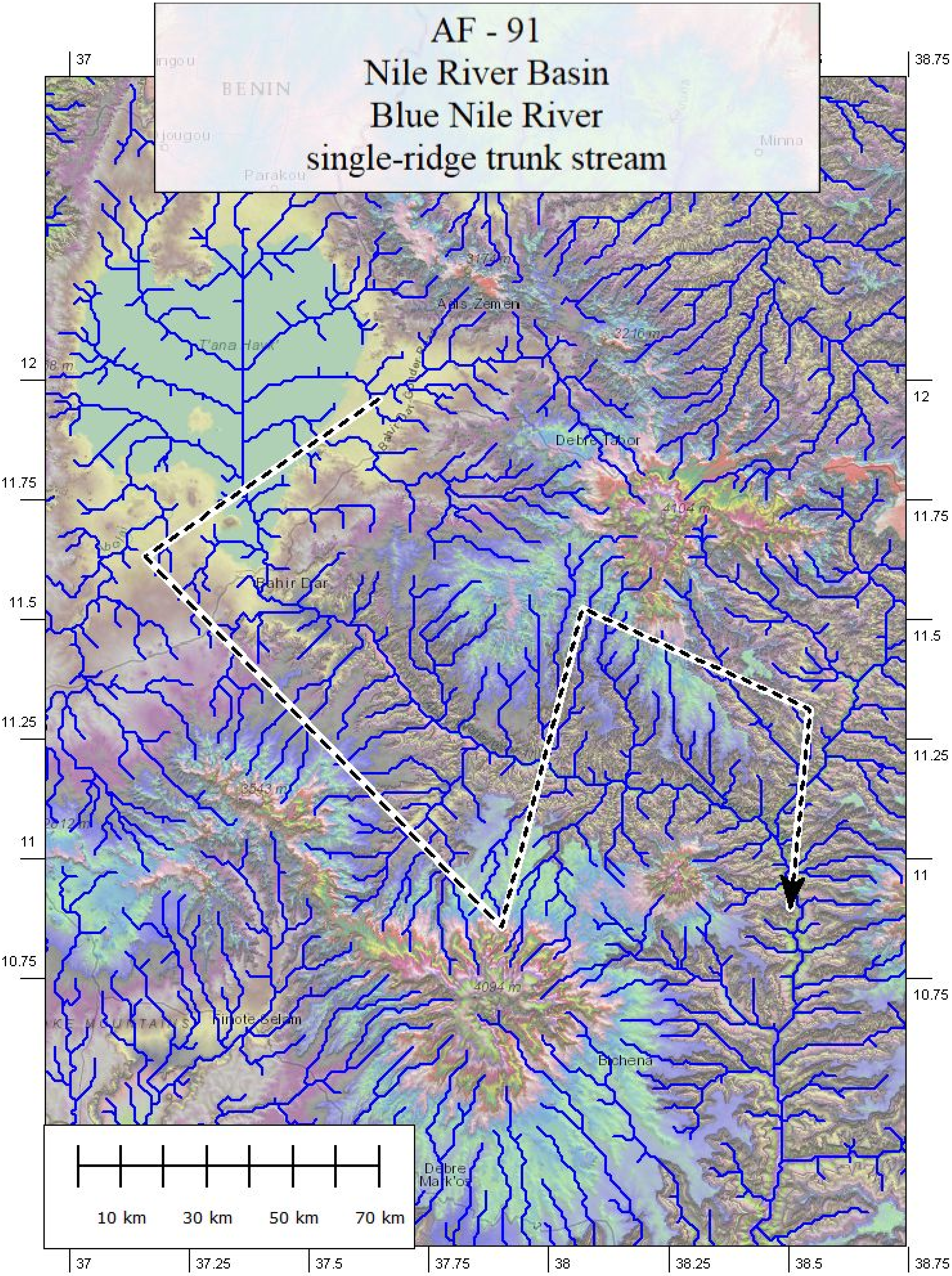
AF - 91
Nile River Basin
Blue Nile River
single-ridge trunk stream
Debre Tabor
Bahir Dar
Addis Zemen
Finote Selam
Bichena
Debre Mark'os
3174 m
3216 m
4104 m
3543 m
4094 m
10 km
30 km
50 km
70 km
37
37.25
37.5
37.75
38
38.25
38.5
38.75
12
11.75
11.5
11.25
11
10.75

# AF - 92
## Jubba River Basin
## Dawa River
## single-ridge trunk stream

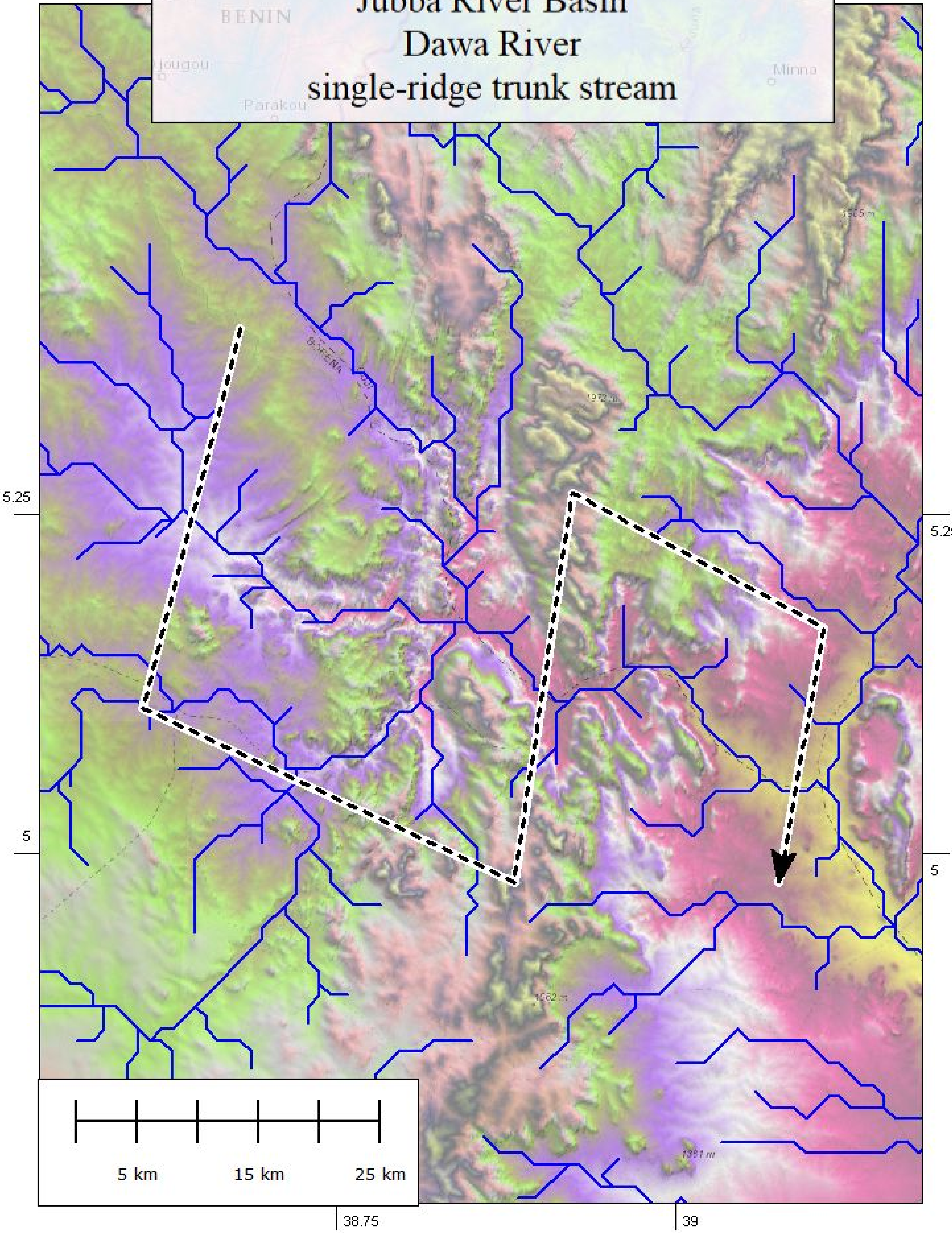

# AF - 106
## Oued Cheliff Basin
## Oued Cheliff
## single-ridge trunk stream

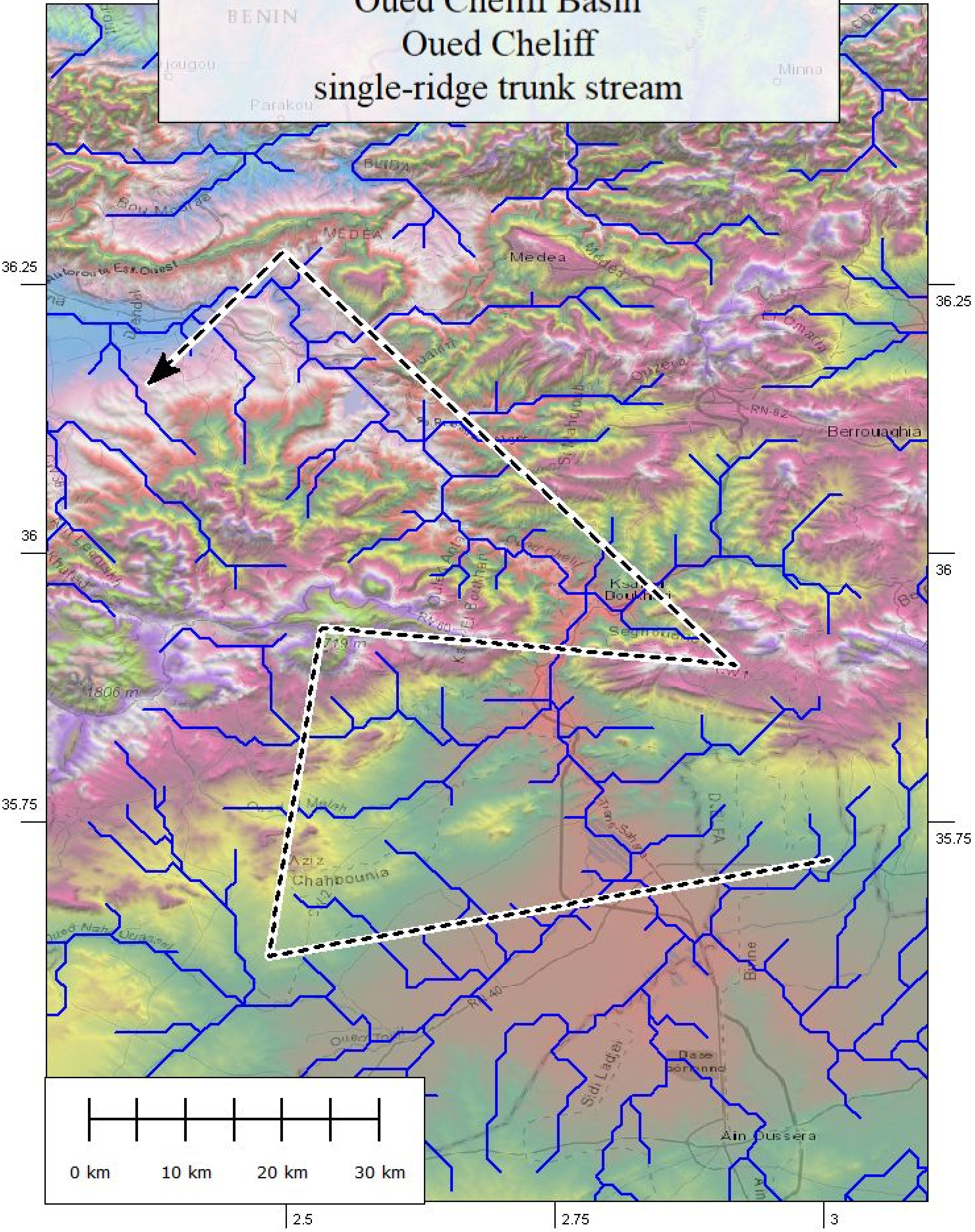

# AF - 122
## Cambongo River Basin
## Cambongo River
## single-ridge trunk stream

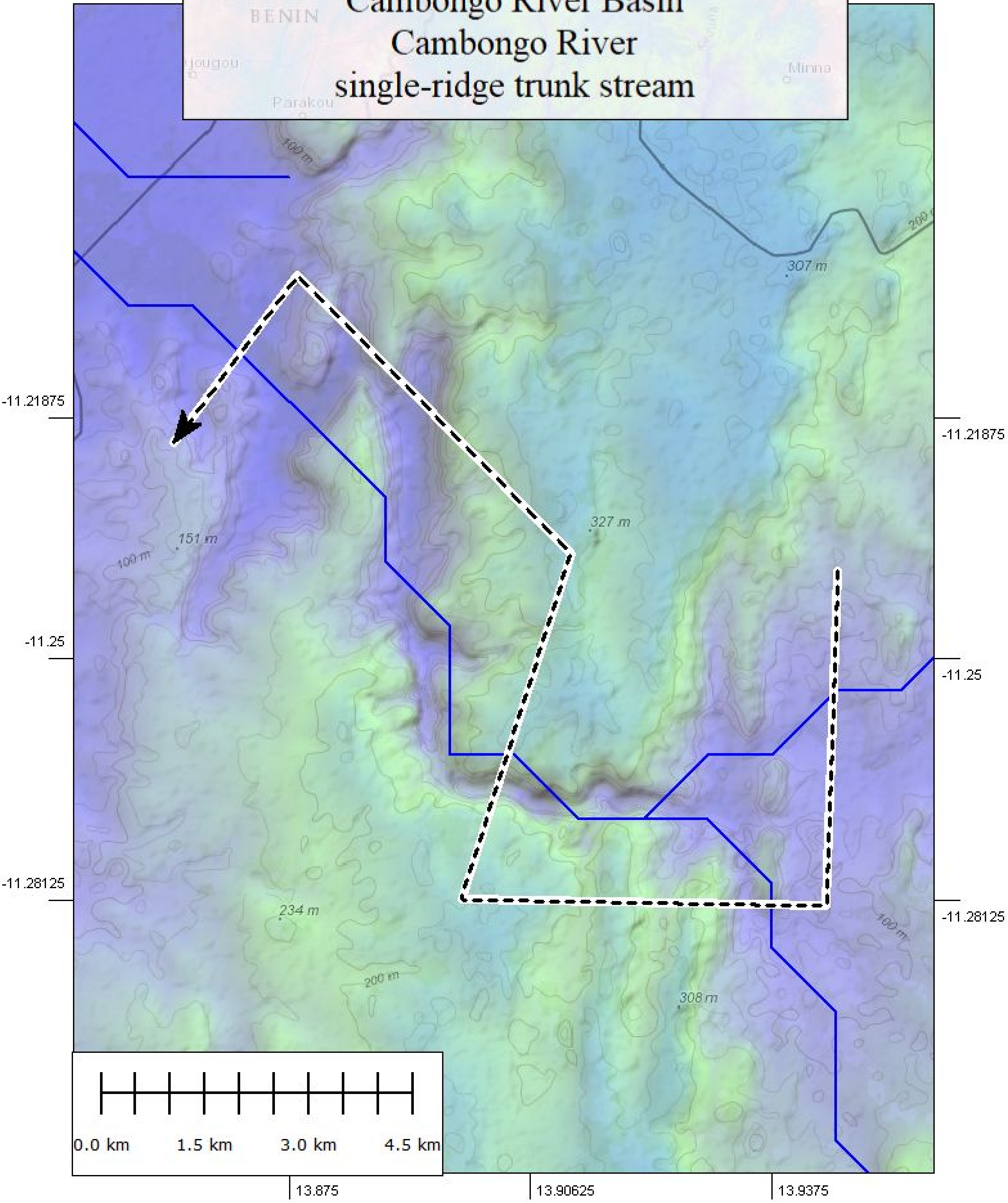

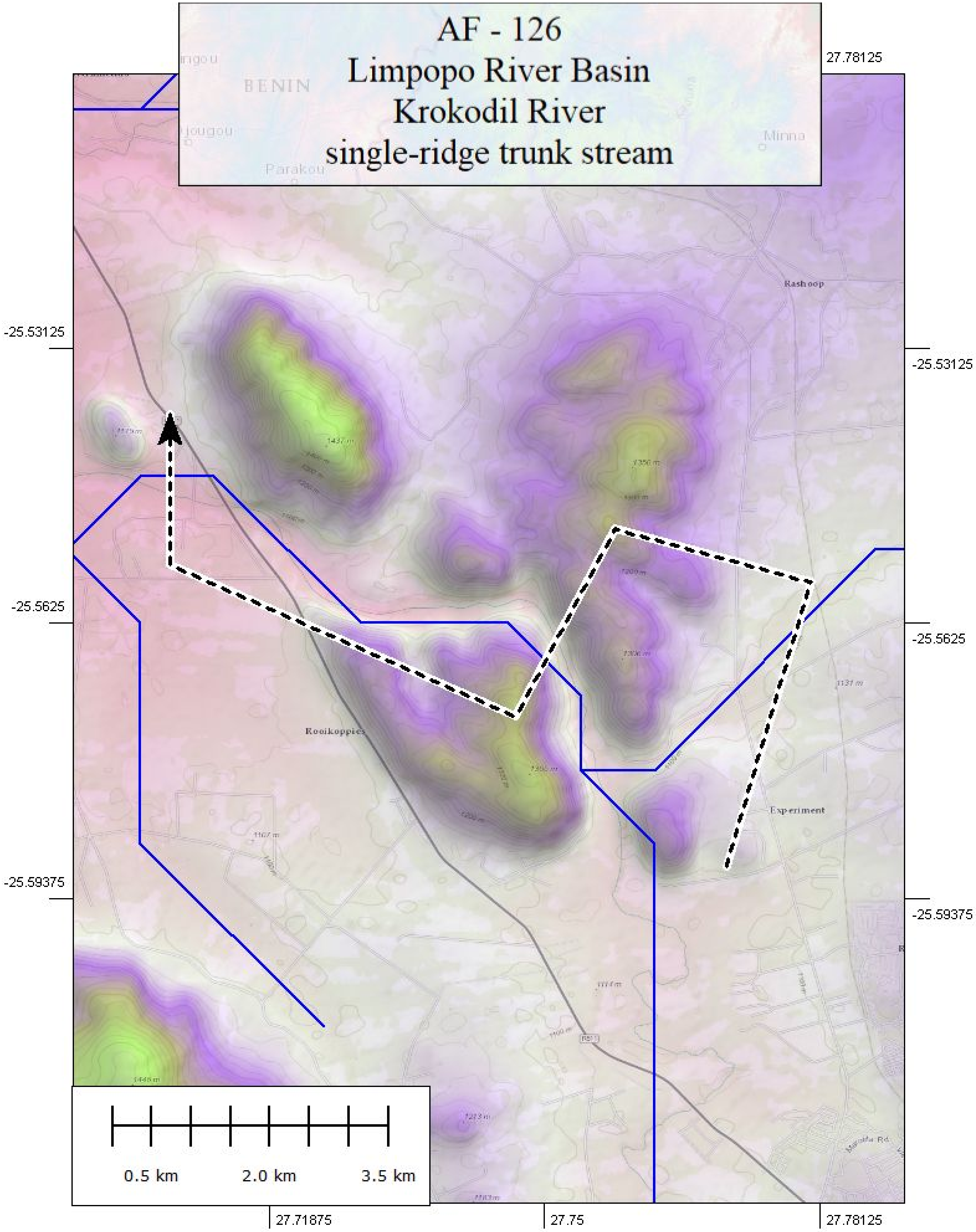

AF - 126
Limpopo River Basin
Krokodil River
single-ridge trunk stream
BENIN
Parakou
Minna
Rashoop
Rooikoppies
Experiment
1437 m
1350 m
1131 m
1114 m
1213 m
27.78125
-25.53125
-25.5625
-25.59375
27.71875
27.75
0.5 km
2.0 km
3.5 km

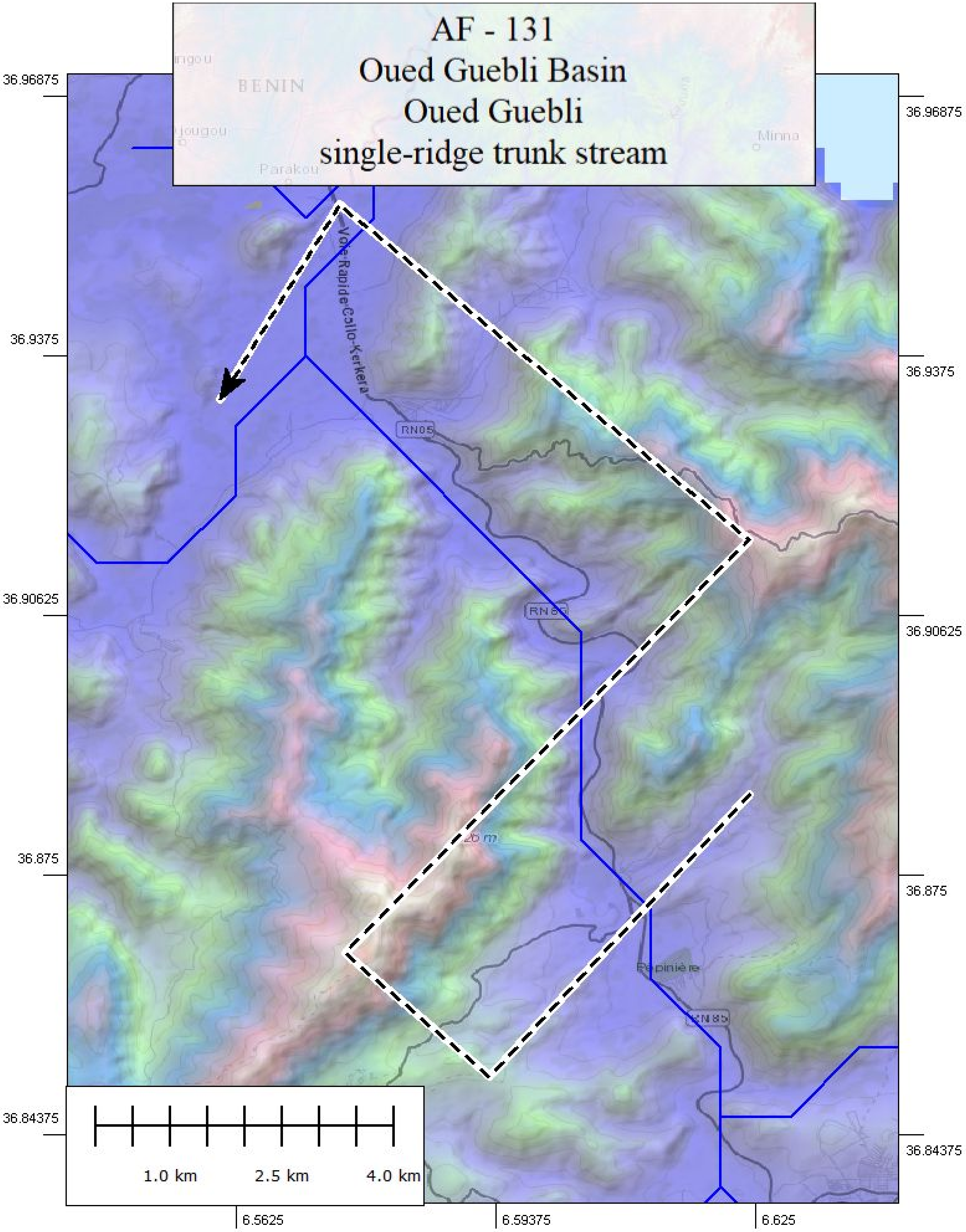
AF - 131
Oued Guebli Basin
Oued Guebli
single-ridge trunk stream
36.96875
36.9375
36.90625
36.875
36.84375
6.5625
6.59375
6.625
Voie Rapide Collo-Kerkera
RN85
Pépinière
1.0 km
2.5 km
4.0 km

# AF - 134
# Oum Er Rbia Basin
# Oued l'abid
# single-ridge trunk stream

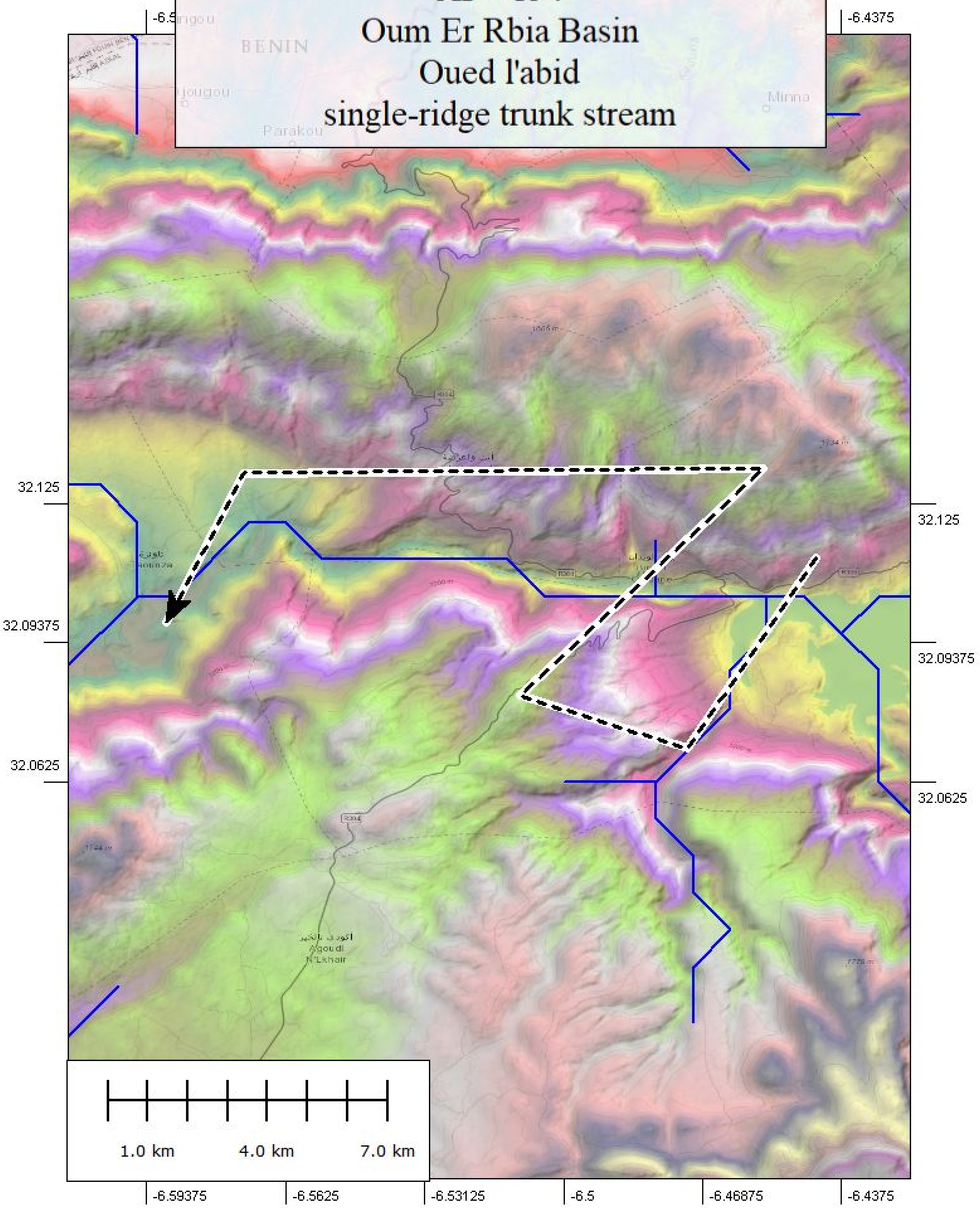

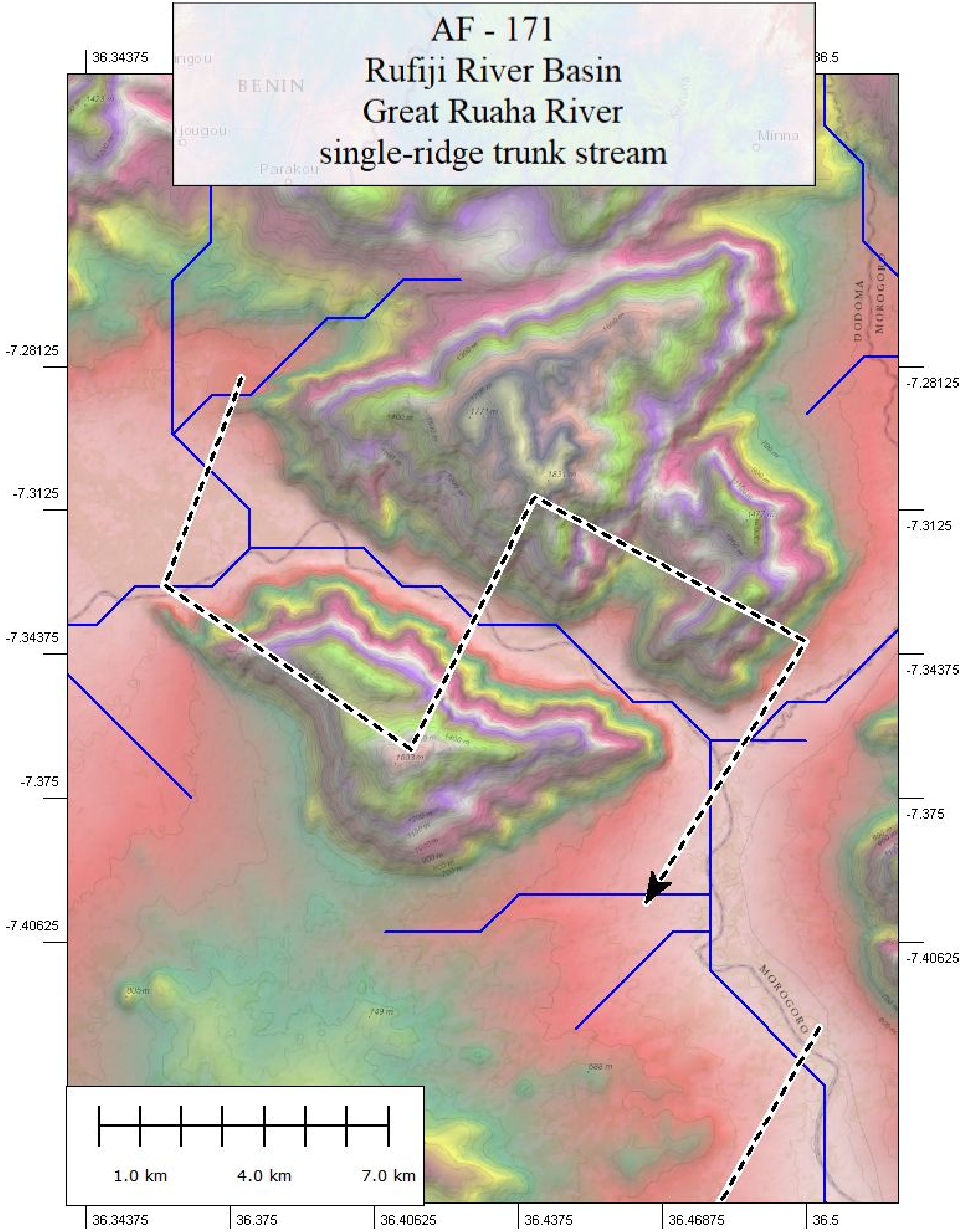
AF - 171
Rufiji River Basin
Great Ruaha River
single-ridge trunk stream
36.34375
36.375
36.40625
36.4375
36.46875
36.5
-7.28125
-7.3125
-7.34375
-7.375
-7.40625
1.0 km
4.0 km
7.0 km
MOROGORO
DODOMA
BENIN
Parakou

# AF - 177
## Morondava River Basin
## Morondava River
## single-ridge trunk stream

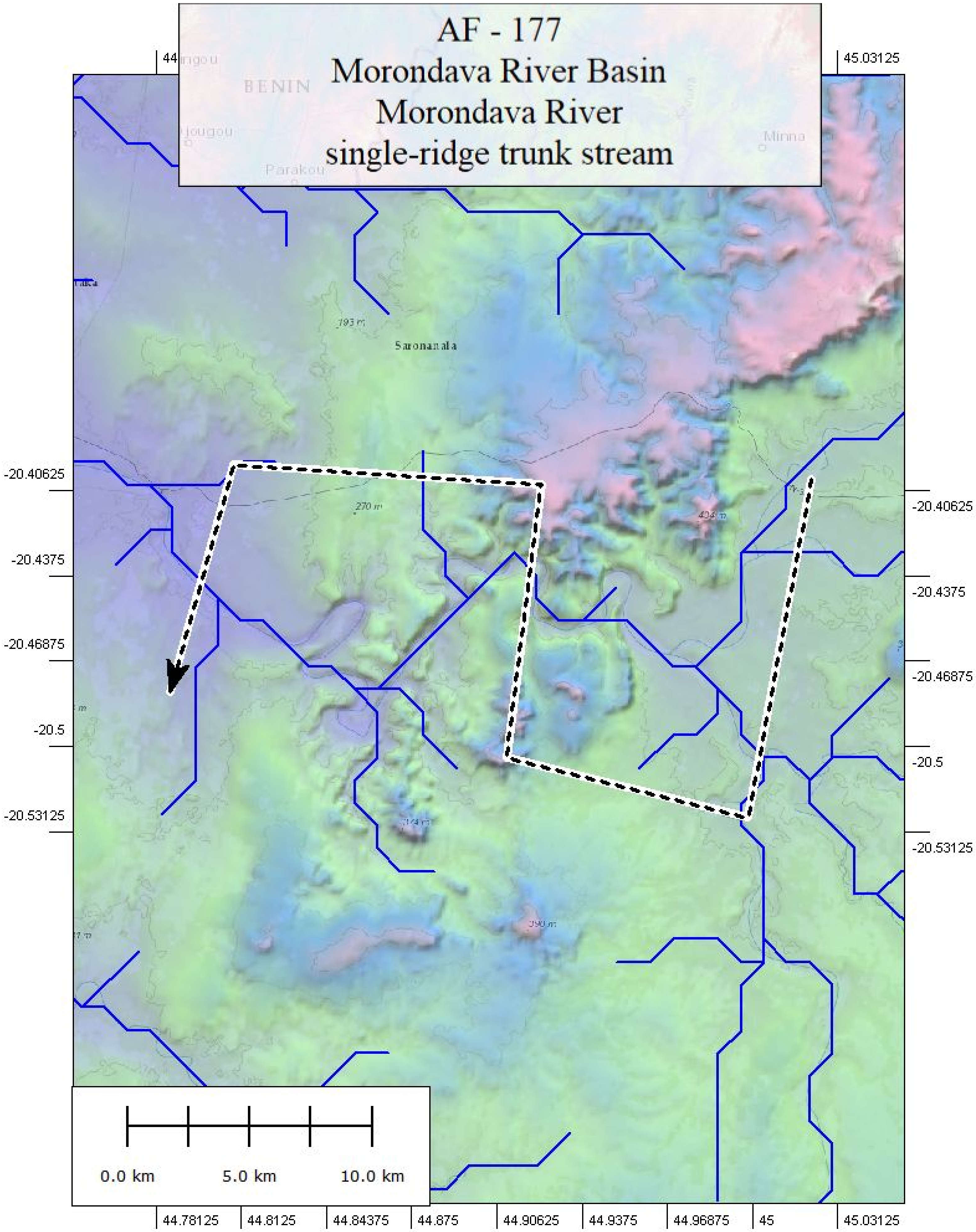

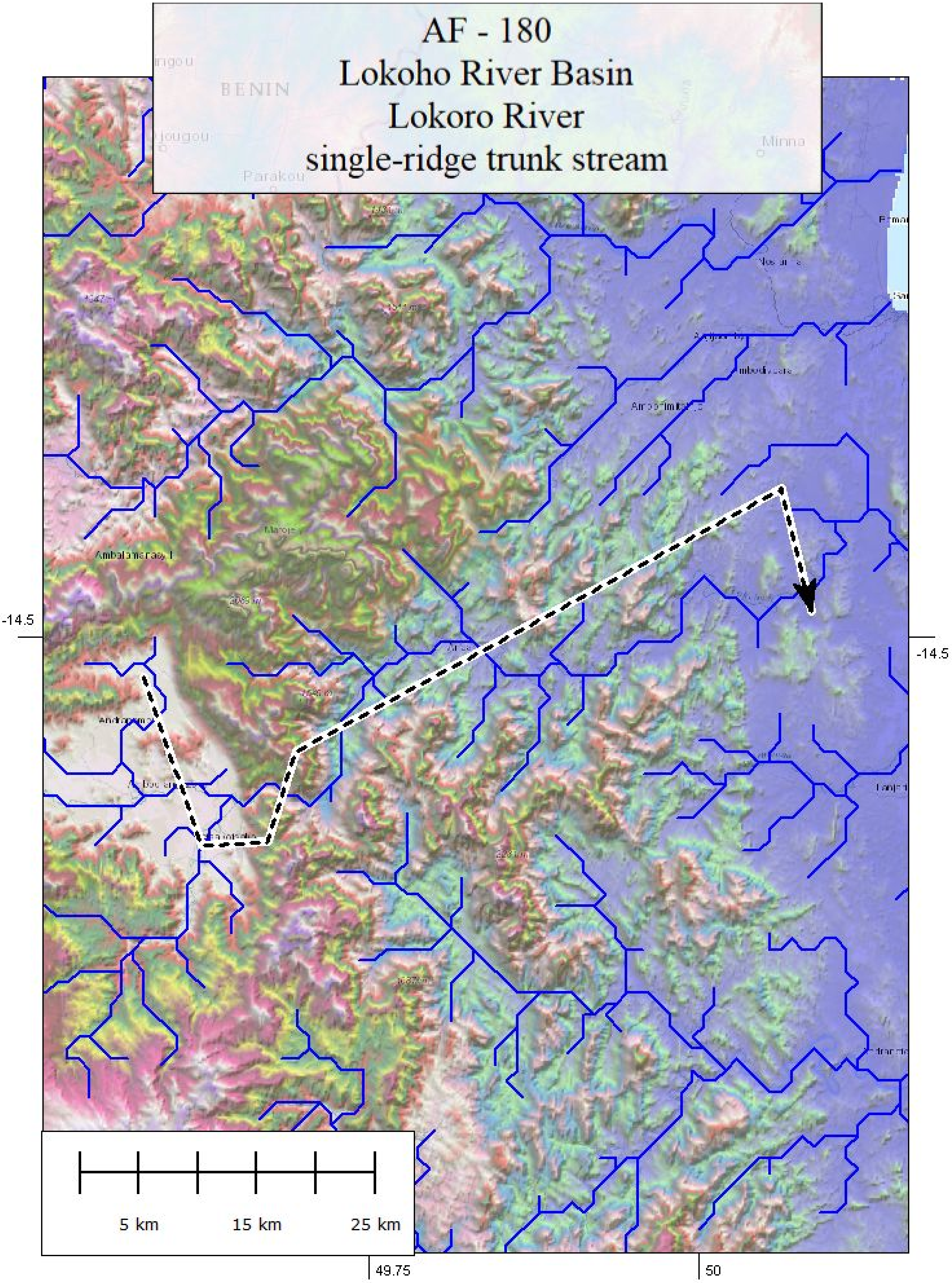
AF - 180
Lokoho River Basin
Lokoro River
single-ridge trunk stream
BENIN
Parakou
Minna
-14.5
-14.5
5 km
15 km
25 km
49.75
50

# AF - 181
## Oued Noun Basin
## Noun River
## single-ridge trunk stream

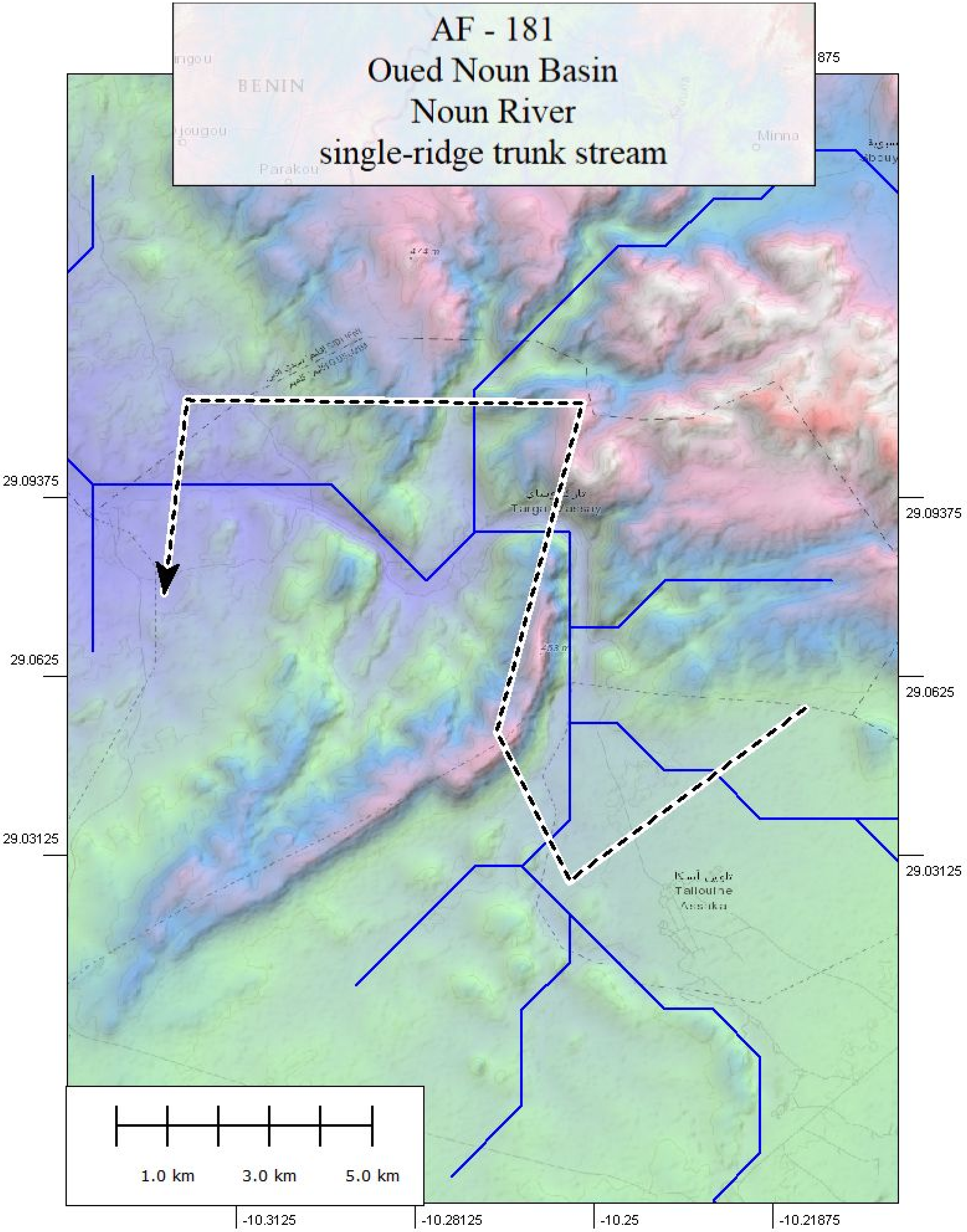

# AF - 190
# Zambezi River Basin
# Minjova River
# single-ridge trunk stream

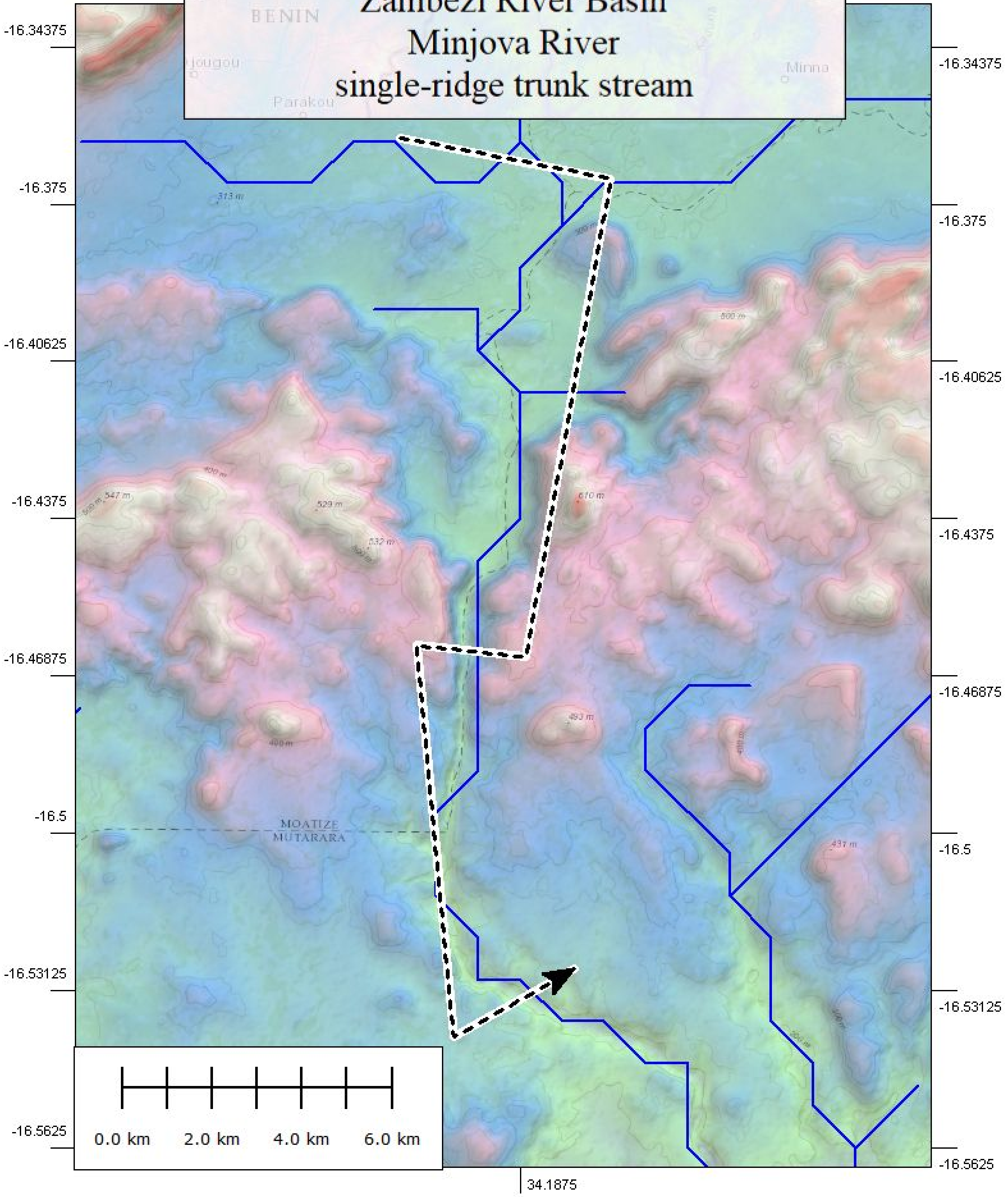

# AF - 199
# Cunene River Basin
# Kunene River
# single-ridge trunk stream

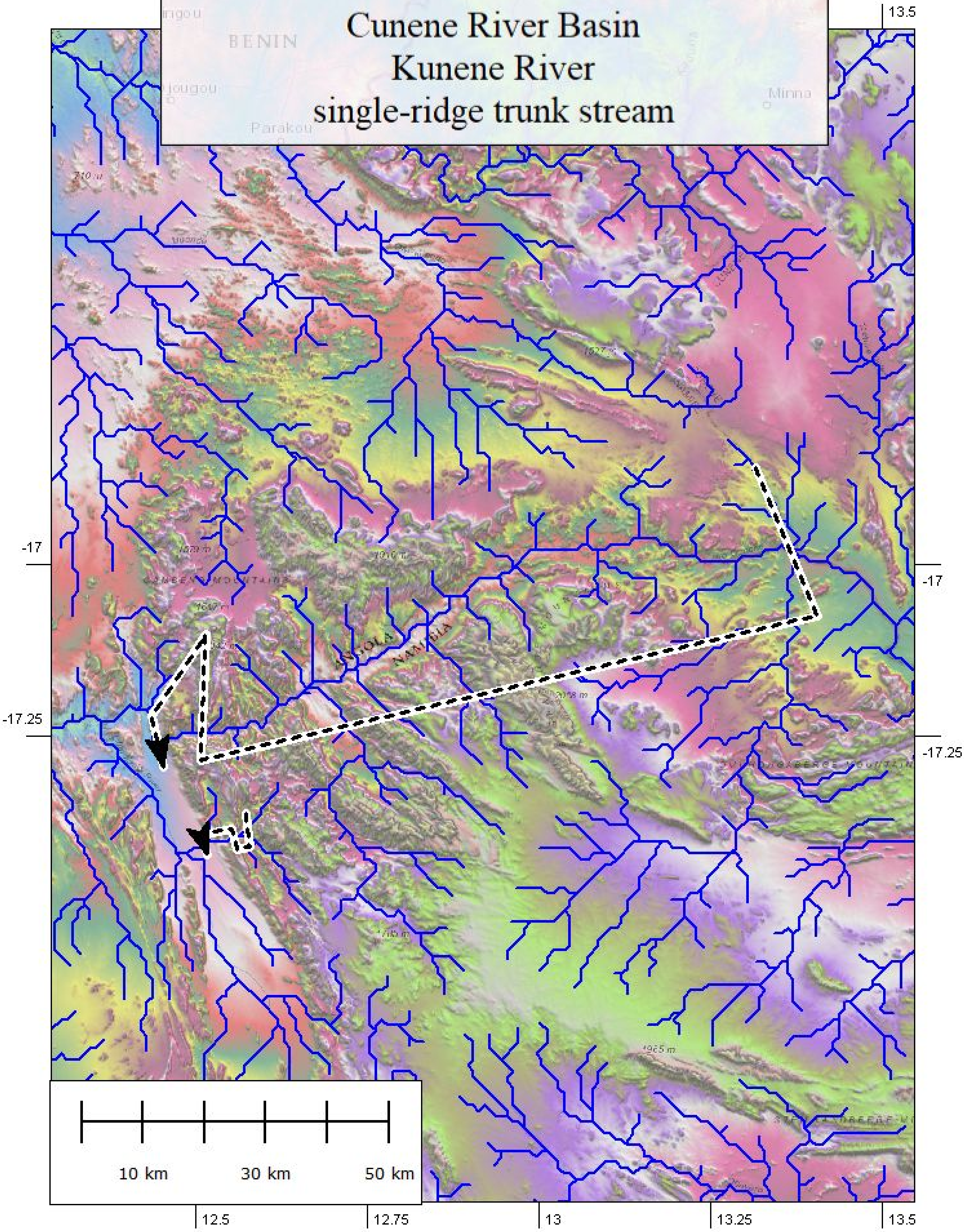

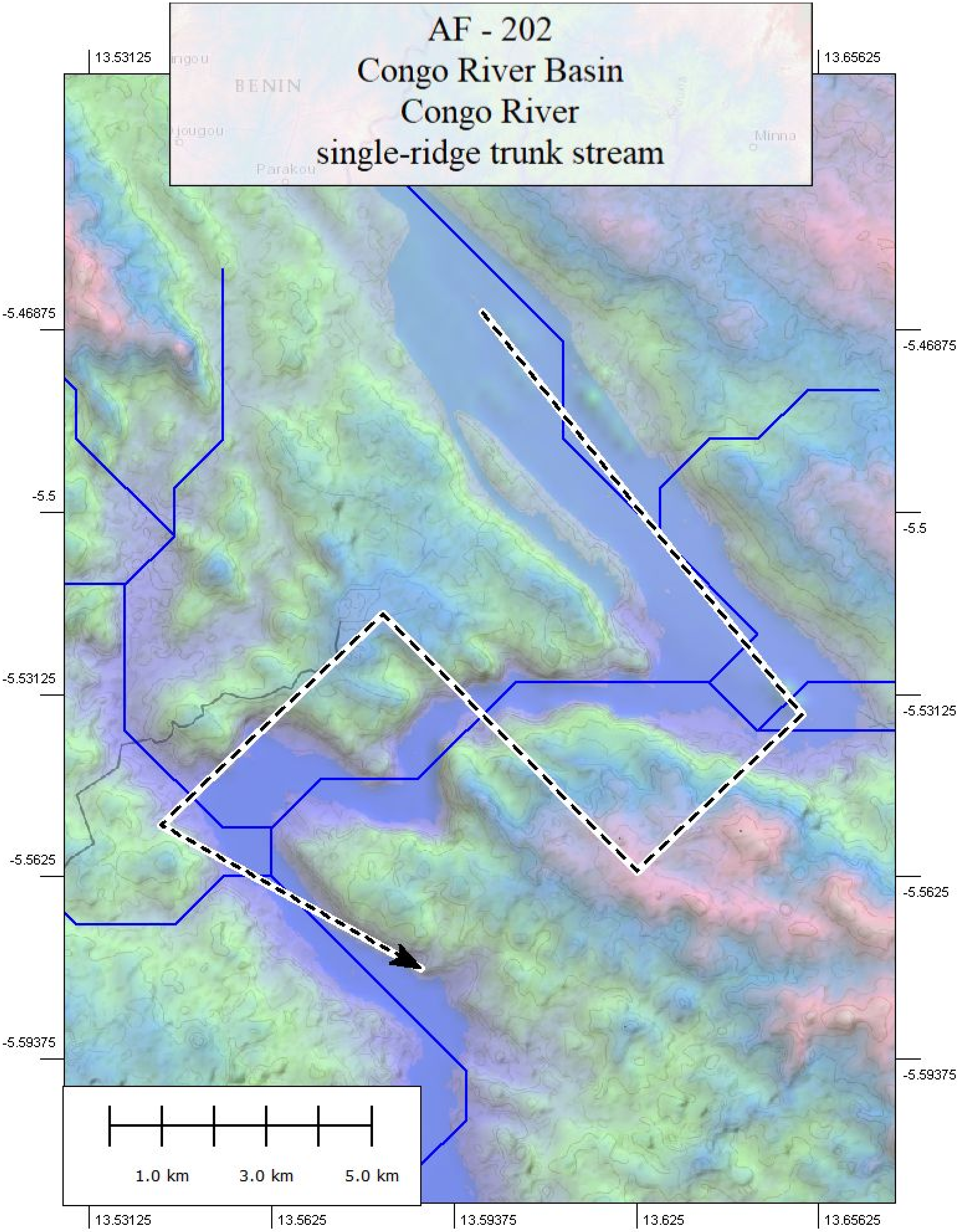
AF - 202
Congo River Basin
Congo River
single-ridge trunk stream
BENIN
Parakou
Minna
13.53125
13.65625
-5.46875
-5.5
-5.53125
-5.5625
-5.59375
13.5625
13.59375
13.625
1.0 km
3.0 km
5.0 km

# AF - 166
## Nile River Basin
## Kagera River
## single-ridge trunk stream

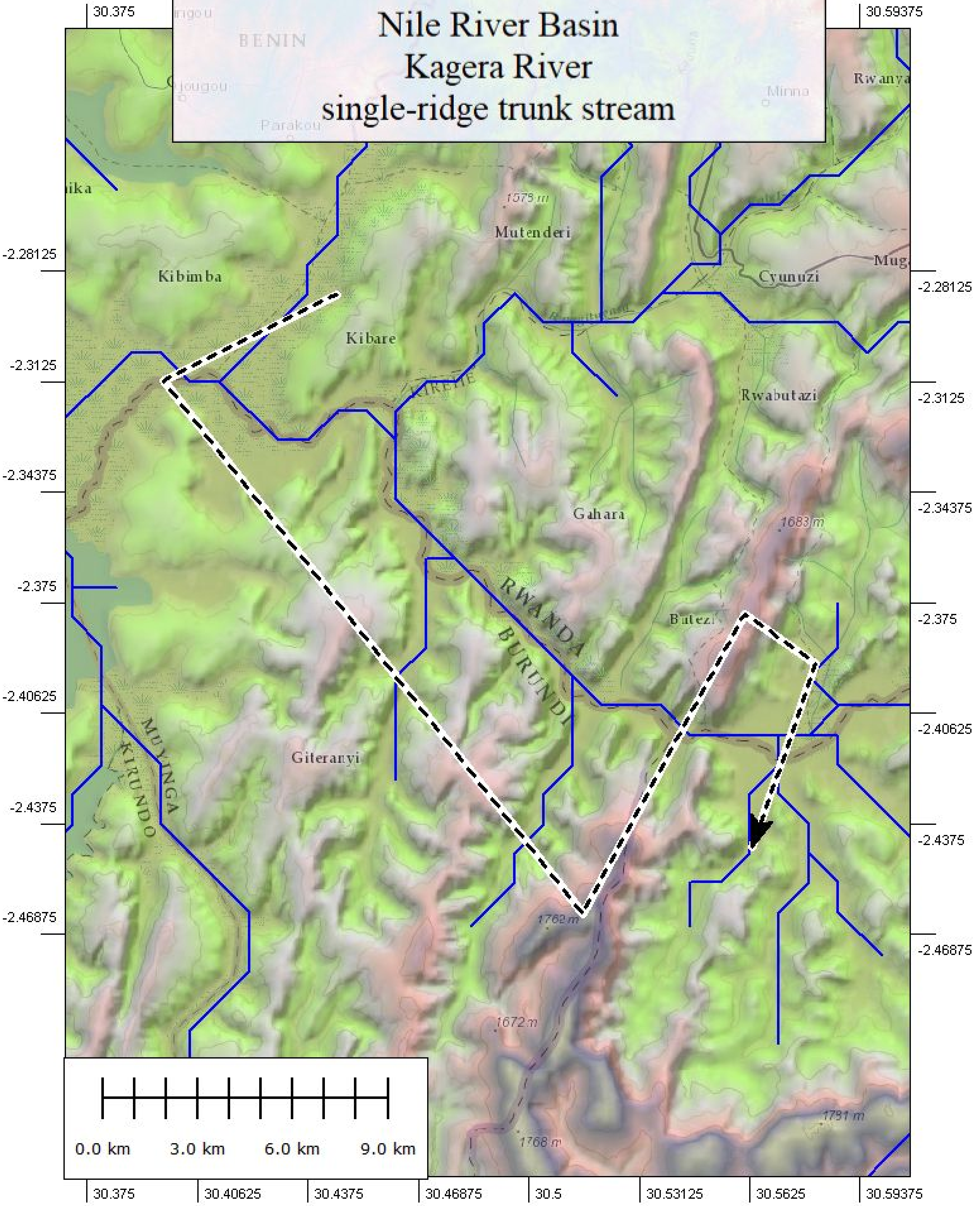

# AF - 185
## Oued Cheliff Basin
## Oued Cheliff
## single-ridge trunk stream

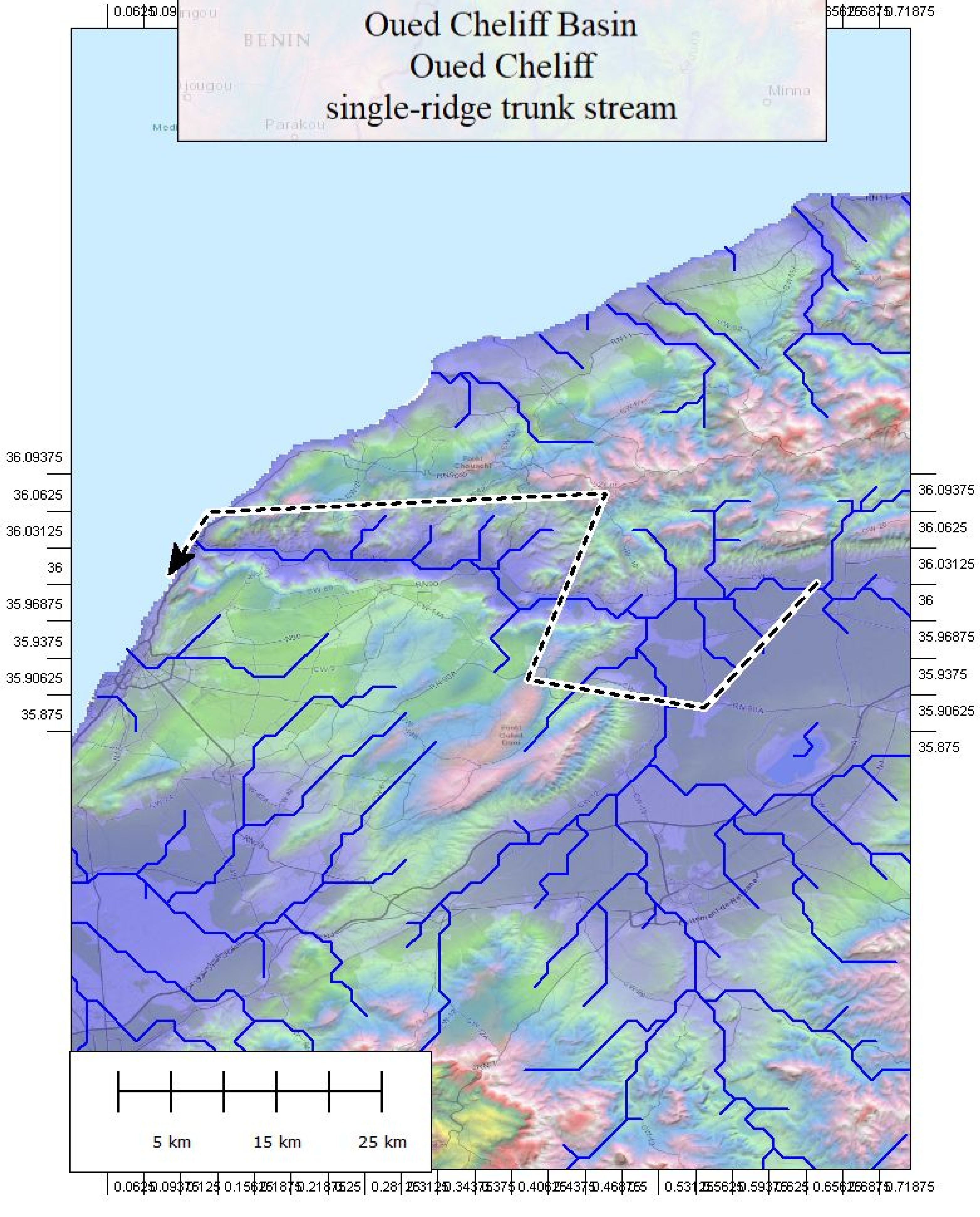

# AF - 146
## Endorheic basin Basin
## single-ridge trunk stream

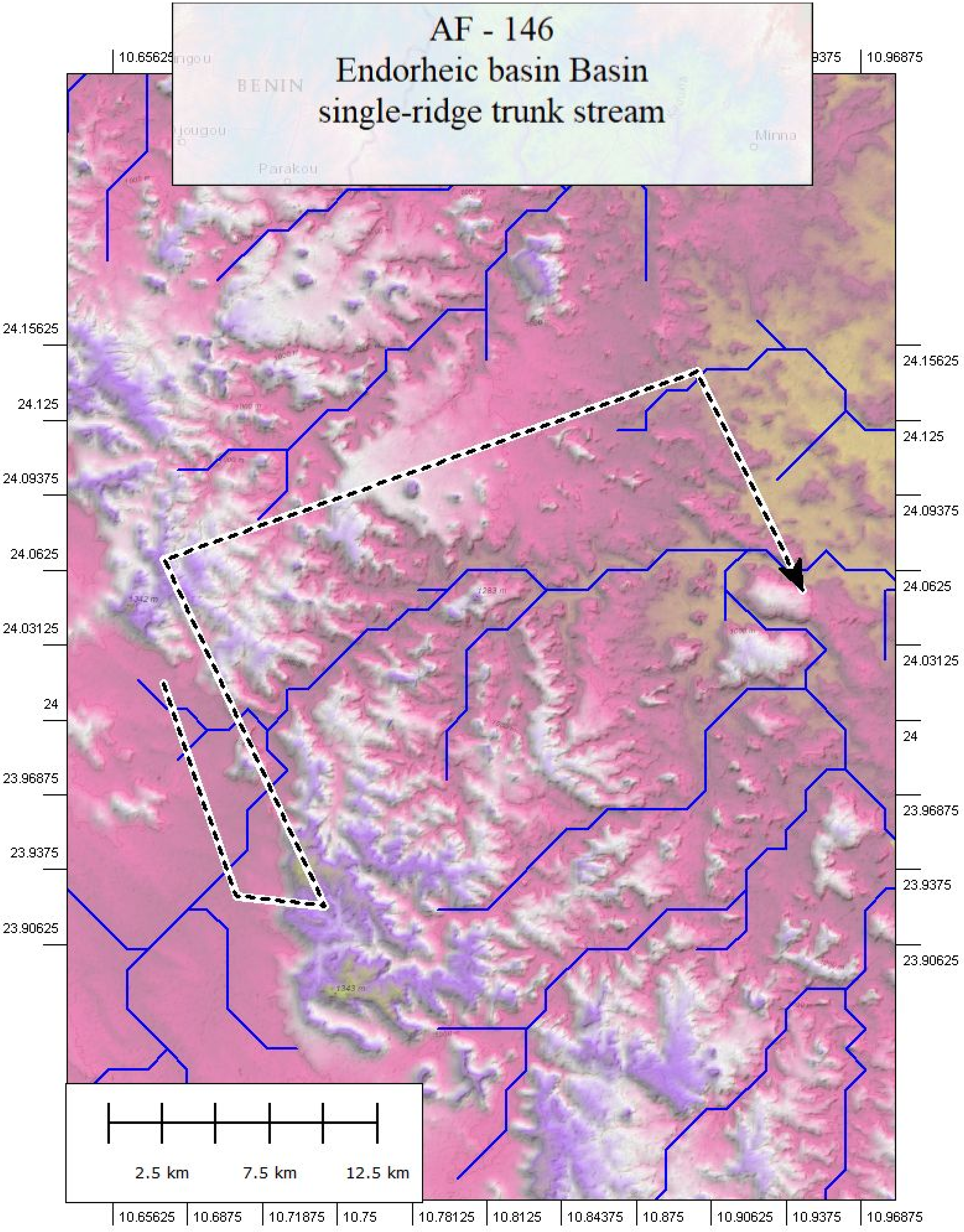

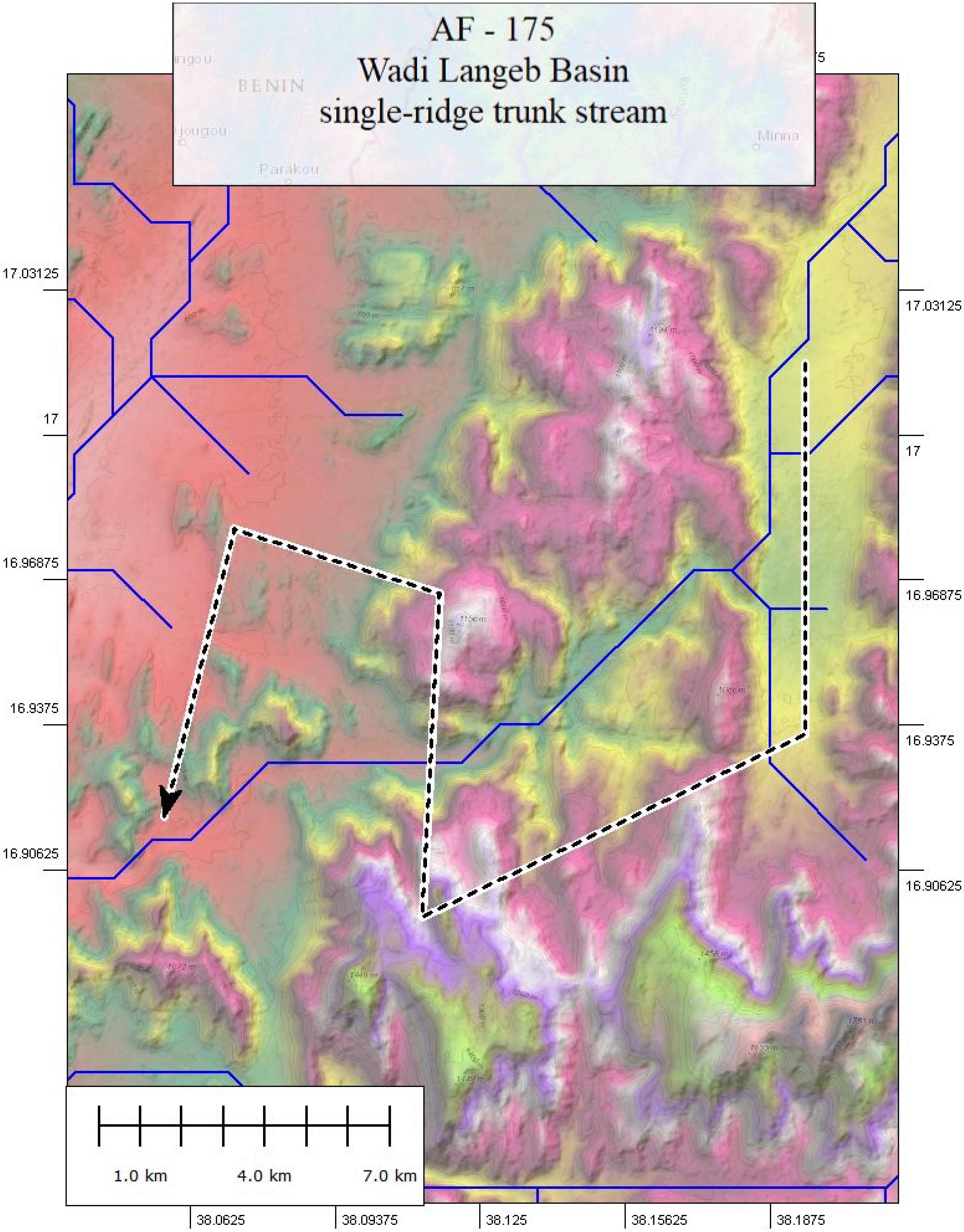

AF - 175
Wadi Langeb Basin
single-ridge trunk stream
BENIN
Parakou
Minna
17.03125
17
16.96875
16.9375
16.90625
38.0625
38.09375
38.125
38.15625
38.1875
1.0 km
4.0 km
7.0 km

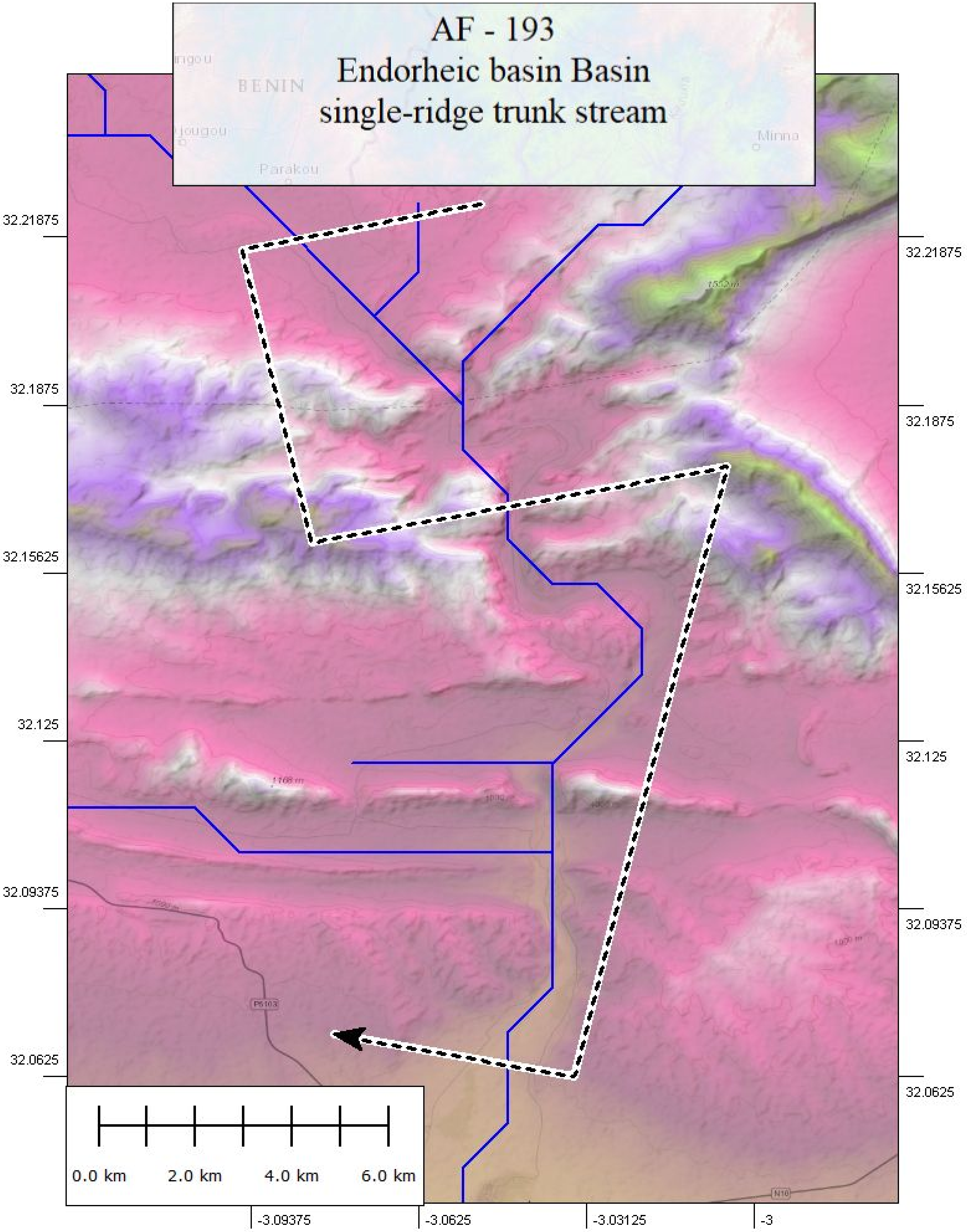
AF - 193
Endorheic basin Basin
single-ridge trunk stream
BENIN
Parakou
Minna
32.21875
32.1875
32.15625
32.125
32.09375
32.0625
-3.09375
-3.0625
-3.03125
-3
0.0 km
2.0 km
4.0 km
6.0 km

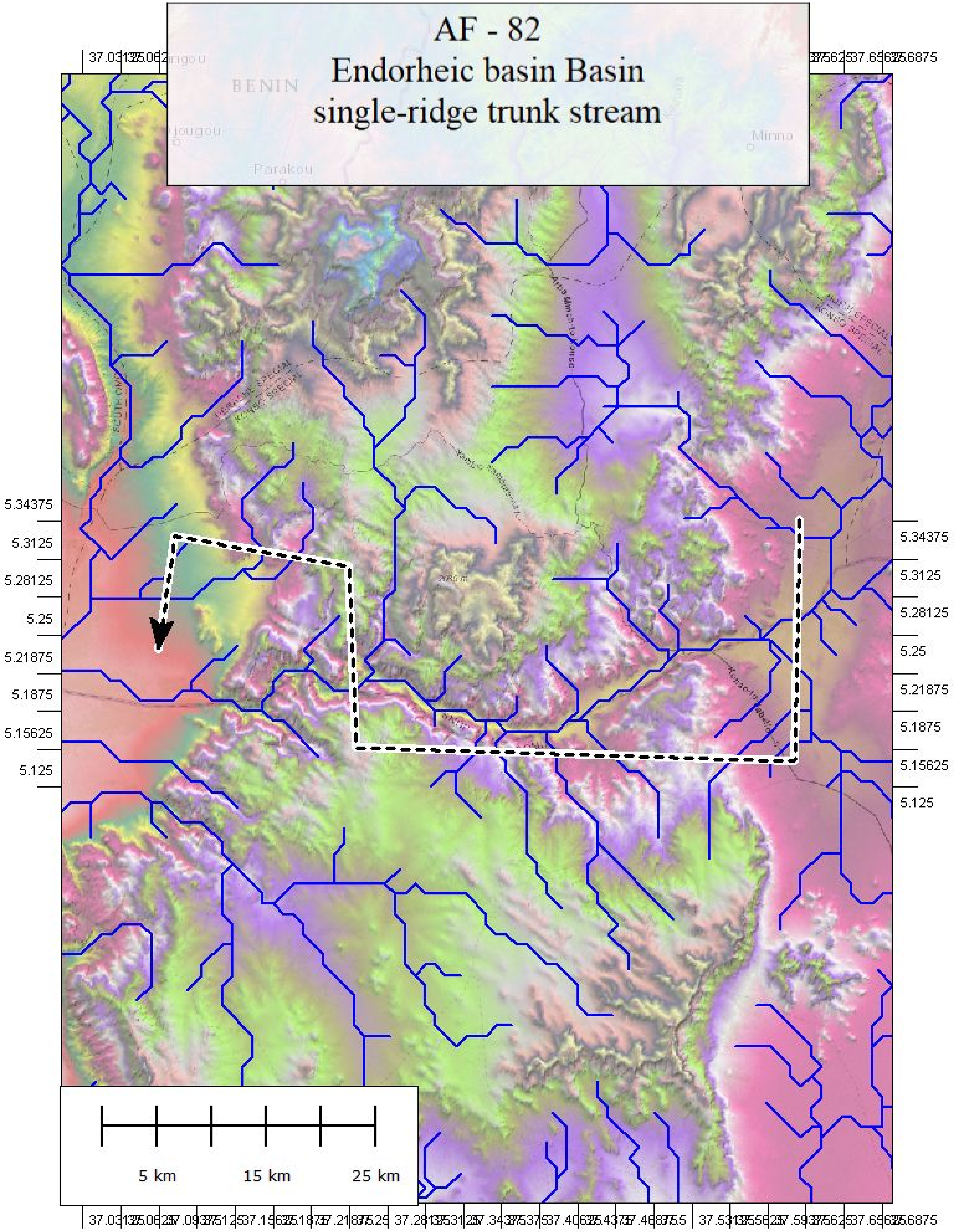
AF - 82
Endorheic basin Basin
single-ridge trunk stream
BENIN
Djougou
Parakou
Minno
5.34375
5.3125
5.28125
5.25
5.21875
5.1875
5.15625
5.125
5 km
15 km
25 km

# AF - 21
# Limpopo River Basin
# Murale River tributary
# single-ridge head stream

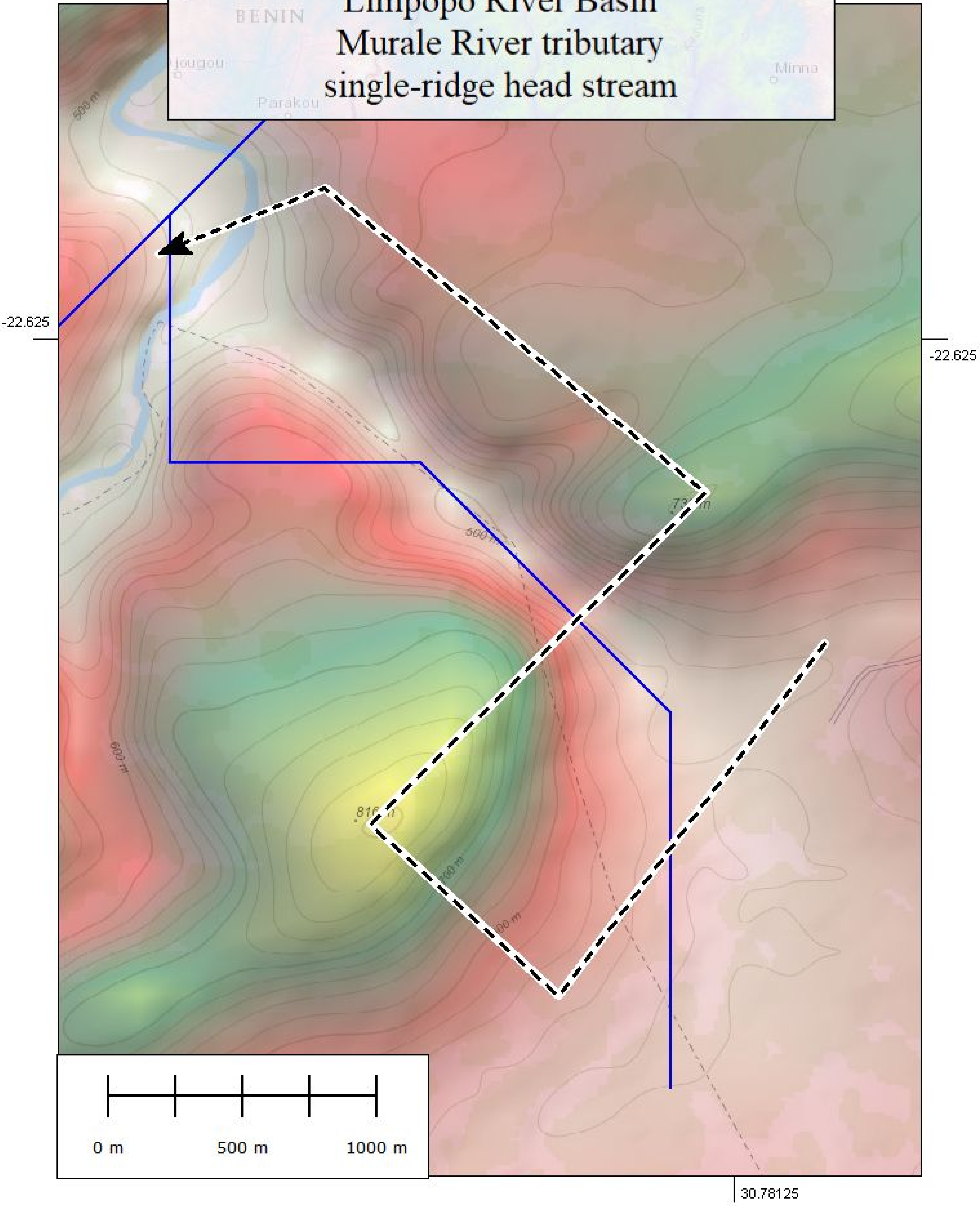

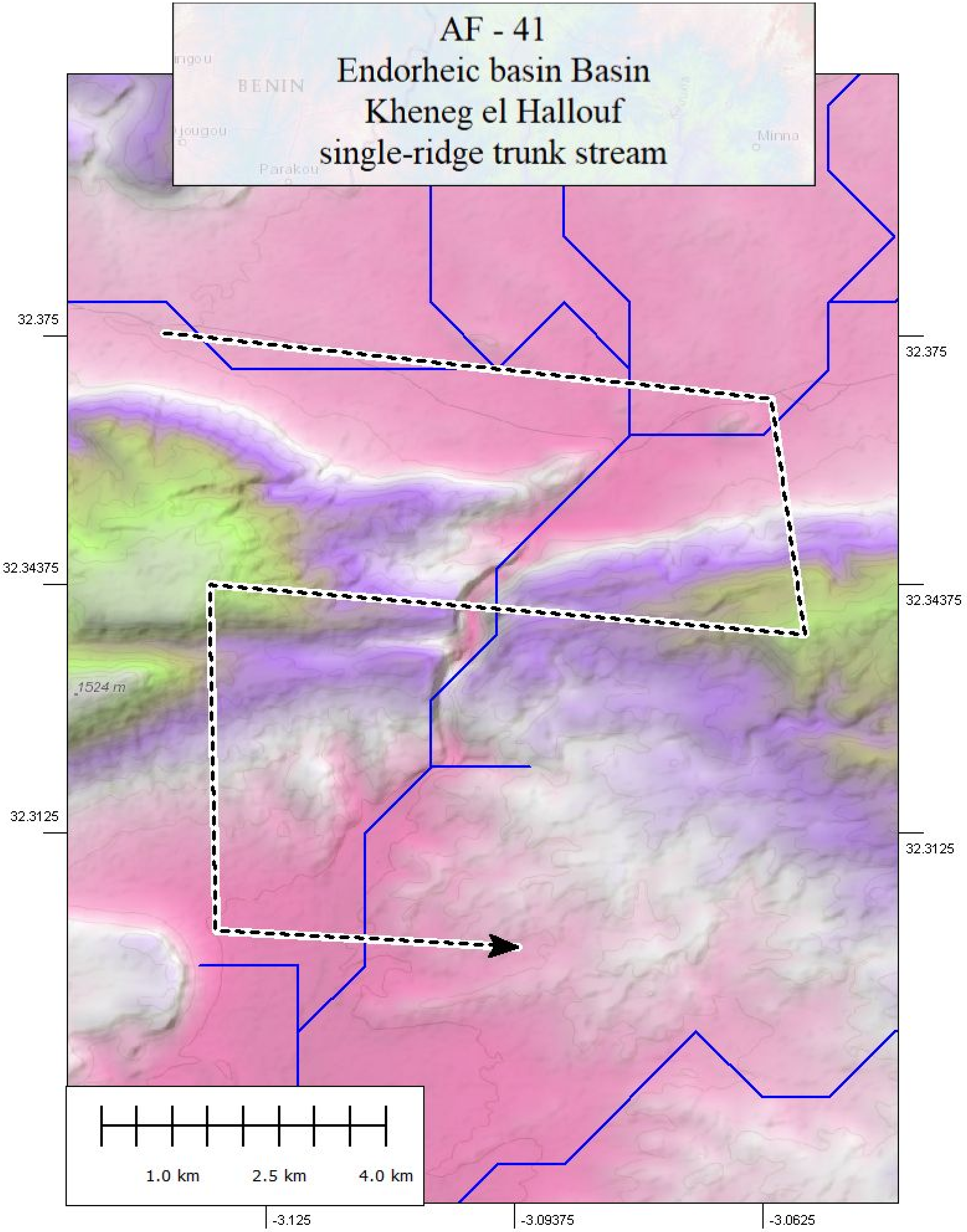
AF - 41
Endorheic basin Basin
Kheneg el Hallouf
single-ridge trunk stream
BENIN
Parakou
Minna
1524 m
32.375
32.34375
32.3125
-3.125
-3.09375
-3.0625
1.0 km
2.5 km
4.0 km

# AF - 45
## Congo River Basin
## Congo River
## single-ridge trunk stream

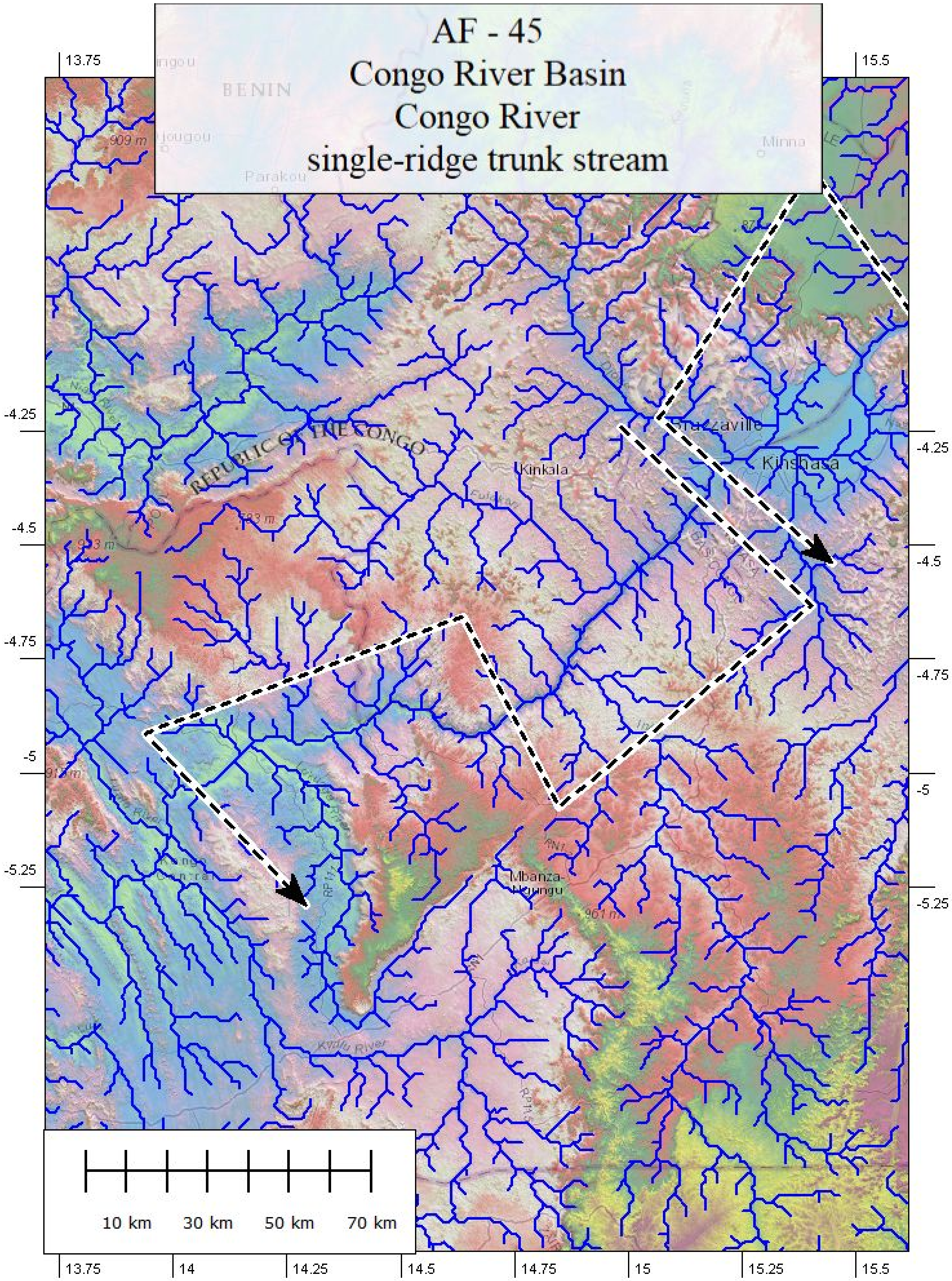

# AF - 66
## Oued Sebou Basin
## Sebou River
## single-ridge trunk stream

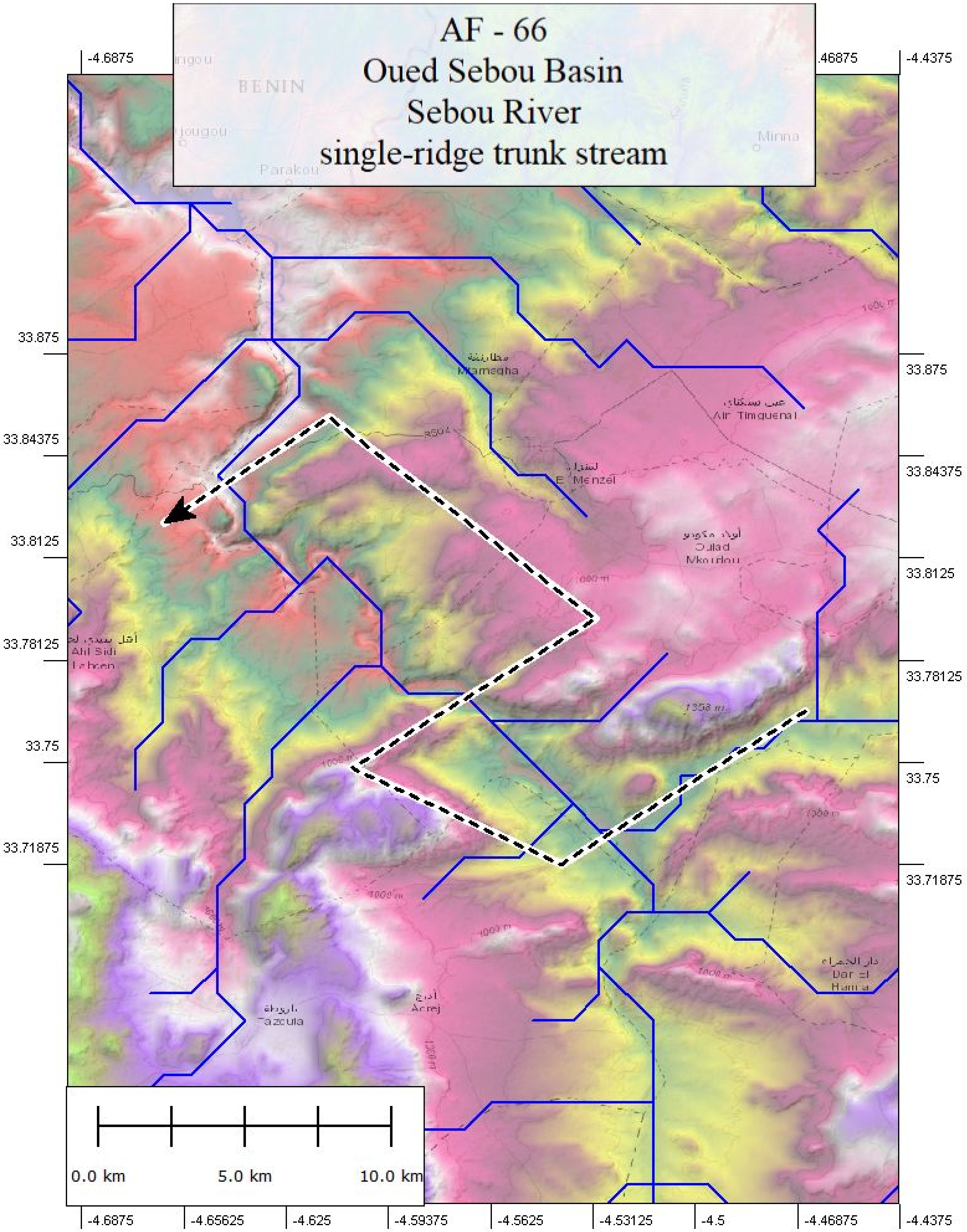

# AF - 67
## Groot-visrivier Basin
## Groot River
## single-ridge trunk stream

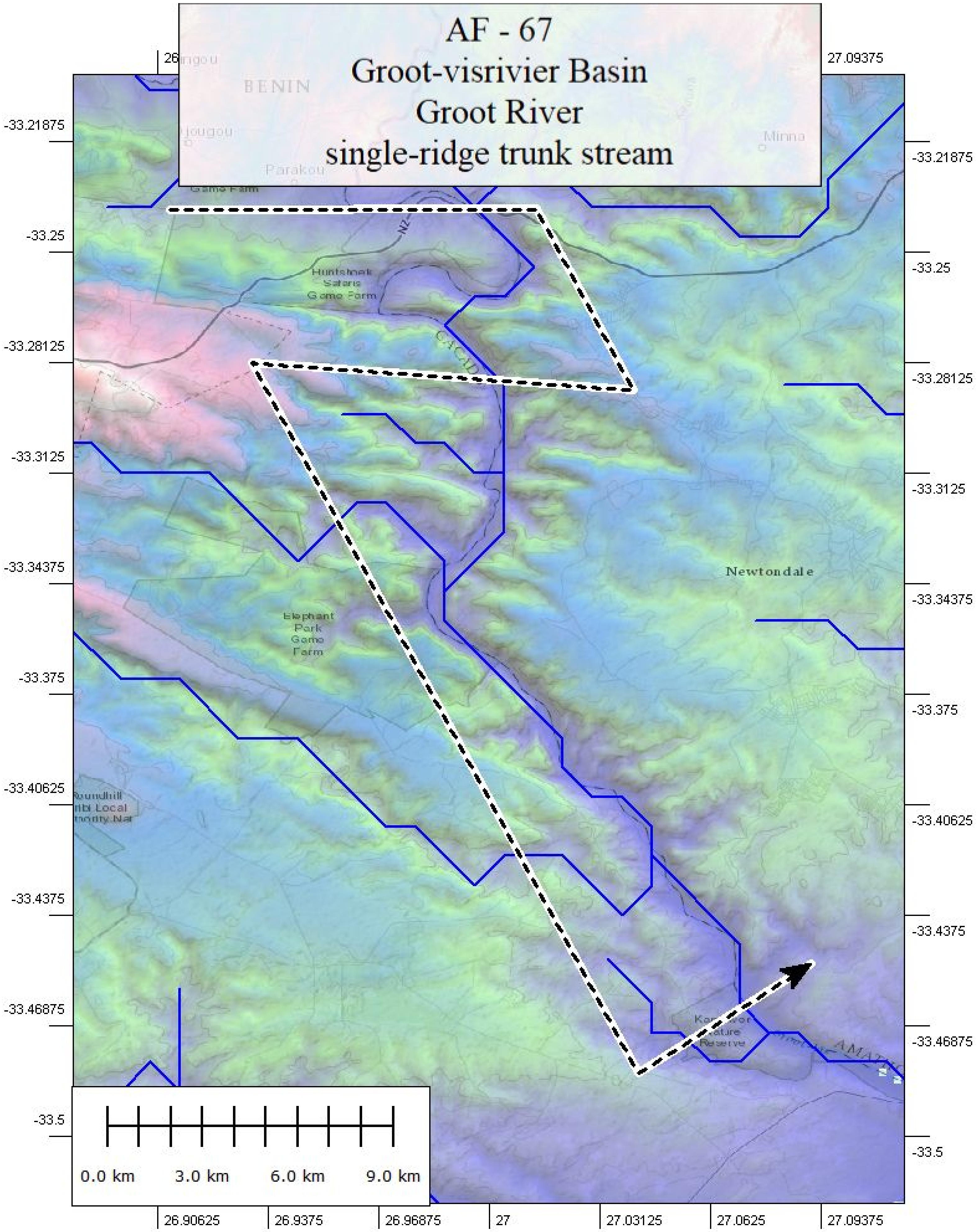

# AF - 88
## Rufiji River Basin
## Great Ruaha River
## single-ridge trunk stream

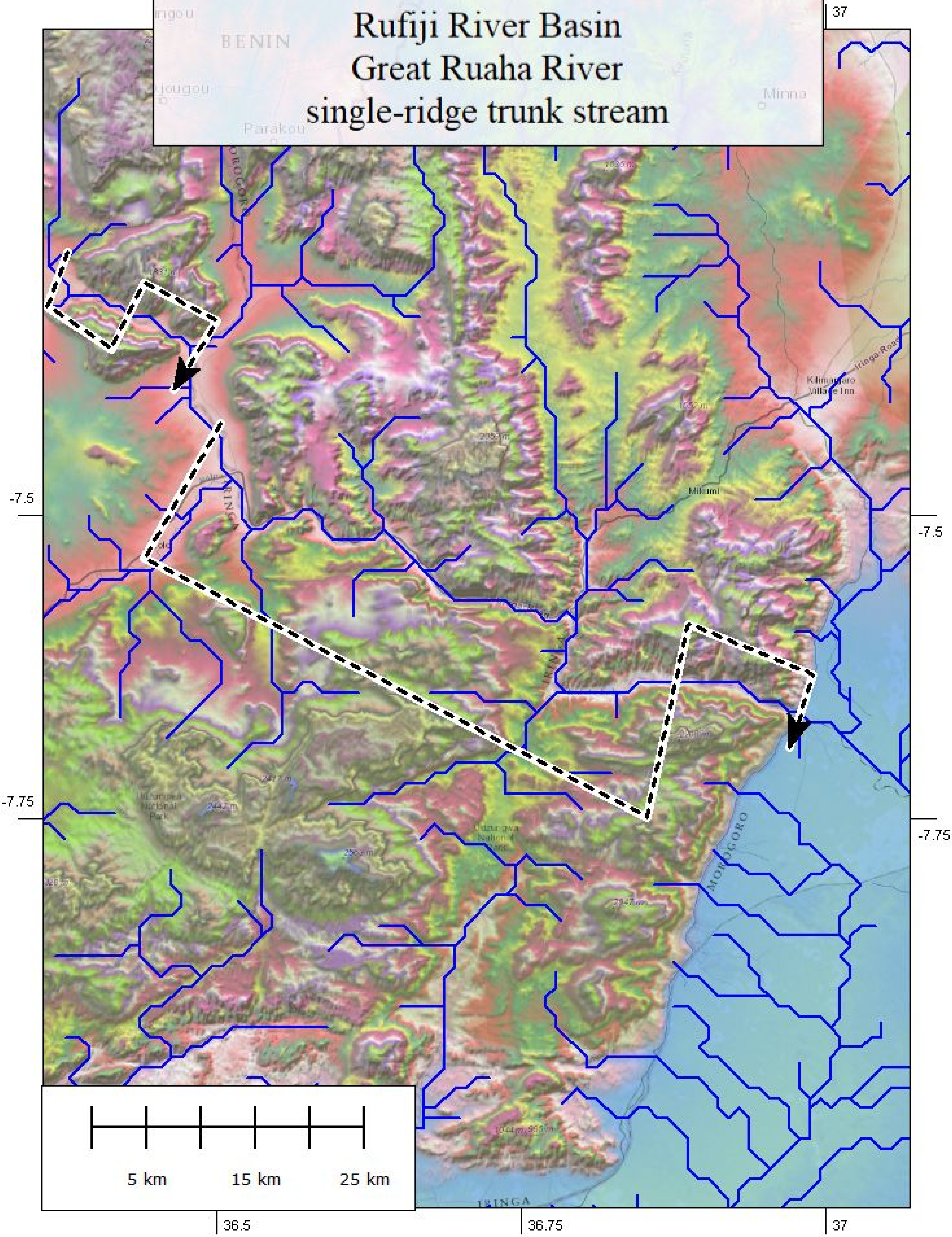

# AF - 93
## Onilahy River Basin
## Onilahy River
## single-ridge trunk stream

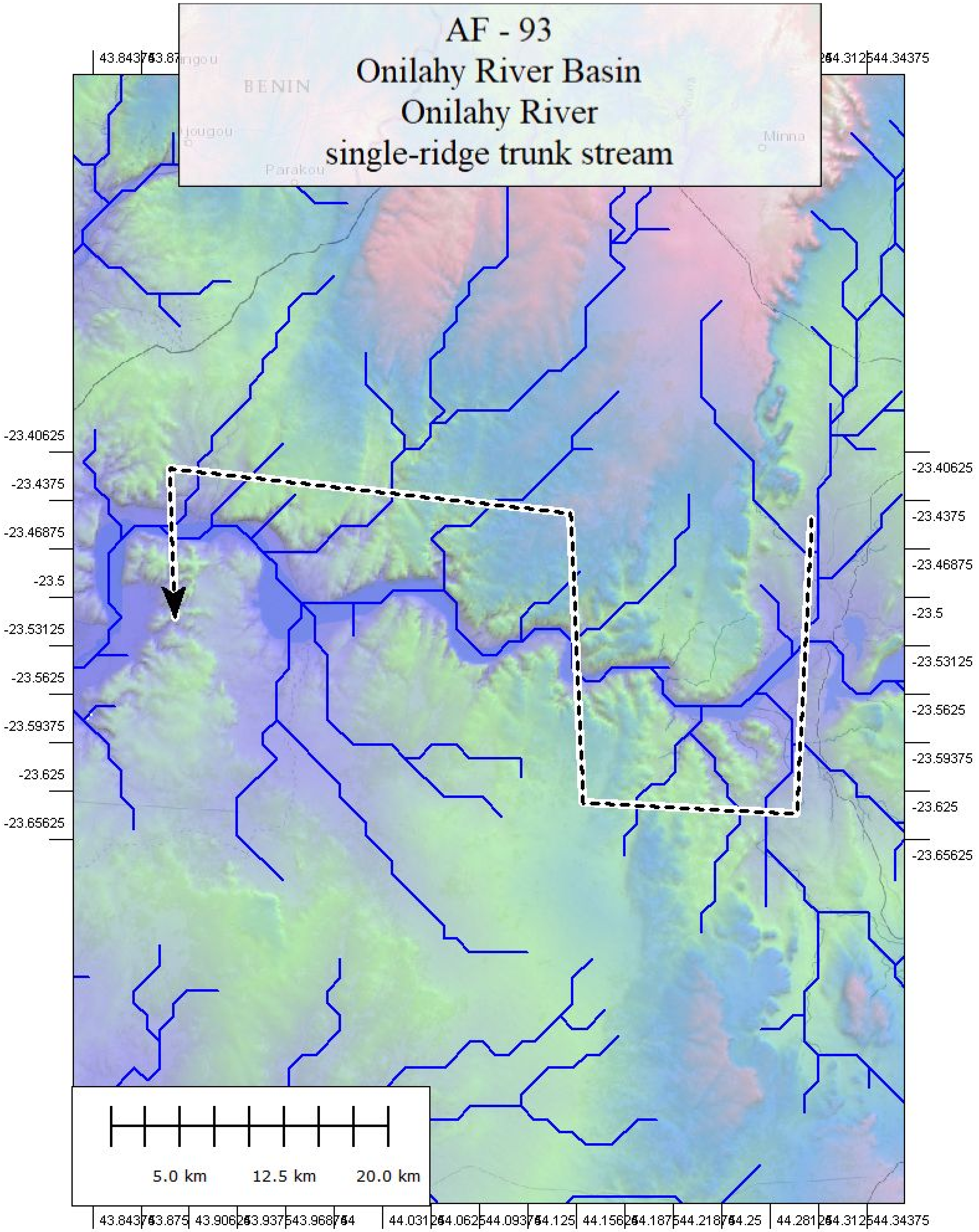

# AF - 105
# Senegal River Basin
# Bakoy River
# single-ridge trunk stream

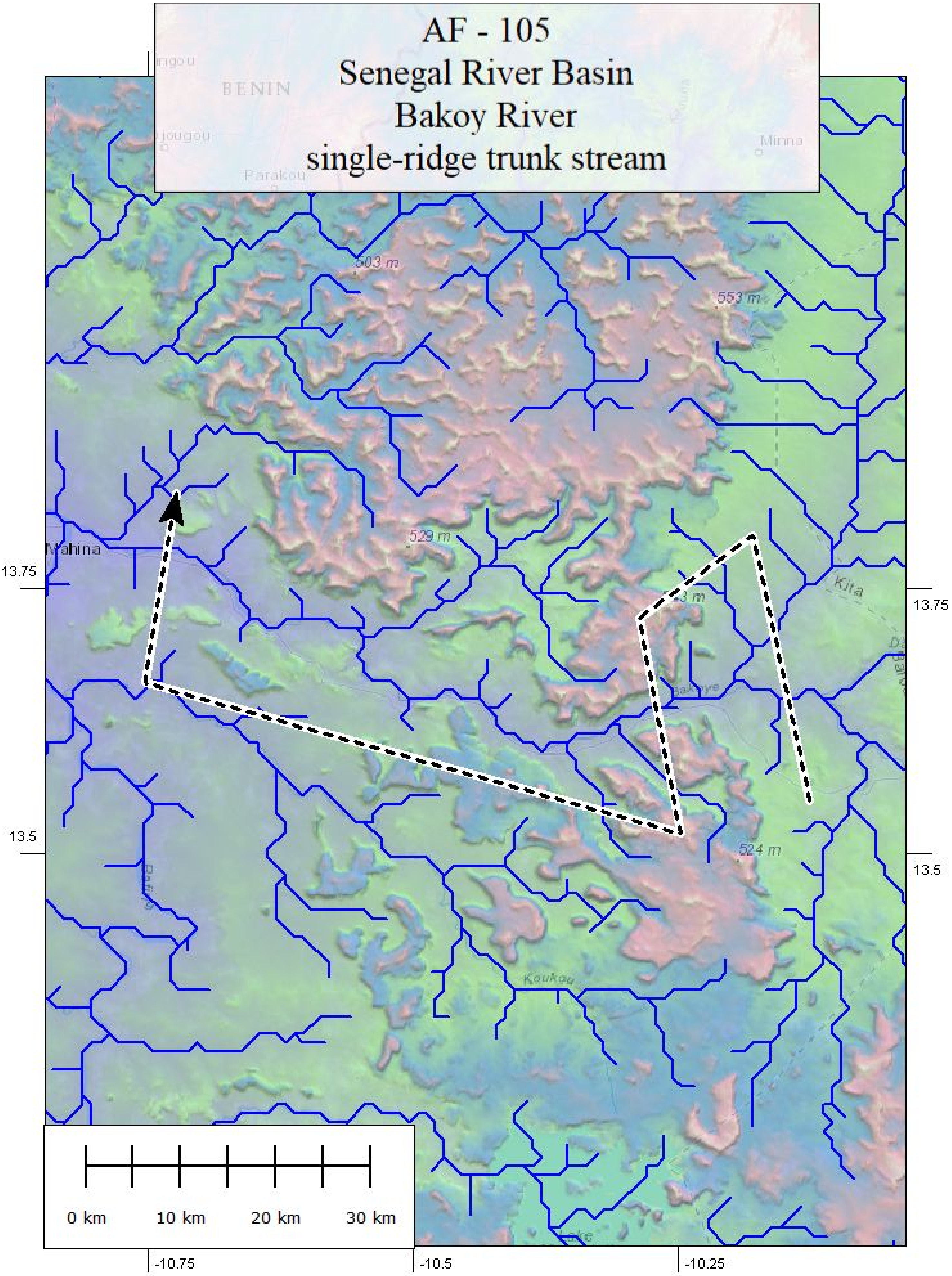

# AF - 116
## Zambezi River Basin
## Kafue River
## single-ridge trunk stream

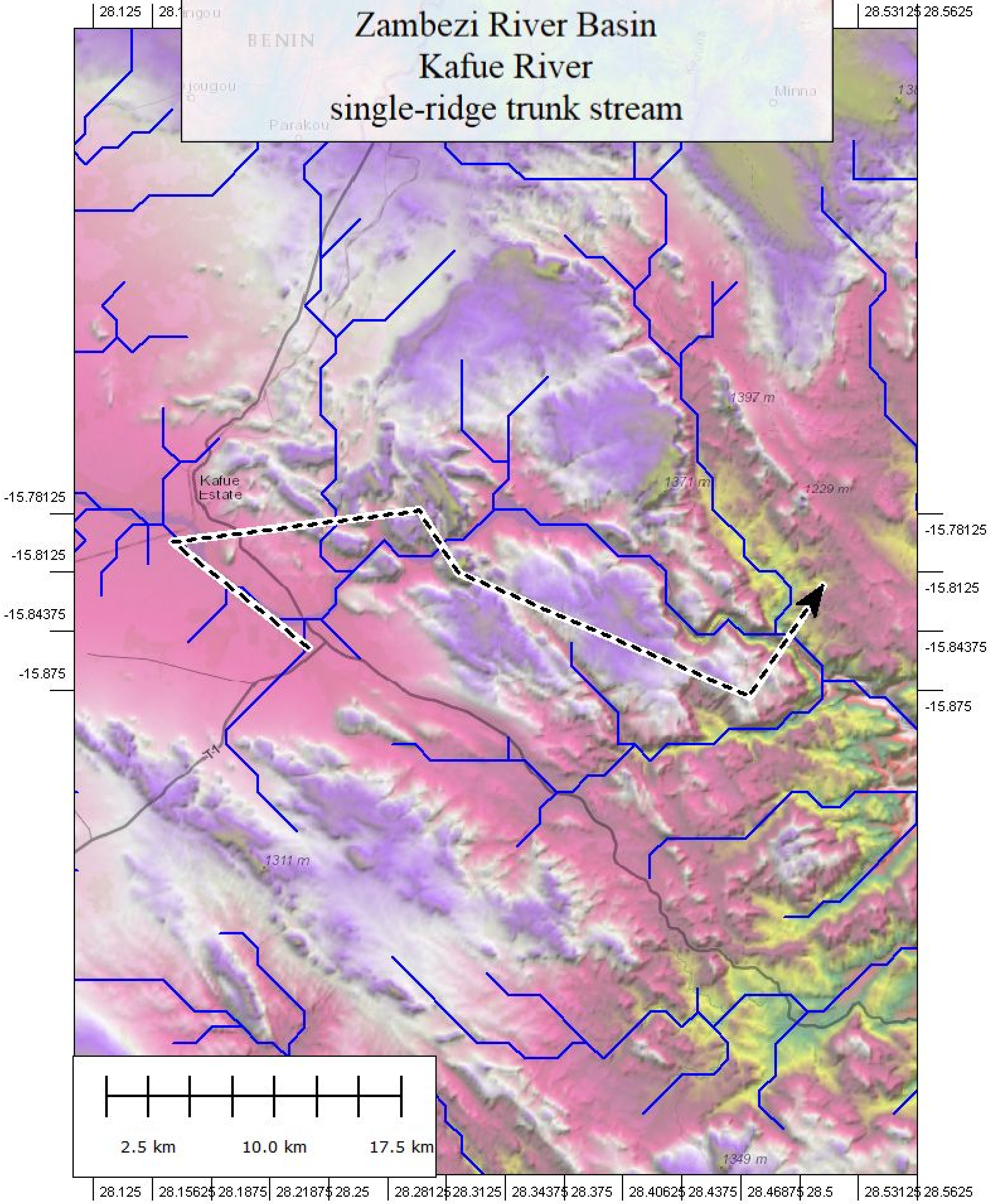

# AF - 124
## Zambezi River Basin
## Zambezi River
## single-ridge trunk stream

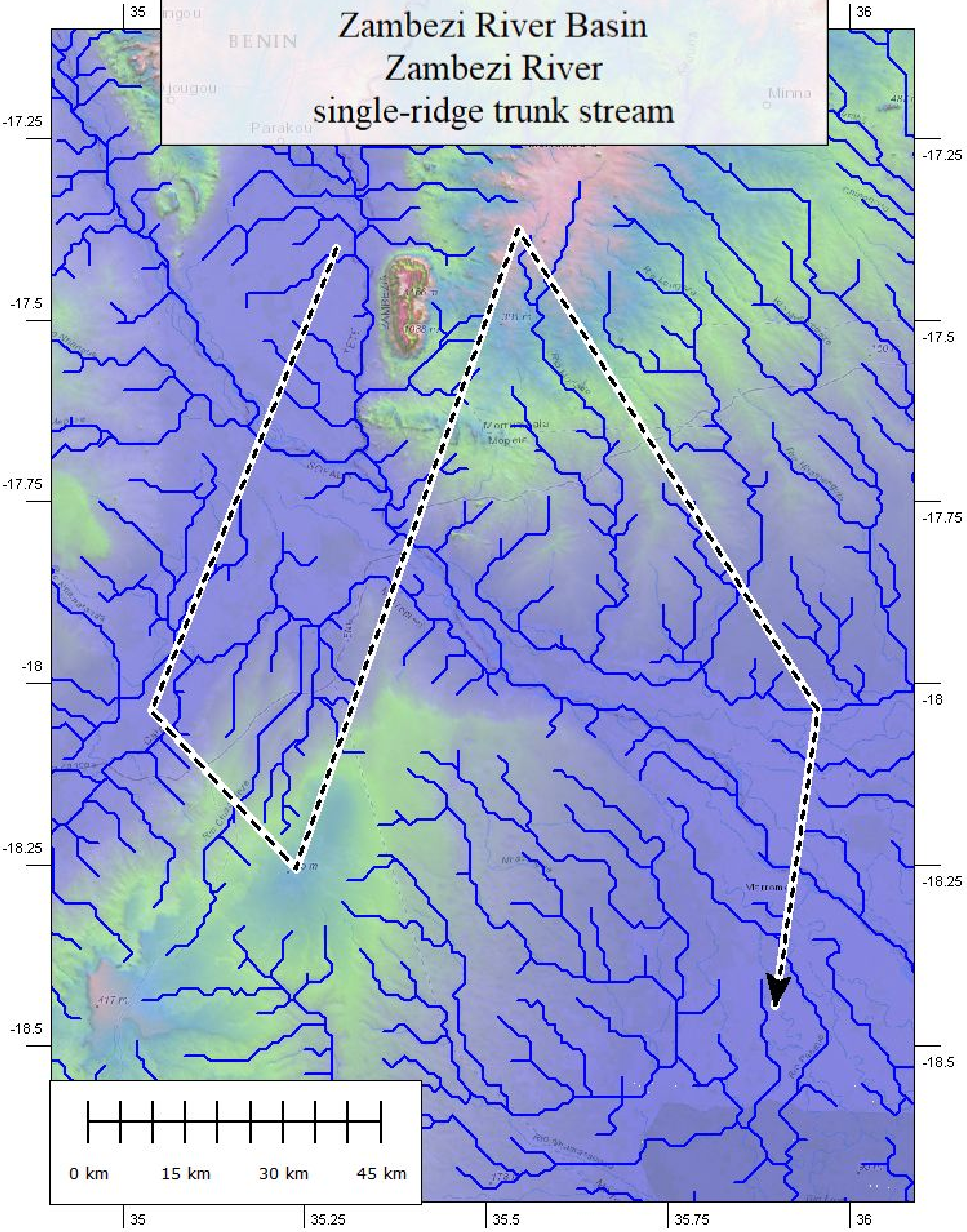

# AF - 130
## Tsiribihina River Basin
## Mania River
## single-ridge trunk stream

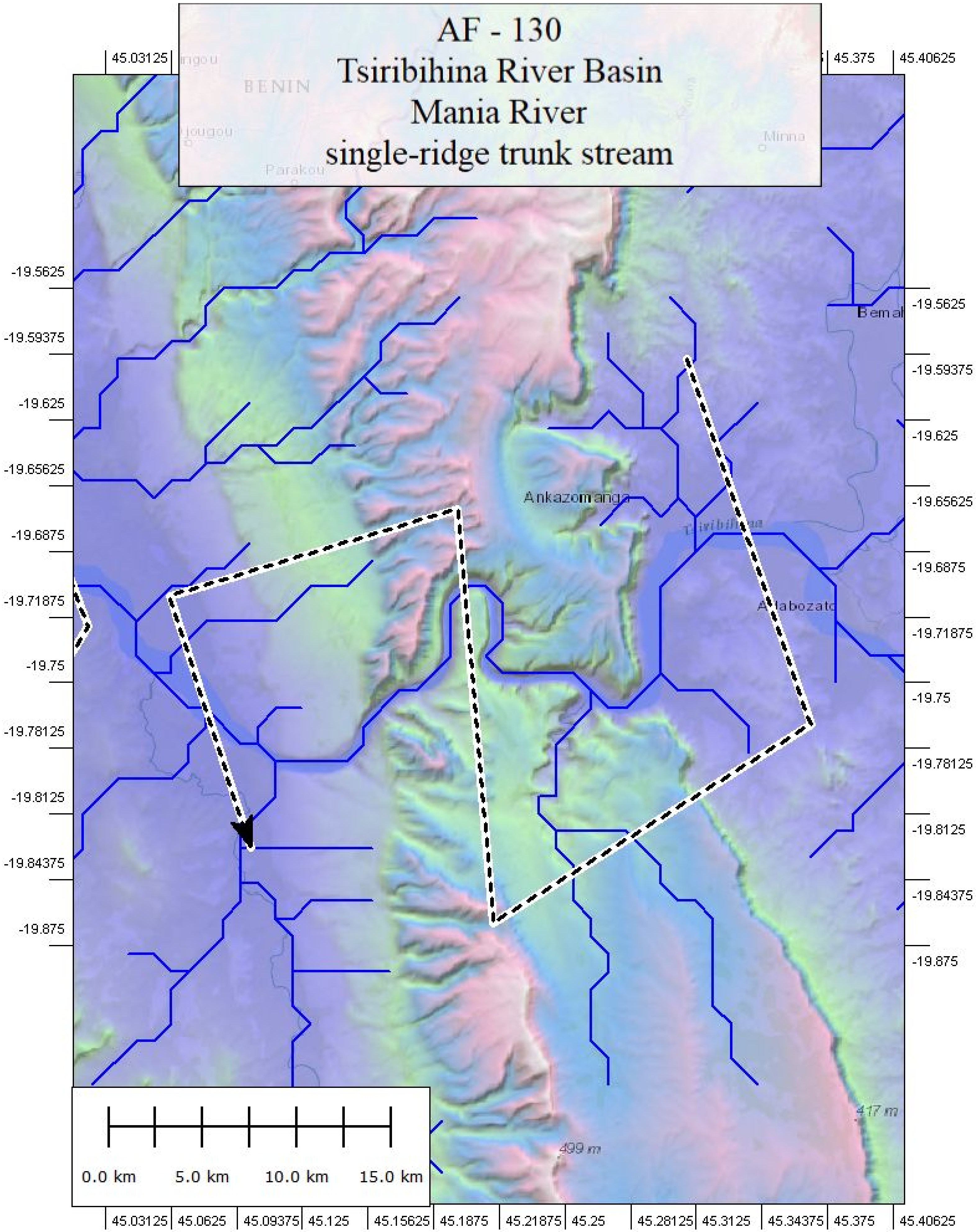

# AF - 132
## Oued Tafna Basin
## Oued Tafna
## single-ridge trunk stream

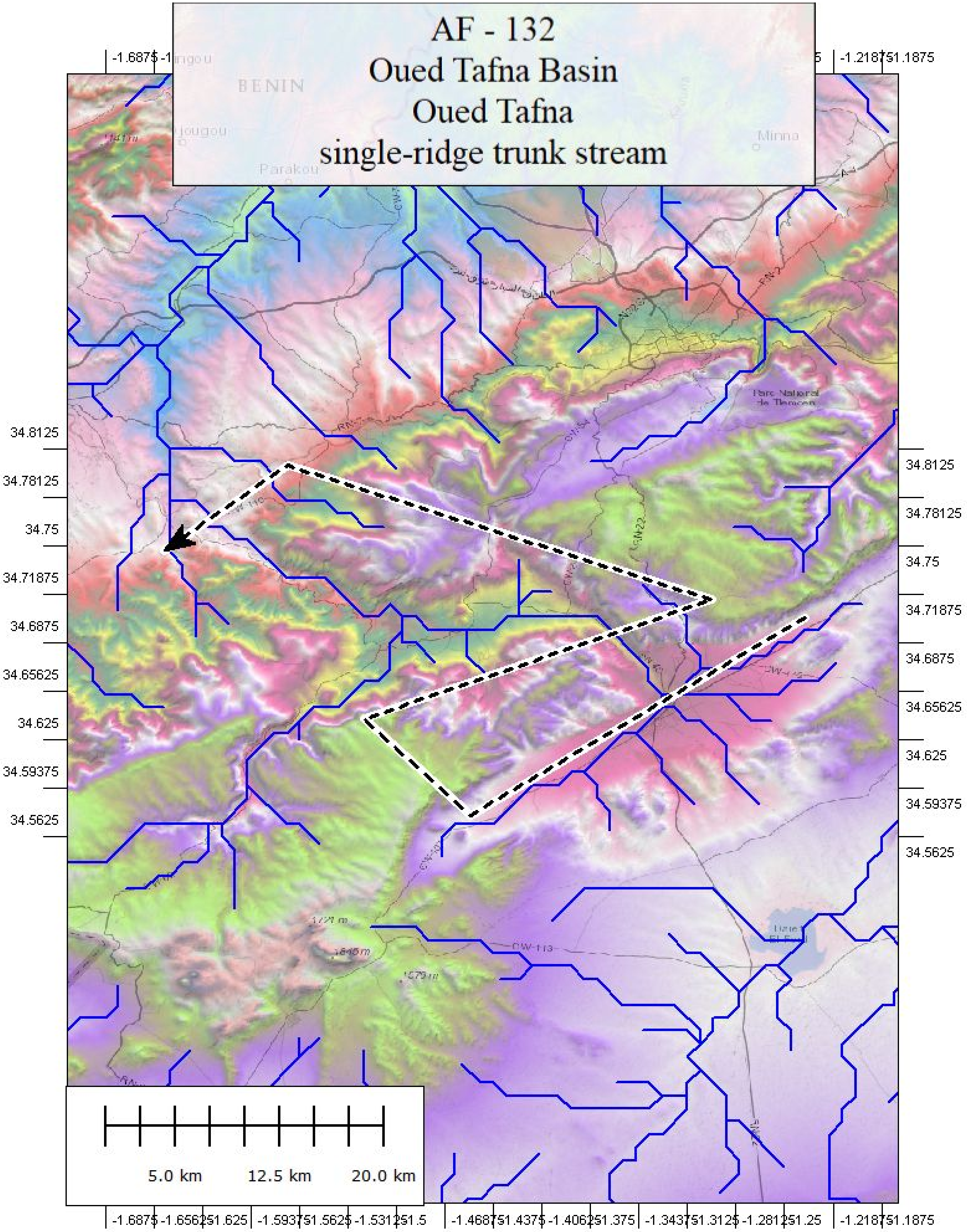

# AF - 154
# Olifants River Basin
# Doring River
# single-ridge trunk stream

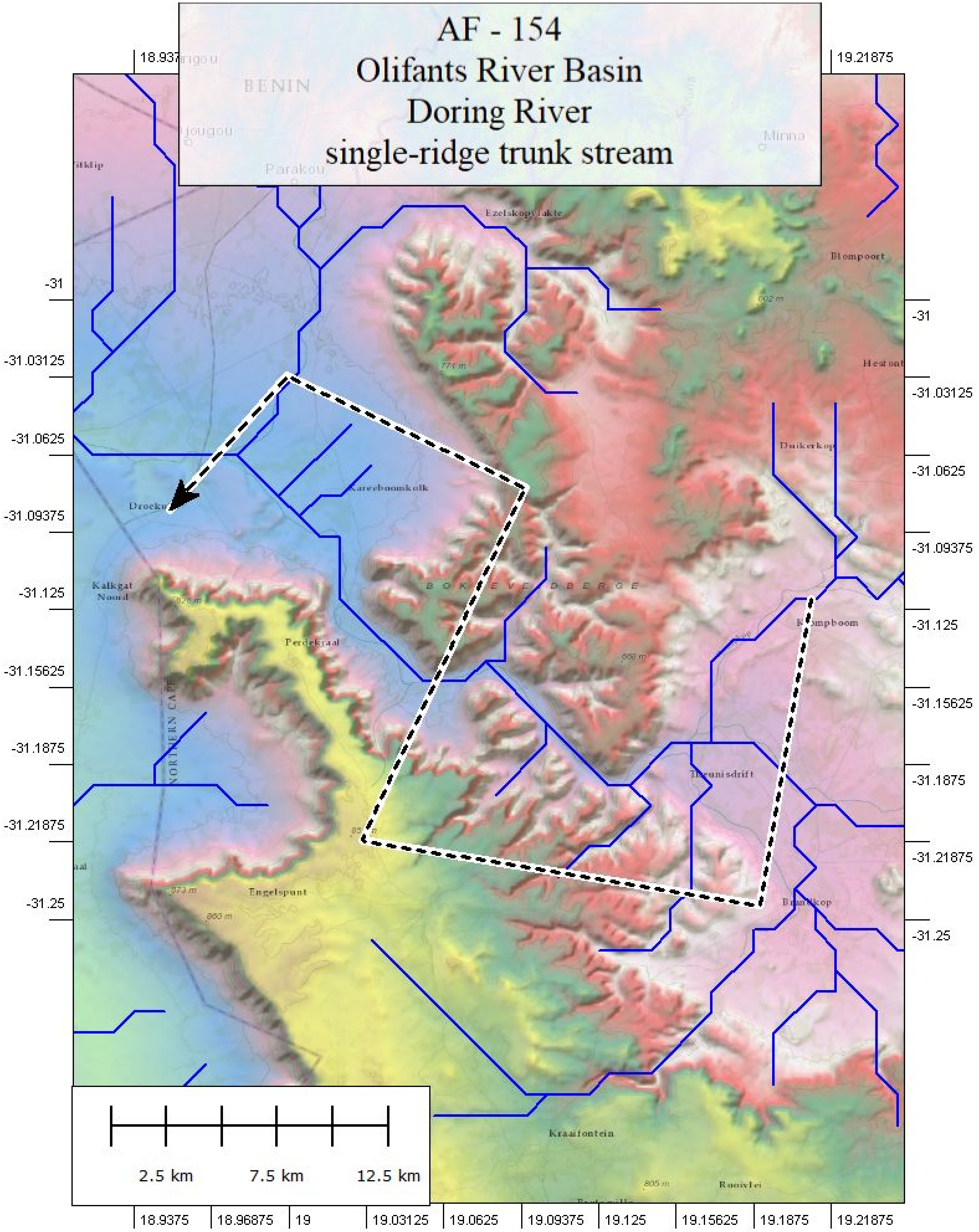

# AF - 161
## Zambezi River Basin
## Lunsemfwa River
## single-ridge trunk stream

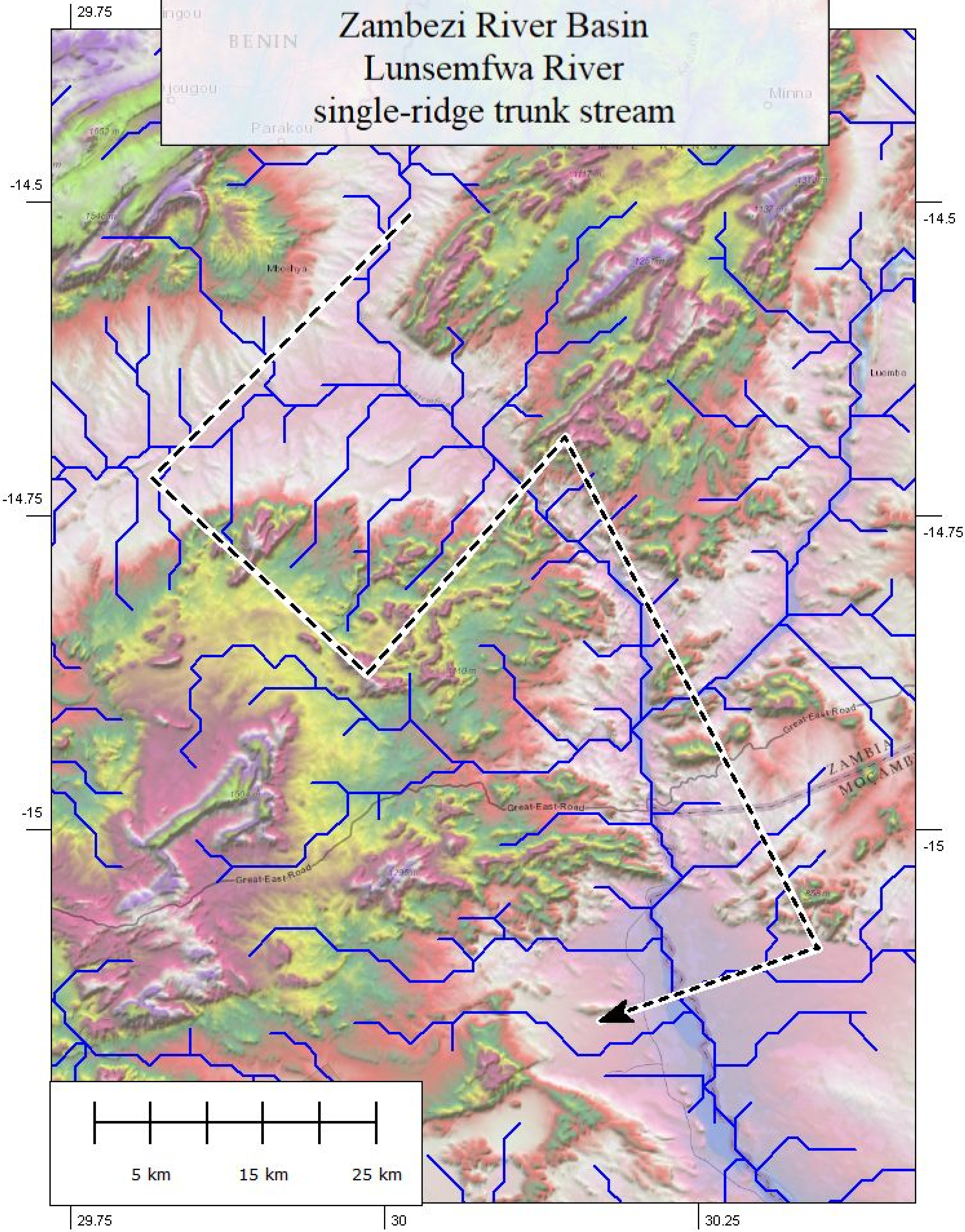

# AF - 162
## Mtamvuna River Basin
## Mtamvuna River
## single-ridge trunk stream

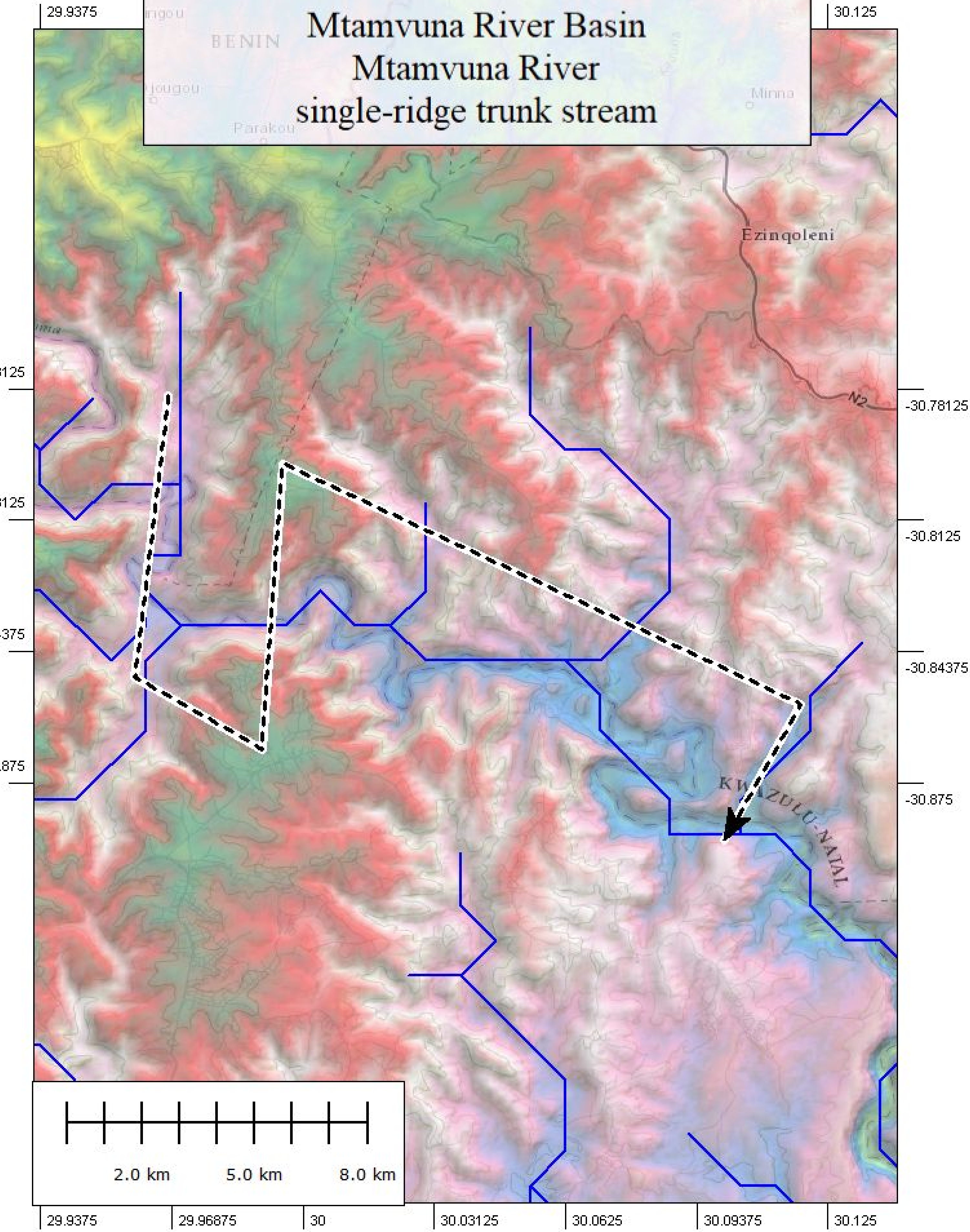

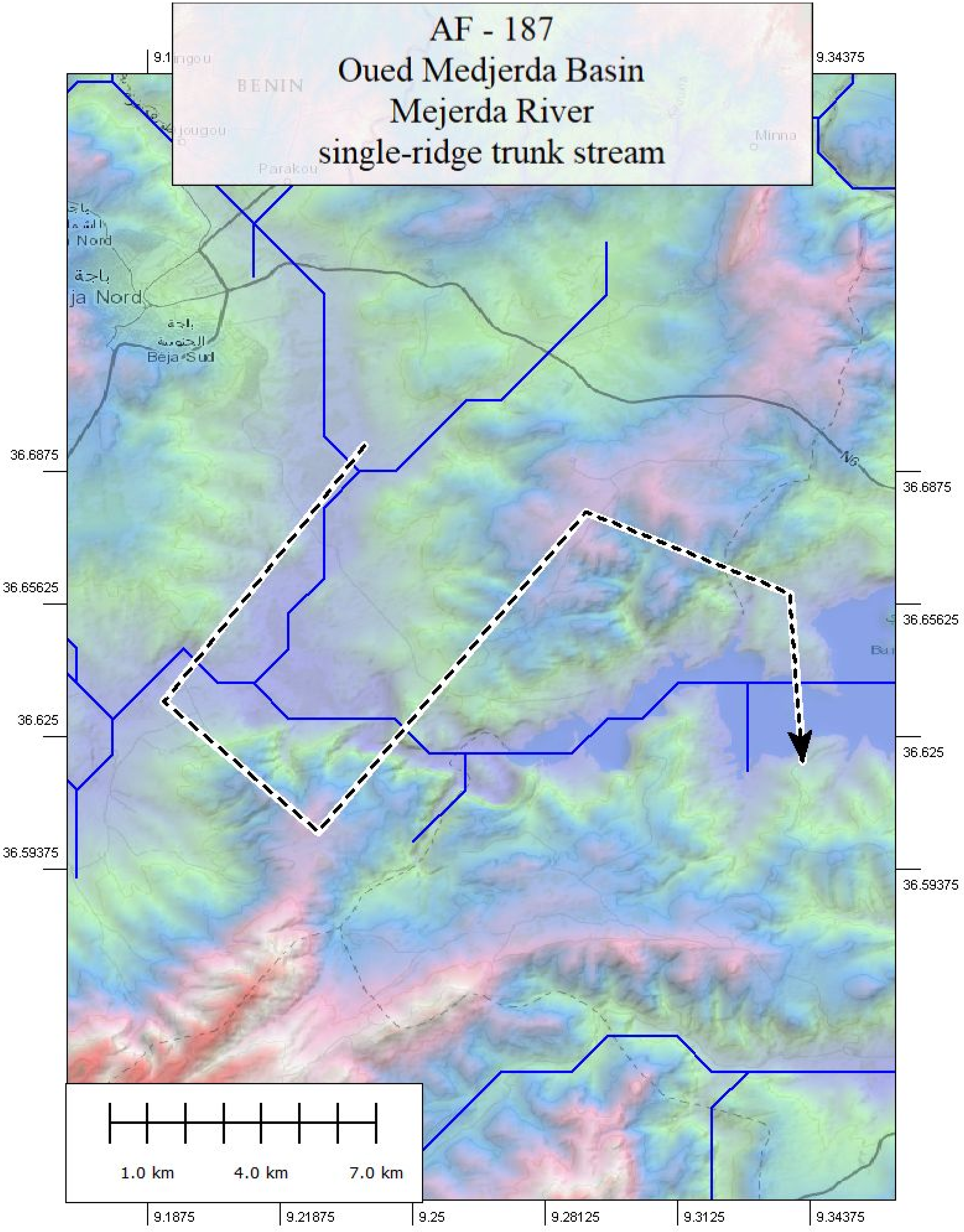
AF - 187
Oued Medjerda Basin
Mejerda River
single-ridge trunk stream
BENIN
Parakou
Minna
Béja Nord
Béja Sud
N6
36.6875
36.65625
36.625
36.59375
9.34375
9.1875
9.21875
9.25
9.28125
9.3125
1.0 km
4.0 km
7.0 km

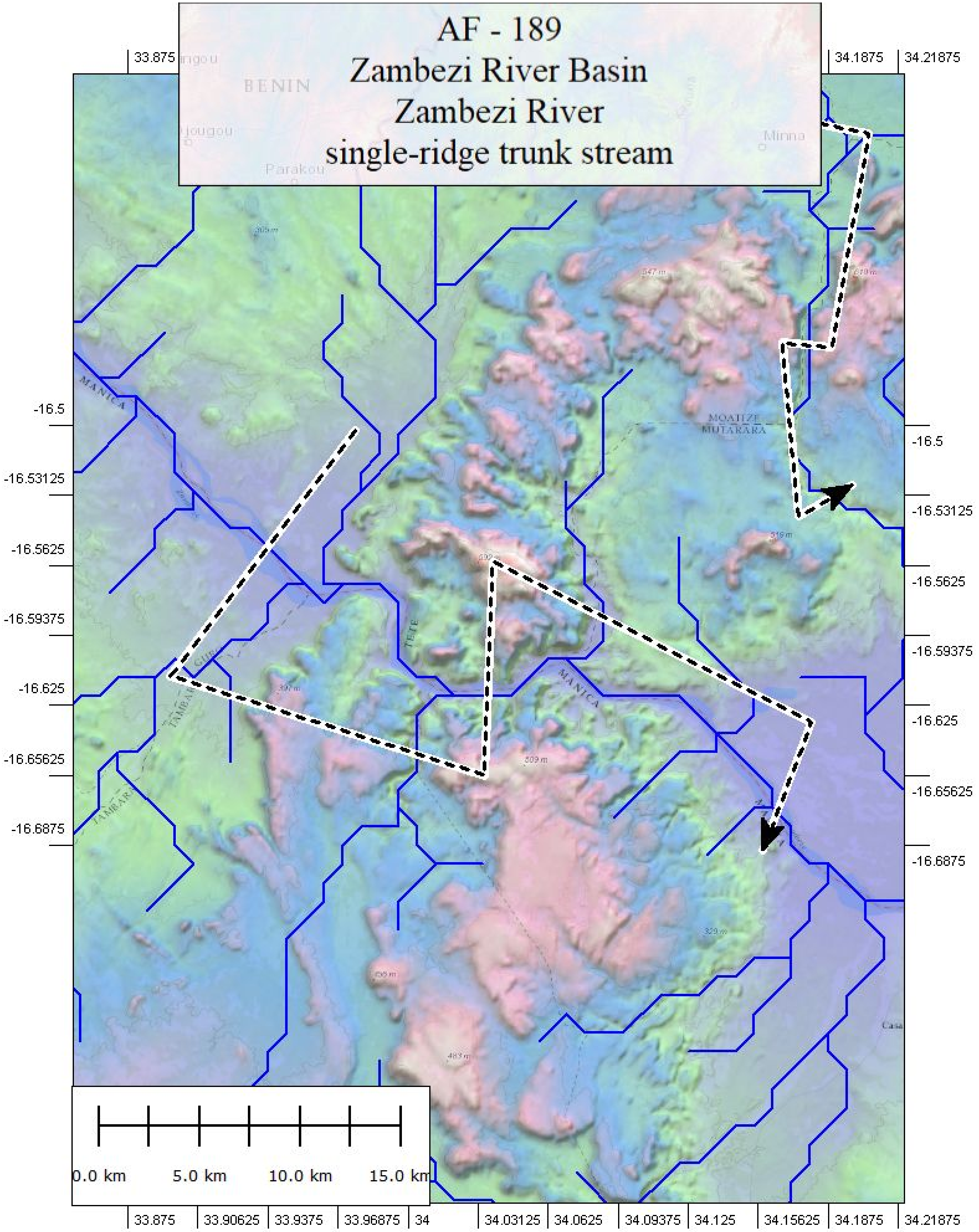
AF - 189
Zambezi River Basin
Zambezi River
single-ridge trunk stream
33.875
34.1875
34.21875
BENIN
Djougou
Parakou
Minna
MANICA
TETE
MOATIZE
MUTARARA
TAMBARA
-16.5
-16.53125
-16.5625
-16.59375
-16.625
-16.65625
-16.6875
0.0 km
5.0 km
10.0 km
15.0 km
33.875
33.90625
33.9375
33.96875
34
34.03125
34.0625
34.09375
34.125
34.15625
34.1875
34.21875
Casa

# AF - 194
## Catumbela River Basin
## Catumbela River
## single-ridge trunk stream

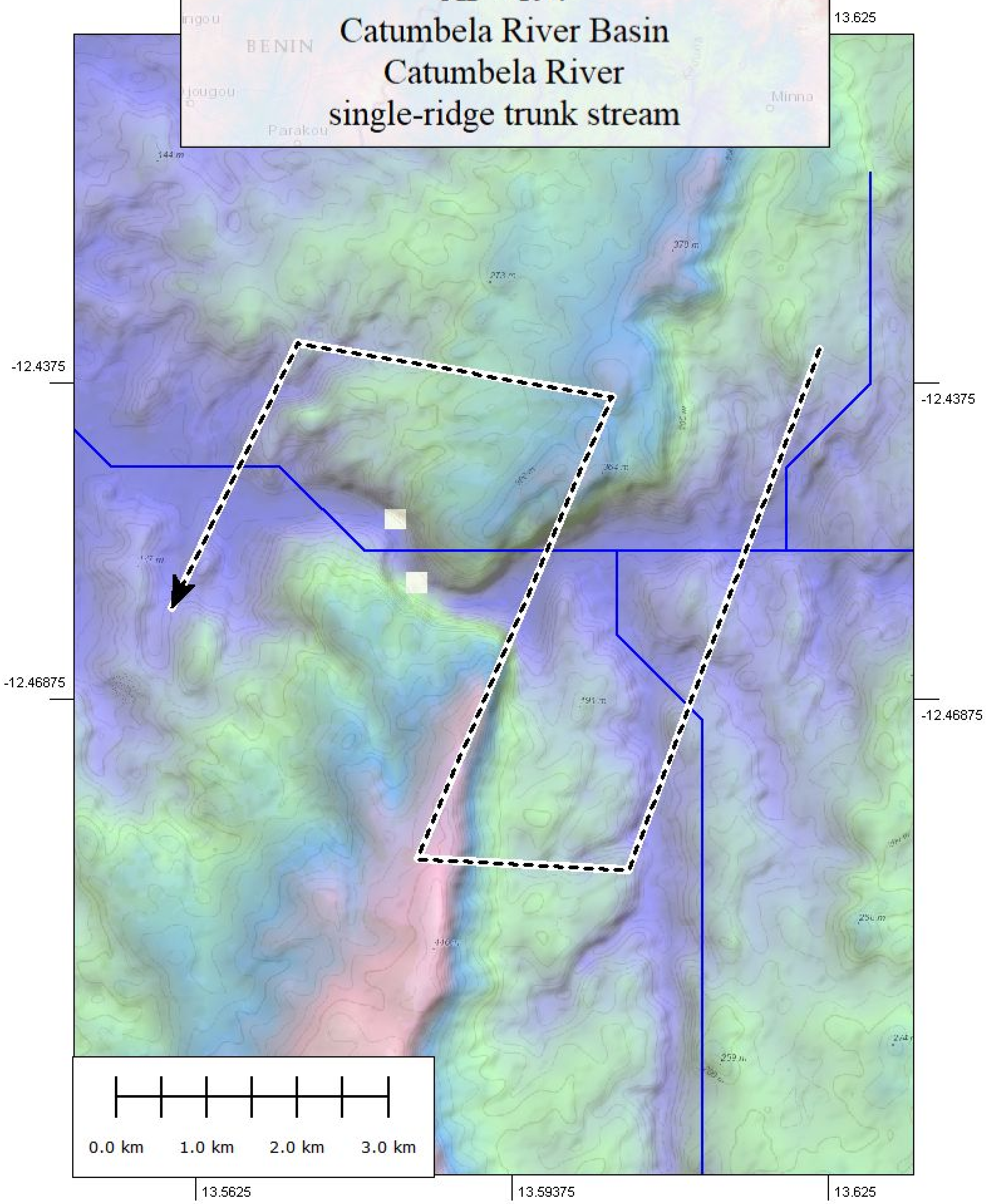

# AF - 197
## Limpopo River Basin
## Luvuvhu River
## single-ridge trunk stream

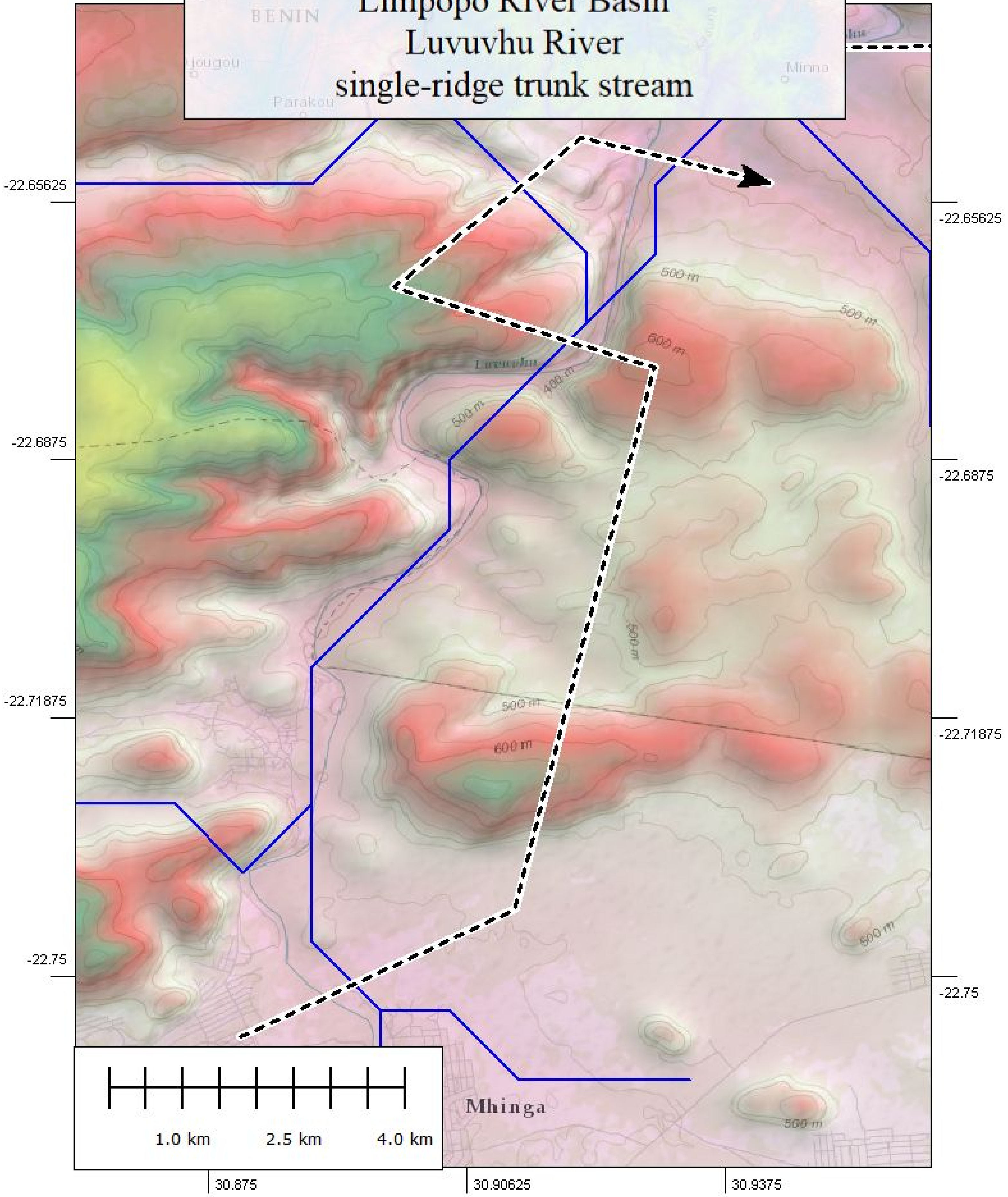

# AF - 129
## Niari River Basin
## Niari River
## single-ridge trunk stream

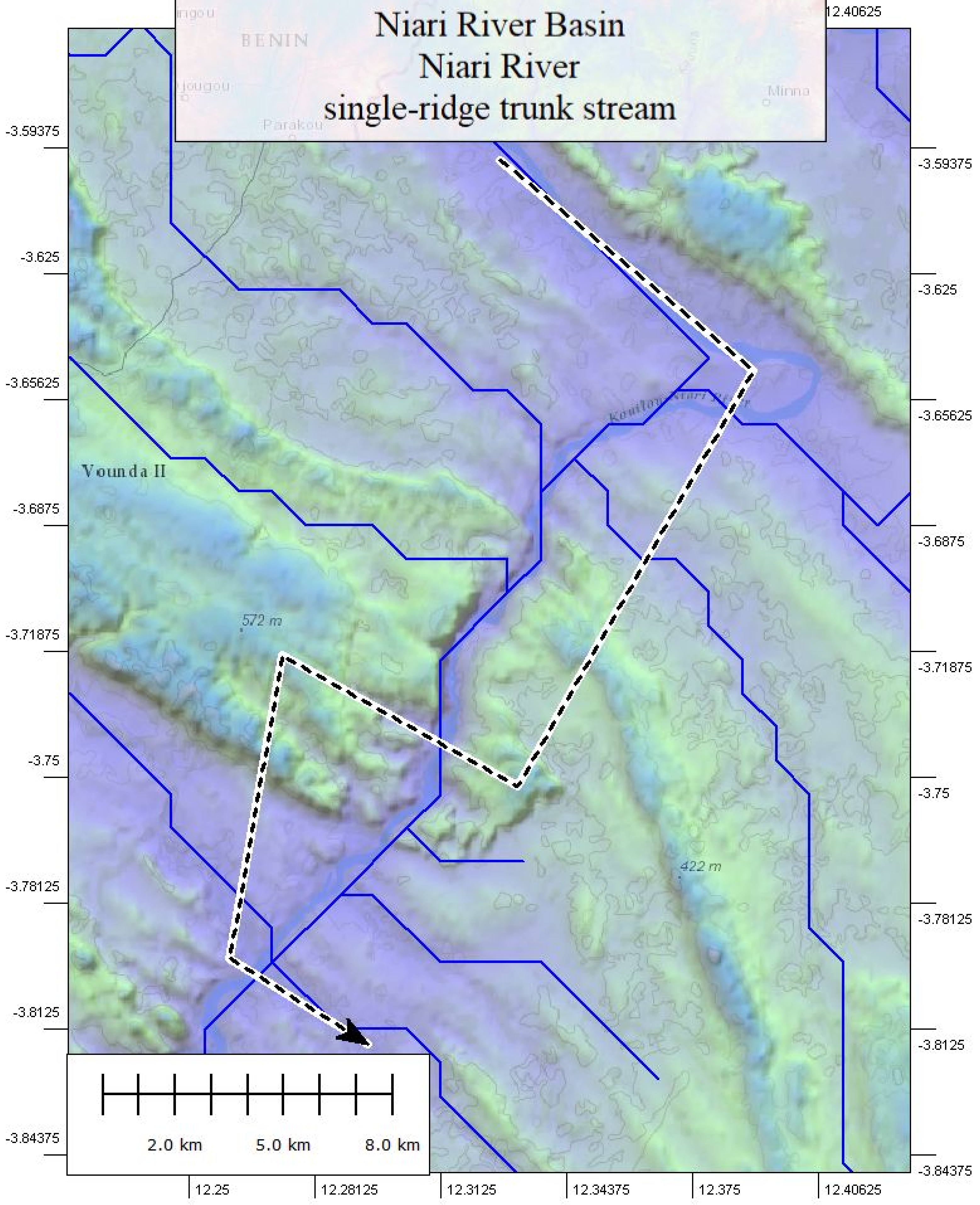

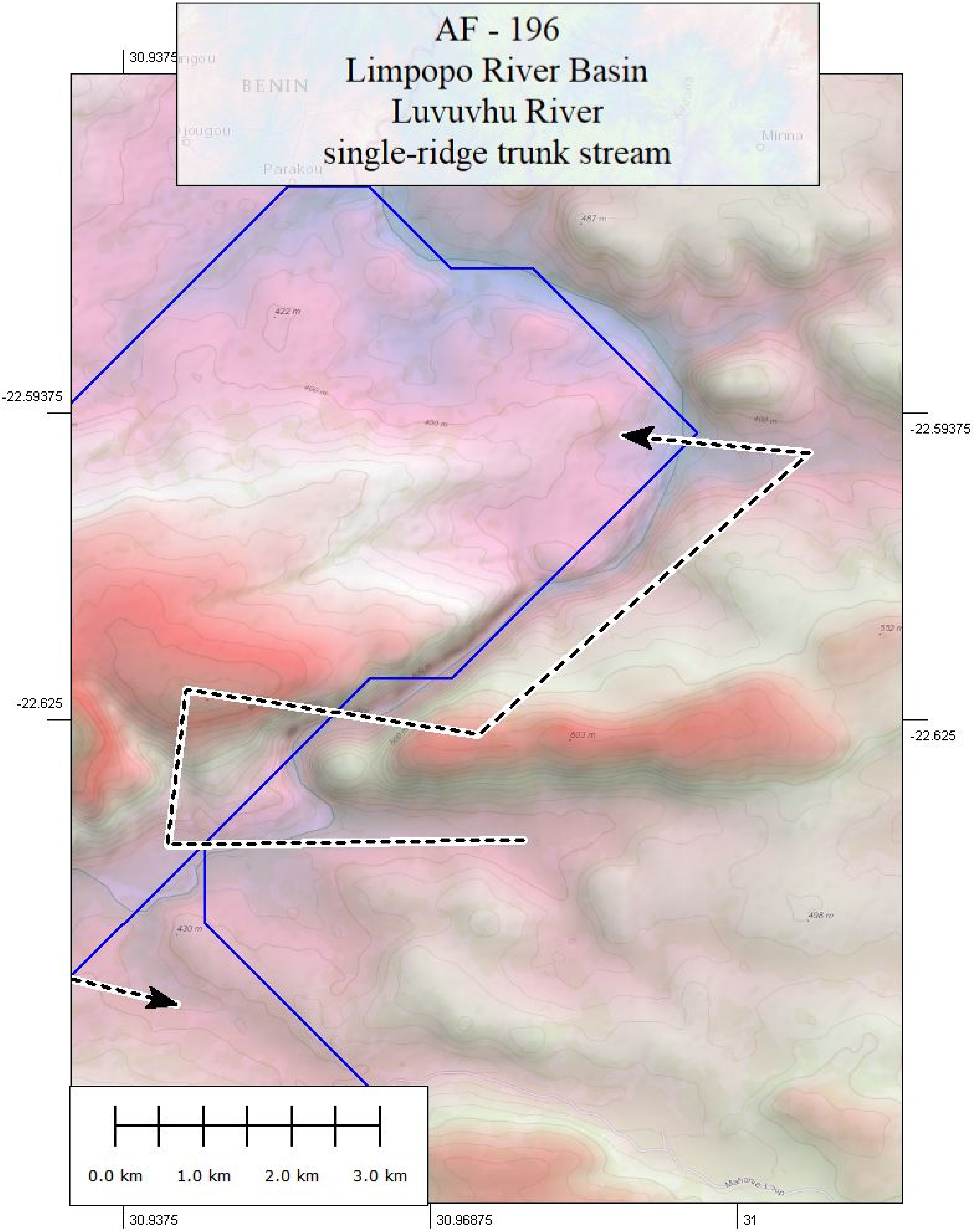
AF - 196
Limpopo River Basin
Luvuvhu River
single-ridge trunk stream
30.9375
30.96875
31
-22.59375
-22.625
487 m
422 m
430 m
498 m
0.0 km
1.0 km
2.0 km
3.0 km

# AF - 52
## Congo River Basin
## Congo River
## single-ridge trunk stream

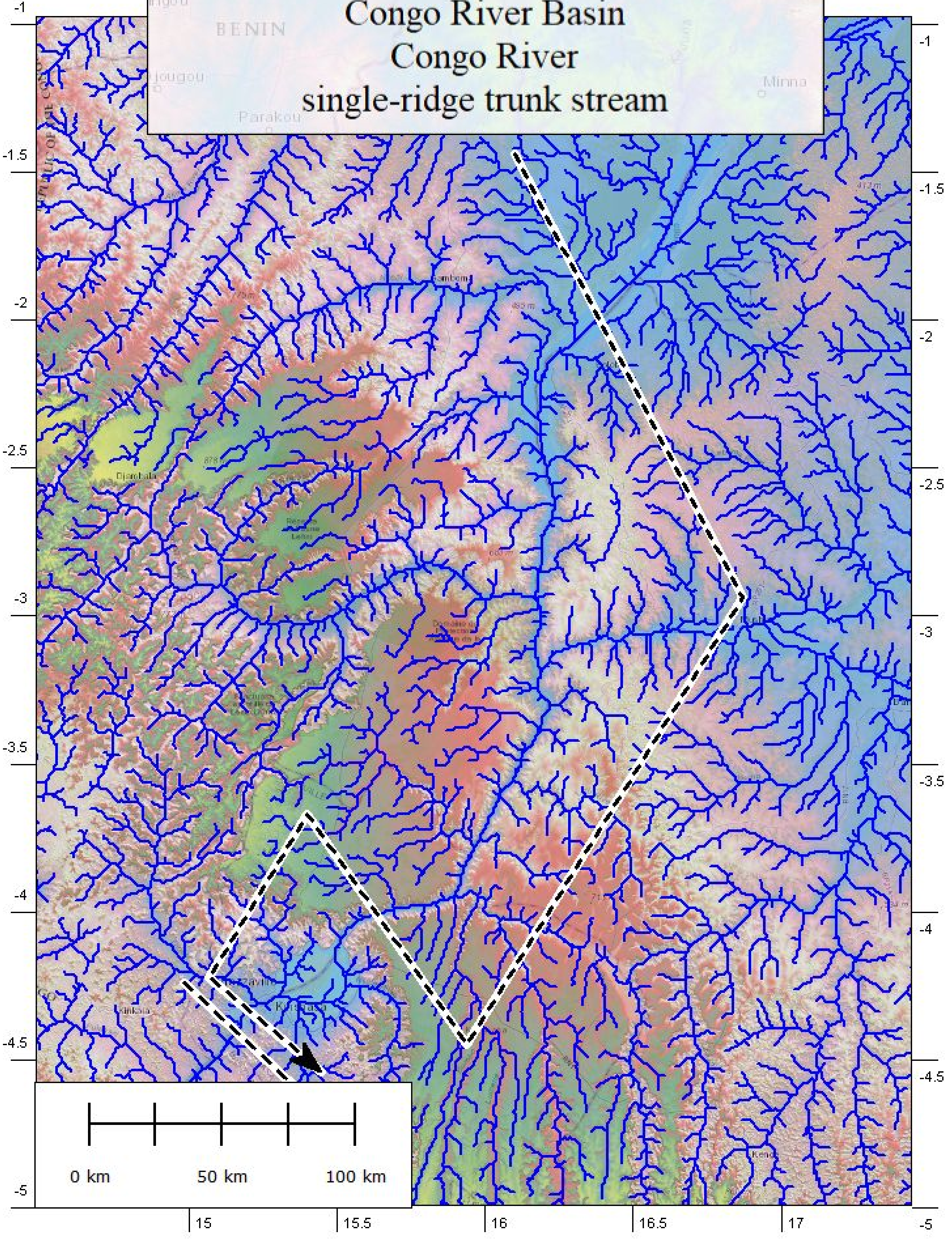

# AF - 59
# Congo River Basin
# Lukuga River
# single-ridge trunk stream

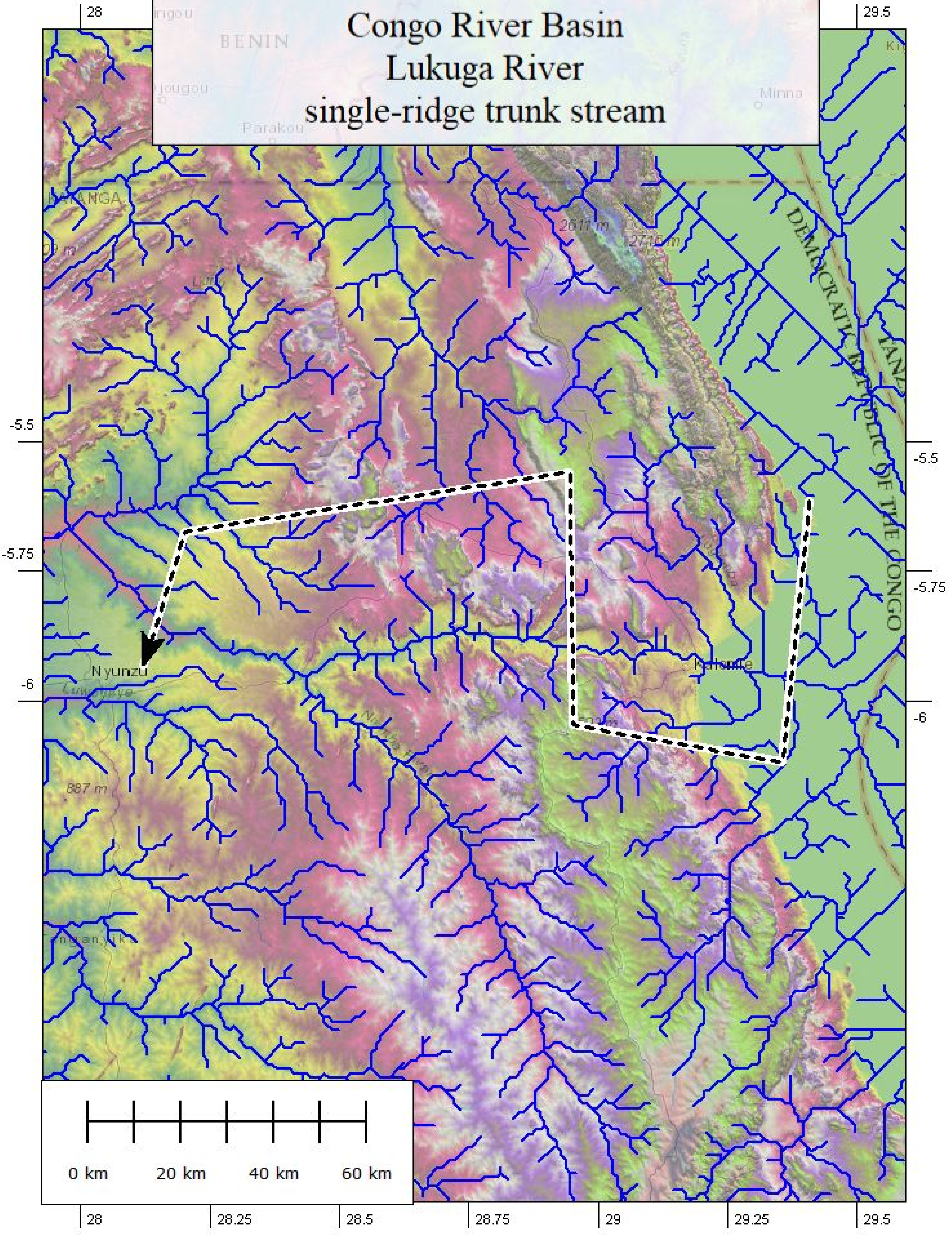

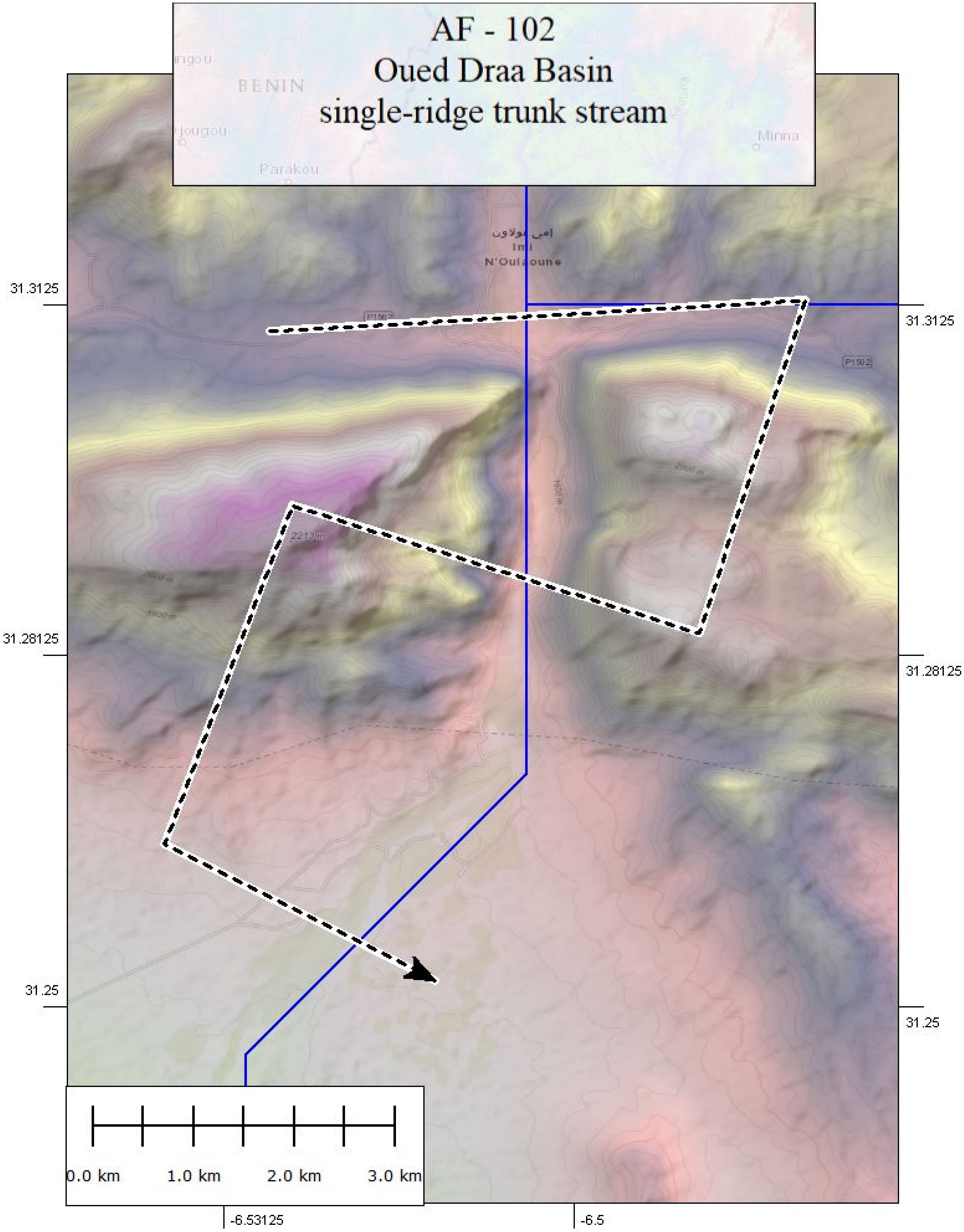
AF - 102
Oued Draa Basin
single-ridge trunk stream
BENIN
Parakou
Minna
Imi
N'Oulaoune
P1502
2213 m
2000 m
1600 m
31.3125
31.28125
31.25
-6.53125
-6.5
0.0 km
1.0 km
2.0 km
3.0 km

# AF - 112
## Rio Cubal de Hanha Basin
## single-ridge trunk stream

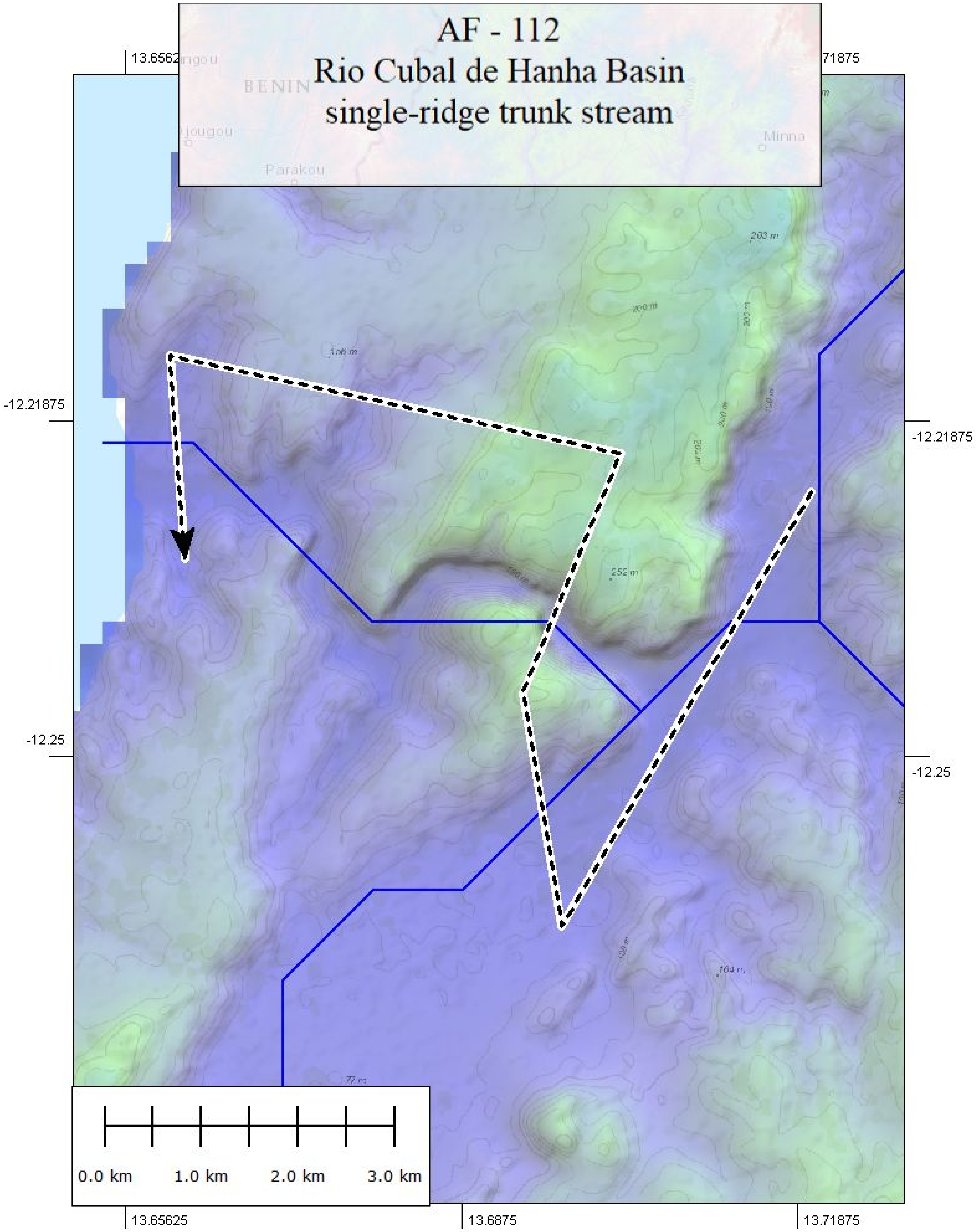

# AF - 120
## Endorheic basin Basin
## single-ridge trunk stream

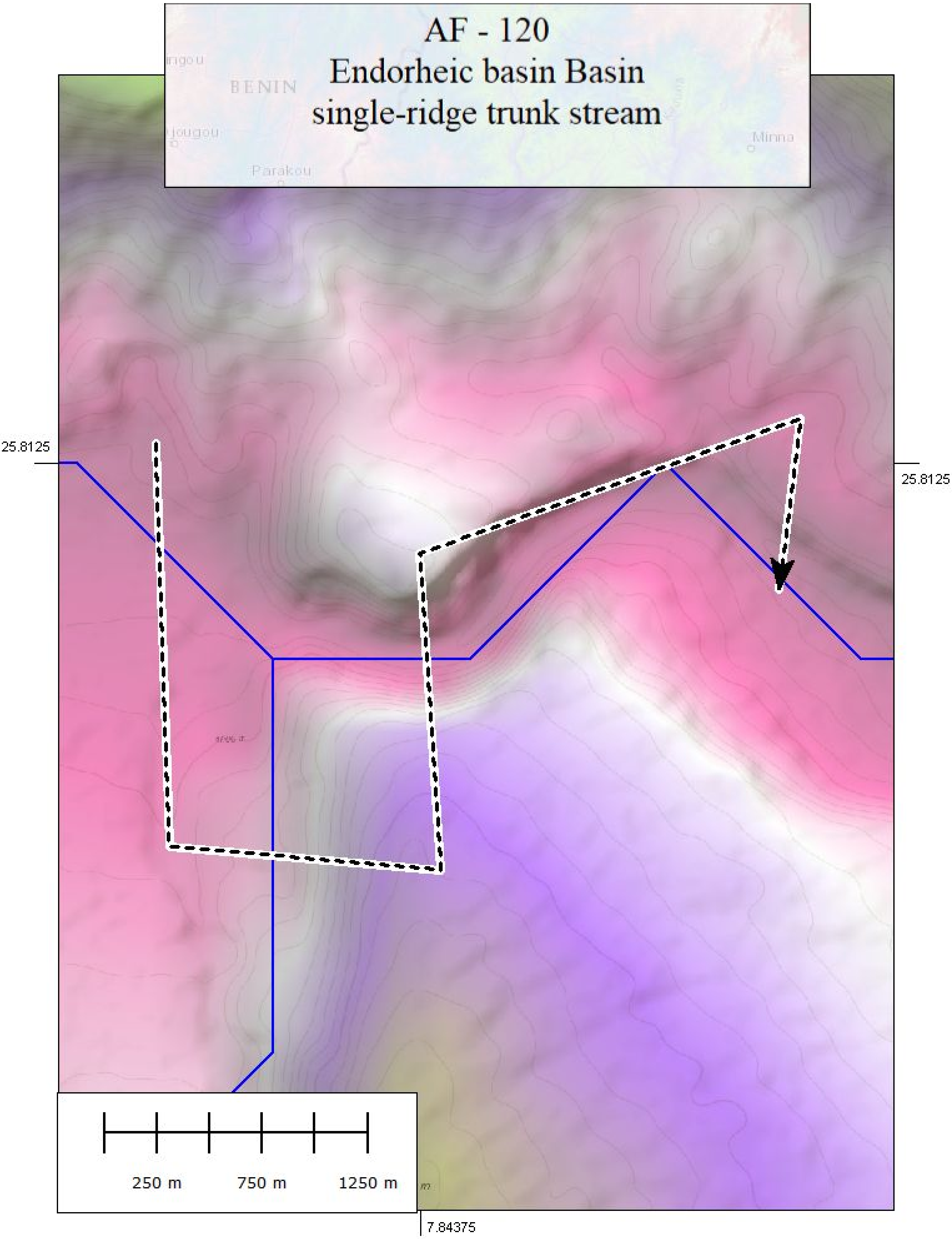

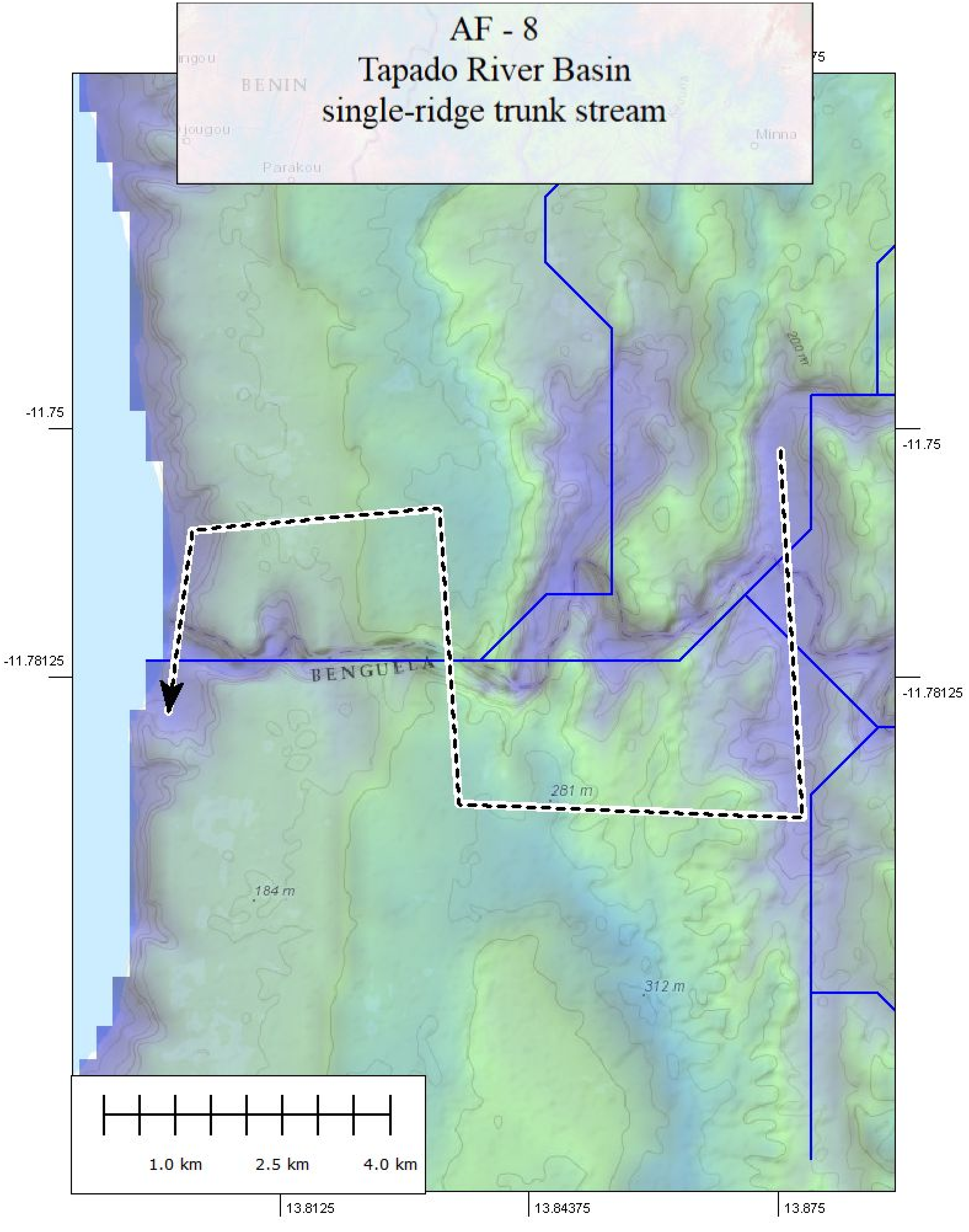
AF - 8
Tapado River Basin
single-ridge trunk stream
BENIN
Parakou
Minna
-11.75
-11.75
-11.78125
-11.78125
BENGUELA
200 m
281 m
184 m
312 m
1.0 km
2.5 km
4.0 km
13.8125
13.84375
13.875

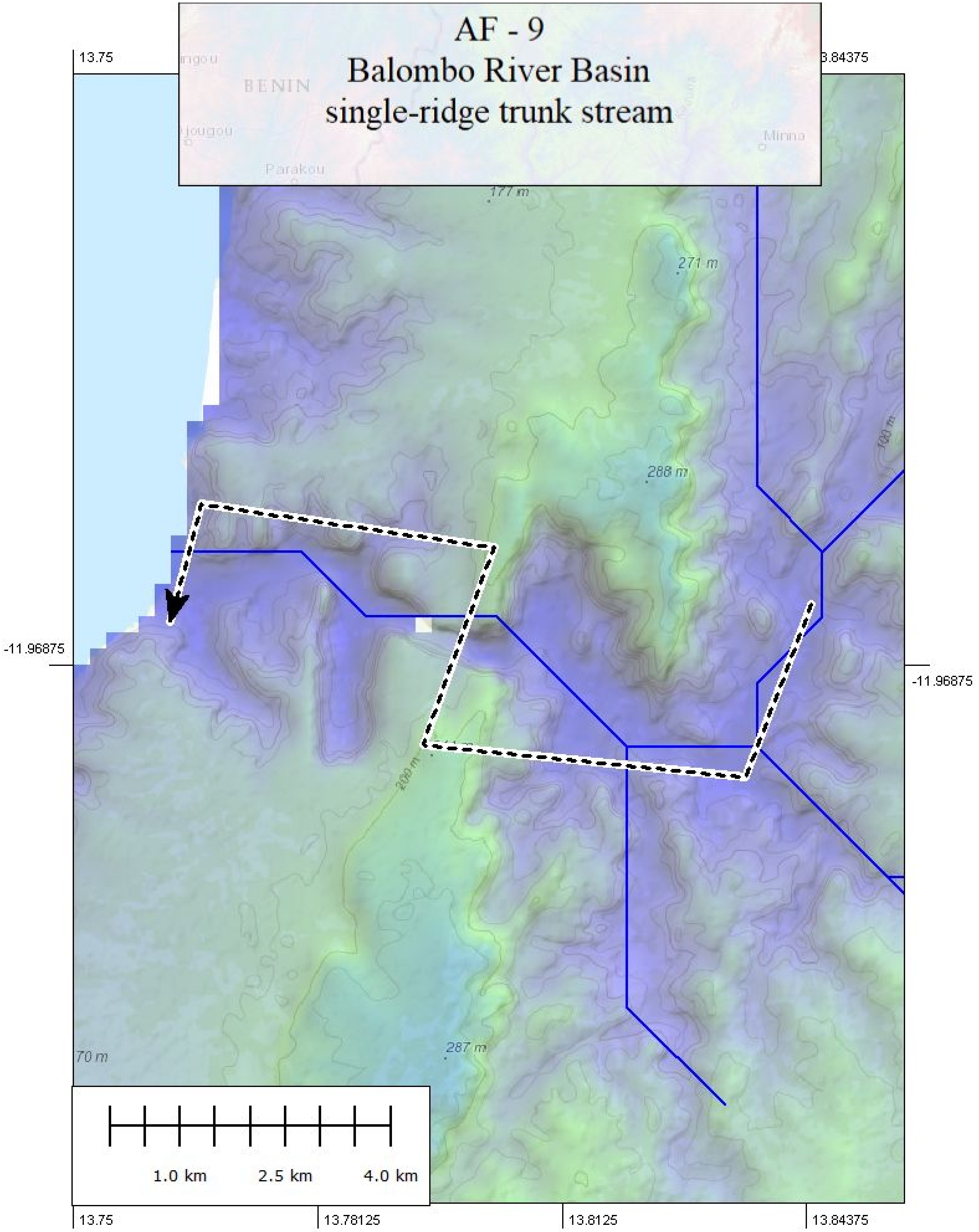
AF - 9
Balombo River Basin
single-ridge trunk stream
13.75
13.84375
BENIN
Parakou
Minna
177 m
271 m
288 m
287 m
200 m
100 m
70 m
-11.96875
-11.96875
1.0 km
2.5 km
4.0 km
13.75
13.78125
13.8125
13.84375

# AF - 35
# Gourits River Basin
# Klip River
# multi-ridge head stream

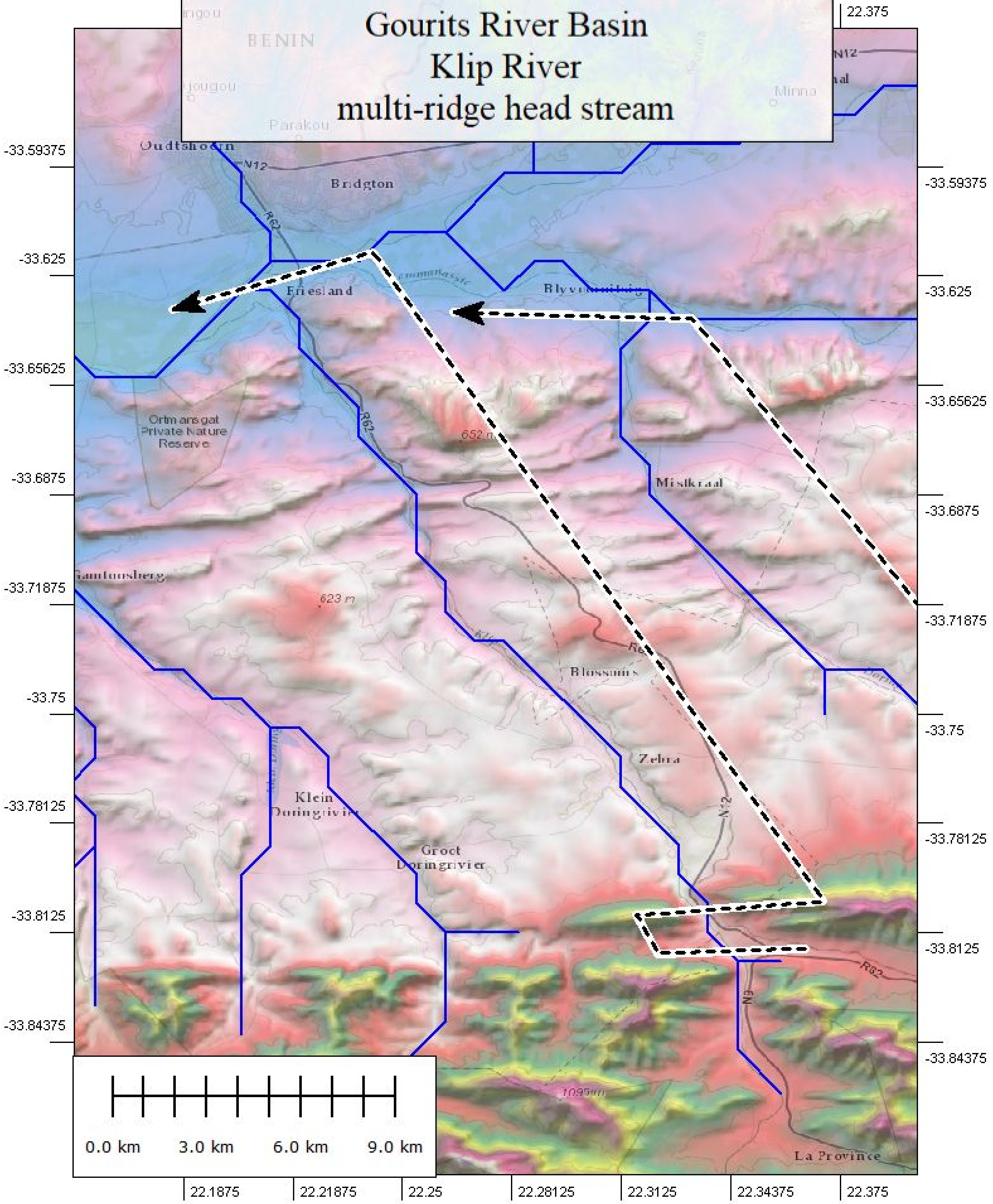

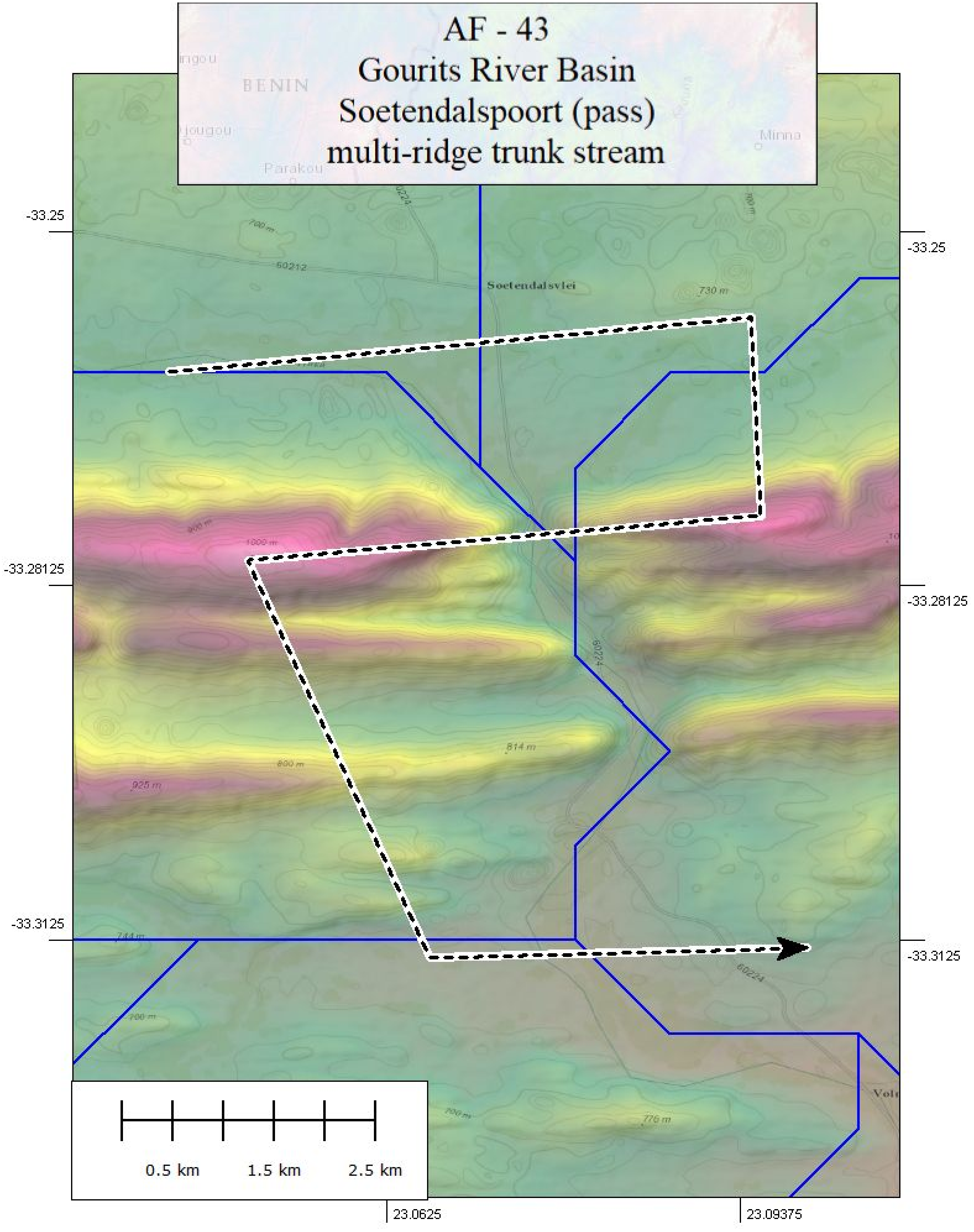

AF - 43
Gourits River Basin
Soetendalspoort (pass)
multi-ridge trunk stream
Soetendalsvlei
-33.25
-33.28125
-33.3125
23.0625
23.09375
0.5 km
1.5 km
2.5 km

# AF - 47
## Gamtoos River Basin
## Swanepoelspoort (pass)
## multi-ridge trunk stream

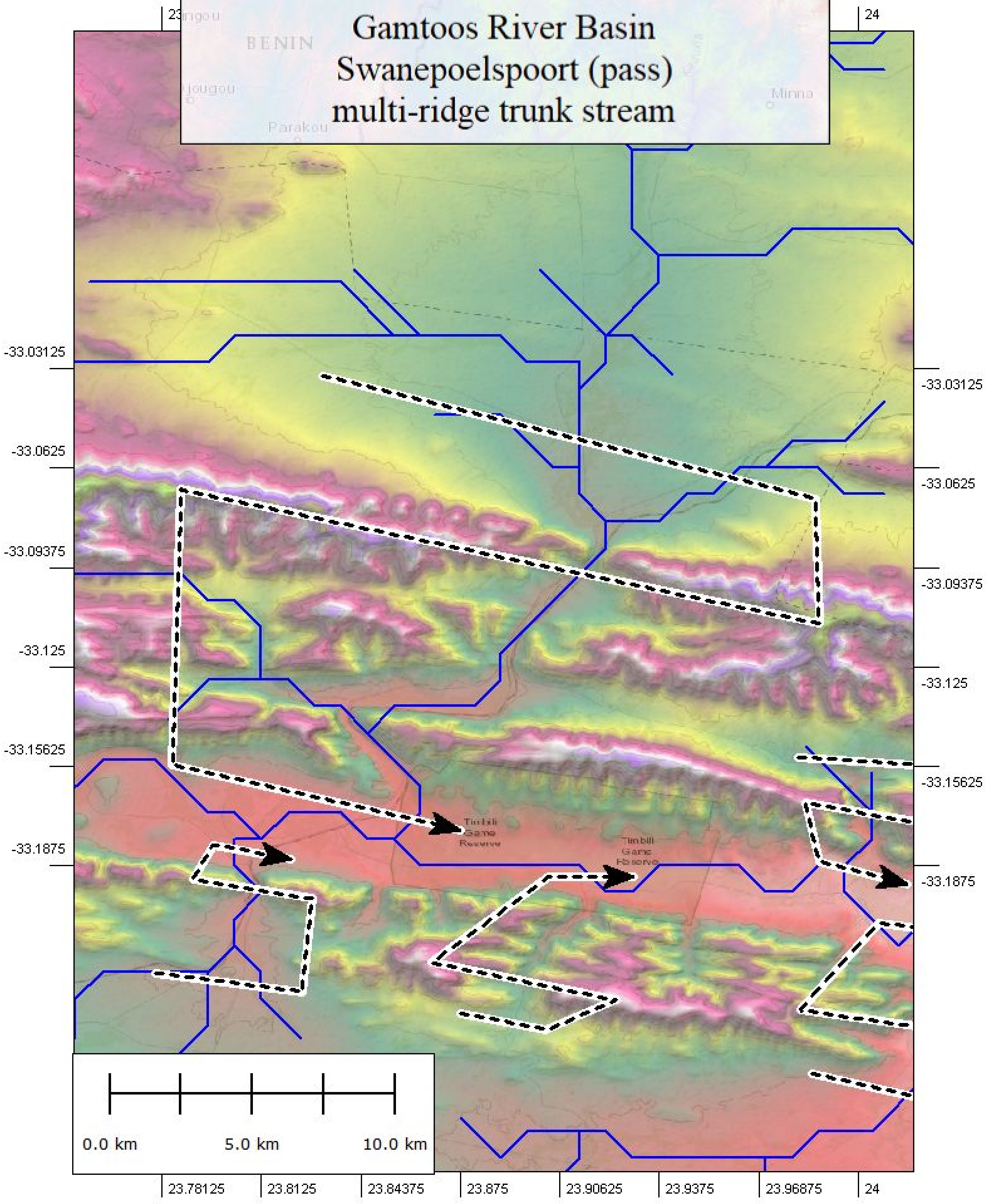

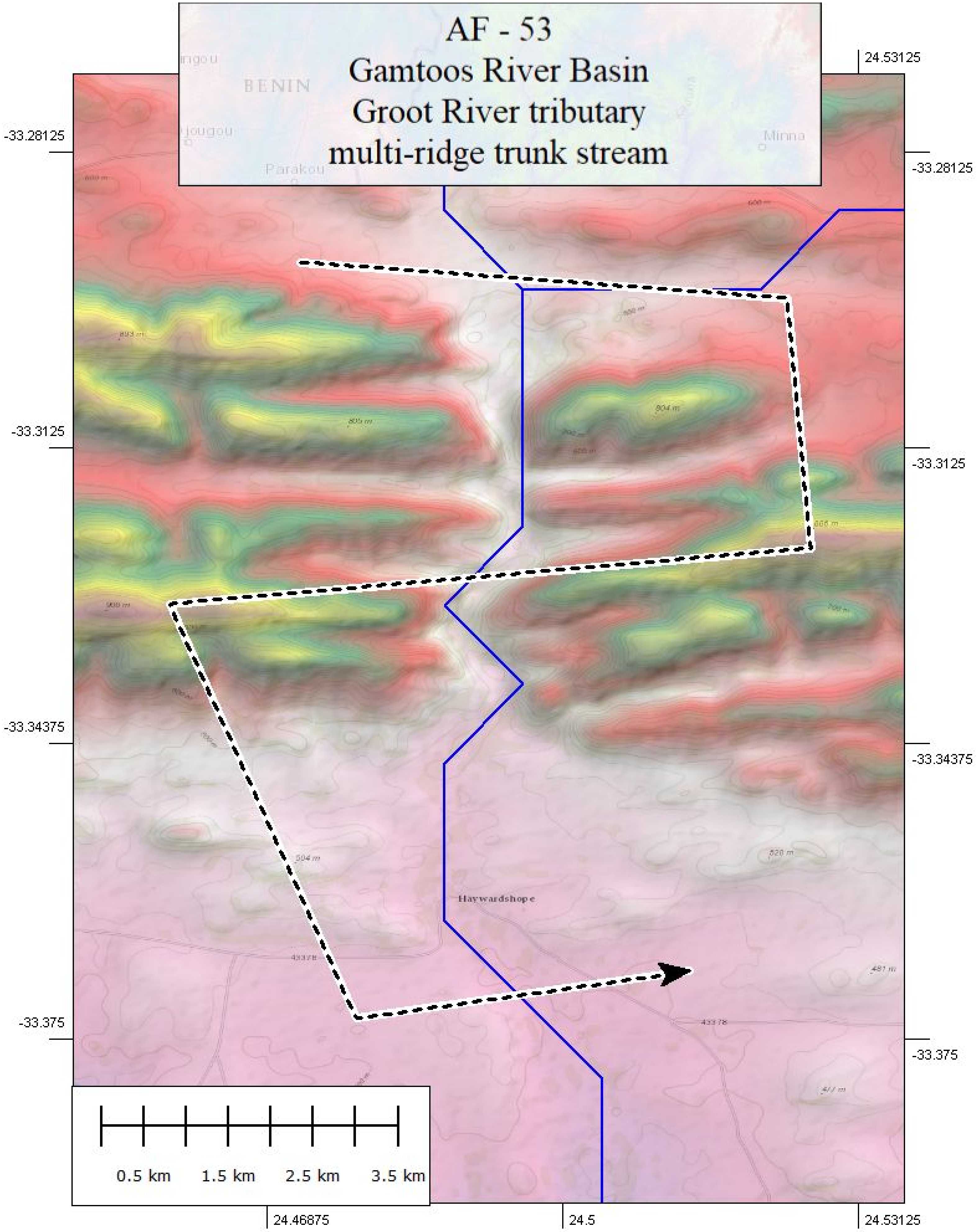
AF - 53
Gamtoos River Basin
Groot River tributary
multi-ridge trunk stream
24.53125
-33.28125
-33.28125
-33.3125
-33.3125
-33.34375
-33.34375
-33.375
-33.375
Haywardshope
0.5 km
1.5 km
2.5 km
3.5 km
24.46875
24.5
24.53125

# AF - 56
## Gamtoos River Basin
## Groot River
## multi-ridge trunk stream

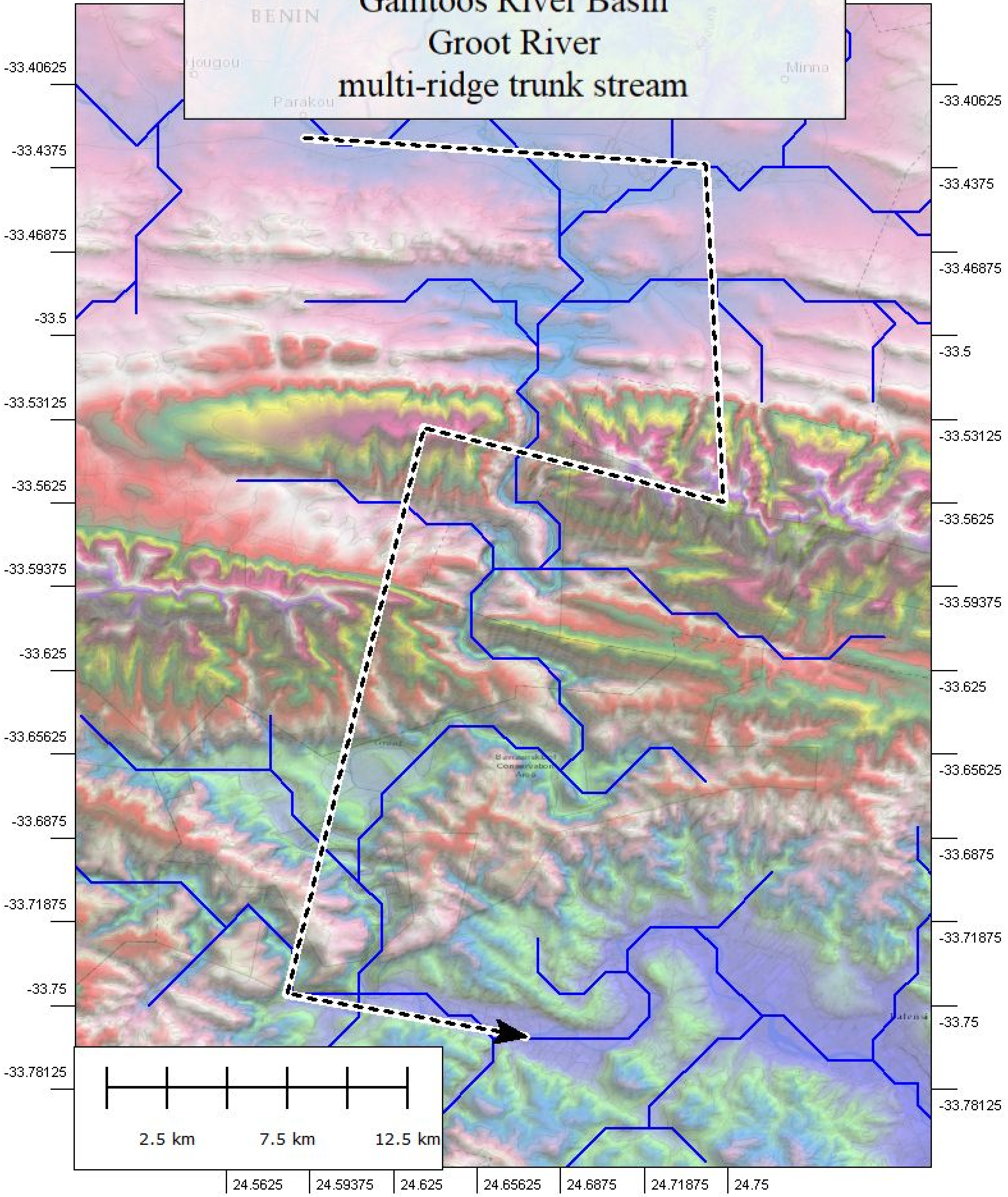

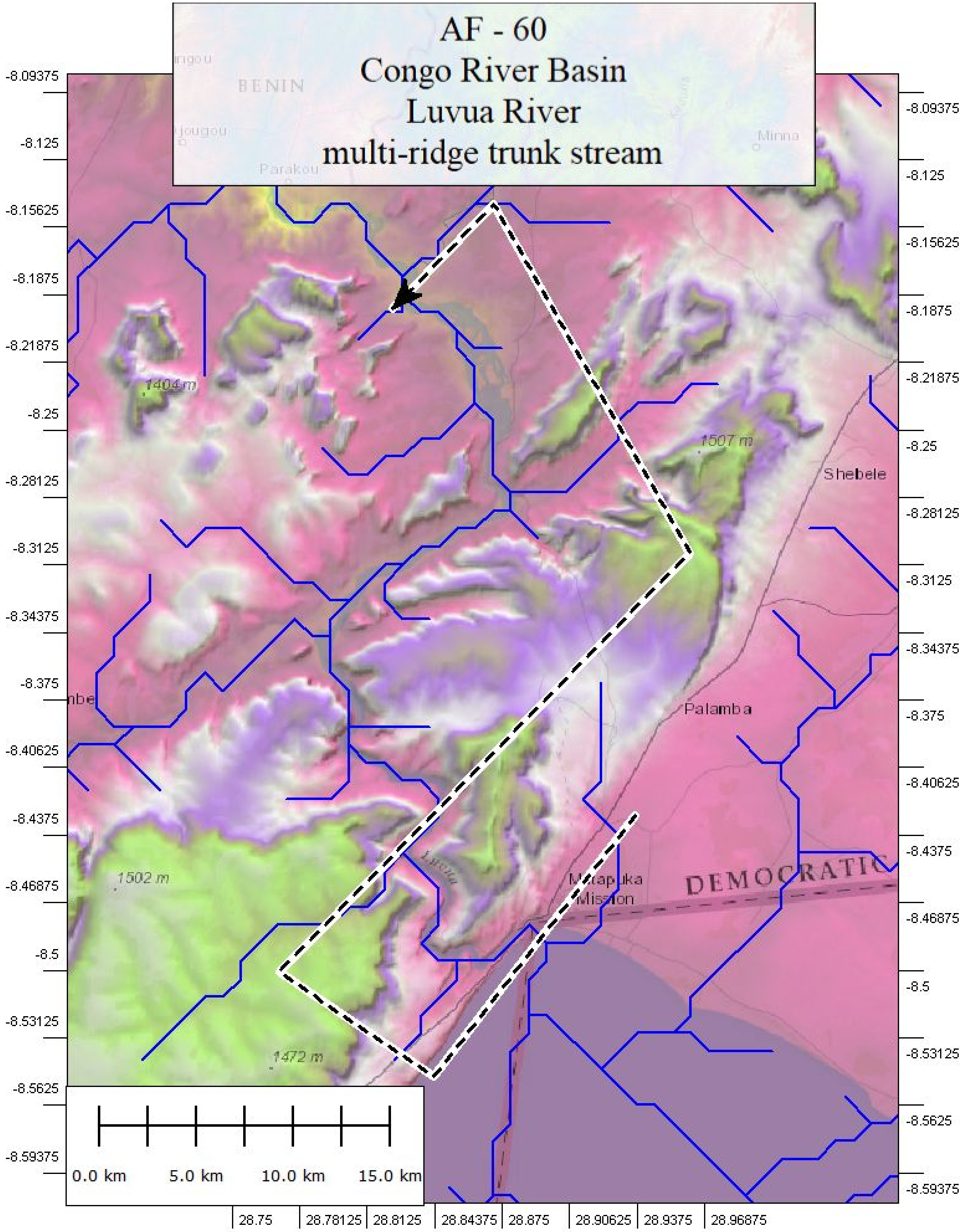
AF - 60
Congo River Basin
Luvua River
multi-ridge trunk stream
BENIN
Parakou
Minna
1404 m
1507 m
1502 m
1472 m
Shebele
Palamba
Luvua
Mitapuka Mission
DEMOCRATIC
0.0 km
5.0 km
10.0 km
15.0 km
-8.09375
-8.125
-8.15625
-8.1875
-8.21875
-8.25
-8.28125
-8.3125
-8.34375
-8.375
-8.40625
-8.4375
-8.46875
-8.5
-8.53125
-8.5625
-8.59375
28.75
28.78125
28.8125
28.84375
28.875
28.90625
28.9375
28.96875

# AF - 77
## Limpopo River Basin
## Krokodil River
## multi-ridge trunk stream

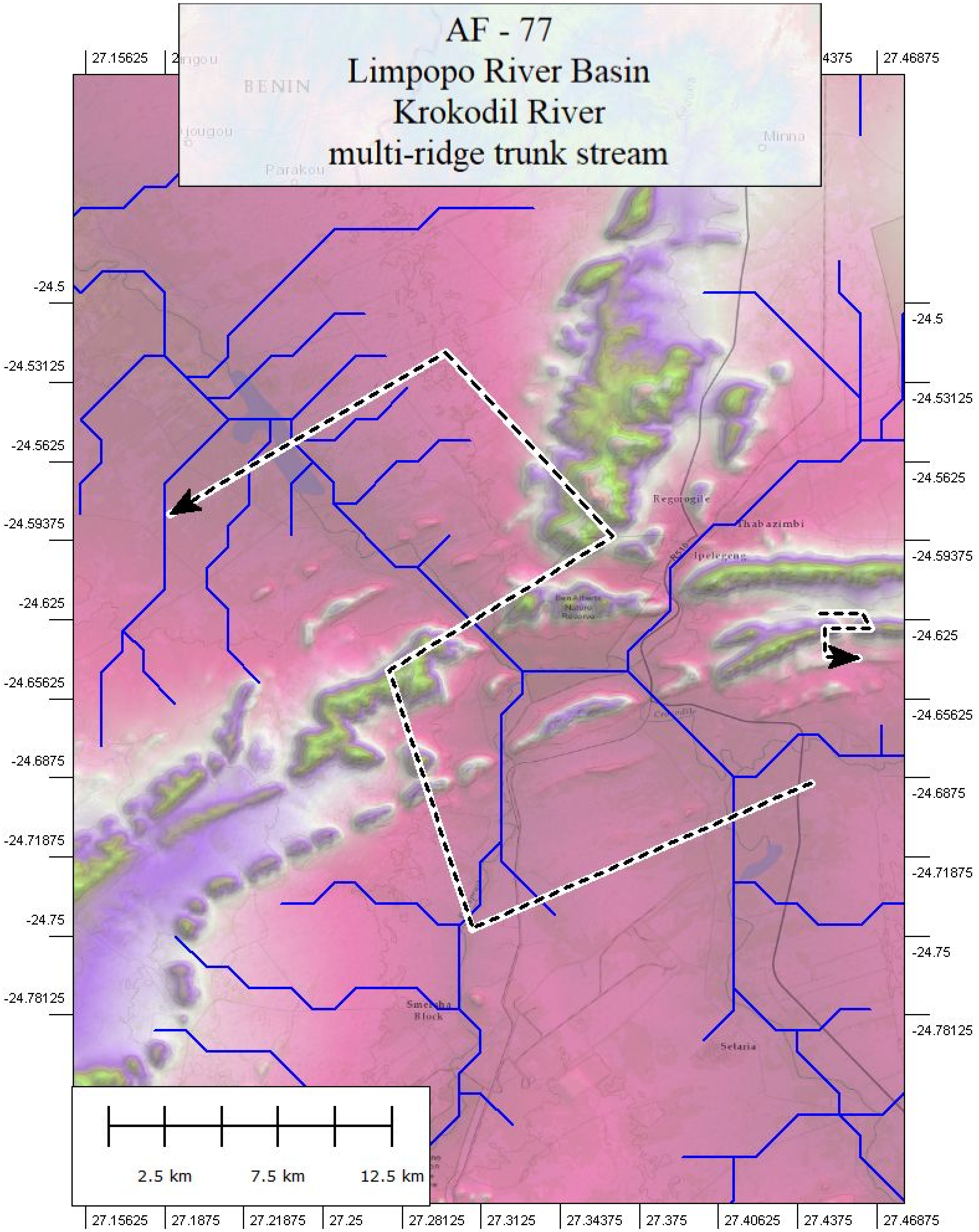

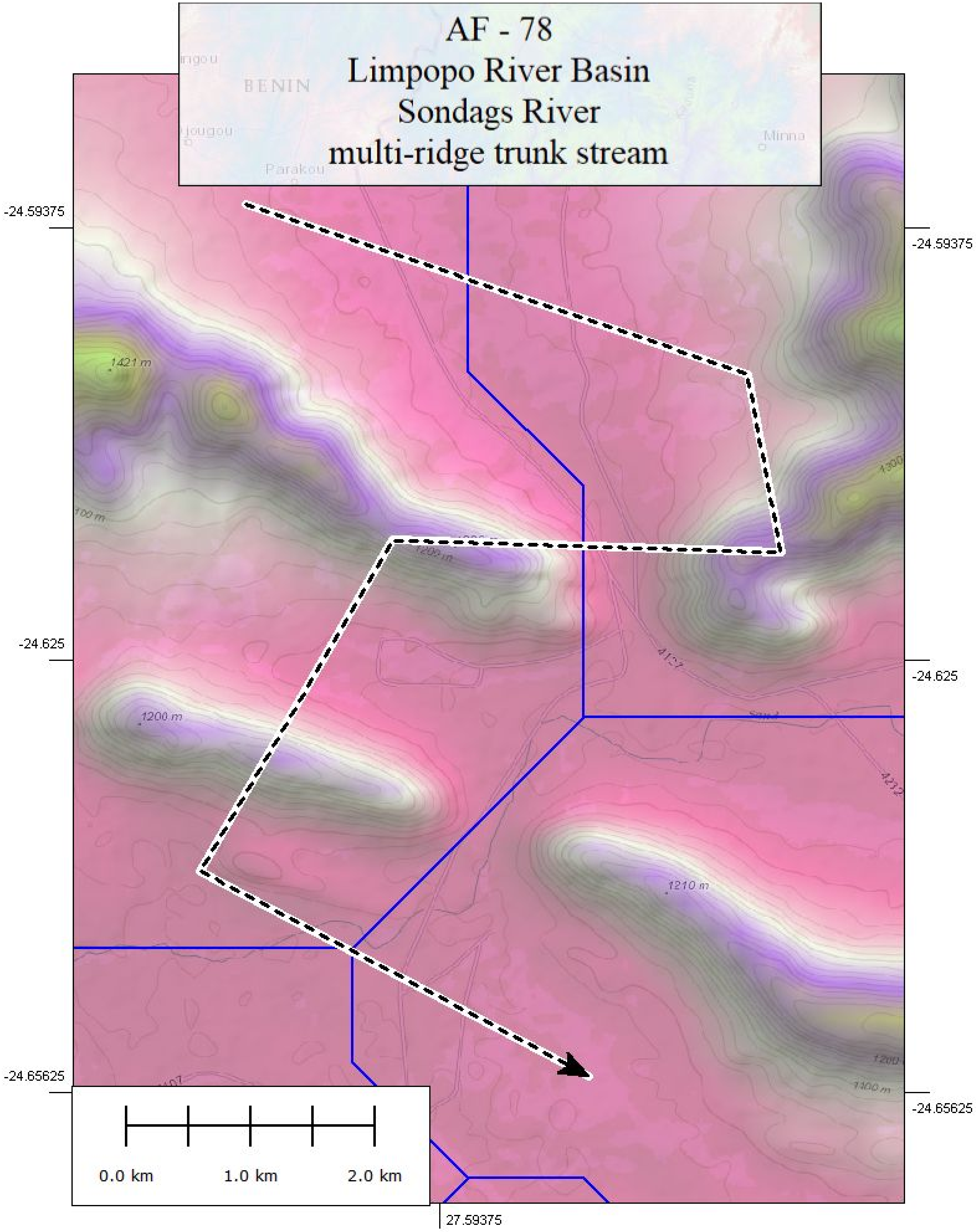

AF - 78
Limpopo River Basin
Sondags River
multi-ridge trunk stream
-24.59375
-24.59375
1421 m
1300
100 m
1200 m
-24.625
-24.625
1200 m
1210 m
-24.65625
-24.65625
0.0 km
1.0 km
2.0 km
27.59375

# AF - 79
## Limpopo River Basin
## Krokodil
## multi-ridge trunk stream

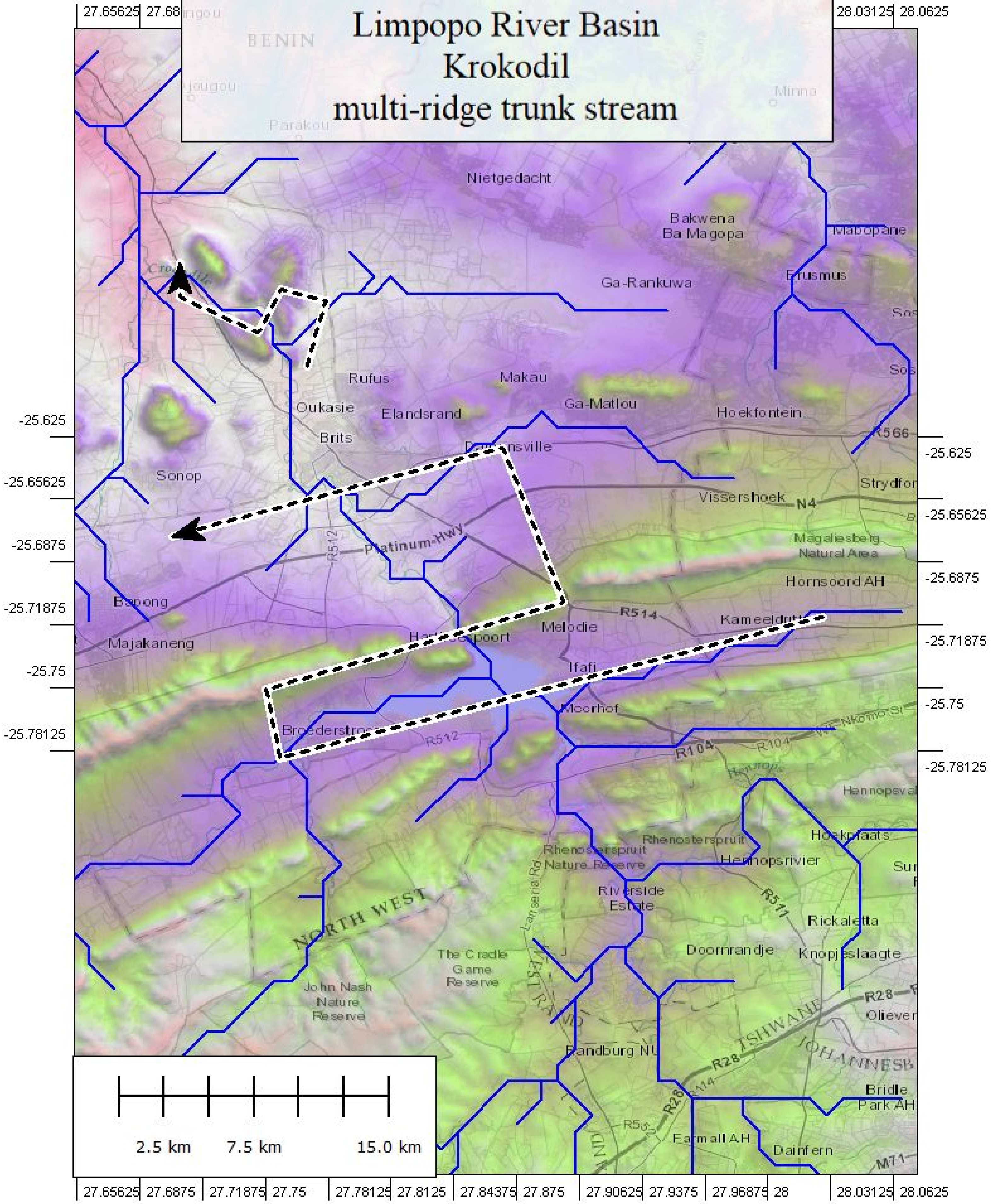

# AF - 147
## Gourits River Basin
## Buffels River
## multi-ridge trunk stream

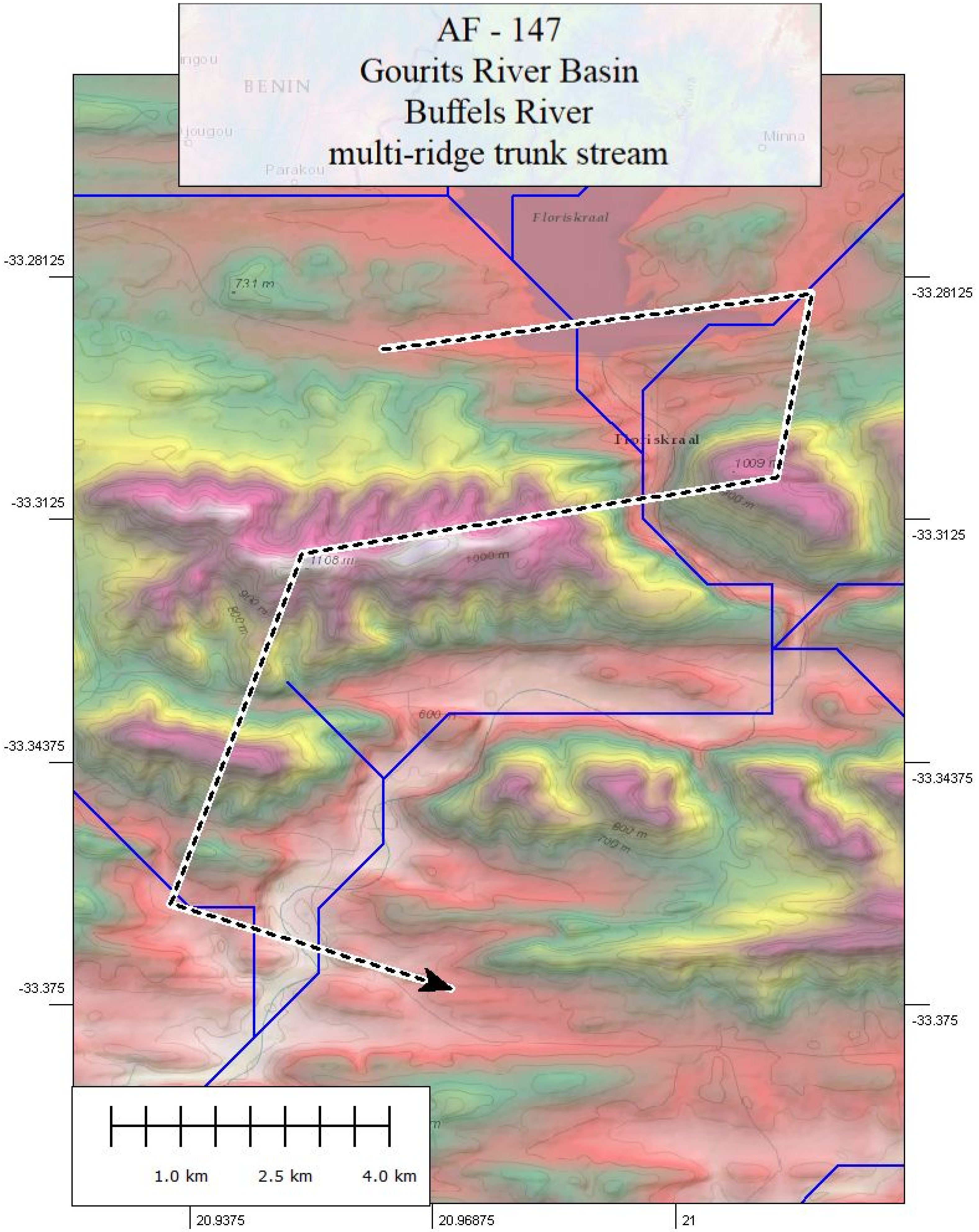

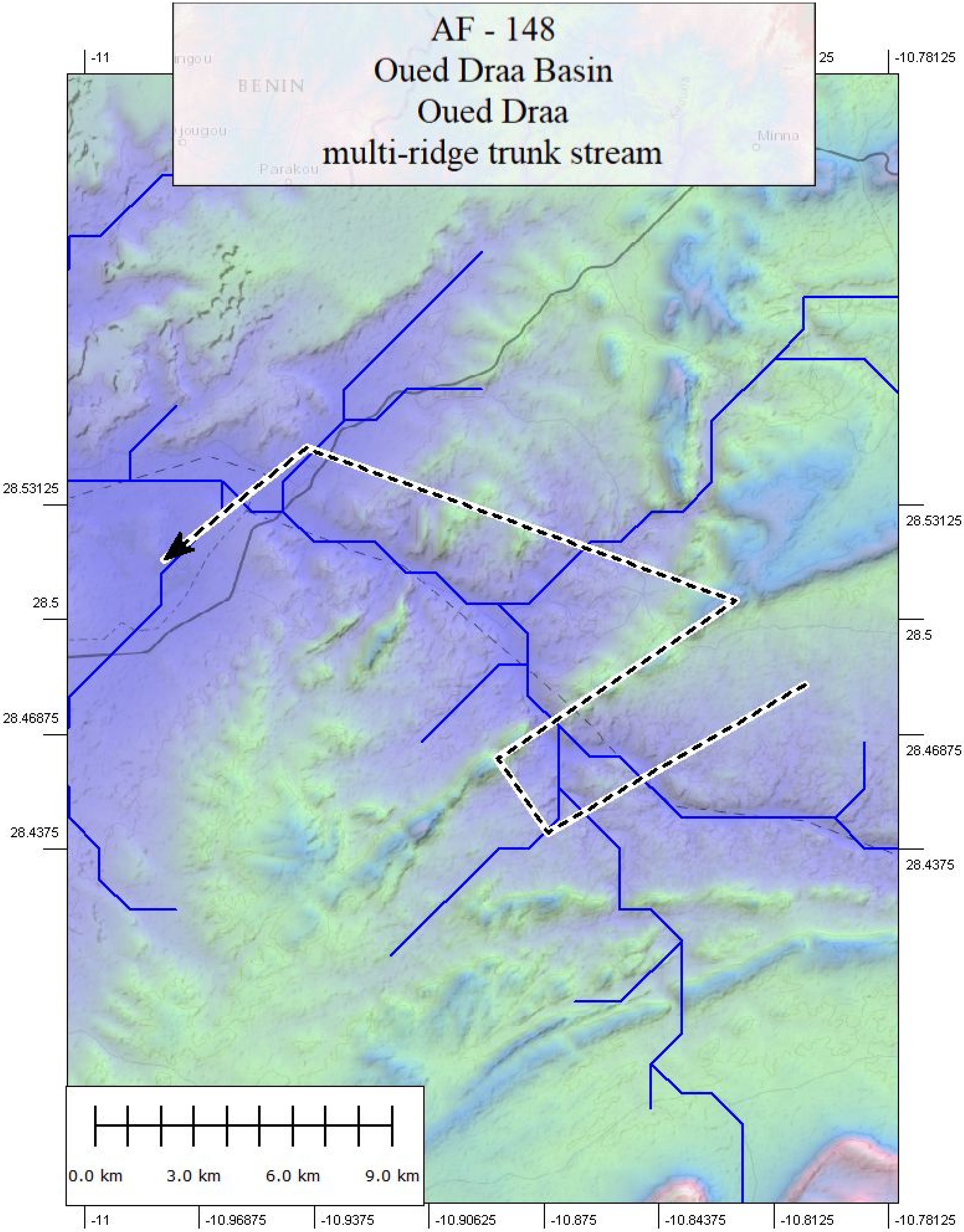
AF - 148
Oued Draa Basin
Oued Draa
multi-ridge trunk stream
BENIN
Parakou
Minna
-11
-10.96875
-10.9375
-10.90625
-10.875
-10.84375
-10.8125
-10.78125
28.53125
28.5
28.46875
28.4375
0.0 km
3.0 km
6.0 km
9.0 km

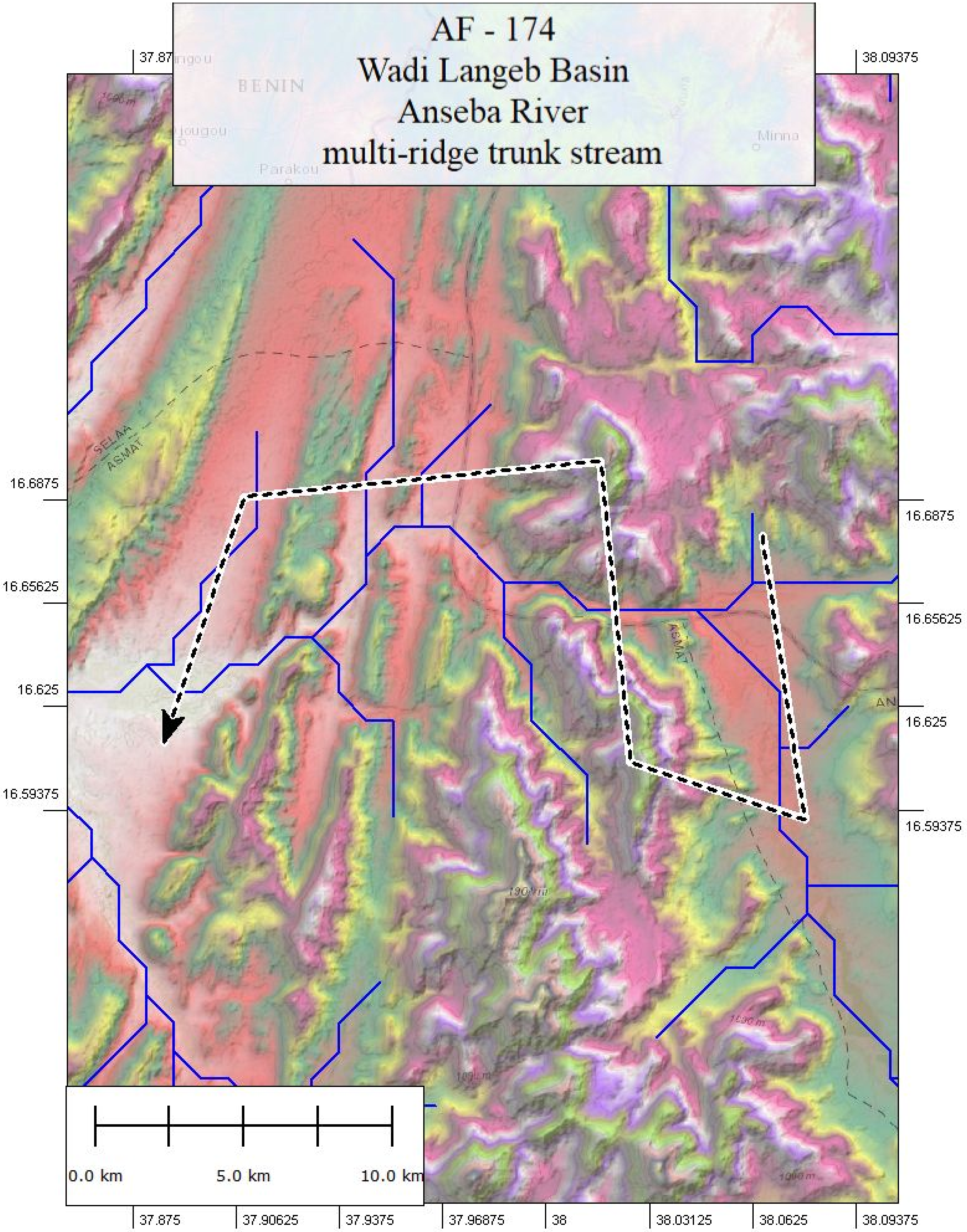
AF - 174
Wadi Langeb Basin
Anseba River
multi-ridge trunk stream
37.875
37.90625
37.9375
37.96875
38
38.03125
38.0625
38.09375
16.6875
16.65625
16.625
16.59375
SELAA
ASMAT
BENIN
Parakou
Minna
0.0 km
5.0 km
10.0 km

# AF - 179
## Gourits River Basin
## Gamkan River
## multi-ridge trunk stream

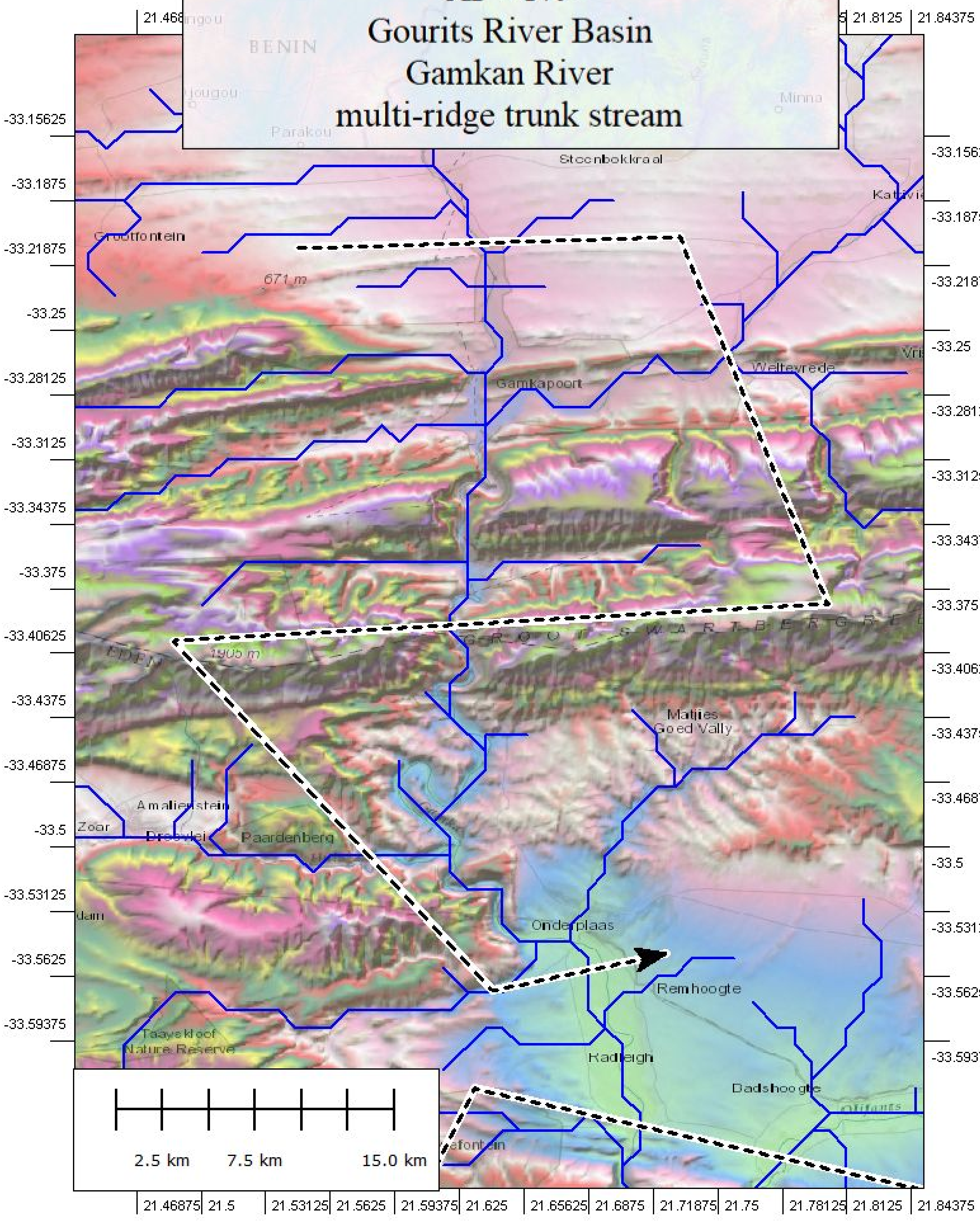

# AF - 191
## Limpopo River Basin
## Sand River
## multi-ridge trunk stream

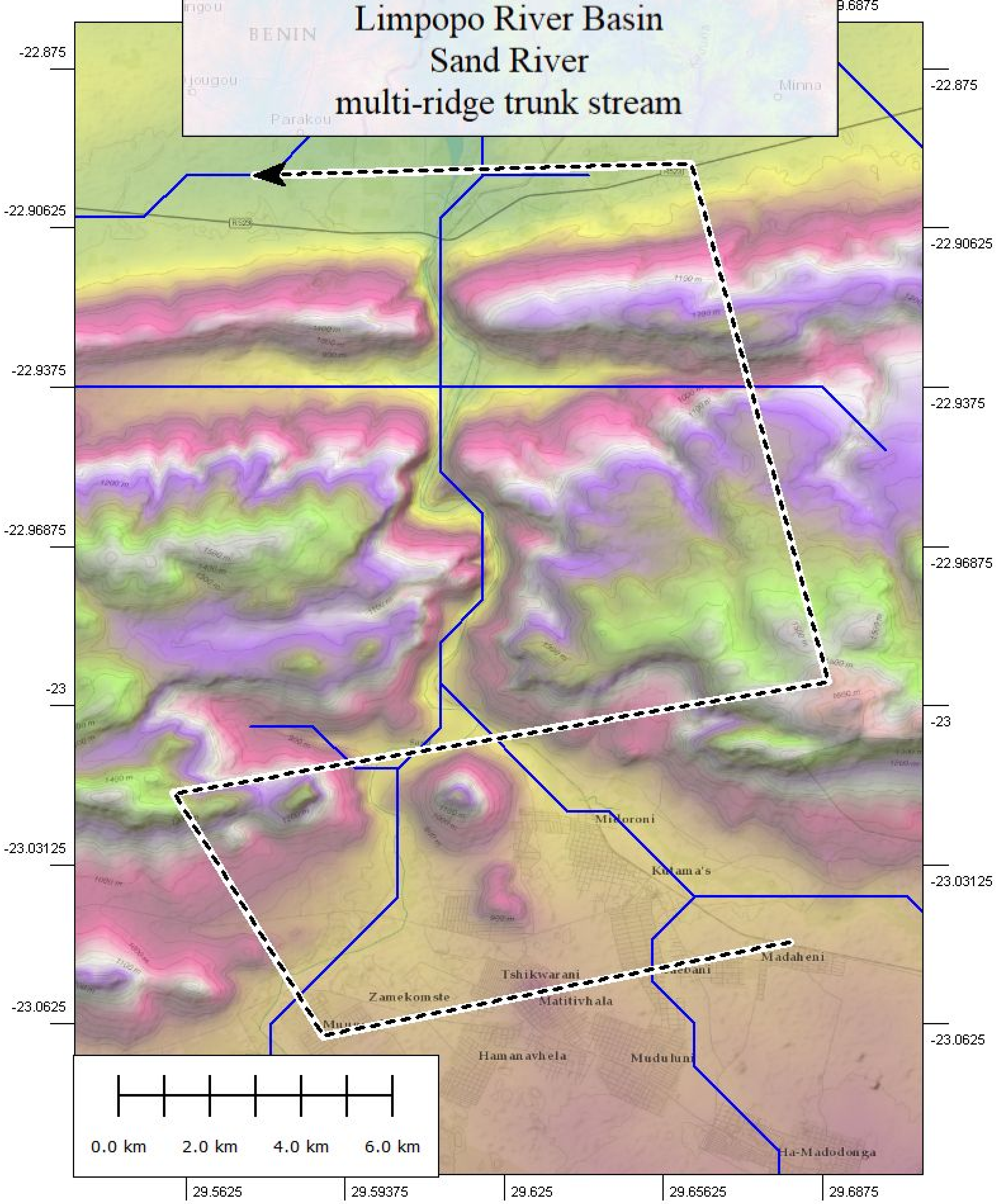

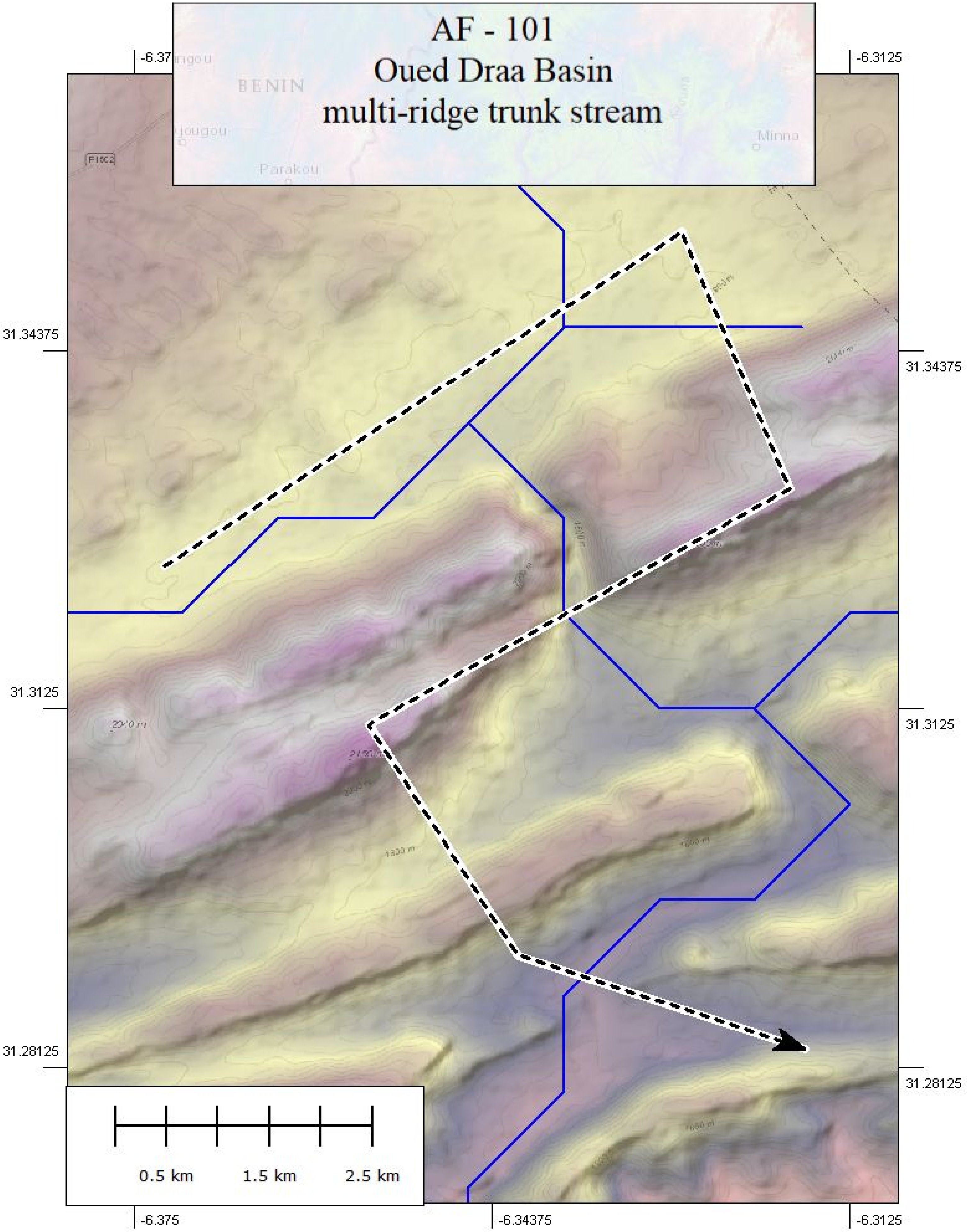
AF - 101
Oued Draa Basin
multi-ridge trunk stream
-6.375
-6.34375
-6.3125
31.34375
31.3125
31.28125
0.5 km
1.5 km
2.5 km

# AF - 69
# Oued Draa Basin
# multi-ridge trunk stream

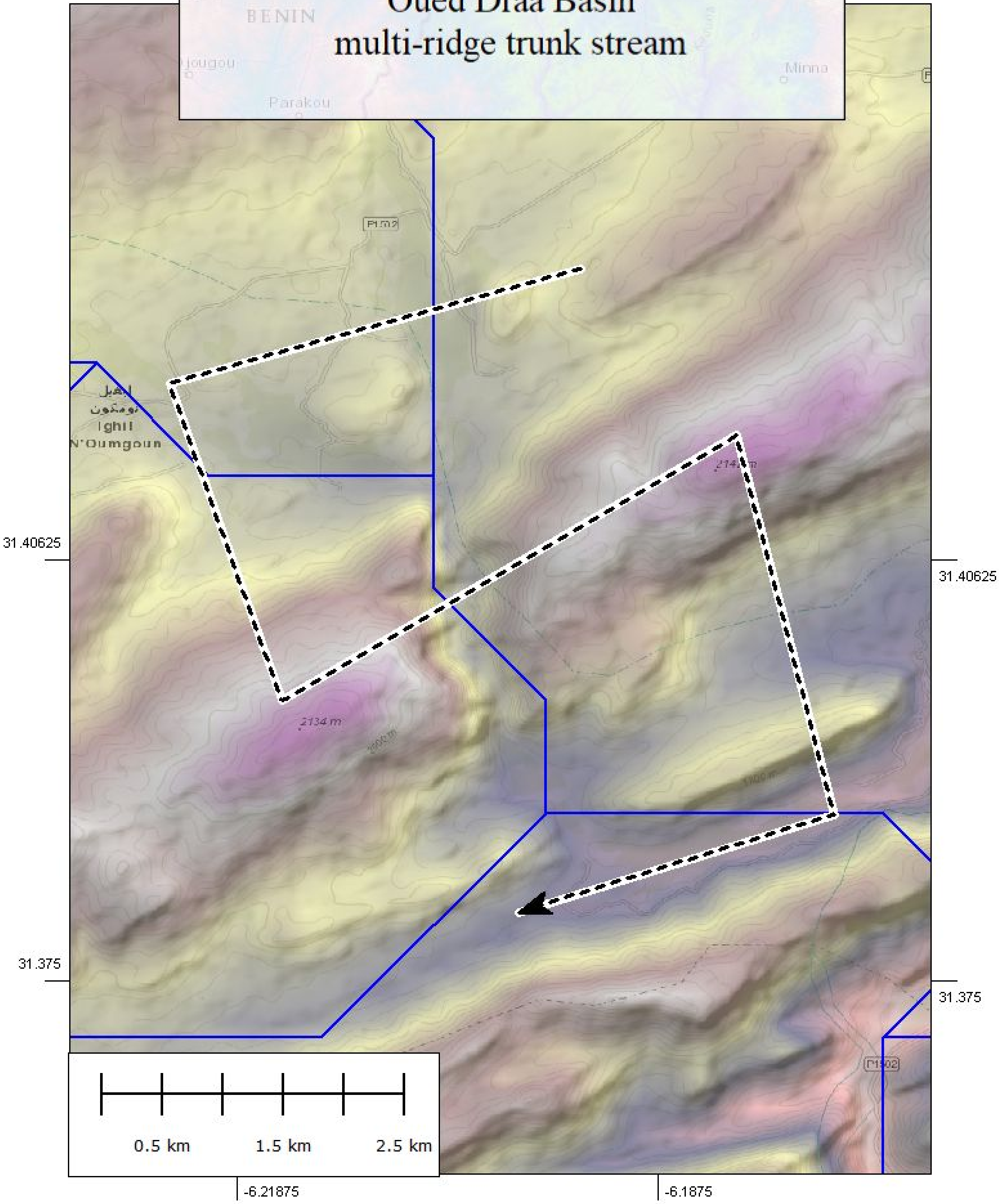

# AF - 96
## Endorheic basin Basin
## multi-ridge trunk stream

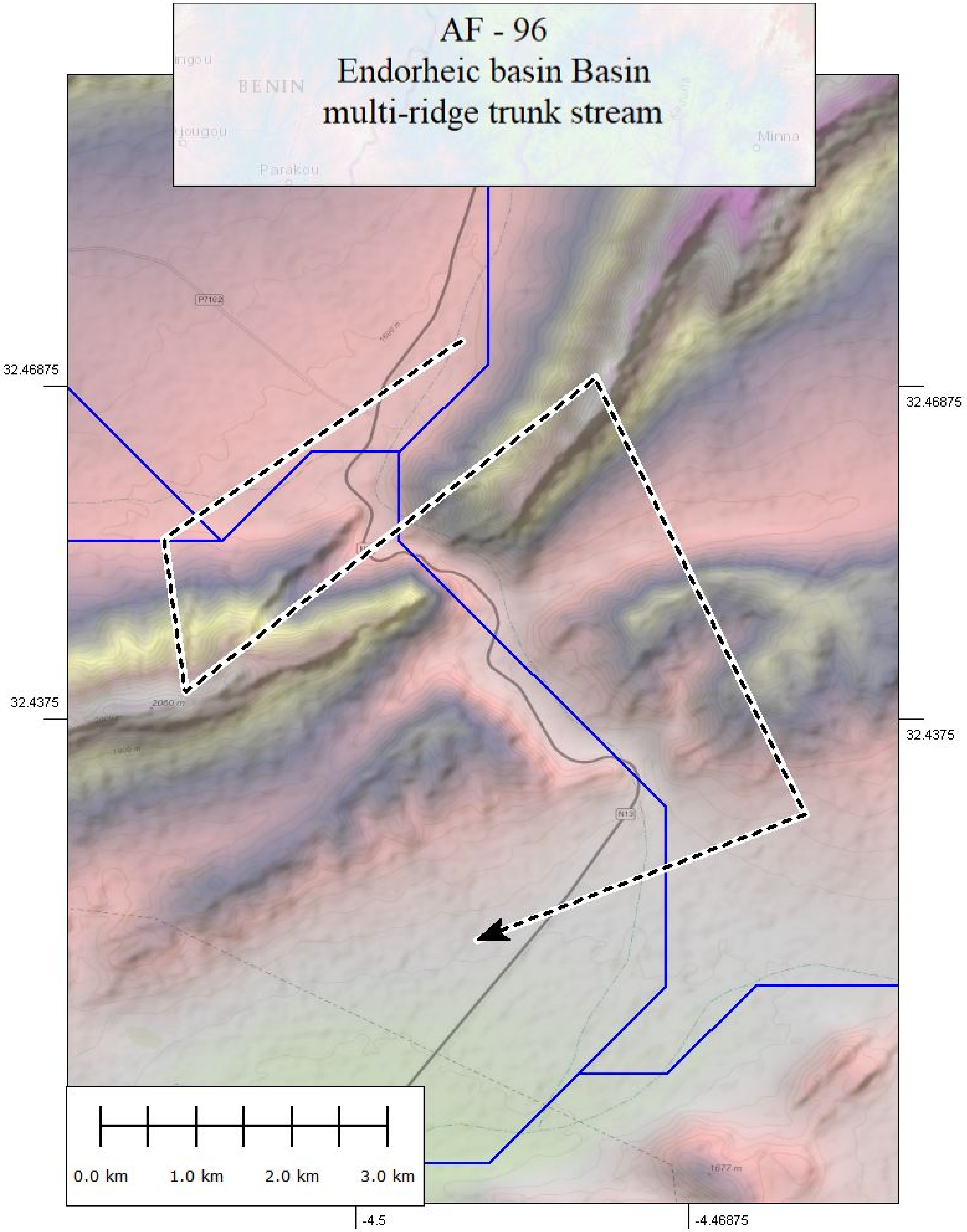

# AF - 98
# Endorheic basin Basin
# multi-ridge trunk stream

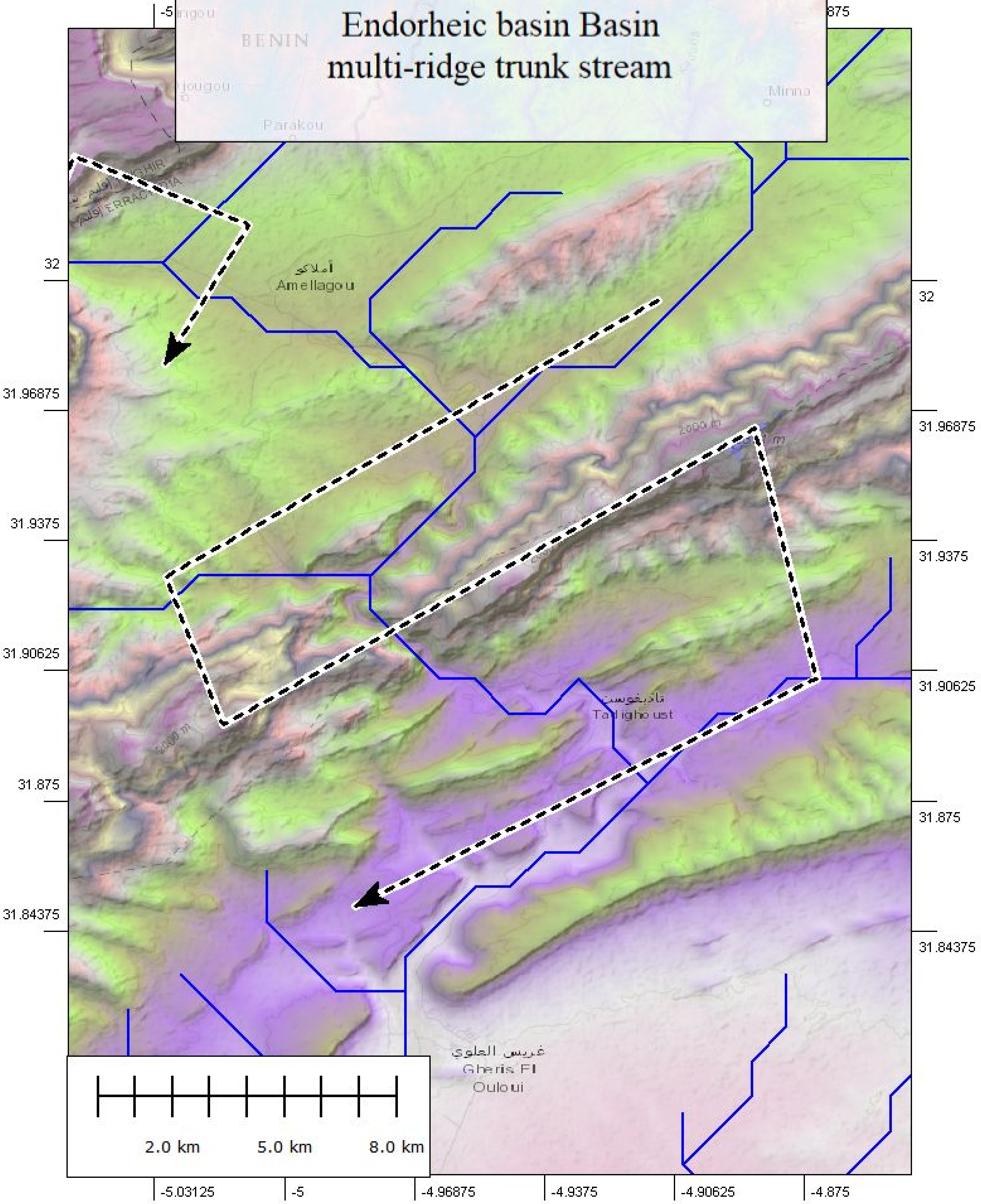

# AF - 0
## Niari River Basin
## Niari River
## single-ridge trunk stream

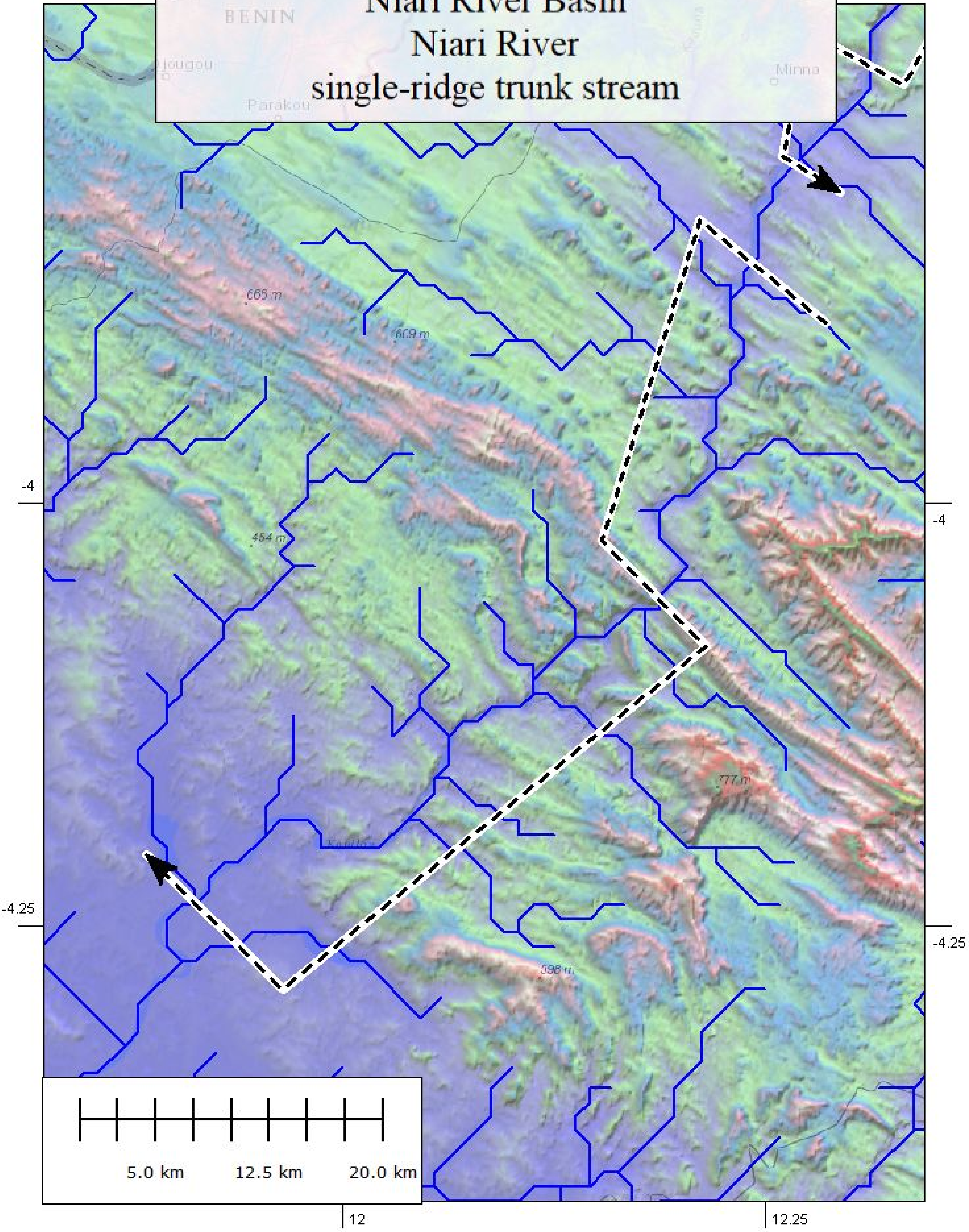

# AF - 1
# Tsiribihina River Basin
# Mania River
# single-ridge trunk stream

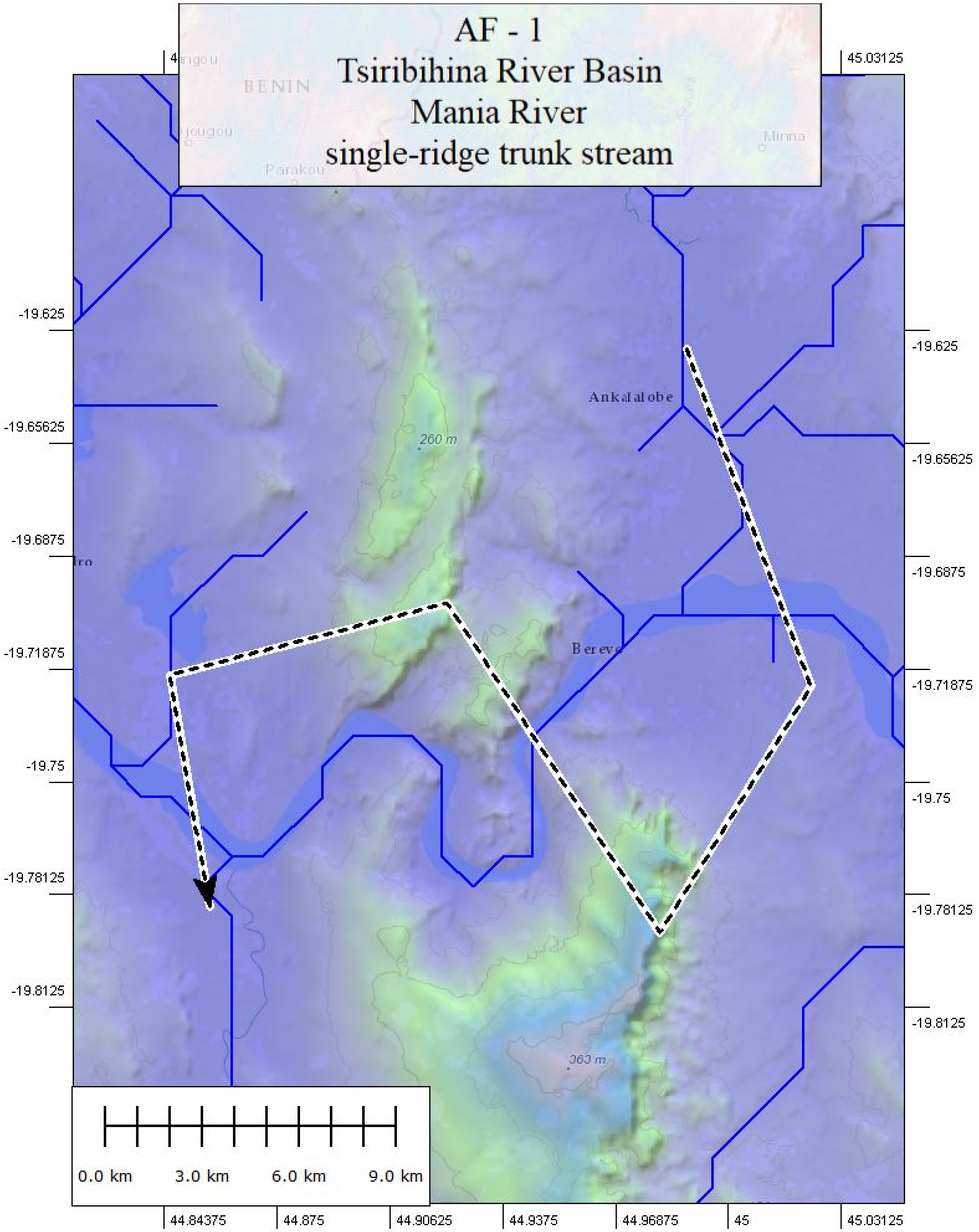

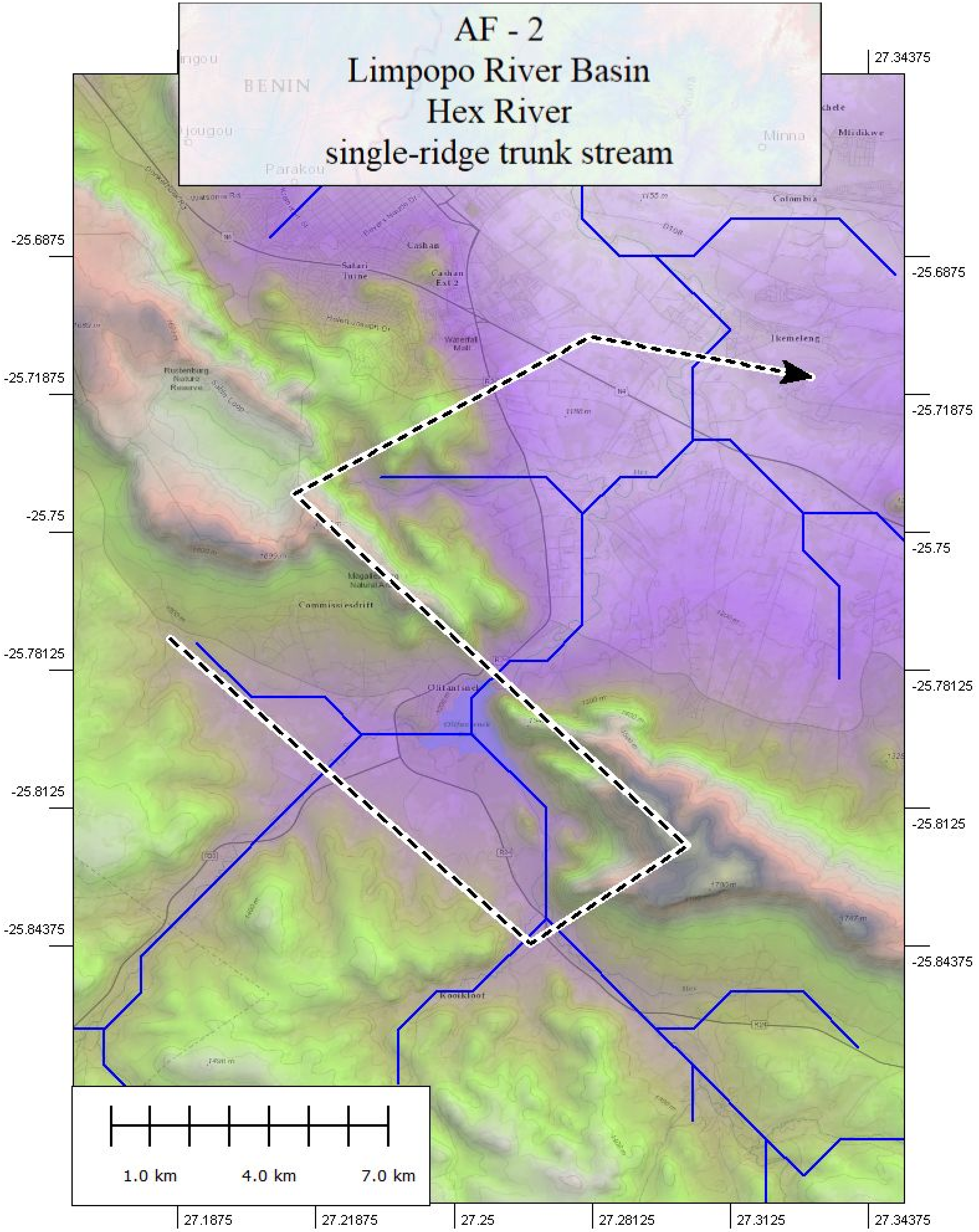
AF - 2
Limpopo River Basin
Hex River
single-ridge trunk stream
27.34375
-25.6875
-25.71875
-25.75
-25.78125
-25.8125
-25.84375
27.1875
27.21875
27.25
27.28125
27.3125
1.0 km
4.0 km
7.0 km
Cashan
Ikemelang
Colombia
Rustenburg Nature Reserve
Commissiesdrift
Olifantsnek
Kooikloof

# AF - 3
## Stillbay River Basin
## Kruis River
## single-ridge head stream

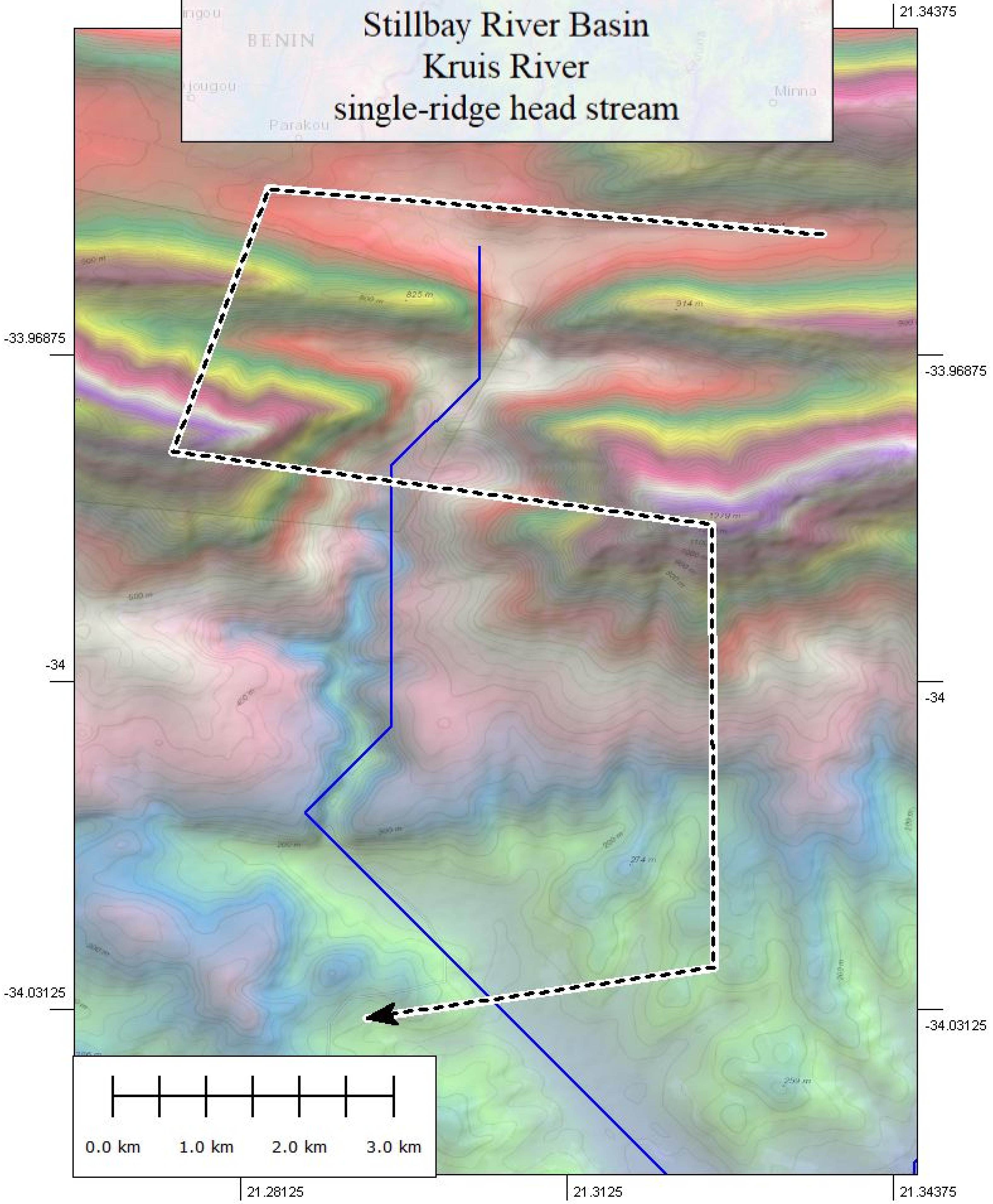

# AF - 5
Stillbay River Basin
Kruis River tributary
single-ridge head stream

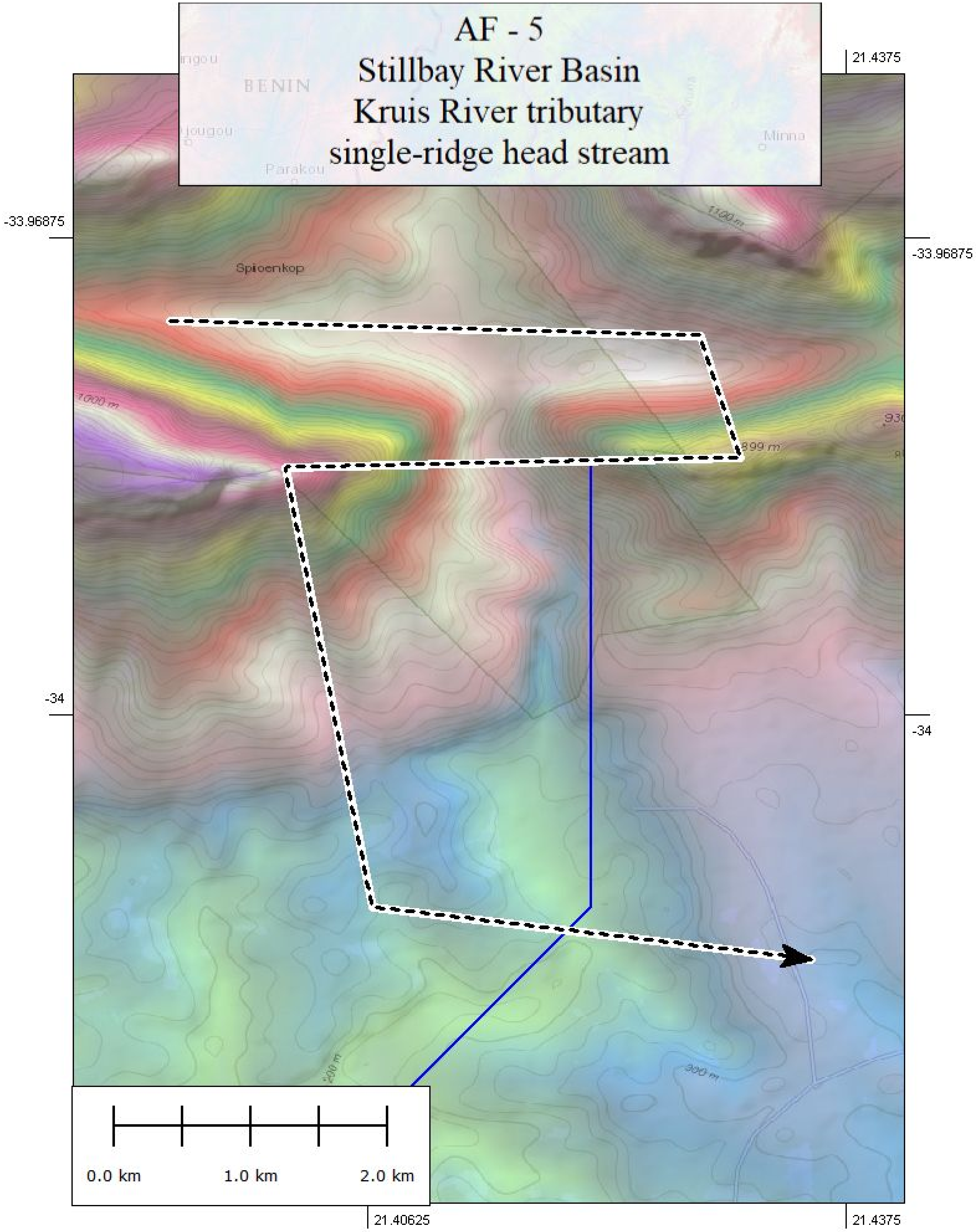

# AF - 10
## Oued Draa Basin
## Foum Meskaou
## single-ridge head stream

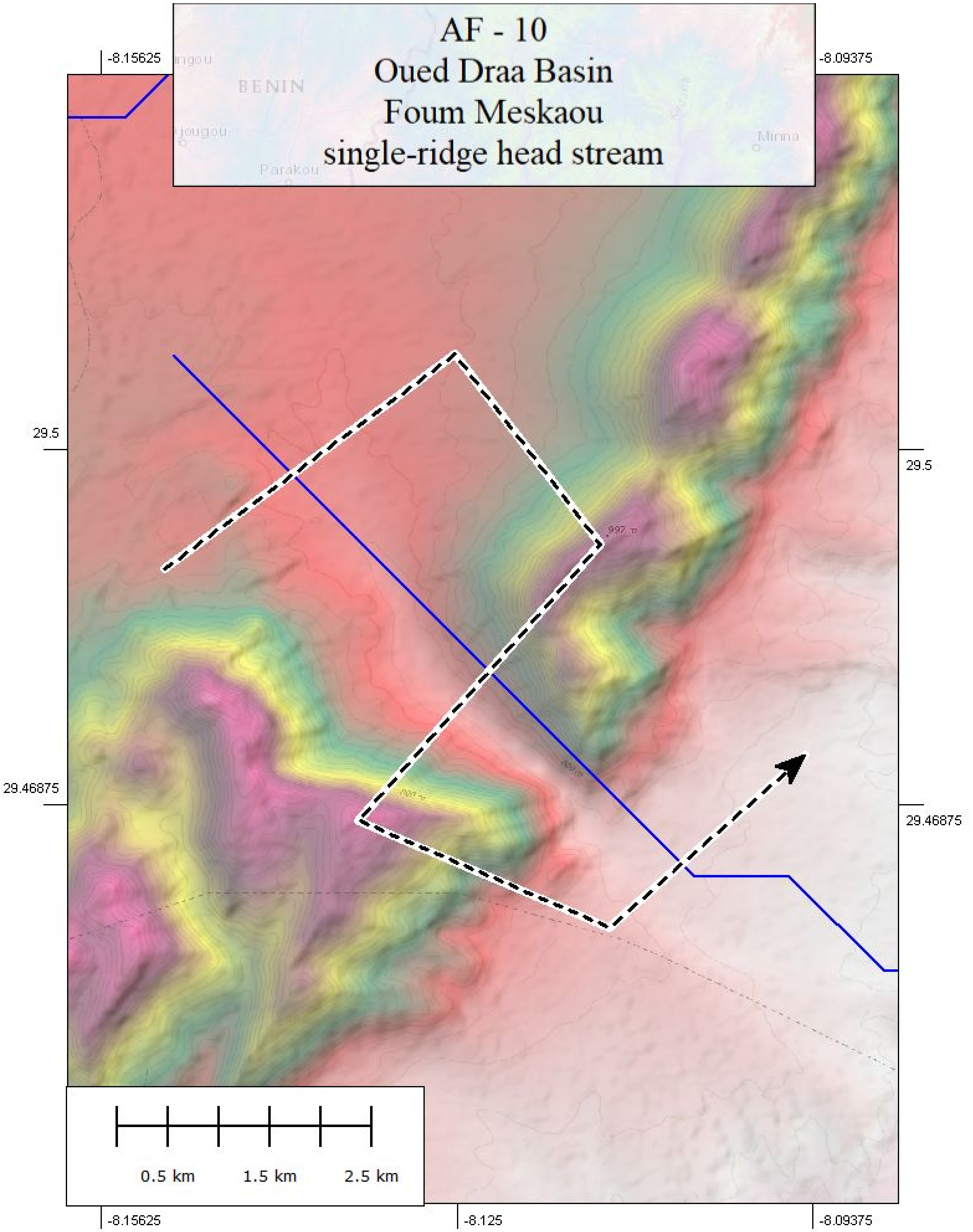

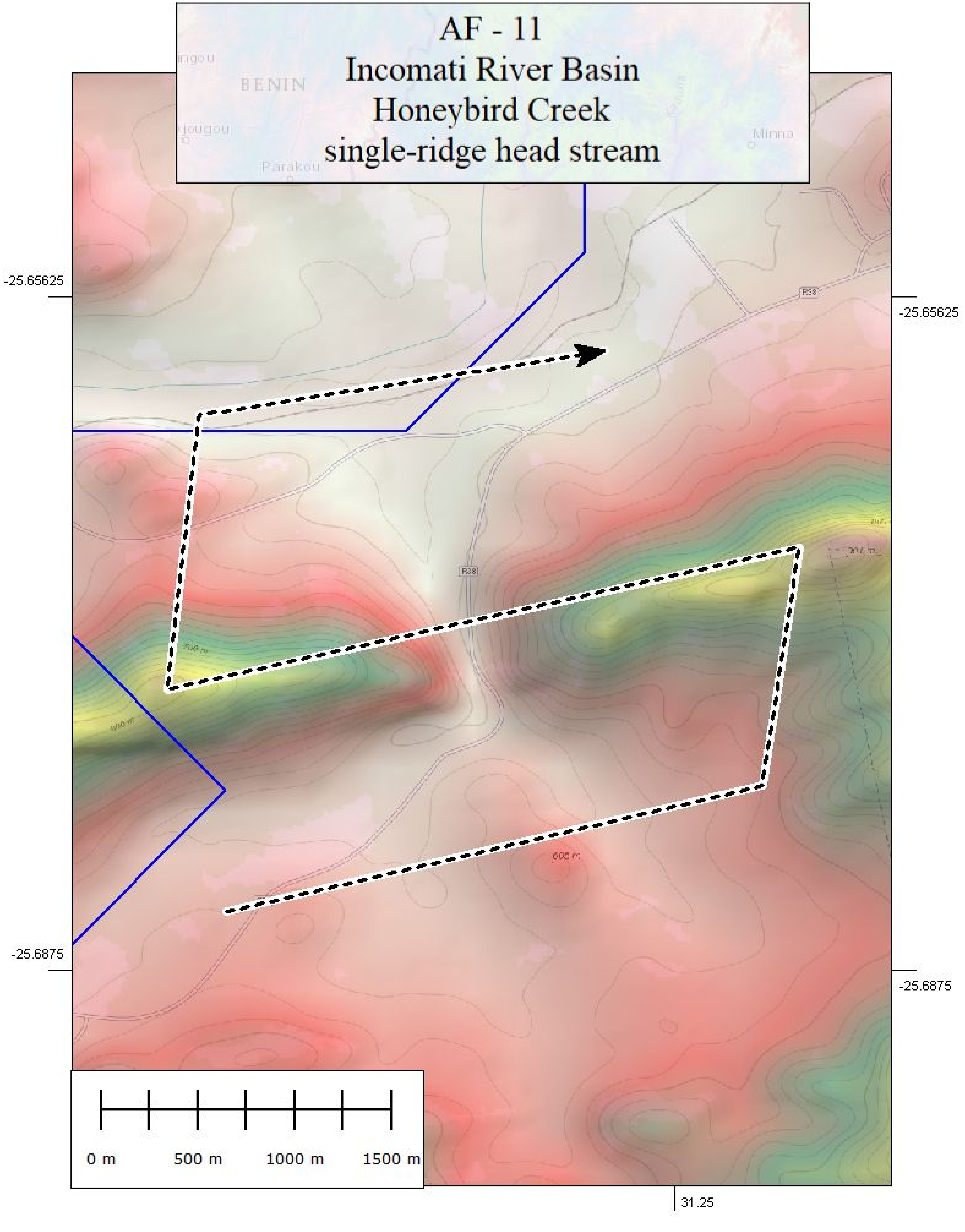
AF - 11
Incomati River Basin
Honeybird Creek
single-ridge head stream
BENIN
Parakou
Minna
-25.65625
-25.65625
R38
R38
600 m
-25.6875
-25.6875
31.25
0 m
500 m
1000 m
1500 m

# AF - 12
## Gourits River Basin
## Potjies River
## single-ridge head stream

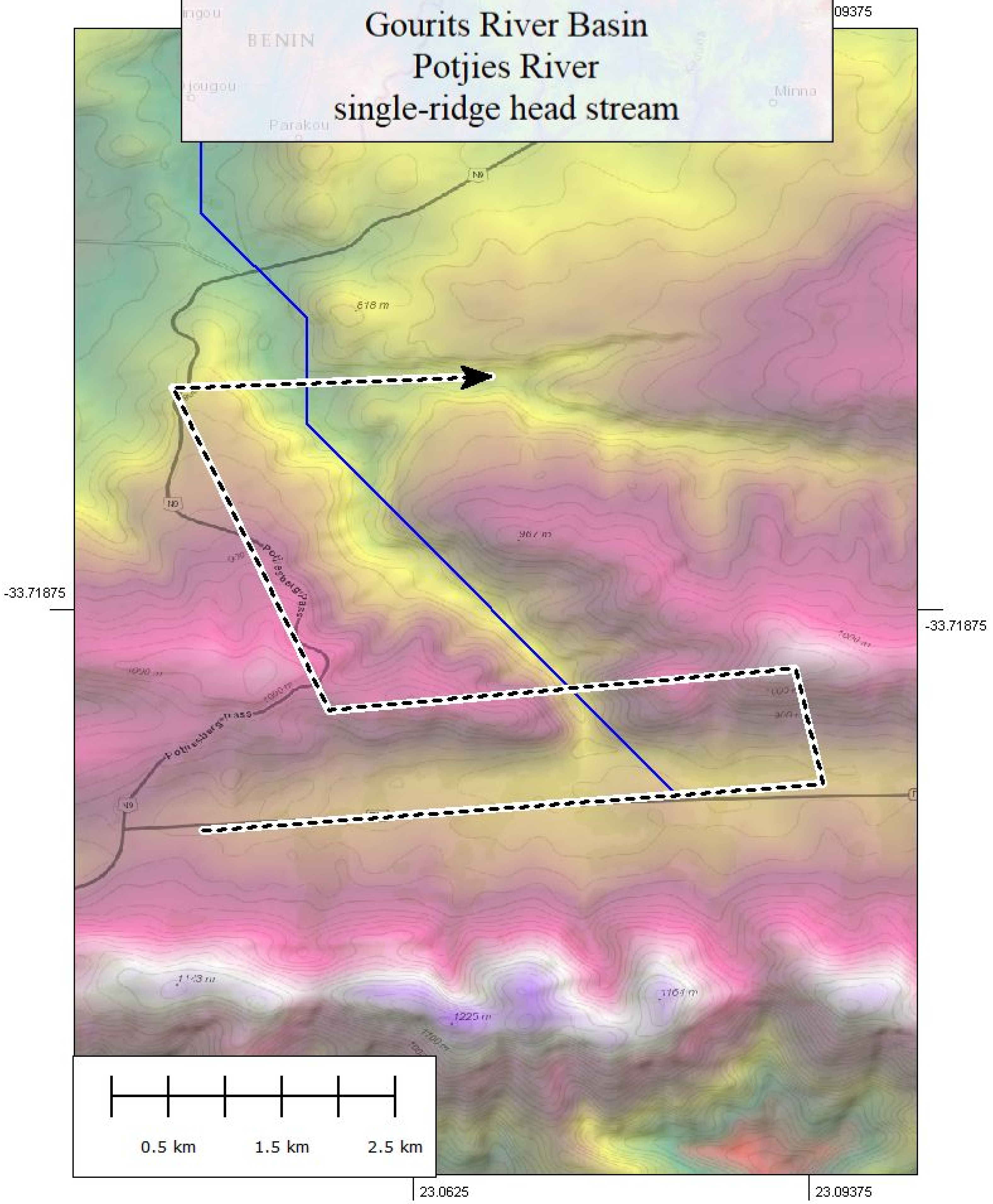

# AF - 14
## Gamtoos River Basin
## Groot River tributary
## single-ridge head stream

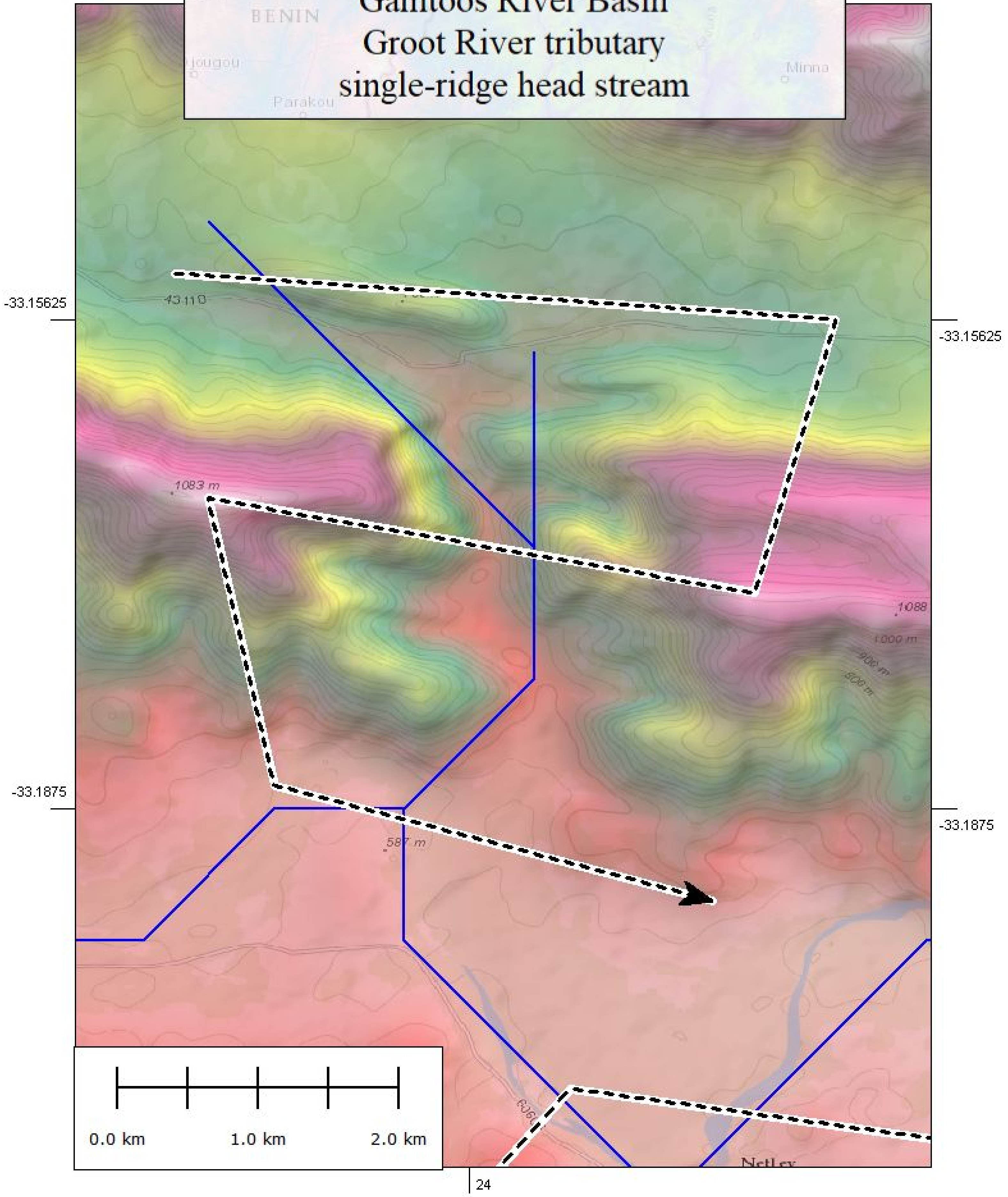

# AF - 15
# Gamtoos River Basin
# Pienaarspoort Pass
# single-ridge head stream

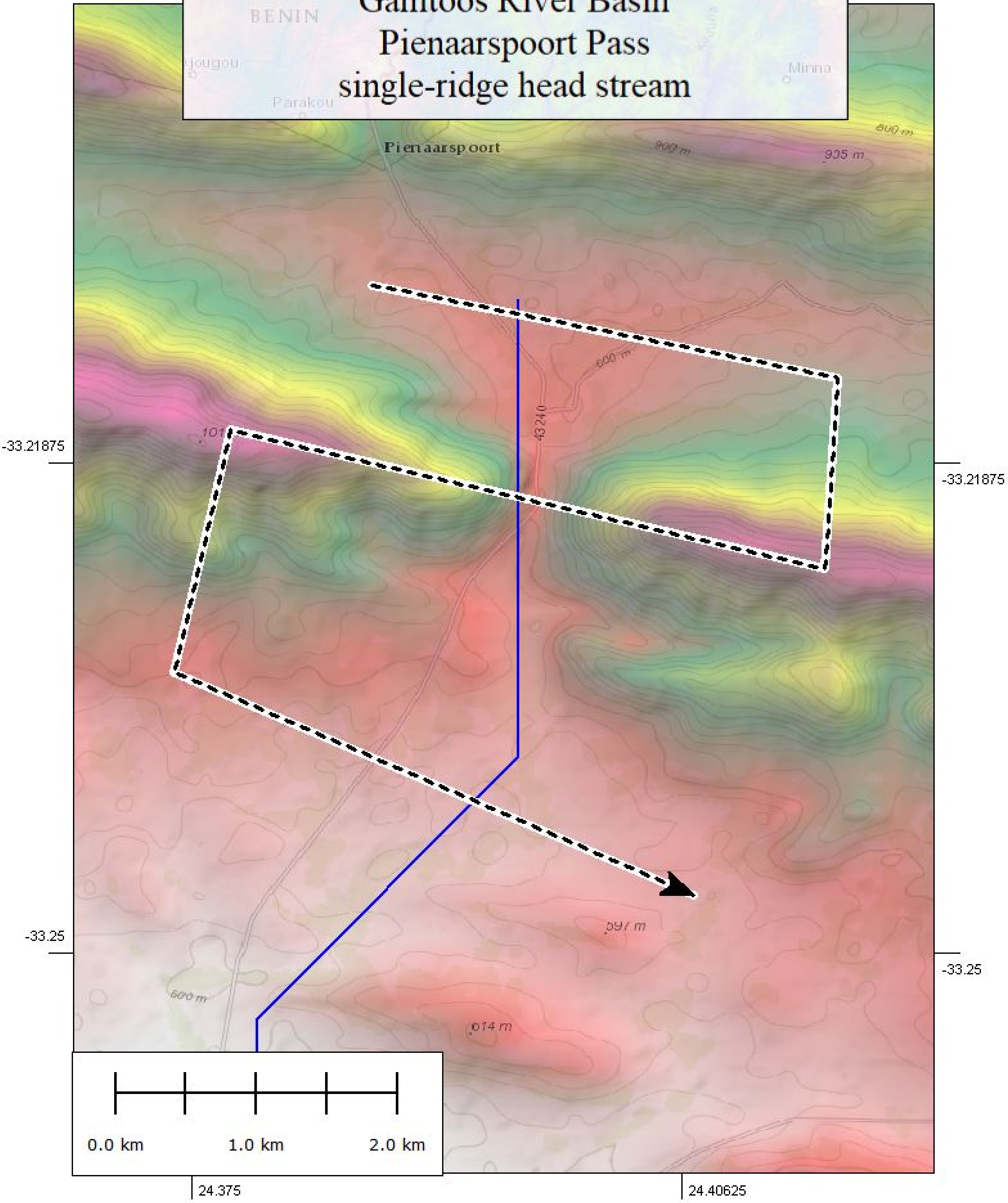

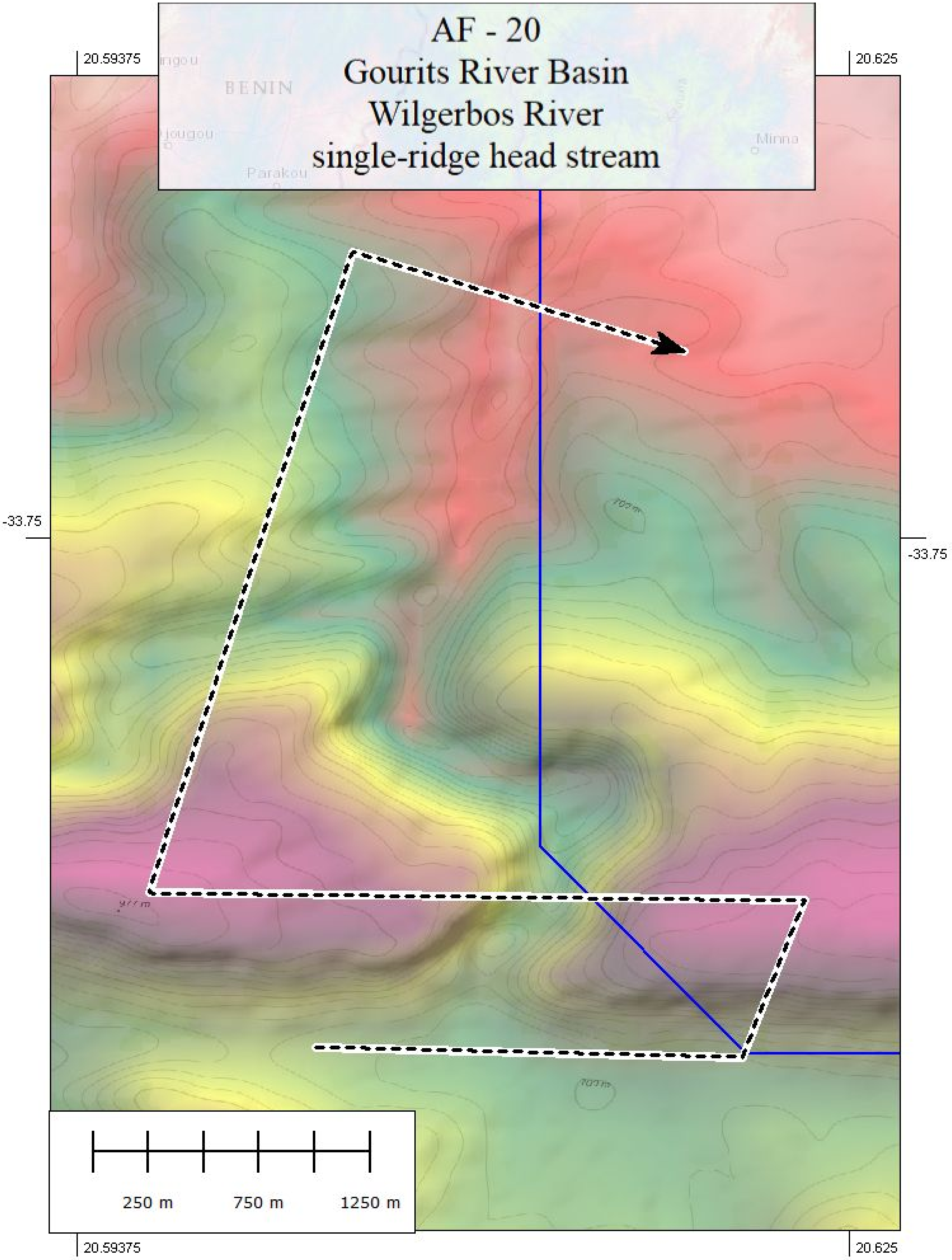
AF - 20
Gourits River Basin
Wilgerbos River
single-ridge head stream
20.59375
20.625
-33.75
-33.75
250 m
750 m
1250 m
20.59375
20.625

# AF - 25
## Gourits River Basin
## Kammanass River tributary
## single-ridge head stream

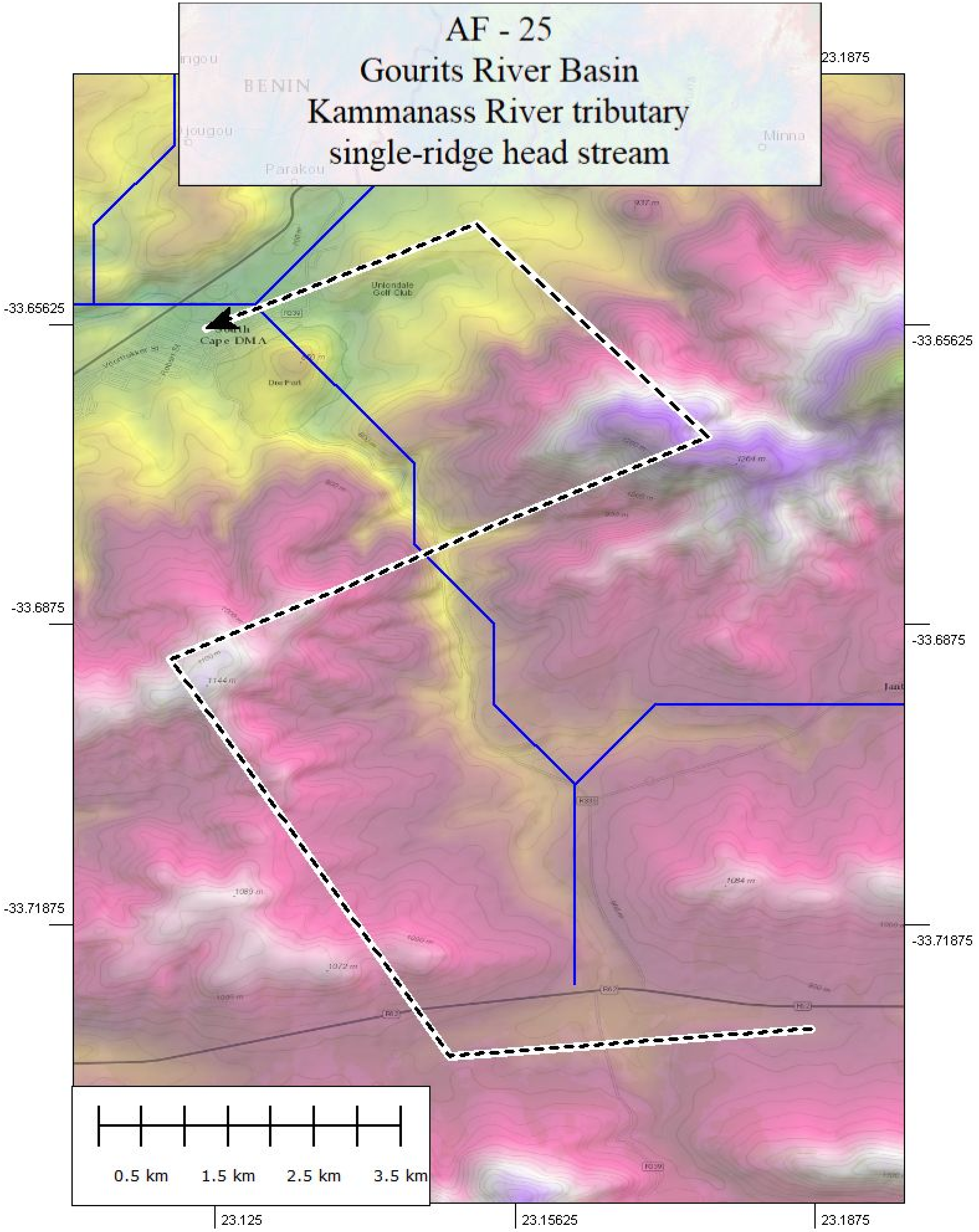

# AF - 26
## Gourits River Basin
## Leeukloof gorge
## single-ridge head stream

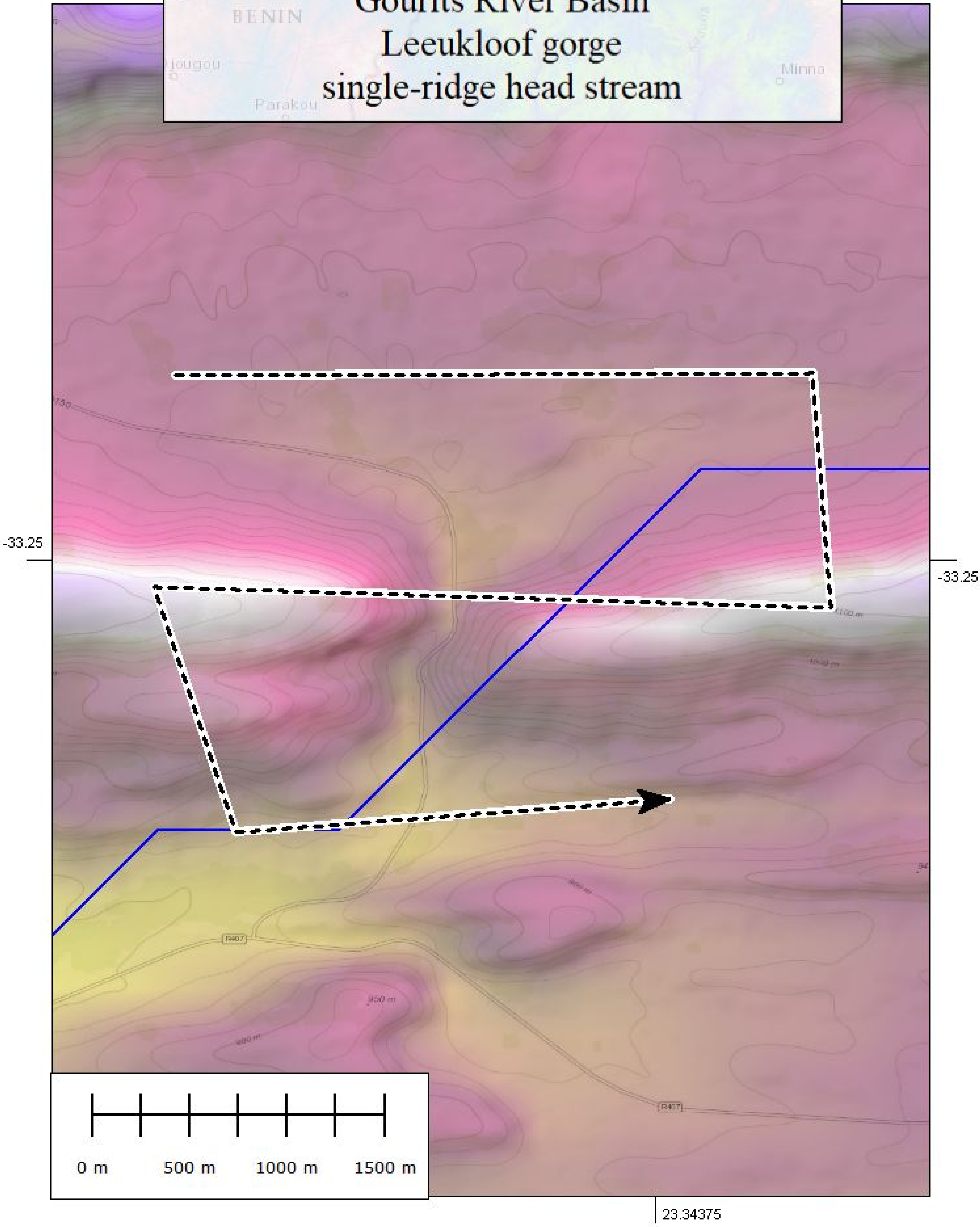

# AF - 27
## Gamtoos River Basin
## Groot River tributary
## single-ridge head stream

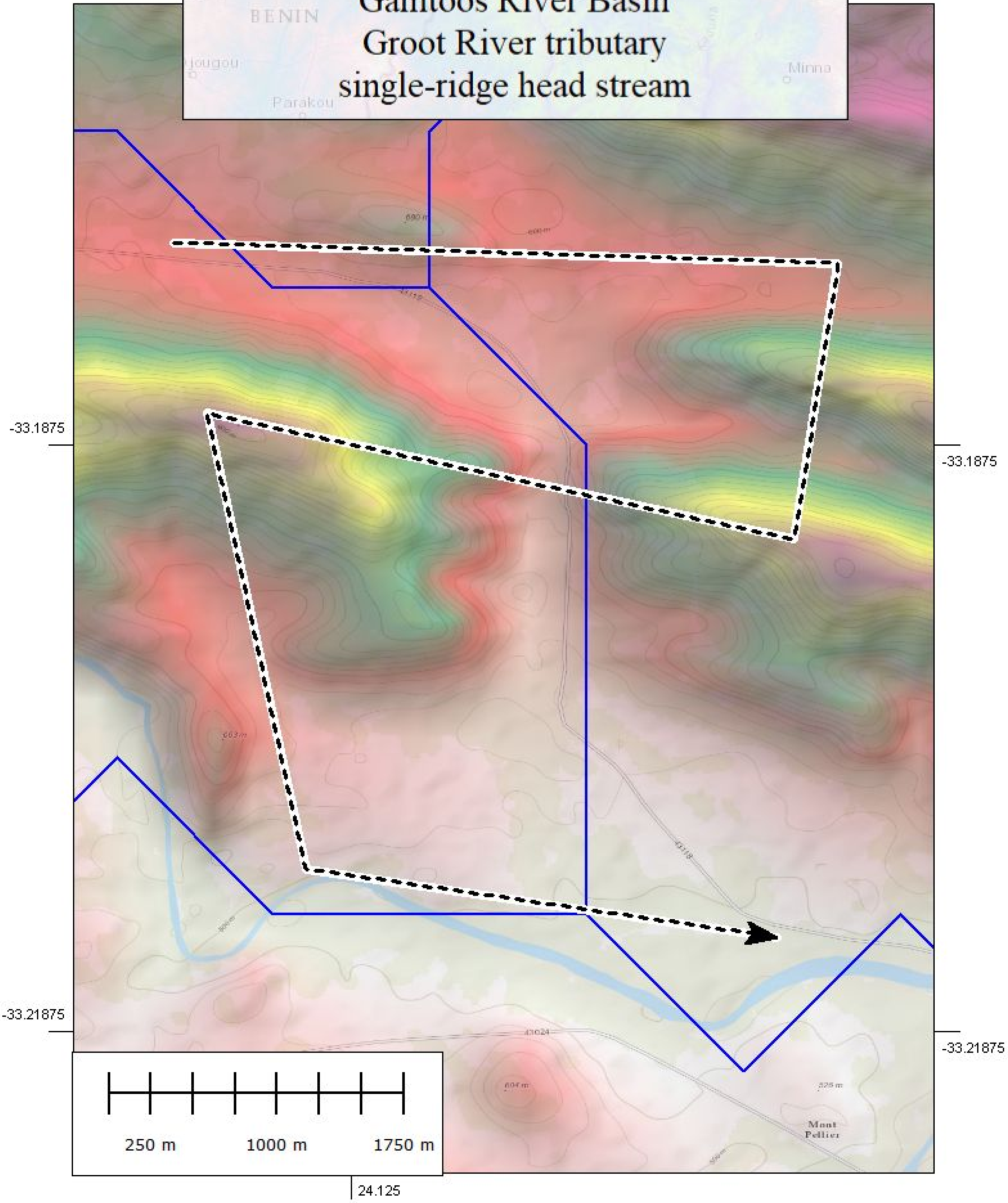

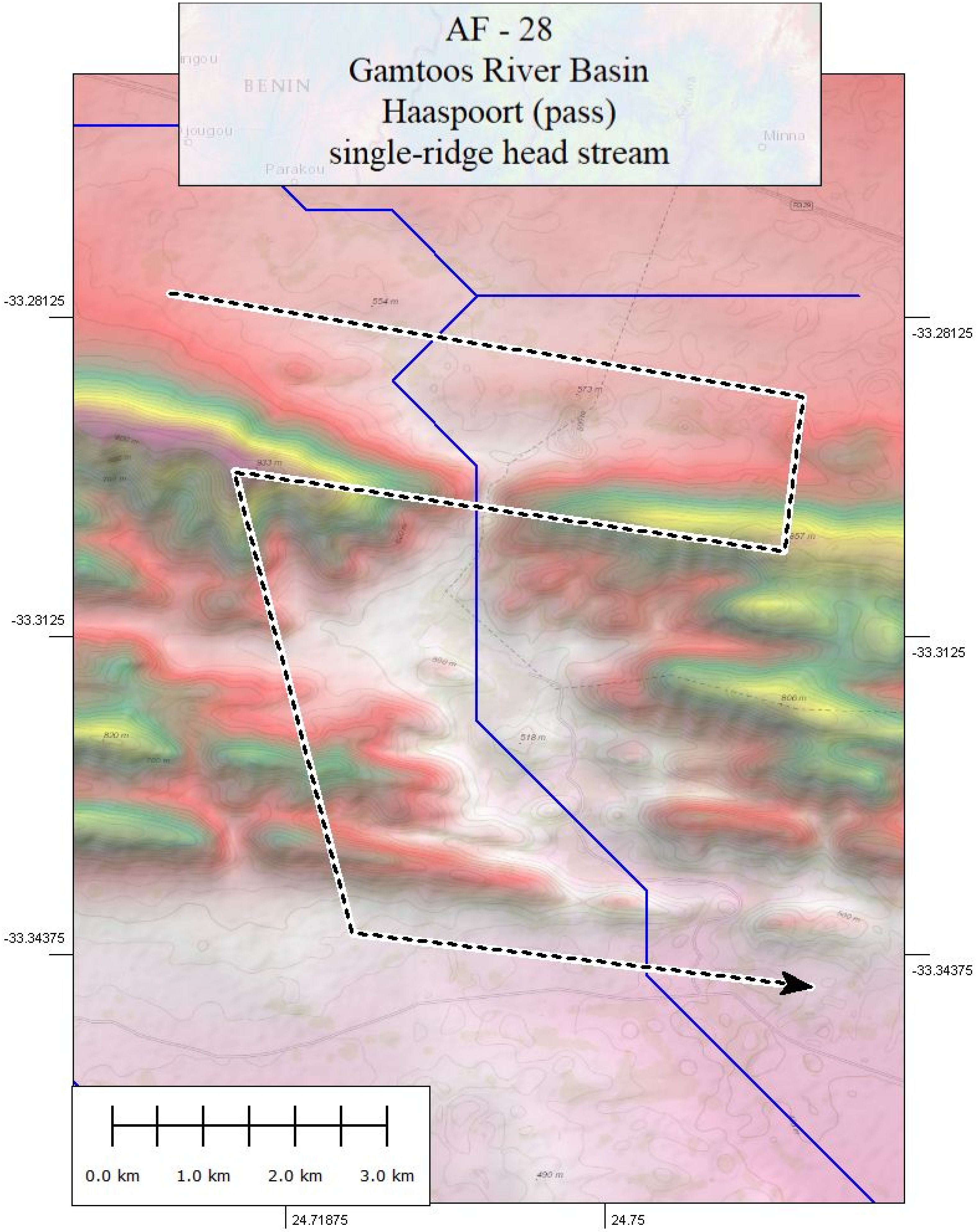
AF - 28
Gamtoos River Basin
Haaspoort (pass)
single-ridge head stream
-33.28125
-33.28125
-33.3125
-33.3125
-33.34375
-33.34375
0.0 km
1.0 km
2.0 km
3.0 km
24.71875
24.75

# AF - 33
# Moulouya River Basin
# Bab el Berd (pass)
# single-ridge head stream

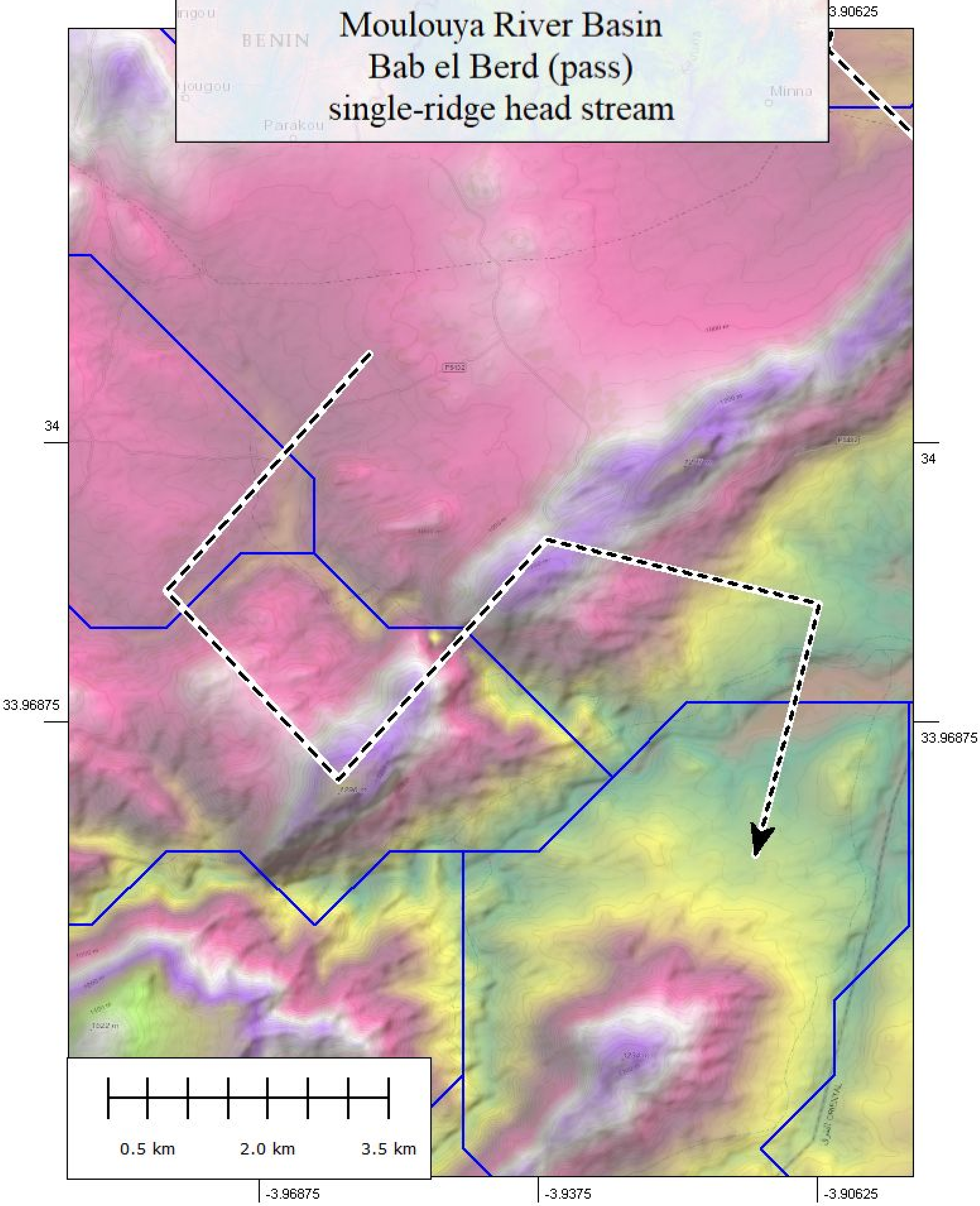

# AF - 34
# Limpopo River Basin
# Sand River tributary
# single-ridge head stream

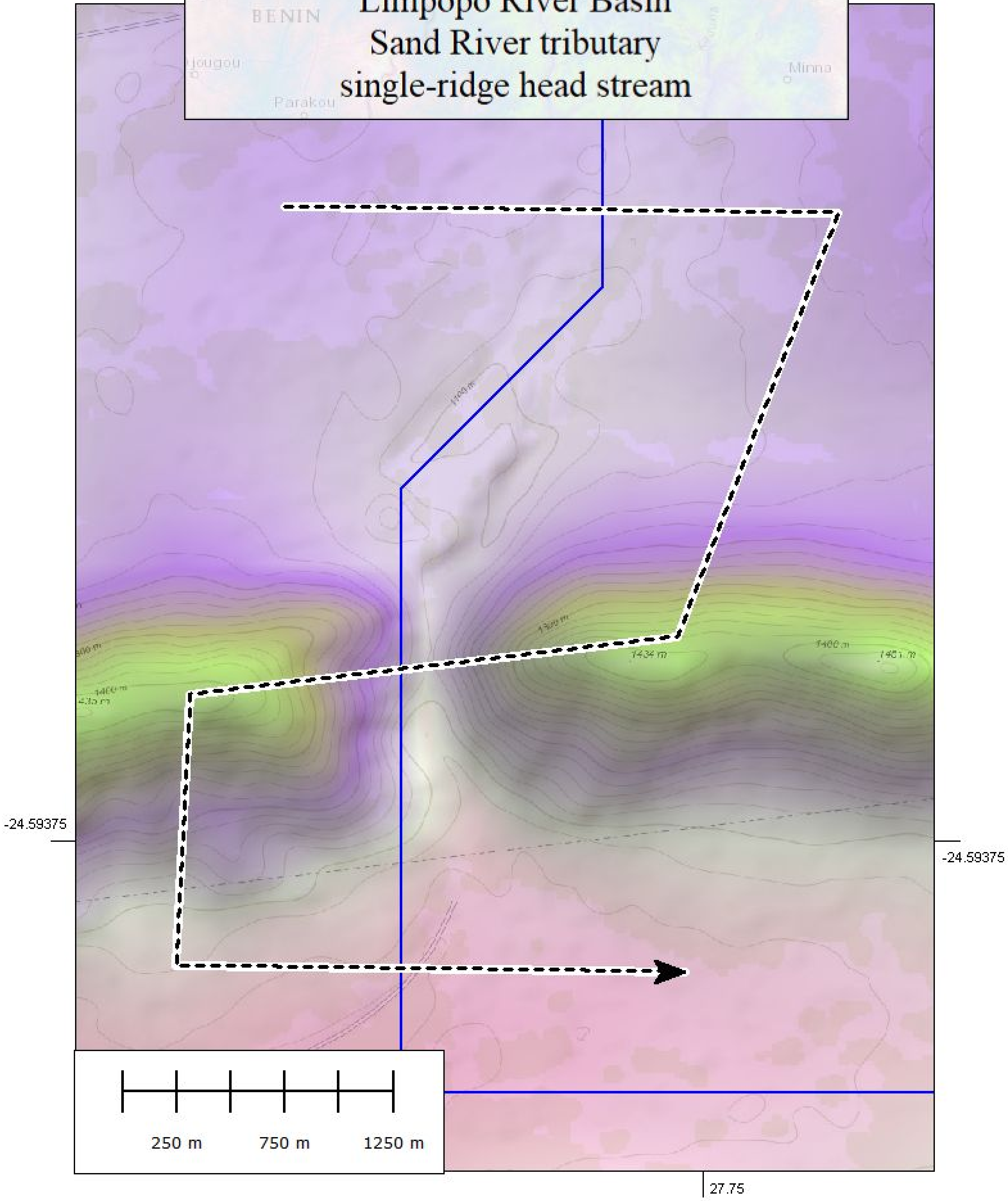

# AF - 36
# Gourits River Basin
# Vet River
# single-ridge head stream

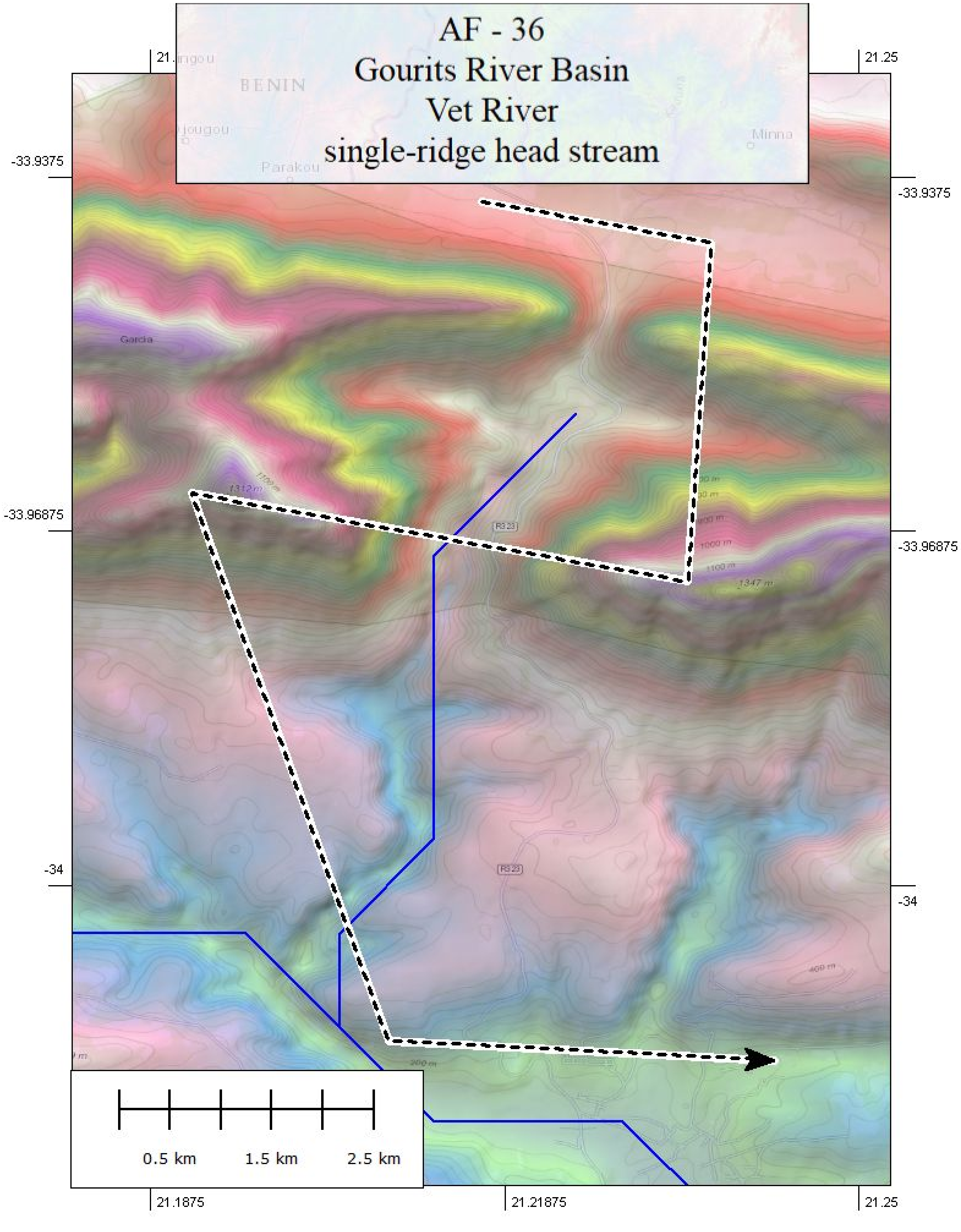

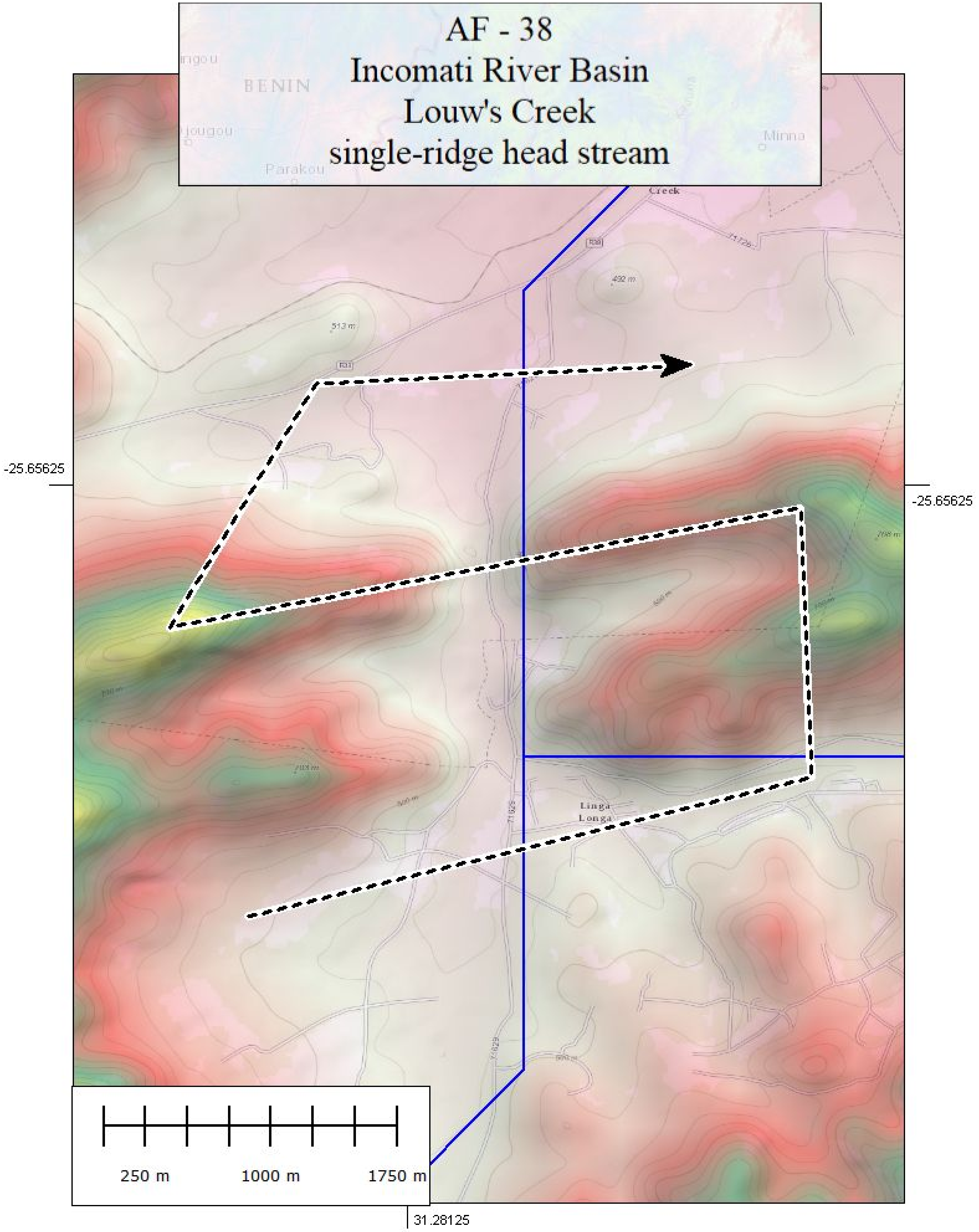
AF - 38
Incomati River Basin
Louw's Creek
single-ridge head stream
-25.65625
-25.65625
31.28125
250 m
1000 m
1750 m
Linga
Longa
513 m
492 m

# AF - 39
## Gourits River Basin
## Seweweekspoort River
## single-ridge head stream

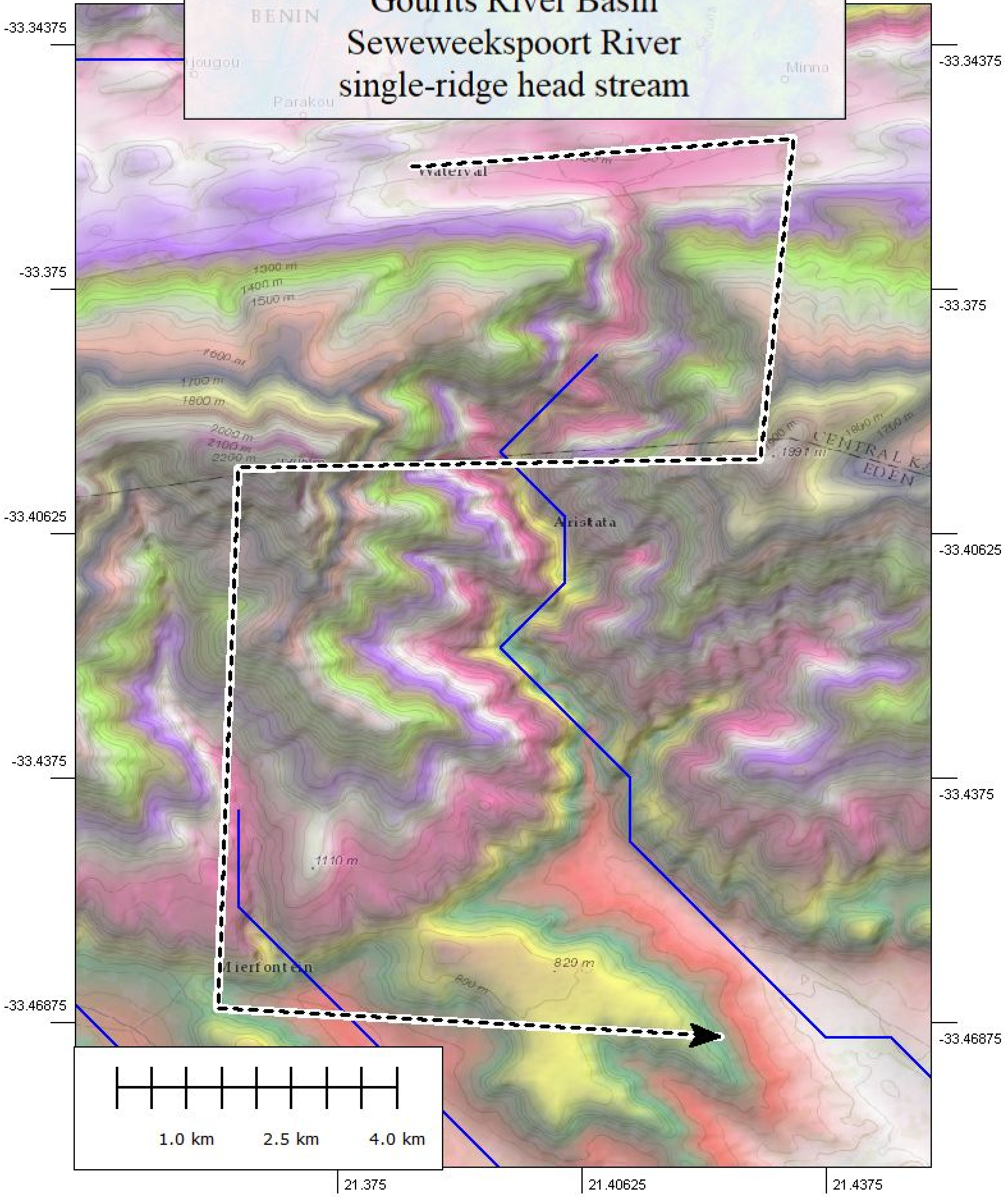

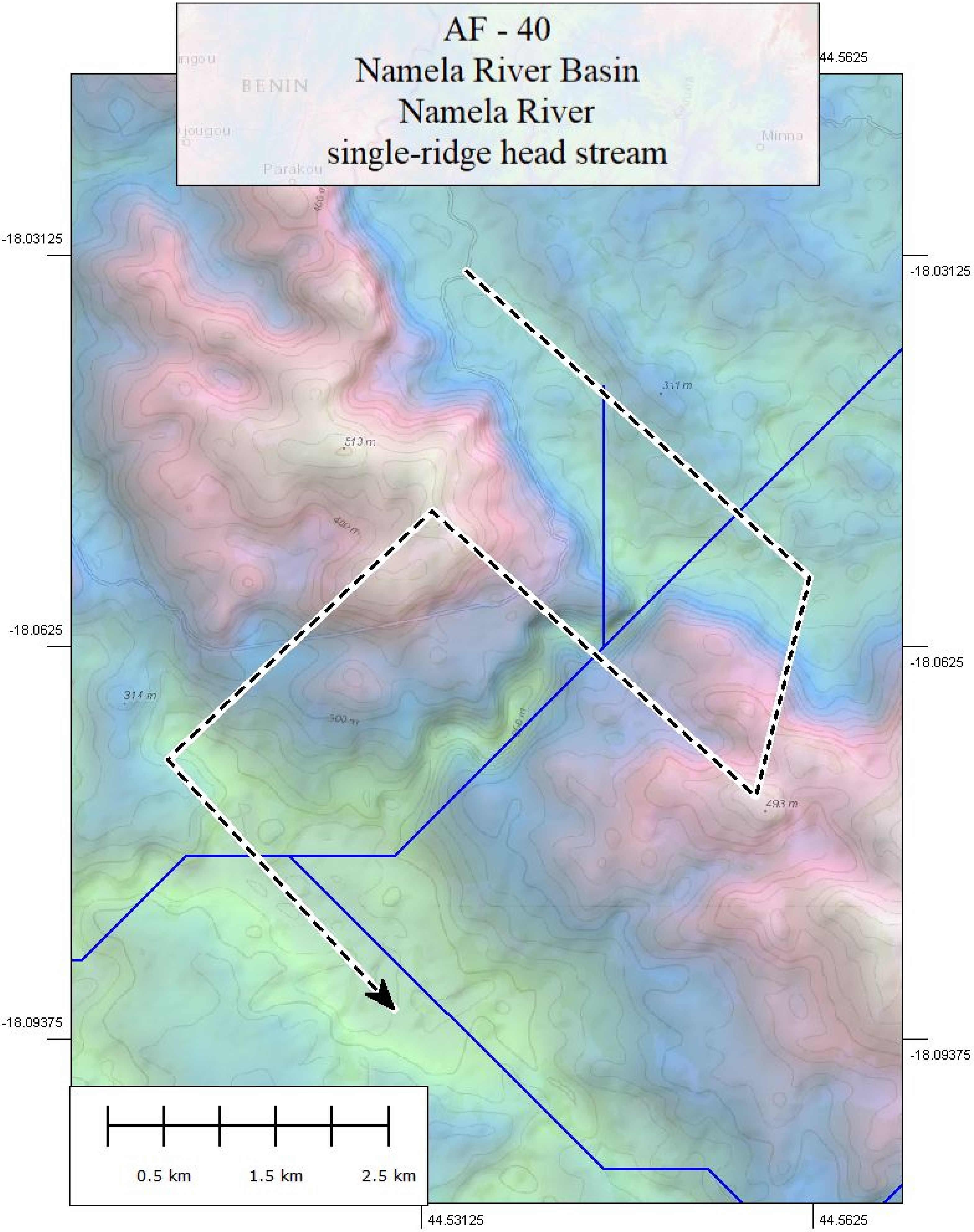
AF - 40
Namela River Basin
Namela River
single-ridge head stream
44.5625
-18.03125
-18.03125
311 m
513 m
400 m
-18.0625
-18.0625
314 m
500 m
493 m
-18.09375
-18.09375
0.5 km
1.5 km
2.5 km
44.53125
44.5625

# AF - 42
# Gourits River Basin
# Groot River
# single-ridge trunk stream

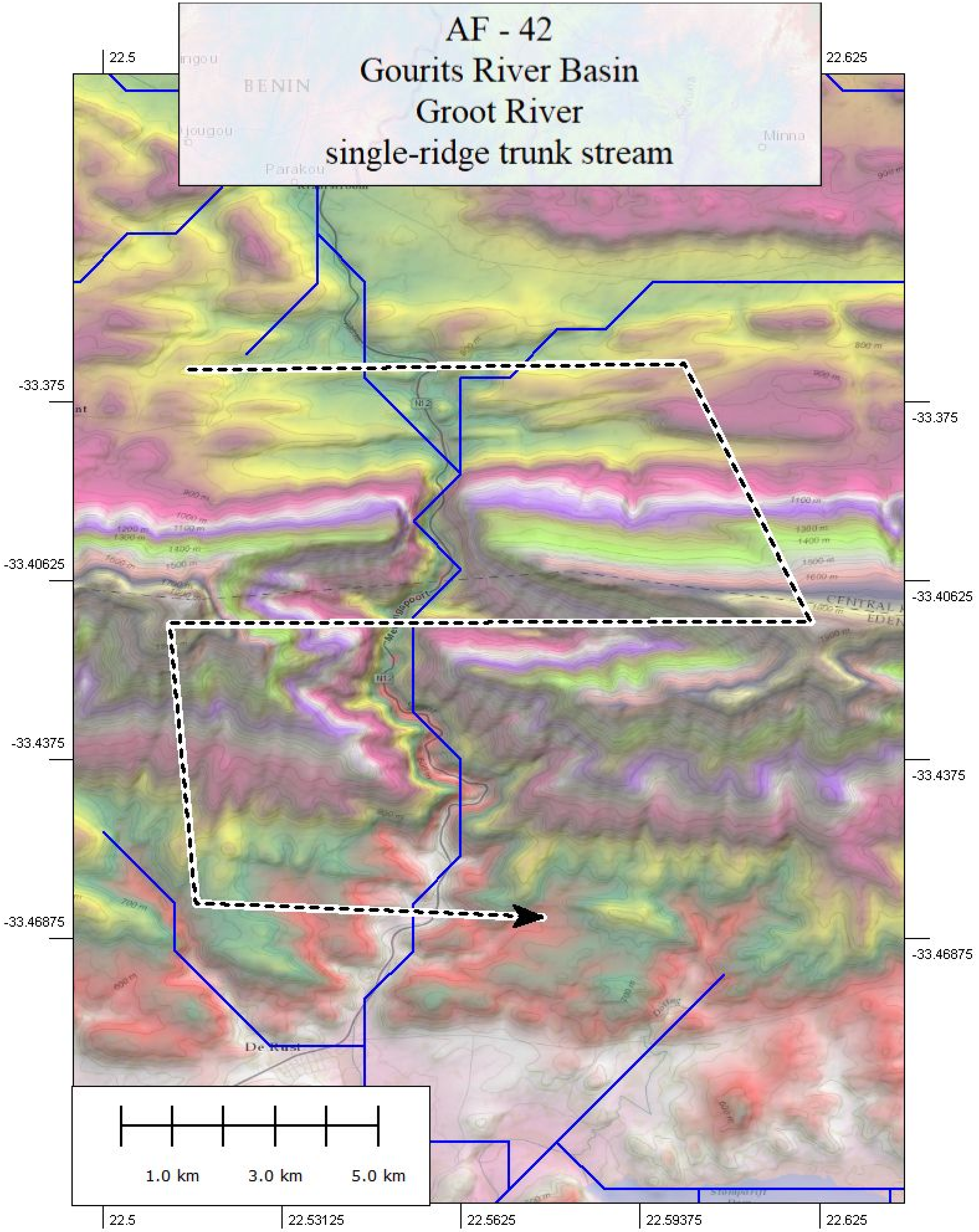

# AF - 44
## Gamtoos River Basin
## Vlermuispoort (pass)
## single-ridge trunk stream

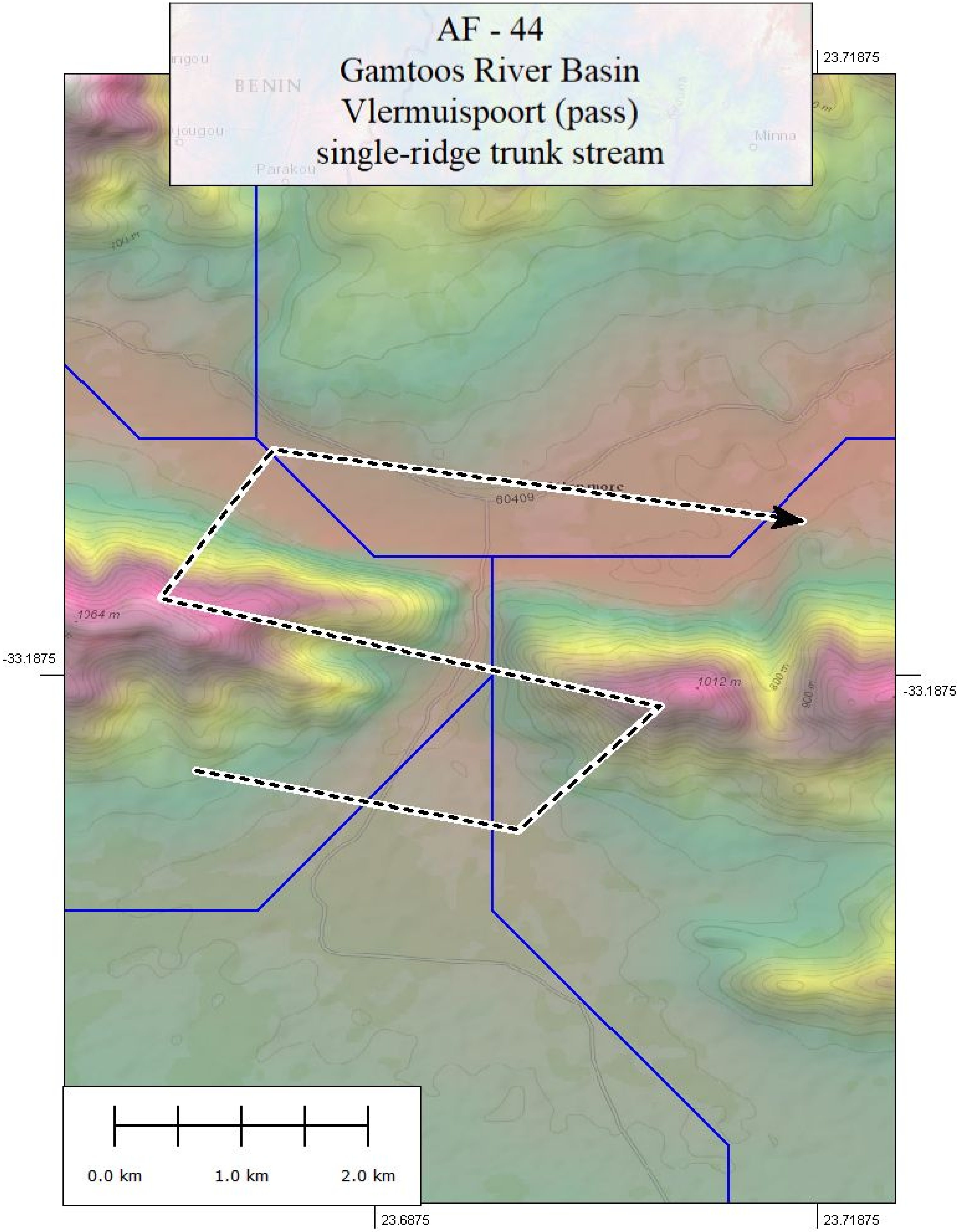

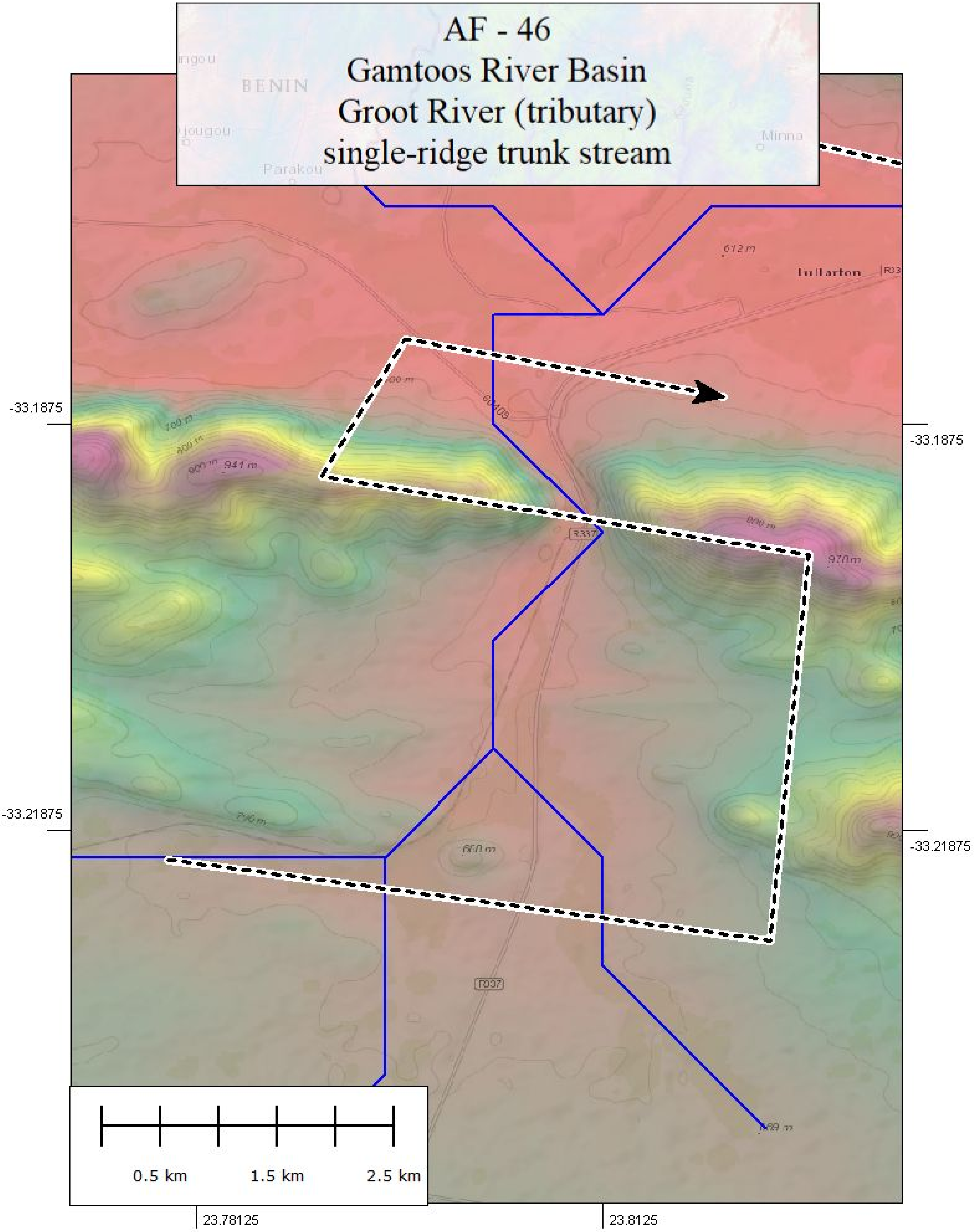
AF - 46
Gamtoos River Basin
Groot River (tributary)
single-ridge trunk stream
-33.1875
-33.1875
-33.21875
-33.21875
23.78125
23.8125
0.5 km
1.5 km
2.5 km
612 m
941 m
970 m
600 m
R337

# AF - 48
## Gourits River Basin
## Prins River
## single-ridge trunk stream

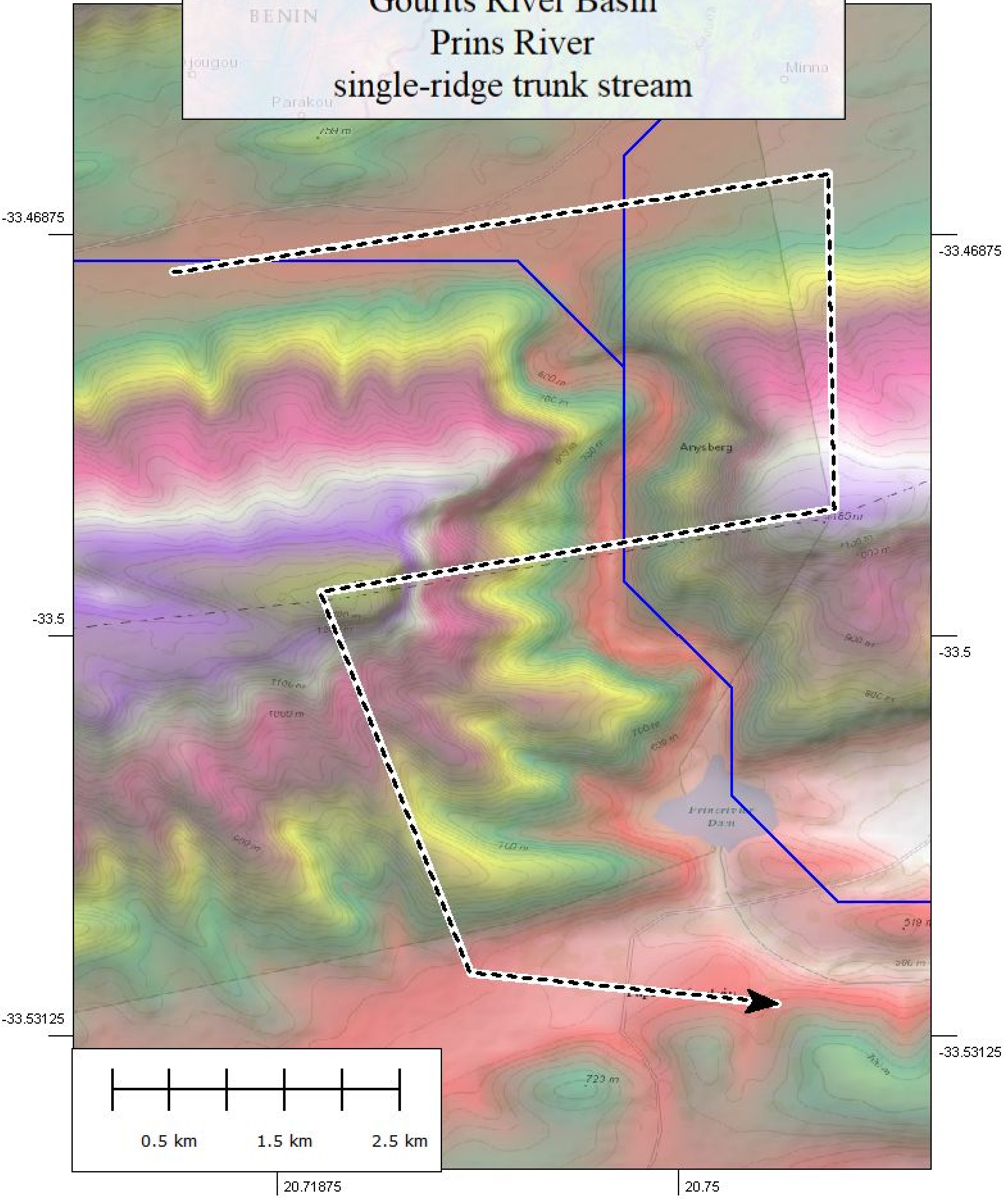

# AF - 49
## Gamtoos River Basin
## Sandkraalspoort (pass)
## single-ridge trunk stream

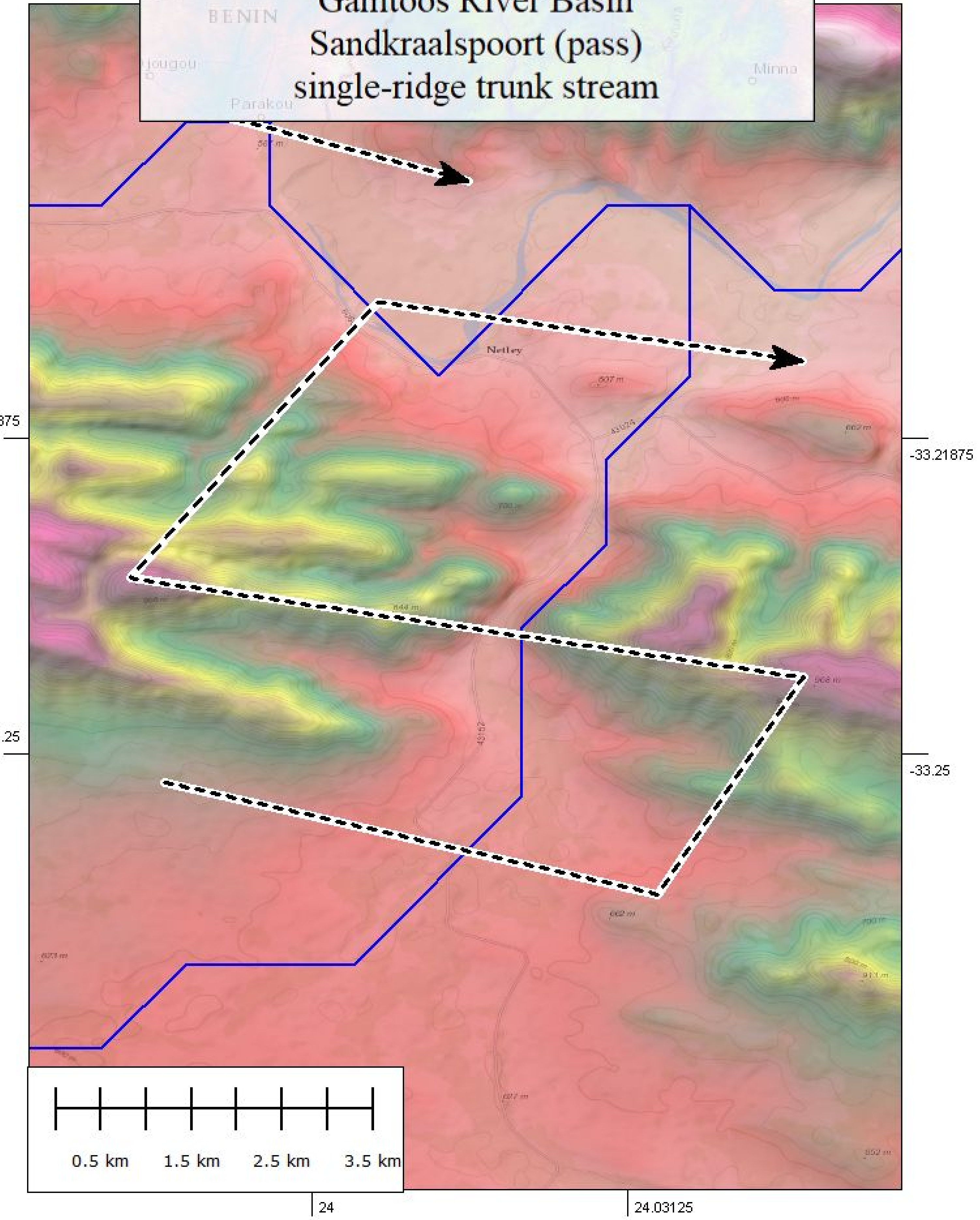

# AF - 50
# Gamtoos River Basin
# Groot River tributary
# single-ridge trunk stream

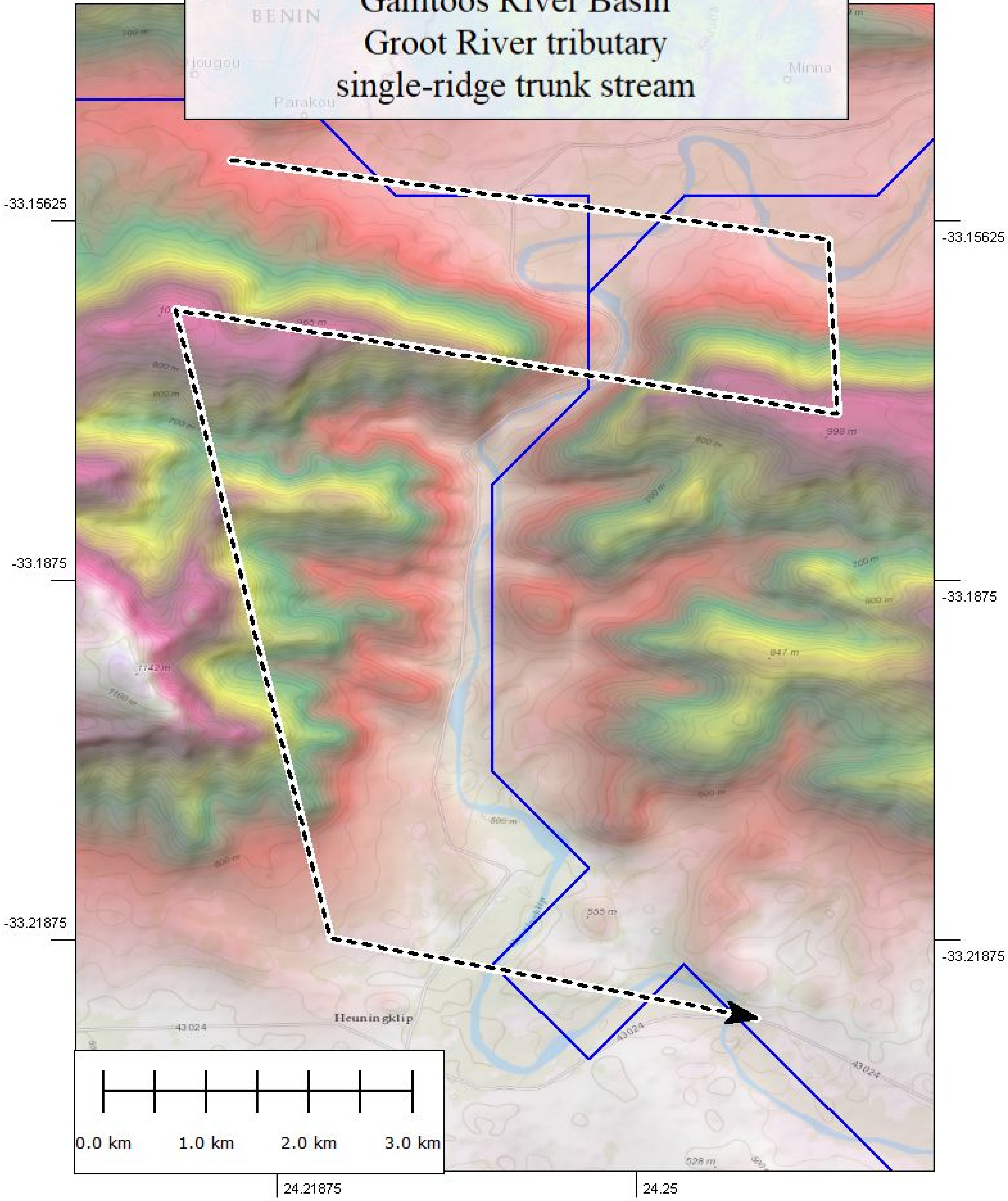

# AF - 51
## Gamtoos River Basin
## Groot River
## single-ridge trunk stream

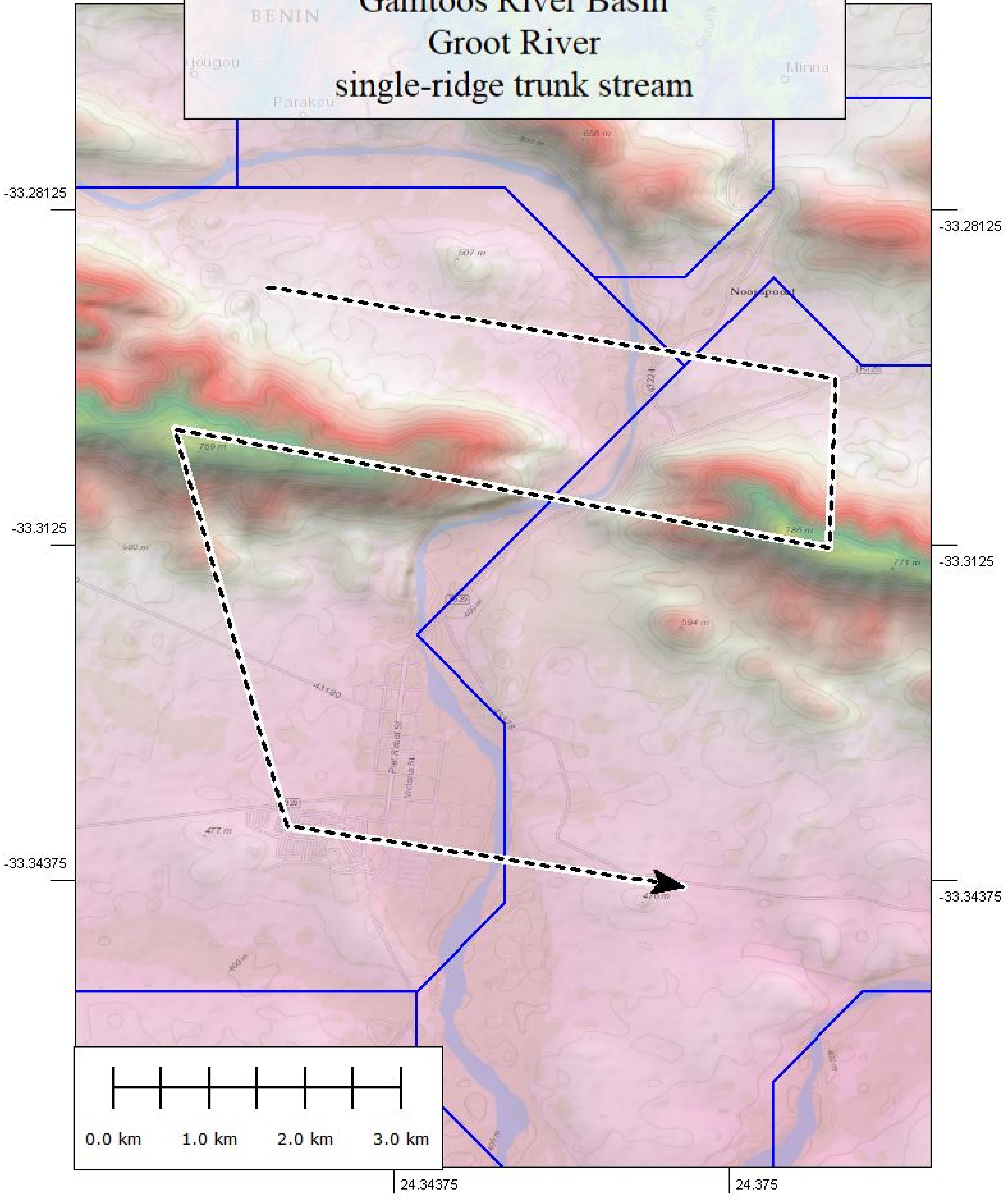

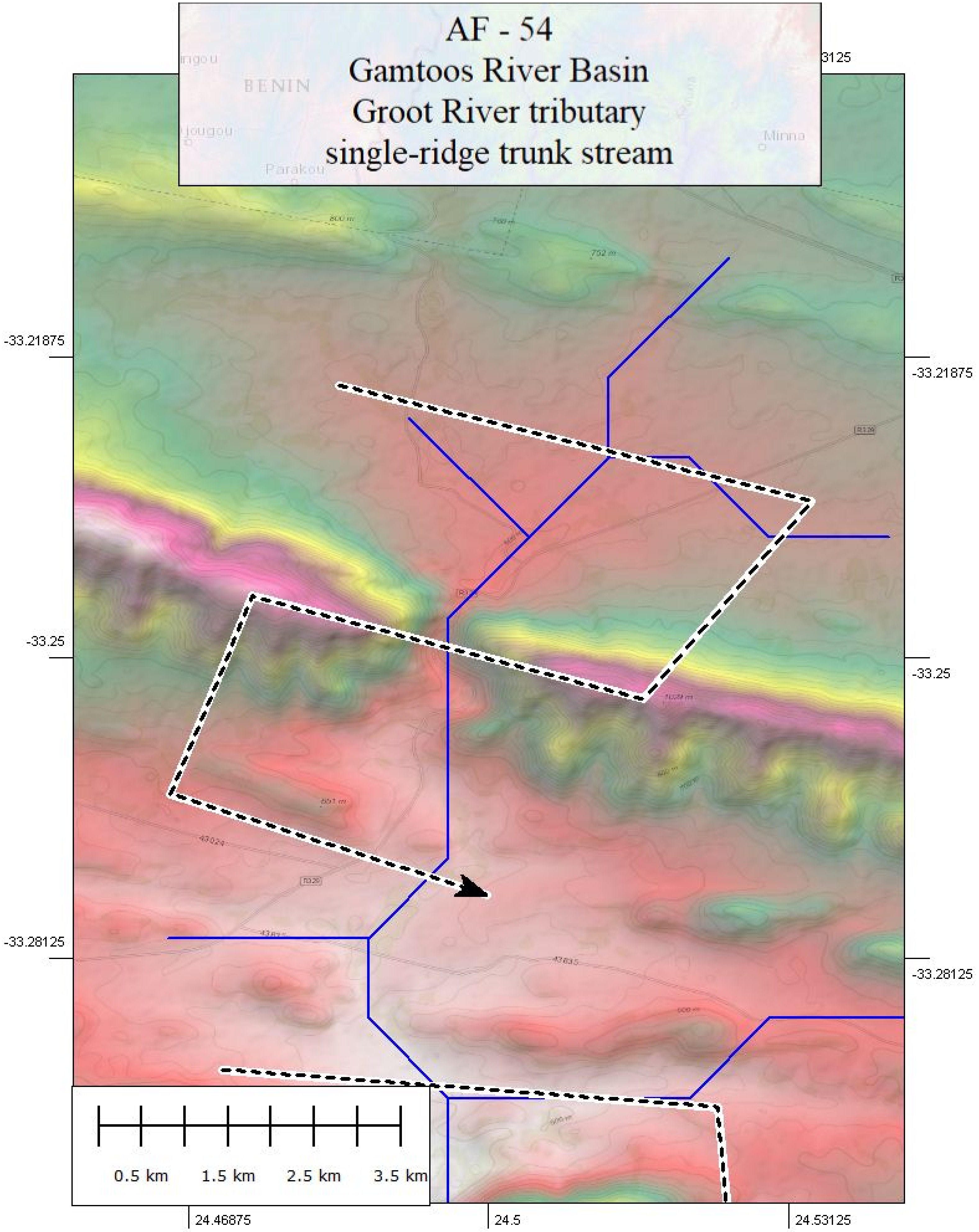
AF - 54
Gamtoos River Basin
Groot River tributary
single-ridge trunk stream
-33.21875
-33.21875
-33.25
-33.25
-33.28125
-33.28125
0.5 km
1.5 km
2.5 km
3.5 km
24.46875
24.5
24.53125

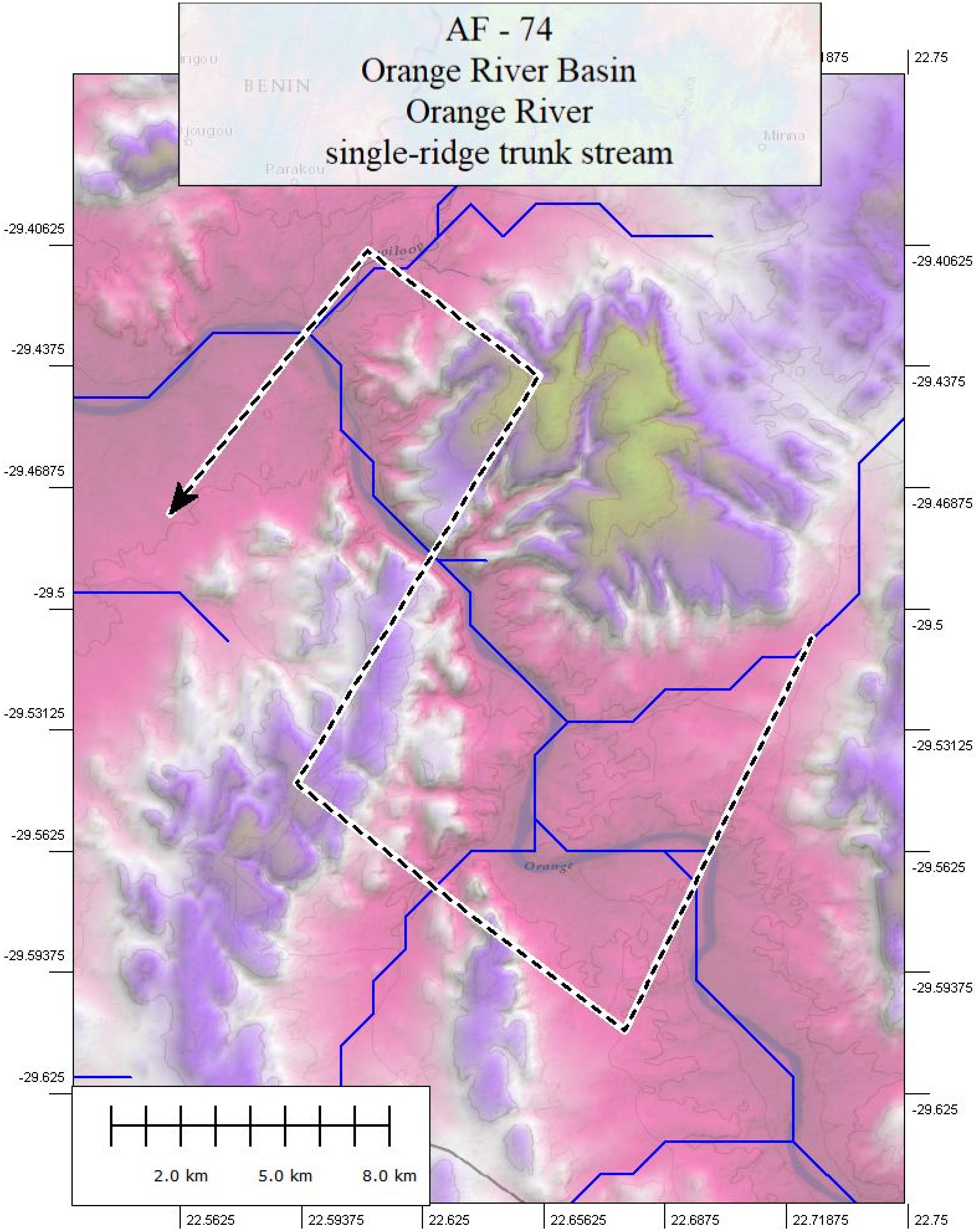
AF - 74
Orange River Basin
Orange River
single-ridge trunk stream
BENIN
Parakou
Minna
Orange
-29.40625
-29.4375
-29.46875
-29.5
-29.53125
-29.5625
-29.59375
-29.625
22.5625
22.59375
22.625
22.65625
22.6875
22.71875
22.75
2.0 km
5.0 km
8.0 km

# AF - 76
## Sonderend River Basin
## Kogmanskloof River
## single-ridge trunk stream

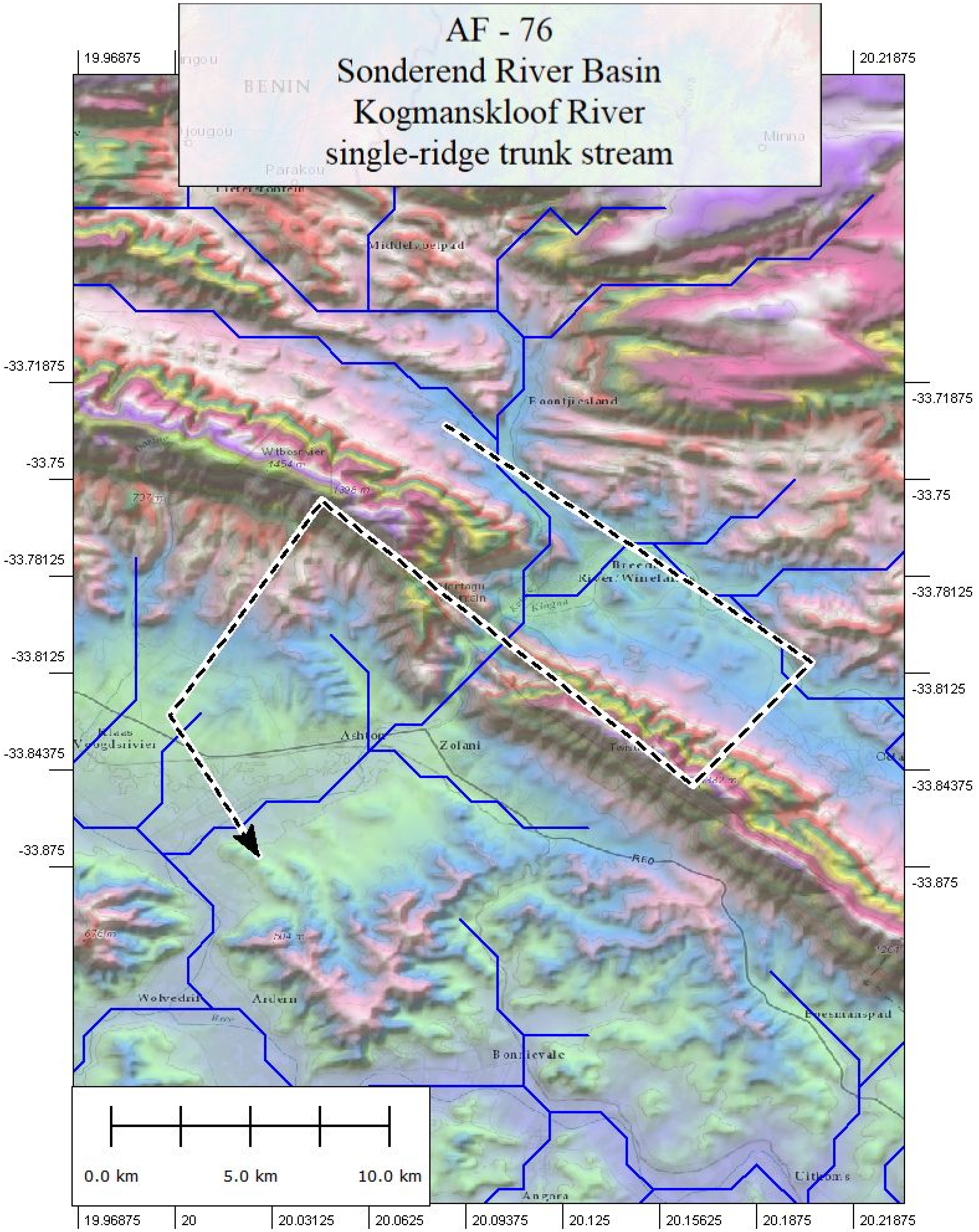

# AF - 81
## Limpopo River Basin
## Apies River
## single-ridge trunk stream

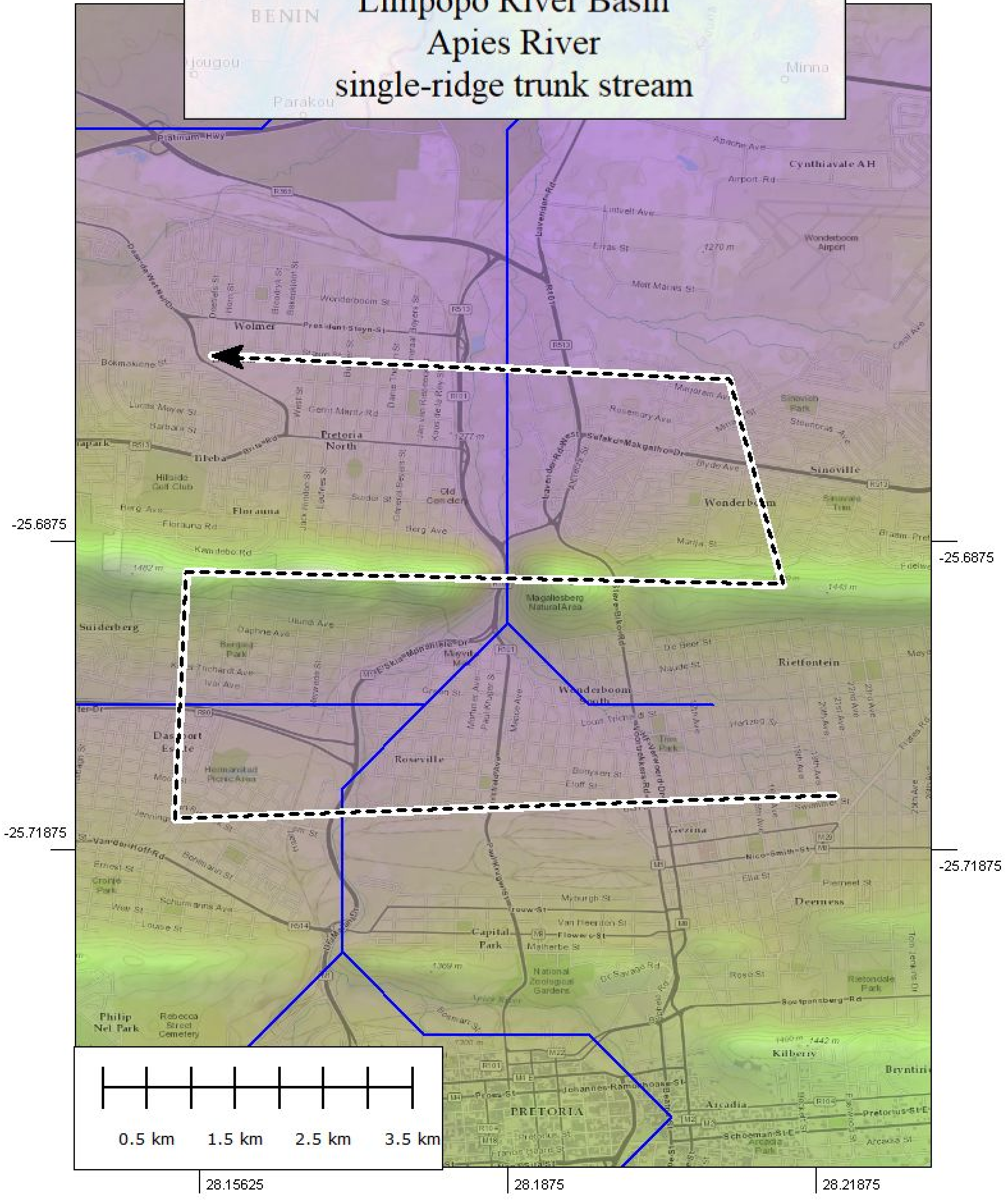

# AF - 83
## Limpopo River Basin
## Mphogodima River
## single-ridge trunk stream

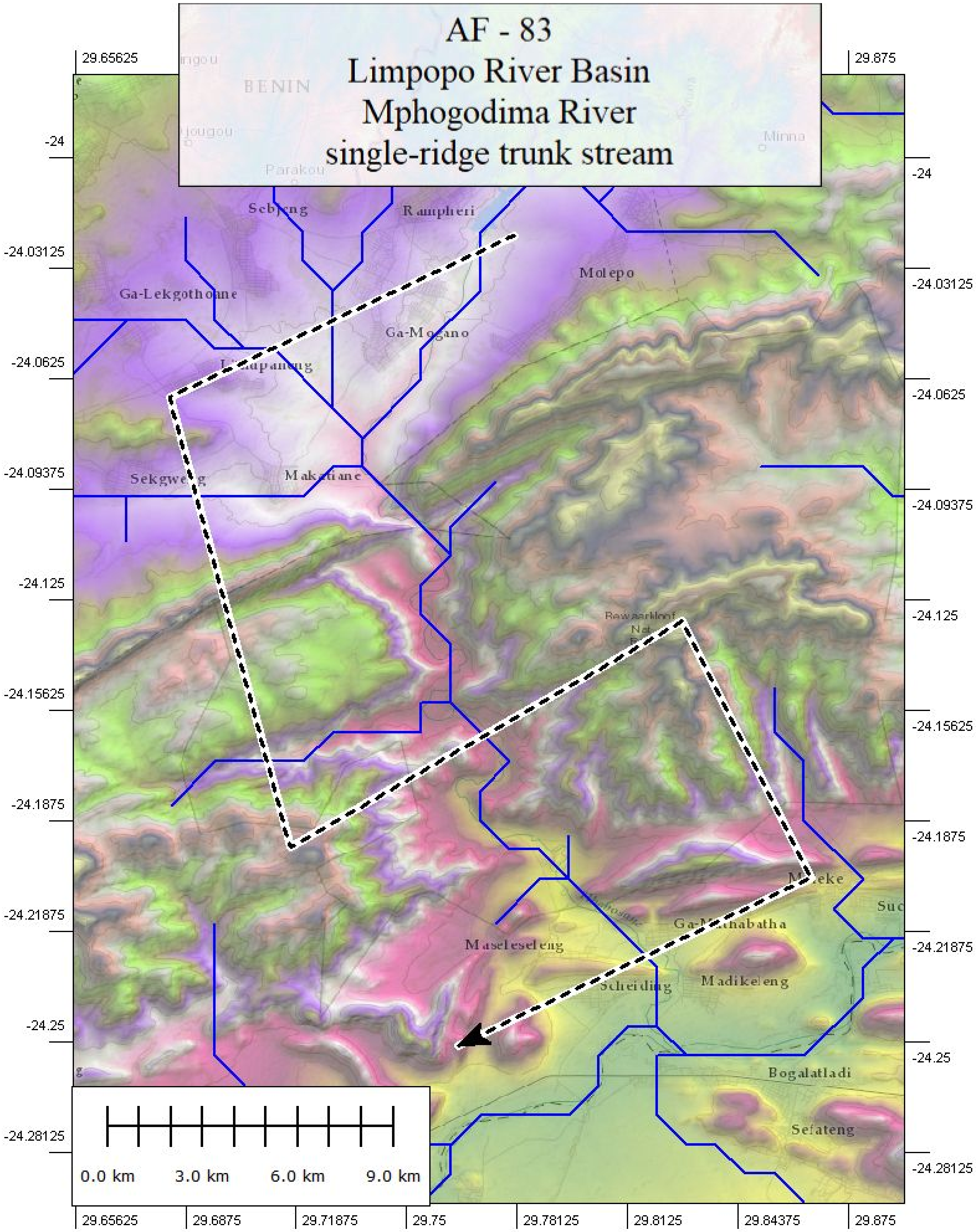

# AF - 85
## Incomatì River Basin
## Mbuluzi River
## single-ridge trunk stream

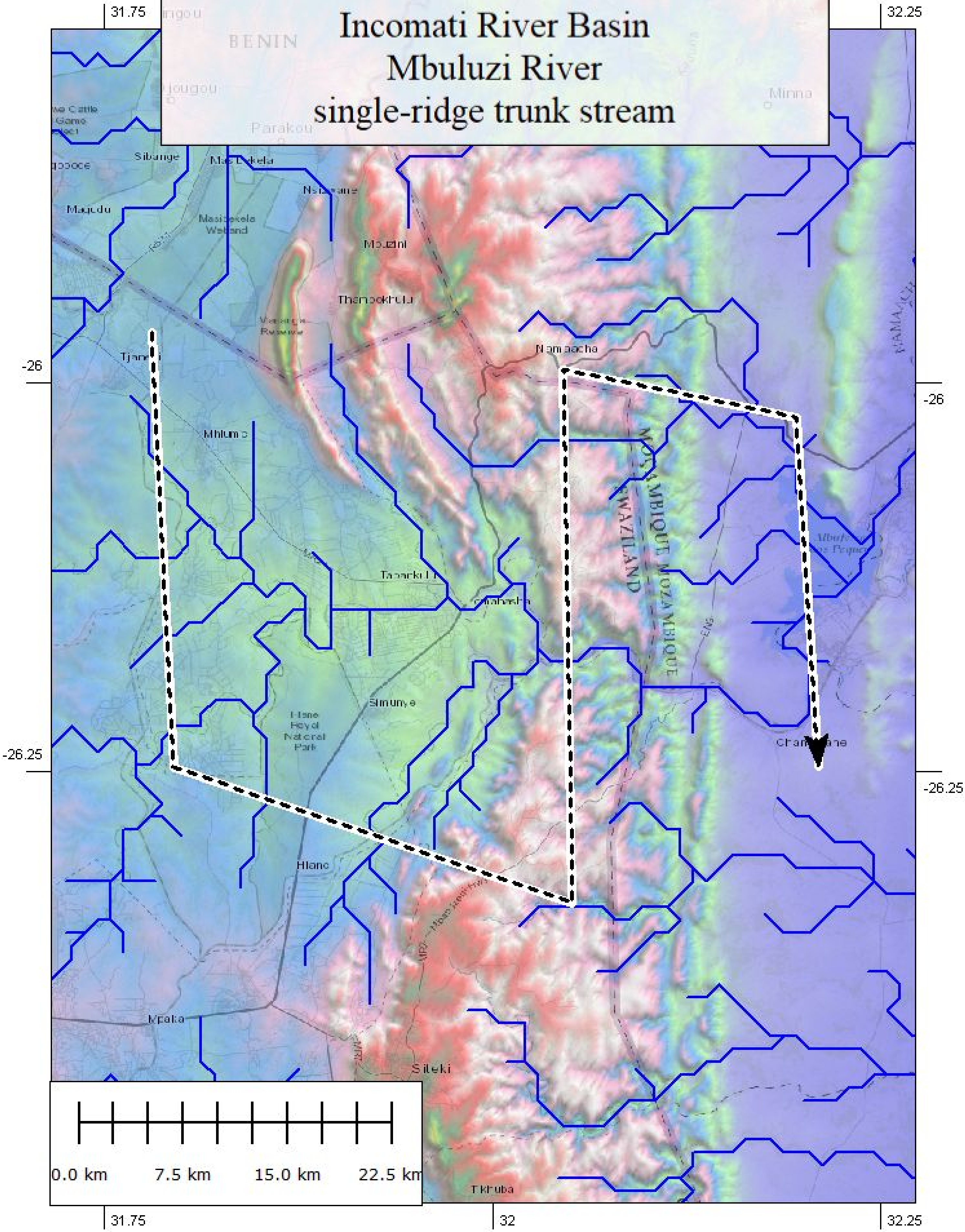

# AF - 86
## Maputo River Basin
## Maputo River
## single-ridge trunk stream

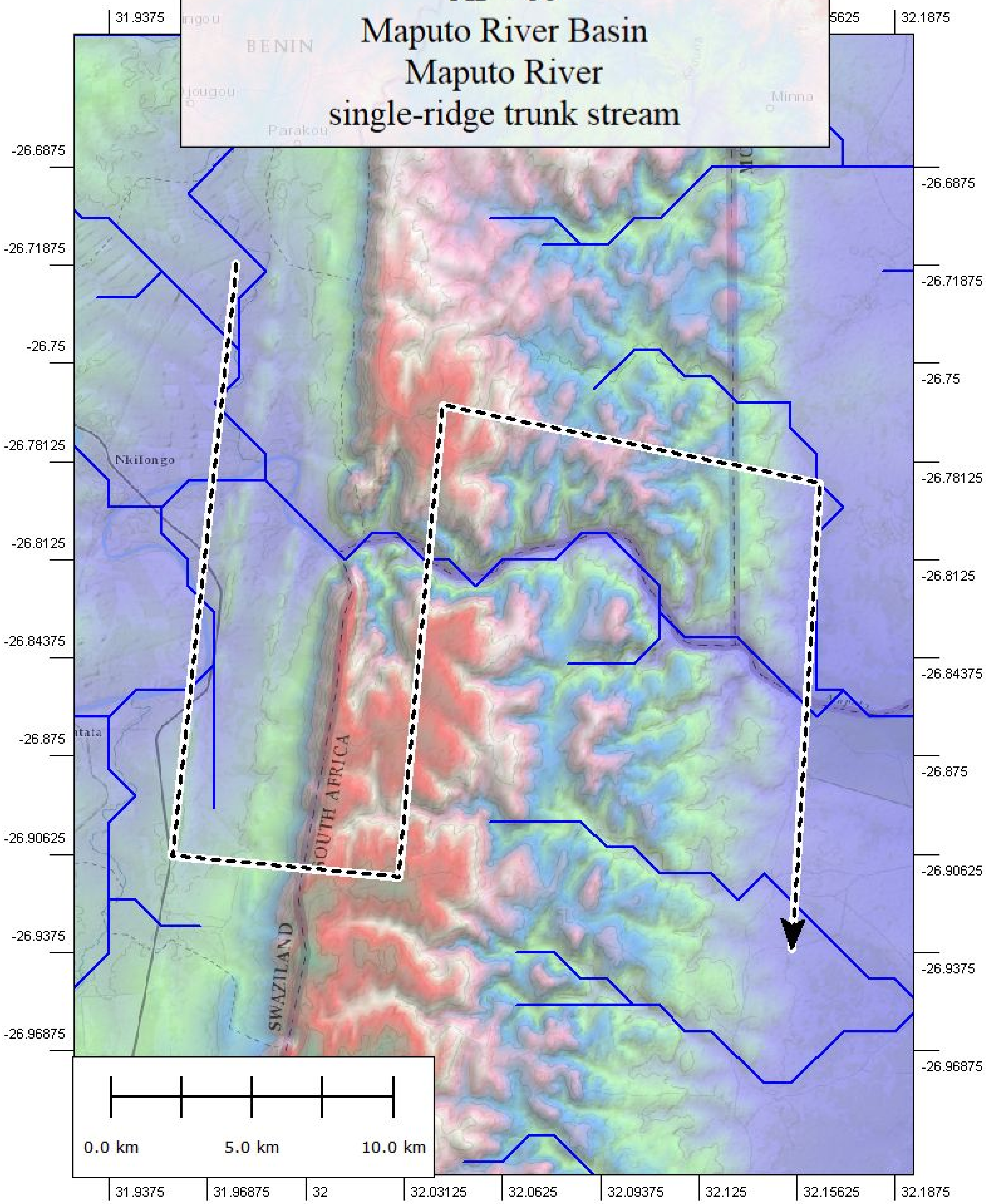

# AF - 89
# Licungo River Basin
# Luo River
# single-ridge trunk stream

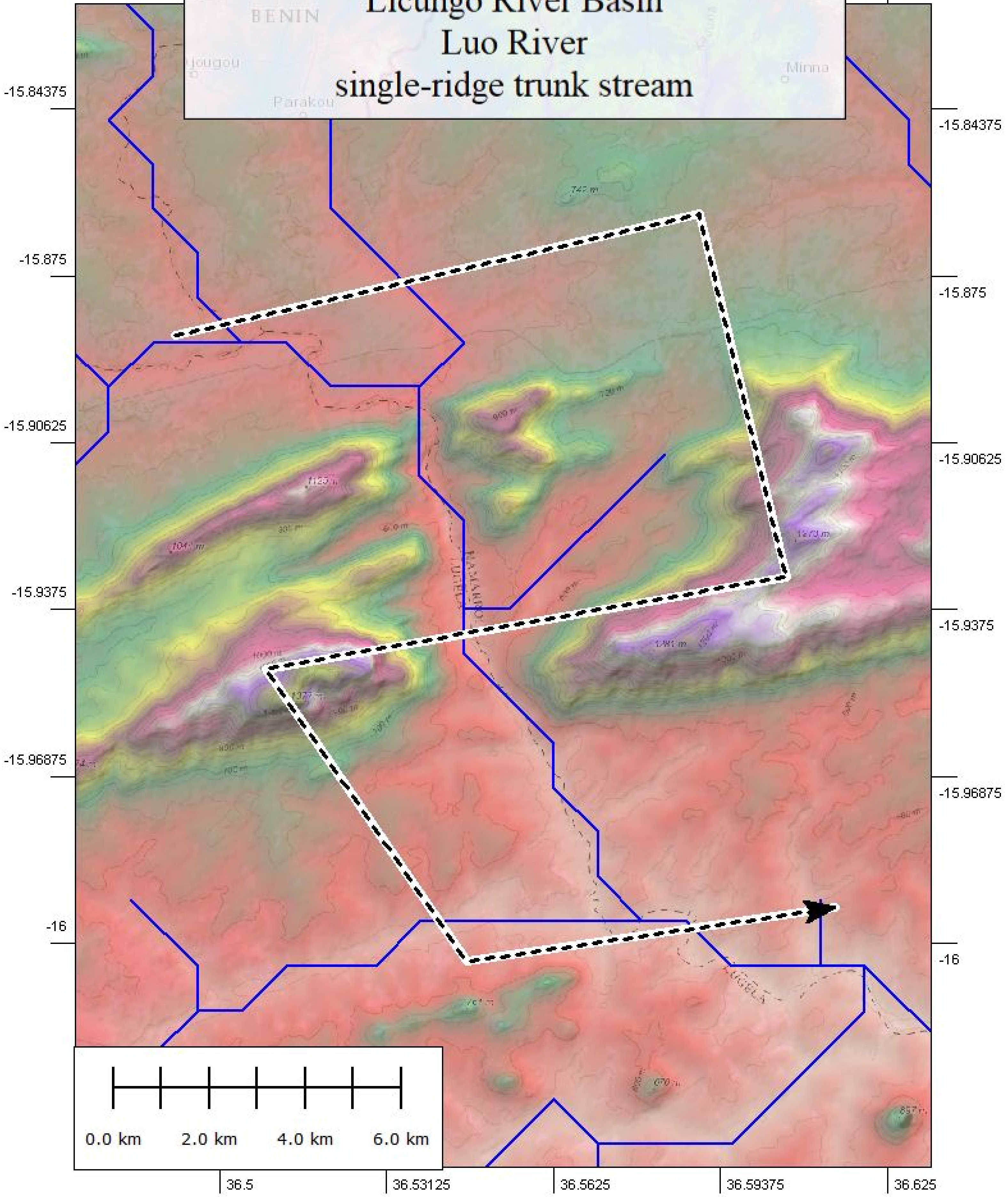

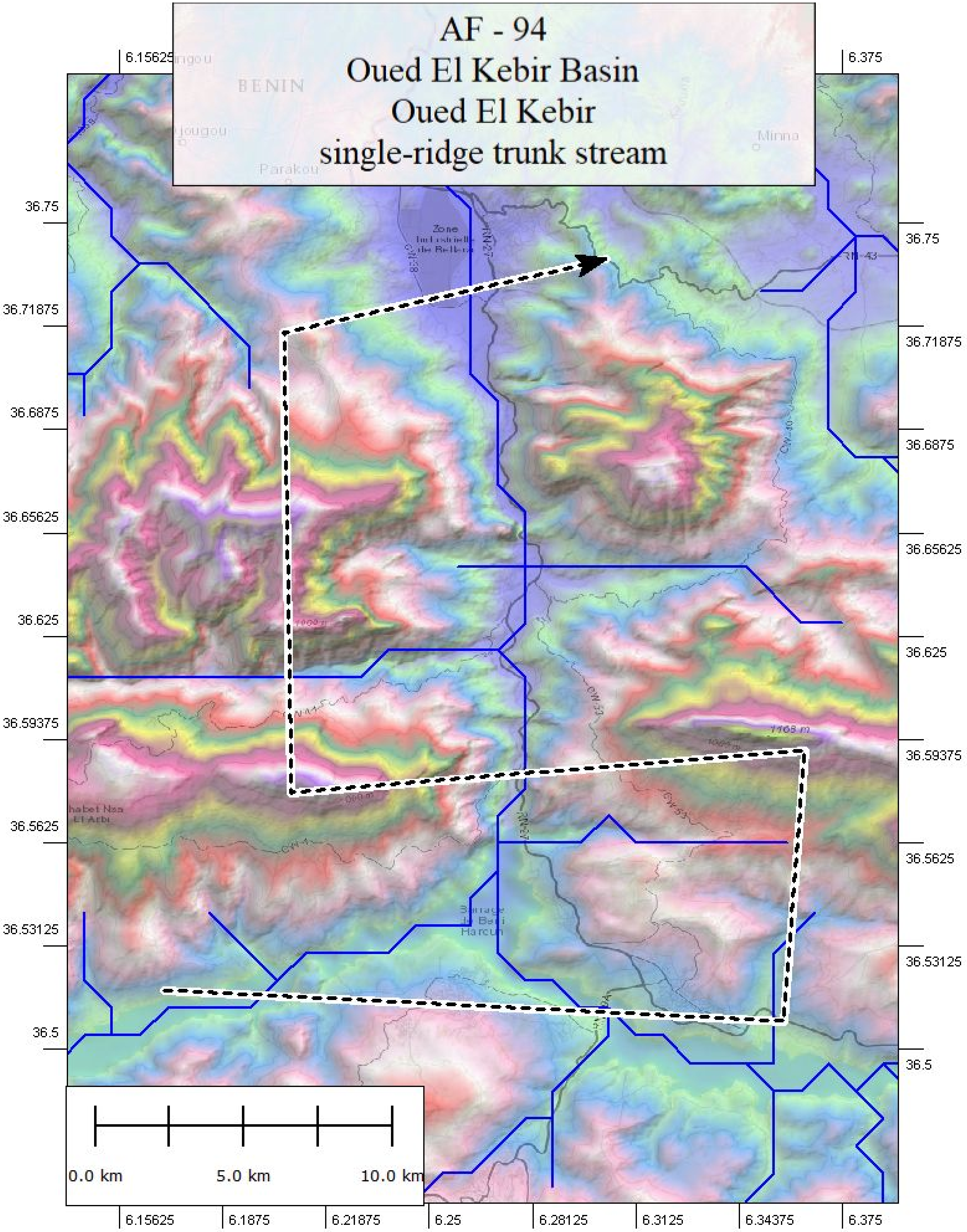

AF - 94
Oued El Kebir Basin
Oued El Kebir
single-ridge trunk stream
6.15625
6.375
36.75
36.71875
36.6875
36.65625
36.625
36.59375
36.5625
36.53125
36.5
6.1875
6.21875
6.25
6.28125
6.3125
6.34375
0.0 km
5.0 km
10.0 km

# AF - 95
# Endorheic basin Basin
# Ziz River
# single-ridge trunk stream

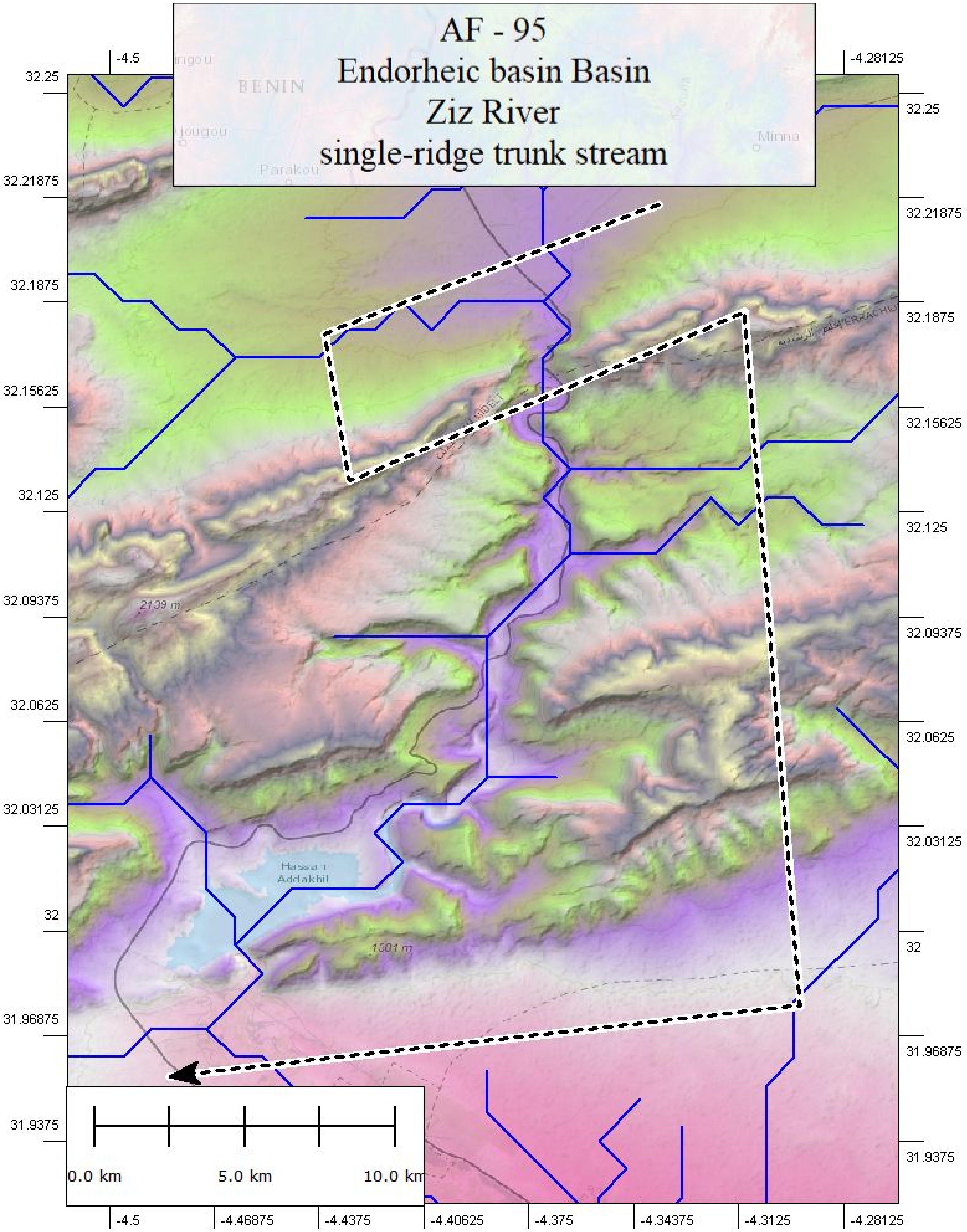

# AF - 97
## Endorheic basin Basin
## Ziz River
## single-ridge trunk stream

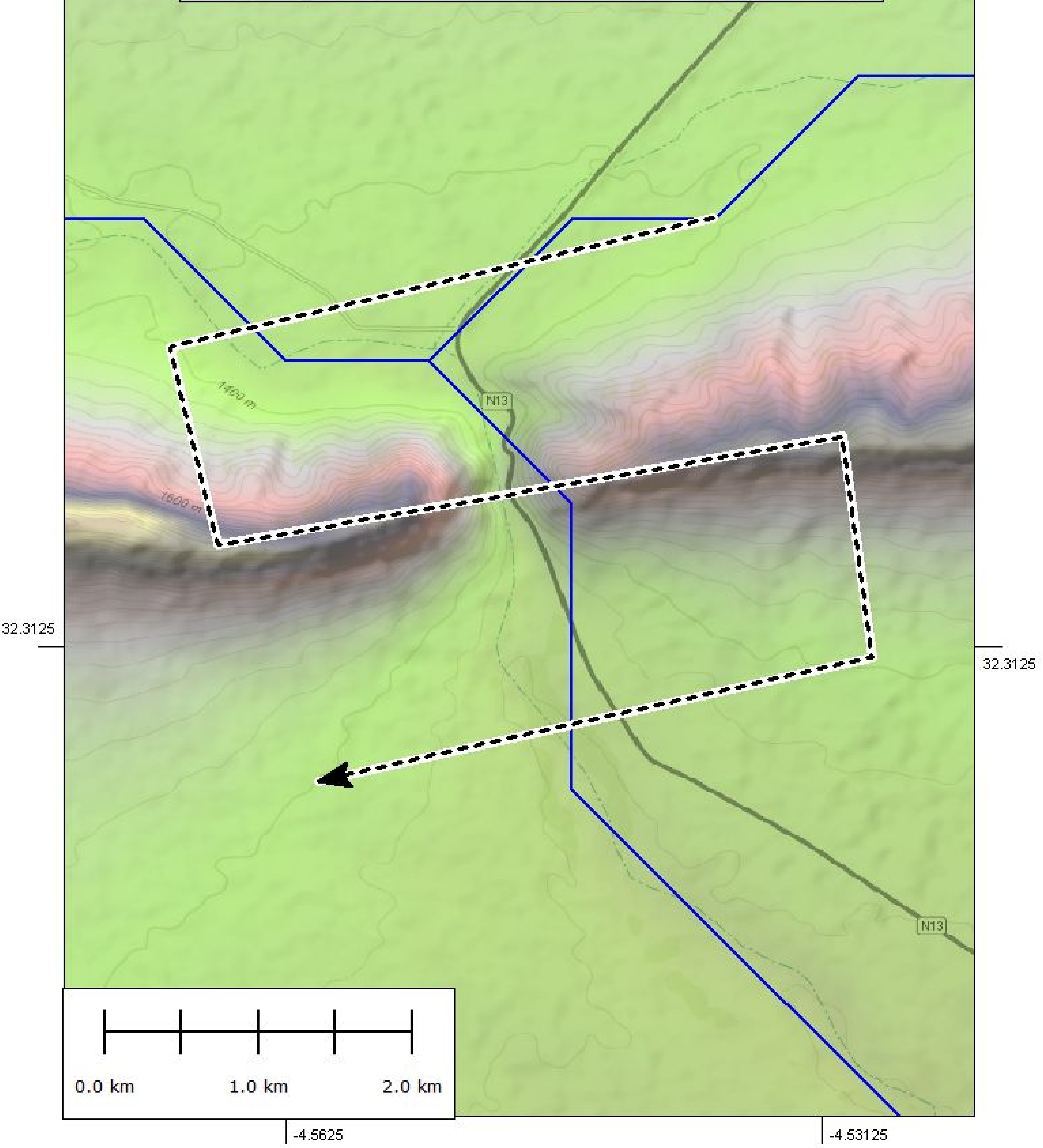

AF - 100
Endorheic basin Basin
Timkite Dam
single-ridge trunk stream

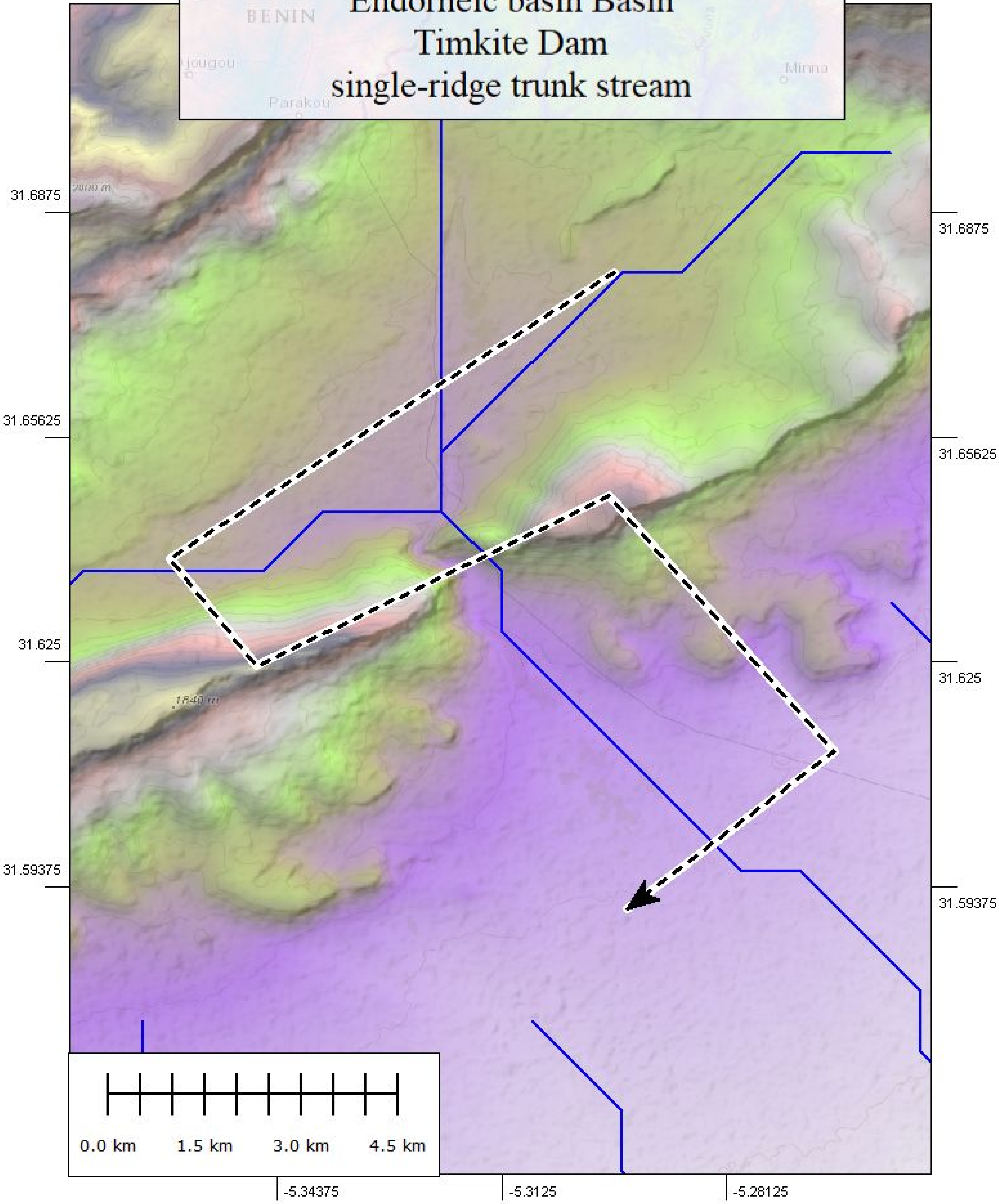

# AF - 109
## Endorheic basin Basin
## Les Gorges d'El Kantara
## single-ridge trunk stream

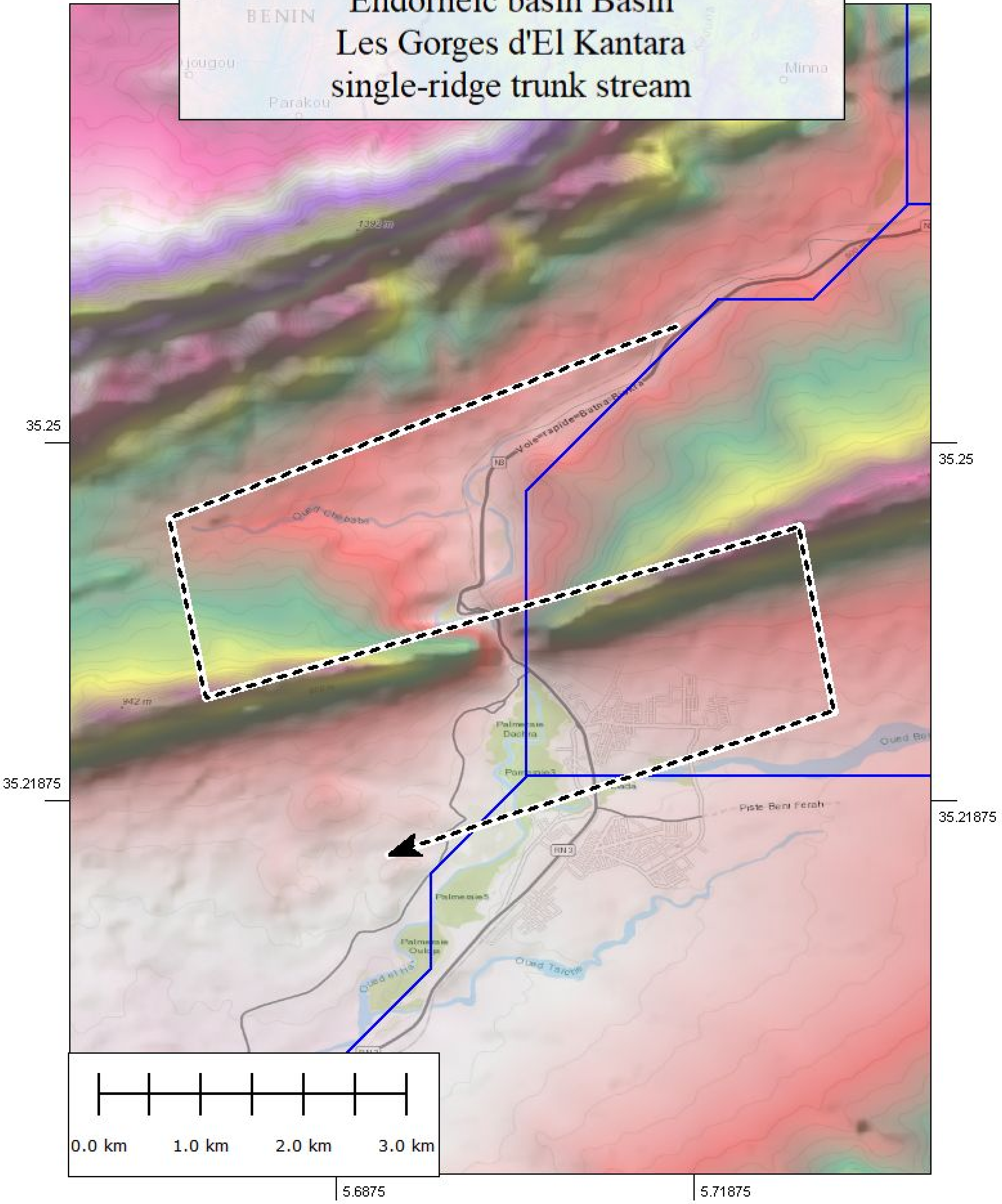

# AF - 113
## Gourits River Basin
## Gourits River
## single-ridge trunk stream

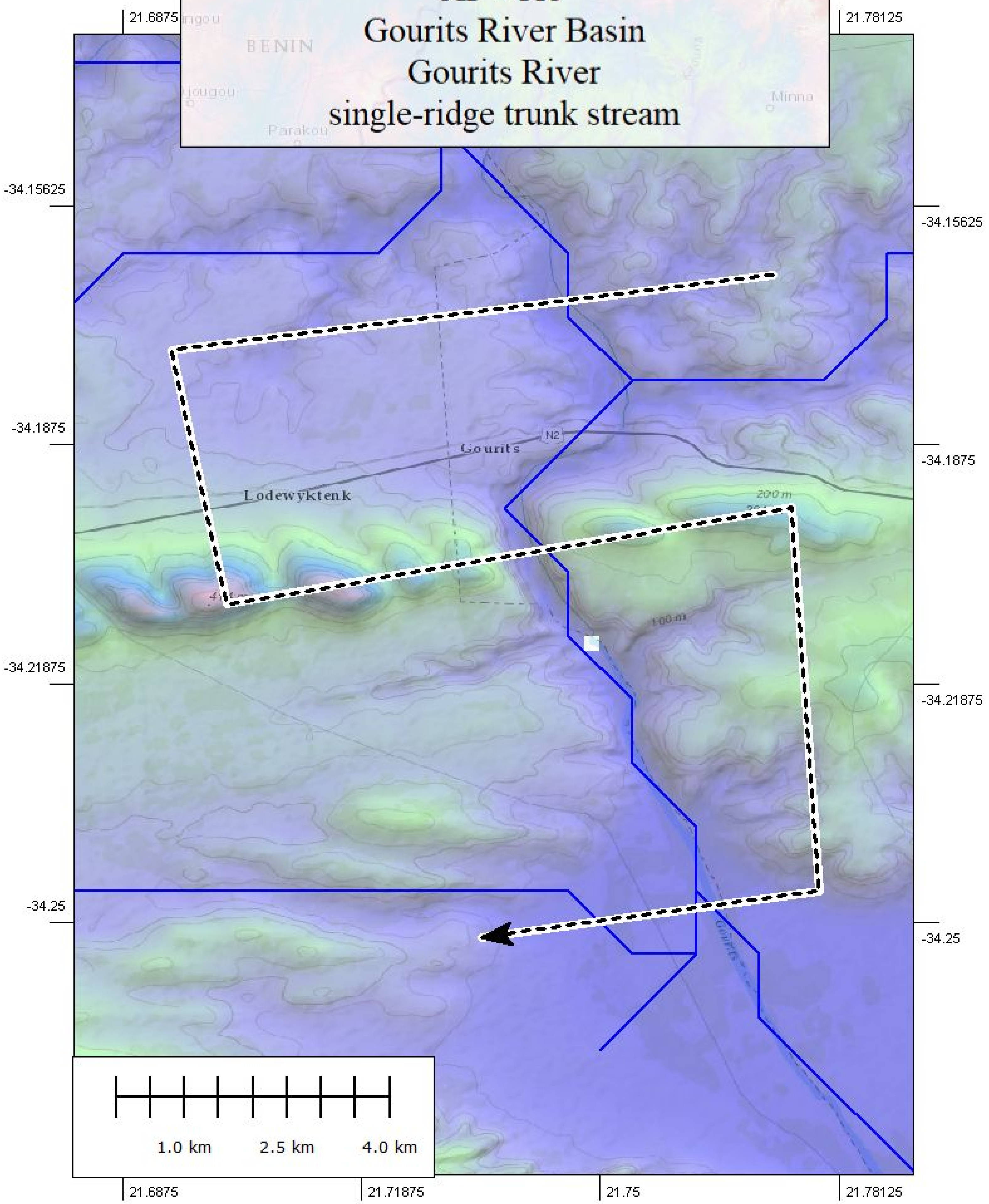

# AF - 117
## Sonderend River Basin
## Sonderend River
## single-ridge trunk stream

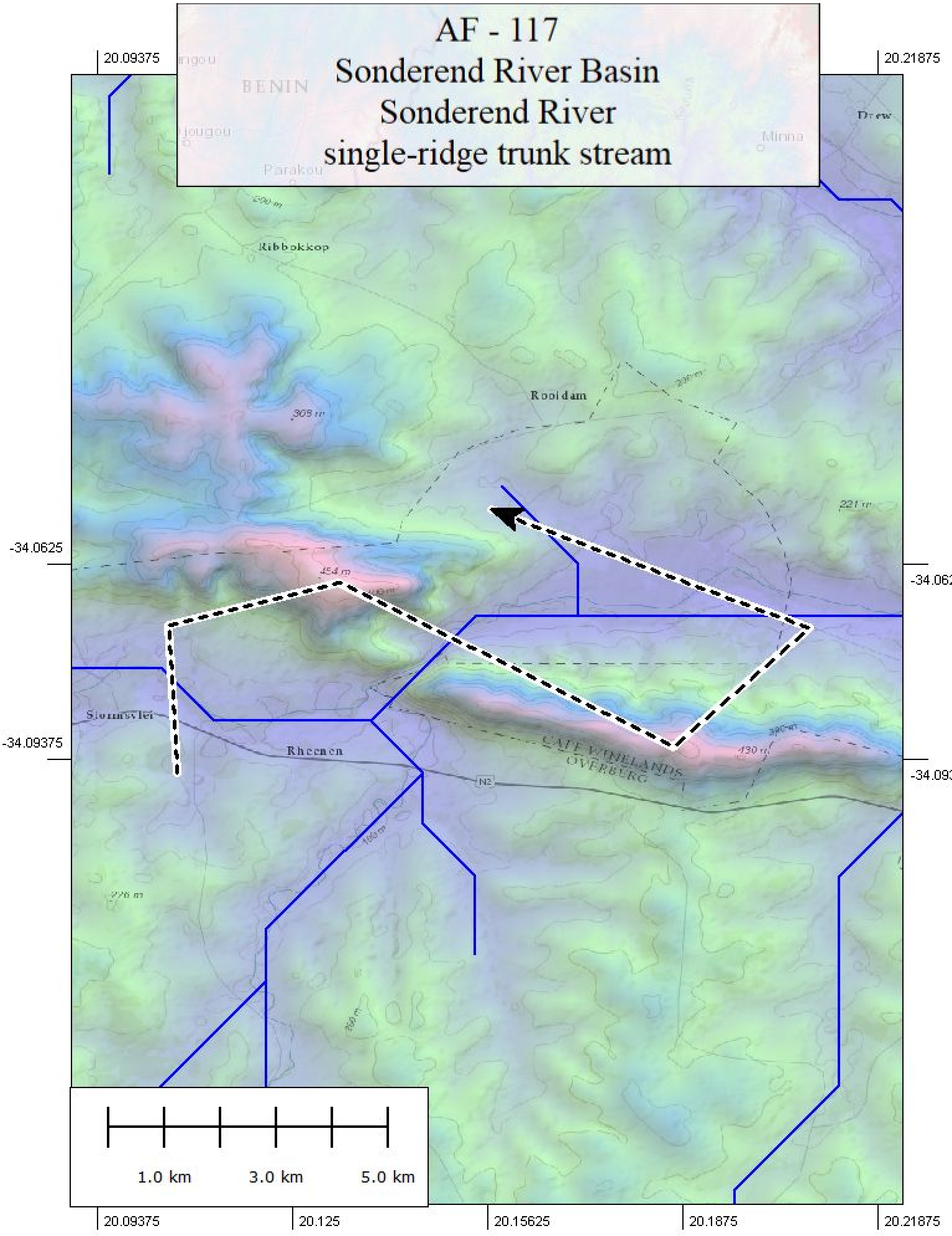

# AF - 123
# Nile River Basin
# Nyabugogo River
# single-ridge trunk stream

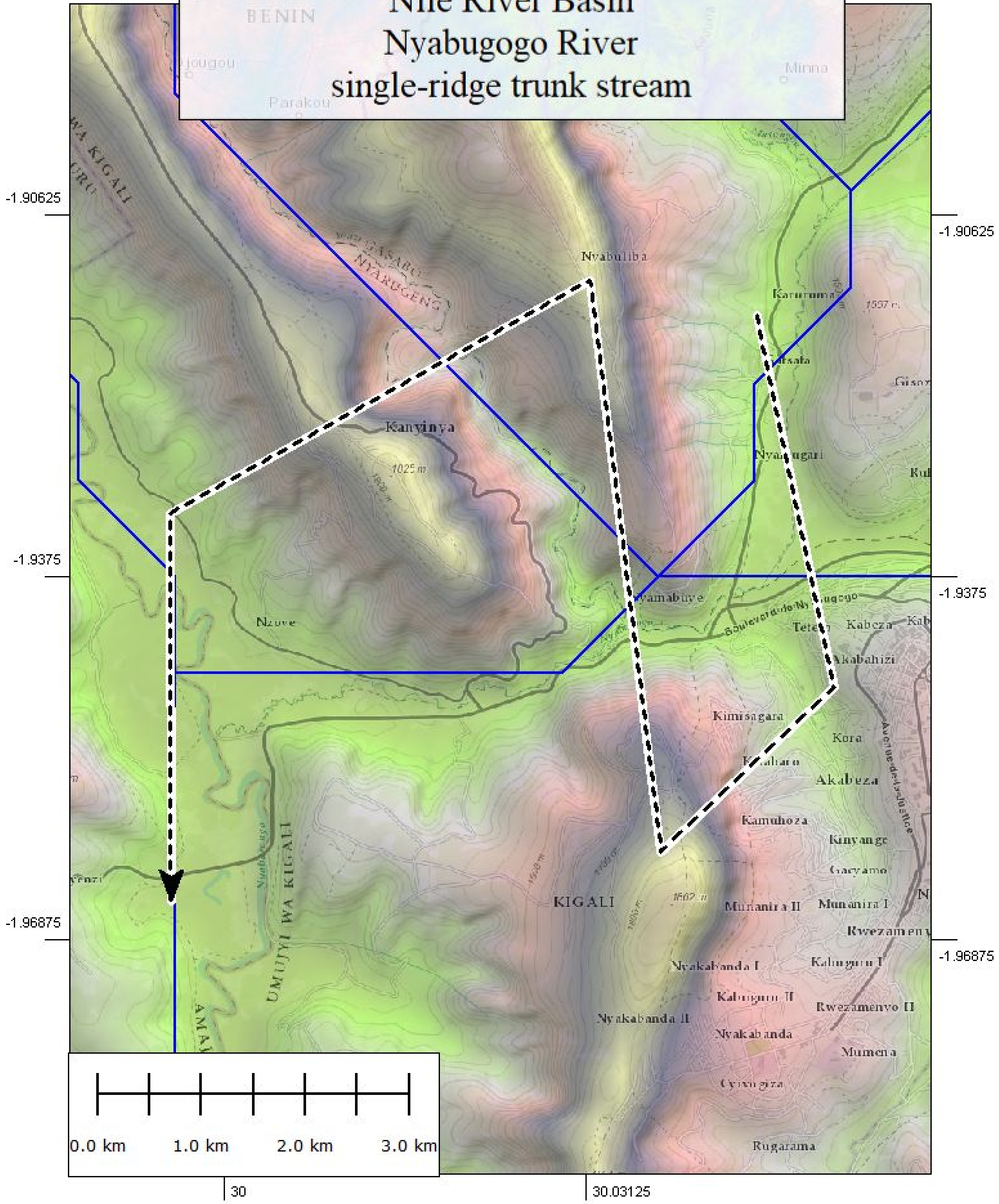

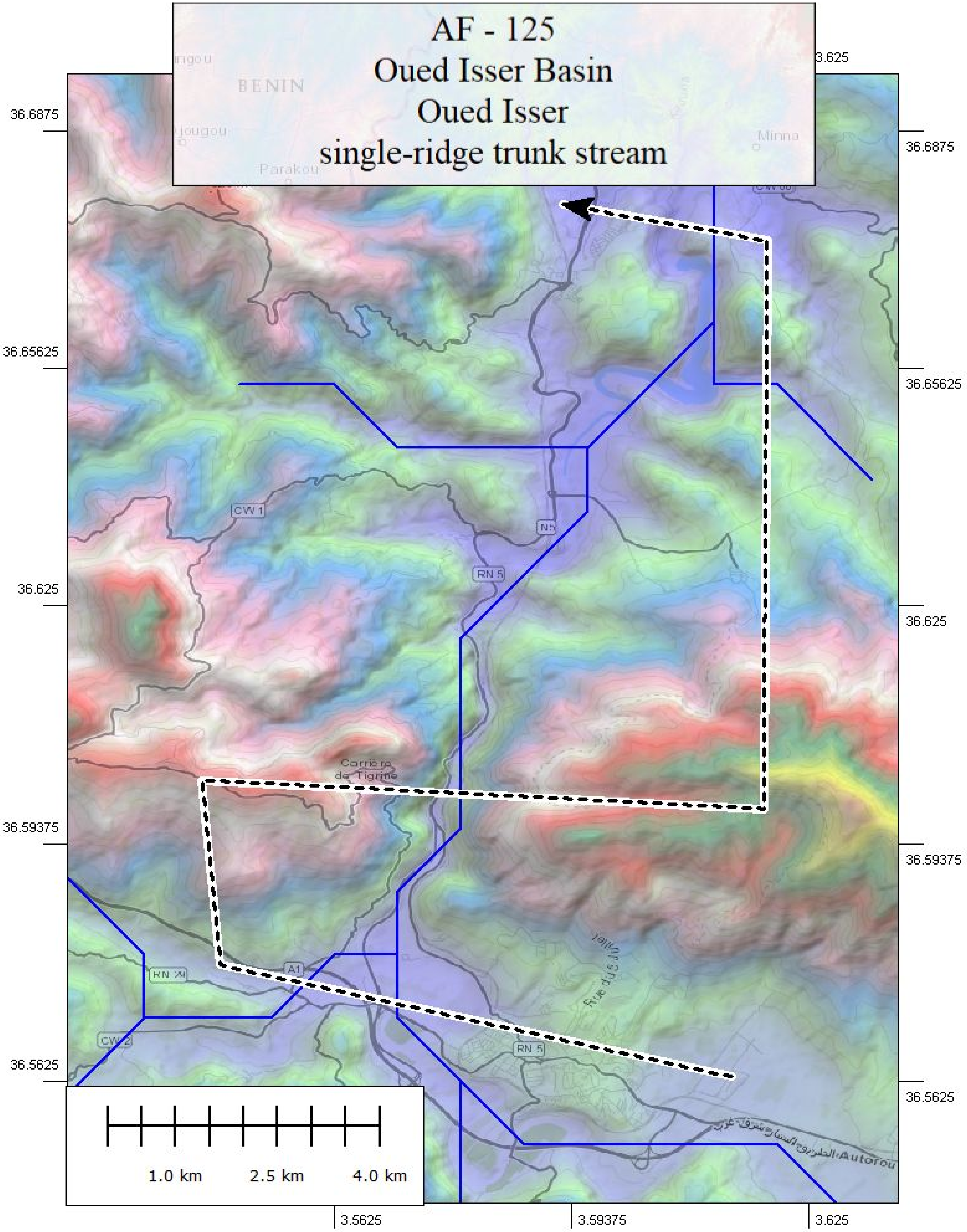
AF - 125
Oued Isser Basin
Oued Isser
single-ridge trunk stream
36.6875
36.65625
36.625
36.59375
36.5625
3.5625
3.59375
3.625
1.0 km
2.5 km
4.0 km
CW 1
RN 5
A1
Corrière de Tigrine

# AF - 127
## Sonderend River Basin
## Tradourivier
## single-ridge trunk stream

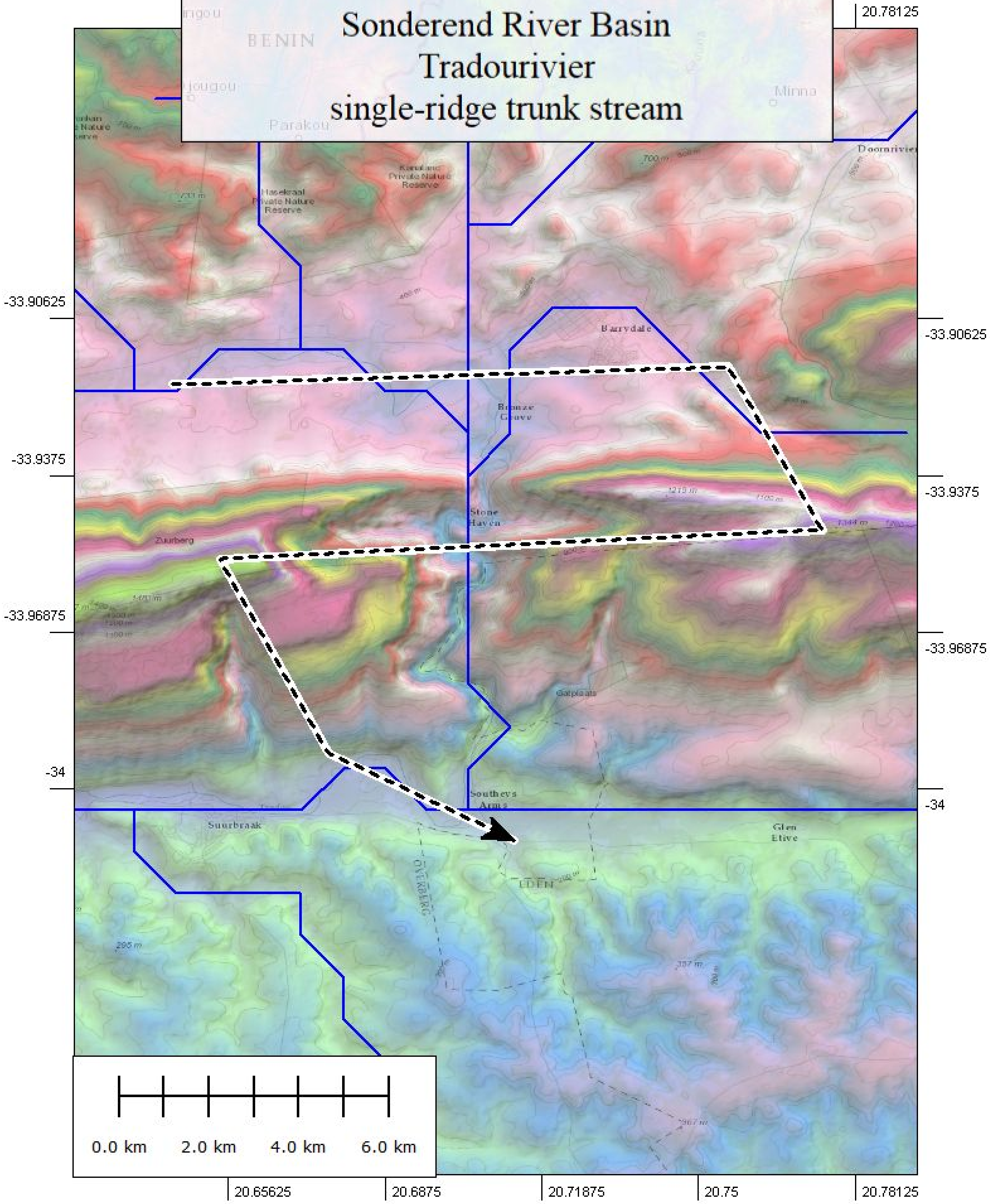

# AF - 128
# Gourits River Basin
# Buffels River
# single-ridge trunk stream

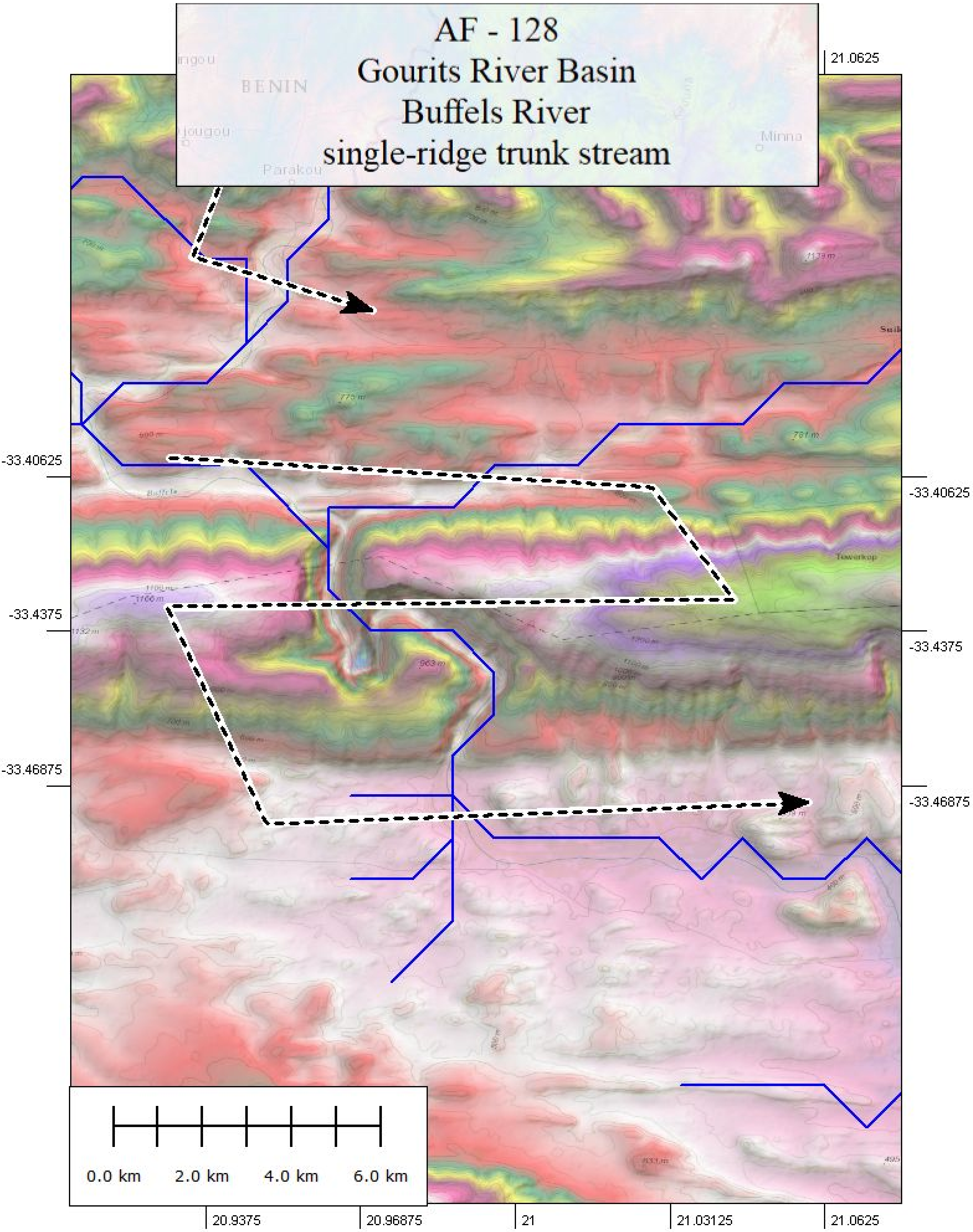

# AF - 133
# Oued Sebou Basin
# Oued Ait Makhchoure
# single-ridge trunk stream

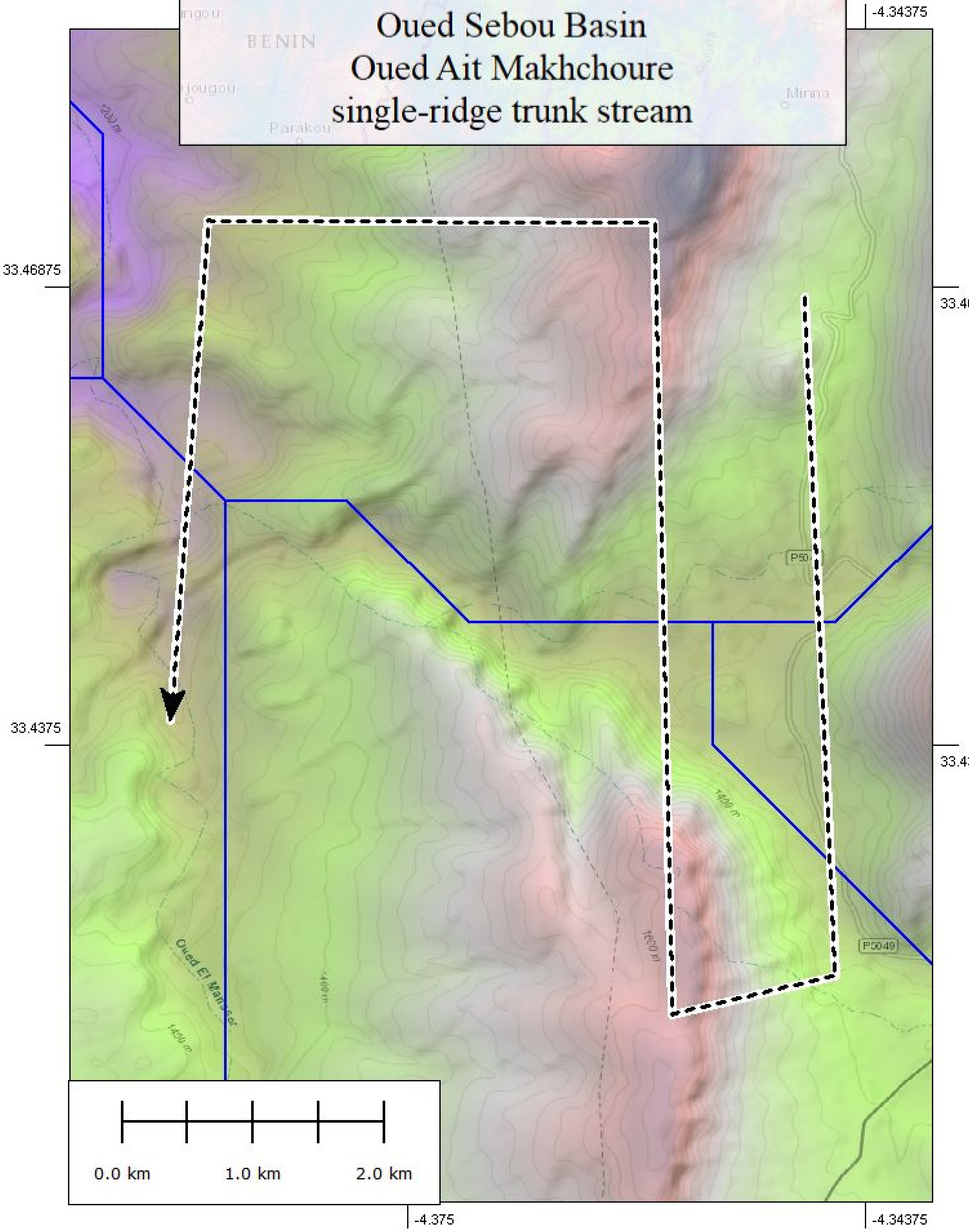

# AF - 136
## Oued Draa Basin
## Kheneg Tarfa (pass)
## single-ridge trunk stream

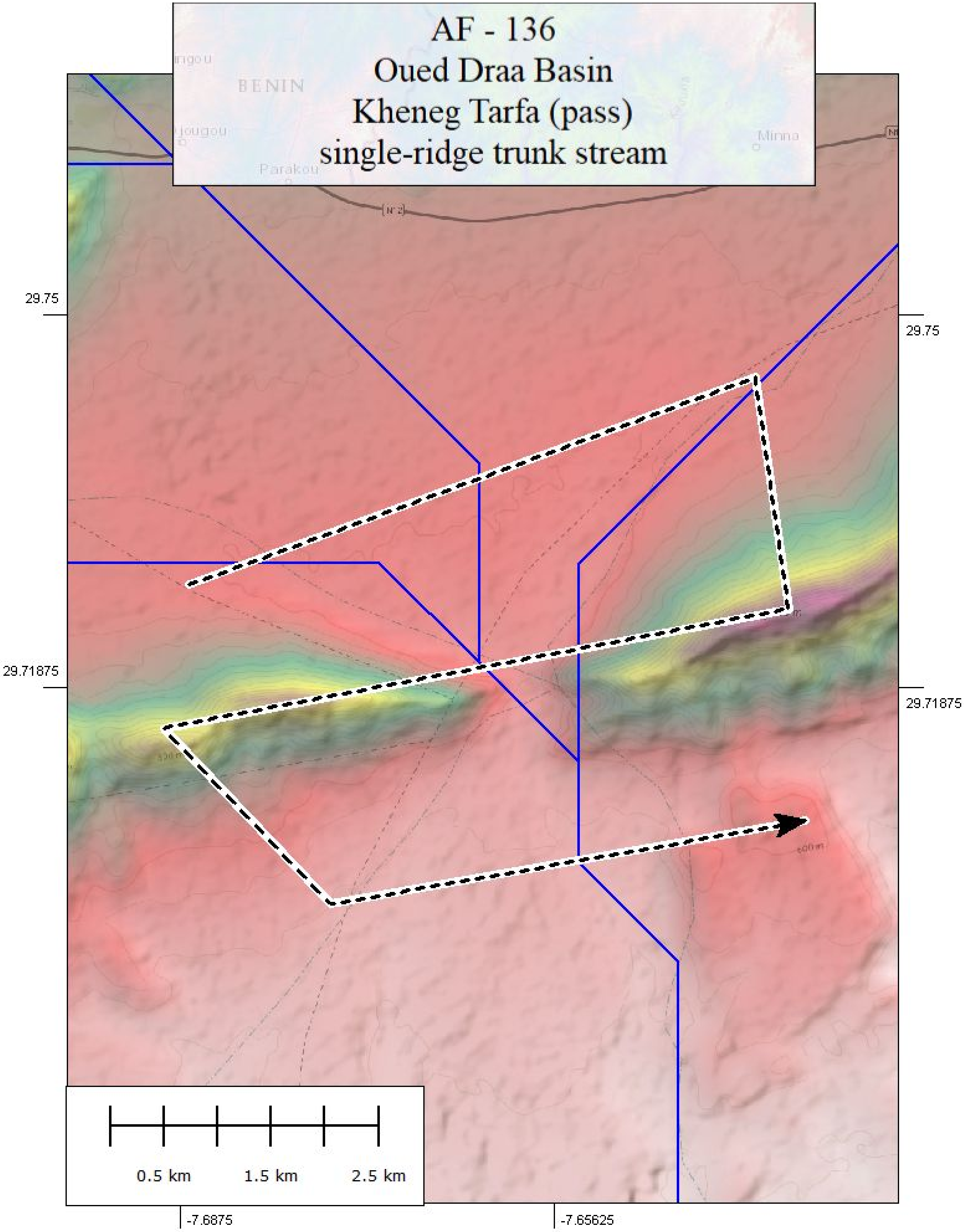

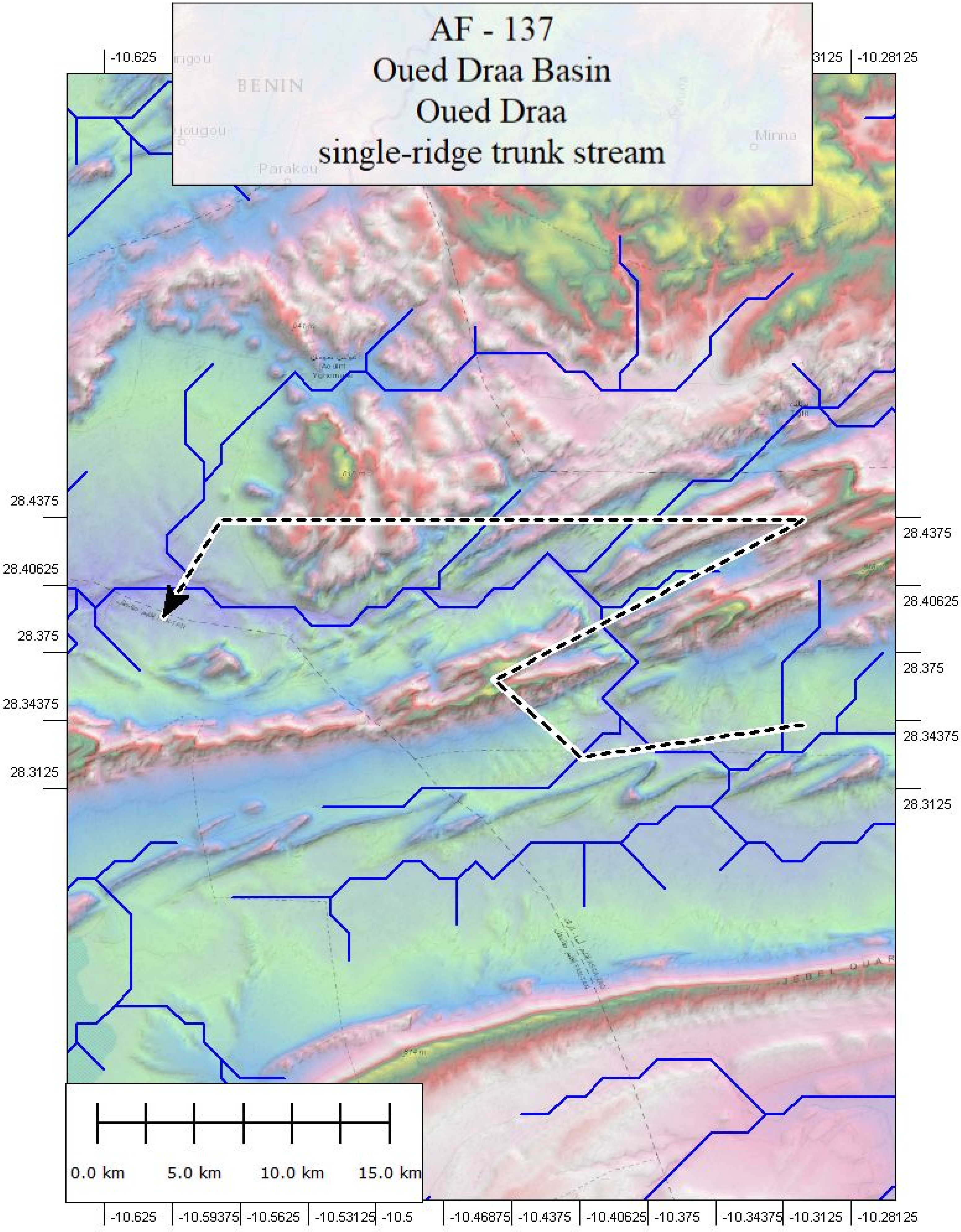
AF - 137
Oued Draa Basin
Oued Draa
single-ridge trunk stream
BENIN
Parakou
Minna
-10.625
-10.59375
-10.5625
-10.53125
-10.5
-10.46875
-10.4375
-10.40625
-10.375
-10.34375
-10.3125
-10.28125
28.4375
28.40625
28.375
28.34375
28.3125
0.0 km
5.0 km
10.0 km
15.0 km

# AF - 138
## Oued Draa Basin
## Imi n'Ou Akka
## single-ridge trunk stream

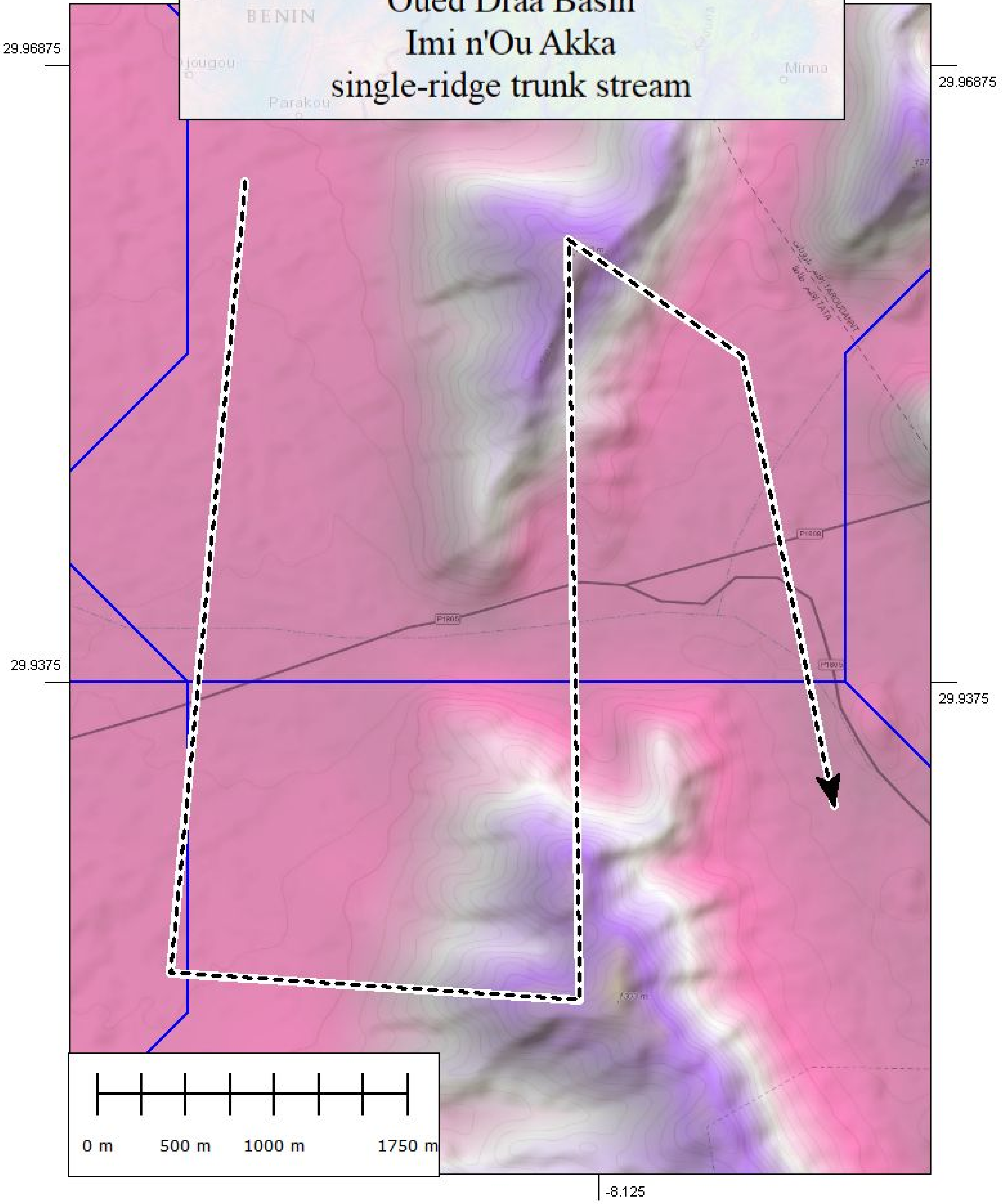

# AF - 139
## Oued Draa Basin
## Amond Oasis
## single-ridge trunk stream

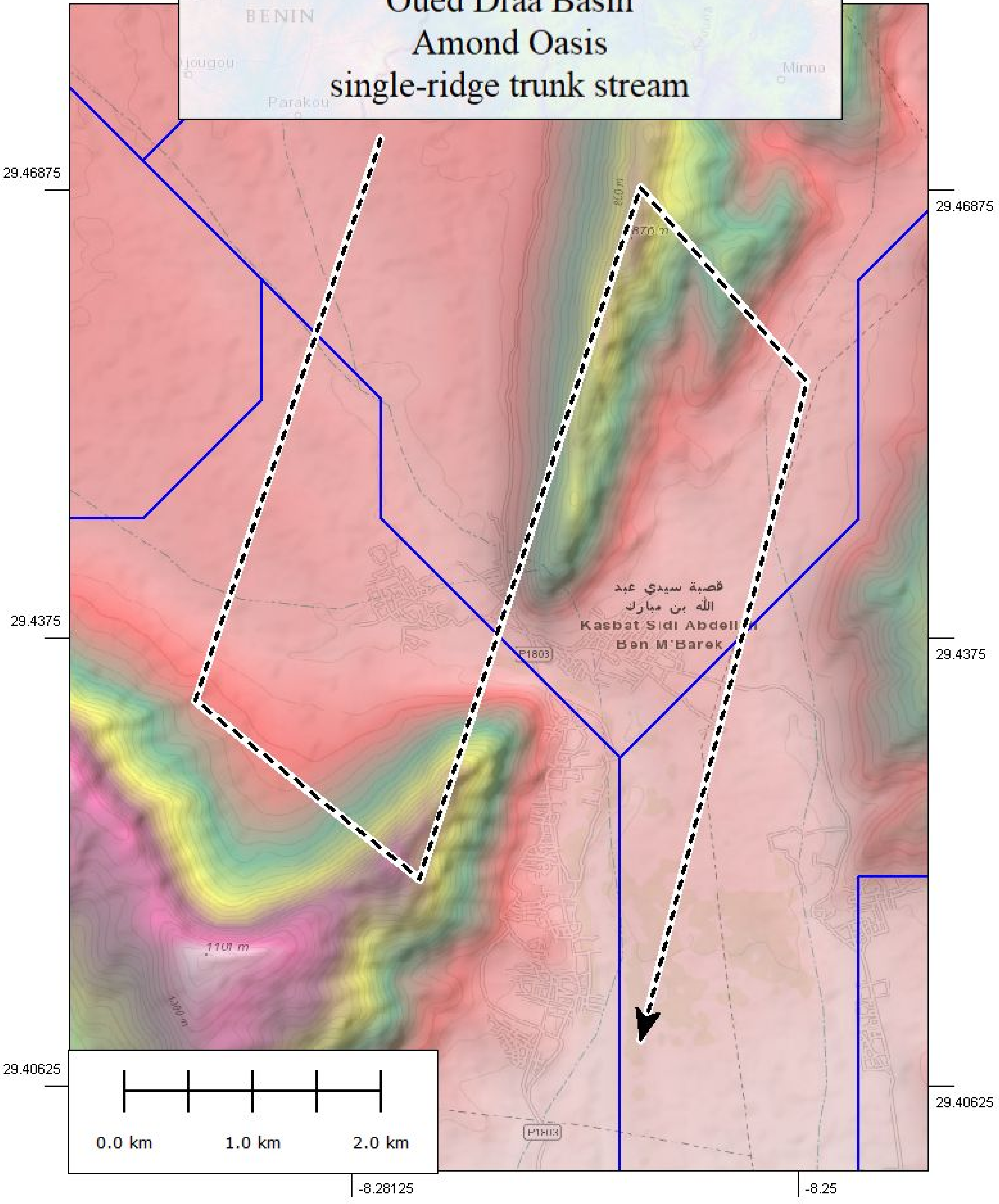

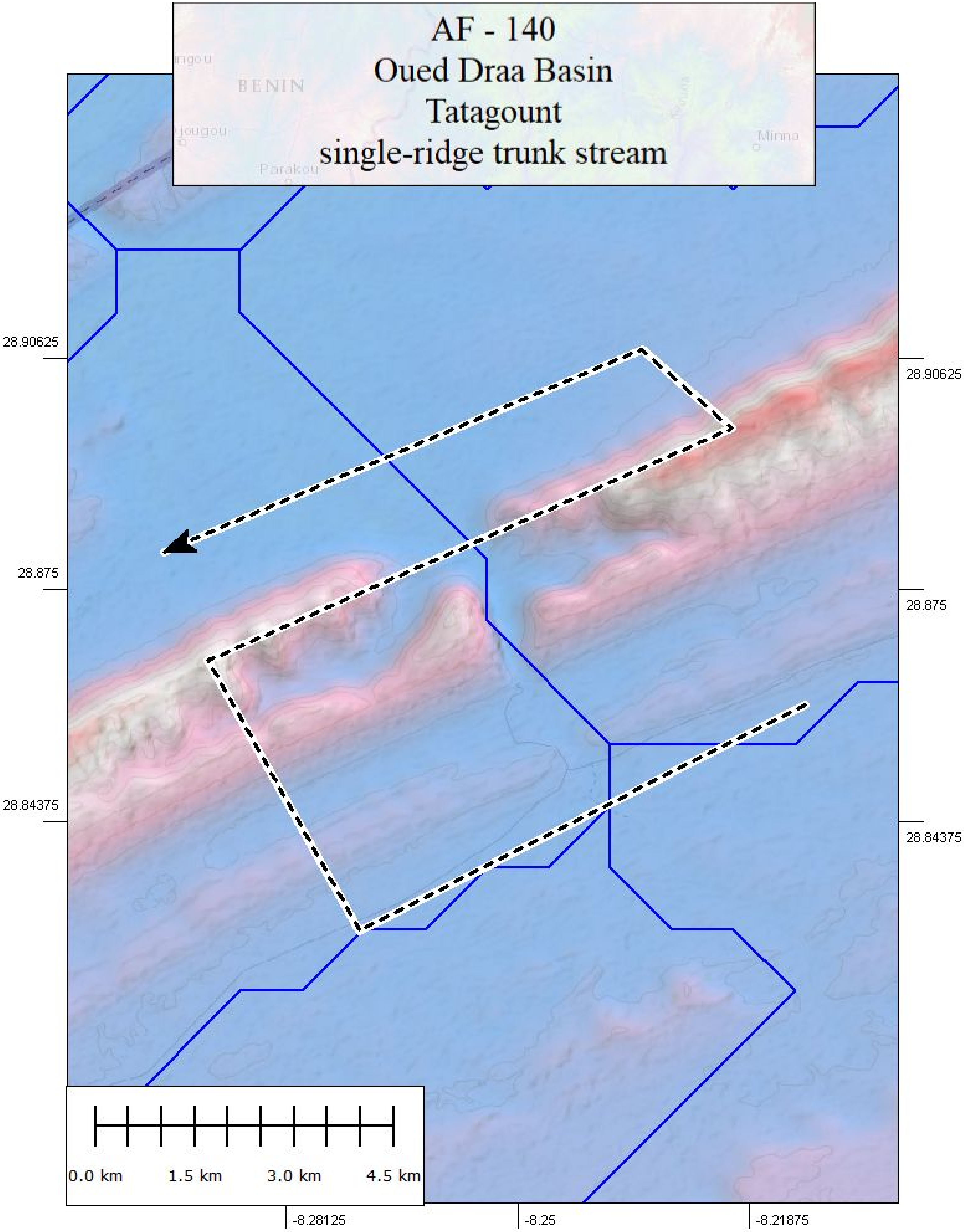
AF - 140
Oued Draa Basin
Tatagount
single-ridge trunk stream
BENIN
Parakou
Minna
28.90625
28.875
28.84375
-8.28125
-8.25
-8.21875
0.0 km
1.5 km
3.0 km
4.5 km

# AF - 141
## Oued Draa Basin
## Kheneg Oua Belli (pass)
## single-ridge trunk stream

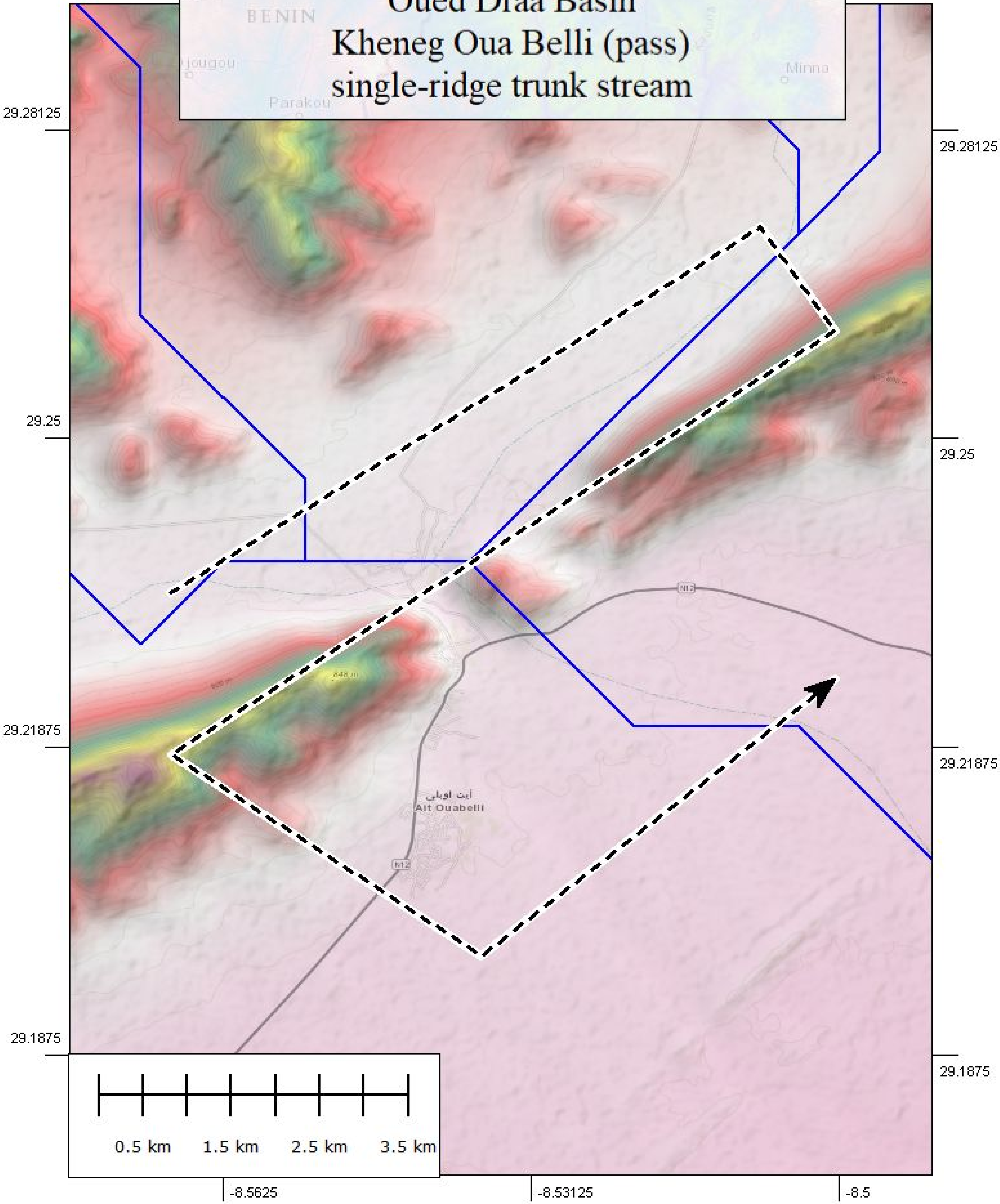

# AF - 142
## Oued Draa Basin
## Oued Draa
## single-ridge trunk stream

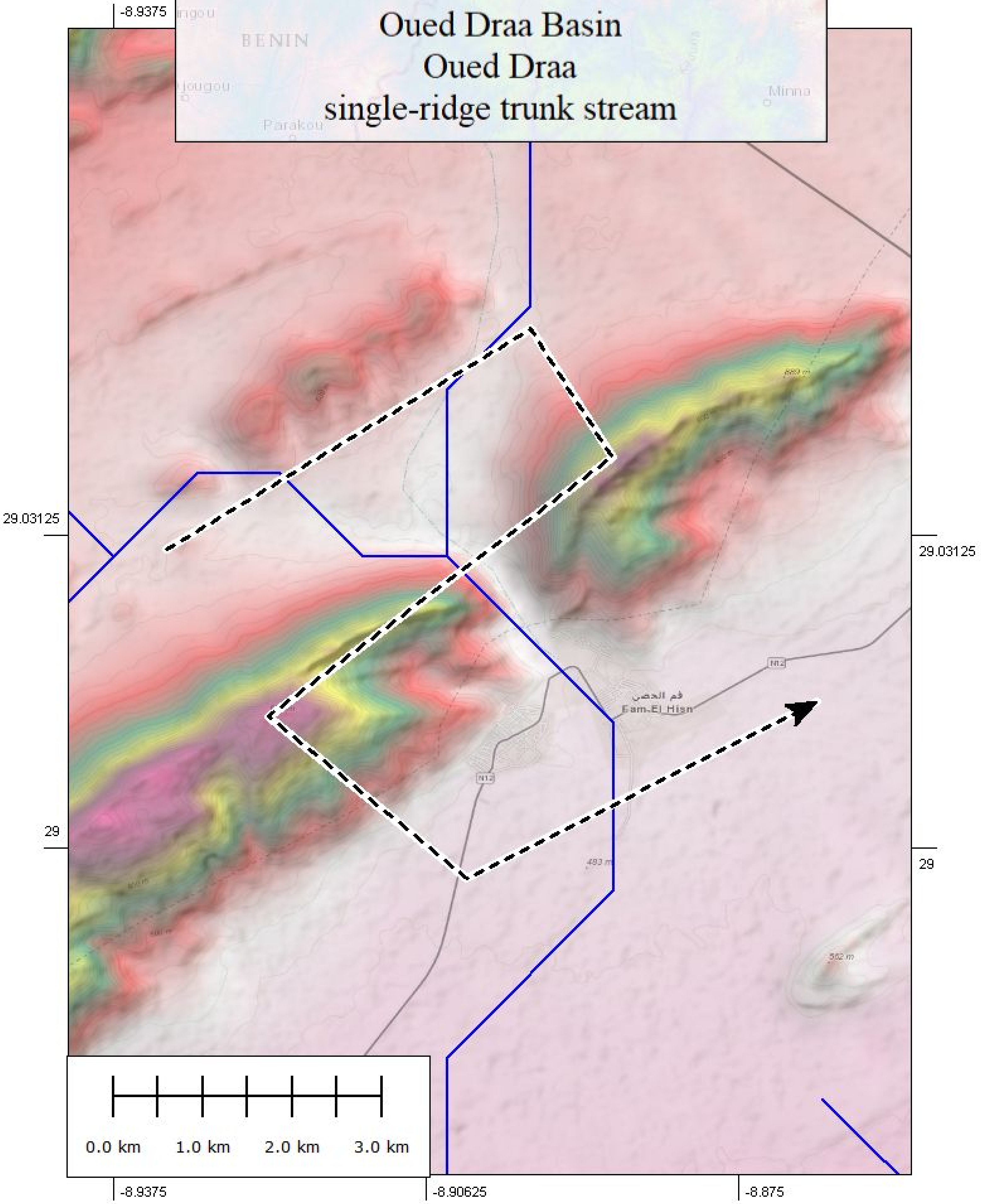

# AF - 144
# Volta River Basin
# Asukawkaw River
# single-ridge trunk stream

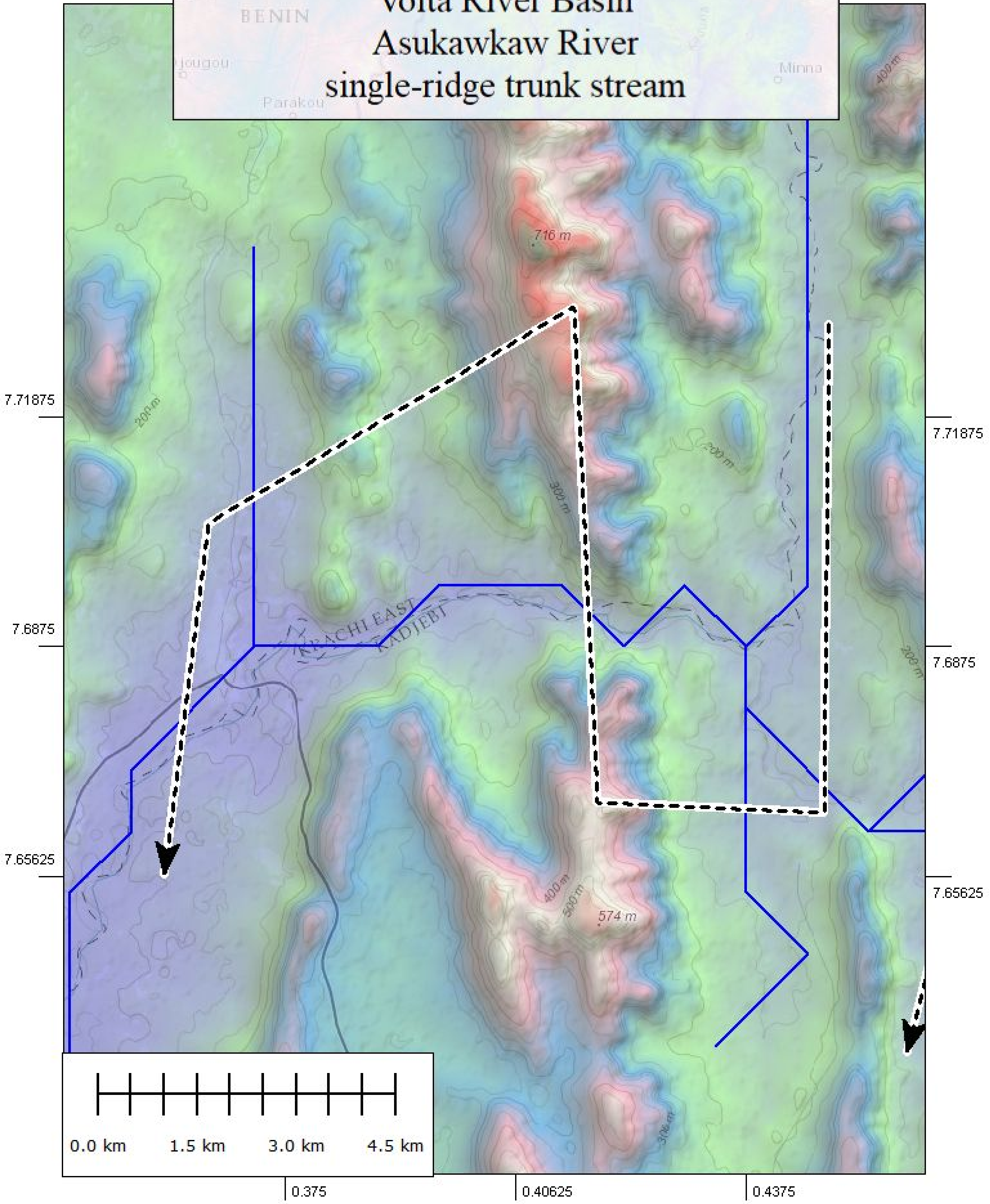

# AF - 145
# Volta River Basin
# Jelen River
# single-ridge trunk stream

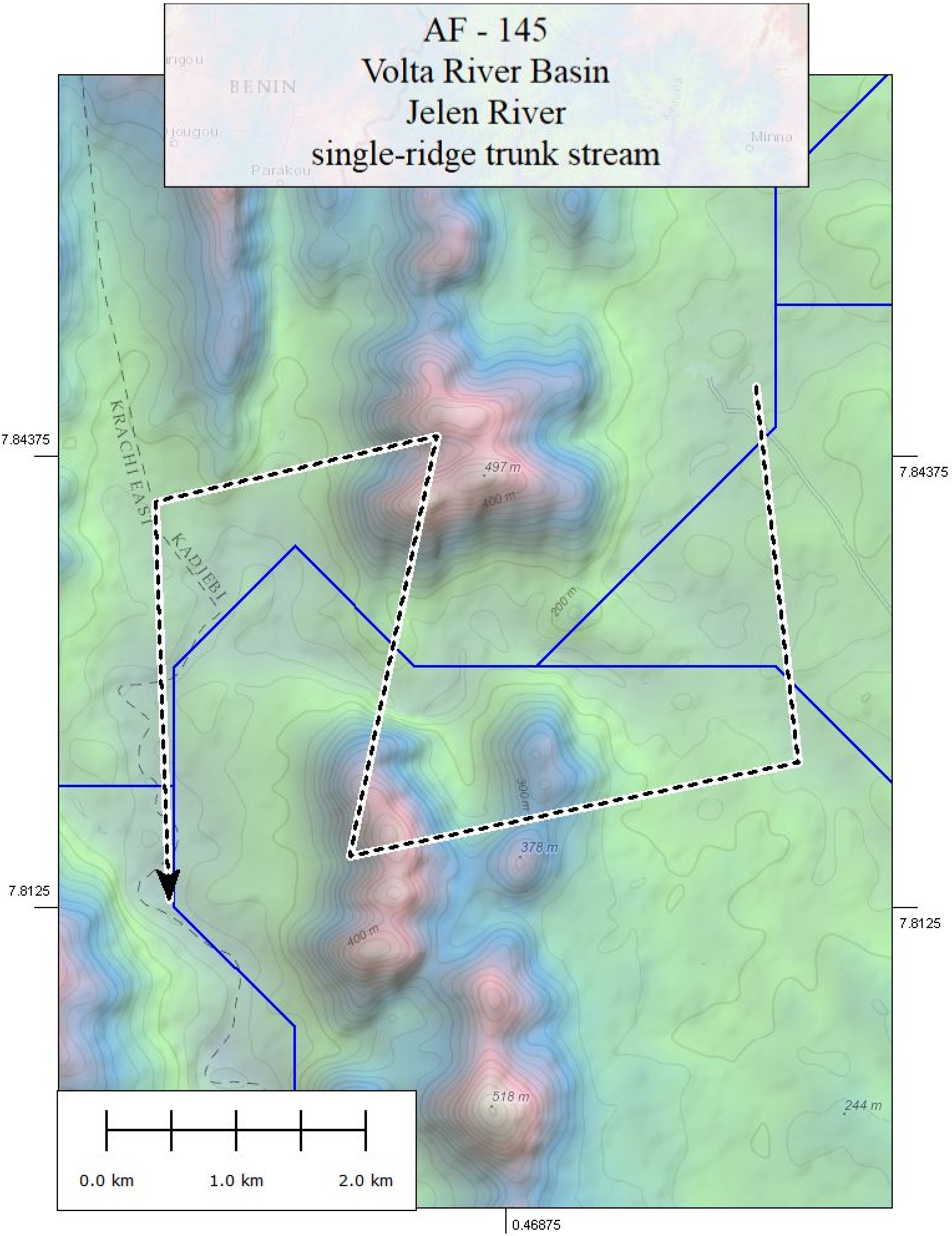

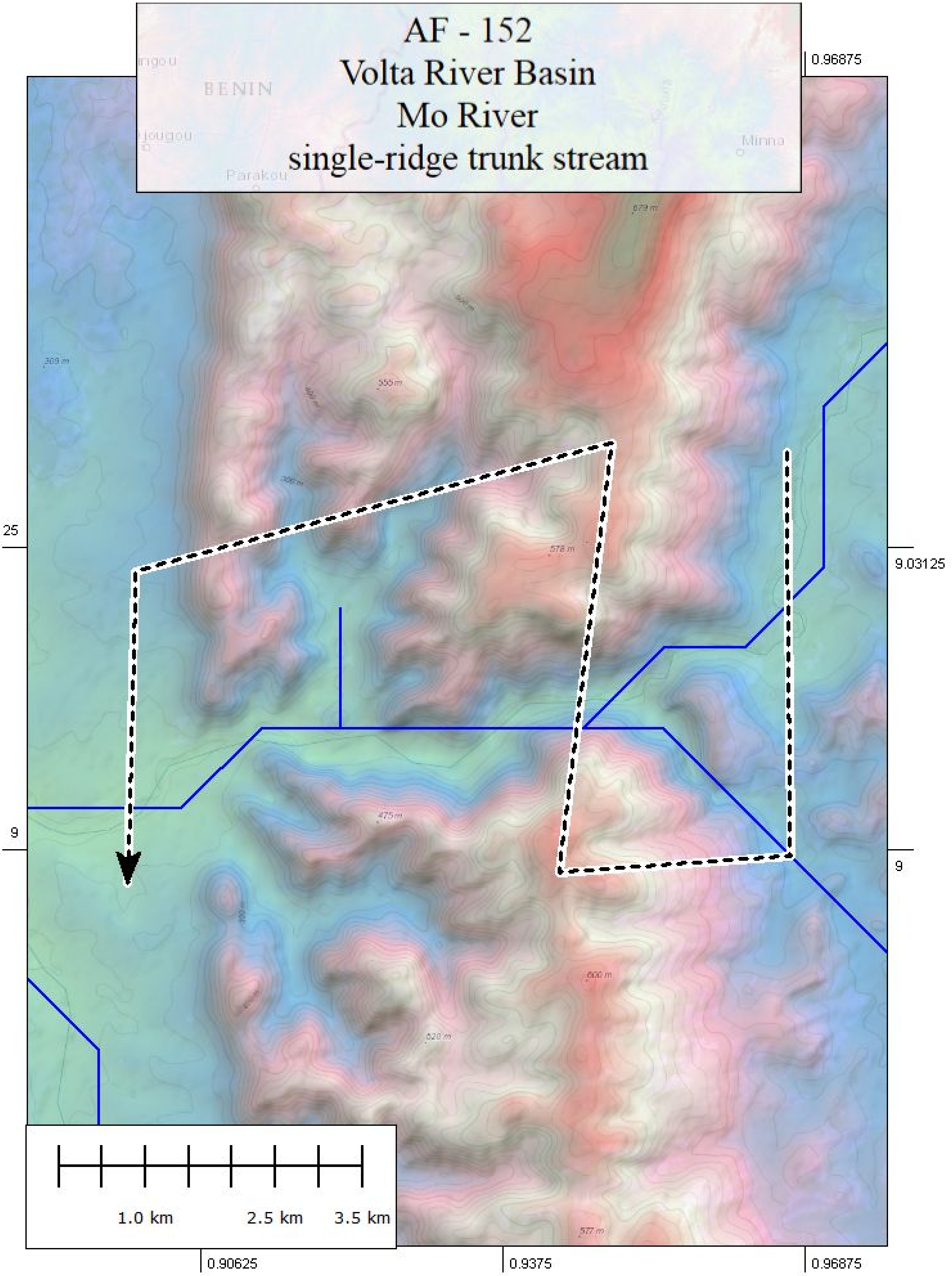
AF - 152
Volta River Basin
Mo River
single-ridge trunk stream
BENIN
Parakou
Minna
9.03125
9.03125
9
9
0.96875
0.90625
0.9375
0.96875
1.0 km
2.5 km
3.5 km
555 m
578 m
475 m
600 m
520 m

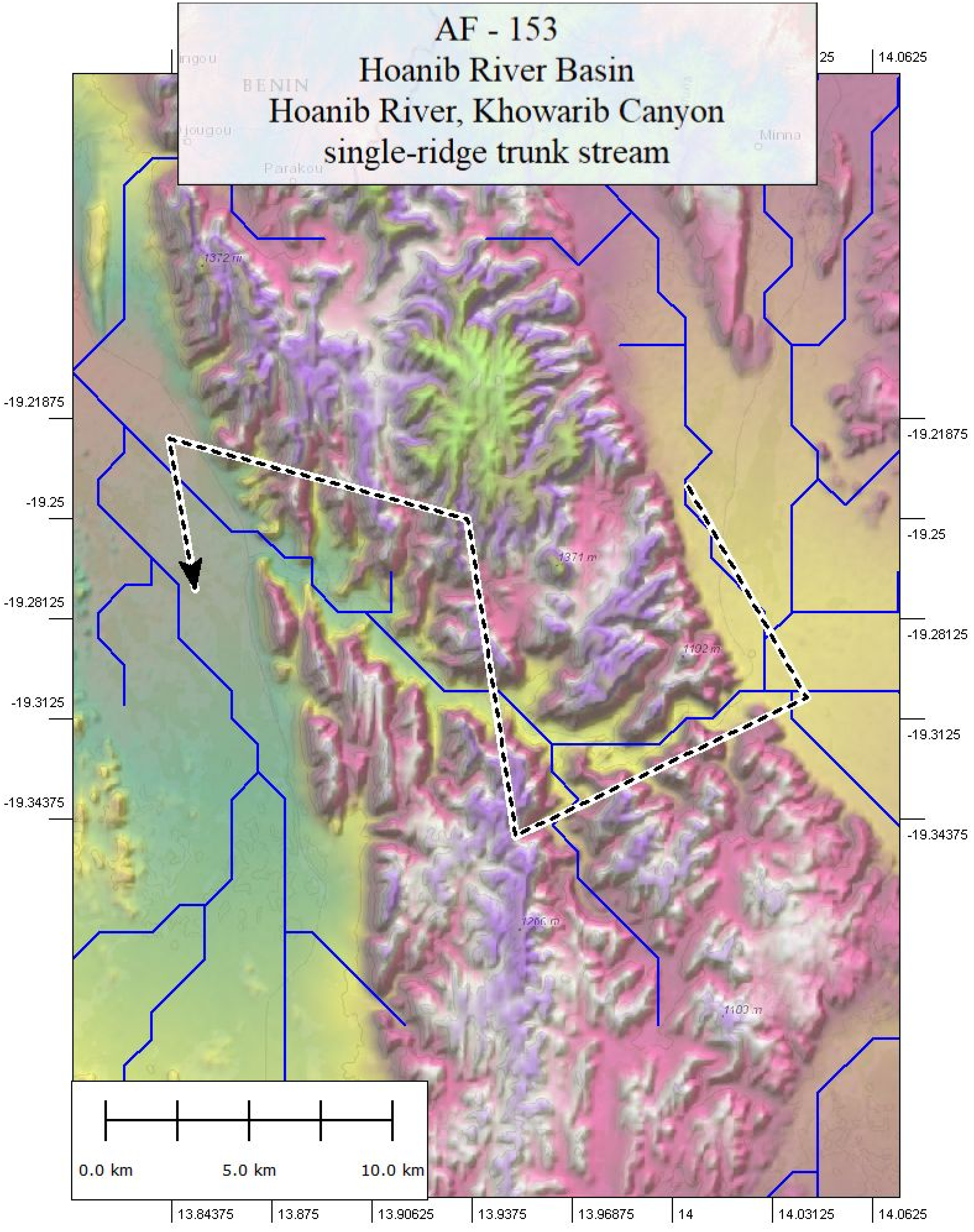
AF - 153
Hoanib River Basin
Hoanib River, Khowarib Canyon
single-ridge trunk stream
BENIN
Parakou
Minna
1372 m
1371 m
1102 m
1266 m
1100 m
-19.21875
-19.25
-19.28125
-19.3125
-19.34375
13.84375
13.875
13.90625
13.9375
13.96875
14
14.03125
14.0625
0.0 km
5.0 km
10.0 km

# AF - 156
# Sonderend River Basin
# Bree RIver
# single-ridge trunk stream

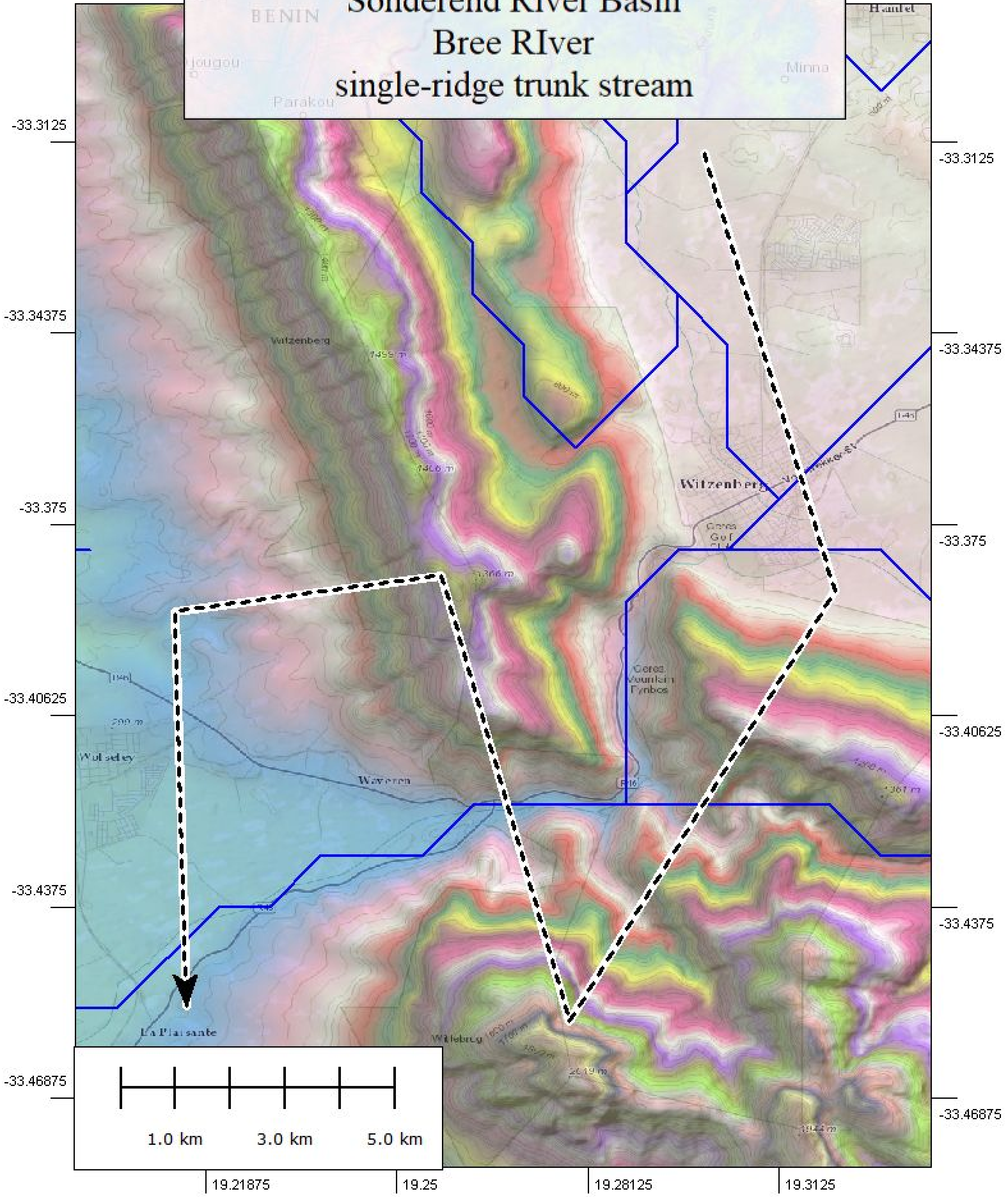

# AF - 159
# Boesmans River Basin
# Boesmans River
# single-ridge trunk stream

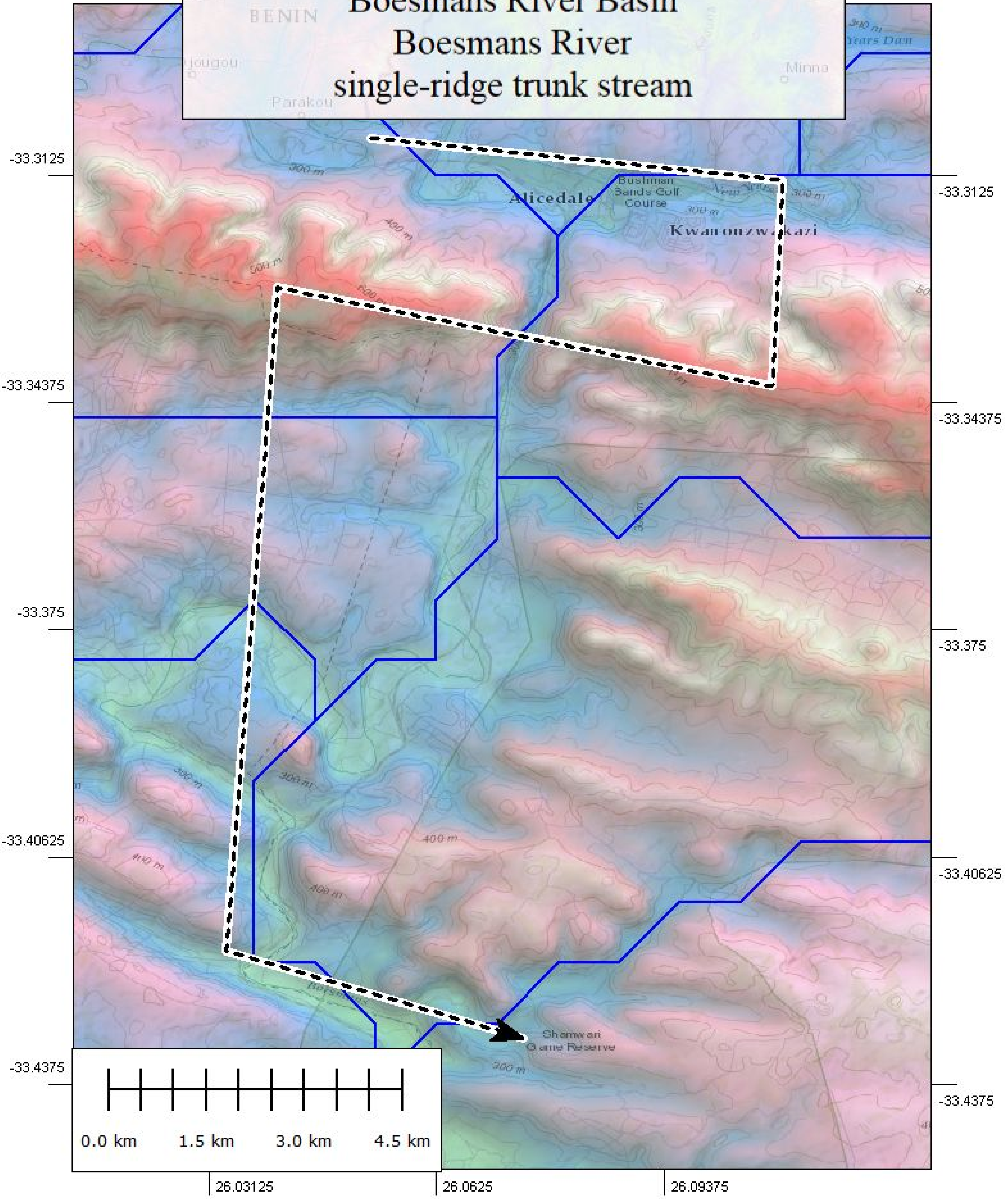

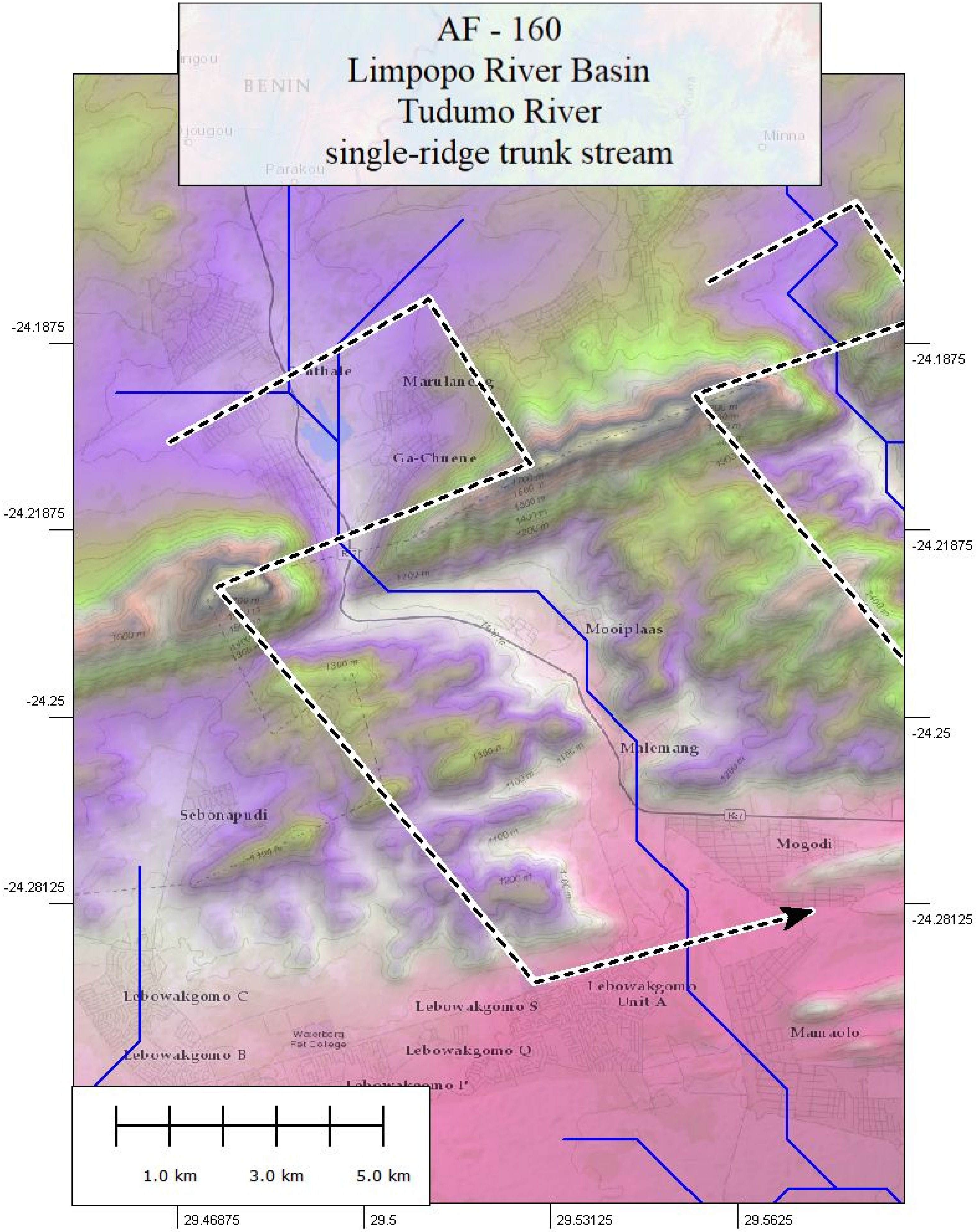
AF - 160
Limpopo River Basin
Tudumo River
single-ridge trunk stream
BENIN
Parakou
Minna
Marulaneng
Ga-Chuene
Mooiplaas
Mphahlele
Mogodi
Sebonapudi
Lebowakgomo C
Lebowakgomo B
Lebowakgomo S
Lebowakgomo Q
Lebowakgomo Unit A
Waterberg Fet College
Mamaolo
1.0 km
3.0 km
5.0 km
-24.1875
-24.21875
-24.25
-24.28125
29.46875
29.5
29.53125
29.5625

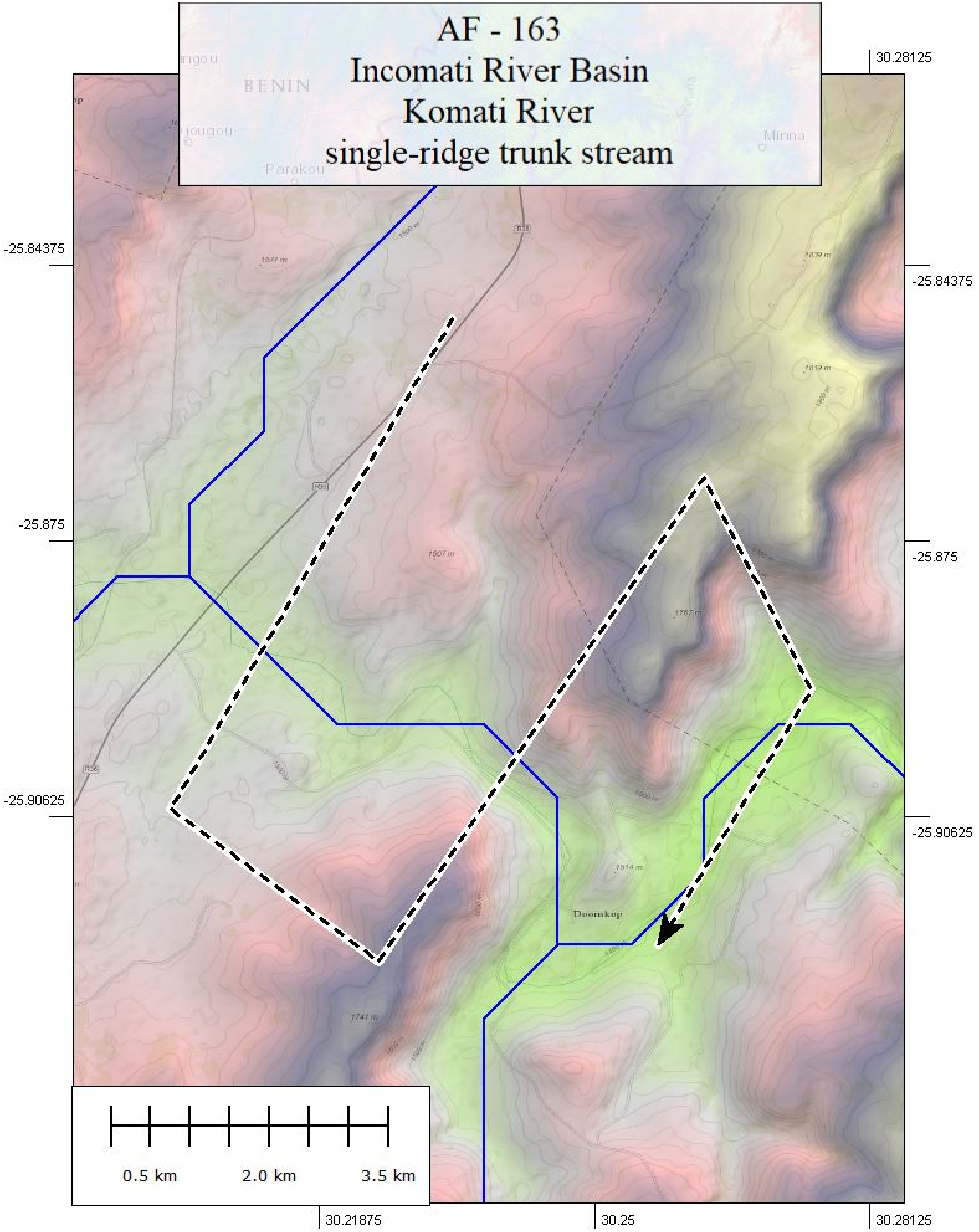
AF - 163
Incomati River Basin
Komati River
single-ridge trunk stream
30.28125
-25.84375
-25.84375
-25.875
-25.875
-25.90625
-25.90625
Doornkop
0.5 km
2.0 km
3.5 km
30.21875
30.25
30.28125

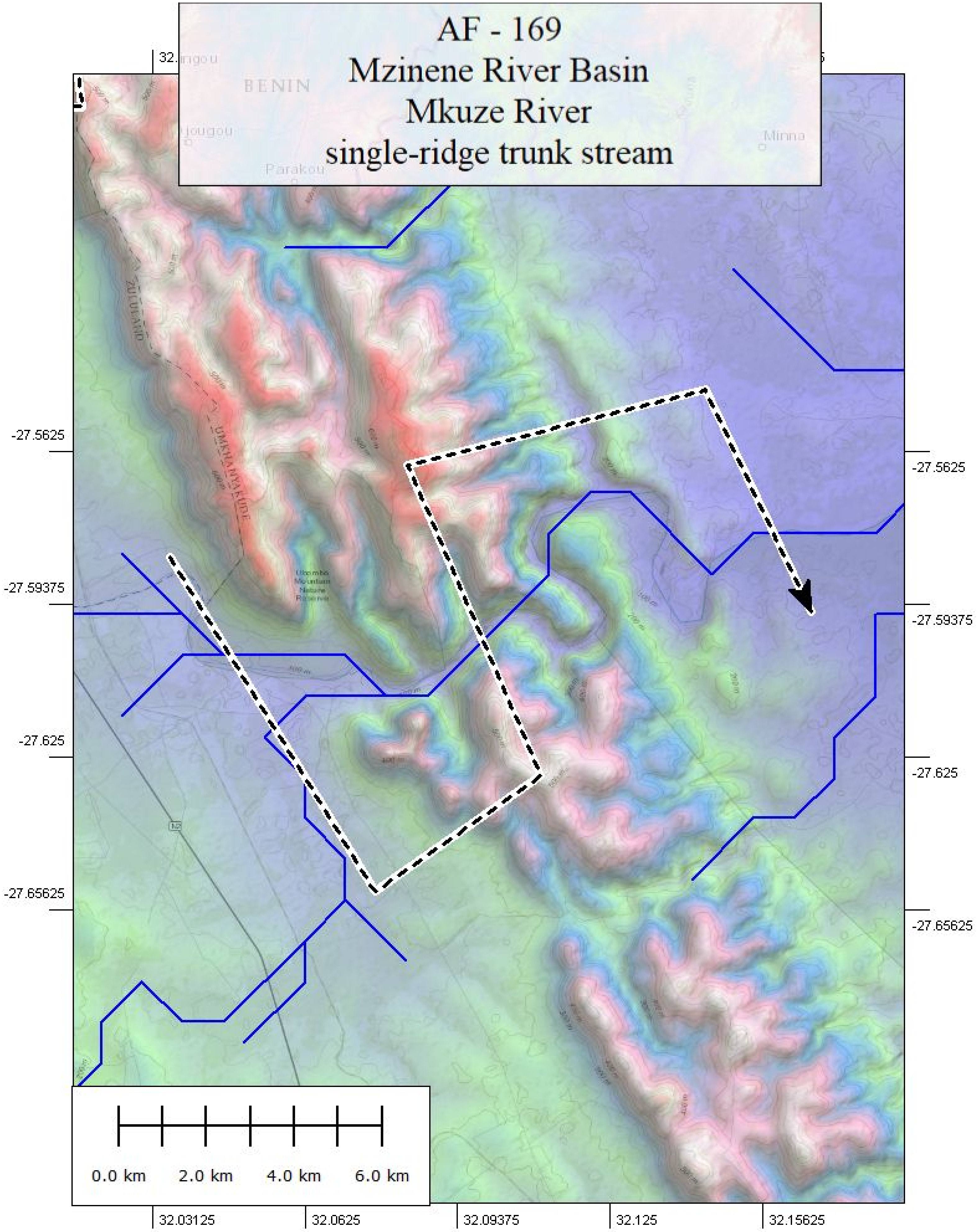
AF - 169
Mzinene River Basin
Mkuze River
single-ridge trunk stream
-27.5625
-27.59375
-27.625
-27.65625
0.0 km
2.0 km
4.0 km
6.0 km
32.03125
32.0625
32.09375
32.125
32.15625

# AF - 170
## Zambezi River Basin
## South Rukuru River
## single-ridge trunk stream

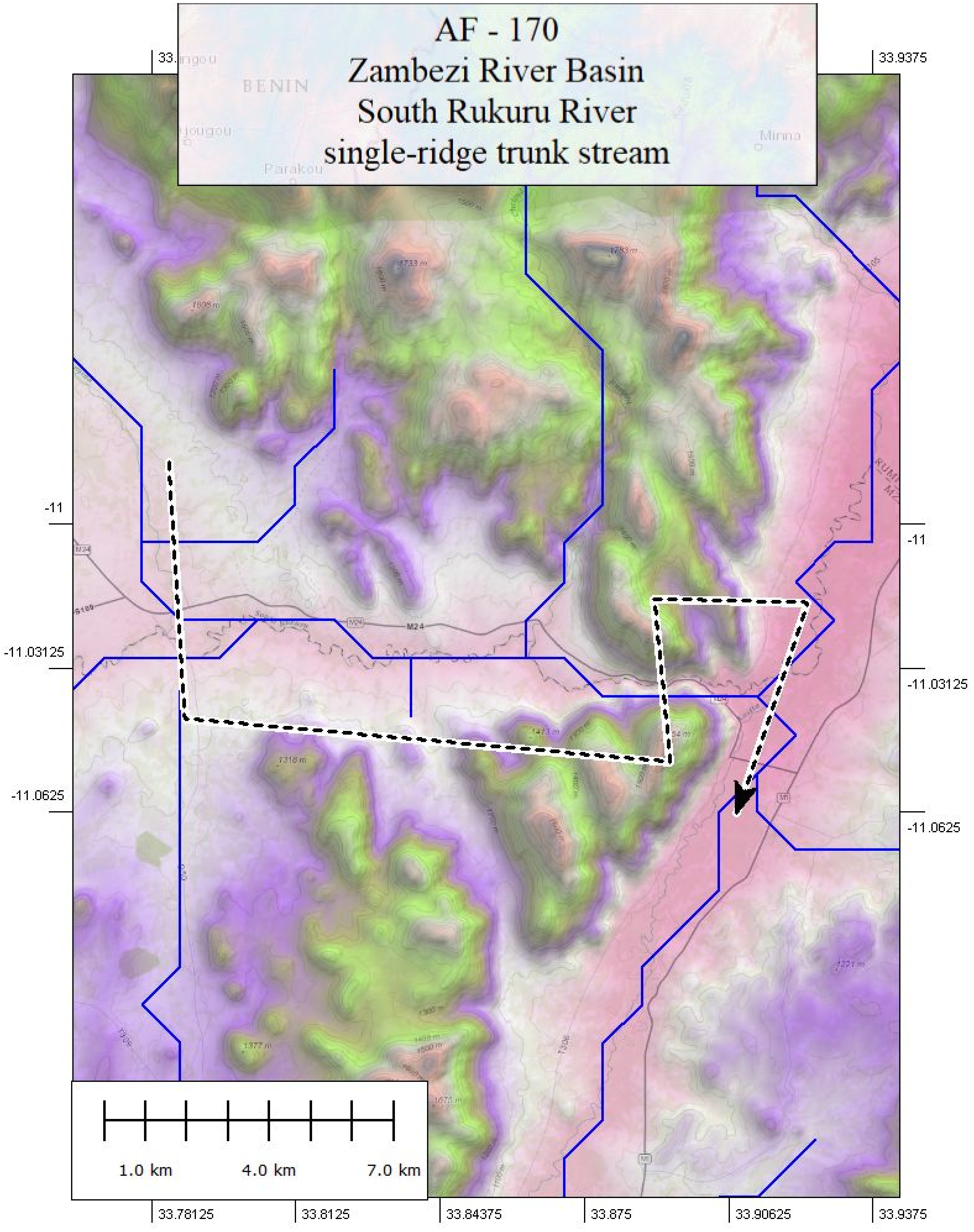

# AF - 172
## Oued Draa Basin
## Foum Akka Ou Tamia (pass)
## single-ridge trunk stream

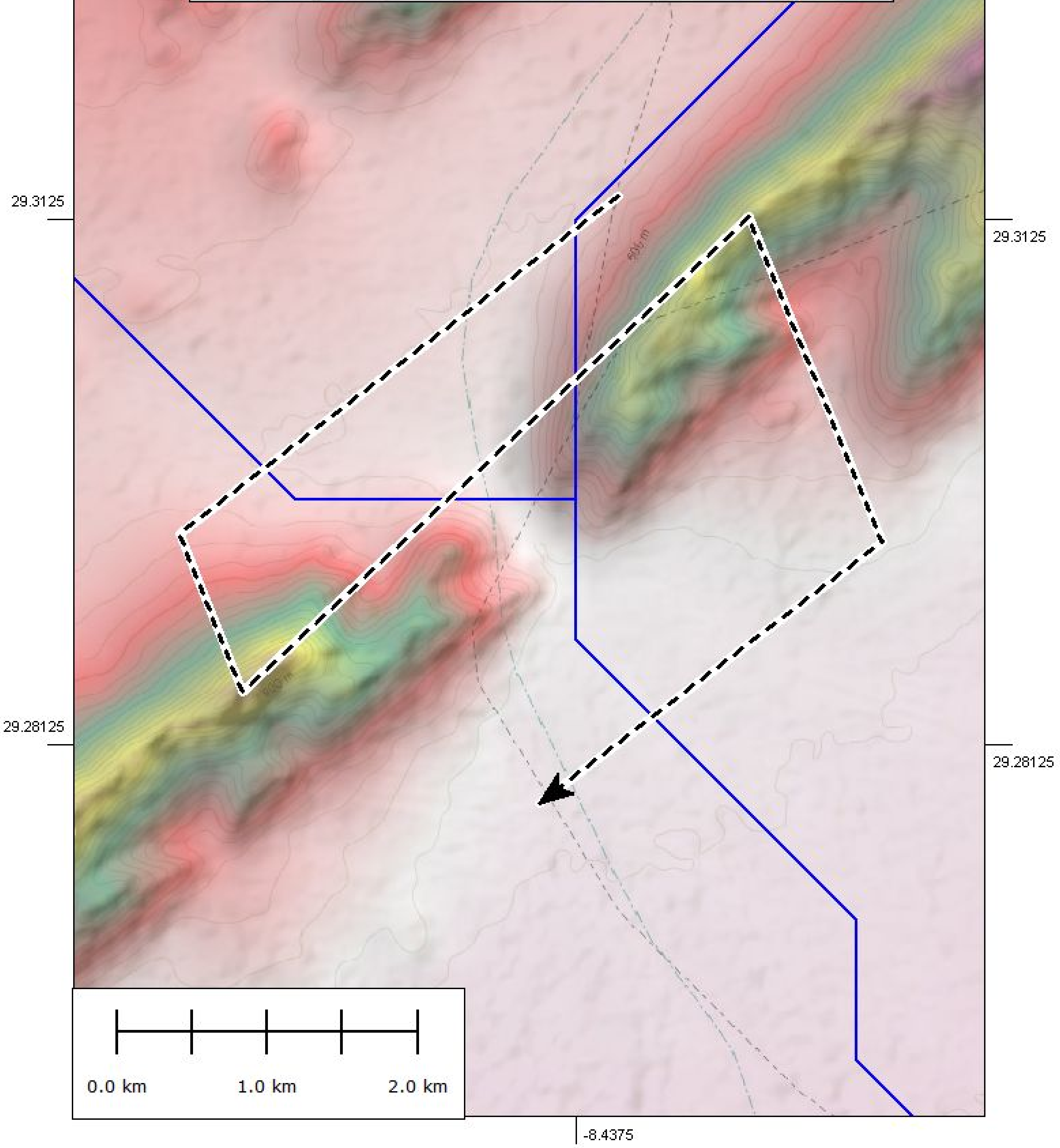

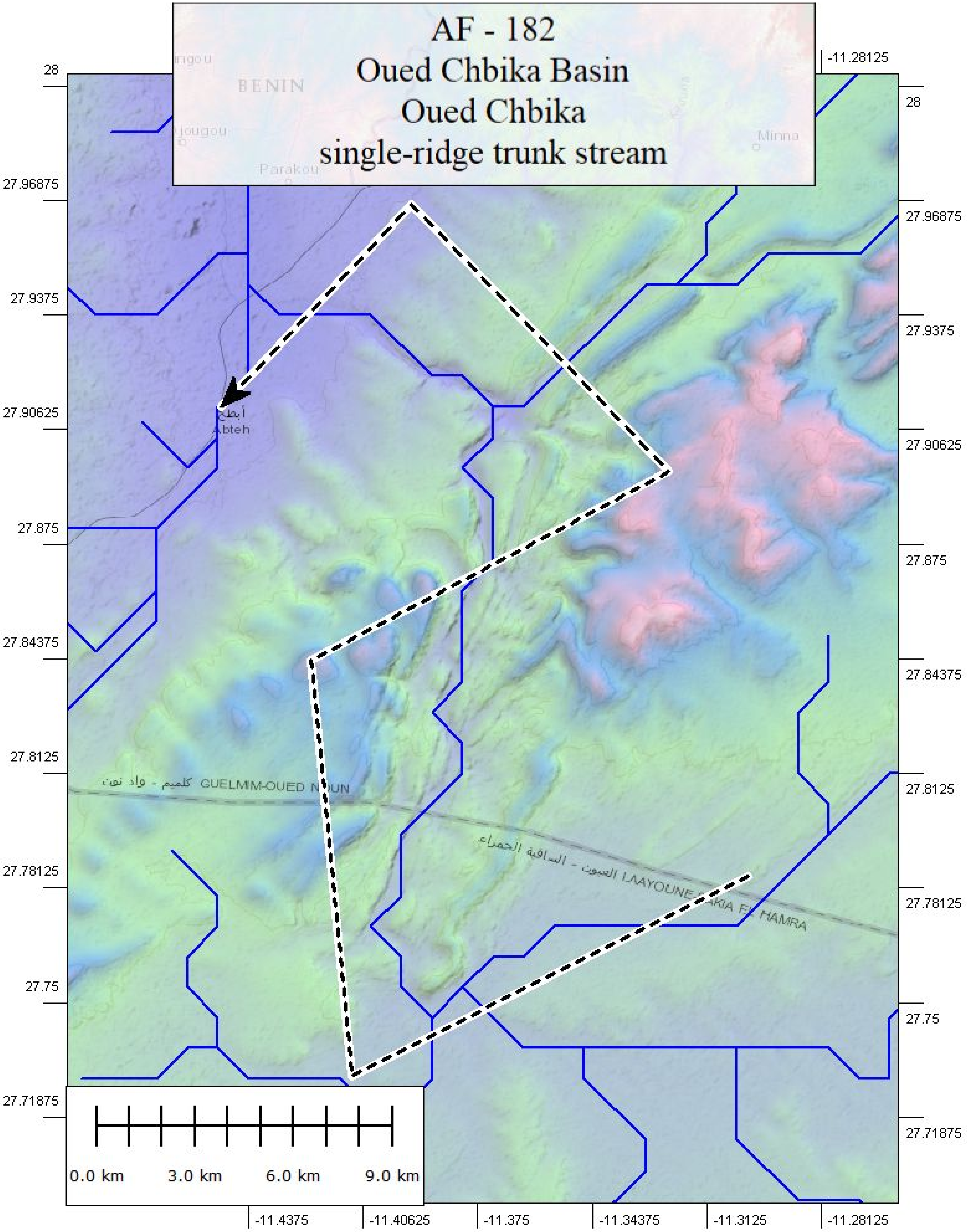

AF - 182
Oued Chbika Basin
Oued Chbika
single-ridge trunk stream
Abteh
أبطح
GUELMIM-OUED NOUN
كلميم - واد نون
LAAYOUNE-SAKIA EL HAMRA
العيون - الساقية الحمراء
BENIN
Djougou
Parakou
Minna
28
27.96875
27.9375
27.90625
27.875
27.84375
27.8125
27.78125
27.75
27.71875
-11.4375
-11.40625
-11.375
-11.34375
-11.3125
-11.28125
0.0 km
3.0 km
6.0 km
9.0 km

# AF - 184
## Oued Massa Basin
## Oued Massa
## single-ridge trunk stream

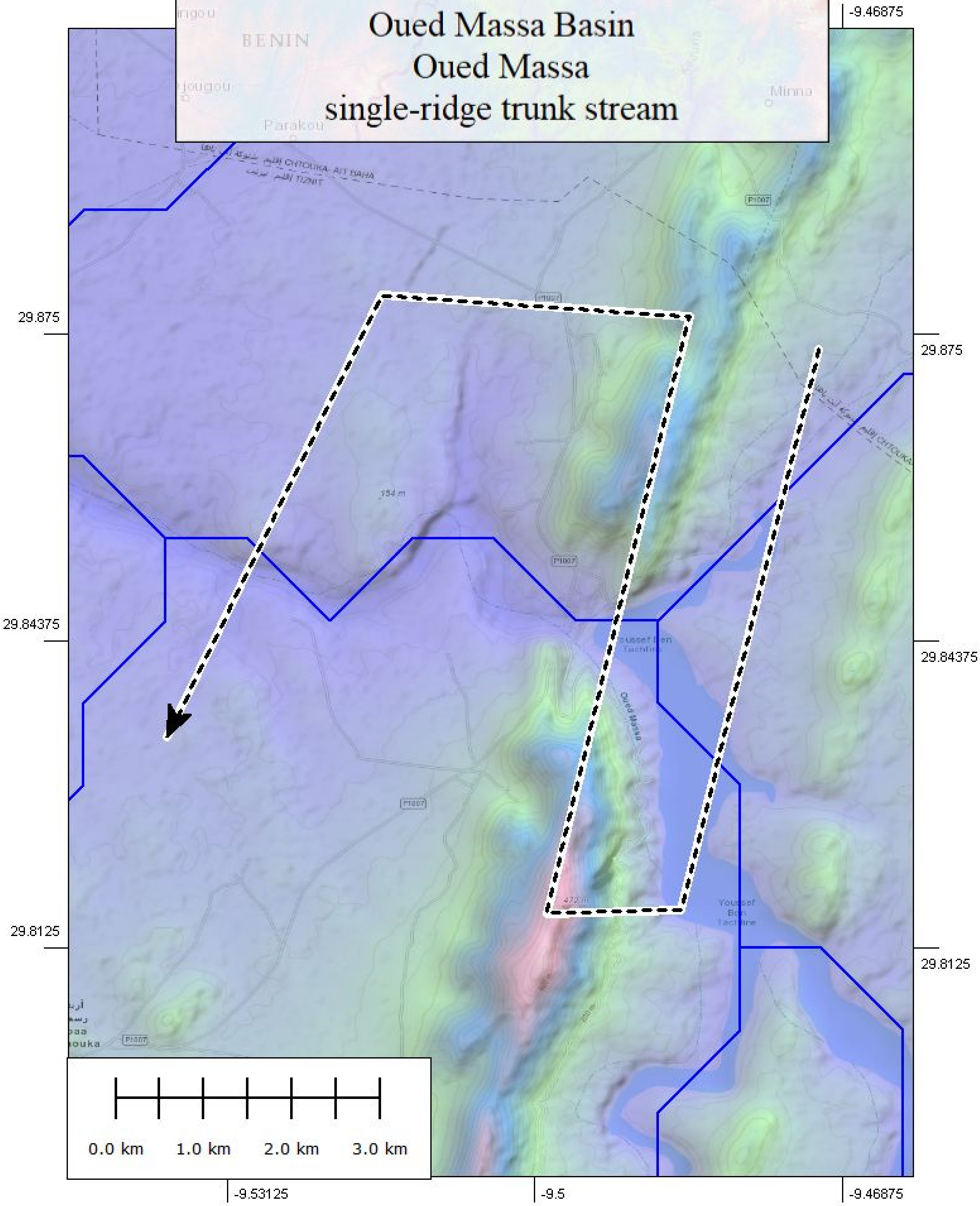

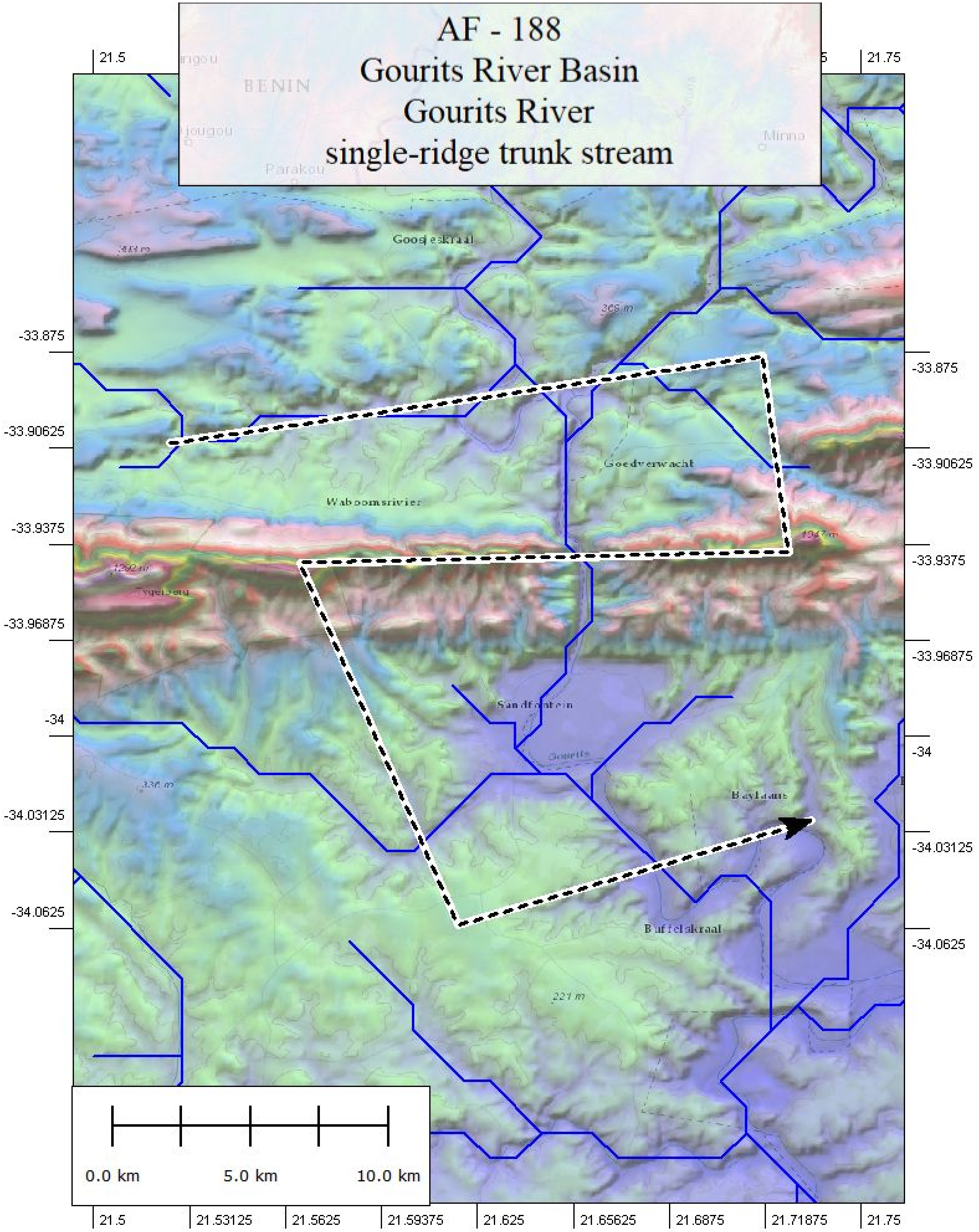
AF - 188
Gourits River Basin
Gourits River
single-ridge trunk stream
21.5
21.75
21.53125
21.5625
21.59375
21.625
21.65625
21.6875
21.71875
-33.875
-33.90625
-33.9375
-33.96875
-34
-34.03125
-34.0625
Goosjeskraal
Goedverwacht
Waboomsrivier
Sandfontein
Gourits
Buffelskraal
221 m
0.0 km
5.0 km
10.0 km

# AF - 192
## Limpopo River Basin
## Nzhelele River
## single-ridge trunk stream

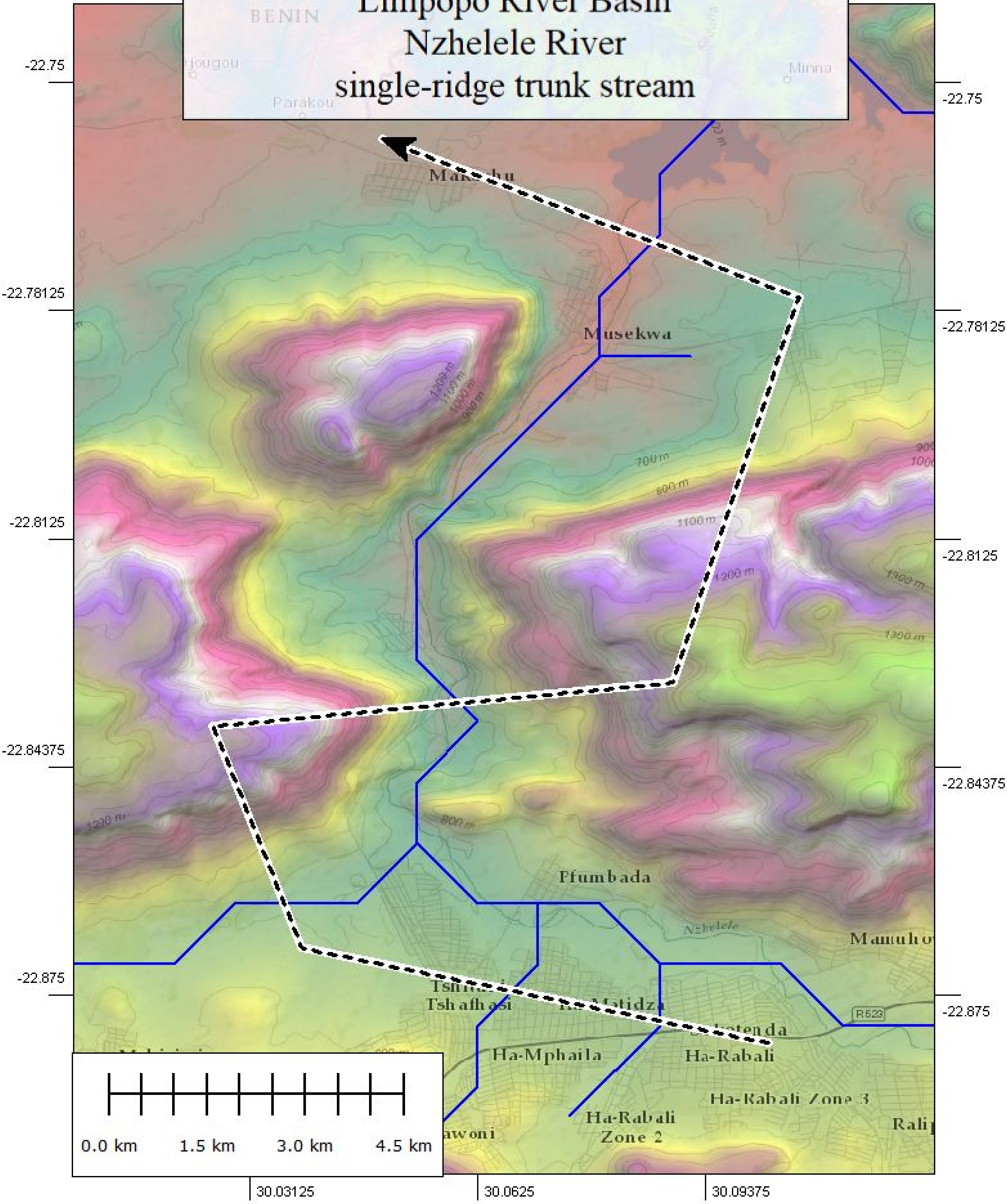

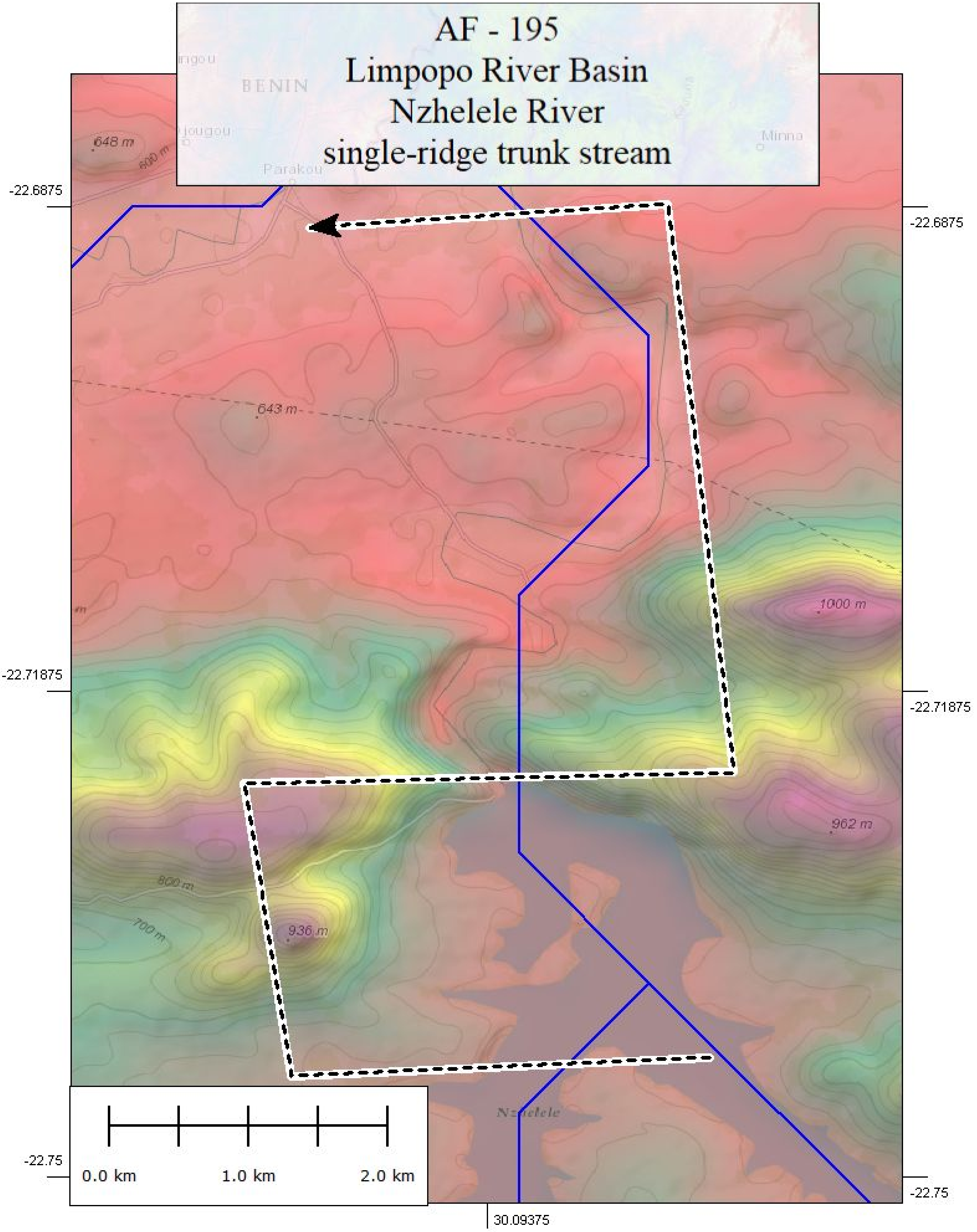
AF - 195
Limpopo River Basin
Nzhelele River
single-ridge trunk stream
BENIN
Parakou
Minna
648 m
600 m
643 m
1000 m
962 m
800 m
700 m
936 m
Nzhelele
-22.6875
-22.71875
-22.75
30.09375
0.0 km
1.0 km
2.0 km

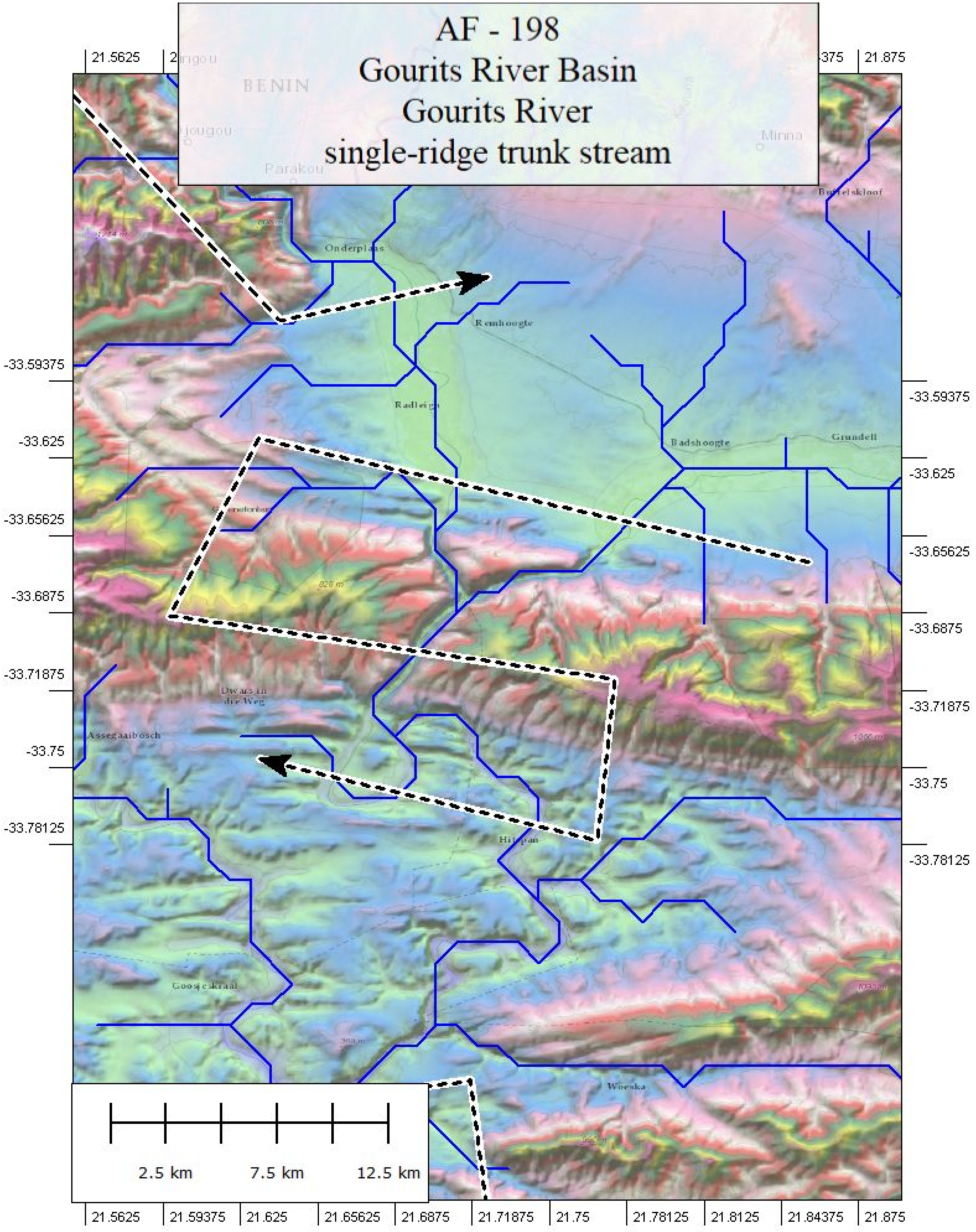
AF - 198
Gourits River Basin
Gourits River
single-ridge trunk stream
BENIN
Parakou
Minna
Onderplaas
Remhoogte
Radleigh
Badshoogte
Grundell
Buffelskloof
Dwars in die Weg
Assegaaibosch
Goosjeskraal
Woeska
2.5 km
7.5 km
12.5 km
21.5625
21.59375
21.625
21.65625
21.6875
21.71875
21.75
21.78125
21.8125
21.84375
21.875
-33.59375
-33.625
-33.65625
-33.6875
-33.71875
-33.75
-33.78125

# AF - 200
# Cunene River Basin
# Marienfluss
# single-ridge trunk stream

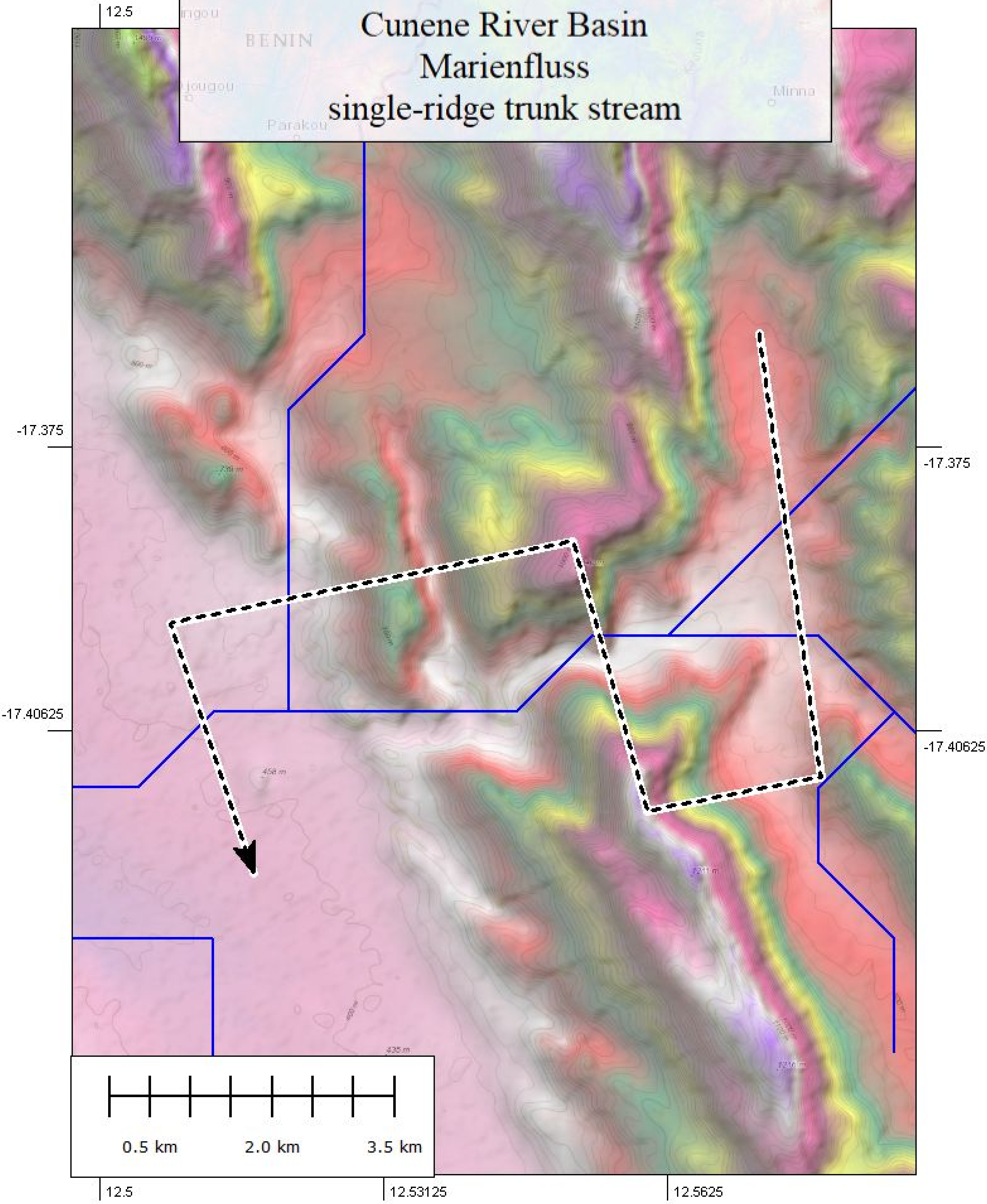

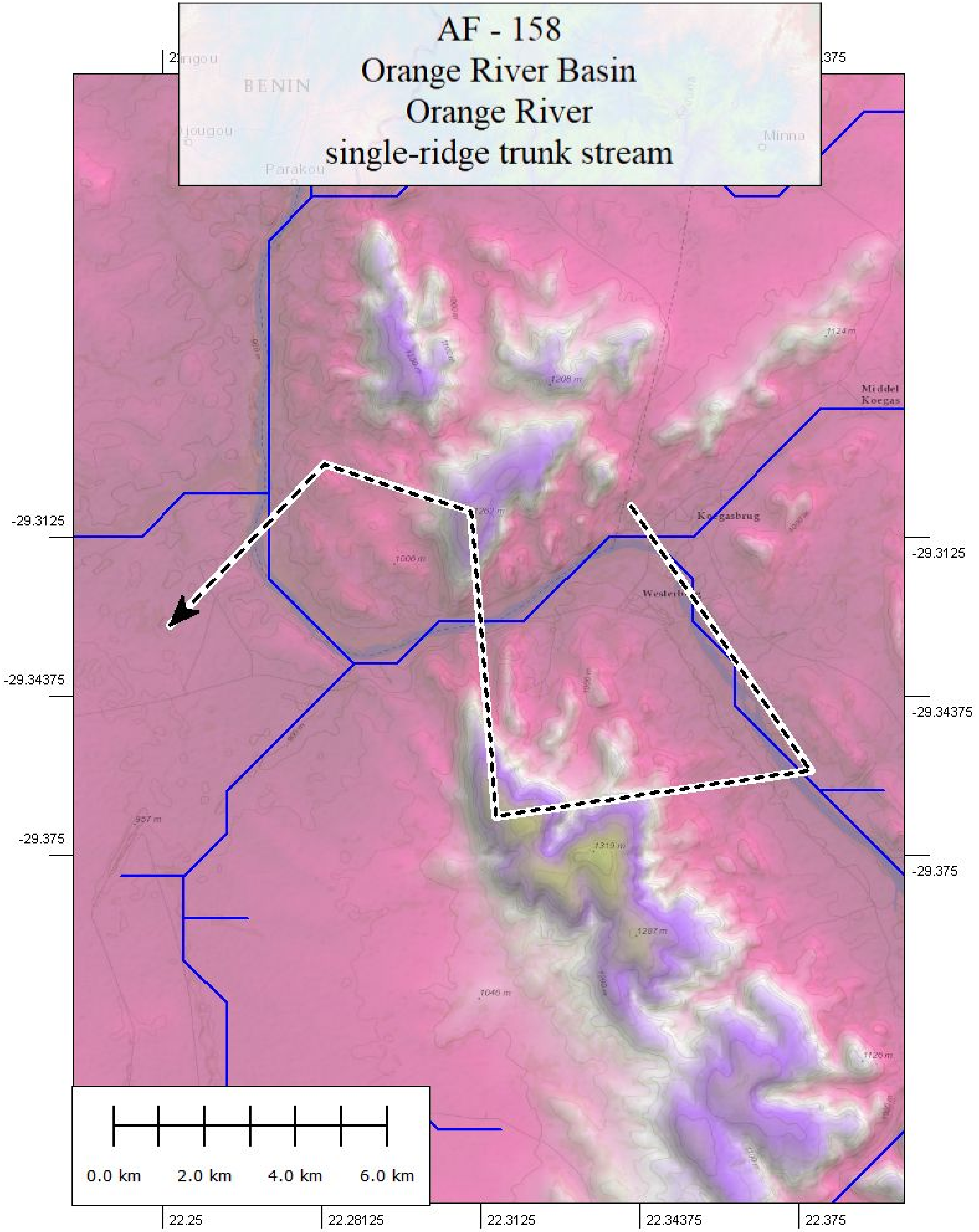

AF - 158
Orange River Basin
Orange River
single-ridge trunk stream
Middel Koegas
Koegasbrug
-29.3125
-29.34375
-29.375
22.25
22.28125
22.3125
22.34375
22.375
0.0 km
2.0 km
4.0 km
6.0 km

# AF - 167
## Nile River Basin
## Kagera River
## single-ridge trunk stream

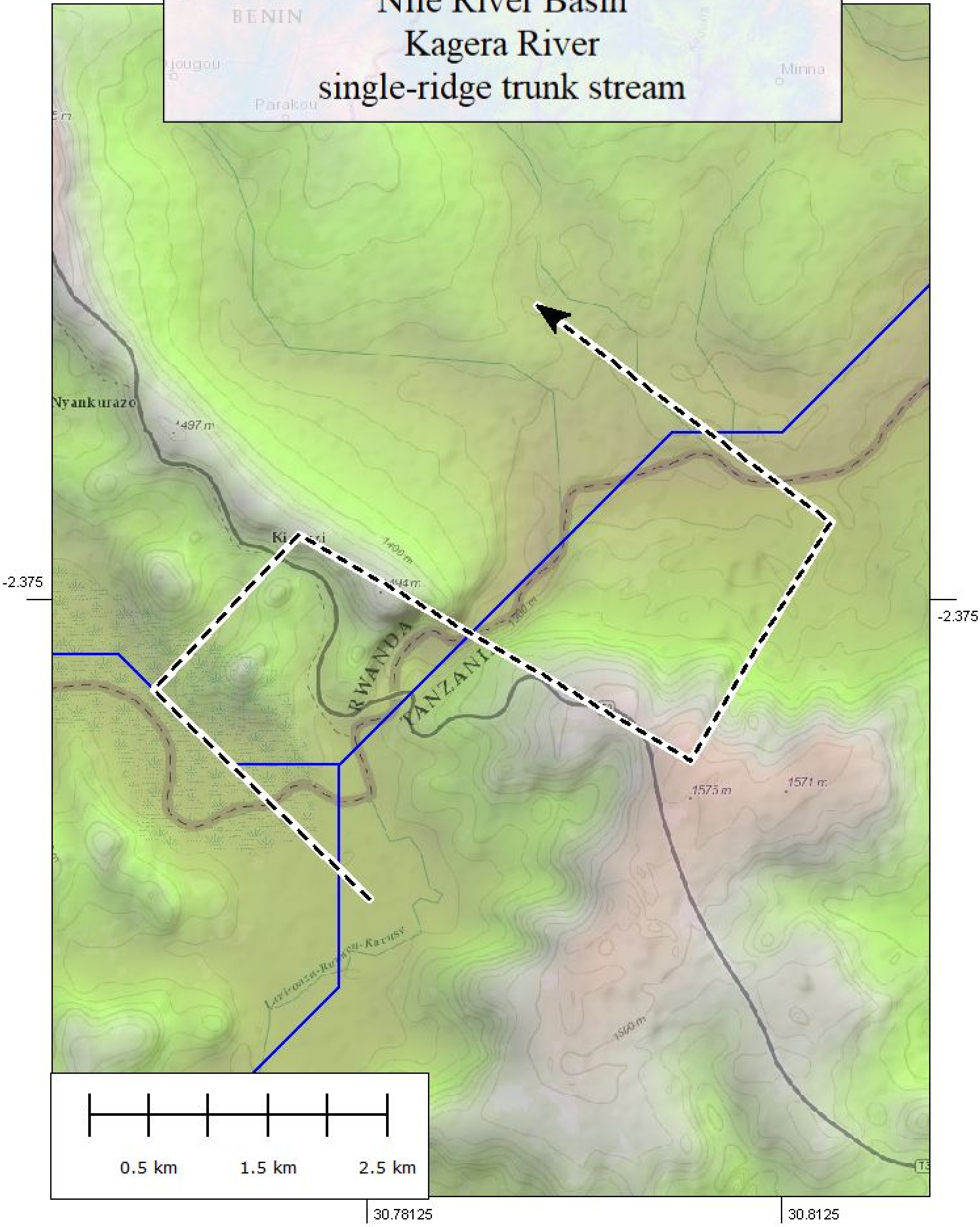

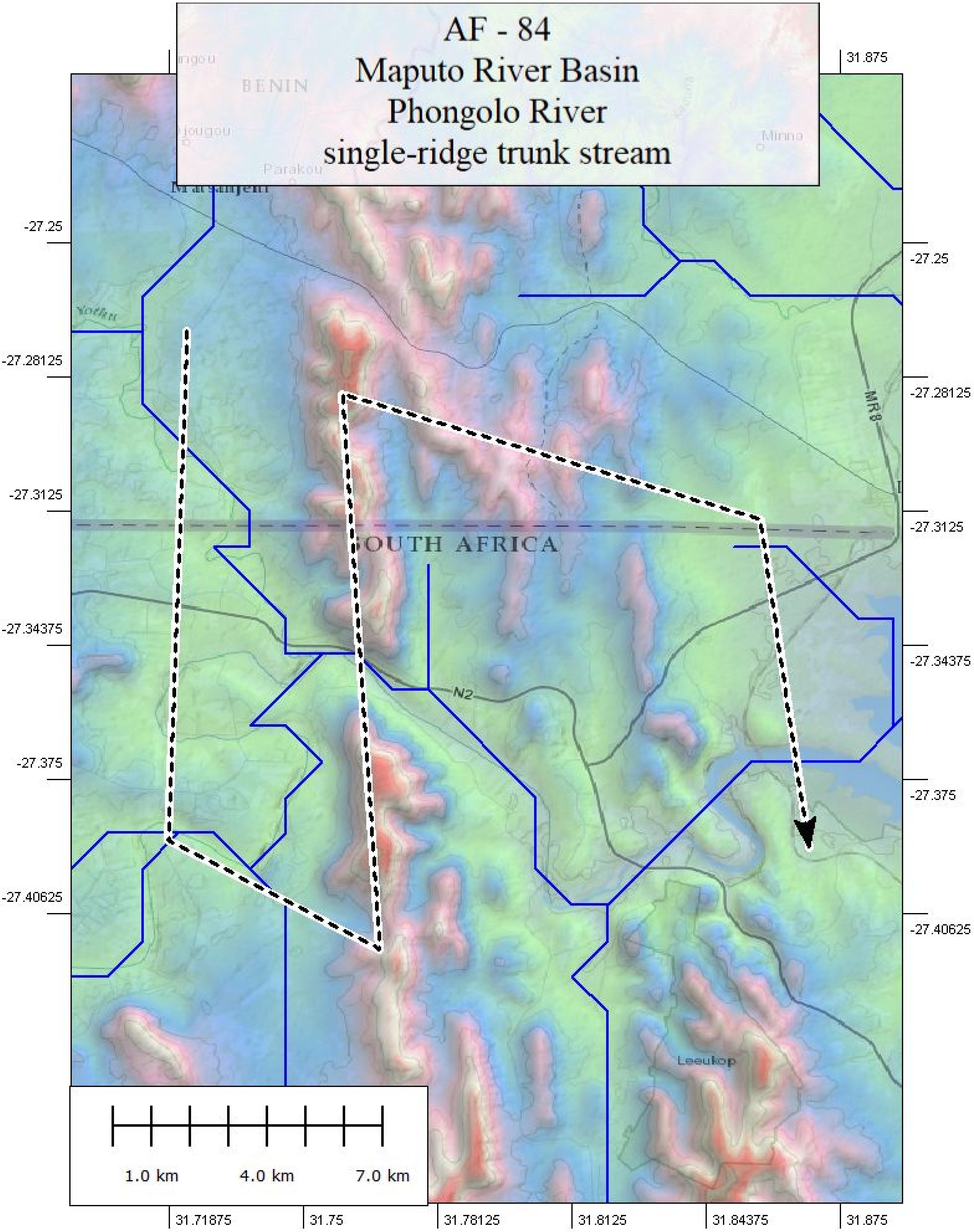
AF - 84
Maputo River Basin
Phongolo River
single-ridge trunk stream
BENIN
Parakou
Minna
Matsanjeni
SOUTH AFRICA
N2
MR8
Leeukop
-27.25
-27.28125
-27.3125
-27.34375
-27.375
-27.40625
31.71875
31.75
31.78125
31.8125
31.84375
31.875
1.0 km
4.0 km
7.0 km

# AF - 87
## Rufiji River Basin
## Great Ruaha River
## single-ridge trunk stream

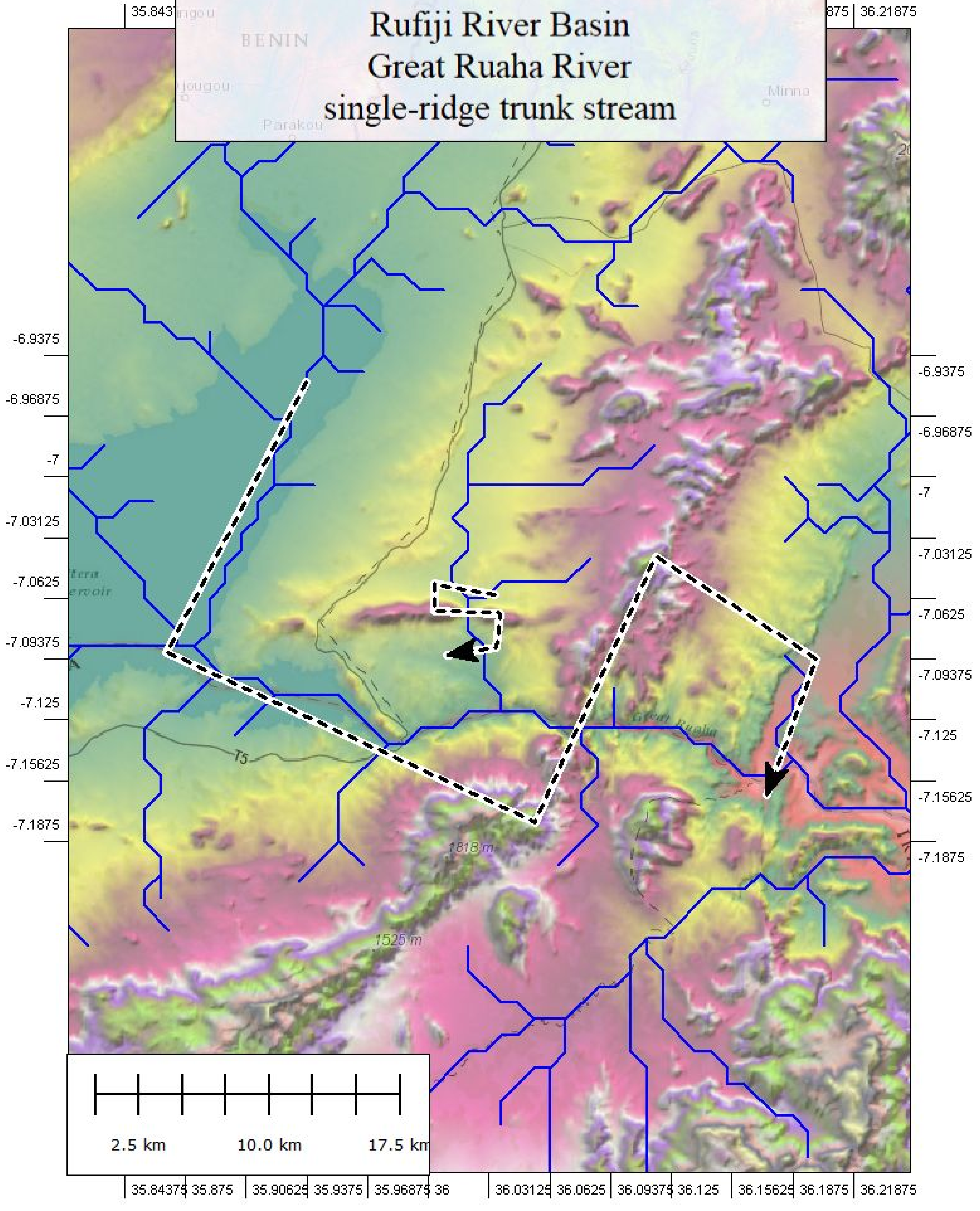

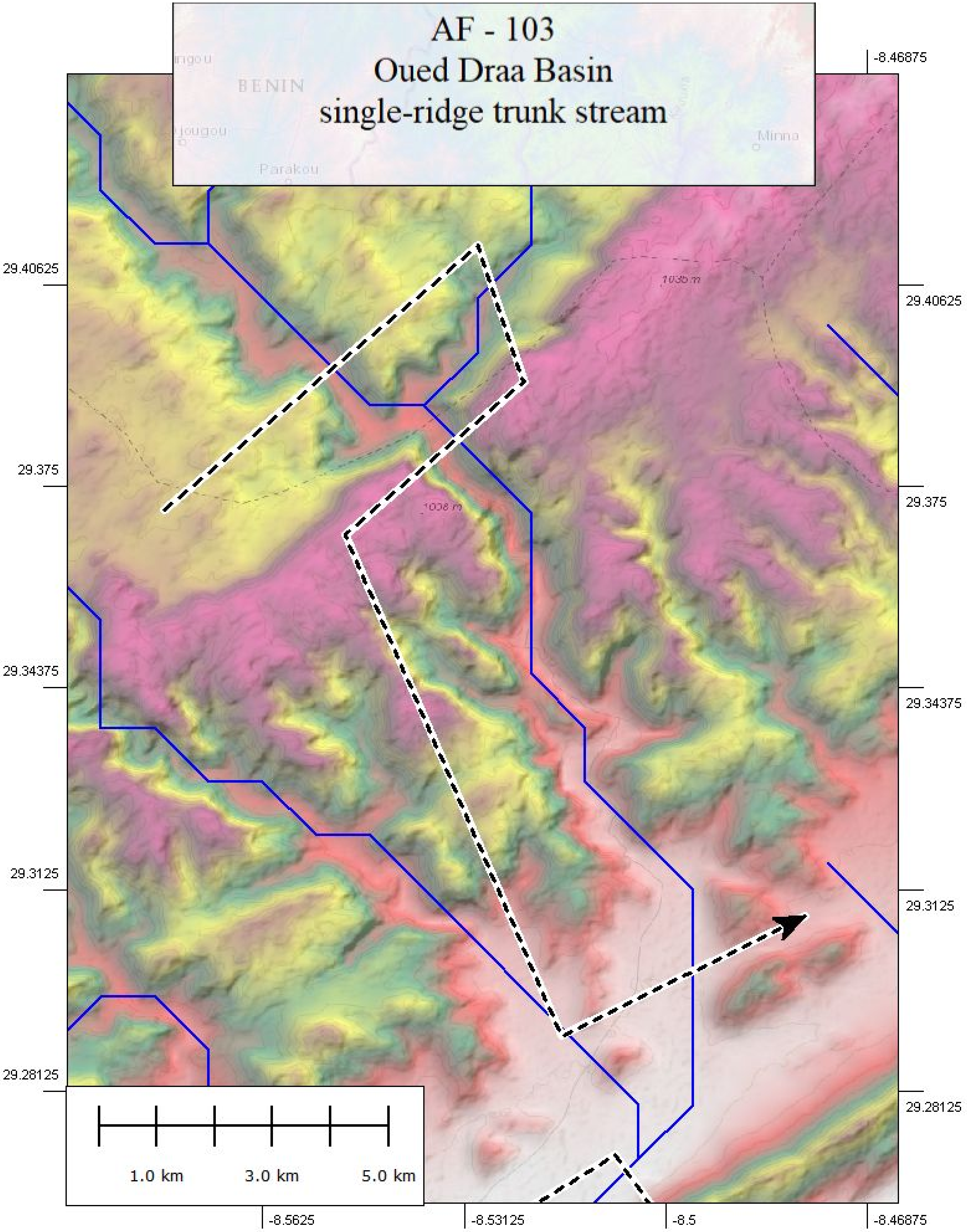

AF - 103
Oued Draa Basin
single-ridge trunk stream
BENIN
Parakou
Minna
1035 m
1008 m
29.40625
29.375
29.34375
29.3125
29.28125
-8.46875
-8.5625
-8.53125
-8.5
1.0 km
3.0 km
5.0 km

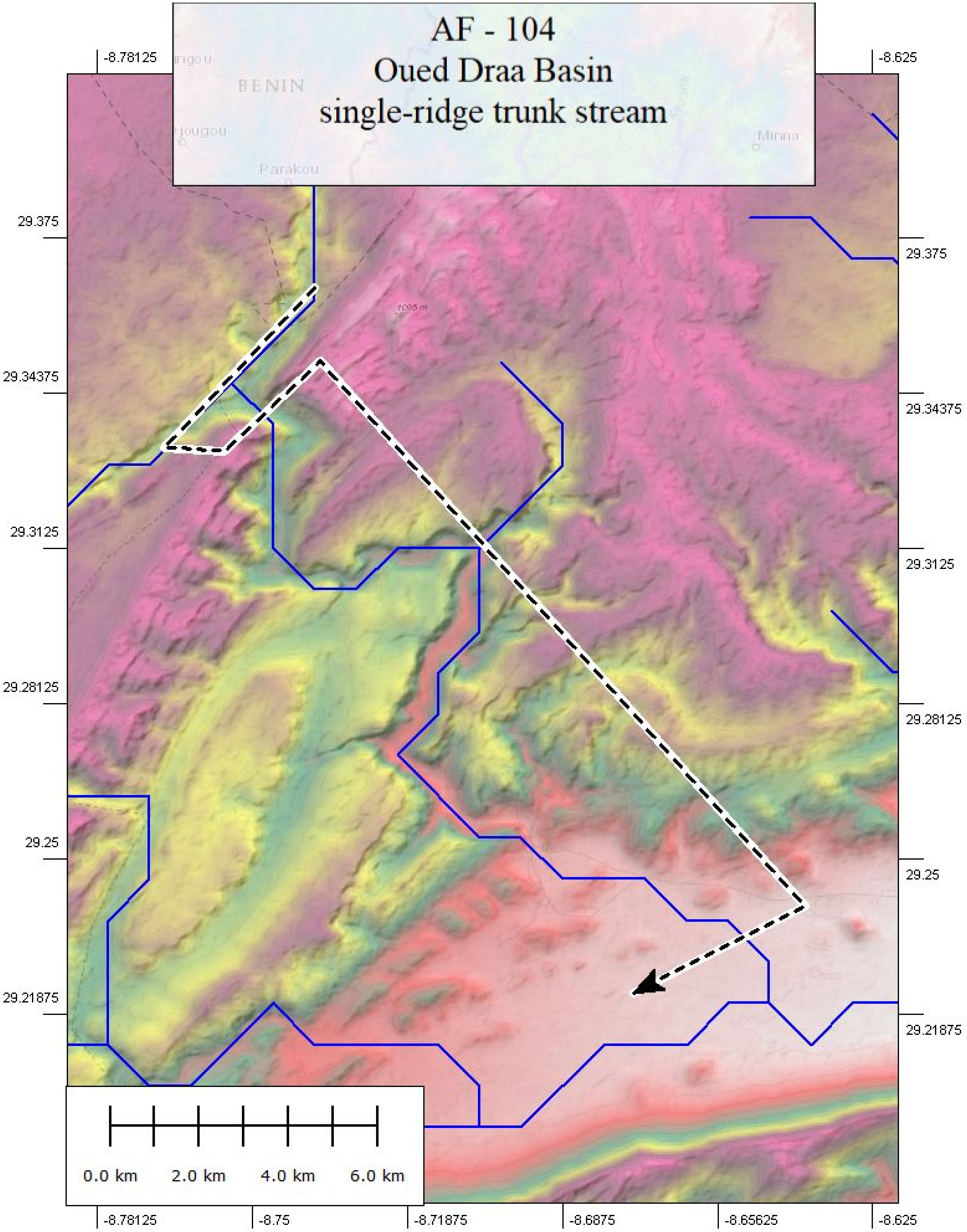

AF - 104
Oued Draa Basin
single-ridge trunk stream
BENIN
Parakou
Minna
-8.78125
-8.75
-8.71875
-8.6875
-8.65625
-8.625
29.375
29.34375
29.3125
29.28125
29.25
29.21875
0.0 km
2.0 km
4.0 km
6.0 km

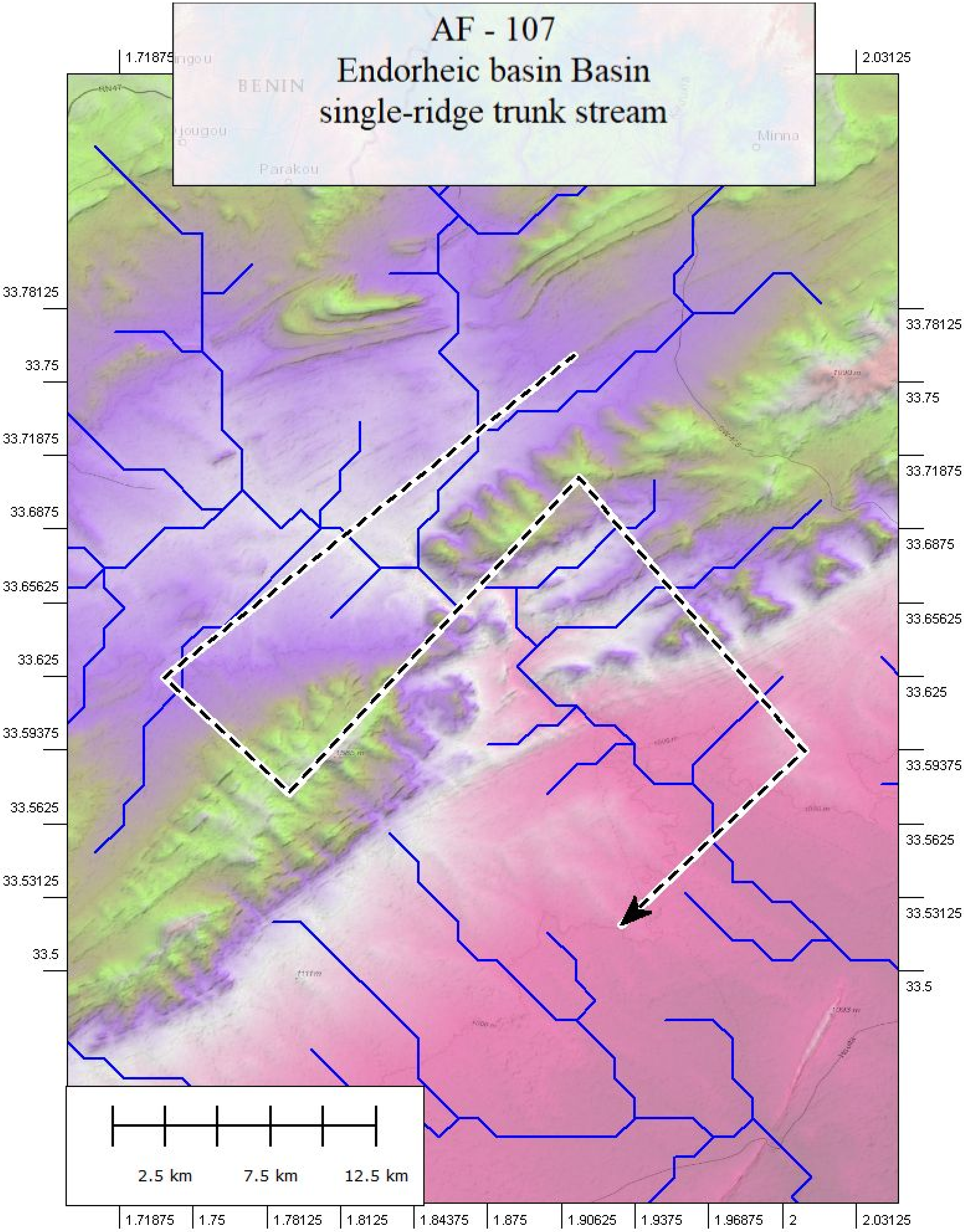
AF - 107
Endorheic basin Basin
single-ridge trunk stream
BENIN
Parakou
Minna
2.5 km
7.5 km
12.5 km
1.71875
1.75
1.78125
1.8125
1.84375
1.875
1.90625
1.9375
1.96875
2
2.03125
33.78125
33.75
33.71875
33.6875
33.65625
33.625
33.59375
33.5625
33.53125
33.5

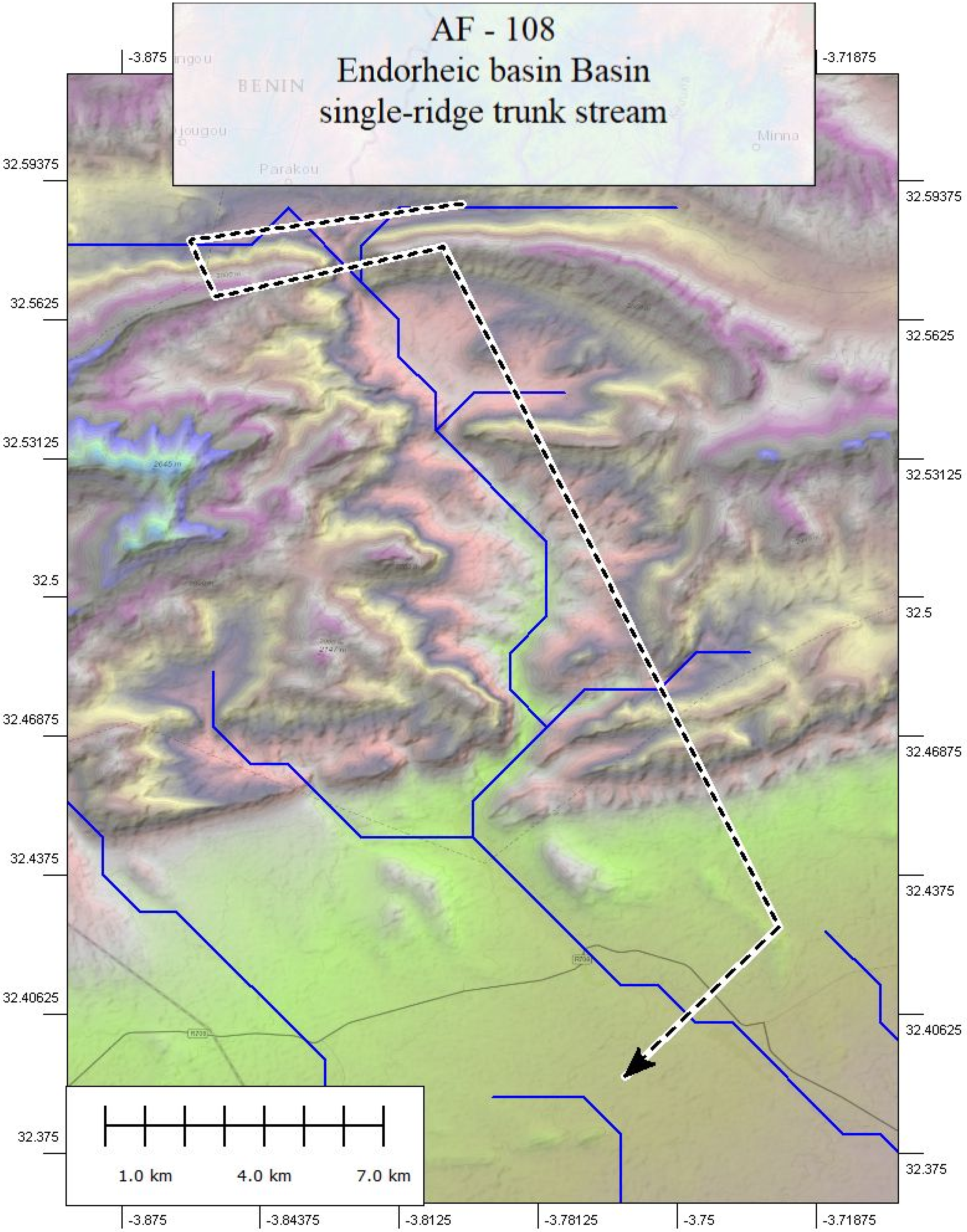
AF - 108
Endorheic basin Basin
single-ridge trunk stream
-3.875
-3.84375
-3.8125
-3.78125
-3.75
-3.71875
32.59375
32.5625
32.53125
32.5
32.46875
32.4375
32.40625
32.375
BENIN
Parakou
Minna
R709
1.0 km
4.0 km
7.0 km

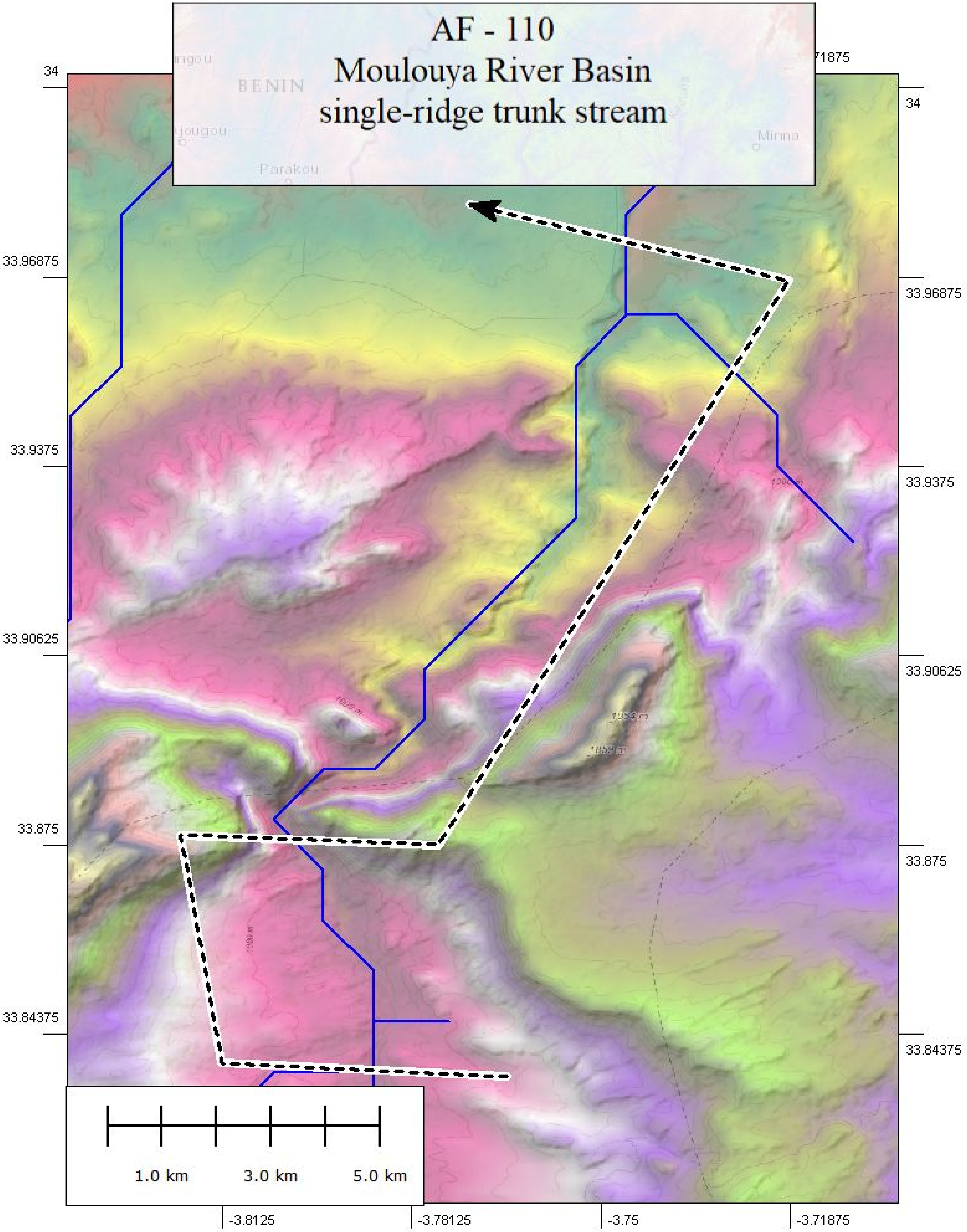
AF - 110
Moulouya River Basin
single-ridge trunk stream
BENIN
Parakou
Minna
34
33.96875
33.9375
33.90625
33.875
33.84375
-3.8125
-3.78125
-3.75
-3.71875
1.0 km
3.0 km
5.0 km

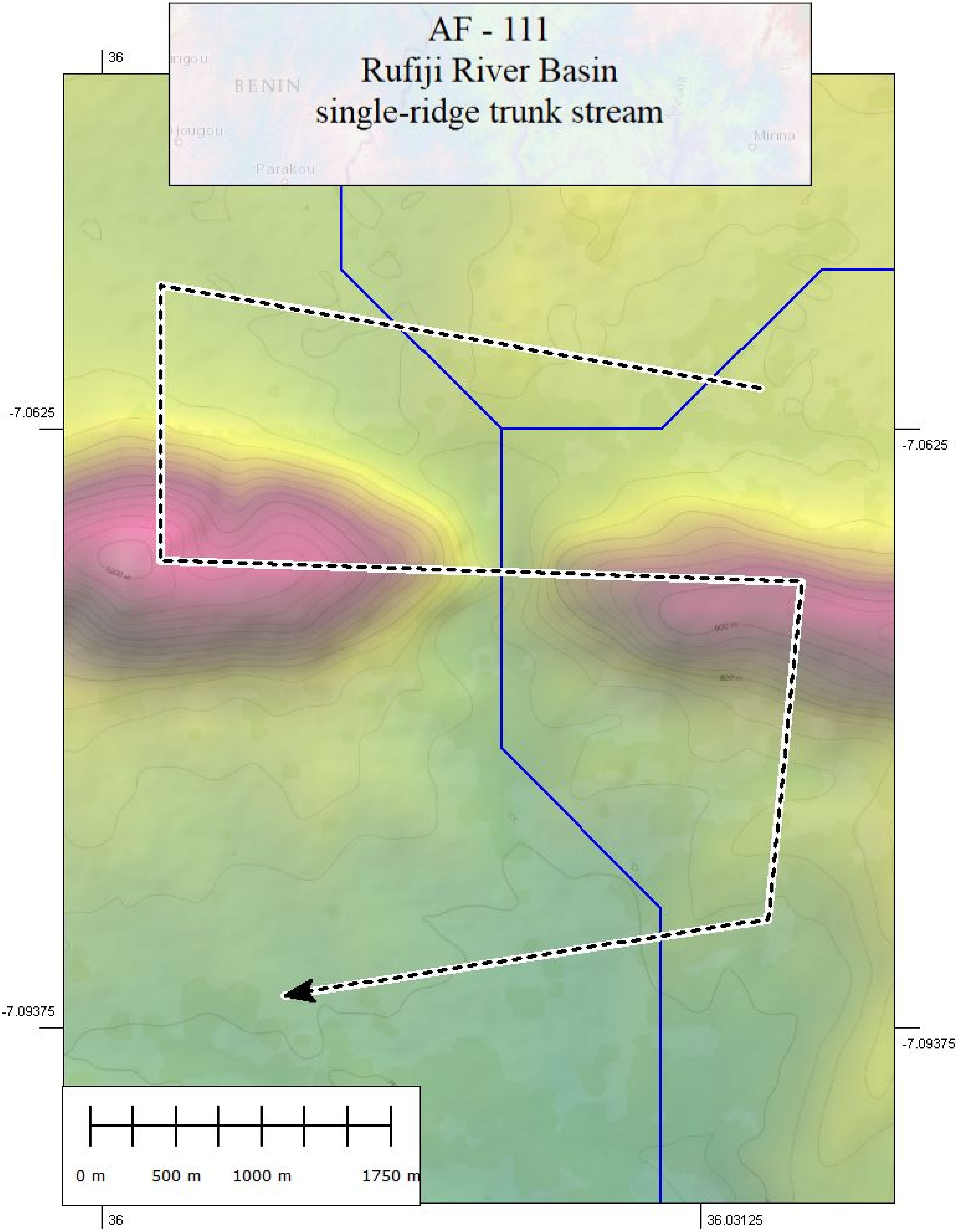
AF - 111
Rufiji River Basin
single-ridge trunk stream
BENIN
Parakou
Minna
36
-7.0625
-7.09375
36.03125
0 m
500 m
1000 m
1750 m

# AF - 119
## West of Gebel Elba Basin
## single-ridge trunk stream

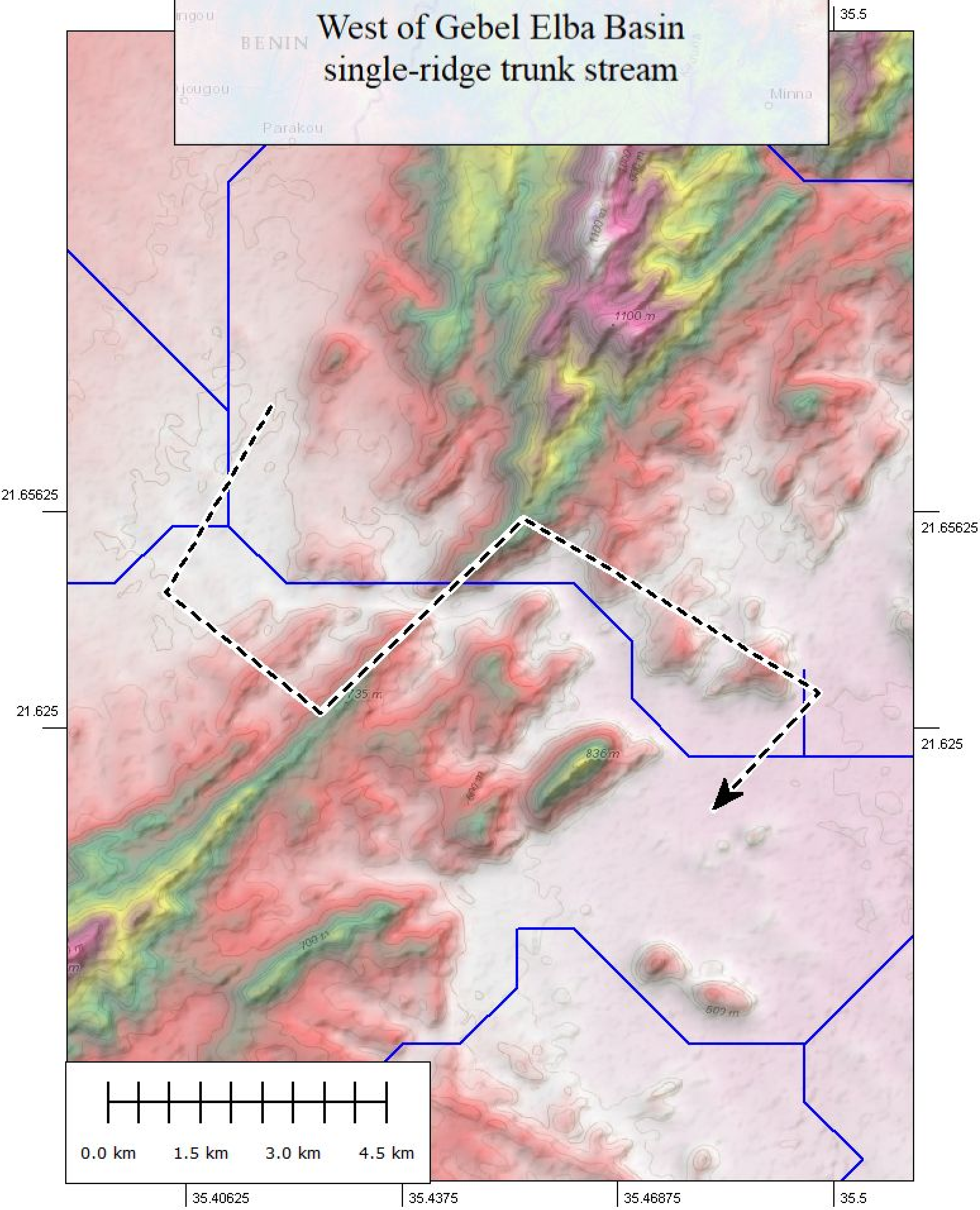

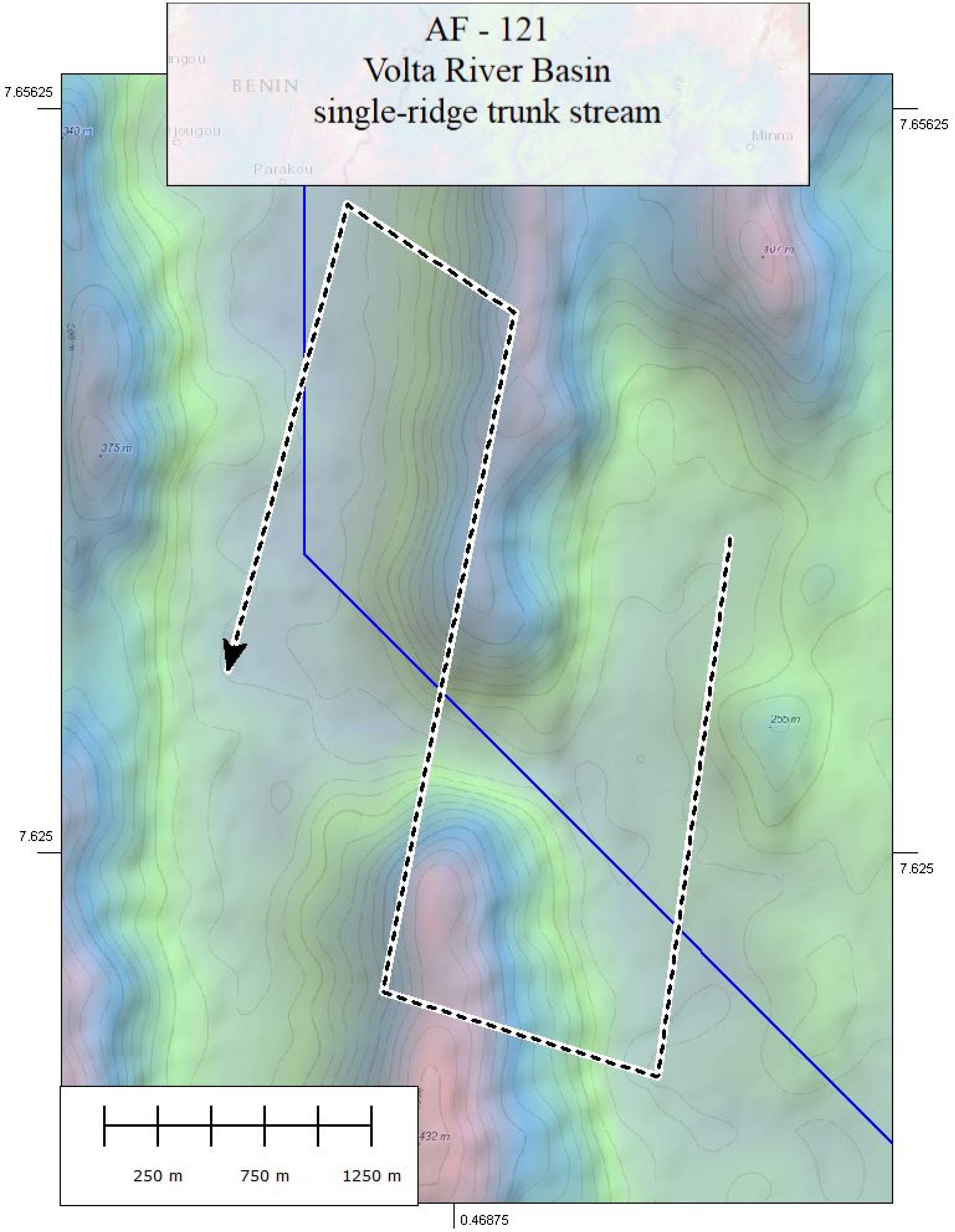
AF - 121
Volta River Basin
single-ridge trunk stream
7.65625
7.65625
BENIN
Parakou
Minna
375 m
255 m
432 m
7.625
7.625
250 m
750 m
1250 m
0.46875

# AF - 13
## Gamtoos River Basin
## single-ridge head stream

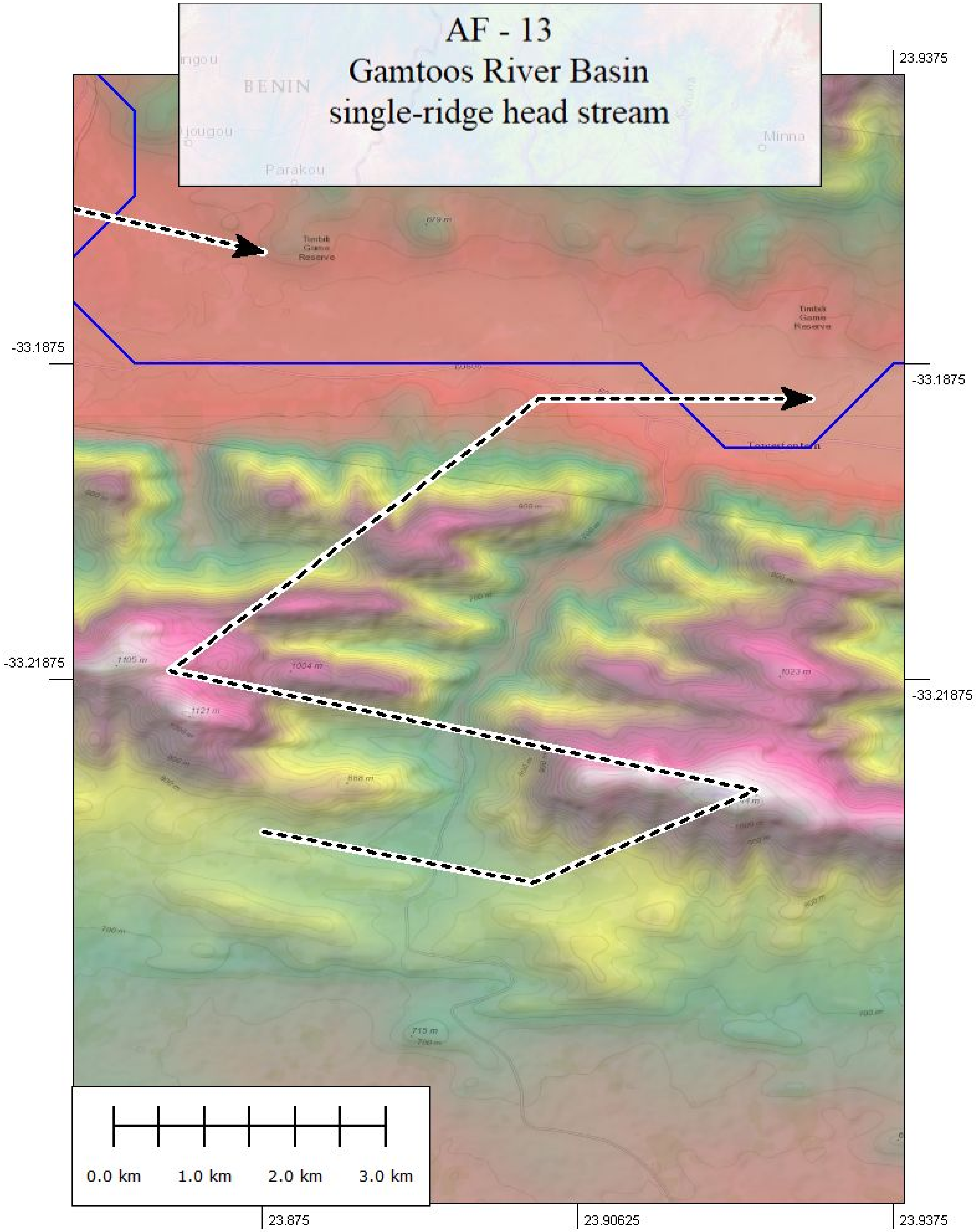

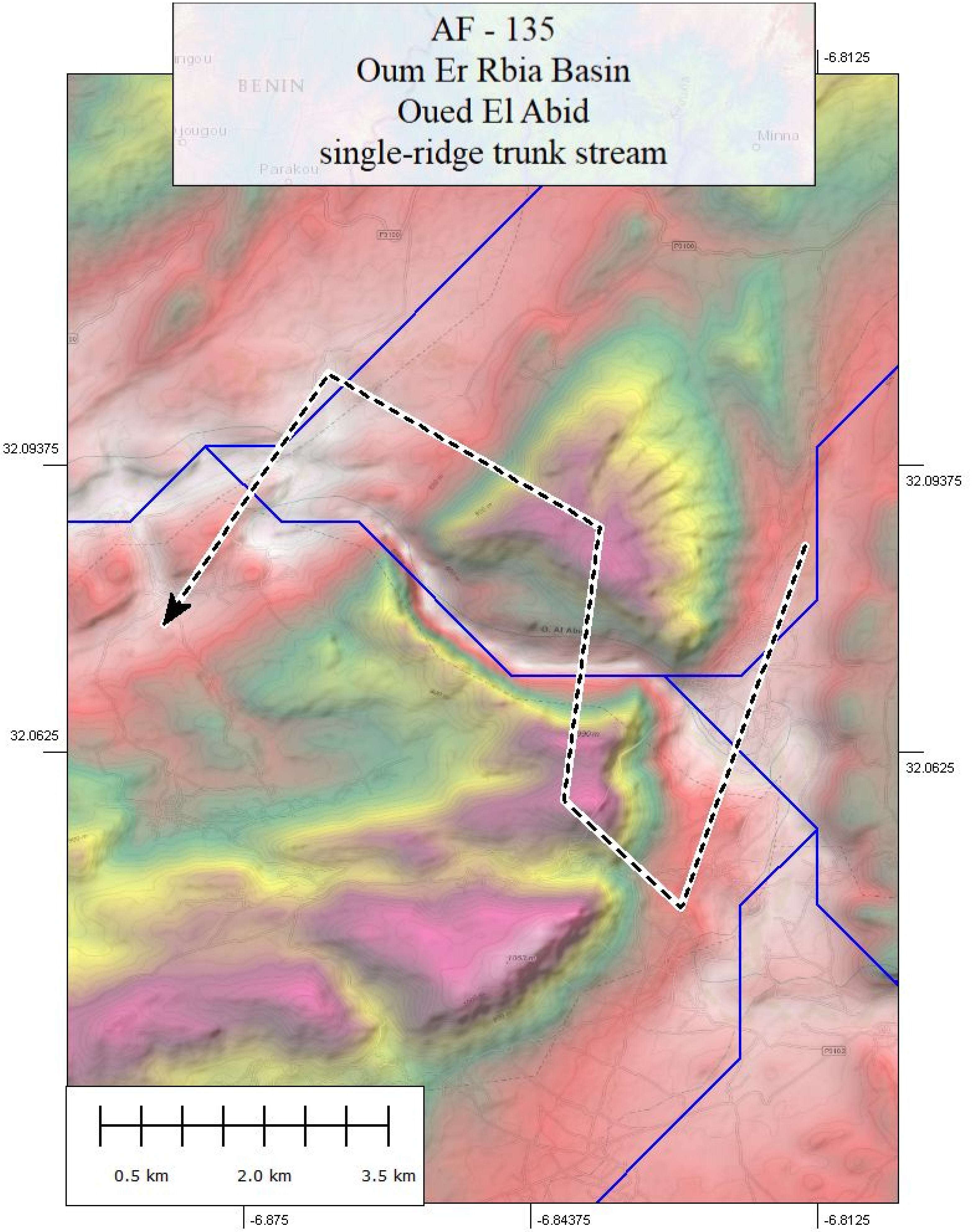

AF - 135
Oum Er Rbia Basin
Oued El Abid
single-ridge trunk stream
BENIN
Parakou
Minna
O. Al Abid
32.09375
32.09375
32.0625
32.0625
-6.8125
-6.875
-6.84375
-6.8125
0.5 km
2.0 km
3.5 km

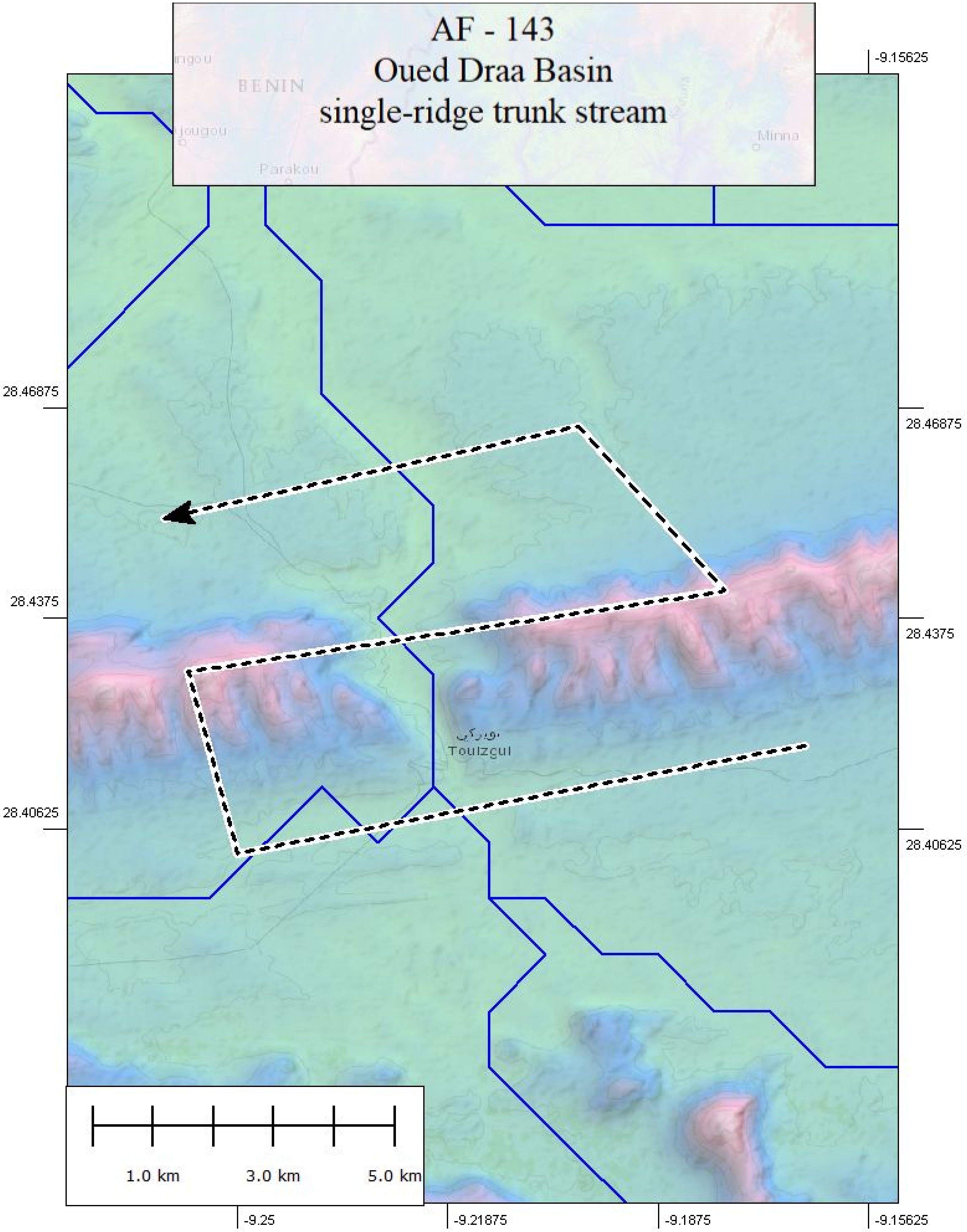

AF - 143
Oued Draa Basin
single-ridge trunk stream
BENIN
Parakou
Minna
Toulzgui
-9.15625
28.46875
28.46875
28.4375
28.4375
28.40625
28.40625
1.0 km
3.0 km
5.0 km
-9.25
-9.21875
-9.1875
-9.15625

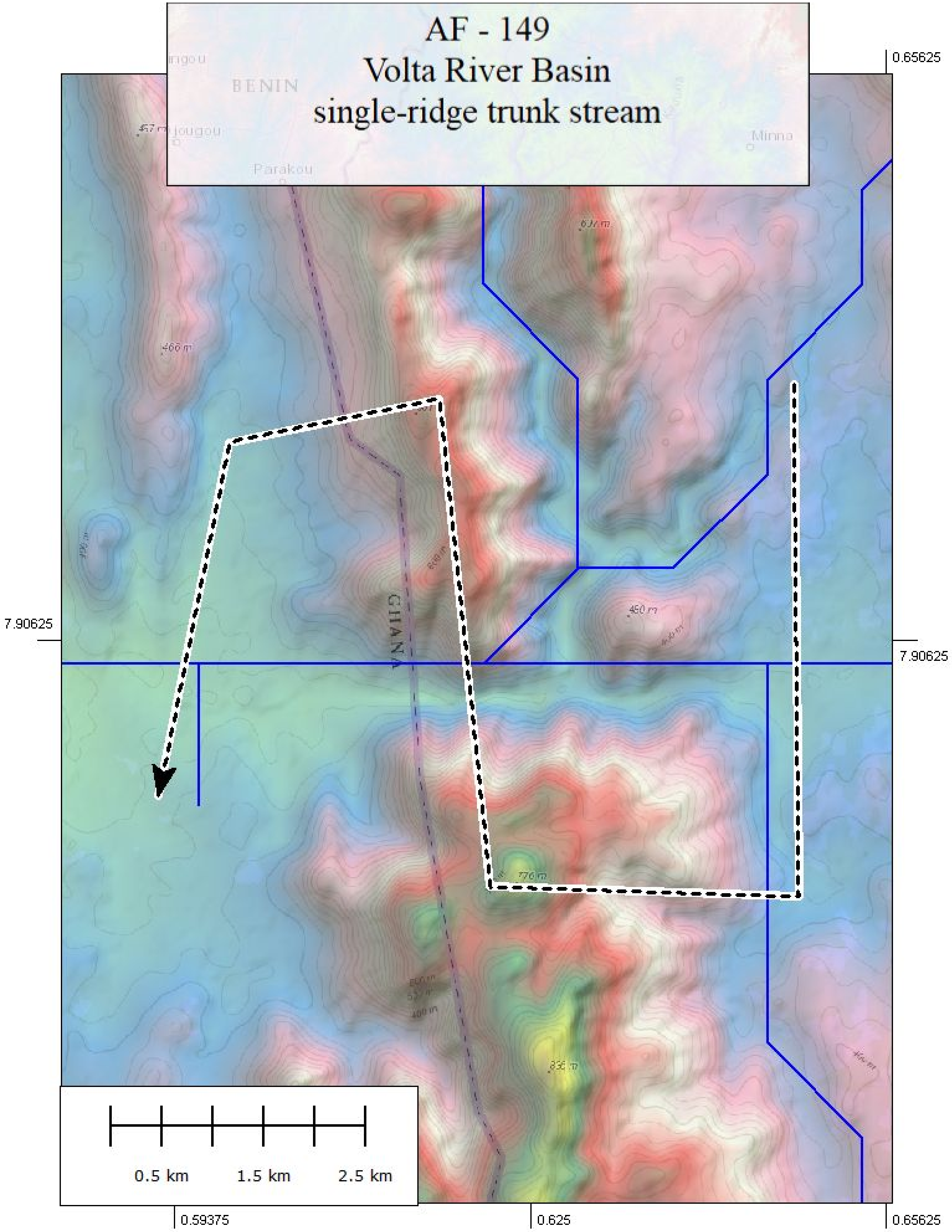
AF - 149
Volta River Basin
single-ridge trunk stream
BENIN
Parakou
Minna
GHANA
0.65625
7.90625
7.90625
0.5 km
1.5 km
2.5 km
0.59375
0.625
0.65625

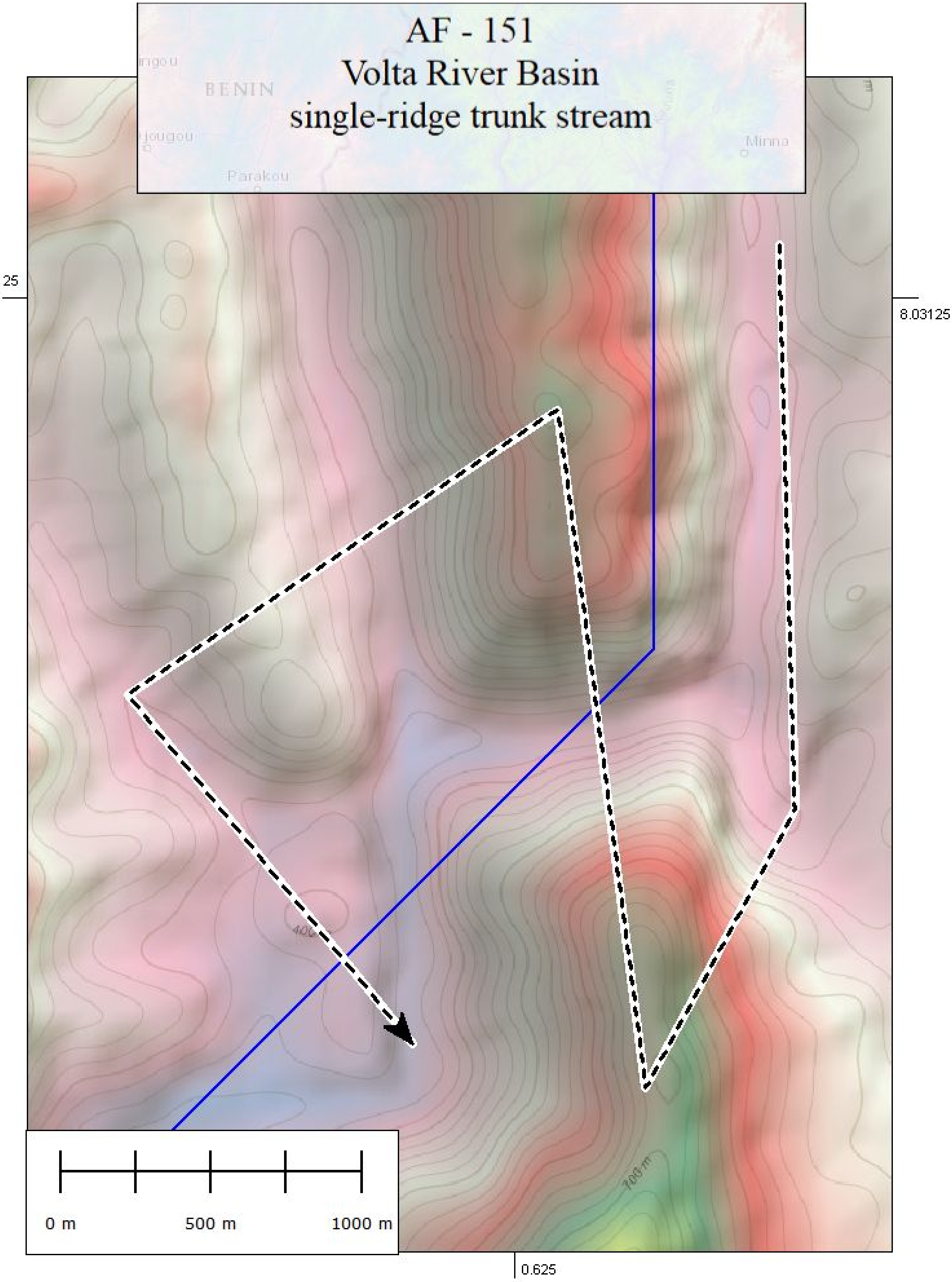
AF - 151
Volta River Basin
single-ridge trunk stream
BENIN
Parakou
Minna
8.03125
8.03125
400
700 m
0 m
500 m
1000 m
0.625

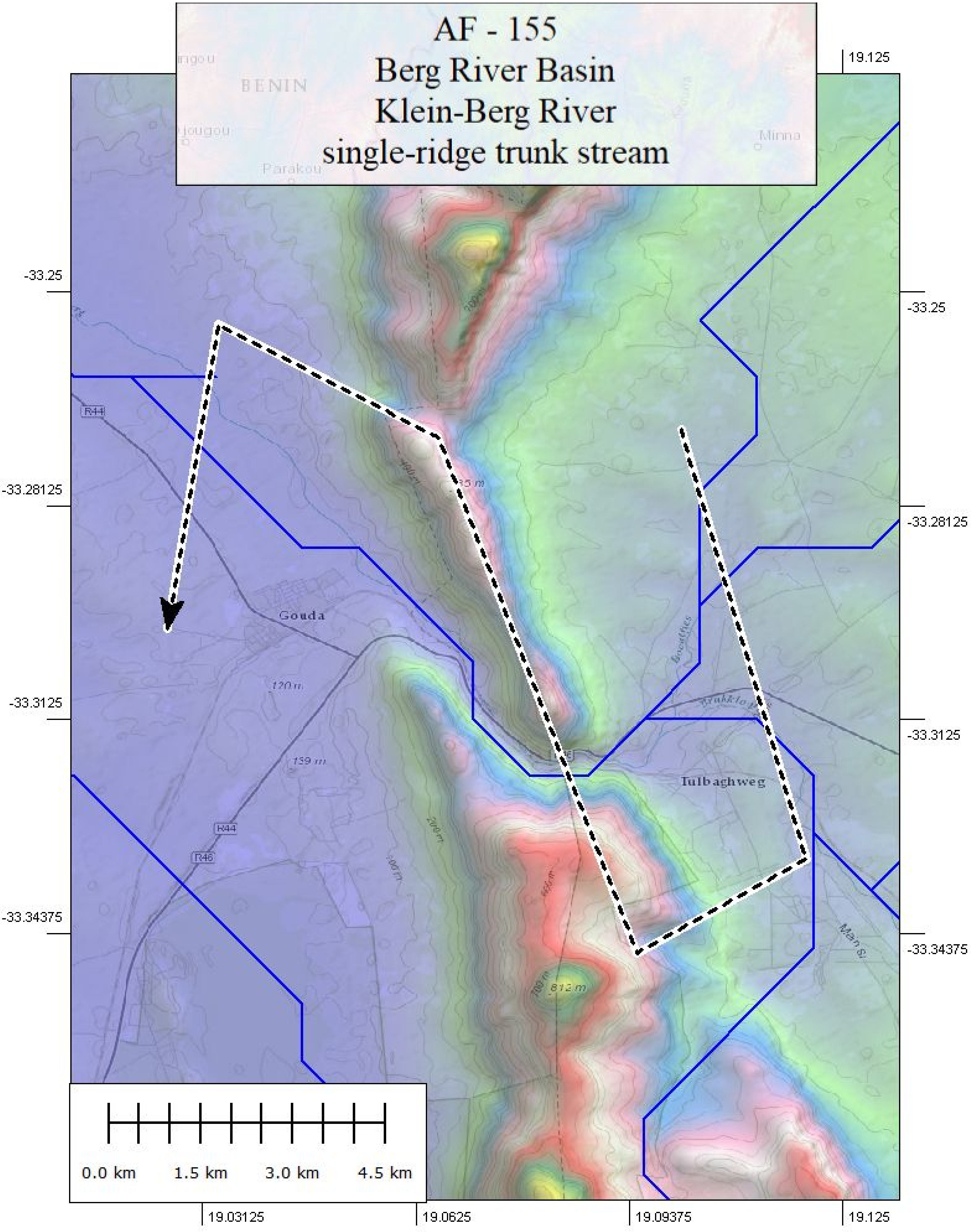
AF - 155
Berg River Basin
Klein-Berg River
single-ridge trunk stream
19.125
-33.25
-33.28125
-33.3125
-33.34375
19.03125
19.0625
19.09375
BENIN
Parakou
Gouda
Tulbaghweg
R44
R46
120 m
159 m
812 m
0.0 km
1.5 km
3.0 km
4.5 km

# AF - 16
# Gamtoos River Basin
# single-ridge head stream

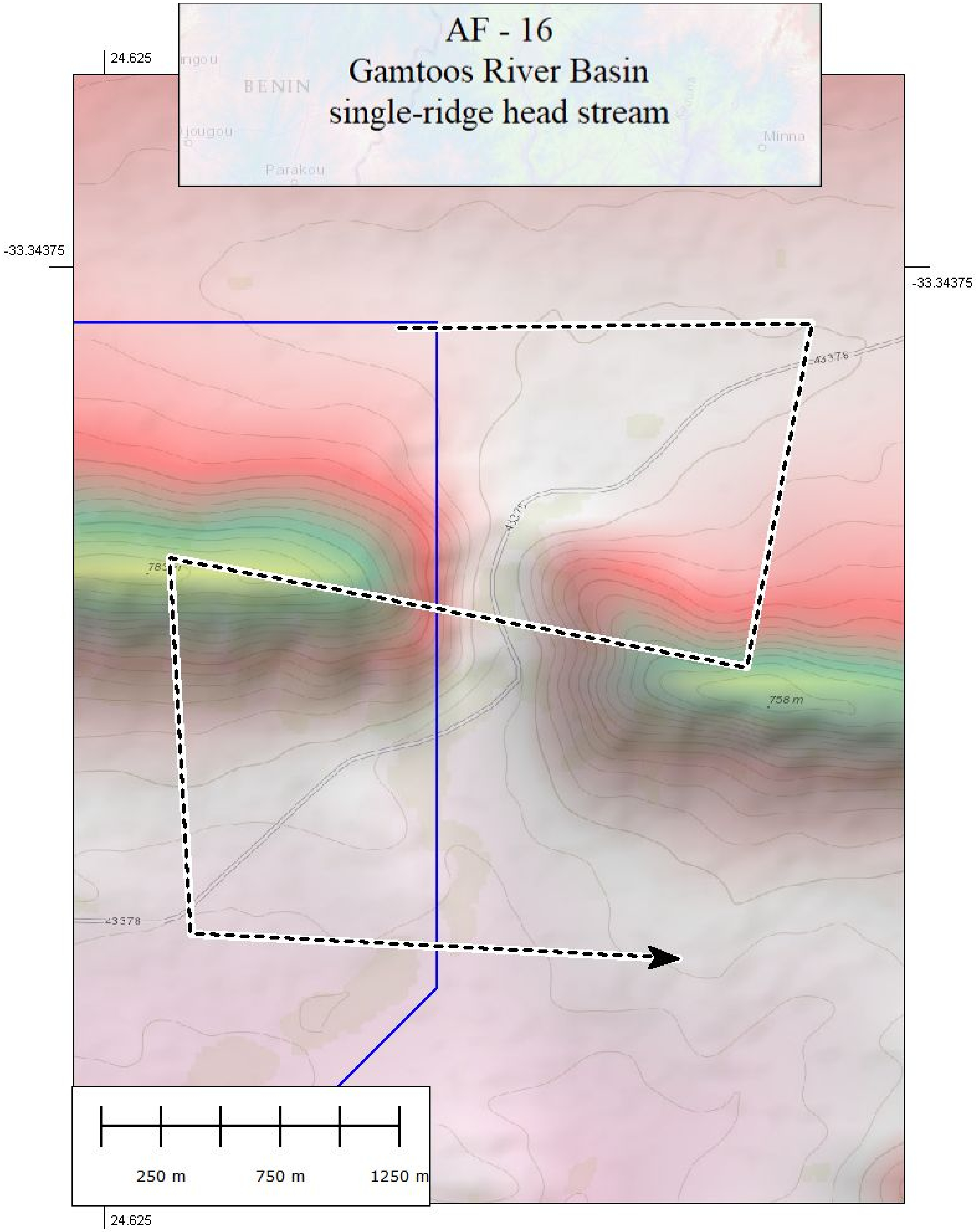

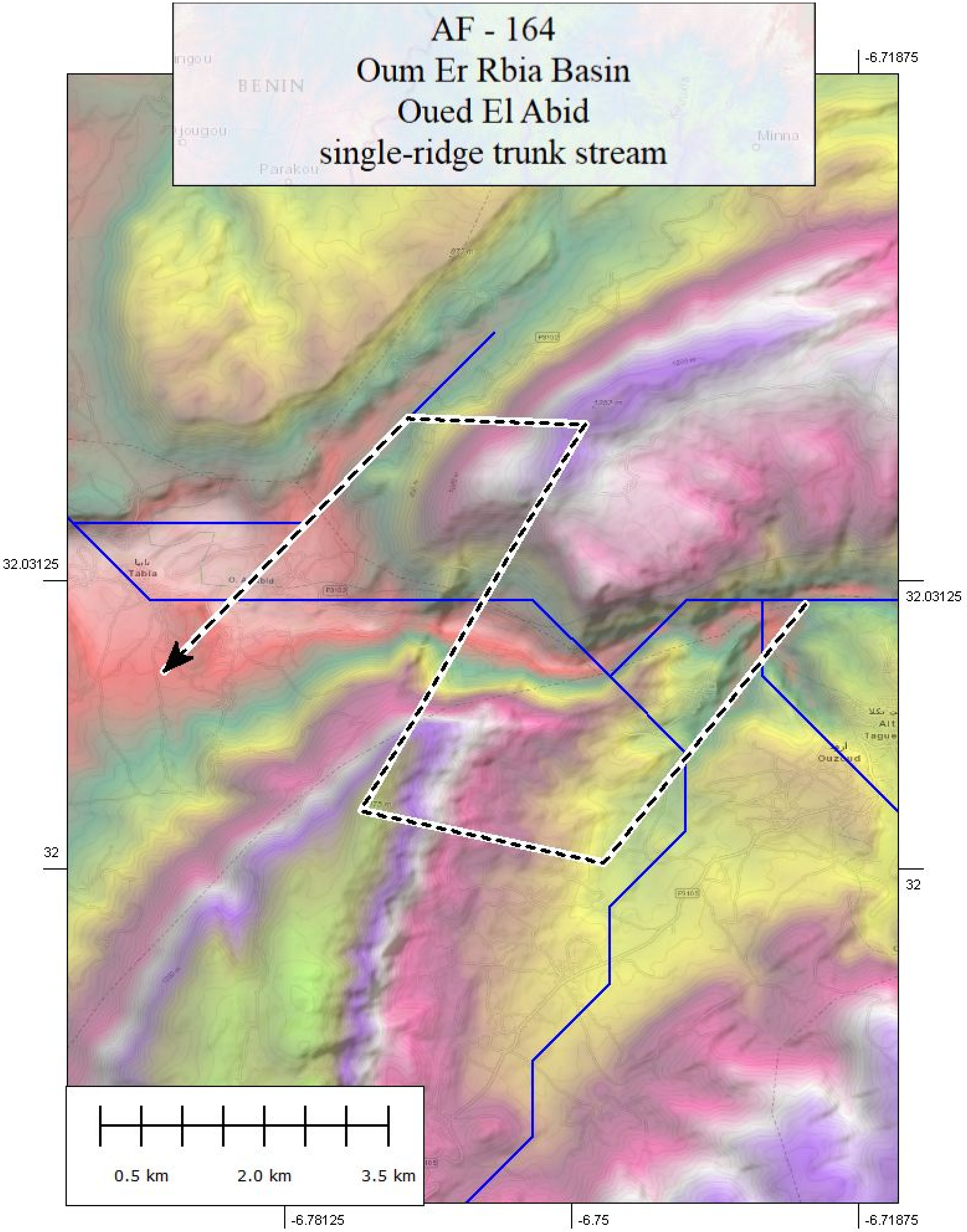
AF - 164
Oum Er Rbia Basin
Oued El Abid
single-ridge trunk stream
-6.71875
32.03125
32.03125
32
32
0.5 km
2.0 km
3.5 km
-6.78125
-6.75
-6.71875
Tabla
Ouzoud

# AF - 17
## Limpopo River Basin
## single-ridge head stream

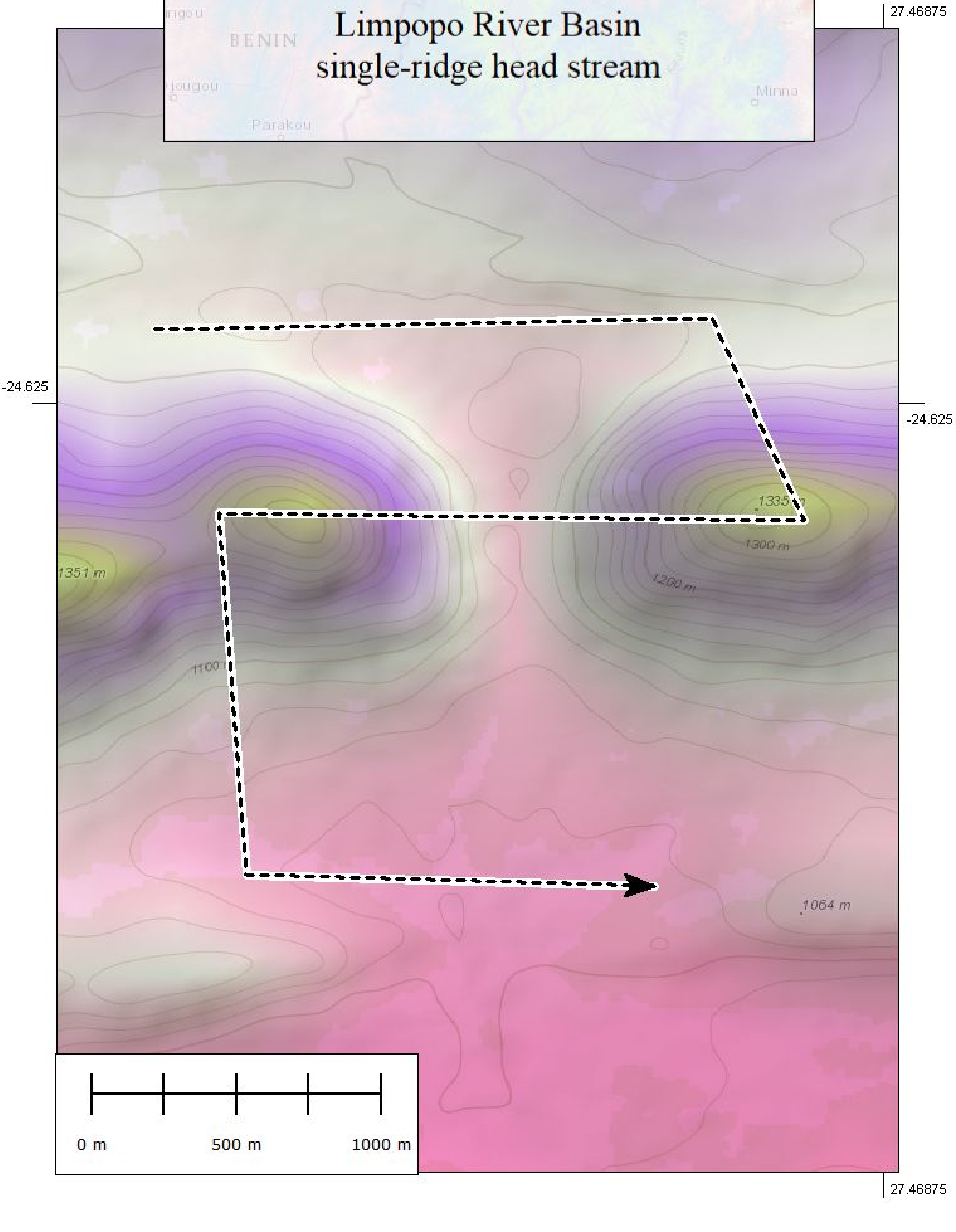

# AF - 178
## Manambolo River Basin
## single-ridge trunk stream

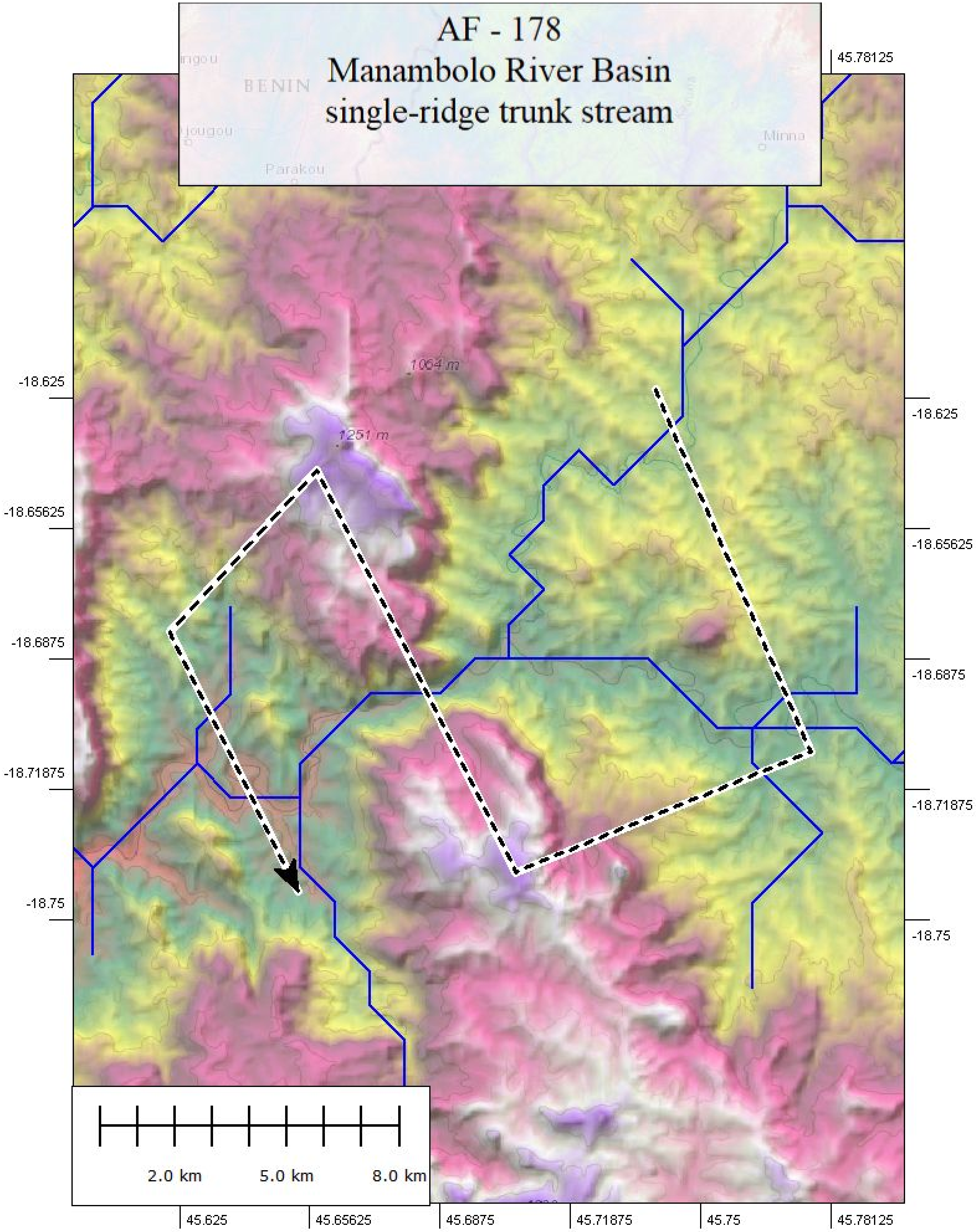

# AF - 18
# Limpopo River Basin
# single-ridge head stream

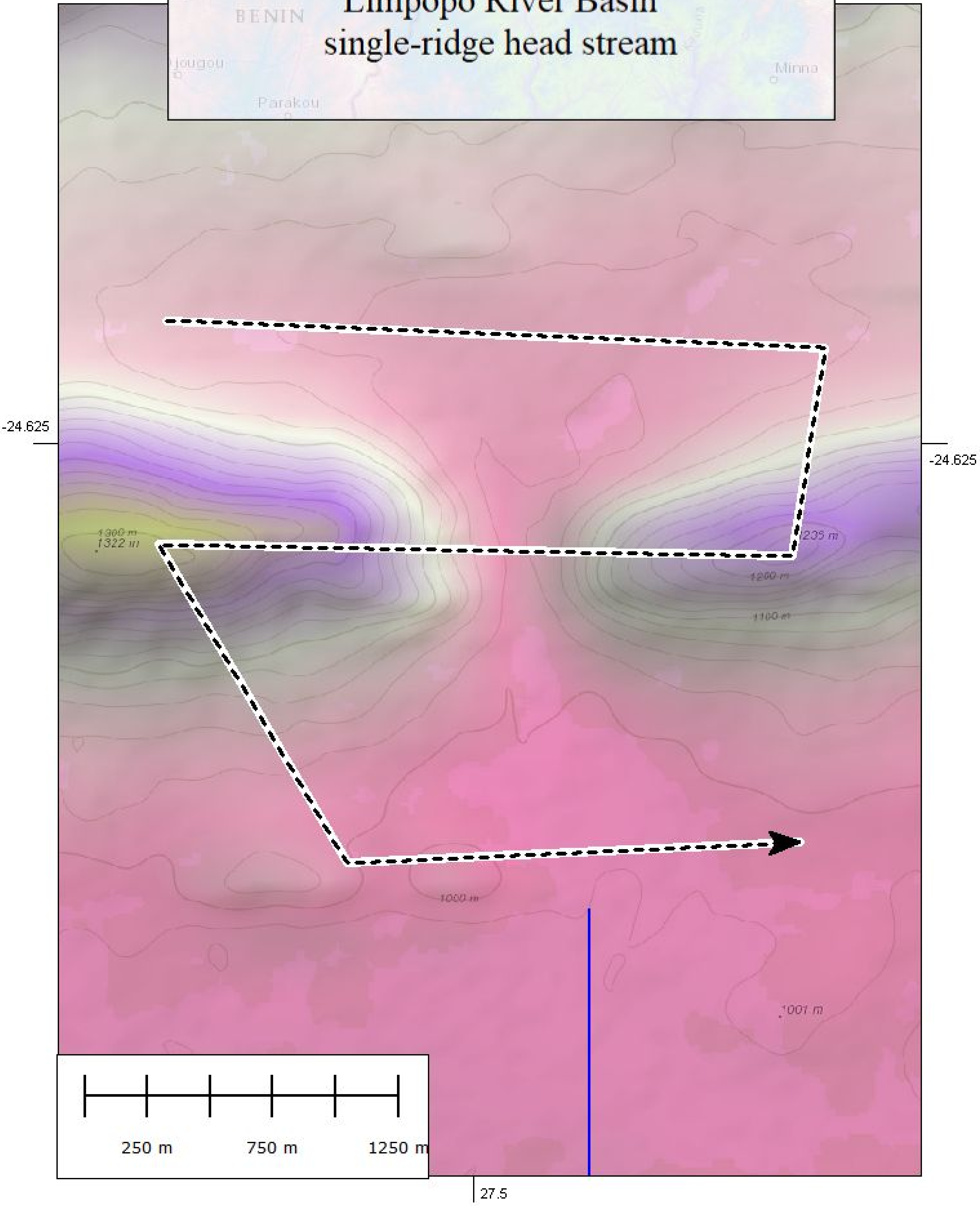

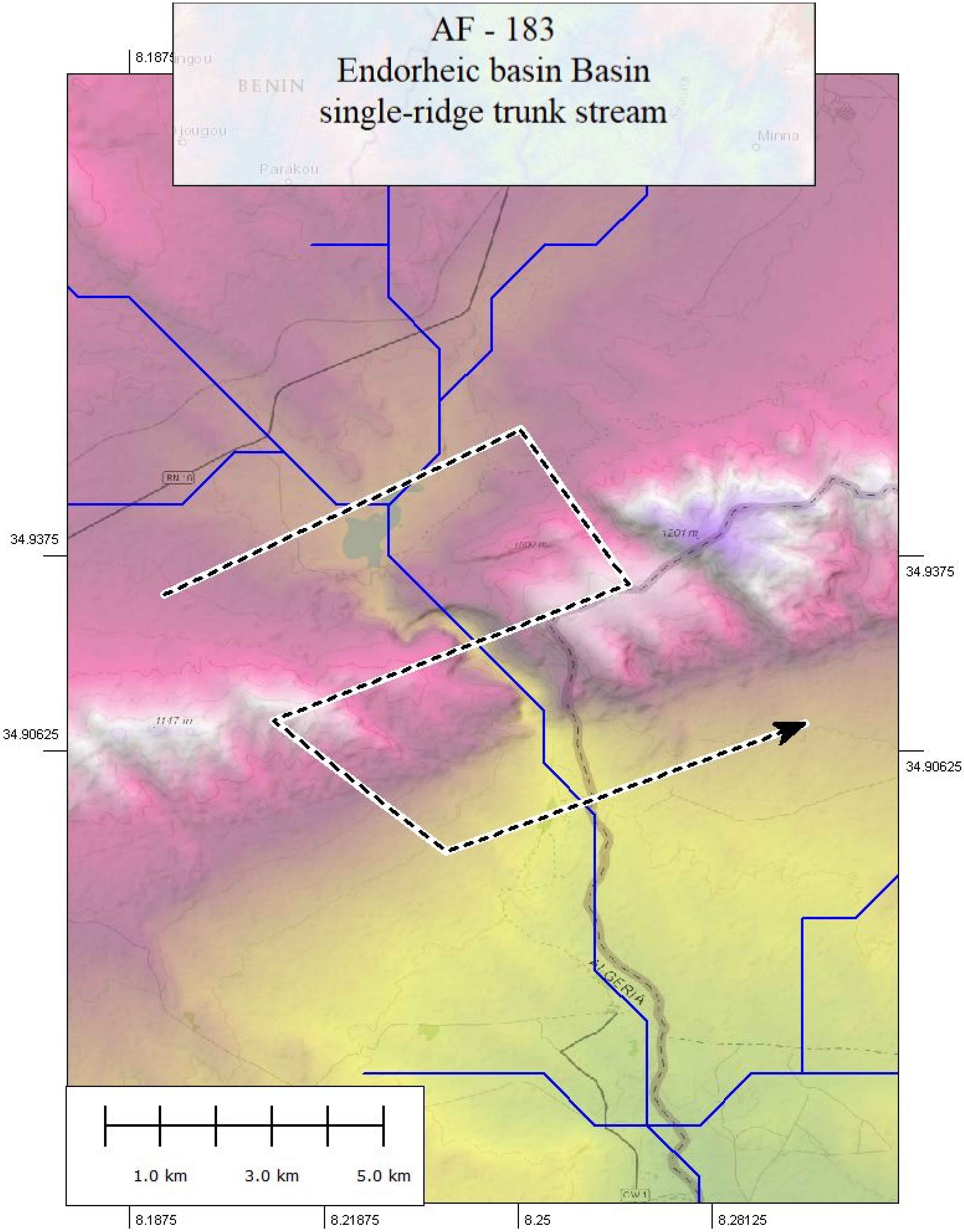
AF - 183
Endorheic basin Basin
single-ridge trunk stream
8.1875
BENIN
Djougou
Parakou
Minna
RN 10
34.9375
1000 m
1201 m
1117 m
34.90625
ALGERIA
CW1
1.0 km
3.0 km
5.0 km
8.21875
8.25
8.28125

# AF - 19
## Limpopo River Basin
## single-ridge head stream

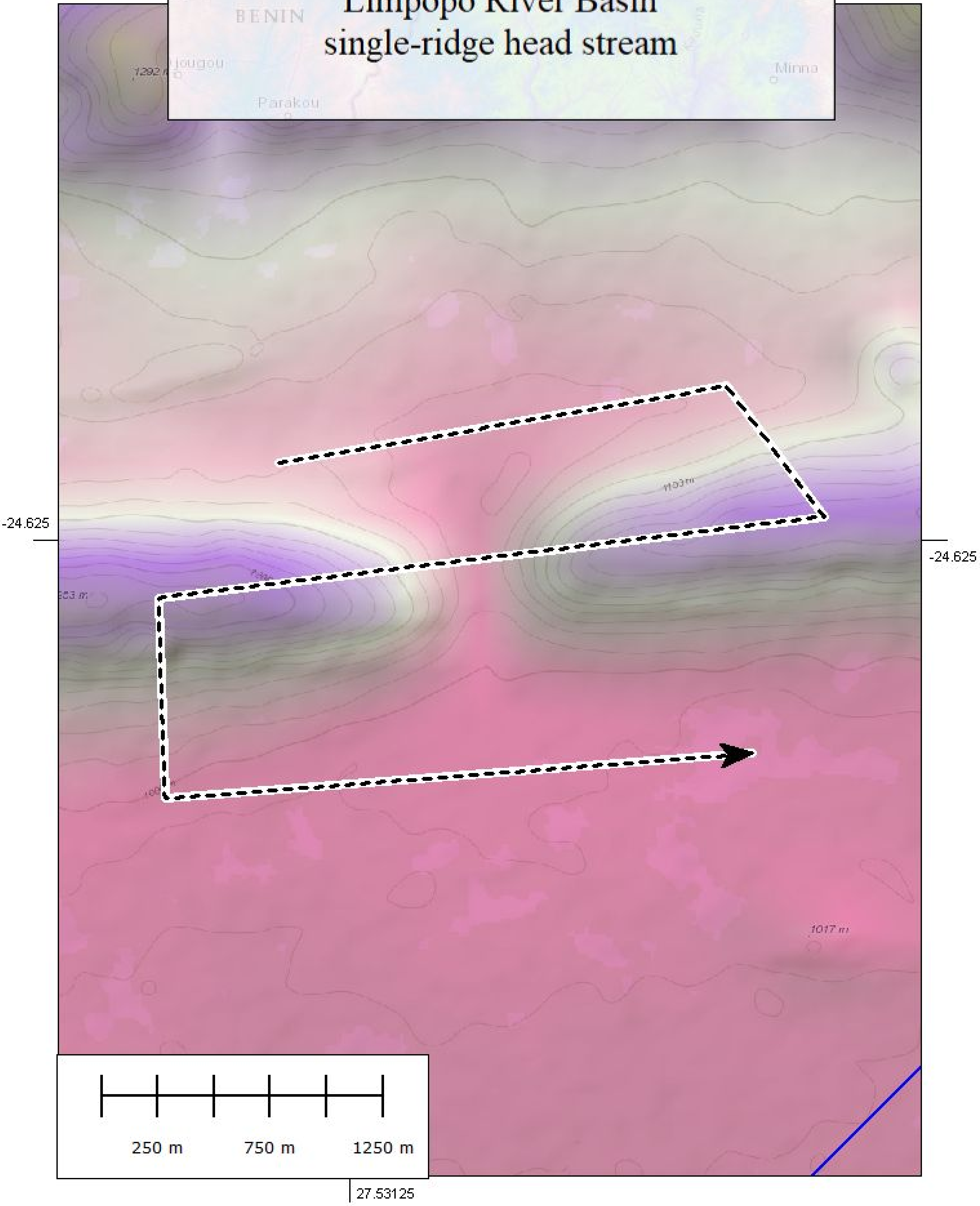

# AF - 22
## Oum Er Rbia Basin
## single-ridge head stream

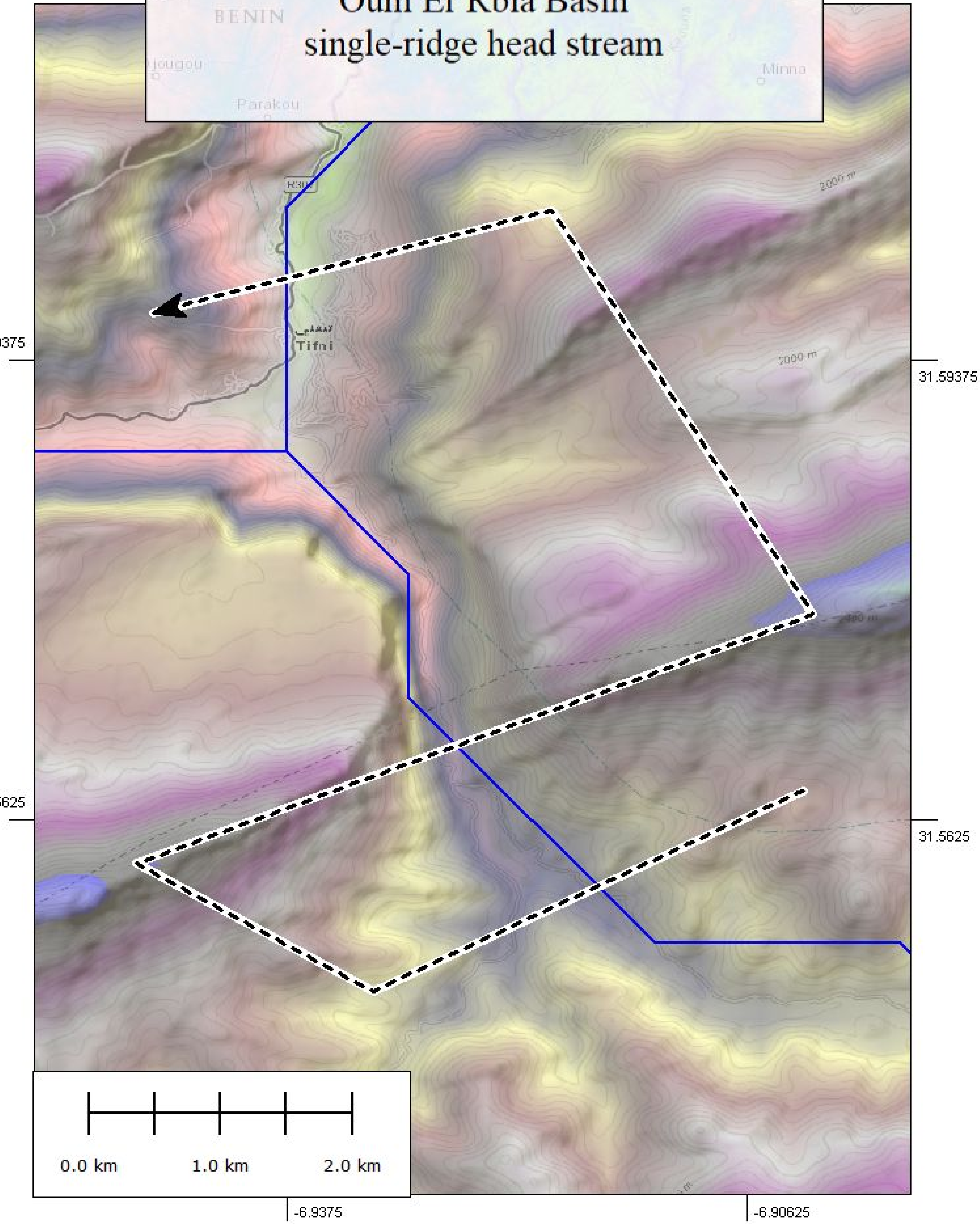

# AF - 23
## Oum Er Rbia Basin
## single-ridge head stream

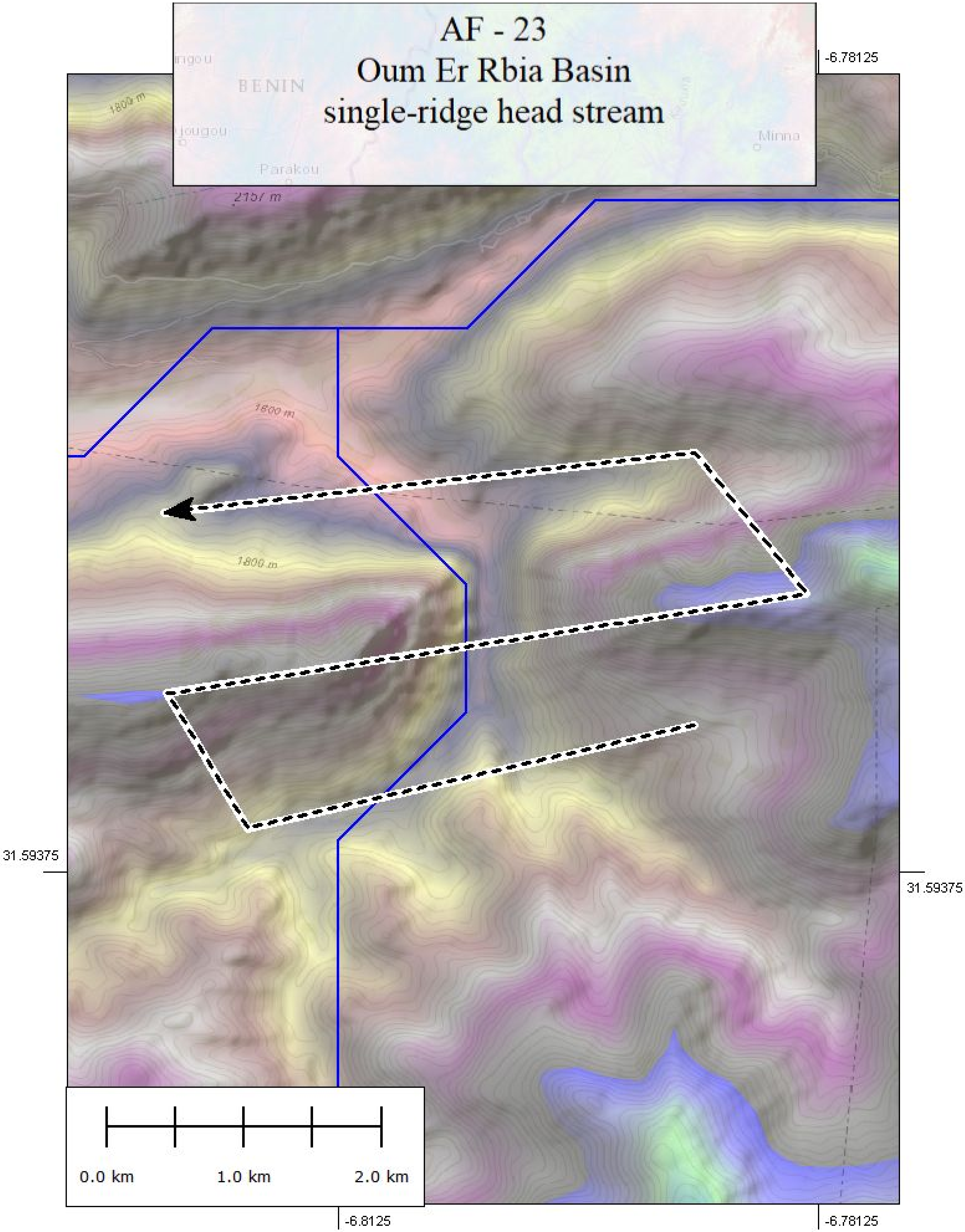

# AF - 29
# Endorheic basin Basin
# single-ridge head stream

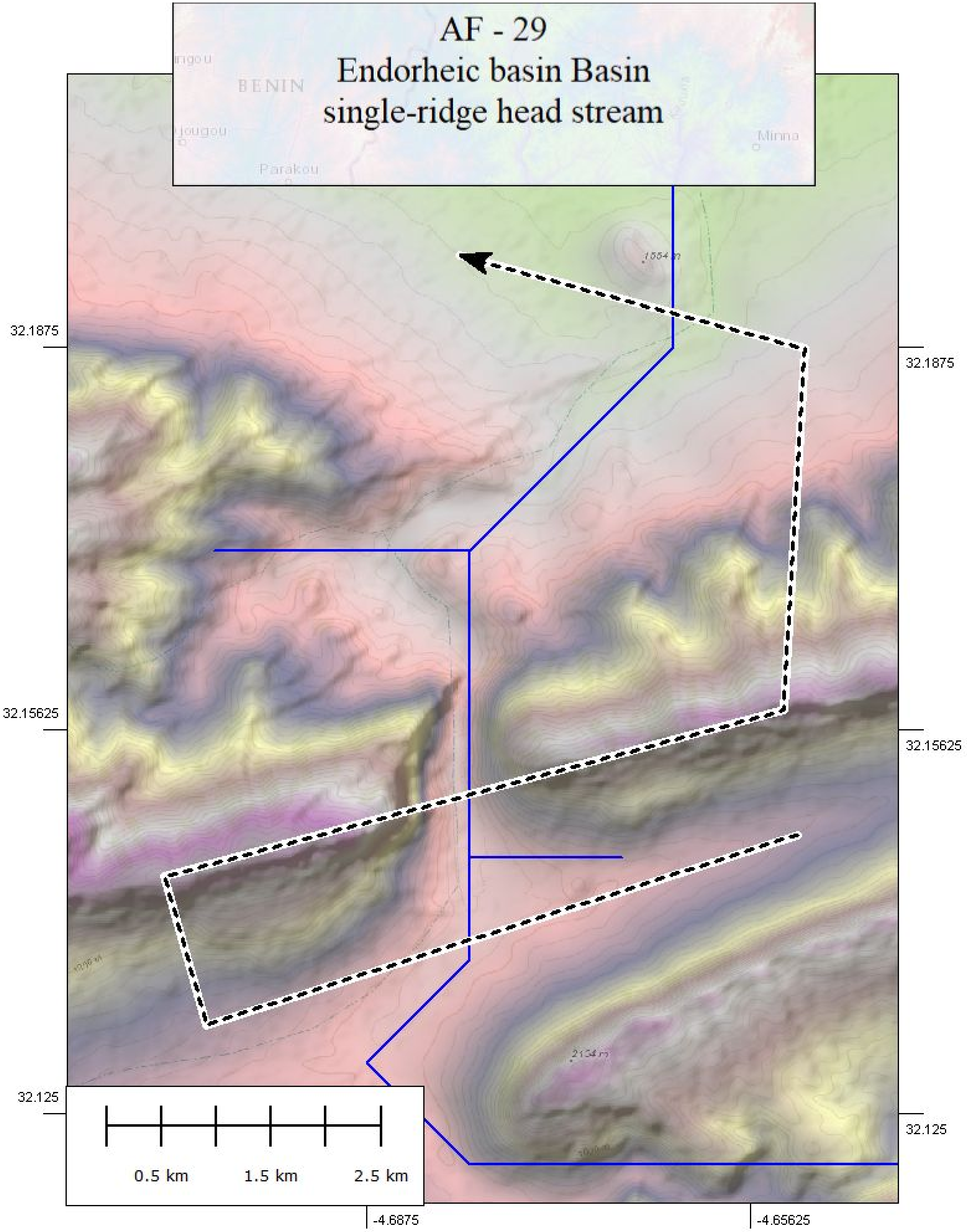

# AF - 30
# Moulouya River Basin
# single-ridge head stream

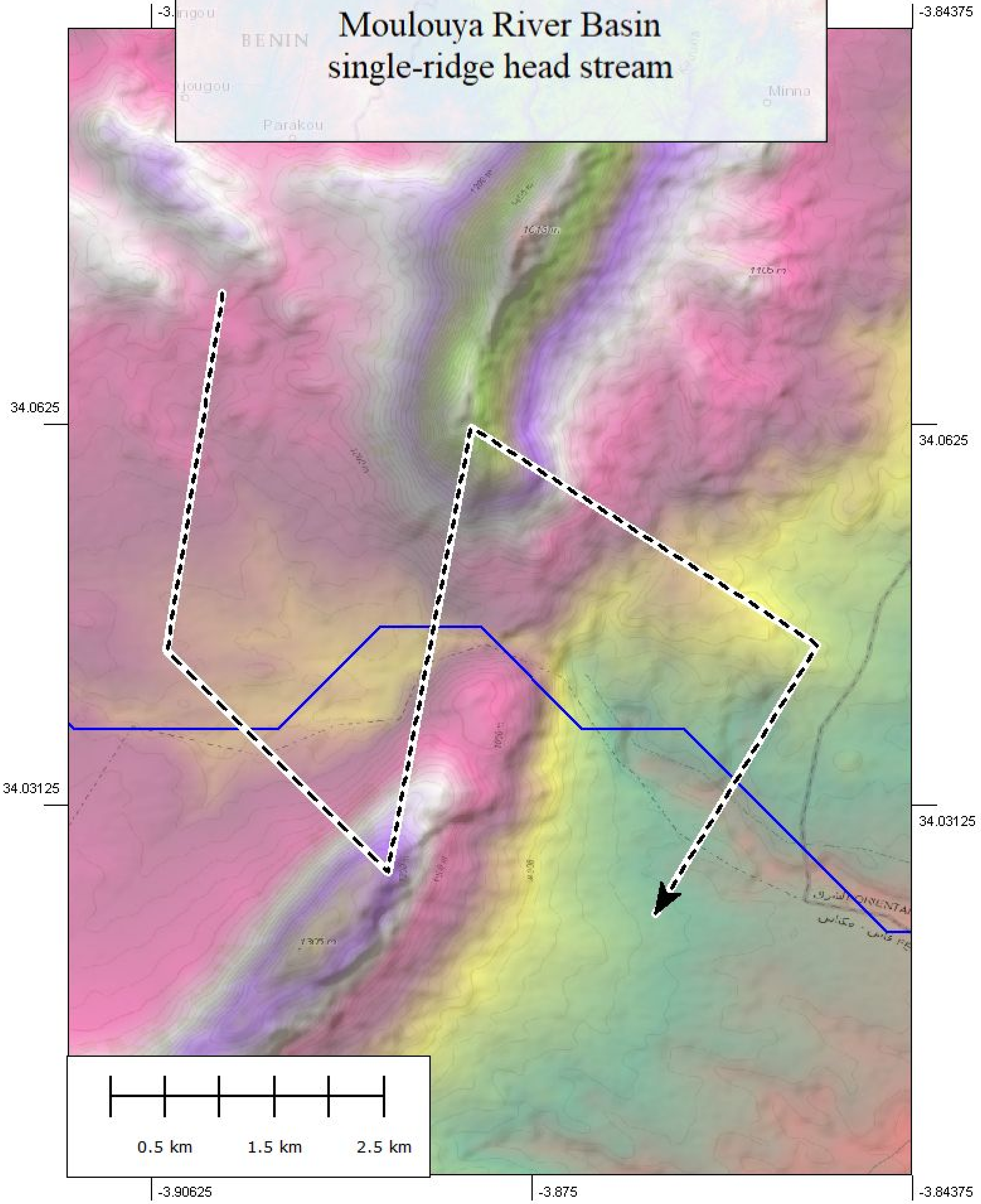

# AF - 31
## Oum Er Rbia Basin
## single-ridge head stream

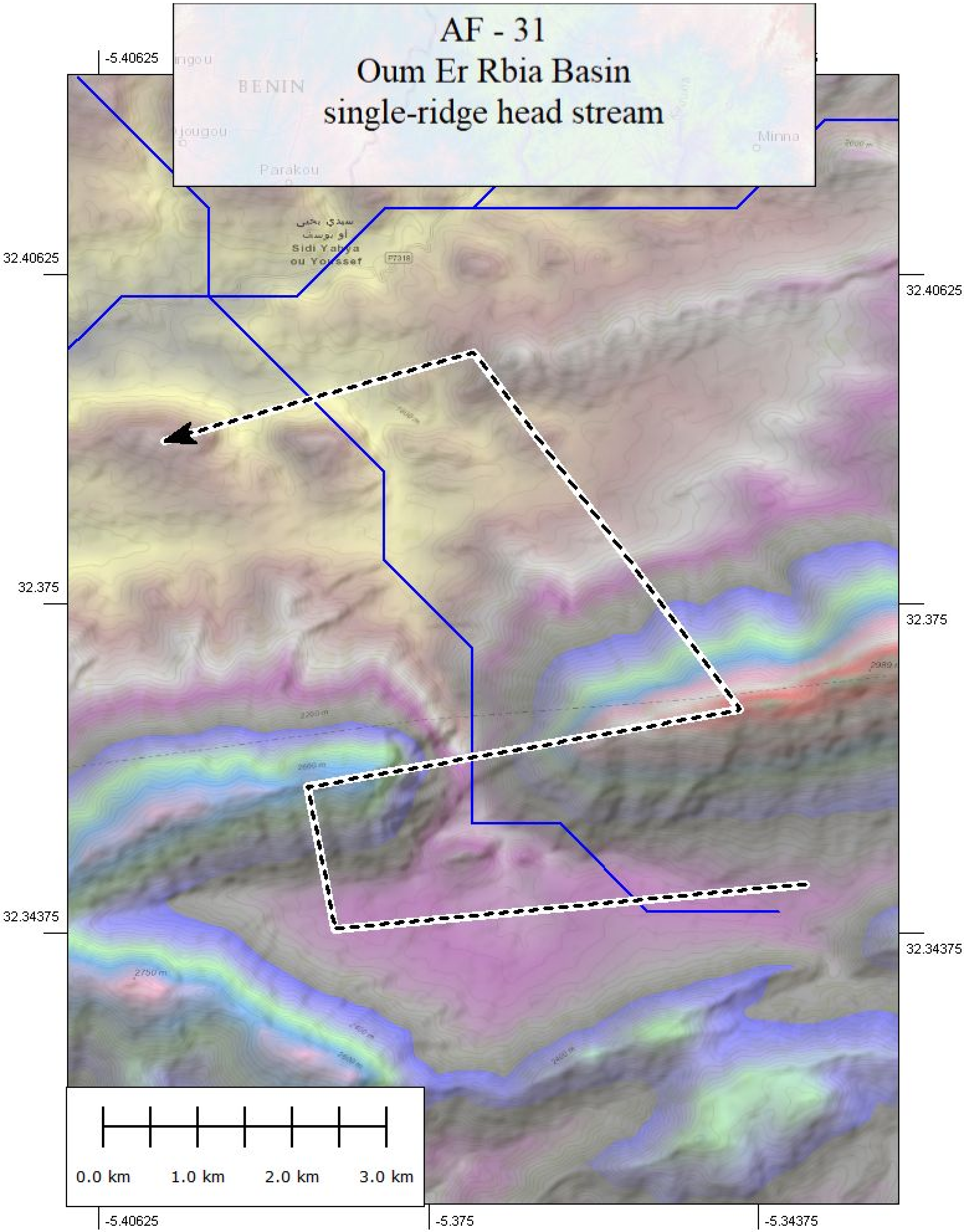

# AF - 32
## Endorheic basin Basin
## single-ridge head stream

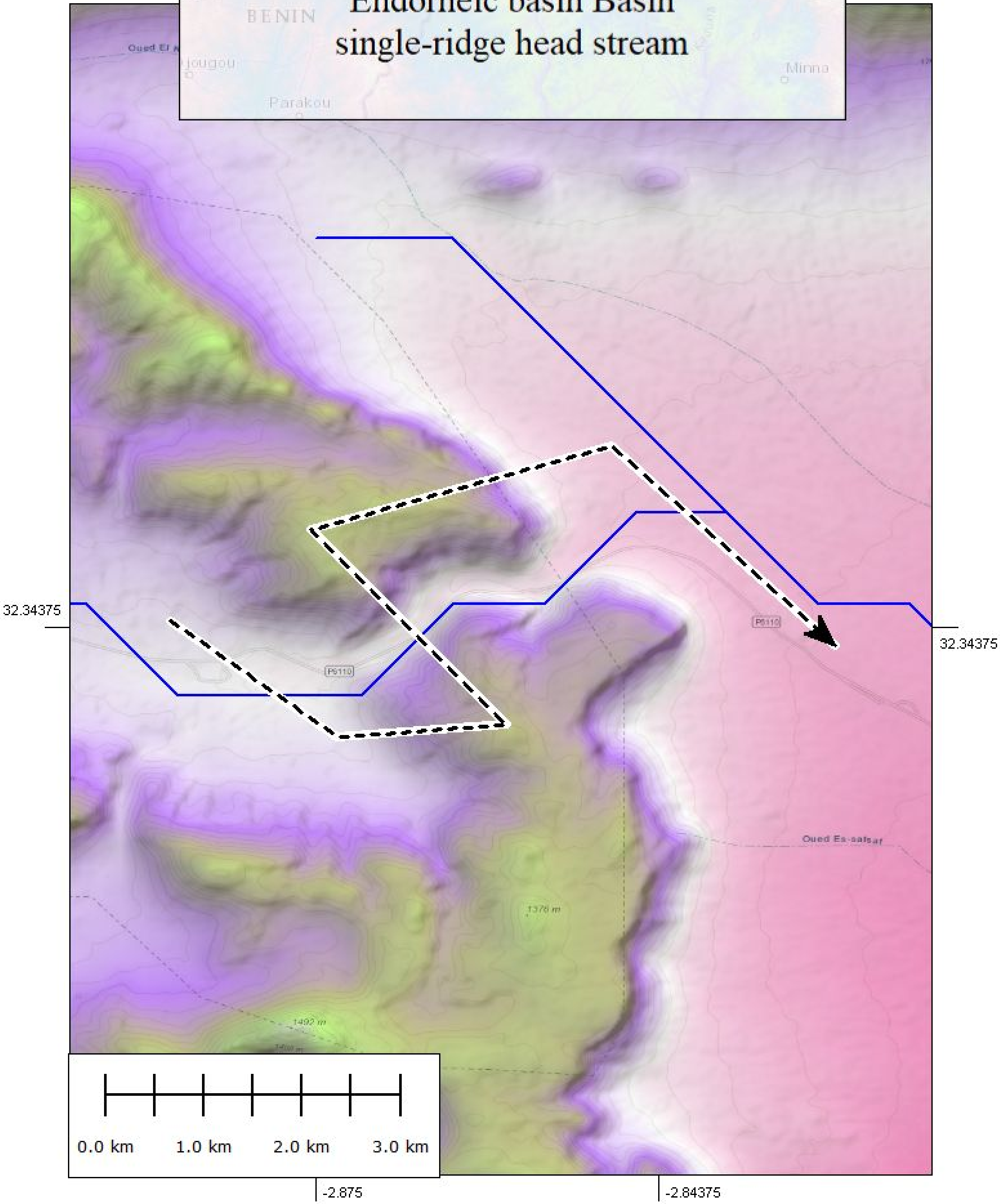

# AF - 37
# Limpopo River Basin
# single-ridge head stream

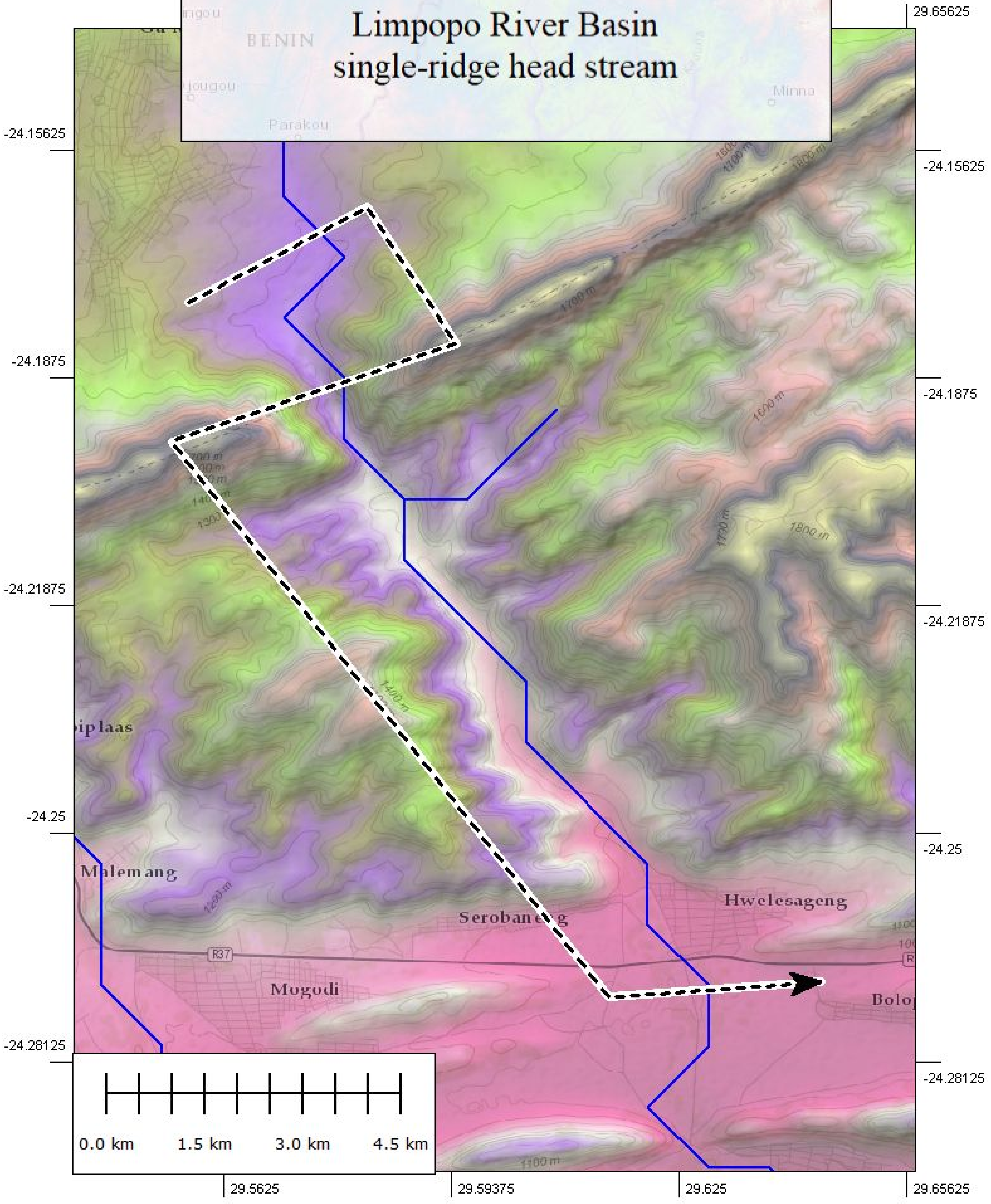

# AF - 6
# Endorheic basin Basin
# single-ridge trunk stream

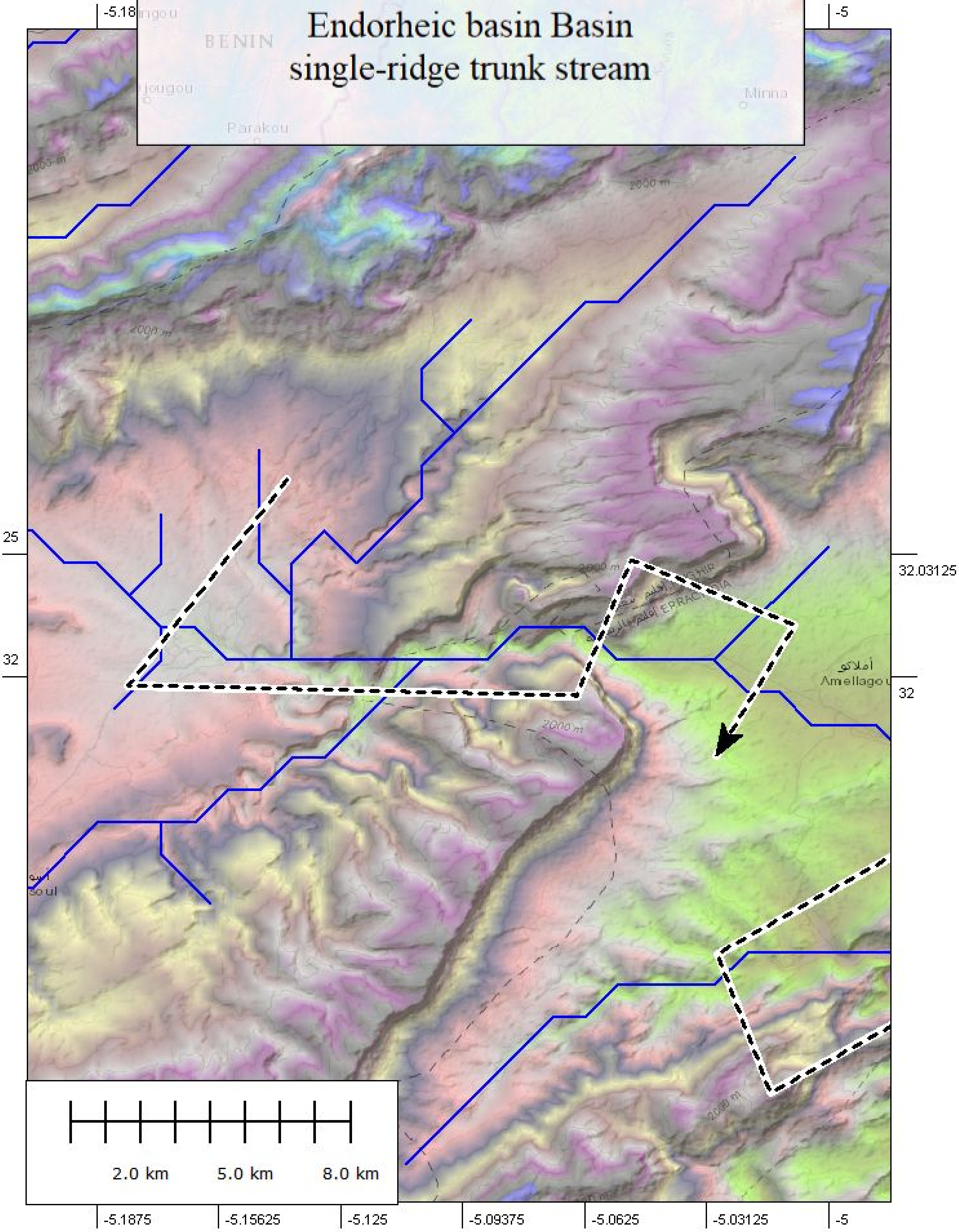

# AF - 63
# Congo River Basin
# single-ridge trunk stream

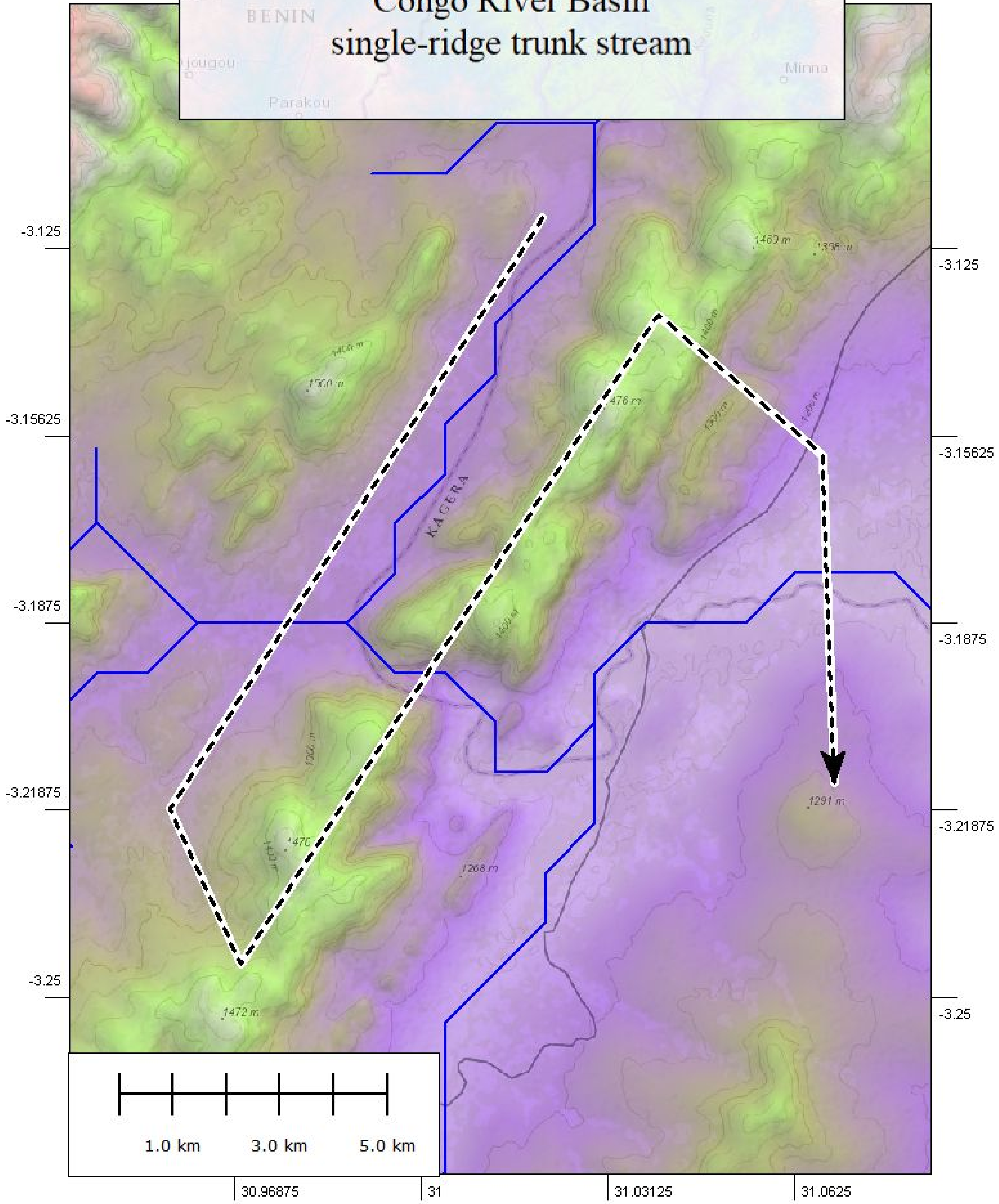

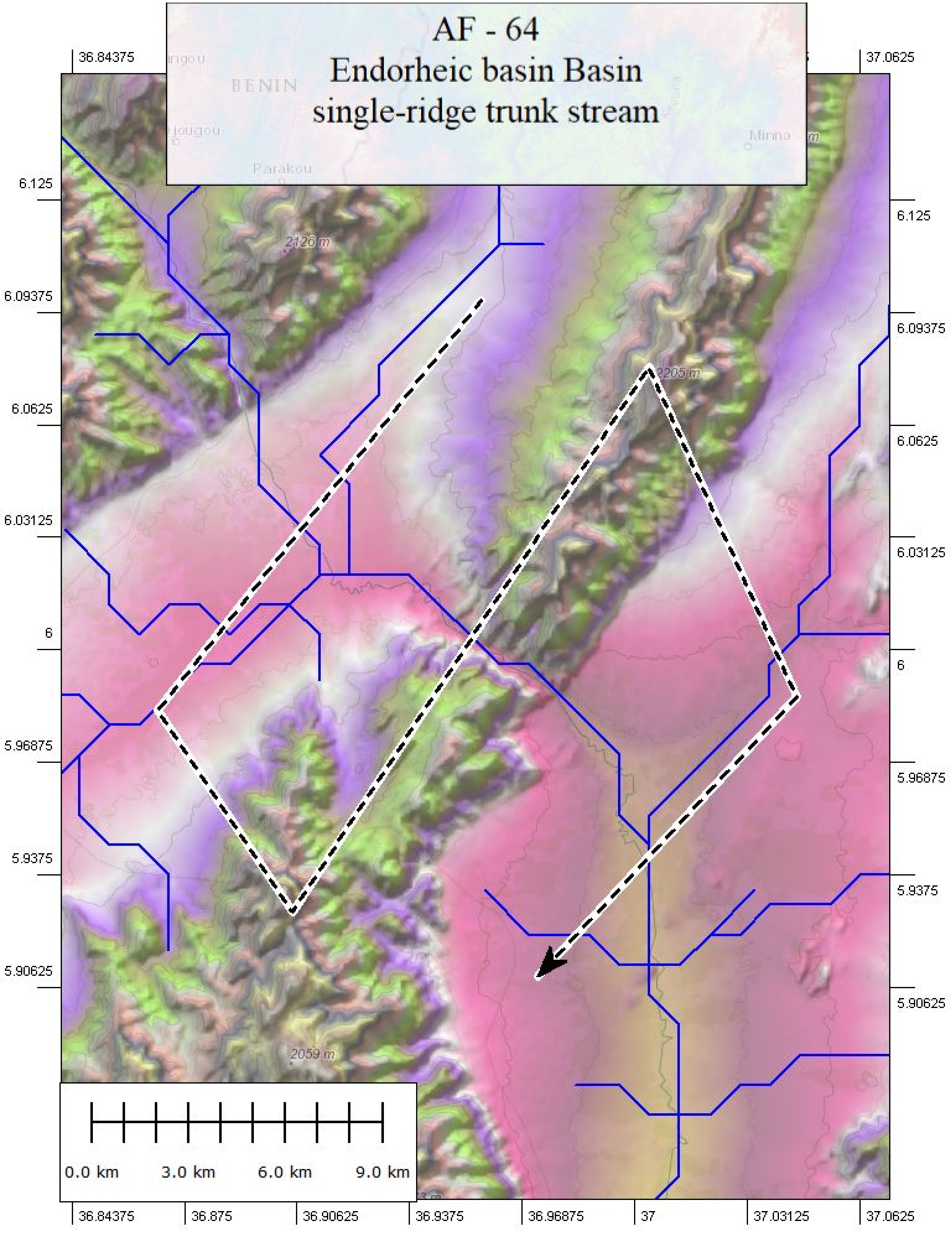
AF - 64
Endorheic basin Basin
single-ridge trunk stream
36.84375
36.875
36.90625
36.9375
36.96875
37
37.03125
37.0625
6.125
6.09375
6.0625
6.03125
6
5.96875
5.9375
5.90625
2126 m
2205 m
2059 m
0.0 km
3.0 km
6.0 km
9.0 km

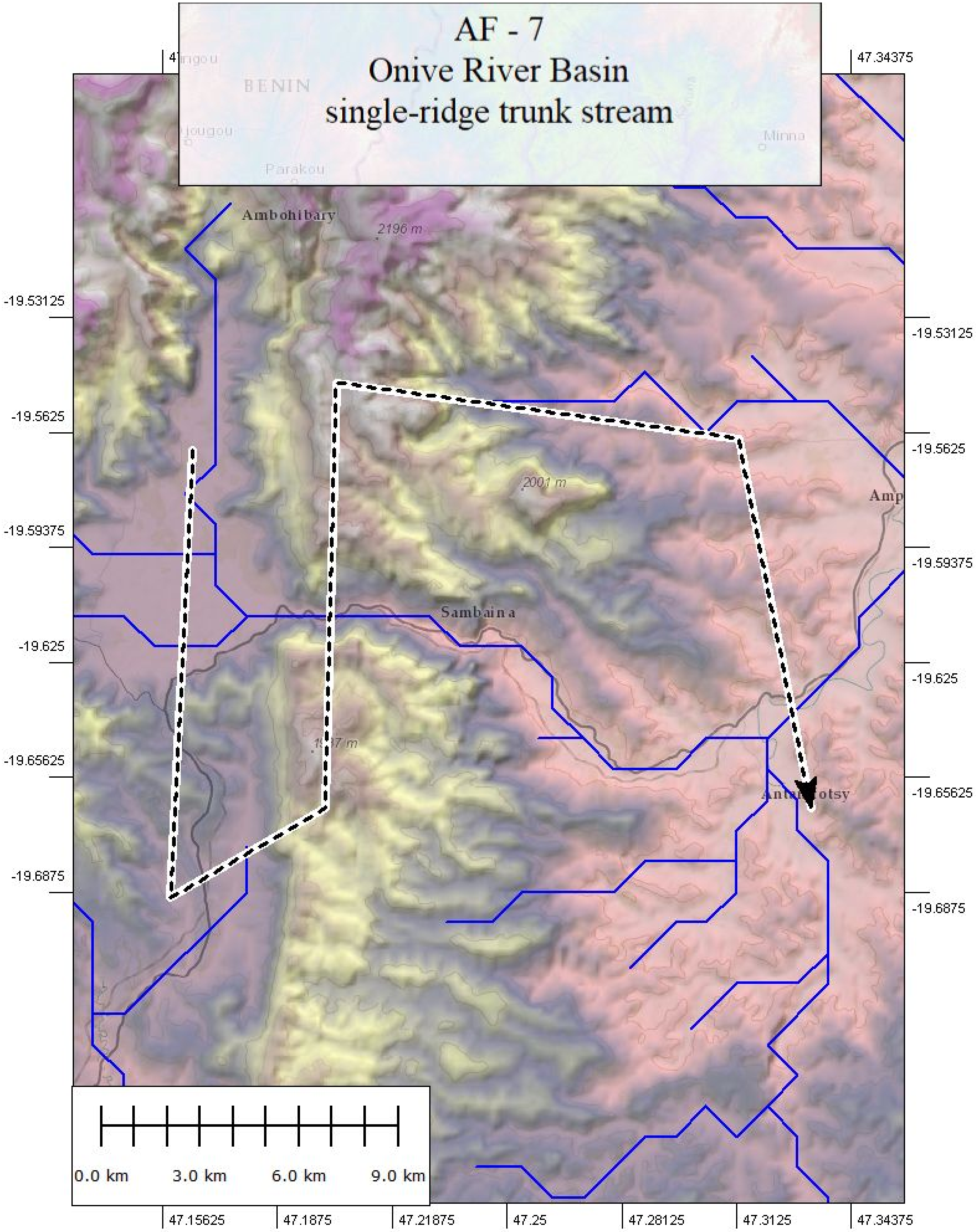
AF - 7
Onive River Basin
single-ridge trunk stream
BENIN
Parakou
Minna
Ambohibary
2196 m
2001 m
Sambaina
47.15625
47.1875
47.21875
47.25
47.28125
47.3125
47.34375
-19.53125
-19.5625
-19.59375
-19.625
-19.65625
-19.6875
0.0 km
3.0 km
6.0 km
9.0 km

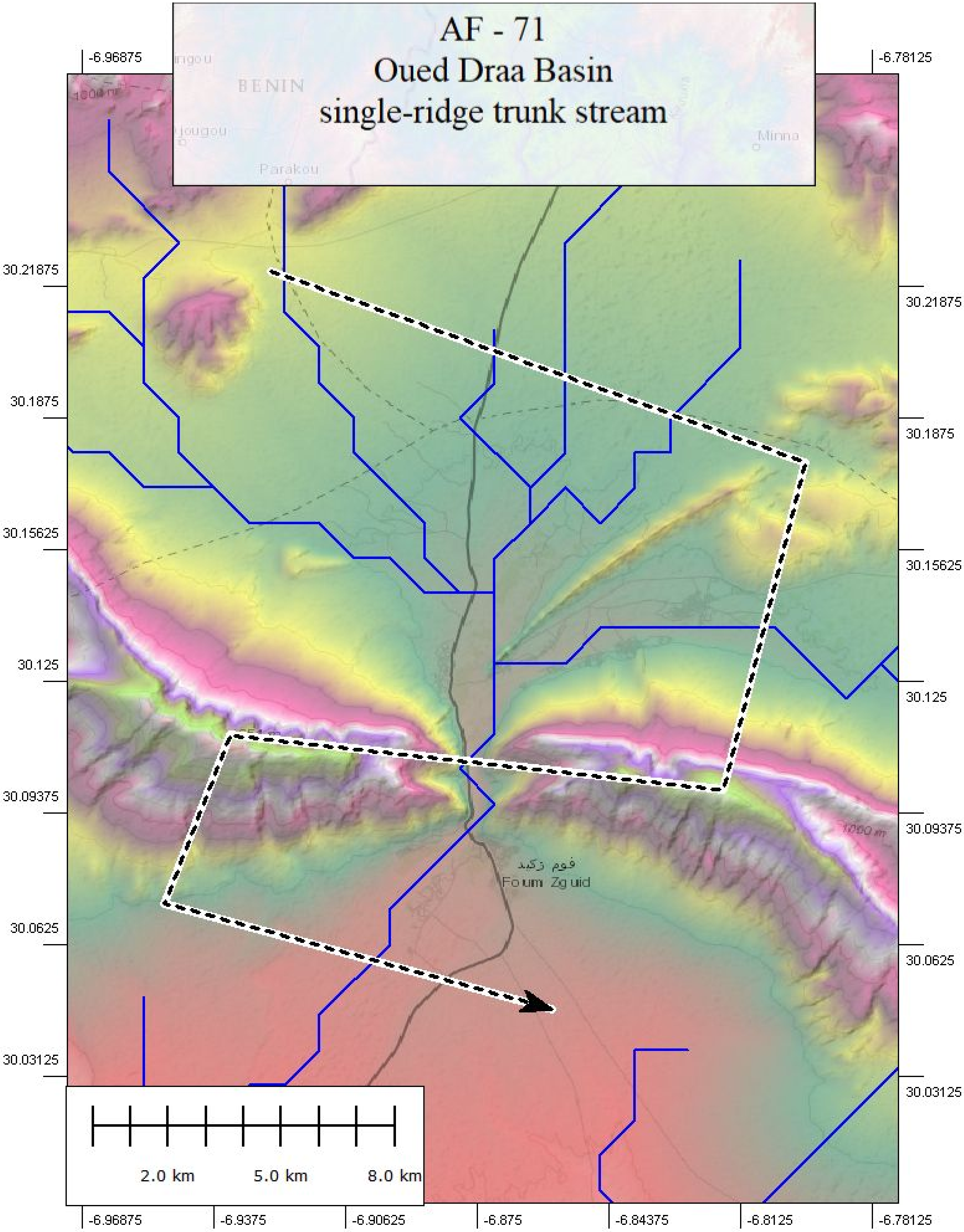
AF - 71
Oued Draa Basin
single-ridge trunk stream
-6.96875
-6.78125
30.21875
30.1875
30.15625
30.125
30.09375
30.0625
30.03125
-6.9375
-6.90625
-6.875
-6.84375
-6.8125
BENIN
Parakou
Minna
1000 m
فوم زكيد
Foum Zguid
2.0 km
5.0 km
8.0 km

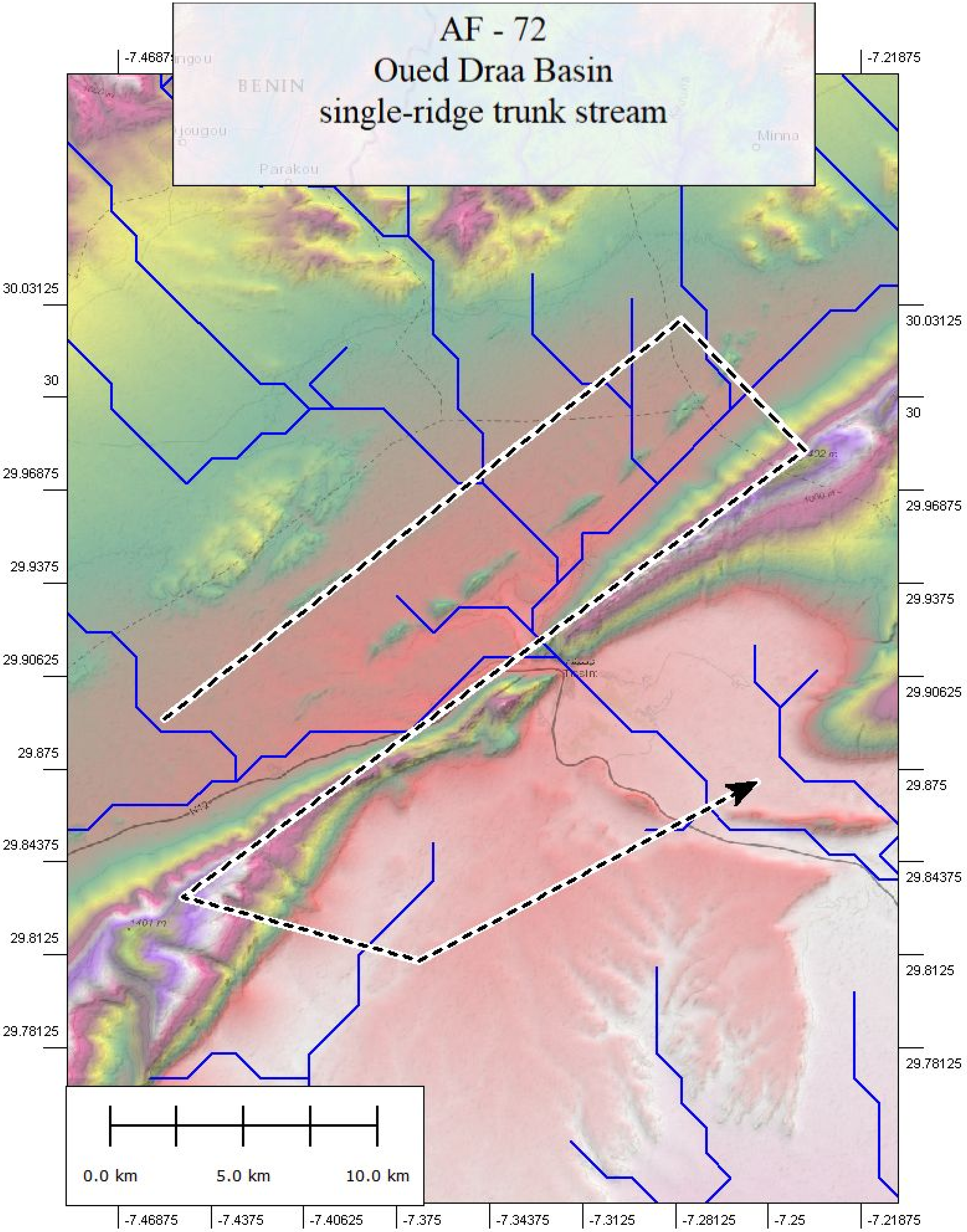


AF - 72
Oued Draa Basin
single-ridge trunk stream

# AF - 90
# Endorheic basin Basin
# single-ridge trunk stream

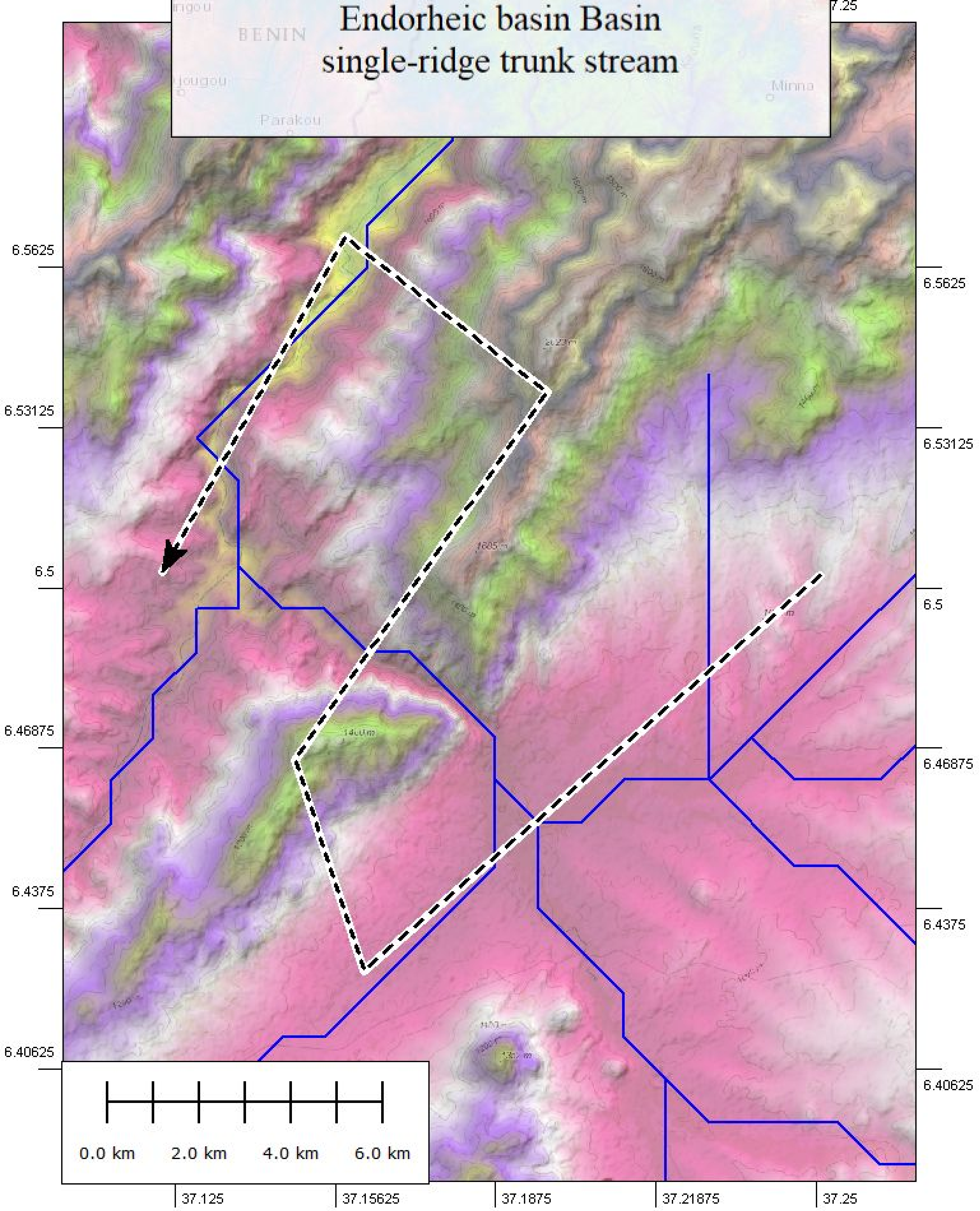

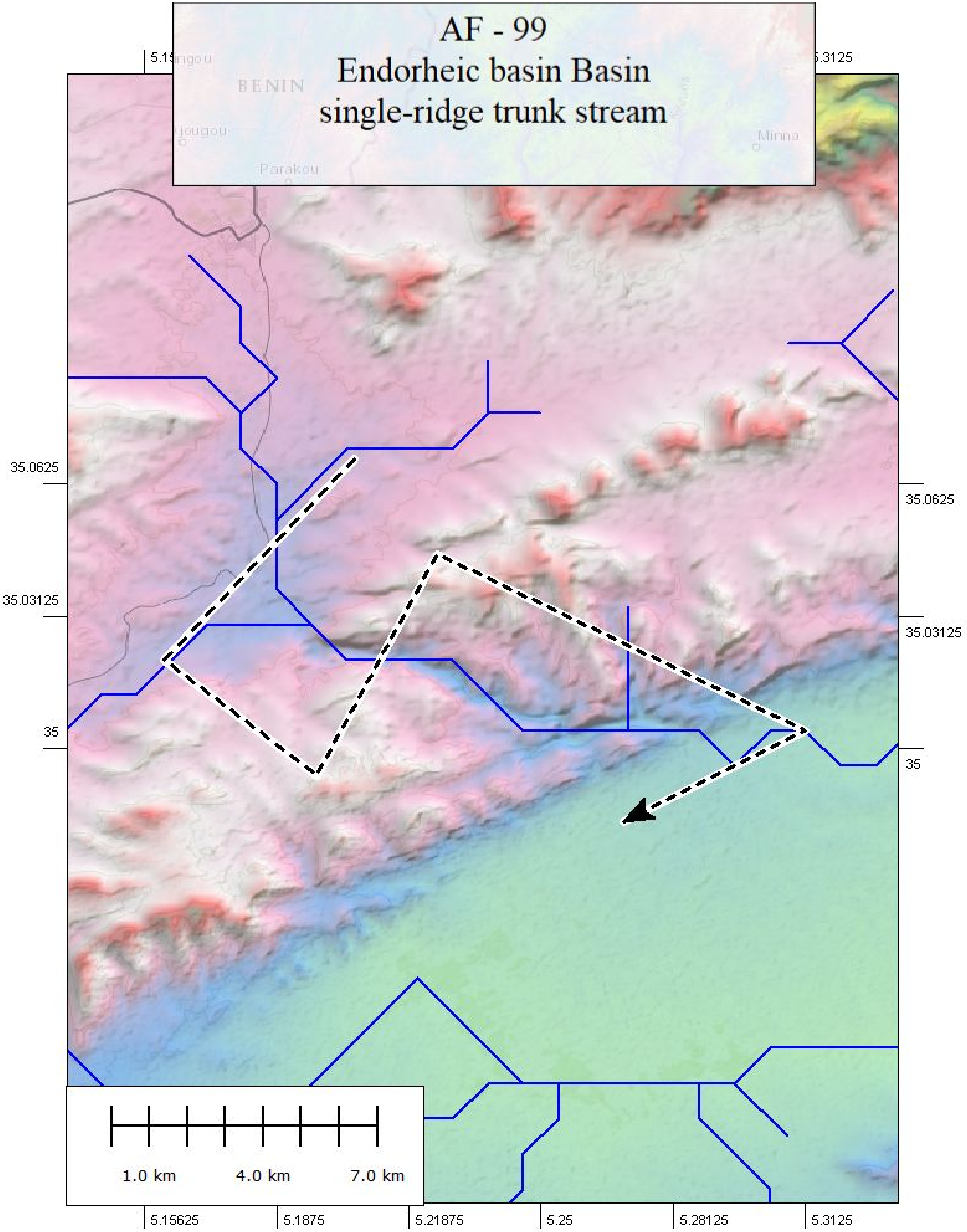
AF - 99
Endorheic basin Basin
single-ridge trunk stream
BENIN
Parakou
Minna
35.0625
35.03125
35
5.15625
5.1875
5.21875
5.25
5.28125
5.3125
1.0 km
4.0 km
7.0 km
