# Supplementary material for: A global database of large-scale transverse drainages
Source: Data Brief. 2019 Jan 14;23:103650. doi: 10.1016/j.dib.2018.12.088 (PMC6369416; doi:10.1016/j.dib.2018.12.088)

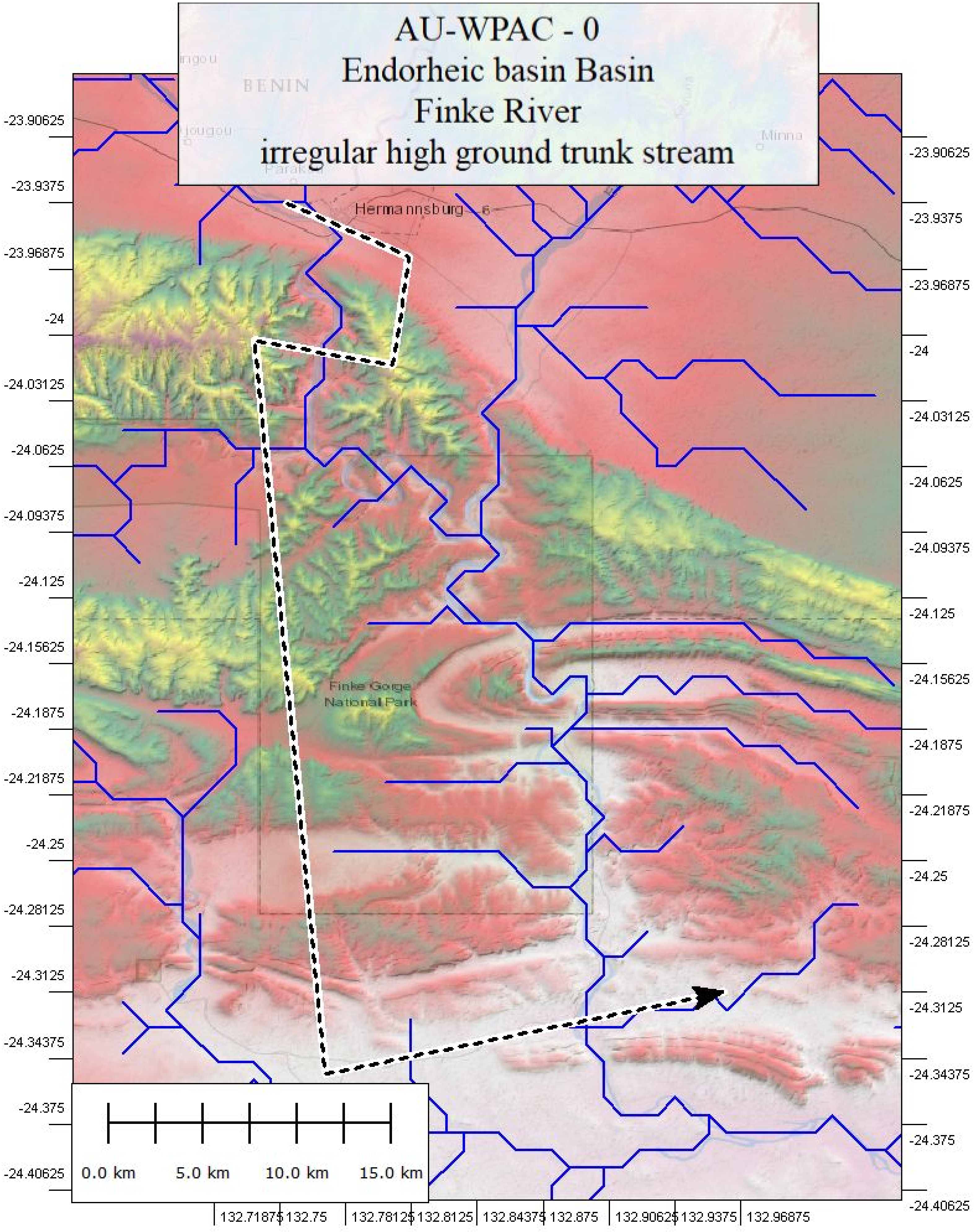
AU-WPAC - 0
Endorheic basin Basin
Finke River
irregular high ground trunk stream
BENIN
Hermannsburg
Finke Gorge National Park
0.0 km
5.0 km
10.0 km
15.0 km
-23.90625
-23.9375
-23.96875
-24
-24.03125
-24.0625
-24.09375
-24.125
-24.15625
-24.1875
-24.21875
-24.25
-24.28125
-24.3125
-24.34375
-24.375
-24.40625
132.71875
132.75
132.78125
132.8125
132.84375
132.875
132.90625
132.9375
132.96875

AU-WPAC - 1
Victoria River Basin
Victoria River
irregular high ground trunk stream

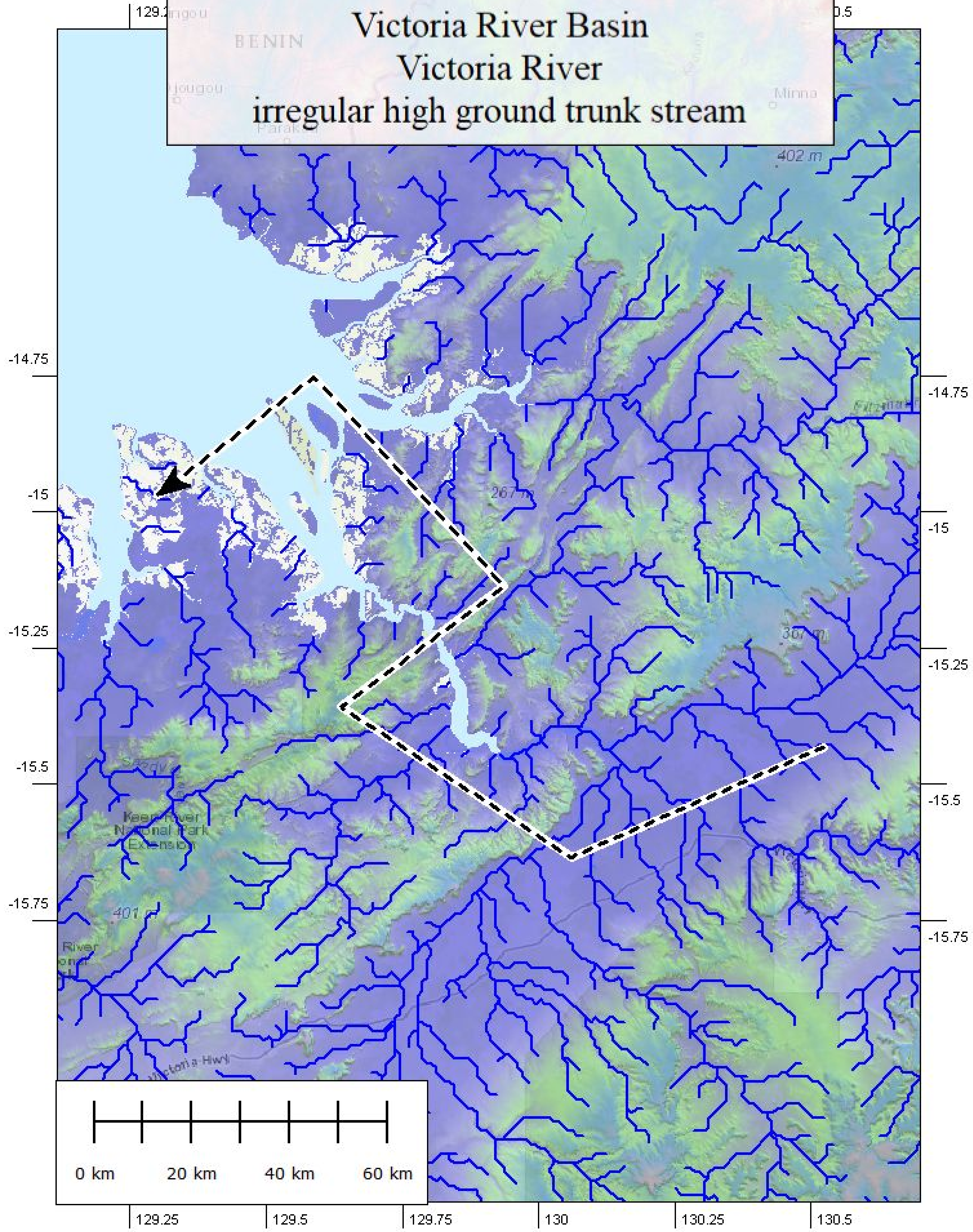

AU-WPAC - 11
Butler River Basin
Buller River
irregular high ground trunk stream

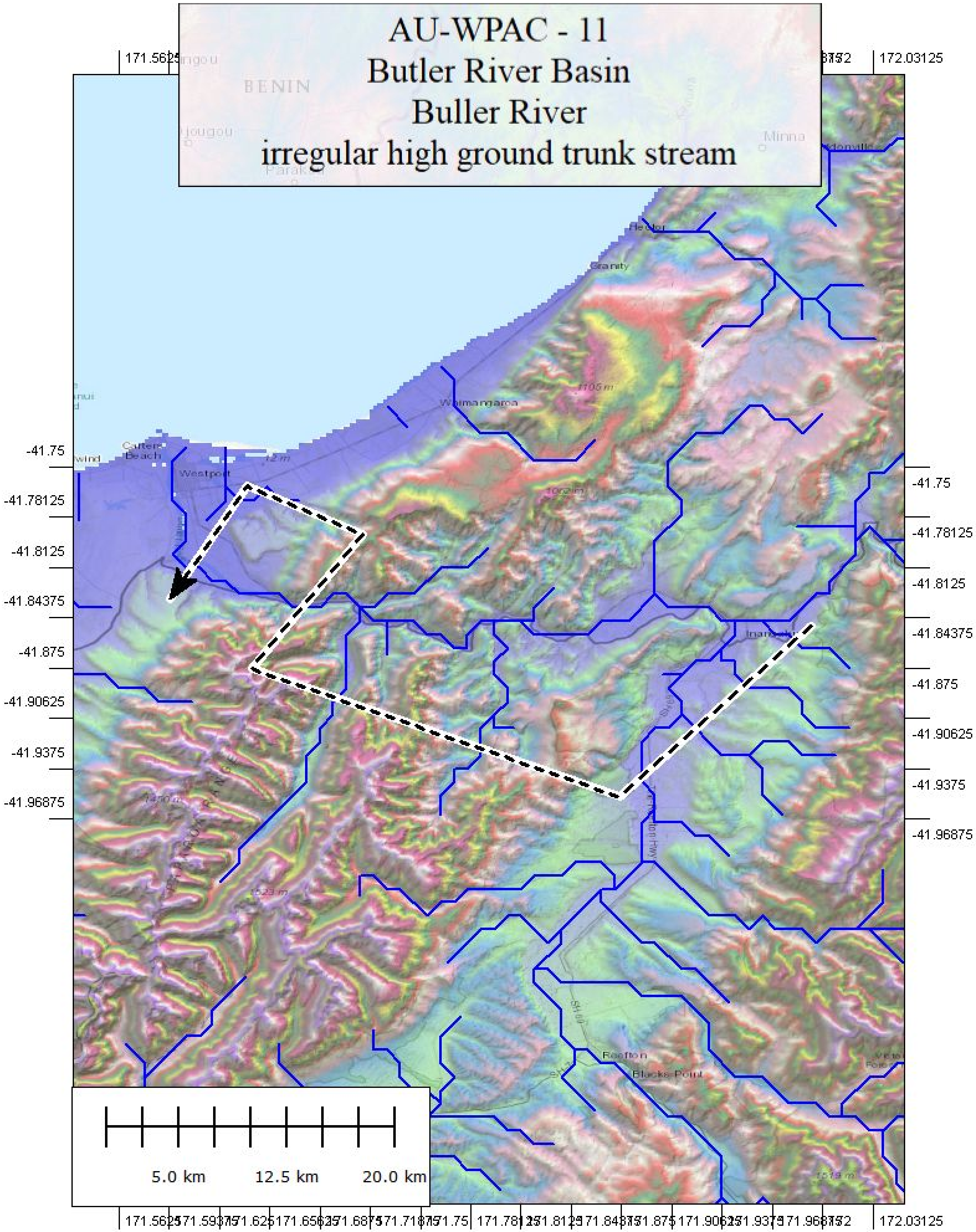

AU-WPAC - 21
Indragiri River Basin
Lake Singkarak tributary
downdip plateau trunk stream

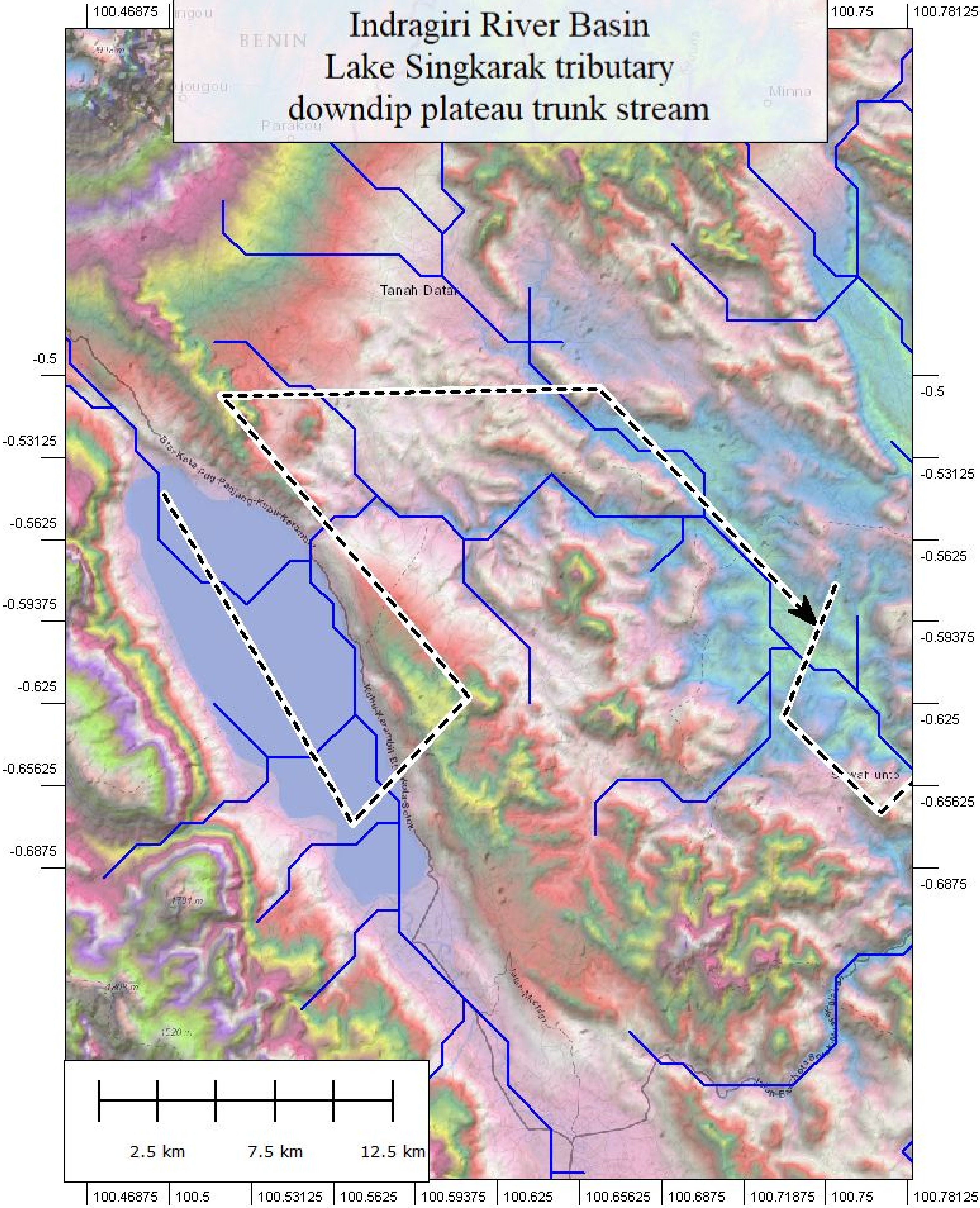

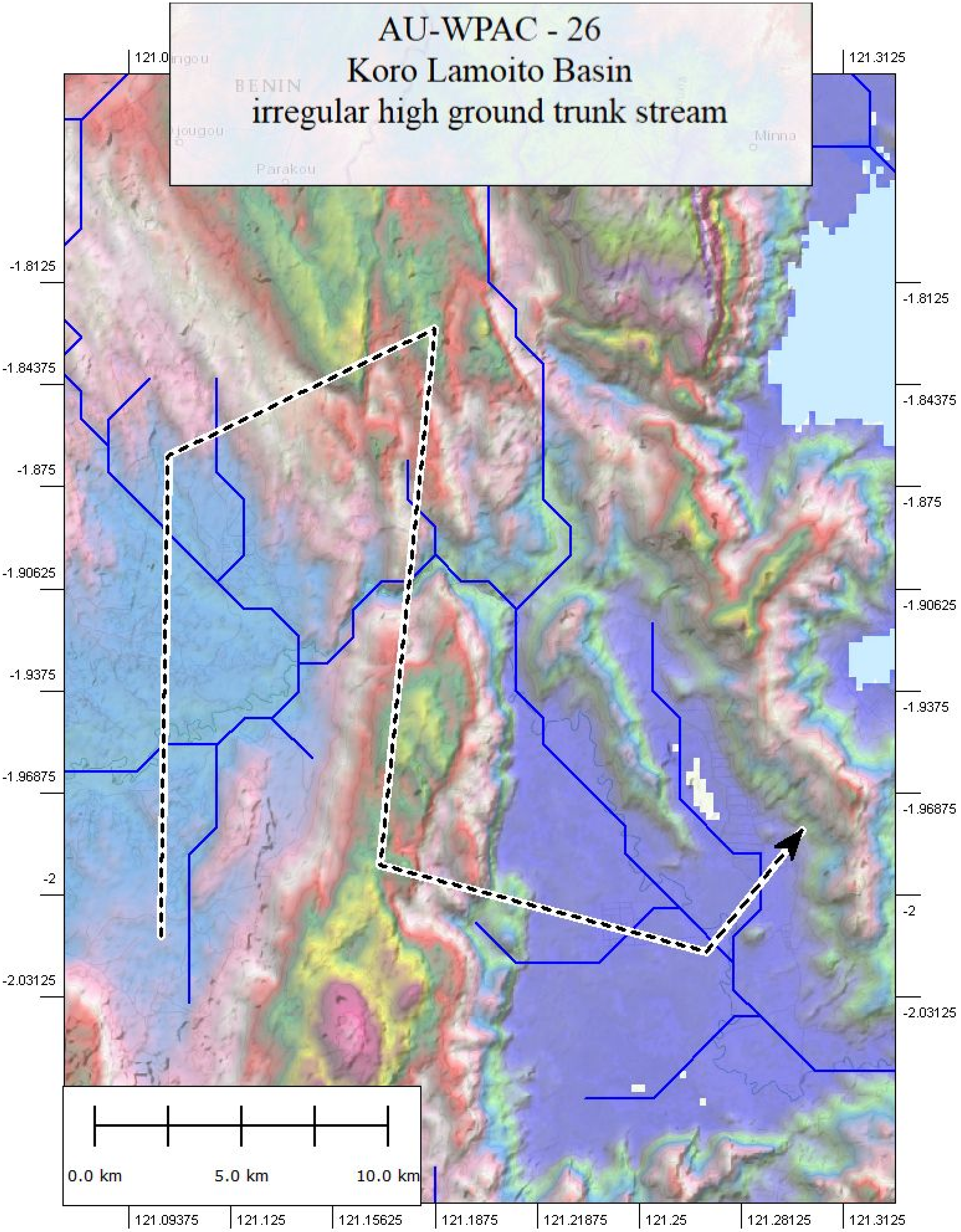
AU-WPAC - 26
Koro Lamoito Basin
irregular high ground trunk stream
121.3125
-1.8125
-1.84375
-1.875
-1.90625
-1.9375
-1.96875
-2
-2.03125
121.09375
121.125
121.15625
121.1875
121.21875
121.25
121.28125
0.0 km
5.0 km
10.0 km
BENIN
Parakou
Minna

AU-WPAC - 29
Palu River Basin
Palu River
irregular high ground trunk stream

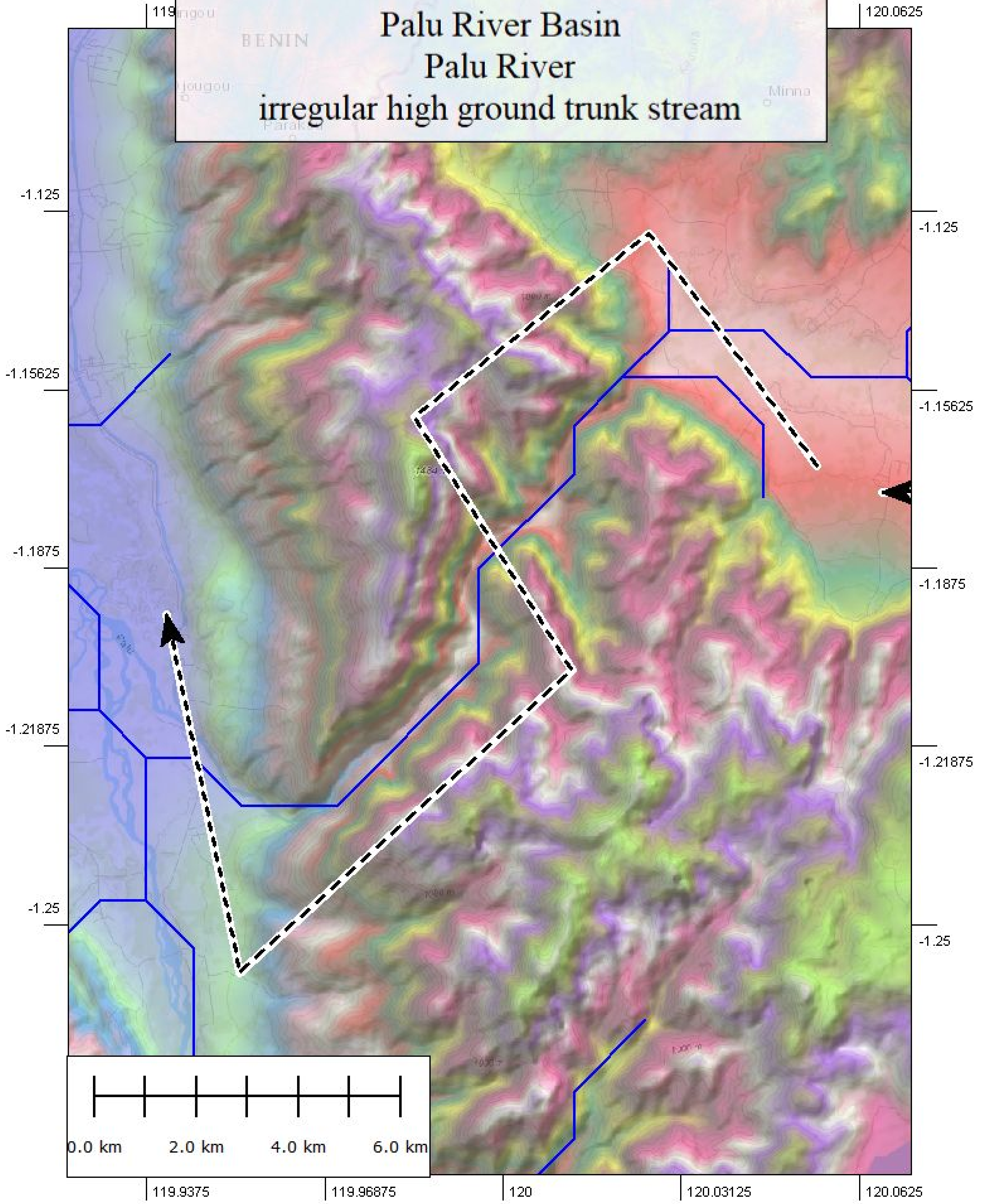

AU-WPAC - 30
Gordon River Basin
Gordon River
plateau trunk stream

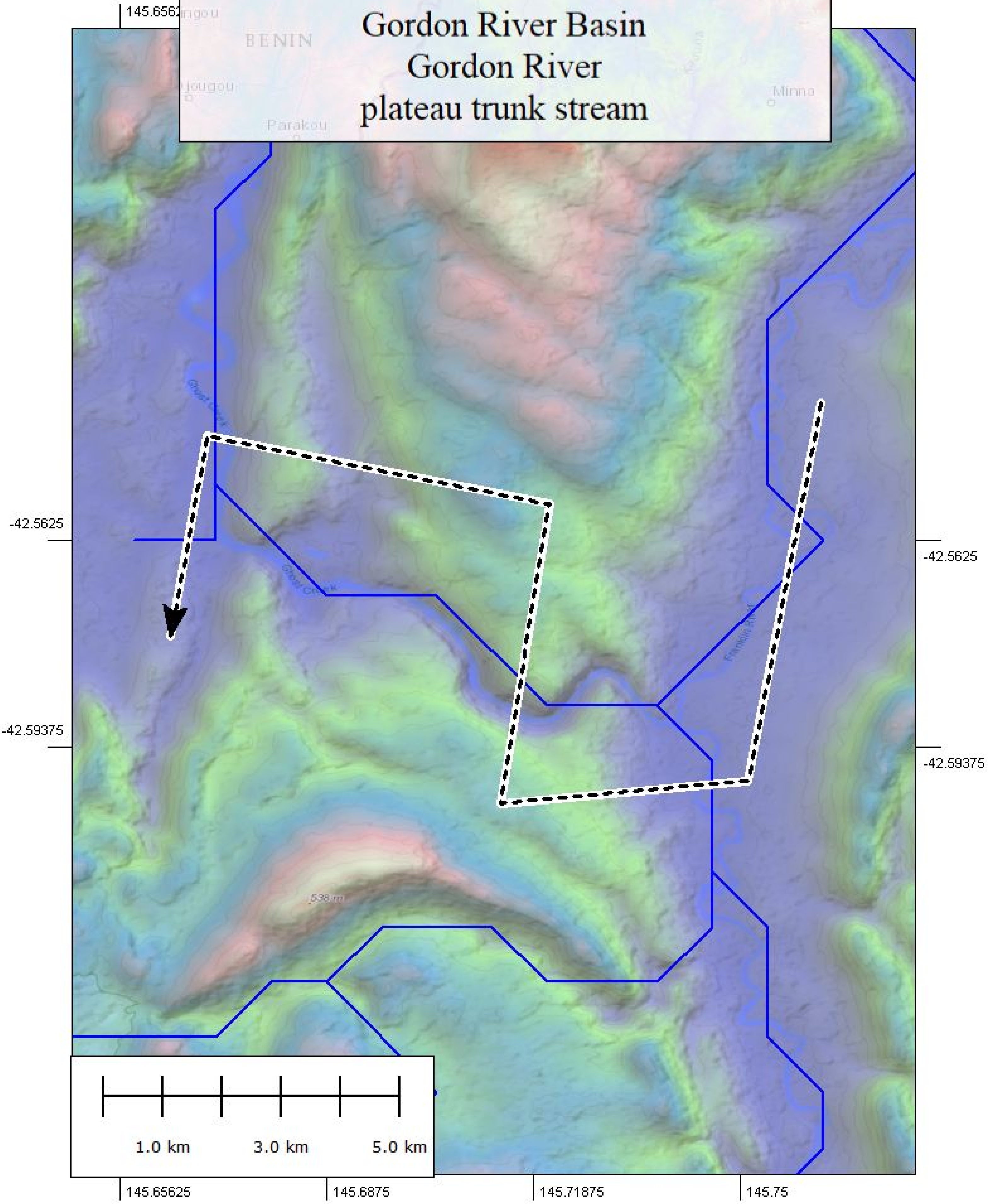

AU-WPAC - 33
Murray-Darling River Basin
Goulborn River
plateau trunk stream

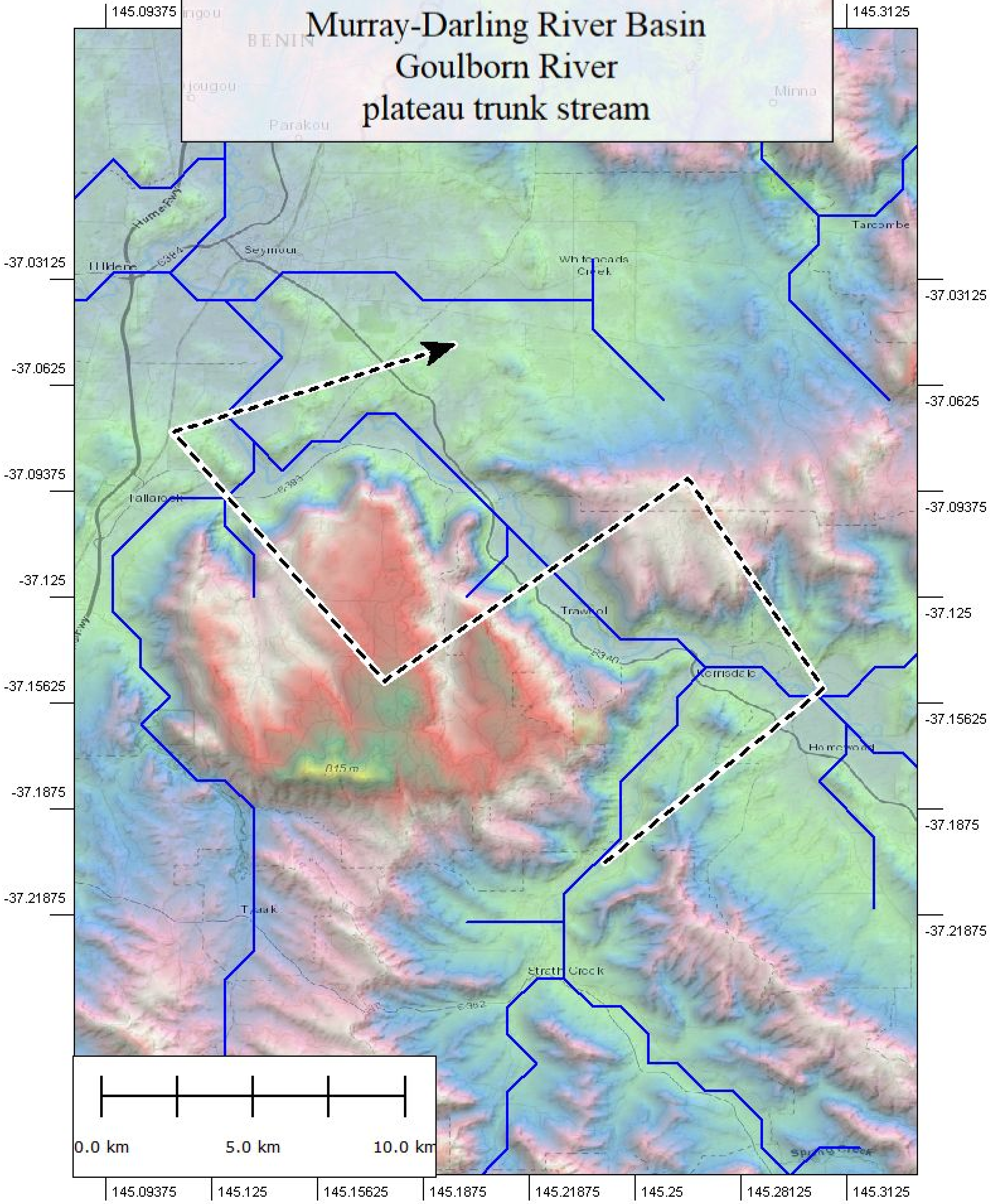

AU-WPAC - 34
Waiau River Basin
Waiau River
irregular high ground trunk stream

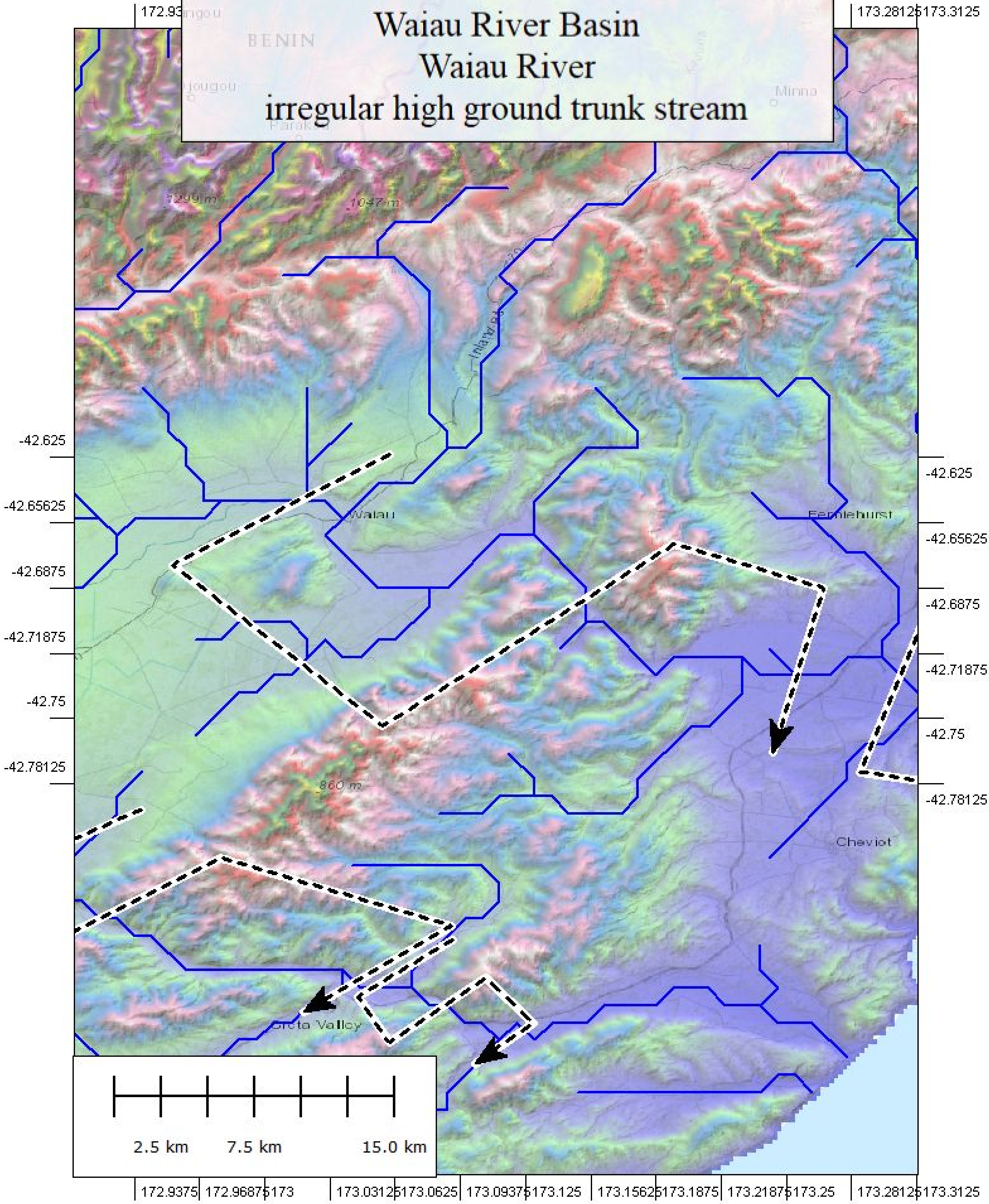

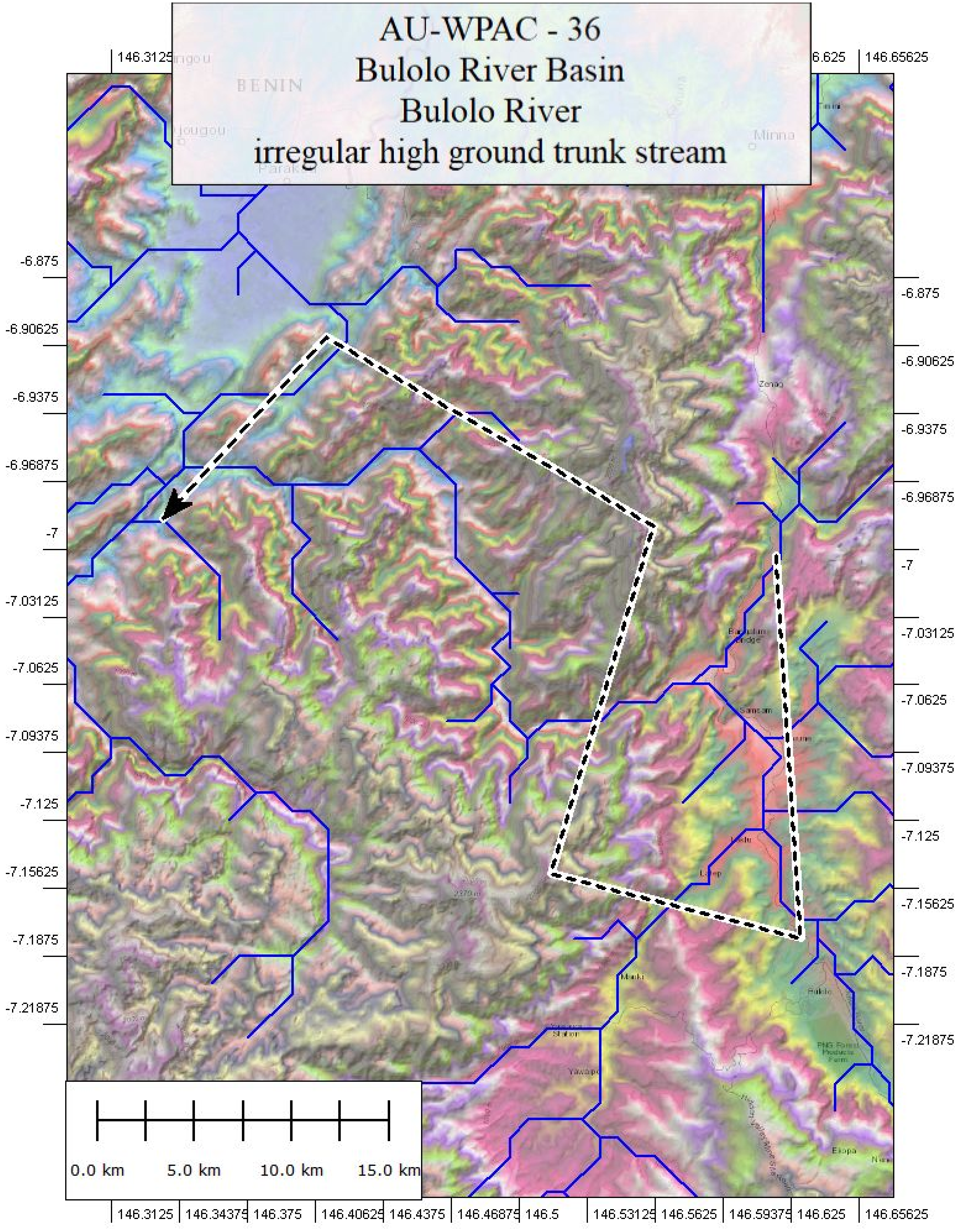
AU-WPAC - 36
Bulolo River Basin
Bulolo River
irregular high ground trunk stream
146.3125
146.34375
146.375
146.40625
146.4375
146.46875
146.5
146.53125
146.5625
146.59375
146.625
146.65625
-6.875
-6.90625
-6.9375
-6.96875
-7
-7.03125
-7.0625
-7.09375
-7.125
-7.15625
-7.1875
-7.21875
0.0 km
5.0 km
10.0 km
15.0 km

AU-WPAC - 38
Clarence River Basin
Timbarra River
irregular high ground trunk stream

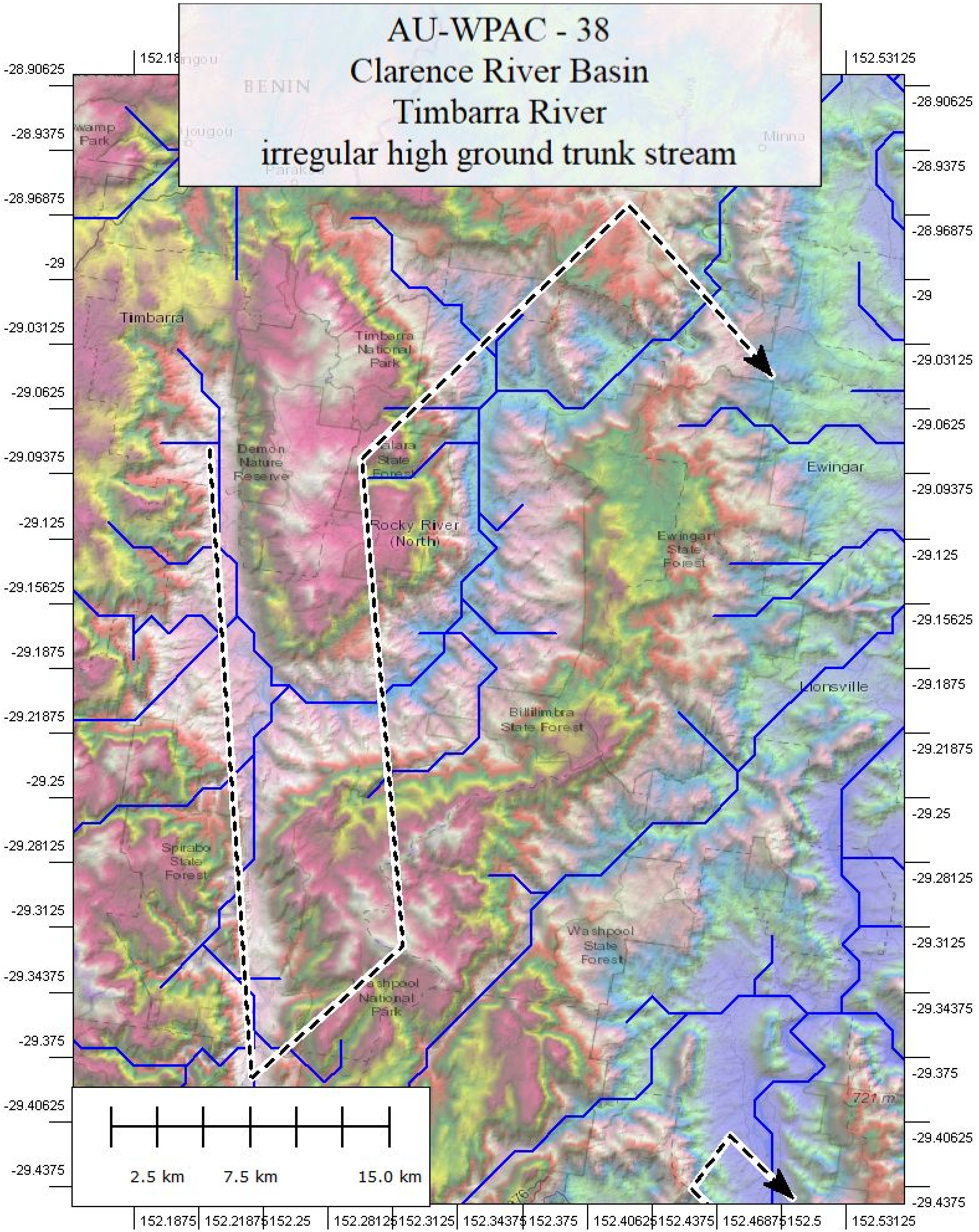

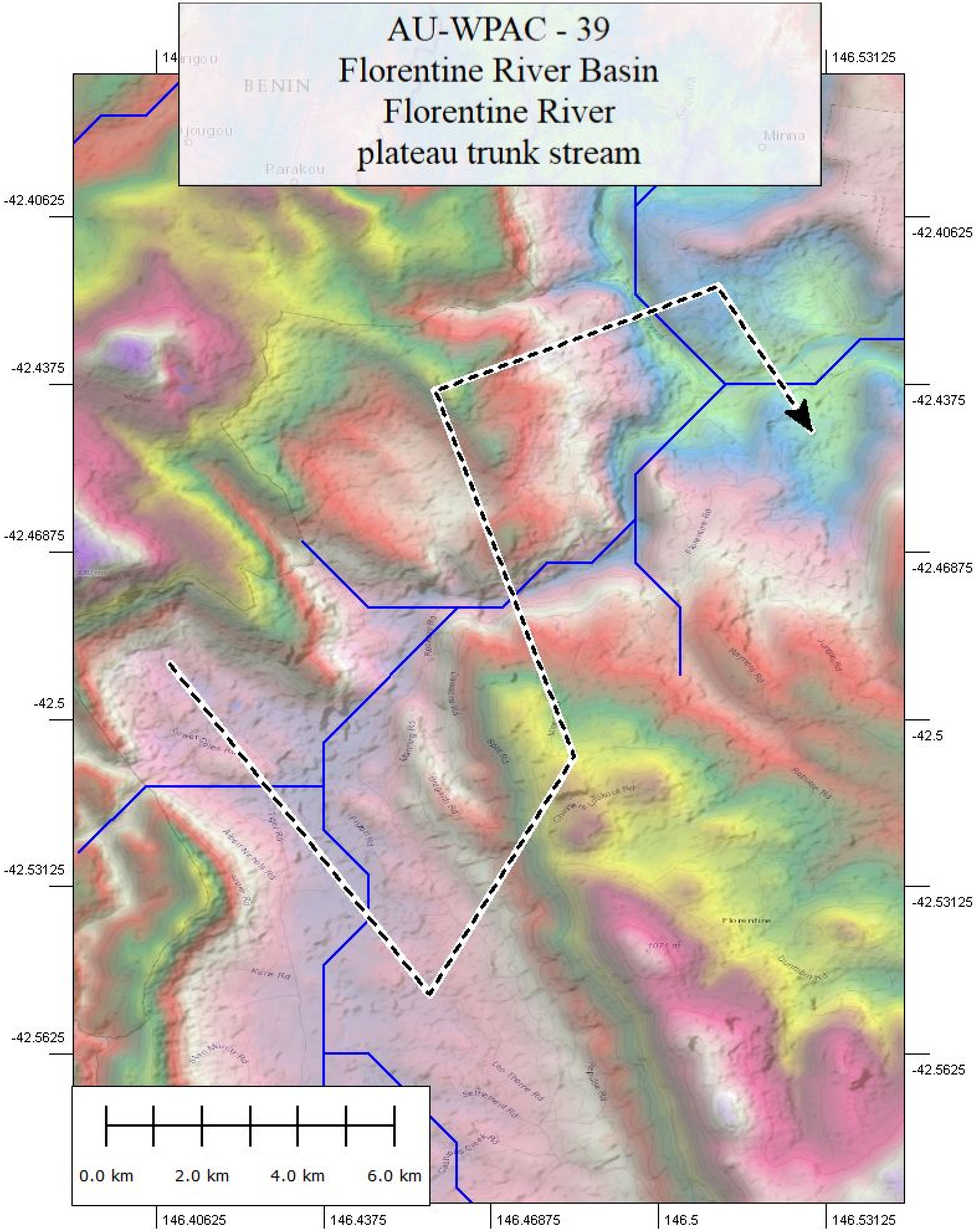
AU-WPAC - 39
Florentine River Basin
Florentine River
plateau trunk stream
146.53125
-42.40625
-42.4375
-42.46875
-42.5
-42.53125
-42.5625
146.40625
146.4375
146.46875
146.5
0.0 km
2.0 km
4.0 km
6.0 km
Florentine
1071 m

AU-WPAC - 43
Baram River Basin
Batang Baram River
irregular high ground trunk stream

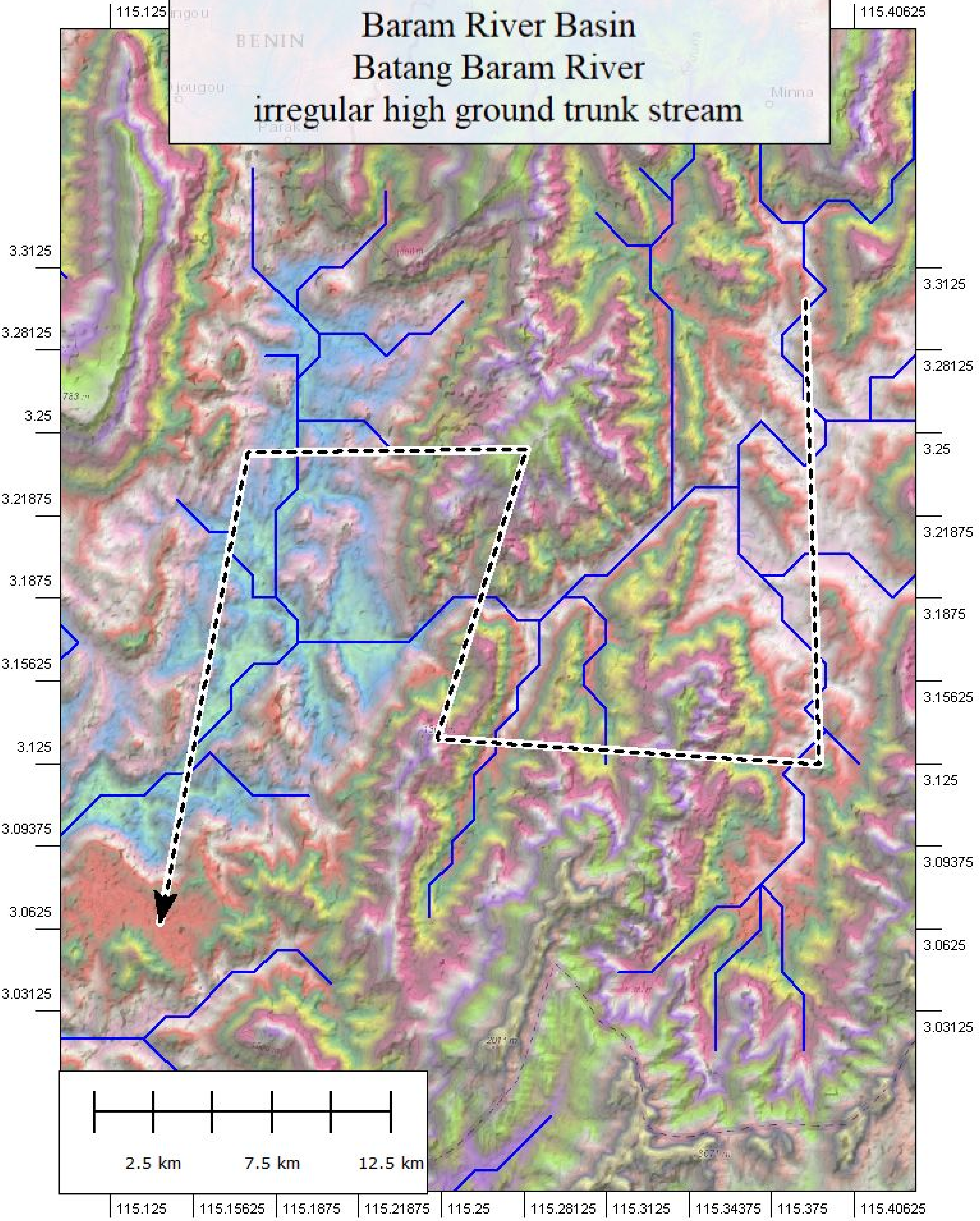

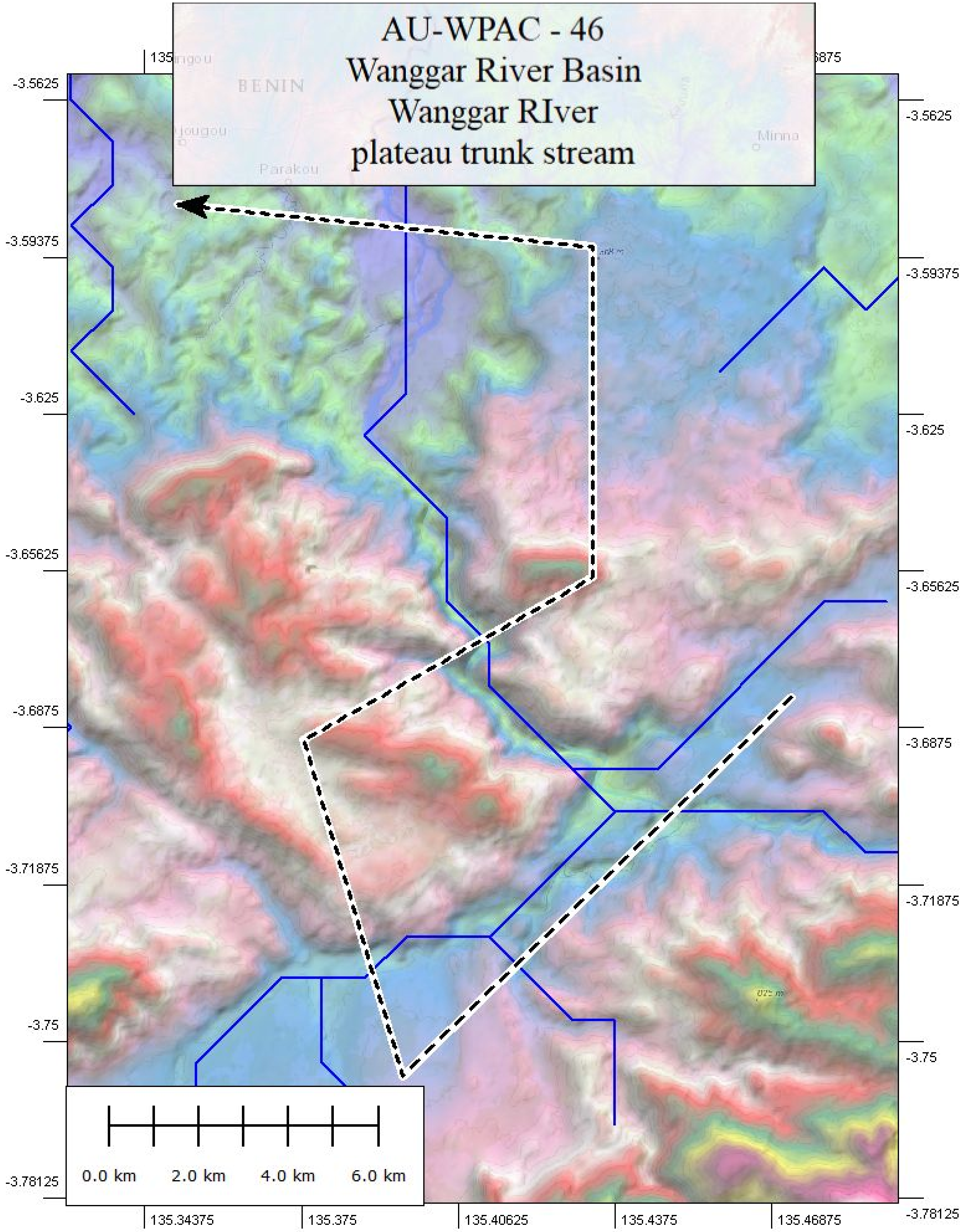
AU-WPAC - 46
Wanggar River Basin
Wanggar RIver
plateau trunk stream
-3.5625
-3.59375
-3.625
-3.65625
-3.6875
-3.71875
-3.75
-3.78125
135.34375
135.375
135.40625
135.4375
135.46875
0.0 km
2.0 km
4.0 km
6.0 km

AU-WPAC - 50
Sungai Kampar Basin
irregular high ground trunk stream

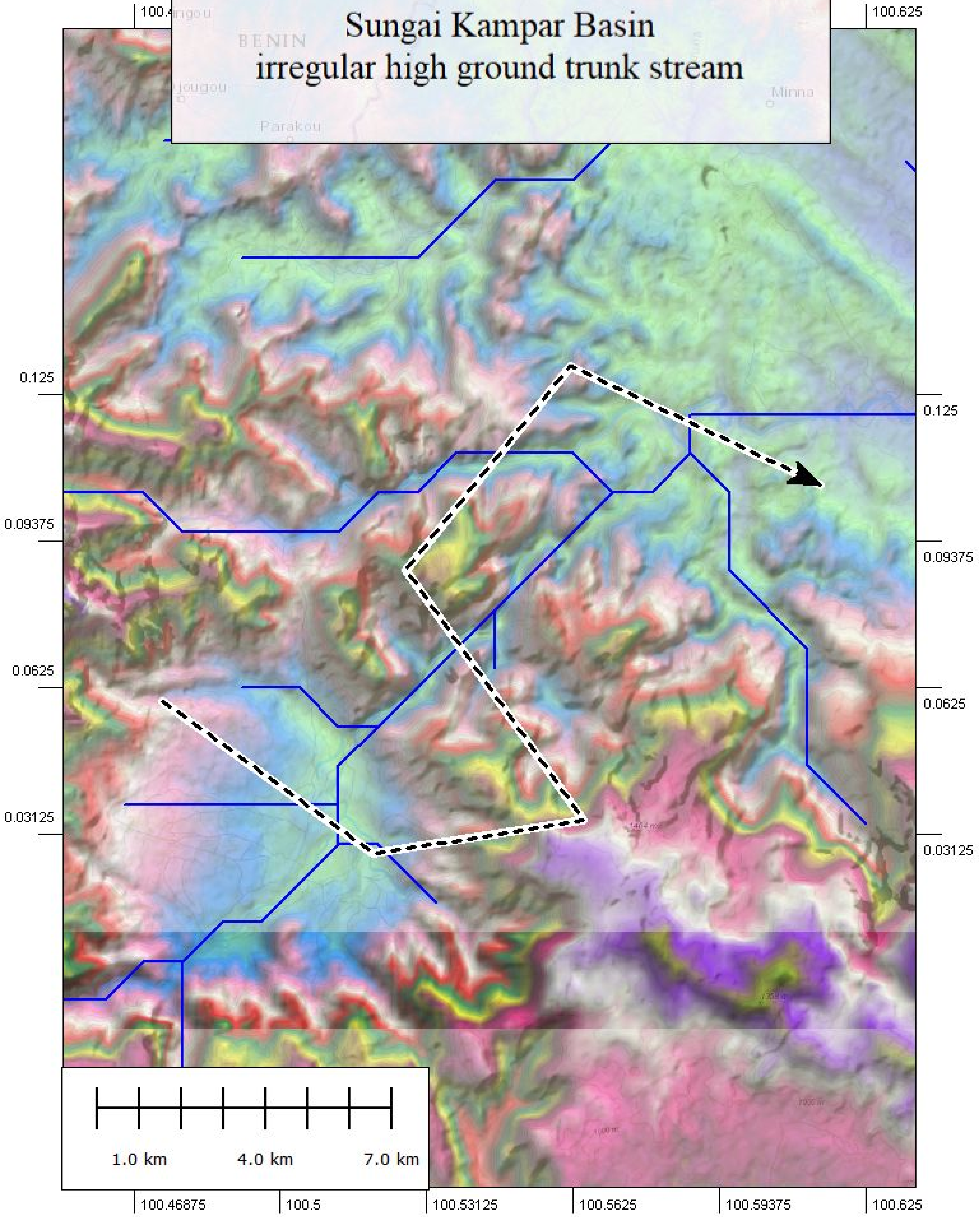

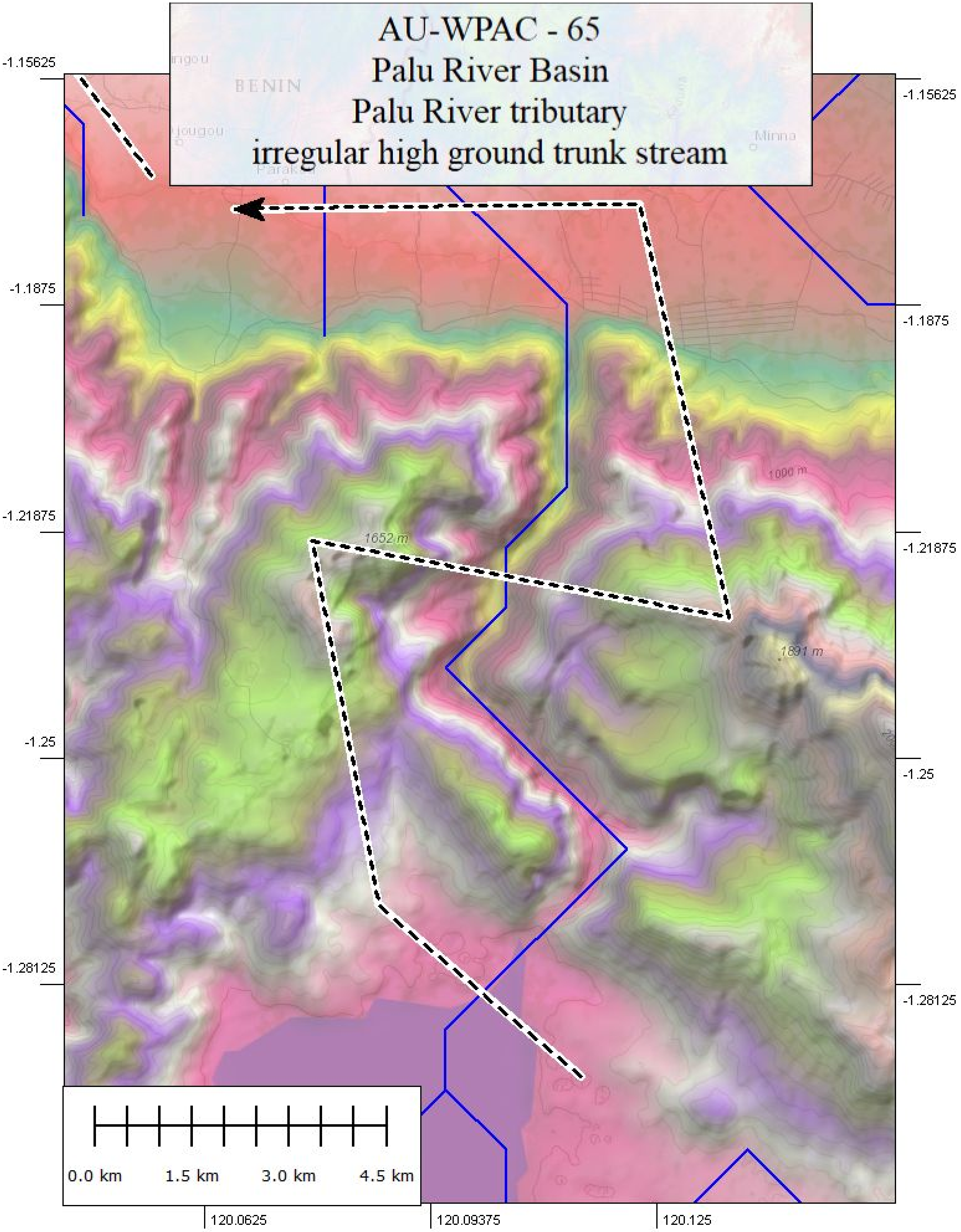
AU-WPAC - 65
Palu River Basin
Palu River tributary
irregular high ground trunk stream
-1.15625
-1.1875
-1.21875
-1.25
-1.28125
1652 m
1891 m
1000 m
BENIN
Minna
0.0 km
1.5 km
3.0 km
4.5 km
120.0625
120.09375
120.125

AU-WPAC - 66
Rokan River Basin
irregular high ground trunk stream

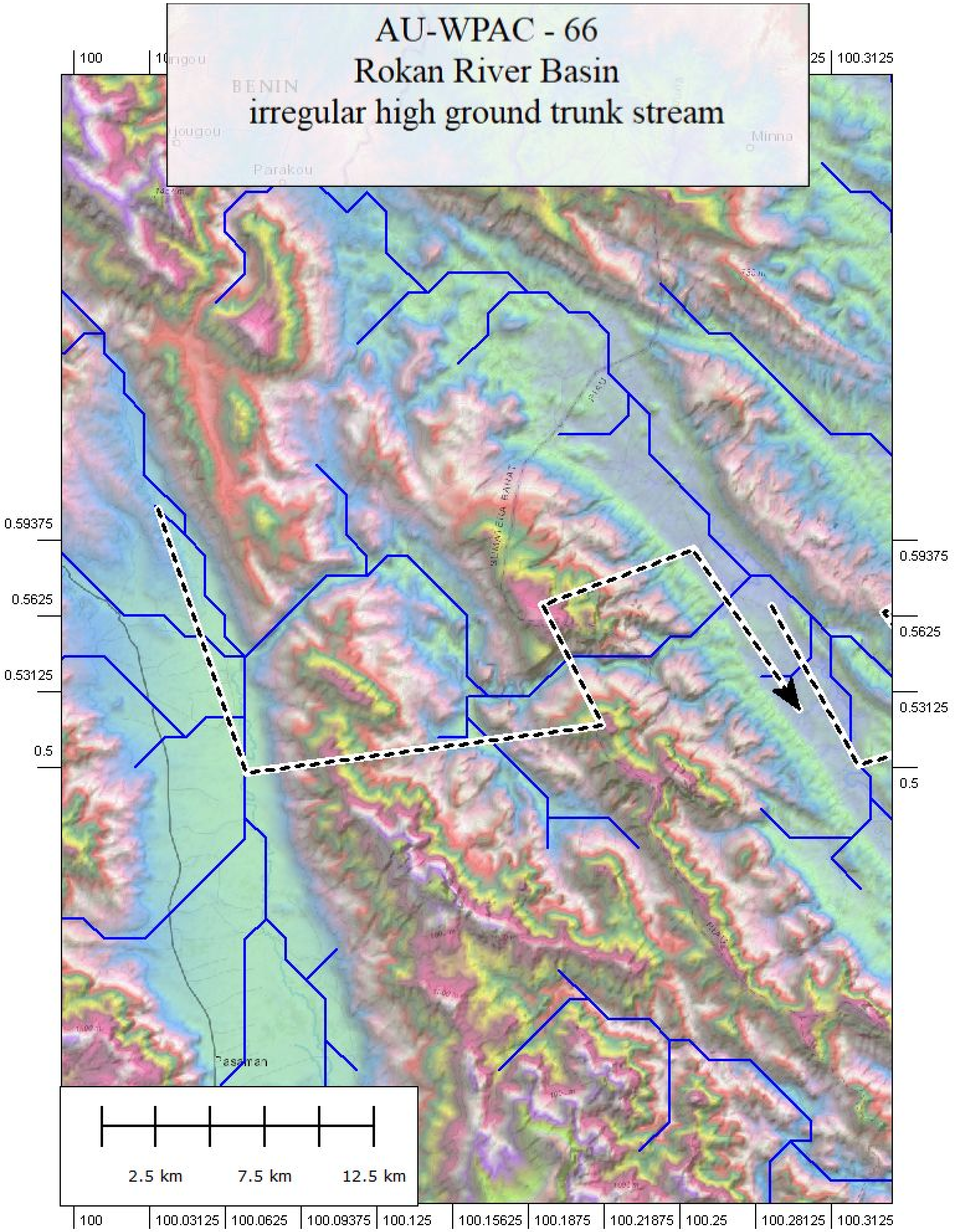

AU-WPAC - 75
Batang Gadis River Basin
irregular high ground trunk stream

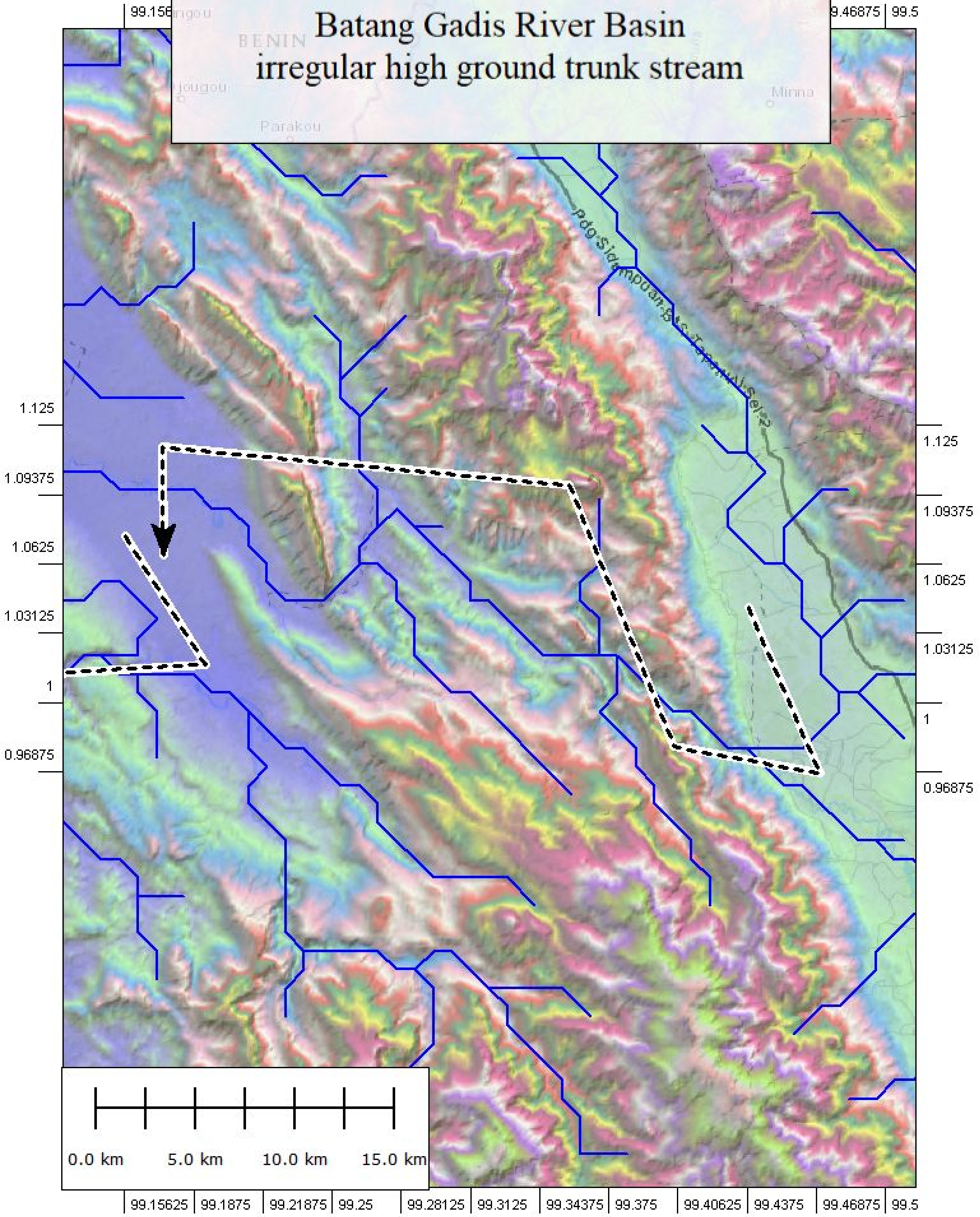

AU-WPAC - 76
Masi River Basin
Musi River
irregular high ground trunk stream

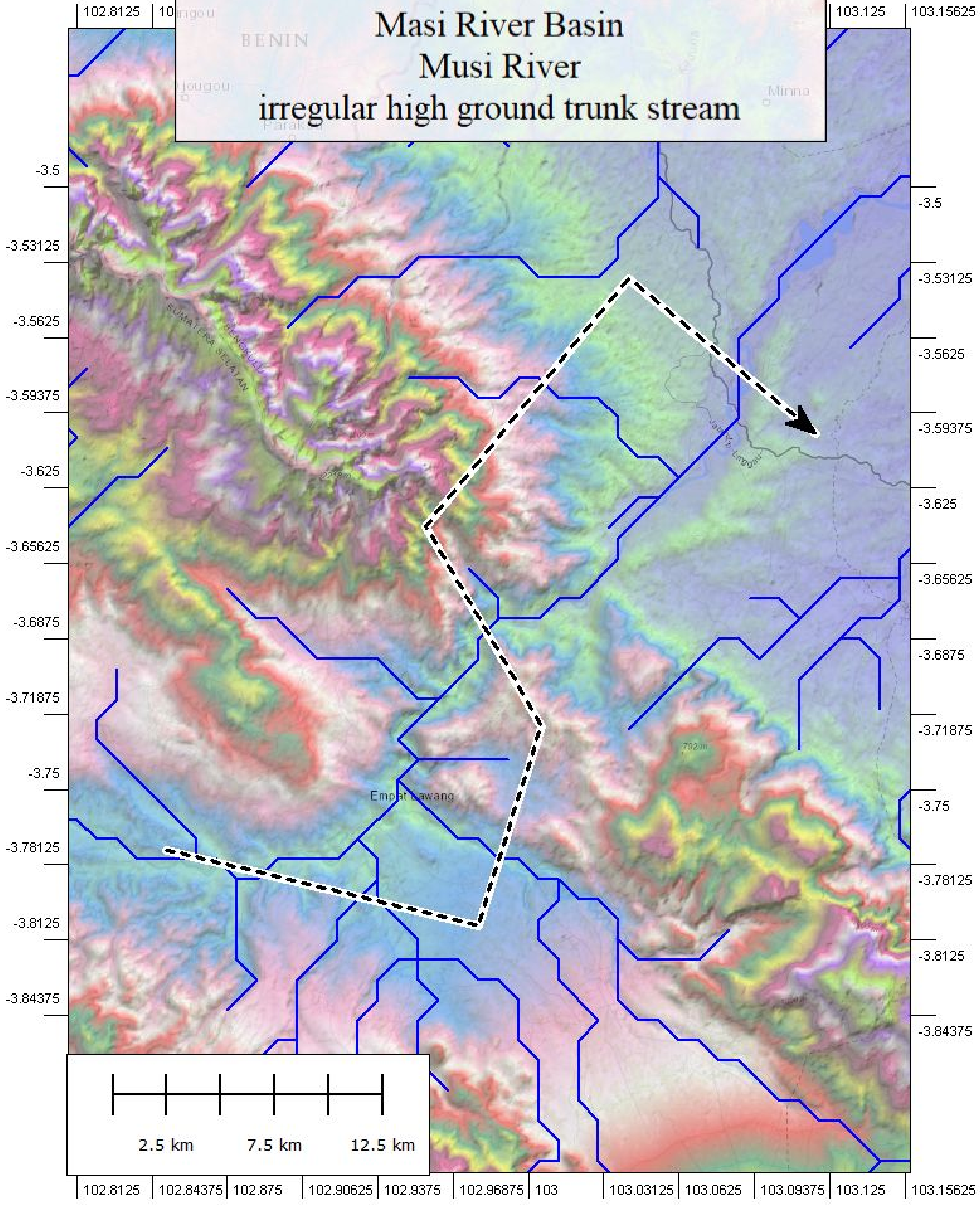

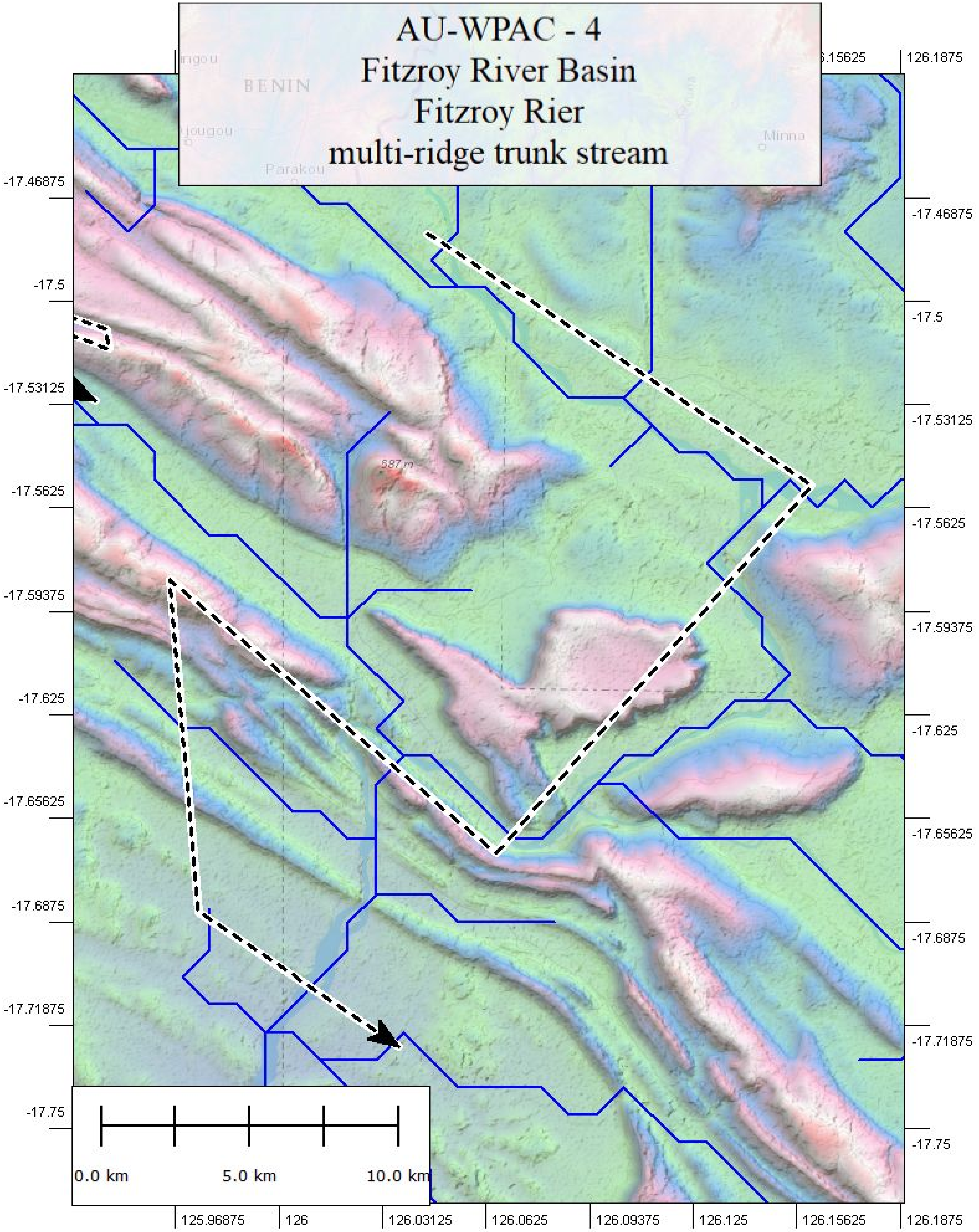
AU-WPAC - 4
Fitzroy River Basin
Fitzroy Rier
multi-ridge trunk stream
587 m
0.0 km
5.0 km
10.0 km
-17.46875
-17.5
-17.53125
-17.5625
-17.59375
-17.625
-17.65625
-17.6875
-17.71875
-17.75
125.96875
126
126.03125
126.0625
126.09375
126.125
126.15625
126.1875

AU-WPAC - 14
King River Basin
King River
multi-ridge trunk stream

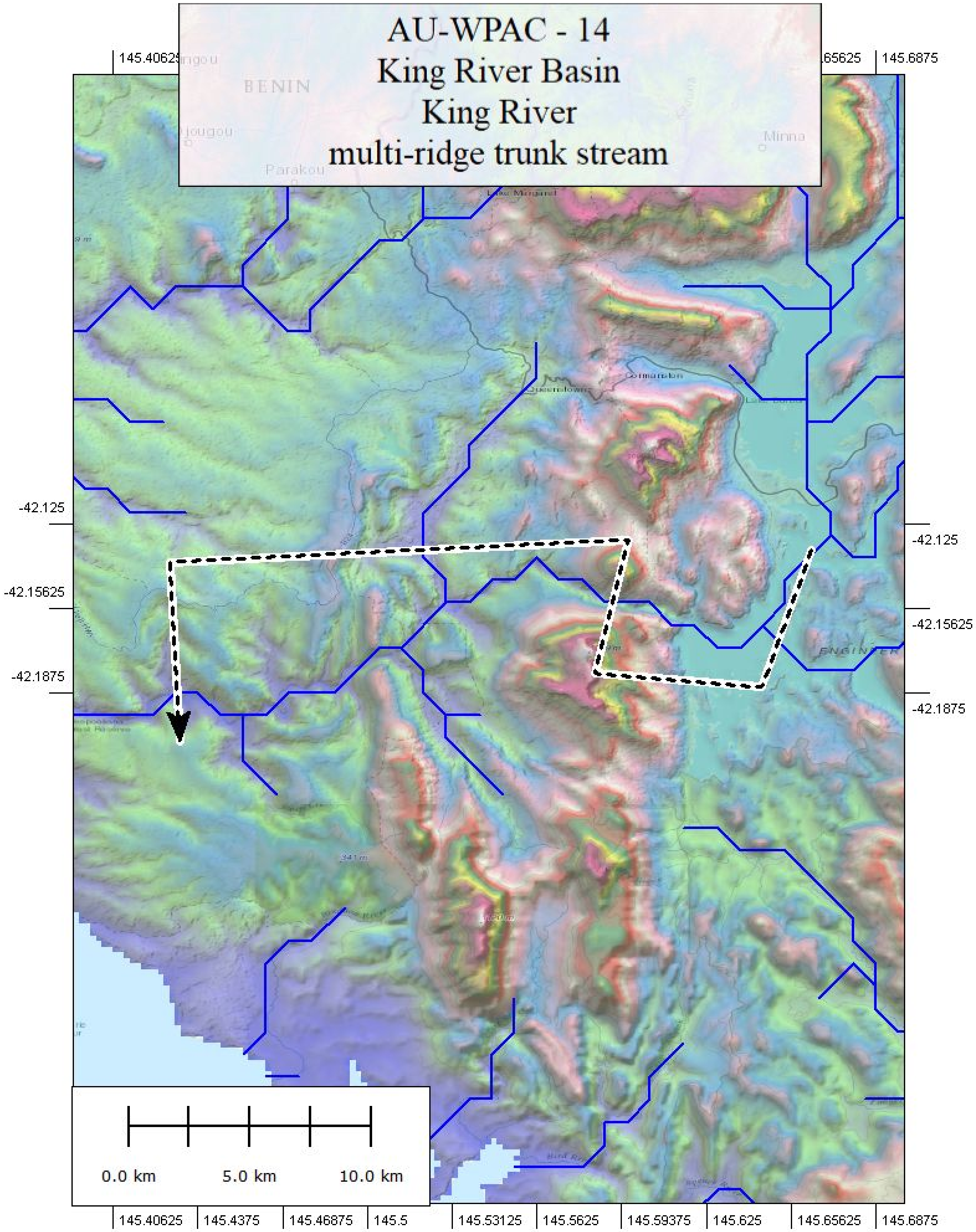

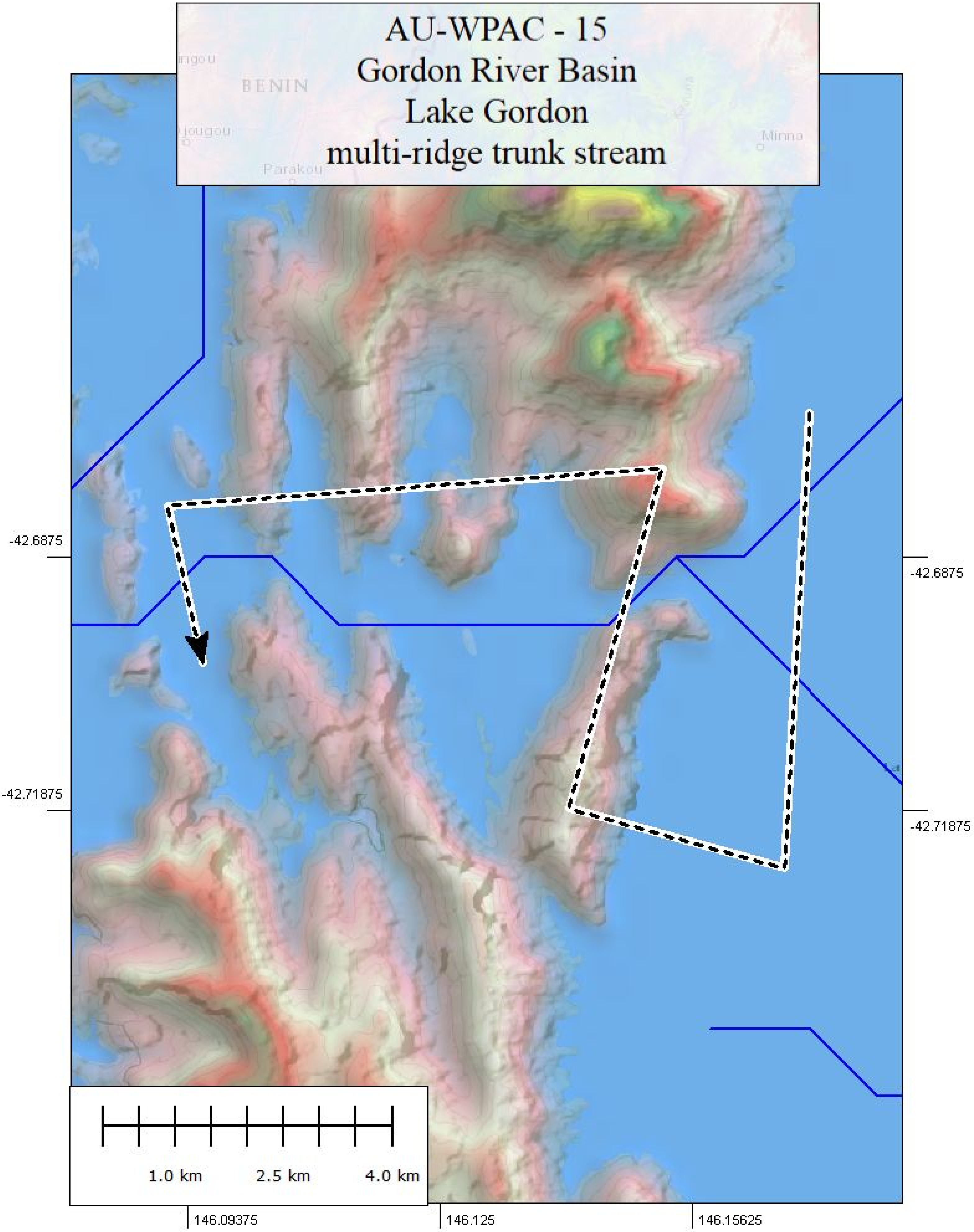
AU-WPAC - 15
Gordon River Basin
Lake Gordon
multi-ridge trunk stream
BENIN
Parakou
Minna
-42.6875
-42.71875
-42.6875
-42.71875
1.0 km
2.5 km
4.0 km
146.09375
146.125
146.15625

AU-WPAC - 20
Kikori River Basin
Waga River
multi-ridge trunk stream

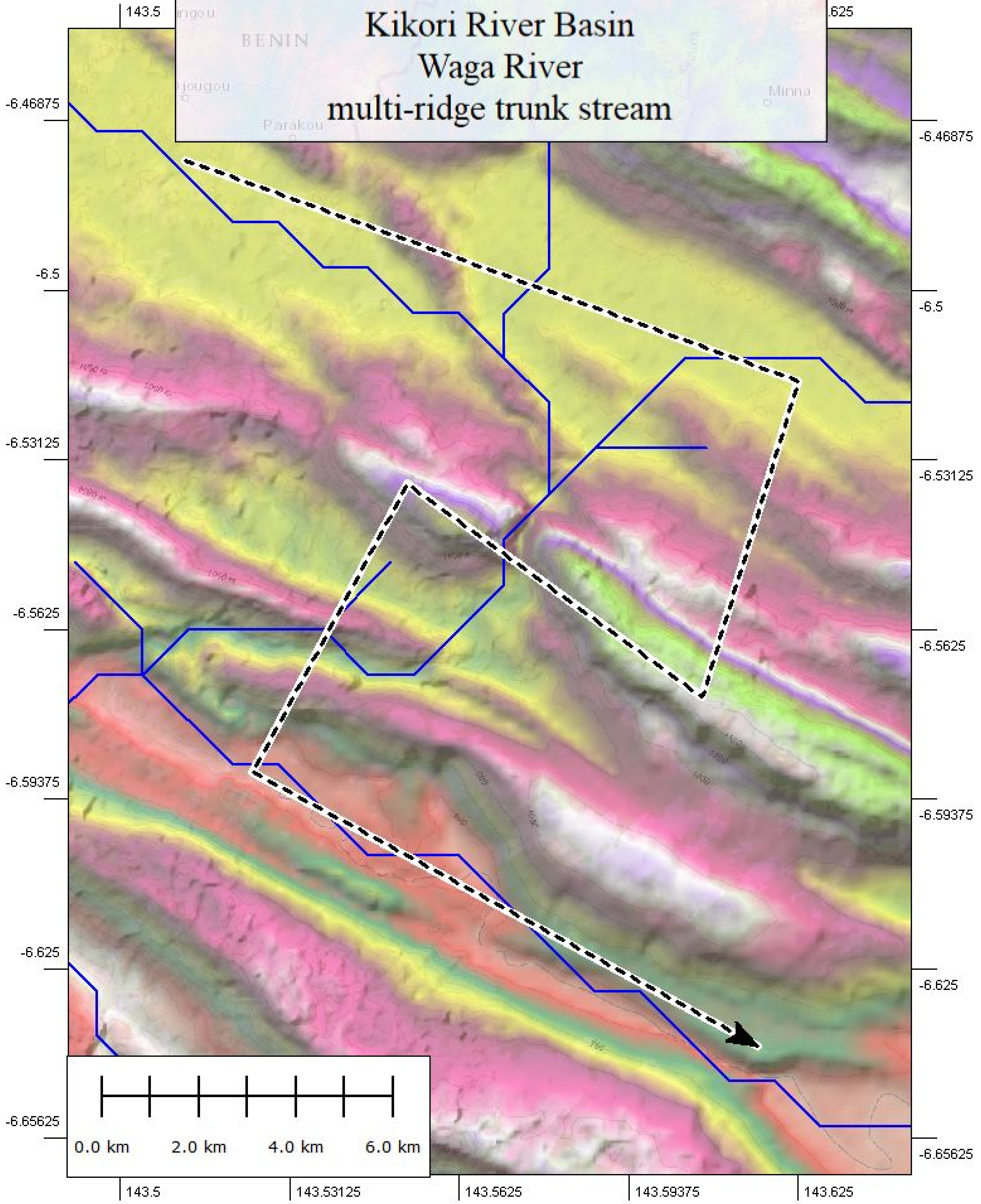

AU-WPAC - 47
Endorheic basin Basin
Finke River
multi-ridge trunk stream

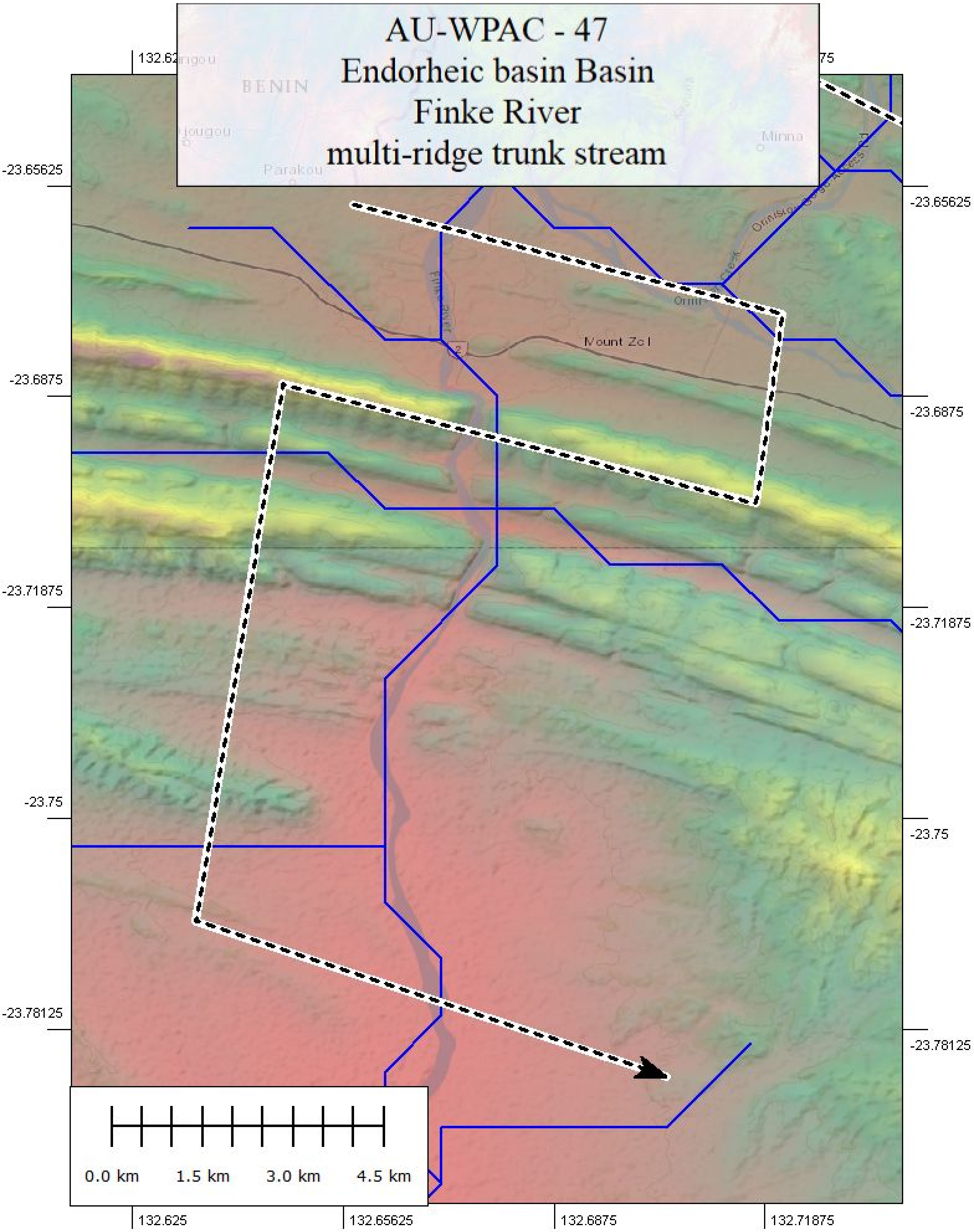

AU-WPAC - 68
Endorheic basin Basin
Finke River
multi-ridge trunk stream

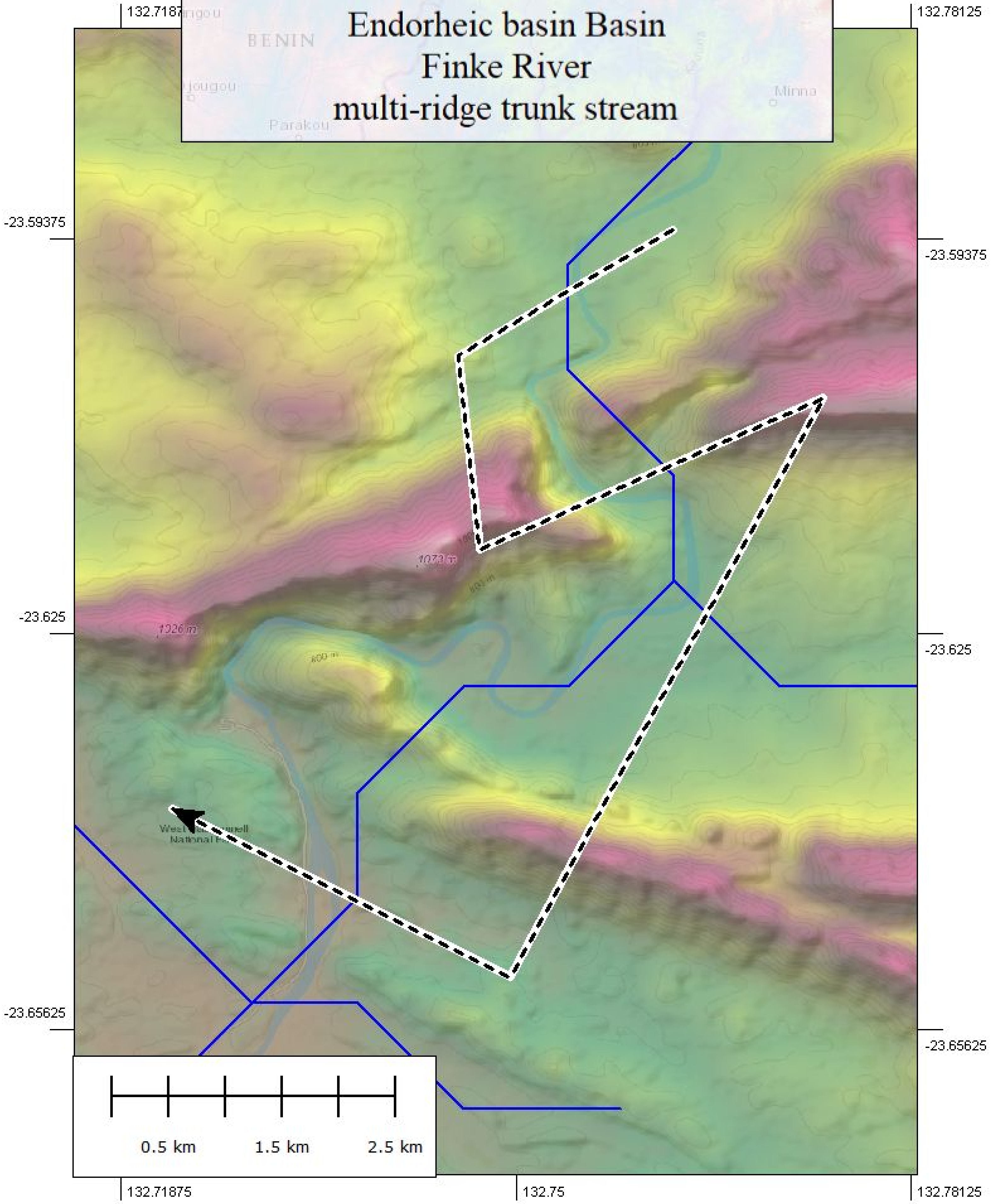

AU-WPAC - 2
Ord River Basin
single-ridge head stream

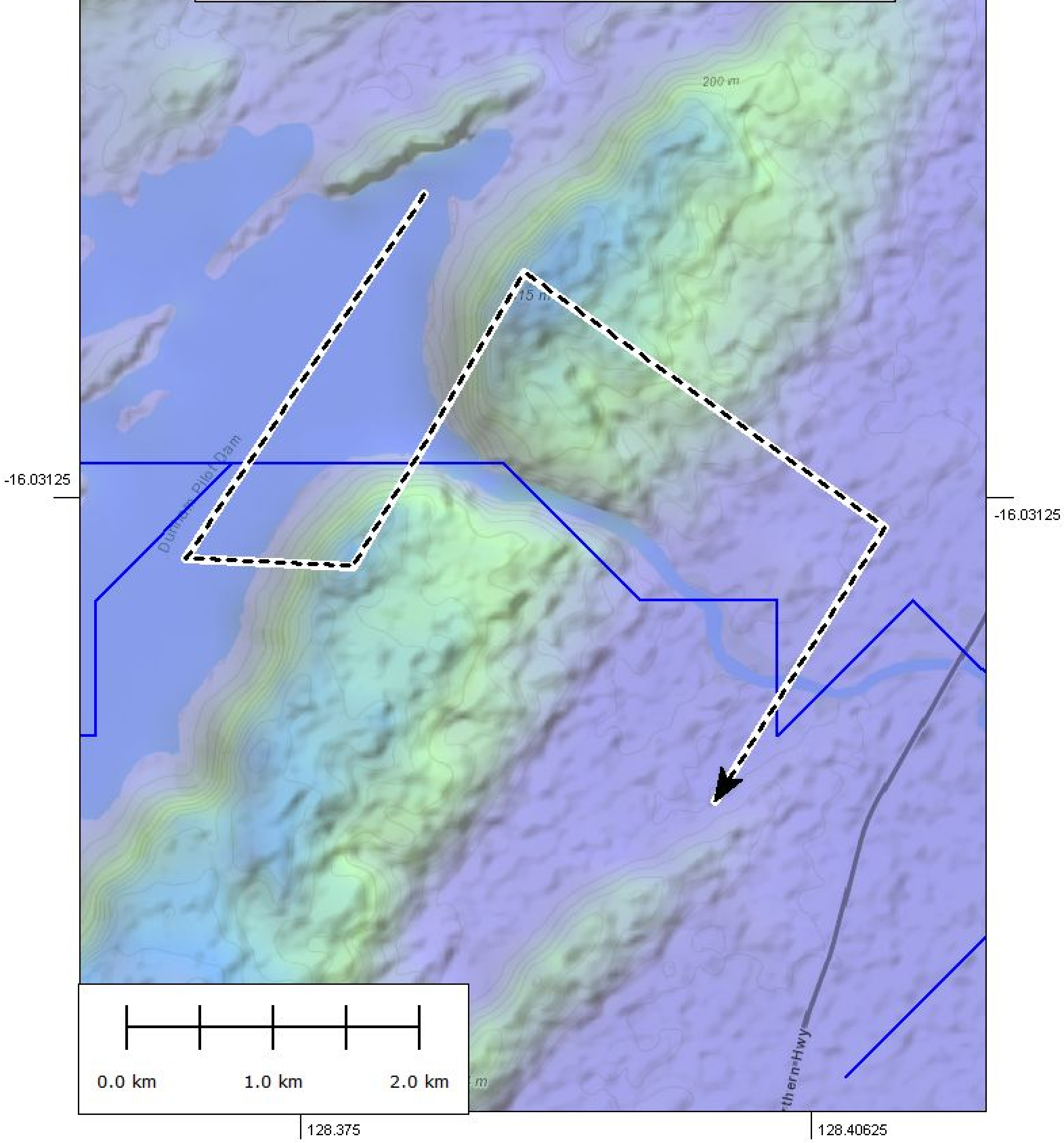

AU-WPAC - 3
Fitzroy River Basin
Fitzroy River tributary
single-ridge head stream

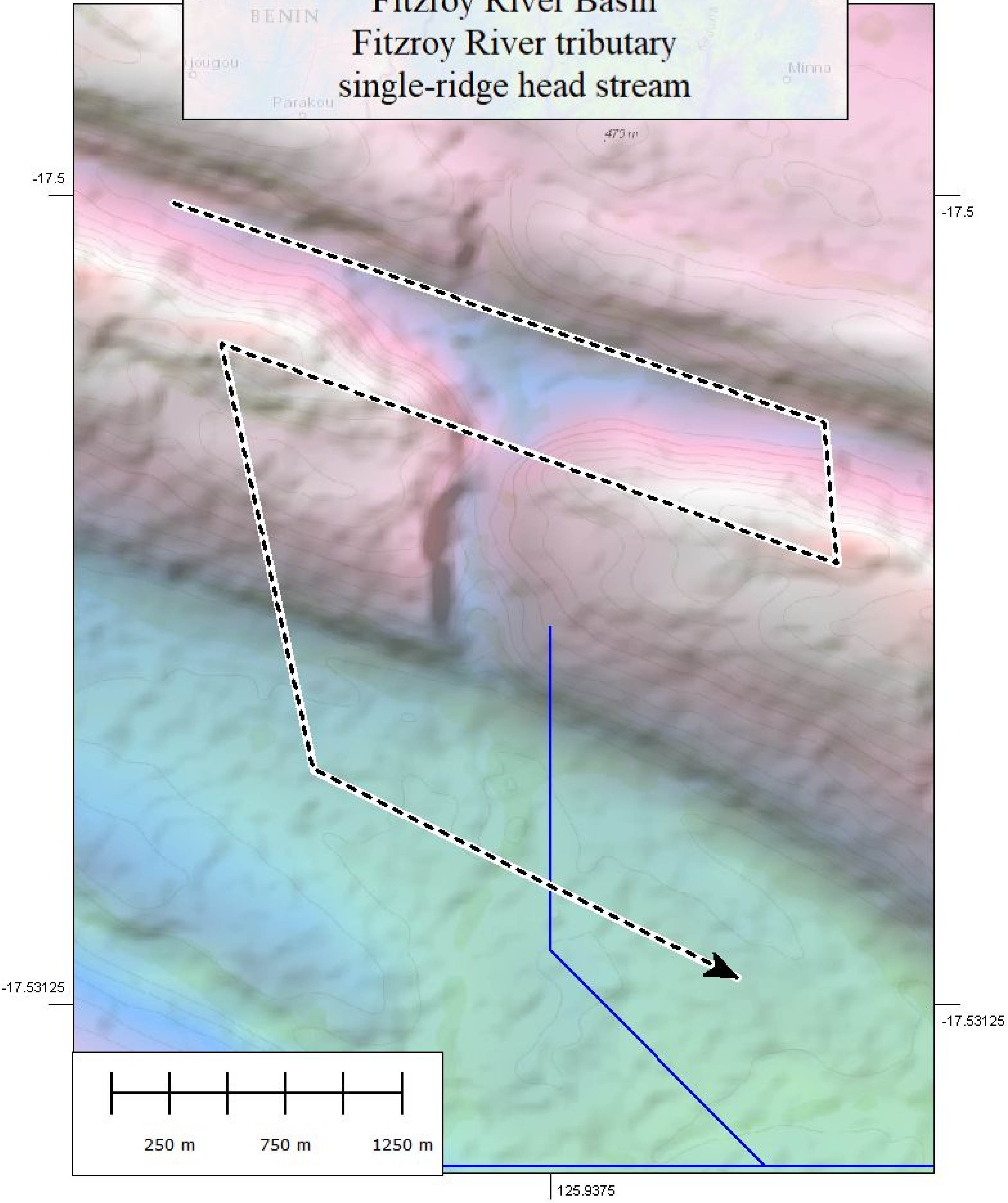

AU-WPAC - 5
Kampar River Basin
single-ridge trunk stream

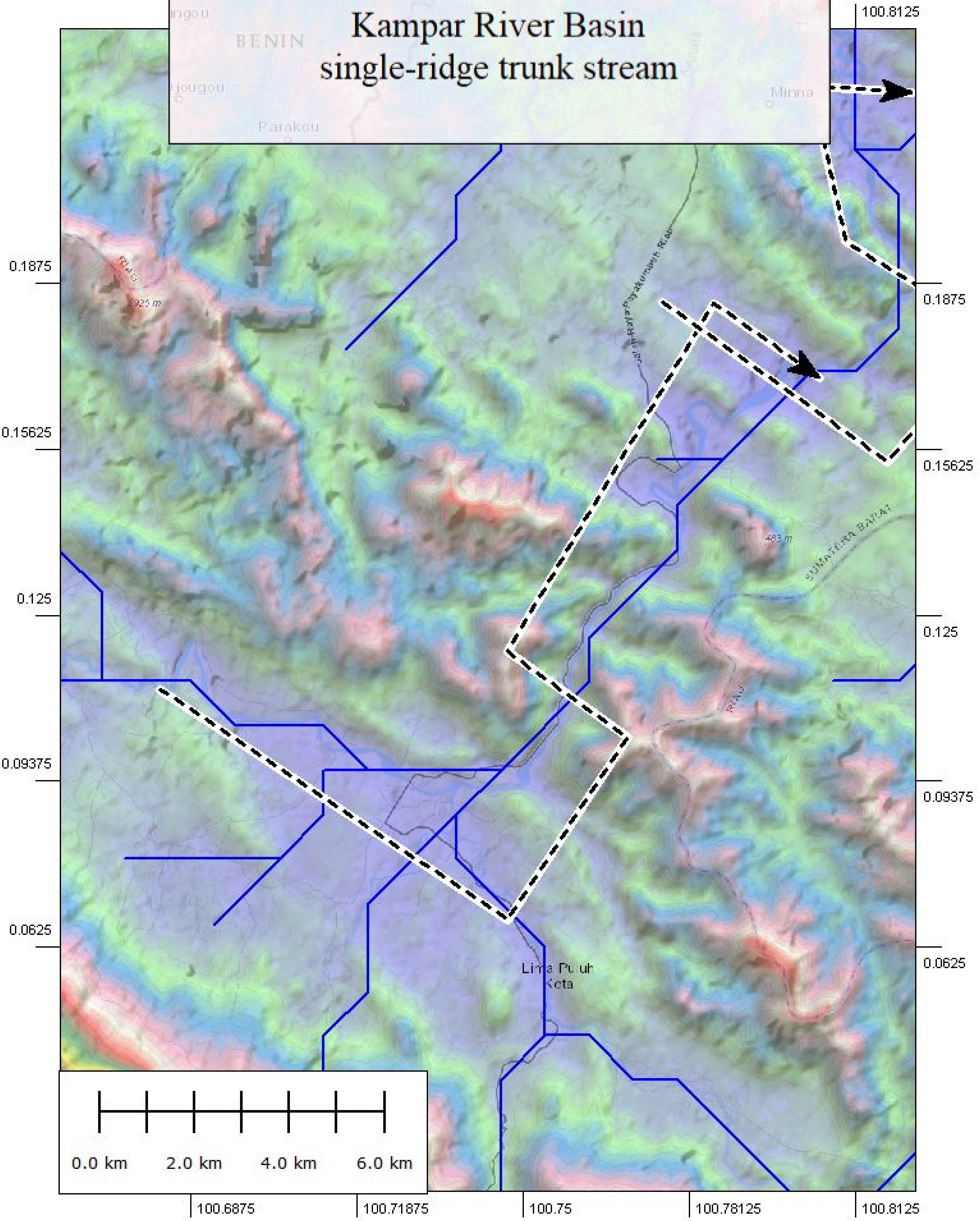

AU-WPAC - 6
Kampar River Basin
single-ridge trunk stream

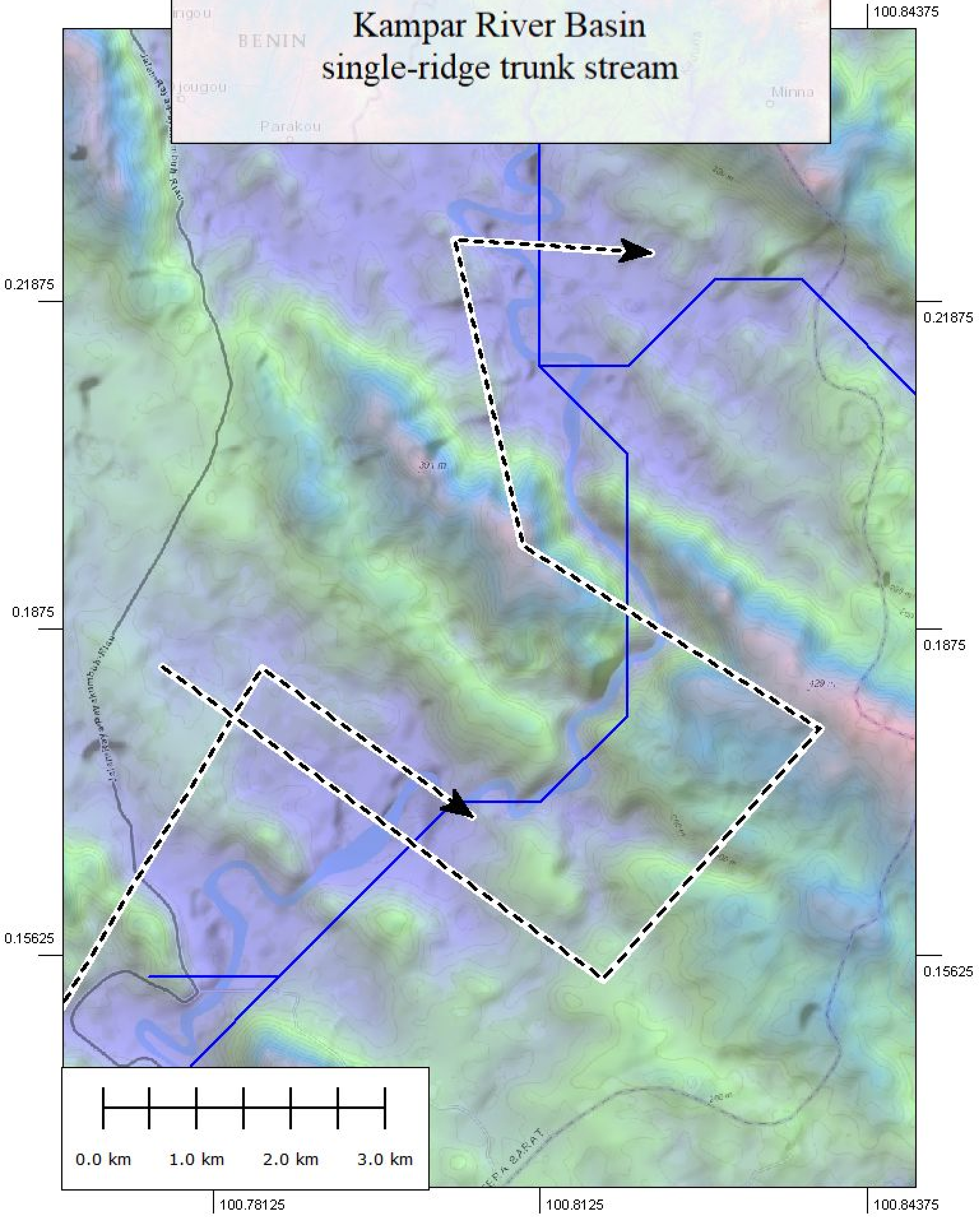

AU-WPAC - 7
Kampar River Basin
single-ridge trunk stream

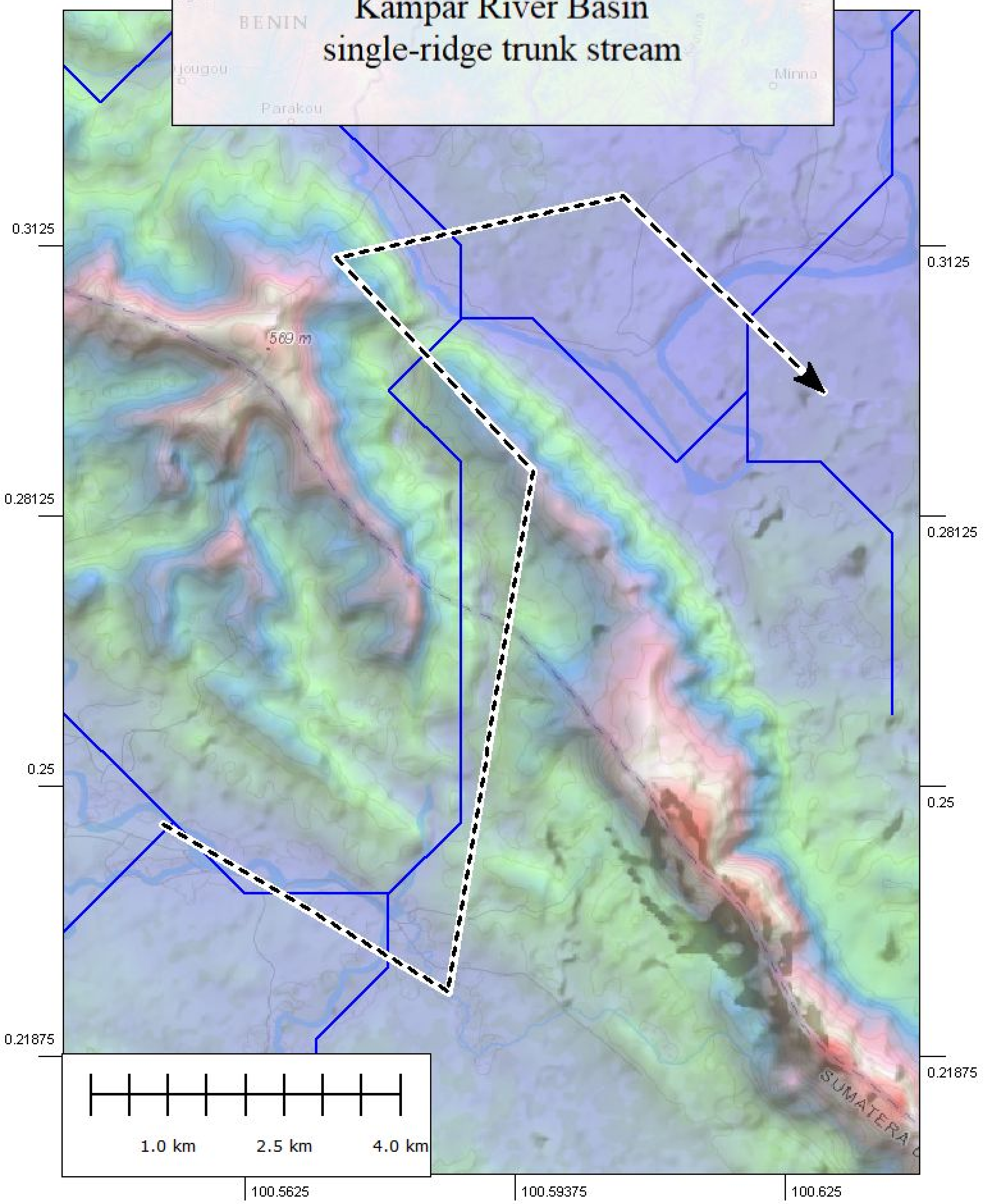

AU-WPAC - 8
Manawatu River Basin
Manawatu River
single-ridge trunk stream

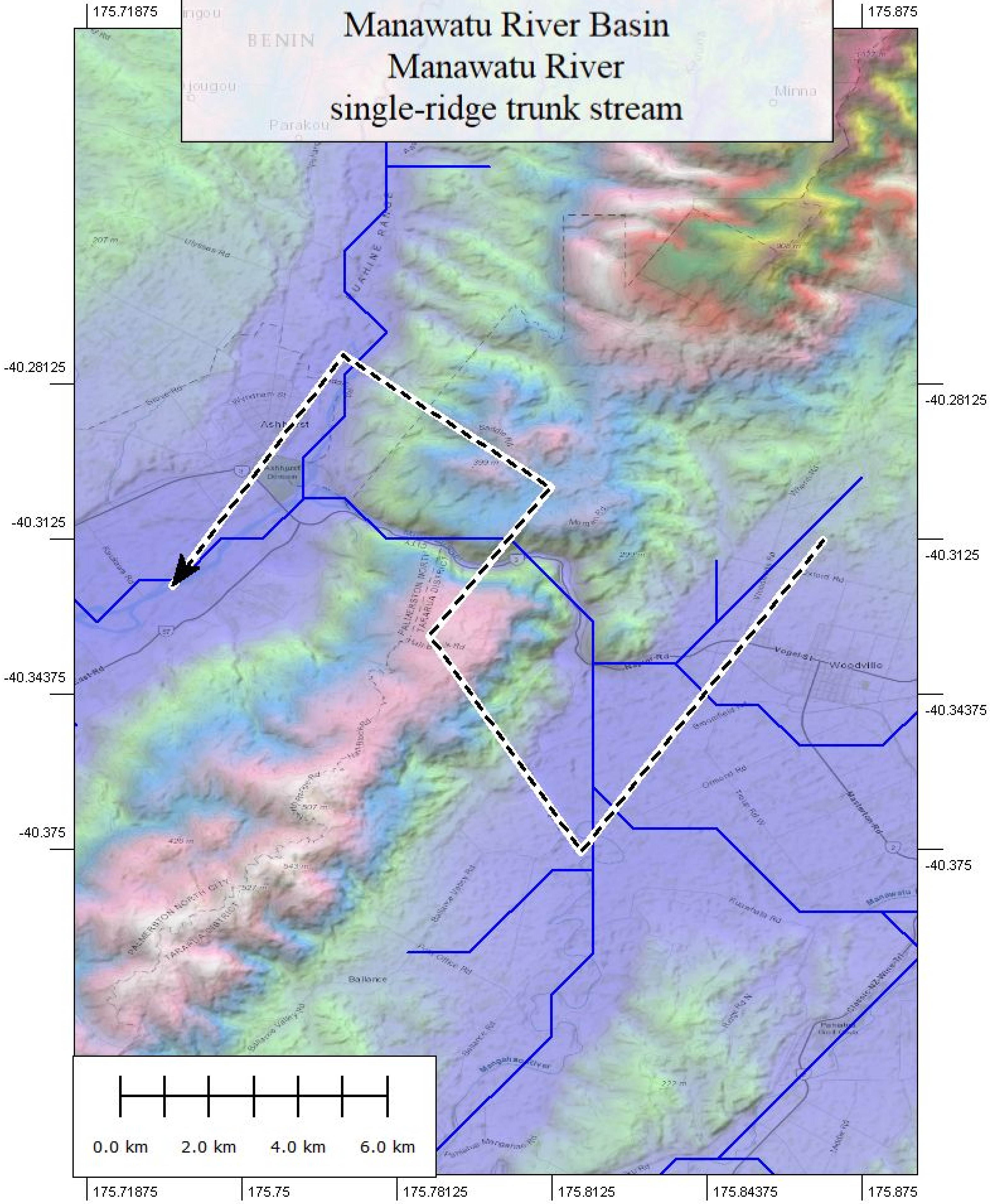

AU-WPAC - 10
Ashley Gorge-Rakahuri River Basin
Ashley River
single-ridge trunk stream

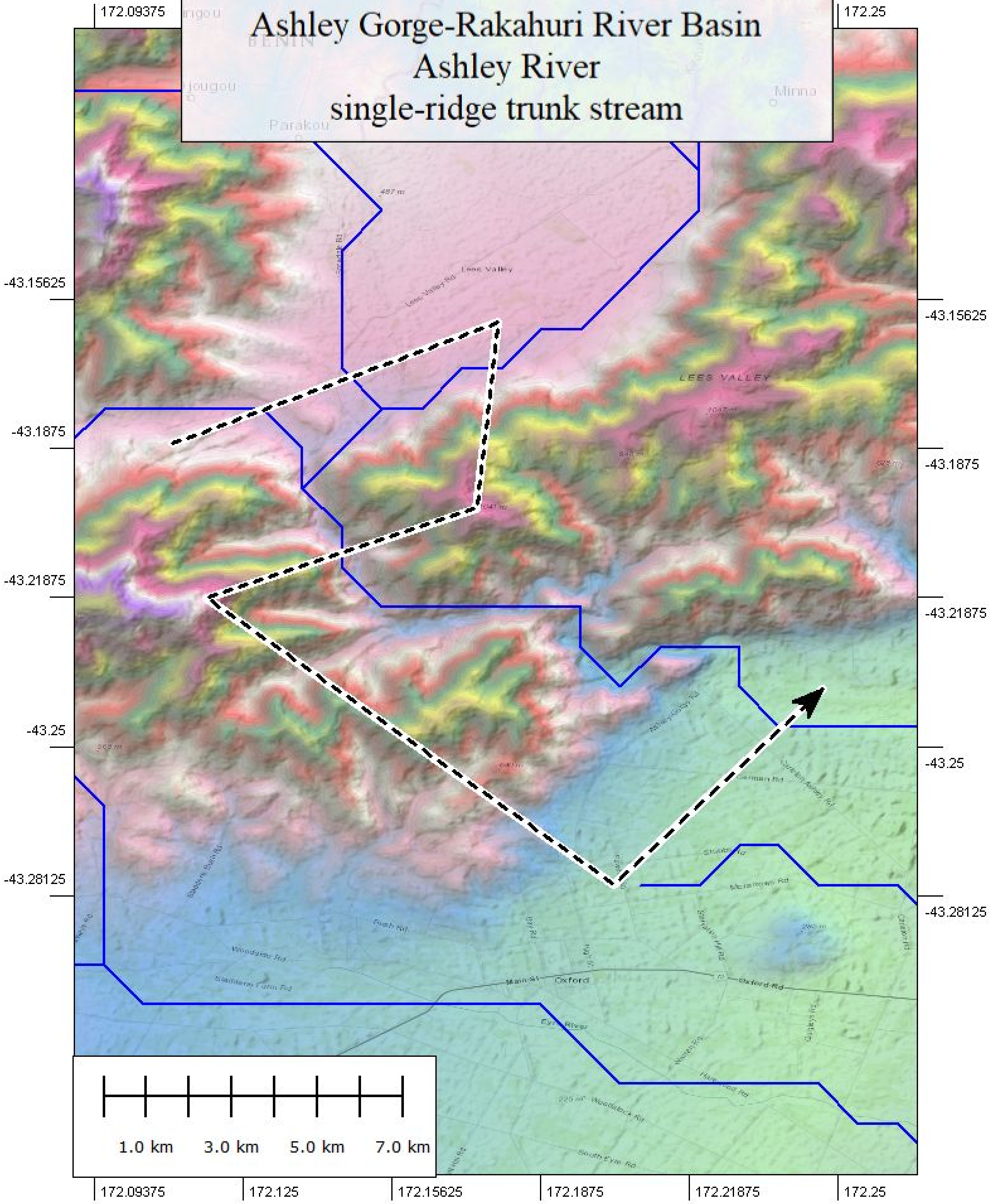

AU-WPAC - 12
Porangahau River Basin
Porangahau River
single-ridge trunk stream

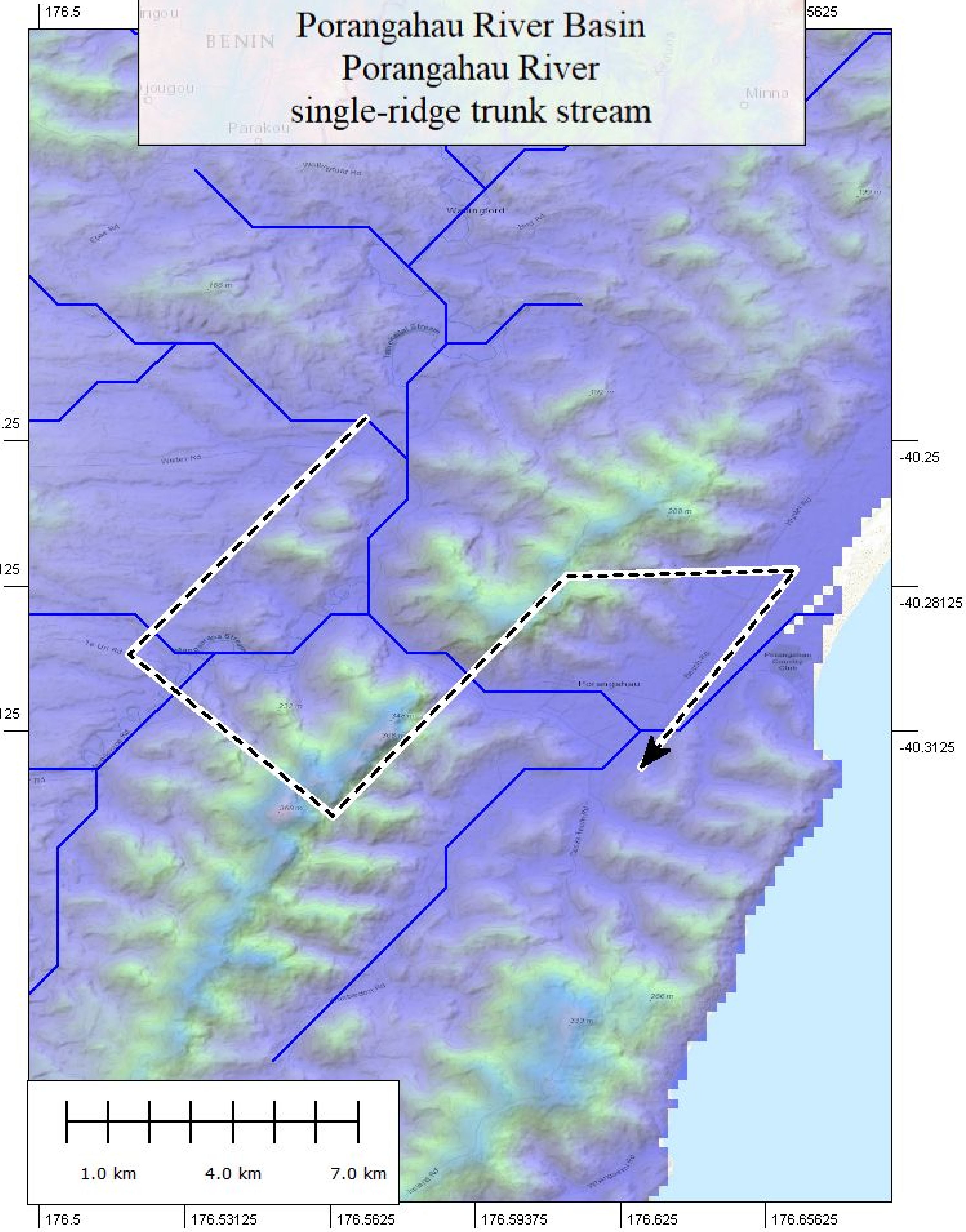

AU-WPAC - 13
Clutha River Basin
Clutha River
single-ridge trunk stream

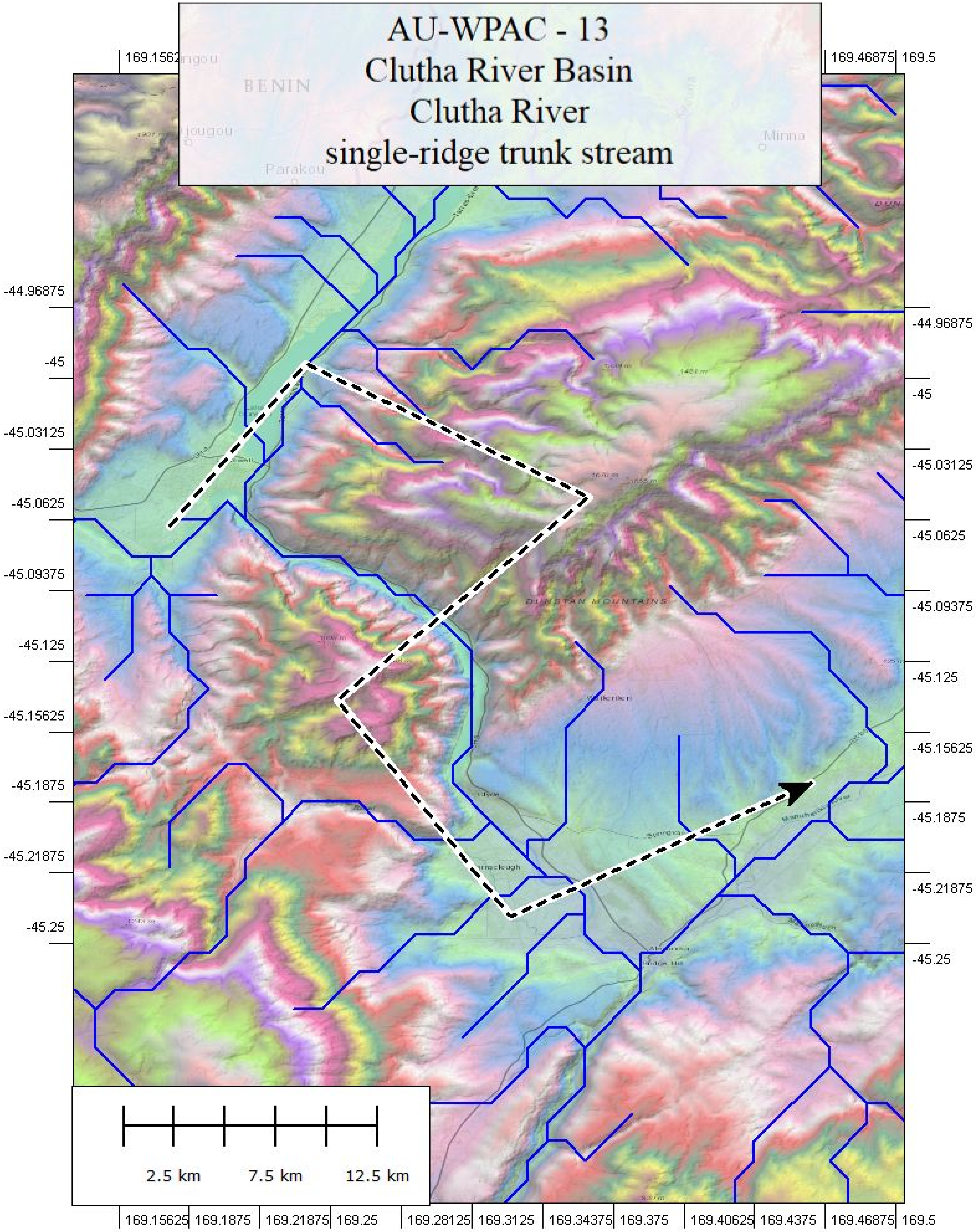

AU-WPAC - 16
Gordon River Basin
Lake Gordon
single-ridge trunk stream

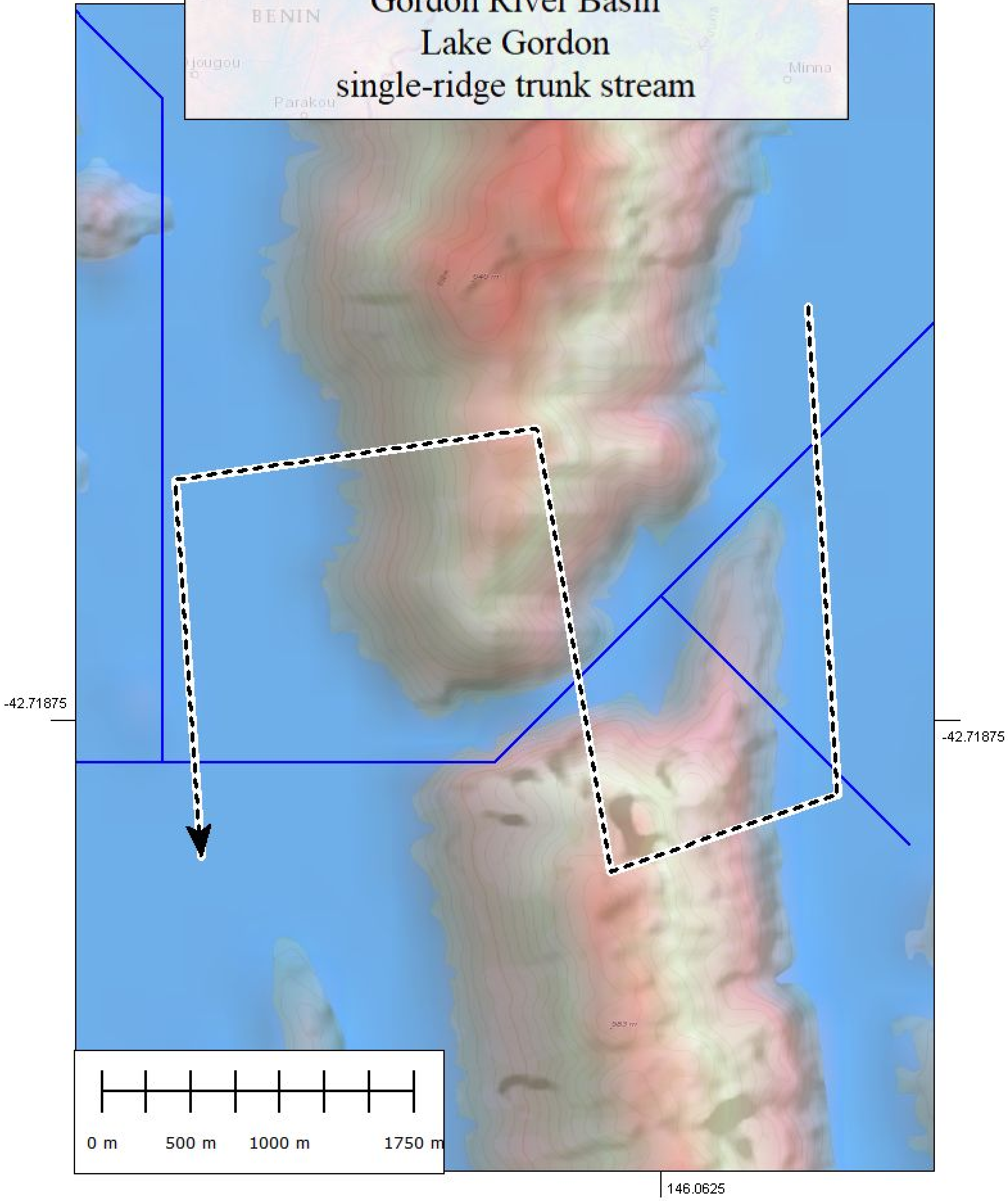

AU-WPAC - 17
Gordon River Basin
Gordon River
single-ridge trunk stream

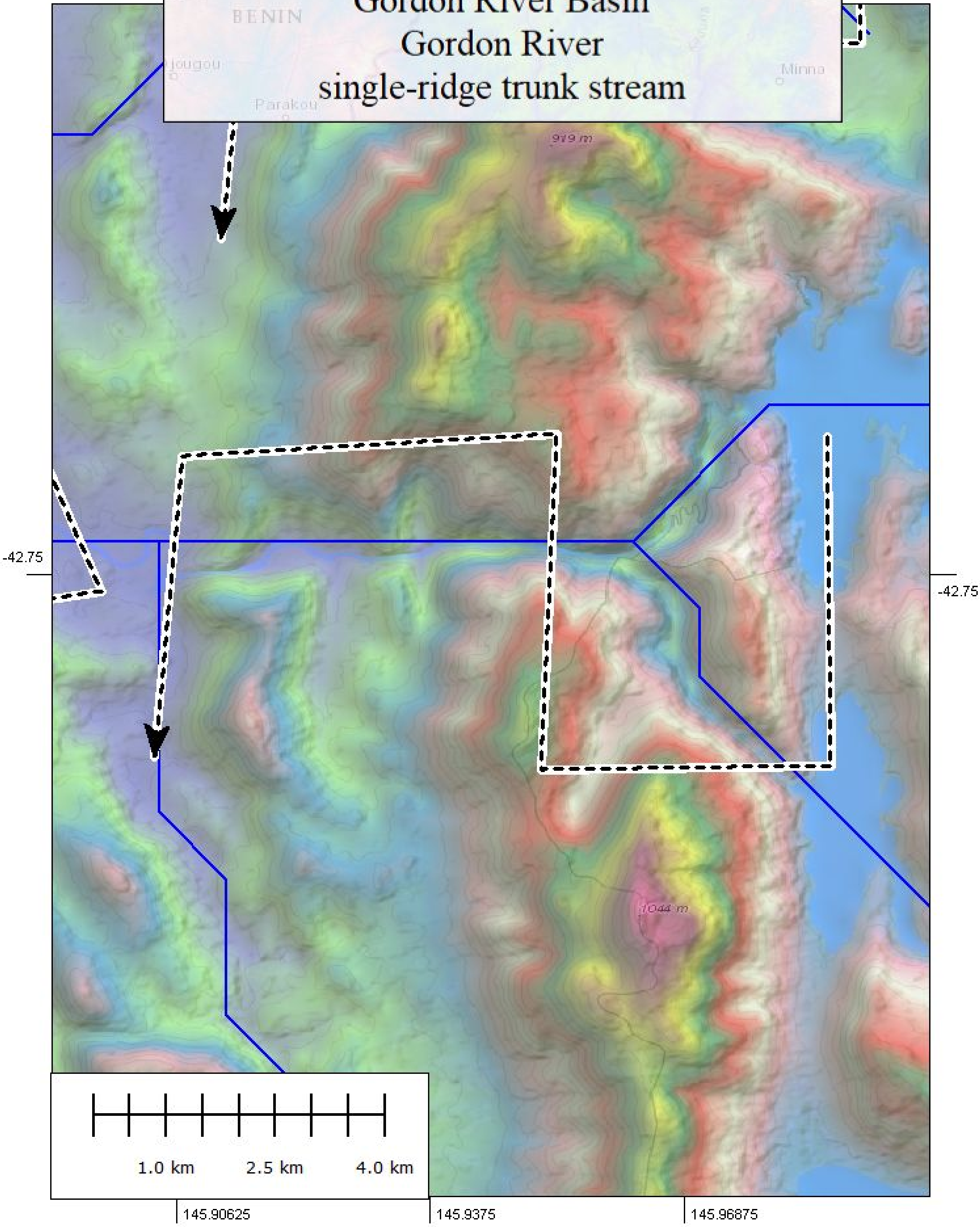

AU-WPAC - 18
Gordon River Basin
Denison River
single-ridge trunk stream

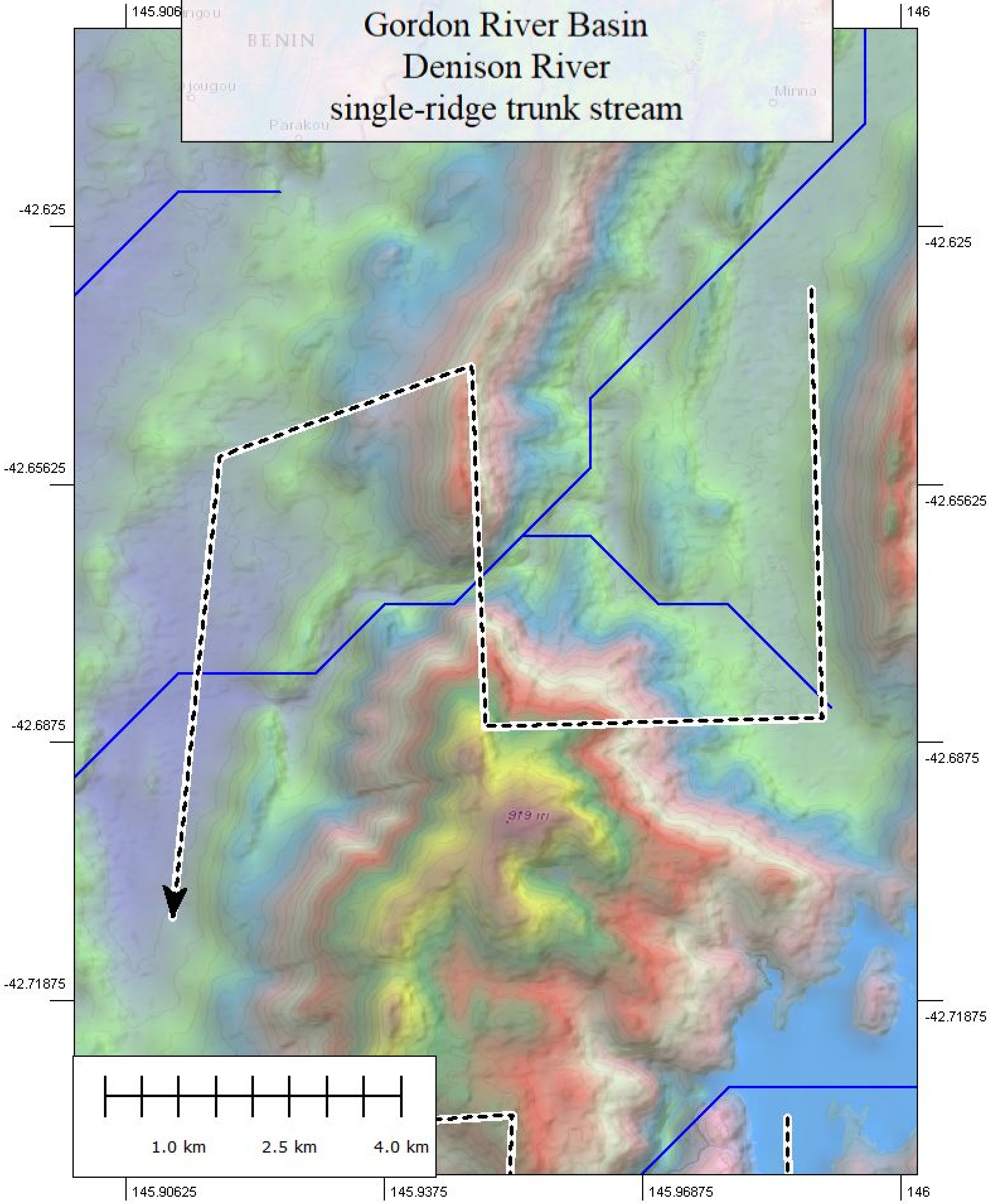

AU-WPAC - 19
Clarence River Basin
Mann River
single-ridge trunk stream

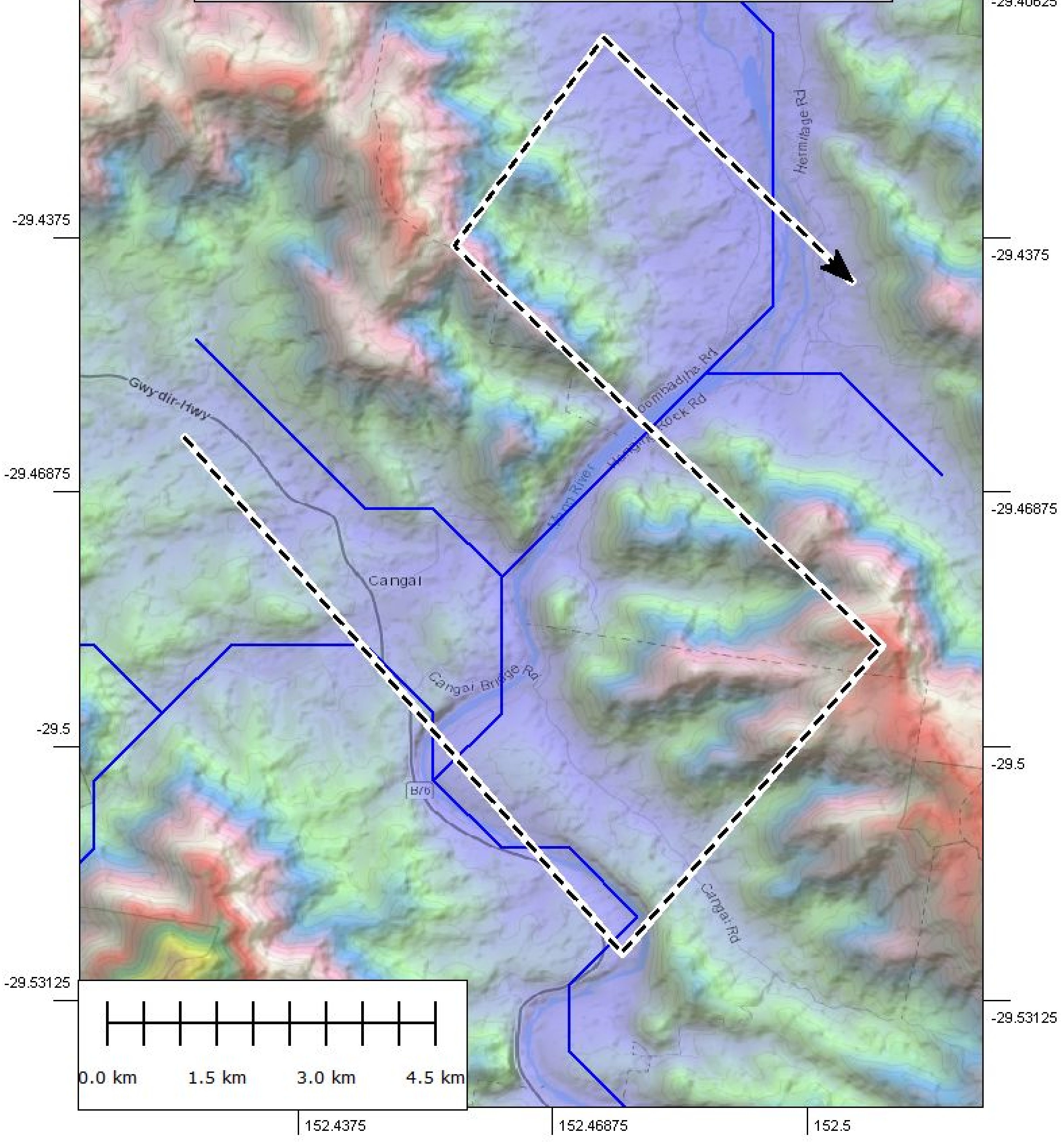

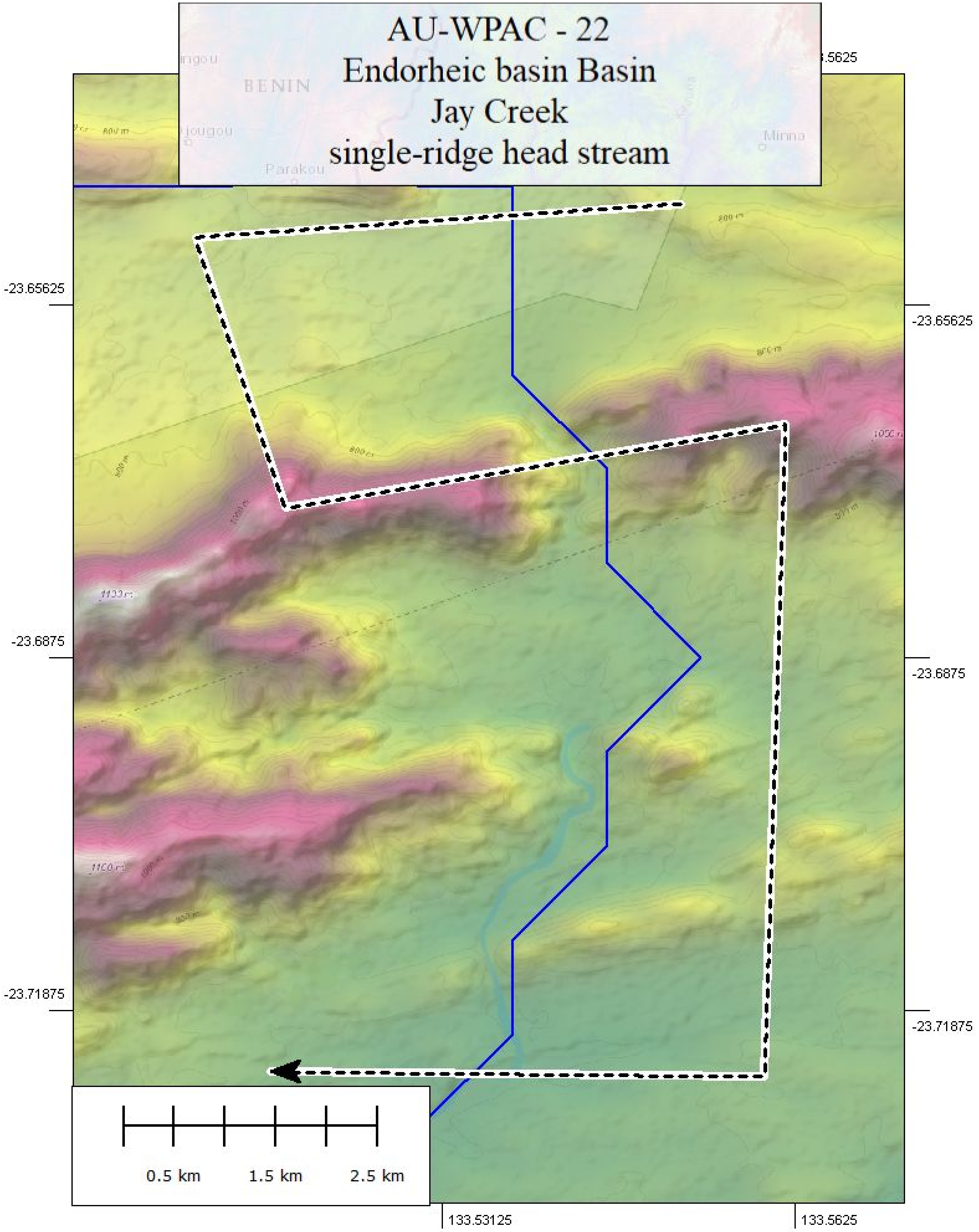
AU-WPAC - 22
Endorheic basin Basin
Jay Creek
single-ridge head stream
-23.65625
-23.6875
-23.71875
-23.65625
-23.6875
-23.71875
133.53125
133.5625
0.5 km
1.5 km
2.5 km

AU-WPAC - 23
Asimi River Basin
single-ridge trunk stream

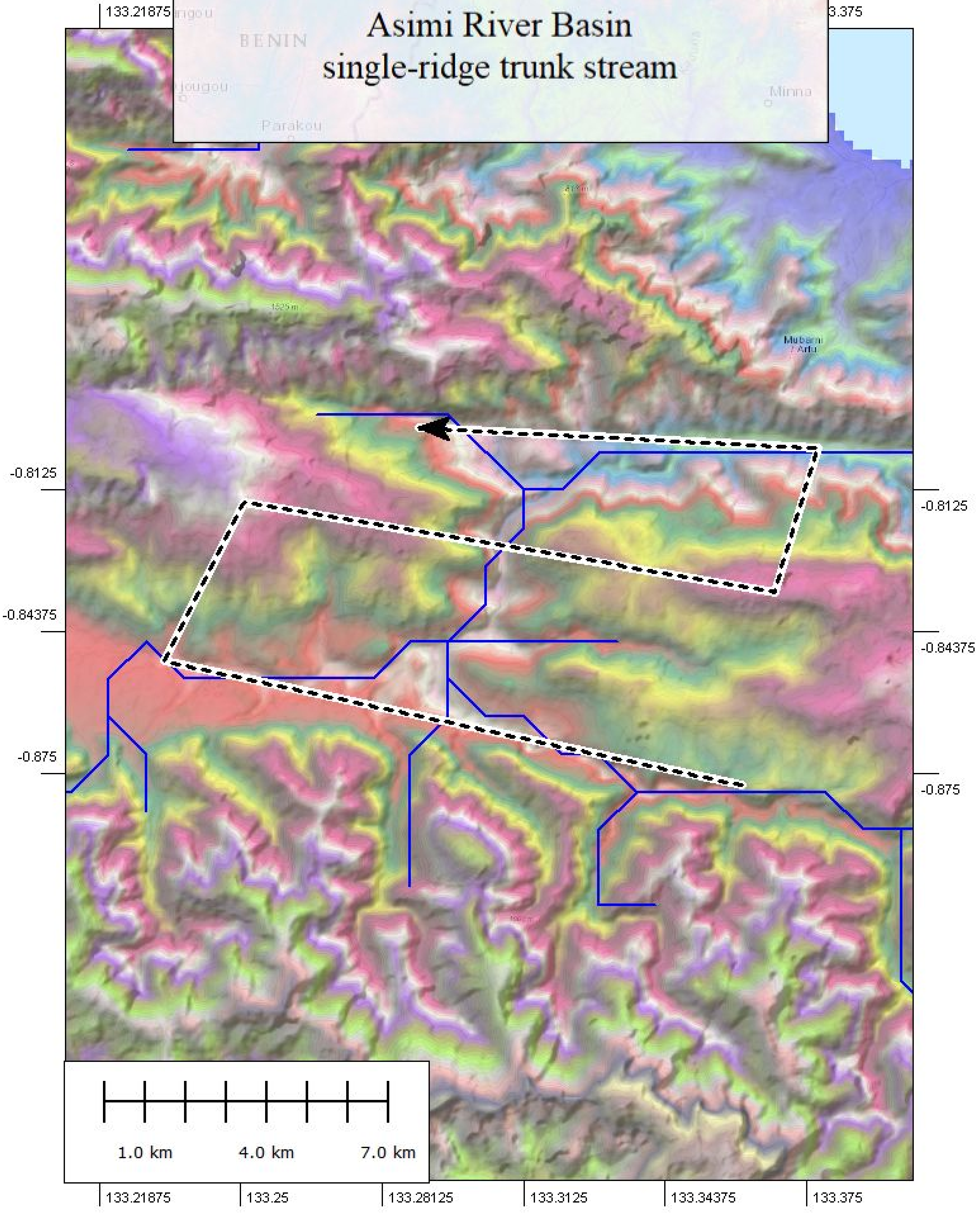

AU-WPAC - 24
Endorheic basin Basin
Hugh River tributary
single-ridge trunk stream

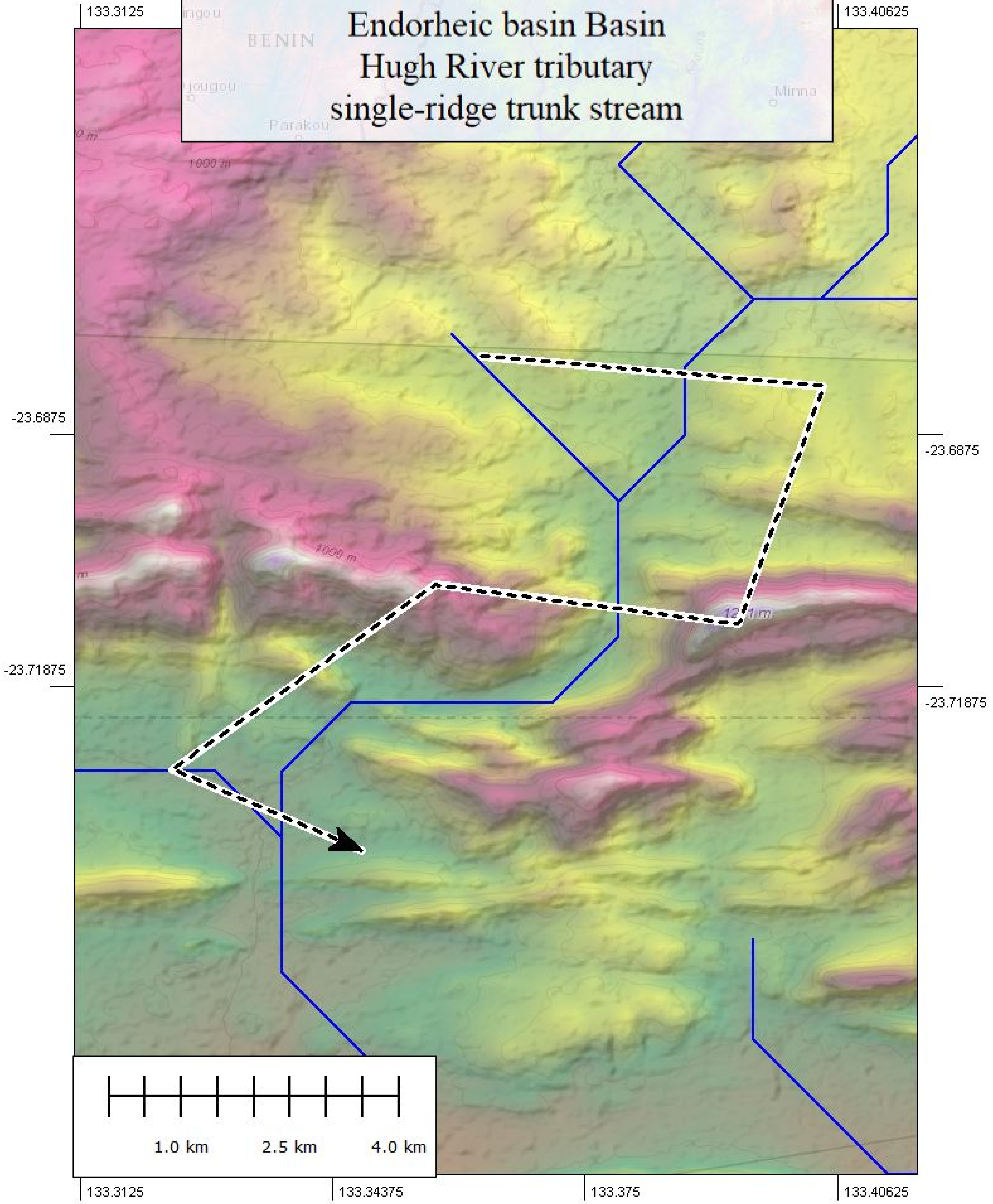

AU-WPAC - 25
Endorheic basin Basin
Todd River
single-ridge trunk stream

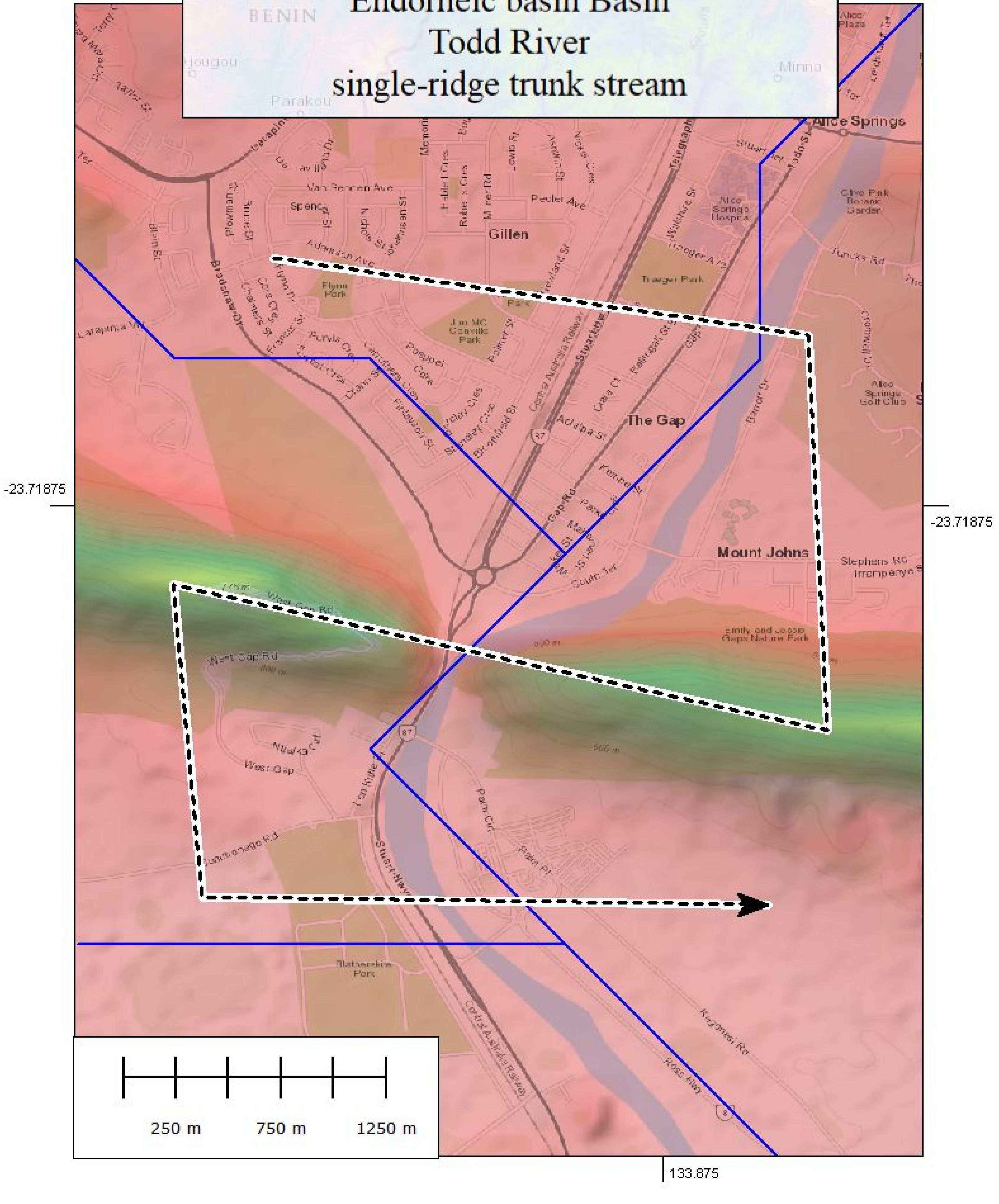

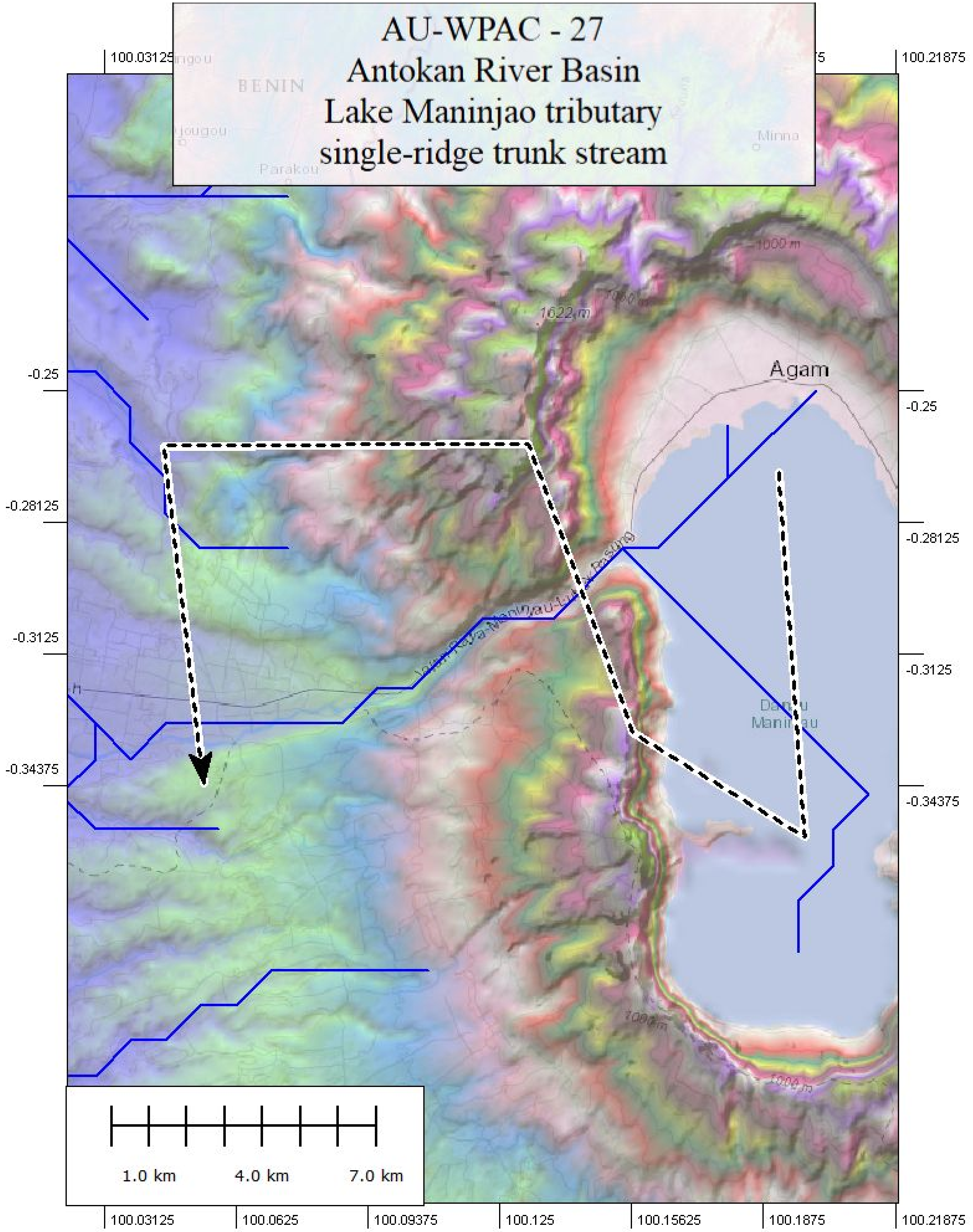

AU-WPAC - 27
Antokan River Basin
Lake Maninjao tributary
single-ridge trunk stream
100.03125
100.0625
100.09375
100.125
100.15625
100.1875
100.21875
-0.25
-0.28125
-0.3125
-0.34375
Agam
1622 m
1000 m
Danau Maninjau
1.0 km
4.0 km
7.0 km

AU-WPAC - 28
Waiau River Basin
Waiau River
single-ridge trunk stream

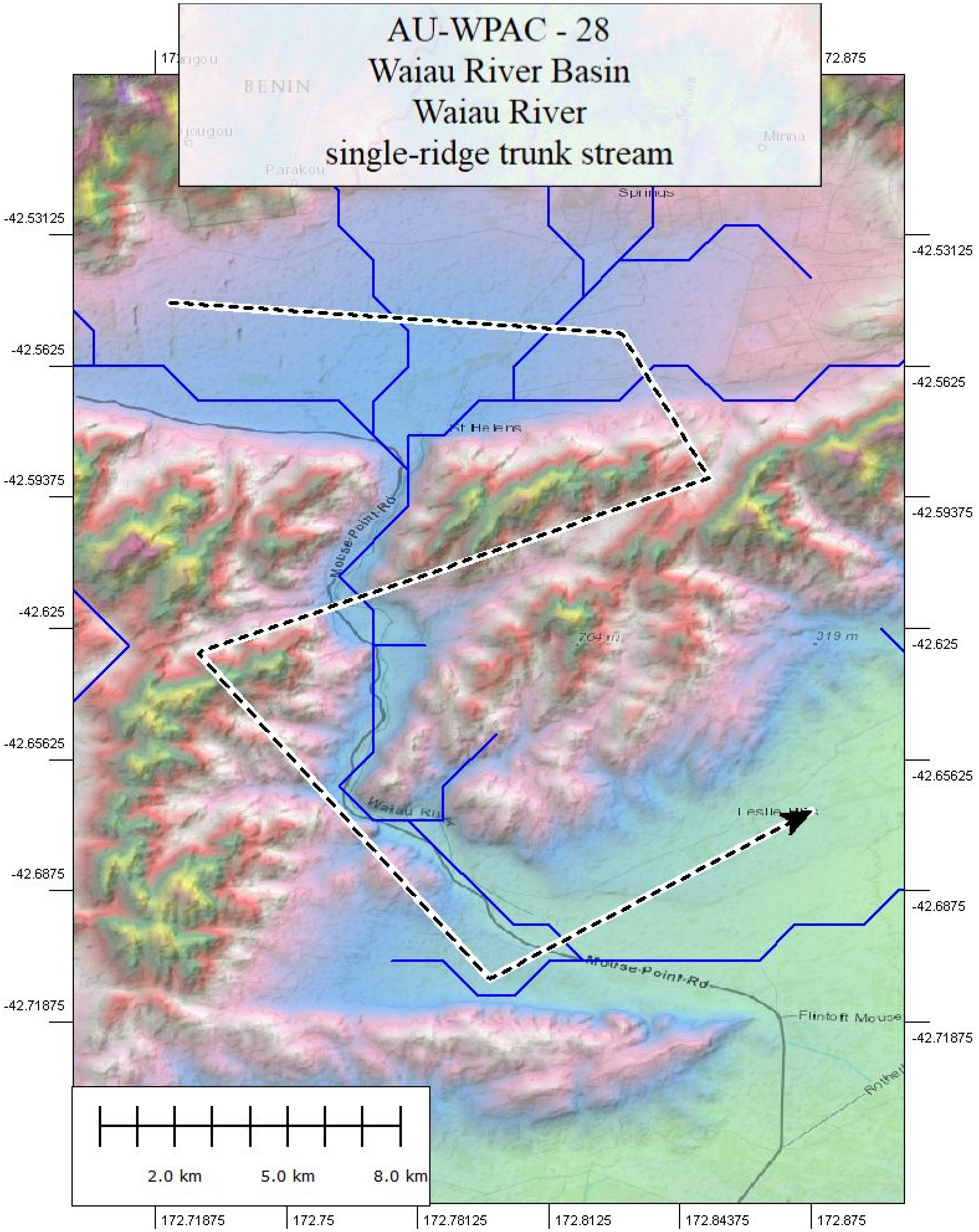

AU-WPAC - 31
Endorheic basin Basin
Ellery Creek
single-ridge head stream

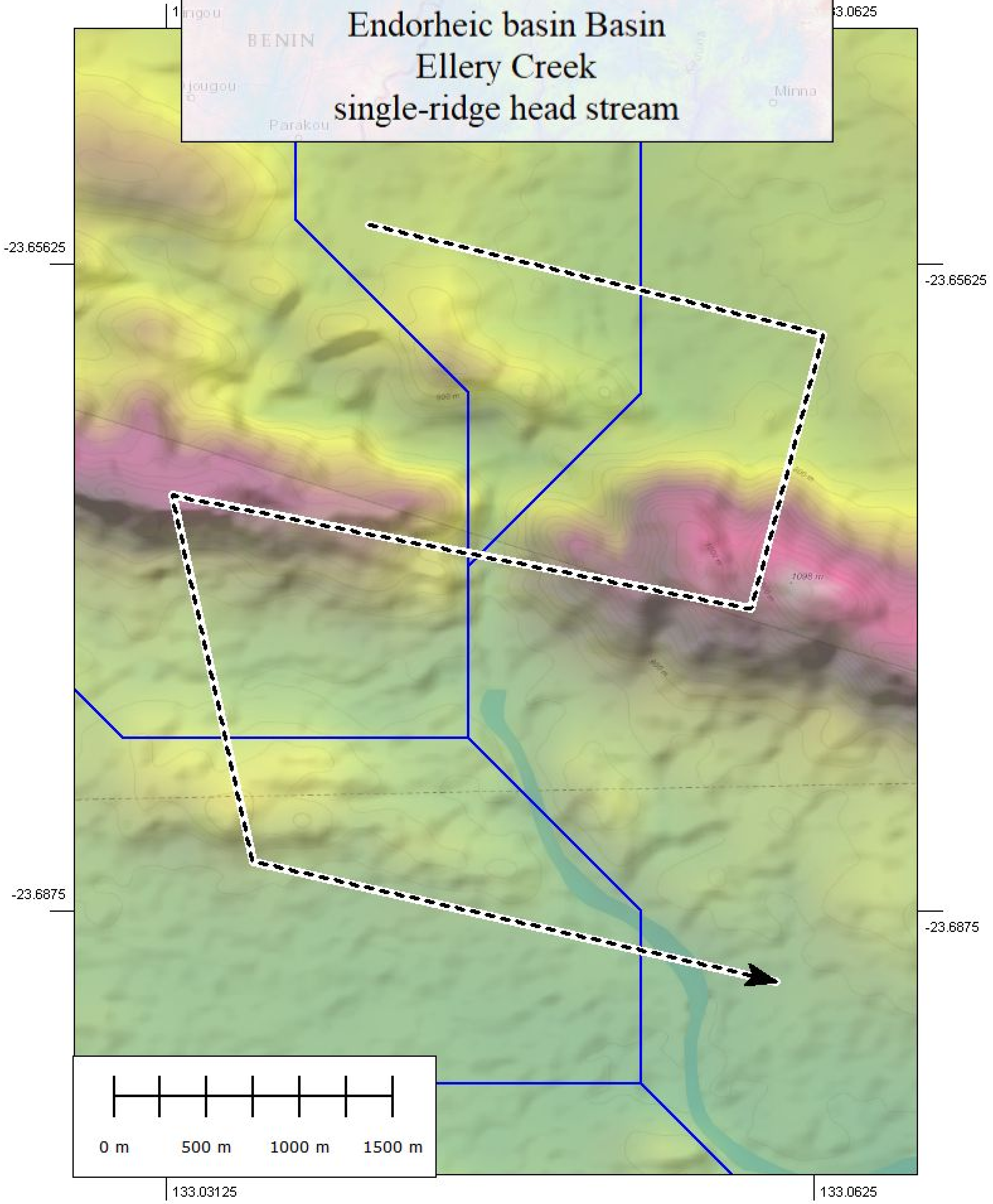

AU-WPAC - 32
Rokan River Basin
single-ridge trunk stream

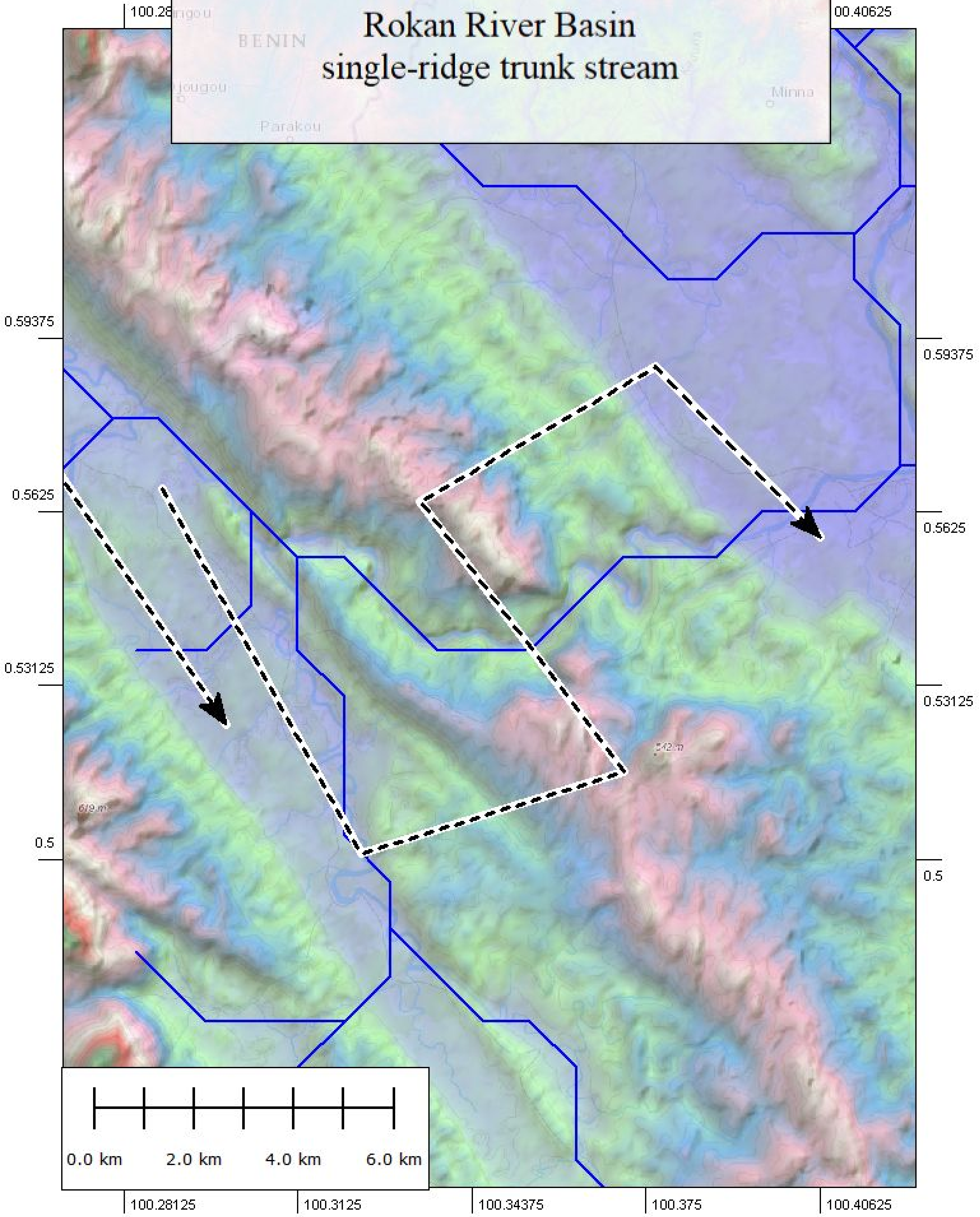

AU-WPAC - 35
Hurunui River Basin
Hurunui River
single-ridge trunk stream

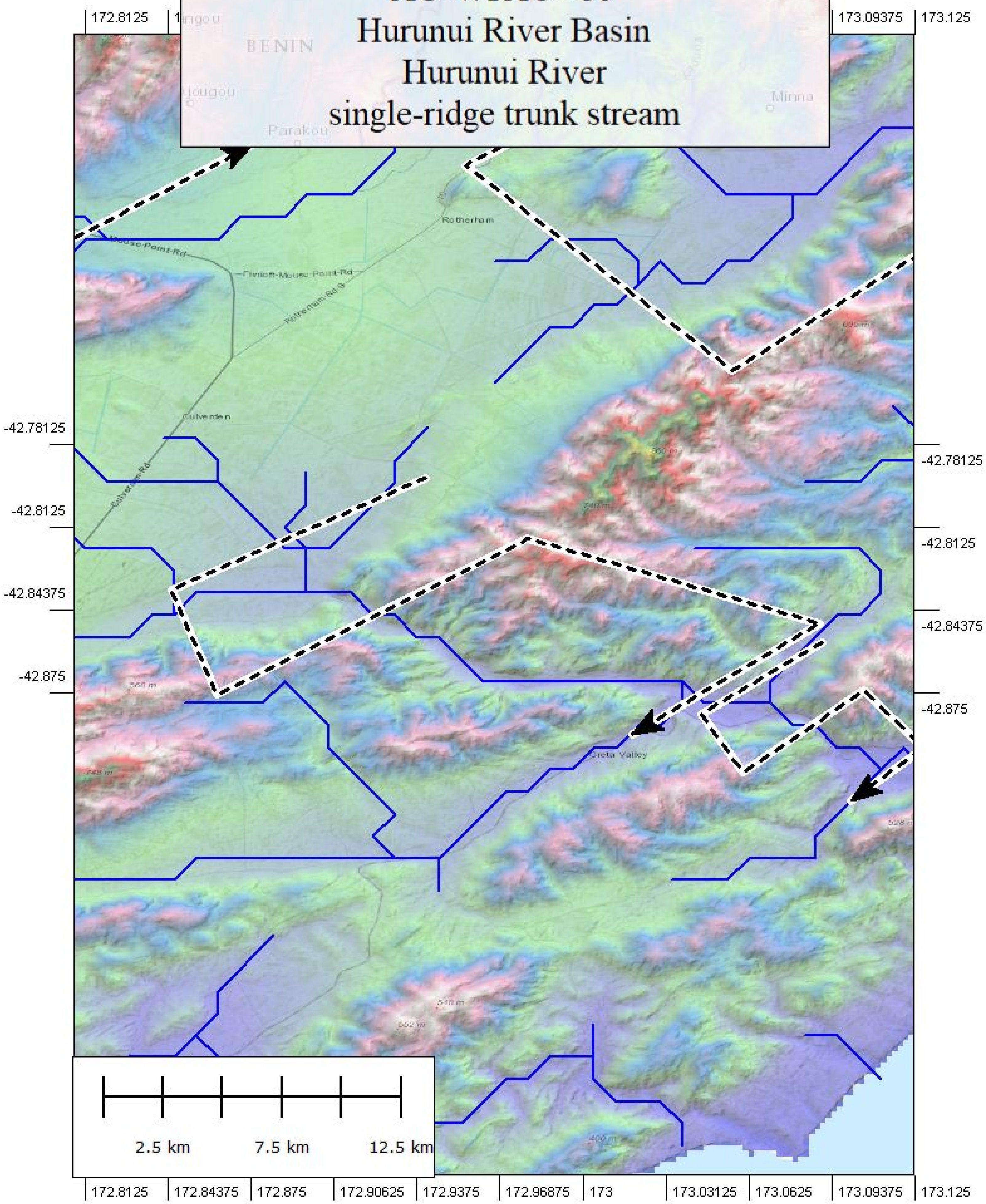

AU-WPAC - 37
Hurunui River Basin
Hurunui River
single-ridge trunk stream

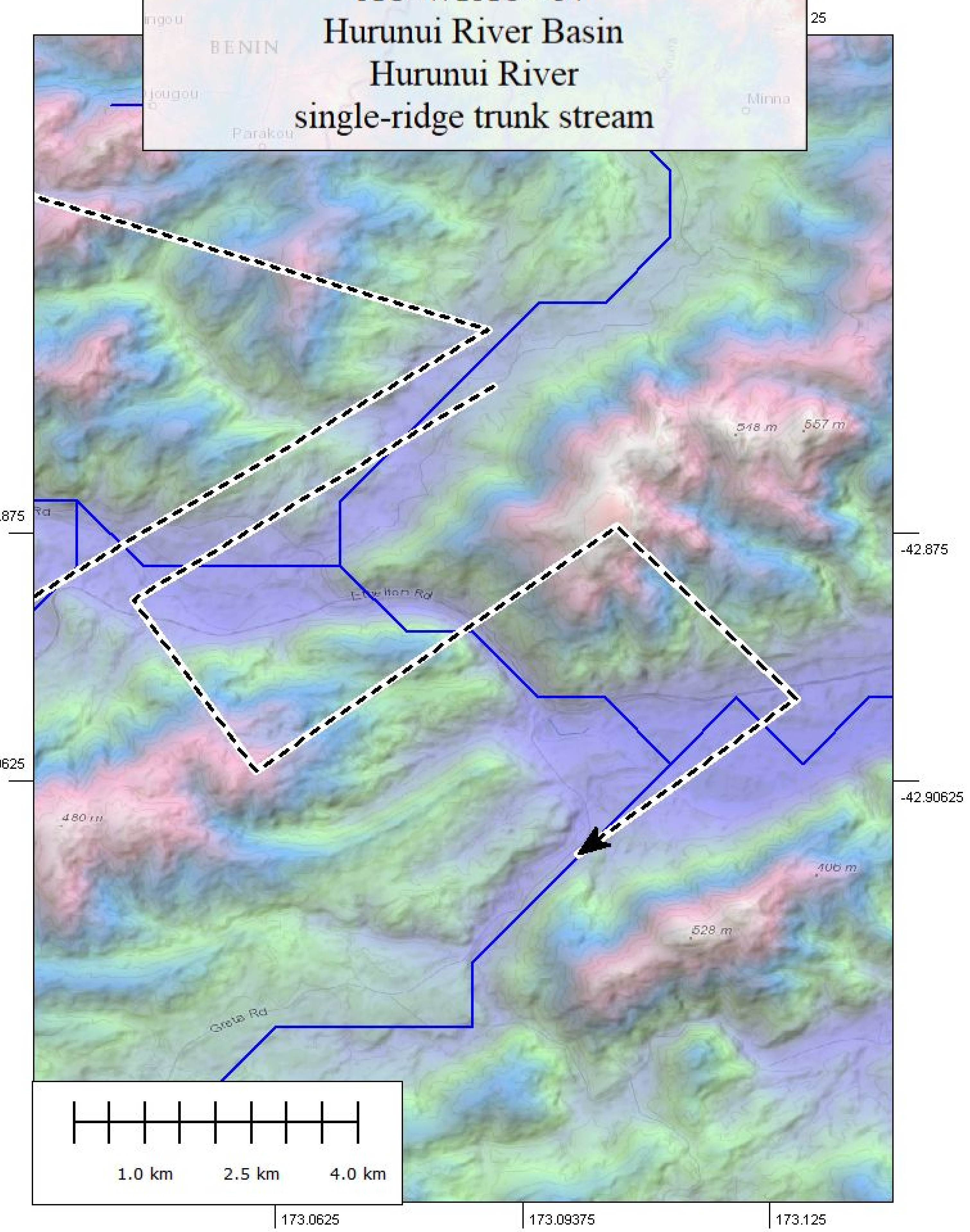

AU-WPAC - 40
Gordon River Basin
Gordon River
single-ridge trunk stream

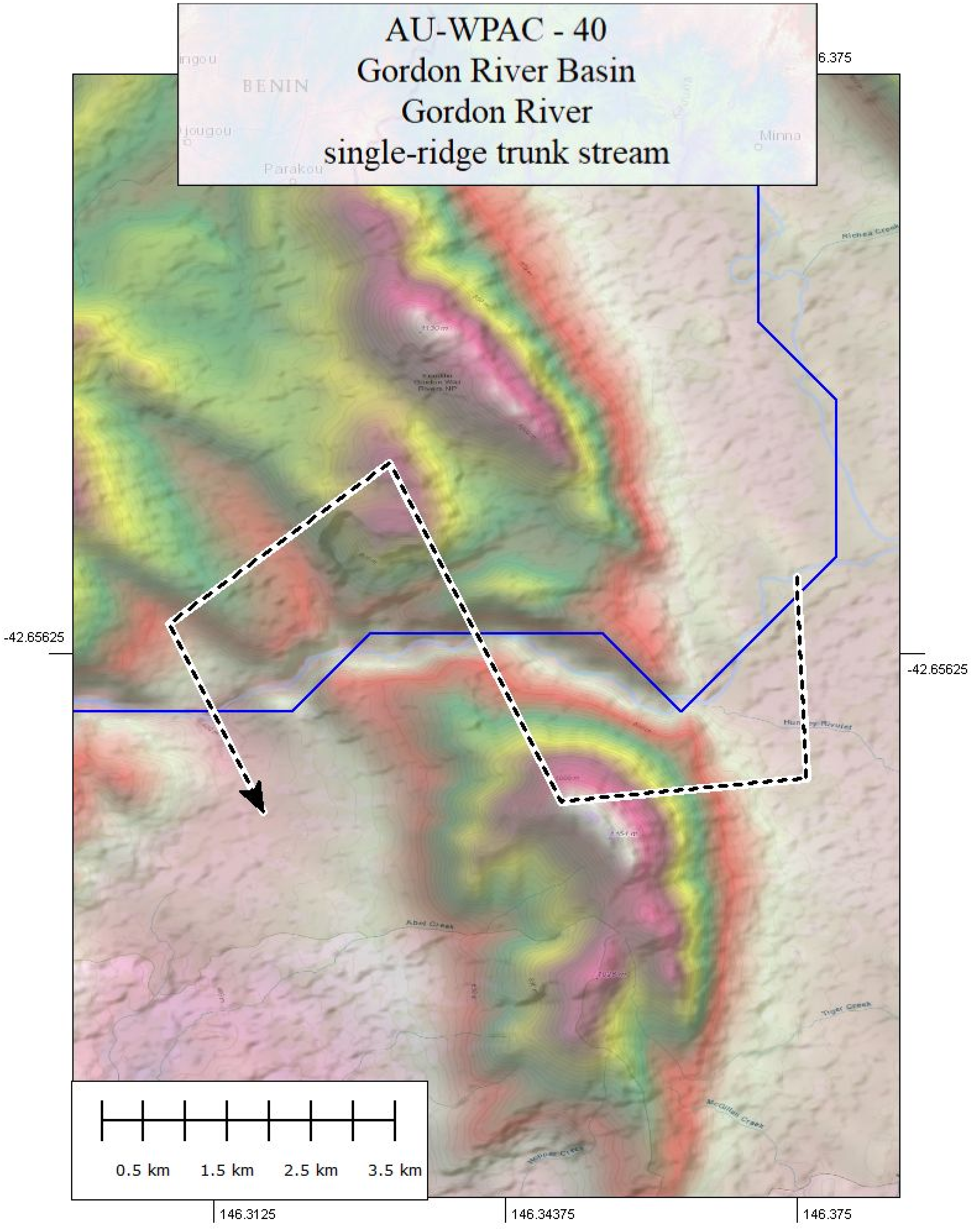

AU-WPAC - 41
Purari River Basin
Purani River
single-ridge trunk stream

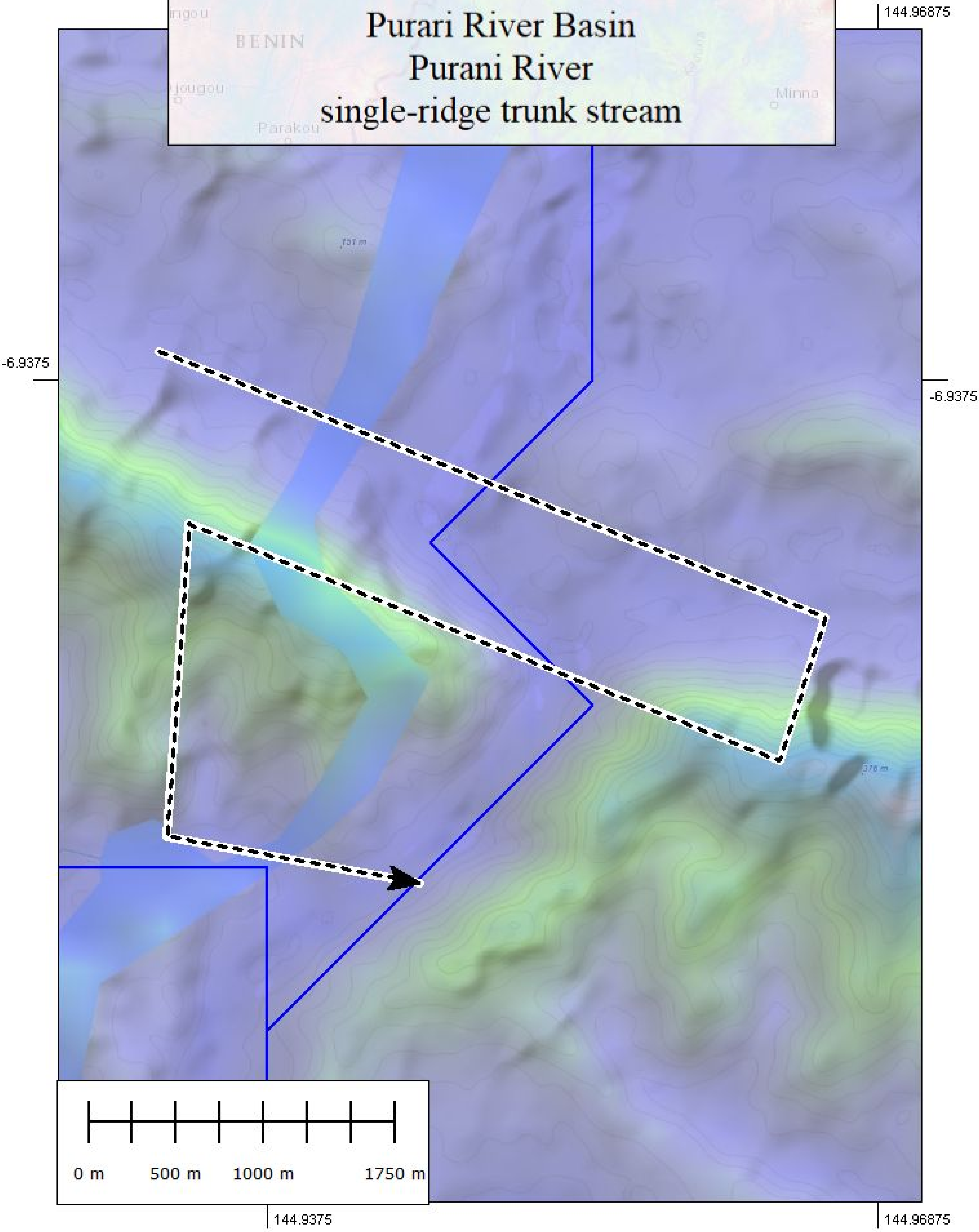

AU-WPAC - 42
Purari River Basin
Purari River
single-ridge trunk stream

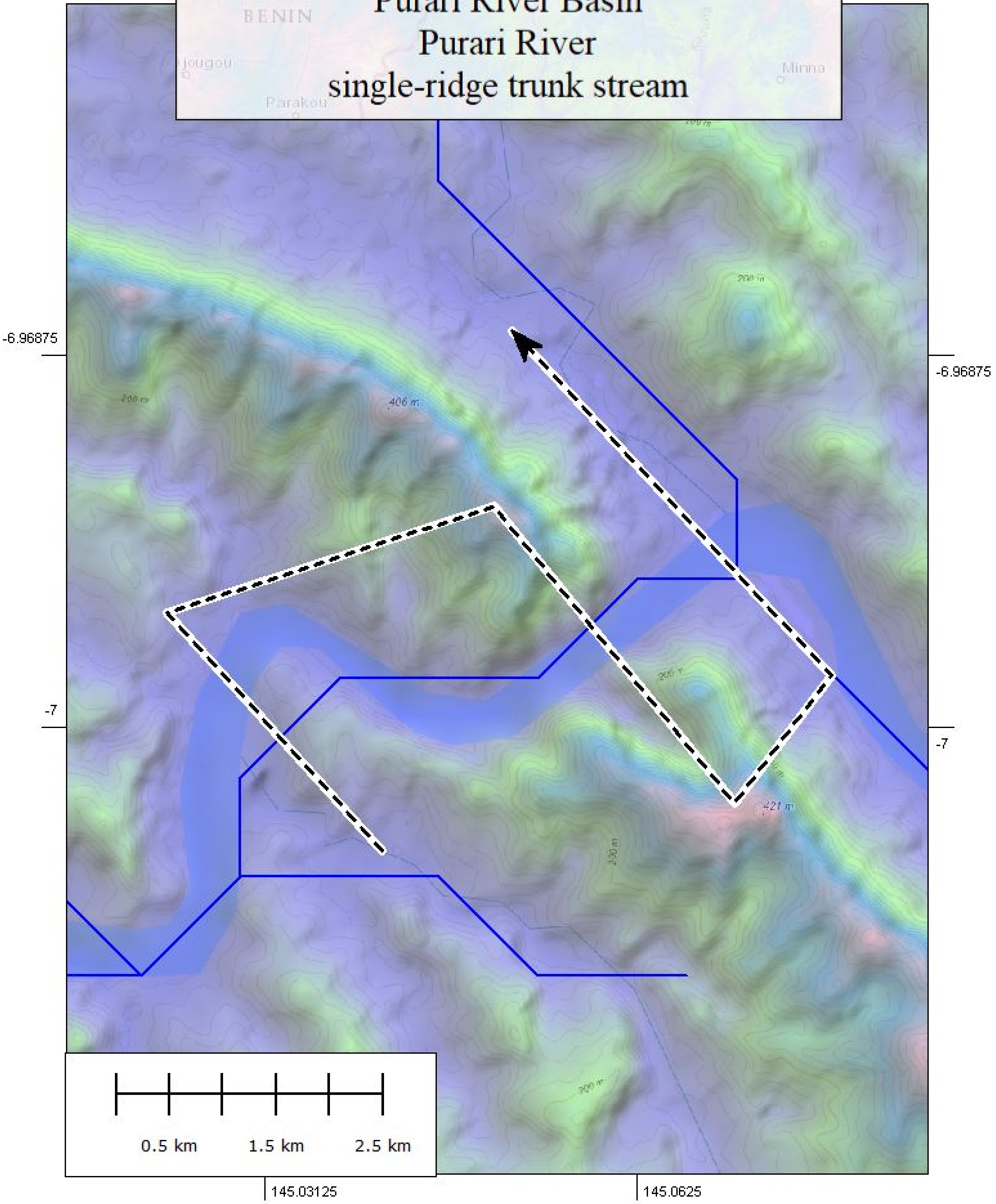

AU-WPAC - 44
Musa River Basin
Musa River
single-ridge trunk stream

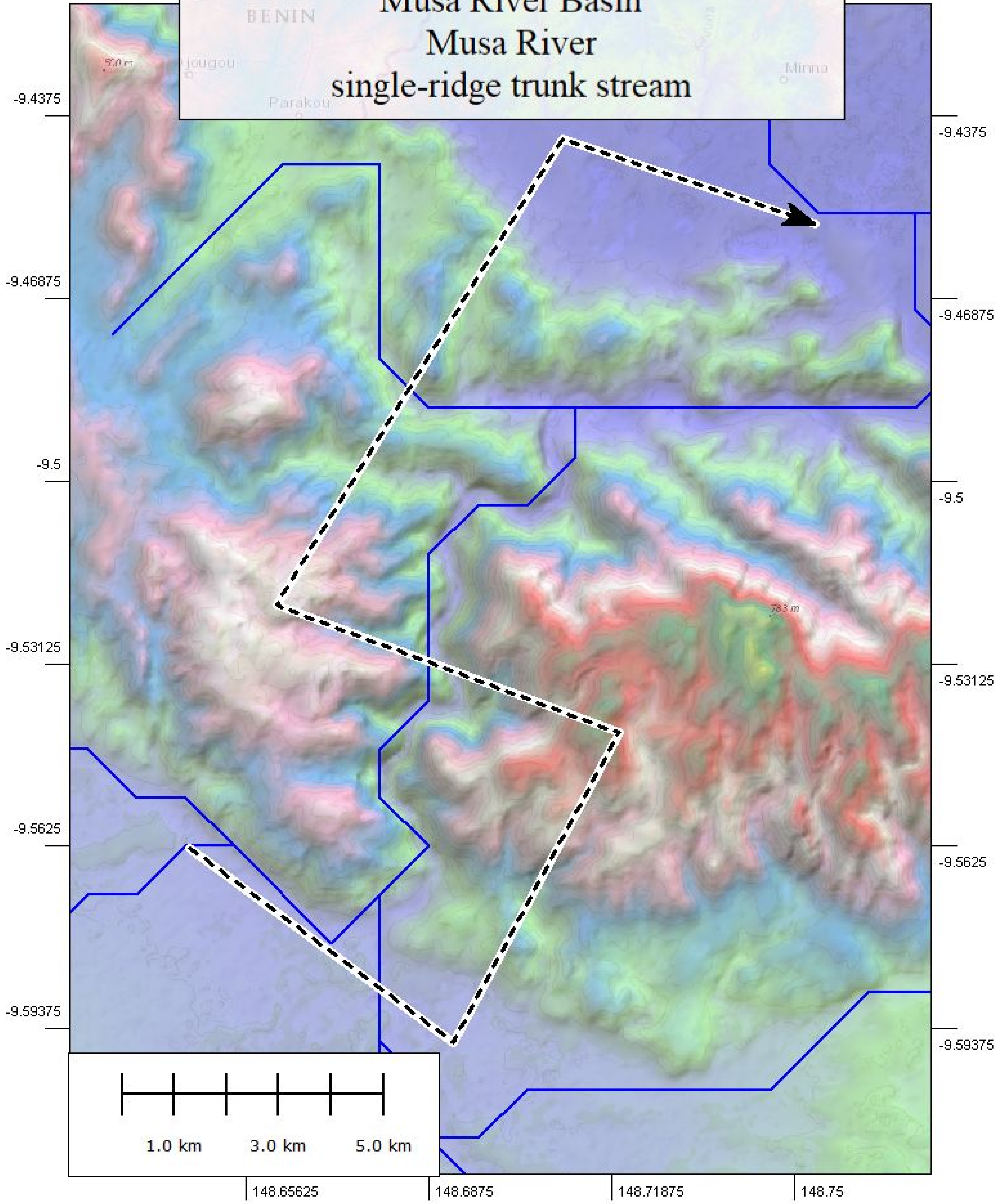

AU-WPAC - 48
Murray-Darling River Basin
Rocky Creek
single-ridge trunk stream

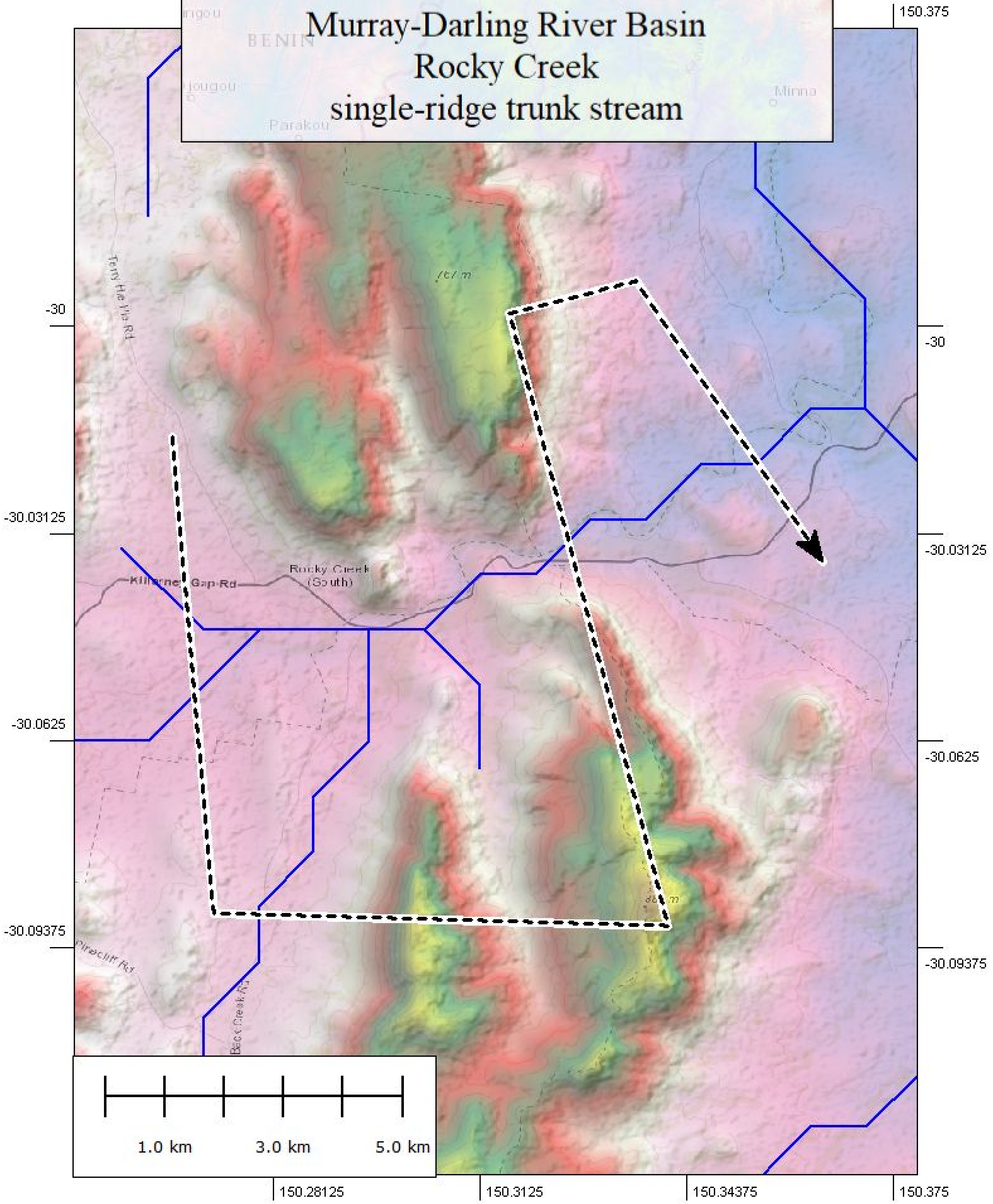

AU-WPAC - 51
Lennard River Basin
Lennard River
single-ridge trunk stream

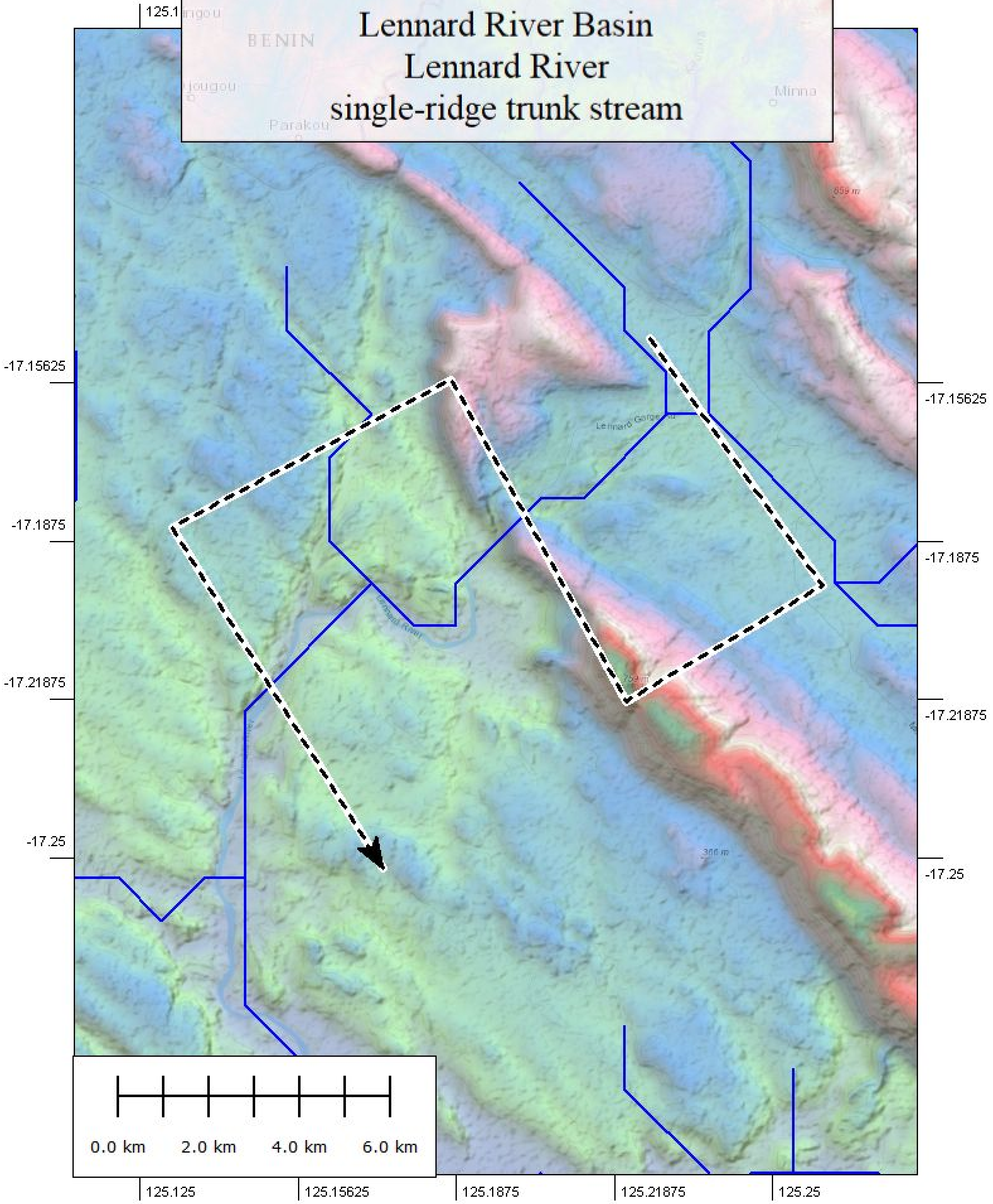

AU-WPAC - 52
Mamberamo River Basin
Baliem River tributary
single-ridge trunk stream

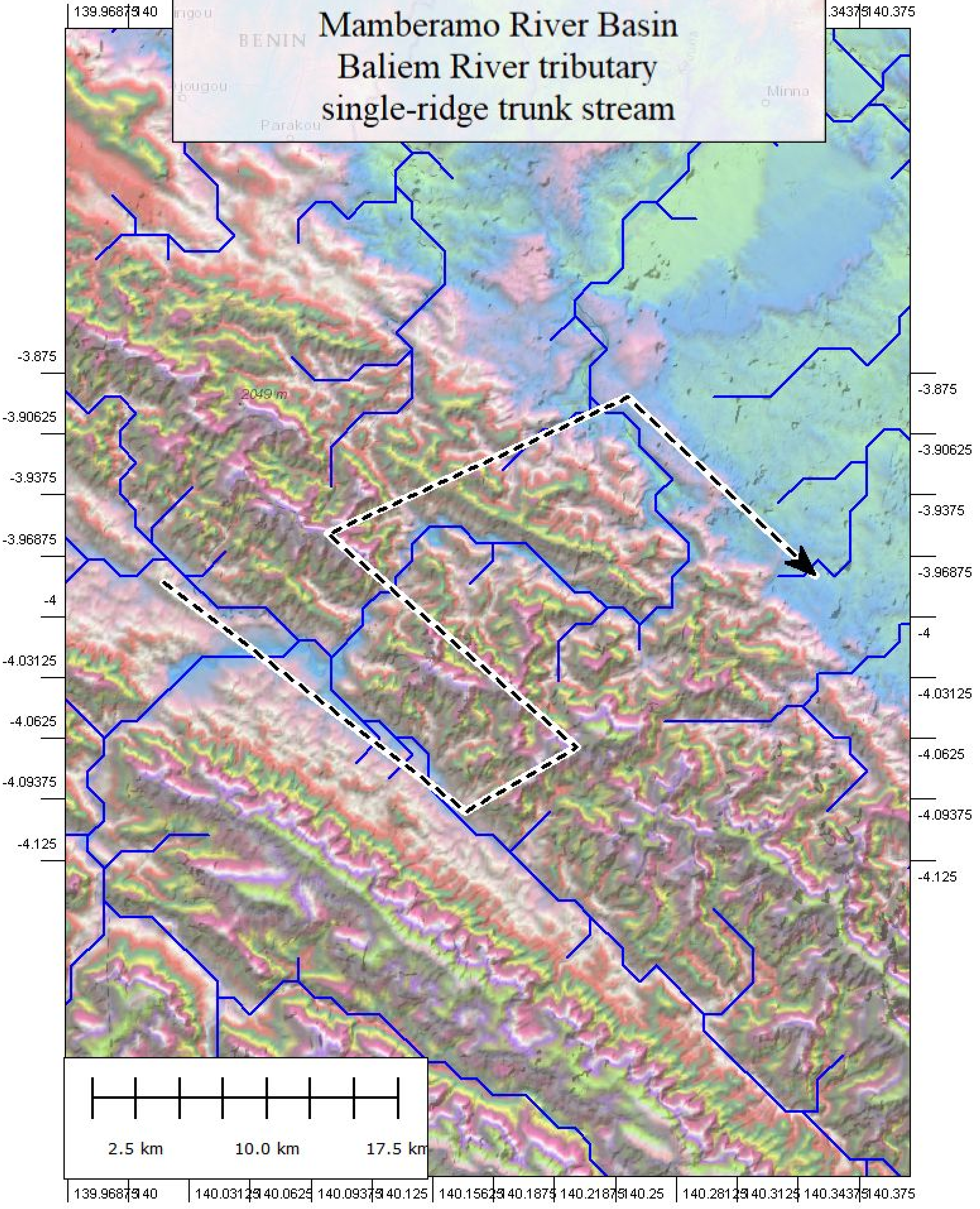

AU-WPAC - 53
Endorheic basin Basin
Ellery Creek
single-ridge trunk stream

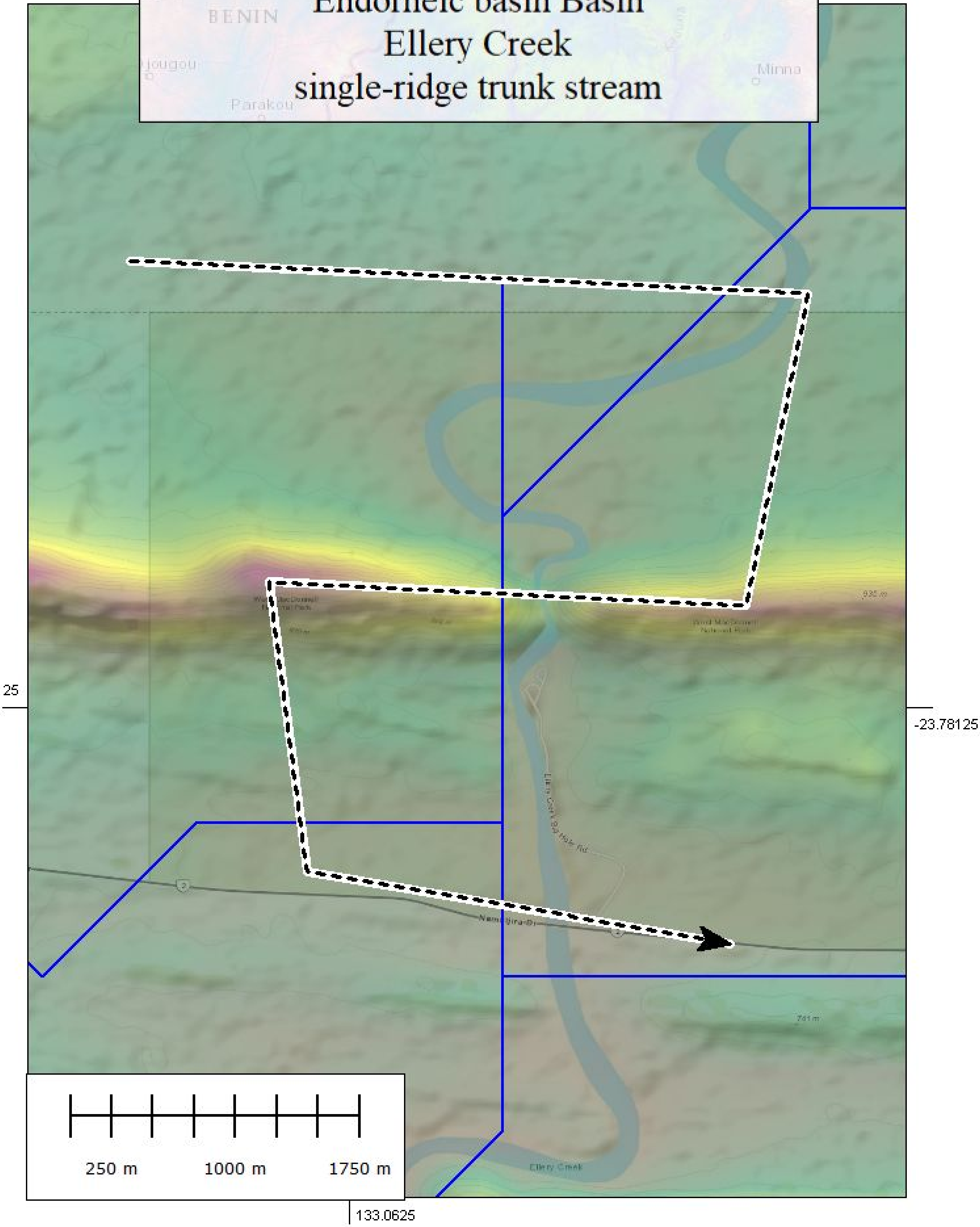

AU-WPAC - 54
Indragiri River Basin
Indragiri River
single-ridge trunk stream

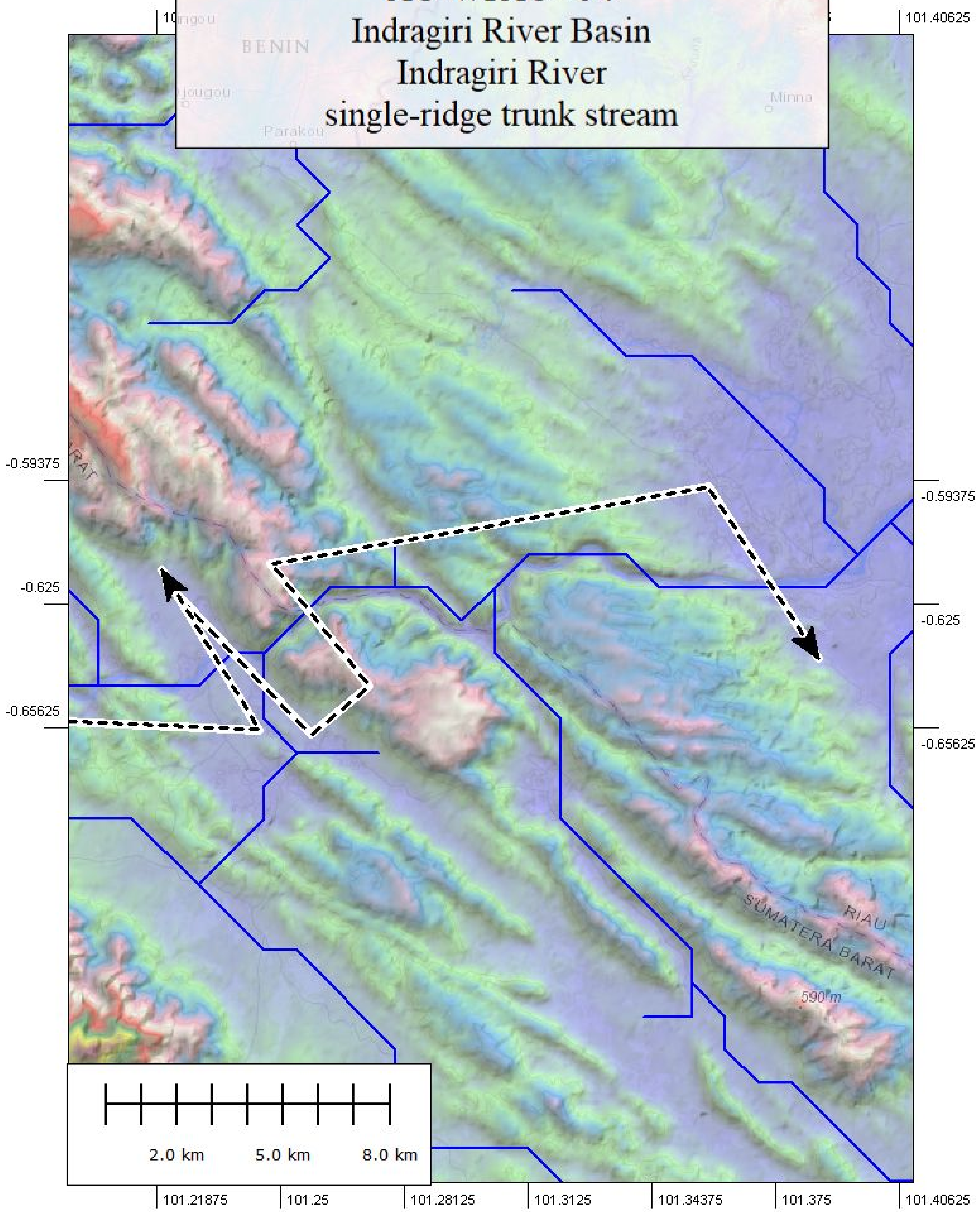

AU-WPAC - 55
Gordon River Basin
Gordon River
single-ridge trunk stream

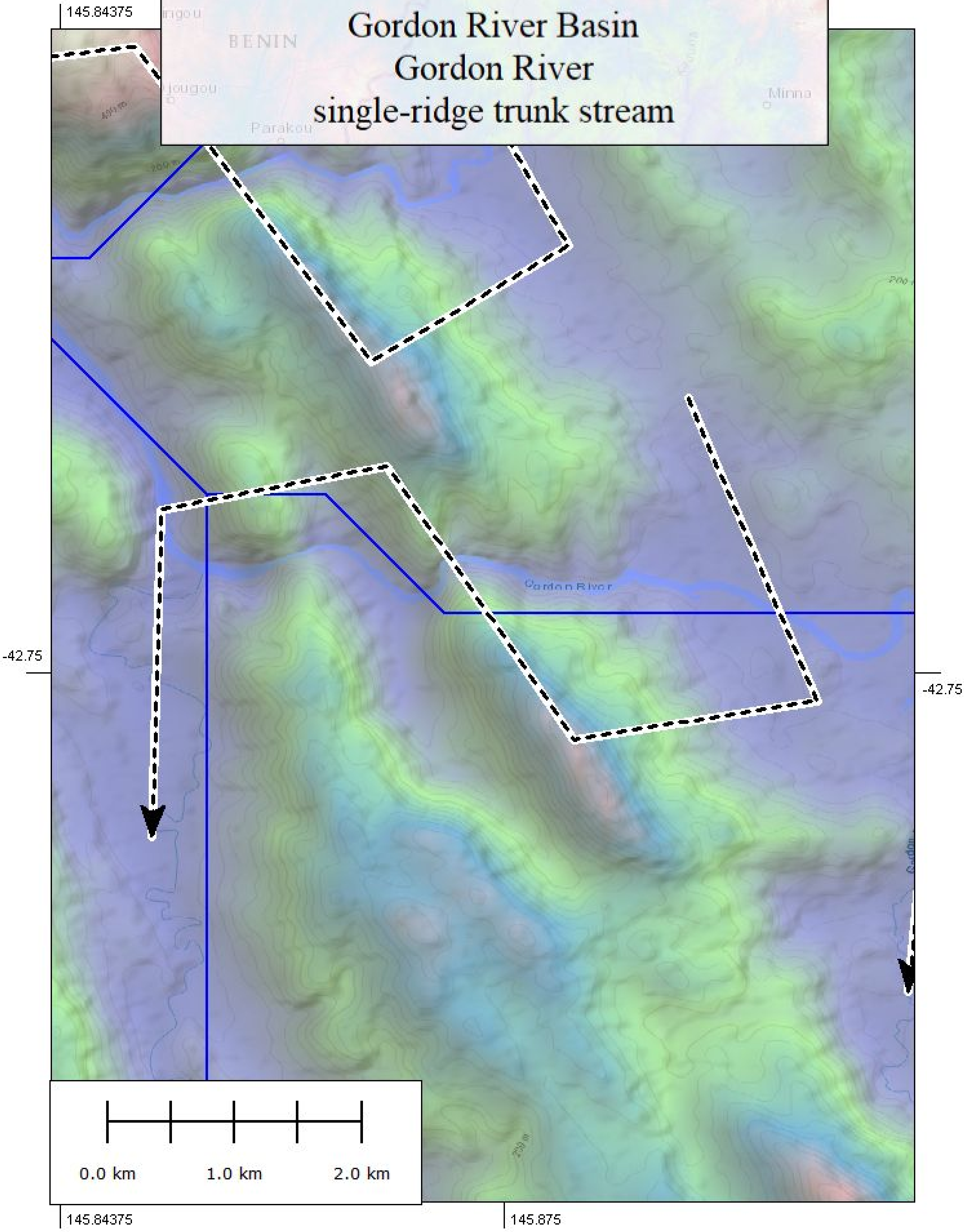

AU-WPAC - 56
Gordon River Basin
Denison River
single-ridge trunk stream

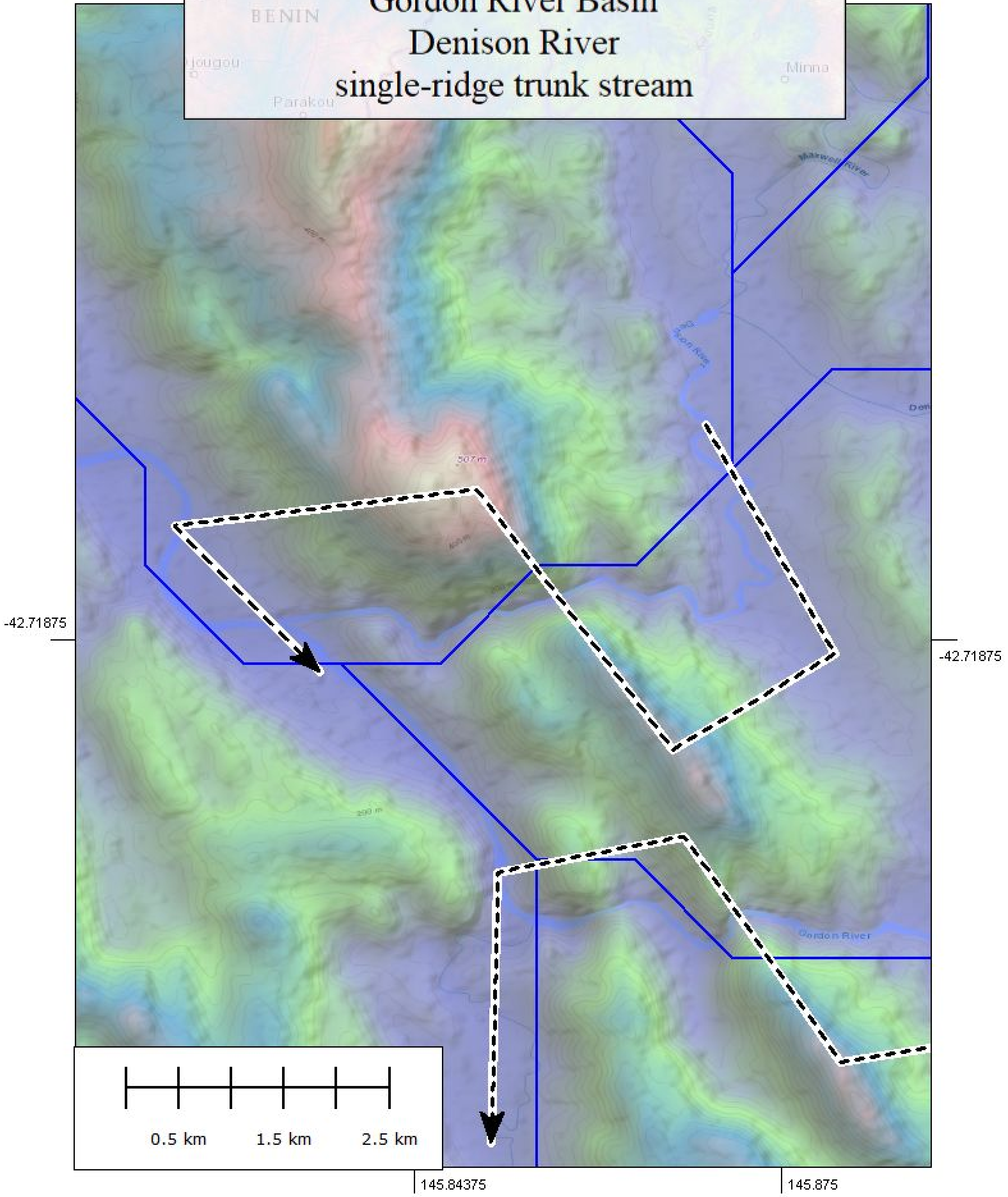

AU-WPAC - 57
Gordon River Basin
Gordon River
single-ridge trunk stream

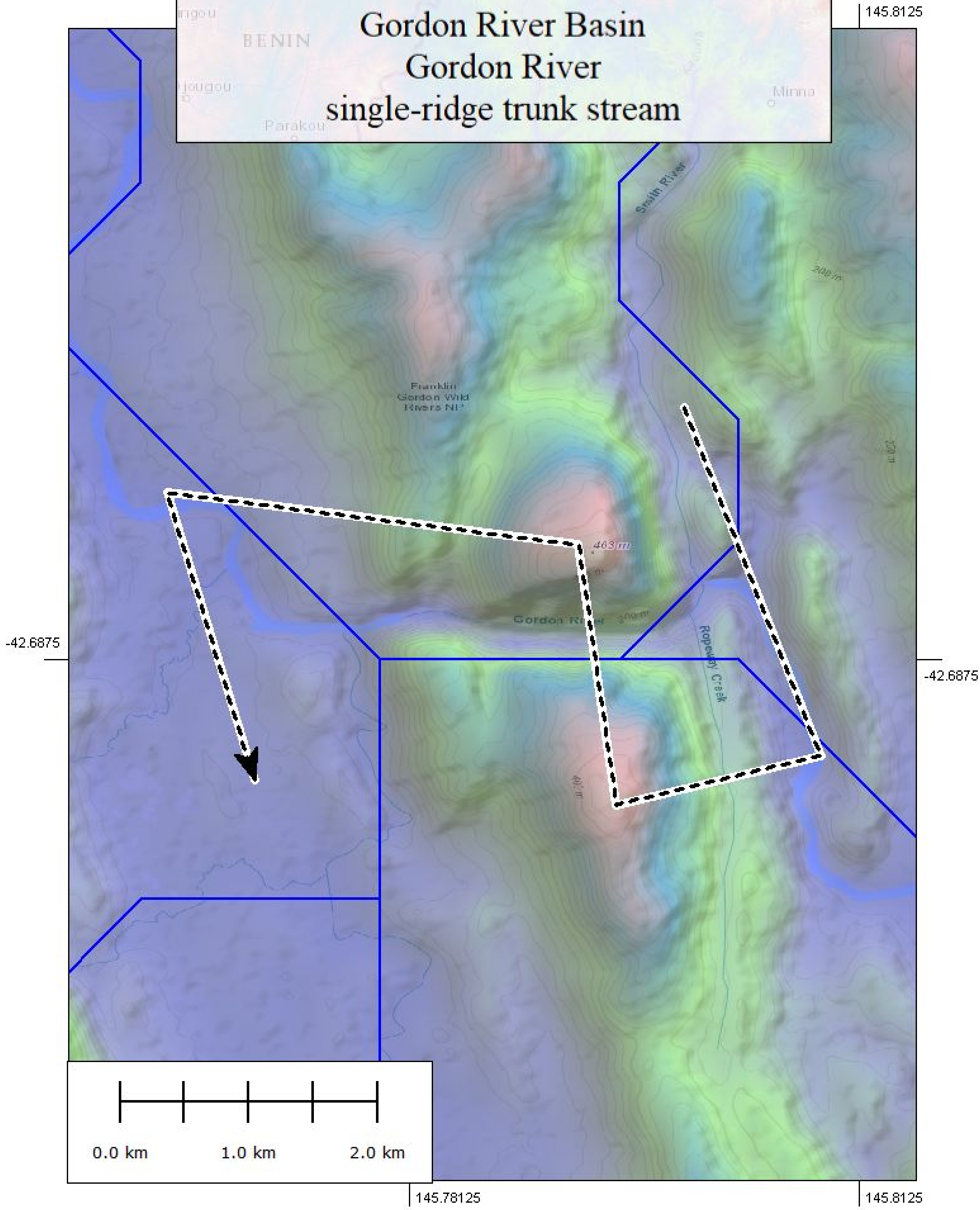

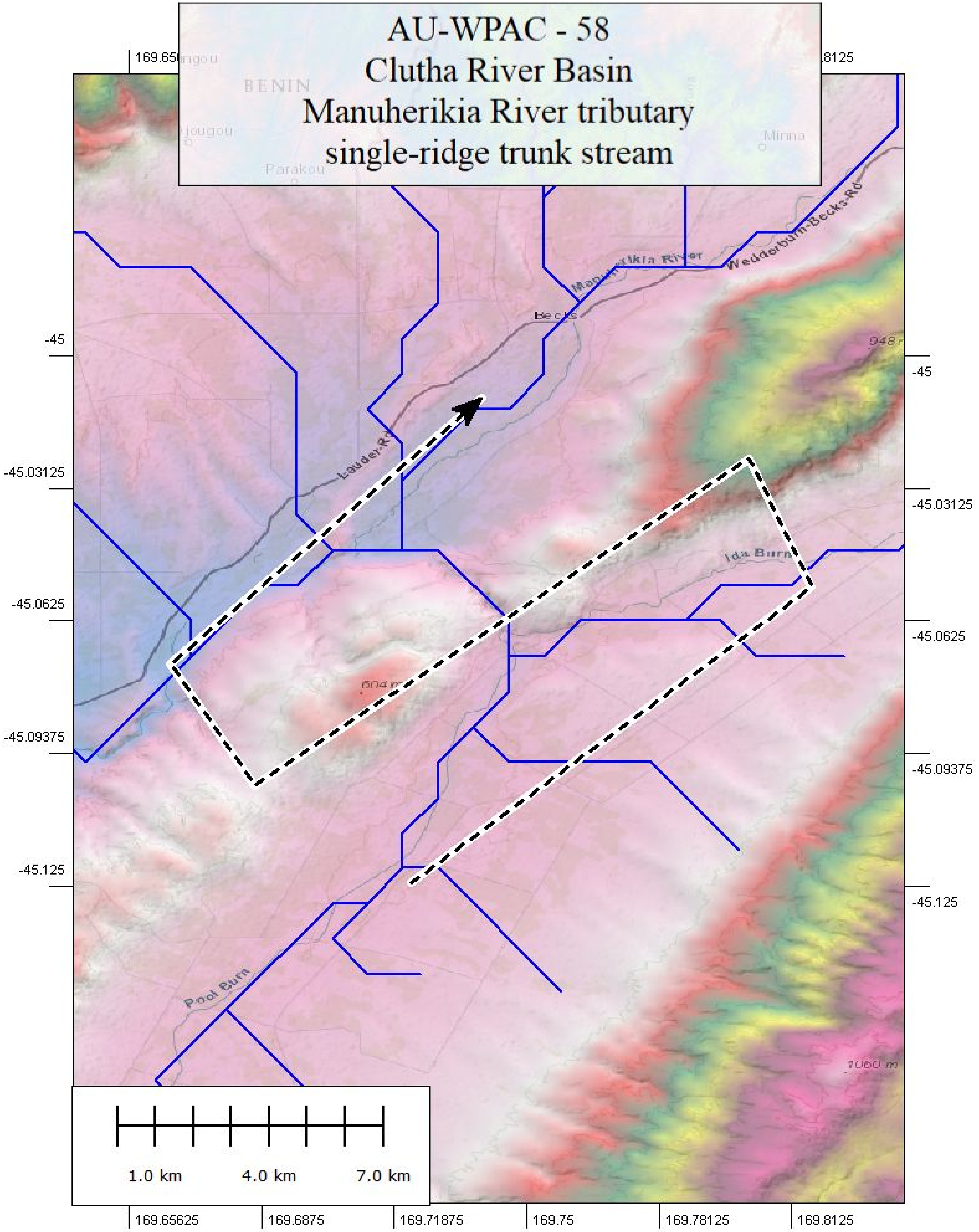
AU-WPAC - 58
Clutha River Basin
Manuherikia River tributary
single-ridge trunk stream
BENIN
Djougou
Parakou
Minna
Manuherikia River
Wedderburn-Becks-Rd
Becks
Lauder-Rd
Ida Burn
Pool Burn
604 m
948 m
1060 m
-45
-45.03125
-45.0625
-45.09375
-45.125
169.65625
169.6875
169.71875
169.75
169.78125
169.8125
1.0 km
4.0 km
7.0 km

AU-WPAC - 59
Kikori River Basin
Waga River
single-ridge trunk stream

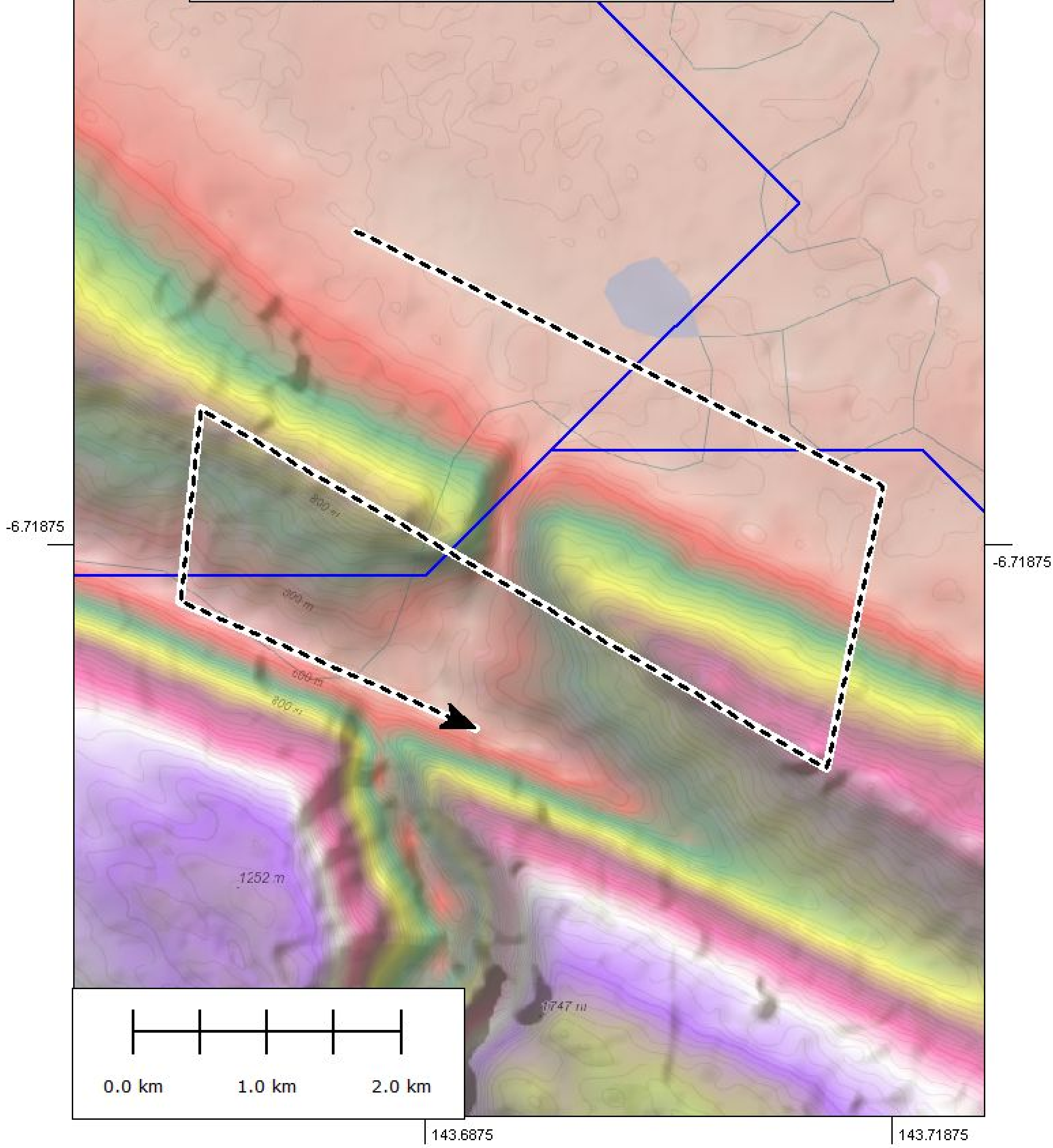

AU-WPAC - 60
Seruway River? Basin
single-ridge trunk stream

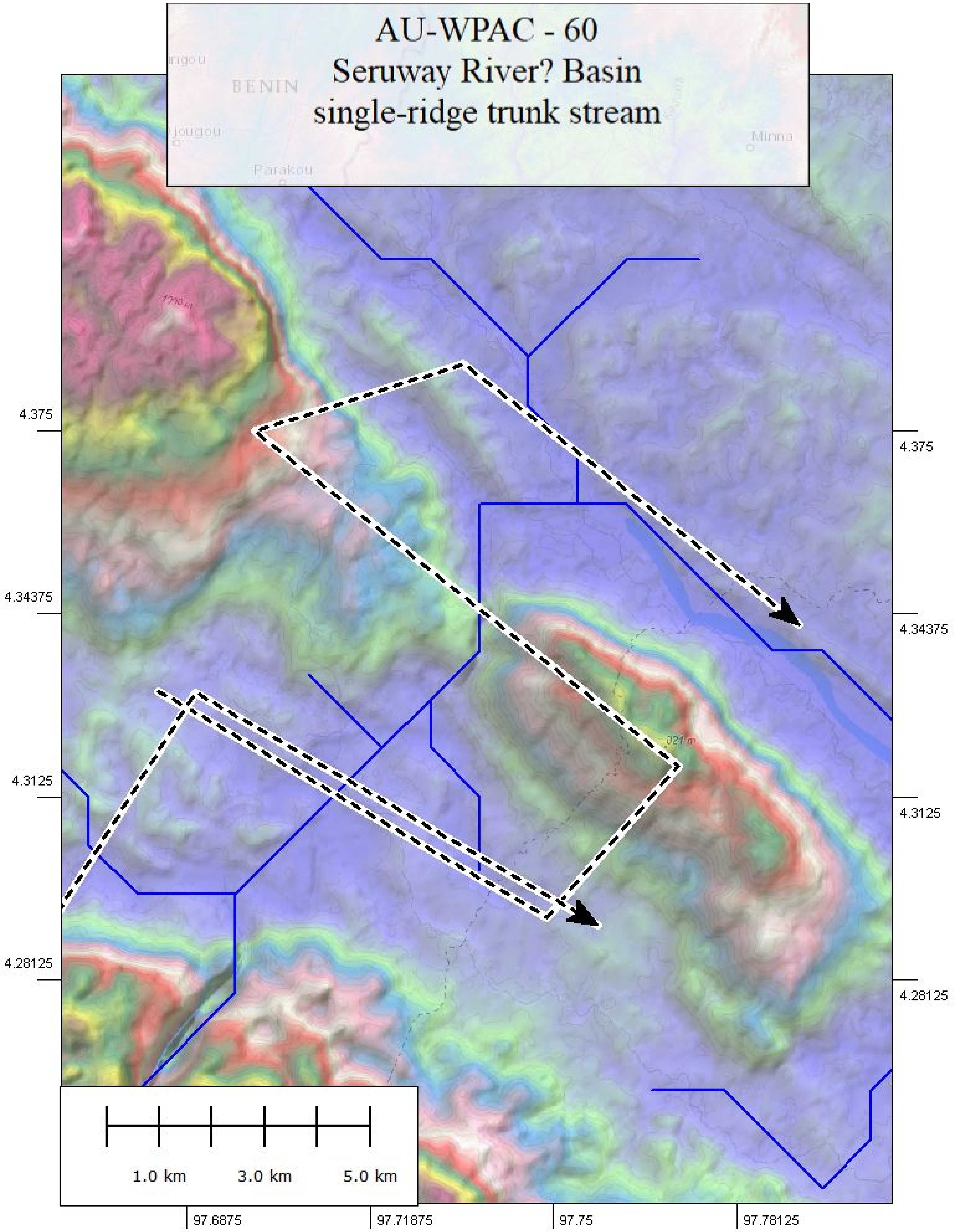

AU-WPAC - 61
Seruway River? Basin
single-ridge trunk stream

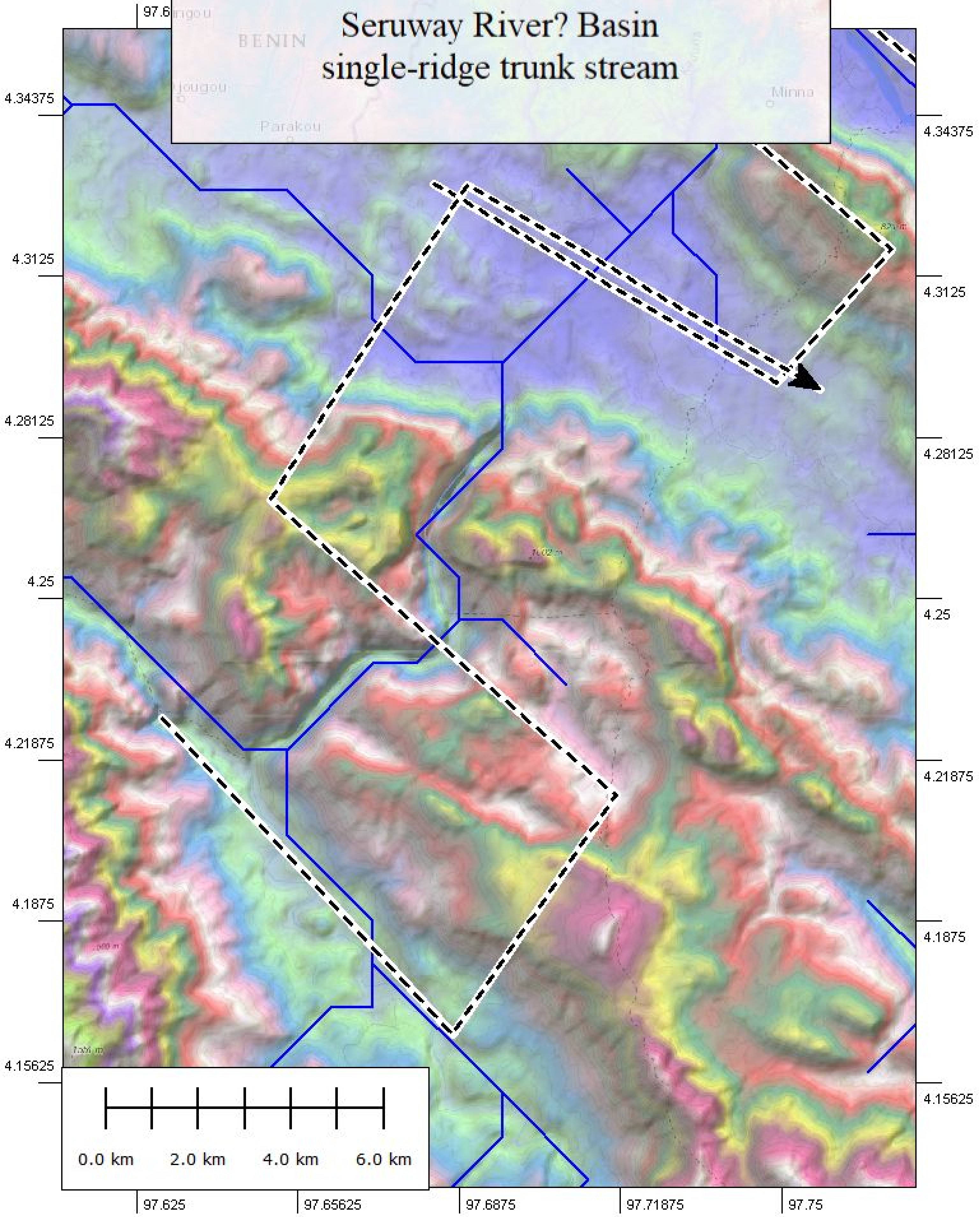

AU-WPAC - 62
Gordon River Basin
Jane River
single-ridge trunk stream

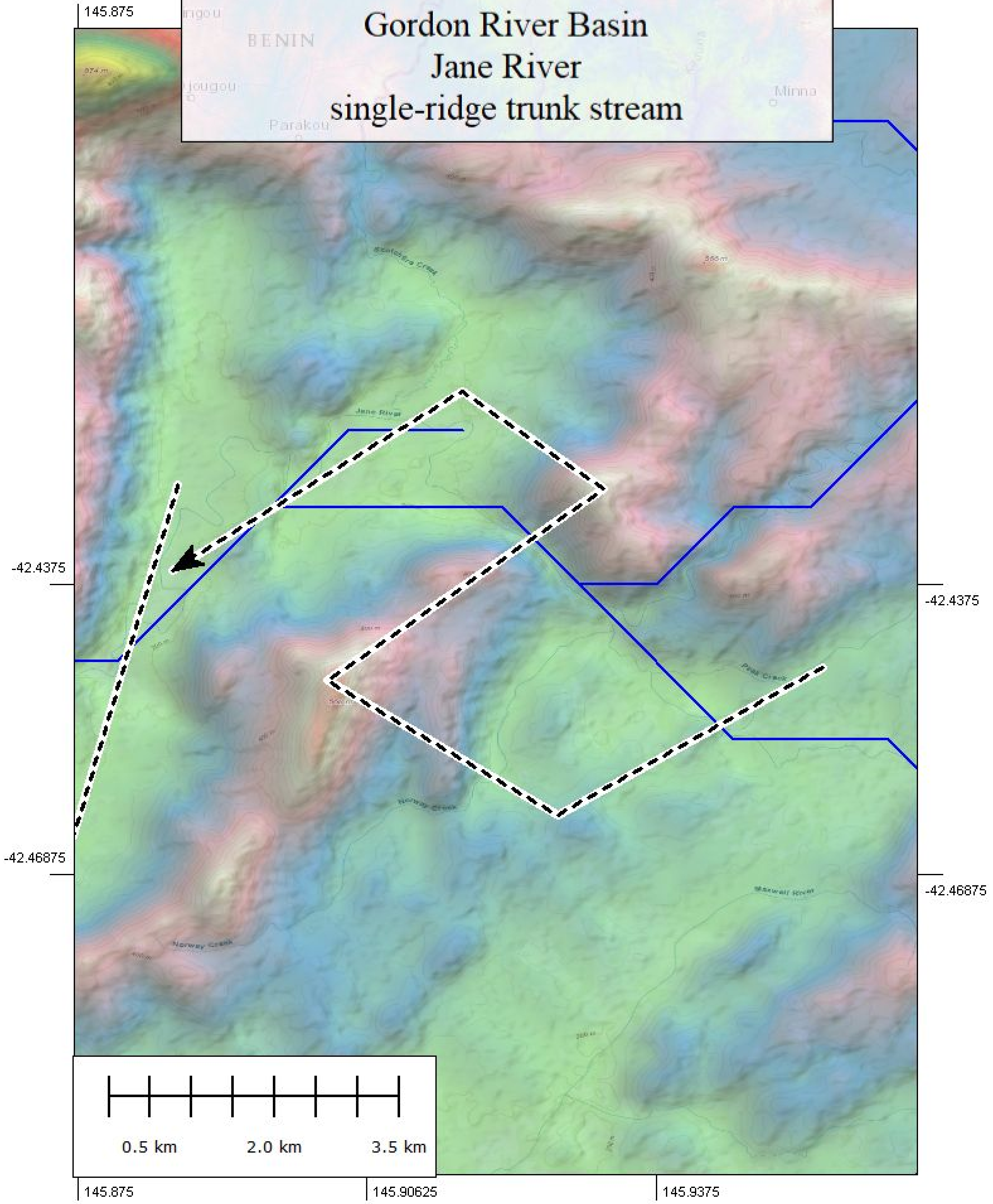

AU-WPAC - 63
Gordon River Basin
Jane River
single-ridge trunk stream

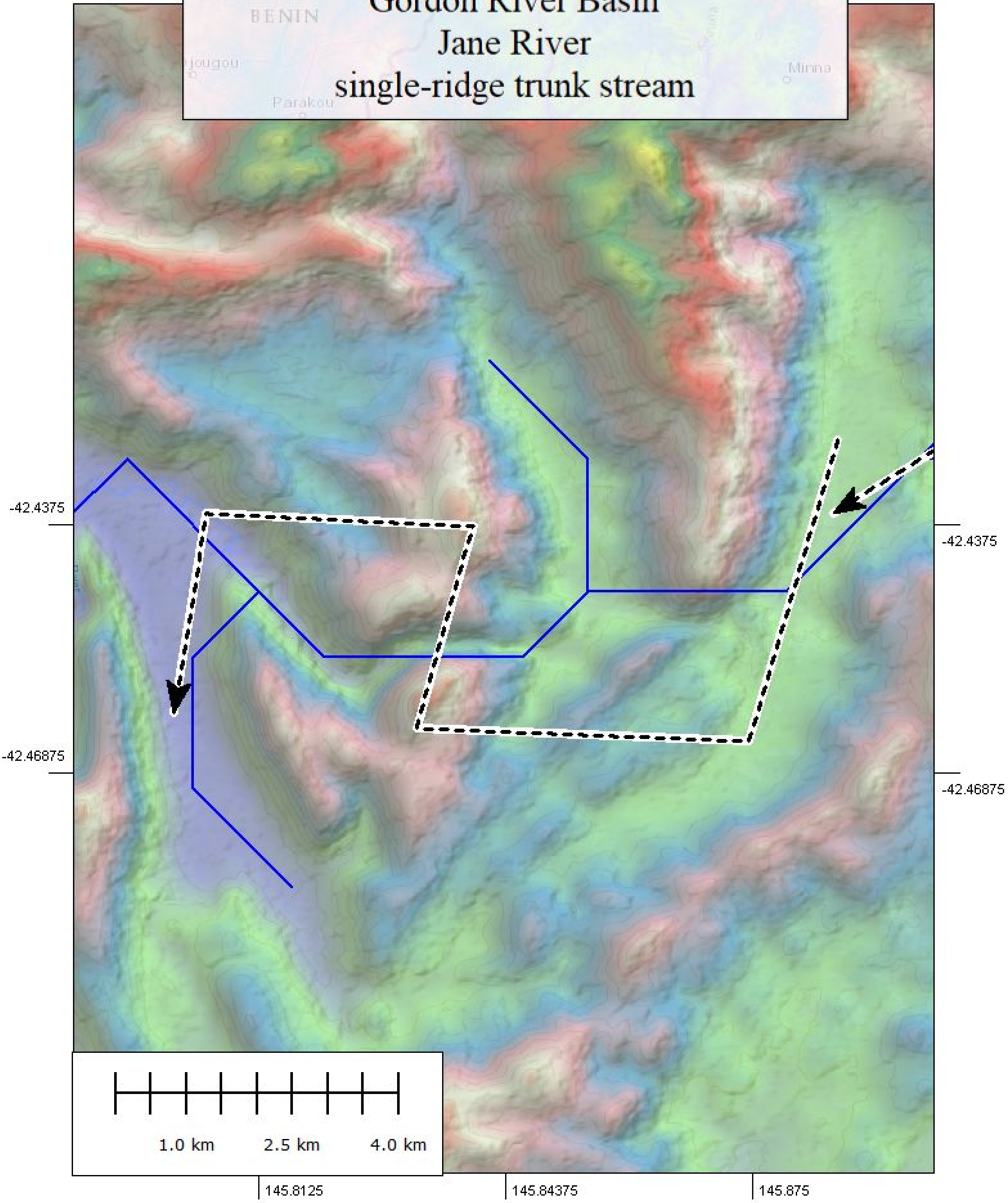

AU-WPAC - 64
Lalindu River Basin
Lasgai Lasolo River
single-ridge trunk stream

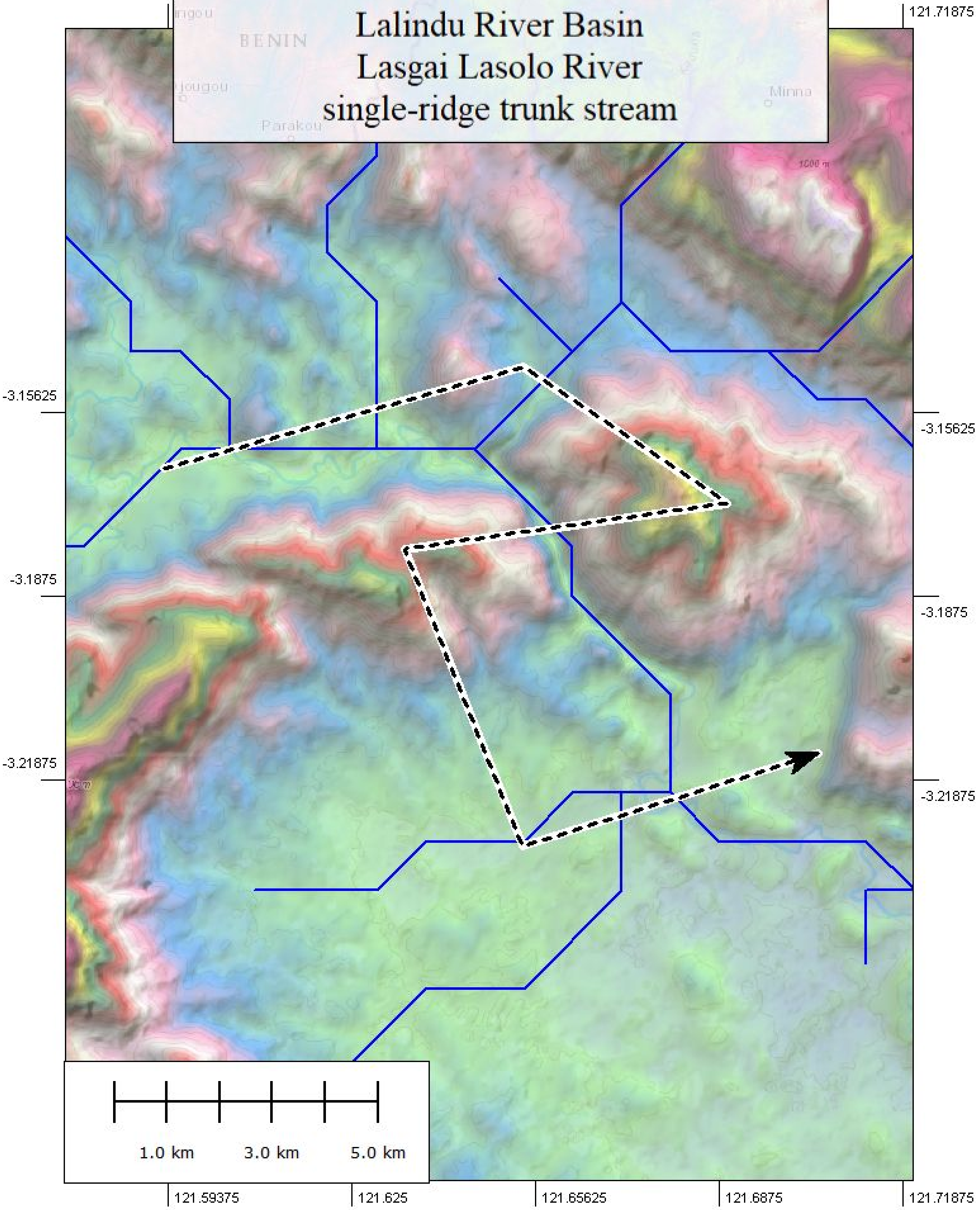

AU-WPAC - 67
Indragiri River Basin
Indragiri River tributary
single-ridge head stream

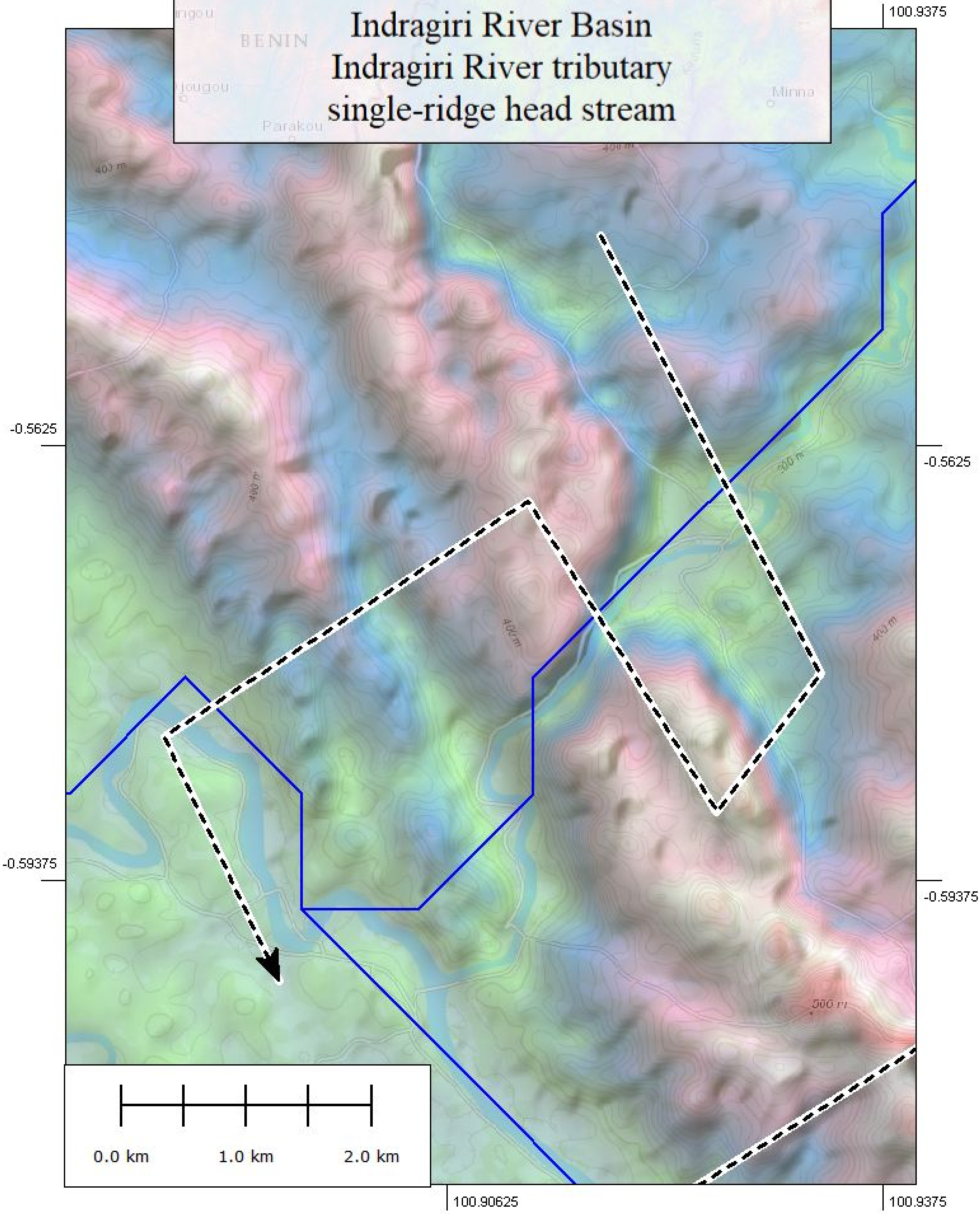

AU-WPAC - 69
Alas River Basin
single-ridge trunk stream

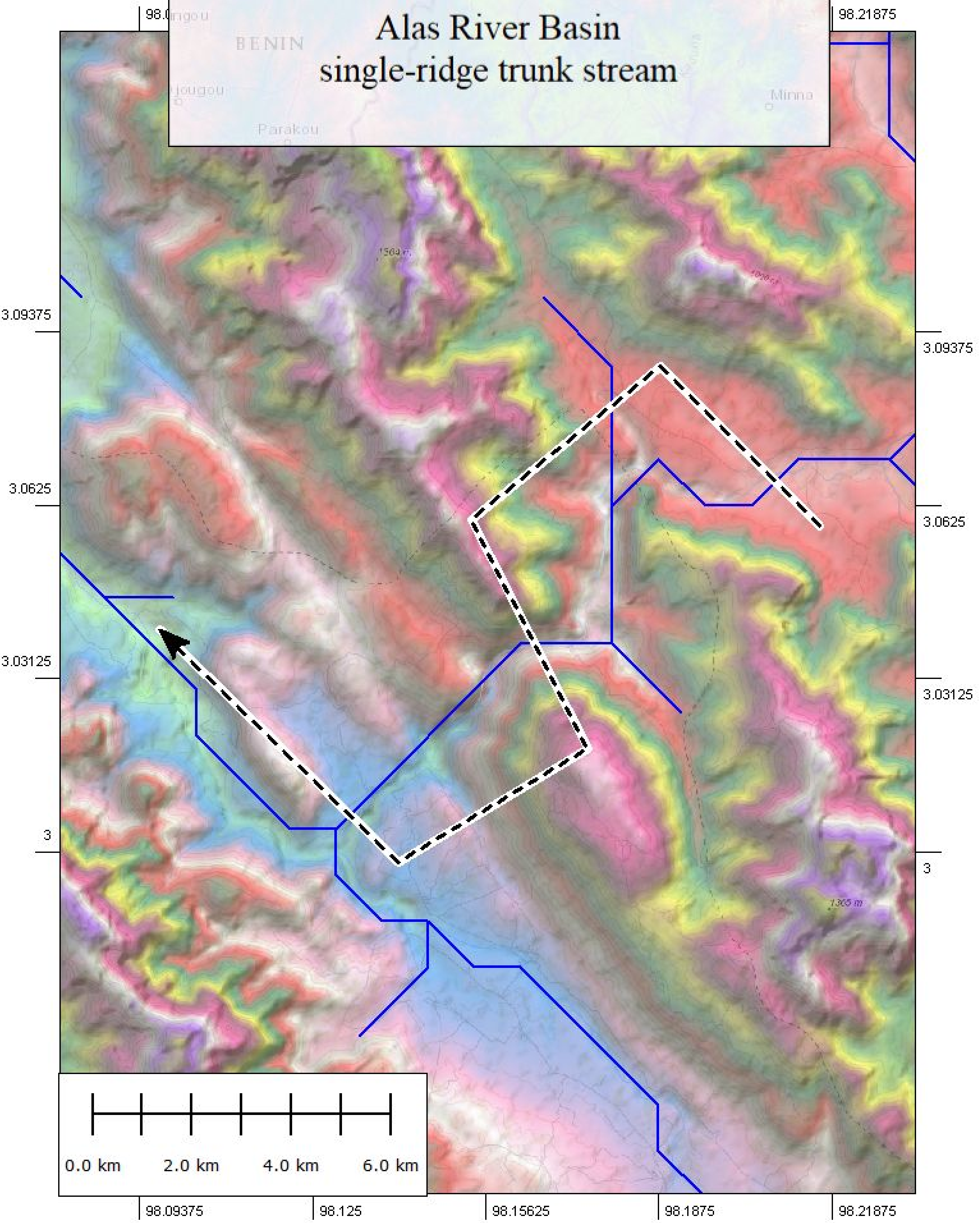

AU-WPAC - 70
Waiau River Basin
Waiau River
single-ridge trunk stream

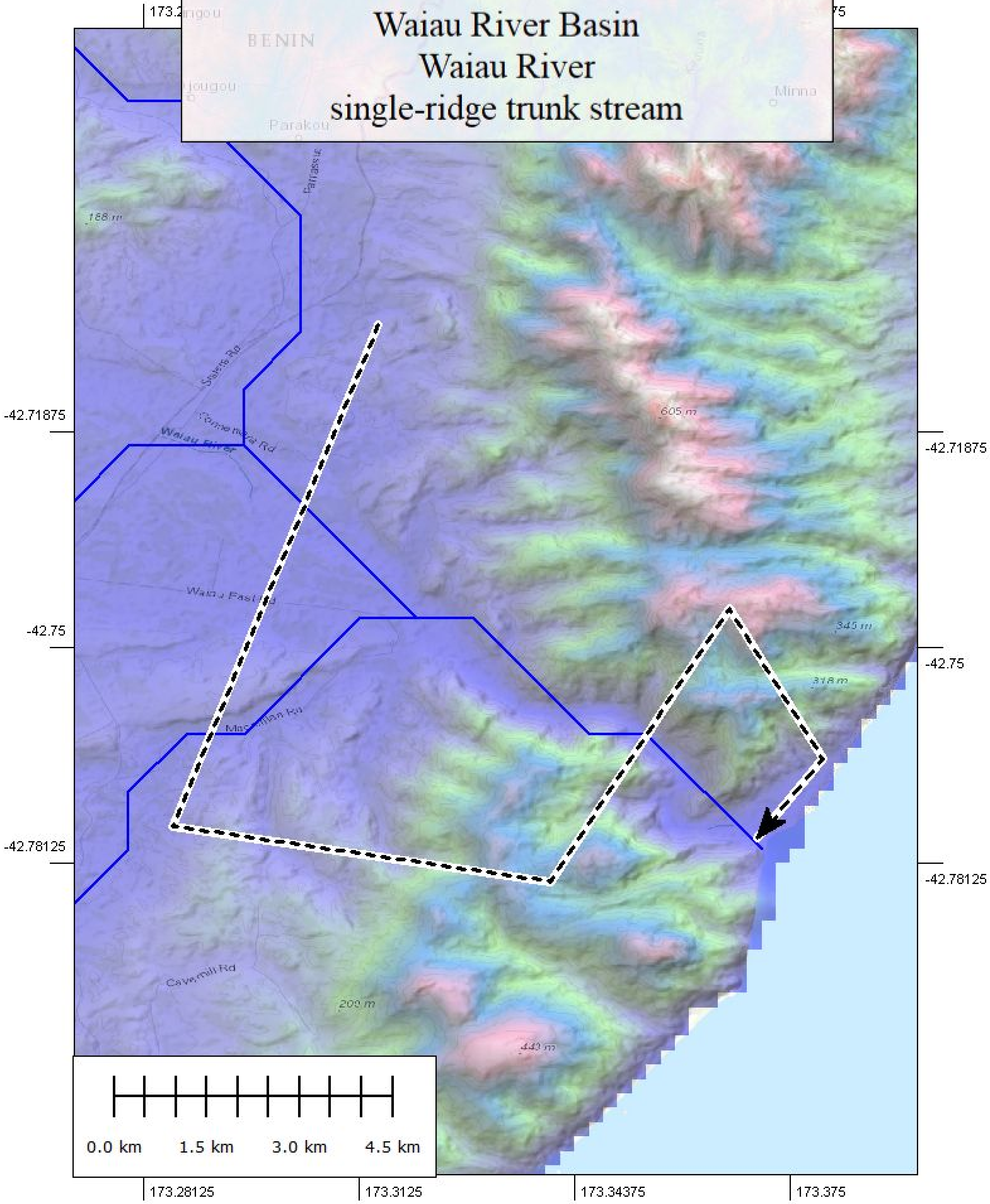

AU-WPAC - 71
Batang Gadis River Basin
single-ridge trunk stream

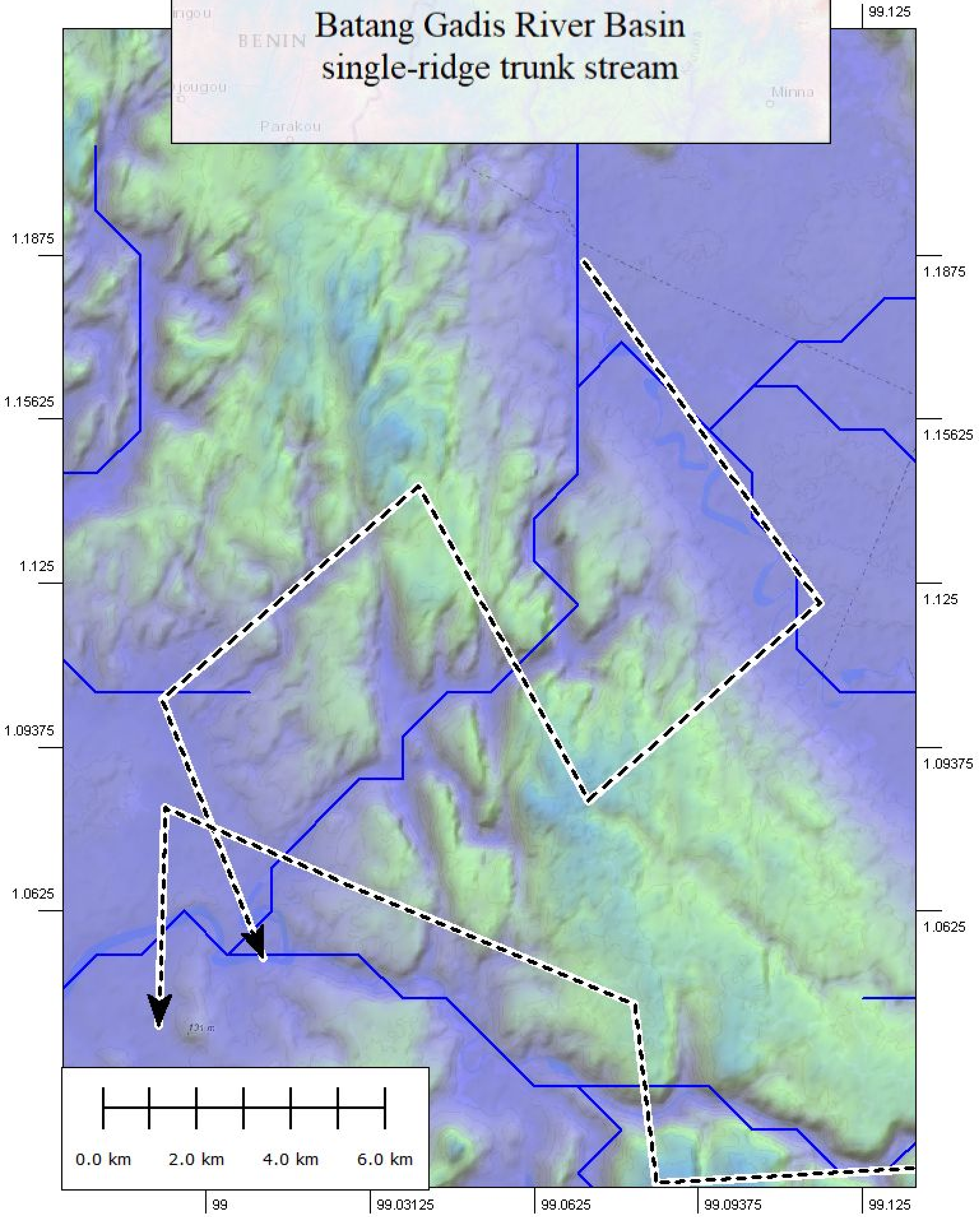

AU-WPAC - 72
Batang Gadis River Basin
single-ridge trunk stream

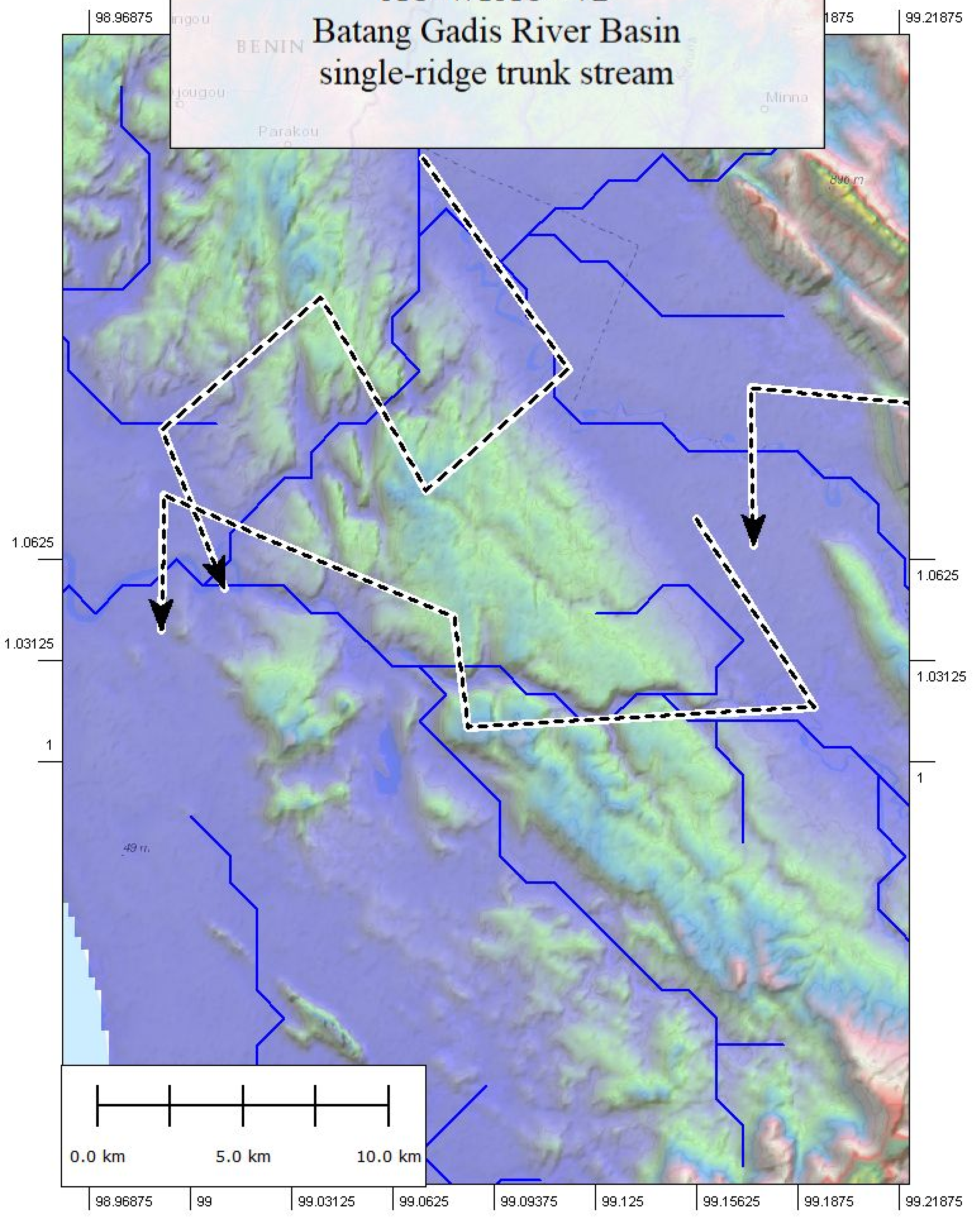

AU-WPAC - 73
Fitzroy River Basin
Leopold River tributary
single-ridge trunk stream

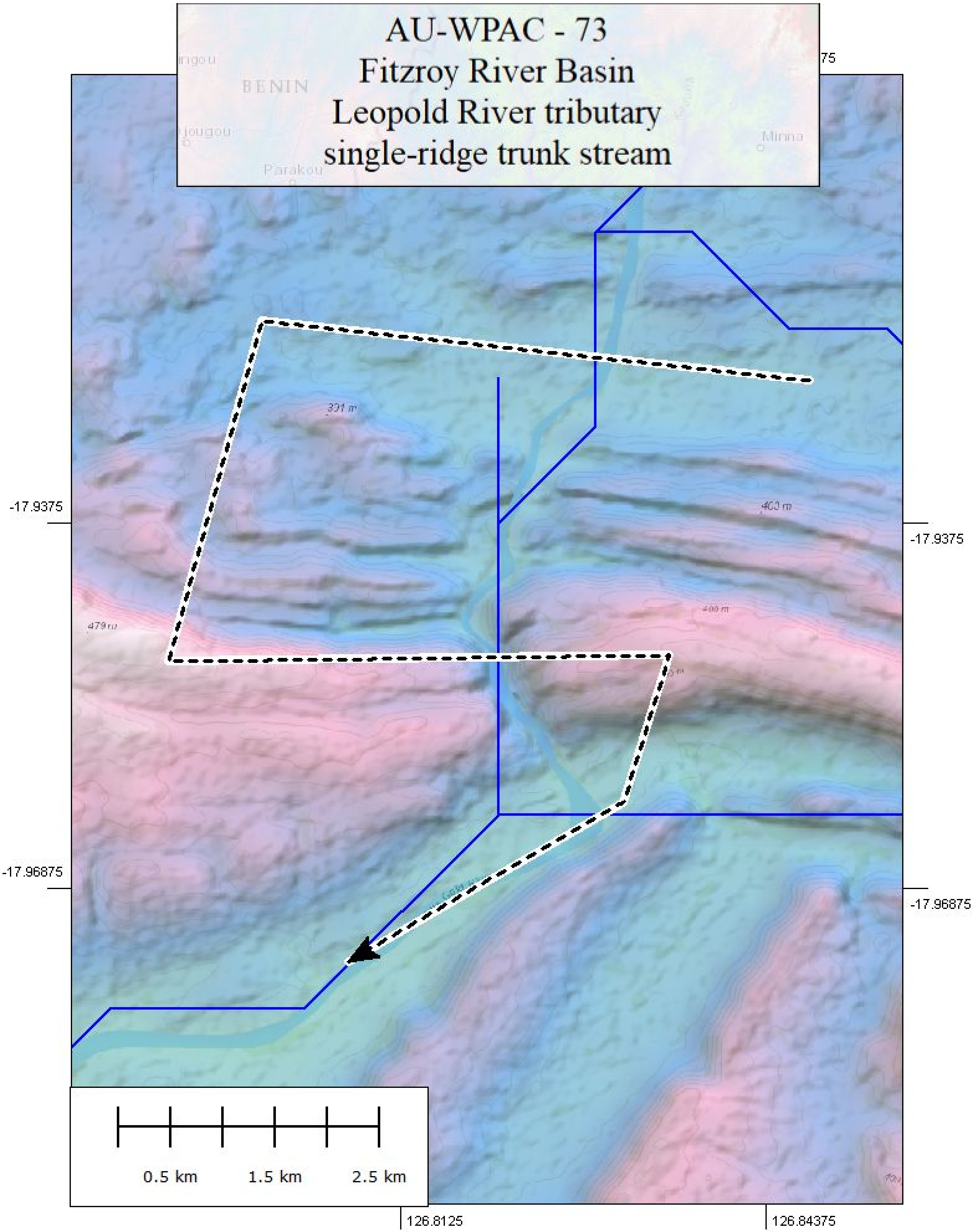

AU-WPAC - 74
Indragiri River Basin
Indragiri River tributary
single-ridge trunk stream

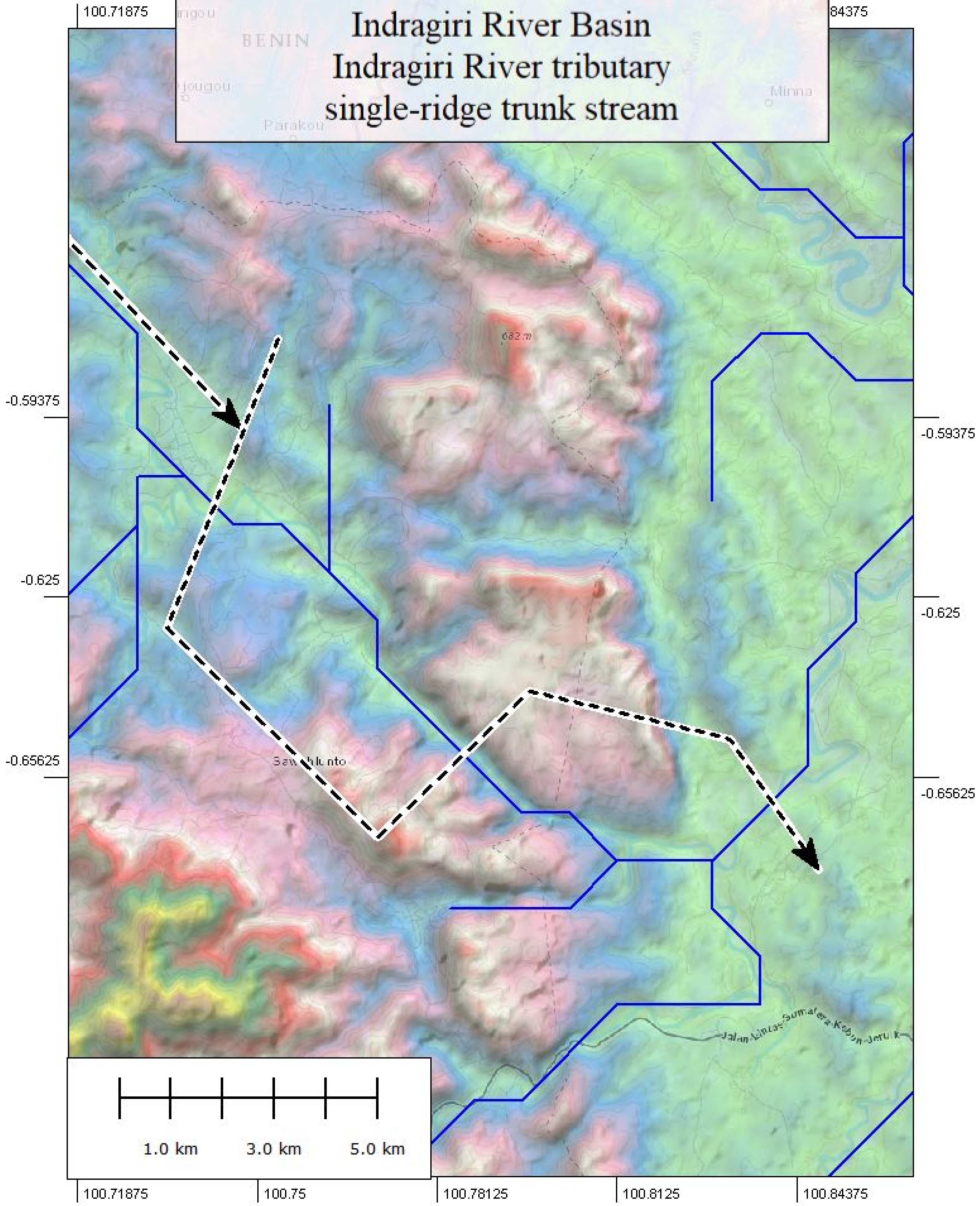

AU-WPAC - 45
Alas River Basin
irregular high ground trunk stream

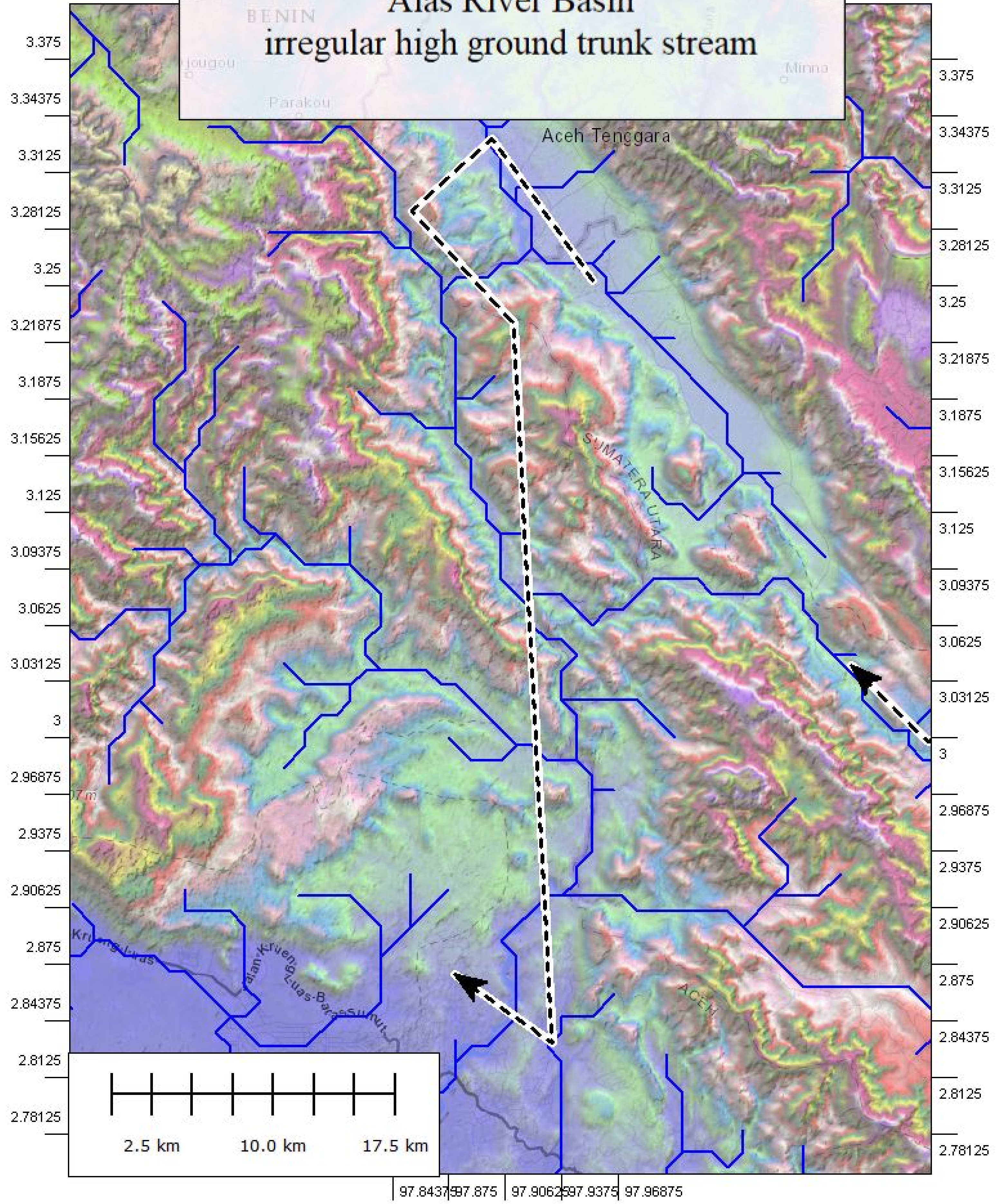

AU-WPAC - 49
Batanghari River Basin
Batanghari River tributary
irregular high ground trunk stream

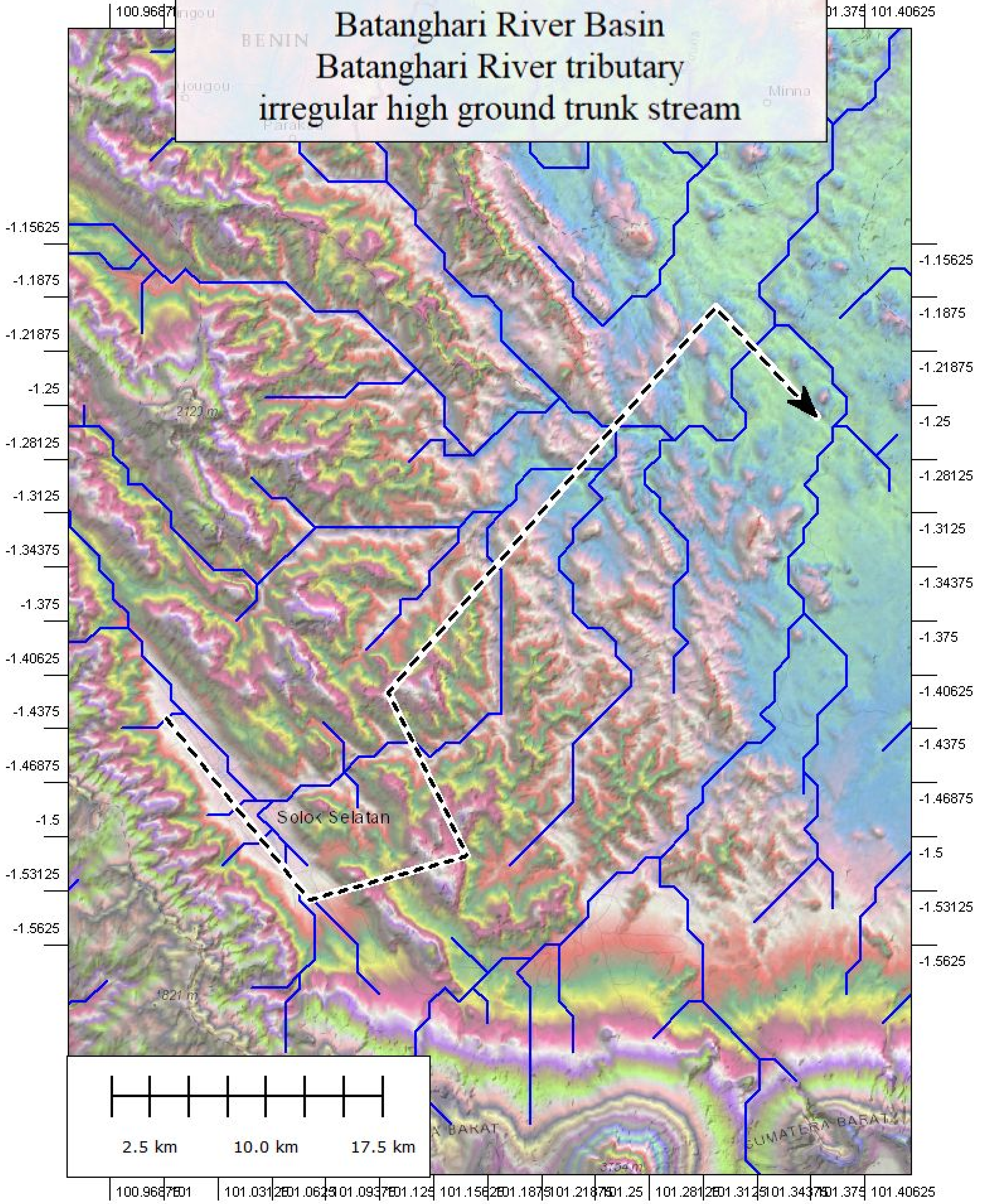

AU-WPAC - 9
Indragiri River Basin
Indragiri River
irregular high ground trunk stream

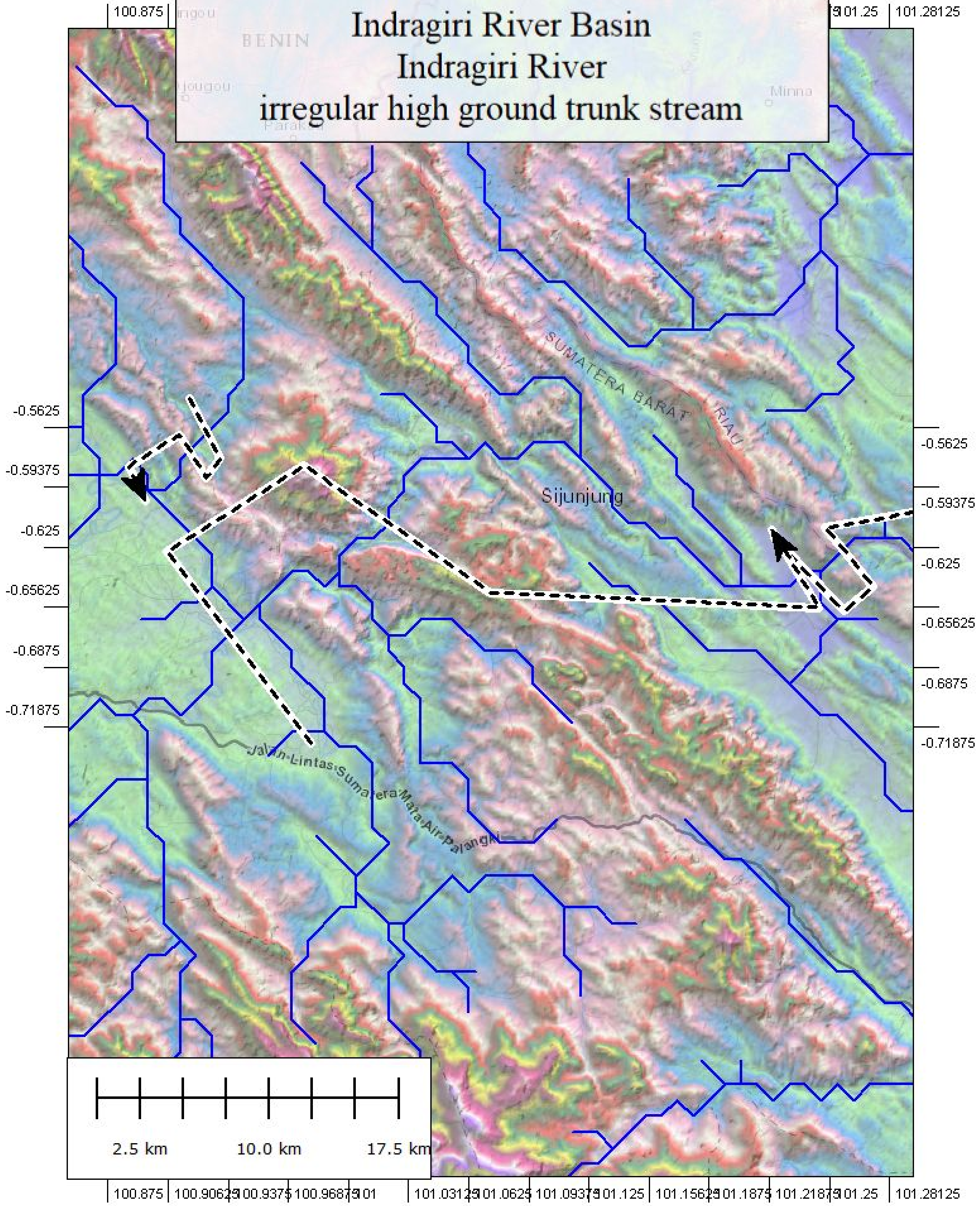

Supplement: Supplementary file 6 — Supplementary material [file mmc6.pdf]
