# Supplementary material for: A global database of large-scale transverse drainages
Source: Data Brief. 2019 Jan 14;23:103650. doi: 10.1016/j.dib.2018.12.088 (PMC6369416; doi:10.1016/j.dib.2018.12.088)
Supplement: Supplementary file 7 — Supplementary material [file mmc7.pdf]

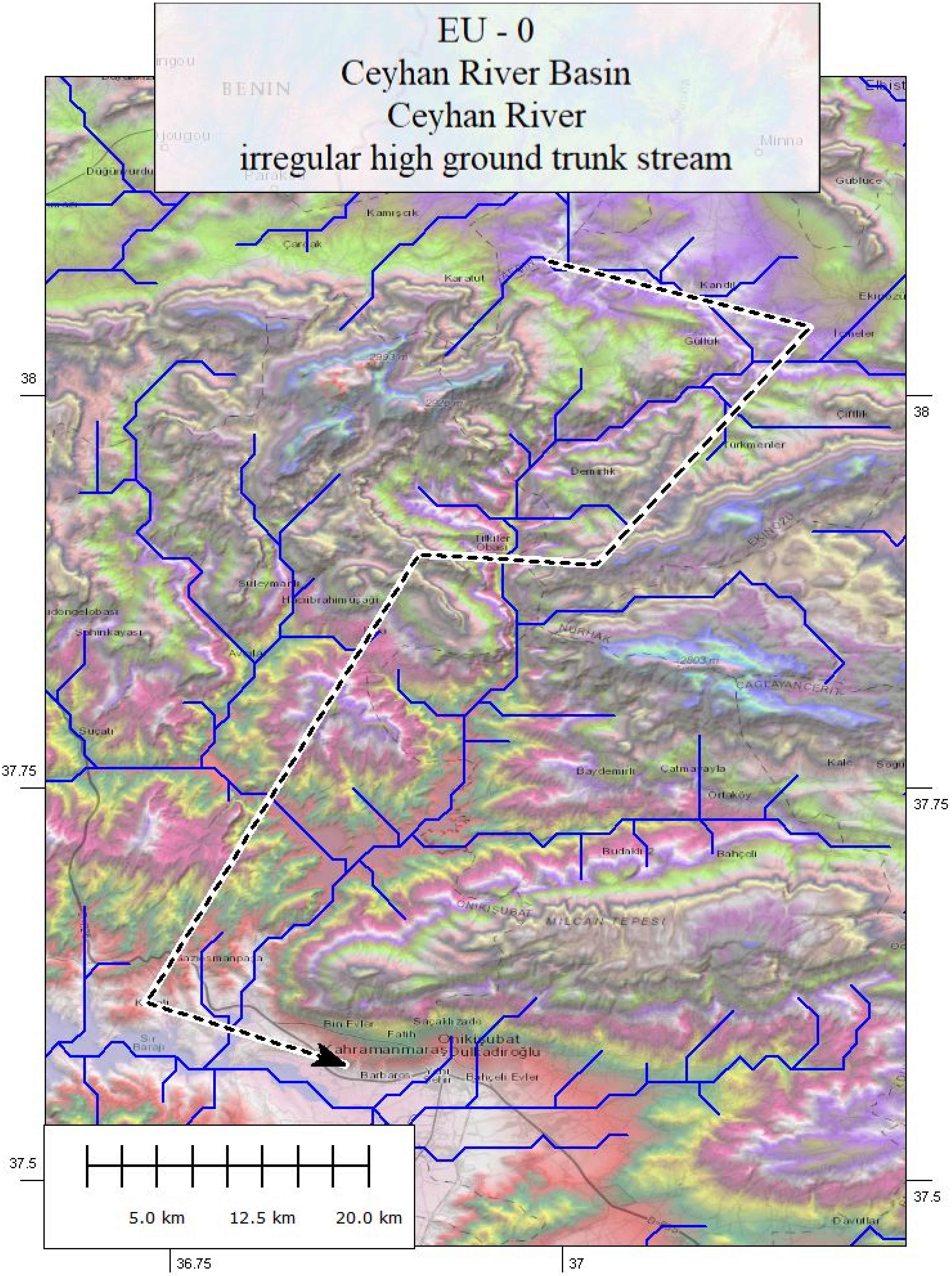
EU - 0
Ceyhan River Basin
Ceyhan River
irregular high ground trunk stream
BENIN
Düğünyurdu
Kamışcık
Çardak
Karatut
Kandil
Güllük
Gubluce
Minna
Ekinözü
Temeler
Çiftlik
Türkmenler
Demirlik
Tilkiler Obası
Süleymanlı
Hacıibrahimuşağı
Şahinkayası
Avcılar
NURHAK
2803 m
ÇAĞLAYANCERİT
Suçatı
Baydemirli
Çatmarayla
Ortaköy
Kale
Budaklı 2
Bahçeli
ONİKİŞUBAT
MILCAN TEPESİ
Gaziosmanpaşa
Sır Barajı
Bin Evler
Saçaklızade
Fatih
Onikişubat
Kahramanmaraş
Dulkadiroğlu
Barbaros
Yeni Şehir
Bahçeli Evler
Davutlar
38
37.75
37.5
36.75
37
5.0 km
12.5 km
20.0 km

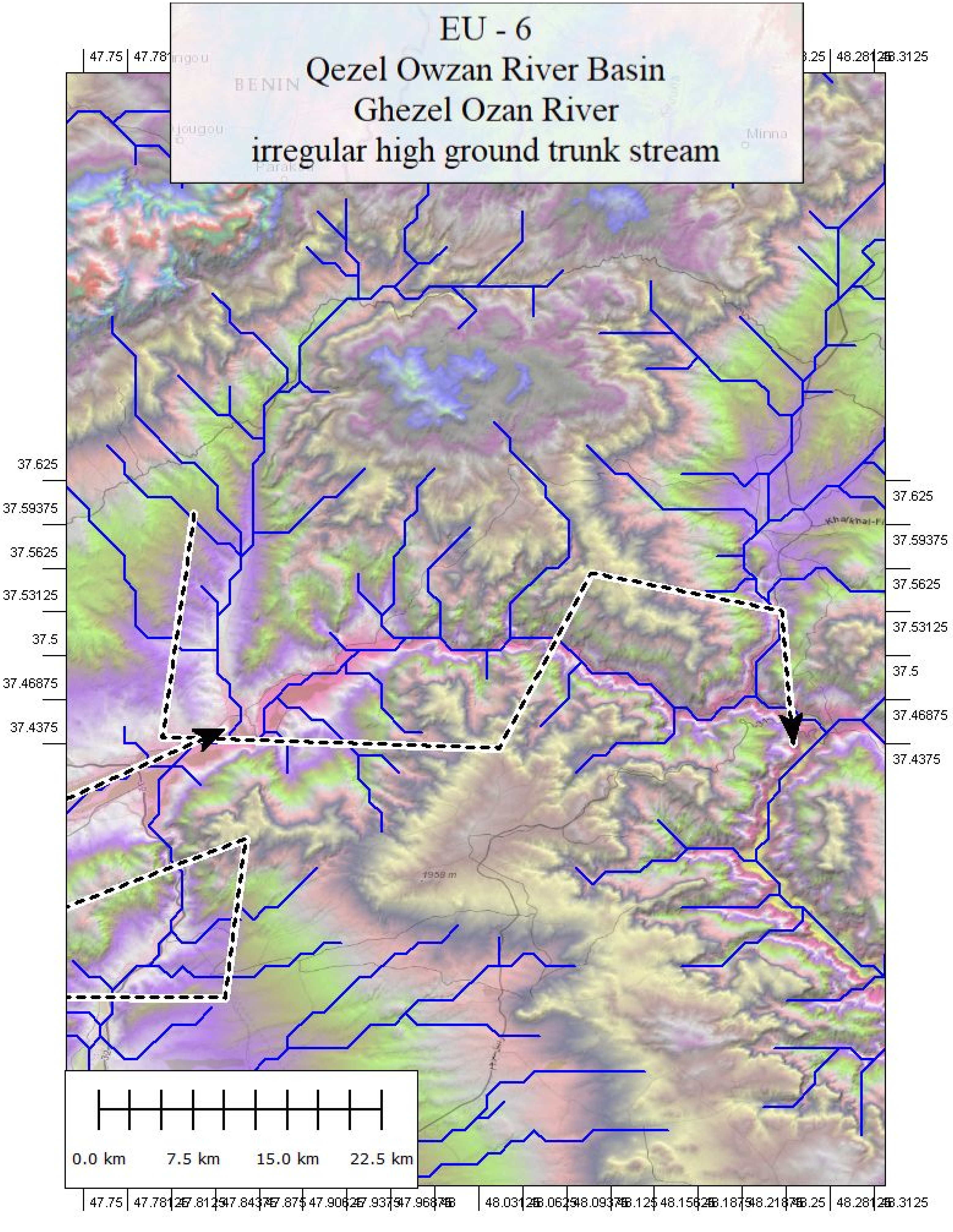
EU - 6
Qezel Owzan River Basin
Ghezel Ozan River
irregular high ground trunk stream
37.625
37.59375
37.5625
37.53125
37.5
37.46875
37.4375
47.75
1958 m
0.0 km
7.5 km
15.0 km
22.5 km

EU - 12
Euphrates River Basin
irregular high ground trunk stream

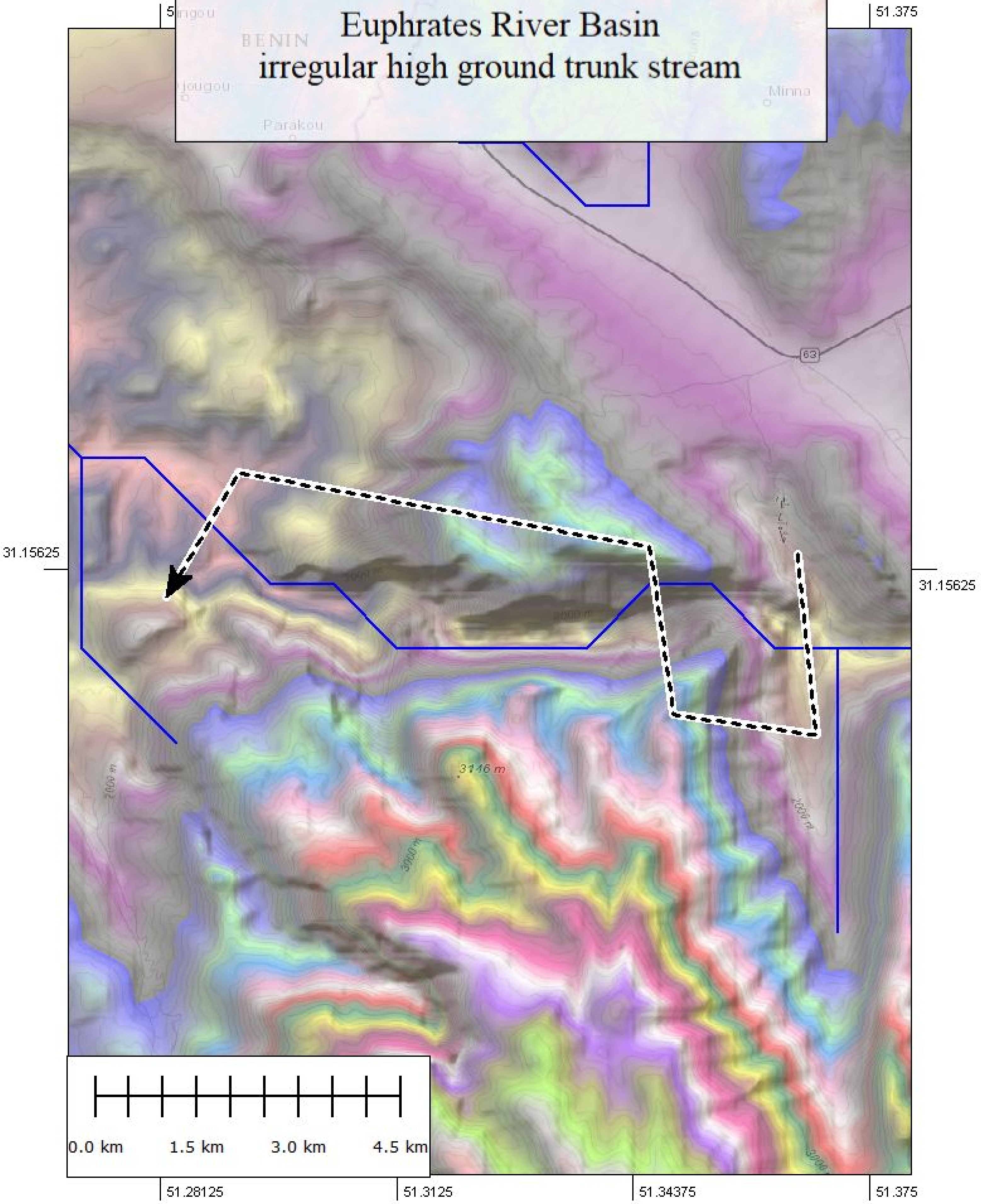

EU - 23
Rhine River Basin
Rhine River
irregular high ground trunk stream

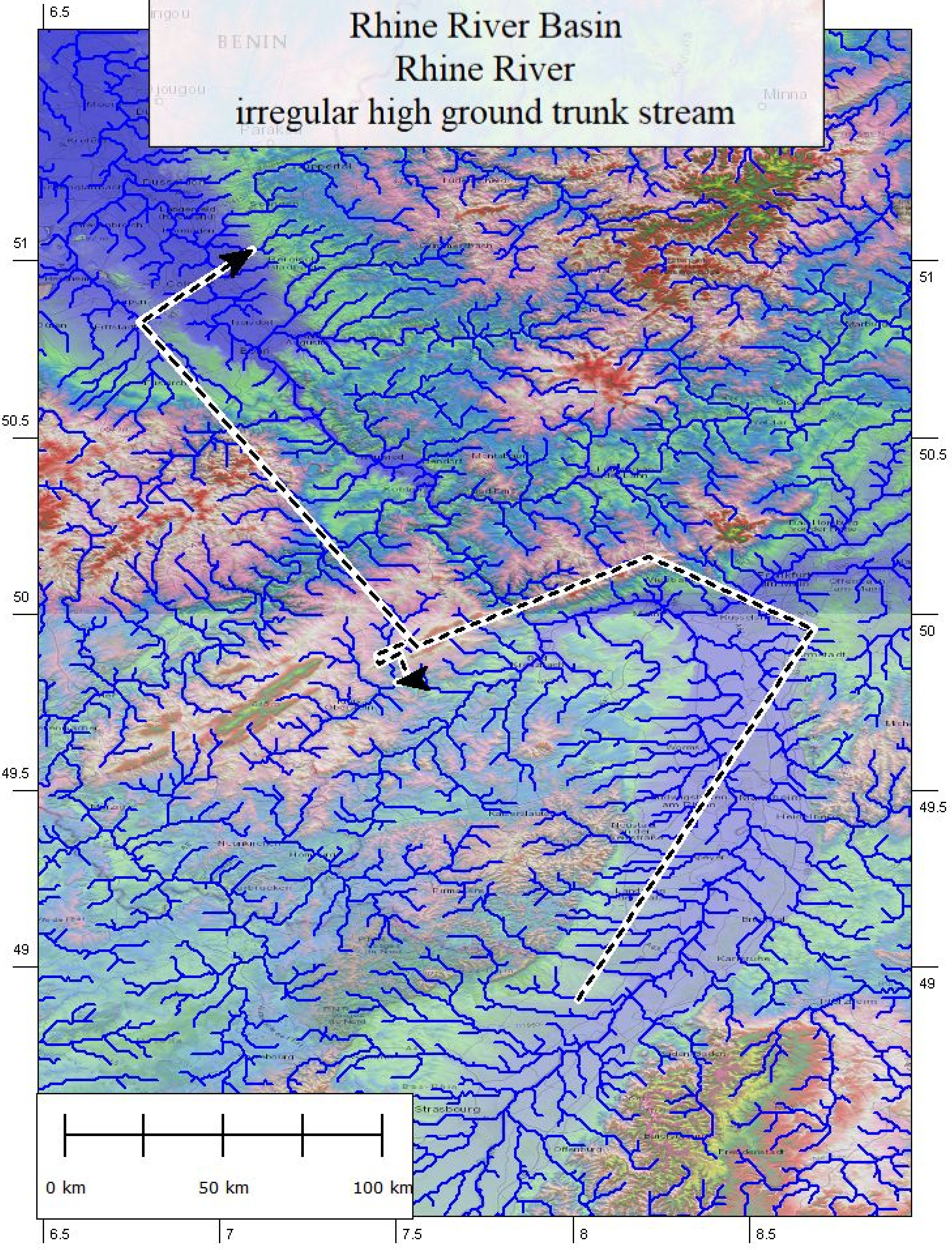

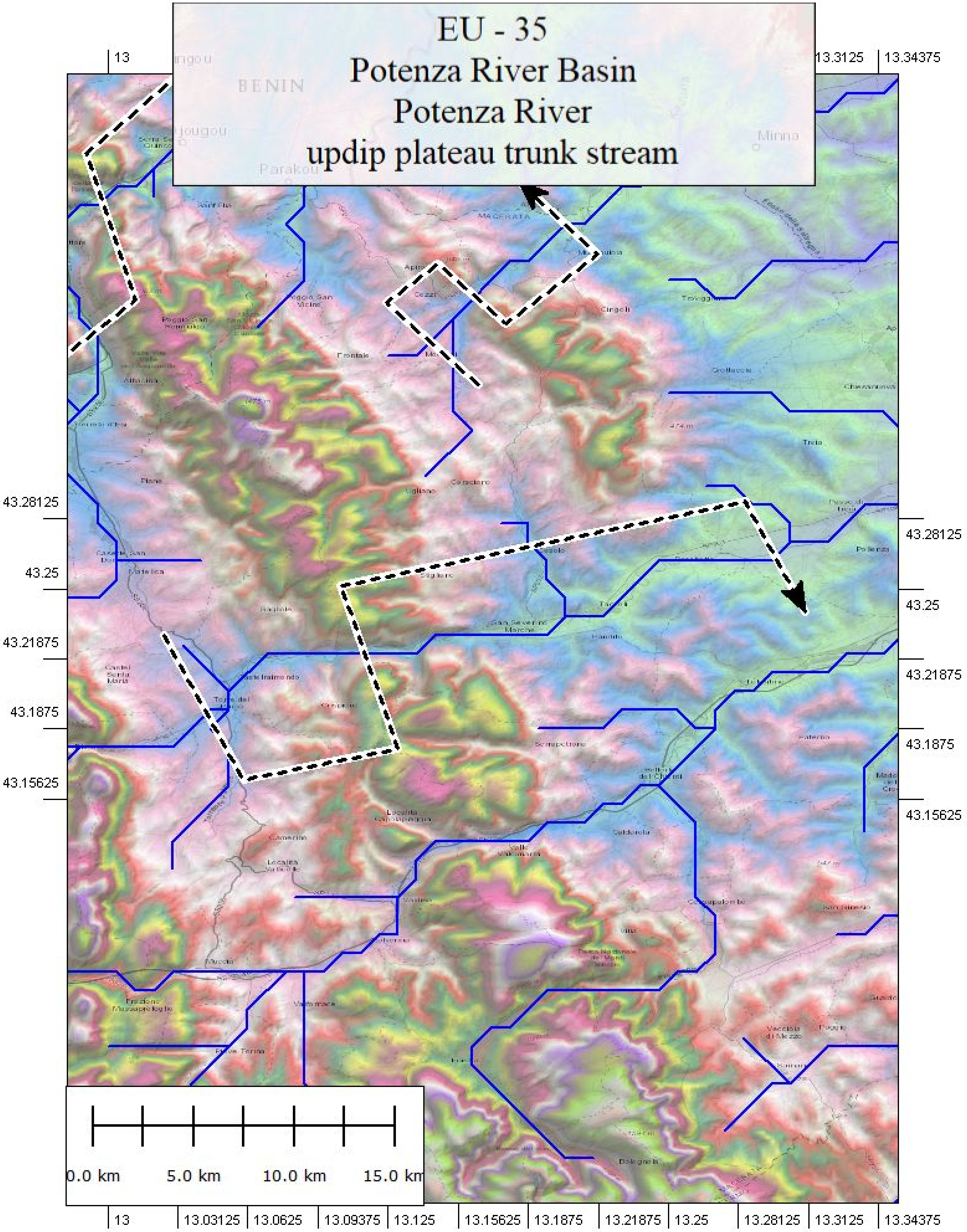
EU - 35
Potenza River Basin
Potenza River
updip plateau trunk stream
0.0 km
5.0 km
10.0 km
15.0 km
13
13.03125
13.0625
13.09375
13.125
13.15625
13.1875
13.21875
13.25
13.28125
13.3125
13.34375
43.28125
43.25
43.21875
43.1875
43.15625

EU - 39
Siah Kaur Basin
Ghaz Ab Kaur River
plateau head stream

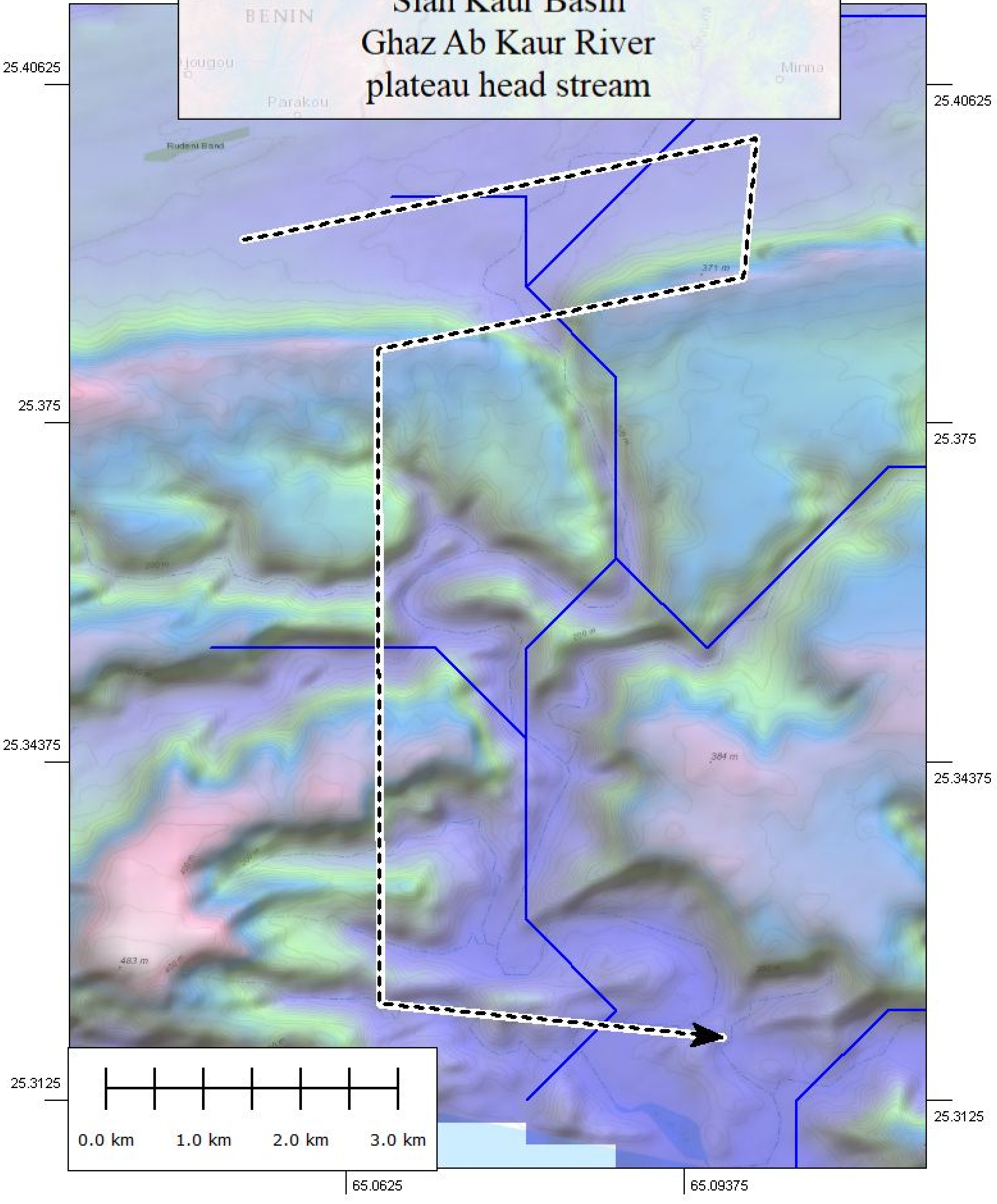

EU - 47
Prut River Basin
Sava River
irregular high ground trunk stream

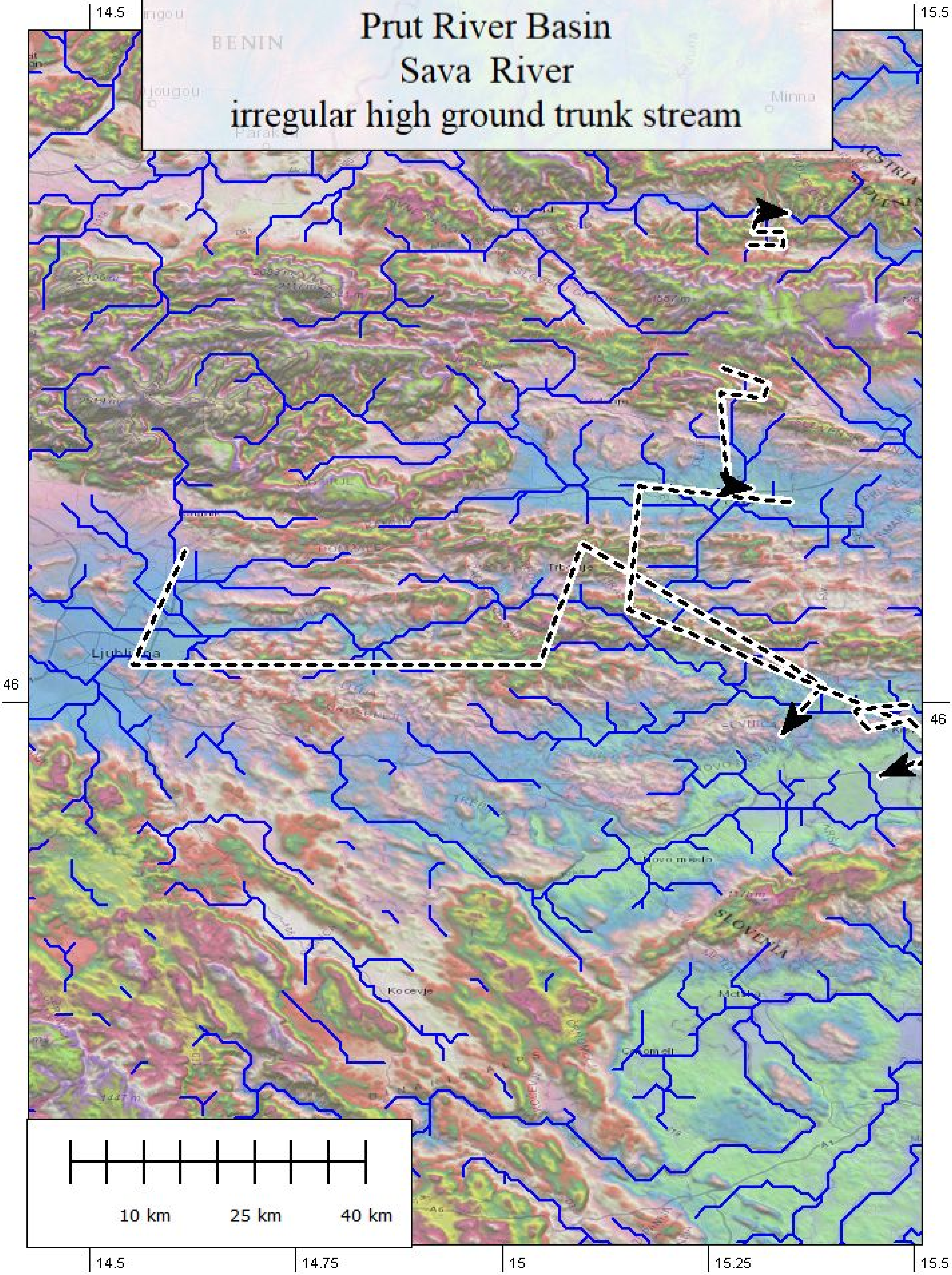

EU - 48
Ebro River Basin
Ebro River
irregular high ground trunk stream

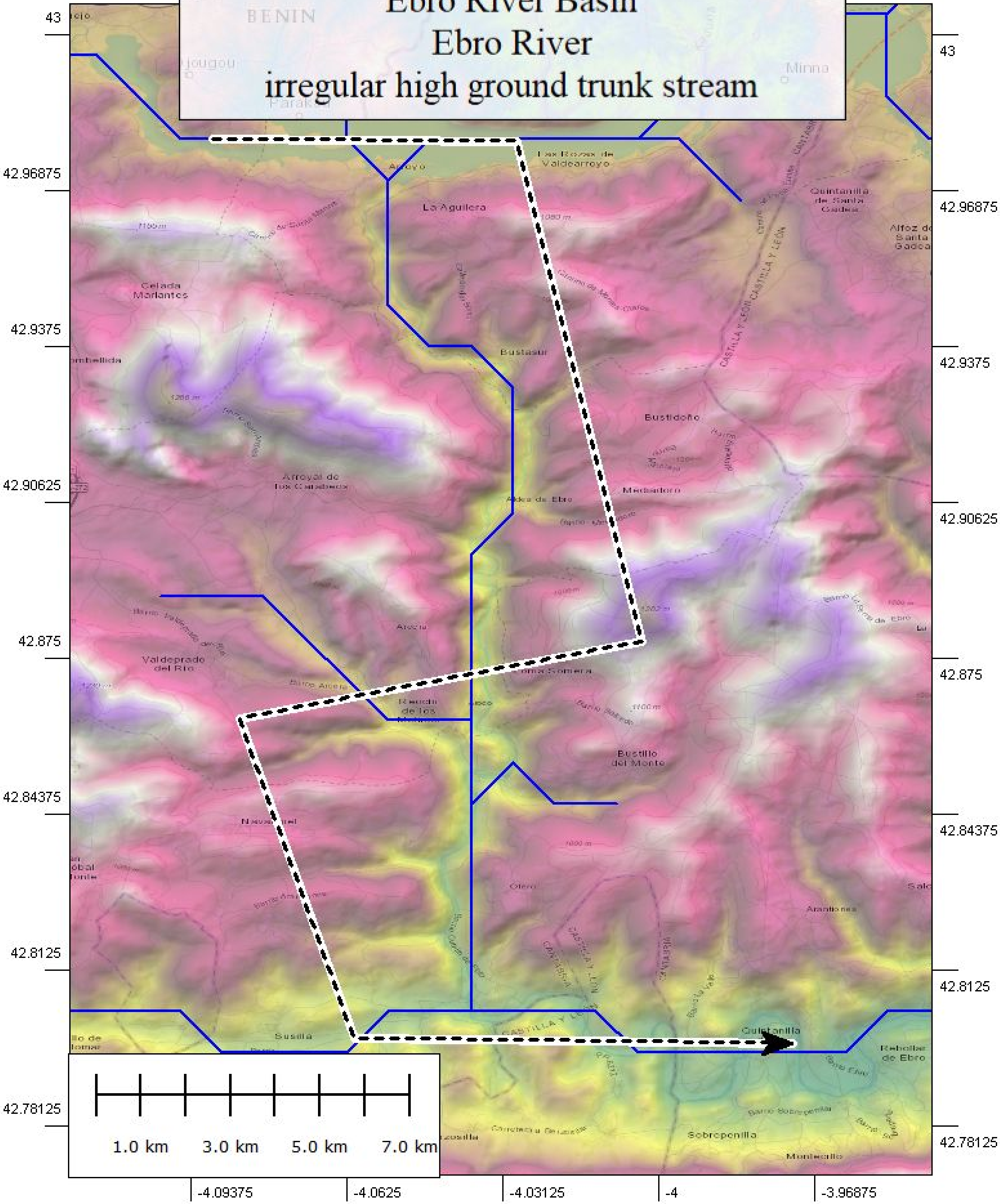

EU - 50
Ural River Basin
Ural River
irregular high ground trunk stream

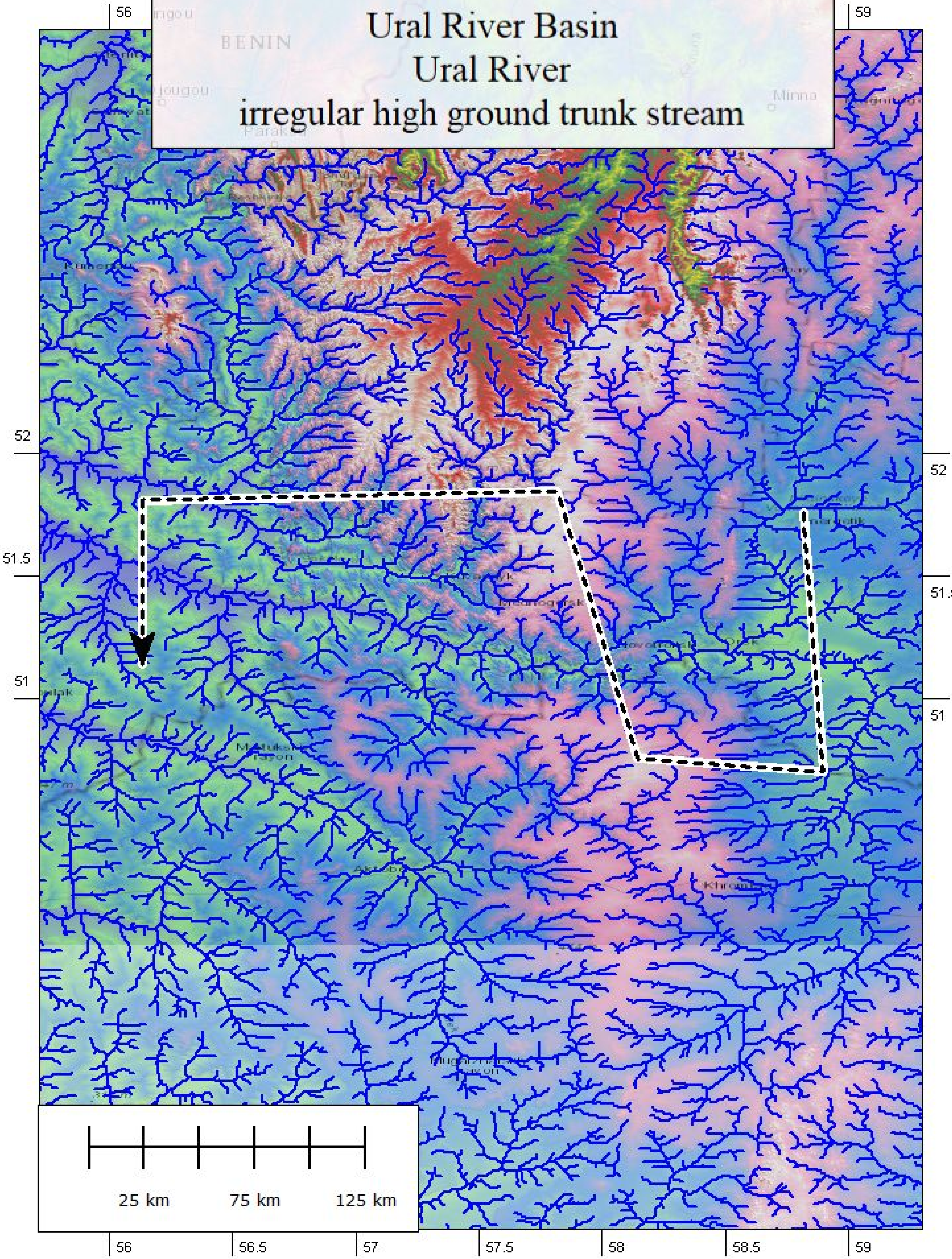

EU - 51
Endorheic basin Basin
irregular high ground trunk stream

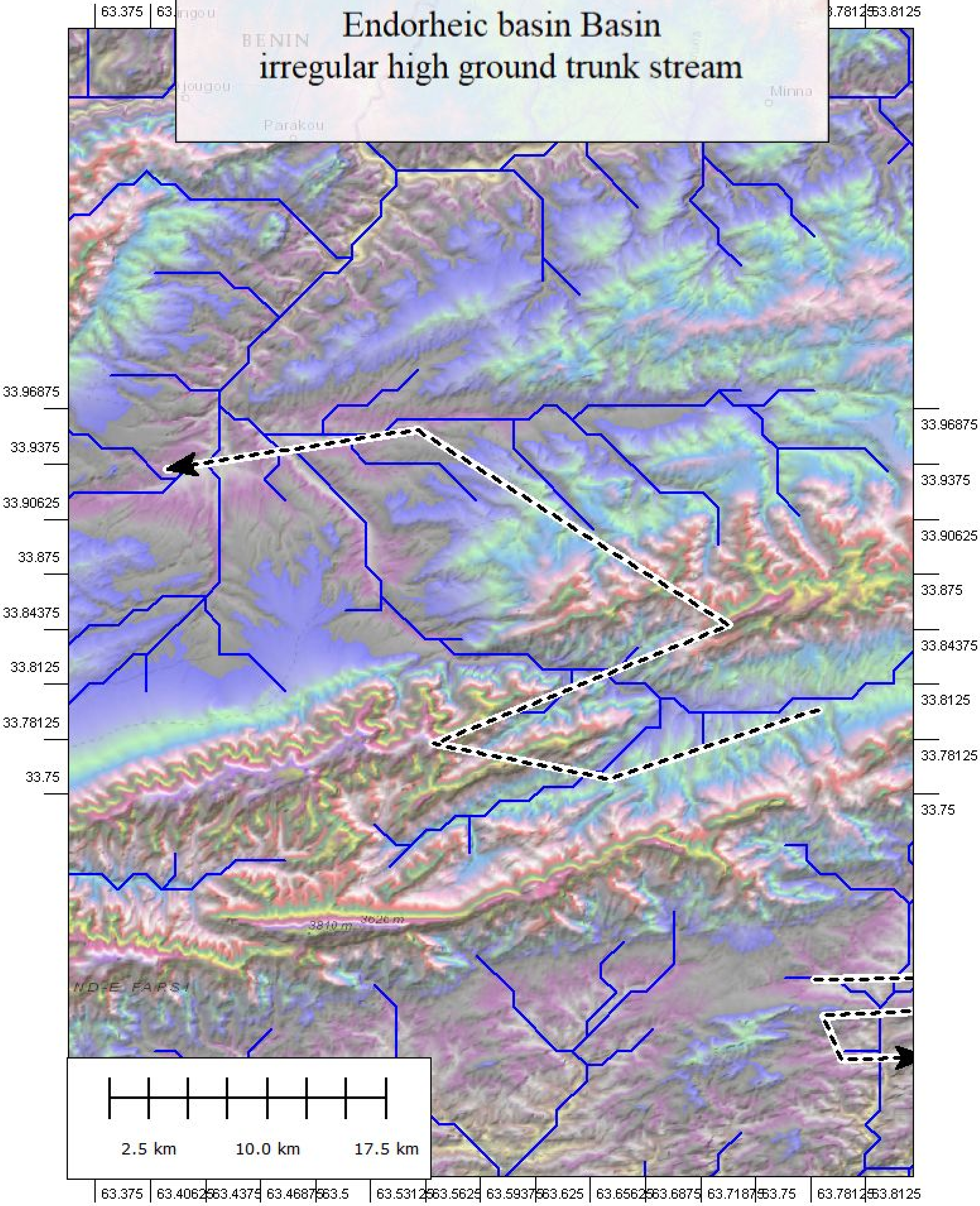

EU - 54
Ebro River Basin
Ebro River
irregular high ground trunk stream

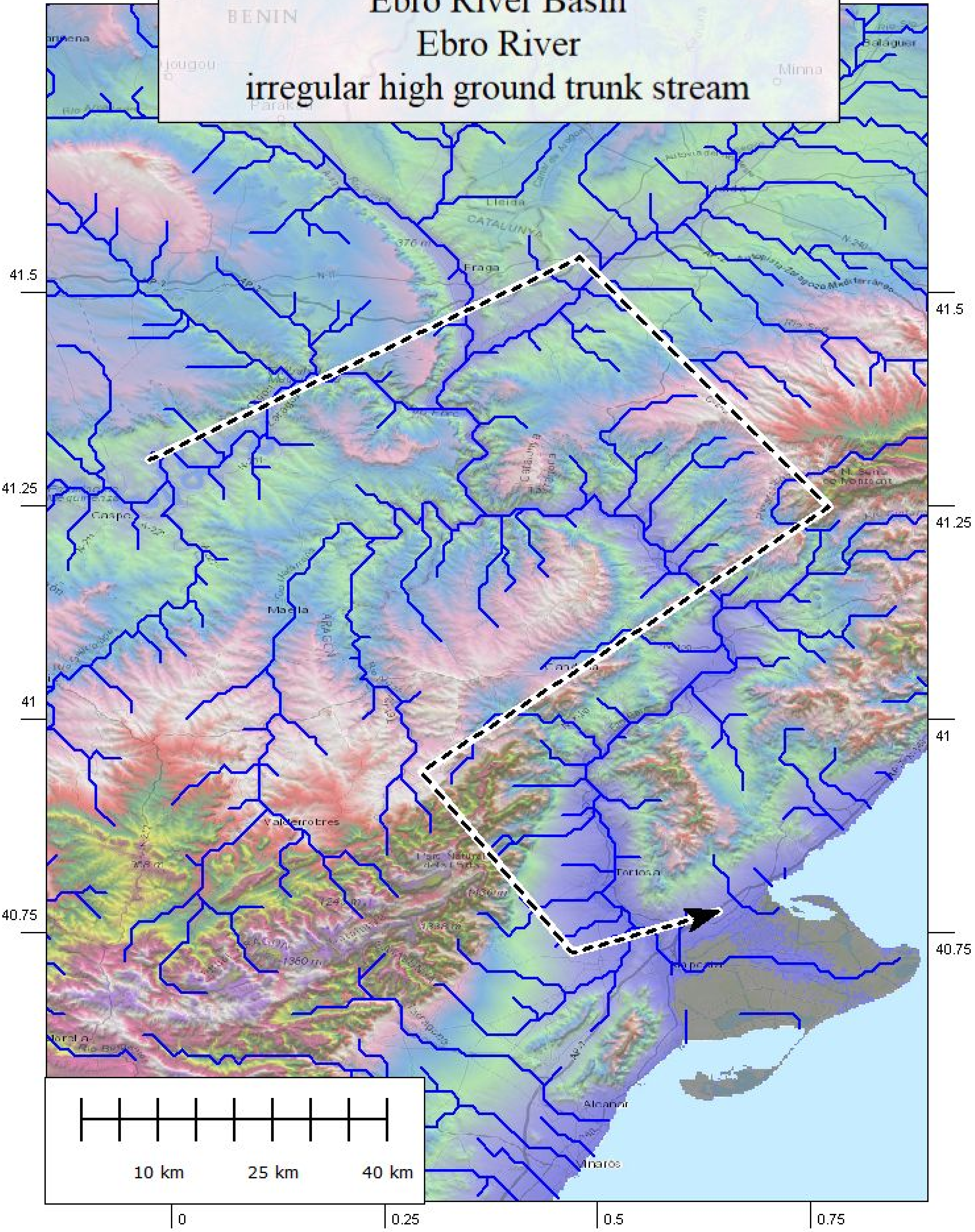

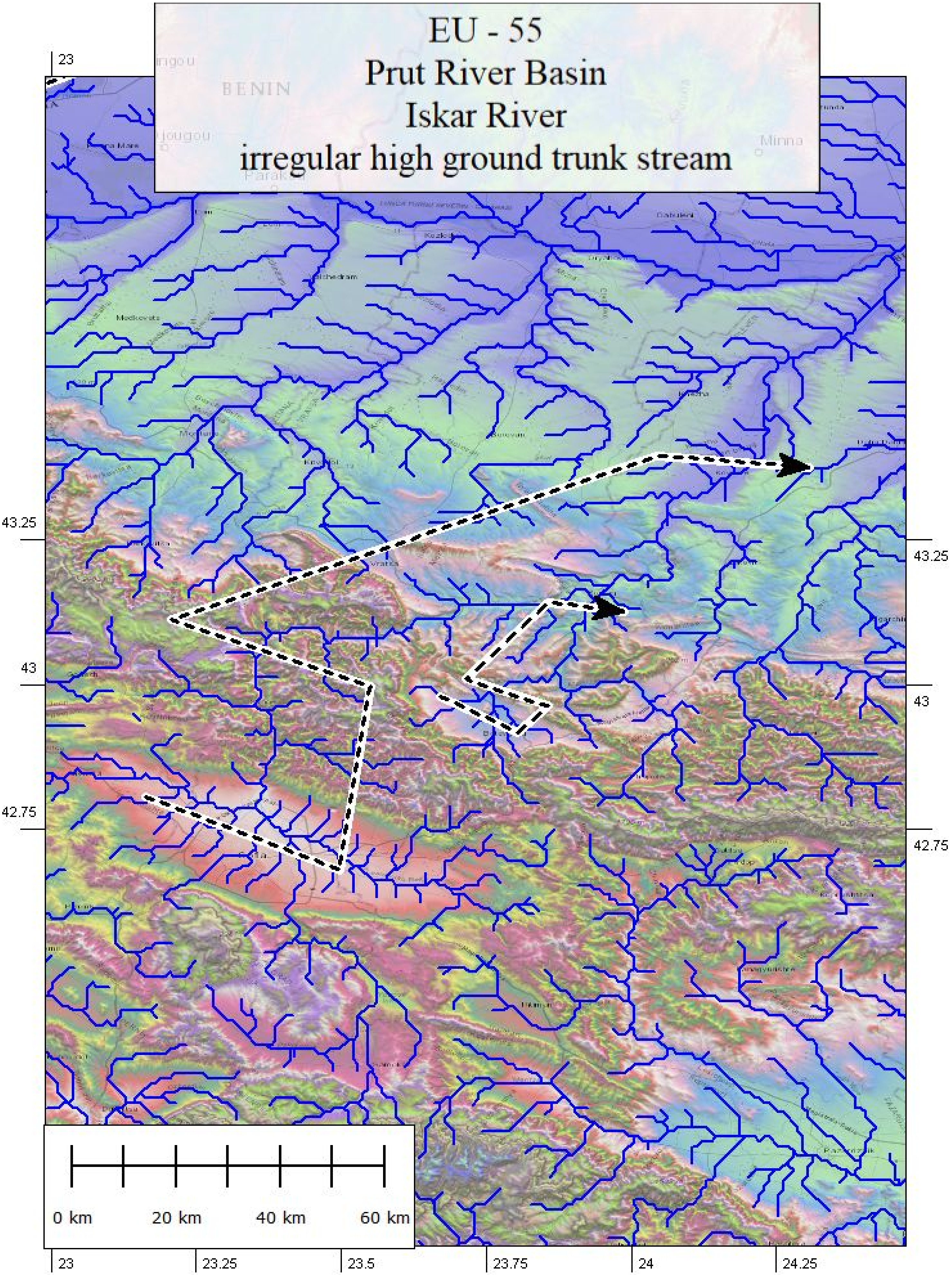
EU - 55
Prut River Basin
Iskar River
irregular high ground trunk stream
23
23.25
23.5
23.75
24
24.25
43.25
43
42.75
0 km
20 km
40 km
60 km

EU - 56
Vardar River Basin
Crna River
irregular high ground trunk stream

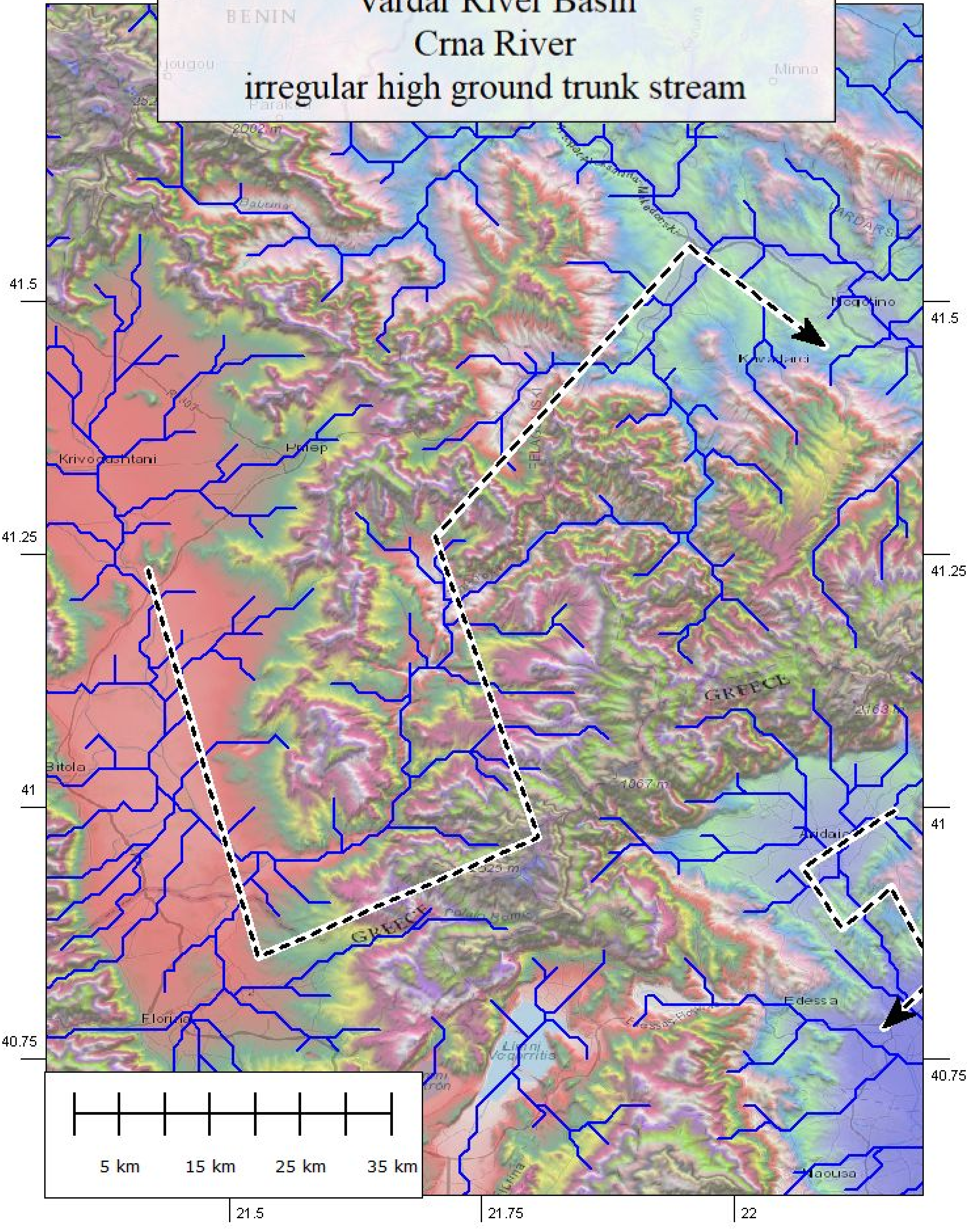

EU - 59
Prut River Basin
Buzau River
irregular high ground trunk stream

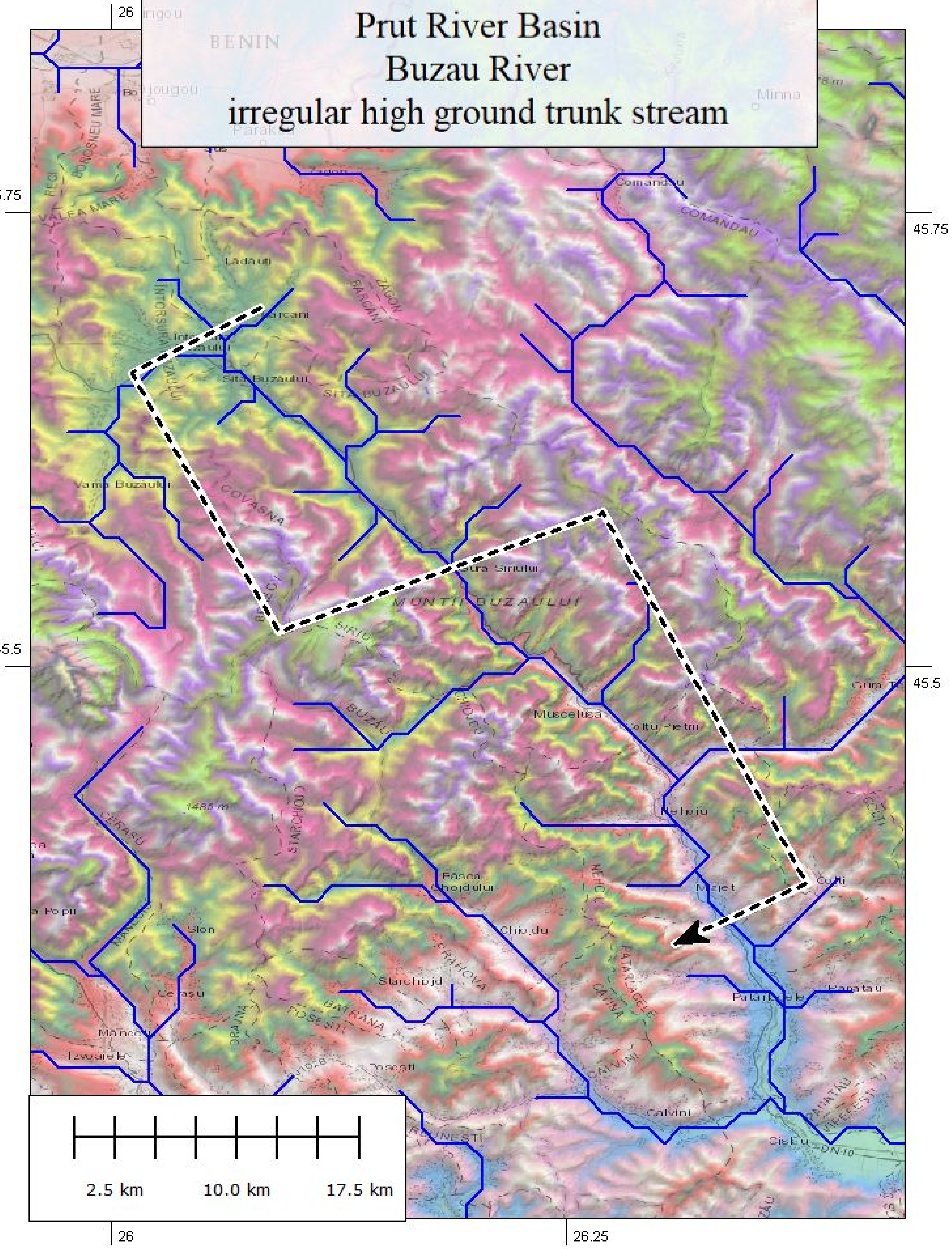

EU - 78
Euphrates River Basin
Euphrates River
irregular high ground trunk stream

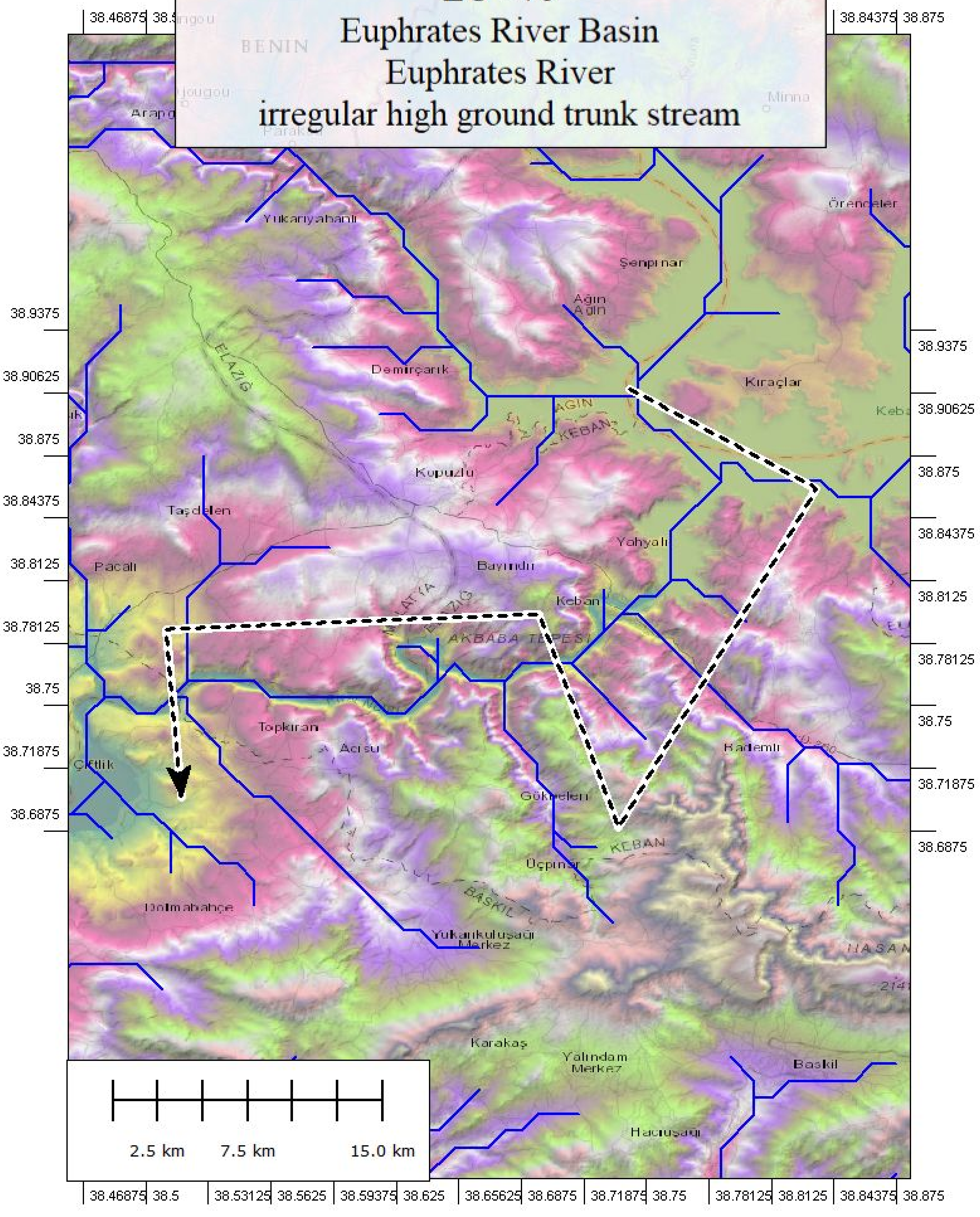

EU - 89
Kr River Basin
Aras River
irregular high ground trunk stream

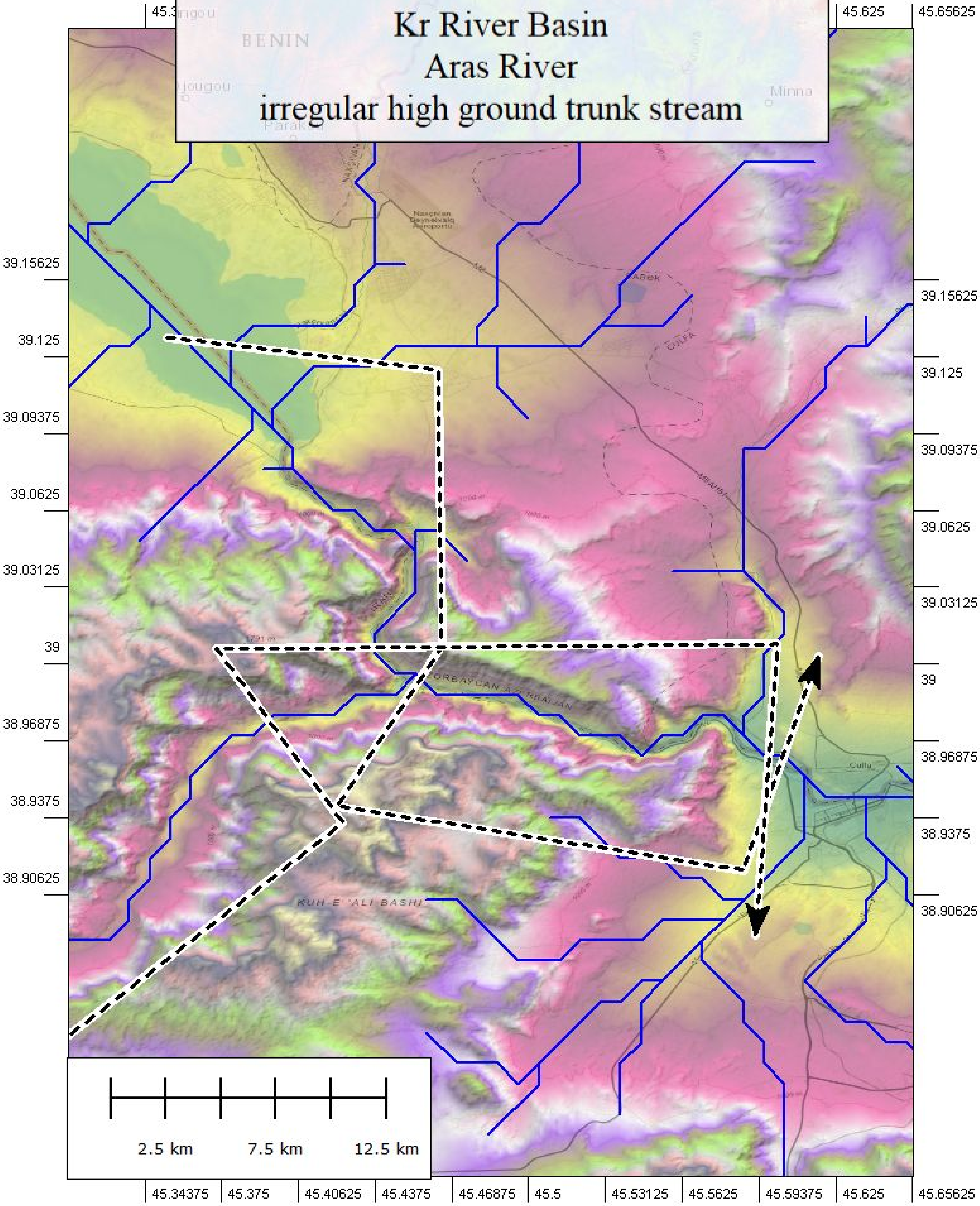

EU - 105
Euphrates River Basin
Seimare River
updip plateau trunk stream

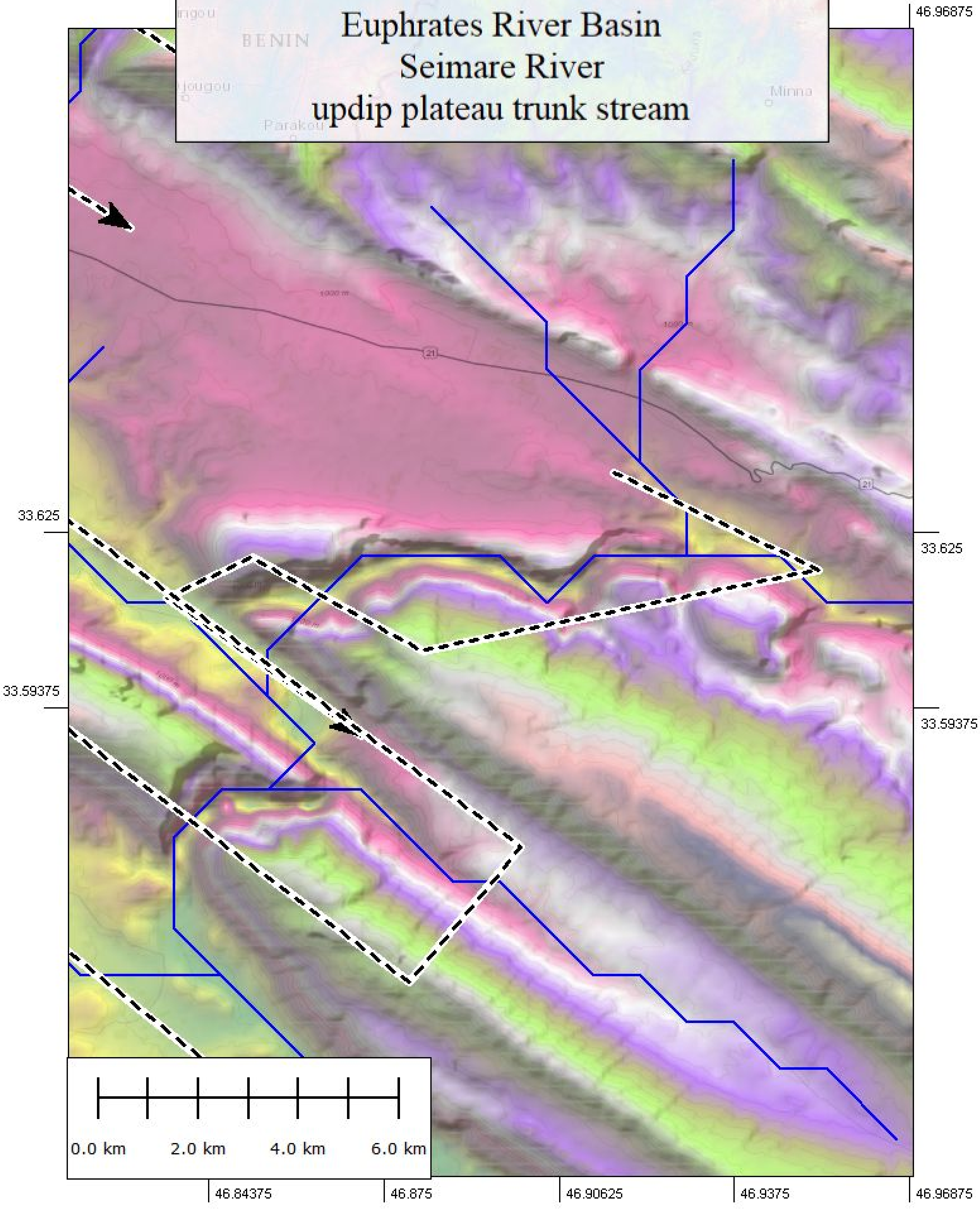

EU - 114
Prut River Basin
Moravska Sazava River
irregular high ground trunk stream

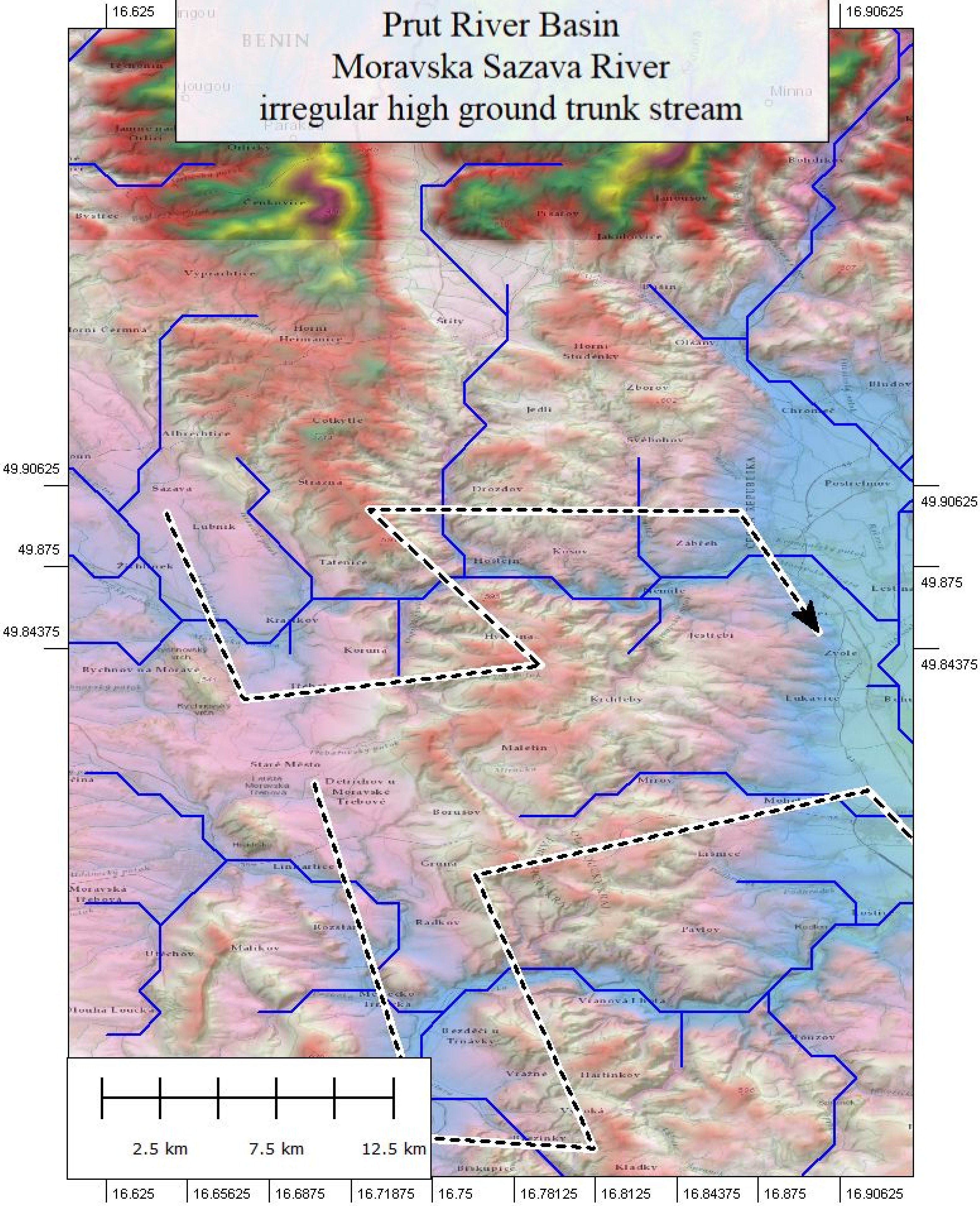

EU - 131
Prut River Basin
Trebuvka River
irregular high ground trunk stream

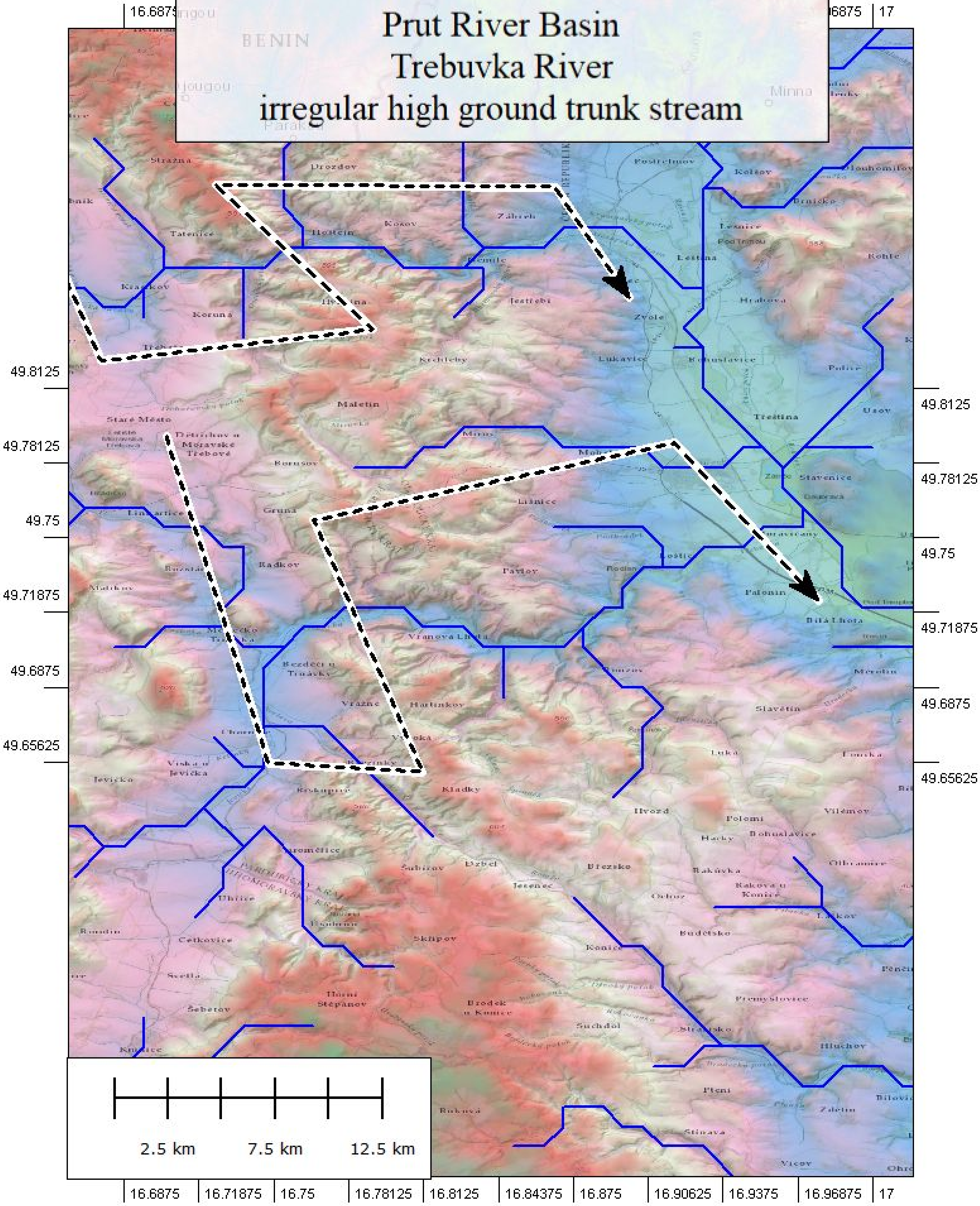

EU - 141
Qezel Owzan River Basin
irregular high ground trunk stream

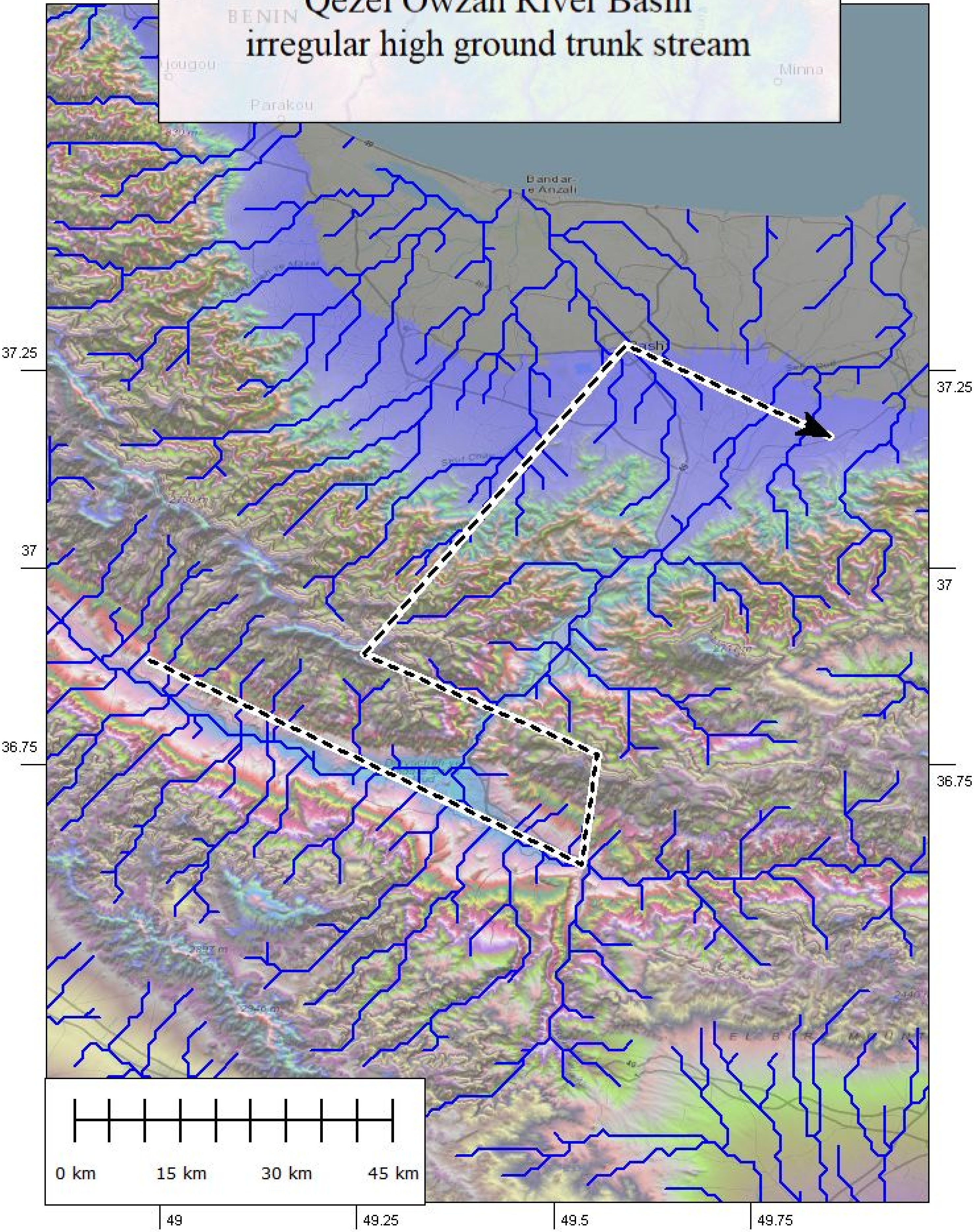

EU - 144
Prut River Basin
Jiu River
irregular high ground trunk stream

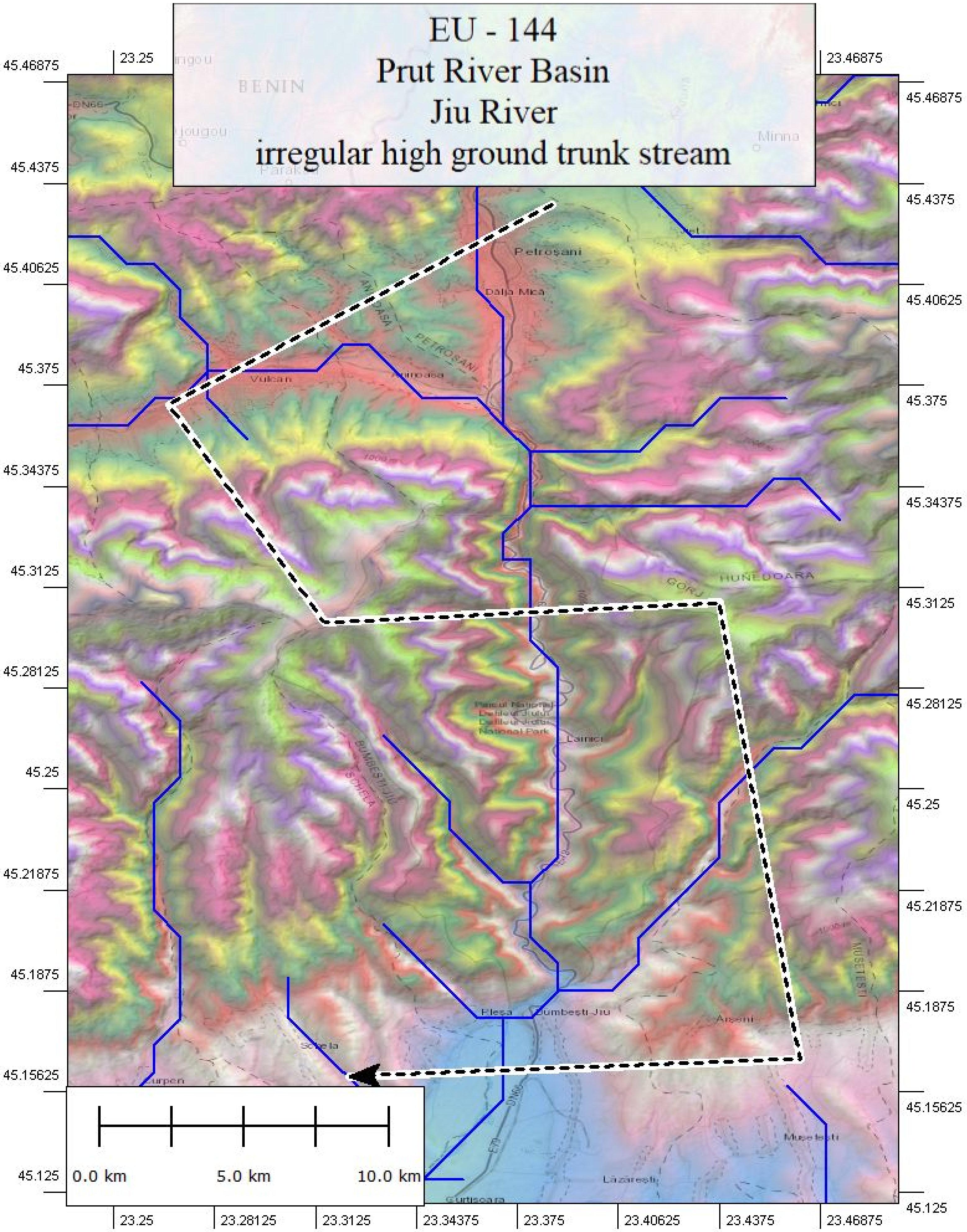

EU - 145
Euphrates River Basin
Karun River
downdip plateau trunk stream

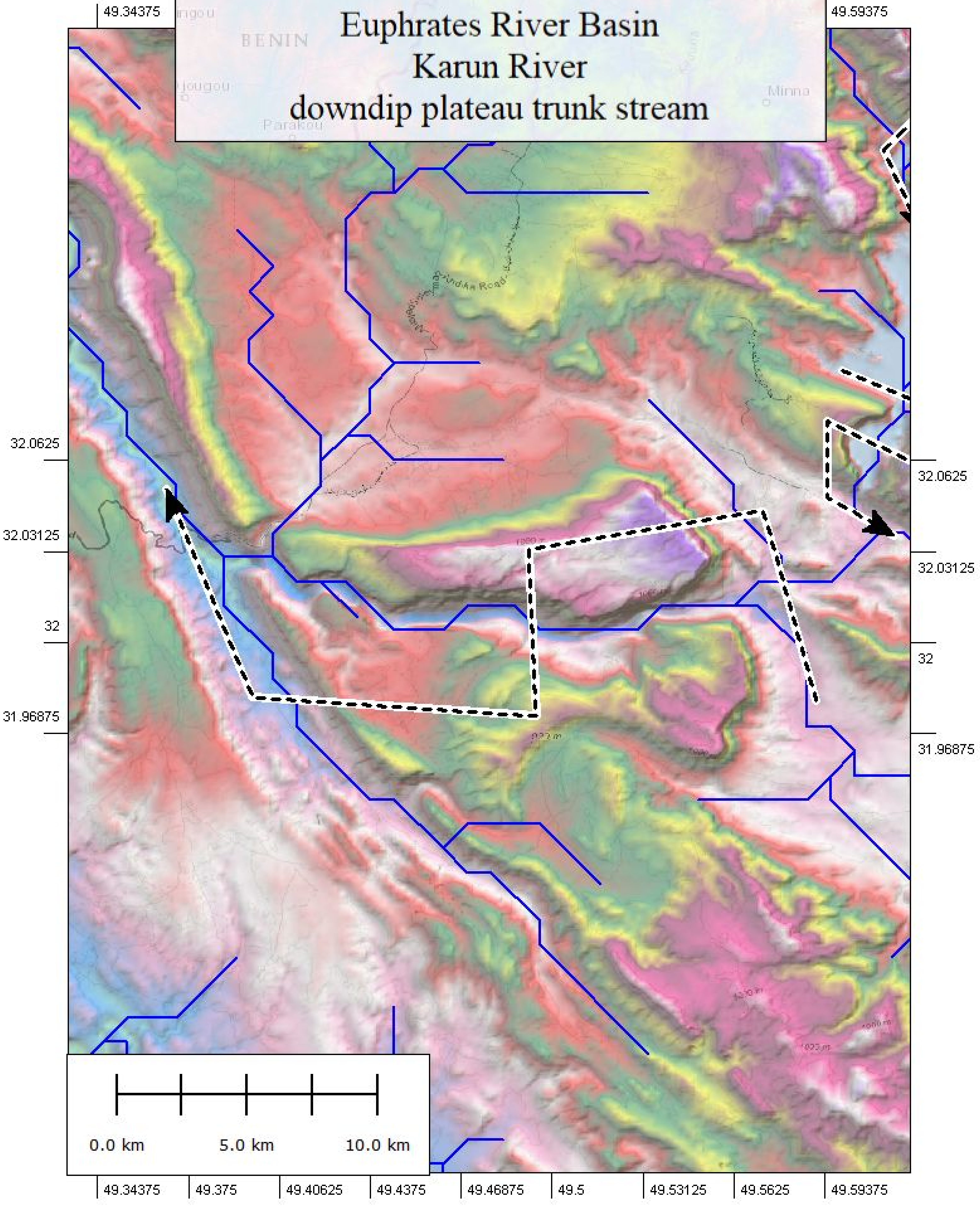

EU - 146
Elbe River Basin
Tricha Orlice River
irregular high ground trunk stream

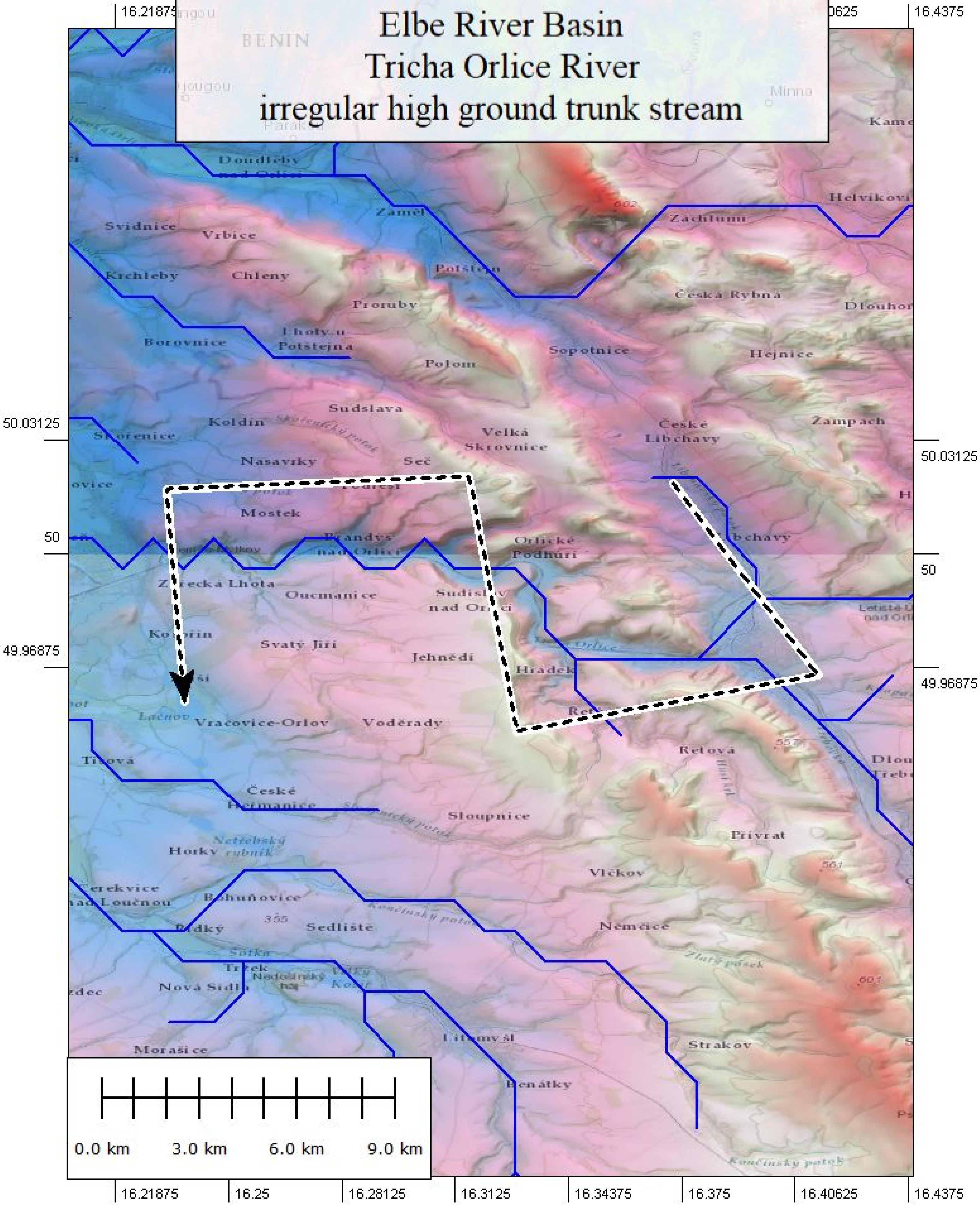

EU - 152
Endorheic basin Basin
Morghab River
plateau trunk stream

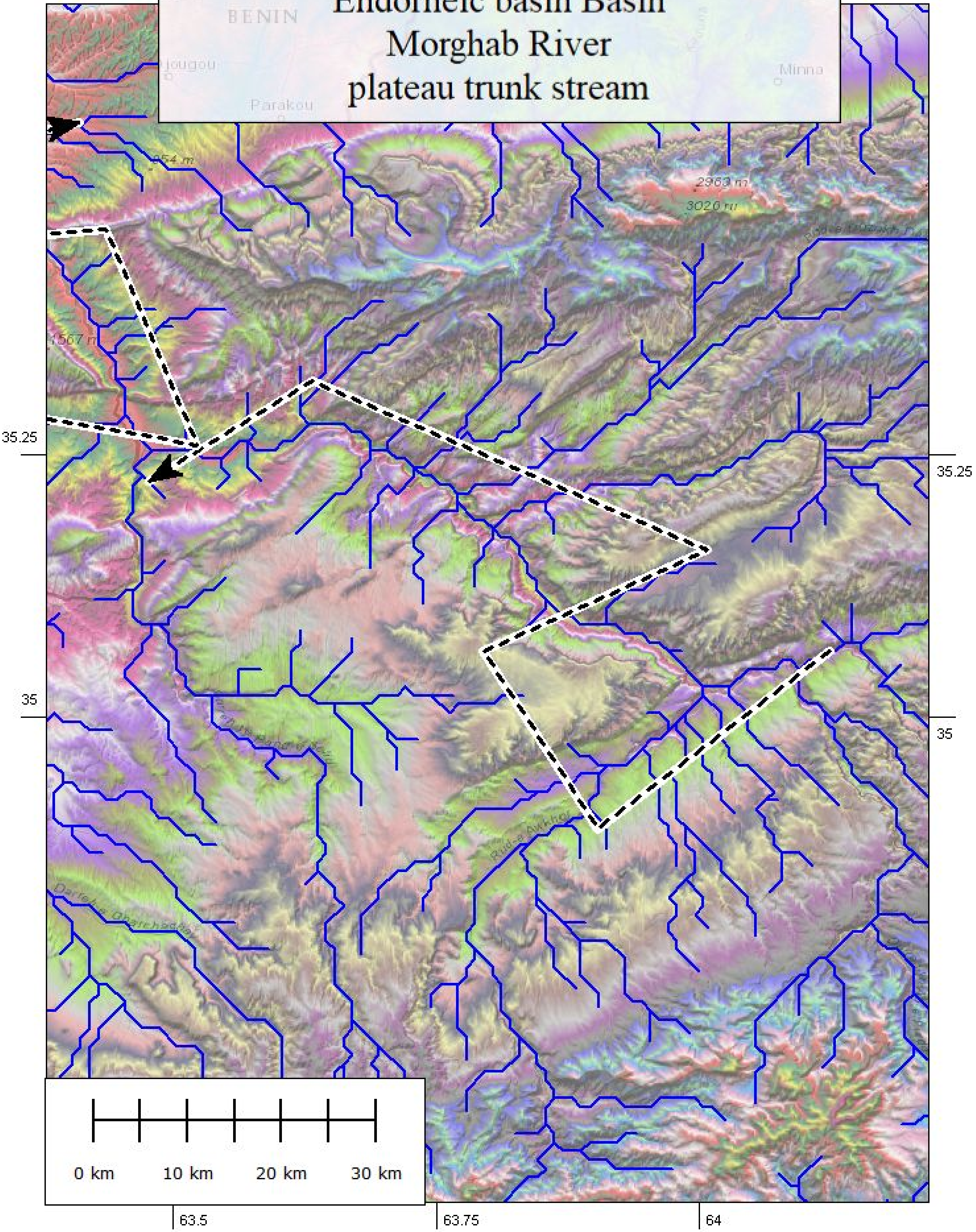

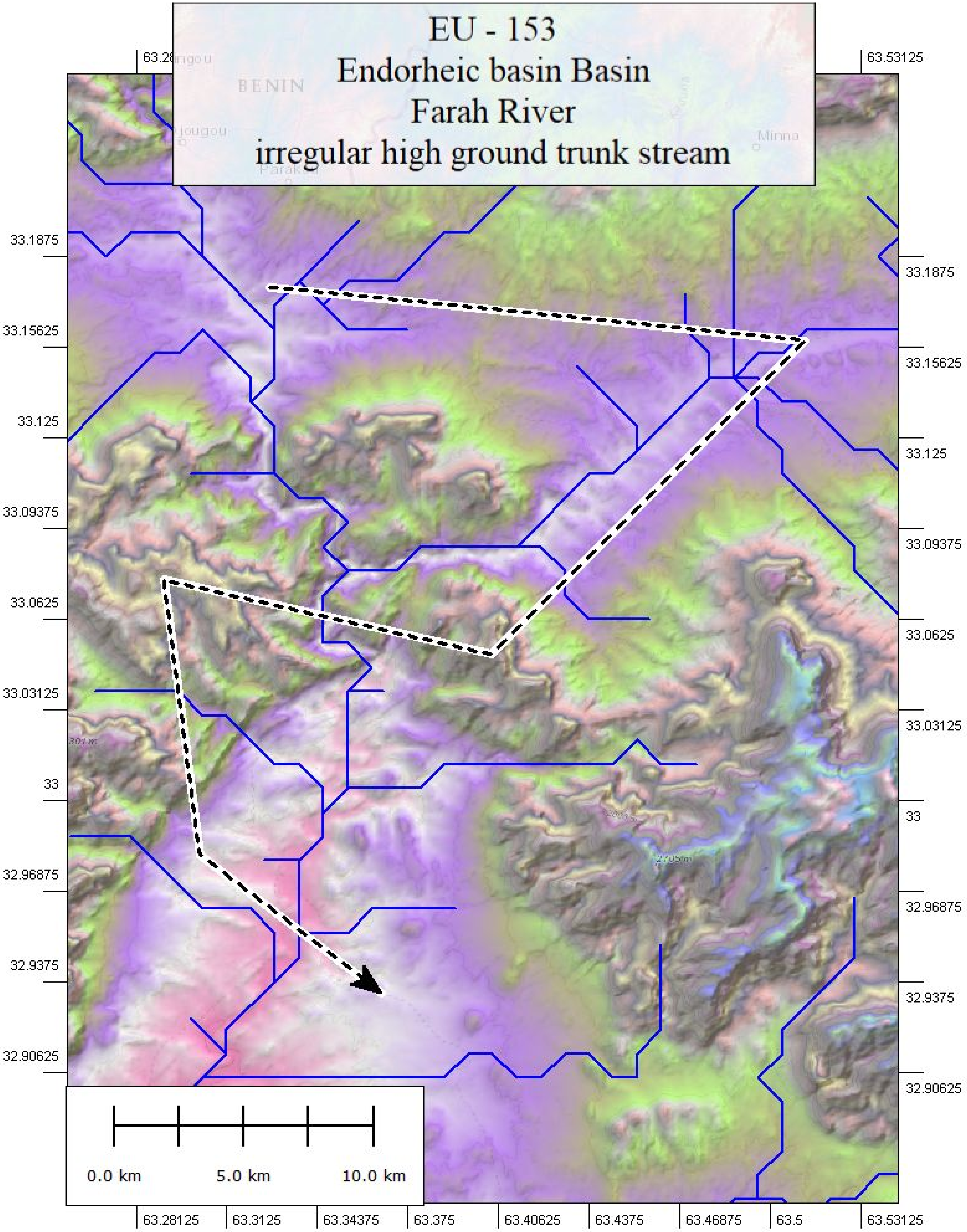
EU - 153
Endorheic basin Basin
Farah River
irregular high ground trunk stream
33.1875
33.15625
33.125
33.09375
33.0625
33.03125
33
32.96875
32.9375
32.90625
63.28125
63.3125
63.34375
63.375
63.40625
63.4375
63.46875
63.5
63.53125
0.0 km
5.0 km
10.0 km

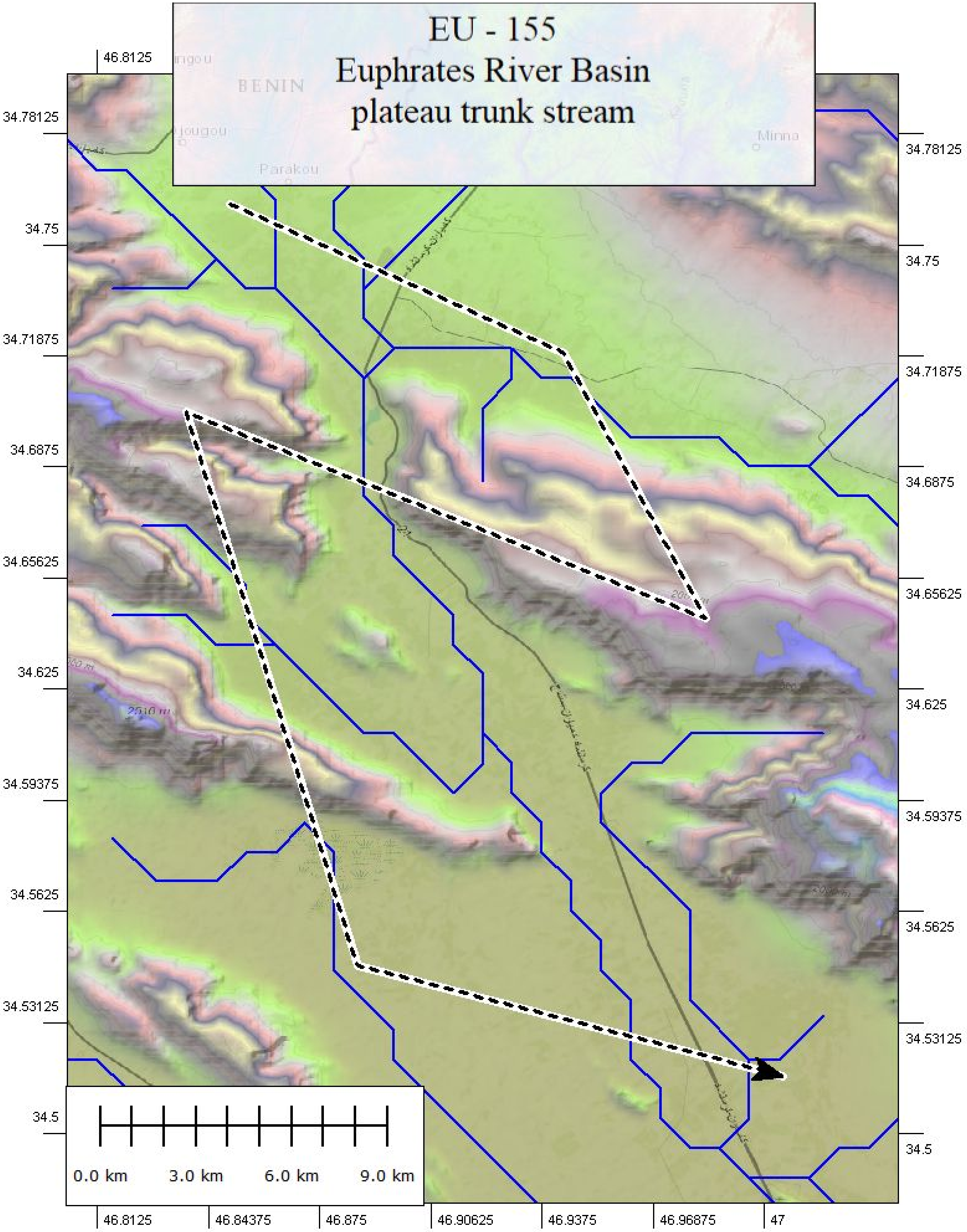

EU - 155
Euphrates River Basin
plateau trunk stream
46.8125
46.84375
46.875
46.90625
46.9375
46.96875
47
34.78125
34.75
34.71875
34.6875
34.65625
34.625
34.59375
34.5625
34.53125
34.5
0.0 km
3.0 km
6.0 km
9.0 km
BENIN
Parakou
Minna
2010 m

EU - 159
Prut River Basin
Bebresh River
irregular high ground trunk stream

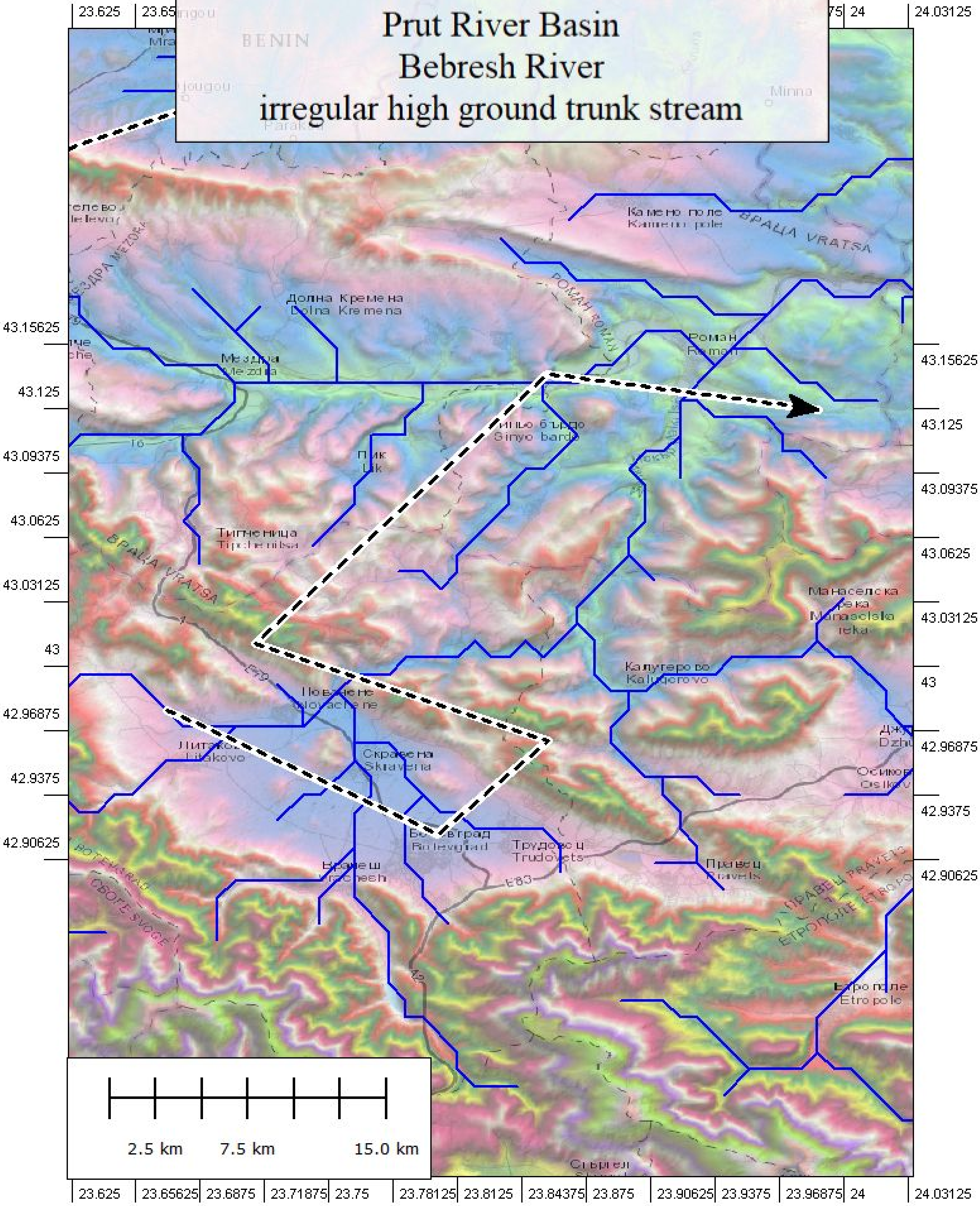

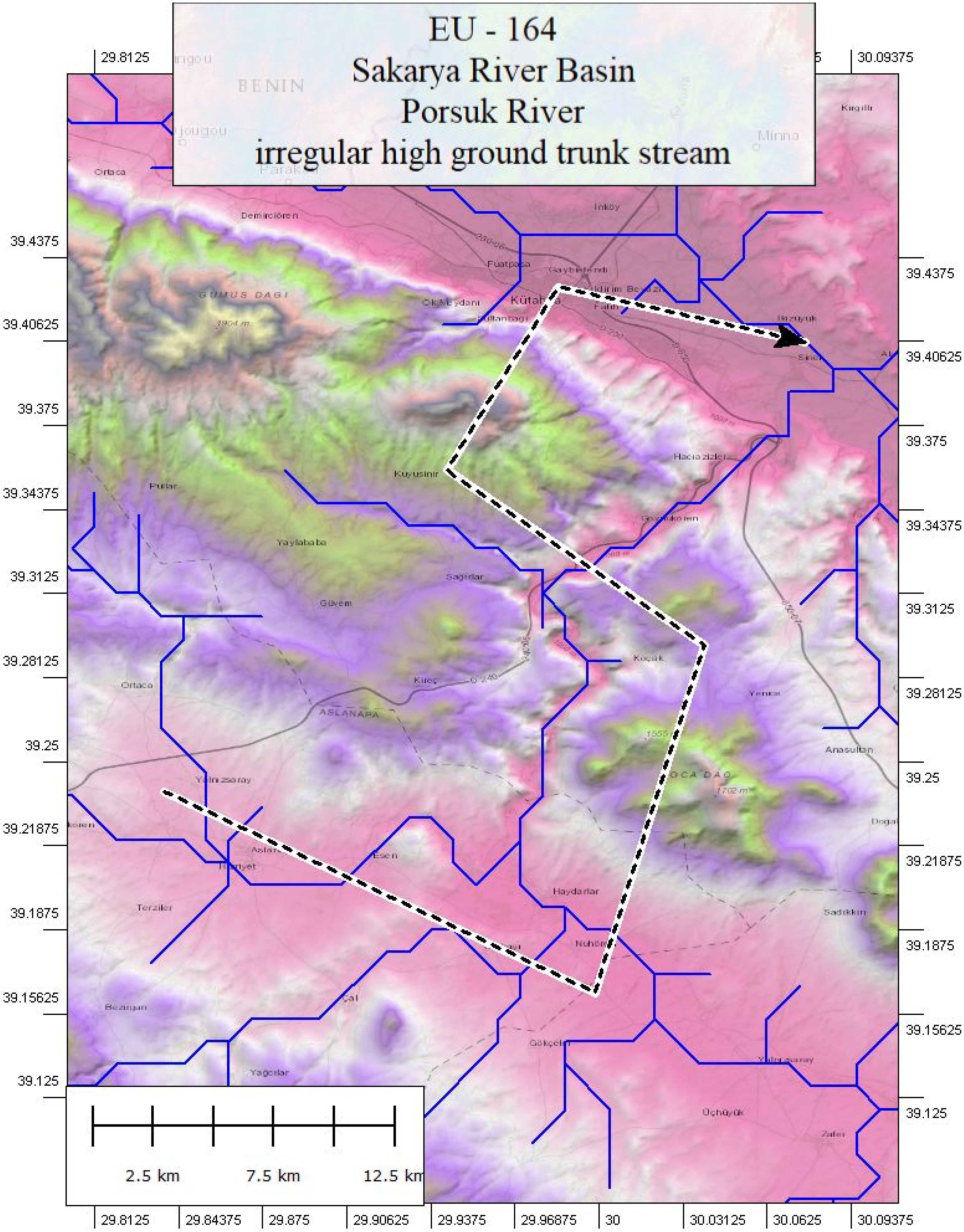
EU - 164
Sakarya River Basin
Porsuk River
irregular high ground trunk stream
29.8125
30.09375
39.4375
39.40625
39.375
39.34375
39.3125
39.28125
39.25
39.21875
39.1875
39.15625
39.125
2.5 km
7.5 km
12.5 km
29.84375
29.875
29.90625
29.9375
29.96875
30
30.03125
30.0625
Ortaca
Demirciören
GUMUS DAGI
Inköy
Fuatpaşa
Okmeydanı
Kütahya
Kuyusinir
Pullar
Yaylababa
Sagirlar
Güvem
Kireç
ASLANAPA
Koçak
Yenice
Anasultan
Yalnizsaray
Esen
Haydarlar
Terziler
Nuhören
Bezirgan
Çal
Gökçeler
Yagorlar
Üçhüyük
Zafer
Sadıkkırı
Hacıazizler
Kırgıllı

EU - 166
Prut River Basin
Danube River
irregular high ground trunk stream

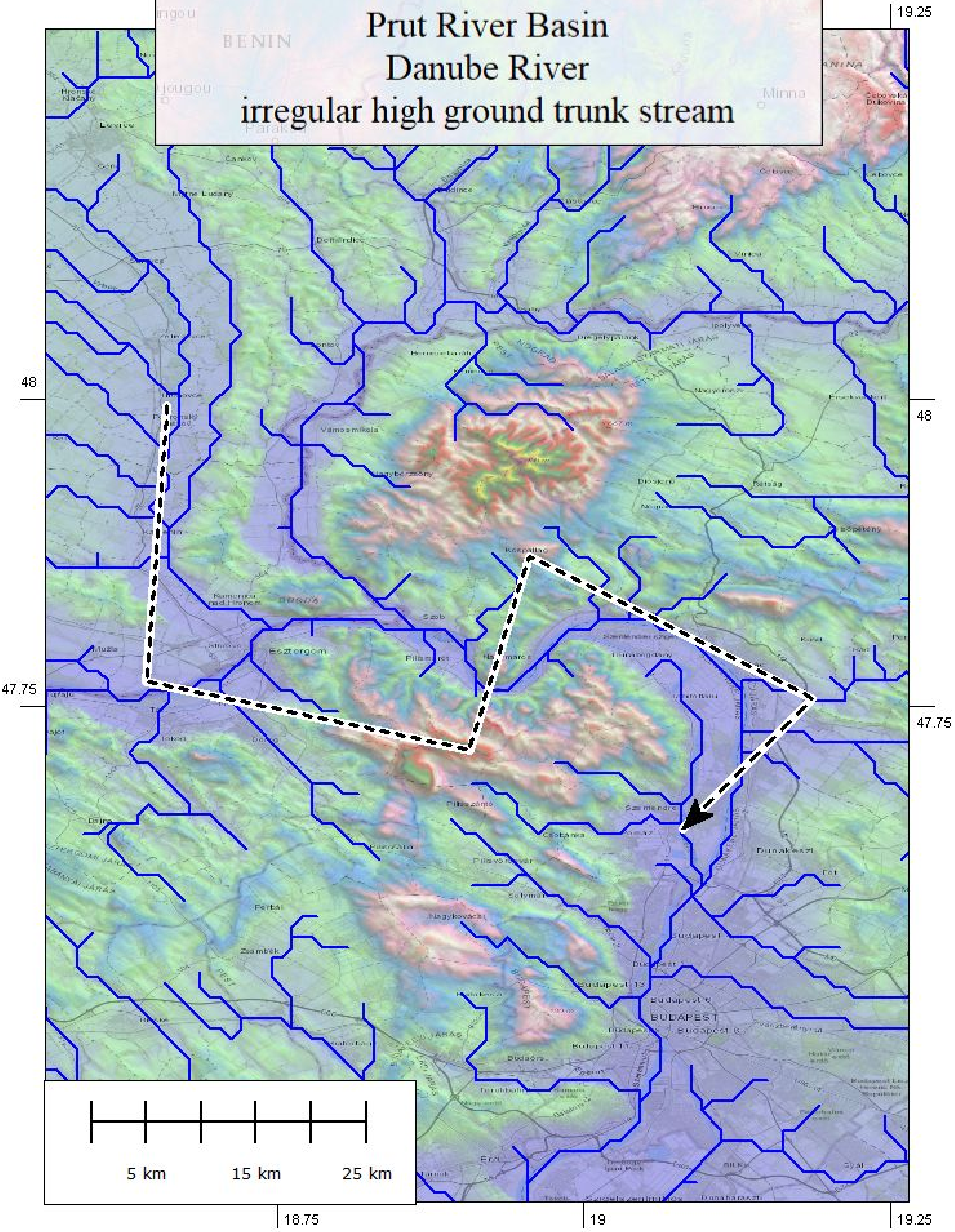

EU - 167
Sakarya River Basin
Sakarya River
updip plateau trunk stream

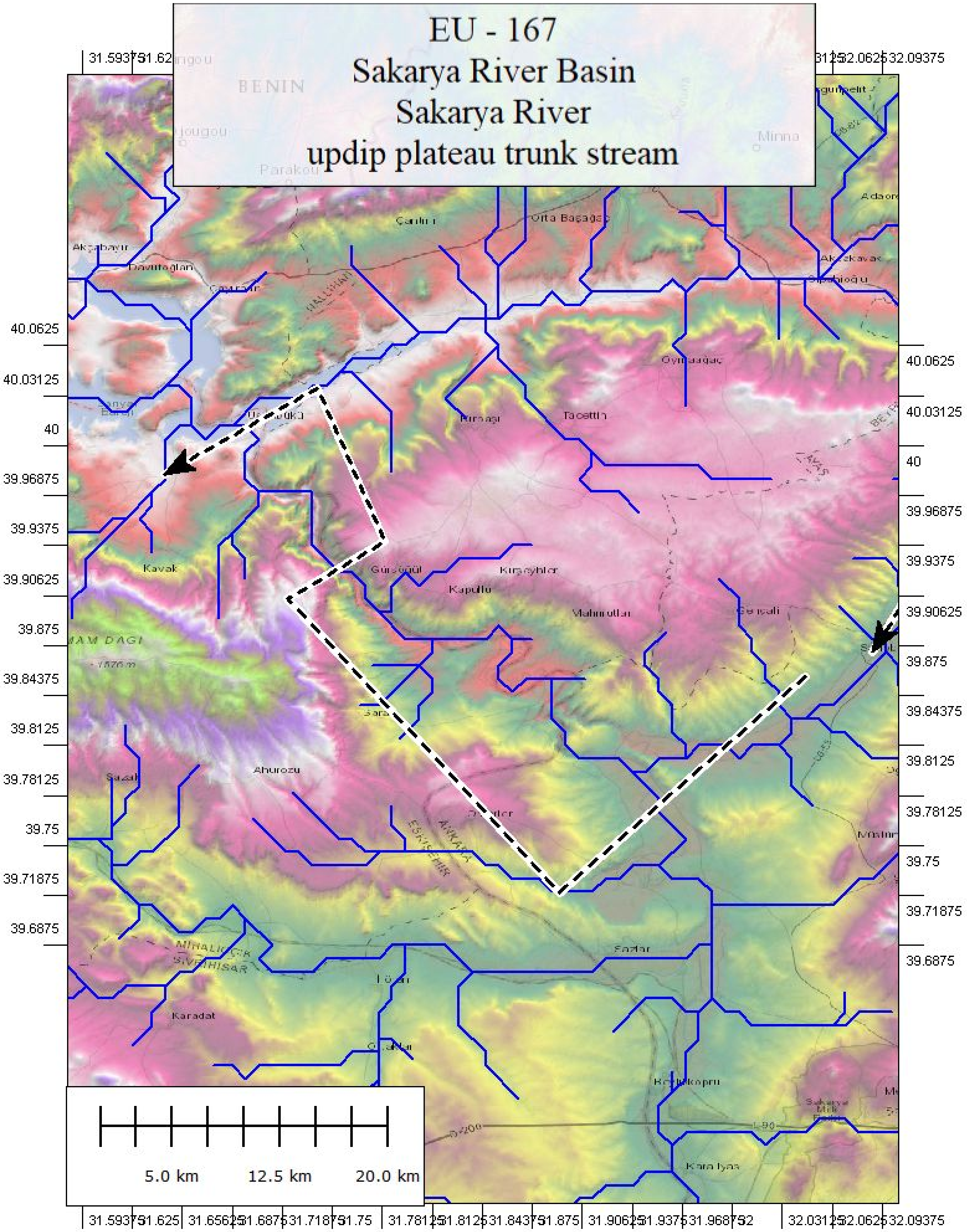

EU - 188
Euphrates River Basin
Karun River
updip plateau trunk stream

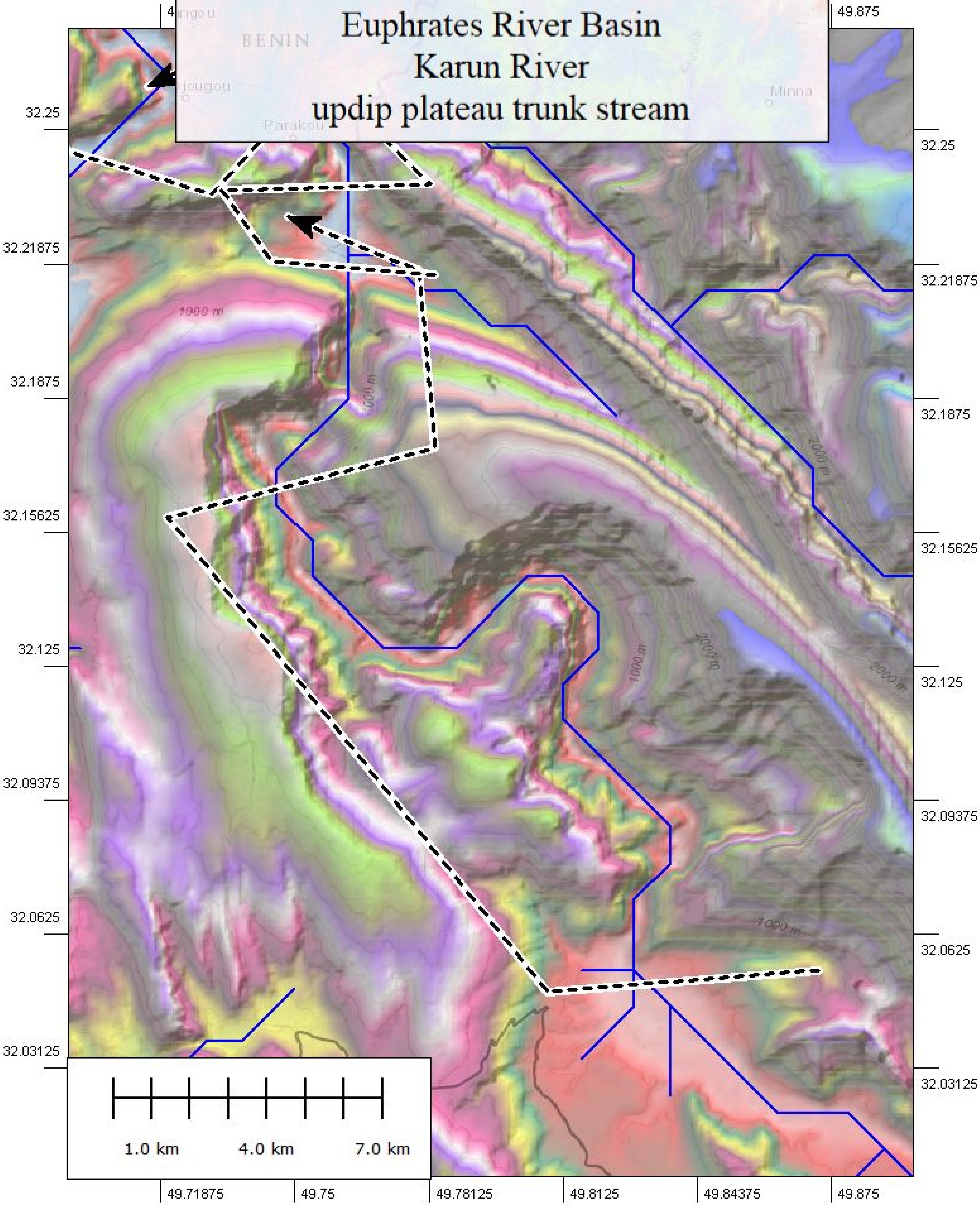

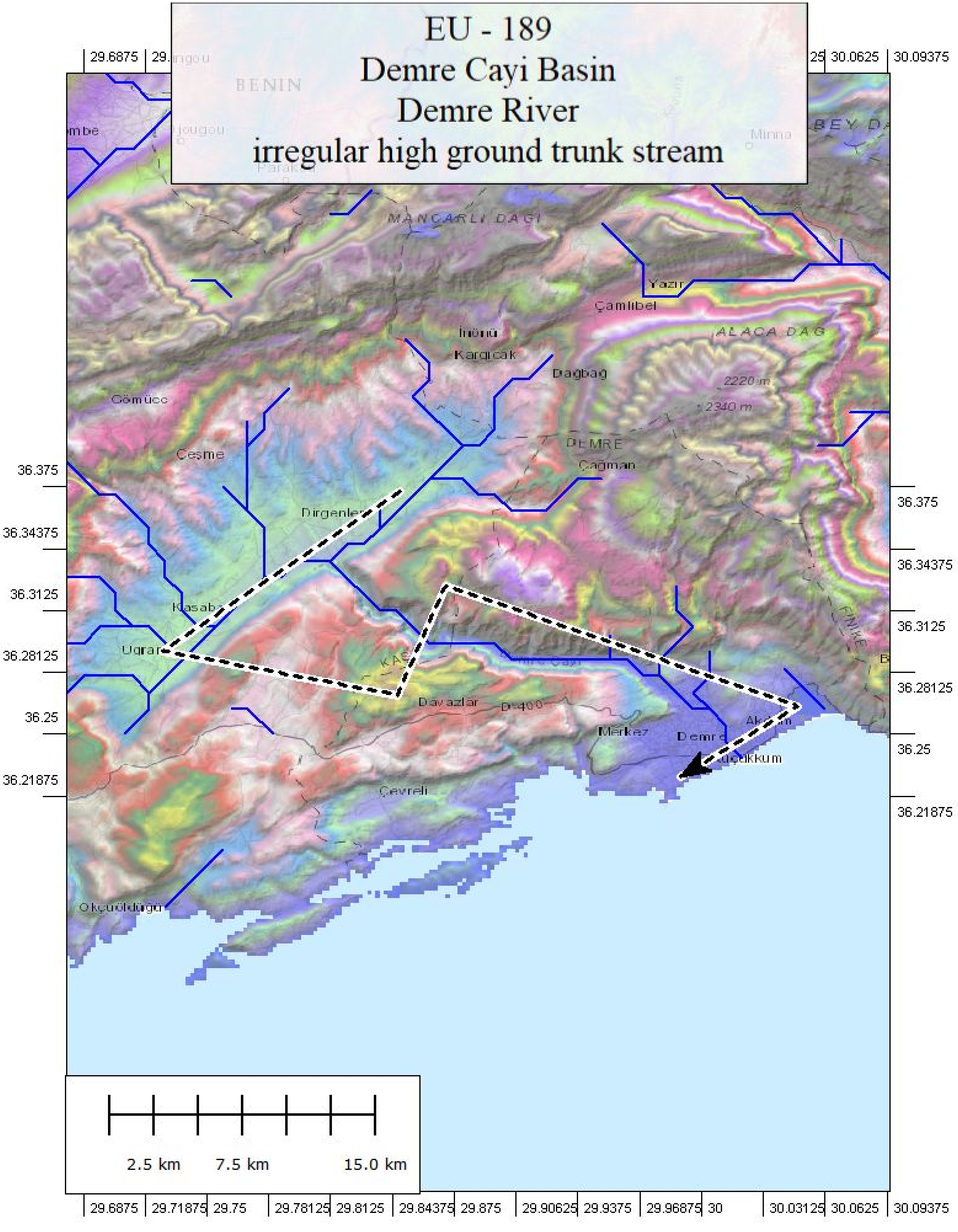

EU - 189
Demre Cayi Basin
Demre River
irregular high ground trunk stream
29.6875
30.0625
30.09375
BENIN
Minna
MANCARLI DAGI
Yazır
Çamlıbel
ALACA DAG
İnönü
Kargıcak
Dagbag
2220 m
2340 m
Gömücc
DEMRE
Çağman
Çeşme
Dirgenler
Kasaba
Uğrar
Davazlar
D-400
Merkez
Demre
Küçükkum
Çevreli
Okçuöldügü
FİNİKE
36.375
36.34375
36.3125
36.28125
36.25
36.21875
2.5 km
7.5 km
15.0 km
29.71875
29.75
29.78125
29.8125
29.84375
29.875
29.90625
29.9375
29.96875
30
30.03125

EU - 192
Euphrates River Basin
Murat River
irregular high ground trunk stream

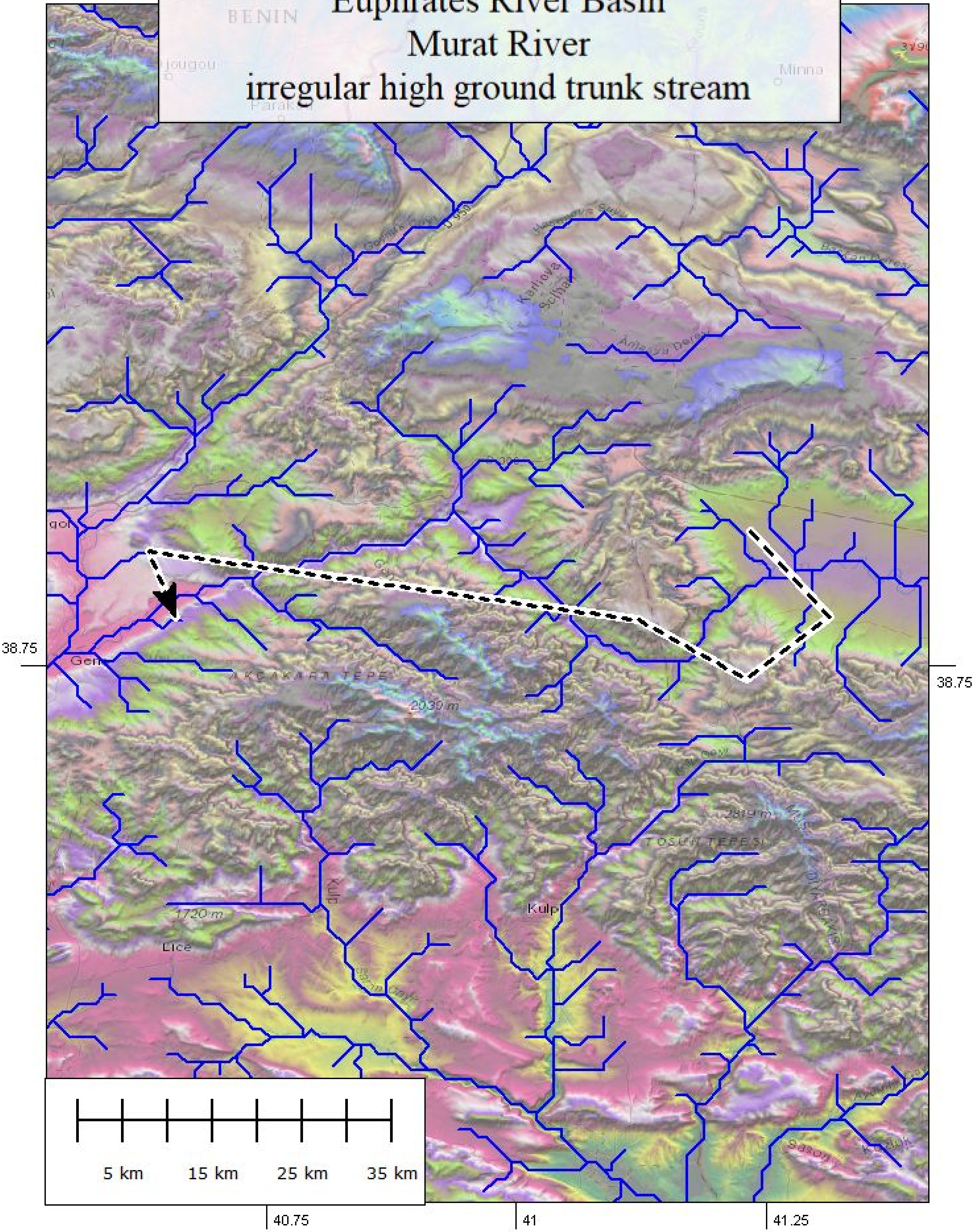

EU - 193
Kr River Basin
Aras River
irregular high ground trunk stream

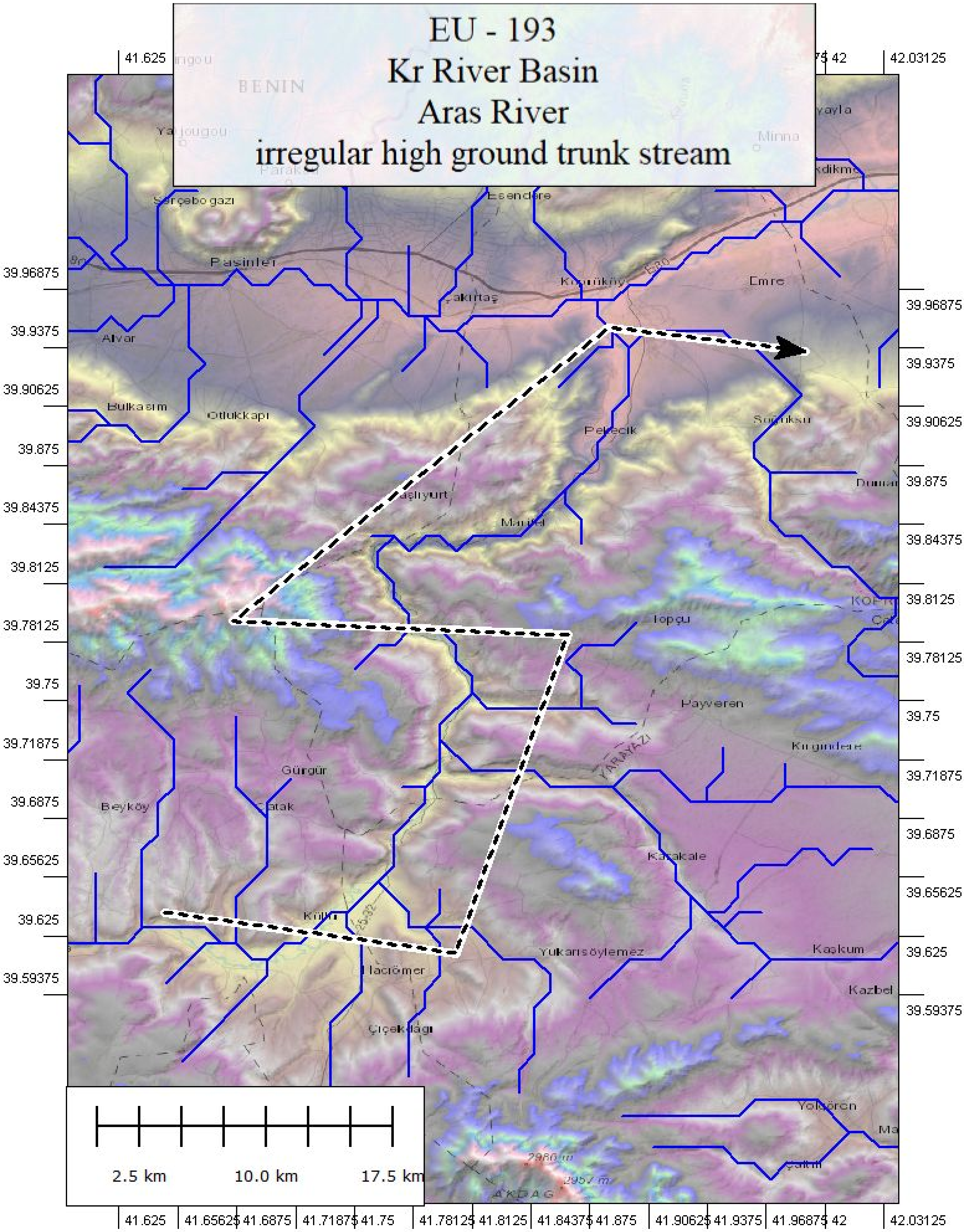

EU - 203
Prut River Basin
Danube River
irregular high ground trunk stream

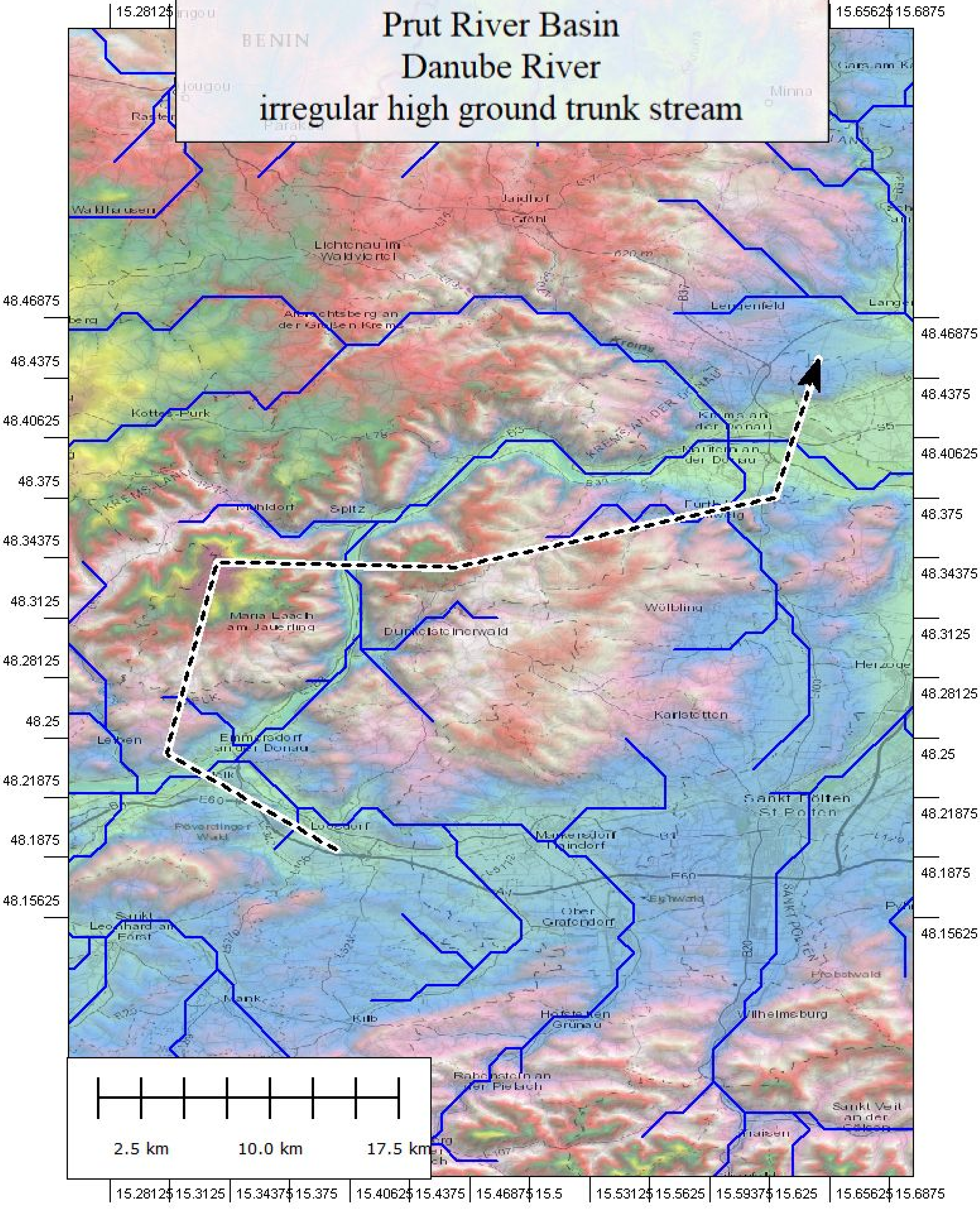

EU - 208
Kr River Basin
Aras River tributary
irregular high ground trunk stream

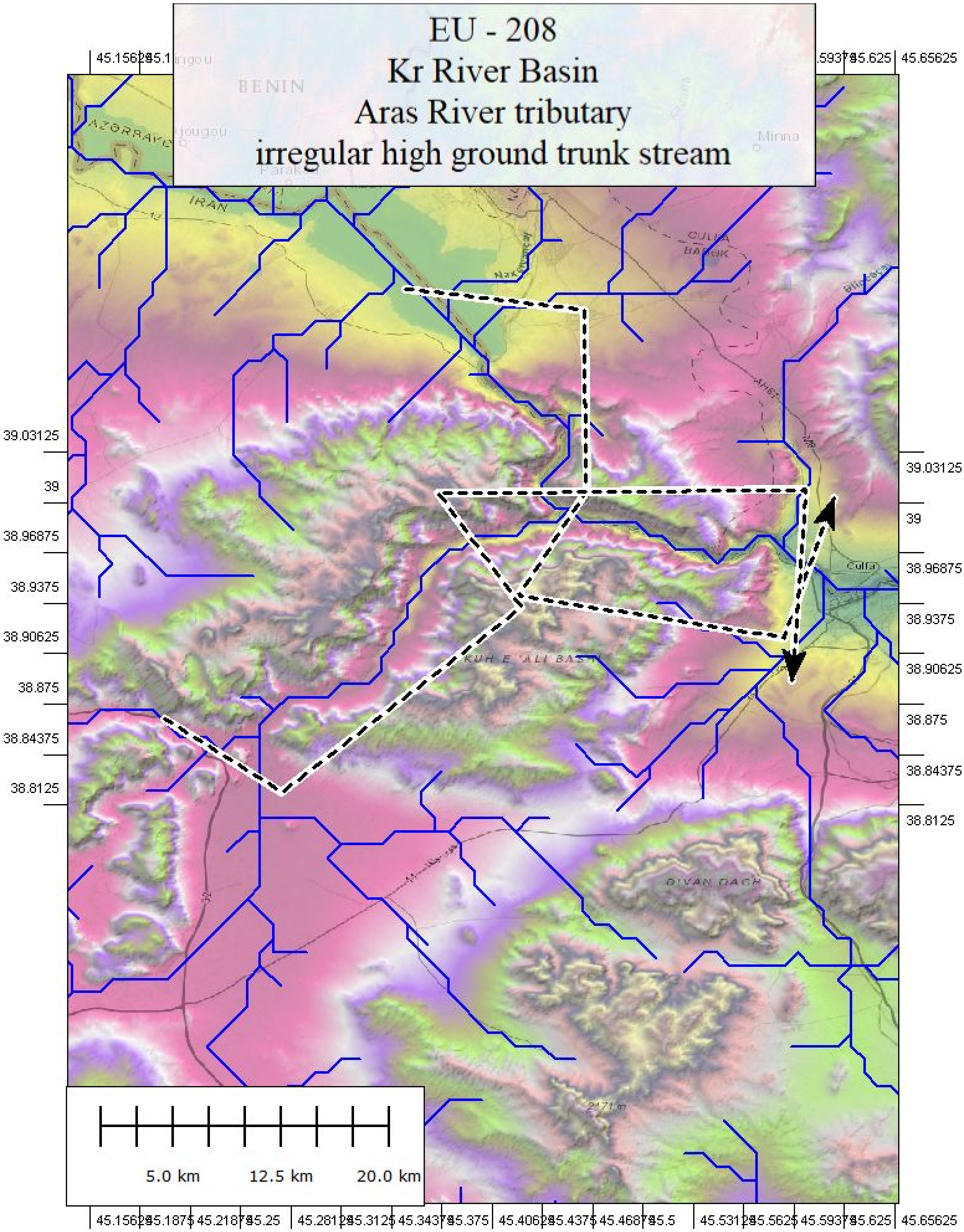

EU - 212
Tevere River Basin
Tevere River
irregular high ground trunk stream

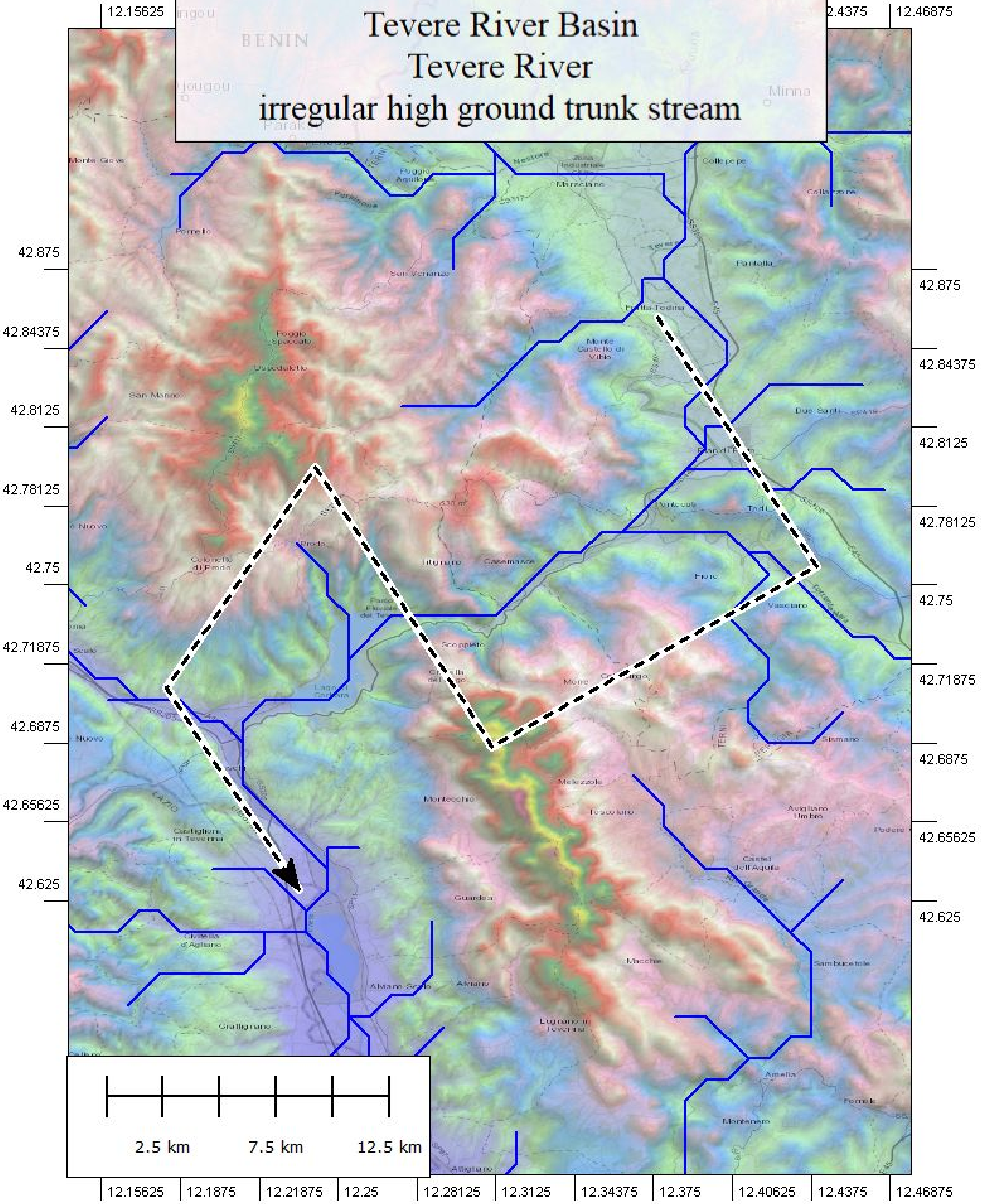

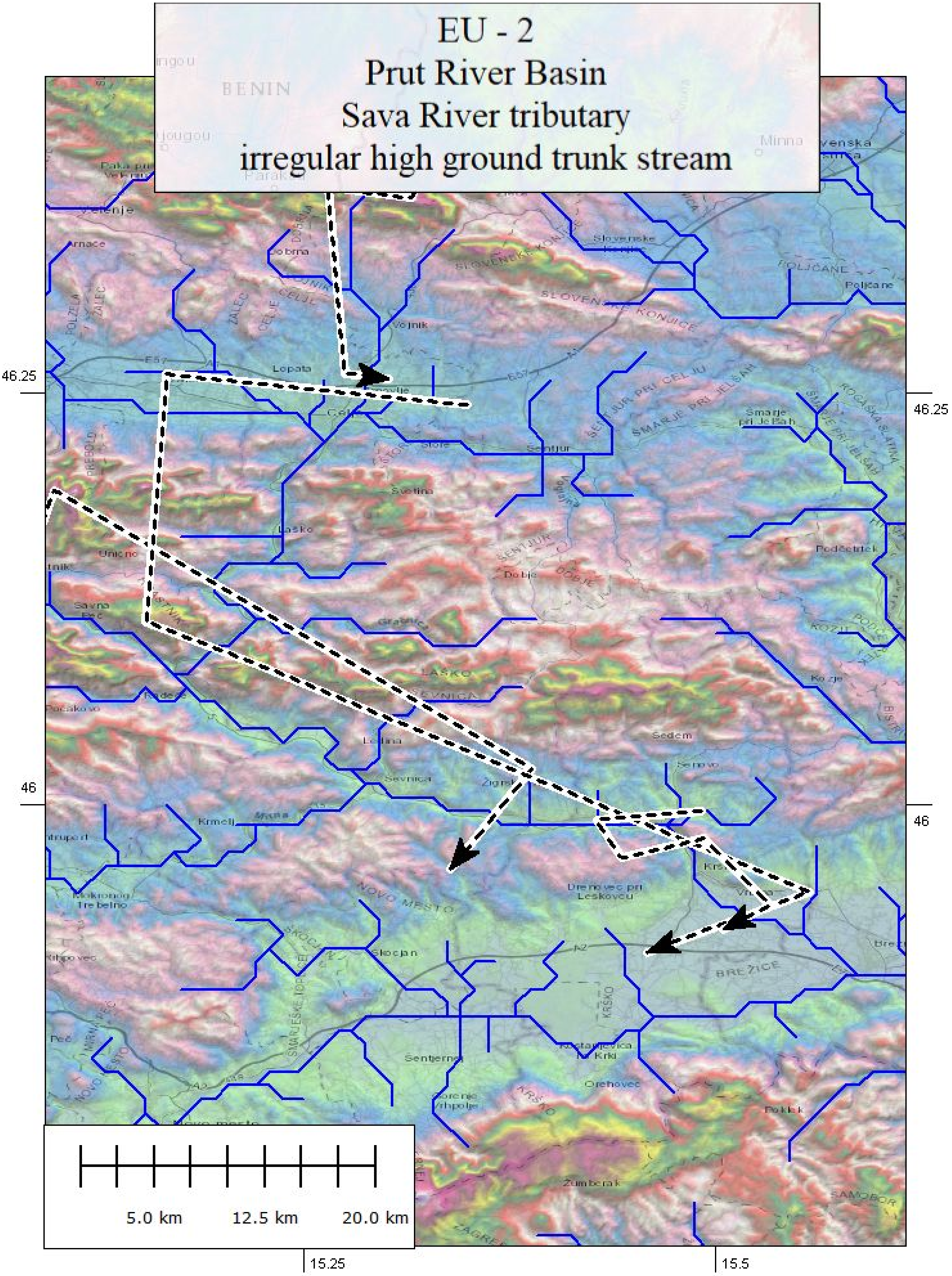
EU - 2
Prut River Basin
Sava River tributary
irregular high ground trunk stream
46.25
46
15.25
15.5
5.0 km
12.5 km
20.0 km

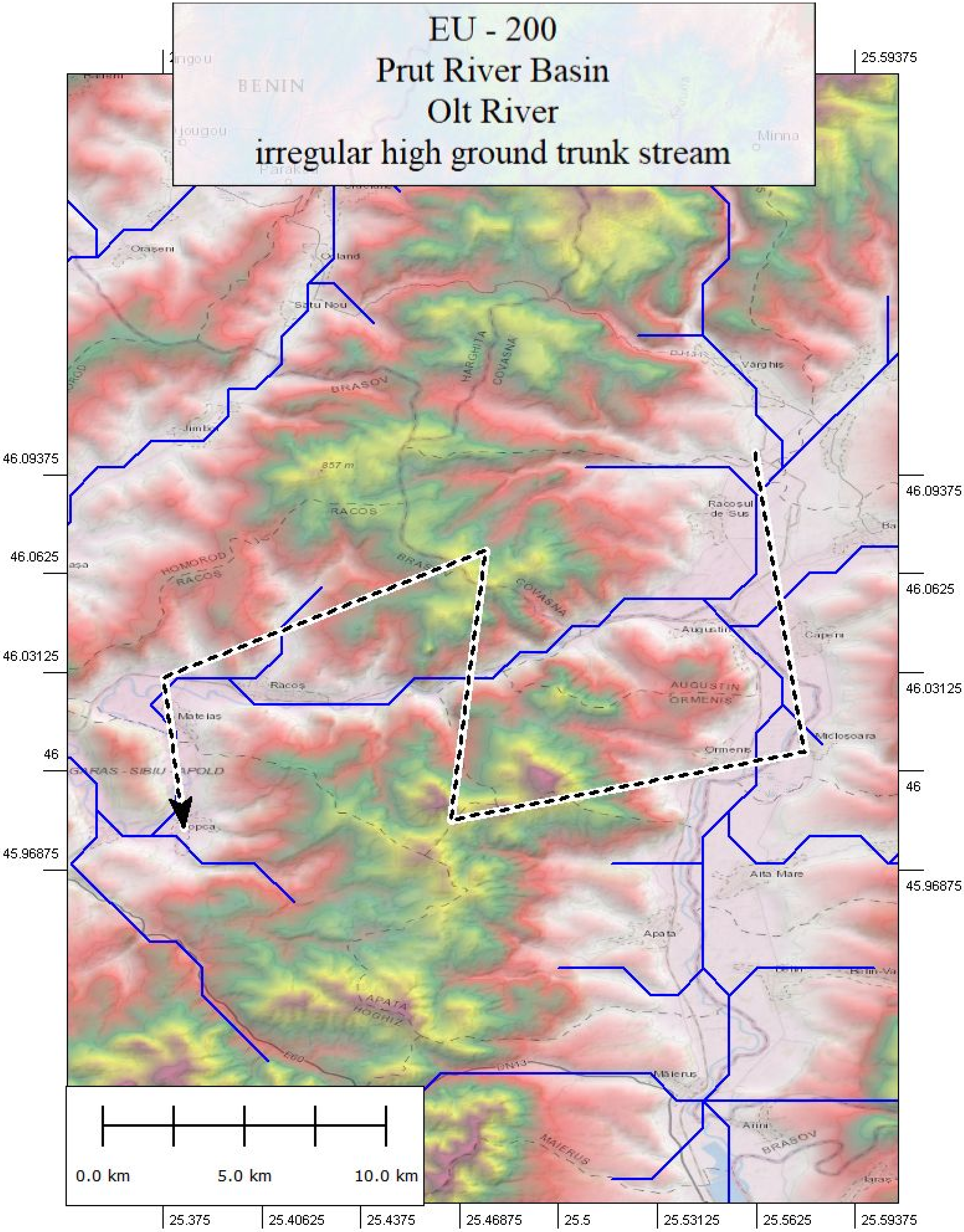
EU - 200
Prut River Basin
Olt River
irregular high ground trunk stream
Orașeni
Orland
Satu Nou
HARGHITA
COVASNA
BRASOV
Varghis
Jimbor
857 m
RACOS
Racoșul de Sus
HOMOROD
RACOS
COVASNA
Augustin
Caperi
Racoș
AUGUSTIN
ORMENIS
Mateias
Miclosoara
Ormeniș
Aita Mare
Apata
APATA
HOGHIZ
DN13
Maierus
Arini
MAIERUS
BRASOV
0.0 km
5.0 km
10.0 km
46.09375
46.0625
46.03125
46
45.96875
25.375
25.40625
25.4375
25.46875
25.5
25.53125
25.5625
25.59375

EU - 49
Prut River Basin
Olt River
irregular high ground trunk stream

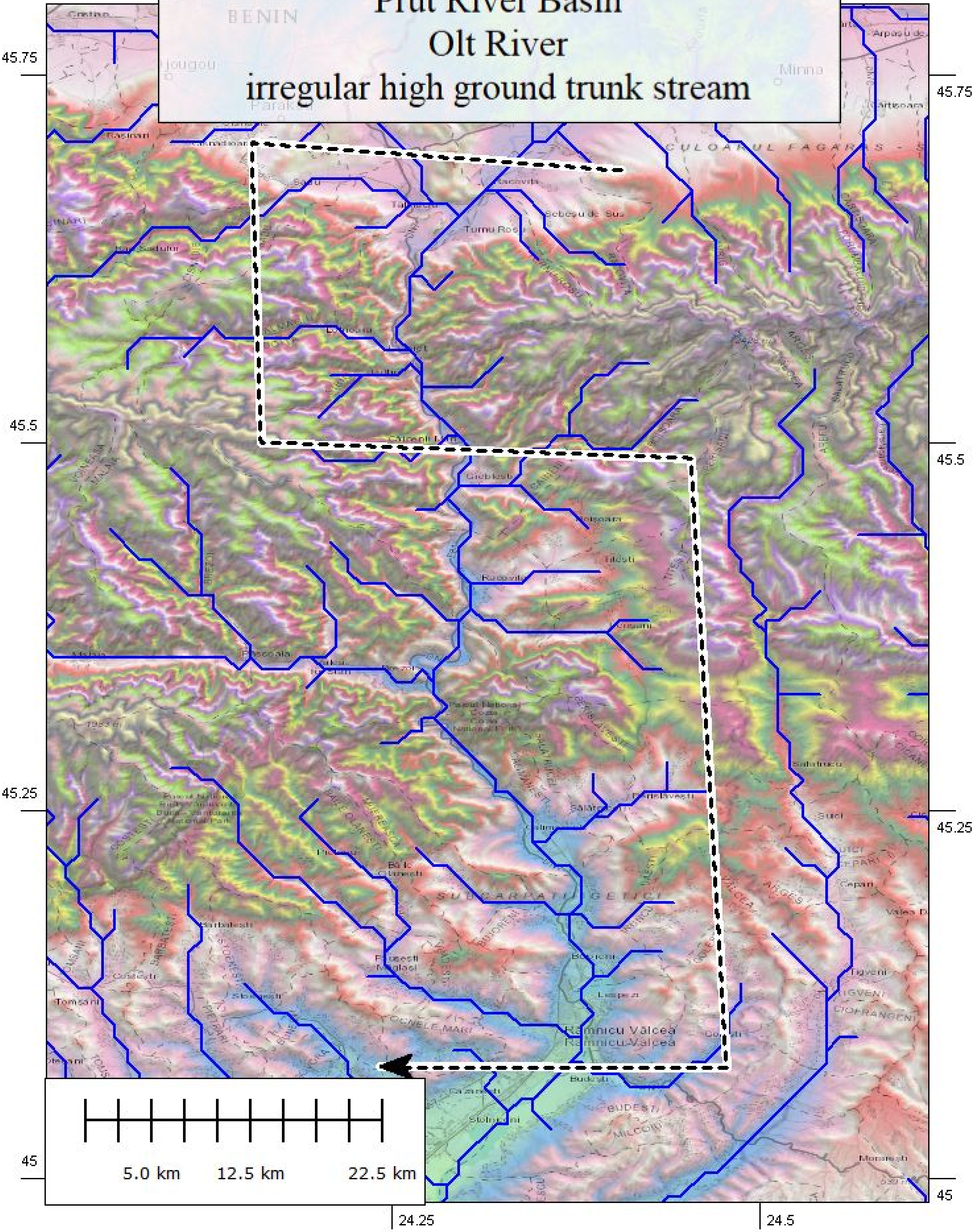

EU - 57
Prut River Basin
Danube River
irregular high ground trunk stream

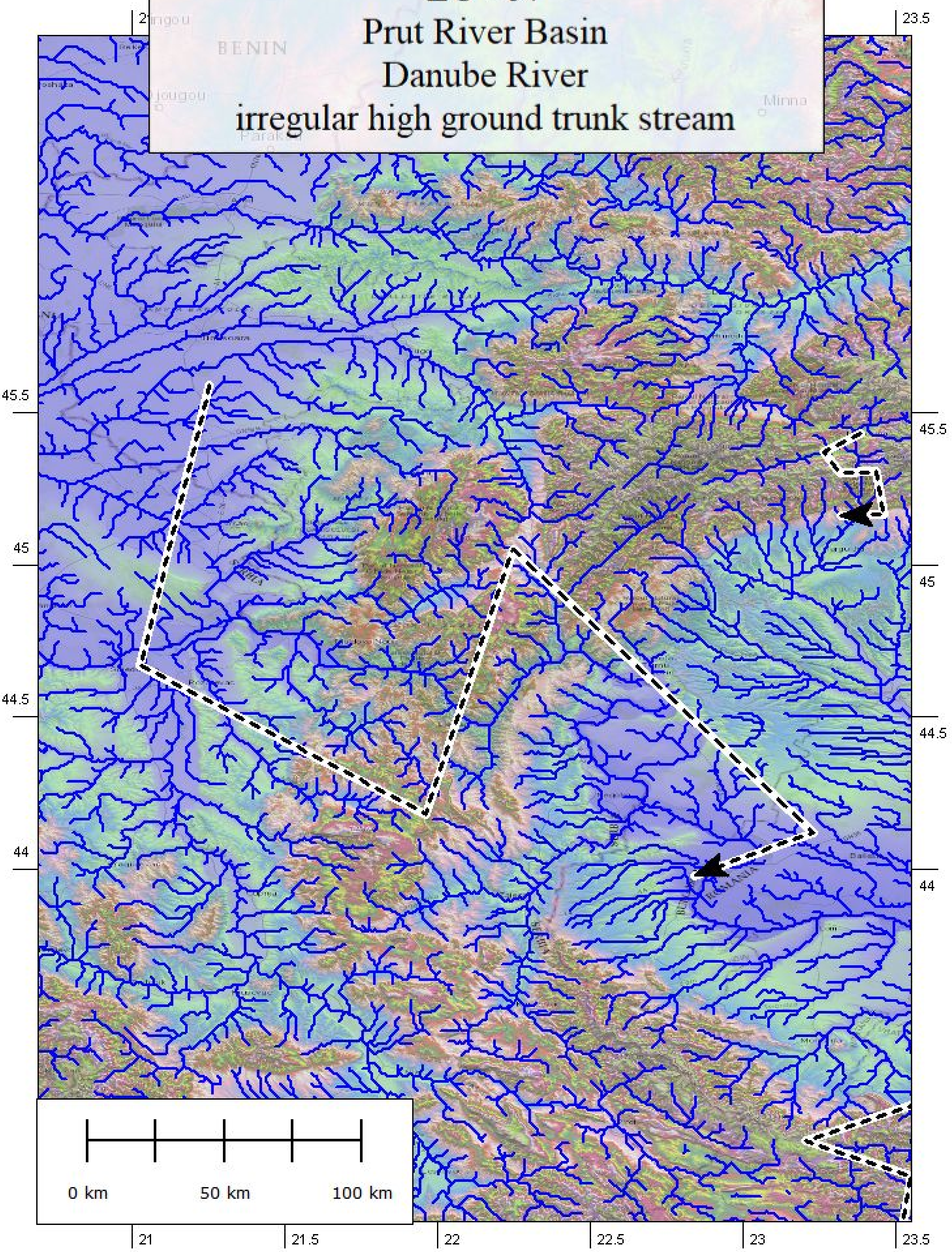

EU - 177
Ebro River Basin
Salazar River
single-ridge trunk stream

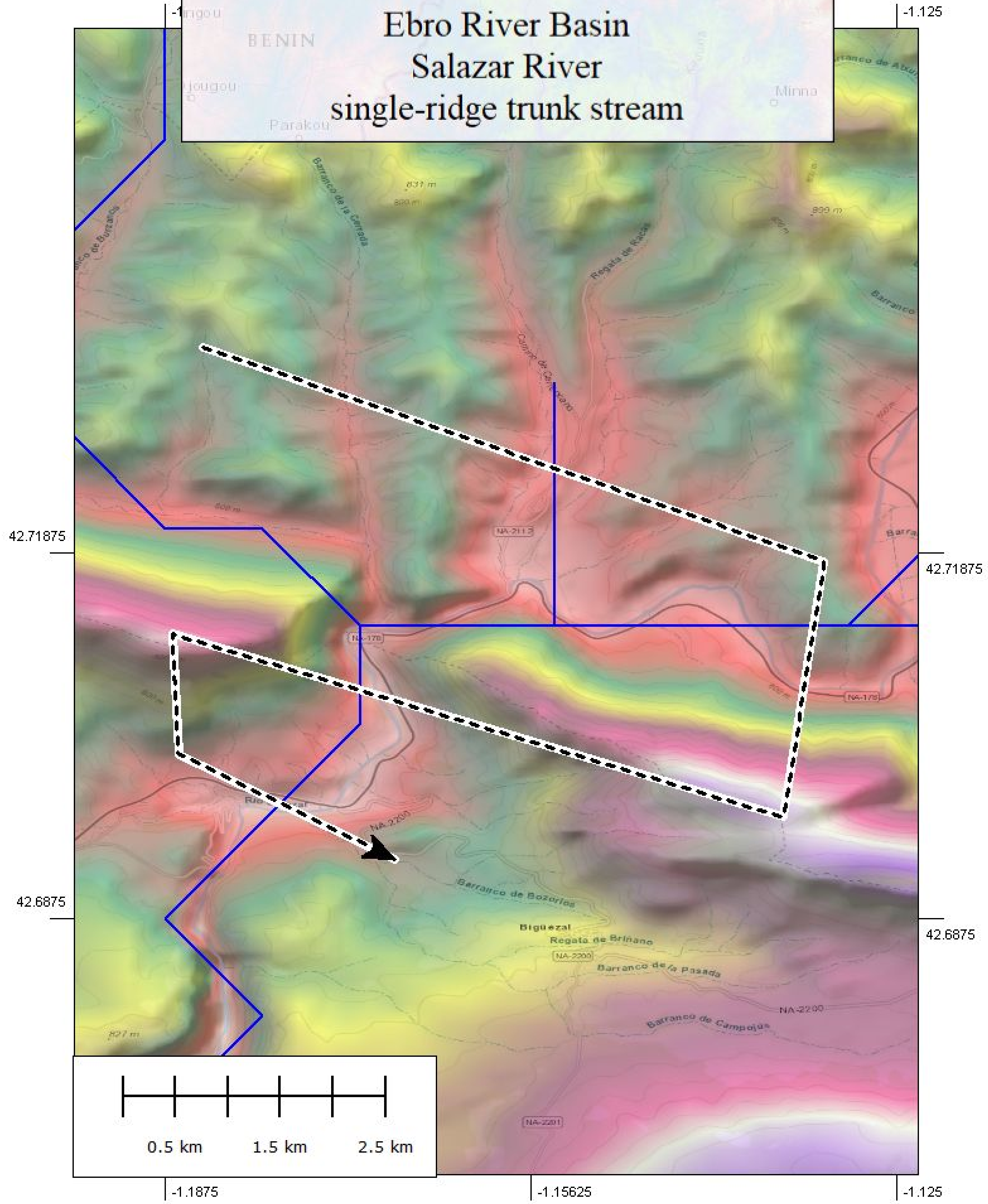

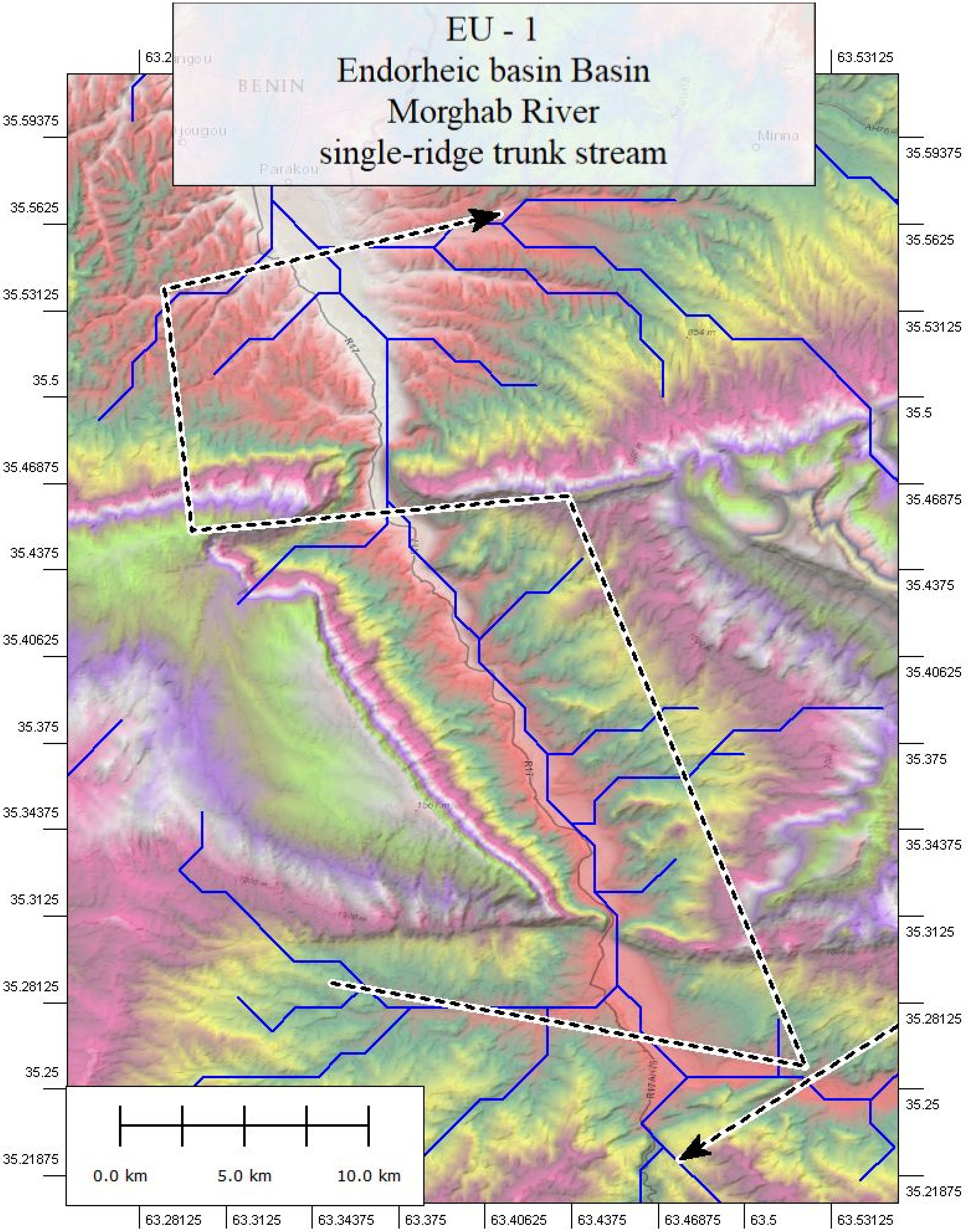

EU - 1
Endorheic basin Basin
Morghab River
single-ridge trunk stream
63.53125
35.59375
35.5625
35.53125
35.5
35.46875
35.4375
35.40625
35.375
35.34375
35.3125
35.28125
35.25
35.21875
0.0 km
5.0 km
10.0 km
63.28125
63.3125
63.34375
63.375
63.40625
63.4375
63.46875
63.5
63.53125

EU - 3
Prut River Basin
Drava River tributary
single-ridge head stream

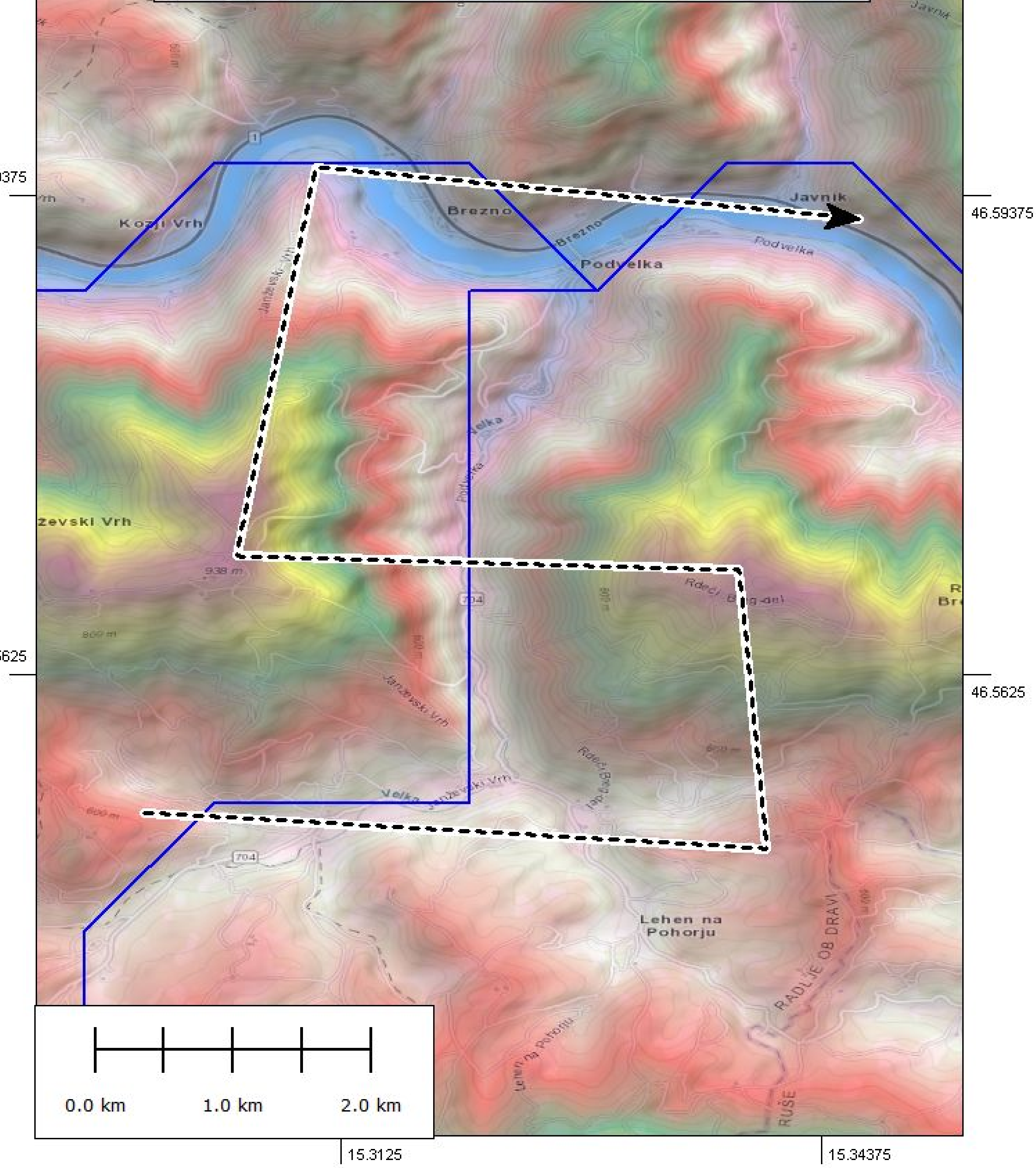

EU - 4
Mand River Basin
Mand River
single-ridge trunk stream

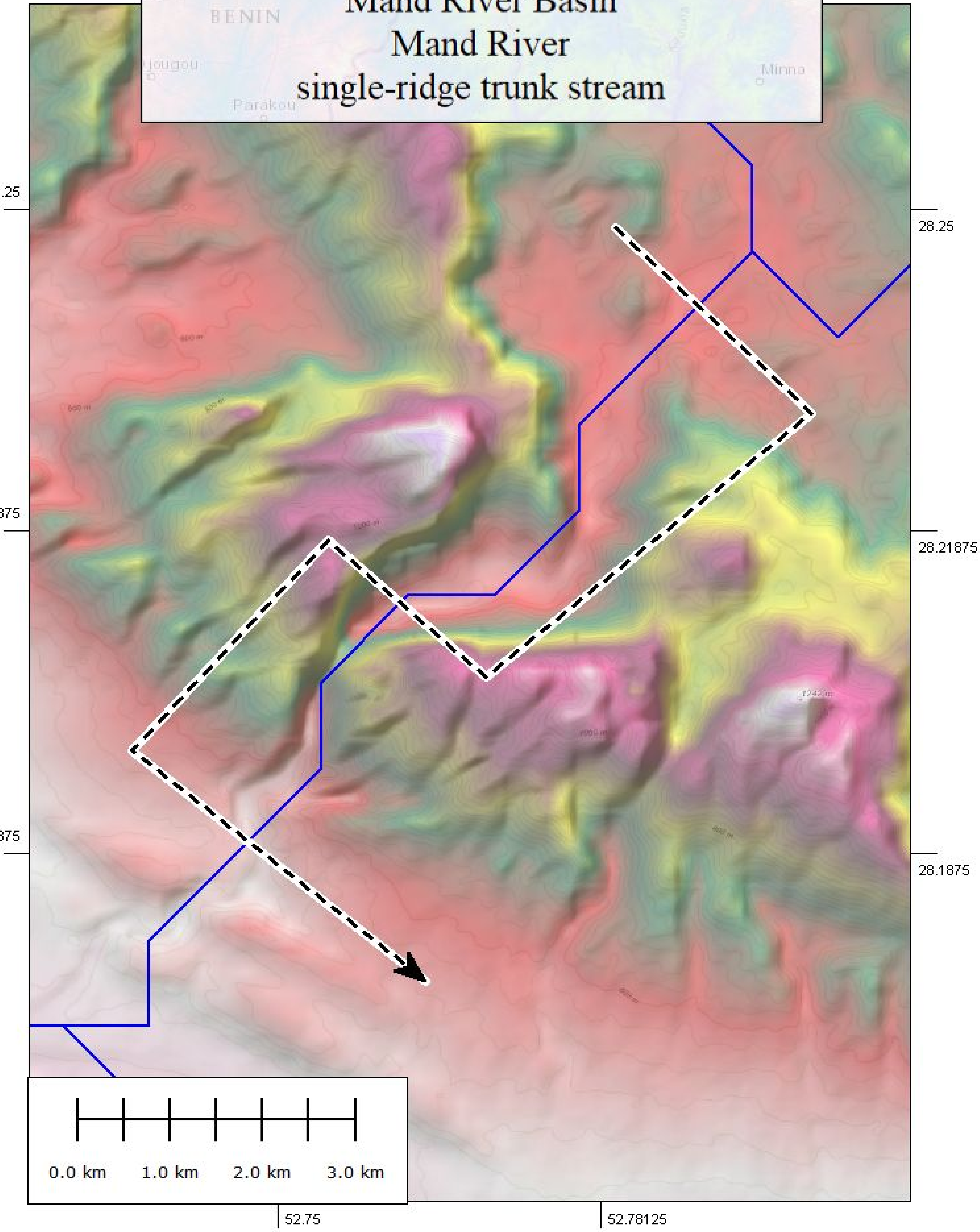

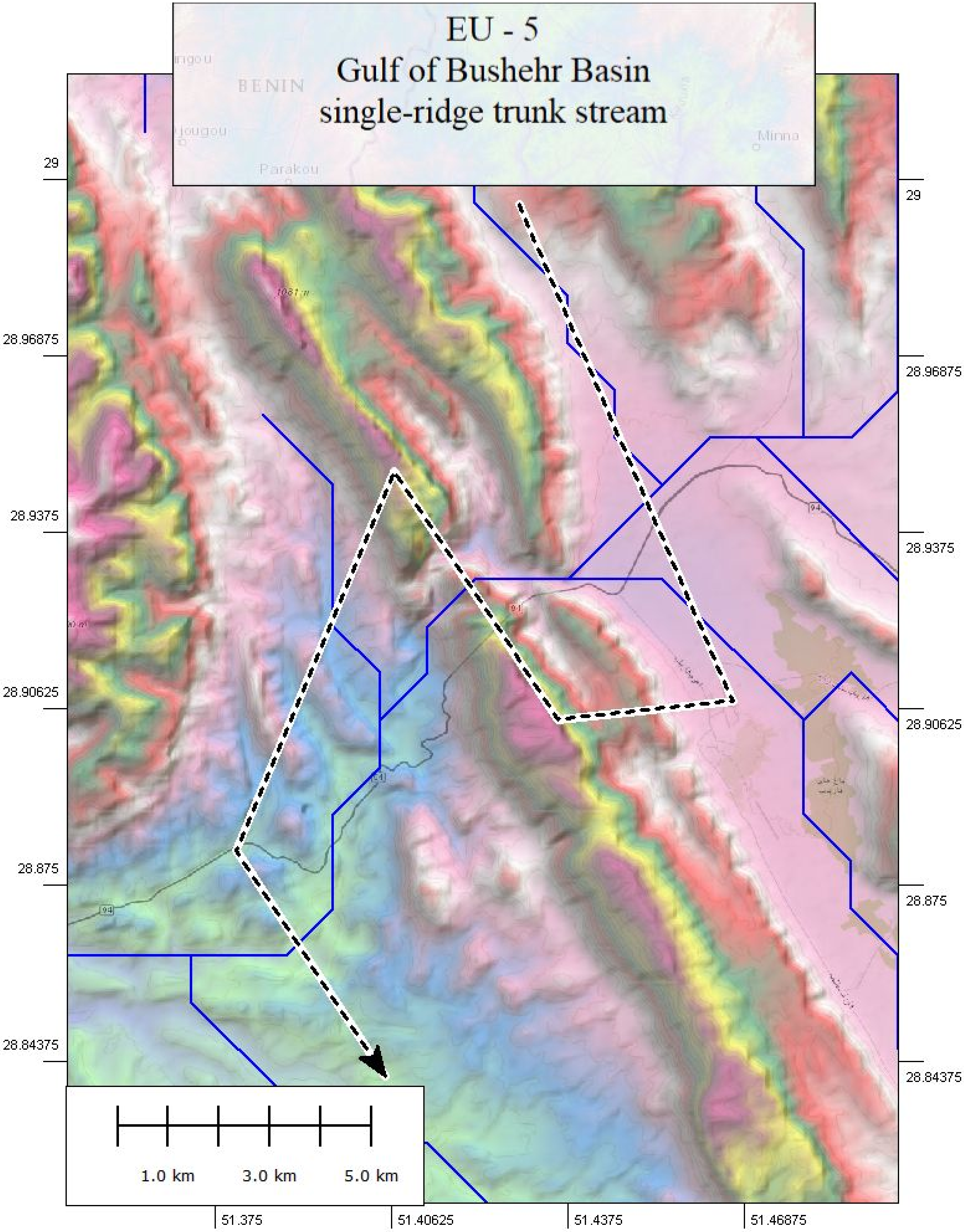
EU - 5
Gulf of Bushehr Basin
single-ridge trunk stream
BENIN
Parakou
Minna
1081 m
94
29
28.96875
28.9375
28.90625
28.875
28.84375
51.375
51.40625
51.4375
51.46875
1.0 km
3.0 km
5.0 km

EU - 7
Euphrates River Basin
single-ridge trunk stream

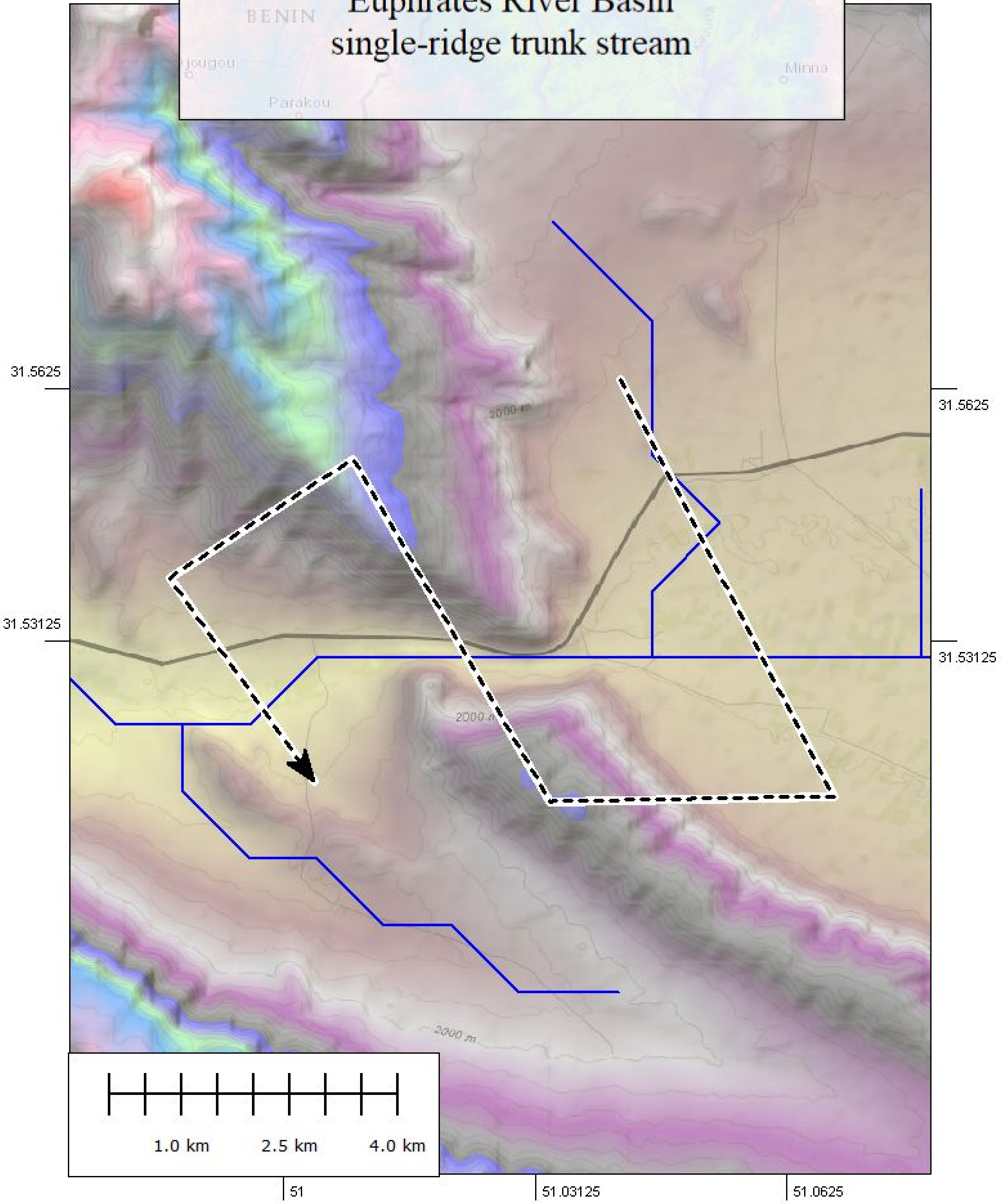

EU - 8
Euphrates River Basin
Karun River tributary
single-ridge head stream

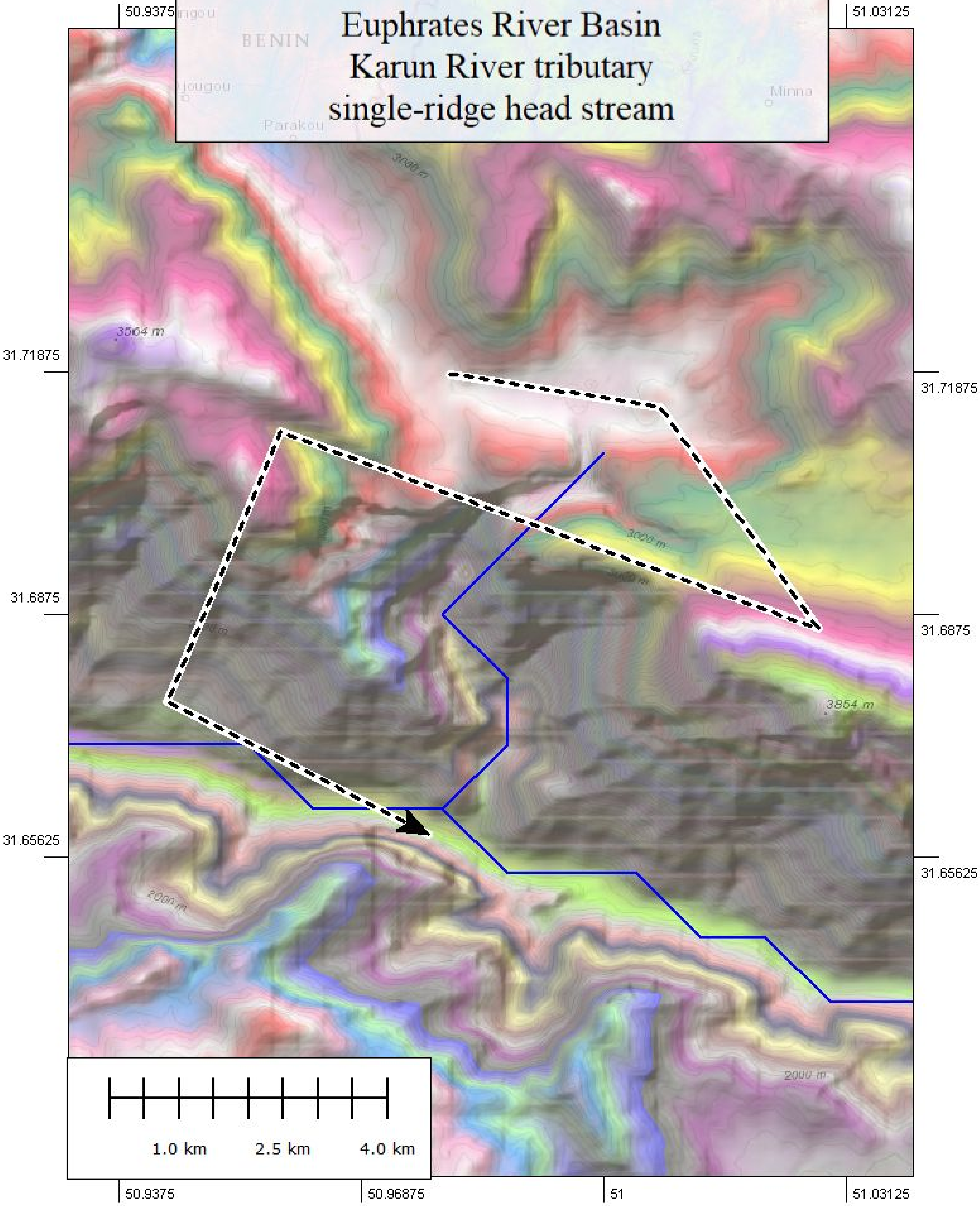

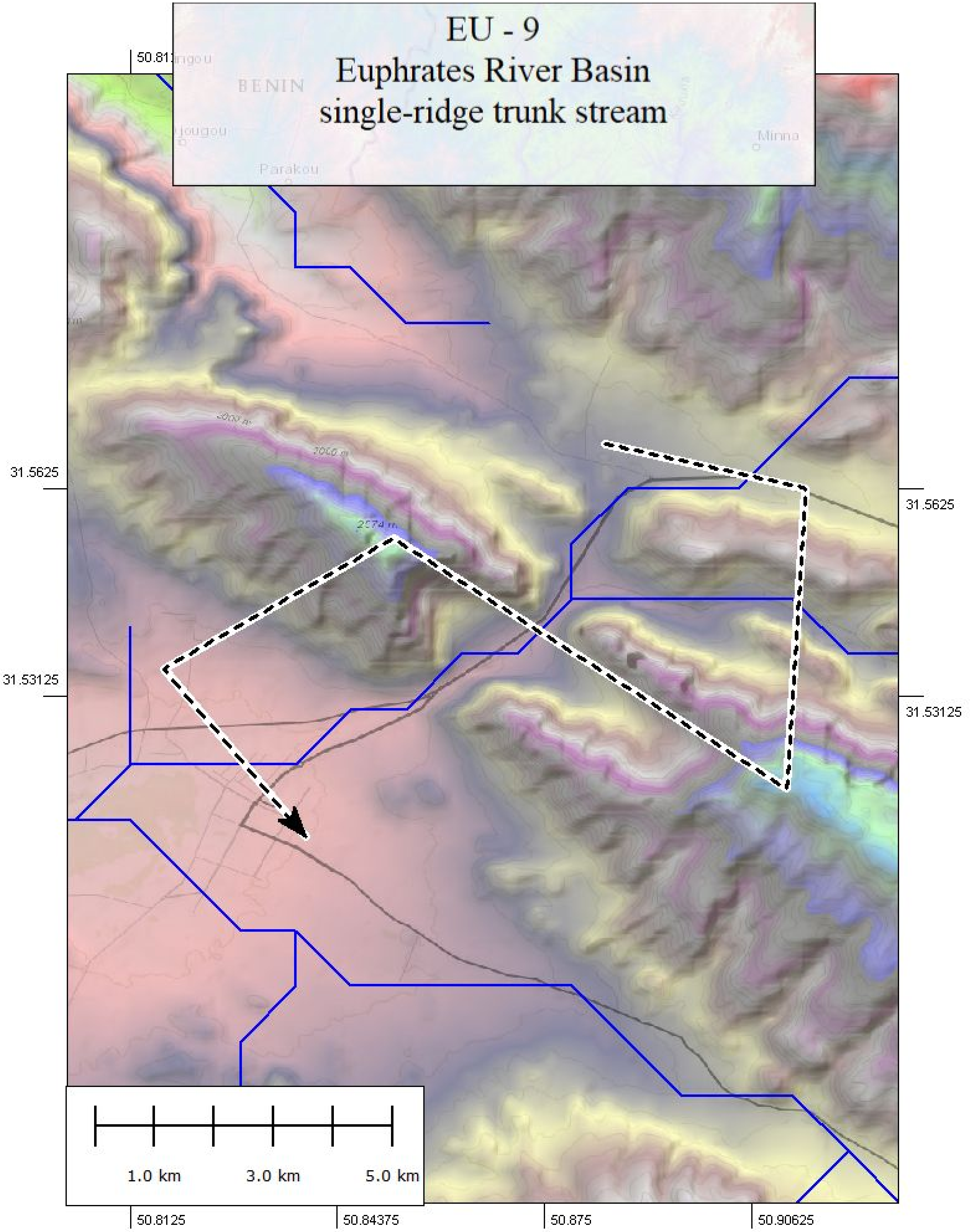

EU - 9
Euphrates River Basin
single-ridge trunk stream
BENIN
Parakou
Minna
2000 m
2050 m
2574 m
31.5625
31.53125
50.8125
50.84375
50.875
50.90625
1.0 km
3.0 km
5.0 km

EU - 10
Euphrates River Basin
single-ridge trunk stream

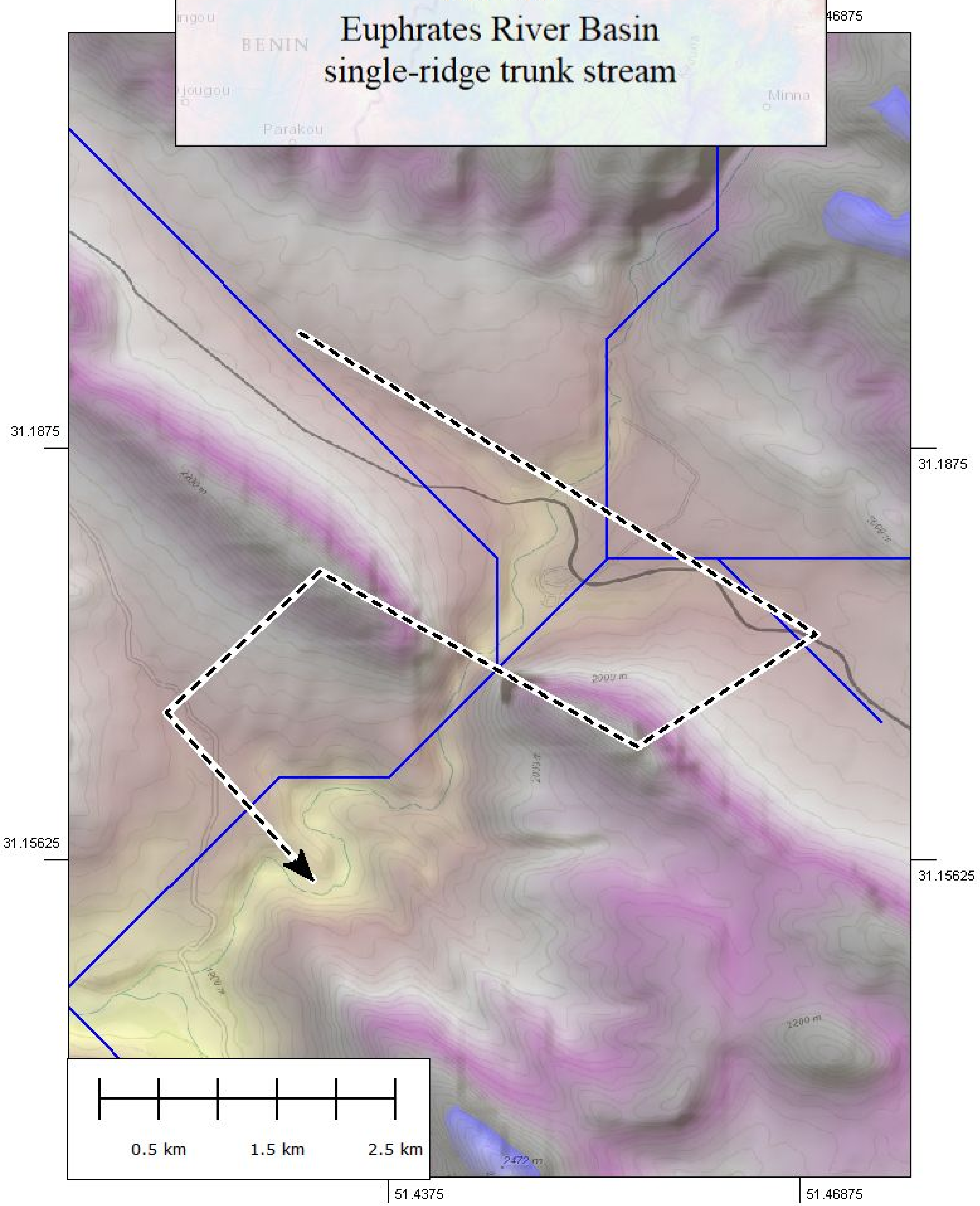

EU - 11
Khowr-e Dowraq Basin
multi-ridge trunk stream

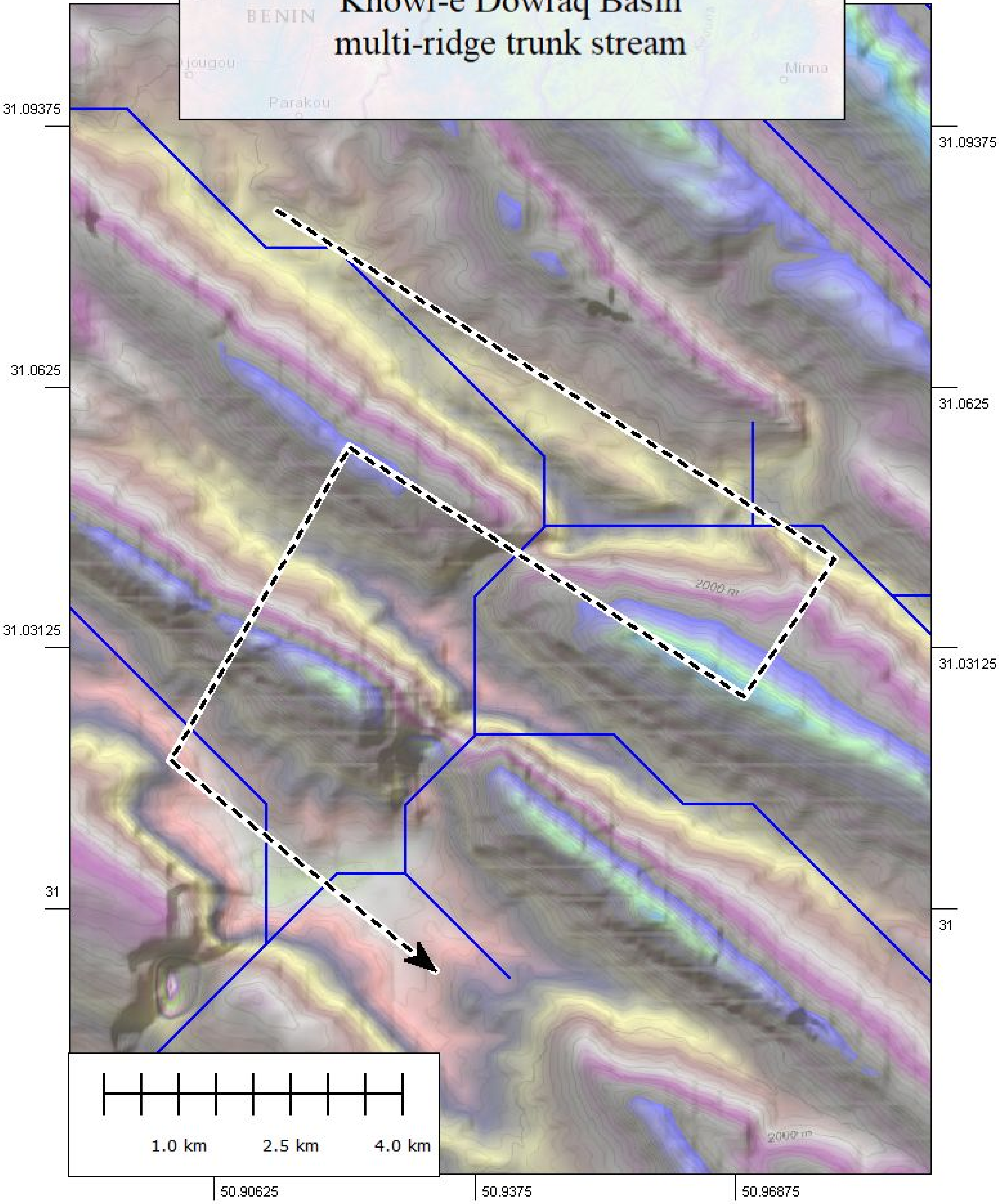

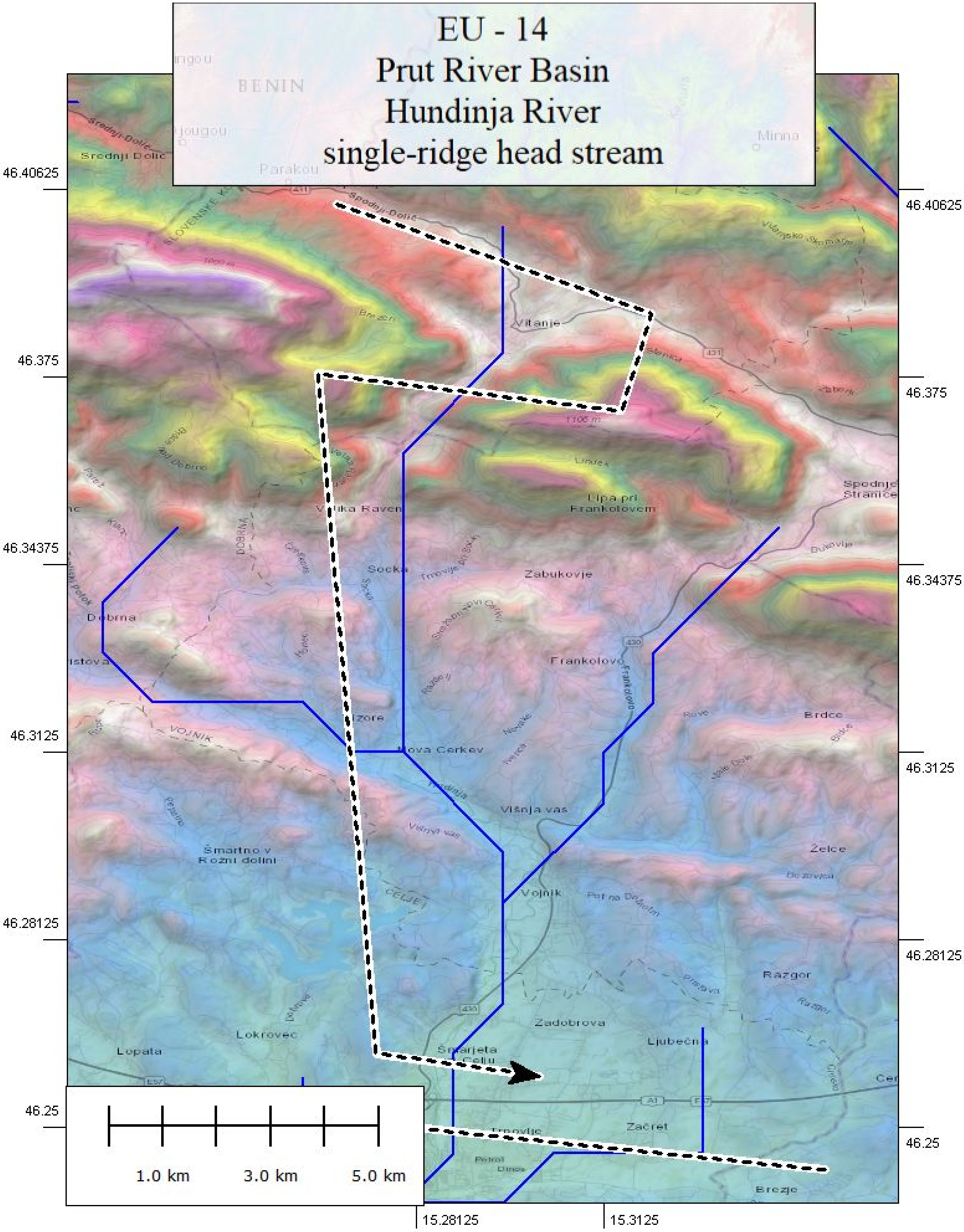
EU - 14
Prut River Basin
Hundinja River
single-ridge head stream
46.40625
46.375
46.34375
46.3125
46.28125
46.25
15.28125
15.3125
1.0 km
3.0 km
5.0 km
Vitanje
Velika Raven
Lipa pri Frankolovem
Socka
Zabukovje
Dobrna
Frankolovo
Nova Cerkev
Višnja vas
Vojnik
Šmartno v Rožni dolini
Razgor
Zadobrova
Ljubečna
Lokrovec
Lopata
Šmarjeta pri Celju
Začret
Trnovlje
Brezje
Brdce
Želce
Spodnje Stranice

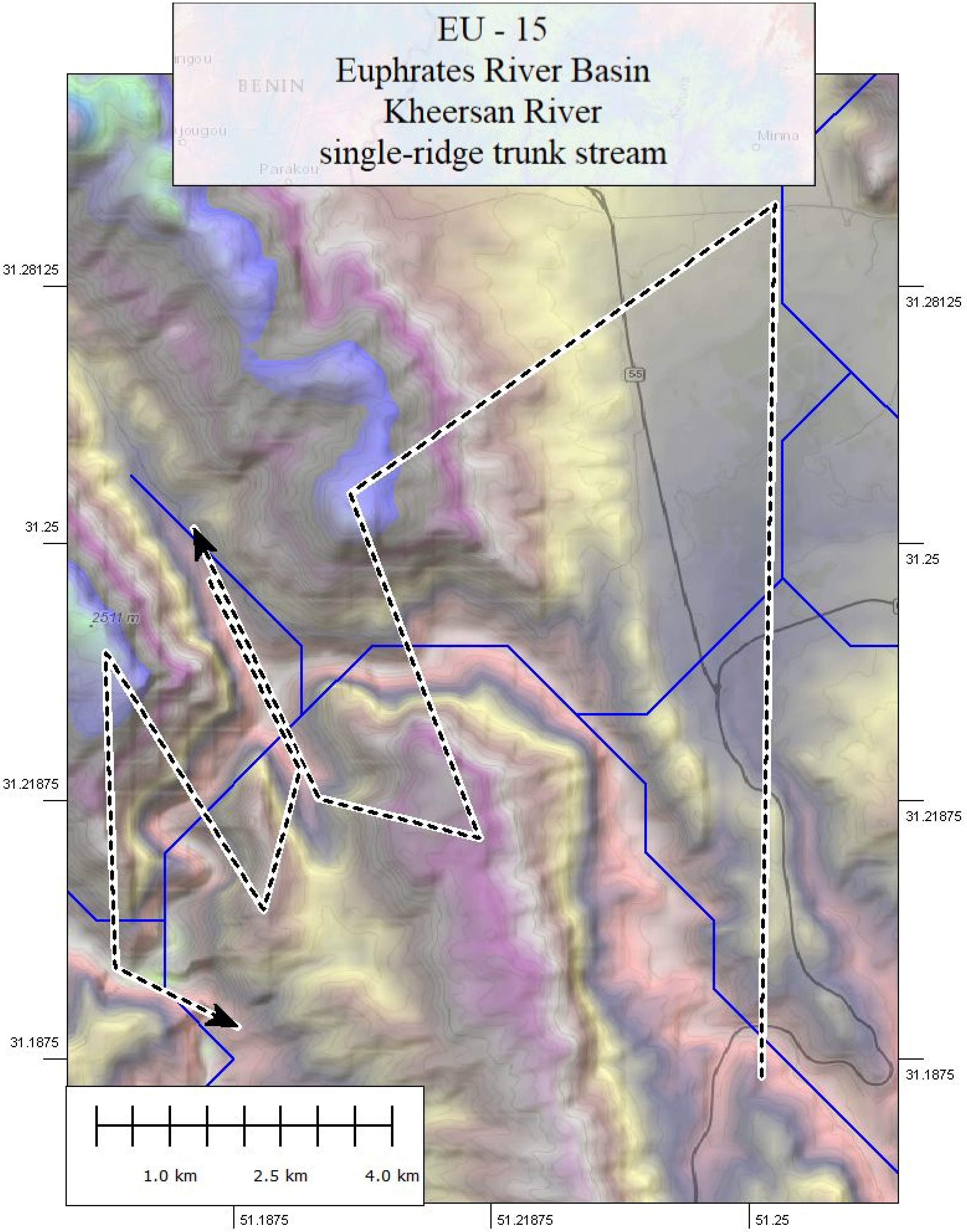
EU - 15
Euphrates River Basin
Kheersan River
single-ridge trunk stream
BENIN
Parakou
Minna
55
2511 m
31.28125
31.25
31.21875
31.1875
51.1875
51.21875
51.25
1.0 km
2.5 km
4.0 km

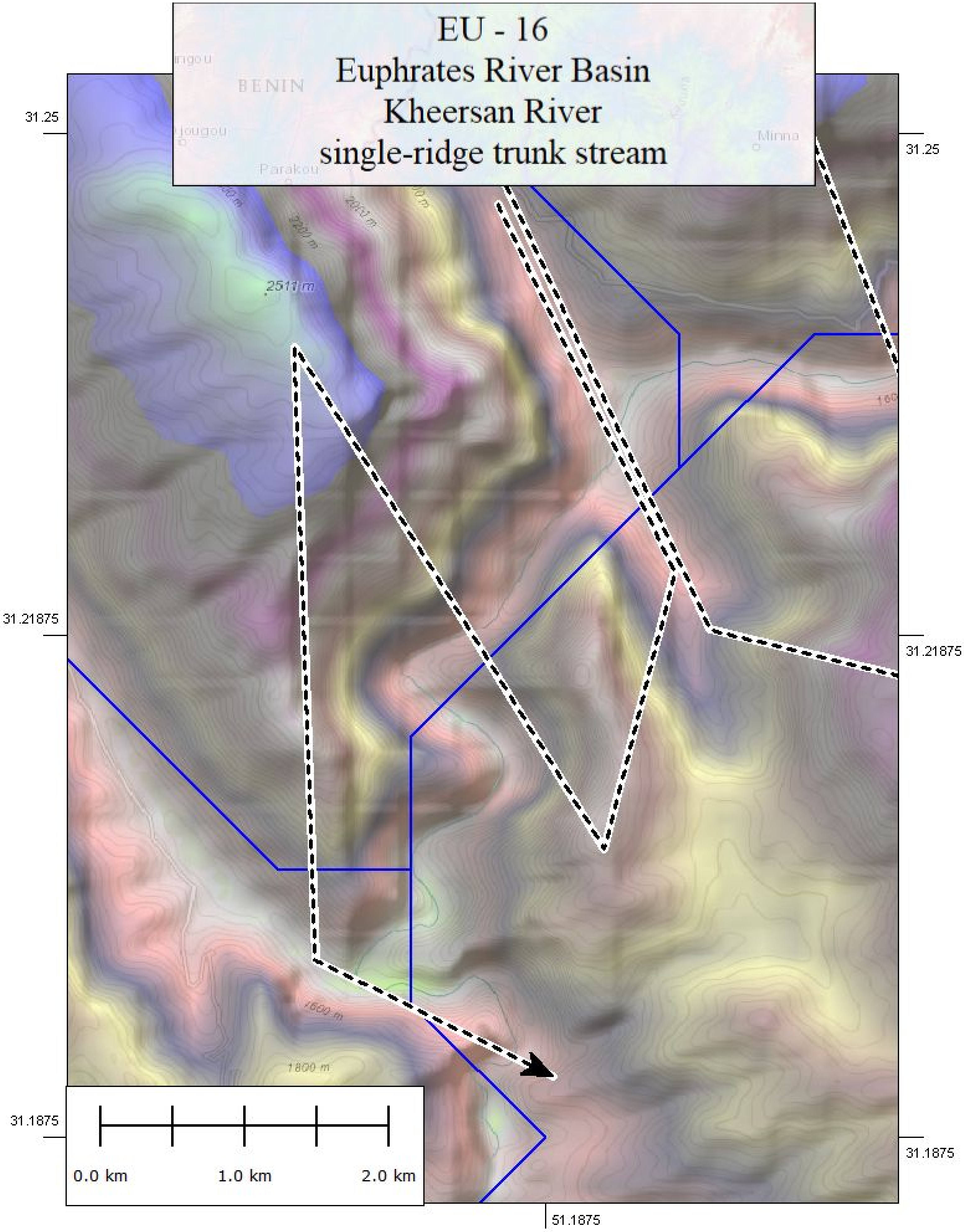
EU - 16
Euphrates River Basin
Kheersan River
single-ridge trunk stream
BENIN
Parakou
Minna
2200 m
2511 m
1600 m
1800 m
31.25
31.25
31.21875
31.21875
31.1875
31.1875
51.1875
0.0 km
1.0 km
2.0 km

EU - 17
Euphrates River Basin
single-ridge trunk stream

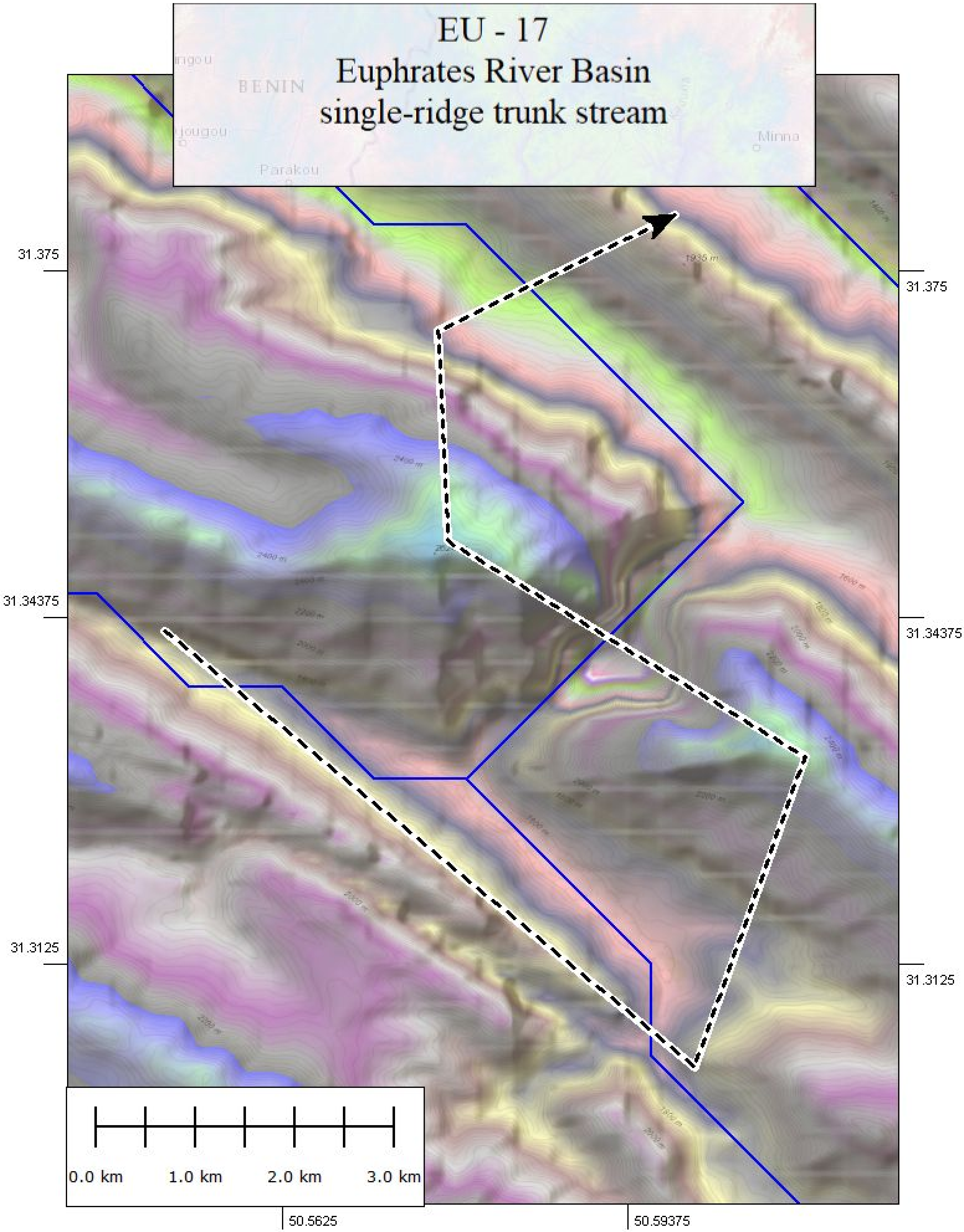

EU - 18
Euphrates River Basin
single-ridge trunk stream

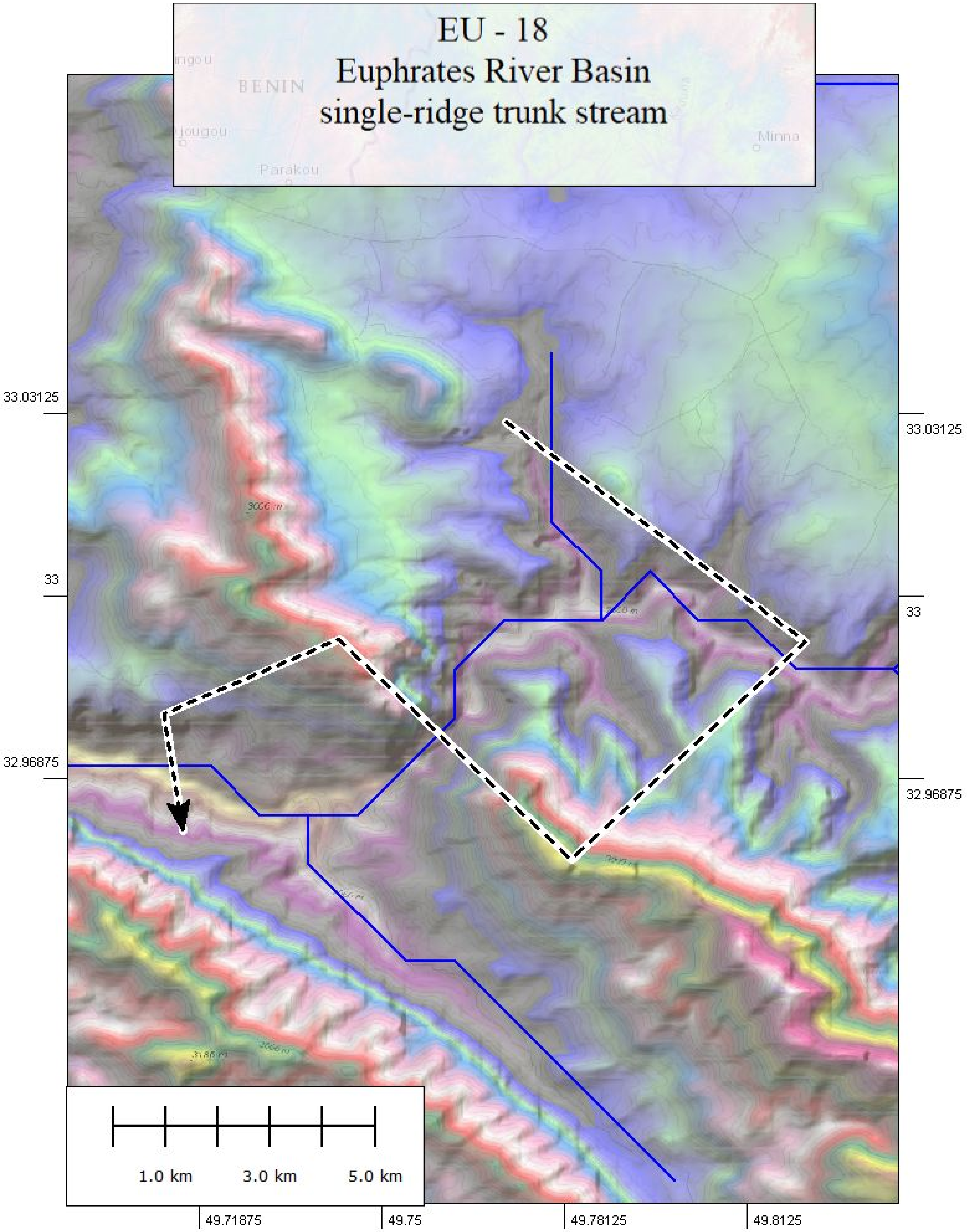

EU - 19
Mand River Basin
Mand River
single-ridge trunk stream

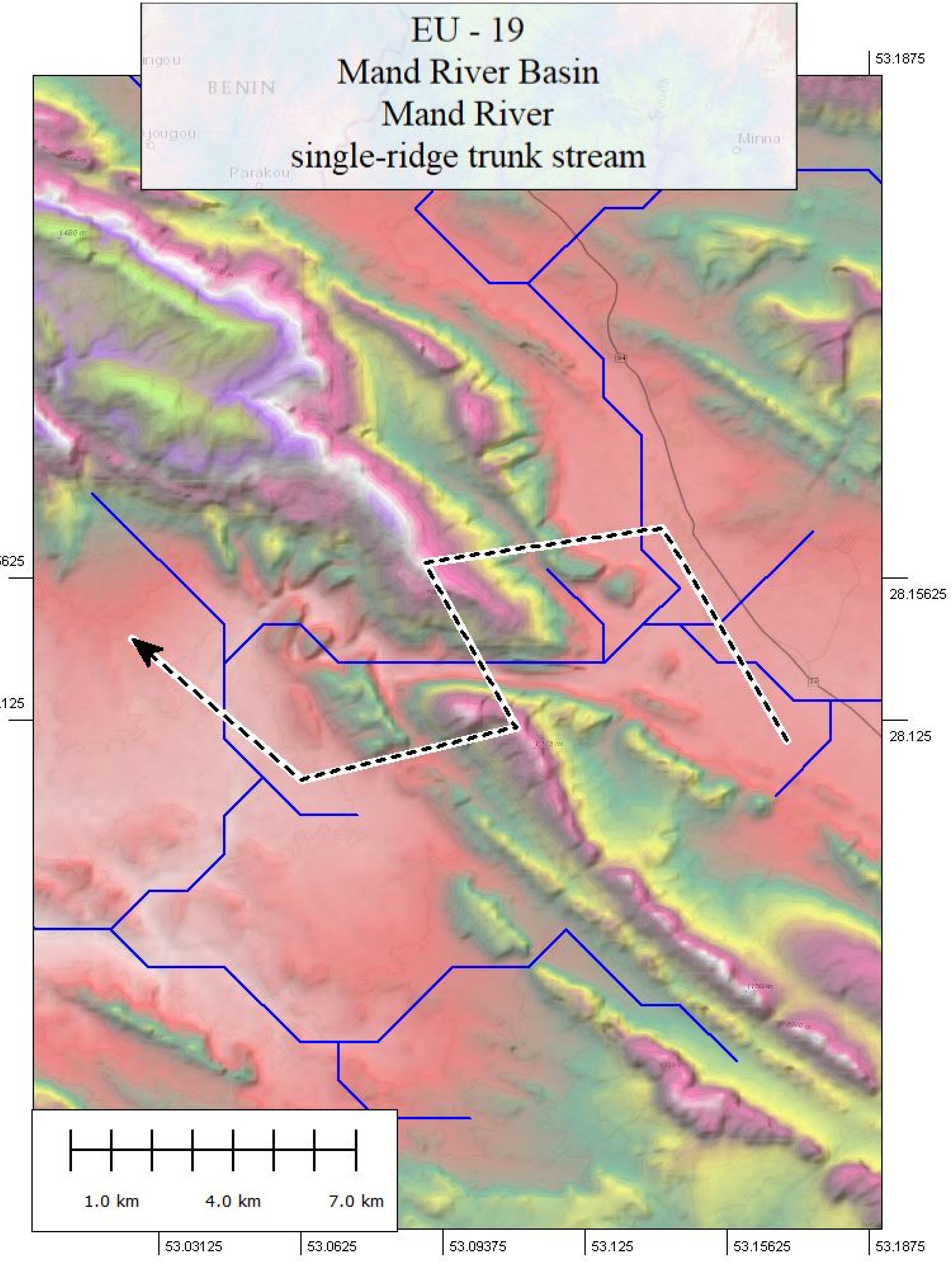

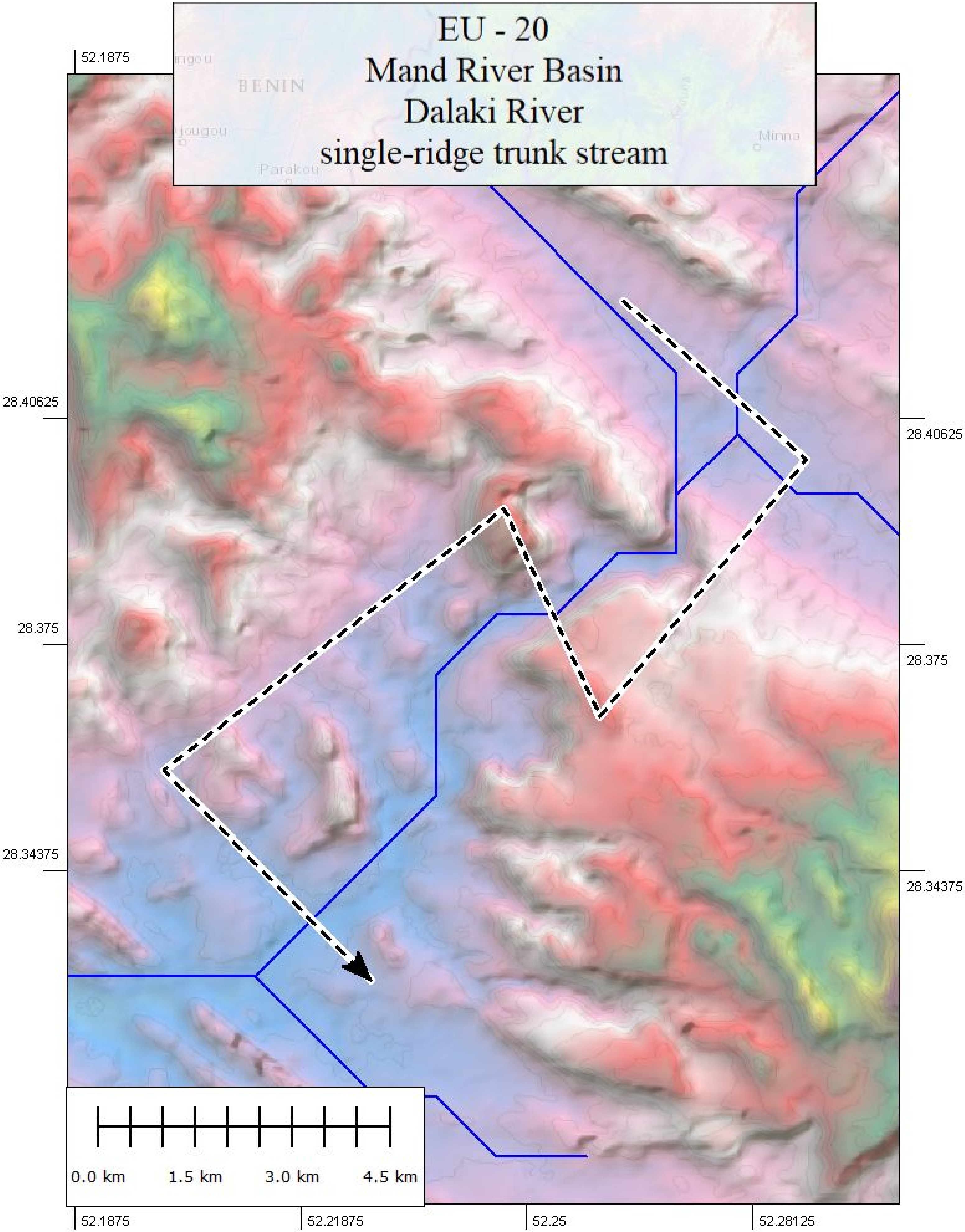
EU - 20
Mand River Basin
Dalaki River
single-ridge trunk stream
52.1875
28.40625
28.375
28.34375
28.40625
28.375
28.34375
0.0 km
1.5 km
3.0 km
4.5 km
52.1875
52.21875
52.25
52.28125

EU - 21
Mand River Basin
Mand River
single-ridge trunk stream

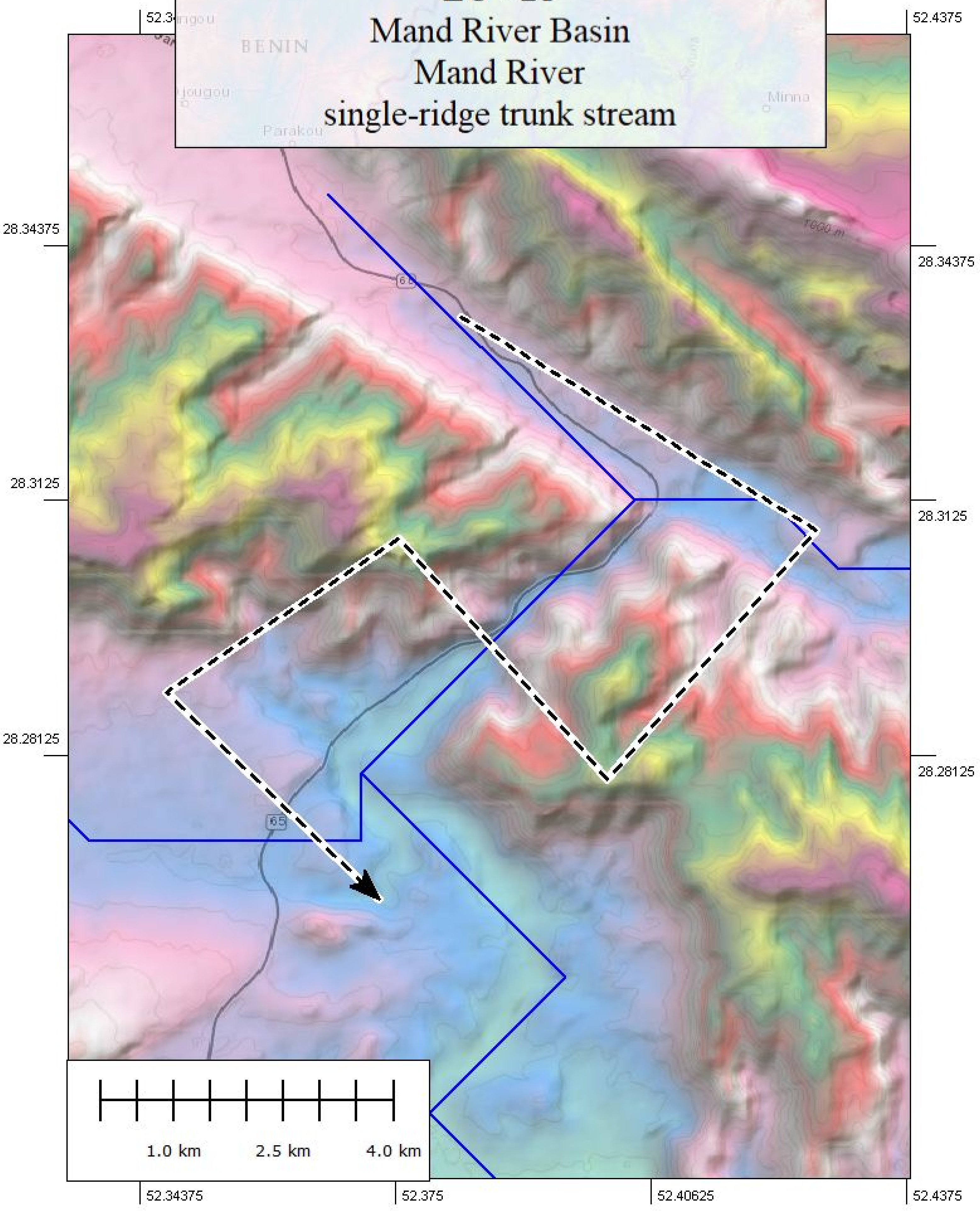

EU - 24
Rhine River Basin
Simmerbach River
single-ridge trunk stream

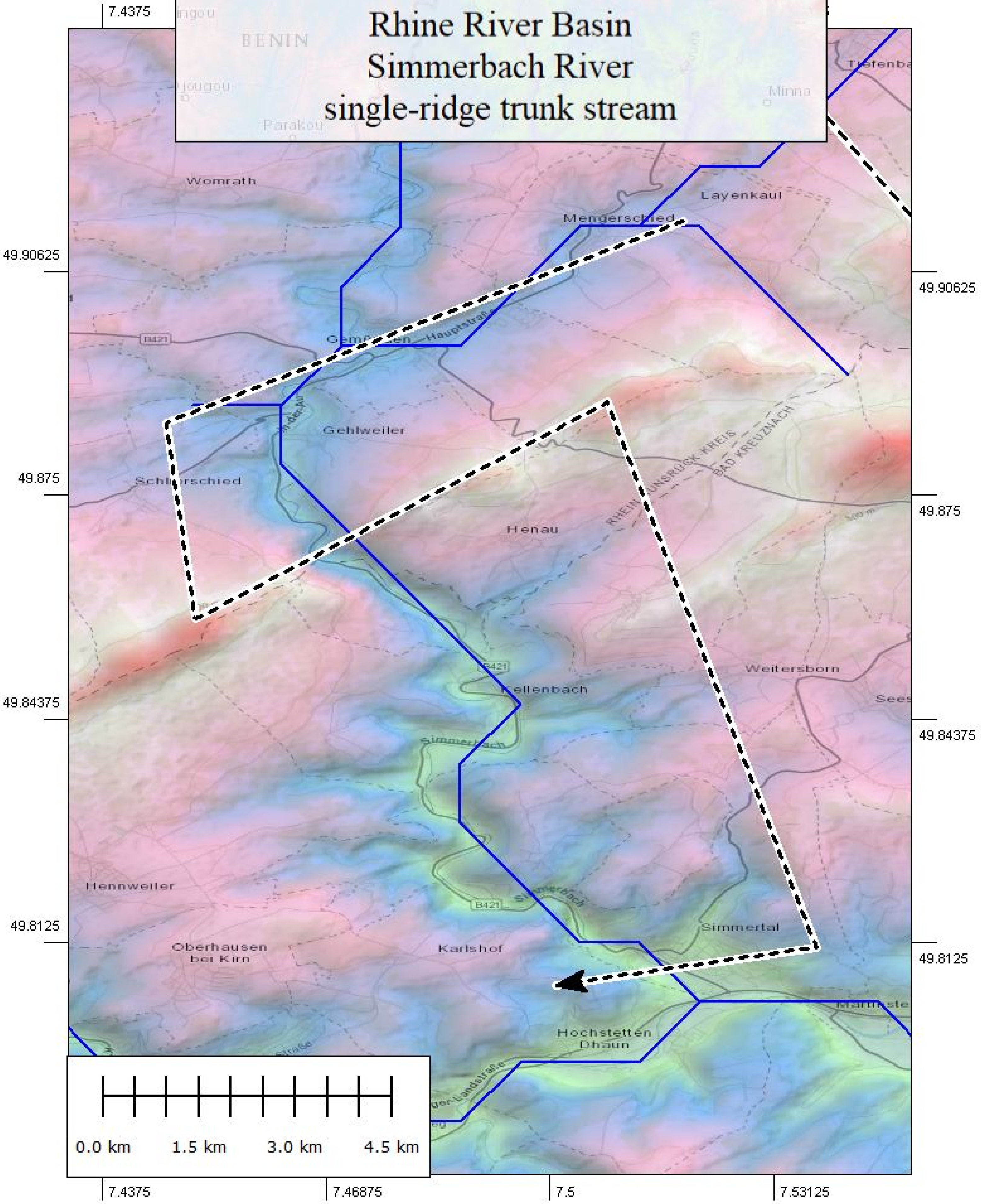

EU - 25
Esino River Basin
Esino River
single-ridge trunk stream

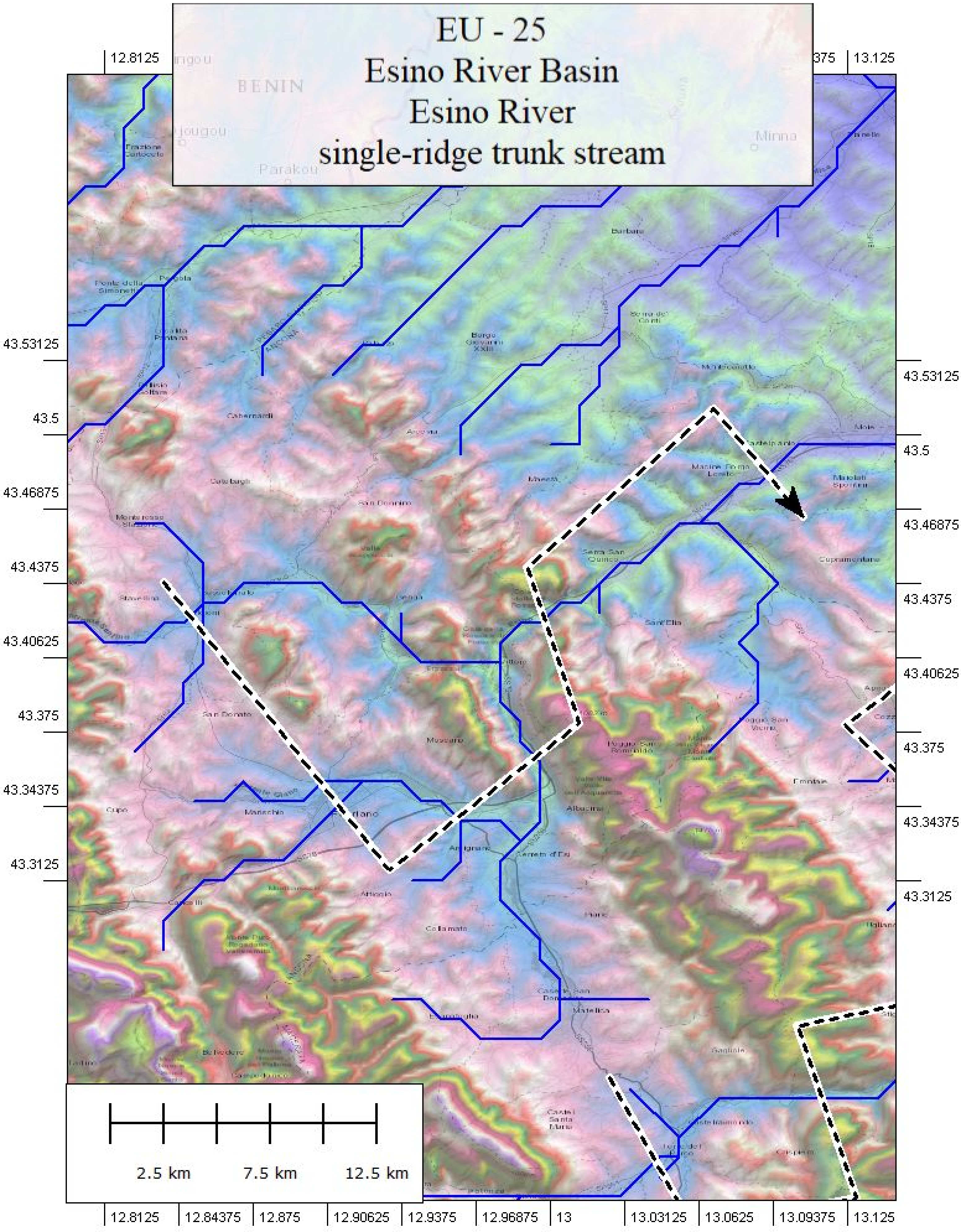

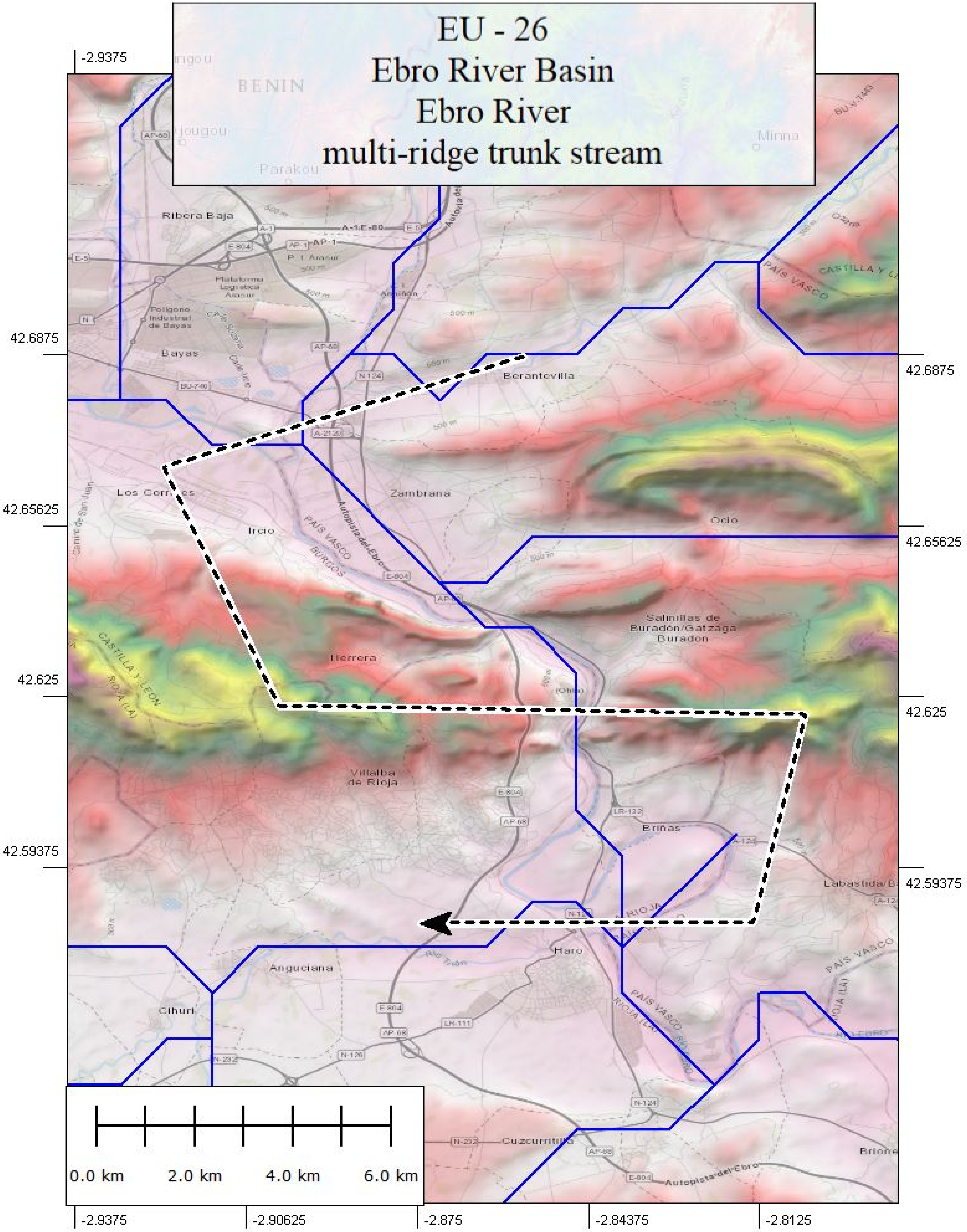

EU - 26
Ebro River Basin
Ebro River
multi-ridge trunk stream
-2.9375
-2.90625
-2.875
-2.84375
-2.8125
42.6875
42.65625
42.625
42.59375
Ribera Baja
Bayas
Berantevilla
Los Corrales
Zambrana
Ircio
Oclo
Salinillas de
Buradón/Gatzaga
Buradon
Herrera
Villalba
de Rioja
Briñas
Haro
Labastida/B
Anguciana
Cihuri
Cuzcurritilla
Briones
PAÍS VASCO
BURGOS
CASTILLA Y LEÓN
RIOJA (LA)
Autopista del Ebro
0.0 km
2.0 km
4.0 km
6.0 km

EU - 27
Mand River Basin
single-ridge head stream

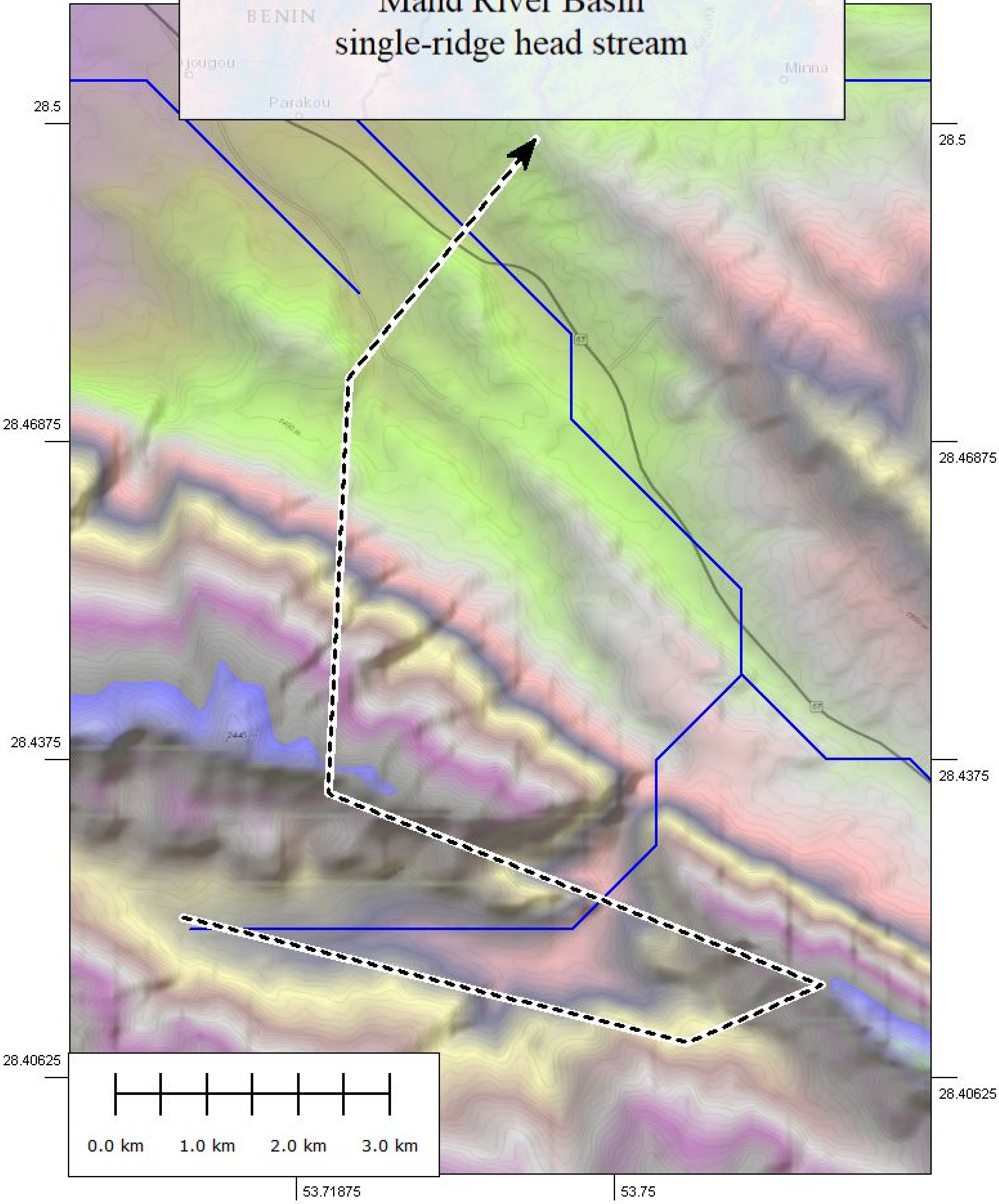

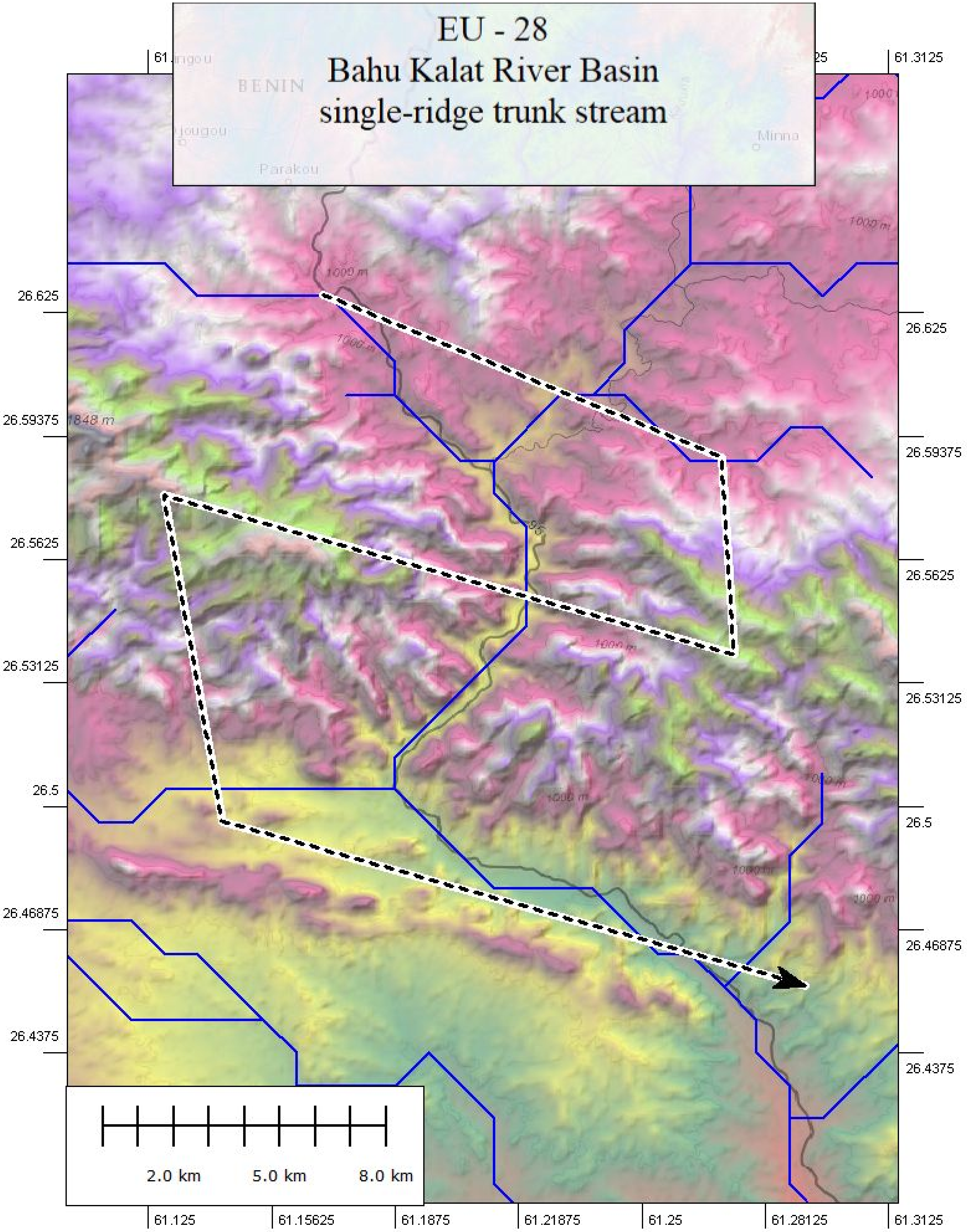
EU - 28
Bahu Kalat River Basin
single-ridge trunk stream
BENIN
Parakou
Minna
1000 m
1848 m
26.625
26.59375
26.5625
26.53125
26.5
26.46875
26.4375
61.125
61.15625
61.1875
61.21875
61.25
61.28125
61.3125
2.0 km
5.0 km
8.0 km

EU - 29
Wadi near Straits of Hormuz Basin
single-ridge head stream

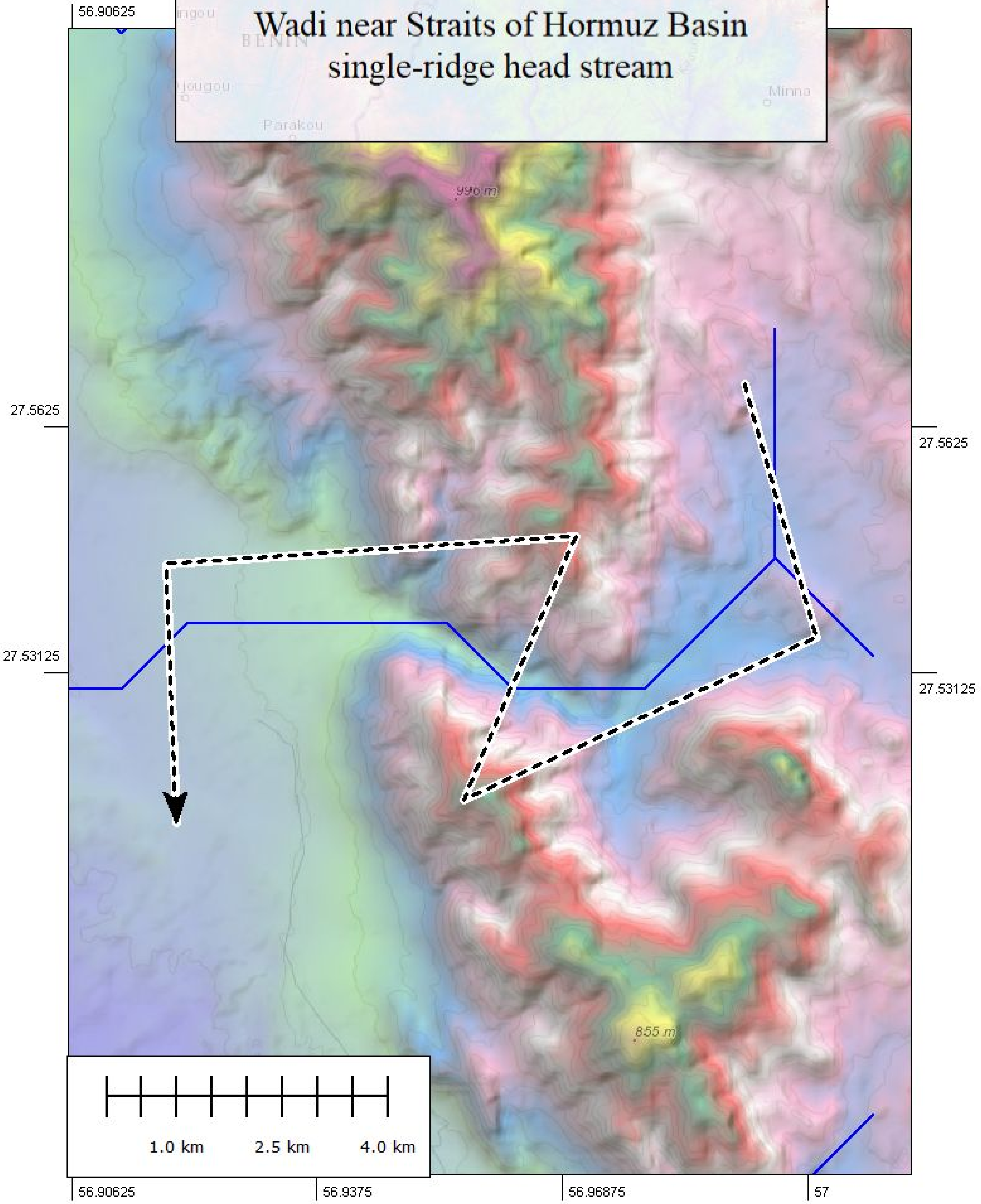

EU - 30
Metauro River Basin
Metauro River tributary
single-ridge trunk stream

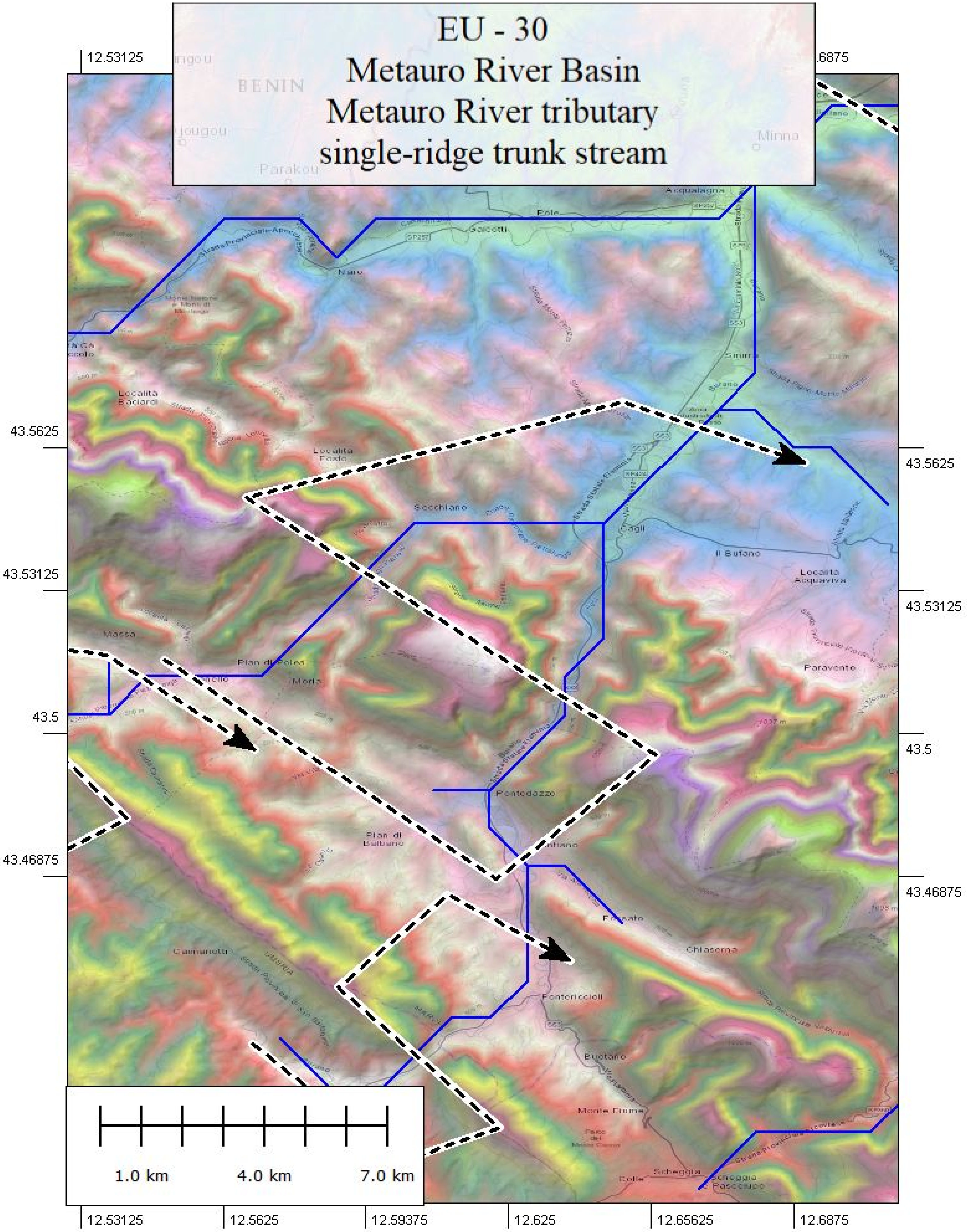

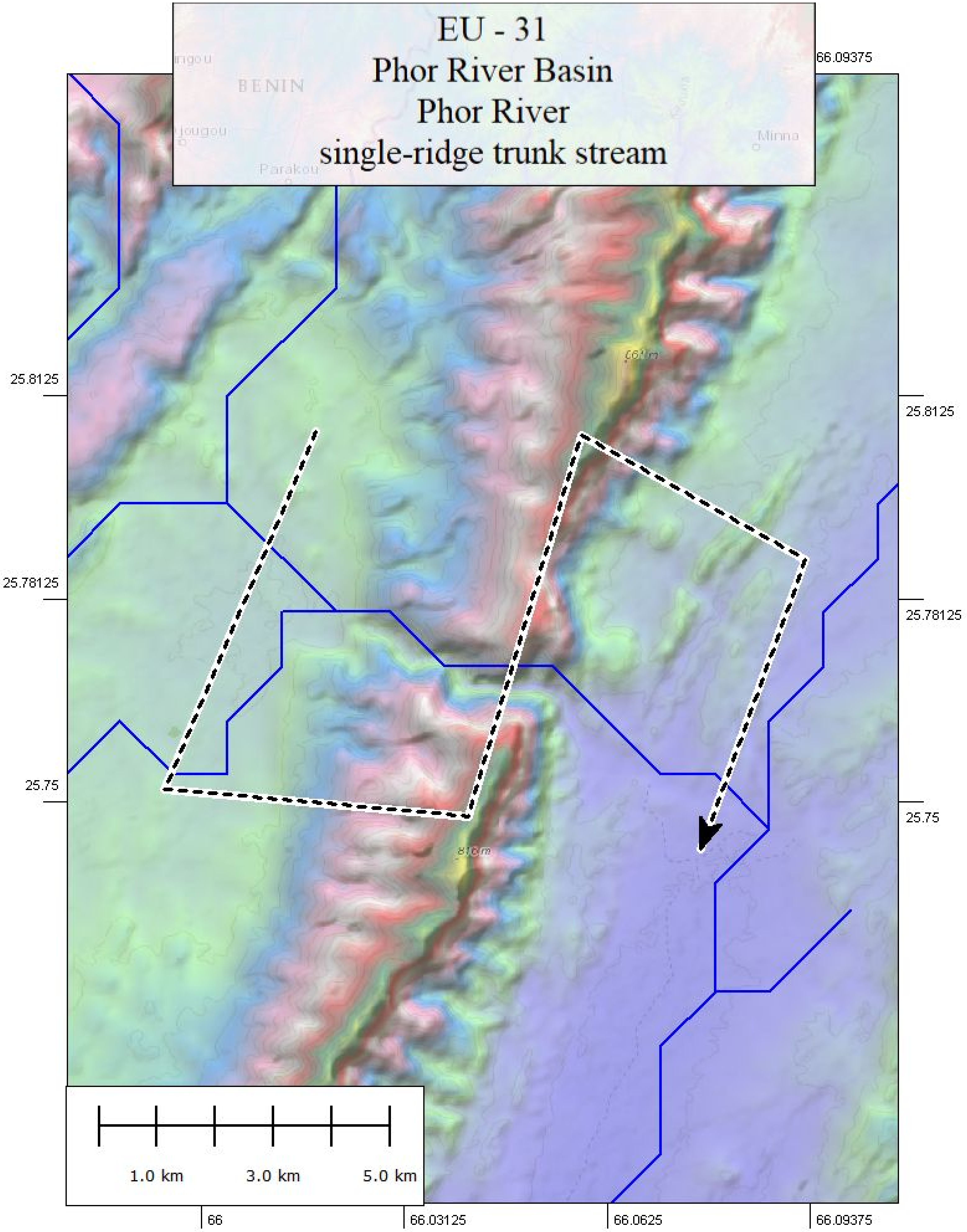

EU - 31
Phor River Basin
Phor River
single-ridge trunk stream
66.09375
BENIN
Parakou
Minna
661m
816m
25.8125
25.78125
25.75
25.8125
25.78125
25.75
1.0 km
3.0 km
5.0 km
66
66.03125
66.0625
66.09375

EU - 32
Metauro River Basin
Metauro River
single-ridge trunk stream

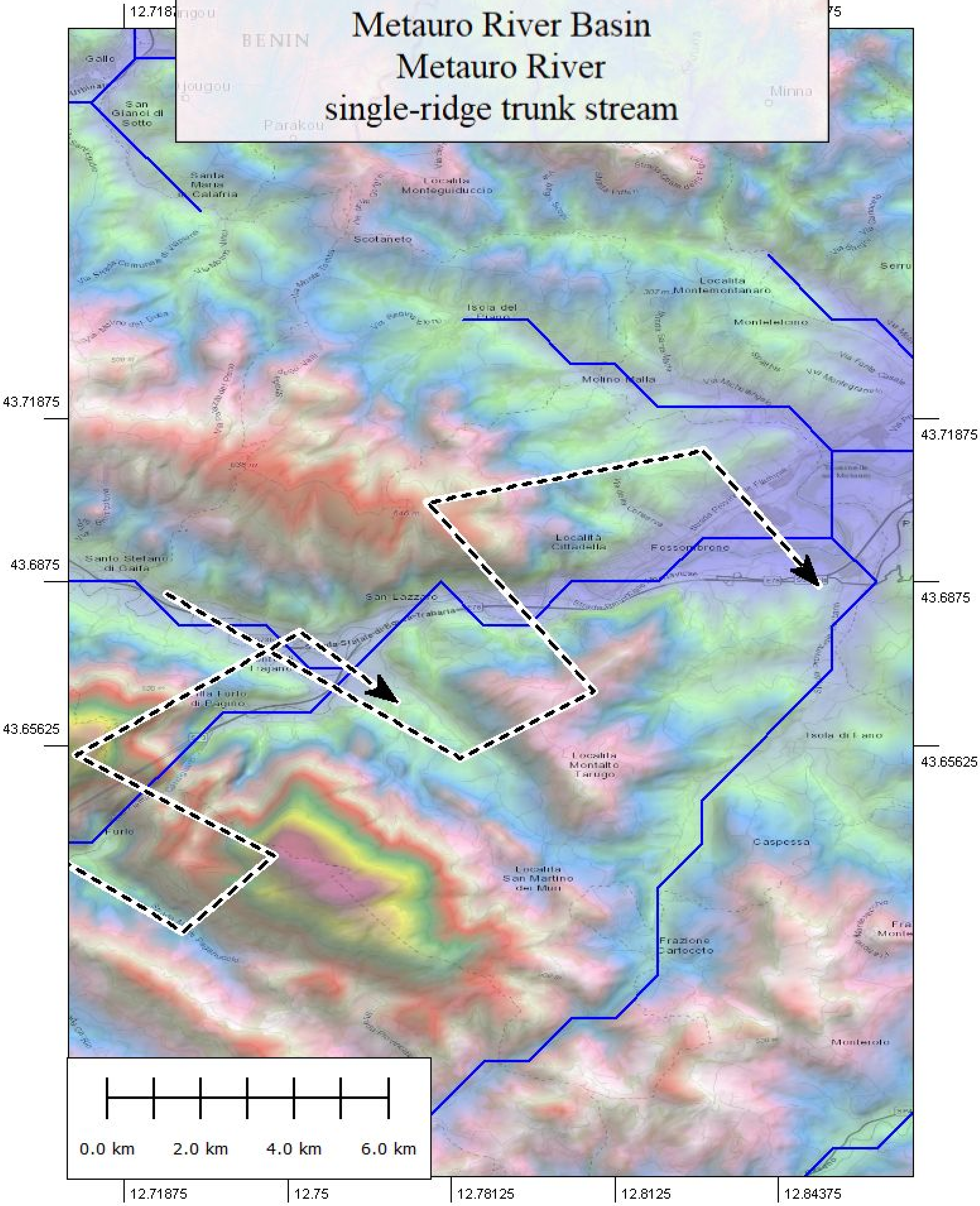

EU - 33
Metauro River Basin
Metauro River
single-ridge trunk stream

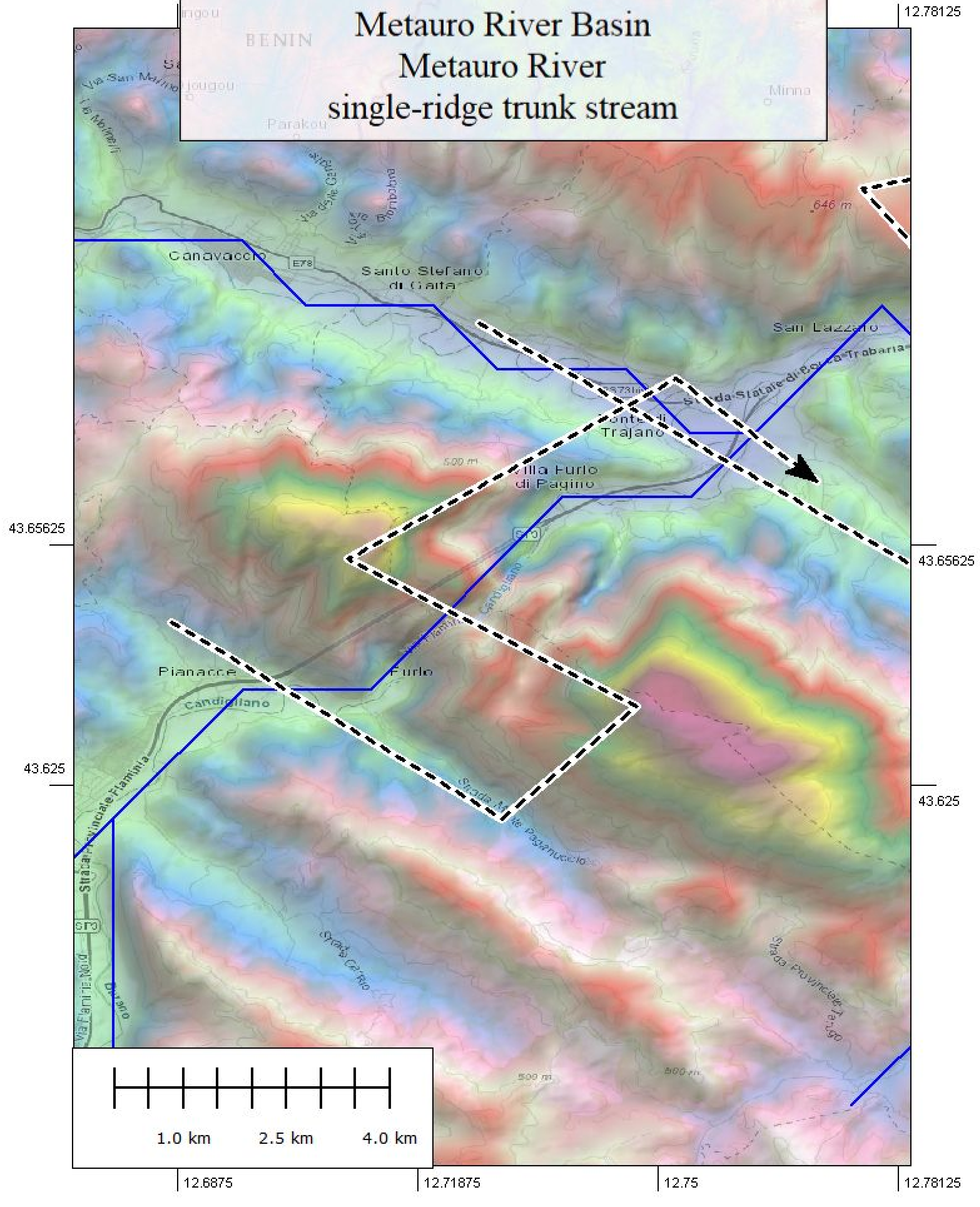

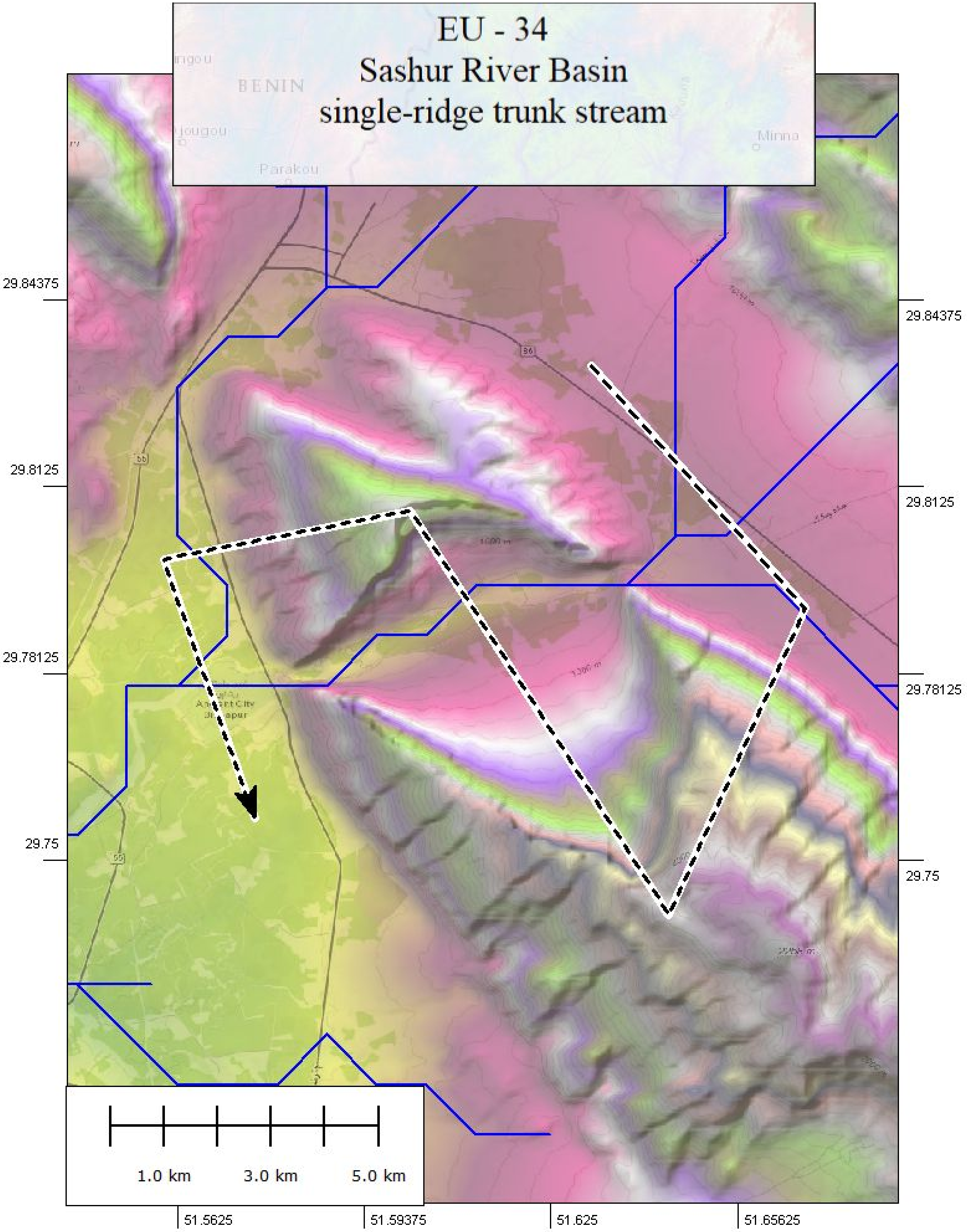
EU - 34
Sashur River Basin
single-ridge trunk stream
BENIN
Parakou
Minna
29.84375
29.8125
29.78125
29.75
29.84375
29.8125
29.78125
29.75
1.0 km
3.0 km
5.0 km
51.5625
51.59375
51.625
51.65625

EU - 36
Metauro River Basin
Metauro River tributary
single-ridge head stream

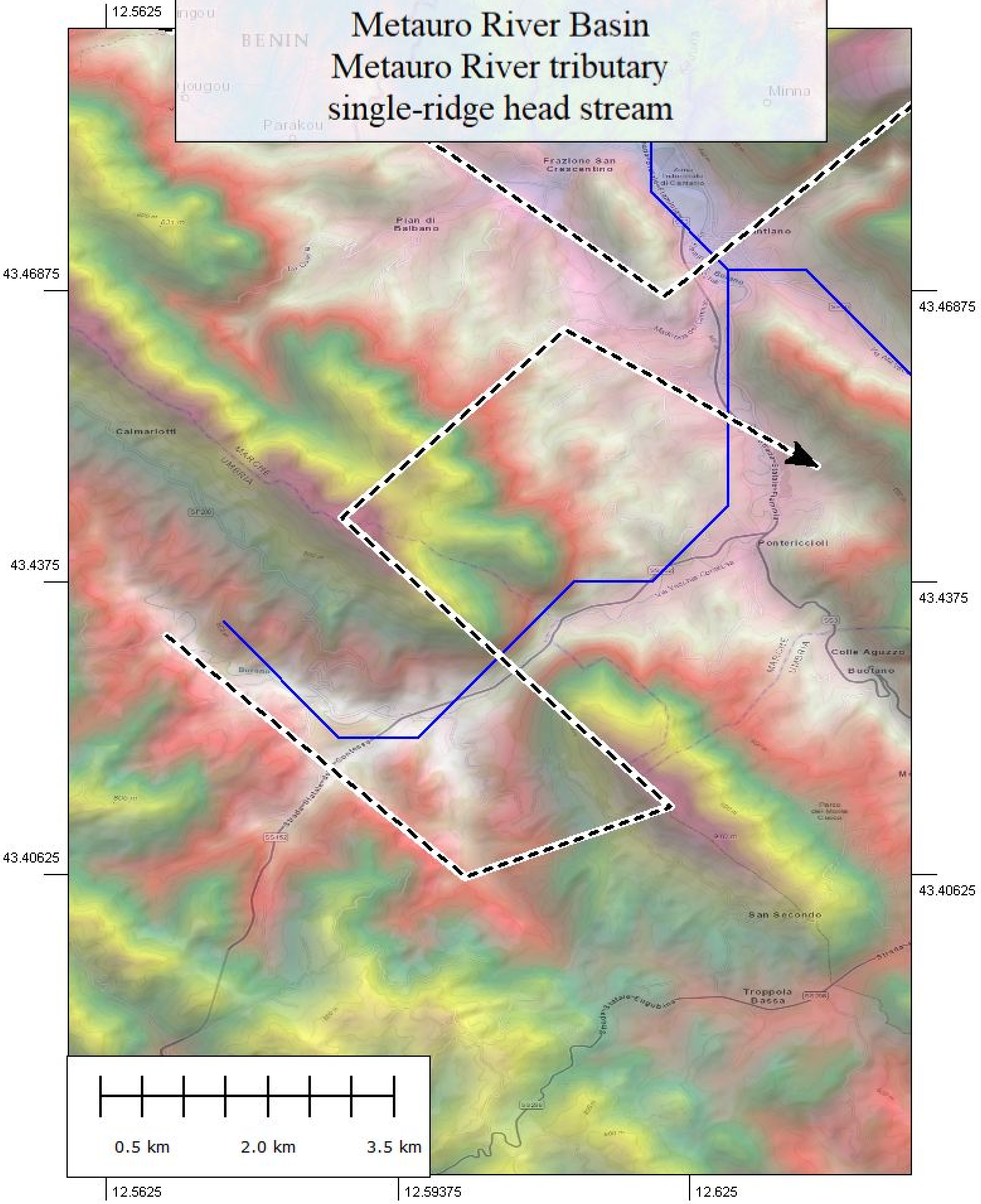

EU - 37
Musone River Basin
Musone River
single-ridge head stream

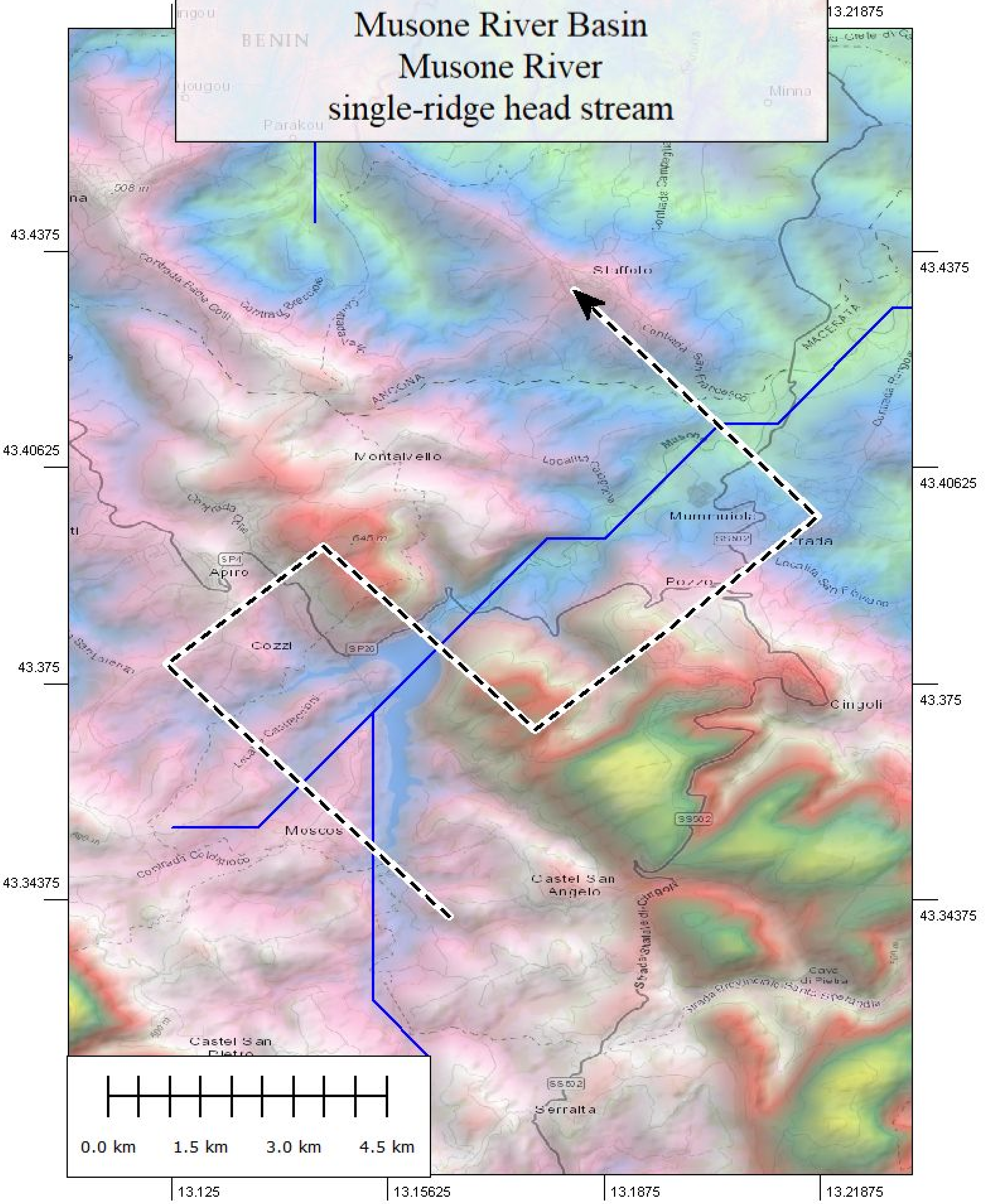

EU - 38
Metauro River Basin
Metauro River tributary single-ridge head stream

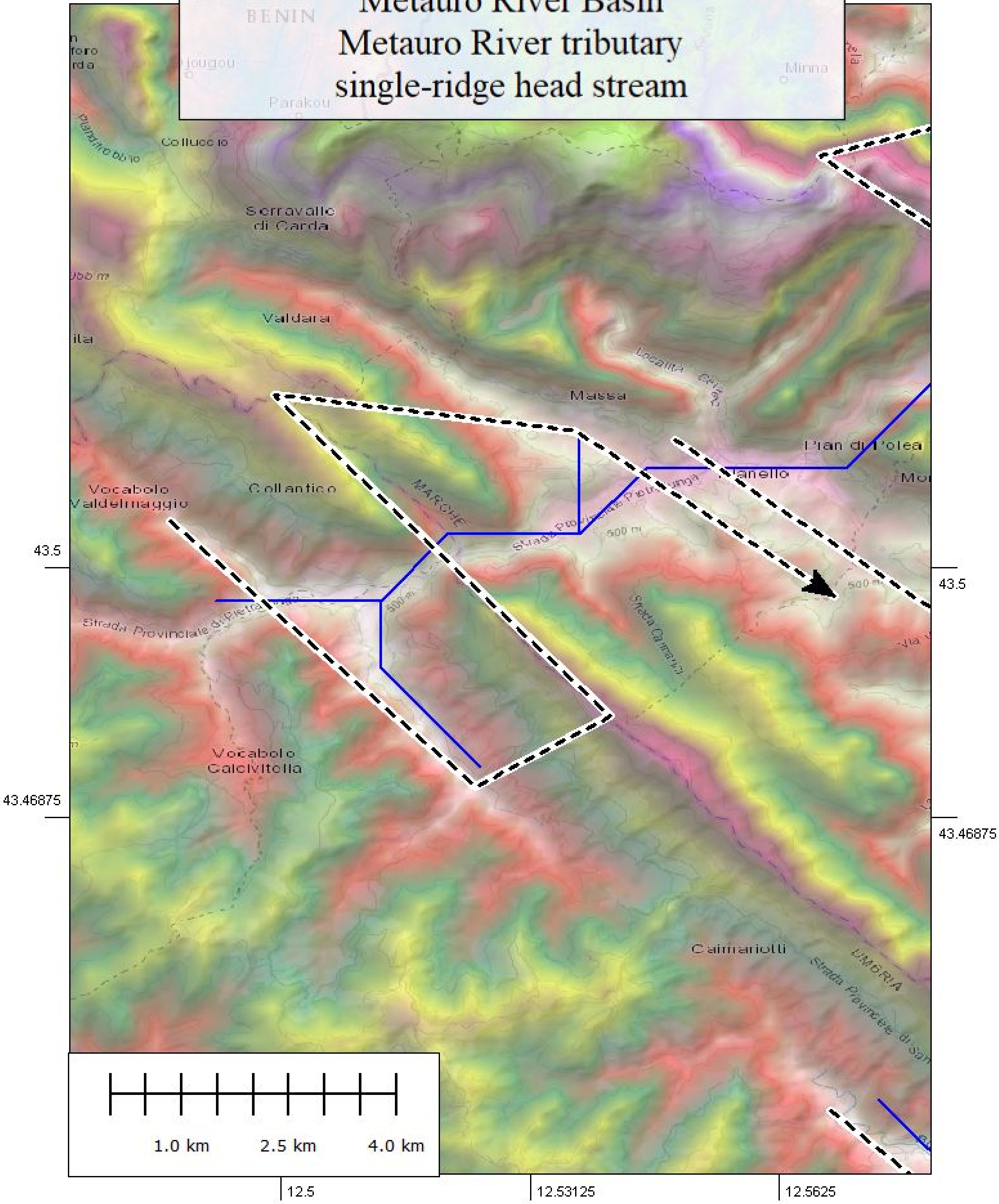

EU - 40
Pechora River Basin
Shchuger River
single-ridge trunk stream

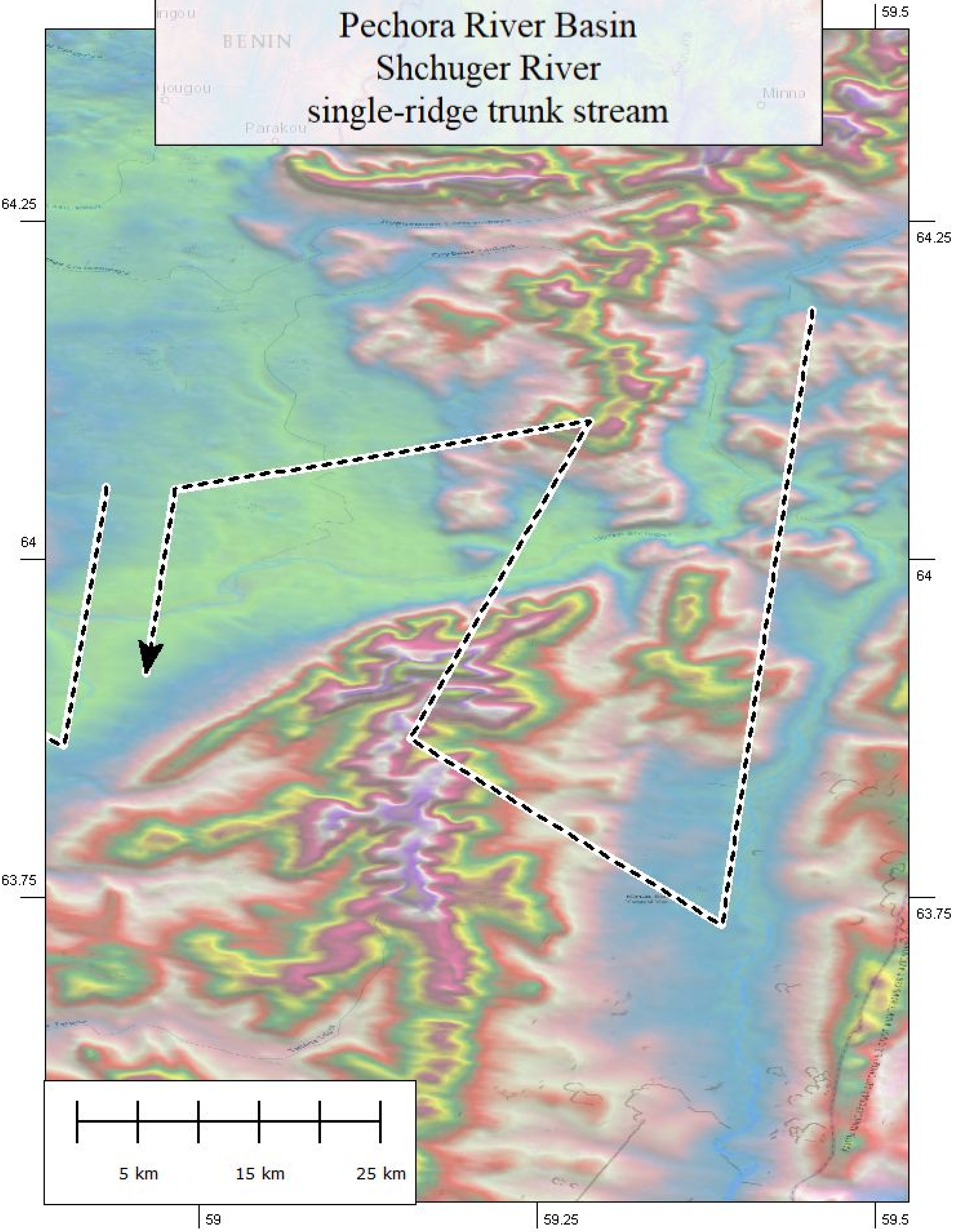

EU - 41
Pechora River Basin
Shchuger River
single-ridge trunk stream

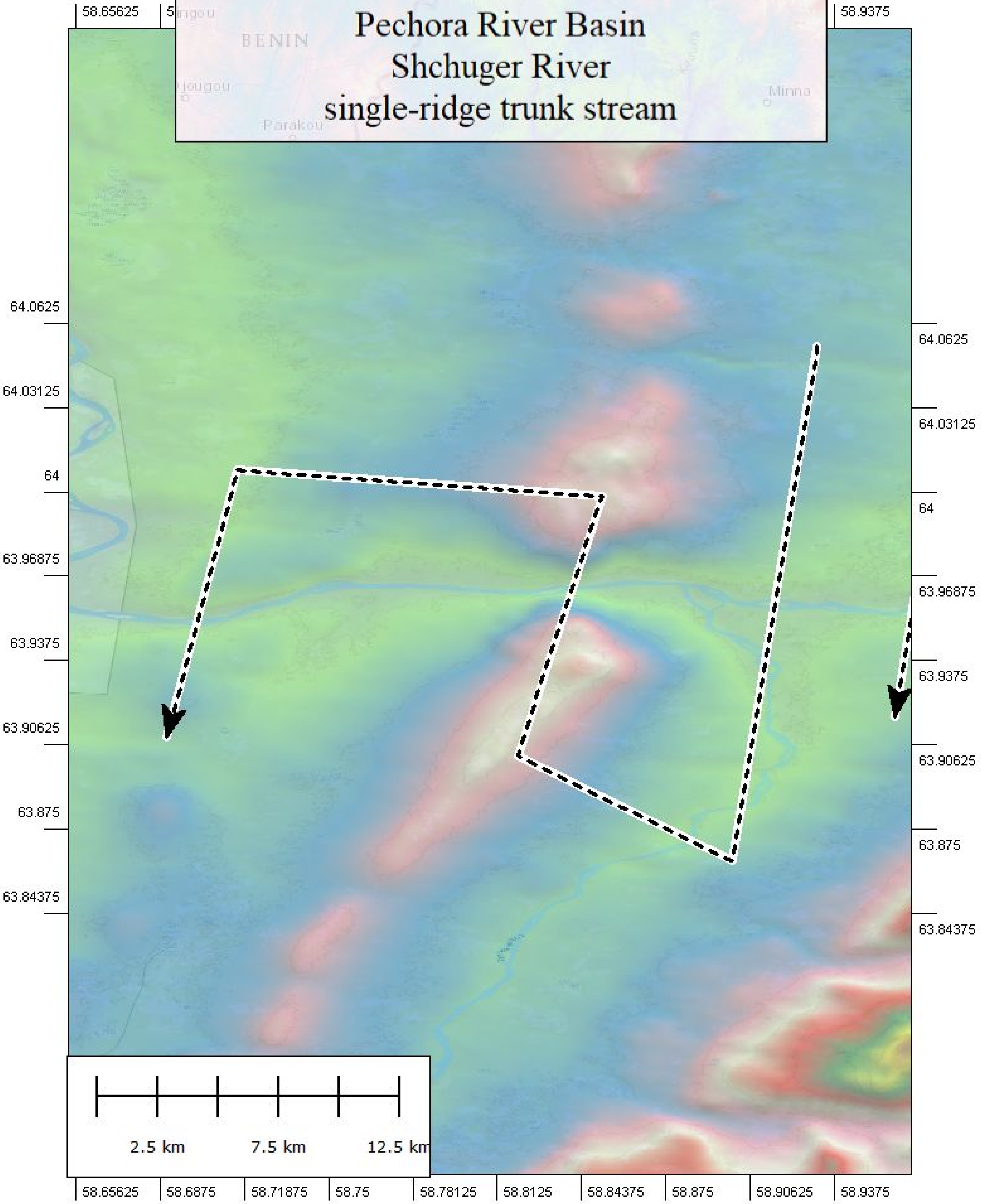

EU - 42
Sakarya River Basin
Sakarya River
single-ridge trunk stream

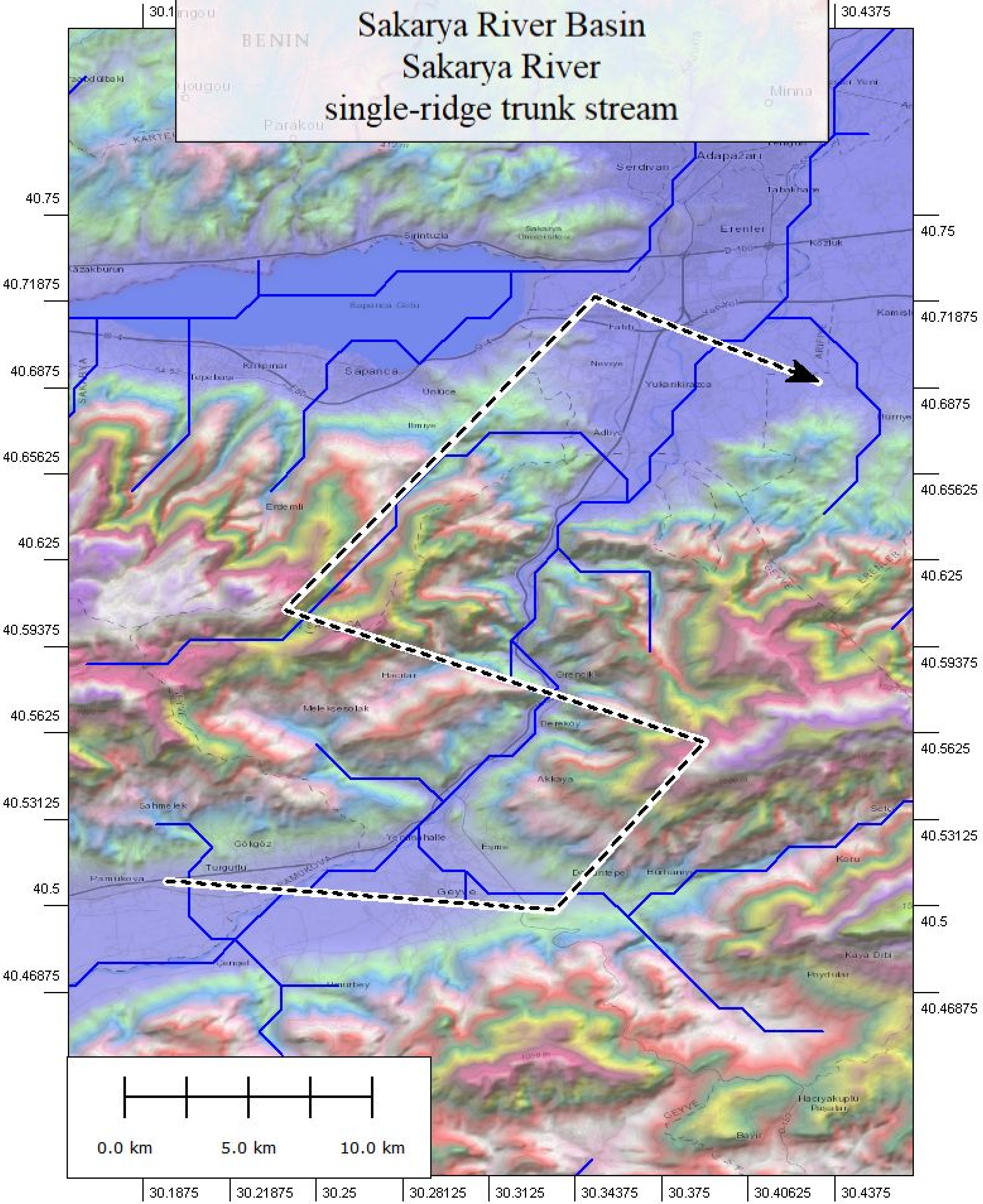

EU - 43
Rio Besaya Basin
Besaya River
single-ridge trunk stream

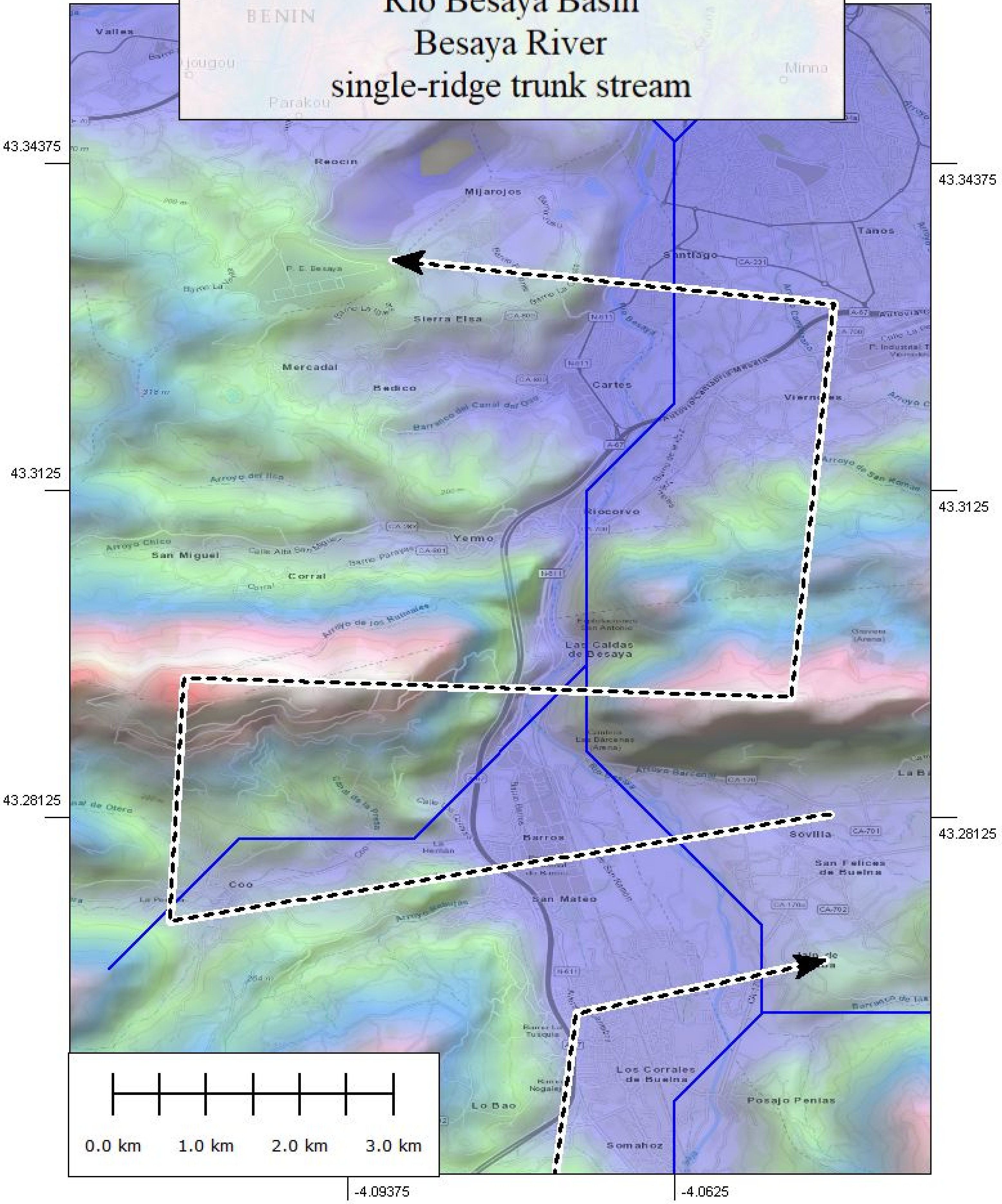

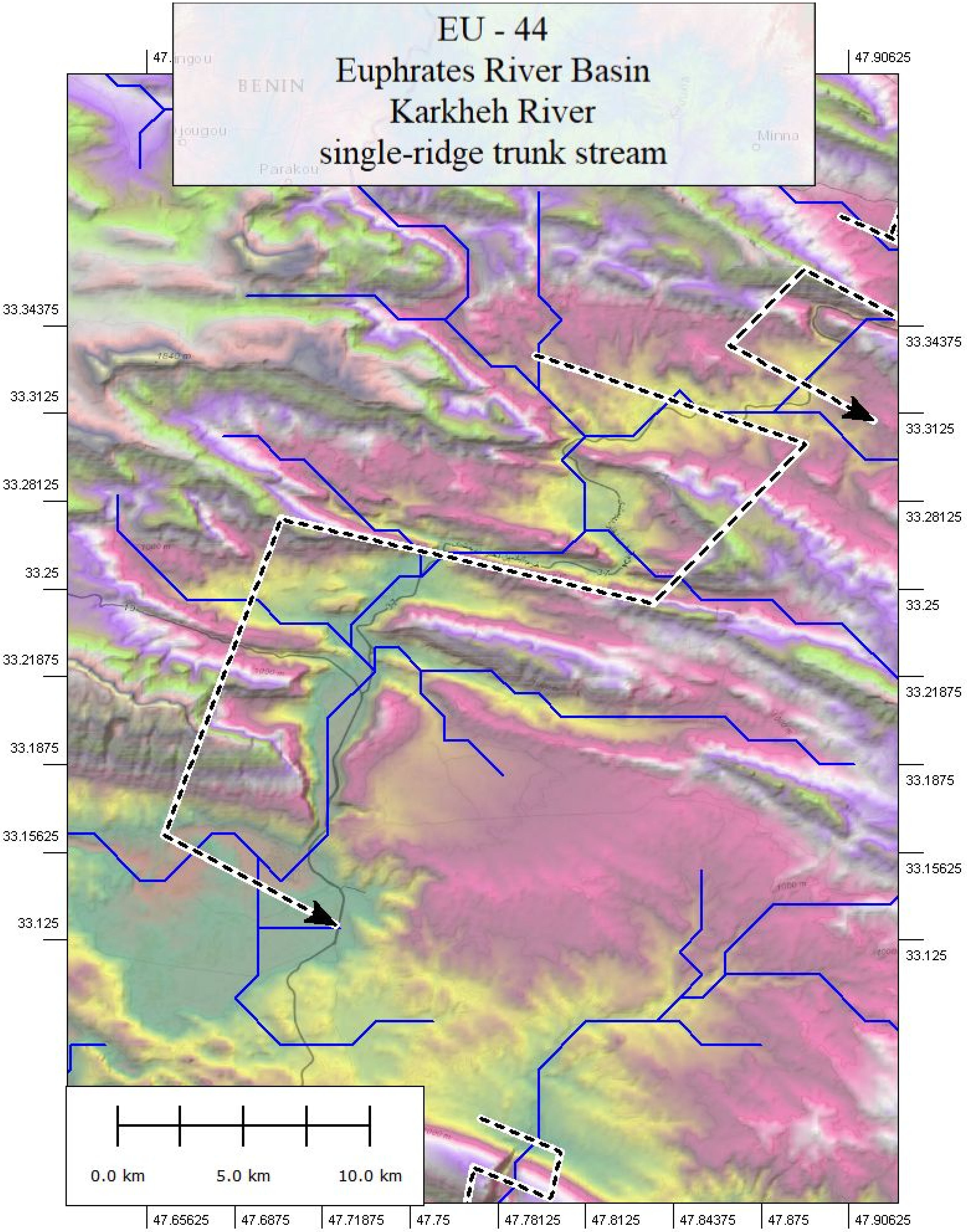
EU - 44
Euphrates River Basin
Karkheh River
single-ridge trunk stream
47.90625
33.34375
33.3125
33.28125
33.25
33.21875
33.1875
33.15625
33.125
0.0 km
5.0 km
10.0 km
47.65625
47.6875
47.71875
47.75
47.78125
47.8125
47.84375
47.875
47.90625
BENIN
Djougou
Parakou
Minna

EU - 45
Mand River Basin
Mand River
multi-ridge trunk stream

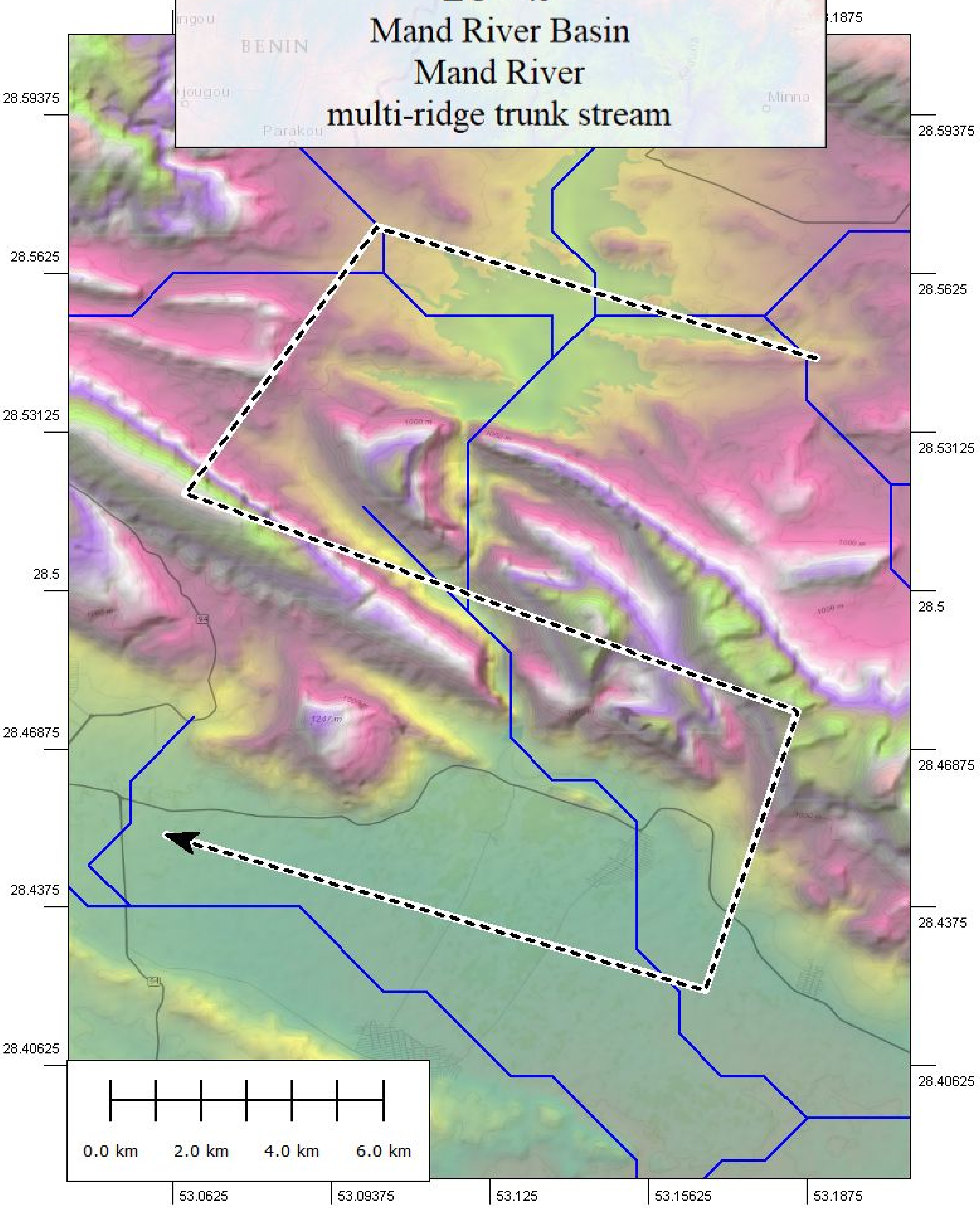

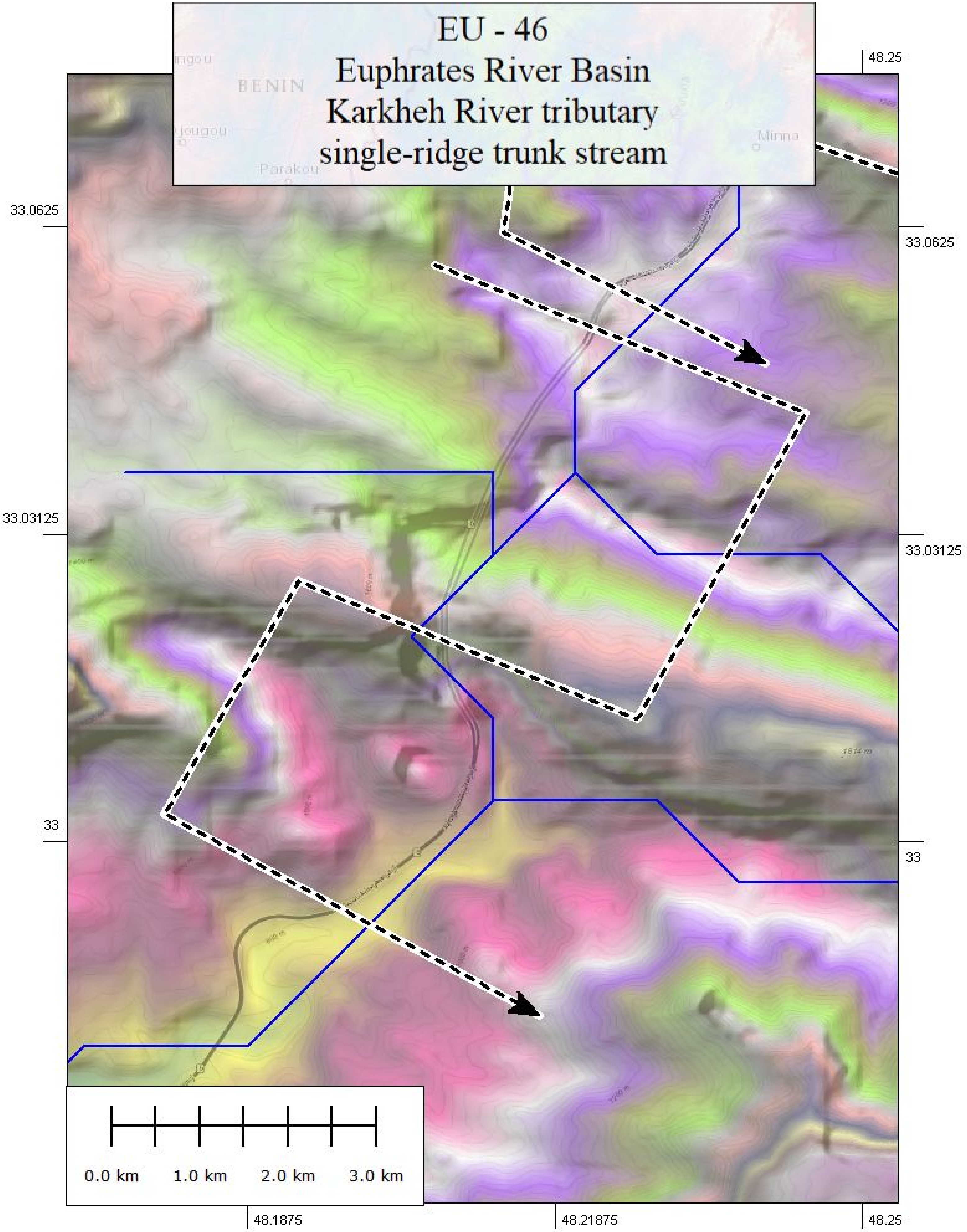

EU - 46
Euphrates River Basin
Karkheh River tributary
single-ridge trunk stream
48.25
33.0625
33.0625
33.03125
33.03125
33
33
0.0 km
1.0 km
2.0 km
3.0 km
48.1875
48.21875
48.25

EU - 52
Euphrates River Basin
Hezil River
single-ridge trunk stream

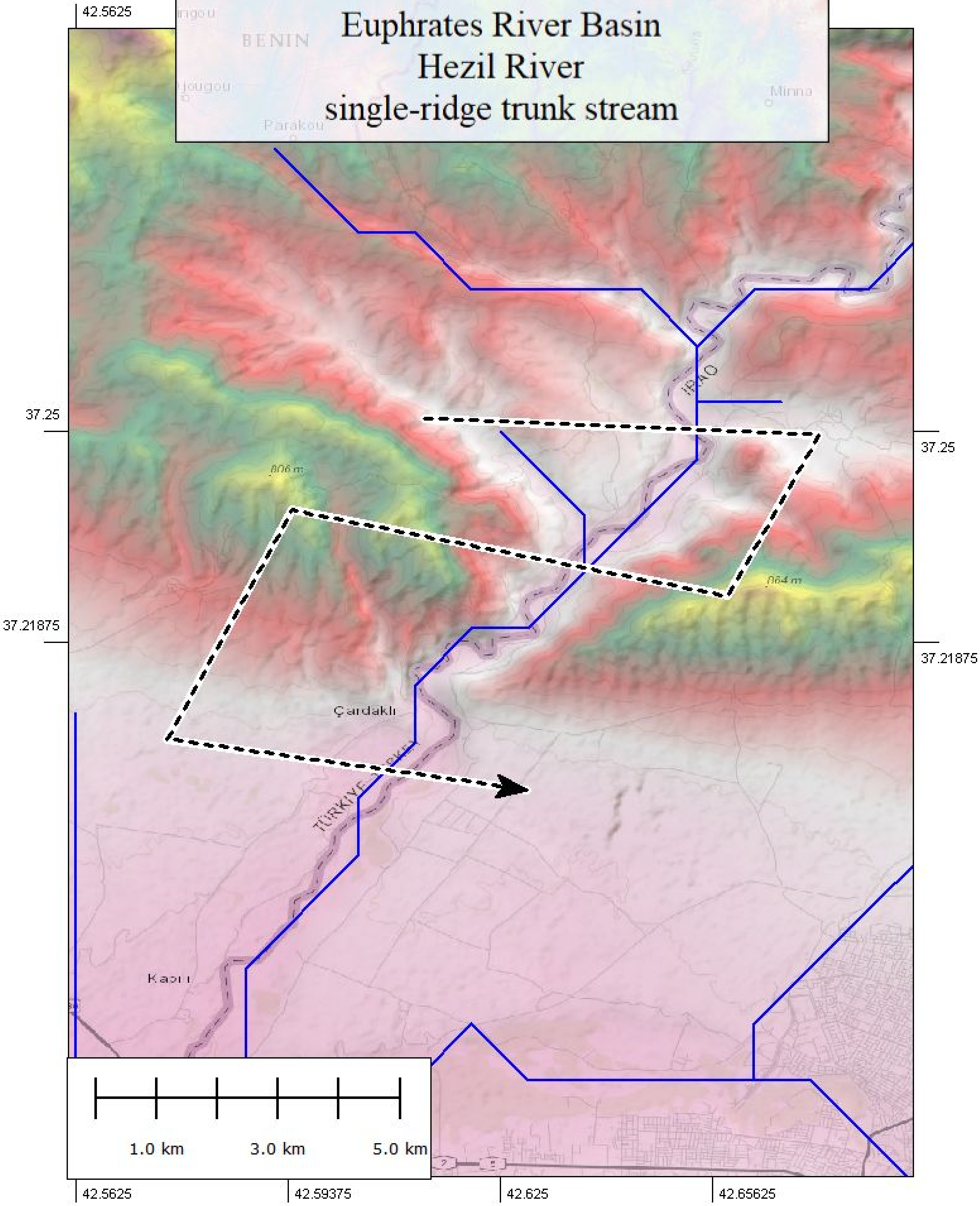

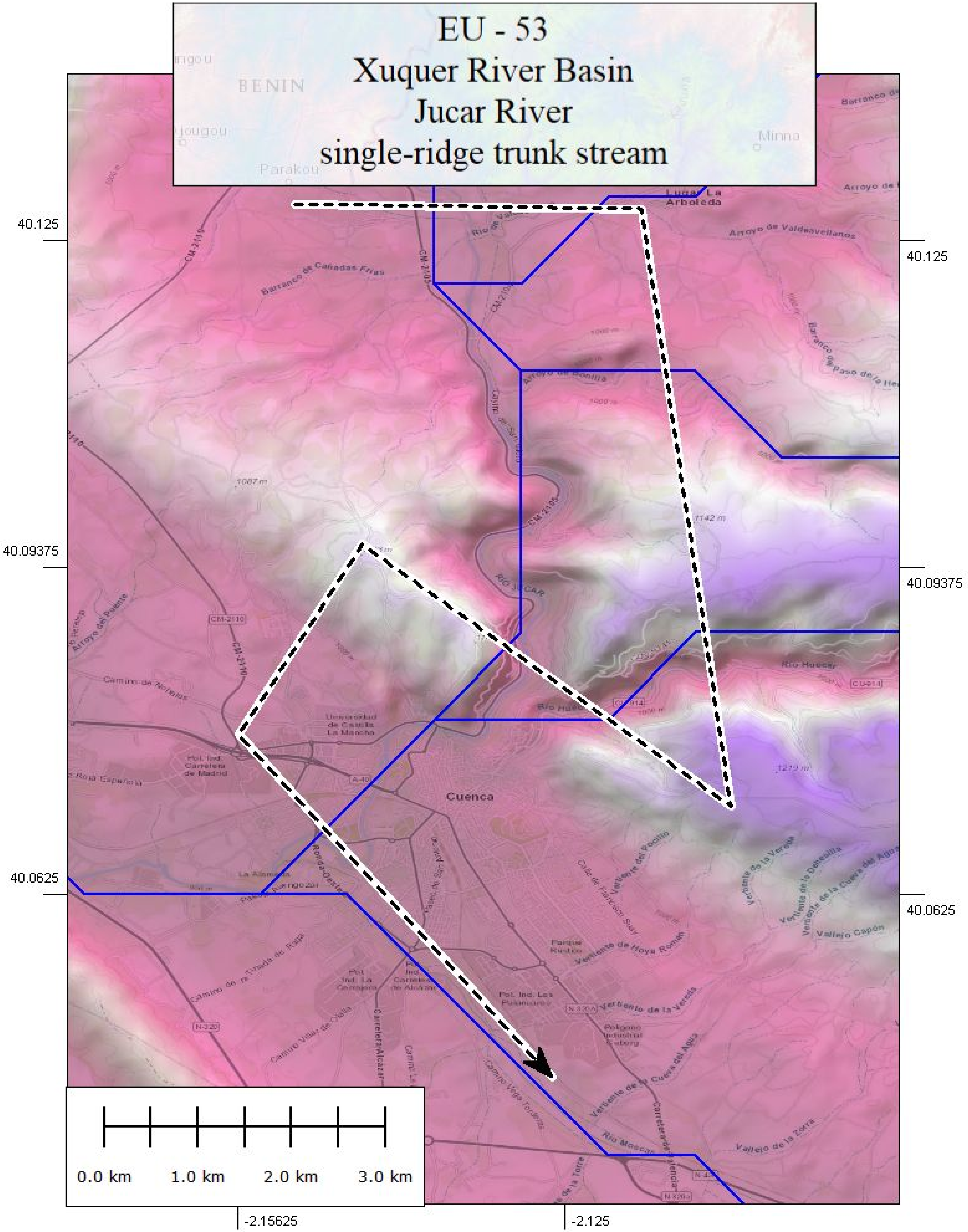
EU - 53
Xuquer River Basin
Jucar River
single-ridge trunk stream
40.125
40.09375
40.0625
-2.15625
-2.125
0.0 km
1.0 km
2.0 km
3.0 km
Cuenca
Rio Jucar
Rio Huecar
Rio Moscas
Universidad de Castilla La Mancha
Arroyo de Valdeavellanos
Barranco de Cañadas Frías
Arroyo de Bonilla
1142 m
1219 m
1087 m

EU - 58
Oder River Basin
Nysa Klodzka River
single-ridge trunk stream

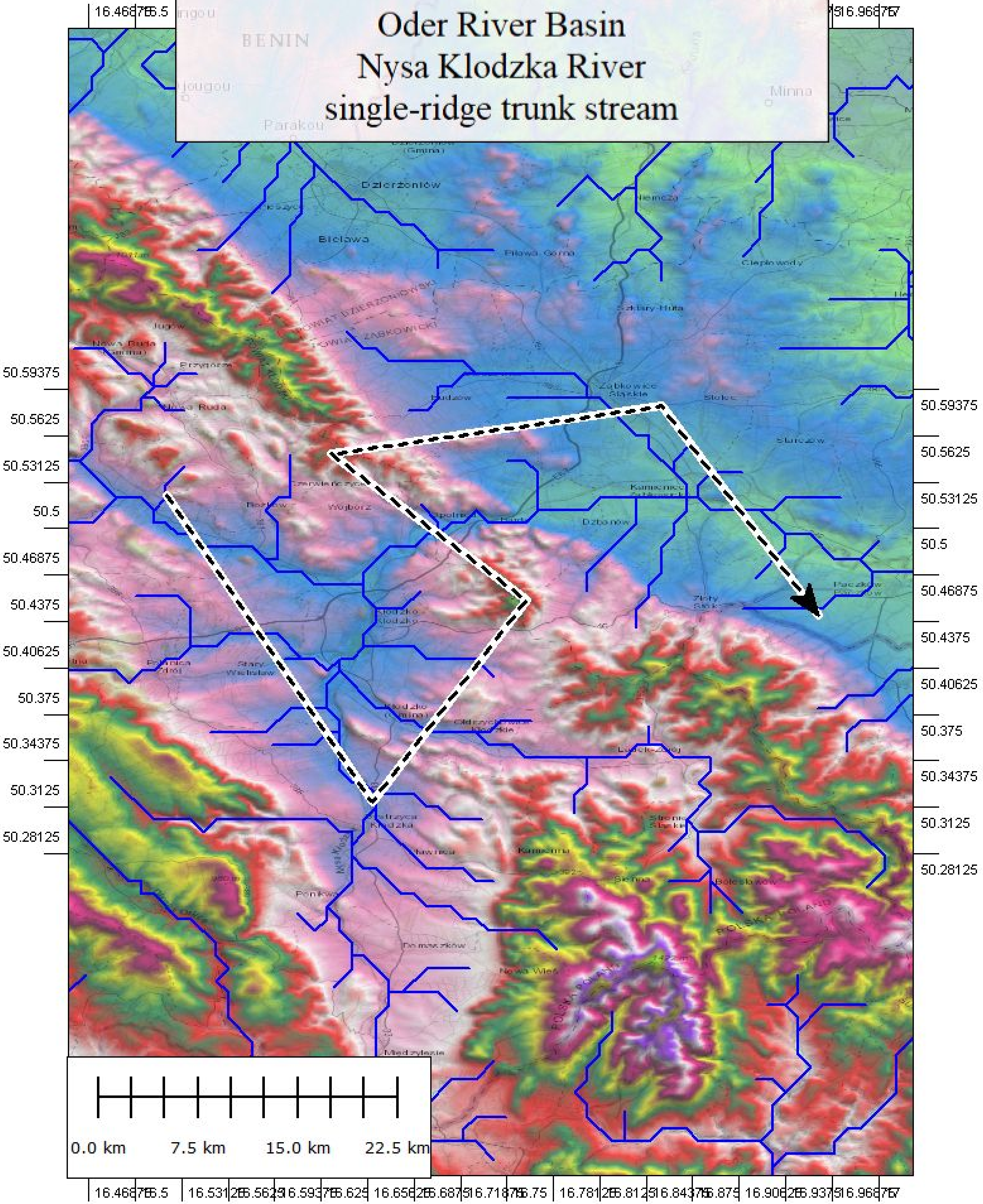

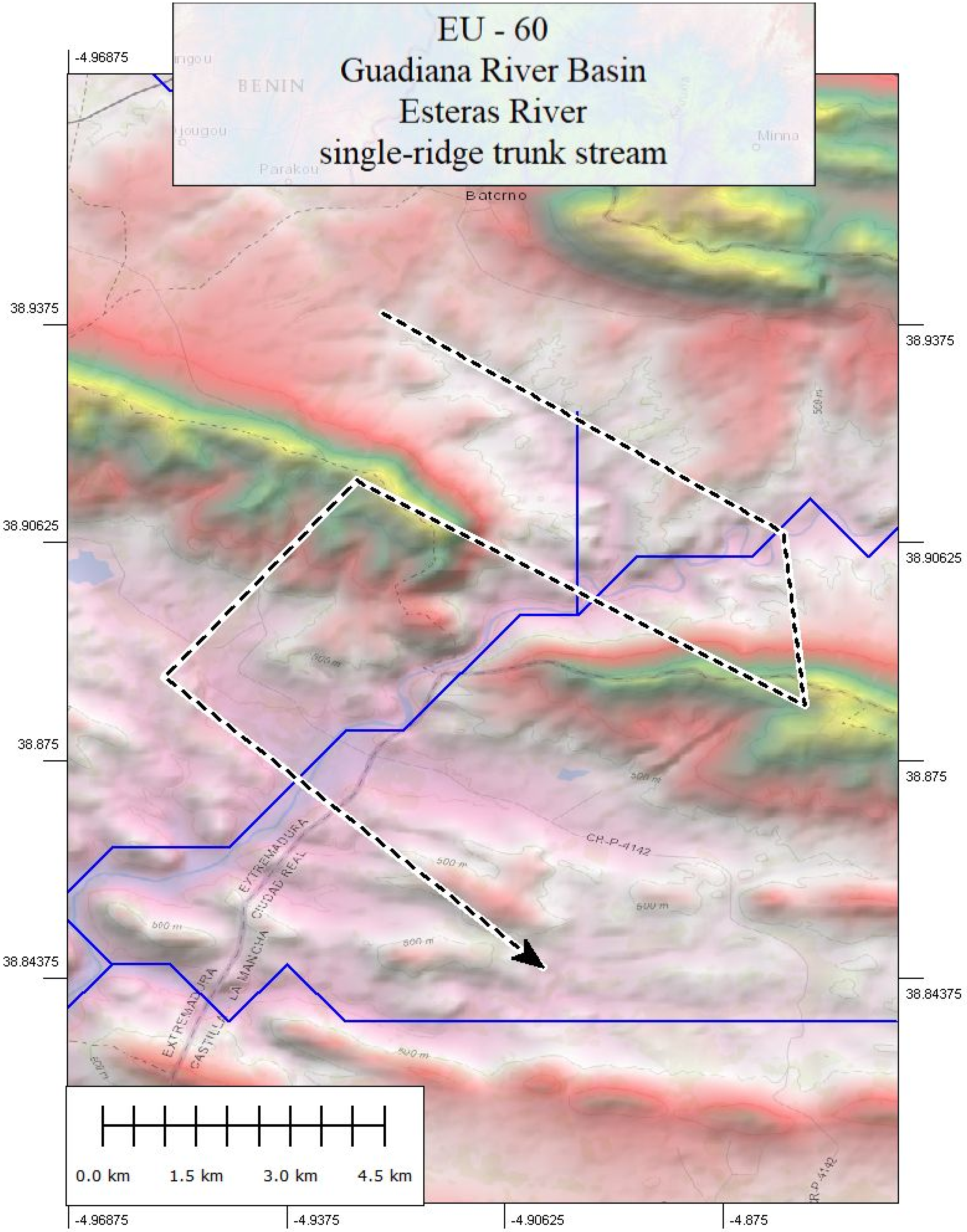
EU - 60
Guadiana River Basin
Esteras River
single-ridge trunk stream
-4.96875
-4.9375
-4.90625
-4.875
38.9375
38.90625
38.875
38.84375
BENIN
Parakou
Batcrno
500 m
CR-P-4142
EXTREMADURA
CIUDAD REAL
CASTILLA LA MANCHA
0.0 km
1.5 km
3.0 km
4.5 km

EU - 61
Ceyhan River Basin
Aksu River
single-ridge trunk stream

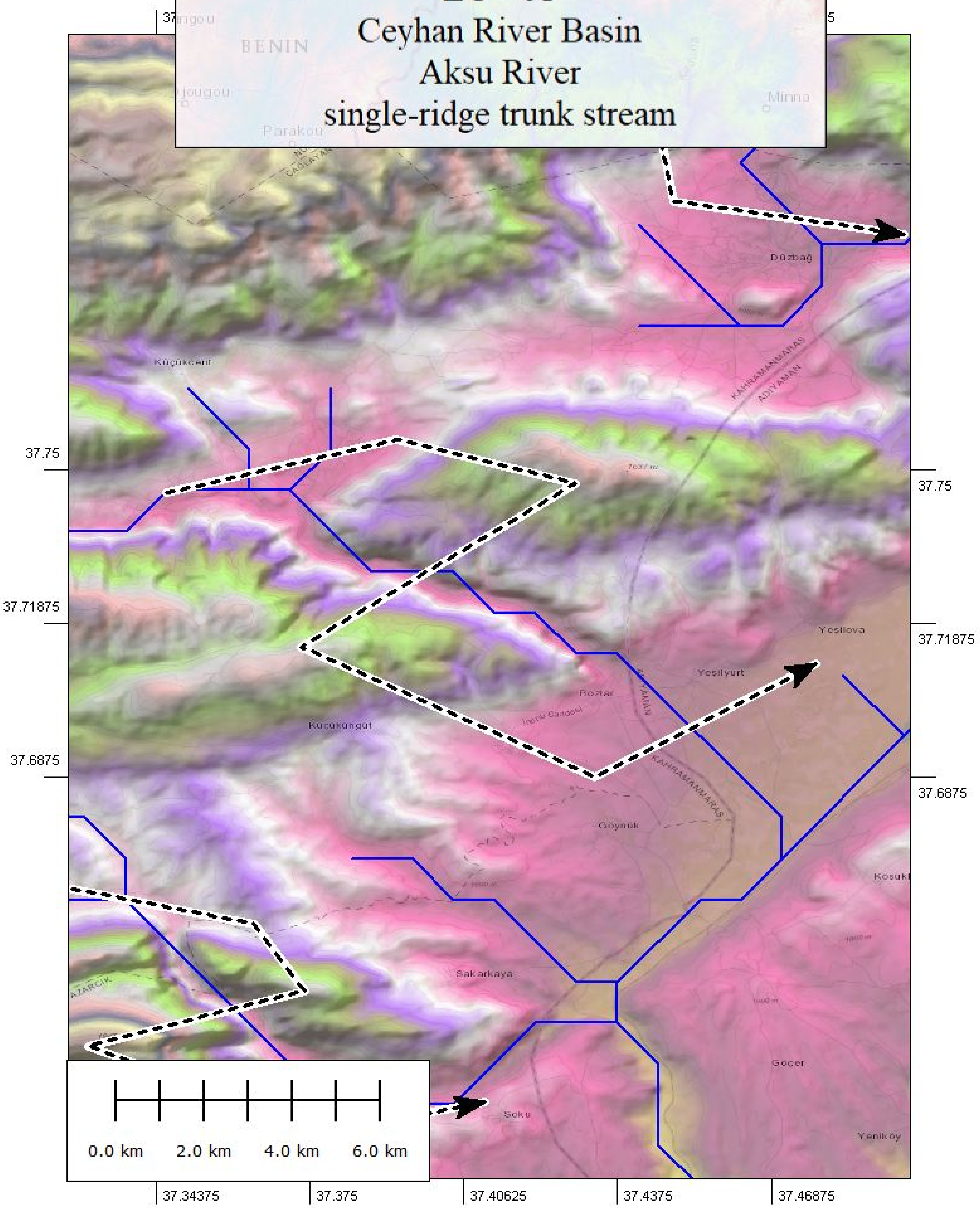

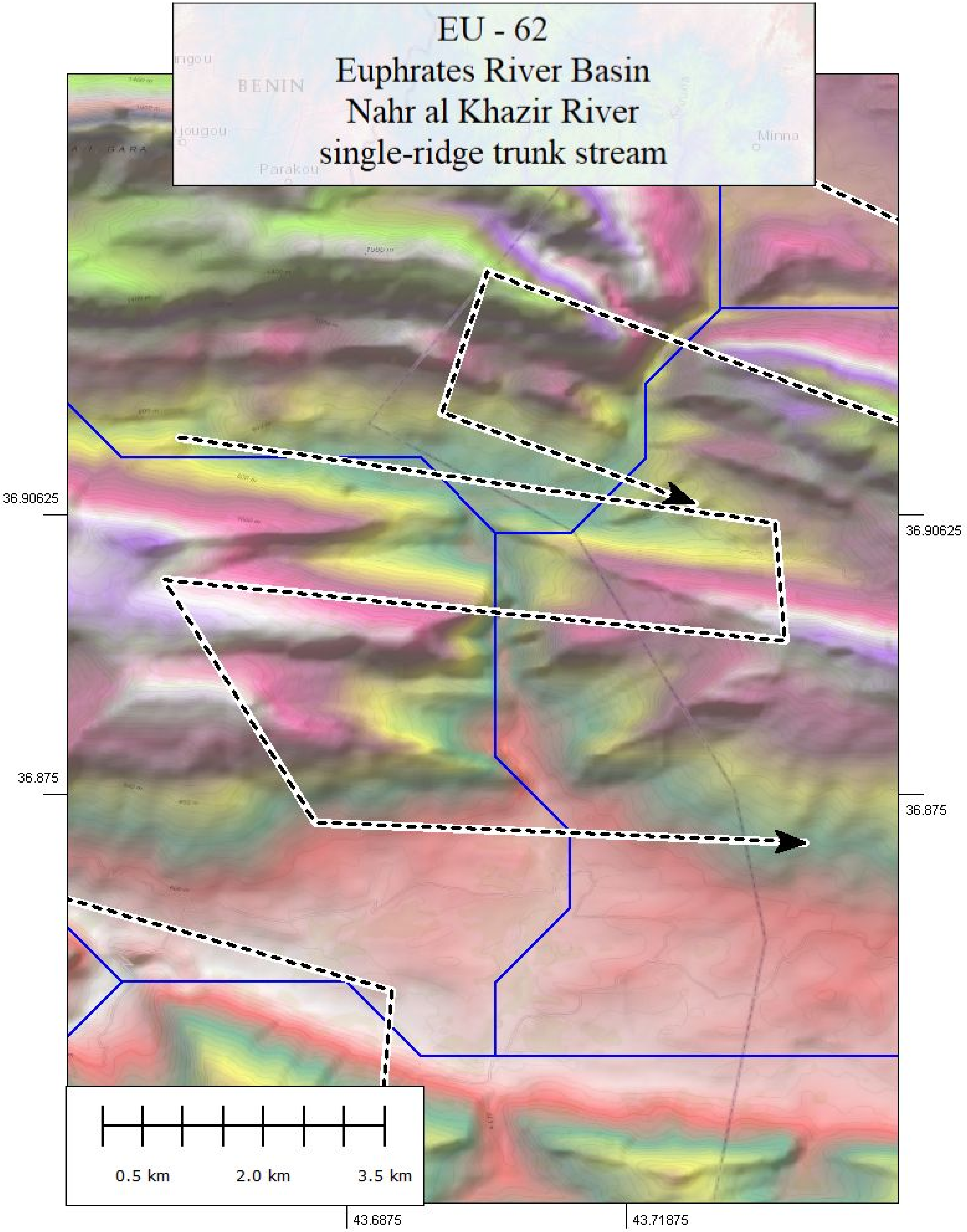

EU - 62
Euphrates River Basin
Nahr al Khazir River
single-ridge trunk stream
36.90625
36.90625
36.875
36.875
0.5 km
2.0 km
3.5 km
43.6875
43.71875

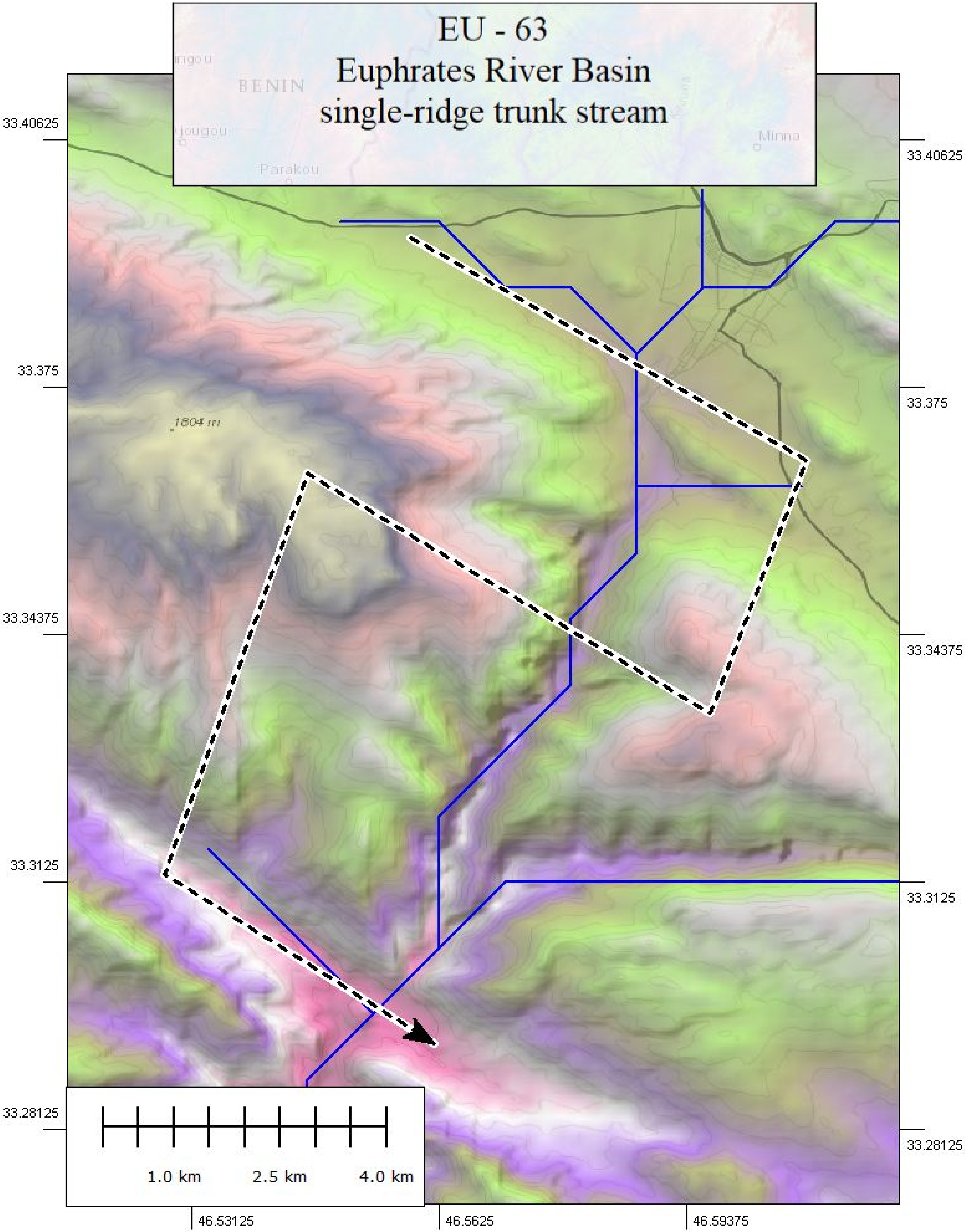
EU - 63
Euphrates River Basin
single-ridge trunk stream
BENIN
Parakou
Minna
1804 m
33.40625
33.375
33.34375
33.3125
33.28125
46.53125
46.5625
46.59375
1.0 km
2.5 km
4.0 km

EU - 64
Rio Pisuena Basin
Pisuena River
single-ridge trunk stream

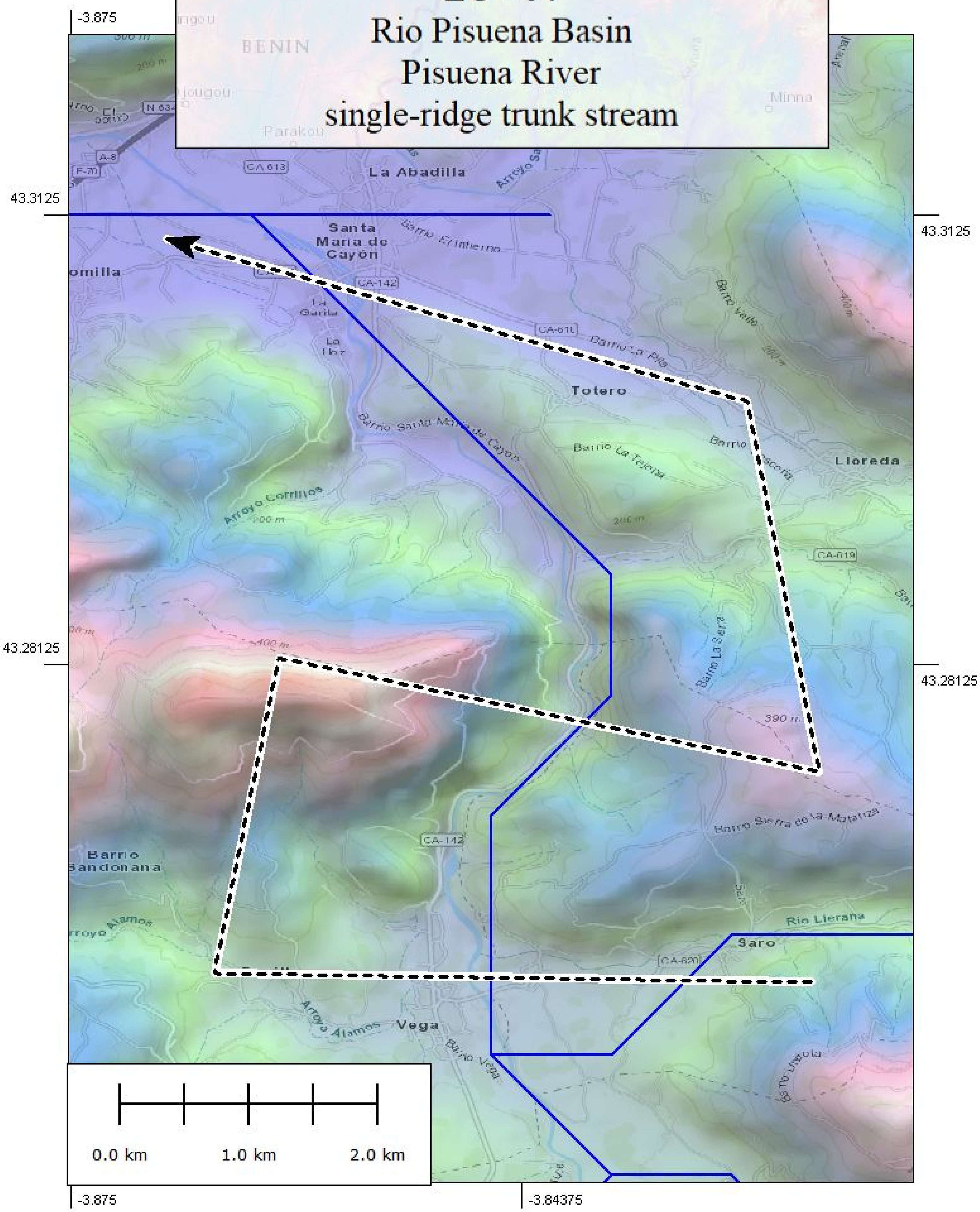

EU - 65
Tagus River Basin
Tagus River
single-ridge trunk stream

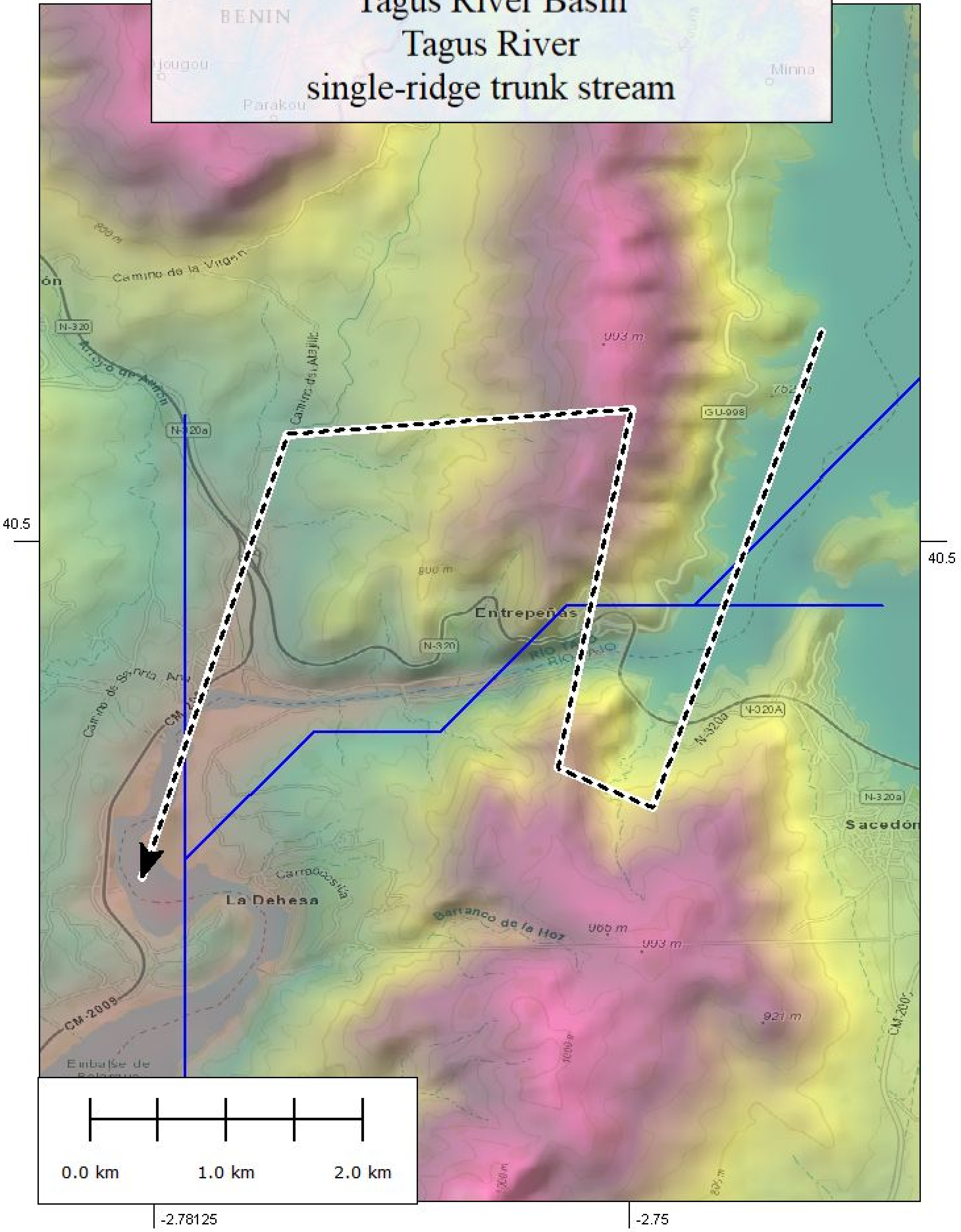

EU - 66
Euphrates River Basin
single-ridge trunk stream

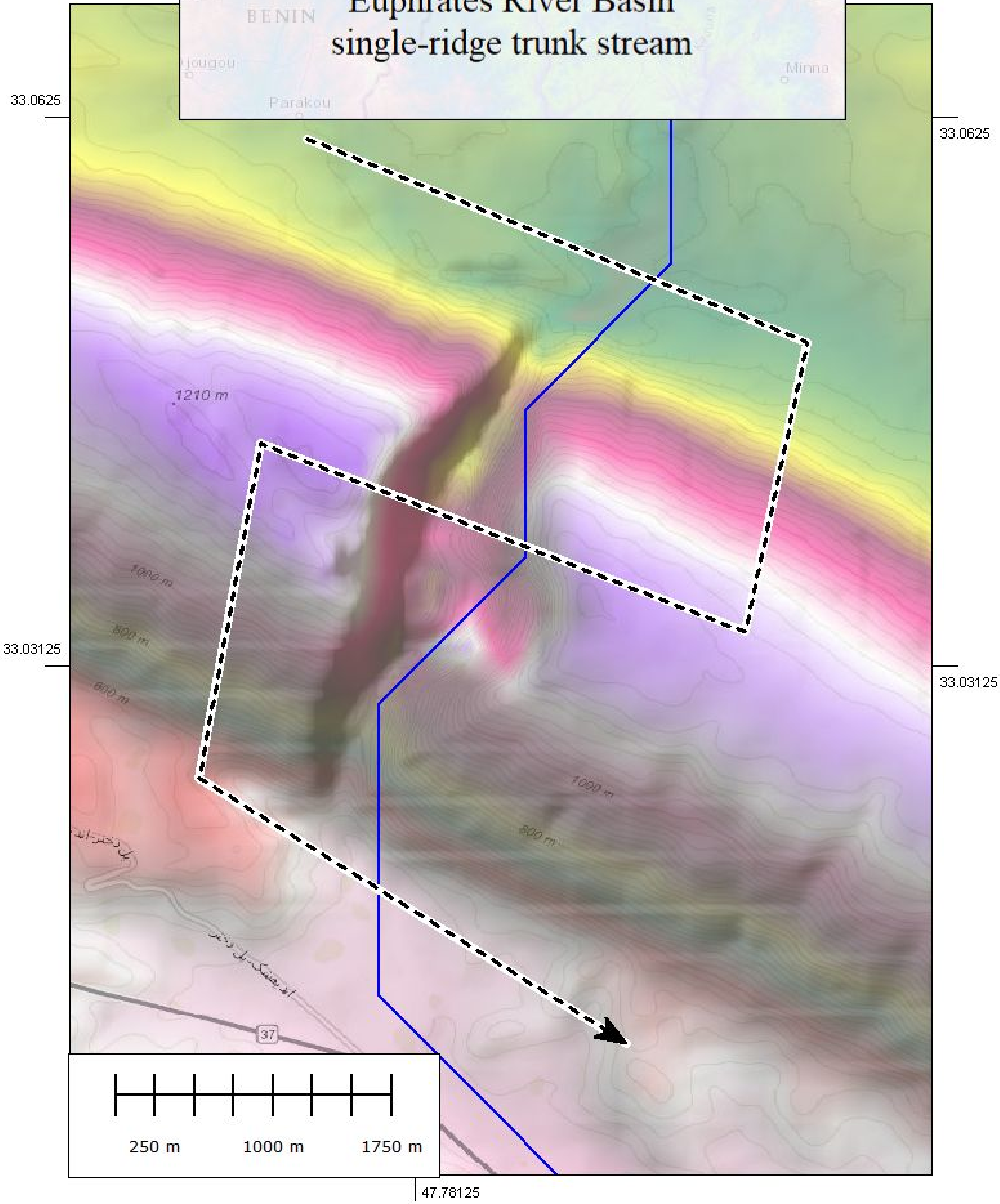

EU - 67
Dasht River Basin
Kil River
single-ridge trunk stream

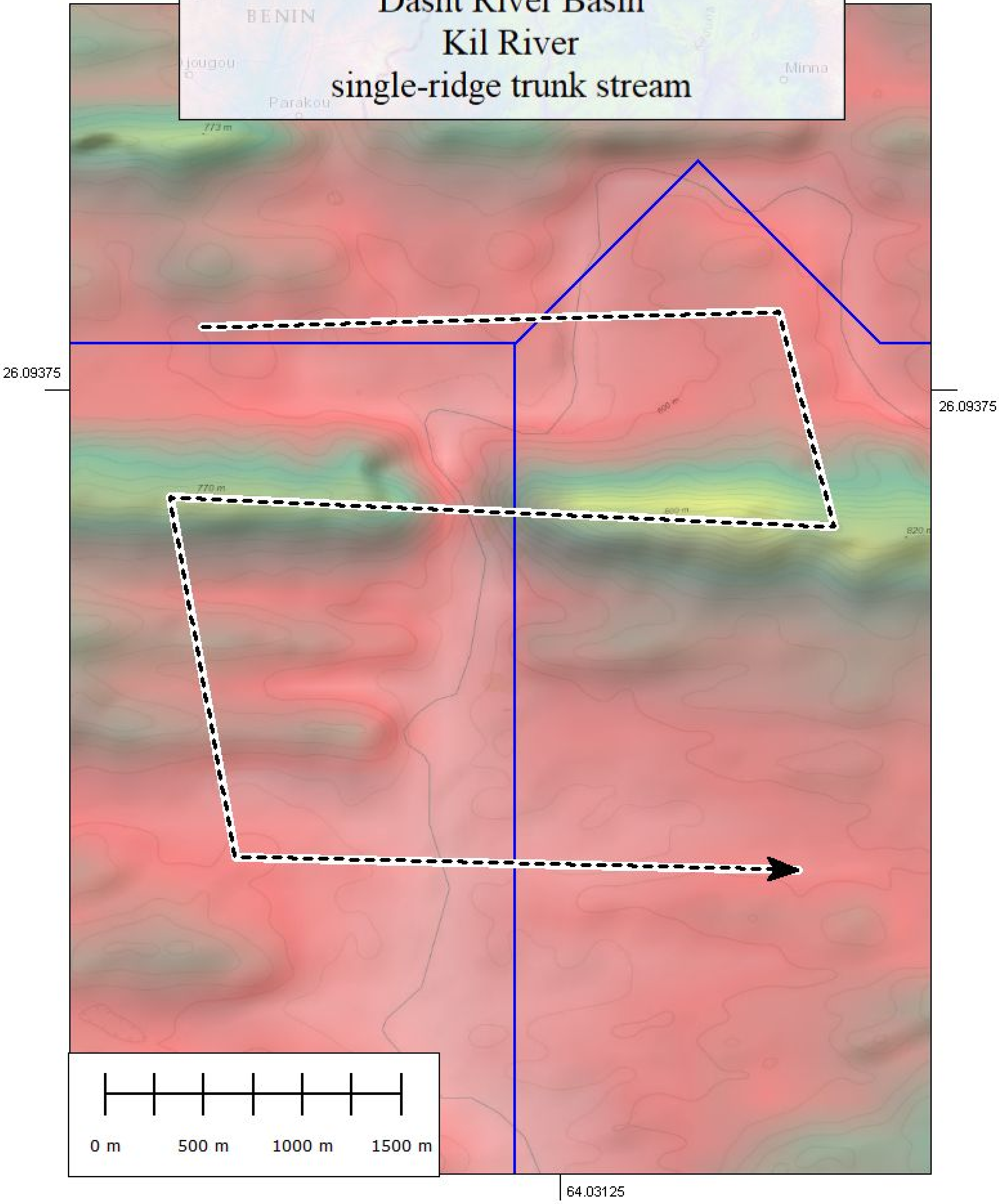

EU - 68
Ebro River Basin
Zadorra River
single-ridge trunk stream

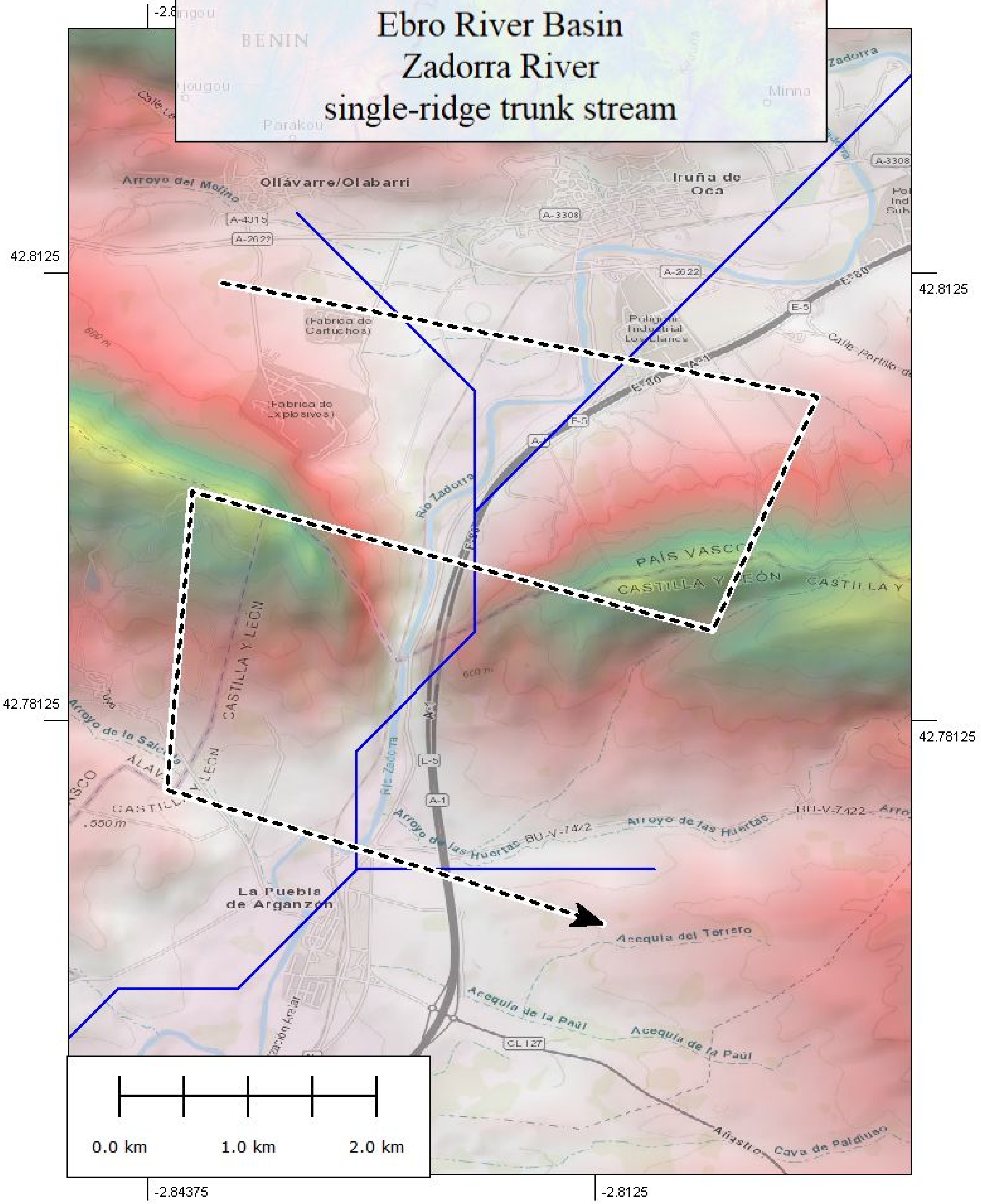

EU - 69
Weser River Basin
Weser River
single-ridge trunk stream

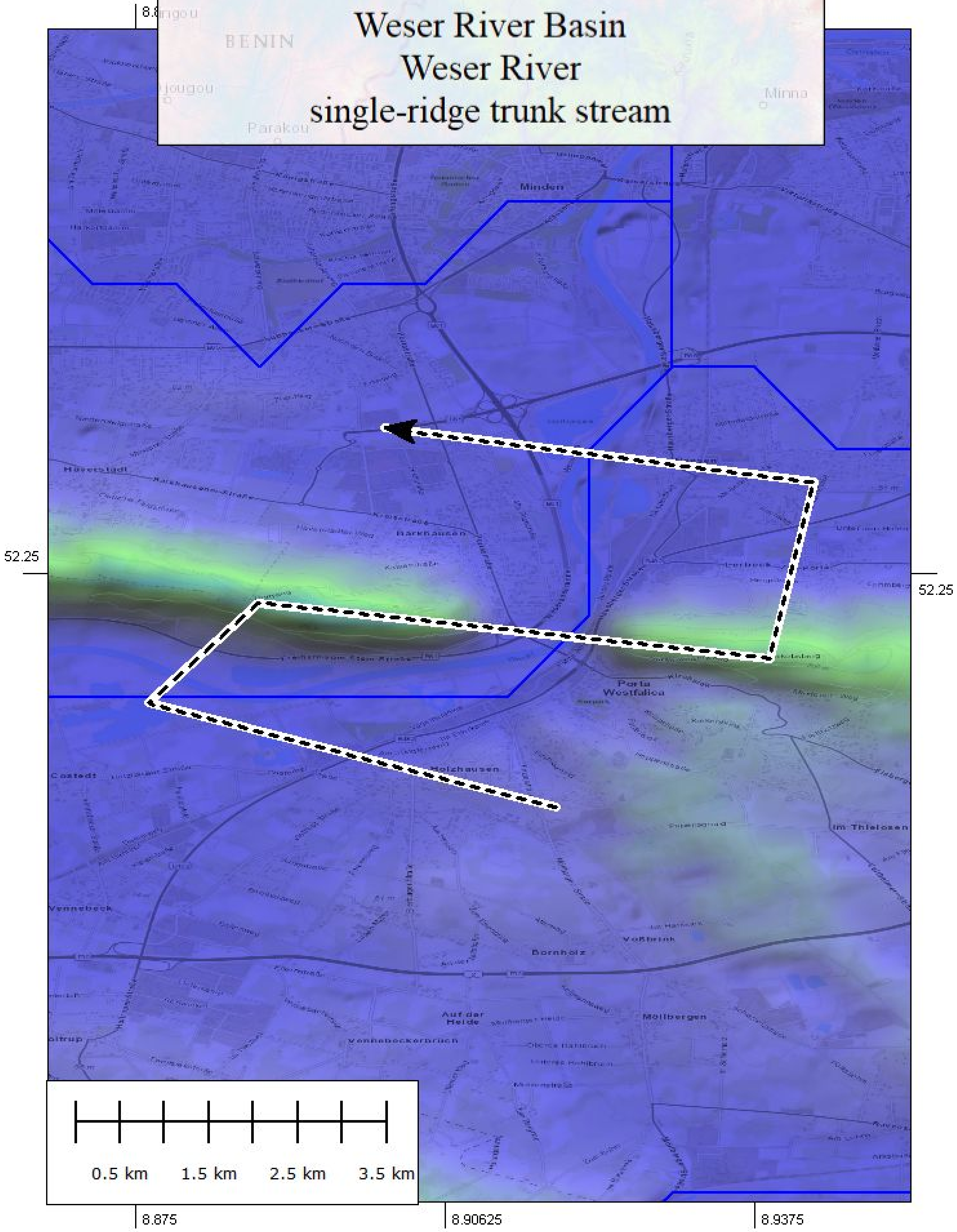

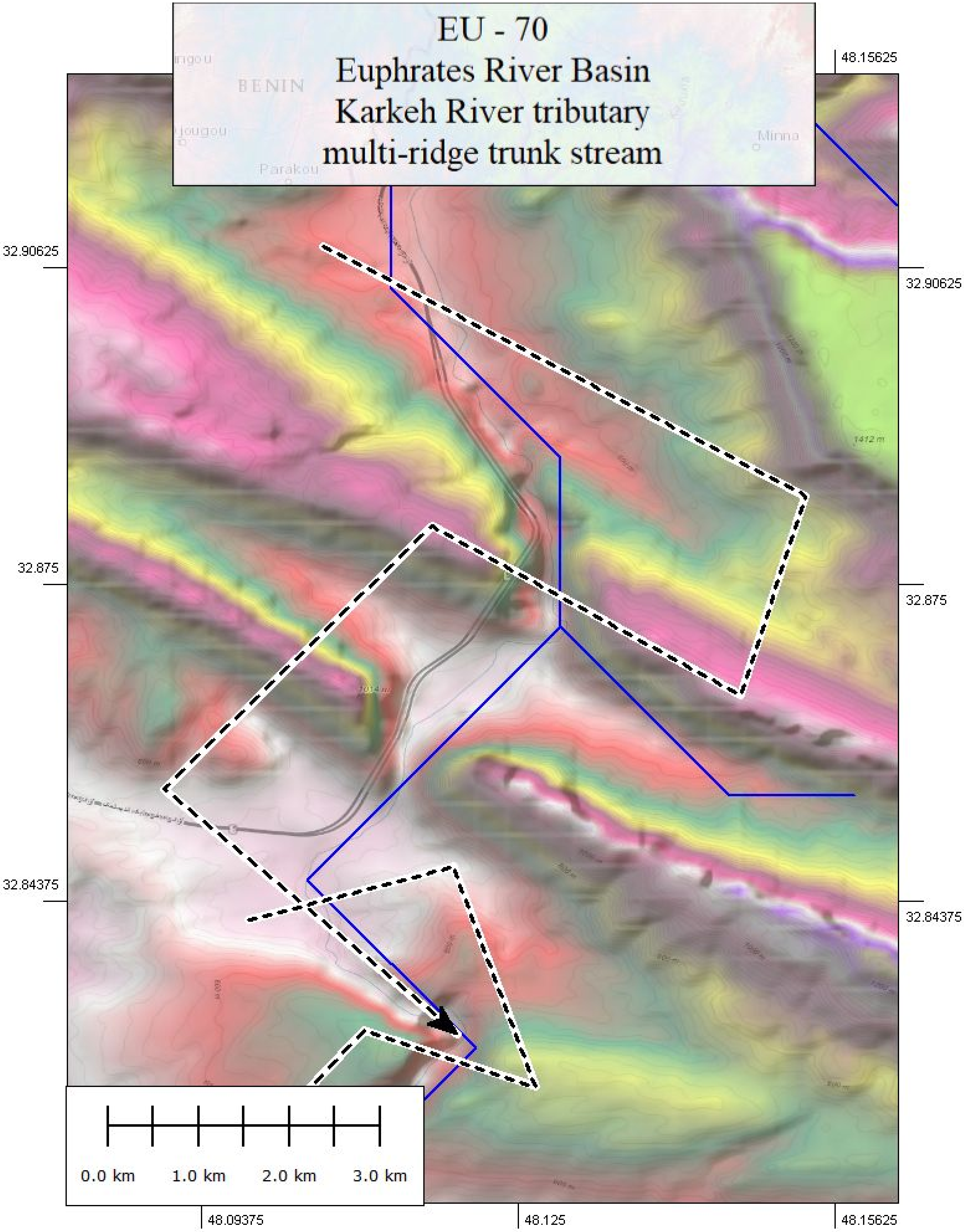
EU - 70
Euphrates River Basin
Karkeh River tributary
multi-ridge trunk stream
BENIN
Parakou
Minna
1412 m
48.15625
32.90625
32.875
32.84375
48.09375
48.125
0.0 km
1.0 km
2.0 km
3.0 km

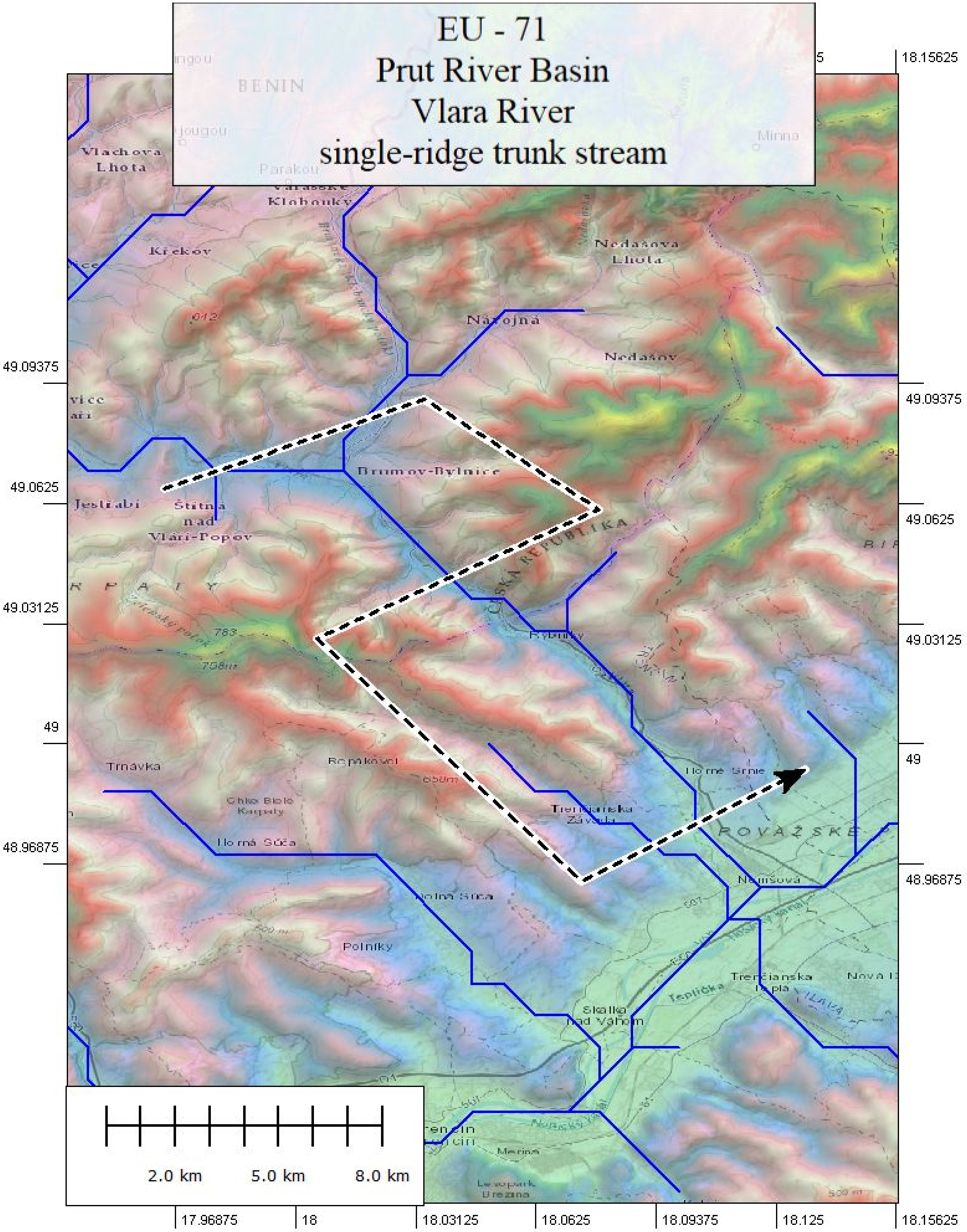
EU - 71
Prut River Basin
Vlara River
single-ridge trunk stream
18.15625
49.09375
49.0625
49.03125
49
48.96875
17.96875
18
18.03125
18.0625
18.09375
18.125
Vlachova Lhota
Křekov
Návojná
Nedašova Lhota
Nedašov
Brumov-Bylnice
Štitná nad Vláří-Popov
Jestřabí
ČESKÁ REPUBLIKA
Rybníky
Trnávka
Horné Srnie
Trenčianska Závada
Horná Súča
Nemšová
Dolná Súča
Polníky
Trenčianska Teplá
Skalka nad Váhom
Teplička
POVAŽSKÉ
2.0 km
5.0 km
8.0 km

EU - 72
Euphrates River Basin
Tigris River
single-ridge trunk stream

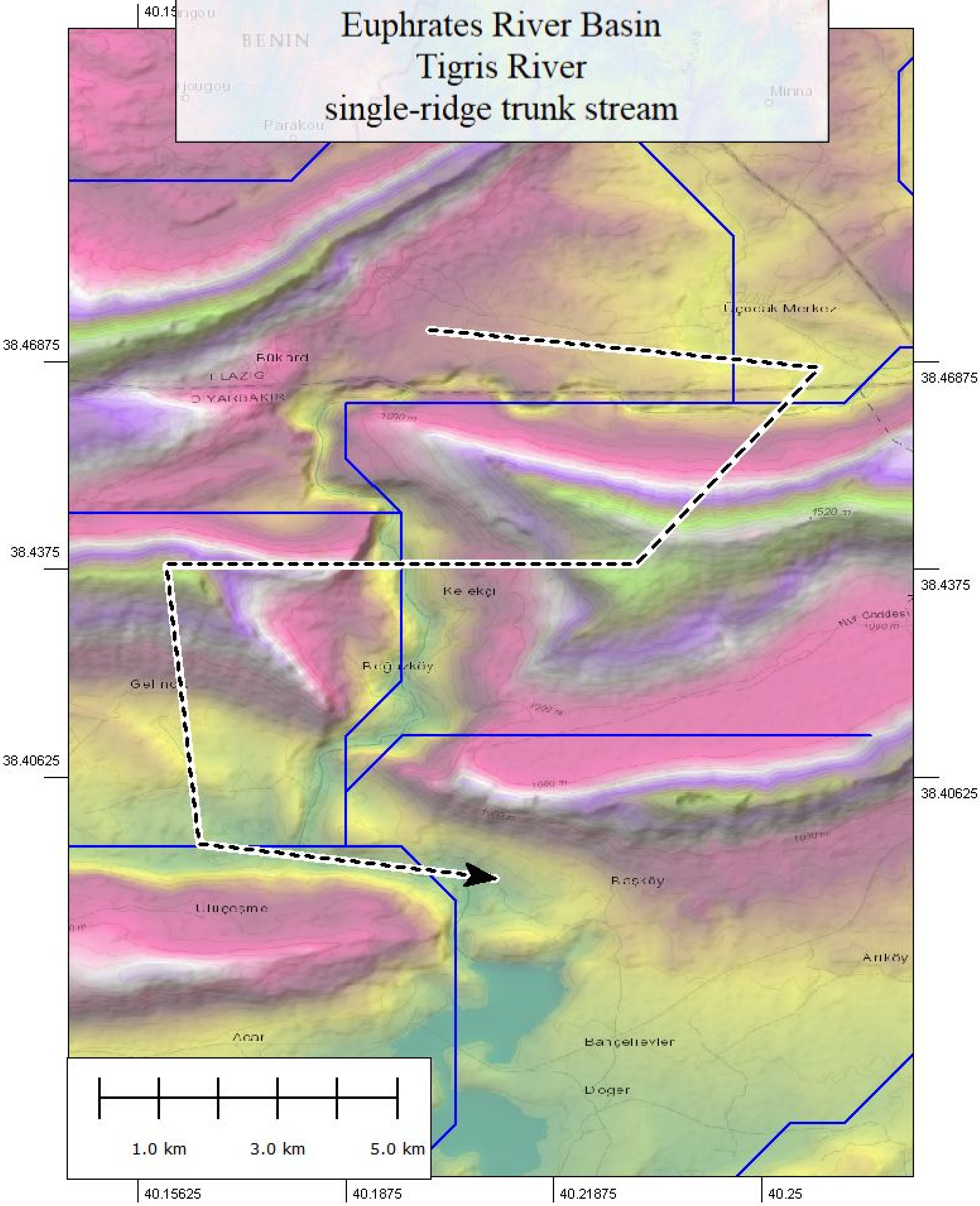

EU - 73
Euphrates River Basin
Seimare River tributary
single-ridge trunk stream

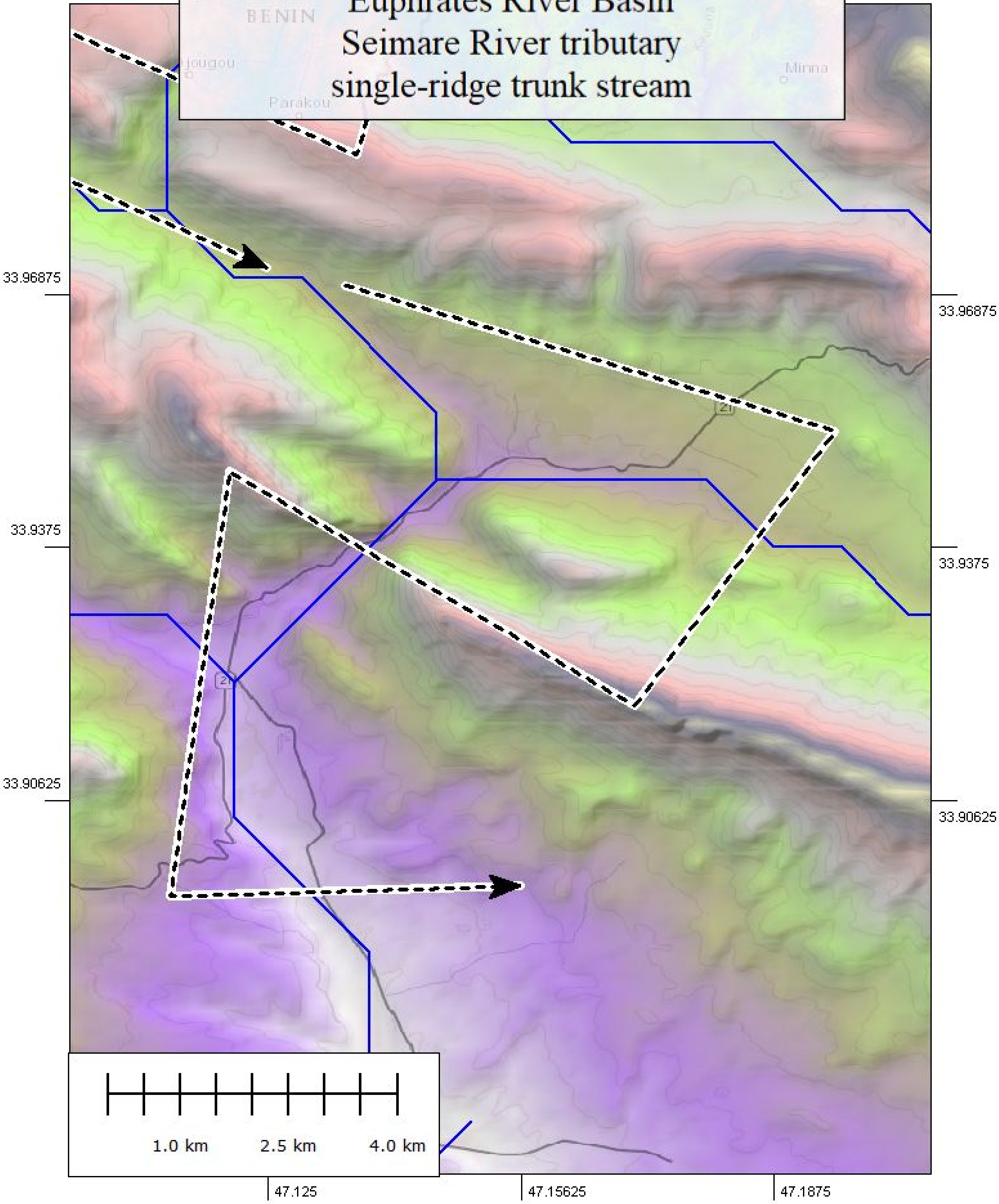

EU - 74
Euphrates River Basin
Seimare River
single-ridge trunk stream

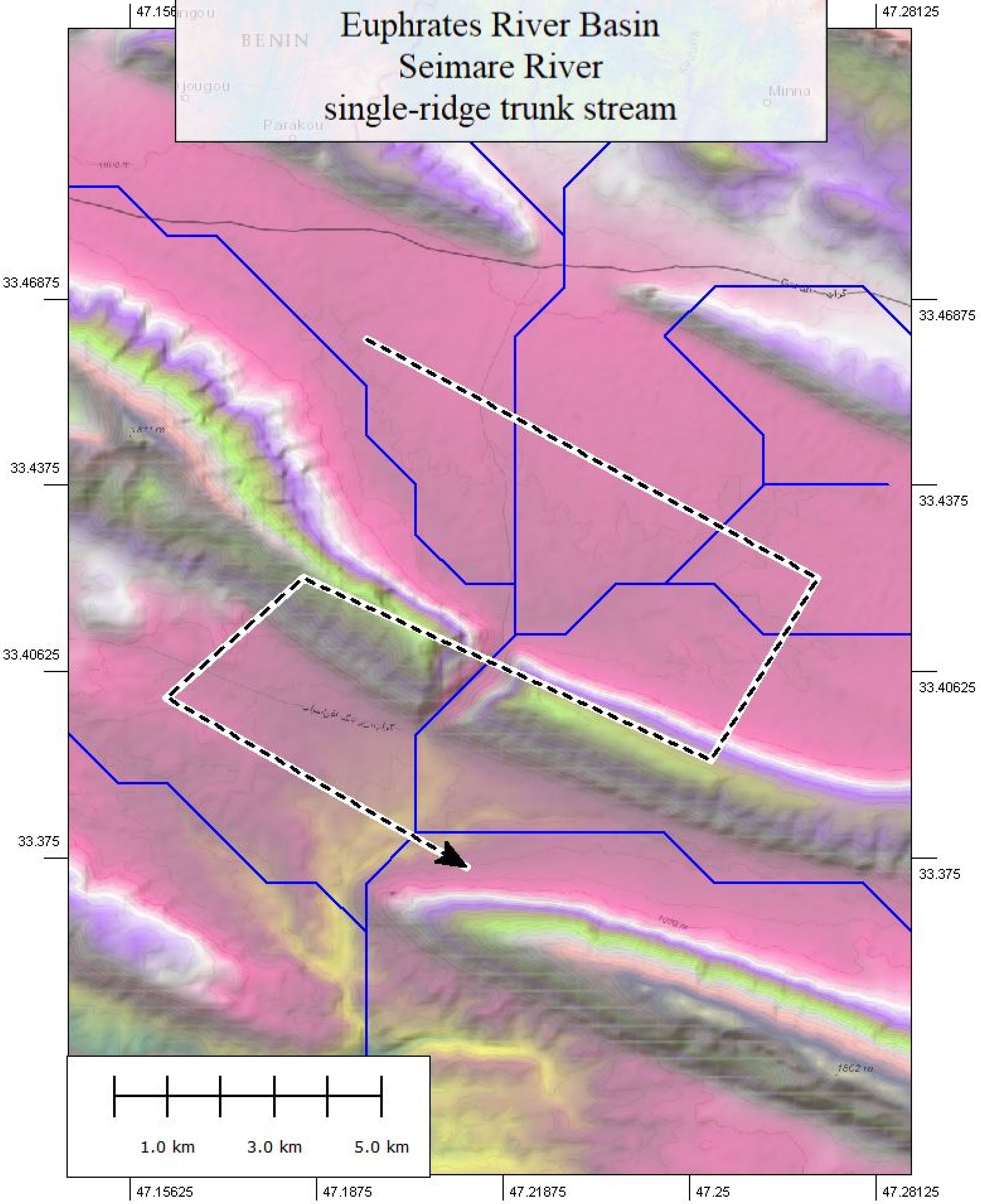

EU - 75
Deva River Basin
Deva River
single-ridge trunk stream

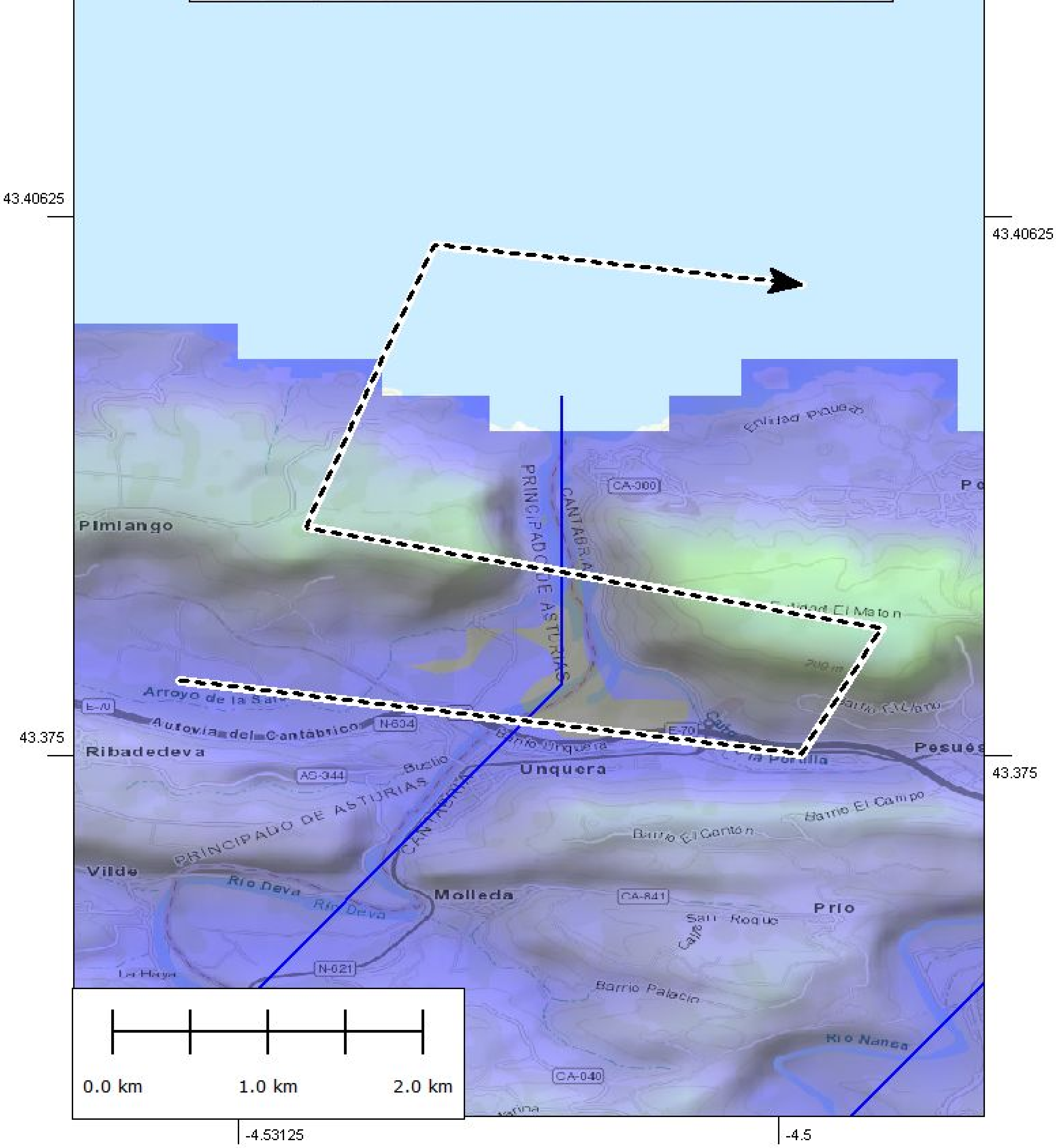

EU - 76
Euphrates River Basin
Karkheh River tributary
single-ridge trunk stream

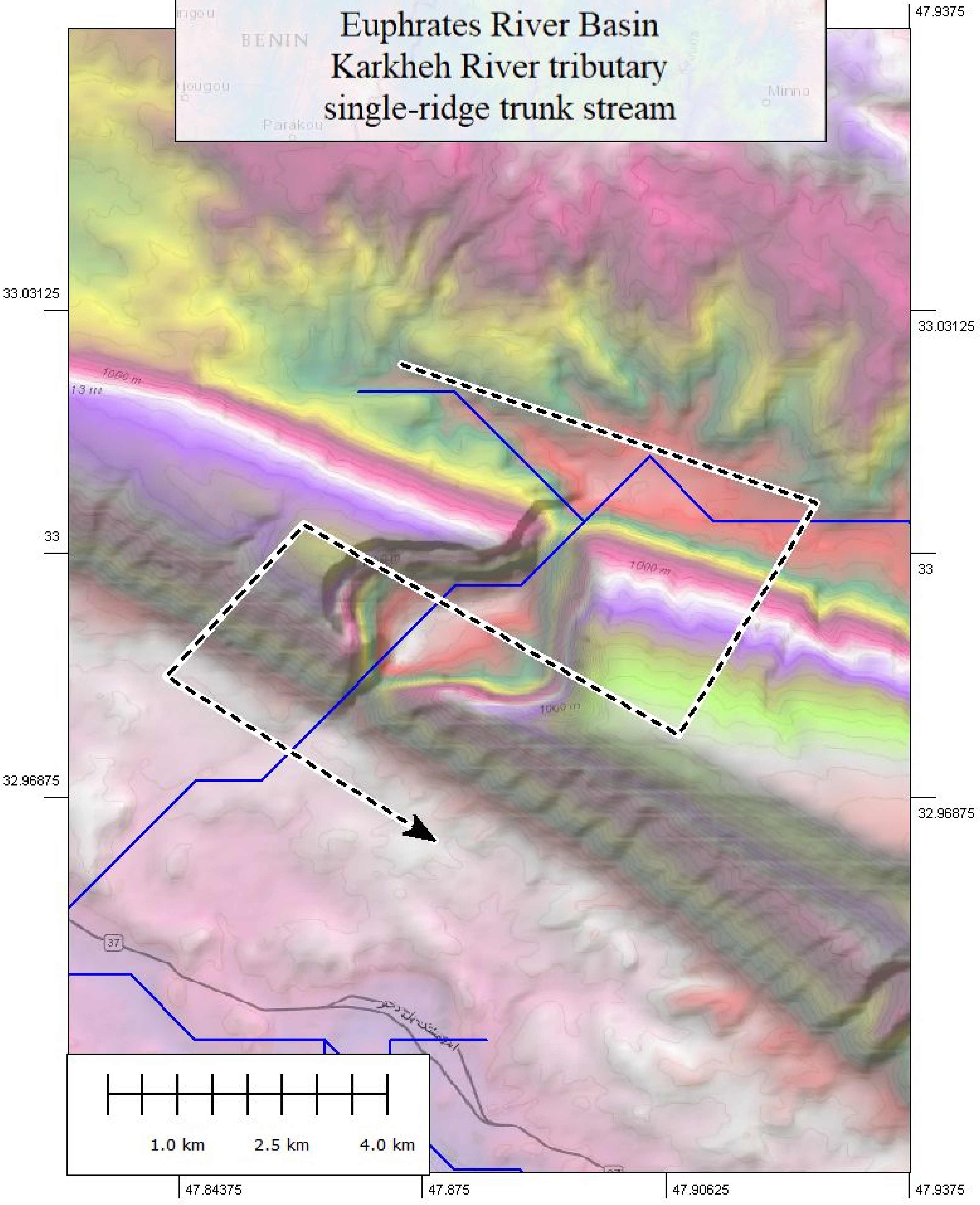

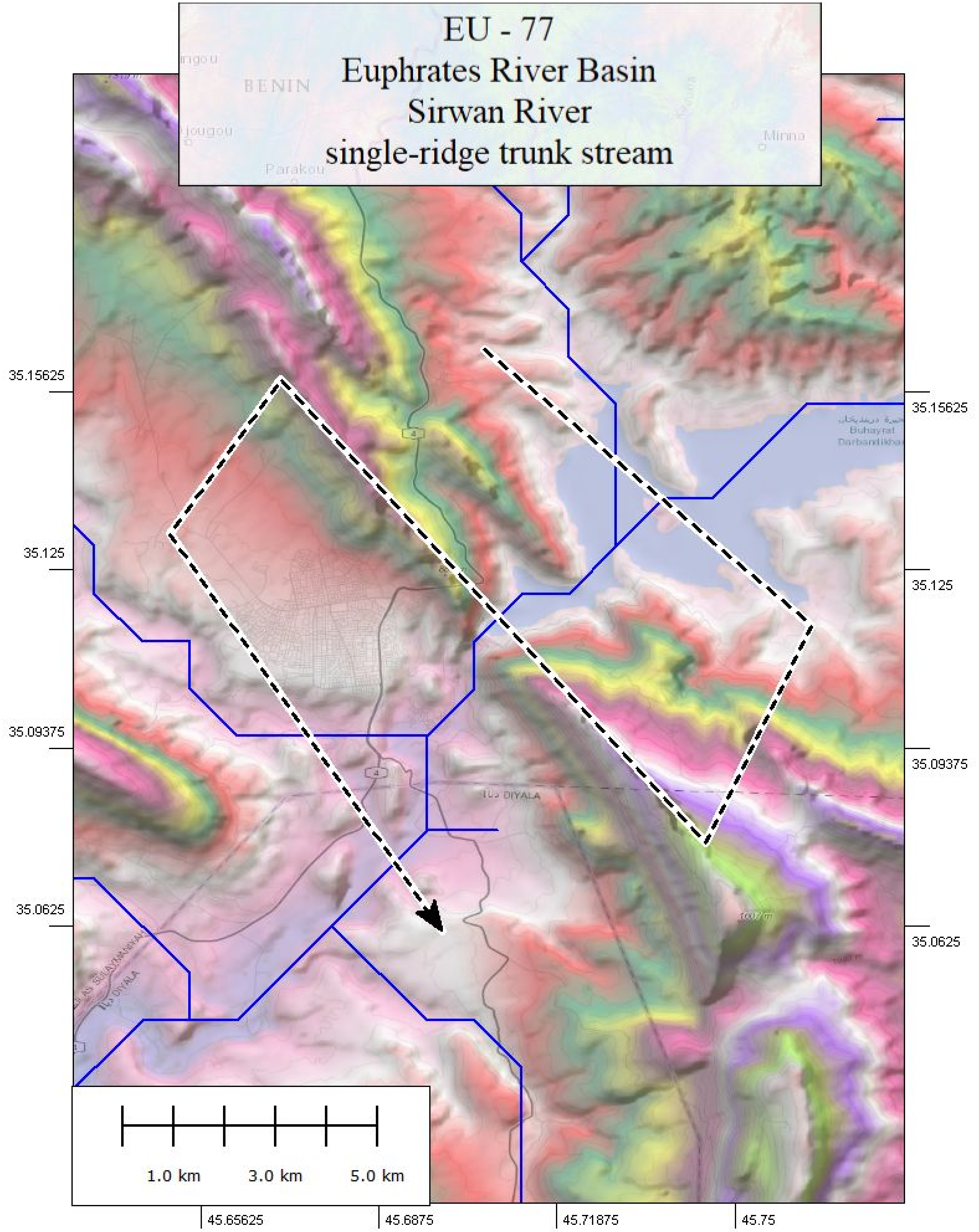

EU - 77
Euphrates River Basin
Sirwan River
single-ridge trunk stream
35.15625
35.125
35.09375
35.0625
45.65625
45.6875
45.71875
45.75
1.0 km
3.0 km
5.0 km
DIYALA
Buhayrat
Darbandikhan

EU - 79
Euphrates River Basin
single-ridge trunk stream

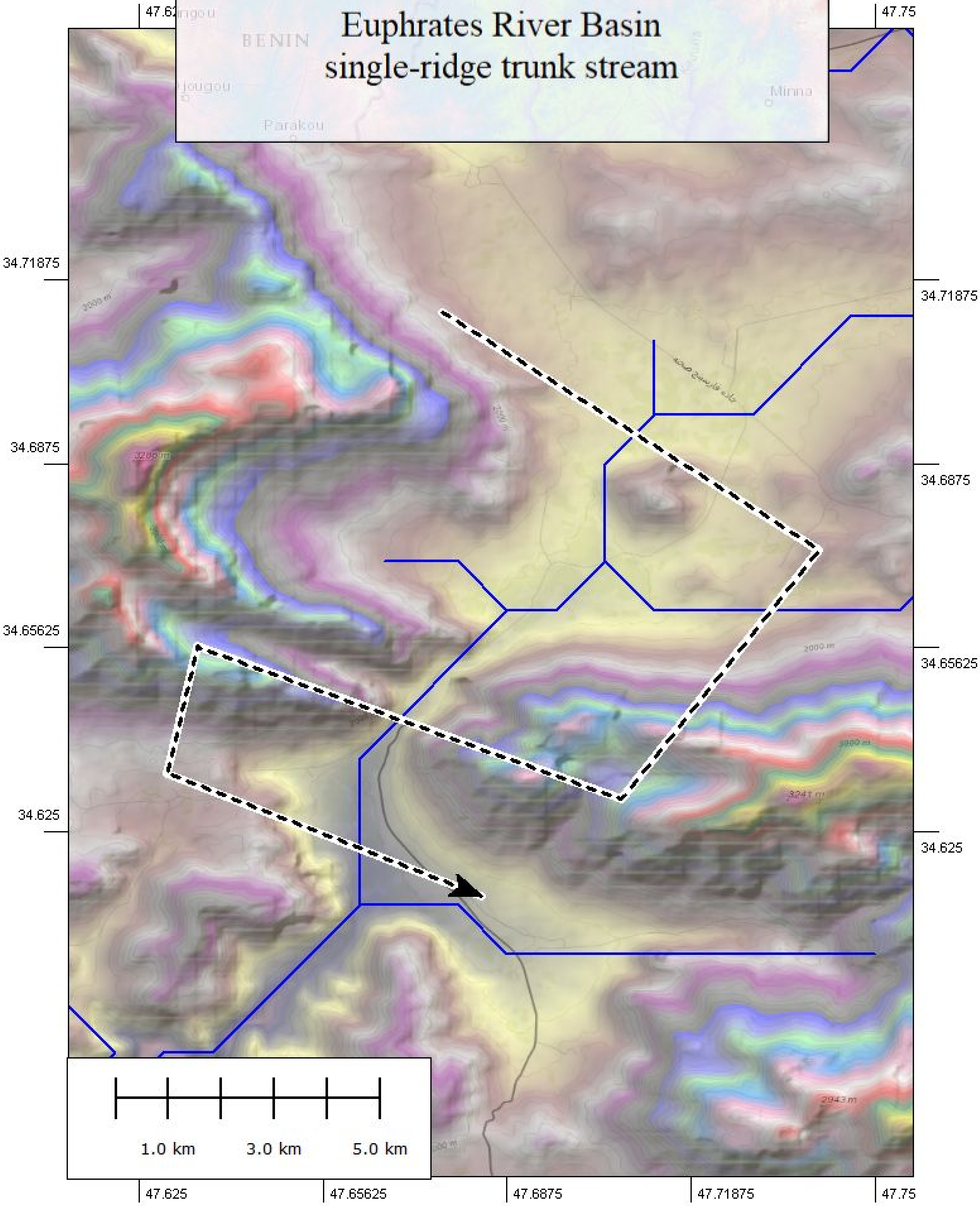

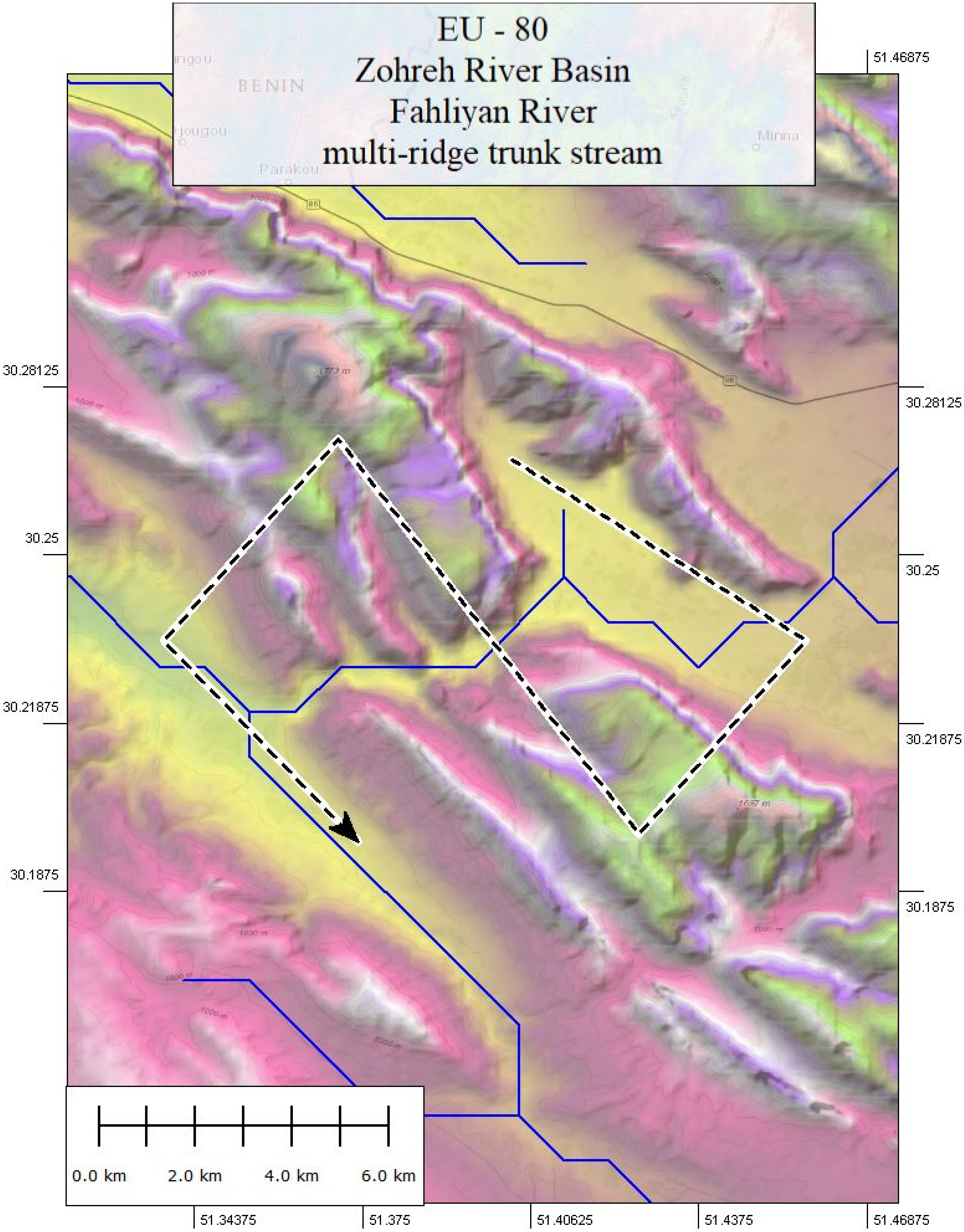
EU - 80
Zohreh River Basin
Fahliyan River
multi-ridge trunk stream
51.46875
30.28125
30.25
30.21875
30.1875
0.0 km
2.0 km
4.0 km
6.0 km
51.34375
51.375
51.40625
51.4375
51.46875

EU - 81
Xuquer River Basin
Xiuquet River
single-ridge trunk stream

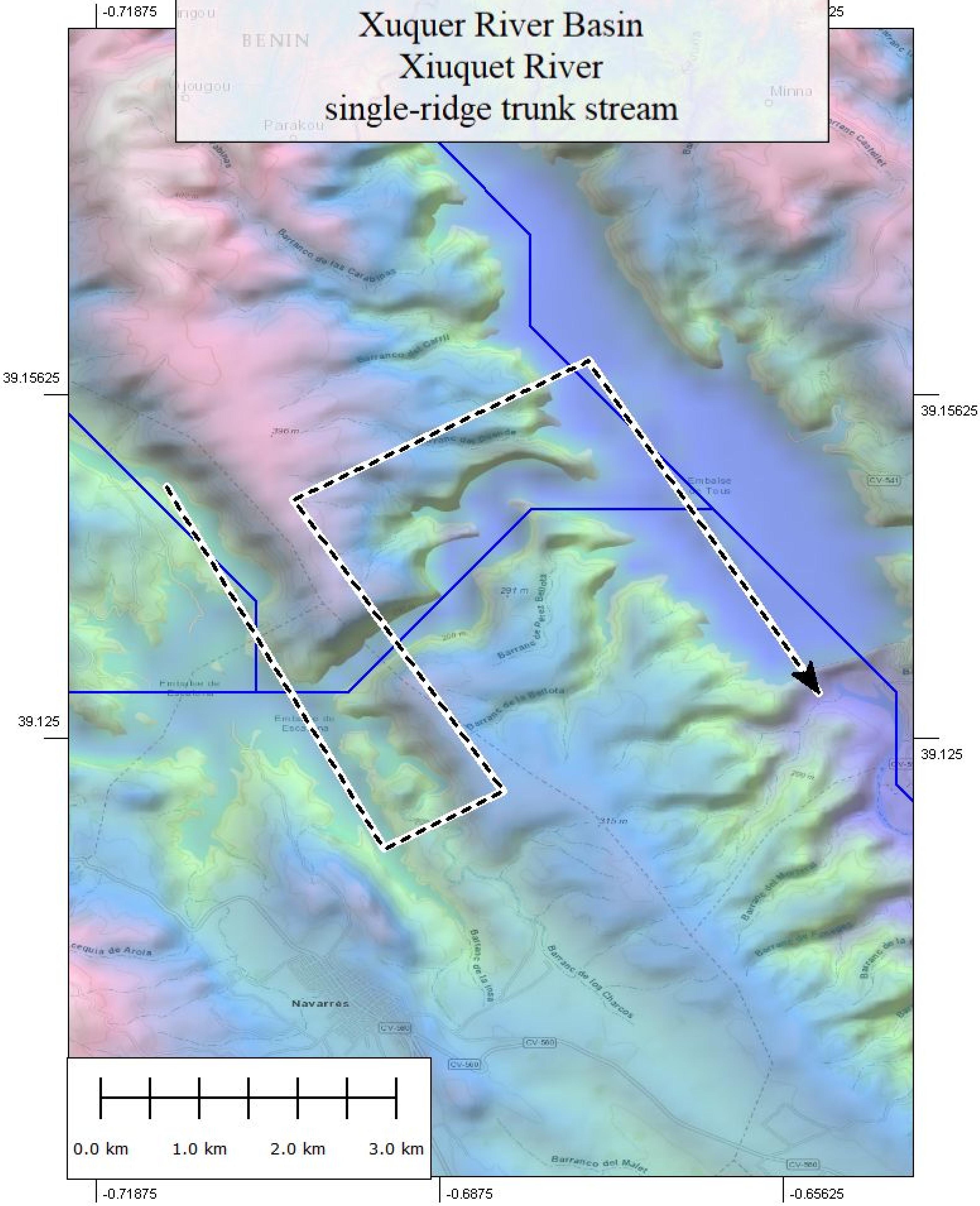

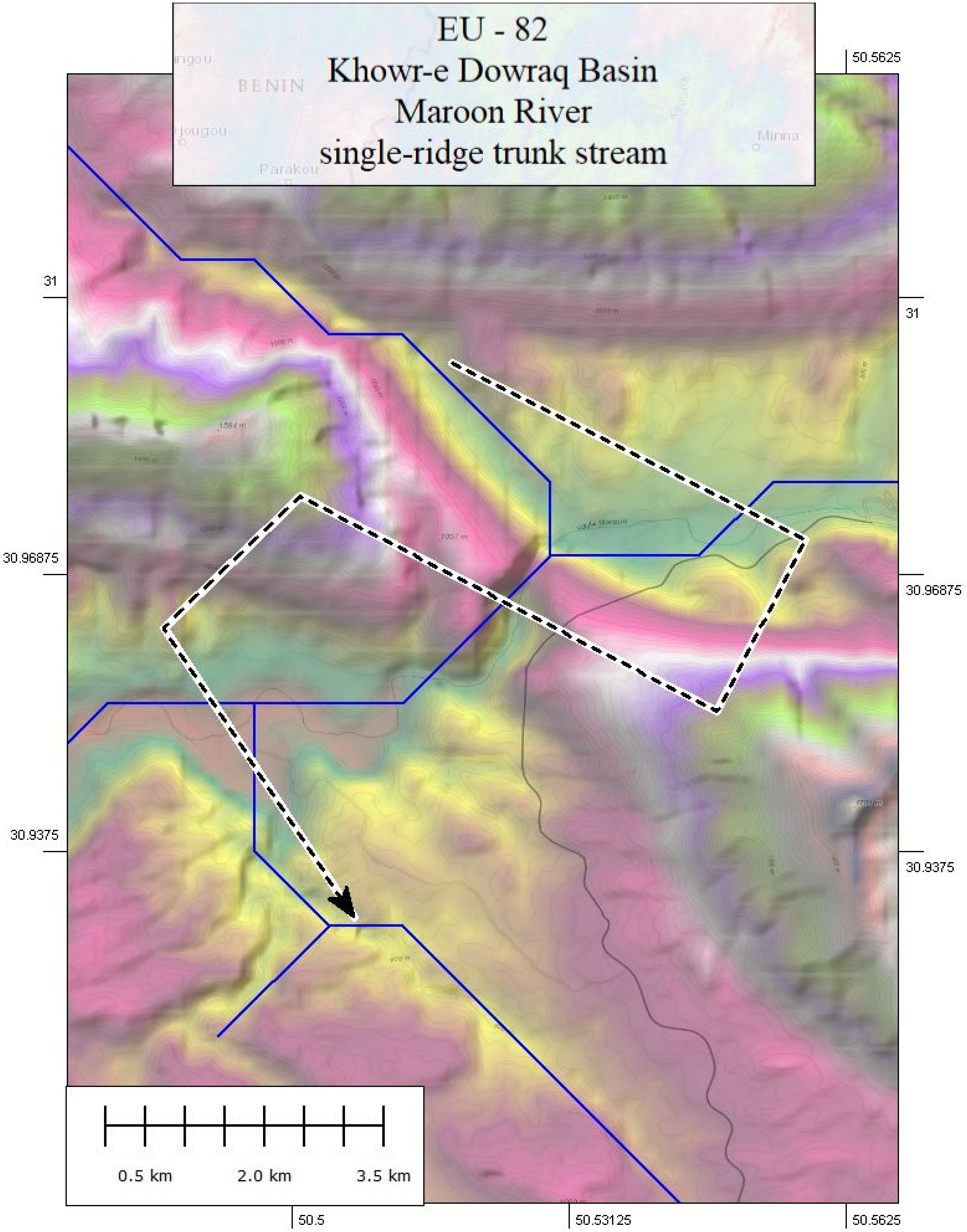

EU - 82
Khowr-e Dowraq Basin
Maroon River
single-ridge trunk stream
BENIN
Parakou
Minna
Maroun
1584 m
1057 m
31
30.96875
30.9375
50.5
50.53125
50.5625
0.5 km
2.0 km
3.5 km

EU - 83
Euphrates River Basin
Karkheh River
single-ridge trunk stream

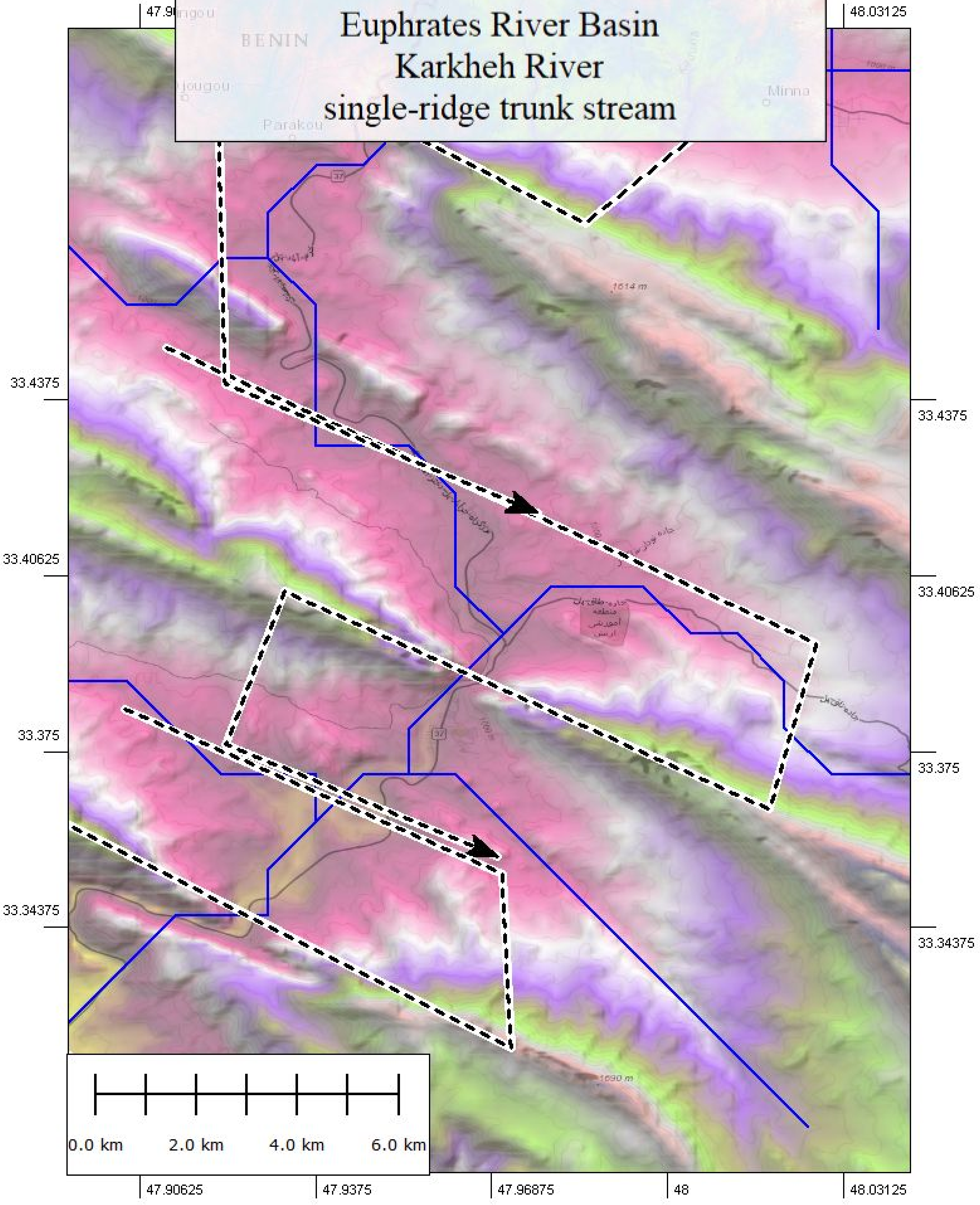

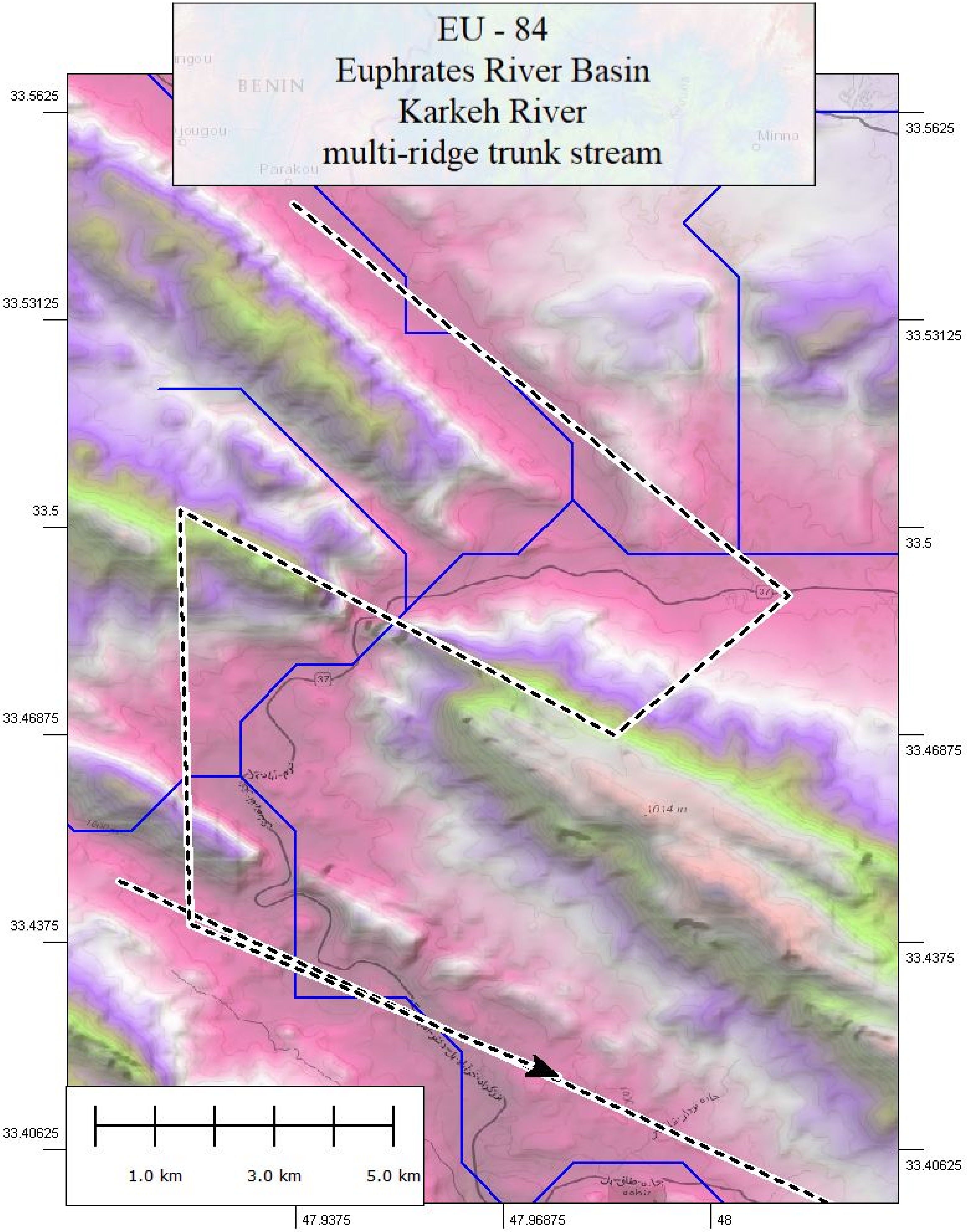

EU - 84
Euphrates River Basin
Karkeh River
multi-ridge trunk stream
33.5625
33.53125
33.5
33.46875
33.4375
33.40625
47.9375
47.96875
48
1.0 km
3.0 km
5.0 km
Parakou
Minna
BENIN

EU - 85
Euphrates River Basin
Karun River
single-ridge trunk stream

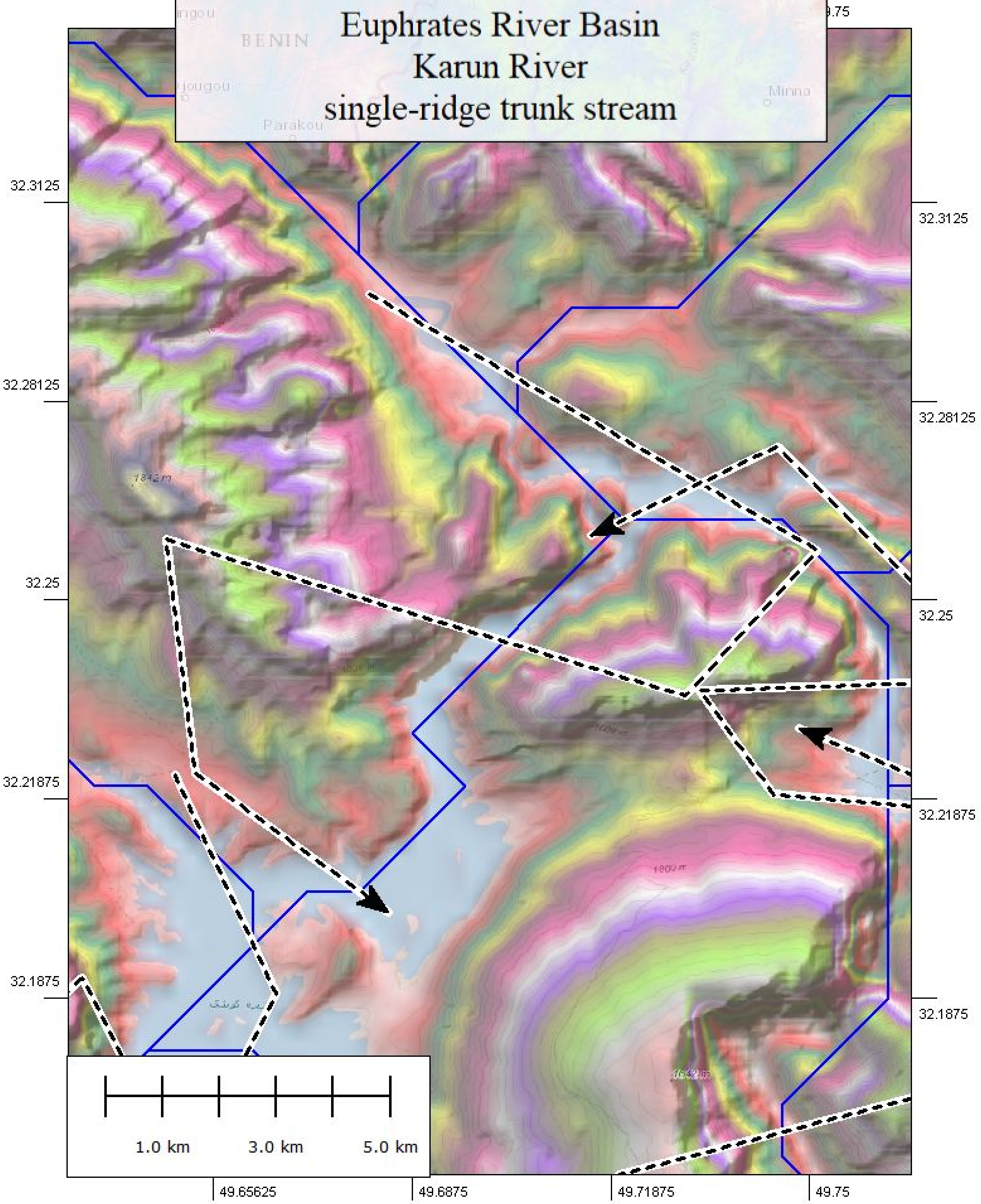

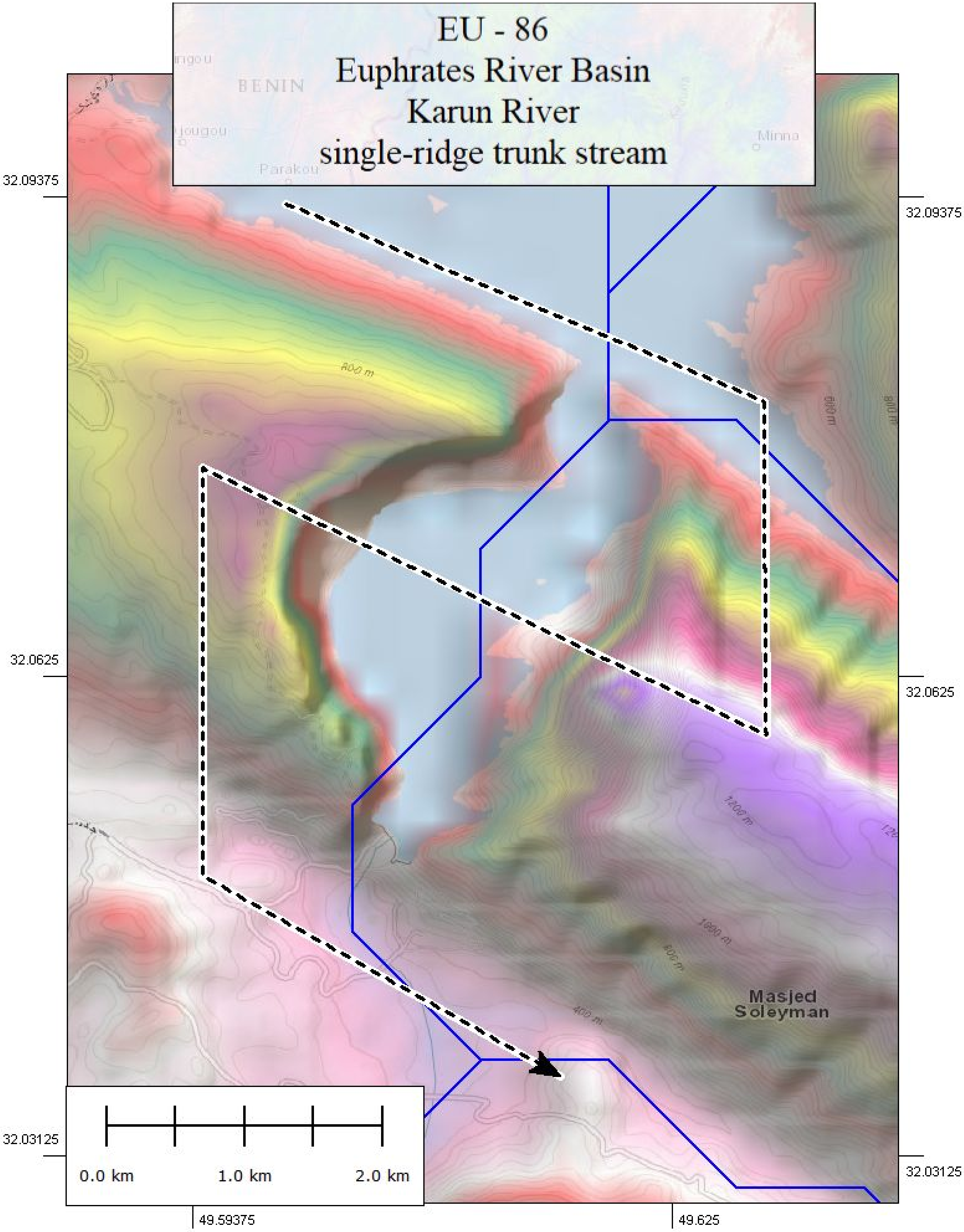
EU - 86
Euphrates River Basin
Karun River
single-ridge trunk stream
BENIN
Parakou
Minna
800 m
1200 m
1000 m
400 m
Masjed Soleyman
32.09375
32.0625
32.03125
49.59375
49.625
0.0 km
1.0 km
2.0 km

EU - 87
Euphrates River Basin
Kezer River
single-ridge trunk stream

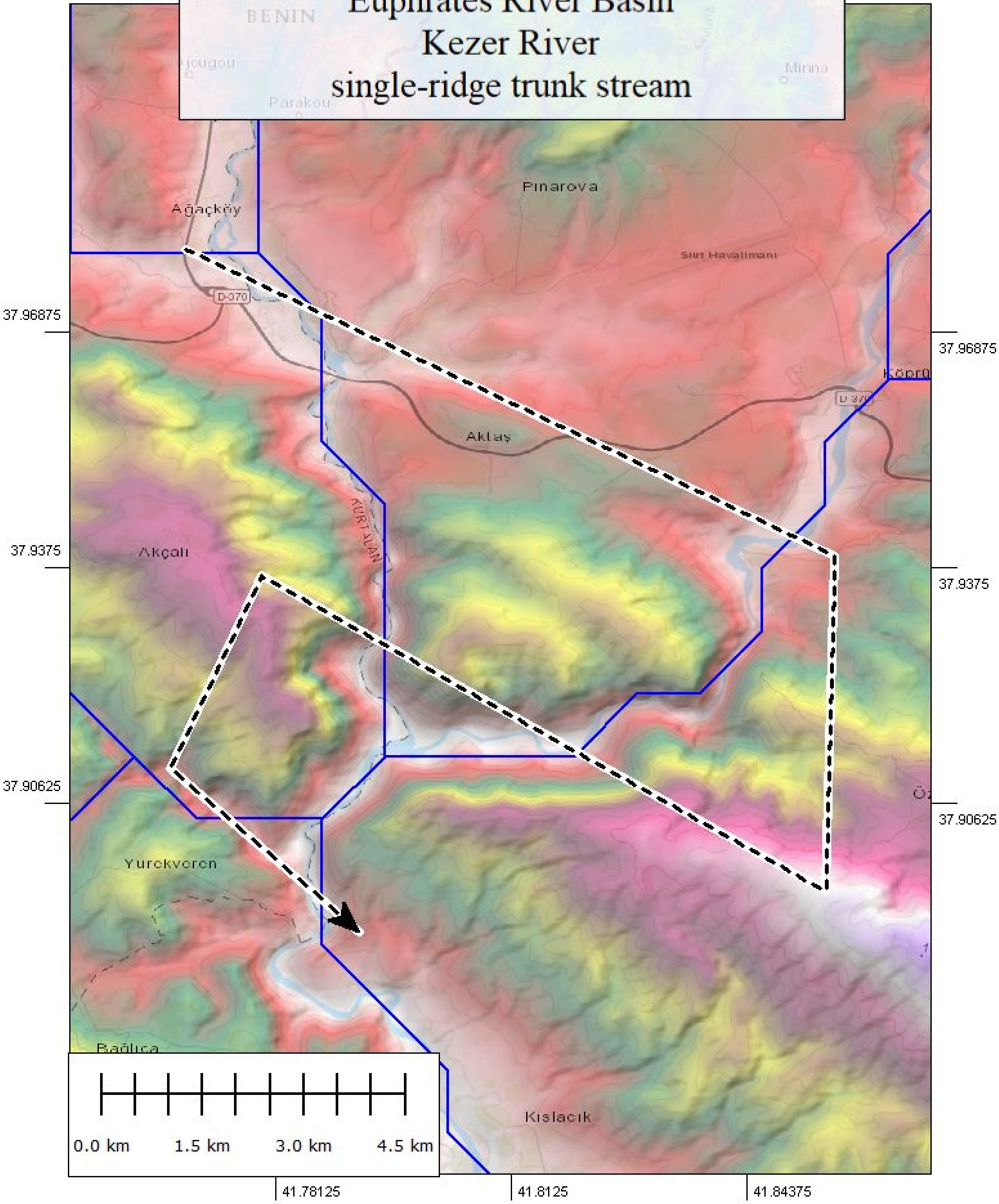

EU - 88
Guadalquivir River Basin
Jandula River
single-ridge trunk stream

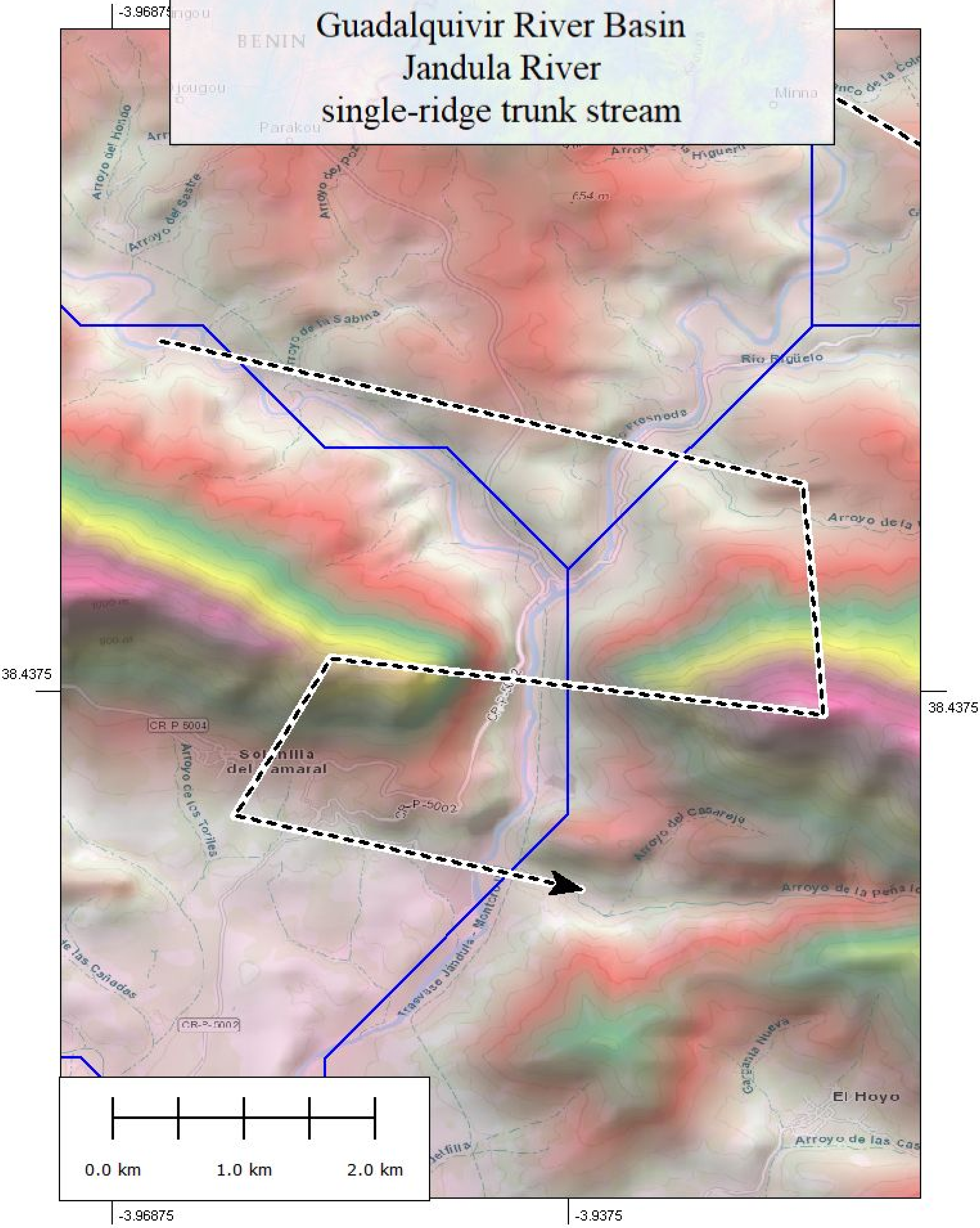

EU - 90
Kr River Basin
Aras River
single-ridge trunk stream

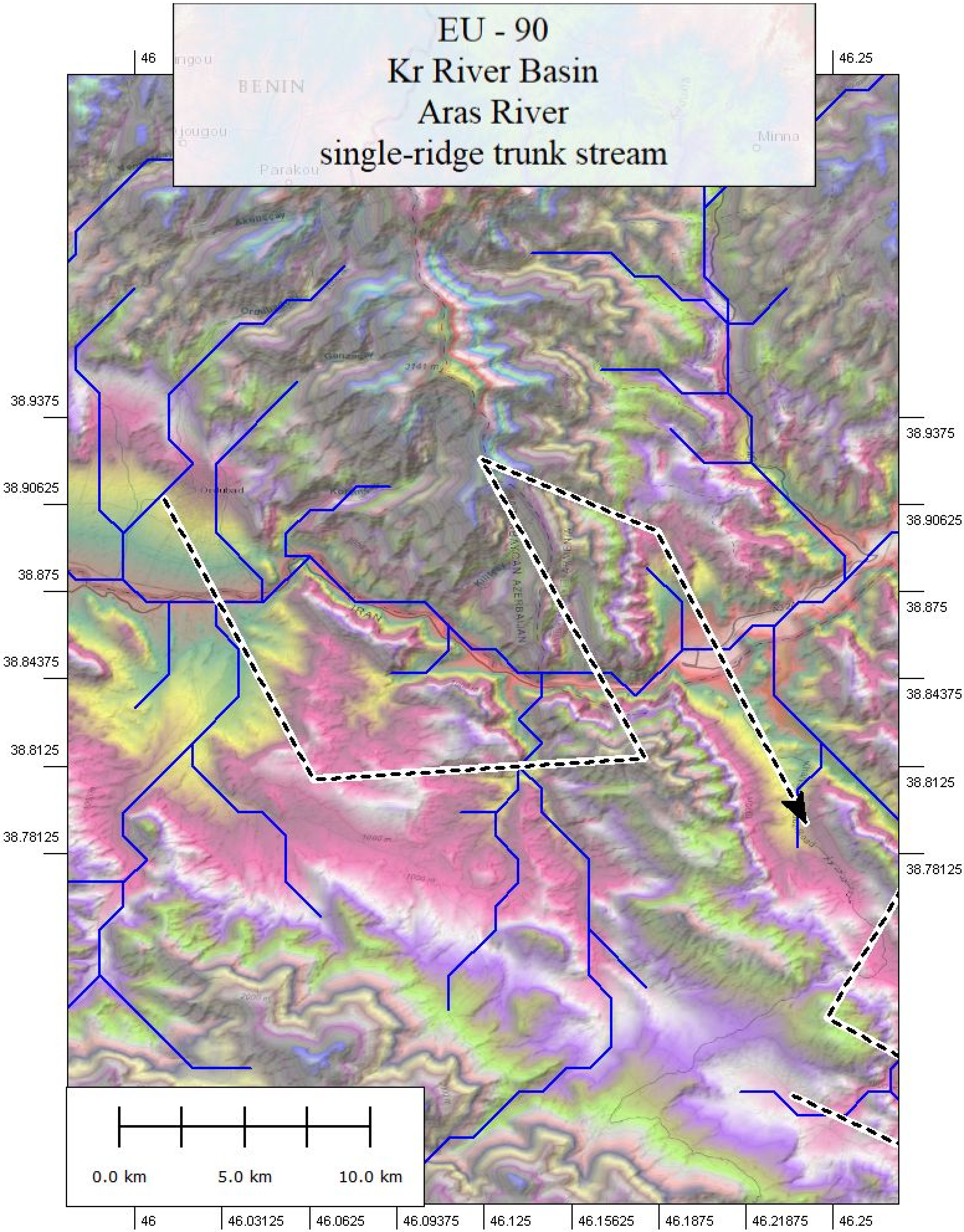

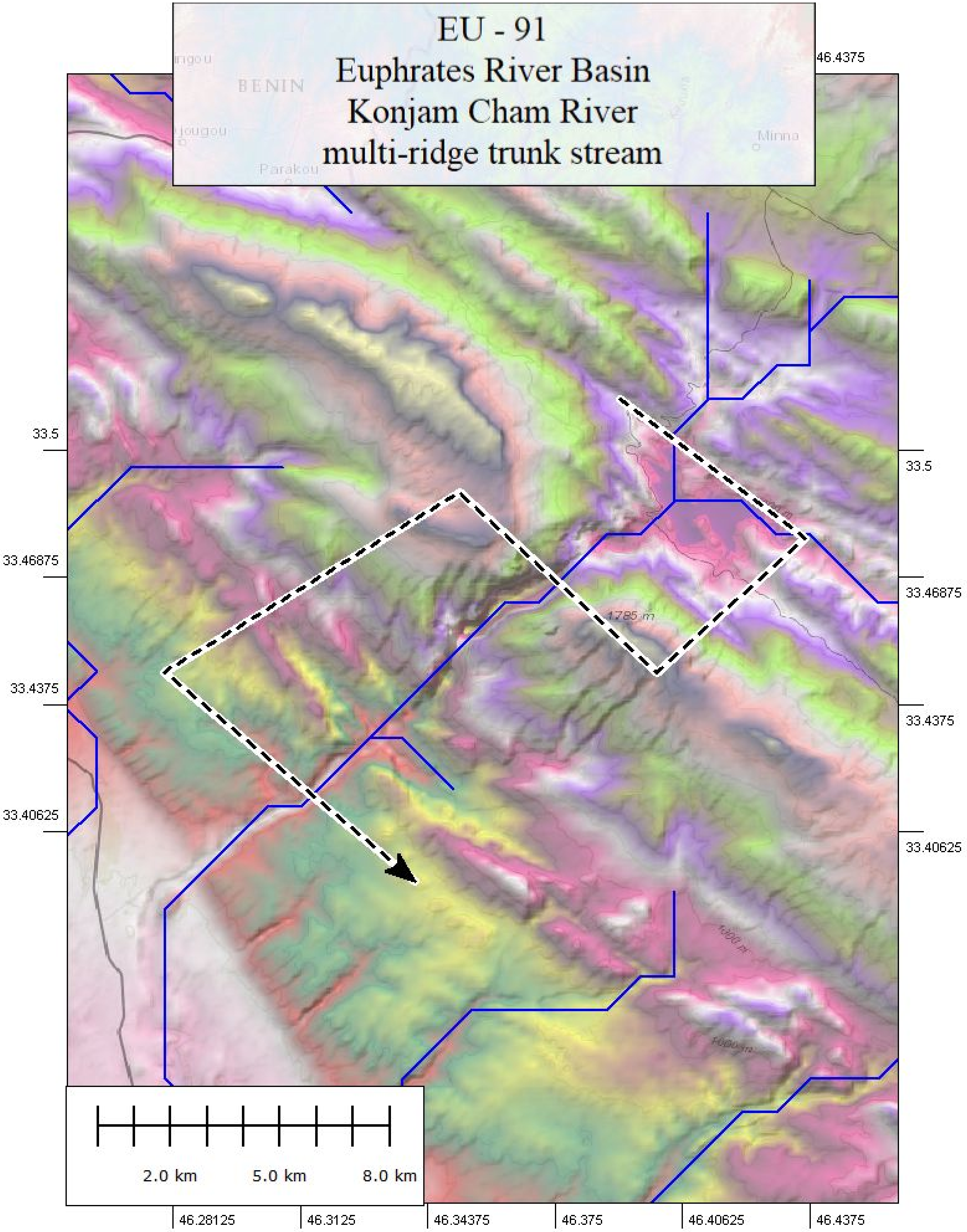
EU - 91
Euphrates River Basin
Konjam Cham River
multi-ridge trunk stream
BENIN
Parakou
Minna
1785 m
1000 m
1000 m
33.5
33.46875
33.4375
33.40625
46.4375
46.28125
46.3125
46.34375
46.375
46.40625
2.0 km
5.0 km
8.0 km

EU - 92
Euphrates River Basin
Murat River
single-ridge trunk stream

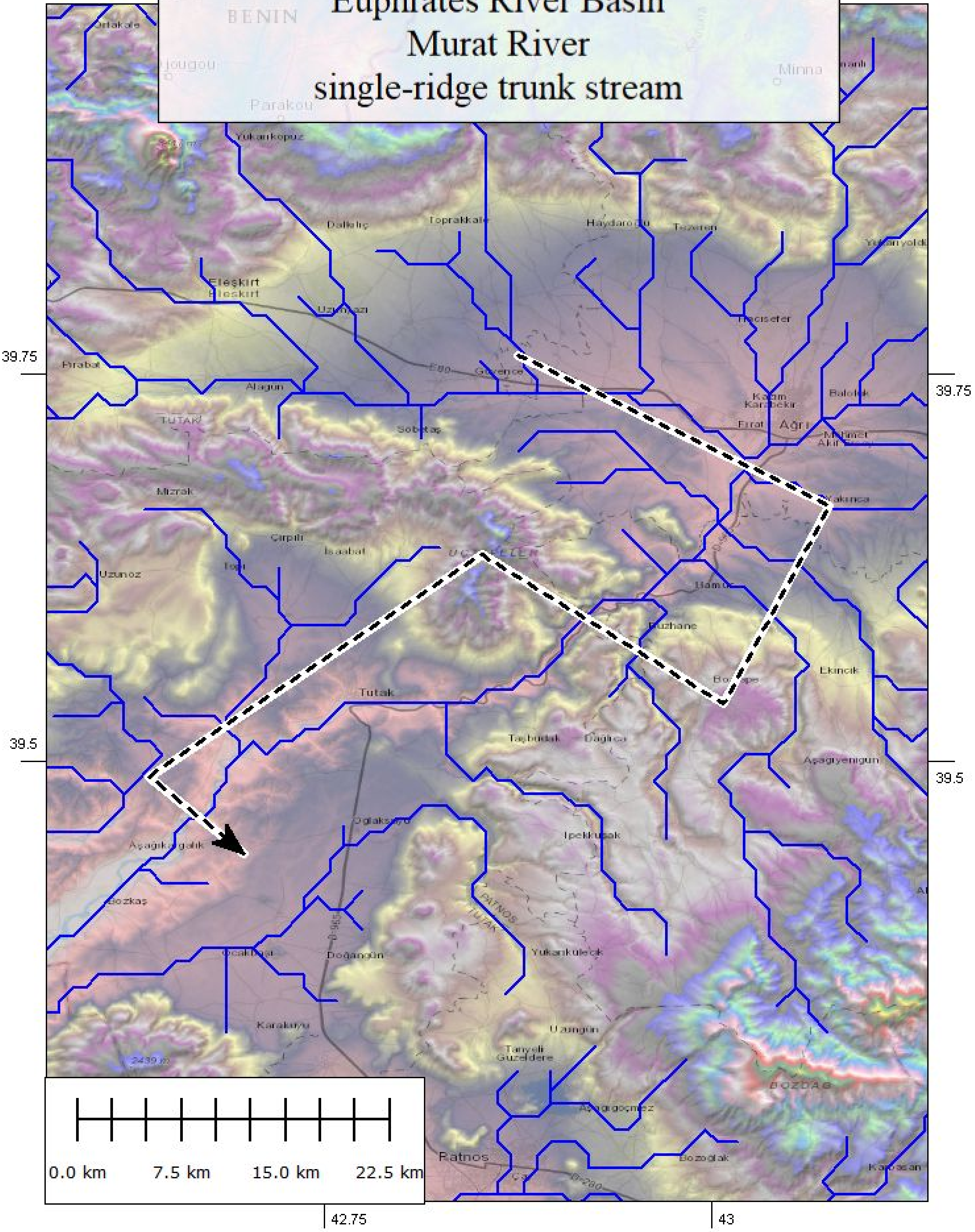

EU - 93
Euphrates River Basin
Euphrates River
single-ridge trunk stream

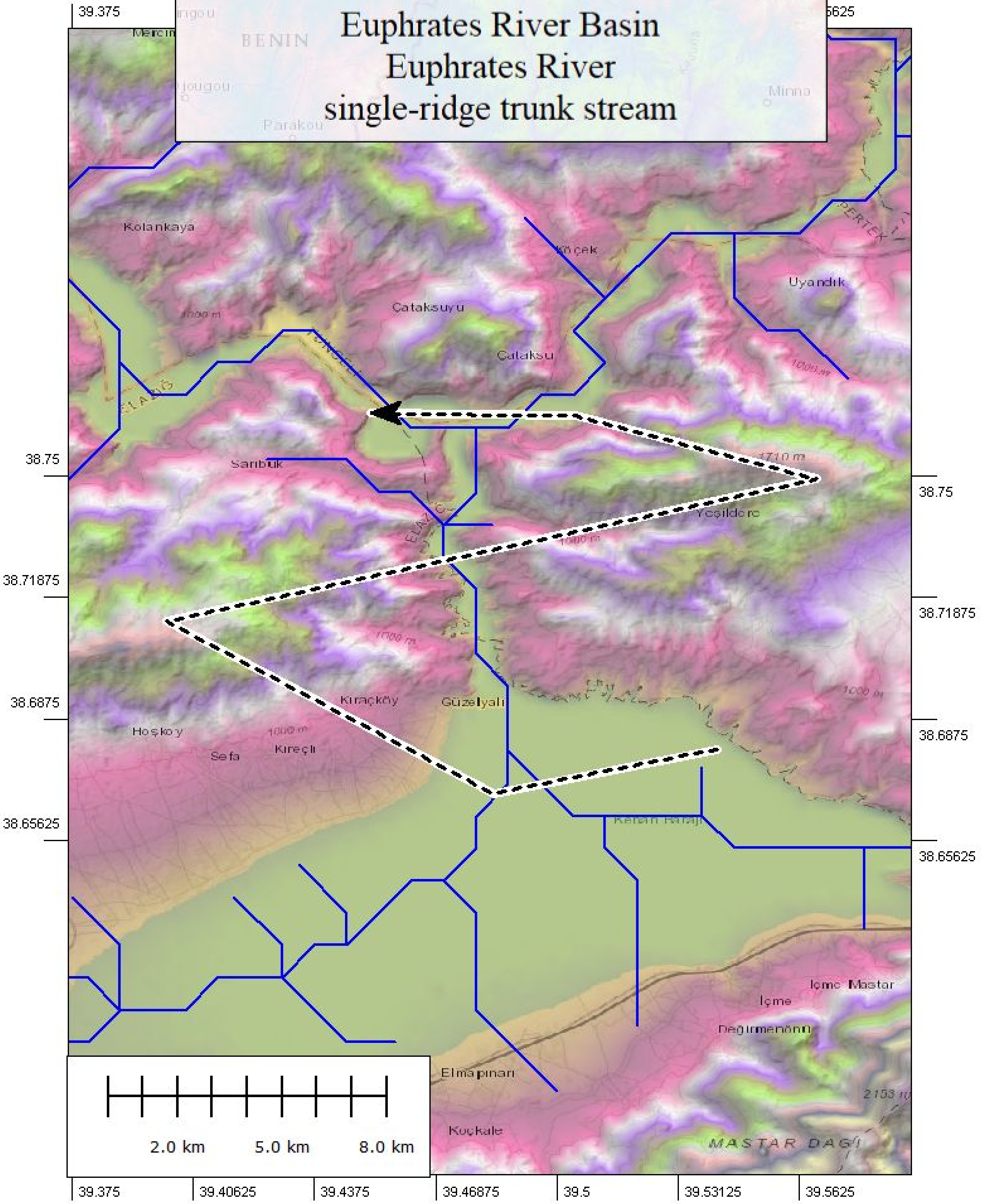

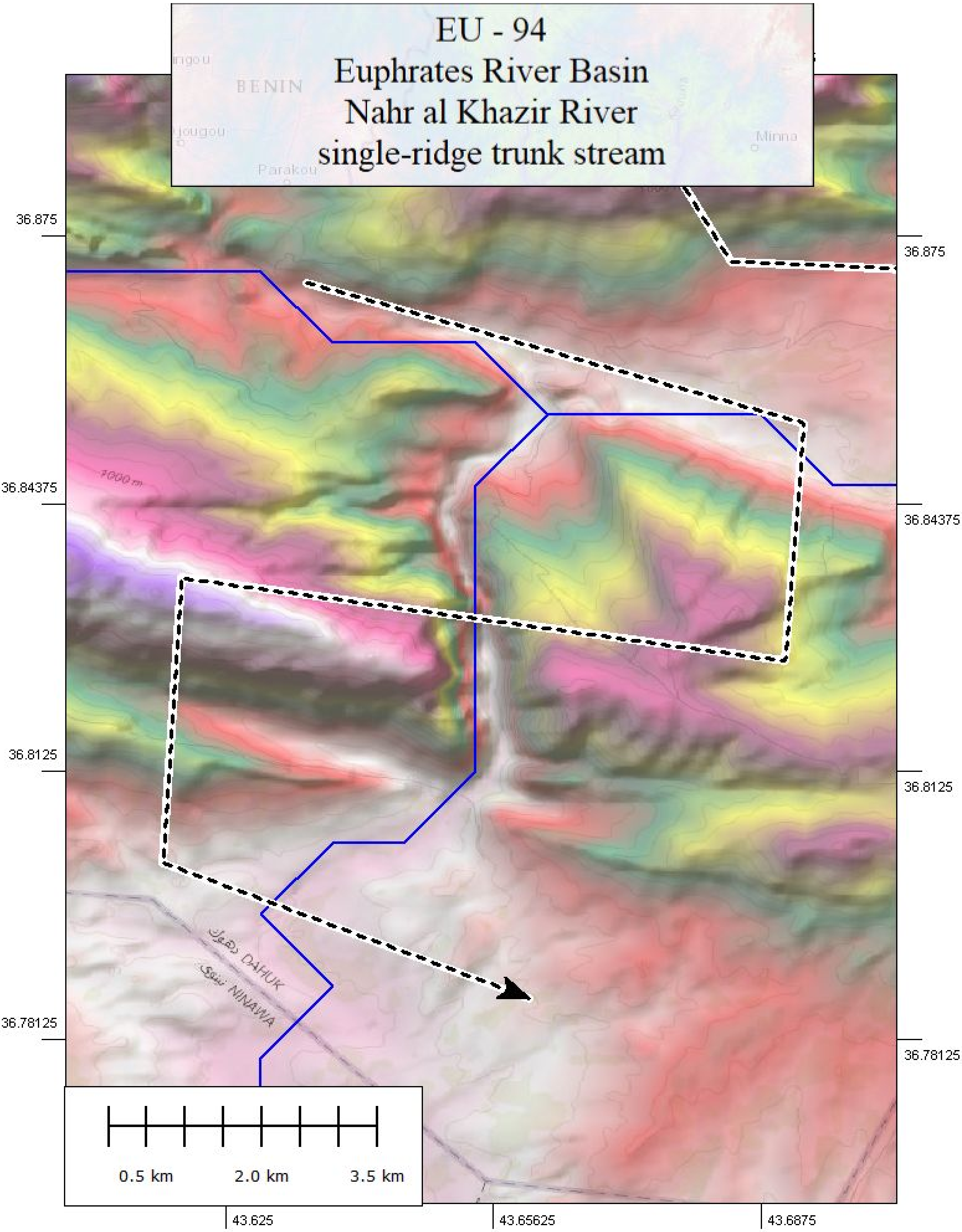
EU - 94
Euphrates River Basin
Nahr al Khazir River
single-ridge trunk stream
36.875
36.875
36.84375
36.84375
36.8125
36.8125
36.78125
36.78125
43.625
43.65625
43.6875
1000 m
DAHUK
NINAWA
0.5 km
2.0 km
3.5 km

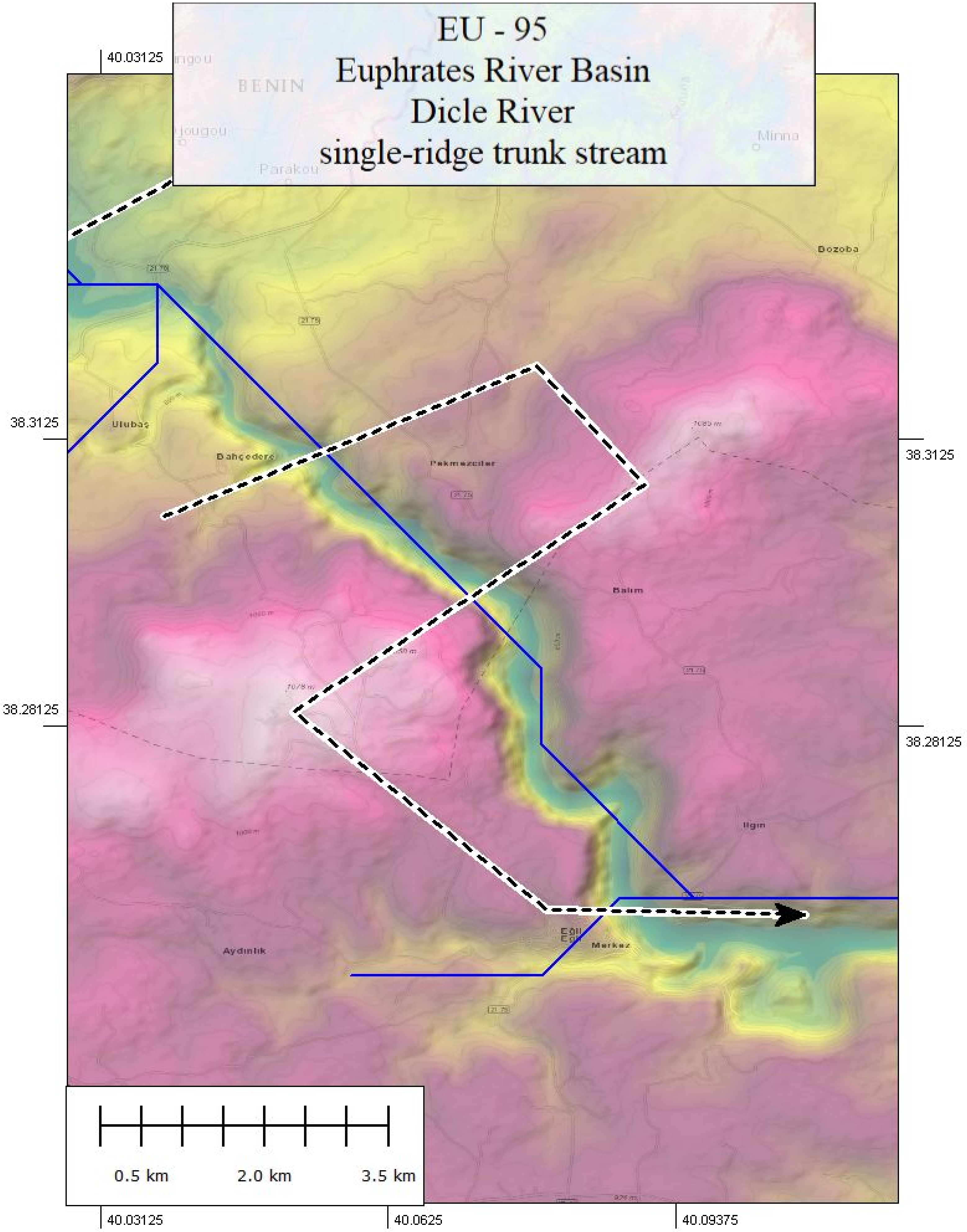
EU - 95
Euphrates River Basin
Dicle River
single-ridge trunk stream
40.03125
38.3125
38.28125
40.0625
40.09375
Dozoba
Ulubaş
Pekmezciler
Balım
Ilgın
Aydınlık
Merkez
0.5 km
2.0 km
3.5 km

EU - 96
Euphrates River Basin
Seimare River
single-ridge trunk stream

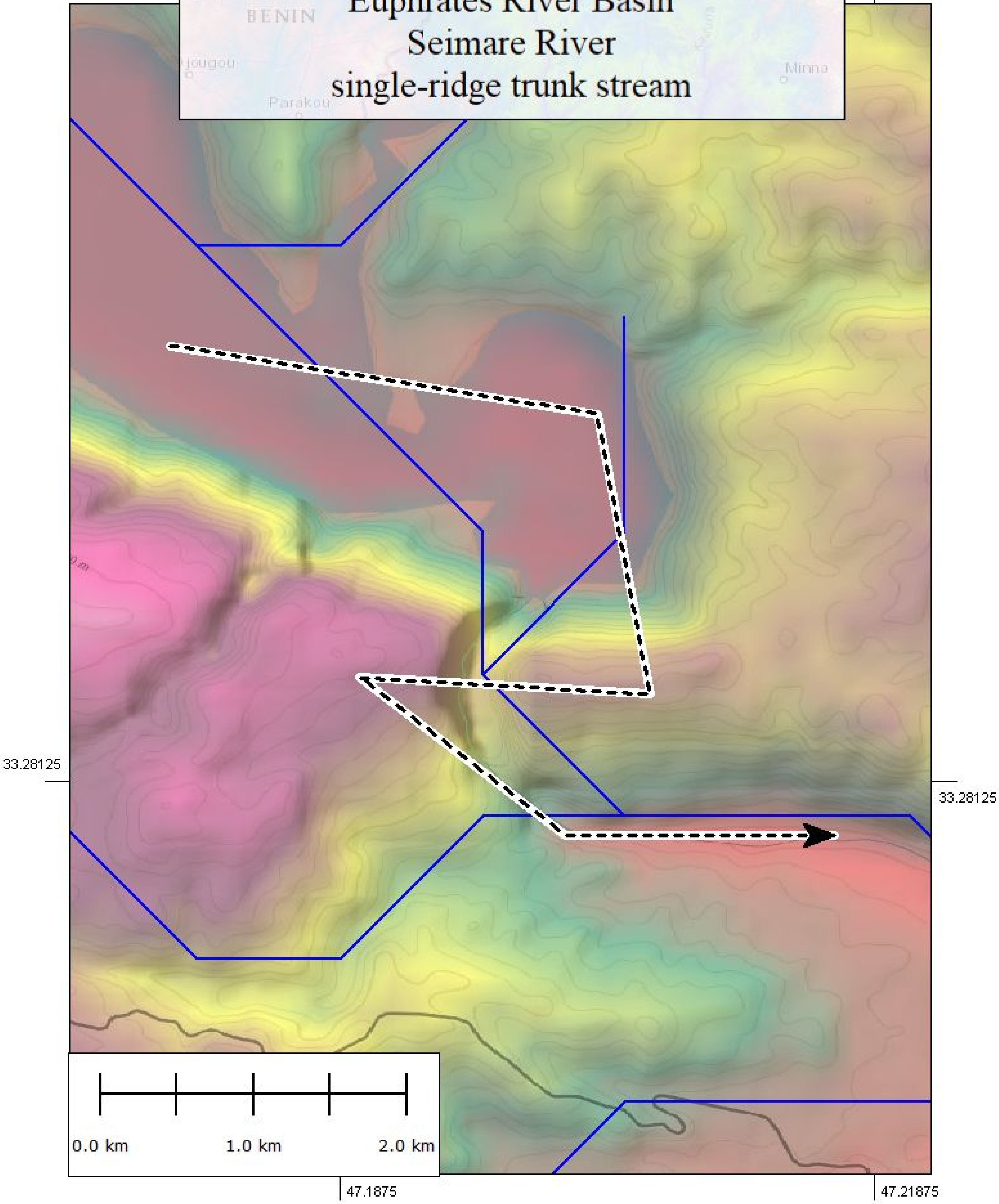

EU - 97
Euphrates River Basin
Seimare River
single-ridge trunk stream

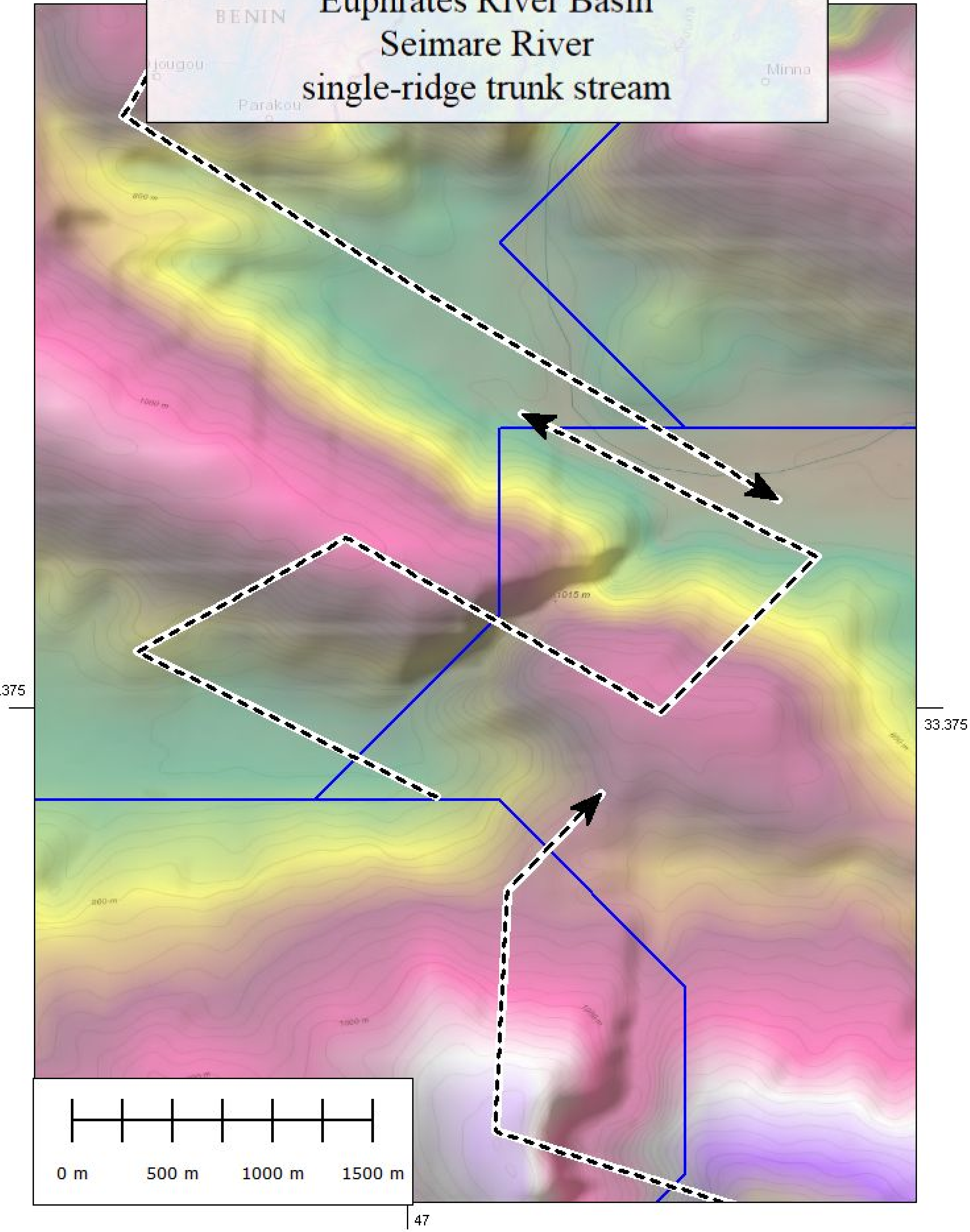

EU - 98
Euphrates River Basin
Great Zab River tributary
single-ridge head stream

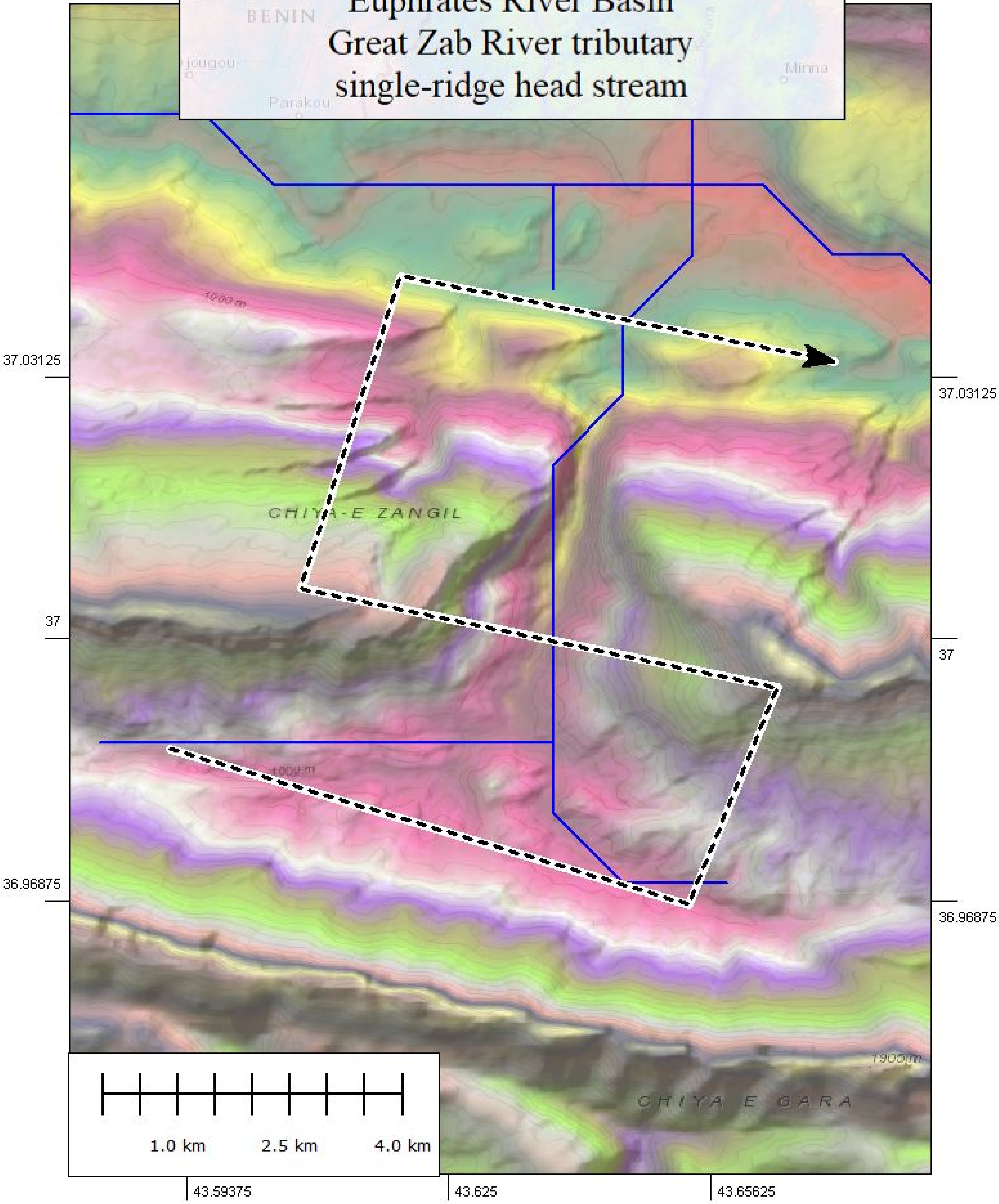

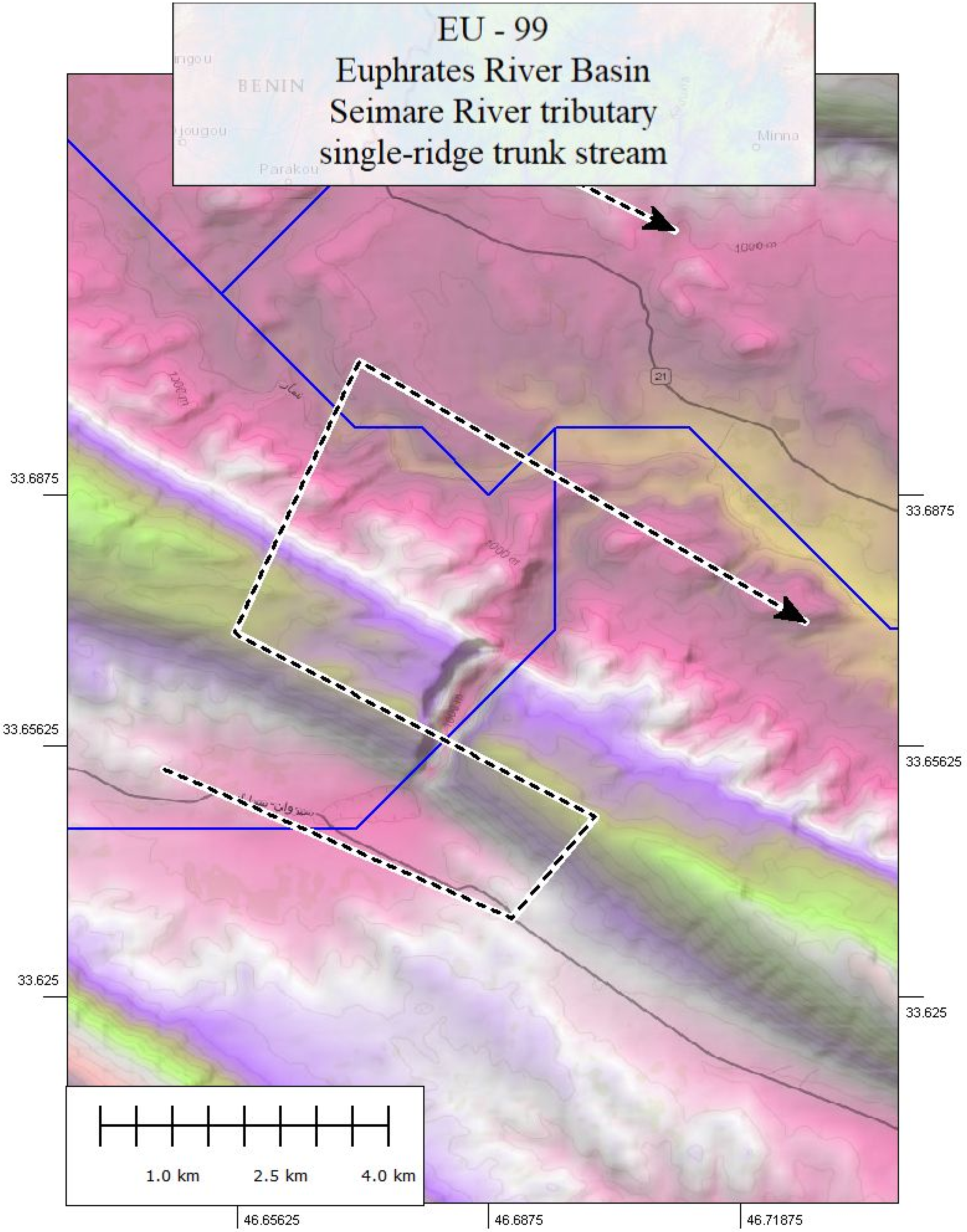
EU - 99
Euphrates River Basin
Seimare River tributary
single-ridge trunk stream
BENIN
Parakou
Minna
1000 m
21
1000 m
1000 m
33.6875
33.65625
33.625
33.6875
33.65625
33.625
1.0 km
2.5 km
4.0 km
46.65625
46.6875
46.71875

EU - 100
Euphrates River Basin
Seimare River
single-ridge trunk stream

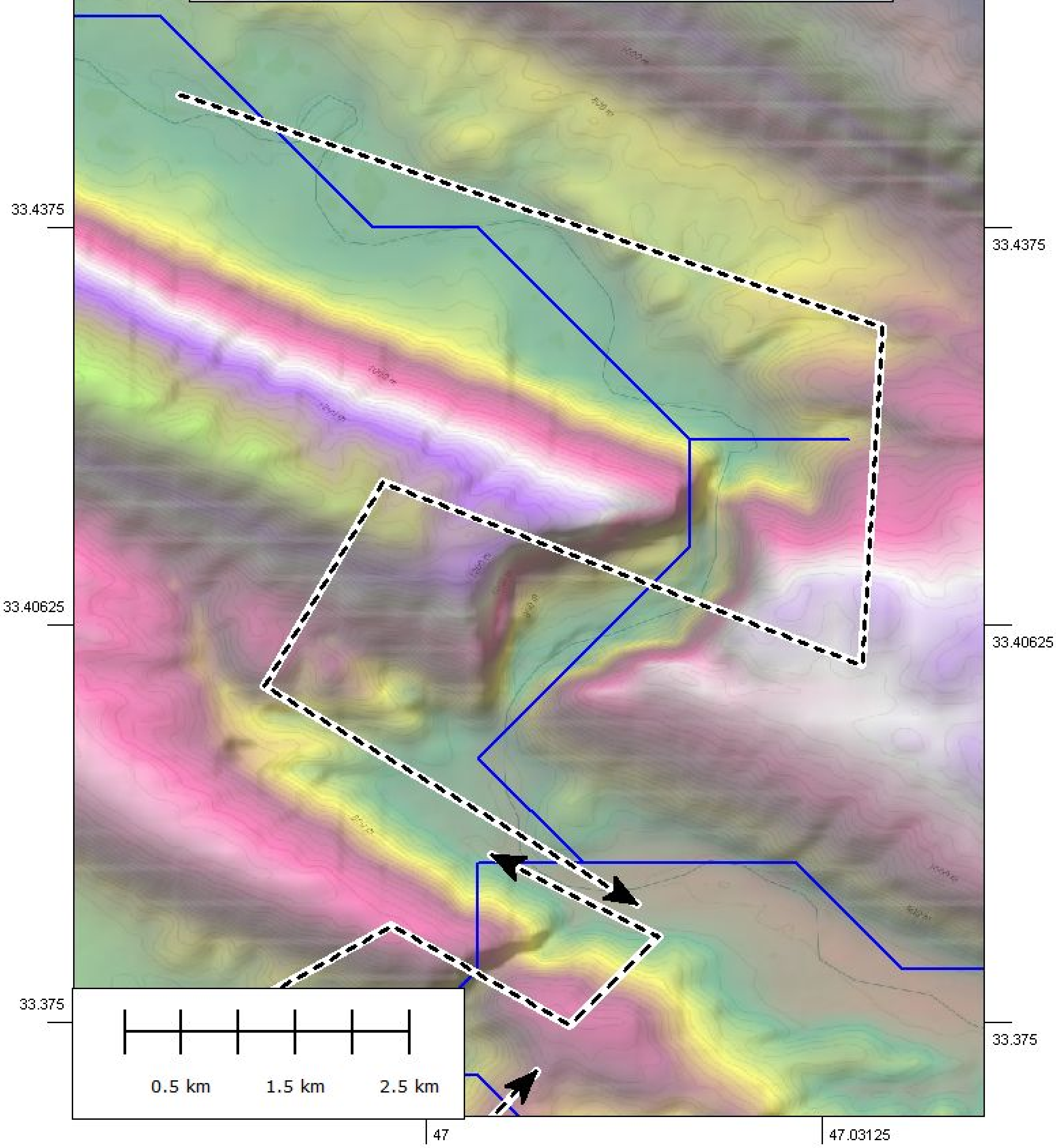

EU - 101
Guadiana River Basin
Estena River
multi-ridge trunk stream

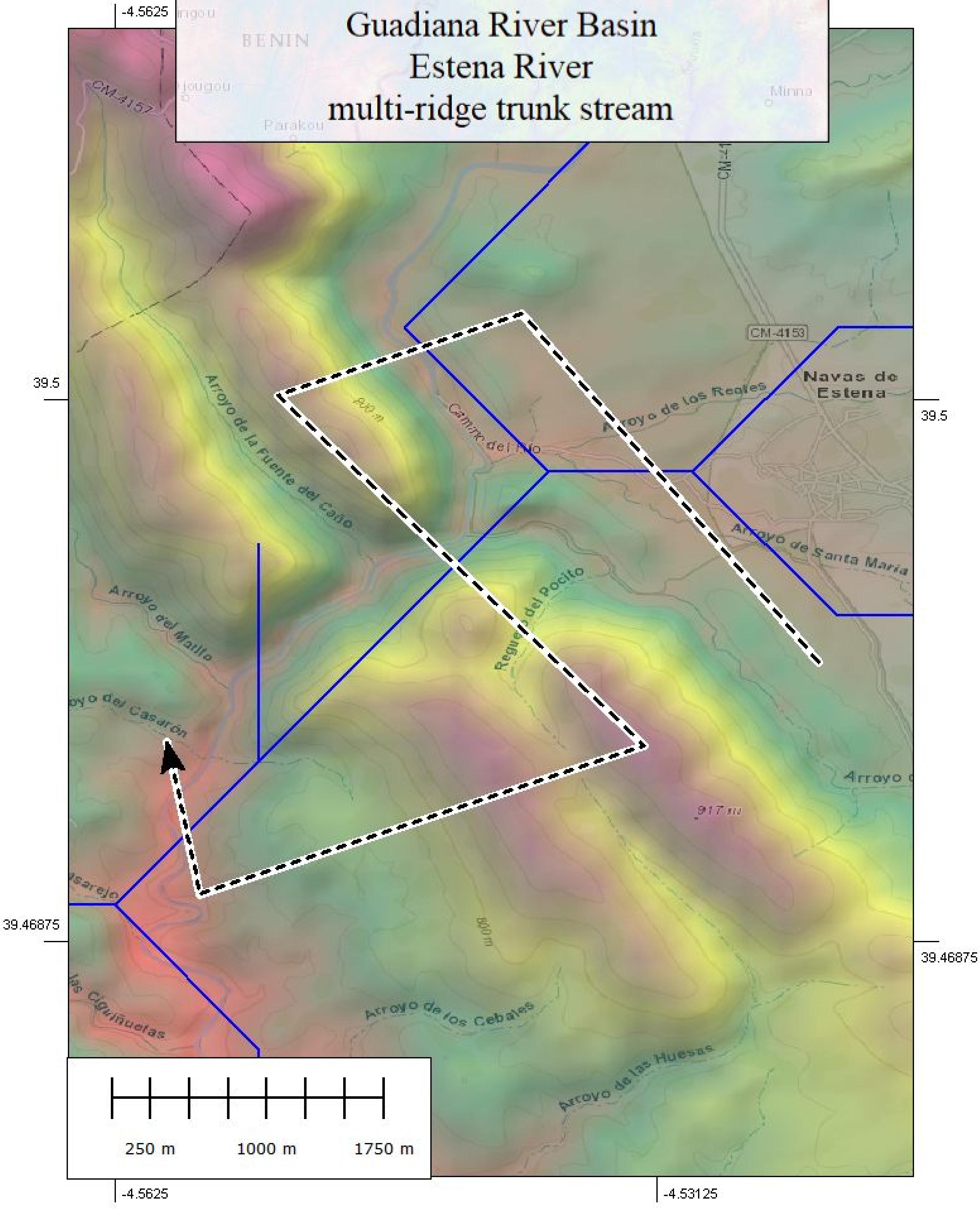

EU - 102
Prut River Basin
Vidima River
single-ridge trunk stream

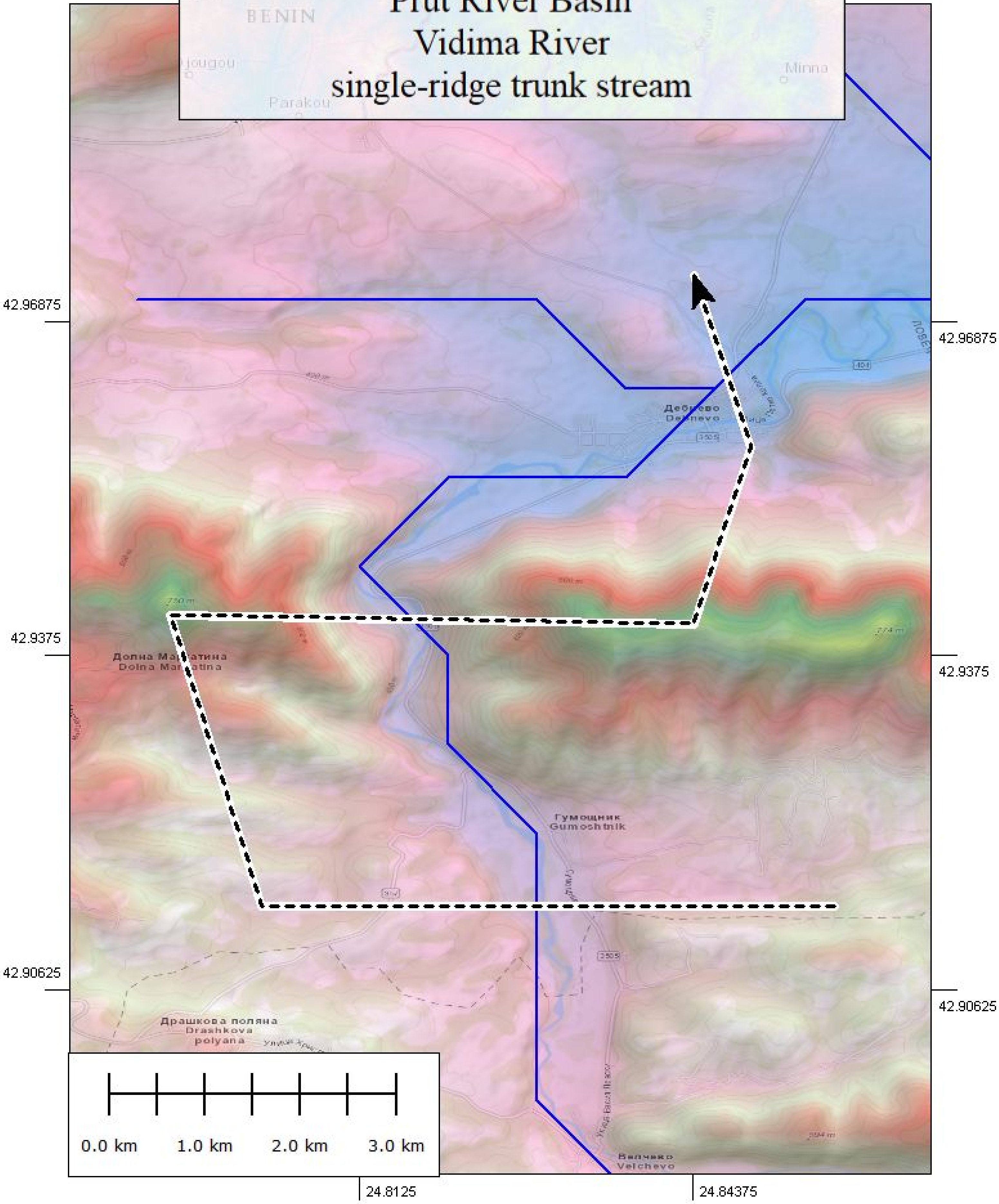

EU - 103
Prut River Basin
Zlatarishka River
multi-ridge trunk stream

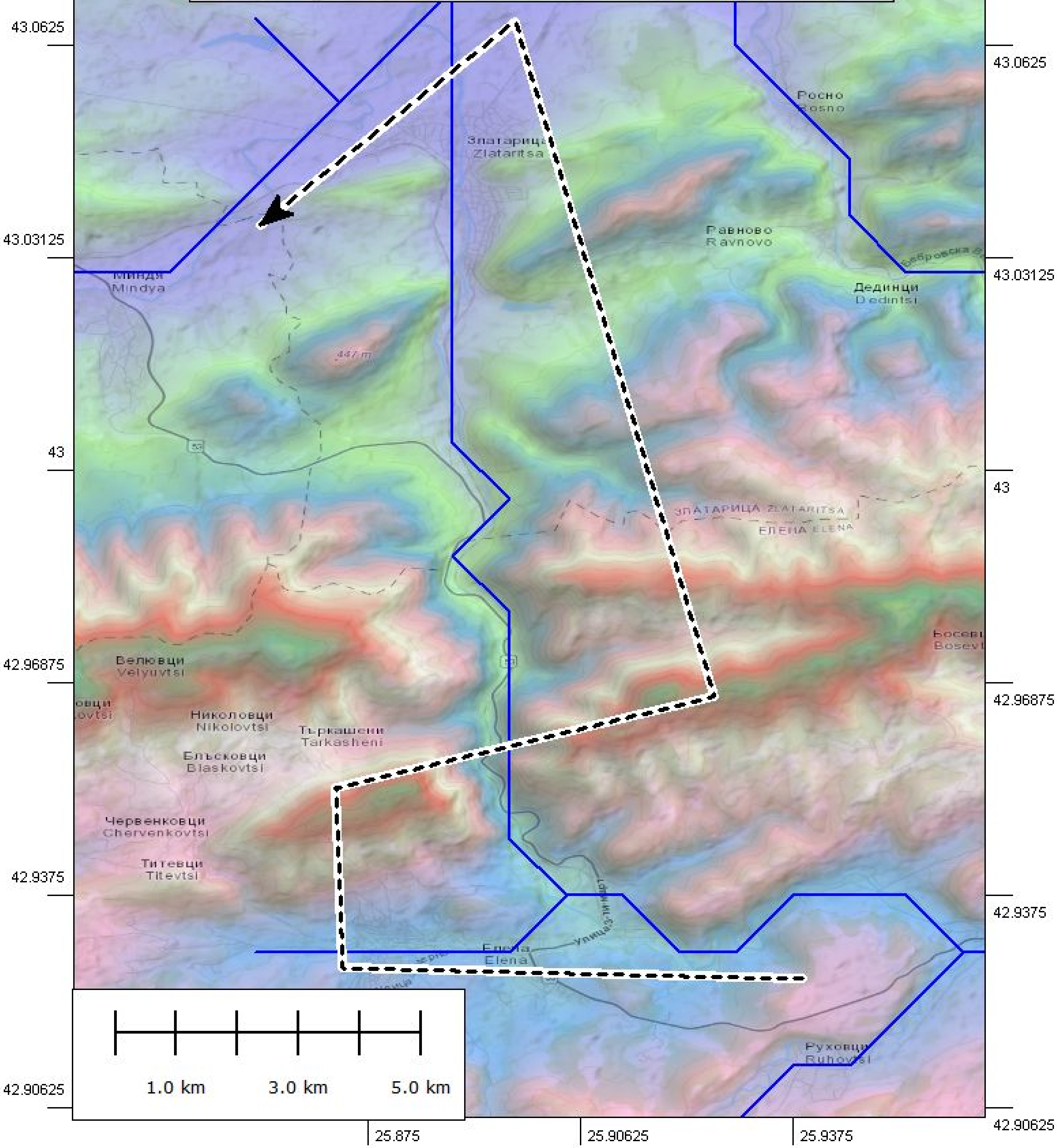

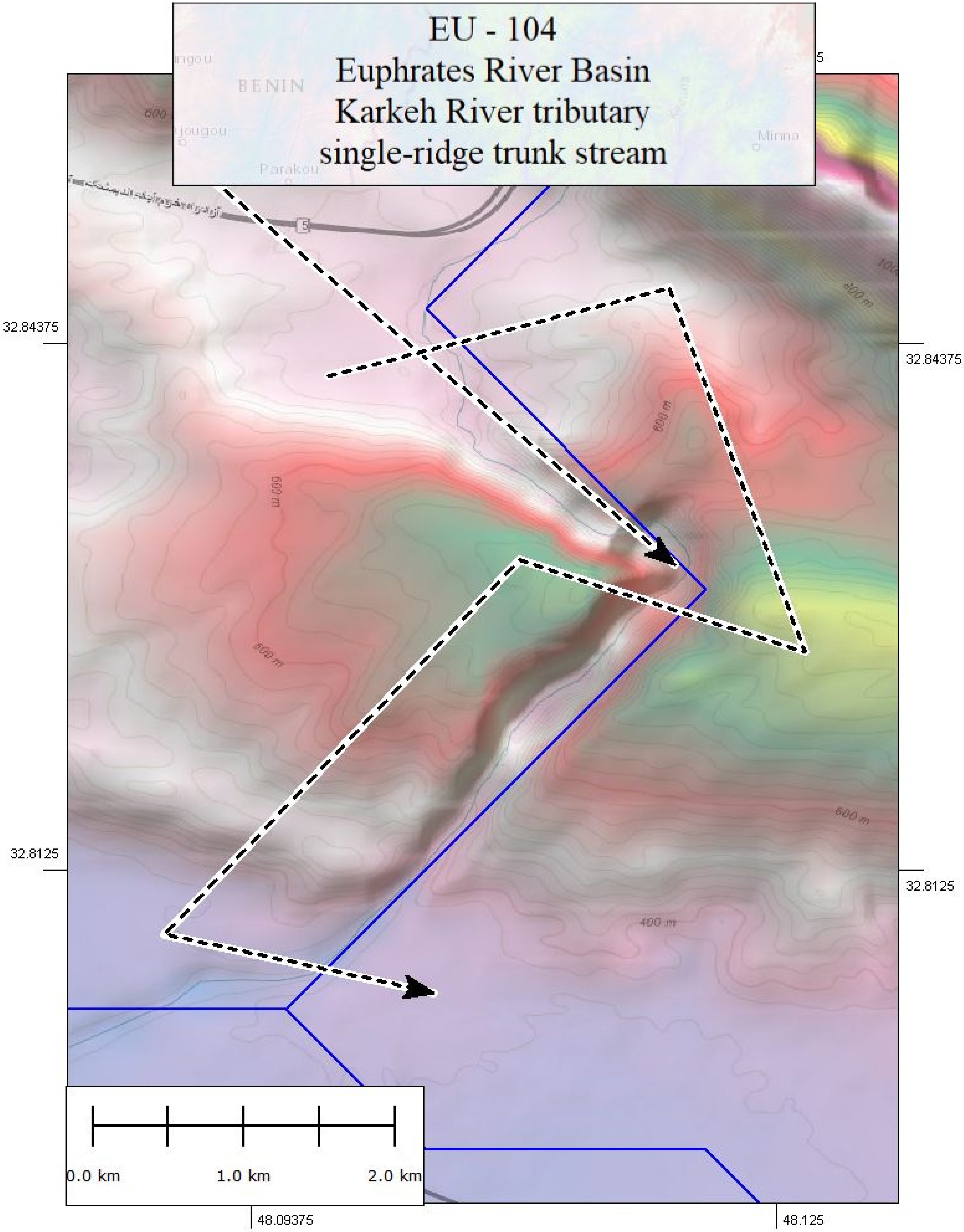
EU - 104
Euphrates River Basin
Karkeh River tributary
single-ridge trunk stream
32.84375
32.84375
32.8125
32.8125
48.09375
48.125
600 m
600 m
600 m
600 m
400 m
0.0 km
1.0 km
2.0 km

EU - 106
Prut River Basin
Veselina River
single-ridge trunk stream

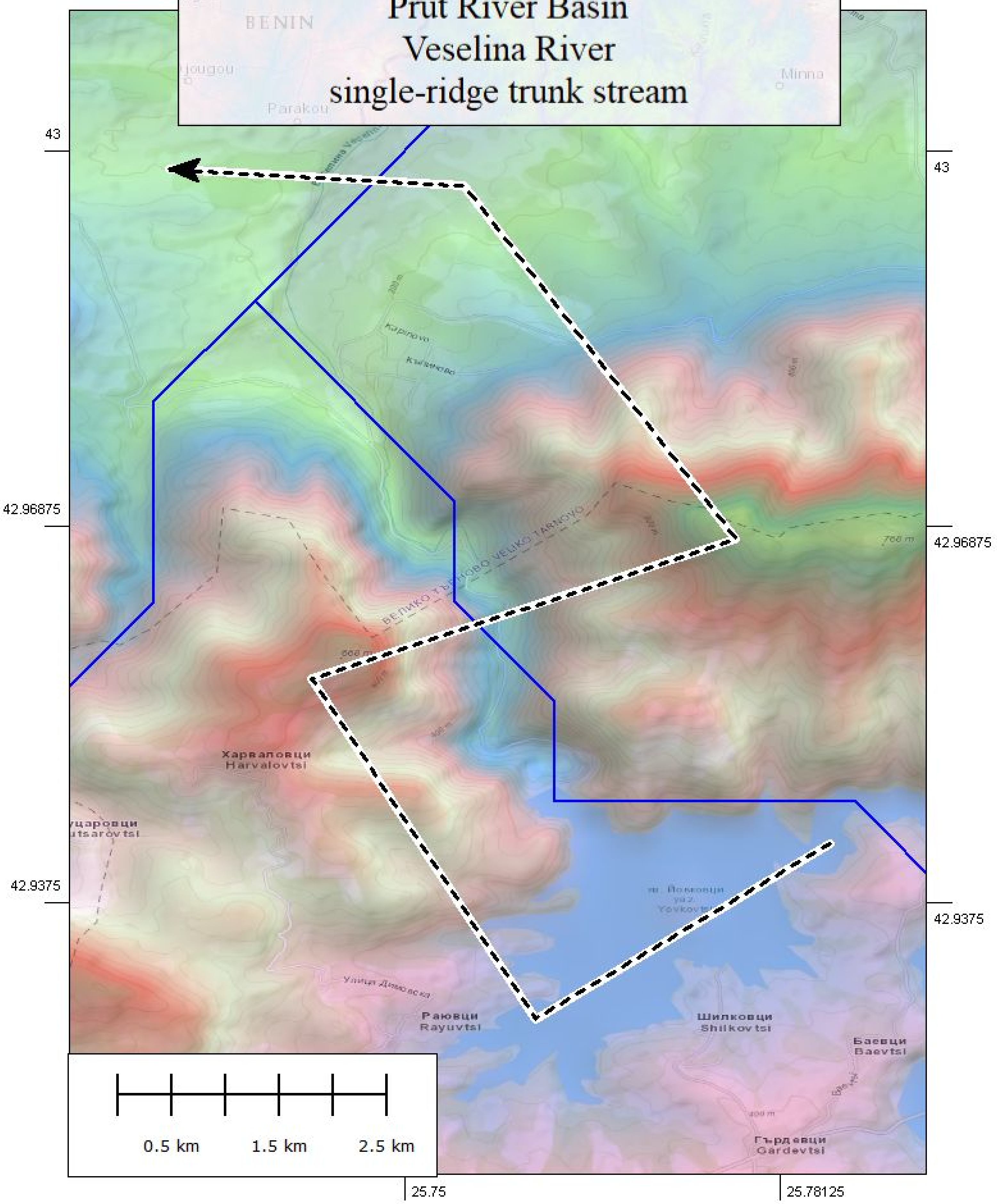

EU - 107
Tagus River Basin
Tagus River
multi-ridge trunk stream

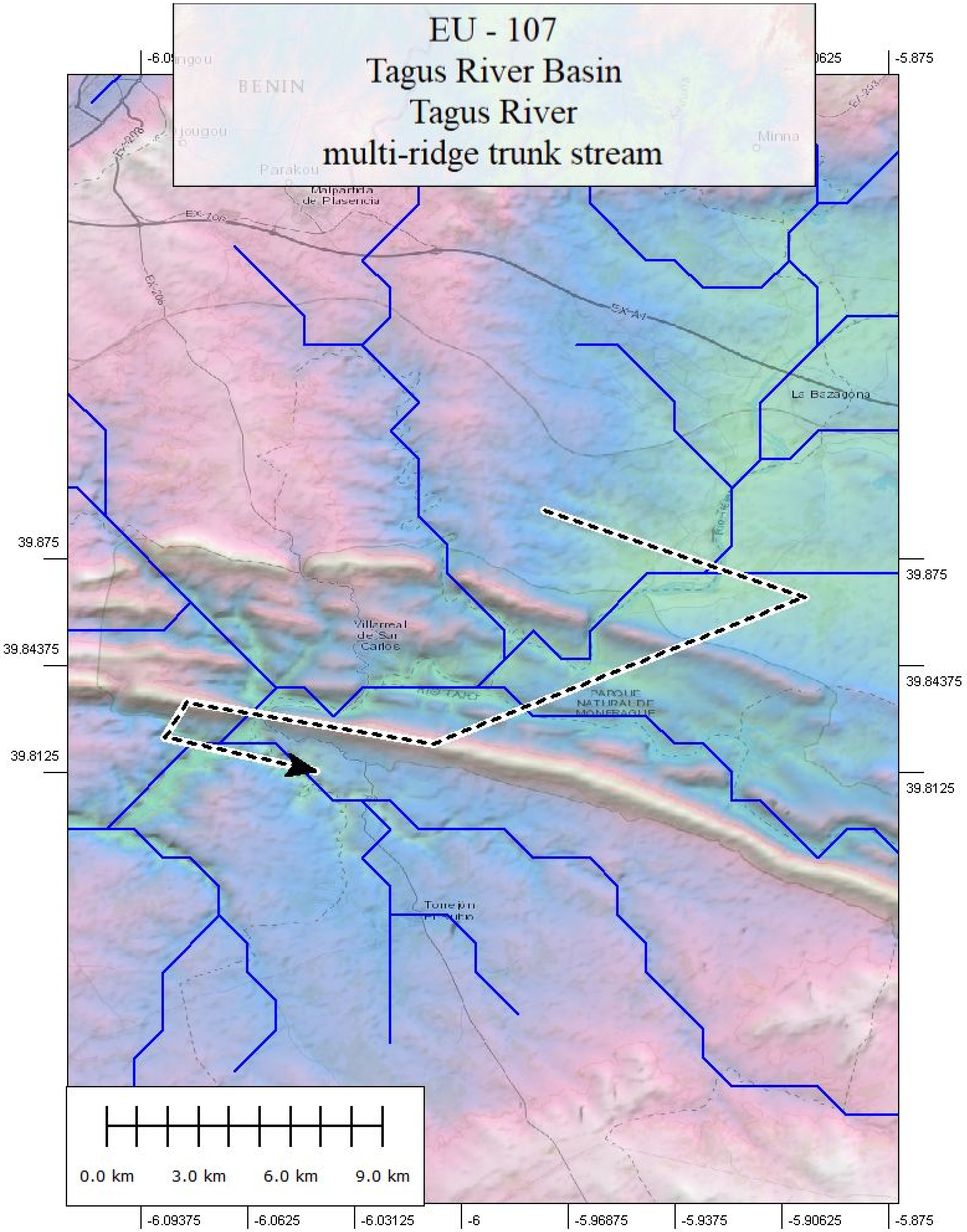

EU - 108
Tagus River Basin
Tagus River
single-ridge trunk stream

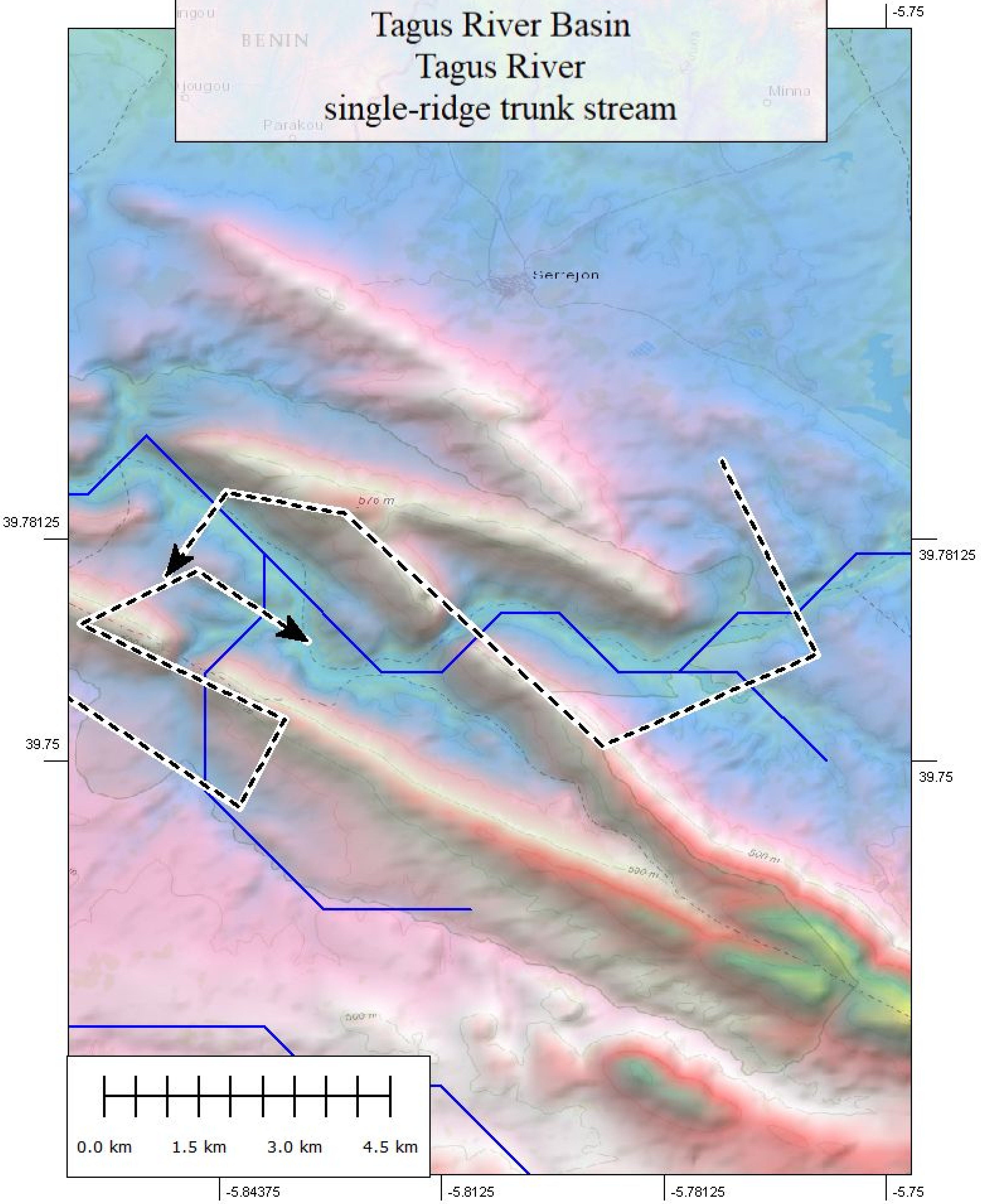

EU - 109
Rhone River Basin
Guiers River
single-ridge trunk stream

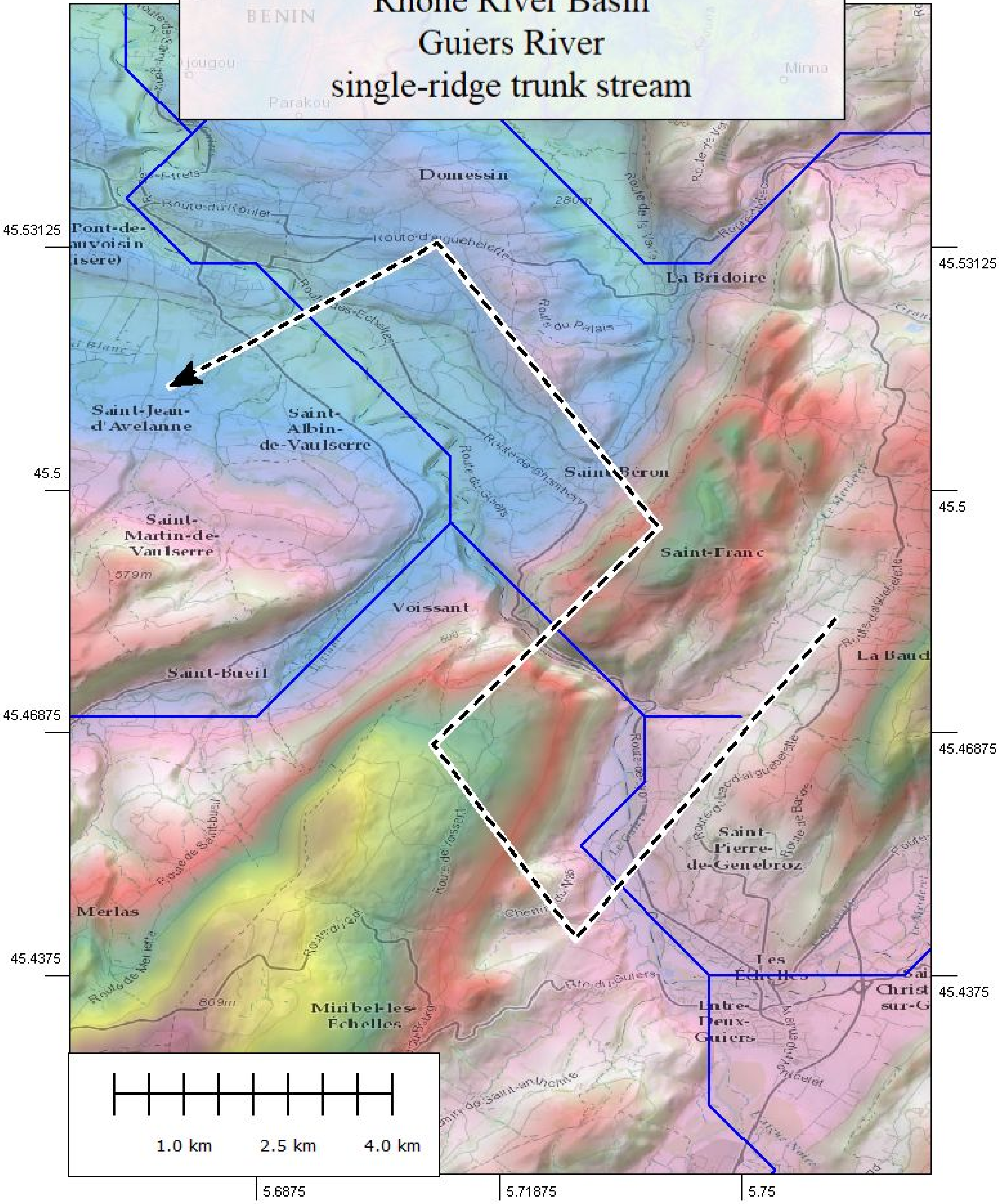

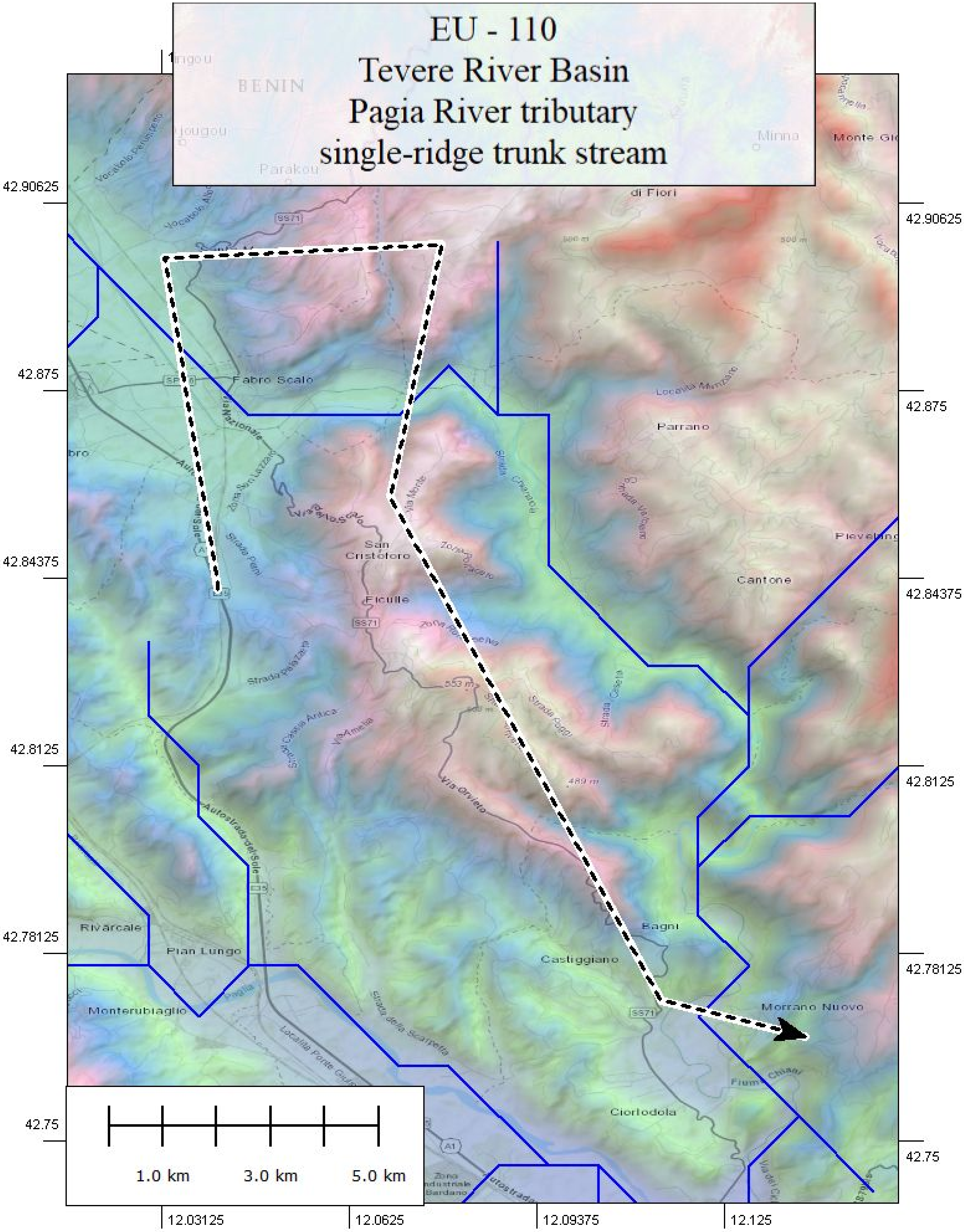
EU - 110
Tevere River Basin
Pagia River tributary
single-ridge trunk stream
42.90625
42.875
42.84375
42.8125
42.78125
42.75
12.03125
12.0625
12.09375
12.125
1.0 km
3.0 km
5.0 km
Fabro Scalo
Parrano
San Cristoforo
Ficulle
Cantone
Bagni
Castiggiano
Rivarcale
Pian Lungo
Monterubiaglio
Morrano Nuovo
Ciorlodola
di Fiori

EU - 111
Euphrates River Basin
Sorkh Ab River
multi-ridge trunk stream

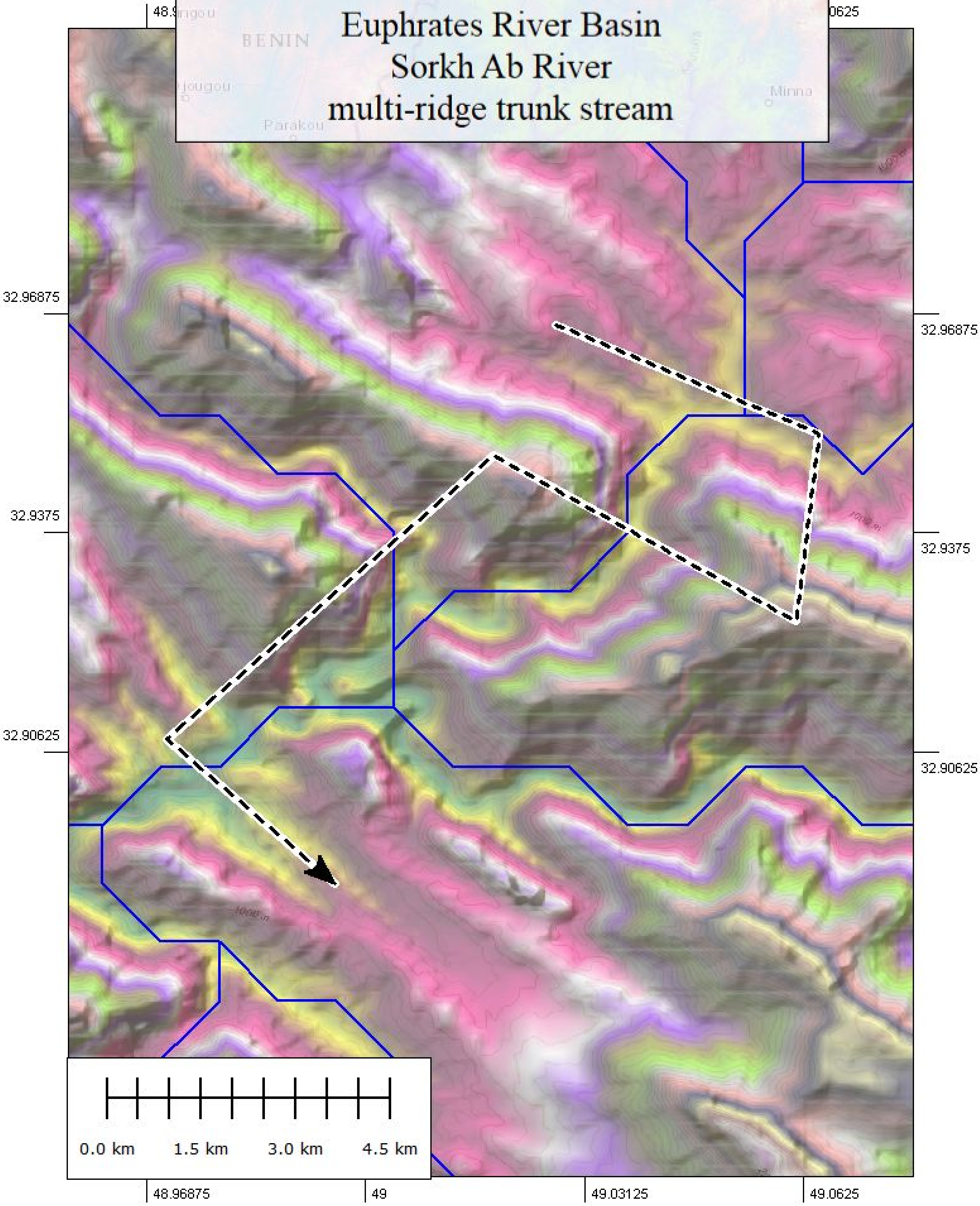

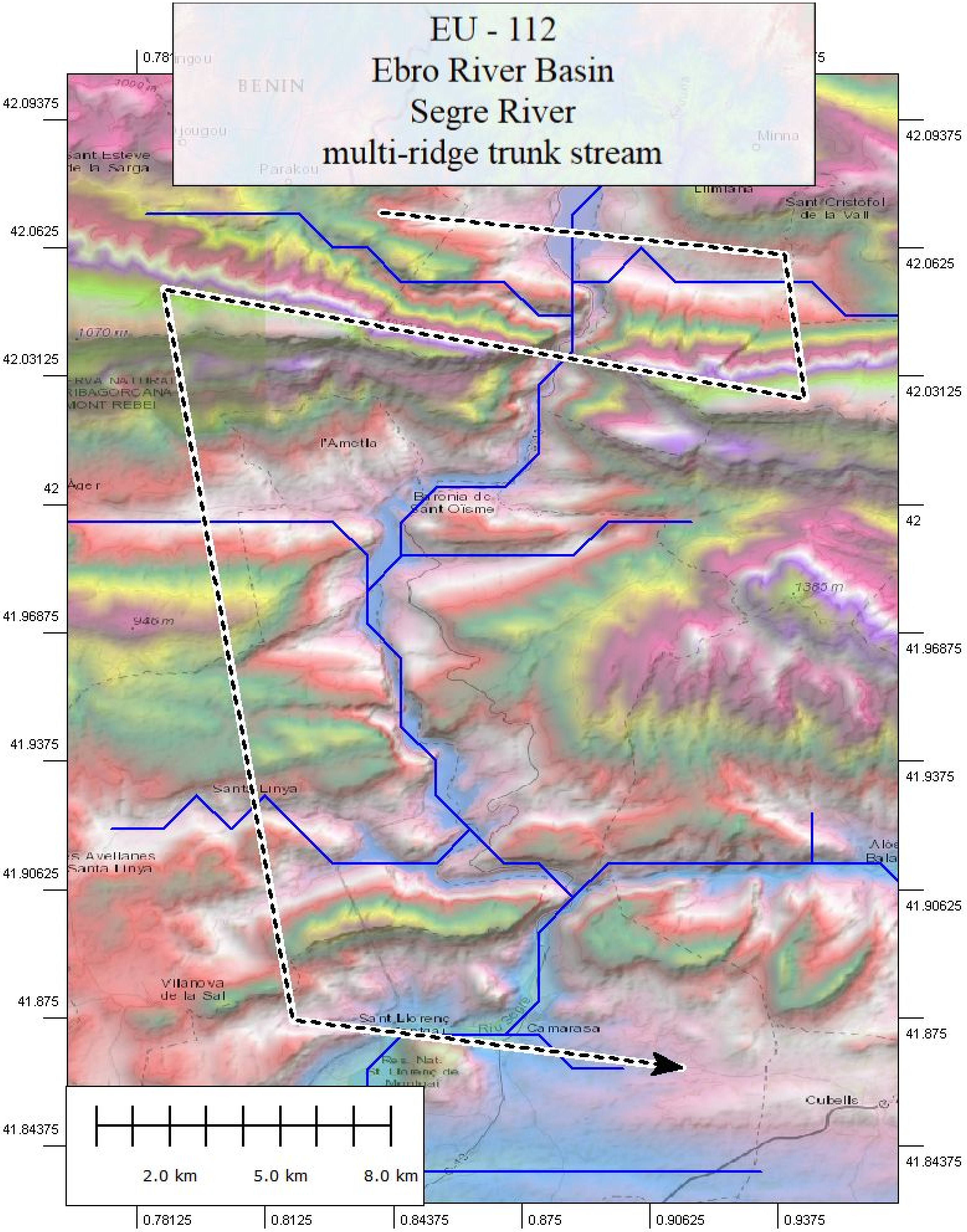
EU - 112
Ebro River Basin
Segre River
multi-ridge trunk stream
42.09375
42.0625
42.03125
42
41.96875
41.9375
41.90625
41.875
41.84375
0.78125
0.8125
0.84375
0.875
0.90625
0.9375
Sant Esteve de la Sarga
Sant Cristòfol de la Vall
1070 m
l'Ametlla
Àger
Baronia de Sant Oïsme
946 m
1365 m
Santa Linya
Vilanova de la Sal
Sant Llorenç
Camarasa
Cubells
2.0 km
5.0 km
8.0 km

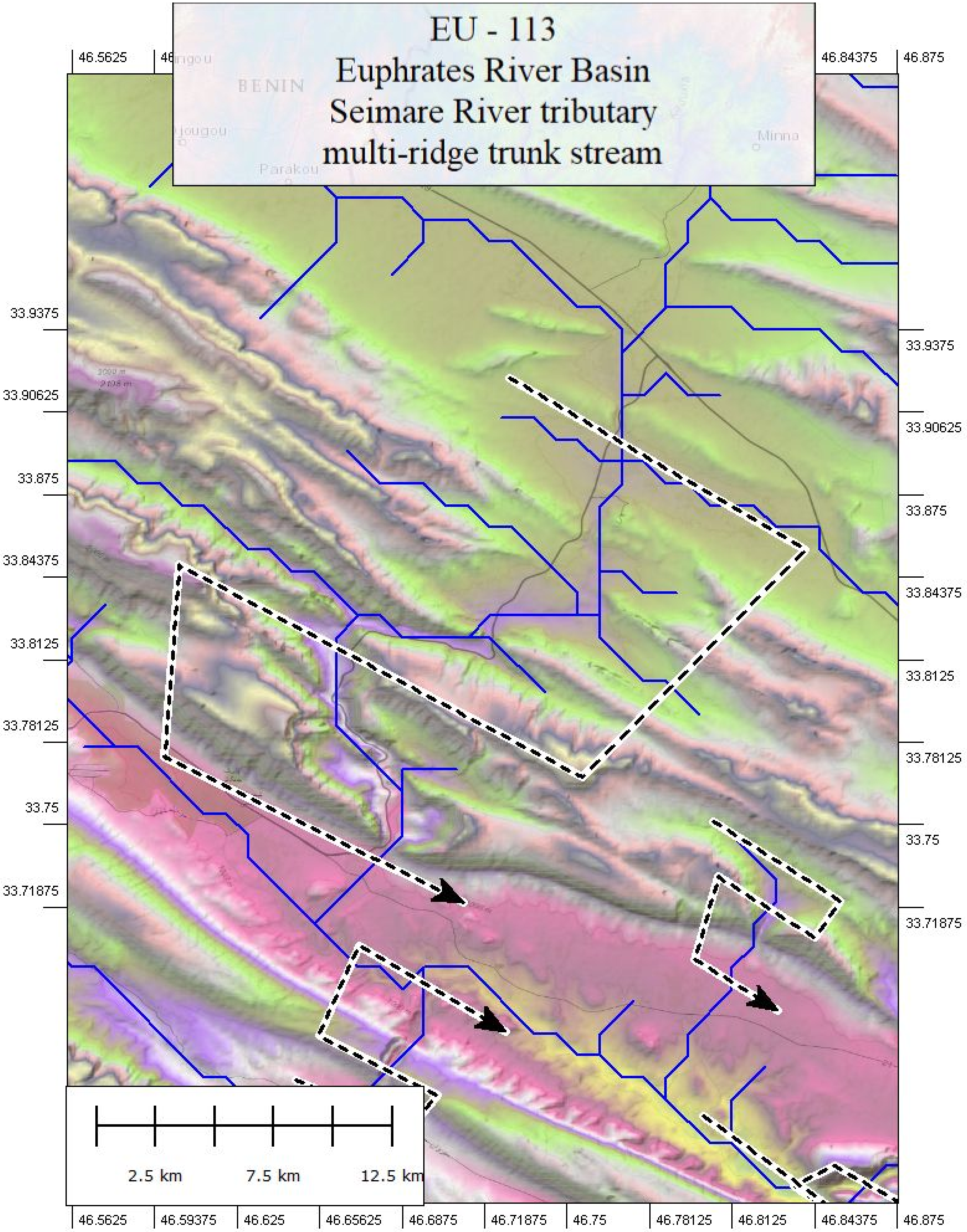
EU - 113
Euphrates River Basin
Seimare River tributary
multi-ridge trunk stream
46.5625
46.59375
46.625
46.65625
46.6875
46.71875
46.75
46.78125
46.8125
46.84375
46.875
33.9375
33.90625
33.875
33.84375
33.8125
33.78125
33.75
33.71875
2.5 km
7.5 km
12.5 km

EU - 115
Ebro River Basin
Ebro River
single-ridge trunk stream

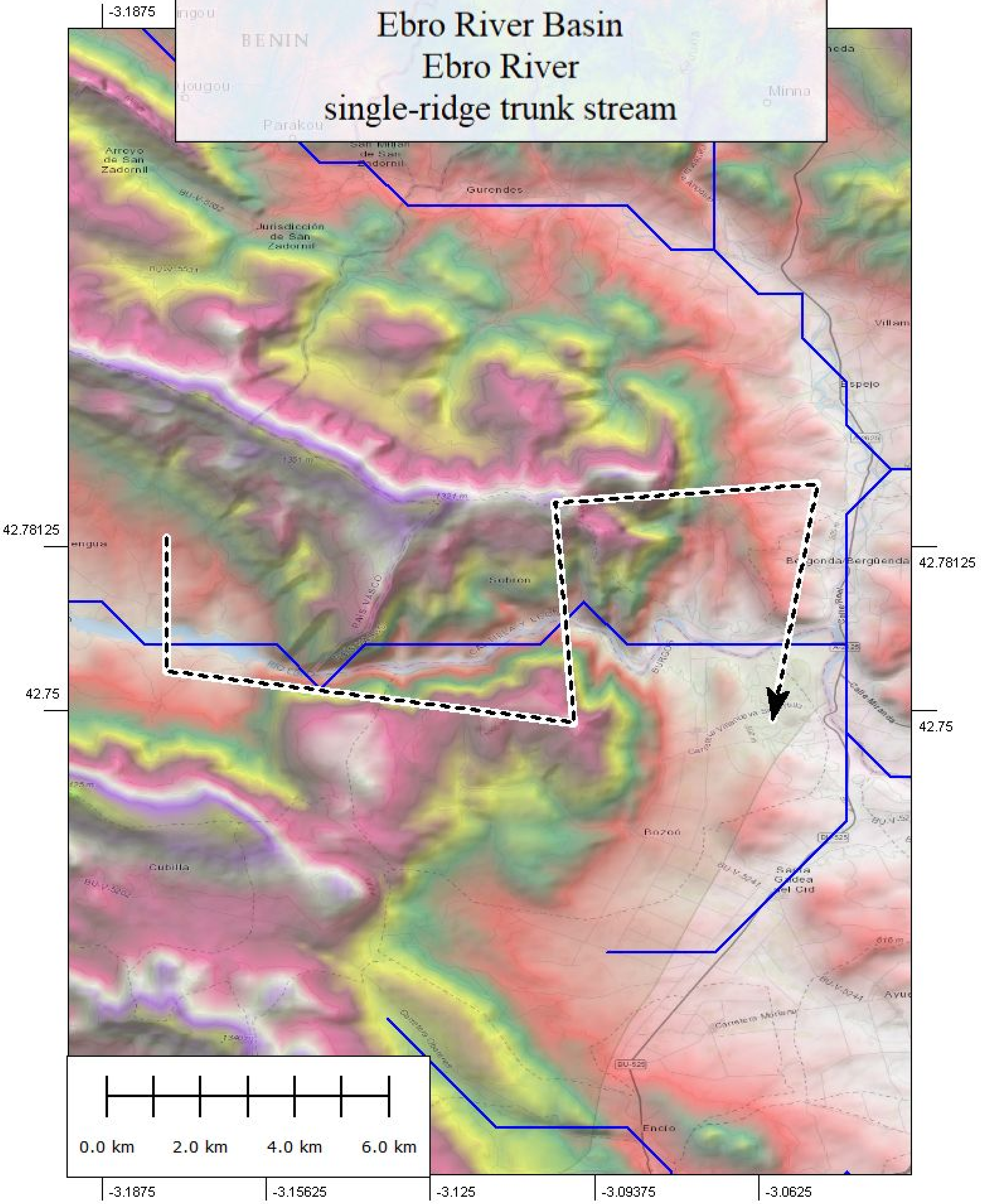

EU - 116
Euphrates River Basin
Beheshtabad River
multi-ridge trunk stream

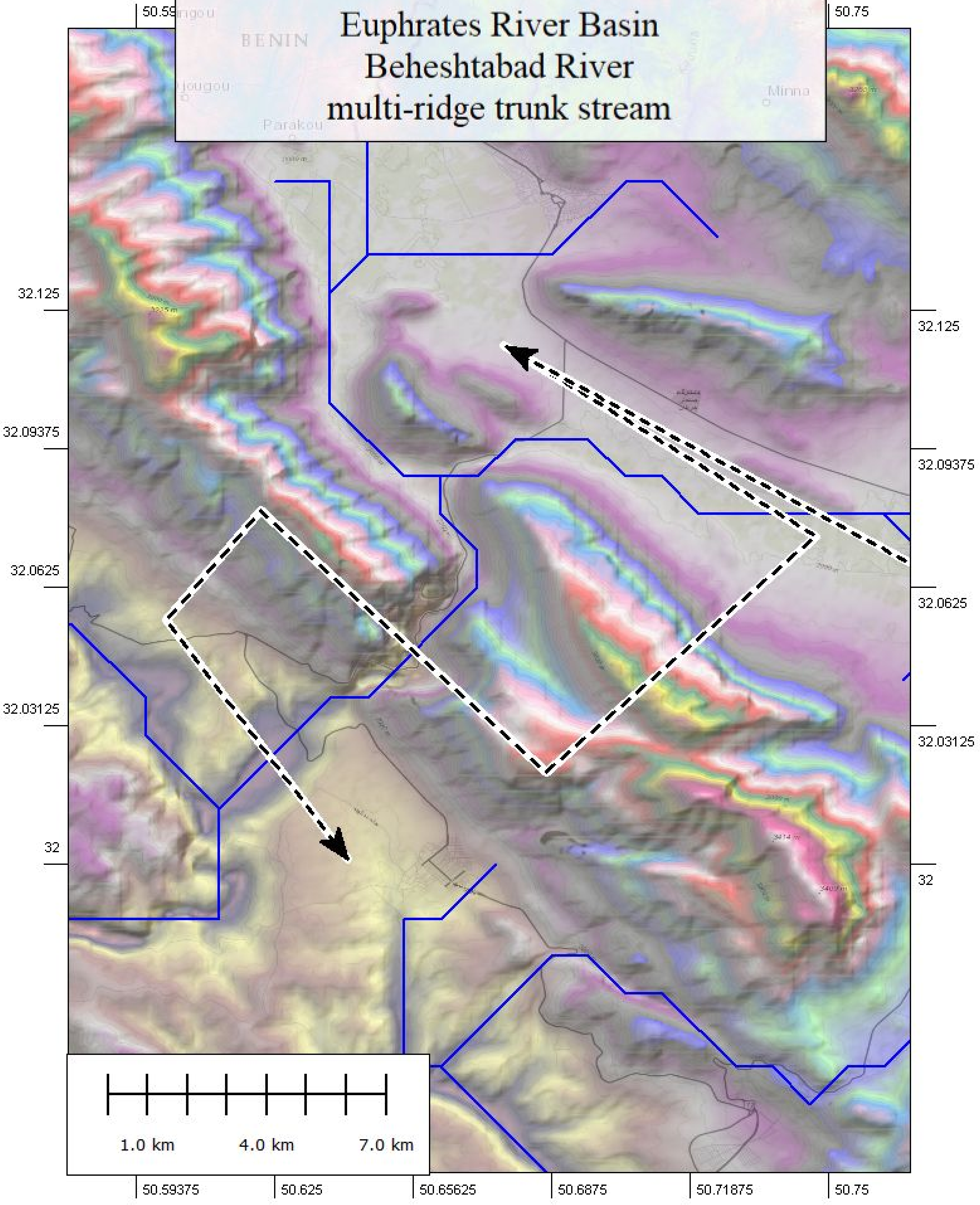

EU - 117
Euphrates River Basin
Dicle River
single-ridge trunk stream

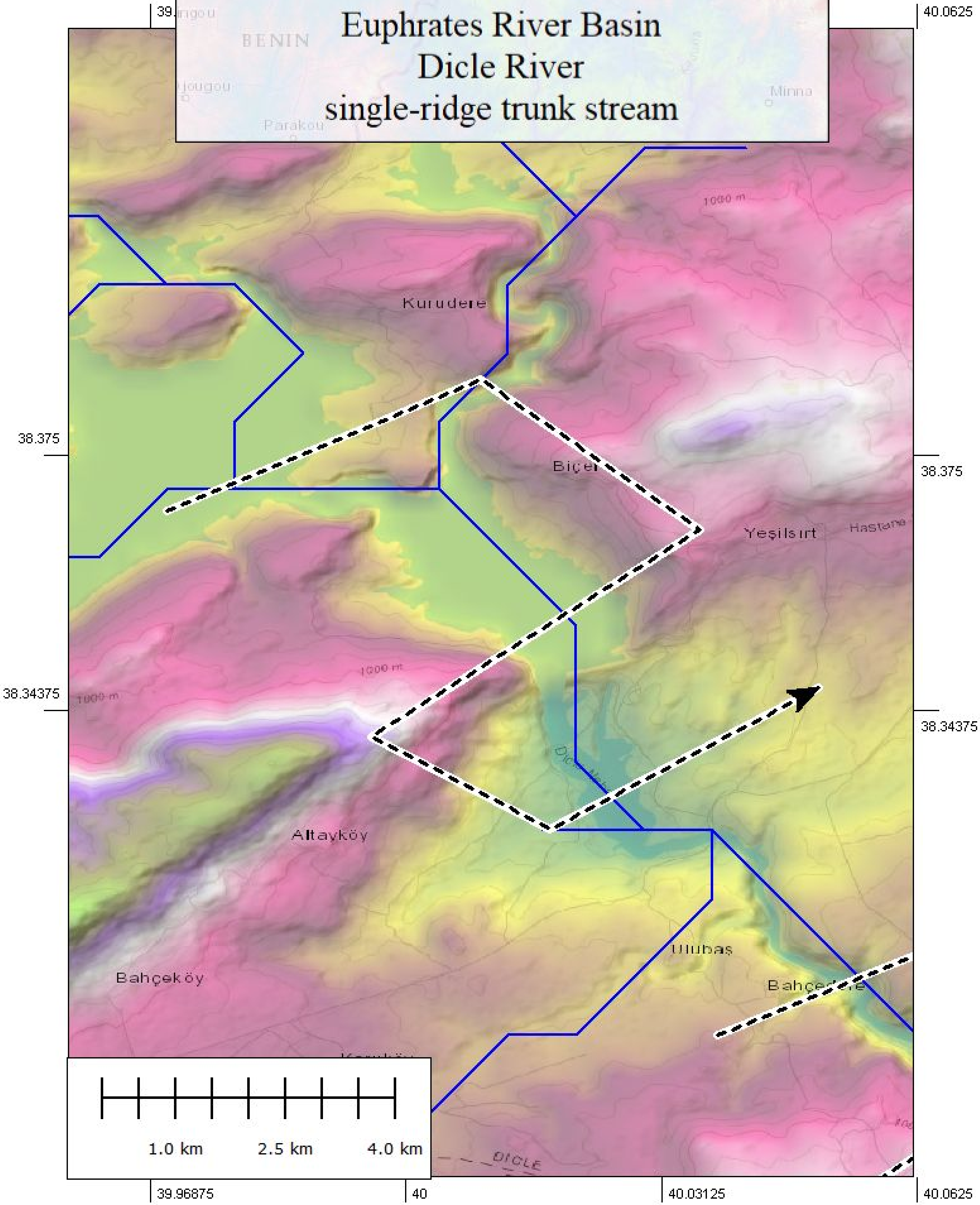

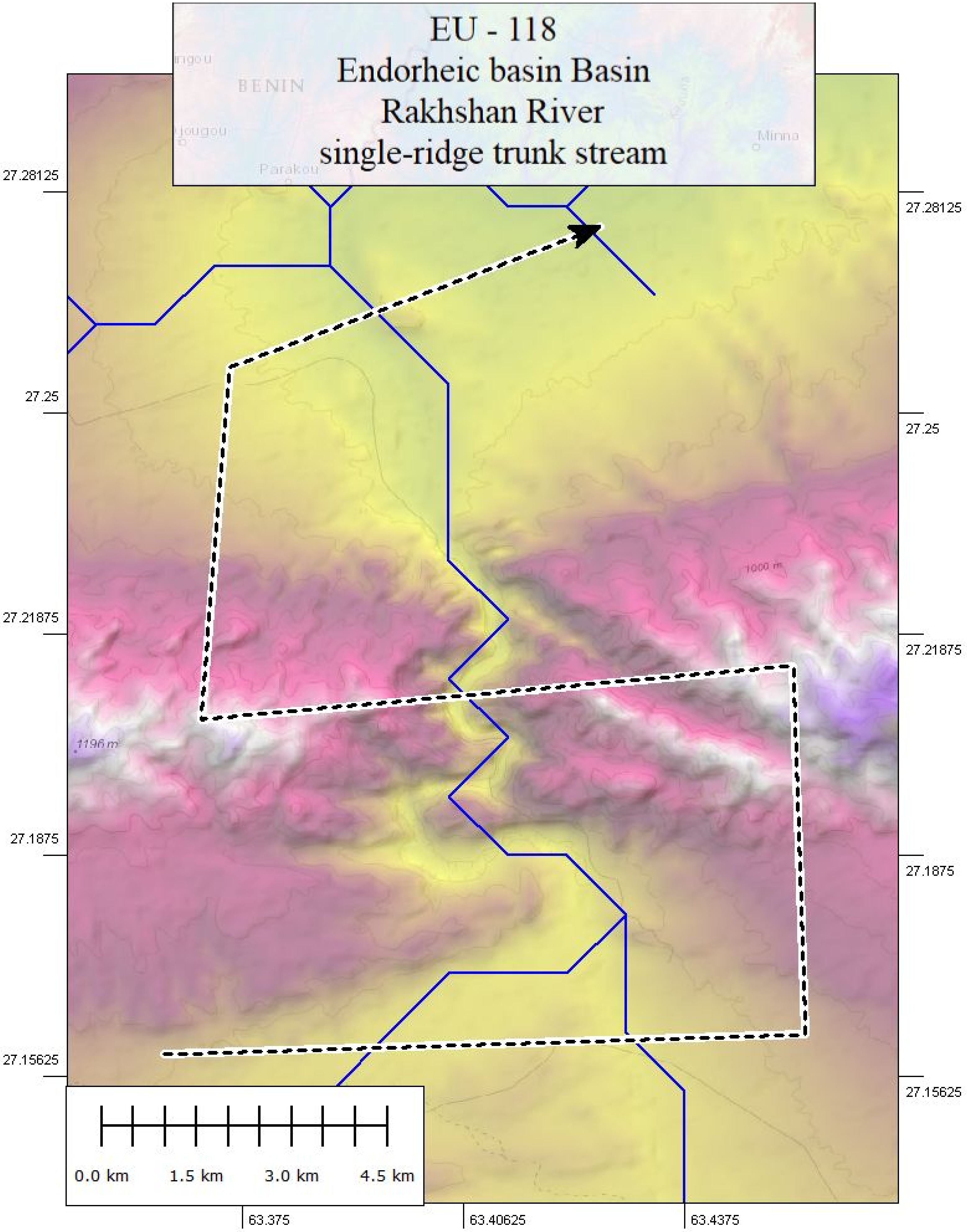
EU - 118
Endorheic basin Basin
Rakhshan River
single-ridge trunk stream
BENIN
Parakou
Minna
1000 m
1196 m
27.28125
27.25
27.21875
27.1875
27.15625
0.0 km
1.5 km
3.0 km
4.5 km
63.375
63.40625
63.4375

EU - 119
Endorheic basin Basin
Rakhshan River
single-ridge trunk stream

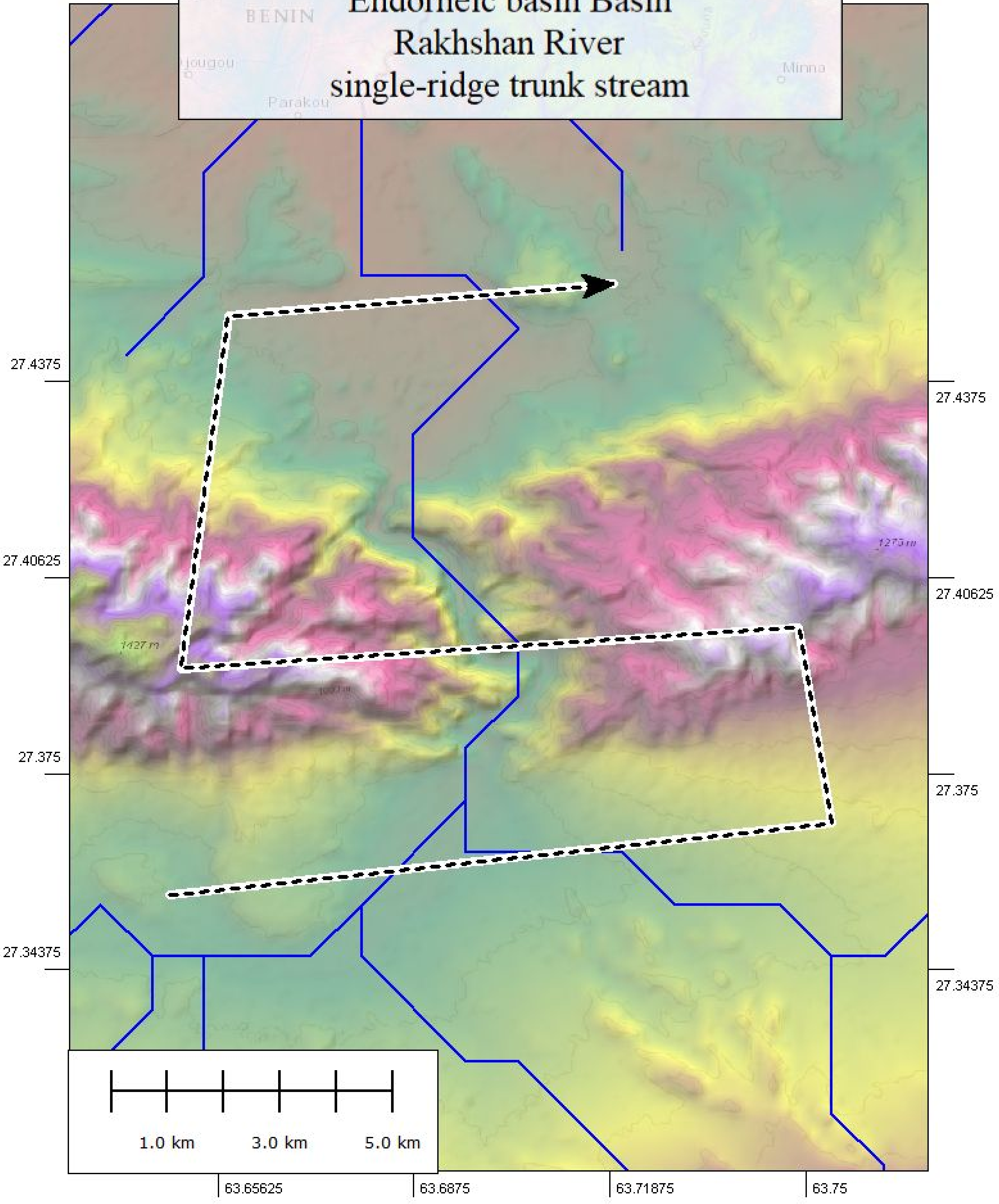

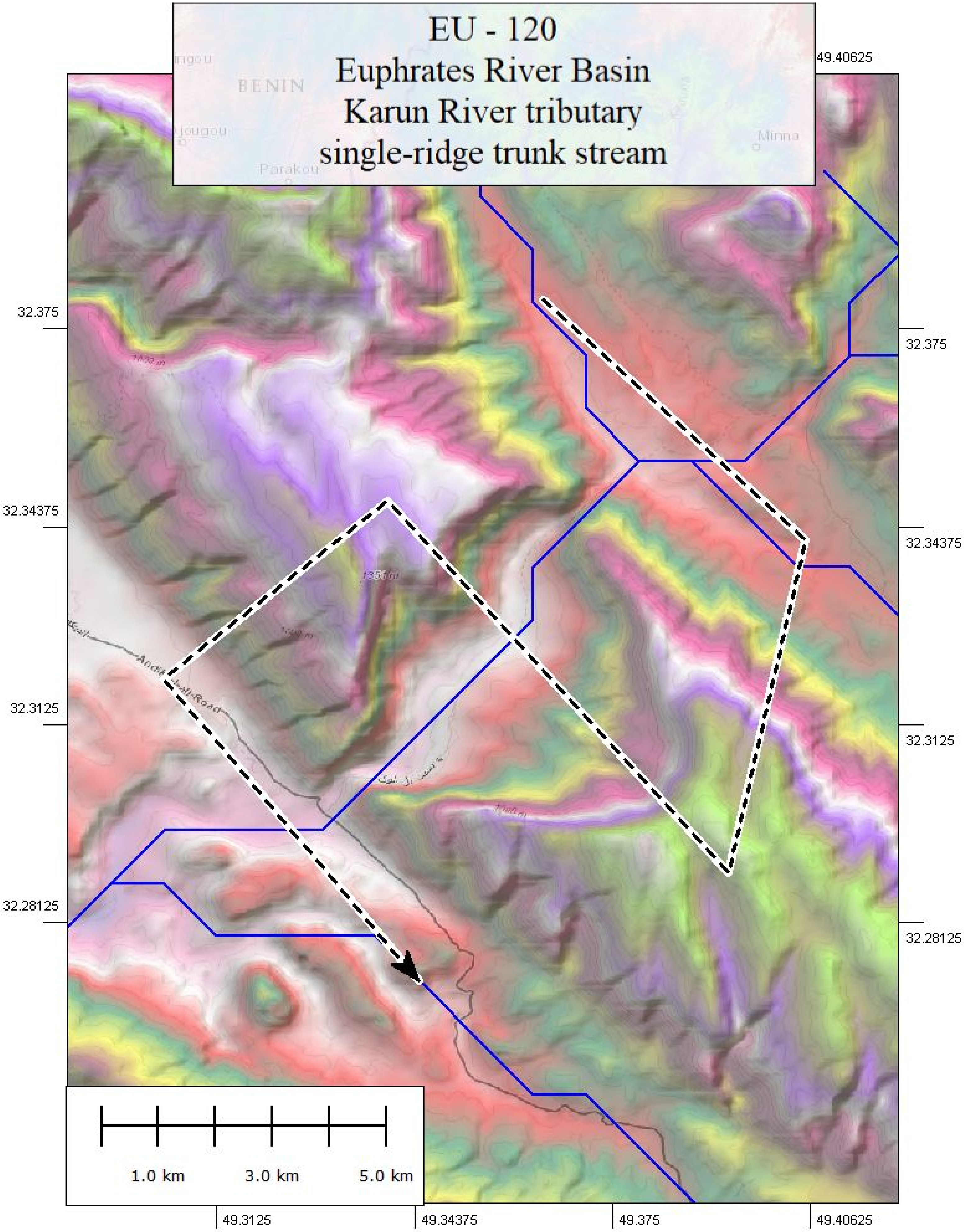
EU - 120
Euphrates River Basin
Karun River tributary
single-ridge trunk stream
BENIN
Parakou
Minna
49.40625
32.375
32.34375
32.3125
32.28125
32.375
32.34375
32.3125
32.28125
1.0 km
3.0 km
5.0 km
49.3125
49.34375
49.375
49.40625

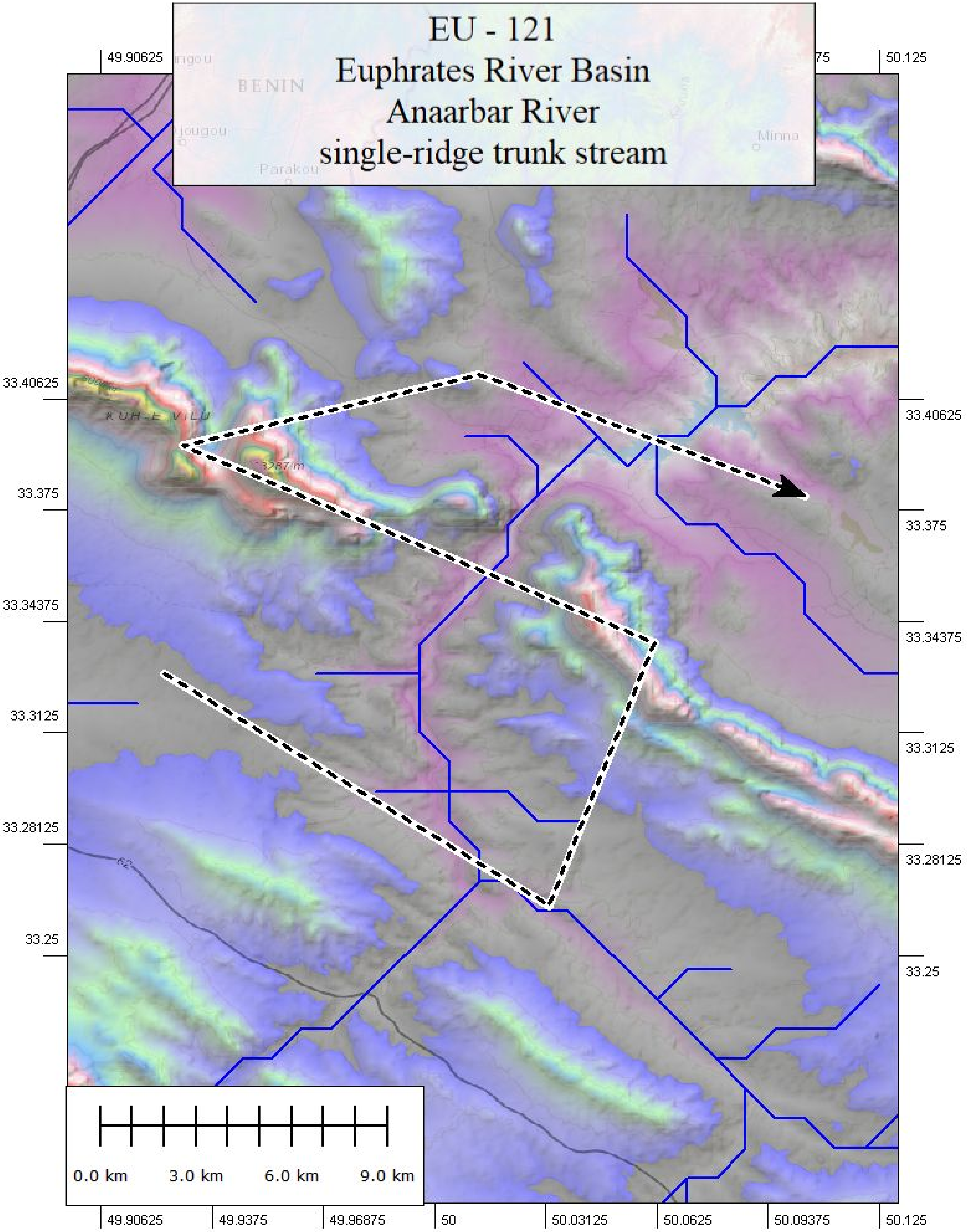
EU - 121
Euphrates River Basin
Anaarbar River
single-ridge trunk stream
49.90625
50.125
33.40625
33.375
33.34375
33.3125
33.28125
33.25
49.9375
49.96875
50
50.03125
50.0625
50.09375
0.0 km
3.0 km
6.0 km
9.0 km

EU - 122
Ebro River Basin
Gallego River
single-ridge trunk stream

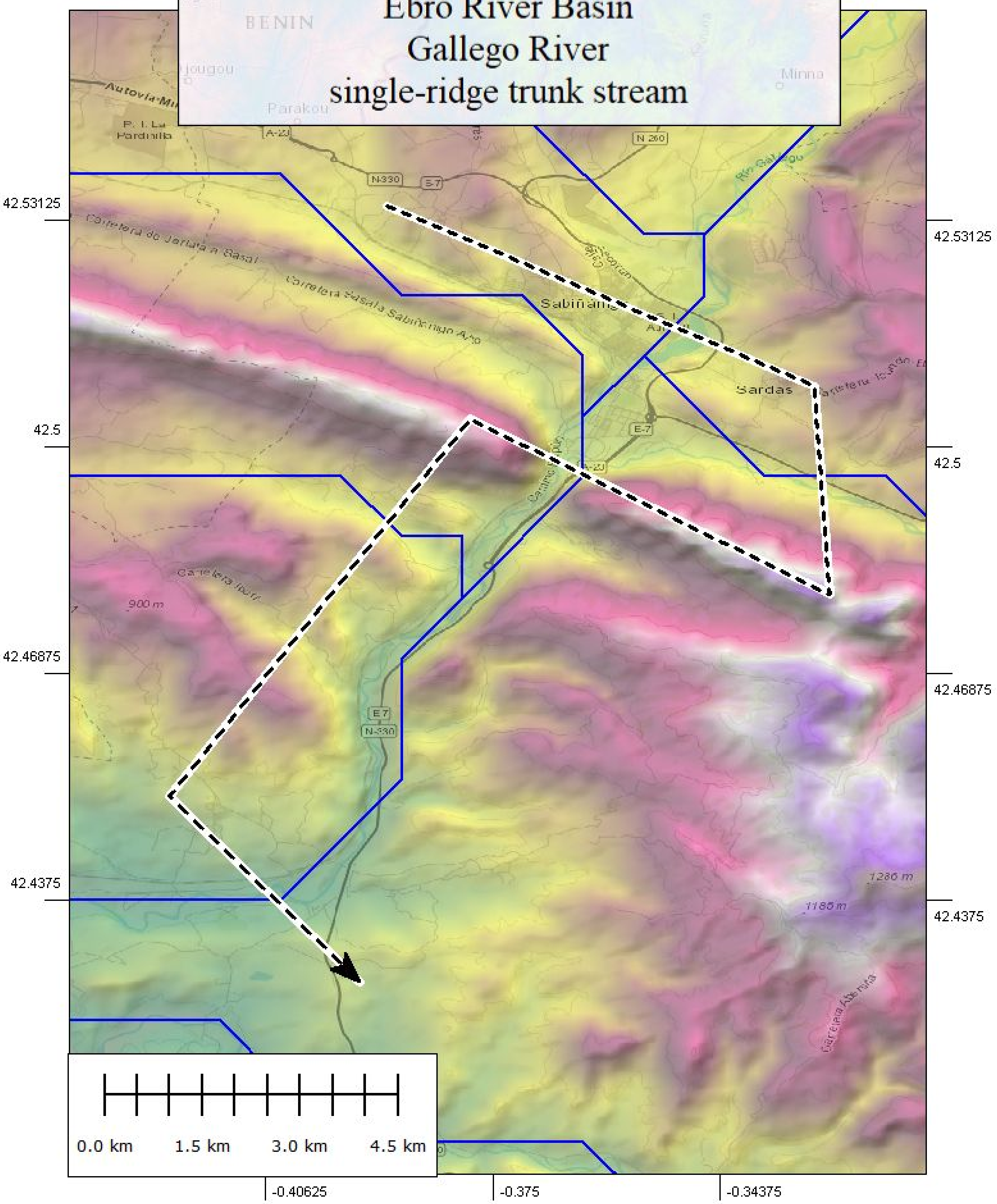

EU - 123
Shur River Basin
single-ridge trunk stream

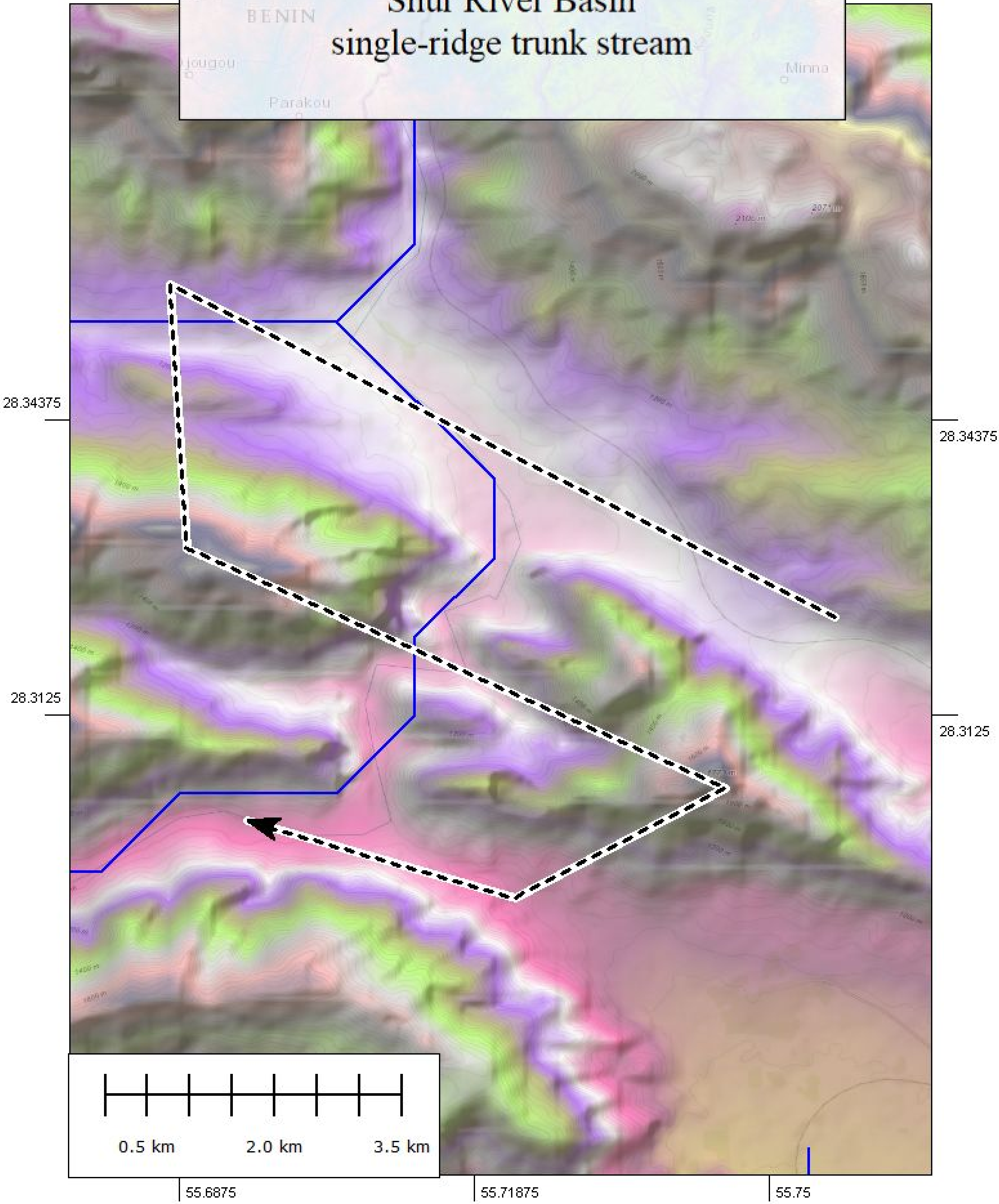

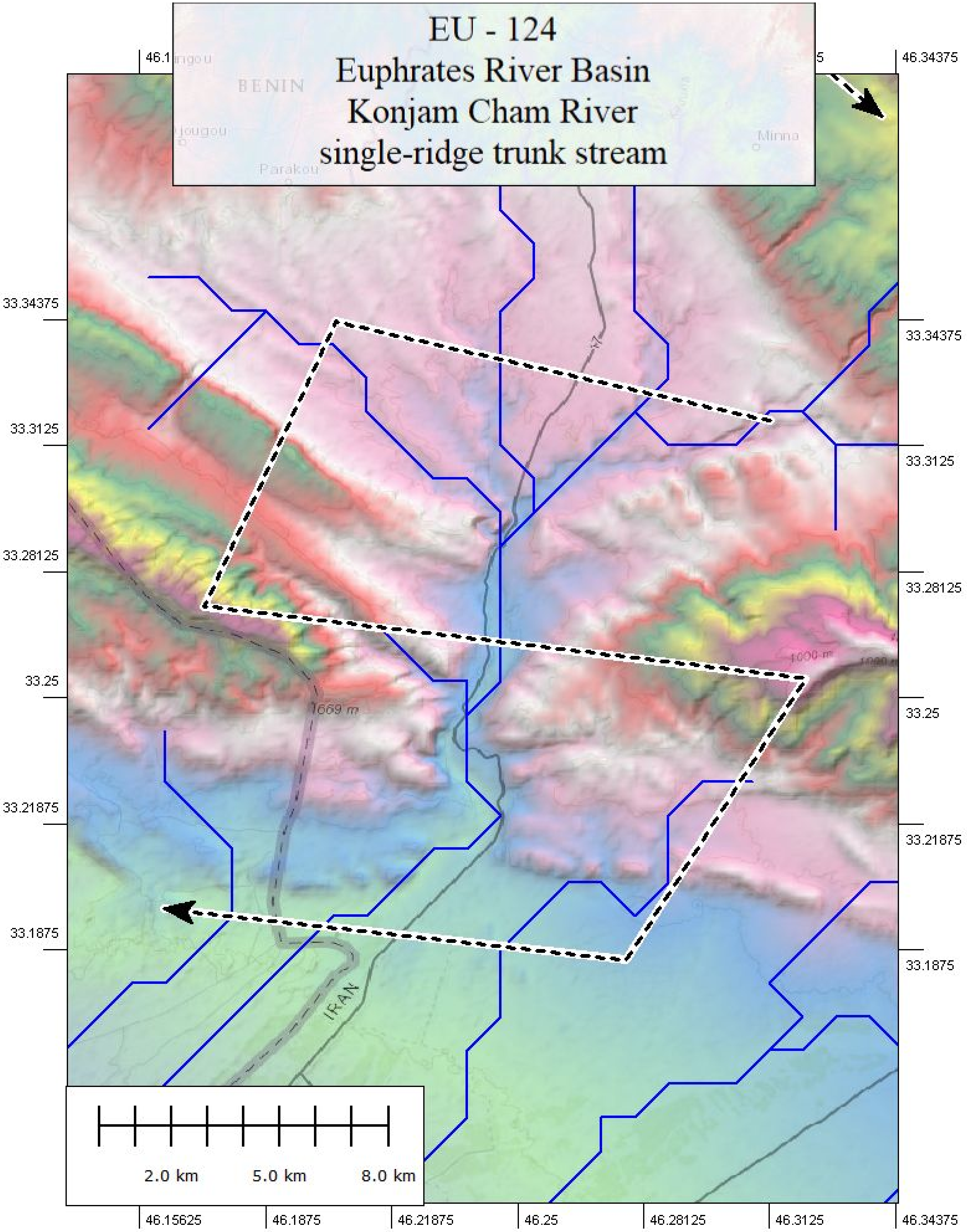
EU - 124
Euphrates River Basin
Konjam Cham River
single-ridge trunk stream
46.34375
BENIN
Parakou
Minna
33.34375
33.3125
33.28125
33.25
33.21875
33.1875
1669 m
1000 m
IRAN
2.0 km
5.0 km
8.0 km
46.15625
46.1875
46.21875
46.25
46.28125
46.3125

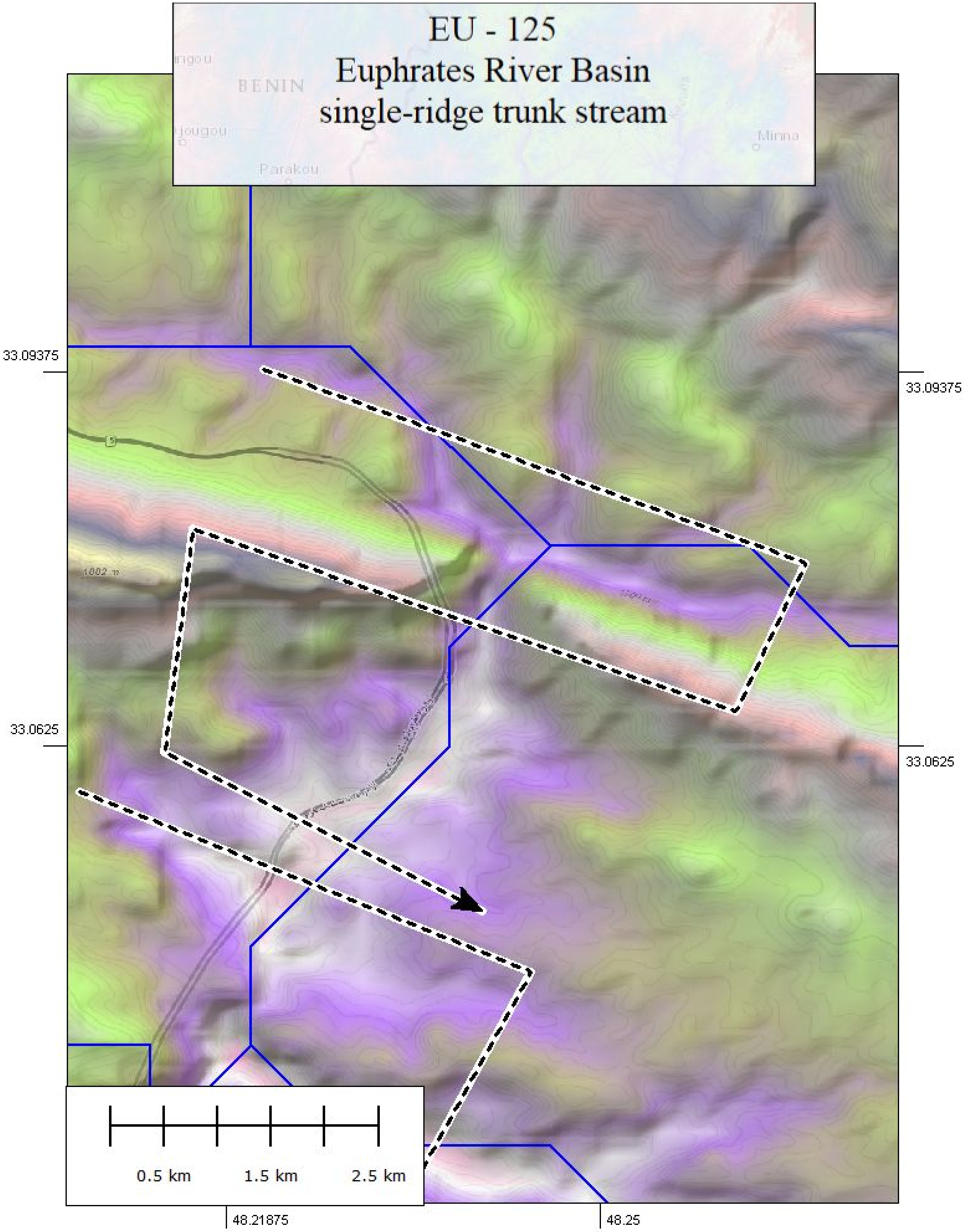
EU - 125
Euphrates River Basin
single-ridge trunk stream
BENIN
Parakou
33.09375
33.09375
33.0625
33.0625
0.5 km
1.5 km
2.5 km
48.21875
48.25

EU - 126
Euphrates River Basin
Kizilsu River
single-ridge trunk stream

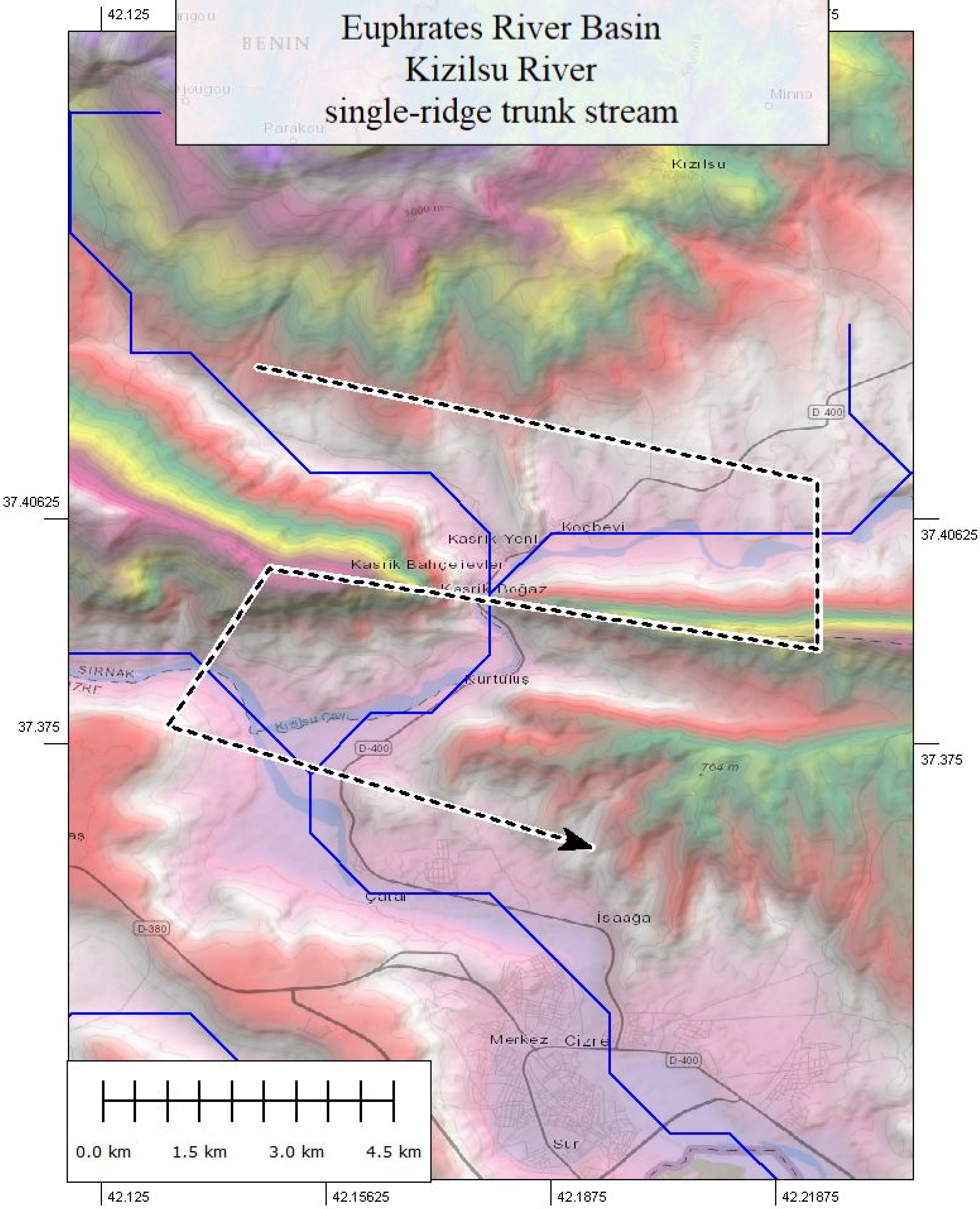

EU - 127
Tagus River Basin
Alberche River
single-ridge trunk stream

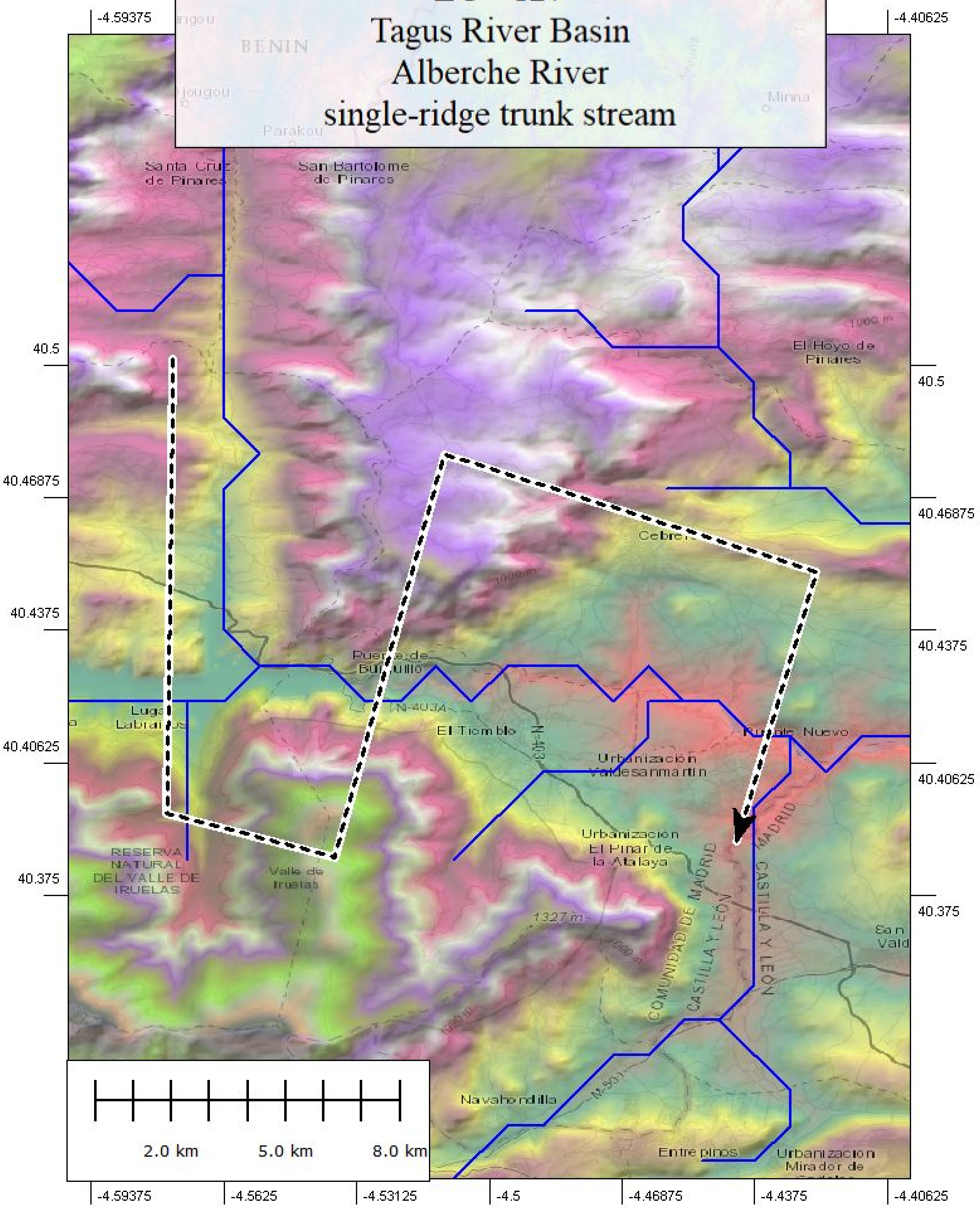

EU - 128
Euphrates River Basin
Sezar River
single-ridge trunk stream

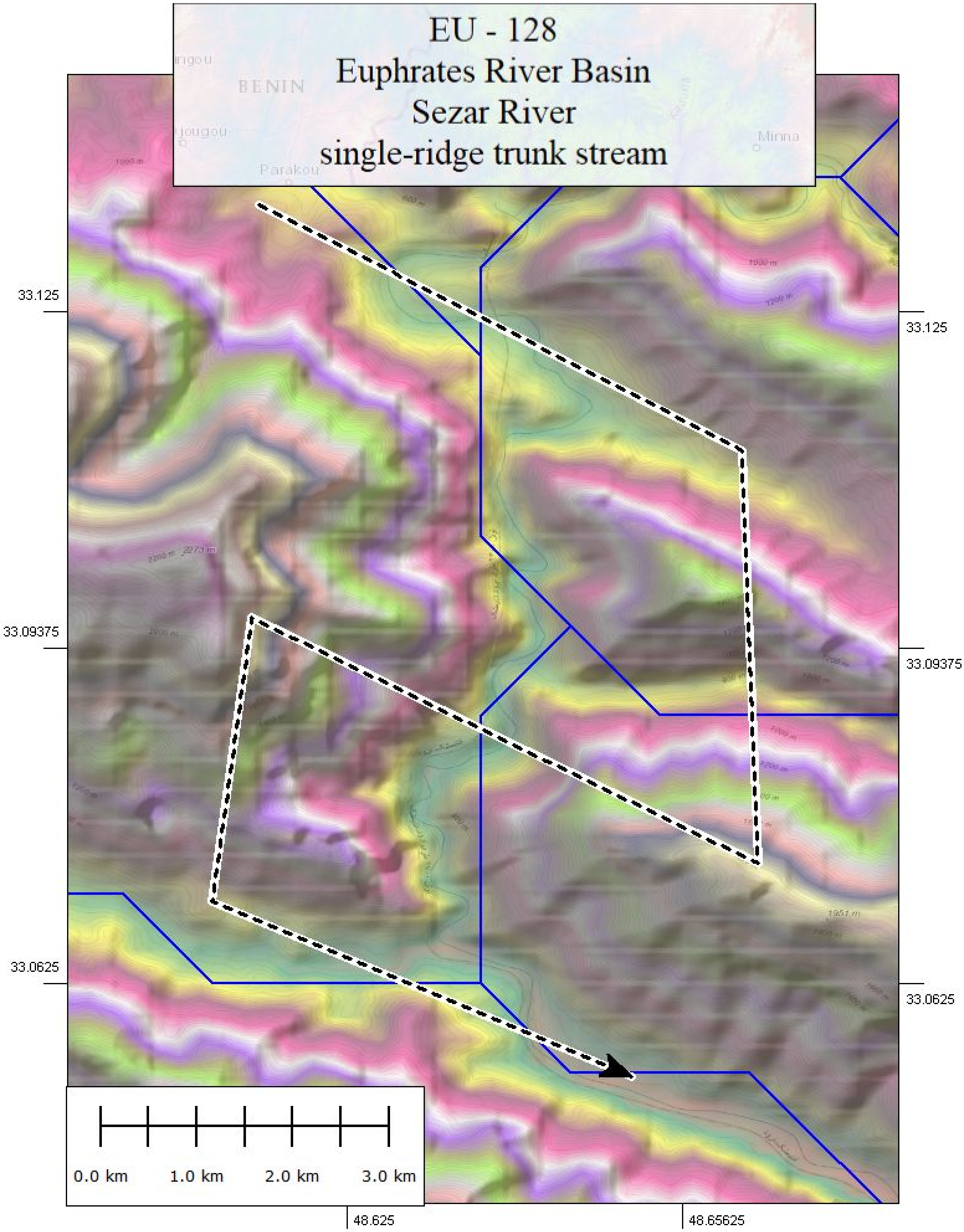

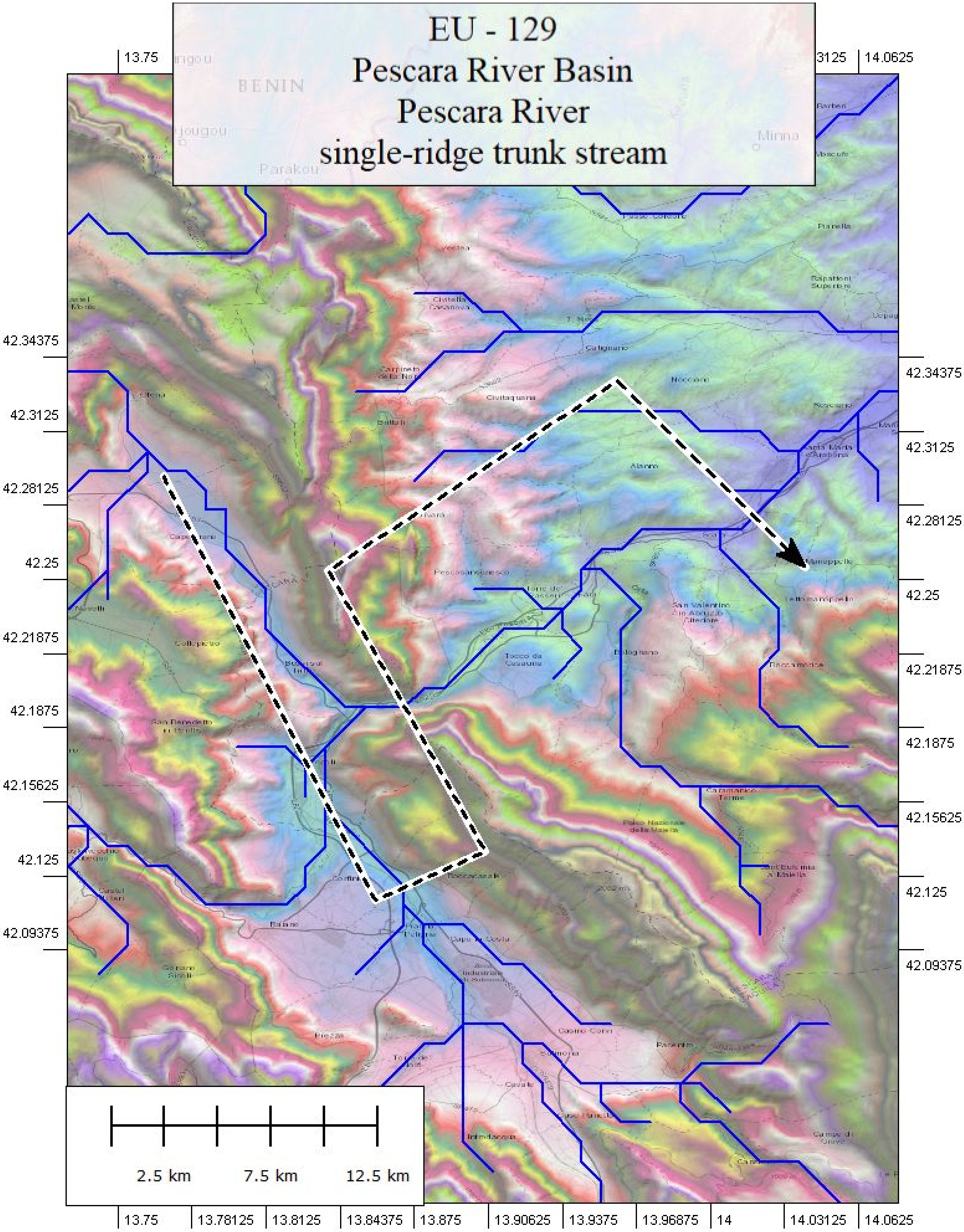
EU - 129
Pescara River Basin
Pescara River
single-ridge trunk stream
13.75
13.78125
13.8125
13.84375
13.875
13.90625
13.9375
13.96875
14
14.03125
14.0625
42.34375
42.3125
42.28125
42.25
42.21875
42.1875
42.15625
42.125
42.09375
2.5 km
7.5 km
12.5 km

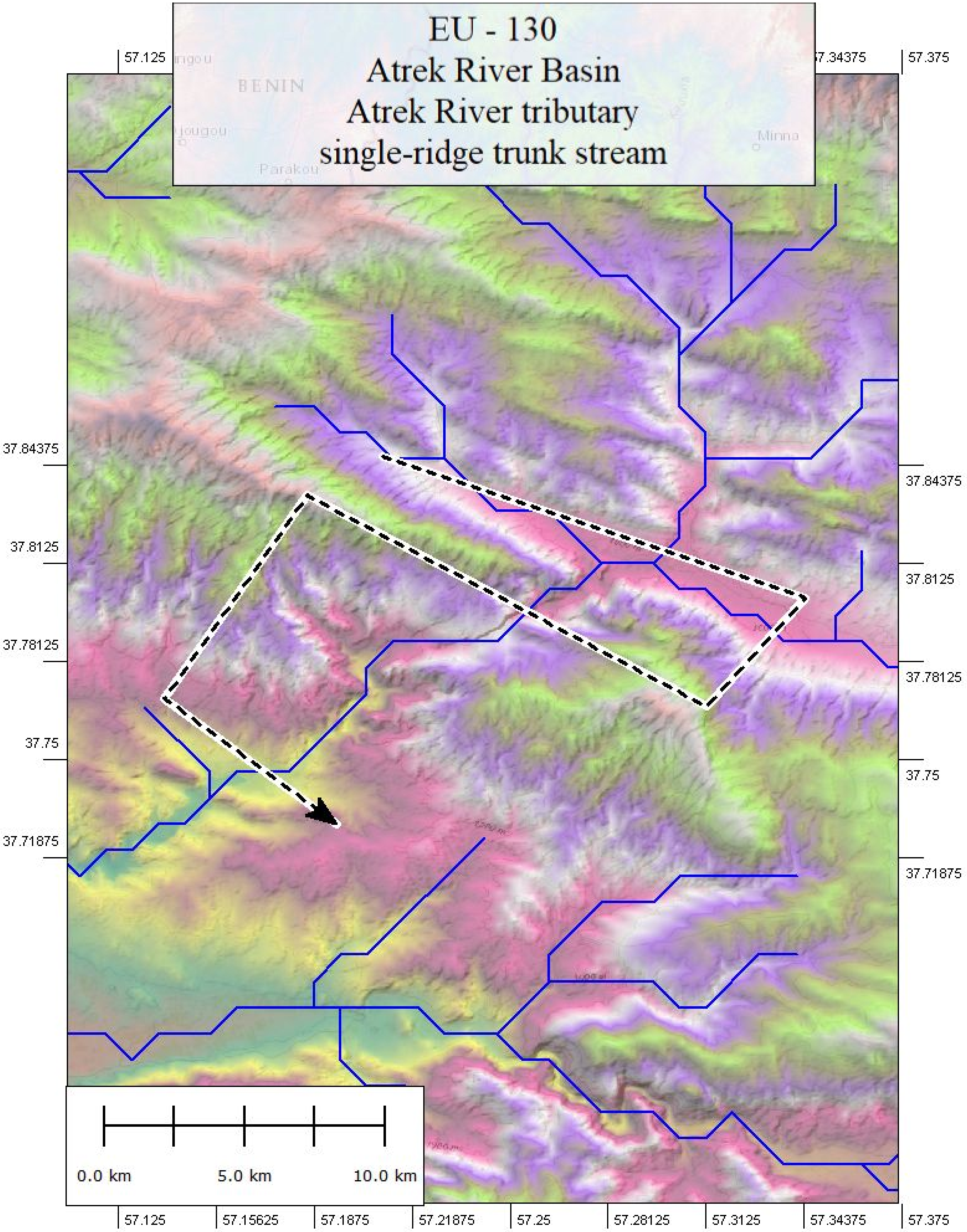
EU - 130
Atrek River Basin
Atrek River tributary
single-ridge trunk stream
BENIN
Parakou
Minna
57.125
57.15625
57.1875
57.21875
57.25
57.28125
57.3125
57.34375
57.375
37.84375
37.8125
37.78125
37.75
37.71875
0.0 km
5.0 km
10.0 km

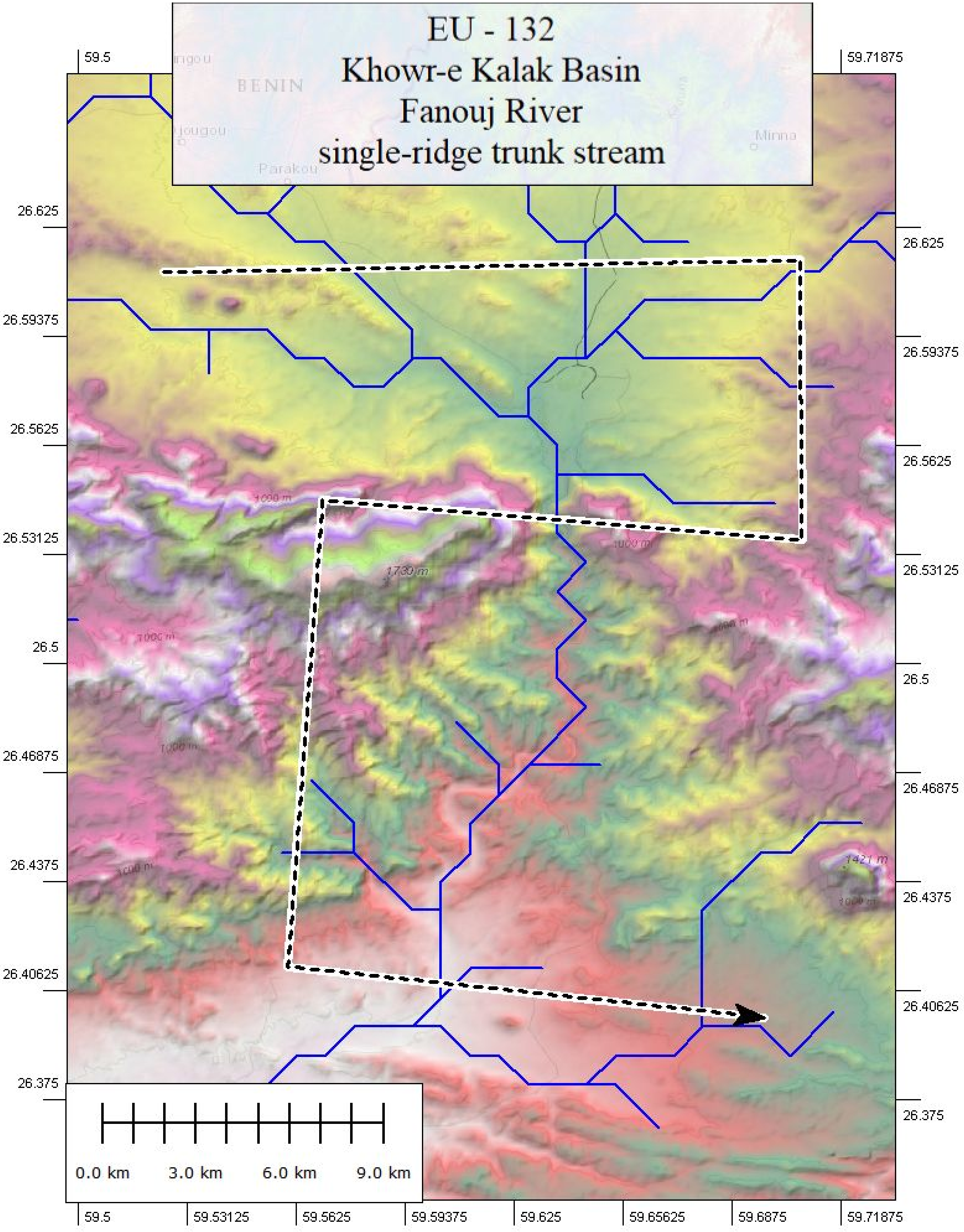
EU - 132
Khowr-e Kalak Basin
Fanouj River
single-ridge trunk stream
BENIN
Parakou
Minna
59.5
59.53125
59.5625
59.59375
59.625
59.65625
59.6875
59.71875
26.625
26.59375
26.5625
26.53125
26.5
26.46875
26.4375
26.40625
26.375
1000 m
1730 m
1421 m
0.0 km
3.0 km
6.0 km
9.0 km

EU - 133
Euphrates River Basin
single-ridge trunk stream

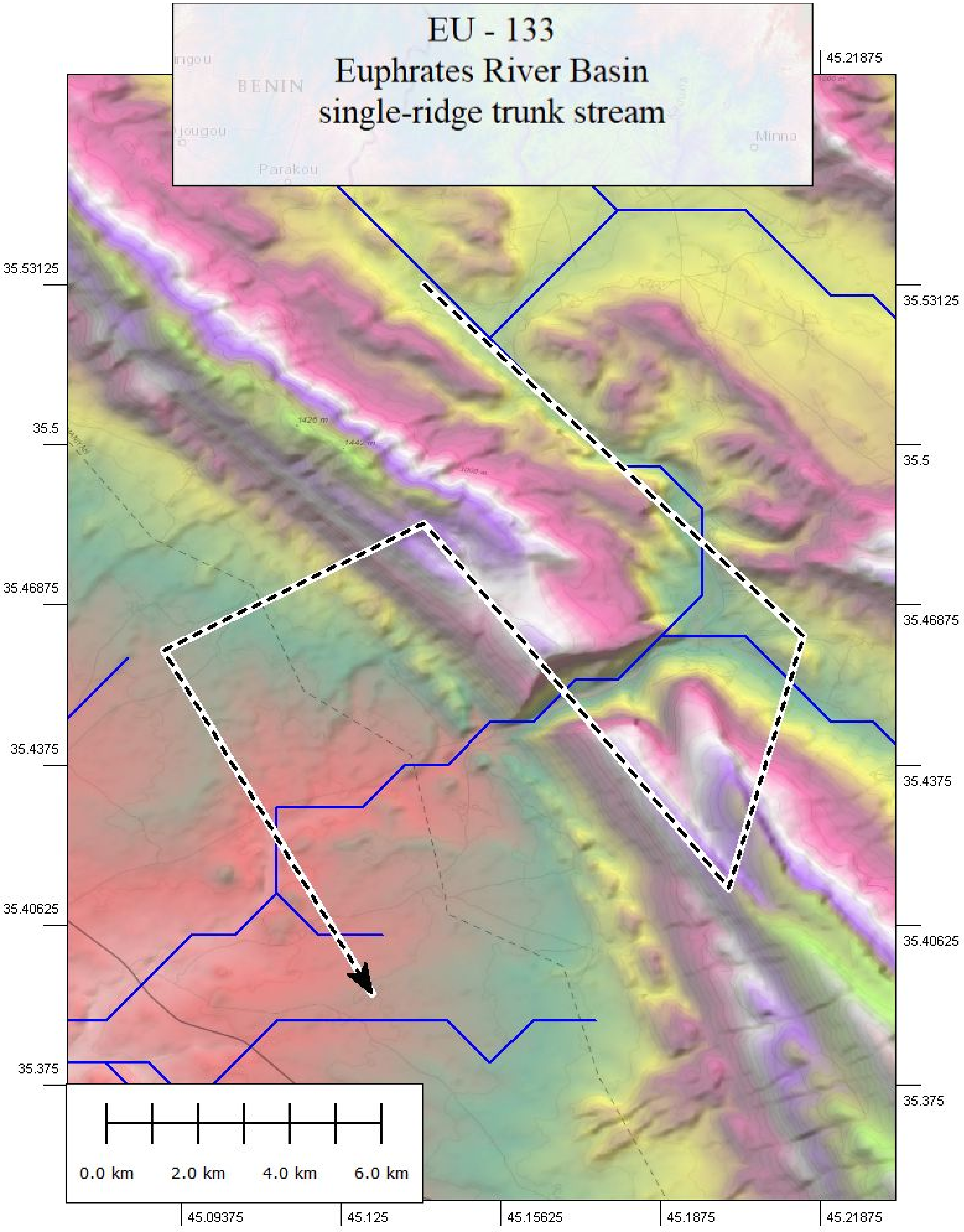

EU - 134
Ebro River Basin
Ebro River
multi-ridge trunk stream

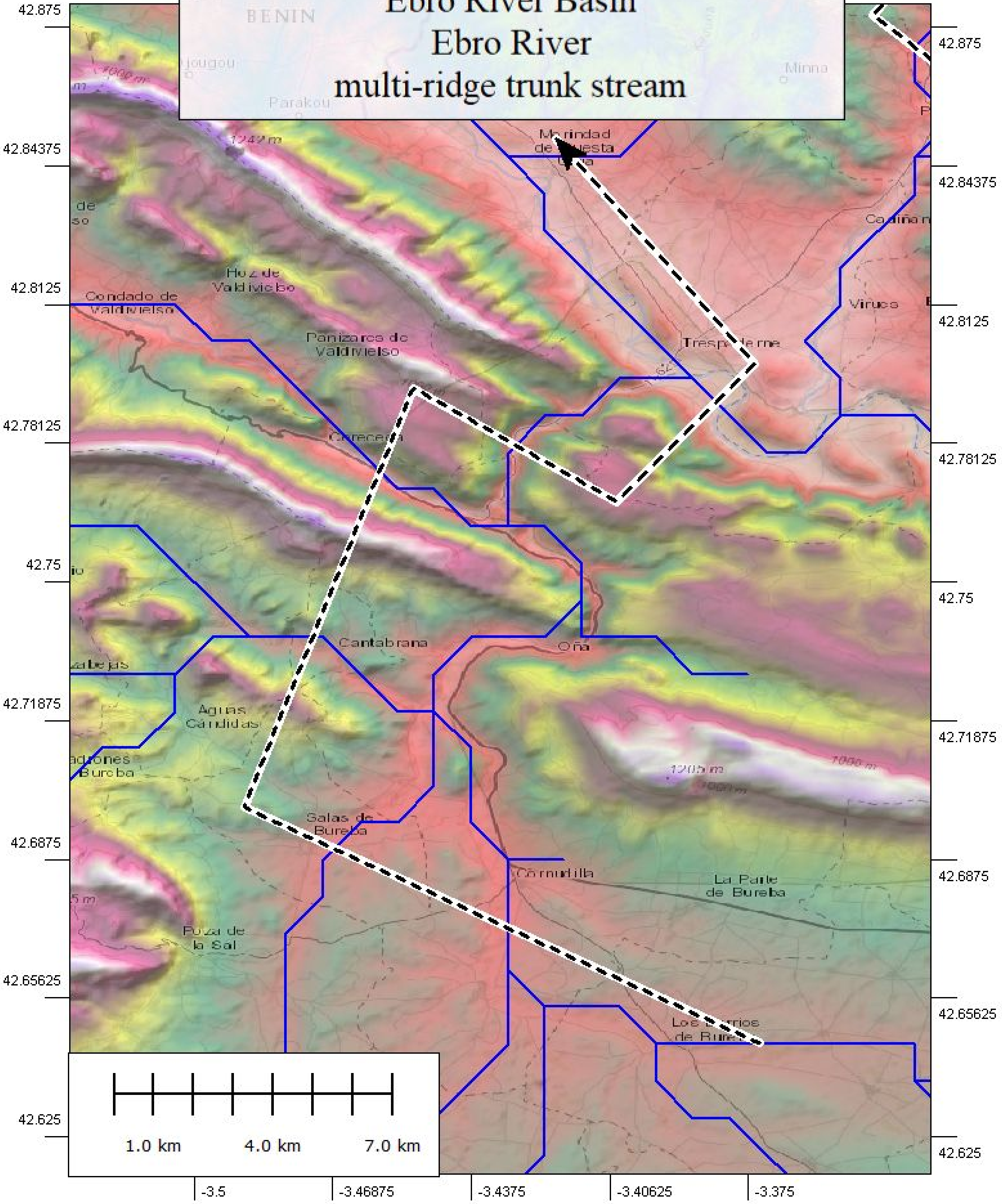

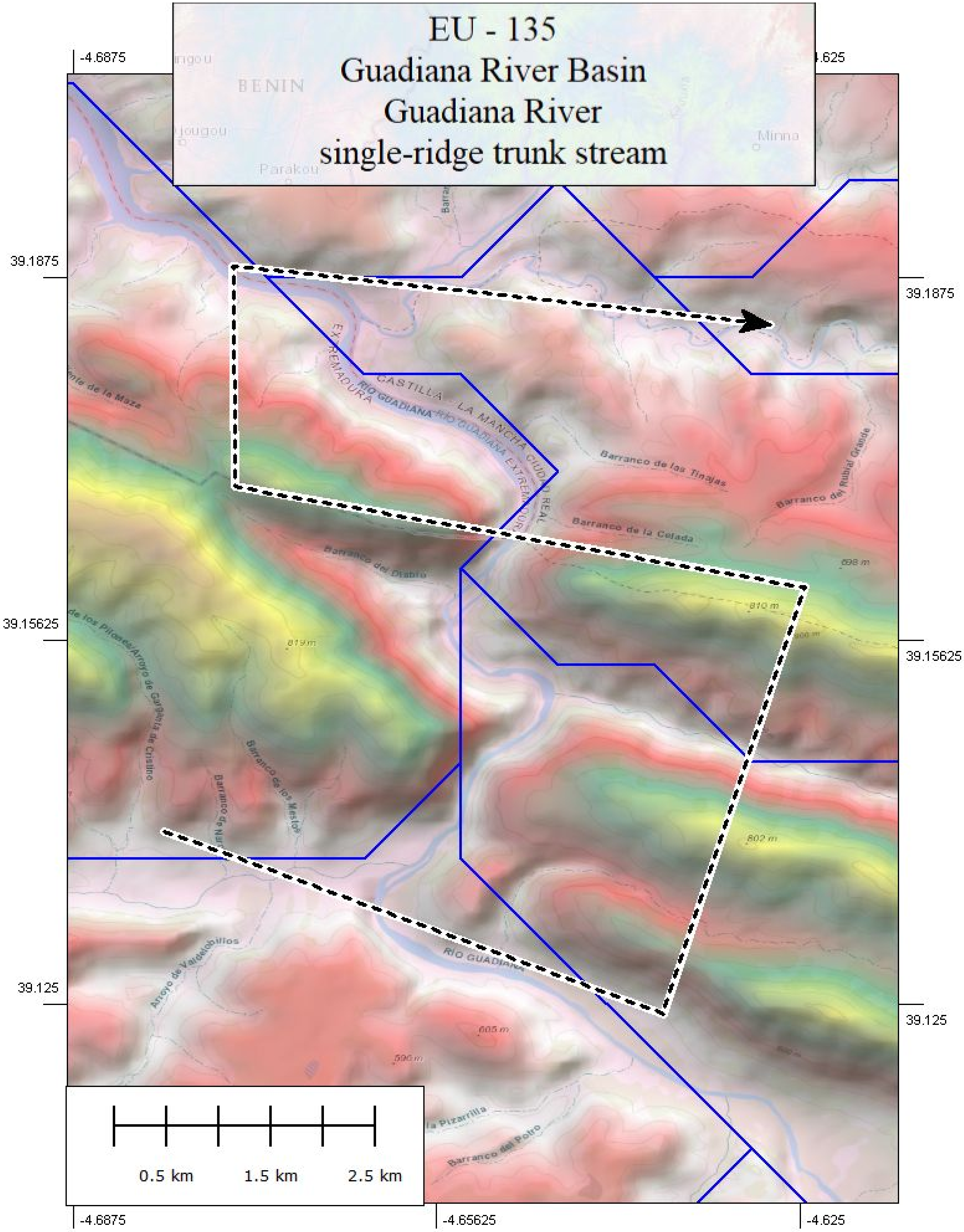
EU - 135
Guadiana River Basin
Guadiana River
single-ridge trunk stream
-4.6875
-4.65625
-4.625
39.1875
39.15625
39.125
RIO GUADIANA
CASTILLA - LA MANCHA
EXTREMADURA
CIUDAD REAL
Barranco de las Tinajas
Barranco de la Celada
Barranco del Rubial Grande
Barranco del Diablo
Barranco de los Mestos
Arroyo de Valdelobillos
Barranco del Potro
810 m
819 m
802 m
605 m
596 m
0.5 km
1.5 km
2.5 km

EU - 136
Atrek River Basin
single-ridge trunk stream

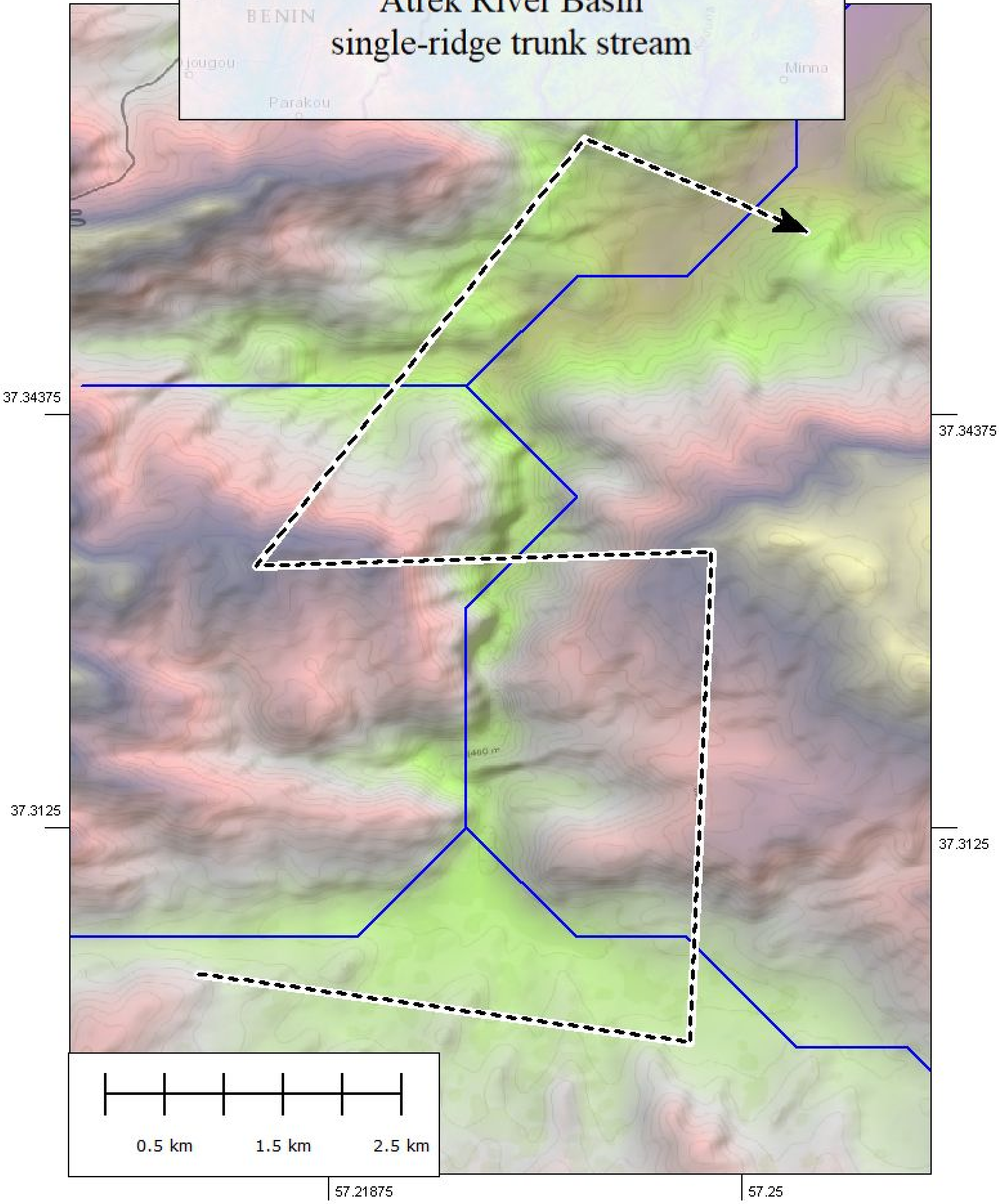

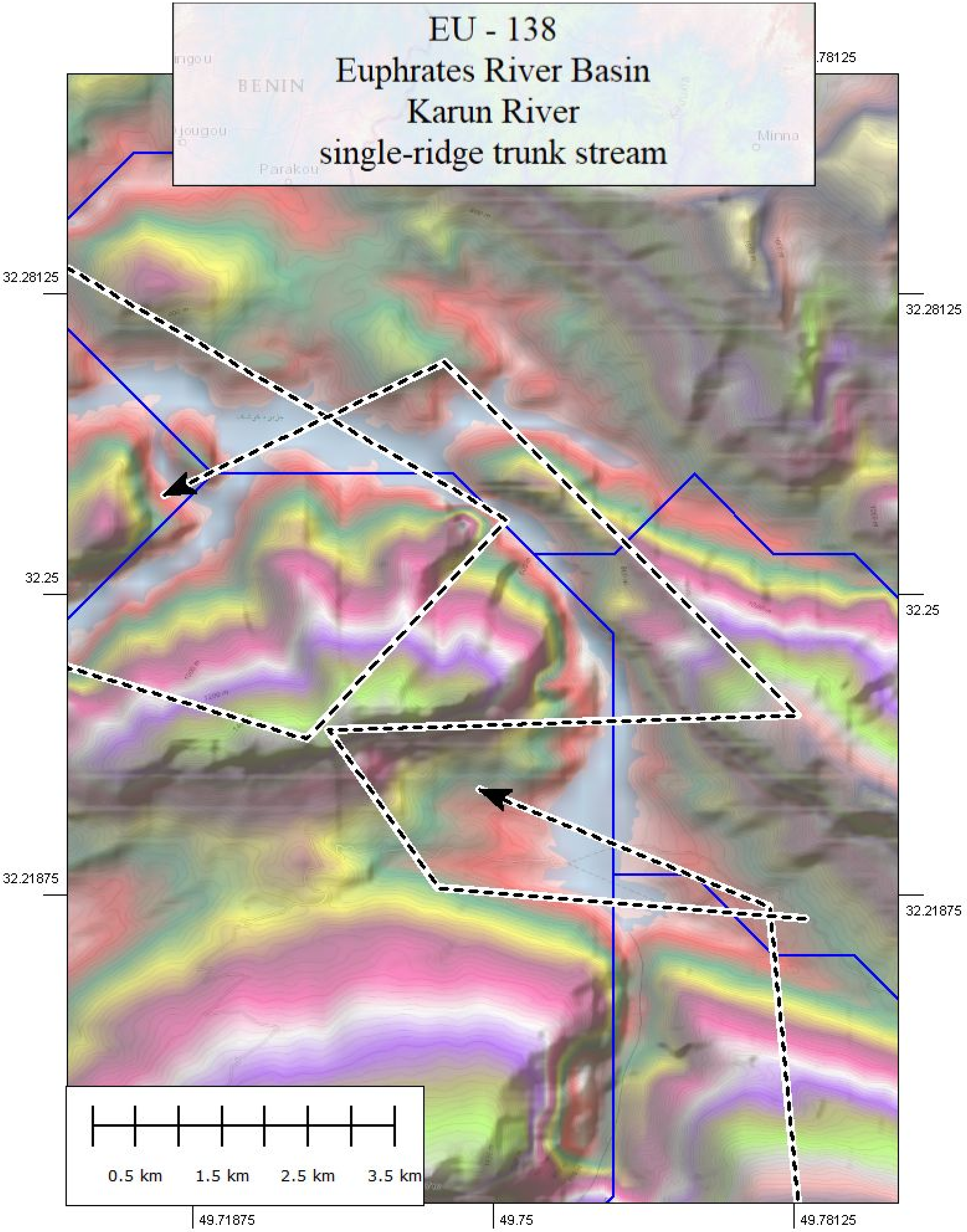
EU - 138
Euphrates River Basin
Karun River
single-ridge trunk stream
BENIN
Parakou
Minna
.78125
32.28125
32.28125
32.25
32.25
32.21875
32.21875
0.5 km
1.5 km
2.5 km
3.5 km
49.71875
49.75
49.78125

EU - 139
Khowr-e Dowraq Basin
Maroun Dam
single-ridge trunk stream

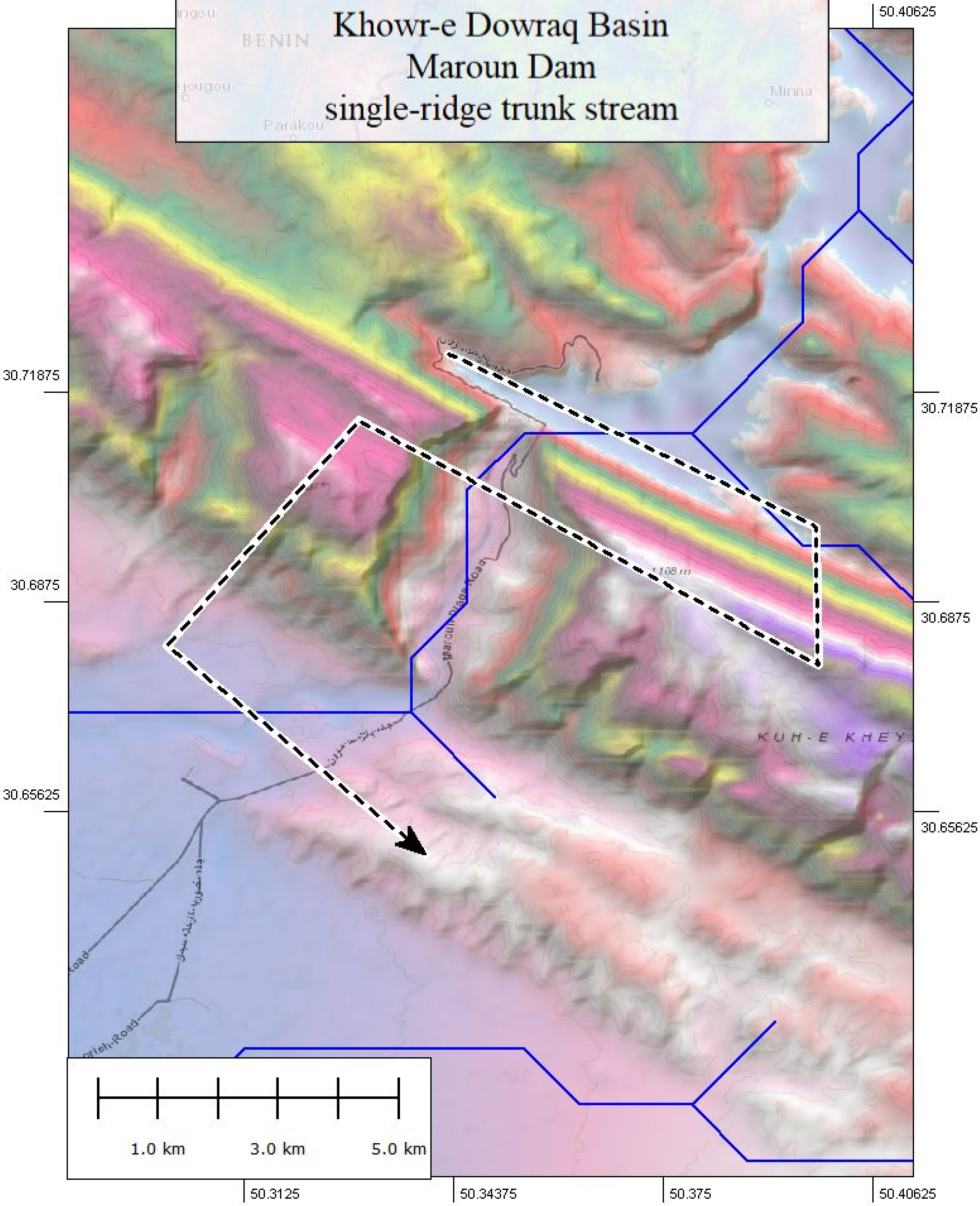

EU - 140
Euphrates River Basin
Karkheh River
single-ridge trunk stream

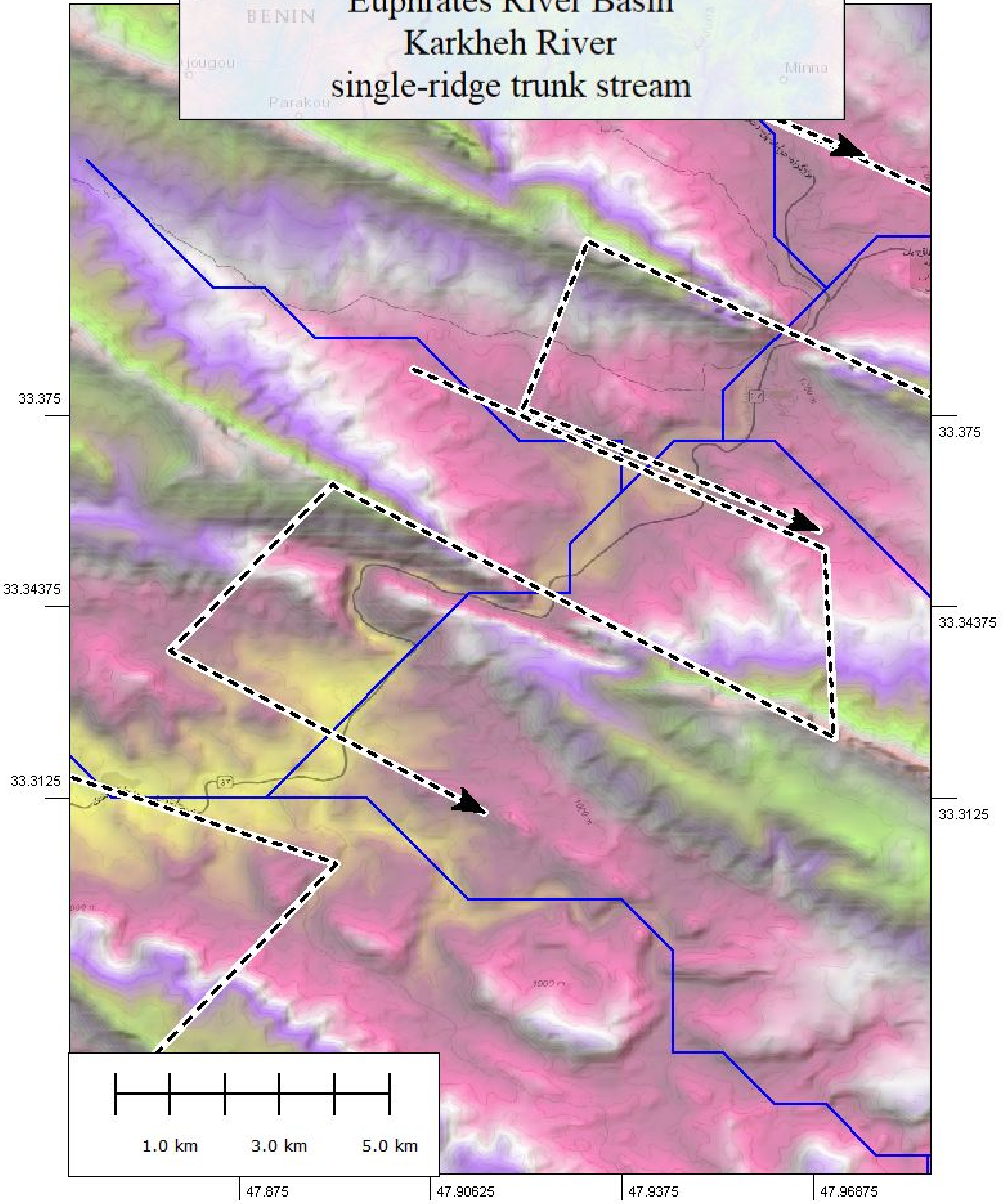

EU - 142
Euphrates River Basin
single-ridge trunk stream

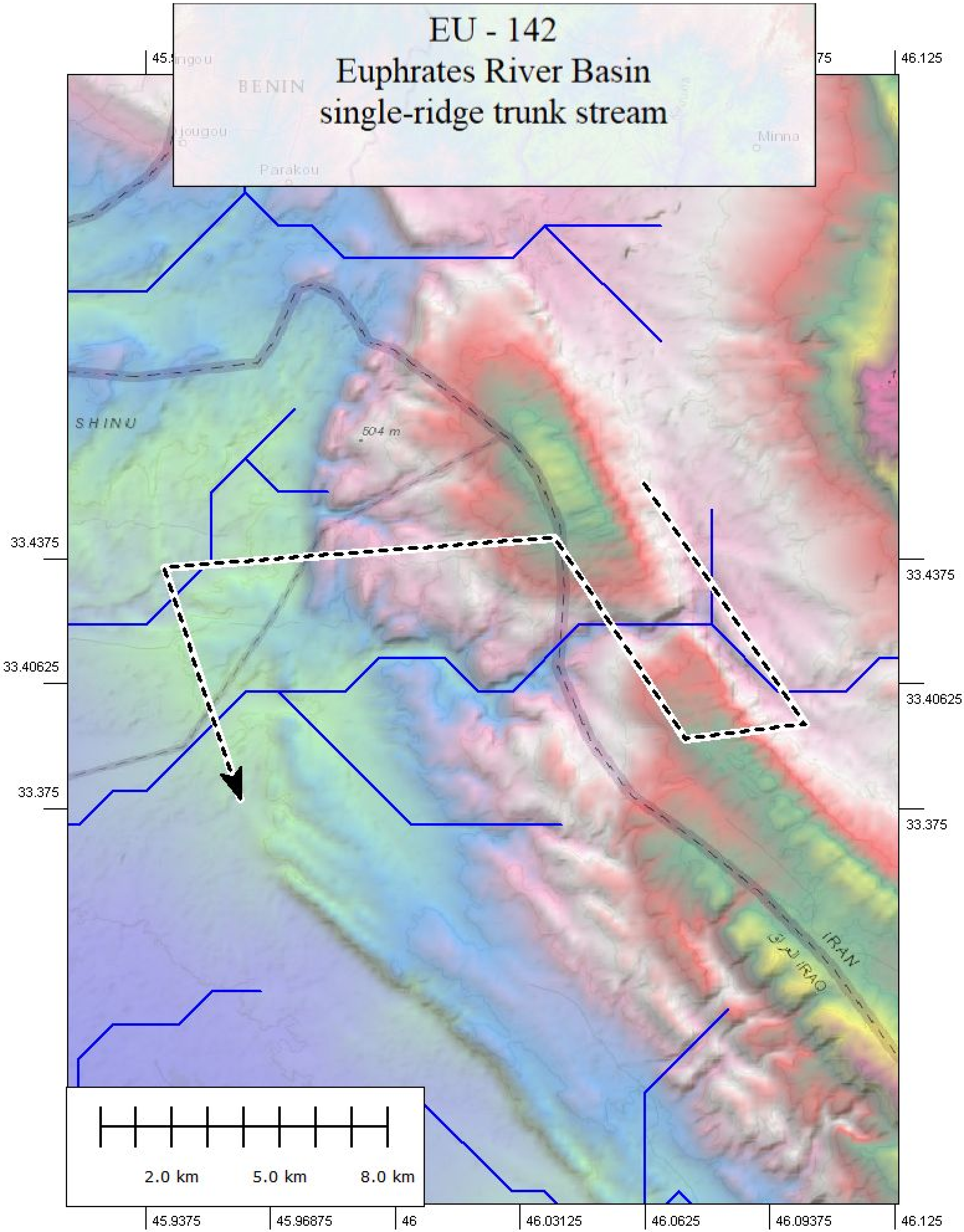

EU - 143
Ebro River Basin
Bayas River
single-ridge trunk stream

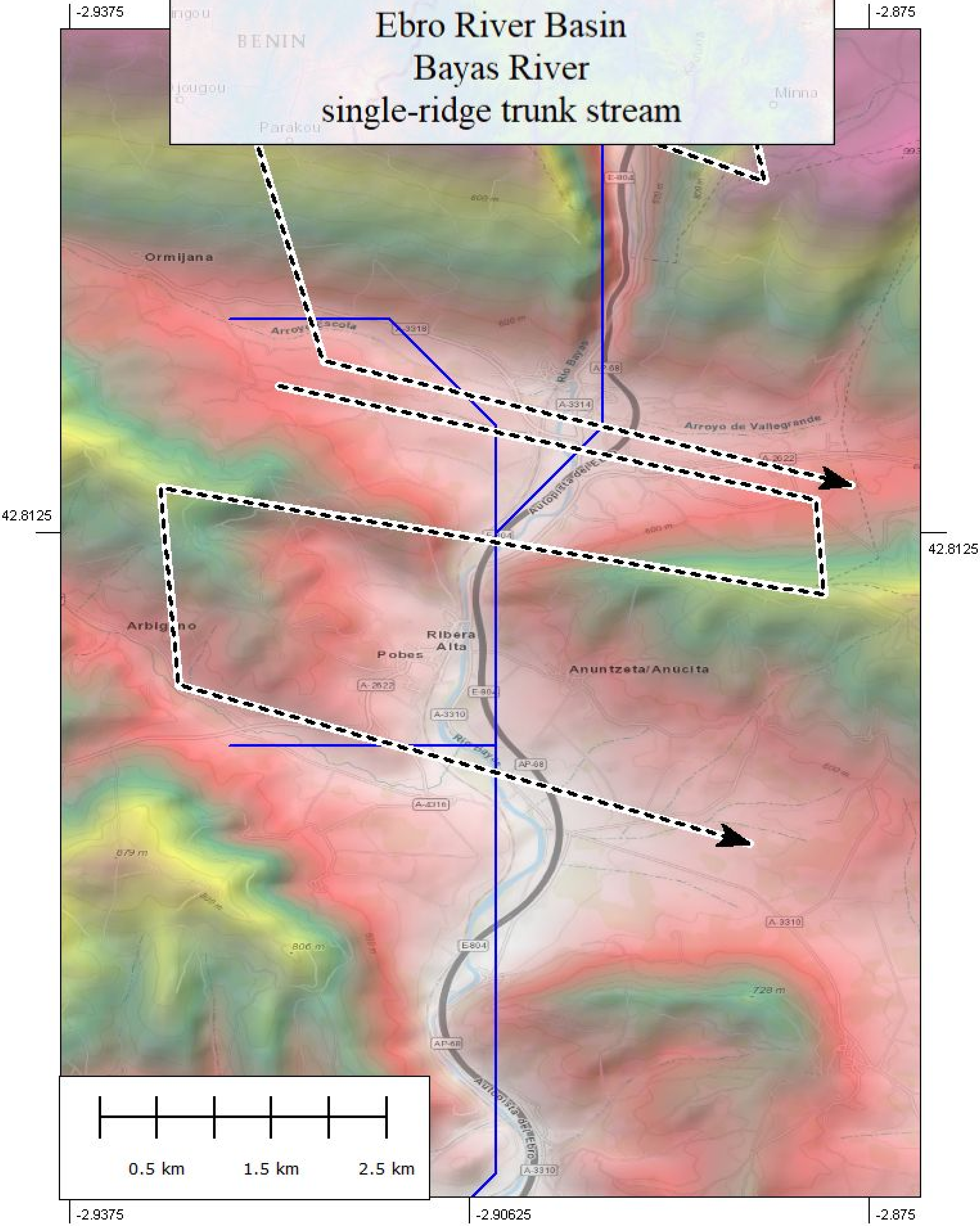

EU - 147
Ebro River Basin
Gallego River
single-ridge trunk stream

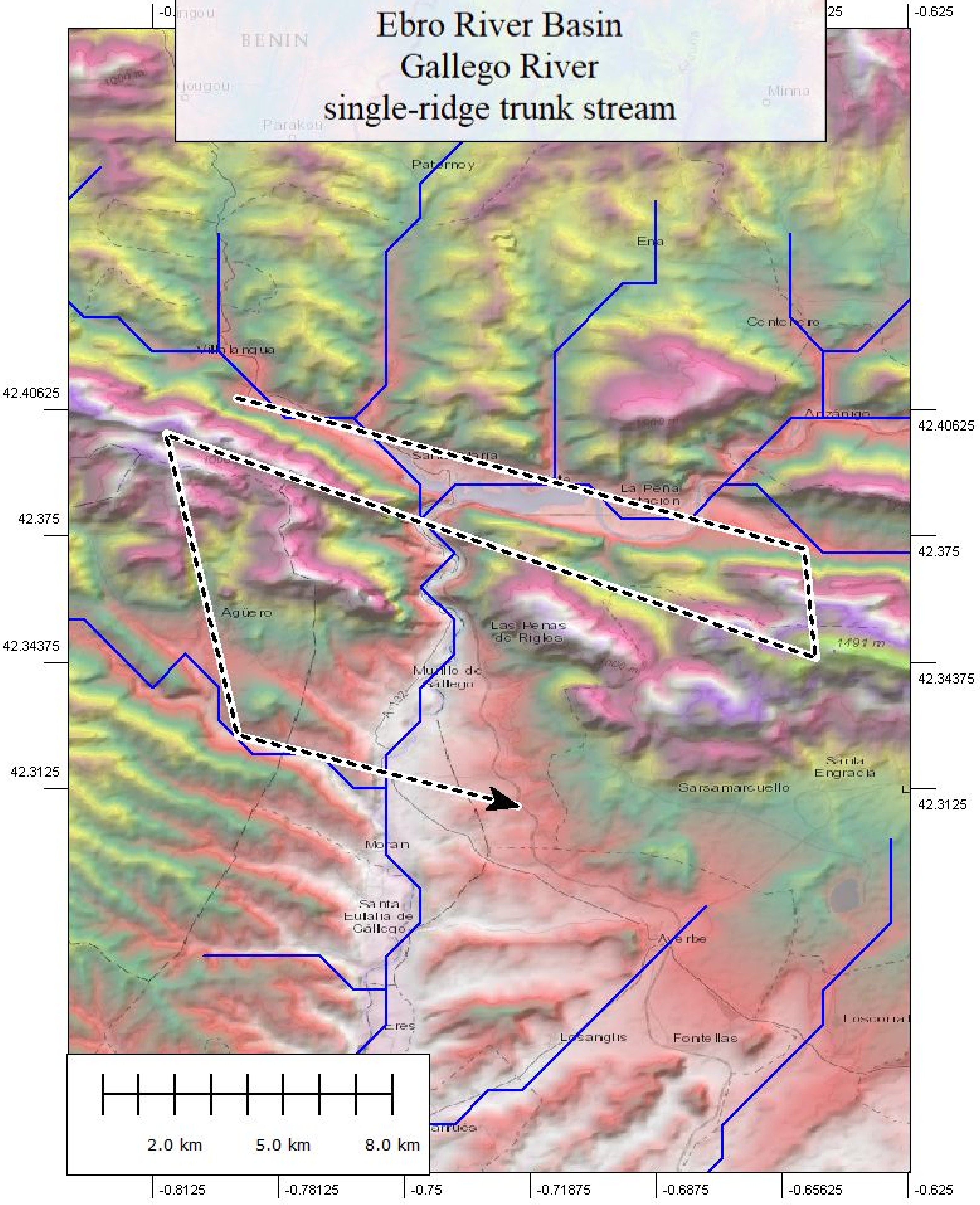

EU - 148
Ebro River Basin
Jerea River
single-ridge trunk stream

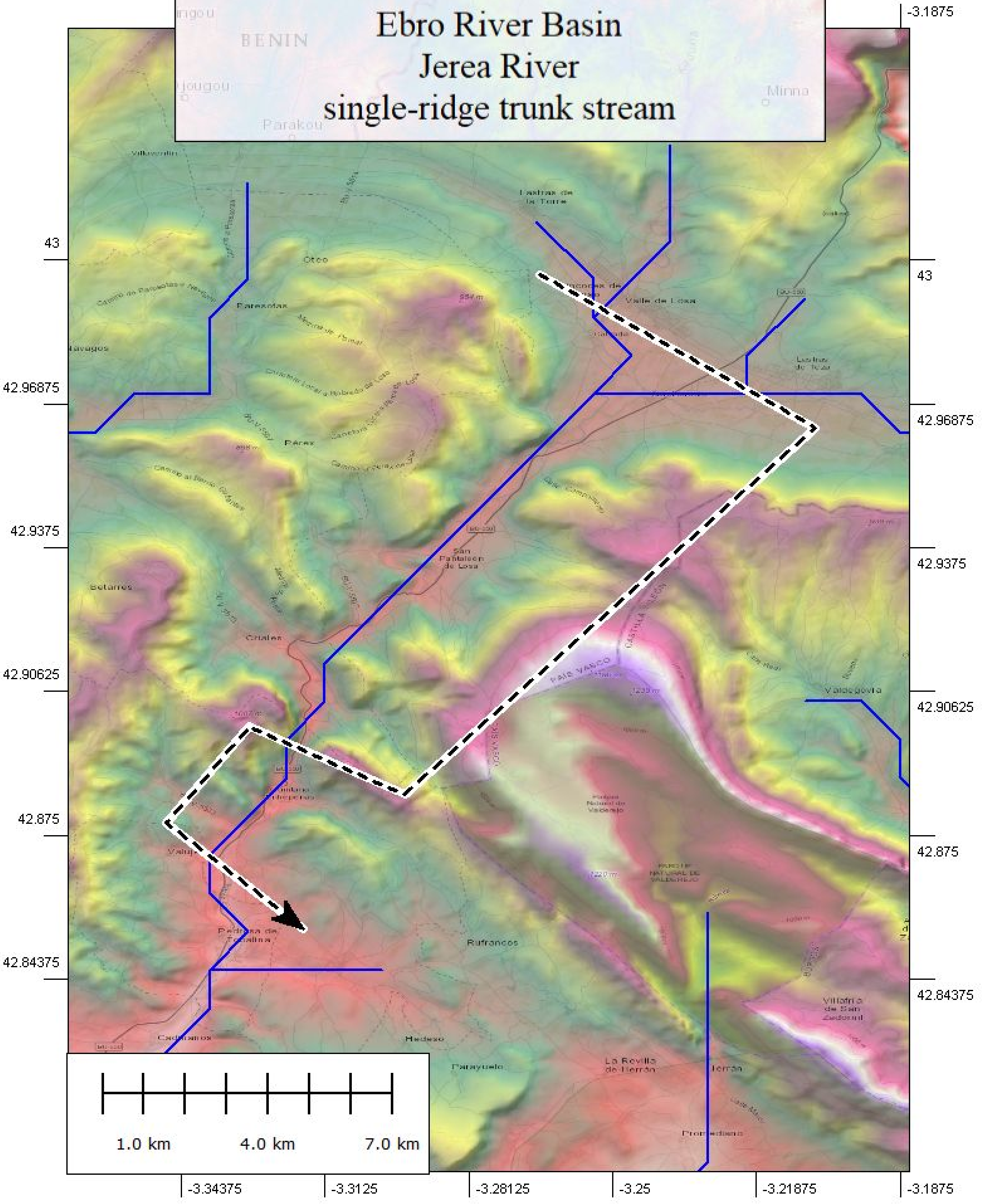

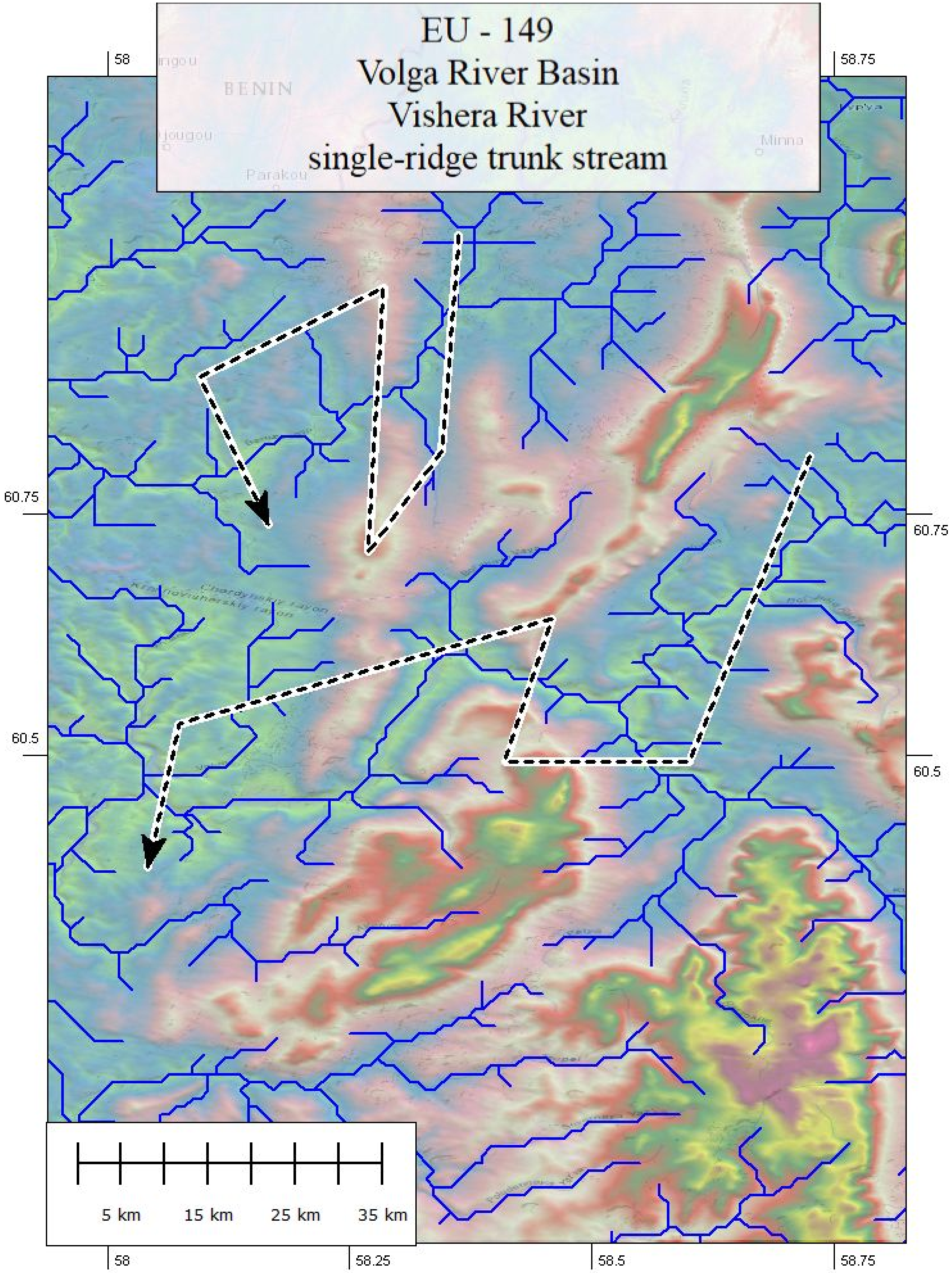
EU - 149
Volga River Basin
Vishera River
single-ridge trunk stream
58
58.25
58.5
58.75
60.75
60.5
5 km
15 km
25 km
35 km

EU - 150
Ebro River Basin
Esoa River
single-ridge trunk stream

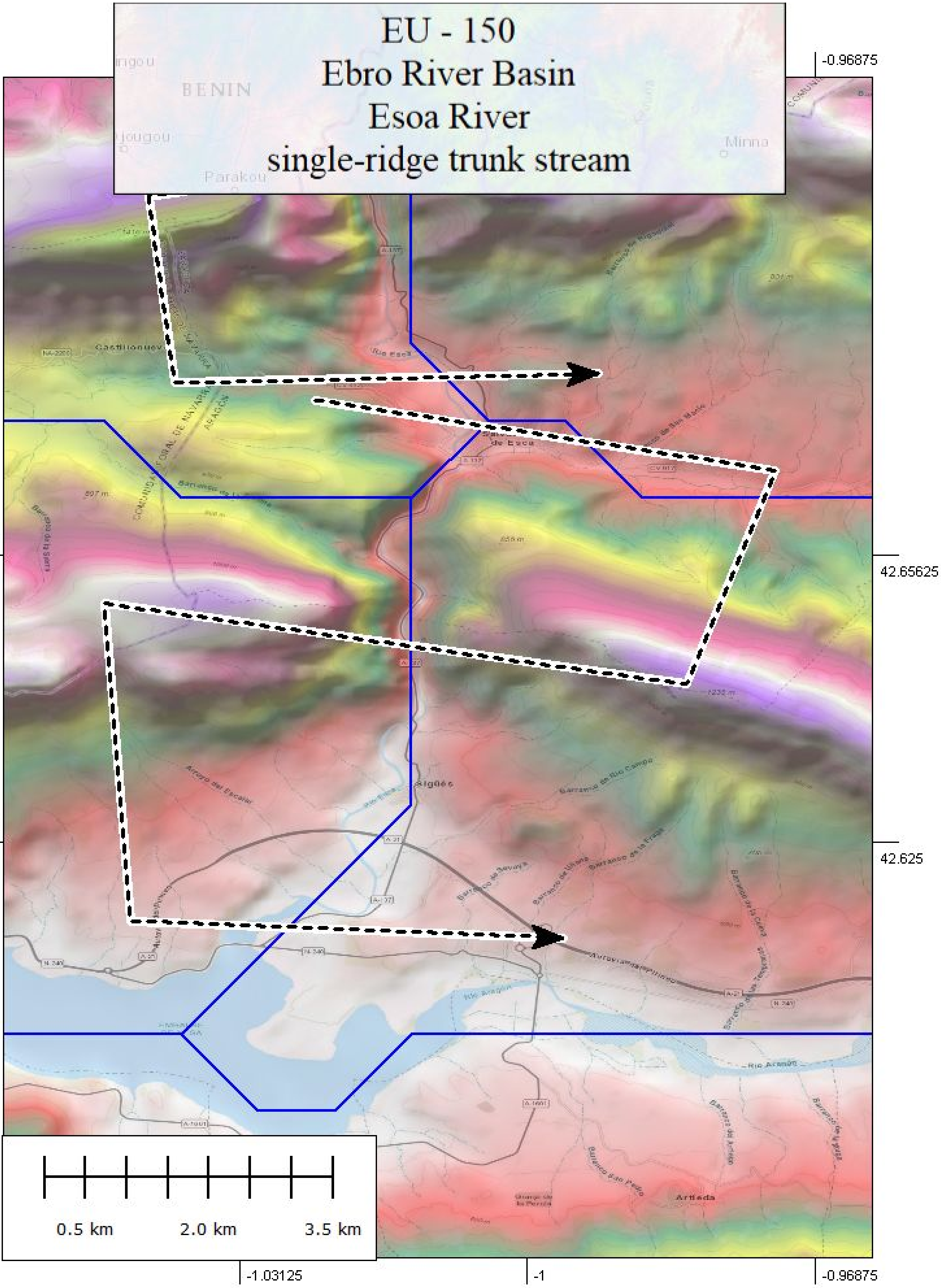

EU - 151
Hingol River Basin
Hingol River
multi-ridge trunk stream

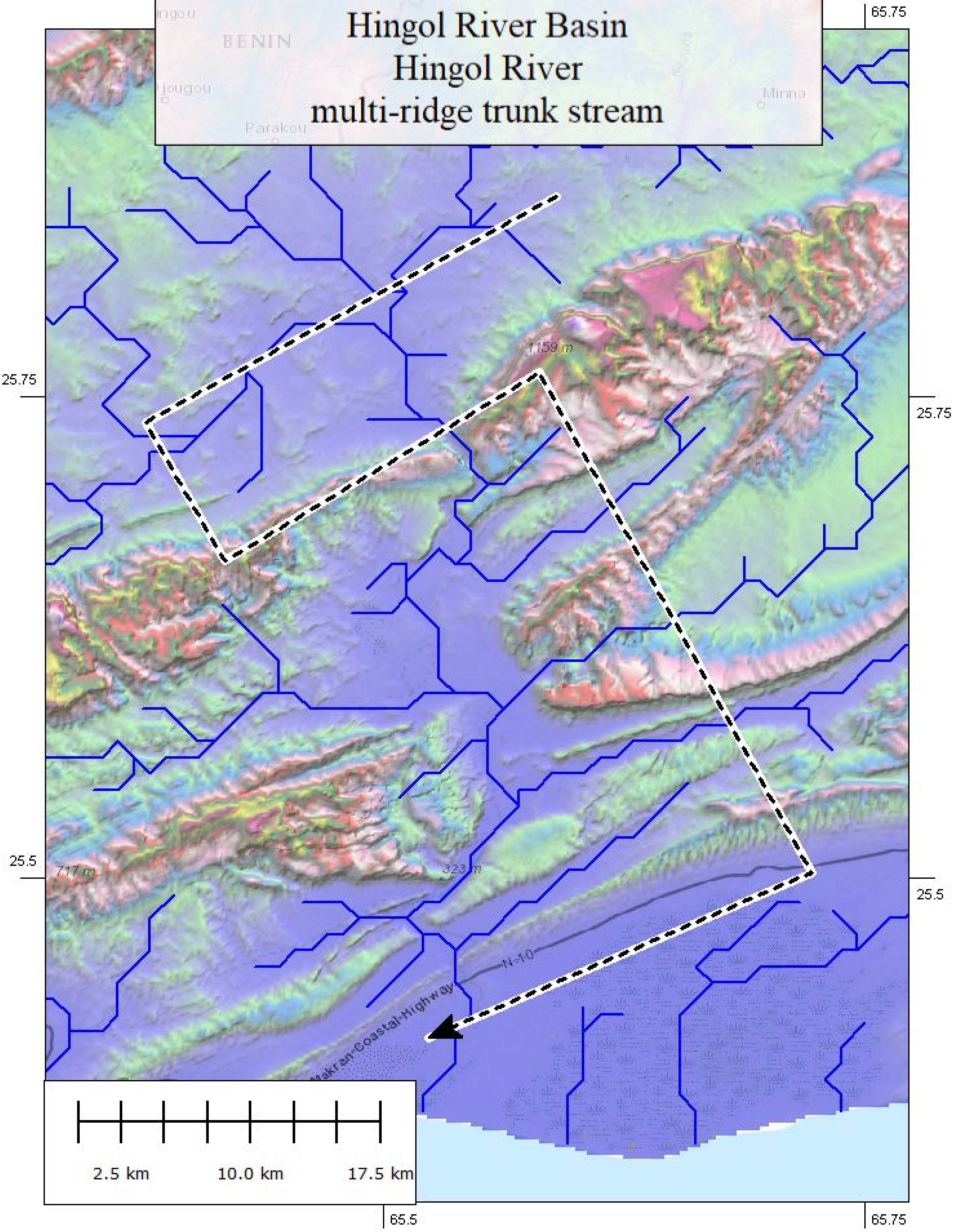

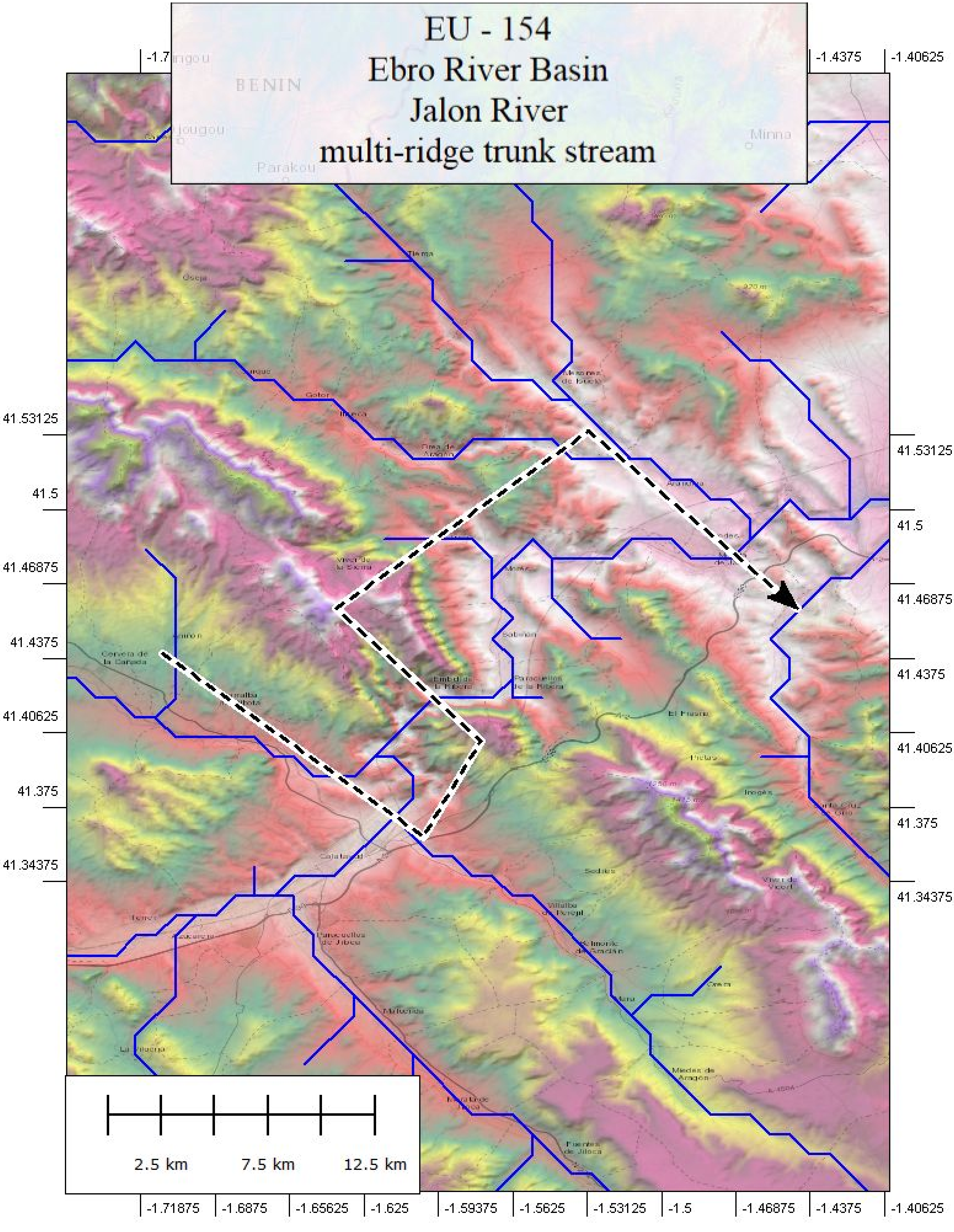
EU - 154
Ebro River Basin
Jalon River
multi-ridge trunk stream
2.5 km
7.5 km
12.5 km
41.53125
41.5
41.46875
41.4375
41.40625
41.375
41.34375
-1.71875
-1.6875
-1.65625
-1.625
-1.59375
-1.5625
-1.53125
-1.5
-1.46875
-1.4375
-1.40625

EU - 156
Euphrates River Basin
Great Zab River
single-ridge trunk stream

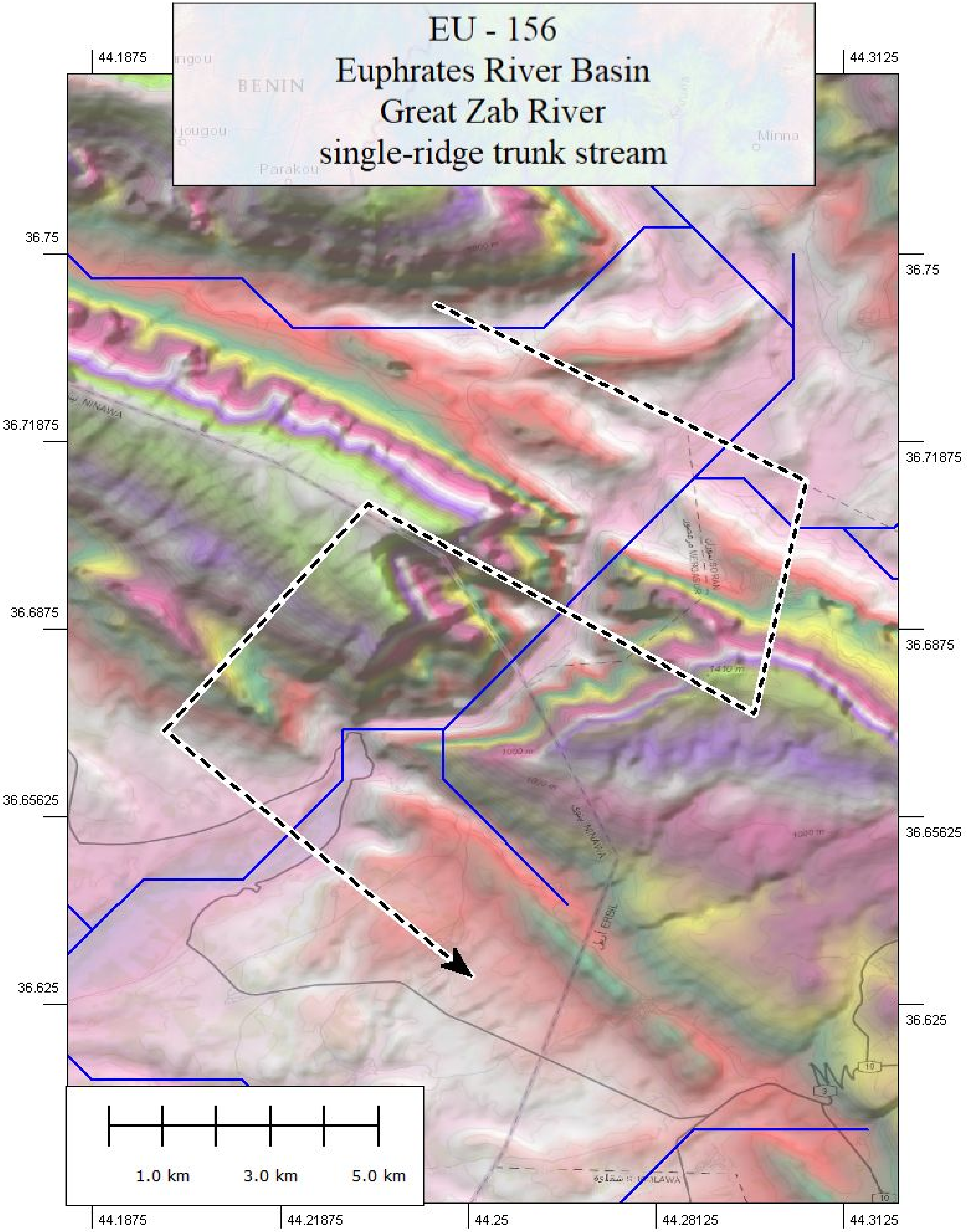

EU - 157
Rhone River Basin
Isere River
single-ridge trunk stream

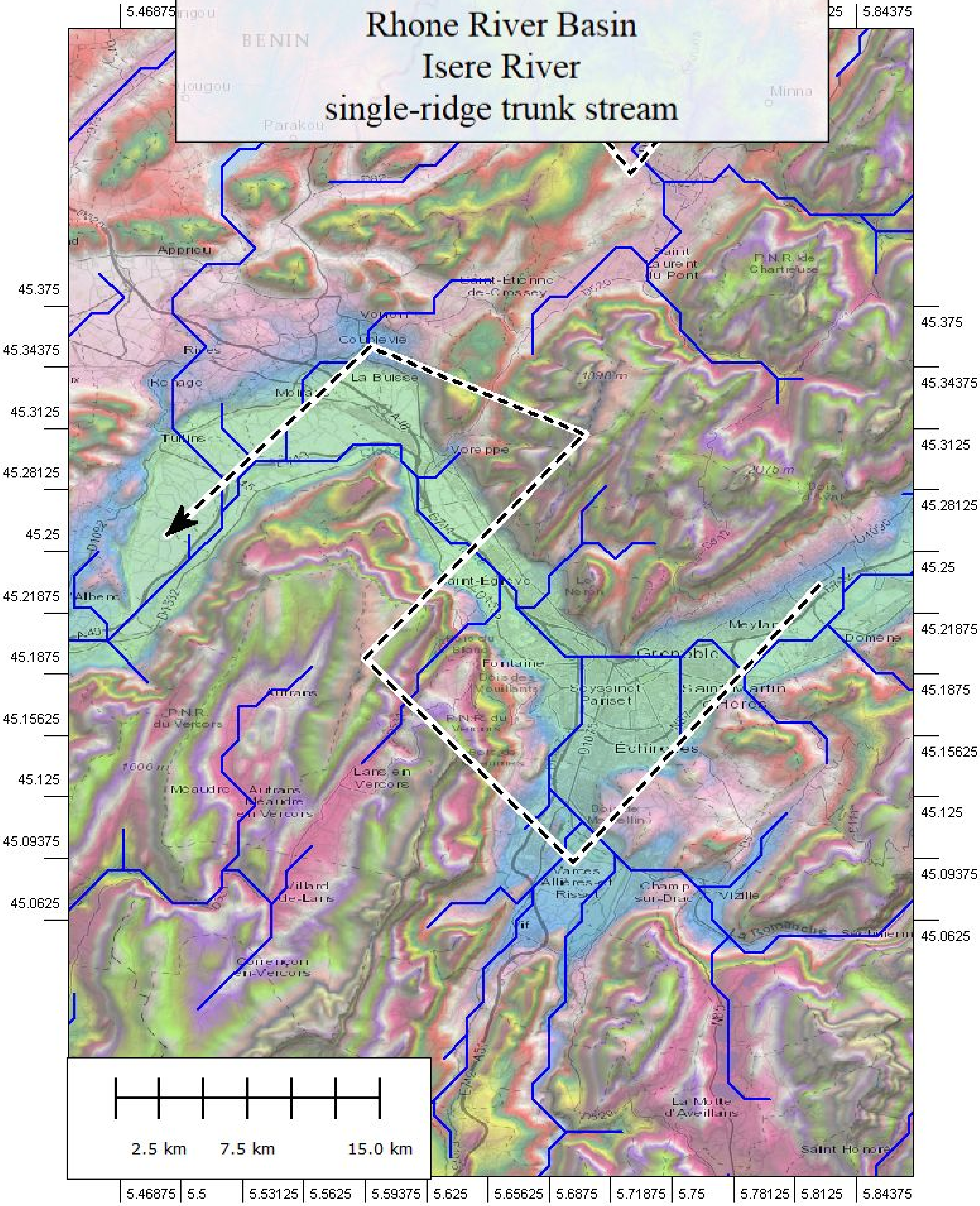

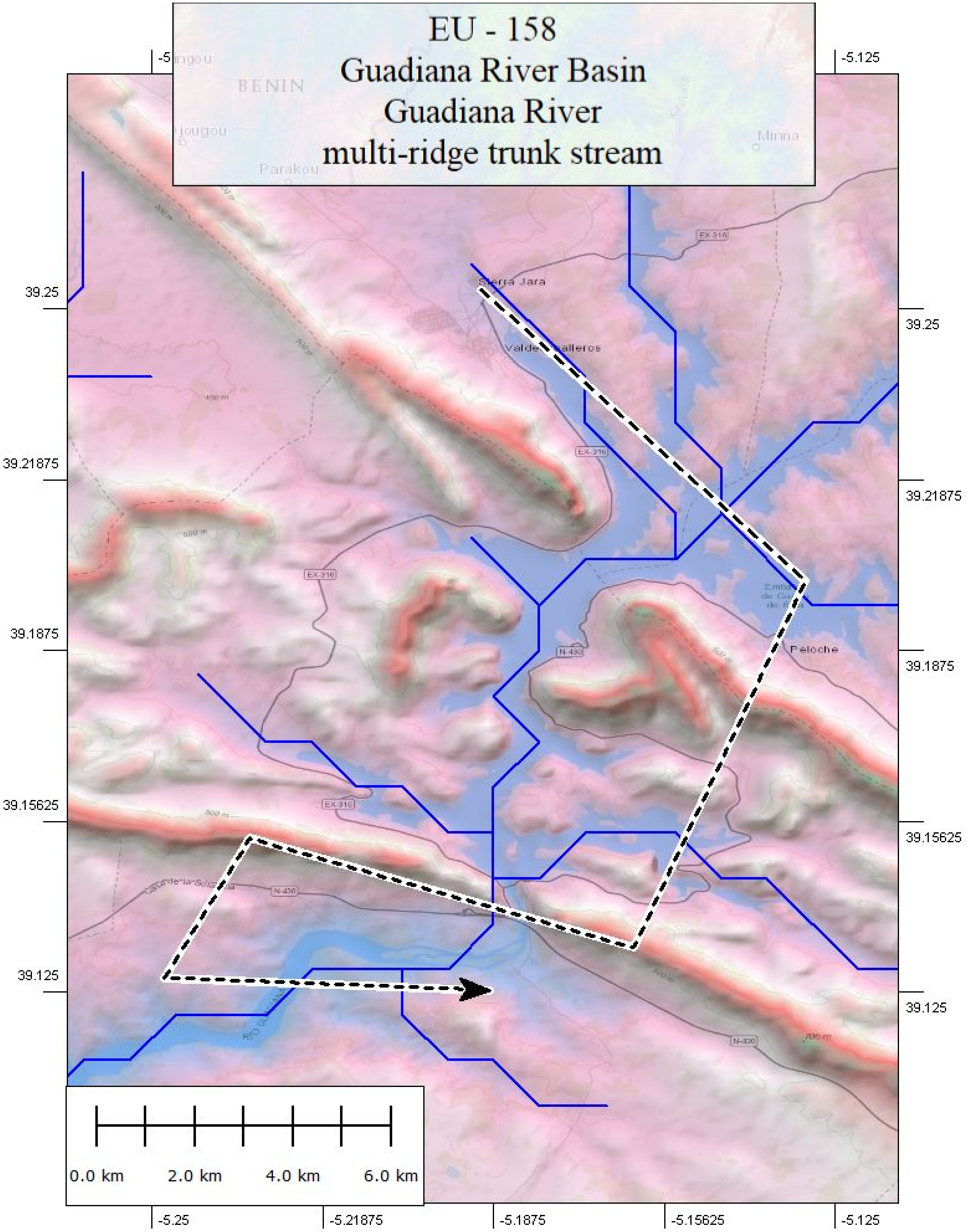

EU - 158
Guadiana River Basin
Guadiana River
multi-ridge trunk stream
Sierra Jara
Peloche
EX-316
N-430
-5.25
-5.21875
-5.1875
-5.15625
-5.125
39.25
39.21875
39.1875
39.15625
39.125
0.0 km
2.0 km
4.0 km
6.0 km

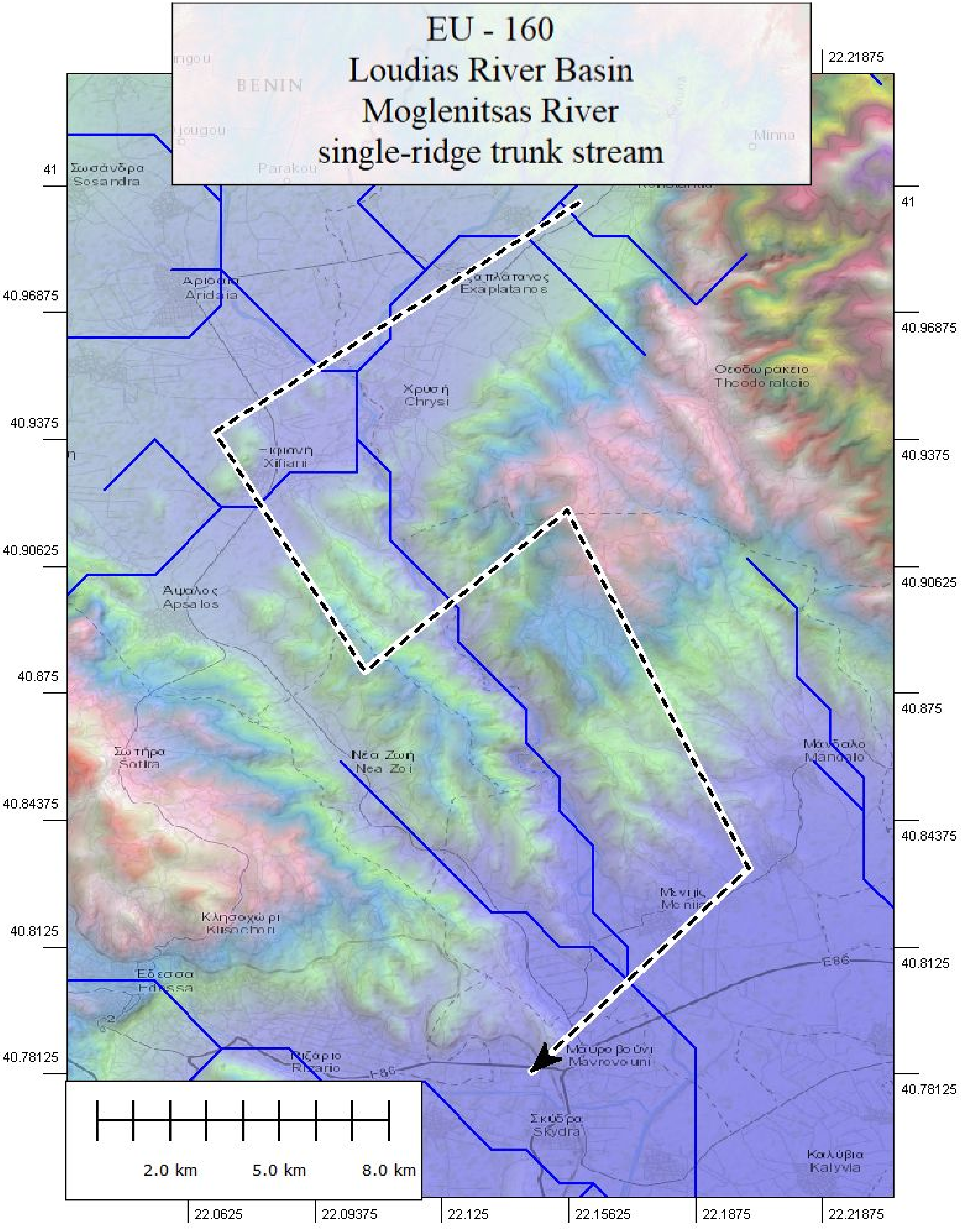
EU - 160
Loudias River Basin
Moglenitsas River
single-ridge trunk stream
Σωσάνδρα
Sosandra
Αριδαία
Aridaia
Εξαπλάτανος
Exaplatanos
Χρυσή
Chrysi
Θεοδωράκειο
Theodorakeio
Xifiani
Αψαλος
Apsalos
Σωτήρα
Sotira
Νέα Ζωή
Nea Zoi
Μάνδαλο
Mandalo
Κλησοχώρι
Klisochori
Έδεσσα
Edessa
Ριζάριο
Rizario
Μαυροβούνι
Mavrovouni
Σκύδρα
Skydra
Καλύβια
Kalyvia
E86
2.0 km
5.0 km
8.0 km
41
40.96875
40.9375
40.90625
40.875
40.84375
40.8125
40.78125
22.0625
22.09375
22.125
22.15625
22.1875
22.21875

EU - 161
Dasht River Basin
Gish River
single-ridge trunk stream

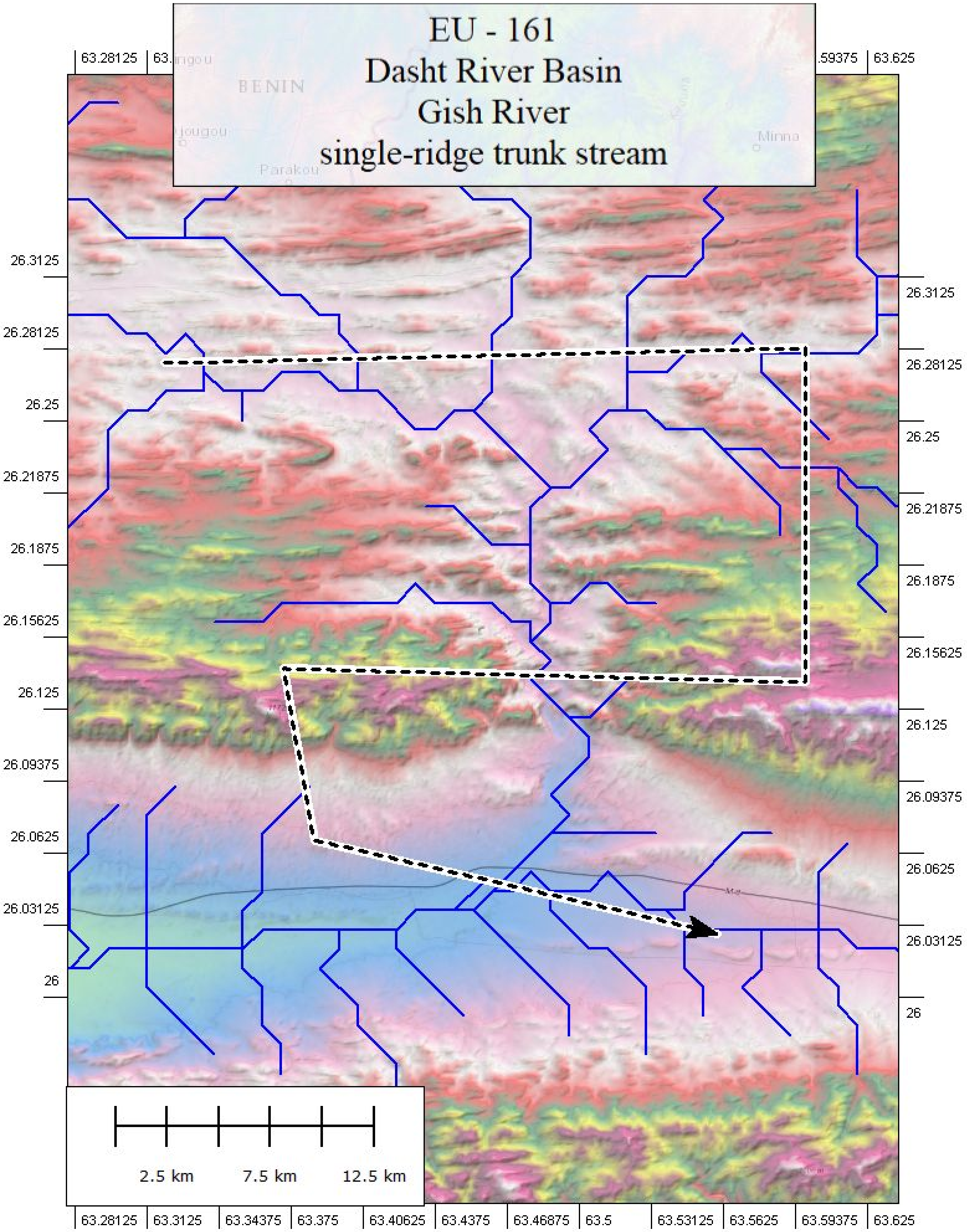

EU - 162
Euphrates River Basin
Sican River
single-ridge trunk stream

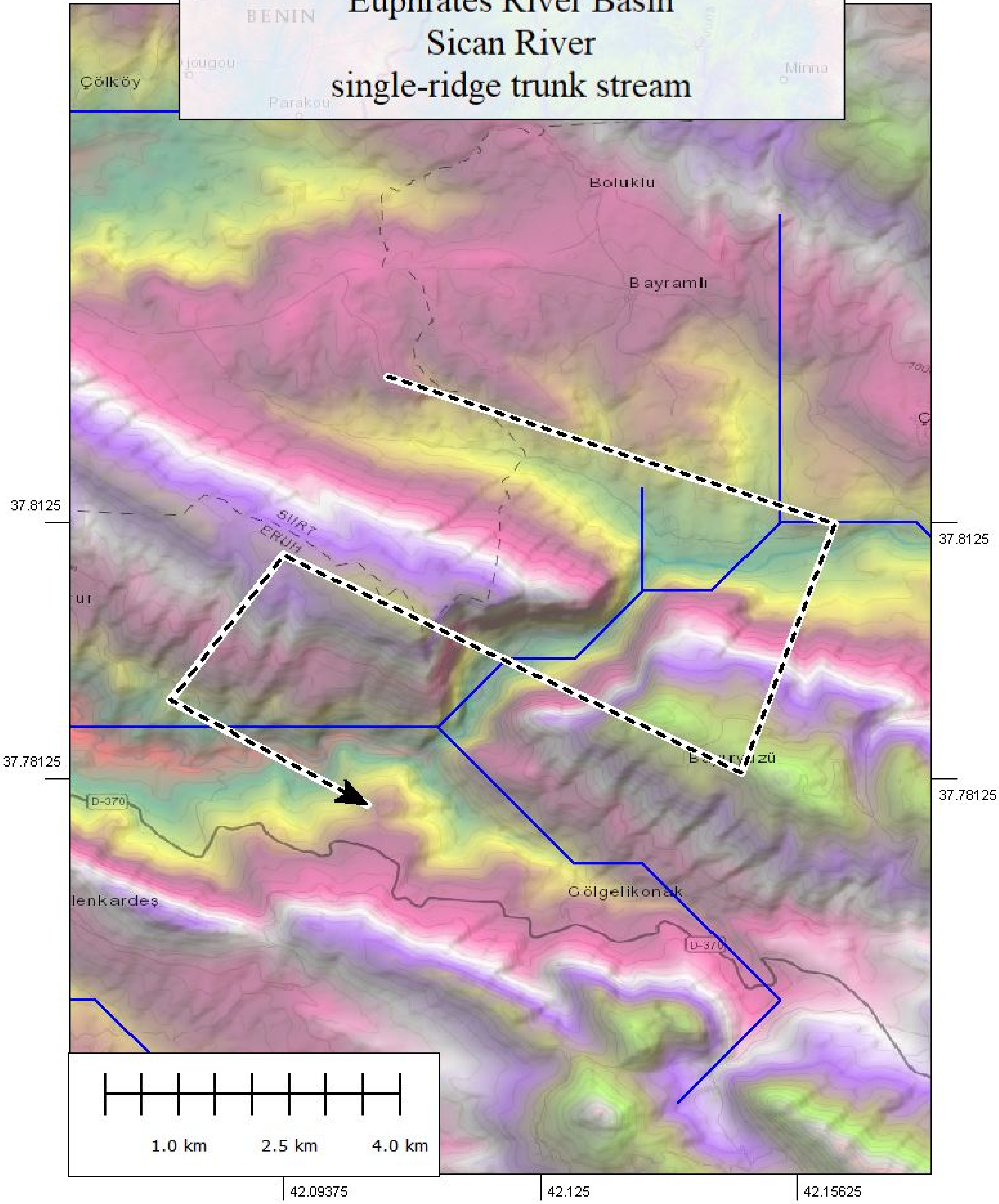

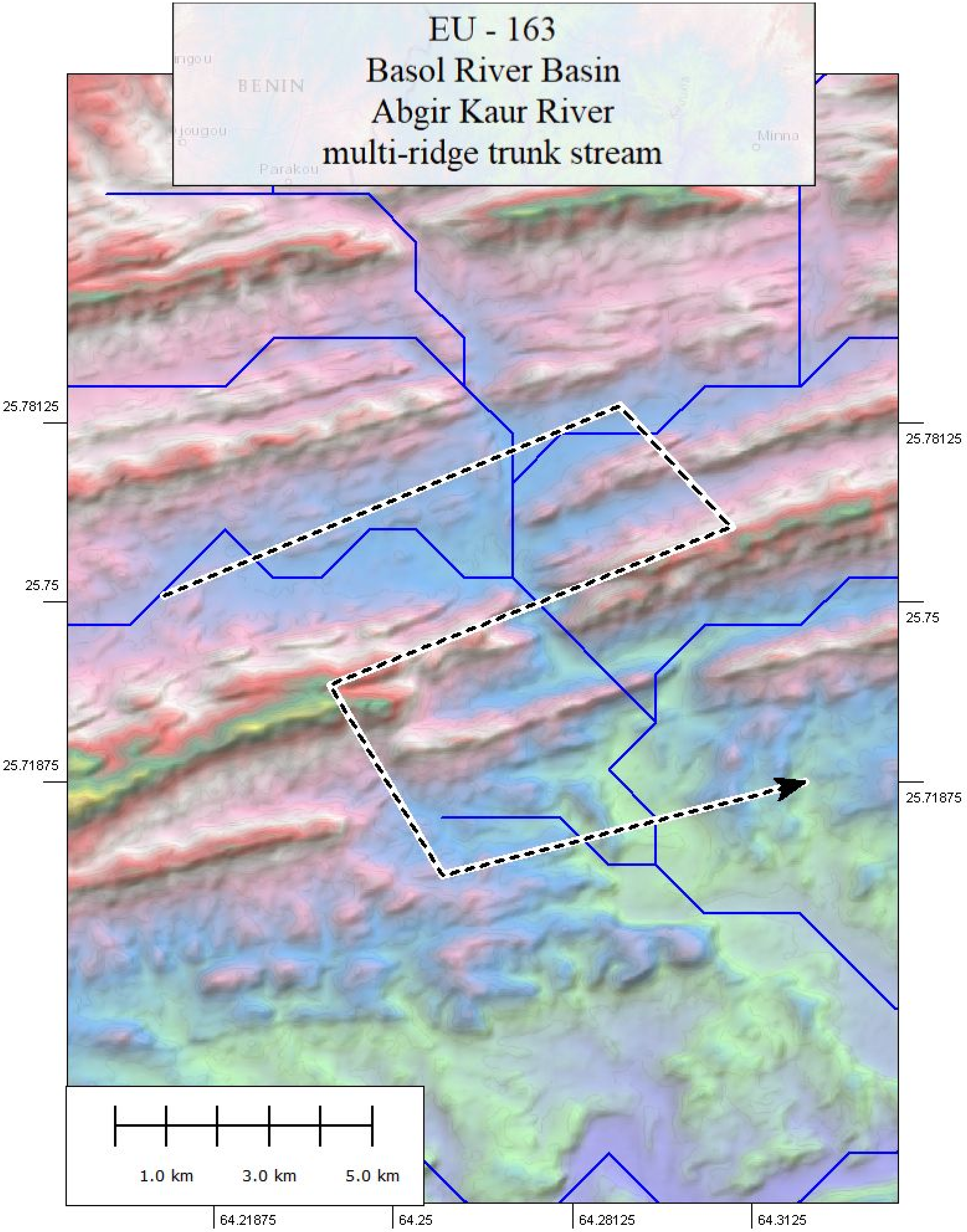

EU - 163
Basol River Basin
Abgir Kaur River
multi-ridge trunk stream
BENIN
Djougou
Parakou
Minna
25.78125
25.75
25.71875
25.78125
25.75
25.71875
1.0 km
3.0 km
5.0 km
64.21875
64.25
64.28125
64.3125

EU - 165
Sakarya River Basin
Goksu River
single-ridge trunk stream

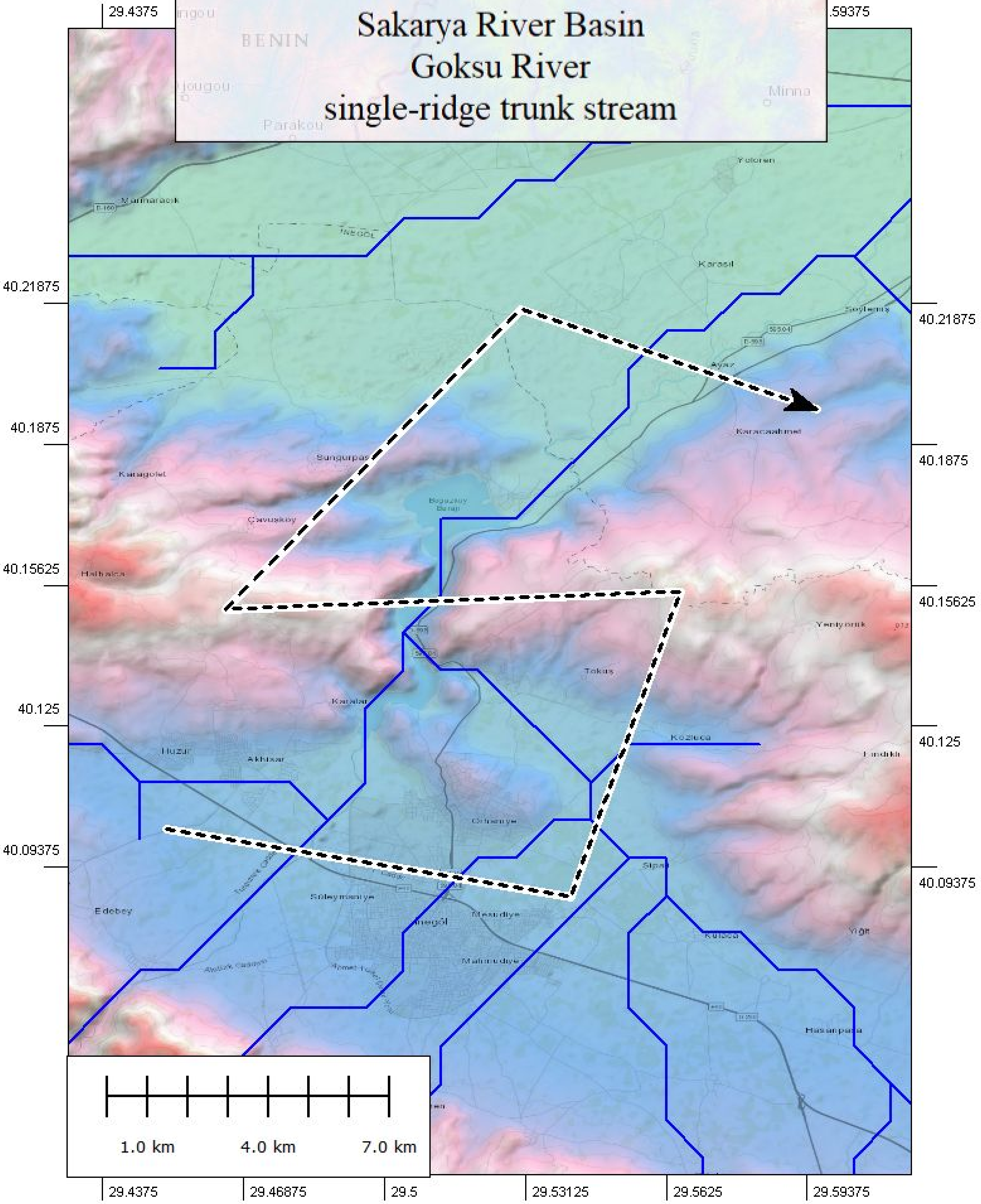

EU - 168
Euphrates River Basin
Goksu River
single-ridge trunk stream

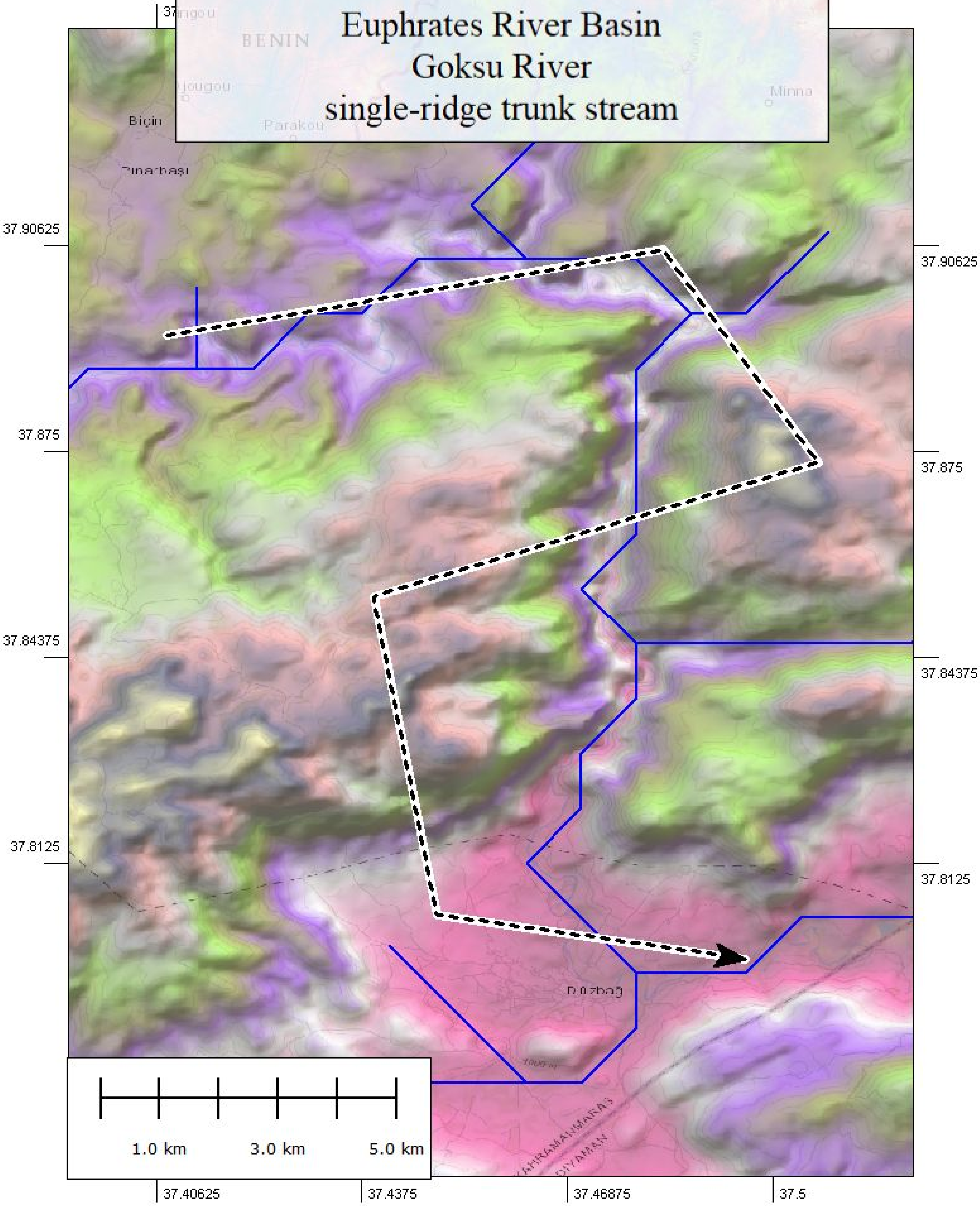

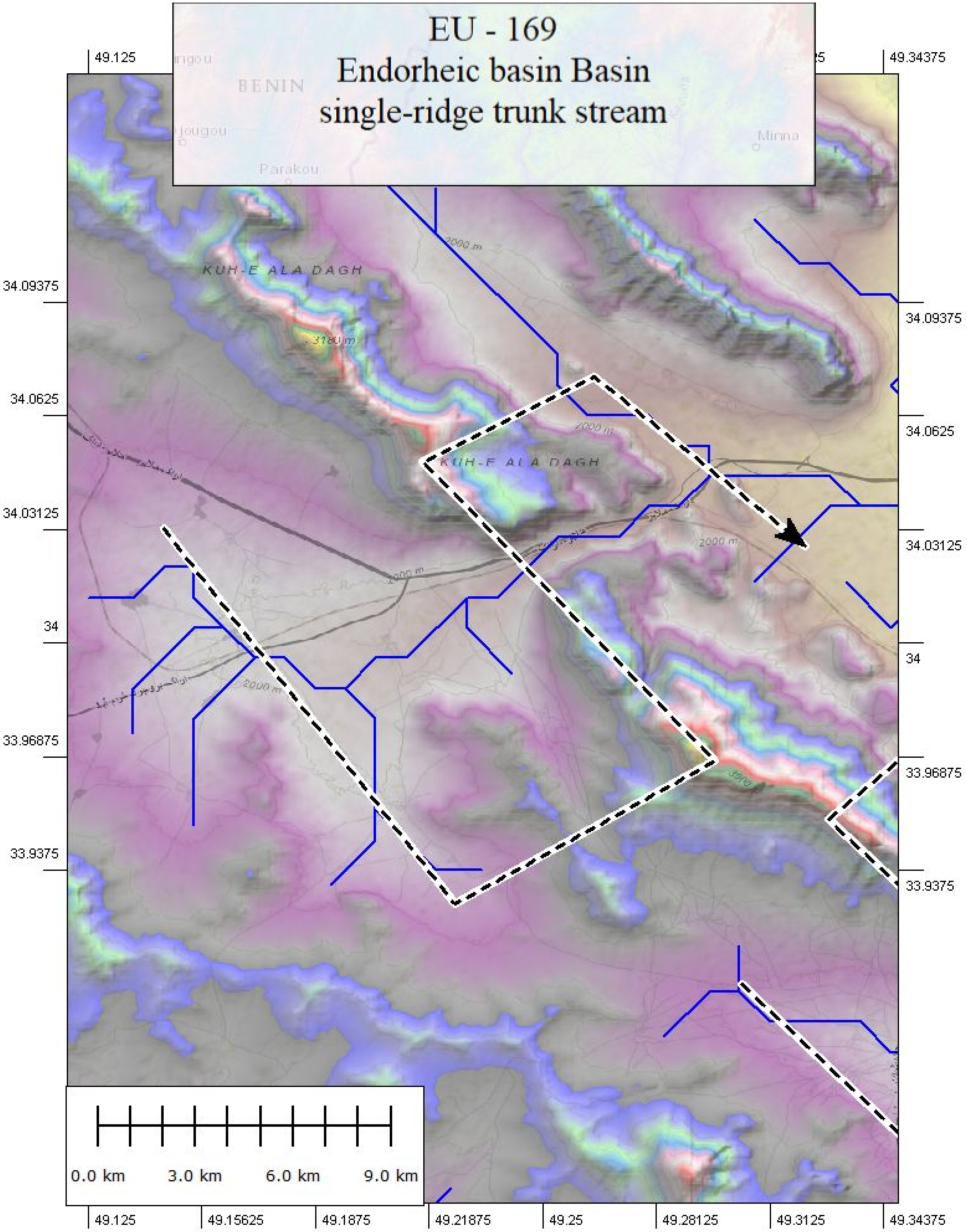

EU - 169
Endorheic basin Basin
single-ridge trunk stream
49.125
49.34375
BENIN
Parakou
Minna
KUH-E ALA DAGH
3180 m
2000 m
34.09375
34.0625
34.03125
34
33.96875
33.9375
0.0 km
3.0 km
6.0 km
9.0 km
49.15625
49.1875
49.21875
49.25
49.28125
49.3125

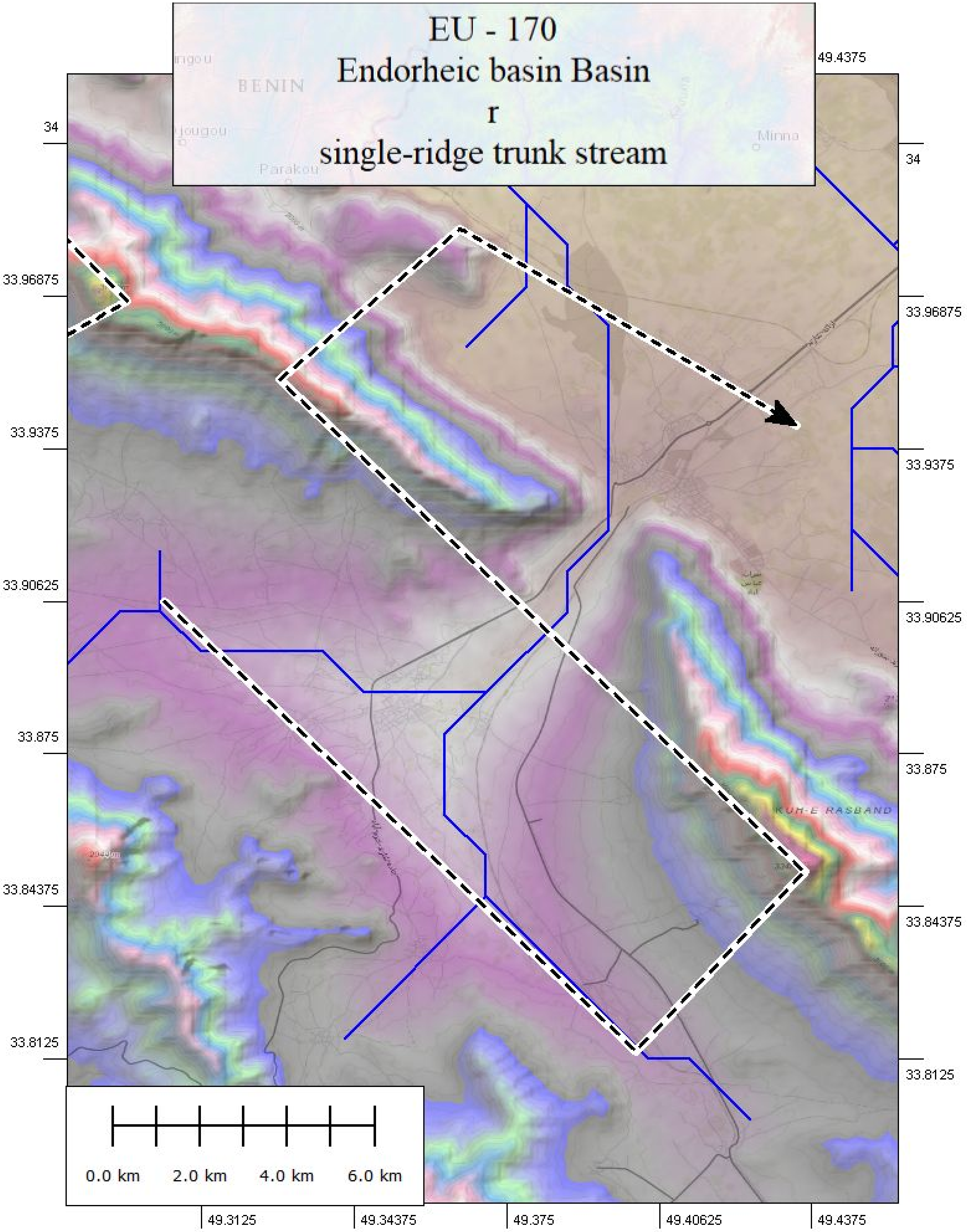
EU - 170
Endorheic basin Basin
r
single-ridge trunk stream
BENIN
Parakou
Minna
KUH-E RASBAND
34
33.96875
33.9375
33.90625
33.875
33.84375
33.8125
49.3125
49.34375
49.375
49.40625
49.4375
0.0 km
2.0 km
4.0 km
6.0 km

EU - 171
Rio Besaya Basin
Besaya River
single-ridge trunk stream

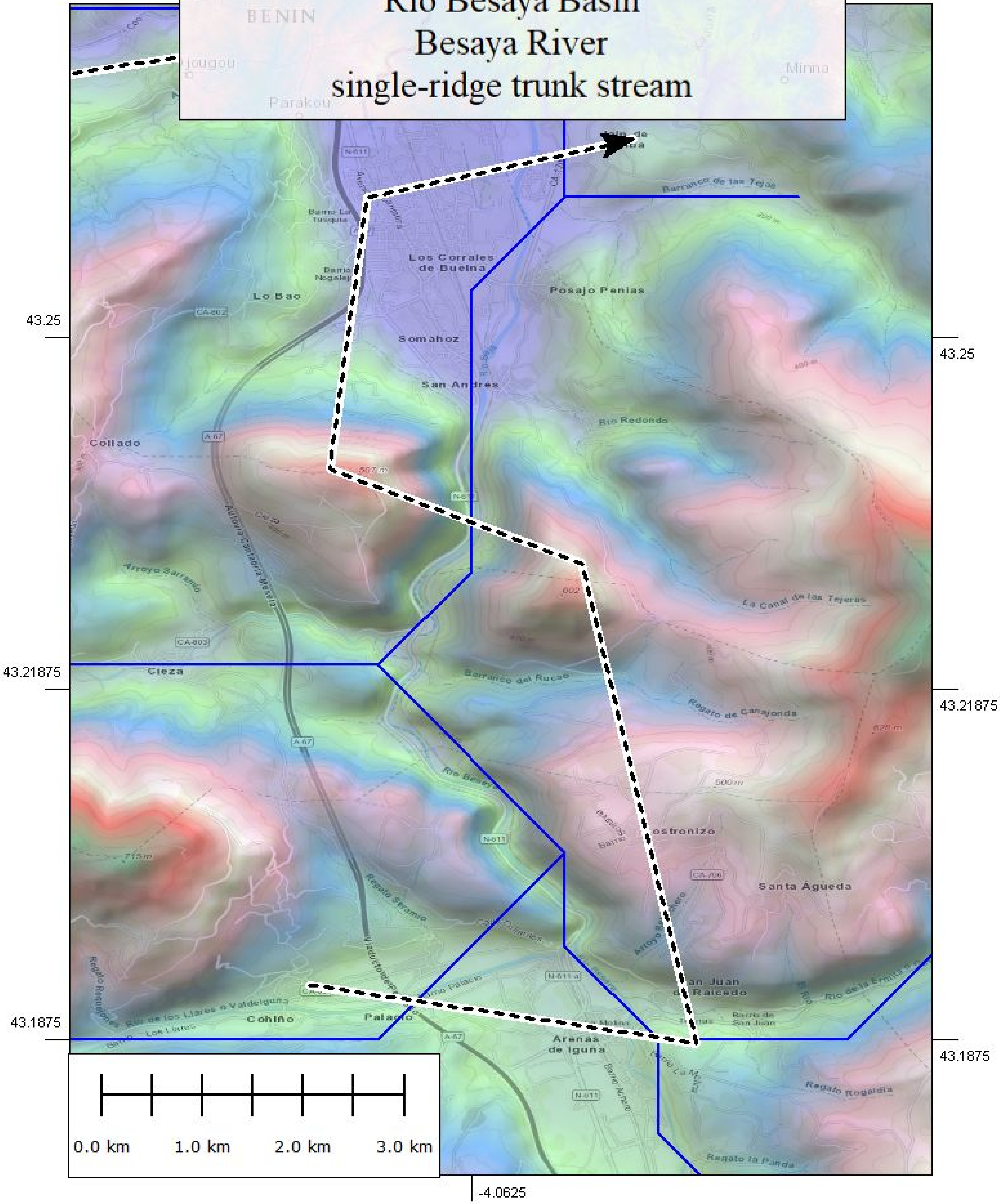

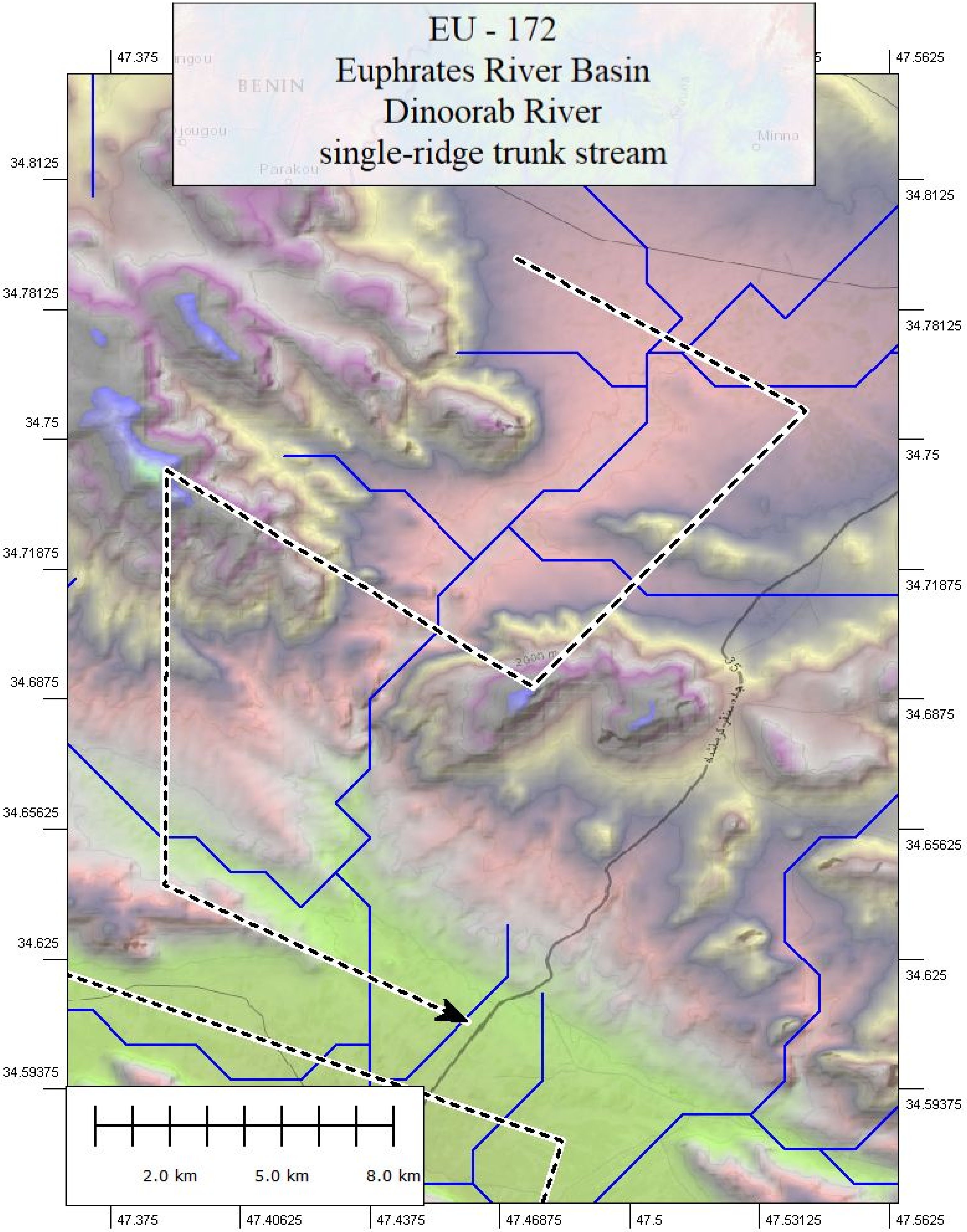

EU - 172
Euphrates River Basin
Dinoorab River
single-ridge trunk stream
47.375
47.40625
47.4375
47.46875
47.5
47.53125
47.5625
34.8125
34.78125
34.75
34.71875
34.6875
34.65625
34.625
34.59375
2.0 km
5.0 km
8.0 km

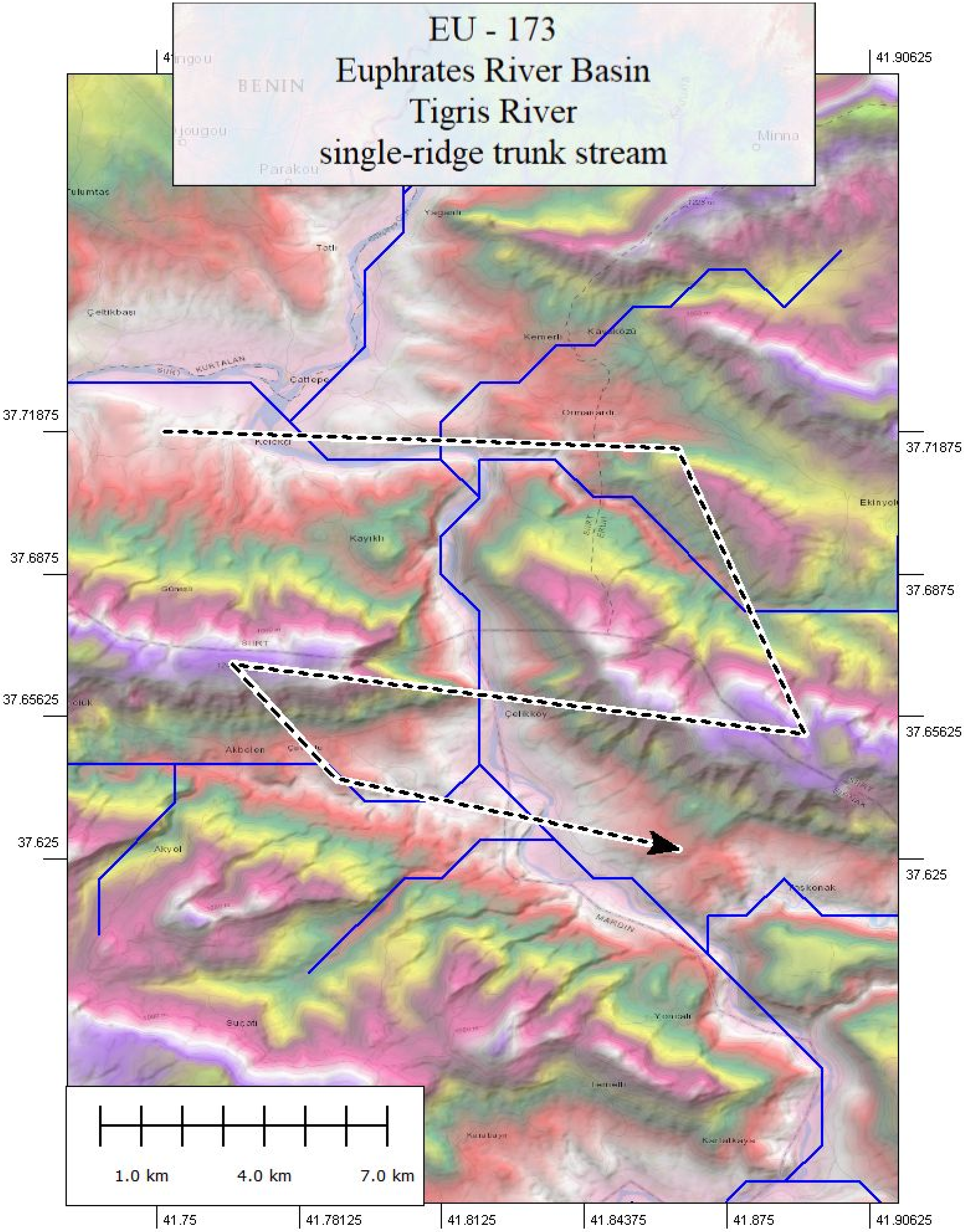
EU - 173
Euphrates River Basin
Tigris River
single-ridge trunk stream
41.90625
37.71875
37.6875
37.65625
37.625
41.75
41.78125
41.8125
41.84375
41.875
1.0 km
4.0 km
7.0 km
Tatlı
Çeltikbası
Kemerli
Çattepe
Kayıklı
Çelikköy
Akbelen
Akyol
Ekinyol
Suçatı
Yemeli
Kurtalan
