# Supplementary material for: A global database of large-scale transverse drainages
Source: Data Brief. 2019 Jan 14;23:103650. doi: 10.1016/j.dib.2018.12.088 (PMC6369416; doi:10.1016/j.dib.2018.12.088)
Supplement: Supplementary file 9 — Supplementary material [file mmc9.pdf]

SA - 6
Rio Magdalena Basin
Magdalena River
irregular high ground trunk stream

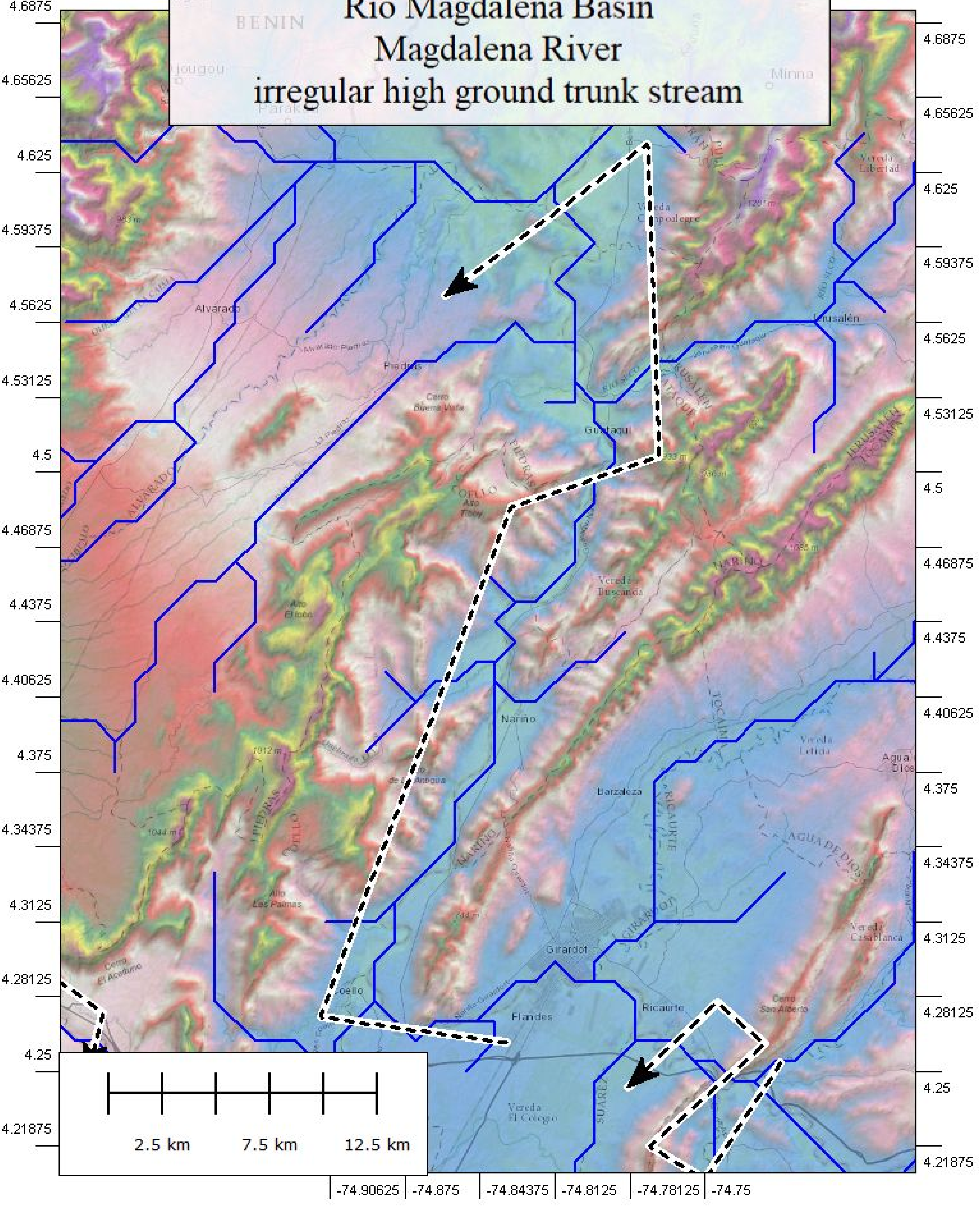

SA - 37
Rio Maule Basin
Reloca River
irregular high ground head stream

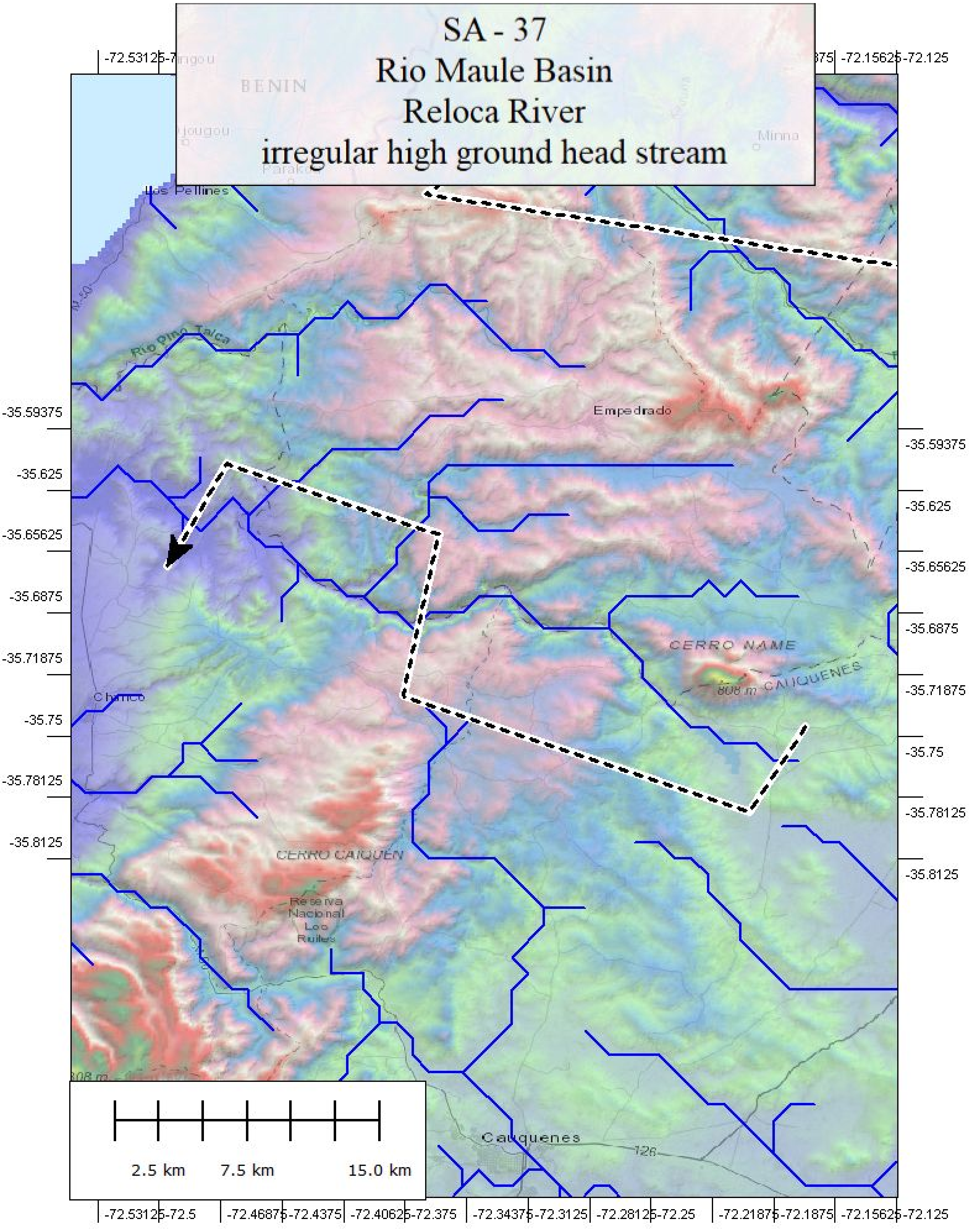

SA - 57
Orinoco River Basin
Caroni River tributary
updip plateau head stream

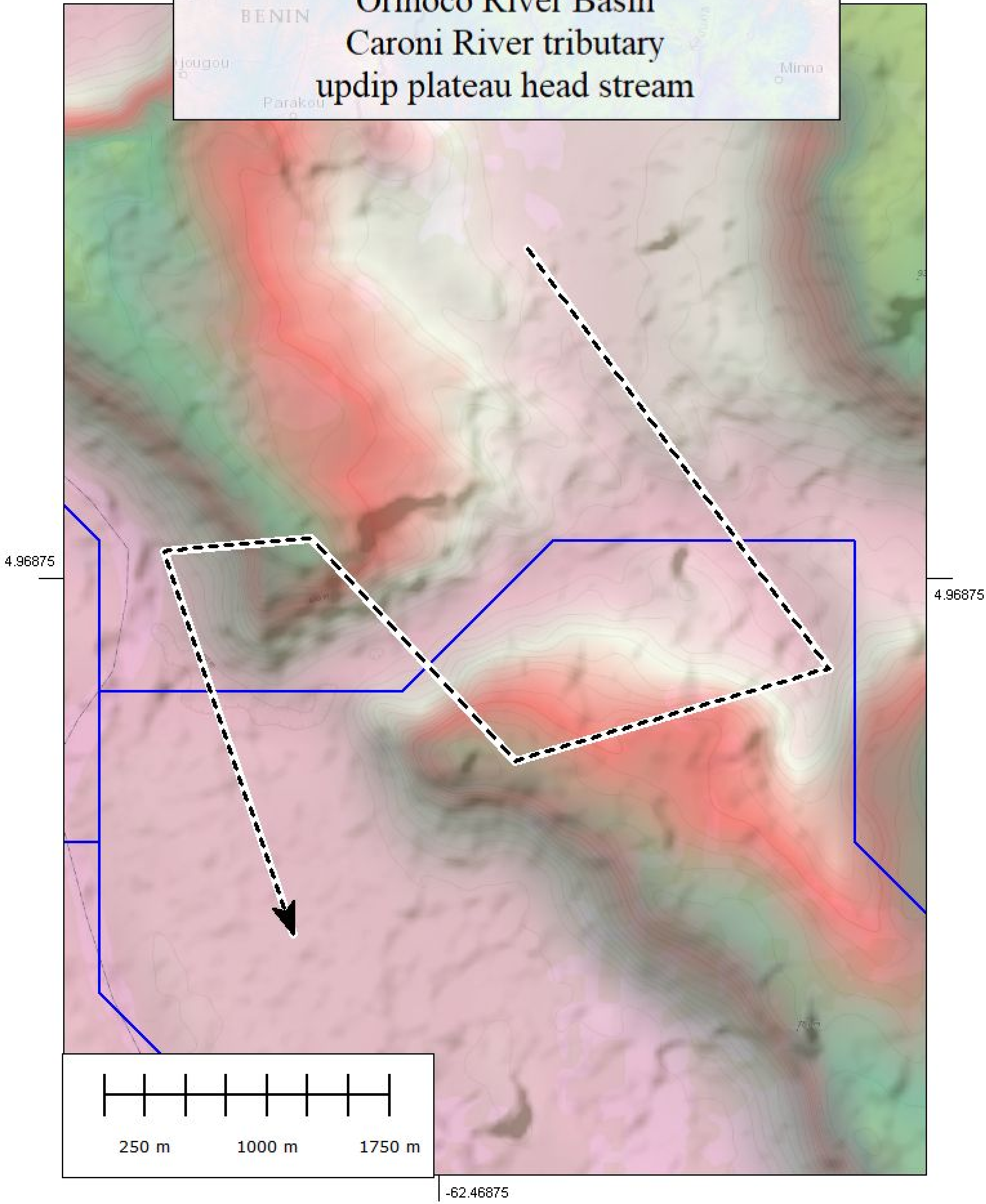

SA - 63
Rio Magdalena Basin
Pomeca River
downdip plateau trunk stream

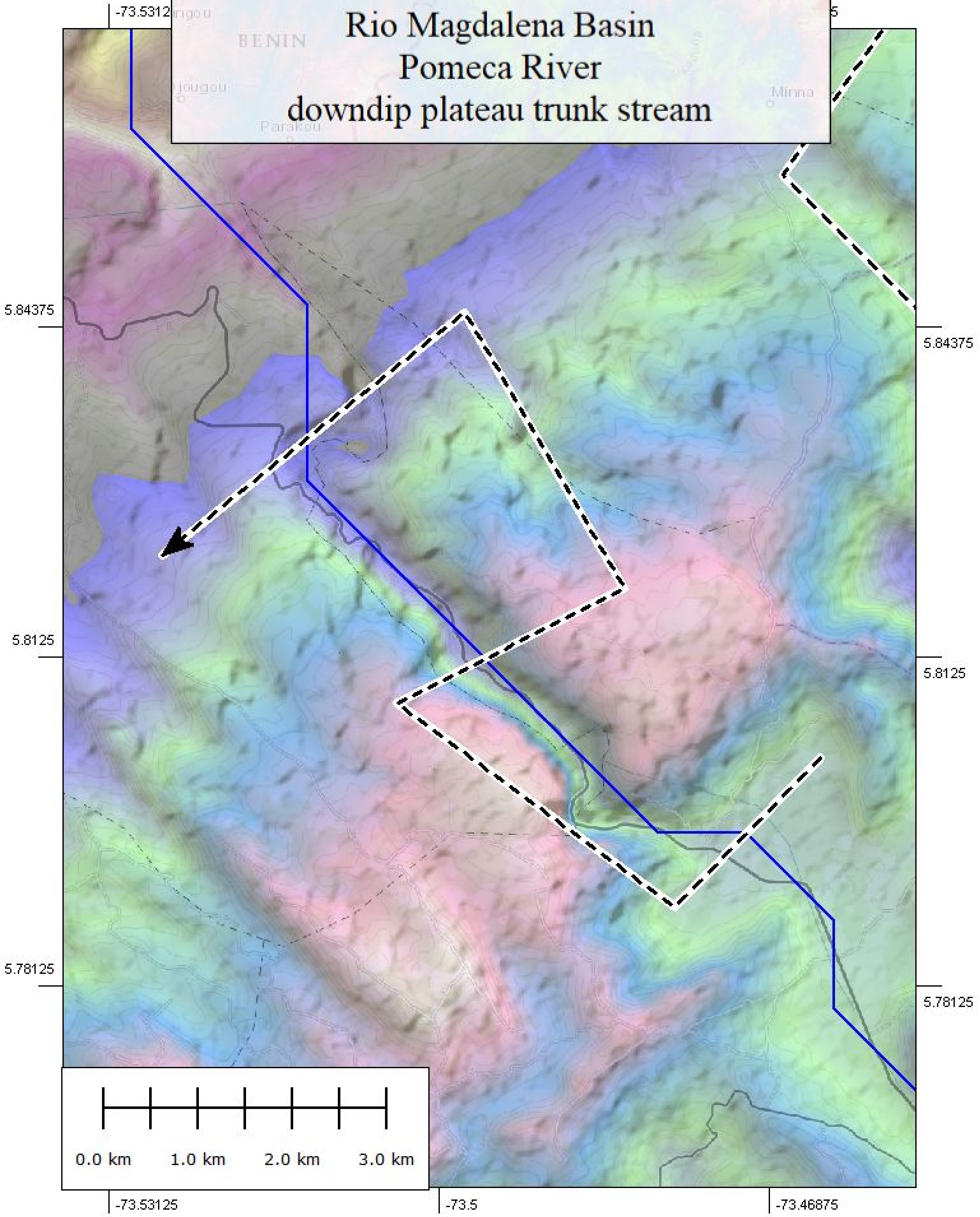

SA - 68
Amazon River Basin
Aguarico River
irregular high ground trunk stream

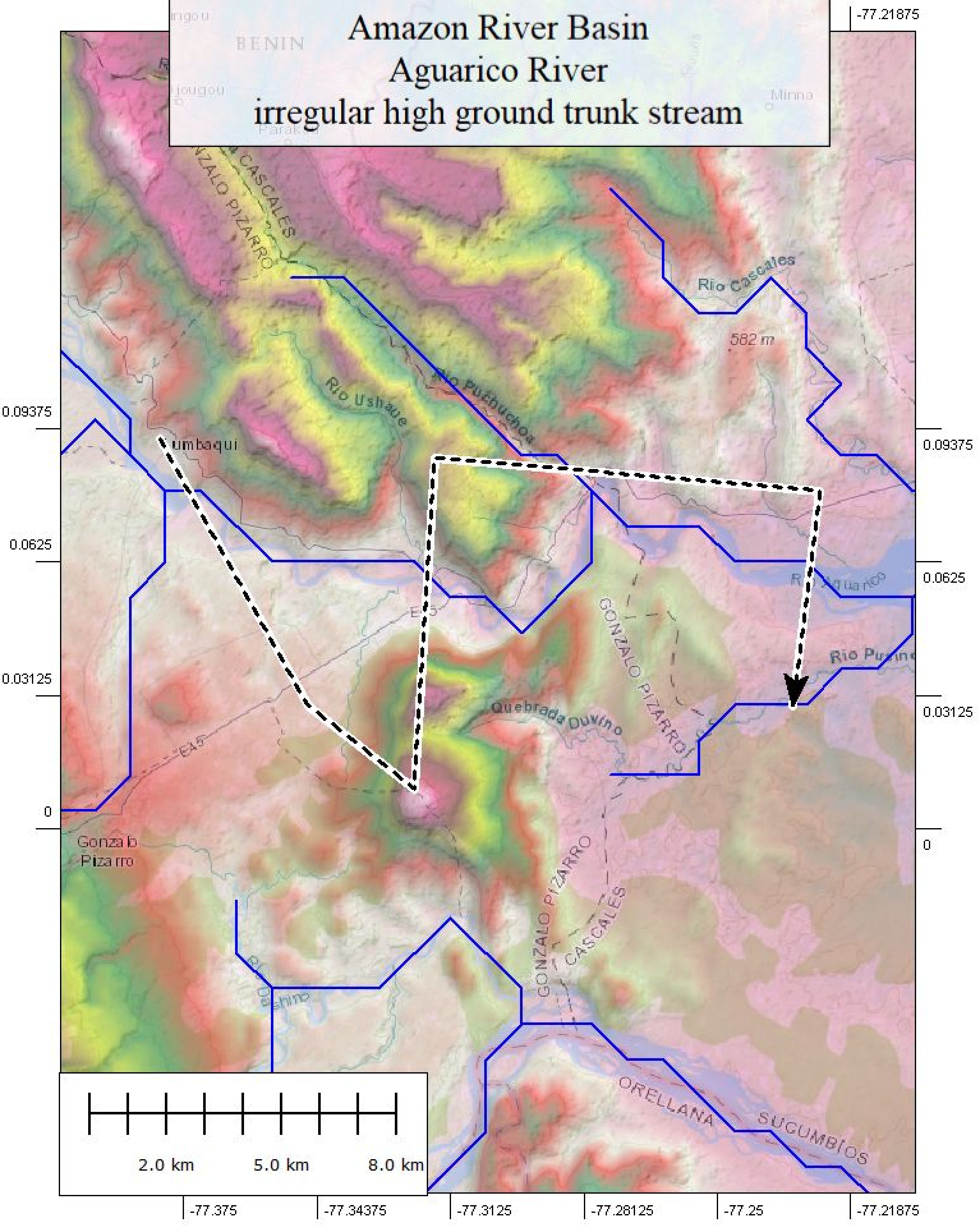

SA - 70
Rio Magdalena Basin
Quebrada Tres Aguas
plateau trunk stream

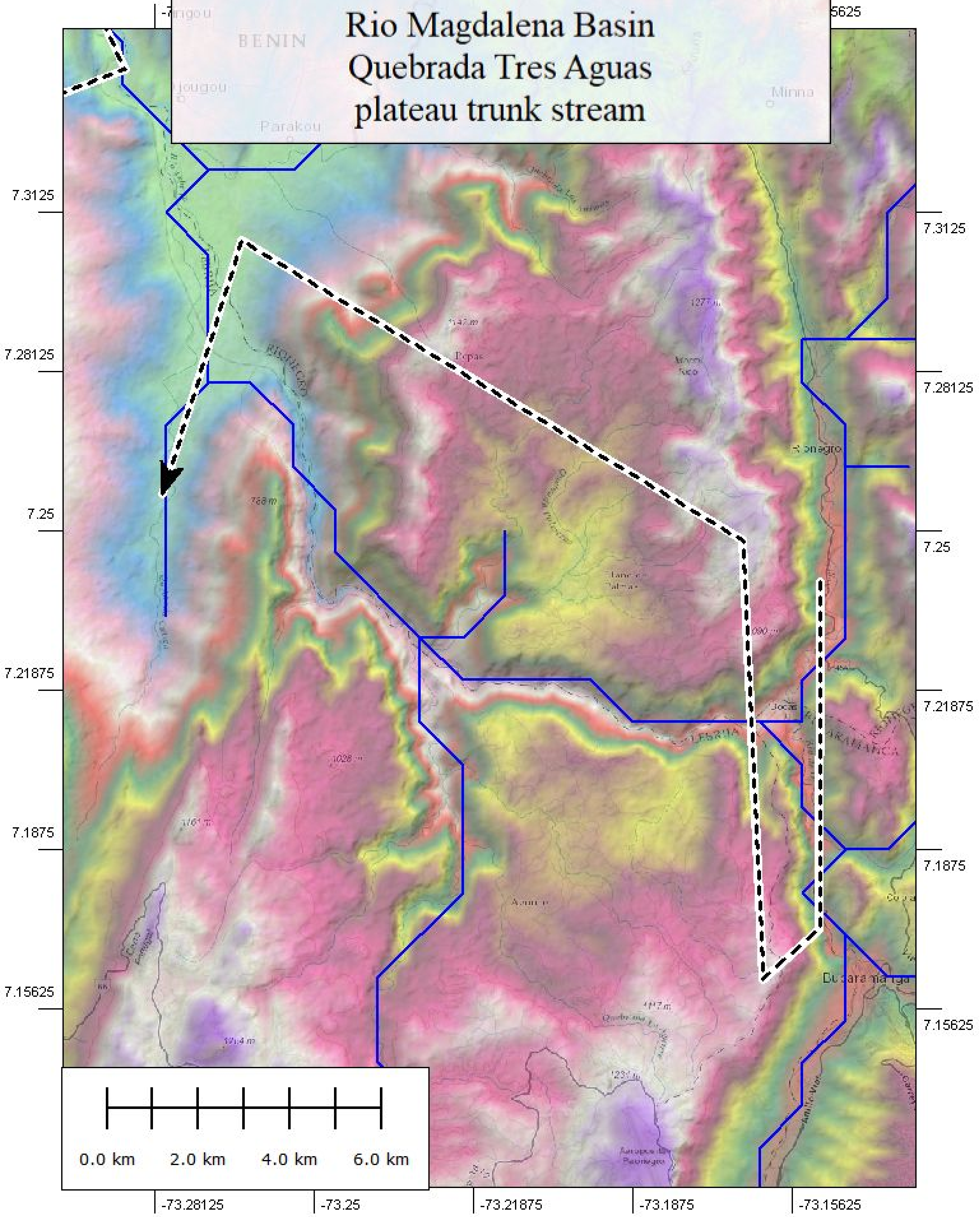

SA - 76
Amazon River Basin
Perene River
irregular high ground trunk stream

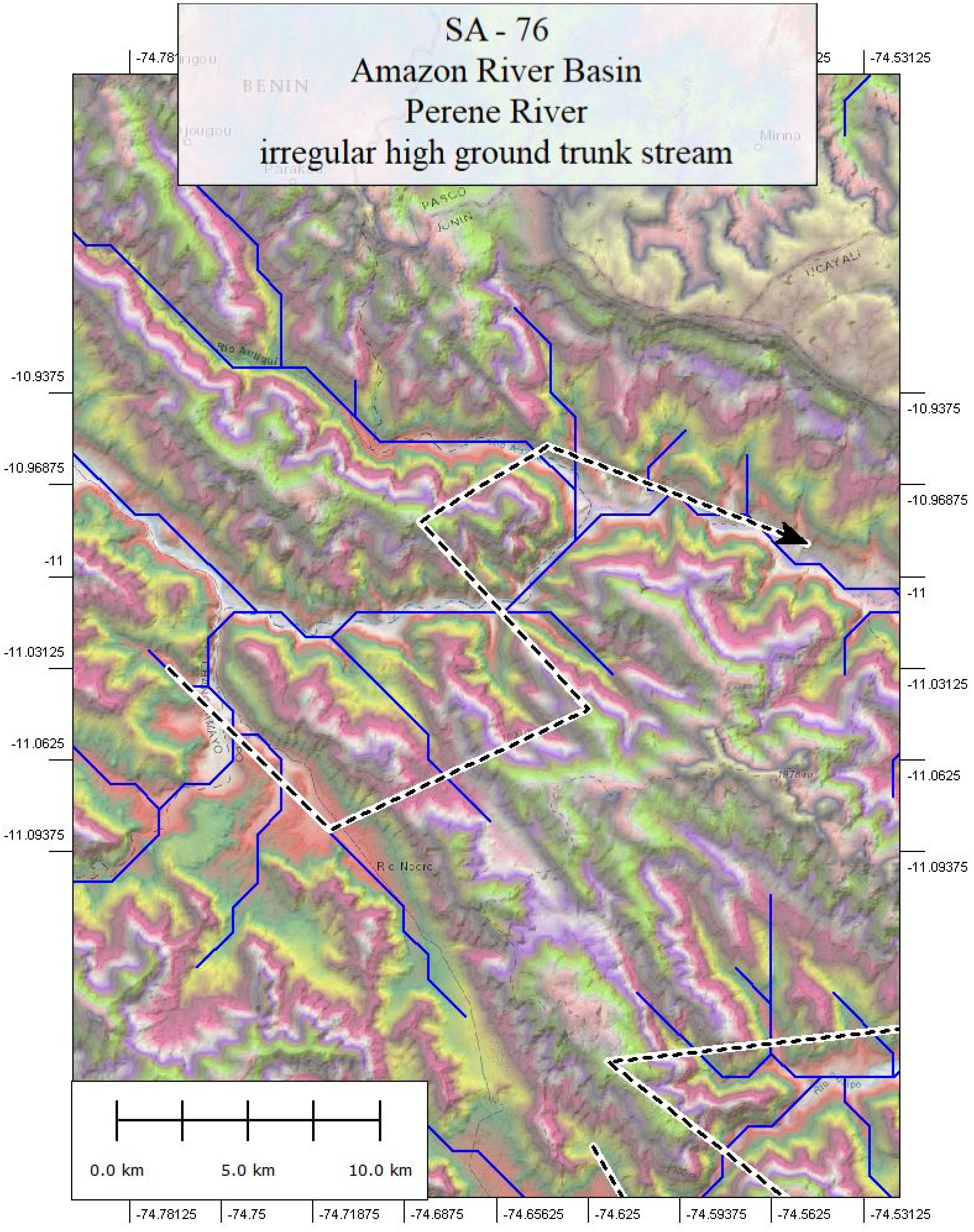

SA - 82
Amazon River Basin
Caguan River
irregular high ground trunk stream

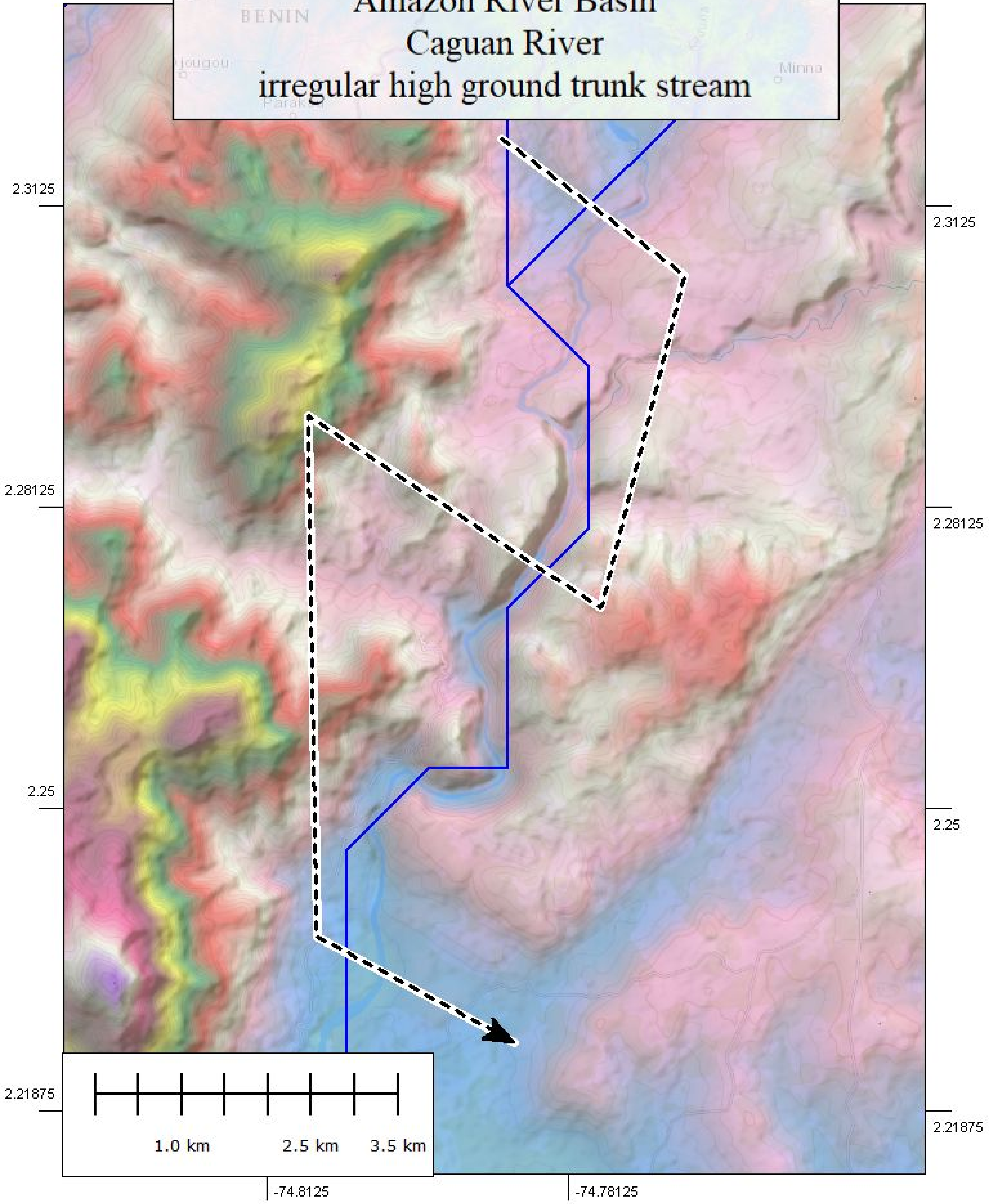

SA - 97
Amazon River Basin
Sonomoro River
irregular high ground trunk stream

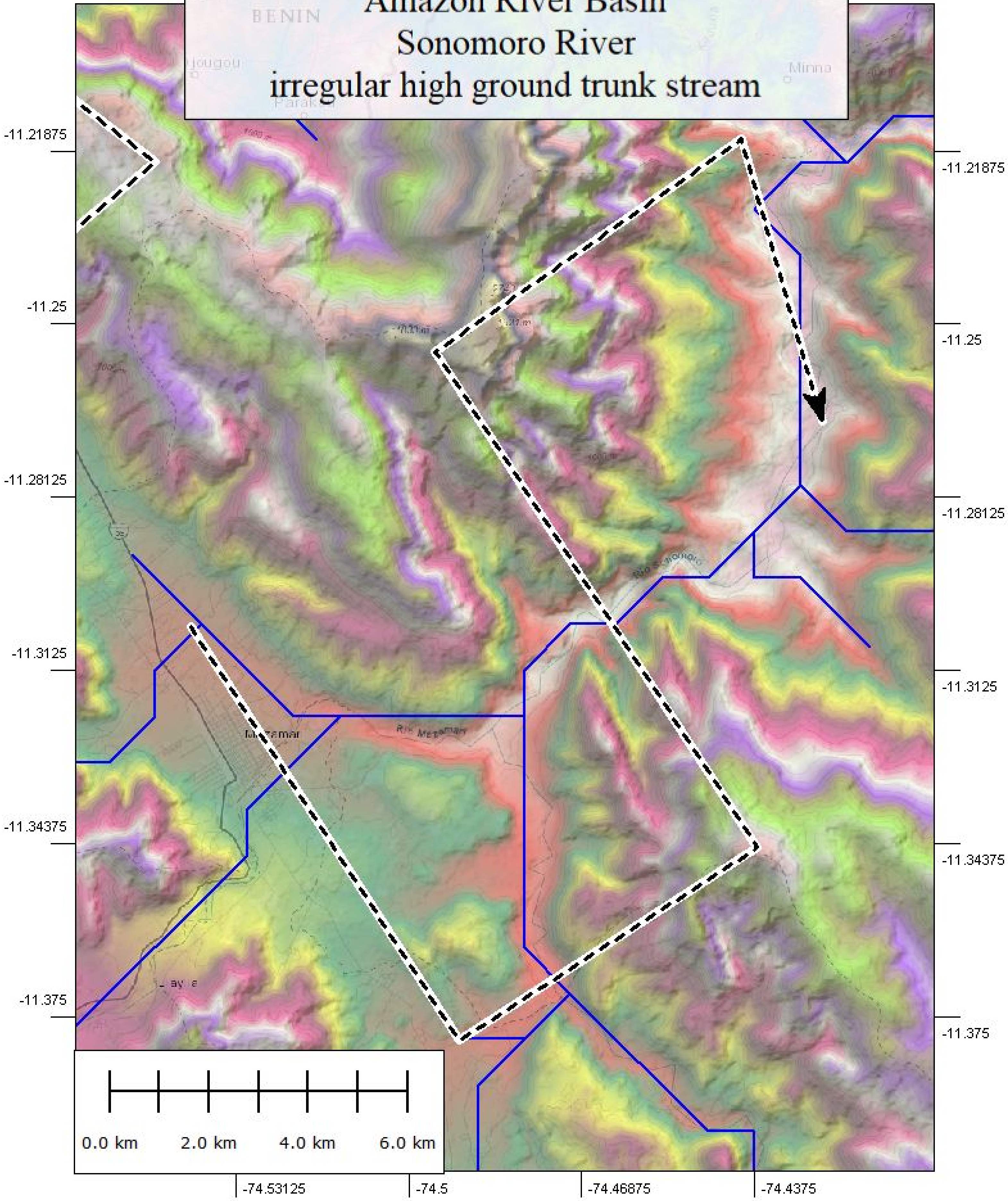

SA - 98
Amazon River Basin
Satipo River
irregular high ground trunk stream

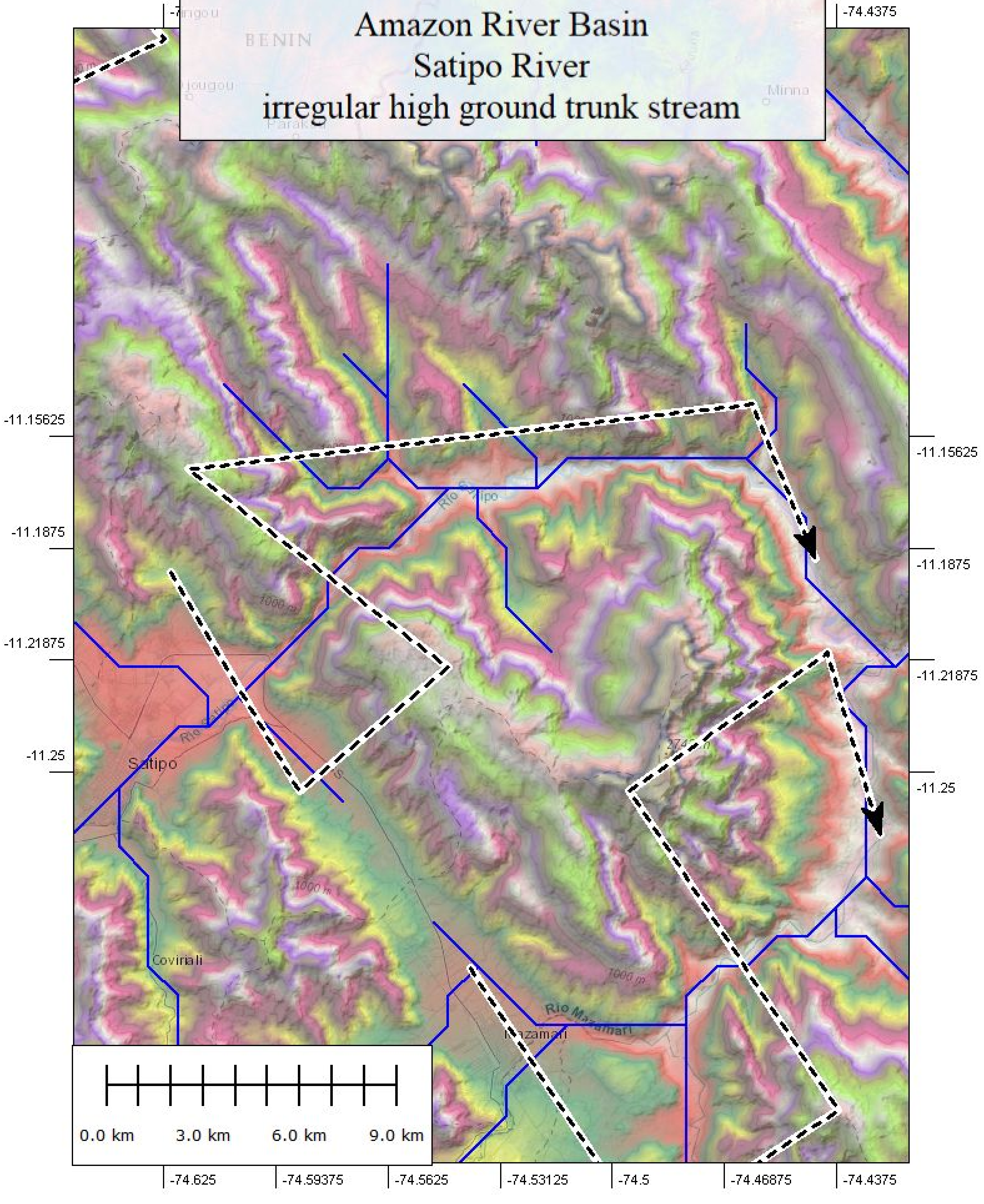

SA - 99

Parana River Basin

Pilcomayo River

irregular high ground trunk stream

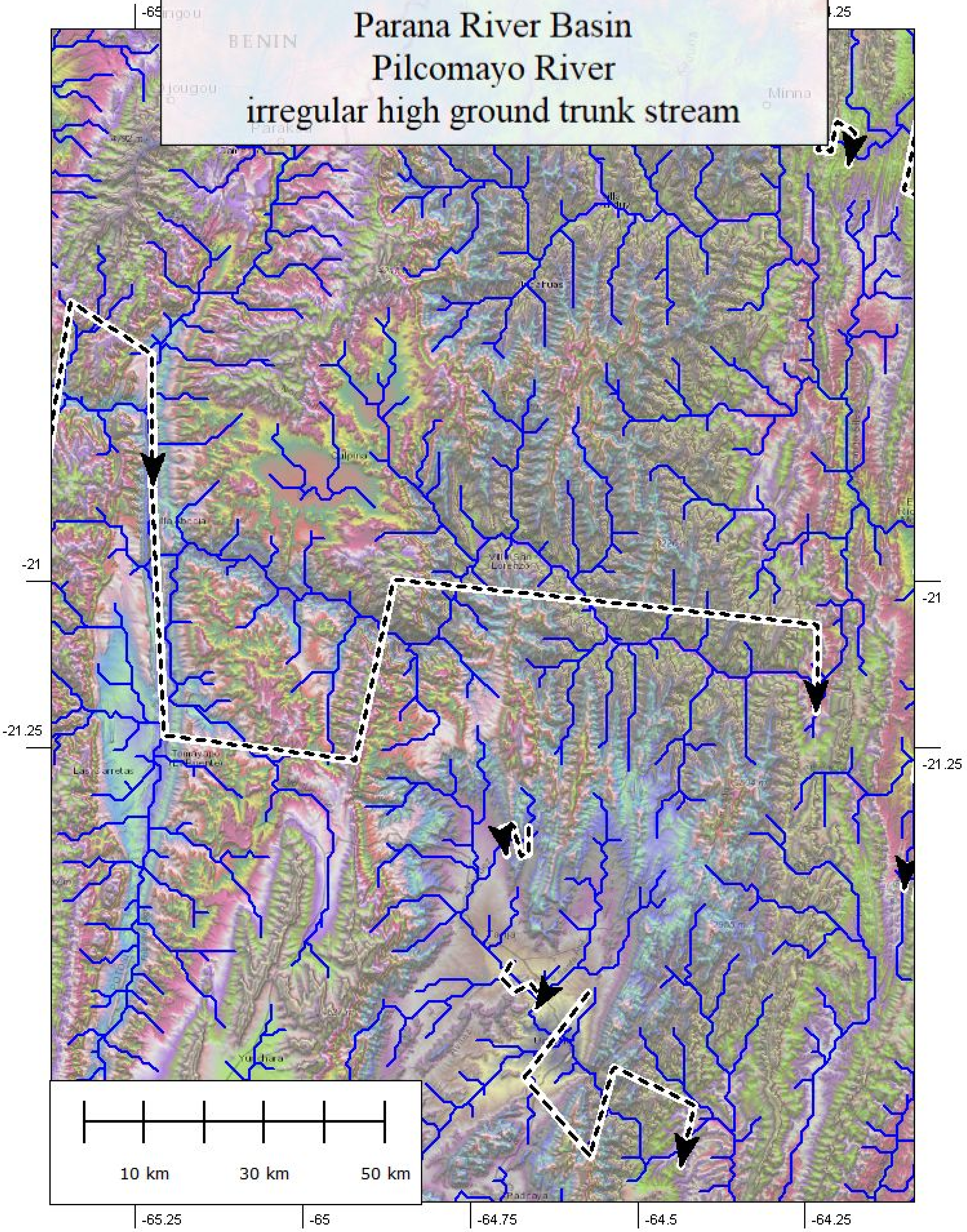

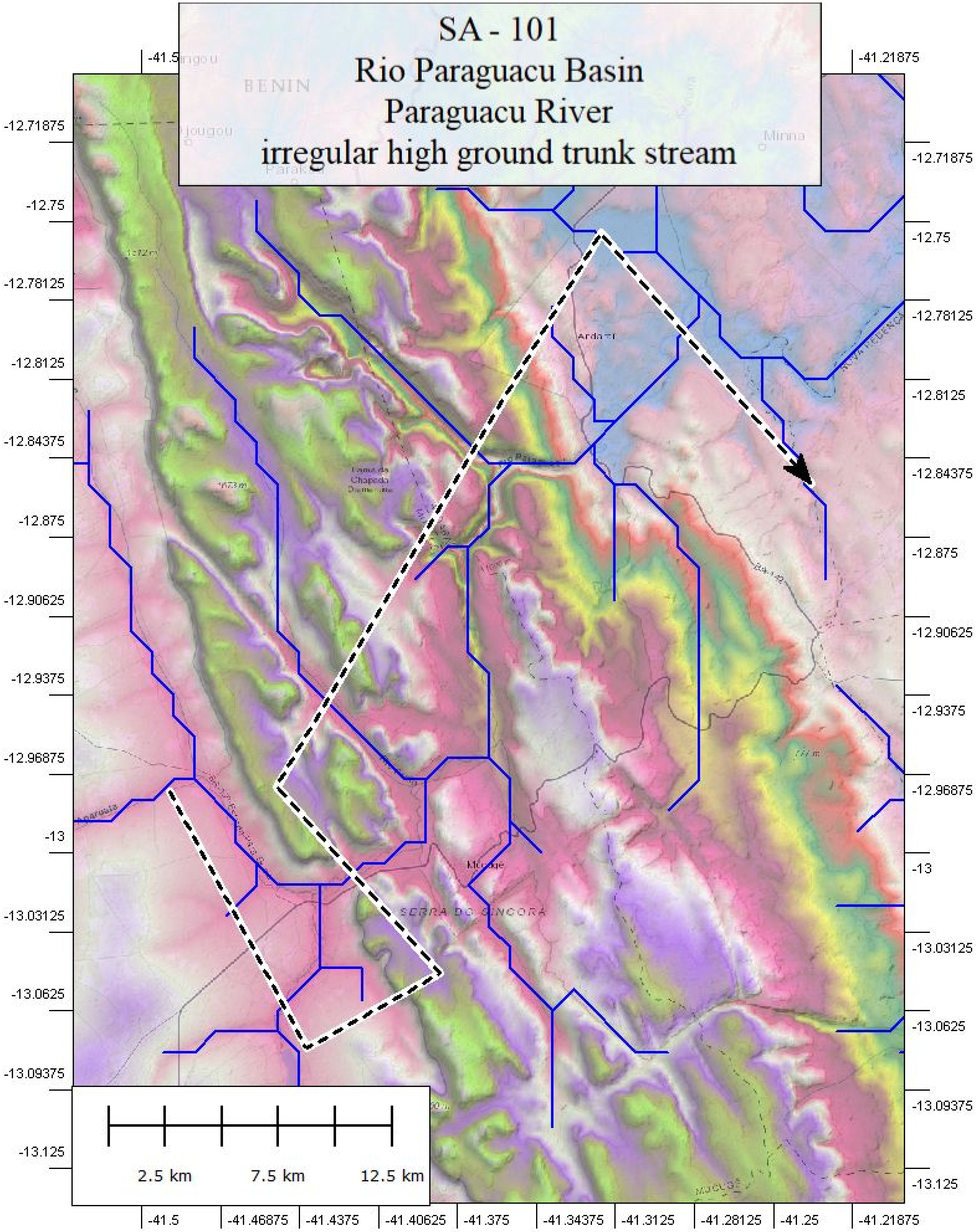
SA - 101
Rio Paraguacu Basin
Paraguacu River
irregular high ground trunk stream
-41.5
-41.21875
-12.71875
-12.75
-12.78125
-12.8125
-12.84375
-12.875
-12.90625
-12.9375
-12.96875
-13
-13.03125
-13.0625
-13.09375
-13.125
-41.46875
-41.4375
-41.40625
-41.375
-41.34375
-41.3125
-41.28125
-41.25
SERRA DO SINCORA
2.5 km
7.5 km
12.5 km

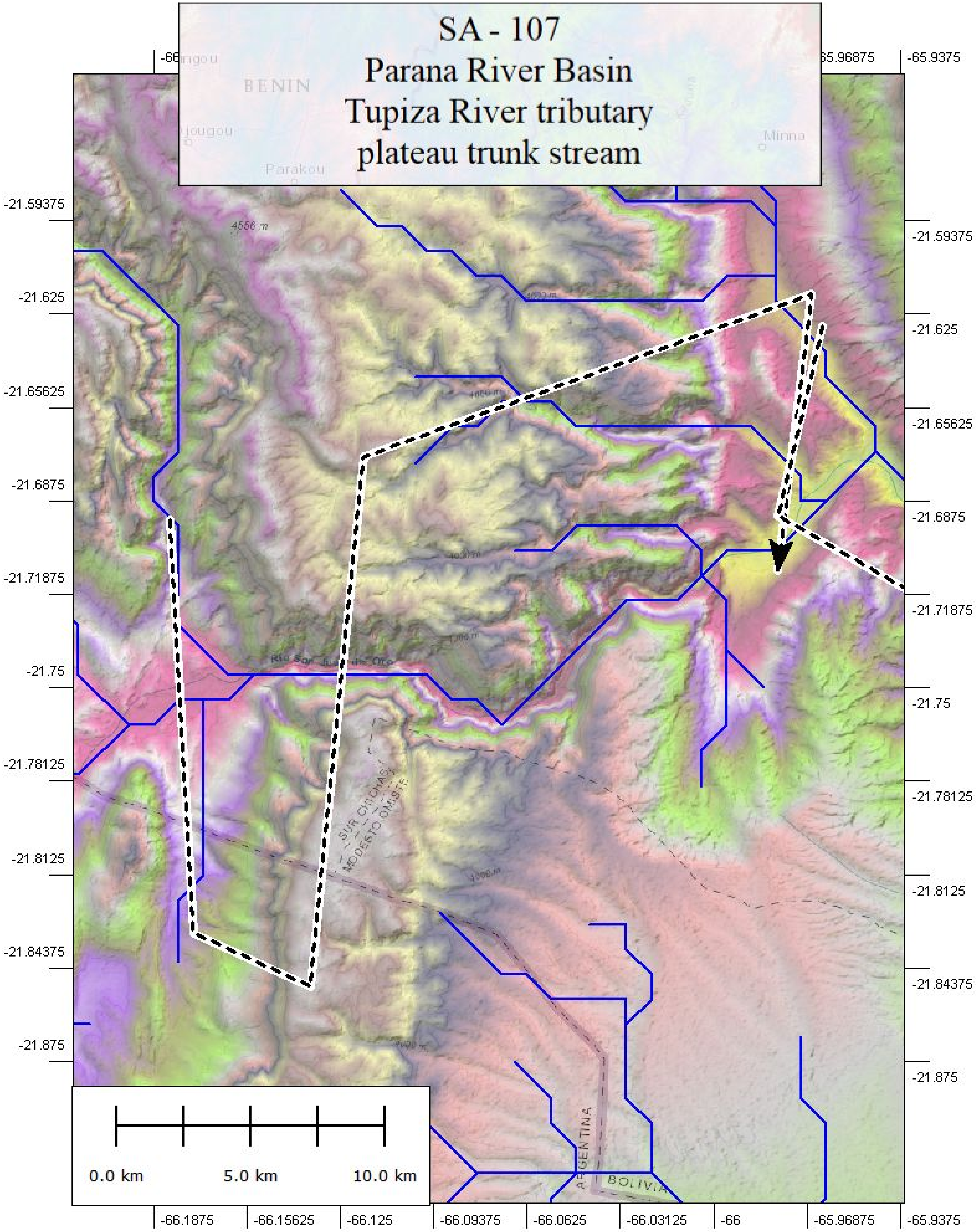
SA - 107
Parana River Basin
Tupiza River tributary
plateau trunk stream
-21.59375
-21.625
-21.65625
-21.6875
-21.71875
-21.75
-21.78125
-21.8125
-21.84375
-21.875
-66.1875
-66.15625
-66.125
-66.09375
-66.0625
-66.03125
-66
-65.96875
-65.9375
4556 m
SUR CHICHAS
MODESTO OMISTE
ARGENTINA
BOLIVIA
BENIN
Parakou
Minna
0.0 km
5.0 km
10.0 km

SA - 115
Amazon River Basin
Maranon River
irregular high ground trunk stream

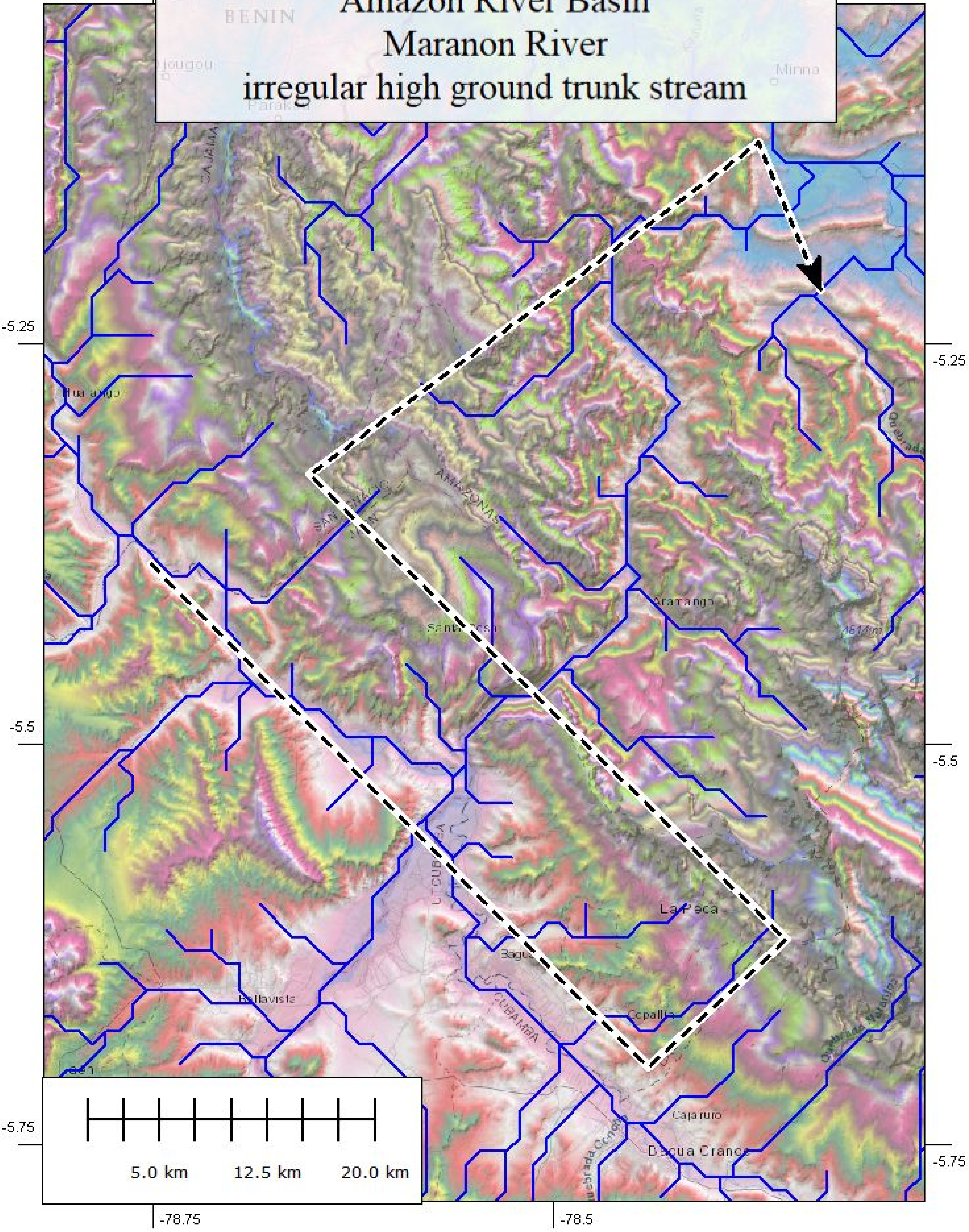

SA - 117
Amazon River Basin
Caqueta River
irregular high ground trunk stream

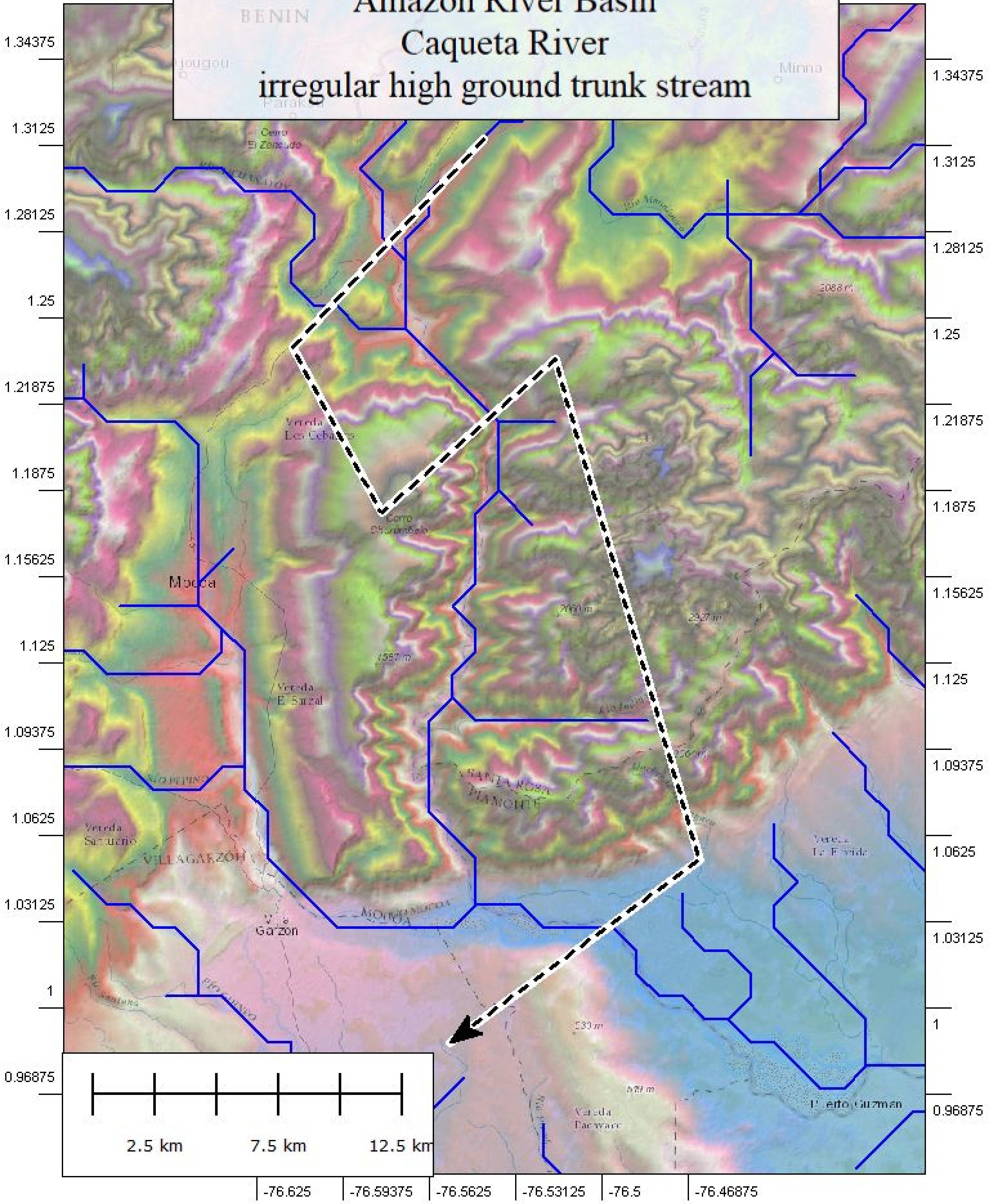

SA - 118
Rio Magdalena Basin
Uvaso River
downdip plateau trunk stream

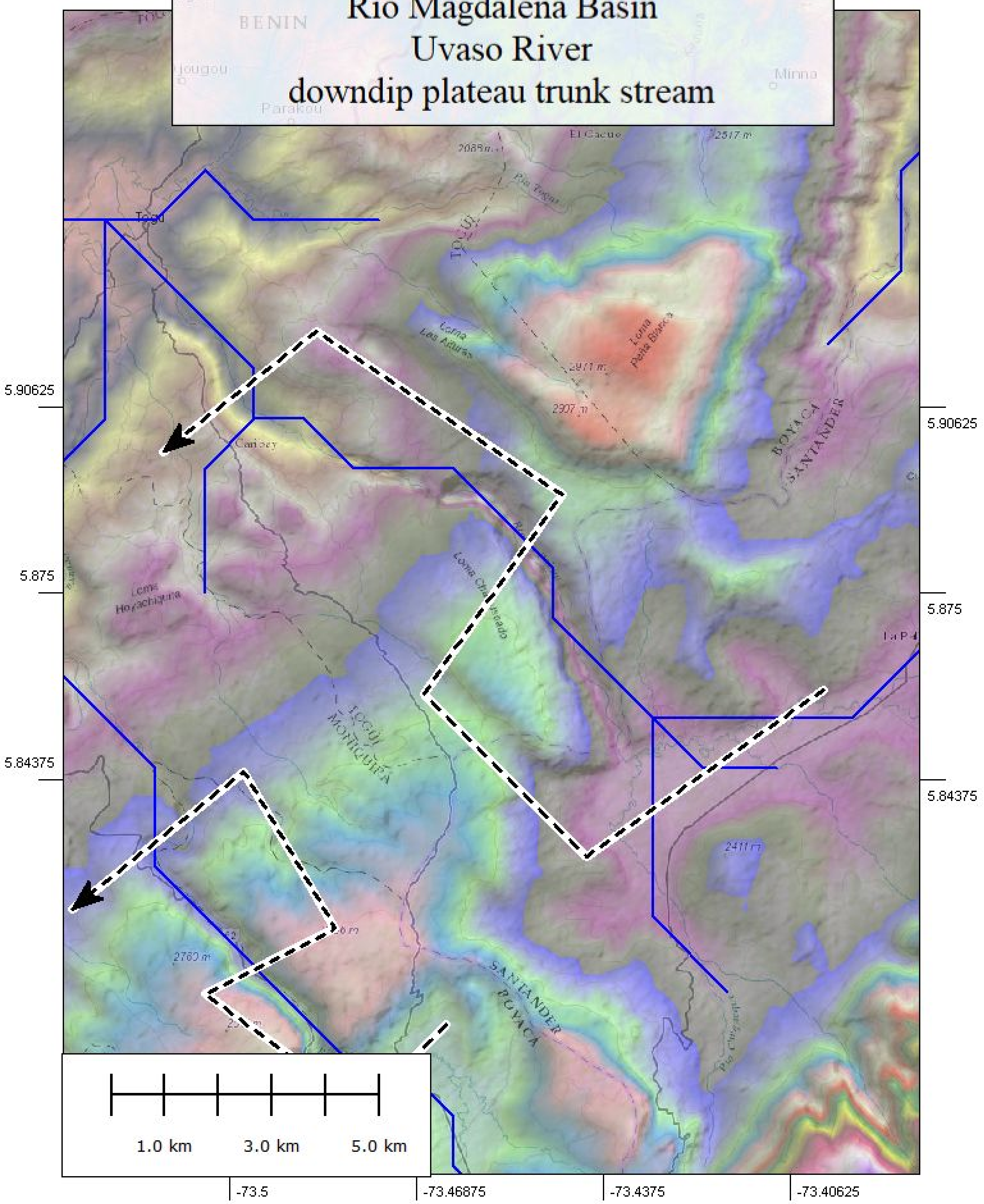

SA - 145
Parana River Basin
Compuel River
irregular high ground trunk stream

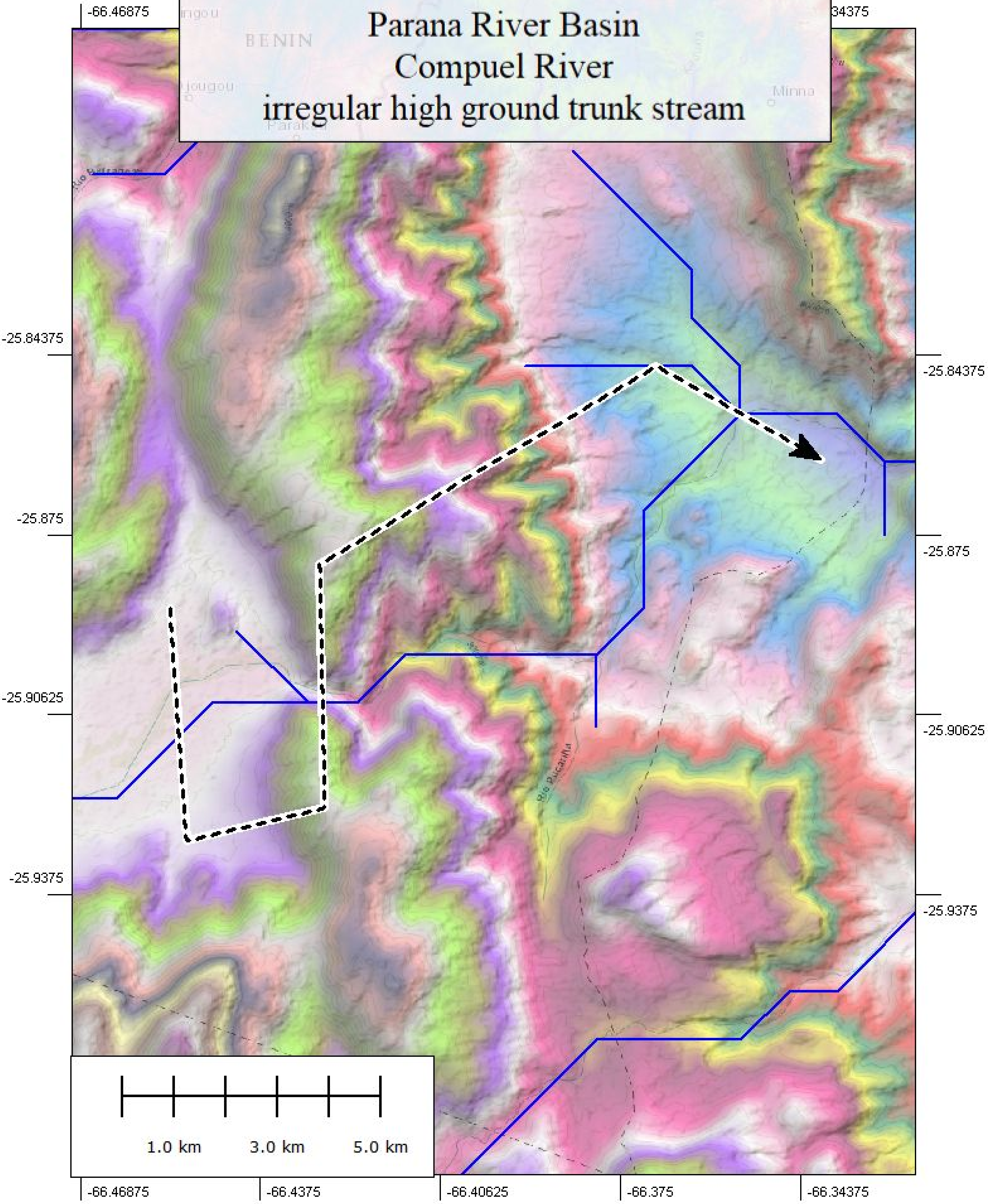

SA - 147
Parana River Basin
Angastaco River
irregular high ground trunk stream

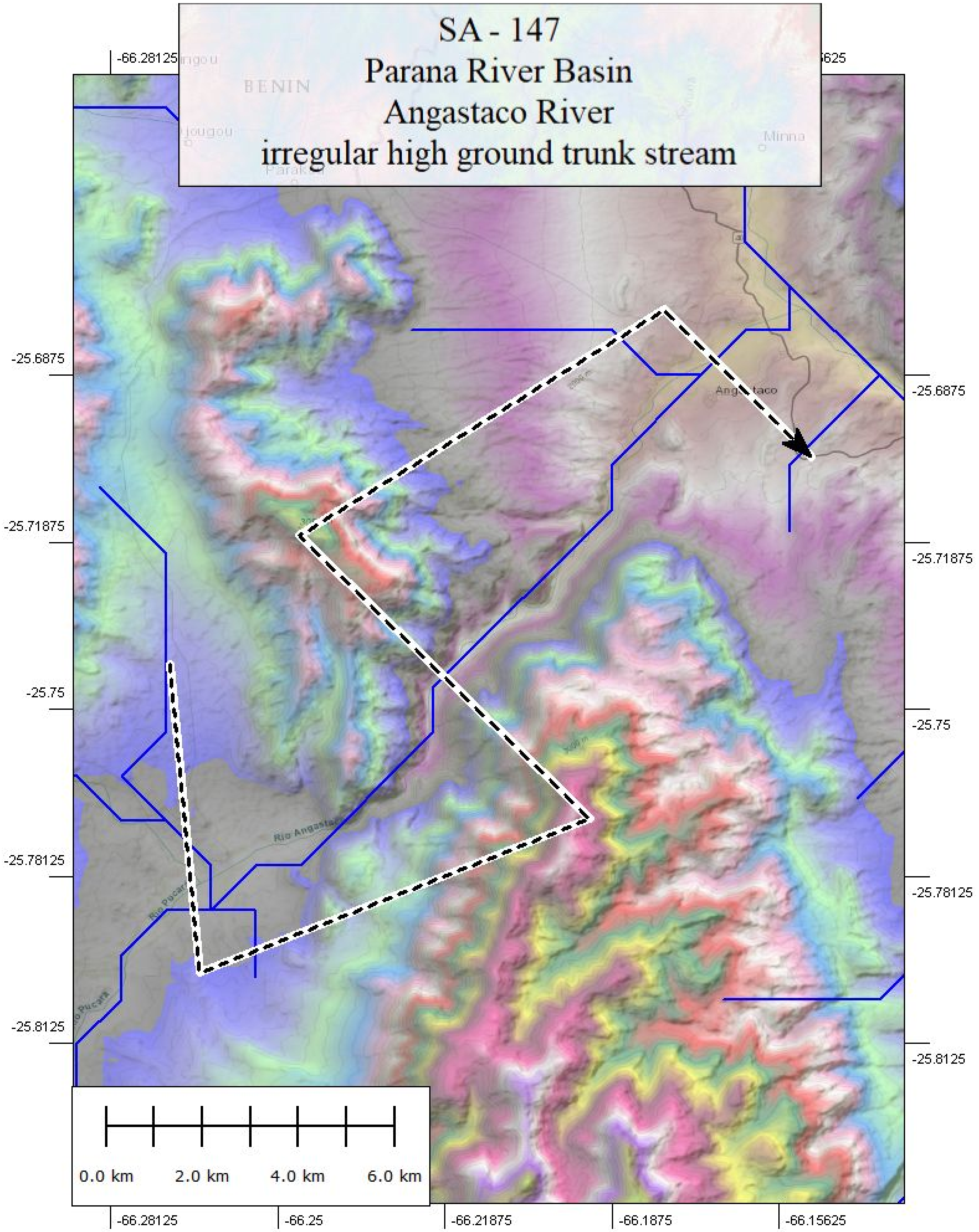

SA - 151
Rio Tocatins Basin
Parana River
irregular high ground trunk stream

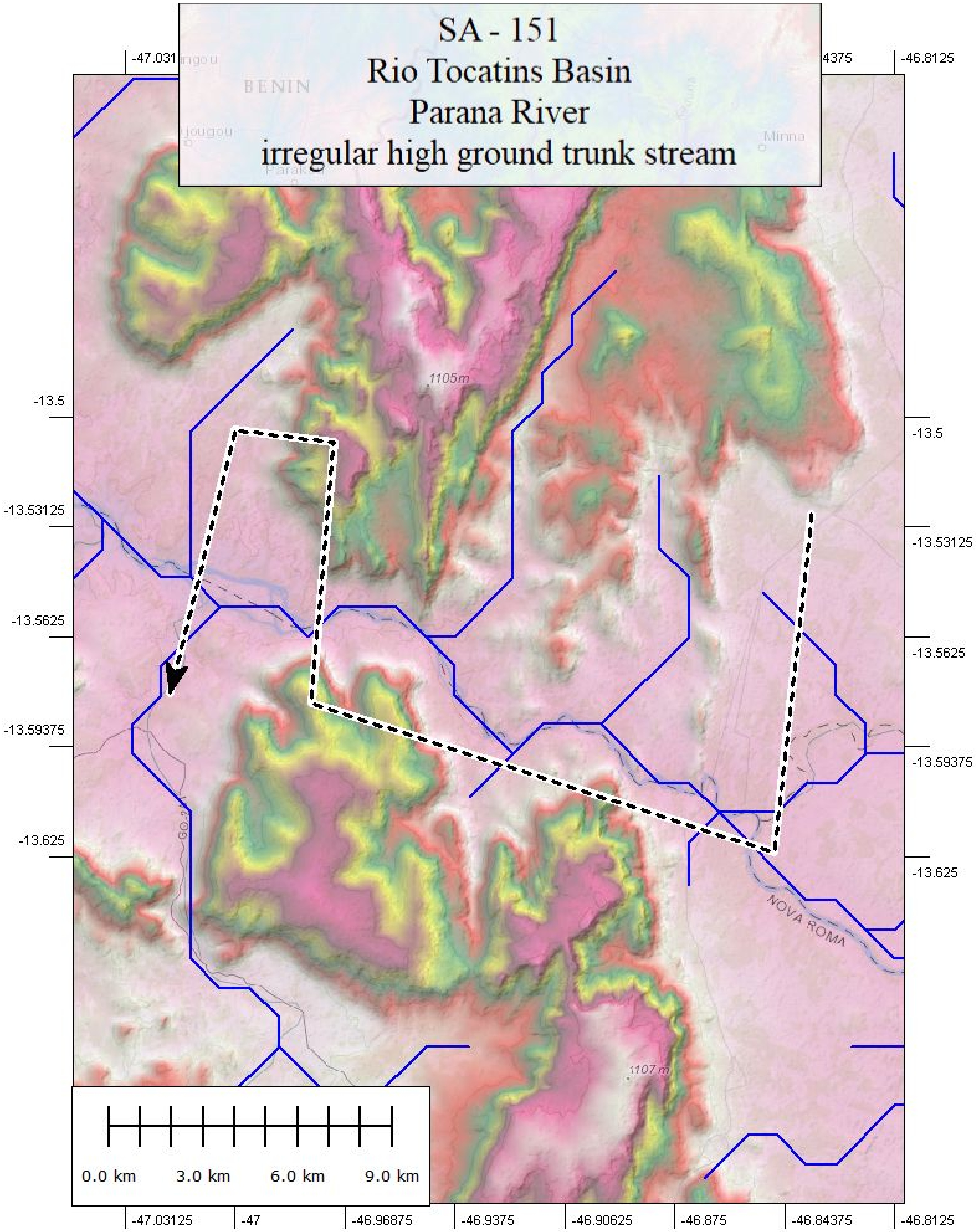

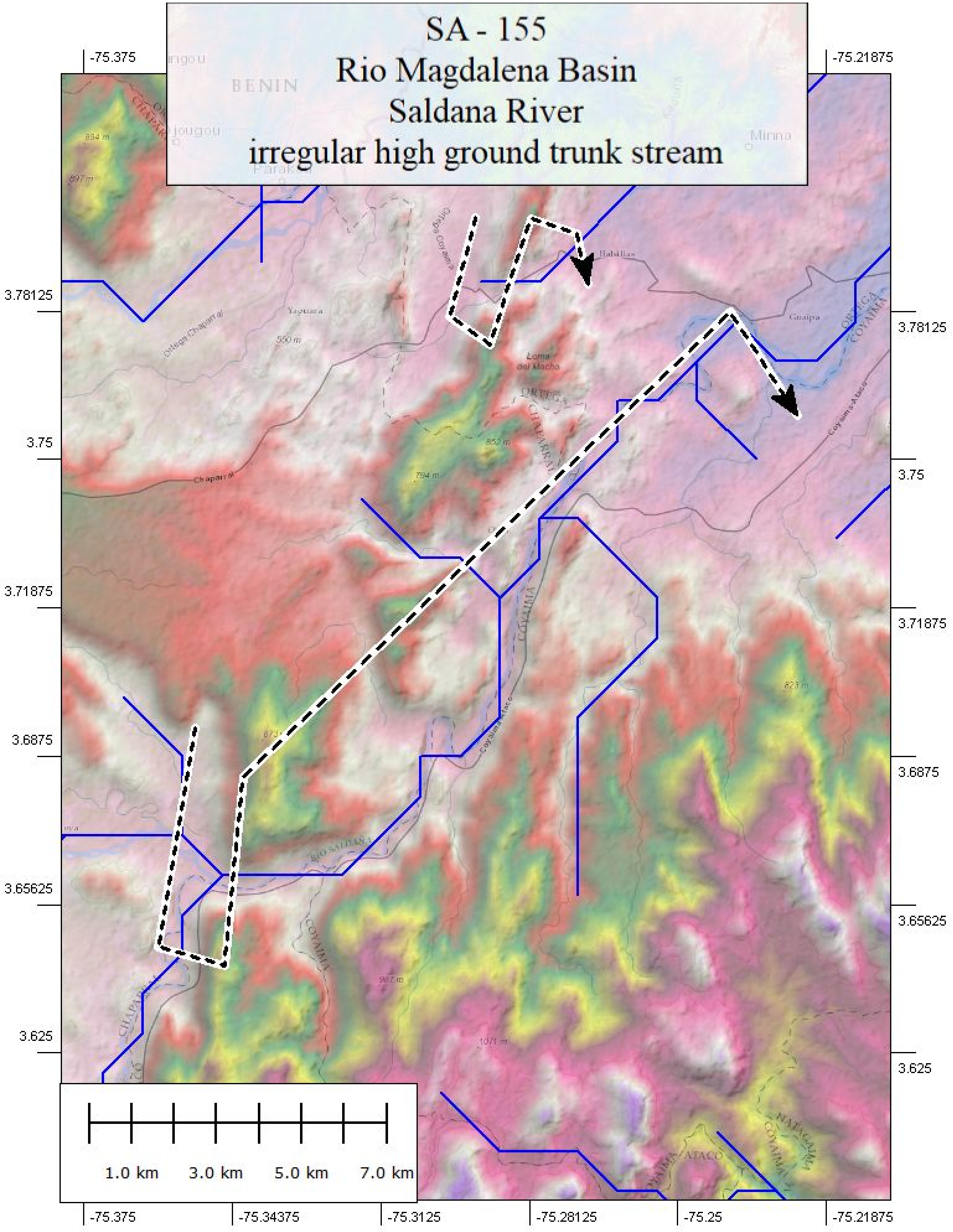
SA - 155
Rio Magdalena Basin
Saldana River
irregular high ground trunk stream
-75.375
-75.21875
-75.34375
-75.3125
-75.28125
-75.25
3.78125
3.75
3.71875
3.6875
3.65625
3.625
1.0 km
3.0 km
5.0 km
7.0 km
Balsillas
Guaipa
Yaguara
Ortega Chaparral
550 m
Loma del Macho
ORTEGA
CHAPARRAL
852 m
794 m
Chaparral
COYAIMA
Coyaima-Ataco
RIO SALDANA
873 m
823 m
967 m
1071 m
ATACO
NATAGAIMA

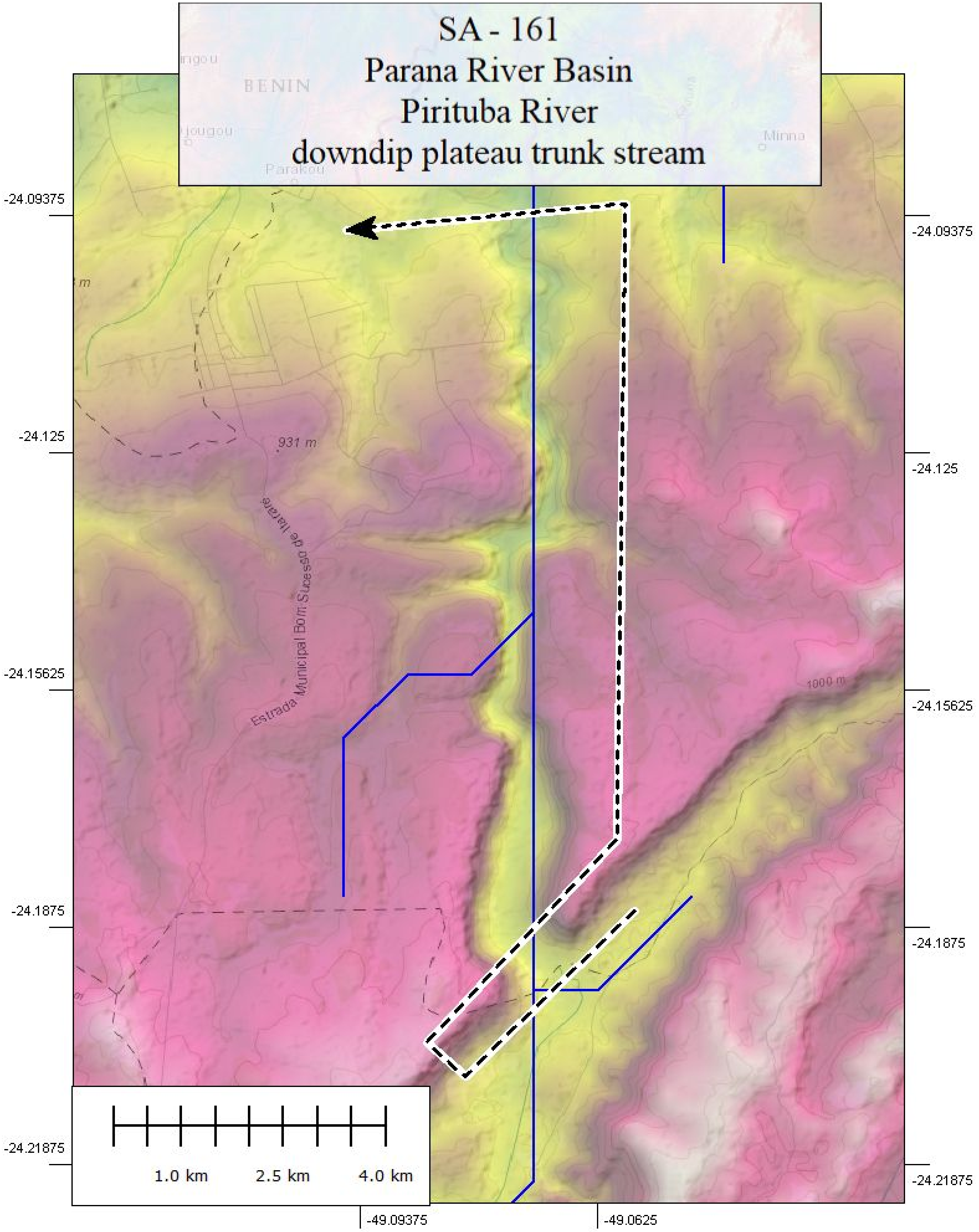

SA - 161
Parana River Basin
Pirituba River
downdip plateau trunk stream
BENIN
Parakou
Minna
-24.09375
-24.125
-24.15625
-24.1875
-24.21875
-49.09375
-49.0625
931 m
1000 m
Estrada Municipal Bom Sucesso de Itarare
1.0 km
2.5 km
4.0 km

SA - 162
Rio Maule Basin
Maule River
irregular high ground trunk stream

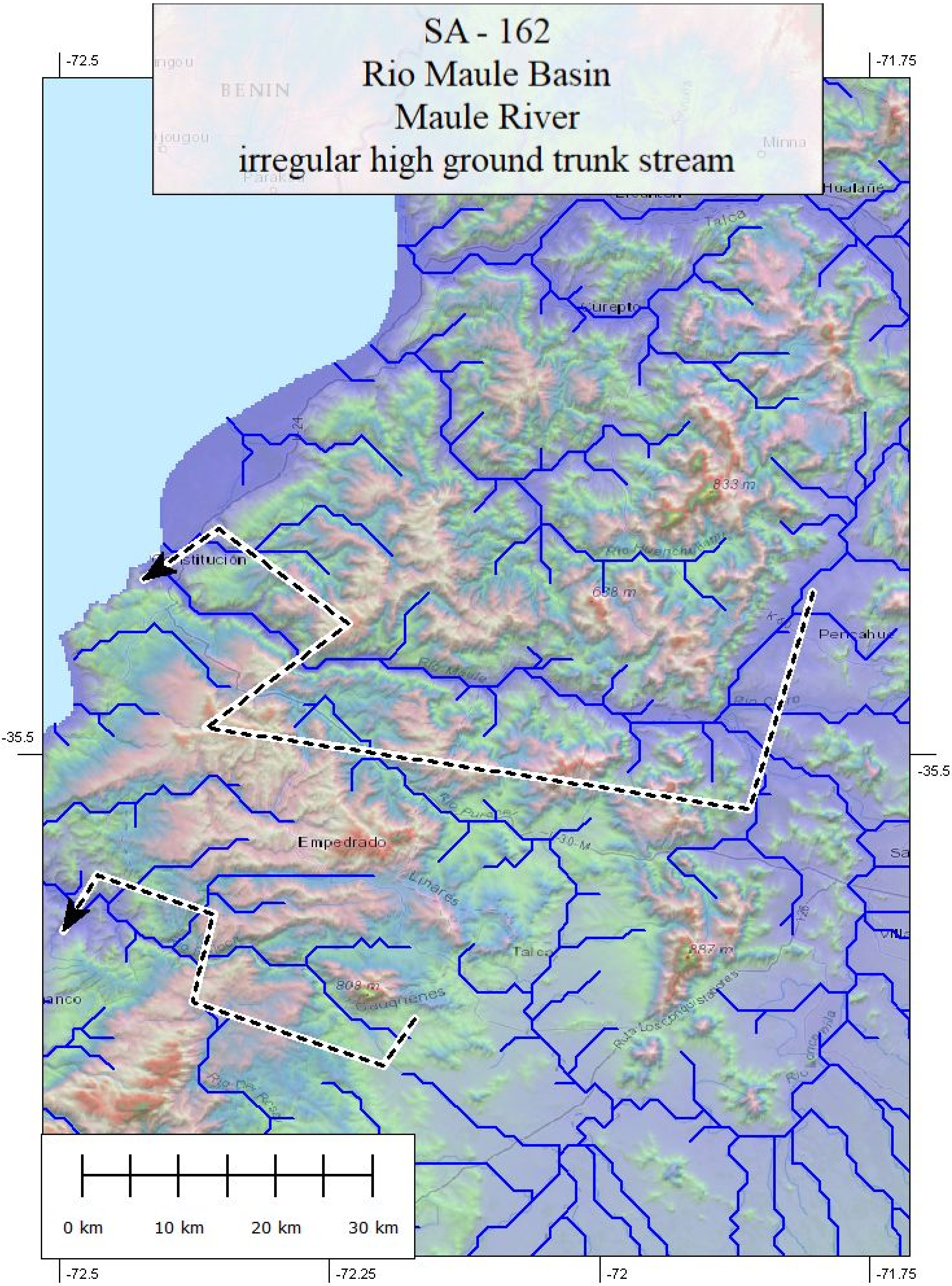

SA - 163
Rio Paraguacu Basin
Santo Antonio River
irregular high ground trunk stream

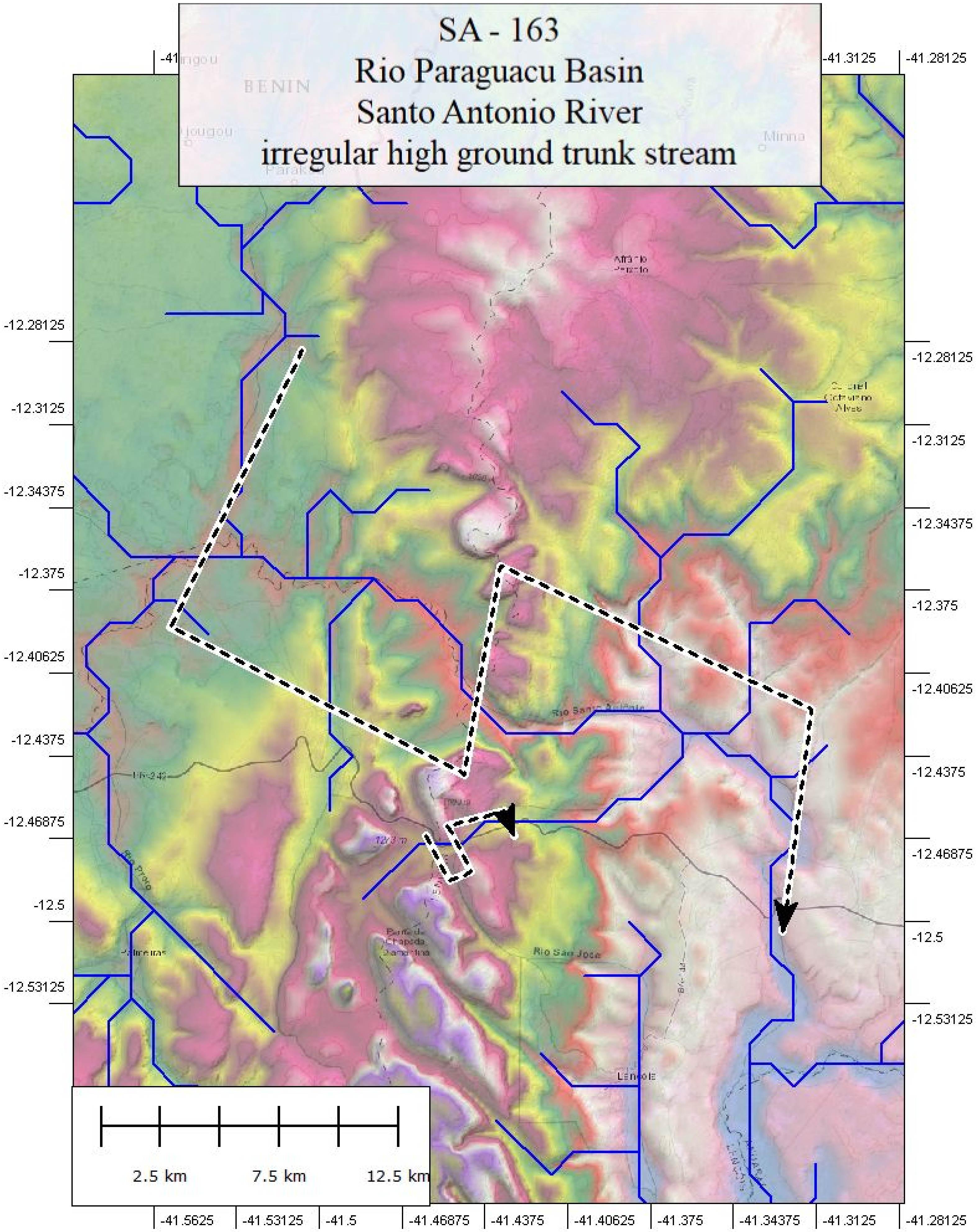

SA - 173
Amazon River Basin
Jari River
plateau trunk stream

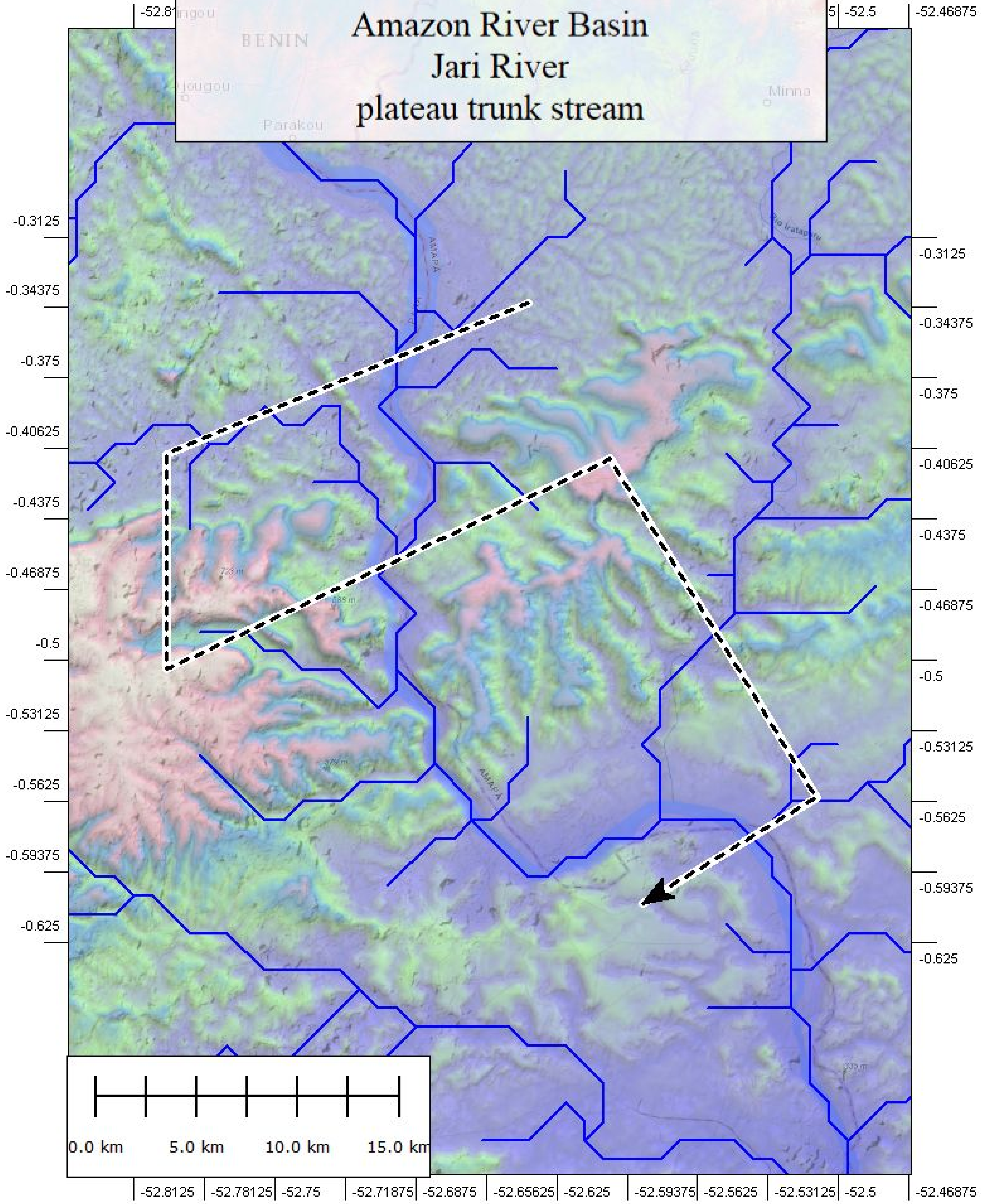

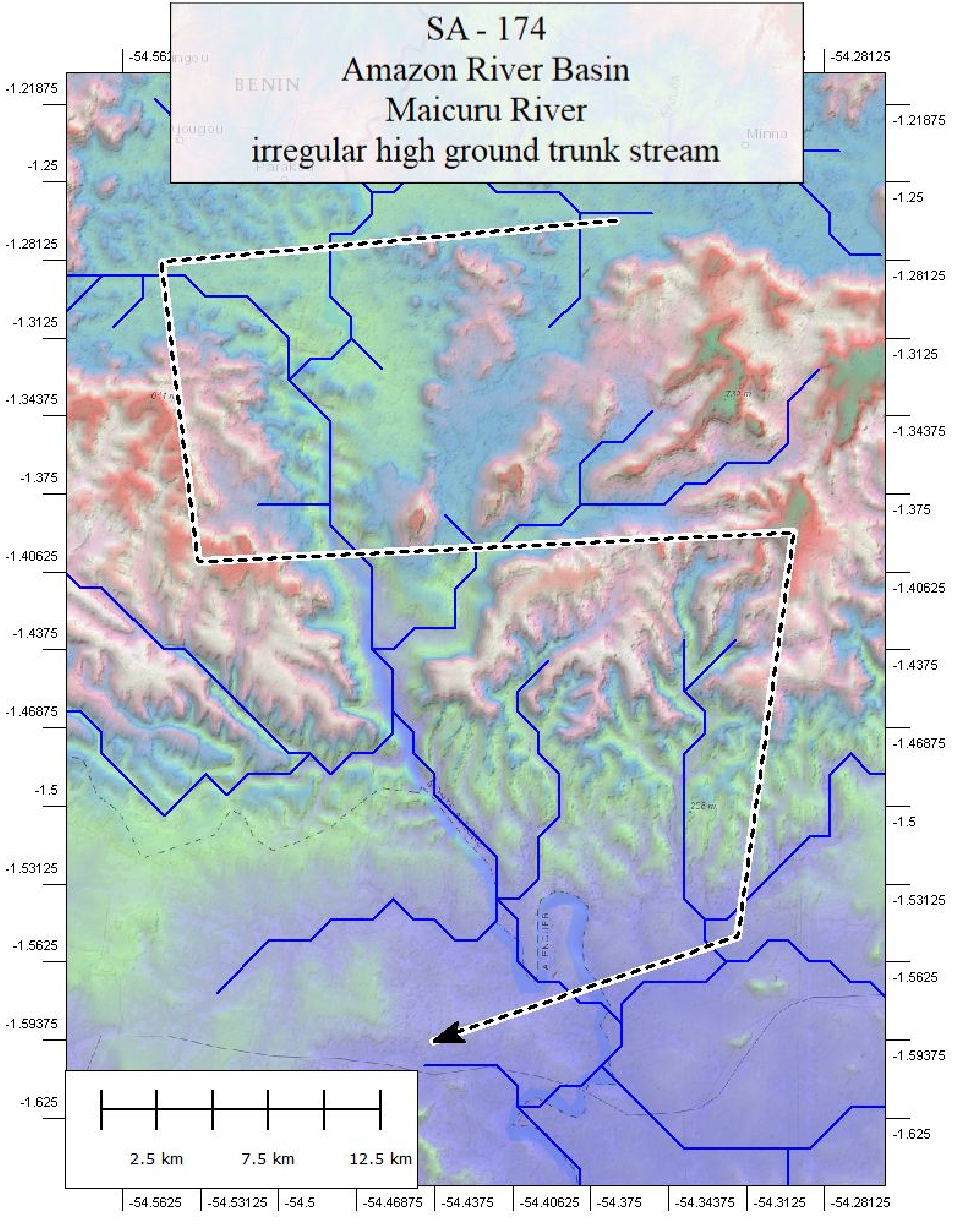
SA - 174
Amazon River Basin
Maicuru River
irregular high ground trunk stream
-54.5625
-54.53125
-54.5
-54.46875
-54.4375
-54.40625
-54.375
-54.34375
-54.3125
-54.28125
-1.21875
-1.25
-1.28125
-1.3125
-1.34375
-1.375
-1.40625
-1.4375
-1.46875
-1.5
-1.53125
-1.5625
-1.59375
-1.625
2.5 km
7.5 km
12.5 km

SA - 179
Rio Parnaiba Basin
Poti River
downdip plateau trunk stream

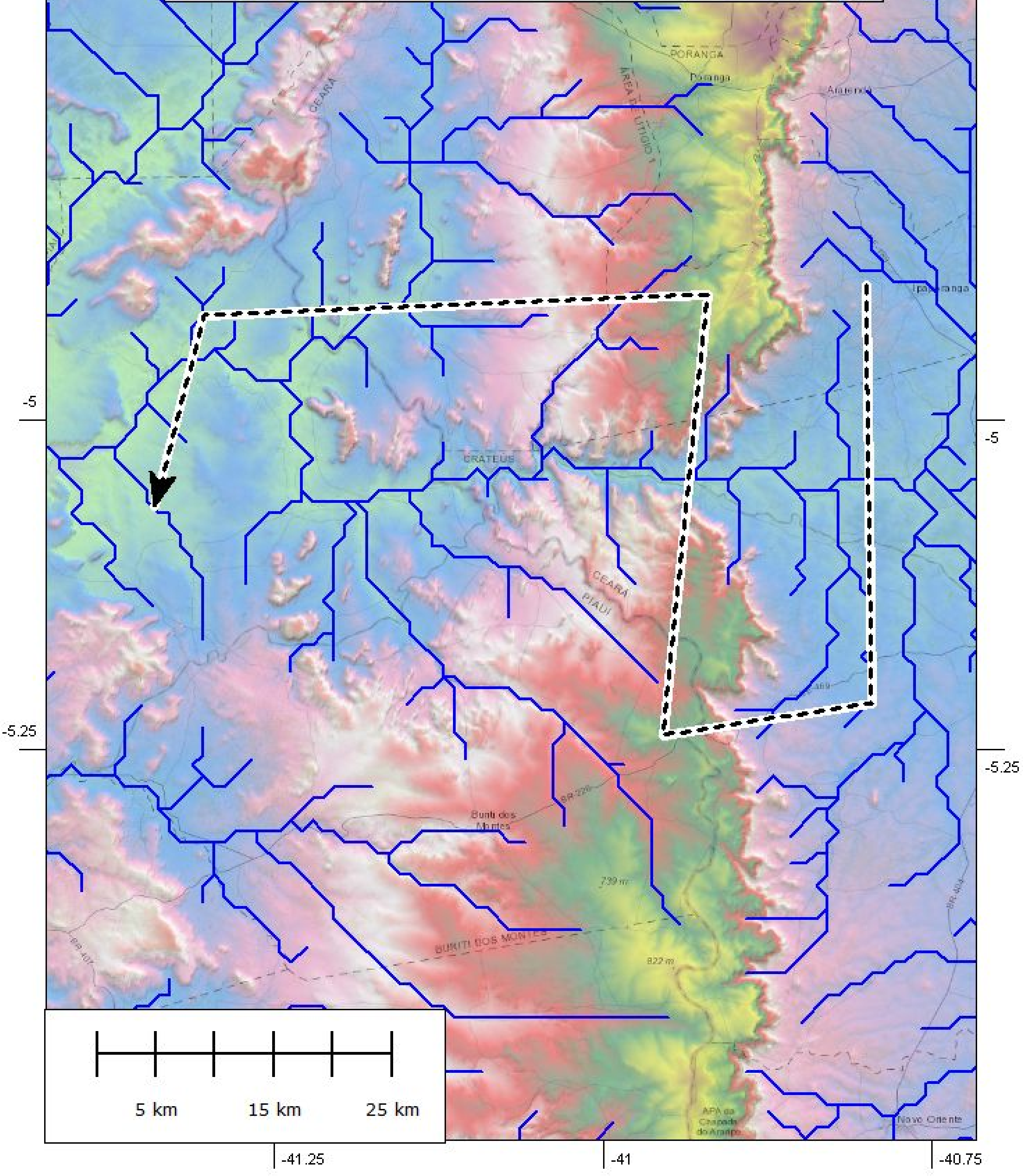

SA - 185
Rio Colorado (South America) Basin
San Juan River (Argentina)
irregular high ground trunk stream

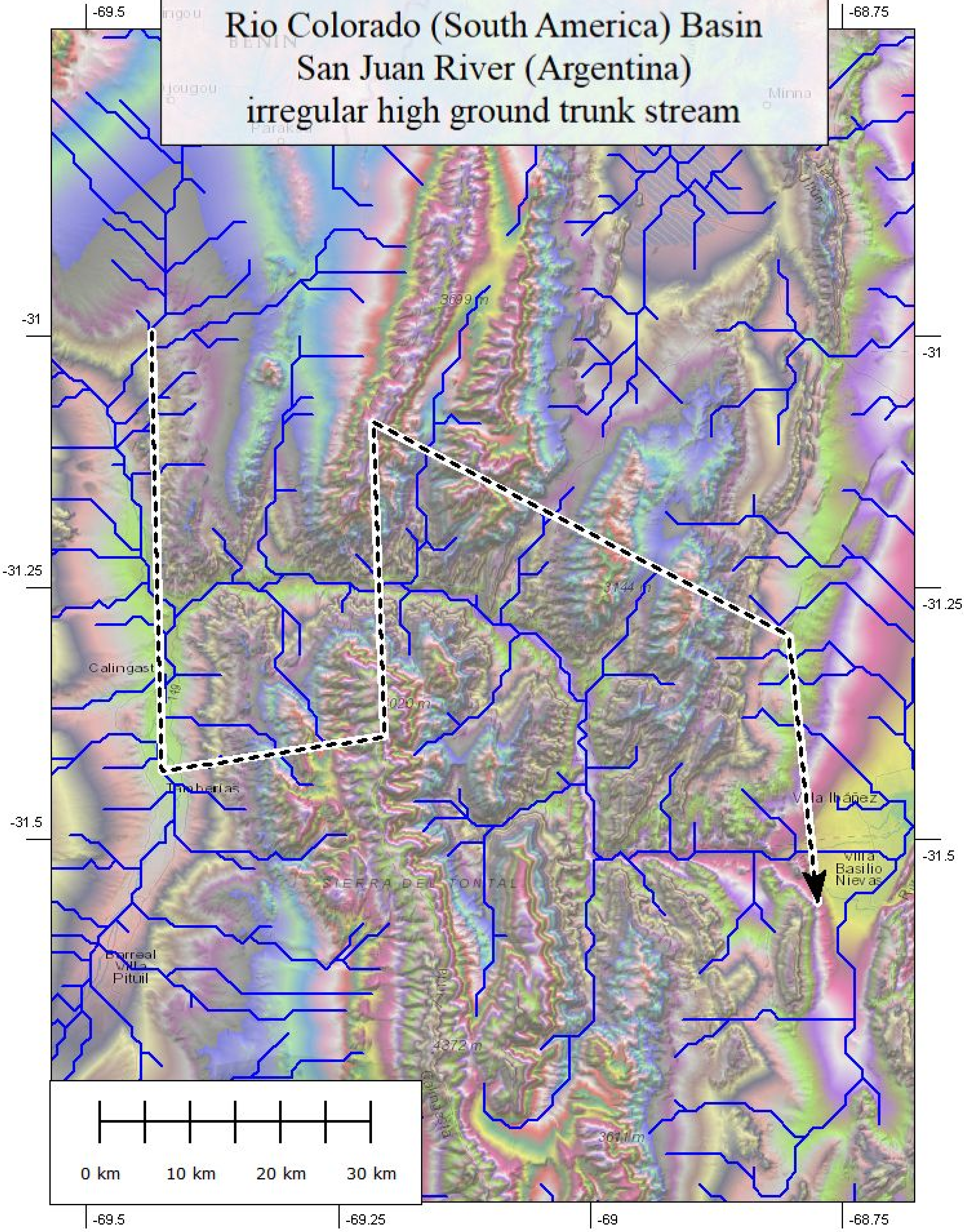

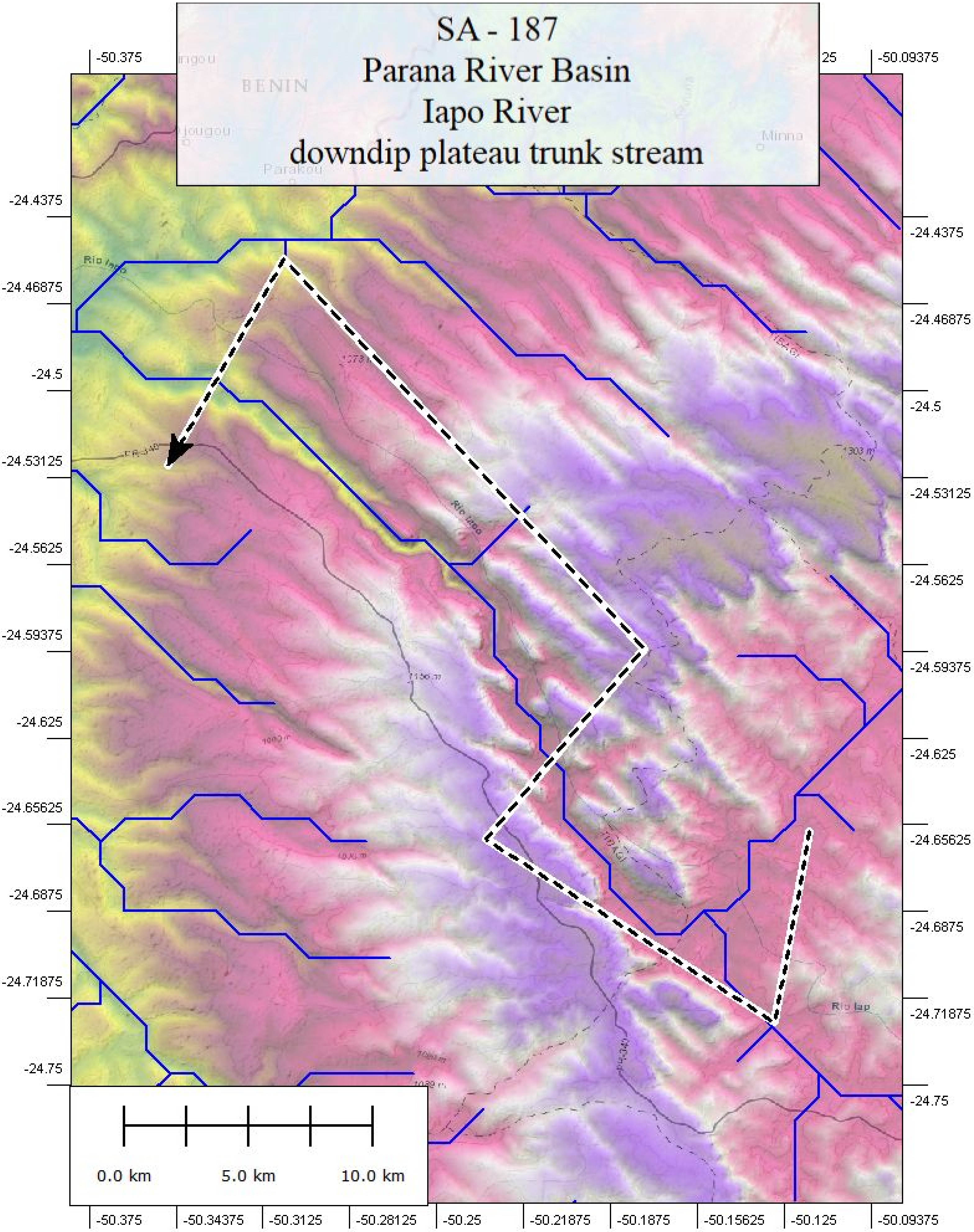
SA - 187
Parana River Basin
Iapo River
downdip plateau trunk stream
-50.375
-50.09375
-24.4375
-24.46875
-24.5
-24.53125
-24.5625
-24.59375
-24.625
-24.65625
-24.6875
-24.71875
-24.75
Rio Iapo
Rio Iap
0.0 km
5.0 km
10.0 km
-50.34375
-50.3125
-50.28125
-50.25
-50.21875
-50.1875
-50.15625
-50.125

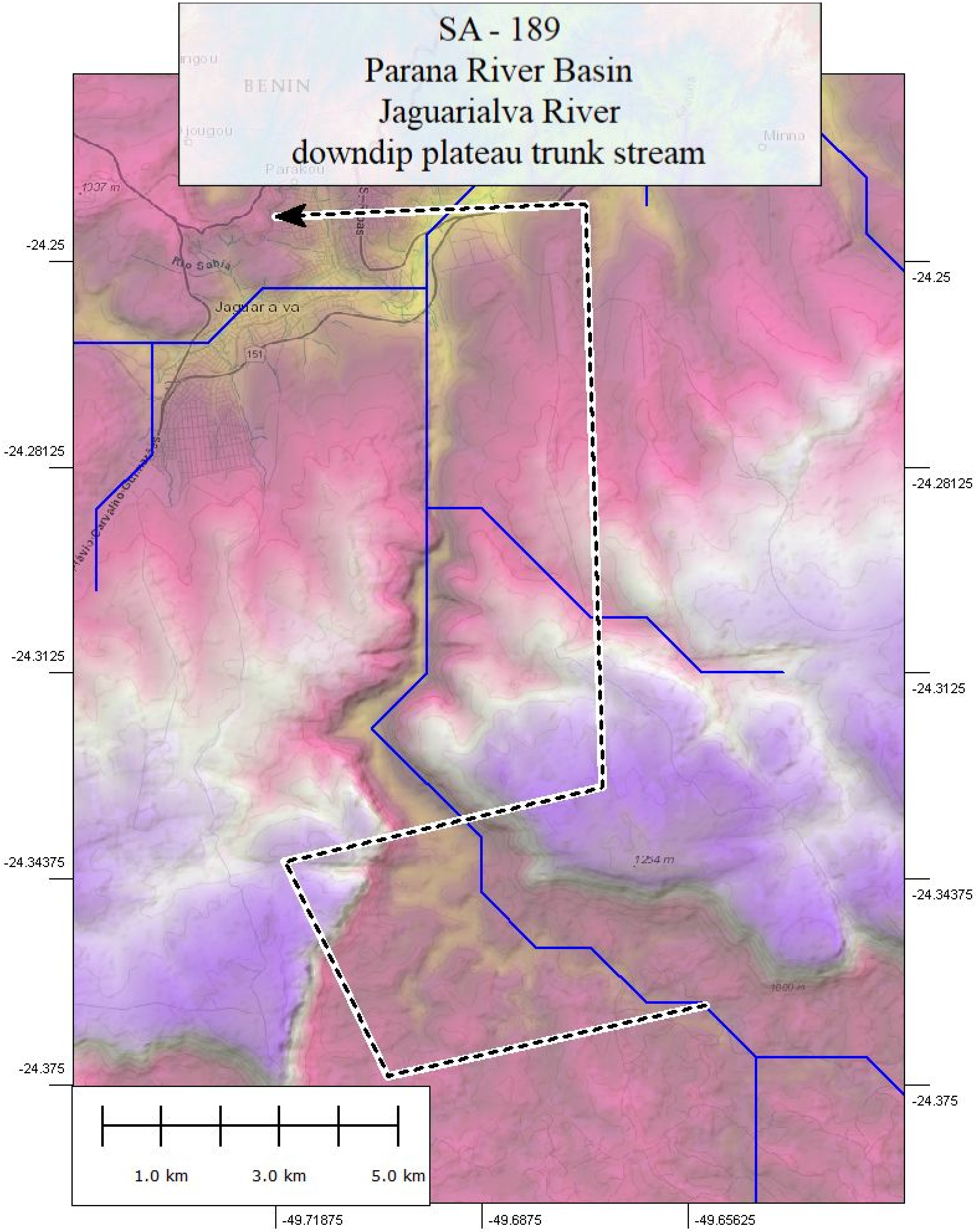
SA - 189
Parana River Basin
Jaguarialva River
downdip plateau trunk stream
-24.25
-24.28125
-24.3125
-24.34375
-24.375
-49.71875
-49.6875
-49.65625
1.0 km
3.0 km
5.0 km
Jaguar a va
Rio Sabia
151
1254 m
1037 m
BENIN
Parakou
Minna

SA - 191
Rio Tocatins Basin
Ribeirao Aguas Claras
plateau trunk stream

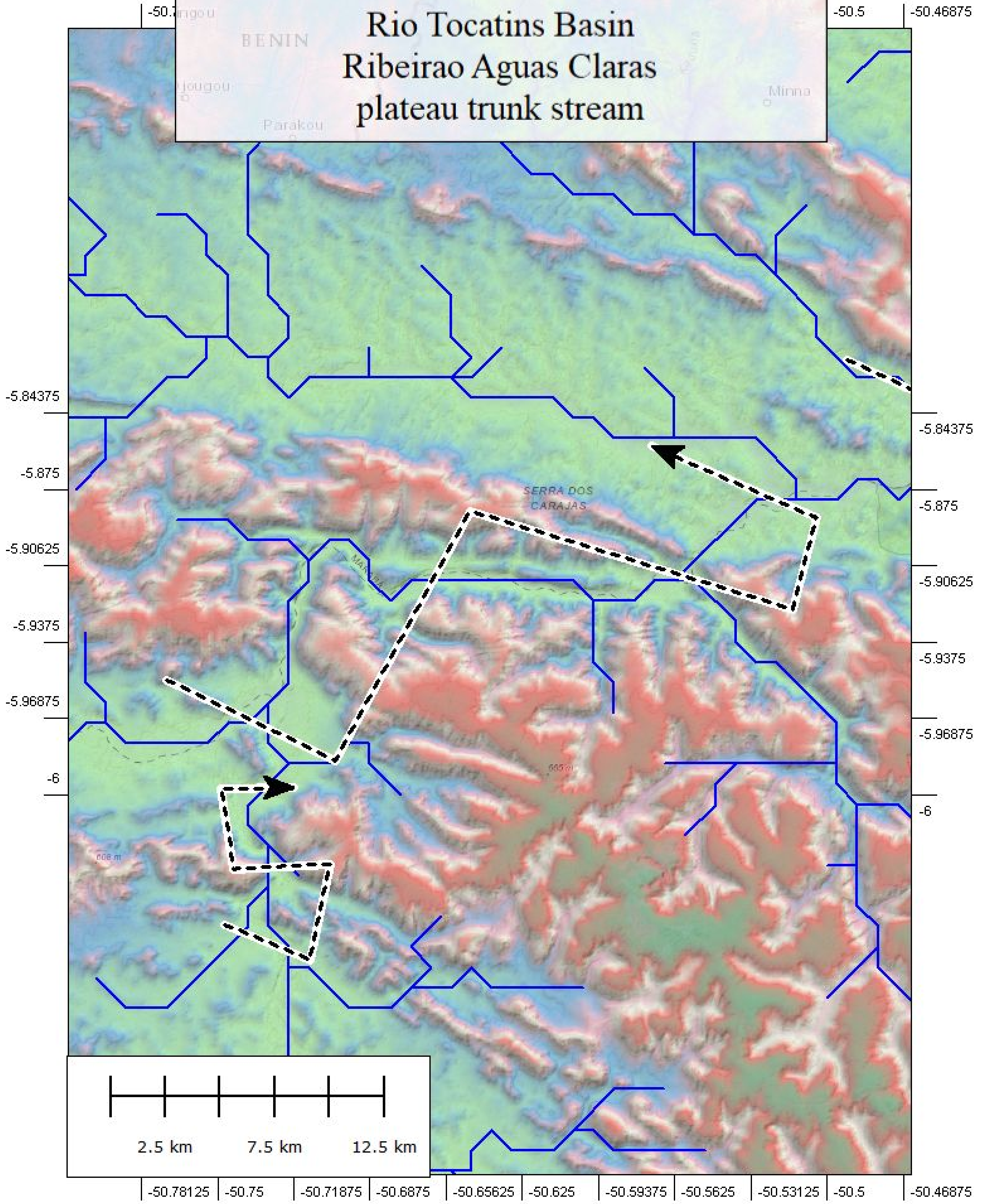

SA - 199
Amazon River Basin
Represa de Agoyan
irregular high ground trunk stream

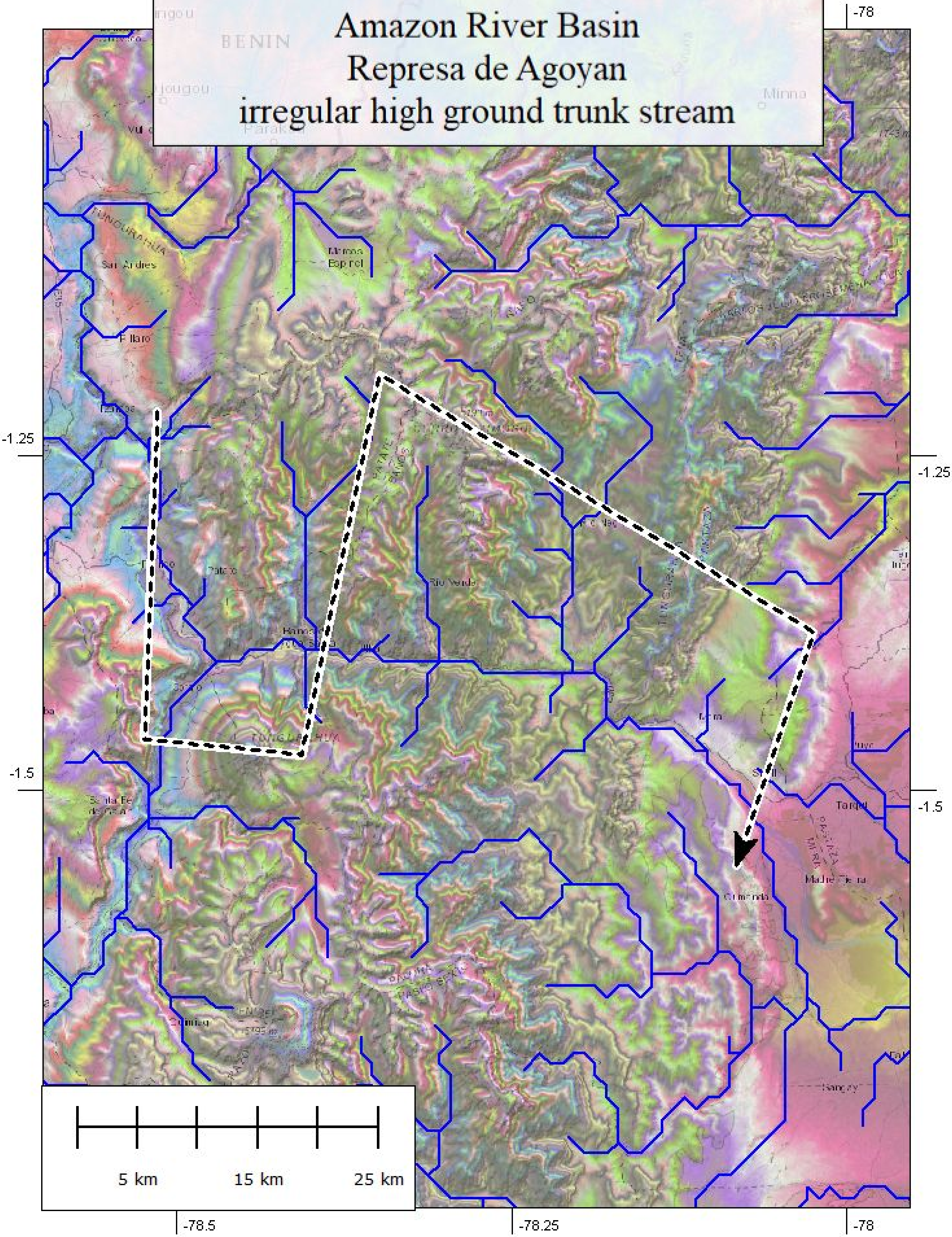

SA - 203
Parana River Basin
Pilcomayo River tributary
irregular high ground trunk stream

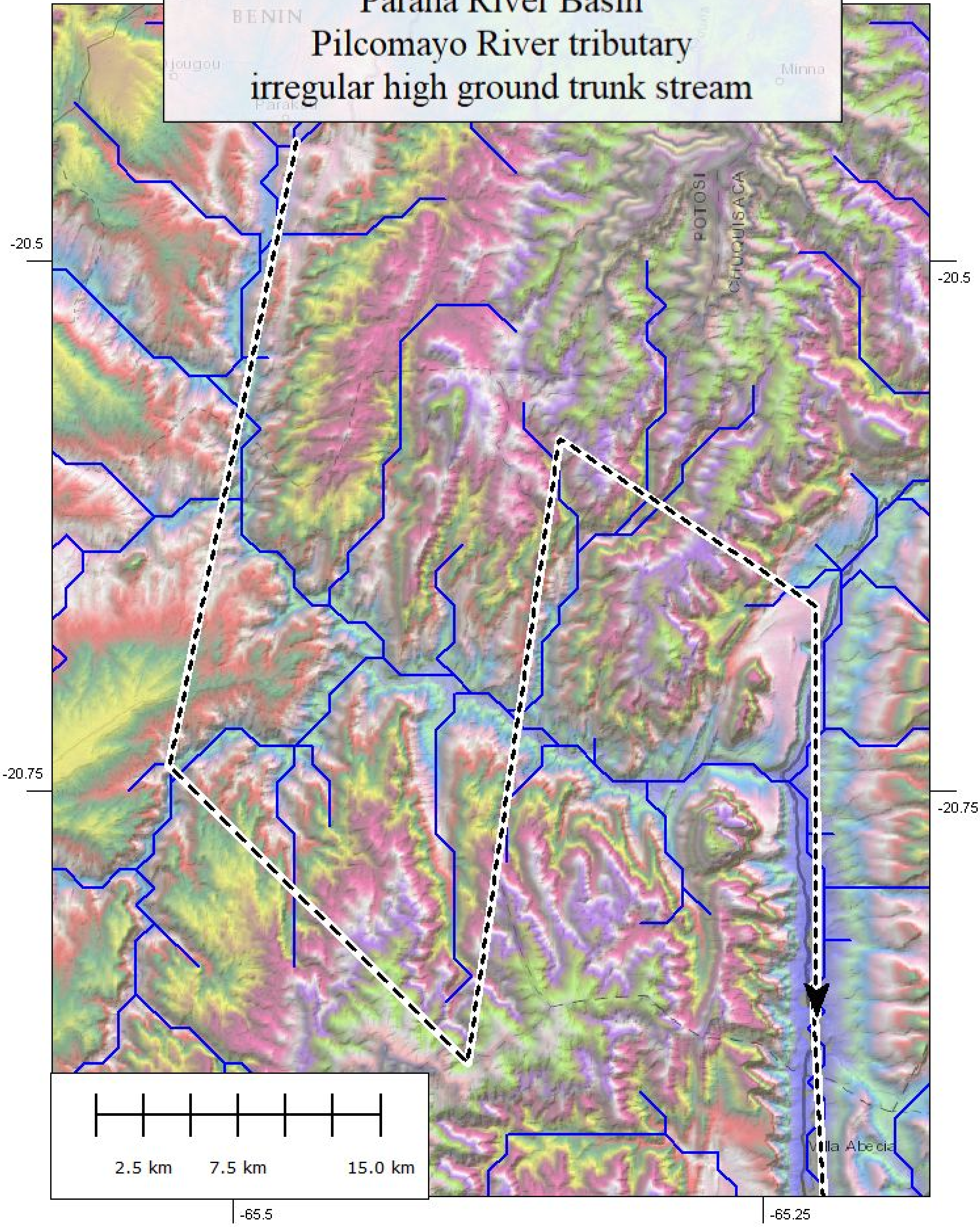

SA - 215
Rio Guaire Basin
Guaire River
irregular high ground trunk stream

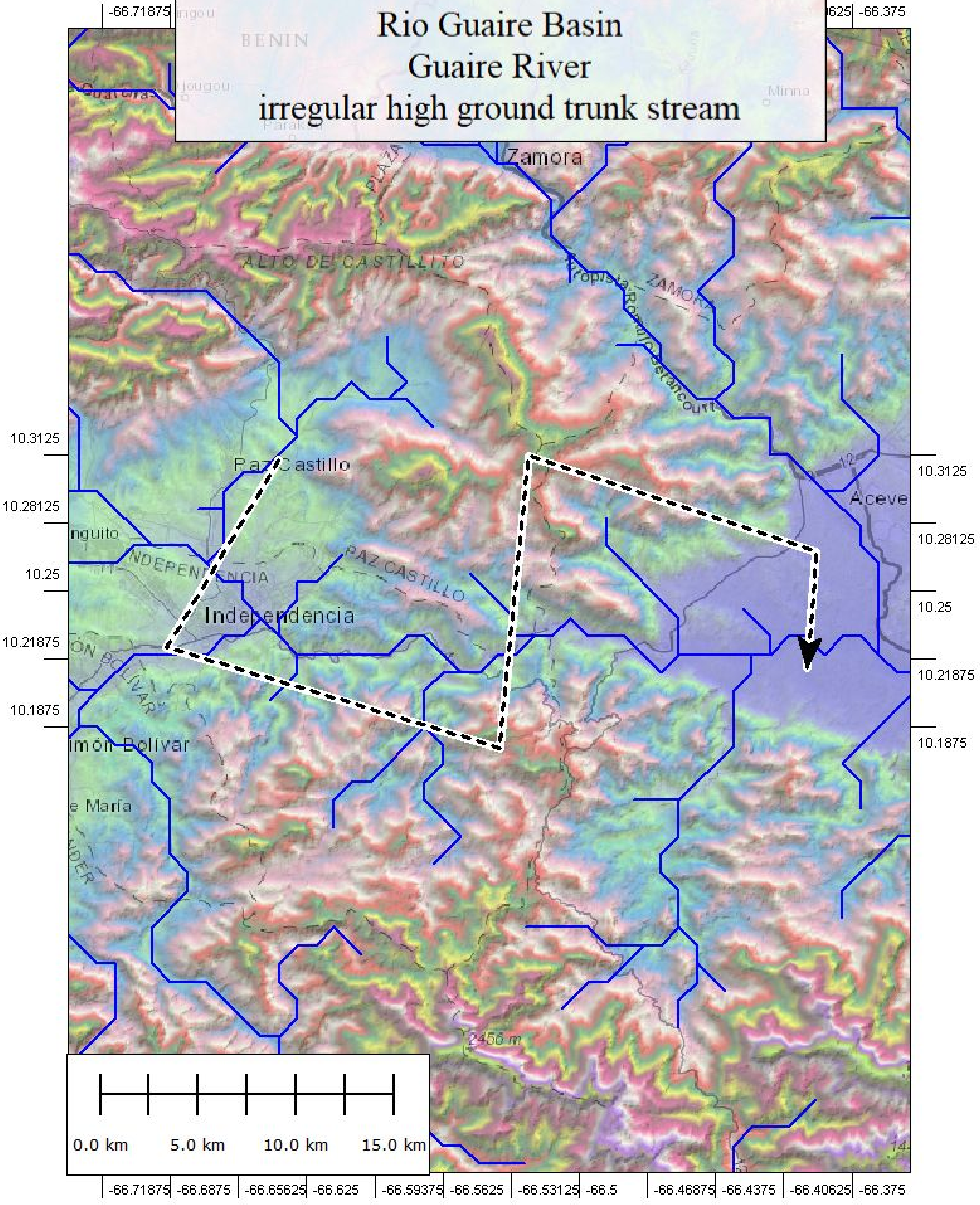

SA - 221
Parana River Basin
Yocoraite River
irregular high ground trunk stream

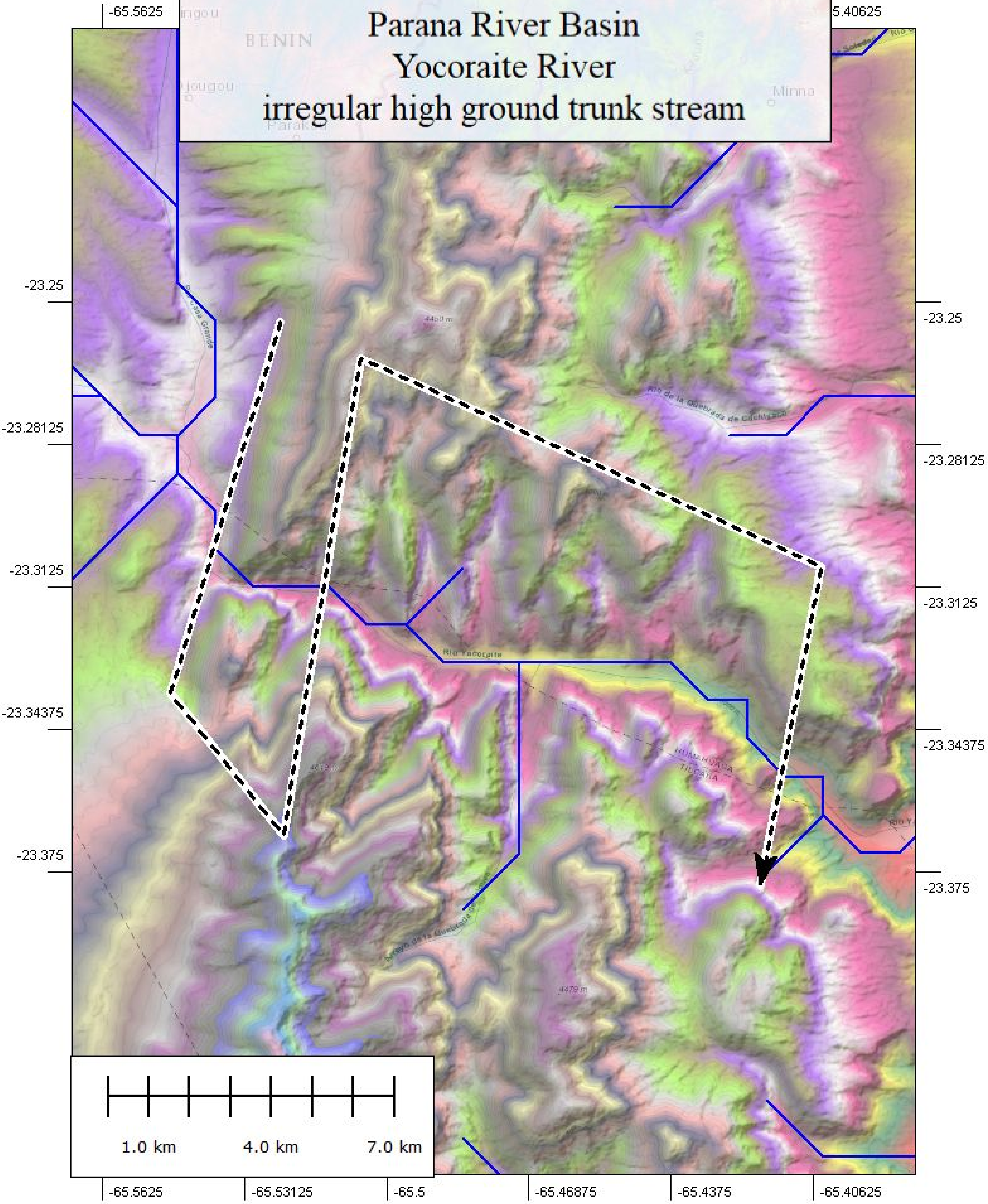

SA - 18
Rio Magdalena Basin
Leiva River tributary
multi-ridge head stream

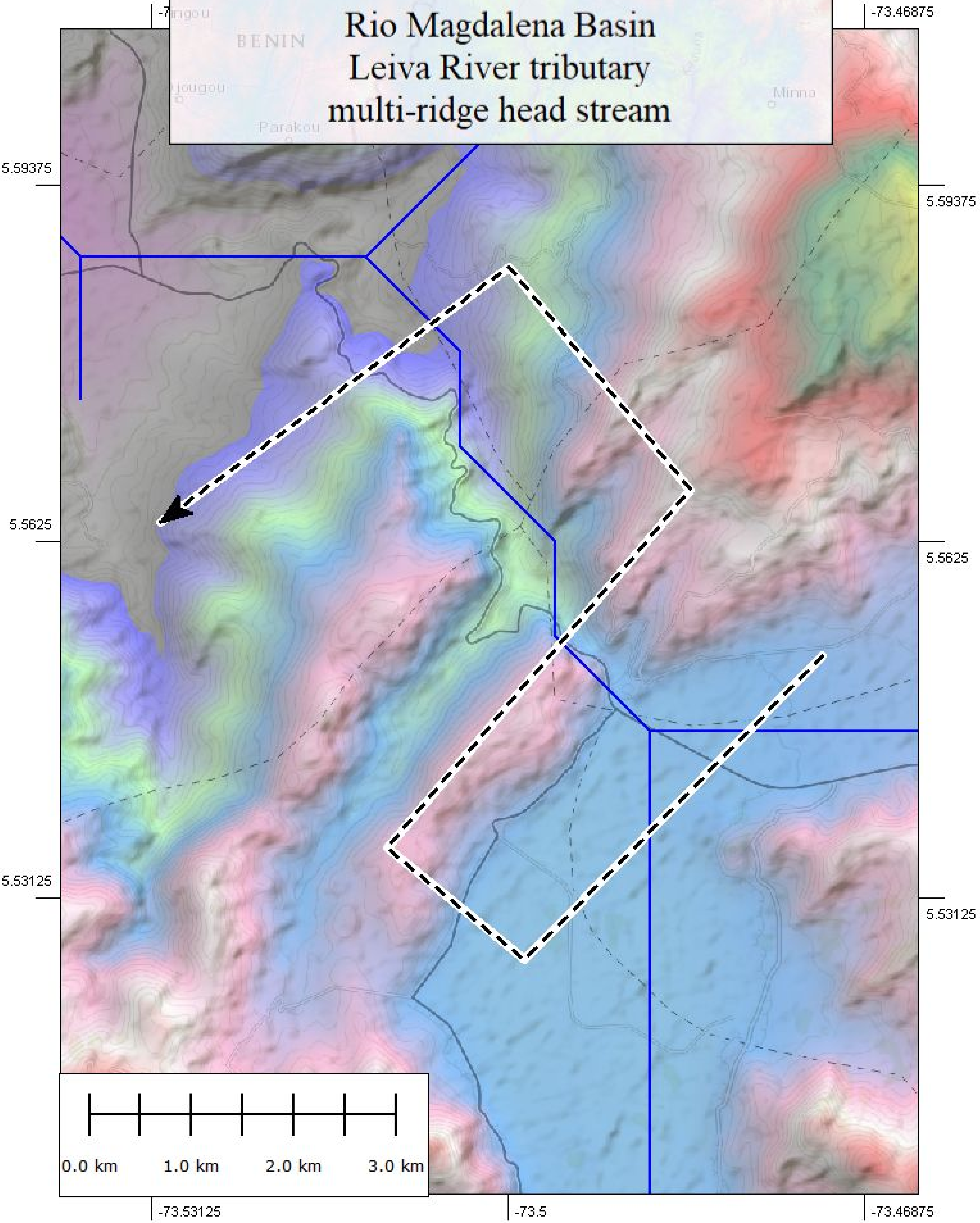

SA - 45
Rio Magdalena Basin
Bache River tributary
multi-ridge head stream

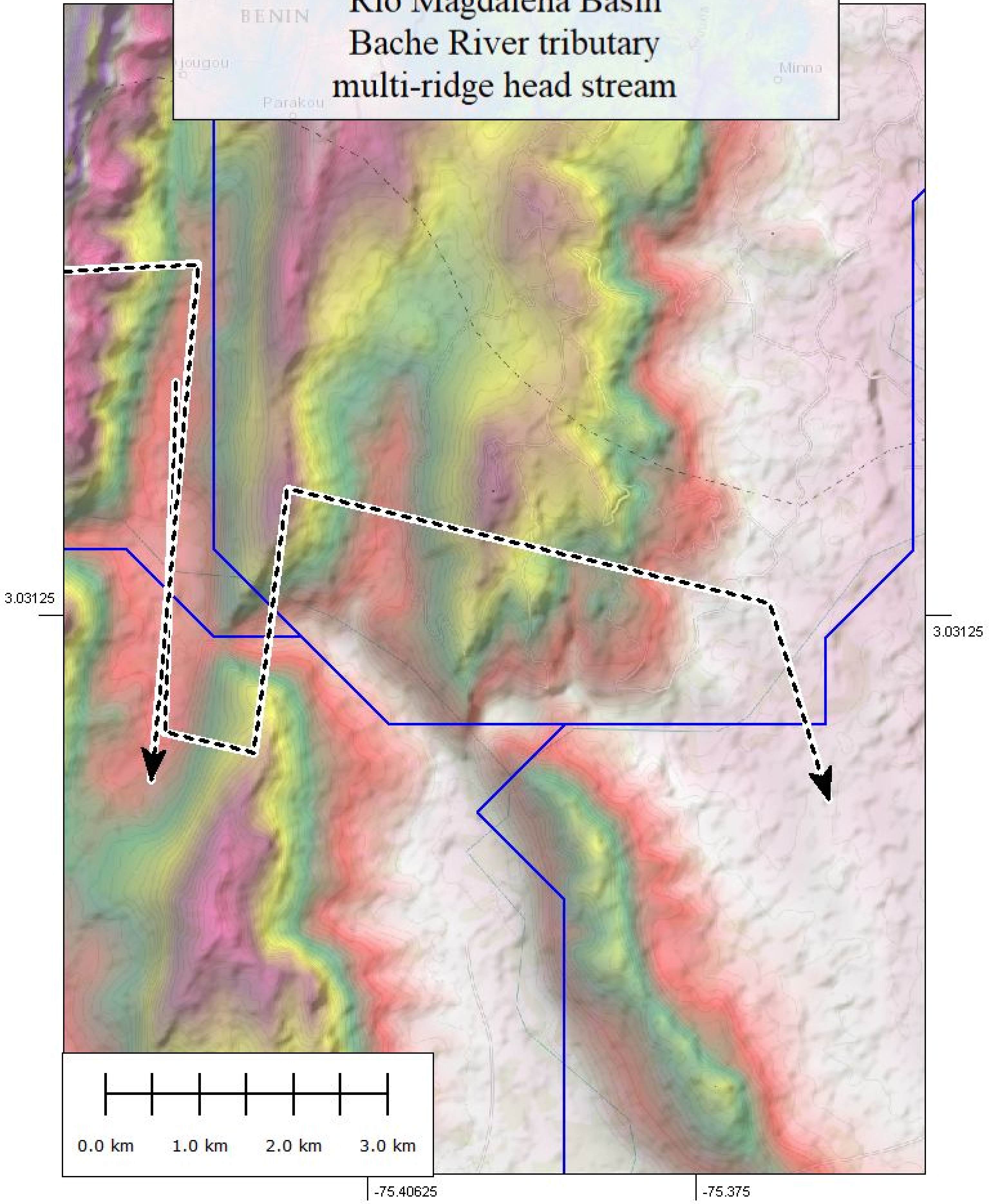

SA - 51
Amazon River Basin
Huallaga River
multi-ridge trunk stream

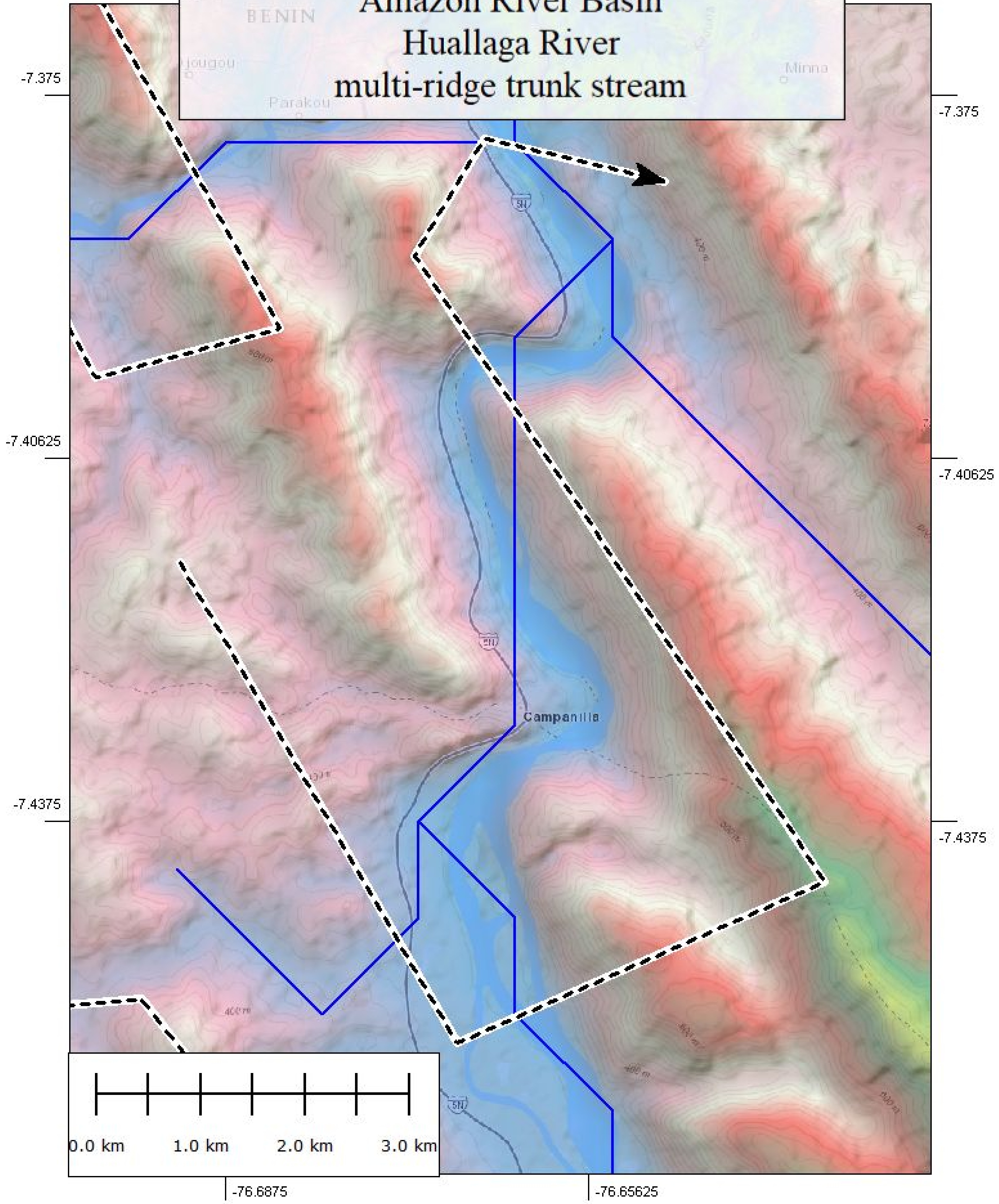

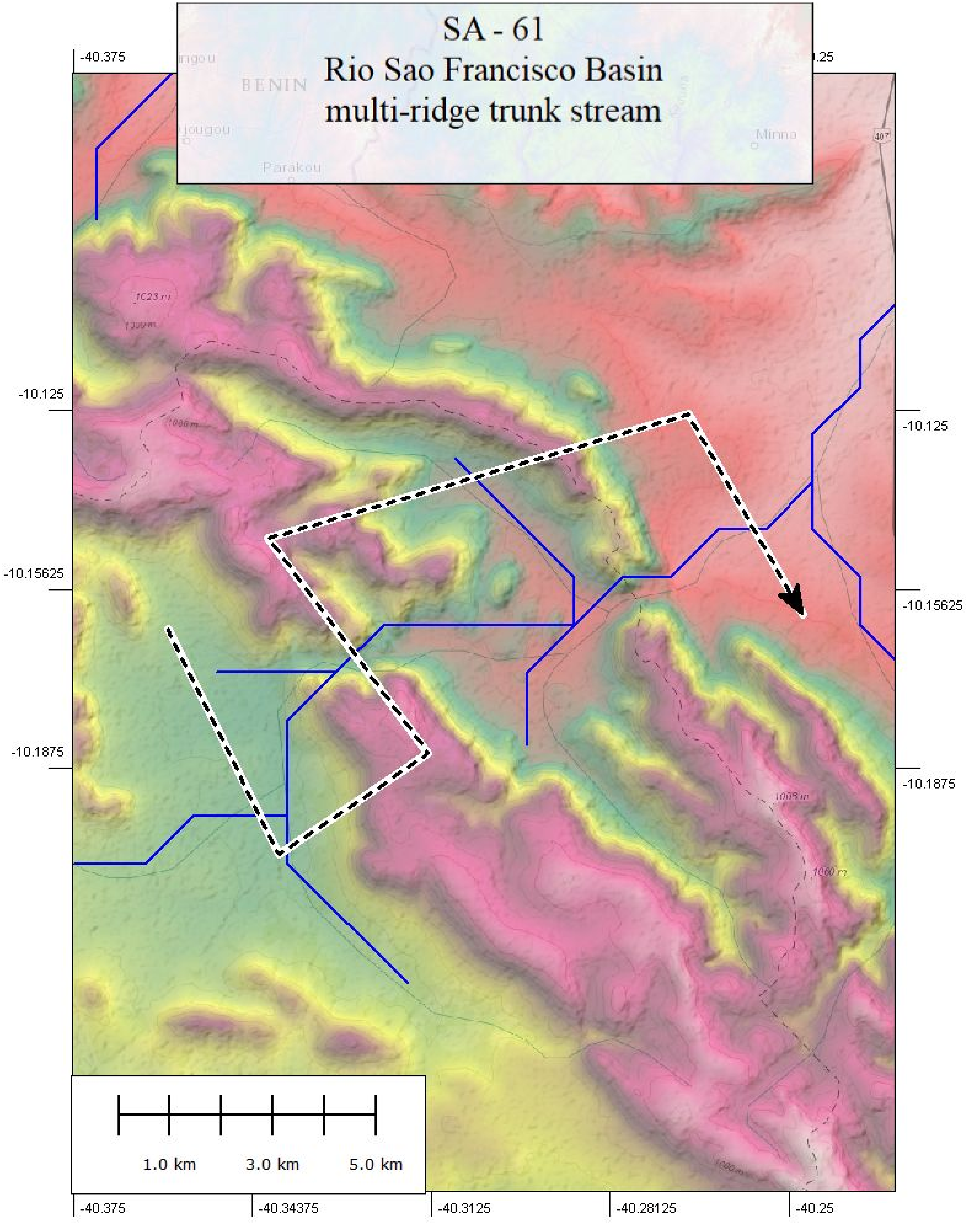
SA - 61
Rio Sao Francisco Basin
multi-ridge trunk stream
-40.375
-40.34375
-40.3125
-40.28125
-40.25
-10.125
-10.15625
-10.1875
1.0 km
3.0 km
5.0 km

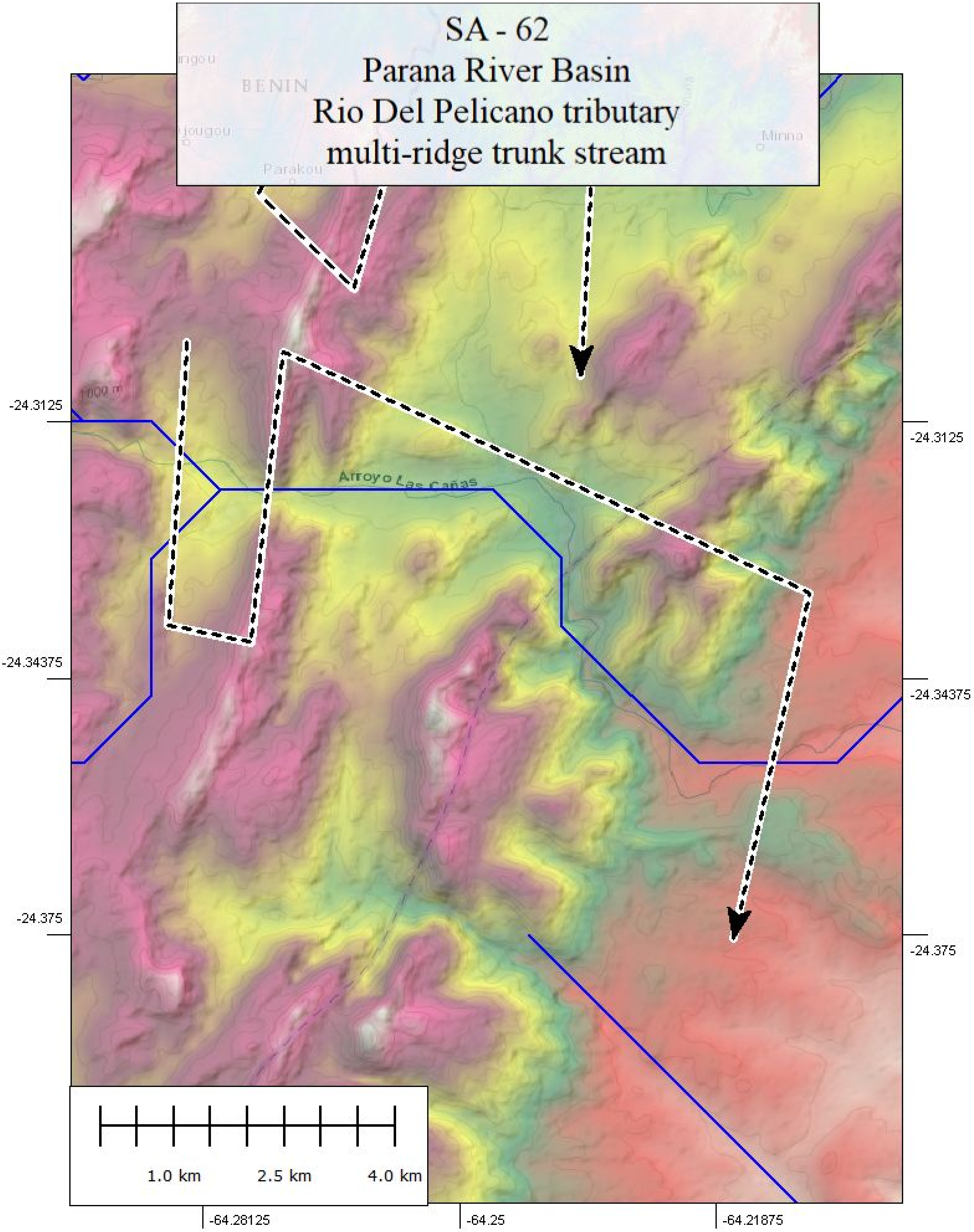
SA - 62
Parana River Basin
Rio Del Pelicano tributary
multi-ridge trunk stream
BENIN
Parakou
Minna
1000 m
Arroyo Las Cañas
-24.3125
-24.34375
-24.375
-64.28125
-64.25
-64.21875
1.0 km
2.5 km
4.0 km

SA - 83
Amazon River Basin
Tupuani River
multi-ridge trunk stream

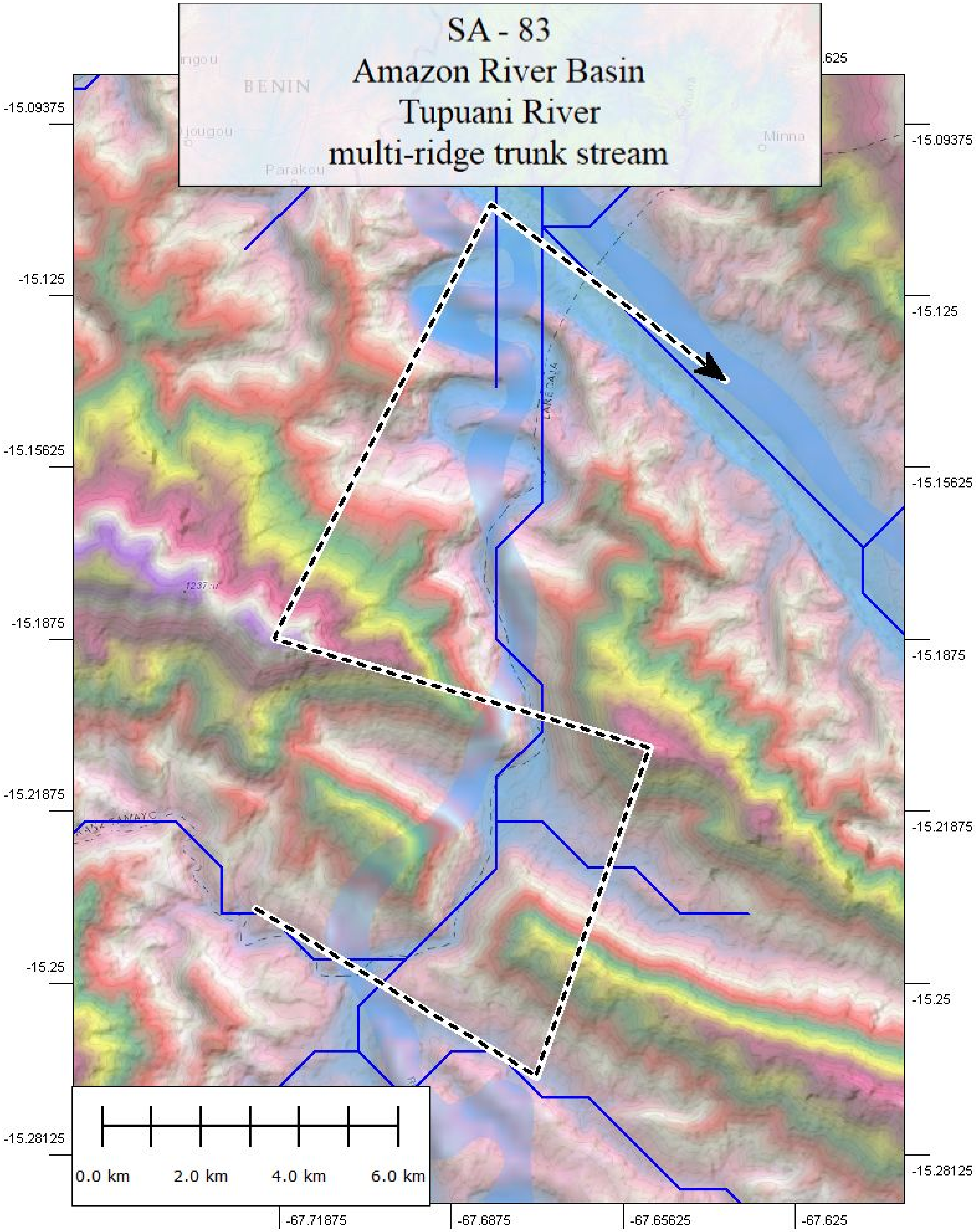

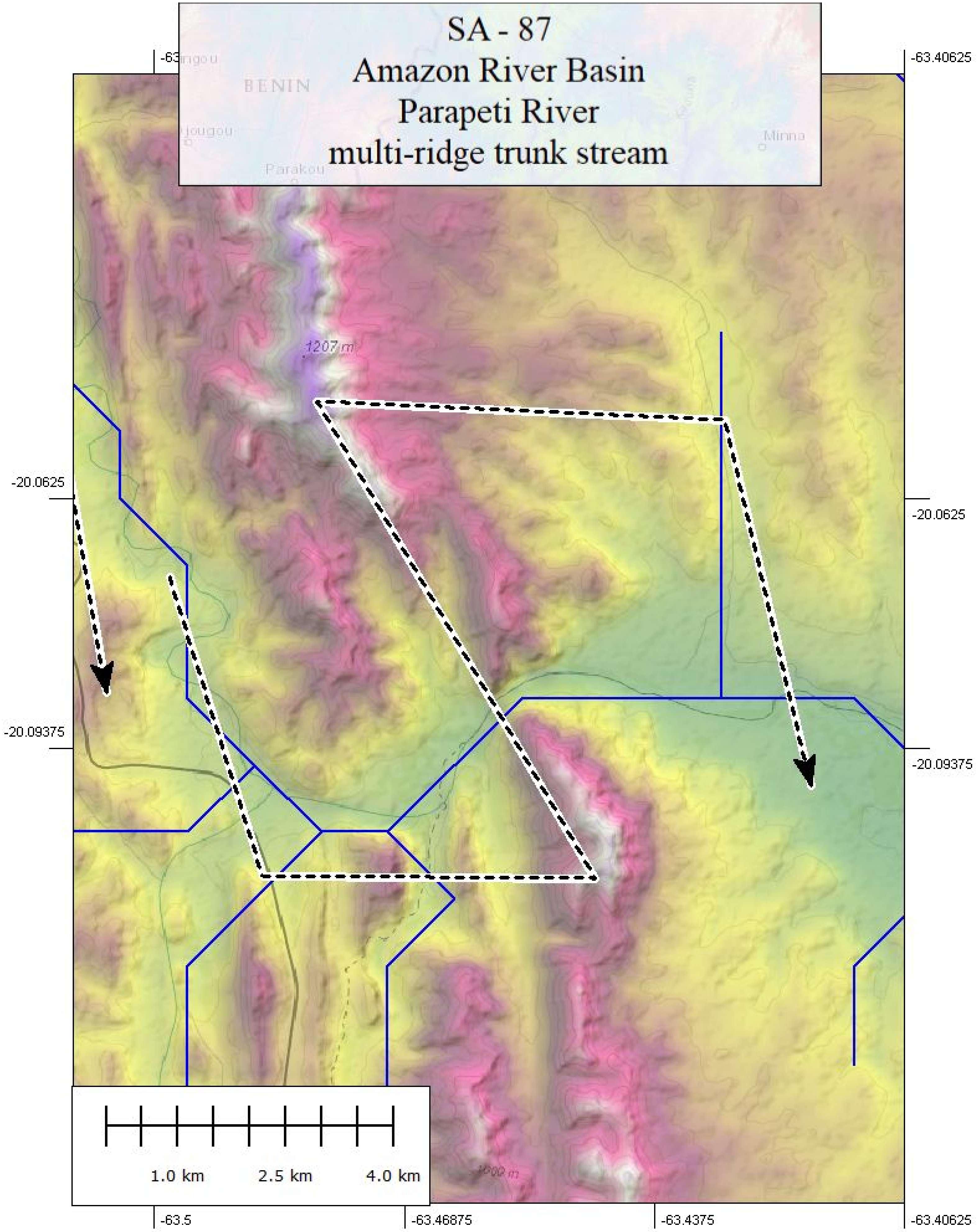

SA - 87
Amazon River Basin
Parapeti River
multi-ridge trunk stream
1207 m
1000 m
-20.0625
-20.09375
-63.5
-63.46875
-63.4375
-63.40625
1.0 km
2.5 km
4.0 km

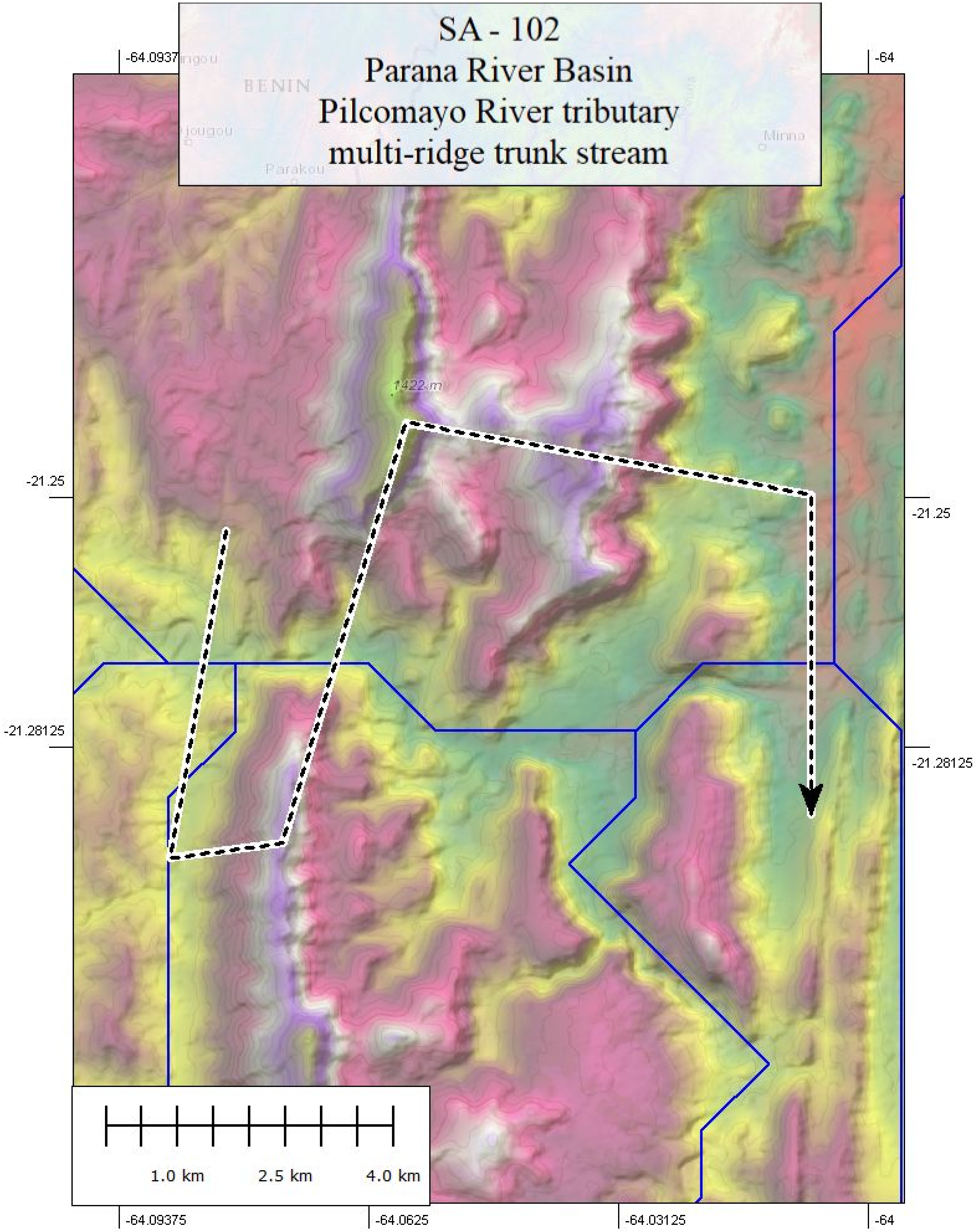

SA - 102
Parana River Basin
Pilcomayo River tributary
multi-ridge trunk stream
-64.09375
-64
-21.25
-21.28125
1422 m
1.0 km
2.5 km
4.0 km
-64.0625
-64.03125

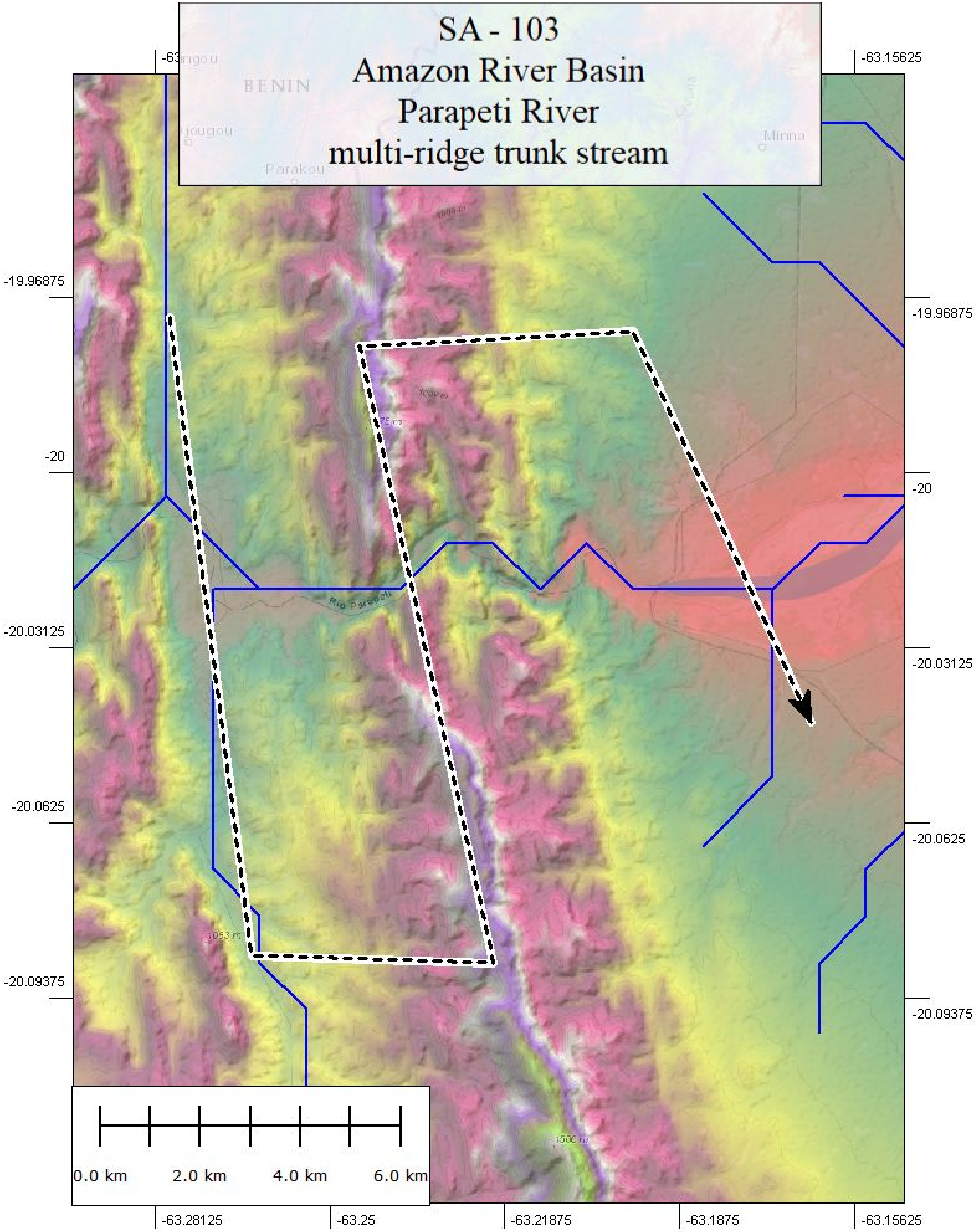

SA - 103
Amazon River Basin
Parapeti River
multi-ridge trunk stream
-19.96875
-20
-20.03125
-20.0625
-20.09375
-63.15625
-63.28125
-63.25
-63.21875
-63.1875
Rio Parapeti
0.0 km
2.0 km
4.0 km
6.0 km

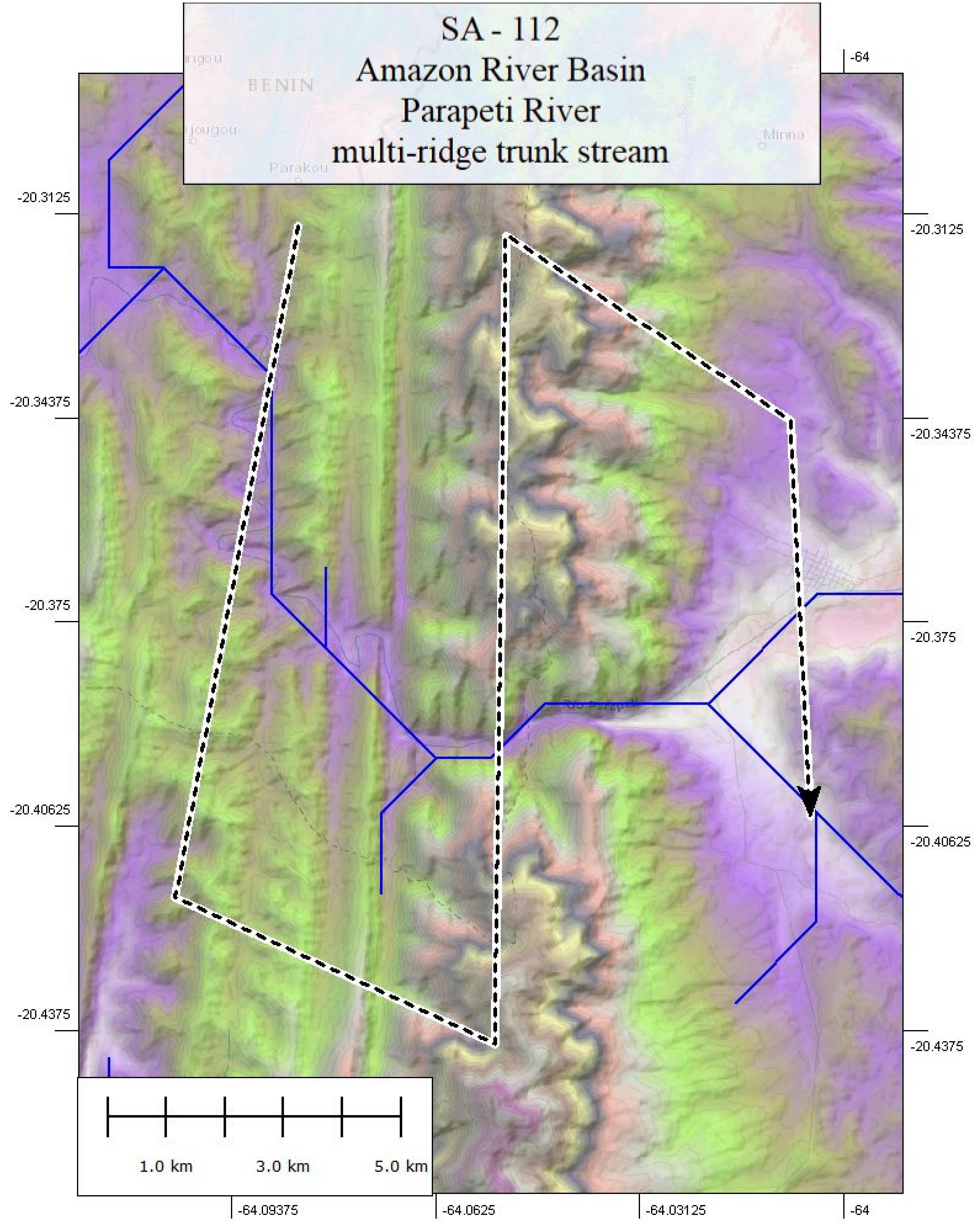
SA - 112
Amazon River Basin
Parapeti River
multi-ridge trunk stream
-64
-20.3125
-20.34375
-20.375
-20.40625
-20.4375
-64.09375
-64.0625
-64.03125
1.0 km
3.0 km
5.0 km

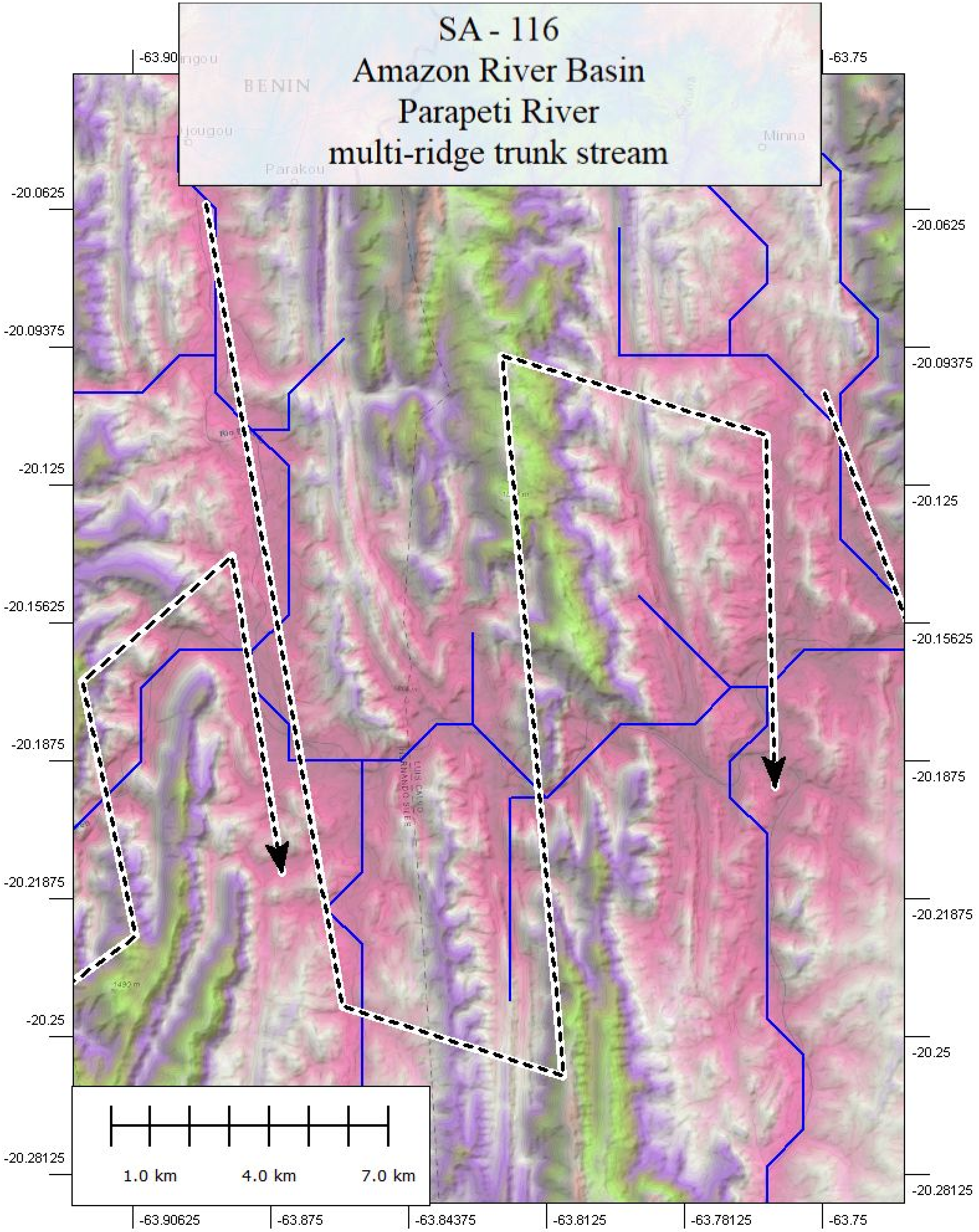
SA - 116
Amazon River Basin
Parapeti River
multi-ridge trunk stream
-63.90
-63.75
-20.0625
-20.09375
-20.125
-20.15625
-20.1875
-20.21875
-20.25
-20.28125
1.0 km
4.0 km
7.0 km
-63.90625
-63.875
-63.84375
-63.8125
-63.78125
-63.75
BENIN
Parakou
Minna

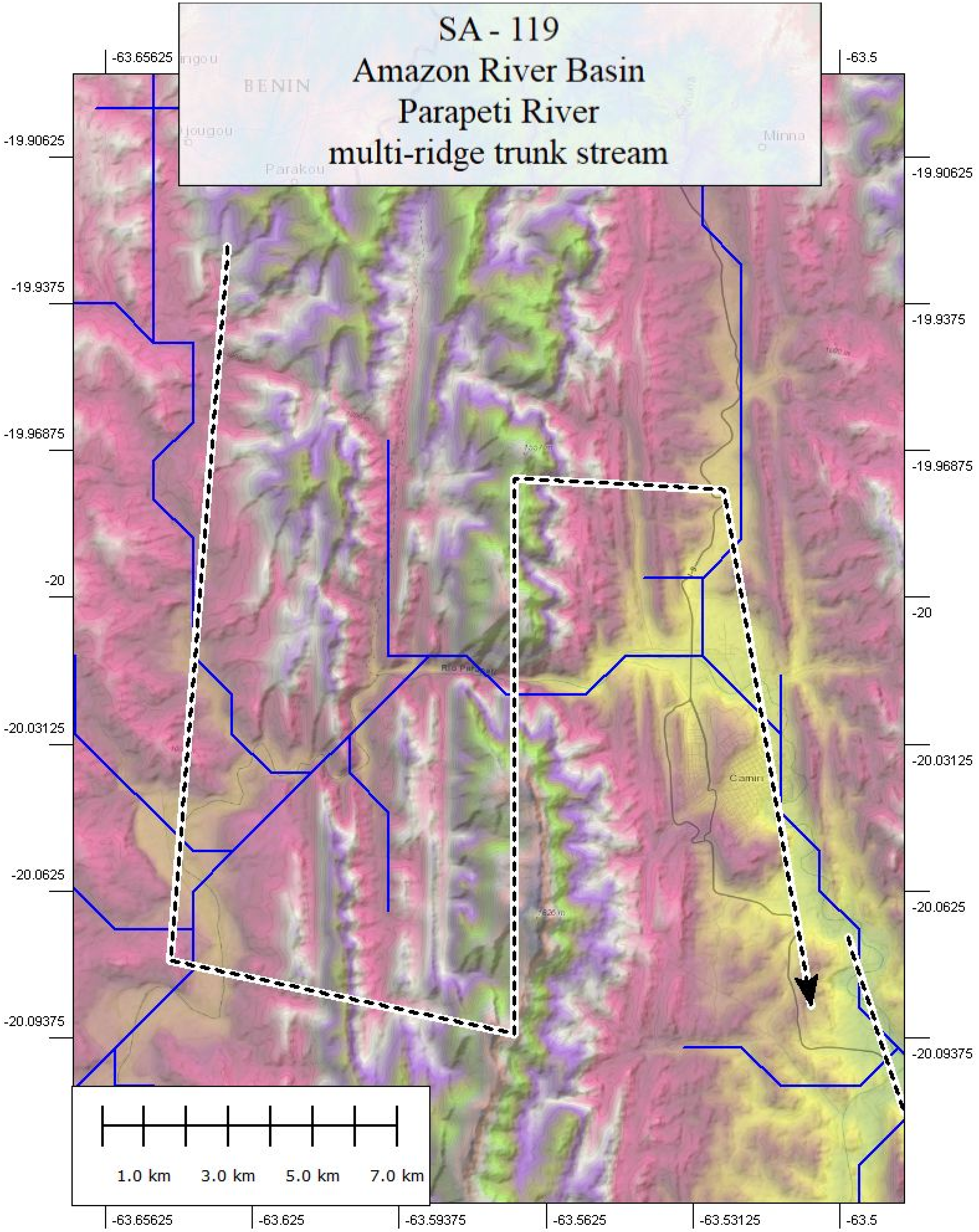
SA - 119
Amazon River Basin
Parapeti River
multi-ridge trunk stream
-63.65625
-63.625
-63.59375
-63.5625
-63.53125
-63.5
-19.90625
-19.9375
-19.96875
-20
-20.03125
-20.0625
-20.09375
BENIN
Parakou
Minna
Rio Parapeti
Camiri
1.0 km
3.0 km
5.0 km
7.0 km

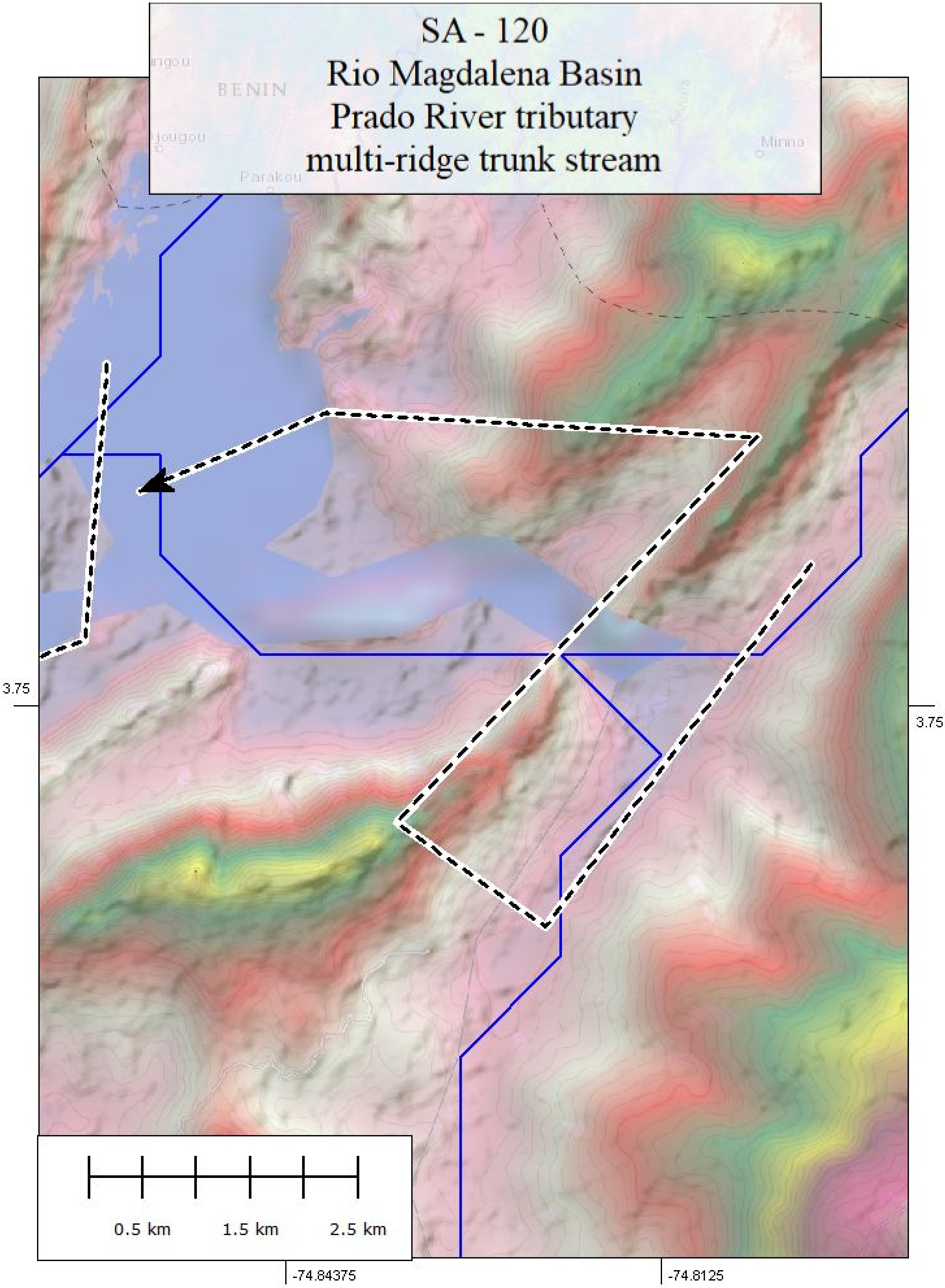
SA - 120
Rio Magdalena Basin
Prado River tributary
multi-ridge trunk stream
BENIN
Parakou
Minna
3.75
3.75
0.5 km
1.5 km
2.5 km
-74.84375
-74.8125

SA - 122
Amazon River Basin
Santiago RIver tributary
multi-ridge trunk stream

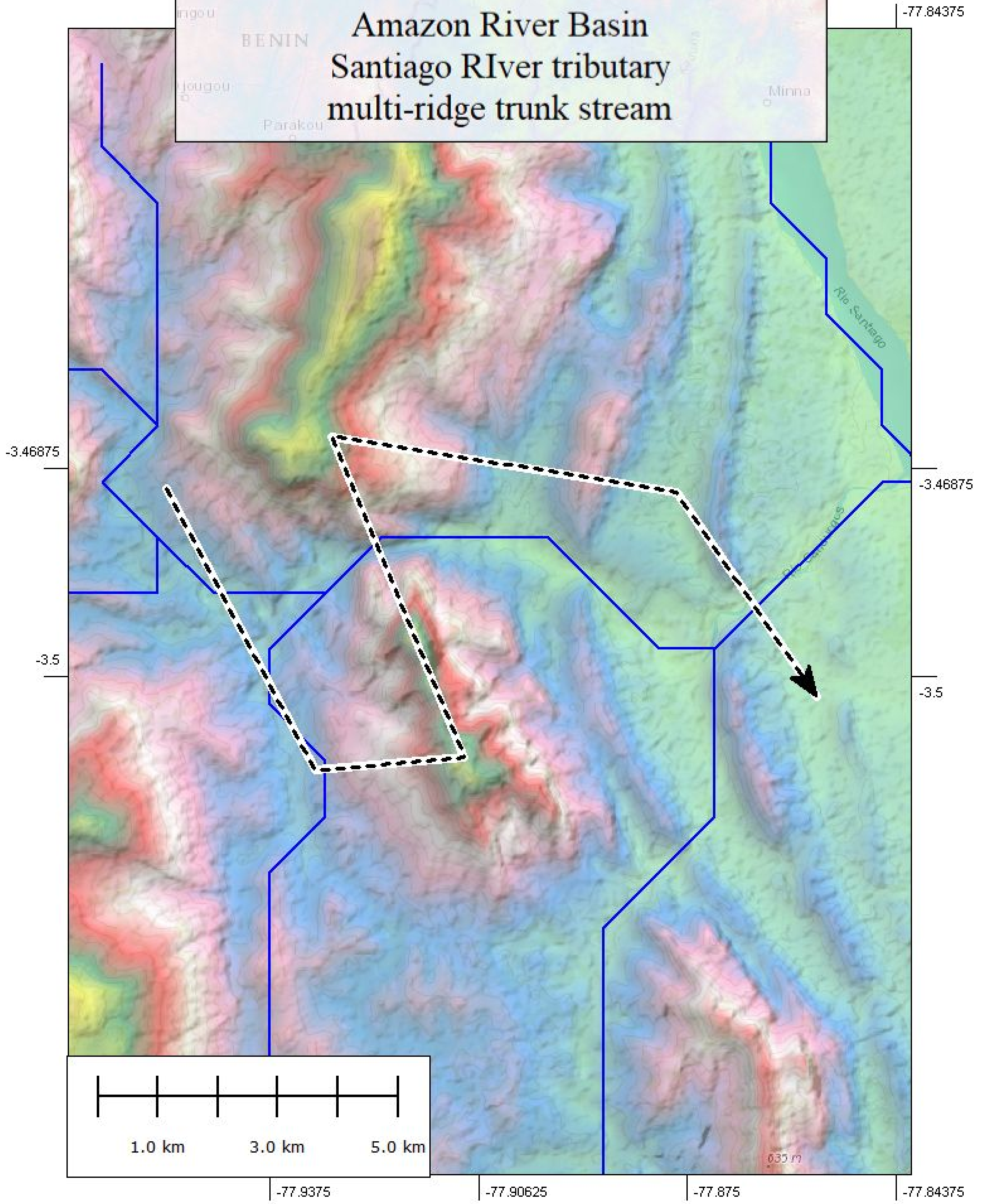

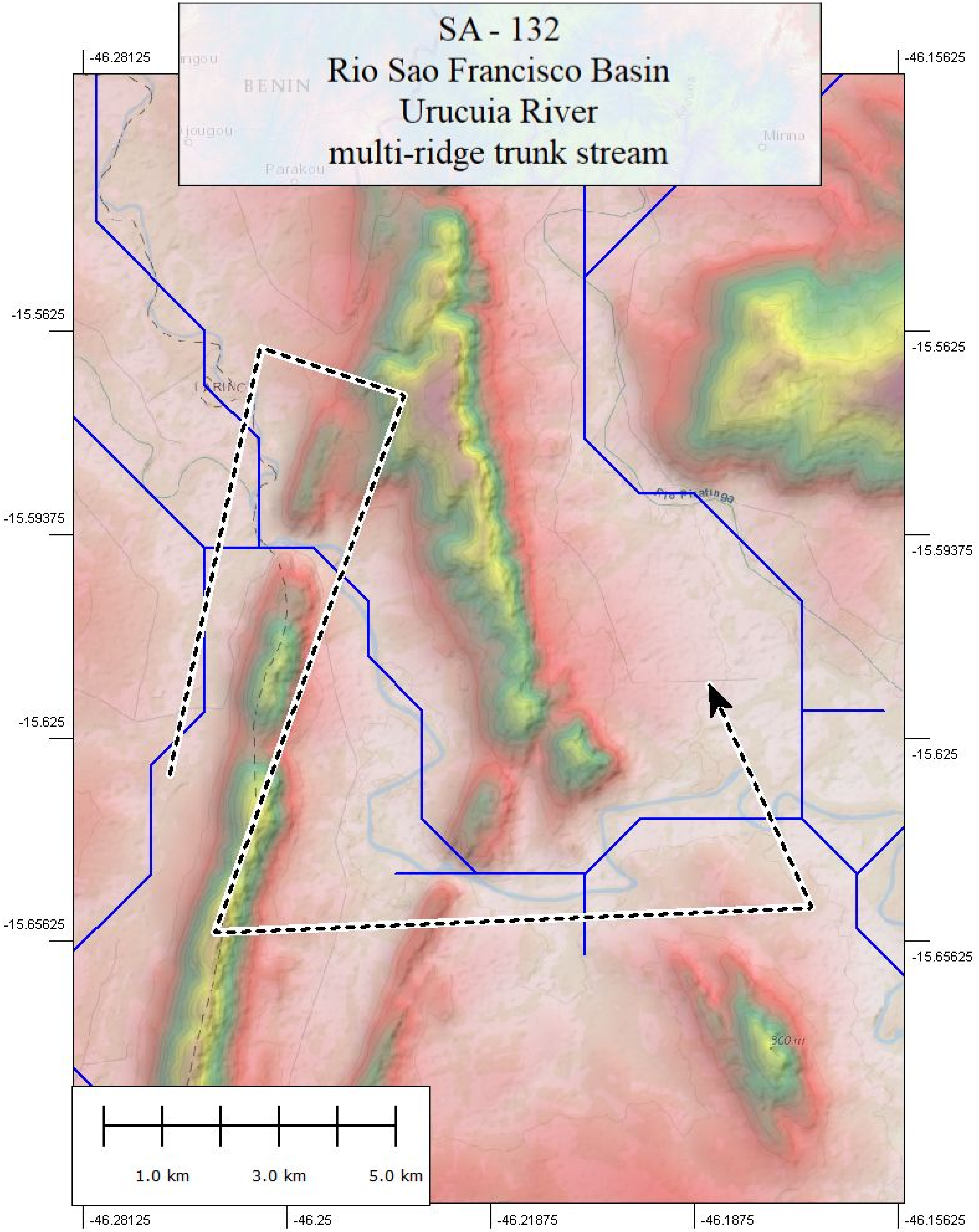
SA - 132
Rio Sao Francisco Basin
Urucuia River
multi-ridge trunk stream
-46.28125
-46.15625
-46.25
-46.21875
-46.1875
-15.5625
-15.59375
-15.625
-15.65625
Rio Piratinga
1.0 km
3.0 km
5.0 km

SA - 153
Rio Colorado (South America) Basin
Huaco River
multi-ridge trunk stream

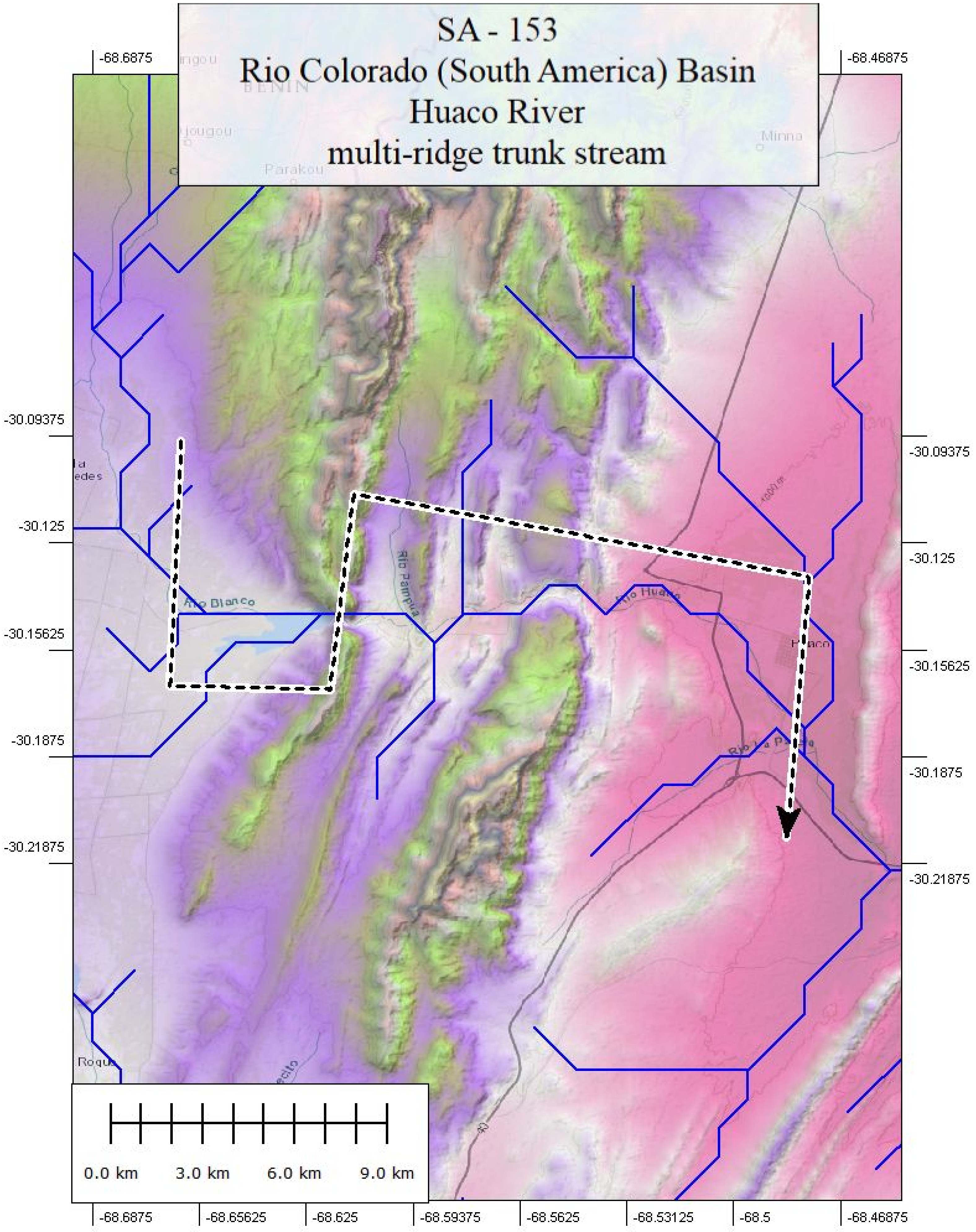

SA - 157
Rio Magdalena Basin
Prado River
multi-ridge trunk stream

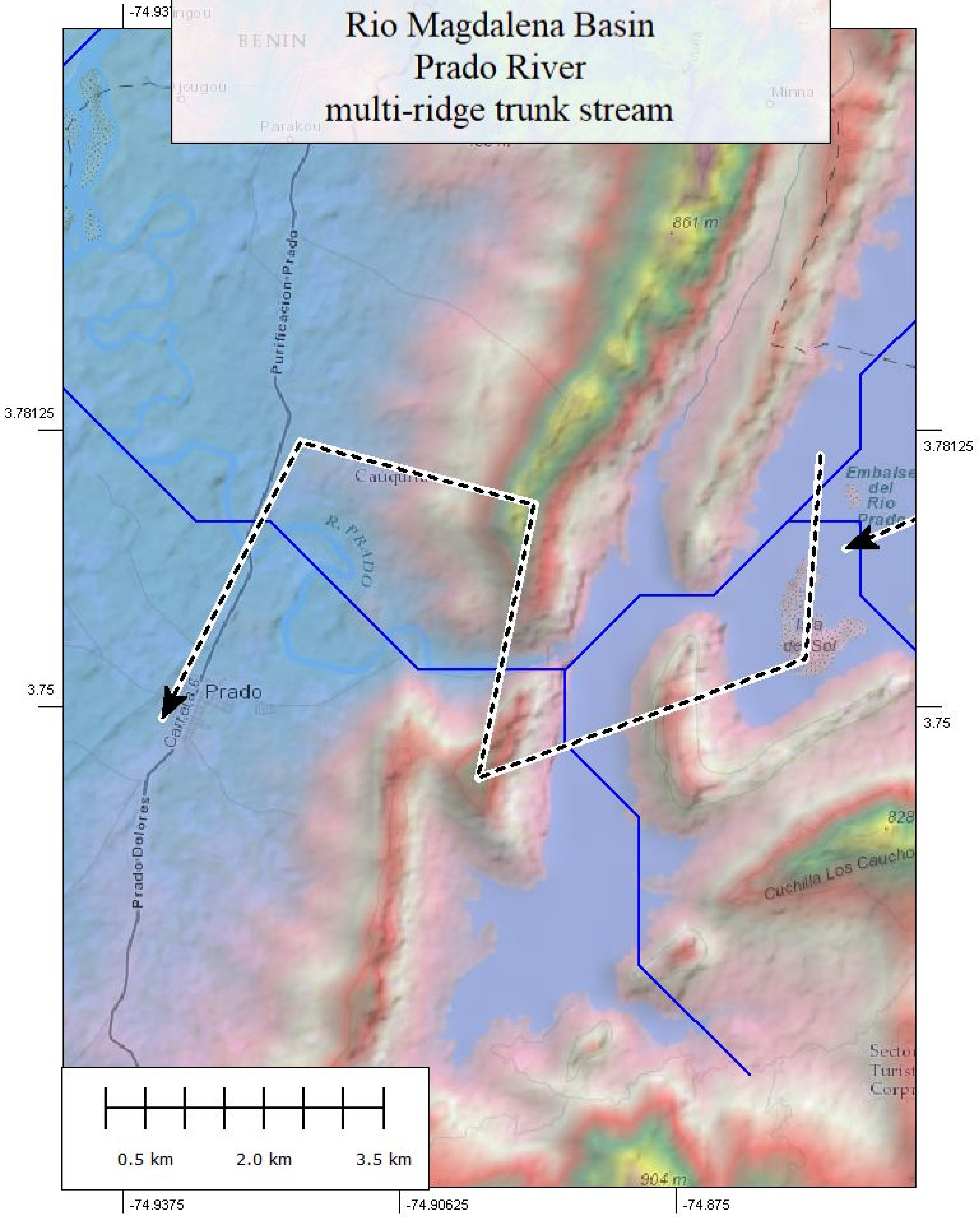

SA - 207
Amazon River Basin
Tupuani River tributary
multi-ridge trunk stream

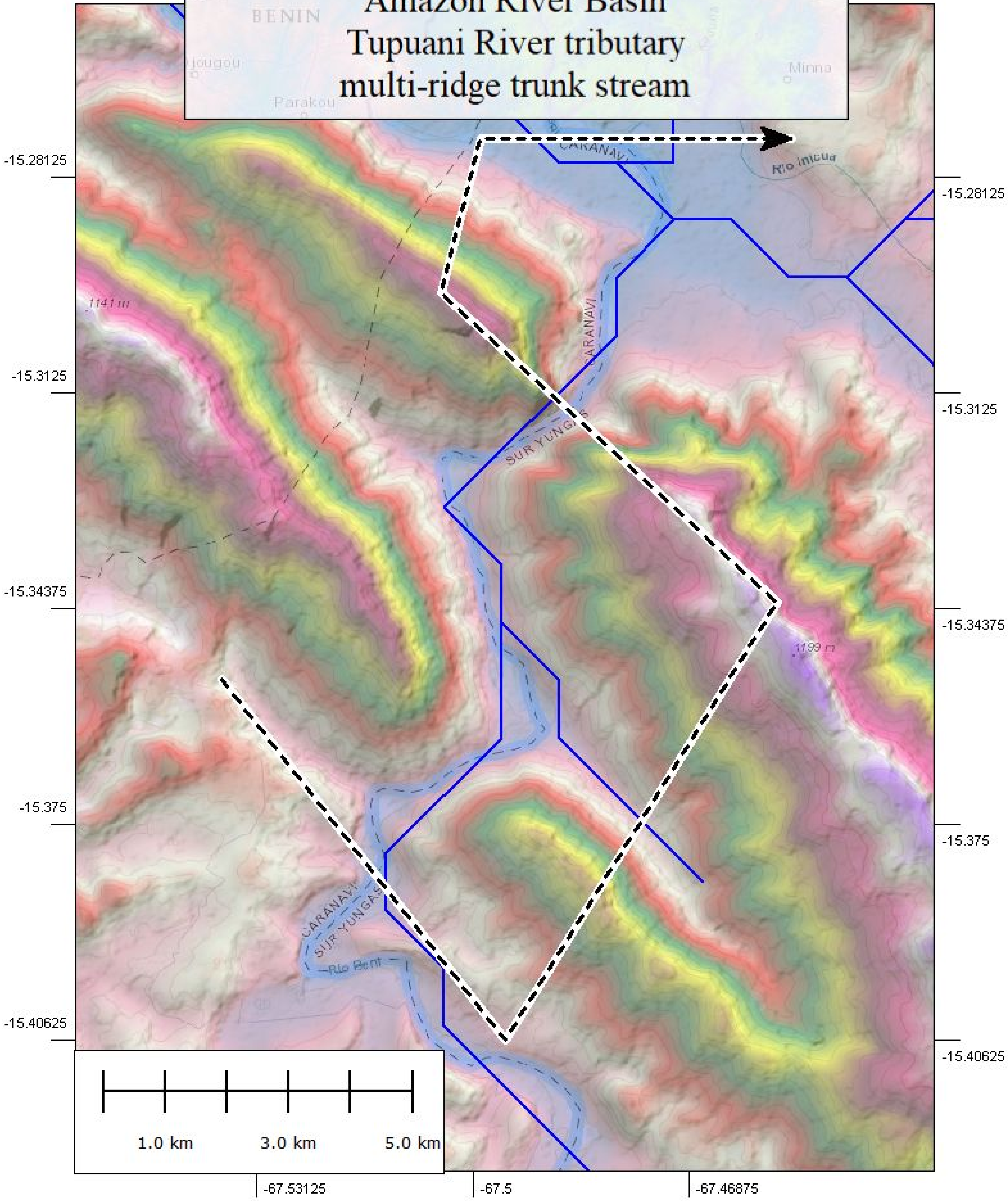

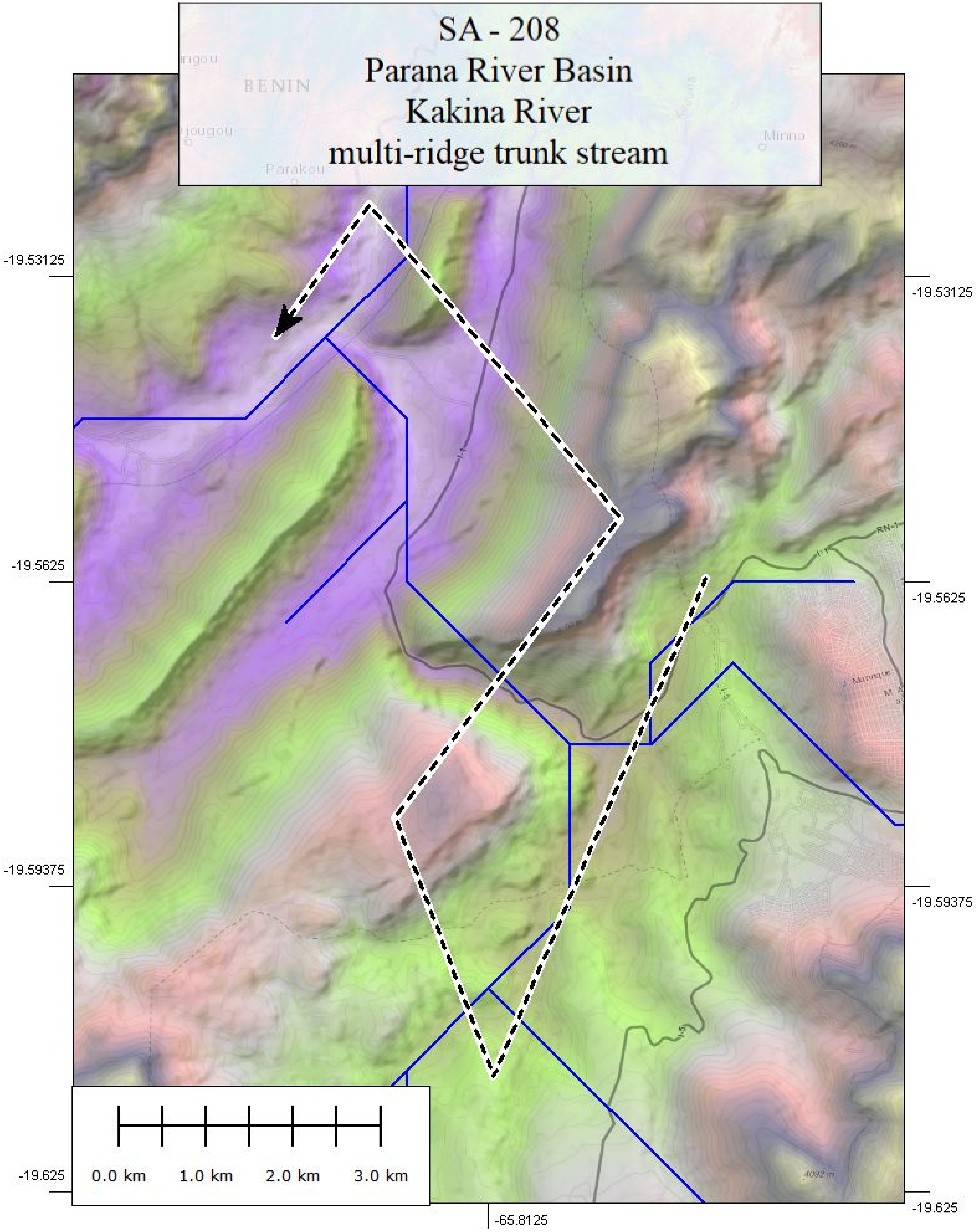

SA - 208
Parana River Basin
Kakina River
multi-ridge trunk stream
BENIN
Parakou
Minna
RN-1
-19.53125
-19.5625
-19.59375
-19.625
-65.8125
0.0 km
1.0 km
2.0 km
3.0 km

SA - 217
Parana River Basin
Bermejo River
multi-ridge trunk stream

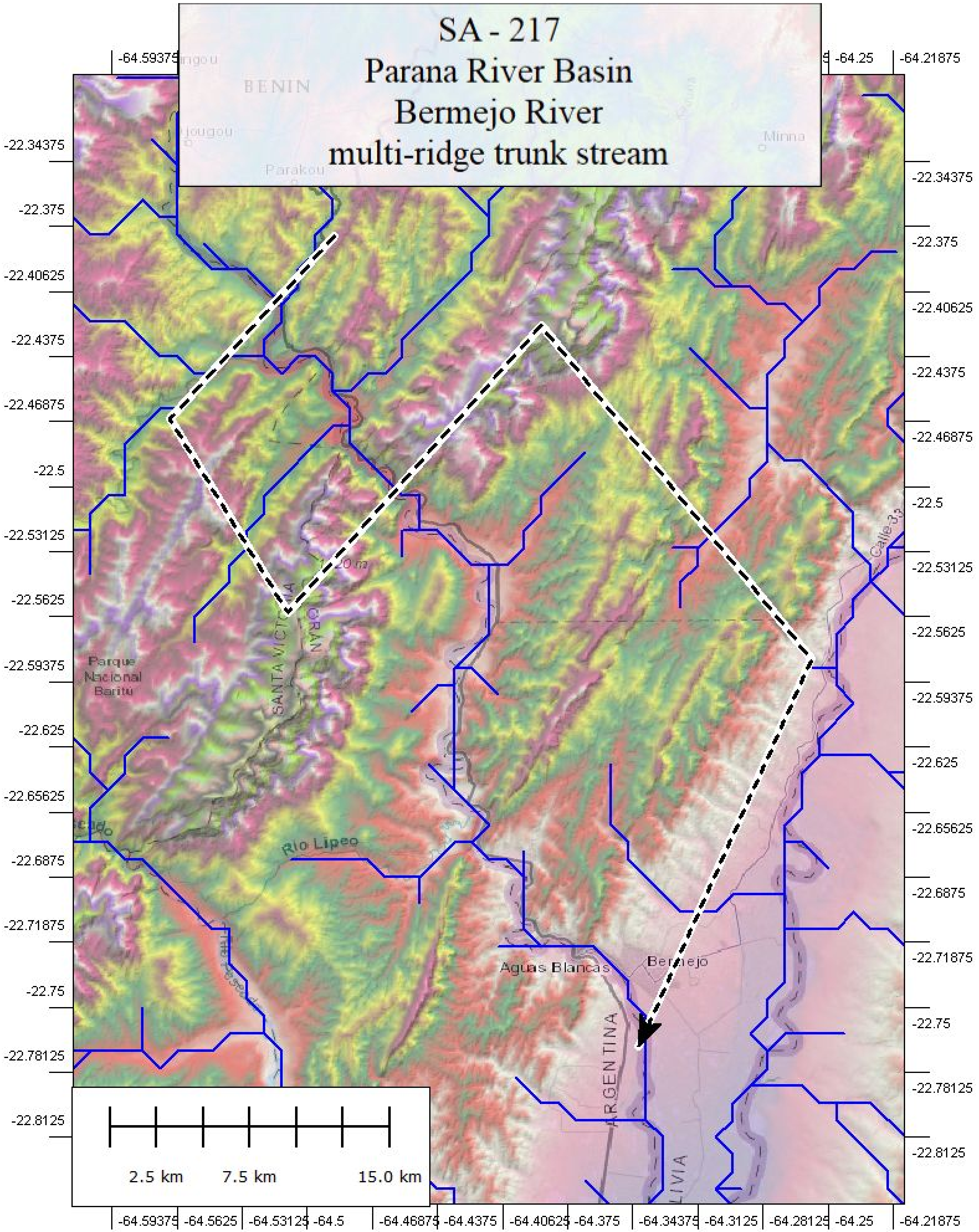

SA - 224
Orinoco River Basin
Unete River
multi-ridge trunk stream

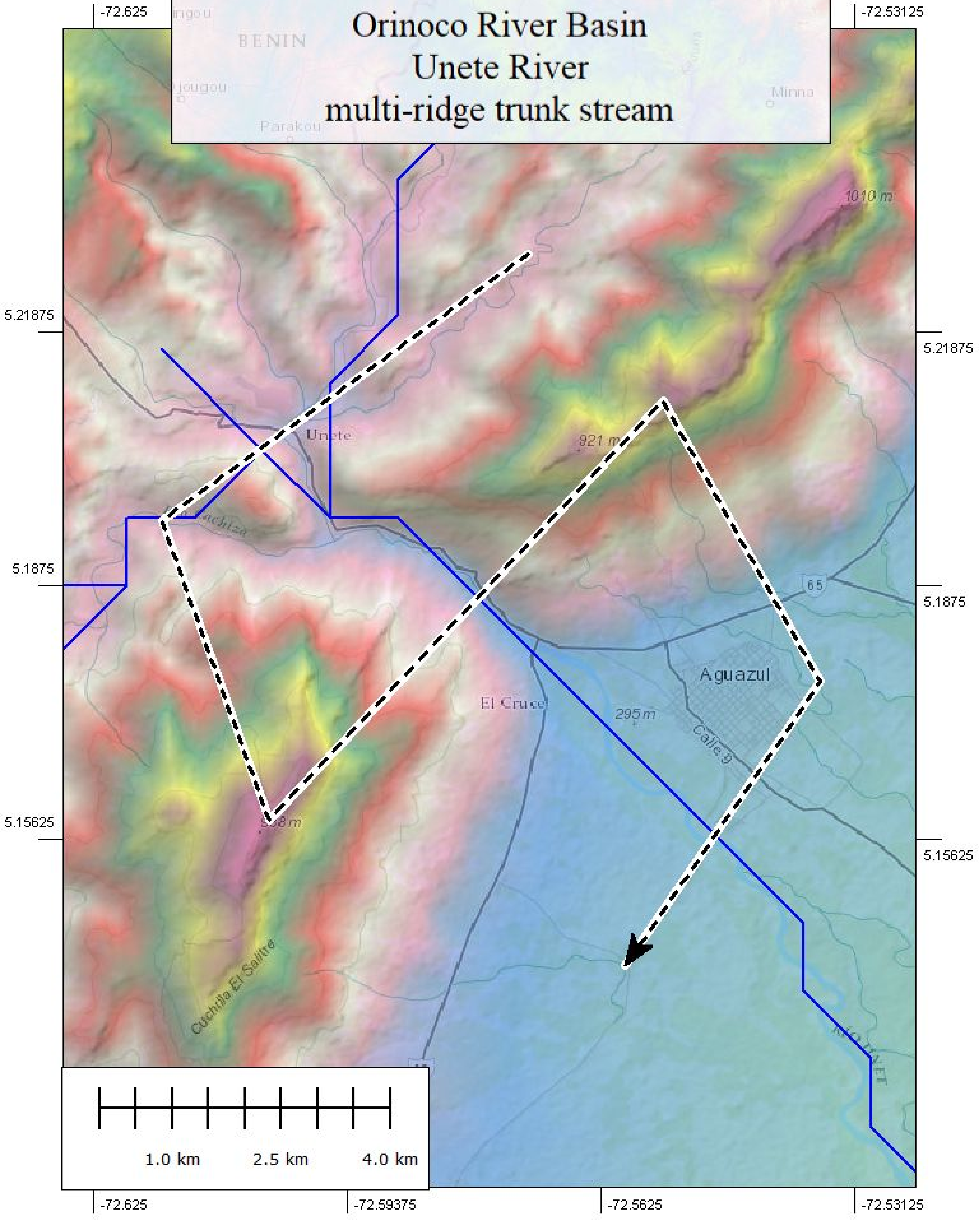

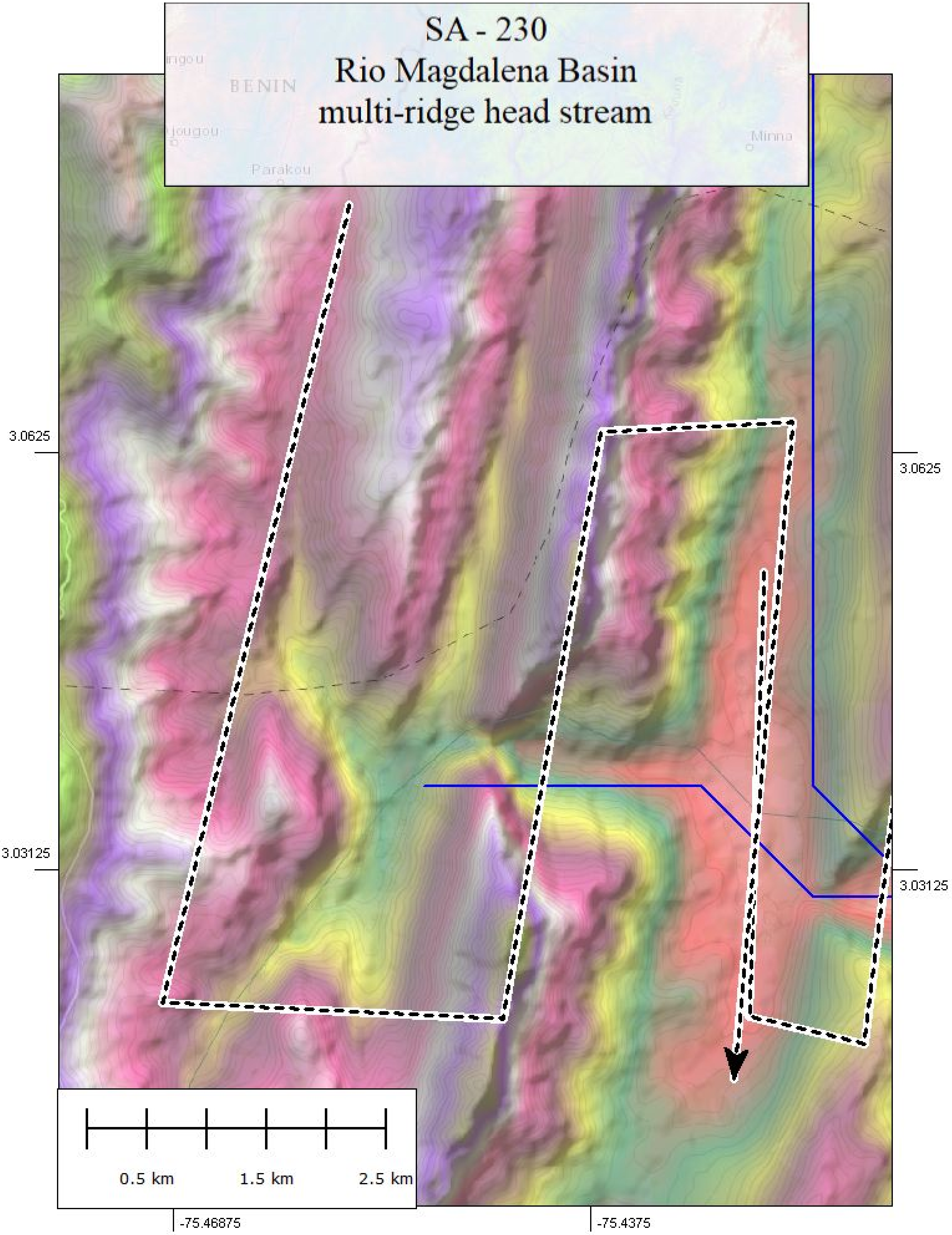
SA - 230
Rio Magdalena Basin
multi-ridge head stream
3.0625
3.0625
3.03125
3.03125
0.5 km
1.5 km
2.5 km
-75.46875
-75.4375

SA - 232
Amazon River Basin
Tupuani River
multi-ridge trunk stream

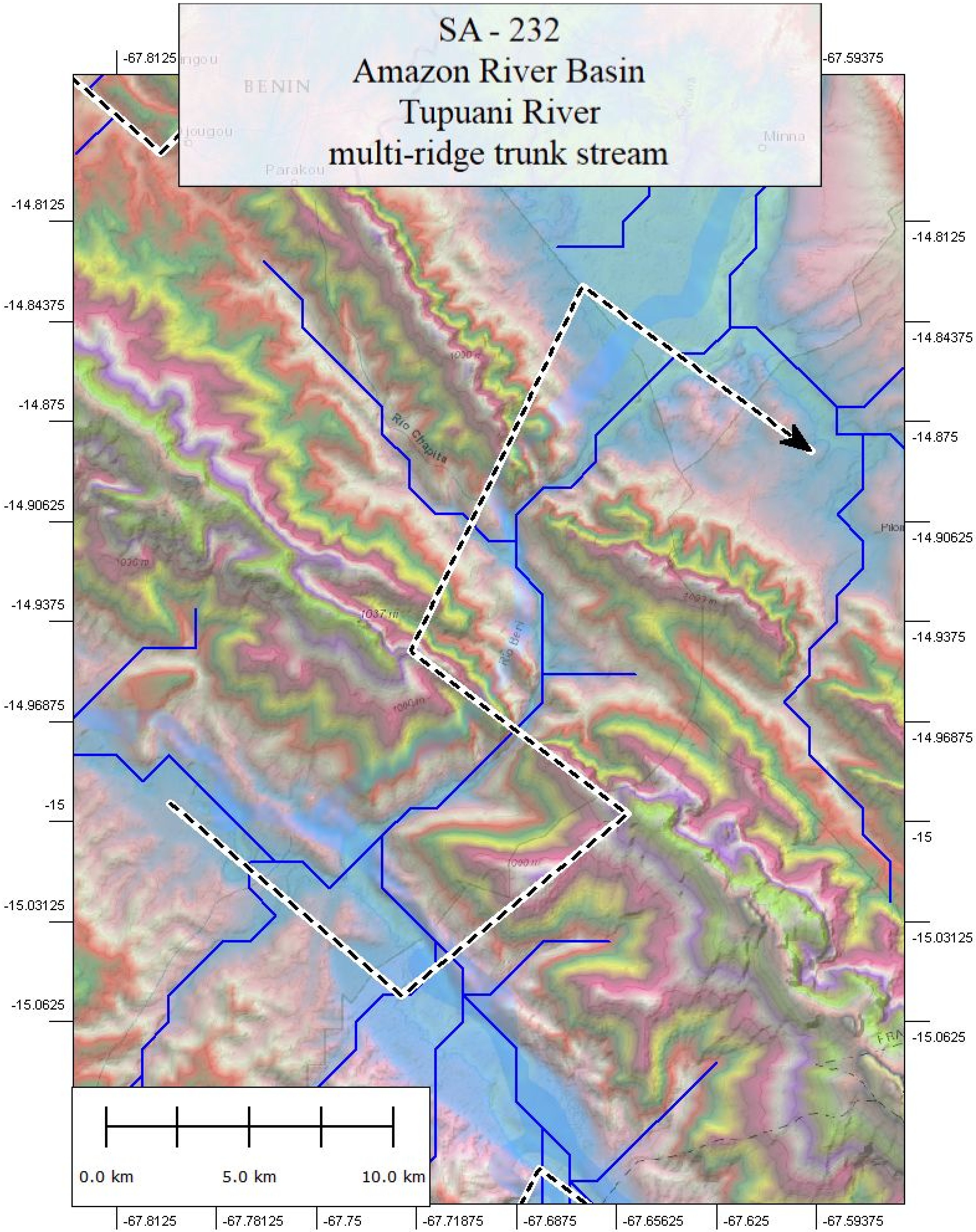

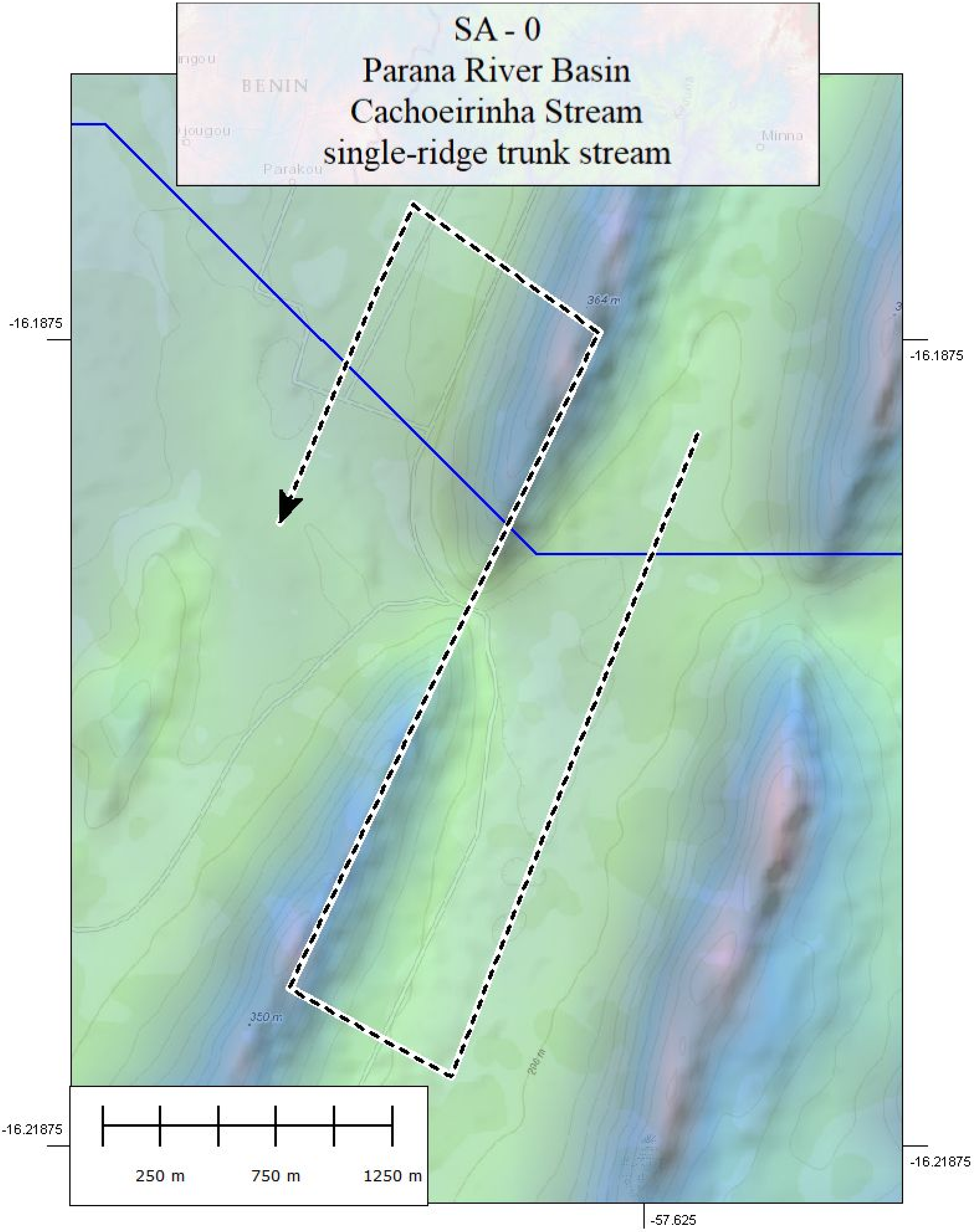
SA - 0
Parana River Basin
Cachoeirinha Stream
single-ridge trunk stream
BENIN
Parakou
Minna
364 m
350 m
-16.1875
-16.1875
-16.21875
-16.21875
-57.625
250 m
750 m
1250 m

SA - 1
Amazon River Basin
Tambo River tributary
single-ridge trunk stream

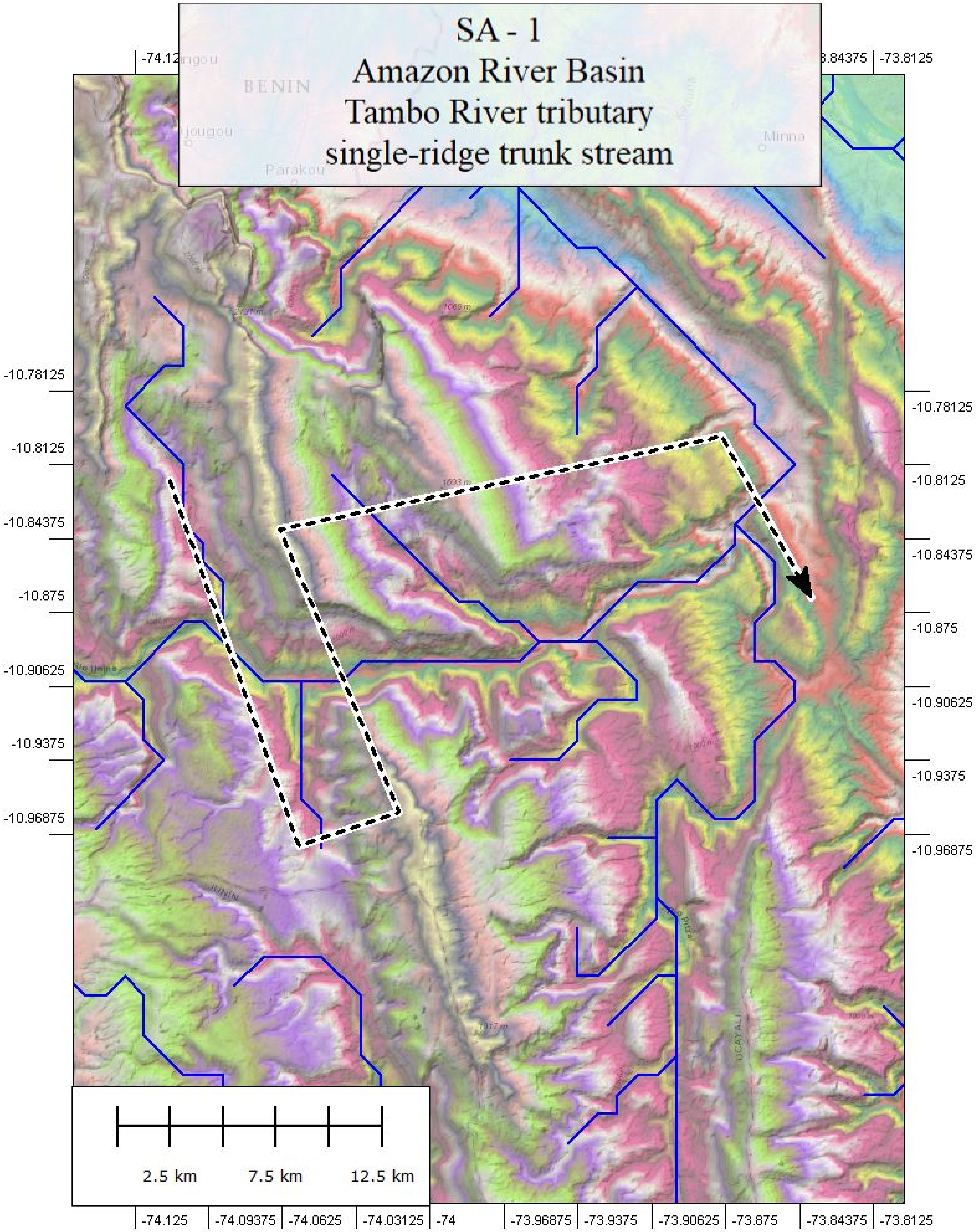

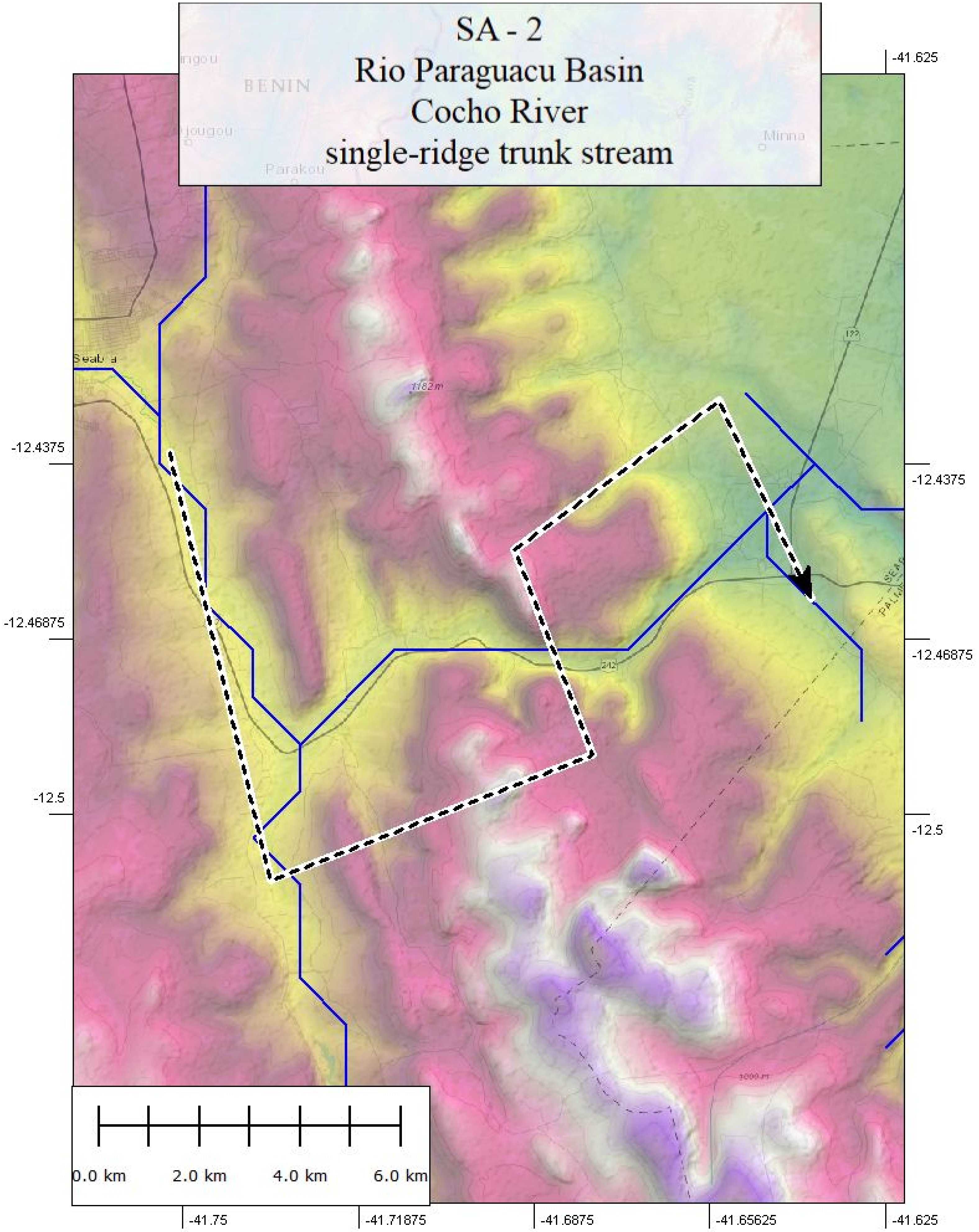
SA - 2
Rio Paraguacu Basin
Cocho River
single-ridge trunk stream
-41.625
-12.4375
-12.46875
-12.5
-12.4375
-12.46875
-12.5
1182 m
0.0 km
2.0 km
4.0 km
6.0 km
-41.75
-41.71875
-41.6875
-41.65625
-41.625

SA - 3
Rio Paraguacu Basin
Sao Jose River
single-ridge head stream

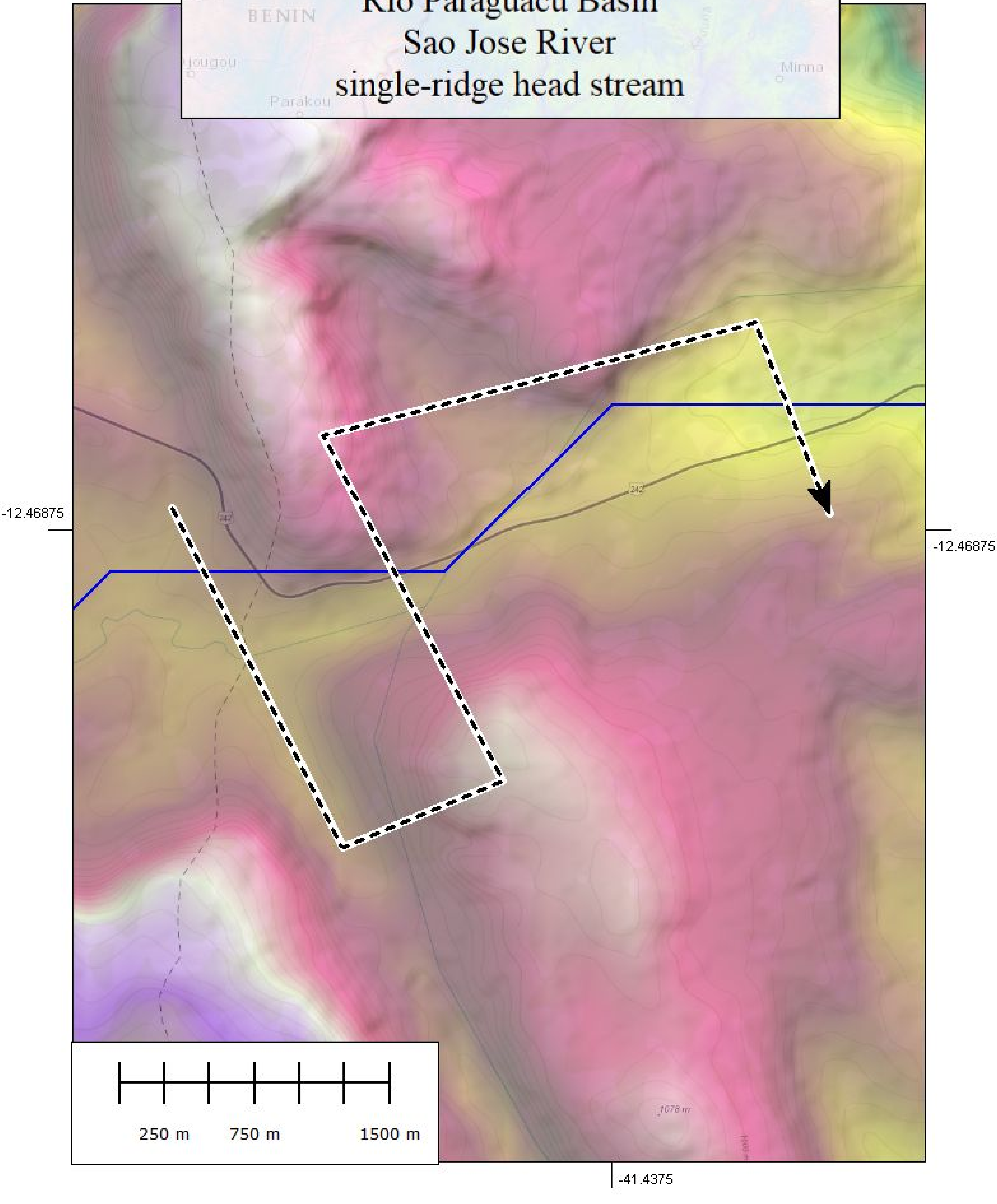

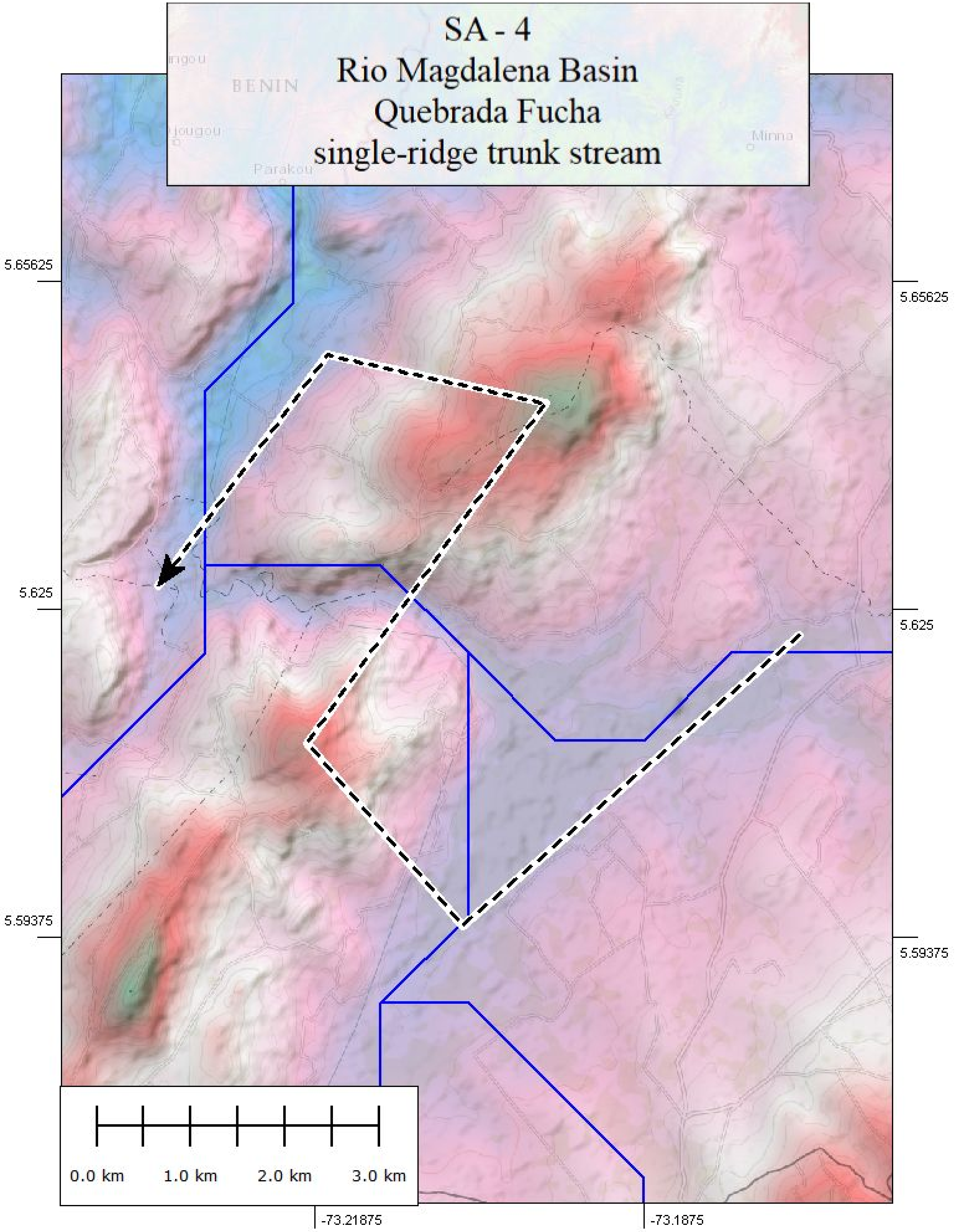
SA - 4
Rio Magdalena Basin
Quebrada Fucha
single-ridge trunk stream
BENIN
Parakou
Minna
5.65625
5.625
5.59375
5.65625
5.625
5.59375
0.0 km
1.0 km
2.0 km
3.0 km
-73.21875
-73.1875

SA - 5
Rio Magdalena Basin
Quebrada Goloso
single-ridge trunk stream

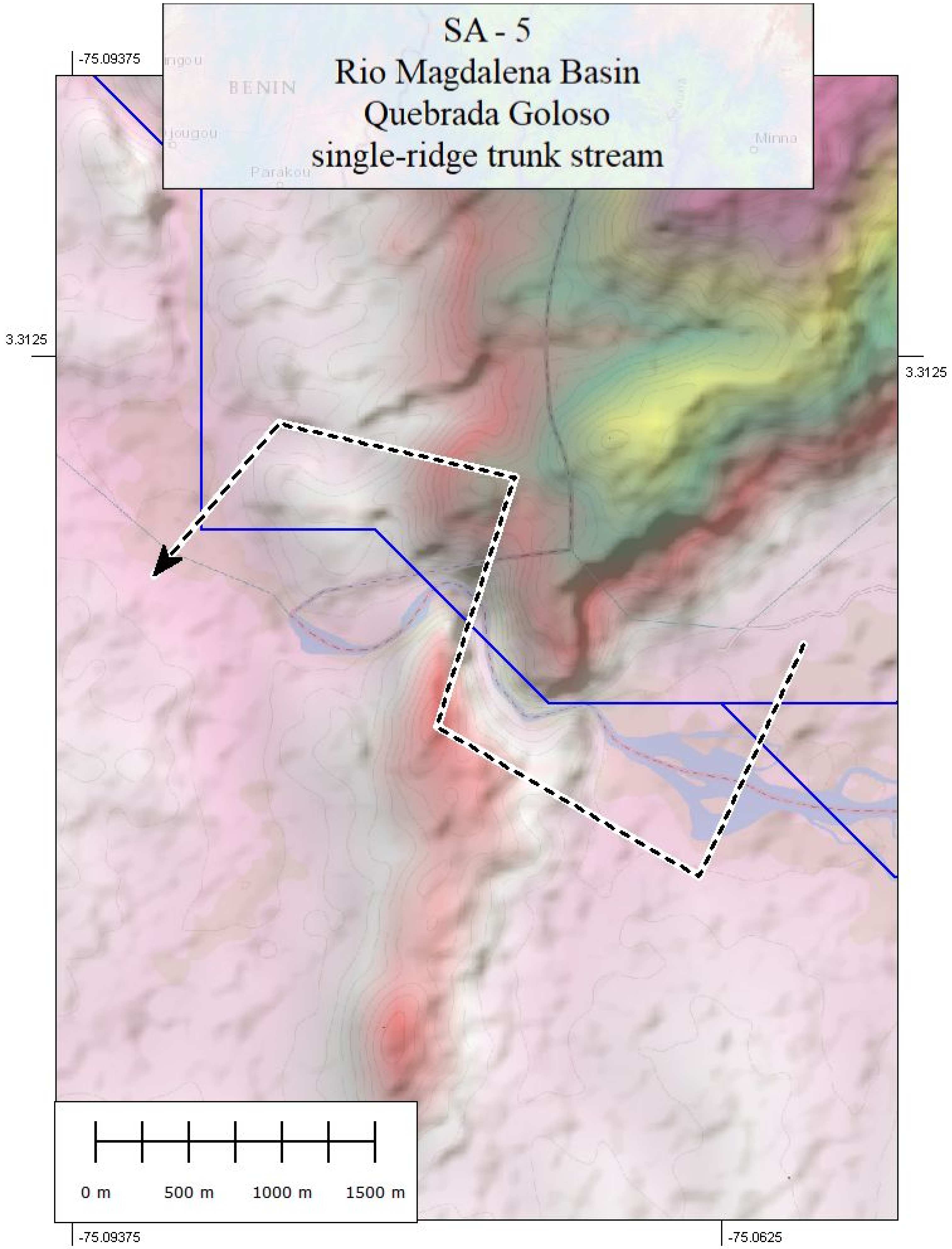

SA - 7
Parana River Basin
Tincuya River tributary
single-ridge head stream

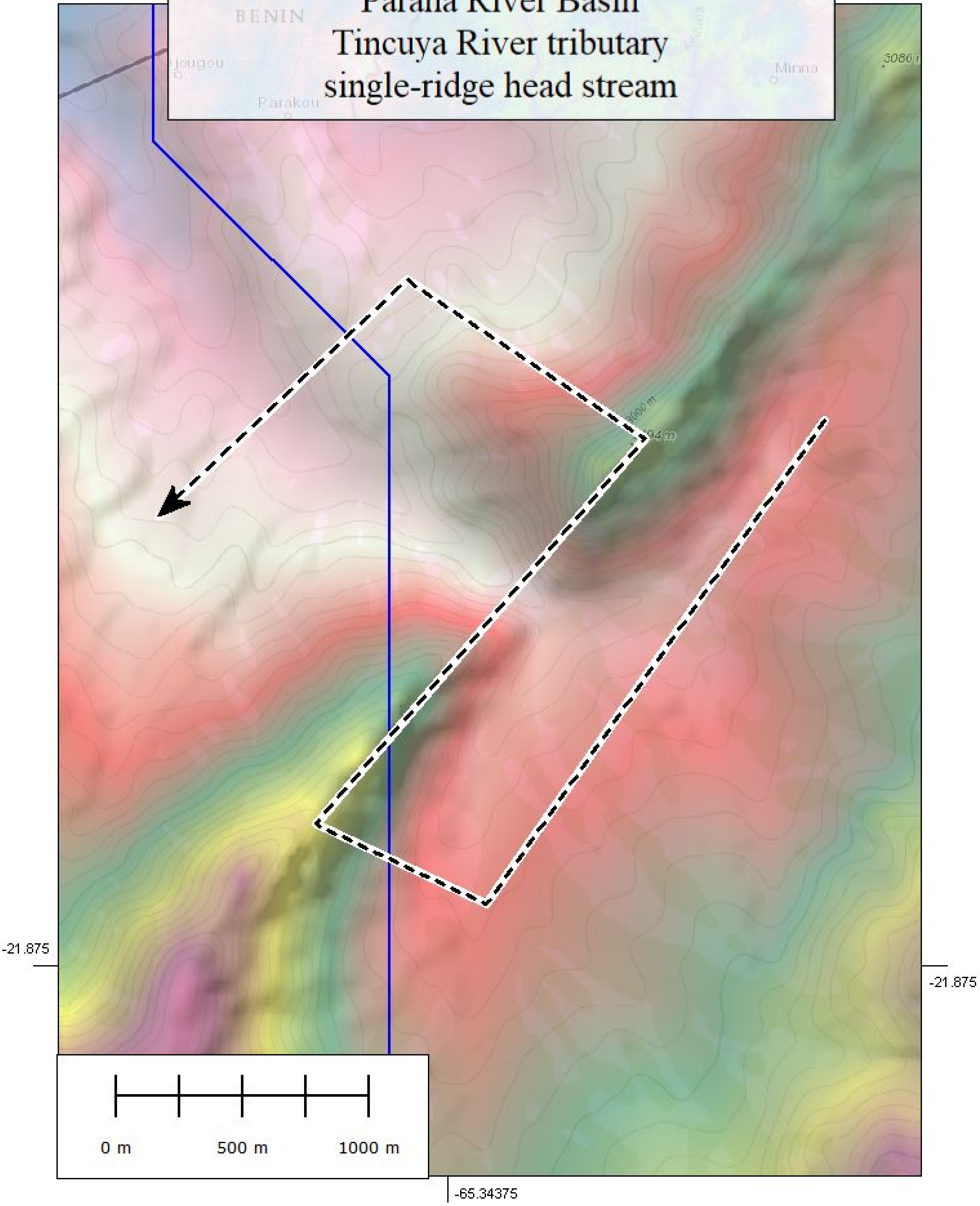

SA - 8
Rio Magdalena Basin
Cachira del Sur River
single-ridge trunk stream

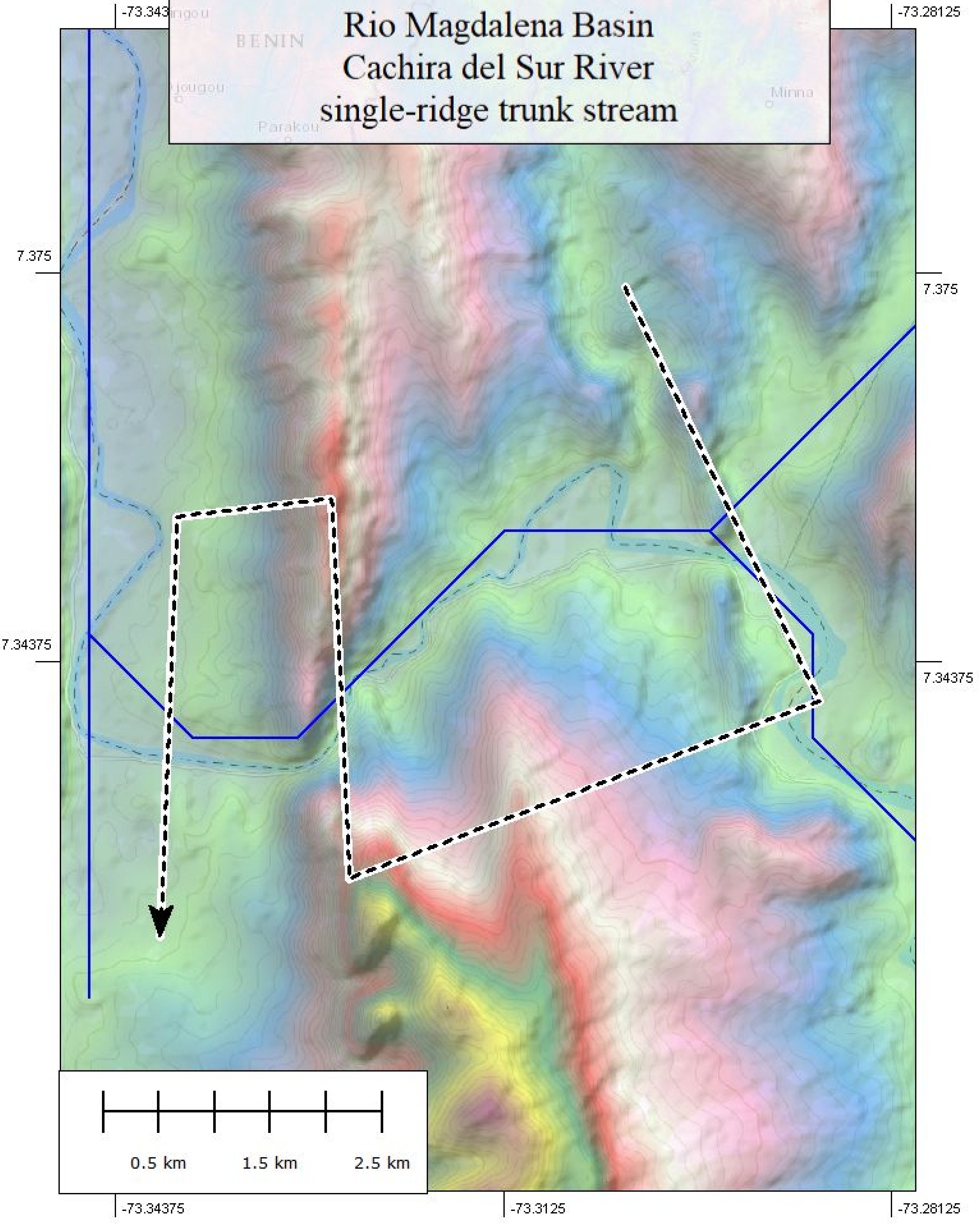

SA - 9
Rio Colorado (South America) Basin
Jachal River
single-ridge trunk stream

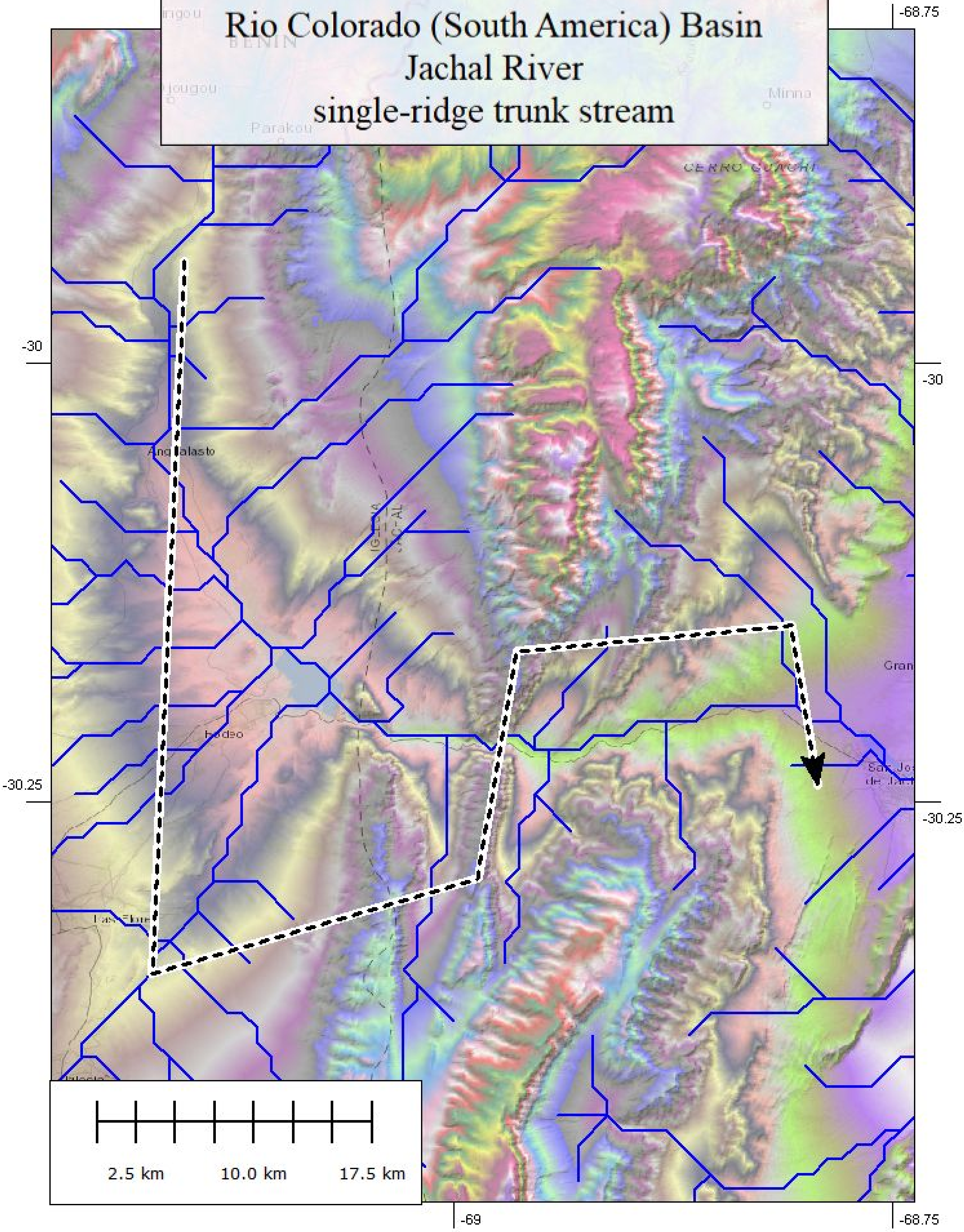

SA - 10
Amazon River Basin
Rio Grande O Guapay tributary
single-ridge head stream

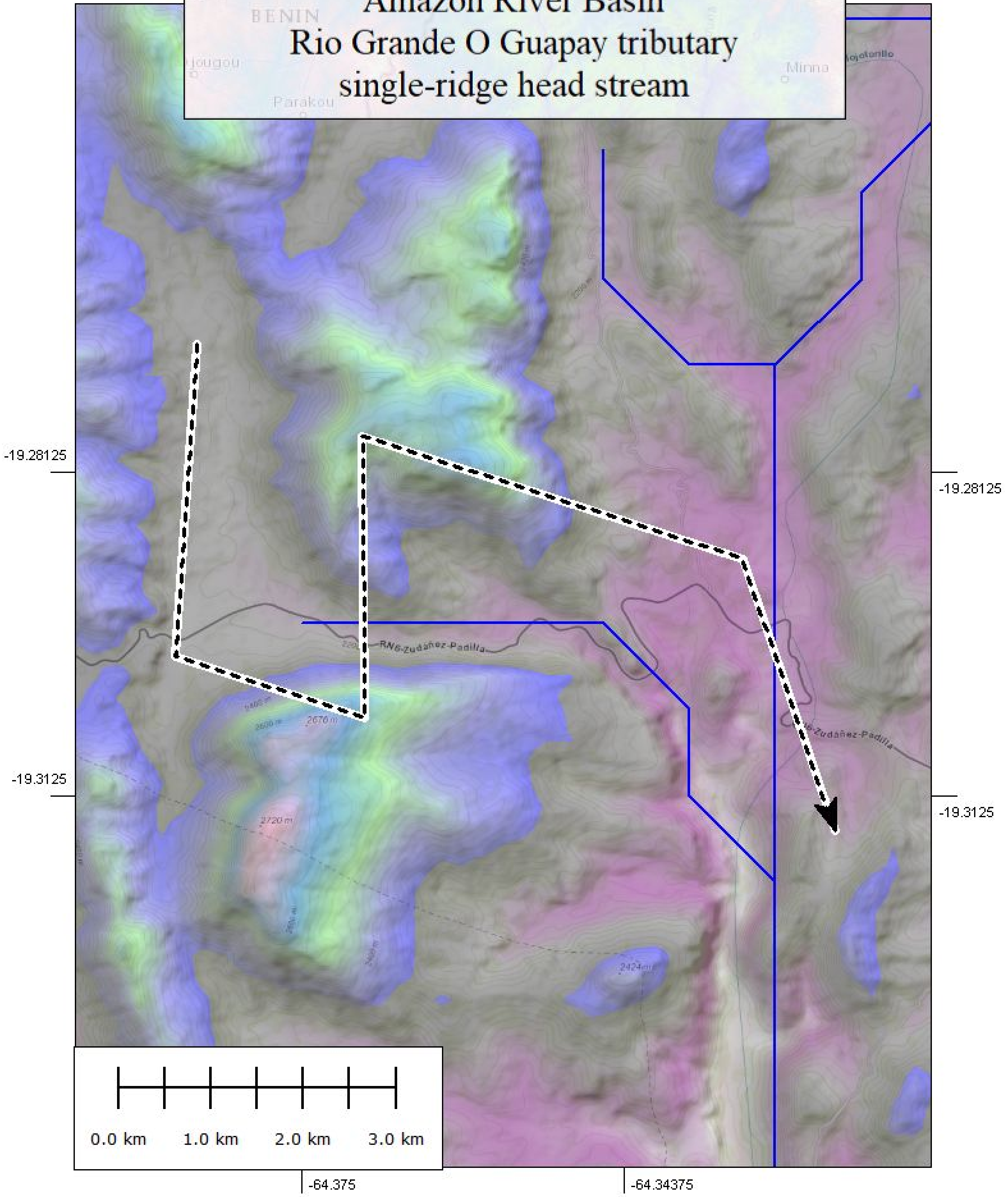

SA - 11
Amazon River Basin
Rio Grande O Guapay
single-ridge trunk stream

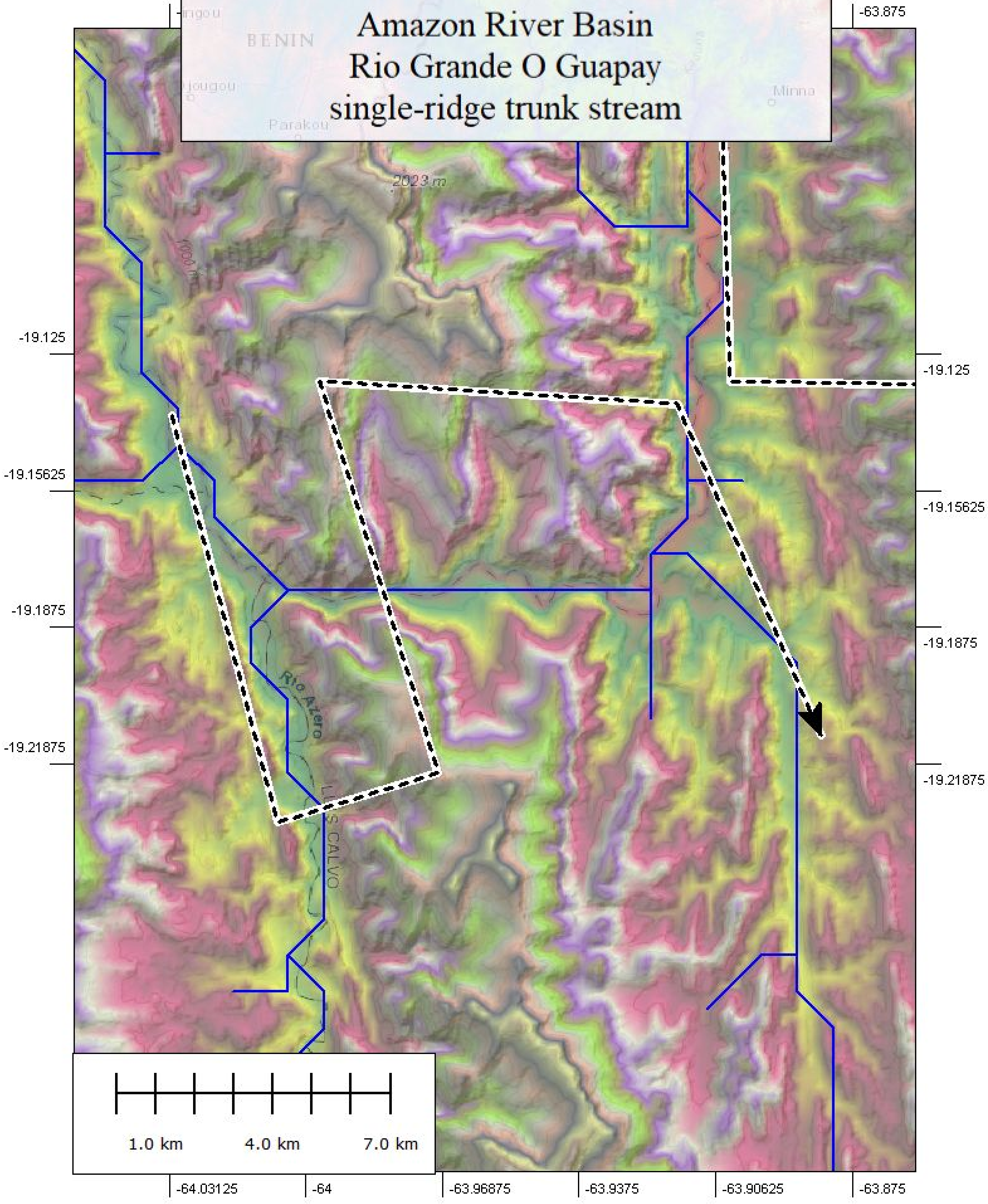

SA - 12
Amazon River Basin
Rio Grande O Guapay tributary
single-ridge trunk stream

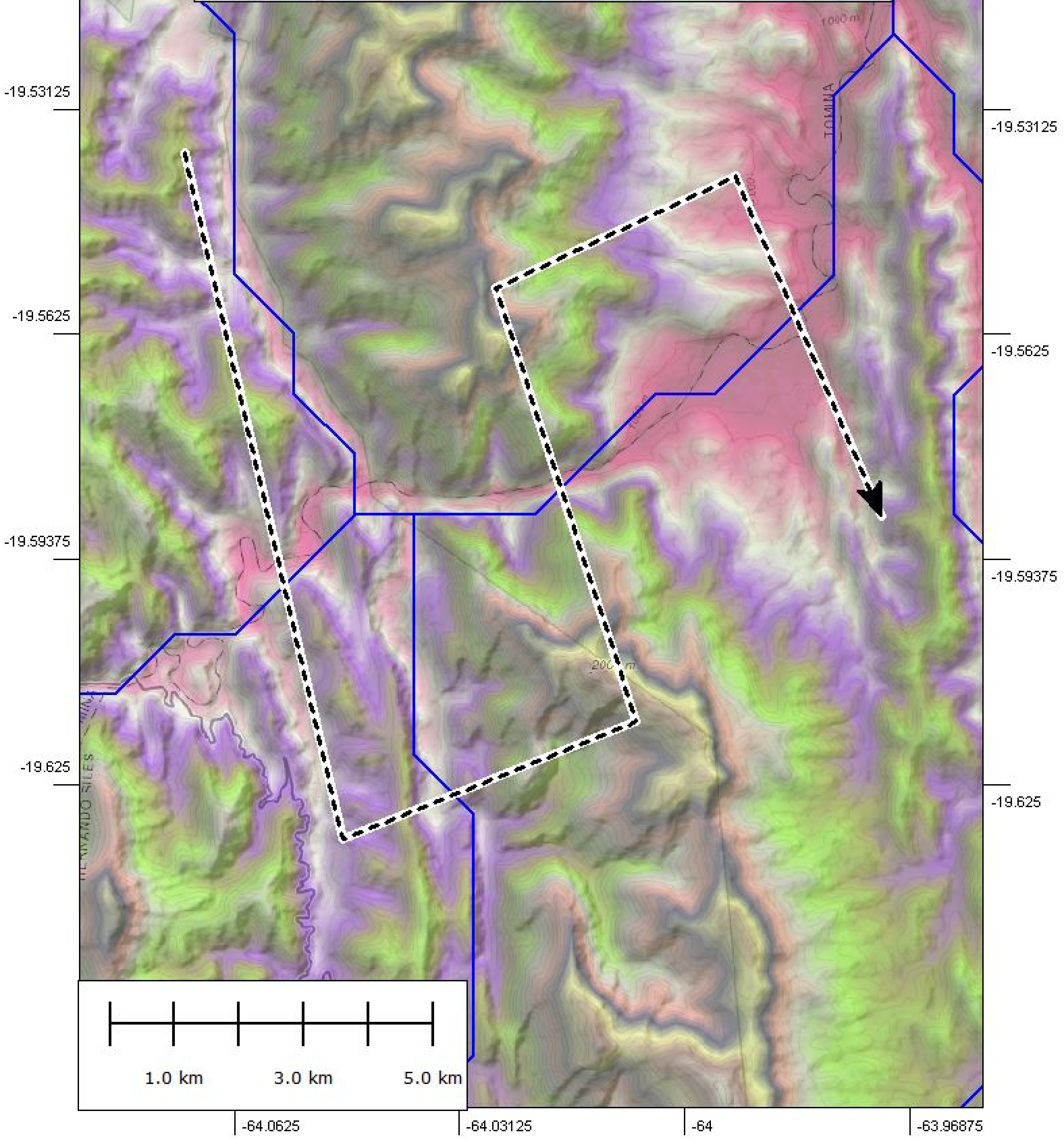

SA - 13
Amazon River Basin
Rio Grande O Guapay tributary
single-ridge trunk stream

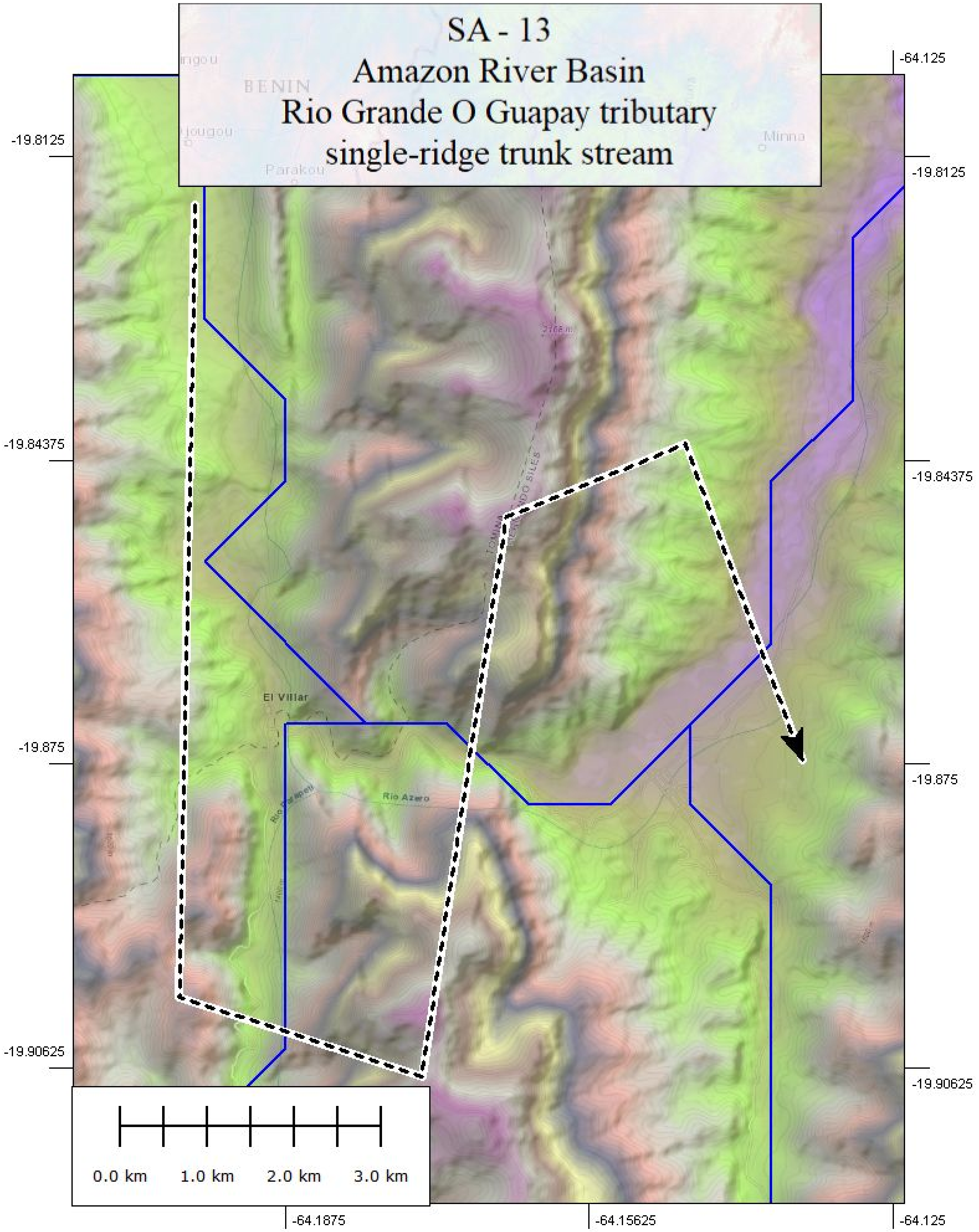

SA - 14
Parana River Basin
Rio Grande (Argentina)
single-ridge trunk stream

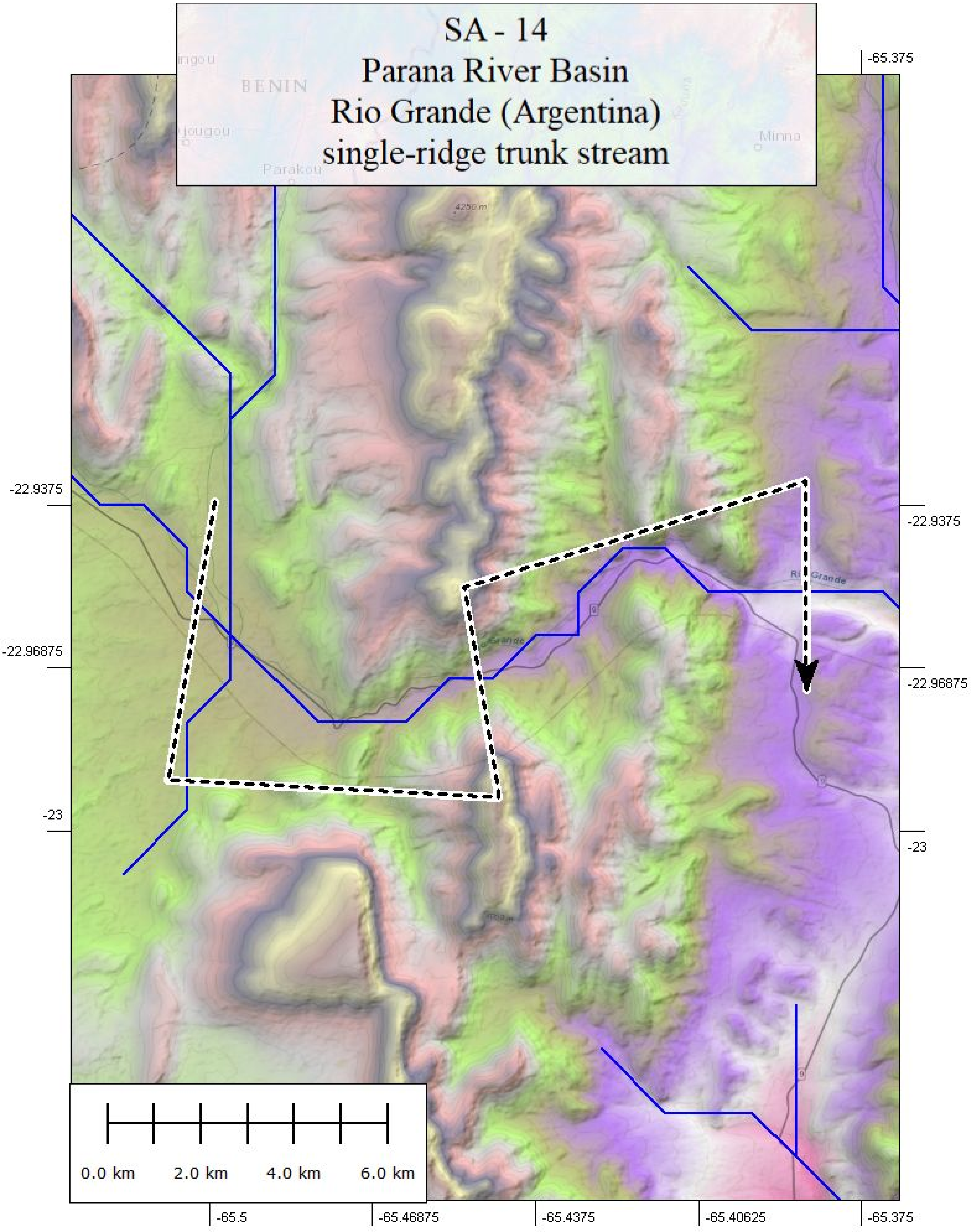

SA - 15
Parana River Basin
Itiyuro River
single-ridge trunk stream

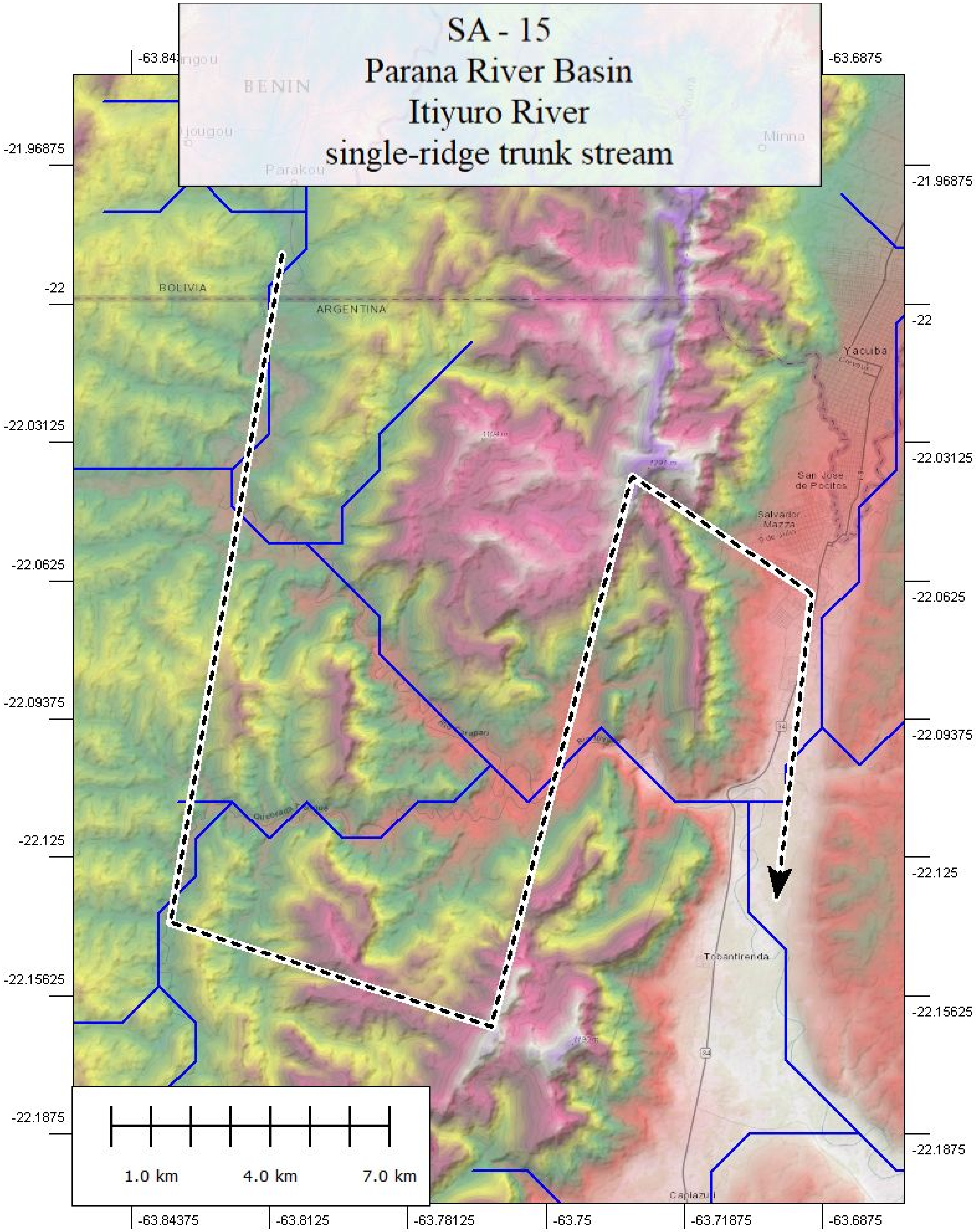

SA - 16
Rio Magdalena Basin
Arcabuco River
single-ridge head stream

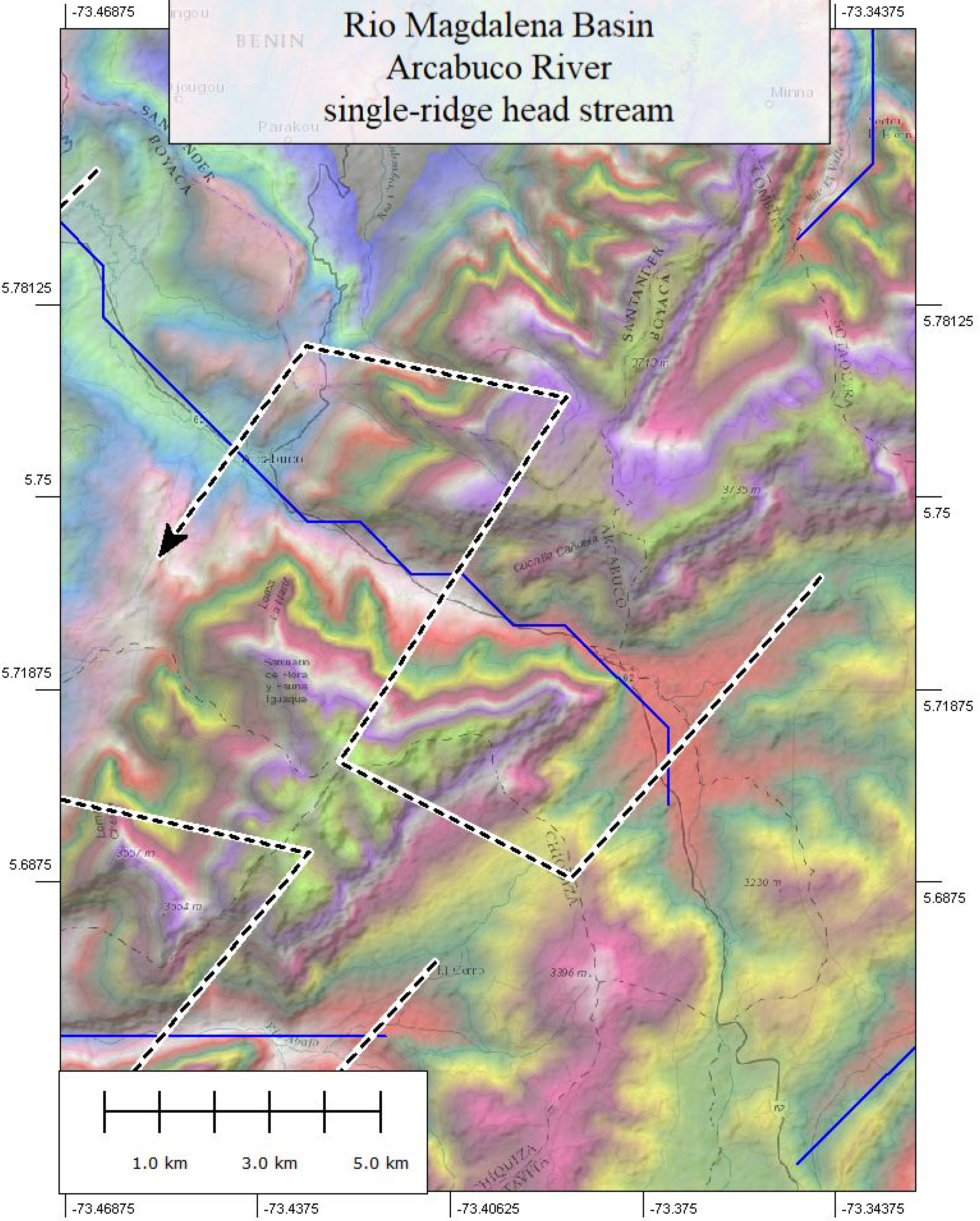

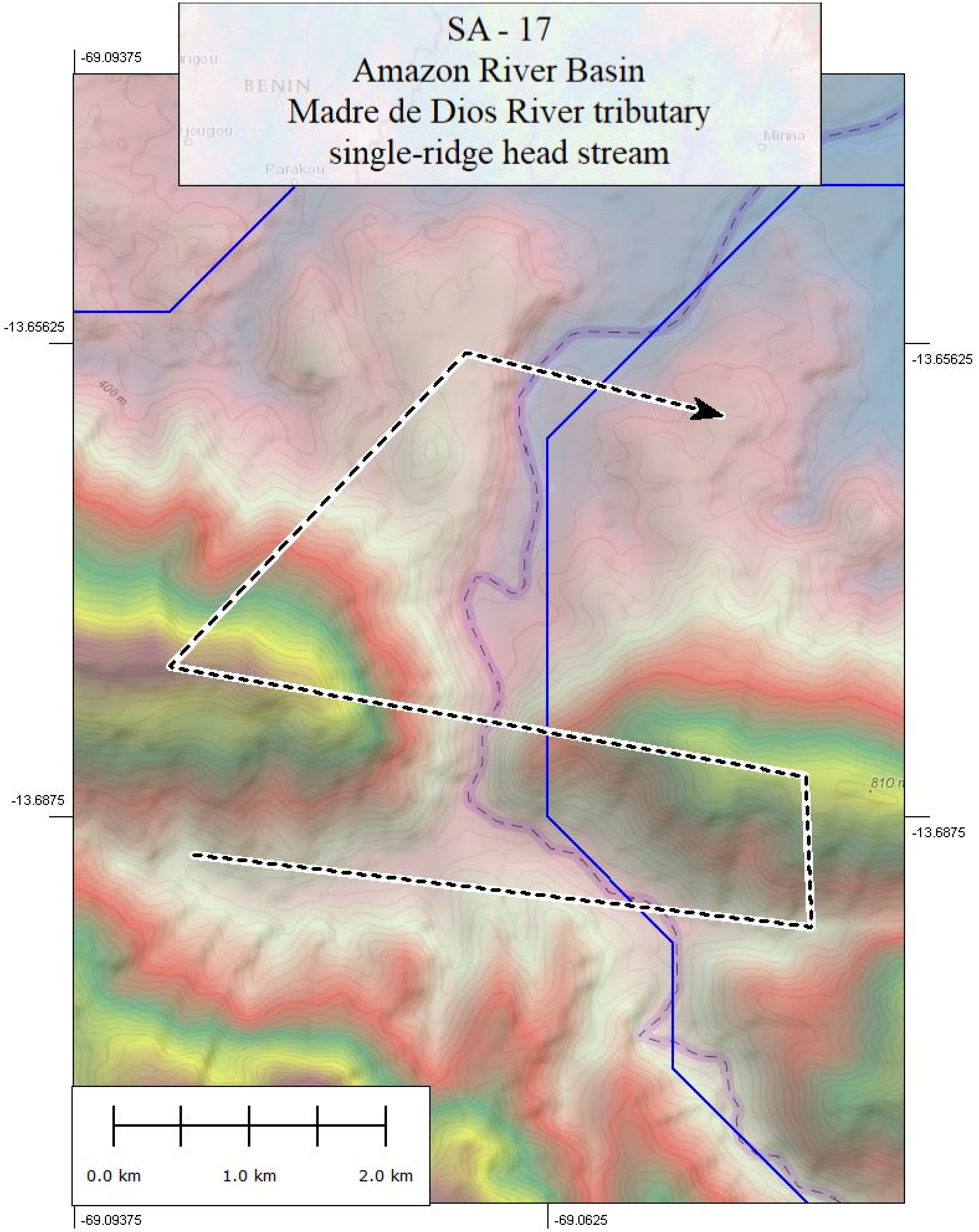
SA - 17
Amazon River Basin
Madre de Dios River tributary
single-ridge head stream
-69.09375
BENIN
Parakou
Minna
-13.65625
-13.65625
400 m
-13.6875
-13.6875
0.0 km
1.0 km
2.0 km
-69.09375
-69.0625

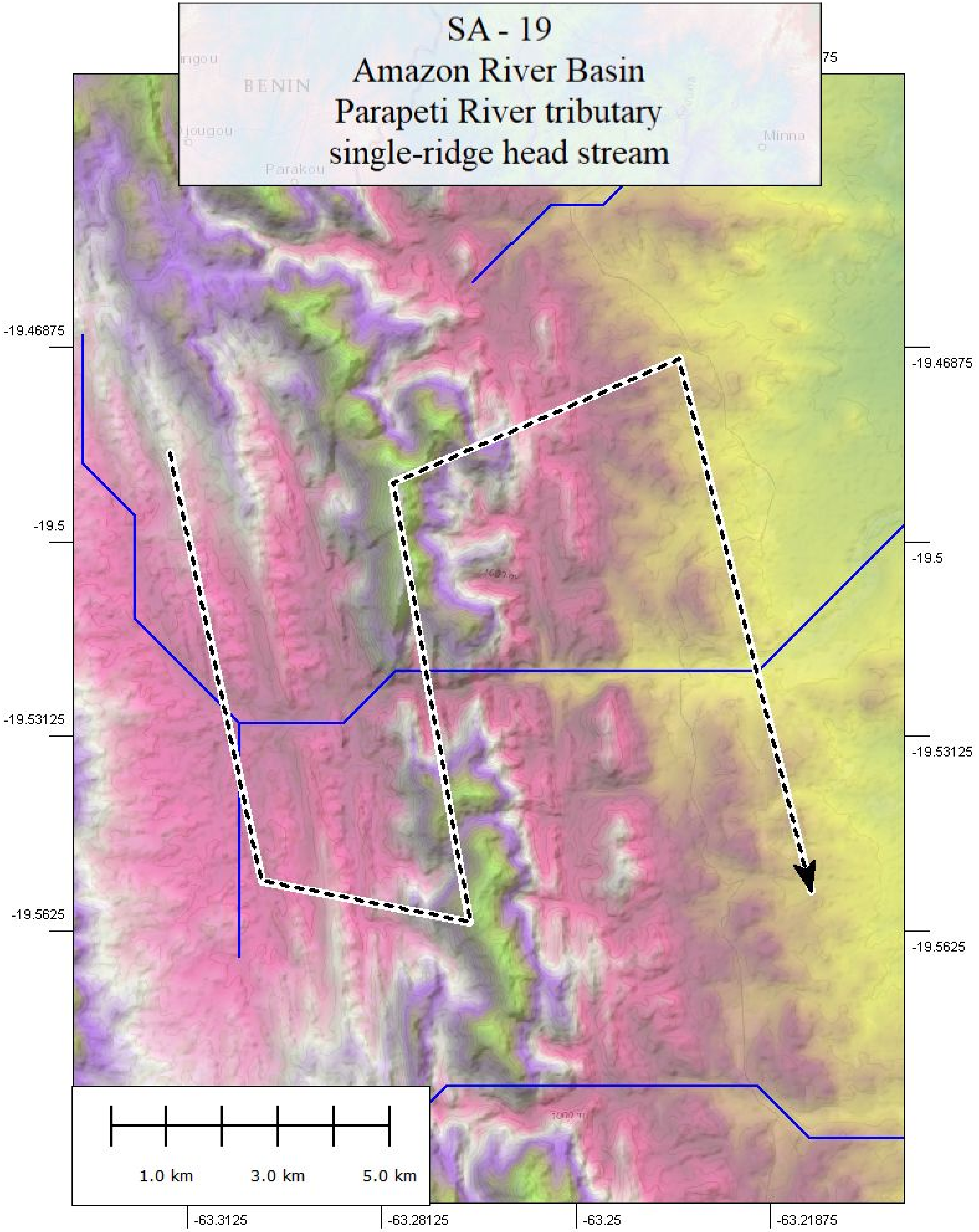

SA - 19
Amazon River Basin
Parapeti River tributary
single-ridge head stream
-19.46875
-19.5
-19.53125
-19.5625
-19.46875
-19.5
-19.53125
-19.5625
-63.3125
-63.28125
-63.25
-63.21875
1.0 km
3.0 km
5.0 km

SA - 20
Parana River Basin
Lavayan River tributary
single-ridge head stream

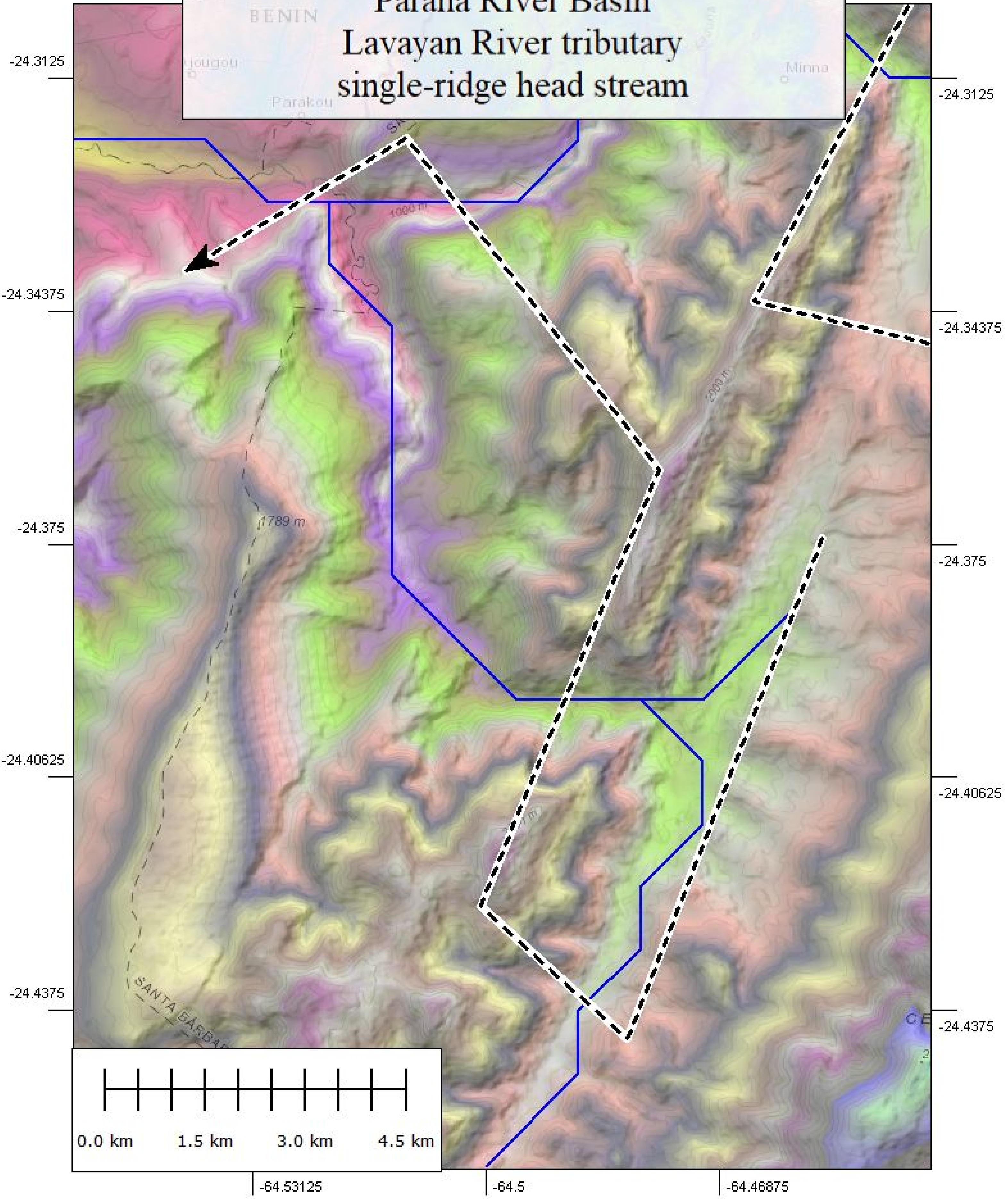

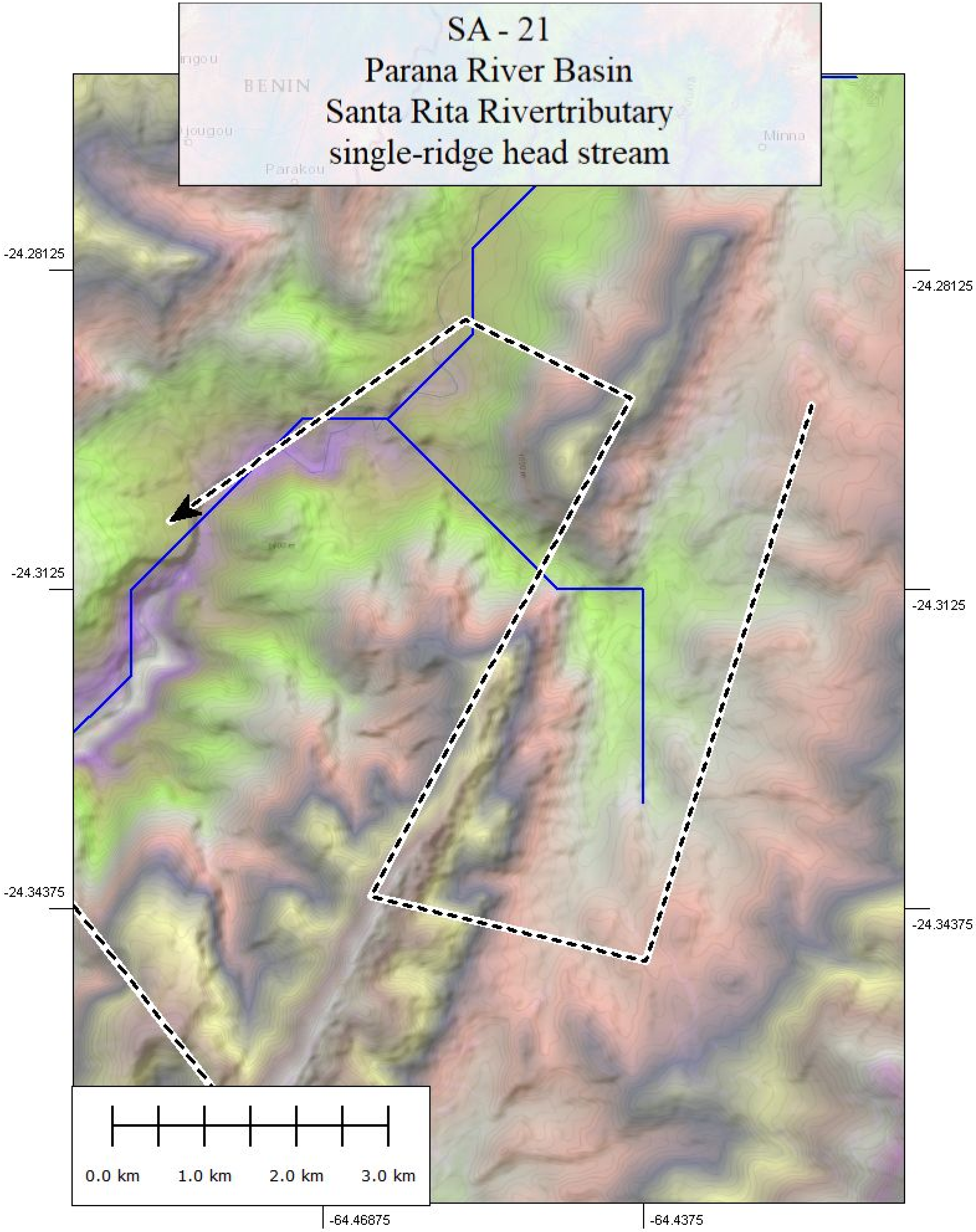
SA - 21
Parana River Basin
Santa Rita Rivertributary
single-ridge head stream
-24.28125
-24.3125
-24.34375
-24.28125
-24.3125
-24.34375
0.0 km
1.0 km
2.0 km
3.0 km
-64.46875
-64.4375

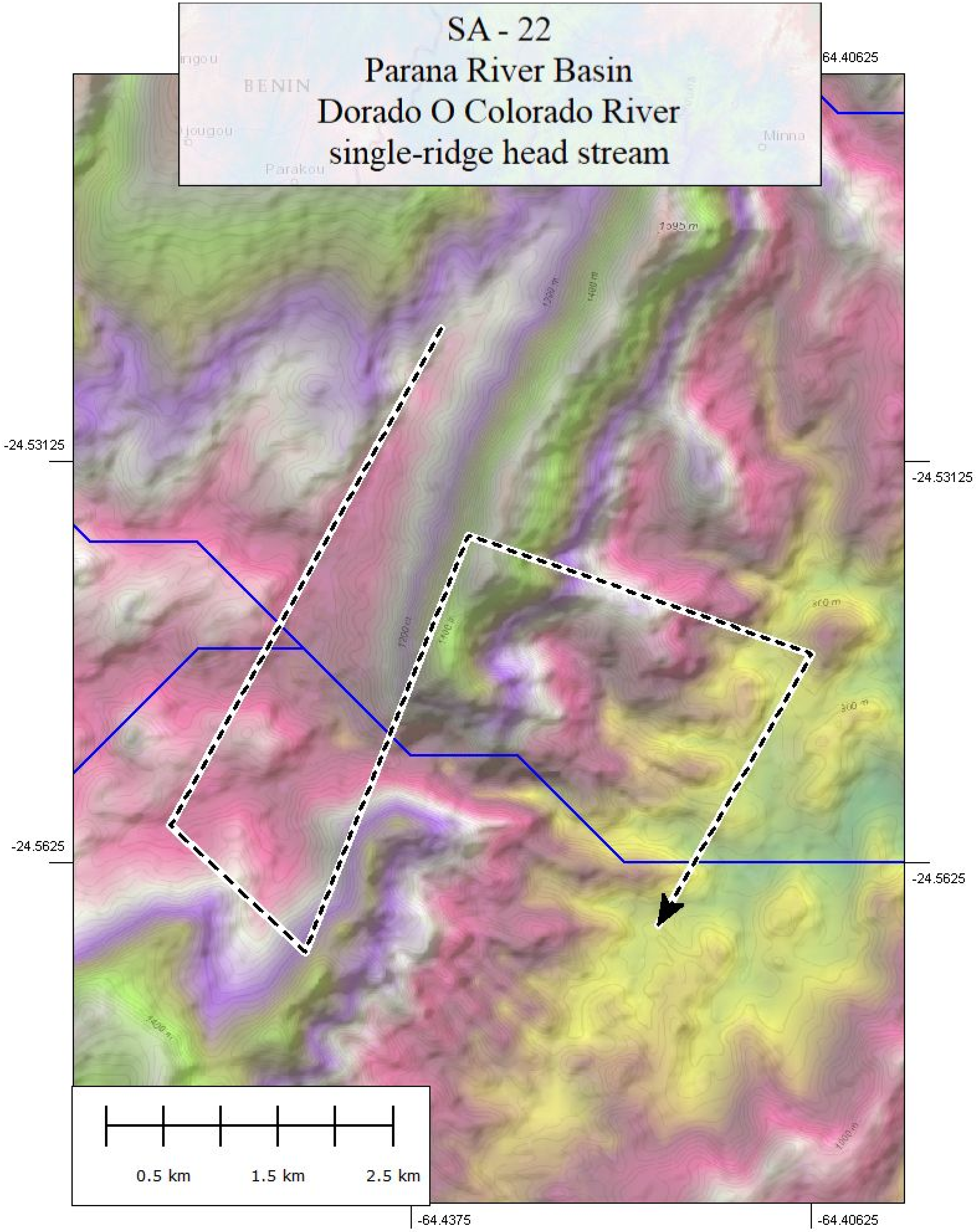

SA - 22
Parana River Basin
Dorado O Colorado River
single-ridge head stream
-64.40625
-24.53125
-24.53125
-24.5625
-24.5625
-64.4375
-64.40625
0.5 km
1.5 km
2.5 km

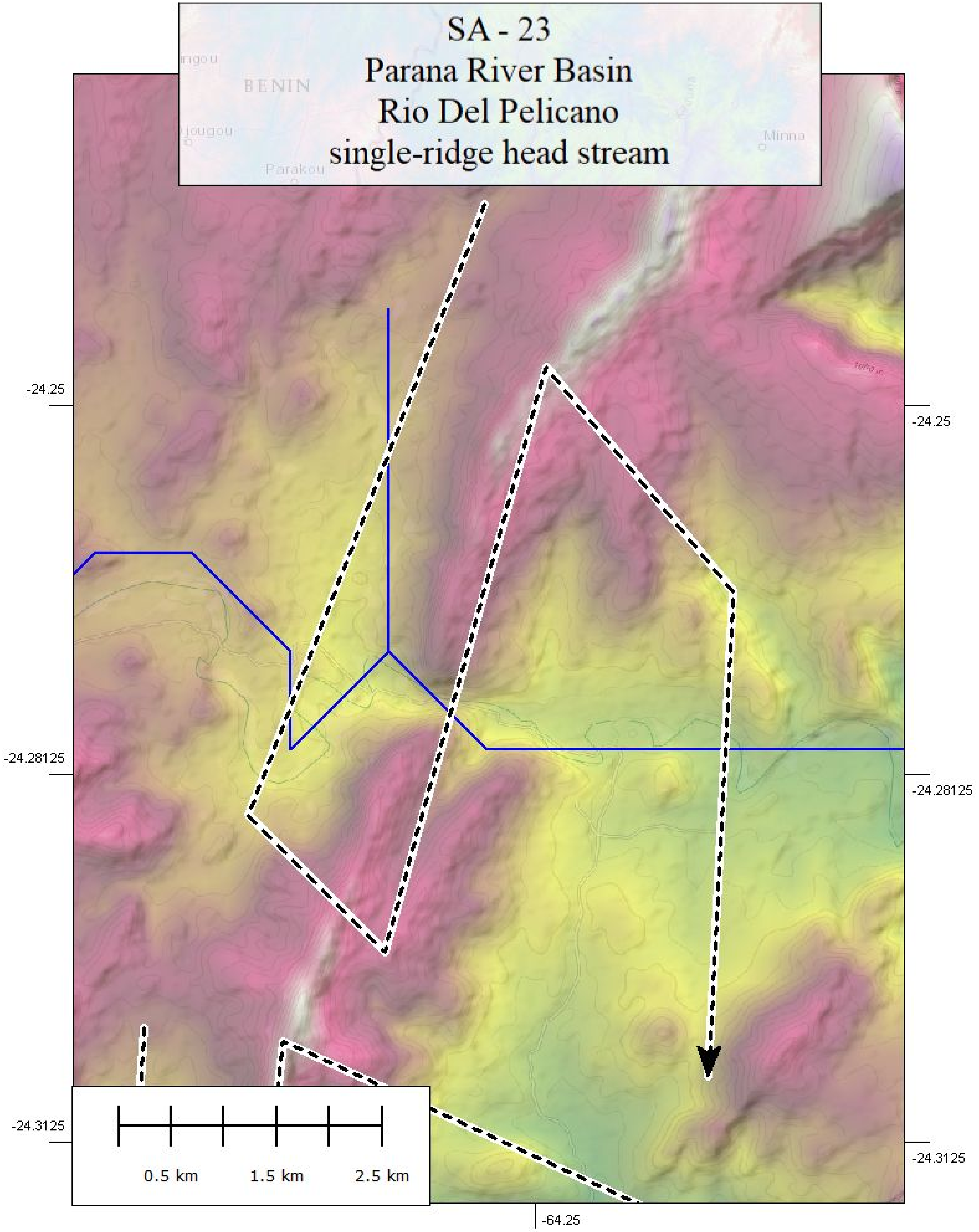
SA - 23
Parana River Basin
Rio Del Pelicano
single-ridge head stream
-24.25
-24.28125
-24.3125
-64.25
0.5 km
1.5 km
2.5 km

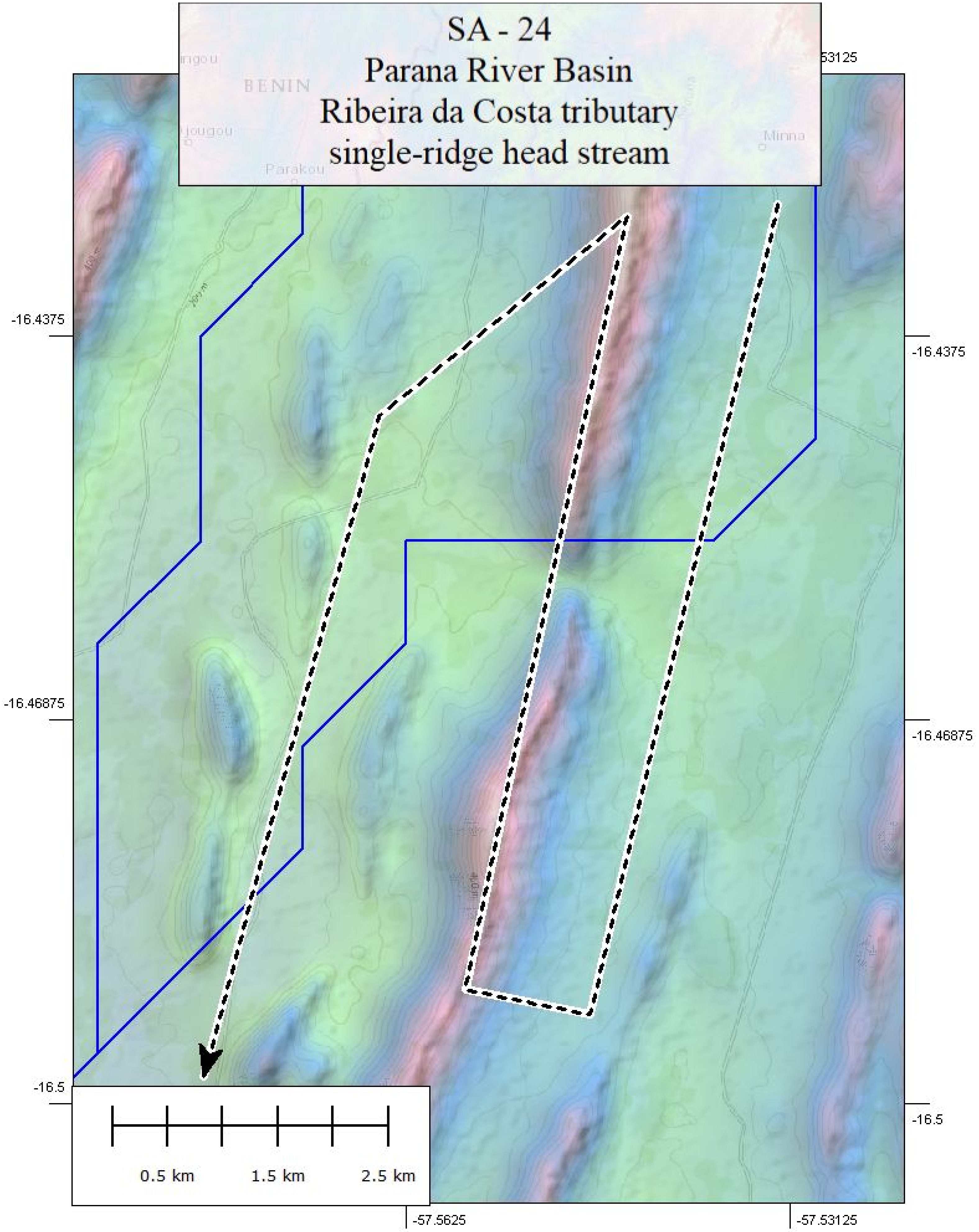
SA - 24
Parana River Basin
Ribeira da Costa tributary
single-ridge head stream
BENIN
Parakou
Minna
-16.4375
-16.4375
-16.46875
-16.46875
-16.5
-16.5
0.5 km
1.5 km
2.5 km
-57.5625
-57.53125

SA - 25
Amazon River Basin
Madre de Dios River tributary
single-ridge head stream

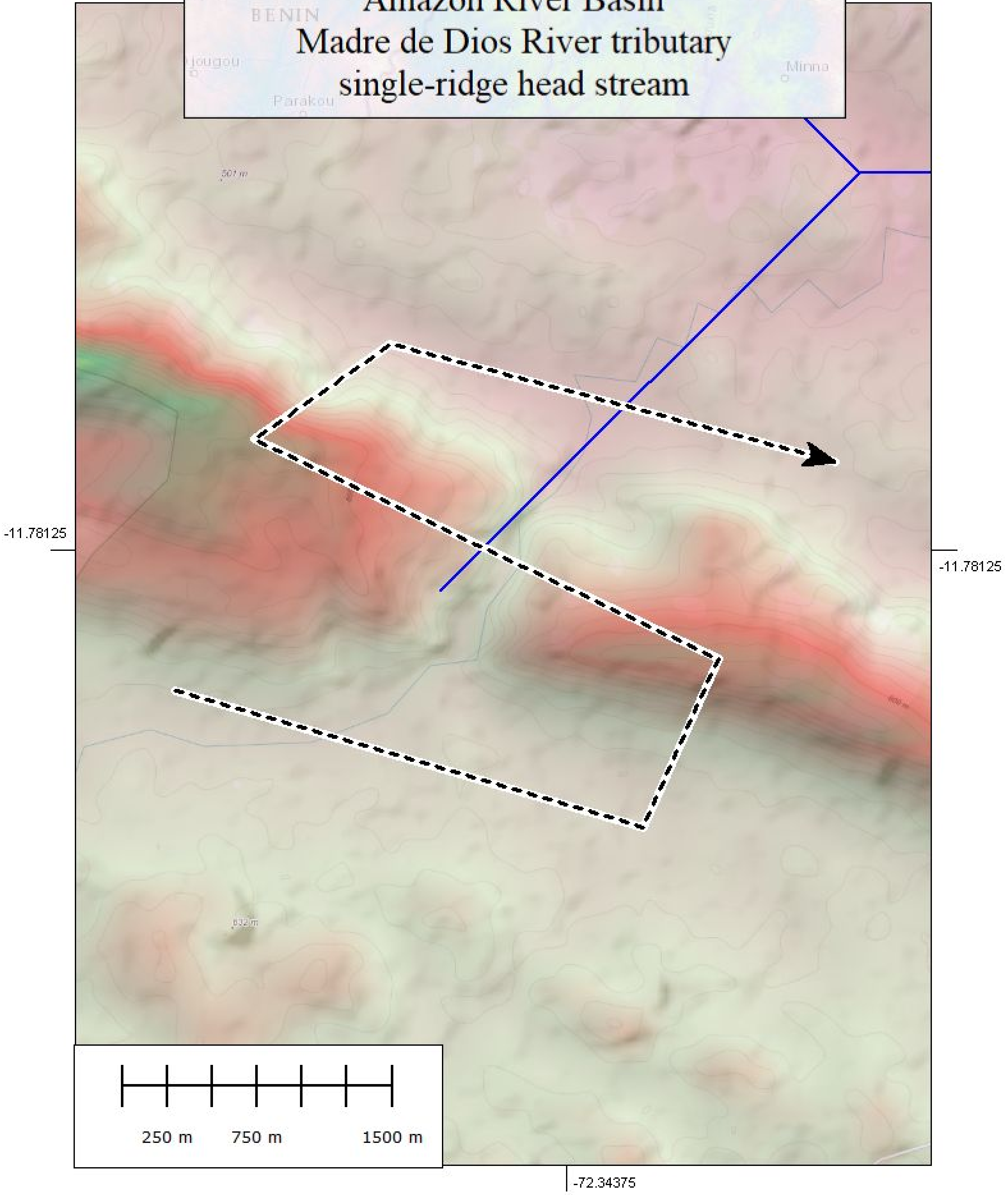

SA - 26
Parana River Basin
Quadalquivir River tributary
single-ridge head stream

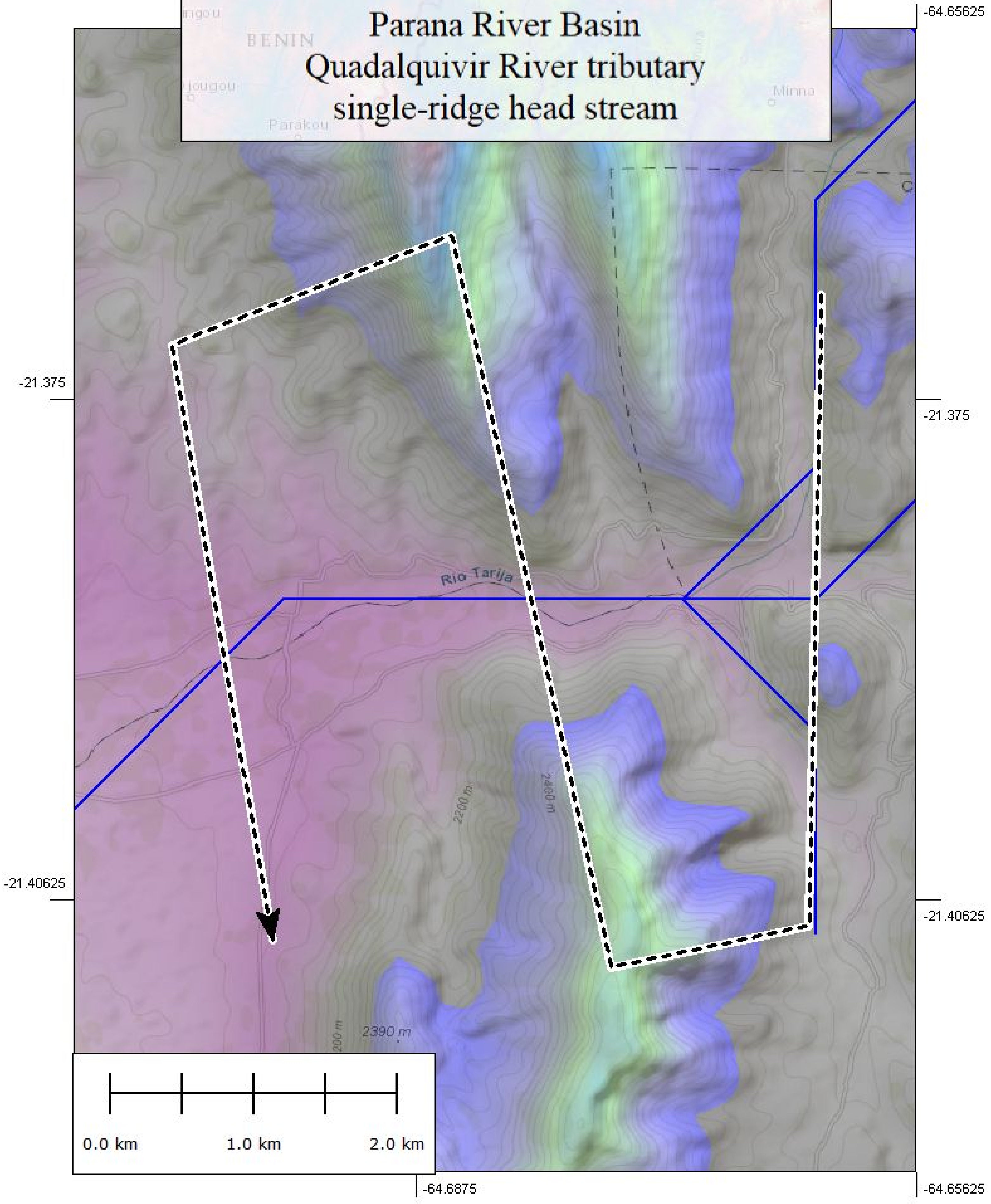

SA - 27
Amazon River Basin
Morono RIver tributary
single-ridge head stream

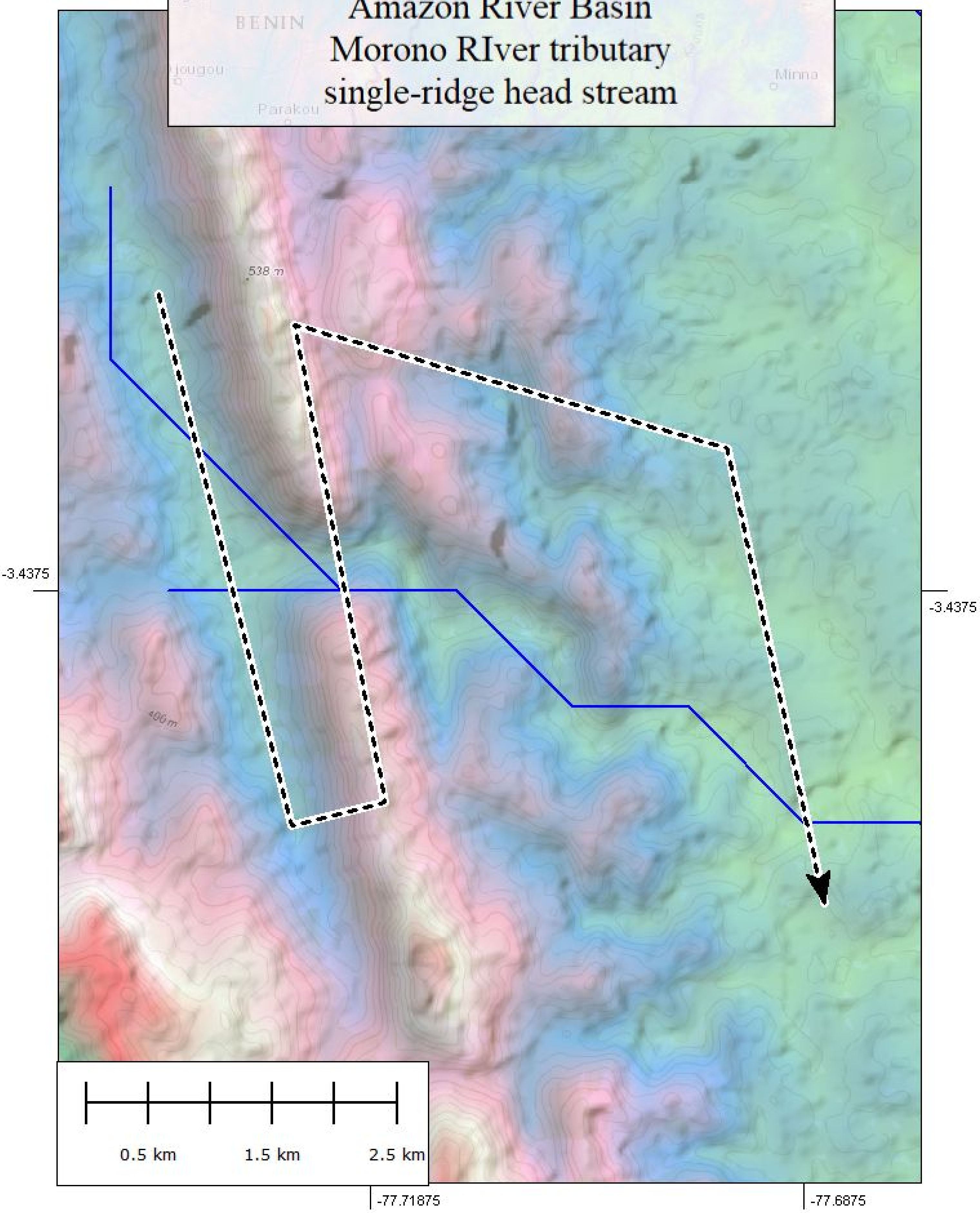

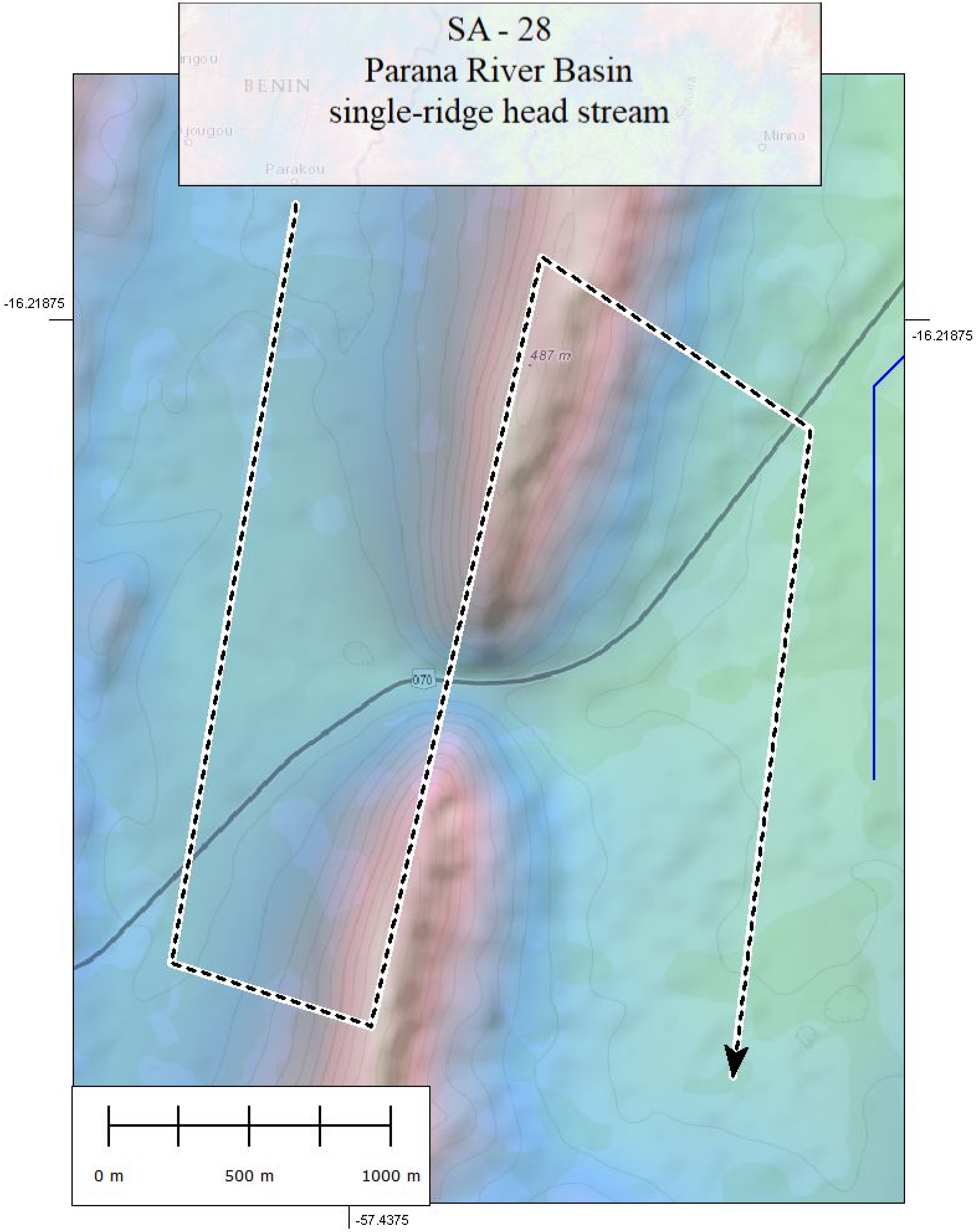
SA - 28
Parana River Basin
single-ridge head stream
BENIN
Djougou
Parakou
Minna
-16.21875
-16.21875
487 m
070
0 m
500 m
1000 m
-57.4375

SA - 29
Mar Pequeno Basin
Mar Pequeno tributary
single-ridge head stream

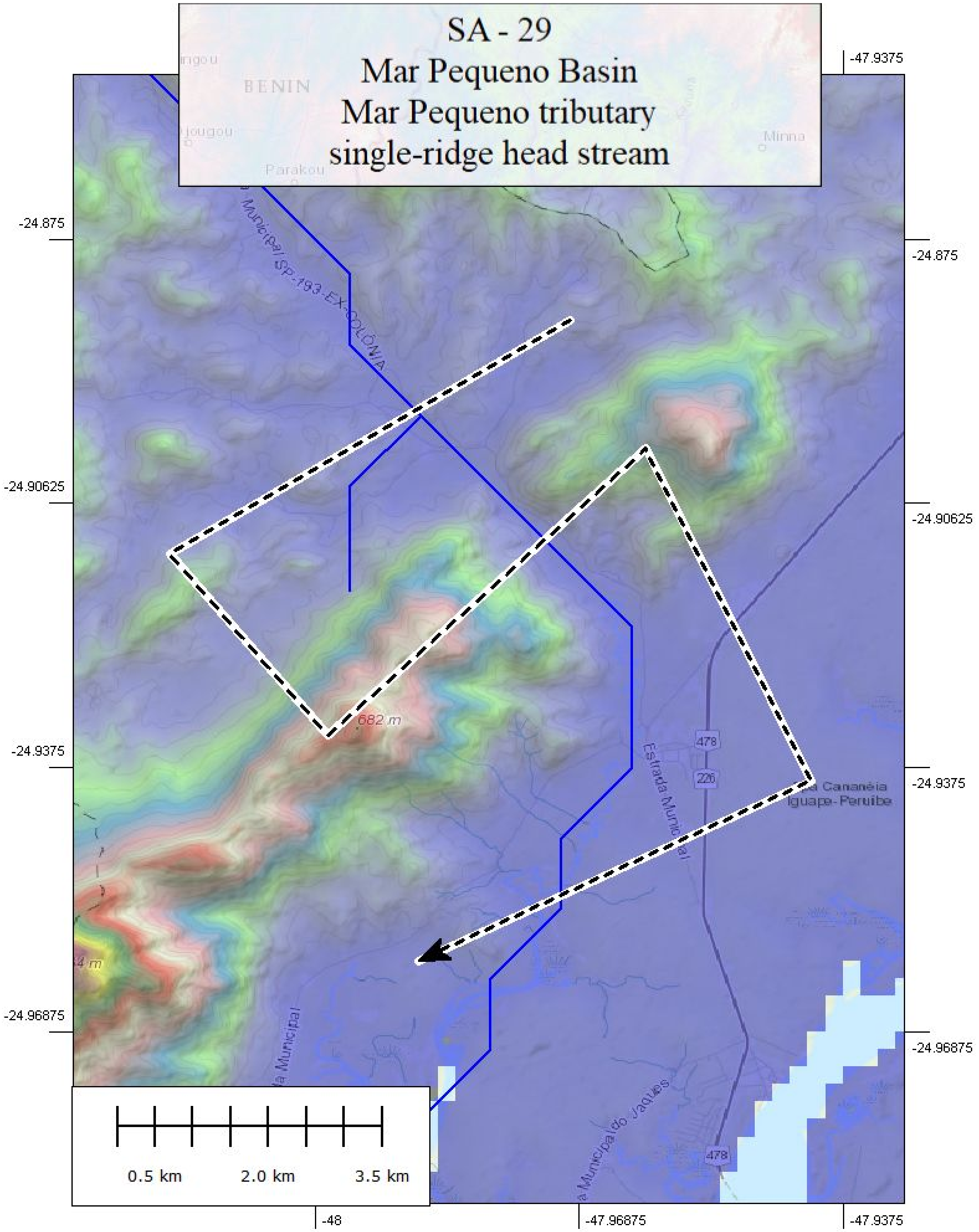

SA - 30
Amazon River Basin
Igarape do Bom Jardim tributary
single-ridge head stream

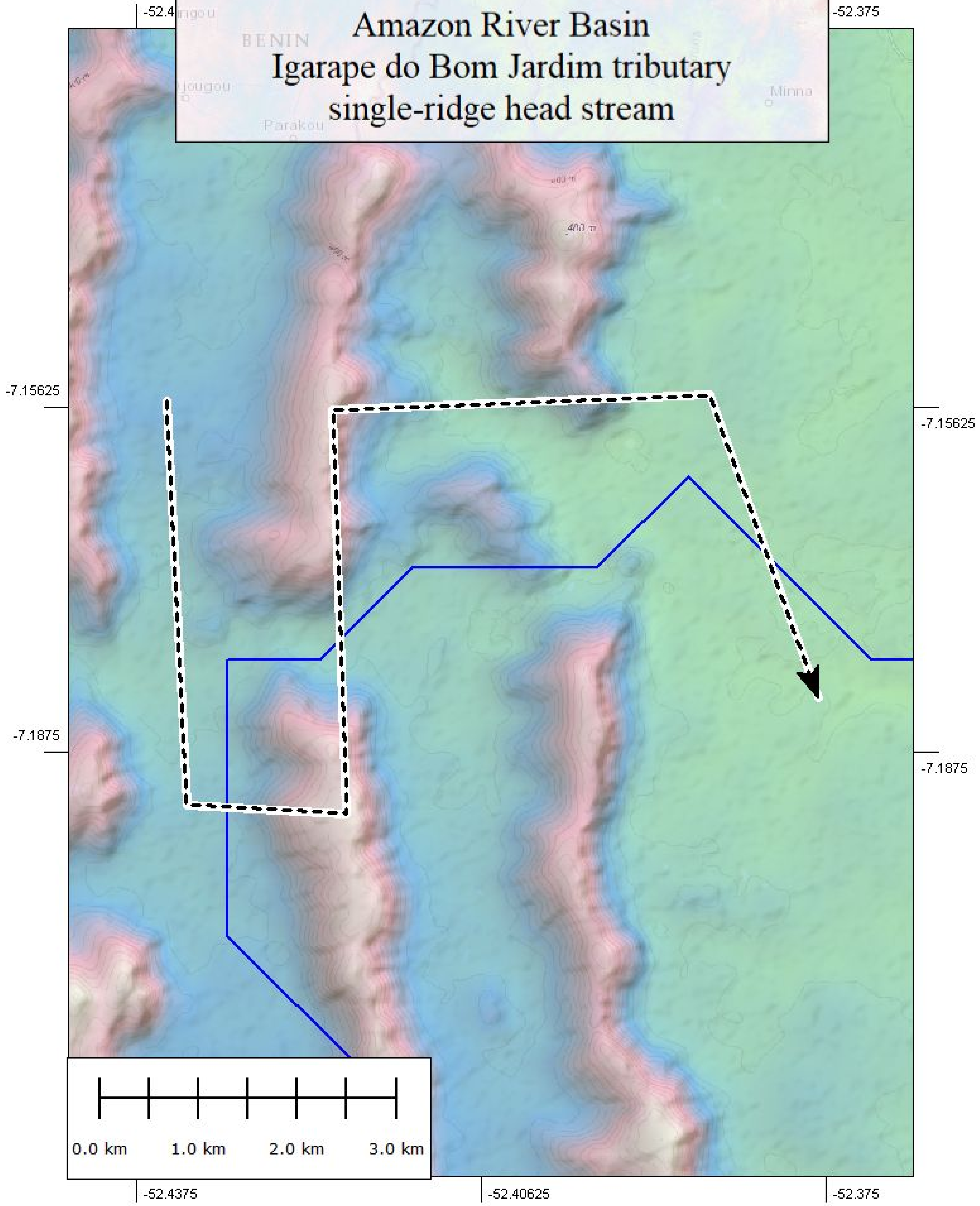

SA - 31
Orinoco River Basin
Paragua River tributary
single-ridge head stream

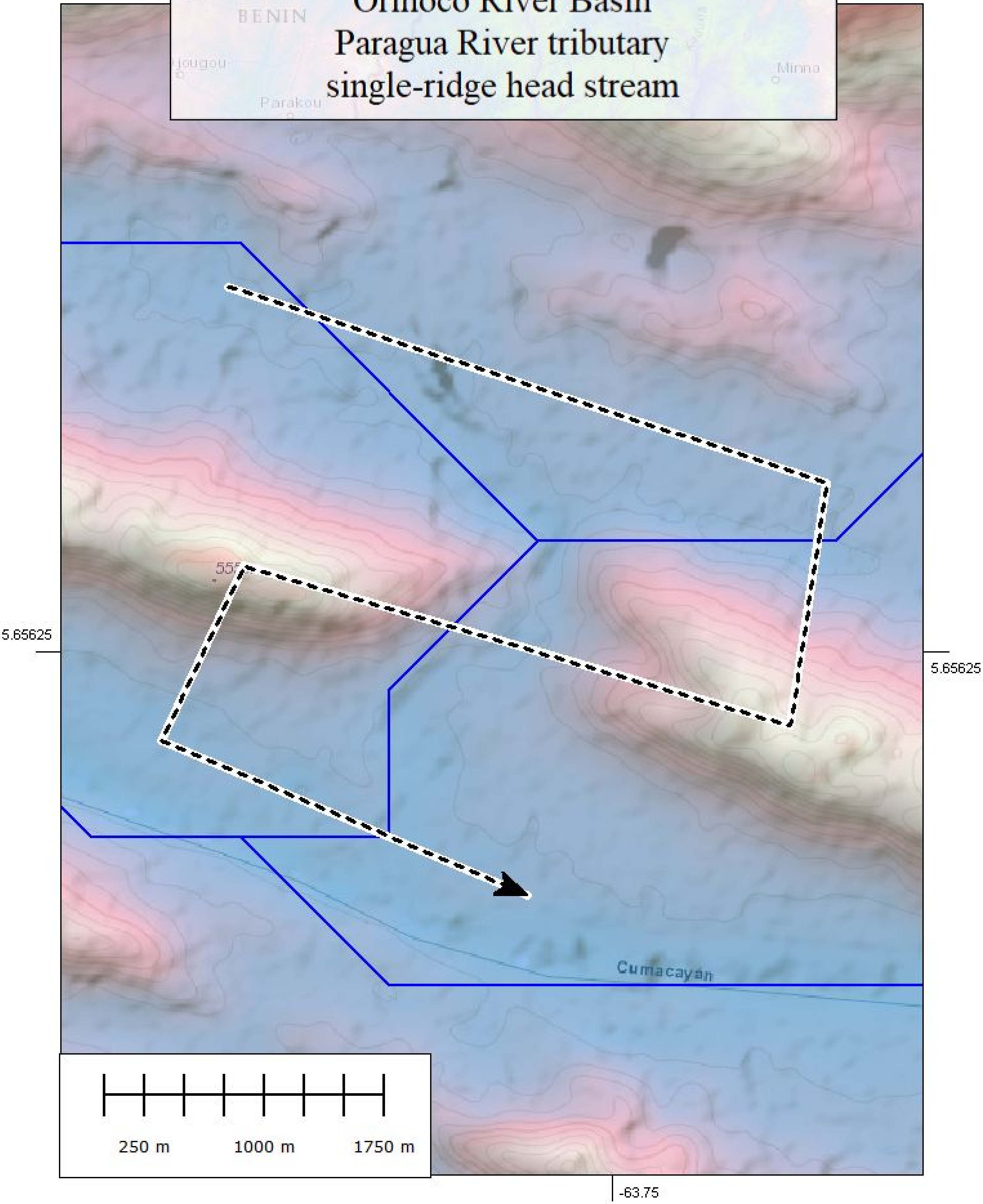

SA - 32
Rio Colorado (South America) Basin
single-ridge head stream

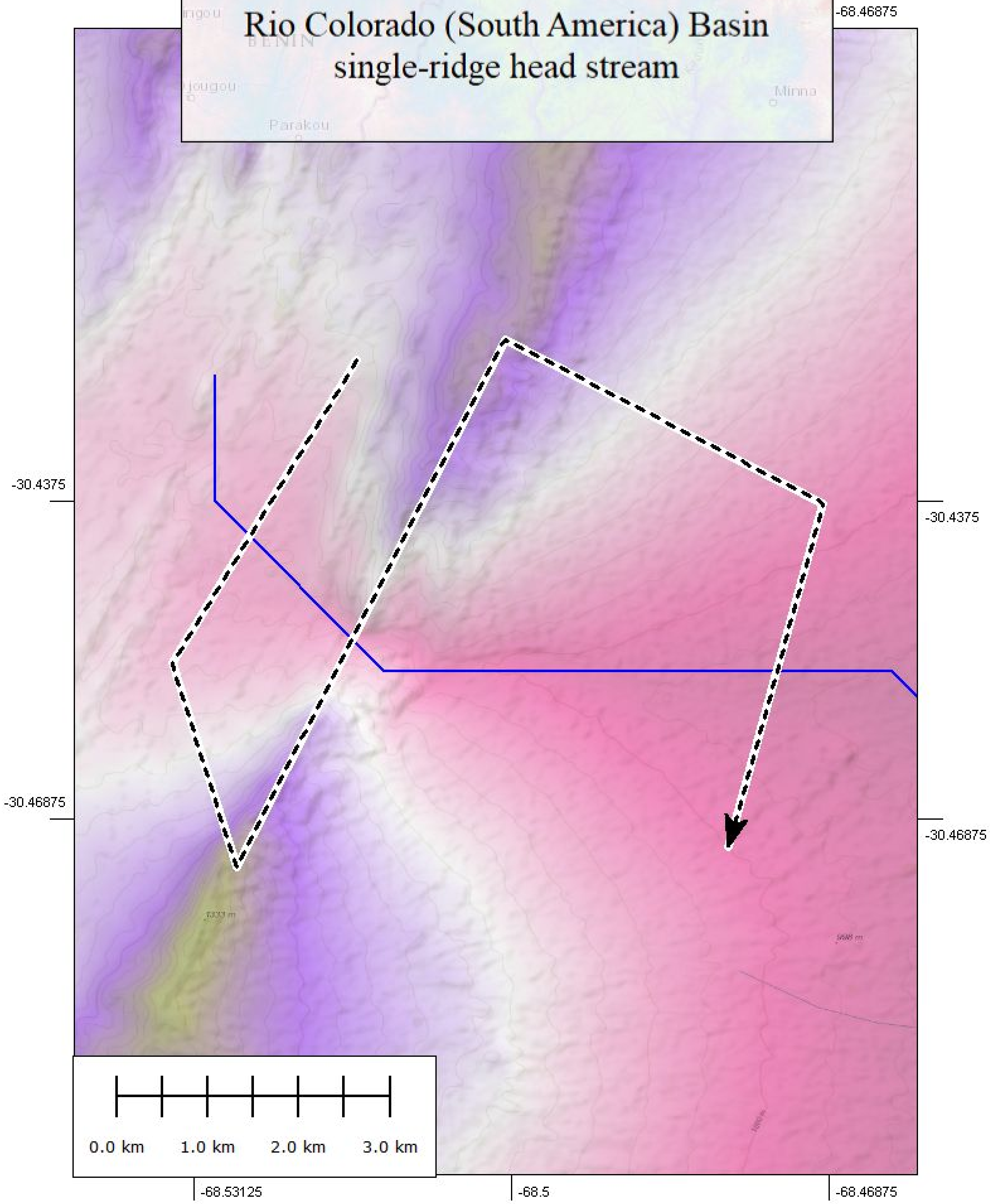

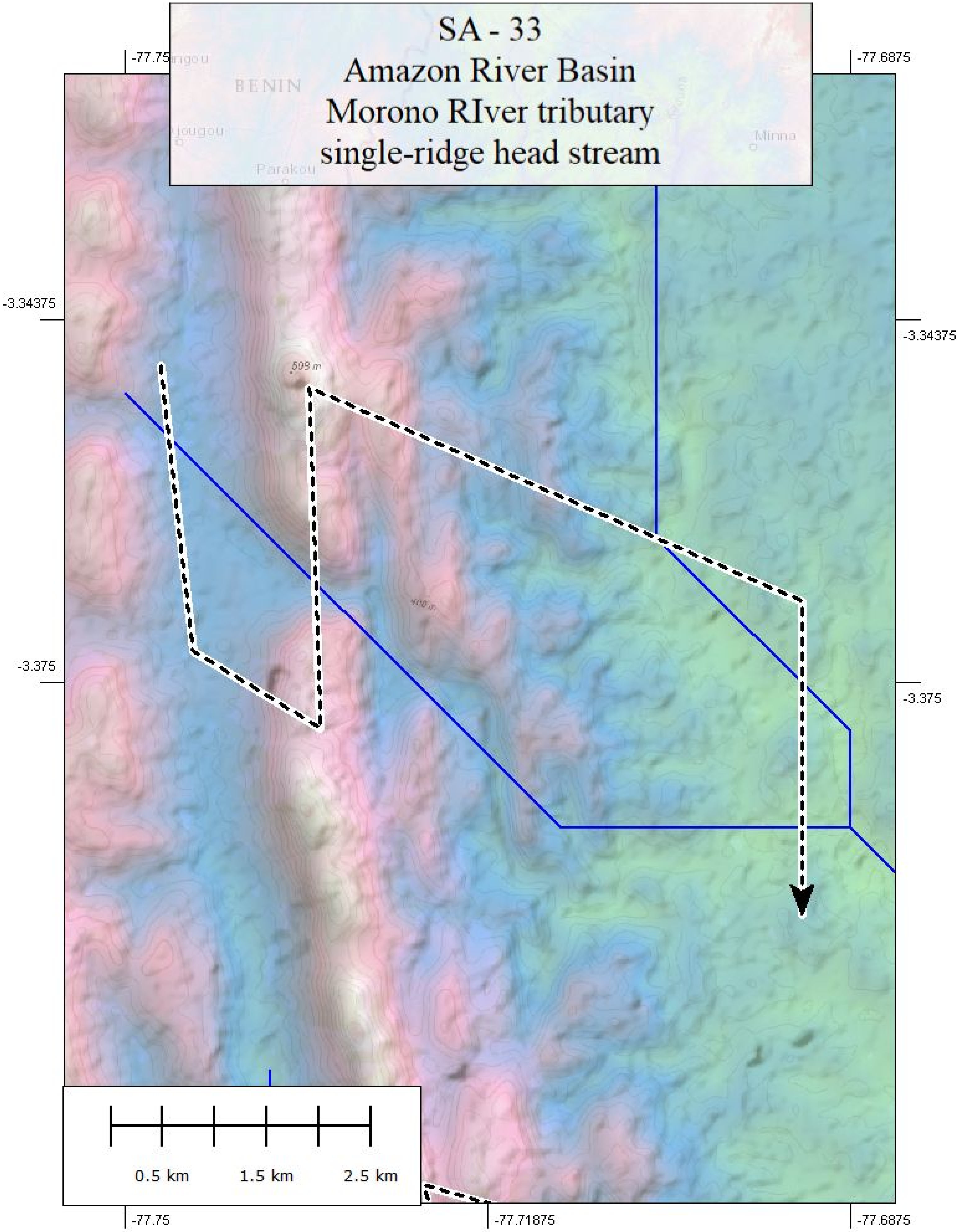
SA - 33
Amazon River Basin
Morono RIver tributary
single-ridge head stream
-77.75
-77.6875
-3.34375
-3.375
599 m
0.5 km
1.5 km
2.5 km
-77.71875

SA - 34
Amazon River Basin
Huallaga River tributary
single-ridge head stream

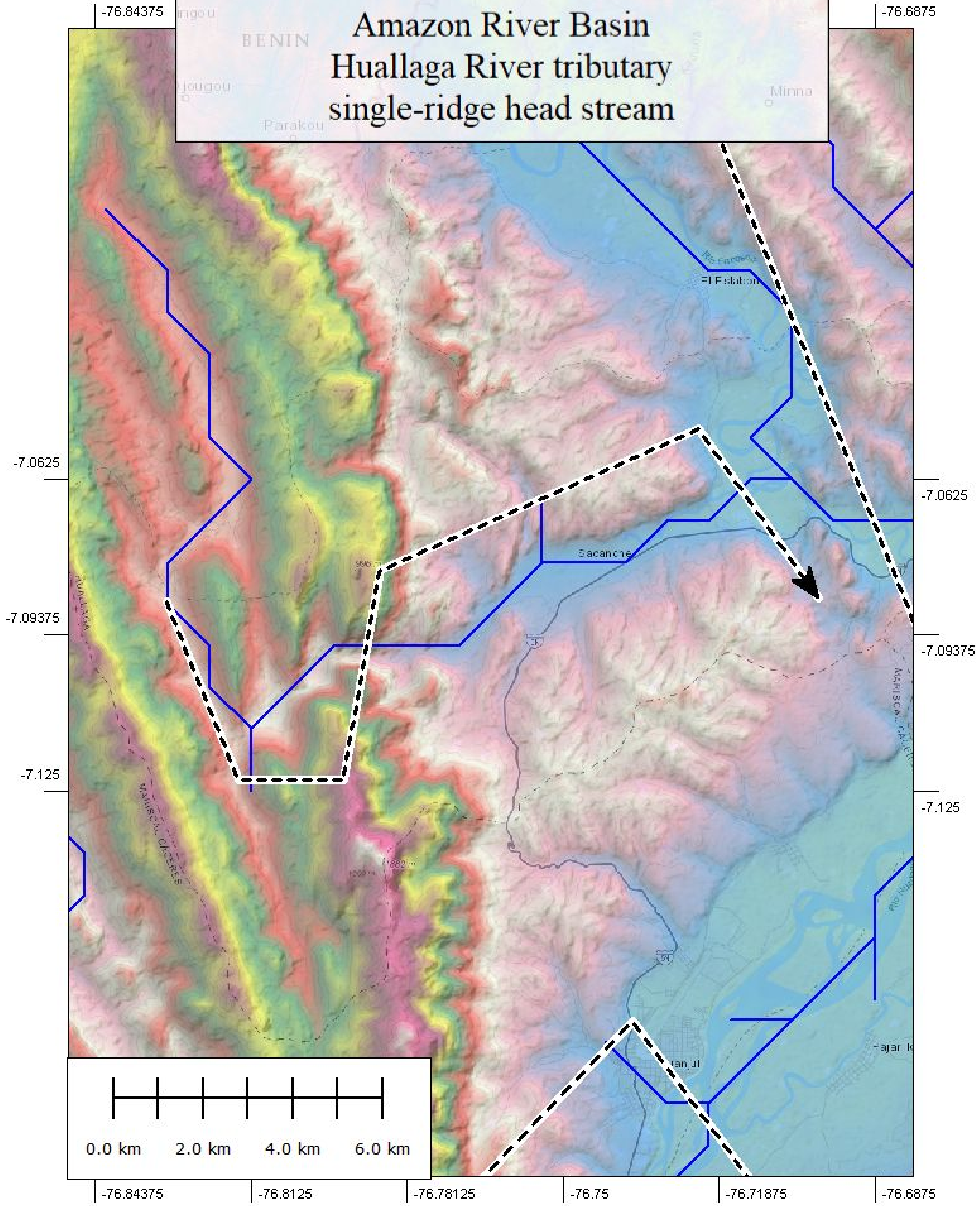

SA - 35
Rio Magdalena Basin
Cane River
single-ridge head stream

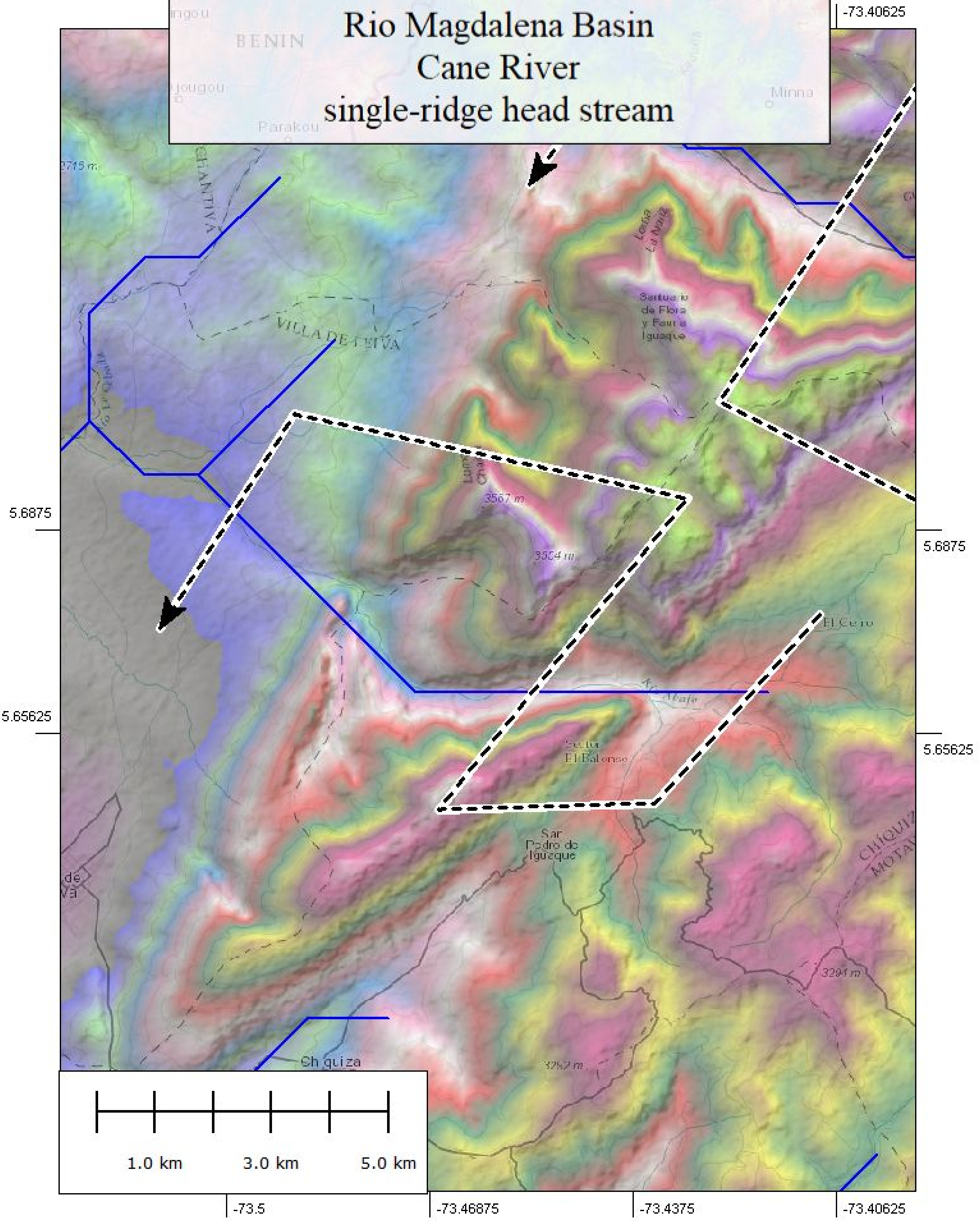

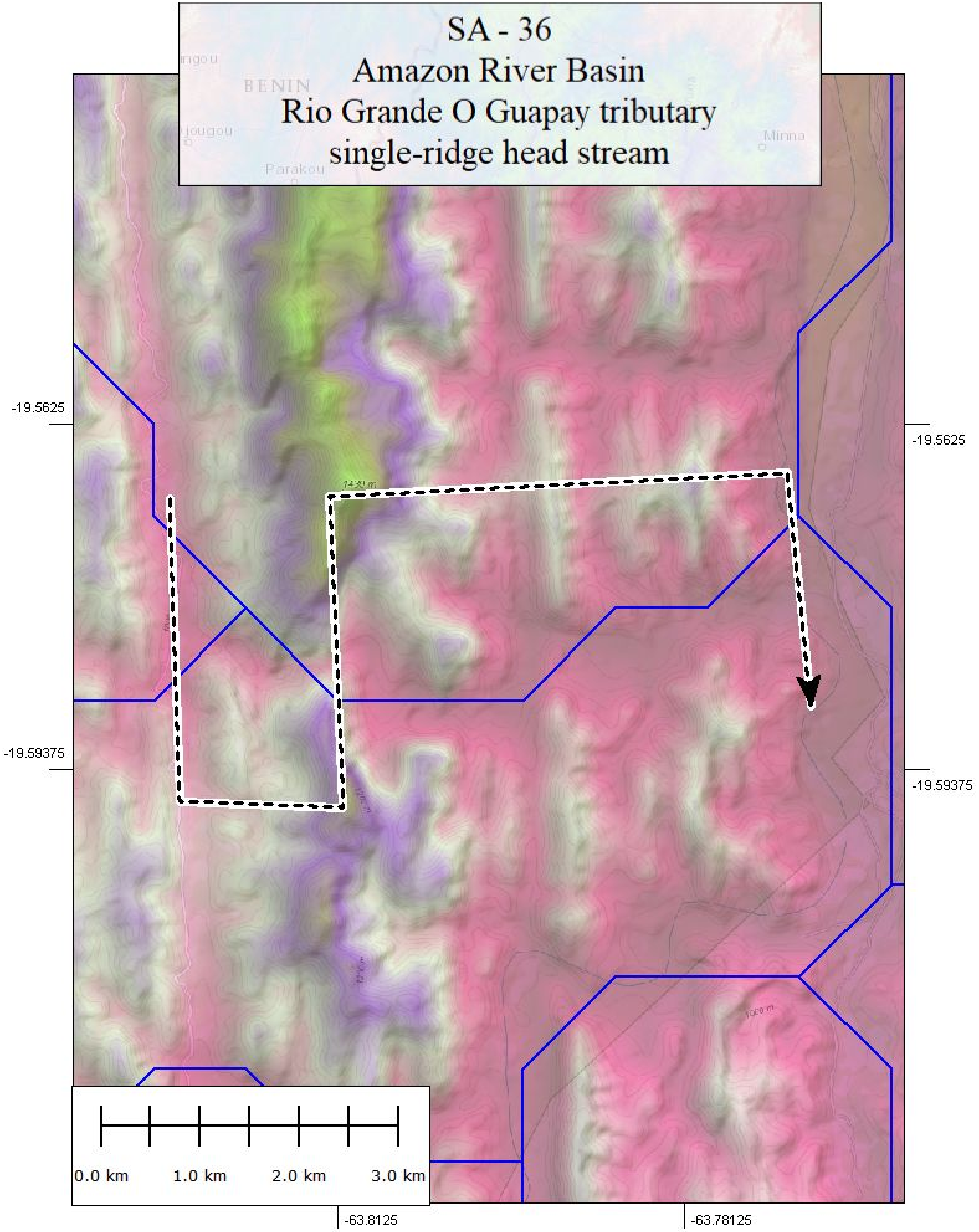
SA - 36
Amazon River Basin
Rio Grande O Guapay tributary
single-ridge head stream
BENIN
Parakou
-19.5625
-19.5625
-19.59375
-19.59375
0.0 km
1.0 km
2.0 km
3.0 km
-63.8125
-63.78125

SA - 38
Parana River Basin
Corrego Cachoeirinha
single-ridge head stream

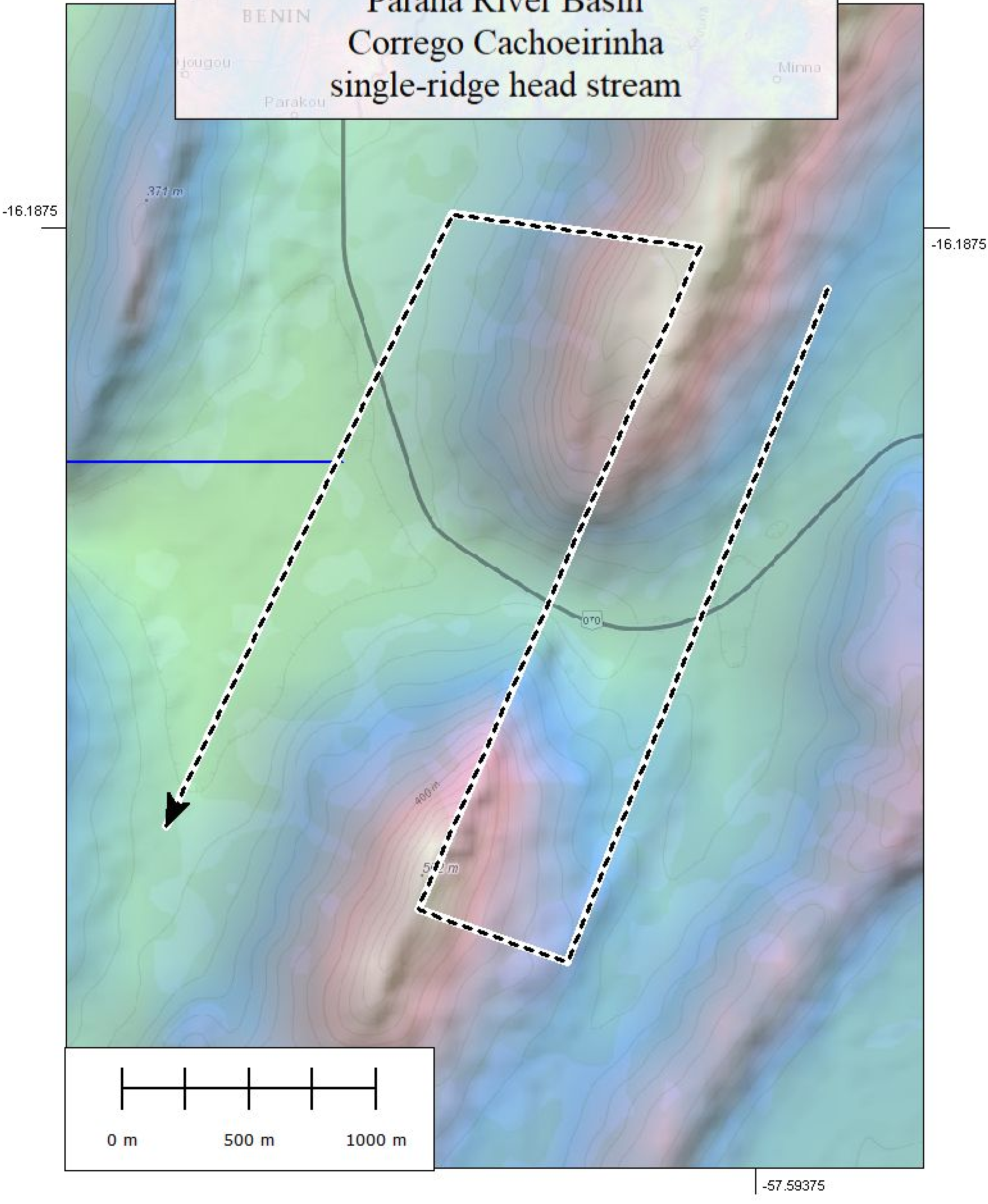

SA - 39
Amazon River Basin
Madidi River tributary
single-ridge head stream

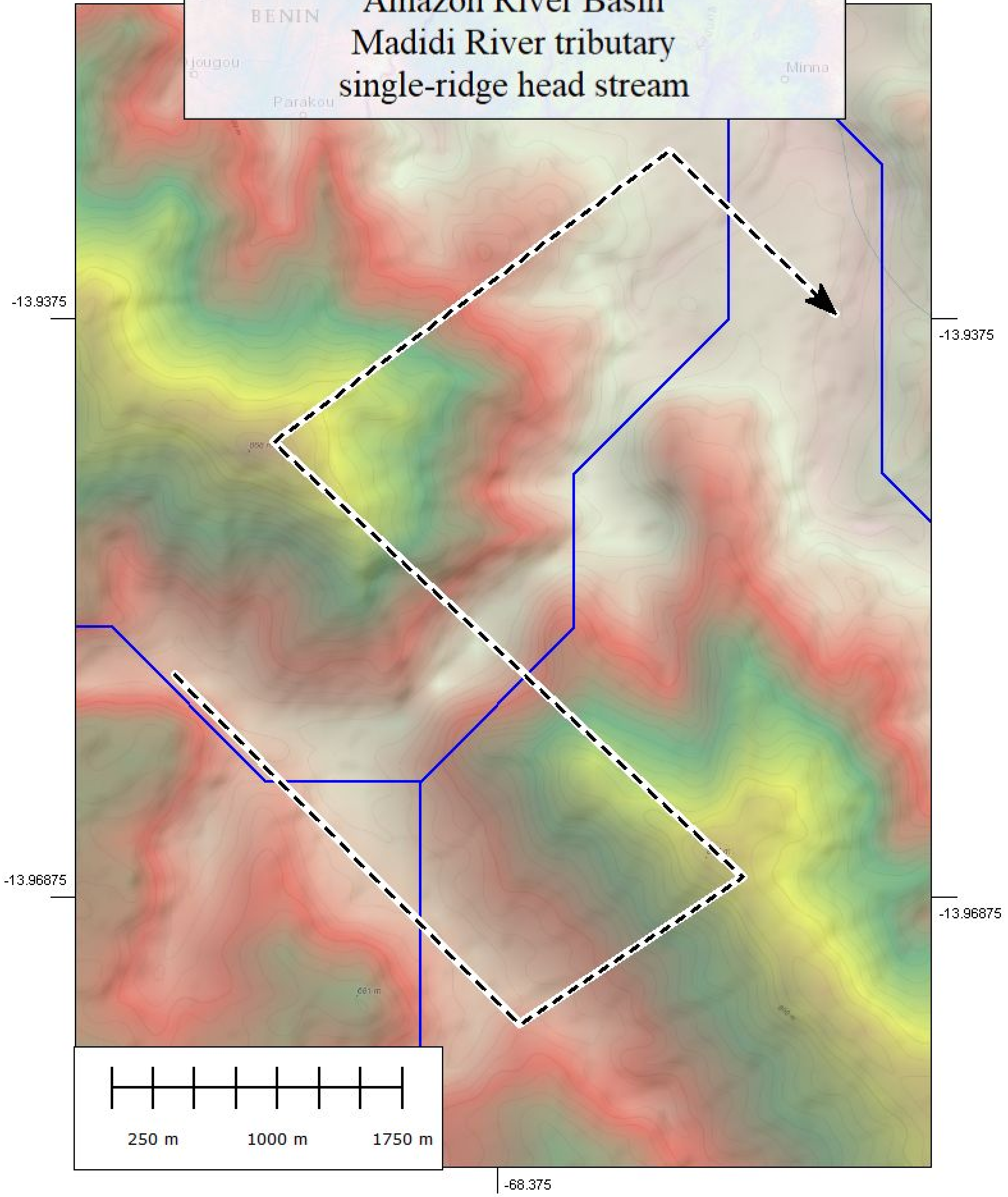

SA - 40
Amazon River Basin
Rio Grande O Guapay tributary
single-ridge head stream

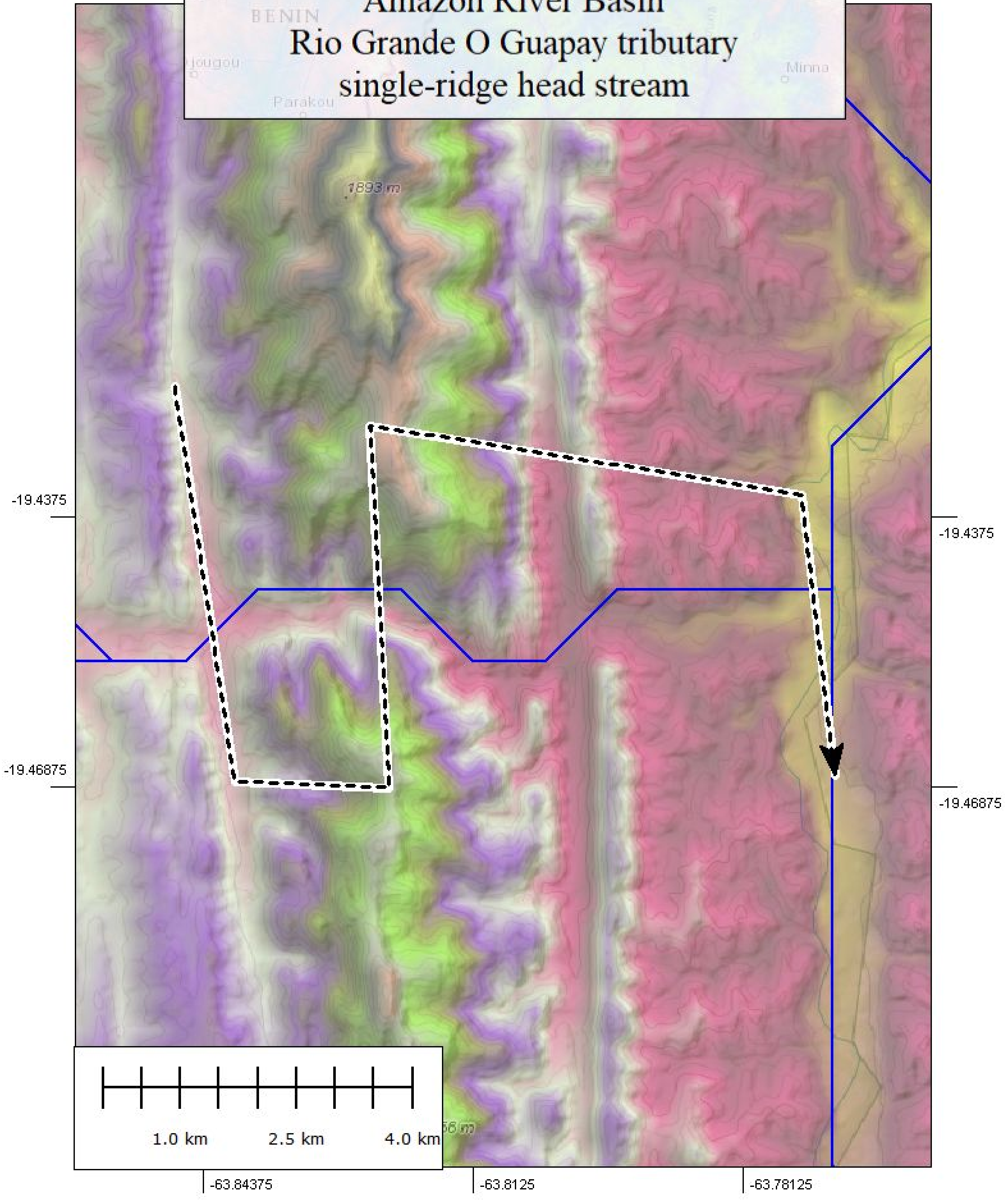

SA - 41
Amazon River Basin
Medidi River tributary
single-ridge head stream

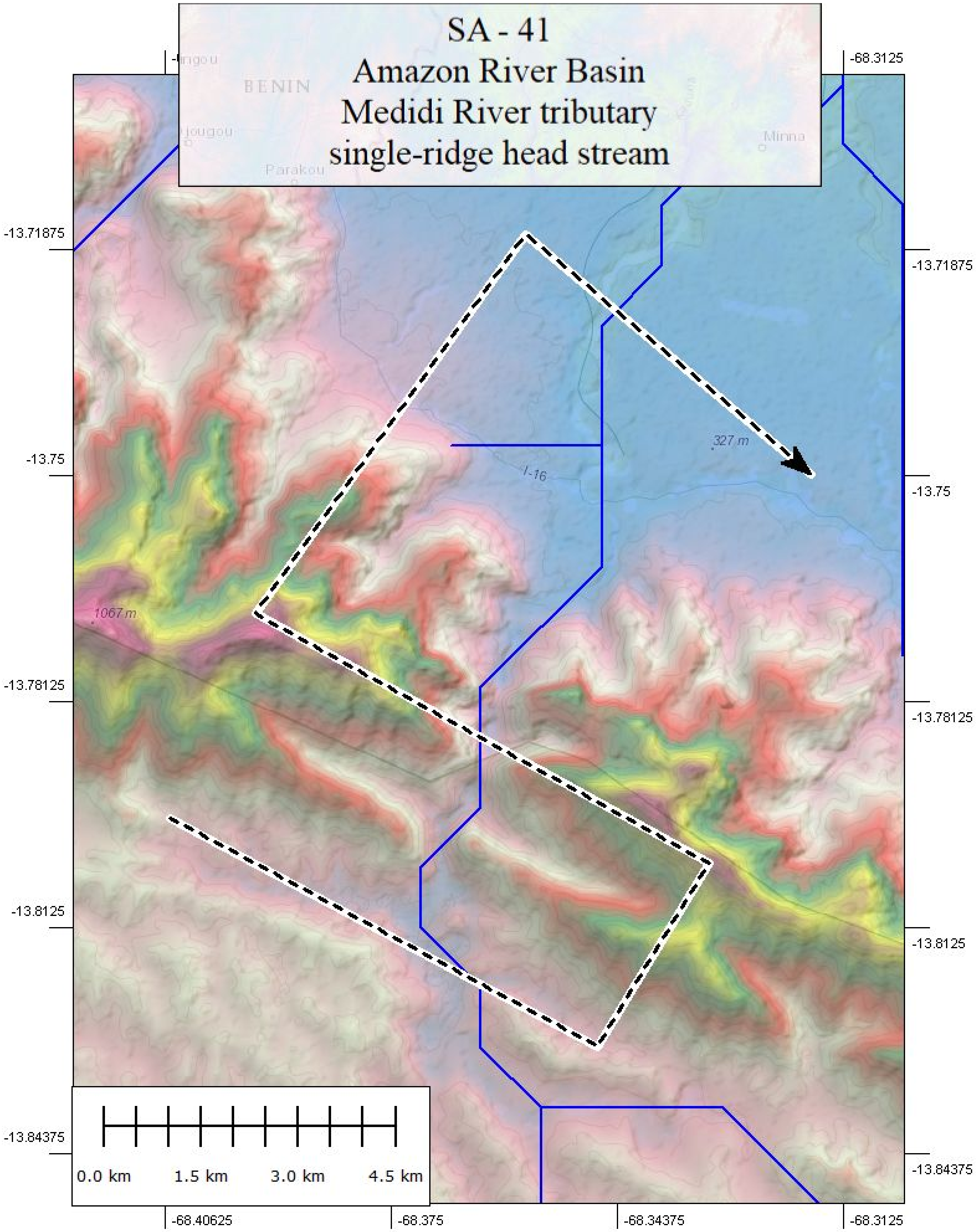

SA - 42
Amazon River Basin
single-ridge head stream

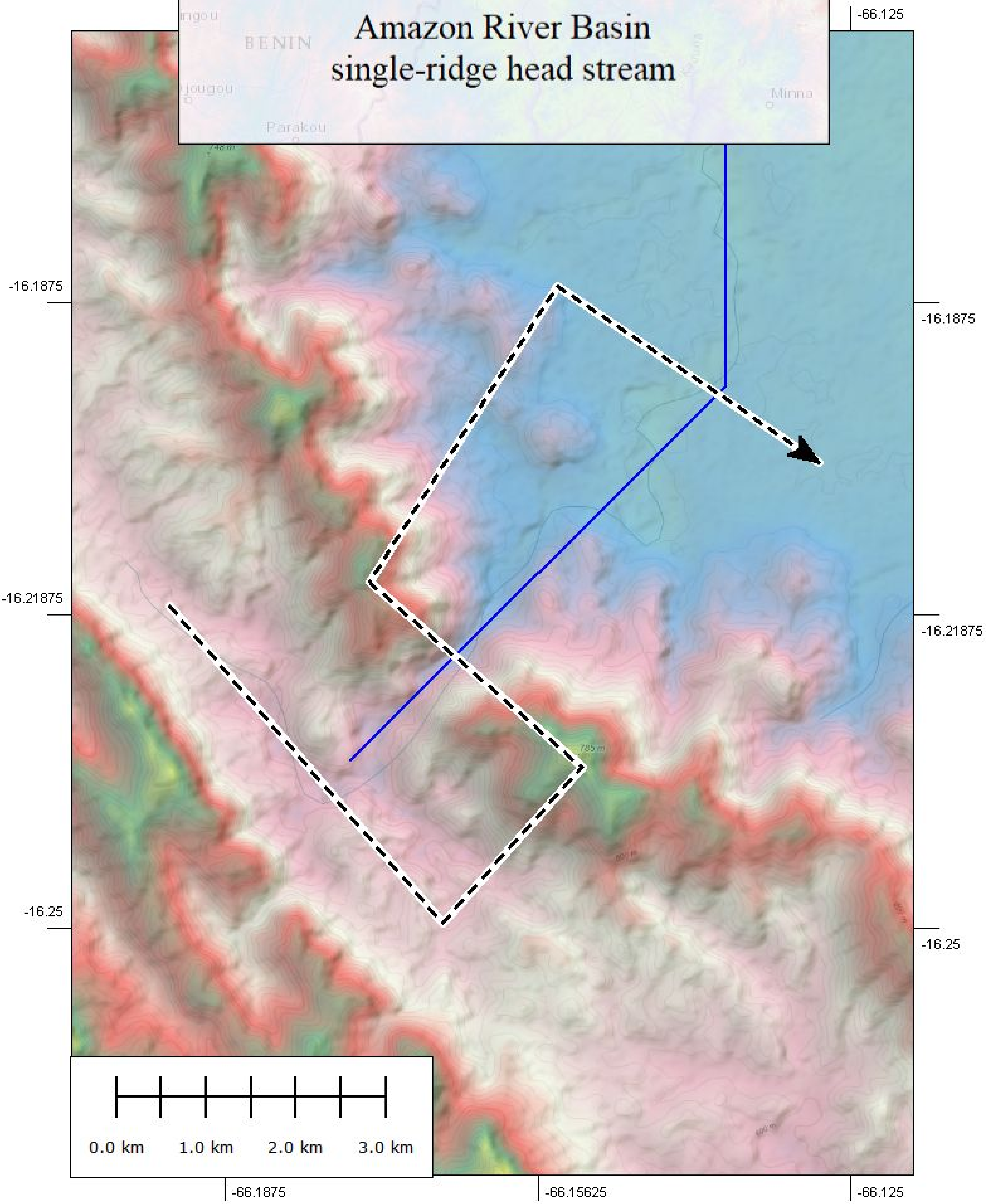

SA - 43
Amazon River Basin
single-ridge head stream

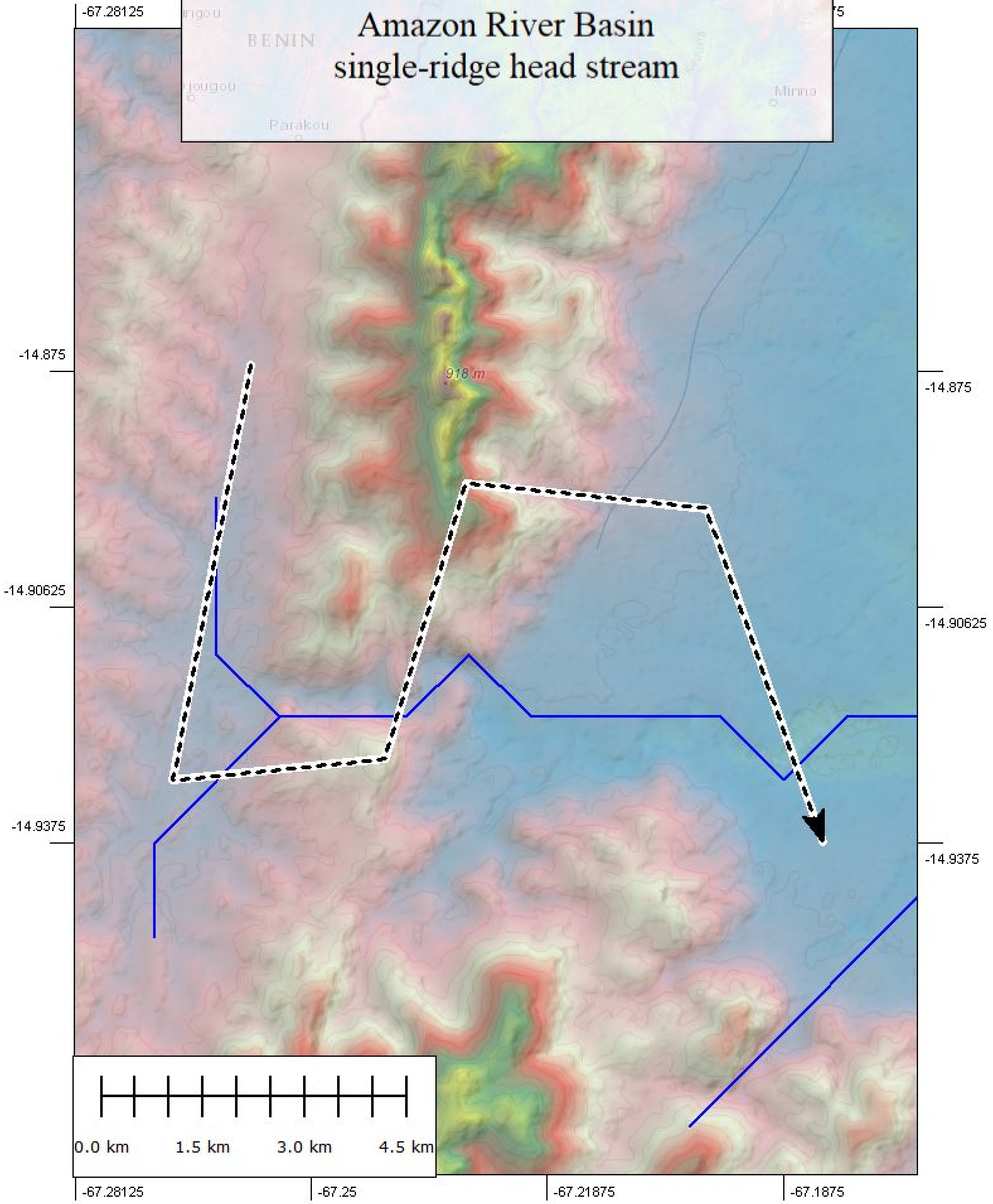

SA - 44
Amazon River Basin
Tupuani River tributary
single-ridge head stream

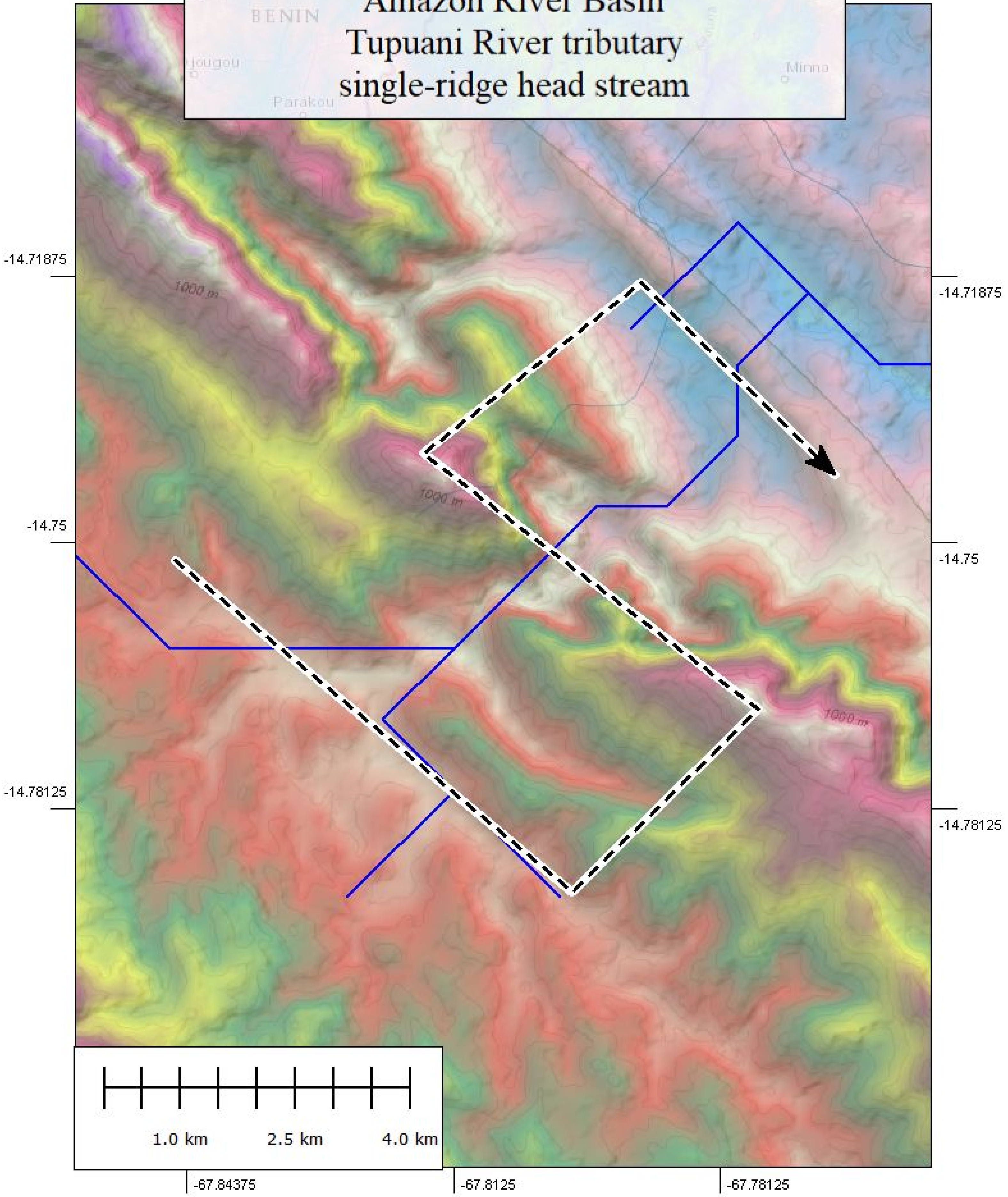

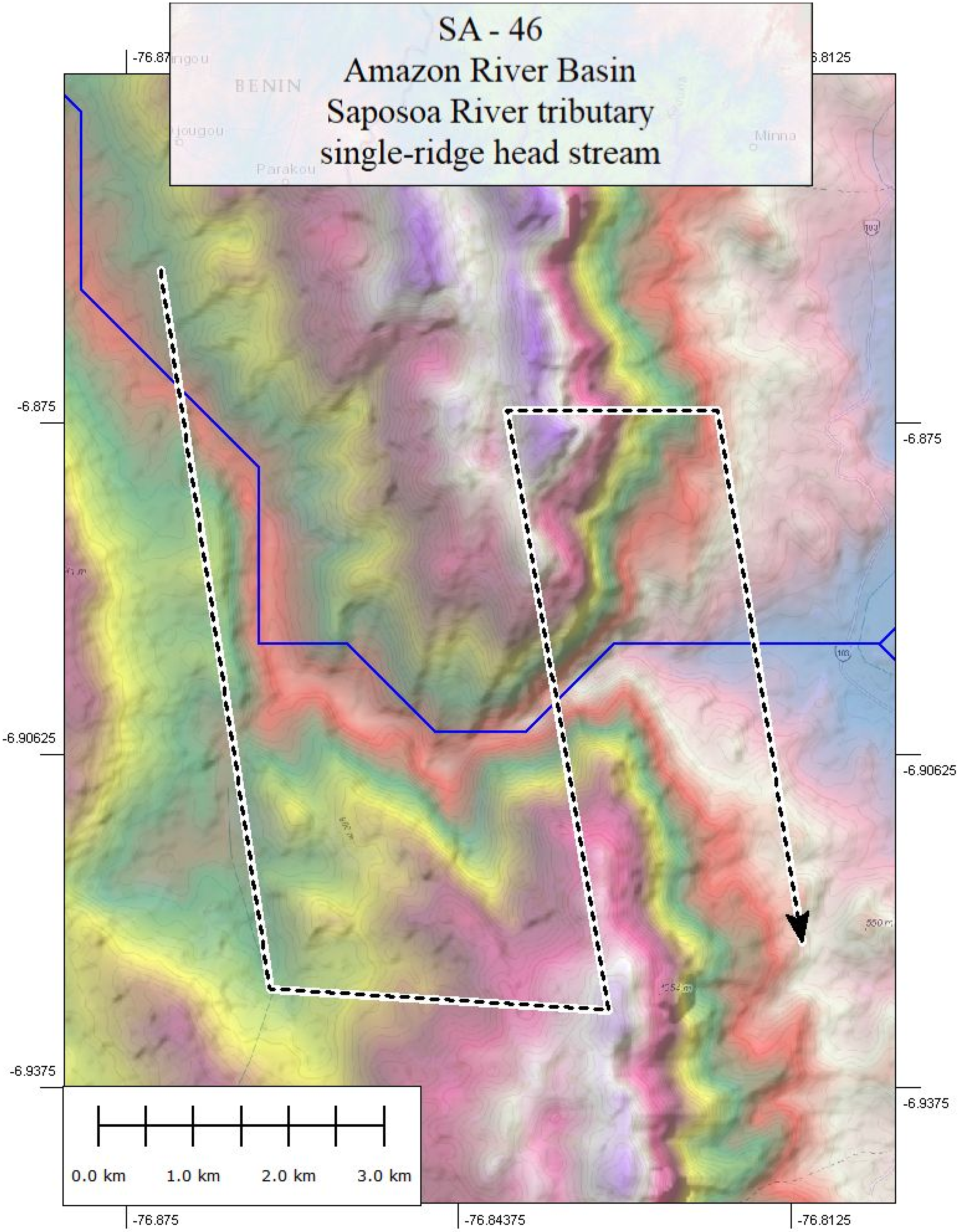
SA - 46
Amazon River Basin
Saposoa River tributary
single-ridge head stream
-76.875
-76.84375
-76.8125
-6.875
-6.90625
-6.9375
0.0 km
1.0 km
2.0 km
3.0 km
BENIN
Parakou
Minna

SA - 47
Amazon River Basin
Moranon River tributary
single-ridge head stream

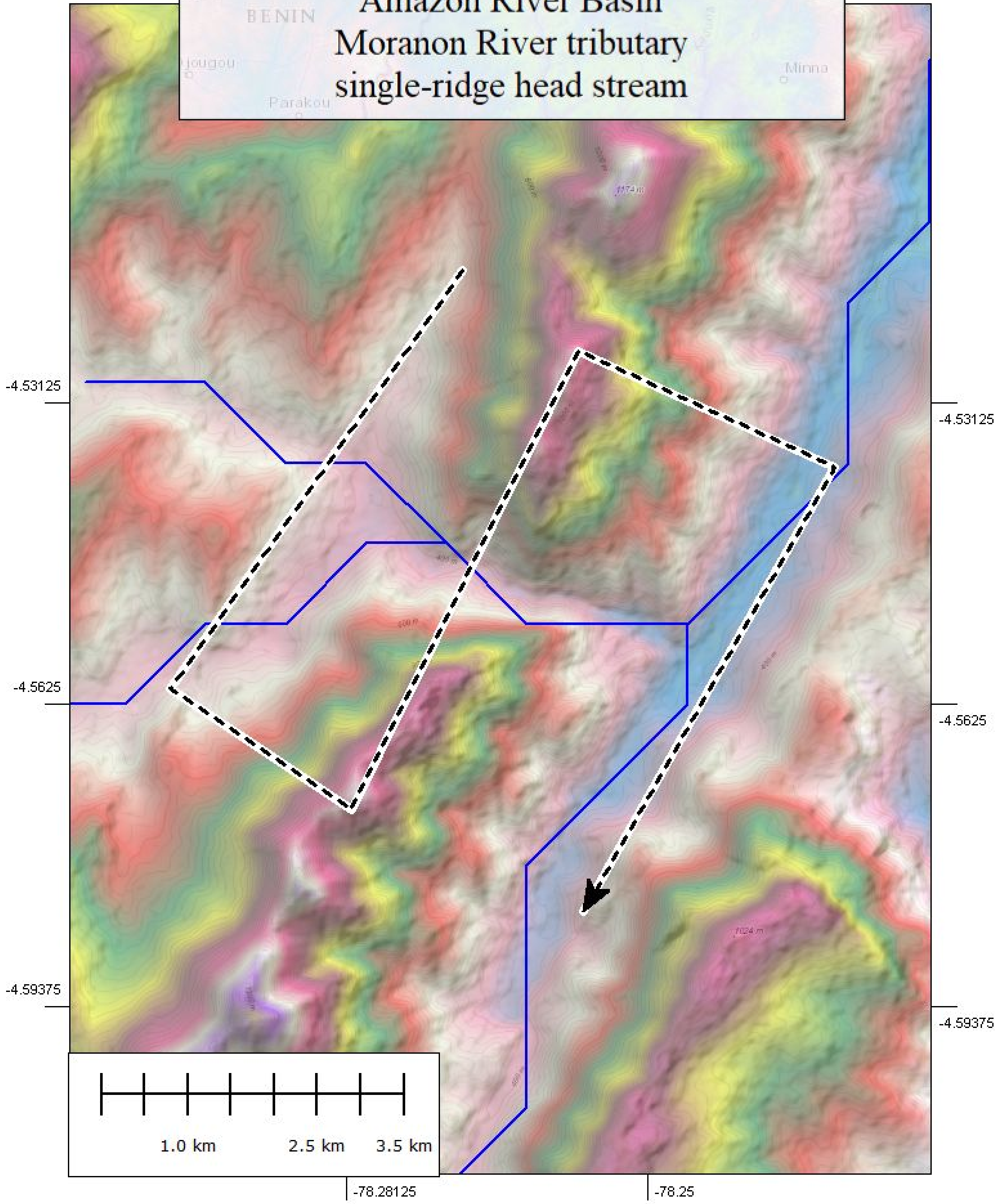

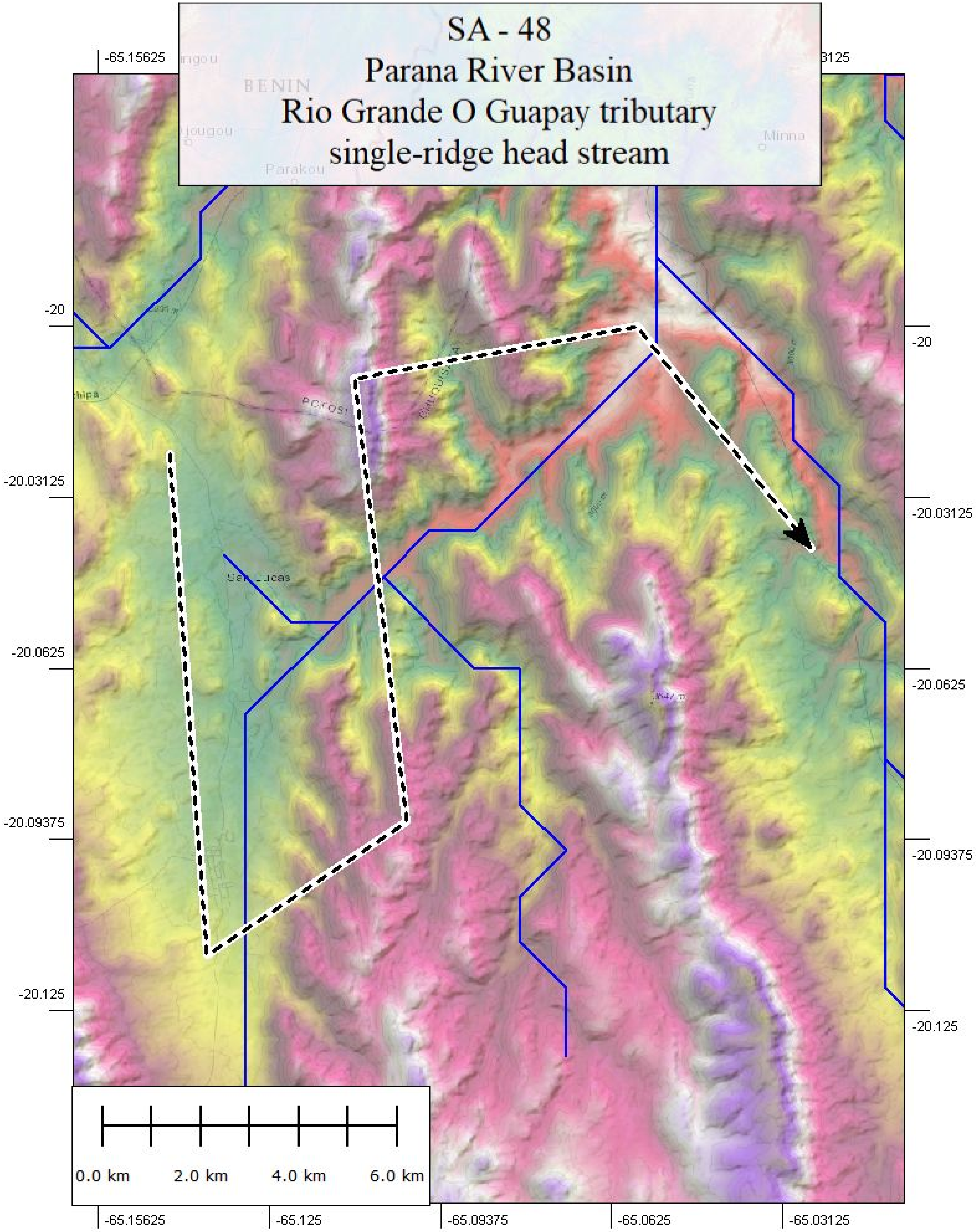
SA - 48
Parana River Basin
Rio Grande O Guapay tributary
single-ridge head stream
-65.15625
-20
-20.03125
-20.0625
-20.09375
-20.125
-65.15625
-65.125
-65.09375
-65.0625
-65.03125
0.0 km
2.0 km
4.0 km
6.0 km

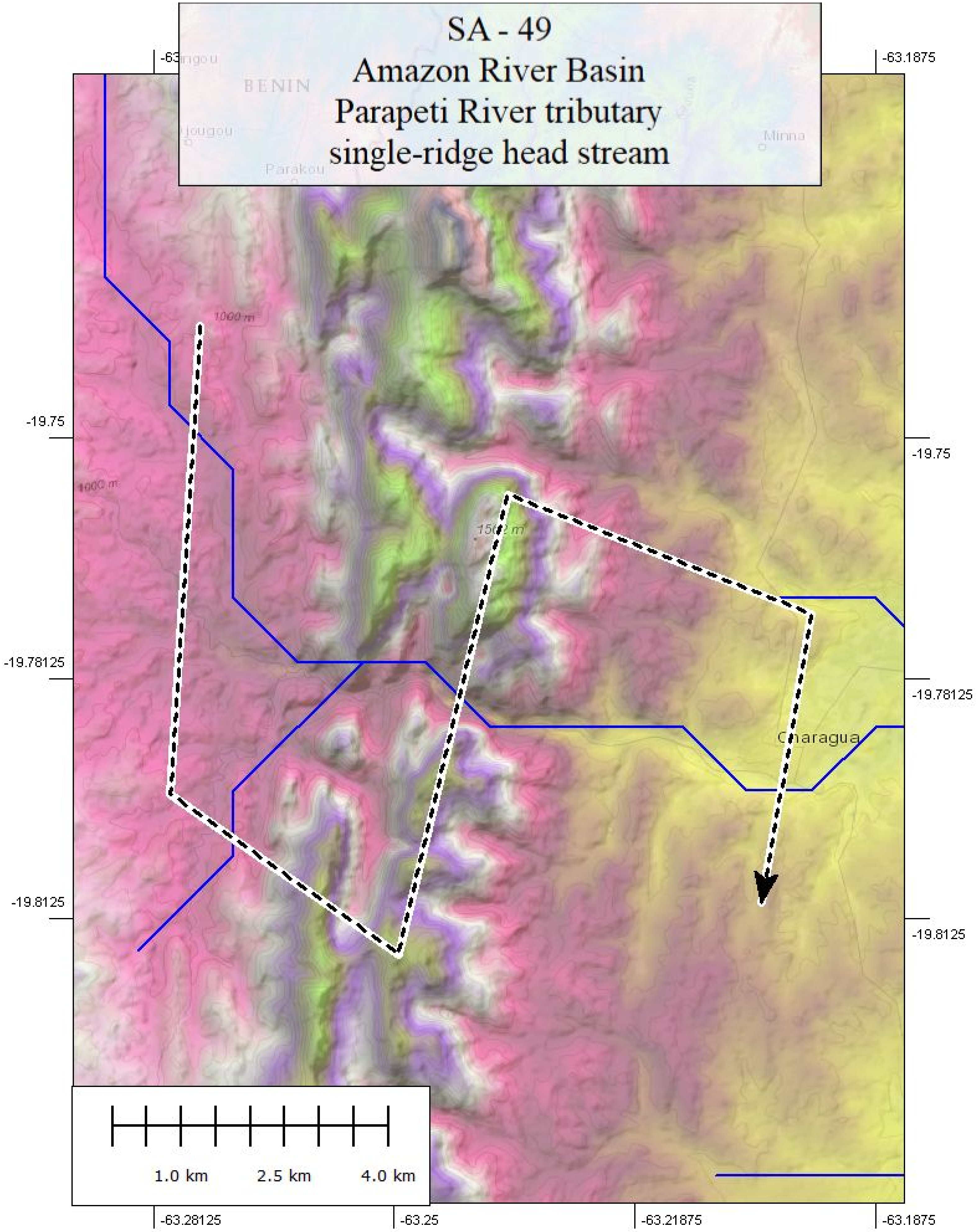
SA - 49
Amazon River Basin
Parapeti River tributary
single-ridge head stream
-63.1875
-19.75
-19.78125
-19.8125
1000 m
1000 m
1502 m
Charagua
1.0 km
2.5 km
4.0 km
-63.28125
-63.25
-63.21875
-63.1875

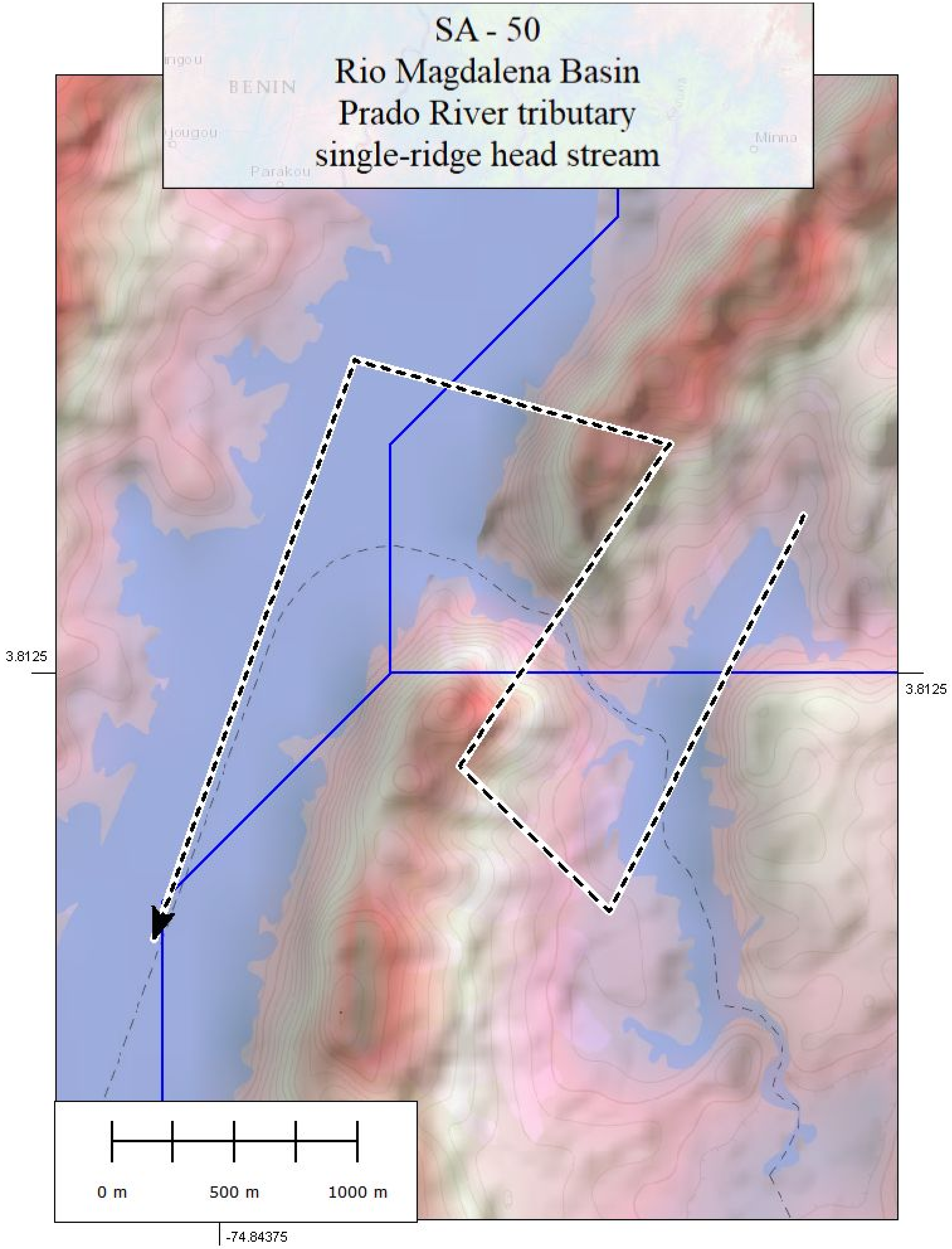
SA - 50
Rio Magdalena Basin
Prado River tributary
single-ridge head stream
BENIN
Parakou
Minna
3.8125
3.8125
0 m
500 m
1000 m
-74.84375

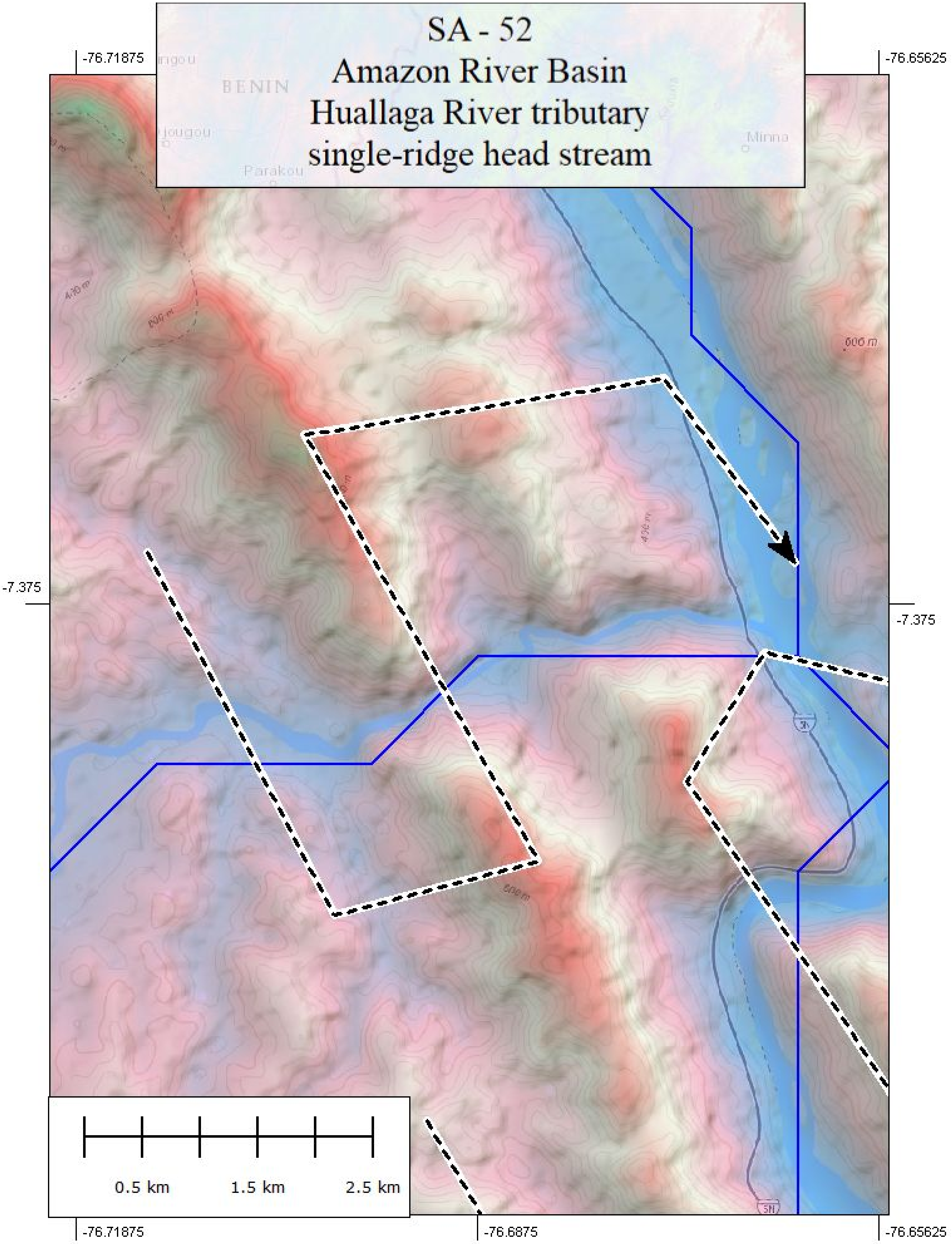
SA - 52
Amazon River Basin
Huallaga River tributary
single-ridge head stream
-76.71875
-76.65625
-7.375
-7.375
-76.71875
-76.6875
-76.65625
BENIN
Parakou
Minna
600 m
0.5 km
1.5 km
2.5 km

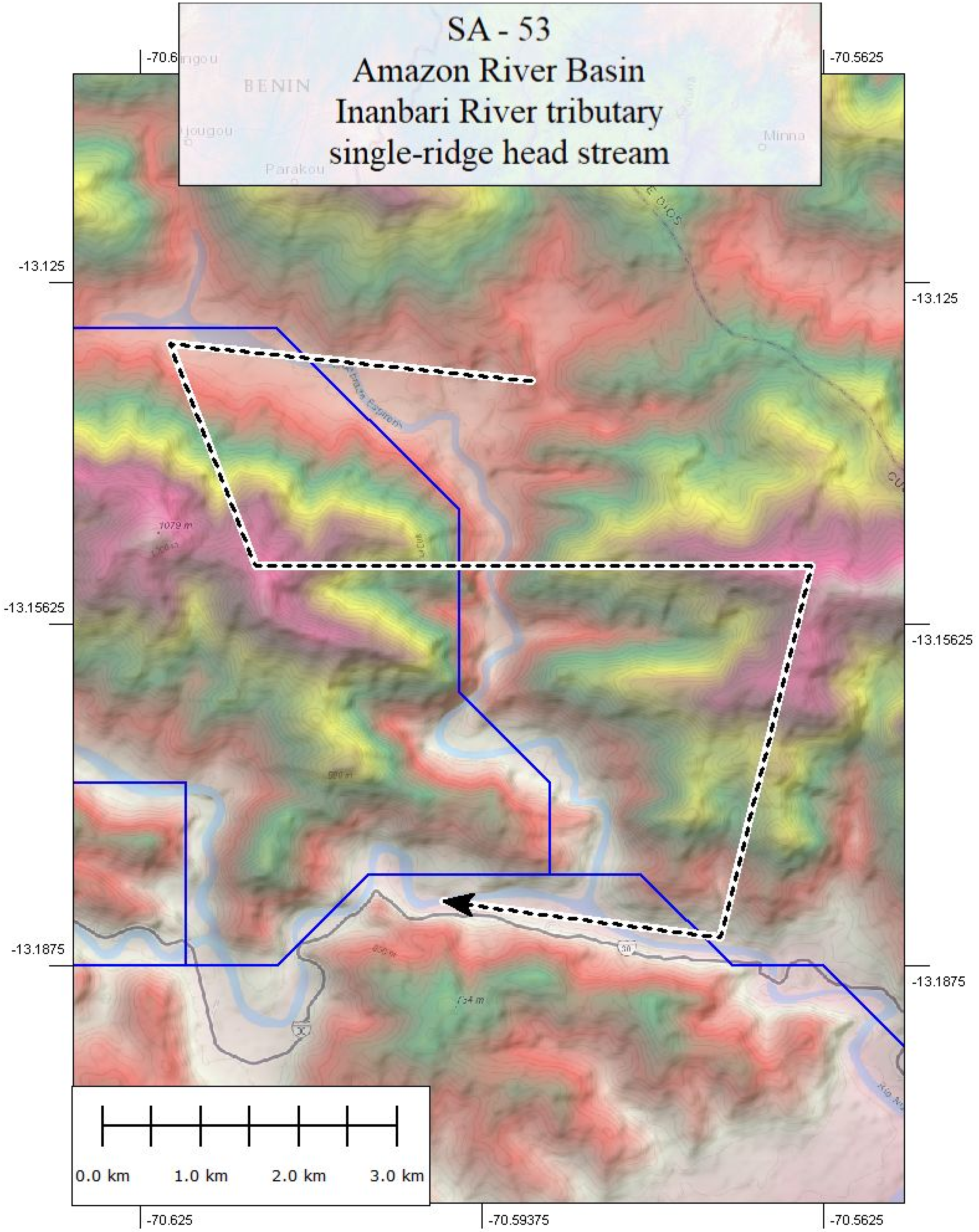
SA - 53
Amazon River Basin
Inanbari River tributary
single-ridge head stream
-70.625
-70.59375
-70.5625
-13.125
-13.15625
-13.1875
1079 m
0.0 km
1.0 km
2.0 km
3.0 km

SA - 54
Amazon River Basin
Madre de Dios River tributary
single-ridge head stream

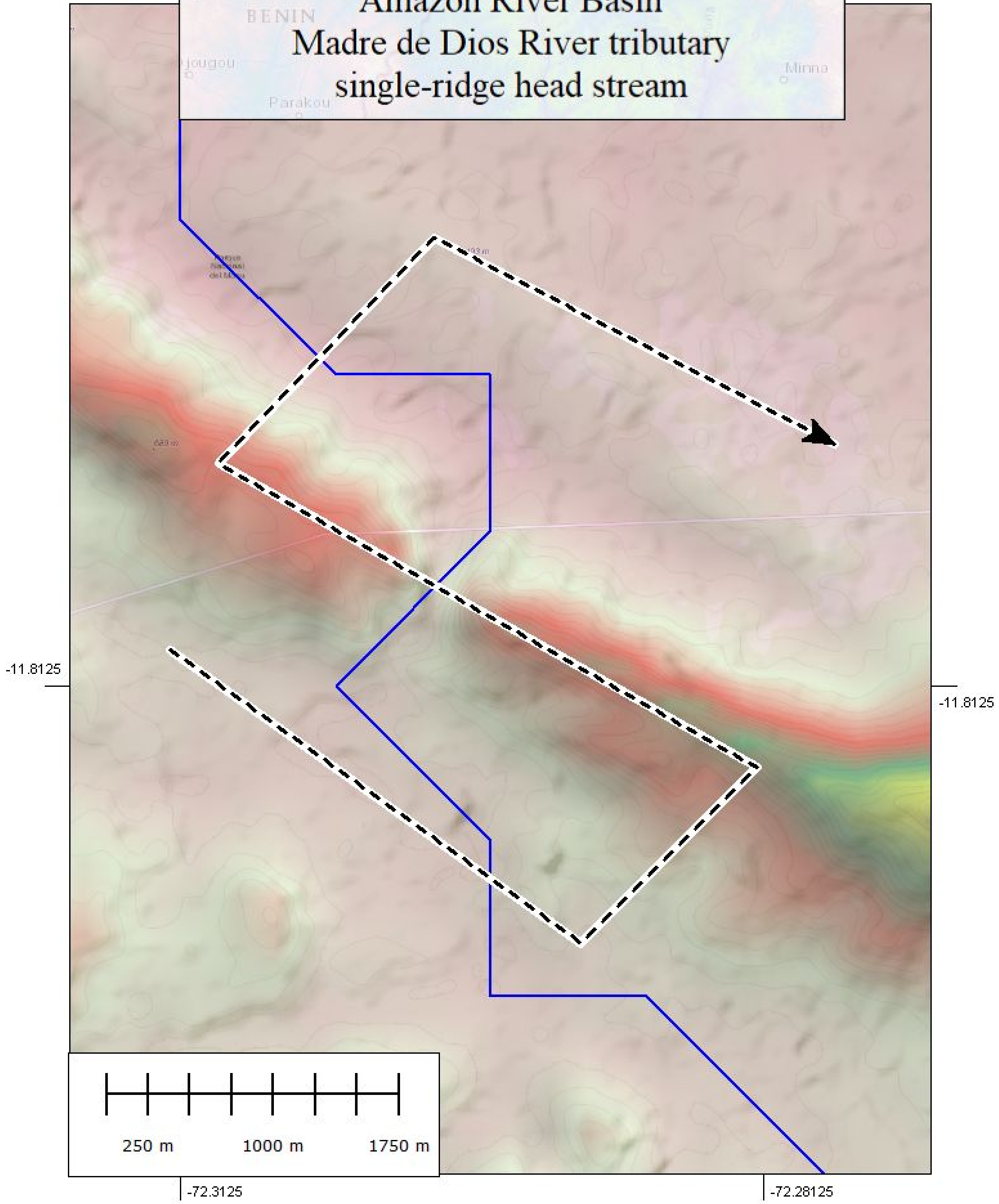

SA - 55
Rio Magdalena Basin
Saldana River tributary
single-ridge head stream

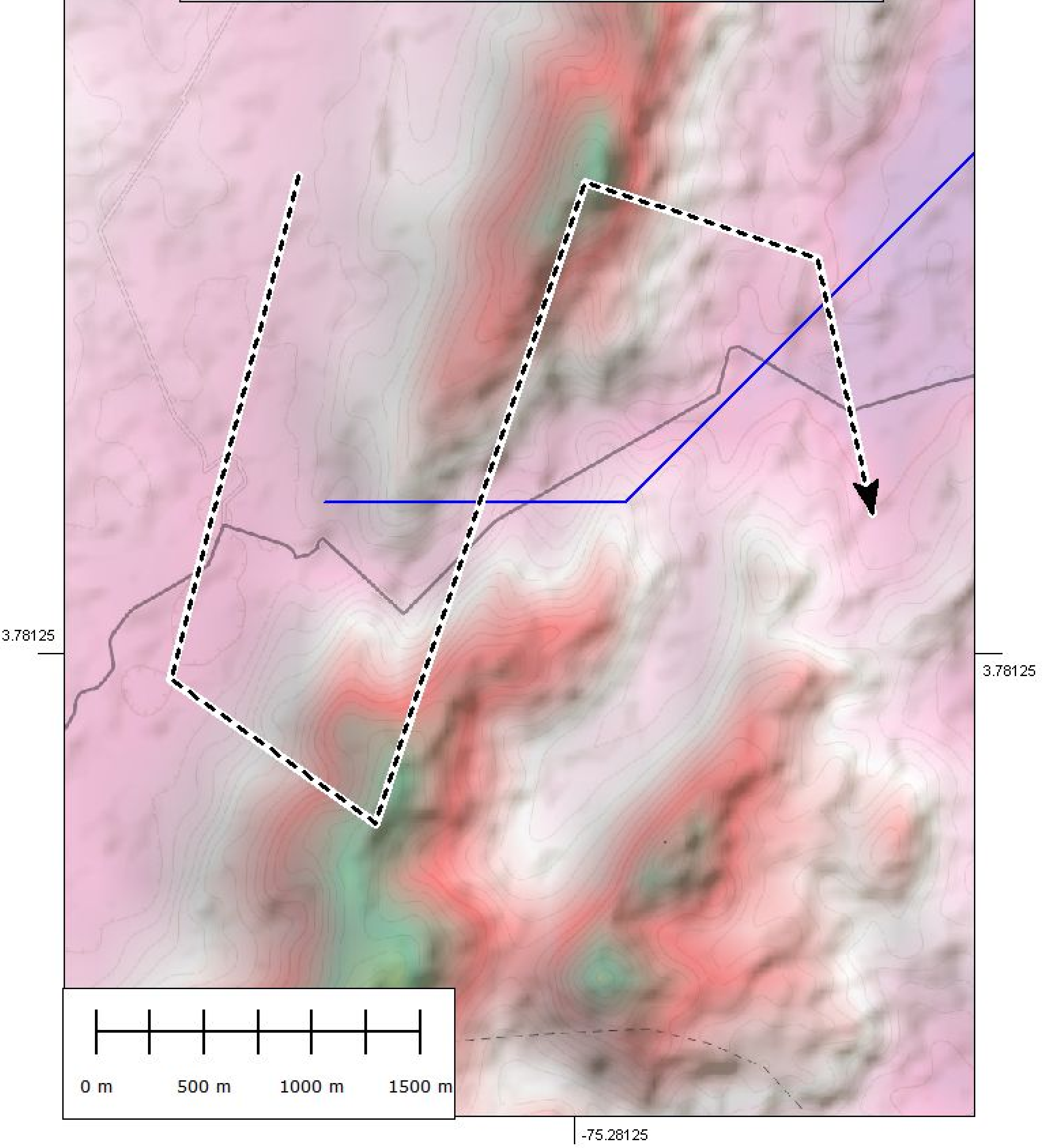

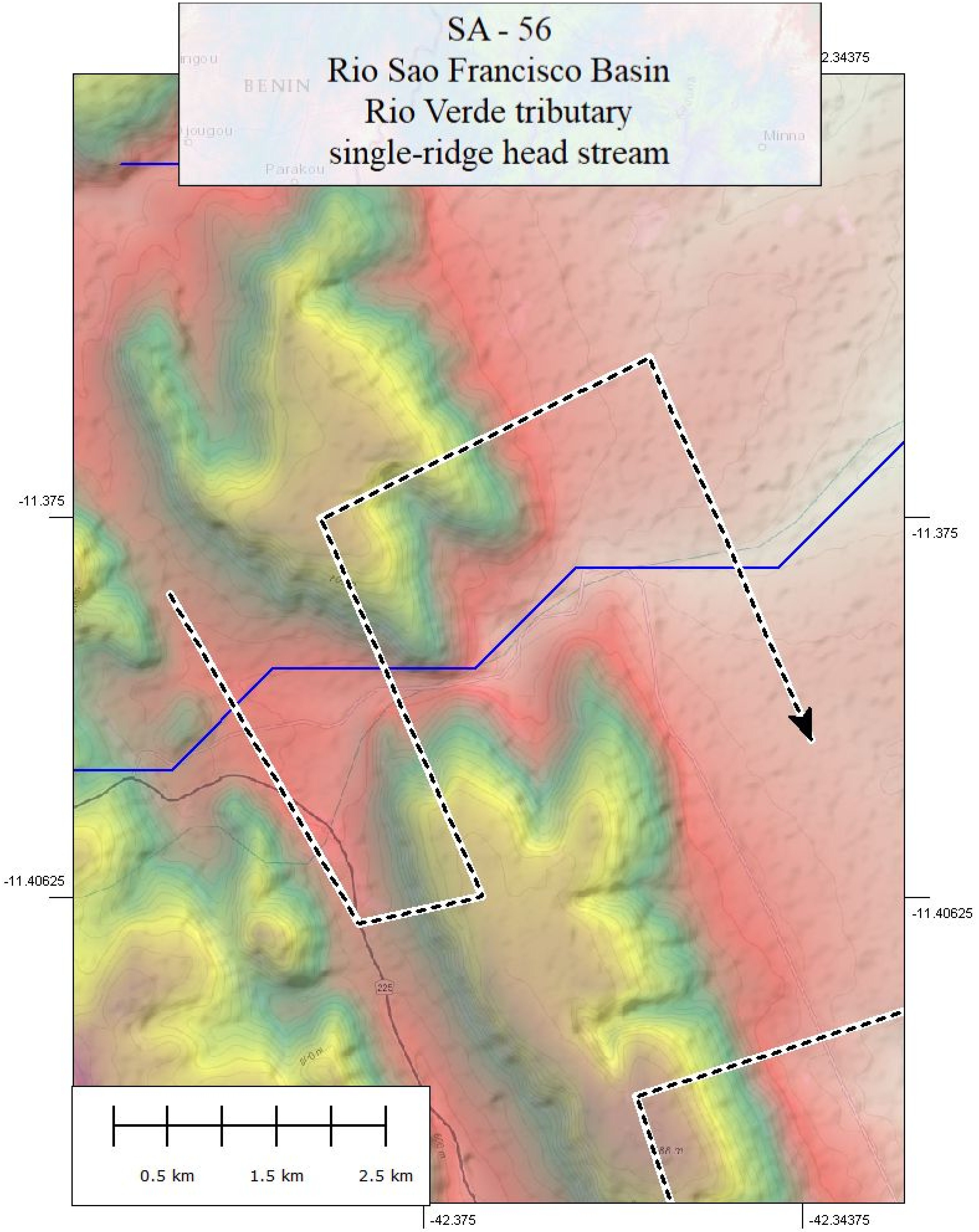
SA - 56
Rio Sao Francisco Basin
Rio Verde tributary
single-ridge head stream
-11.375
-11.375
-11.40625
-11.40625
-42.375
-42.34375
0.5 km
1.5 km
2.5 km

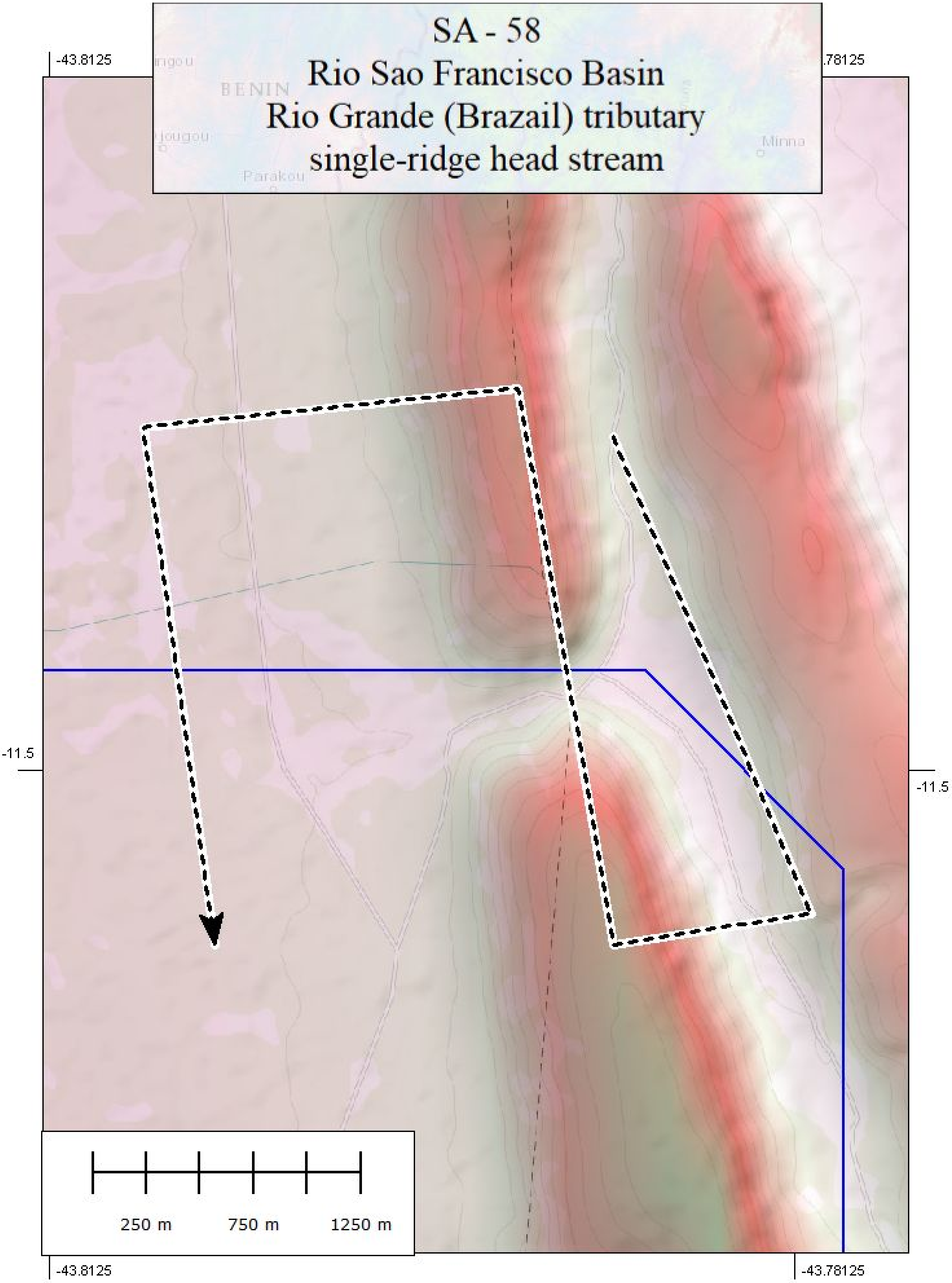
SA - 58
Rio Sao Francisco Basin
Rio Grande (Brazail) tributary
single-ridge head stream
-43.8125
.78125
-11.5
-11.5
250 m
750 m
1250 m
-43.8125
-43.78125

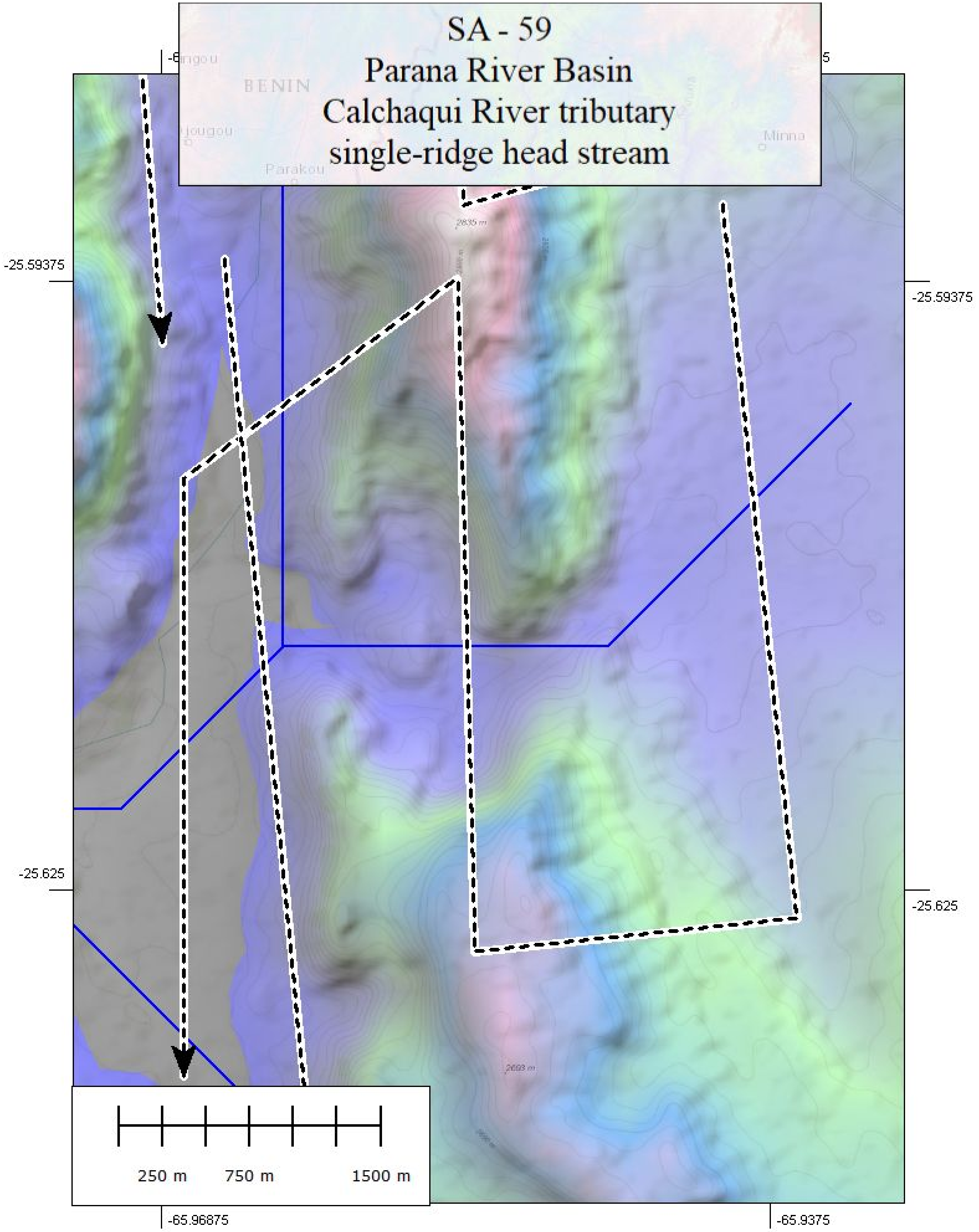
SA - 59
Parana River Basin
Calchaqui River tributary
single-ridge head stream
-25.59375
-25.59375
-25.625
-25.625
-65.96875
-65.9375
250 m
750 m
1500 m

SA - 60
Parana River Basin
Pilcomayo River tributary
single-ridge trunk stream

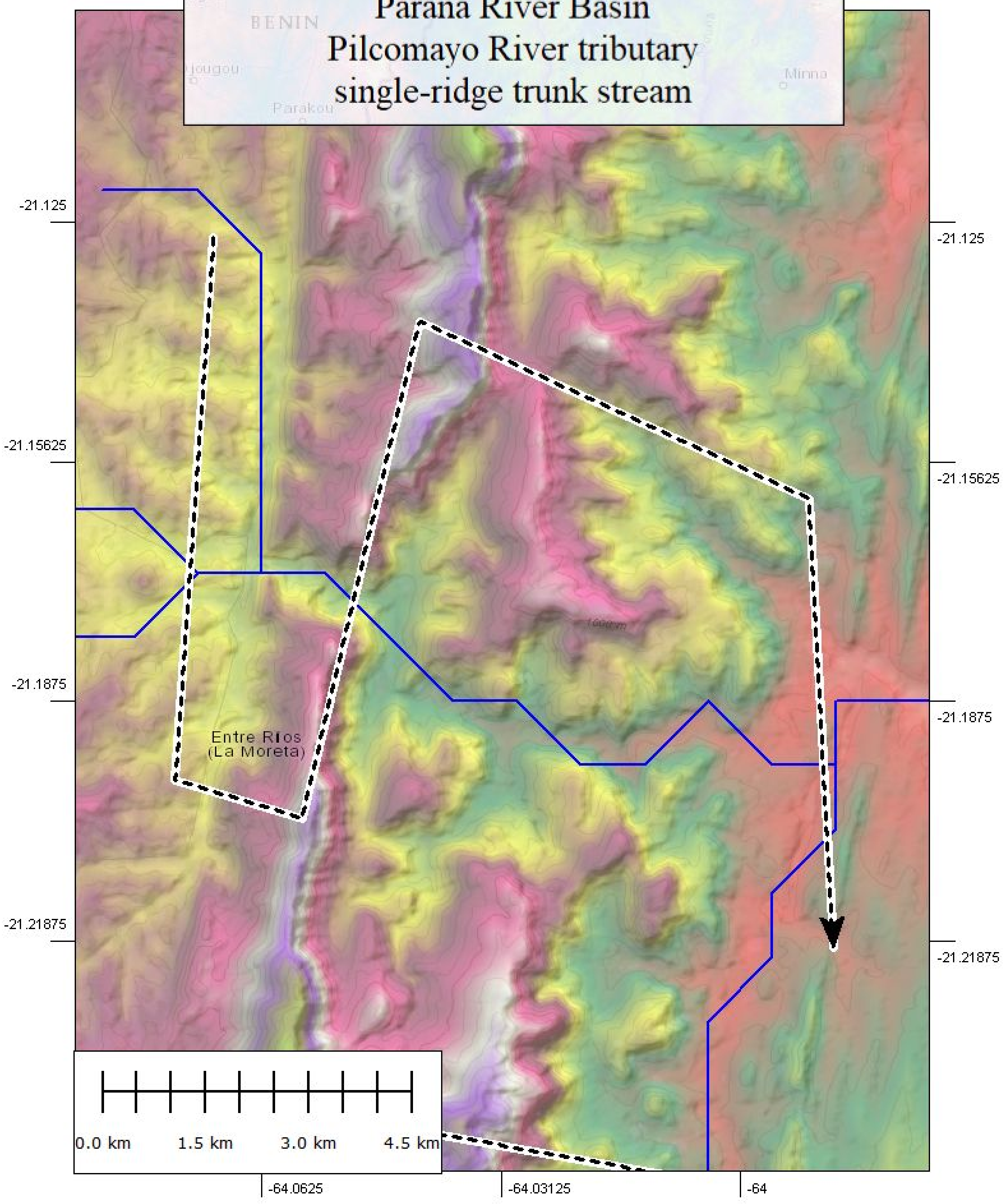

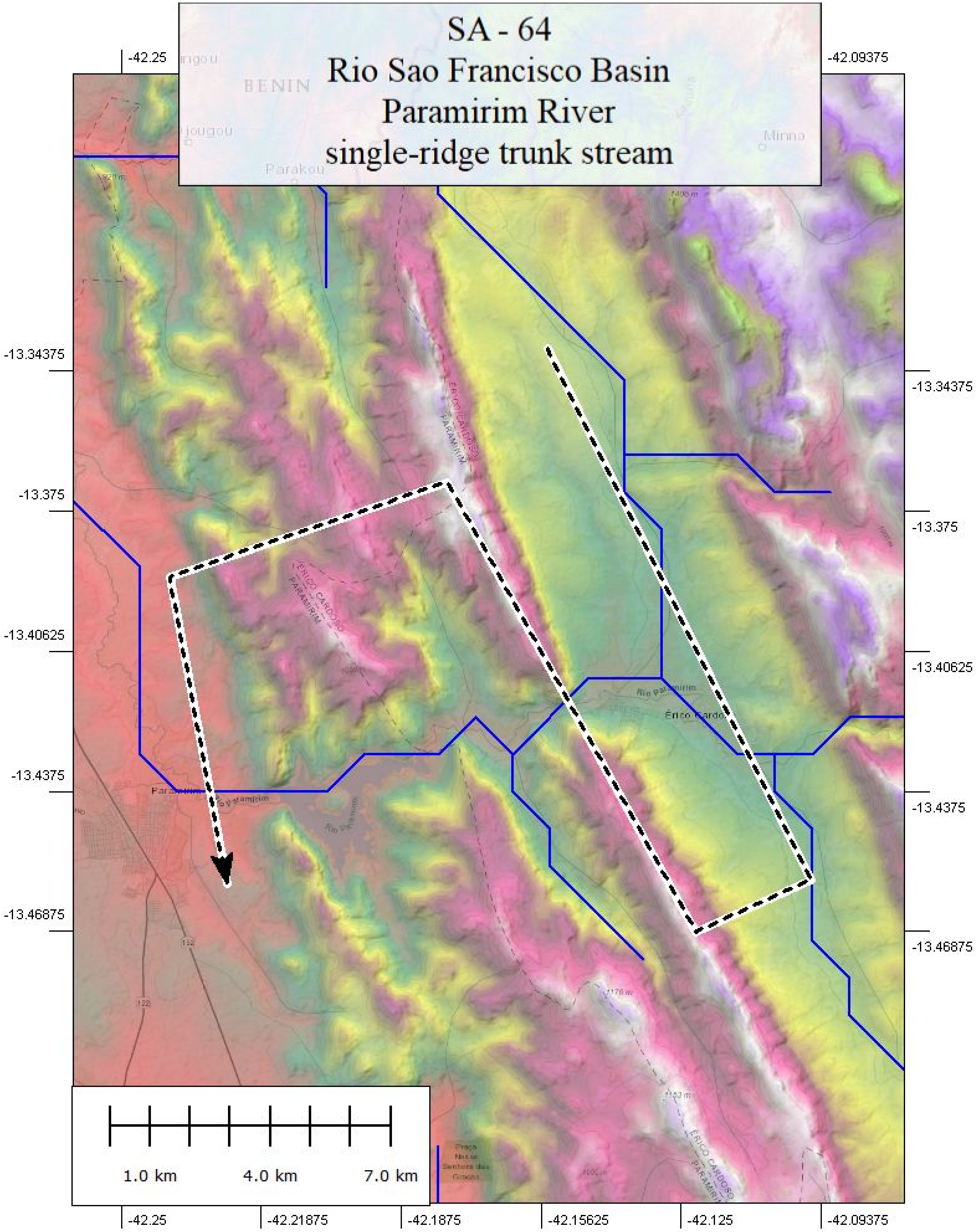
SA - 64
Rio Sao Francisco Basin
Paramirim River
single-ridge trunk stream
-42.25
-42.21875
-42.1875
-42.15625
-42.125
-42.09375
-13.34375
-13.375
-13.40625
-13.4375
-13.46875
1.0 km
4.0 km
7.0 km
BENIN
Parakou
Rio Paramirim
Érico Cardoso

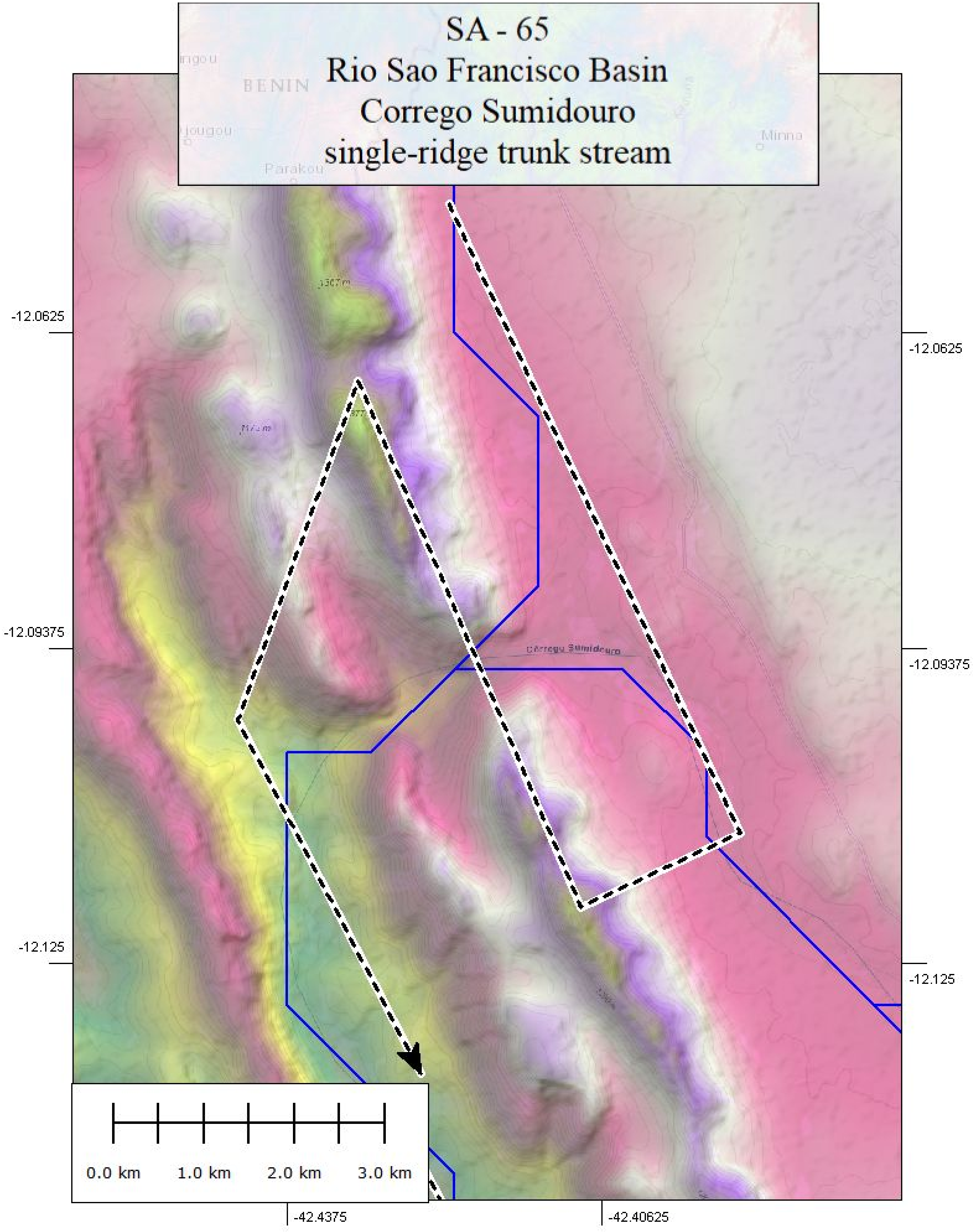
SA - 65
Rio Sao Francisco Basin
Corrego Sumidouro
single-ridge trunk stream
Corrego Sumidouro
-12.0625
-12.09375
-12.125
-42.4375
-42.40625
0.0 km
1.0 km
2.0 km
3.0 km

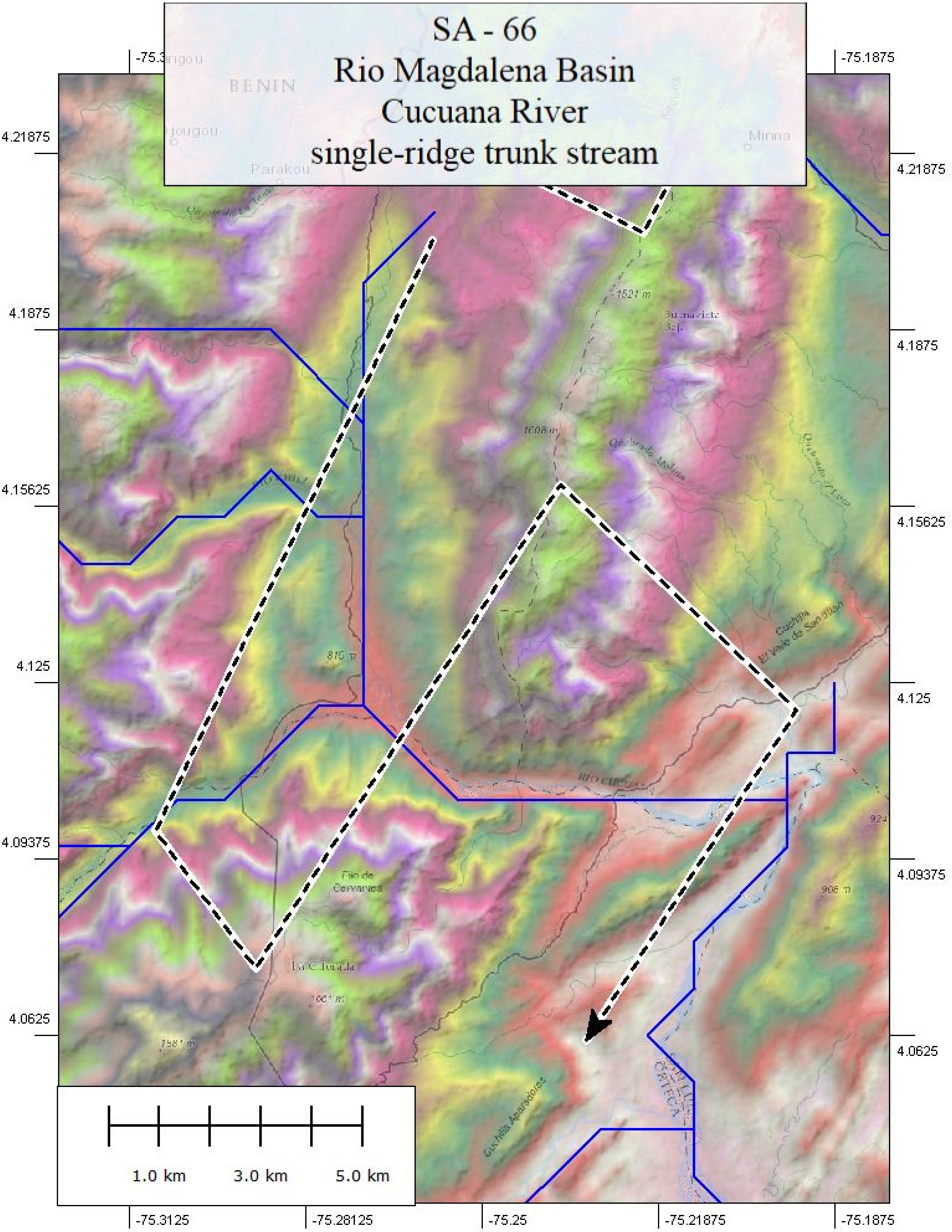
SA - 66
Rio Magdalena Basin
Cucuana River
single-ridge trunk stream
-75.3125
-75.28125
-75.25
-75.21875
-75.1875
4.21875
4.1875
4.15625
4.125
4.09375
4.0625
1.0 km
3.0 km
5.0 km

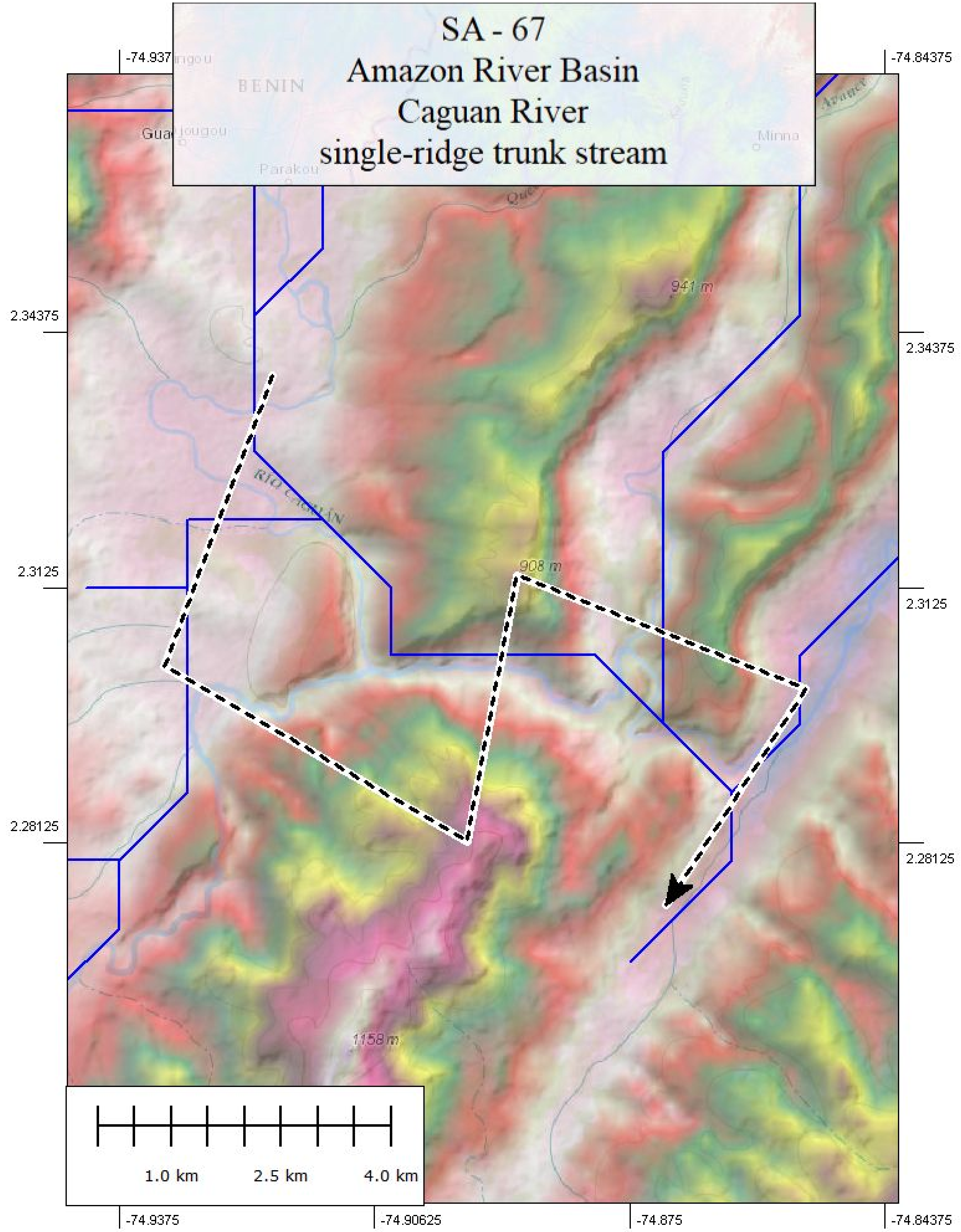

SA - 67
Amazon River Basin
Caguan River
single-ridge trunk stream
-74.9375
-74.90625
-74.875
-74.84375
2.34375
2.3125
2.28125
BENIN
Parakou
Minna
RÍO CAGUÁN
941 m
908 m
1158 m
1.0 km
2.5 km
4.0 km

SA - 69
Orinoco River Basin
Sioca River
single-ridge trunk stream

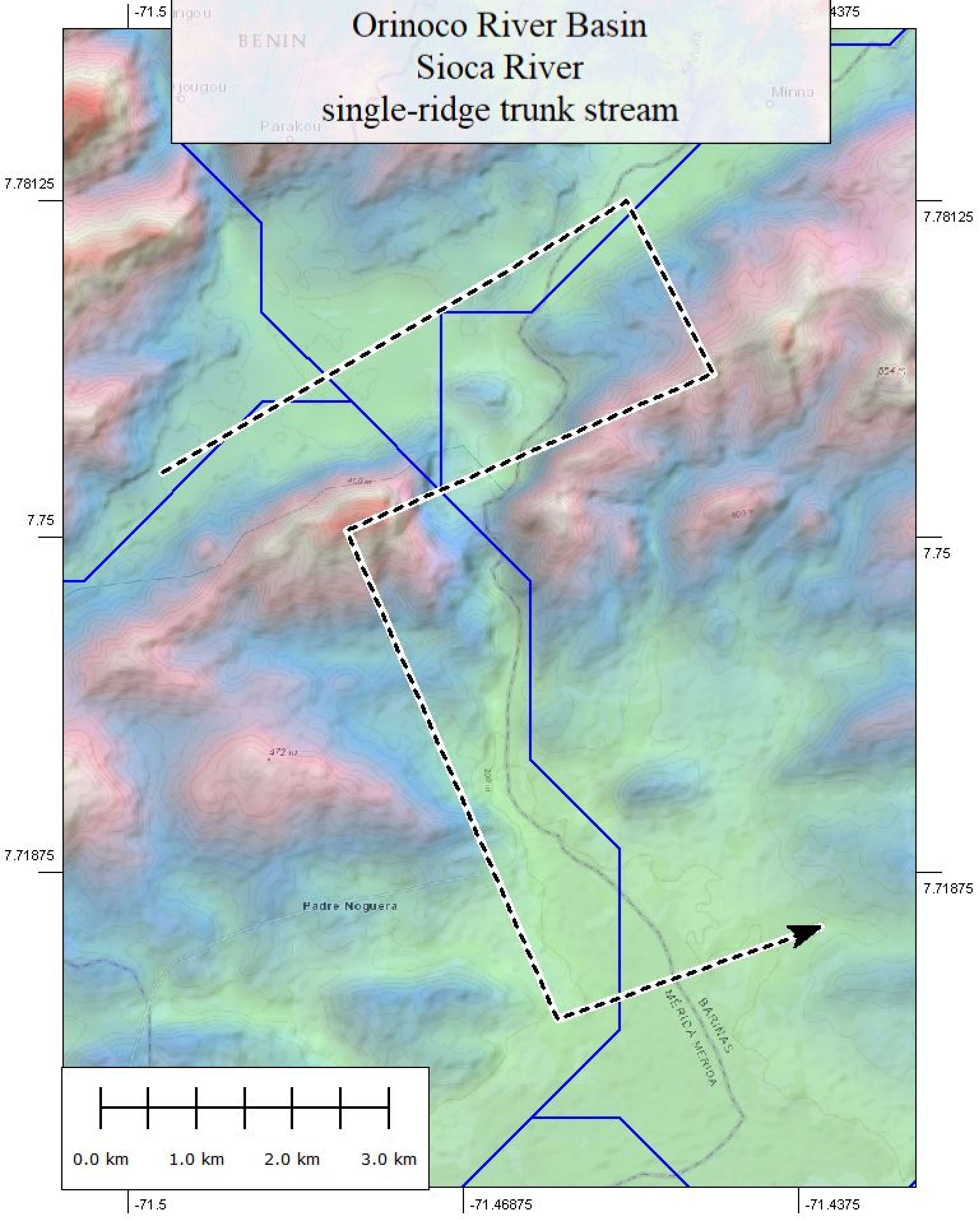

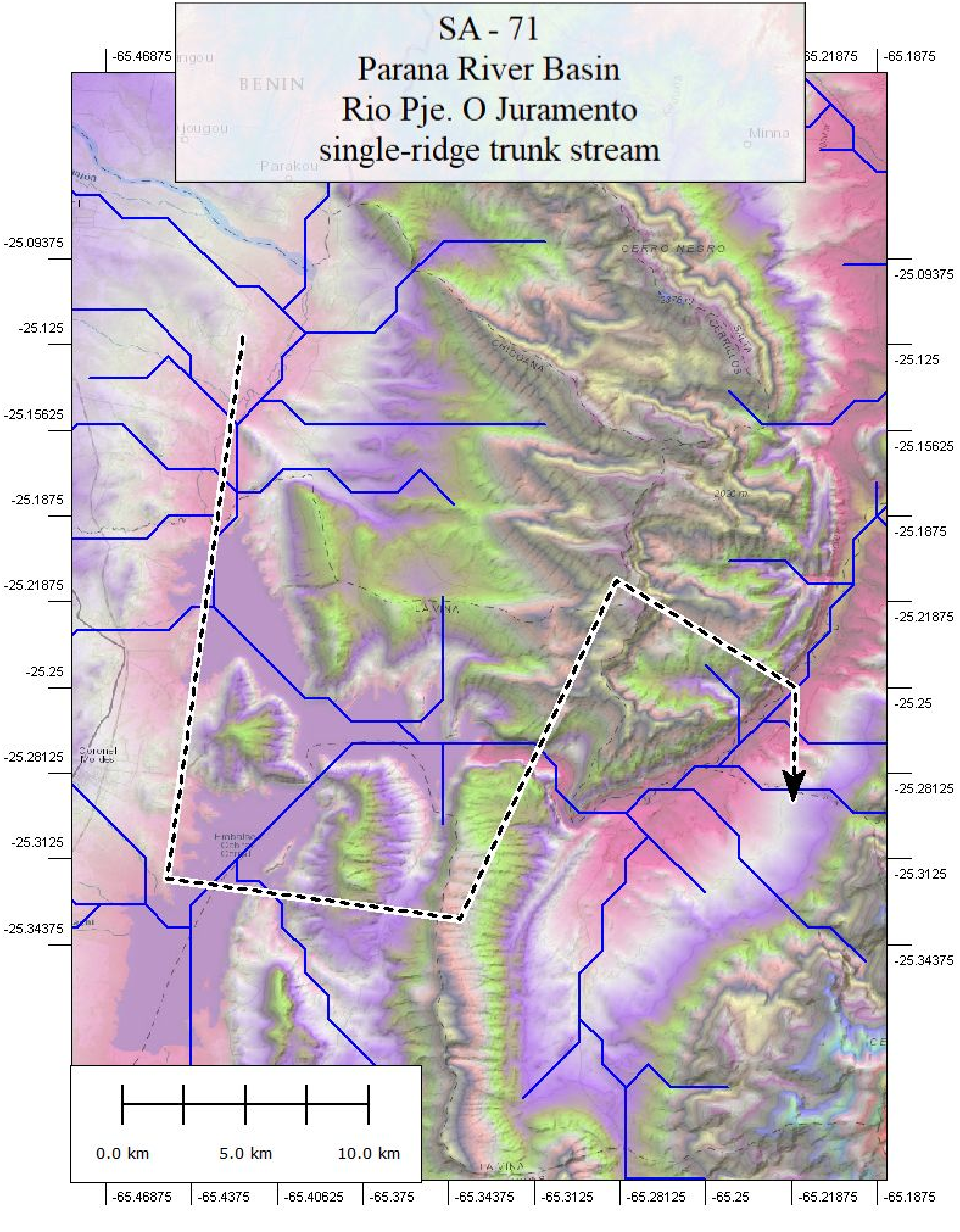

SA - 71
Parana River Basin
Rio Pje. O Juramento
single-ridge trunk stream
-65.46875
-65.21875
-65.1875
-25.09375
-25.125
-25.15625
-25.1875
-25.21875
-25.25
-25.28125
-25.3125
-25.34375
-65.4375
-65.40625
-65.375
-65.34375
-65.3125
-65.28125
-65.25
CERRO NEGRO
CHICOANA
LA VIÑA
0.0 km
5.0 km
10.0 km

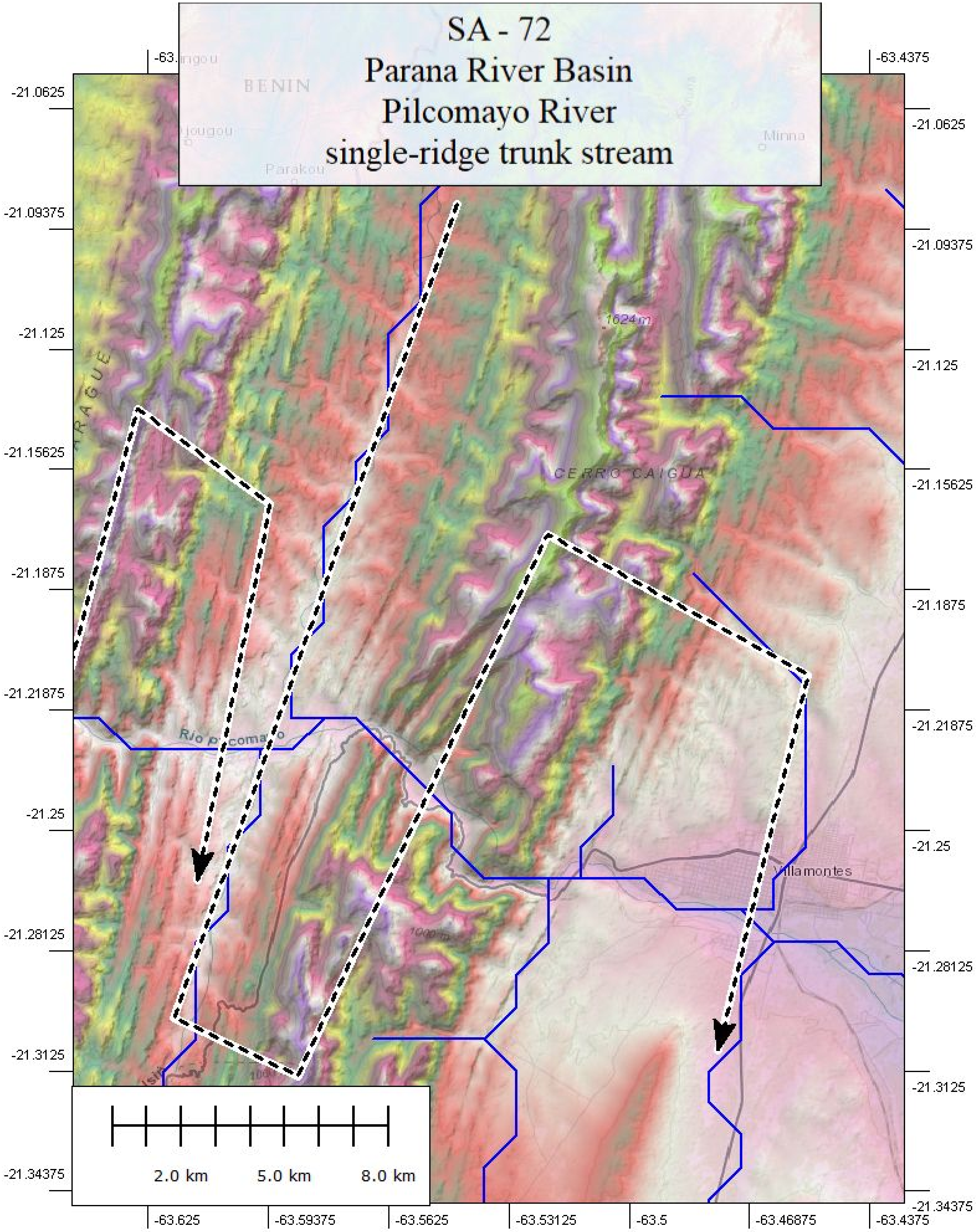
SA - 72
Parana River Basin
Pilcomayo River
single-ridge trunk stream
-21.0625
-21.09375
-21.125
-21.15625
-21.1875
-21.21875
-21.25
-21.28125
-21.3125
-21.34375
-63.625
-63.59375
-63.5625
-63.53125
-63.5
-63.46875
-63.4375
1624m
CERRO CAIGUA
Rio Pilcomayo
Villamontes
1000m
2.0 km
5.0 km
8.0 km

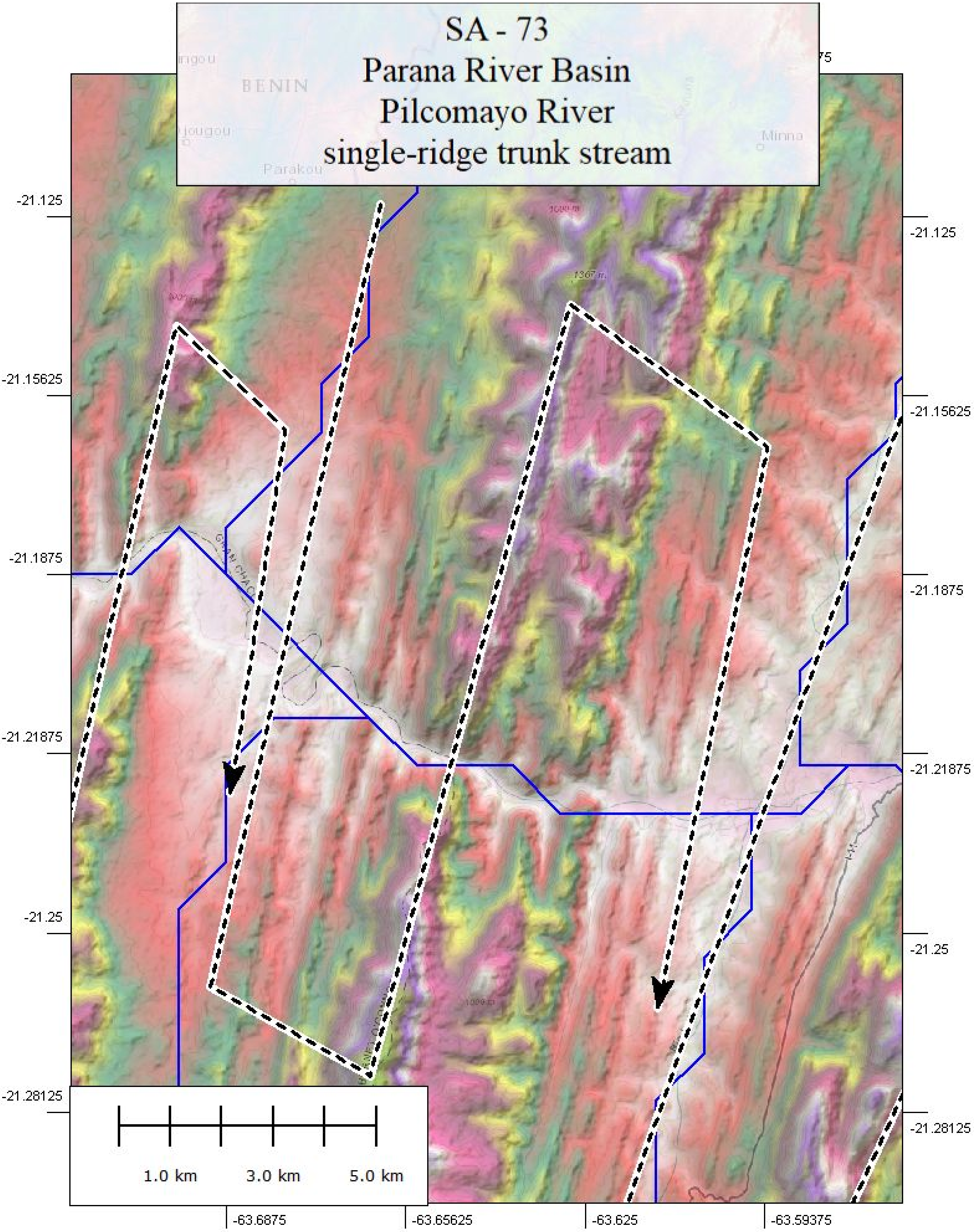
SA - 73
Parana River Basin
Pilcomayo River
single-ridge trunk stream
-21.125
-21.15625
-21.1875
-21.21875
-21.25
-21.28125
-63.6875
-63.65625
-63.625
-63.59375
1.0 km
3.0 km
5.0 km
1367 m
BENIN
Parakou
Minna

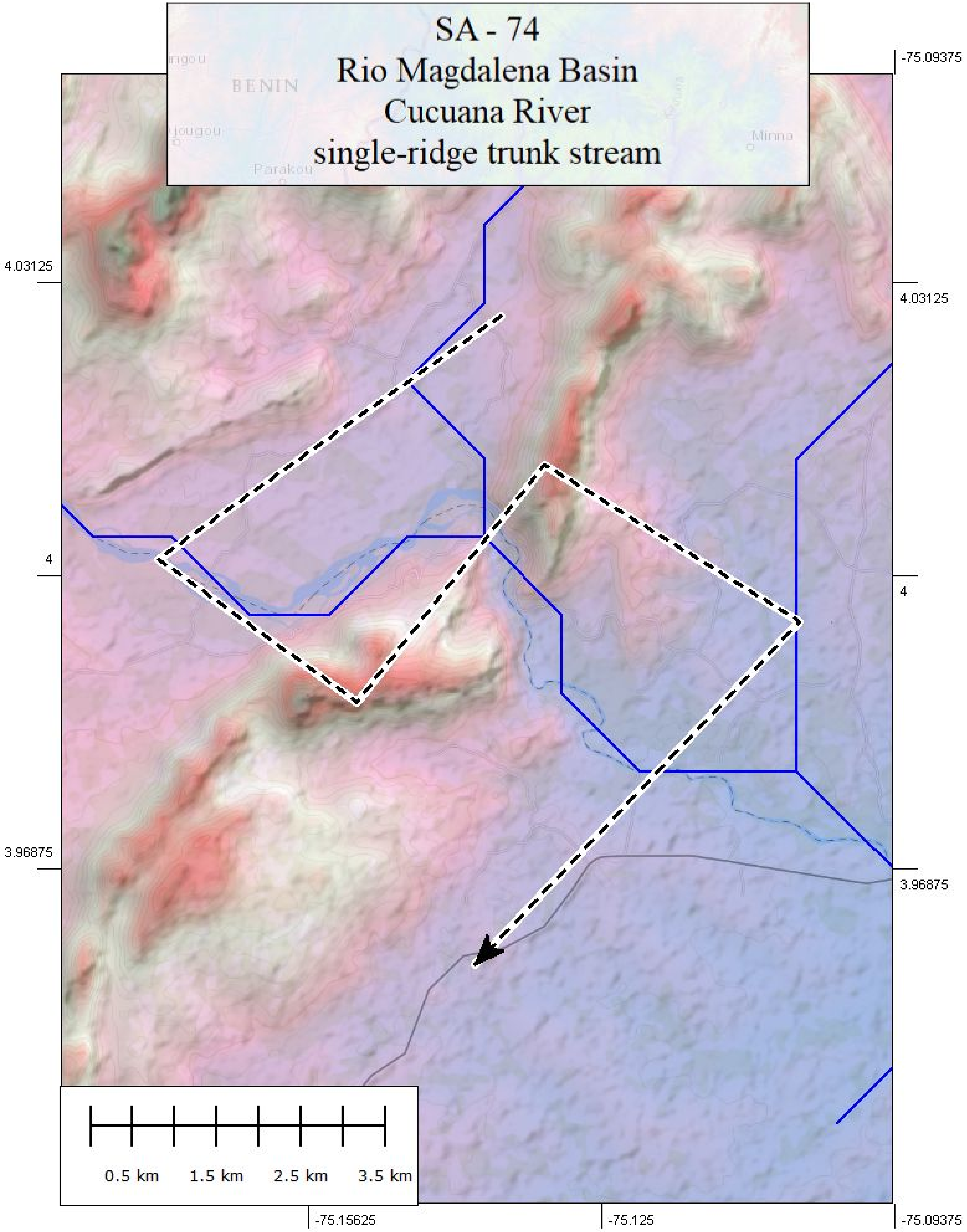

SA - 74
Rio Magdalena Basin
Cucuana River
single-ridge trunk stream
BENIN
Parakou
Minna
4.03125
4
3.96875
-75.09375
-75.15625
-75.125
0.5 km
1.5 km
2.5 km
3.5 km

SA - 75
Rio Magdalena Basin
Saldana River
single-ridge trunk stream

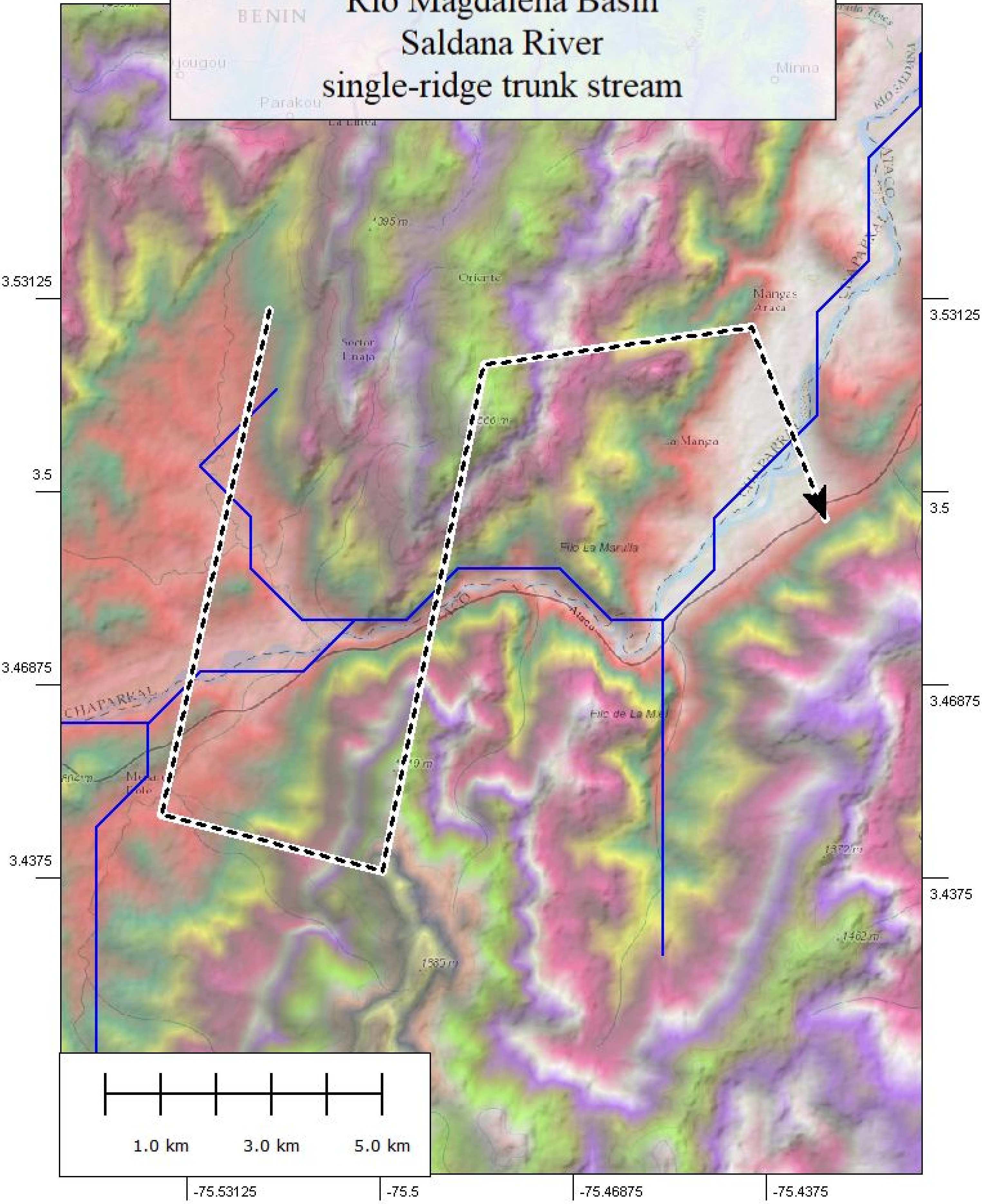

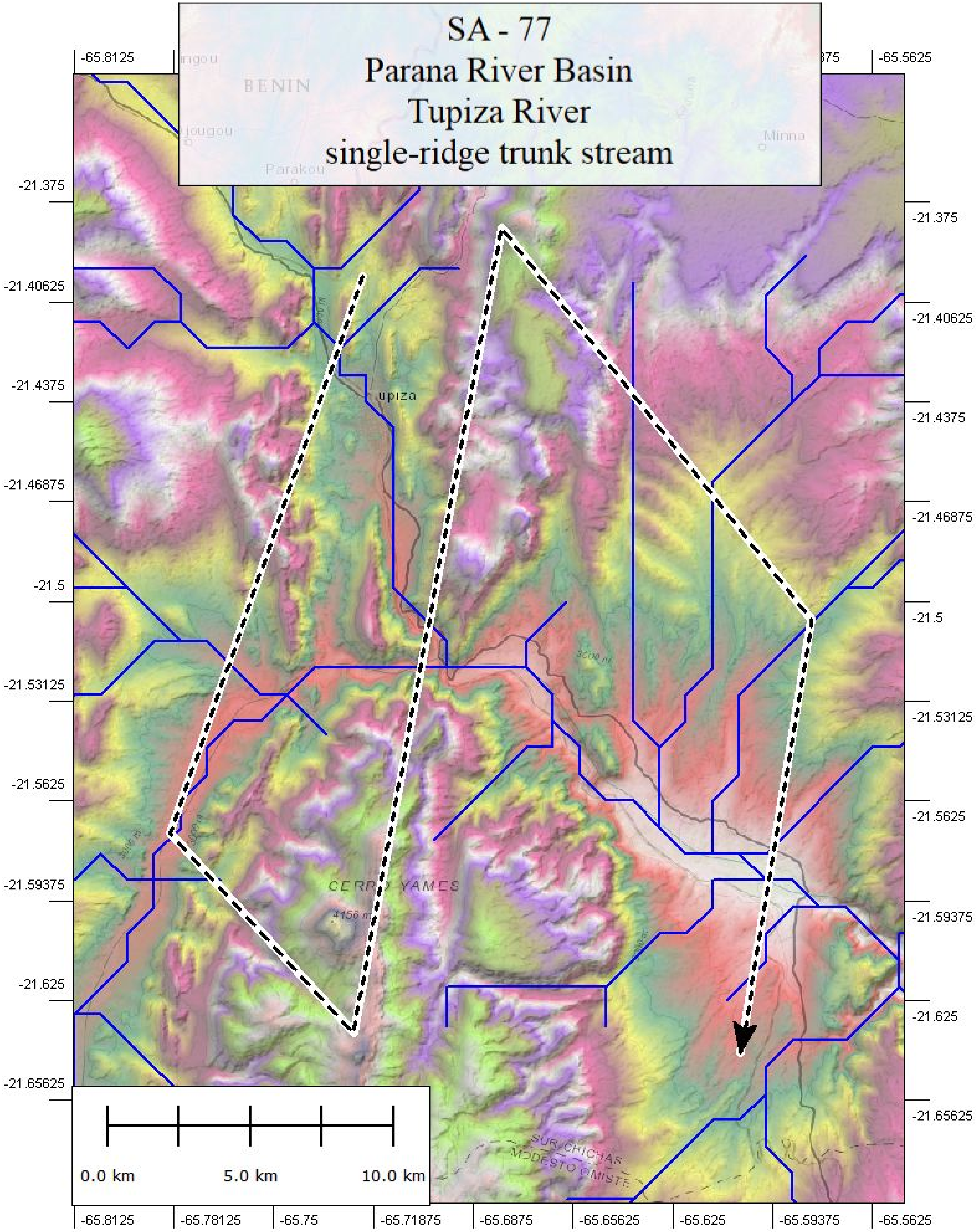
SA - 77
Parana River Basin
Tupiza River
single-ridge trunk stream
-65.8125
-65.5625
-65.78125
-65.75
-65.71875
-65.6875
-65.65625
-65.625
-65.59375
-21.375
-21.40625
-21.4375
-21.46875
-21.5
-21.53125
-21.5625
-21.59375
-21.625
-21.65625
CERRO YAMES
0.0 km
5.0 km
10.0 km

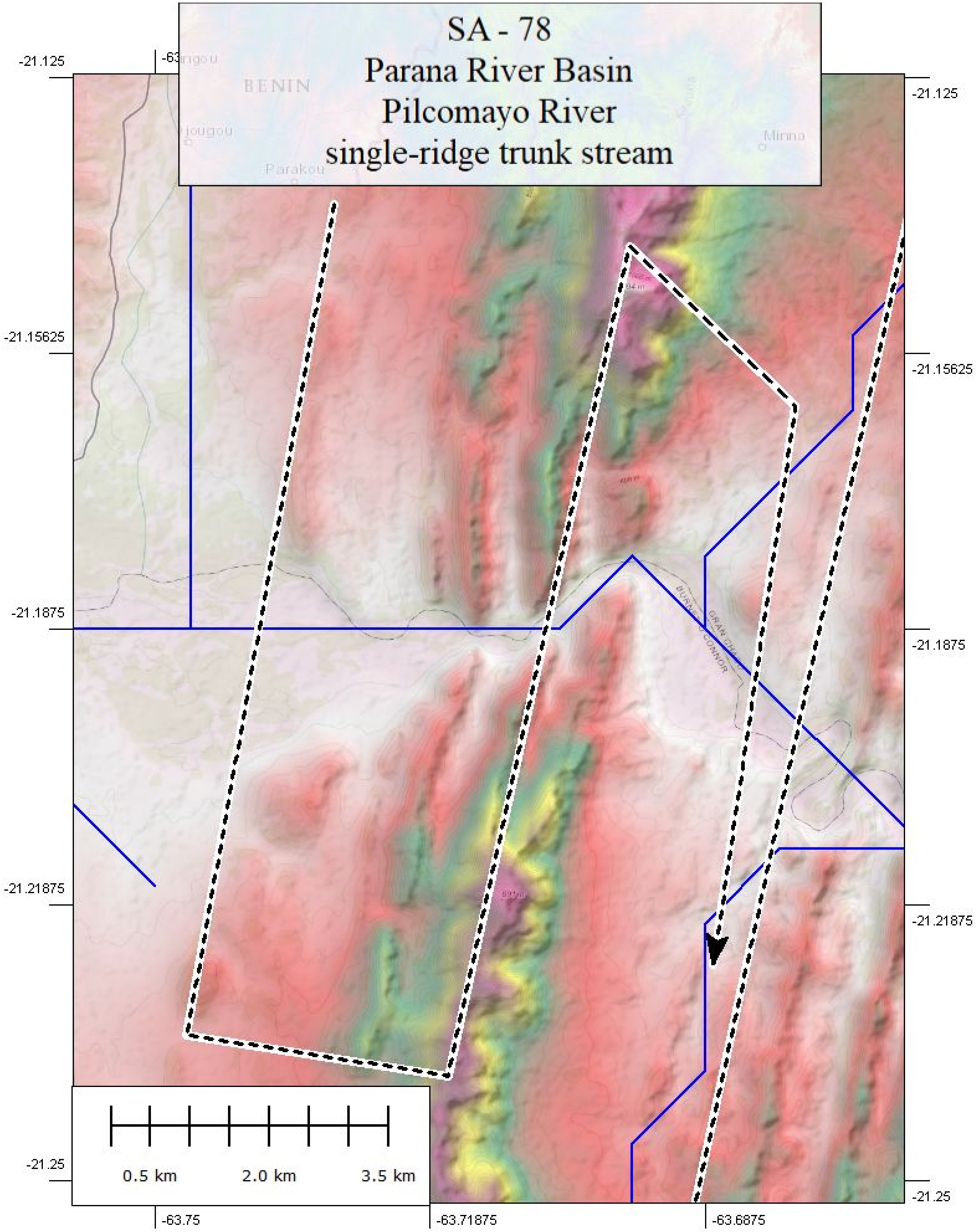
SA - 78
Parana River Basin
Pilcomayo River
single-ridge trunk stream
-21.125
-21.15625
-21.1875
-21.21875
-21.25
-63.75
-63.71875
-63.6875
0.5 km
2.0 km
3.5 km

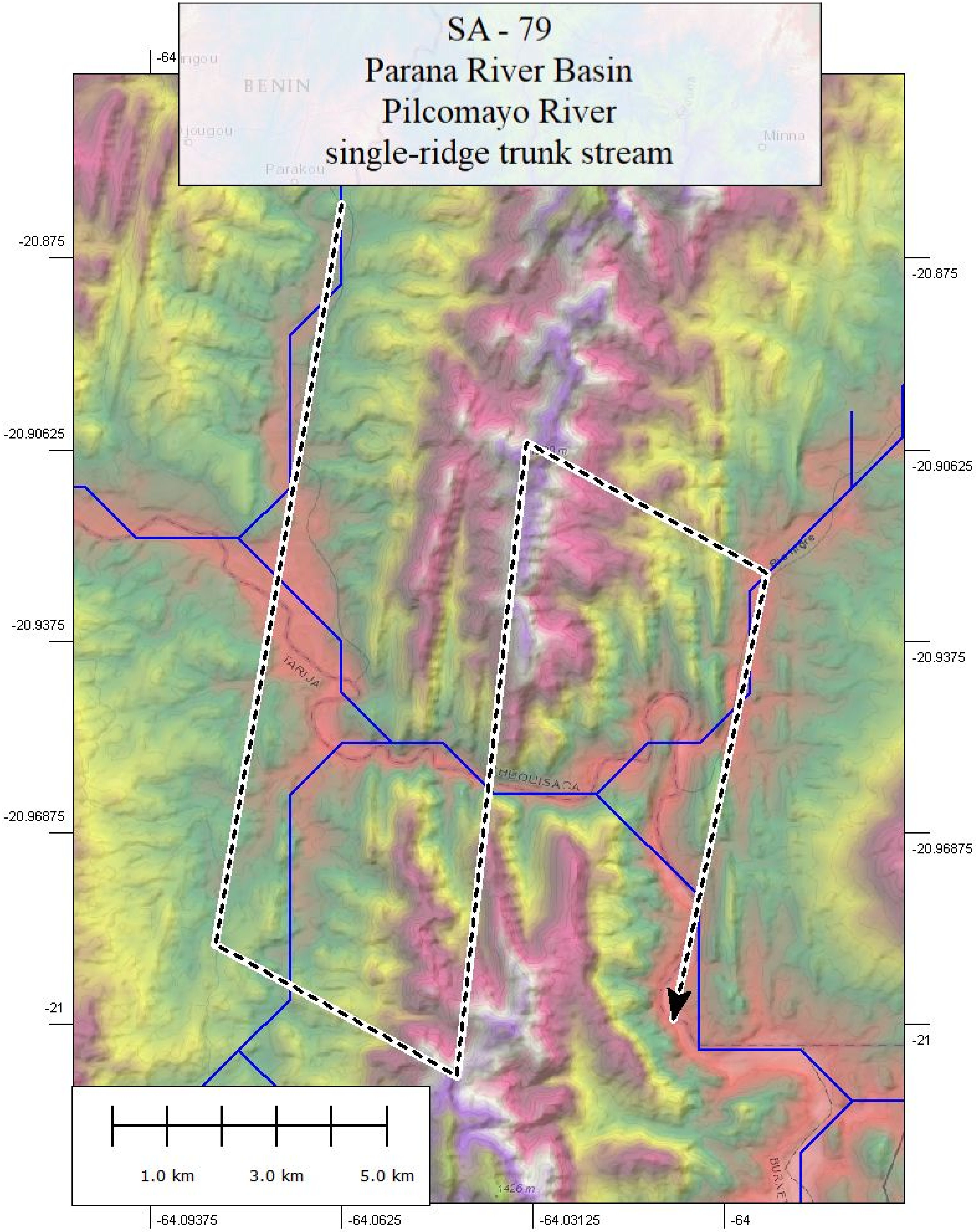
SA - 79
Parana River Basin
Pilcomayo River
single-ridge trunk stream
-64
-64.09375
-64.0625
-64.03125
-20.875
-20.90625
-20.9375
-20.96875
-21
TARIJA
CHUQUISACA
BENIN
Parakou
Minna
1.0 km
3.0 km
5.0 km

SA - 80
Amazon River Basin
Paute River
single-ridge trunk stream

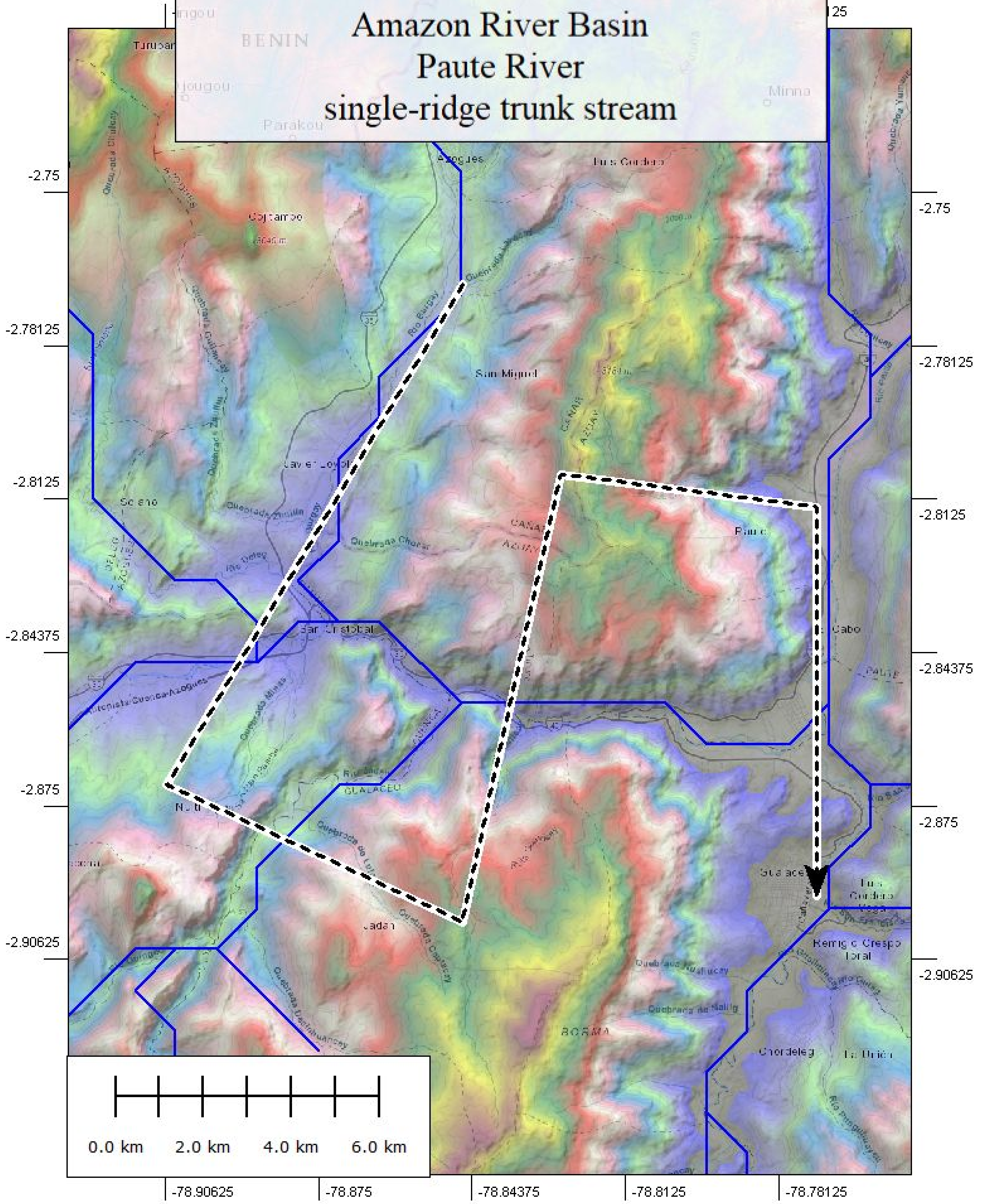

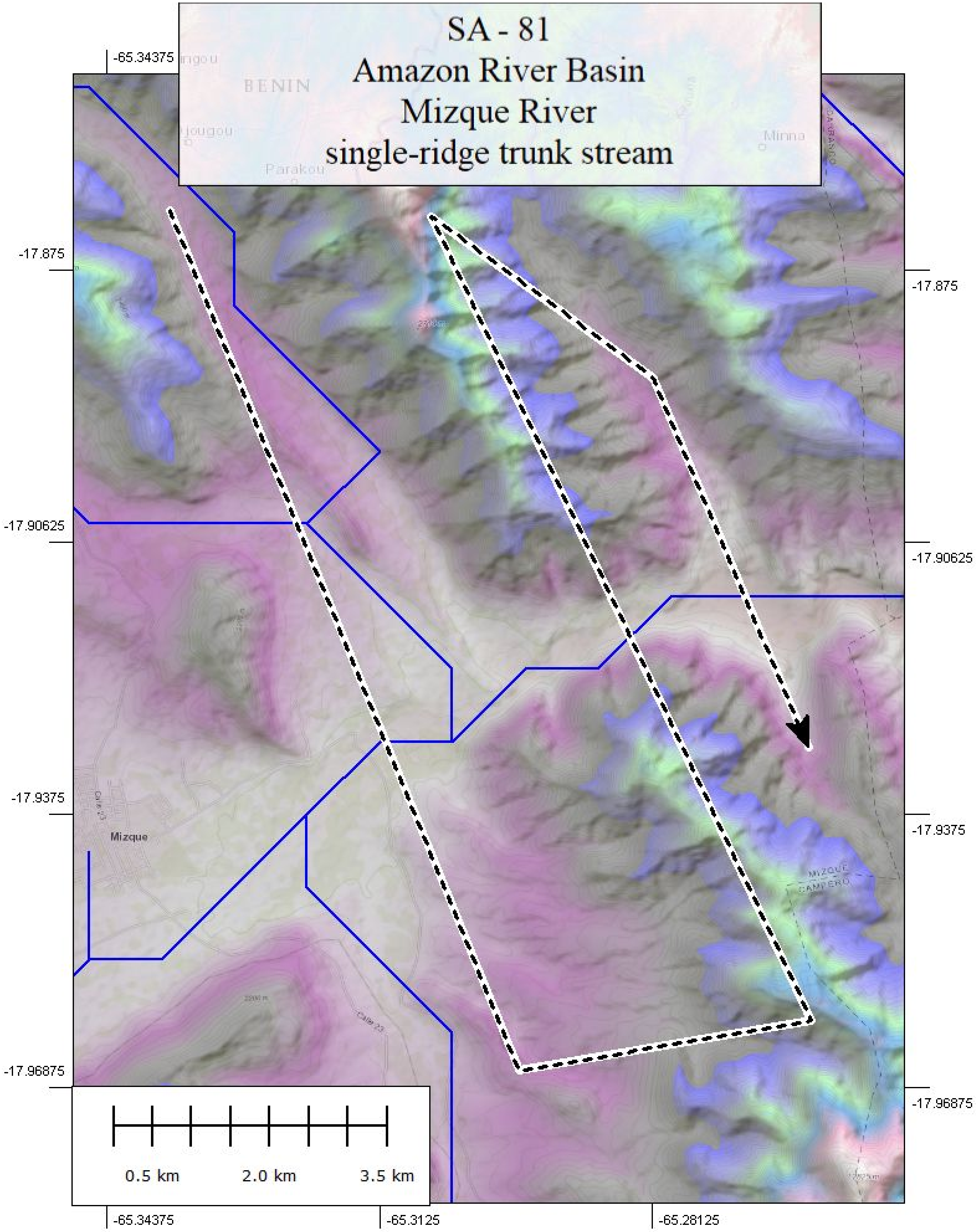
SA - 81
Amazon River Basin
Mizque River
single-ridge trunk stream
-65.34375
-65.3125
-65.28125
-17.875
-17.90625
-17.9375
-17.96875
Mizque
0.5 km
2.0 km
3.5 km

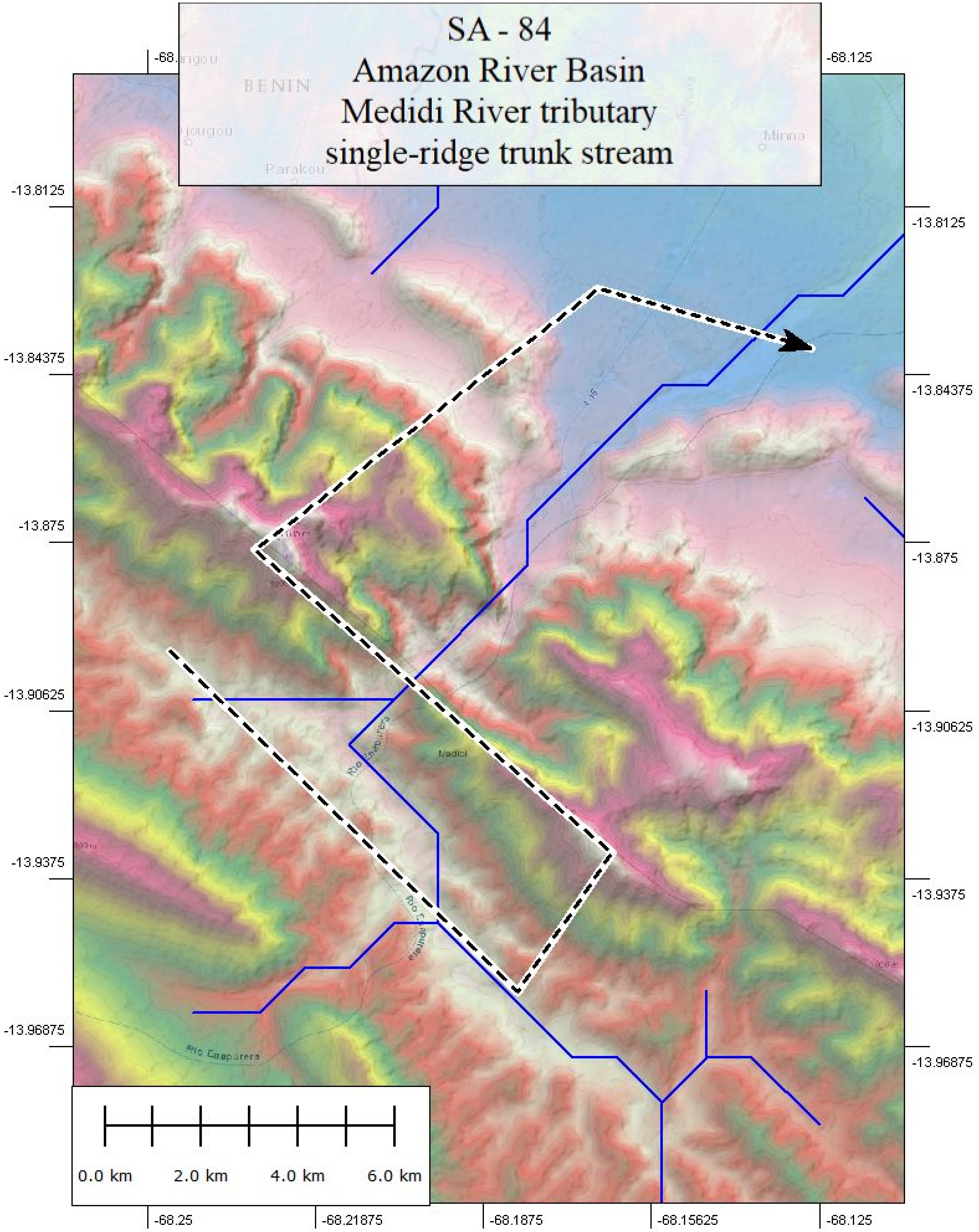
SA - 84
Amazon River Basin
Medidi River tributary
single-ridge trunk stream
-68.125
-68.125
-13.8125
-13.8125
-13.84375
-13.84375
-13.875
-13.875
-13.90625
-13.90625
-13.9375
-13.9375
-13.96875
-13.96875
-68.25
-68.21875
-68.1875
-68.15625
-68.125
0.0 km
2.0 km
4.0 km
6.0 km
BENIN
Parakou
Medidi
Rio Chapurera

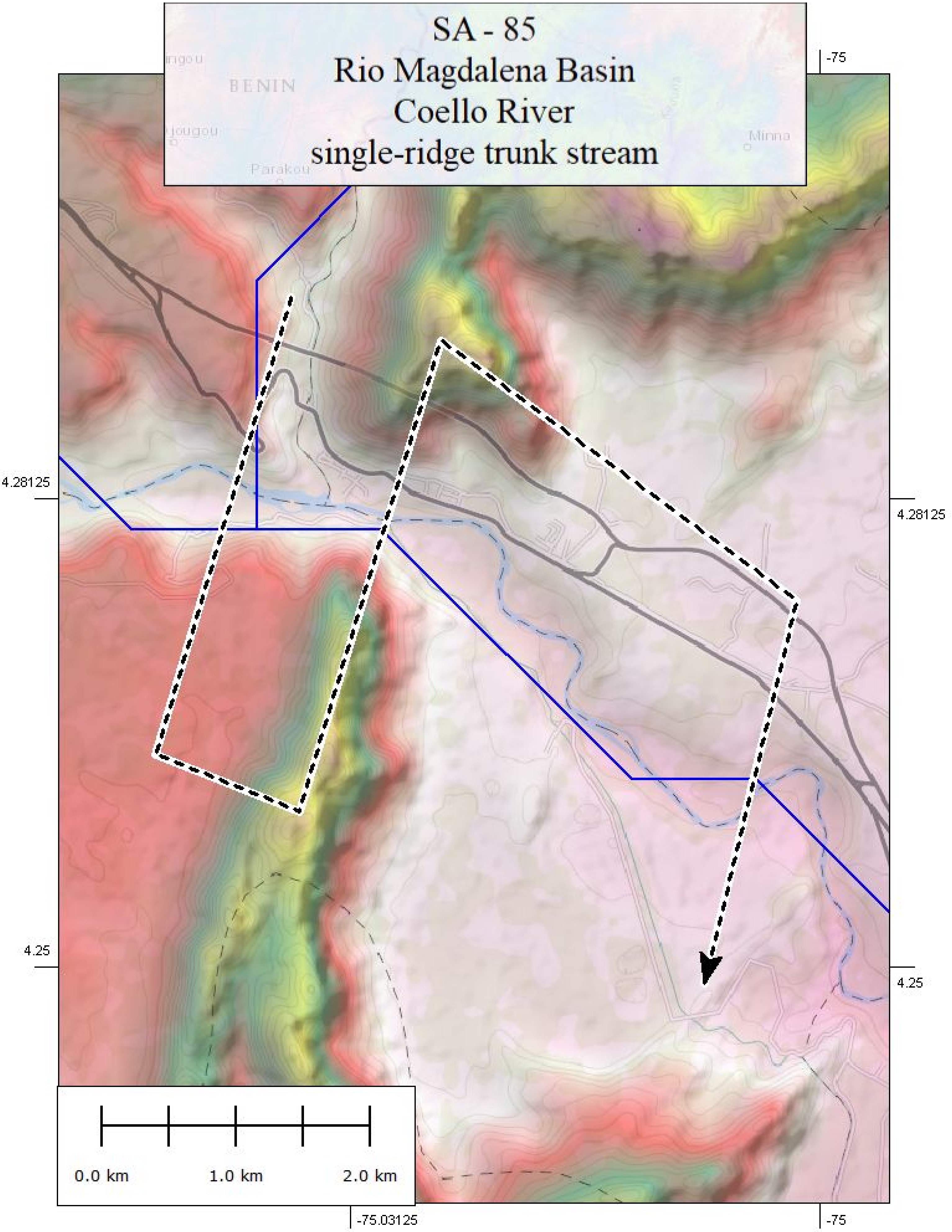
SA - 85
Rio Magdalena Basin
Coello River
single-ridge trunk stream
-75
4.28125
4.28125
4.25
4.25
-75.03125
-75
0.0 km
1.0 km
2.0 km

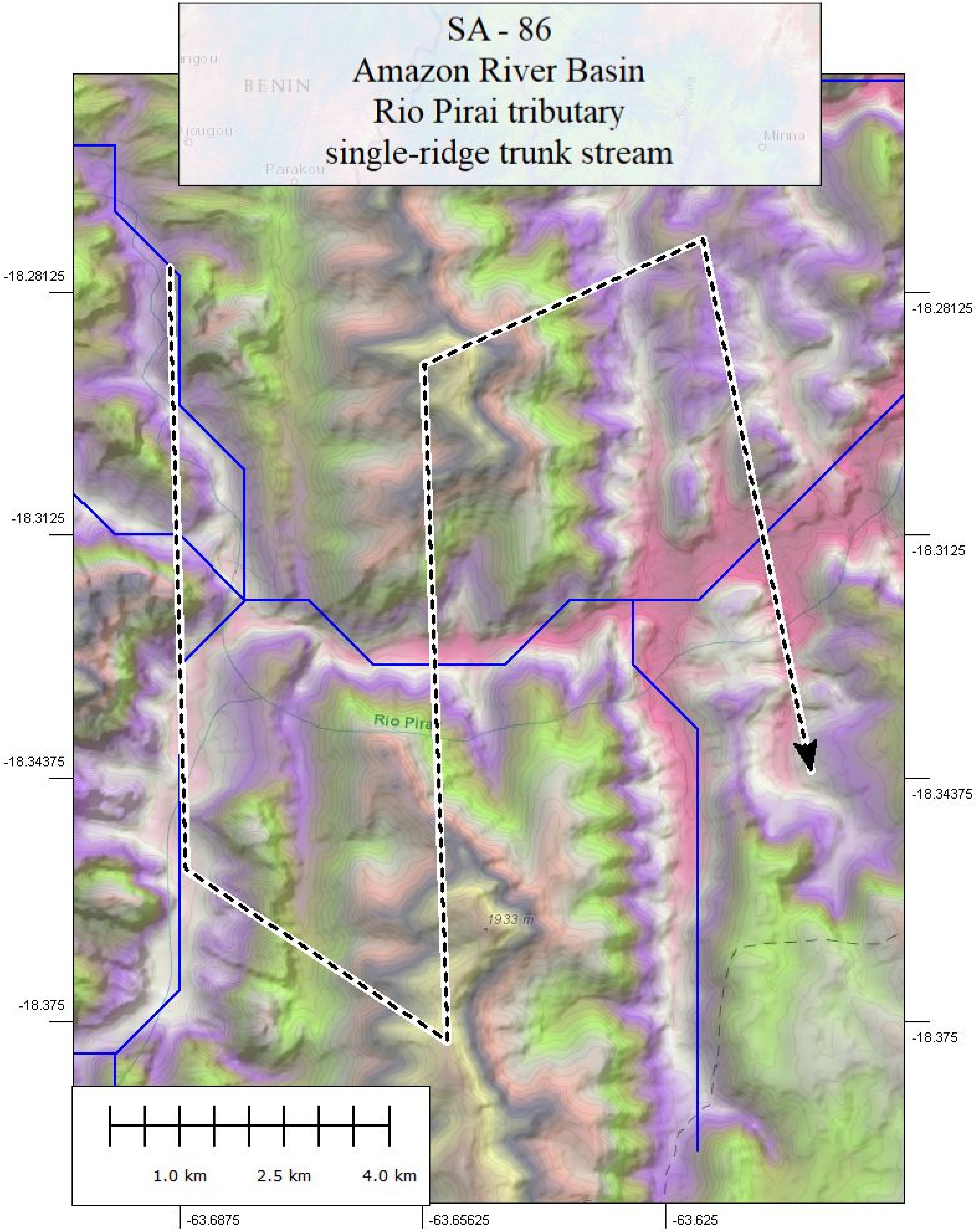

SA - 86
Amazon River Basin
Rio Pirai tributary
single-ridge trunk stream
BENIN
Parakou
Minna
Rio Pirai
1933 m
1.0 km
2.5 km
4.0 km
-18.28125
-18.3125
-18.34375
-18.375
-63.6875
-63.65625
-63.625

SA - 88
Quebrada Maporal Basin
Agua Viva Reservoir
single-ridge trunk stream

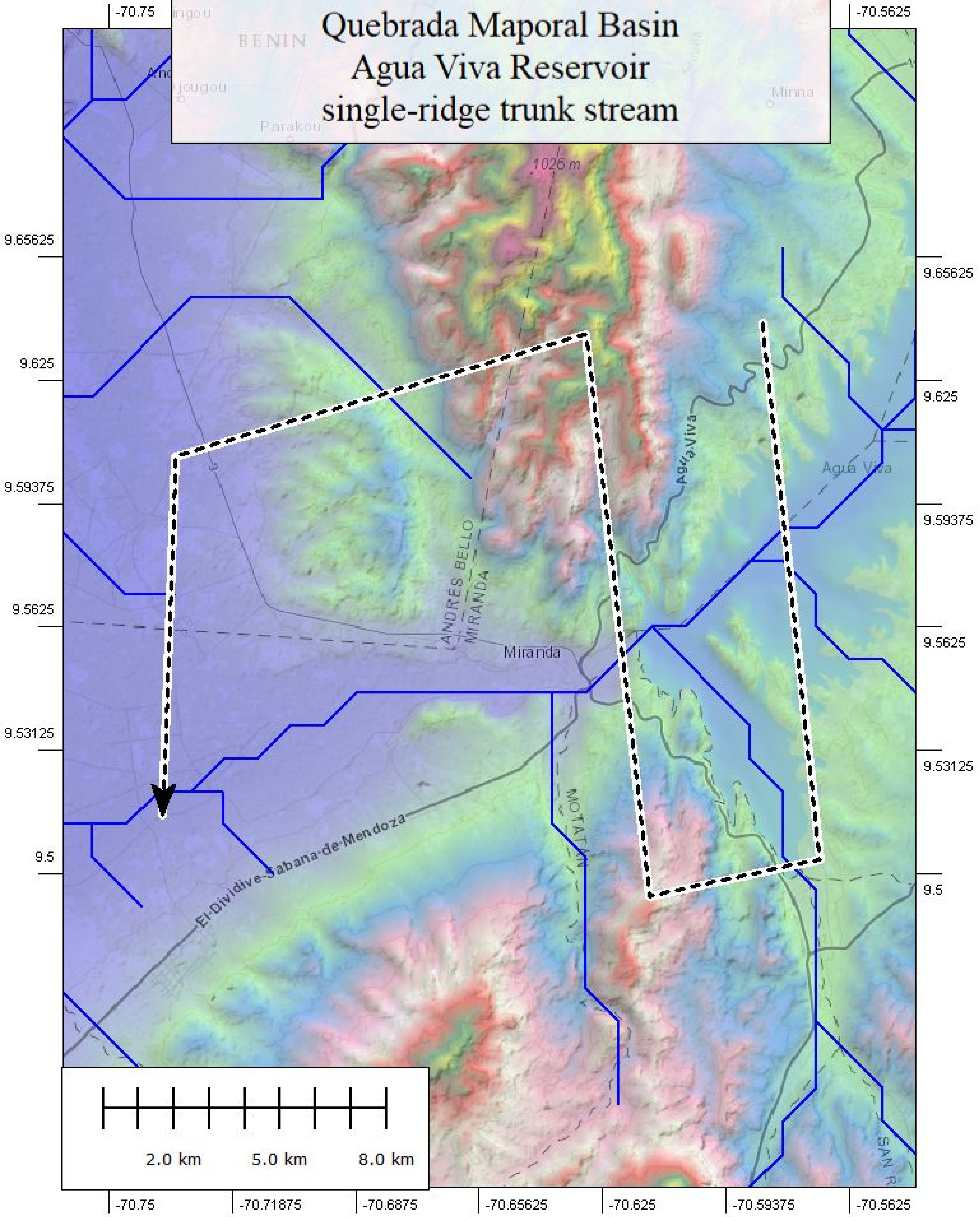

SA - 89
Orinoco River Basin
Bocono River
single-ridge trunk stream

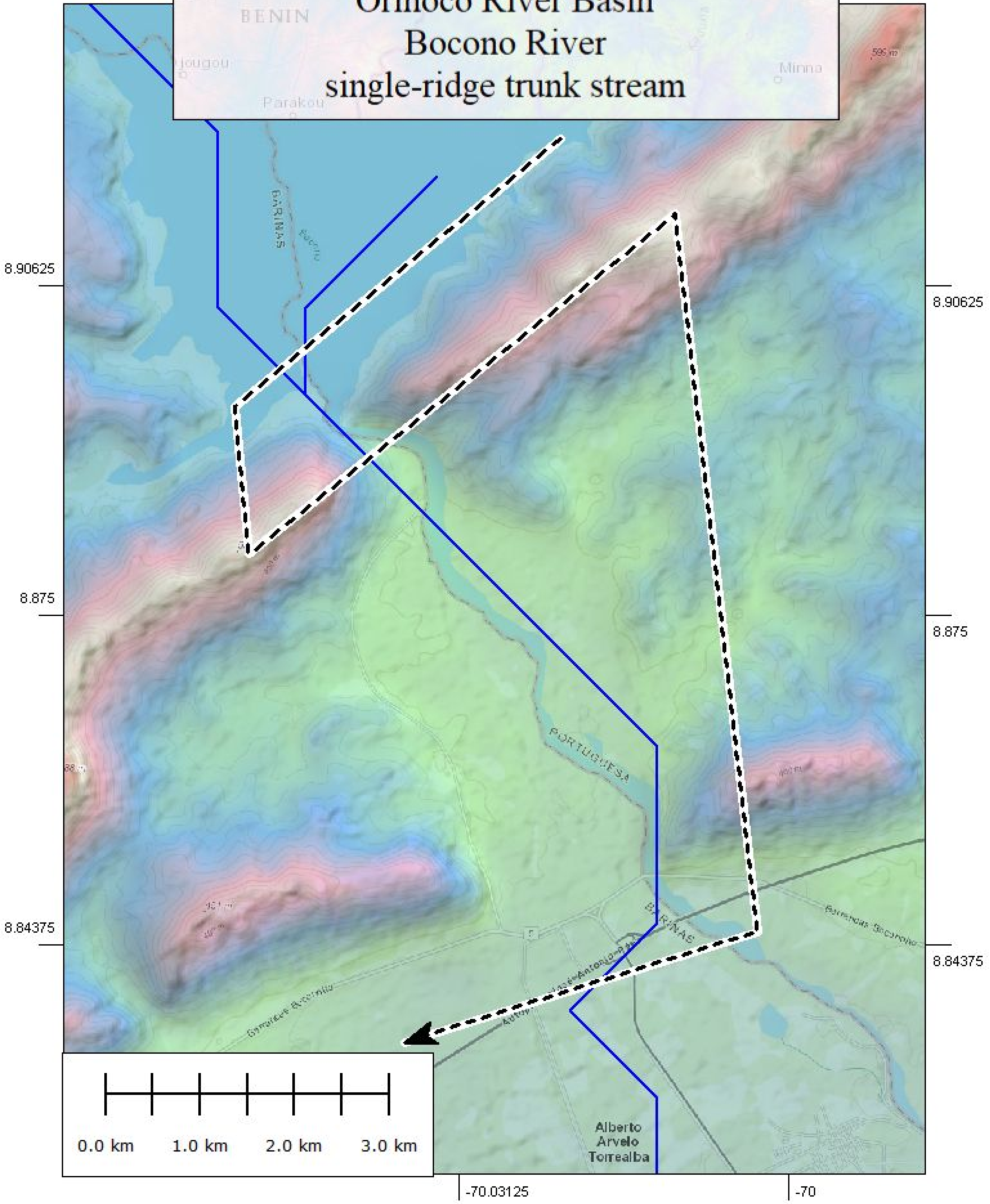

SA - 90
Rio Magdalena Basin
Sogamoso River
single-ridge trunk stream

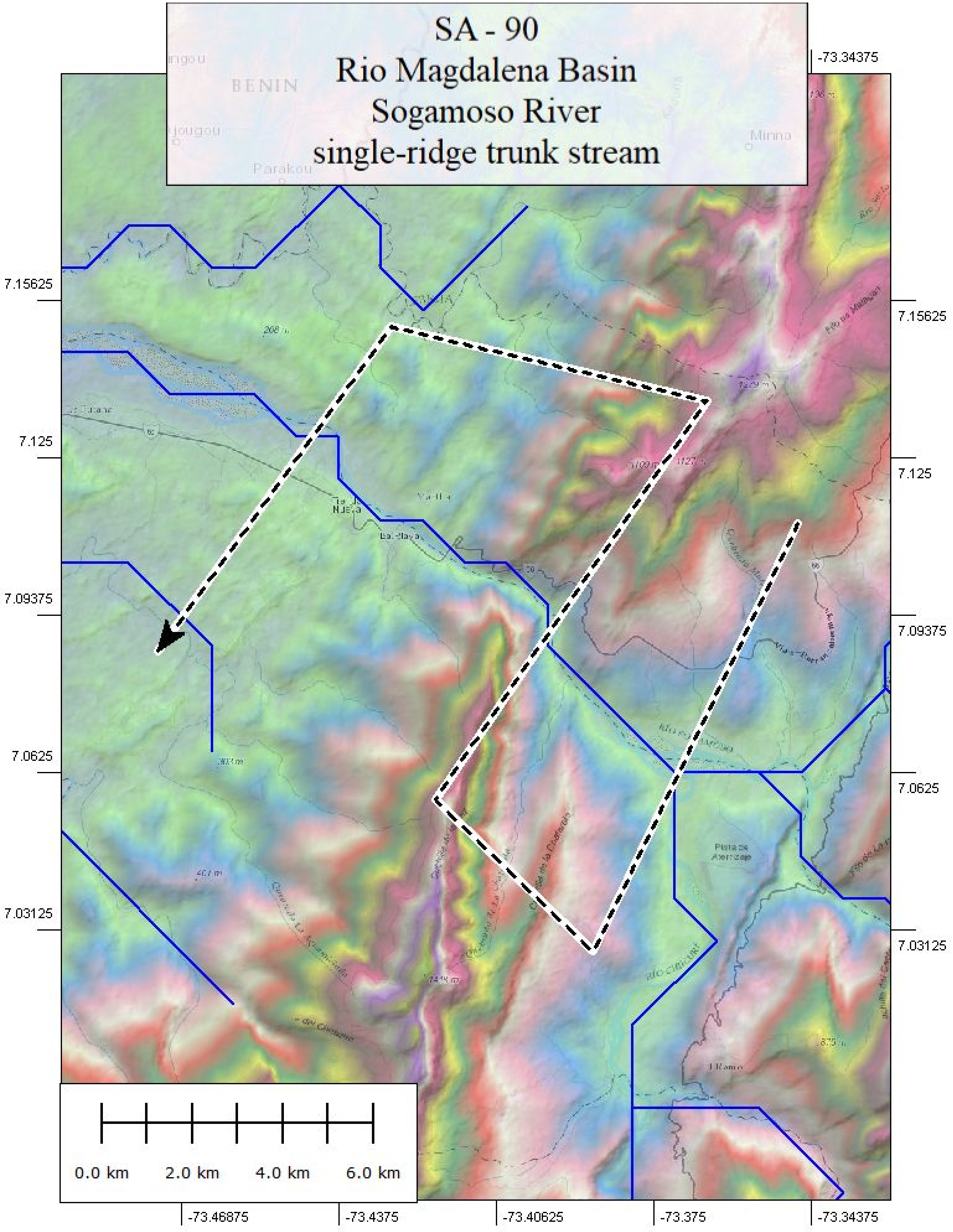

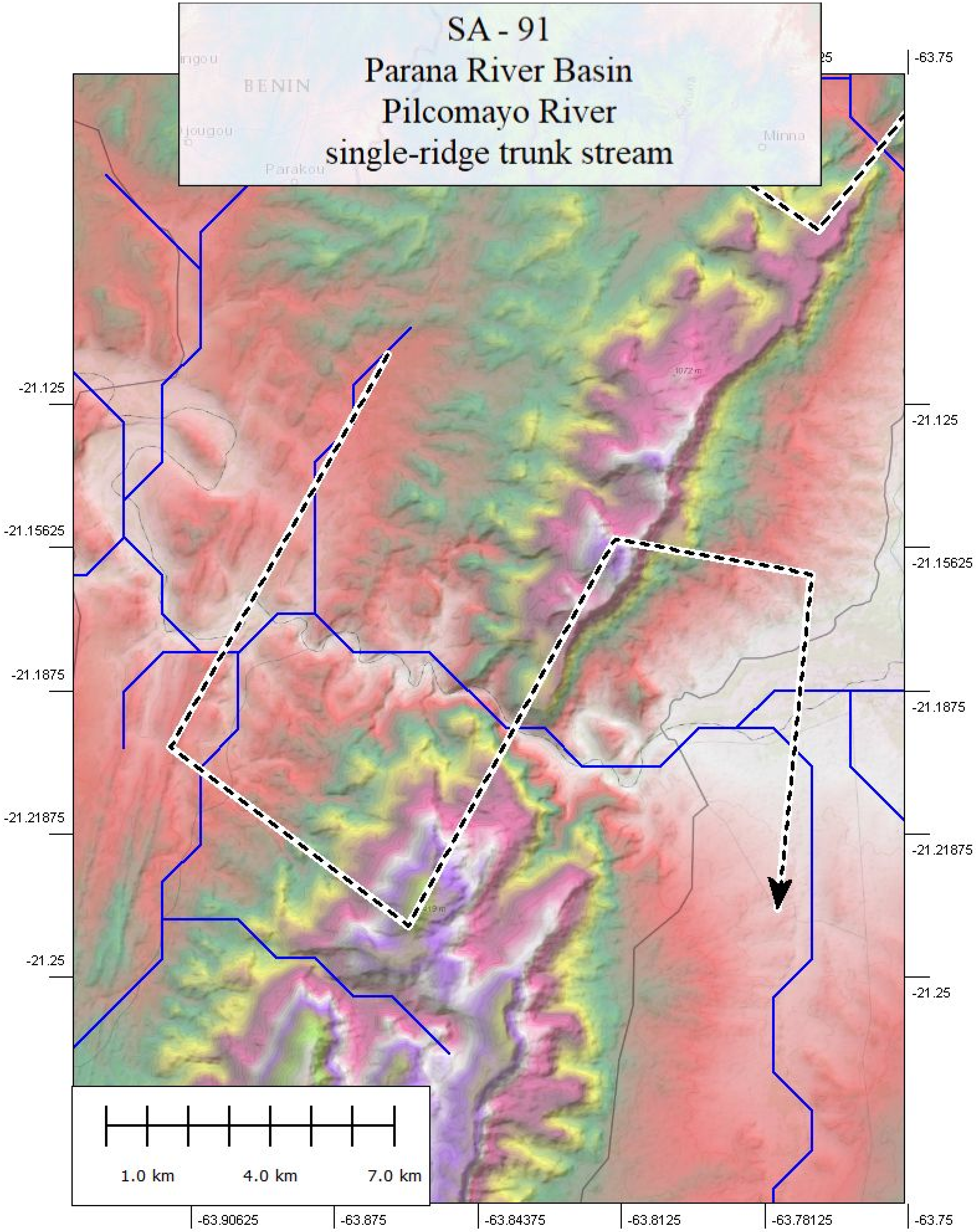
SA - 91
Parana River Basin
Pilcomayo River
single-ridge trunk stream
BENIN
Parakou
Minna
1072 m
-63.75
-21.125
-21.15625
-21.1875
-21.21875
-21.25
1.0 km
4.0 km
7.0 km
-63.90625
-63.875
-63.84375
-63.8125
-63.78125
-63.75

SA - 92
Amazon River Basin
Rio Grande O Guapay tributary
single-ridge trunk stream

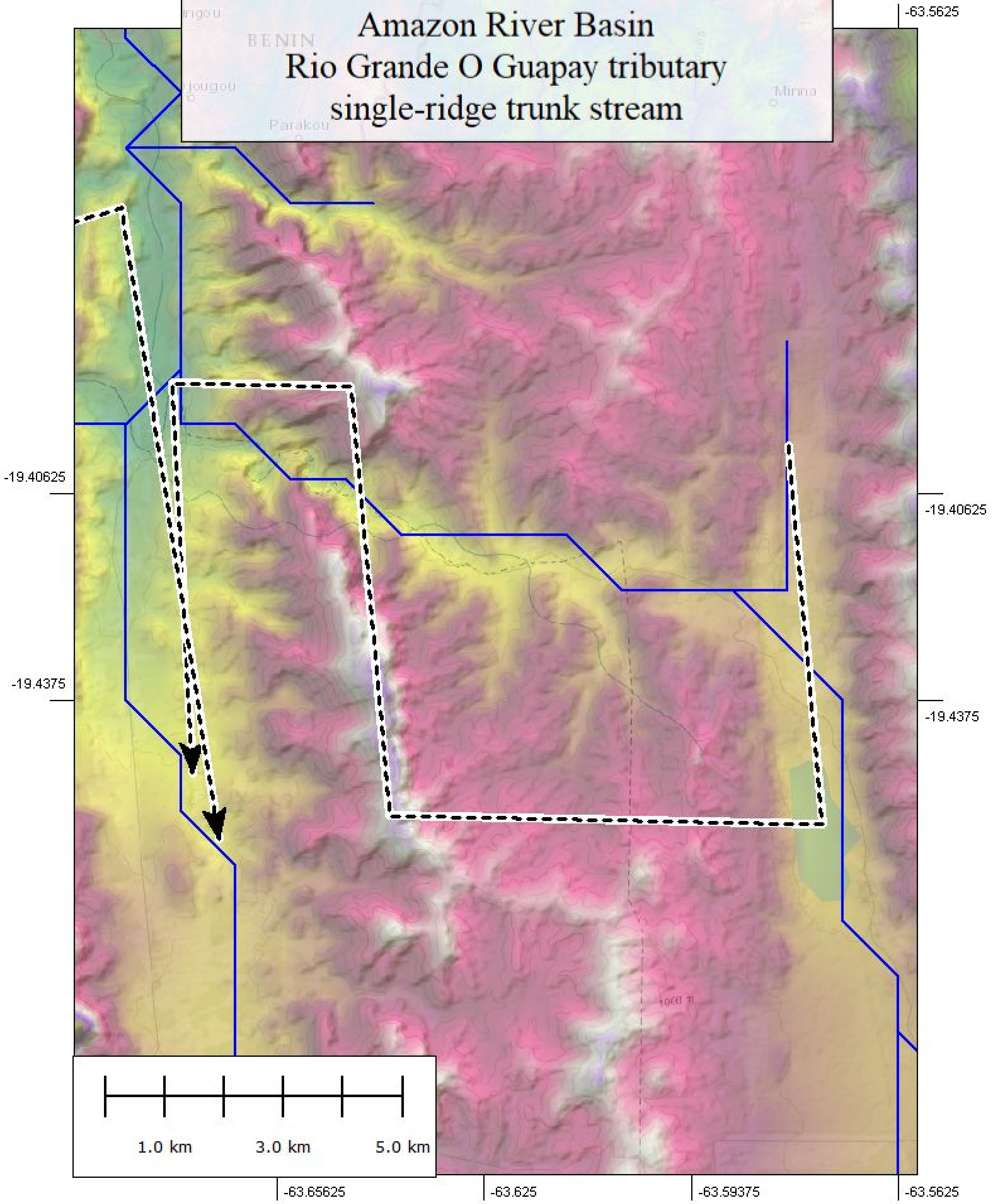

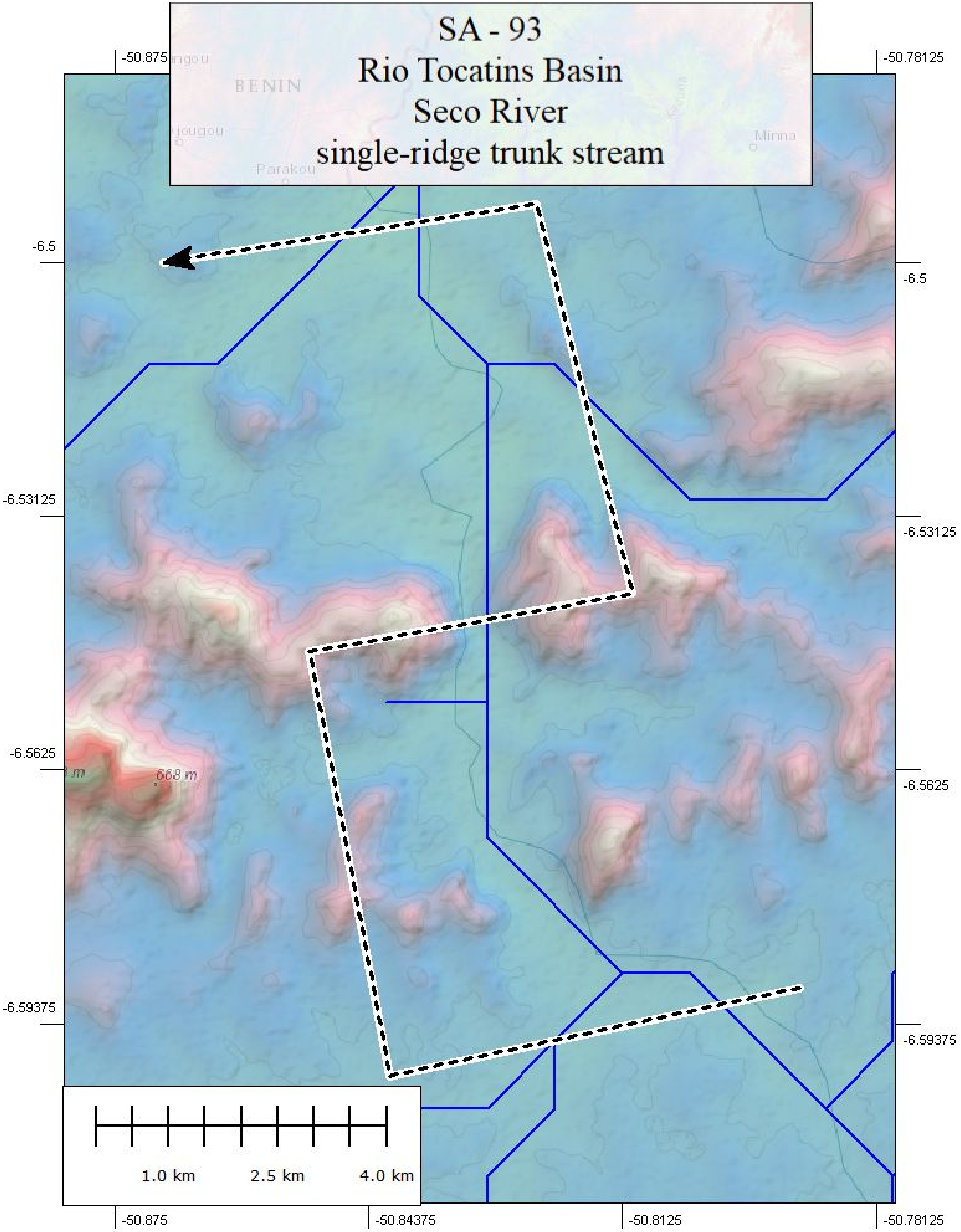

SA - 93
Rio Tocatins Basin
Seco River
single-ridge trunk stream
-50.875
-50.78125
-6.5
-6.53125
-6.5625
-6.59375
-50.84375
-50.8125
668 m
1.0 km
2.5 km
4.0 km
BENIN
Parakou
Minna

SA - 94
Orinoco River Basin
Quebrada La Gritona
single-ridge trunk stream

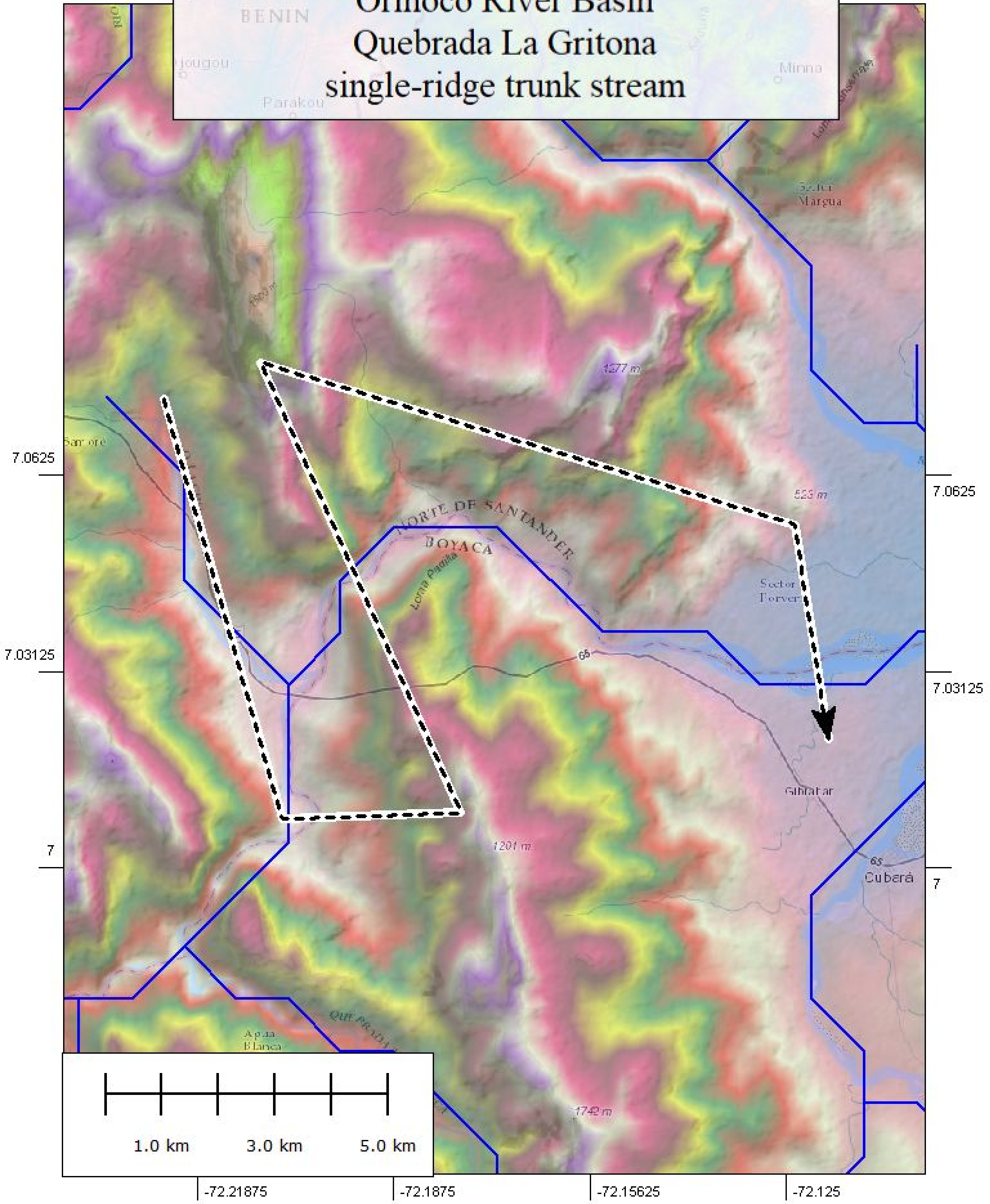

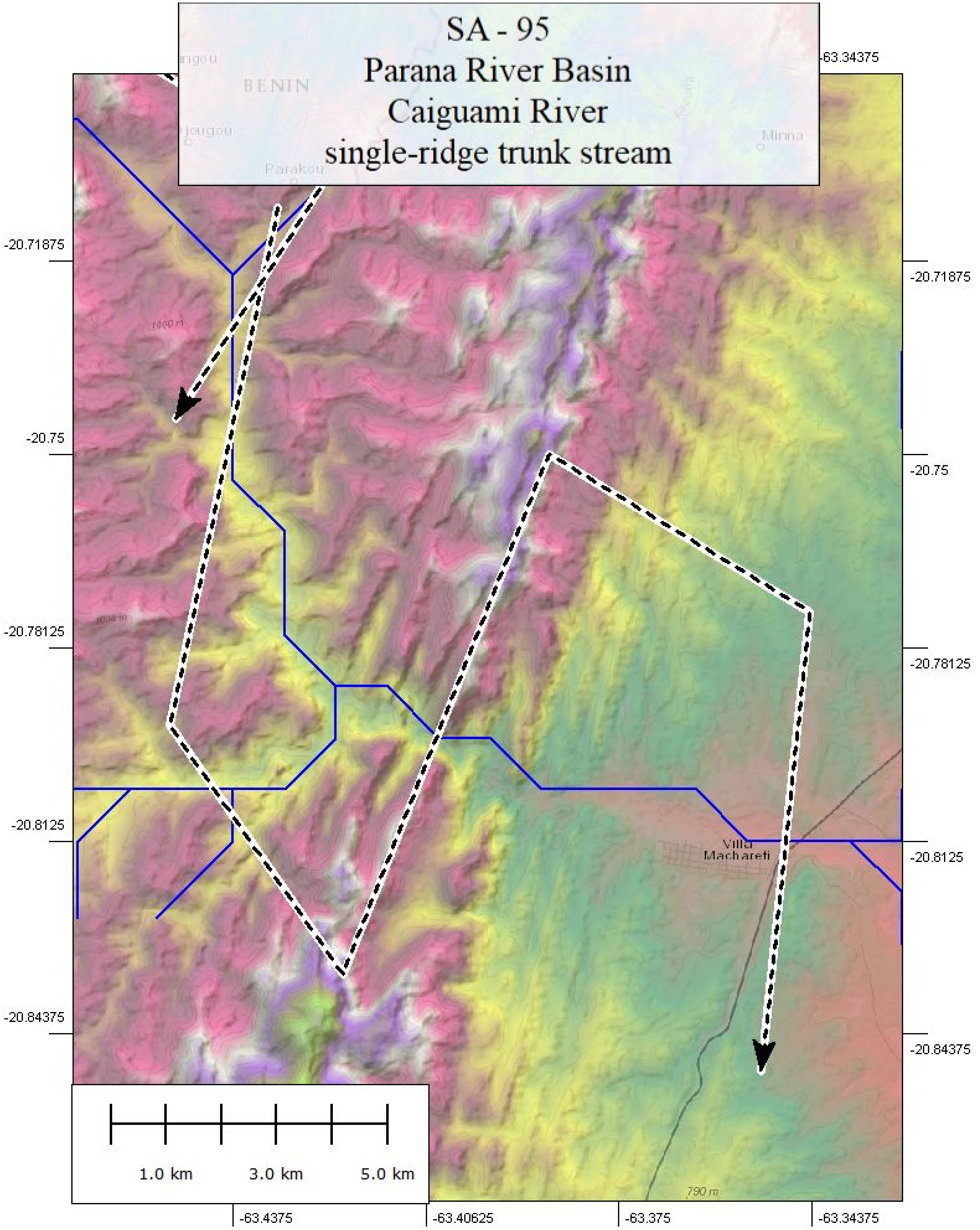
SA - 95
Parana River Basin
Caiguami River
single-ridge trunk stream
BENIN
Parakou
Minna
-63.34375
-20.71875
-20.75
-20.78125
-20.8125
-20.84375
1000 m
Villa
Machareti
790 m
1.0 km
3.0 km
5.0 km
-63.4375
-63.40625
-63.375
-63.34375

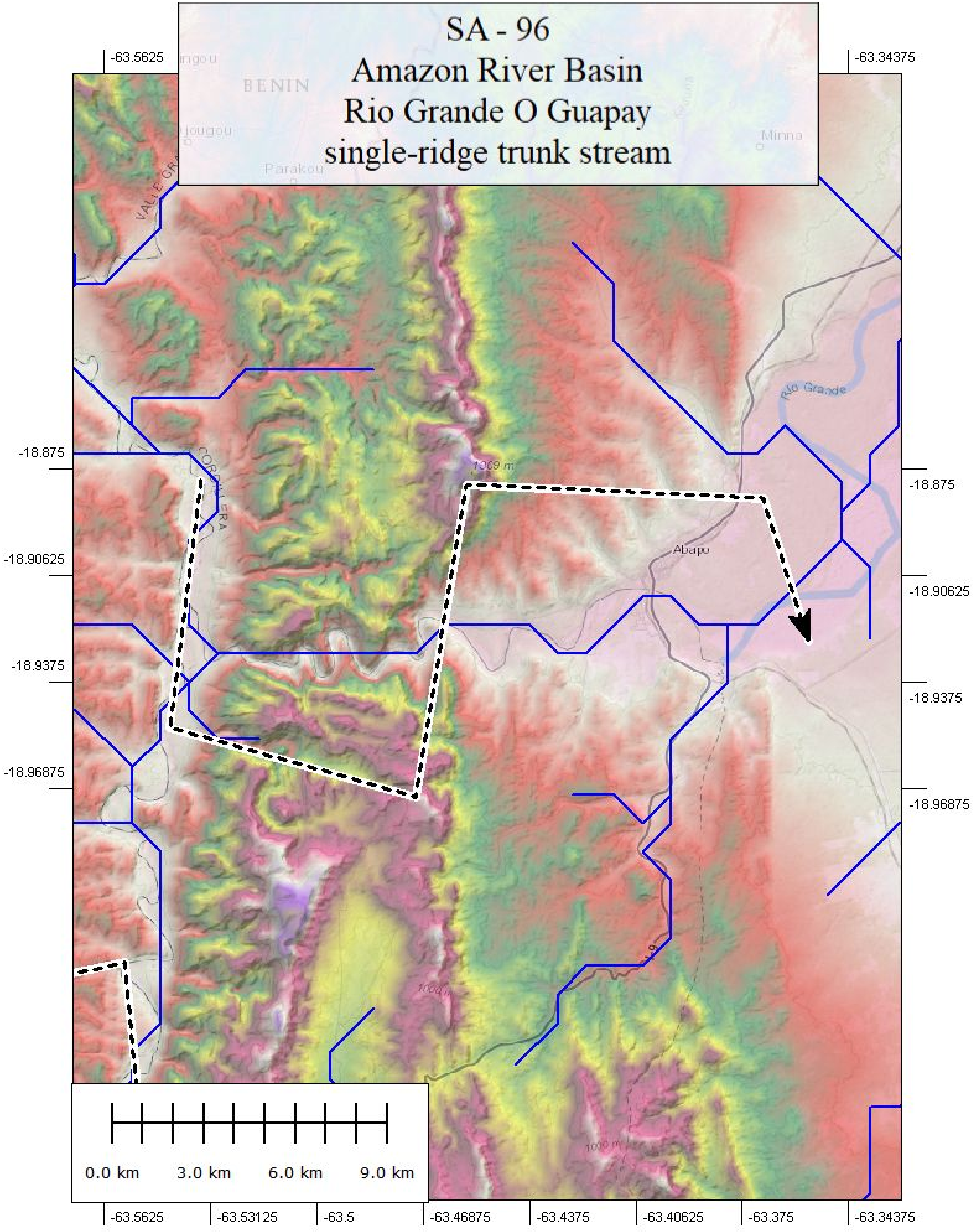

SA - 96
Amazon River Basin
Rio Grande O Guapay
single-ridge trunk stream
-63.5625
-63.34375
BENIN
Parakou
Minna
Rio Grande
1009 m
Abapo
1000 m
-18.875
-18.90625
-18.9375
-18.96875
0.0 km
3.0 km
6.0 km
9.0 km
-63.5625
-63.53125
-63.5
-63.46875
-63.4375
-63.40625
-63.375
-63.34375

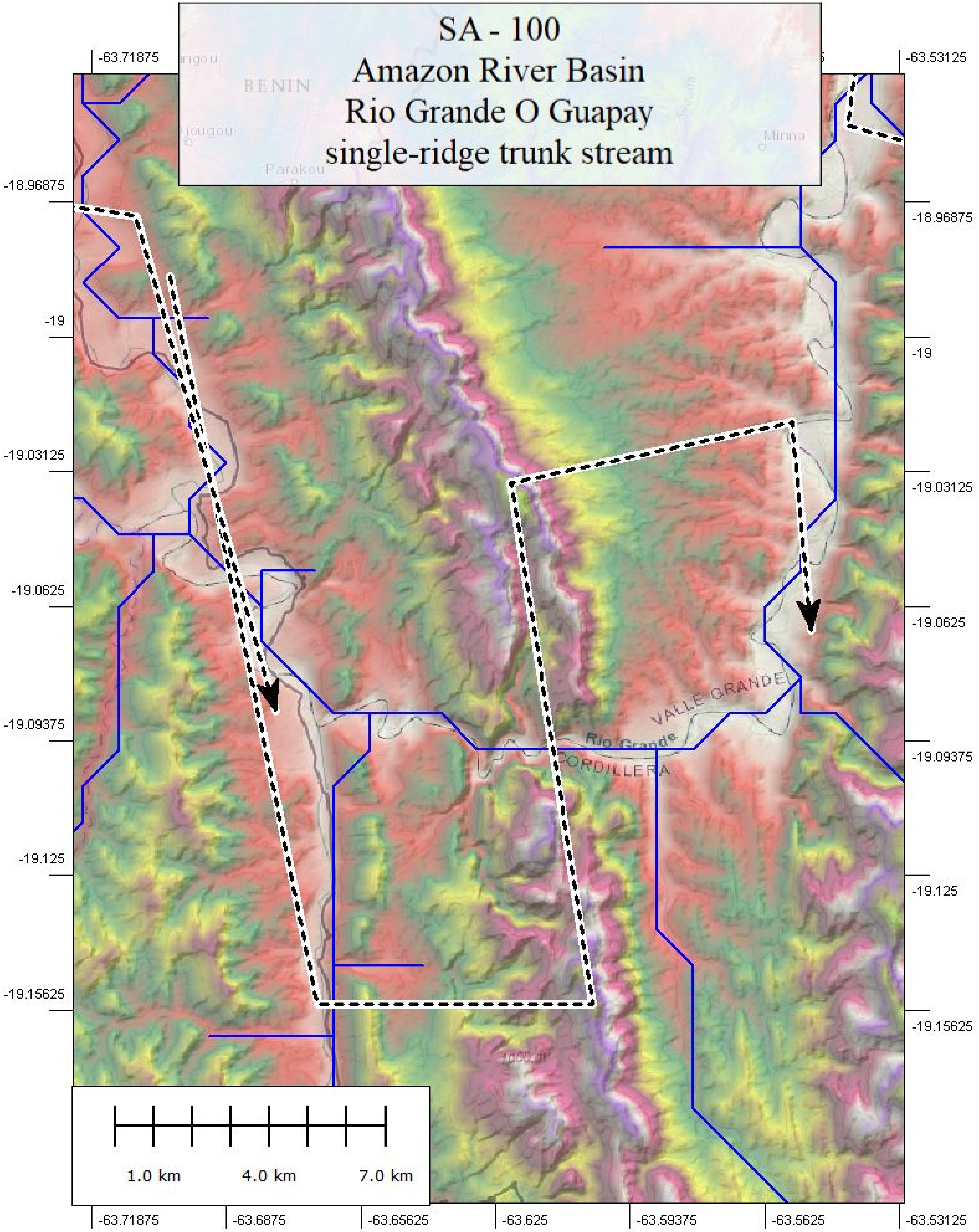
SA - 100
Amazon River Basin
Rio Grande O Guapay
single-ridge trunk stream
-63.71875
-63.53125
-18.96875
-19
-19.03125
-19.0625
-19.09375
-19.125
-19.15625
VALLE GRANDE
Rio Grande
CORDILLERA
1.0 km
4.0 km
7.0 km
-63.6875
-63.65625
-63.625
-63.59375
-63.5625

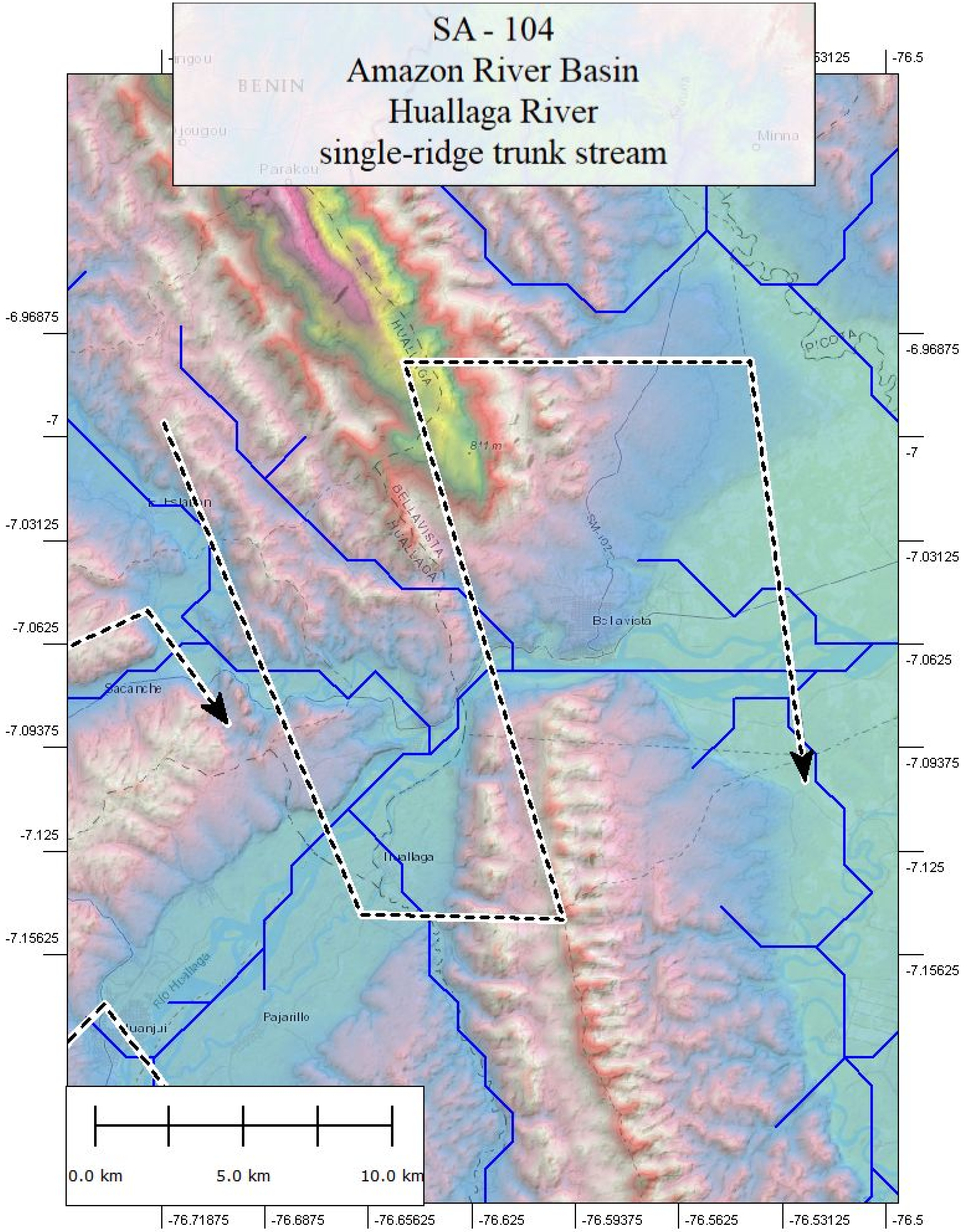
SA - 104
Amazon River Basin
Huallaga River
single-ridge trunk stream
BENIN
Parakou
Minna
HUALLAGA
811 m
BELLAVISTA
HUALLAGA
Bellavista
Sacanche
Huallaga
Pajarillo
Juanjui
Rio Huallaga
PICOTA
SM-102
-6.96875
-7
-7.03125
-7.0625
-7.09375
-7.125
-7.15625
-76.71875
-76.6875
-76.65625
-76.625
-76.59375
-76.5625
-76.53125
-76.5
0.0 km
5.0 km
10.0 km

SA - 105
Amazon River Basin
Huallaga River
single-ridge trunk stream

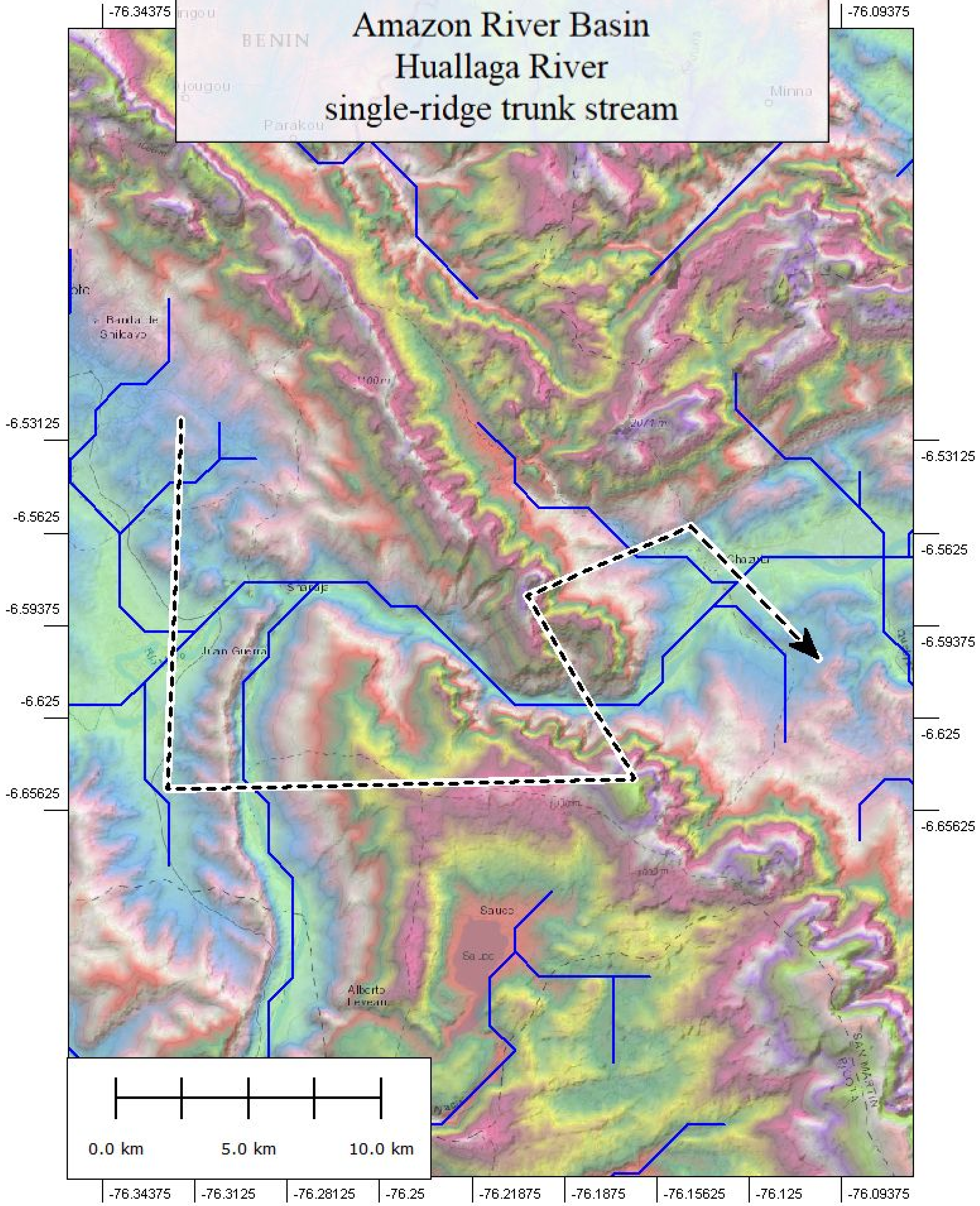

SA - 106
Amazon River Basin
Tupuani River tributary
single-ridge trunk stream

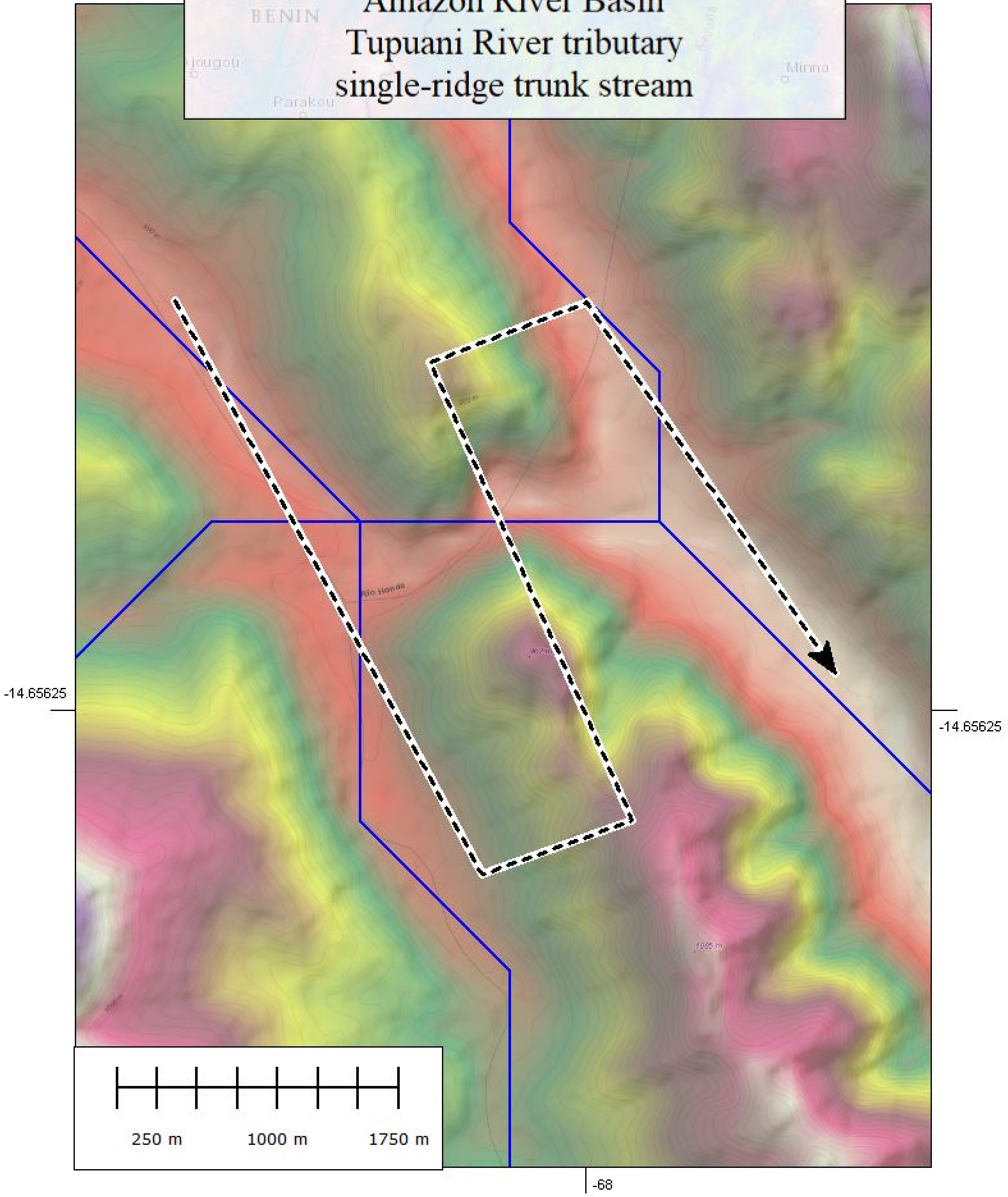

SA - 108
Amazon River Basin
Madre de Dios River tributary
single-ridge trunk stream

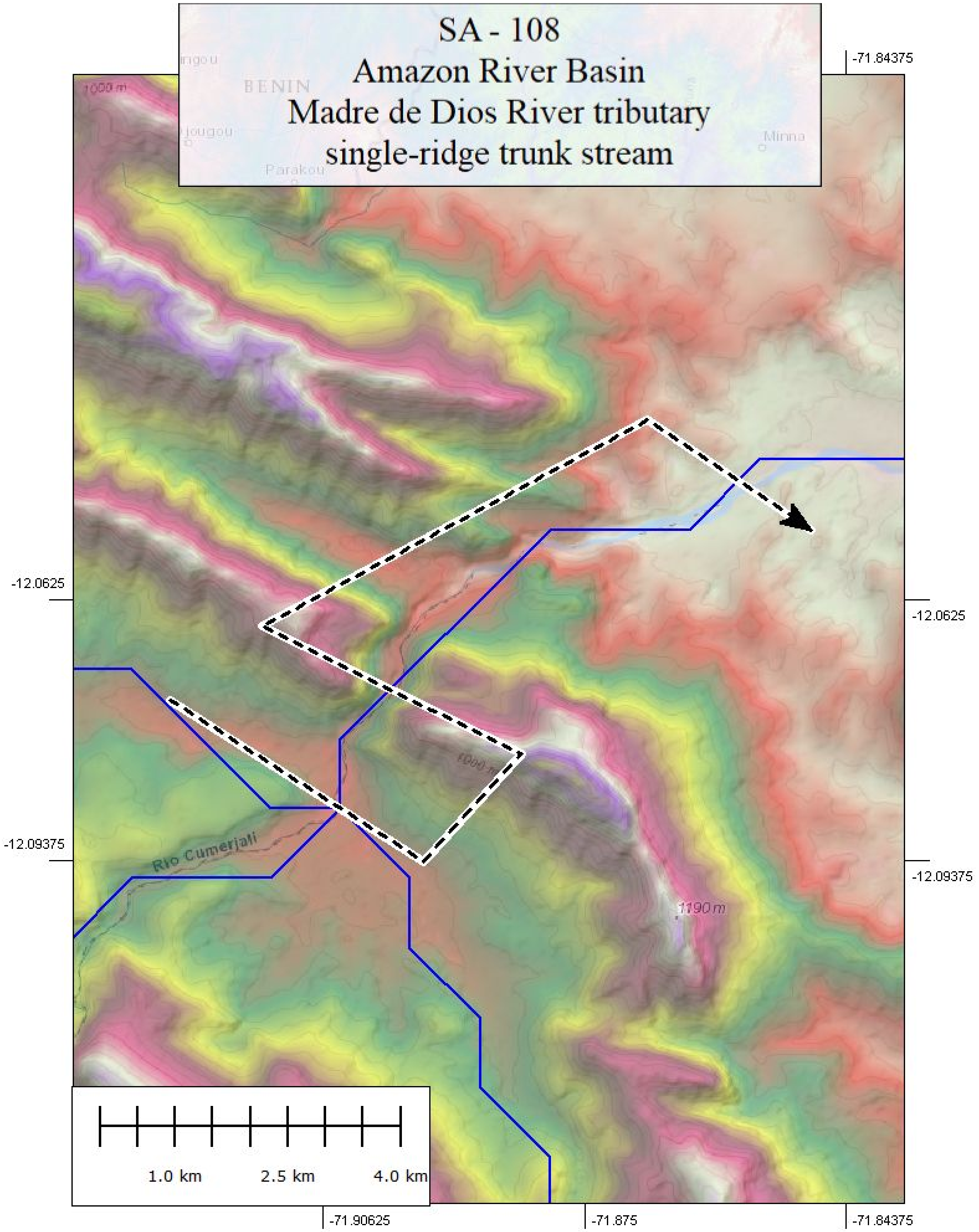

SA - 109
Amazon River Basin
Pini Pini River
single-ridge trunk stream

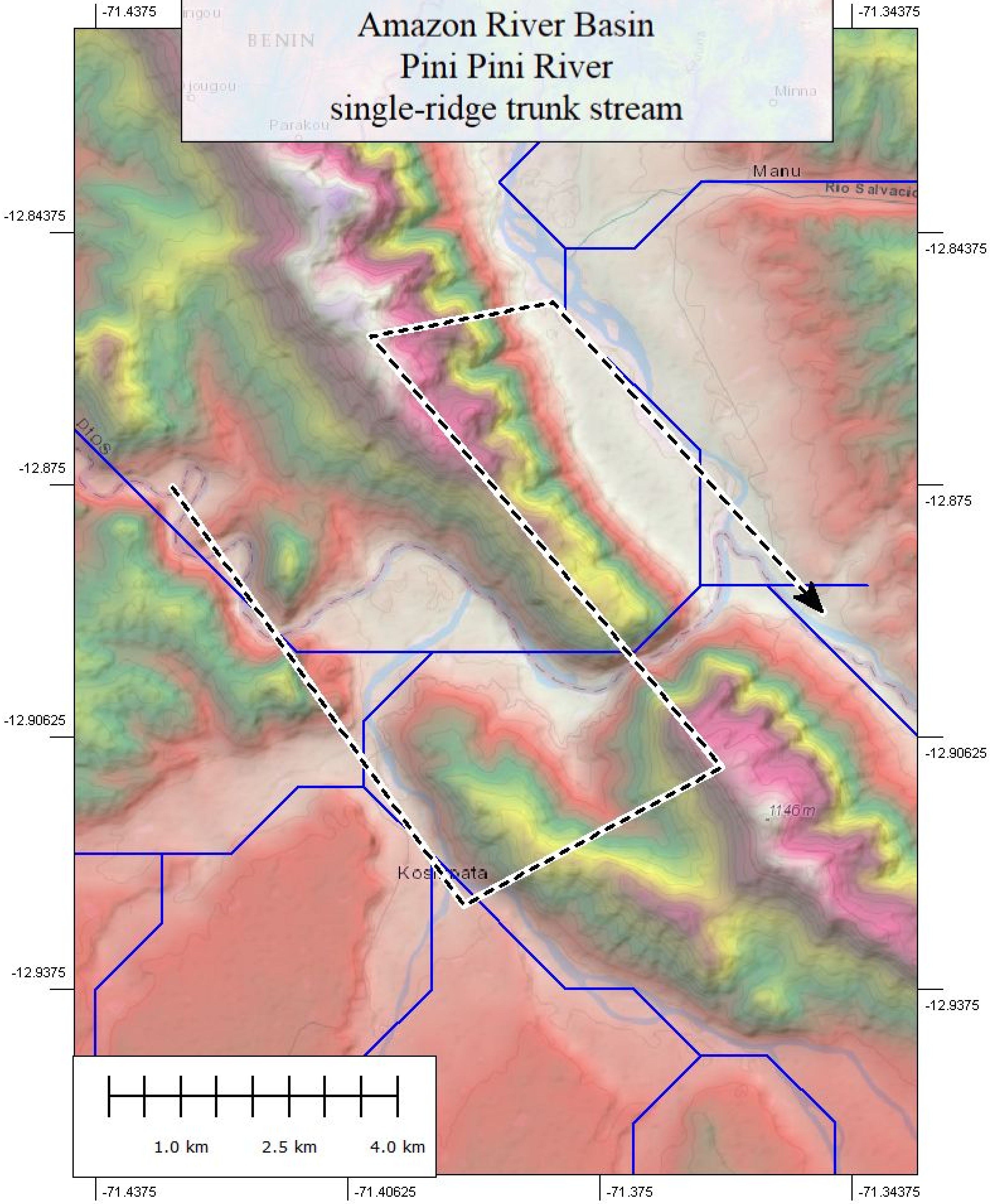

SA - 110
Amazon River Basin
Ichoa River tributary
single-ridge trunk stream

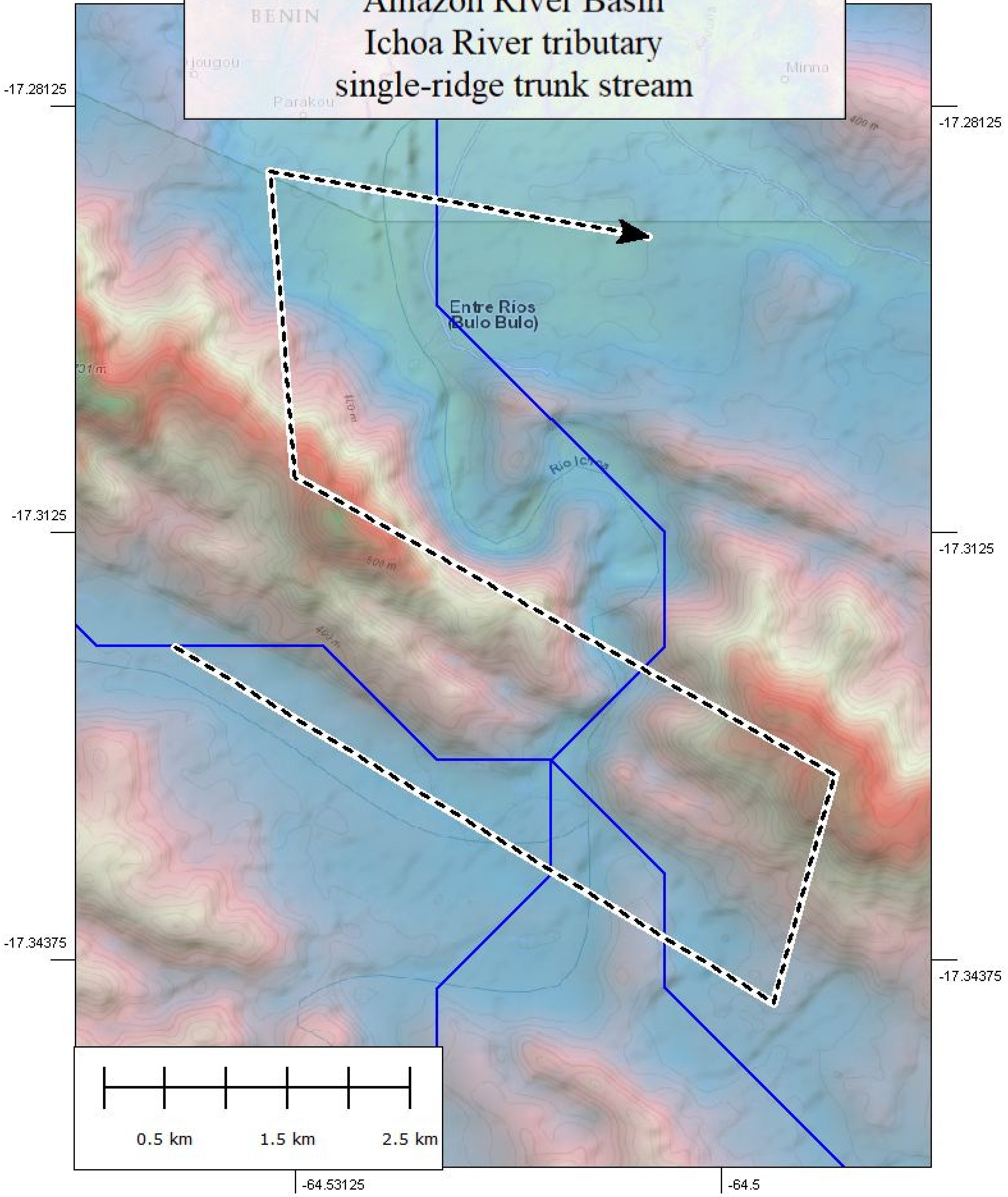

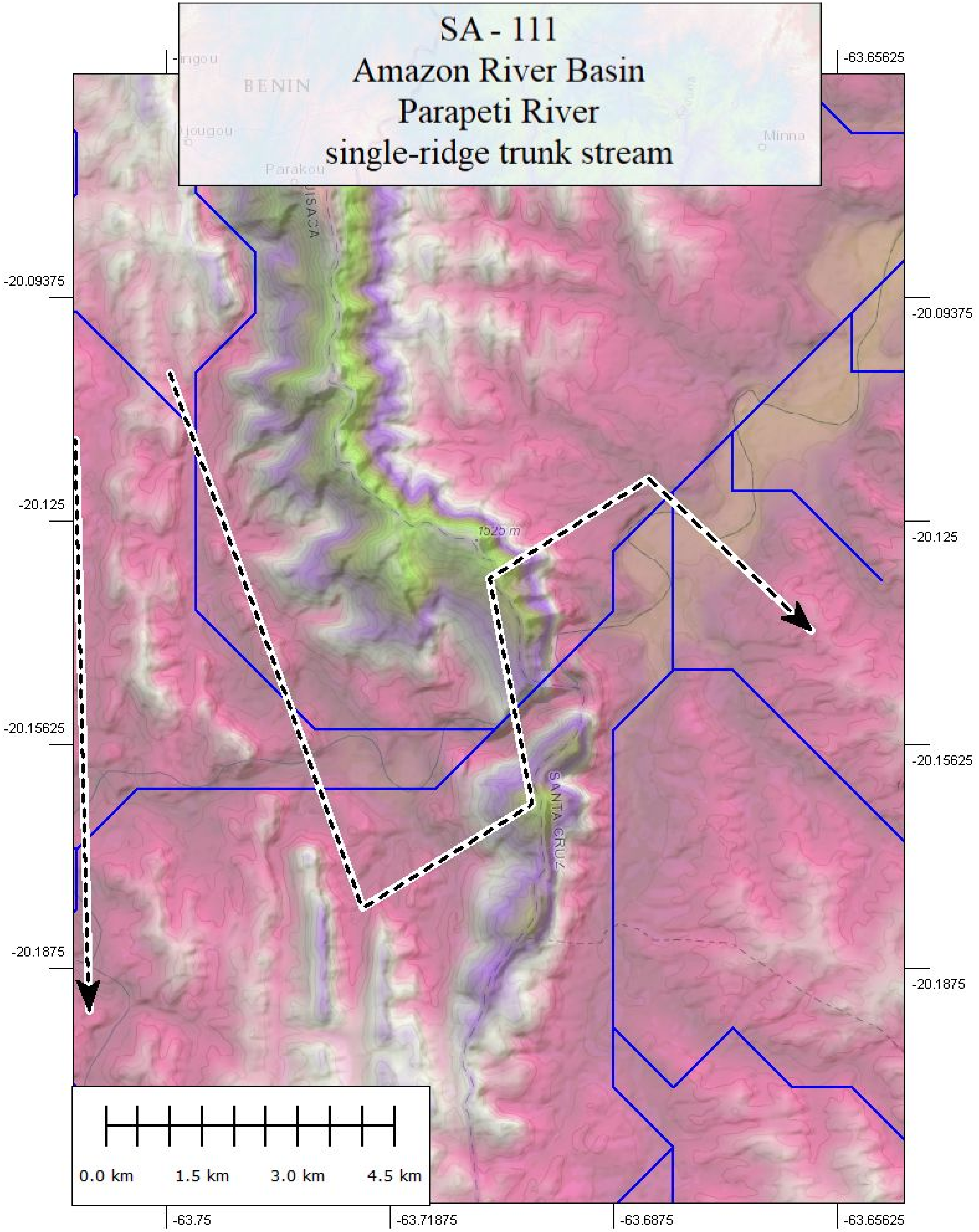
SA - 111
Amazon River Basin
Parapeti River
single-ridge trunk stream
BENIN
Parakou
Minna
1525 m
SANTA CRUZ
-63.65625
-20.09375
-20.125
-20.15625
-20.1875
-63.75
-63.71875
-63.6875
0.0 km
1.5 km
3.0 km
4.5 km

SA - 113
Orinoco River Basin
Upia River
single-ridge trunk stream

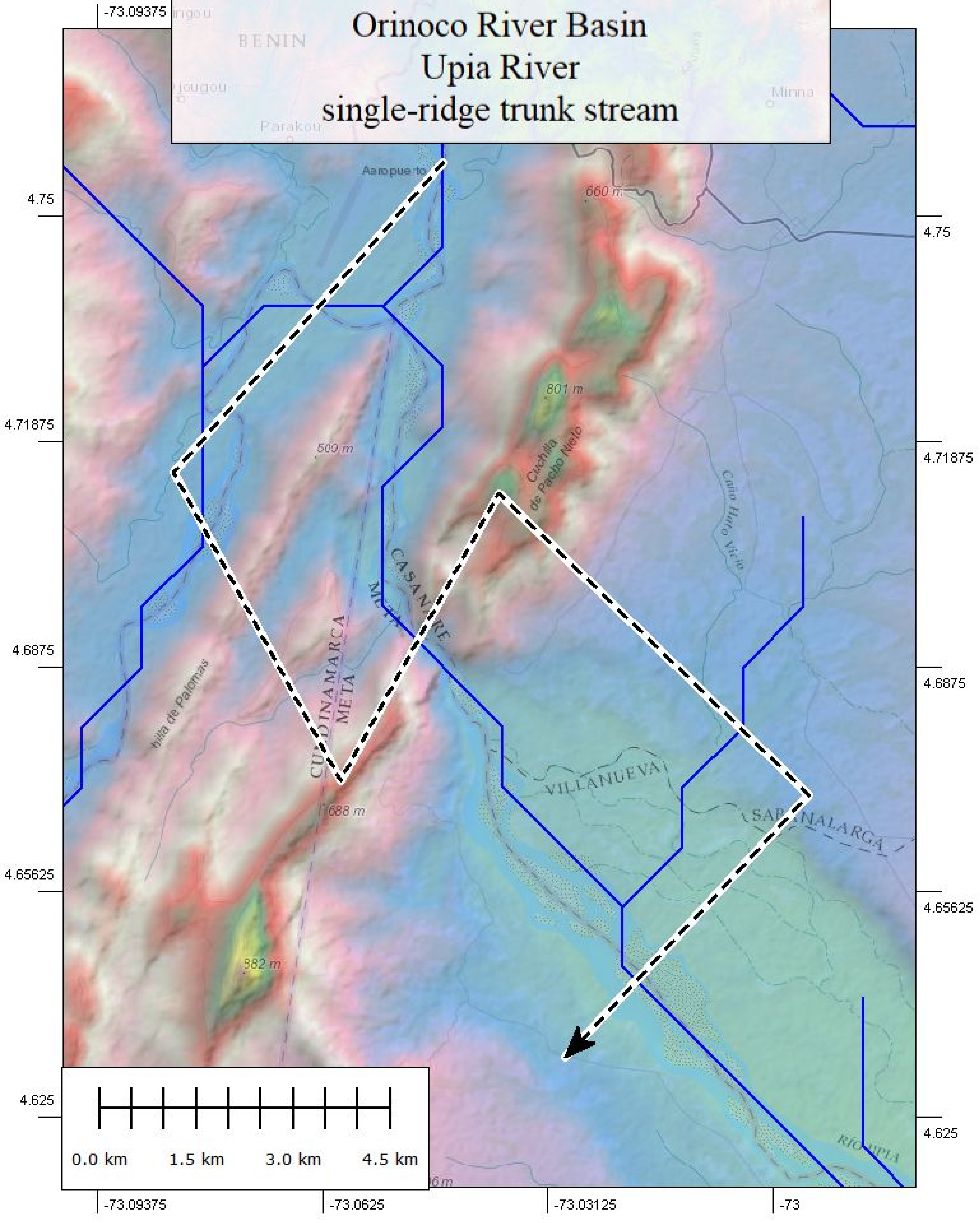

SA - 114
Rio Magdalena Basin
Tetuan River
single-ridge trunk stream

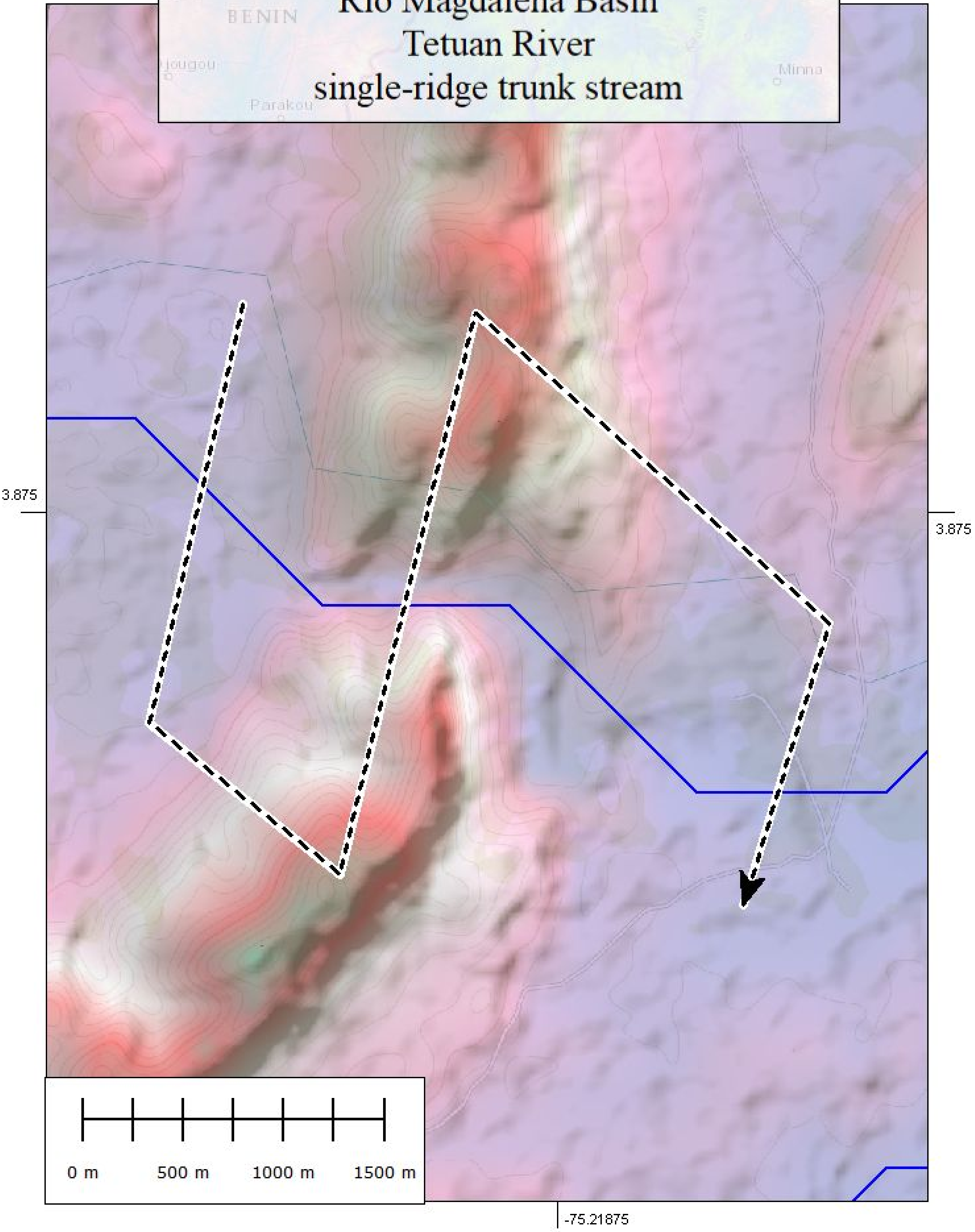

SA - 121
Amazon River Basin
Pozuzo River
single-ridge trunk stream

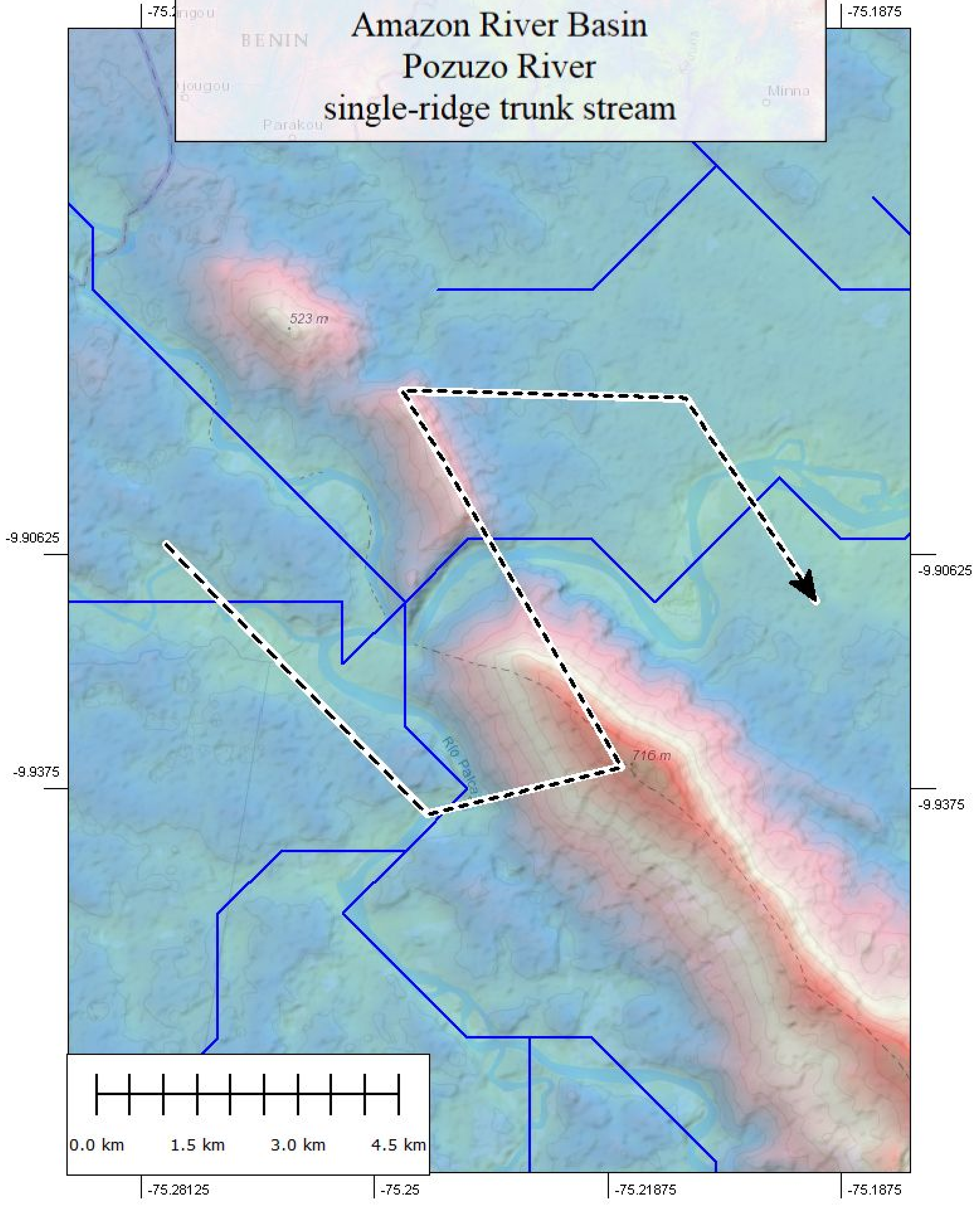

SA - 123
Amazon River Basin
Maranon River
single-ridge trunk stream

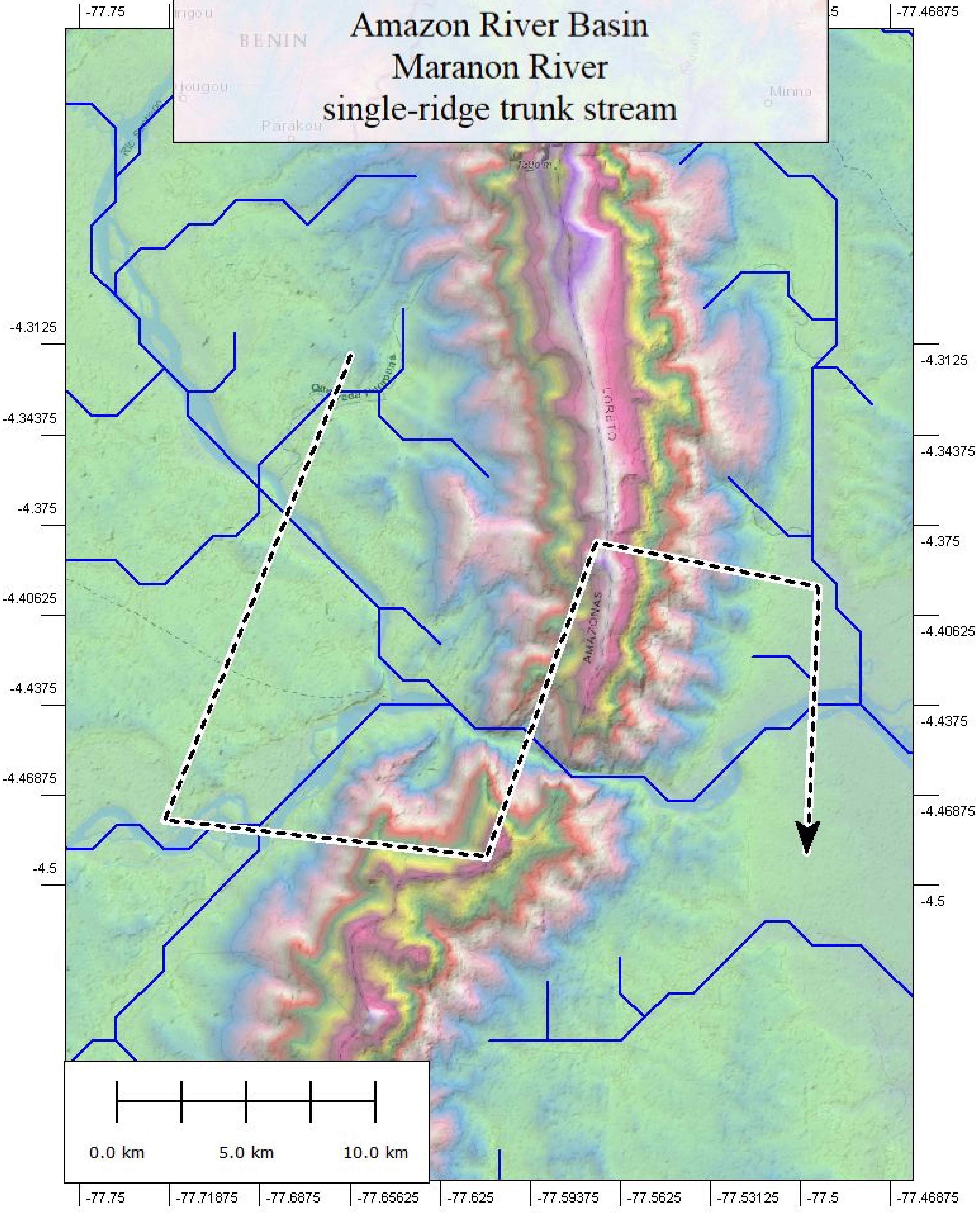

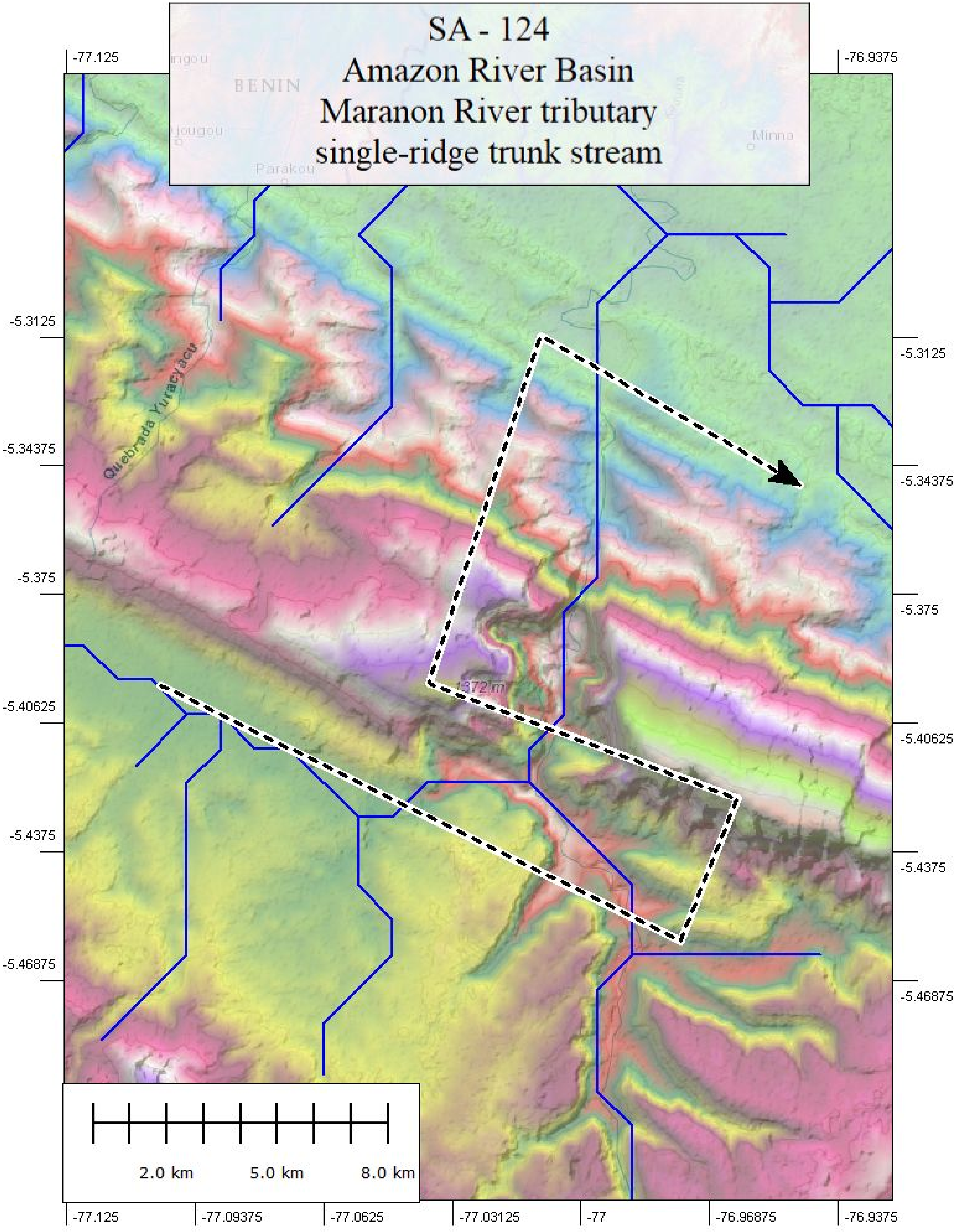
SA - 124
Amazon River Basin
Maranon River tributary
single-ridge trunk stream
BENIN
Parakou
Minna
Quebrada Yuracyacu
1372 m
-77.125
-77.09375
-77.0625
-77.03125
-77
-76.96875
-76.9375
-5.3125
-5.34375
-5.375
-5.40625
-5.4375
-5.46875
2.0 km
5.0 km
8.0 km

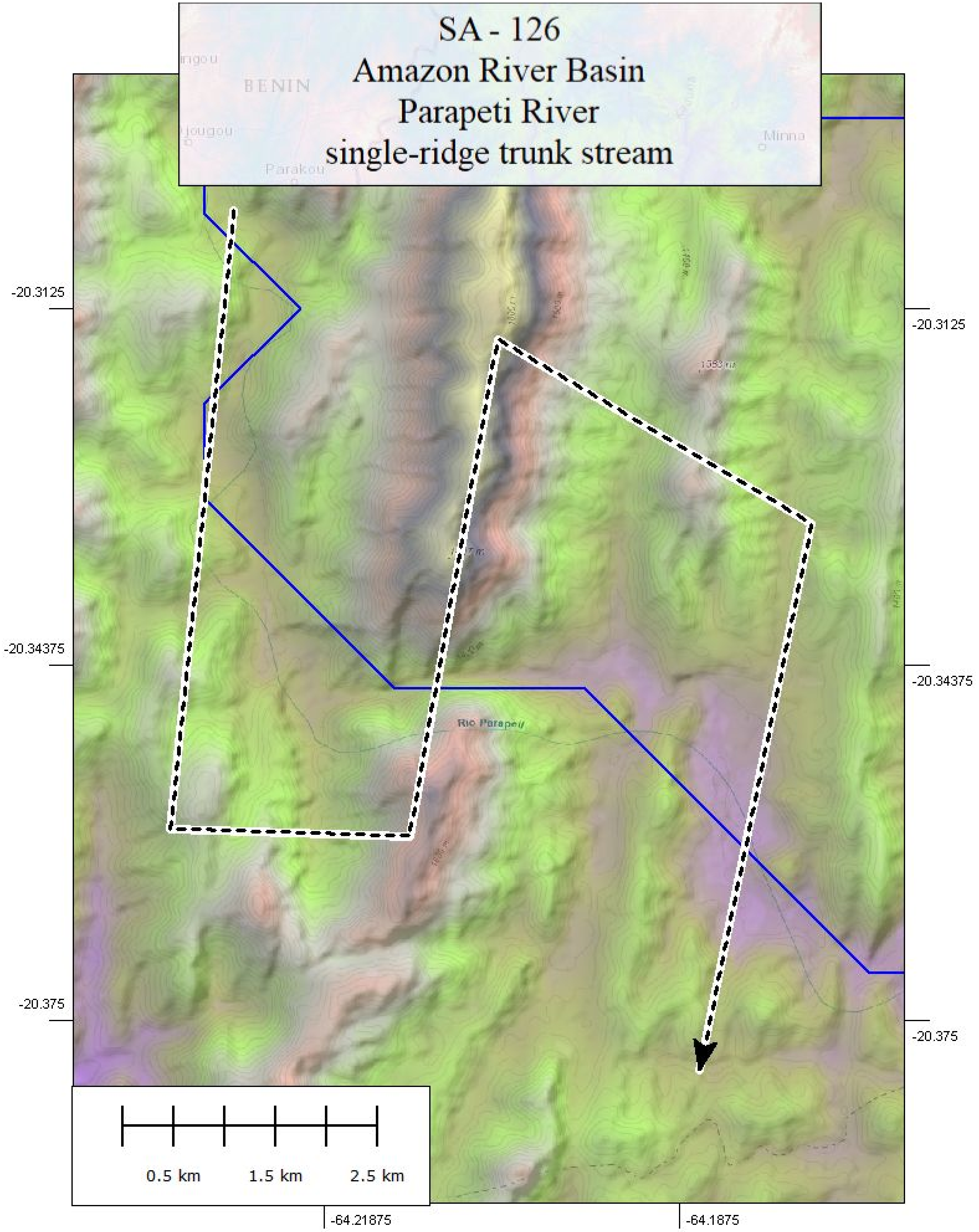
SA - 126
Amazon River Basin
Parapeti River
single-ridge trunk stream
BENIN
Parakou
Minna
Rio Parapeti
-20.3125
-20.34375
-20.375
-64.21875
-64.1875
0.5 km
1.5 km
2.5 km

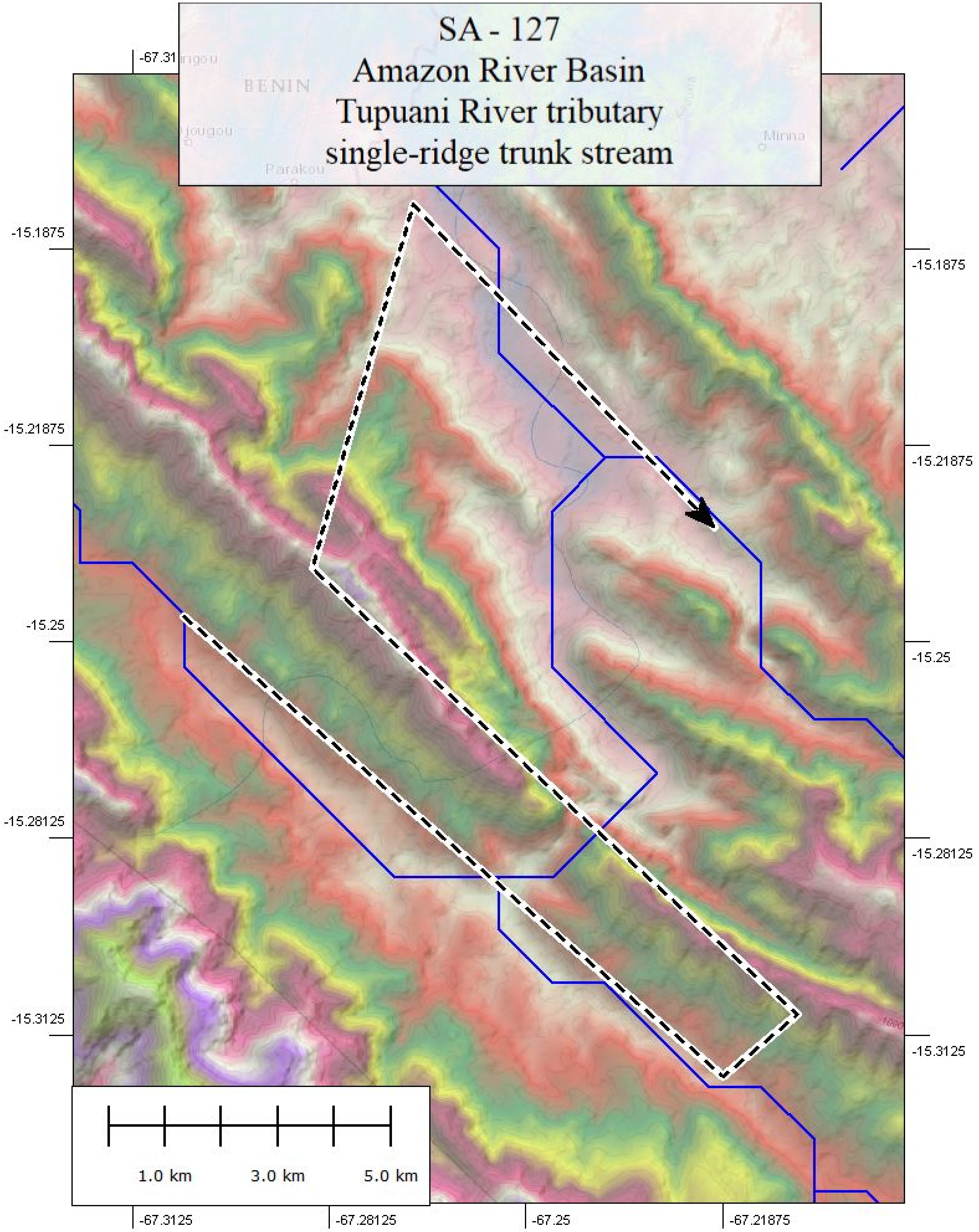
SA - 127
Amazon River Basin
Tupuani River tributary
single-ridge trunk stream
-67.31
-15.1875
-15.21875
-15.25
-15.28125
-15.3125
-67.3125
-67.28125
-67.25
-67.21875
1.0 km
3.0 km
5.0 km

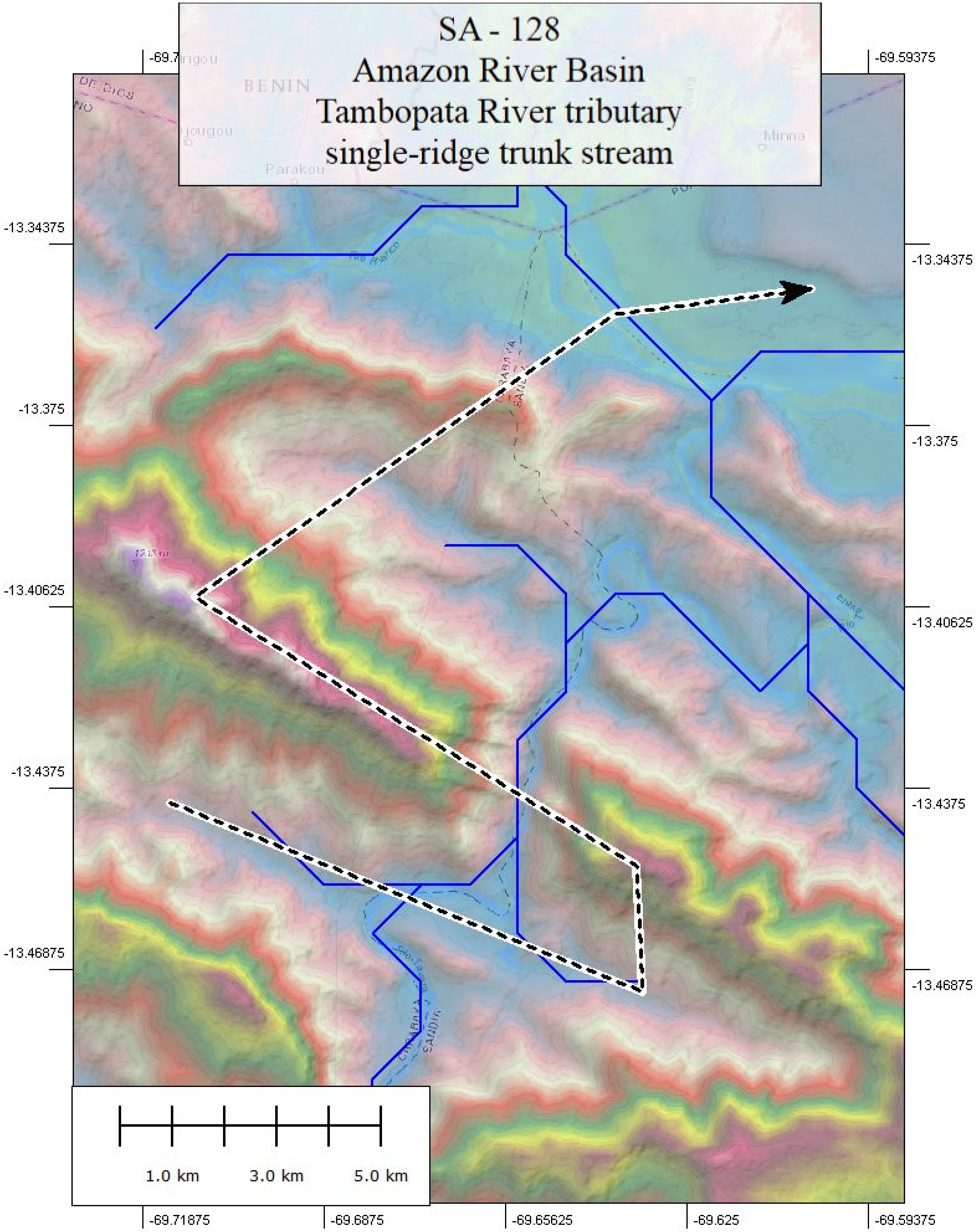
SA - 128
Amazon River Basin
Tambopata River tributary
single-ridge trunk stream
-69.59375
-13.34375
-13.375
-13.40625
-13.4375
-13.46875
-69.71875
-69.6875
-69.65625
-69.625
1.0 km
3.0 km
5.0 km

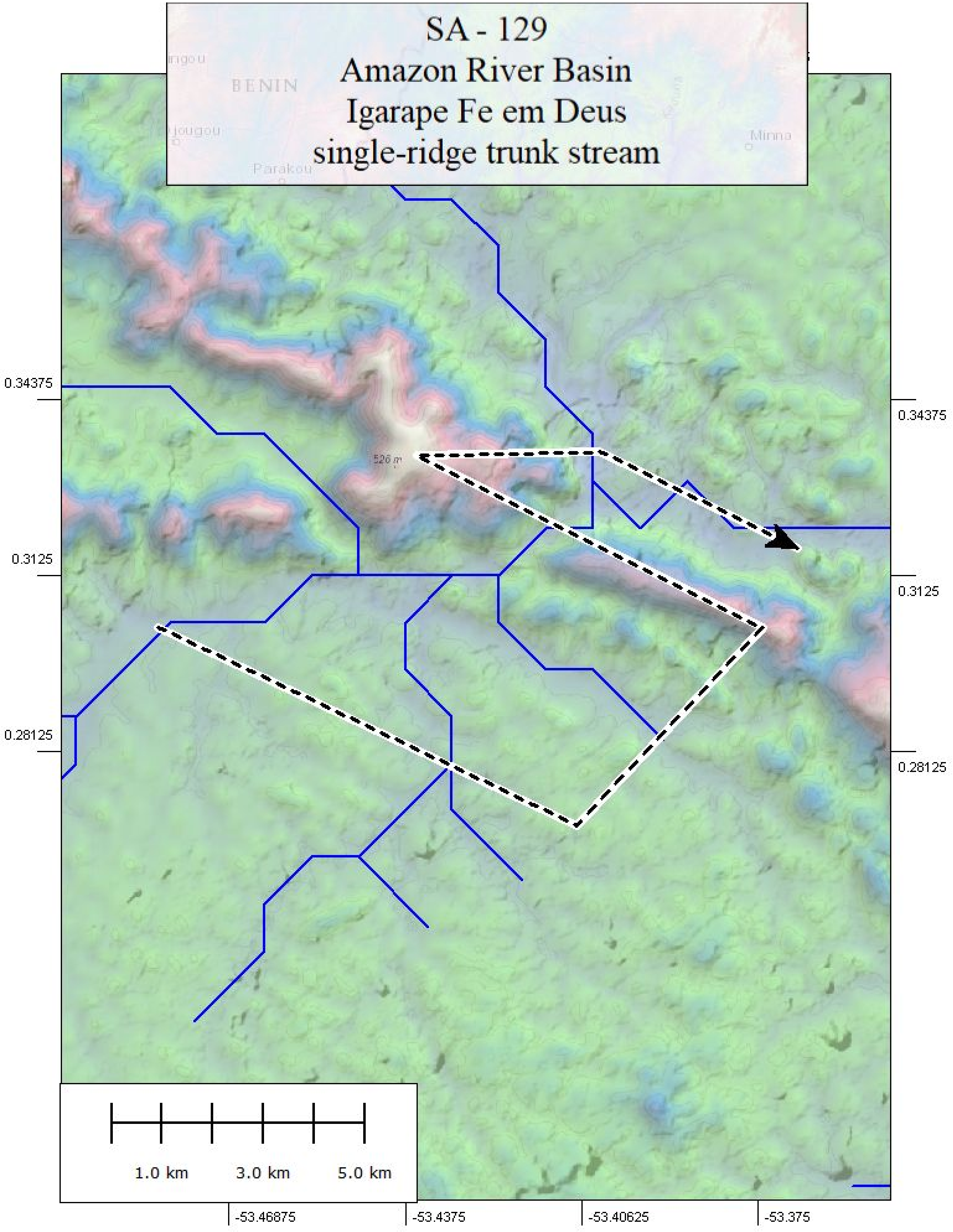
SA - 129
Amazon River Basin
Igarape Fe em Deus
single-ridge trunk stream
526 m
0.34375
0.3125
0.28125
0.34375
0.3125
0.28125
1.0 km
3.0 km
5.0 km
-53.46875
-53.4375
-53.40625
-53.375

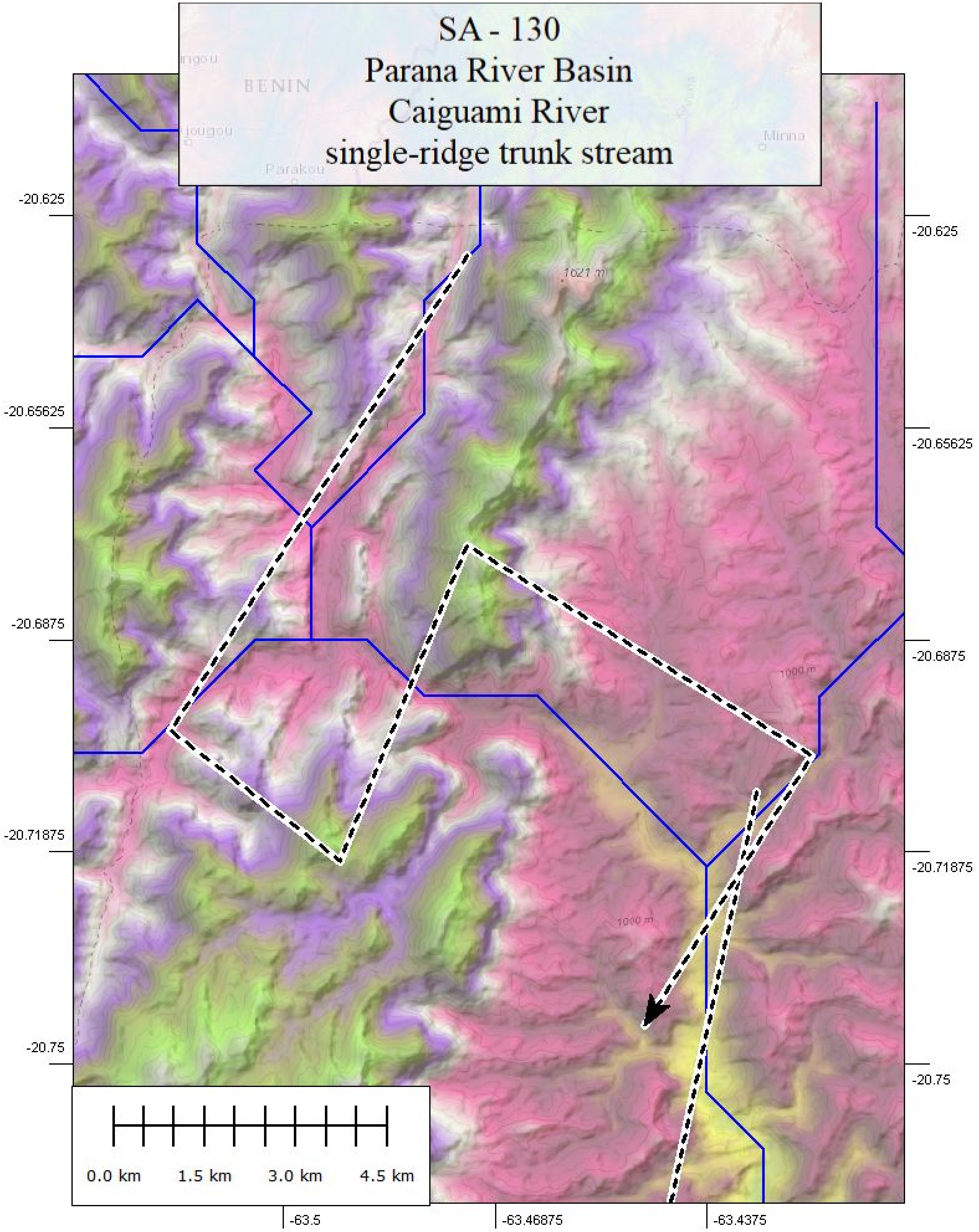
SA - 130
Parana River Basin
Caiguami River
single-ridge trunk stream
BENIN
Parakou
Minna
1621 m
1000 m
1000 m
-20.625
-20.65625
-20.6875
-20.71875
-20.75
-63.5
-63.46875
-63.4375
0.0 km
1.5 km
3.0 km
4.5 km

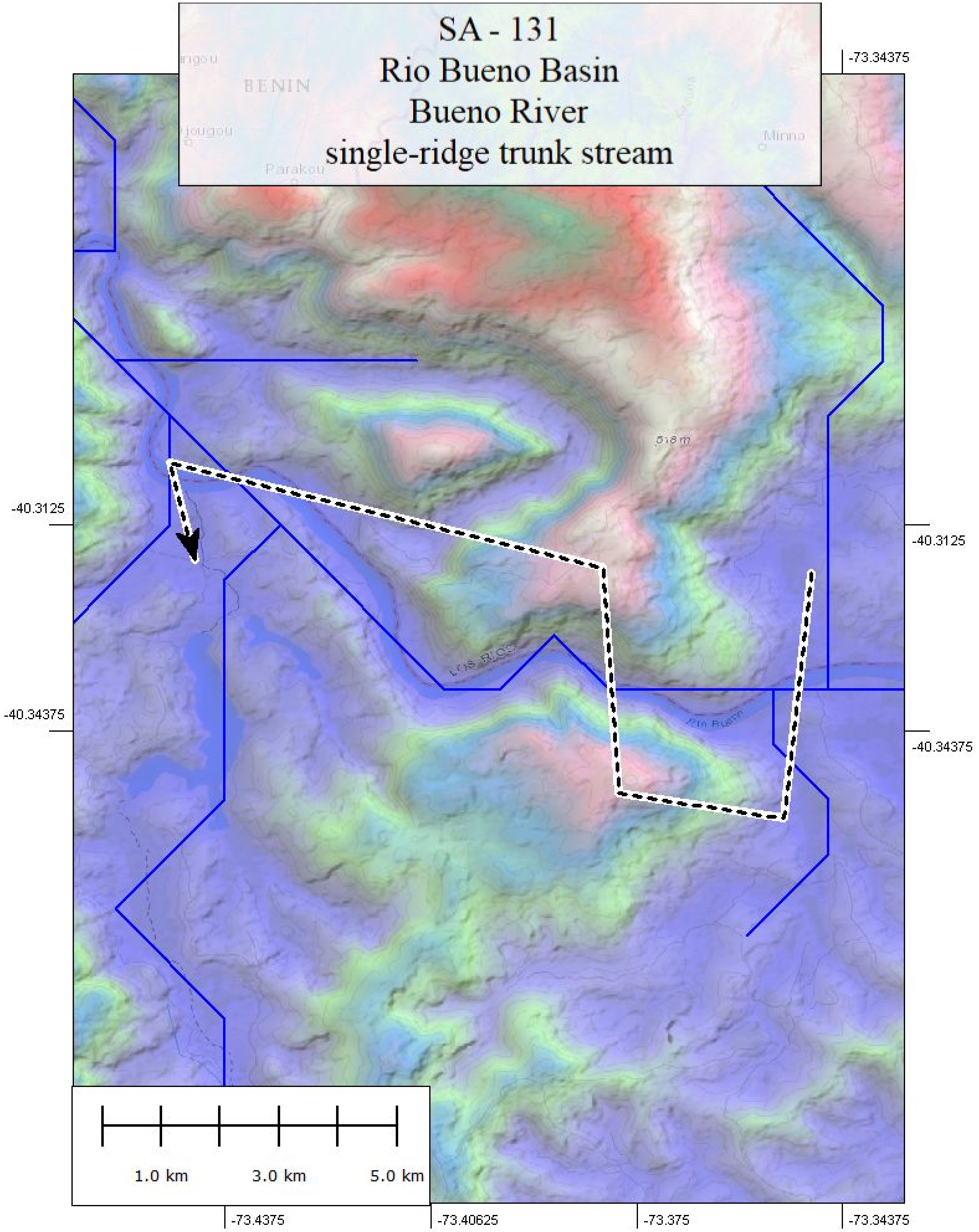
SA - 131
Rio Bueno Basin
Bueno River
single-ridge trunk stream
BENIN
Parakou
518 m
LOS RIOS
Rio Bueno
-40.3125
-40.34375
-73.34375
-73.4375
-73.40625
-73.375
1.0 km
3.0 km
5.0 km

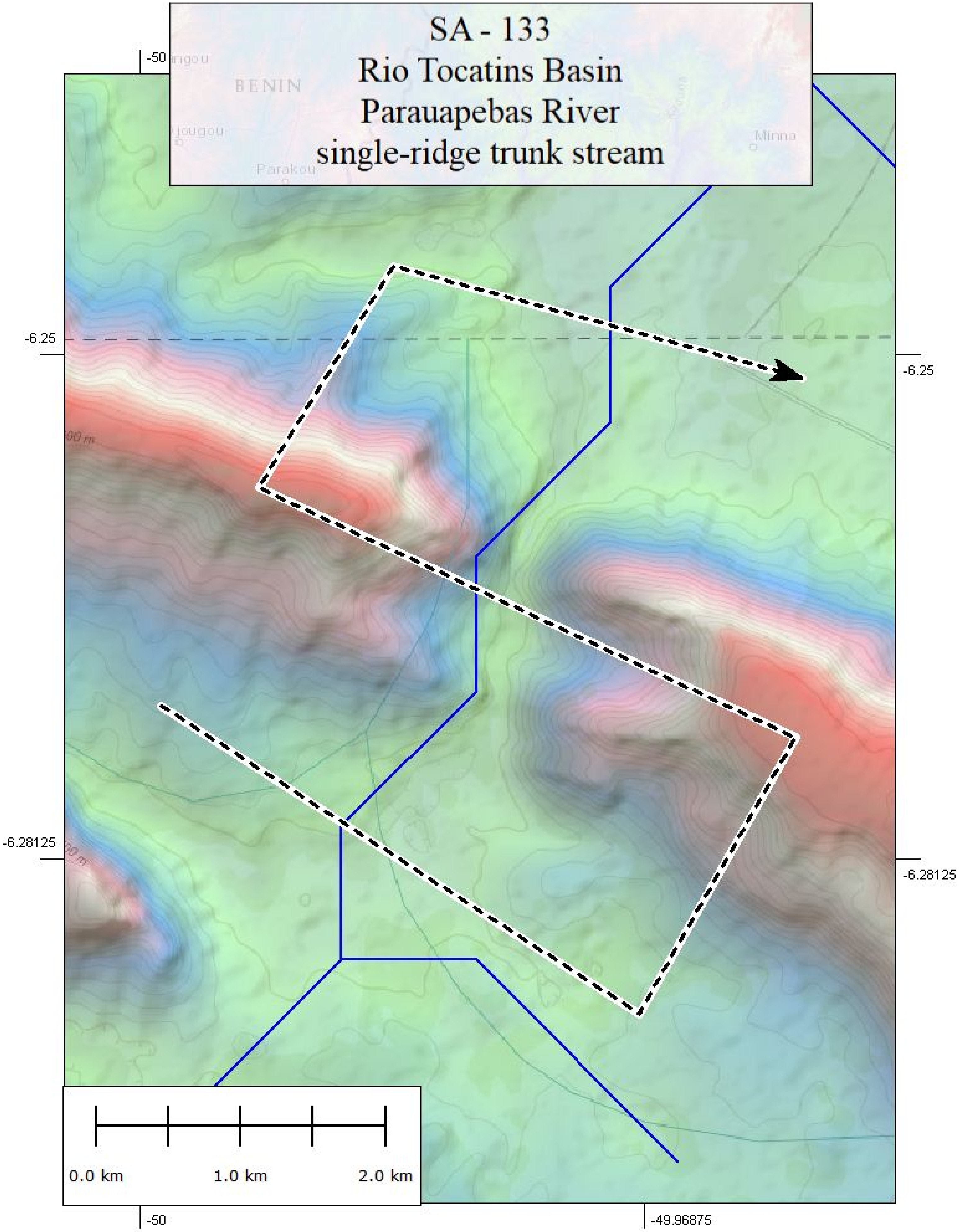
SA - 133
Rio Tocatins Basin
Parauapebas River
single-ridge trunk stream
-50
-6.25
-6.25
-6.28125
-6.28125
0.0 km
1.0 km
2.0 km
-50
-49.96875

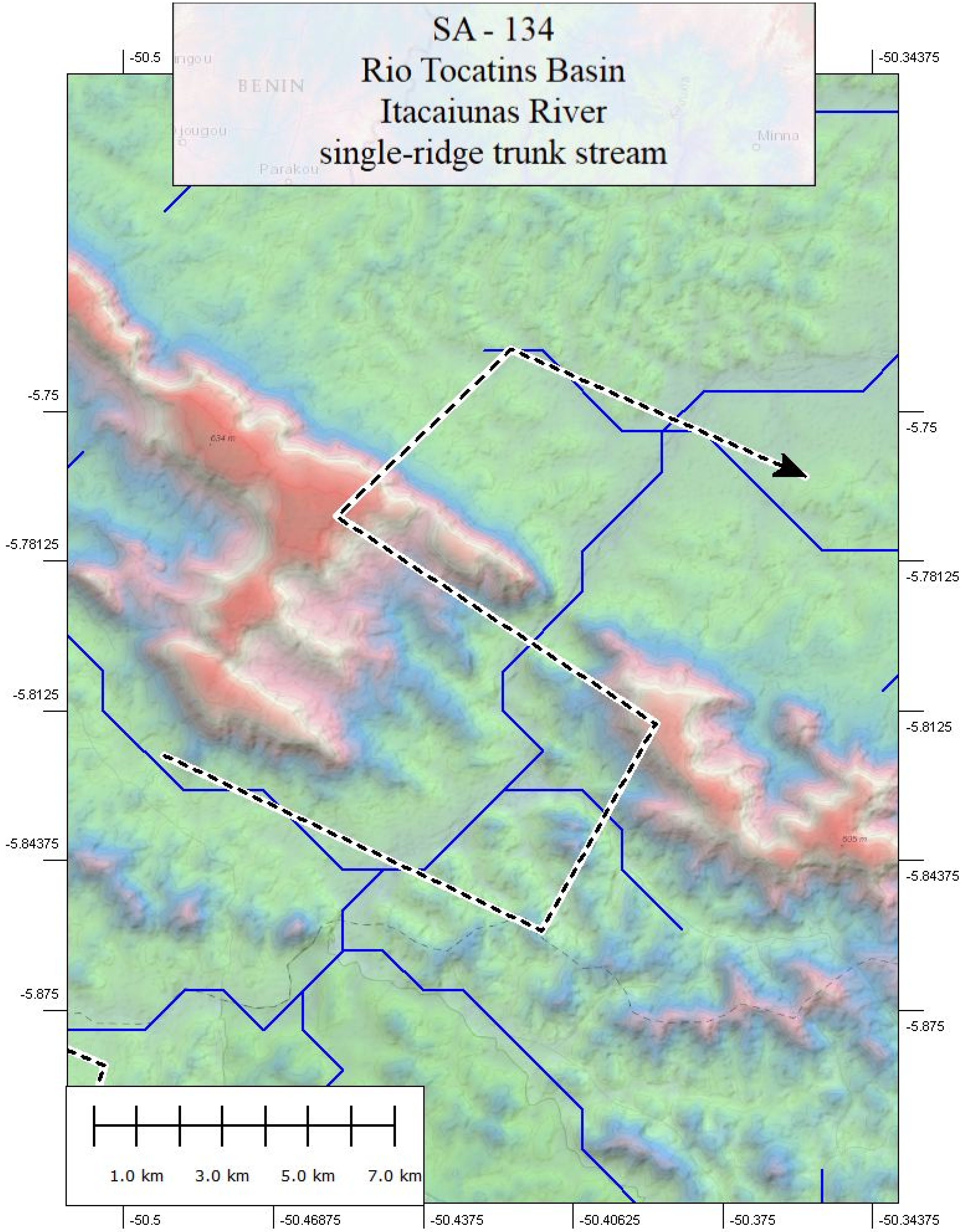
SA - 134
Rio Tocatins Basin
Itacaiunas River
single-ridge trunk stream
-50.5
-50.34375
-5.75
-5.78125
-5.8125
-5.84375
-5.875
634 m
505 m
1.0 km
3.0 km
5.0 km
7.0 km
-50.5
-50.46875
-50.4375
-50.40625
-50.375
-50.34375

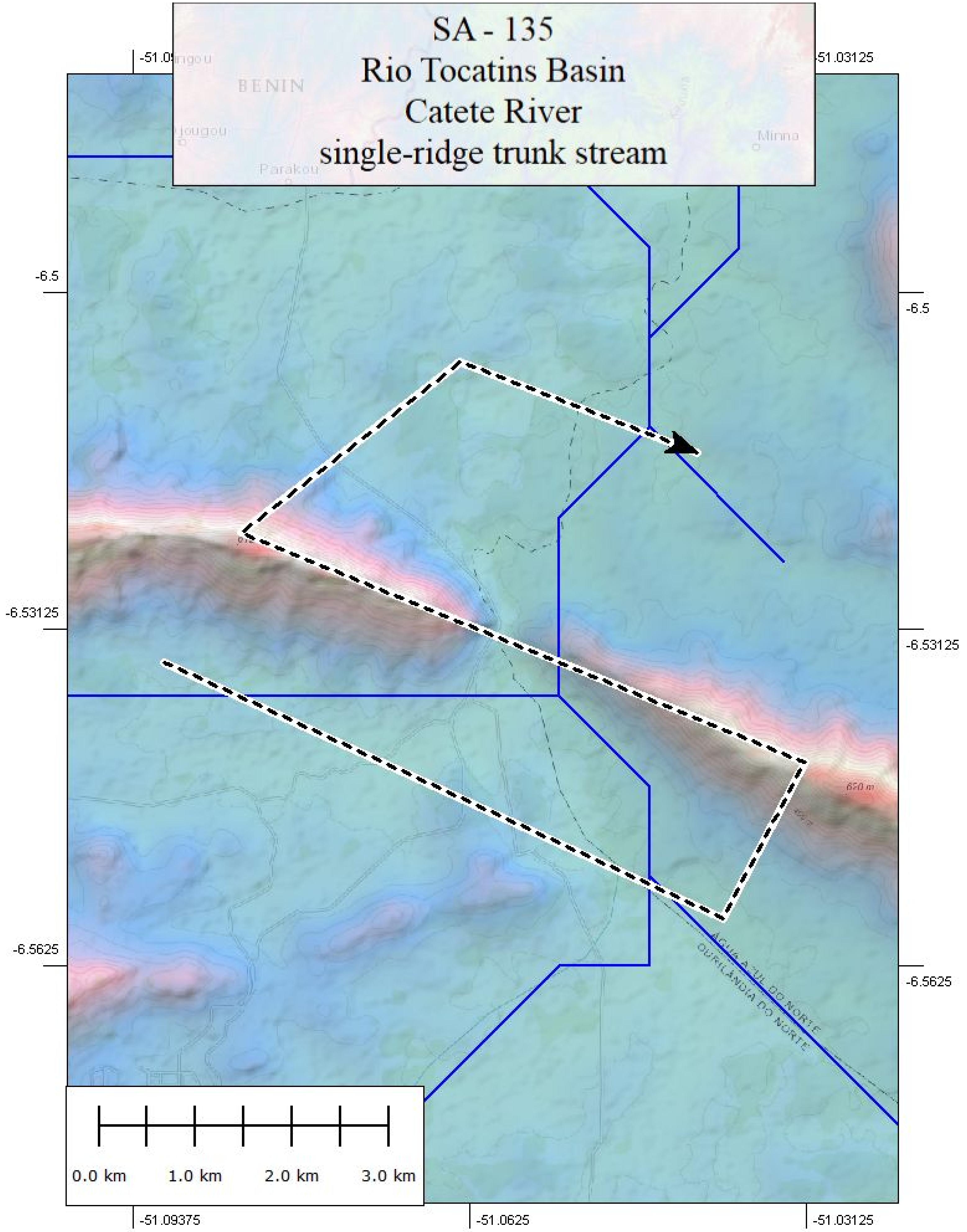
SA - 135
Rio Tocatins Basin
Catete River
single-ridge trunk stream
-51.03125
-6.5
-6.53125
-6.5625
-51.09375
-51.0625
650 m
AGUA AZUL DO NORTE
OURILANDIA DO NORTE
0.0 km
1.0 km
2.0 km
3.0 km

SA - 136
Amazon River Basin
Branco River
single-ridge trunk stream

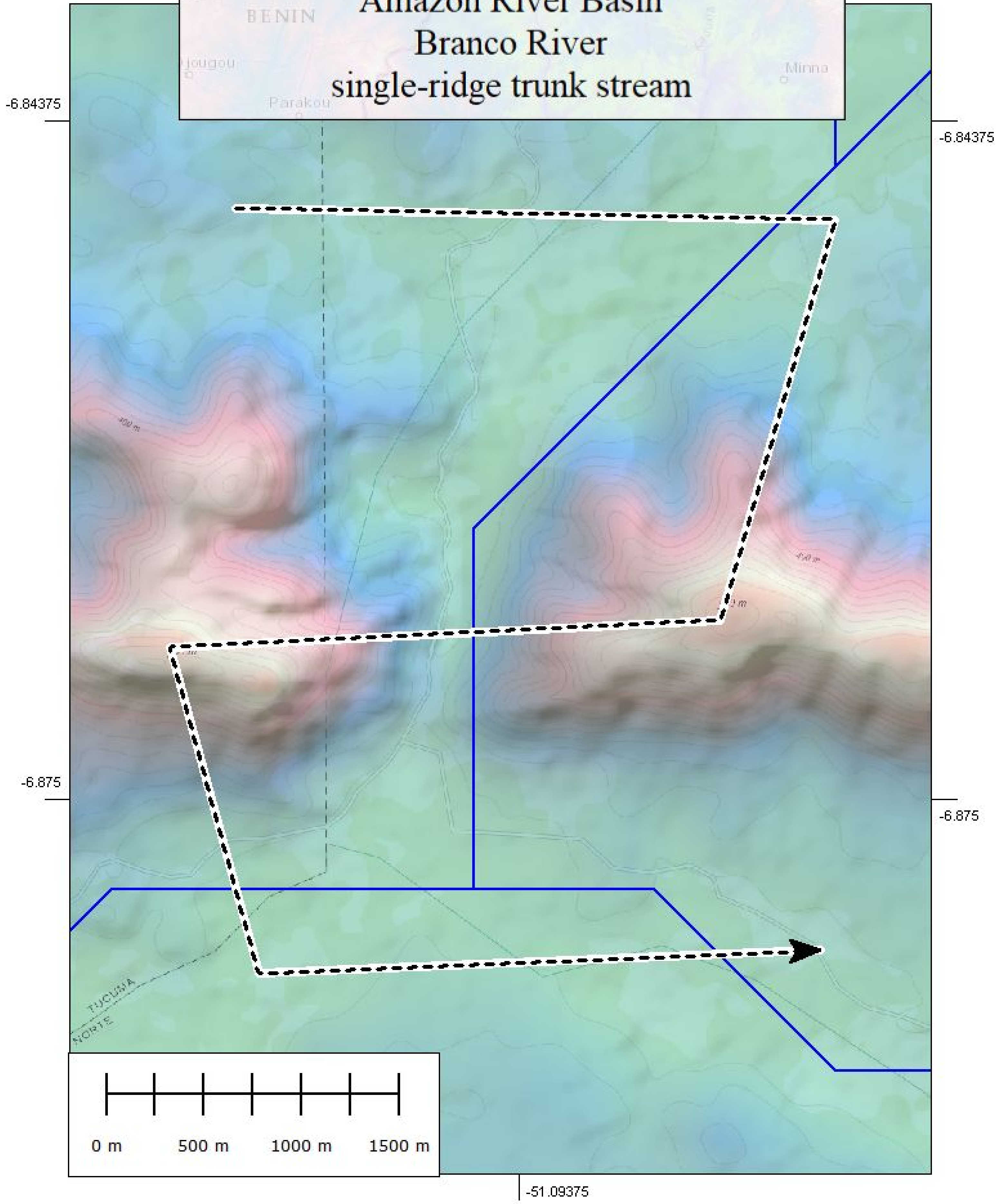

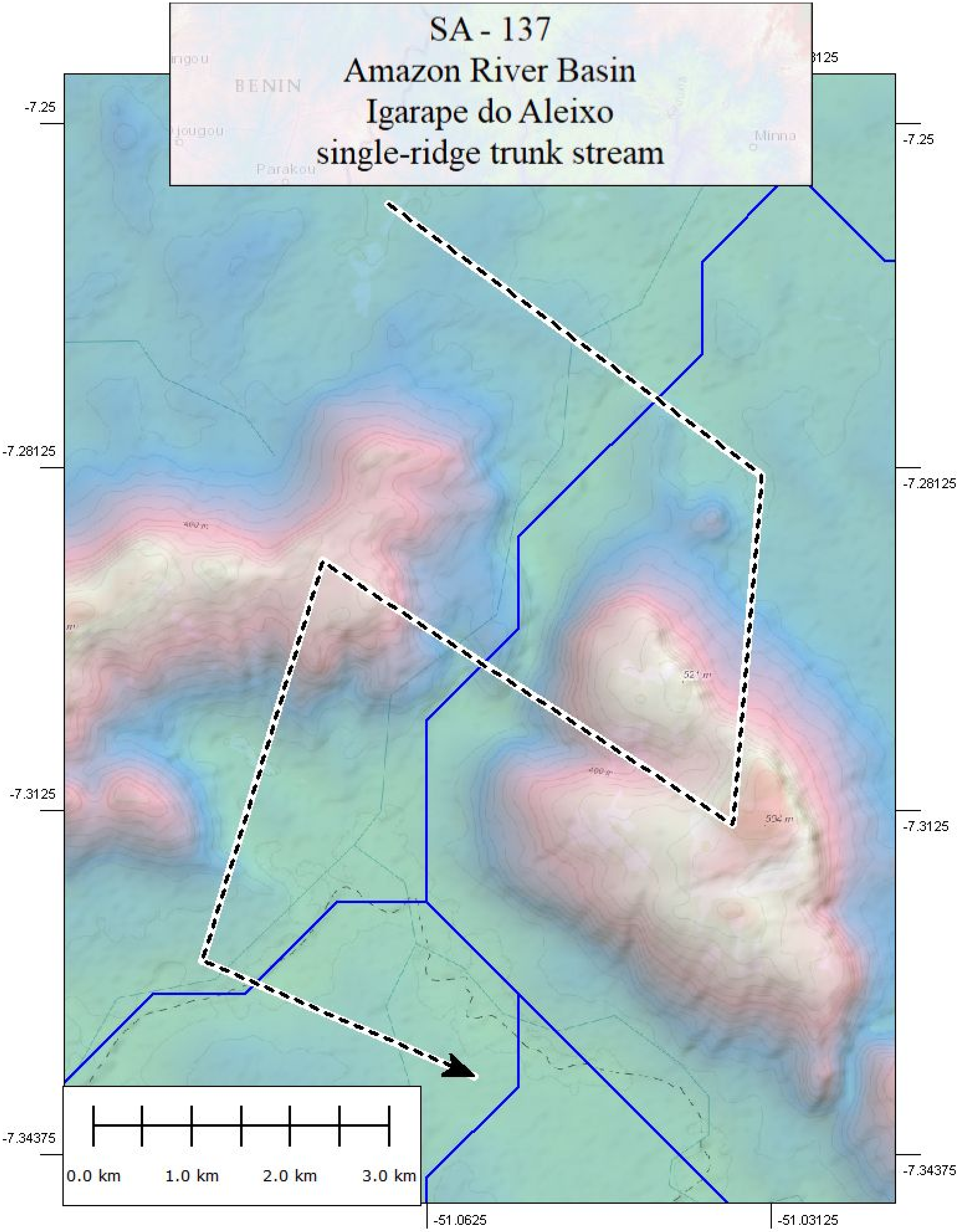
SA - 137
Amazon River Basin
Igarape do Aleixo
single-ridge trunk stream
BENIN
Parakou
Minna
-7.25
-7.25
-7.28125
-7.28125
-7.3125
-7.3125
-7.34375
-7.34375
-51.0625
-51.03125
0.0 km
1.0 km
2.0 km
3.0 km

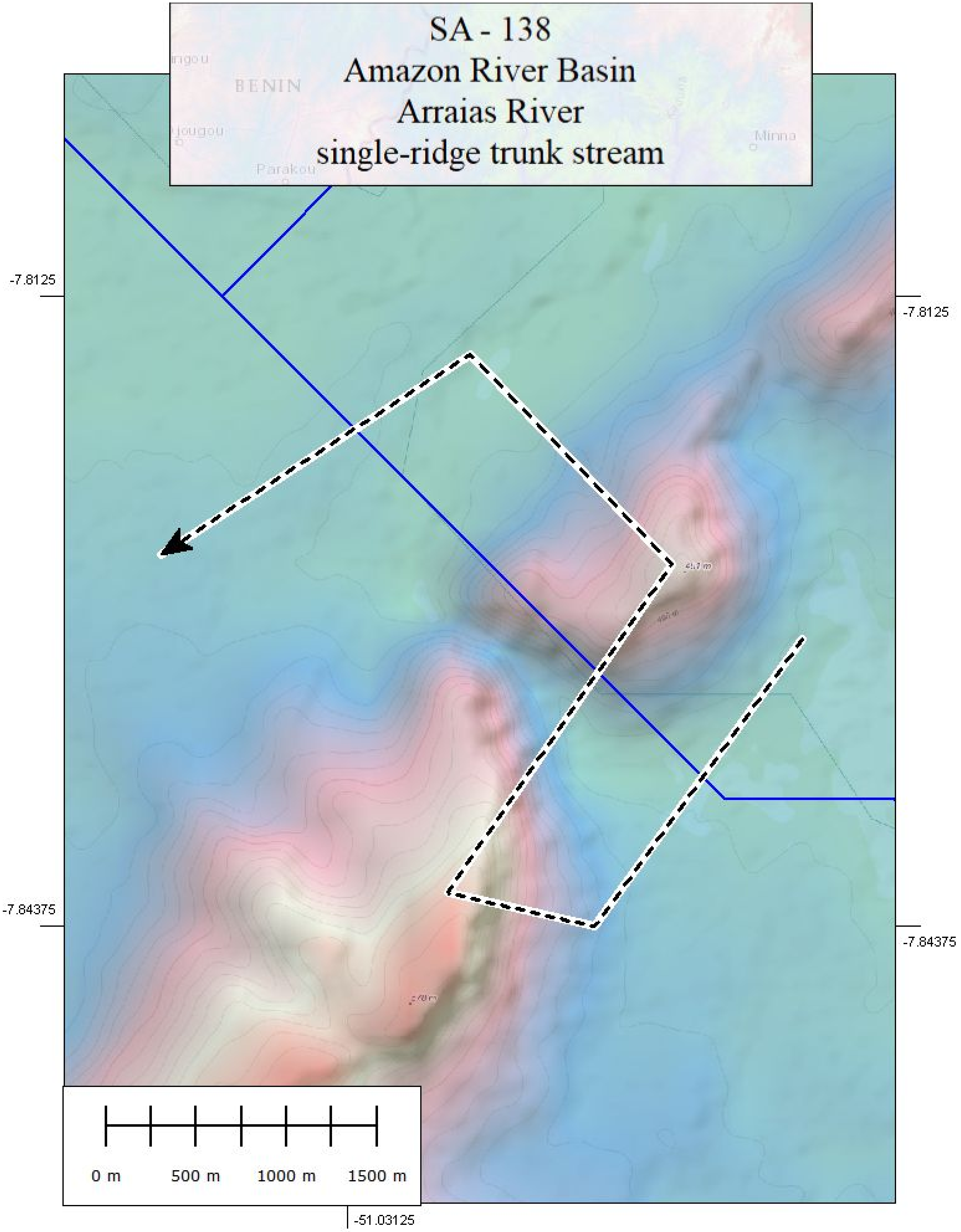
SA - 138
Amazon River Basin
Arraias River
single-ridge trunk stream
-7.8125
-7.8125
-7.84375
-7.84375
-51.03125
0 m
500 m
1000 m
1500 m

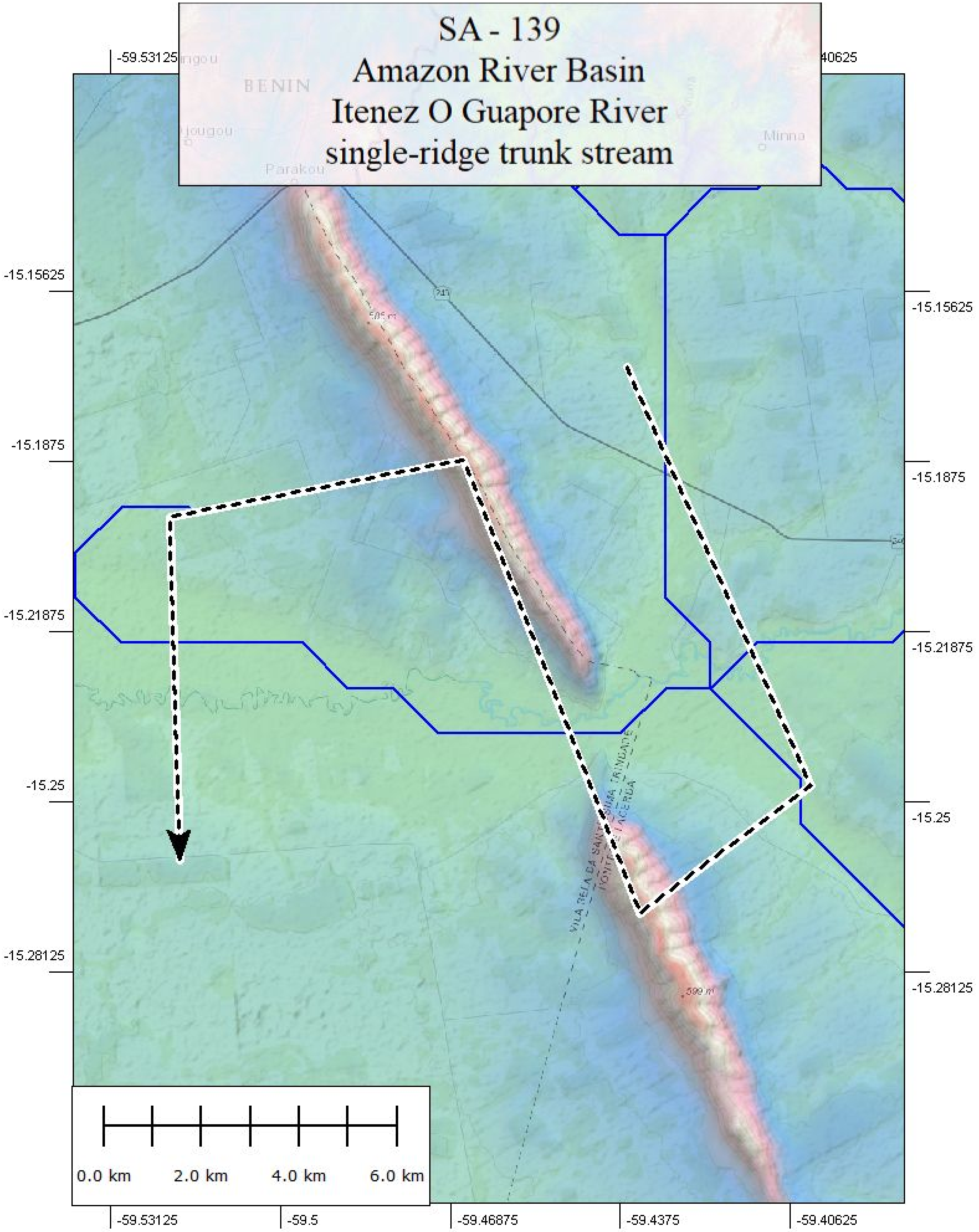
SA - 139
Amazon River Basin
Itenez O Guapore River
single-ridge trunk stream
-59.53125
-59.40625
BENIN
Parakou
Minna
-15.15625
-15.1875
-15.21875
-15.25
-15.28125
0.0 km
2.0 km
4.0 km
6.0 km
-59.53125
-59.5
-59.46875
-59.4375
-59.40625

SA - 140
Rio Colorado (South America) Basin
single-ridge trunk stream

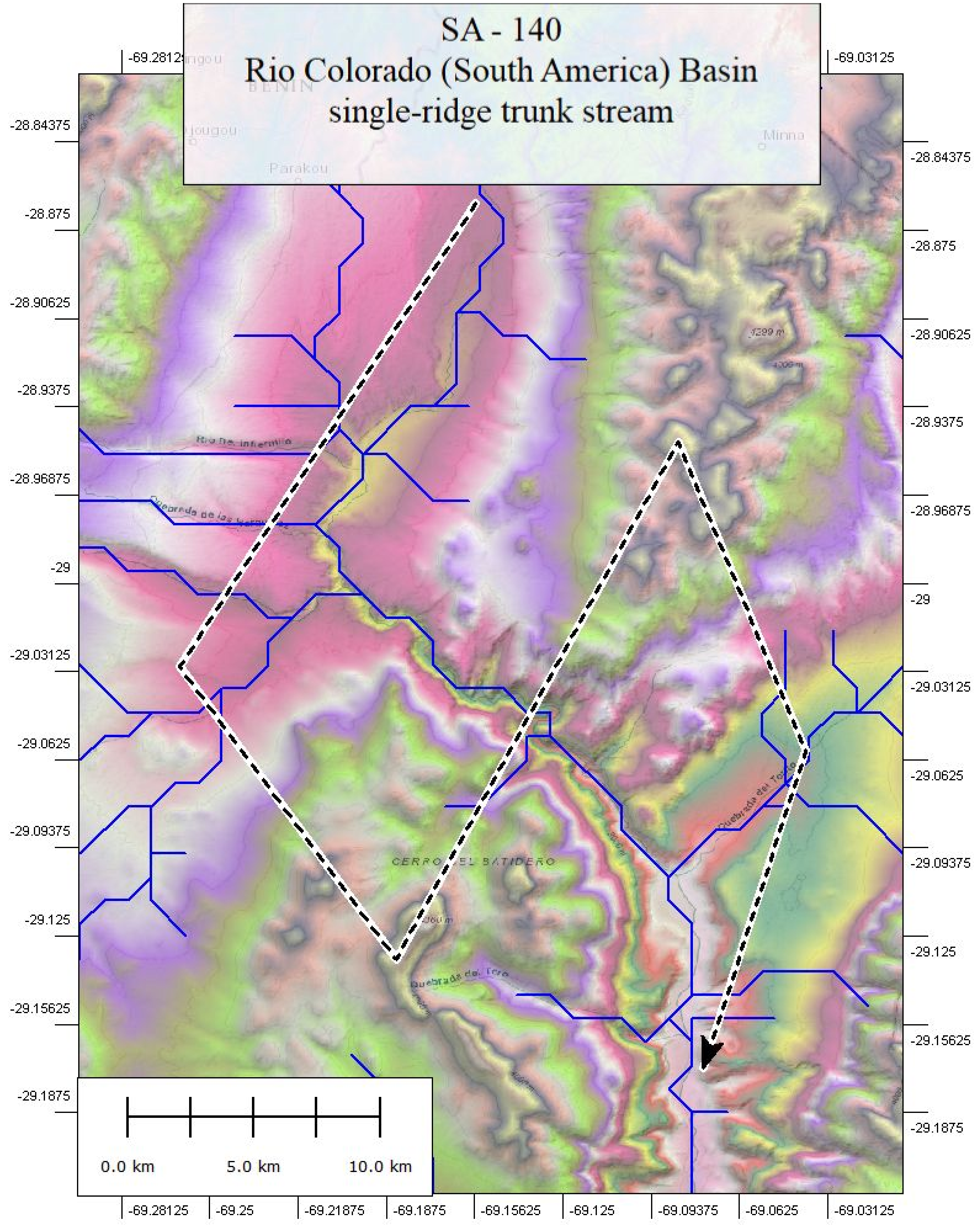

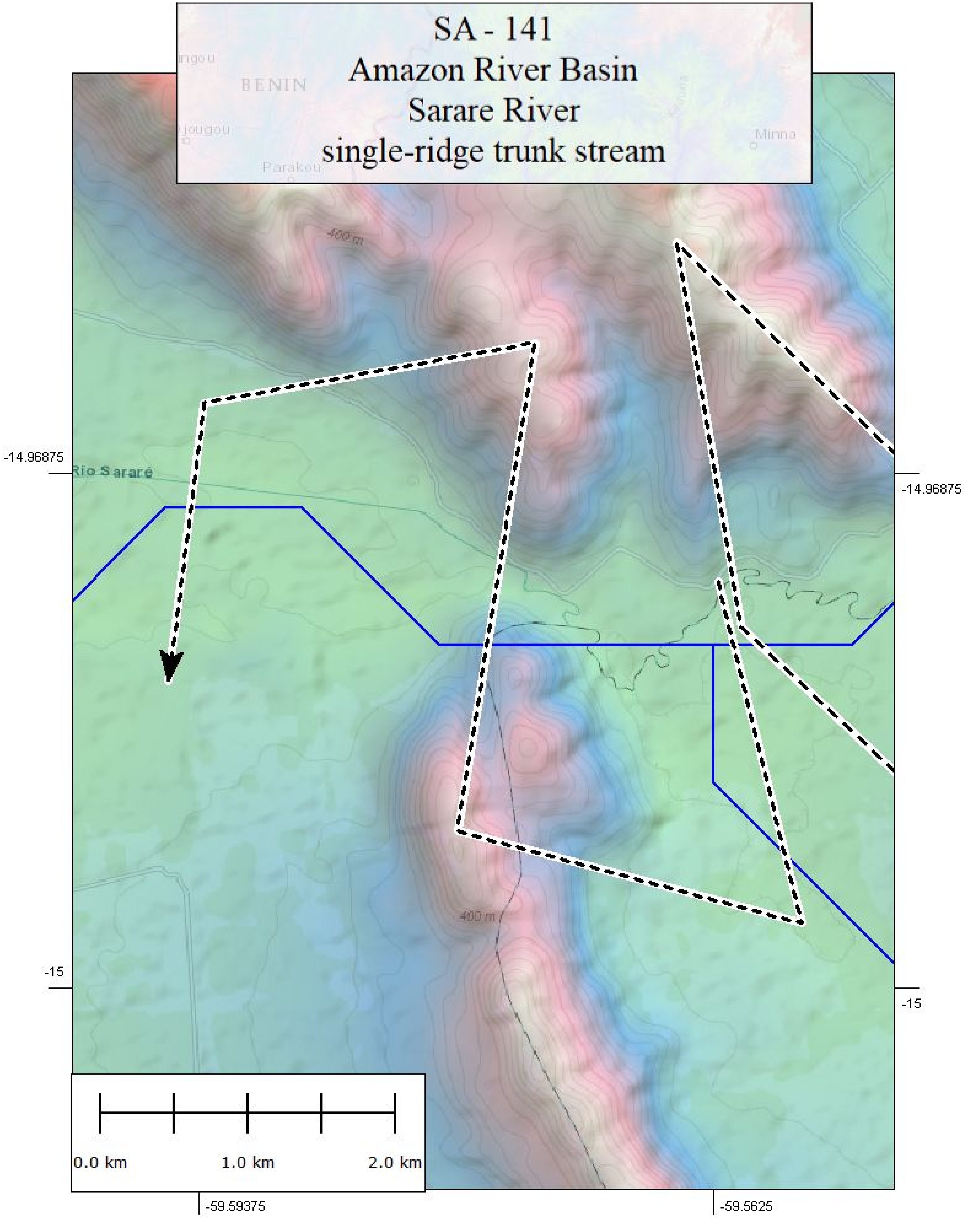
SA - 141
Amazon River Basin
Sarare River
single-ridge trunk stream
BENIN
Parakou
Minna
400 m
Rio Sararé
-14.96875
-14.96875
400 m
-15
-15
0.0 km
1.0 km
2.0 km
-59.59375
-59.5625

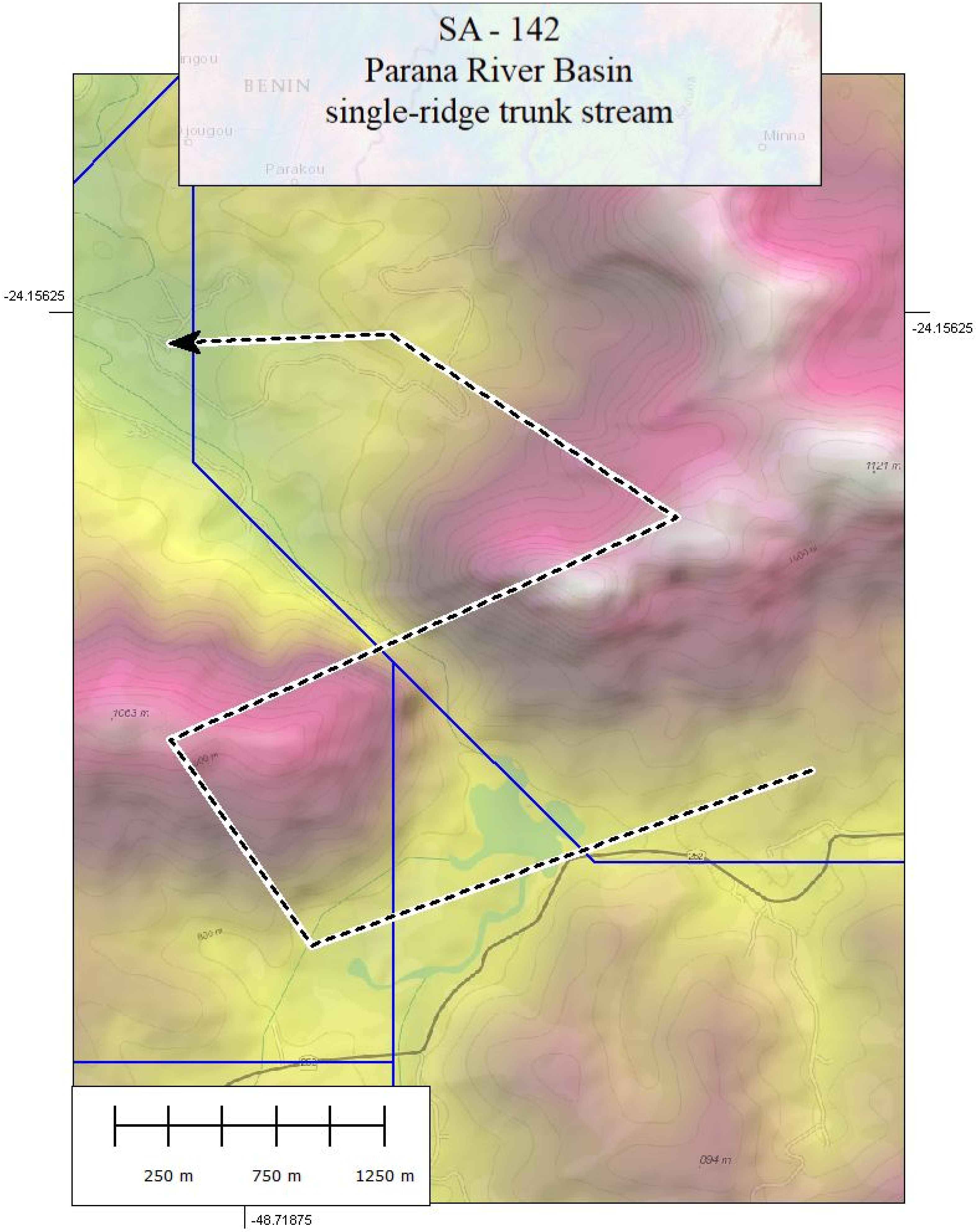
SA - 142
Parana River Basin
single-ridge trunk stream
BENIN
Parakou
Minna
-24.15625
-24.15625
1121 m
1063 m
250 m
750 m
1250 m
-48.71875

SA - 143
Parana River Basin
Paraguay River tributary
single-ridge trunk stream

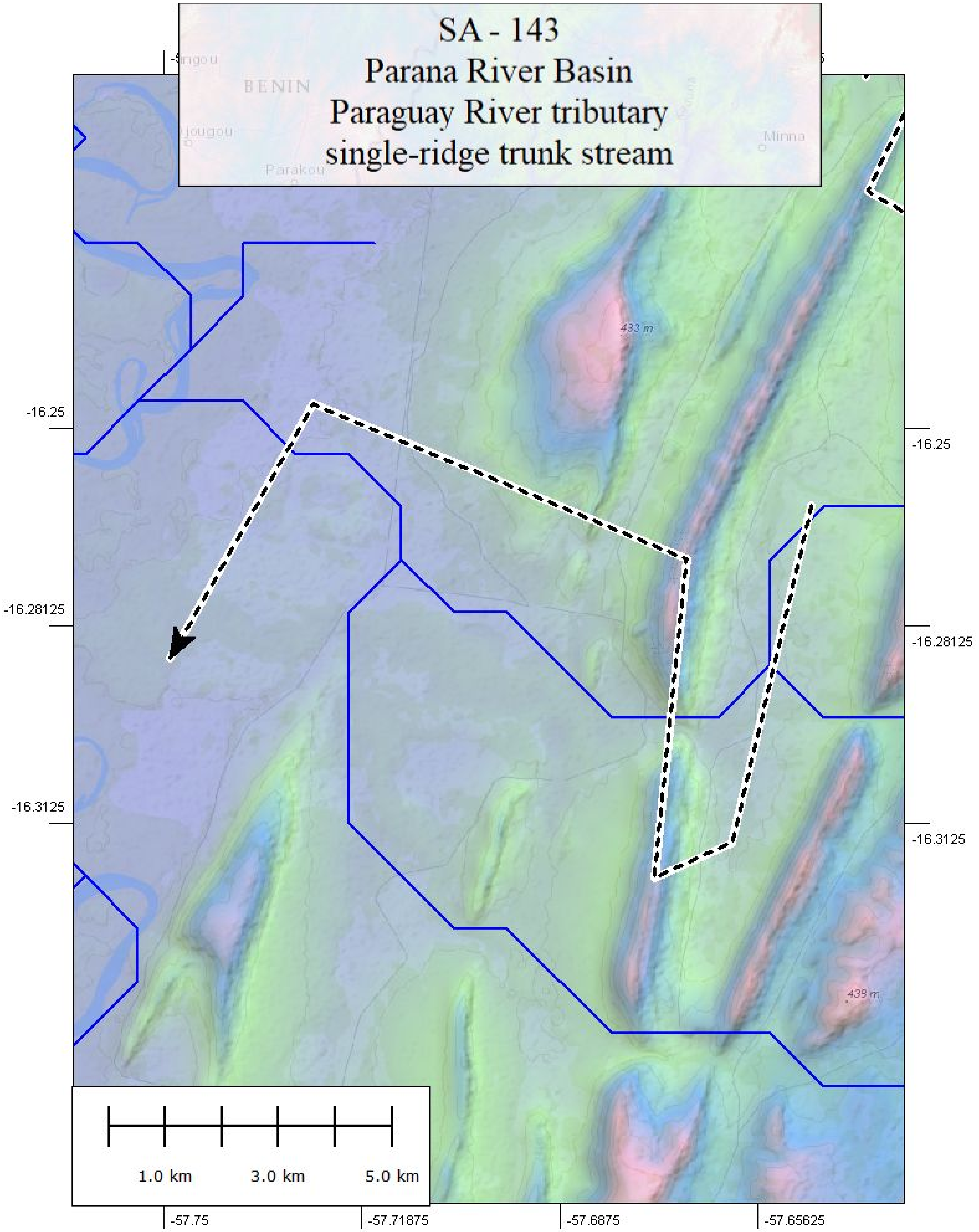

SA - 144
Parana River Basin
Hualfui River
single-ridge trunk stream

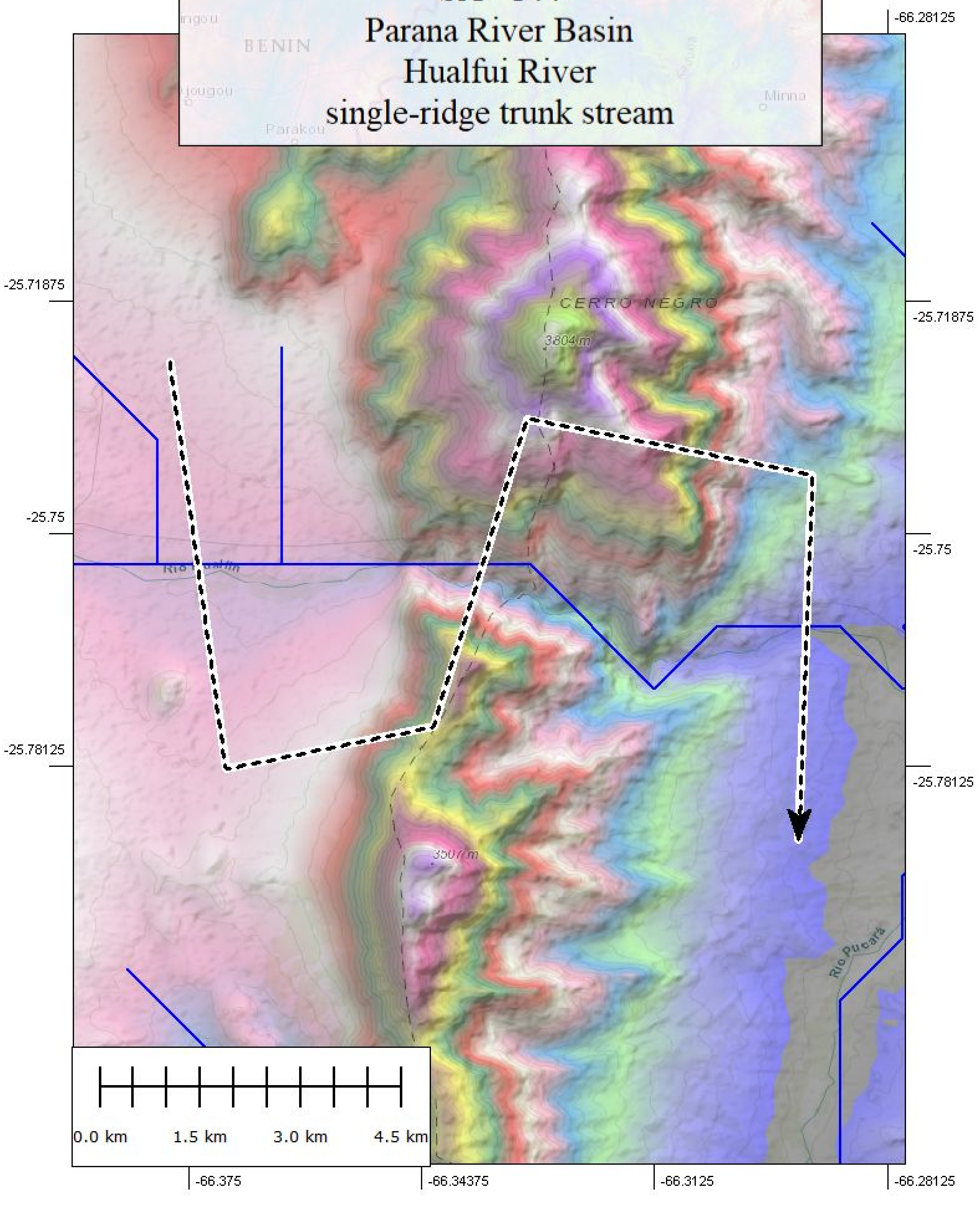

SA - 146
6000040460 Basin
single-ridge trunk stream

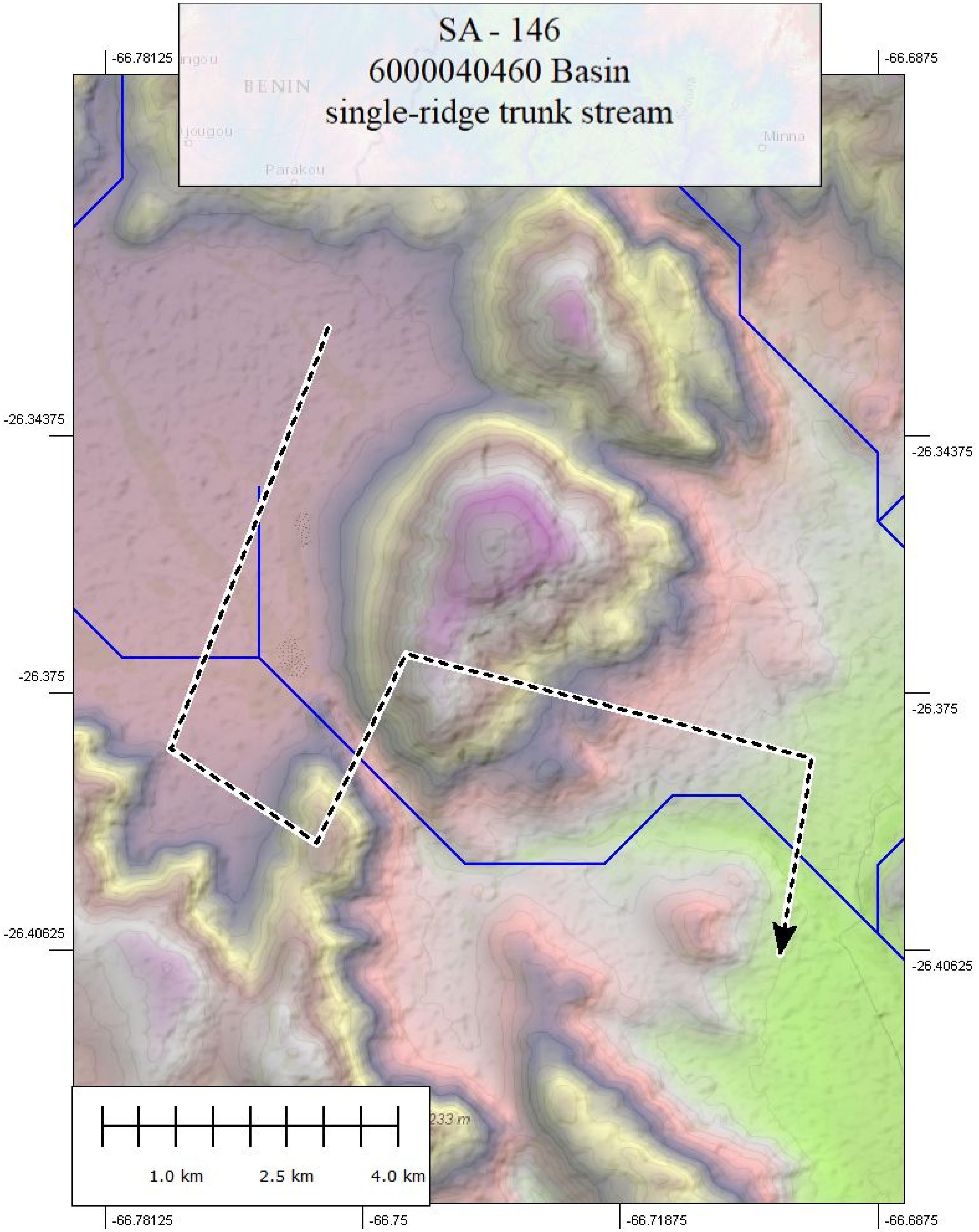

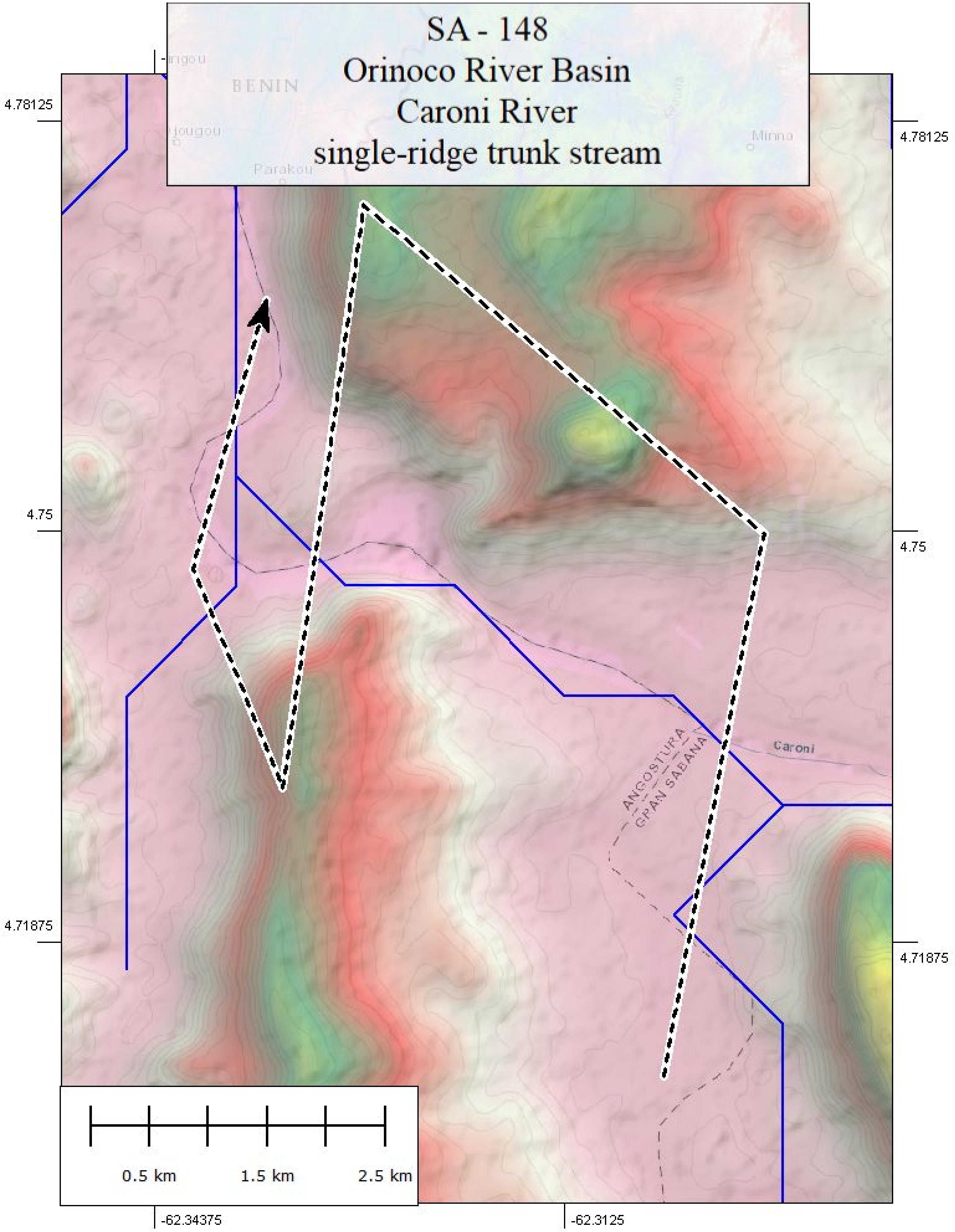

SA - 148
Orinoco River Basin
Caroni River
single-ridge trunk stream
4.78125
4.75
4.71875
-62.34375
-62.3125
Caroni
ANGOSTURA
GRAN SABANA
BENIN
Parakou
Minna
0.5 km
1.5 km
2.5 km

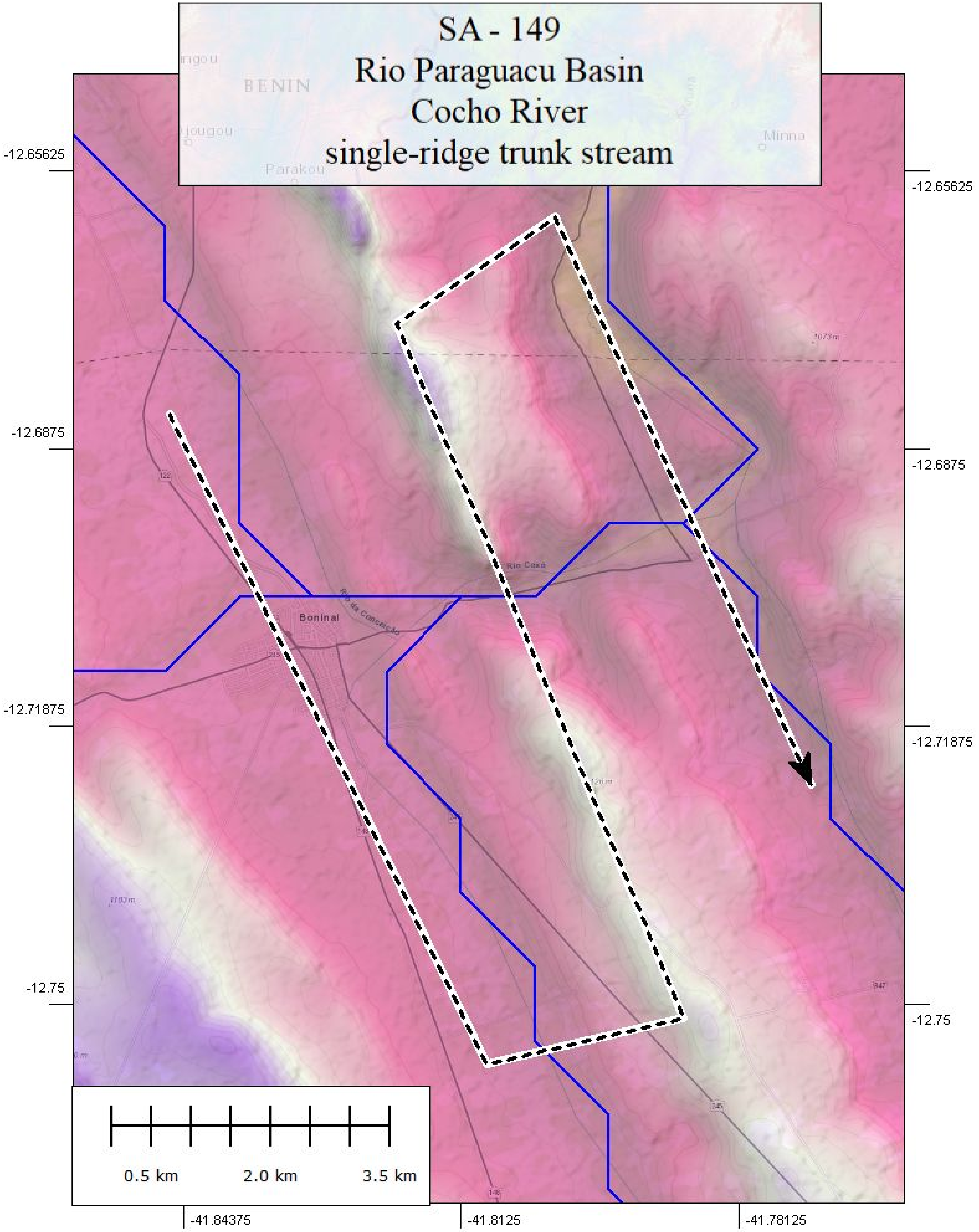
SA - 149
Rio Paraguacu Basin
Cocho River
single-ridge trunk stream
BENIN
Parakou
Boninal
Rio Coxó
-12.65625
-12.6875
-12.71875
-12.75
-41.84375
-41.8125
-41.78125
0.5 km
2.0 km
3.5 km

SA - 150
Rio Sao Francisco Basin
Sao Francisco River tributary
single-ridge trunk stream

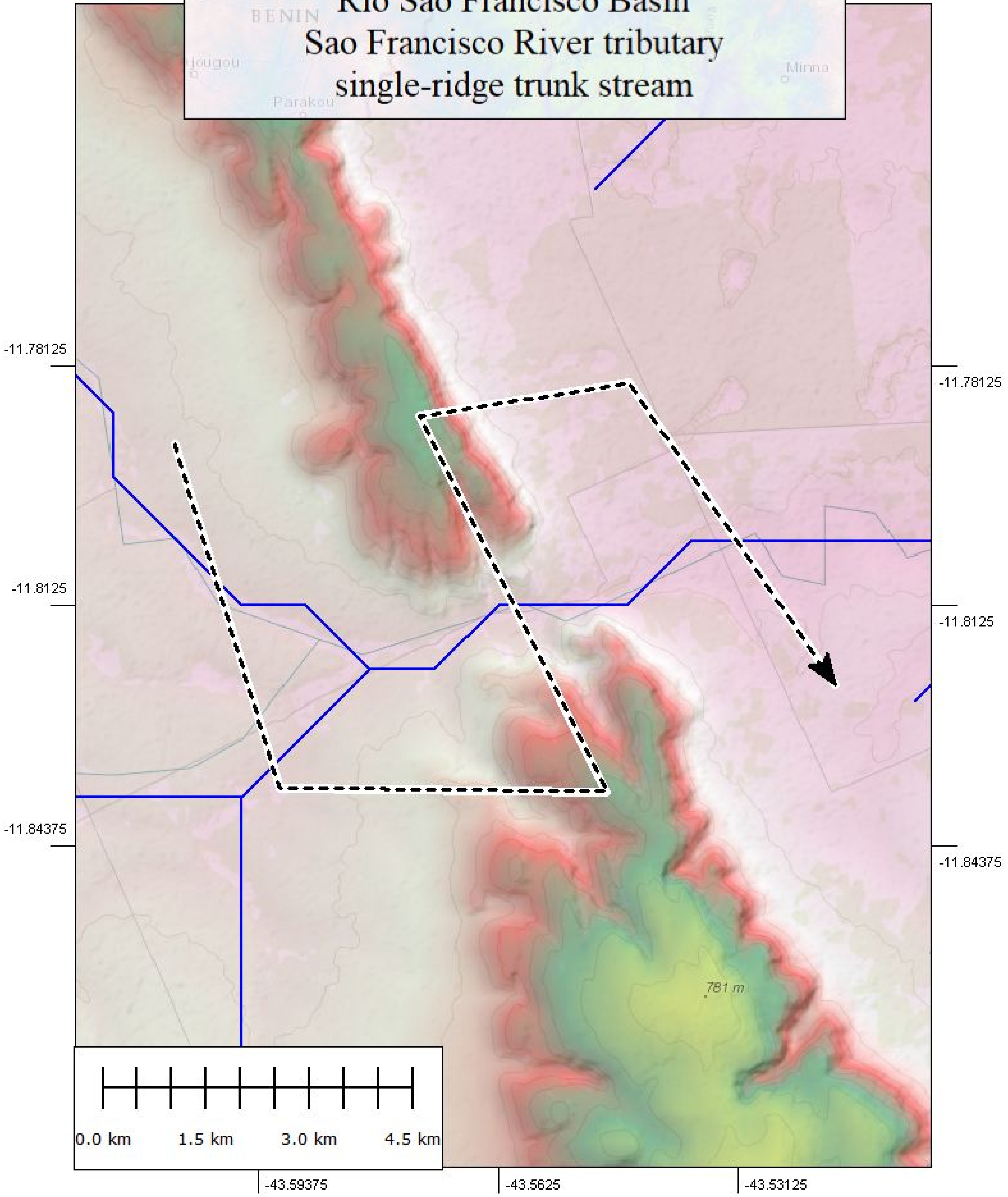

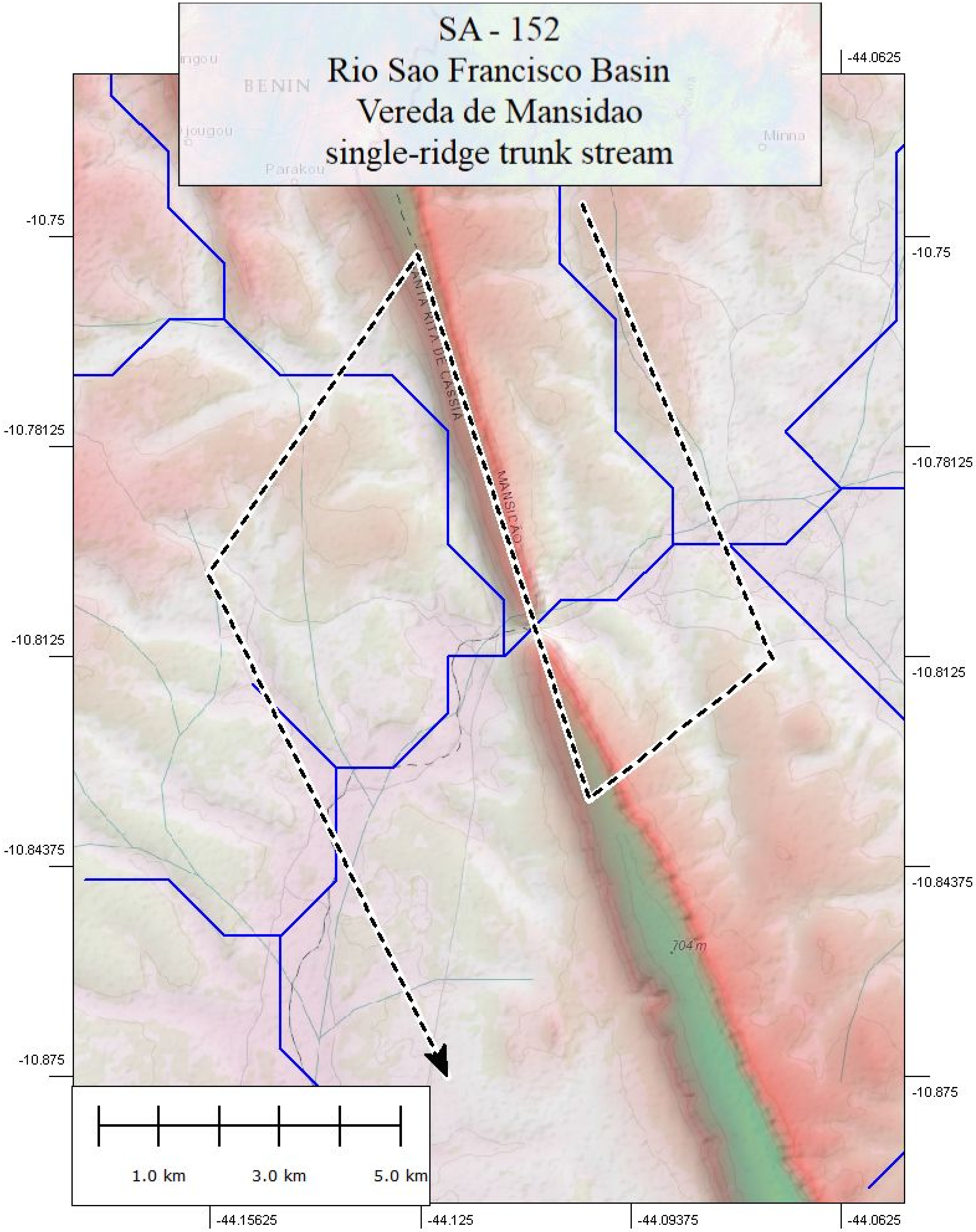
SA - 152
Rio Sao Francisco Basin
Vereda de Mansidao
single-ridge trunk stream
BENIN
Parakou
Minna
SANTA RITA DE CASSIA
MANSIDÃO
704 m
-44.0625
-10.75
-10.78125
-10.8125
-10.84375
-10.875
-44.15625
-44.125
-44.09375
1.0 km
3.0 km
5.0 km

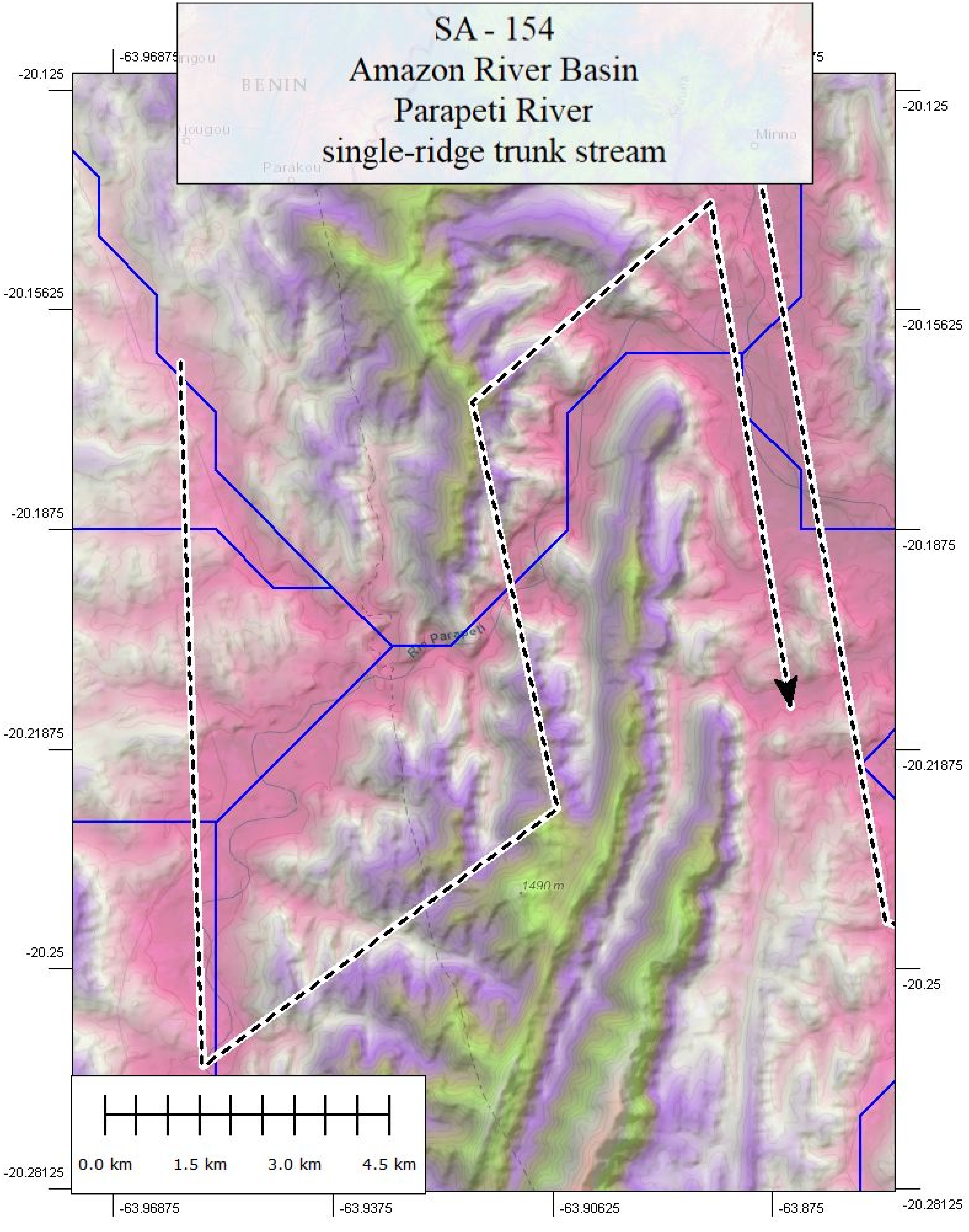
SA - 154
Amazon River Basin
Parapeti River
single-ridge trunk stream
-63.96875
-63.9375
-63.90625
-63.875
-20.125
-20.15625
-20.1875
-20.21875
-20.25
-20.28125
Rio Parapeti
1490 m
0.0 km
1.5 km
3.0 km
4.5 km

SA - 156
Rio Sao Francisco Basin
Rio Grande (Brazil)
single-ridge trunk stream

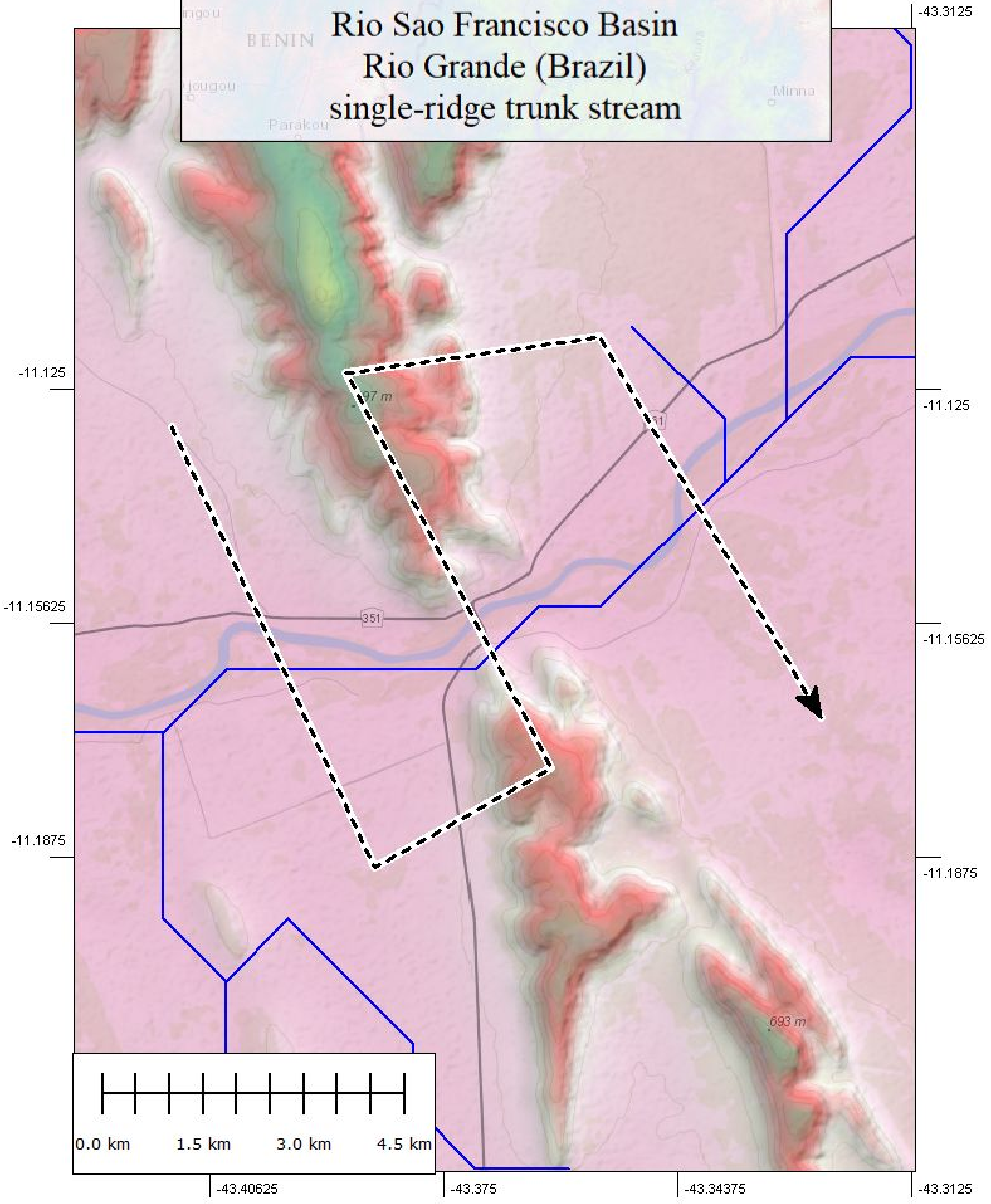

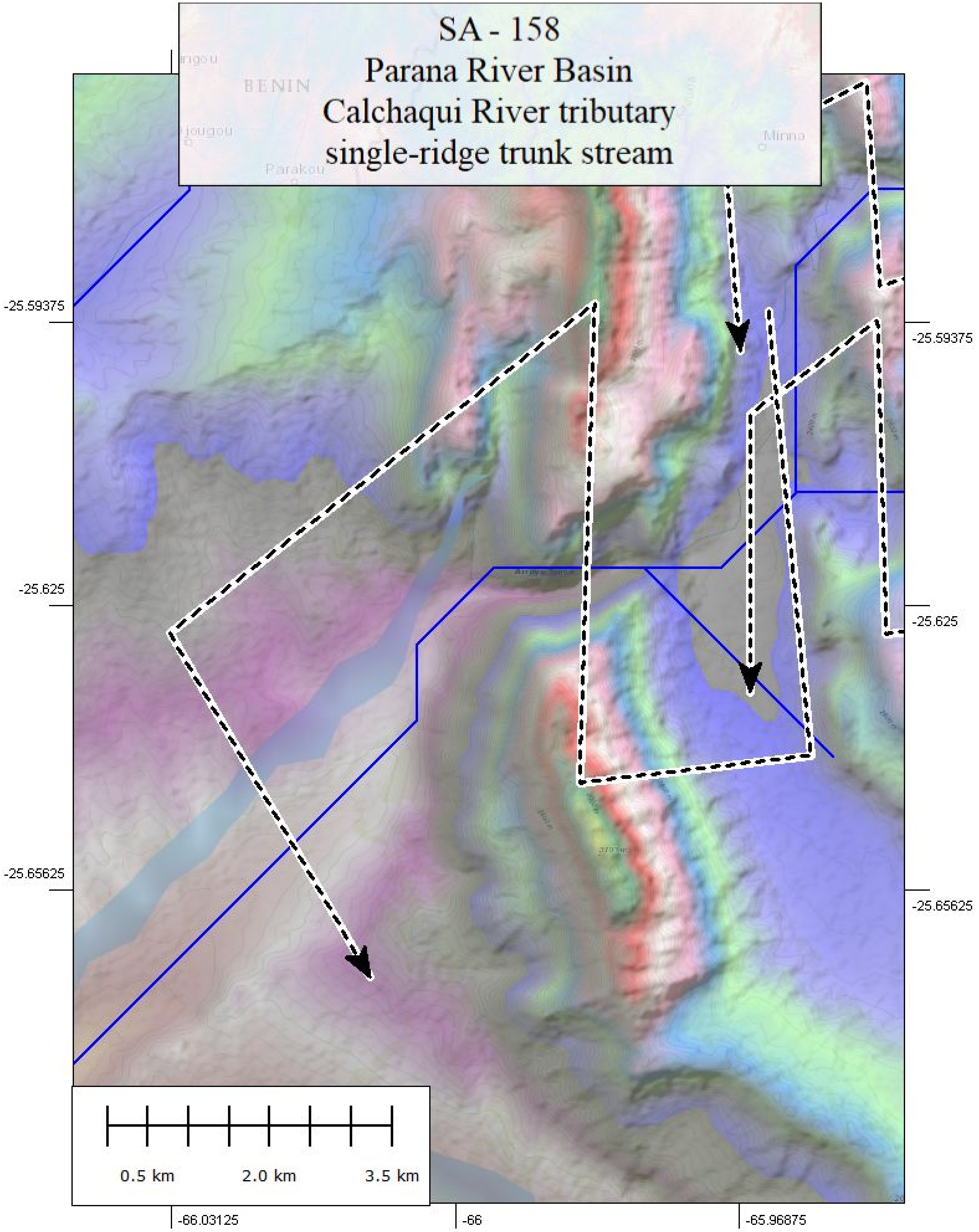
SA - 158
Parana River Basin
Calchaqui River tributary
single-ridge trunk stream
-25.59375
-25.625
-25.65625
-66.03125
-66
-65.96875
0.5 km
2.0 km
3.5 km

SA - 159
Parana River Basin
Calchaqui River tributary
single-ridge trunk stream

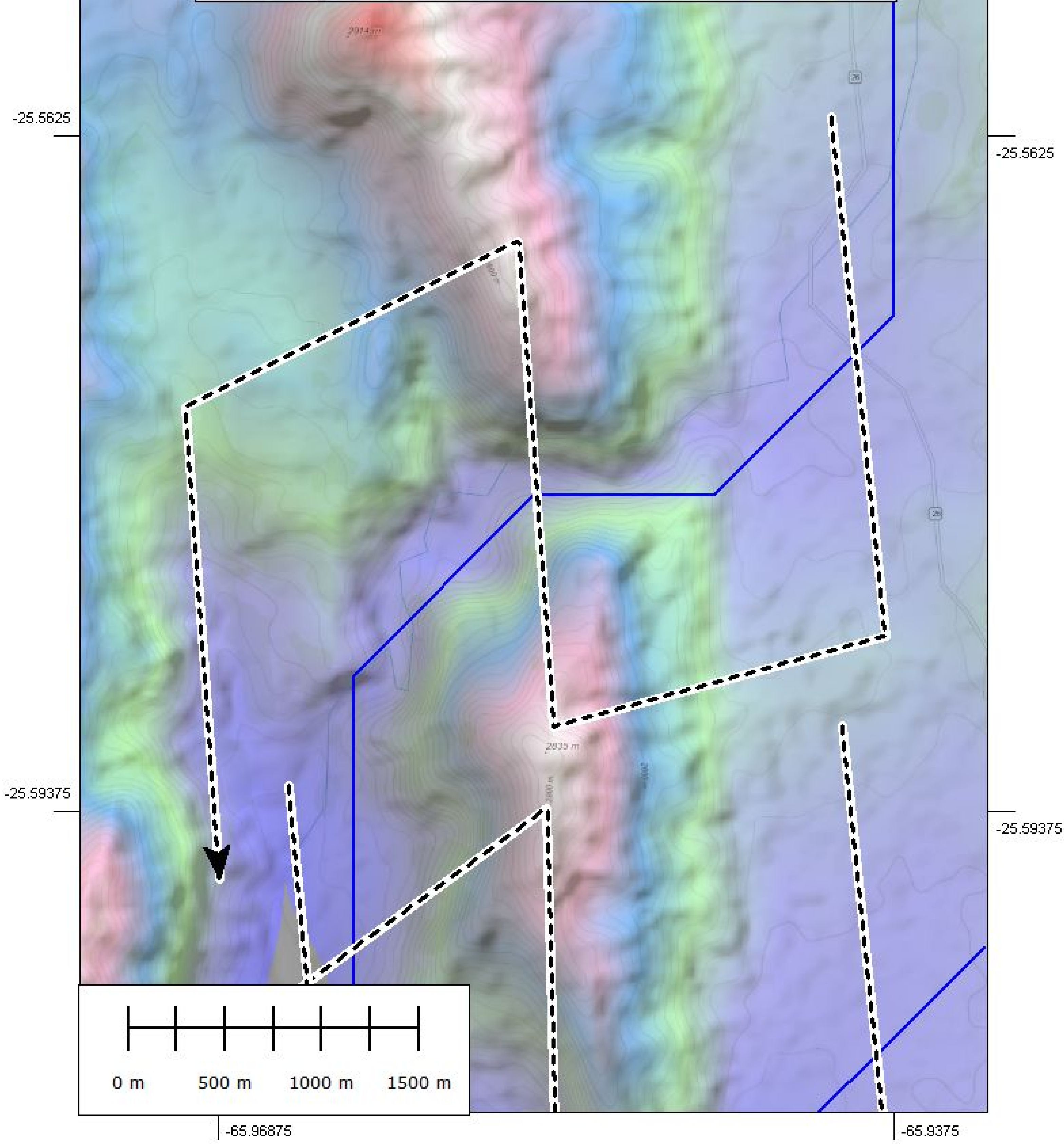

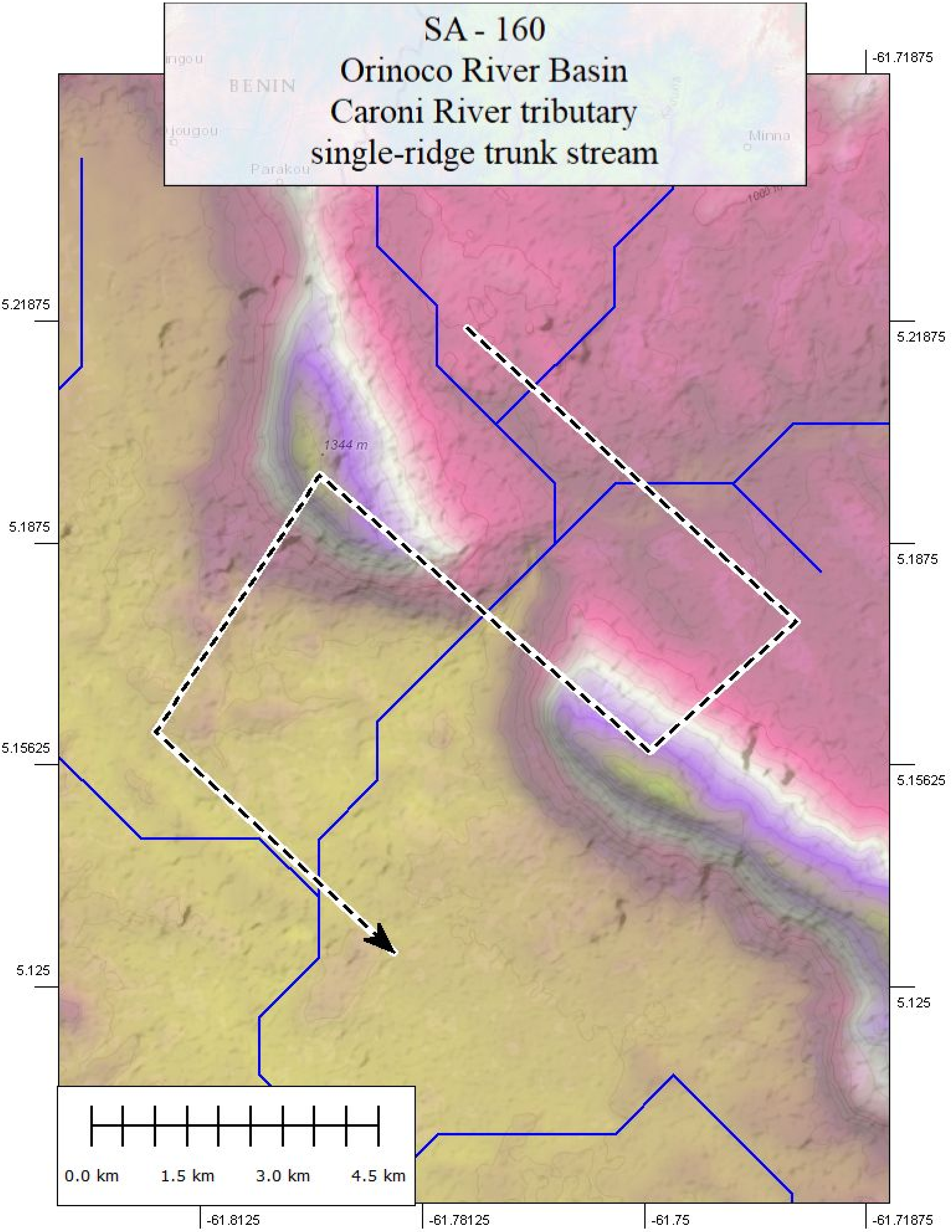
SA - 160
Orinoco River Basin
Caroni River tributary
single-ridge trunk stream
1344 m
5.21875
5.1875
5.15625
5.125
-61.71875
-61.8125
-61.78125
-61.75
0.0 km
1.5 km
3.0 km
4.5 km
BENIN
Parakou
Minna

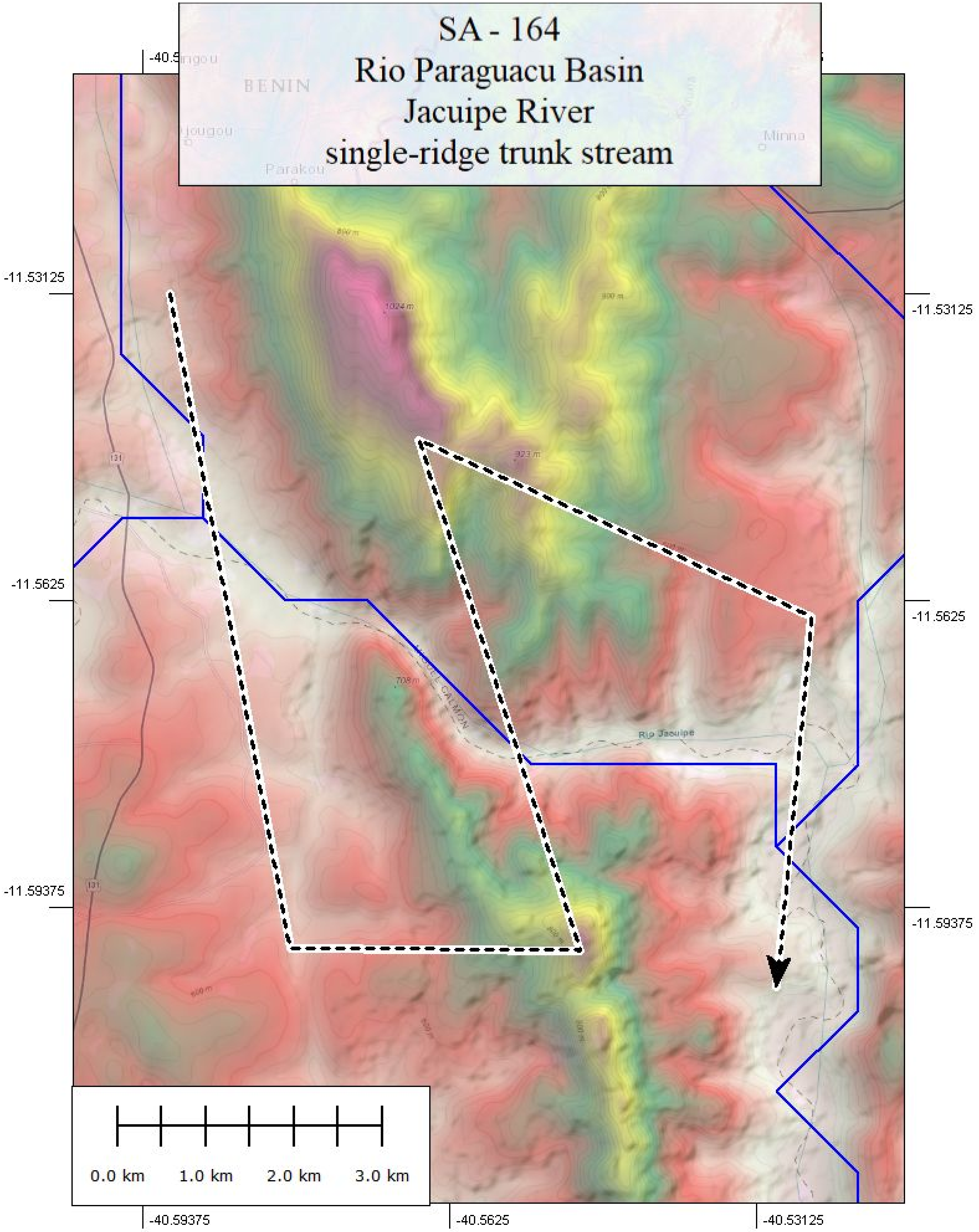

SA - 164
Rio Paraguacu Basin
Jacuipe River
single-ridge trunk stream
-11.53125
-11.5625
-11.59375
-40.59375
-40.5625
-40.53125
Rio Jacuipe
1024 m
923 m
708 m
0.0 km
1.0 km
2.0 km
3.0 km

SA - 165
Rio Itapicuru Basin
single-ridge trunk stream

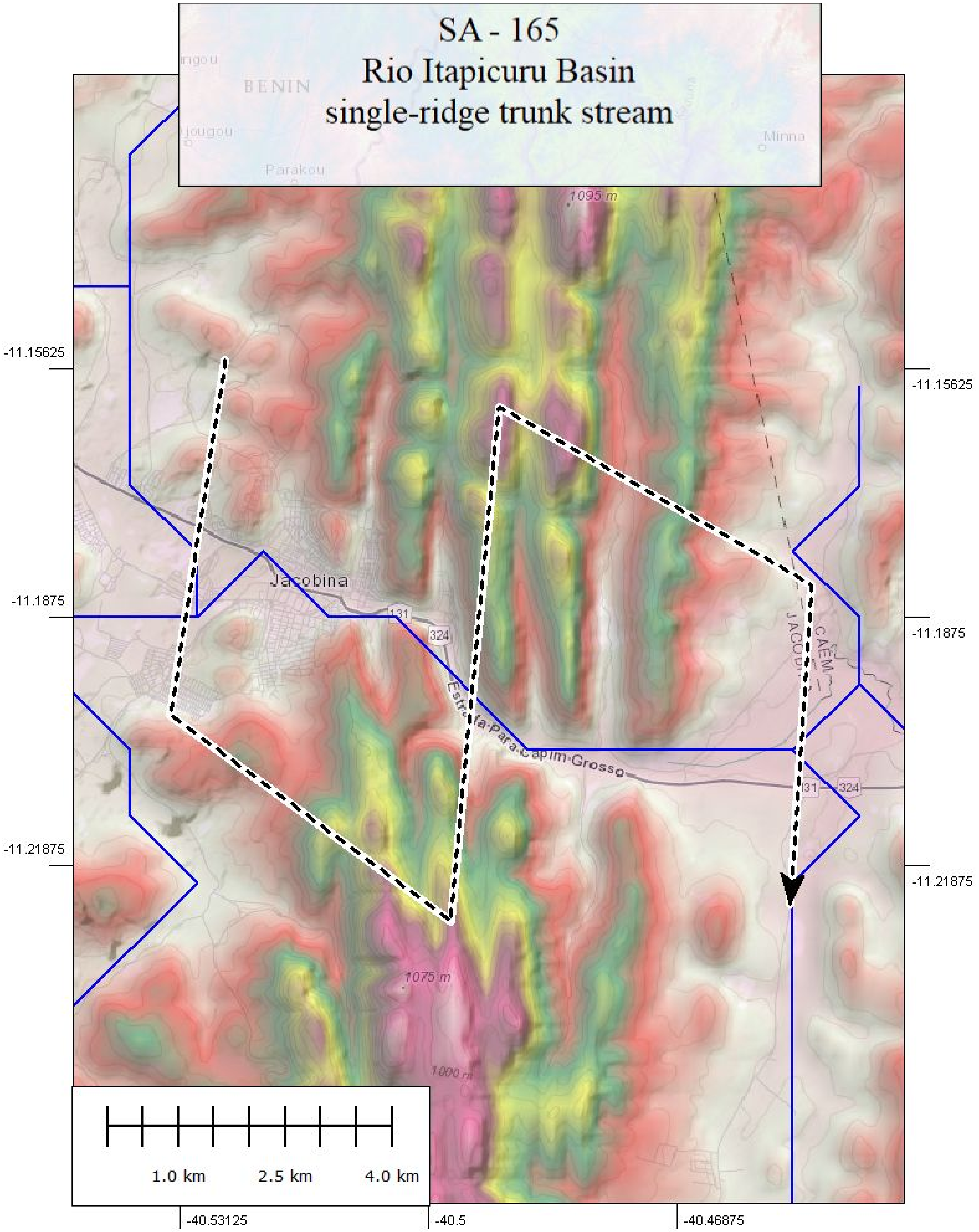

SA - 166
Rio Itapicuru Basin
single-ridge trunk stream

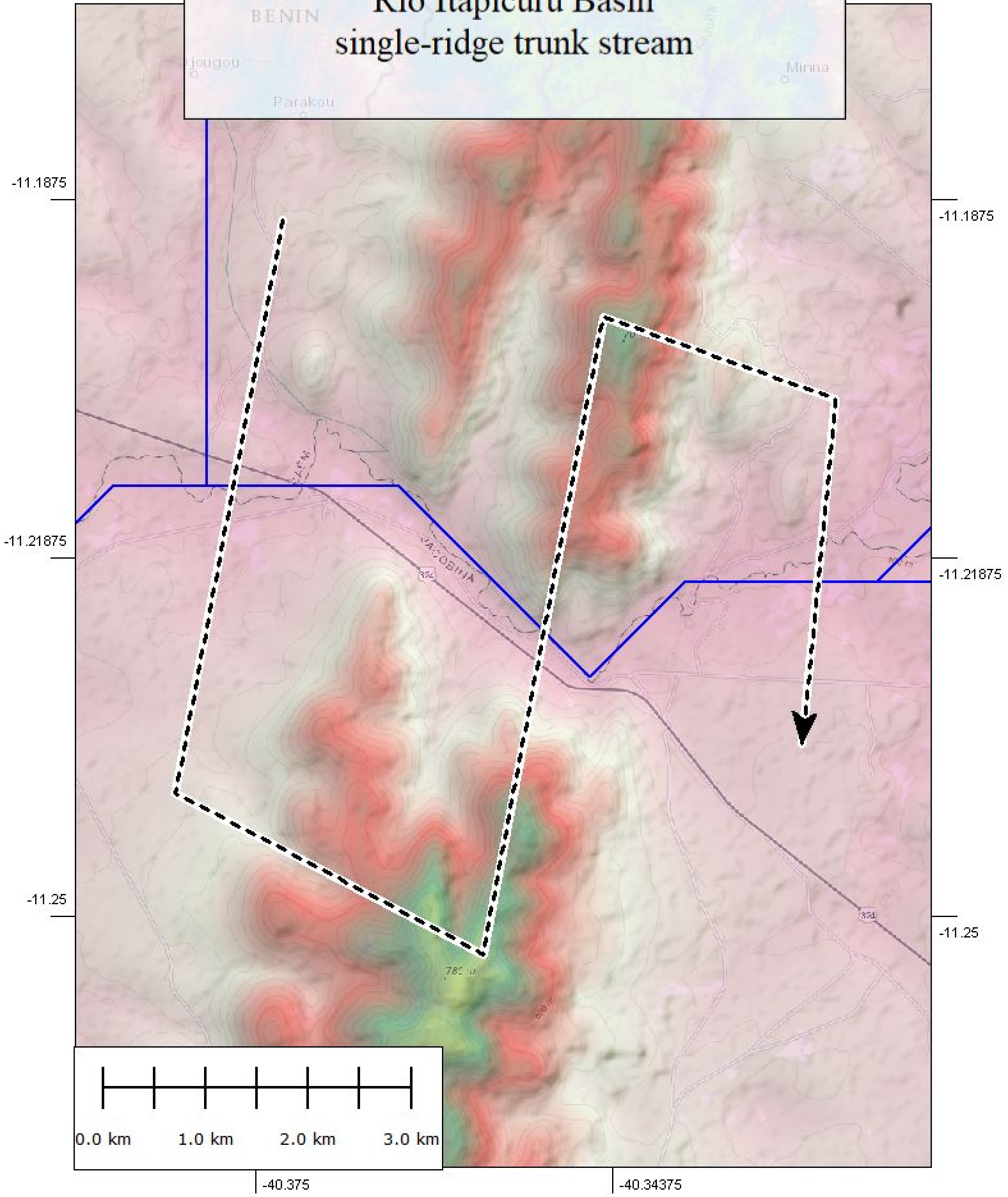

SA - 167
Rio Itapicuru Basin
single-ridge trunk stream

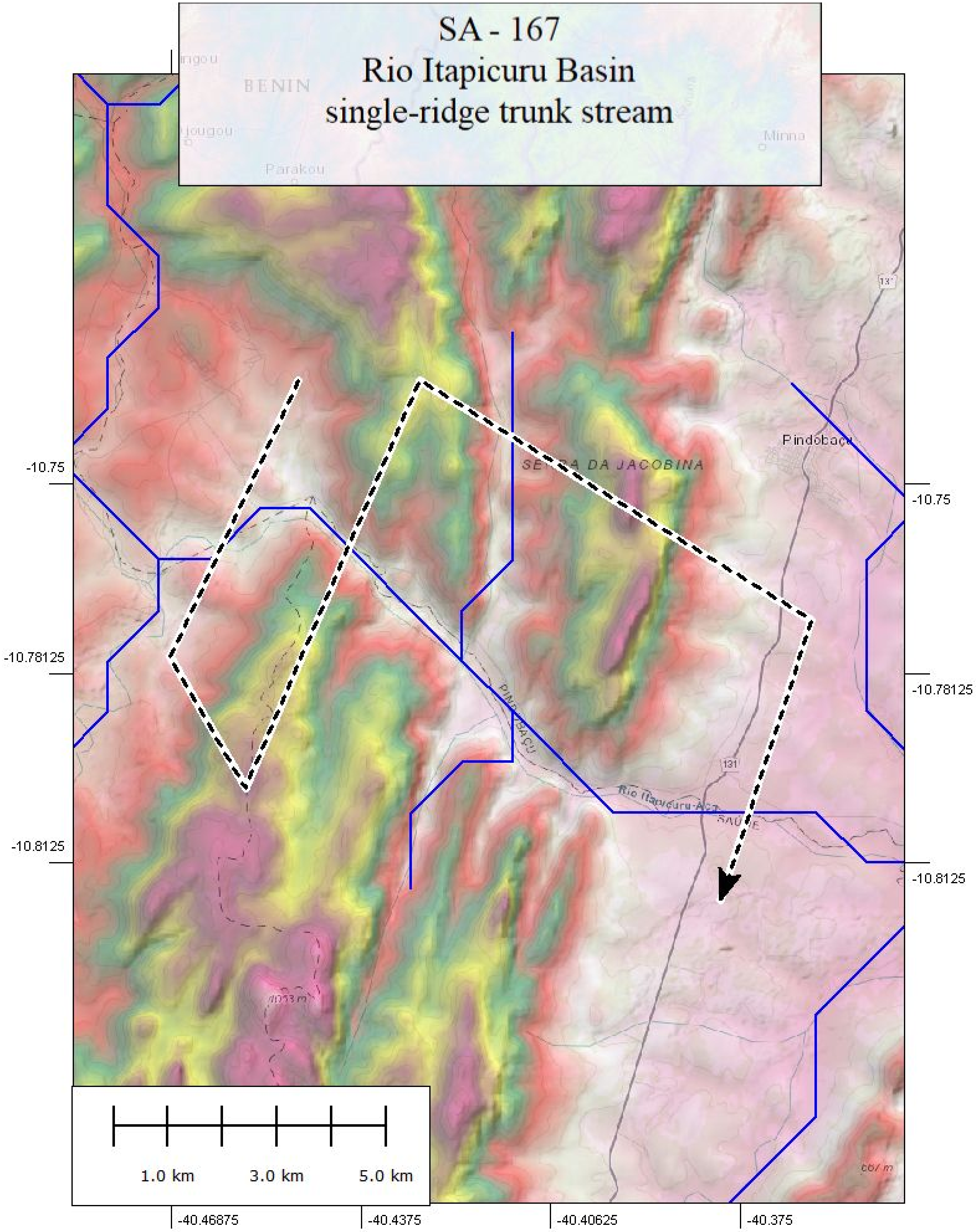

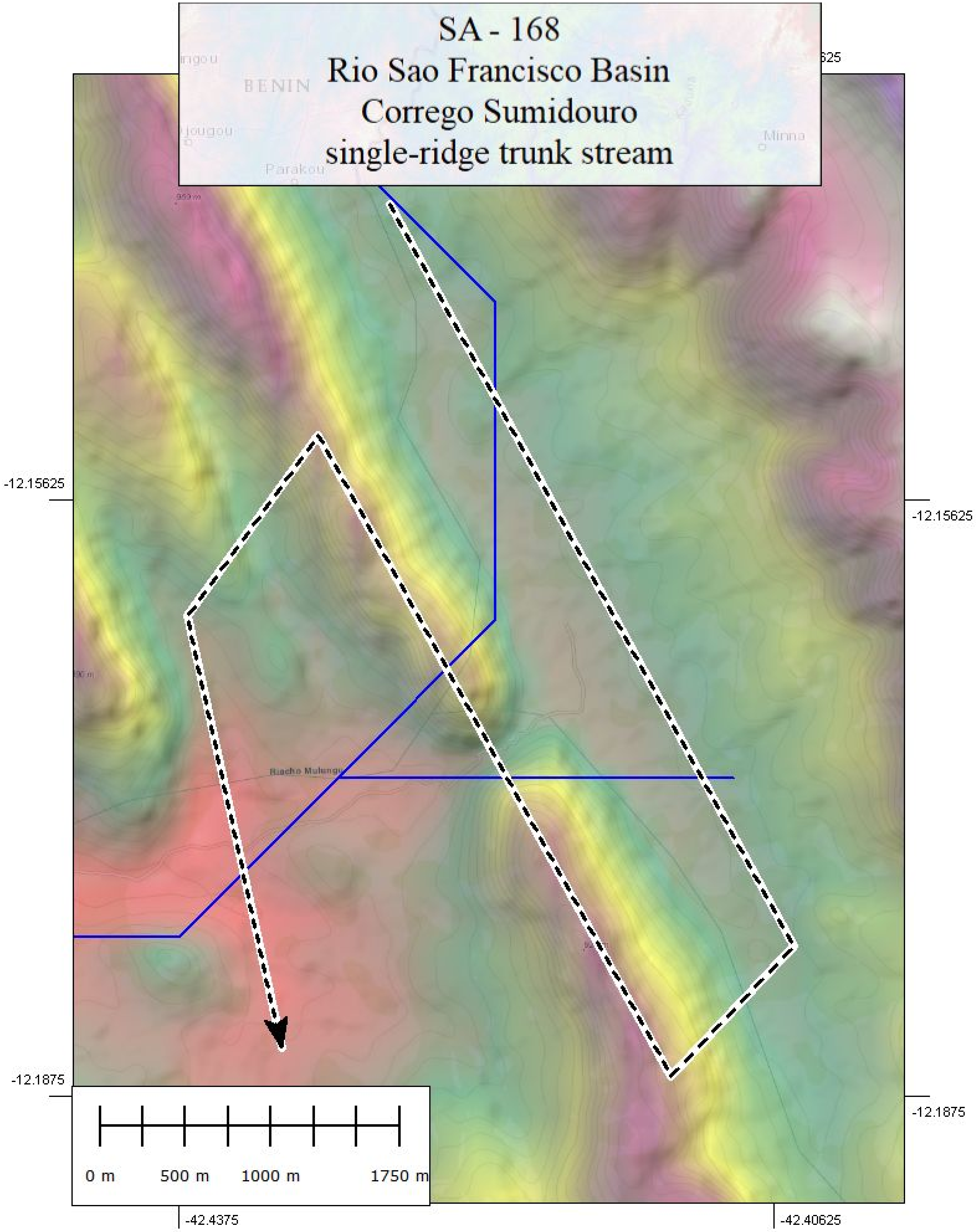
SA - 168
Rio Sao Francisco Basin
Corrego Sumidouro
single-ridge trunk stream
Riacho Mulungu
-12.15625
-12.15625
-12.1875
-12.1875
-42.4375
-42.40625
0 m
500 m
1000 m
1750 m

SA - 169
Rio Itapicuru Basin
Riacho do Aipim
single-ridge trunk stream

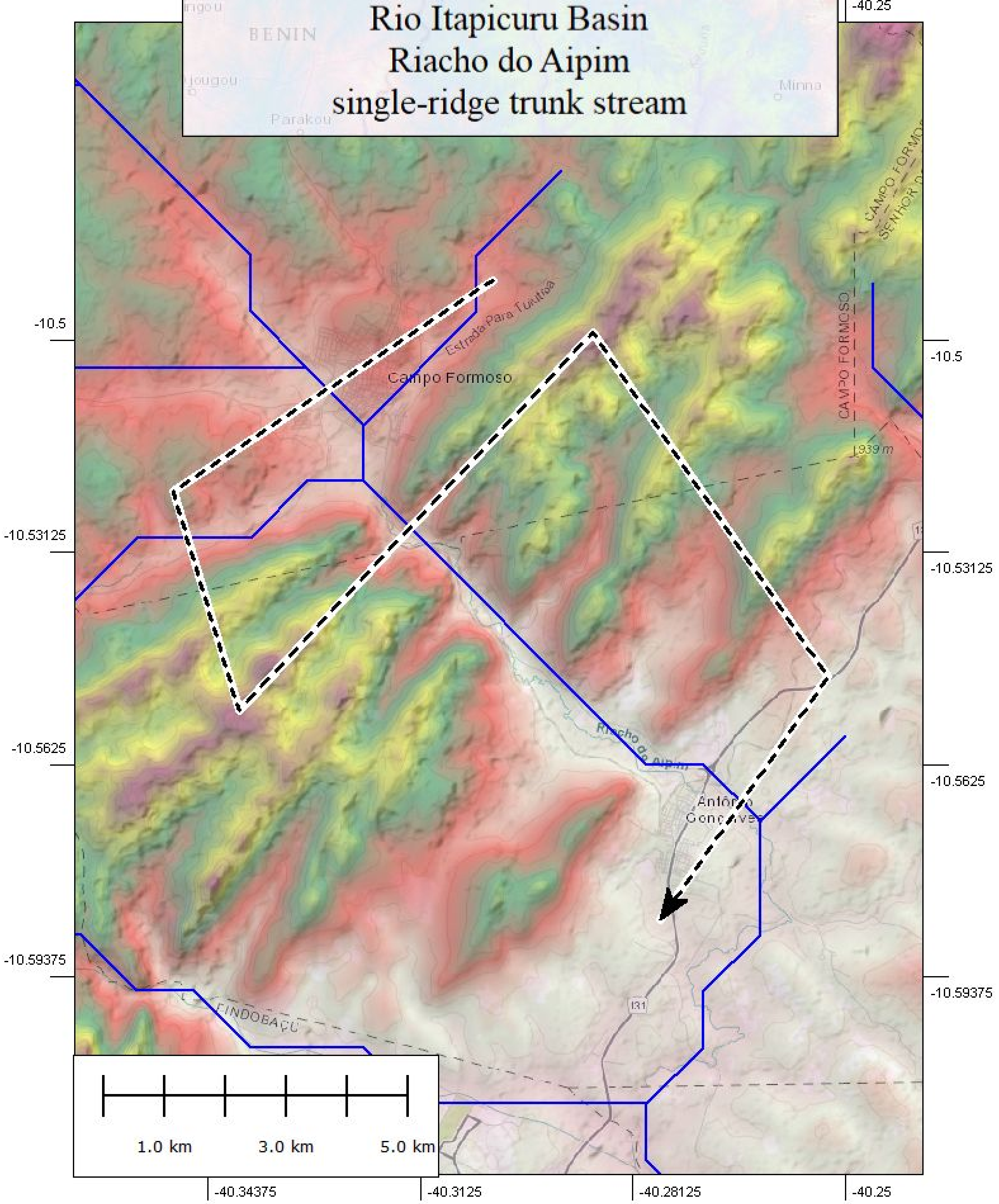

SA - 170
Rio Itapicuru Basin
Itapicuru River
single-ridge trunk stream

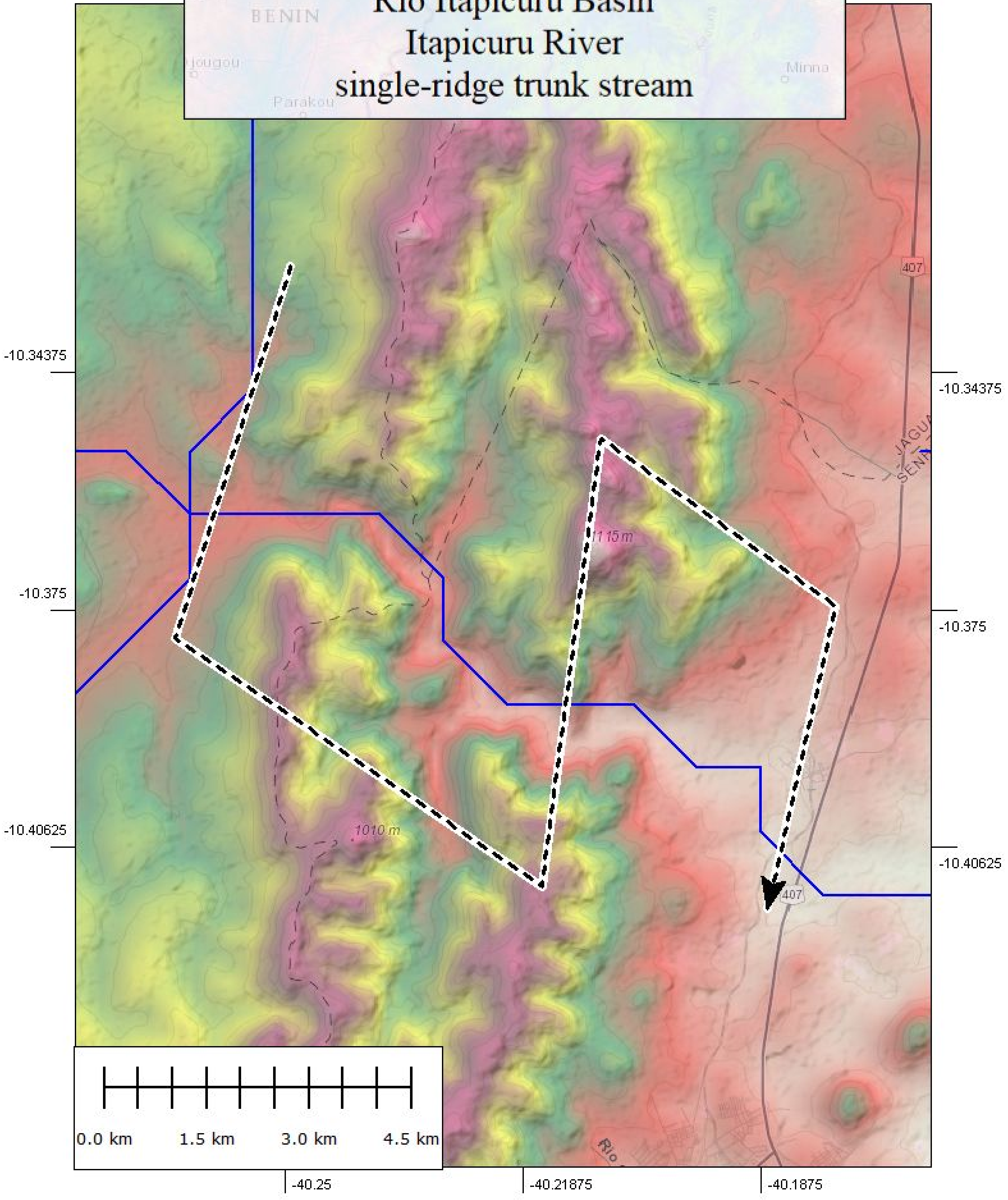

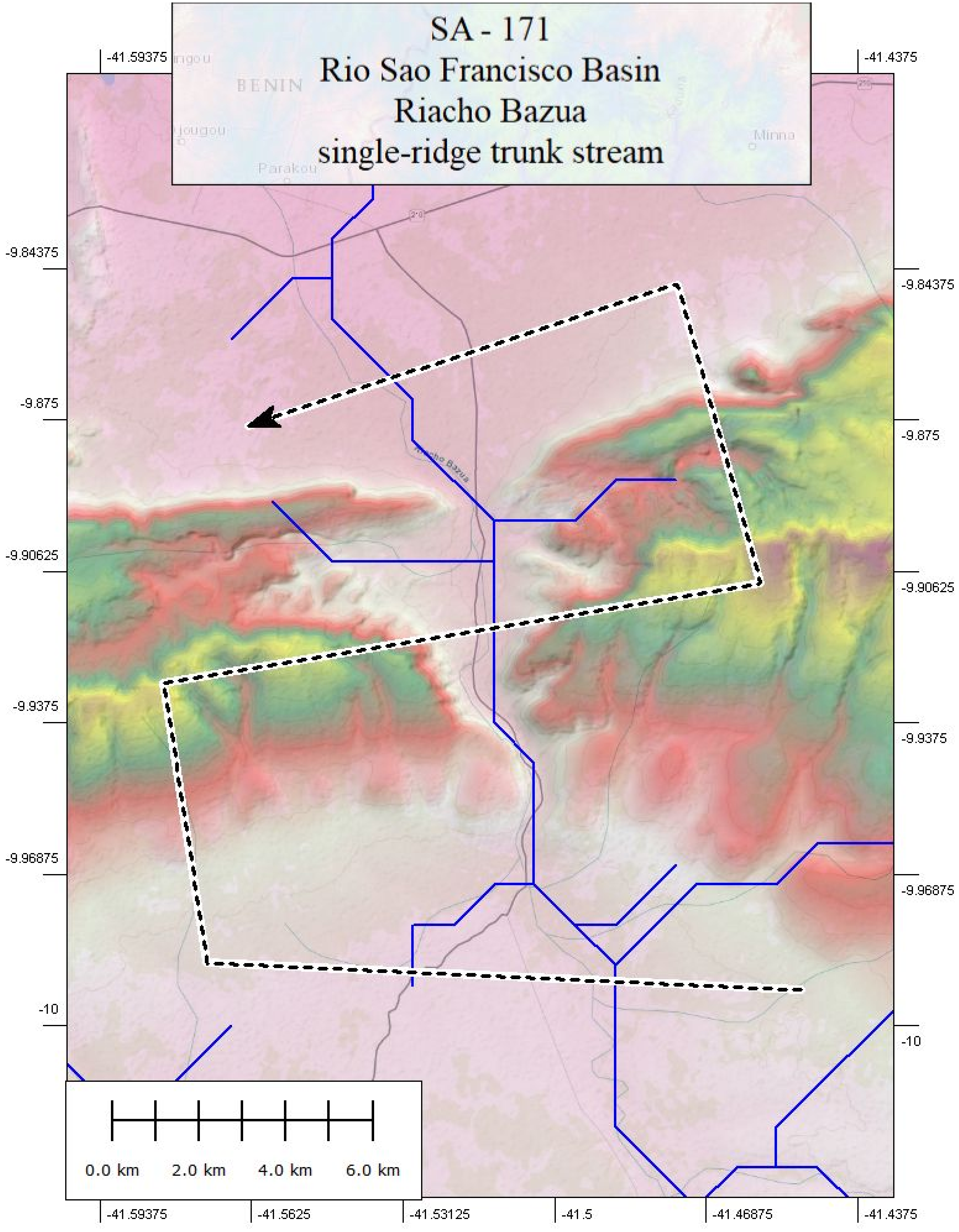
SA - 171
Rio Sao Francisco Basin
Riacho Bazua
single-ridge trunk stream
-41.59375
-41.5625
-41.53125
-41.5
-41.46875
-41.4375
-9.84375
-9.875
-9.90625
-9.9375
-9.96875
-10
BENIN
Djougou
Parakou
Minna
Riacho Bazua
0.0 km
2.0 km
4.0 km
6.0 km

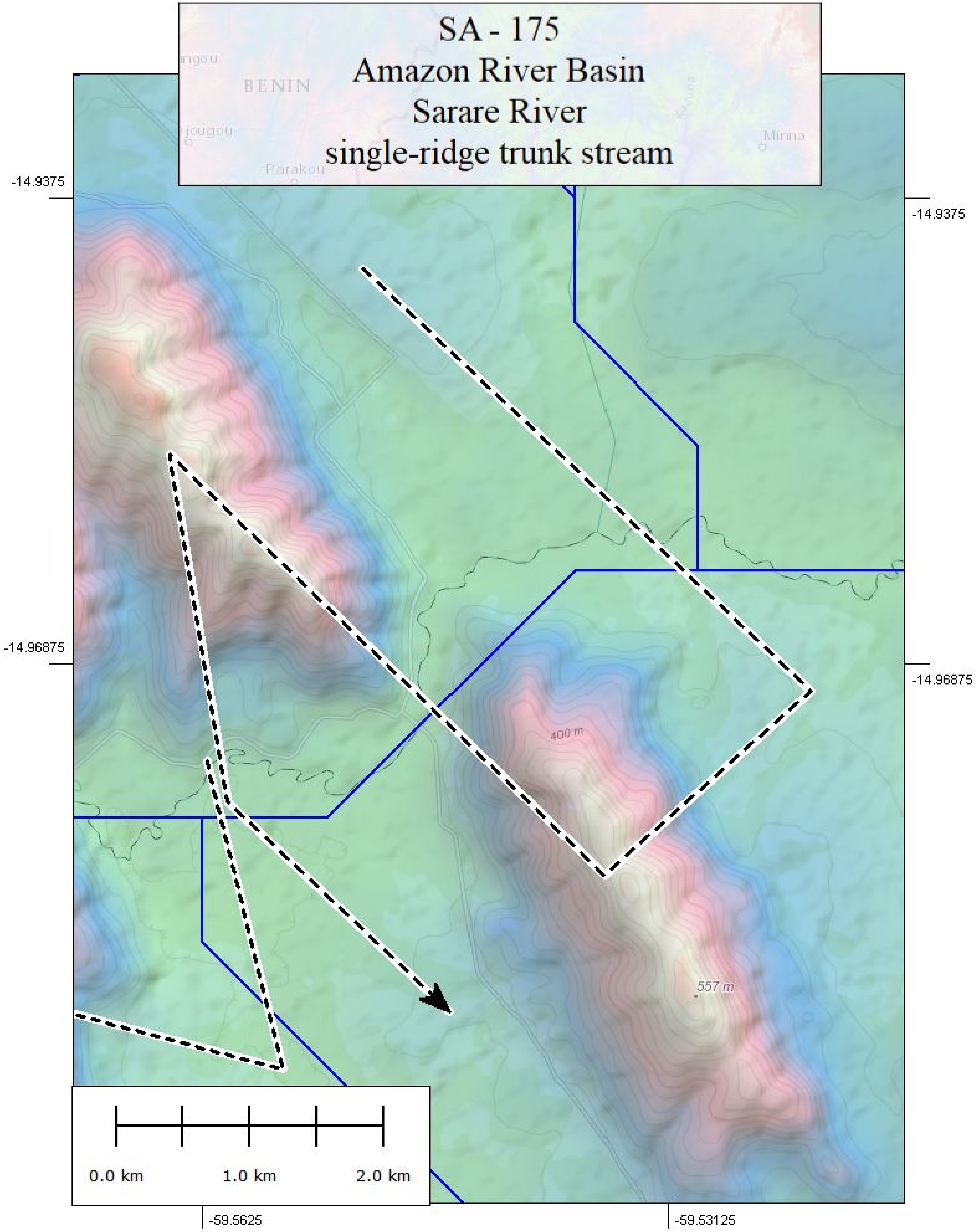
SA - 175
Amazon River Basin
Sarare River
single-ridge trunk stream
-14.9375
-14.9375
-14.96875
-14.96875
400 m
557 m
0.0 km
1.0 km
2.0 km
-59.5625
-59.53125

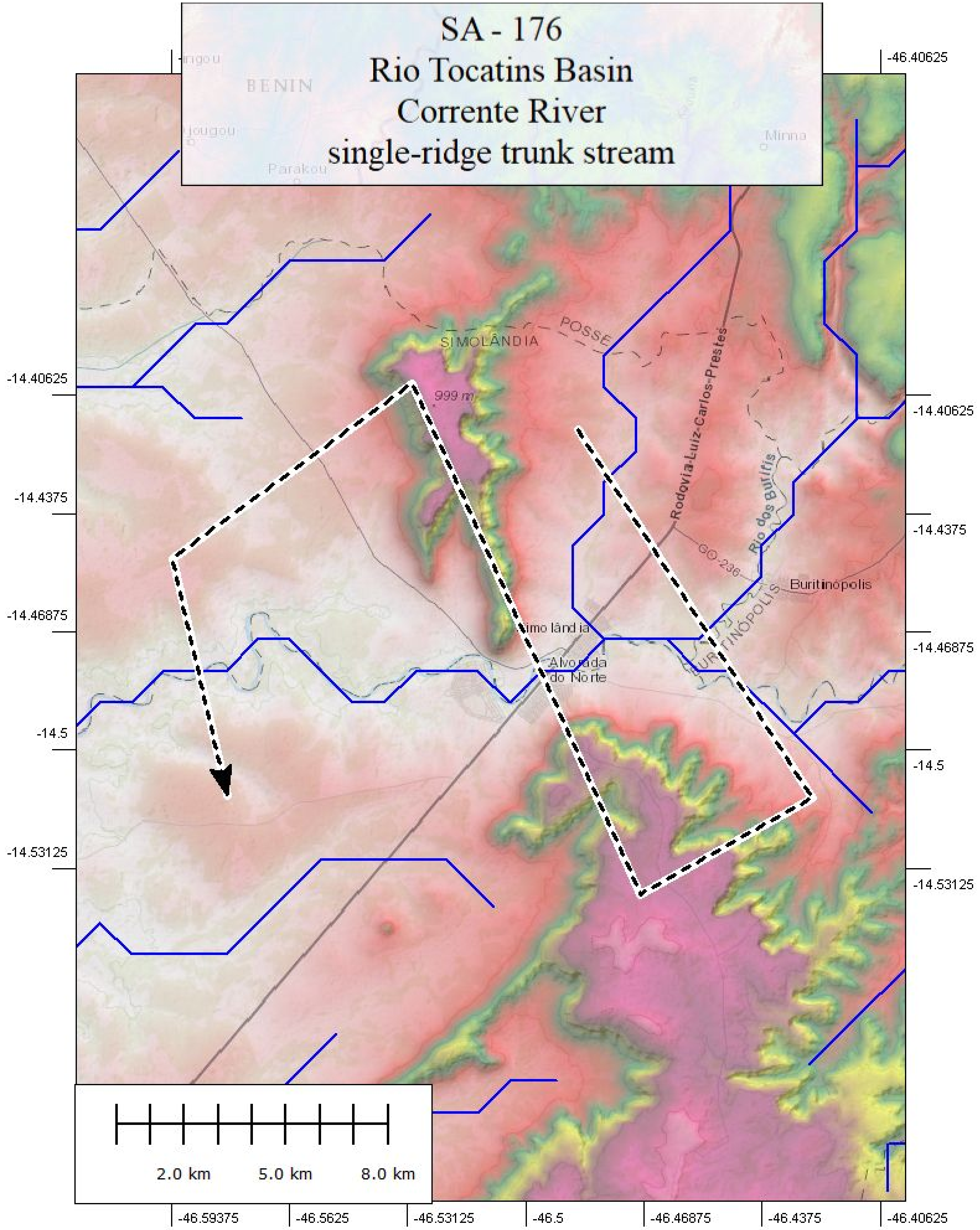
SA - 176
Rio Tocatins Basin
Corrente River
single-ridge trunk stream
BENIN
Parakou
Minna
SIMOLÂNDIA
POSSE
999 m
Rodovia-Luiz-Carlos-Prestes
Rio dos Buritis
GO-236
Buritinópolis
Simolândia
Alvorada do Norte
2.0 km
5.0 km
8.0 km
-46.40625
-14.40625
-14.4375
-14.46875
-14.5
-14.53125
-46.59375
-46.5625
-46.53125
-46.5
-46.46875
-46.4375

SA - 177
Rio Sao Francisco Basin
Jeguitai River
single-ridge trunk stream

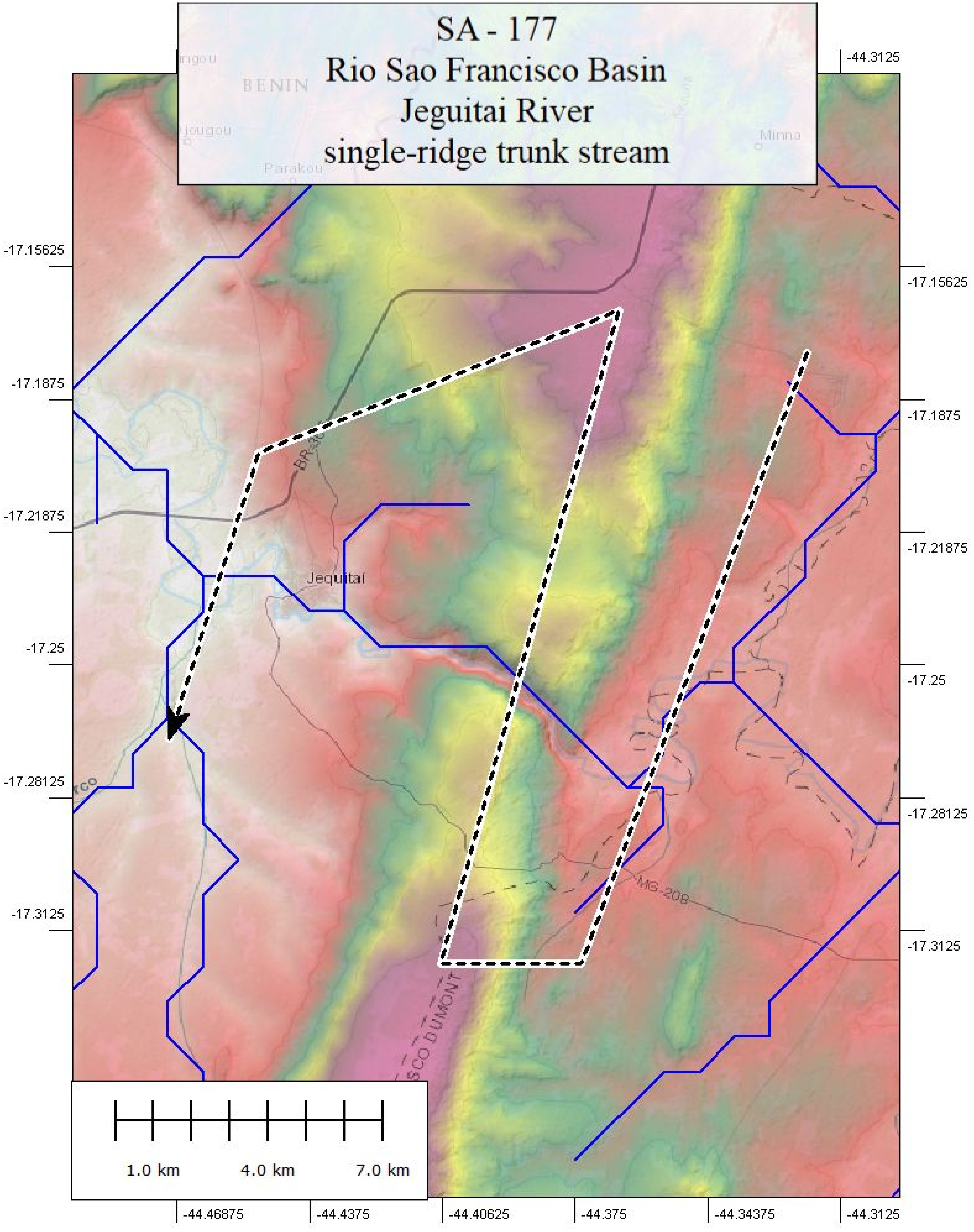

SA - 178
Rio Sao Francisco Basin
Rio Verde
single-ridge trunk stream

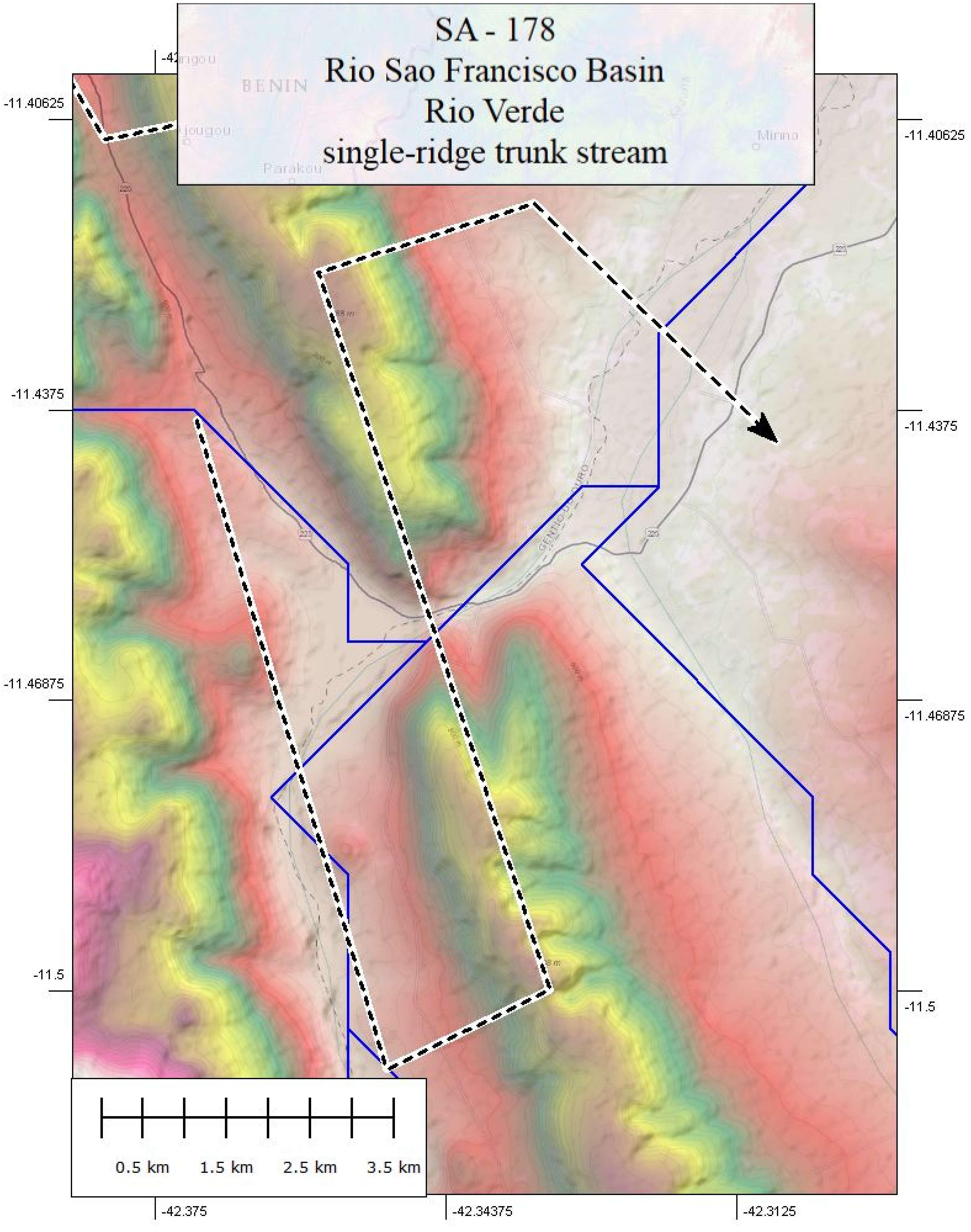

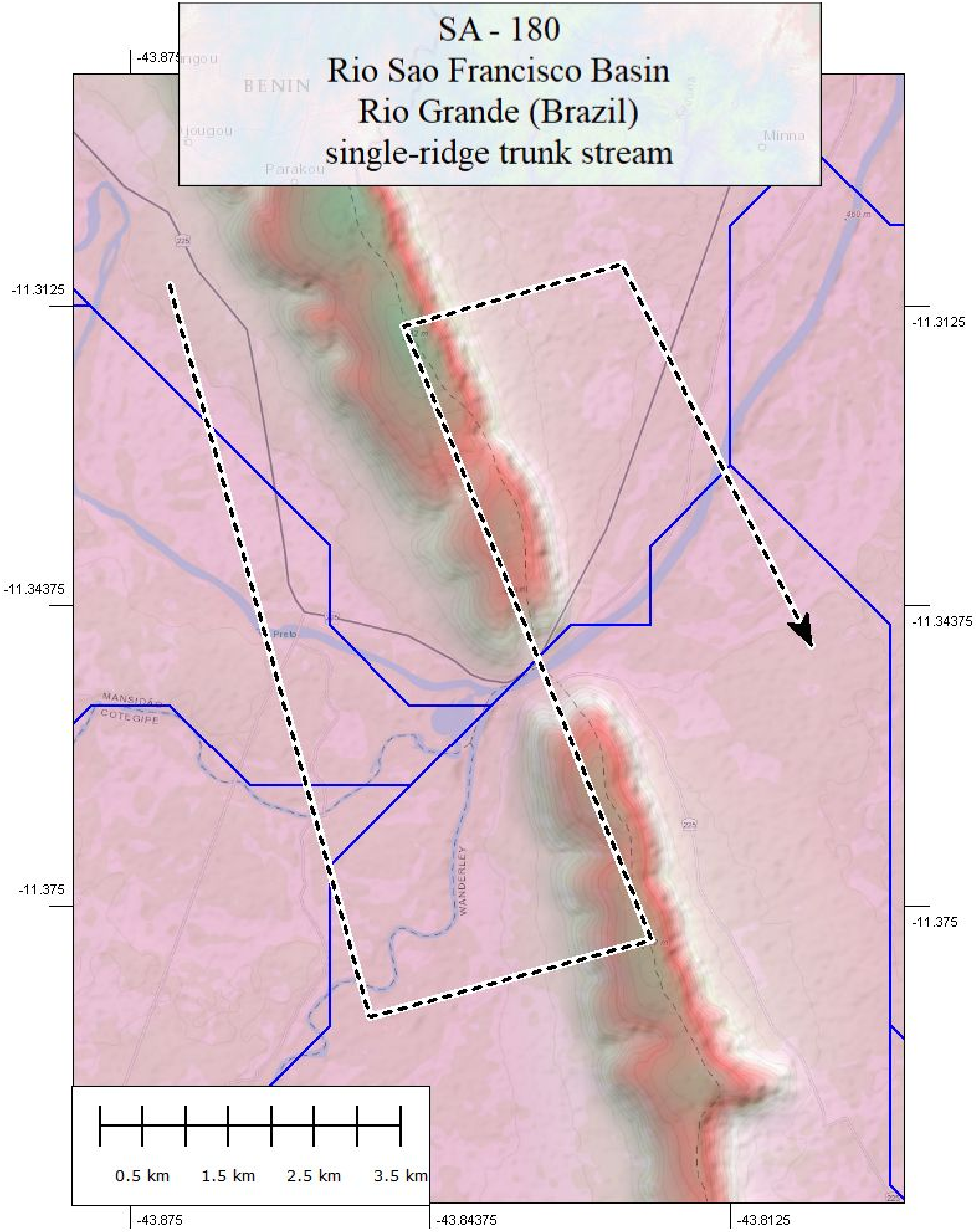
SA - 180
Rio Sao Francisco Basin
Rio Grande (Brazil)
single-ridge trunk stream
-43.875
-43.84375
-43.8125
-11.3125
-11.34375
-11.375
BENIN
Parakou
Minna
Preto
MANSIDÃO
COTEGIPE
WANDERLEY
225
0.5 km
1.5 km
2.5 km
3.5 km

SA - 181
Rio Sao Francisco Basin
Riacho Negro
single-ridge trunk stream

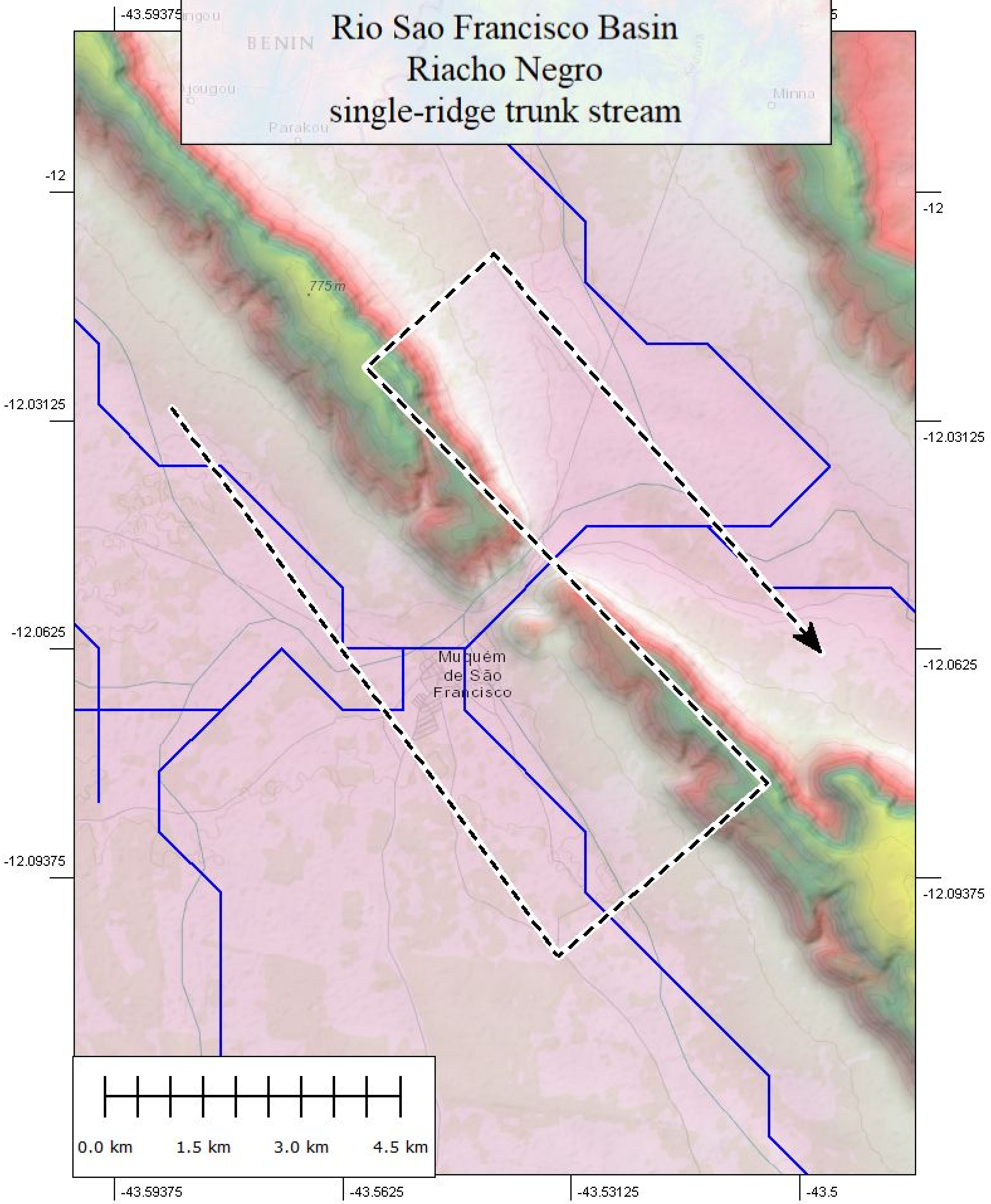

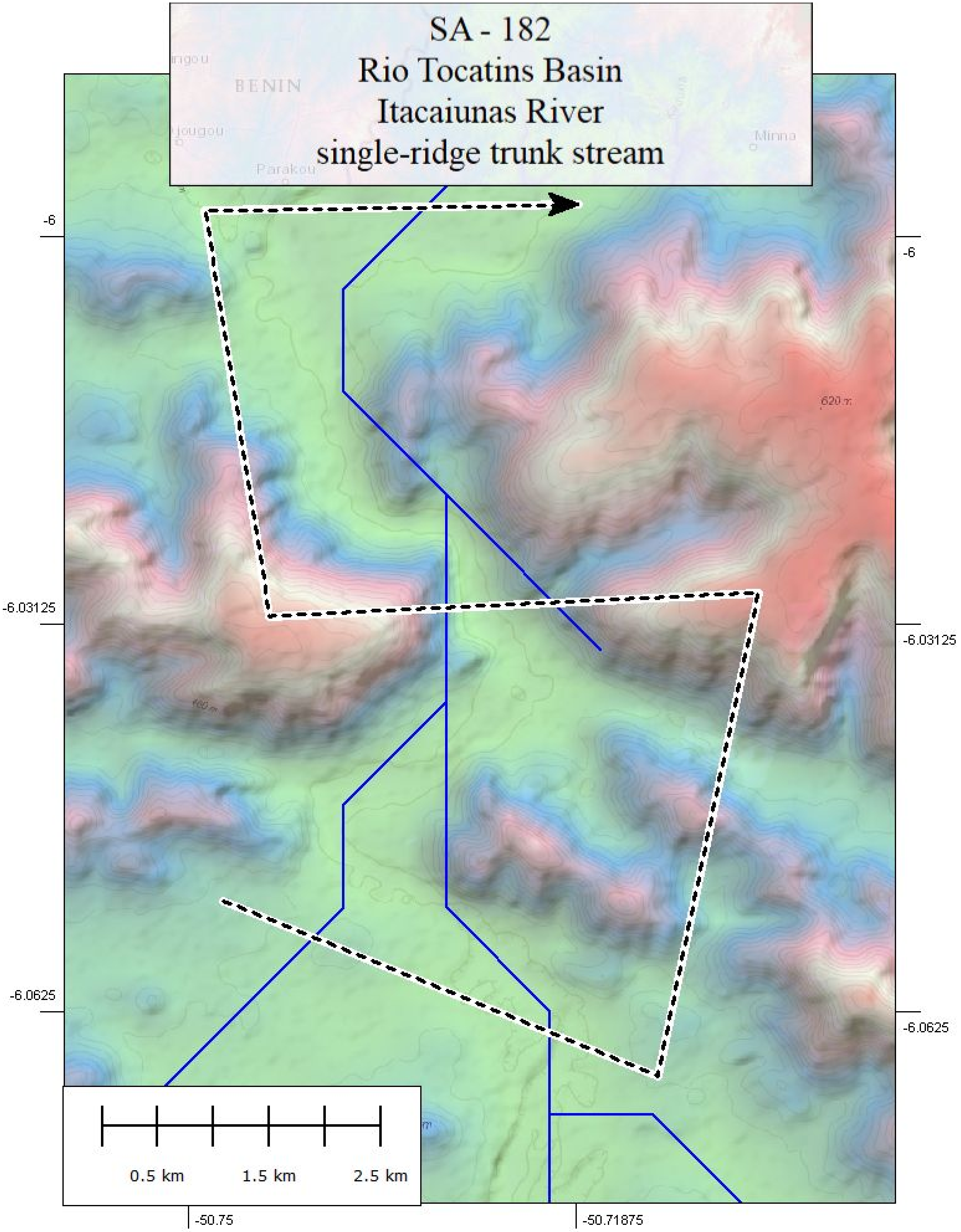
SA - 182
Rio Tocatins Basin
Itacaiunas River
single-ridge trunk stream
BENIN
Parakou
Minna
620 m
-6
-6
-6.03125
-6.03125
-6.0625
-6.0625
0.5 km
1.5 km
2.5 km
-50.75
-50.71875

SA - 183
Amazon River Basin
Juari River
single-ridge trunk stream

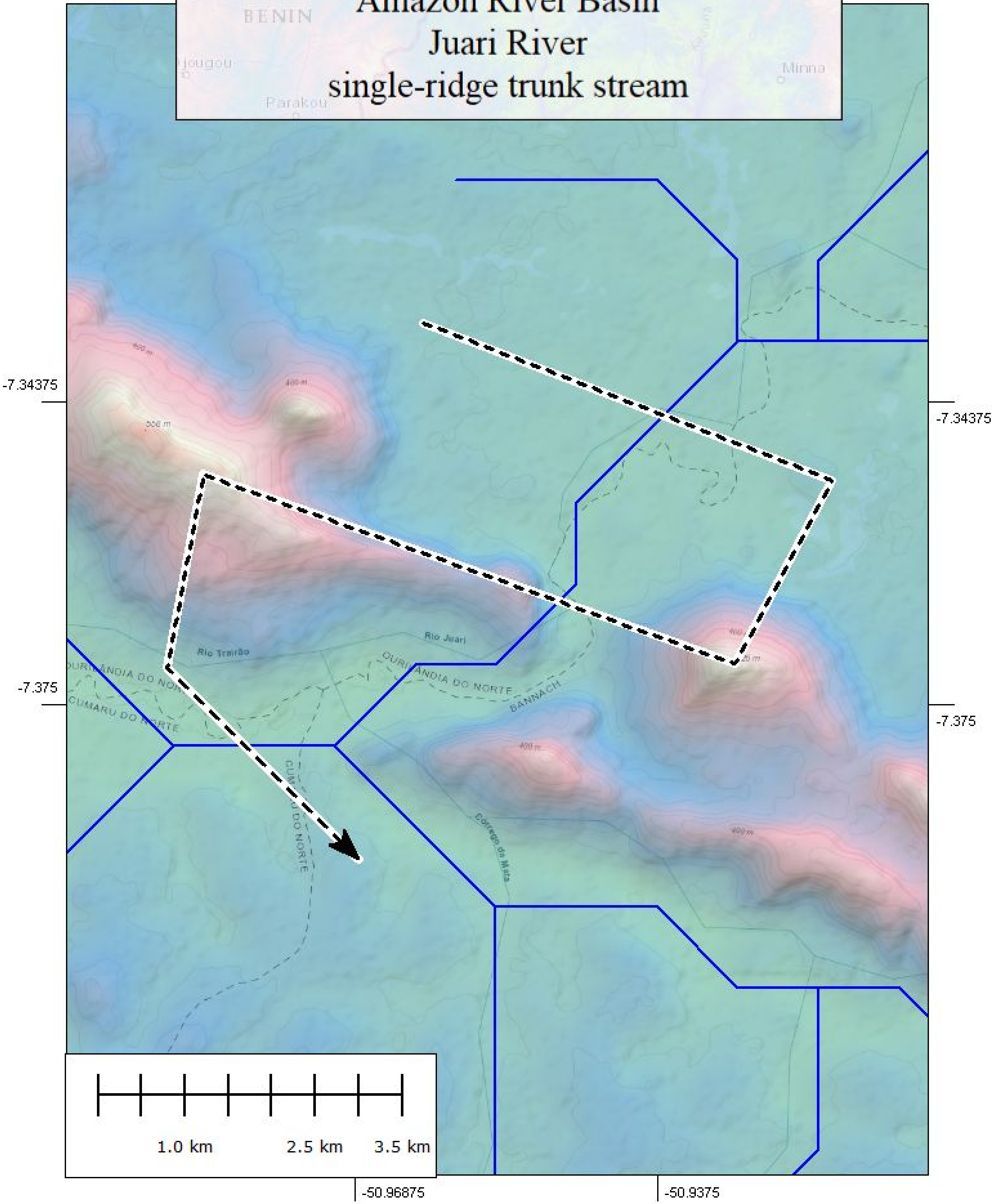

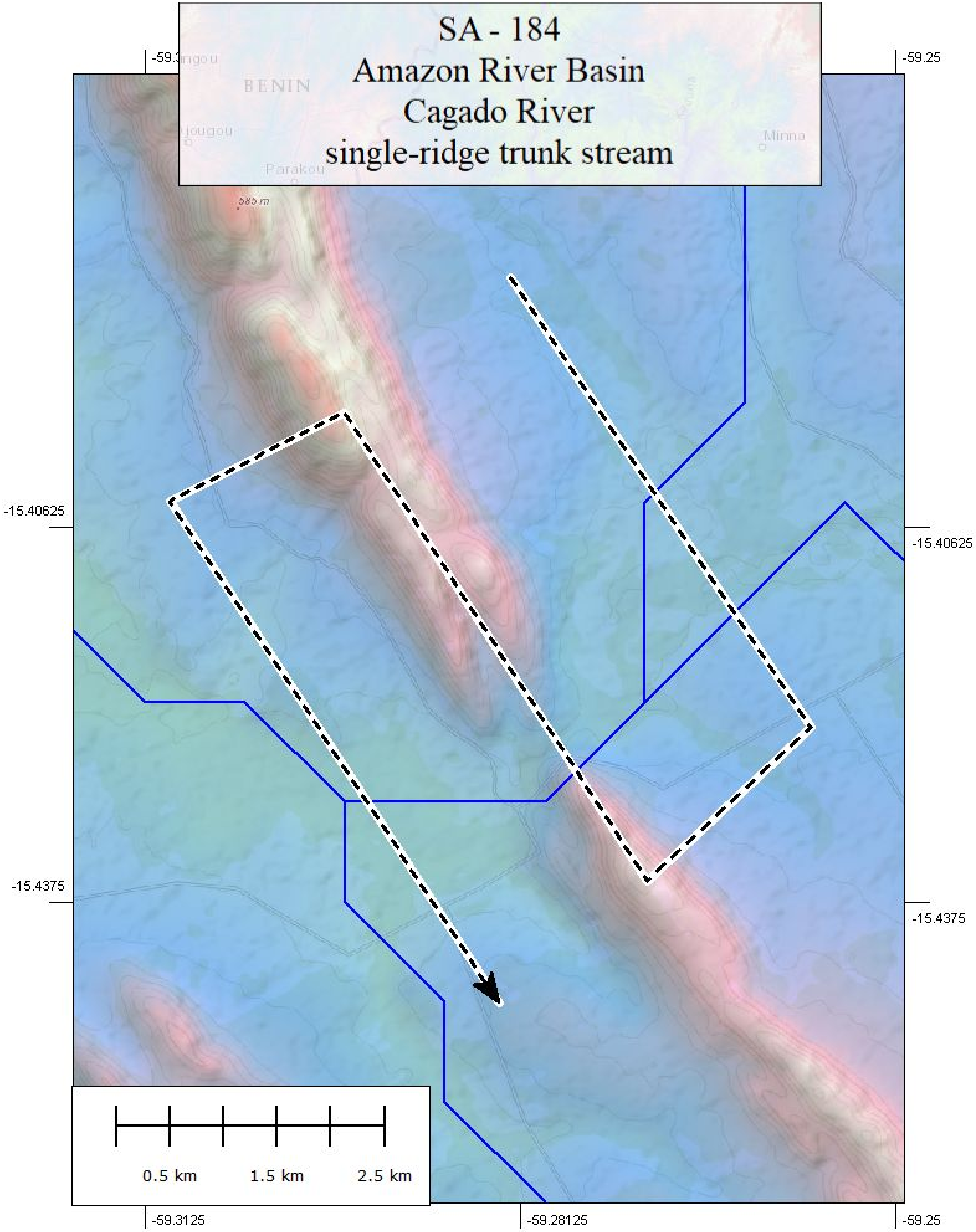
SA - 184
Amazon River Basin
Cagado River
single-ridge trunk stream
-59.3125
-59.25
-15.40625
-15.40625
-15.4375
-15.4375
0.5 km
1.5 km
2.5 km
-59.3125
-59.28125
-59.25

SA - 186
Rio Negro (Golfo San Matias) Basin
Tralalhue River
single-ridge trunk stream

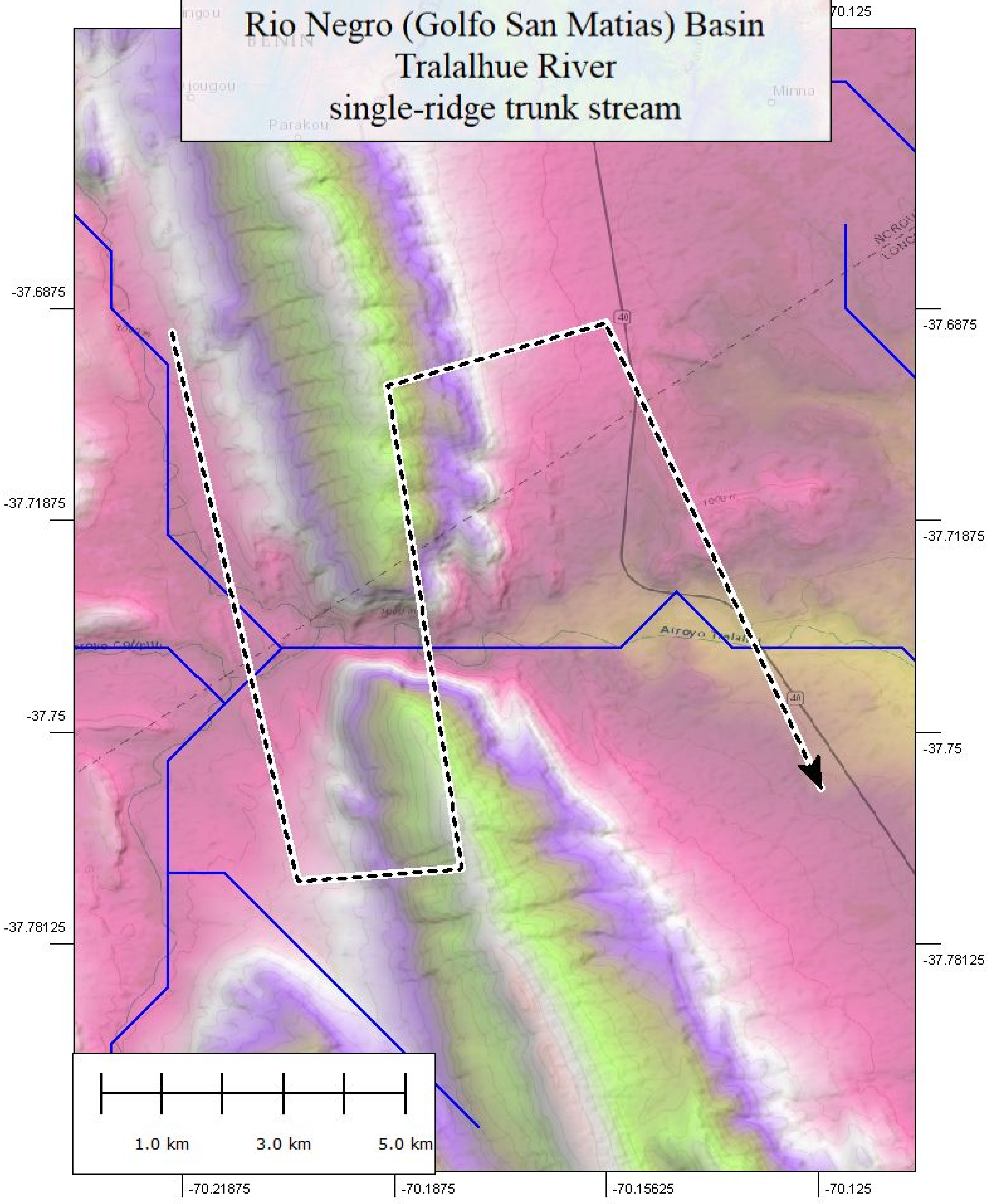

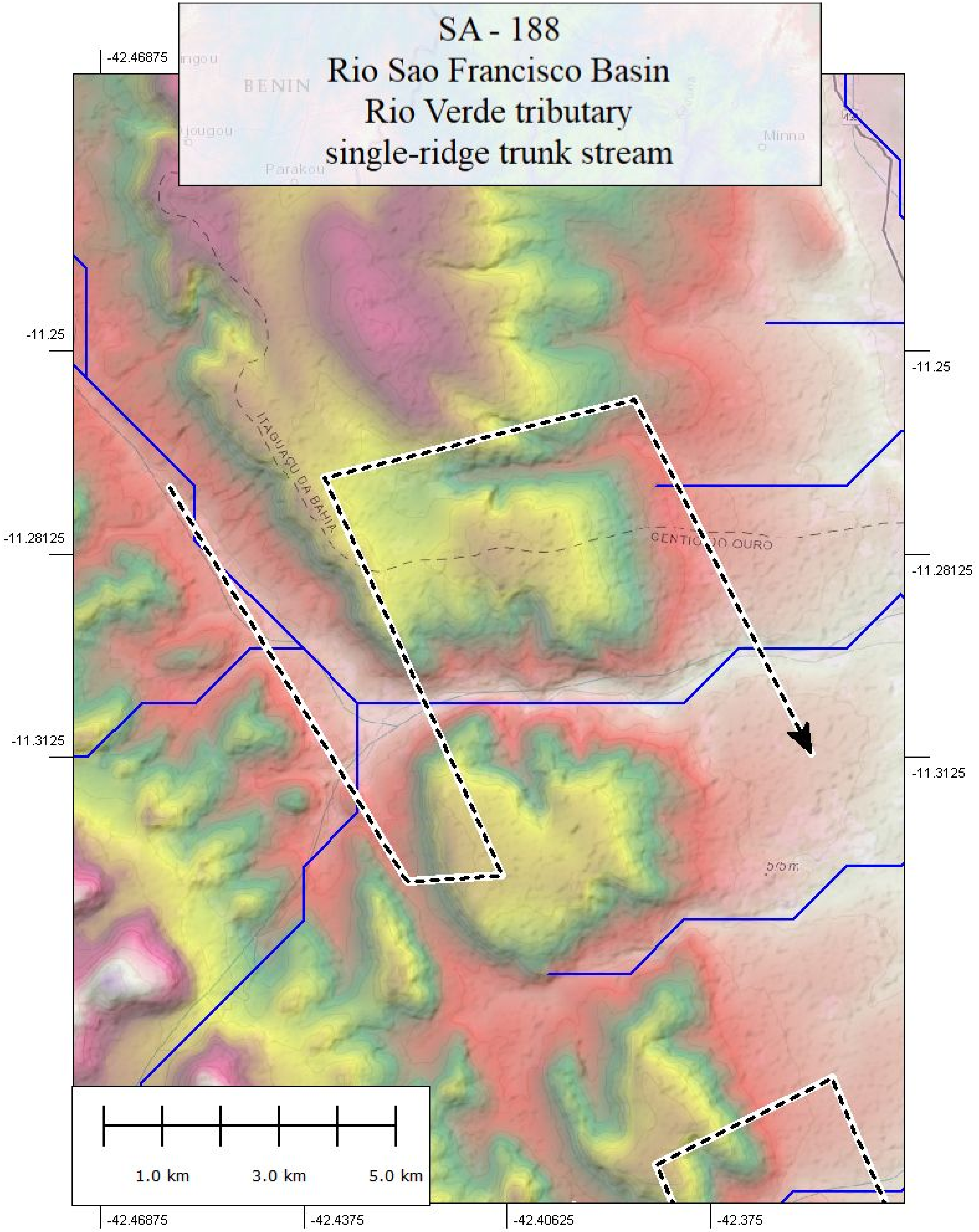

SA - 188
Rio Sao Francisco Basin
Rio Verde tributary
single-ridge trunk stream
-42.46875
-42.4375
-42.40625
-42.375
-11.25
-11.28125
-11.3125
BENIN
Djougou
Parakou
ITAGUAÇU DA BAHIA
GENTIO DO OURO
1.0 km
3.0 km
5.0 km

SA - 190
Parana River Basin
Salobra River
single-ridge trunk stream

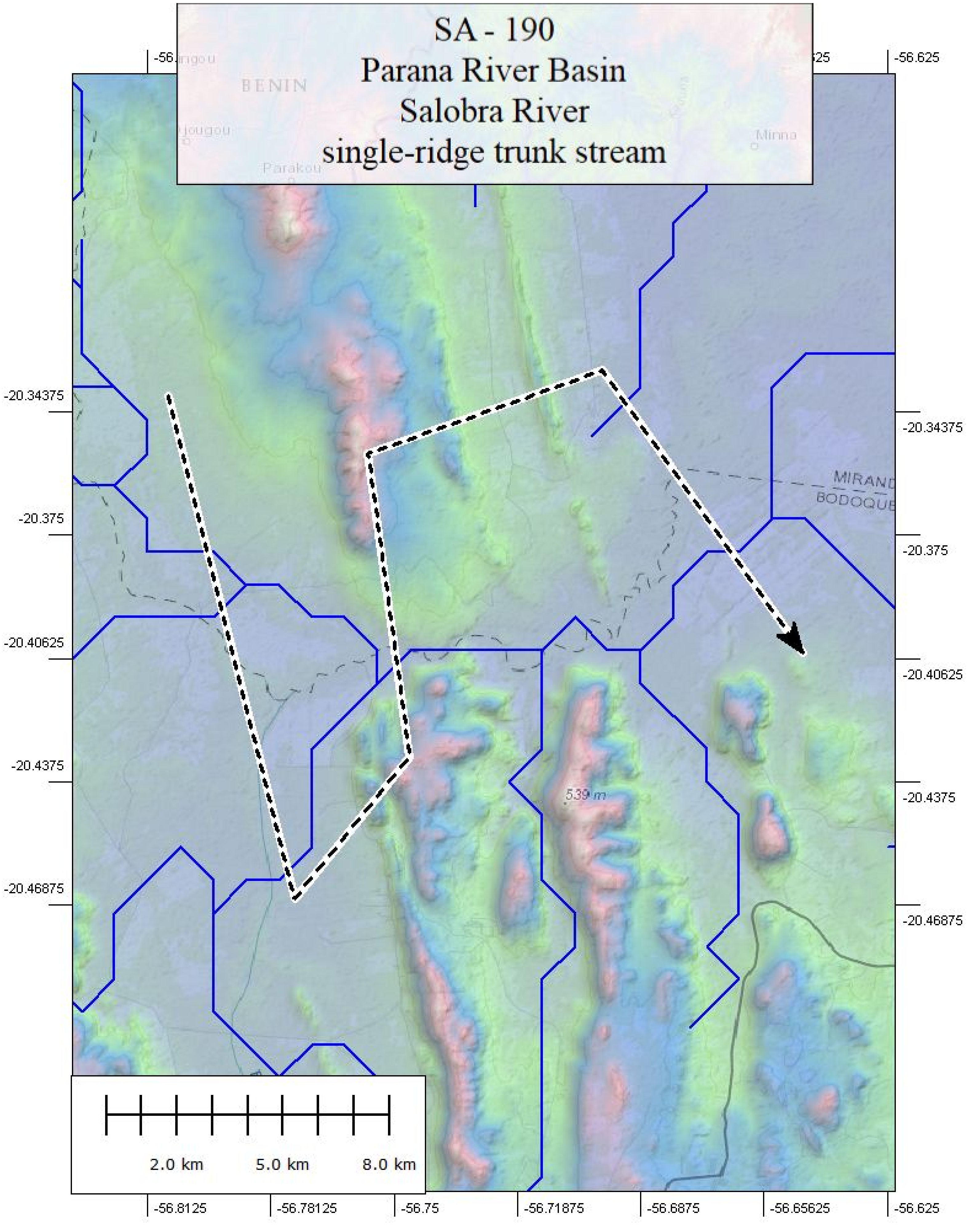

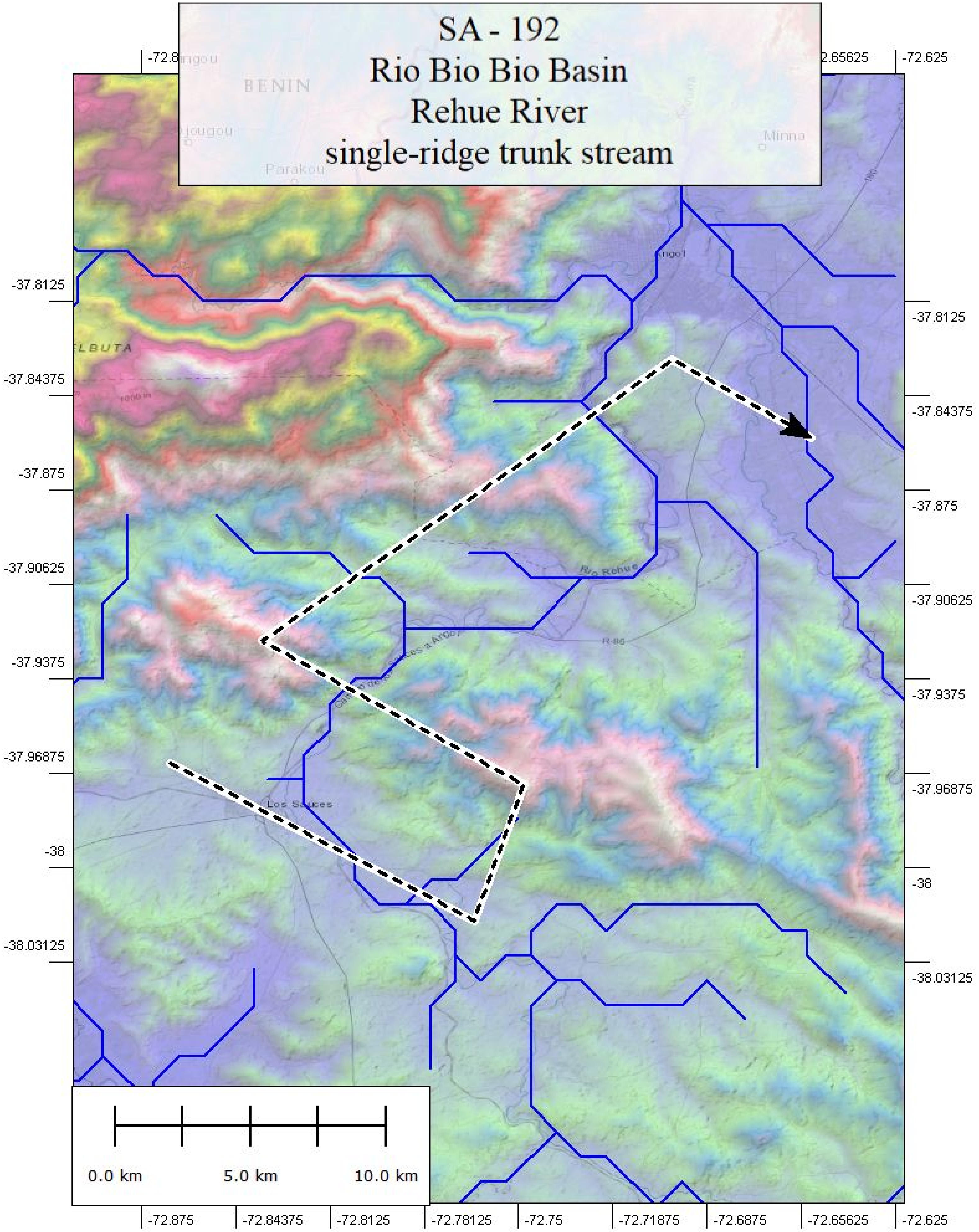
SA - 192
Rio Bio Bio Basin
Rehue River
single-ridge trunk stream
-72.625
-72.65625
-37.8125
-37.84375
-37.875
-37.90625
-37.9375
-37.96875
-38
-38.03125
Rio Rehue
Los Sauces
0.0 km
5.0 km
10.0 km
-72.875
-72.84375
-72.8125
-72.78125
-72.75
-72.71875
-72.6875

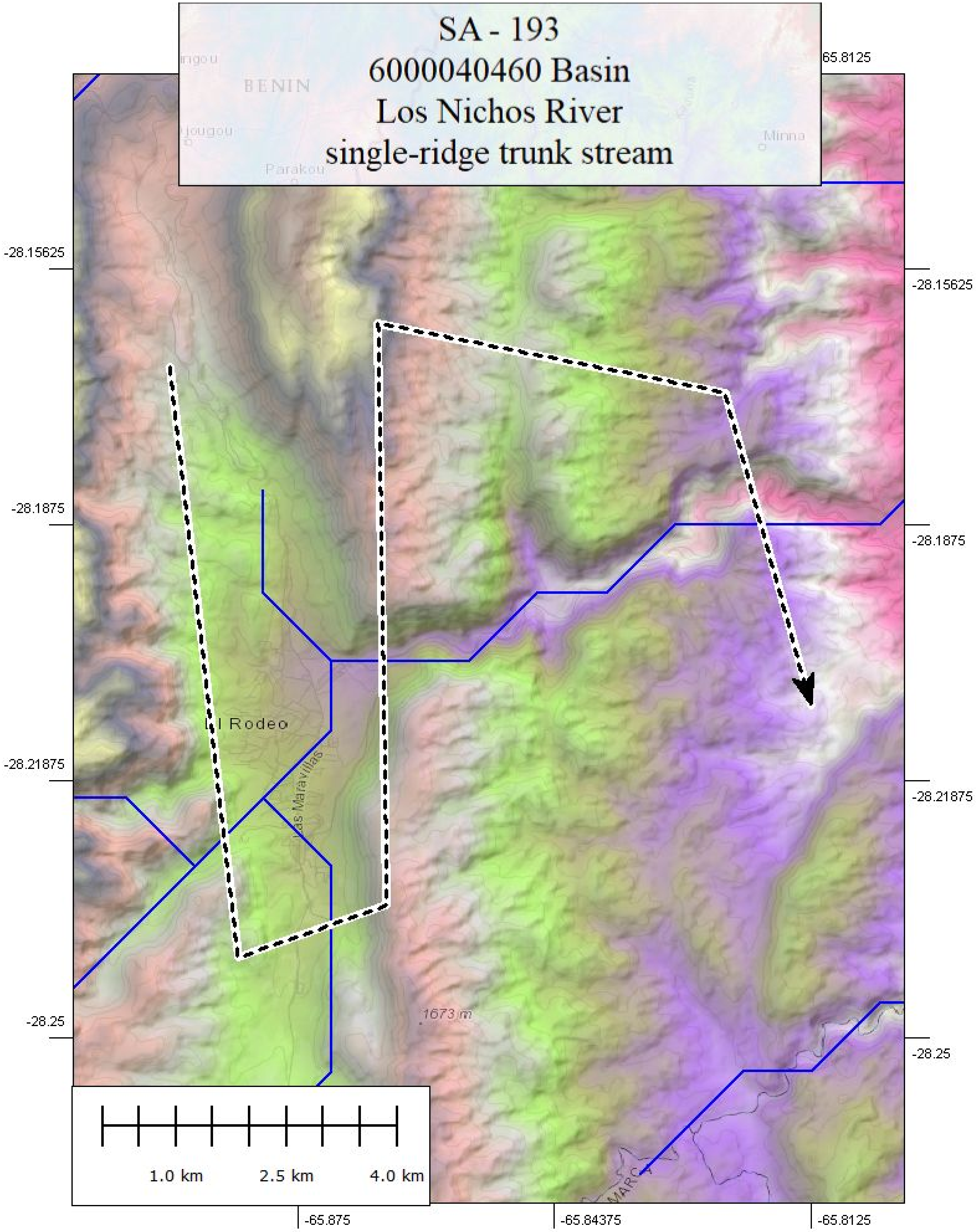
SA - 193
6000040460 Basin
Los Nichos River
single-ridge trunk stream
BENIN
Parakou
Minna
El Rodeo
Las Maravillas
1673 m
-65.8125
-28.15625
-28.1875
-28.21875
-28.25
-65.875
-65.84375
1.0 km
2.5 km
4.0 km

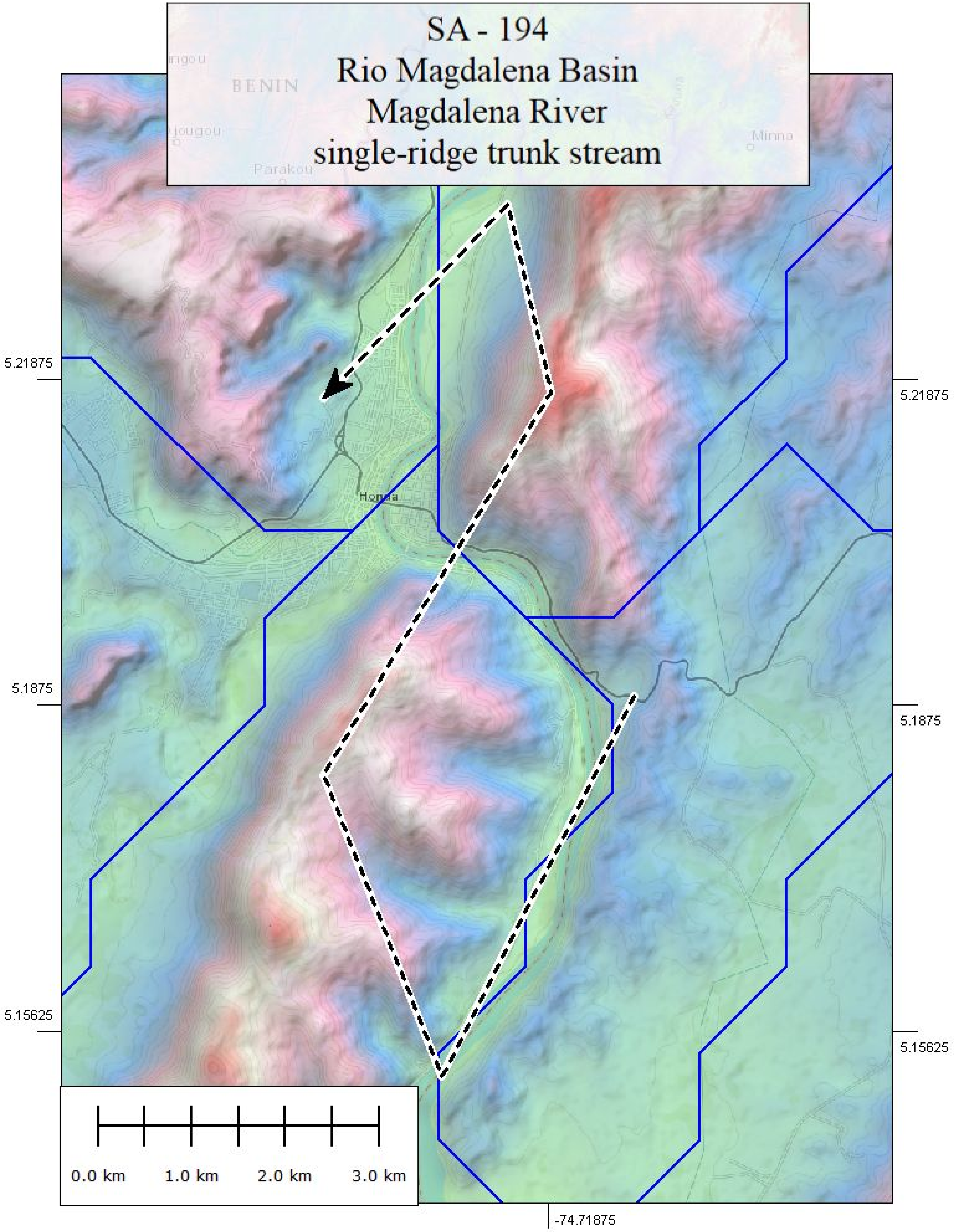
SA - 194
Rio Magdalena Basin
Magdalena River
single-ridge trunk stream
BENIN
Parakou
Minna
Honda
5.21875
5.1875
5.15625
-74.71875
0.0 km
1.0 km
2.0 km
3.0 km

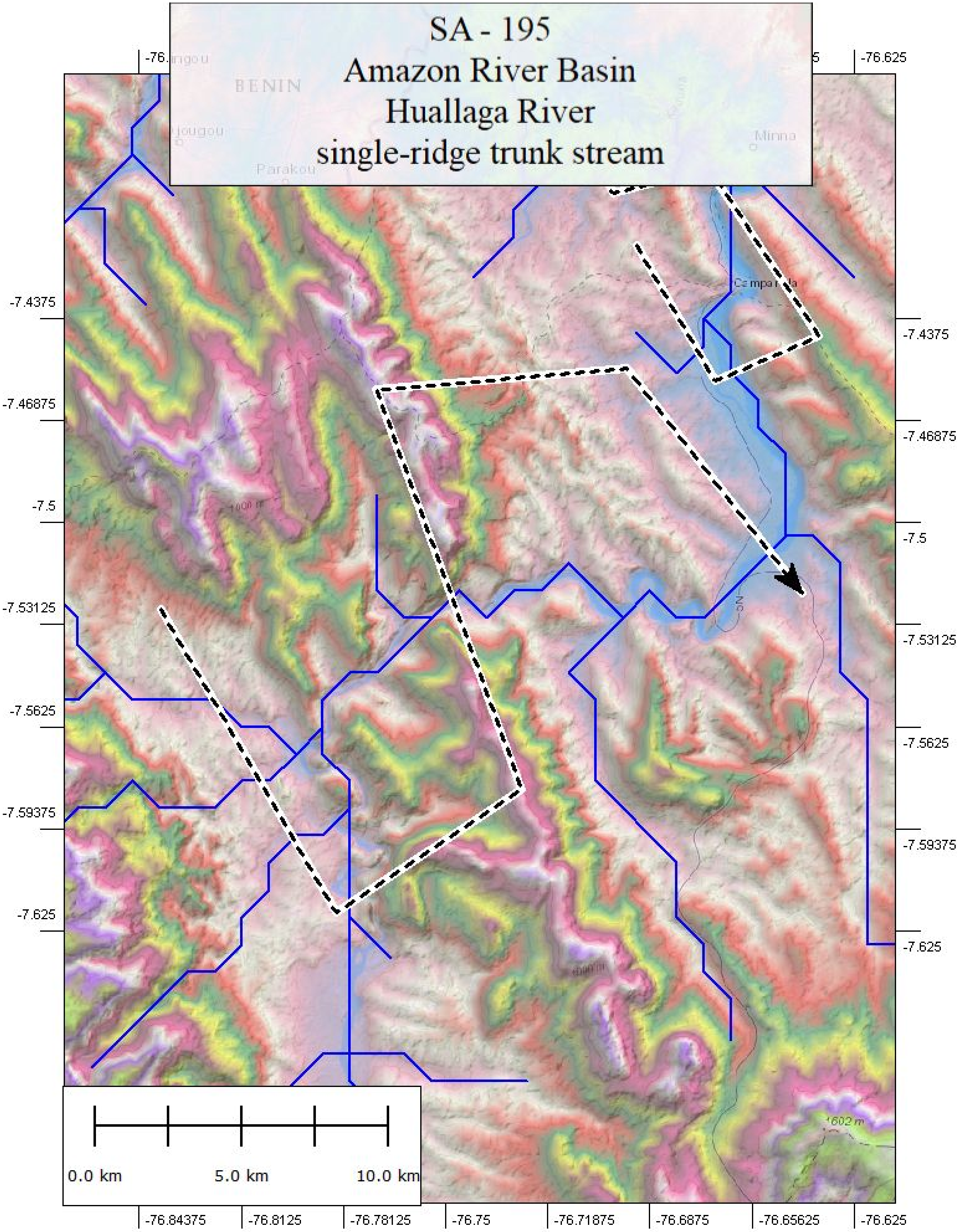
SA - 195
Amazon River Basin
Huallaga River
single-ridge trunk stream
-76.625
-7.4375
-7.46875
-7.5
-7.53125
-7.5625
-7.59375
-7.625
-76.84375
-76.8125
-76.78125
-76.75
-76.71875
-76.6875
-76.65625
-76.625
BENIN
Parakou
Minna
1602 m
0.0 km
5.0 km
10.0 km

SA - 196
Amazon River Basin
Tupuani River
single-ridge trunk stream

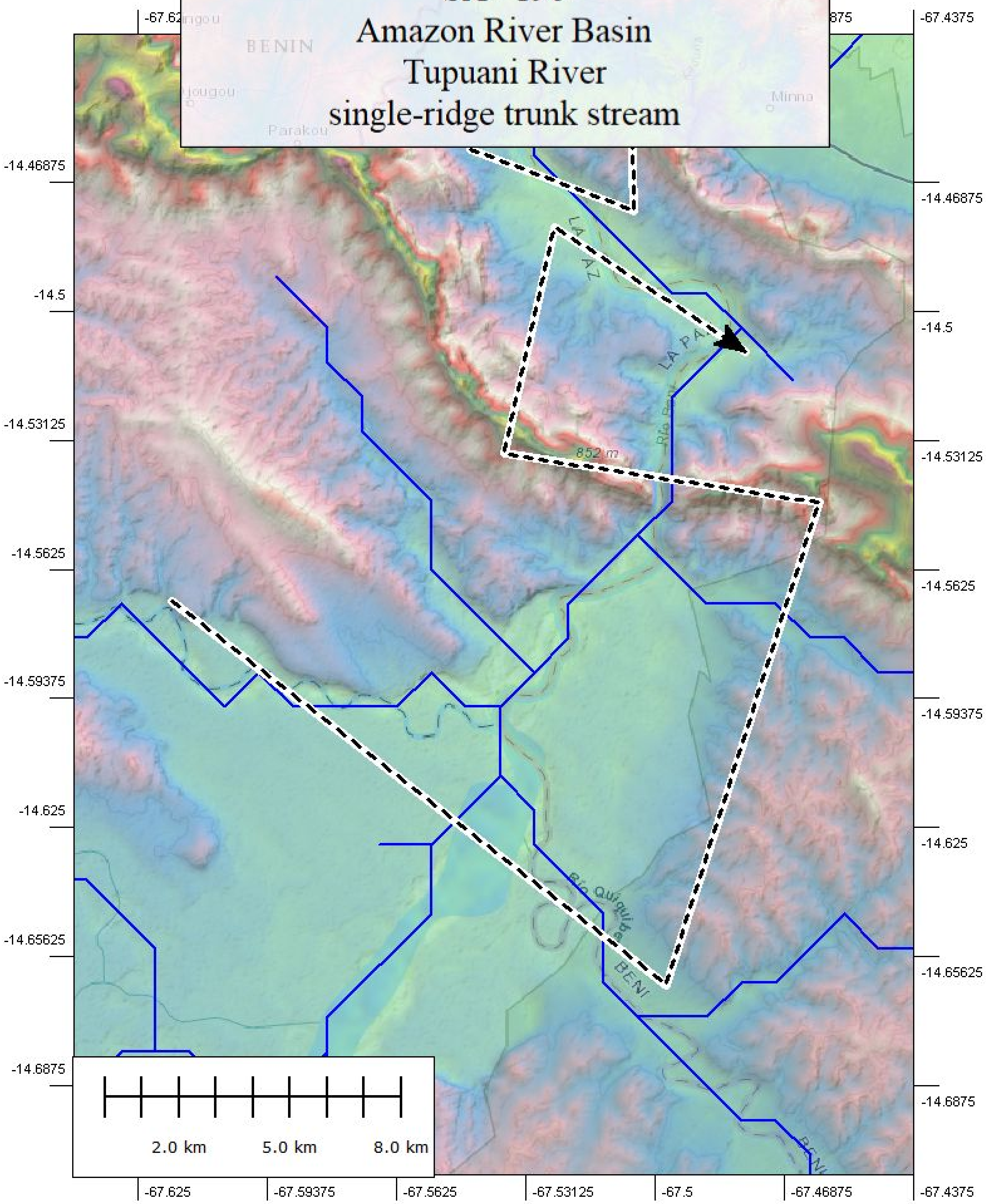

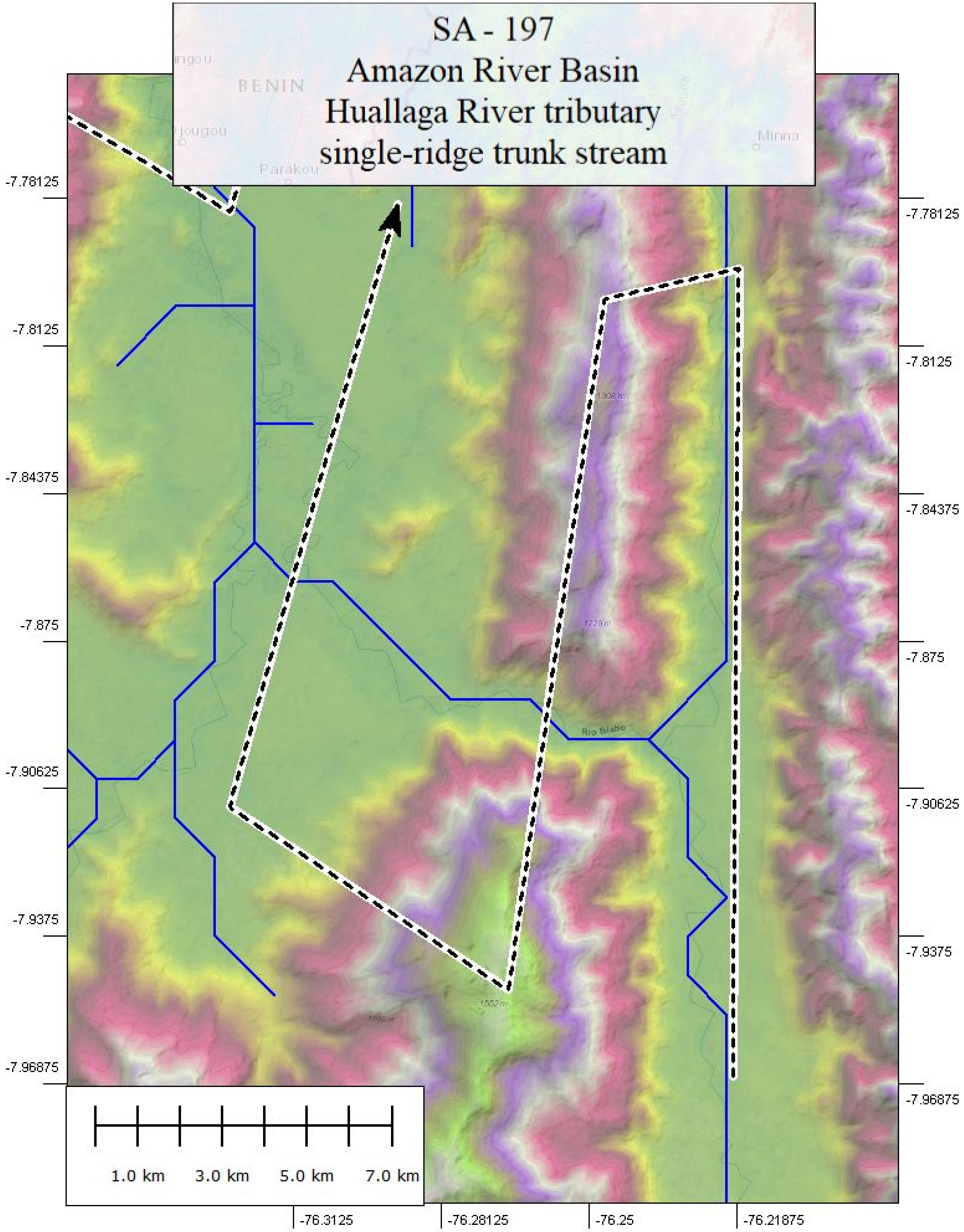

SA - 197
Amazon River Basin
Huallaga River tributary
single-ridge trunk stream
BENIN
Parakou
Minna
Rio Biabo
-7.78125
-7.8125
-7.84375
-7.875
-7.90625
-7.9375
-7.96875
-7.78125
-7.8125
-7.84375
-7.875
-7.90625
-7.9375
-7.96875
-76.3125
-76.28125
-76.25
-76.21875
1.0 km
3.0 km
5.0 km
7.0 km

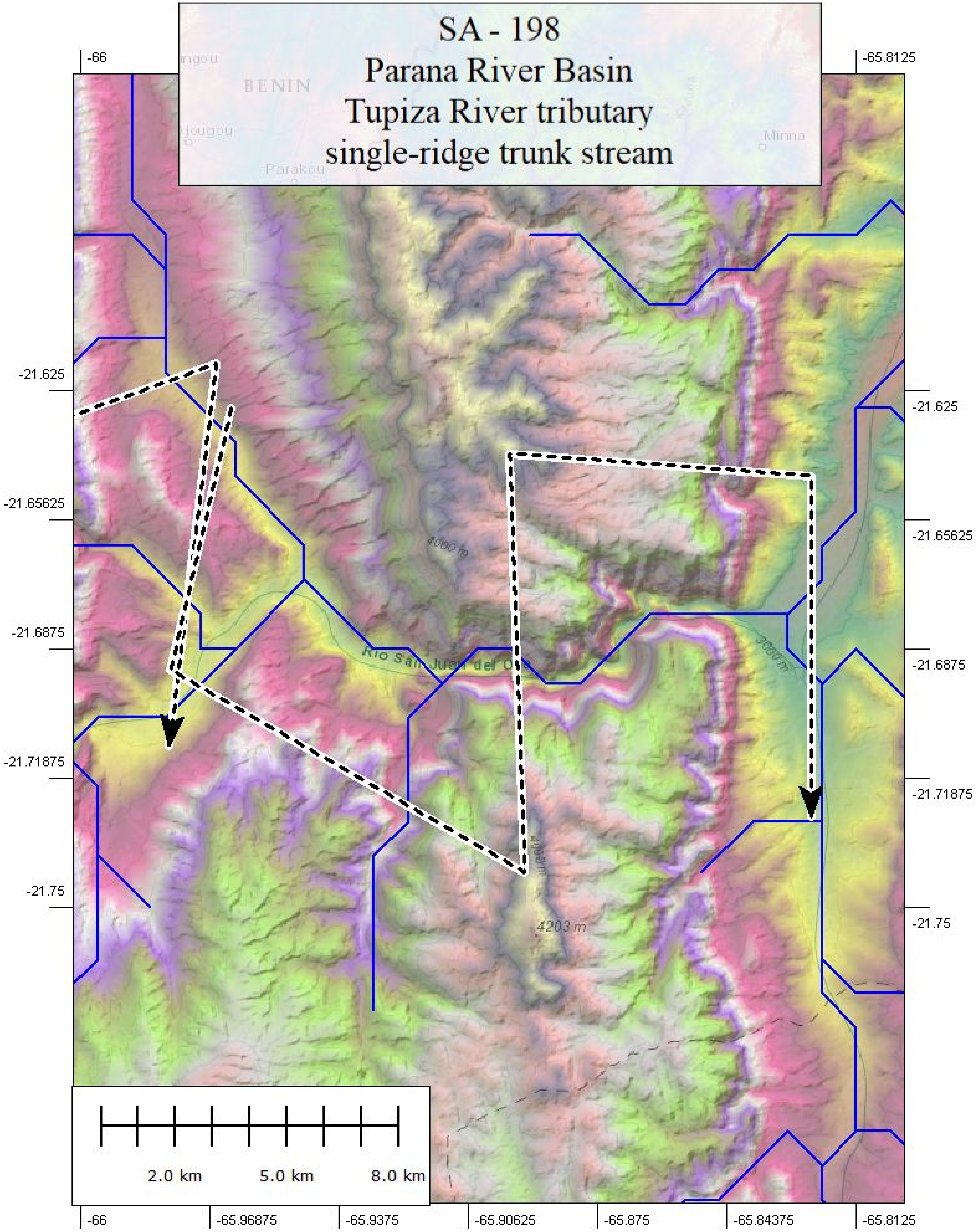
SA - 198
Parana River Basin
Tupiza River tributary
single-ridge trunk stream
-66
-65.8125
-21.625
-21.65625
-21.6875
-21.71875
-21.75
-65.96875
-65.9375
-65.90625
-65.875
-65.84375
Rio San Juan del Oro
4060 m
4203 m
2.0 km
5.0 km
8.0 km

SA - 200
Amazon River Basin
Madre de Dios River tributary
single-ridge trunk stream

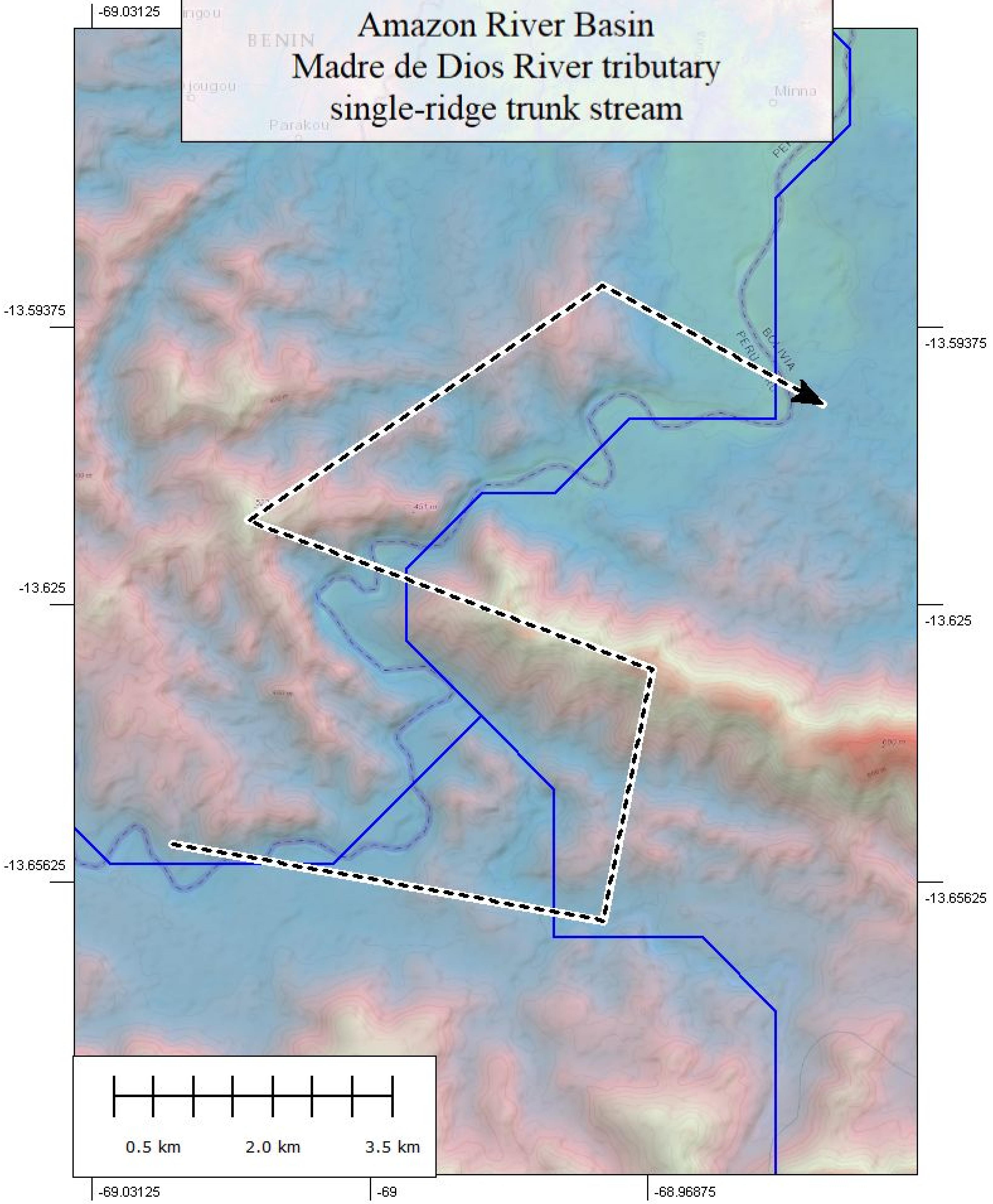

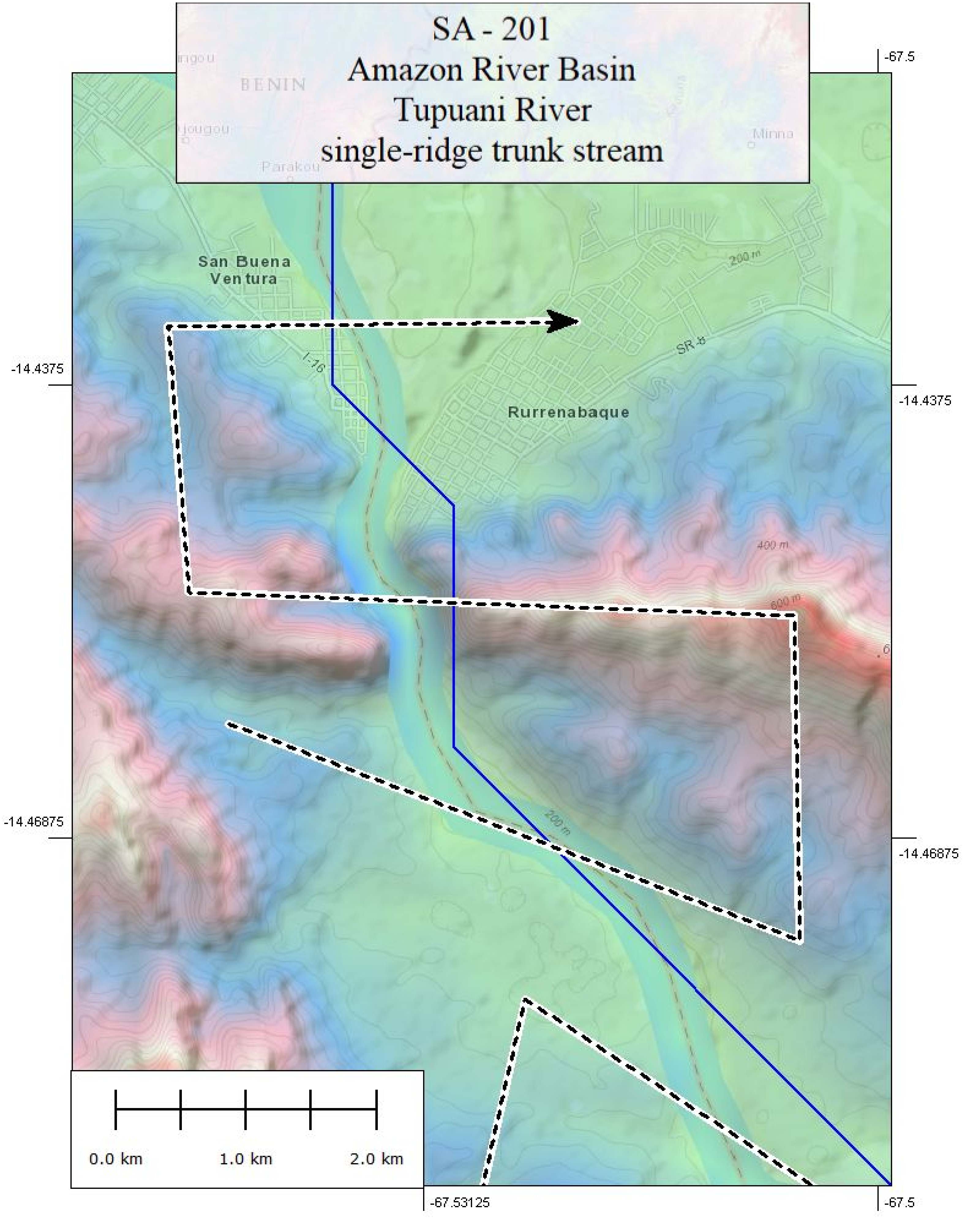
SA - 201
Amazon River Basin
Tupuani River
single-ridge trunk stream
BENIN
Djougou
Parakou
Minna
San Buena
Ventura
Rurrenabaque
I-16
SR 8
200 m
400 m
600 m
200 m
-67.5
-14.4375
-14.4375
-14.46875
-14.46875
0.0 km
1.0 km
2.0 km
-67.53125
-67.5

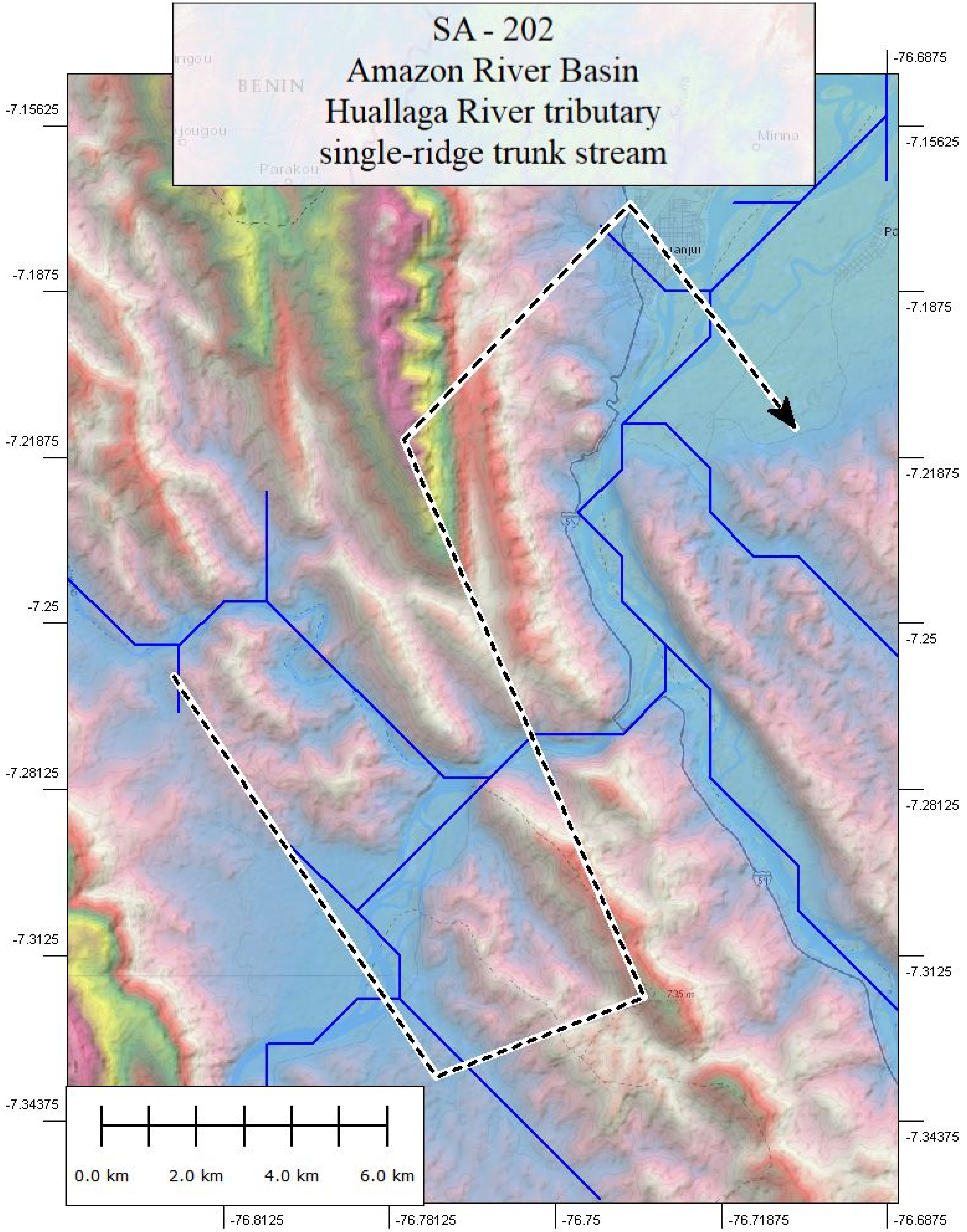
SA - 202
Amazon River Basin
Huallaga River tributary
single-ridge trunk stream
BENIN
Parakou
Minna
-7.15625
-7.1875
-7.21875
-7.25
-7.28125
-7.3125
-7.34375
-76.8125
-76.78125
-76.75
-76.71875
-76.6875
0.0 km
2.0 km
4.0 km
6.0 km

SA - 204
Amazon River Basin
Huallaga River tributary
single-ridge trunk stream

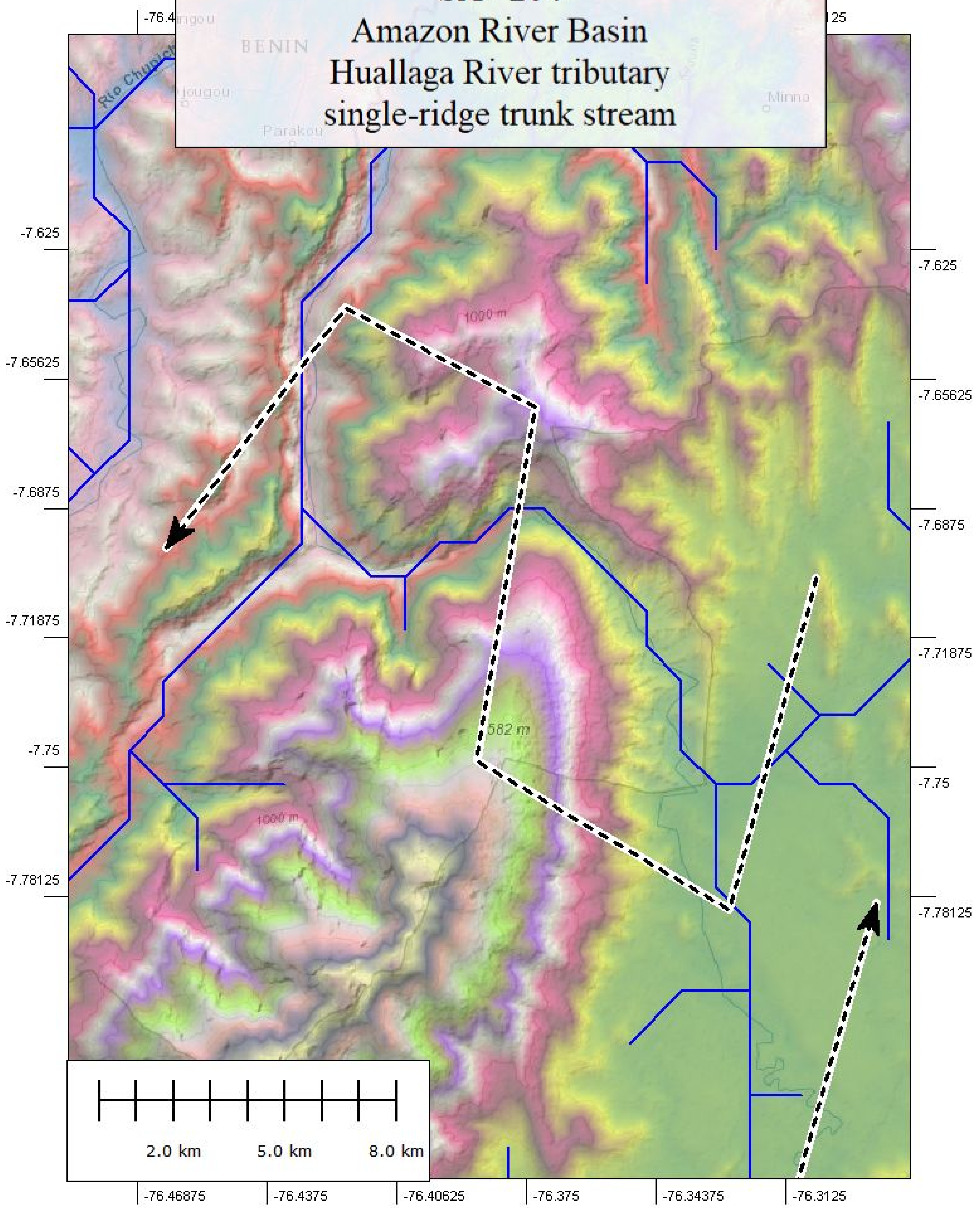

SA - 205
Amazon River Basin
Inanbari River
single-ridge trunk stream

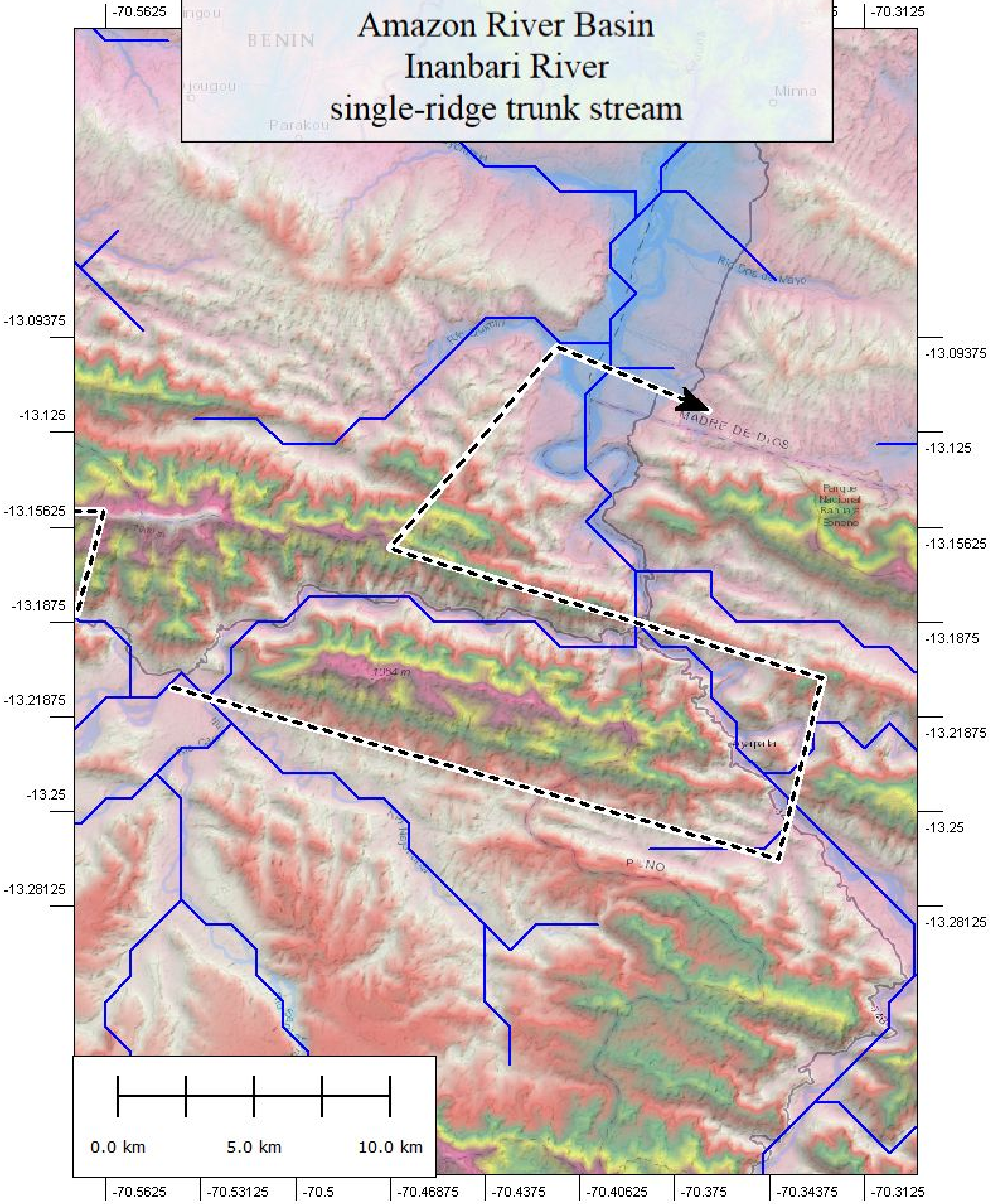

SA - 206
Amazon River Basin
Rio Grande O Guapay
single-ridge trunk stream

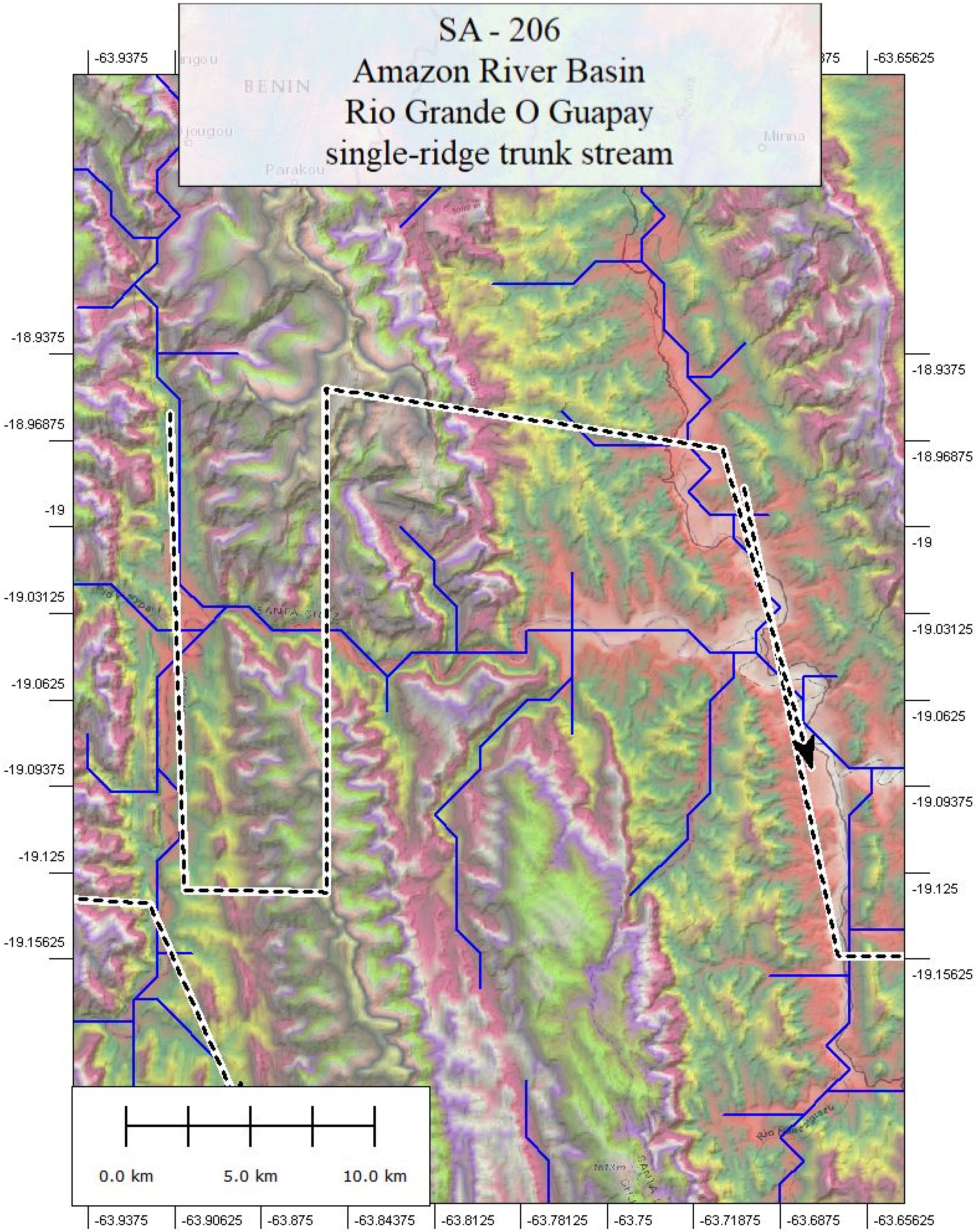

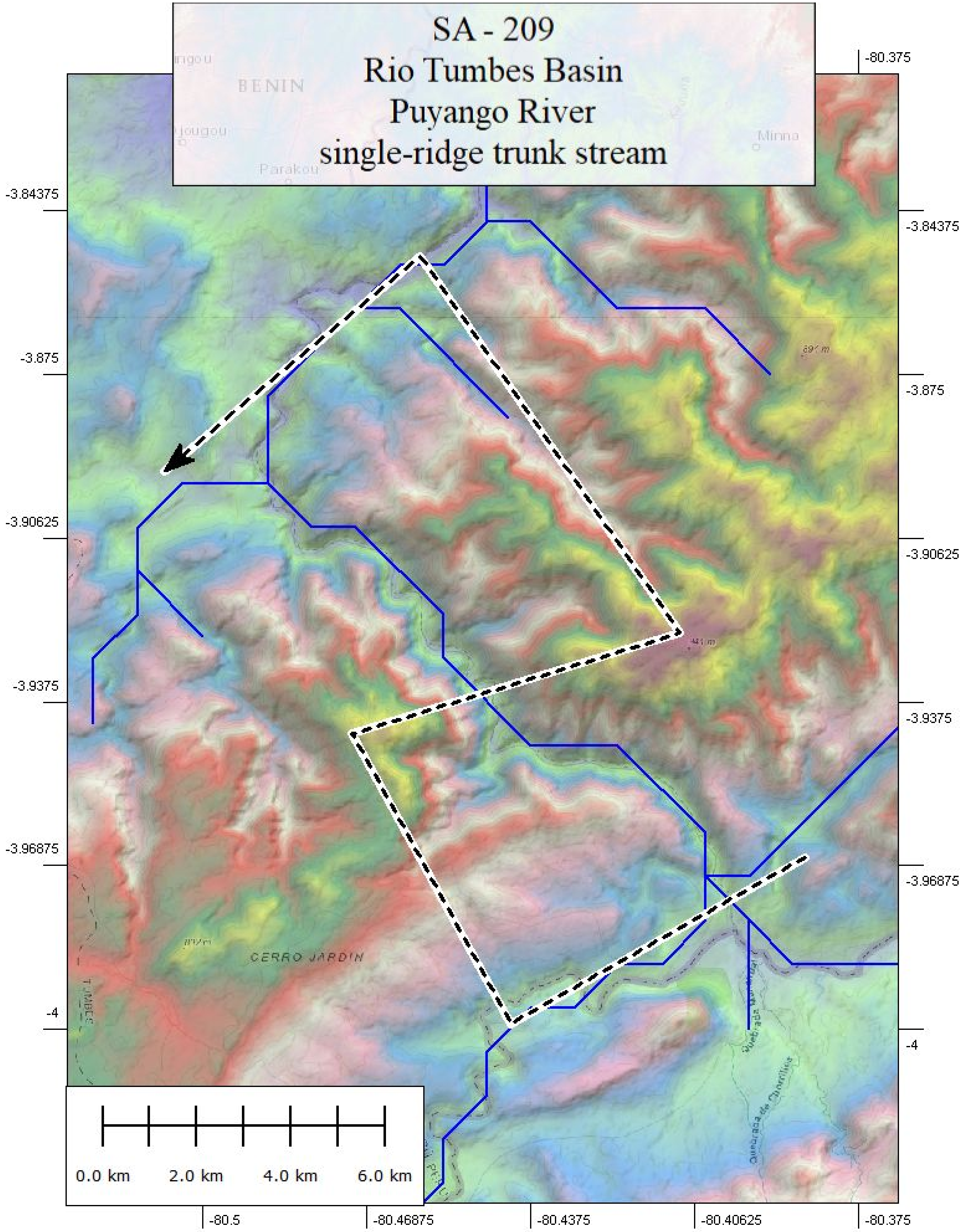
SA - 209
Rio Tumbes Basin
Puyango River
single-ridge trunk stream
-80.375
-3.84375
-3.875
-3.90625
-3.9375
-3.96875
-4
CERRO JARDIN
0.0 km
2.0 km
4.0 km
6.0 km
-80.5
-80.46875
-80.4375
-80.40625
-80.375

SA - 210
Parana River Basin
Las Conchas River
single-ridge trunk stream

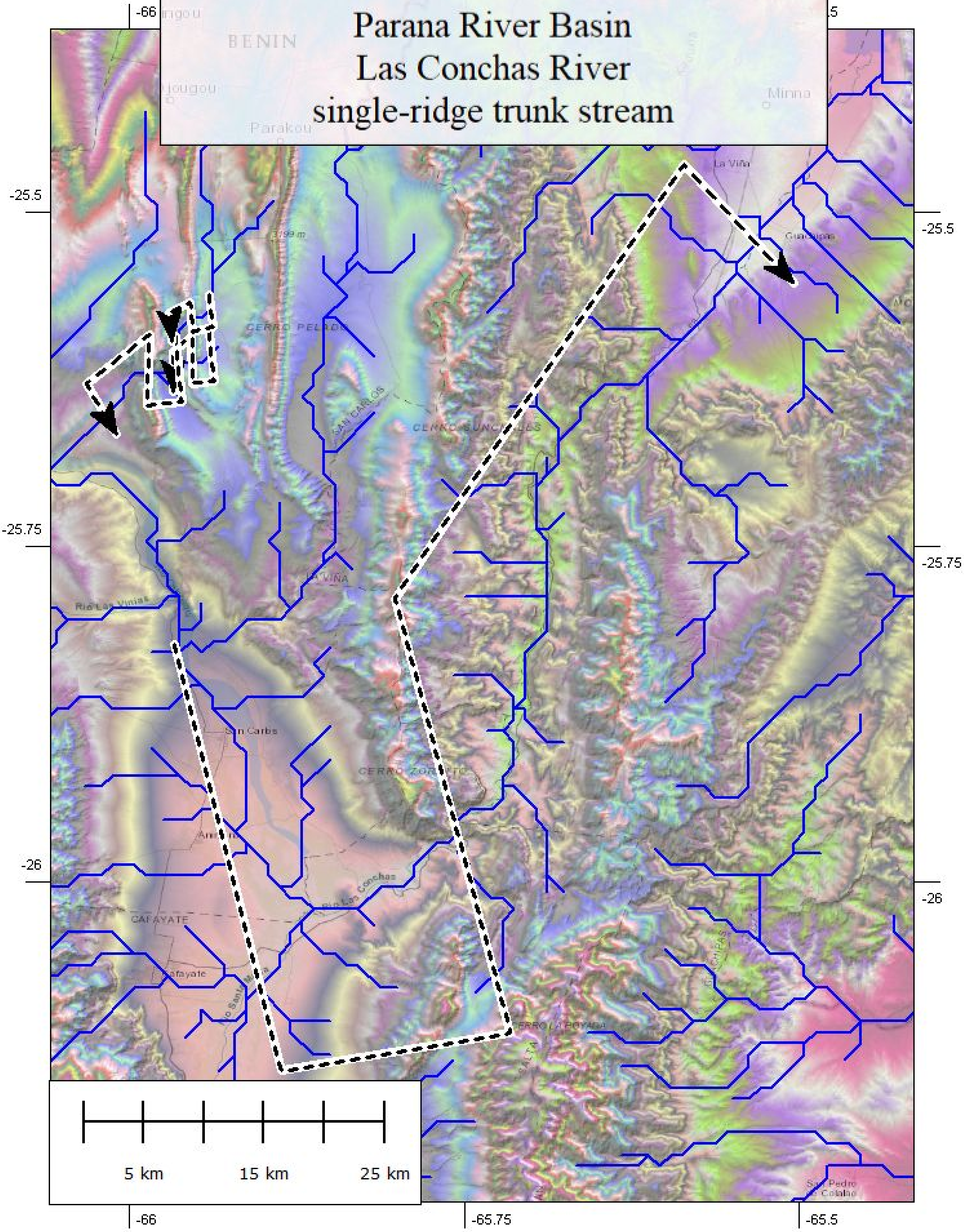

SA - 211
Amazon River Basin
Ichoa River tributary
single-ridge trunk stream

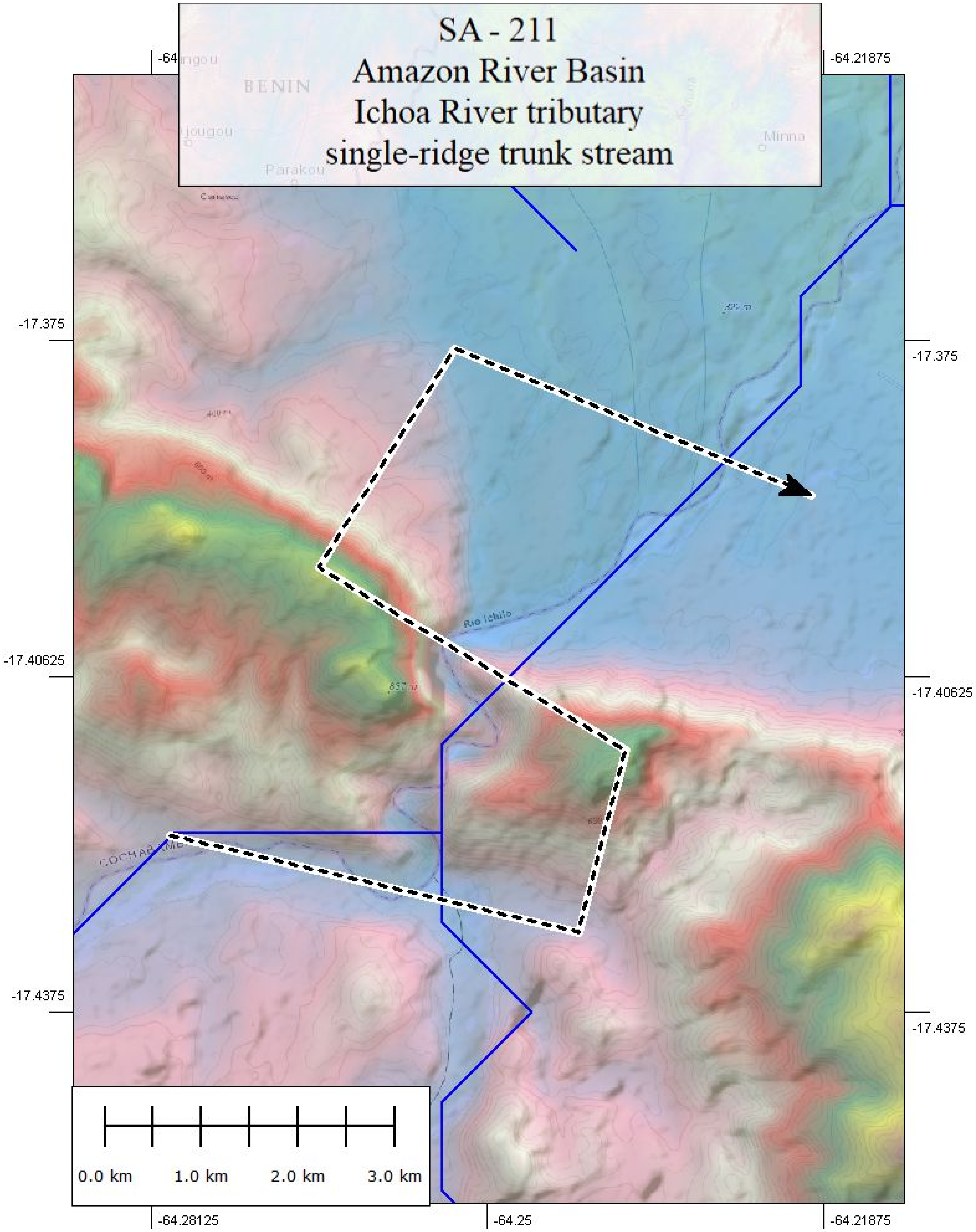

SA - 212
Rio Magdalena Basin
Luisa River
single-ridge trunk stream

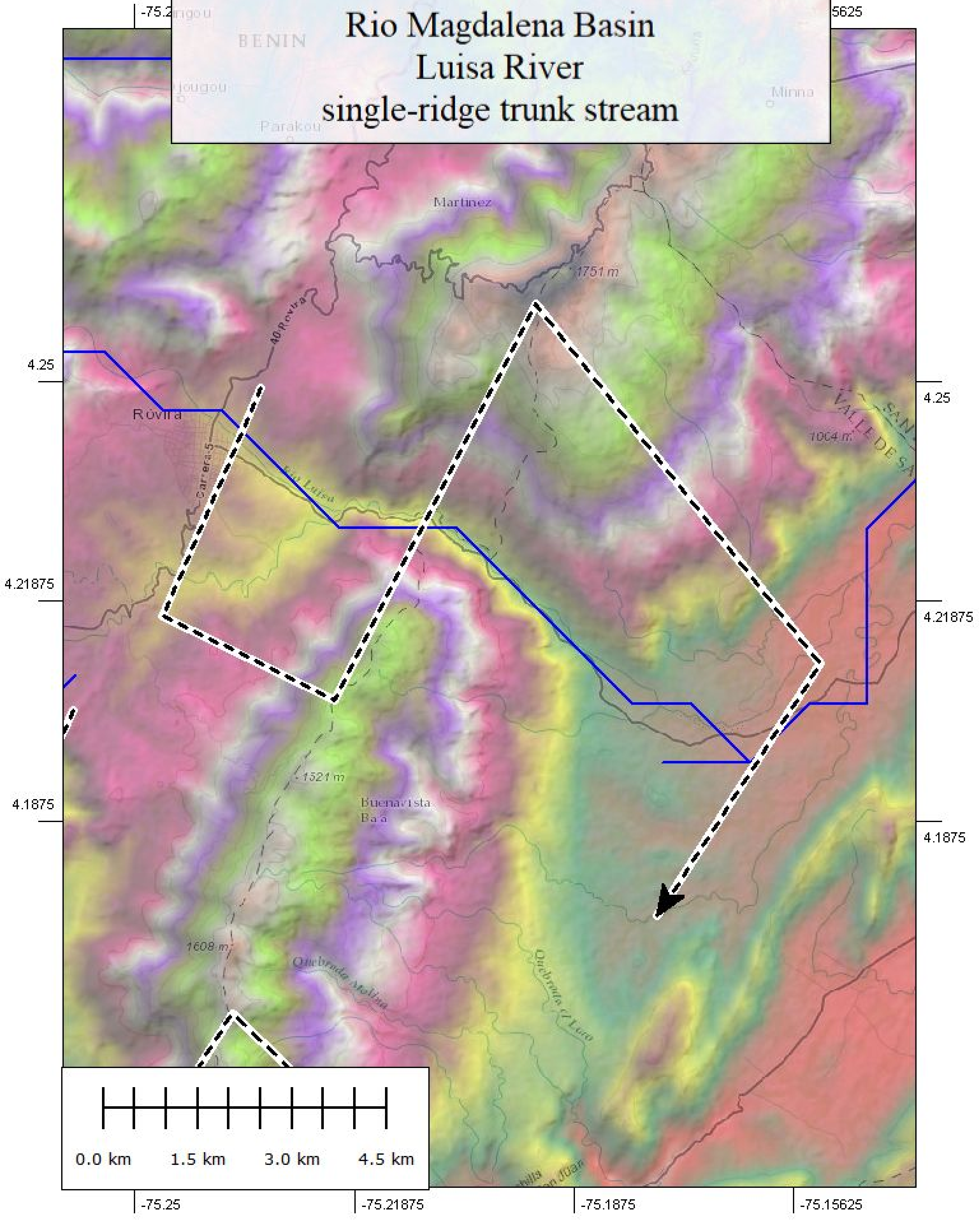

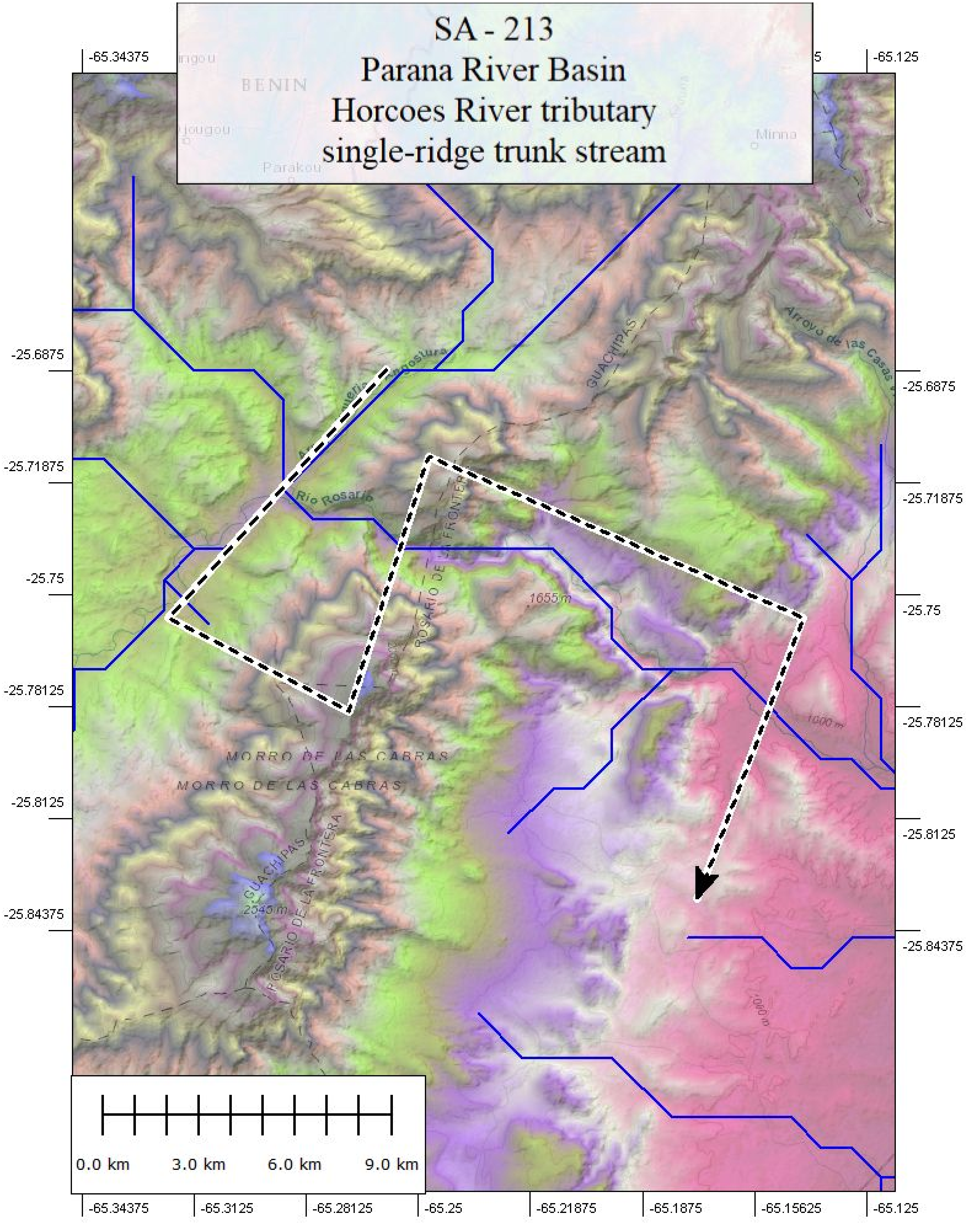
SA - 213
Parana River Basin
Horcoes River tributary
single-ridge trunk stream
-65.34375
-65.125
-25.6875
-25.71875
-25.75
-25.78125
-25.8125
-25.84375
Rio Rosario
GUACHIPAS
Arroyo de las Casas
1655 m
1000 m
ROSARIO DE LA FRONTERA
MORRO DE LAS CABRAS
MORRO DE LAS CABRAS
GUACHIPAS
2545 m
ROSARIO DE LA FRONTERA
0.0 km
3.0 km
6.0 km
9.0 km
-65.34375
-65.3125
-65.28125
-65.25
-65.21875
-65.1875
-65.15625
-65.125

SA - 214
Amazon River Basin
Rio Grande O Guapay tributary
single-ridge trunk stream

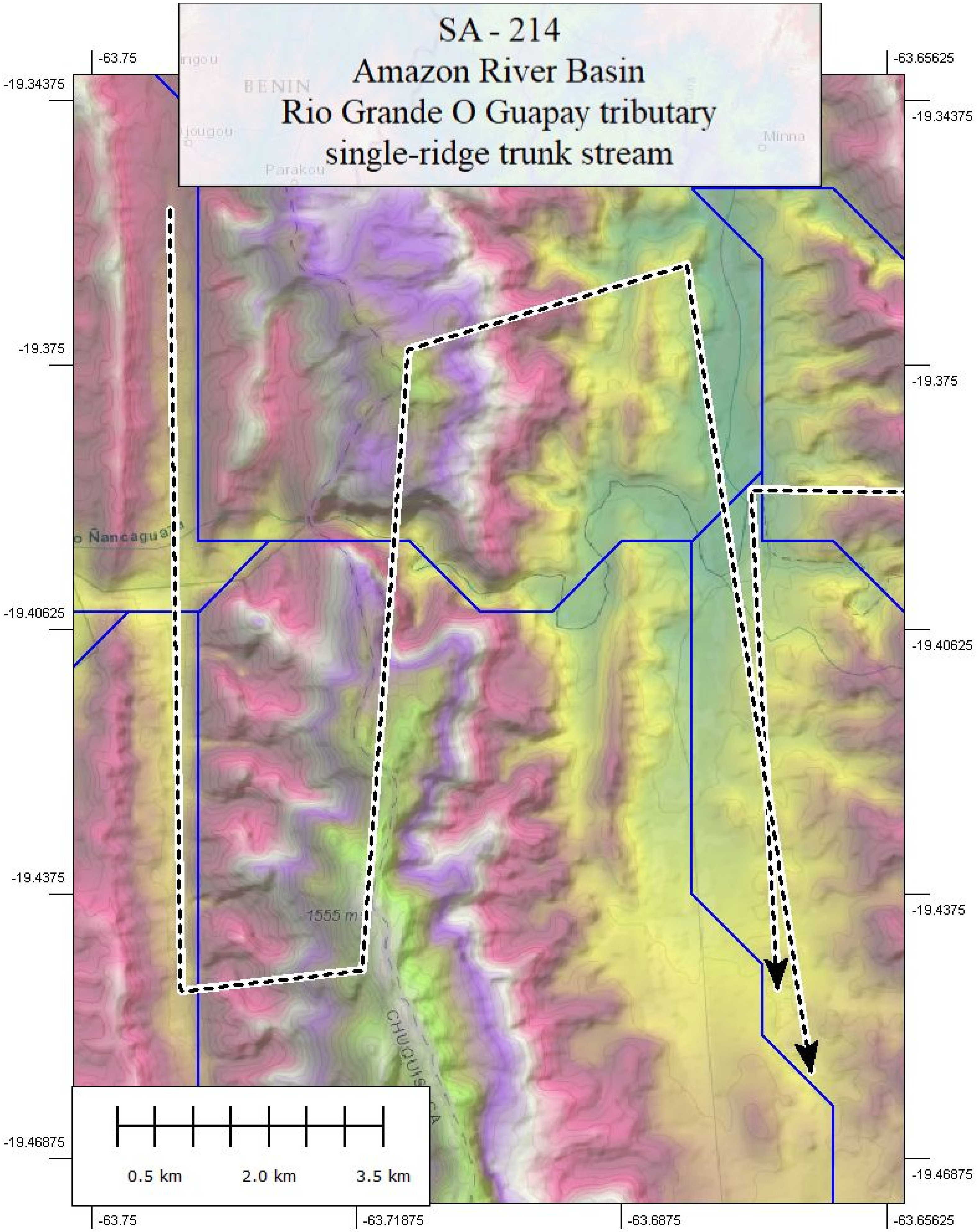

SA - 216
Parana River Basin
single-ridge trunk stream

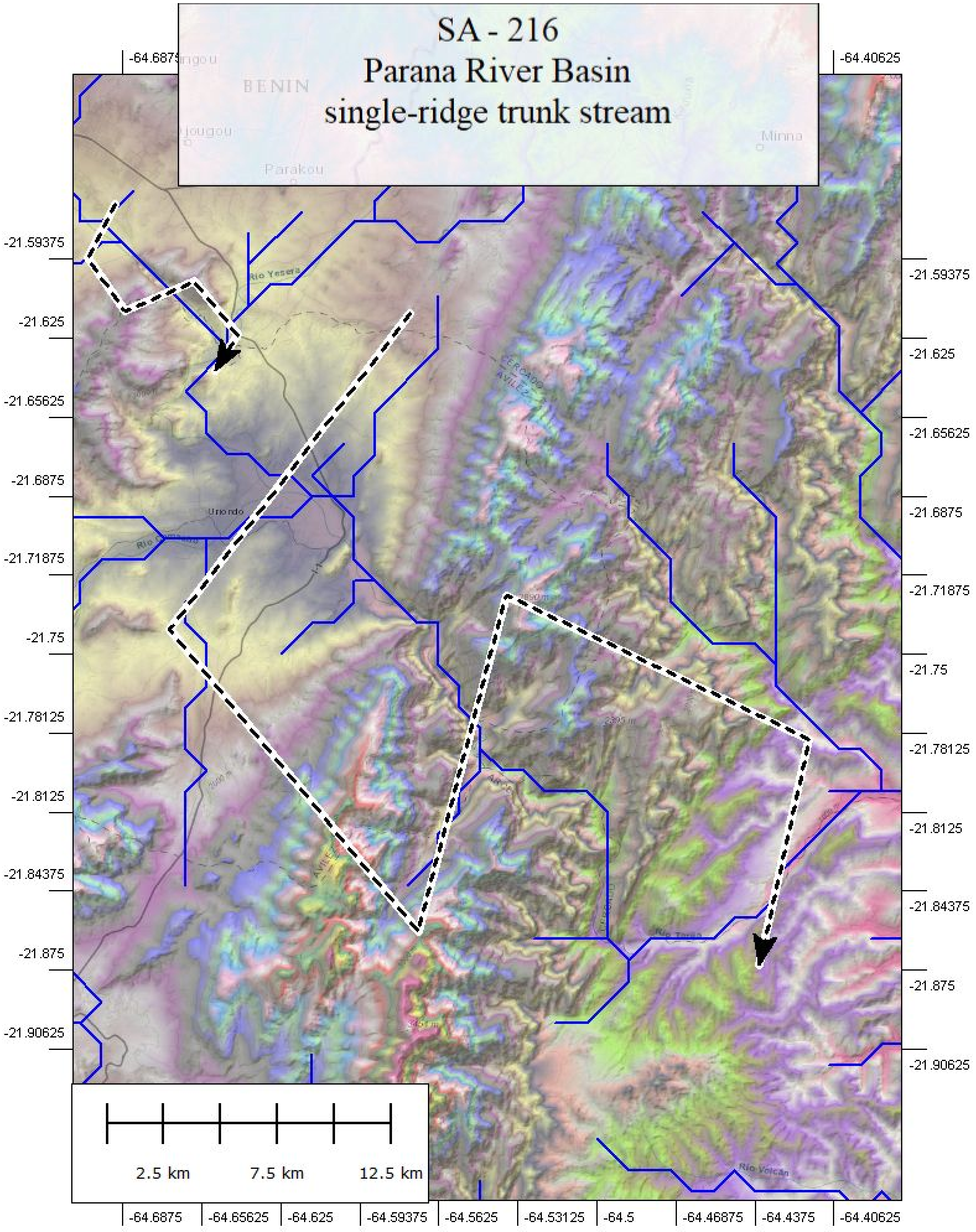

SA - 218
Orinoco River Basin
Tocaria River
single-ridge trunk stream

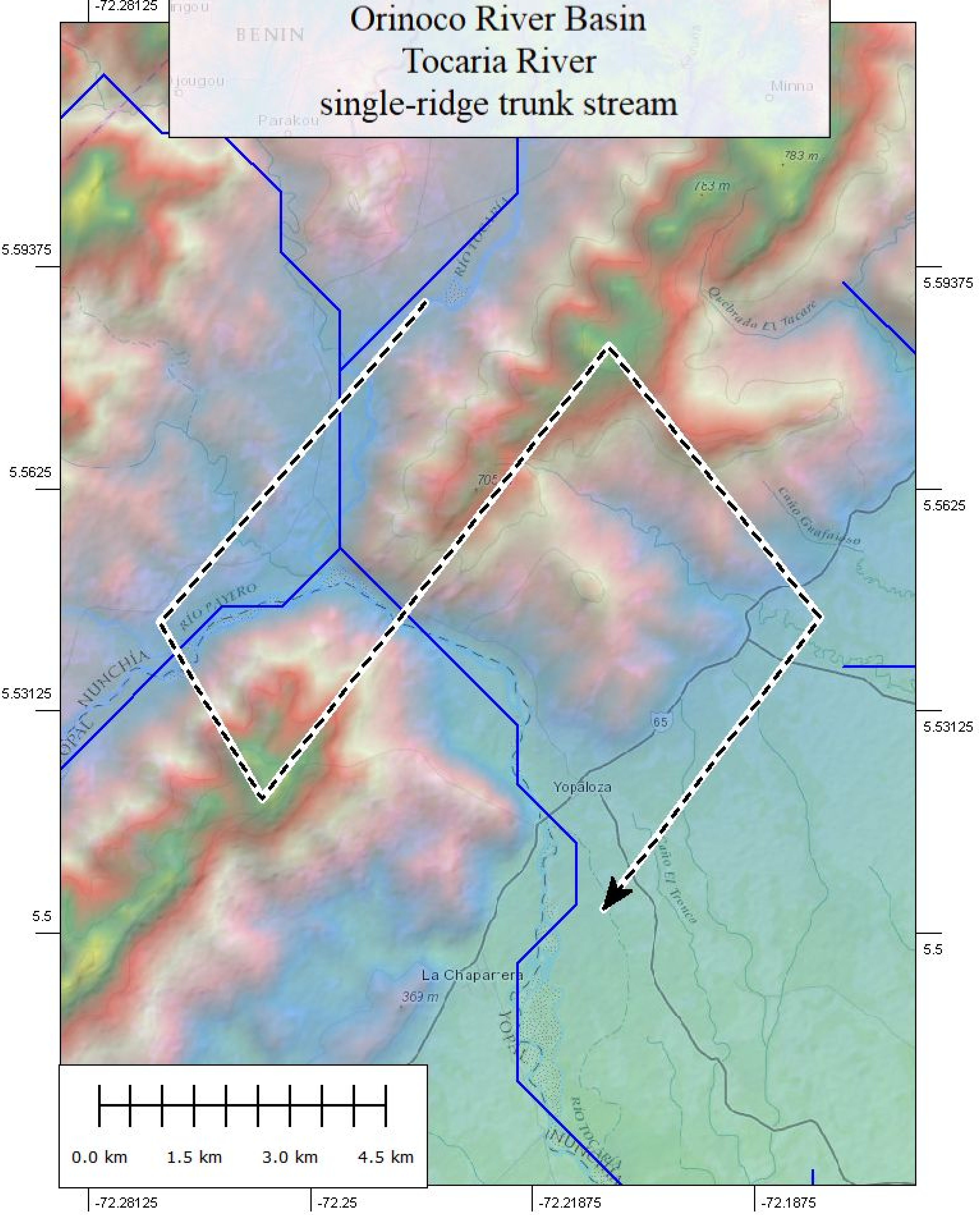

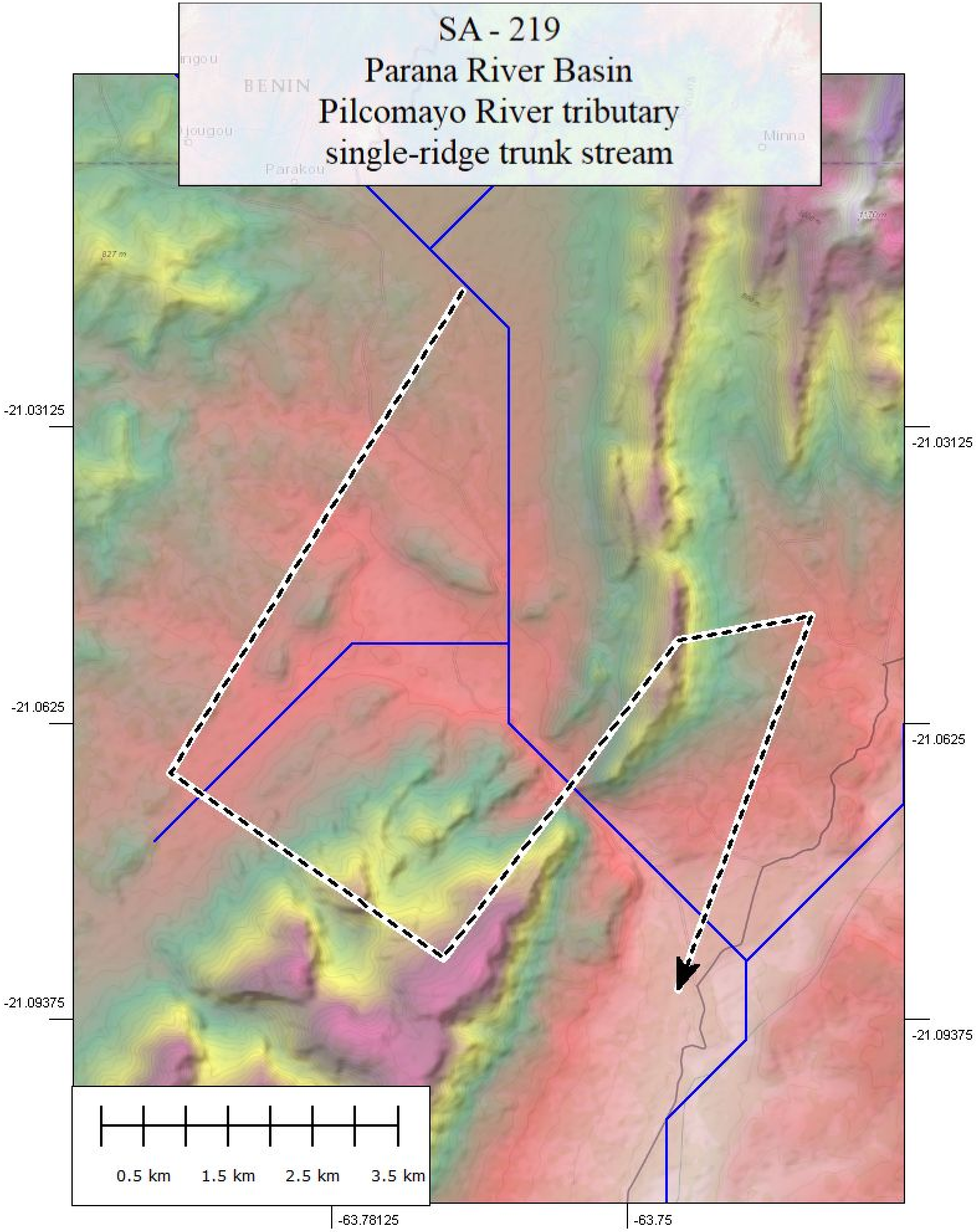
SA - 219
Parana River Basin
Pilcomayo River tributary
single-ridge trunk stream
-21.03125
-21.0625
-21.09375
-21.03125
-21.0625
-21.09375
-63.78125
-63.75
0.5 km 1.5 km 2.5 km 3.5 km

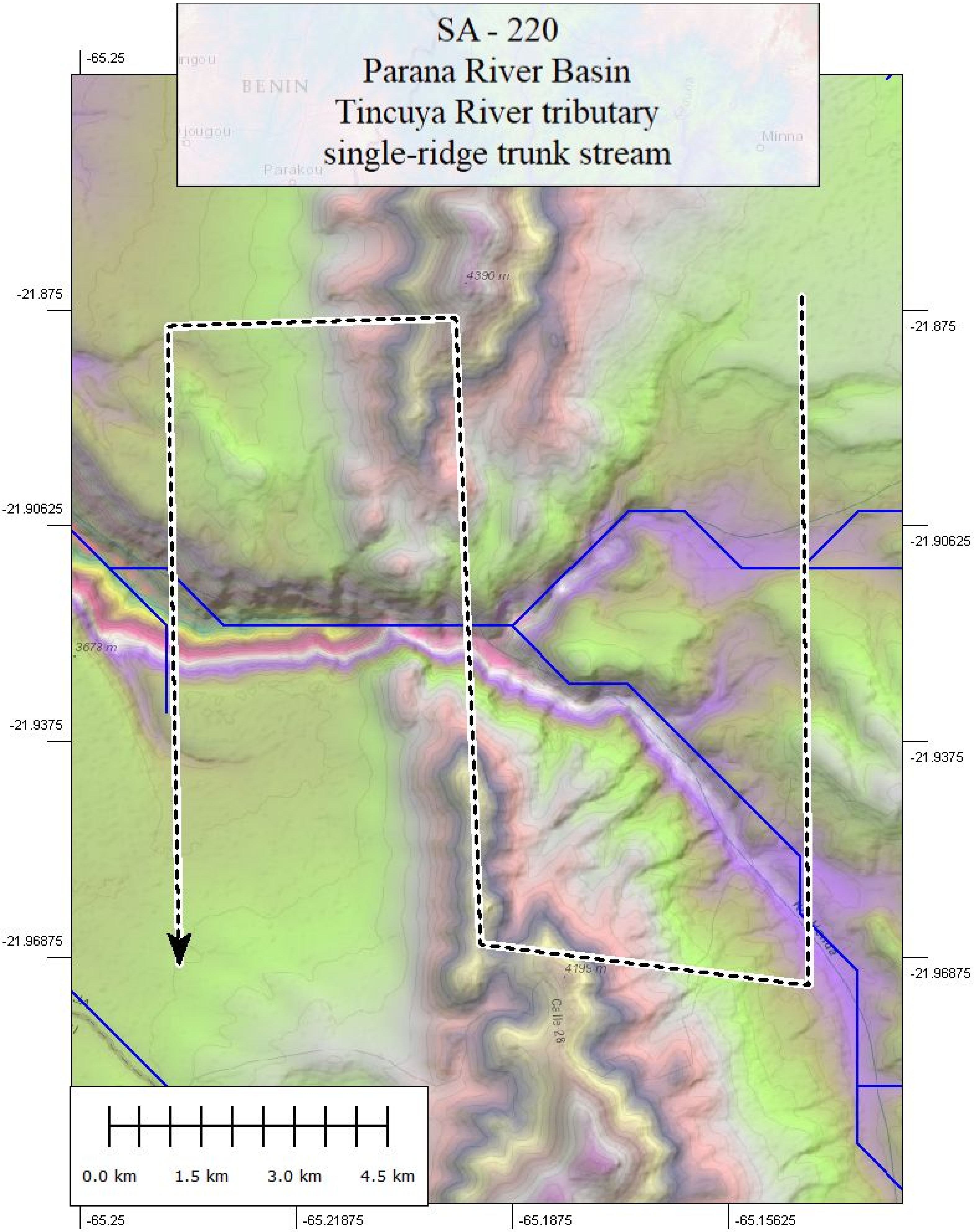
SA - 220
Parana River Basin
Tincuya River tributary
single-ridge trunk stream
-65.25
-65.21875
-65.1875
-65.15625
-21.875
-21.90625
-21.9375
-21.96875
4390 m
3673 m
4195 m
0.0 km
1.5 km
3.0 km
4.5 km

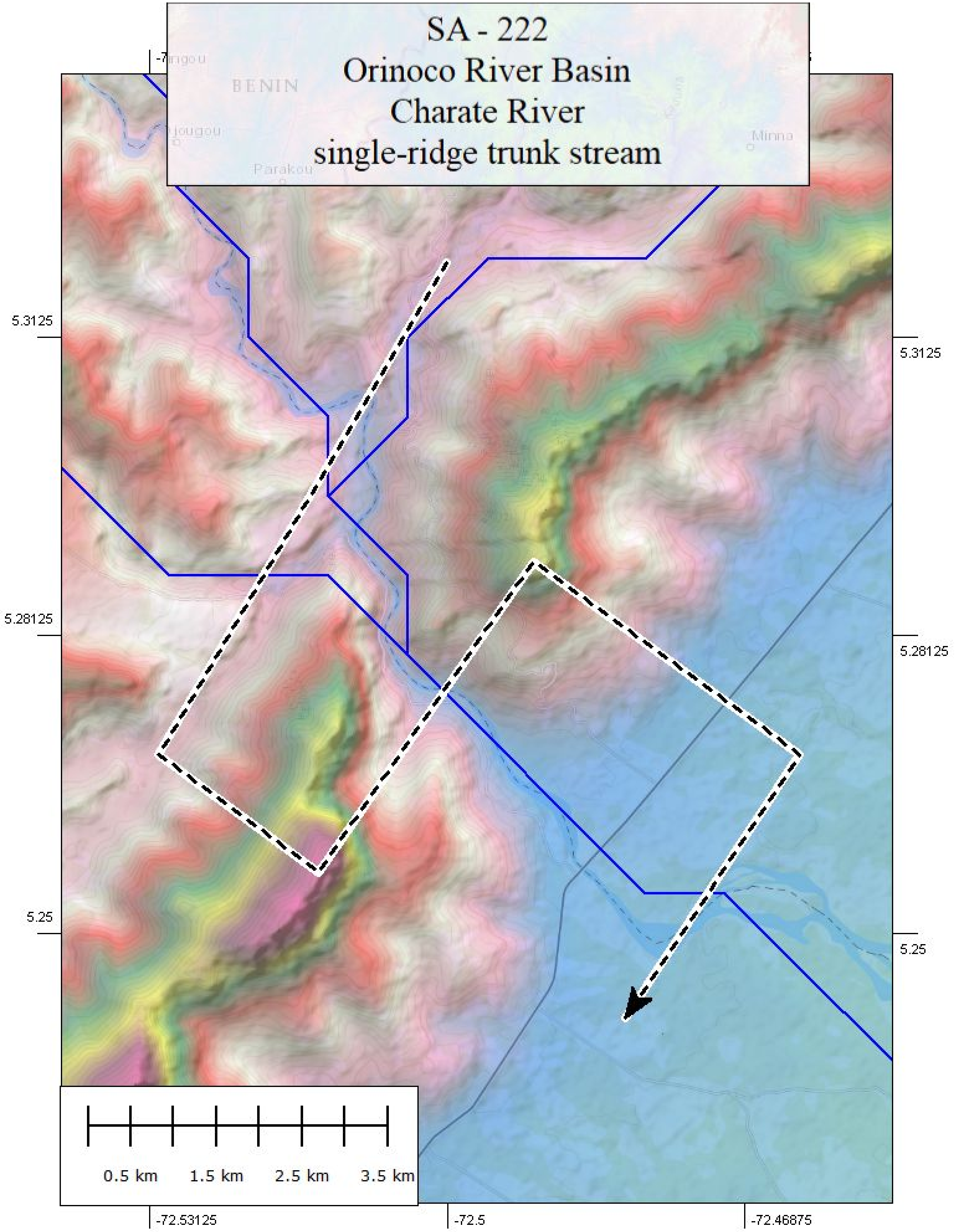

SA - 222
Orinoco River Basin
Charate River
single-ridge trunk stream

SA - 223
6000040470 Basin
single-ridge trunk stream

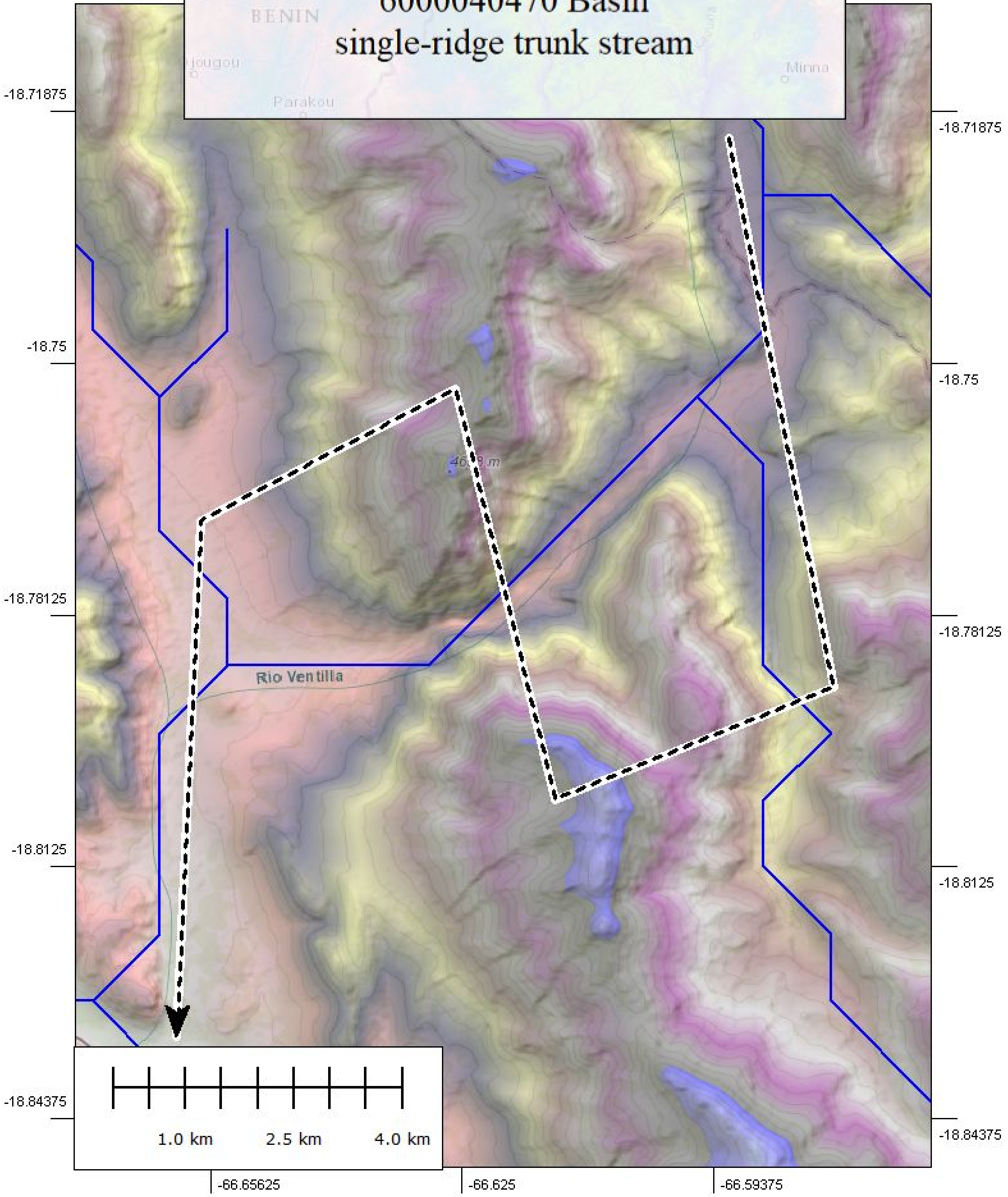

SA - 225
Orinoco River Basin
Guavio River
single-ridge trunk stream

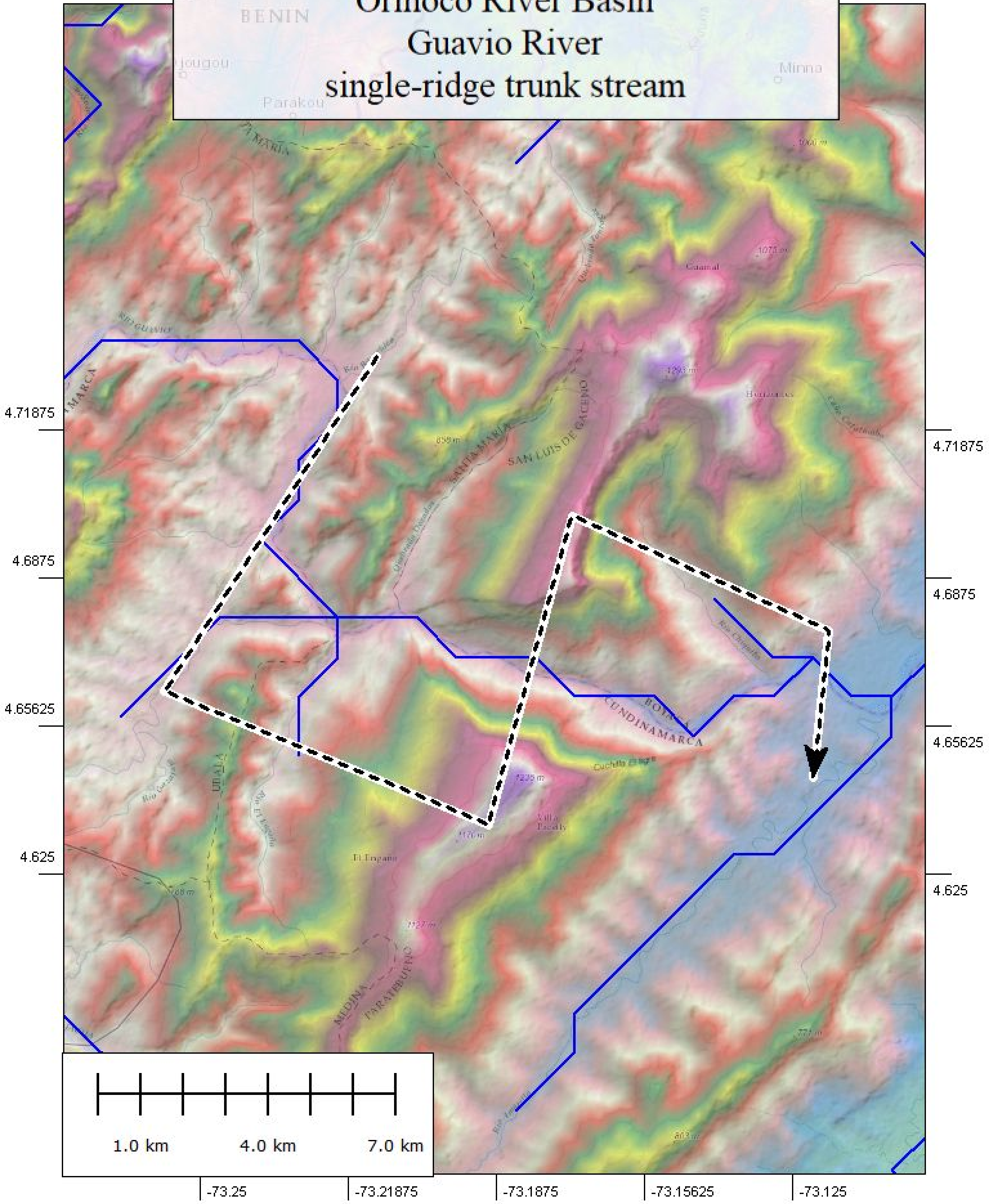

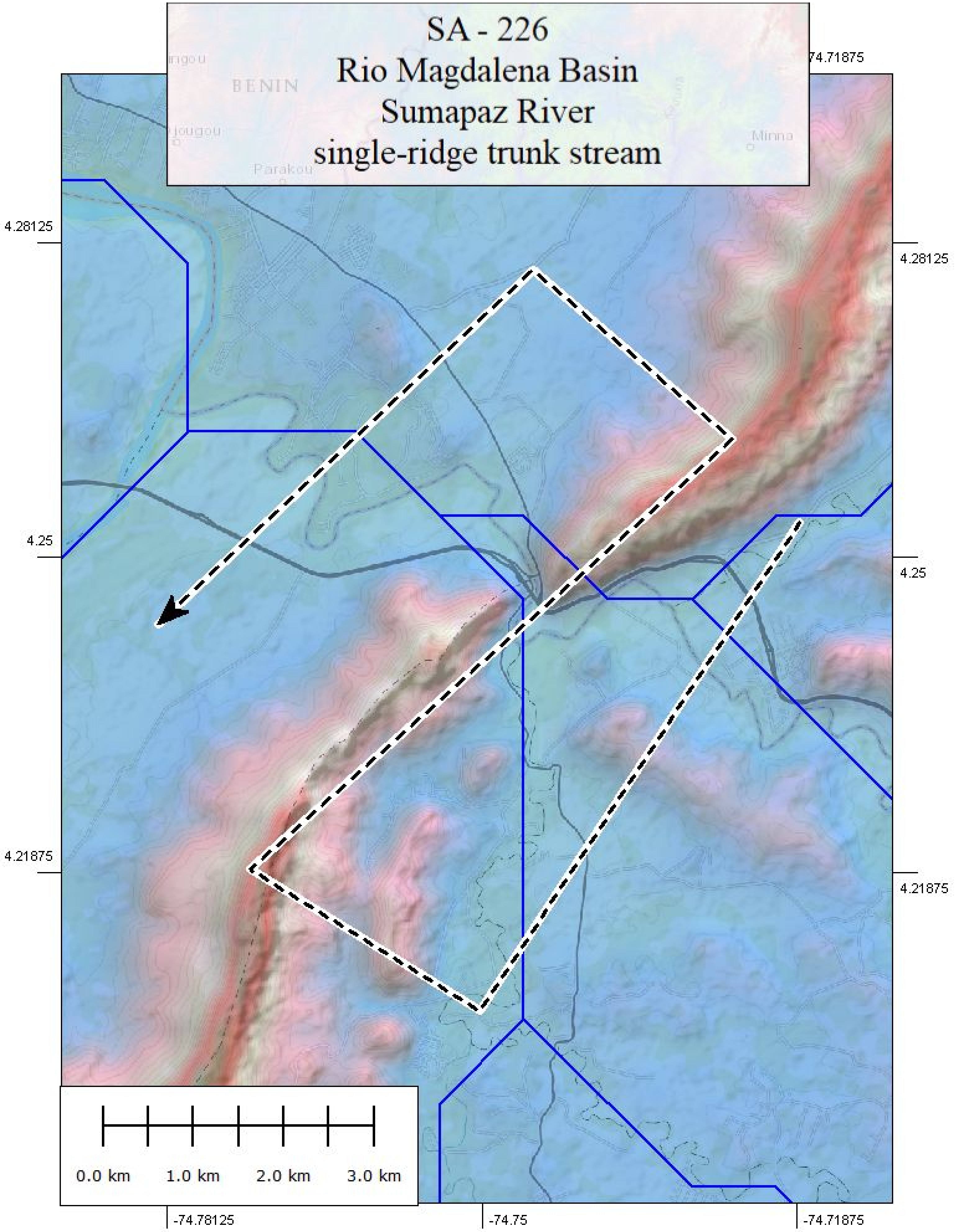
SA - 226
Rio Magdalena Basin
Sumapaz River
single-ridge trunk stream
4.28125
4.25
4.21875
-74.78125
-74.75
-74.71875
0.0 km
1.0 km
2.0 km
3.0 km

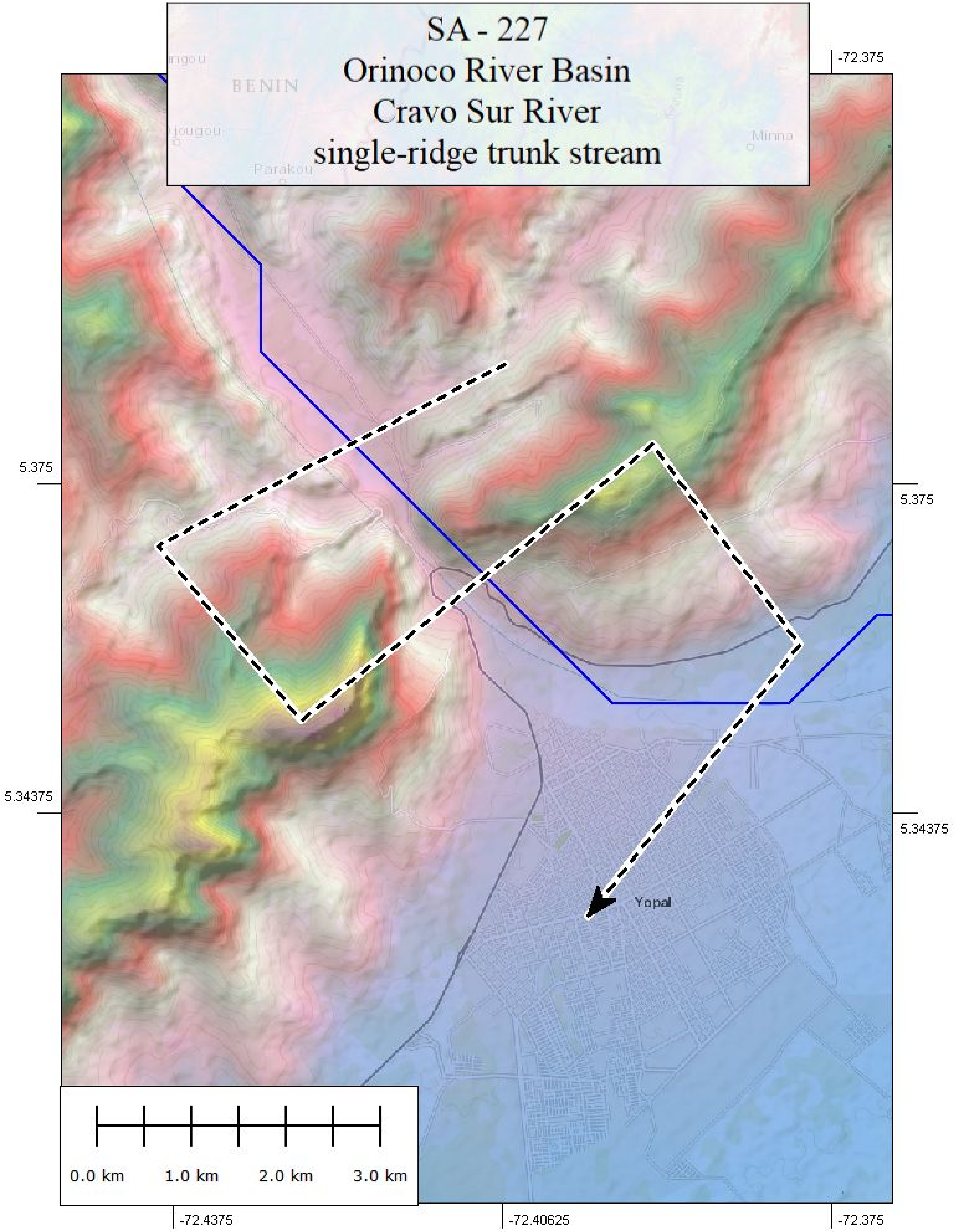
SA - 227
Orinoco River Basin
Cravo Sur River
single-ridge trunk stream
BENIN
Parakou
Minna
Yopal
-72.375
5.375
5.375
5.34375
5.34375
-72.4375
-72.40625
-72.375
0.0 km
1.0 km
2.0 km
3.0 km

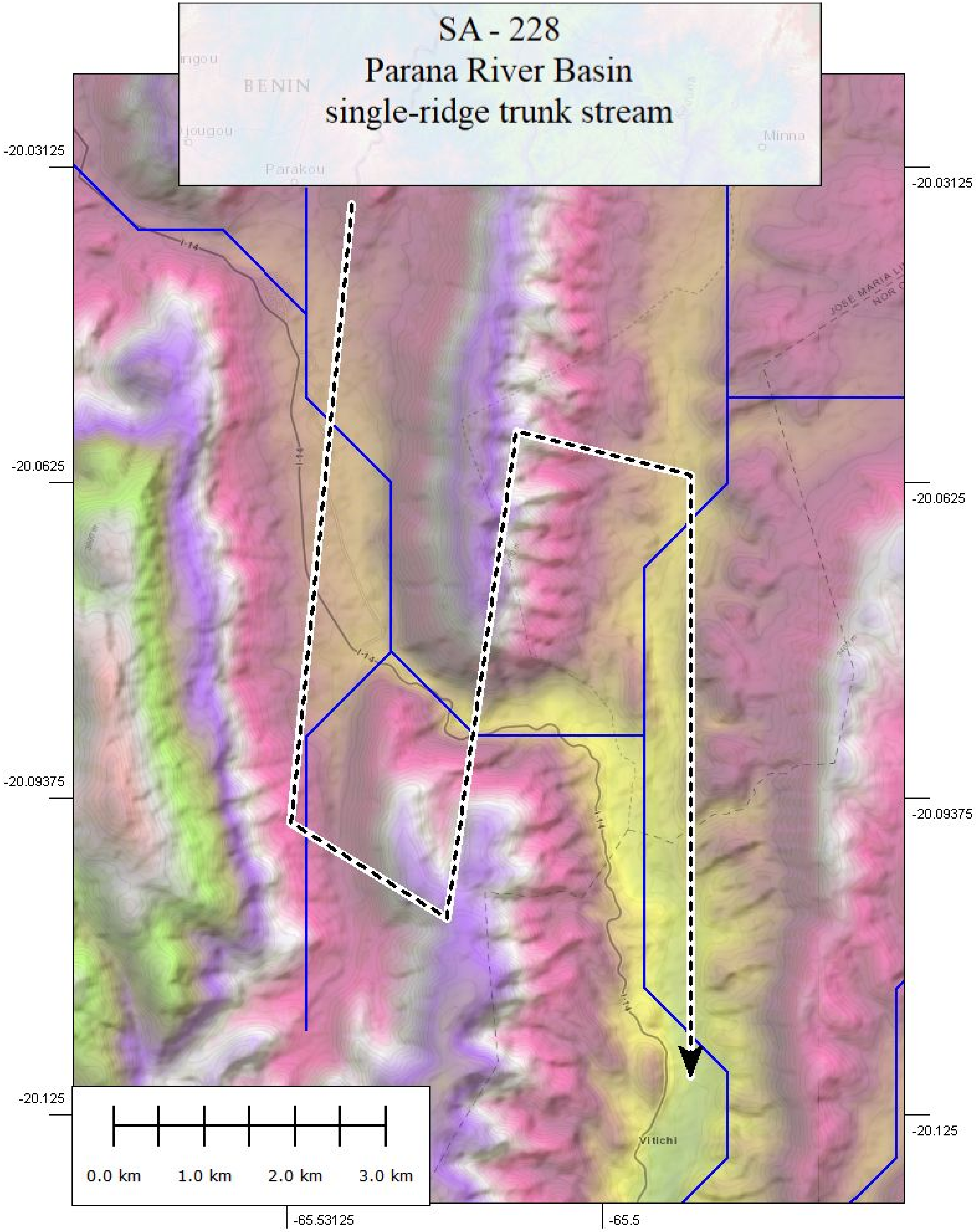
SA - 228
Parana River Basin
single-ridge trunk stream
-20.03125
-20.0625
-20.09375
-20.125
-65.53125
-65.5
0.0 km
1.0 km
2.0 km
3.0 km
BENIN
Parakou
Minna
Viтichi
I-14

SA - 229
Parana River Basin
single-ridge head stream

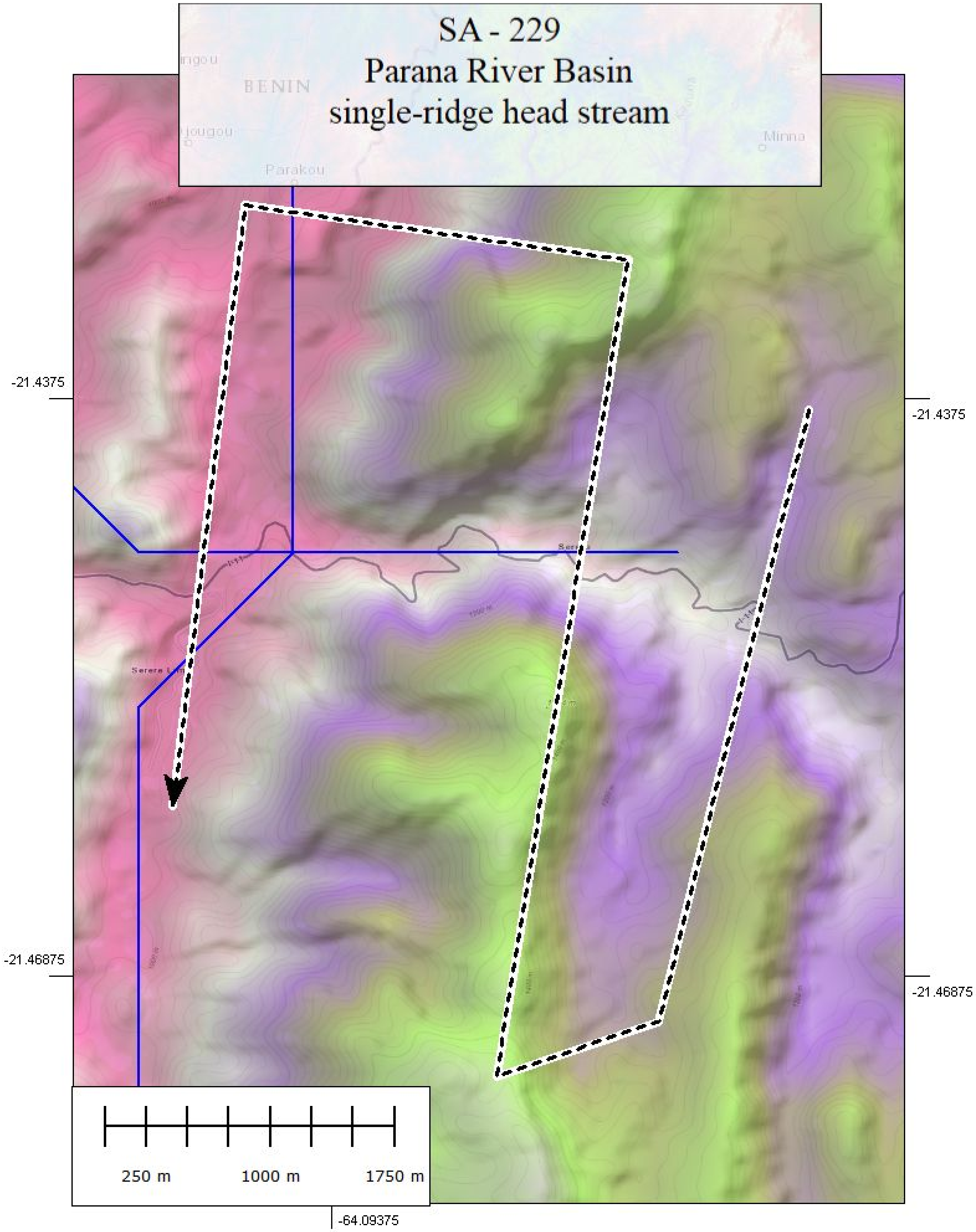

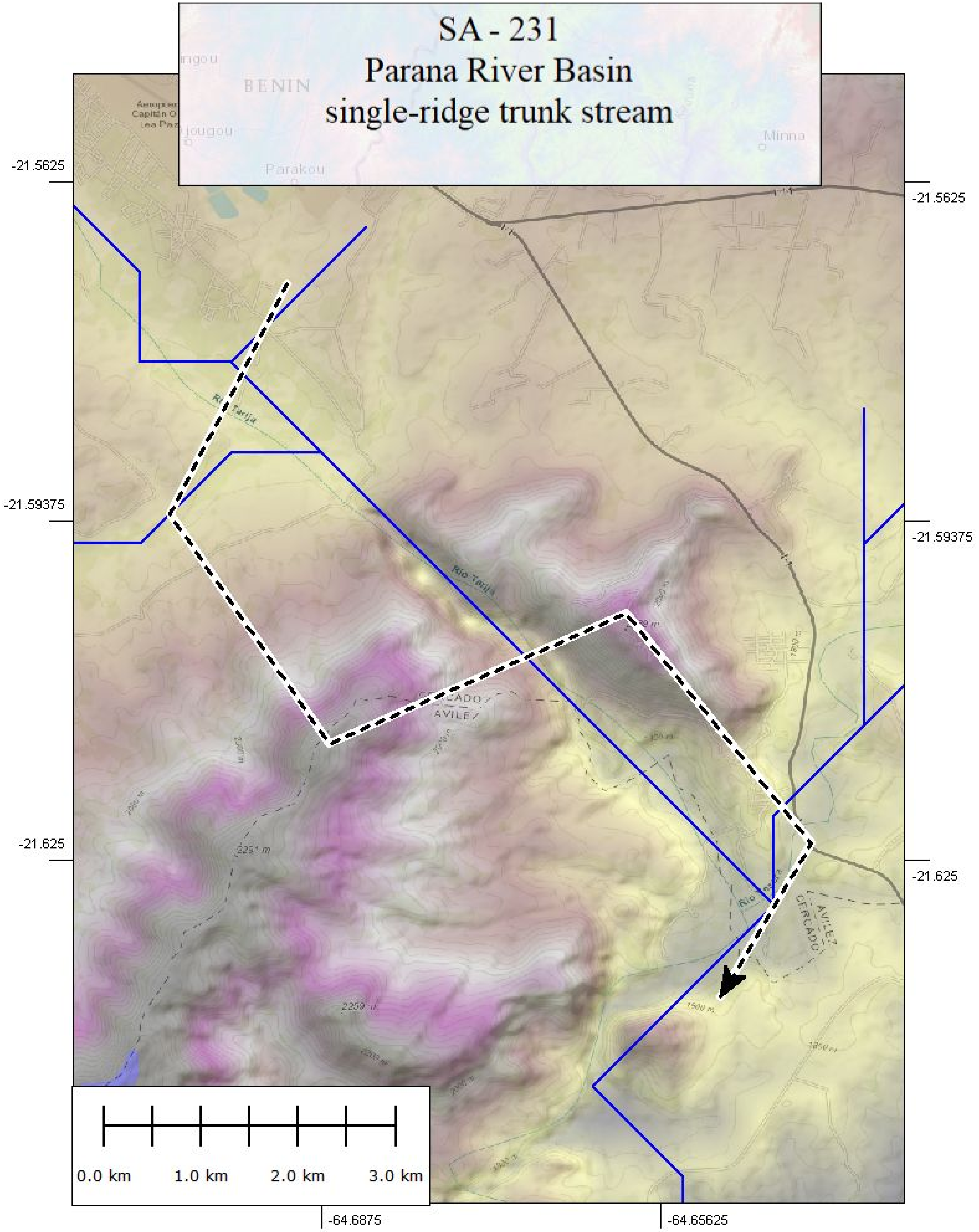
SA - 231
Parana River Basin
single-ridge trunk stream
BENIN
Parakou
Minna
Rio Tarija
Rio Tarija
CERCADO / AVILEZ
AVILEZ CERCADO
2251 m
2259 m
-21.5625
-21.59375
-21.625
-21.5625
-21.59375
-21.625
-64.6875
-64.65625
0.0 km
1.0 km
2.0 km
3.0 km

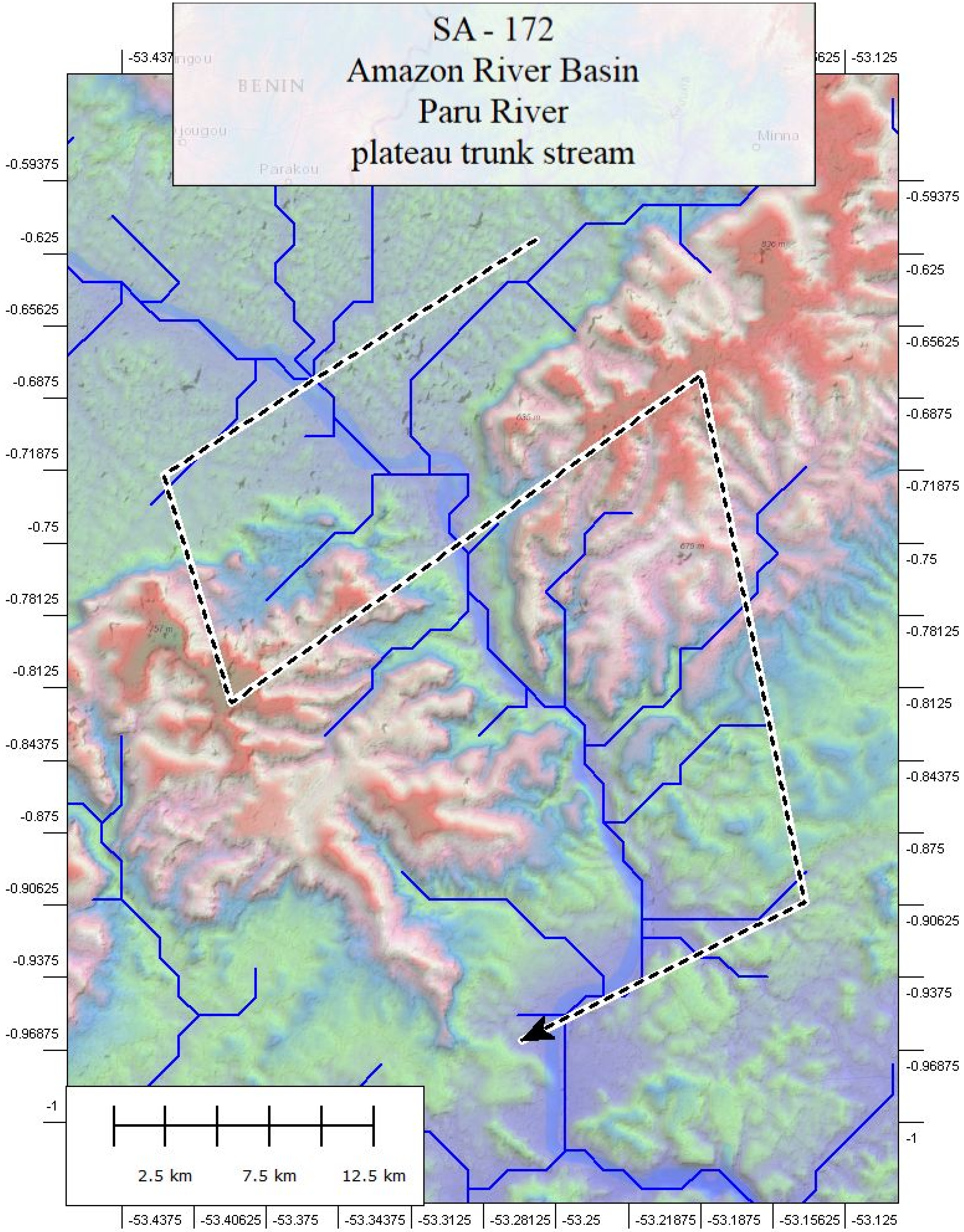
SA - 172
Amazon River Basin
Paru River
plateau trunk stream
2.5 km
7.5 km
12.5 km
-53.4375
-53.40625
-53.375
-53.34375
-53.3125
-53.28125
-53.25
-53.21875
-53.1875
-53.15625
-53.125
-0.59375
-0.625
-0.65625
-0.6875
-0.71875
-0.75
-0.78125
-0.8125
-0.84375
-0.875
-0.90625
-0.9375
-0.96875
-1
