# Supplementary material for: A global database of large-scale transverse drainages
Source: Data Brief. 2019 Jan 14;23:103650. doi: 10.1016/j.dib.2018.12.088 (PMC6369416; doi:10.1016/j.dib.2018.12.088)
Supplement: Supplementary file 5 — Supplementary material [file mmc5.pdf]

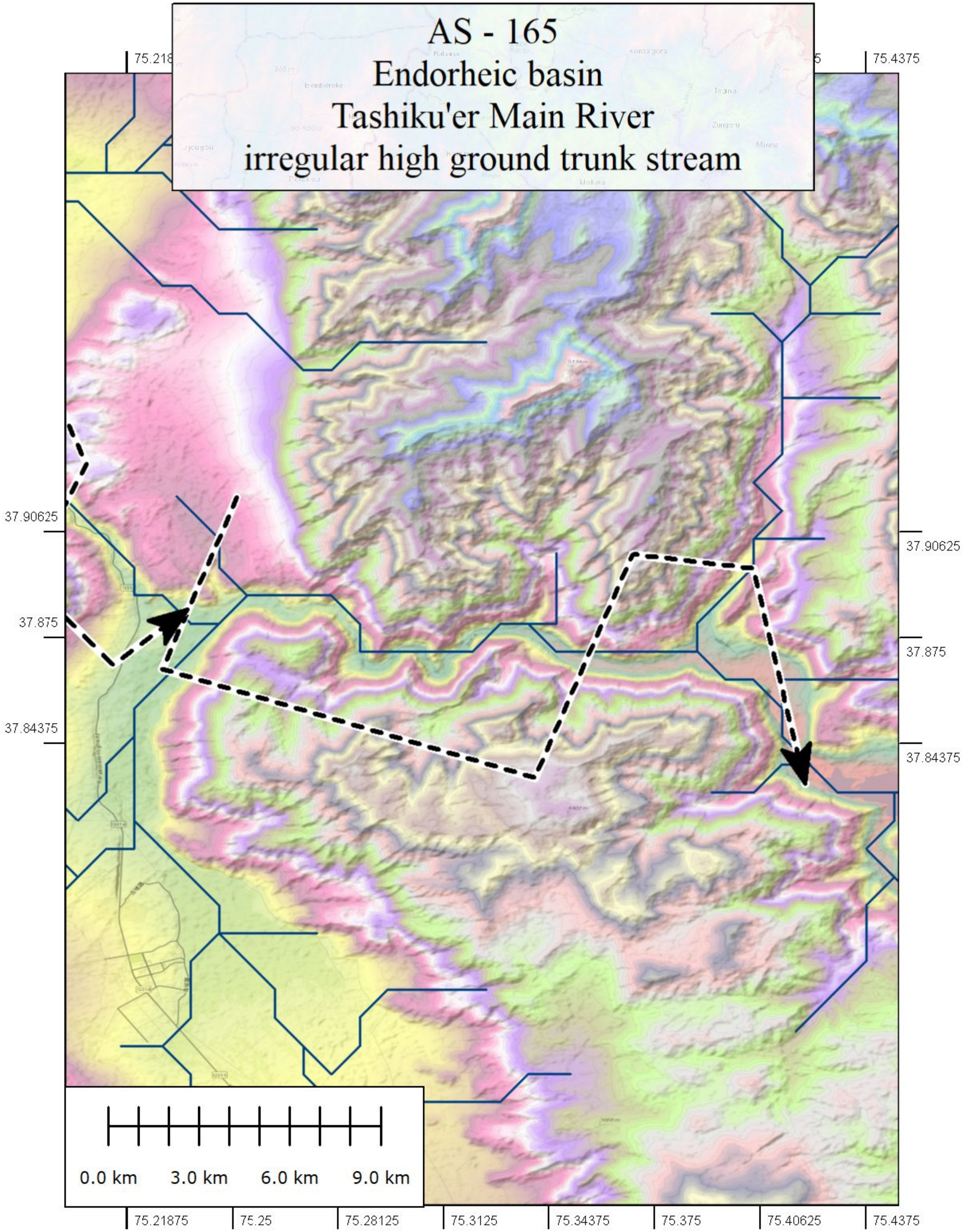
AS - 165
Endorheic basin
Tashiku'er Main River
irregular high ground trunk stream
0.0 km
3.0 km
6.0 km
9.0 km
75.21875
75.25
75.28125
75.3125
75.34375
75.375
75.40625
75.4375
37.90625
37.875
37.84375

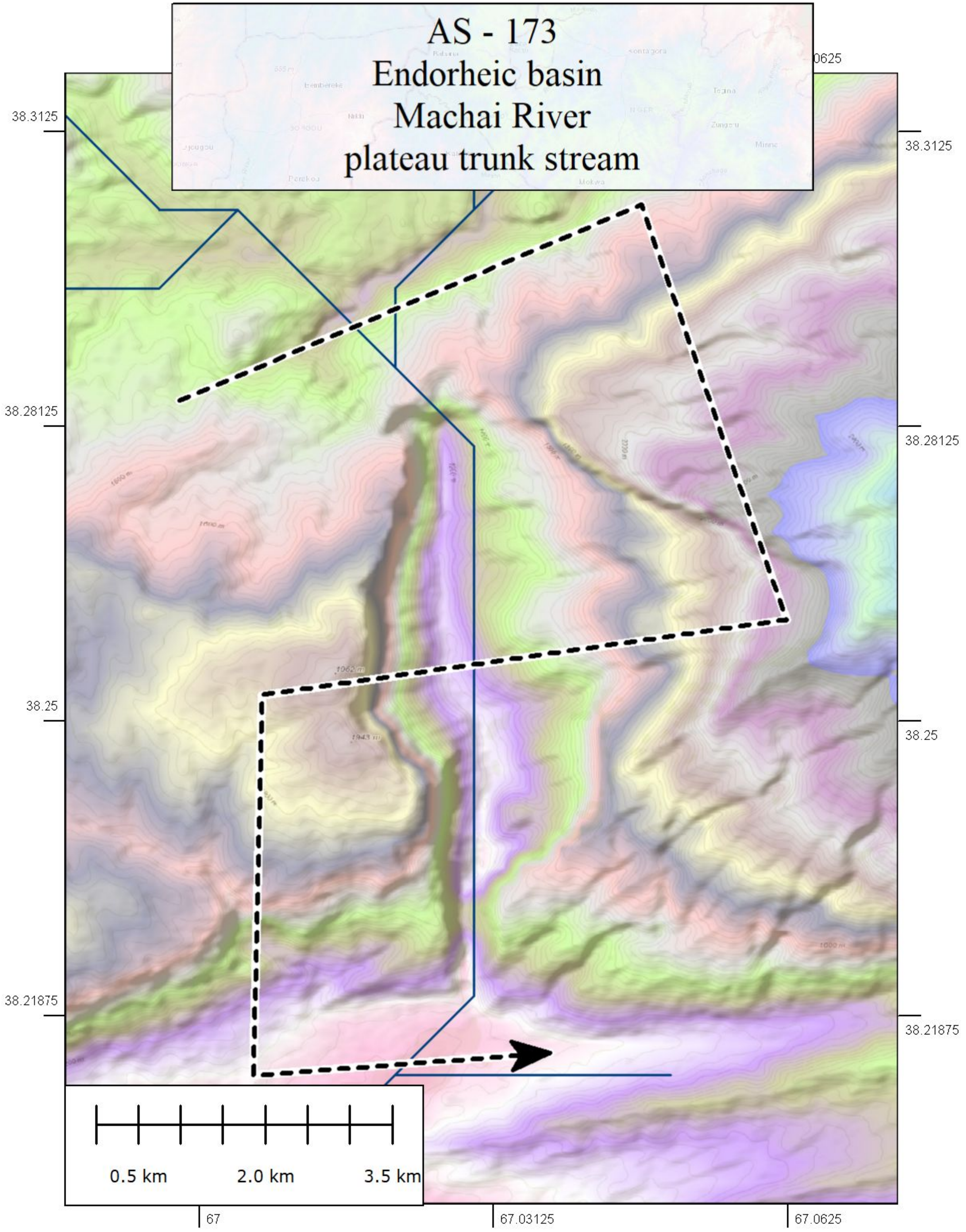
AS - 173
Endorheic basin
Machai River
plateau trunk stream
38.3125
38.28125
38.25
38.21875
67
67.03125
67.0625
0.5 km
2.0 km
3.5 km

AS - 24
Endorheic basin
Nalinggele River tributary
irregular high ground trunk stream

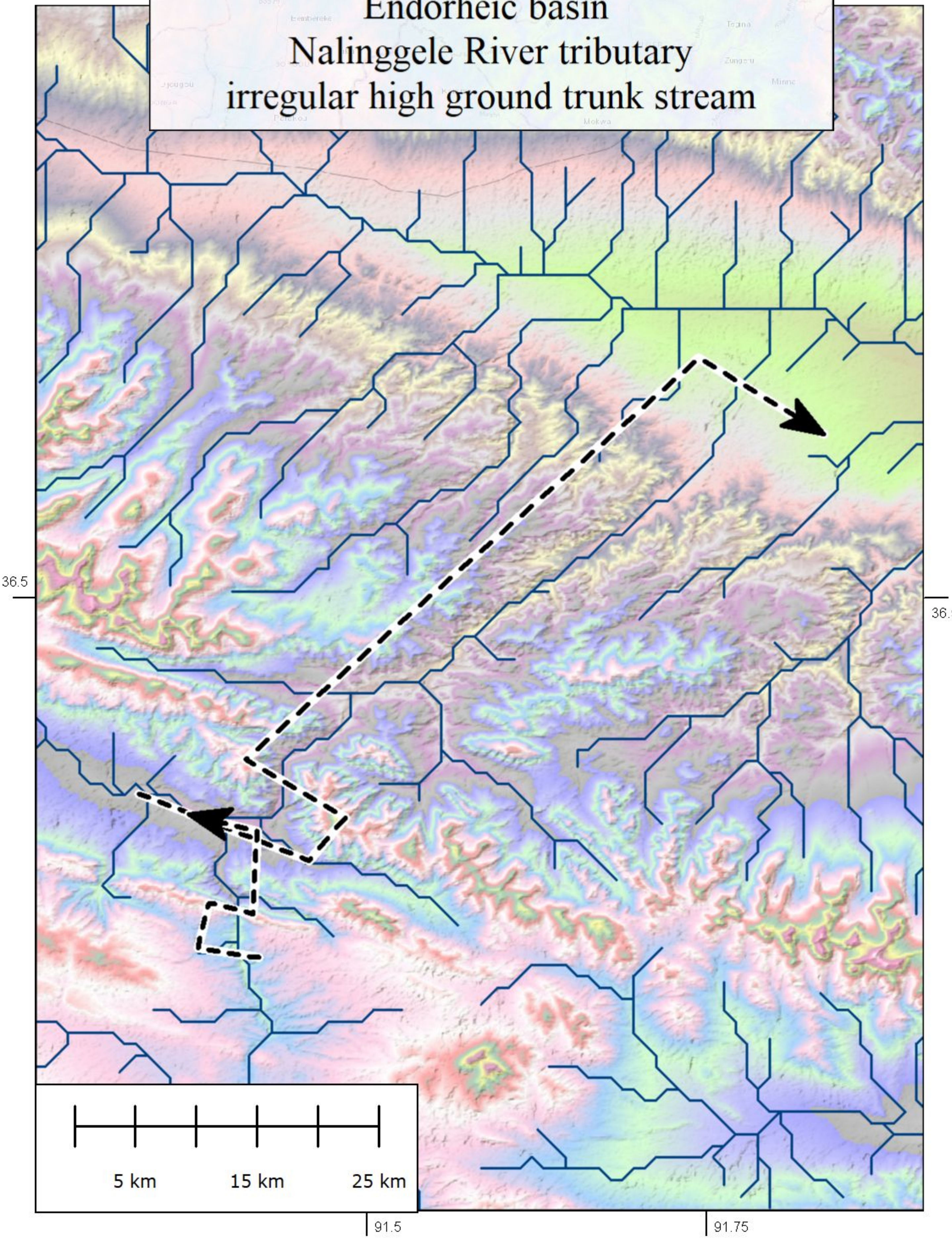

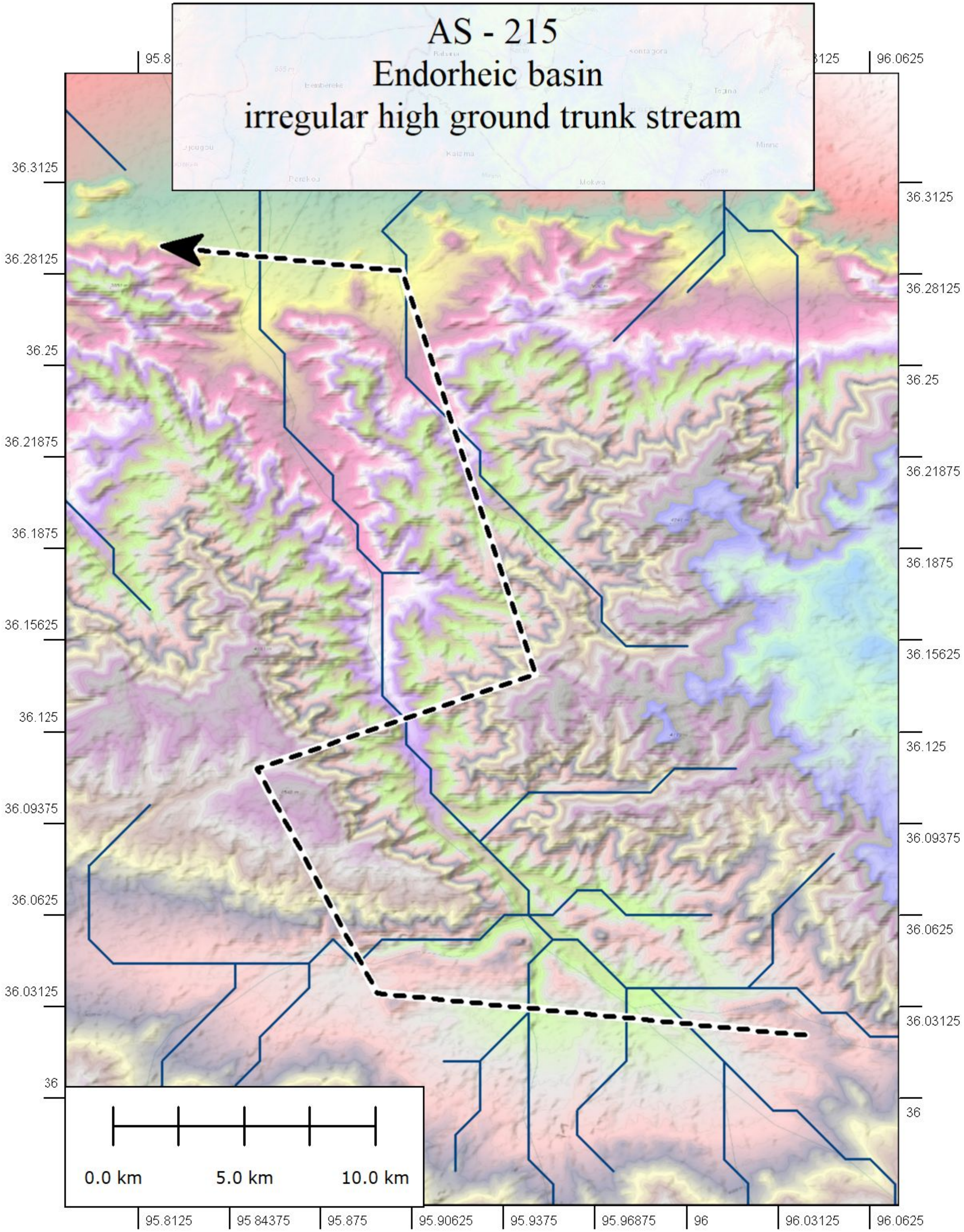
AS - 215
Endorheic basin
irregular high ground trunk stream
95.8125
95.84375
95.875
95.90625
95.9375
95.96875
96
96.03125
96.0625
36.3125
36.28125
36.25
36.21875
36.1875
36.15625
36.125
36.09375
36.0625
36.03125
36
0.0 km
5.0 km
10.0 km

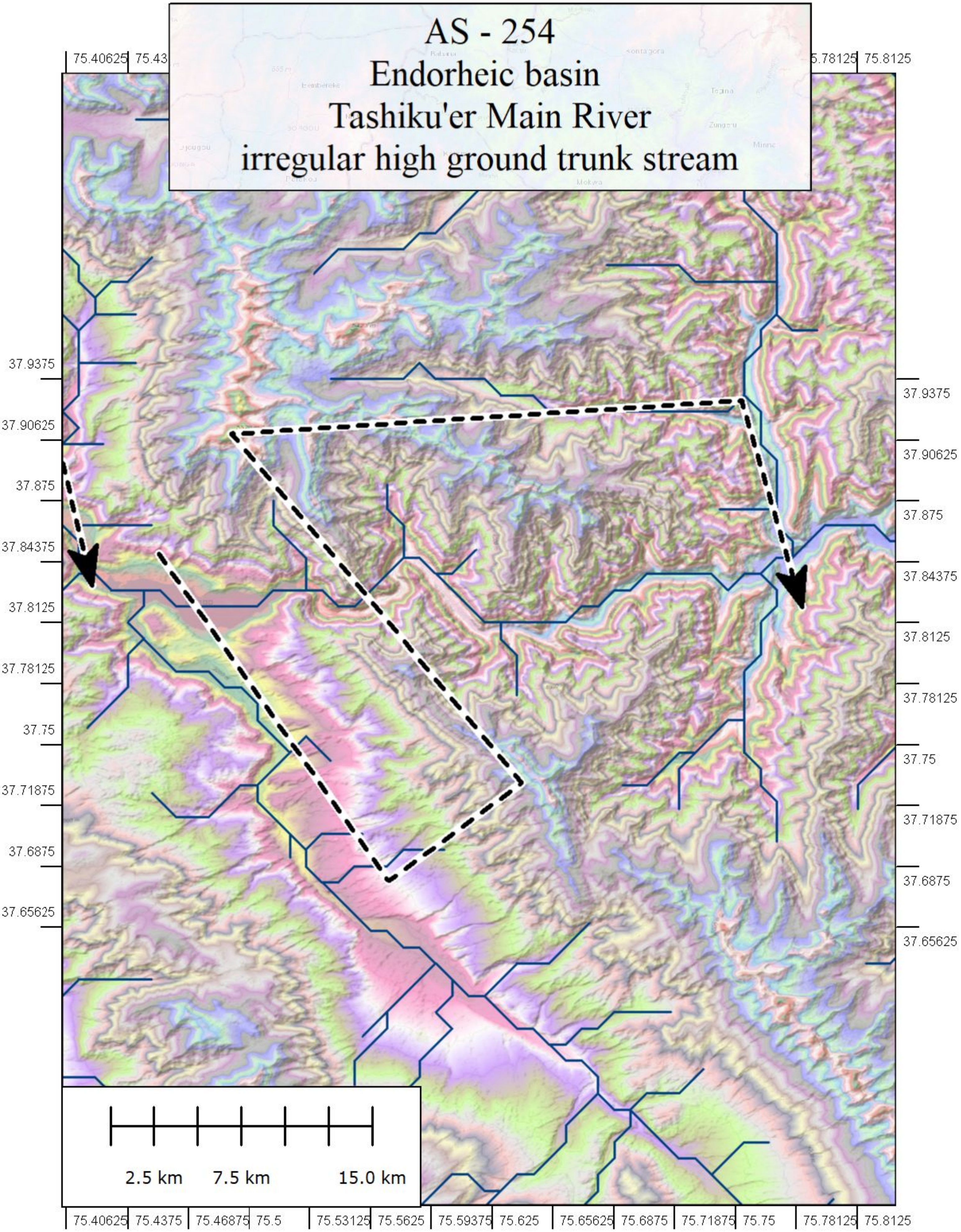
AS - 254
Endorheic basin
Tashiku'er Main River
irregular high ground trunk stream
75.40625 75.4375 75.46875 75.5 75.53125 75.5625 75.59375 75.625 75.65625 75.6875 75.71875 75.75 75.78125 75.8125
37.9375 37.90625 37.875 37.84375 37.8125 37.78125 37.75 37.71875 37.6875 37.65625
2.5 km 7.5 km 15.0 km

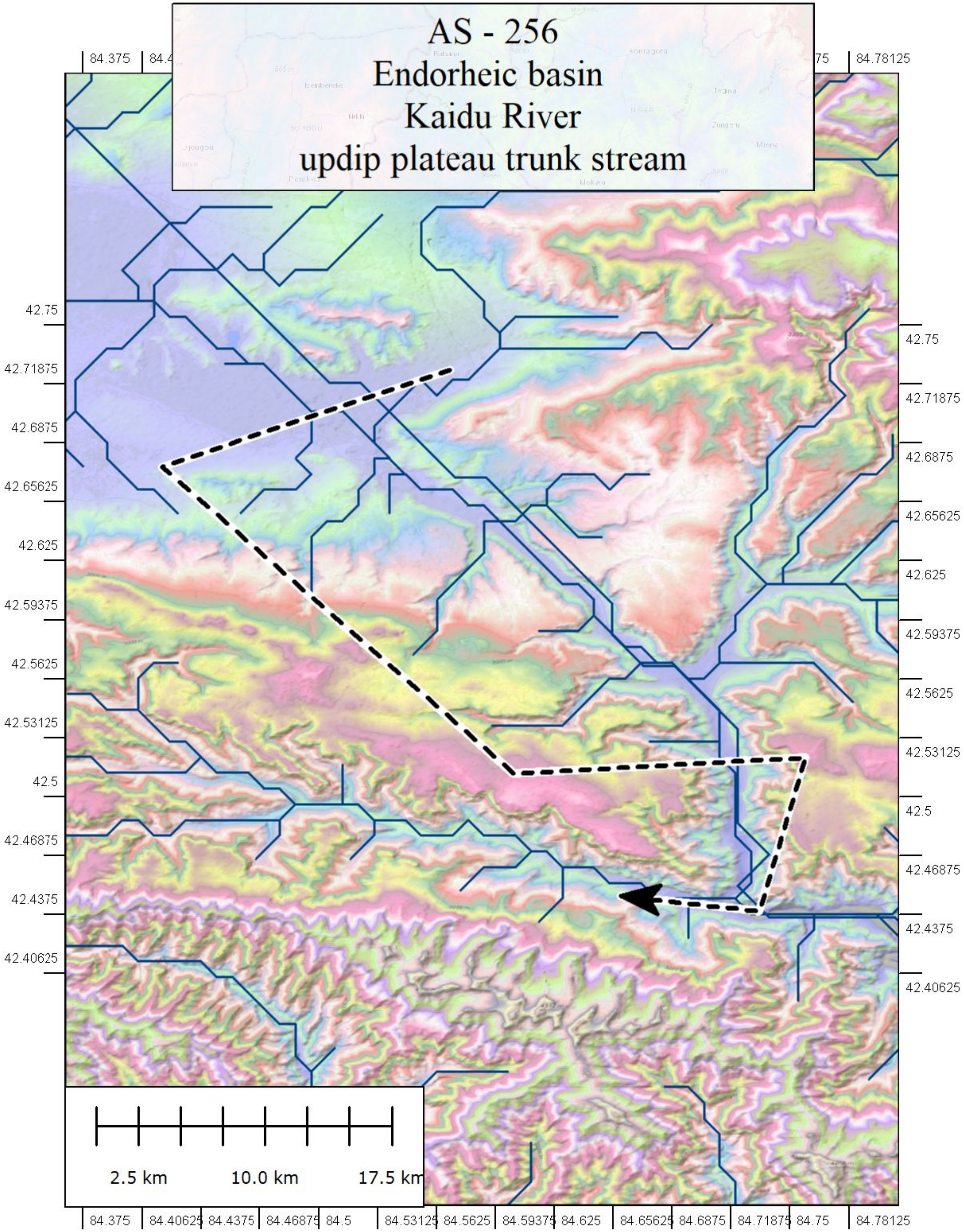
AS - 256
Endorheic basin
Kaidu River
updip plateau trunk stream
84.375
84.40625
84.4375
84.46875
84.5
84.53125
84.5625
84.59375
84.625
84.65625
84.6875
84.71875
84.75
84.78125
42.75
42.71875
42.6875
42.65625
42.625
42.59375
42.5625
42.53125
42.5
42.46875
42.4375
42.40625
2.5 km
10.0 km
17.5 km

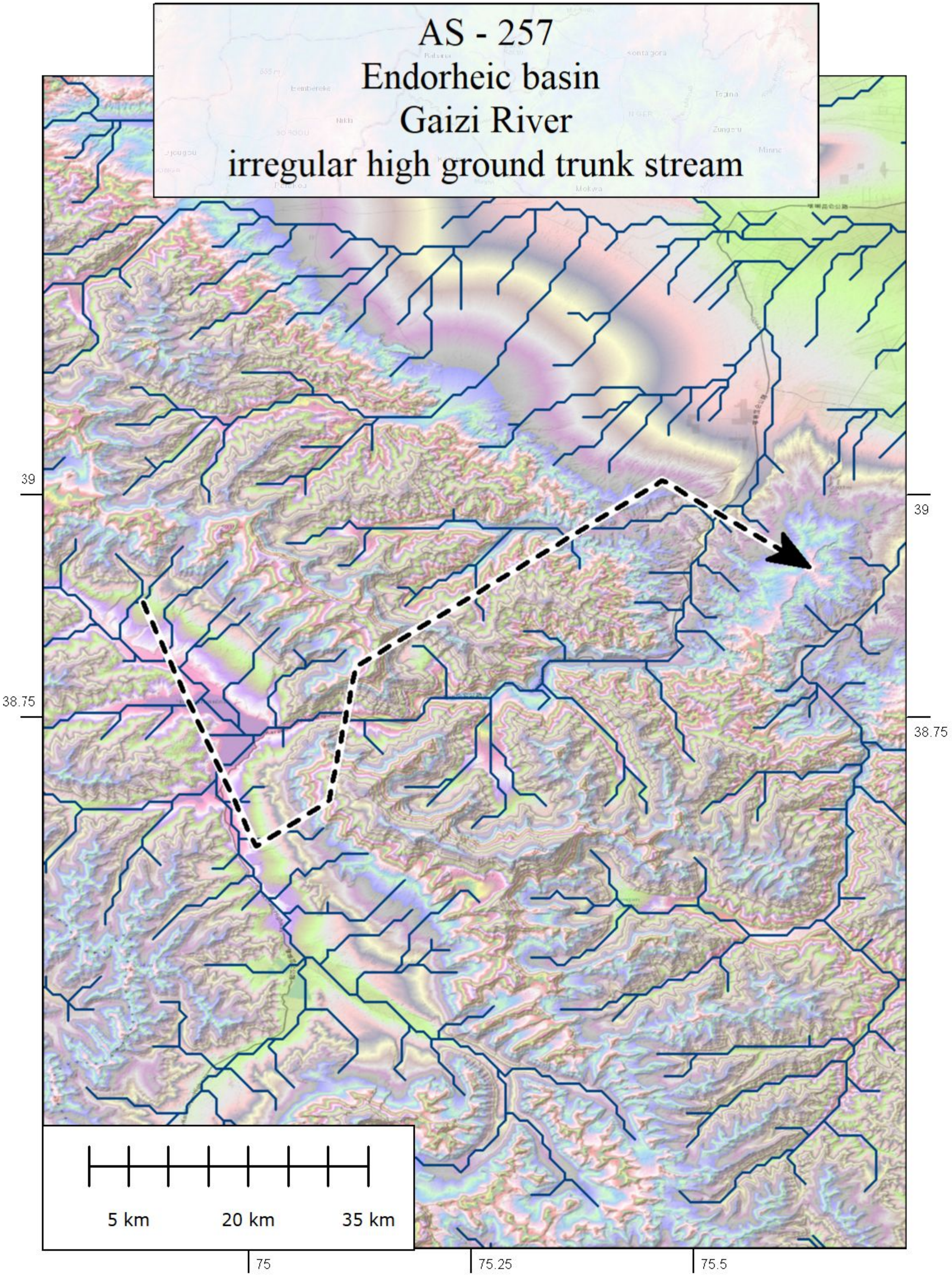
AS - 257
Endorheic basin
Gaizi River
irregular high ground trunk stream
39
39
38.75
38.75
5 km
20 km
35 km
75
75.25
75.5

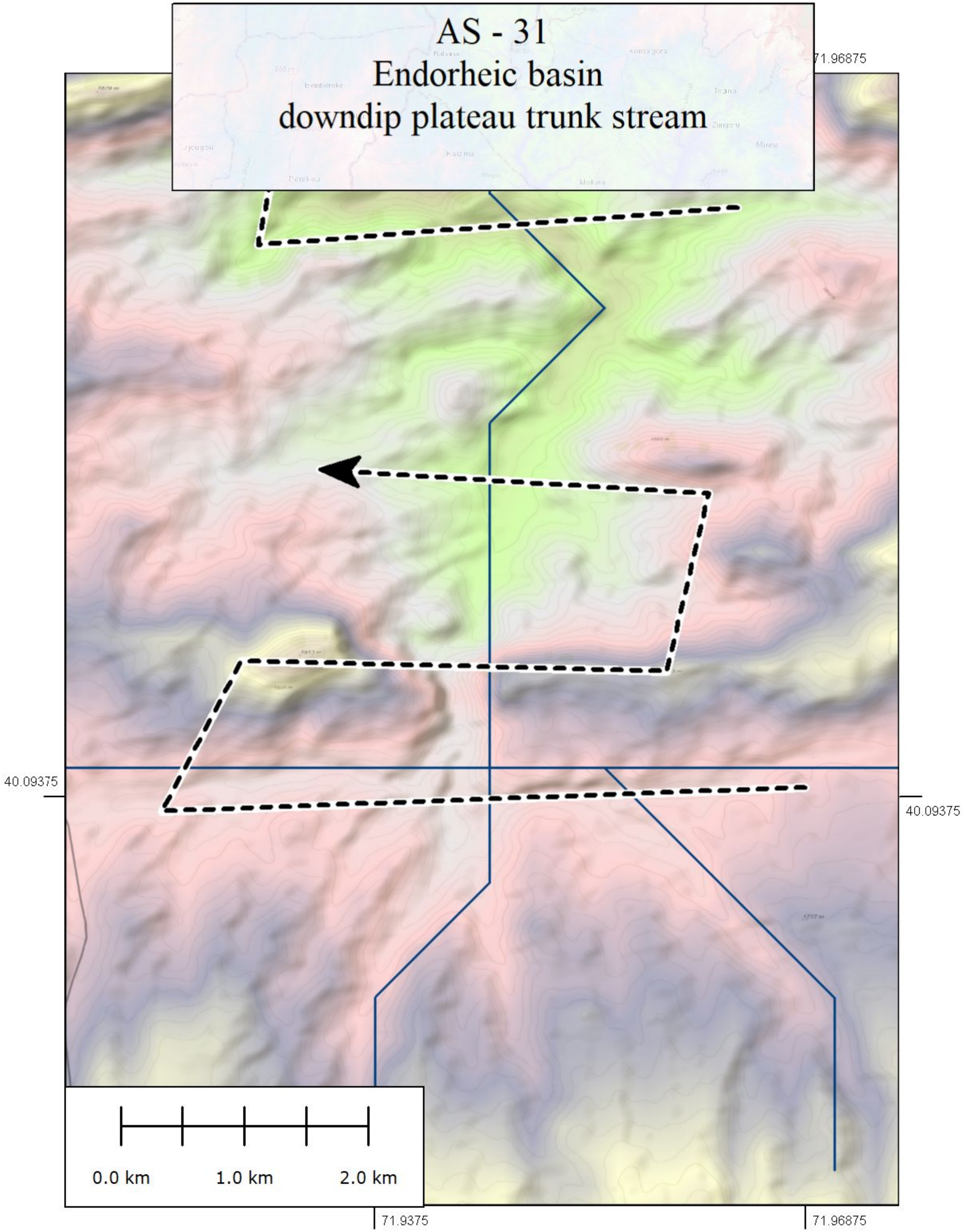
AS - 31
Endorheic basin
downdip plateau trunk stream
71.96875
40.09375
40.09375
0.0 km
1.0 km
2.0 km
71.9375
71.96875

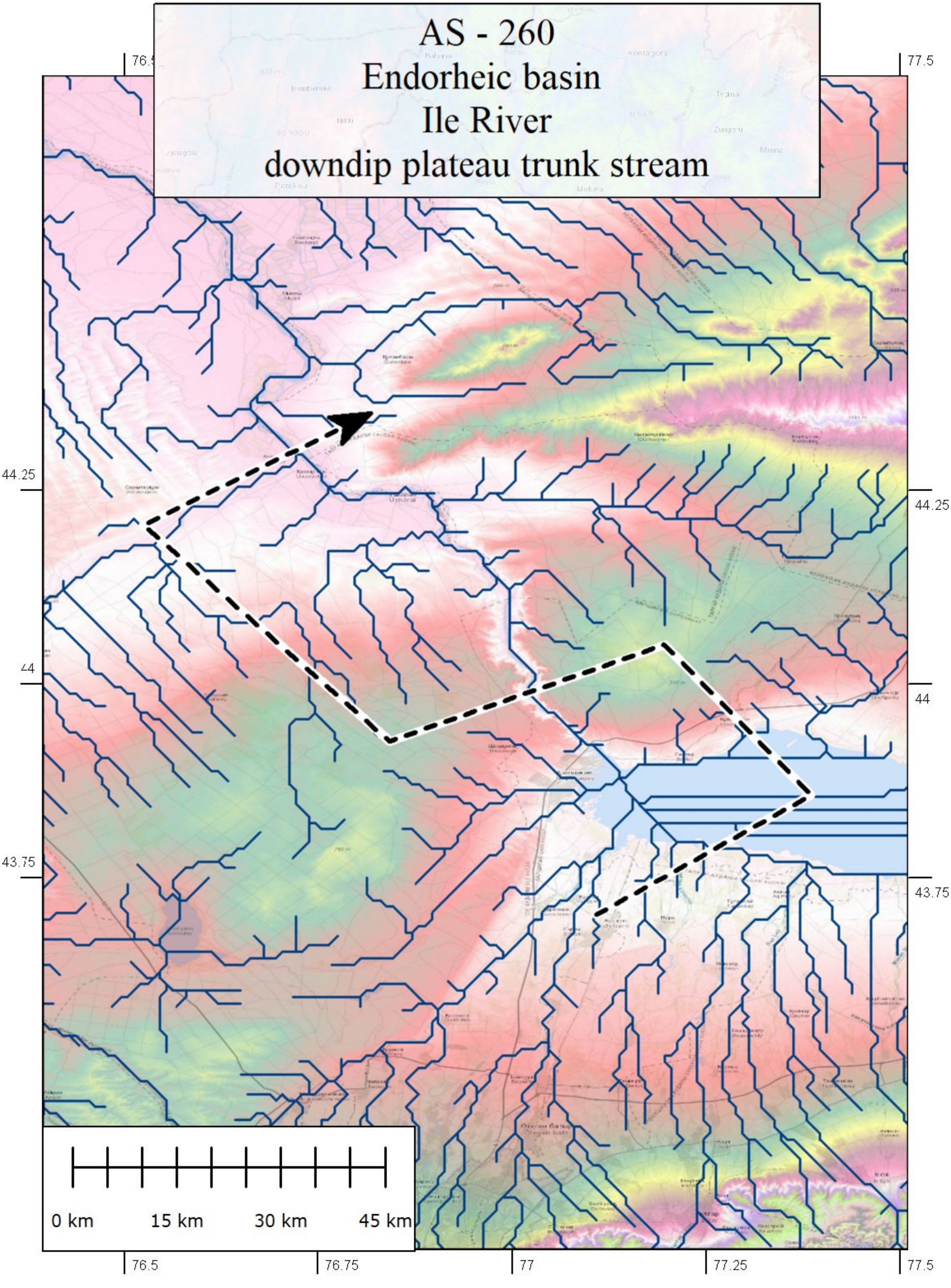
AS - 260
Endorheic basin
Ile River
downdip plateau trunk stream
76.5
76.75
77
77.25
77.5
44.25
44
43.75
0 km
15 km
30 km
45 km

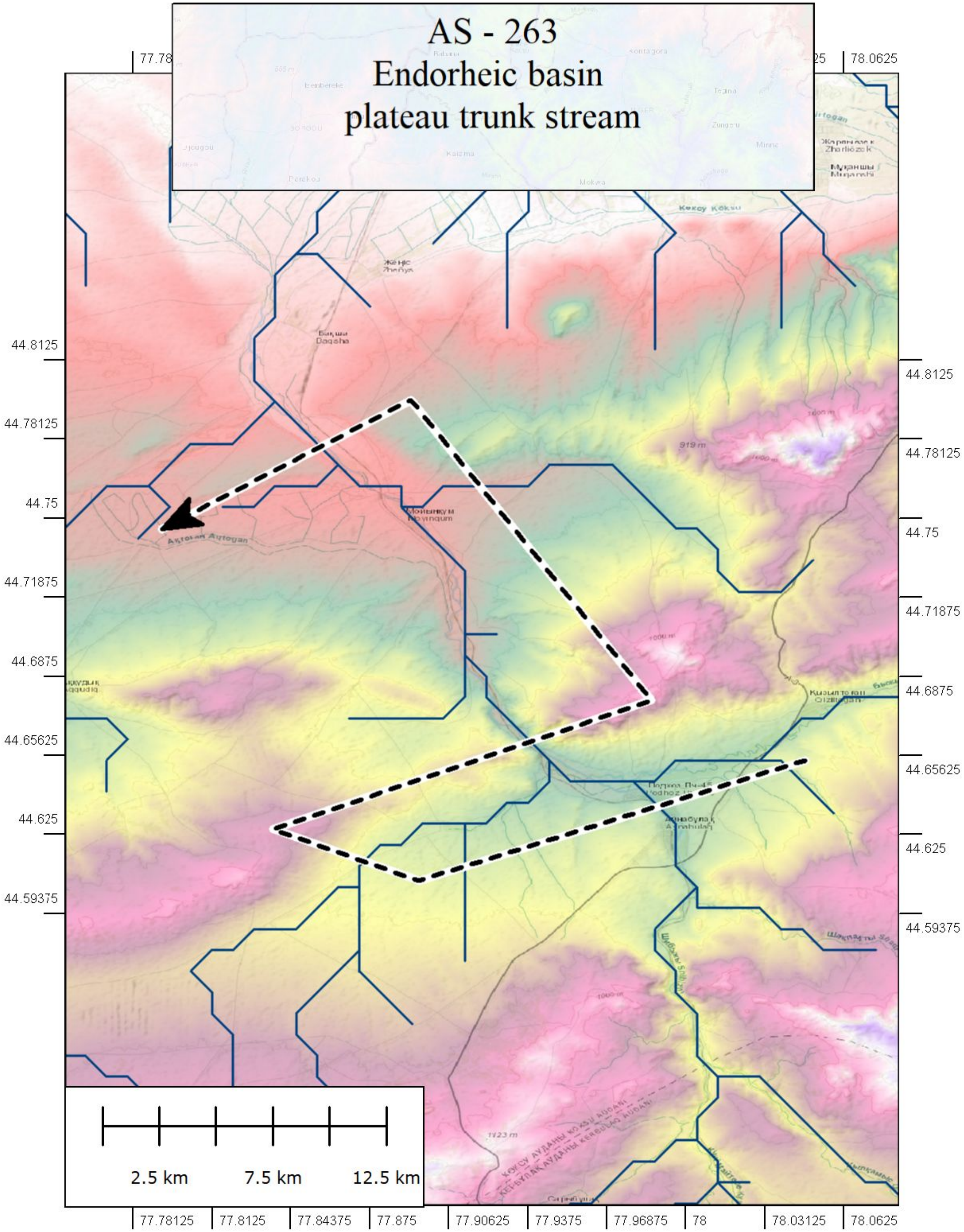

AS - 263
Endorheic basin
plateau trunk stream
77.78125
77.8125
77.84375
77.875
77.90625
77.9375
77.96875
78
78.03125
78.0625
44.8125
44.78125
44.75
44.71875
44.6875
44.65625
44.625
44.59375
2.5 km
7.5 km
12.5 km

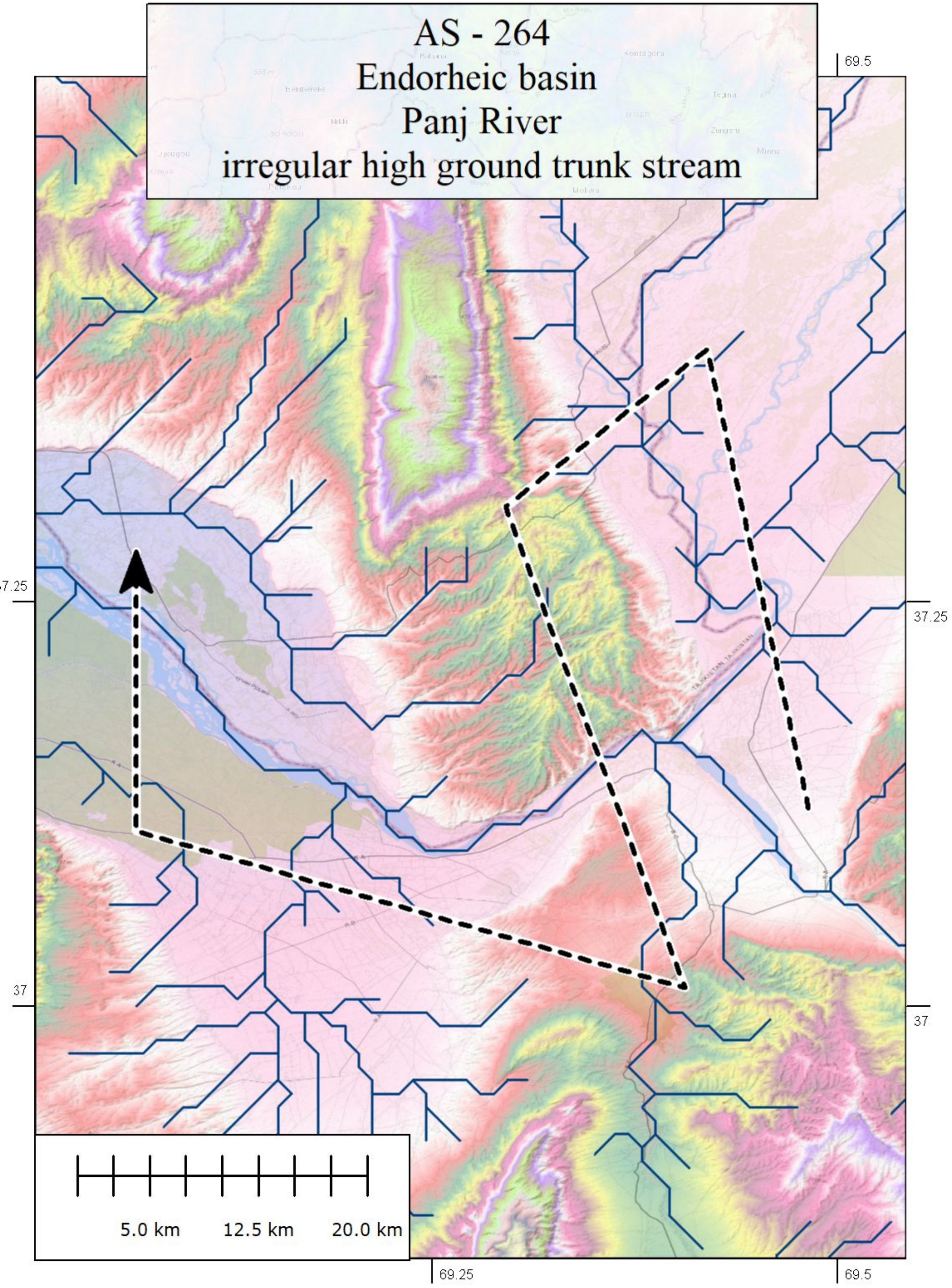
AS - 264
Endorheic basin
Panj River
irregular high ground trunk stream
69.5
37.25
37.25
37
37
5.0 km
12.5 km
20.0 km
69.25
69.5

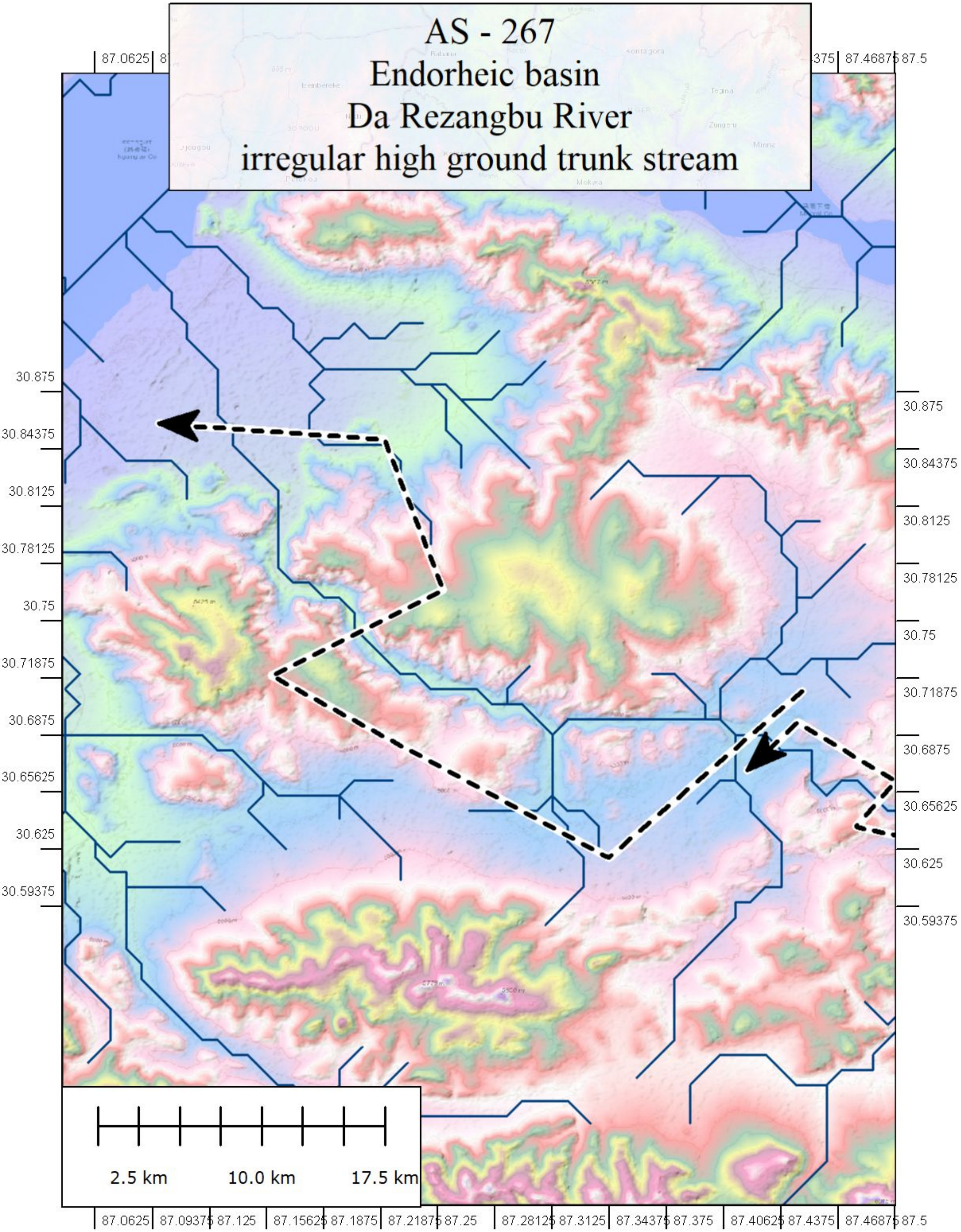
AS - 267
Endorheic basin
Da Rezangbu River
irregular high ground trunk stream
2.5 km
10.0 km
17.5 km
87.0625
87.09375
87.125
87.15625
87.1875
87.21875
87.25
87.28125
87.3125
87.34375
87.375
87.40625
87.4375
87.46875
87.5
30.875
30.84375
30.8125
30.78125
30.75
30.71875
30.6875
30.65625
30.625
30.59375

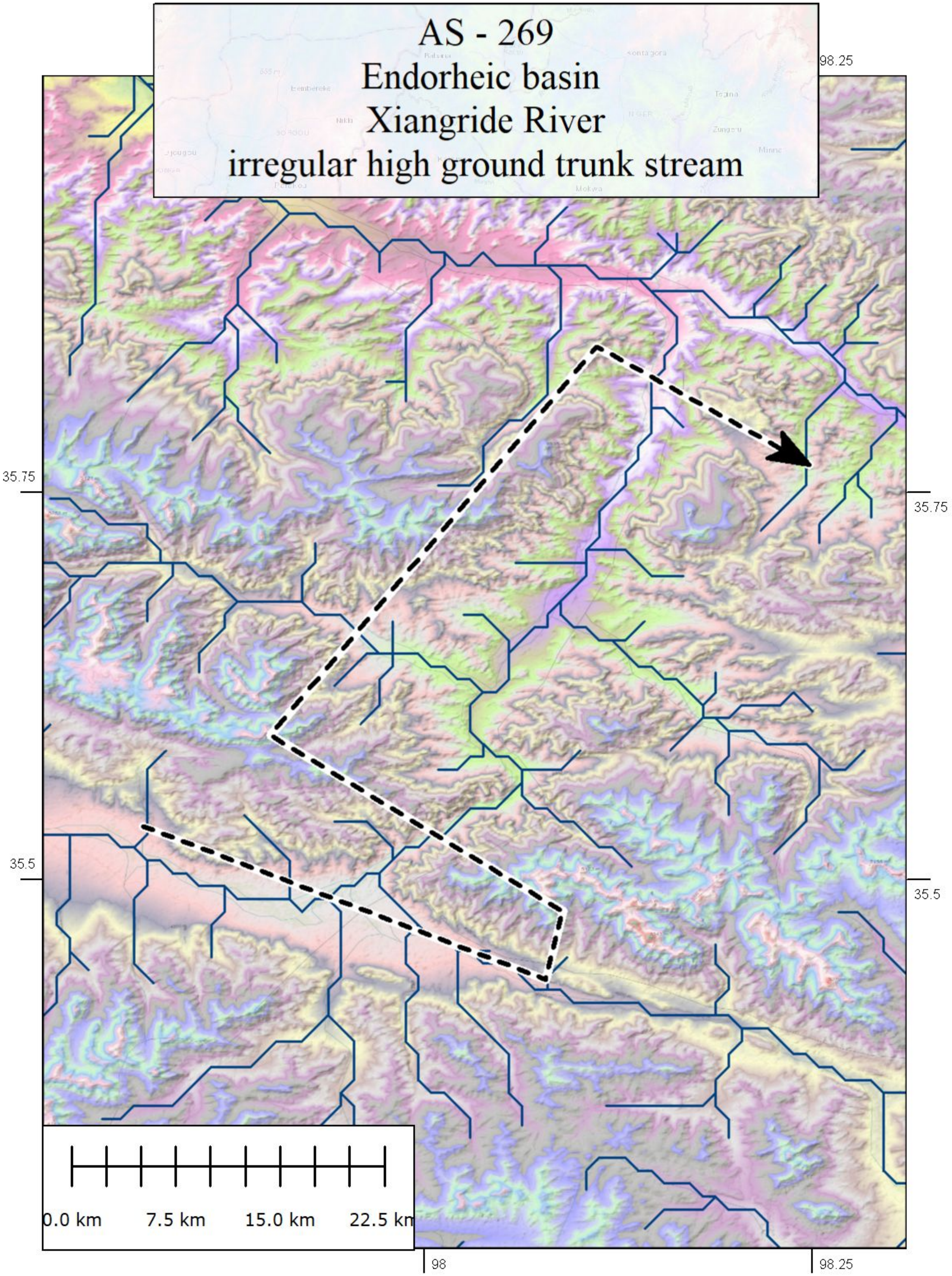
AS - 269
Endorheic basin
Xiangride River
irregular high ground trunk stream
98.25
35.75
35.75
35.5
35.5
0.0 km
7.5 km
15.0 km
22.5 km
98
98.25

AS - 277
Endorheic basin
Chu RIver
irregular high ground trunk stream

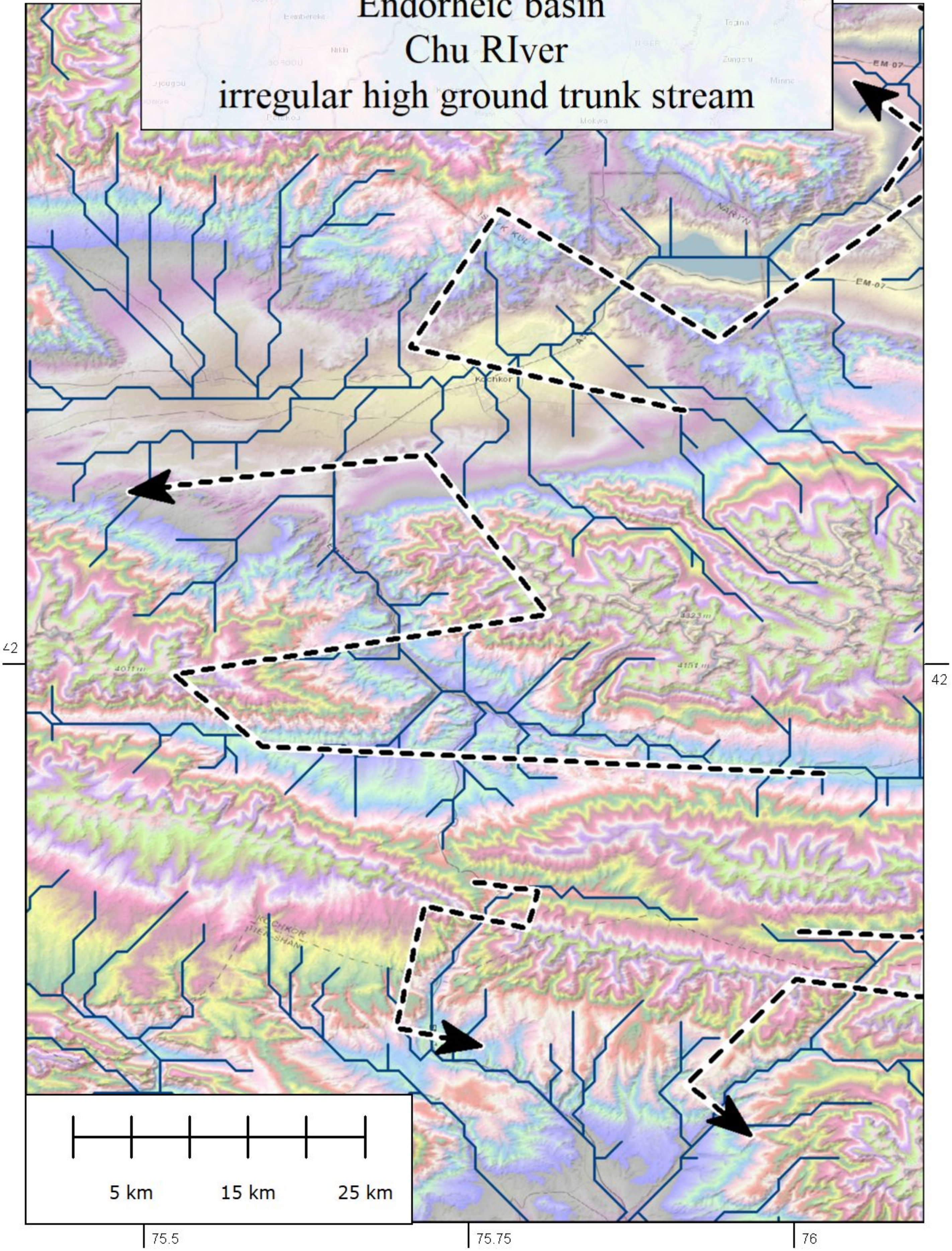

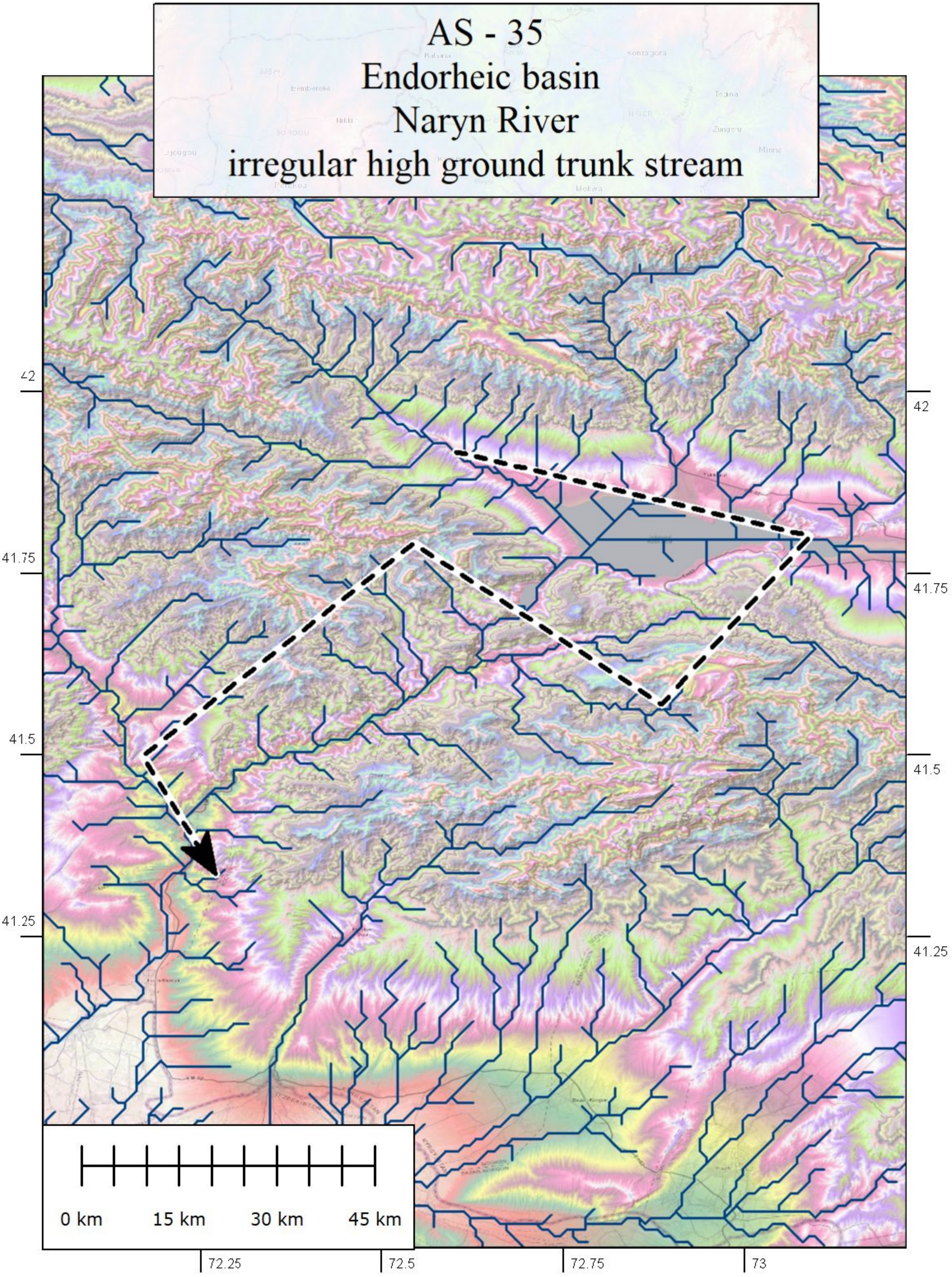
AS - 35
Endorheic basin
Naryn River
irregular high ground trunk stream
42
41.75
41.5
41.25
0 km
15 km
30 km
45 km
72.25
72.5
72.75
73

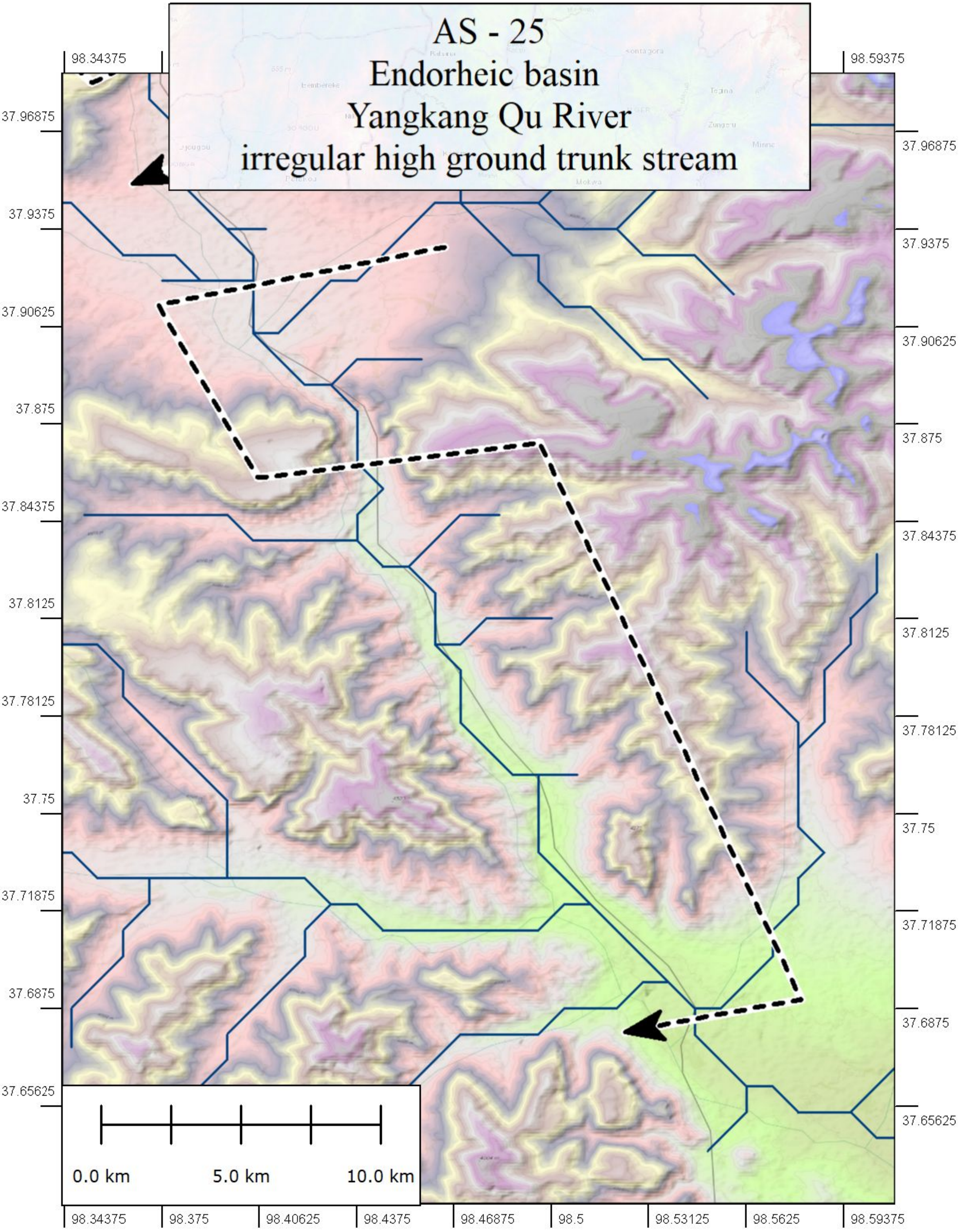
AS - 25
Endorheic basin
Yangkang Qu River
irregular high ground trunk stream
98.34375
98.375
98.40625
98.4375
98.46875
98.5
98.53125
98.5625
98.59375
37.96875
37.9375
37.90625
37.875
37.84375
37.8125
37.78125
37.75
37.71875
37.6875
37.65625
0.0 km
5.0 km
10.0 km

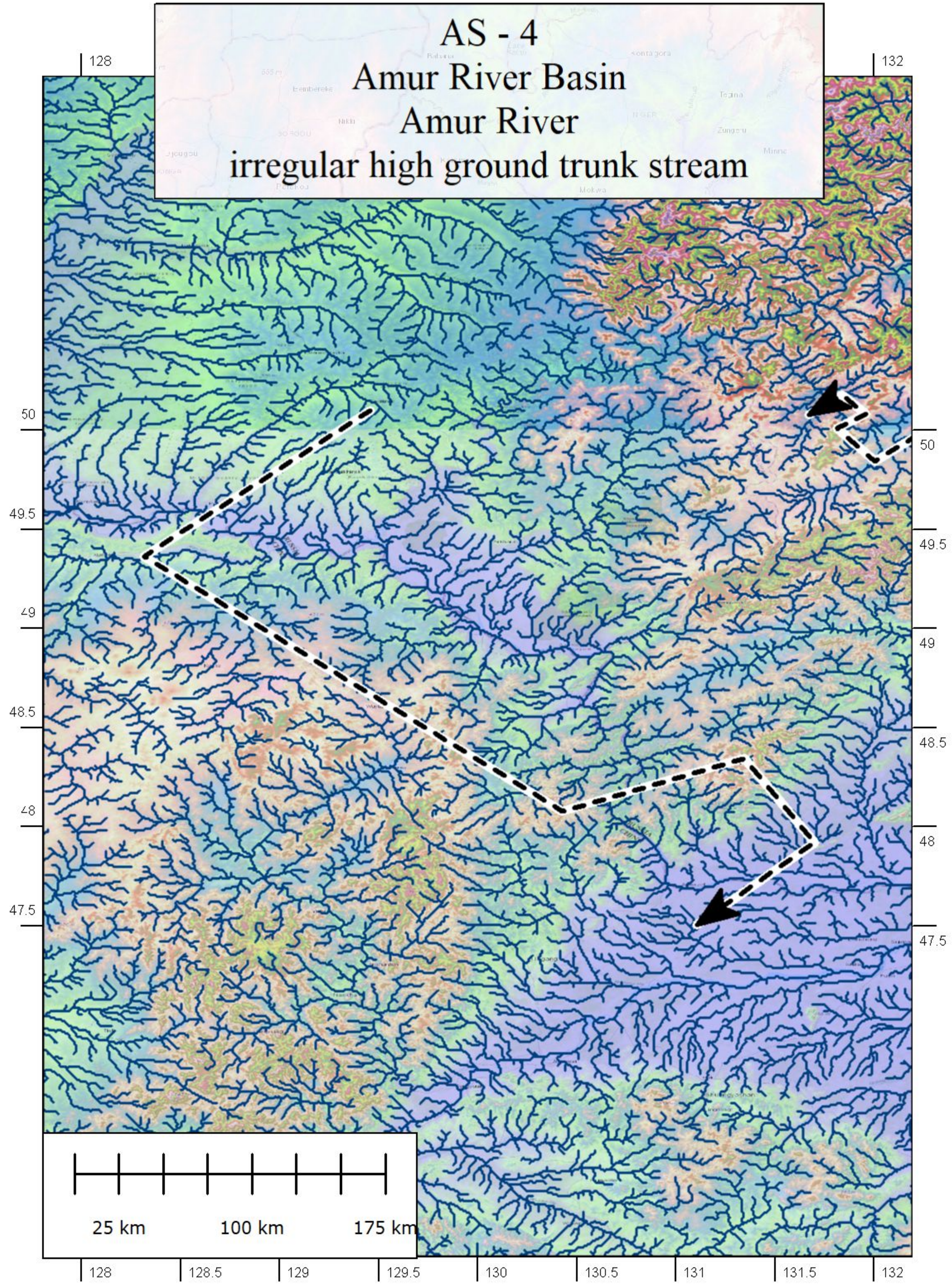
AS - 4
Amur River Basin
Amur River
irregular high ground trunk stream
128
132
50
49.5
49
48.5
48
47.5
25 km
100 km
175 km
128
128.5
129
129.5
130
130.5
131
131.5
132

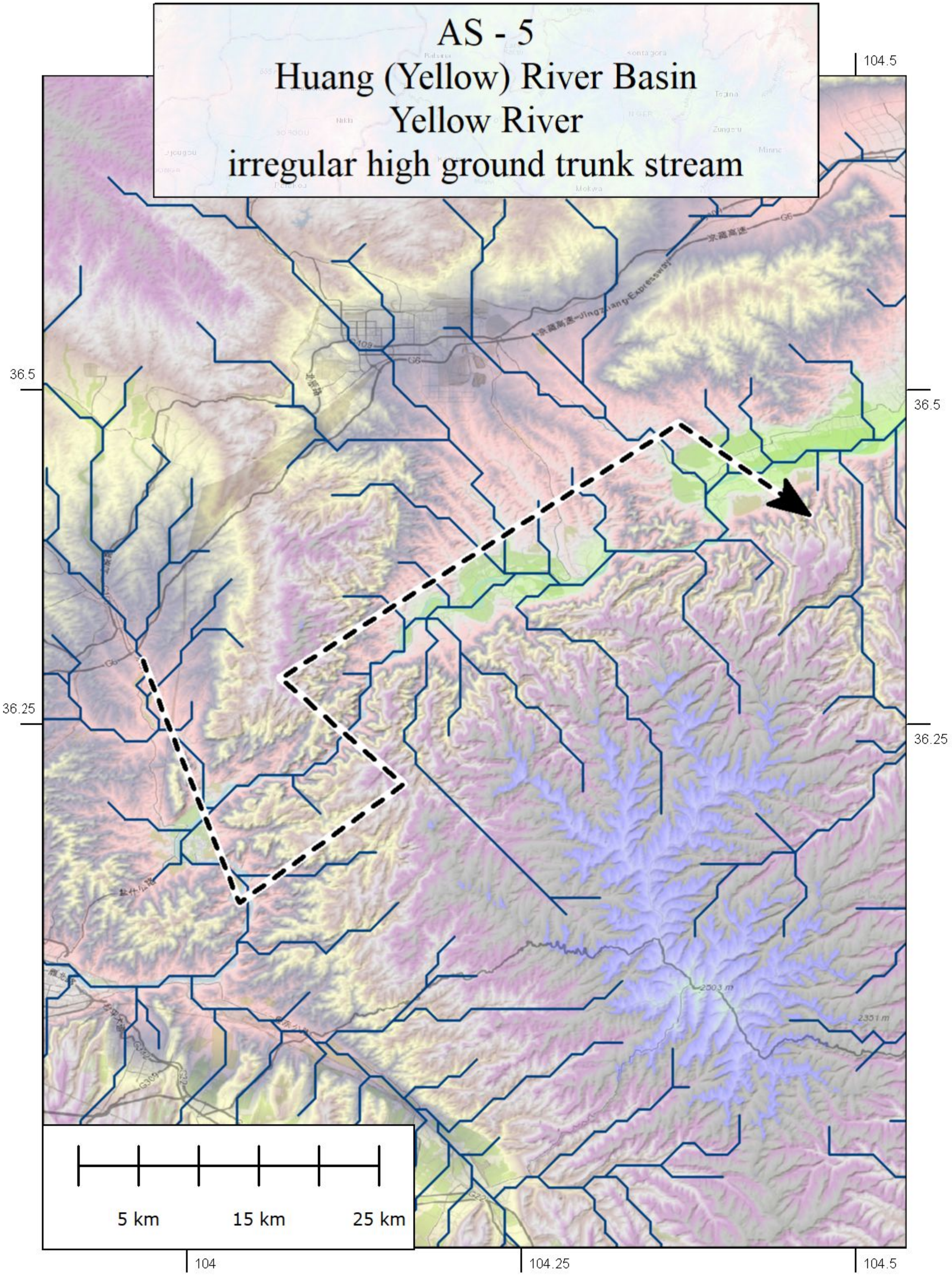
AS - 5
Huang (Yellow) River Basin
Yellow River
irregular high ground trunk stream
104.5
36.5
36.5
36.25
36.25
5 km
15 km
25 km
104
104.25
104.5

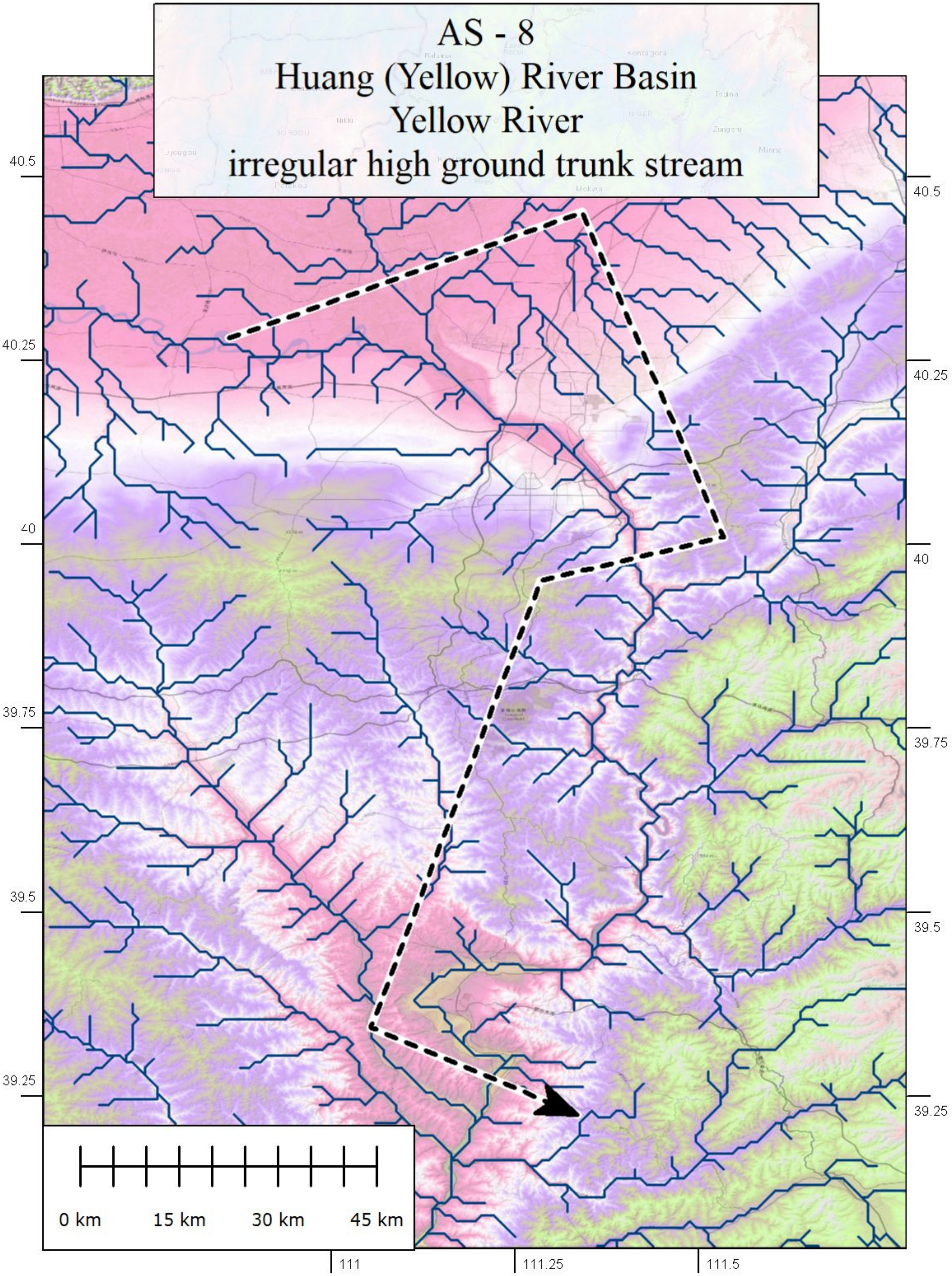
AS - 8
Huang (Yellow) River Basin
Yellow River
irregular high ground trunk stream
40.5
40.25
40
39.75
39.5
39.25
111
111.25
111.5
0 km
15 km
30 km
45 km

AS - 9
Mekong River Basin
Mekong River
downdip plateau trunk stream

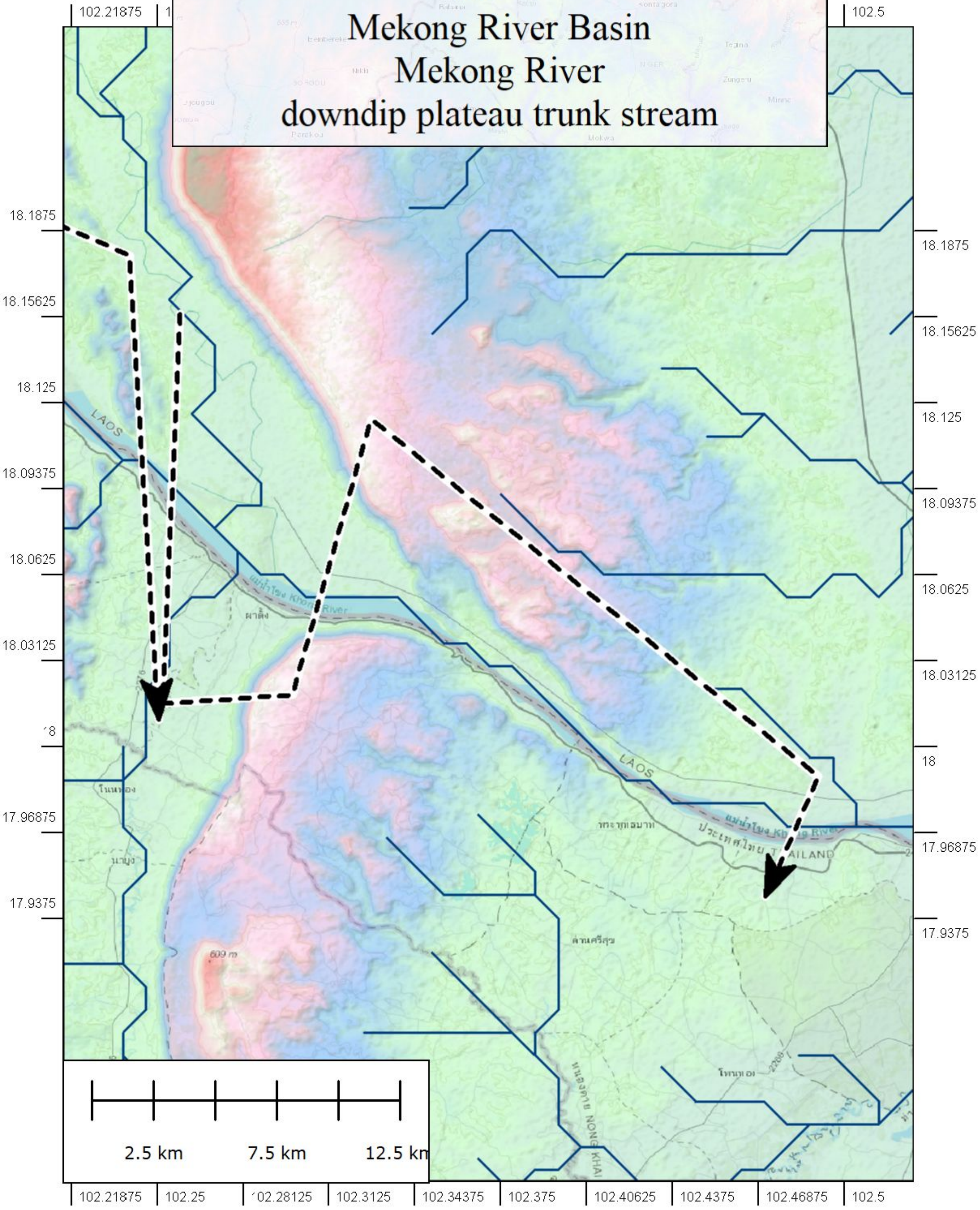

AS - 11
Mekong River Basin
Mekong River
irregular high ground trunk stream

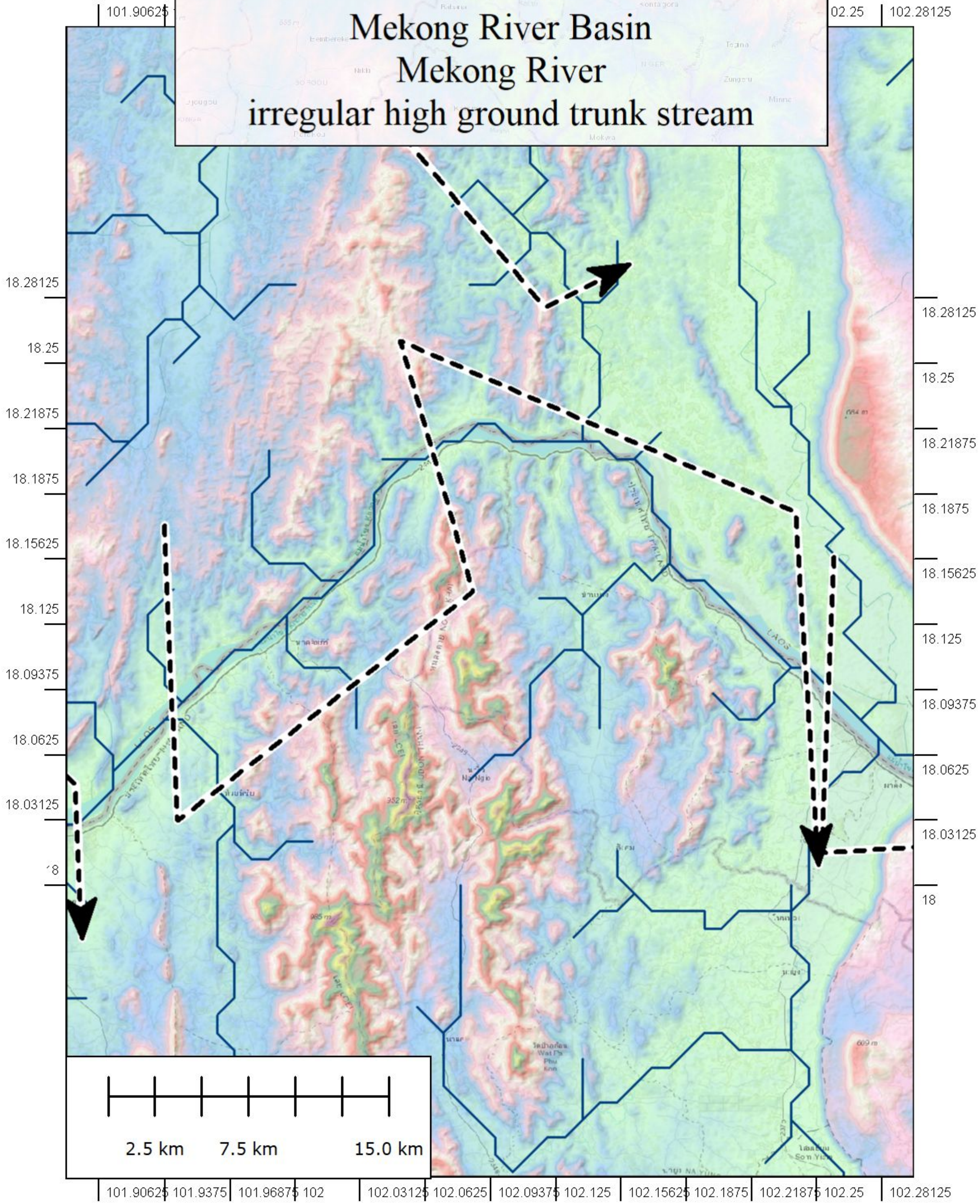

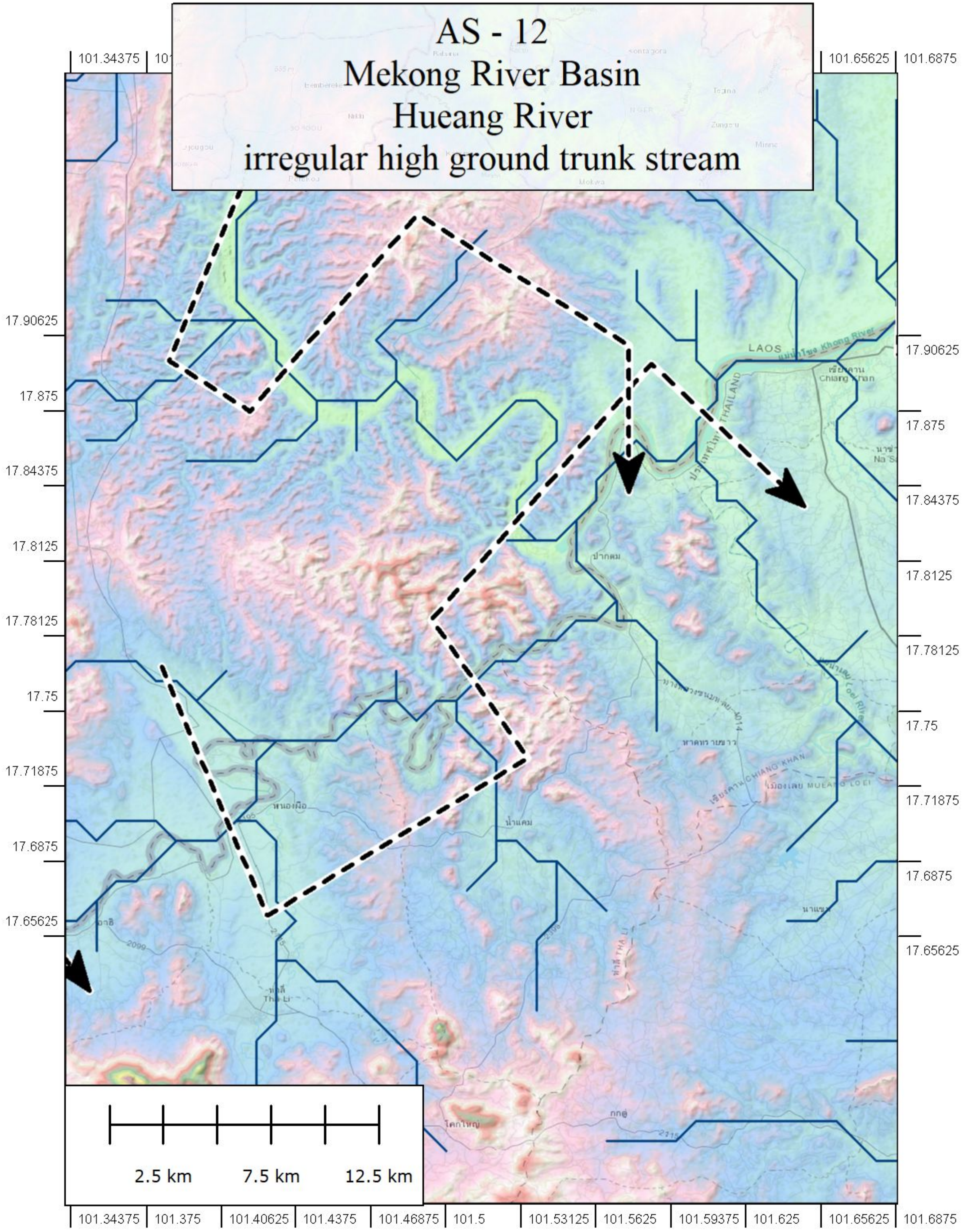
AS - 12
Mekong River Basin
Hueang River
irregular high ground trunk stream
LAOS
THAILAND
Chiang Khan
Loei River
CHIANG KHAN
MUEANG LOEI
Tha Li
2.5 km
7.5 km
12.5 km
101.34375
101.375
101.40625
101.4375
101.46875
101.5
101.53125
101.5625
101.59375
101.625
101.65625
101.6875
17.90625
17.875
17.84375
17.8125
17.78125
17.75
17.71875
17.6875
17.65625

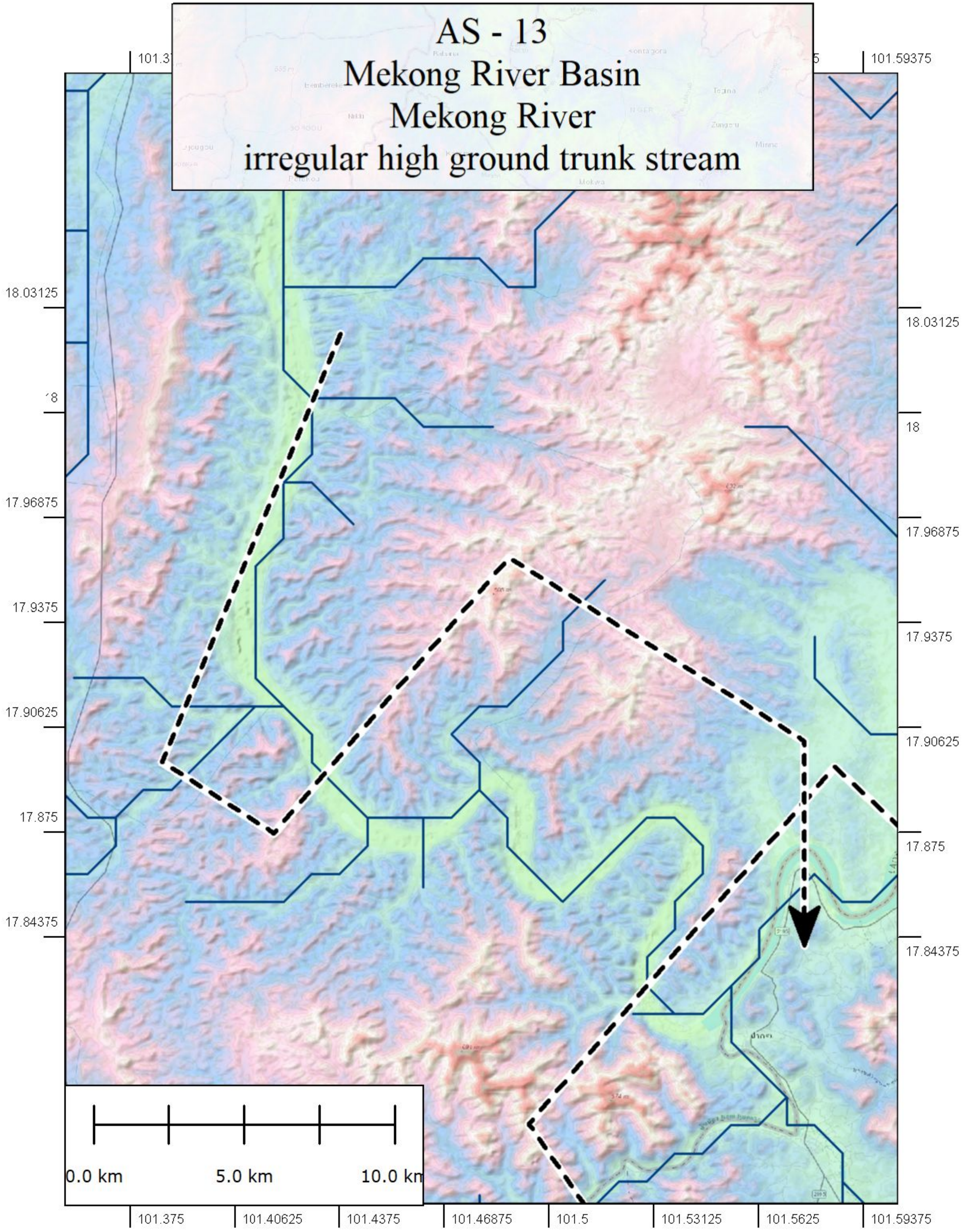
AS - 13
Mekong River Basin
Mekong River
irregular high ground trunk stream
101.59375
18.03125
18
17.96875
17.9375
17.90625
17.875
17.84375
0.0 km
5.0 km
10.0 km
101.375
101.40625
101.4375
101.46875
101.5
101.53125
101.5625

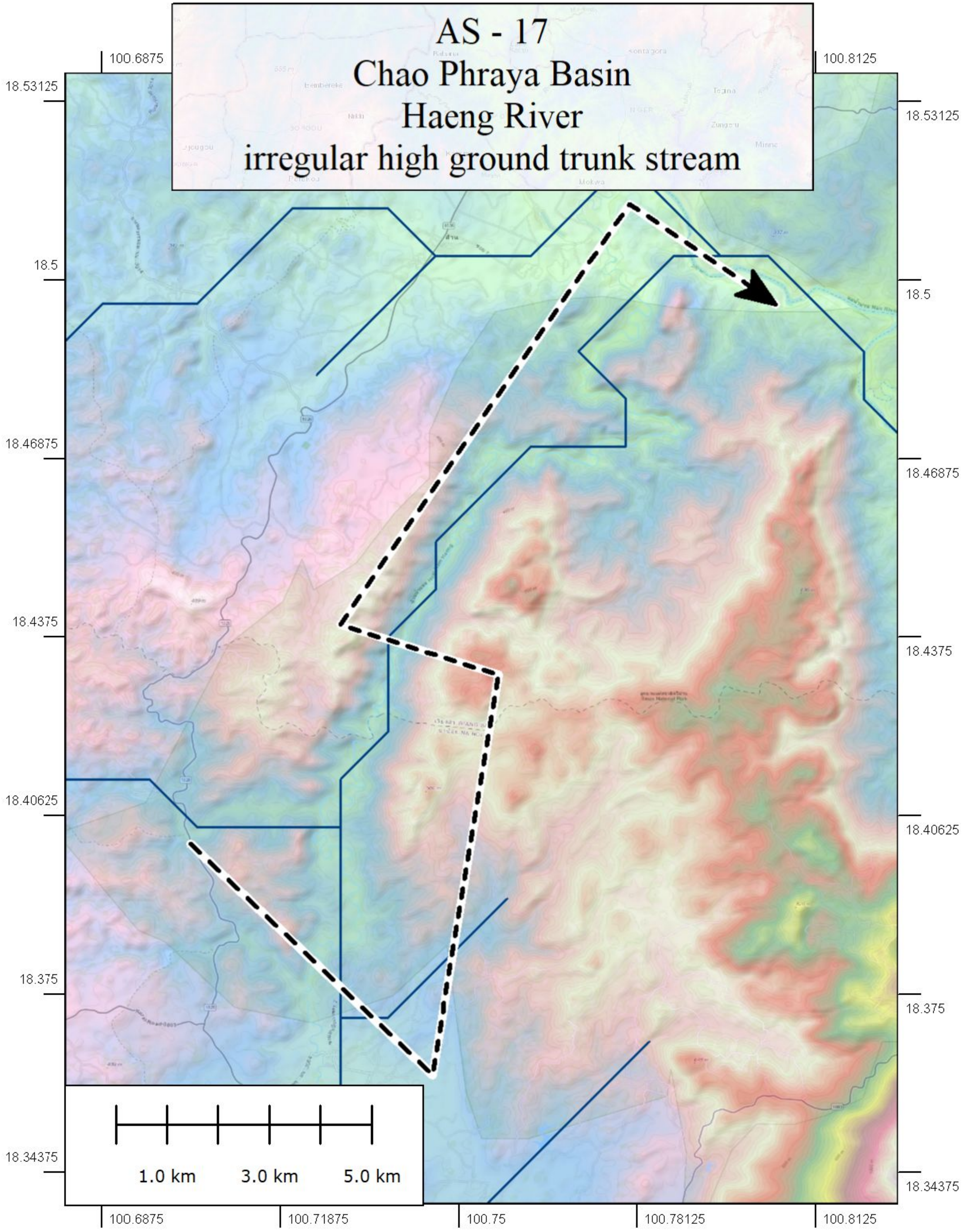
AS - 17
Chao Phraya Basin
Haeng River
irregular high ground trunk stream
100.6875
100.71875
100.75
100.78125
100.8125
18.53125
18.5
18.46875
18.4375
18.40625
18.375
18.34375
1.0 km
3.0 km
5.0 km

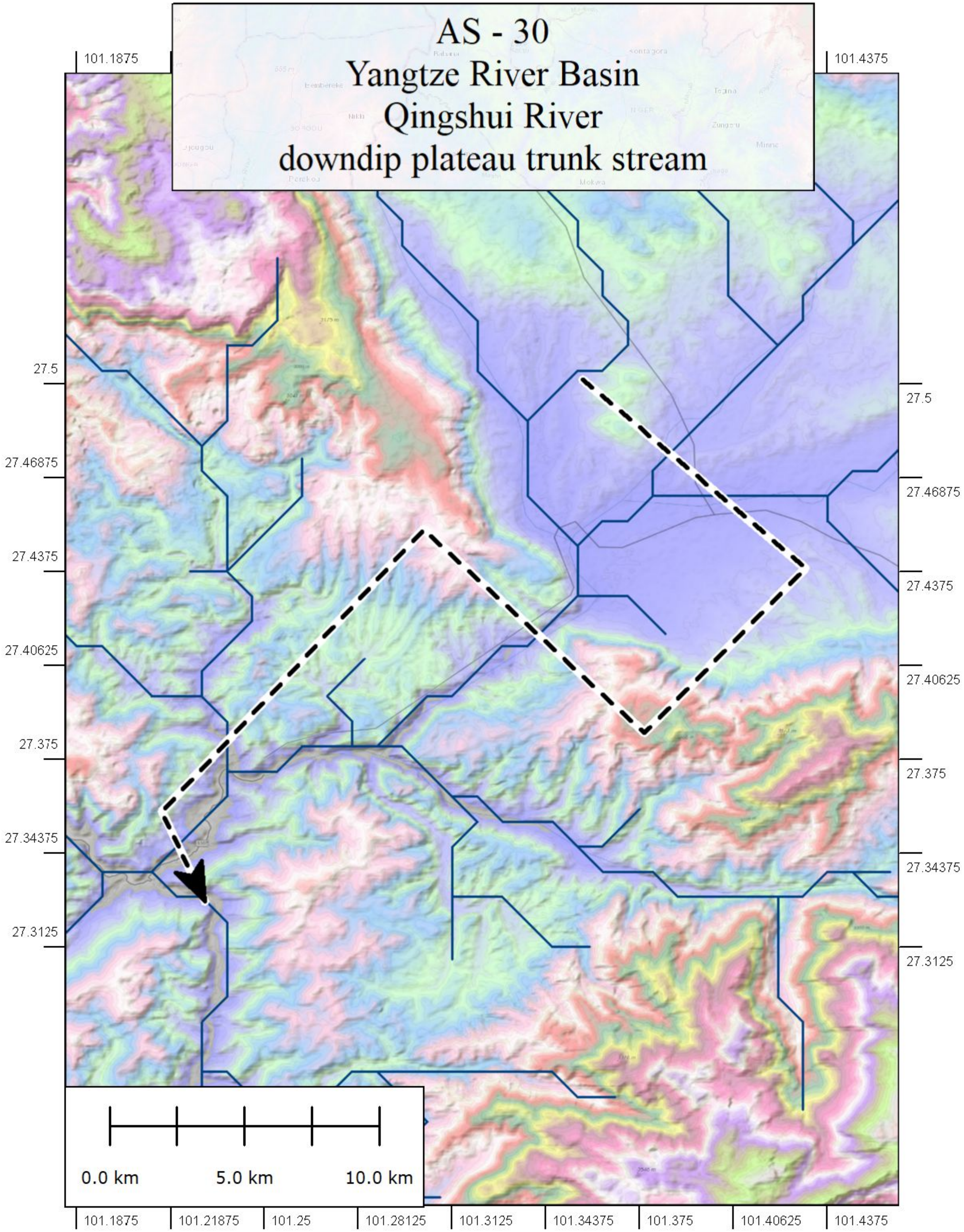
AS - 30
Yangtze River Basin
Qingshui River
downdip plateau trunk stream
101.1875
101.4375
27.5
27.46875
27.4375
27.40625
27.375
27.34375
27.3125
0.0 km
5.0 km
10.0 km
101.1875
101.21875
101.25
101.28125
101.3125
101.34375
101.375
101.40625
101.4375

AS - 45
Amur River Basin
Zeya River
irregular high ground trunk stream

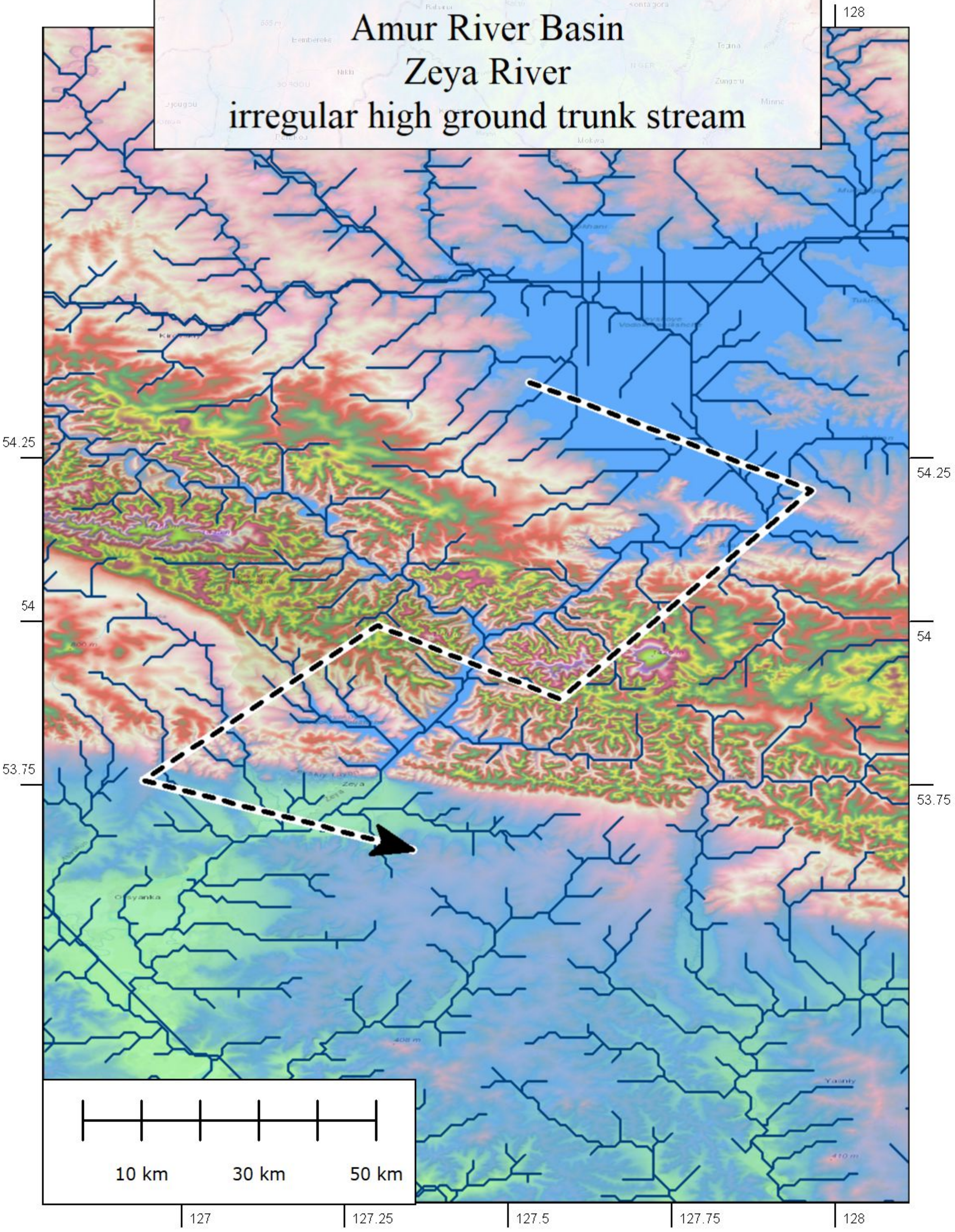

AS - 47
Indus River Basin
Indus River
updip plateau trunk stream

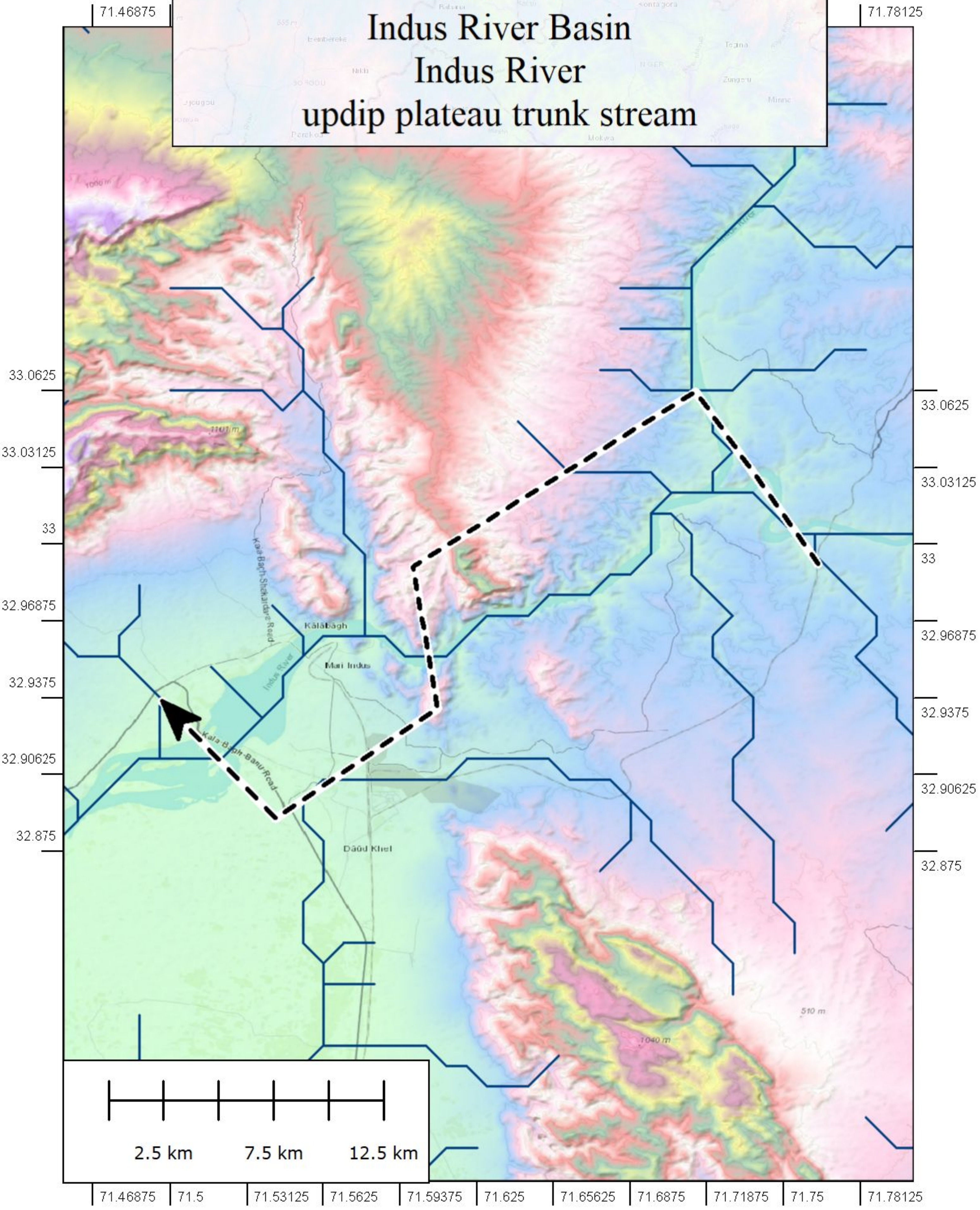

AS - 48
Ganges River Basin
Yarlung Zangbo River
irregular high ground trunk stream

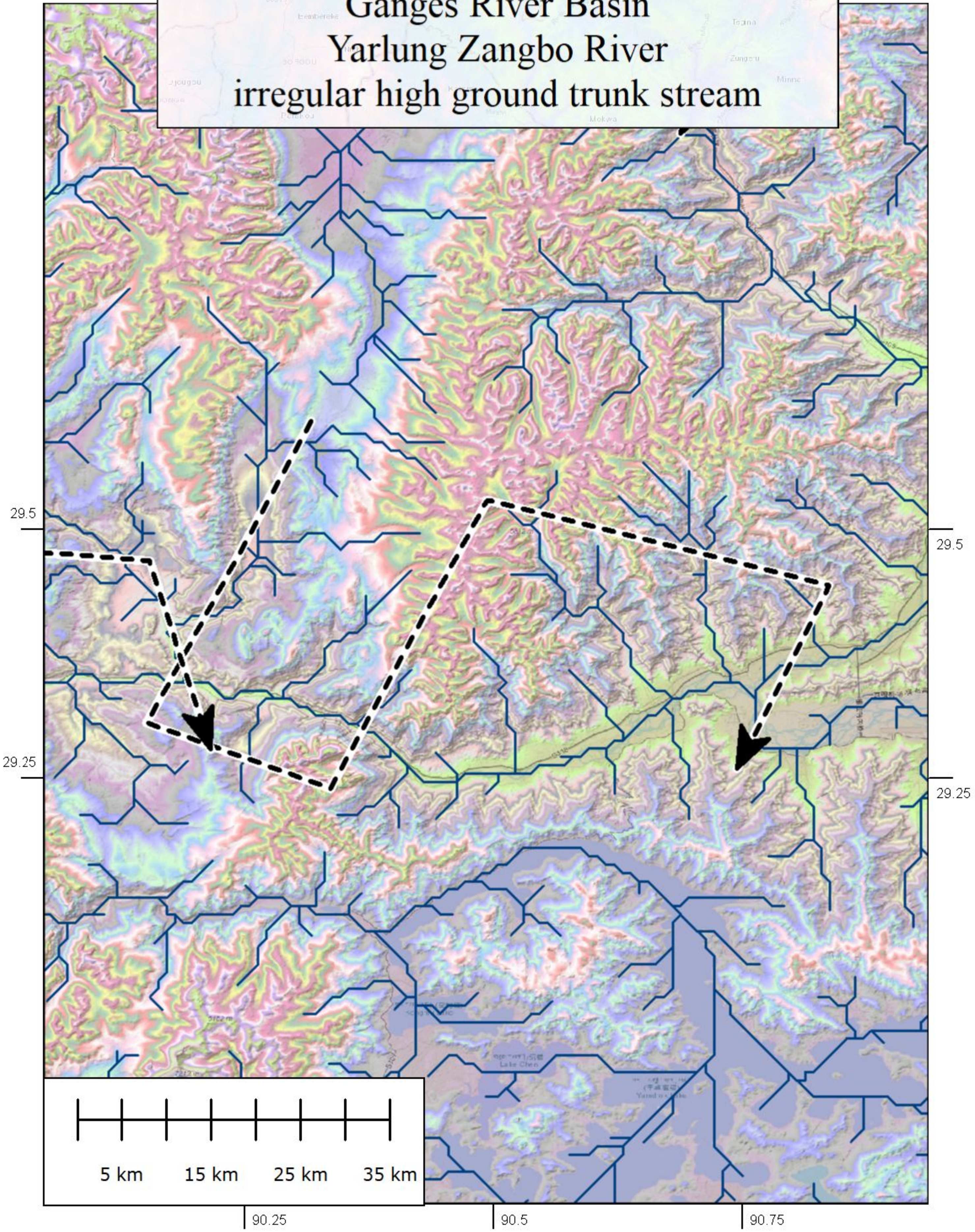

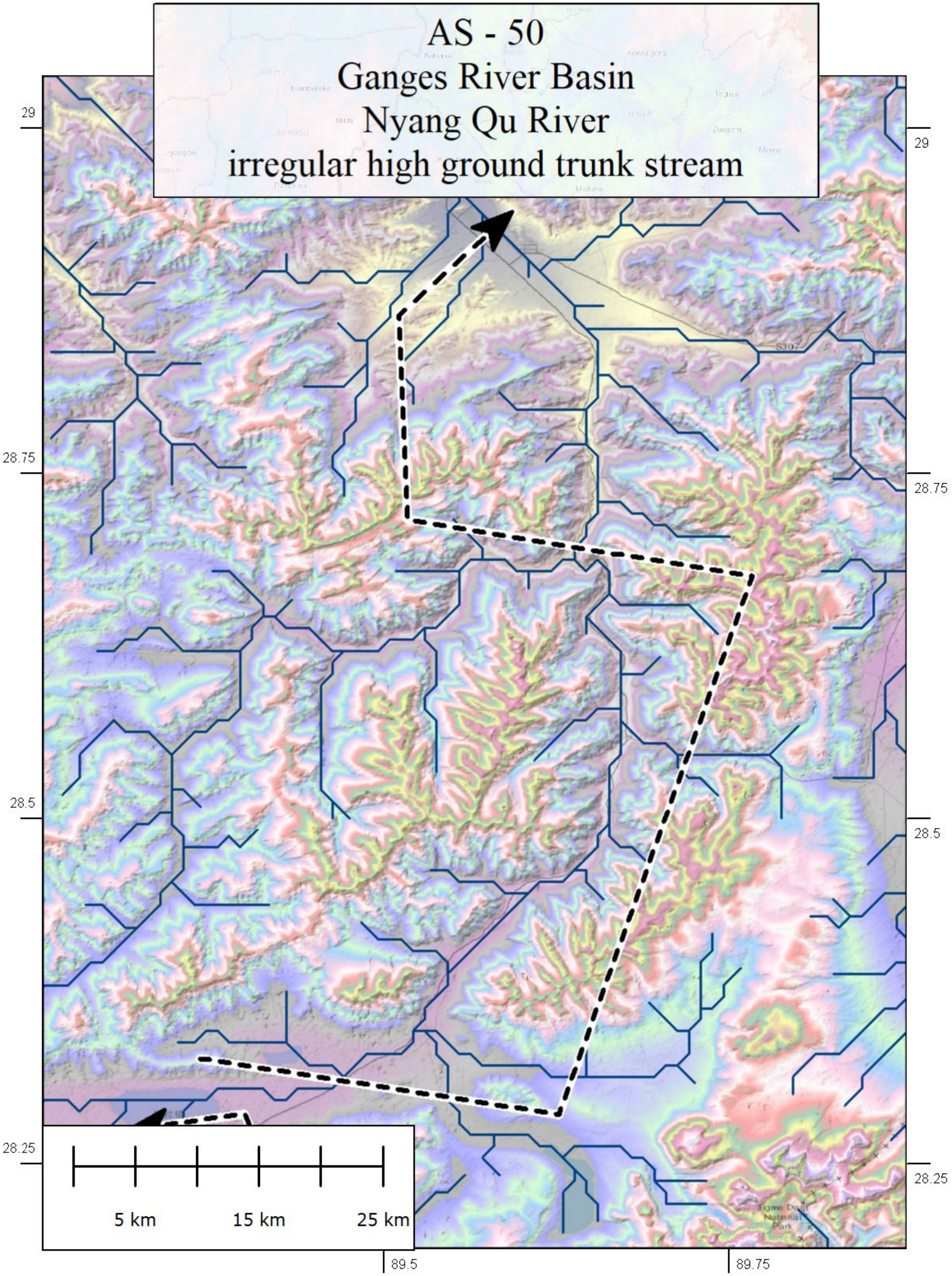
AS - 50
Ganges River Basin
Nyang Qu River
irregular high ground trunk stream
29
29
28.75
28.75
28.5
28.5
28.25
28.25
89.5
89.75
5 km
15 km
25 km

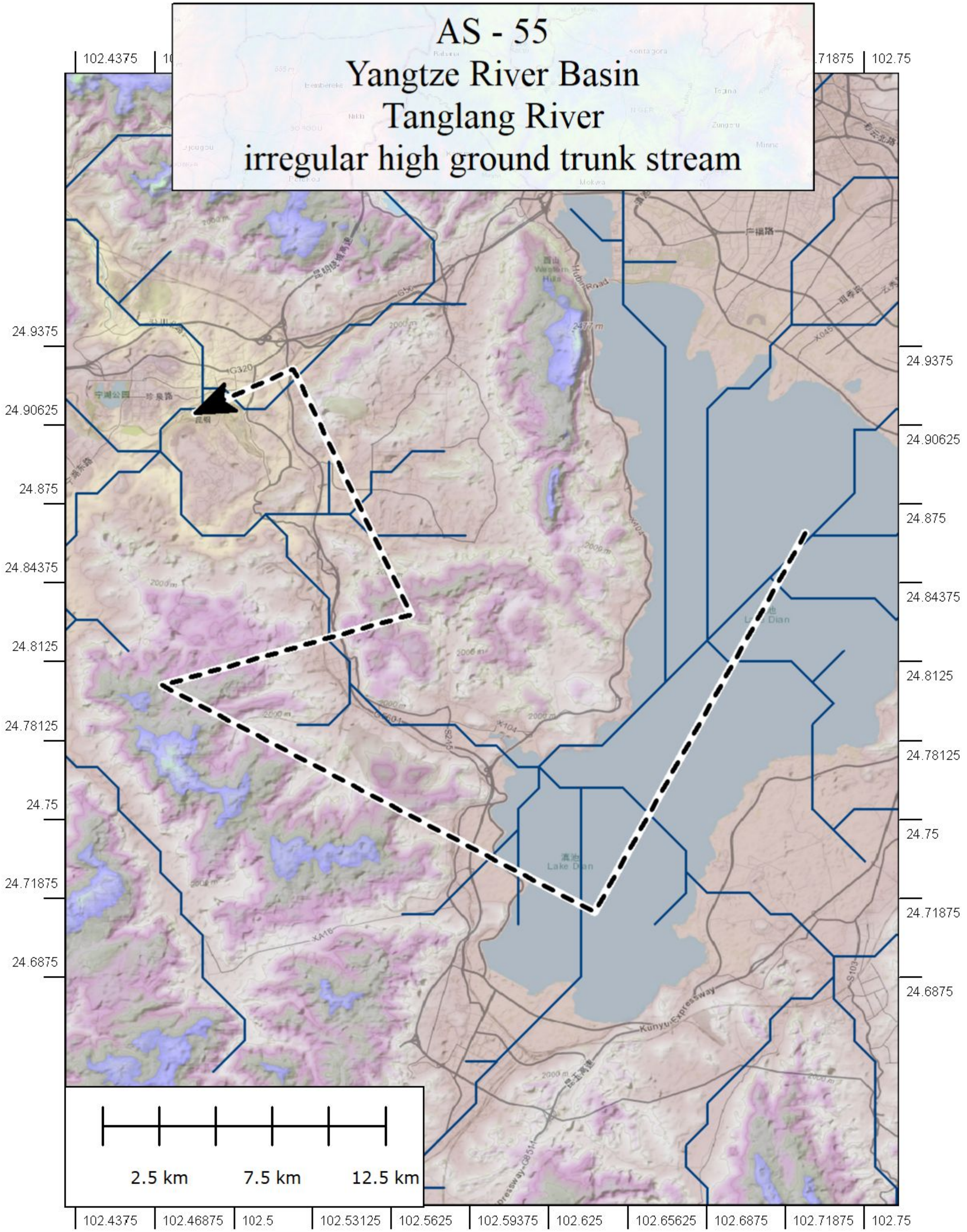
AS - 55
Yangtze River Basin
Tanglang River
irregular high ground trunk stream
2.5 km
7.5 km
12.5 km
102.4375
102.46875
102.5
102.53125
102.5625
102.59375
102.625
102.65625
102.6875
102.71875
102.75
24.9375
24.90625
24.875
24.84375
24.8125
24.78125
24.75
24.71875
24.6875
Lake Dian
Kunyu Expressway
Western Hills
Hubin Road

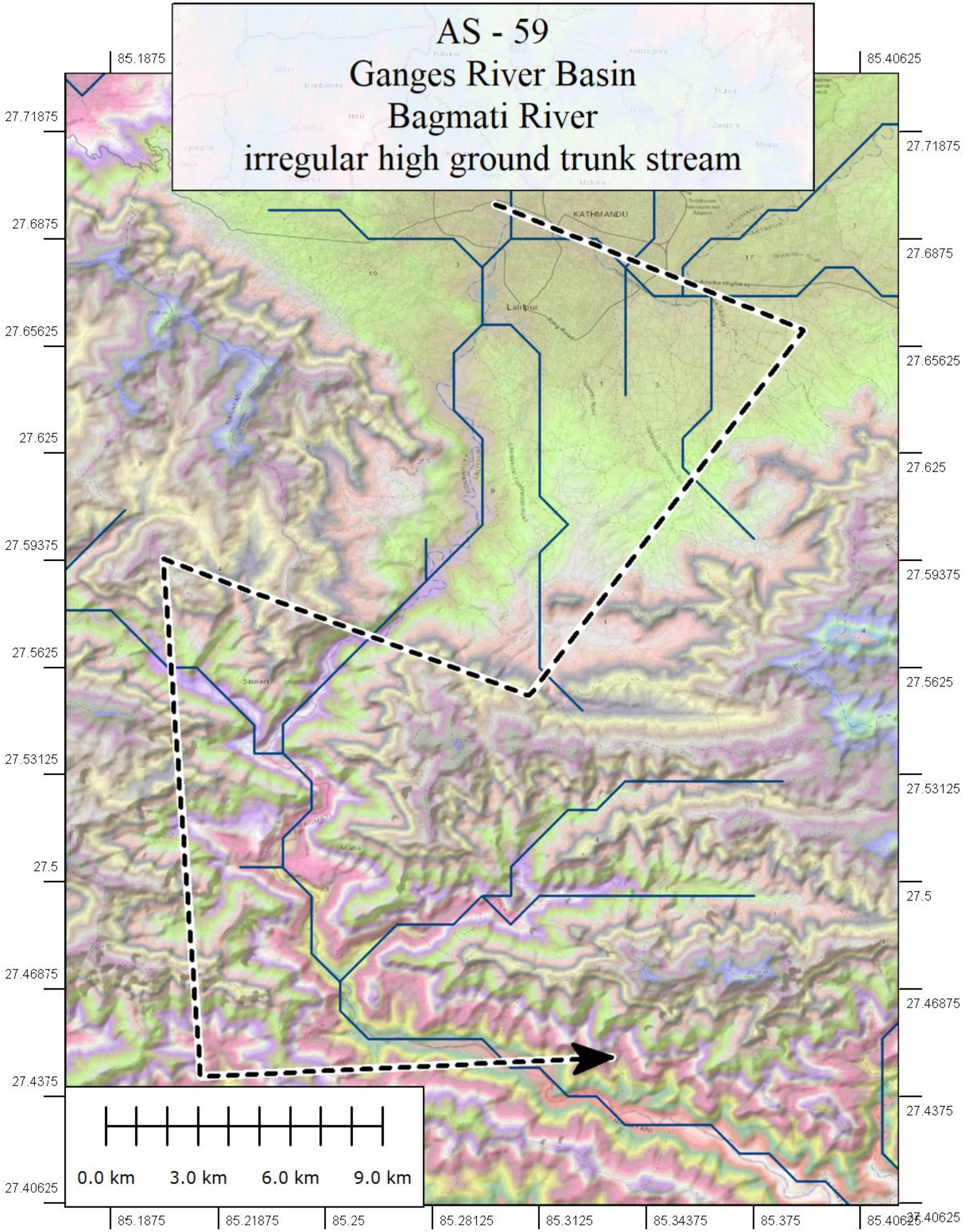
AS - 59
Ganges River Basin
Bagmati River
irregular high ground trunk stream
85.1875
85.21875
85.25
85.28125
85.3125
85.34375
85.375
85.40625
27.71875
27.6875
27.65625
27.625
27.59375
27.5625
27.53125
27.5
27.46875
27.4375
27.40625
KATHMANDU
Lalitpur
0.0 km
3.0 km
6.0 km
9.0 km

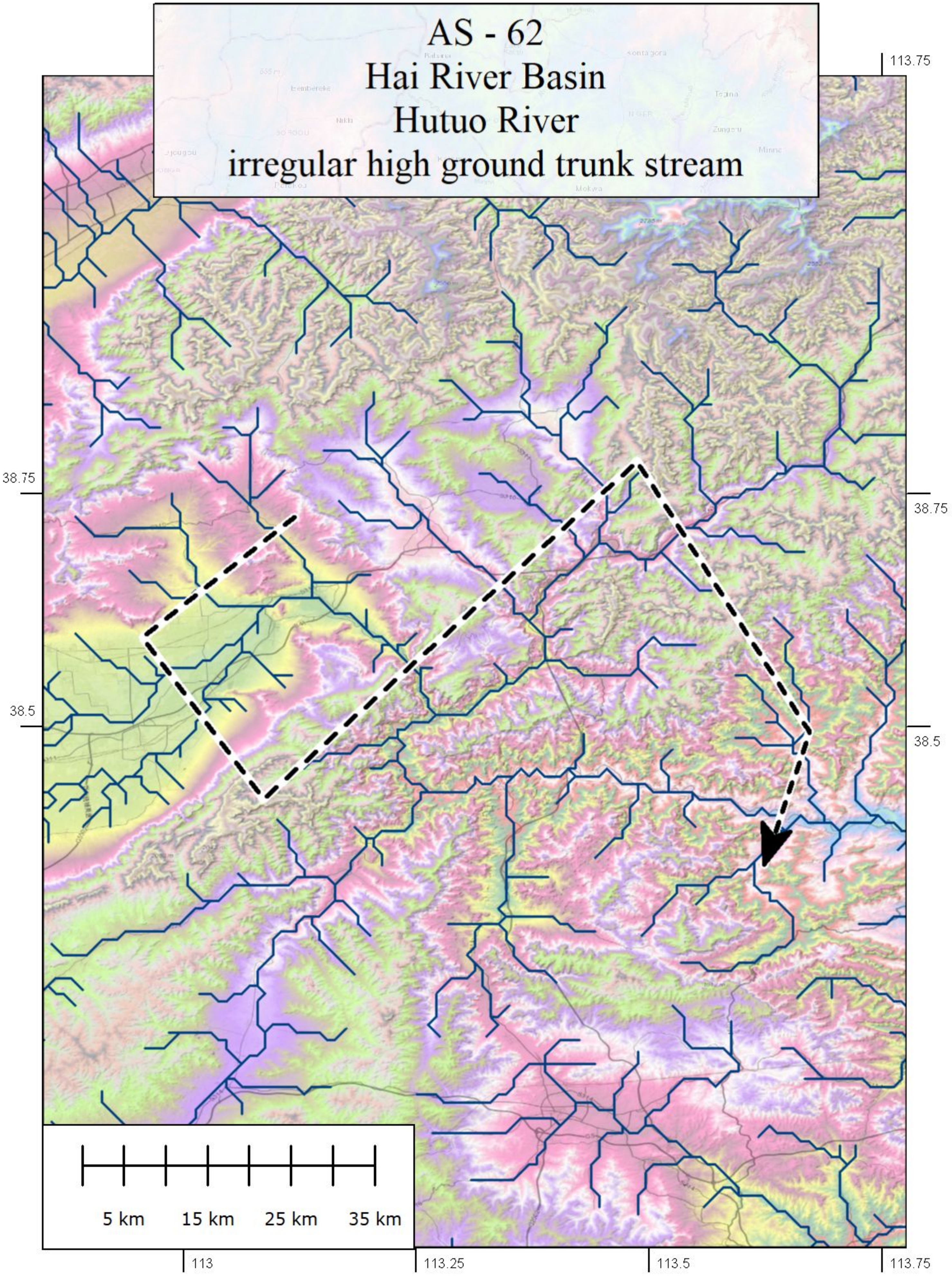
AS - 62
Hai River Basin
Hutuo River
irregular high ground trunk stream
113.75
38.75
38.75
38.5
38.5
5 km
15 km
25 km
35 km
113
113.25
113.5
113.75

AS - 63
Amur River Basin
Herlen River
irregular high ground trunk stream

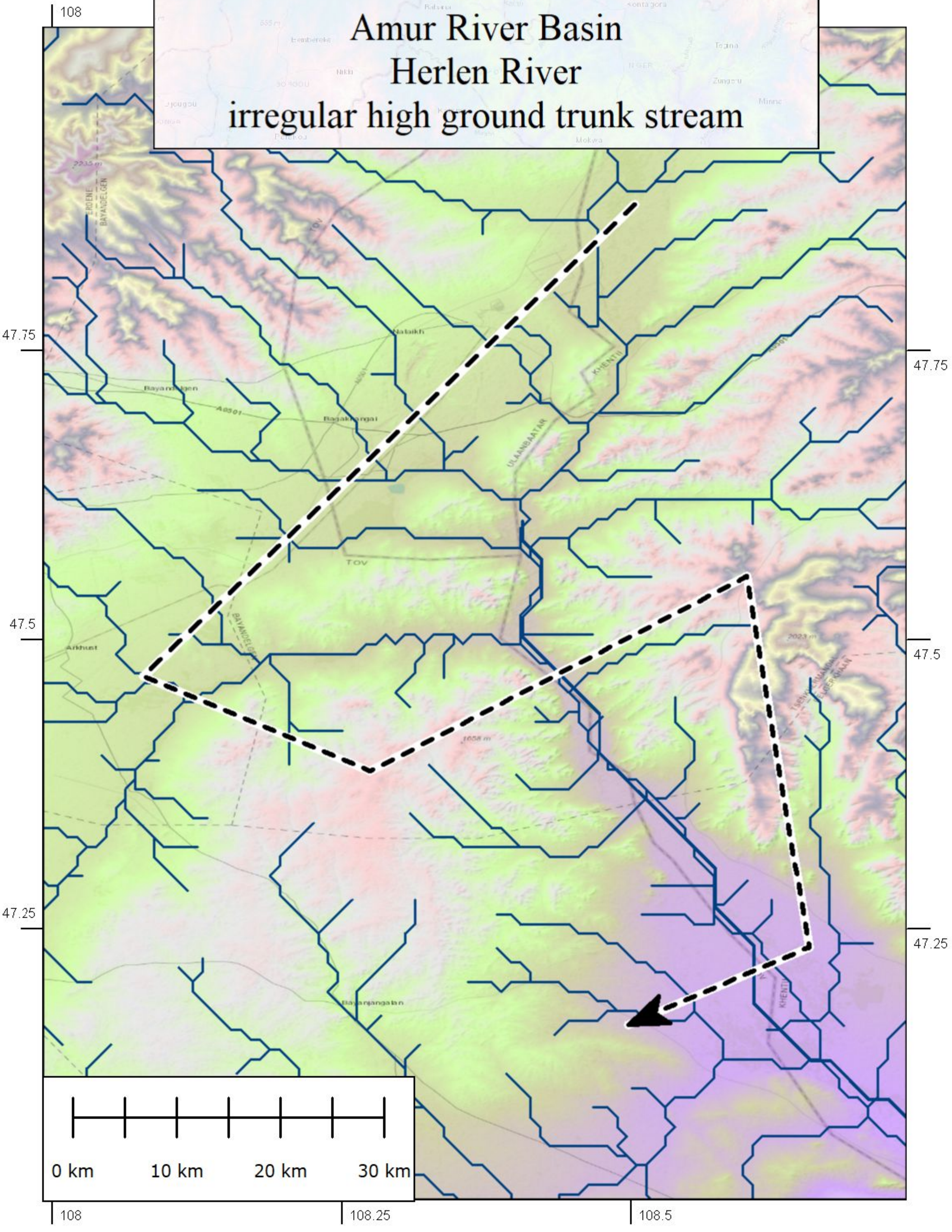

AS - 67
Irrawaddy River Basin
Myitnge River
updip plateau trunk stream

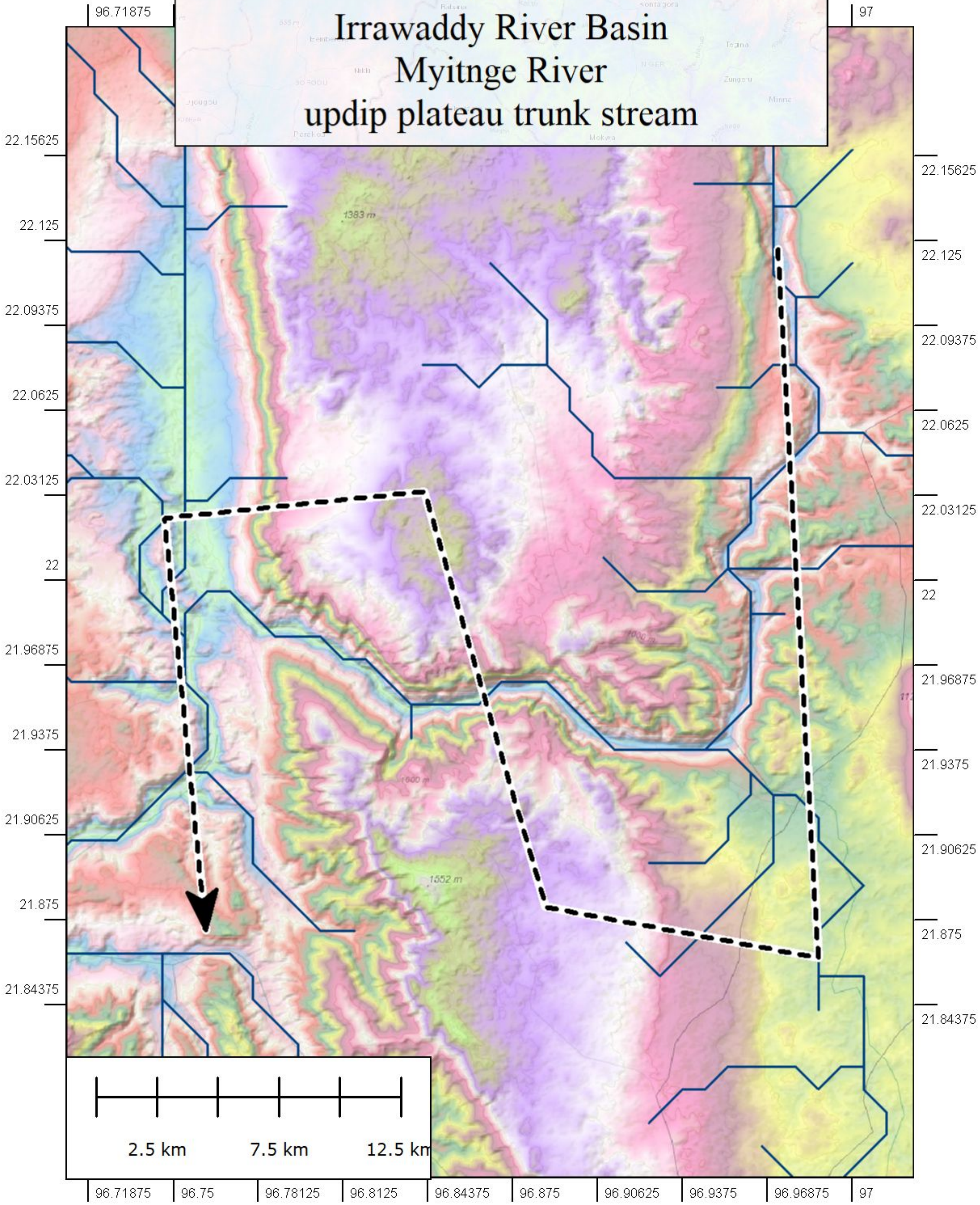

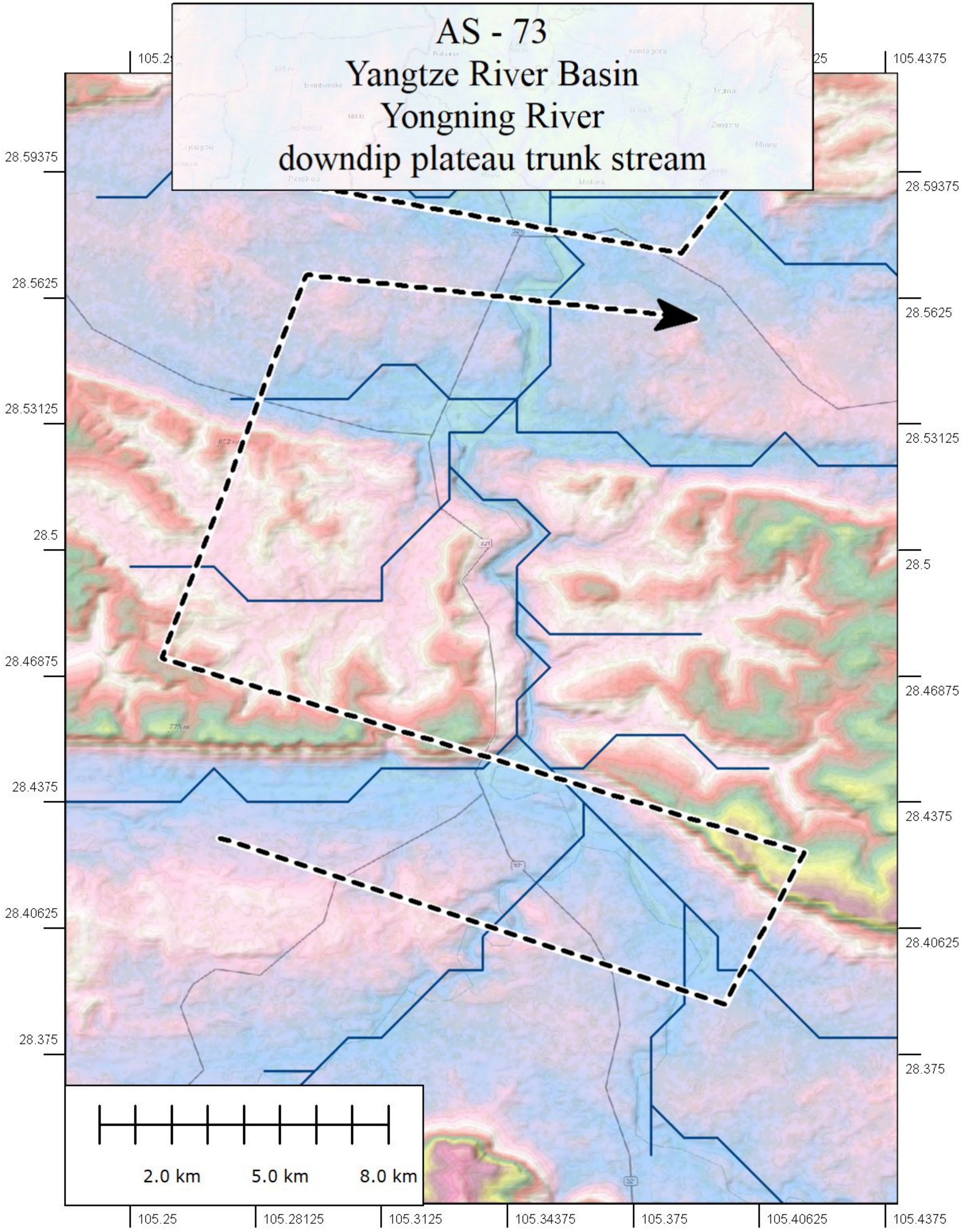
AS - 73
Yangtze River Basin
Yongning River
downdip plateau trunk stream
105.25
105.28125
105.3125
105.34375
105.375
105.40625
105.4375
28.59375
28.5625
28.53125
28.5
28.46875
28.4375
28.40625
28.375
2.0 km
5.0 km
8.0 km

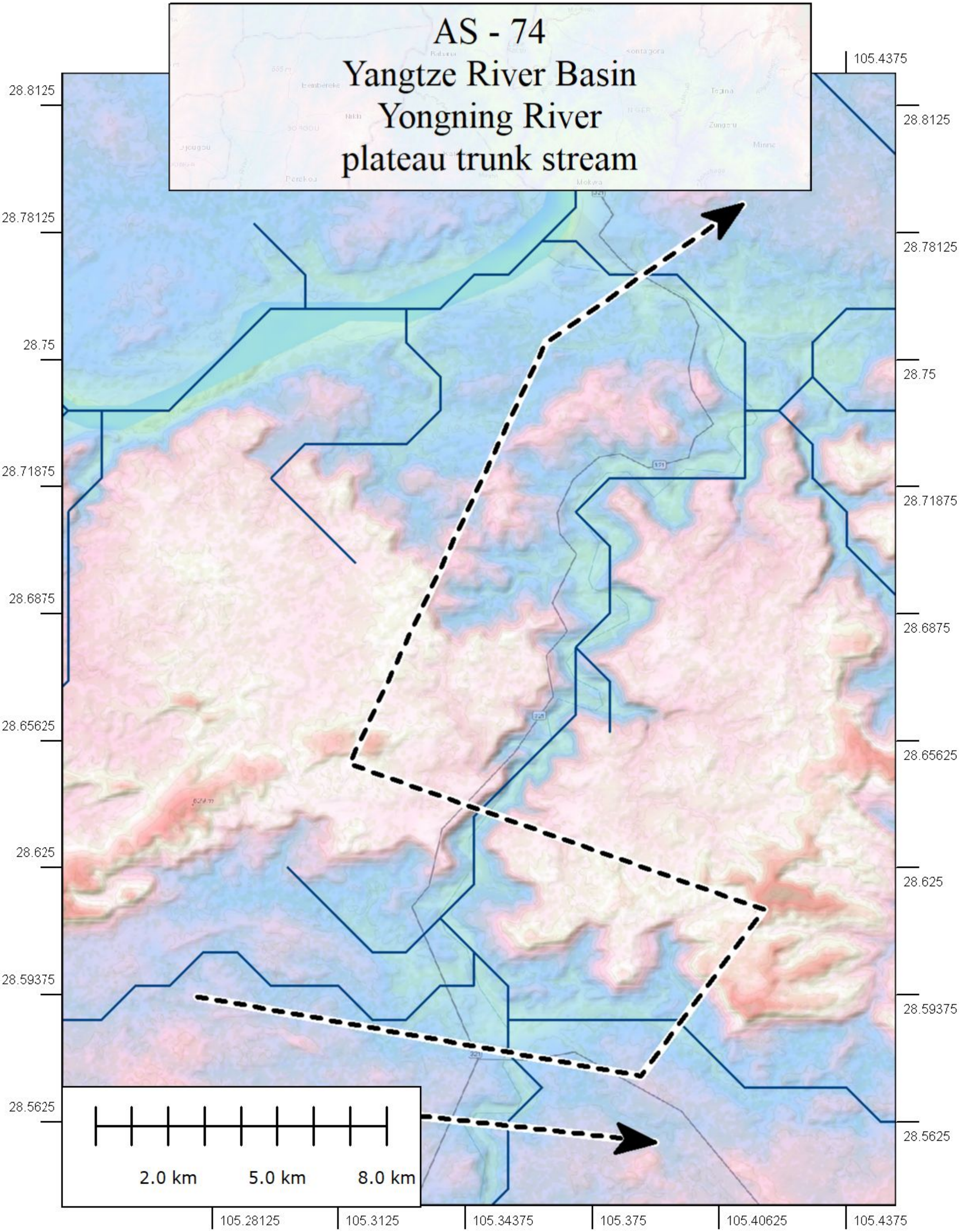
AS - 74
Yangtze River Basin
Yongning River
plateau trunk stream
28.8125
28.78125
28.75
28.71875
28.6875
28.65625
28.625
28.59375
28.5625
105.4375
105.28125
105.3125
105.34375
105.375
105.40625
2.0 km
5.0 km
8.0 km

AS - 77
Indus River Basin
Daro Jhal River
irregular high ground trunk stream

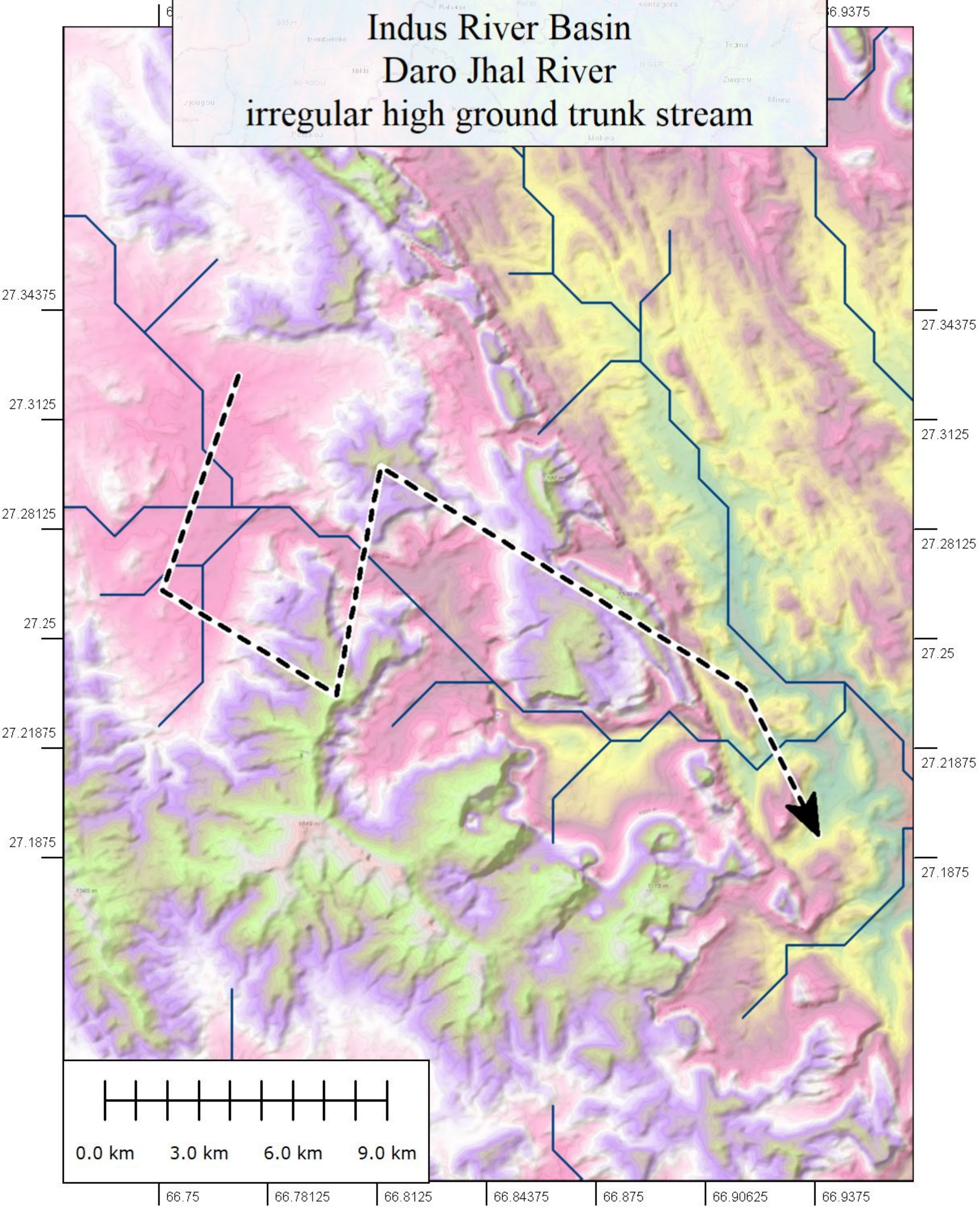

AS - 82
Xi River Basin
Hongshui River
downdip plateau trunk stream

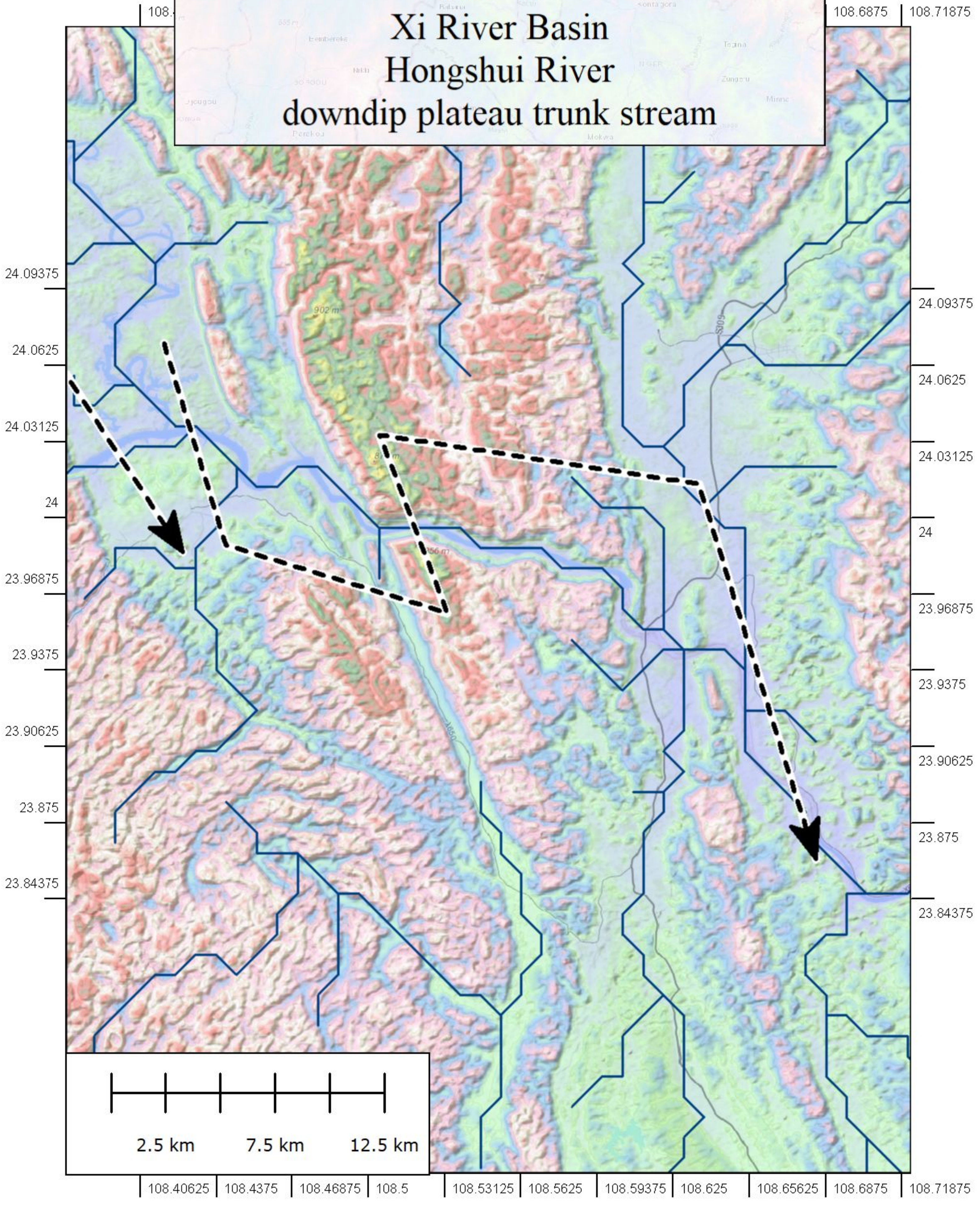

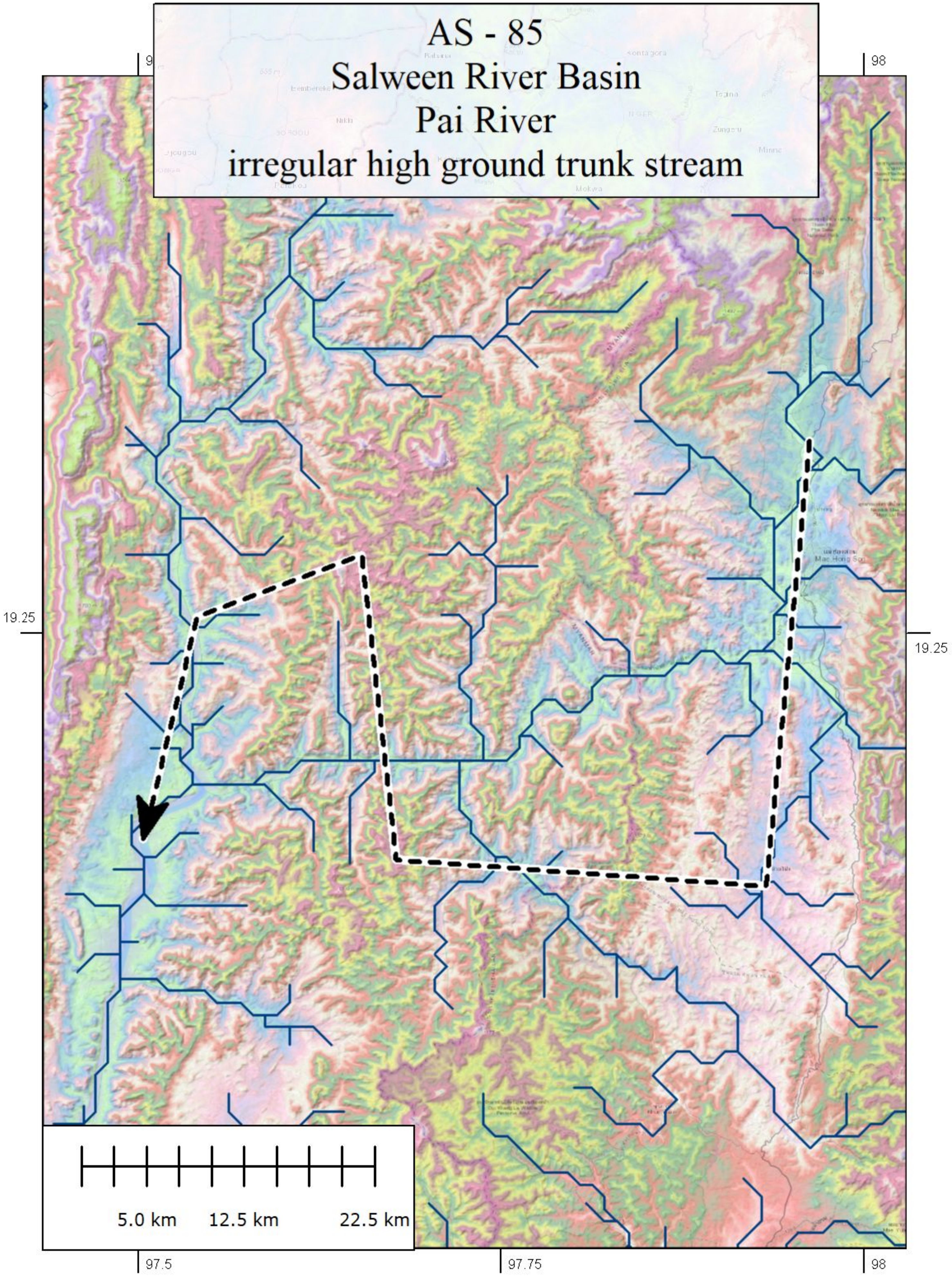
AS - 85
Salween River Basin
Pai River
irregular high ground trunk stream
98
19.25
19.25
5.0 km
12.5 km
22.5 km
97.5
97.75
98

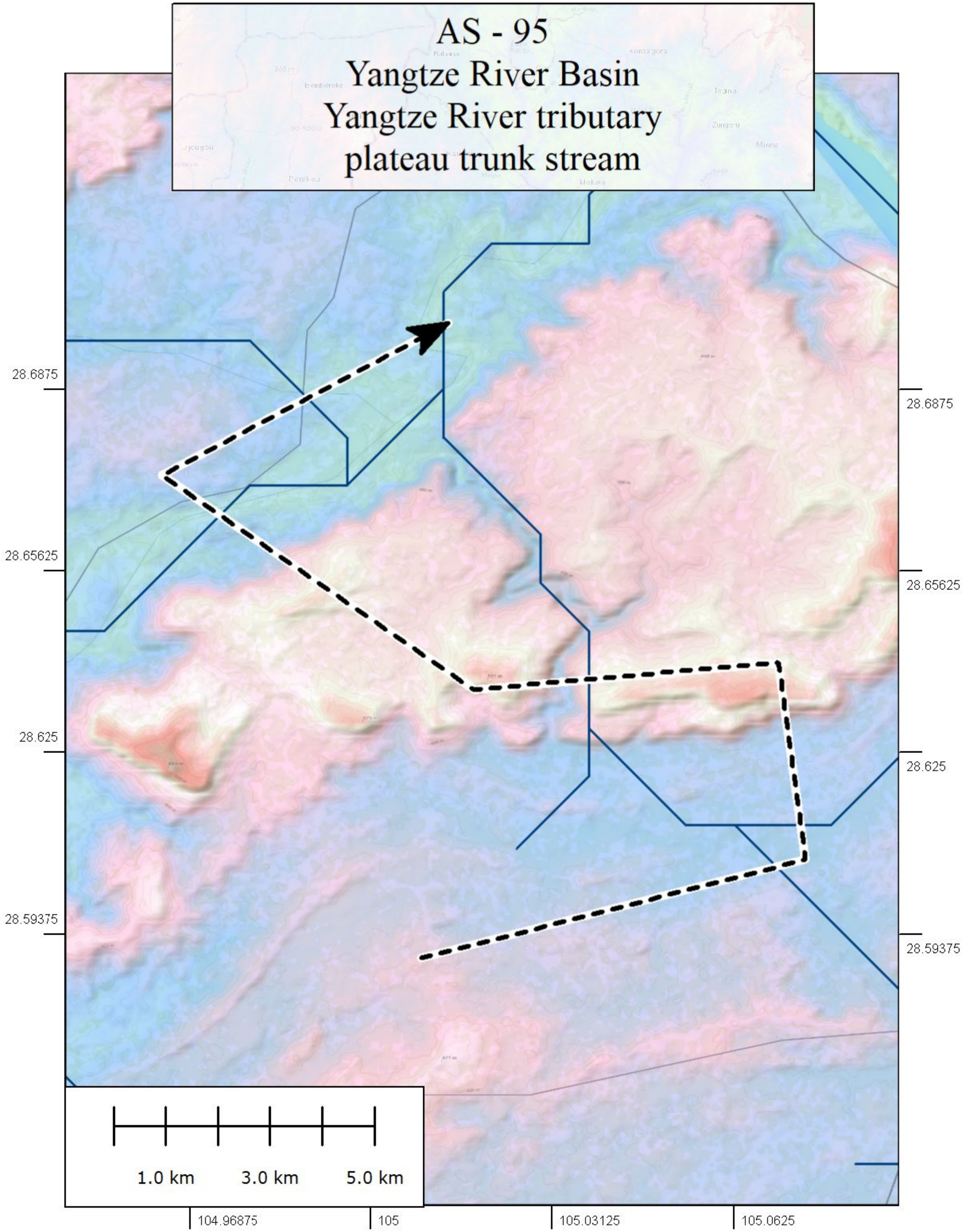
AS - 95
Yangtze River Basin
Yangtze River tributary
plateau trunk stream
28.6875
28.65625
28.625
28.59375
1.0 km
3.0 km
5.0 km
104.96875
105
105.03125
105.0625

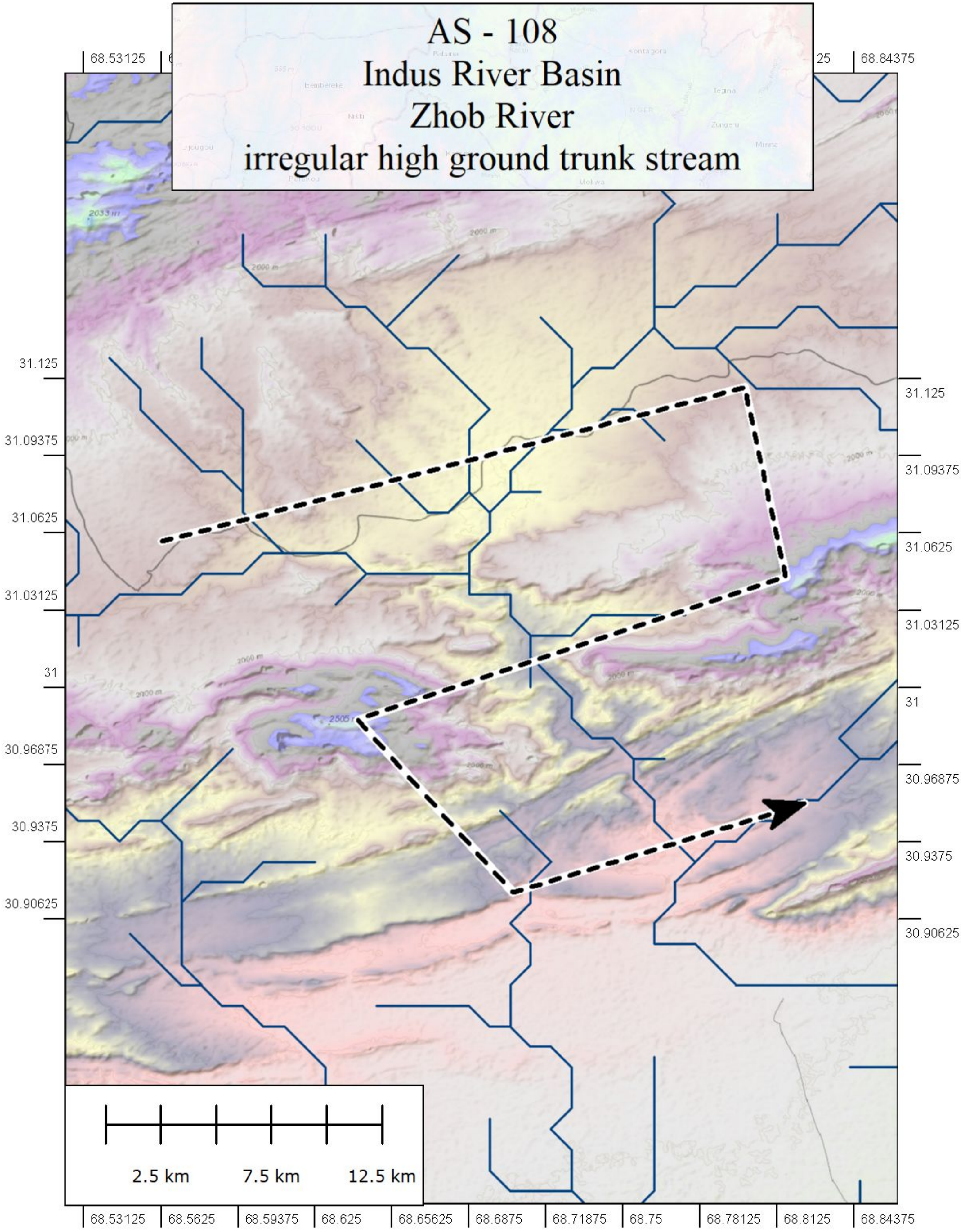

AS - 108
Indus River Basin
Zhob River
irregular high ground trunk stream
68.53125
68.5625
68.59375
68.625
68.65625
68.6875
68.71875
68.75
68.78125
68.8125
68.84375
31.125
31.09375
31.0625
31.03125
31
30.96875
30.9375
30.90625
2033 m
2000 m
2505 m
2.5 km
7.5 km
12.5 km

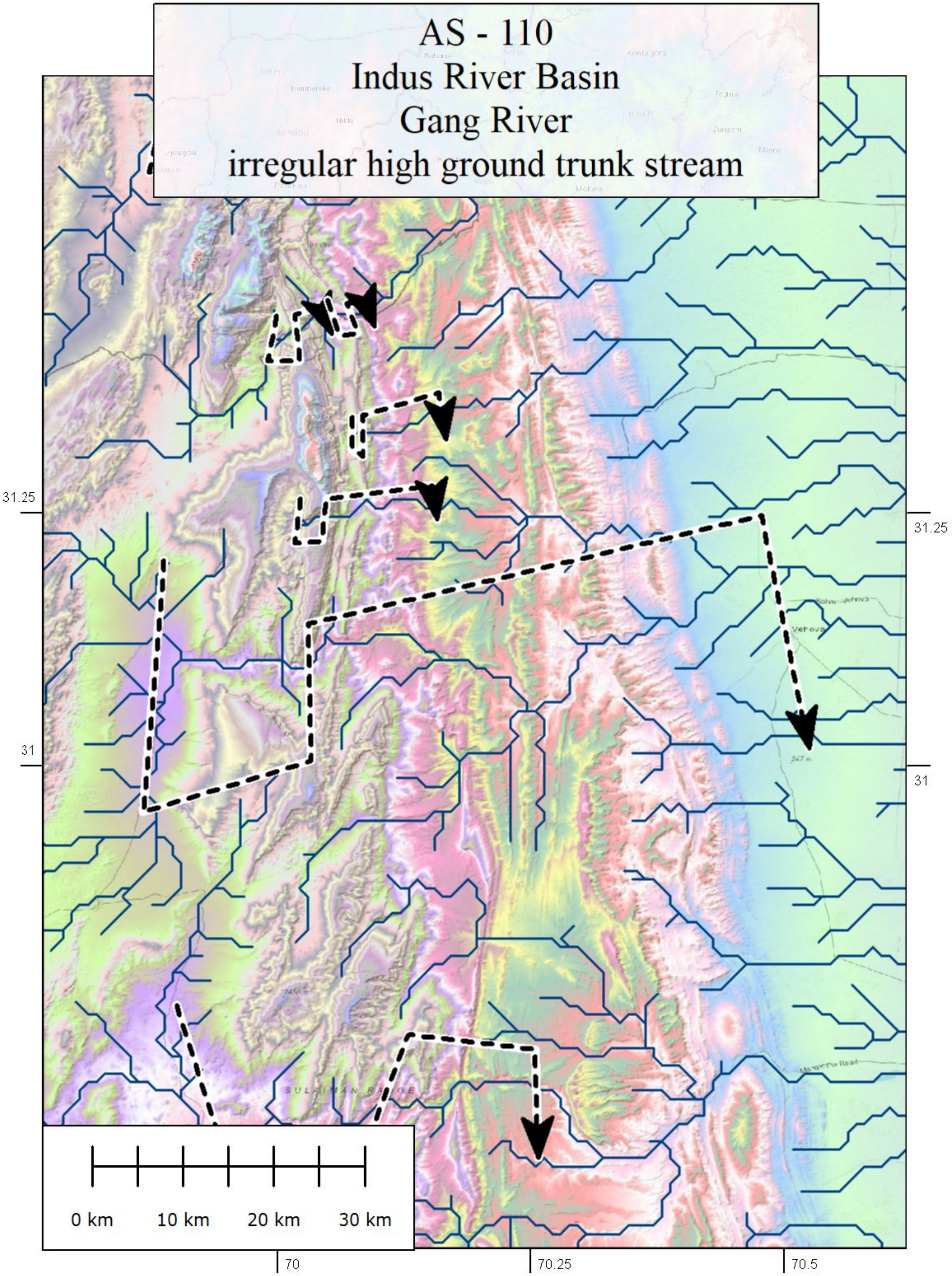
AS - 110
Indus River Basin
Gang River
irregular high ground trunk stream
31.25
31
70
70.25
70.5
0 km
10 km
20 km
30 km

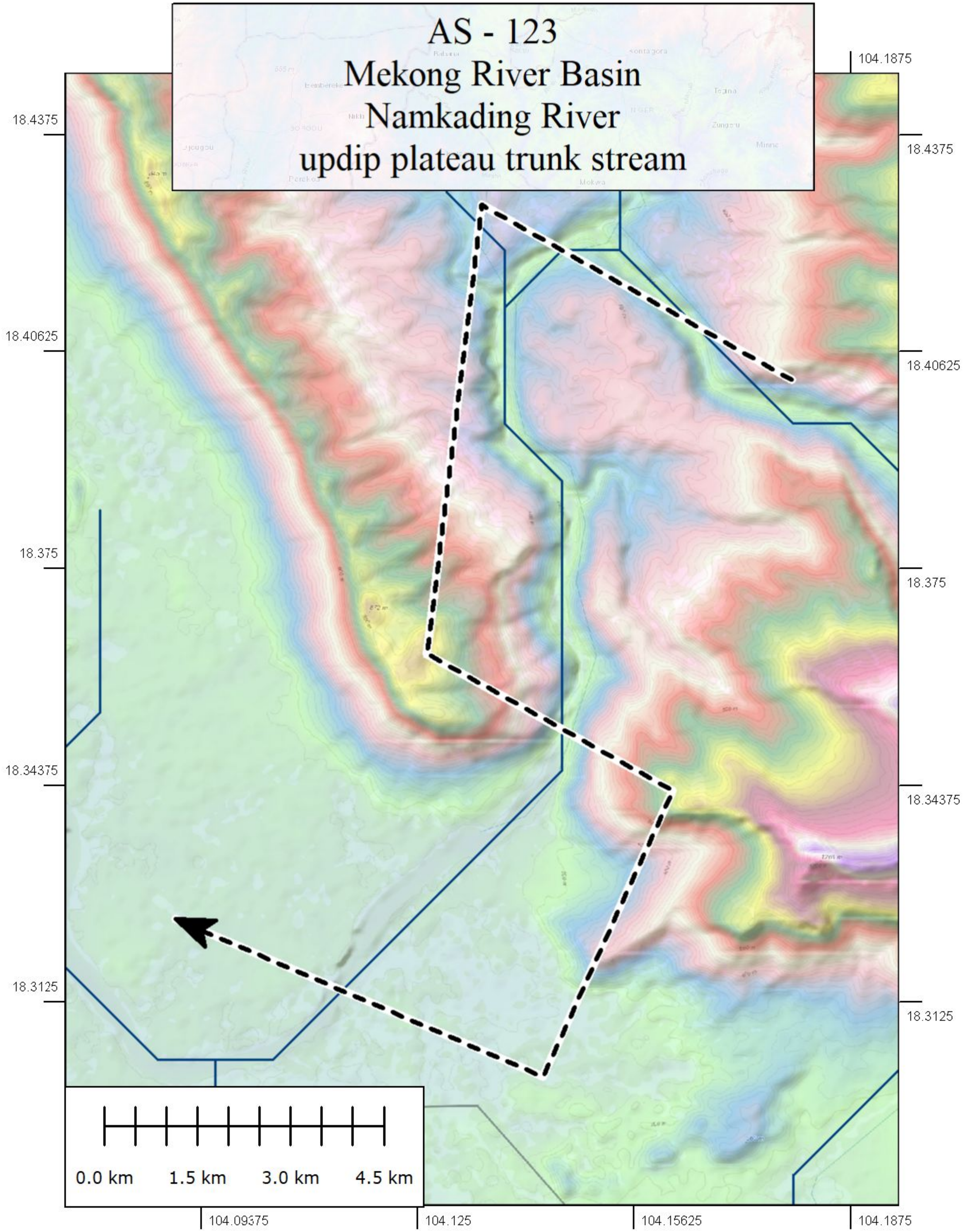
AS - 123
Mekong River Basin
Namkading River
updip plateau trunk stream
104.1875
18.4375
18.40625
18.375
18.34375
18.3125
0.0 km
1.5 km
3.0 km
4.5 km
104.09375
104.125
104.15625
104.1875

AS - 124
Mekong River Basin
Namkading River tributary
downdip plateau trunk stream

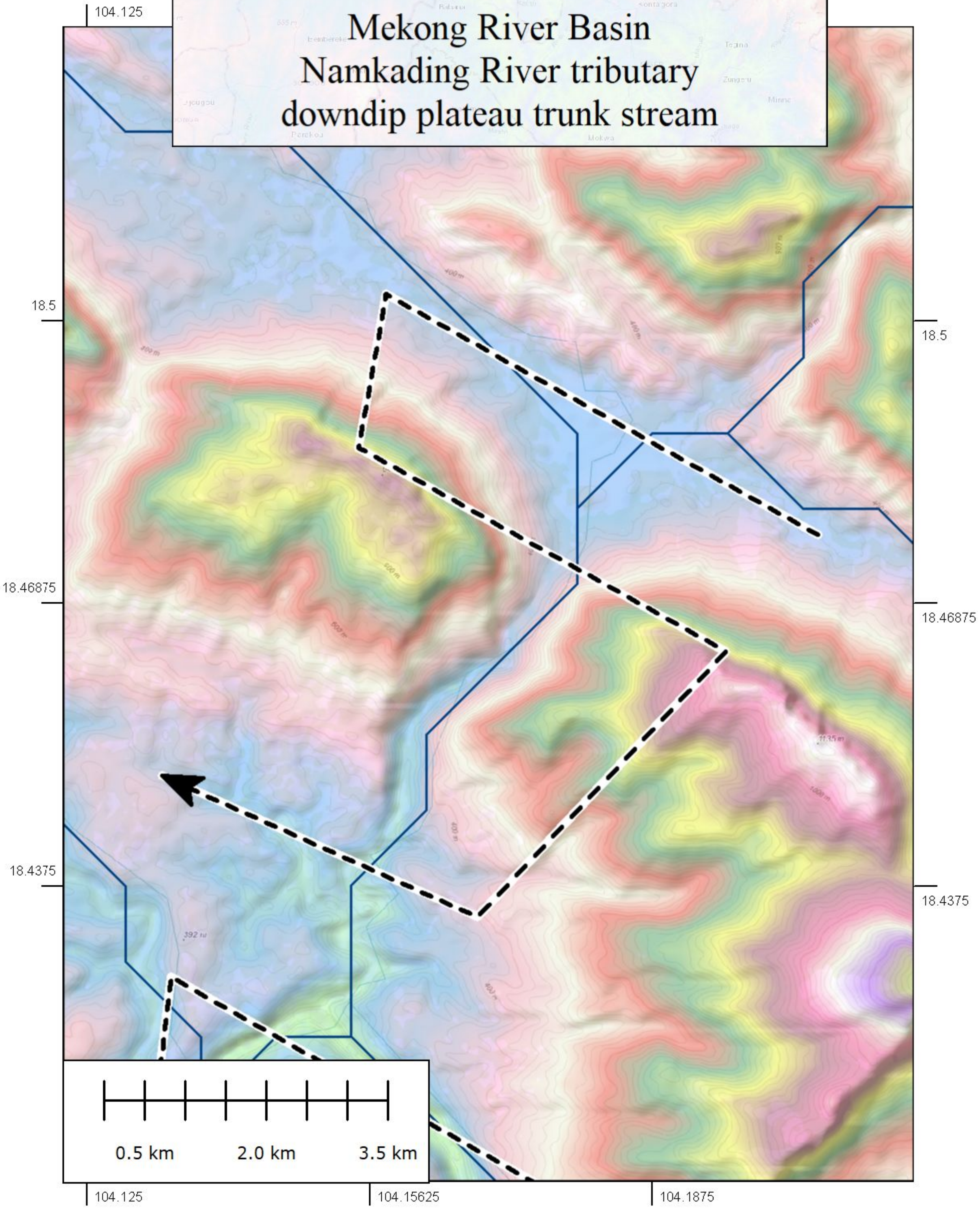

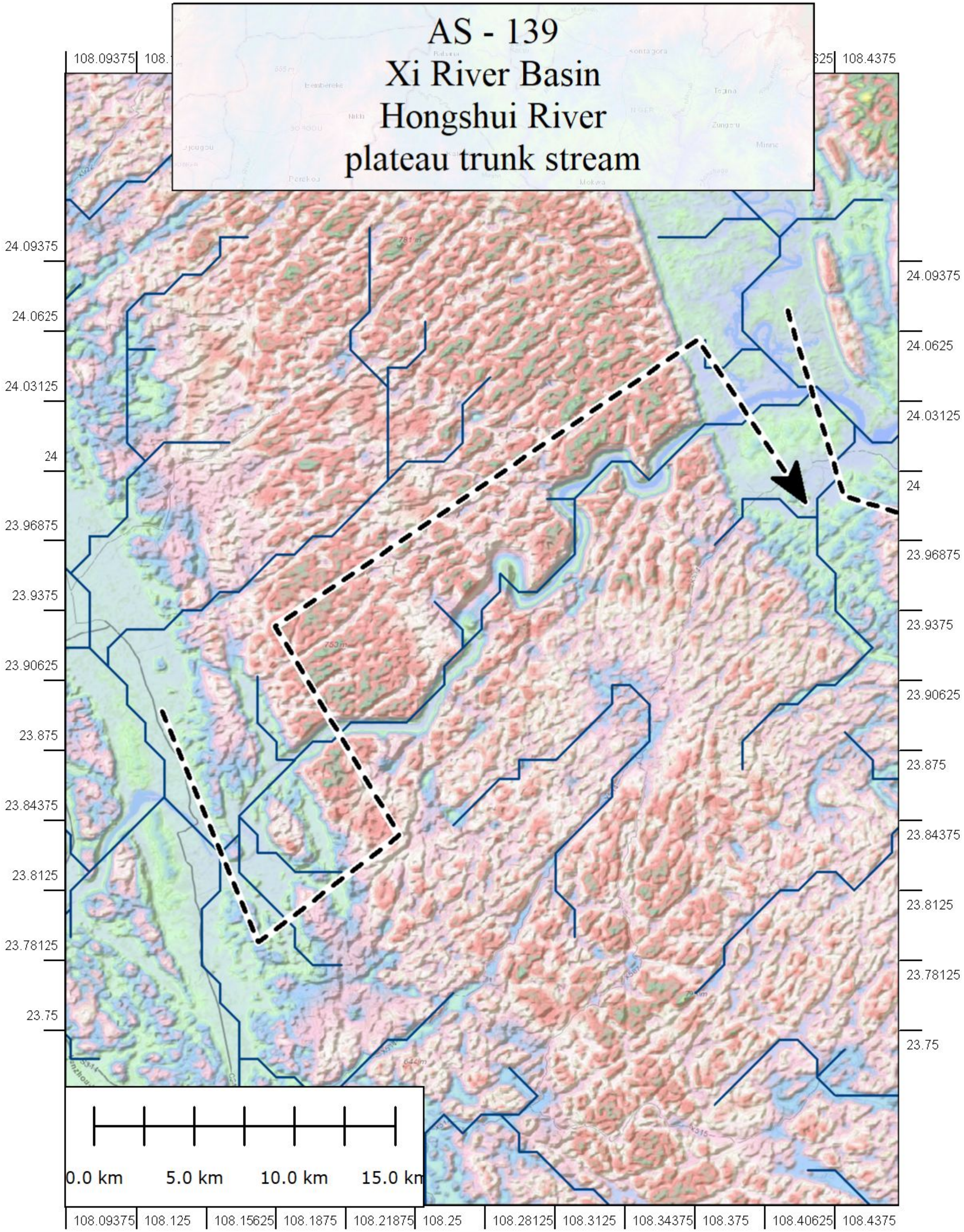
AS - 139
Xi River Basin
Hongshui River
plateau trunk stream
108.09375
108.125
108.15625
108.1875
108.21875
108.25
108.28125
108.3125
108.34375
108.375
108.40625
108.4375
24.09375
24.0625
24.03125
24
23.96875
23.9375
23.90625
23.875
23.84375
23.8125
23.78125
23.75
0.0 km
5.0 km
10.0 km
15.0 km

AS - 142
Chao Phraya Basin
Khwae Noi River
downdip plateau trunk stream

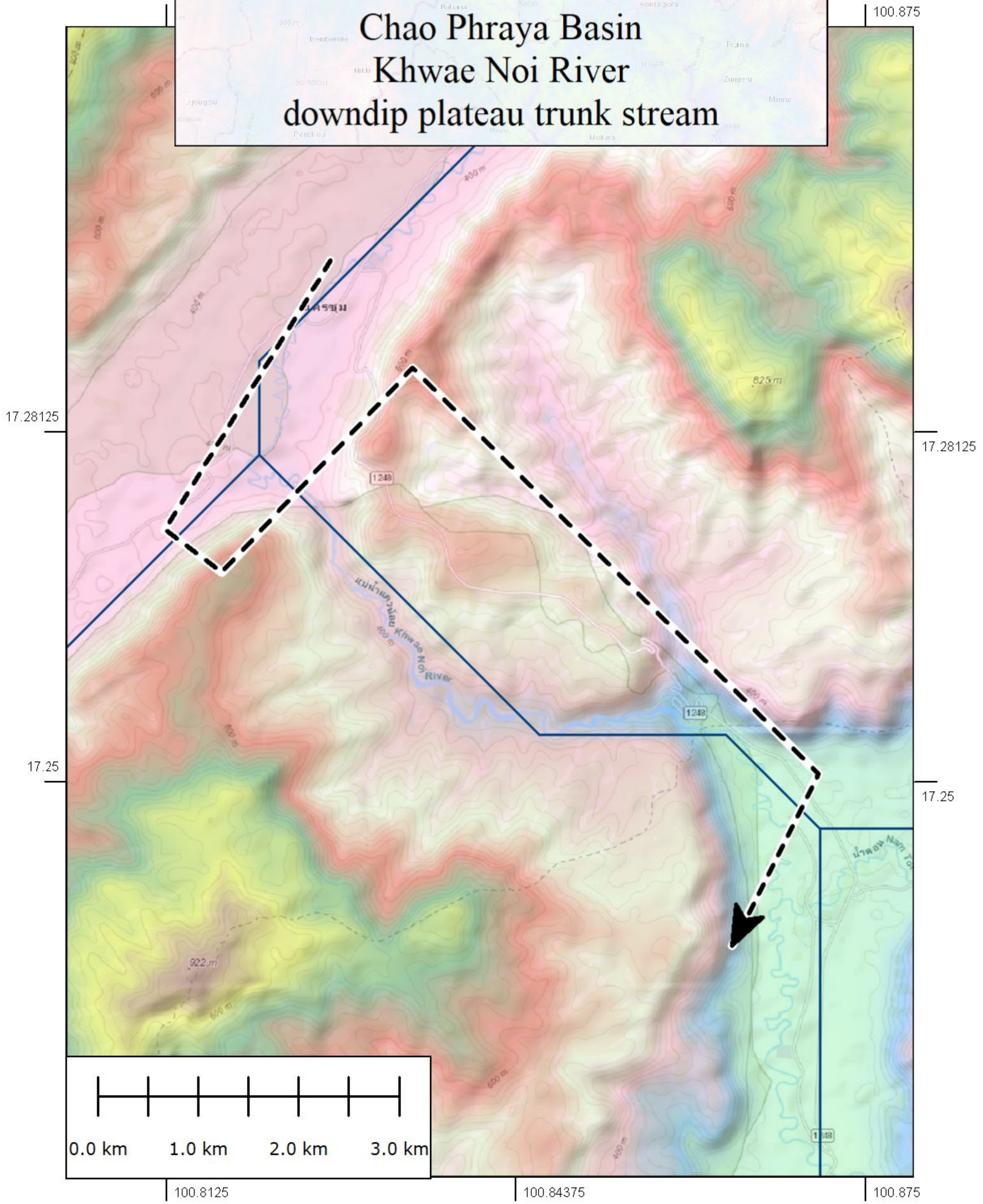

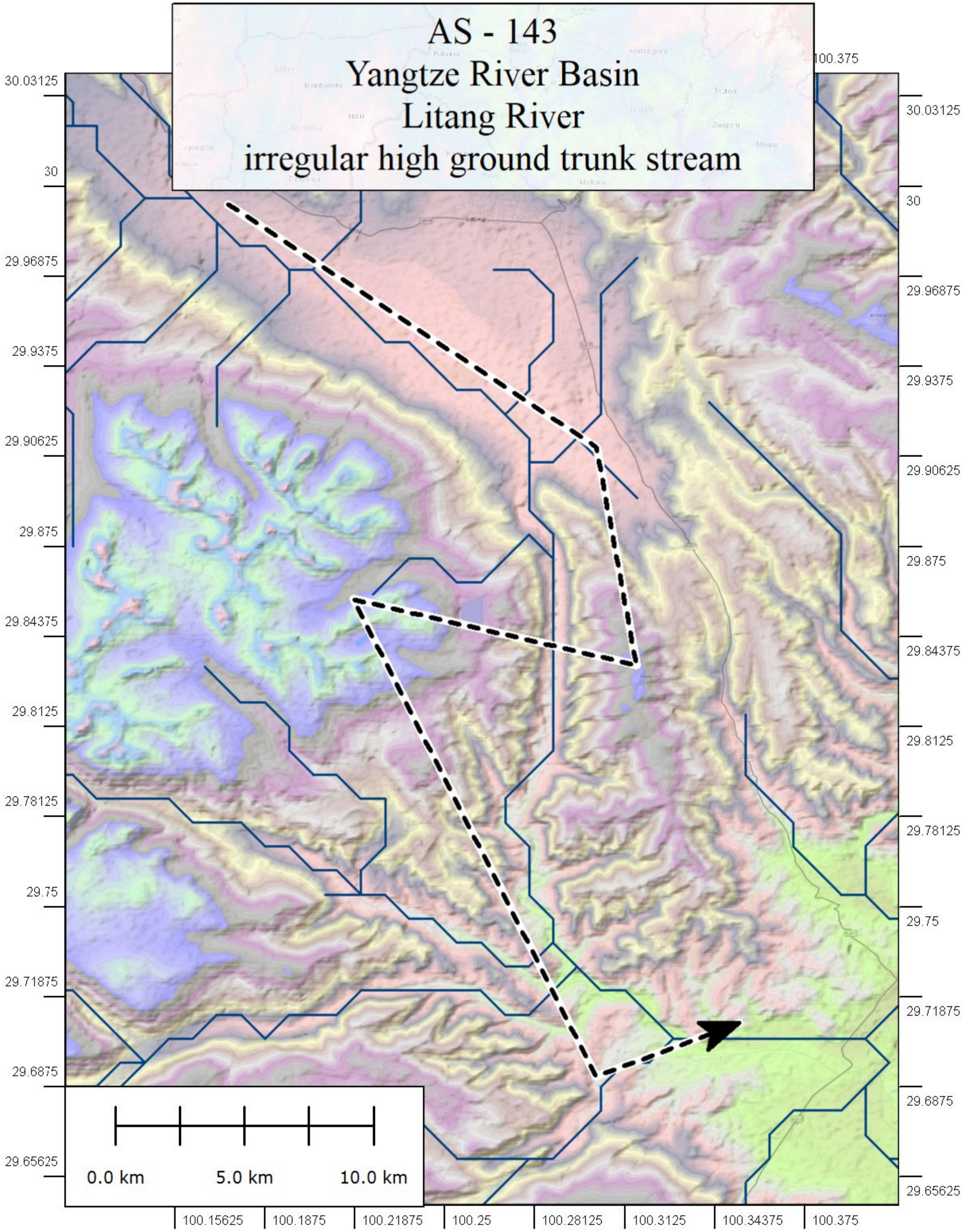
AS - 143
Yangtze River Basin
Litang River
irregular high ground trunk stream
30.03125
30
29.96875
29.9375
29.90625
29.875
29.84375
29.8125
29.78125
29.75
29.71875
29.6875
29.65625
100.15625
100.1875
100.21875
100.25
100.28125
100.3125
100.34375
100.375
0.0 km
5.0 km
10.0 km

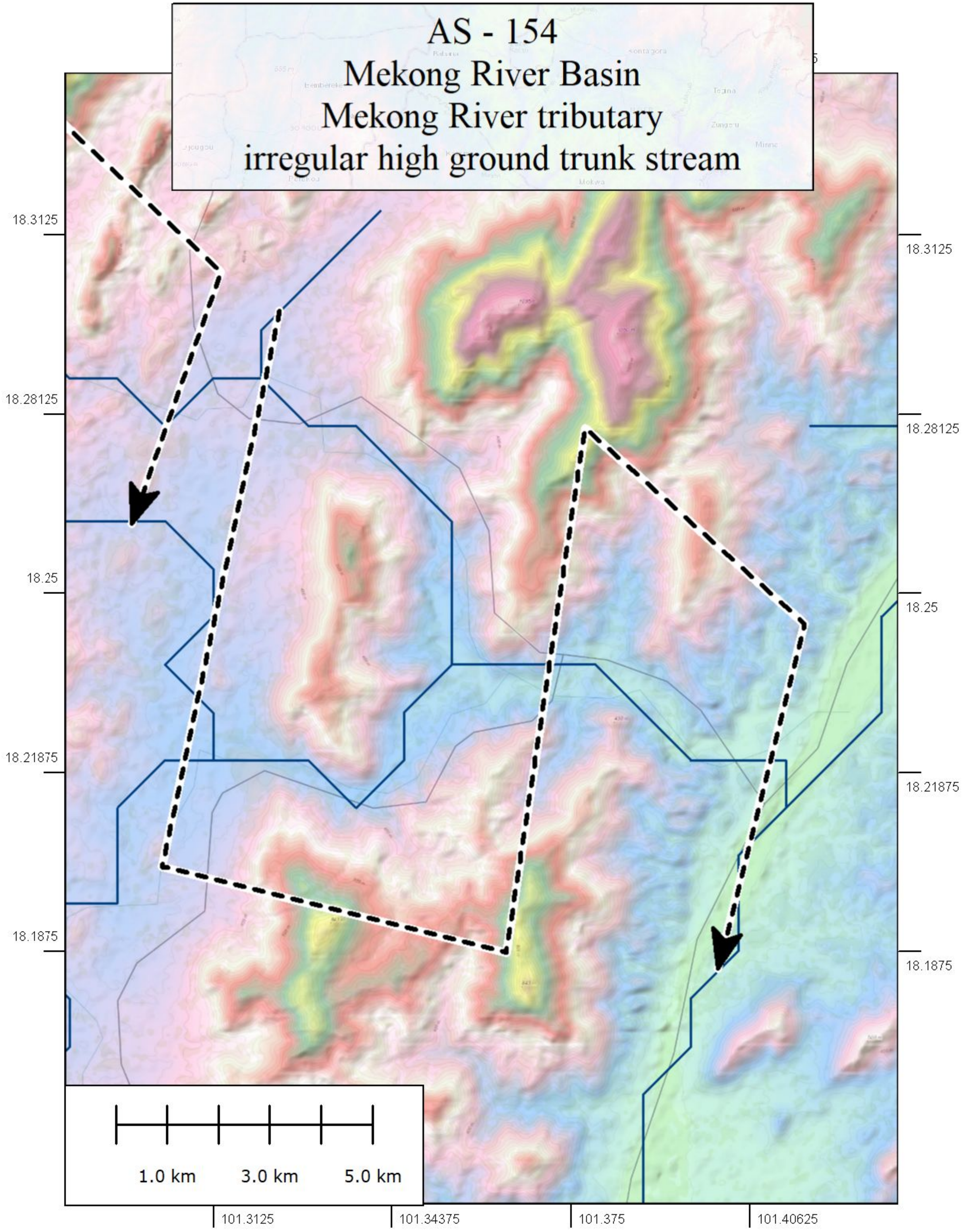

AS - 154
Mekong River Basin
Mekong River tributary
irregular high ground trunk stream
18.3125
18.28125
18.25
18.21875
18.1875
1.0 km
3.0 km
5.0 km
101.3125
101.34375
101.375
101.40625

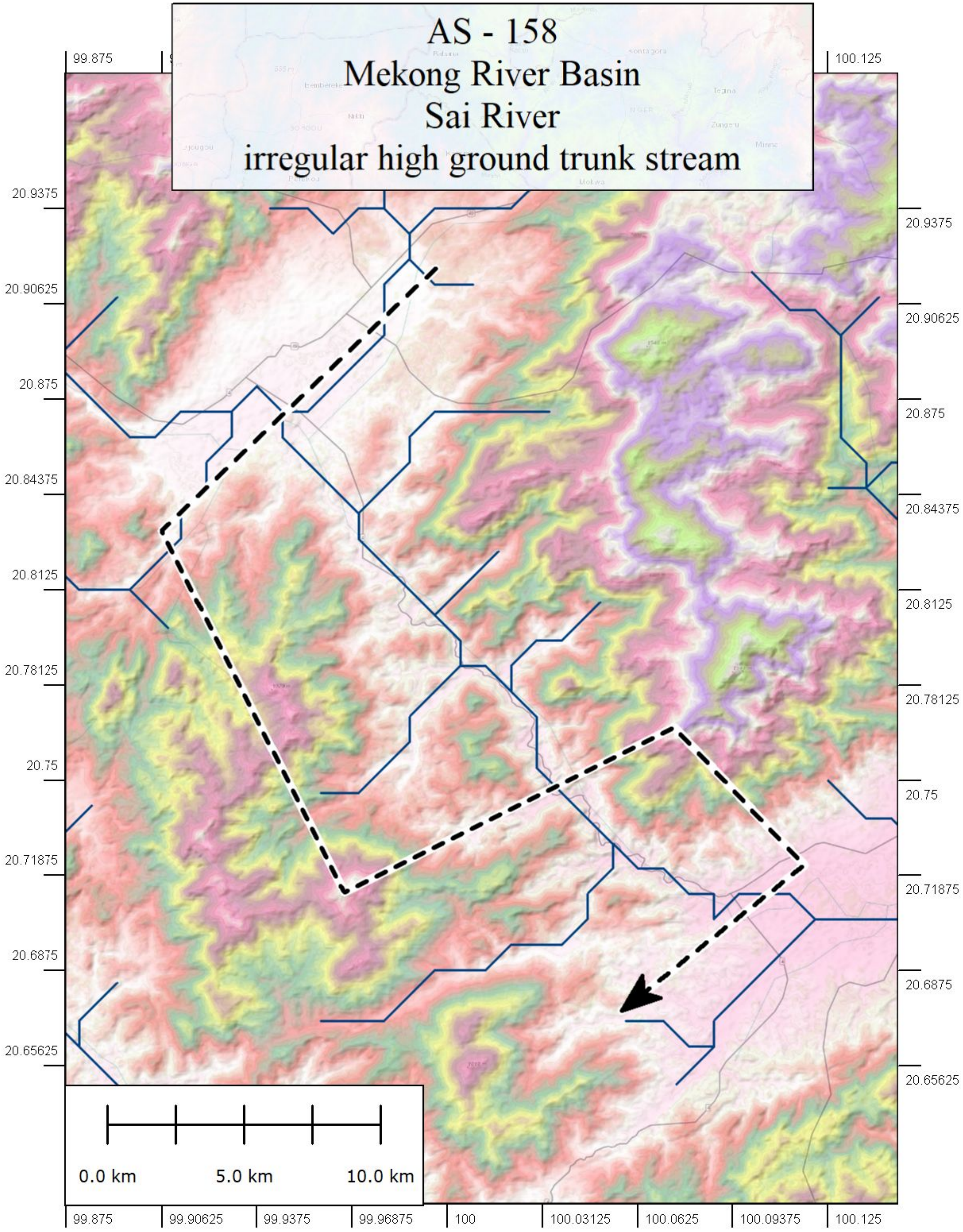
AS - 158
Mekong River Basin
Sai River
irregular high ground trunk stream
99.875
100.125
20.9375
20.90625
20.875
20.84375
20.8125
20.78125
20.75
20.71875
20.6875
20.65625
0.0 km
5.0 km
10.0 km
99.875
99.90625
99.9375
99.96875
100
100.03125
100.0625
100.09375
100.125

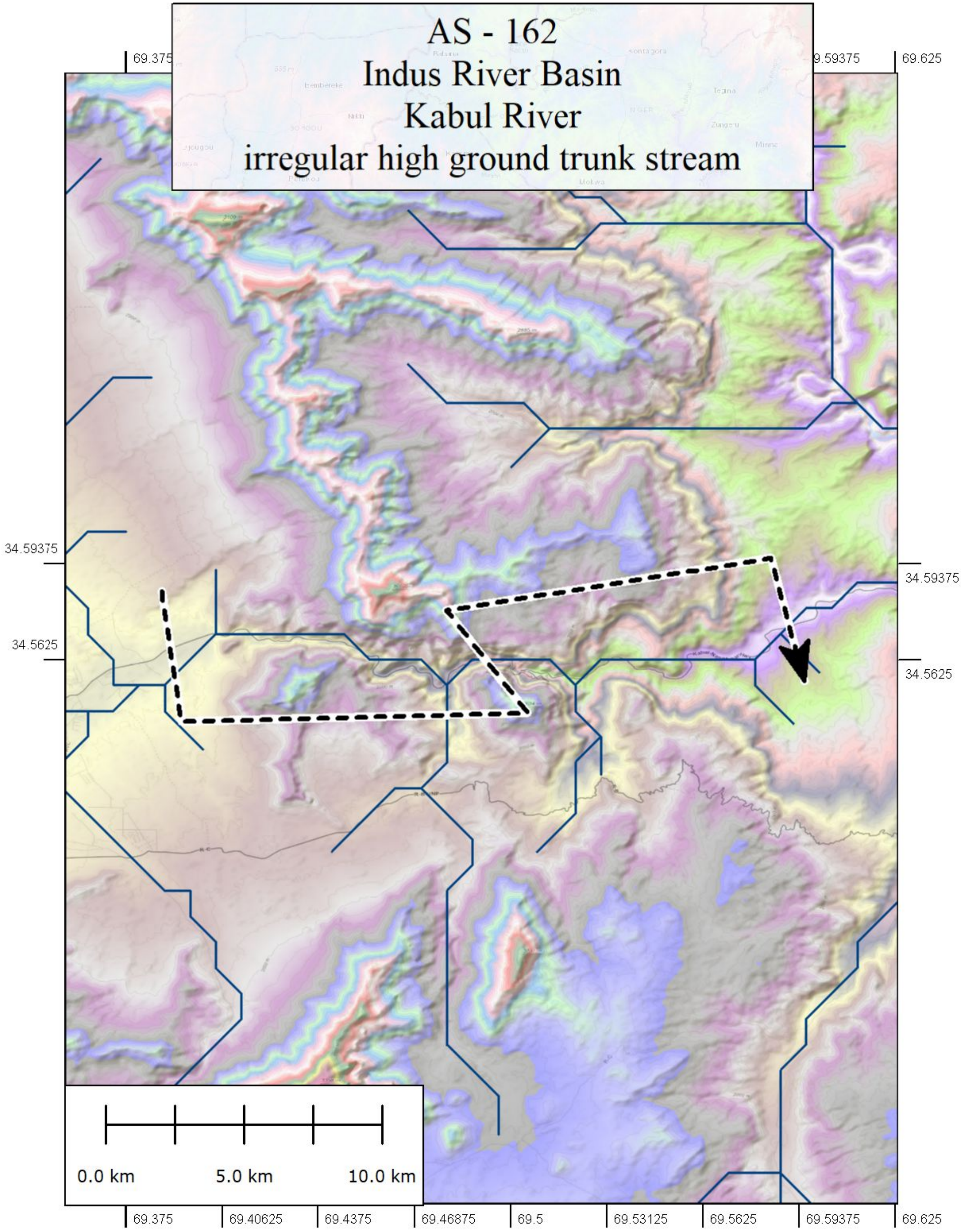
AS - 162
Indus River Basin
Kabul River
irregular high ground trunk stream
69.375
69.40625
69.4375
69.46875
69.5
69.53125
69.5625
69.59375
69.625
34.59375
34.5625
0.0 km
5.0 km
10.0 km

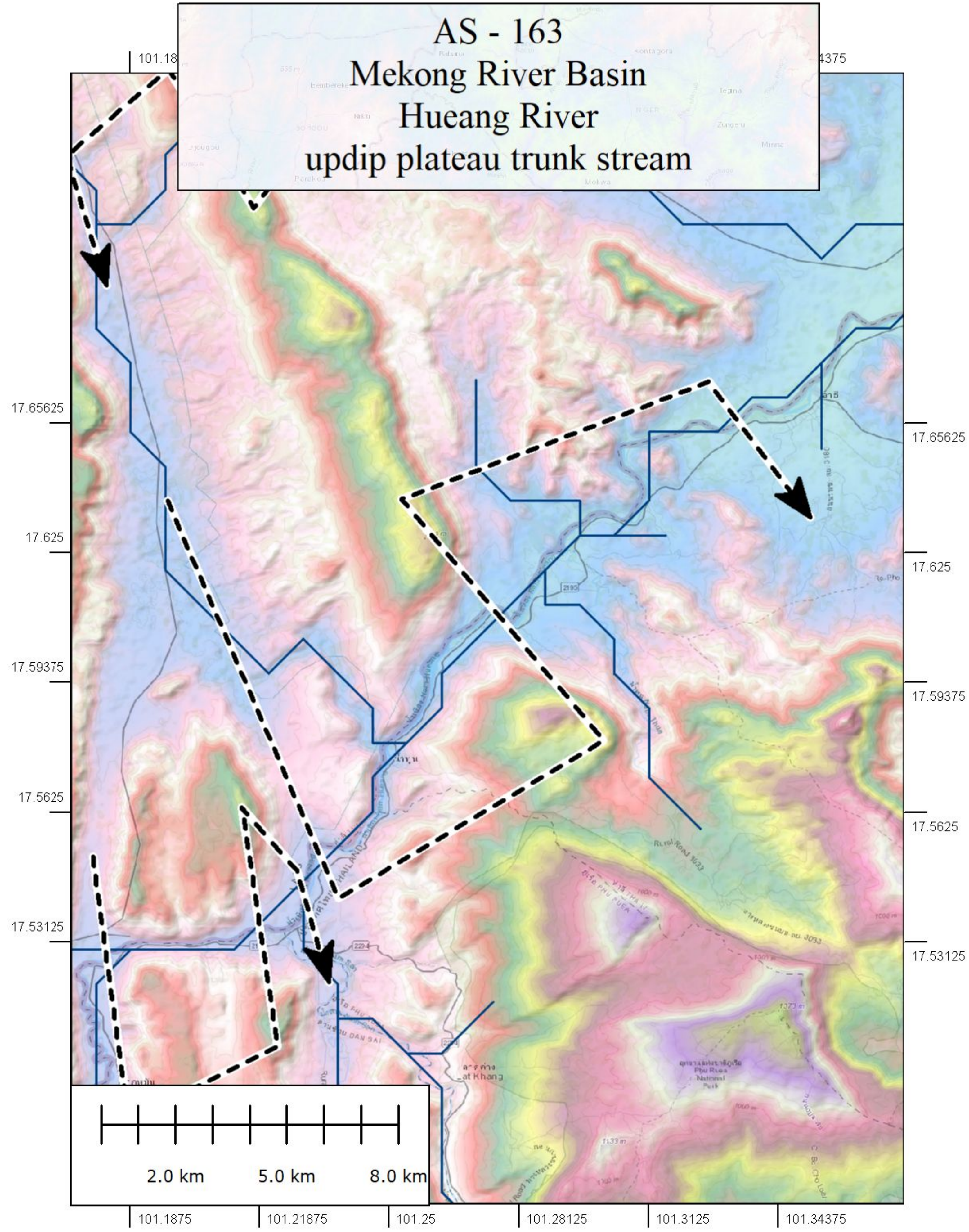
AS - 163
Mekong River Basin
Hueang River
updip plateau trunk stream
101.1875
101.21875
101.25
101.28125
101.3125
101.34375
17.65625
17.625
17.59375
17.5625
17.53125
2.0 km
5.0 km
8.0 km
Rural Road 1033

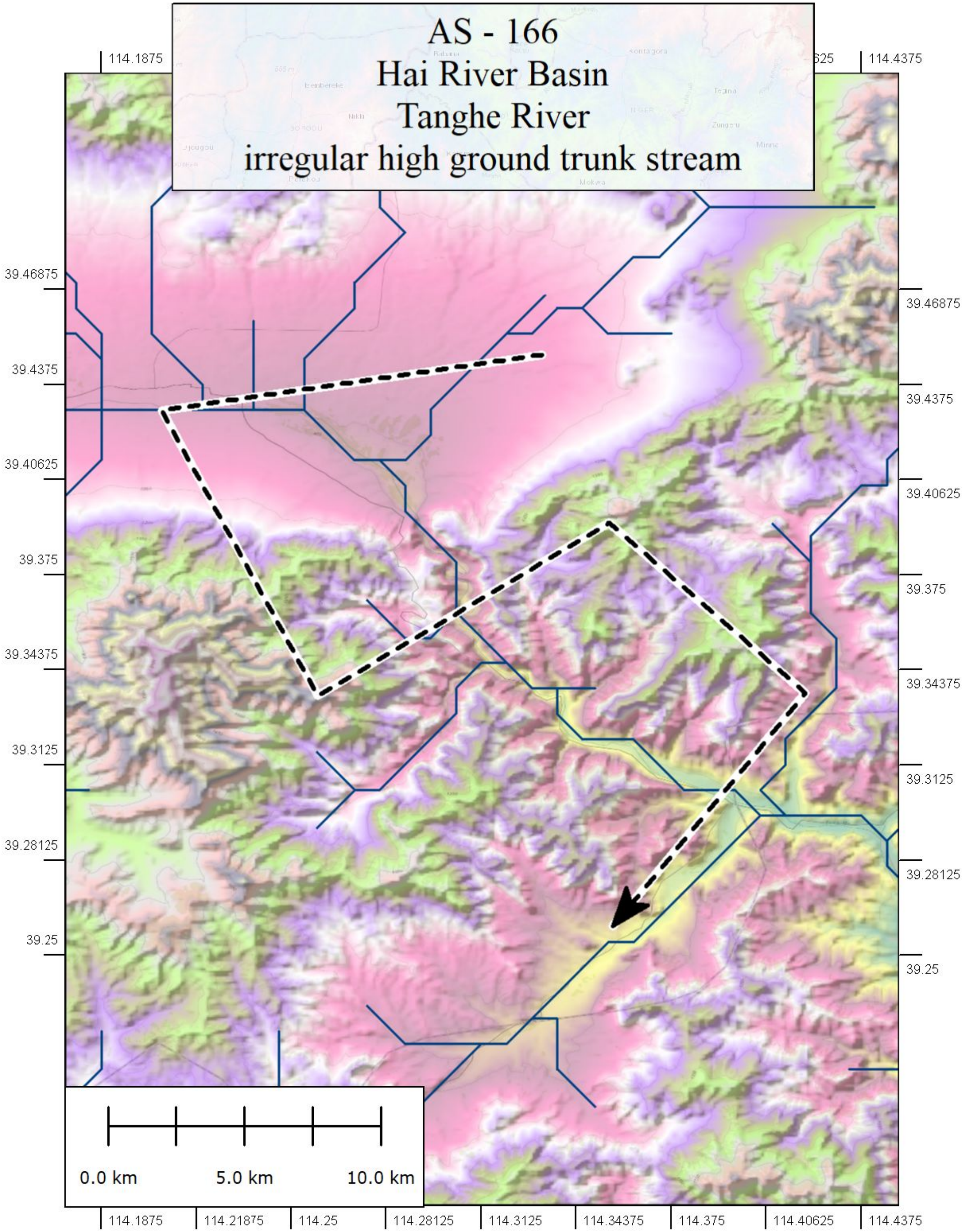

AS - 166
Hai River Basin
Tanghe River
irregular high ground trunk stream
114.1875
114.21875
114.25
114.28125
114.3125
114.34375
114.375
114.40625
114.4375
39.46875
39.4375
39.40625
39.375
39.34375
39.3125
39.28125
39.25
0.0 km
5.0 km
10.0 km

AS - 184
Yangtze River Basin
Yinjiang River
updip plateau trunk stream

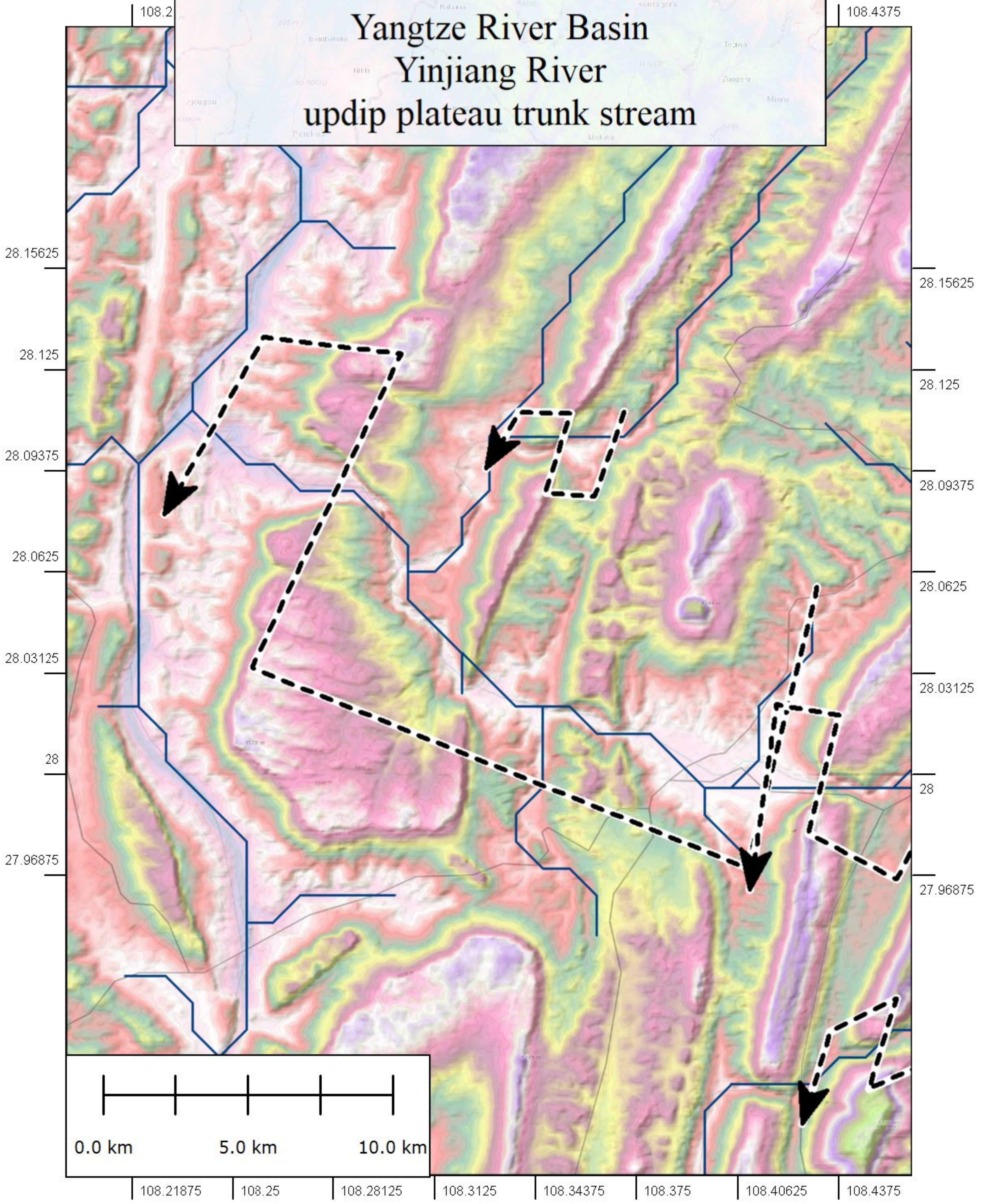

AS - 189
Ganges River Basin
Jatinga River
irregular high ground trunk stream

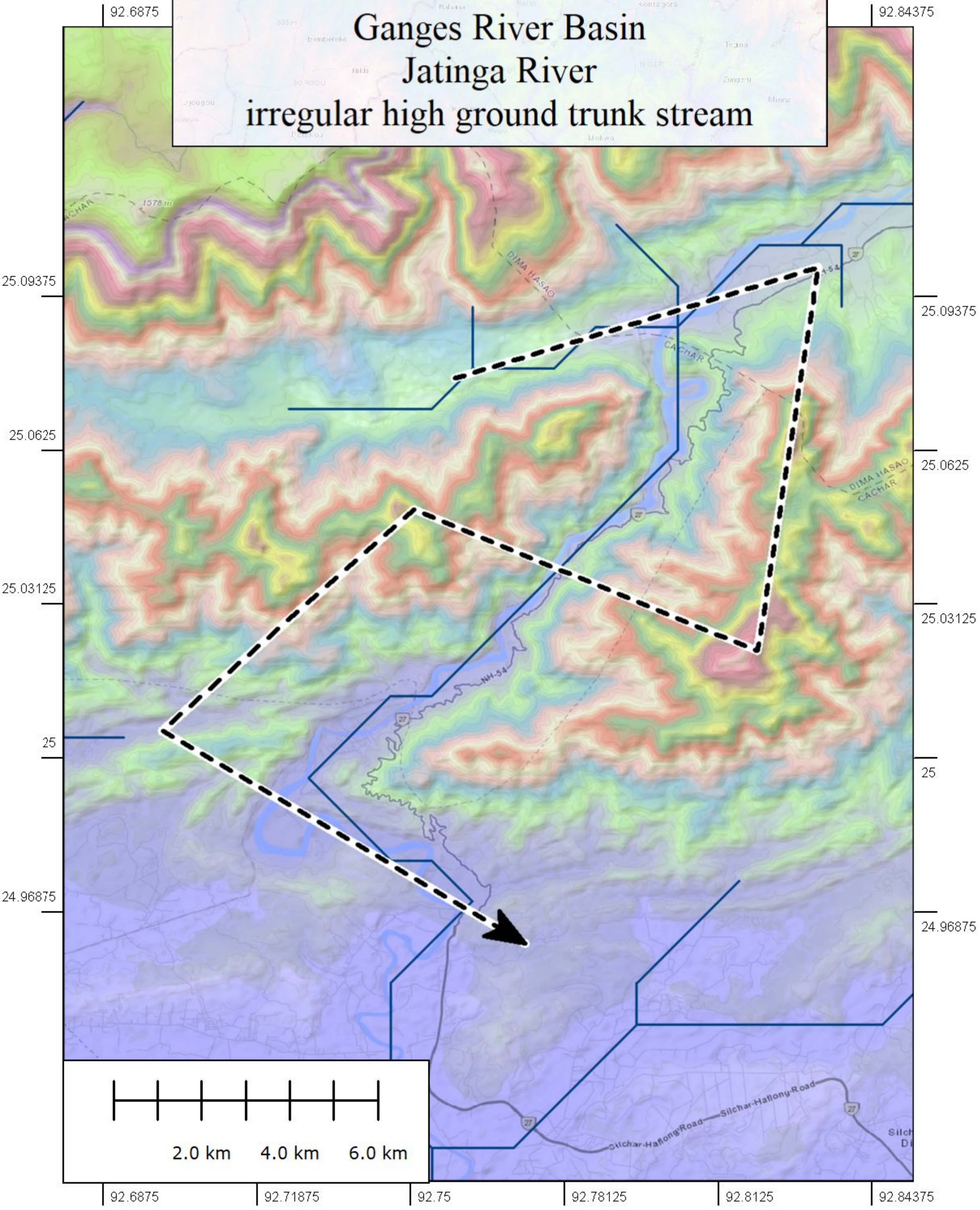

AS - 192
Yangtze River Basin
Daning River
irregular high ground trunk stream

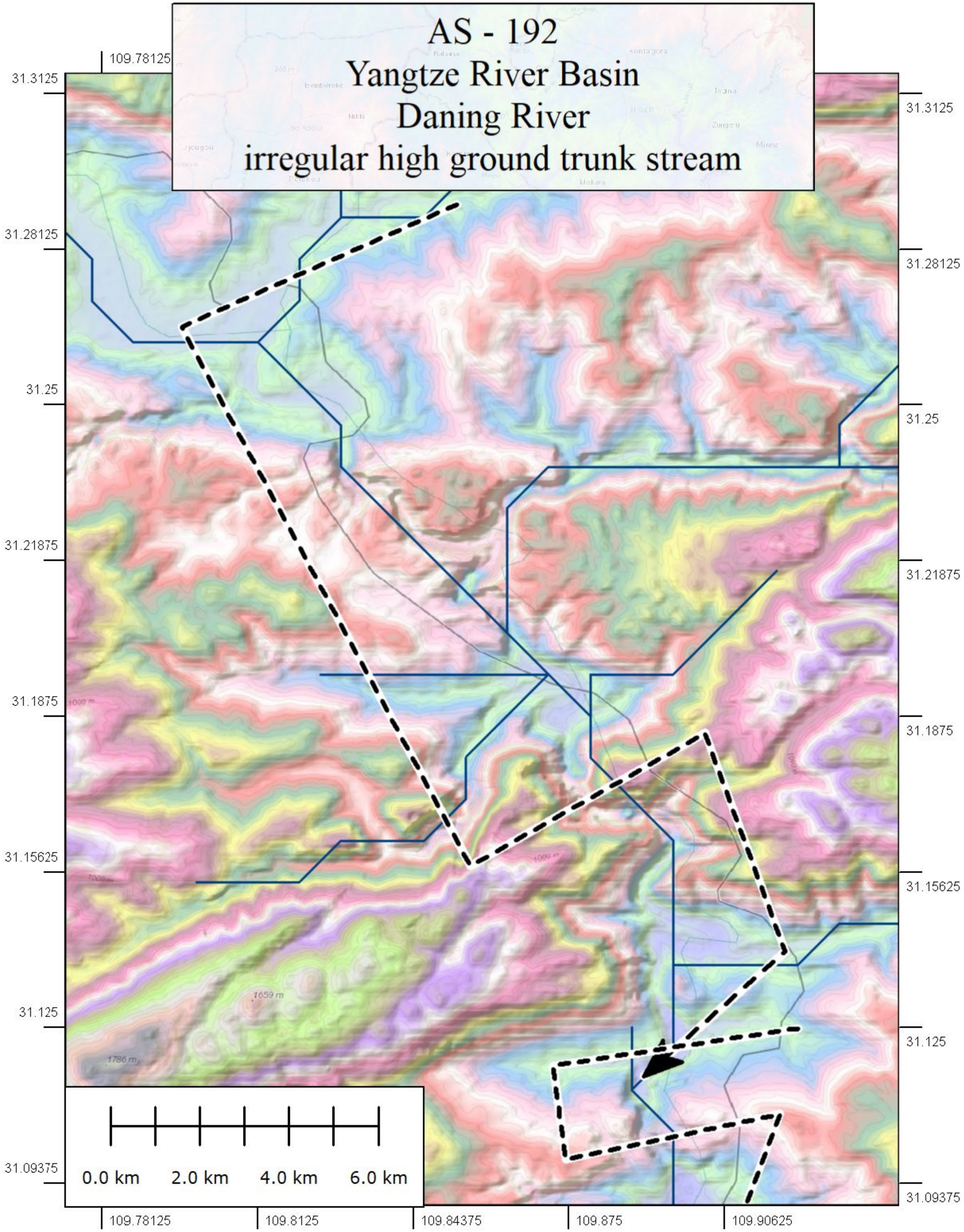

AS - 205
Mekong River Basin
Nem Leuak
irregular high ground trunk stream

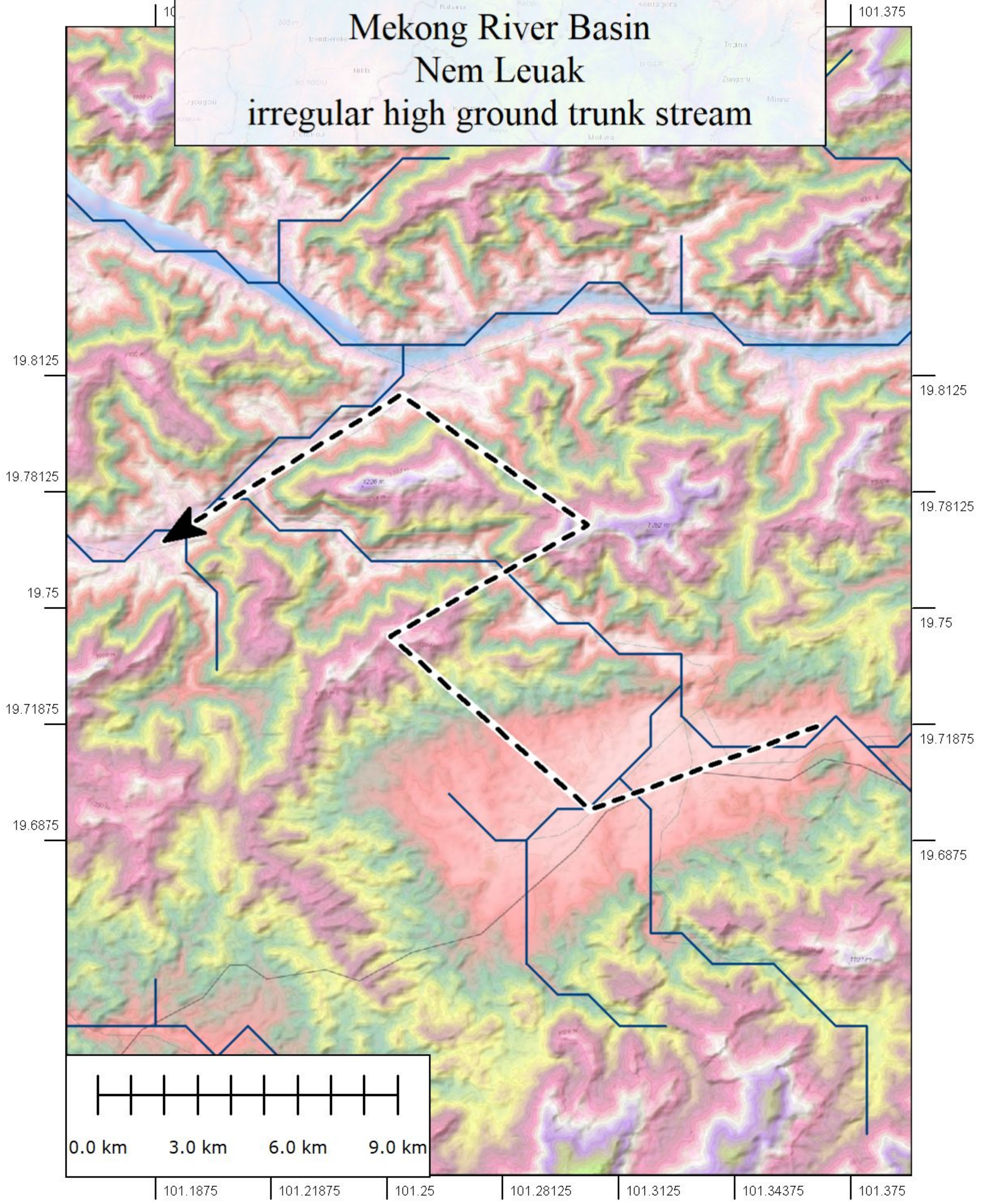

AS - 210
Mekong River Basin
Mekong River tributary
irregular high ground trunk stream

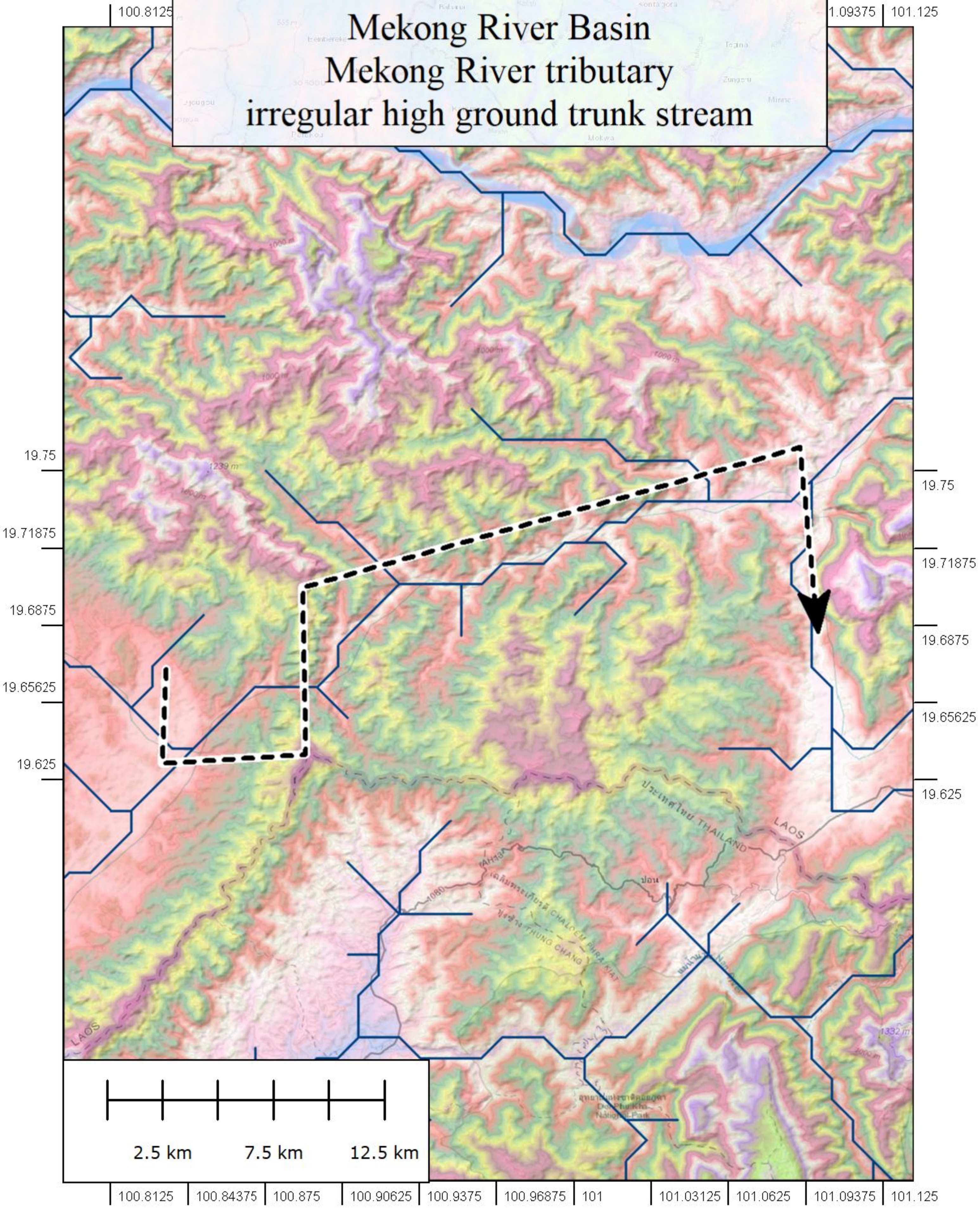

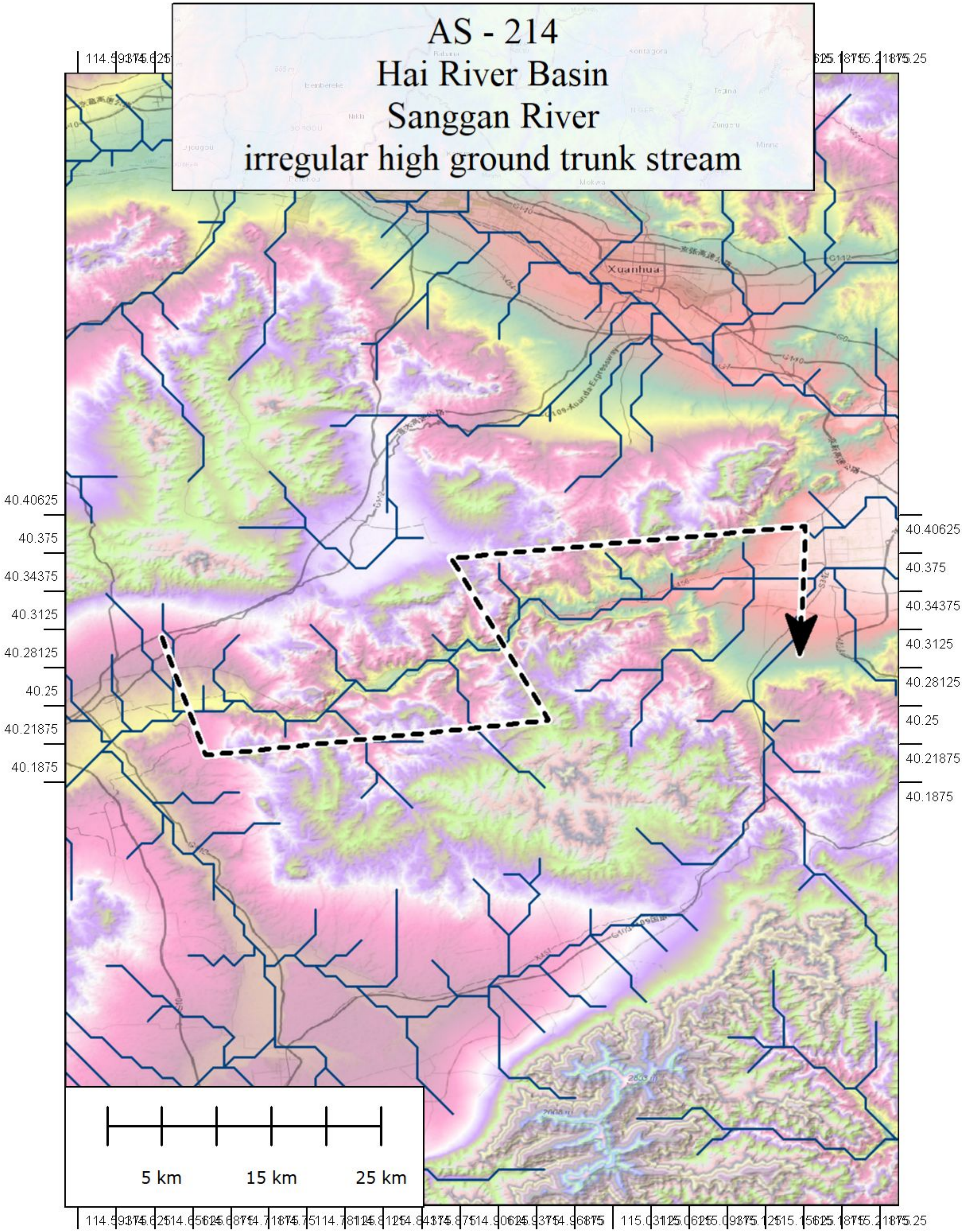
AS - 214
Hai River Basin
Sanggan River
irregular high ground trunk stream
Xuanhua
40.40625
40.375
40.34375
40.3125
40.28125
40.25
40.21875
40.1875
5 km
15 km
25 km

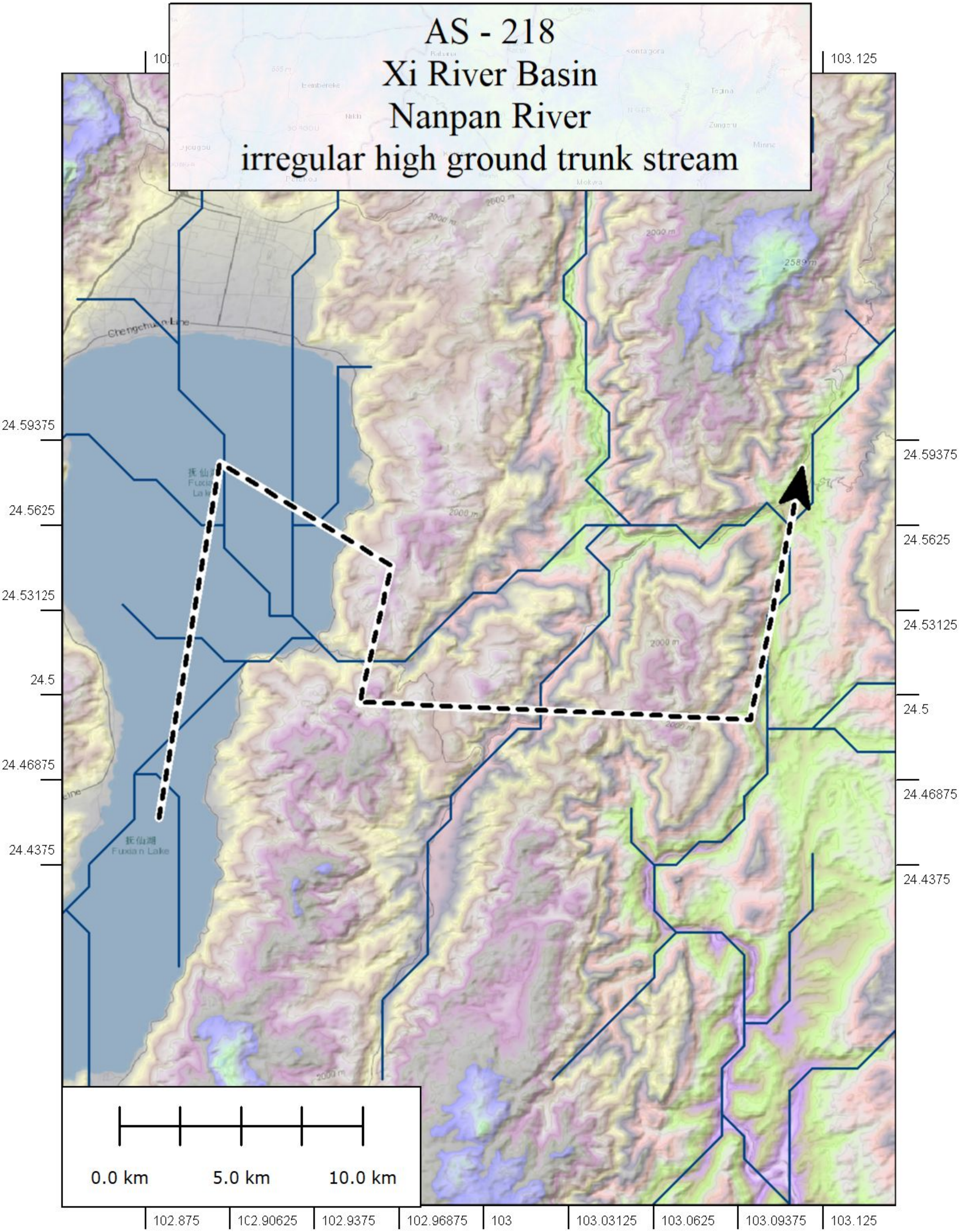

AS - 218
Xi River Basin
Nanpan River
irregular high ground trunk stream
103.125
24.59375
24.5625
24.53125
24.5
24.46875
24.4375
102.875
102.90625
102.9375
102.96875
103
103.03125
103.0625
103.09375
0.0 km
5.0 km
10.0 km
Chengchuan Line
2589 m
2000 m
抚仙湖
Fuxian Lake

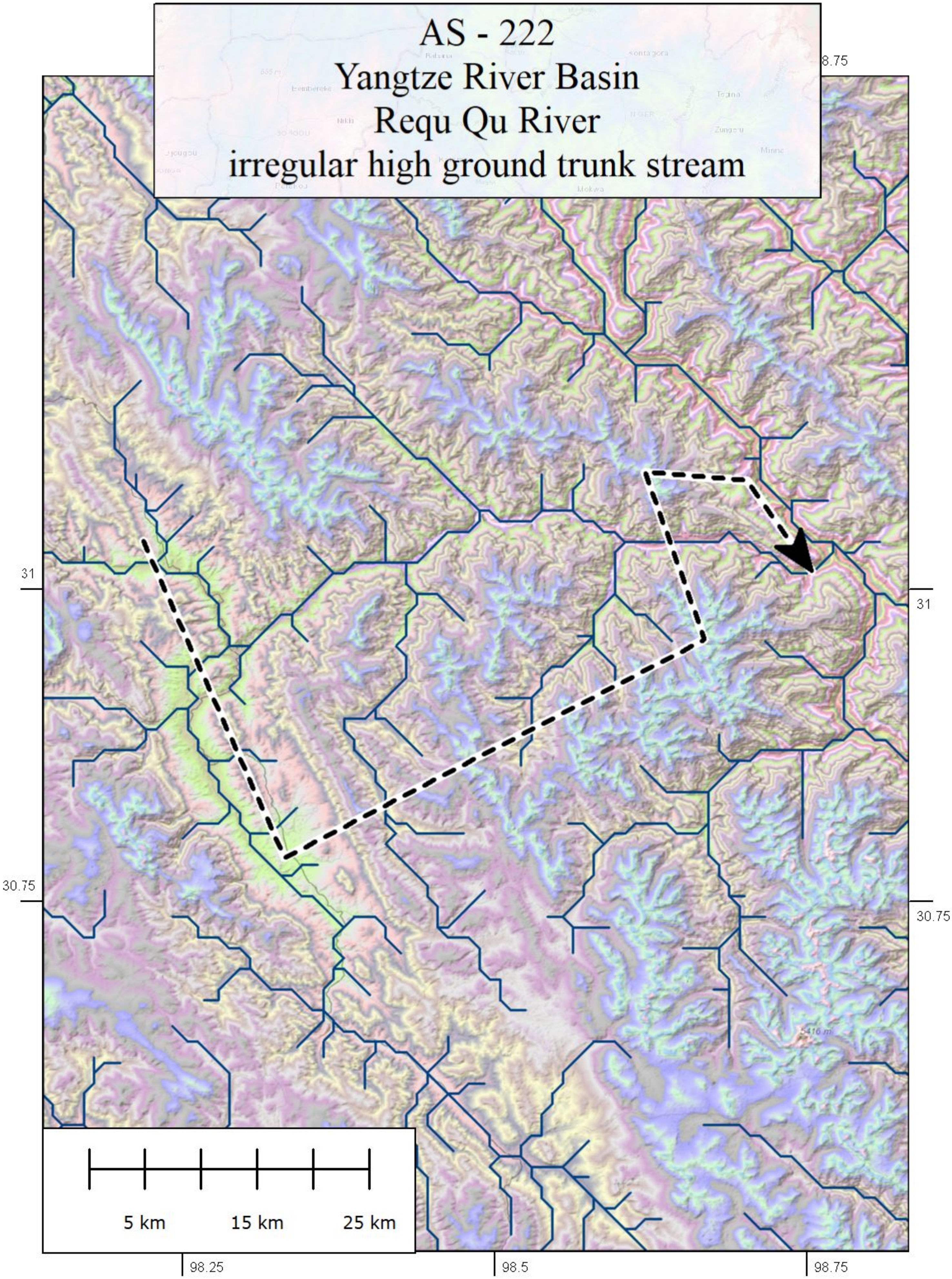
AS - 222
Yangtze River Basin
Requ Qu River
irregular high ground trunk stream
31
30.75
98.25
98.5
98.75
5 km
15 km
25 km

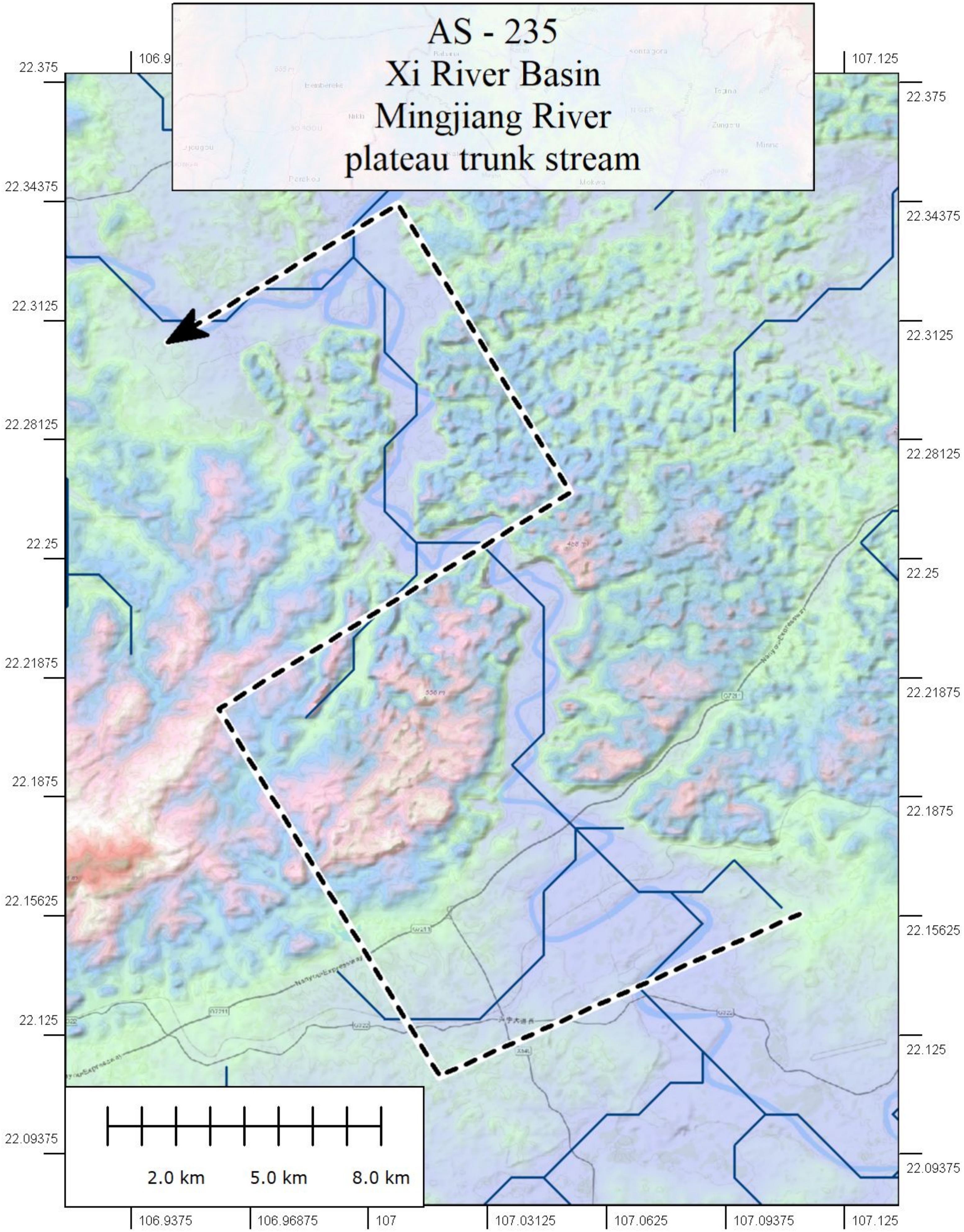

AS - 235
Xi River Basin
Mingjiang River
plateau trunk stream
22.375
22.34375
22.3125
22.28125
22.25
22.21875
22.1875
22.15625
22.125
22.09375
106.9375
106.96875
107
107.03125
107.0625
107.09375
107.125
2.0 km
5.0 km
8.0 km

AS - 239
Mekong River Basin
Kok River
irregular high ground trunk stream

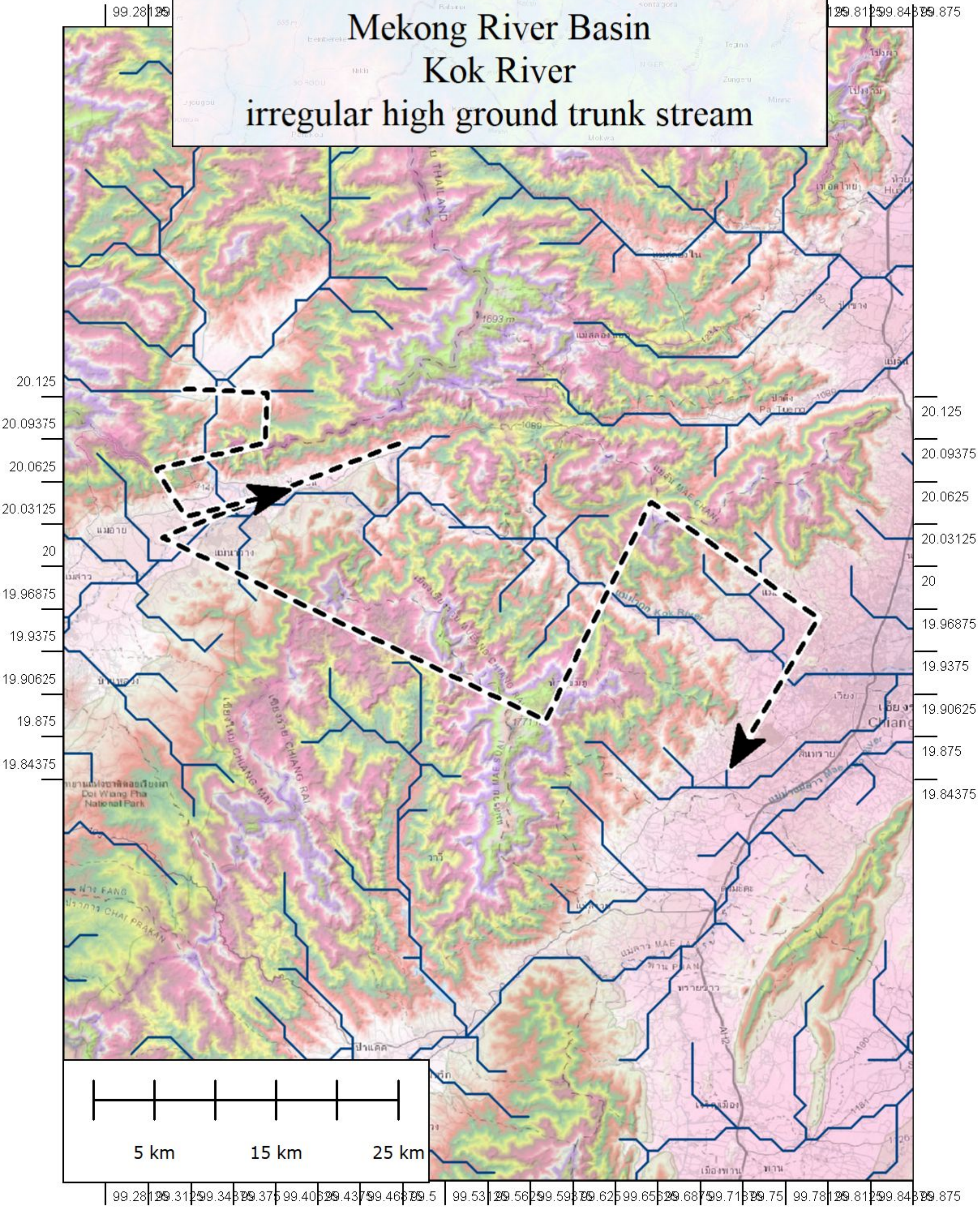

AS - 244
Indus River Basin
Jhelum River
irregular high ground trunk stream

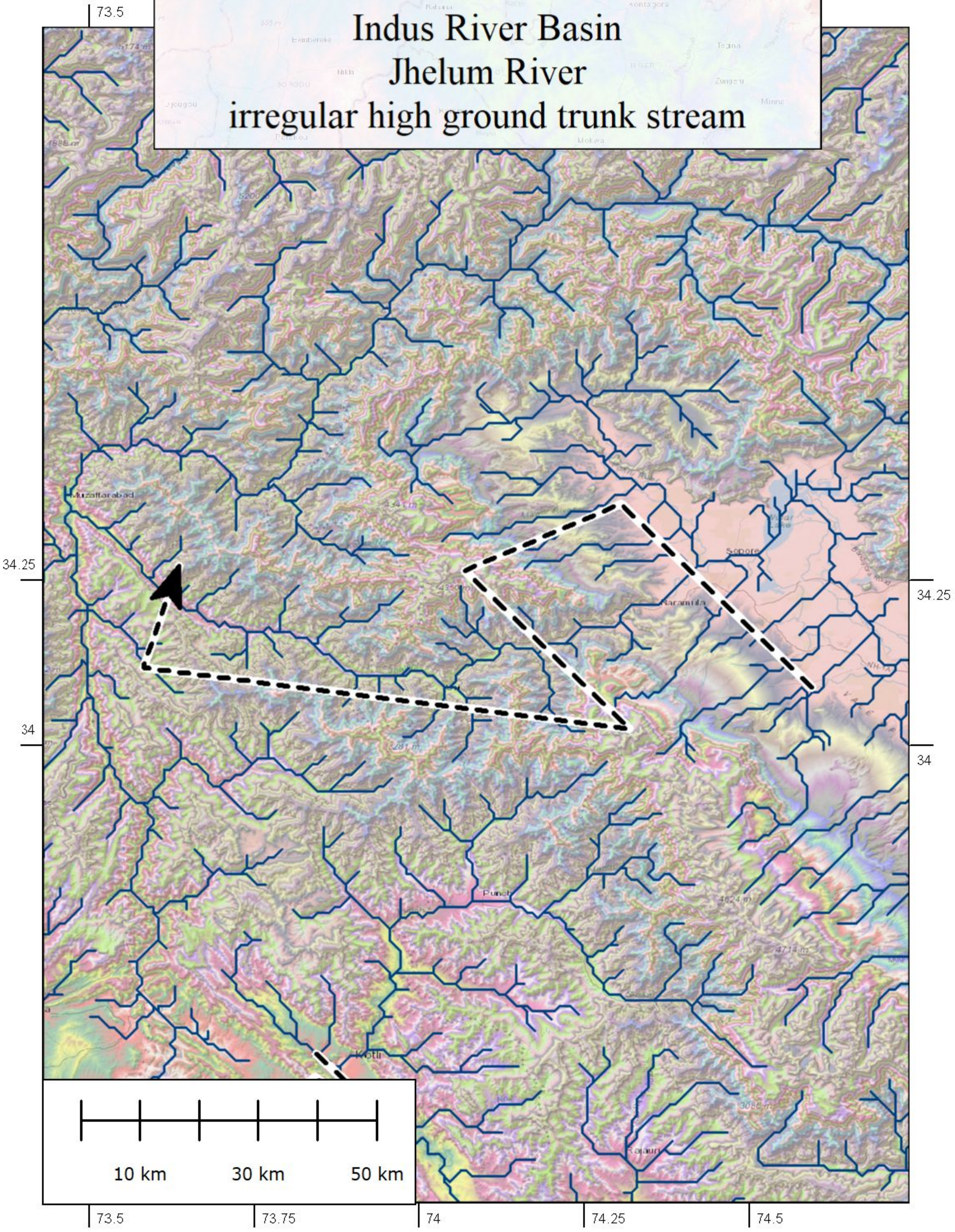

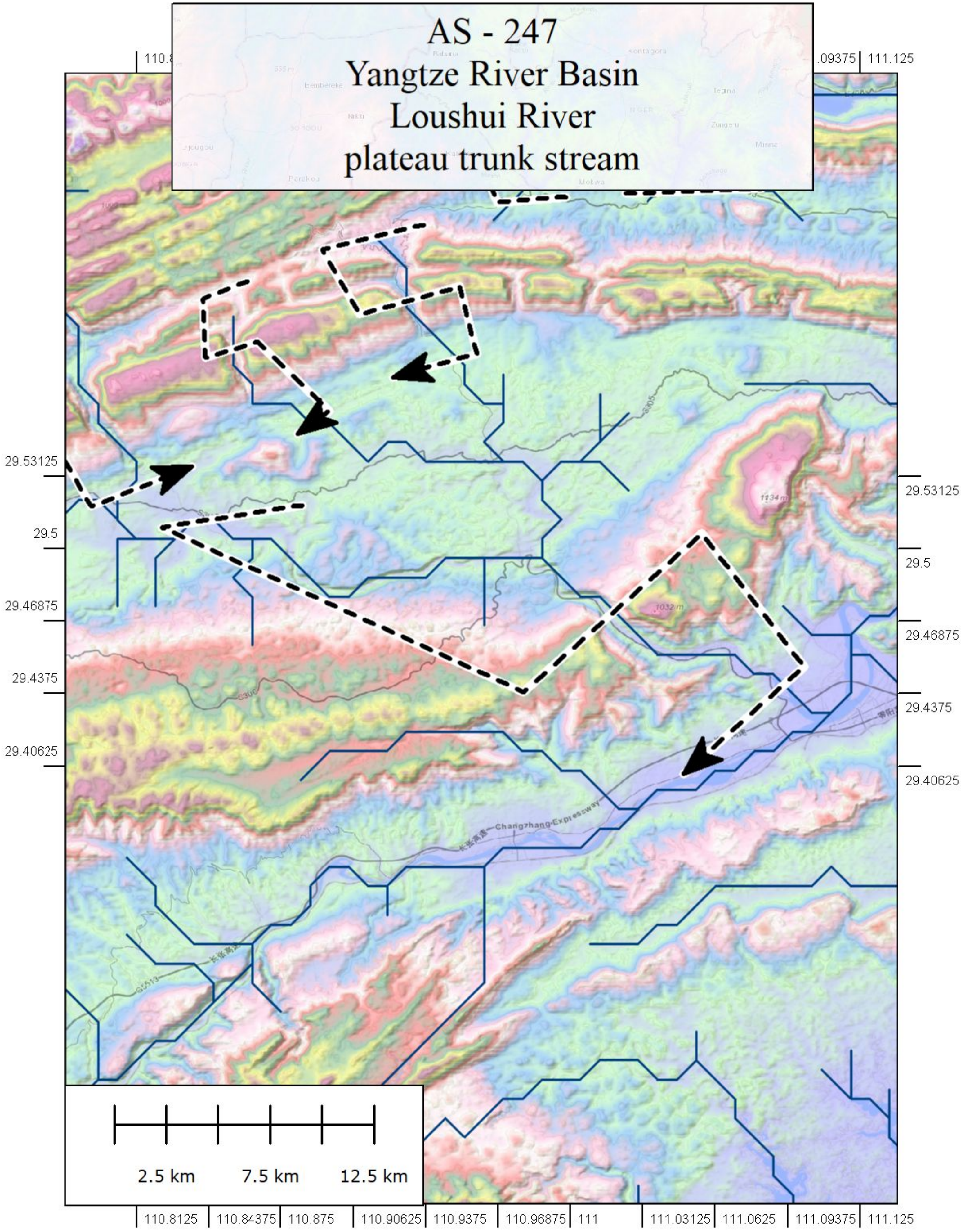
AS - 247
Yangtze River Basin
Loushui River
plateau trunk stream
111.125
29.53125
29.5
29.46875
29.4375
29.40625
1134 m
1032 m
Changzhang Expressway
2.5 km
7.5 km
12.5 km
110.8125
110.84375
110.875
110.90625
110.9375
110.96875
111
111.03125
111.0625
111.09375

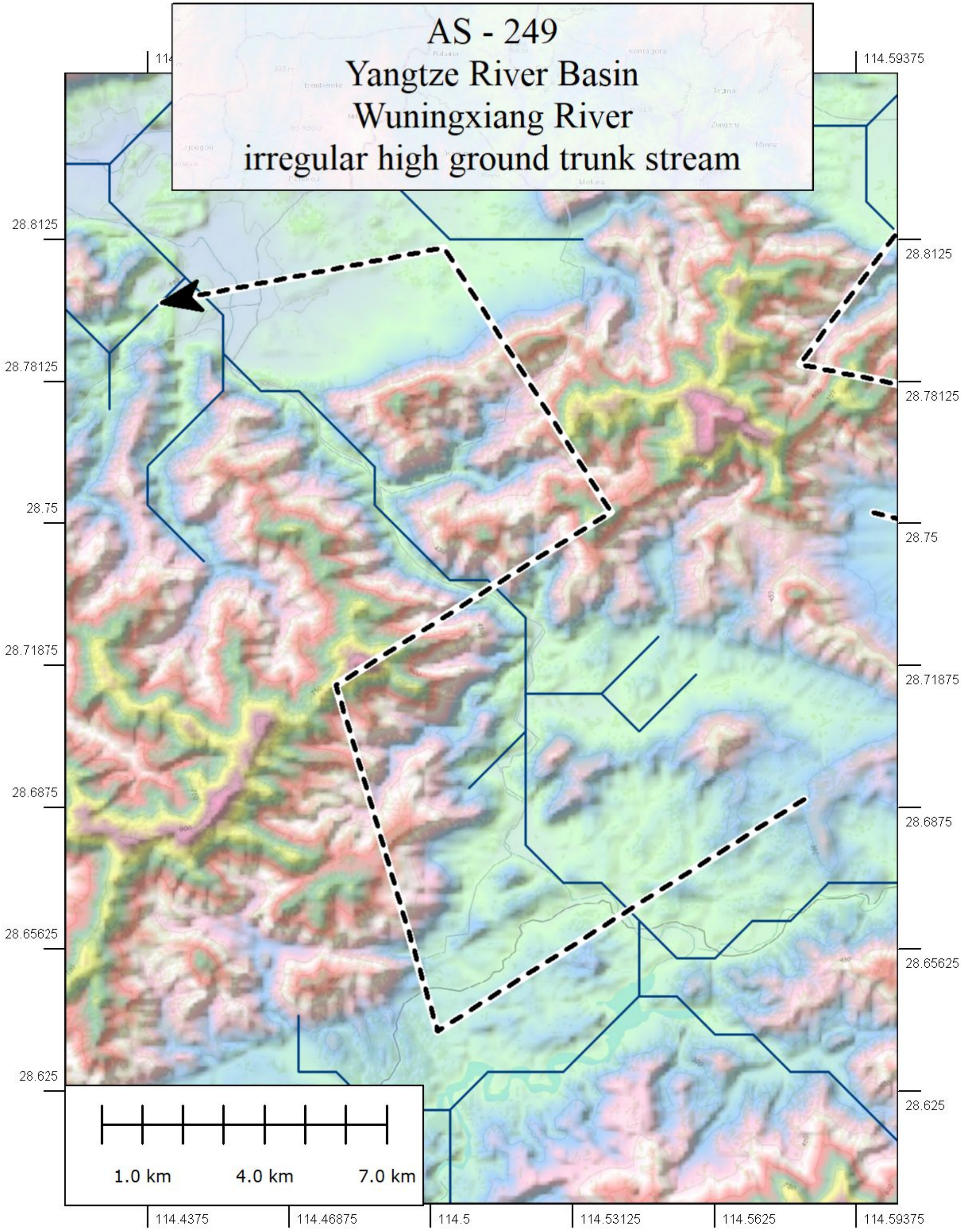
AS - 249
Yangtze River Basin
Wuningxiang River
irregular high ground trunk stream
28.8125
28.78125
28.75
28.71875
28.6875
28.65625
28.625
114.4375
114.46875
114.5
114.53125
114.5625
114.59375
1.0 km
4.0 km
7.0 km

AS - 250
Xi River Basin
Qian River
irregular high ground trunk stream

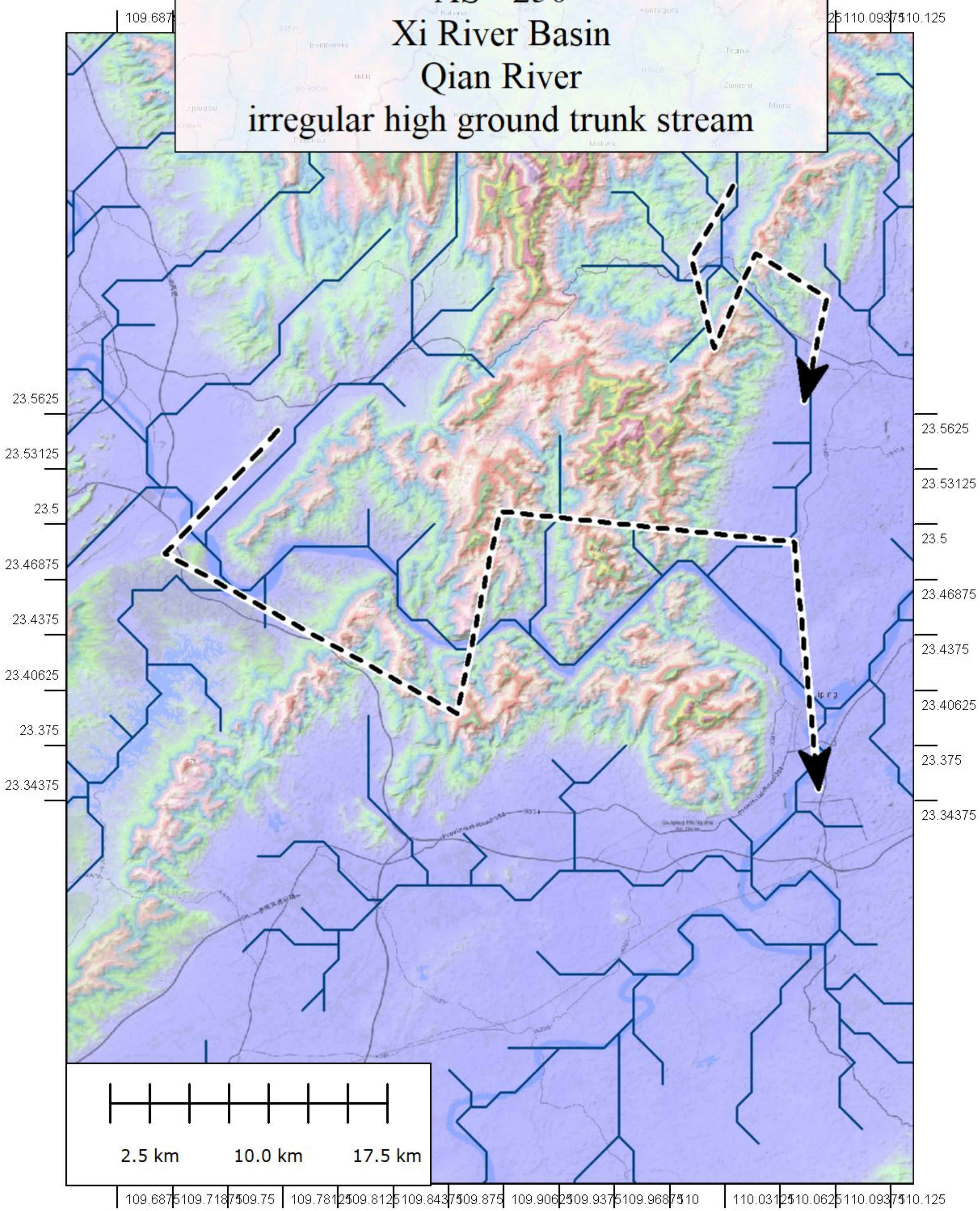

AS - 284
Yenisey River Basin
irregular high ground trunk stream

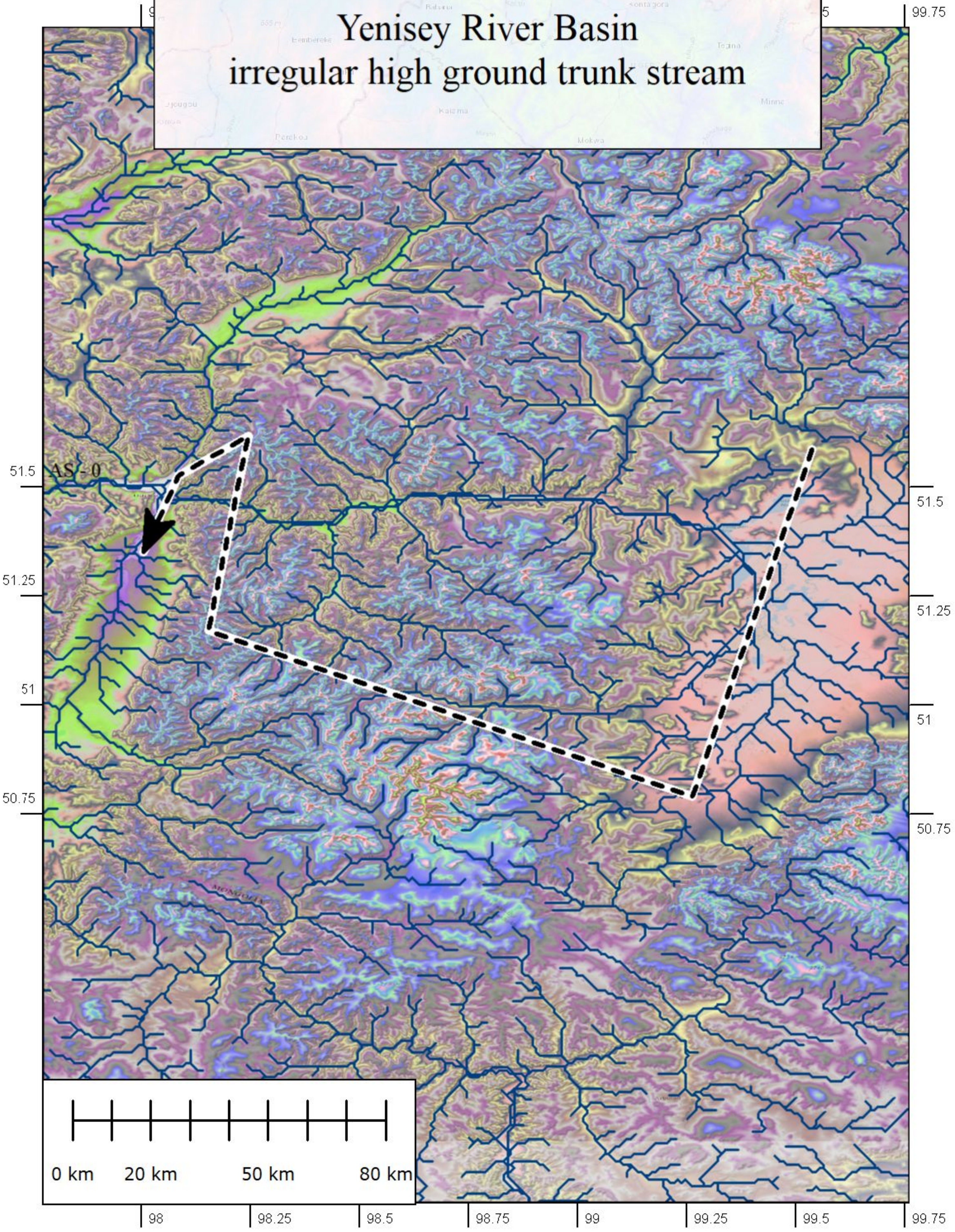

AS - 285
Yenisey River Basin
Yenesei River
irregular high ground trunk stream

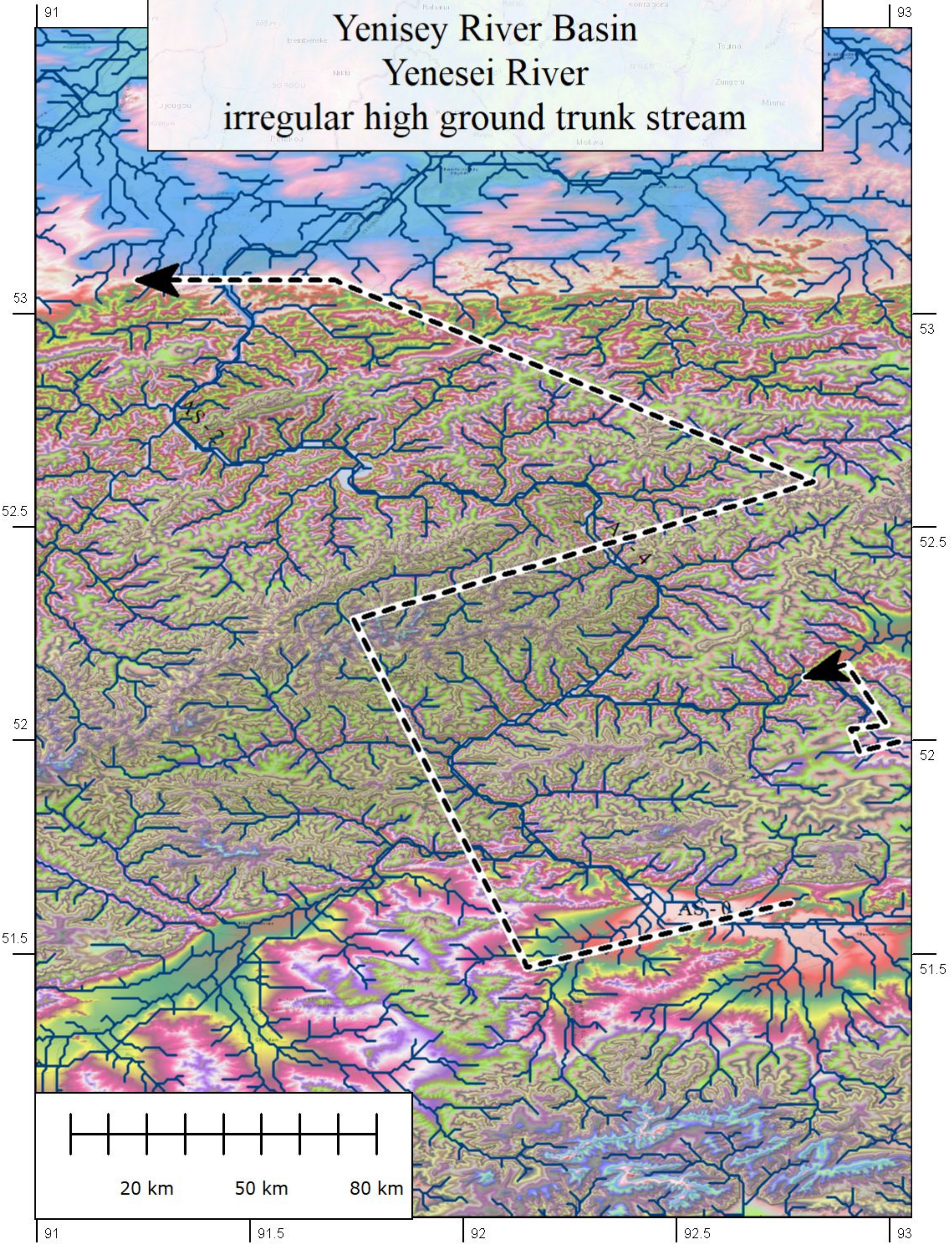

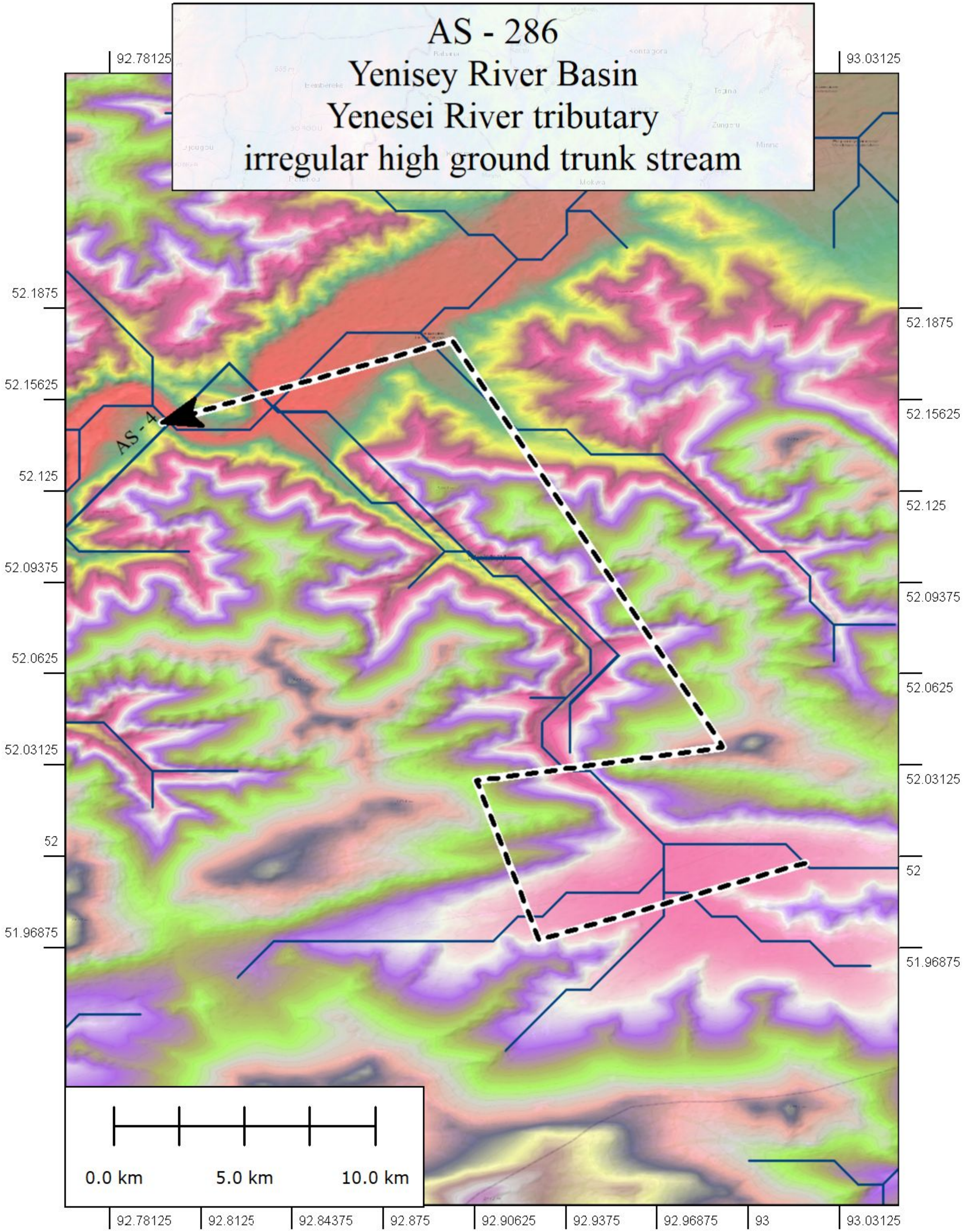
AS - 286
Yenisey River Basin
Yenesei River tributary
irregular high ground trunk stream
AS - 4
92.78125
93.03125
52.1875
52.15625
52.125
52.09375
52.0625
52.03125
52
51.96875
0.0 km
5.0 km
10.0 km
92.8125
92.84375
92.875
92.90625
92.9375
92.96875
93

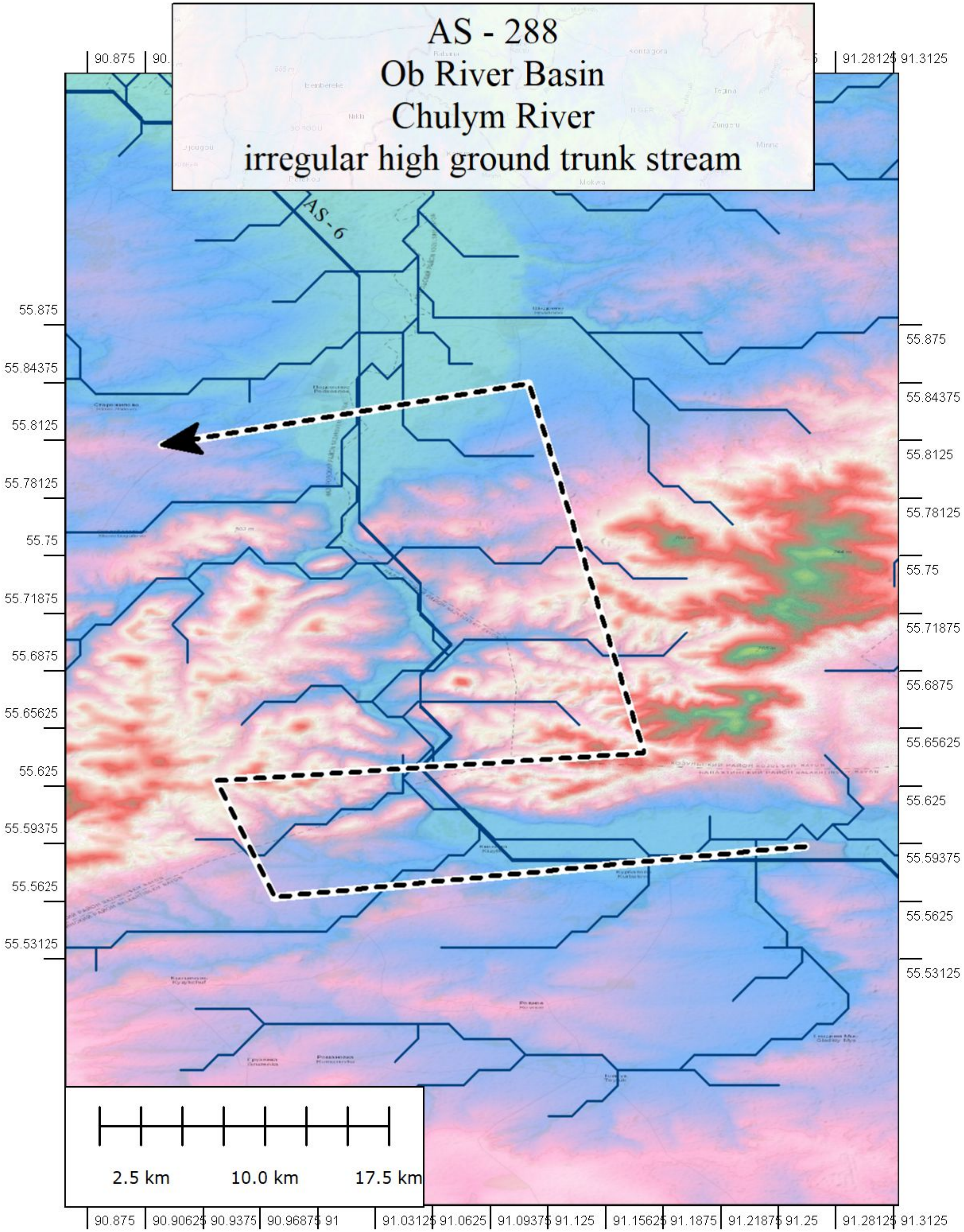
AS - 288
Ob River Basin
Chulym River
irregular high ground trunk stream
AS - 6
90.875
91.28125
91.3125
55.875
55.84375
55.8125
55.78125
55.75
55.71875
55.6875
55.65625
55.625
55.59375
55.5625
55.53125
2.5 km
10.0 km
17.5 km
90.90625
90.9375
90.96875
91
91.03125
91.0625
91.09375
91.125
91.15625
91.1875
91.21875
91.25

AS - 289
Yenisey River Basin
Angara River
downdip plateau trunk stream

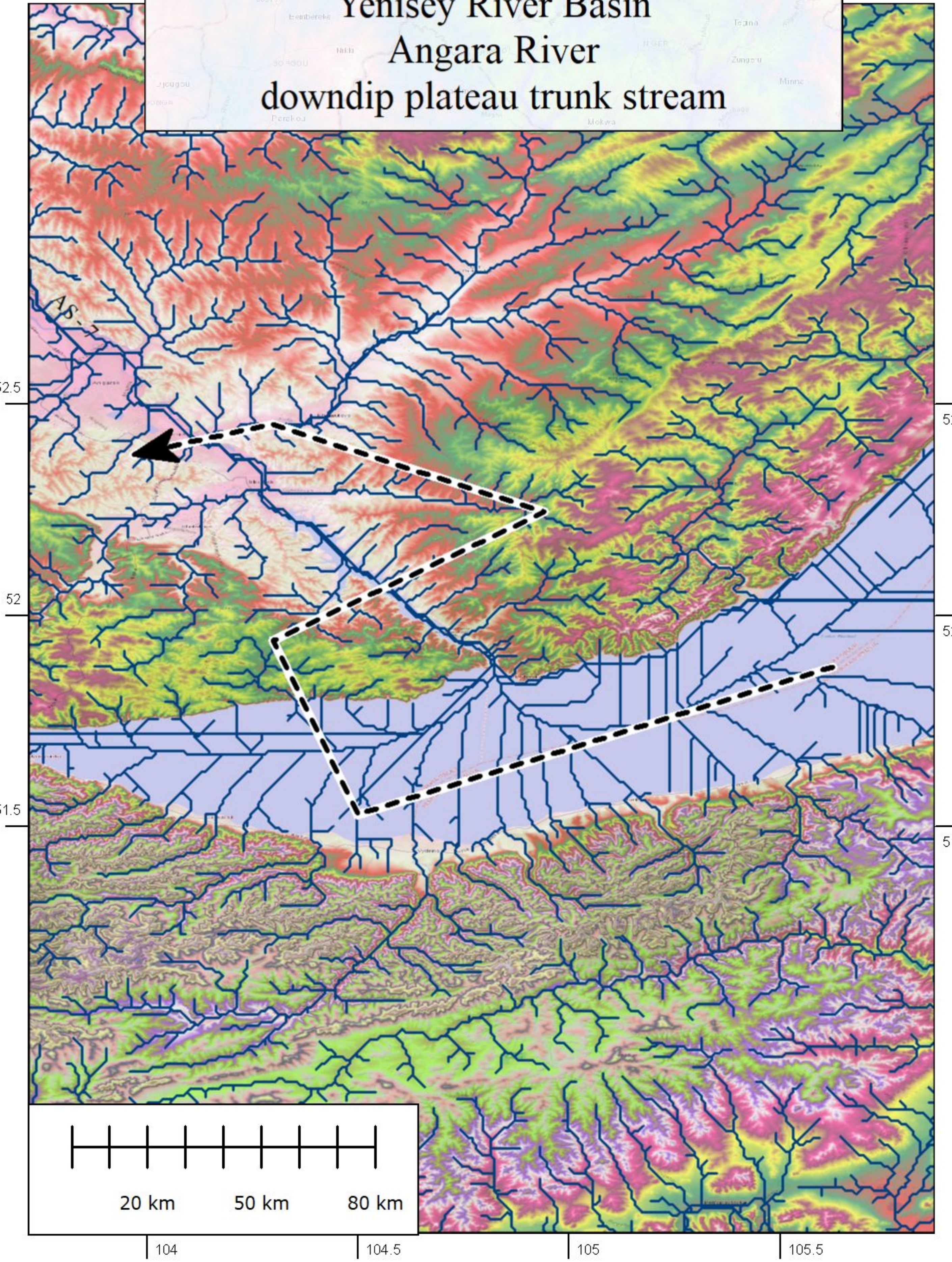

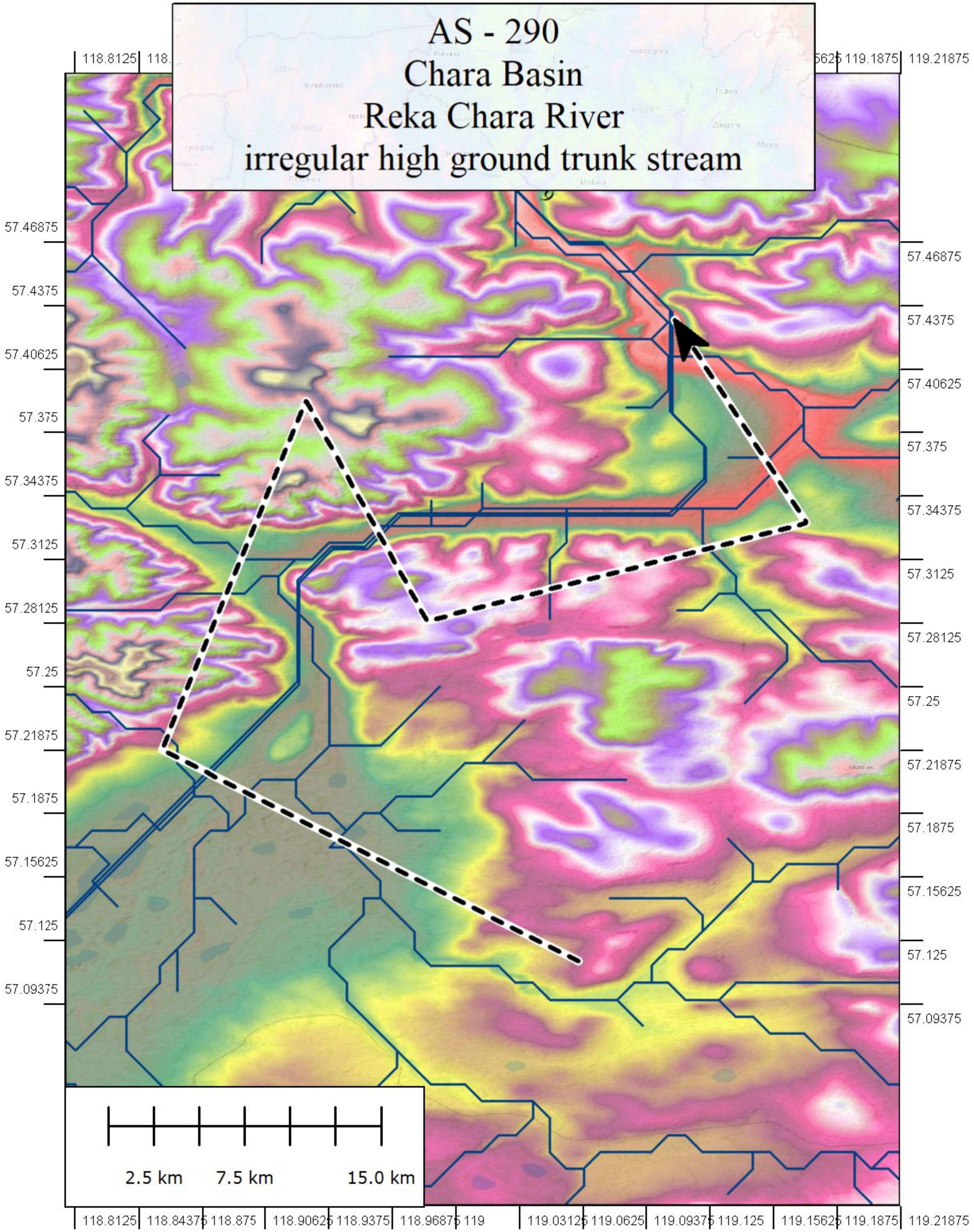

AS - 290
Chara Basin
Reka Chara River
irregular high ground trunk stream
2.5 km
7.5 km
15.0 km
118.8125
118.84375
118.875
118.90625
118.9375
118.96875
119
119.03125
119.0625
119.09375
119.125
119.15625
119.1875
119.21875
57.46875
57.4375
57.40625
57.375
57.34375
57.3125
57.28125
57.25
57.21875
57.1875
57.15625
57.125
57.09375

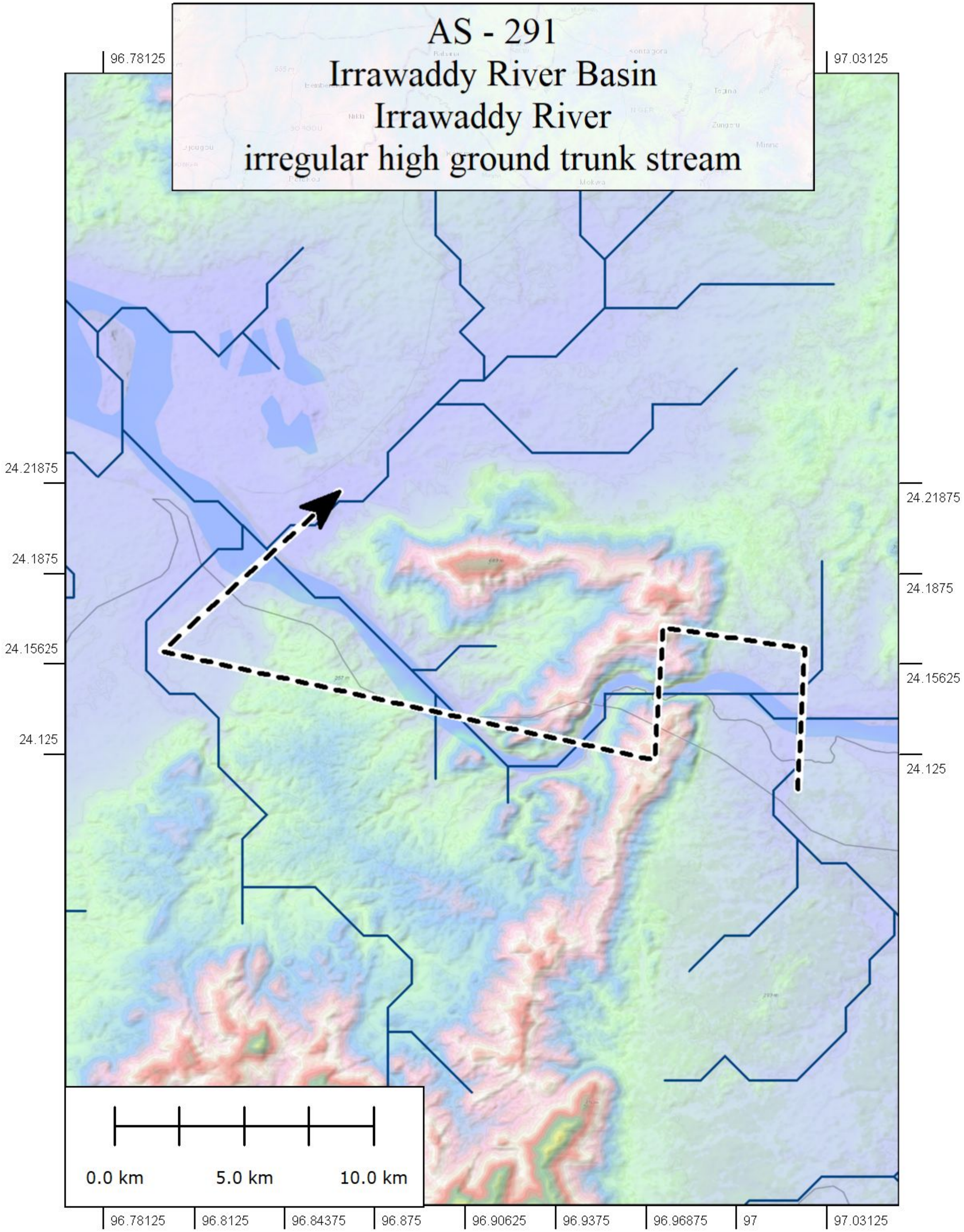
AS - 291
Irrawaddy River Basin
Irrawaddy River
irregular high ground trunk stream
96.78125
97.03125
24.21875
24.1875
24.15625
24.125
24.21875
24.1875
24.15625
24.125
0.0 km
5.0 km
10.0 km
96.78125
96.8125
96.84375
96.875
96.90625
96.9375
96.96875
97
97.03125

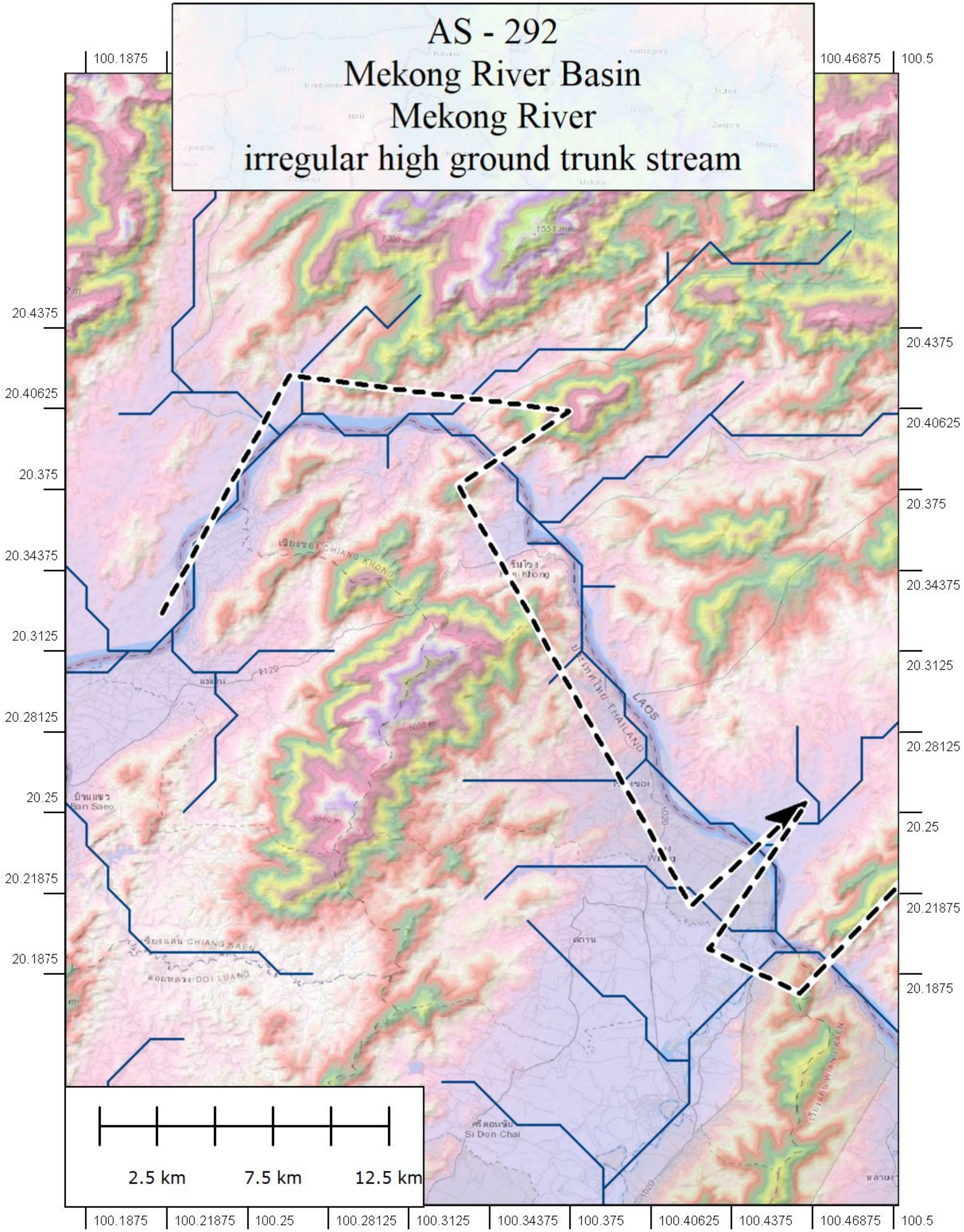

AS - 292
Mekong River Basin
Mekong River
irregular high ground trunk stream
100.1875
100.21875
100.25
100.28125
100.3125
100.34375
100.375
100.40625
100.4375
100.46875
100.5
20.4375
20.40625
20.375
20.34375
20.3125
20.28125
20.25
20.21875
20.1875
2.5 km
7.5 km
12.5 km
1551 m
CHIANG KHONG
LAOS
THAILAND
Ban Saeo
CHIANG SAEN
DOI LUANG
Si Don Chai

AS - 294
Mekong River Basin
Mekong River tributary
irregular high ground trunk stream

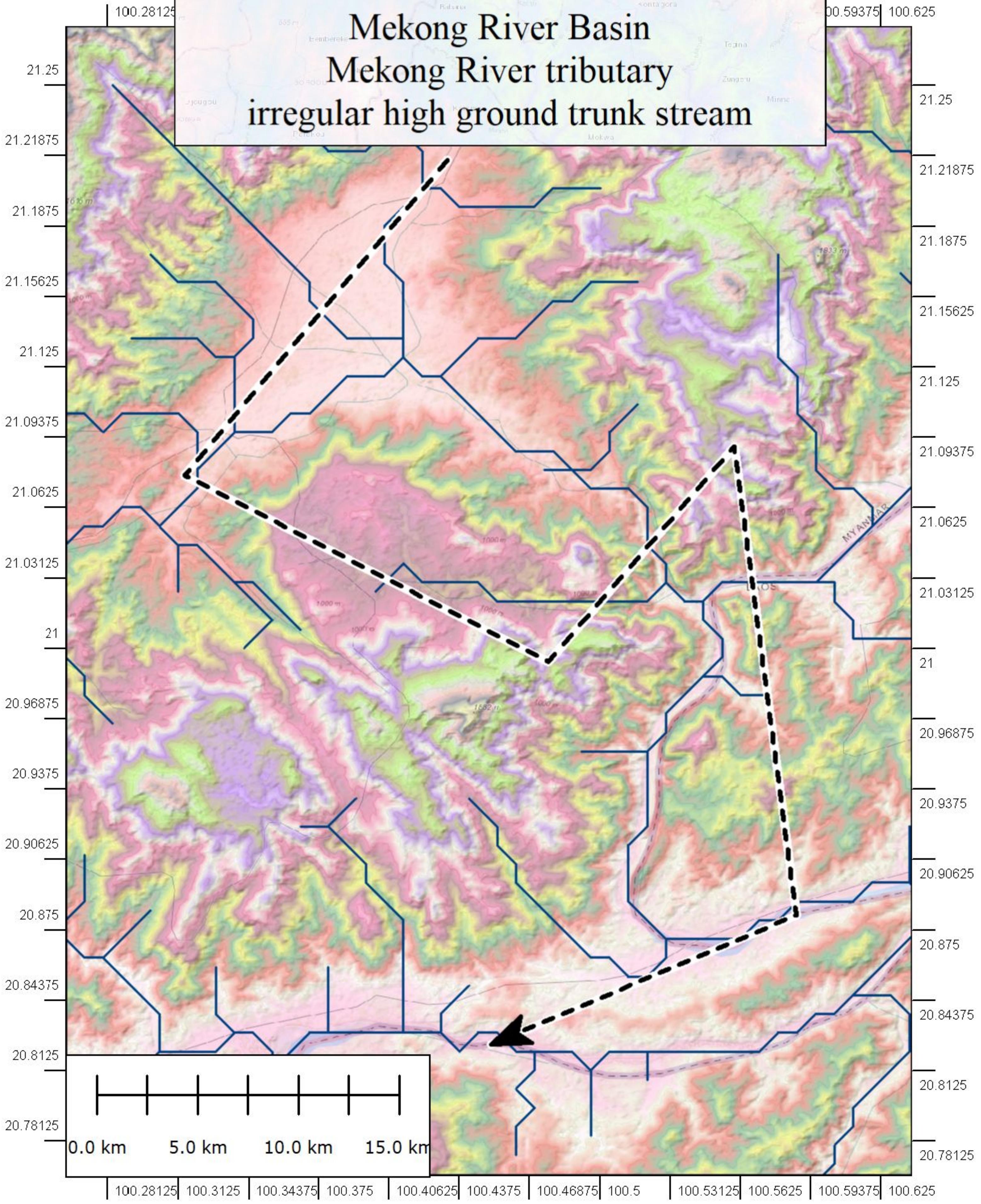

AS - 295
Mekong River Basin
Lancang River
irregular high ground trunk stream

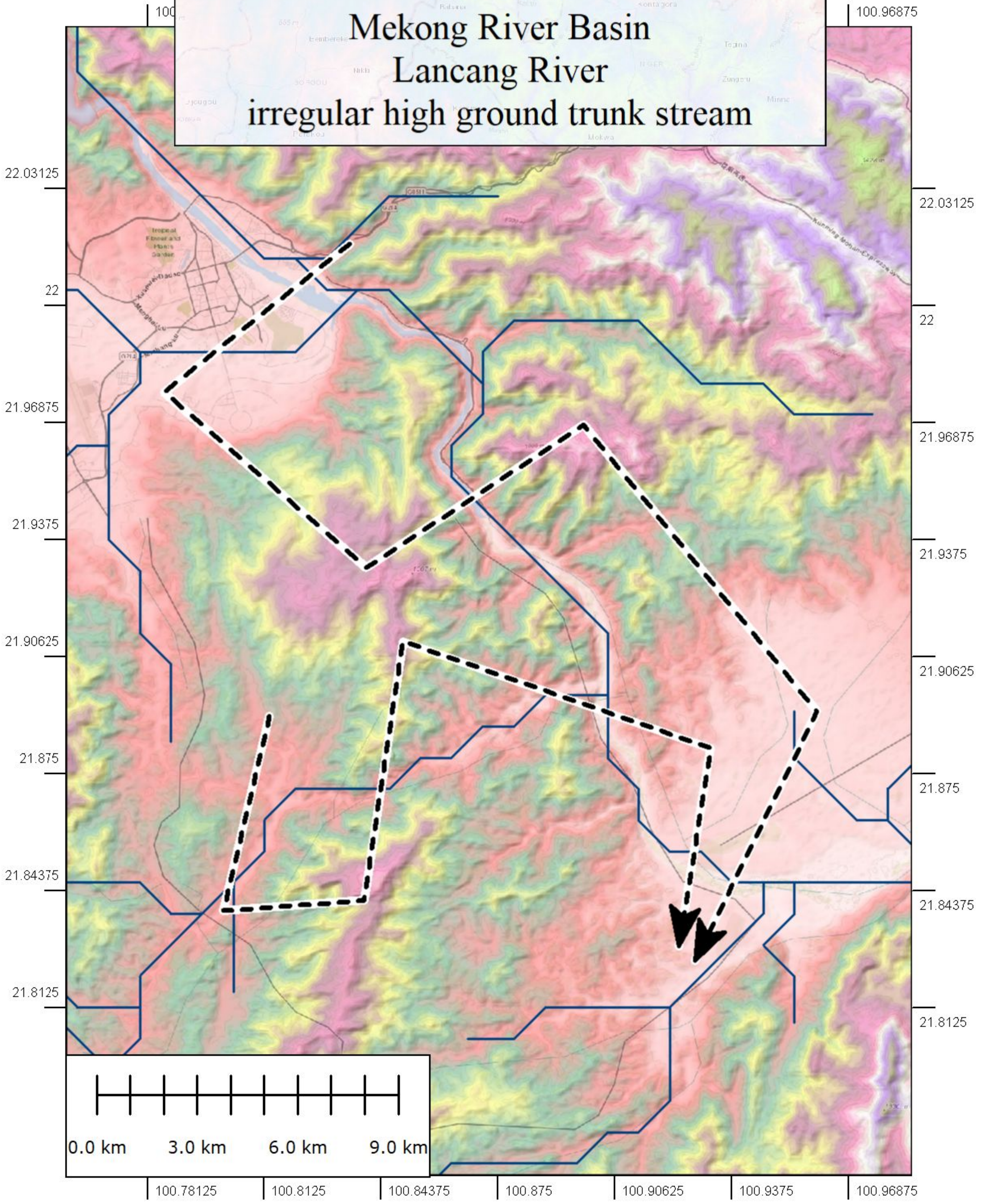

AS - 296
Mekong River Basin
Mannanhui River
irregular high ground trunk stream

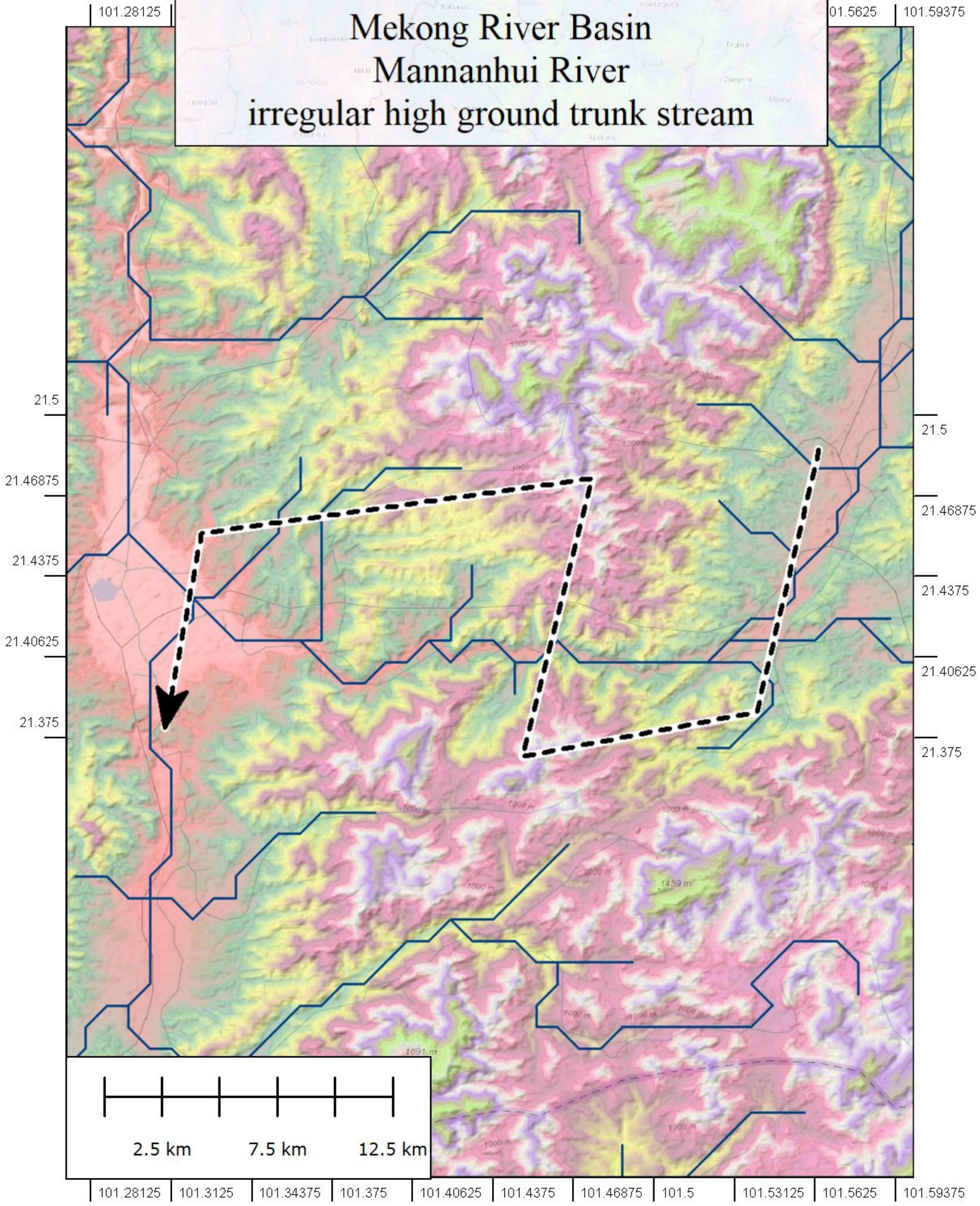

AS - 304
Amur River Basin
Reka Tyrma
irregular high ground trunk stream

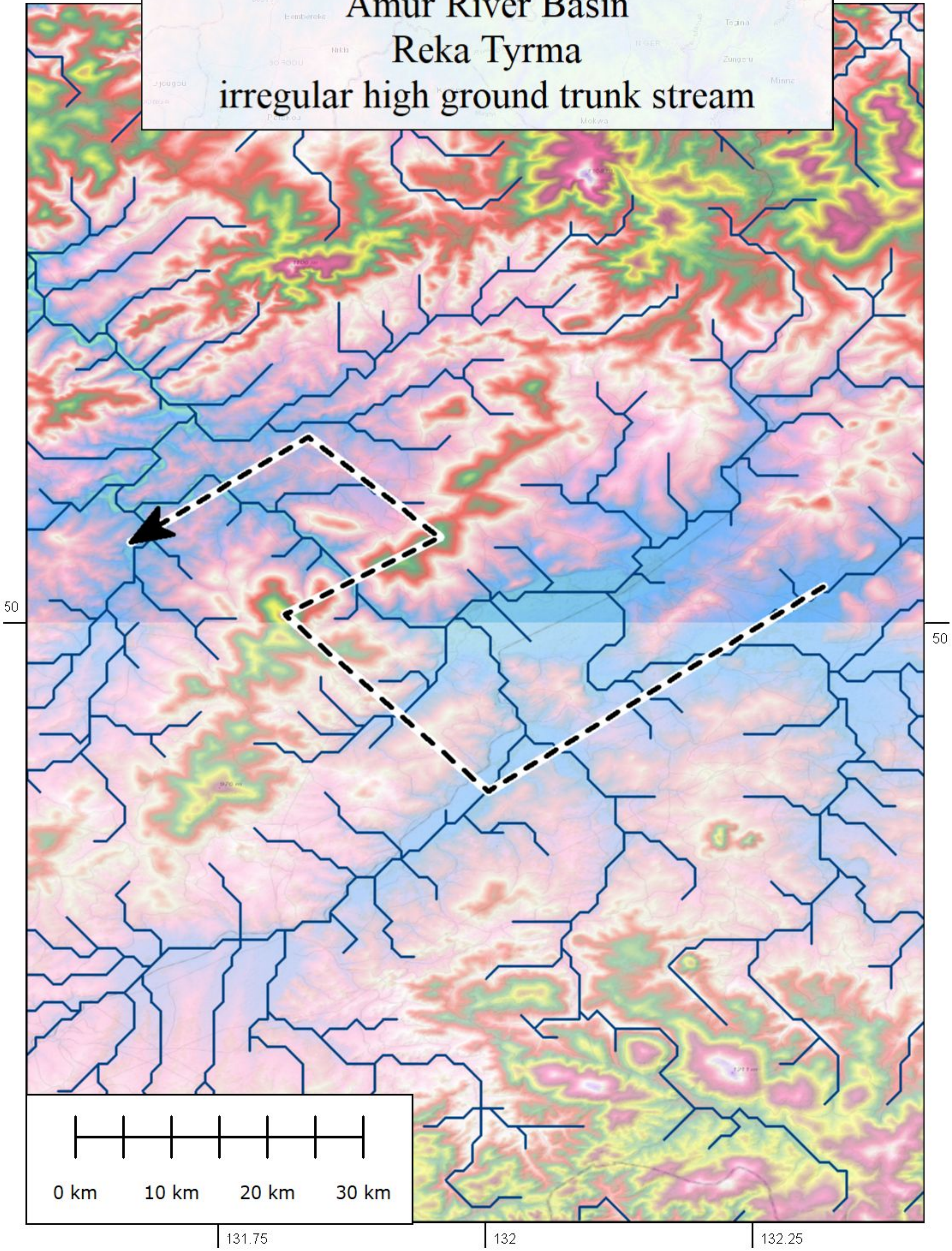

AS - 305
Indus River Basin
Kabul River
irregular high ground trunk stream

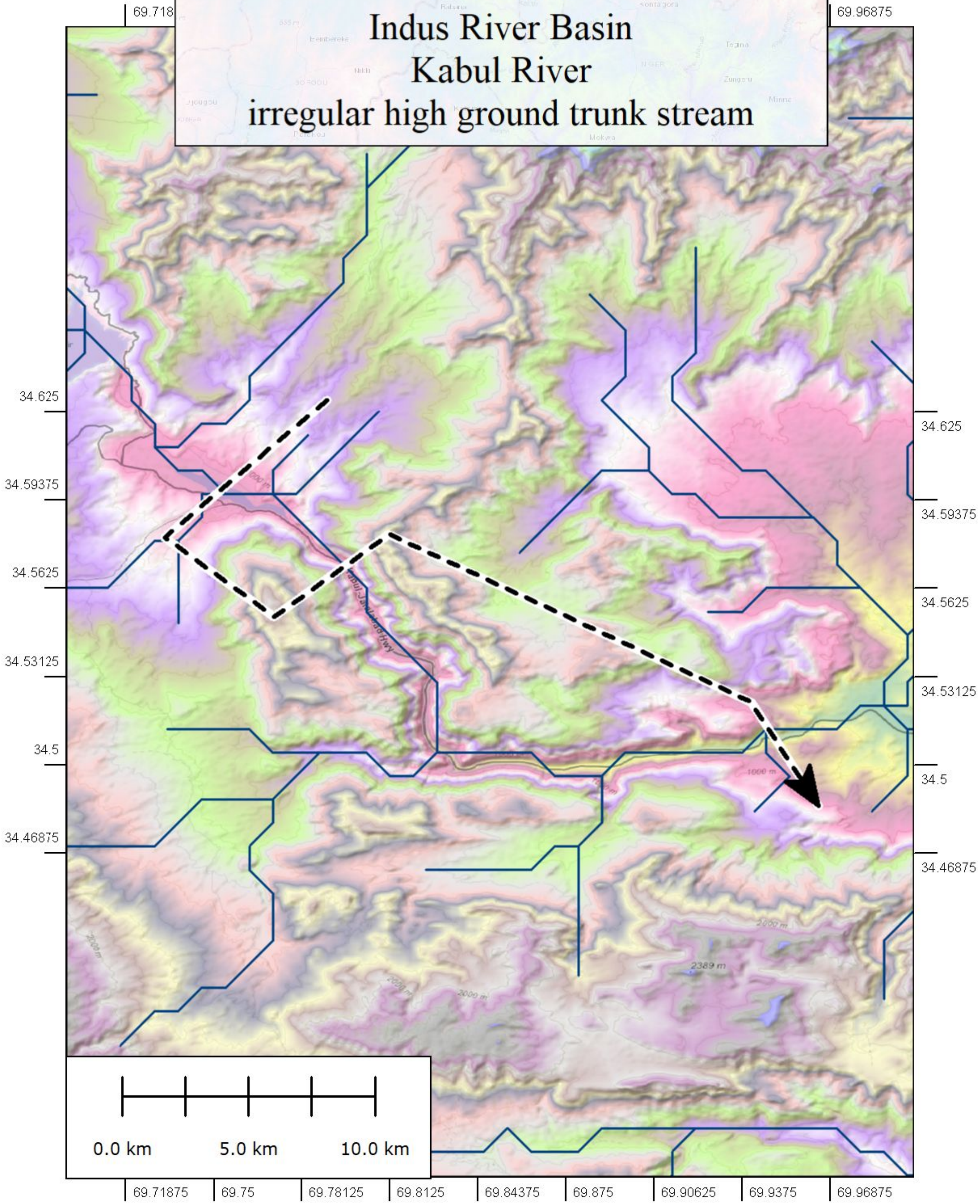

AS - 306
Yenisey River Basin
Selenge River
irregular high ground trunk stream

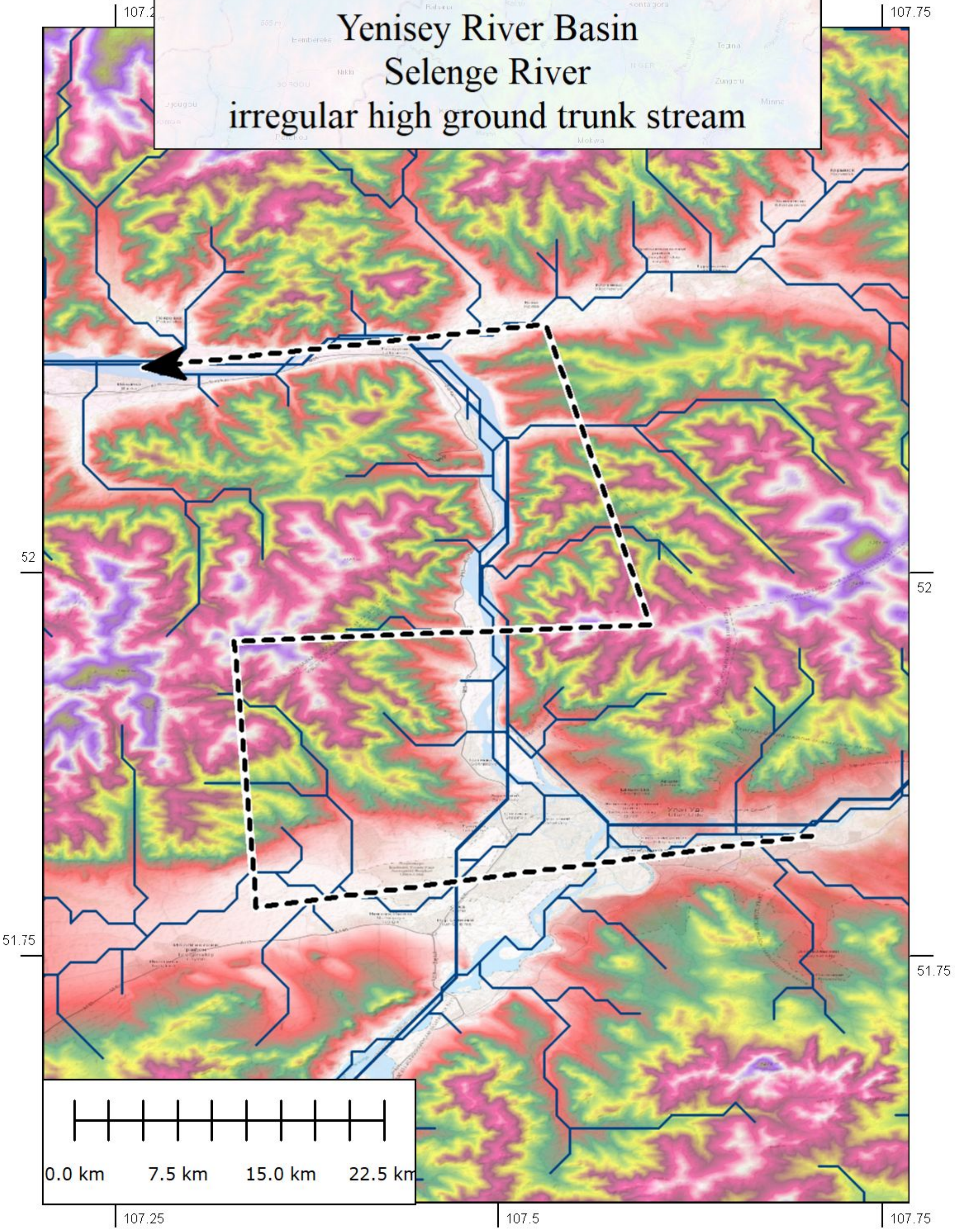

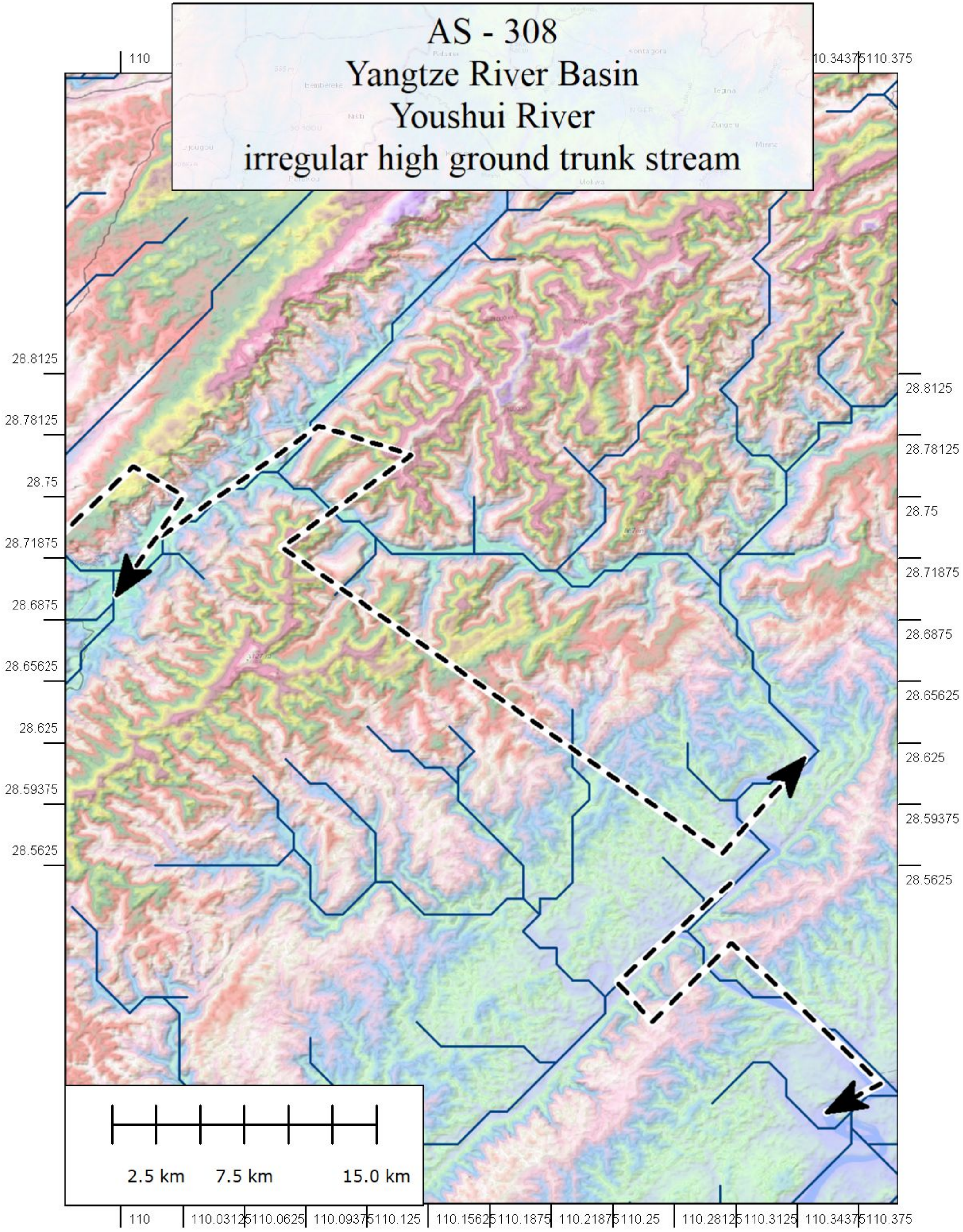
AS - 308
Yangtze River Basin
Youshui River
irregular high ground trunk stream
110
10.34375110.375
28.8125
28.78125
28.75
28.71875
28.6875
28.65625
28.625
28.59375
28.5625
2.5 km
7.5 km
15.0 km
110
110.03125110.0625
110.09375110.125
110.15625110.1875
110.21875110.25
110.28125110.3125
110.34375110.375

AS - 309
Mekong River Basin
Mekong River
irregular high ground trunk stream

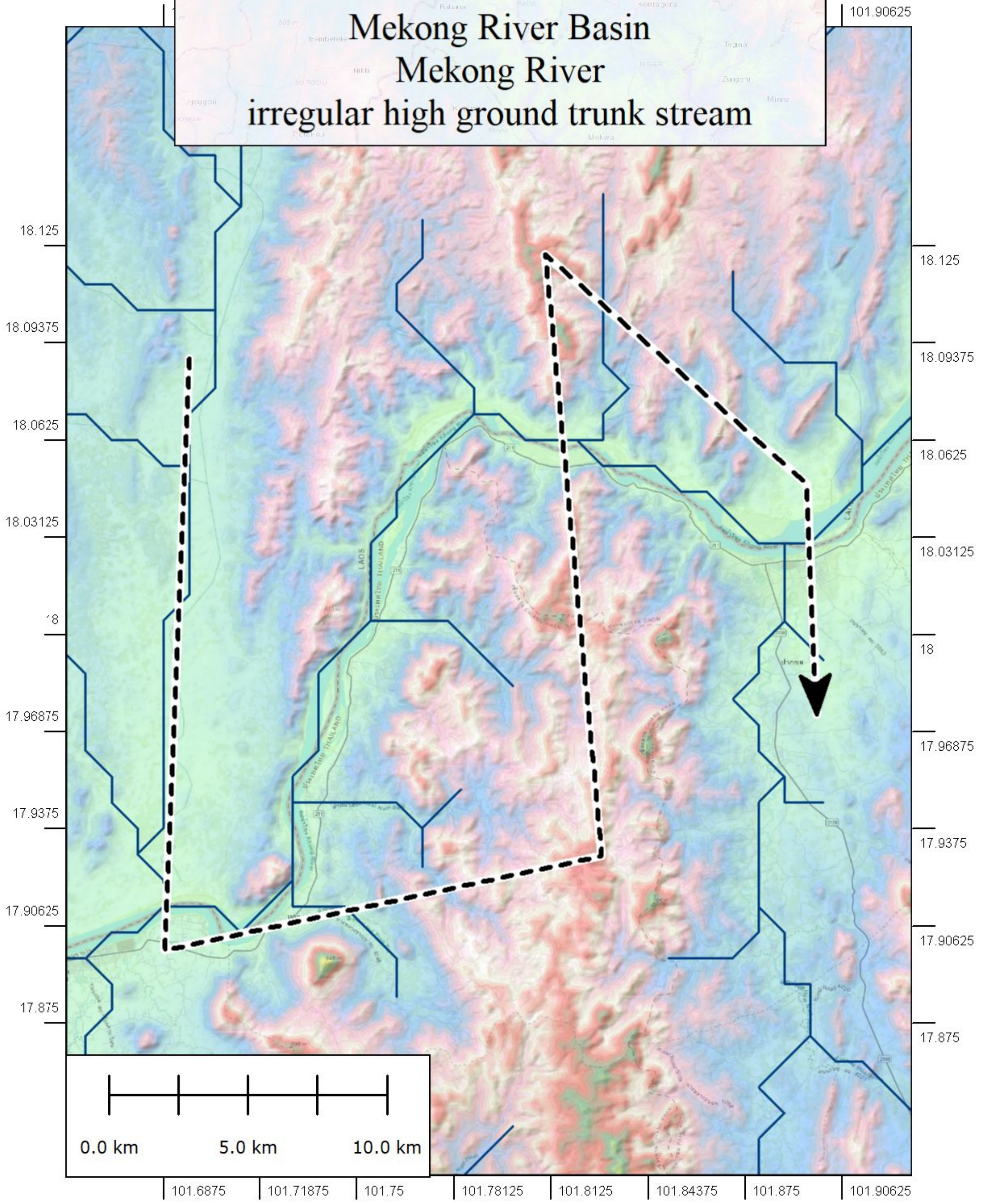

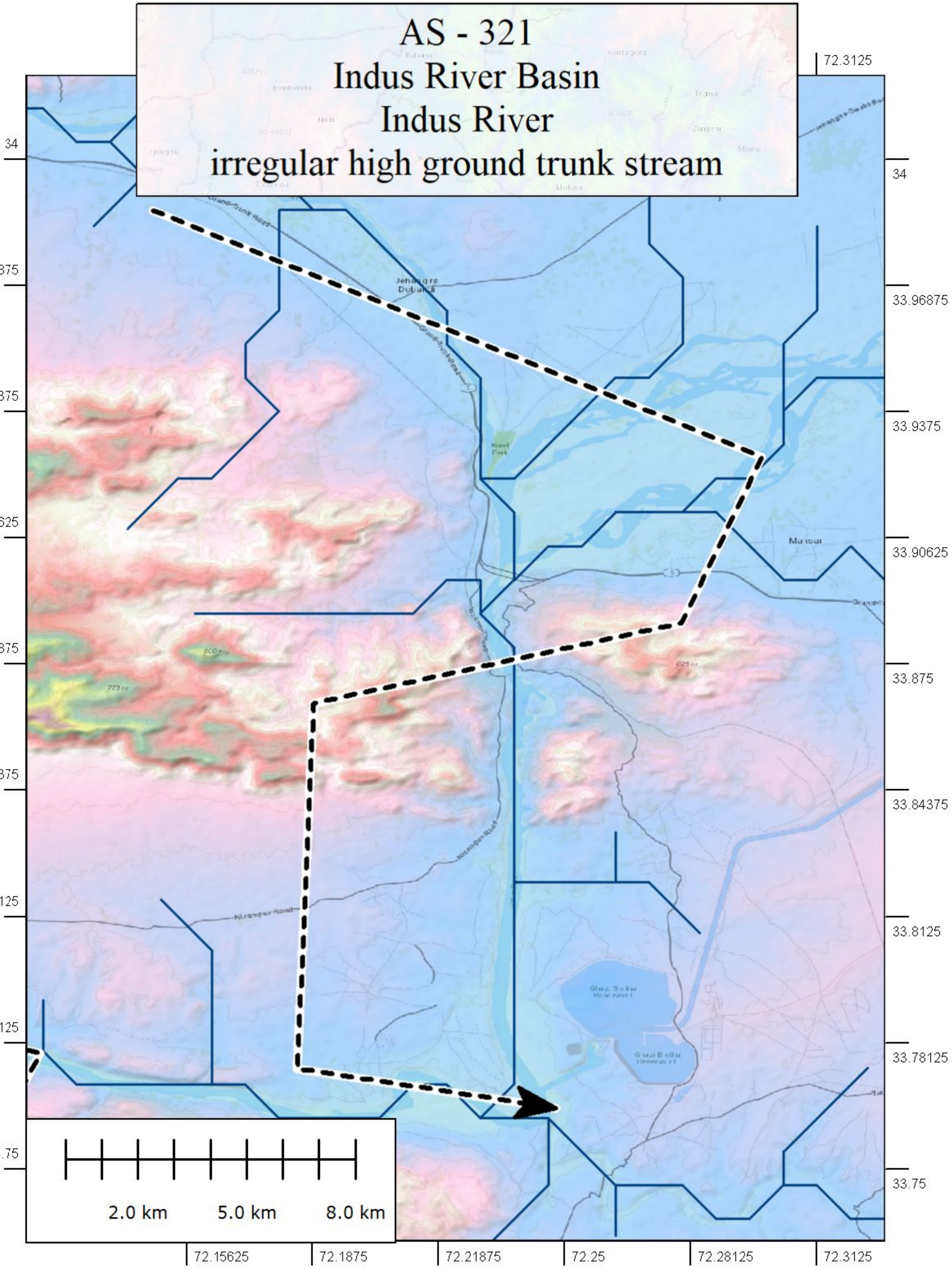
AS - 321
Indus River Basin
Indus River
irregular high ground trunk stream
34
33.96875
33.9375
33.90625
33.875
33.84375
33.8125
33.78125
33.75
72.15625
72.1875
72.21875
72.25
72.28125
72.3125
2.0 km
5.0 km
8.0 km

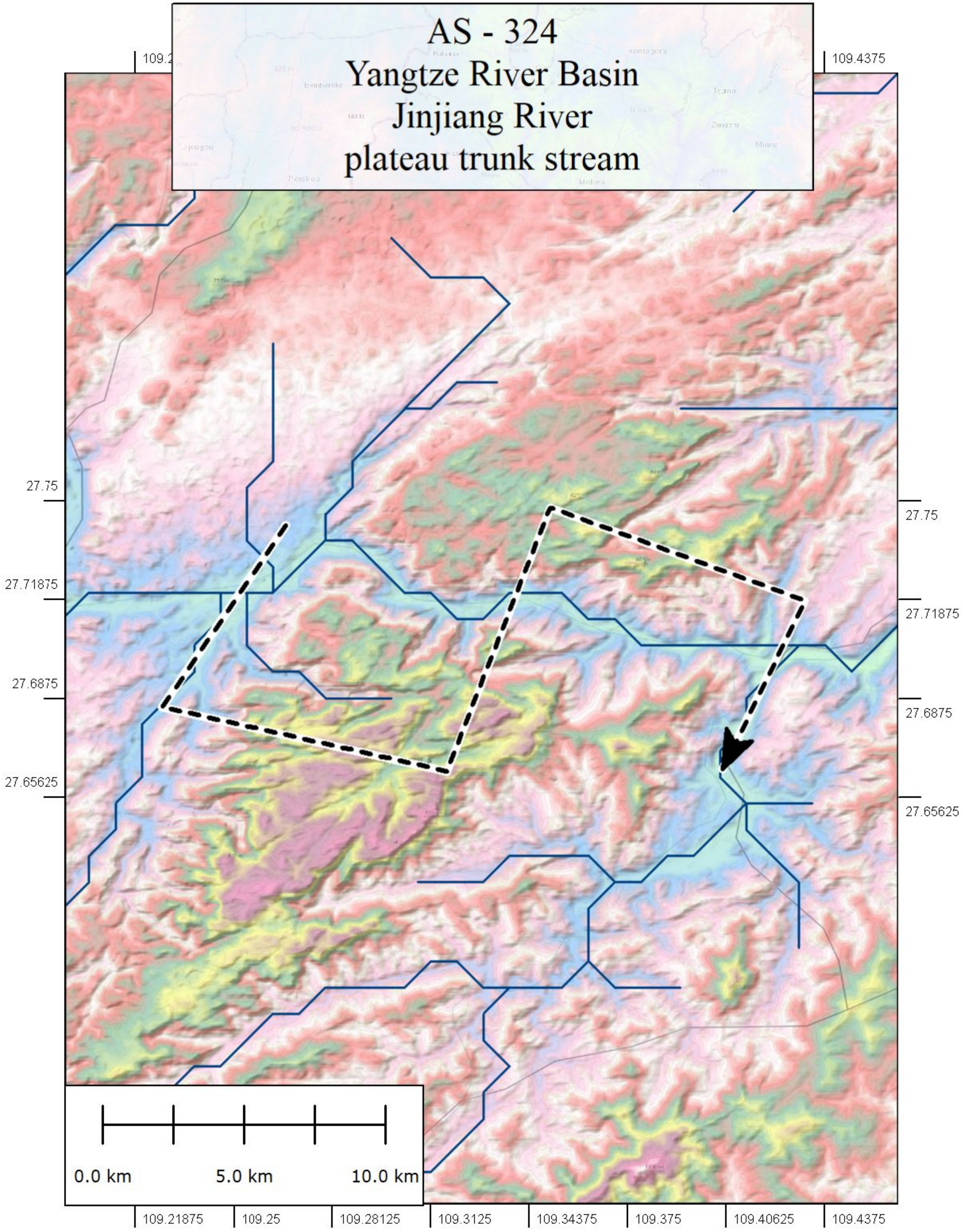
AS - 324
Yangtze River Basin
Jinjiang River
plateau trunk stream
109.4375
27.75
27.71875
27.6875
27.65625
0.0 km
5.0 km
10.0 km
109.21875
109.25
109.28125
109.3125
109.34375
109.375
109.40625
109.4375

AS - 326
Yenisey River Basin
Reka Uda River
irregular high ground trunk stream

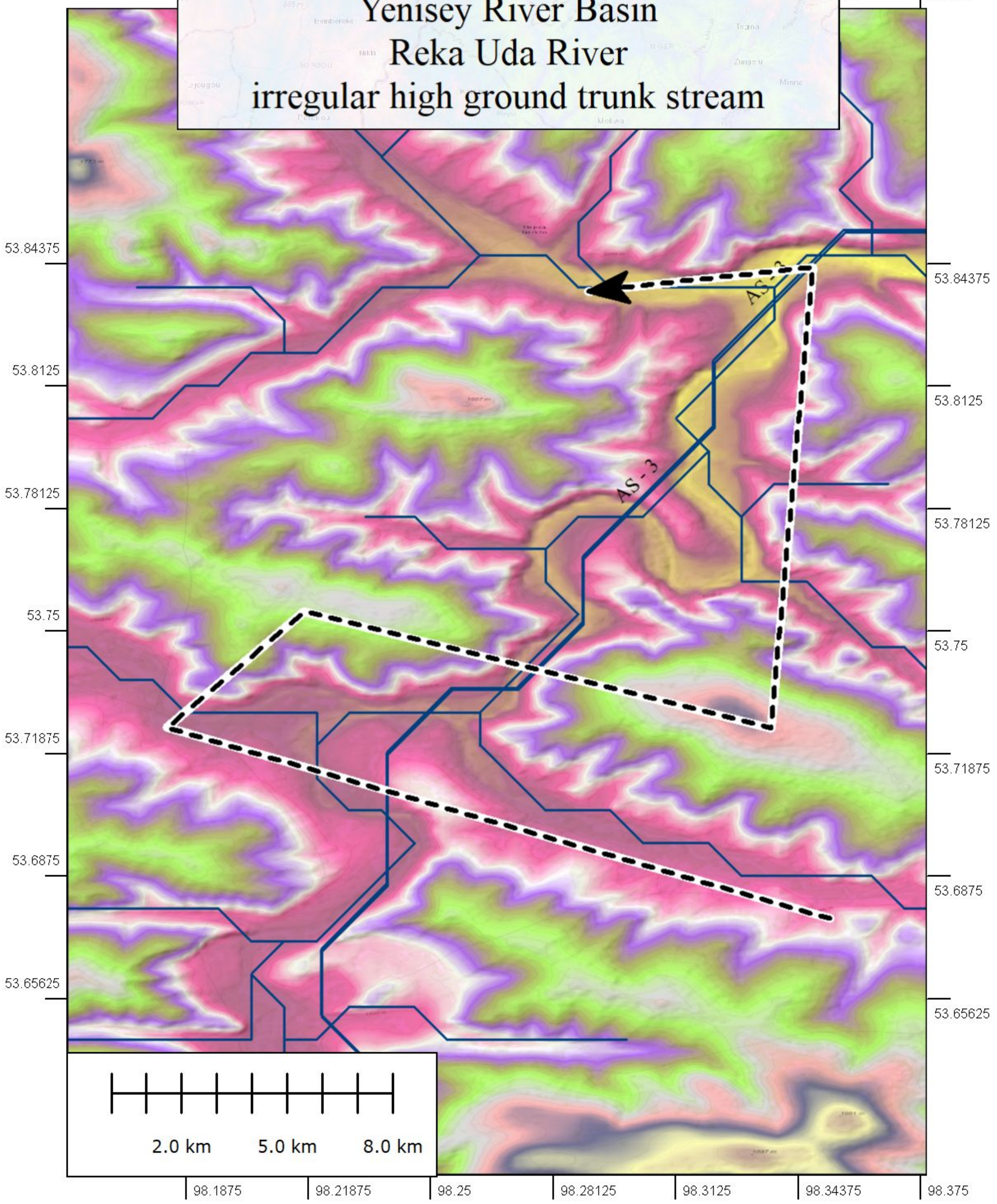

AS - 335
Mekong River Basin
Mekong River tributary
irregular high ground trunk stream

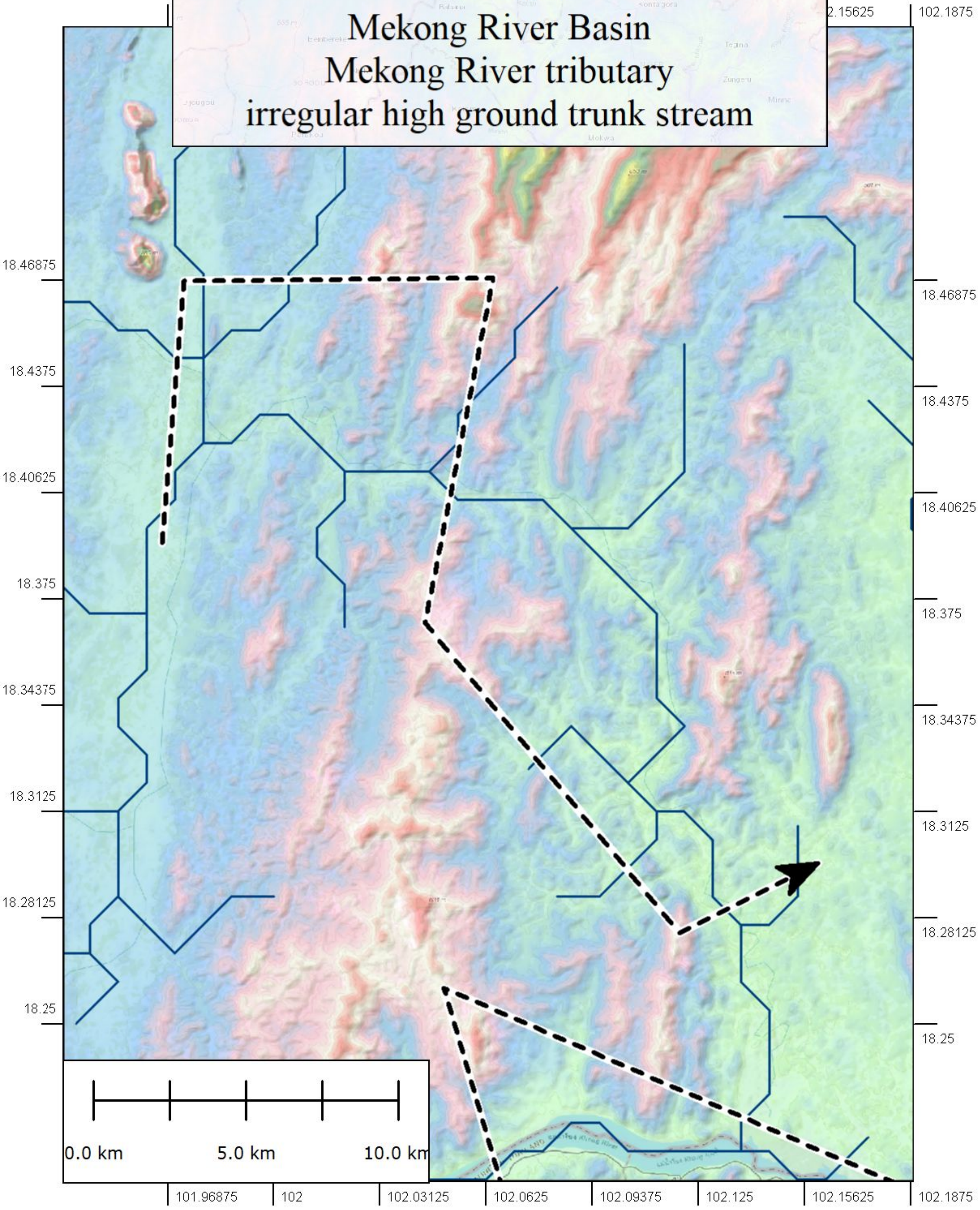

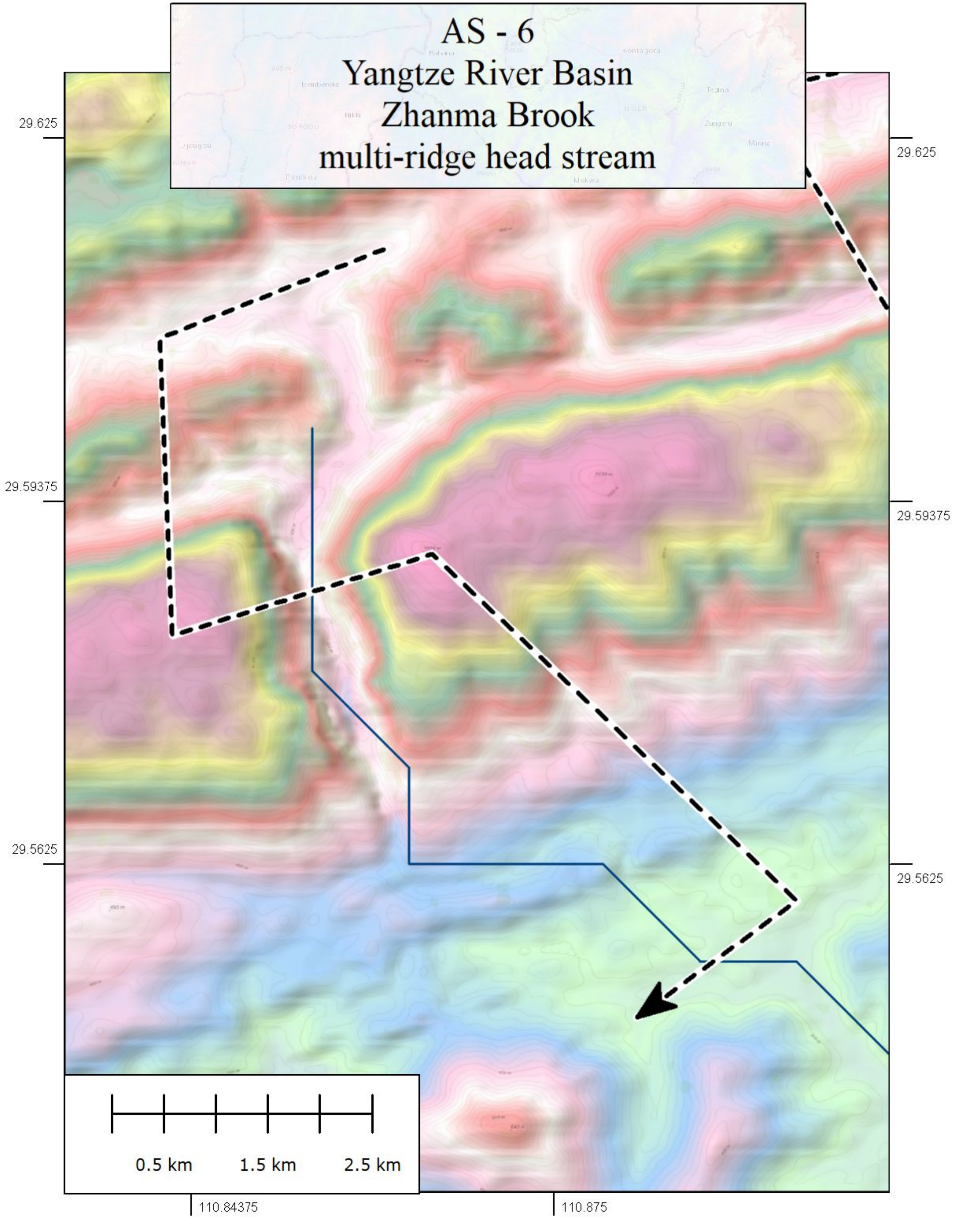
AS - 6
Yangtze River Basin
Zhanma Brook
multi-ridge head stream
29.625
29.625
29.59375
29.59375
29.5625
29.5625
0.5 km
1.5 km
2.5 km
110.84375
110.875

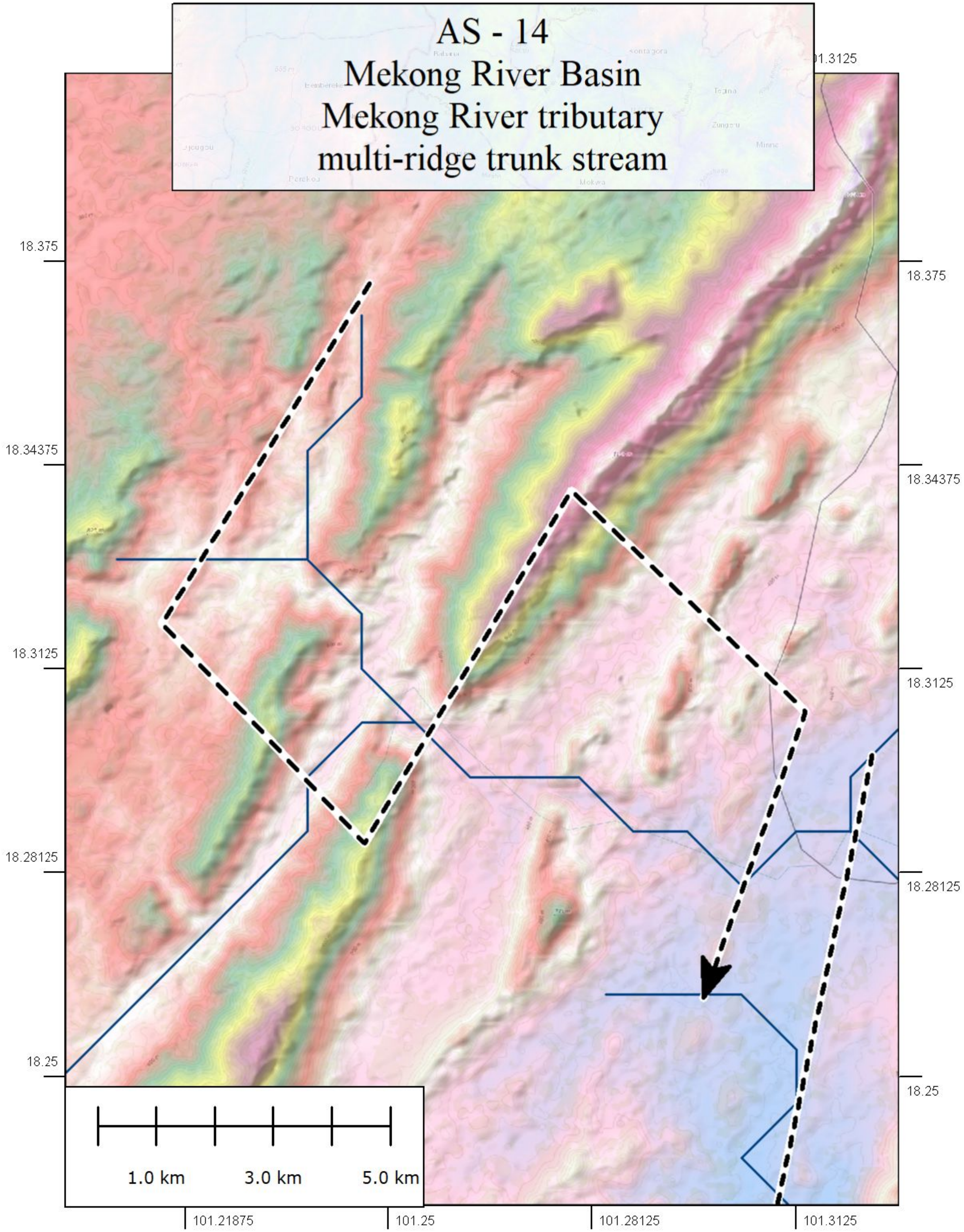
AS - 14
Mekong River Basin
Mekong River tributary
multi-ridge trunk stream
18.375
18.34375
18.3125
18.28125
18.25
101.21875
101.25
101.28125
101.3125
1.0 km
3.0 km
5.0 km

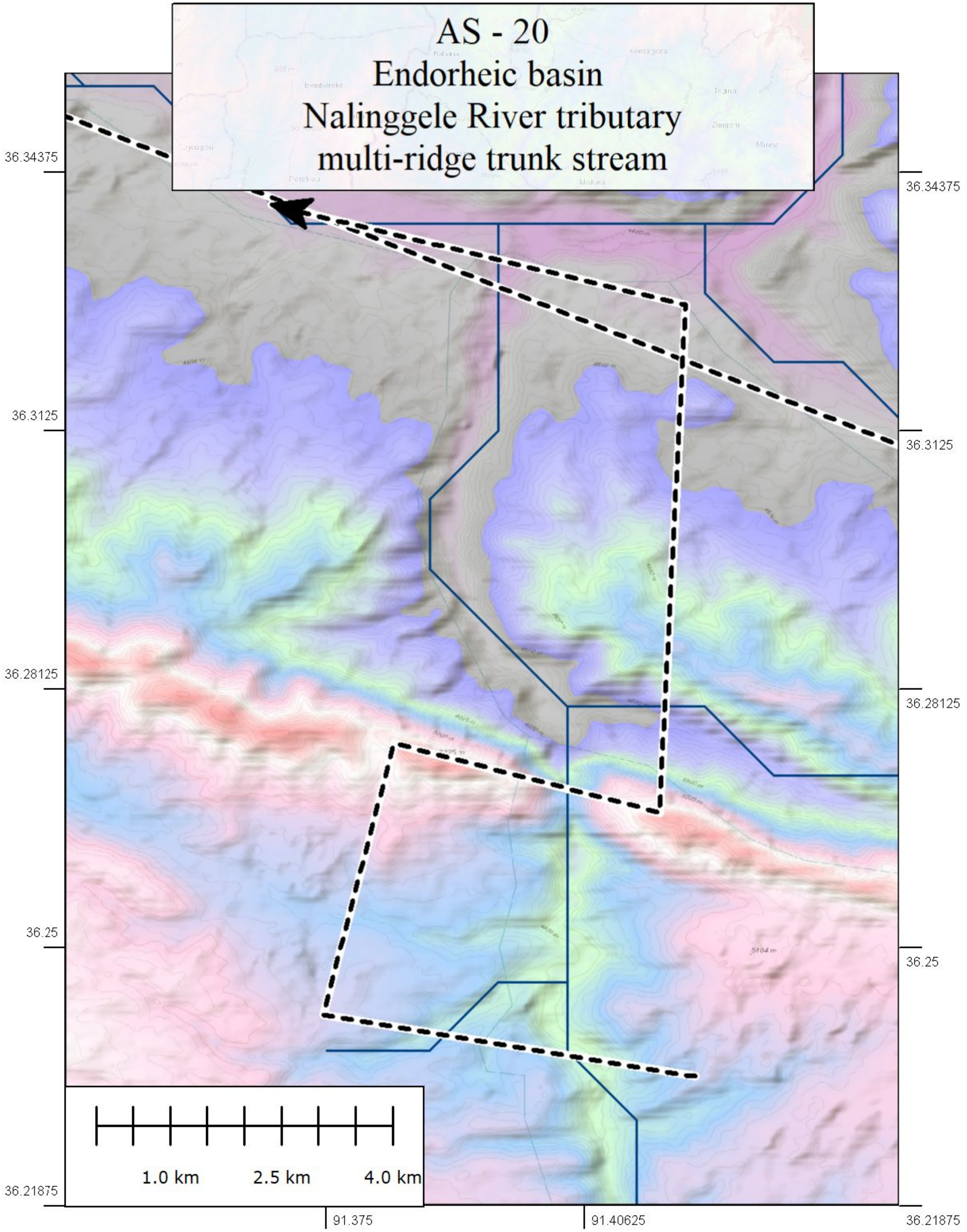
AS - 20
Endorheic basin
Nalinggele River tributary
multi-ridge trunk stream
36.34375
36.3125
36.28125
36.25
36.21875
91.375
91.40625
1.0 km
2.5 km
4.0 km

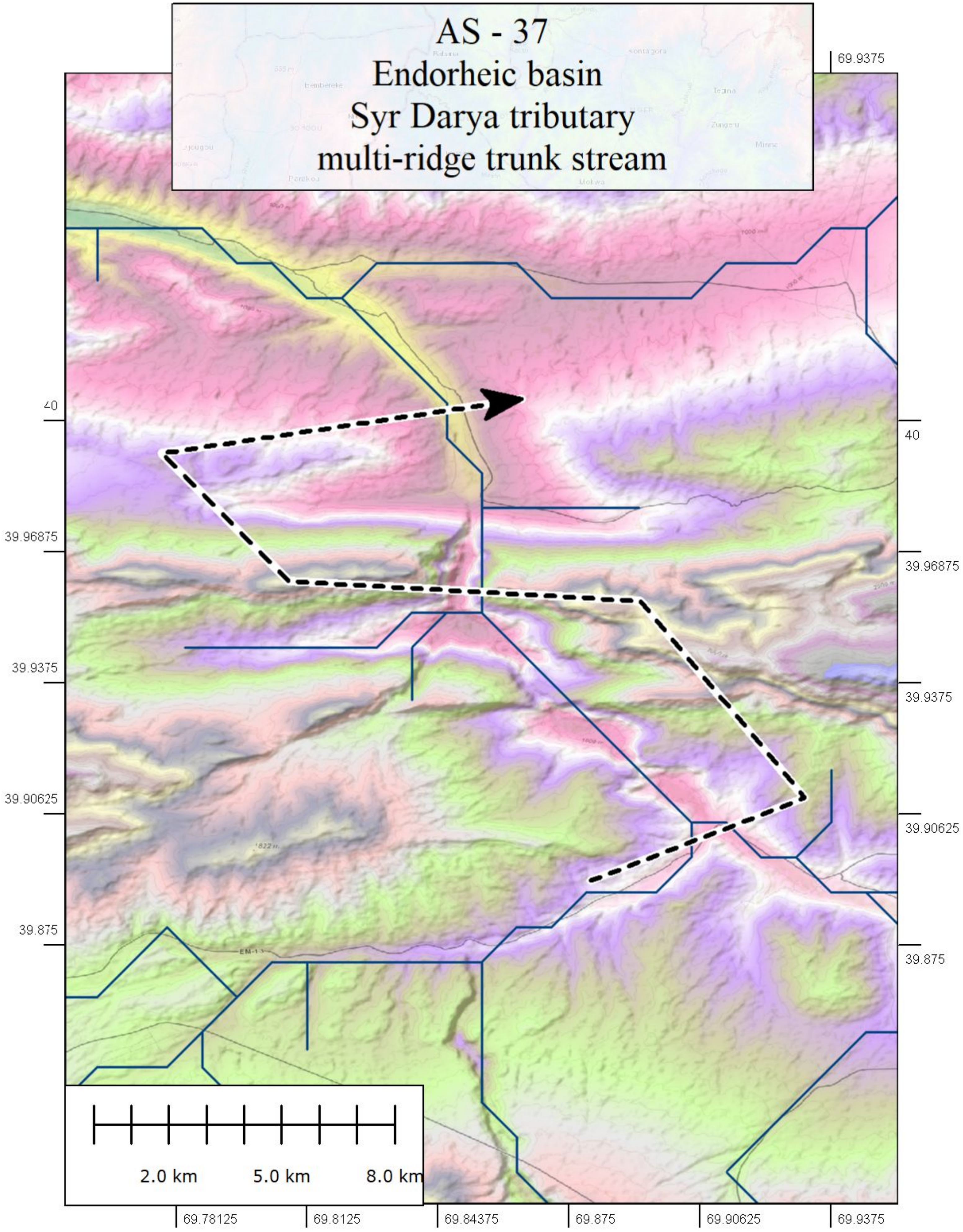
AS - 37
Endorheic basin
Syr Darya tributary
multi-ridge trunk stream
69.9375
40
39.96875
39.9375
39.90625
39.875
69.78125
69.8125
69.84375
69.875
69.90625
69.9375
2.0 km
5.0 km
8.0 km

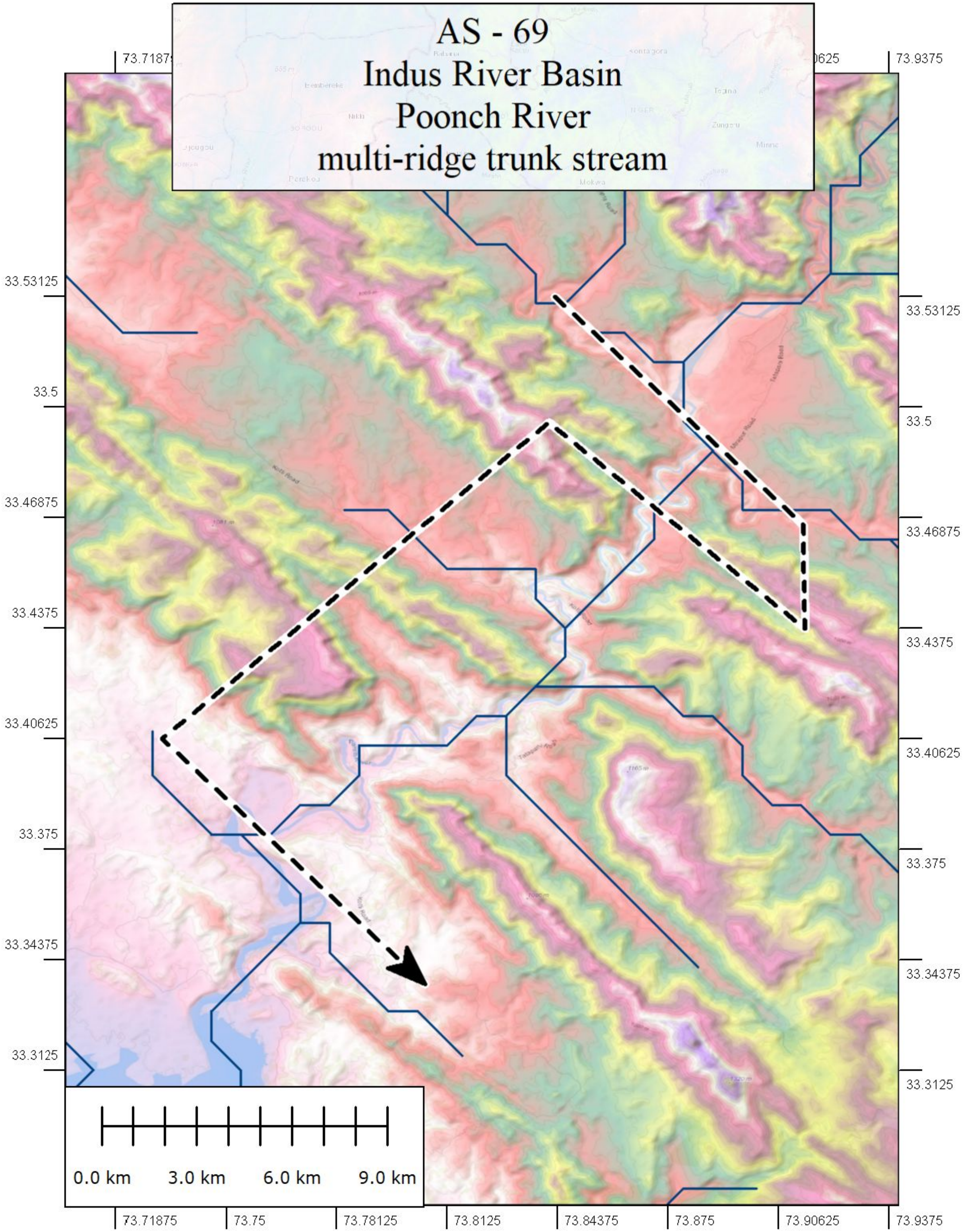
AS - 69
Indus River Basin
Poonch River
multi-ridge trunk stream
73.71875
73.75
73.78125
73.8125
73.84375
73.875
73.90625
73.9375
33.53125
33.5
33.46875
33.4375
33.40625
33.375
33.34375
33.3125
0.0 km
3.0 km
6.0 km
9.0 km

AS - 79
Yangtze River Basin
Huaxi River
multi-ridge trunk stream

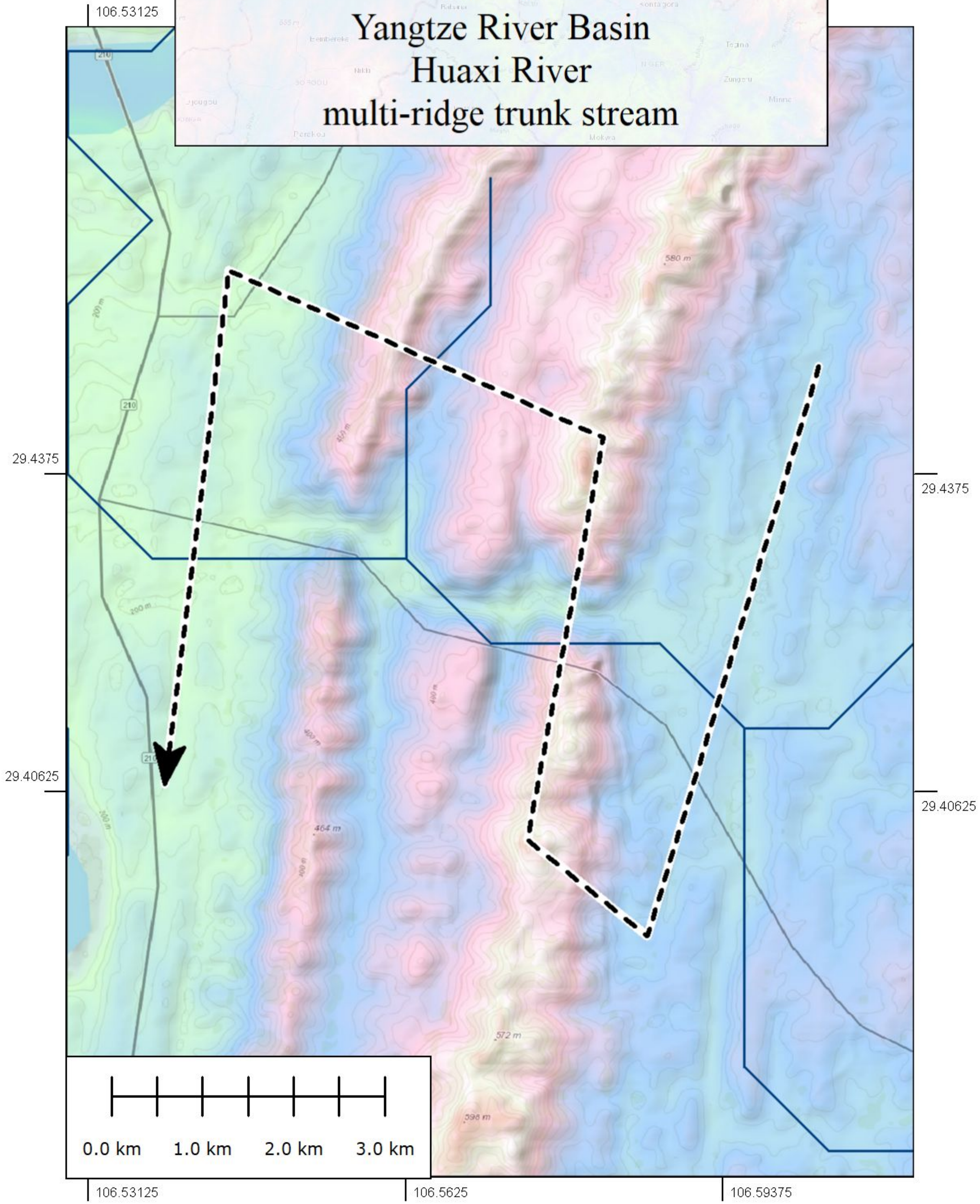

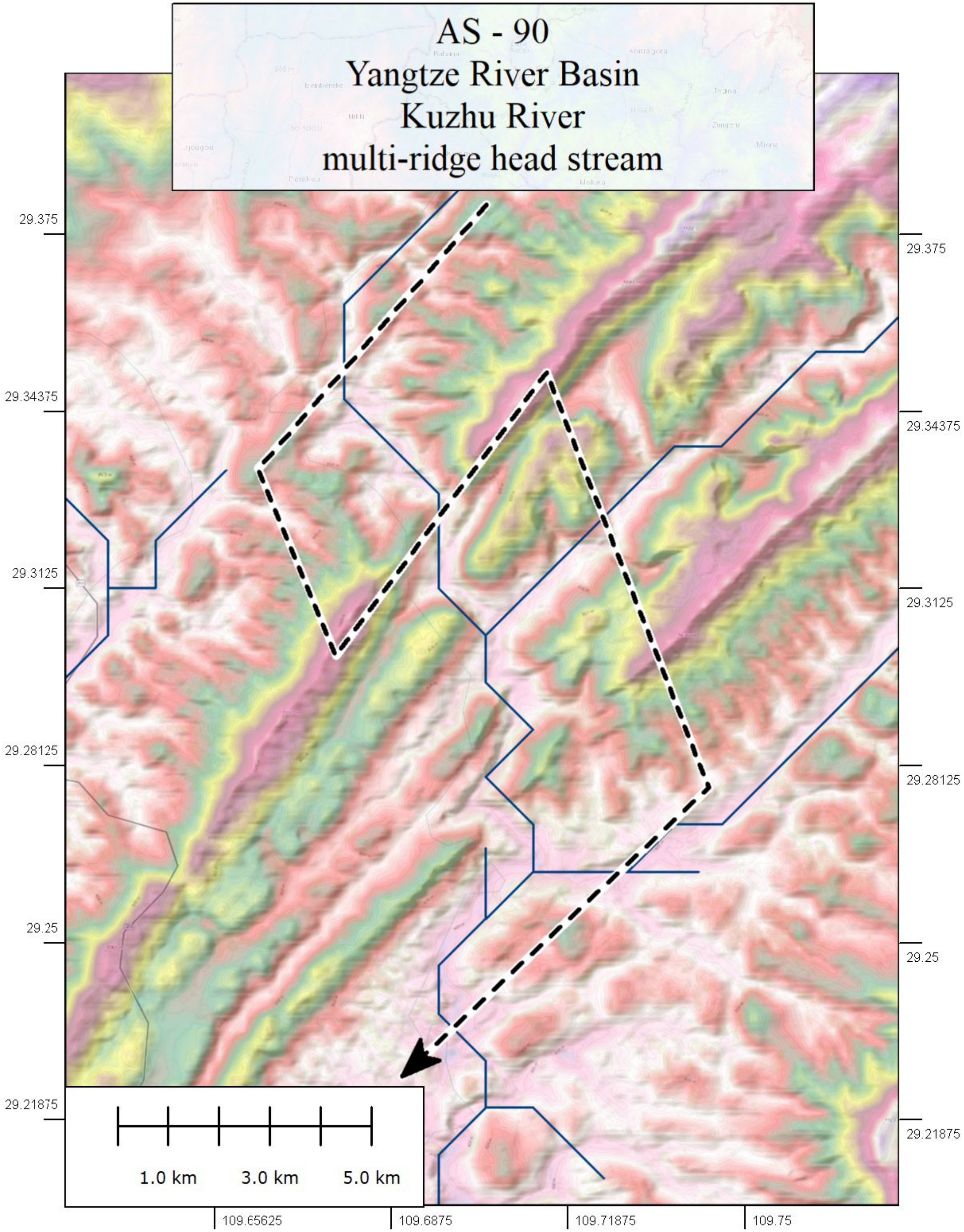

AS - 90
Yangtze River Basin
Kuzhu River
multi-ridge head stream
29.375
29.34375
29.3125
29.28125
29.25
29.21875
1.0 km
3.0 km
5.0 km
109.65625
109.6875
109.71875
109.75

AS - 92
Indus River Basin
multi-ridge trunk stream

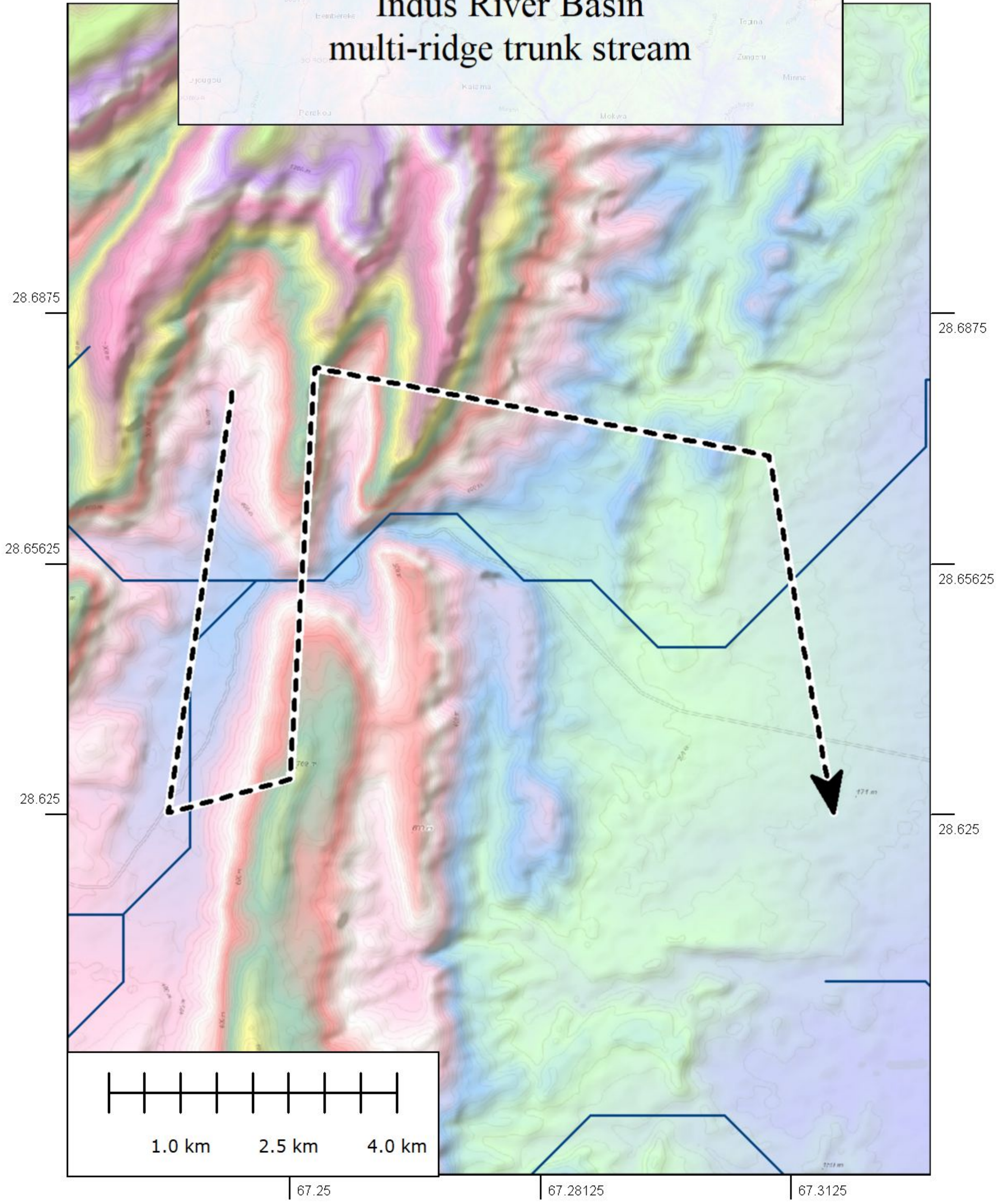

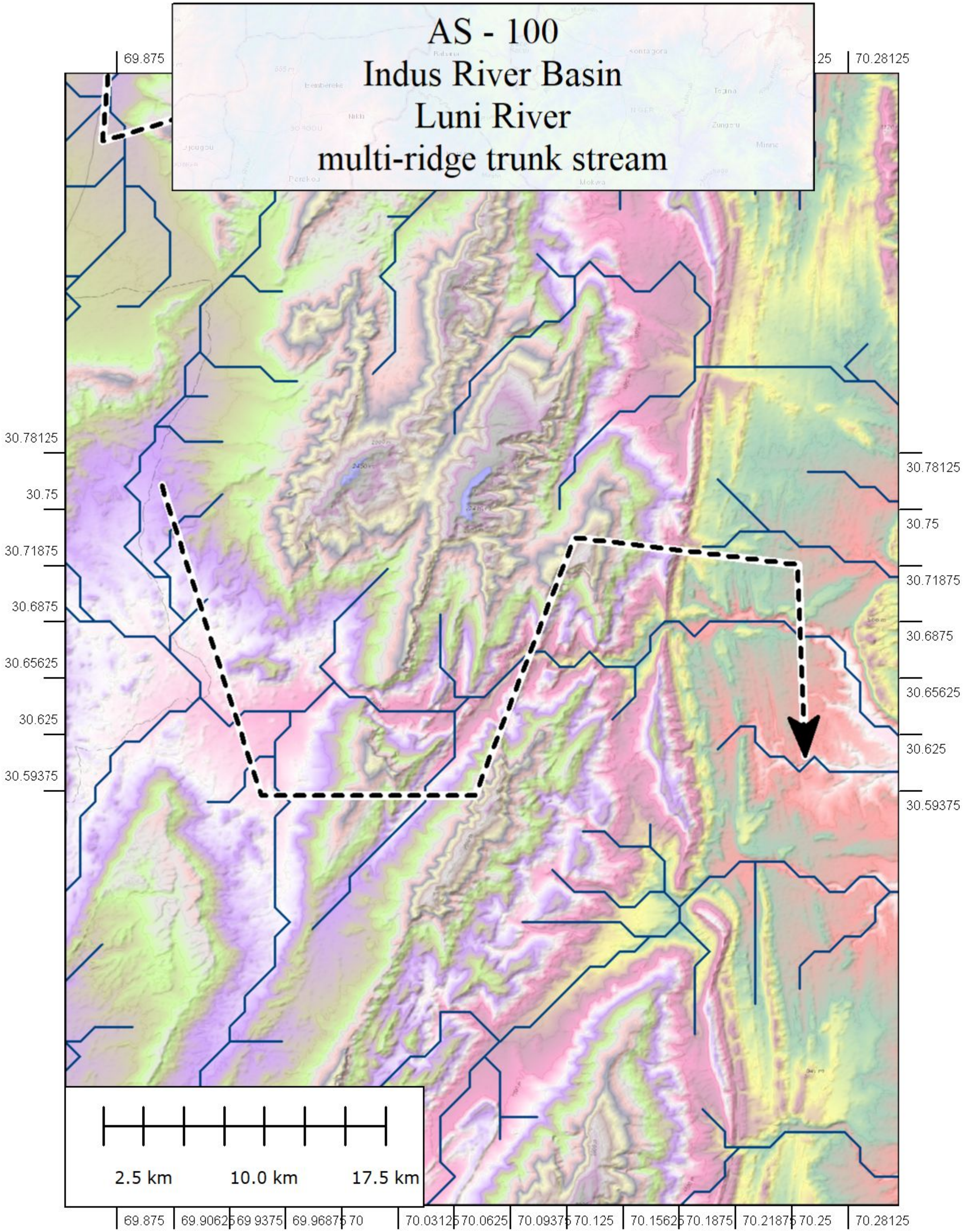
AS - 100
Indus River Basin
Luni River
multi-ridge trunk stream
69.875
70.28125
30.78125
30.75
30.71875
30.6875
30.65625
30.625
30.59375
2.5 km
10.0 km
17.5 km
69.875
69.90625
69.9375
69.96875
70
70.03125
70.0625
70.09375
70.125
70.15625
70.1875
70.21875
70.25
70.28125

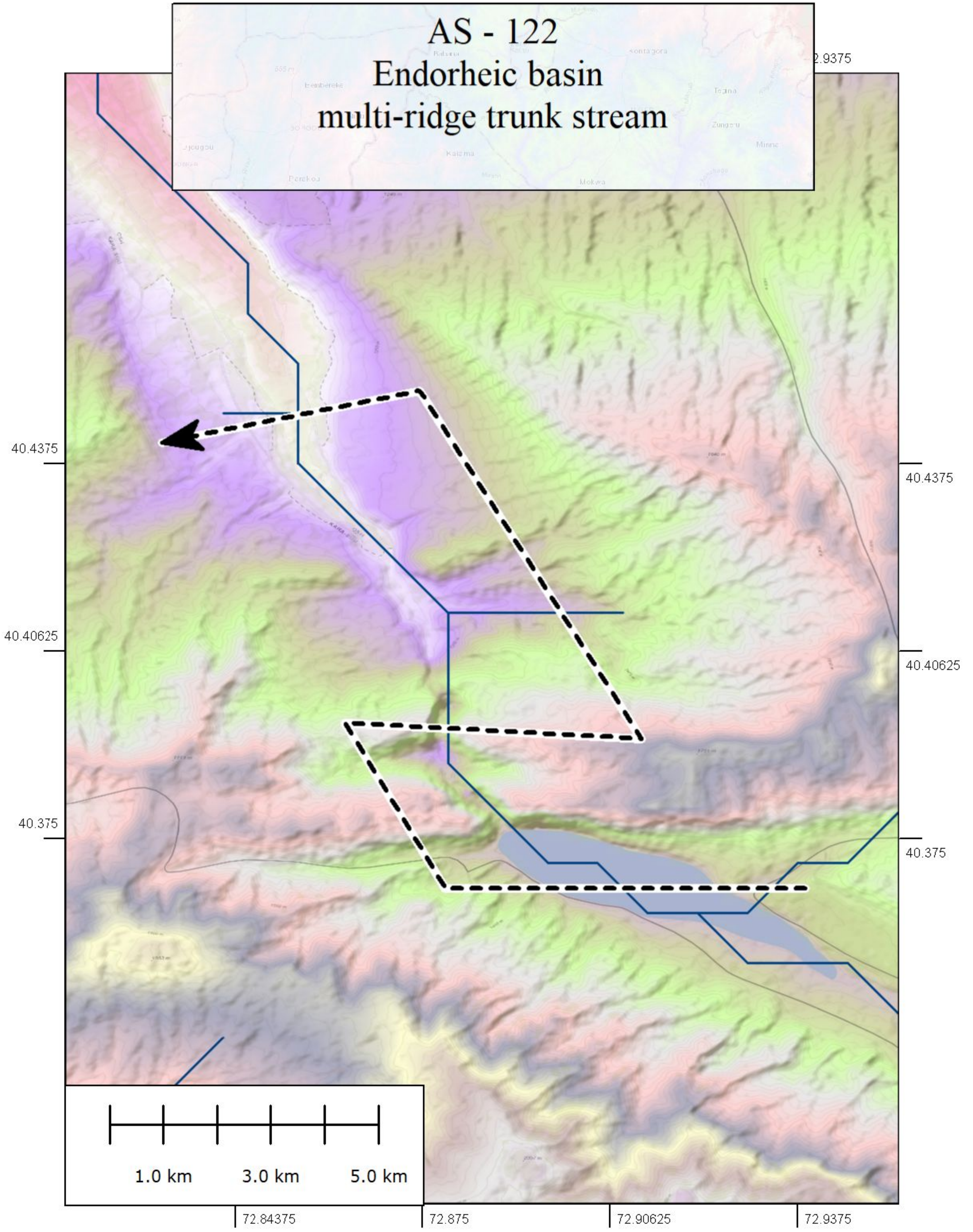

AS - 122
Endorheic basin
multi-ridge trunk stream
40.4375
40.40625
40.375
40.4375
40.40625
40.375
72.84375
72.875
72.90625
72.9375
72.9375
1.0 km
3.0 km
5.0 km

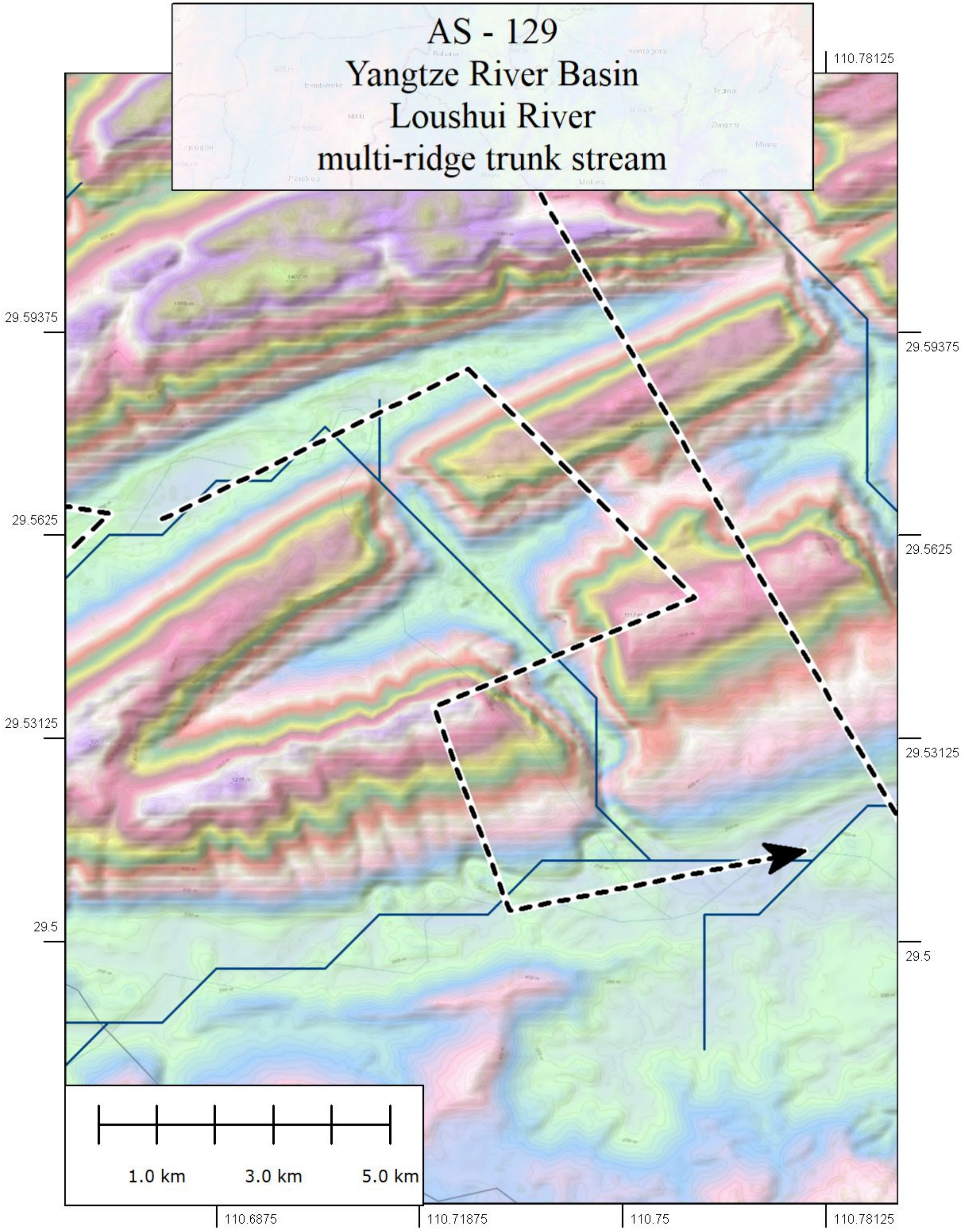
AS - 129
Yangtze River Basin
Loushui River
multi-ridge trunk stream
110.78125
29.59375
29.5625
29.53125
29.5
1.0 km
3.0 km
5.0 km
110.6875
110.71875
110.75
110.78125

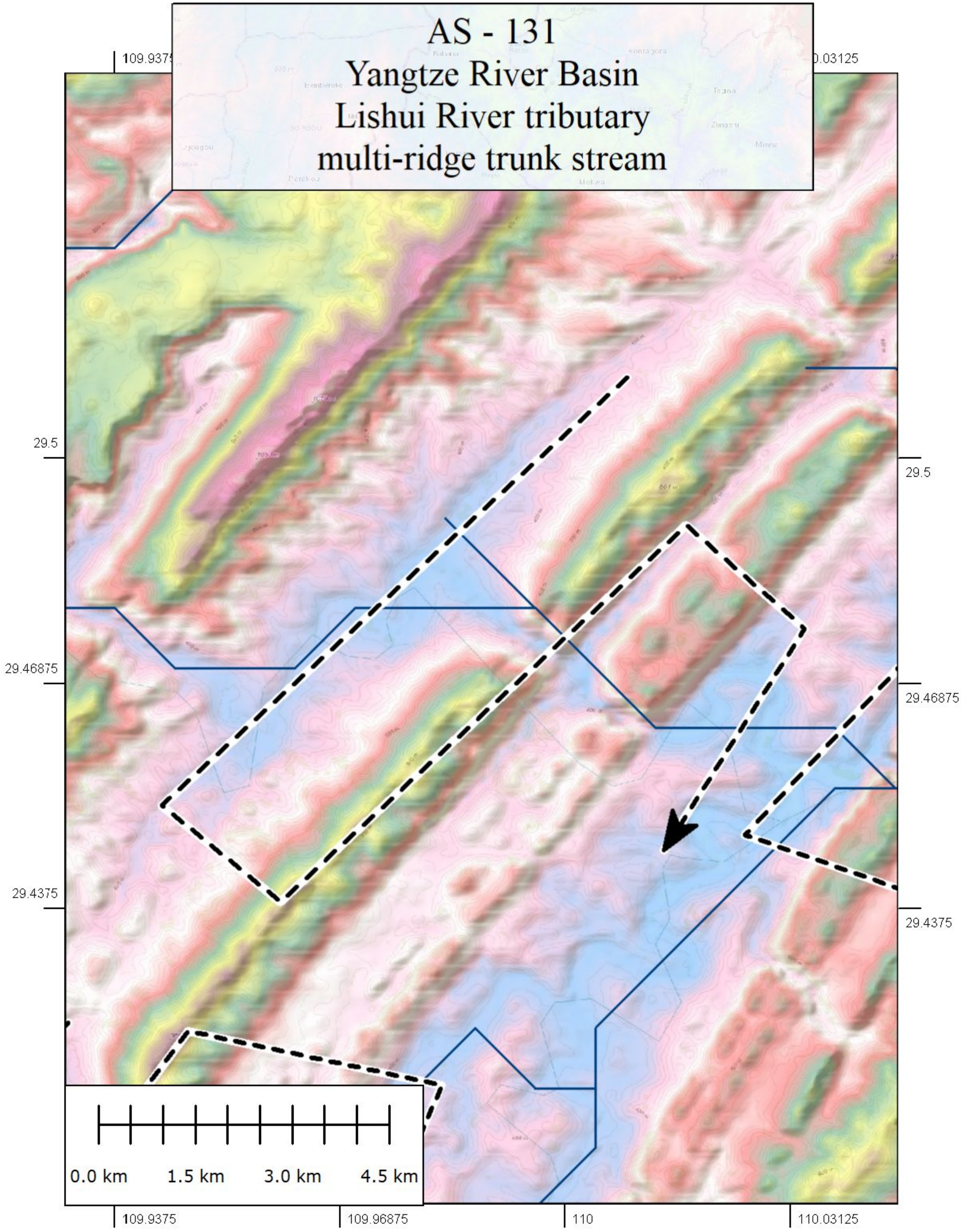

AS - 131
Yangtze River Basin
Lishui River tributary
multi-ridge trunk stream
109.9375
0.03125
29.5
29.46875
29.4375
0.0 km
1.5 km
3.0 km
4.5 km
109.96875
110
110.03125

AS - 144
Yangtze River Basin
Lishui River tributary
multi-ridge head stream

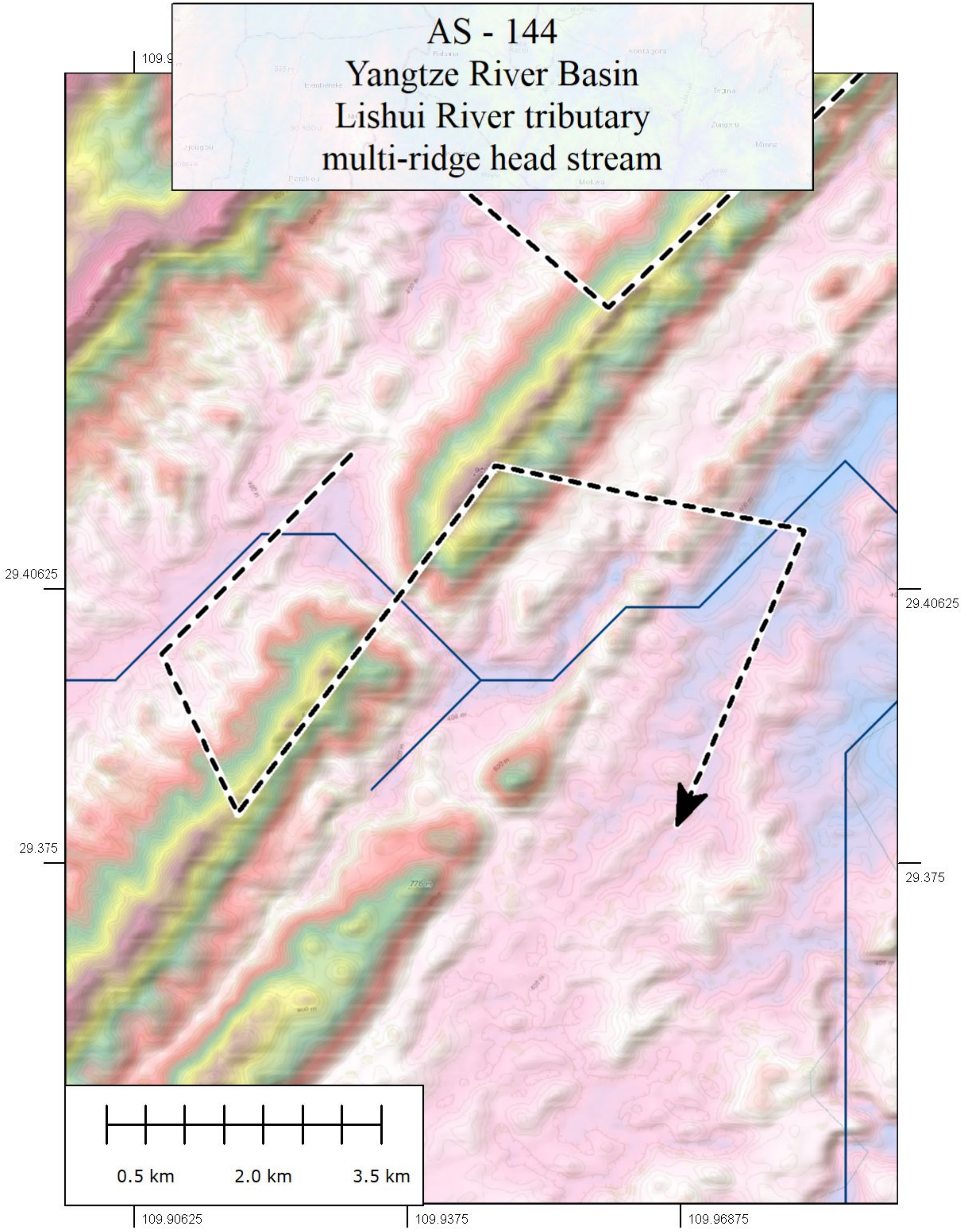

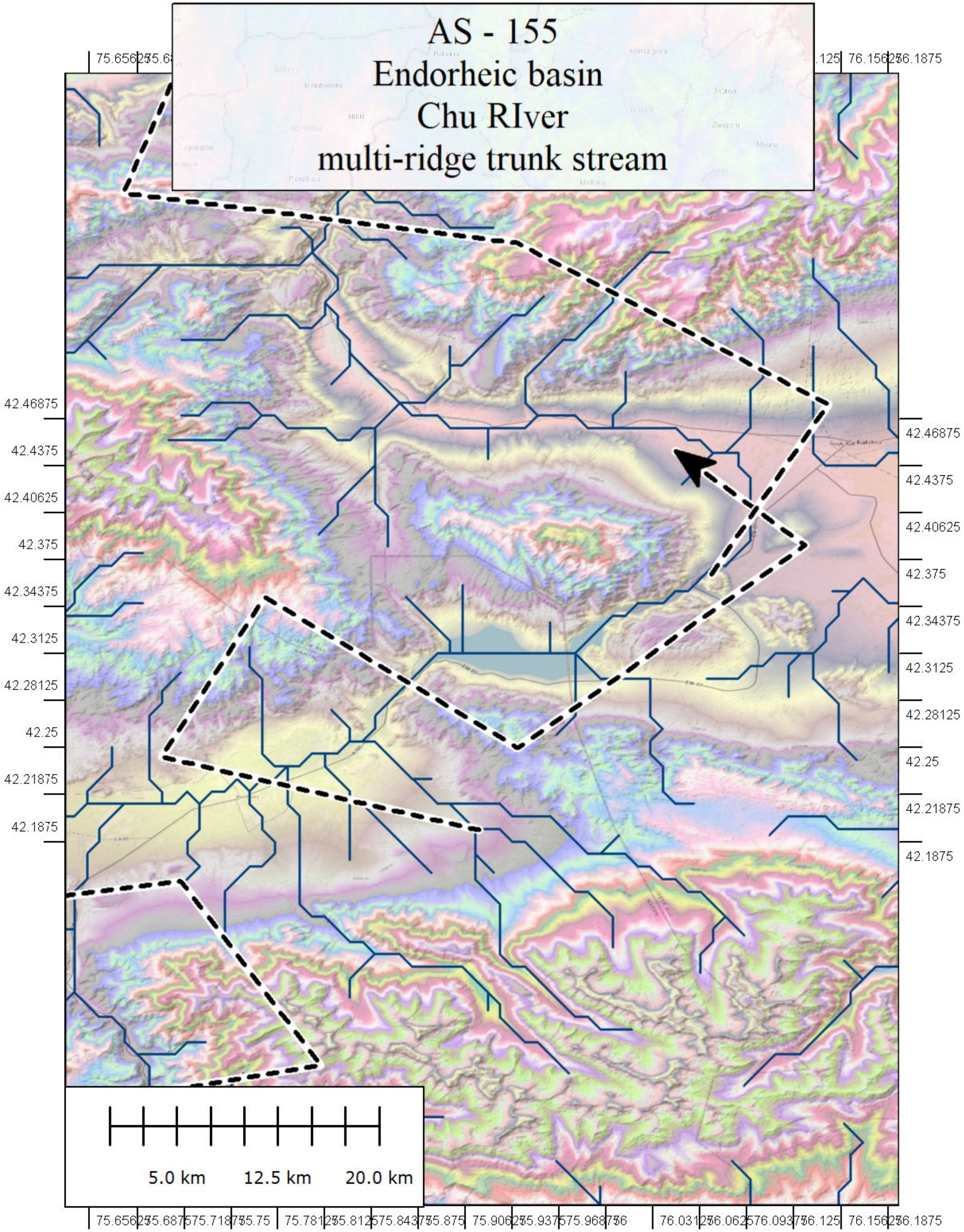
AS - 155
Endorheic basin
Chu RIver
multi-ridge trunk stream
5.0 km
12.5 km
20.0 km
42.46875
42.4375
42.40625
42.375
42.34375
42.3125
42.28125
42.25
42.21875
42.1875
75.65625
75.6875
75.71875
75.75
75.78125
75.8125
75.84375
75.875
75.90625
75.9375
75.96875
76
76.03125
76.0625
76.09375
76.125
76.15625
76.1875

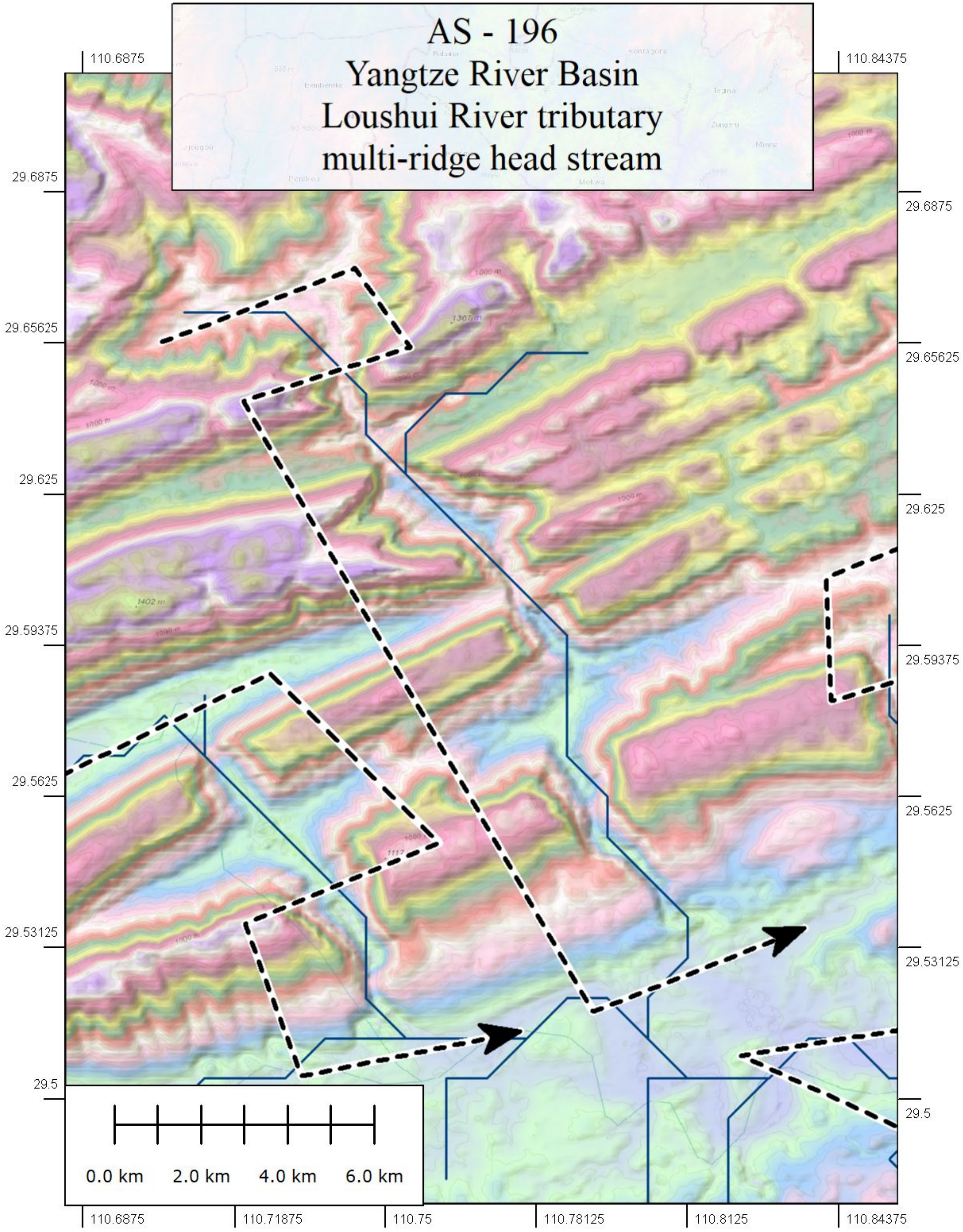
AS - 196
Yangtze River Basin
Loushui River tributary
multi-ridge head stream
110.6875
110.71875
110.75
110.78125
110.8125
110.84375
29.6875
29.65625
29.625
29.59375
29.5625
29.53125
29.5
1000 m
1367 m
1402 m
1117
0.0 km
2.0 km
4.0 km
6.0 km

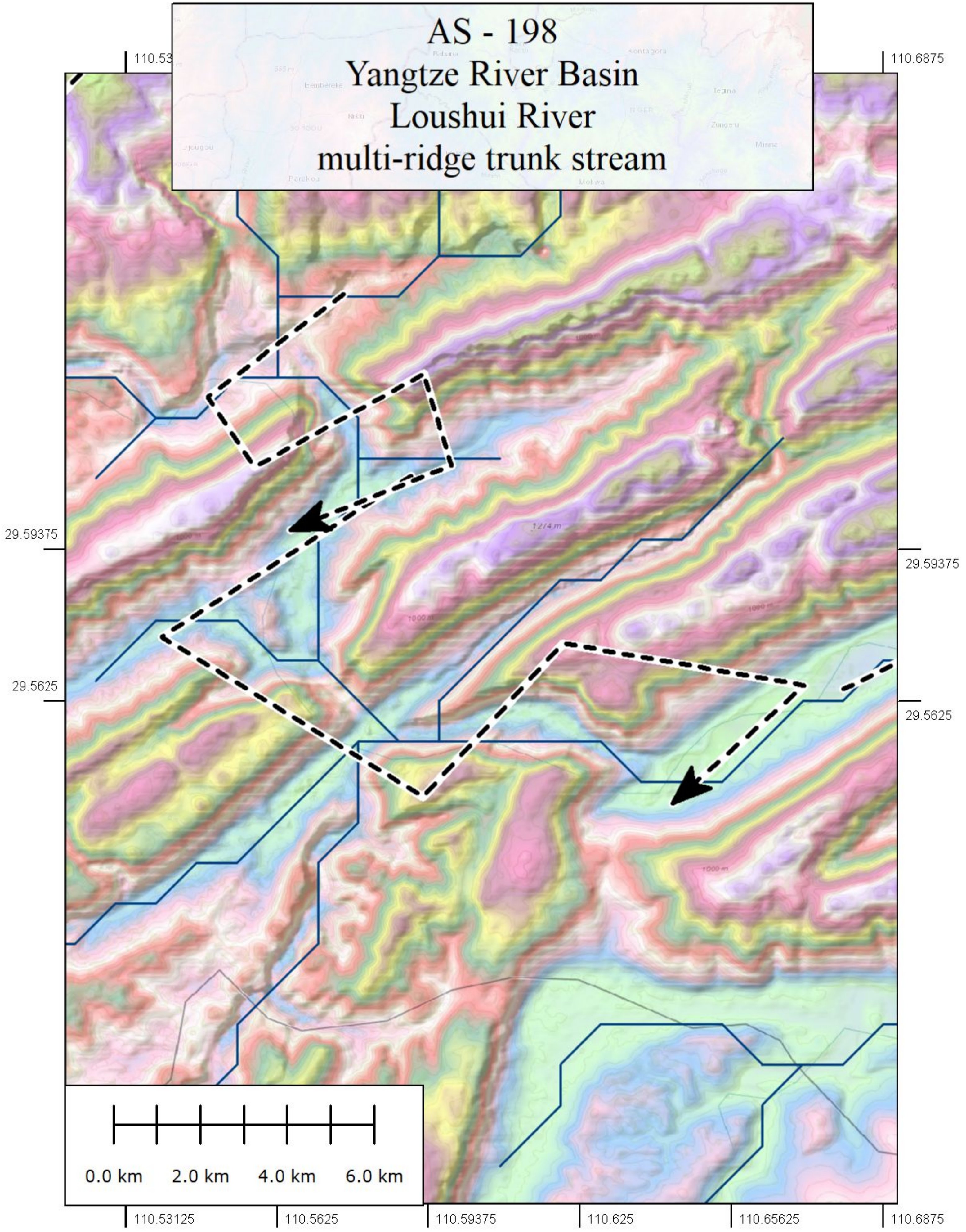
AS - 198
Yangtze River Basin
Loushui River
multi-ridge trunk stream
110.53125
110.5625
110.59375
110.625
110.65625
110.6875
29.59375
29.5625
1274 m
1000 m
0.0 km
2.0 km
4.0 km
6.0 km

AS - 199
Yangtze River Basin
Zhunagta Resrvoir
multi-ridge head stream

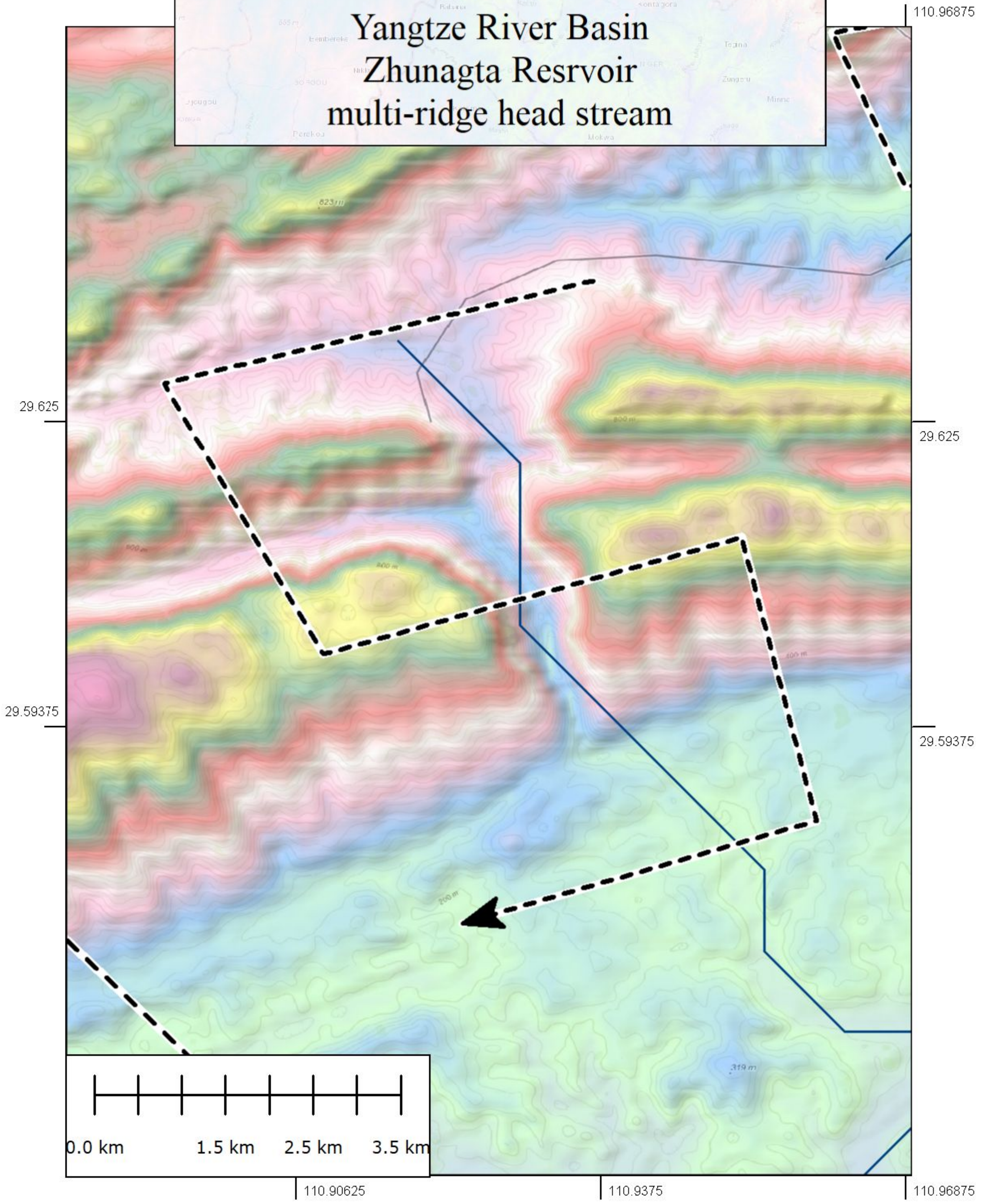

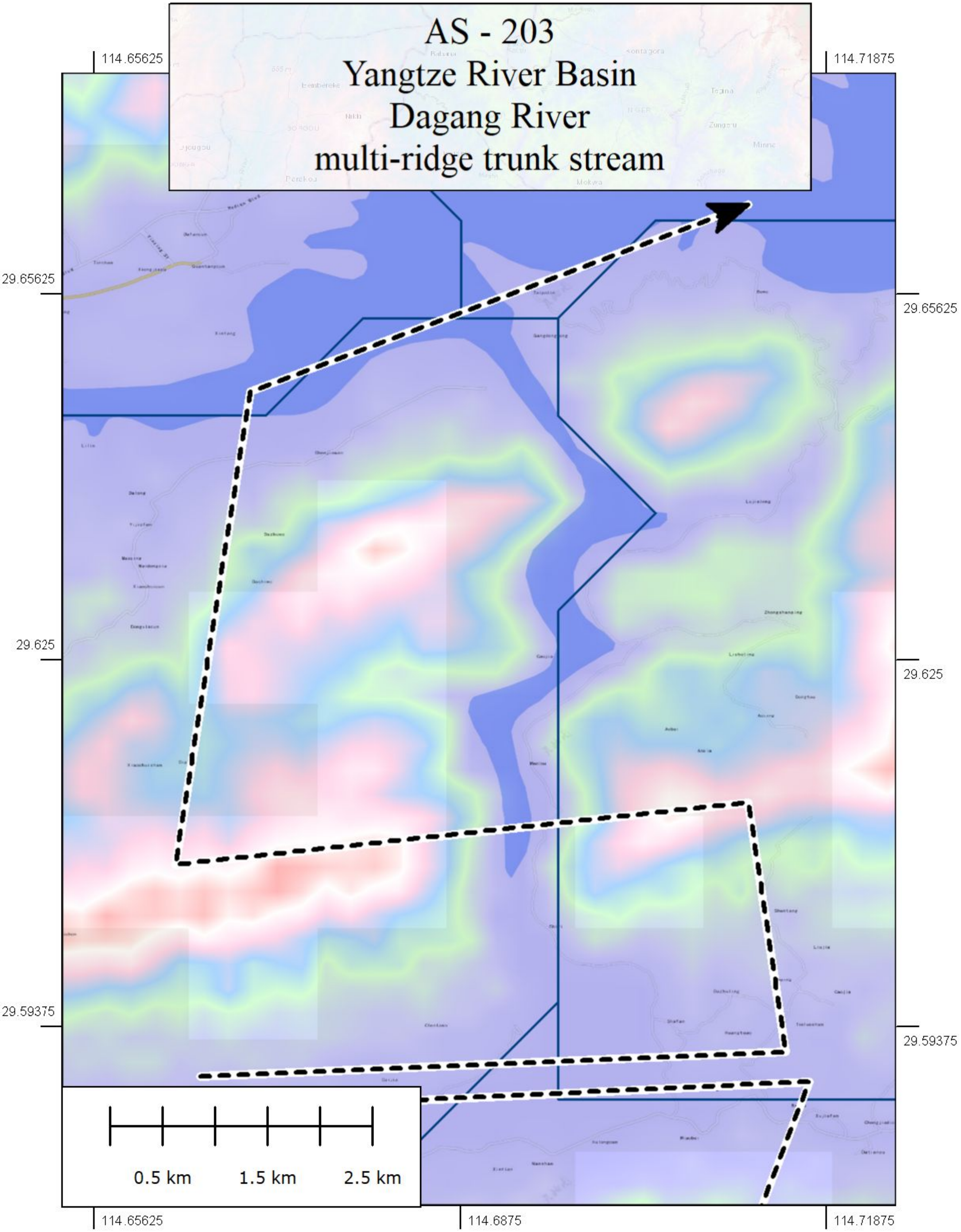
AS - 203
Yangtze River Basin
Dagang River
multi-ridge trunk stream
114.65625
114.71875
29.65625
29.625
29.59375
114.6875
0.5 km
1.5 km
2.5 km

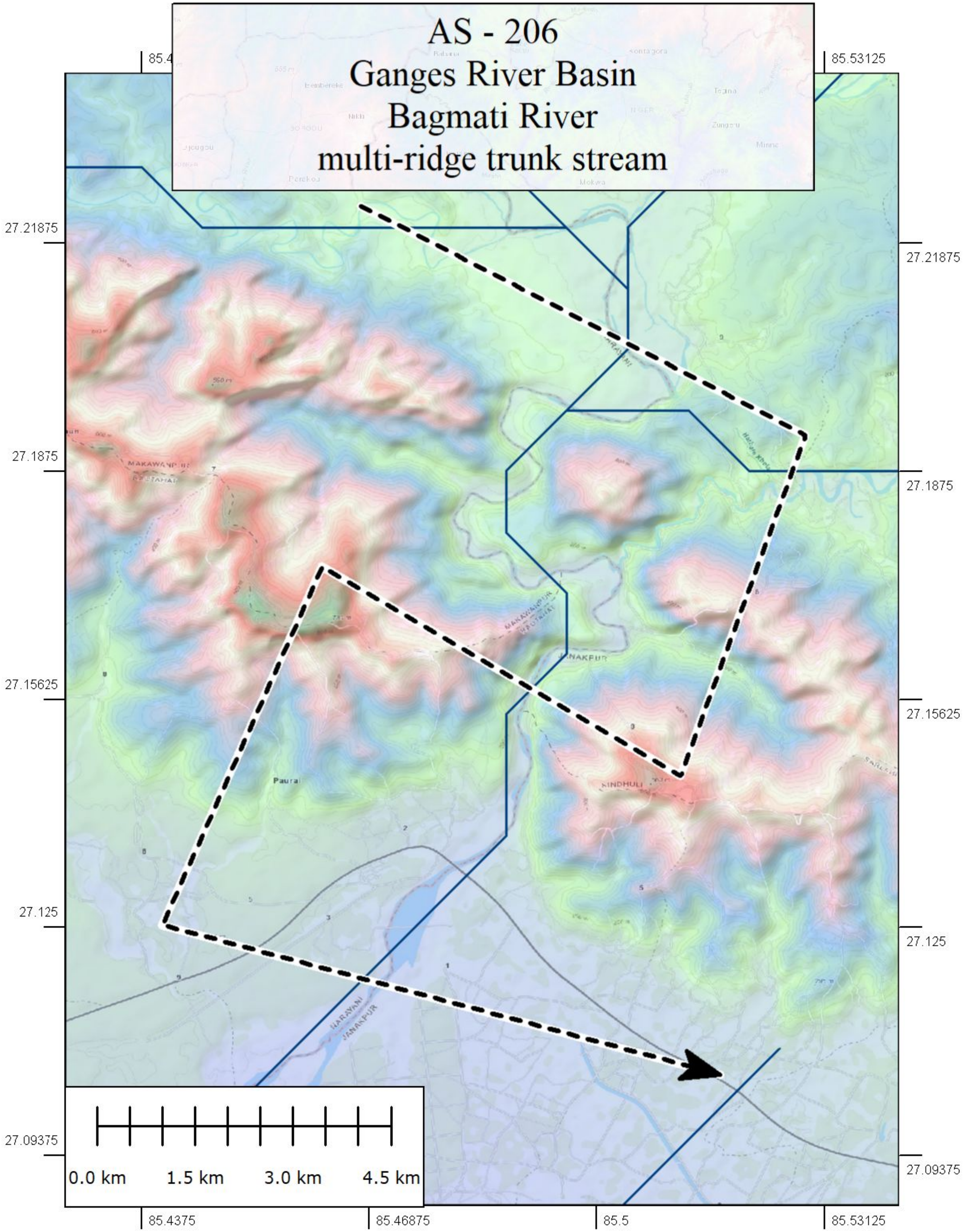
AS - 206
Ganges River Basin
Bagmati River
multi-ridge trunk stream
85.53125
27.21875
27.1875
27.15625
27.125
27.09375
85.4375
85.46875
85.5
MAKAWANPUR
JANAKPUR
SINDHULI
Paurai
NARAYANI
0.0 km
1.5 km
3.0 km
4.5 km

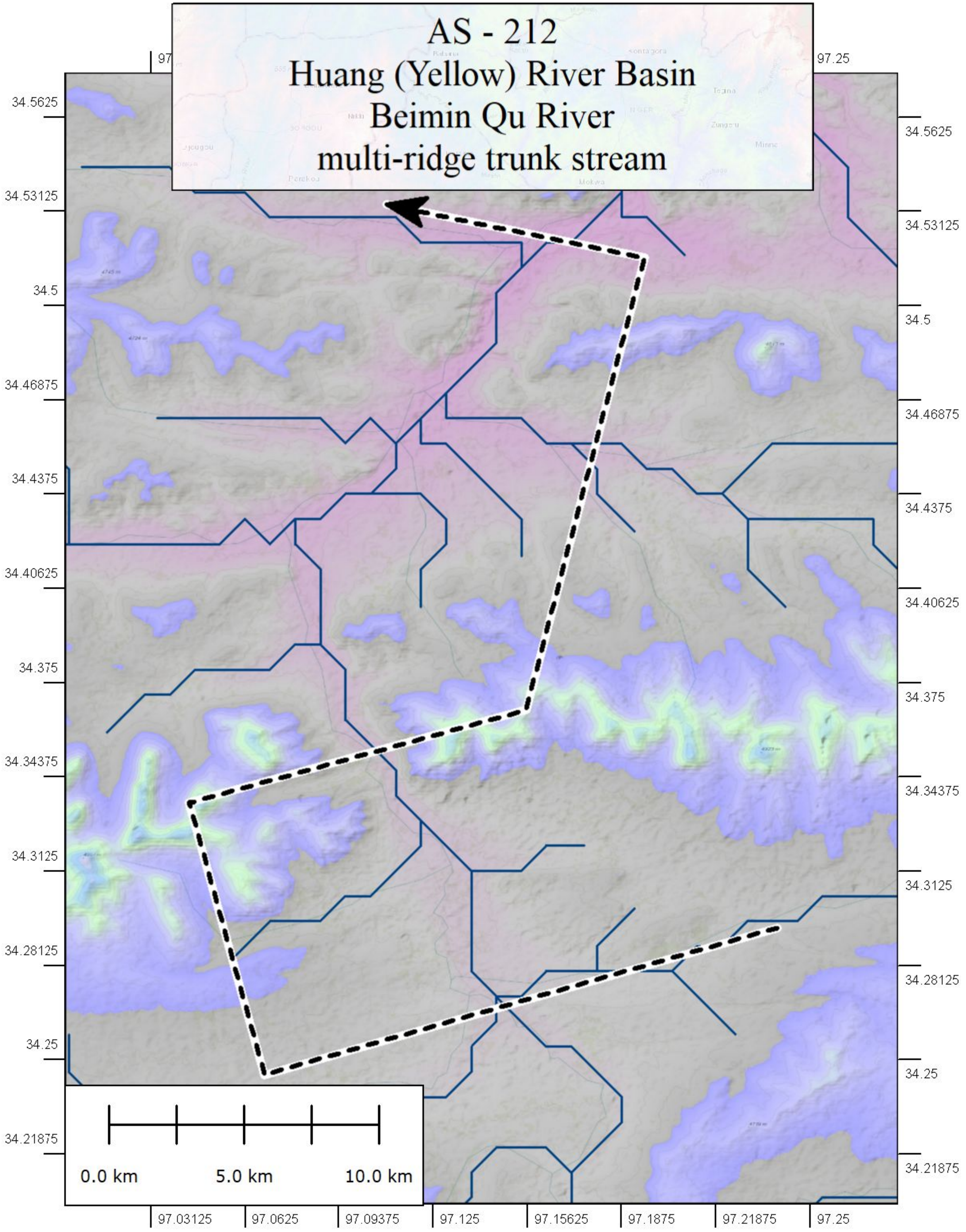
AS - 212
Huang (Yellow) River Basin
Beimin Qu River
multi-ridge trunk stream
0.0 km
5.0 km
10.0 km
34.5625
34.53125
34.5
34.46875
34.4375
34.40625
34.375
34.34375
34.3125
34.28125
34.25
34.21875
97.03125
97.0625
97.09375
97.125
97.15625
97.1875
97.21875
97.25

AS - 228
Ganges River Basin
multi-ridge trunk stream

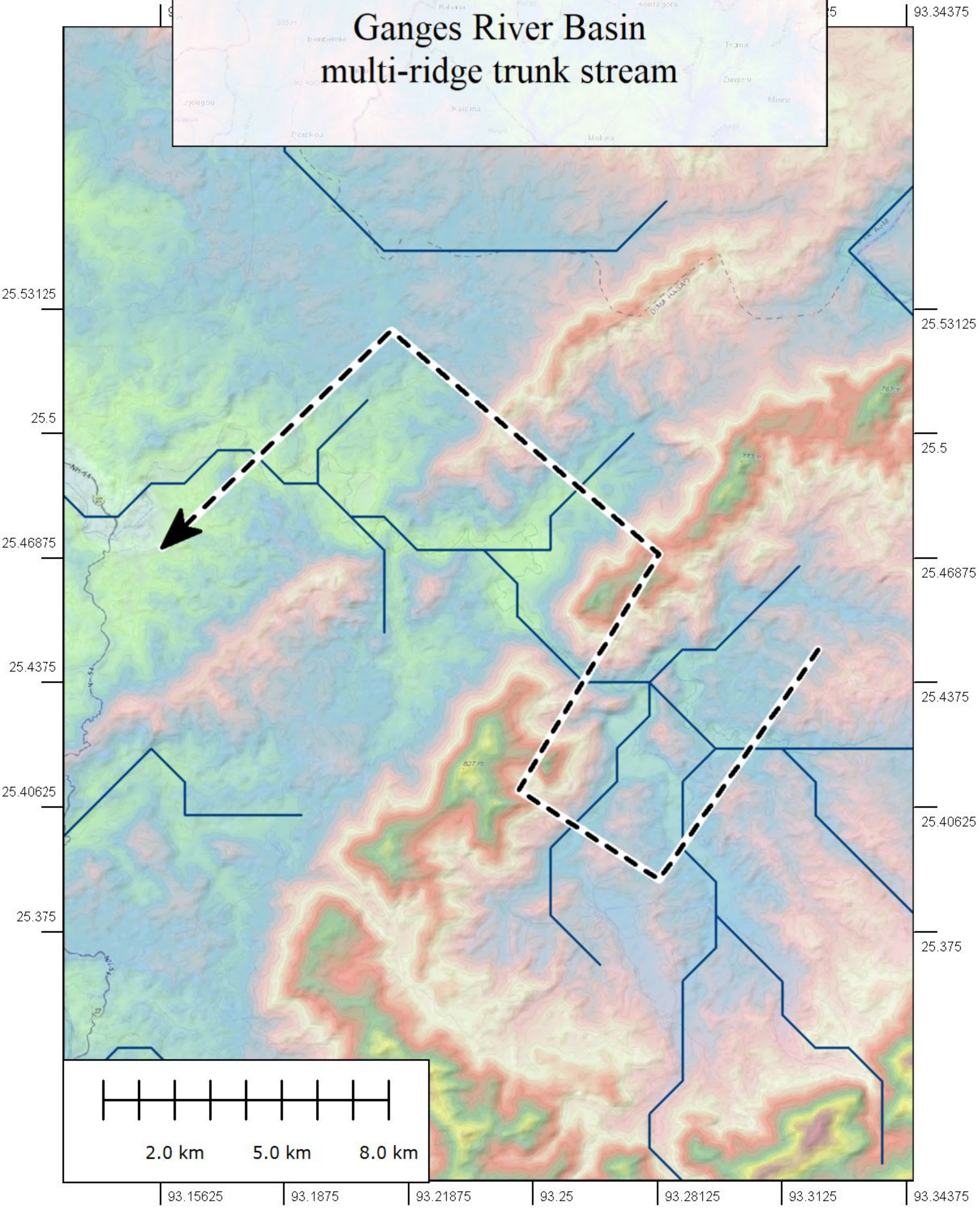

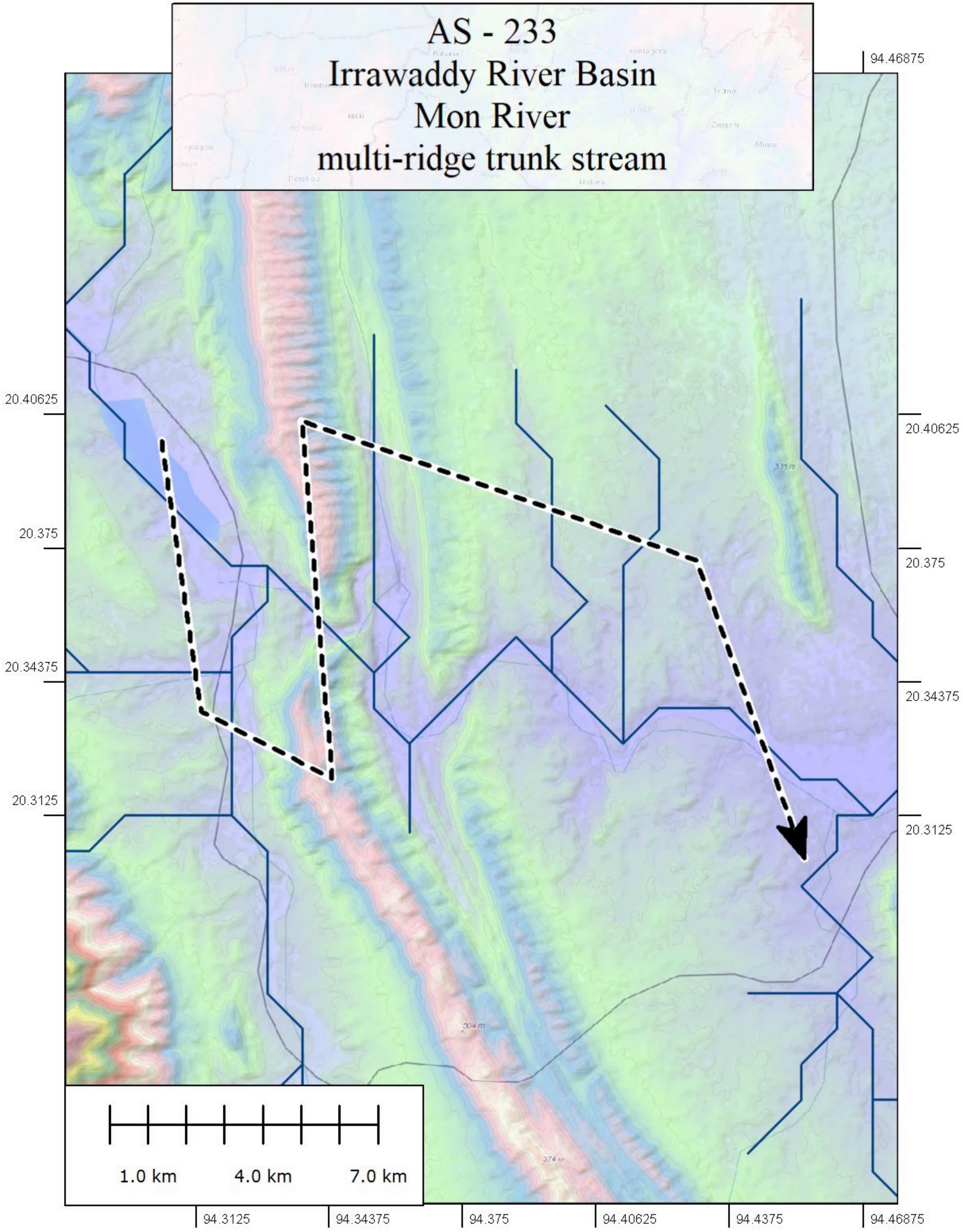

AS - 233
Irrawaddy River Basin
Mon River
multi-ridge trunk stream
20.40625
20.375
20.34375
20.3125
94.46875
94.3125
94.34375
94.375
94.40625
94.4375
1.0 km
4.0 km
7.0 km

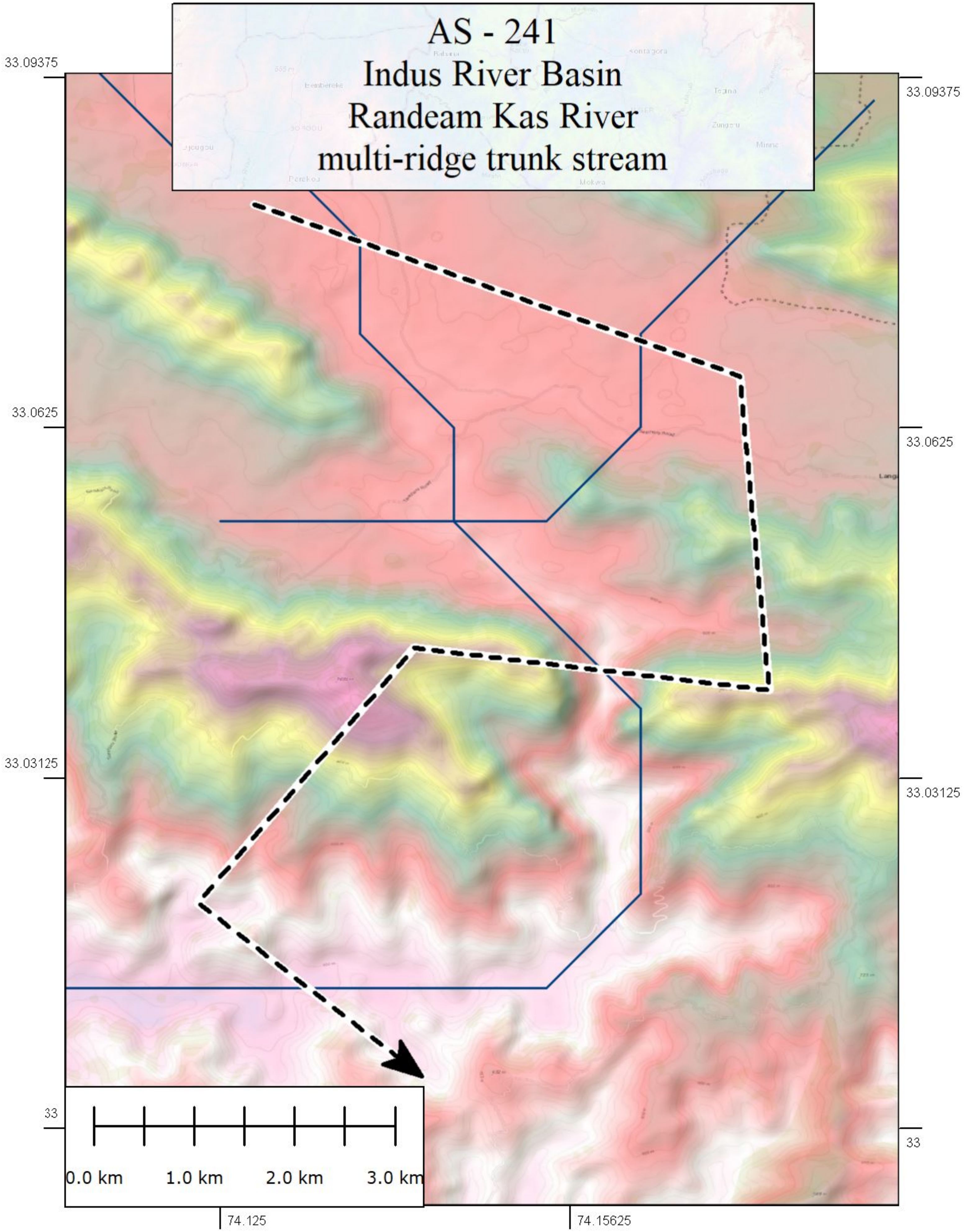
AS - 241
Indus River Basin
Randeam Kas River
multi-ridge trunk stream
33.09375
33.09375
33.0625
33.0625
33.03125
33.03125
33
33
0.0 km
1.0 km
2.0 km
3.0 km
74.125
74.15625

AS - 302
Yangtze River Basin
Jialing River
multi-ridge trunk stream

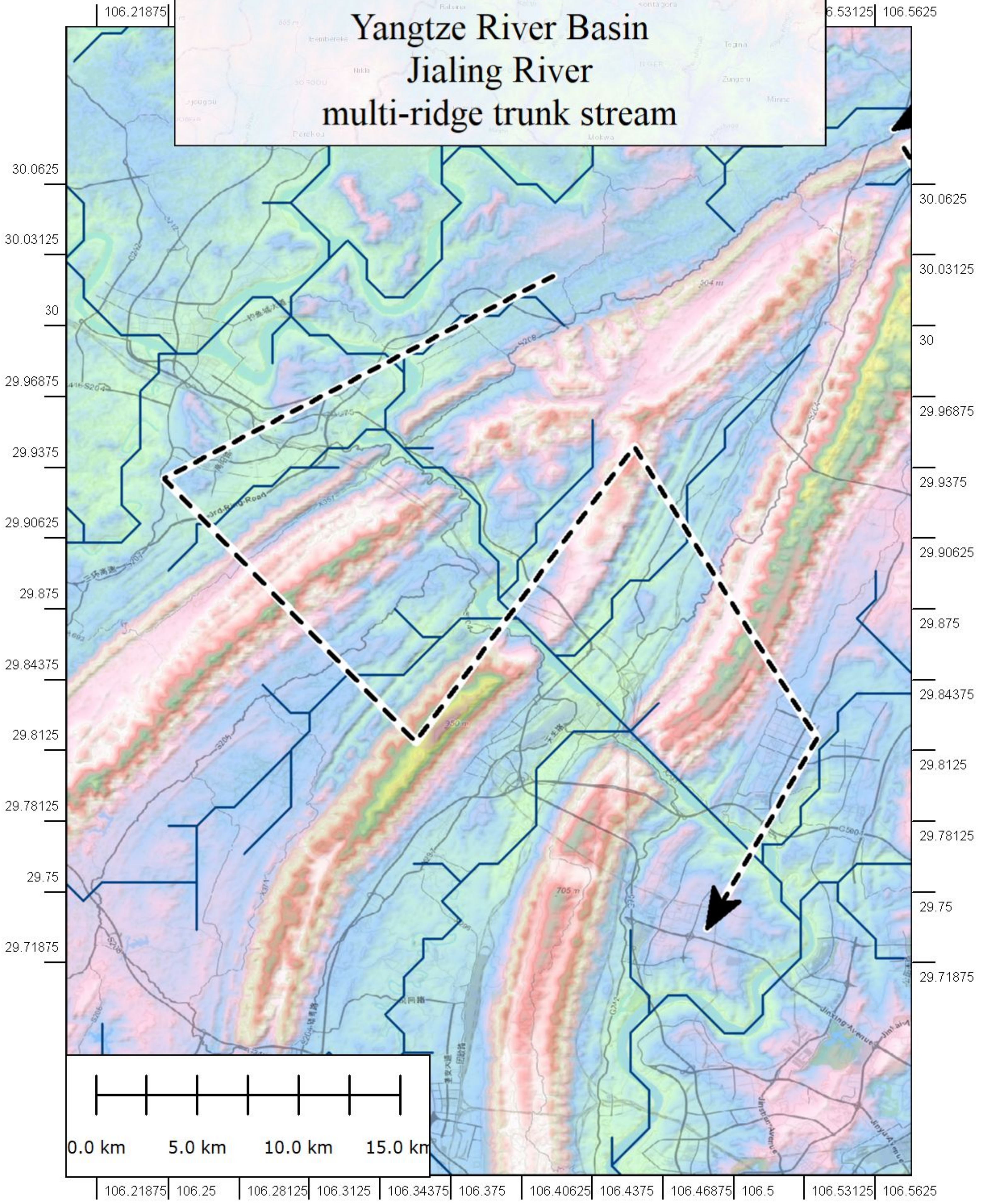

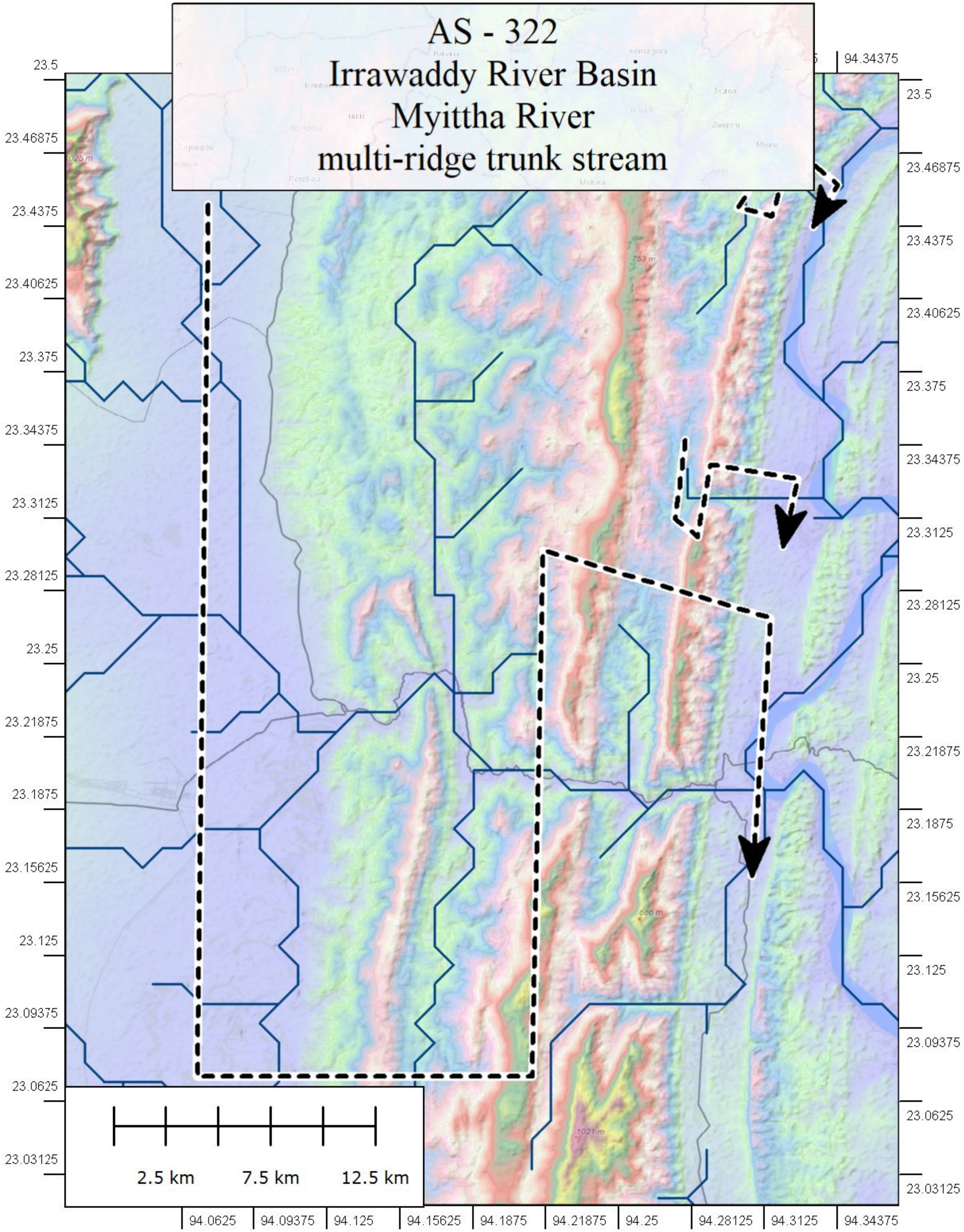
AS - 322
Irrawaddy River Basin
Myittha River
multi-ridge trunk stream
23.5
23.46875
23.4375
23.40625
23.375
23.34375
23.3125
23.28125
23.25
23.21875
23.1875
23.15625
23.125
23.09375
23.0625
23.03125
94.0625
94.09375
94.125
94.15625
94.1875
94.21875
94.25
94.28125
94.3125
94.34375
2.5 km
7.5 km
12.5 km

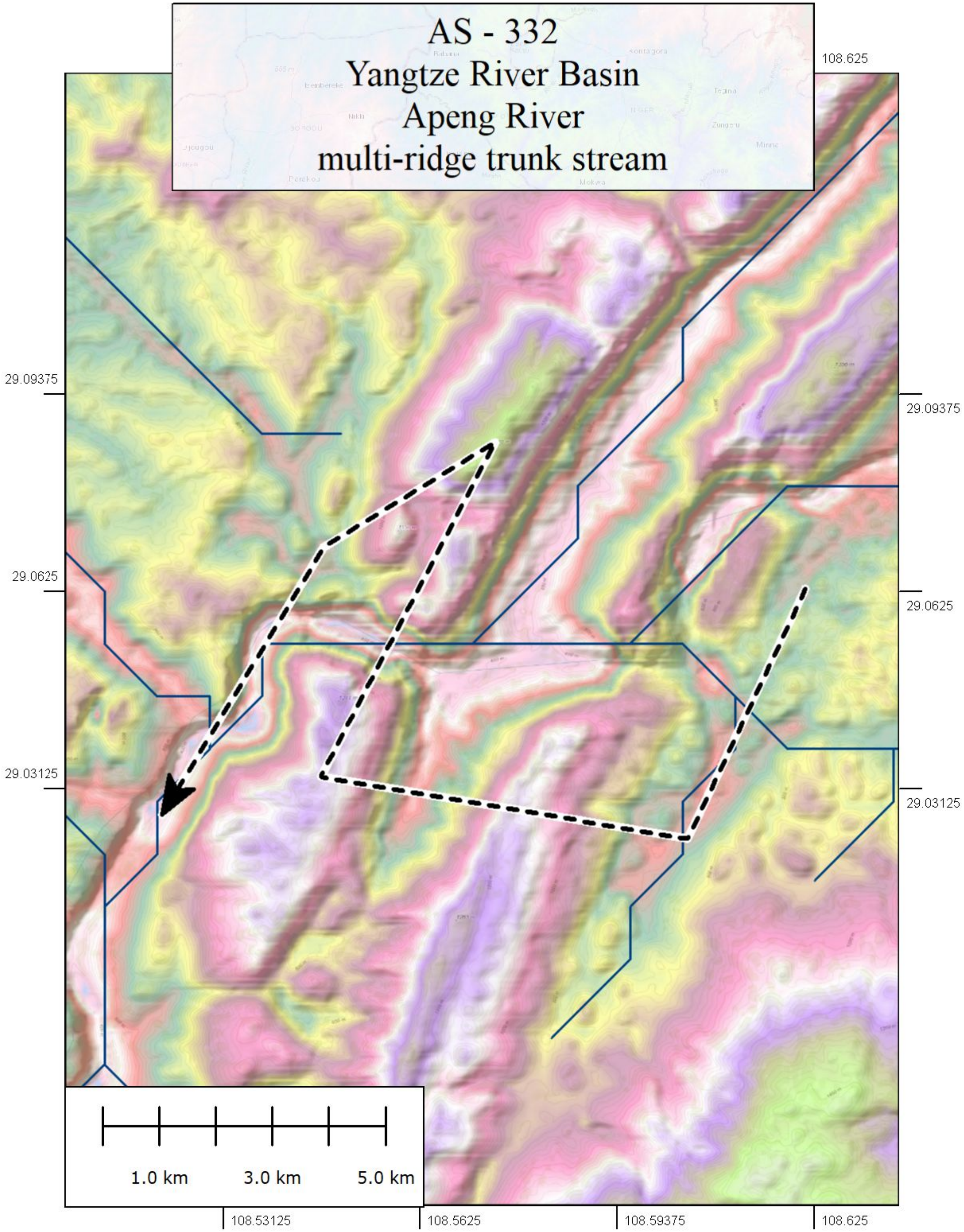
AS - 332
Yangtze River Basin
Apeng River
multi-ridge trunk stream
108.625
29.09375
29.09375
29.0625
29.0625
29.03125
29.03125
1.0 km
3.0 km
5.0 km
108.53125
108.5625
108.59375
108.625

AS - 1
Yangtze River Basin
single-ridge head stream

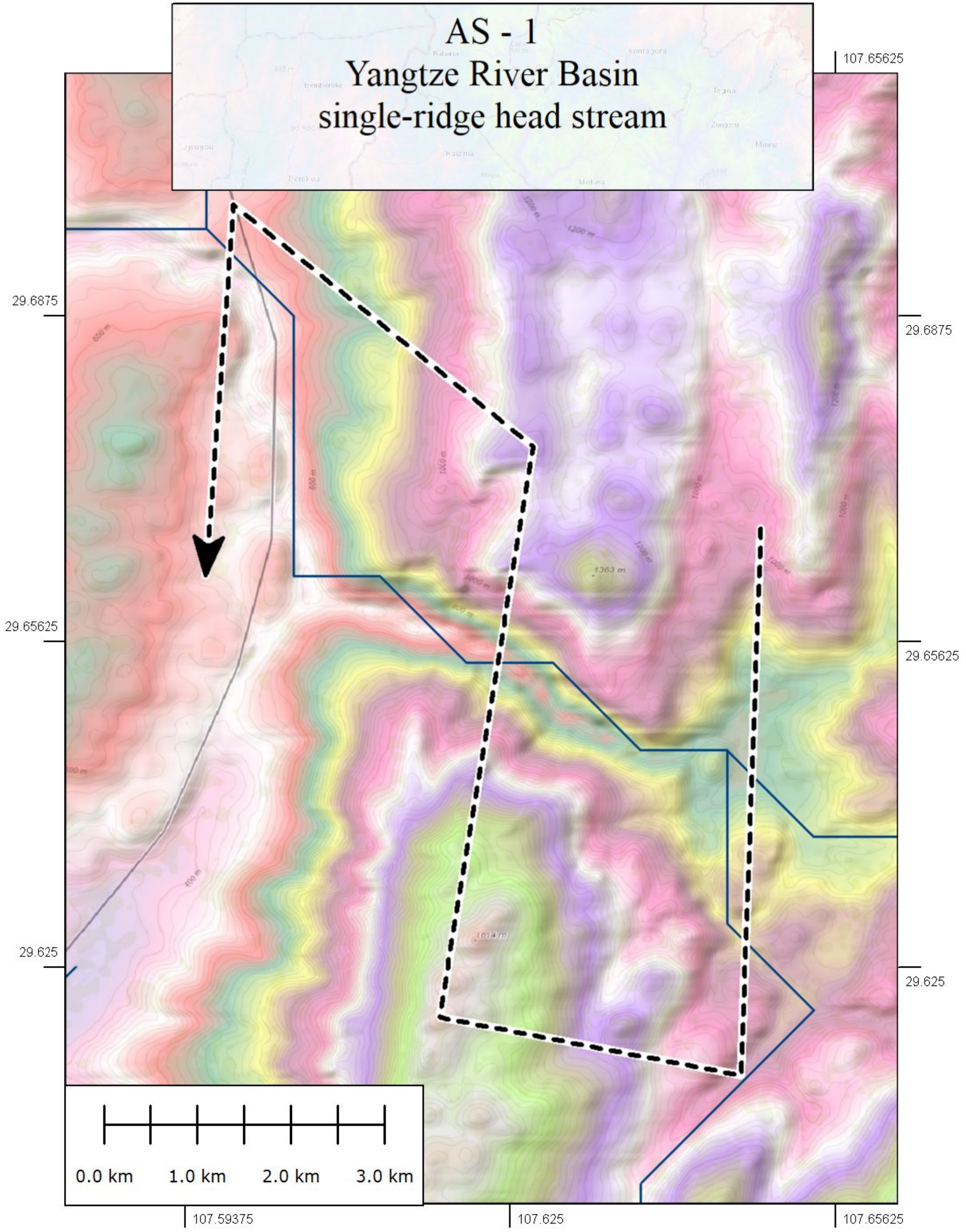

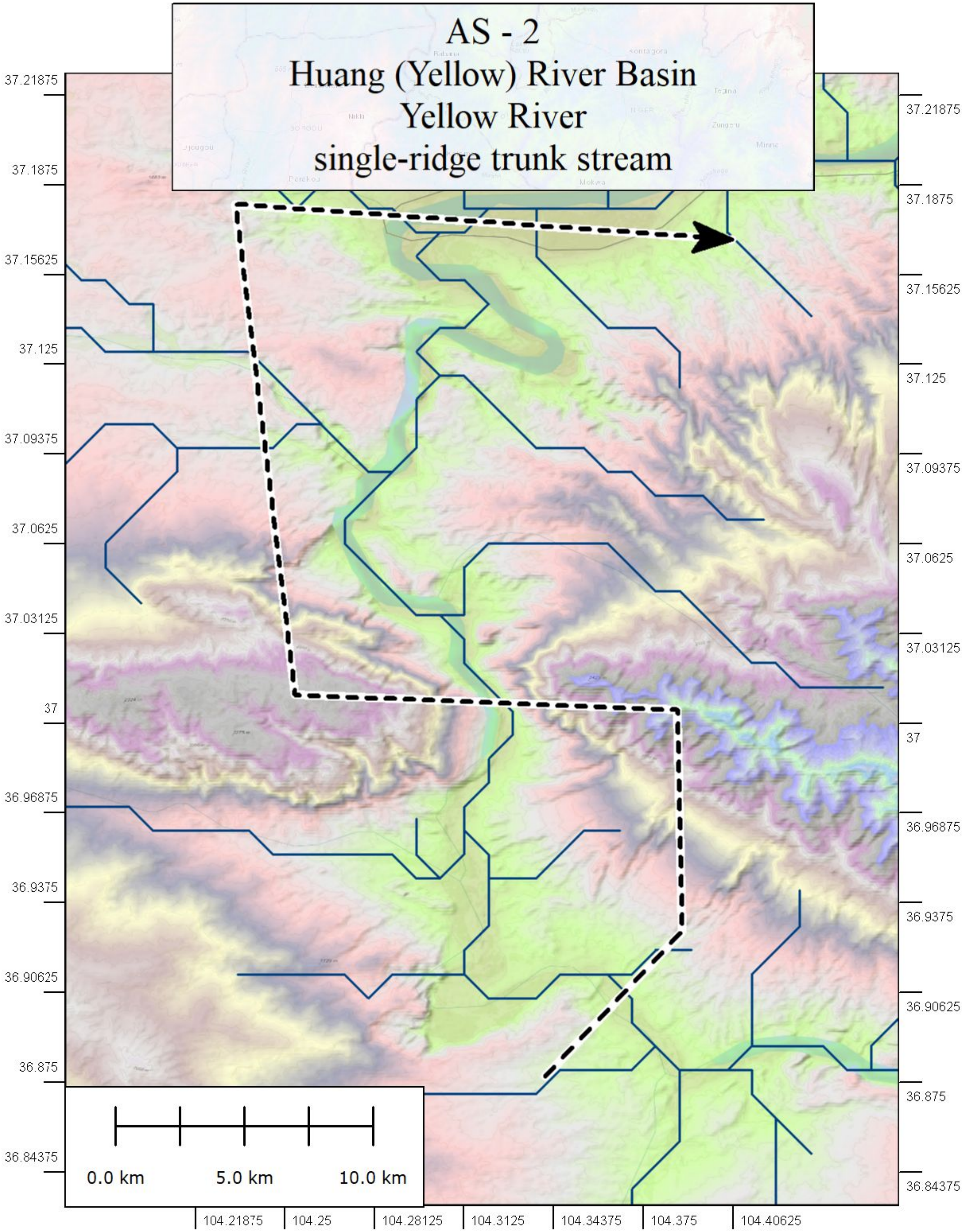
AS - 2
Huang (Yellow) River Basin
Yellow River
single-ridge trunk stream
37.21875
37.1875
37.15625
37.125
37.09375
37.0625
37.03125
37
36.96875
36.9375
36.90625
36.875
36.84375
104.21875
104.25
104.28125
104.3125
104.34375
104.375
104.40625
0.0 km
5.0 km
10.0 km

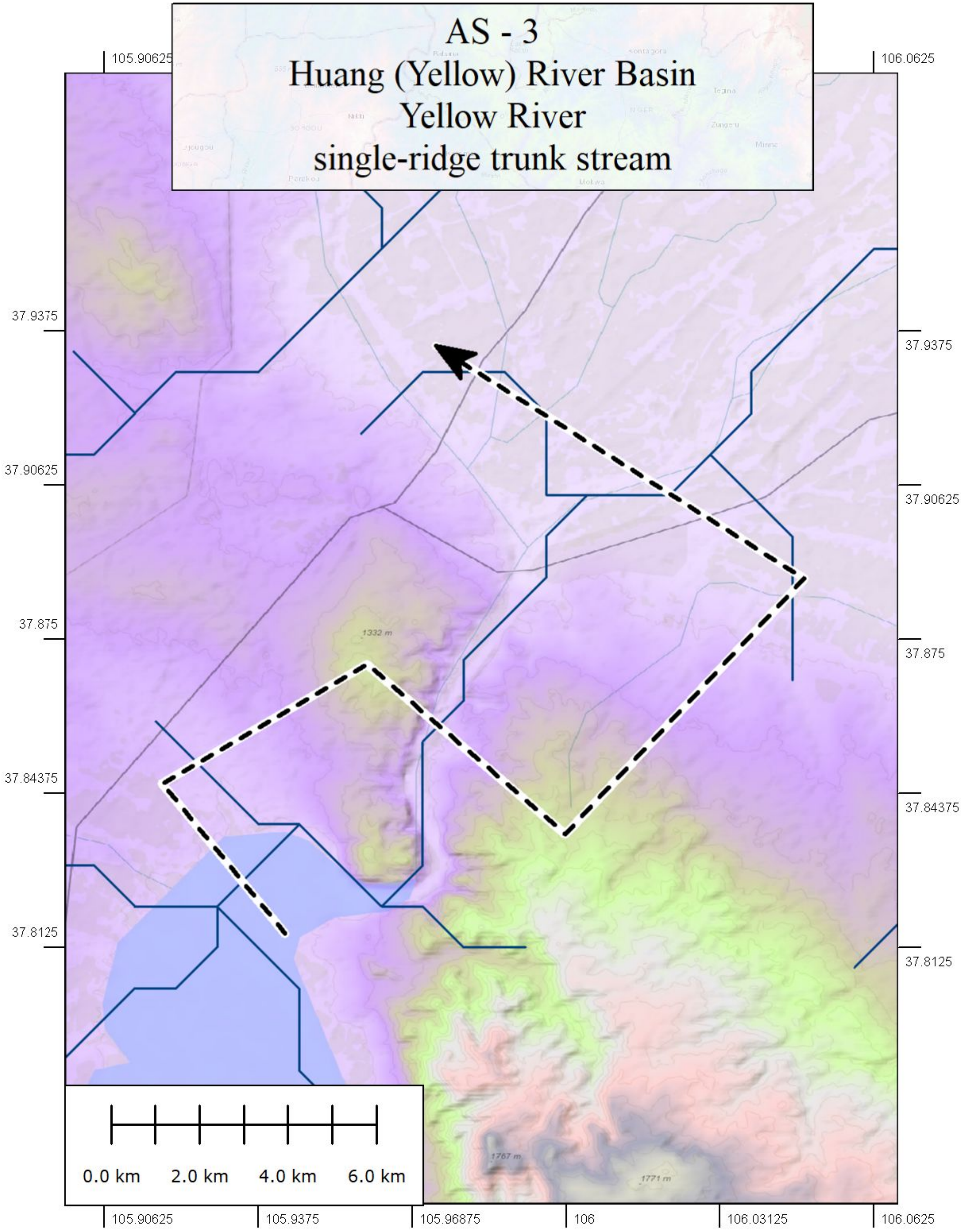

AS - 3
Huang (Yellow) River Basin
Yellow River
single-ridge trunk stream
105.90625
106.0625
37.9375
37.90625
37.875
37.84375
37.8125
1332 m
1767 m
1771 m
0.0 km
2.0 km
4.0 km
6.0 km
105.90625
105.9375
105.96875
106
106.03125
106.0625

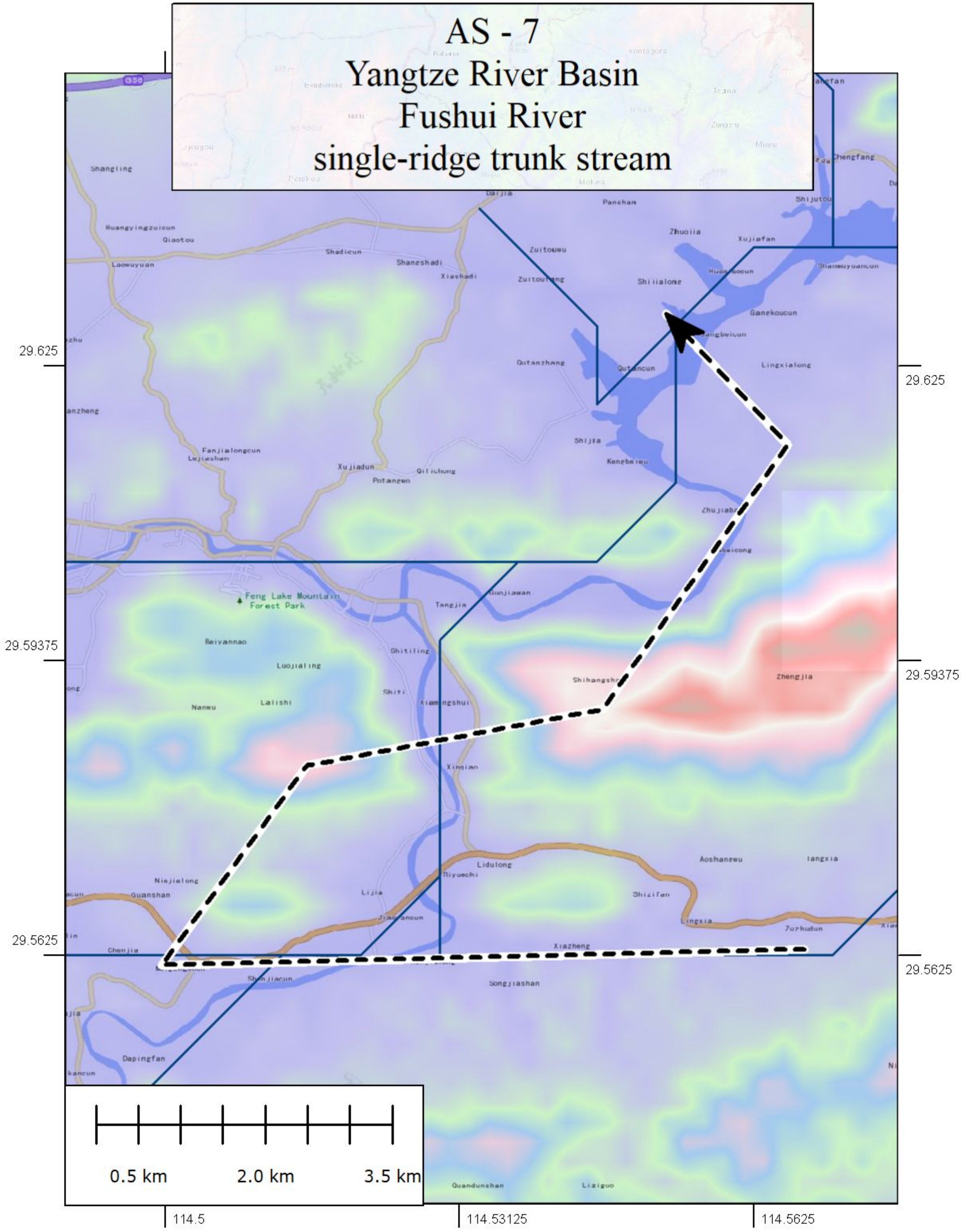
AS - 7
Yangtze River Basin
Fushui River
single-ridge trunk stream
29.625
29.59375
29.5625
114.5
114.53125
114.5625
0.5 km
2.0 km
3.5 km
Shangling
Huangyingzuicun
Qiaotou
Laowuyuan
Shadicun
Shangshadi
Xiashadi
Daijia
Panshan
Zhuojia
Xujiafan
Zuitouwu
Shijialong
Gangkoucun
Qutanzhang
Qutancun
Lingxialong
Shijutou
Chengfang
Fanjialongcun
Lujiashan
Xujiadun
Qichong
Potangwo
Shijia
Kengbeiwu
Feng Lake Mountain
Forest Park
Tangjia
Hunjiawan
Beivannao
Luojialing
Shitiling
Shiti
Xiamingshui
Shihangshu
Zhengjia
Nanwu
Lalishi
Xinqiao
Lidulong
Riyuechi
Aoshanwu
Niejialong
Guanshan
Lijia
Jiaowancun
Shizifan
Lingxia
Zuzhudun
Chenjia
Xiazheng
Shenjiacun
Songjiashan
Dapingfan
Quandunshan
Liziguo

AS - 10
Chao Phraya Basin
Sum River
single-ridge trunk stream

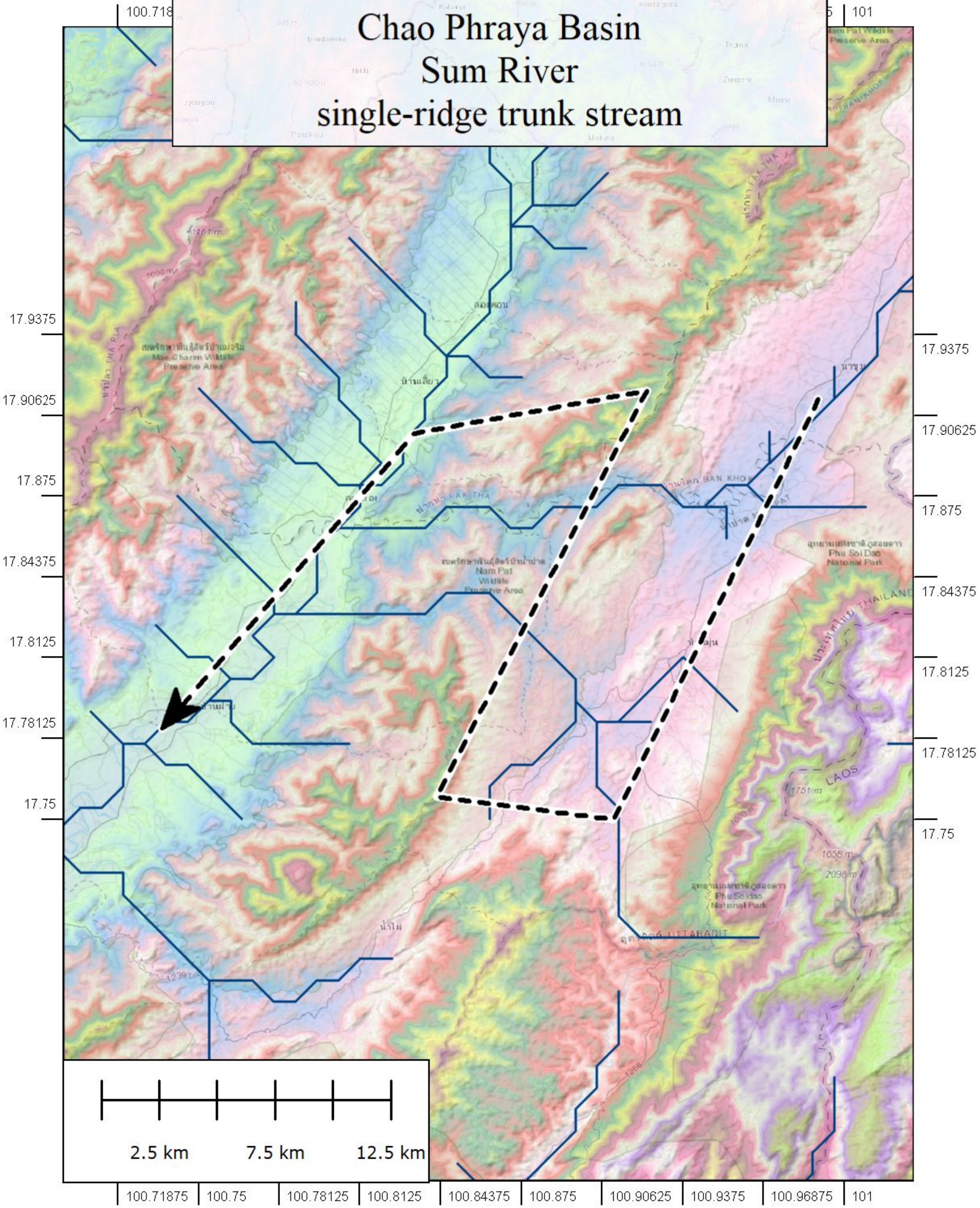

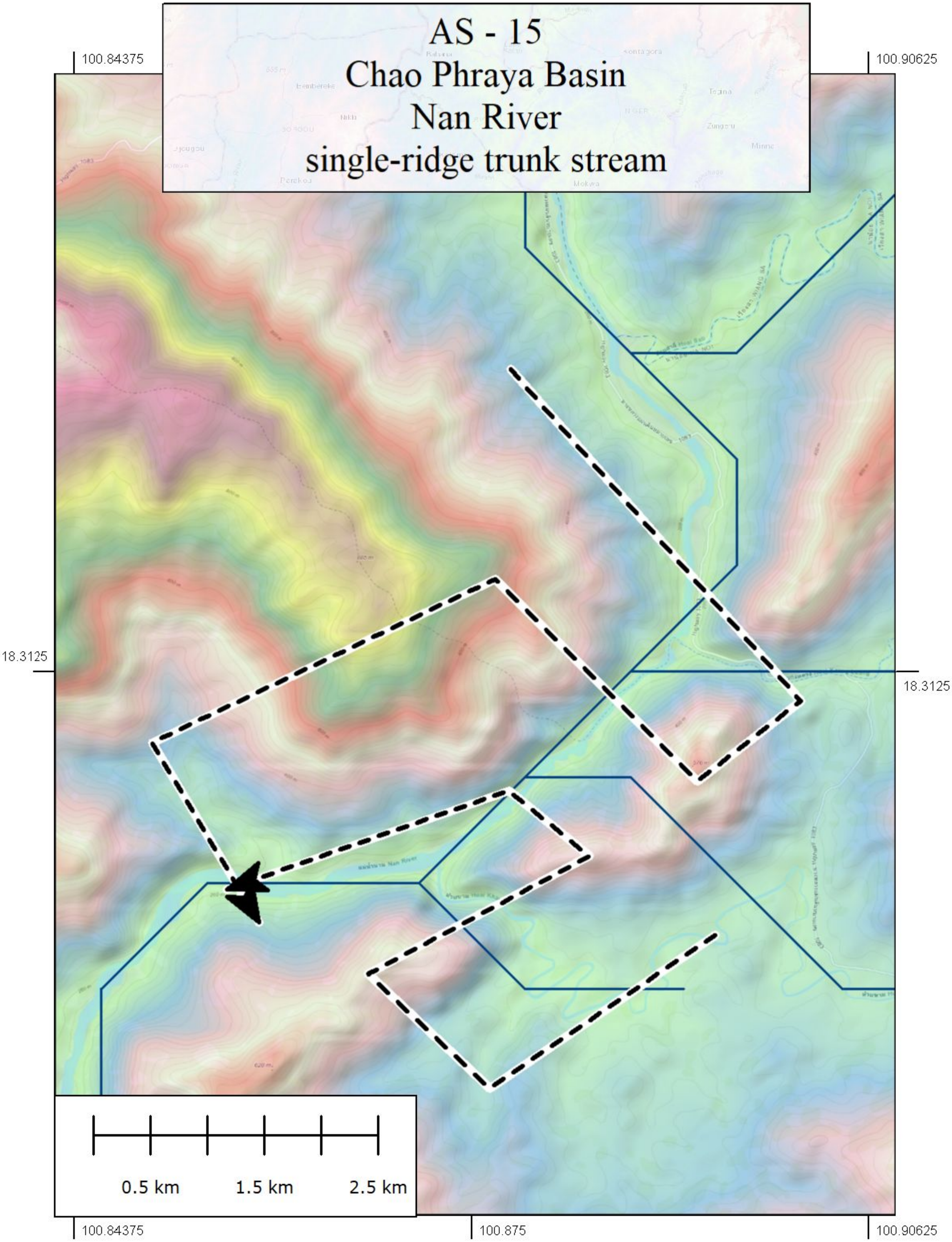
AS - 15
Chao Phraya Basin
Nan River
single-ridge trunk stream
100.84375
100.90625
100.875
18.3125
0.5 km
1.5 km
2.5 km

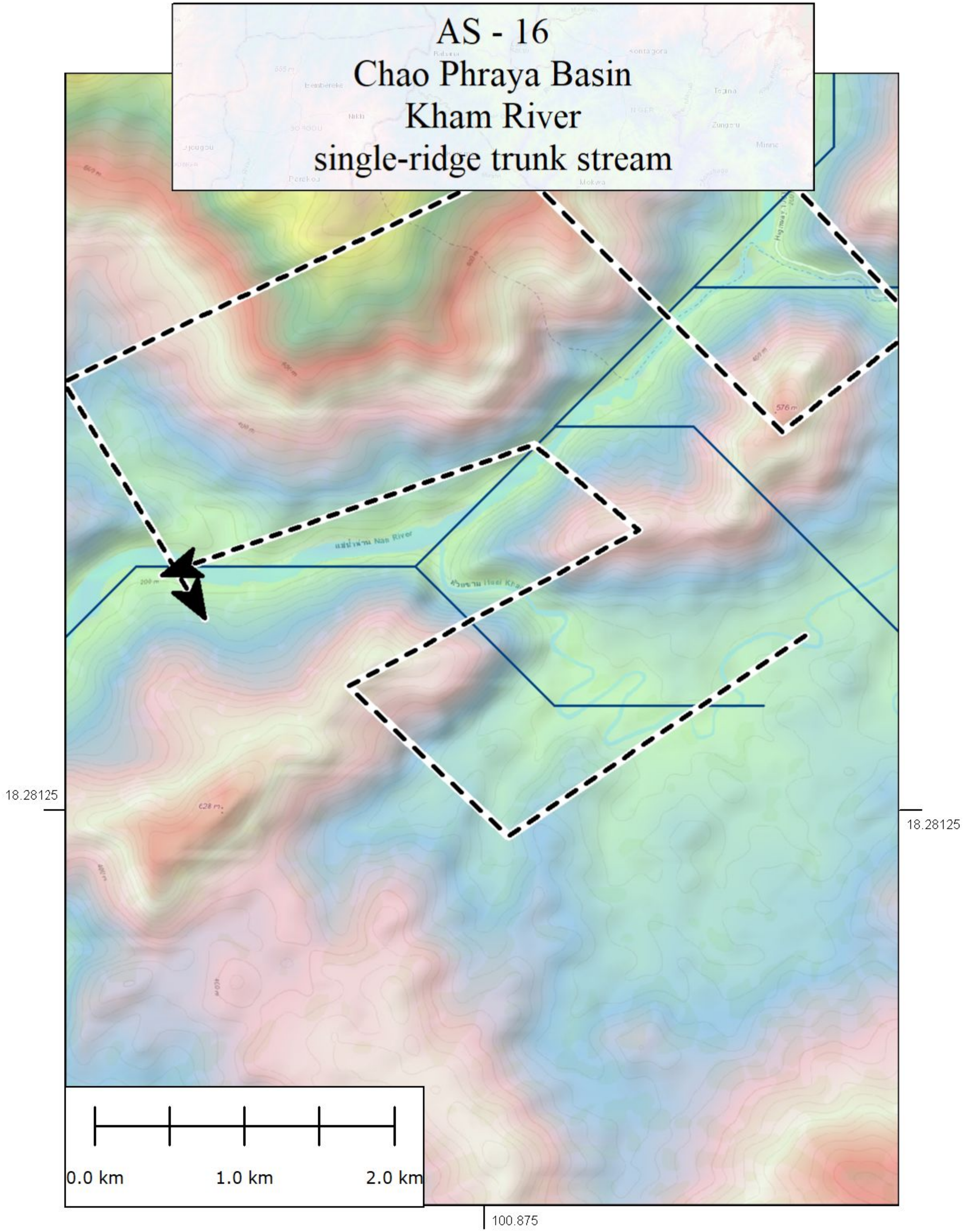
AS - 16
Chao Phraya Basin
Kham River
single-ridge trunk stream
18.28125
18.28125
100.875
0.0 km
1.0 km
2.0 km

AS - 18
Chao Phraya Basin
Nan River Sirikit Reservoir
single-ridge trunk stream

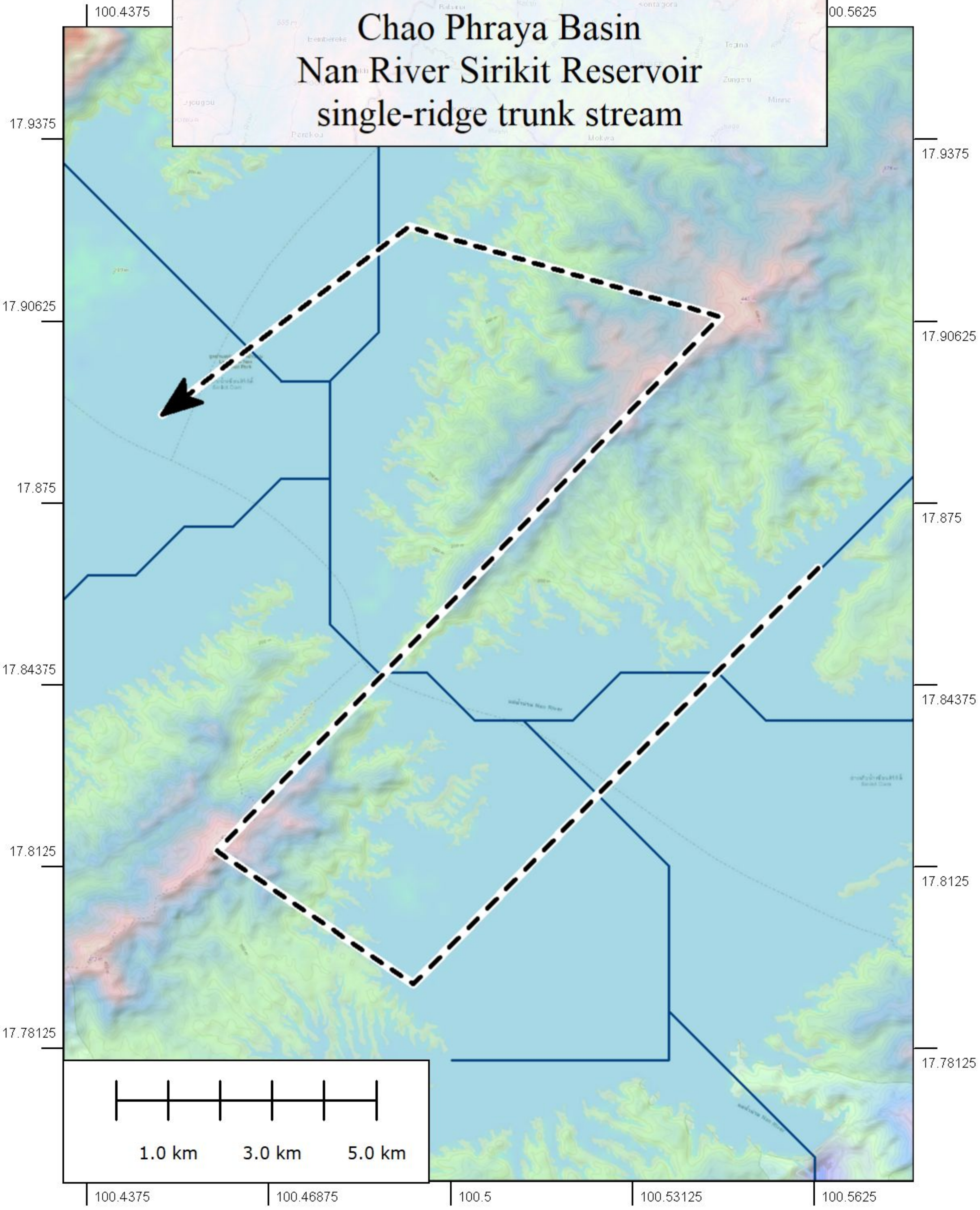

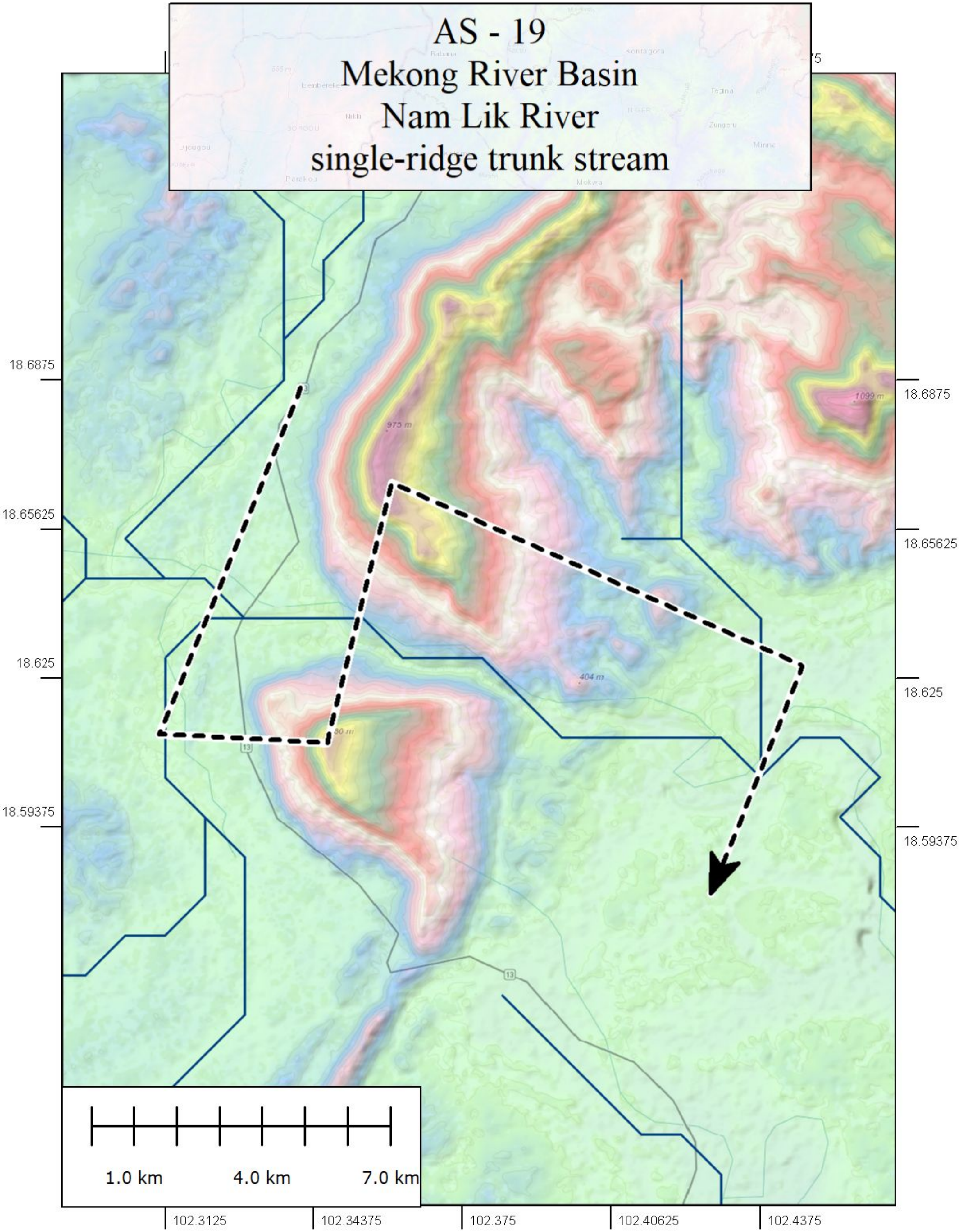
AS - 19
Mekong River Basin
Nam Lik River
single-ridge trunk stream
18.6875
18.65625
18.625
18.59375
102.3125
102.34375
102.375
102.40625
102.4375
975 m
1099 m
404 m
1.0 km
4.0 km
7.0 km

AS - 21
Endorheic basin single-ridge trunk stream

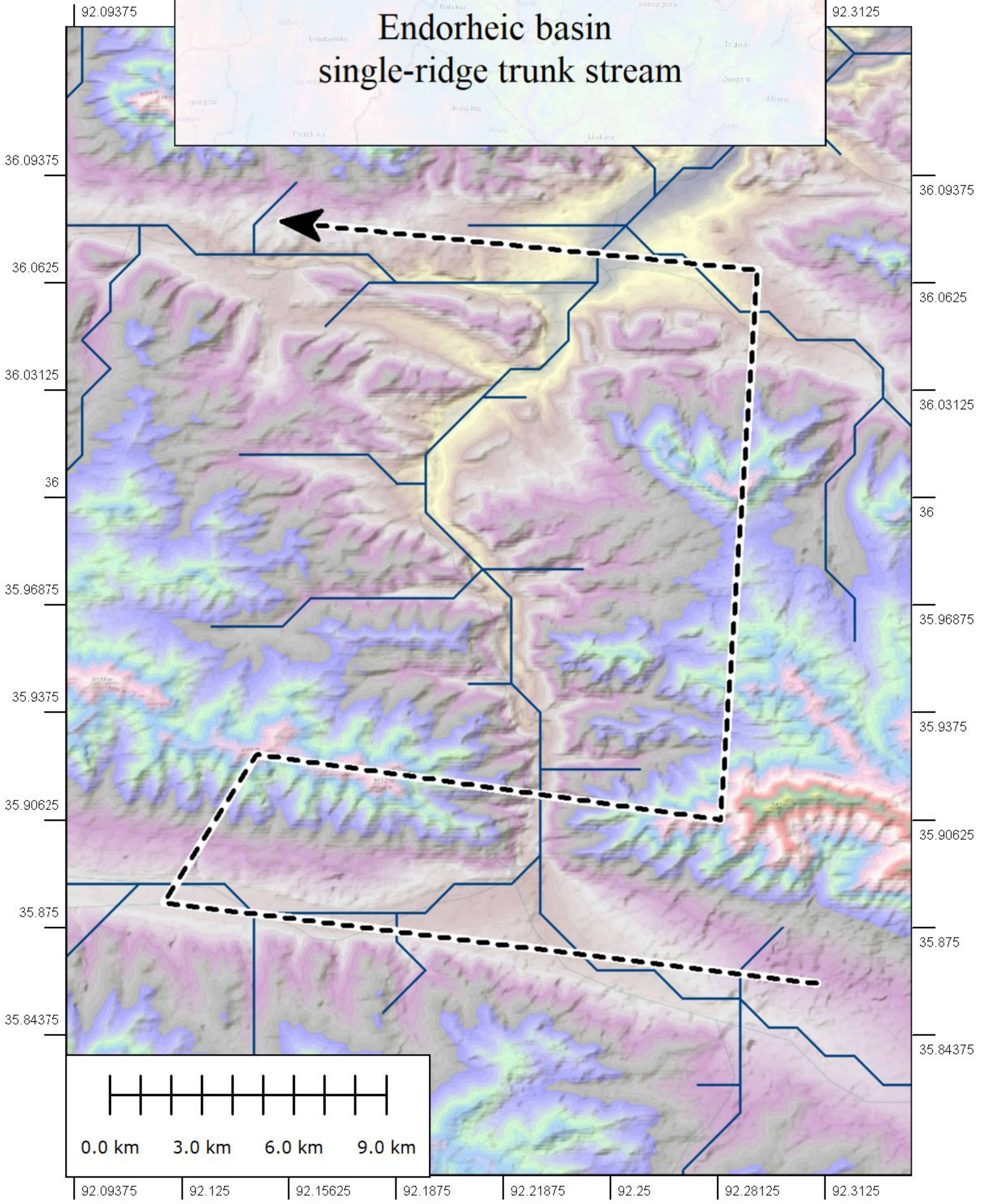

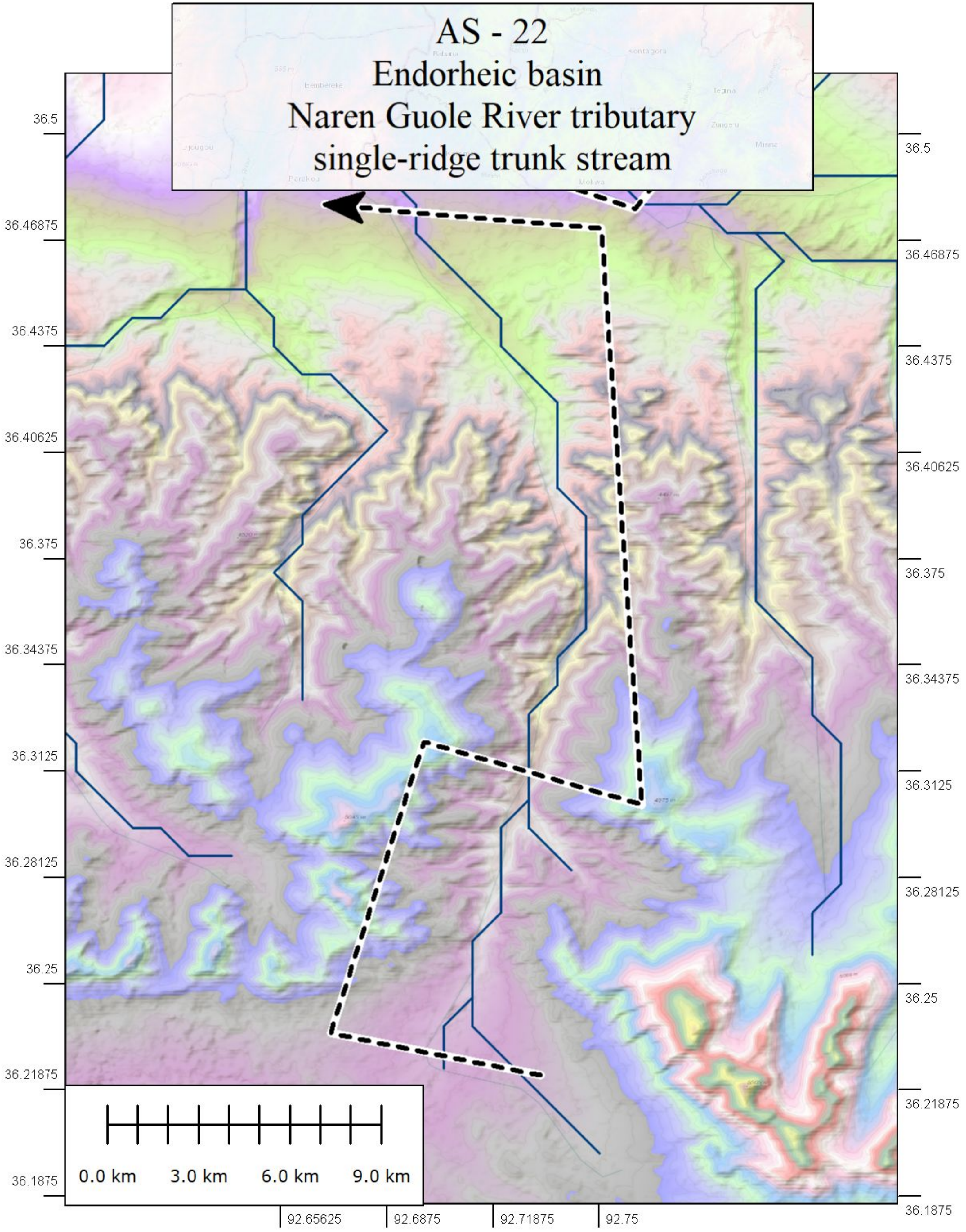
AS - 22
Endorheic basin
Naren Guole River tributary
single-ridge trunk stream
36.5
36.46875
36.4375
36.40625
36.375
36.34375
36.3125
36.28125
36.25
36.21875
36.1875
0.0 km
3.0 km
6.0 km
9.0 km
92.65625
92.6875
92.71875
92.75

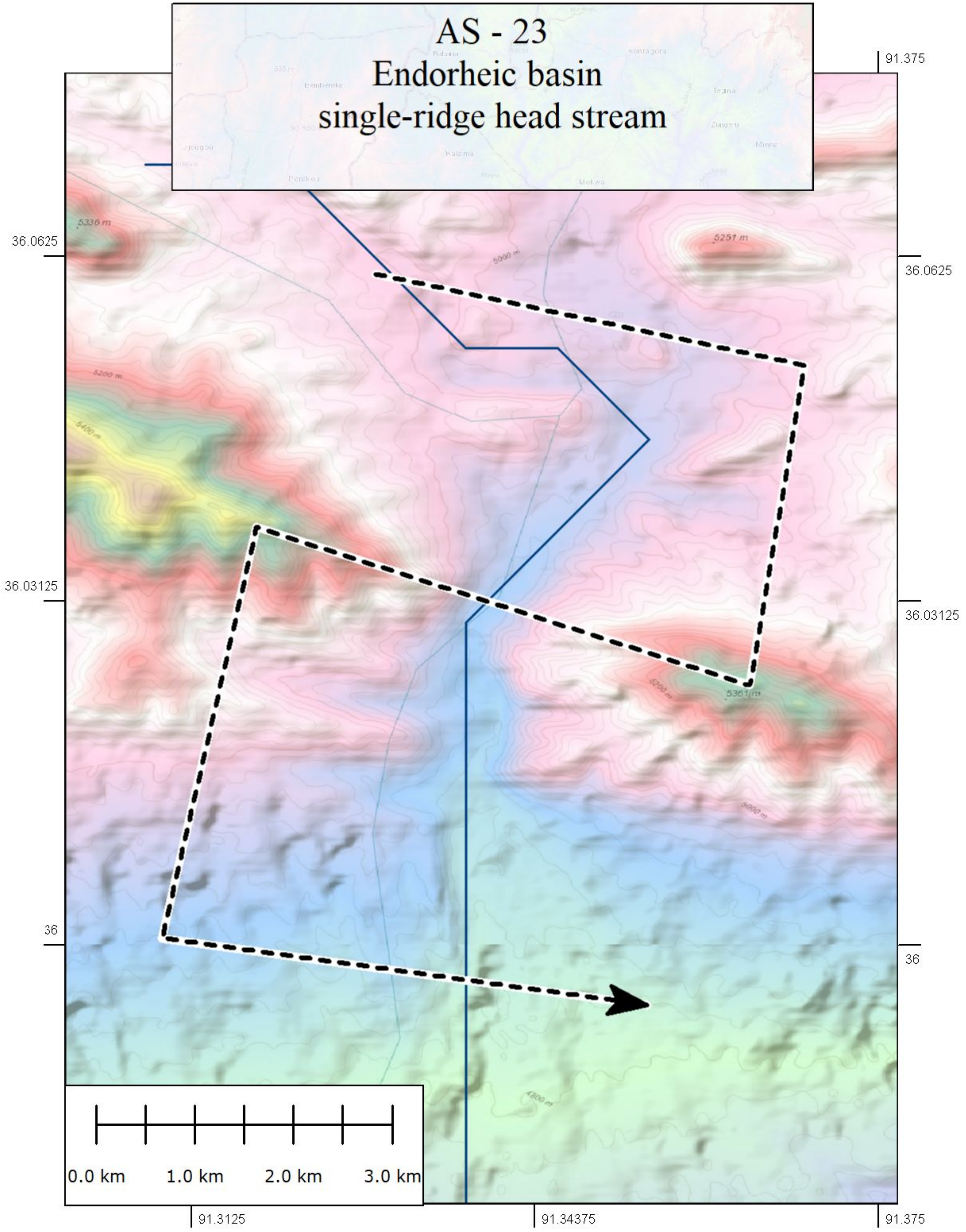
AS - 23
Endorheic basin
single-ridge head stream
91.375
36.0625
36.0625
5336 m
5251 m
5000 m
5200 m
5400 m
36.03125
36.03125
5200 m
5361 m
5000 m
36
36
4800 m
0.0 km
1.0 km
2.0 km
3.0 km
91.3125
91.34375
91.375

AS - 26
Endorheic basin
Yangkang Qu River
single-ridge trunk stream

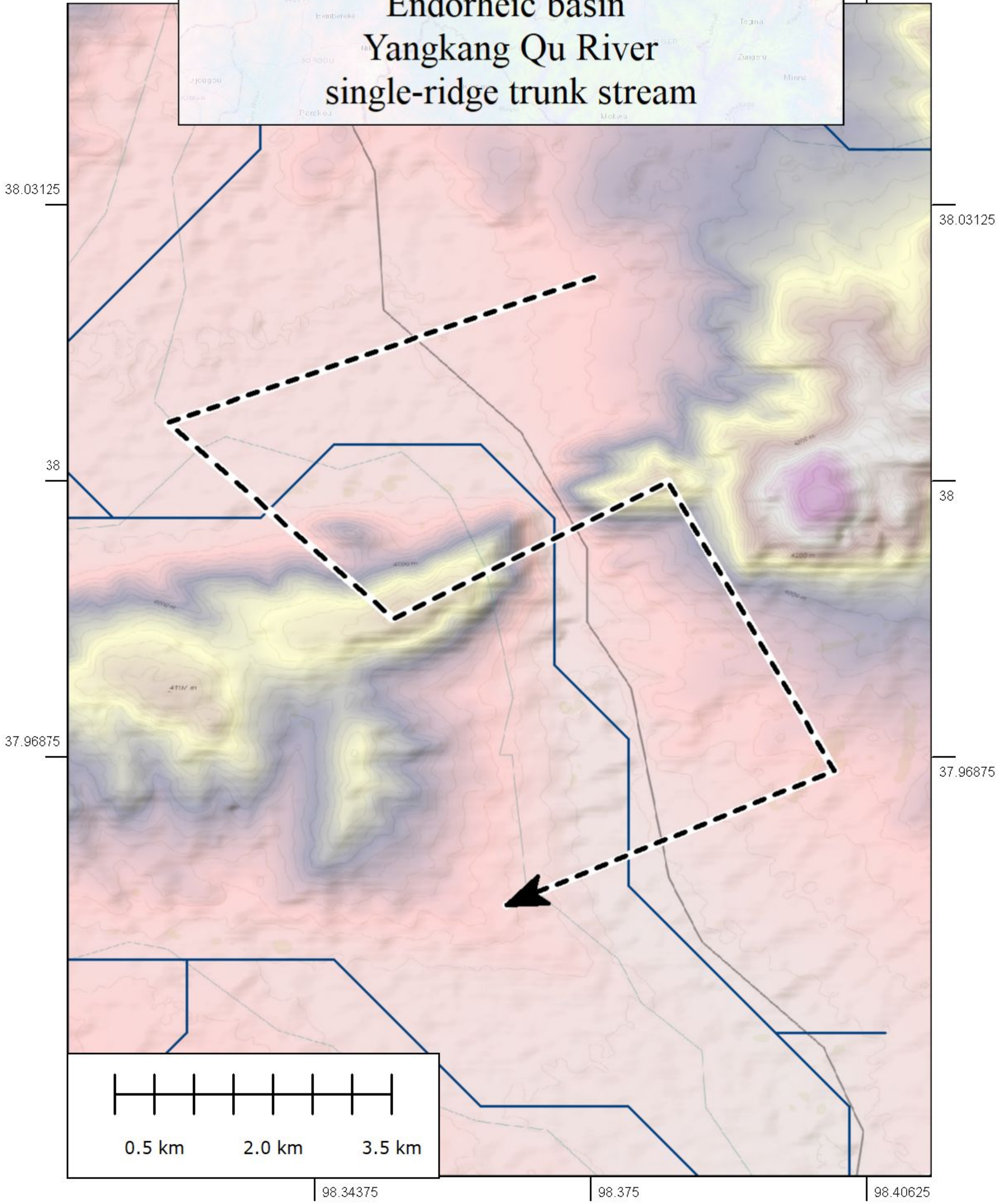

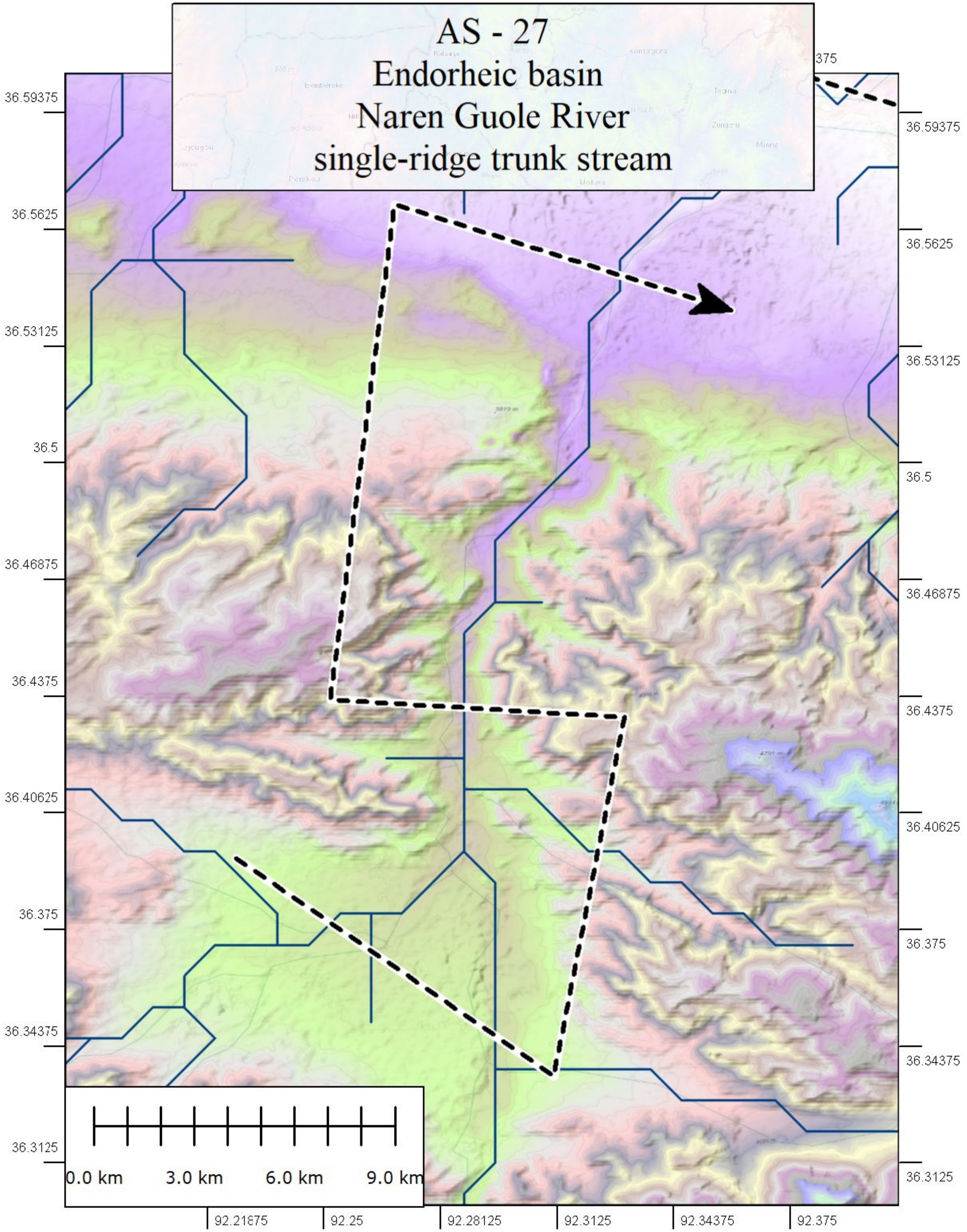
AS - 27
Endorheic basin
Naren Guole River
single-ridge trunk stream
36.59375
36.5625
36.53125
36.5
36.46875
36.4375
36.40625
36.375
36.34375
36.3125
92.21875
92.25
92.28125
92.3125
92.34375
92.375
0.0 km
3.0 km
6.0 km
9.0 km

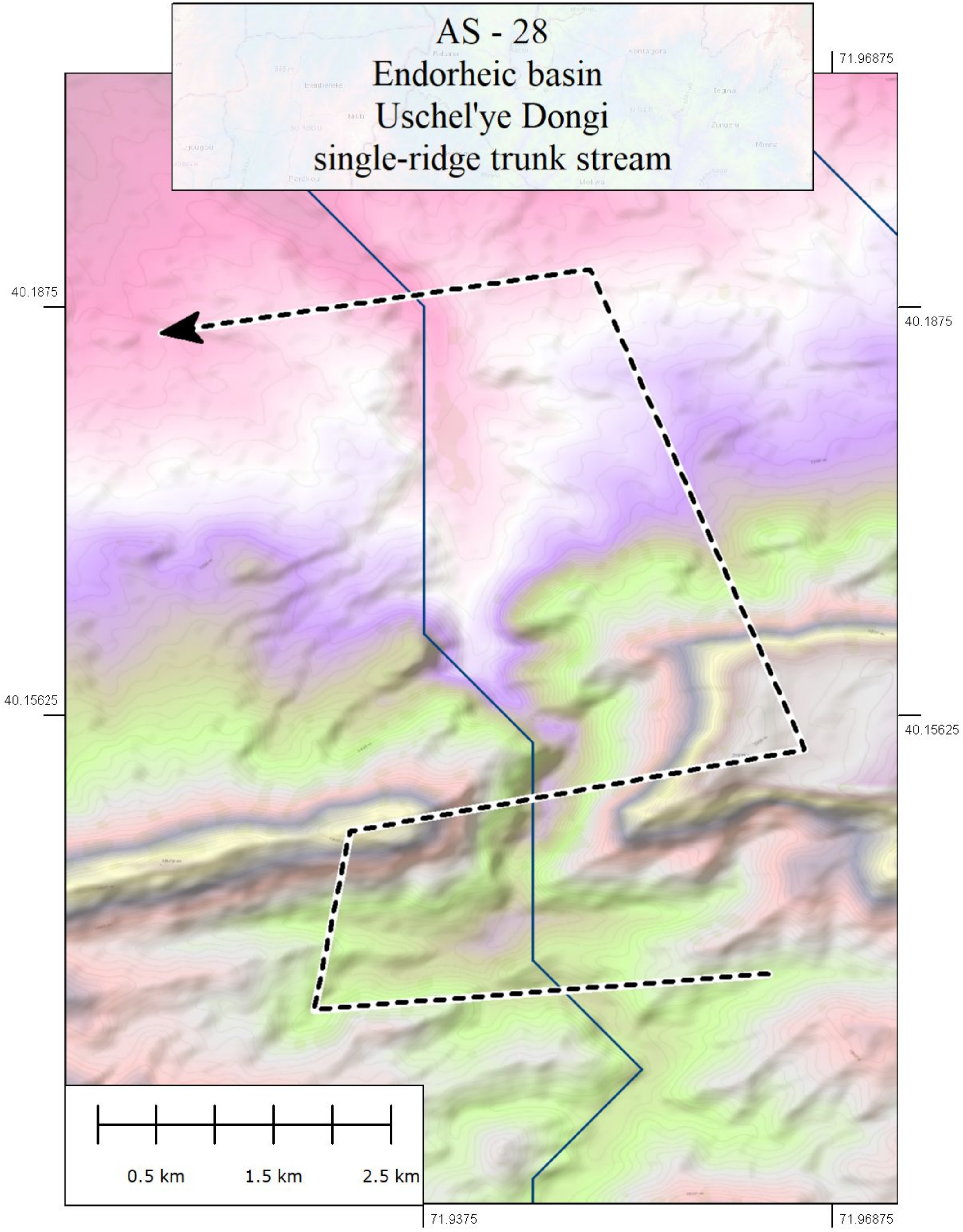
AS - 28
Endorheic basin
Uschel'ye Dongi
single-ridge trunk stream
71.96875
40.1875
40.1875
40.15625
40.15625
0.5 km
1.5 km
2.5 km
71.9375
71.96875

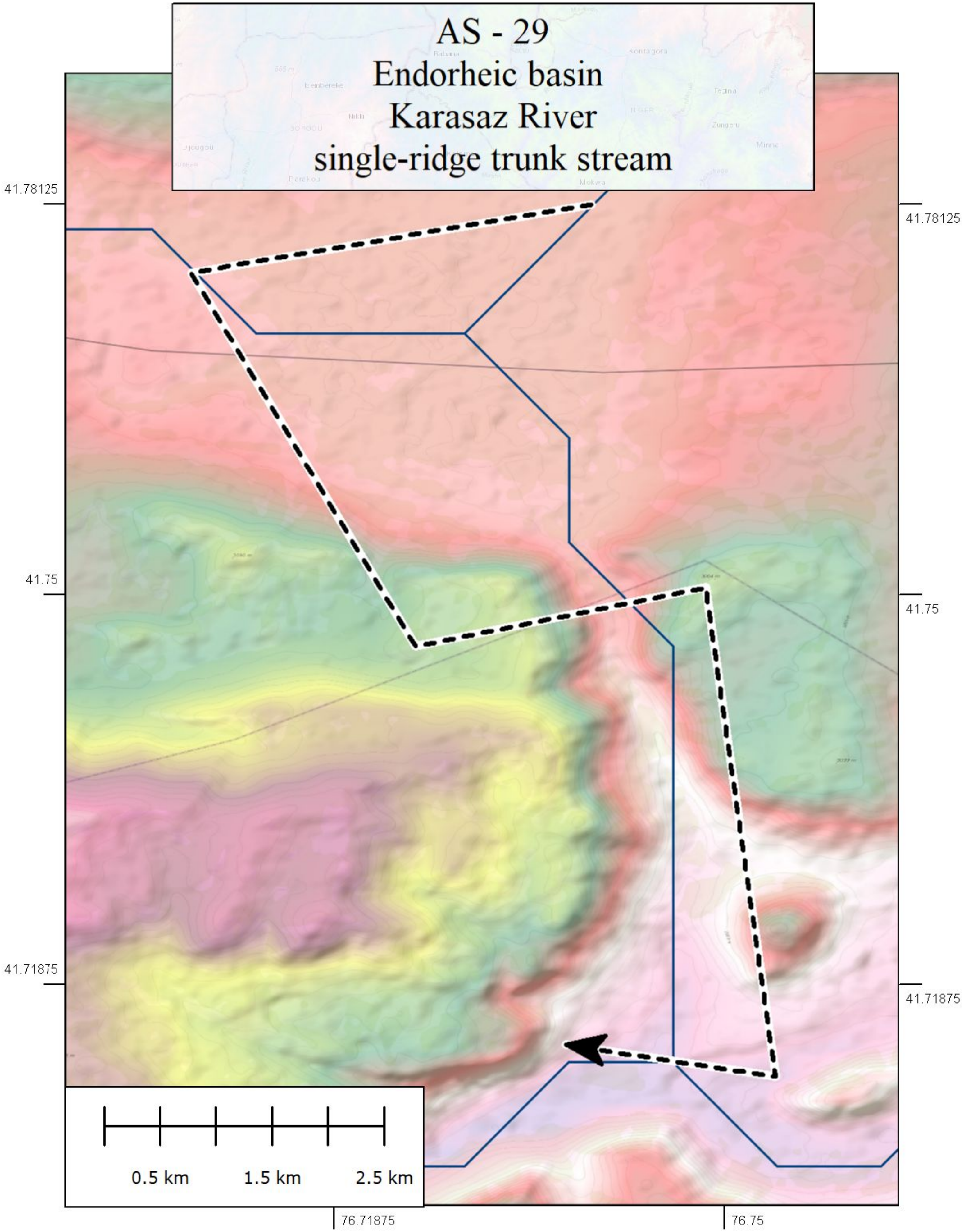

AS - 29
Endorheic basin
Karasaz River
single-ridge trunk stream
41.78125
41.78125
41.75
41.75
41.71875
41.71875
0.5 km
1.5 km
2.5 km
76.71875
76.75

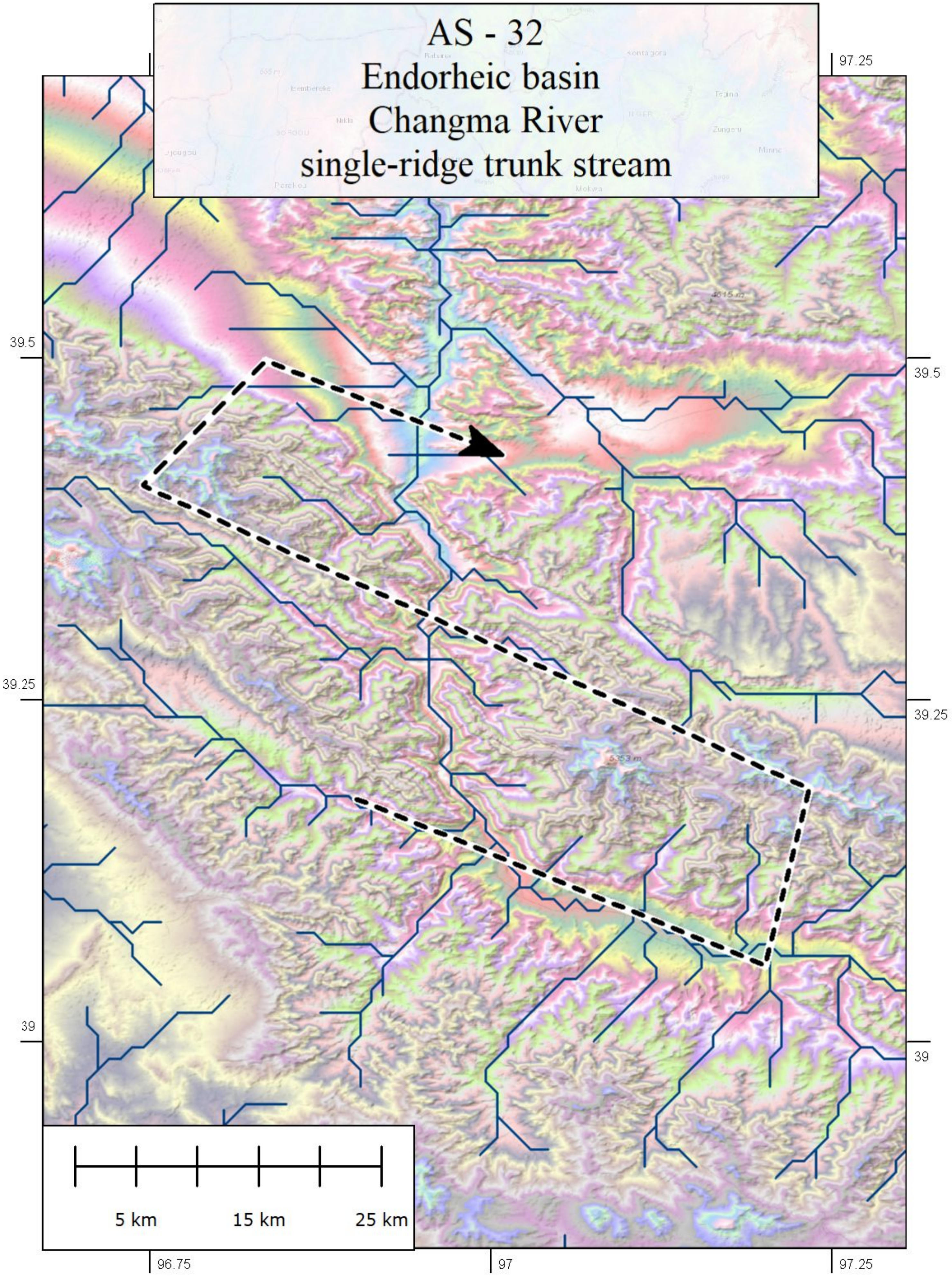
AS - 32
Endorheic basin
Changma River
single-ridge trunk stream
97.25
39.5
39.5
39.25
39.25
39
39
5 km
15 km
25 km
96.75
97
97.25

AS - 33
Endorheic basin
Kyrgyz Ata River
single-ridge trunk stream

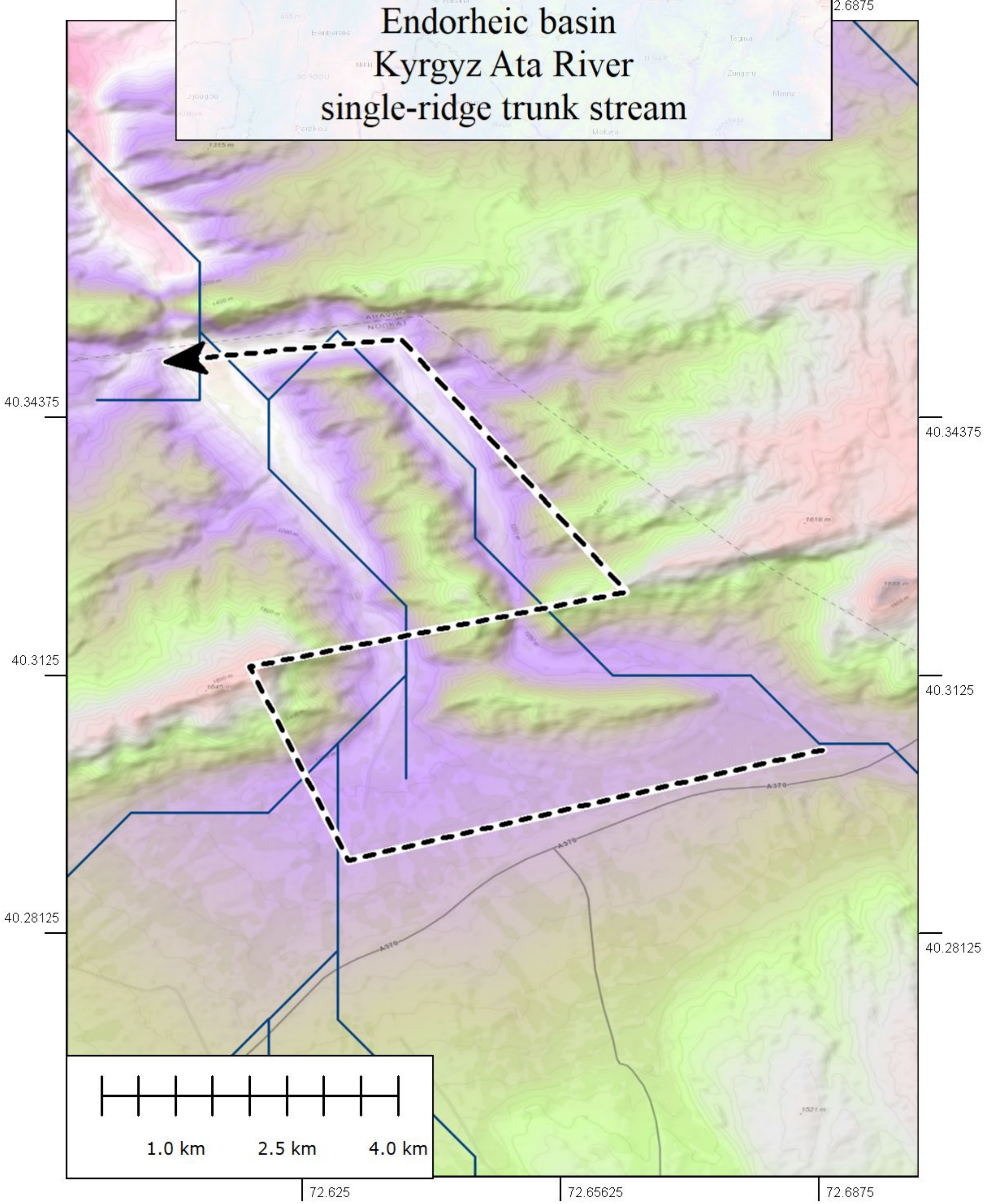

AS - 34
Mekong River Basin
Namkading River
single-ridge trunk stream

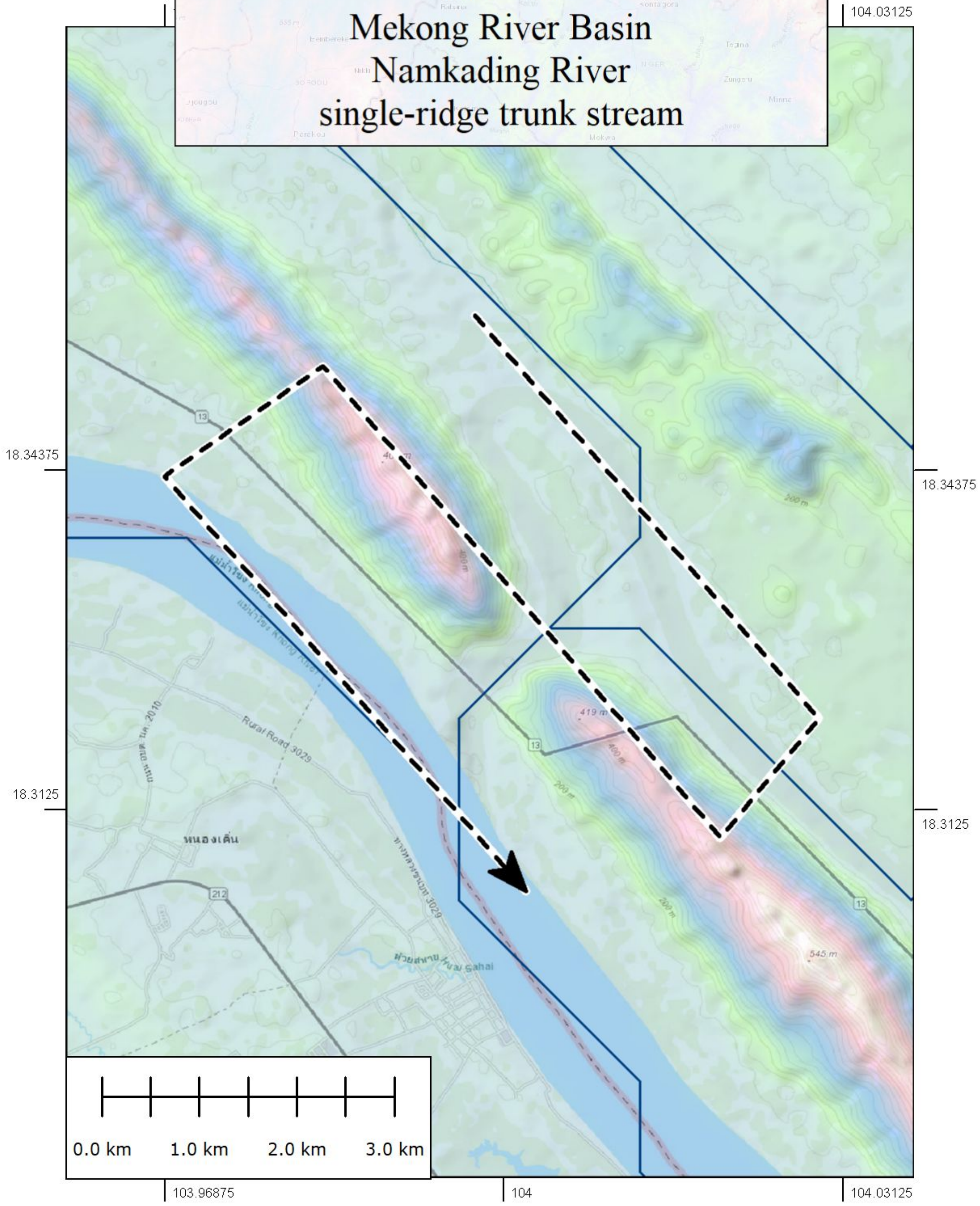

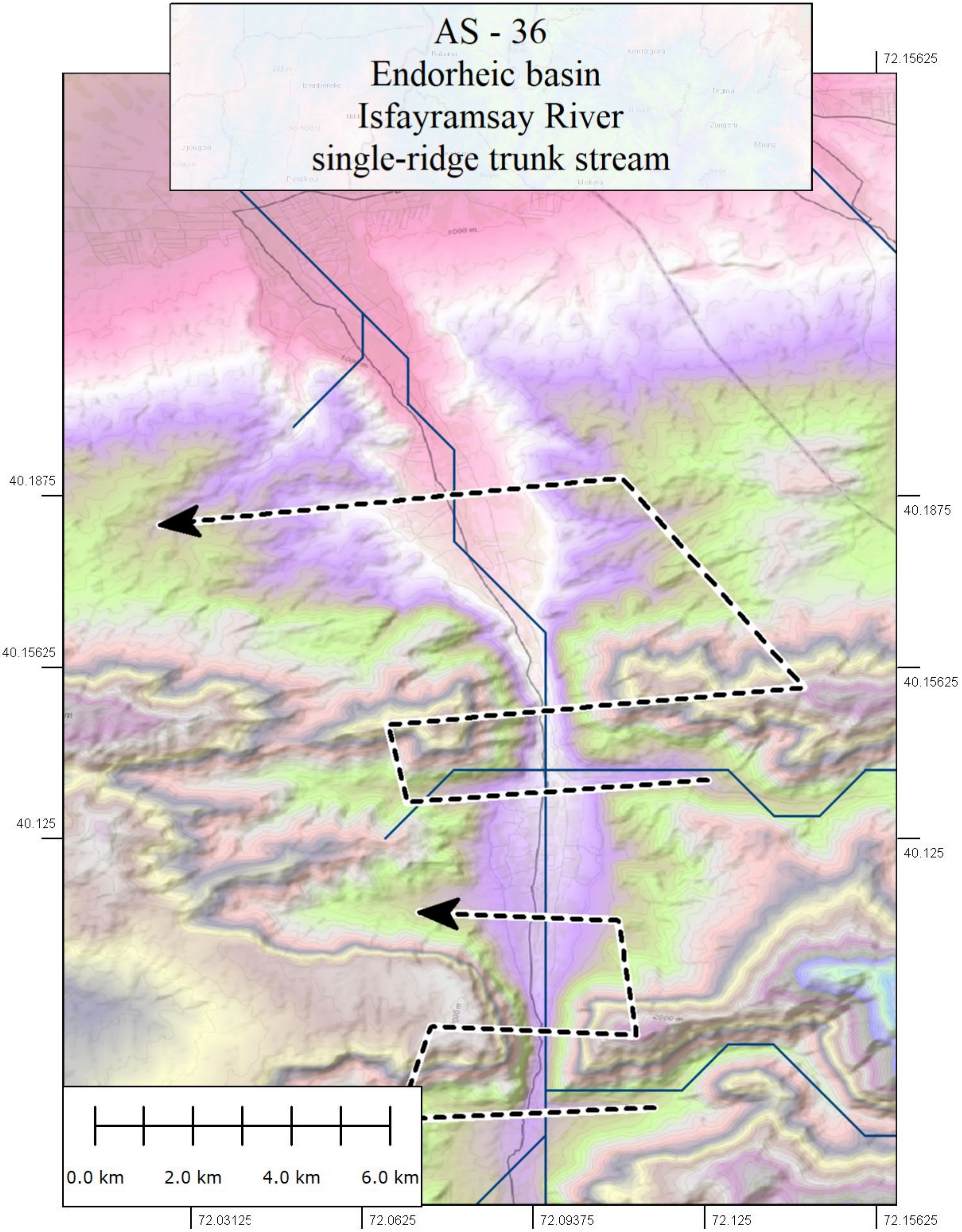
AS - 36
Endorheic basin
Isfayramsay River
single-ridge trunk stream
72.15625
40.1875
40.15625
40.125
40.1875
40.15625
40.125
1000 m
2000 m
0.0 km
2.0 km
4.0 km
6.0 km
72.03125
72.0625
72.09375
72.125
72.15625

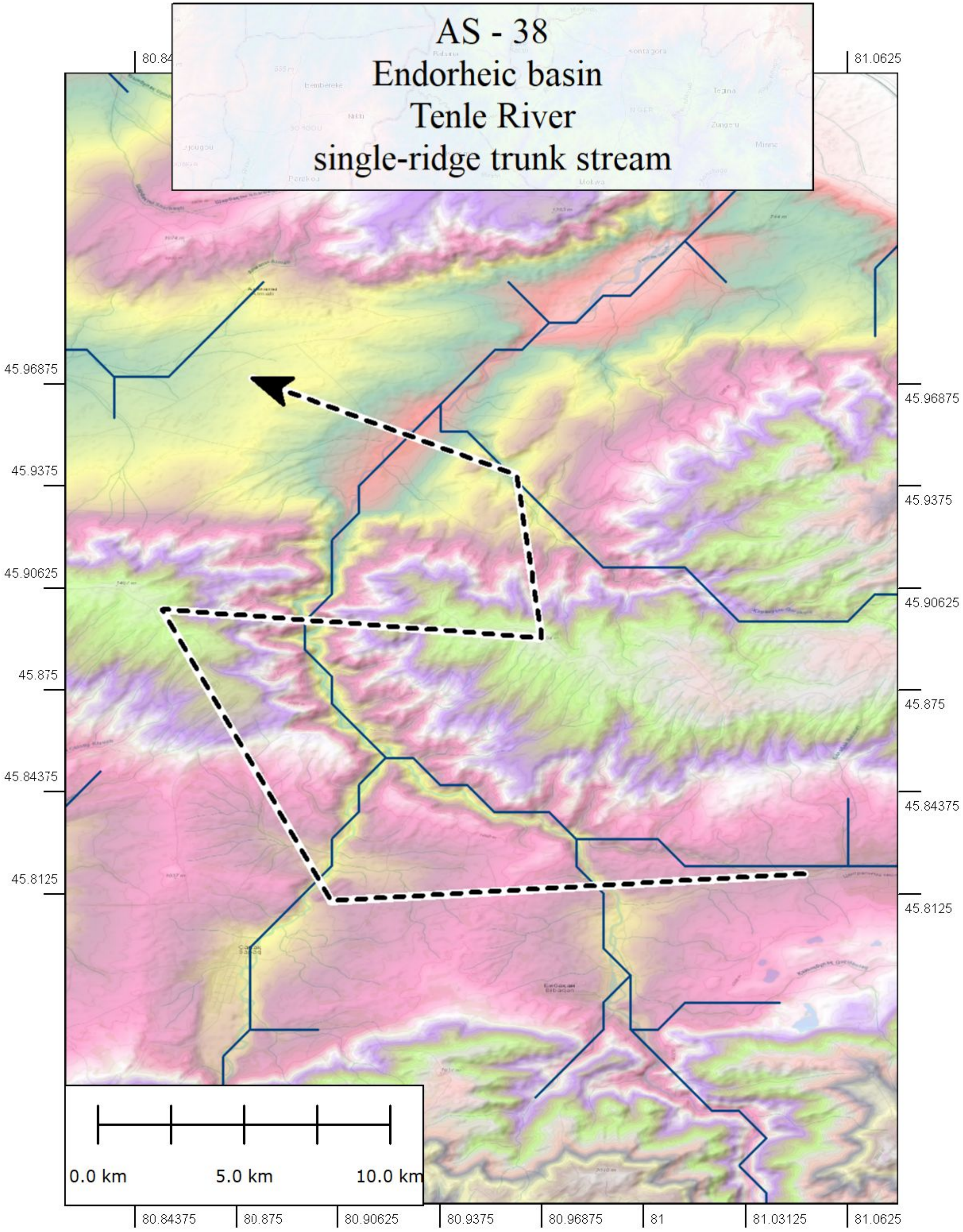

AS - 38
Endorheic basin
Tenle River
single-ridge trunk stream
81.0625
45.96875
45.9375
45.90625
45.875
45.84375
45.8125
0.0 km
5.0 km
10.0 km
80.84375
80.875
80.90625
80.9375
80.96875
81
81.03125
81.0625

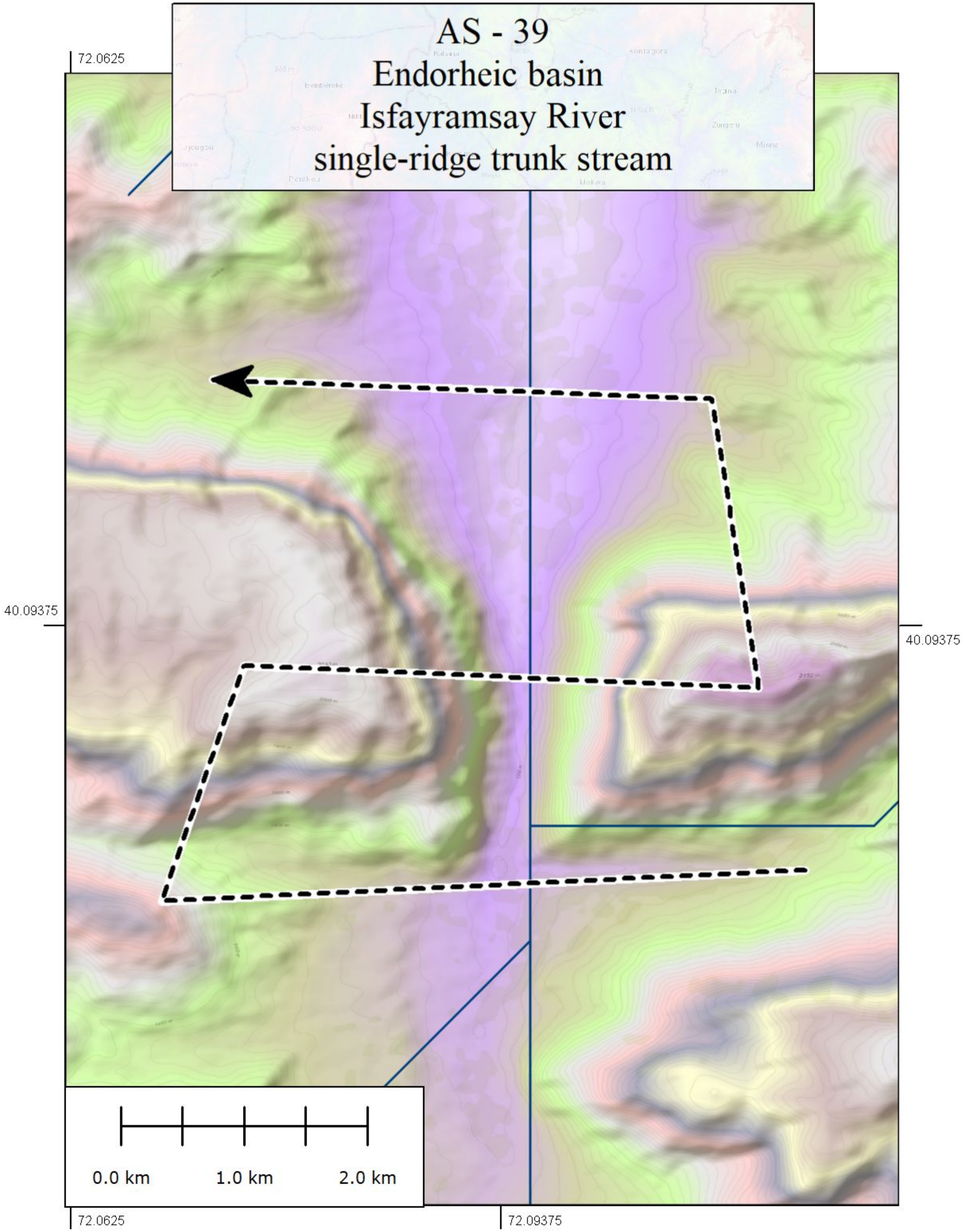
AS - 39
Endorheic basin
Isfayramsay River
single-ridge trunk stream
72.0625
40.09375
40.09375
72.0625
72.09375
0.0 km
1.0 km
2.0 km

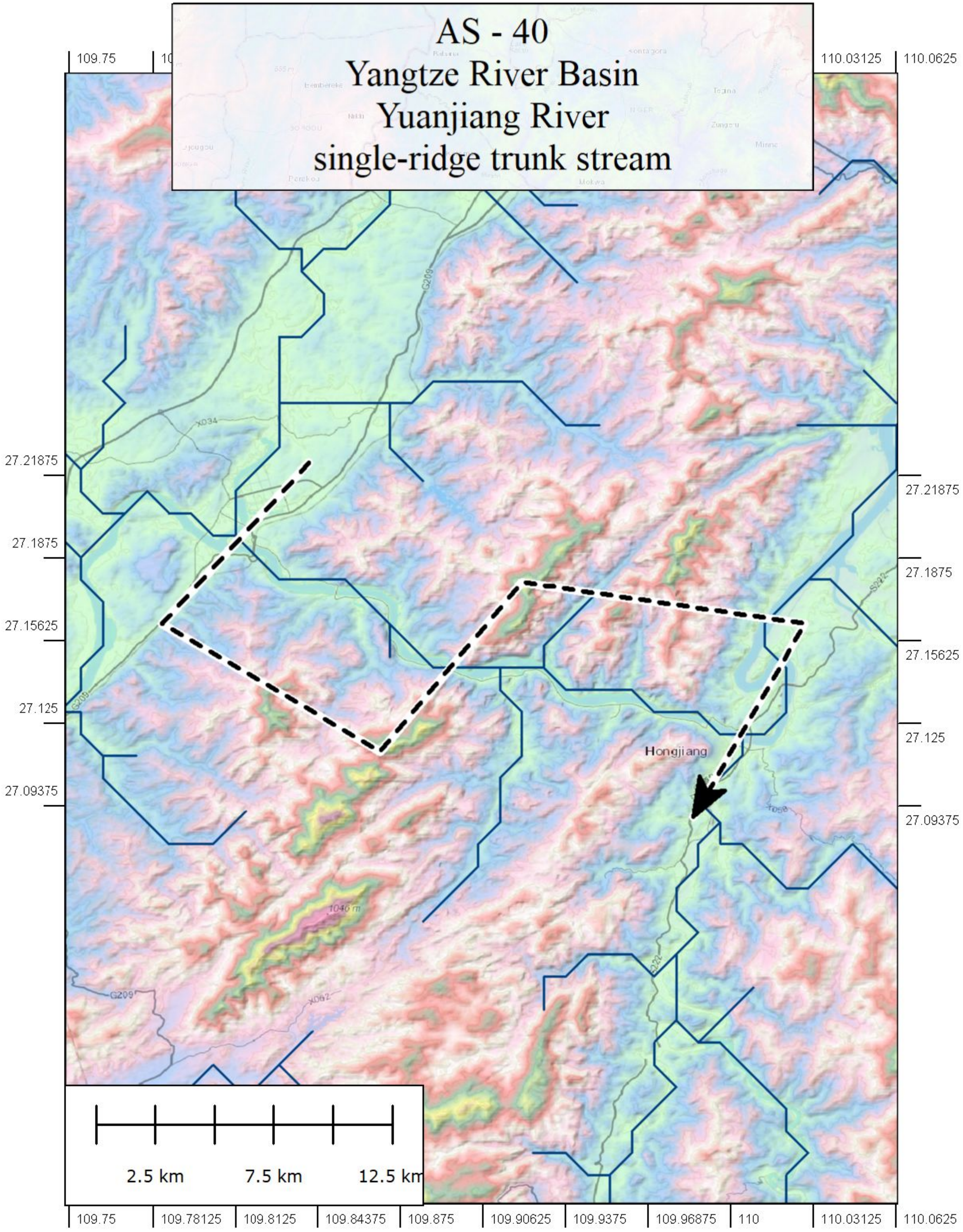
AS - 40
Yangtze River Basin
Yuanjiang River
single-ridge trunk stream
109.75
110.03125
110.0625
27.21875
27.1875
27.15625
27.125
27.09375
Hongjiang
1040 m
G209
X034
S222
X062
2.5 km
7.5 km
12.5 km
109.78125
109.8125
109.84375
109.875
109.90625
109.9375
109.96875
110

AS - 41
Ganges River Basin
Qialuozangbu River
single-ridge trunk stream

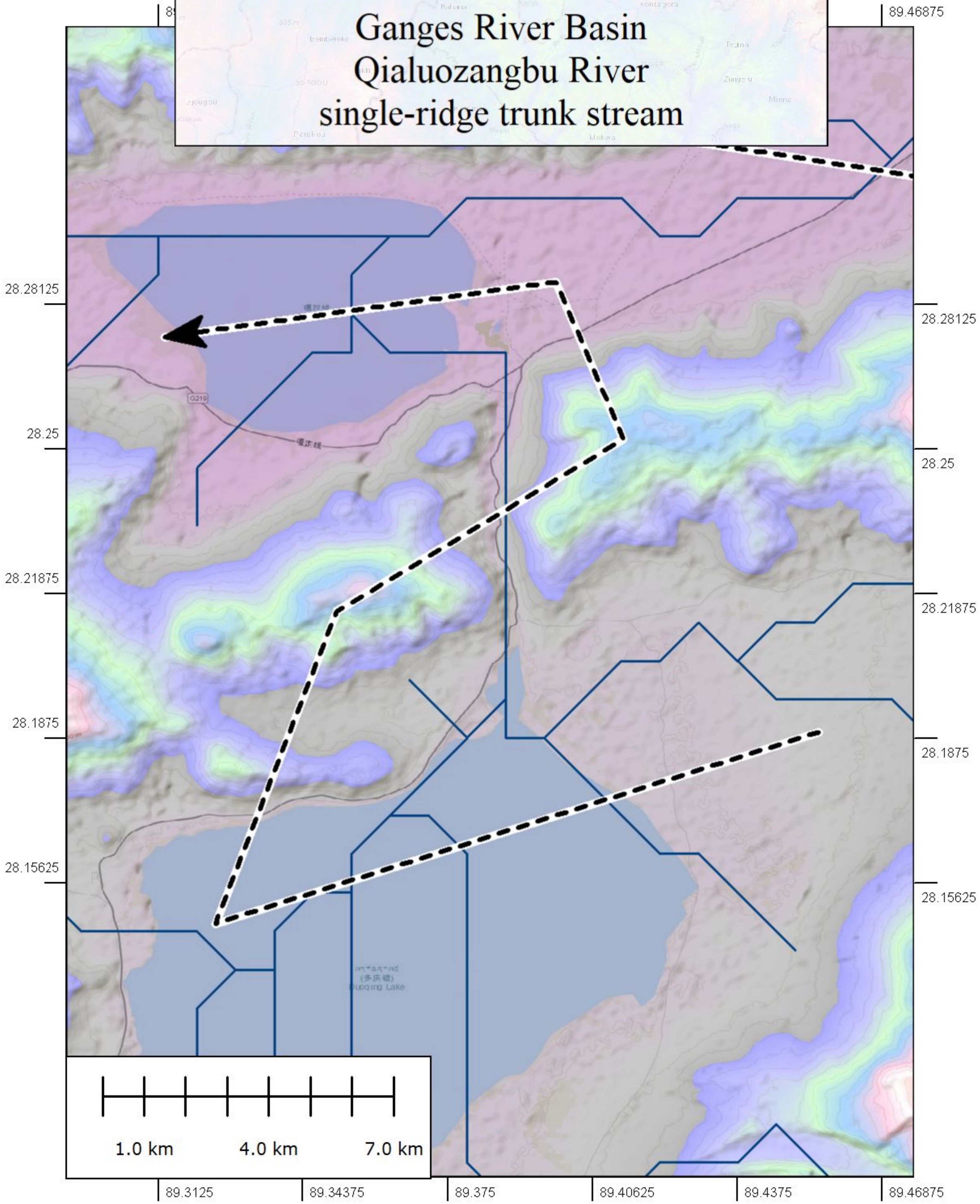

AS - 42
Yangtze River Basin
Yinjiang River
single-ridge trunk stream

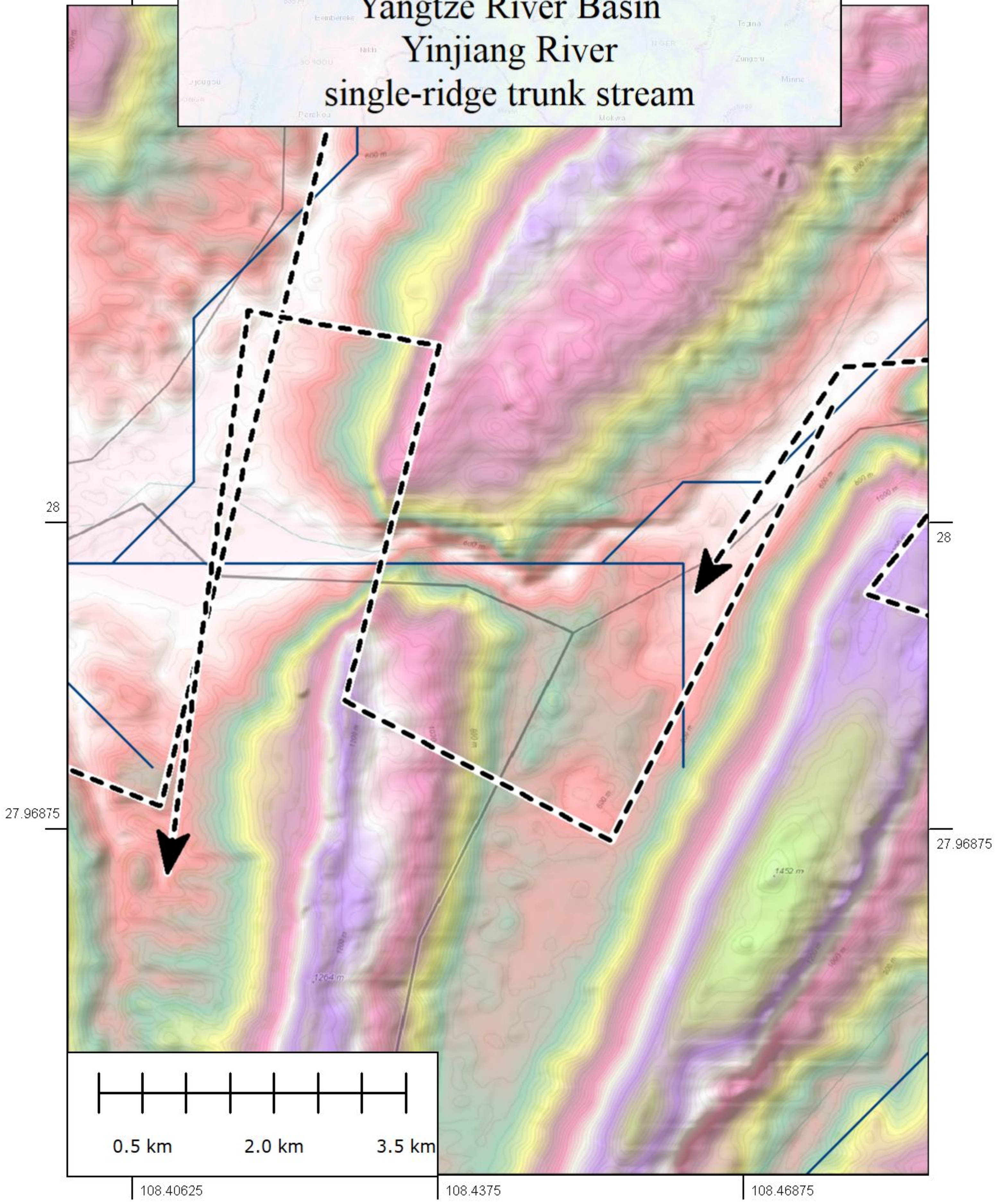

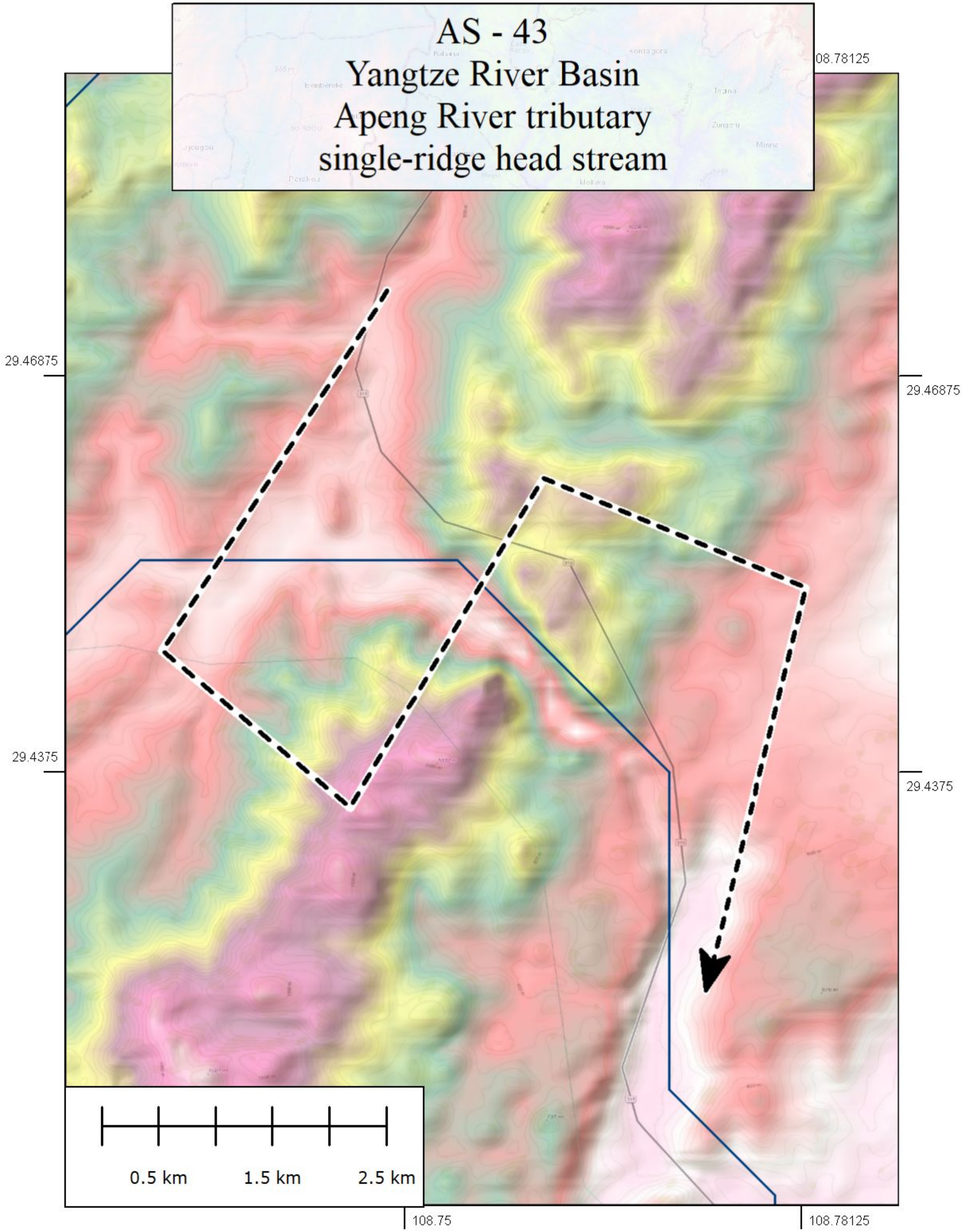
AS - 43
Yangtze River Basin
Apeng River tributary
single-ridge head stream
108.78125
29.46875
29.46875
29.4375
29.4375
0.5 km
1.5 km
2.5 km
108.75
108.78125

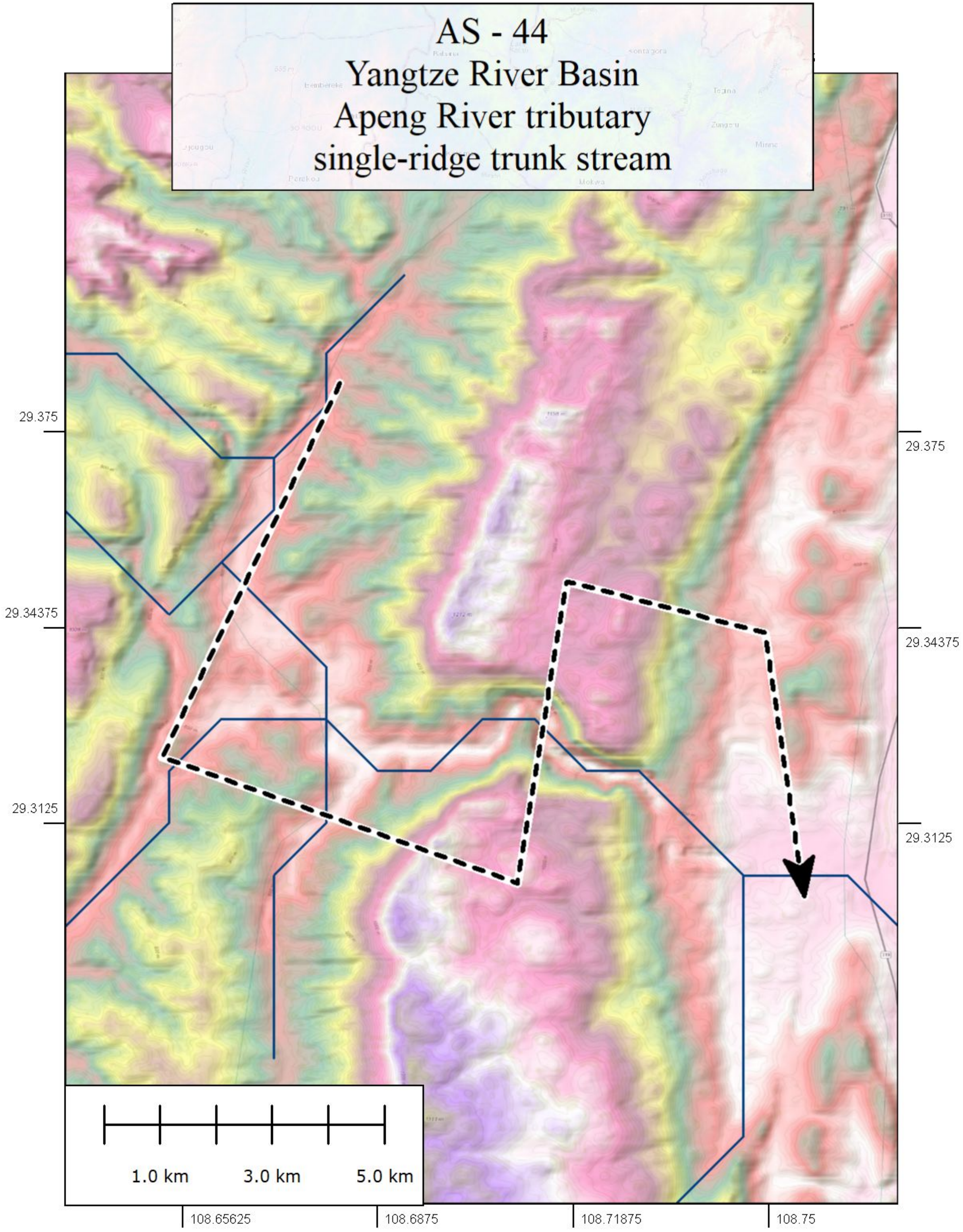

AS - 44
Yangtze River Basin
Apeng River tributary
single-ridge trunk stream
29.375
29.34375
29.3125
1.0 km
3.0 km
5.0 km
108.65625
108.6875
108.71875
108.75

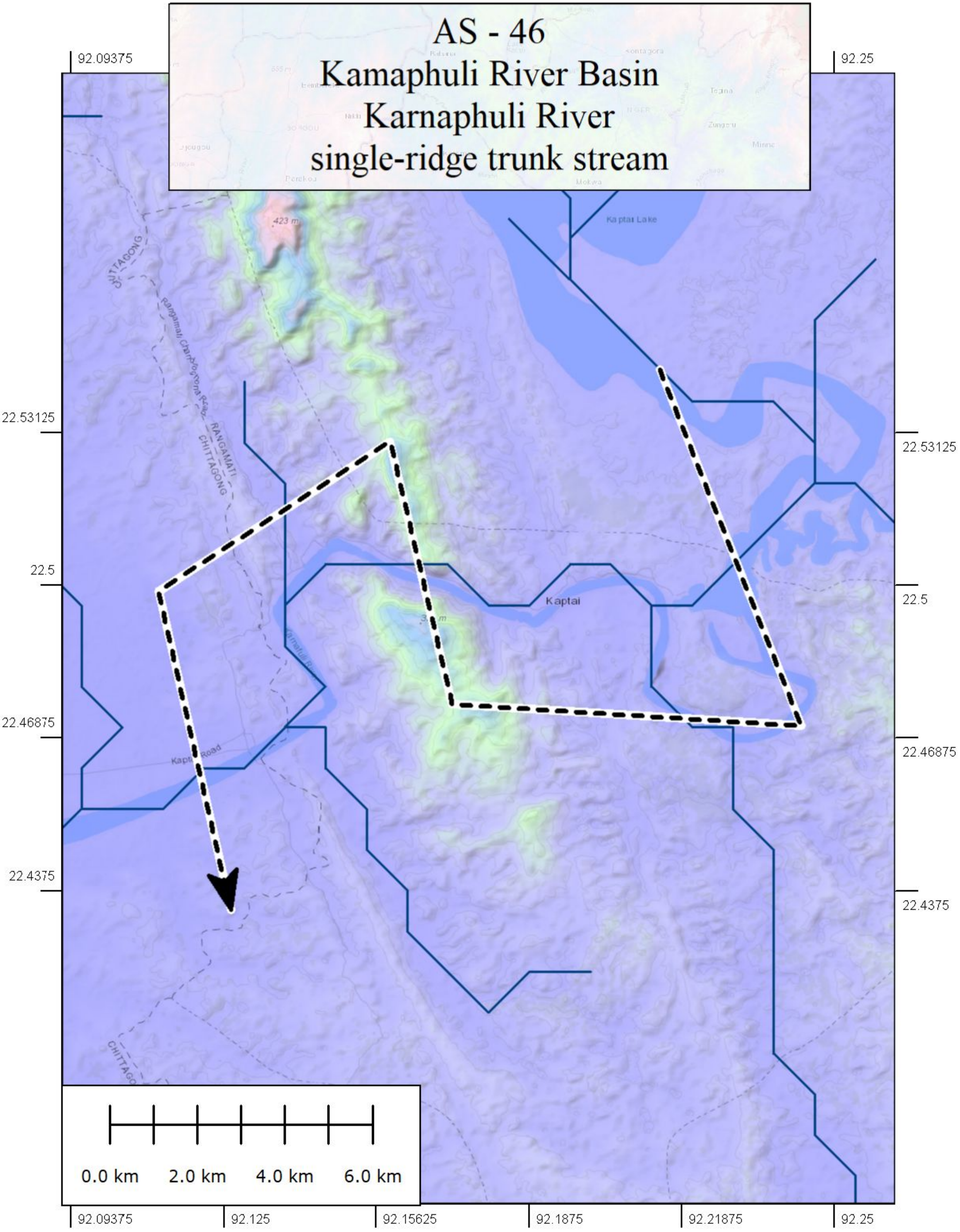

AS - 46
Kamaphuli River Basin
Karnaphuli River
single-ridge trunk stream
92.09375
92.25
22.53125
22.5
22.46875
22.4375
92.125
92.15625
92.1875
92.21875
Kaptai Lake
Kaptai
423 m
CHITTAGONG
RANGAMATI
Kaptai Road
0.0 km
2.0 km
4.0 km
6.0 km

AS - 49
Yangtze River Basin
Zhouhe River
single-ridge trunk stream

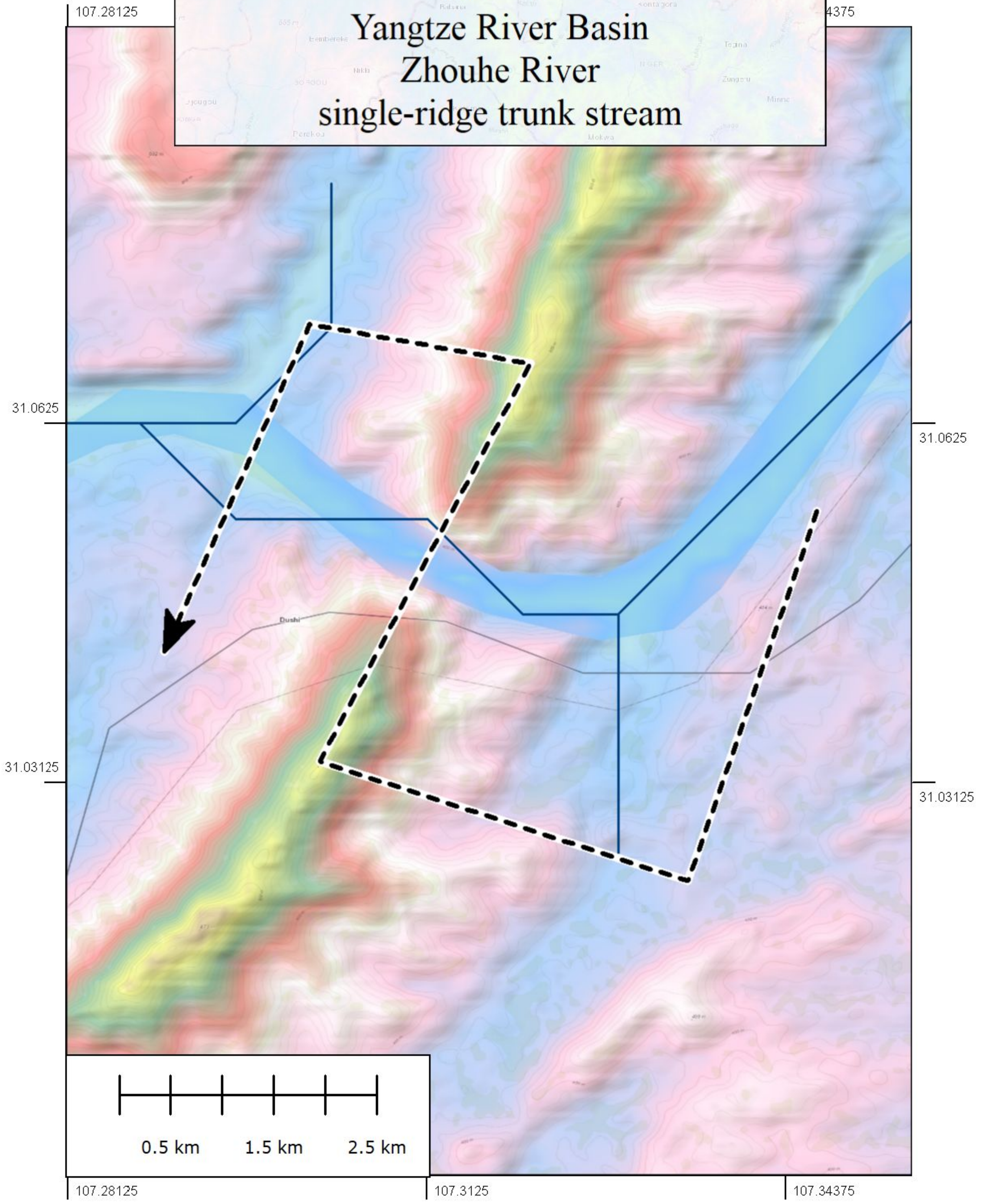

AS - 51
Yangtze River Basin
Pengxi Brook
single-ridge trunk stream

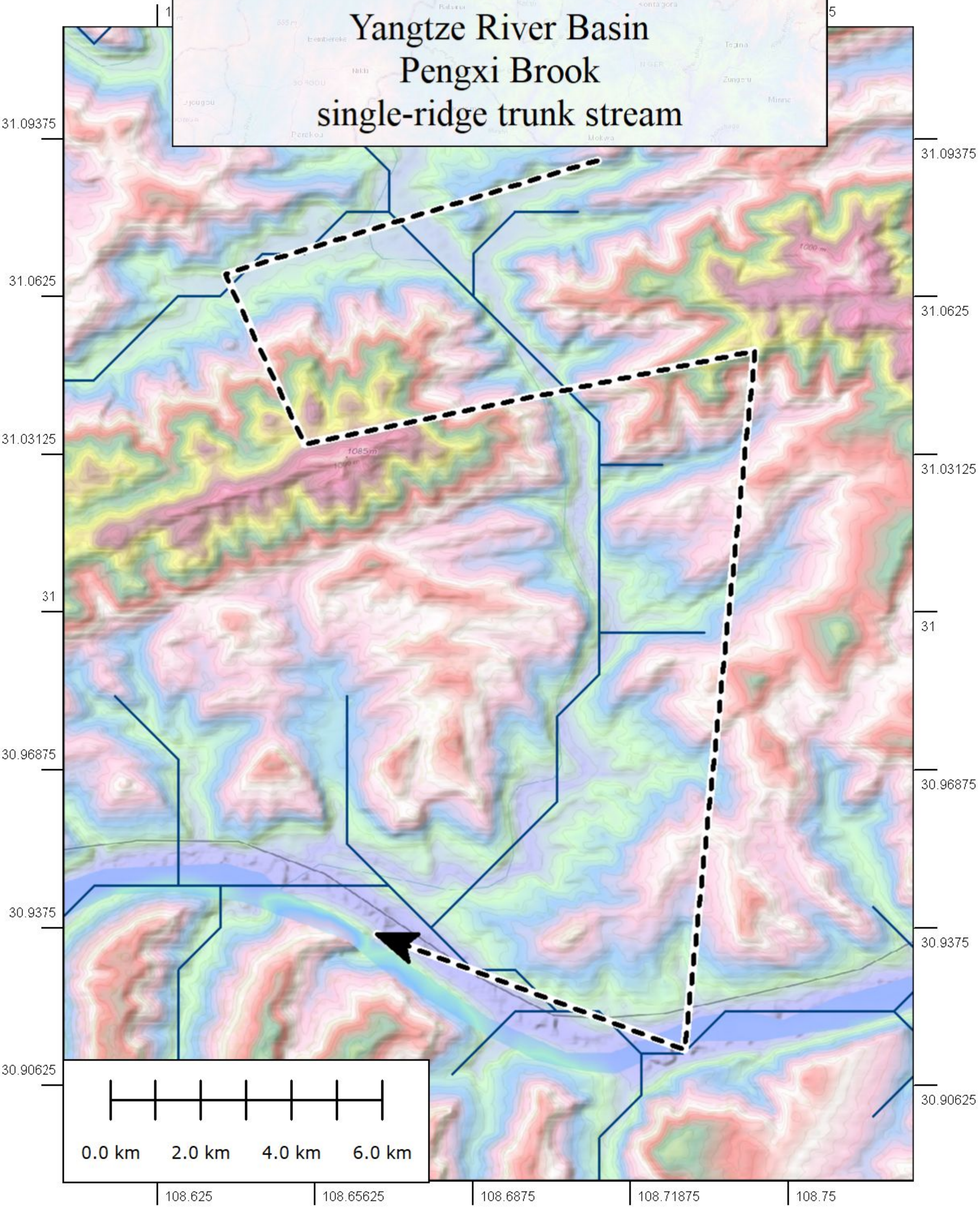

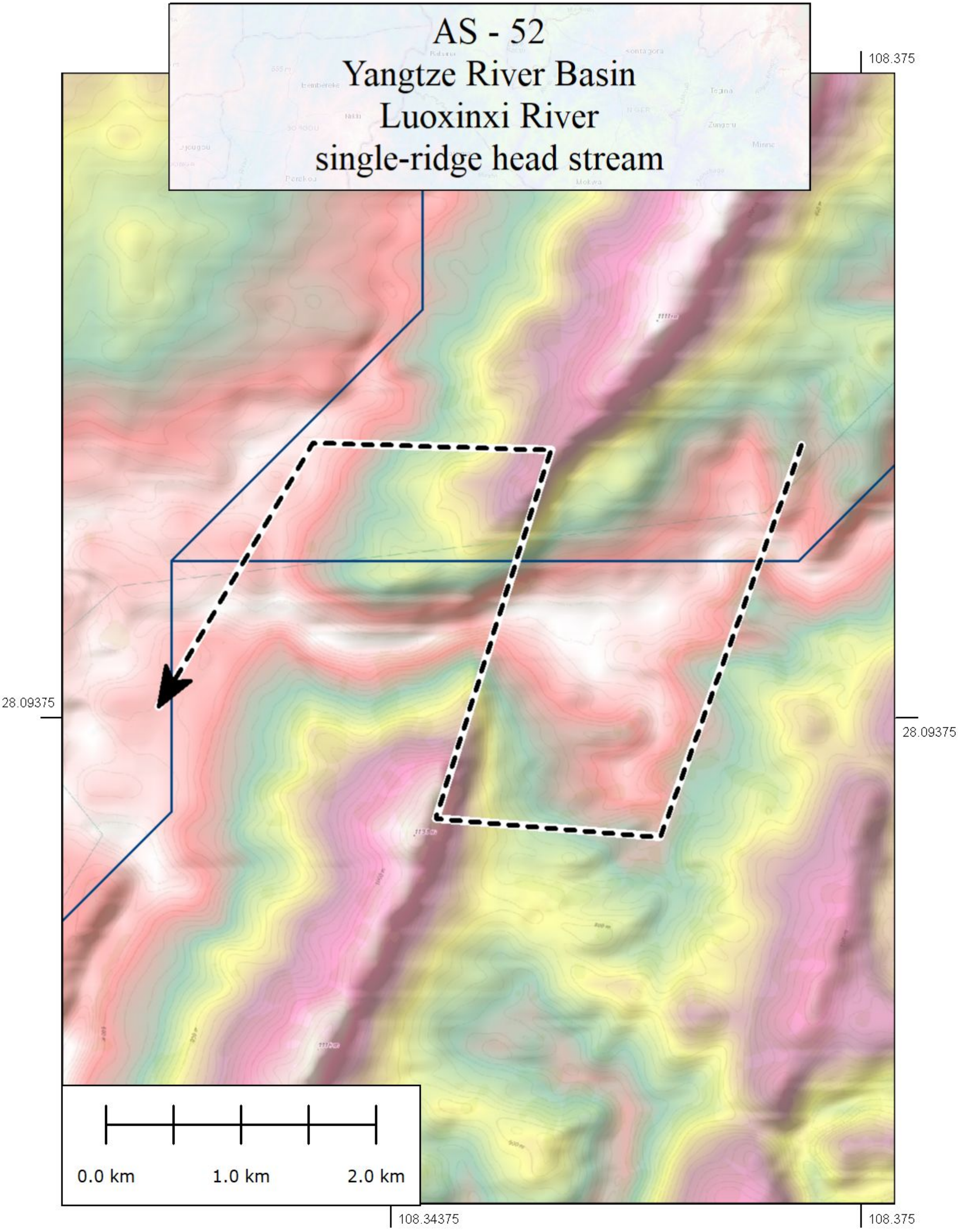
AS - 52
Yangtze River Basin
Luoxinxi River
single-ridge head stream
108.375
28.09375
28.09375
0.0 km
1.0 km
2.0 km
108.34375
108.375

AS - 53
Yangtze River Basin
Yinjiang River
single-ridge trunk stream

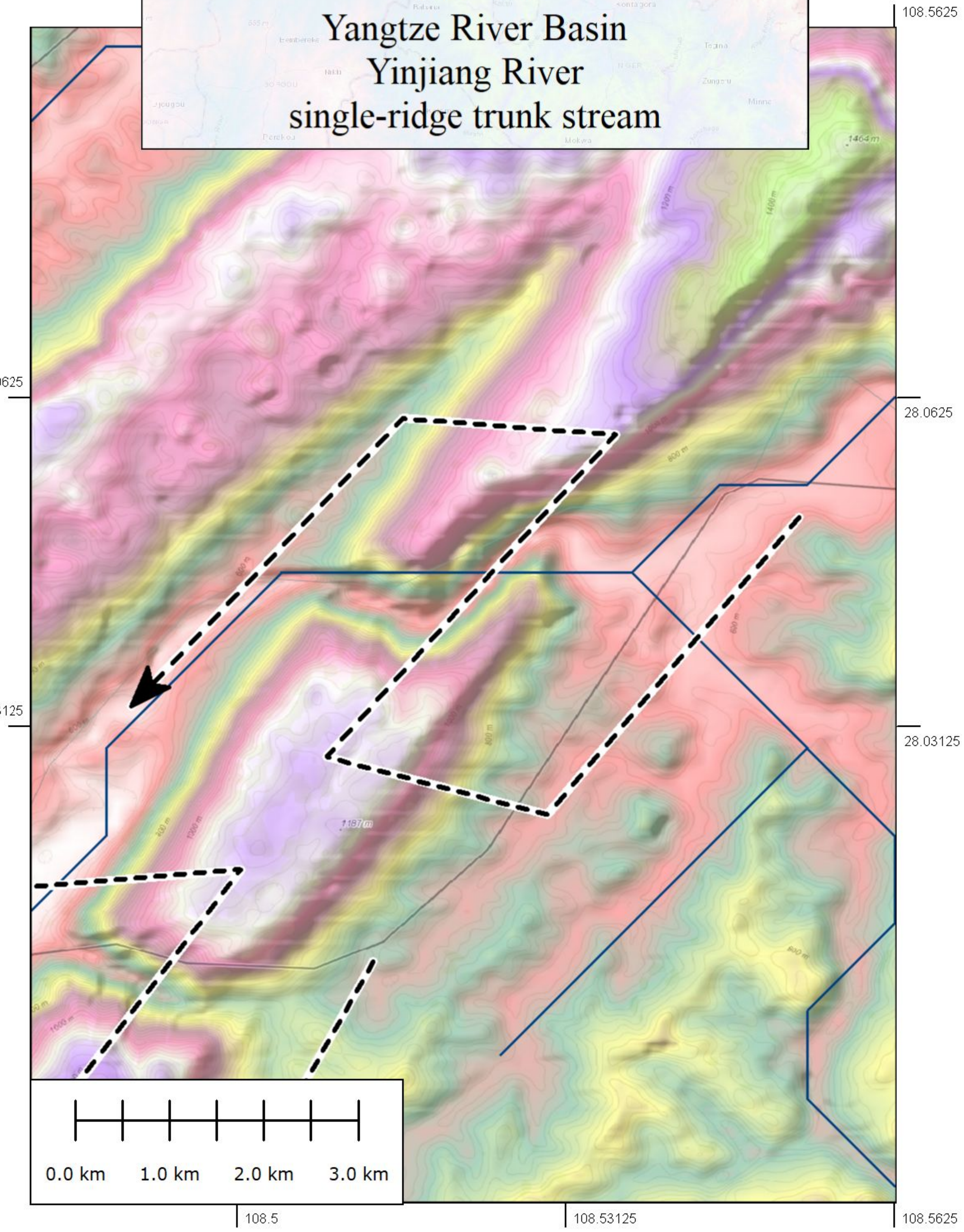

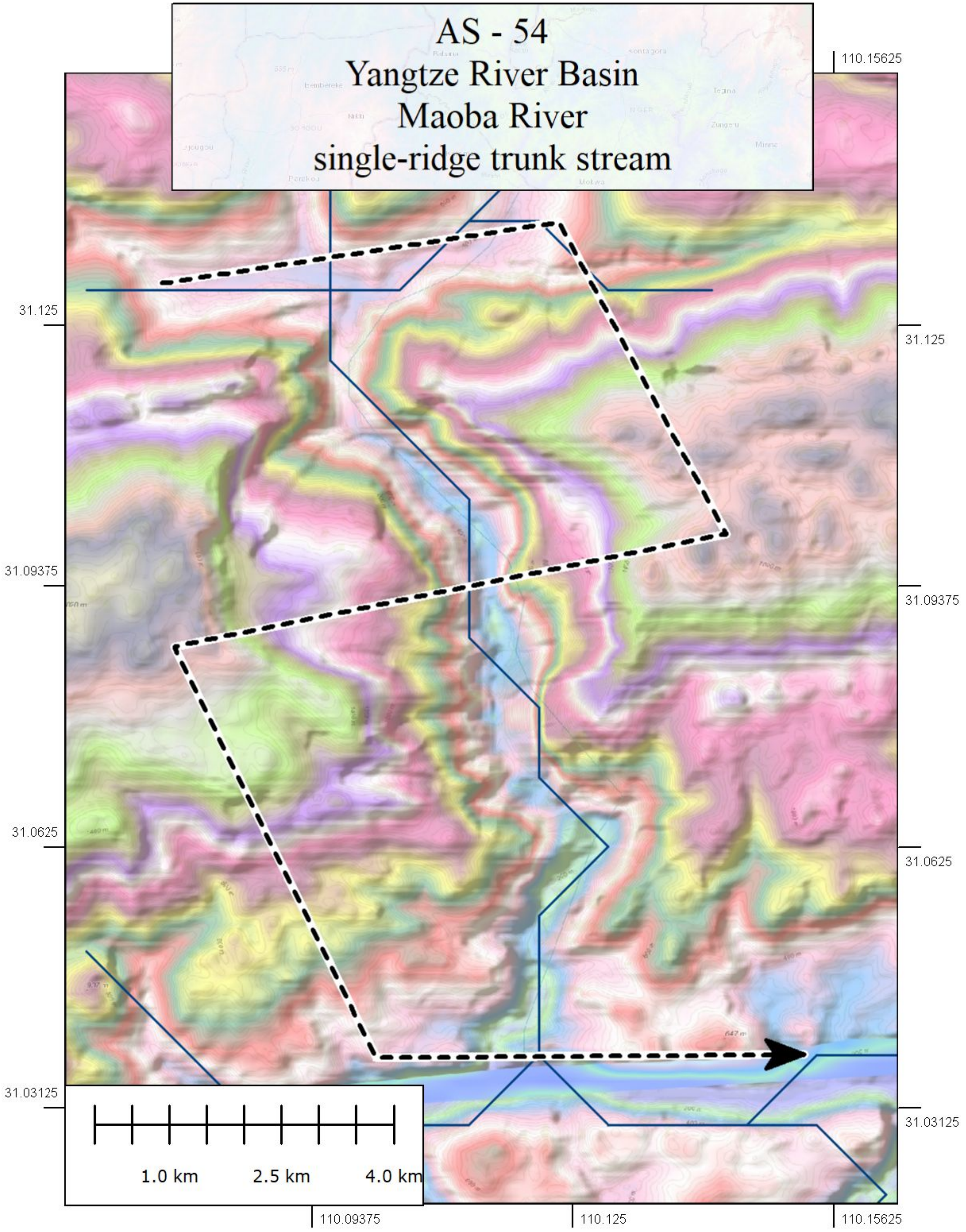
AS - 54
Yangtze River Basin
Maoba River
single-ridge trunk stream
110.15625
31.125
31.125
31.09375
31.09375
31.0625
31.0625
31.03125
31.03125
1.0 km
2.5 km
4.0 km
110.09375
110.125
110.15625

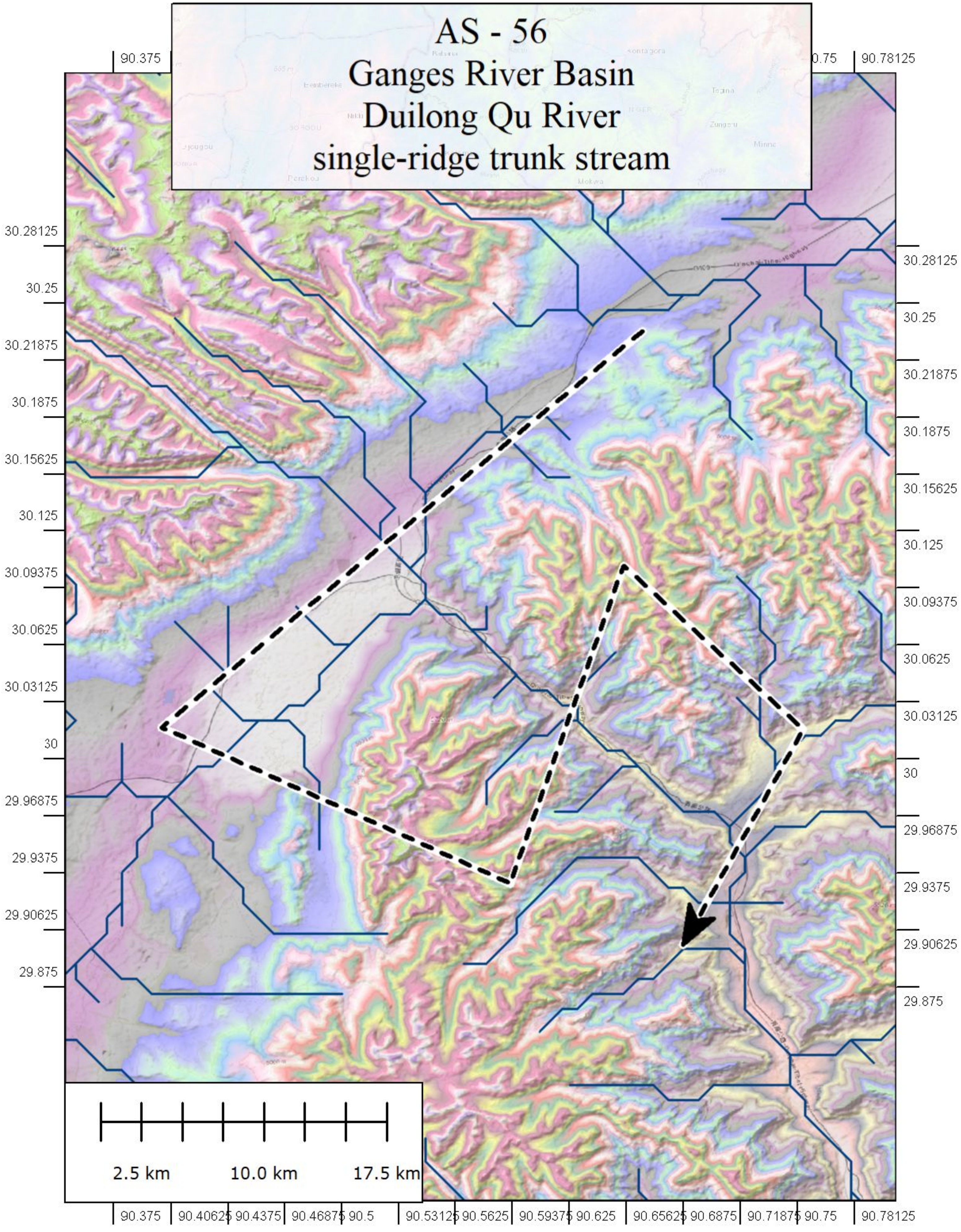
AS - 56
Ganges River Basin
Duilong Qu River
single-ridge trunk stream
2.5 km
10.0 km
17.5 km
90.375
90.40625
90.4375
90.46875
90.5
90.53125
90.5625
90.59375
90.625
90.65625
90.6875
90.71875
90.75
90.78125
30.28125
30.25
30.21875
30.1875
30.15625
30.125
30.09375
30.0625
30.03125
30
29.96875
29.9375
29.90625
29.875

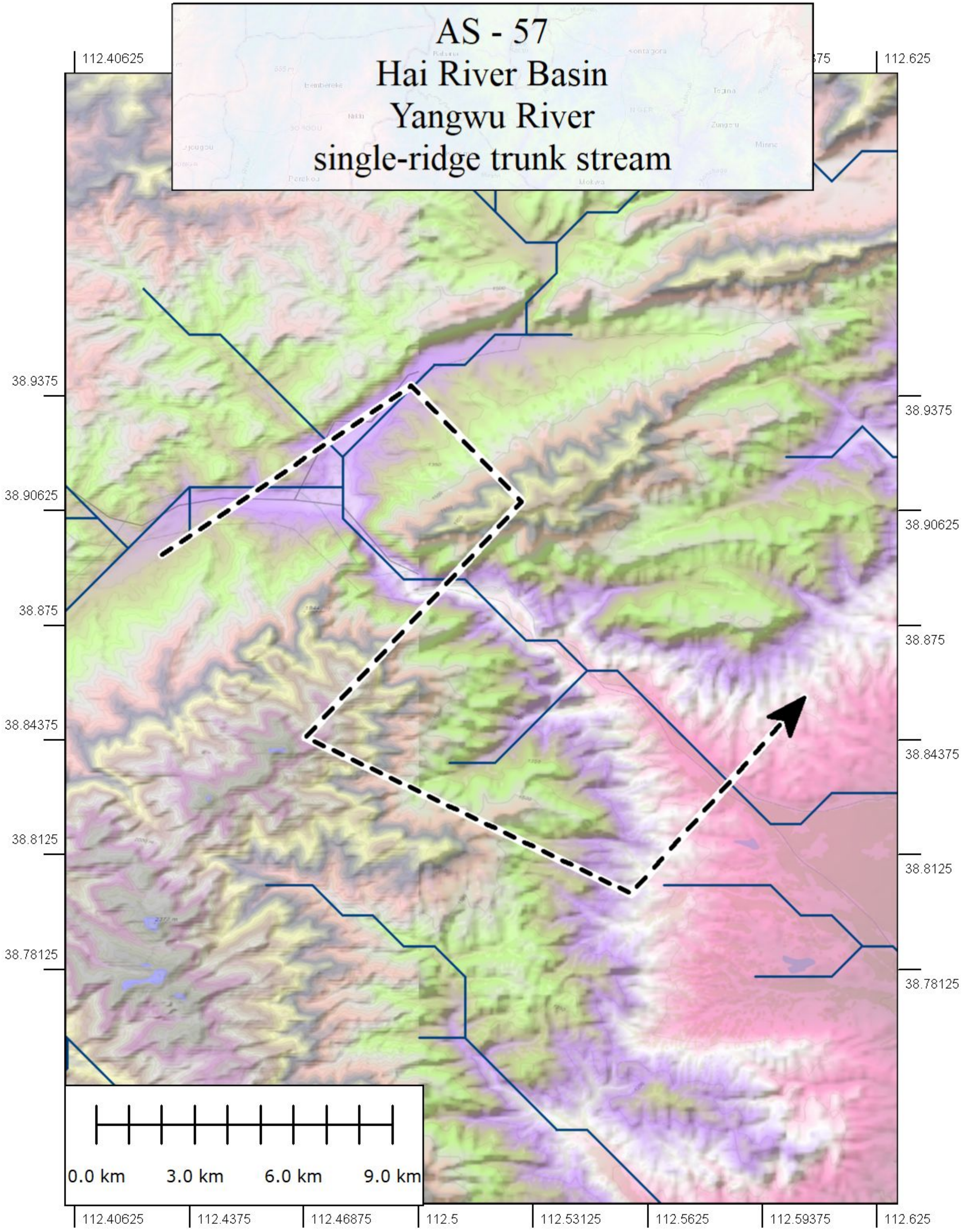

AS - 57
Hai River Basin
Yangwu River
single-ridge trunk stream
112.40625
112.625
112.4375
112.46875
112.5
112.53125
112.5625
112.59375
38.9375
38.90625
38.875
38.84375
38.8125
38.78125
0.0 km
3.0 km
6.0 km
9.0 km

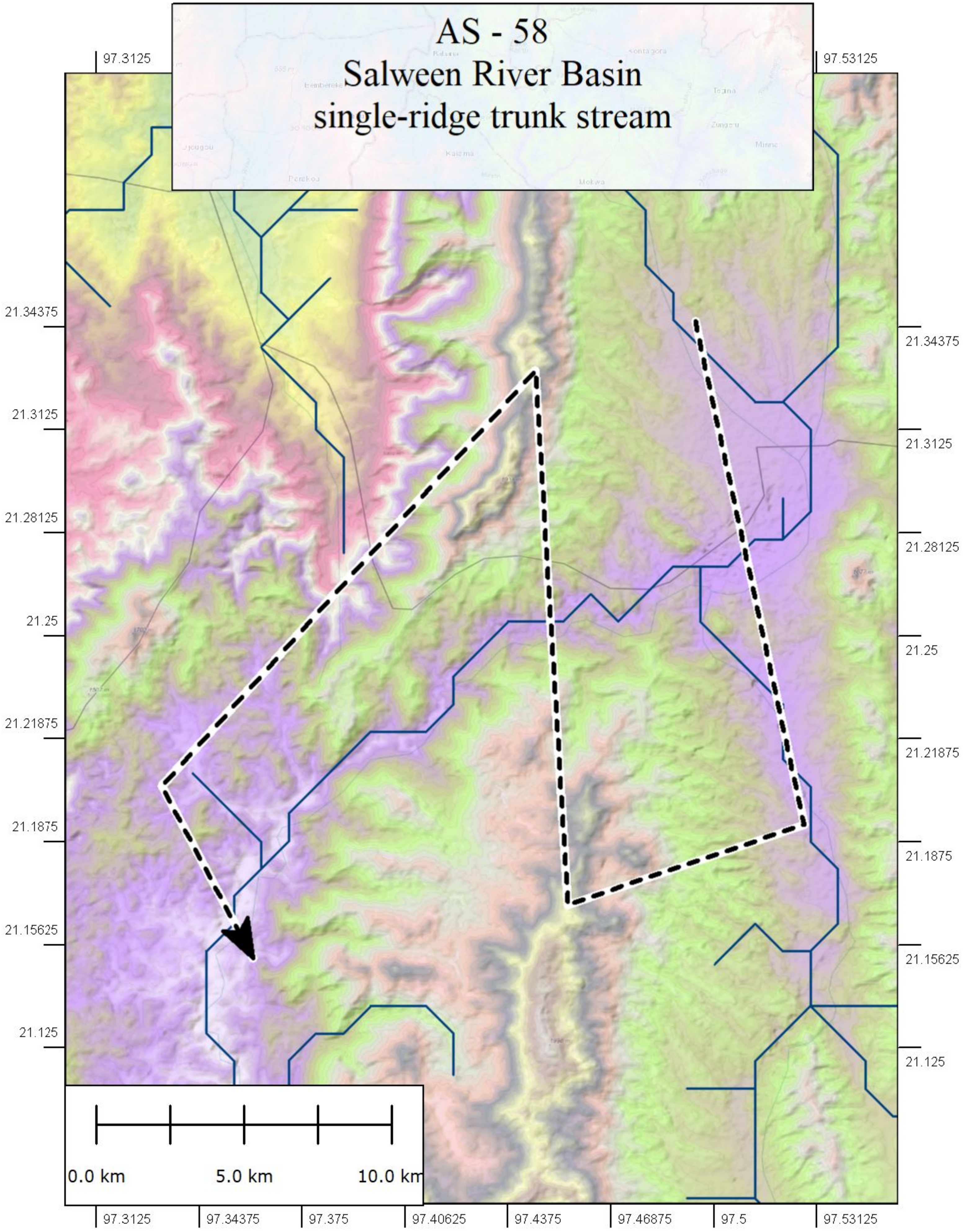
AS - 58
Salween River Basin
single-ridge trunk stream
97.3125
97.53125
21.34375
21.3125
21.28125
21.25
21.21875
21.1875
21.15625
21.125
97.3125
97.34375
97.375
97.40625
97.4375
97.46875
97.5
97.53125
0.0 km
5.0 km
10.0 km

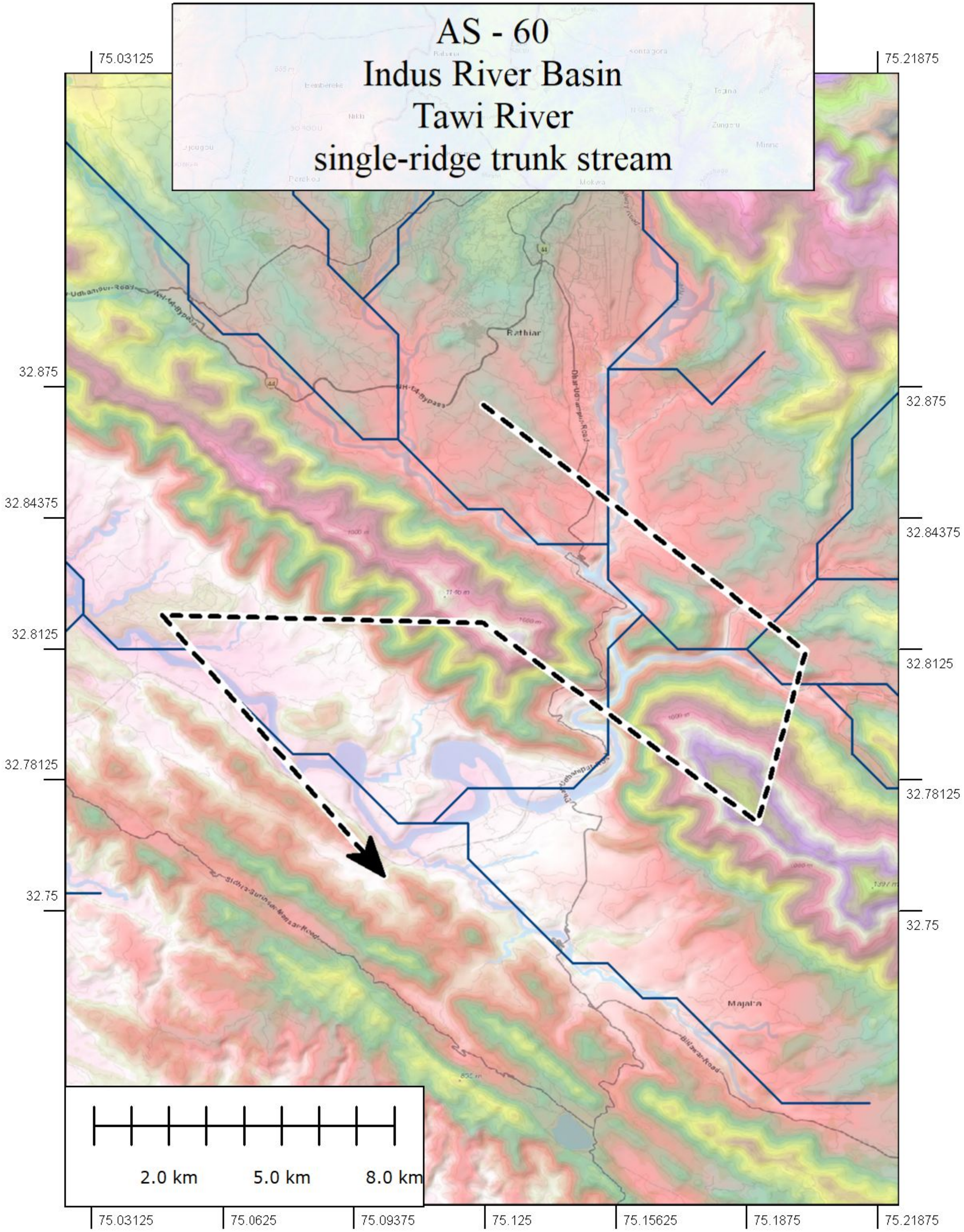
AS - 60
Indus River Basin
Tawi River
single-ridge trunk stream
75.03125
75.0625
75.09375
75.125
75.15625
75.1875
75.21875
32.875
32.84375
32.8125
32.78125
32.75
Rathiar
Majalta
2.0 km
5.0 km
8.0 km

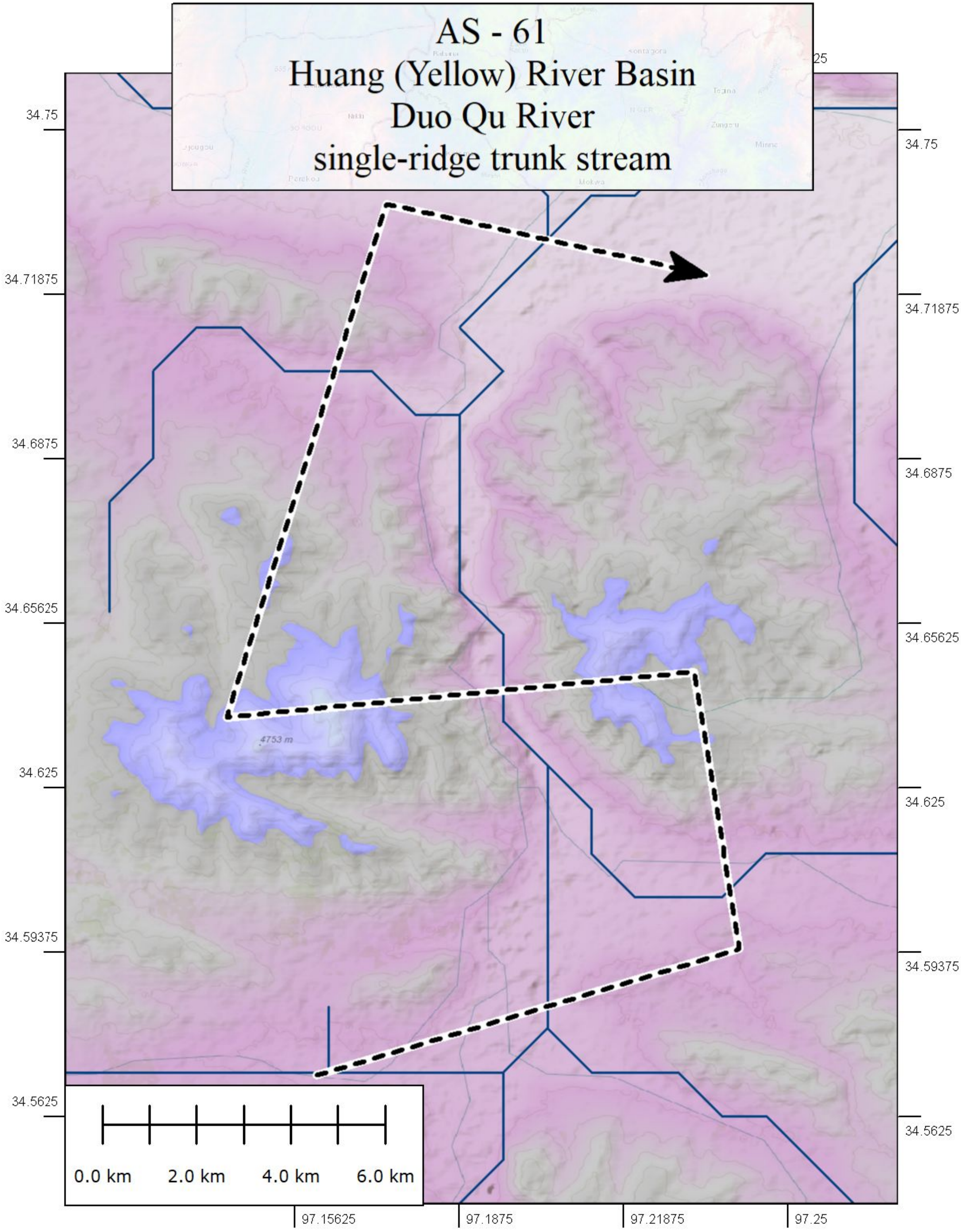
AS - 61
Huang (Yellow) River Basin
Duo Qu River
single-ridge trunk stream
34.75
34.71875
34.6875
34.65625
34.625
34.59375
34.5625
4753 m
0.0 km
2.0 km
4.0 km
6.0 km
97.15625
97.1875
97.21875
97.25

AS - 64
Mekong River Basin
Nam Xuang River
single-ridge trunk stream

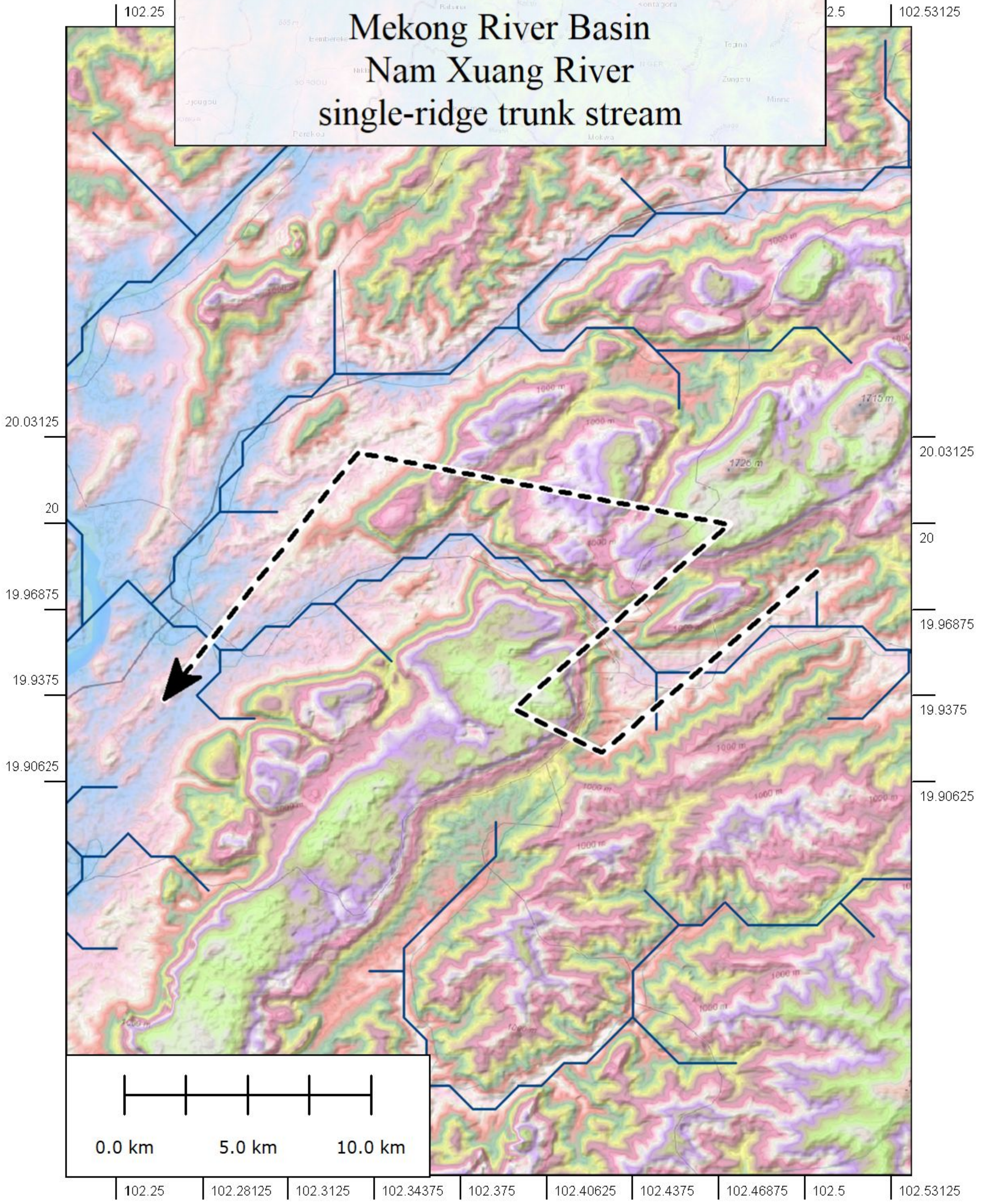

AS - 65
Ganges River Basin
Kali Gandaki River
single-ridge trunk stream

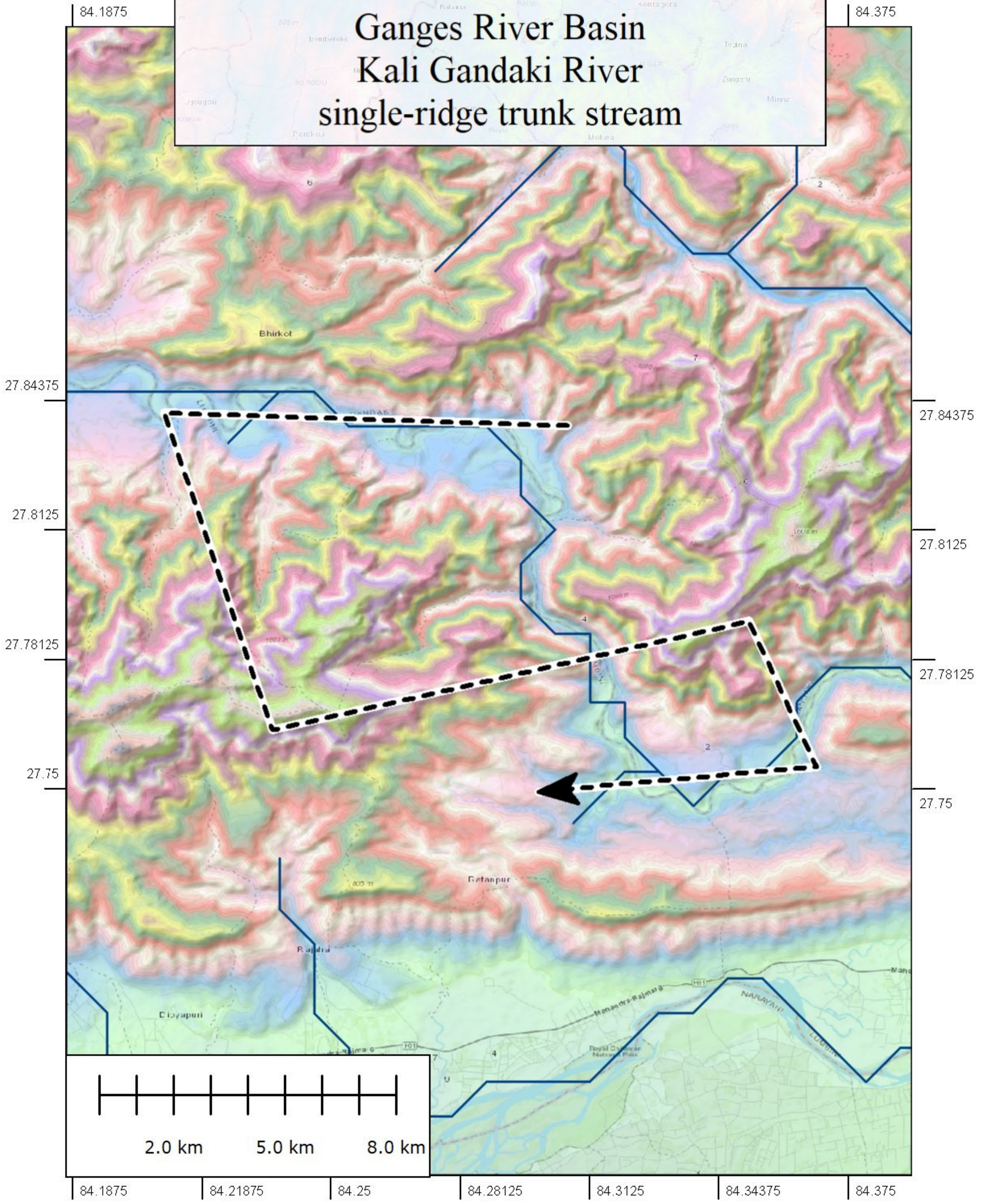

AS - 66
Ganges River Basin
Kamali River
single-ridge trunk stream

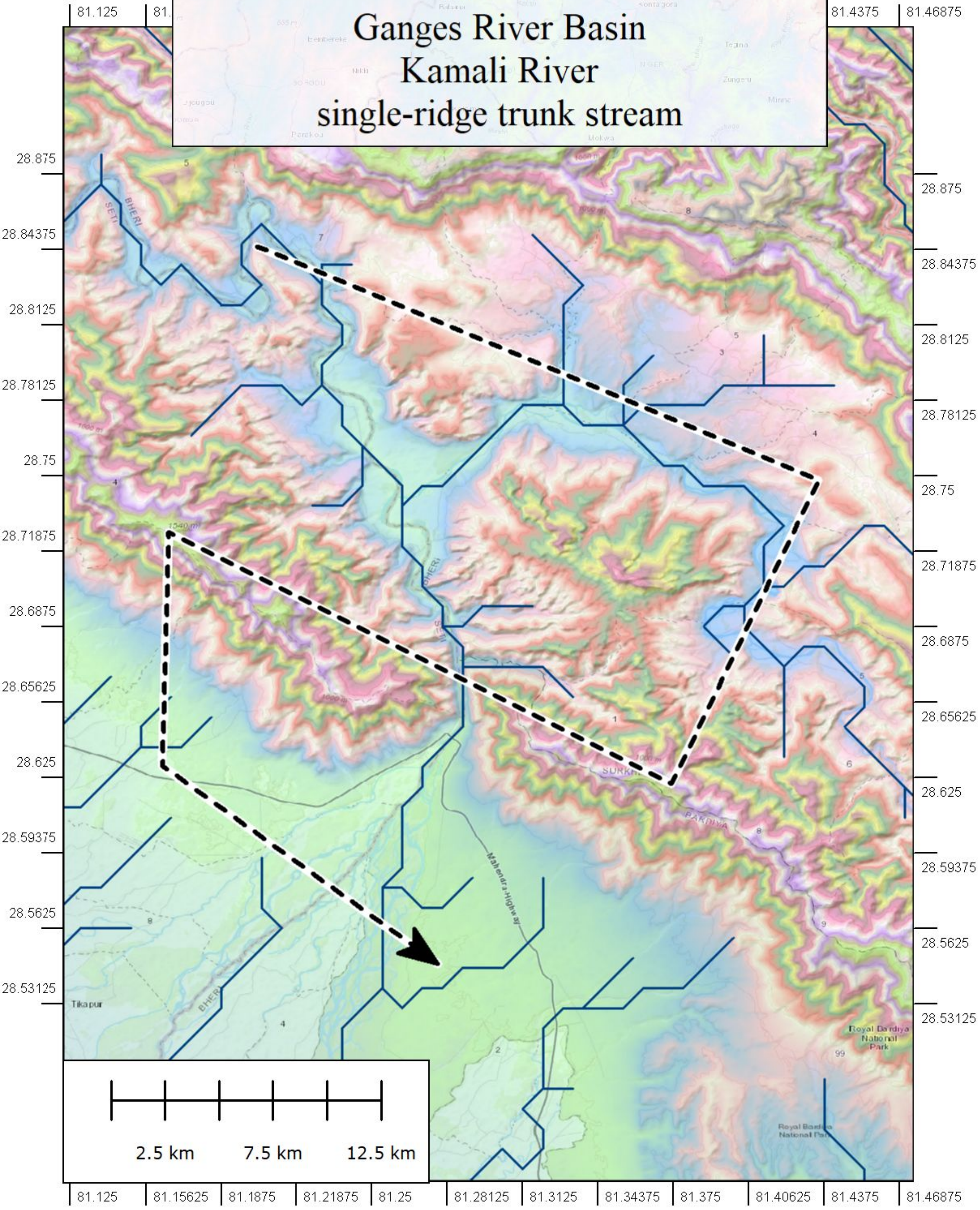

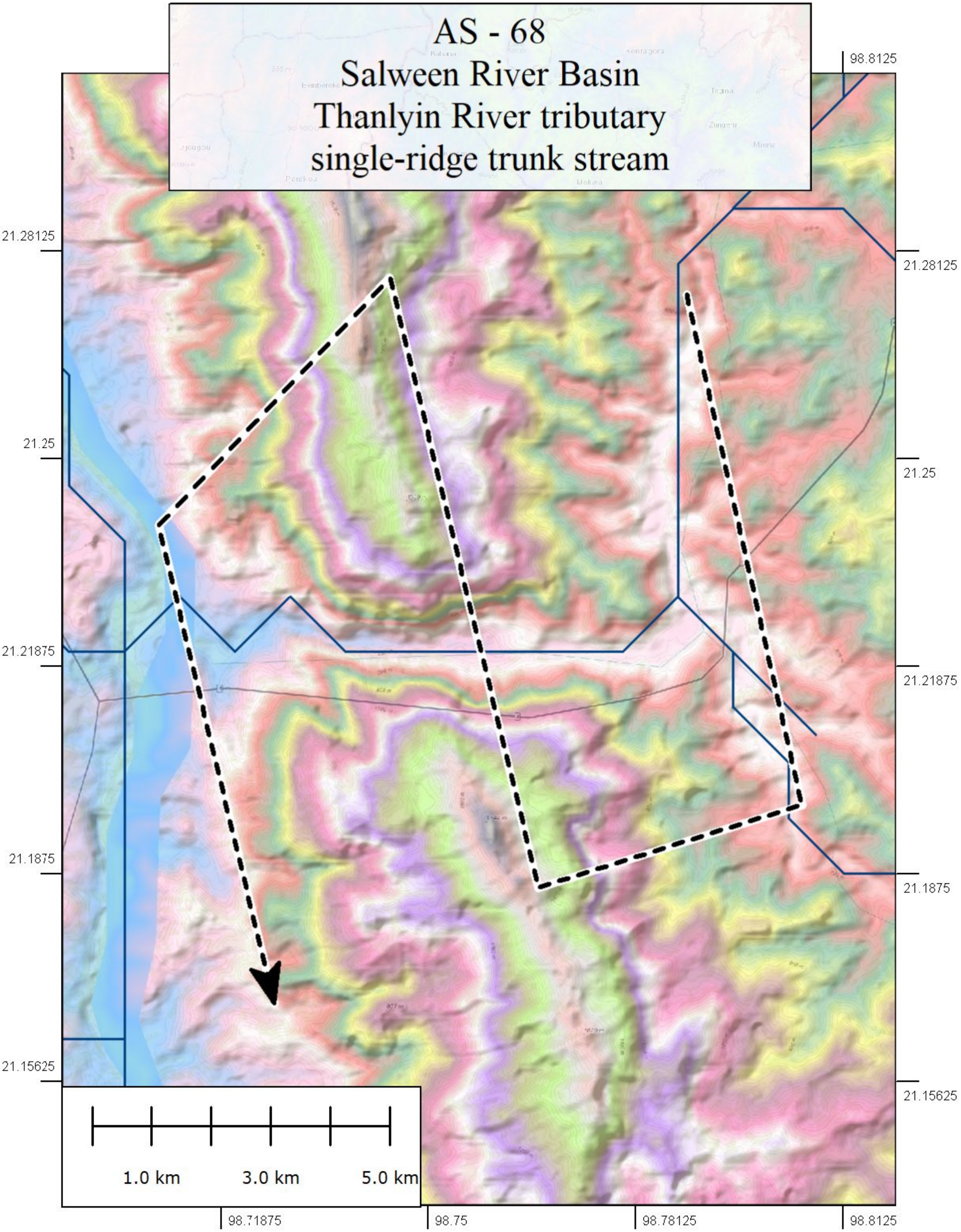
AS - 68
Salween River Basin
Thanlyin River tributary
single-ridge trunk stream
98.8125
21.28125
21.25
21.21875
21.1875
21.15625
1.0 km
3.0 km
5.0 km
98.71875
98.75
98.78125
98.8125

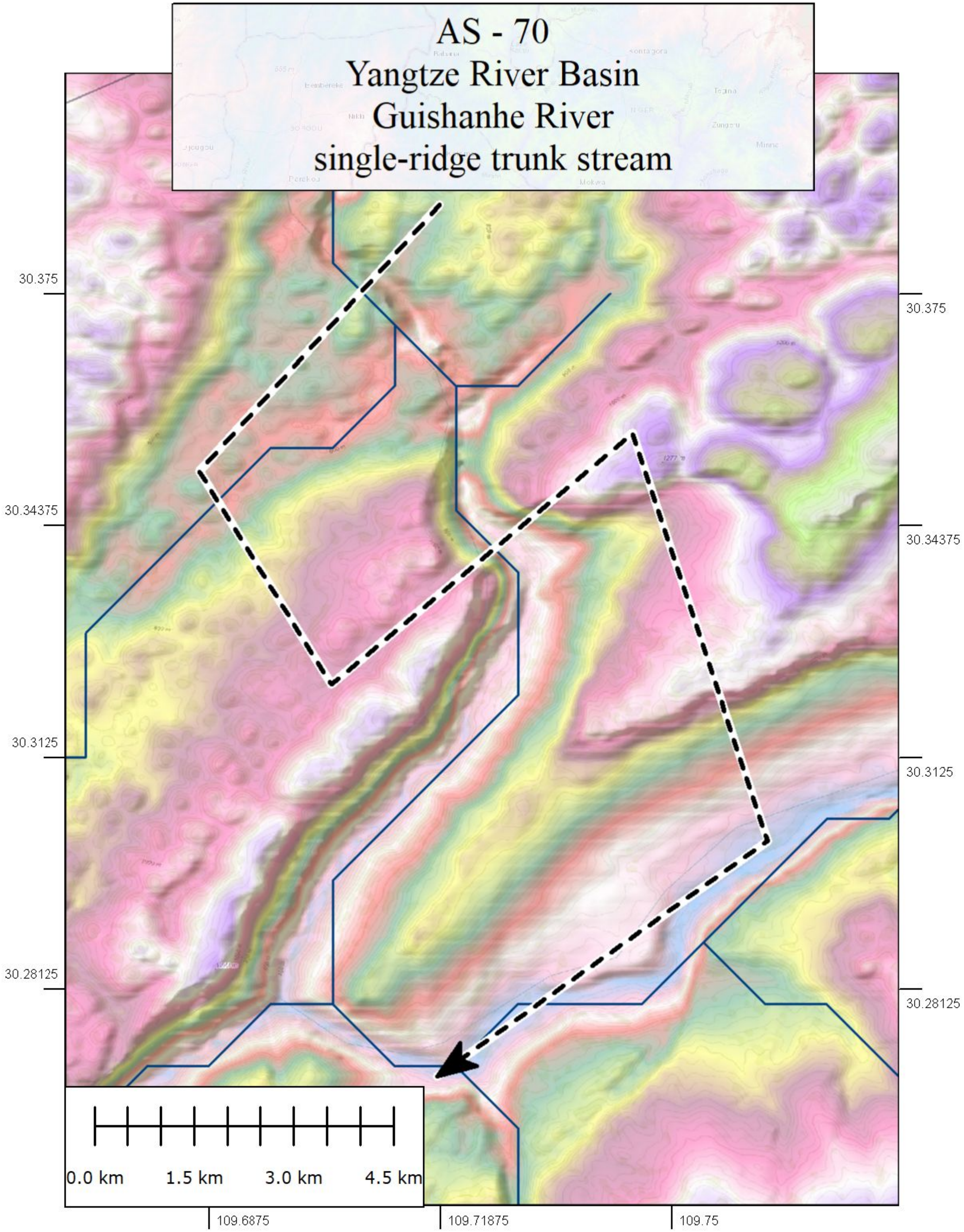
AS - 70
Yangtze River Basin
Guishanhe River
single-ridge trunk stream
30.375
30.34375
30.3125
30.28125
0.0 km
1.5 km
3.0 km
4.5 km
109.6875
109.71875
109.75

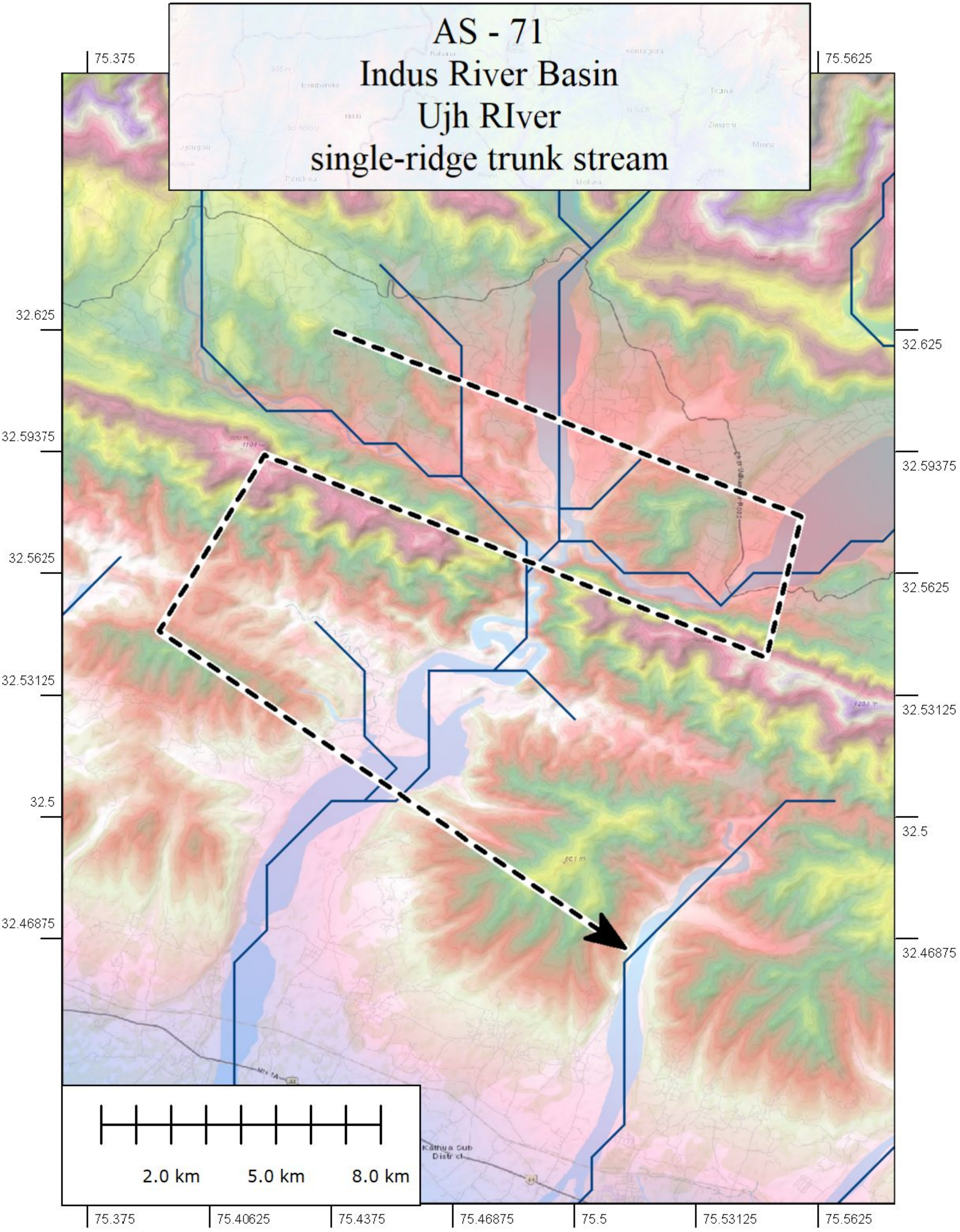
AS - 71
Indus River Basin
Ujh RIver
single-ridge trunk stream
75.375
75.40625
75.4375
75.46875
75.5
75.53125
75.5625
32.625
32.59375
32.5625
32.53125
32.5
32.46875
2.0 km
5.0 km
8.0 km

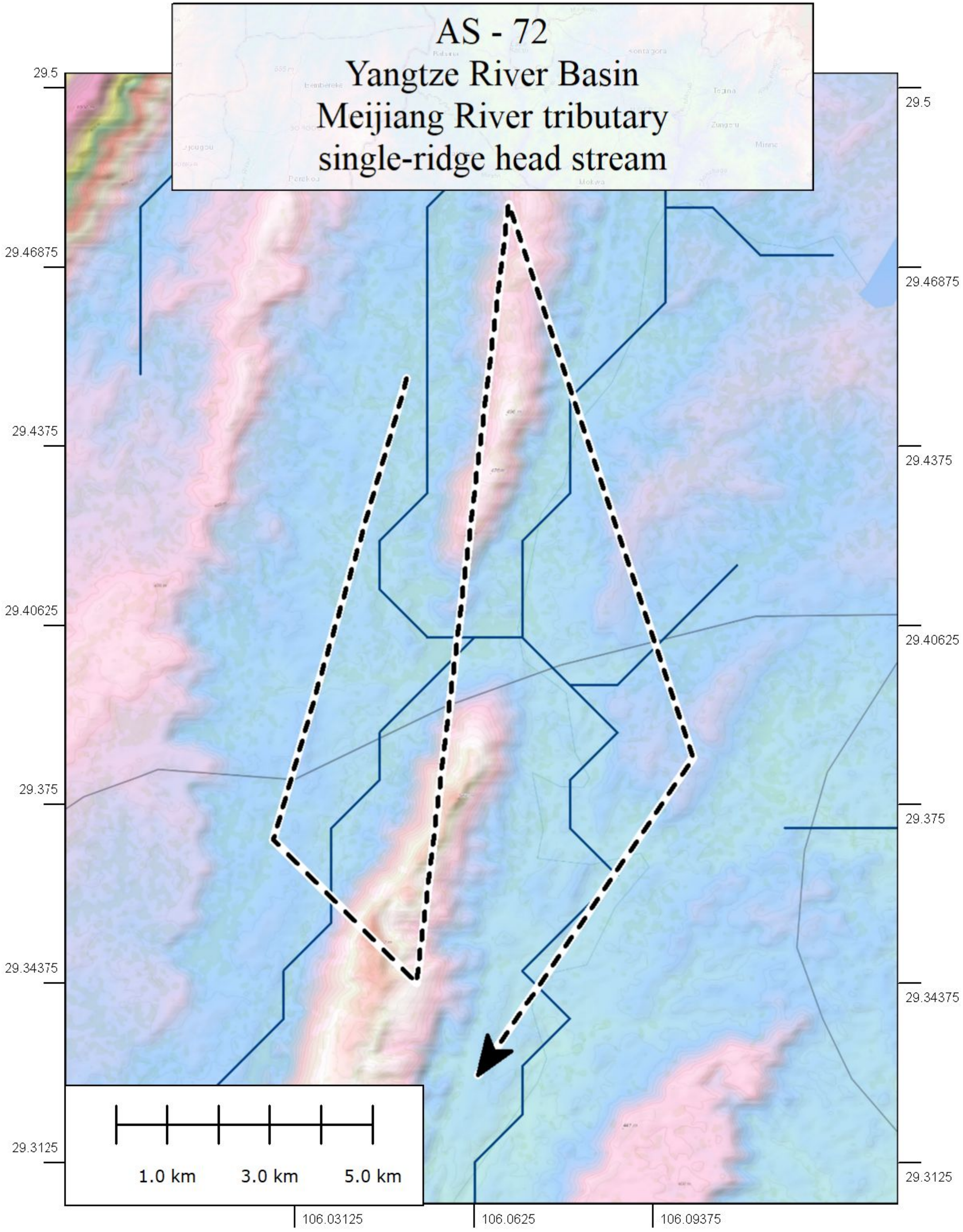
AS - 72
Yangtze River Basin
Meijiang River tributary
single-ridge head stream
29.5
29.46875
29.4375
29.40625
29.375
29.34375
29.3125
106.03125
106.0625
106.09375
1.0 km
3.0 km
5.0 km

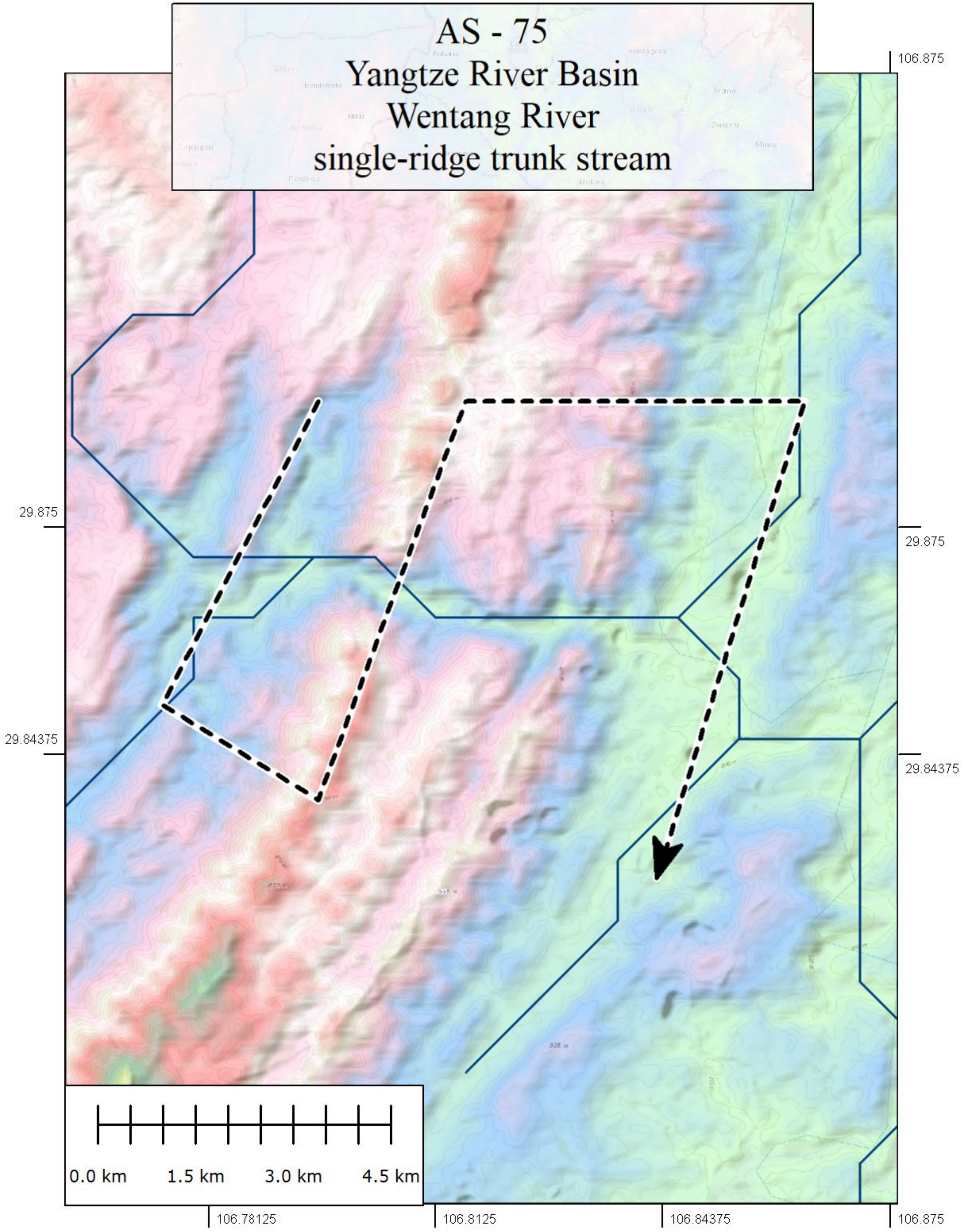
AS - 75
Yangtze River Basin
Wentang River
single-ridge trunk stream
106.875
29.875
29.875
29.84375
29.84375
0.0 km
1.5 km
3.0 km
4.5 km
106.78125
106.8125
106.84375
106.875

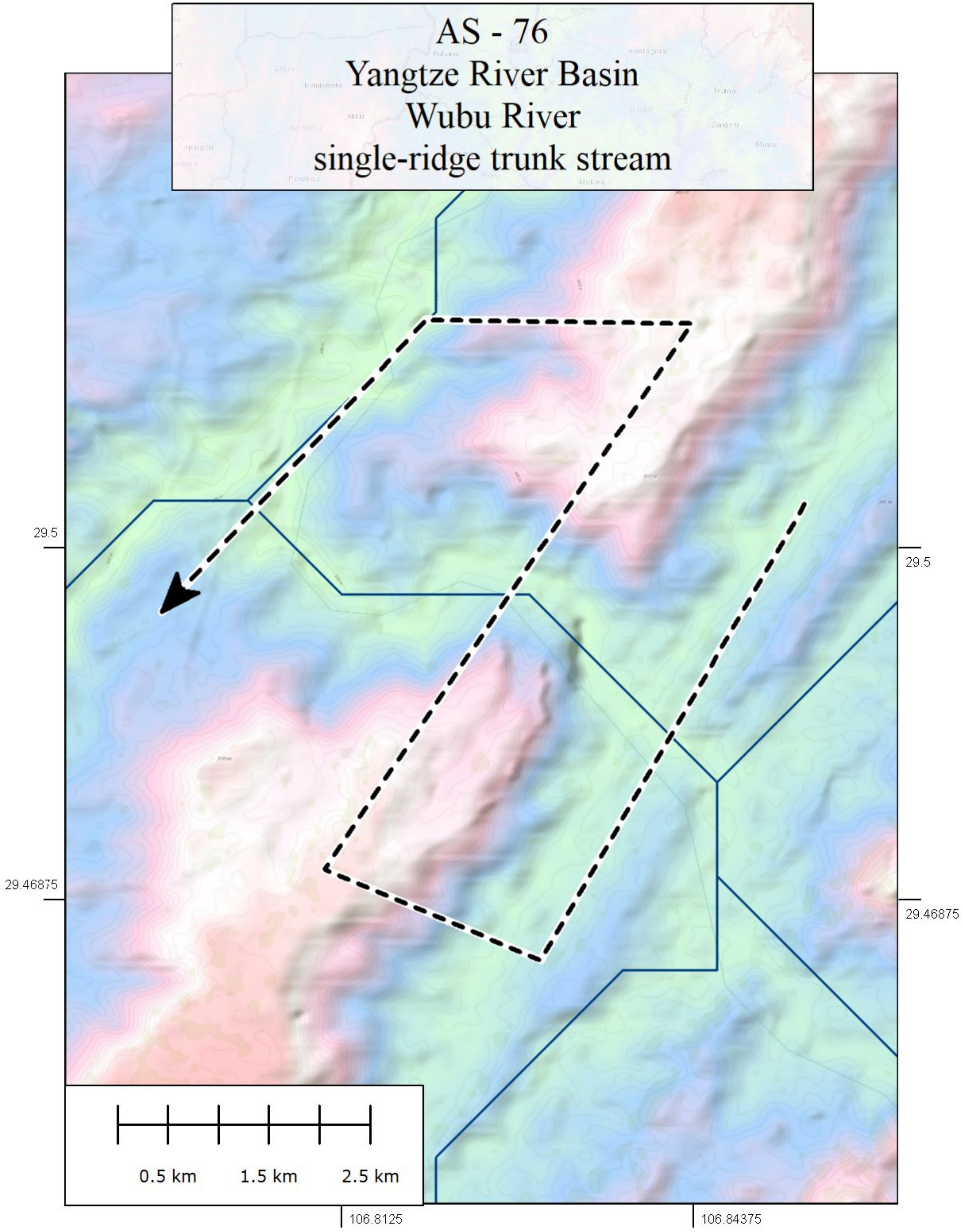

AS - 76
Yangtze River Basin
Wubu River
single-ridge trunk stream
29.5
29.5
29.46875
29.46875
0.5 km
1.5 km
2.5 km
106.8125
106.84375

AS - 78
Yangtze River Basin
Xhouhe River tributary
single-ridge trunk stream

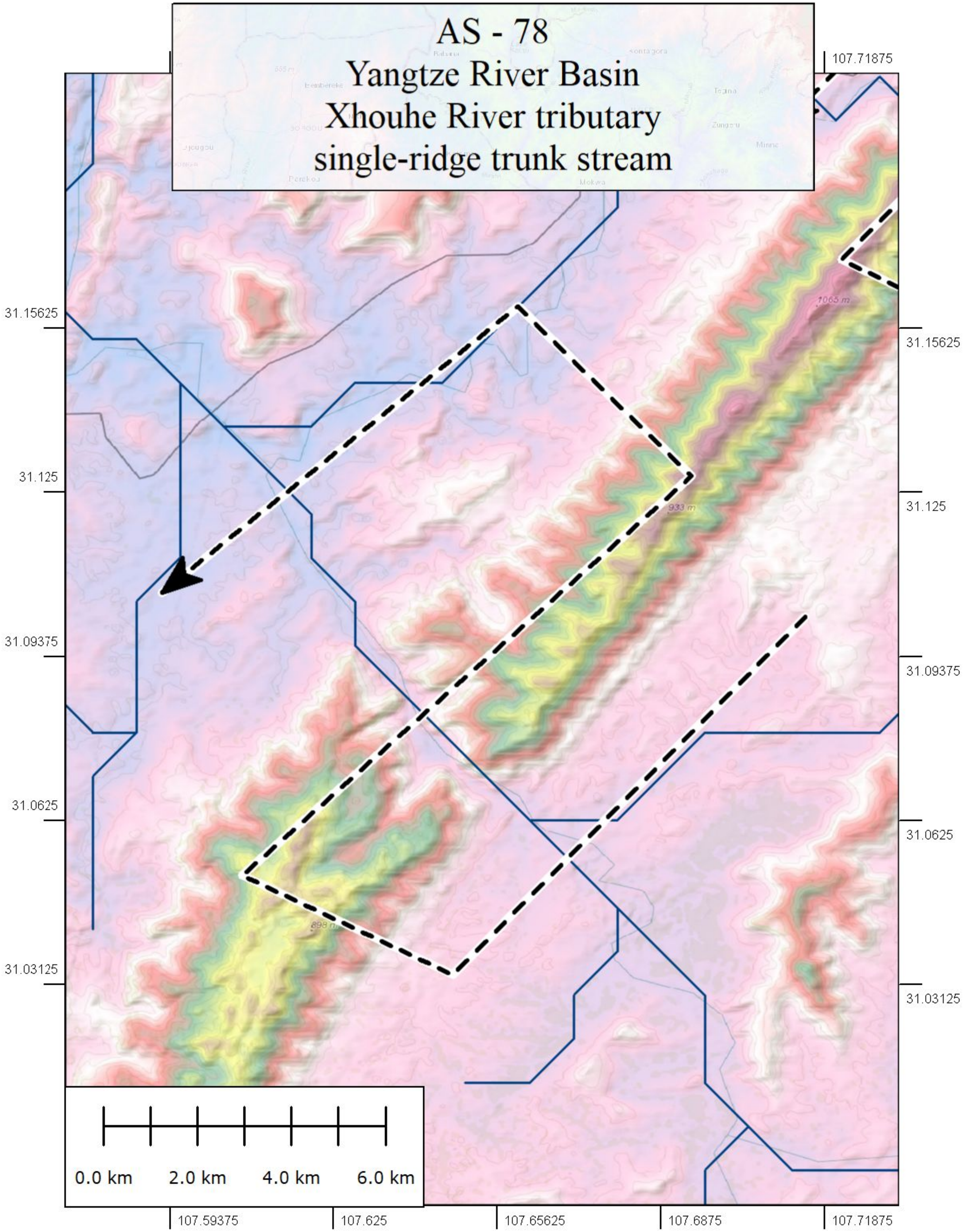

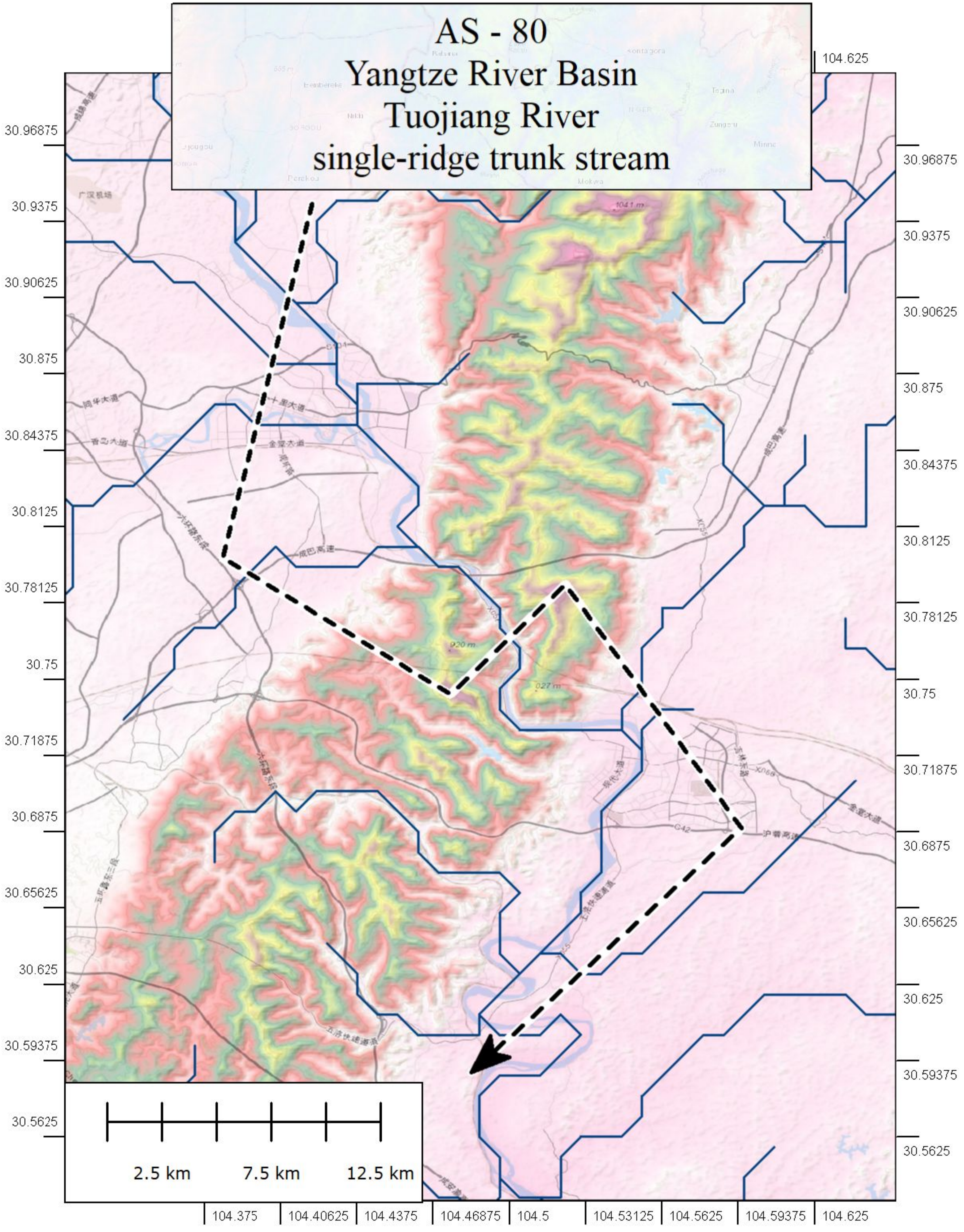
AS - 80
Yangtze River Basin
Tuojiang River
single-ridge trunk stream
2.5 km
7.5 km
12.5 km
30.96875
30.9375
30.90625
30.875
30.84375
30.8125
30.78125
30.75
30.71875
30.6875
30.65625
30.625
30.59375
30.5625
104.375
104.40625
104.4375
104.46875
104.5
104.53125
104.5625
104.59375
104.625

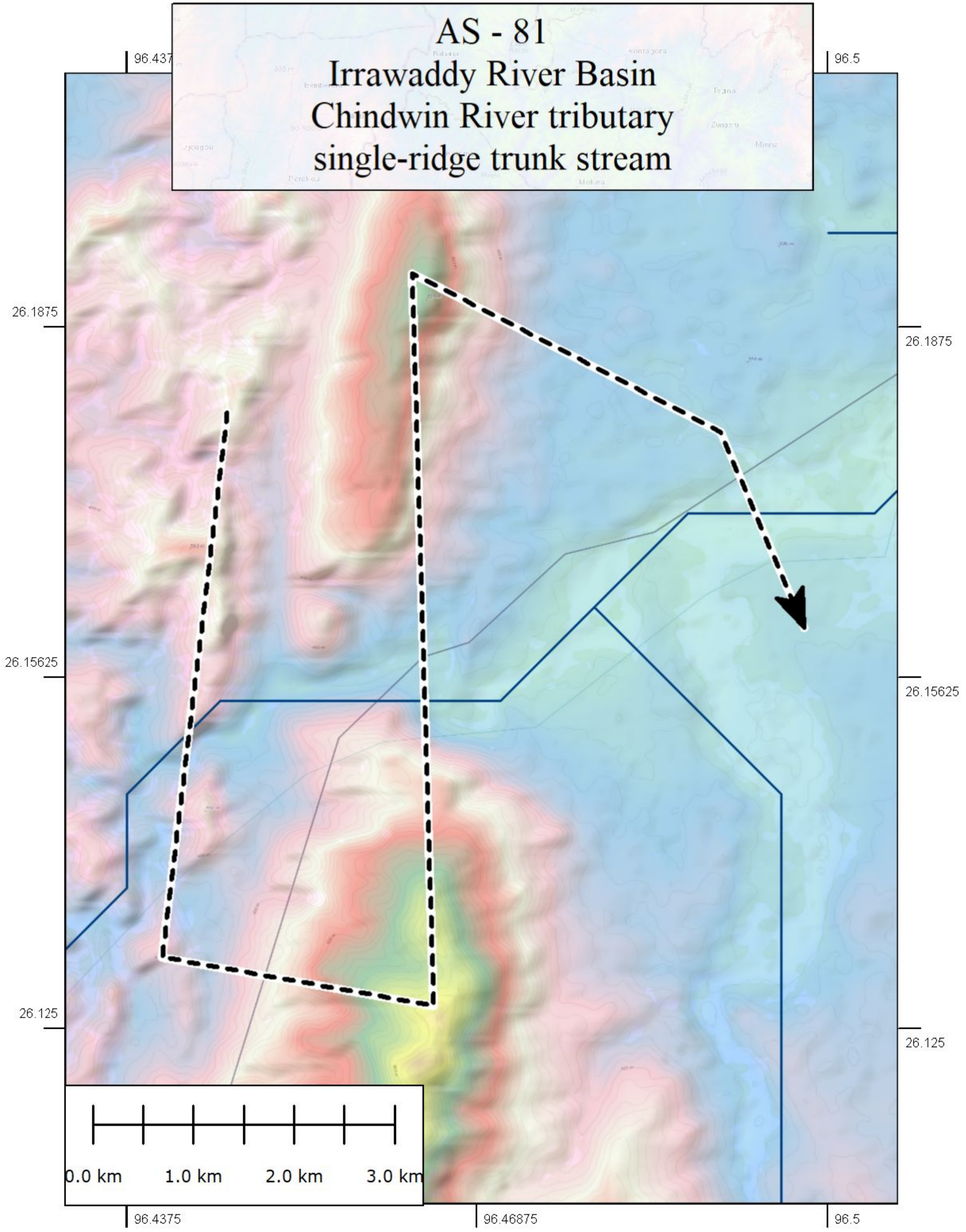
AS - 81
Irrawaddy River Basin
Chindwin River tributary
single-ridge trunk stream
96.4375
96.46875
96.5
26.1875
26.15625
26.125
0.0 km
1.0 km
2.0 km
3.0 km

AS - 83
Chao Phraya Basin
Ping River
single-ridge trunk stream

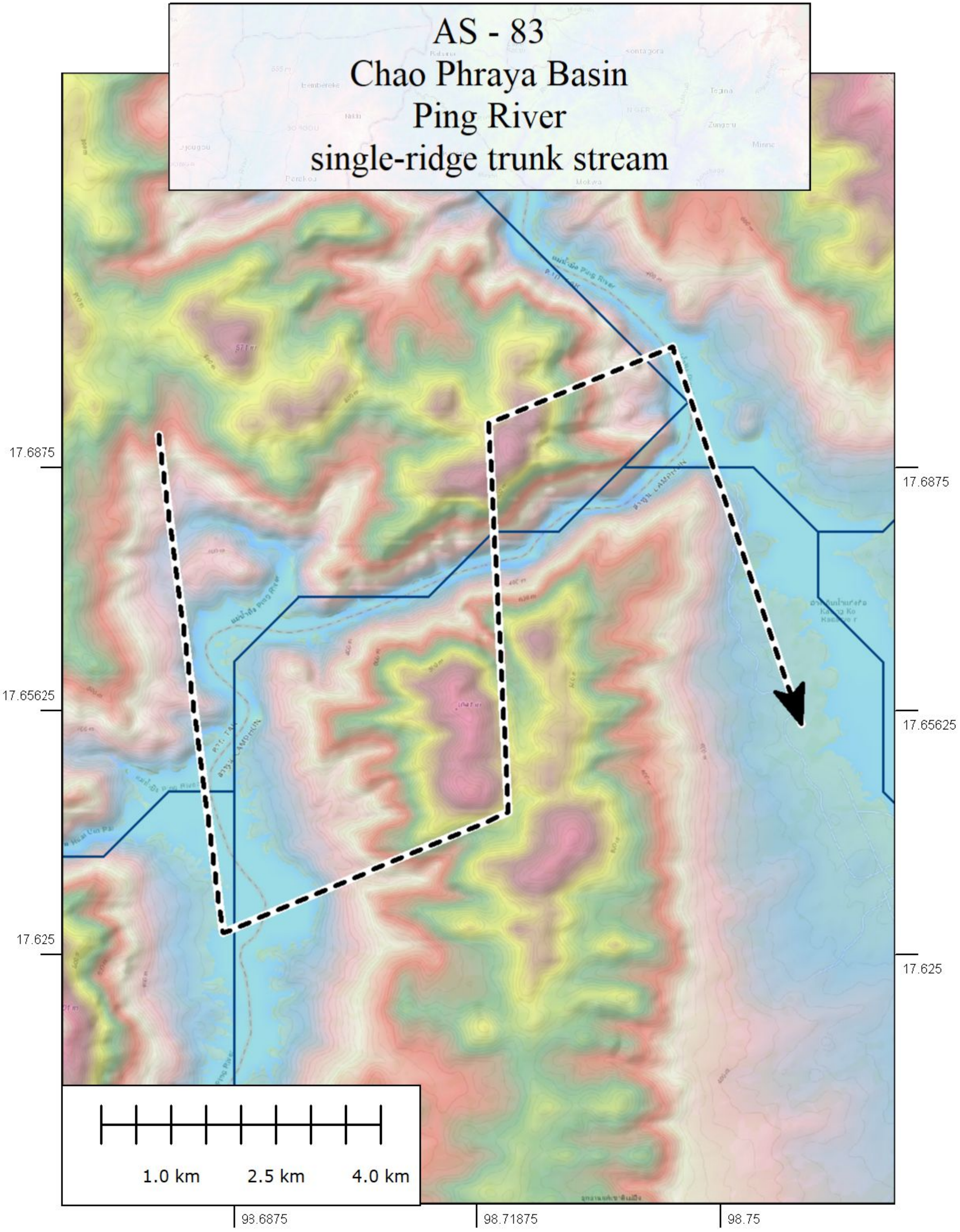

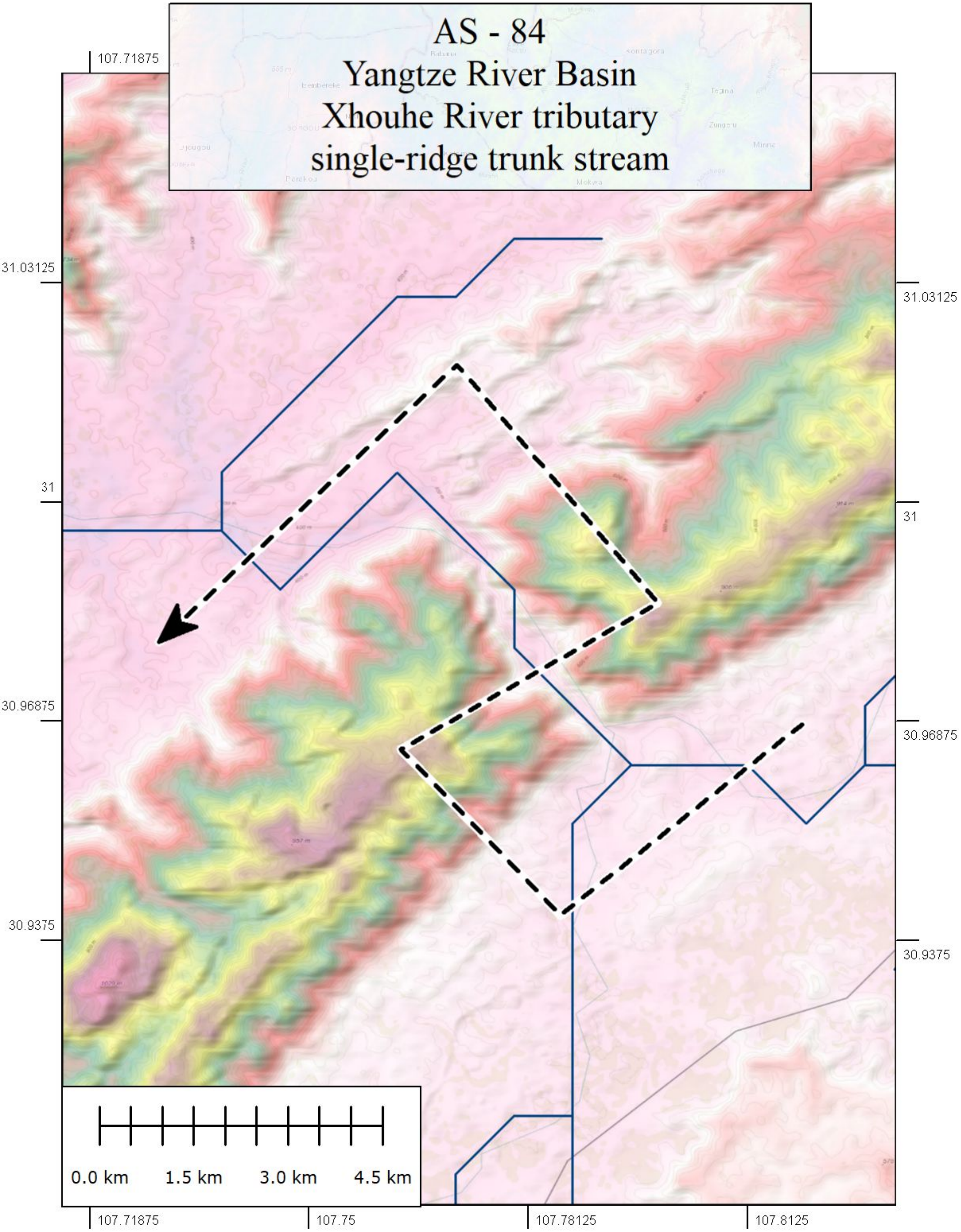
AS - 84
Yangtze River Basin
Xhouhe River tributary
single-ridge trunk stream
107.71875
107.75
107.78125
107.8125
31.03125
31
30.96875
30.9375
0.0 km
1.5 km
3.0 km
4.5 km

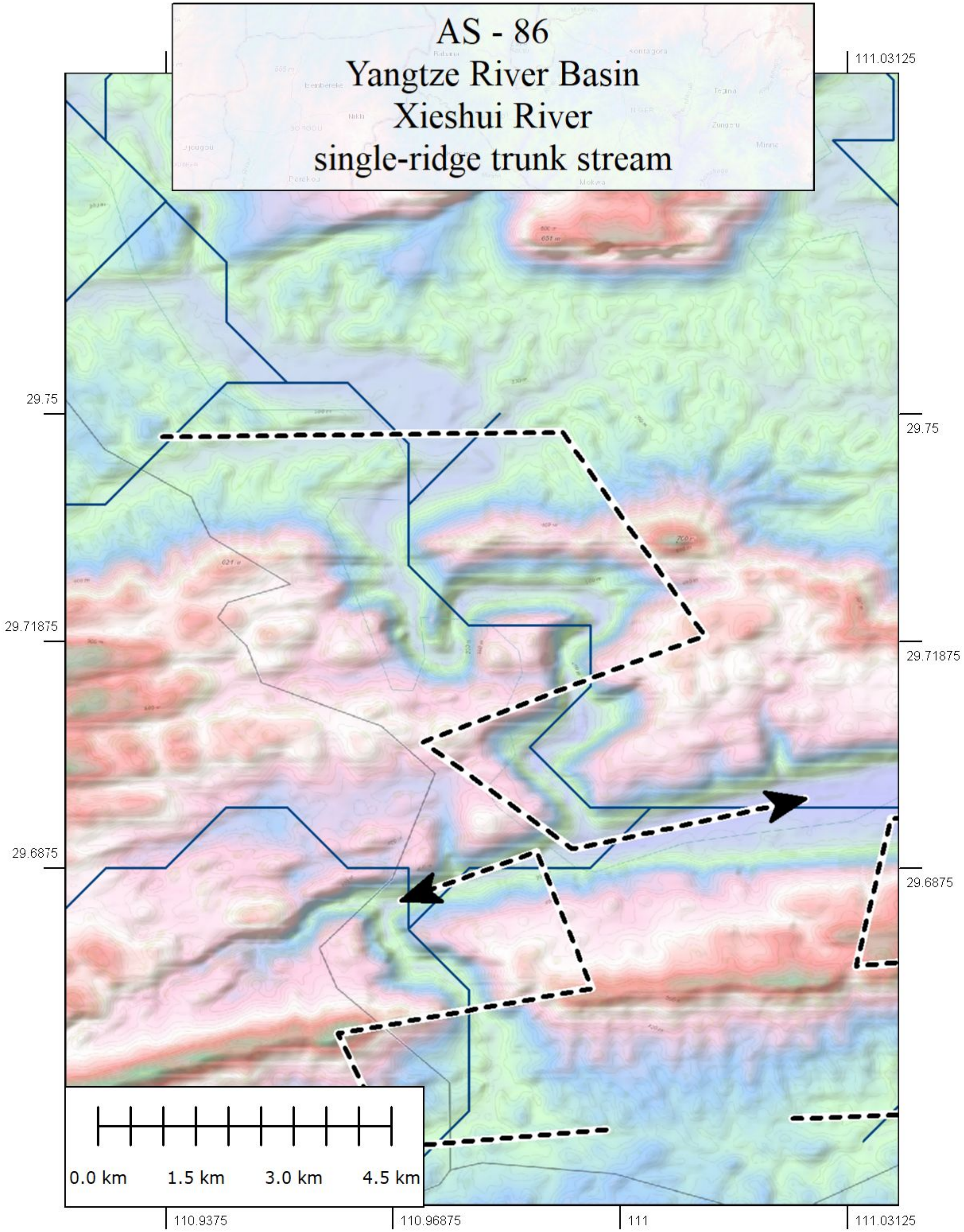
AS - 86
Yangtze River Basin
Xieshui River
single-ridge trunk stream
111.03125
29.75
29.75
29.71875
29.71875
29.6875
29.6875
110.9375
110.96875
111
111.03125
0.0 km
1.5 km
3.0 km
4.5 km

AS - 87
Yangtze River Basin
Apeng River tributary
single-ridge trunk stream

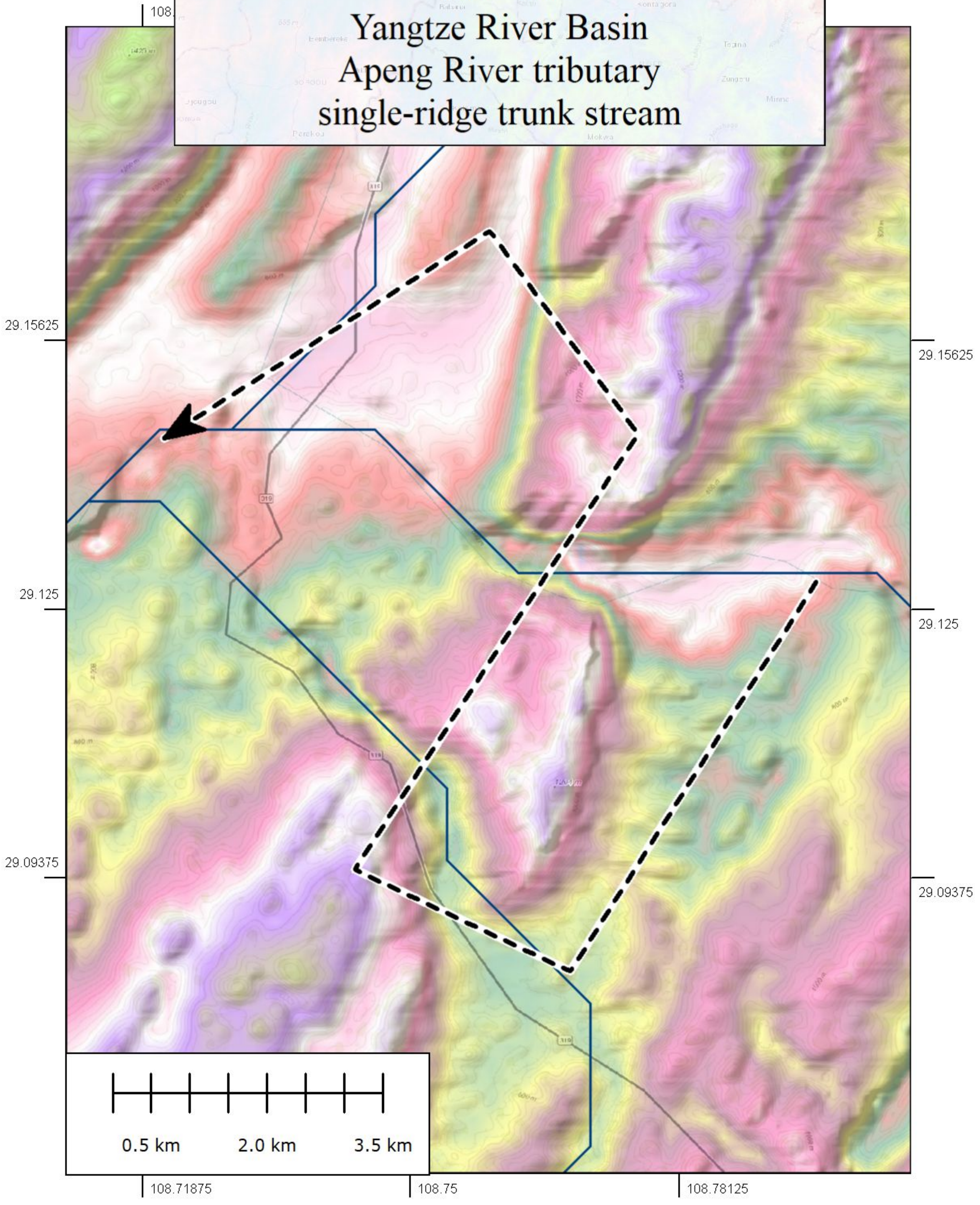

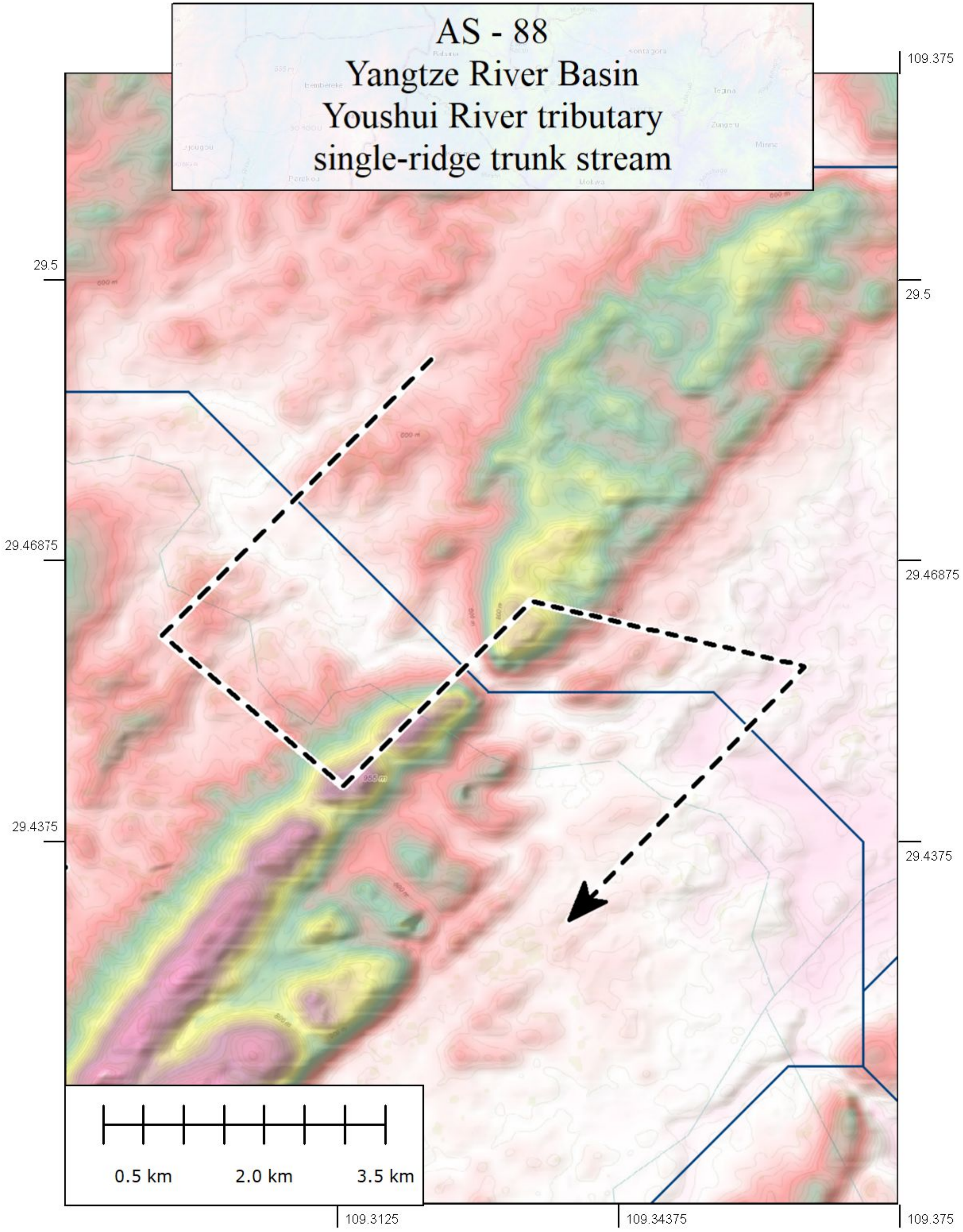
AS - 88
Yangtze River Basin
Youshui River tributary
single-ridge trunk stream
109.375
29.5
29.5
29.46875
29.46875
29.4375
29.4375
0.5 km
2.0 km
3.5 km
109.3125
109.34375
109.375

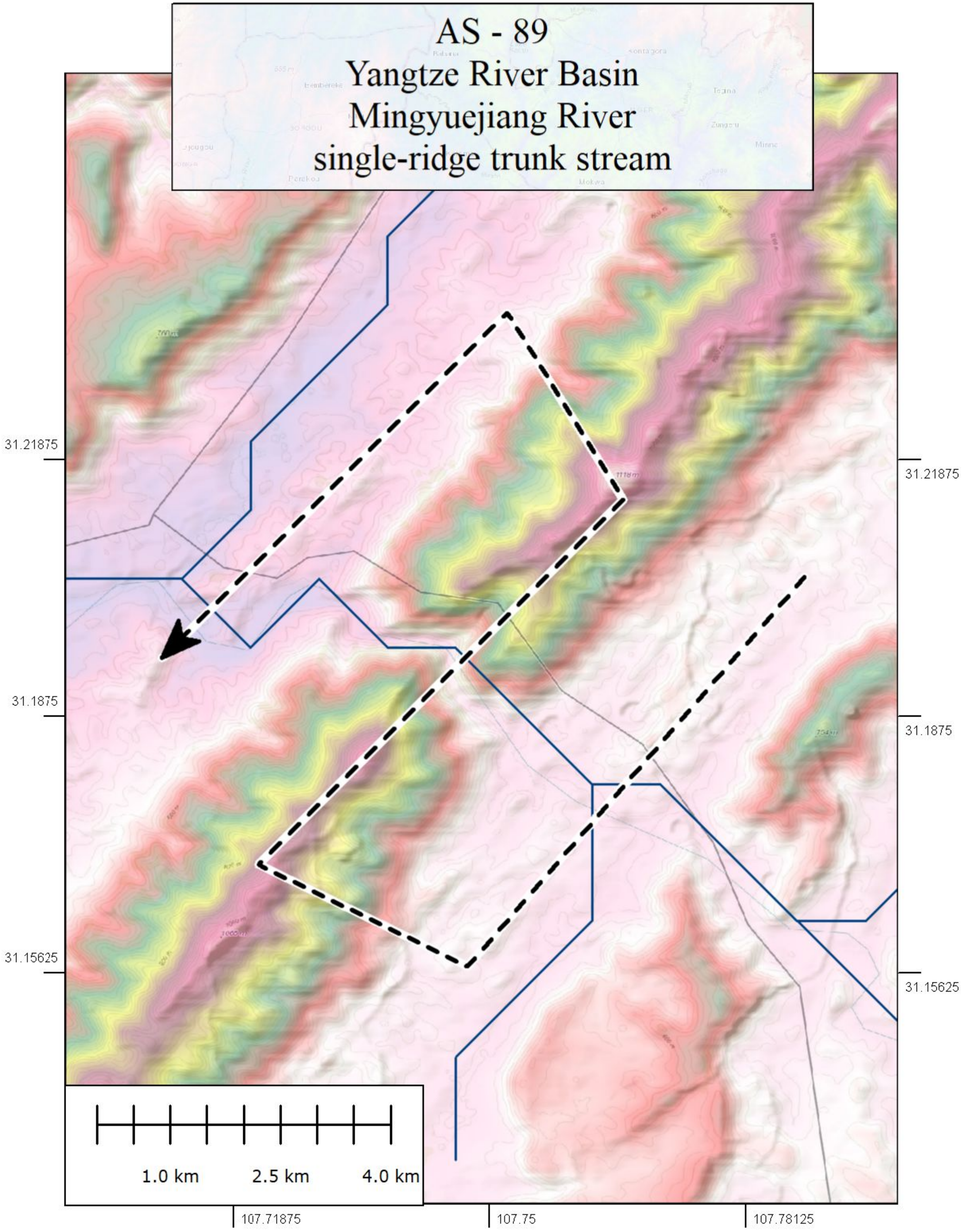

AS - 89
Yangtze River Basin
Mingyuejiang River
single-ridge trunk stream
31.21875
31.1875
31.15625
107.71875
107.75
107.78125
1.0 km
2.5 km
4.0 km

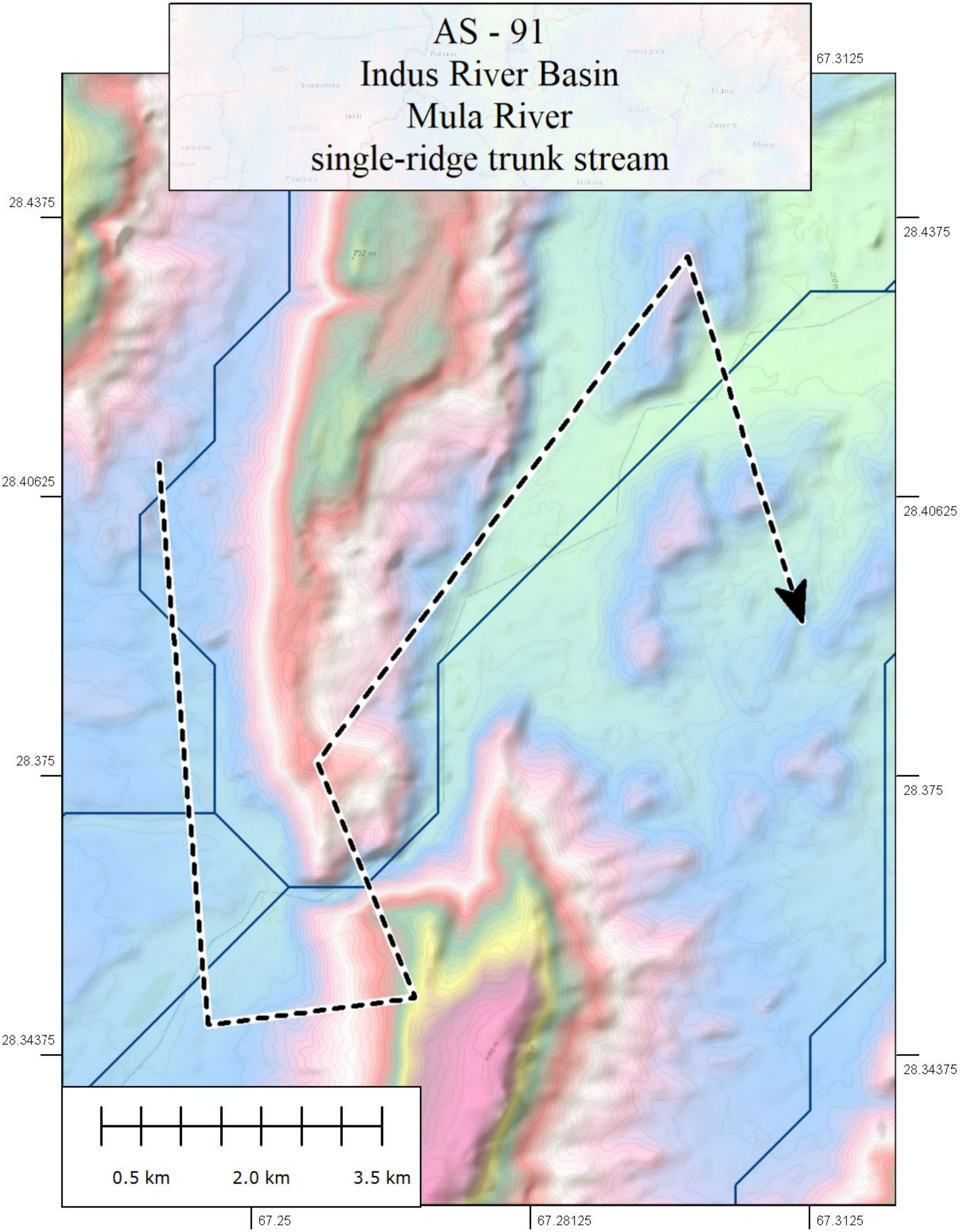
AS - 91
Indus River Basin
Mula River
single-ridge trunk stream
67.3125
28.4375
28.4375
28.40625
28.40625
28.375
28.375
28.34375
28.34375
0.5 km
2.0 km
3.5 km
67.25
67.28125
67.3125

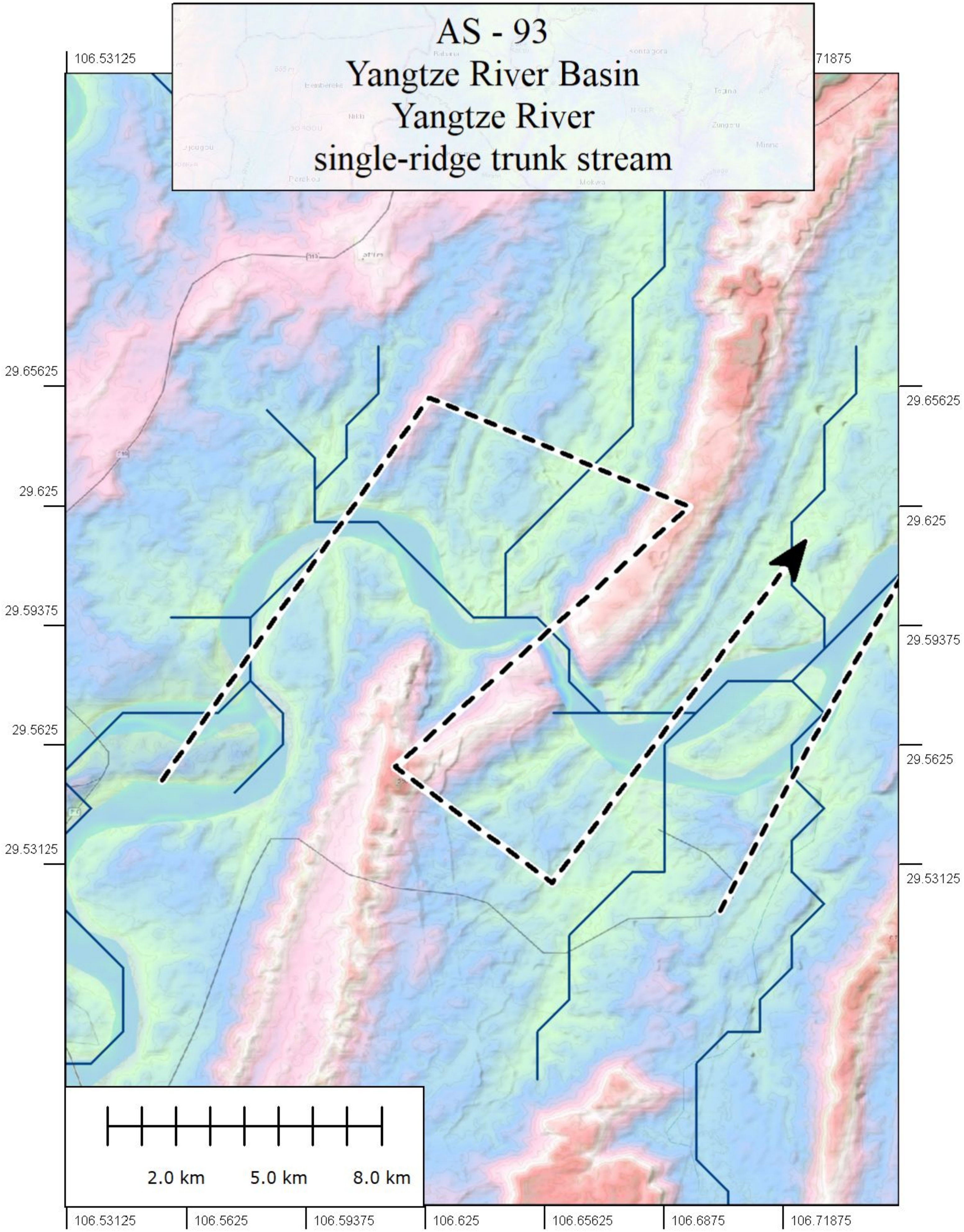
AS - 93
Yangtze River Basin
Yangtze River
single-ridge trunk stream
106.53125
71875
29.65625
29.625
29.59375
29.5625
29.53125
29.65625
29.625
29.59375
29.5625
29.53125
2.0 km
5.0 km
8.0 km
106.53125
106.5625
106.59375
106.625
106.65625
106.6875
106.71875

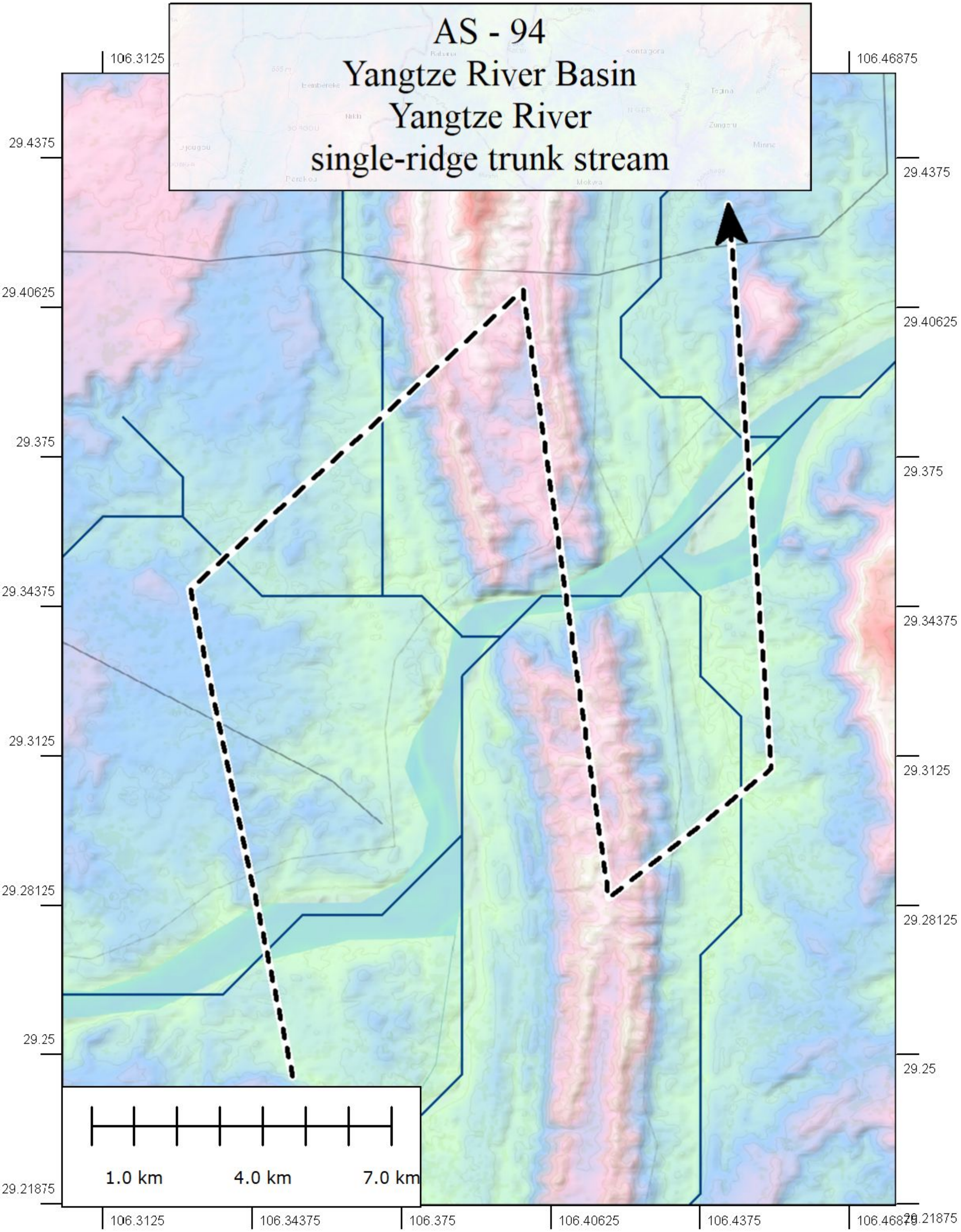
AS - 94
Yangtze River Basin
Yangtze River
single-ridge trunk stream
106.3125
106.34375
106.375
106.40625
106.4375
106.46875
29.4375
29.40625
29.375
29.34375
29.3125
29.28125
29.25
29.21875
1.0 km
4.0 km
7.0 km

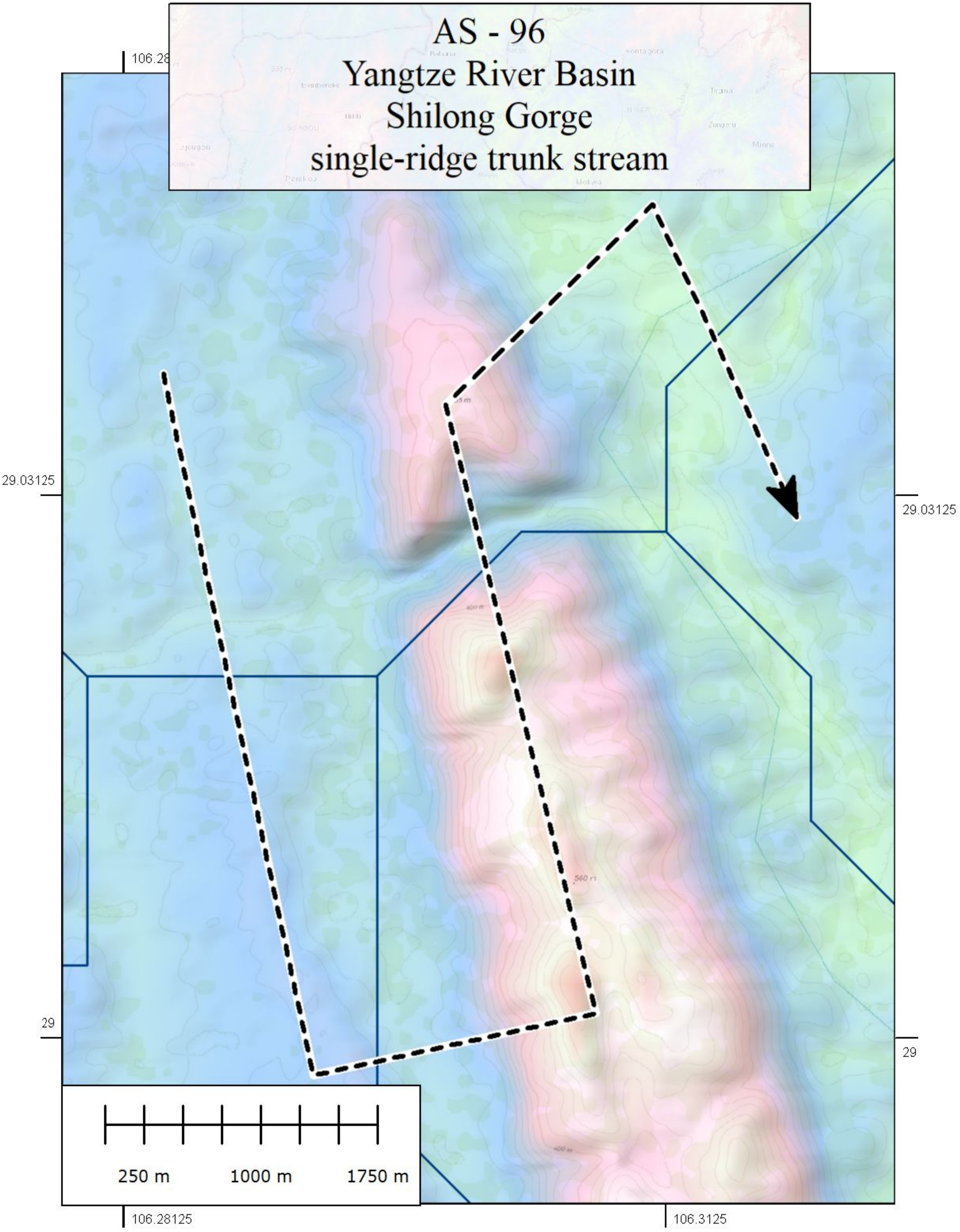
AS - 96
Yangtze River Basin
Shilong Gorge
single-ridge trunk stream
29.03125
29.03125
29
29
106.28125
106.3125
250 m
1000 m
1750 m

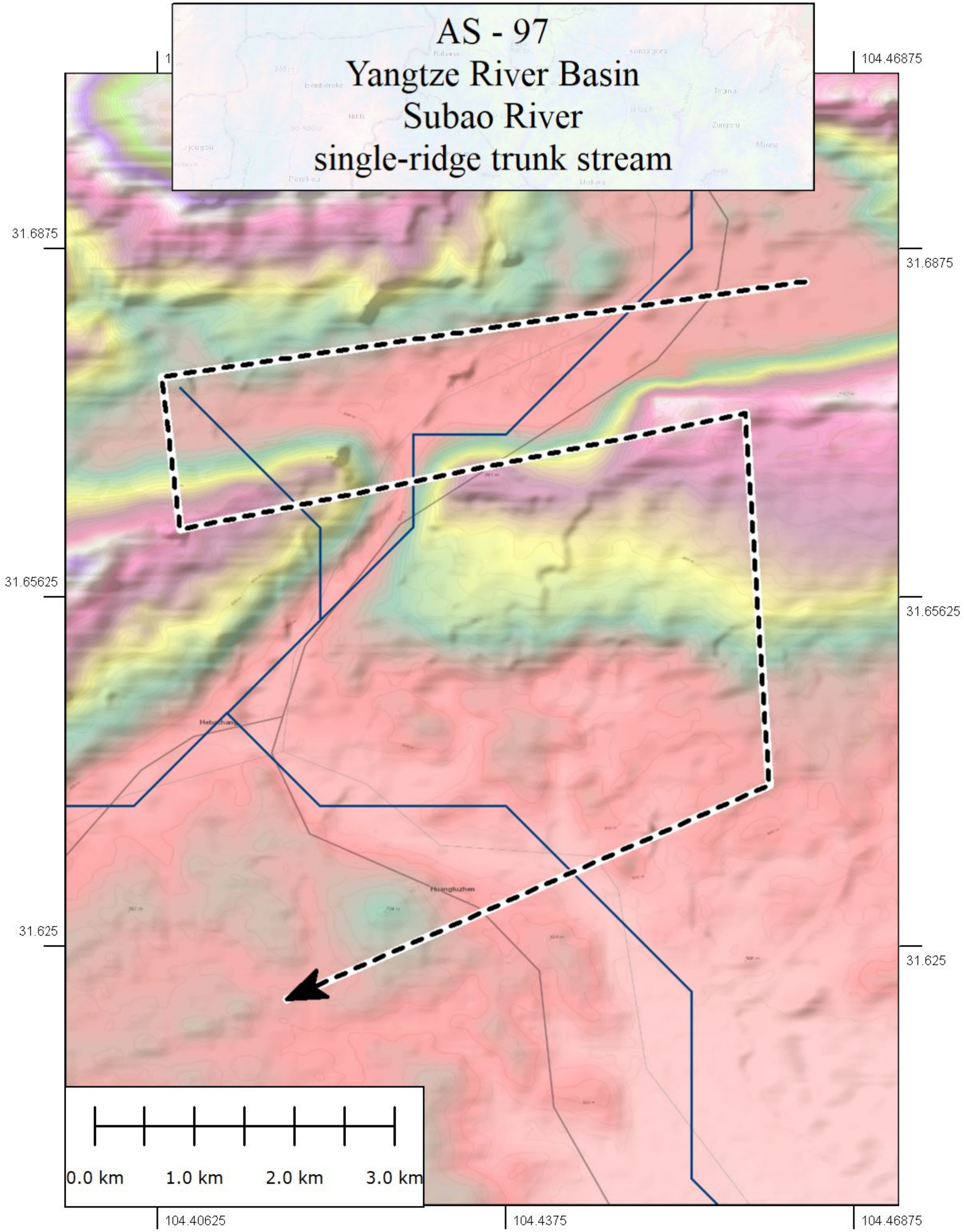
AS - 97
Yangtze River Basin
Subao River
single-ridge trunk stream
104.46875
31.6875
31.6875
31.65625
31.65625
31.625
31.625
Huangtuzhen
0.0 km
1.0 km
2.0 km
3.0 km
104.40625
104.4375
104.46875

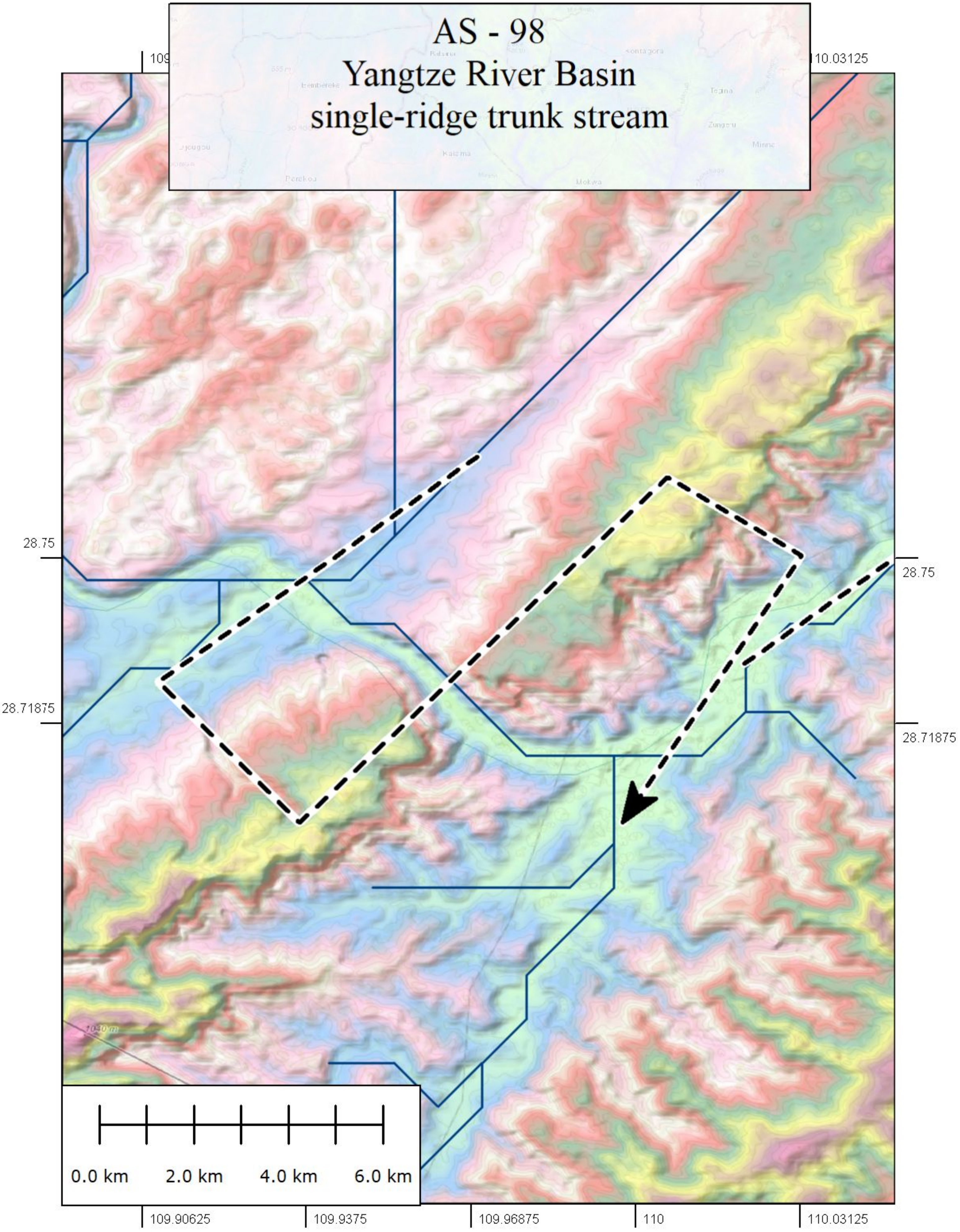

AS - 98
Yangtze River Basin
single-ridge trunk stream
110.03125
28.75
28.71875
28.75
28.71875
0.0 km
2.0 km
4.0 km
6.0 km
109.90625
109.9375
109.96875
110
110.03125

AS - 99
Indus River Basin
Indus River tributary
single-ridge trunk stream

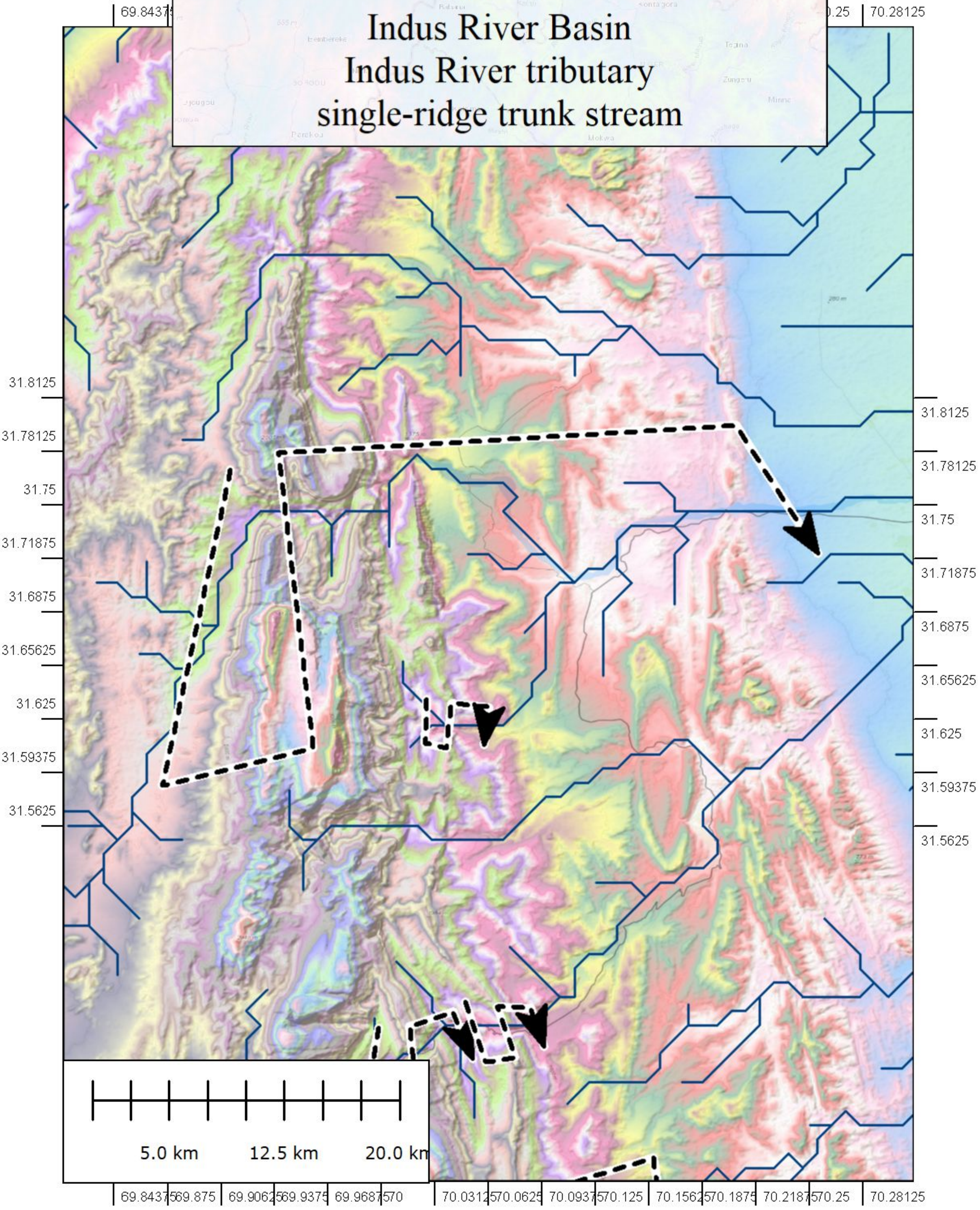

AS - 101
Indus River Basin
Indus River tributary
single-ridge head stream

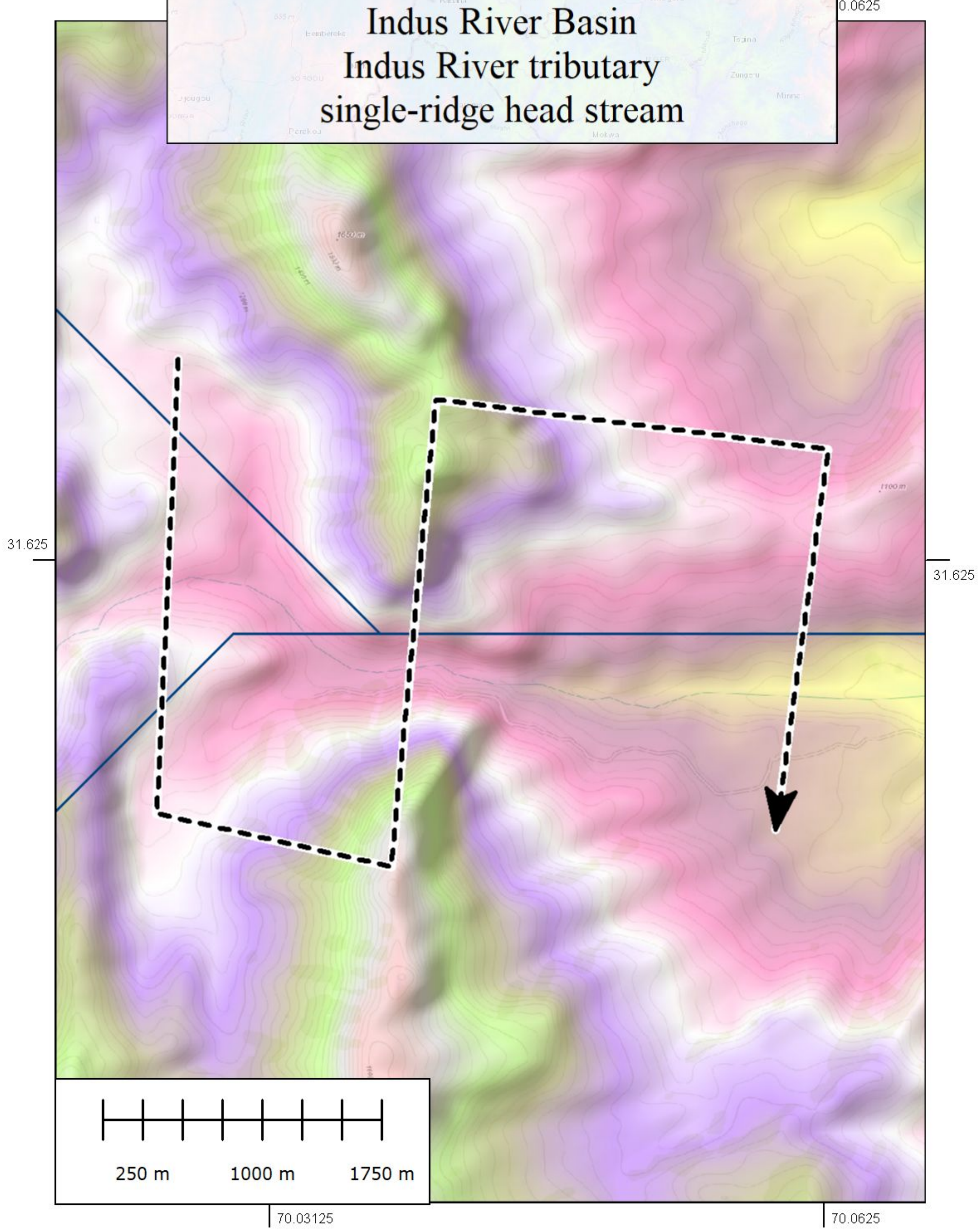

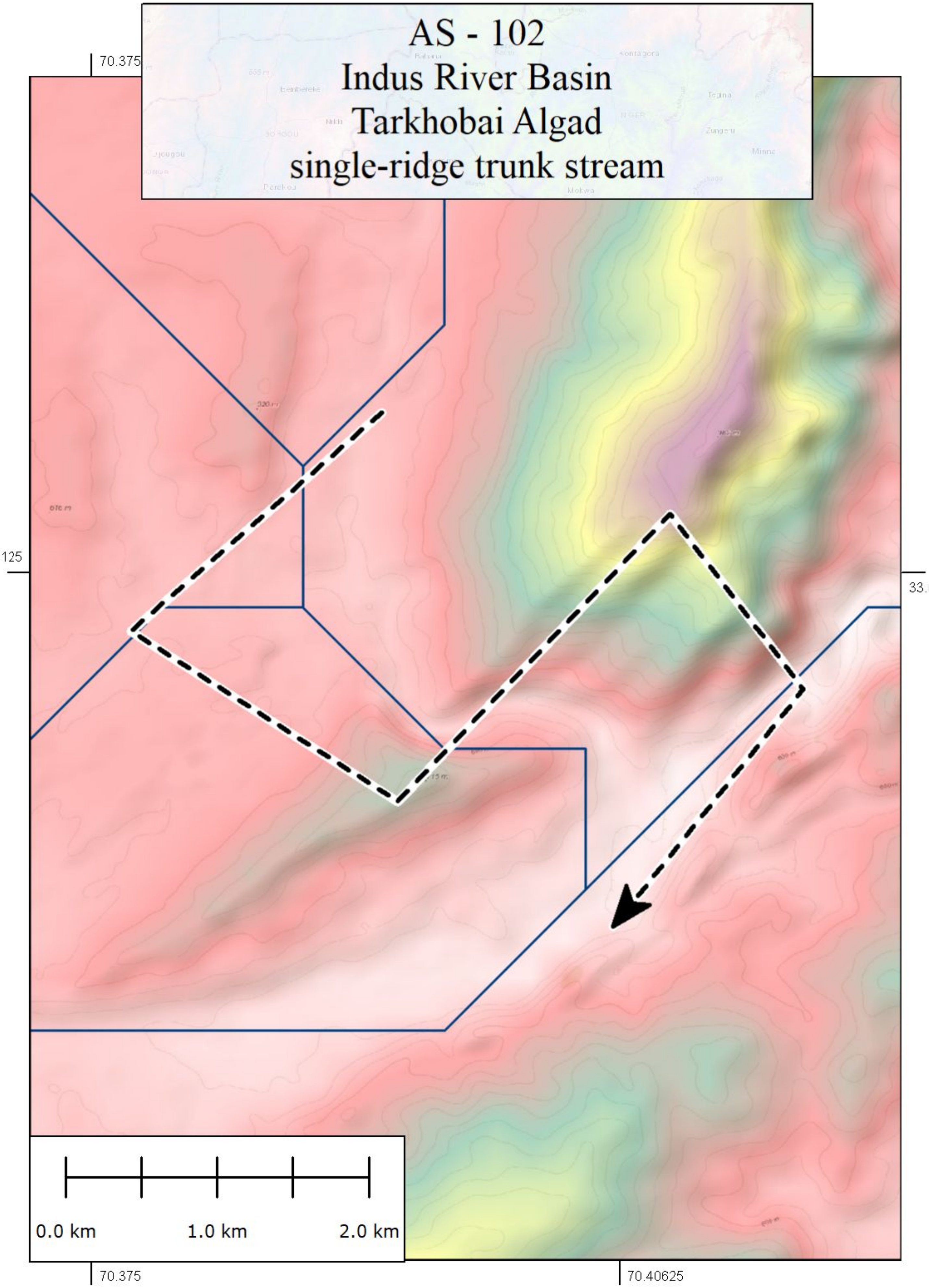
AS - 102
Indus River Basin
Tarkhobai Algad
single-ridge trunk stream
70.375
33.03125
33.03125
0.0 km
1.0 km
2.0 km
70.375
70.40625

AS - 103
Indus River Basin
Walwast Nala
single-ridge trunk stream

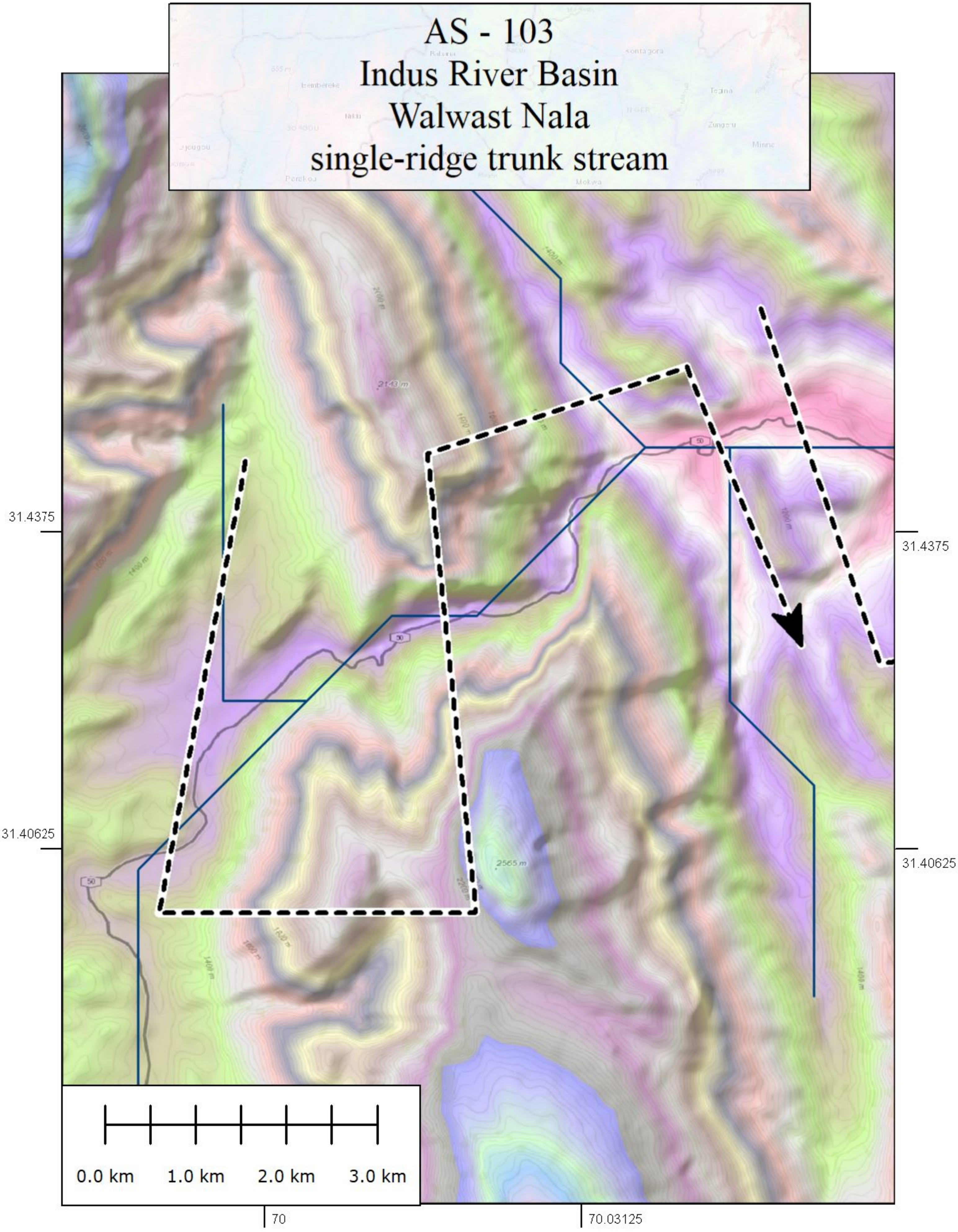

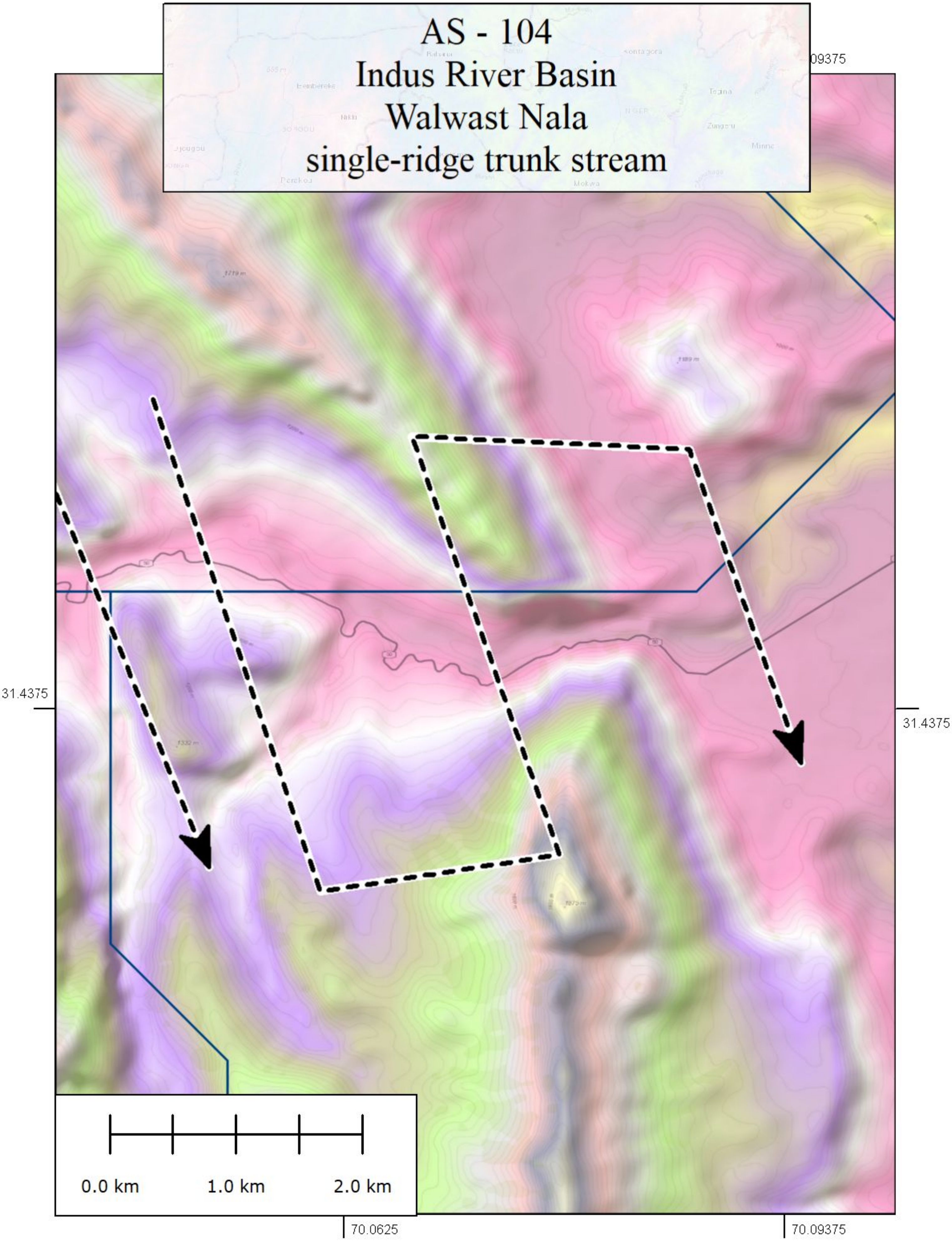
AS - 104
Indus River Basin
Walwast Nala
single-ridge trunk stream
09375
31.4375
31.4375
0.0 km
1.0 km
2.0 km
70.0625
70.09375

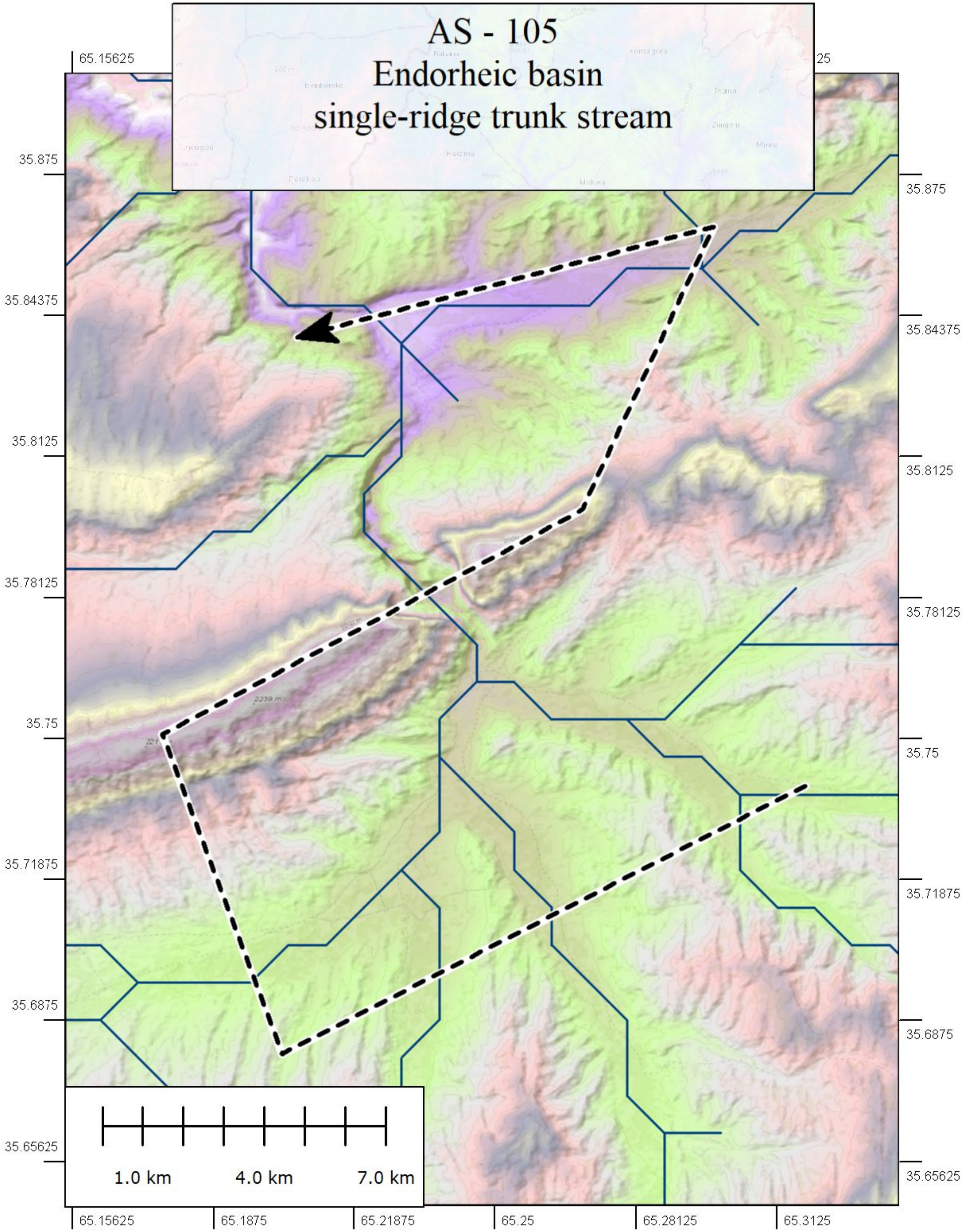

AS - 105
Endorheic basin
single-ridge trunk stream
65.15625
35.875
35.84375
35.8125
35.78125
35.75
35.71875
35.6875
35.65625
2239 m
1.0 km
4.0 km
7.0 km
65.1875
65.21875
65.25
65.28125
65.3125

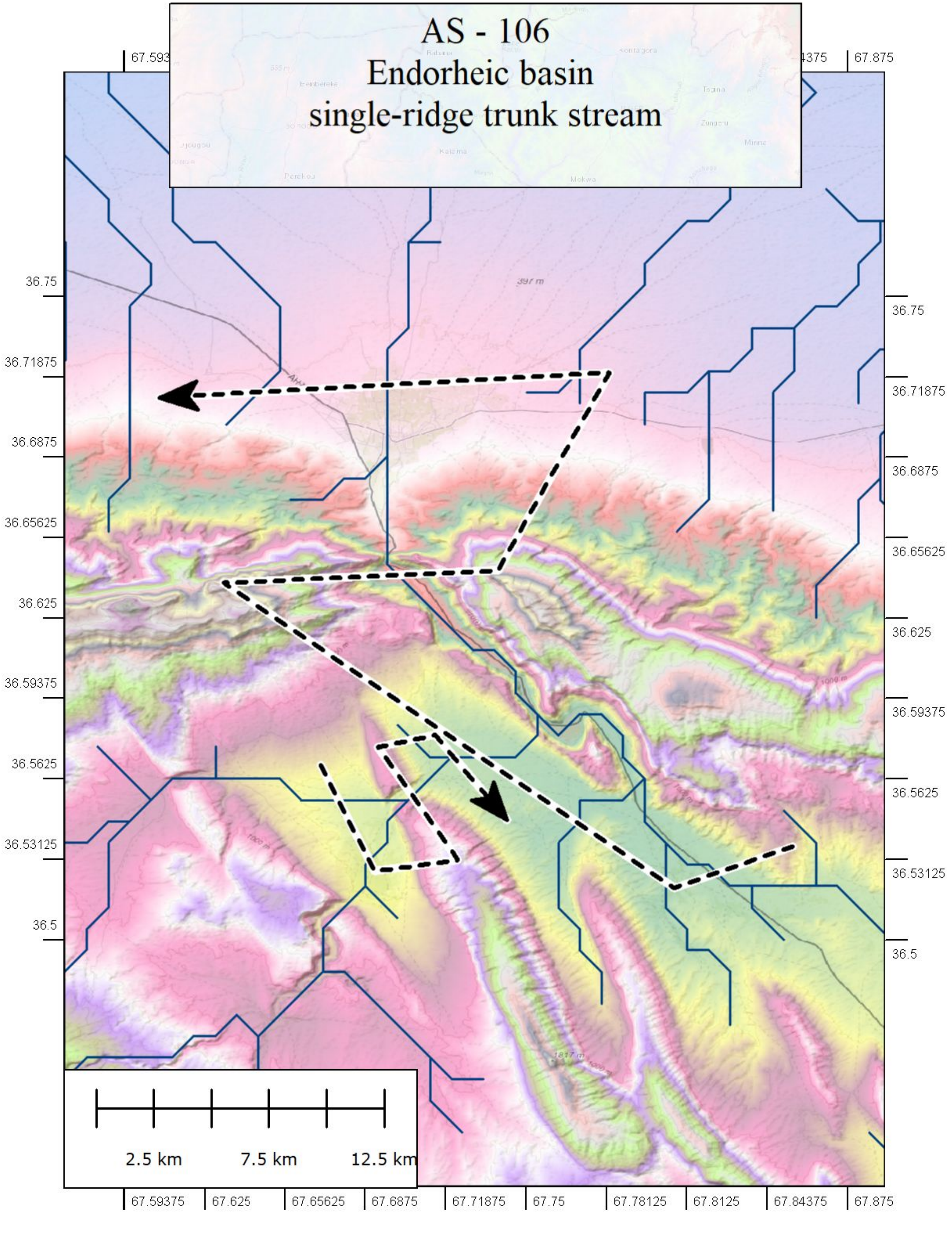
AS - 106
Endorheic basin
single-ridge trunk stream
36.75
36.71875
36.6875
36.65625
36.625
36.59375
36.5625
36.53125
36.5
67.59375
67.625
67.65625
67.6875
67.71875
67.75
67.78125
67.8125
67.84375
67.875
2.5 km
7.5 km
12.5 km

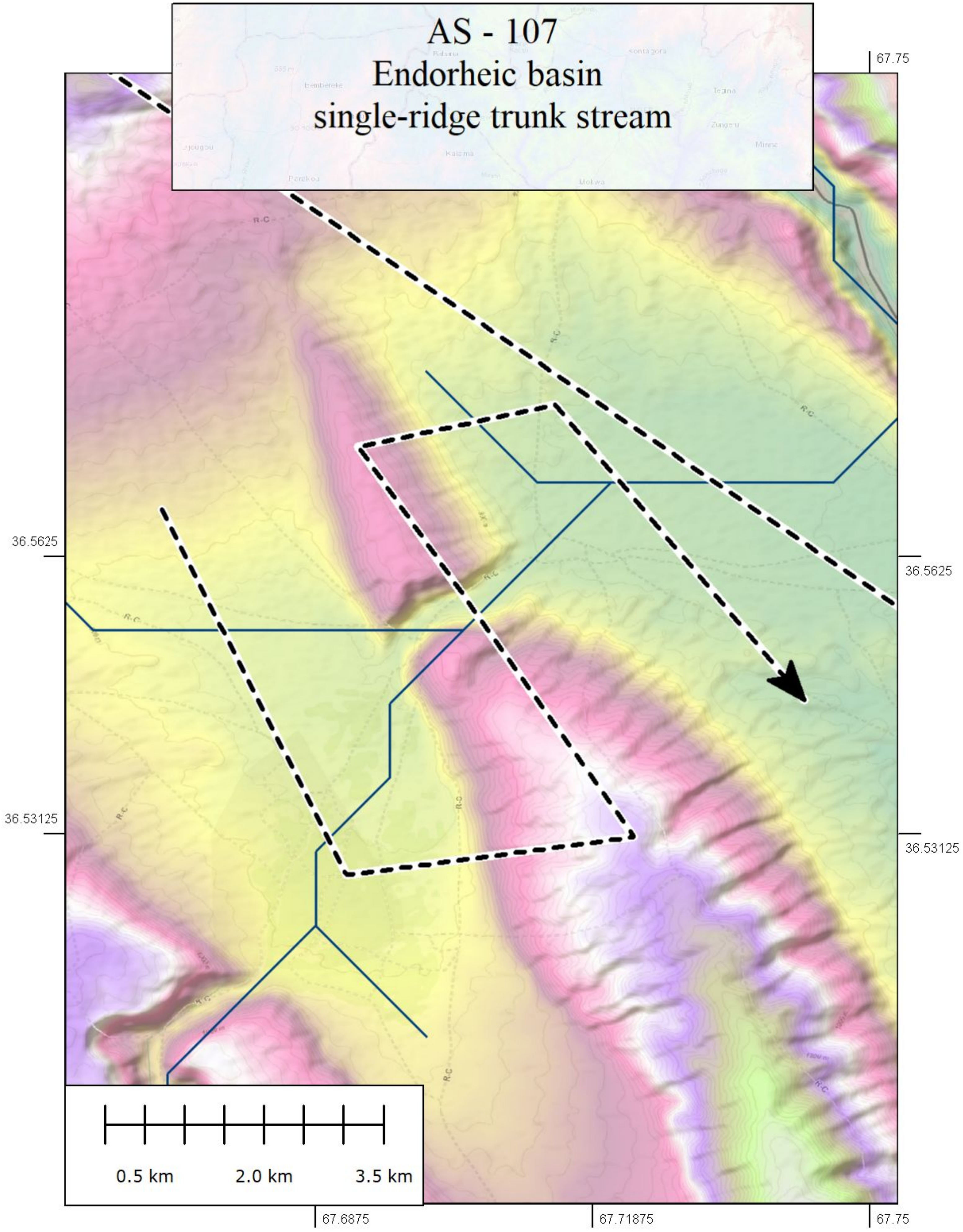

AS - 107
Endorheic basin
single-ridge trunk stream
67.75
36.5625
36.5625
36.53125
36.53125
0.5 km
2.0 km
3.5 km
67.6875
67.71875
67.75

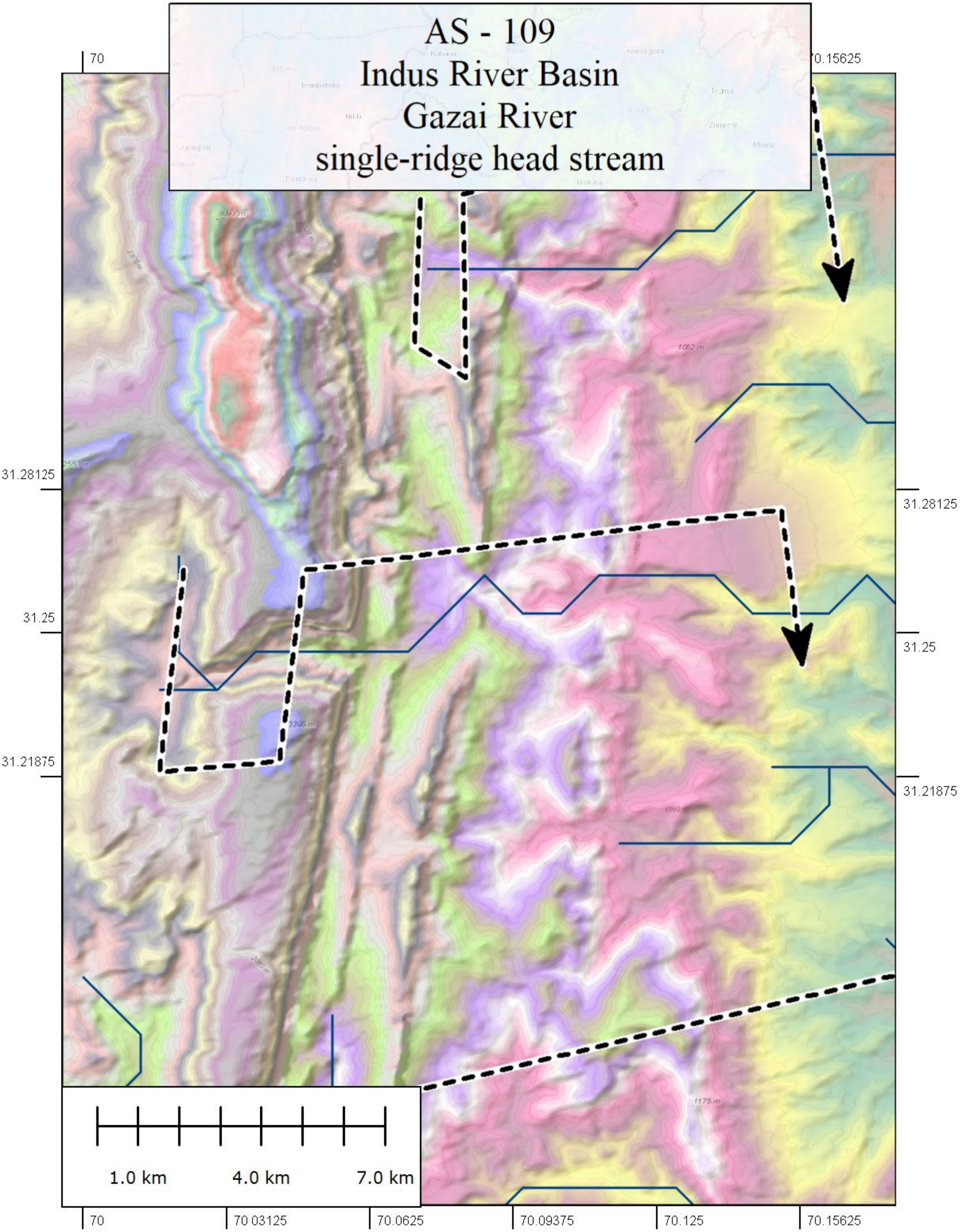
AS - 109
Indus River Basin
Gazai River
single-ridge head stream
70
70.15625
31.28125
31.25
31.21875
1062 m
1175 m
1.0 km
4.0 km
7.0 km
70 03125
70.0625
70.09375
70.125

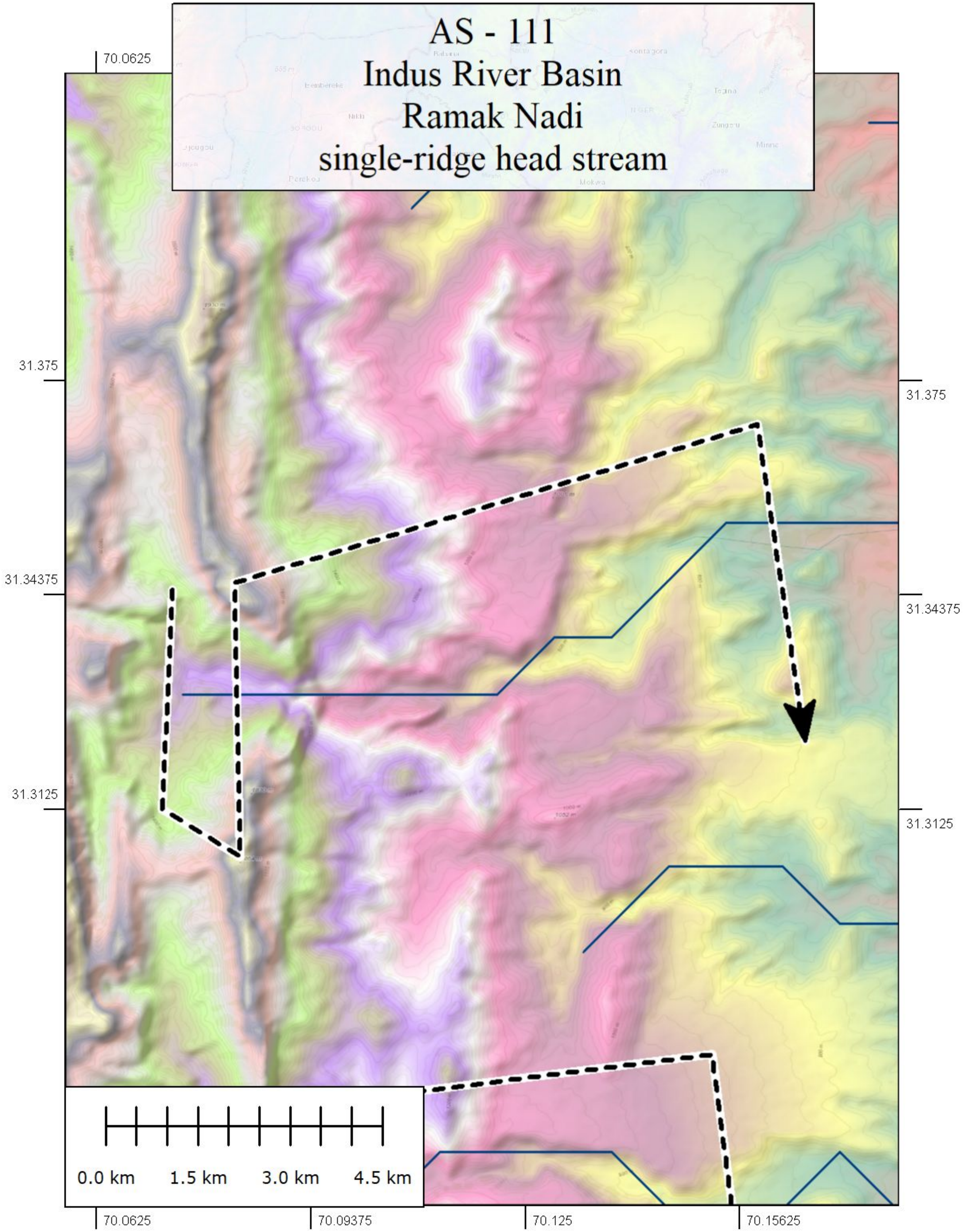
AS - 111
Indus River Basin
Ramak Nadi
single-ridge head stream
70.0625
31.375
31.34375
31.3125
0.0 km
1.5 km
3.0 km
4.5 km
70.0625
70.09375
70.125
70.15625

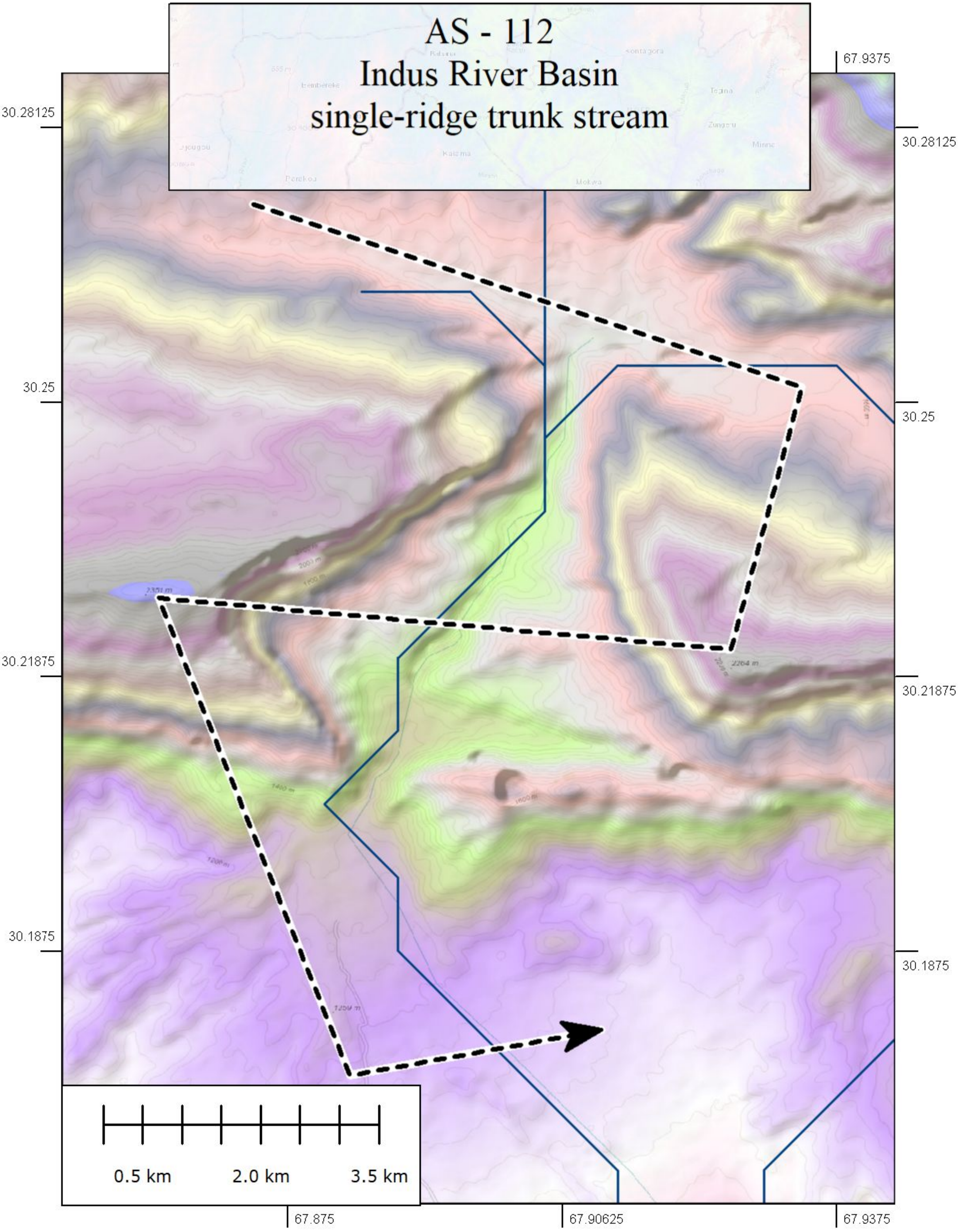
AS - 112
Indus River Basin
single-ridge trunk stream
67.9375
30.28125
30.28125
30.25
30.25
30.21875
30.21875
30.1875
30.1875
2264 m
0.5 km
2.0 km
3.5 km
67.875
67.90625
67.9375

AS - 113
Indus River Basin
Tangi Sar
single-ridge trunk stream

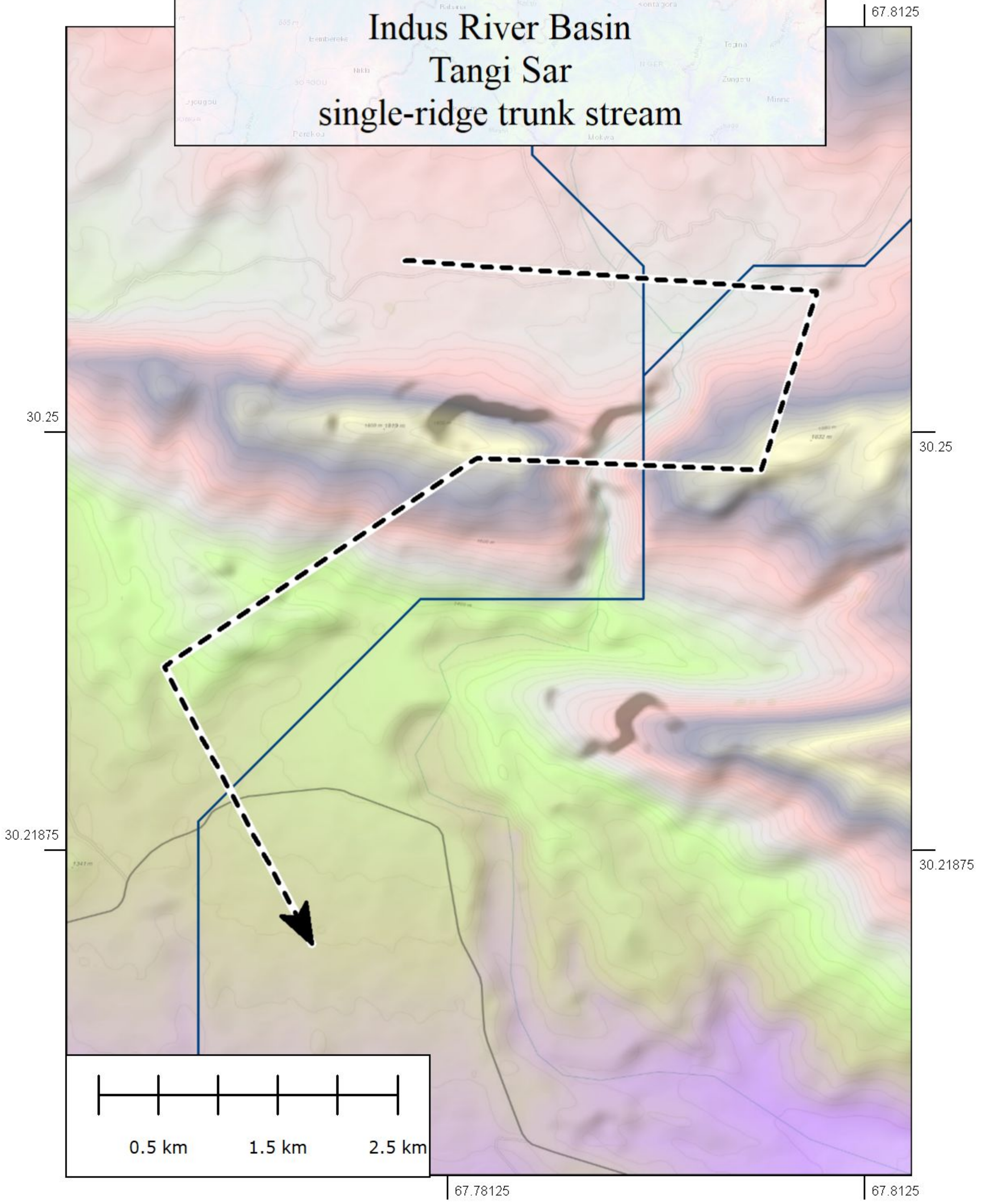

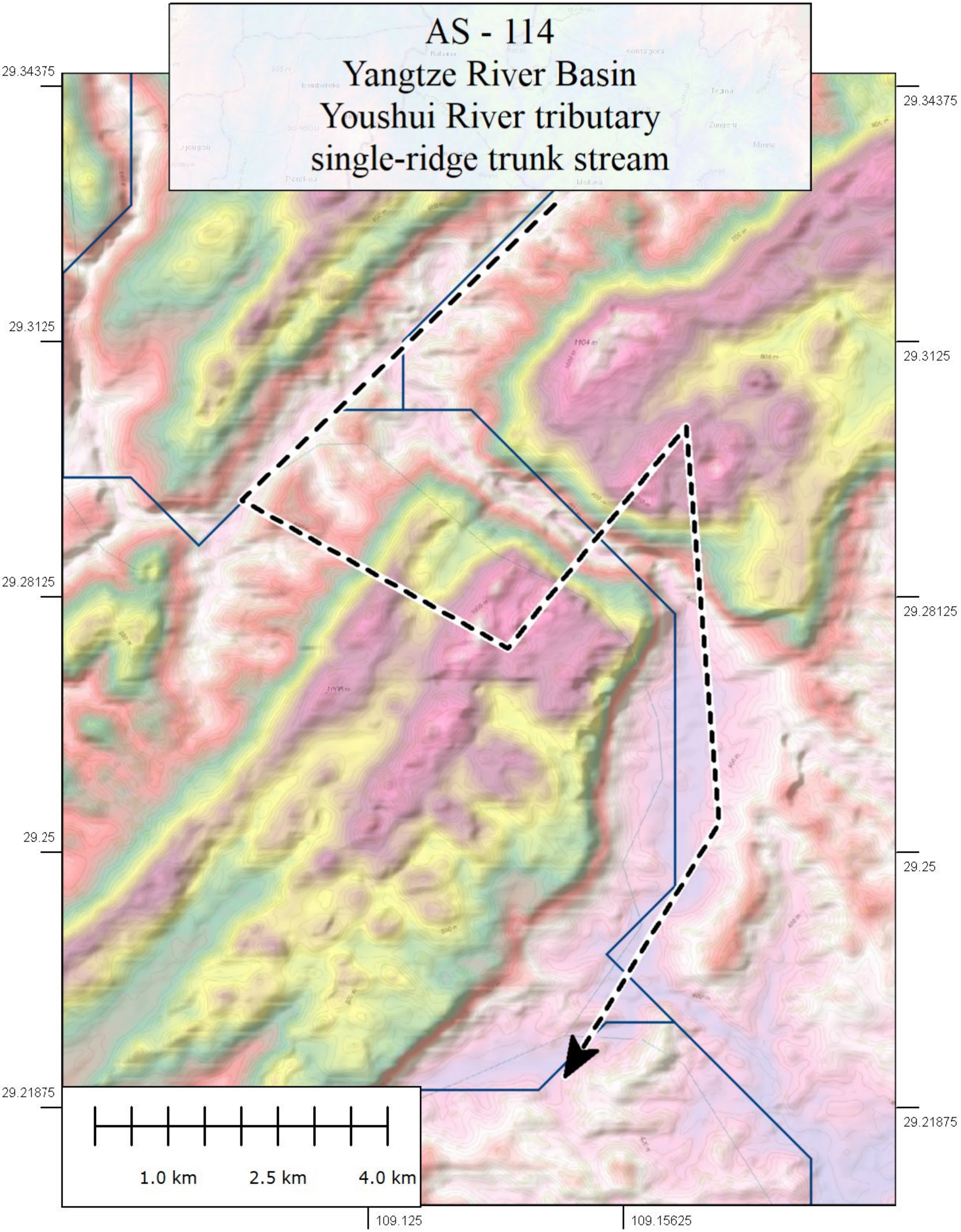
AS - 114
Yangtze River Basin
Youshui River tributary
single-ridge trunk stream
29.34375
29.3125
29.28125
29.25
29.21875
1.0 km
2.5 km
4.0 km
109.125
109.15625

AS - 115
Mekong River Basin
Hueang River
single-ridge trunk stream

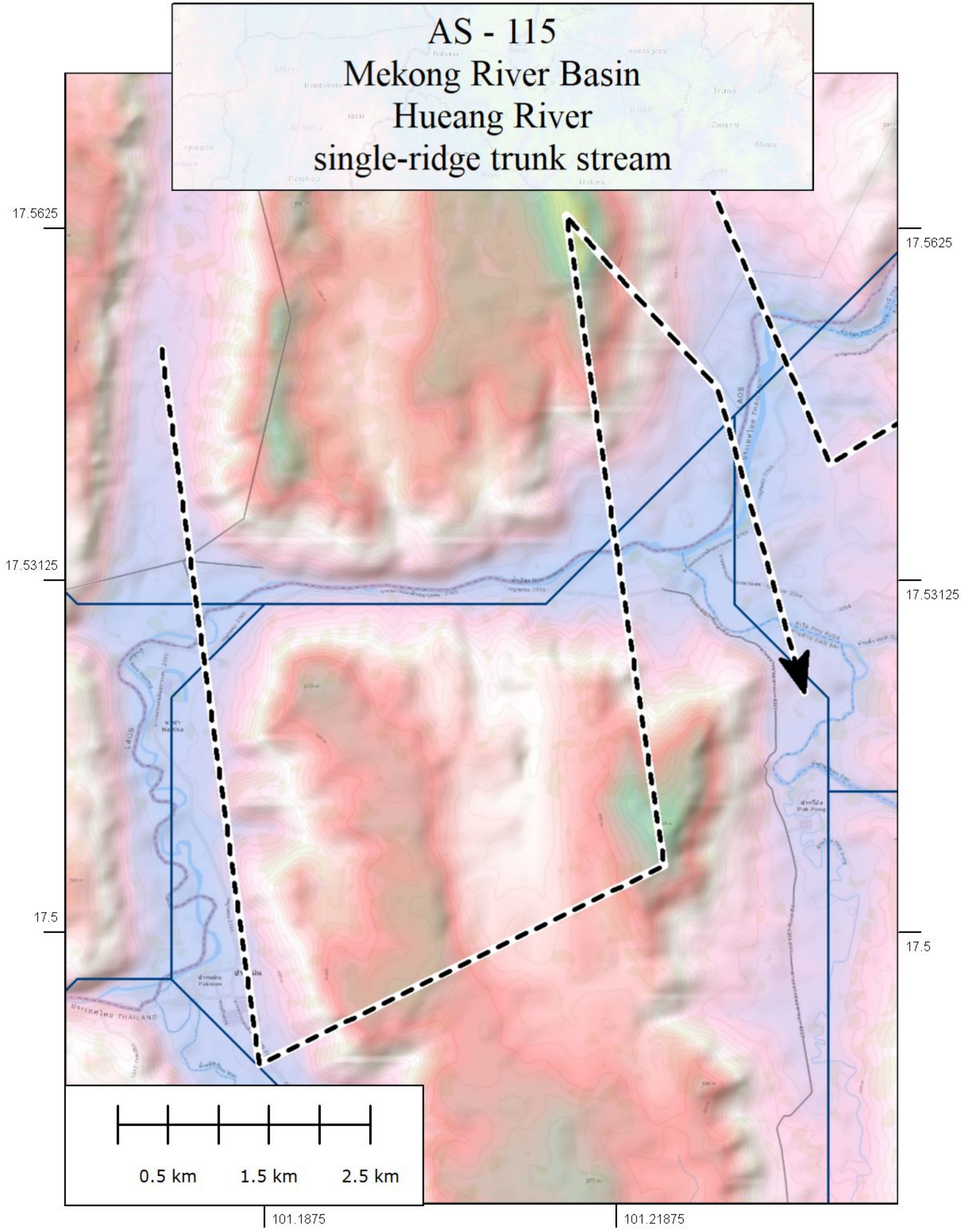

AS - 116
Yangtze River Basin
Lishui River tributary
single-ridge head stream

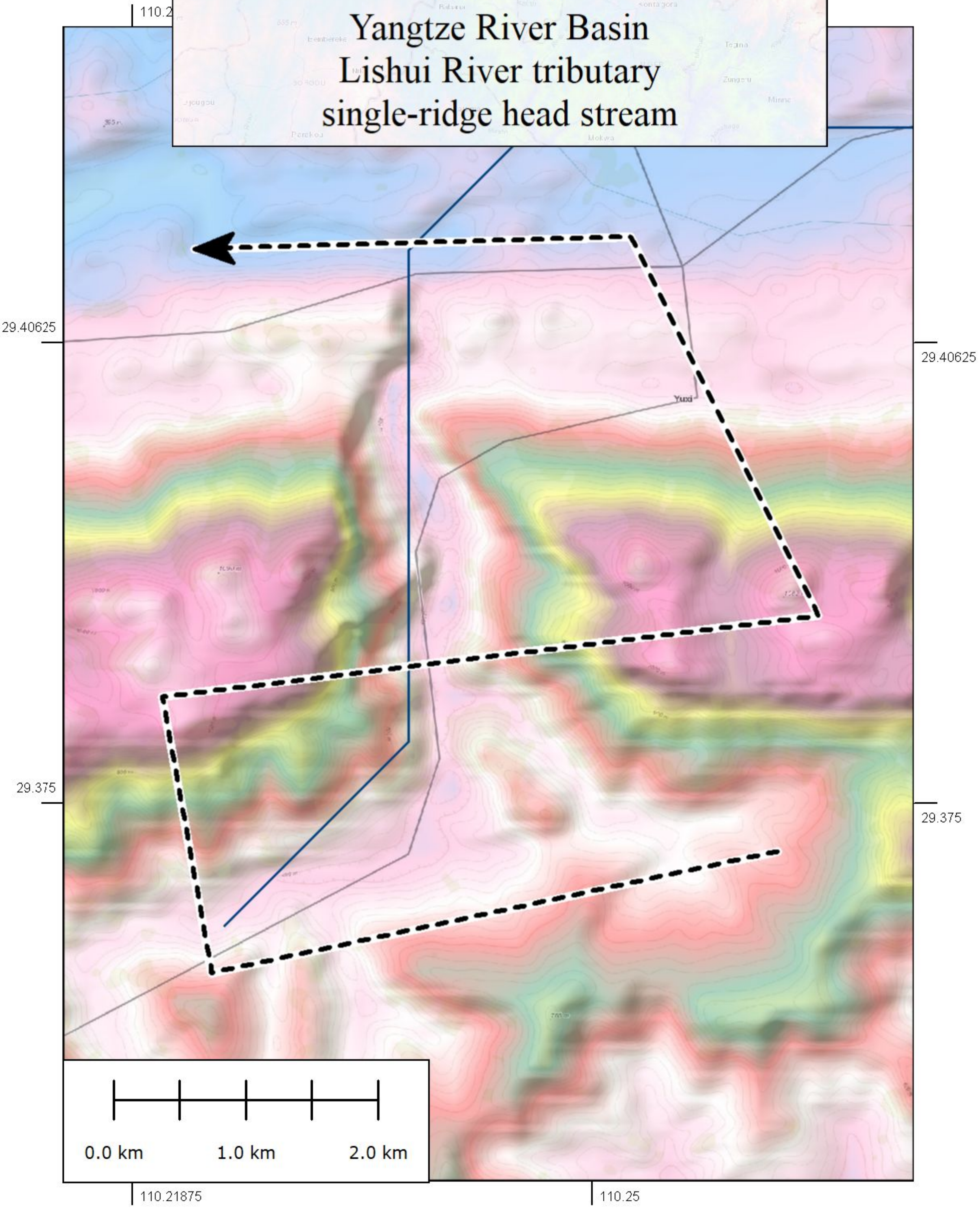

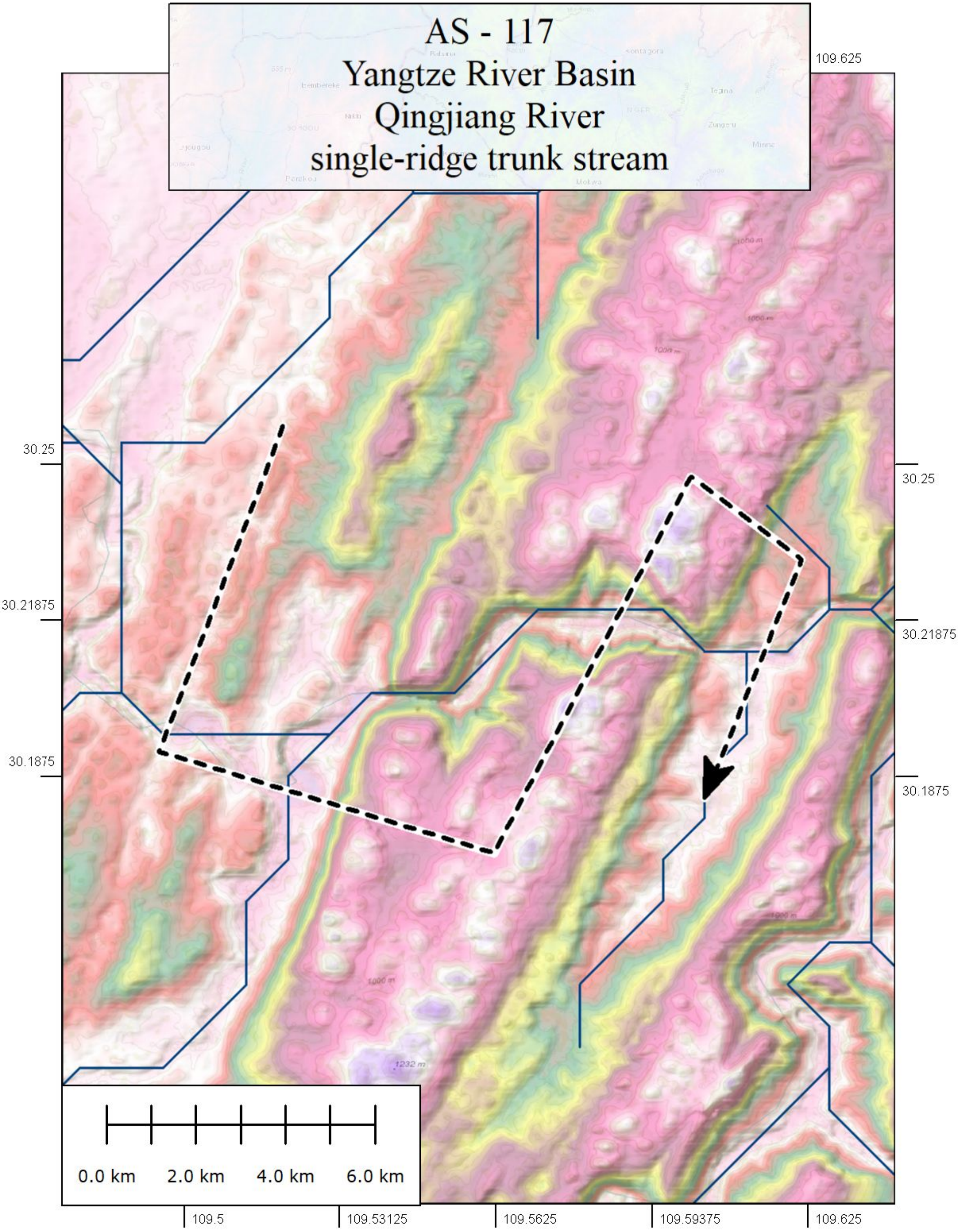
AS - 117
Yangtze River Basin
Qingjiang River
single-ridge trunk stream
109.625
30.25
30.25
30.21875
30.21875
30.1875
30.1875
1000 m
1000 m
1000 m
1000 m
1000 m
1232 m
0.0 km
2.0 km
4.0 km
6.0 km
109.5
109.53125
109.5625
109.59375
109.625

AS - 118
Huang (Yellow) River Basin
Qinggen River
single-ridge trunk stream

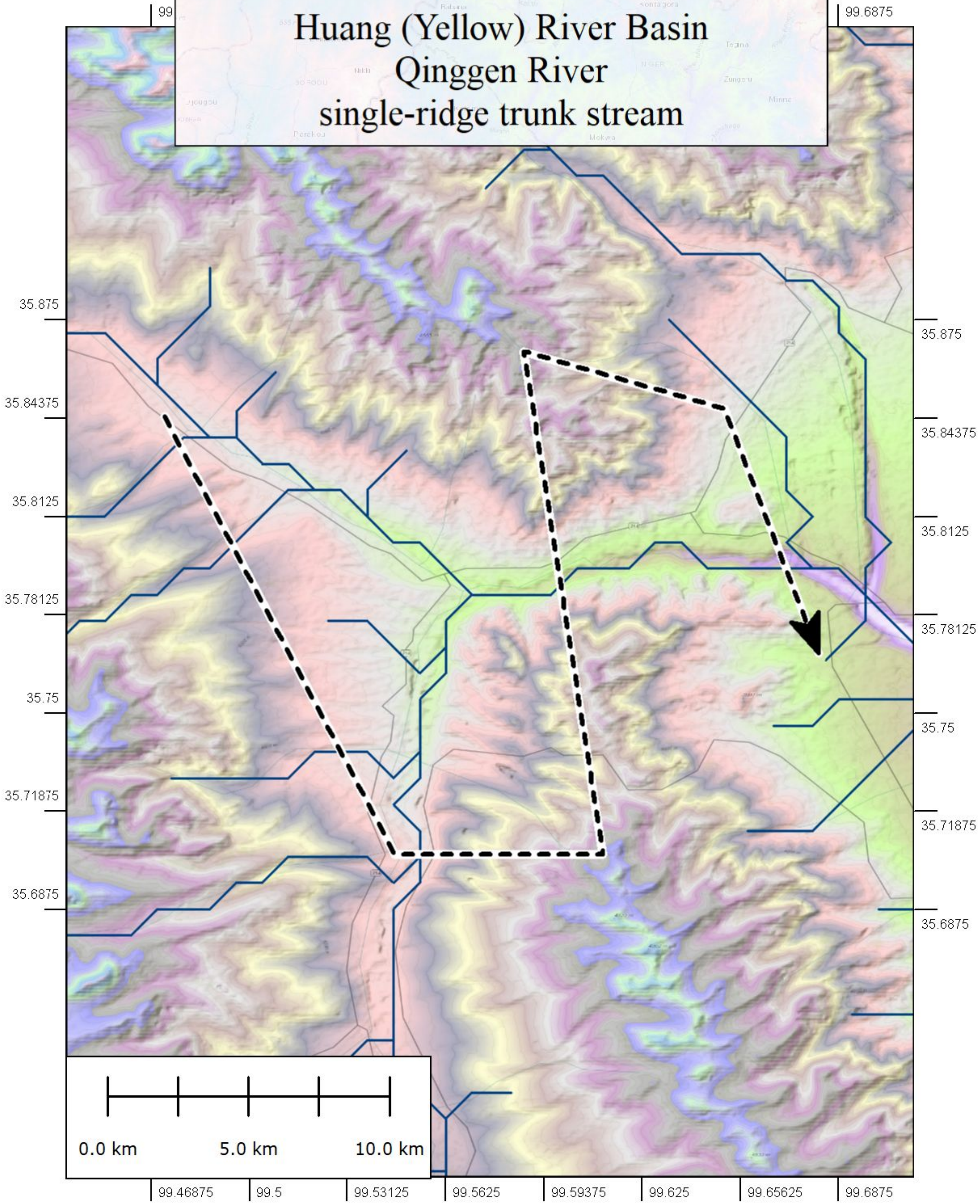

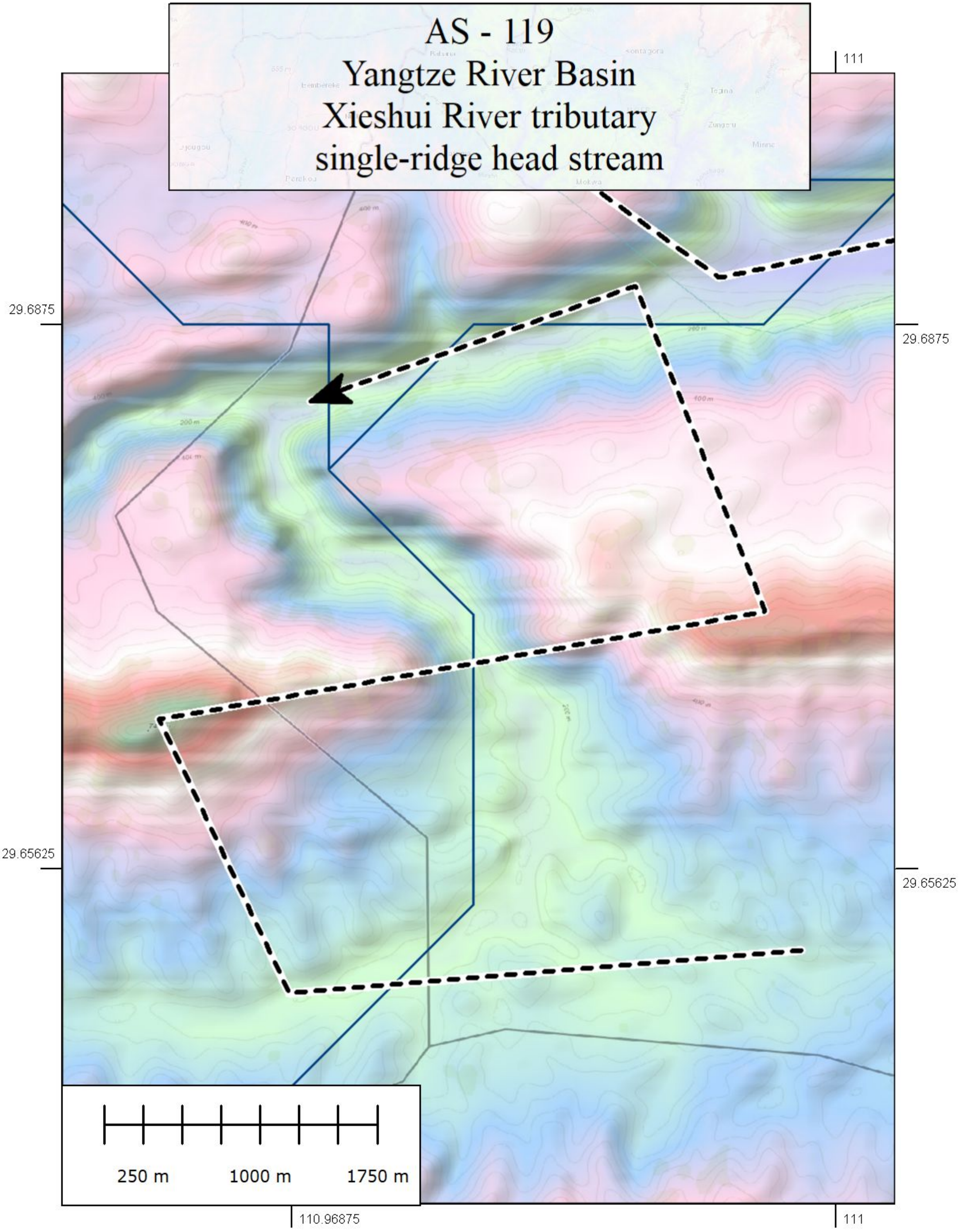
AS - 119
Yangtze River Basin
Xieshui River tributary
single-ridge head stream
111
29.6875
29.6875
29.65625
29.65625
250 m
1000 m
1750 m
110.96875
111

AS - 120
Mekong River Basin
Nam Mang River
single-ridge trunk stream

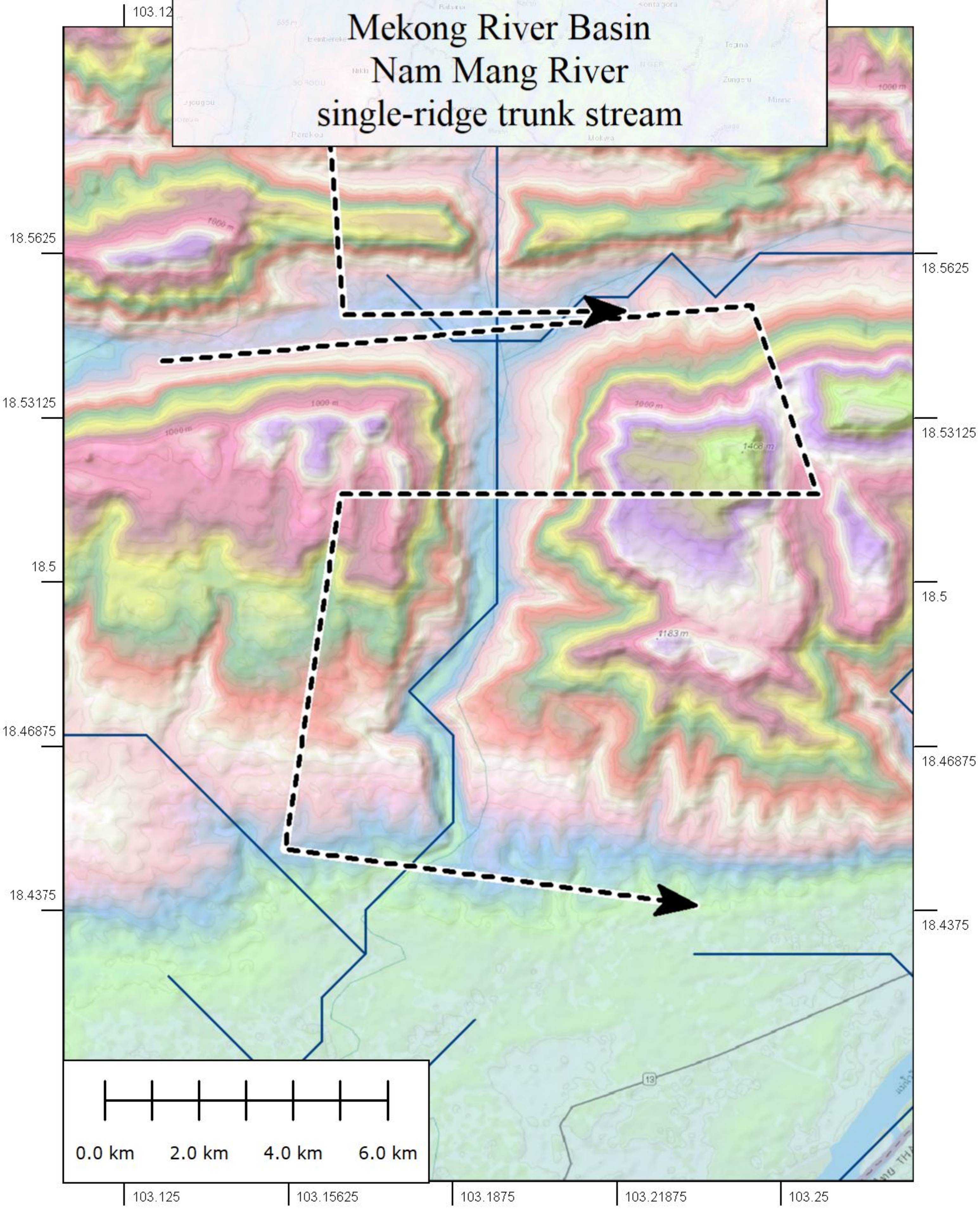

AS - 121
Endorheic basin
single-ridge trunk stream

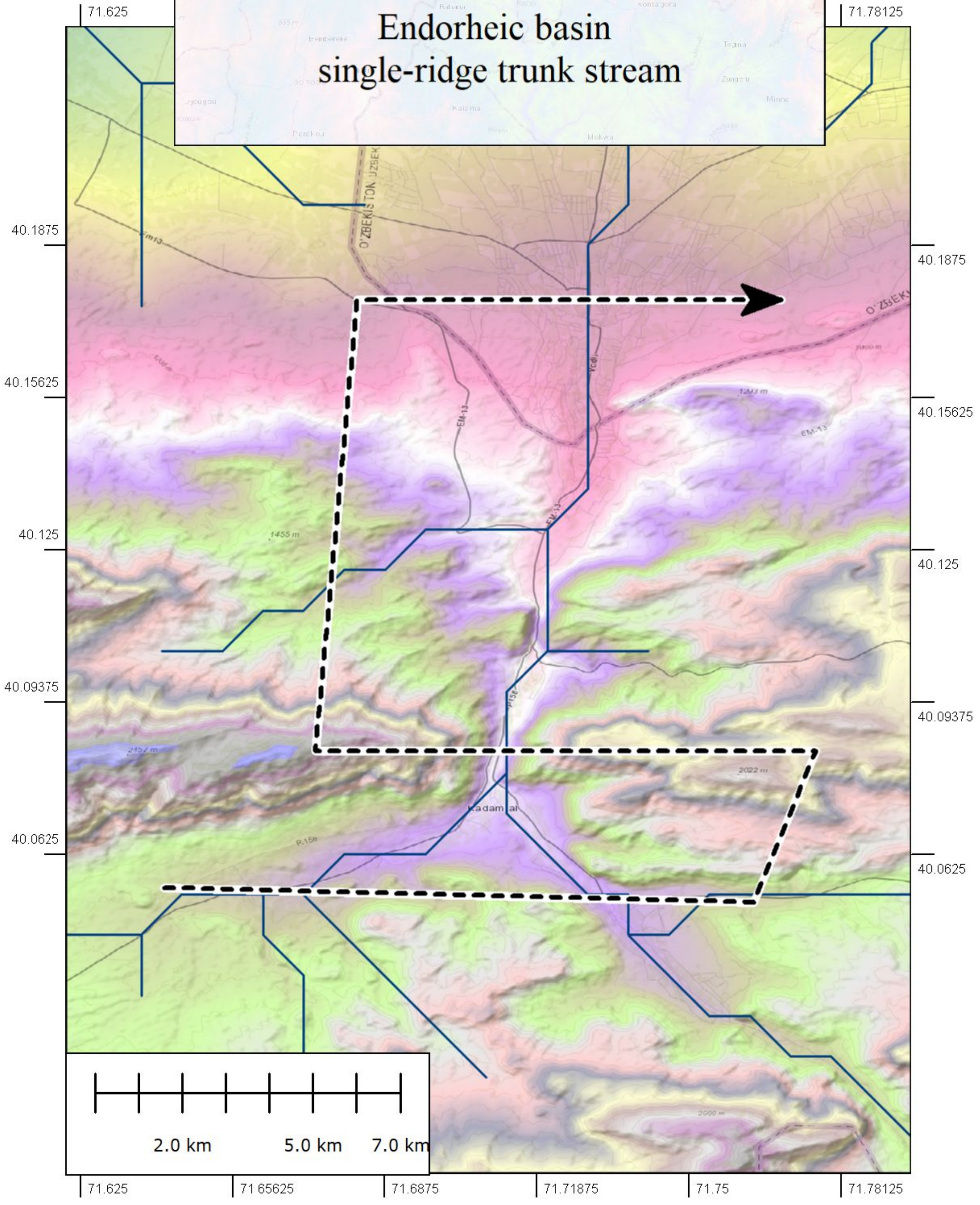

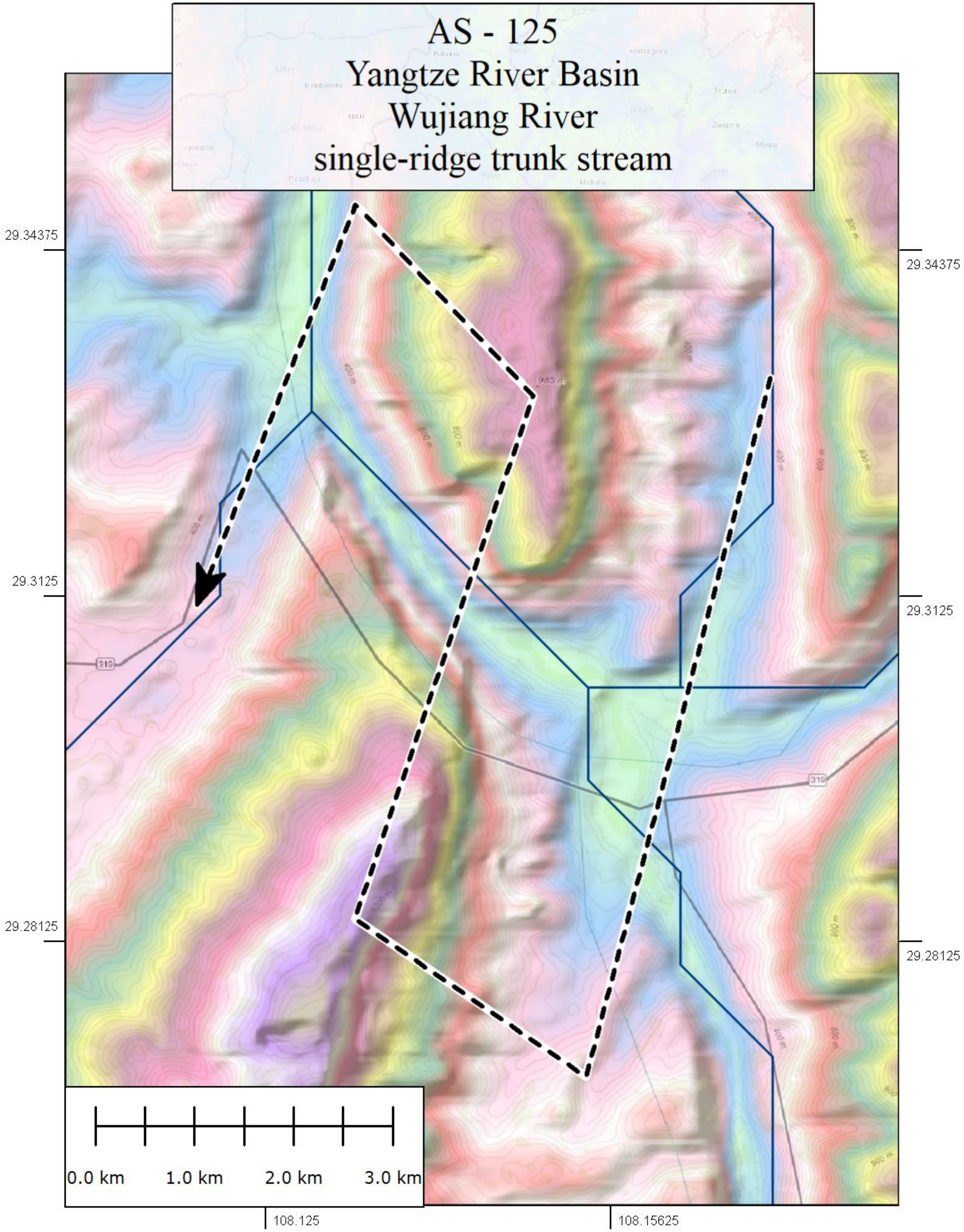
AS - 125
Yangtze River Basin
Wujiang River
single-ridge trunk stream
29.34375
29.34375
29.3125
29.3125
29.28125
29.28125
985 m
400 m
600 m
800 m
319
0.0 km
1.0 km
2.0 km
3.0 km
108.125
108.15625

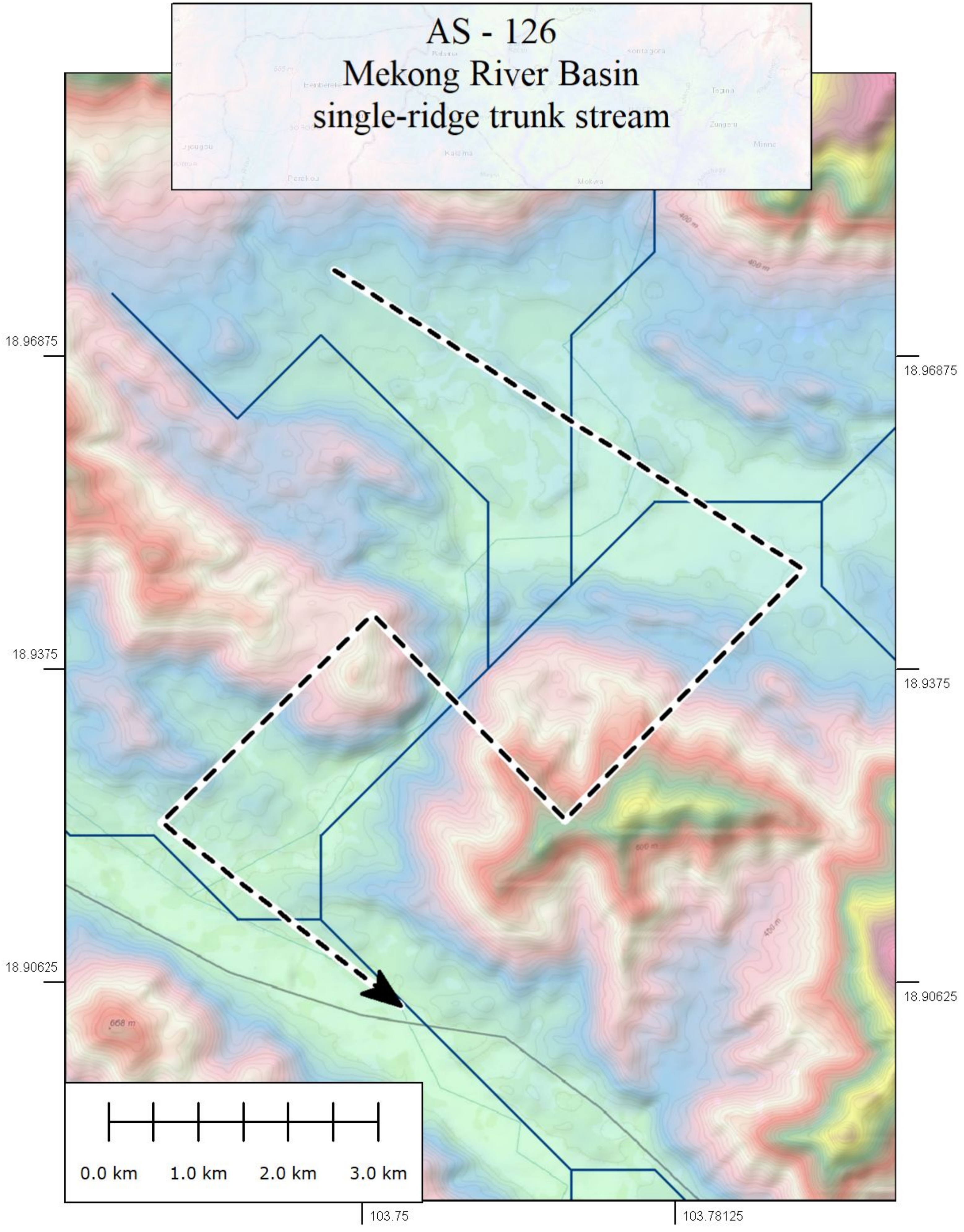
AS - 126
Mekong River Basin
single-ridge trunk stream
18.96875
18.96875
18.9375
18.9375
18.90625
18.90625
103.75
103.78125
0.0 km
1.0 km
2.0 km
3.0 km
400 m
600 m
450 m
668 m

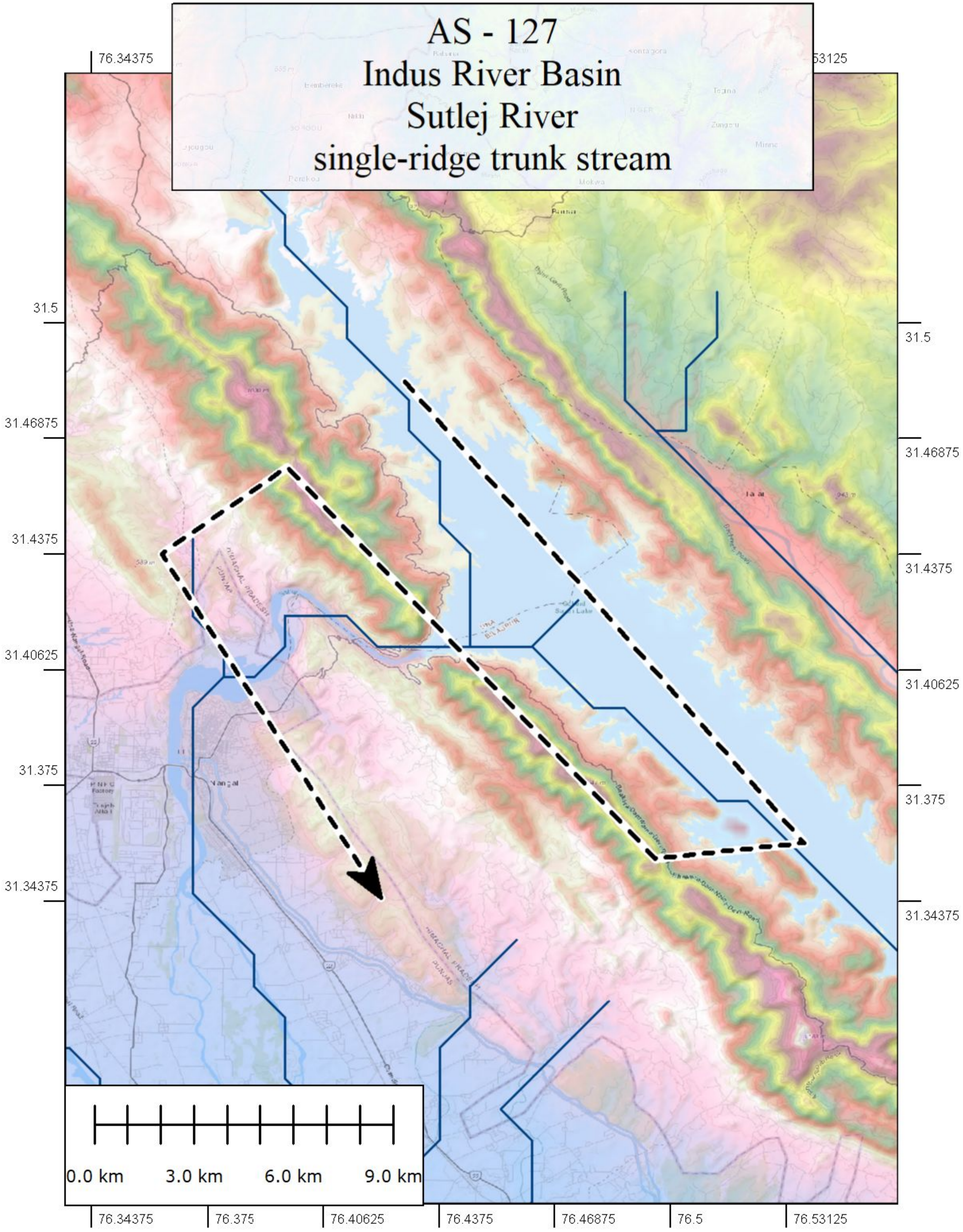
AS - 127
Indus River Basin
Sutlej River
single-ridge trunk stream
76.34375
76.375
76.40625
76.4375
76.46875
76.5
76.53125
31.5
31.46875
31.4375
31.40625
31.375
31.34375
Nangal
0.0 km
3.0 km
6.0 km
9.0 km

AS - 128
Ganges River Basin
Tipong River
single-ridge head stream

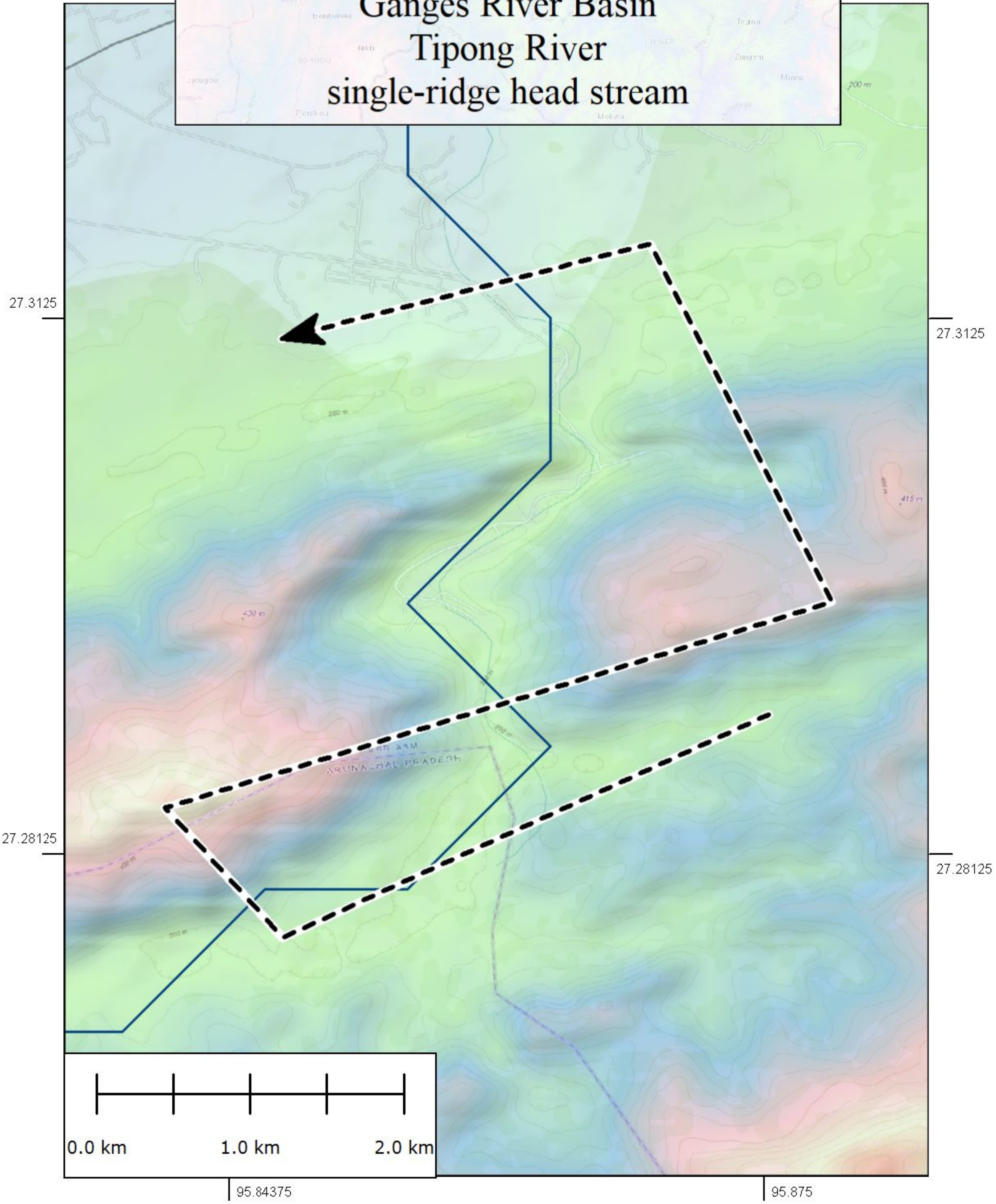

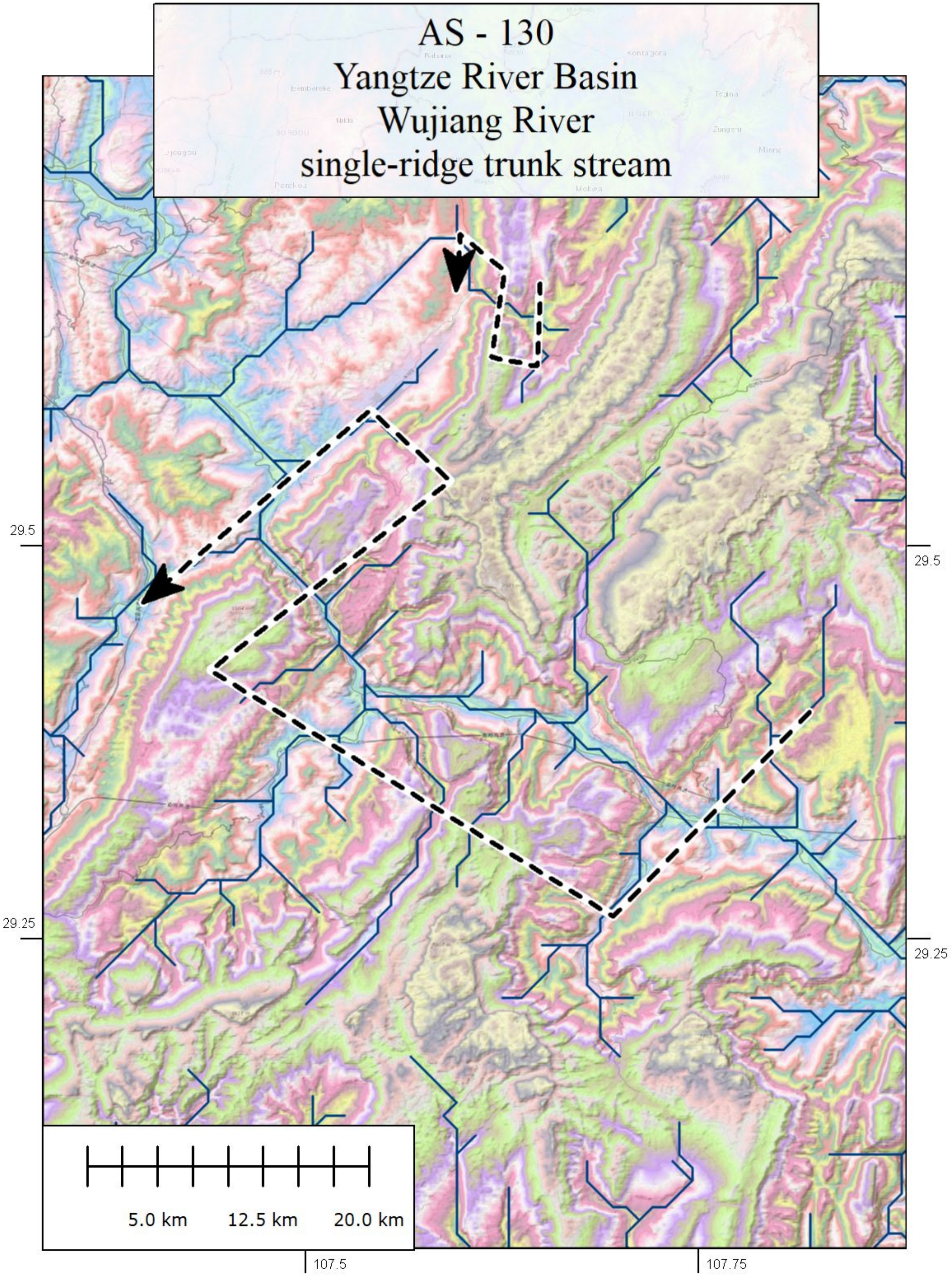
AS - 130
Yangtze River Basin
Wujiang River
single-ridge trunk stream
29.5
29.5
29.25
29.25
5.0 km
12.5 km
20.0 km
107.5
107.75

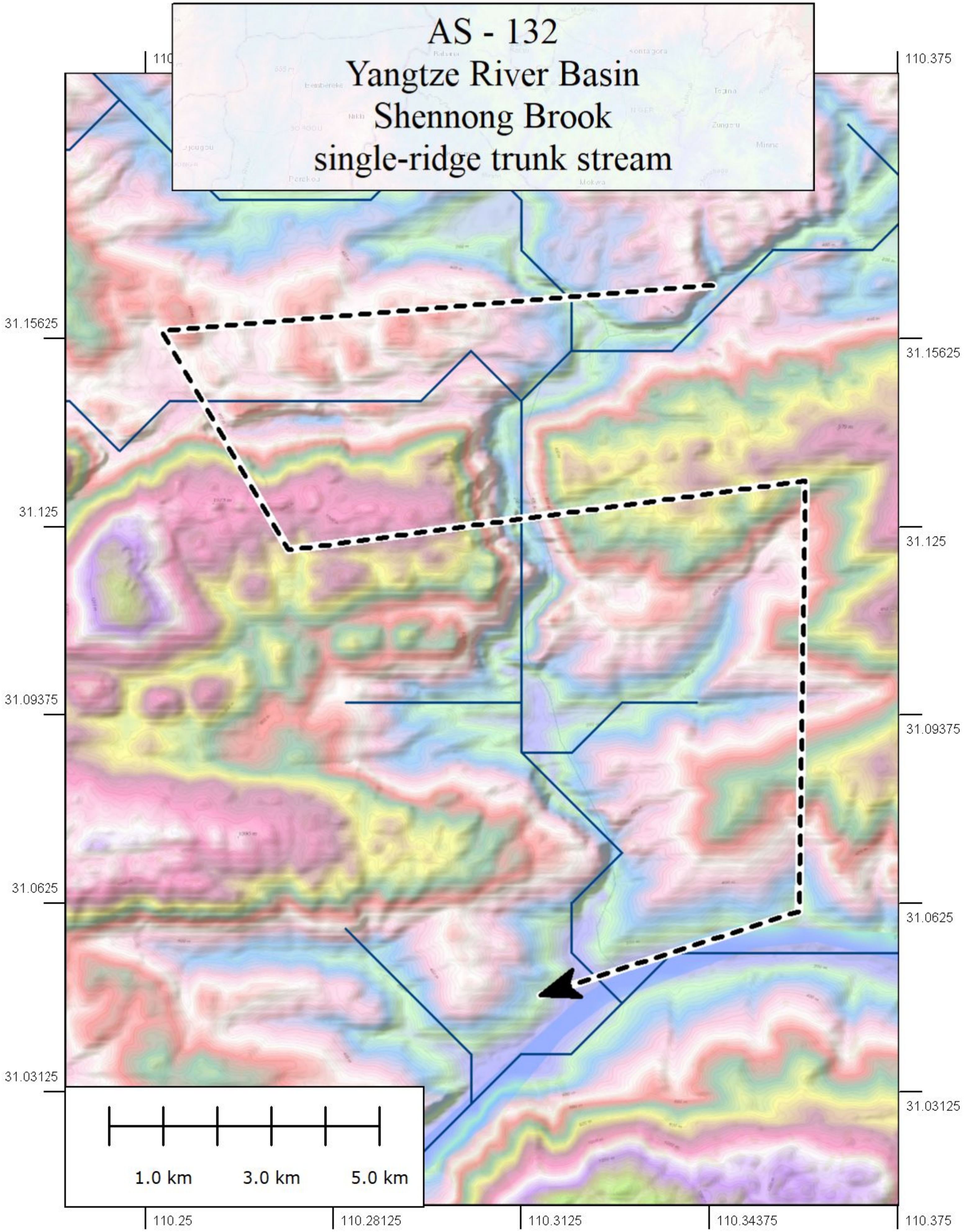
AS - 132
Yangtze River Basin
Shennong Brook
single-ridge trunk stream
110.375
31.15625
31.125
31.09375
31.0625
31.03125
1.0 km
3.0 km
5.0 km
110.25
110.28125
110.3125
110.34375

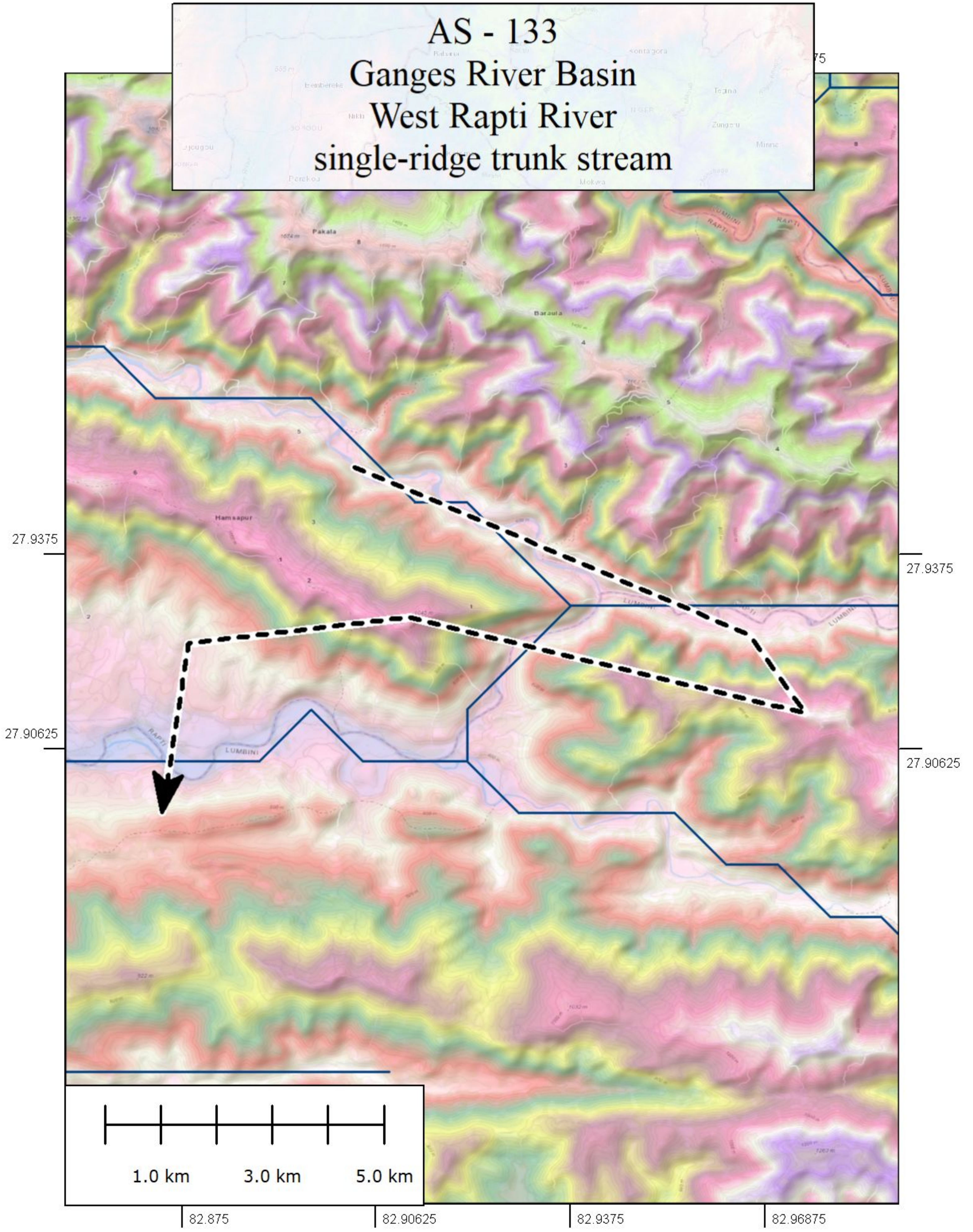

AS - 133
Ganges River Basin
West Rapti River
single-ridge trunk stream
27.9375
27.90625
82.875
82.90625
82.9375
82.96875
1.0 km
3.0 km
5.0 km
Hamsapur
Pakala
Baraula
LUMBINI
RAPTI

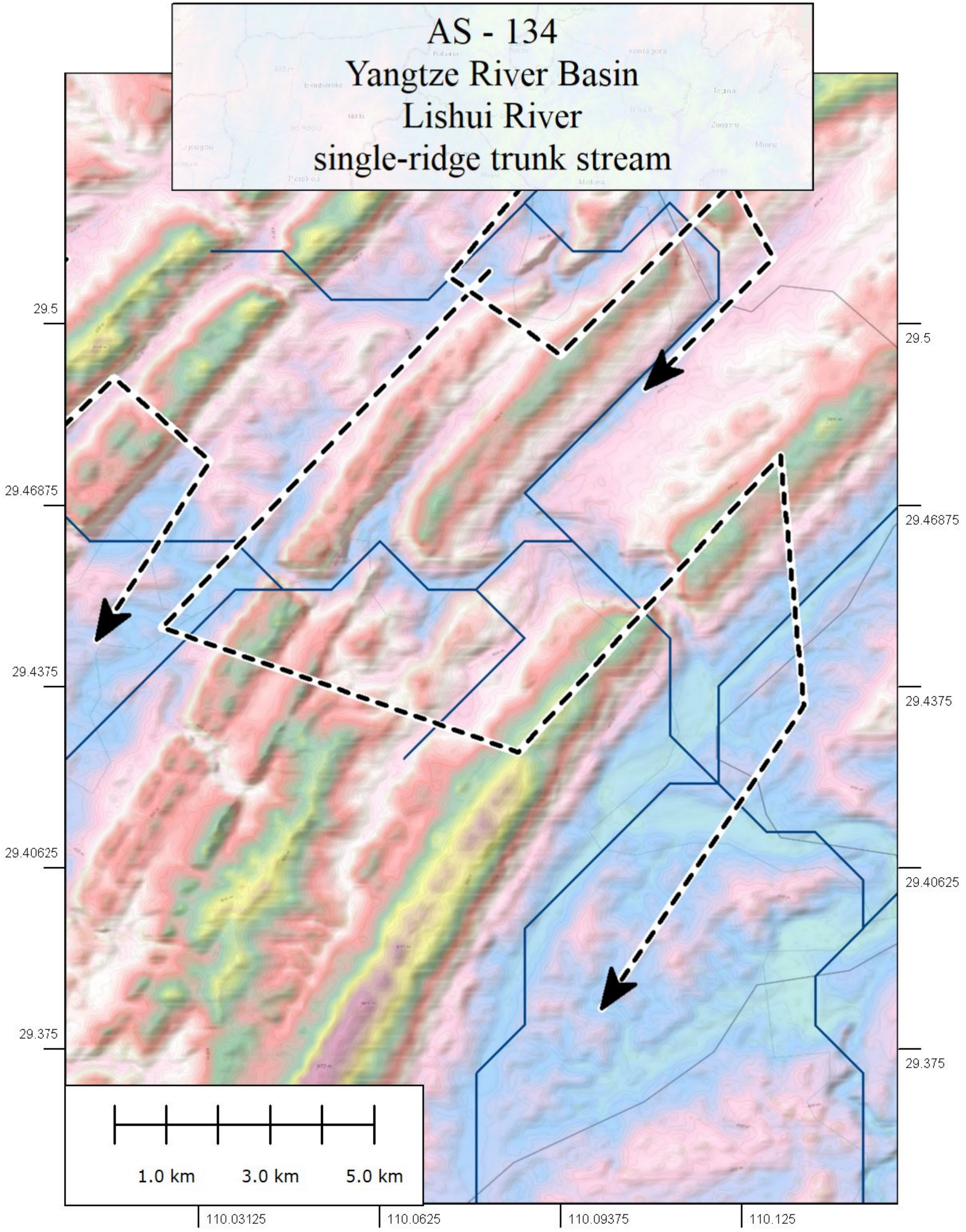
AS - 134
Yangtze River Basin
Lishui River
single-ridge trunk stream
29.5
29.46875
29.4375
29.40625
29.375
29.5
29.46875
29.4375
29.40625
29.375
1.0 km
3.0 km
5.0 km
110.03125
110.0625
110.09375
110.125

AS - 135
Indus River Basin
Ravi River
single-ridge trunk stream

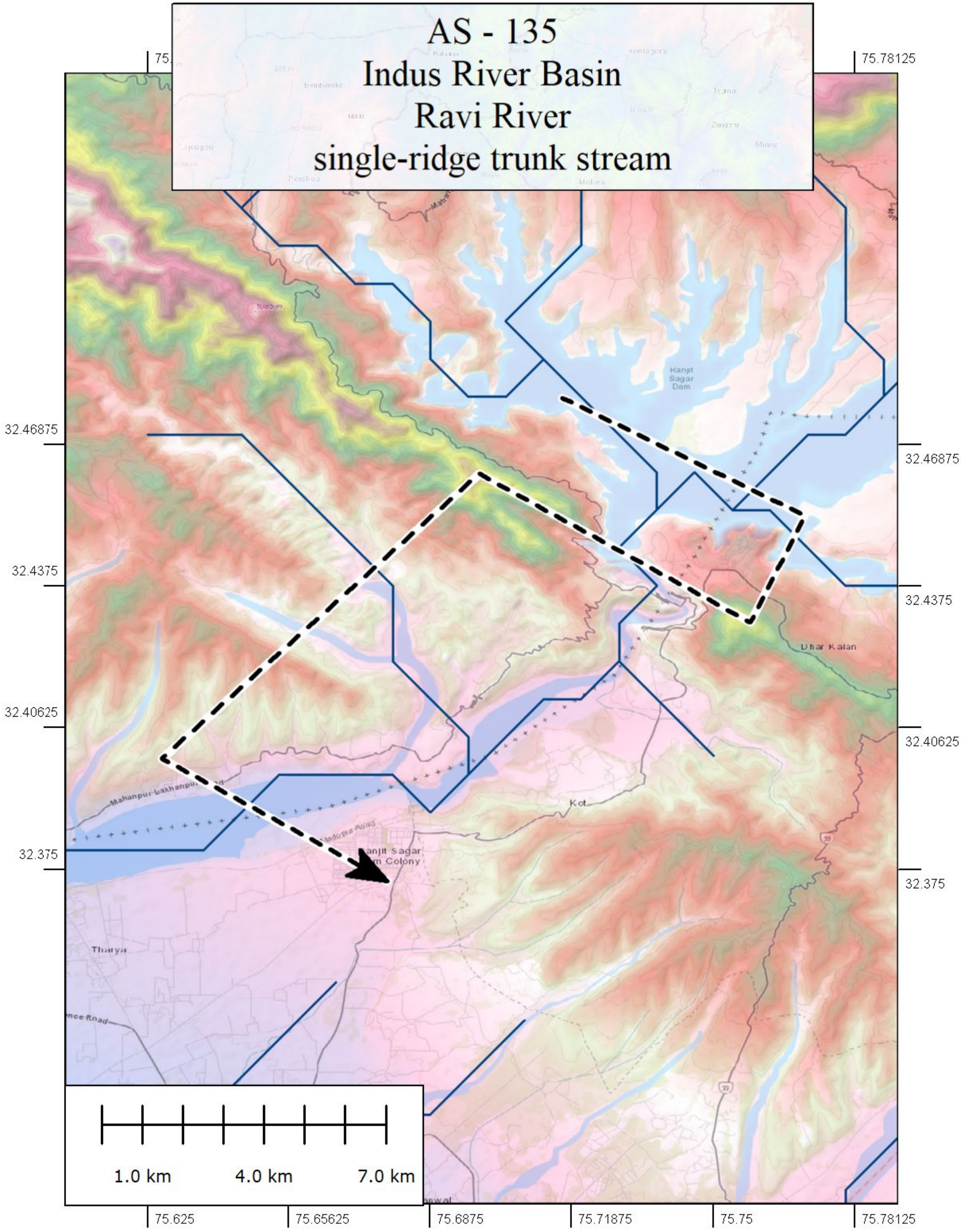

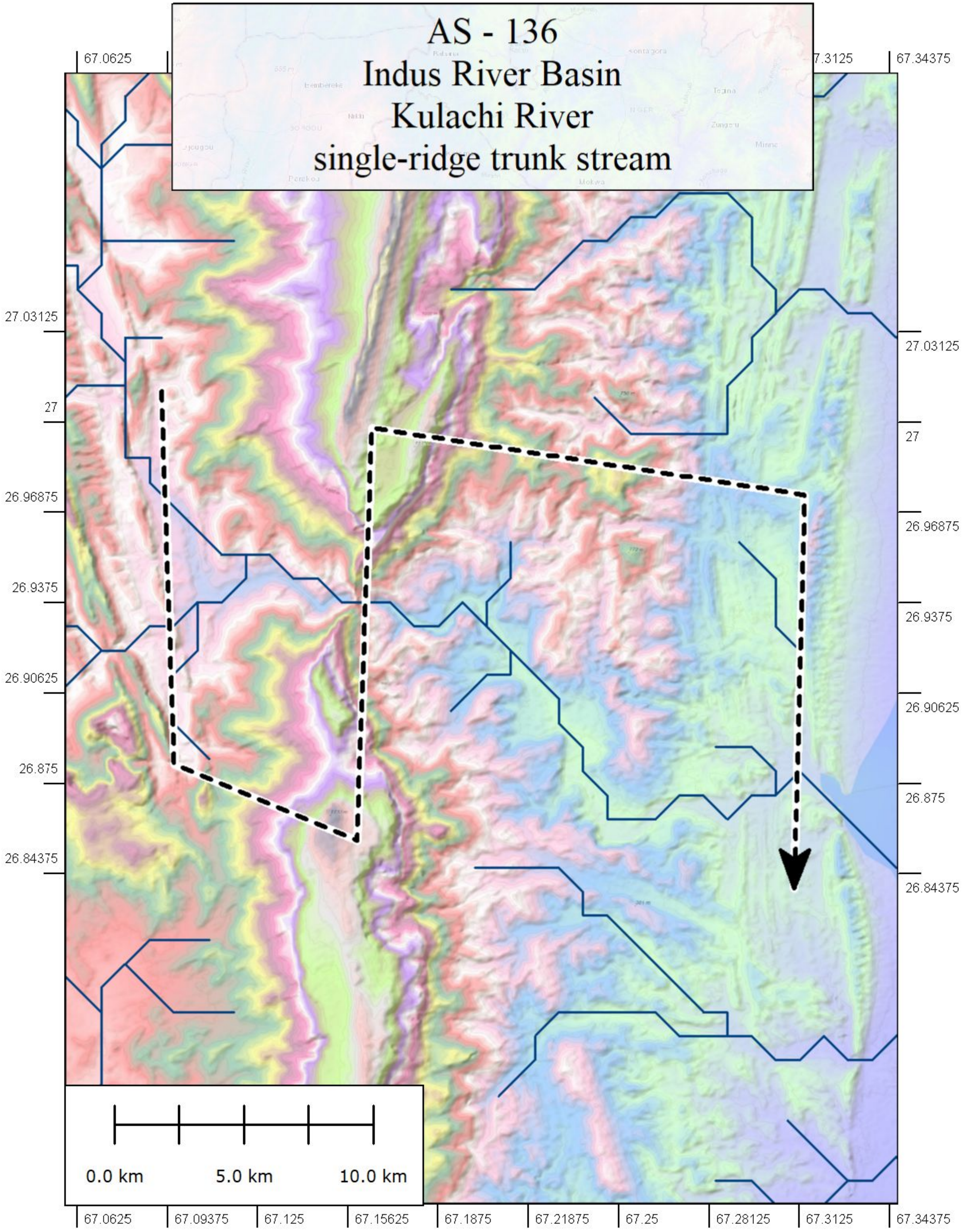
AS - 136
Indus River Basin
Kulachi River
single-ridge trunk stream
67.0625
67.09375
67.125
67.15625
67.1875
67.21875
67.25
67.28125
67.3125
67.34375
27.03125
27
26.96875
26.9375
26.90625
26.875
26.84375
0.0 km
5.0 km
10.0 km

AS - 137
Mekong River Basin
Kok River
single-ridge trunk stream

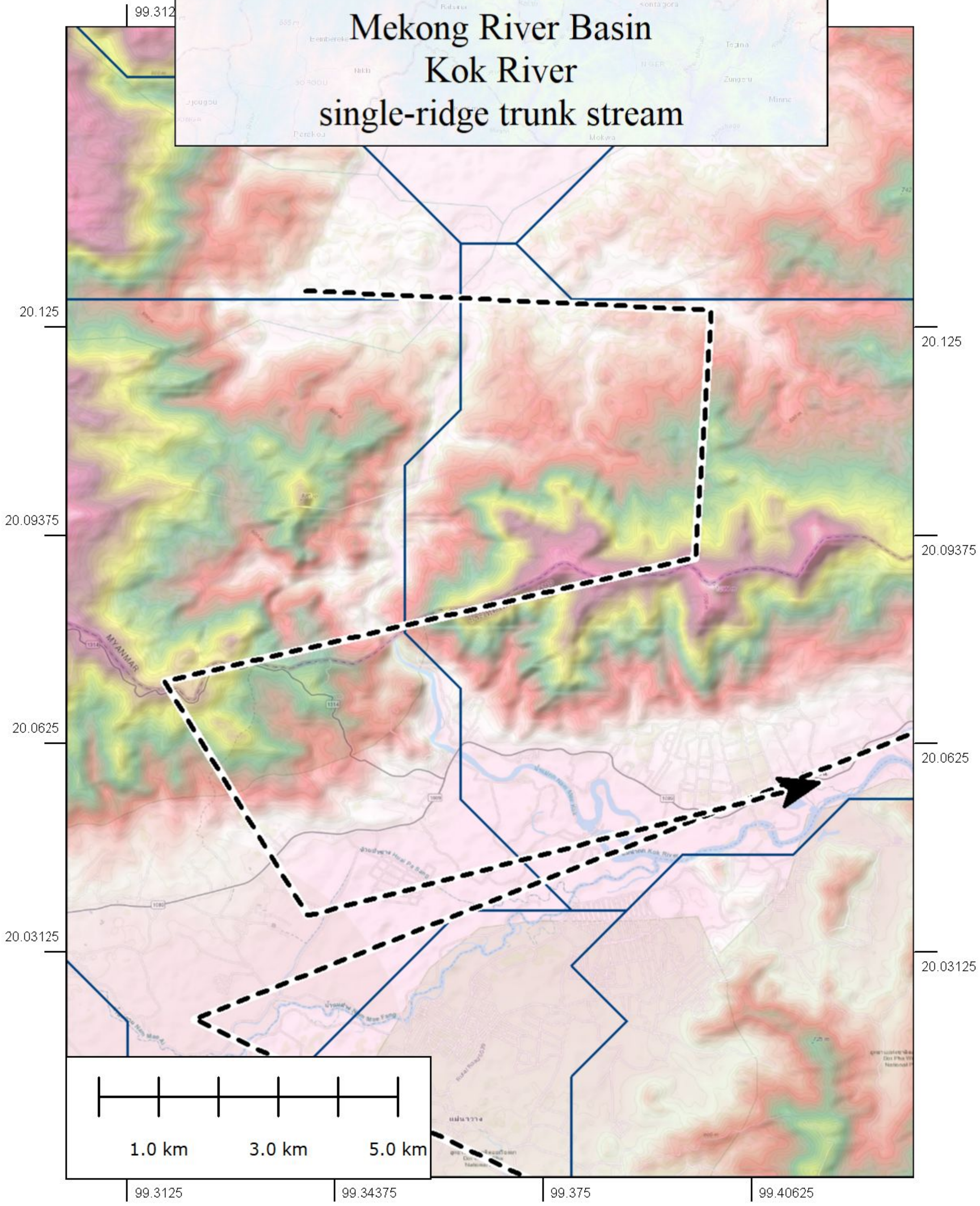

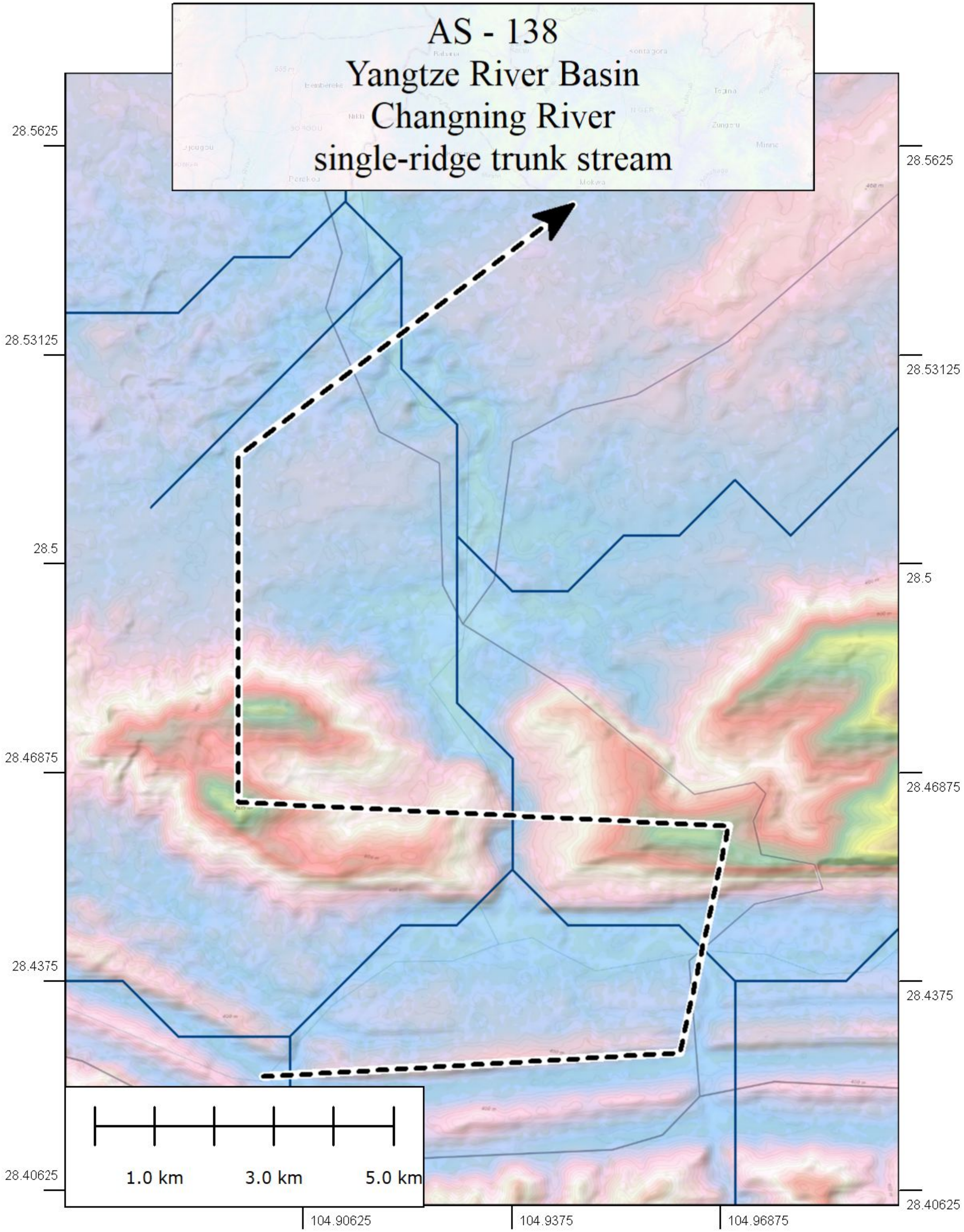
AS - 138
Yangtze River Basin
Changning River
single-ridge trunk stream
28.5625
28.53125
28.5
28.46875
28.4375
28.40625
104.90625
104.9375
104.96875
1.0 km
3.0 km
5.0 km

AS - 140
Mekong River Basin
single-ridge head stream

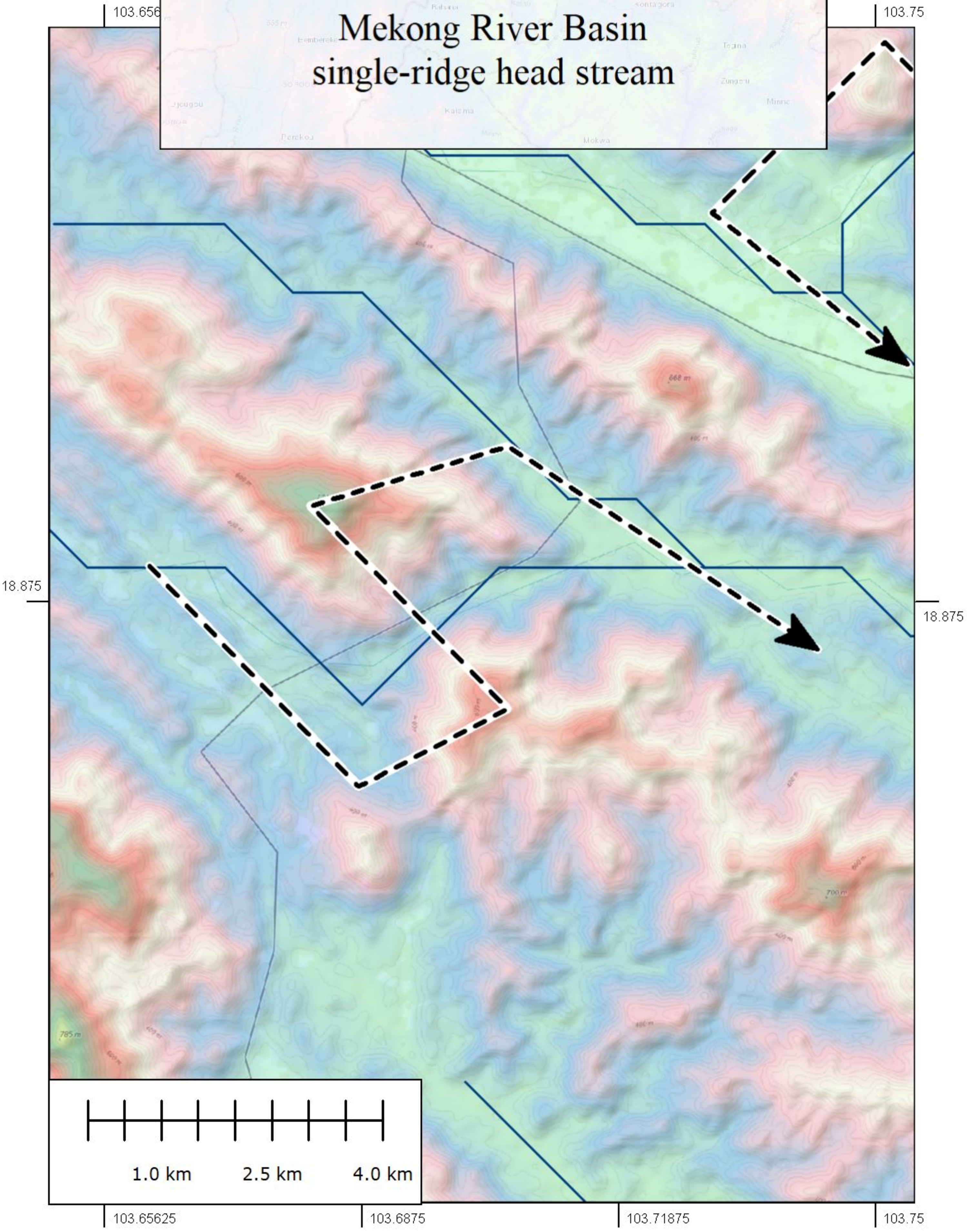

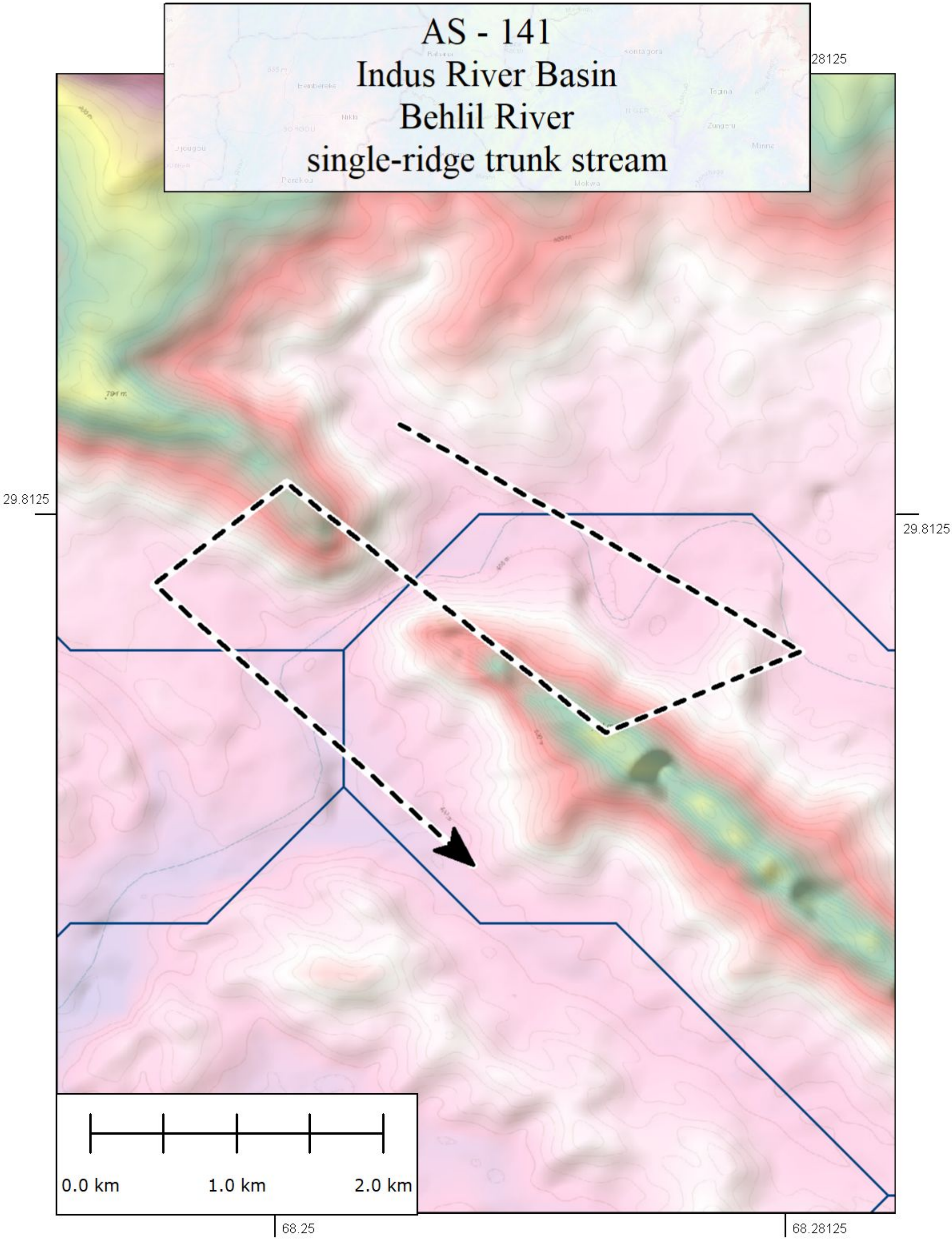
AS - 141
Indus River Basin
Behlil River
single-ridge trunk stream
28125
29.8125
29.8125
0.0 km
1.0 km
2.0 km
68.25
68.28125

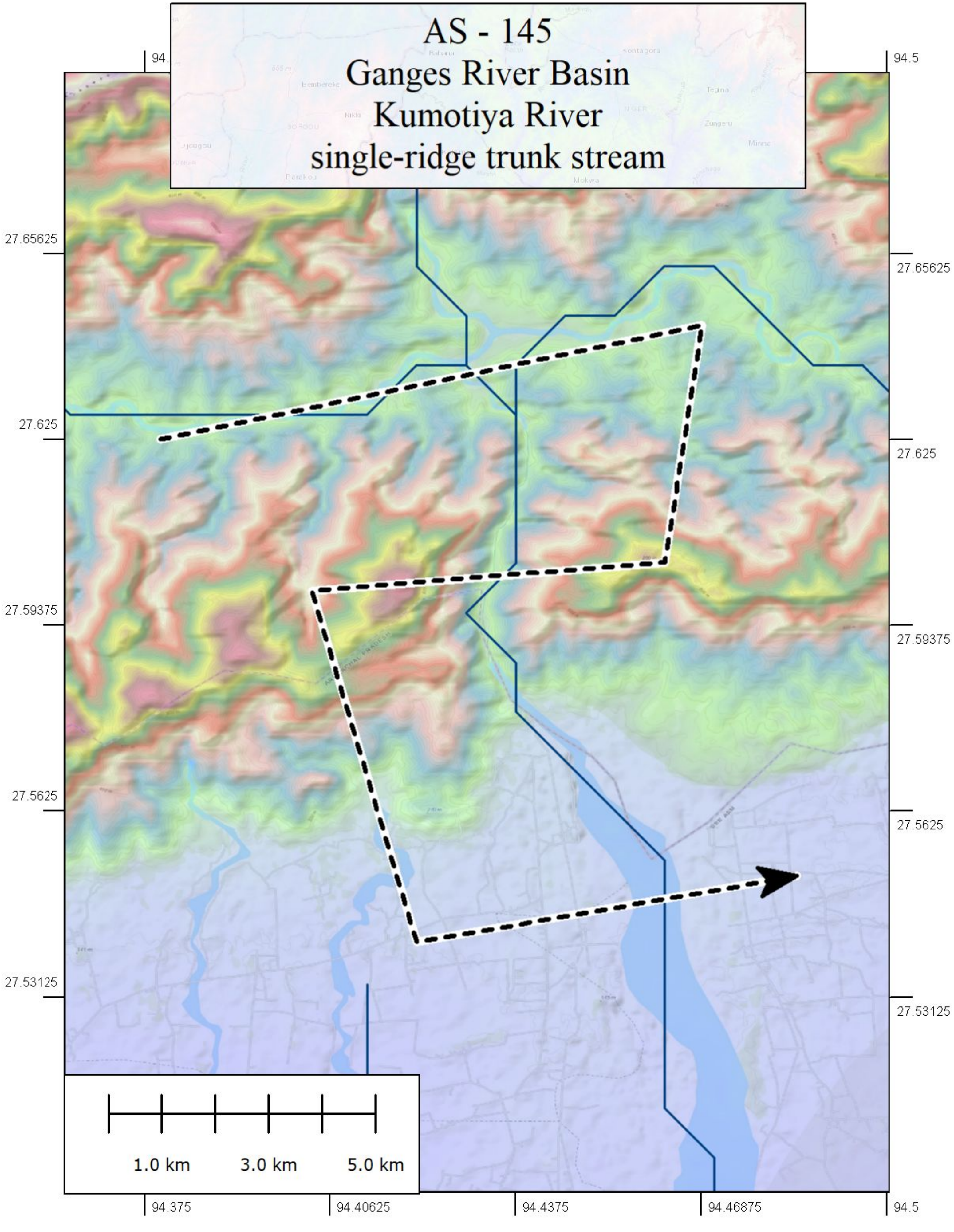
AS - 145
Ganges River Basin
Kumotiya River
single-ridge trunk stream
94.5
27.65625
27.625
27.59375
27.5625
27.53125
27.65625
27.625
27.59375
27.5625
27.53125
94.375
94.40625
94.4375
94.46875
94.5
1.0 km
3.0 km
5.0 km

AS - 146
Kamaphuli River Basin
single-ridge trunk stream

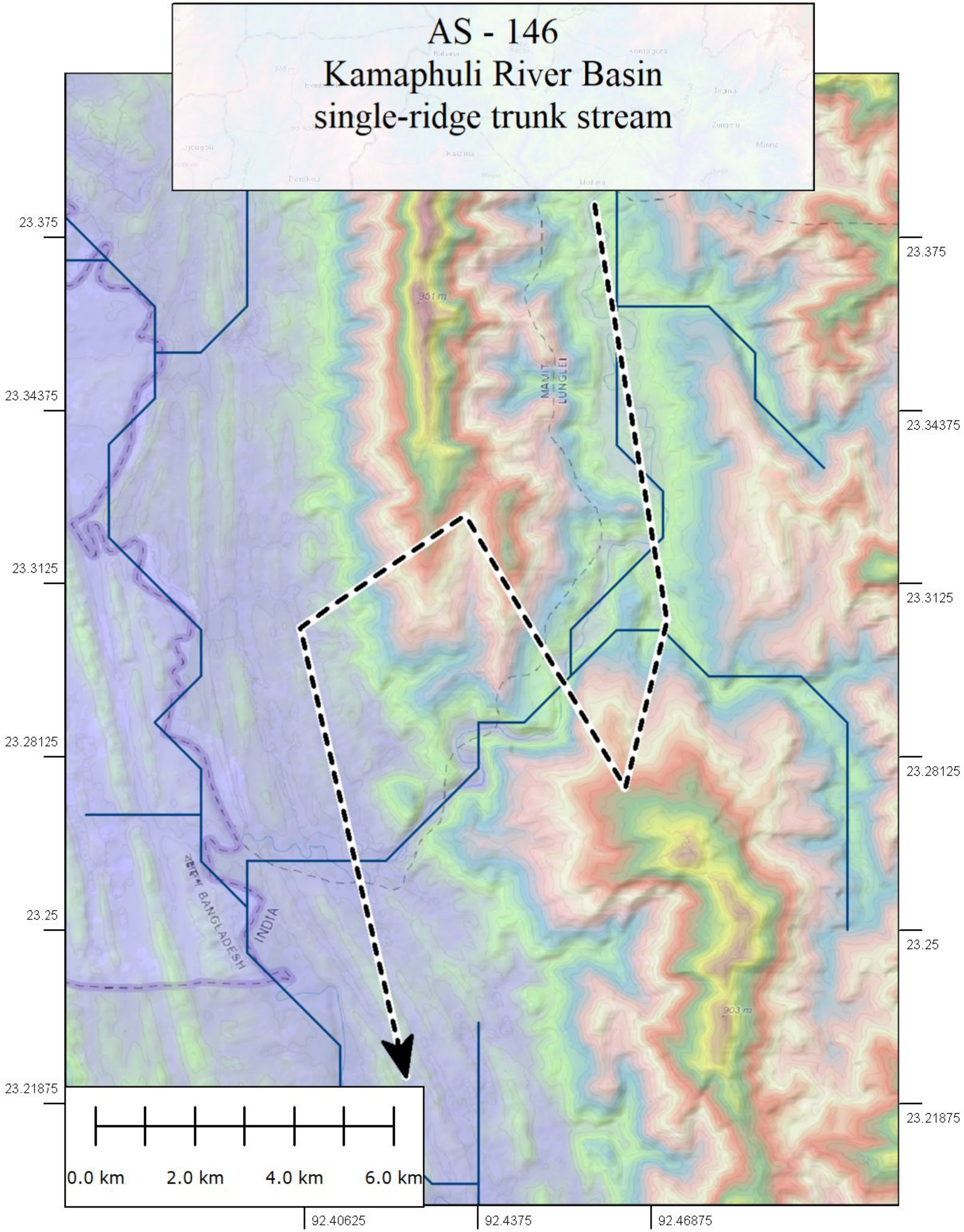

AS - 147
Irrawaddy River Basin
single-ridge trunk stream

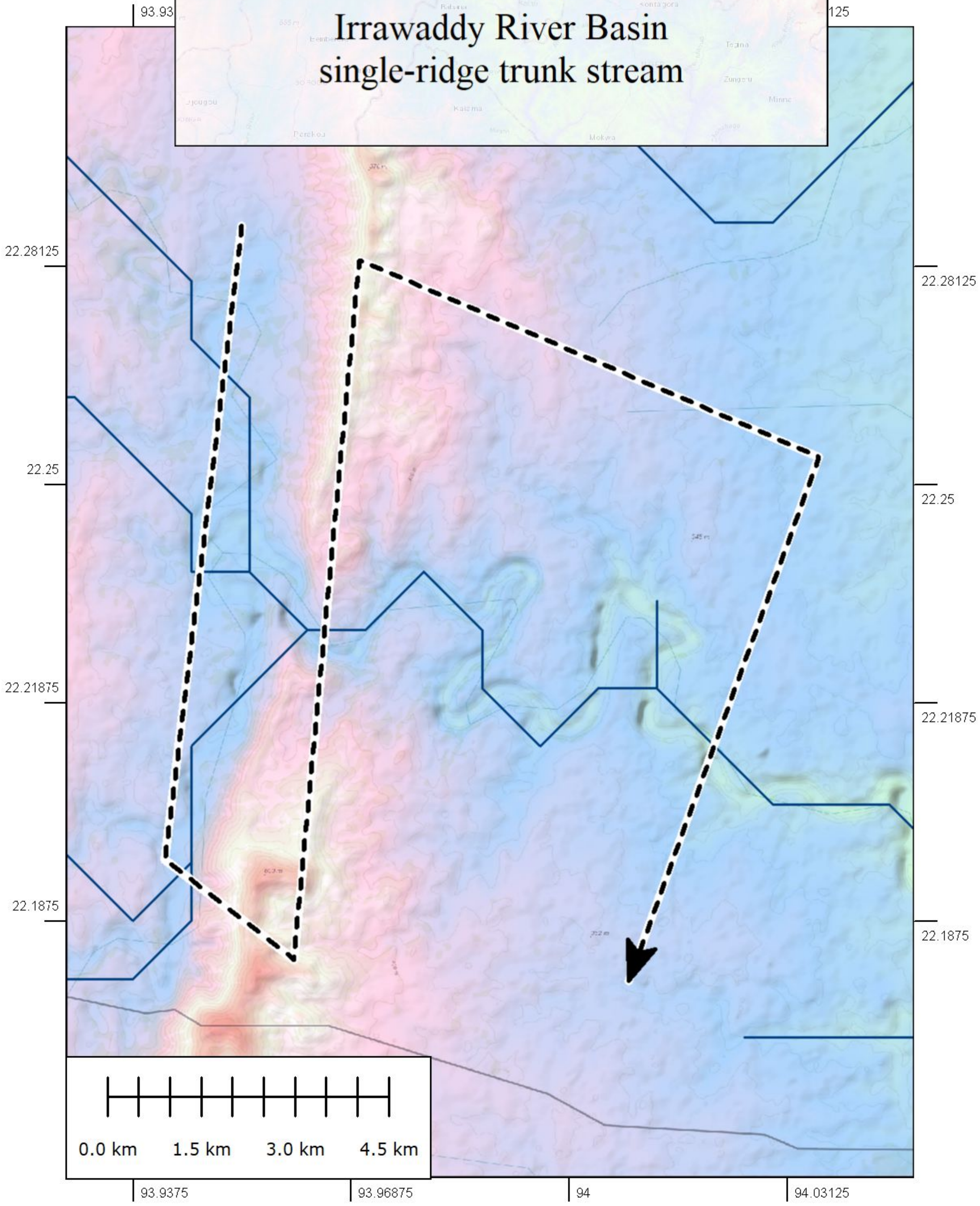

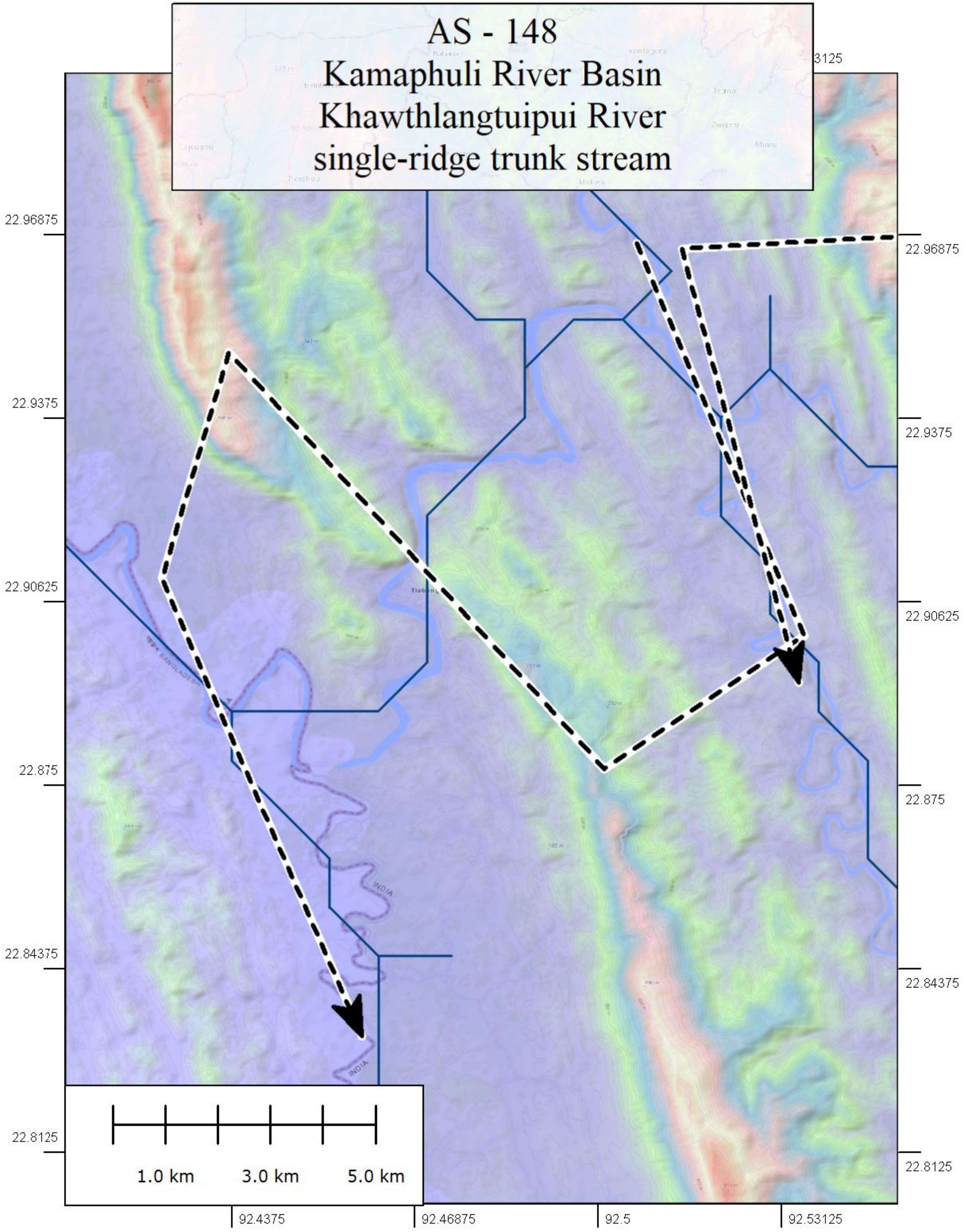
AS - 148
Kamaphuli River Basin
Khawthlangtuipui River
single-ridge trunk stream
22.96875
22.9375
22.90625
22.875
22.84375
22.8125
3125
22.96875
22.9375
22.90625
22.875
22.84375
22.8125
92.4375
92.46875
92.5
92.53125
1.0 km
3.0 km
5.0 km
BANGLADESH
INDIA
INDIA

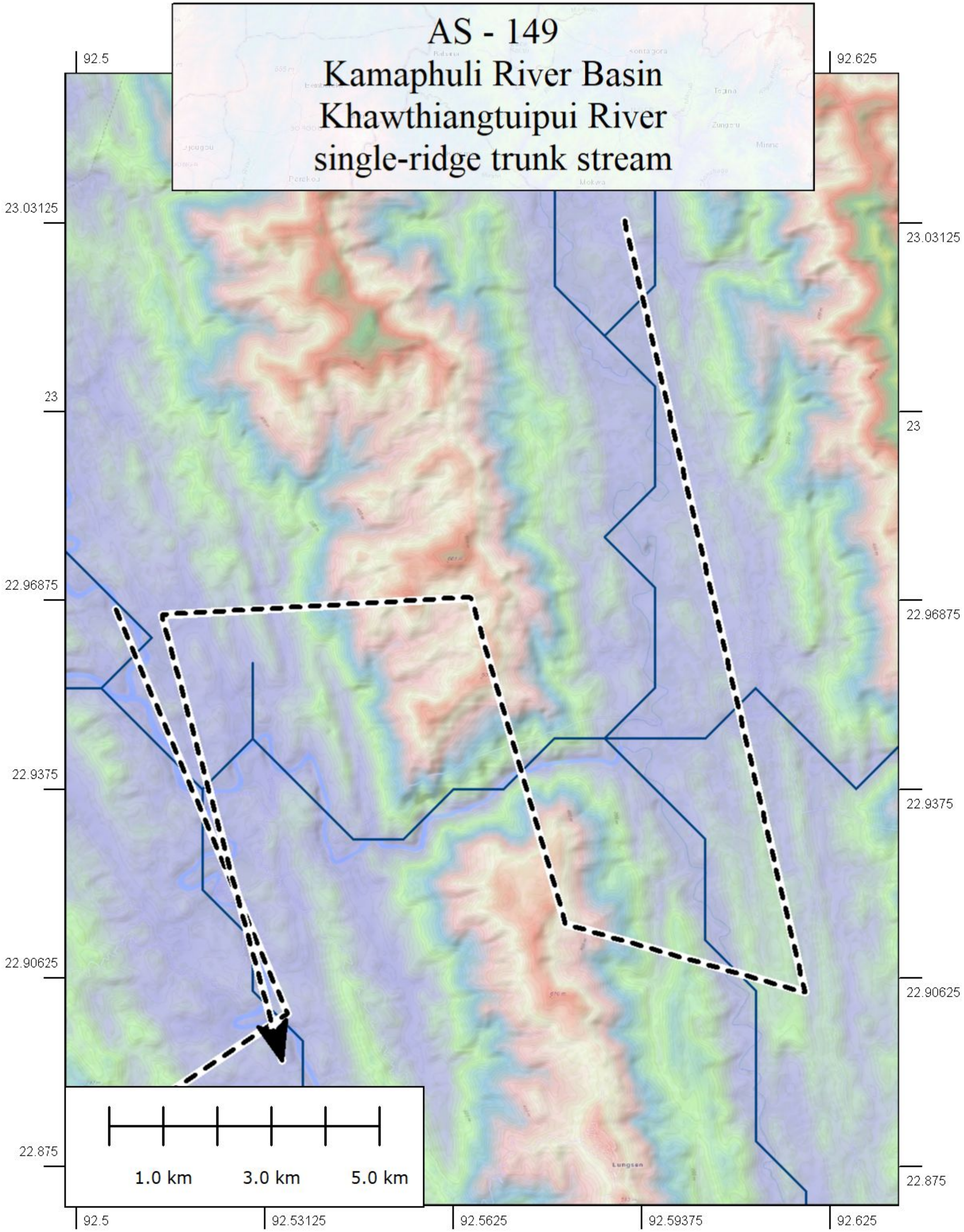
AS - 149
Kamaphuli River Basin
Khawthiangtuipui River
single-ridge trunk stream
92.5
92.53125
92.5625
92.59375
92.625
23.03125
23
22.96875
22.9375
22.90625
22.875
1.0 km
3.0 km
5.0 km

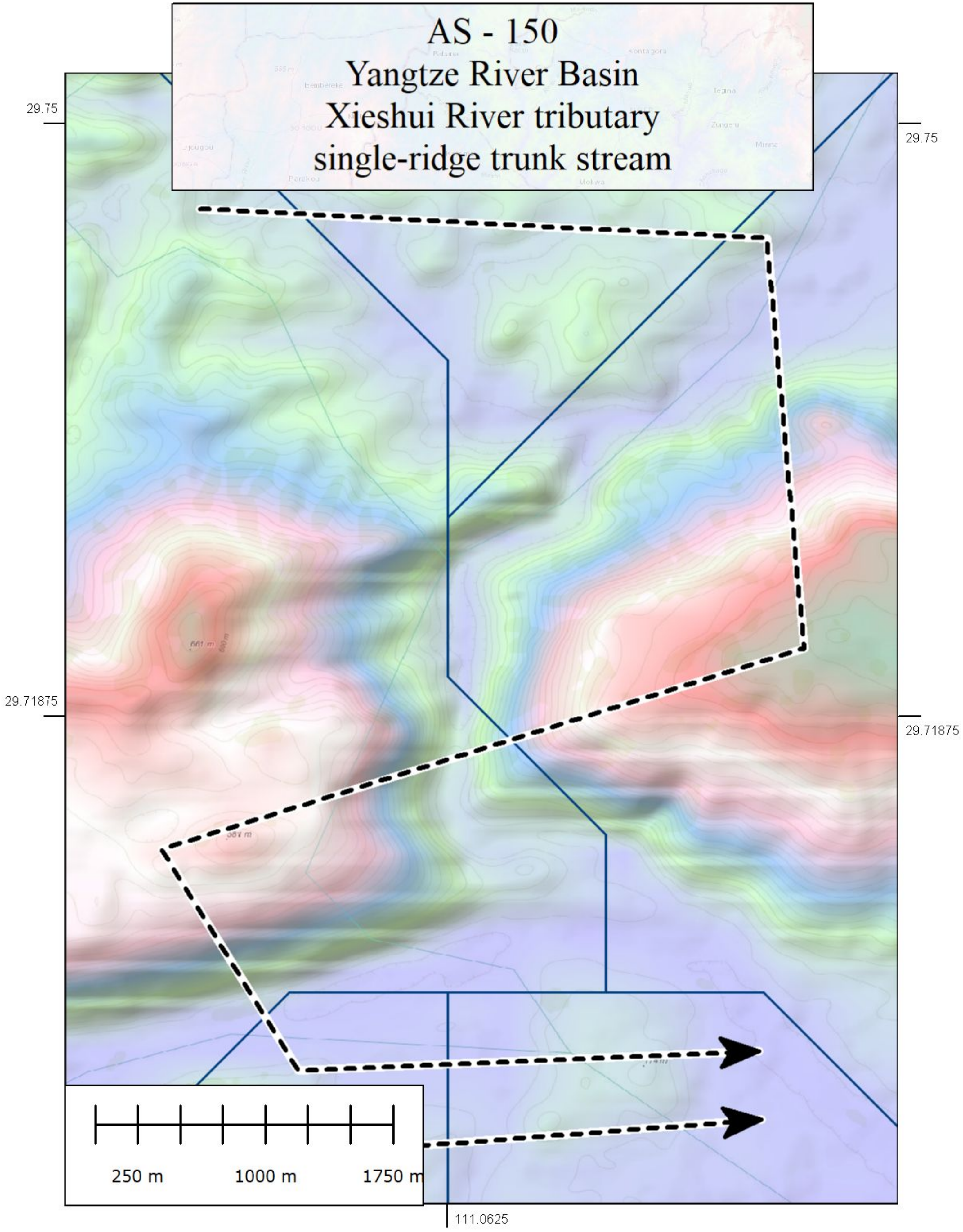
AS - 150
Yangtze River Basin
Xieshui River tributary
single-ridge trunk stream
29.75
29.75
29.71875
29.71875
111.0625
250 m
1000 m
1750 m

AS - 151
Mekong River Basin
Nam Ngiap River tributary
single-ridge trunk stream

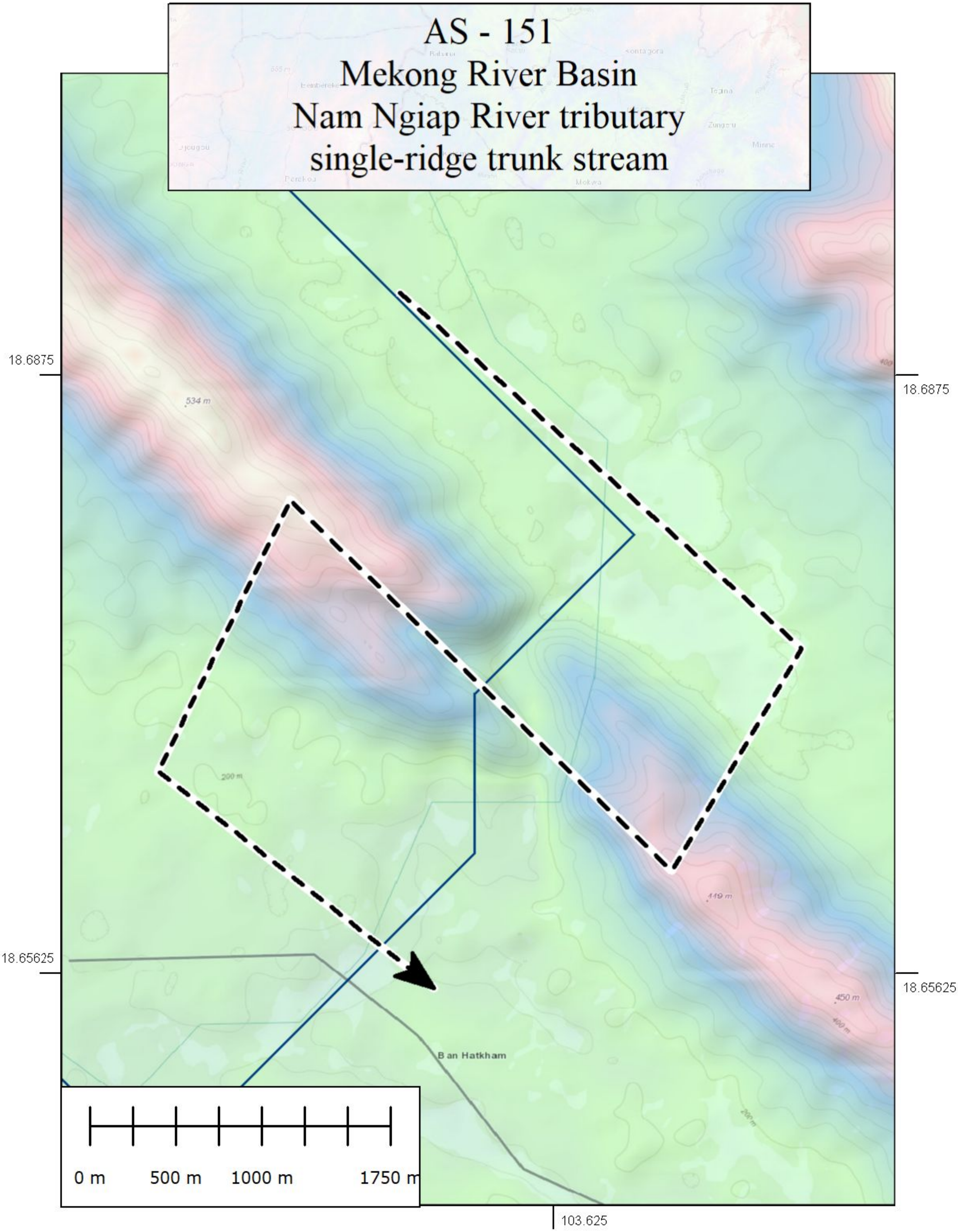

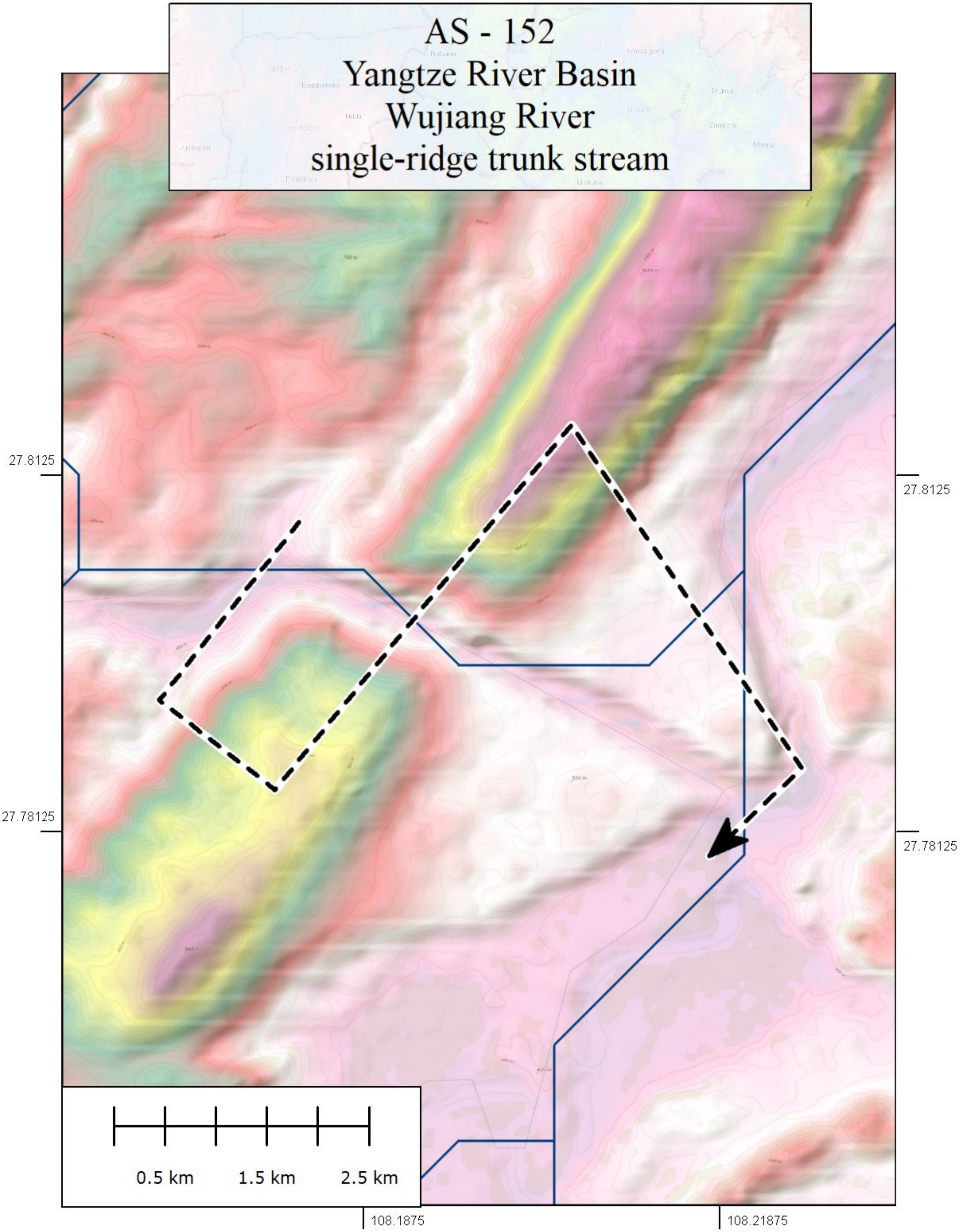
AS - 152
Yangtze River Basin
Wujiang River
single-ridge trunk stream
27.8125
27.8125
27.78125
27.78125
0.5 km
1.5 km
2.5 km
108.1875
108.21875

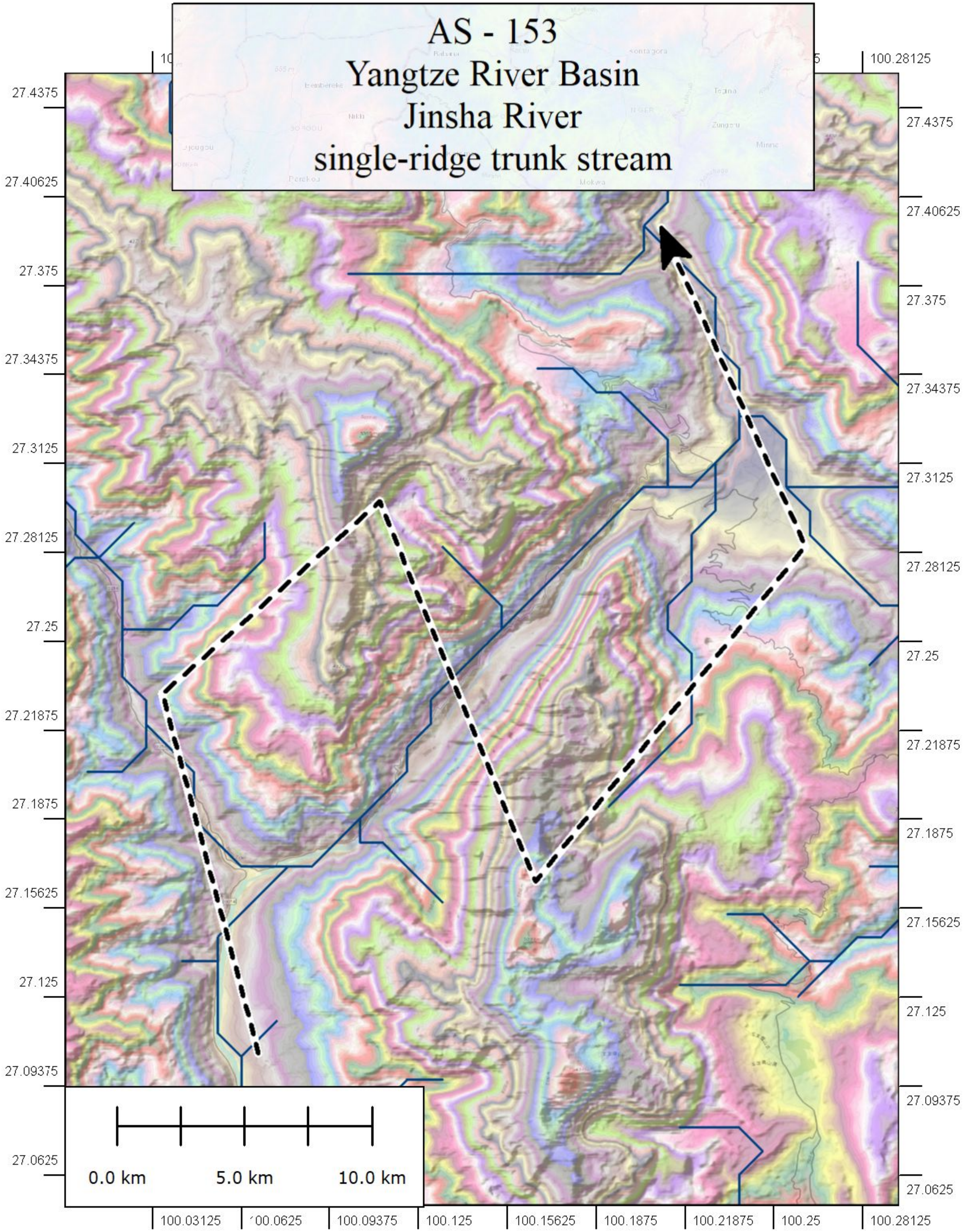

AS - 153
Yangtze River Basin
Jinsha River
single-ridge trunk stream
27.4375
27.40625
27.375
27.34375
27.3125
27.28125
27.25
27.21875
27.1875
27.15625
27.125
27.09375
27.0625
100.03125
100.0625
100.09375
100.125
100.15625
100.1875
100.21875
100.25
100.28125
0.0 km
5.0 km
10.0 km

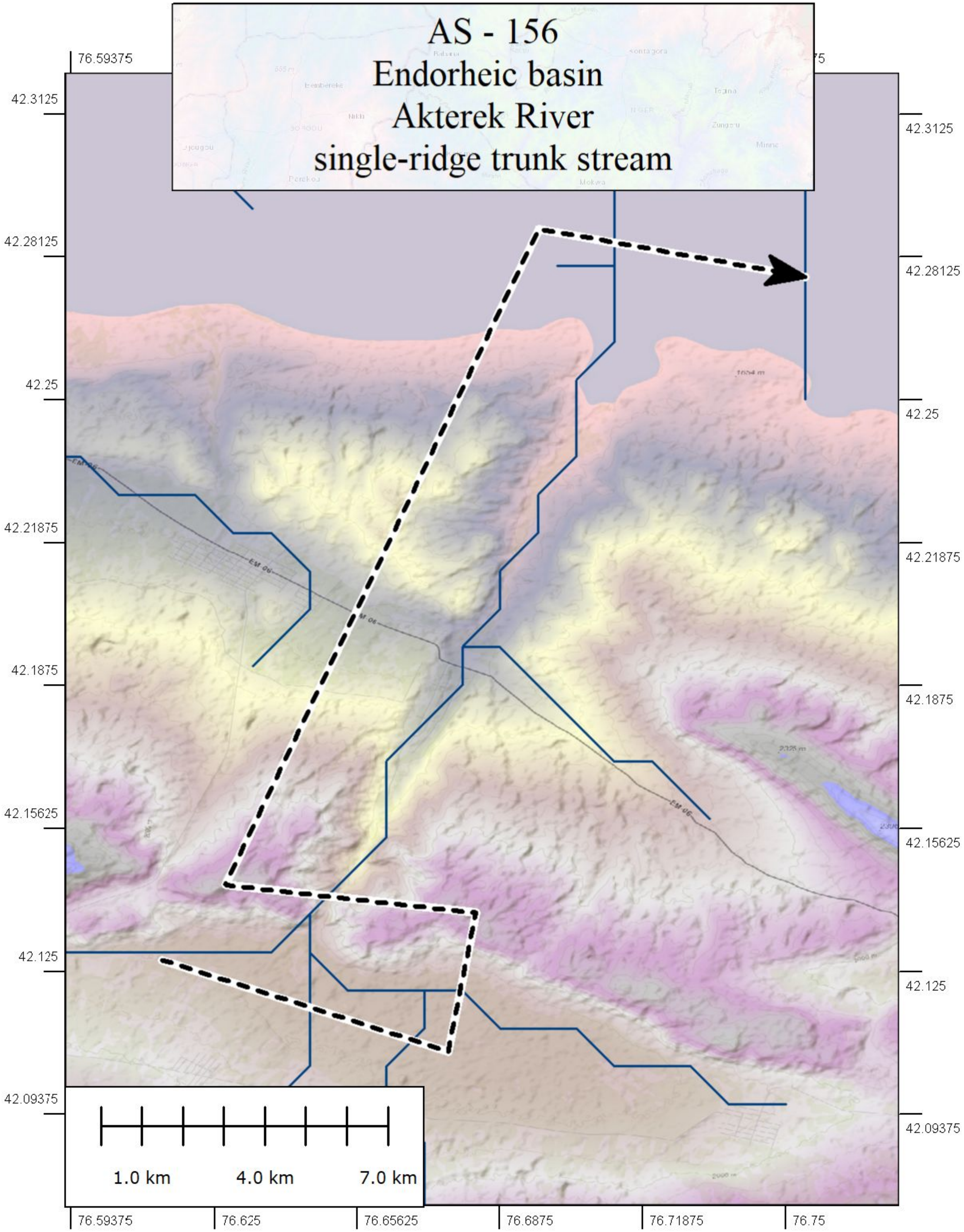
AS - 156
Endorheic basin
Akterek River
single-ridge trunk stream
76.59375
76.625
76.65625
76.6875
76.71875
76.75
42.3125
42.28125
42.25
42.21875
42.1875
42.15625
42.125
42.09375
1.0 km
4.0 km
7.0 km
EM 06

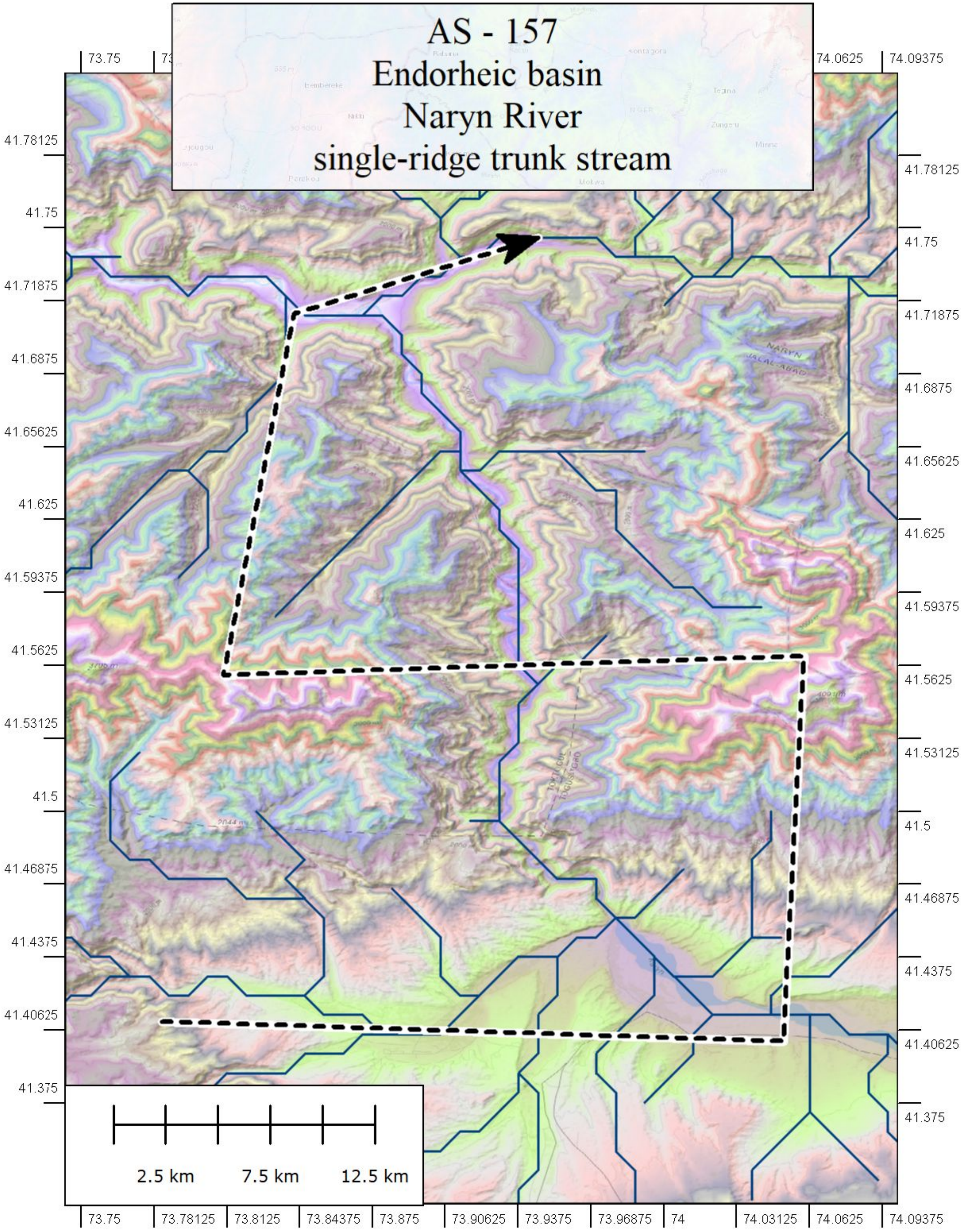
AS - 157
Endorheic basin
Naryn River
single-ridge trunk stream
73.75
73.78125
73.8125
73.84375
73.875
73.90625
73.9375
73.96875
74
74.03125
74.0625
74.09375
41.78125
41.75
41.71875
41.6875
41.65625
41.625
41.59375
41.5625
41.53125
41.5
41.46875
41.4375
41.40625
41.375
NARYN
JALAL-ABAD
2.5 km
7.5 km
12.5 km

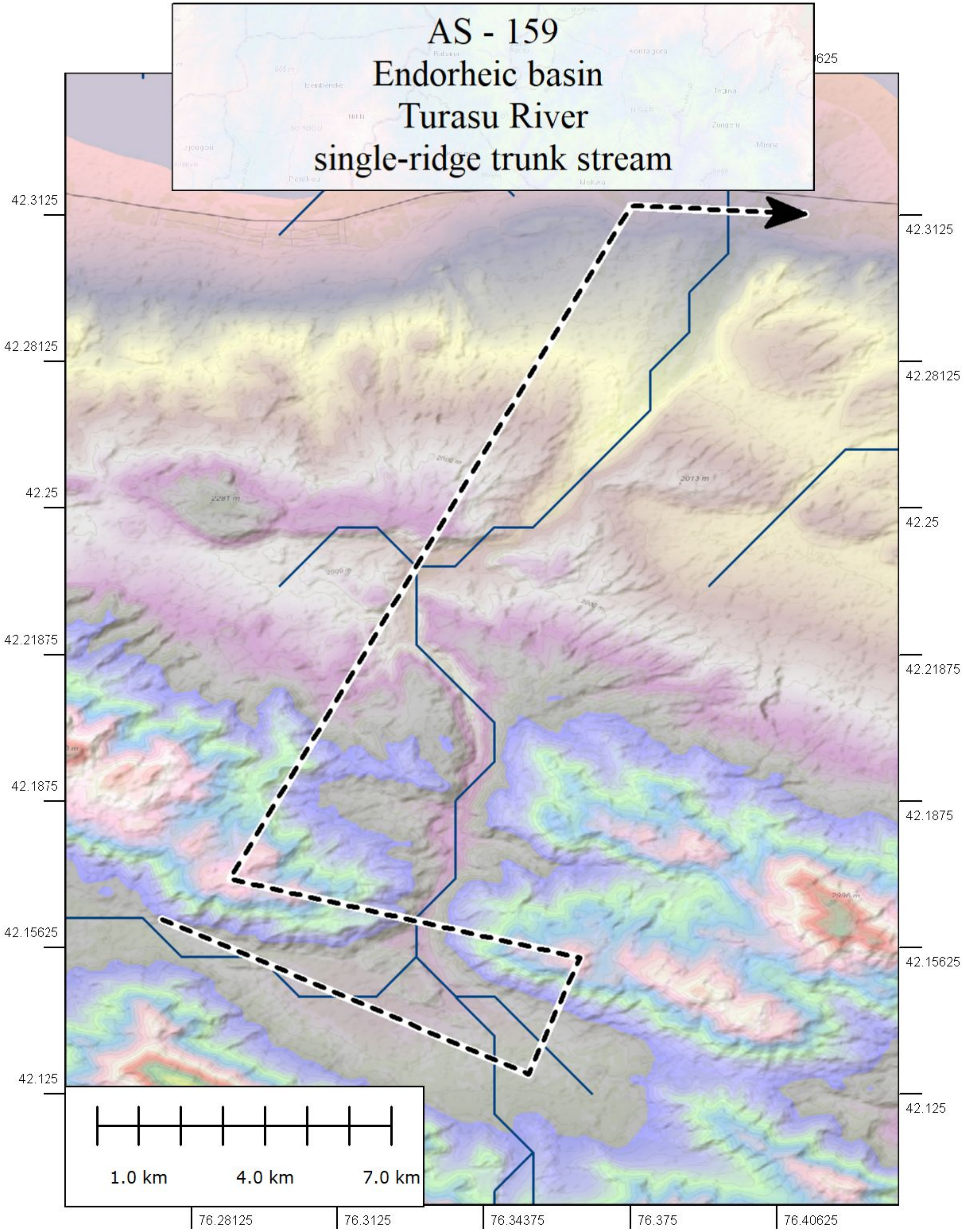
AS - 159
Endorheic basin
Turasu River
single-ridge trunk stream
42.3125
42.28125
42.25
42.21875
42.1875
42.15625
42.125
2281 m
2000 m
2013 m
2996 m
1.0 km
4.0 km
7.0 km
76.28125
76.3125
76.34375
76.375
76.40625

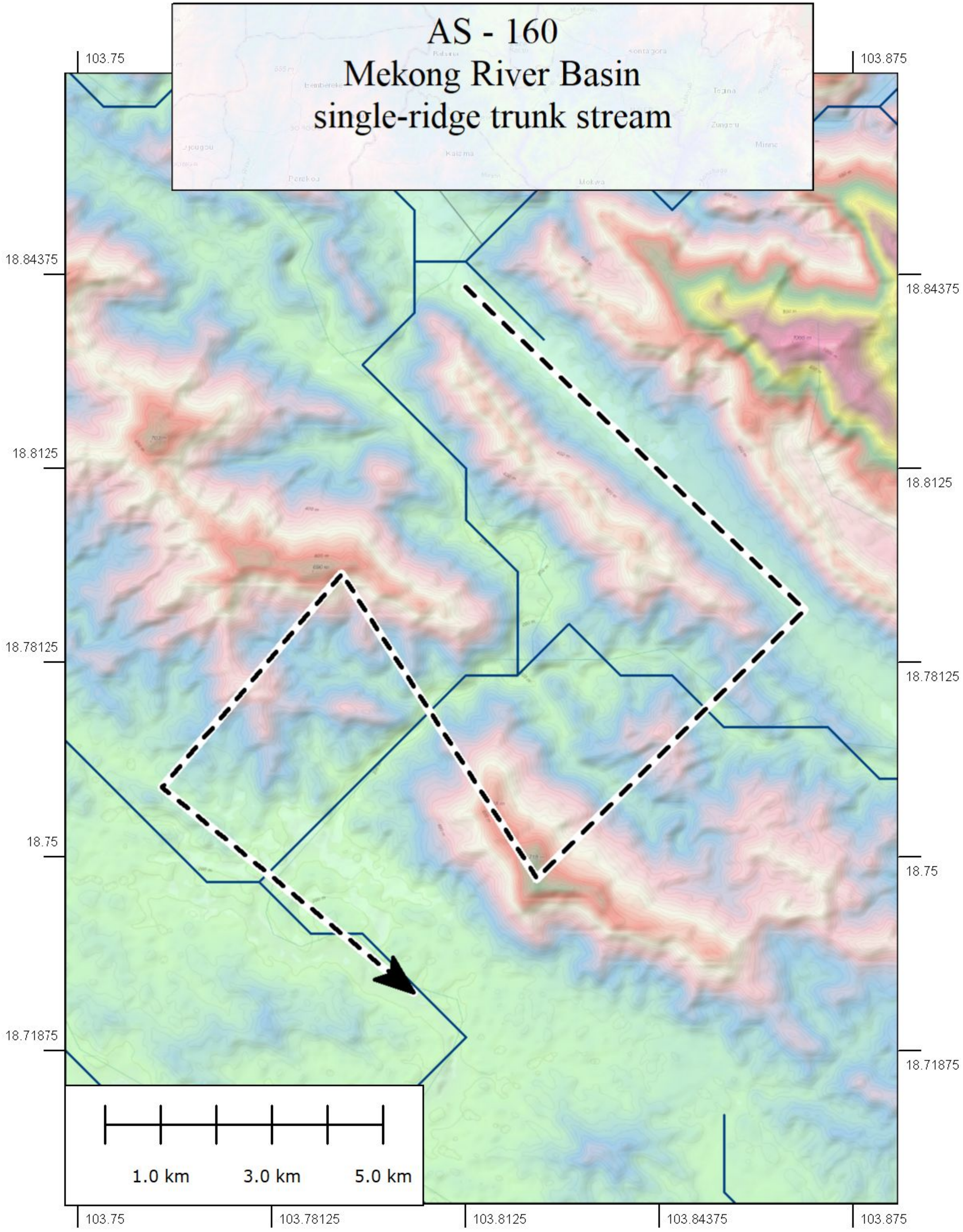
AS - 160
Mekong River Basin
single-ridge trunk stream
103.75
103.875
18.84375
18.8125
18.78125
18.75
18.71875
1.0 km
3.0 km
5.0 km
103.75
103.78125
103.8125
103.84375
103.875

AS - 161
Brahmani River Basin
Brahmani River tributary
single-ridge head stream

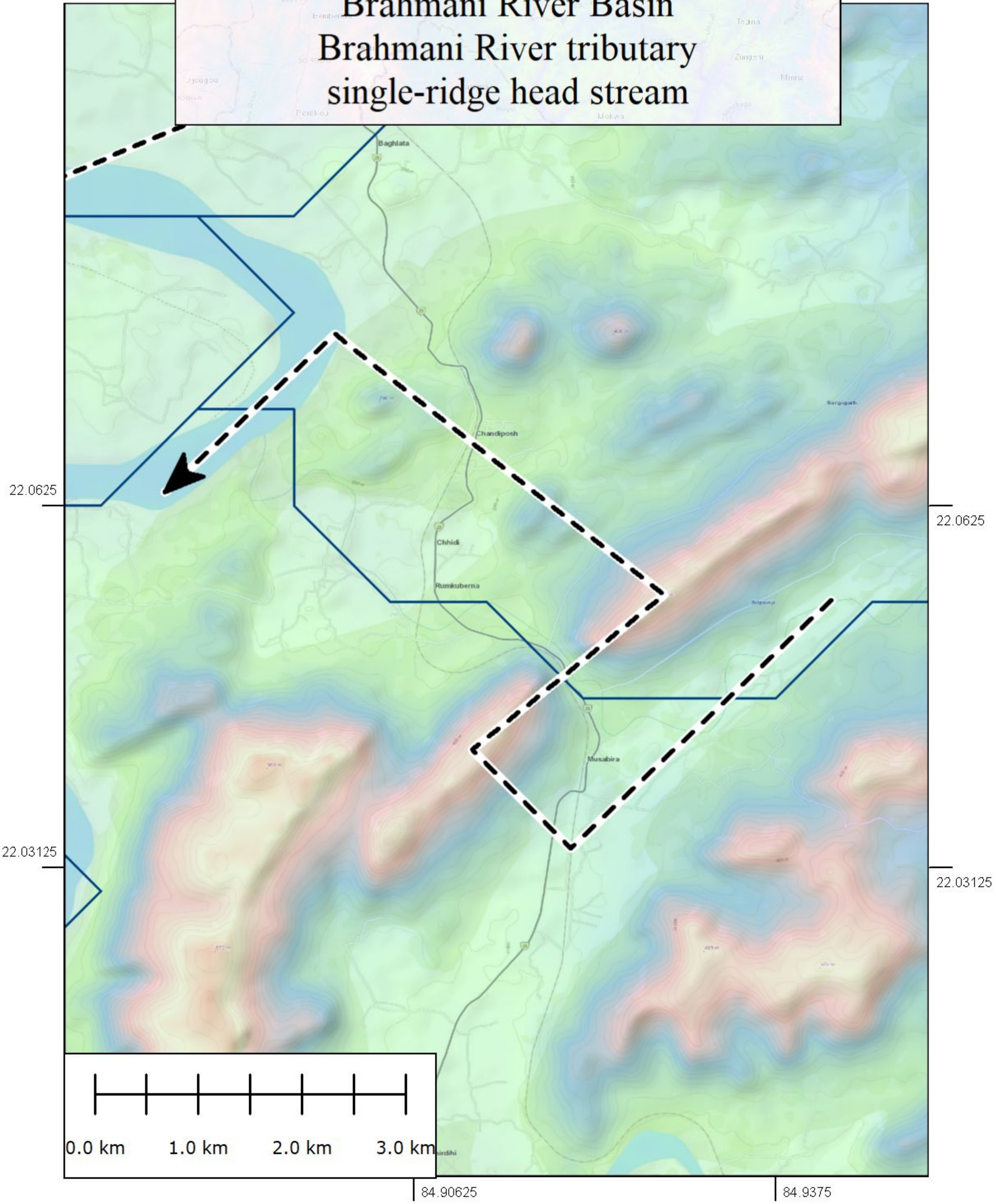

AS - 164
Hai River Basin
Nanyang River
single-ridge trunk stream

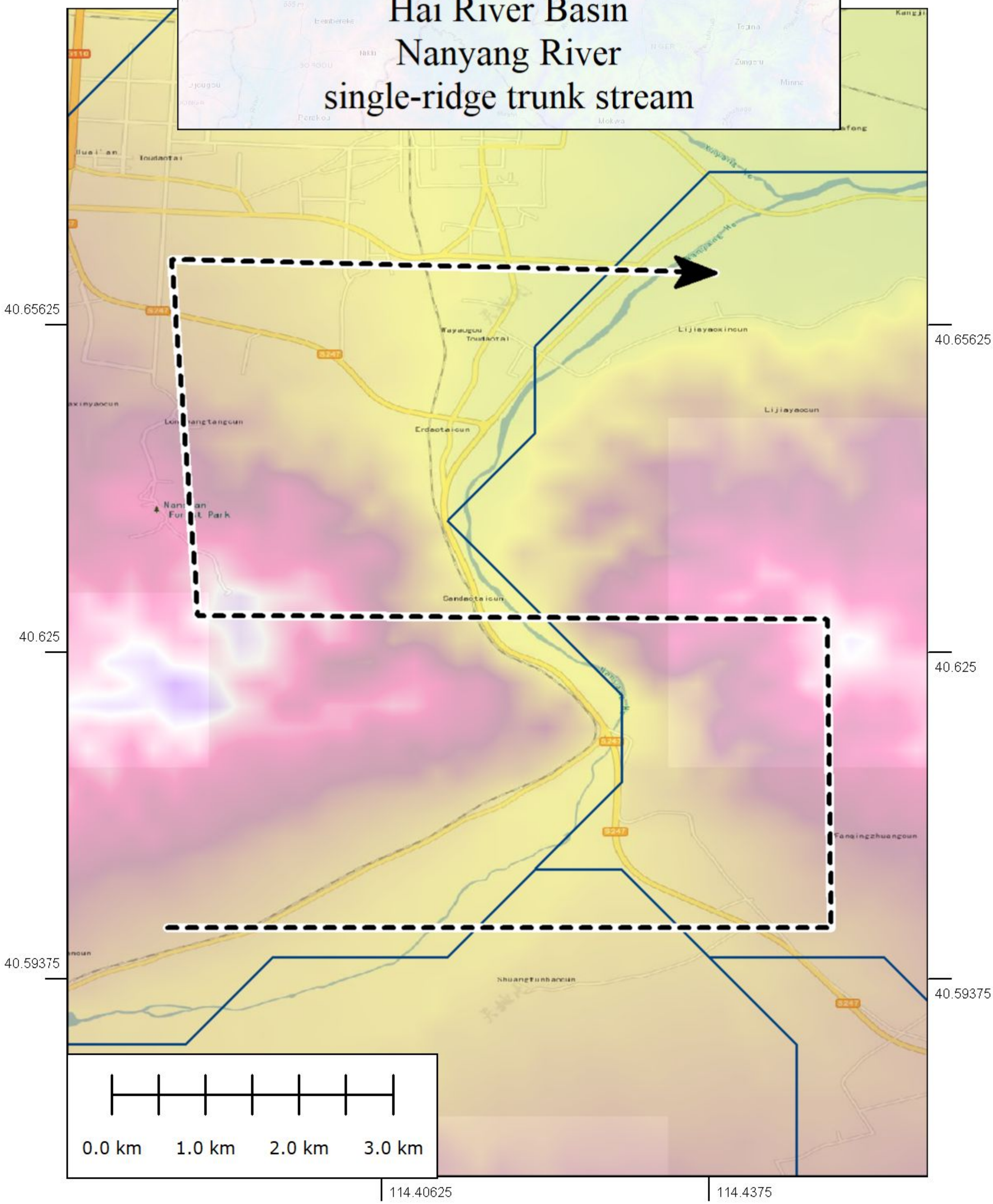

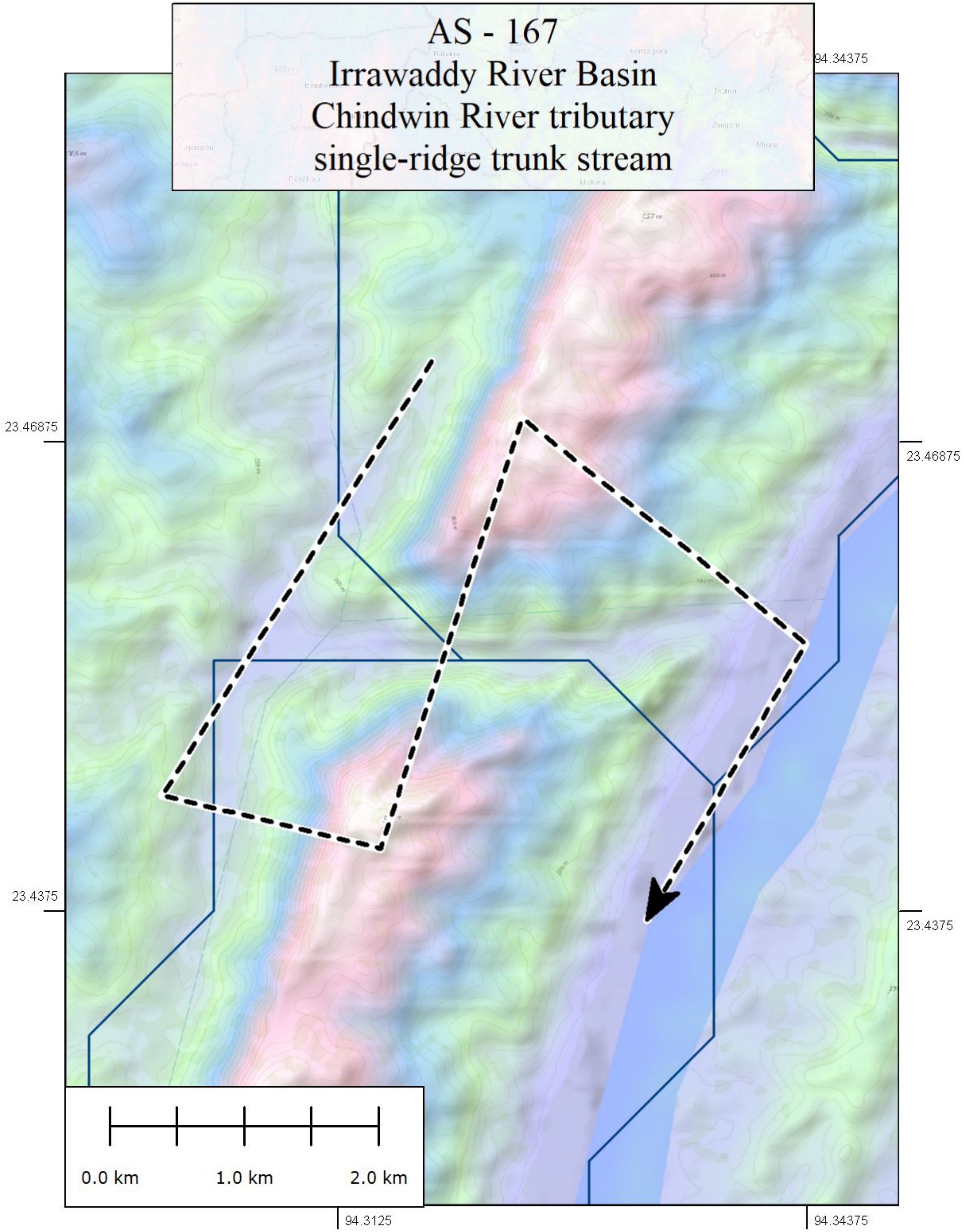
AS - 167
Irrawaddy River Basin
Chindwin River tributary
single-ridge trunk stream
94.34375
23.46875
23.46875
23.4375
23.4375
0.0 km
1.0 km
2.0 km
94.3125
94.34375

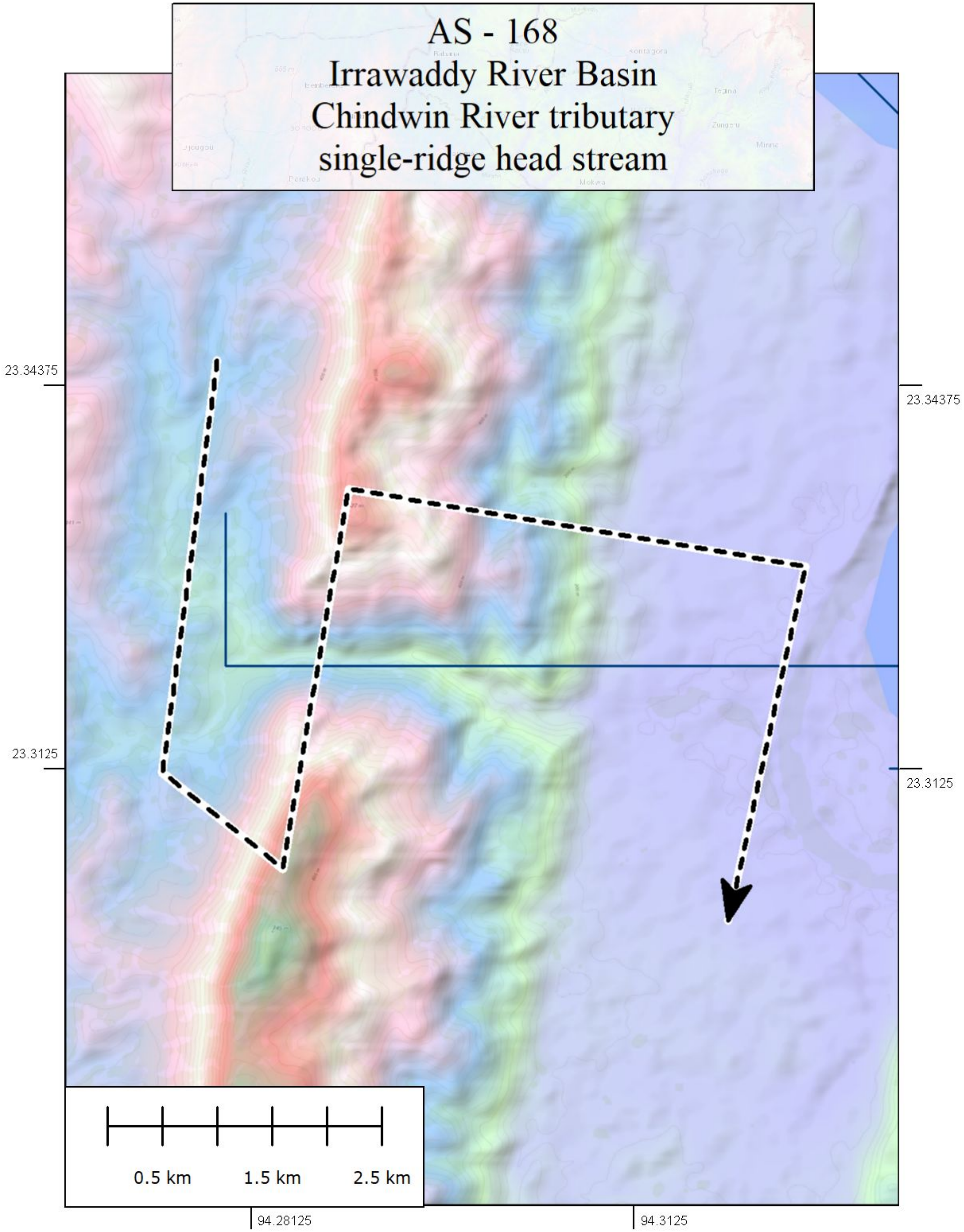
AS - 168
Irrawaddy River Basin
Chindwin River tributary
single-ridge head stream
23.34375
23.34375
23.3125
23.3125
0.5 km
1.5 km
2.5 km
94.28125
94.3125

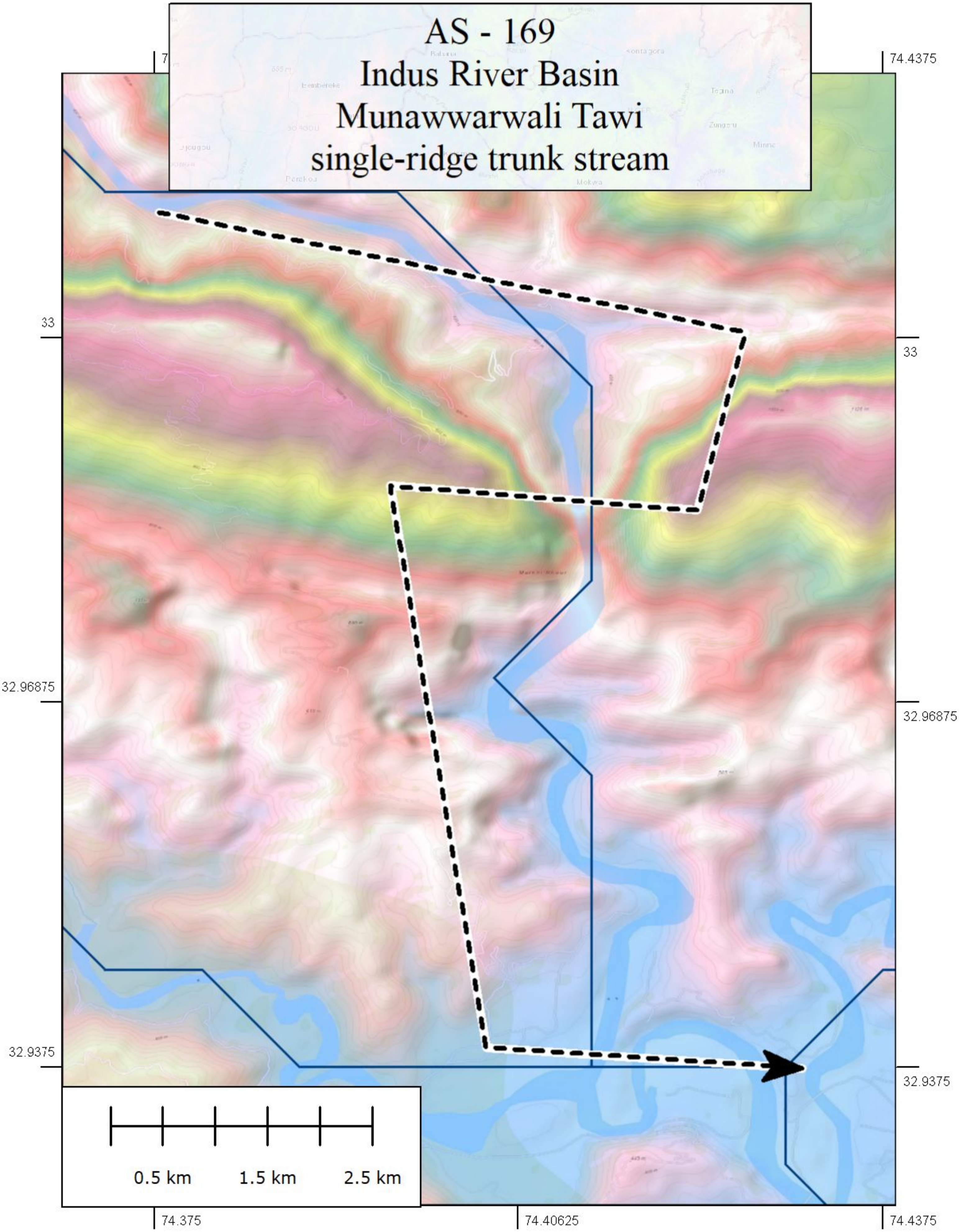
AS - 169
Indus River Basin
Munawwarwali Tawi
single-ridge trunk stream
74.4375
33
33
32.96875
32.96875
32.9375
32.9375
0.5 km
1.5 km
2.5 km
74.375
74.40625
74.4375

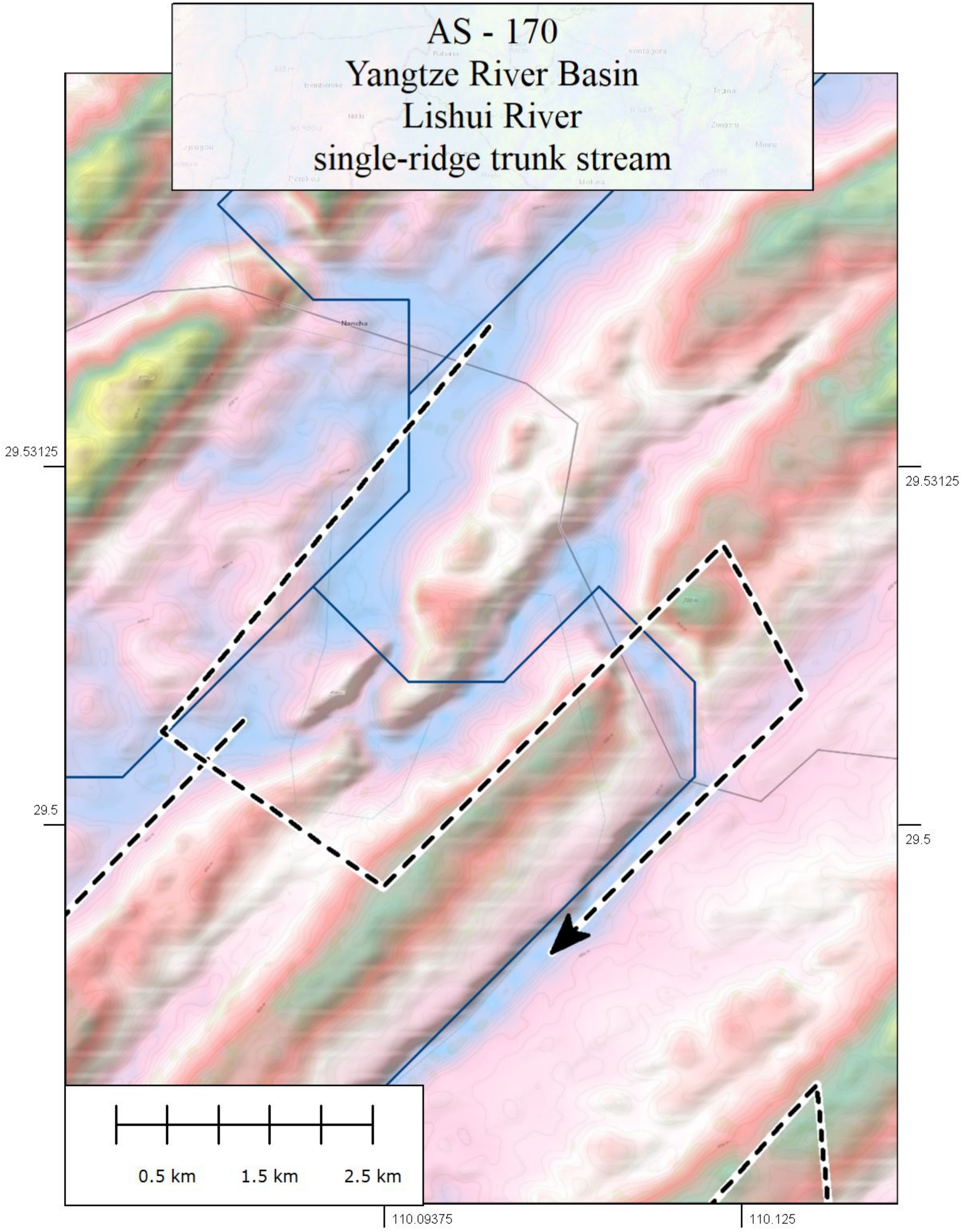
AS - 170
Yangtze River Basin
Lishui River
single-ridge trunk stream
Nancha
29.53125
29.53125
29.5
29.5
0.5 km
1.5 km
2.5 km
110.09375
110.125

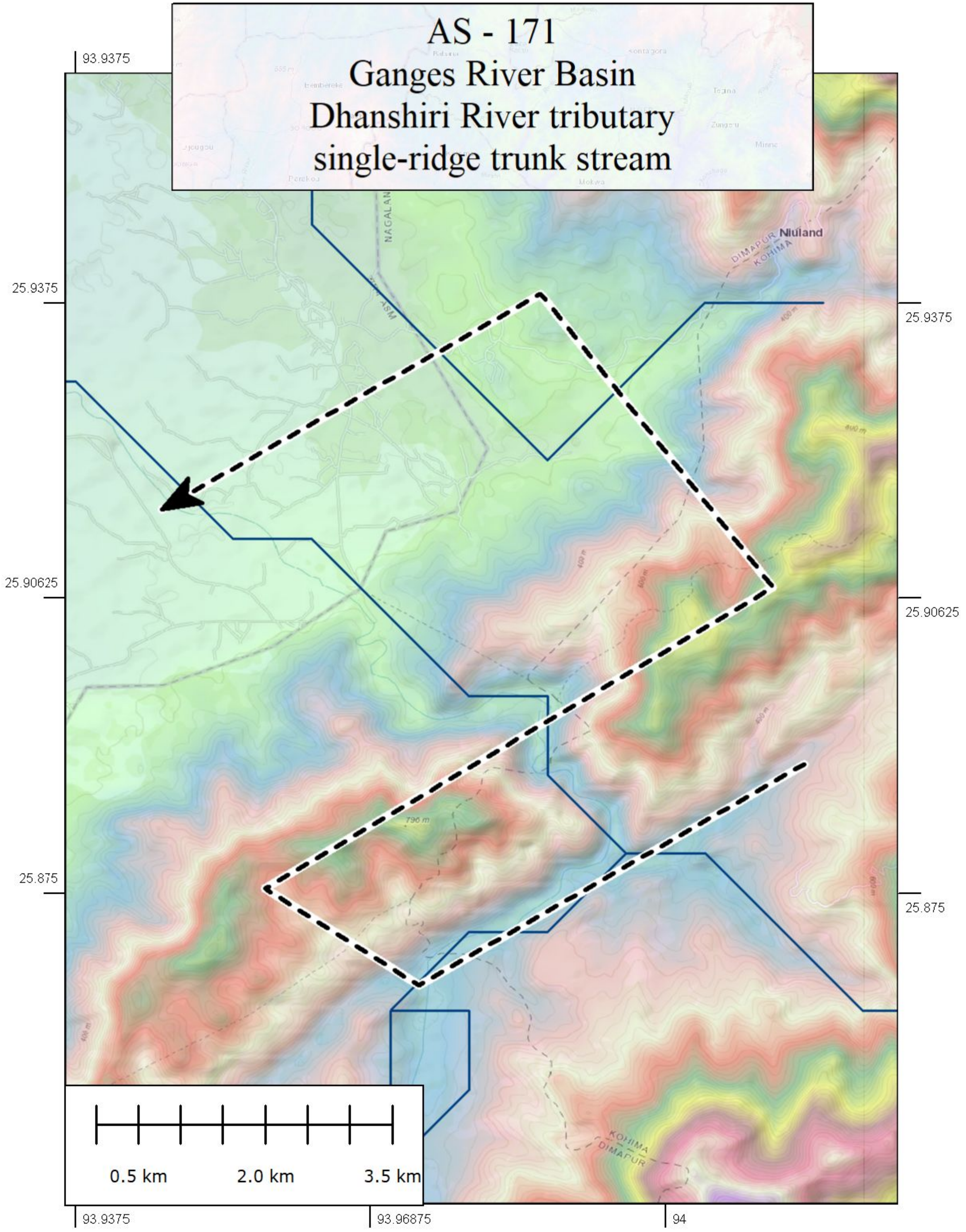

AS - 171
Ganges River Basin
Dhanshiri River tributary
single-ridge trunk stream
93.9375
93.96875
94
25.9375
25.90625
25.875
0.5 km
2.0 km
3.5 km
Niuland
DIMAPUR KOHIMA
KOHIMA DIMAPUR
NAGALAND
796 m

AS - 172
Yangtze River Basin
Youshui River
single-ridge trunk stream

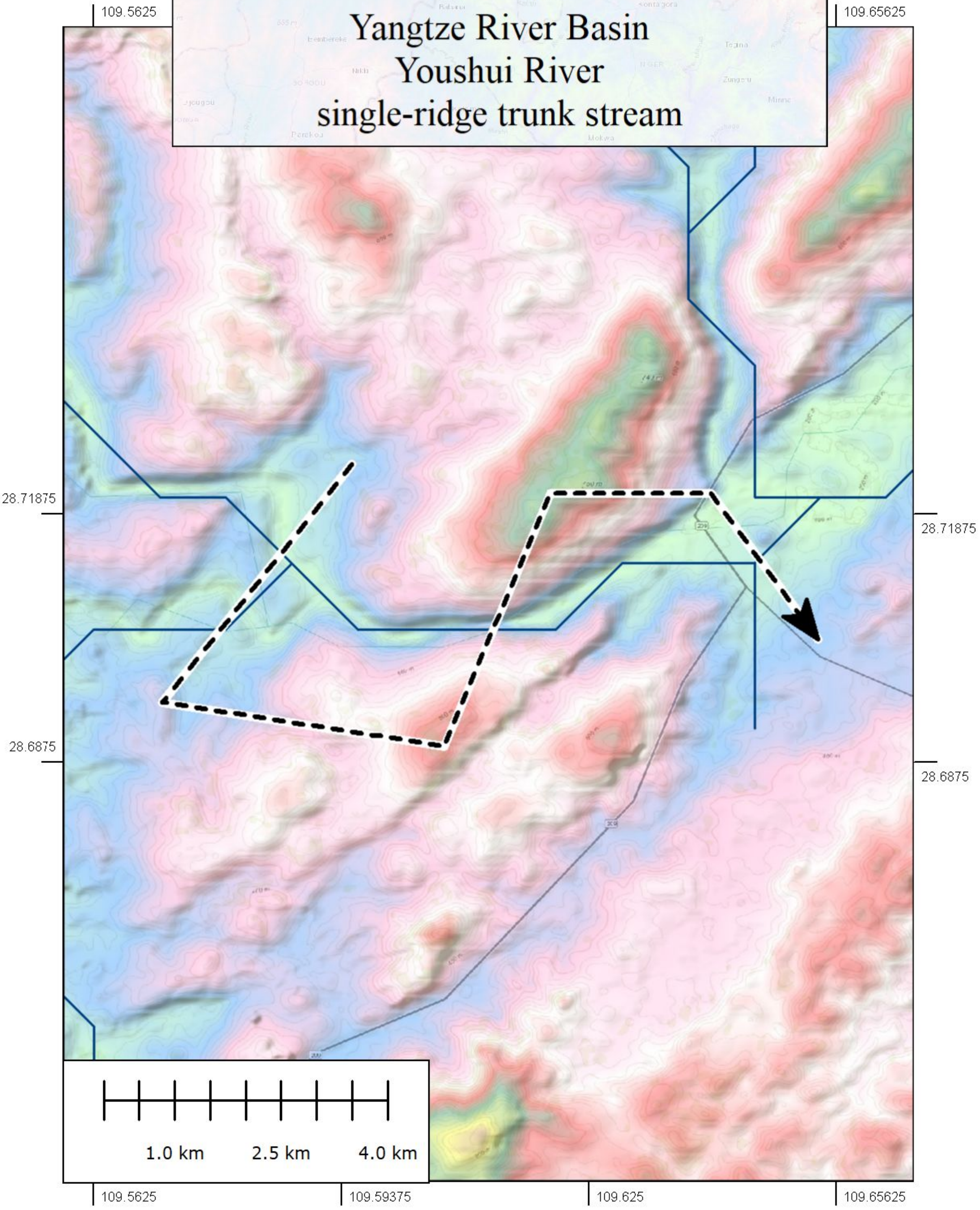

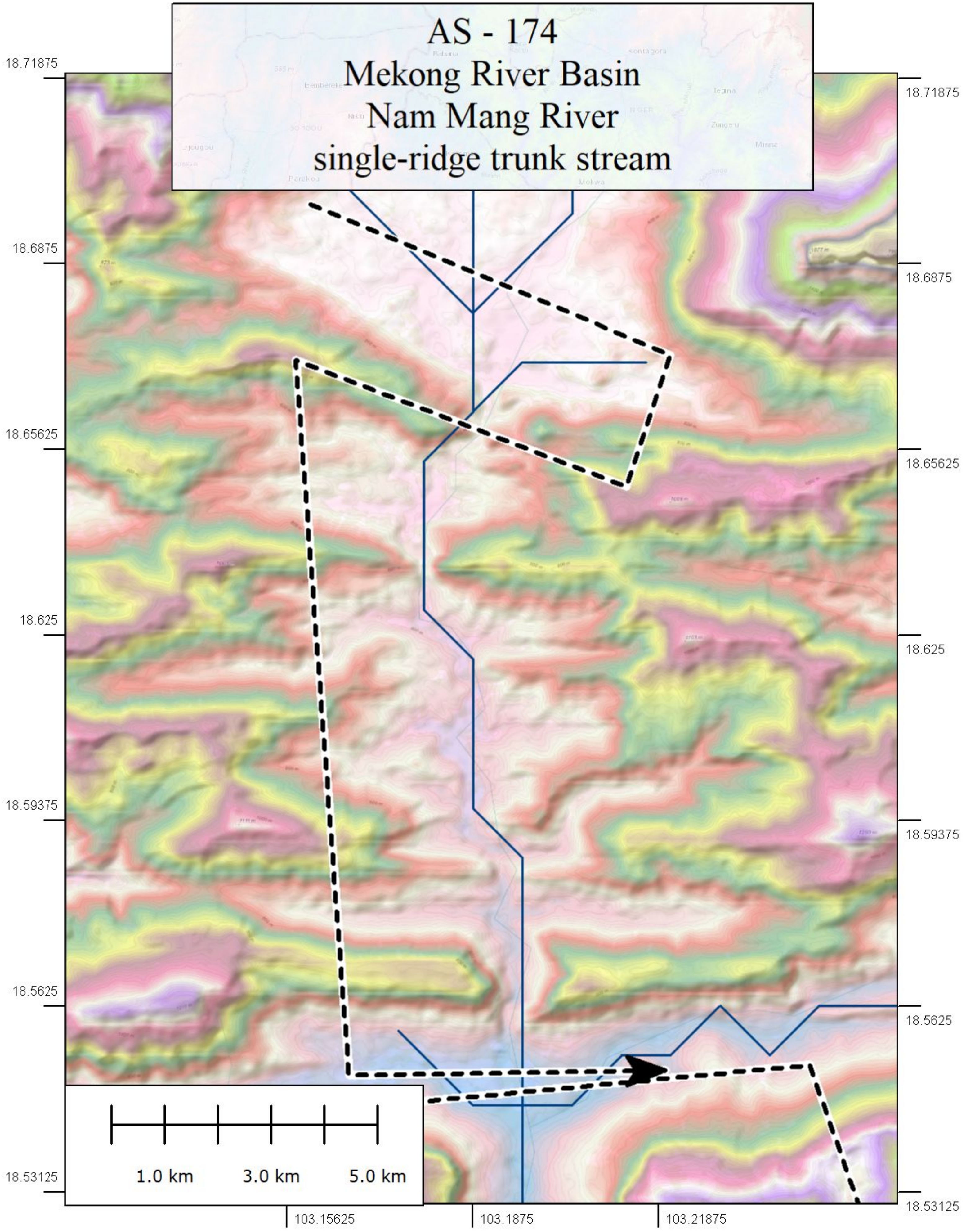
AS - 174
Mekong River Basin
Nam Mang River
single-ridge trunk stream
18.71875
18.6875
18.65625
18.625
18.59375
18.5625
18.53125
103.15625
103.1875
103.21875
1.0 km
3.0 km
5.0 km

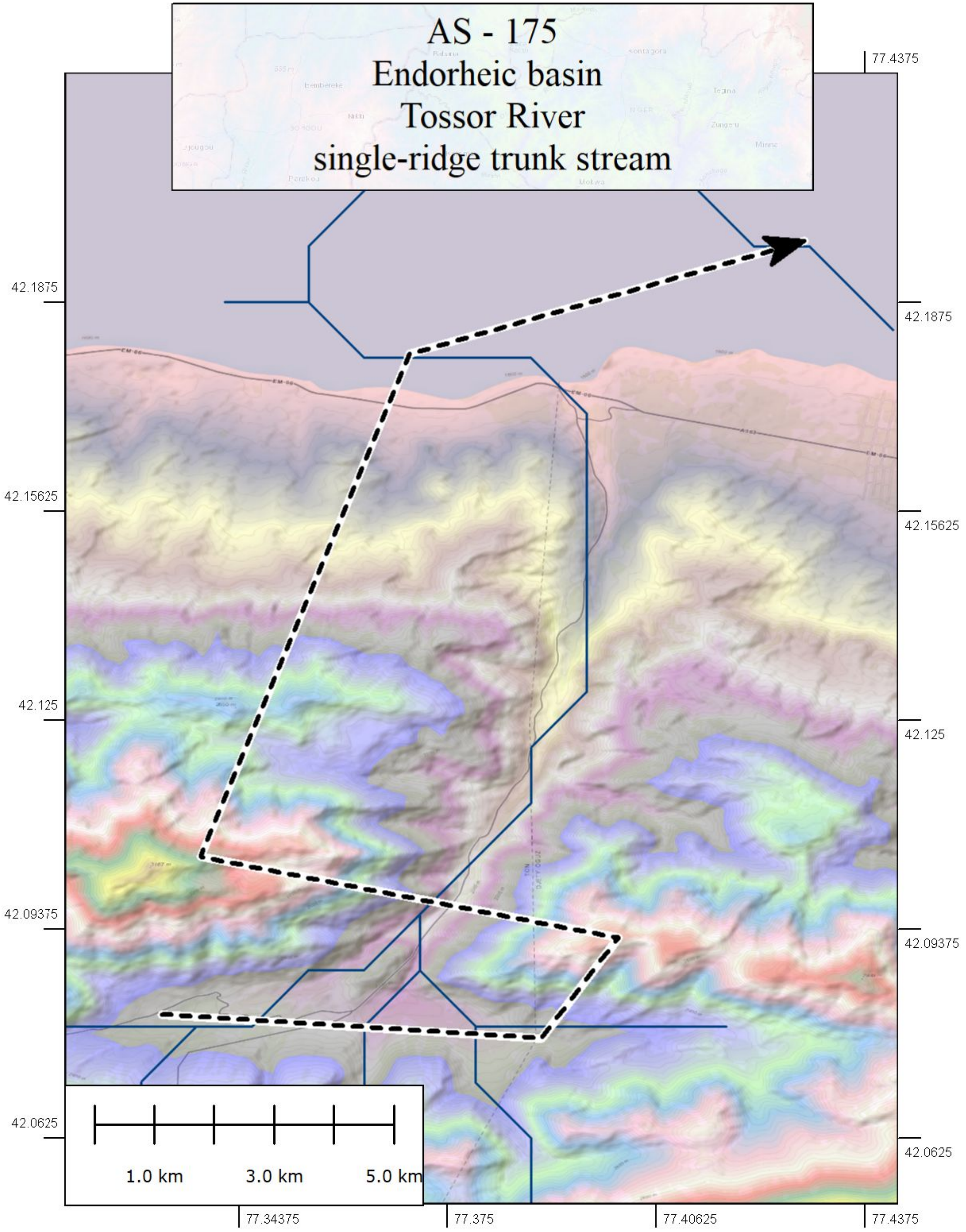

AS - 175
Endorheic basin
Tossor River
single-ridge trunk stream
42.1875
42.15625
42.125
42.09375
42.0625
77.4375
77.34375
77.375
77.40625
1.0 km
3.0 km
5.0 km

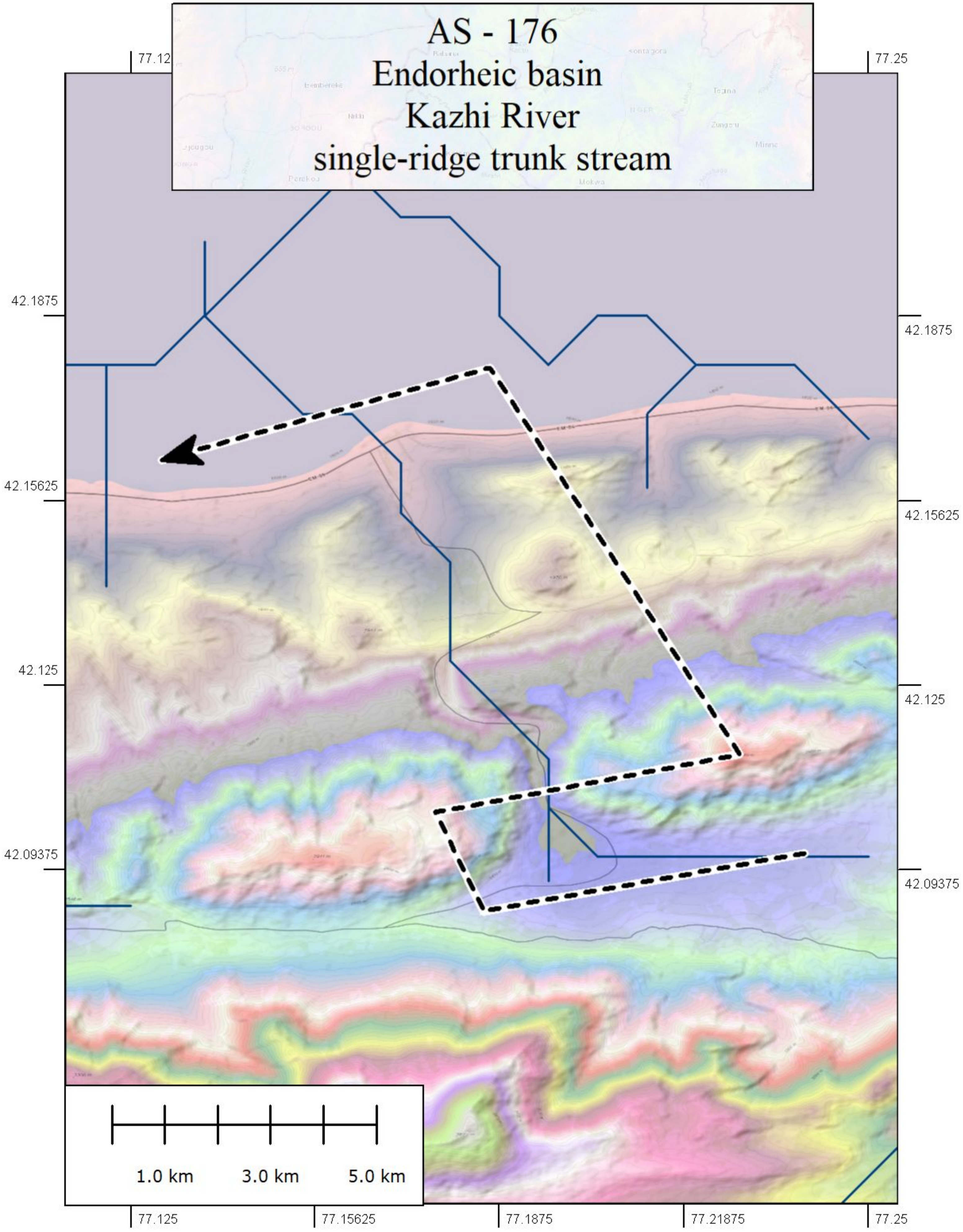
AS - 176
Endorheic basin
Kazhi River
single-ridge trunk stream
77.125
77.15625
77.1875
77.21875
77.25
42.1875
42.15625
42.125
42.09375
1.0 km
3.0 km
5.0 km

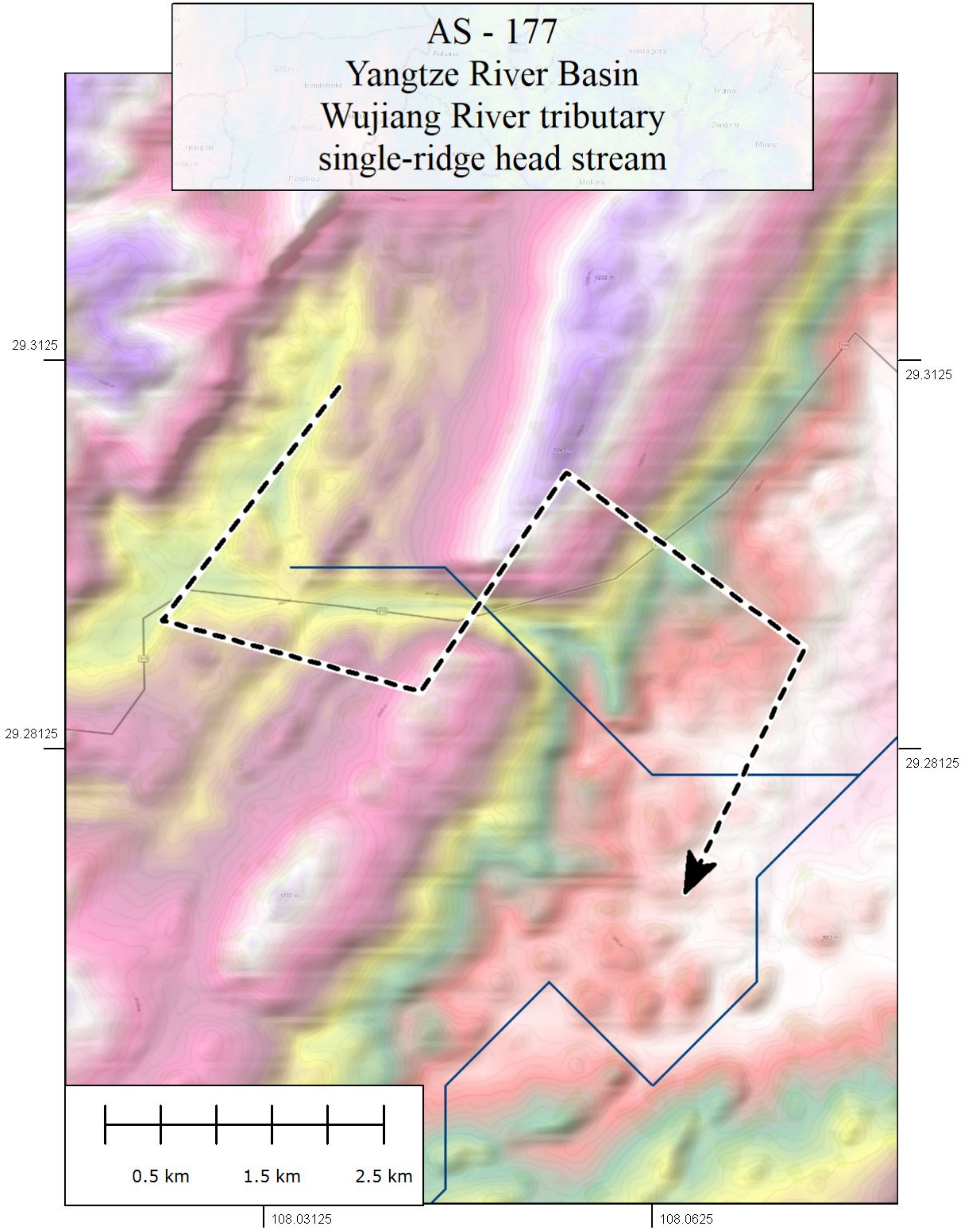

AS - 177
Yangtze River Basin
Wujiang River tributary
single-ridge head stream
29.3125
29.3125
29.28125
29.28125
0.5 km
1.5 km
2.5 km
108.03125
108.0625

AS - 178
Yangtze River Basin
Ganlong River
single-ridge trunk stream

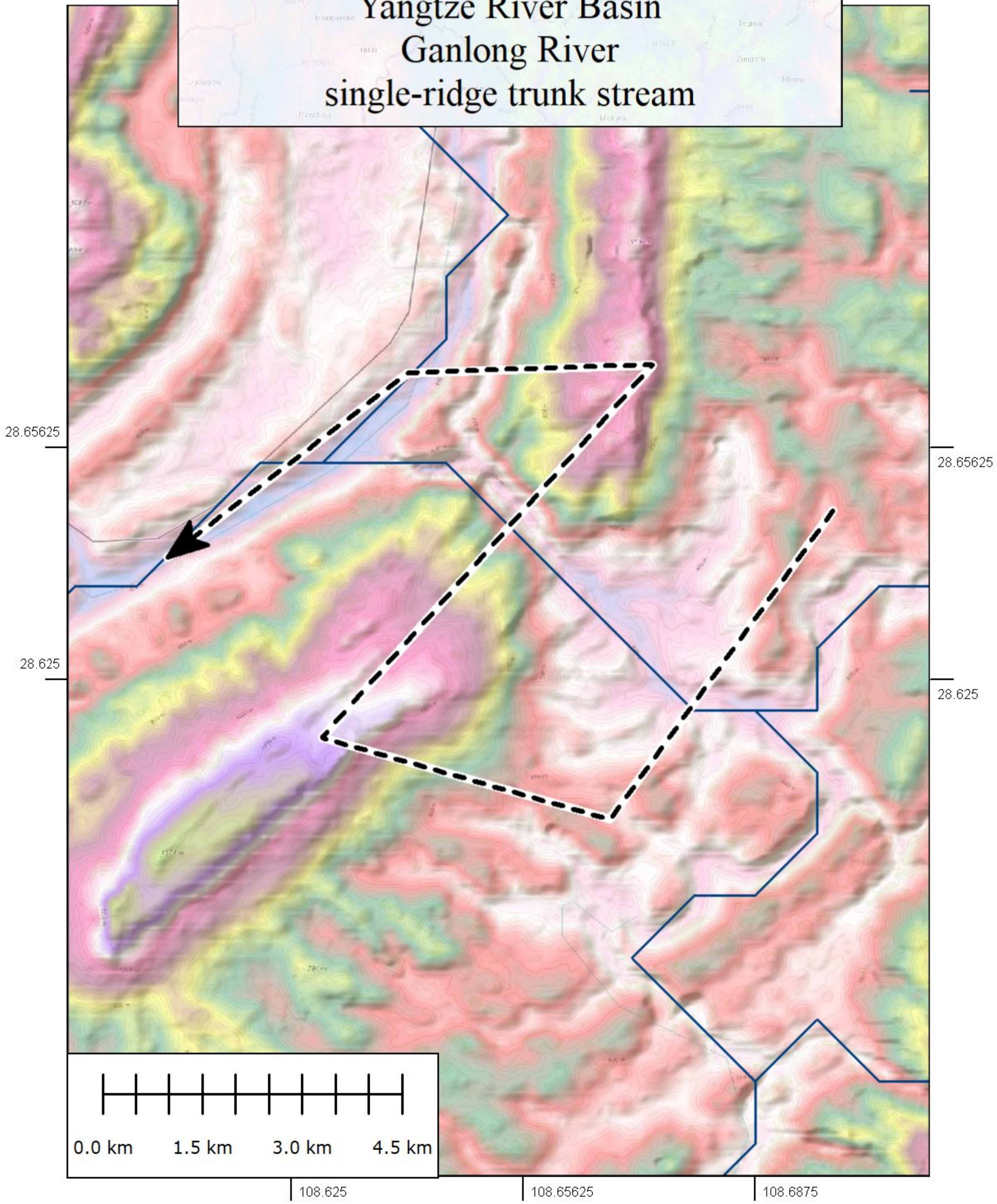

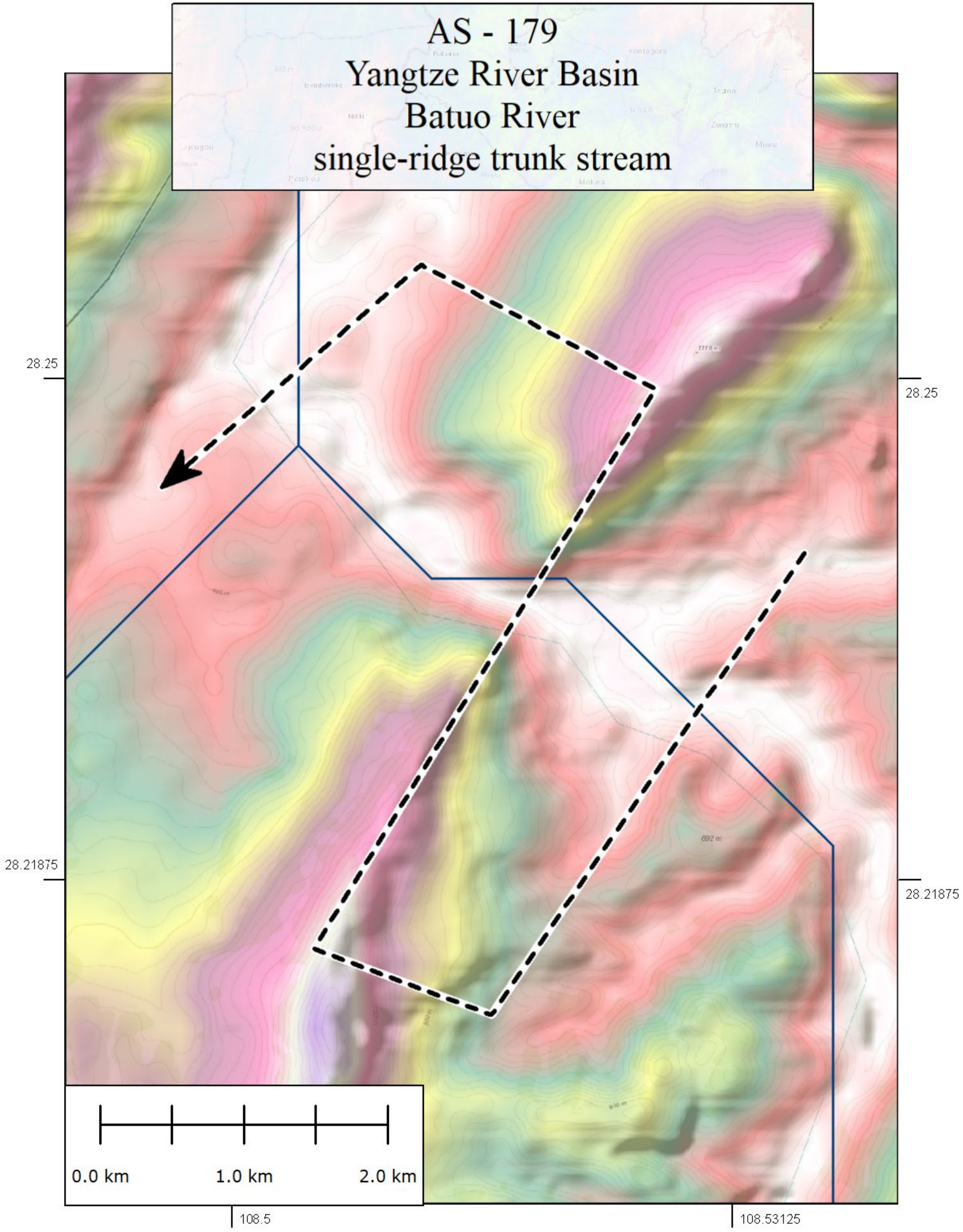
AS - 179
Yangtze River Basin
Batuo River
single-ridge trunk stream
28.25
28.25
28.21875
28.21875
0.0 km
1.0 km
2.0 km
108.5
108.53125

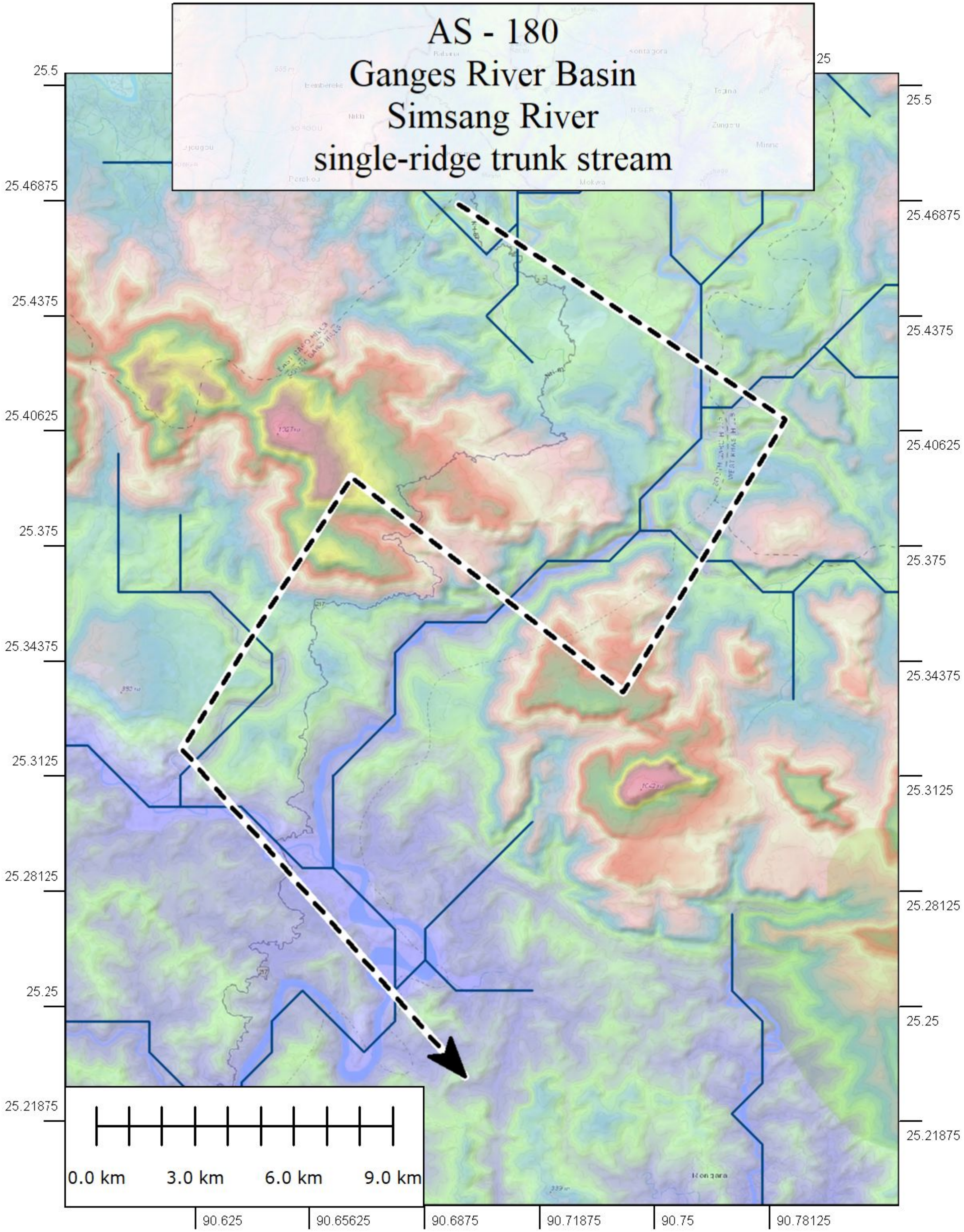
AS - 180
Ganges River Basin
Simsang River
single-ridge trunk stream
25.5
25.46875
25.4375
25.40625
25.375
25.34375
25.3125
25.28125
25.25
25.21875
90.625
90.65625
90.6875
90.71875
90.75
90.78125
0.0 km
3.0 km
6.0 km
9.0 km

AS - 181
Yangtze River Basin
Shiqian River tributary
single-ridge head stream

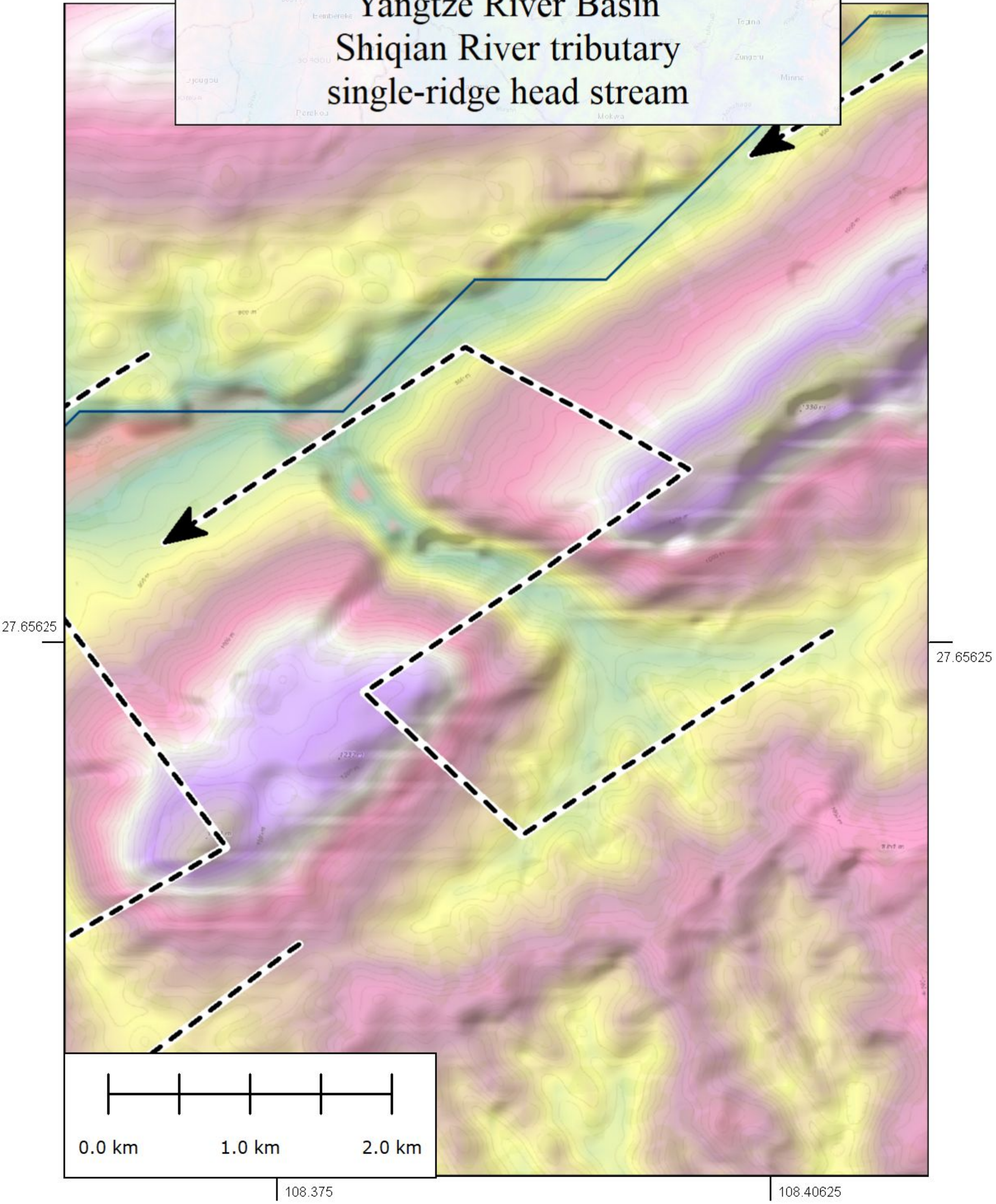

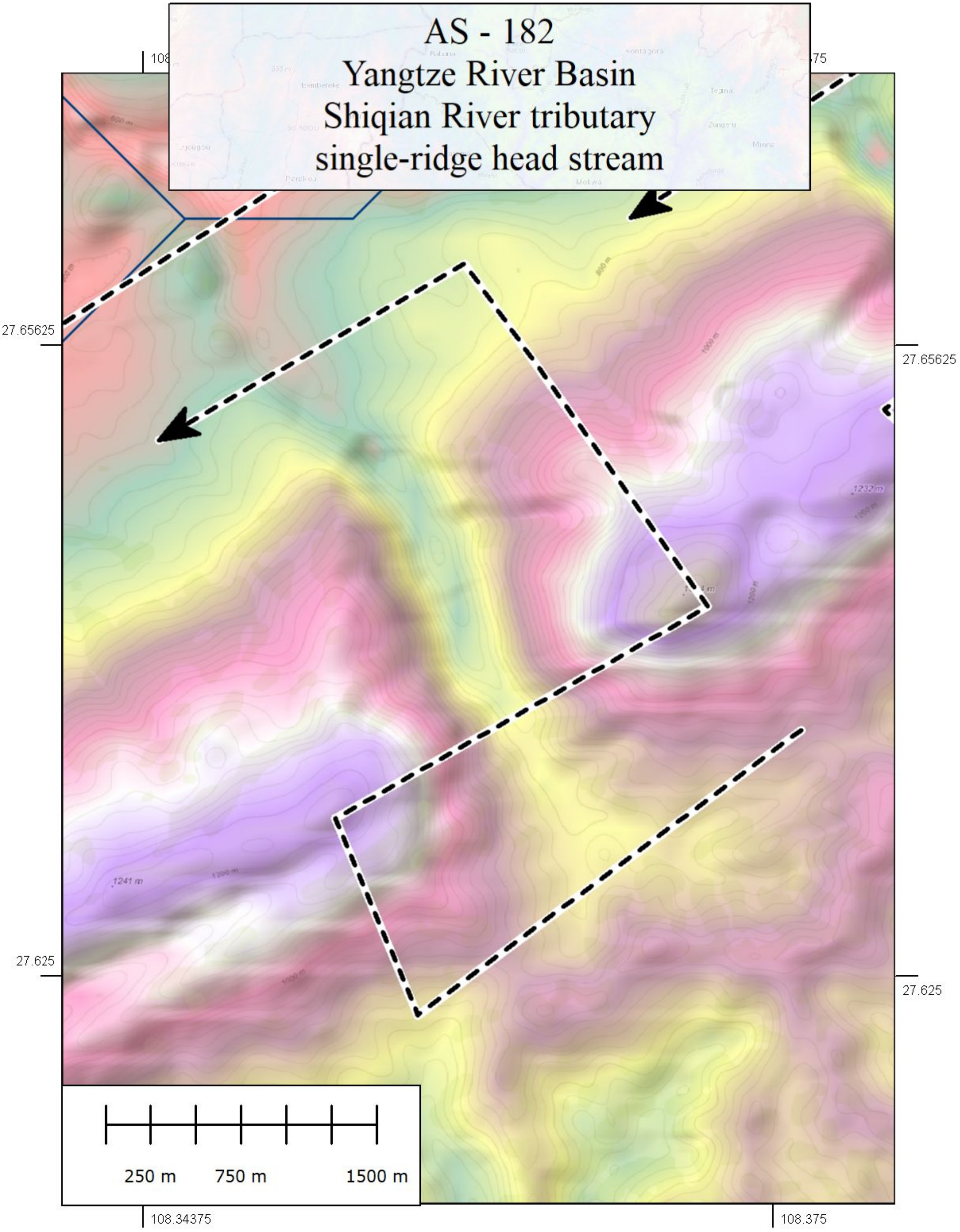
AS - 182
Yangtze River Basin
Shiqian River tributary
single-ridge head stream
27.65625
27.65625
27.625
27.625
108.34375
108.375
250 m
750 m
1500 m
1232 m
1241 m
1200 m
1000 m
800 m

AS - 183
Yangtze River Basin
Shiqian River tributary
single-ridge trunk stream

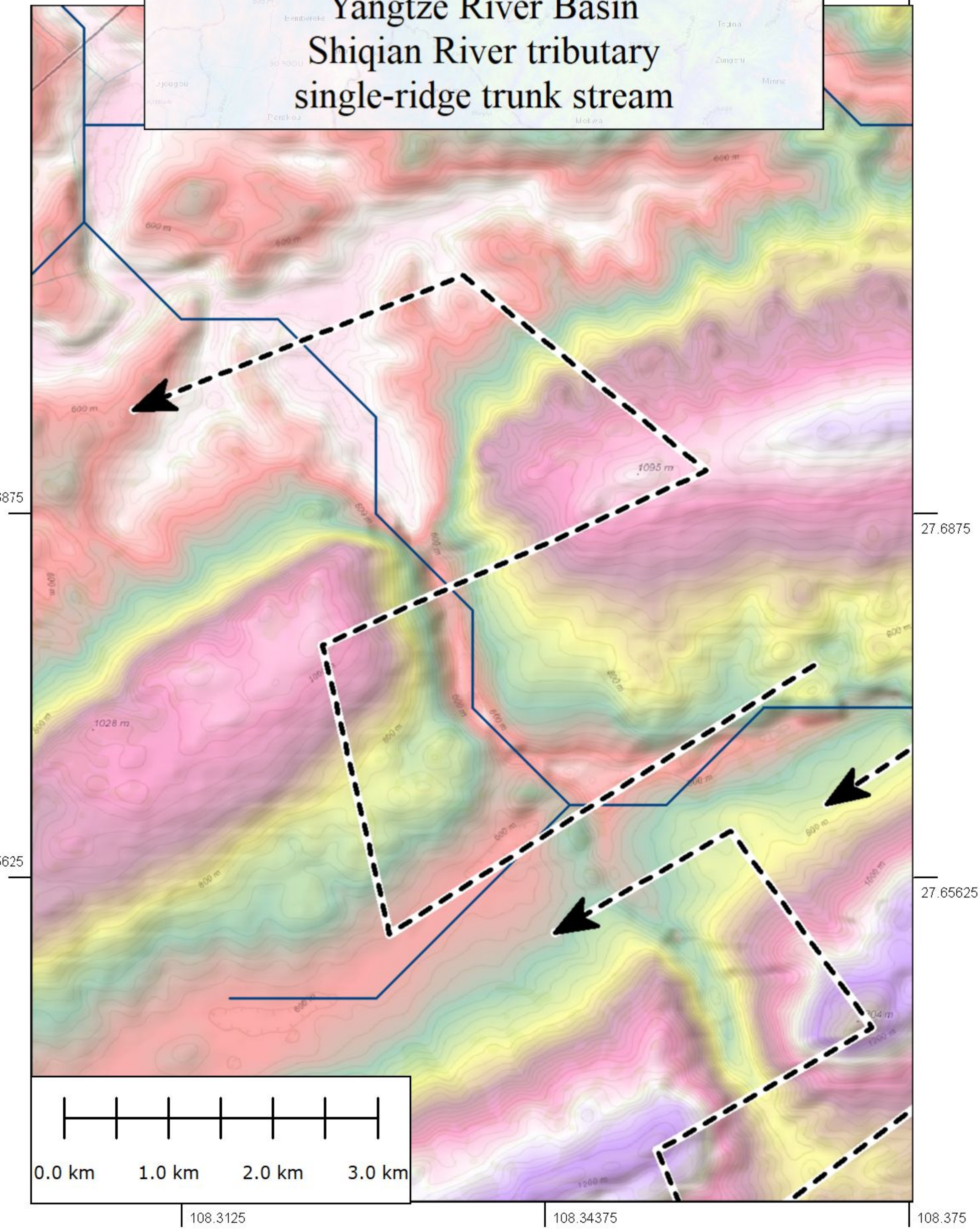

AS - 185
Yangtze River Basin
Leqijiang Rier
single-ridge head stream

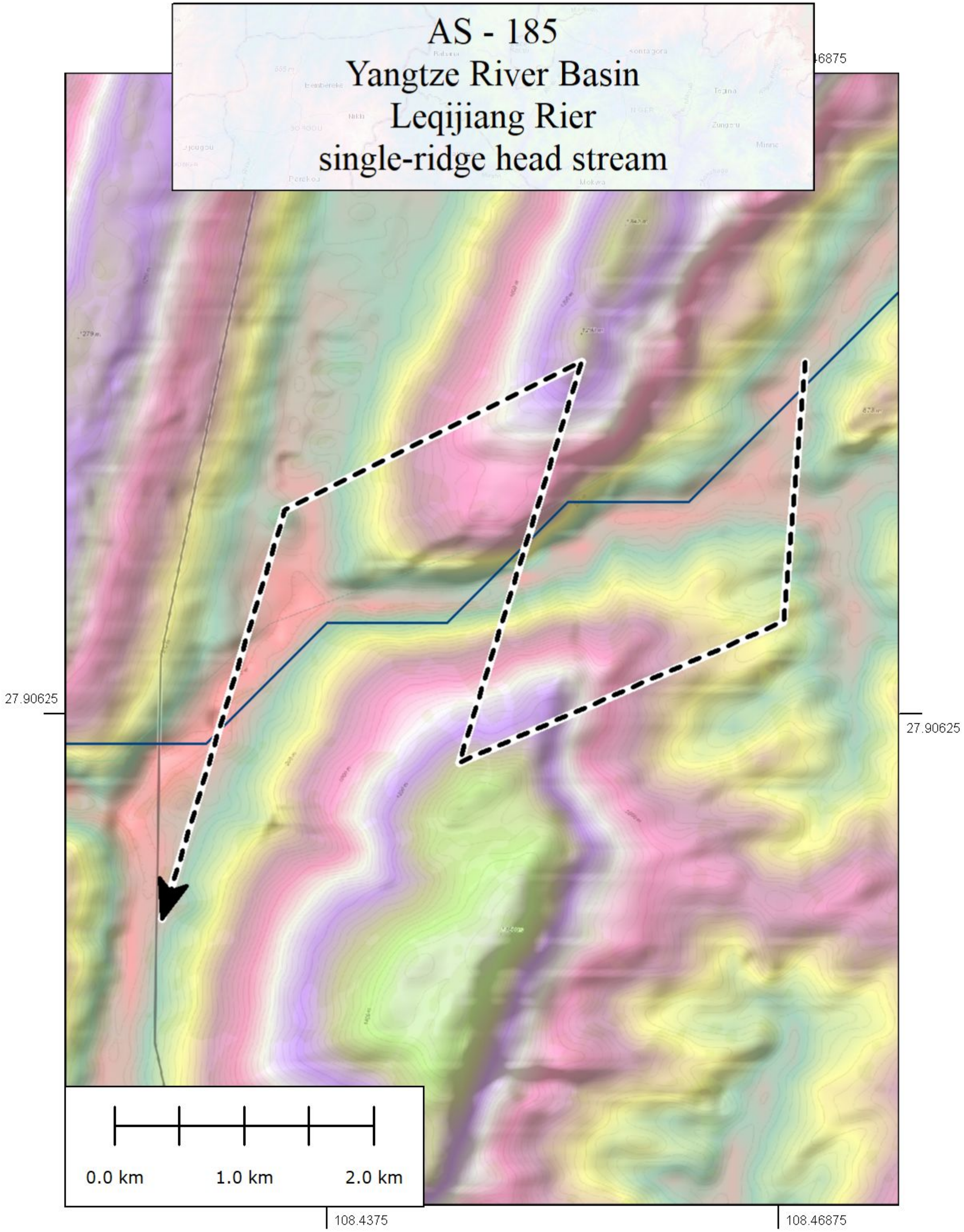

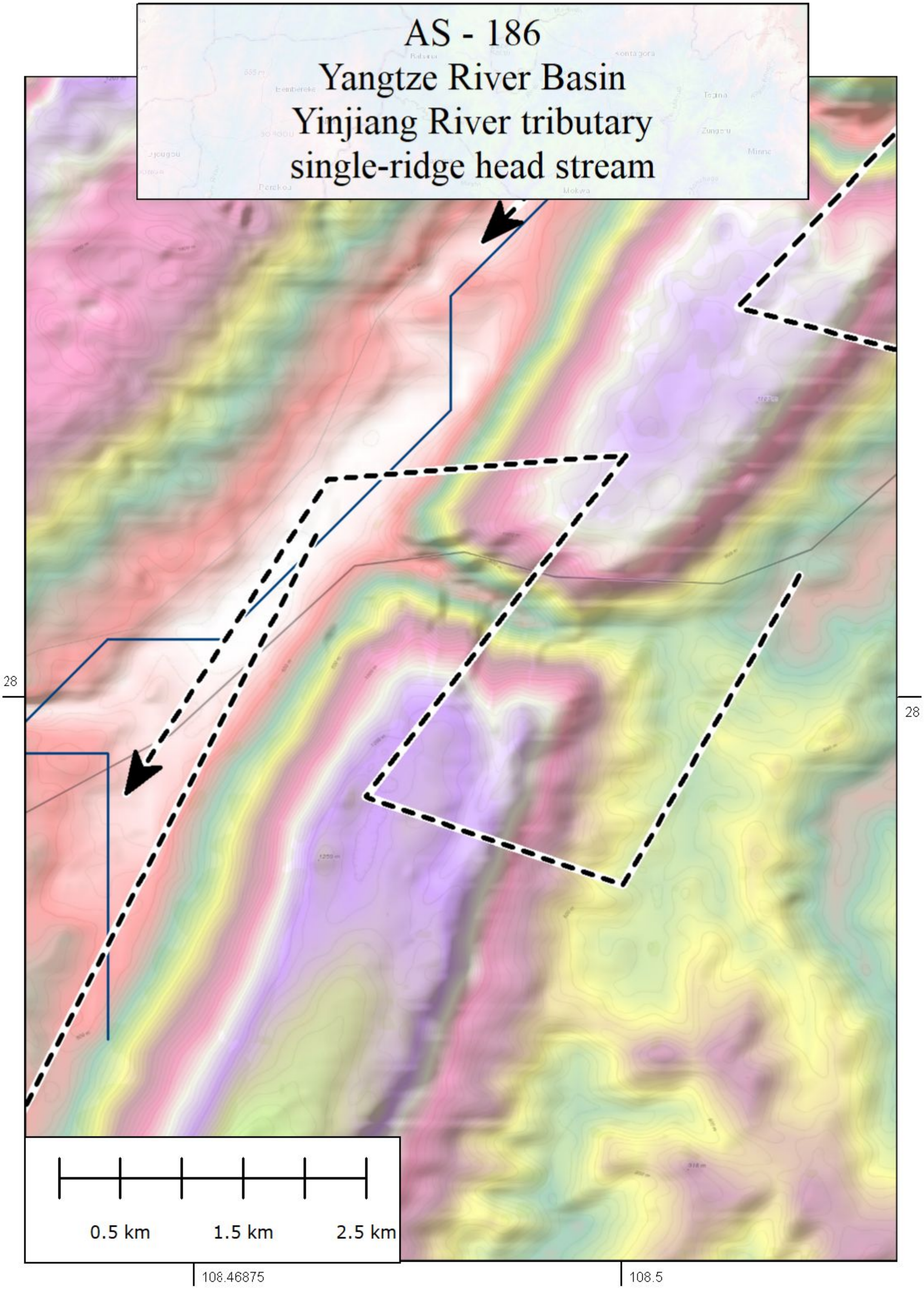
AS - 186
Yangtze River Basin
Yinjiang River tributary
single-ridge head stream
28
28
0.5 km
1.5 km
2.5 km
108.46875
108.5

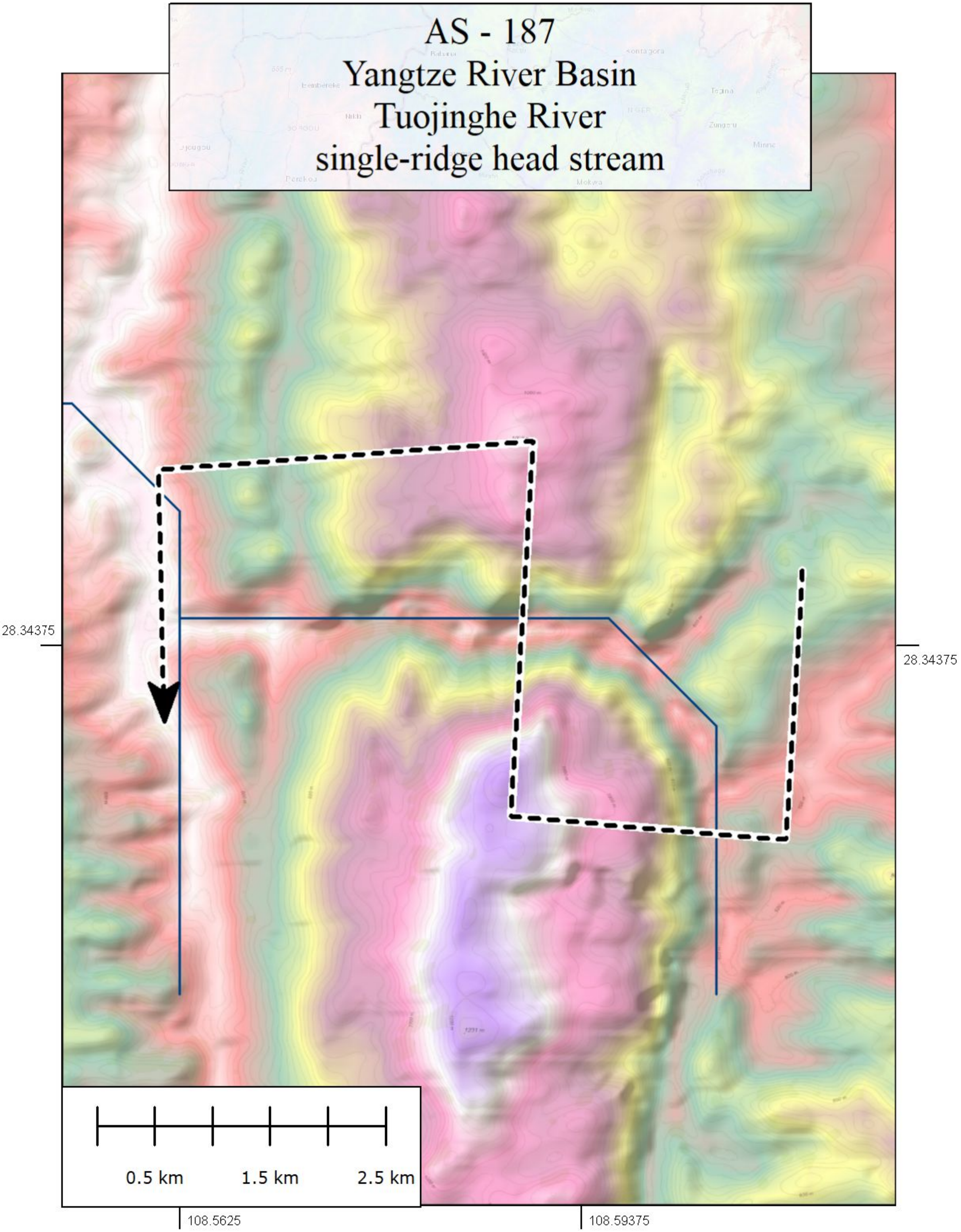
AS - 187
Yangtze River Basin
Tuojinghe River
single-ridge head stream
28.34375
28.34375
0.5 km
1.5 km
2.5 km
108.5625
108.59375

AS - 188
Yangtze River Basin
Qujiang River tributary
single-ridge head stream

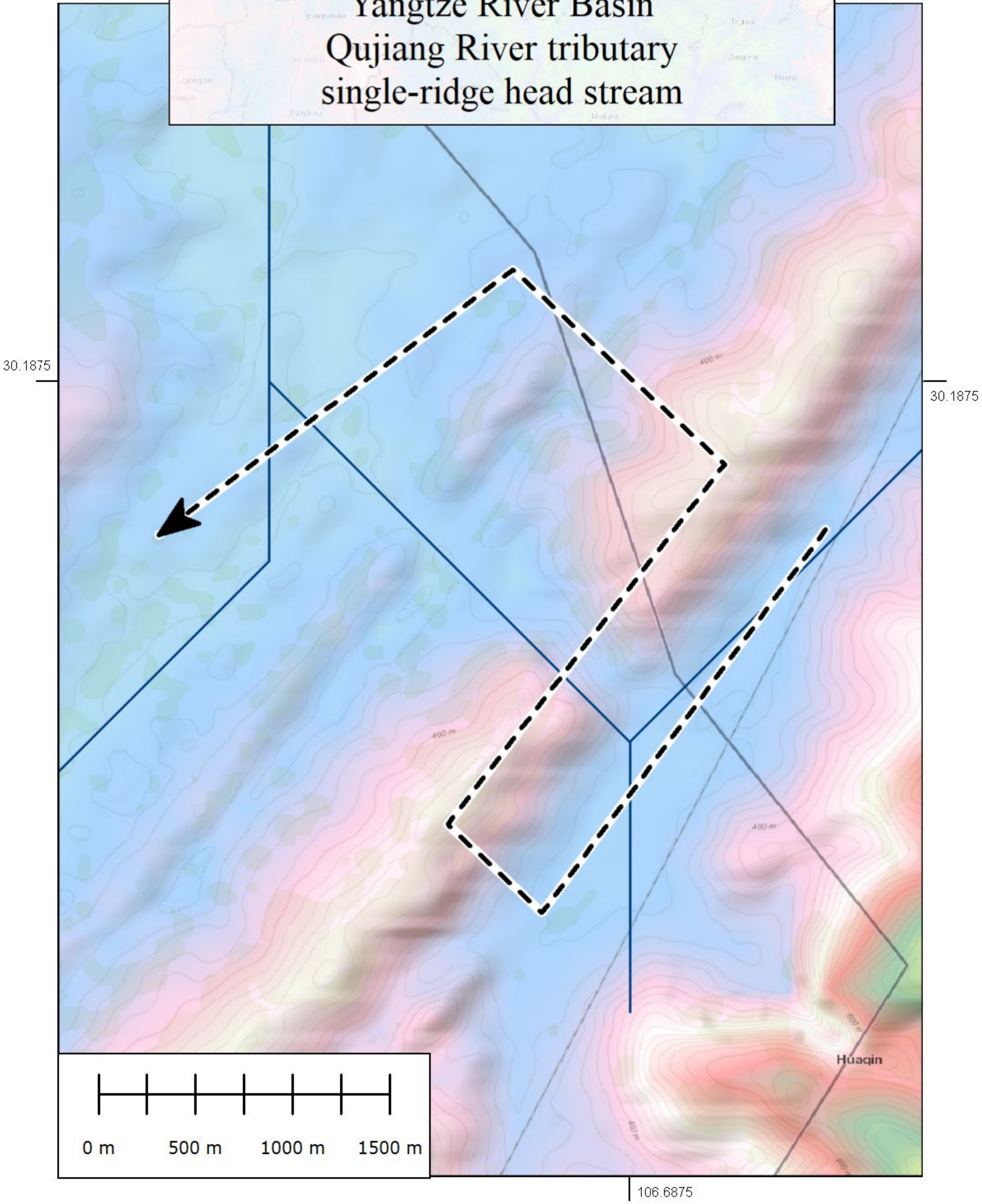

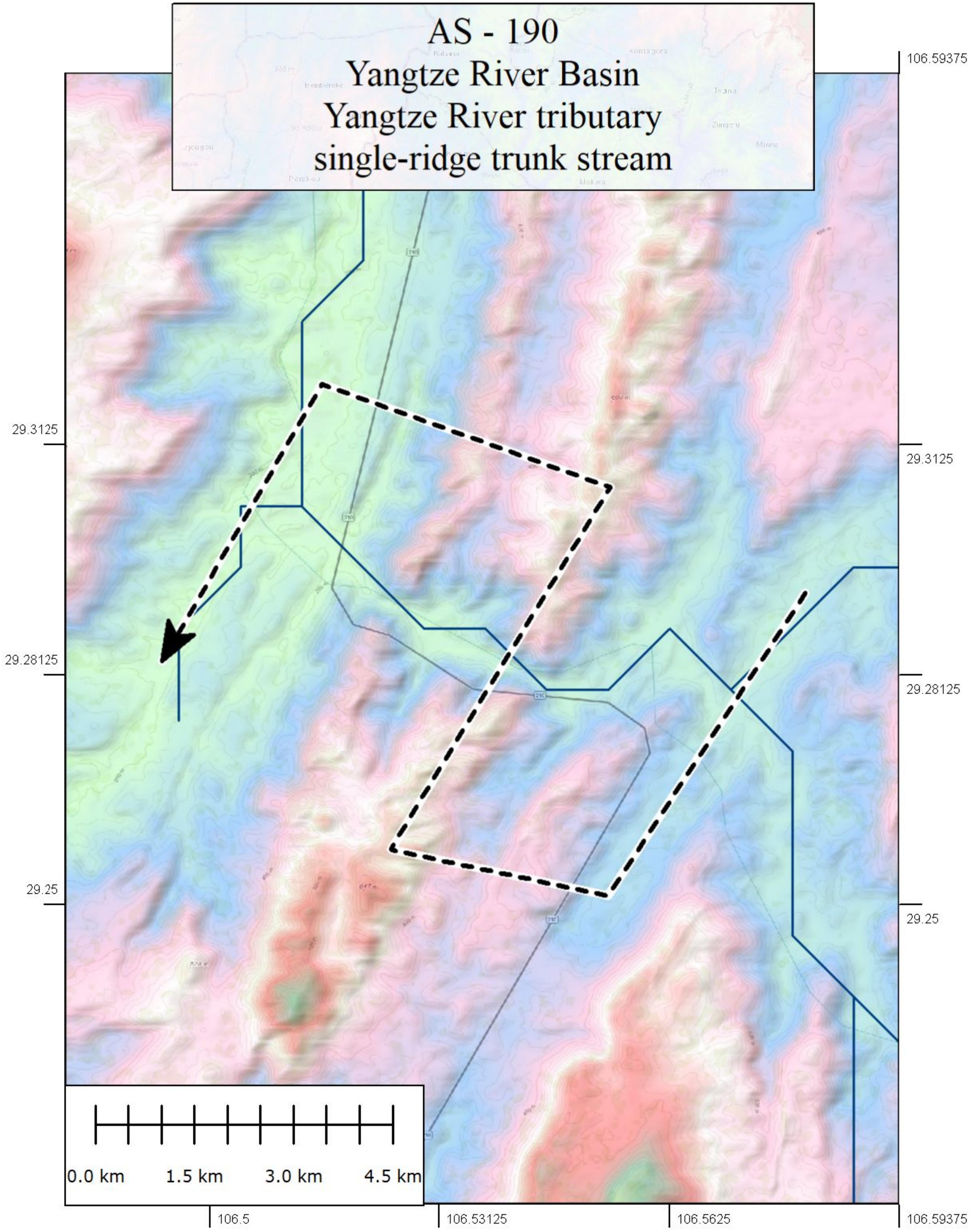
AS - 190
Yangtze River Basin
Yangtze River tributary
single-ridge trunk stream
106.59375
29.3125
29.3125
29.28125
29.28125
29.25
29.25
0.0 km
1.5 km
3.0 km
4.5 km
106.5
106.53125
106.5625
106.59375

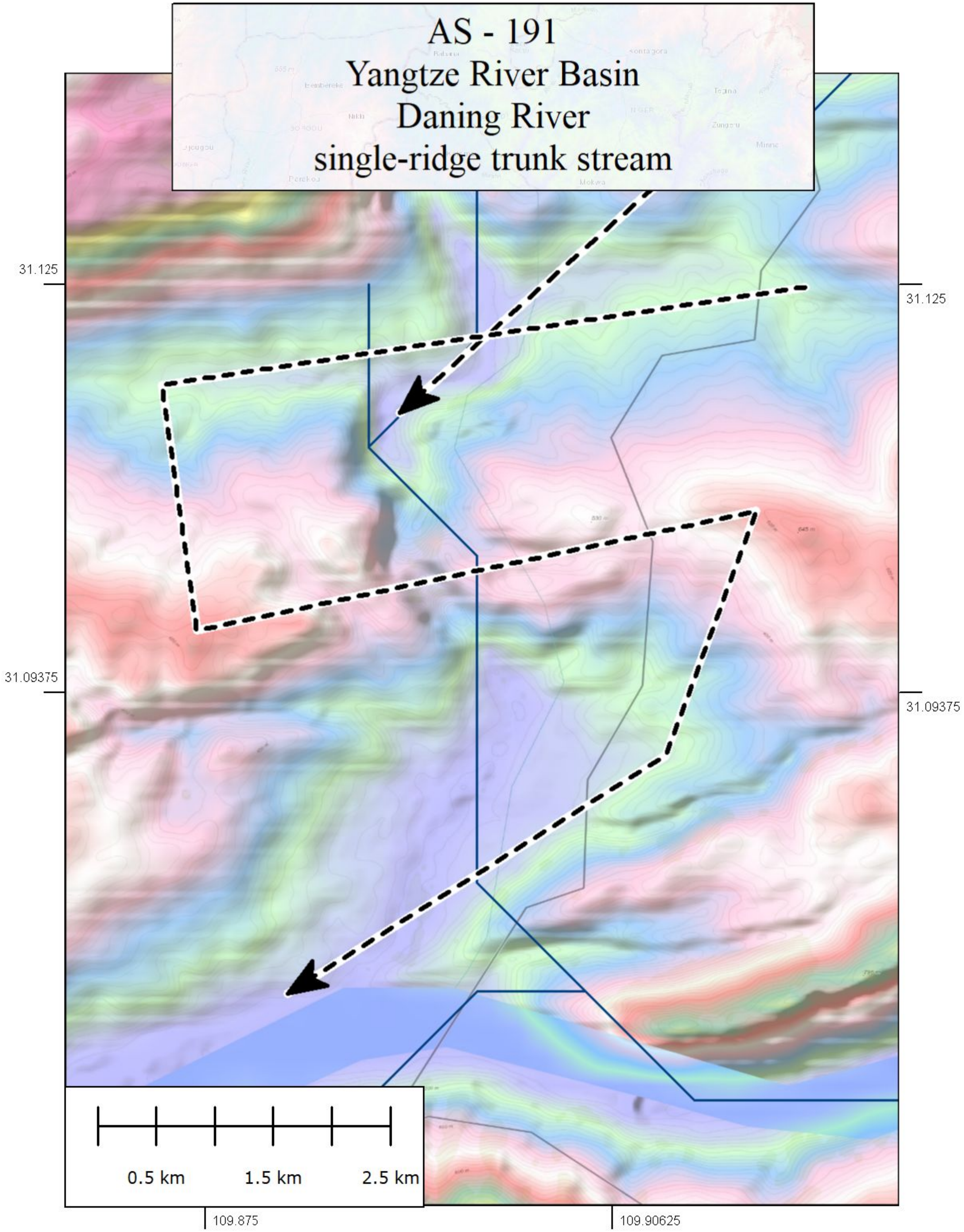
AS - 191
Yangtze River Basin
Daning River
single-ridge trunk stream
31.125
31.125
31.09375
31.09375
0.5 km
1.5 km
2.5 km
109.875
109.90625

AS - 193
Kamaphuli River Basin
single-ridge head stream

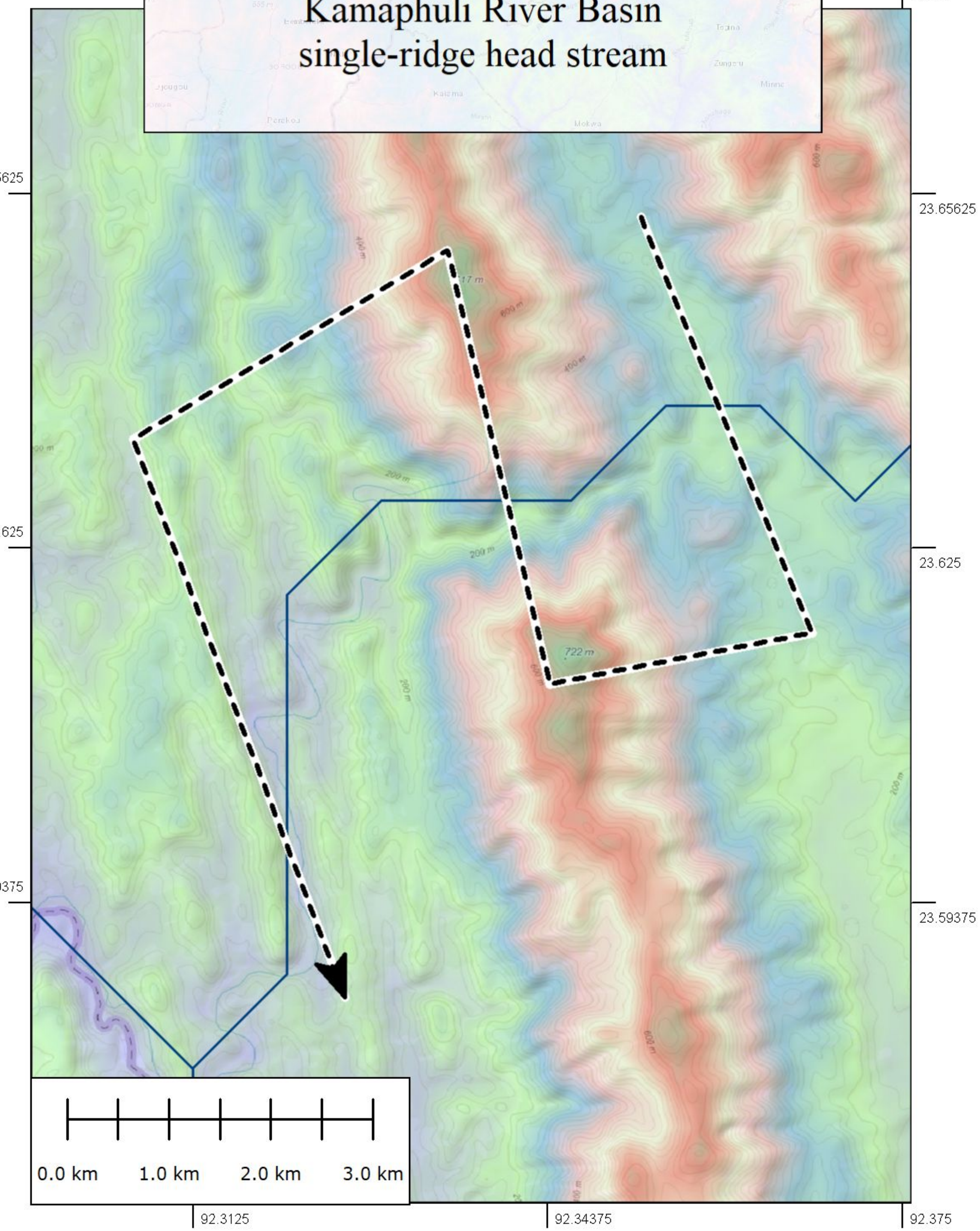

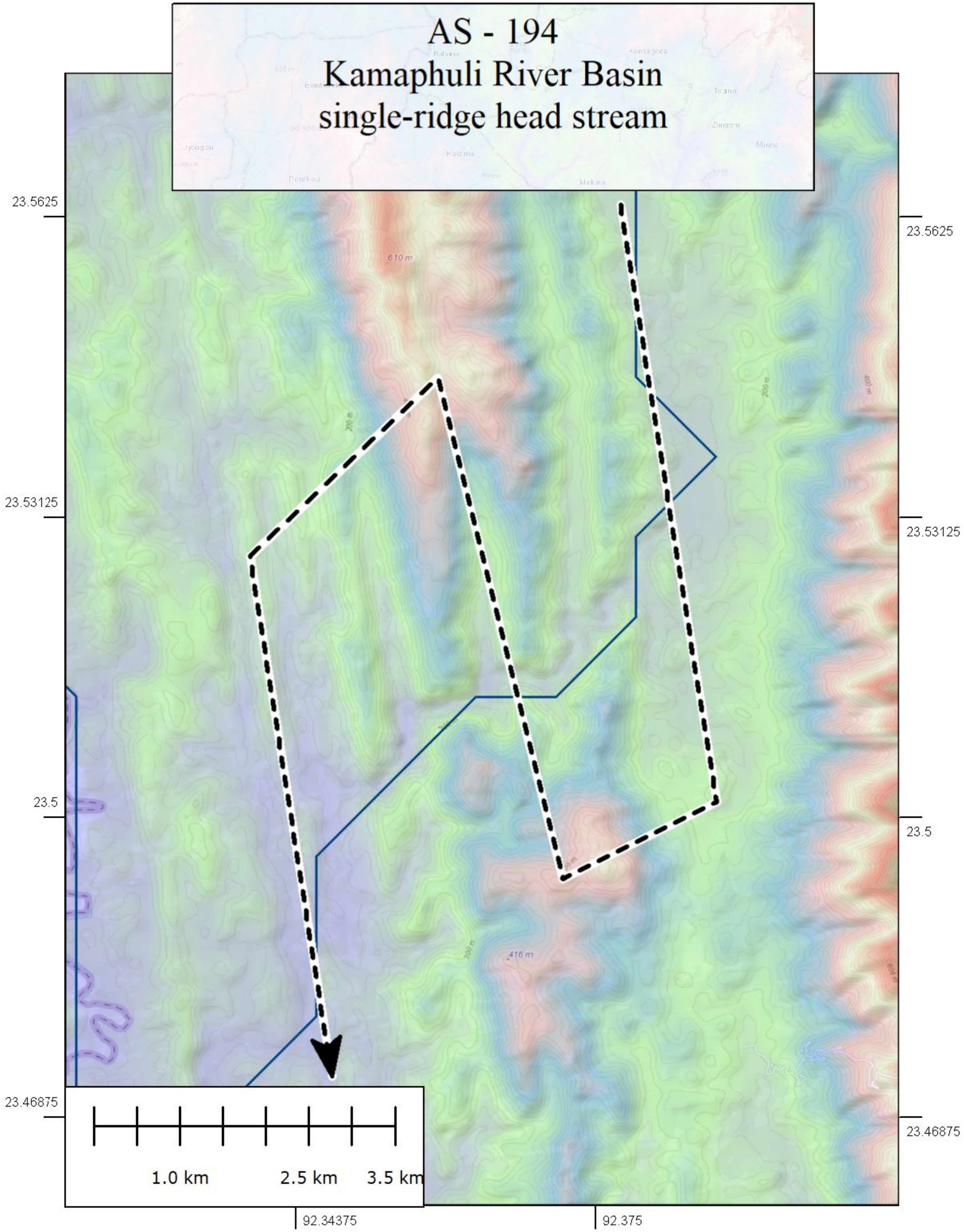
AS - 194
Kamaphuli River Basin
single-ridge head stream
23.5625
23.53125
23.5
23.46875
92.34375
92.375
1.0 km
2.5 km
3.5 km
610 m
416 m

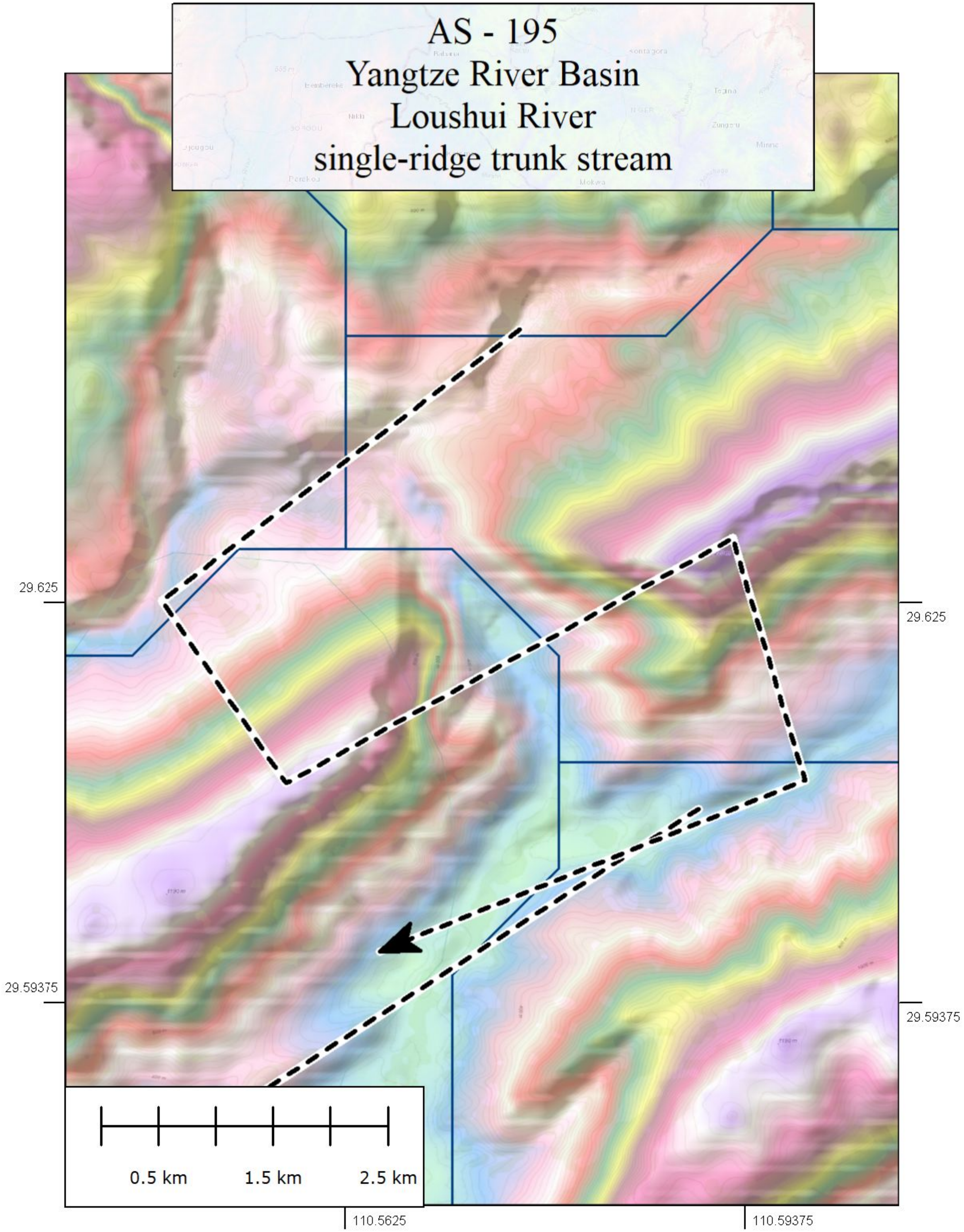
AS - 195
Yangtze River Basin
Loushui River
single-ridge trunk stream
29.625
29.625
29.59375
29.59375
0.5 km
1.5 km
2.5 km
110.5625
110.59375

AS - 197
Ganges River Basin
single-ridge trunk stream

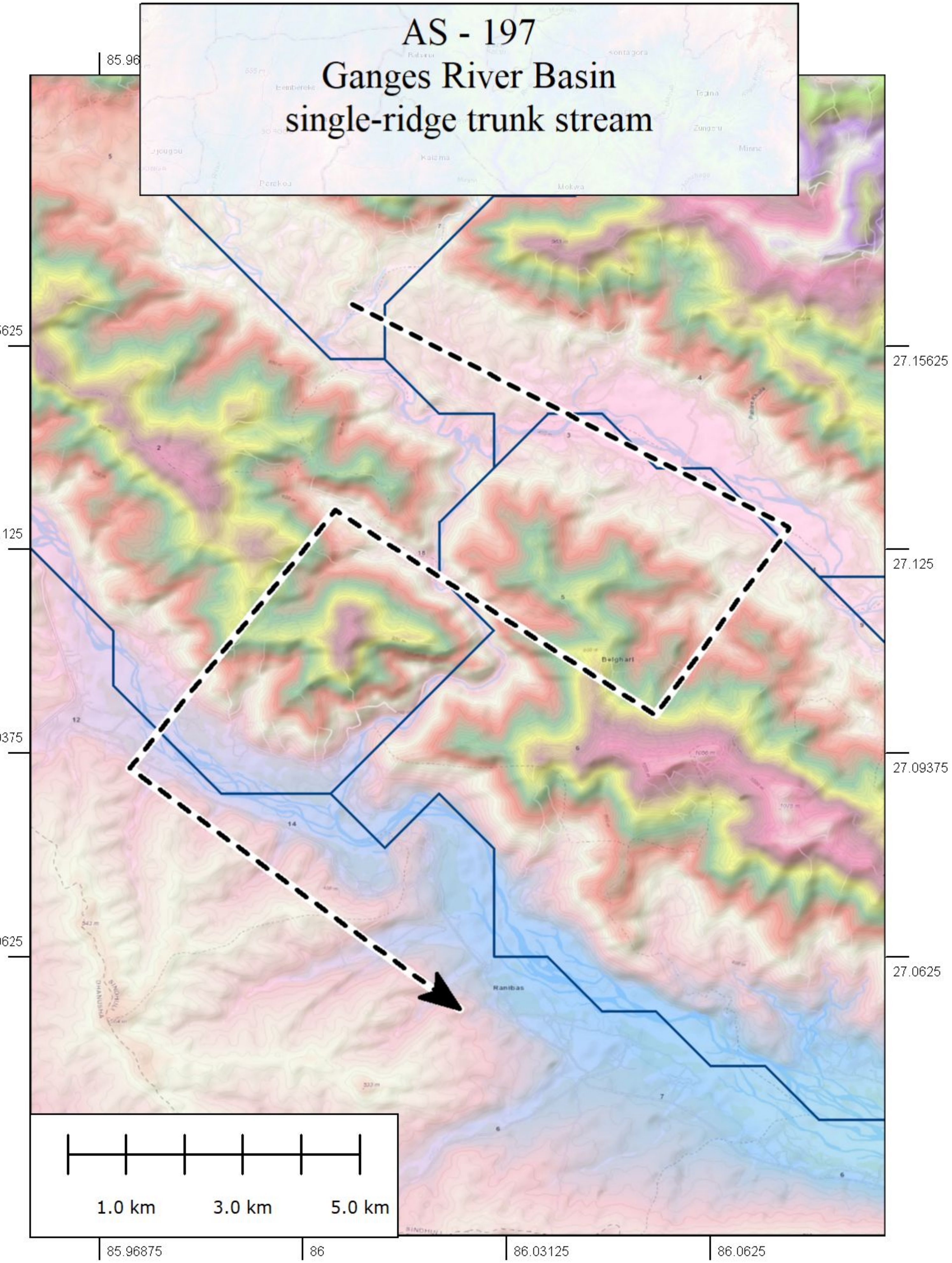

AS - 200
Yangtze River Basin
Yuanjiang River tributary
single-ridge trunk stream

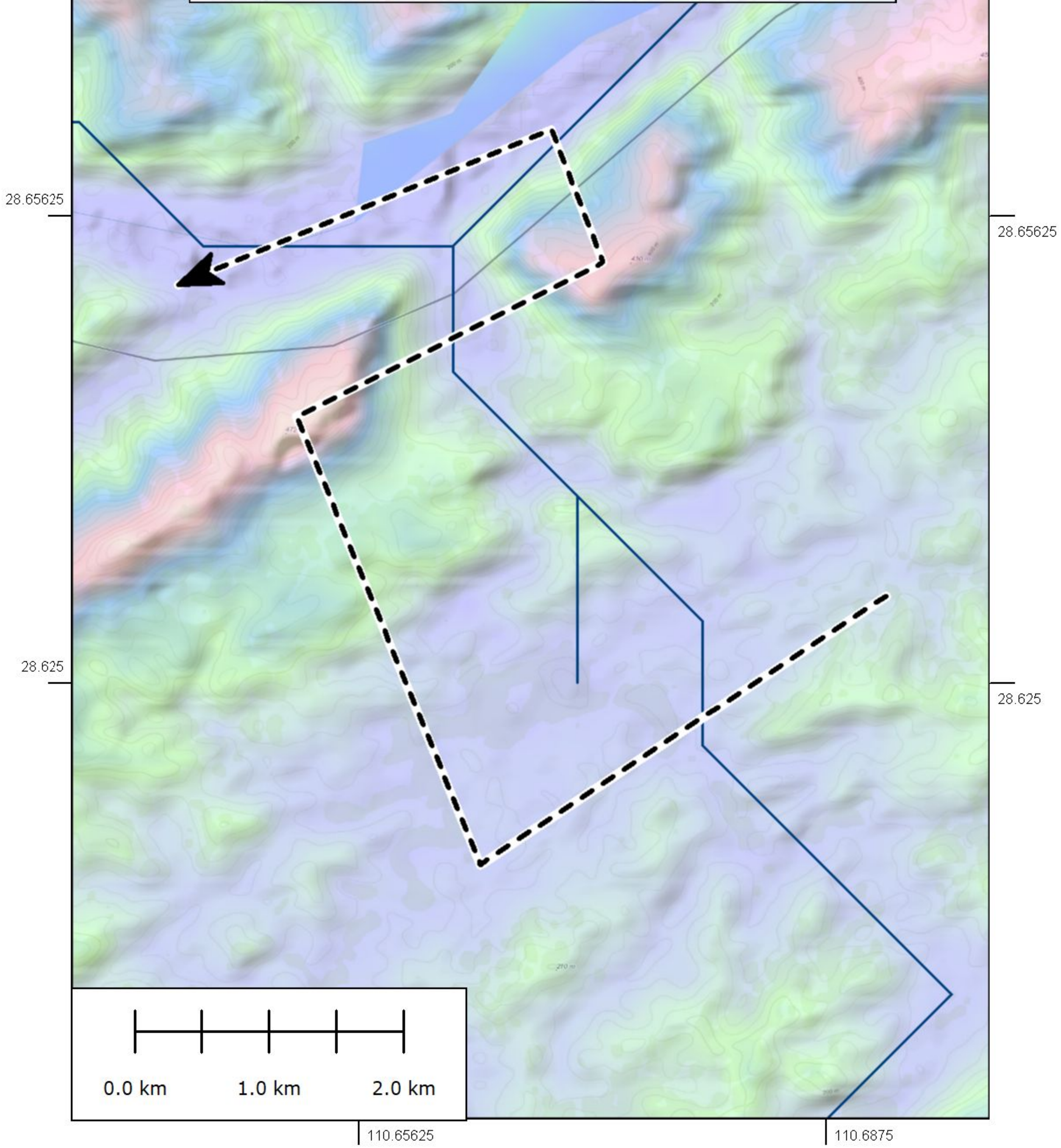

AS - 201 Yangtze River Basin Longgang River single-ridge trunk stream

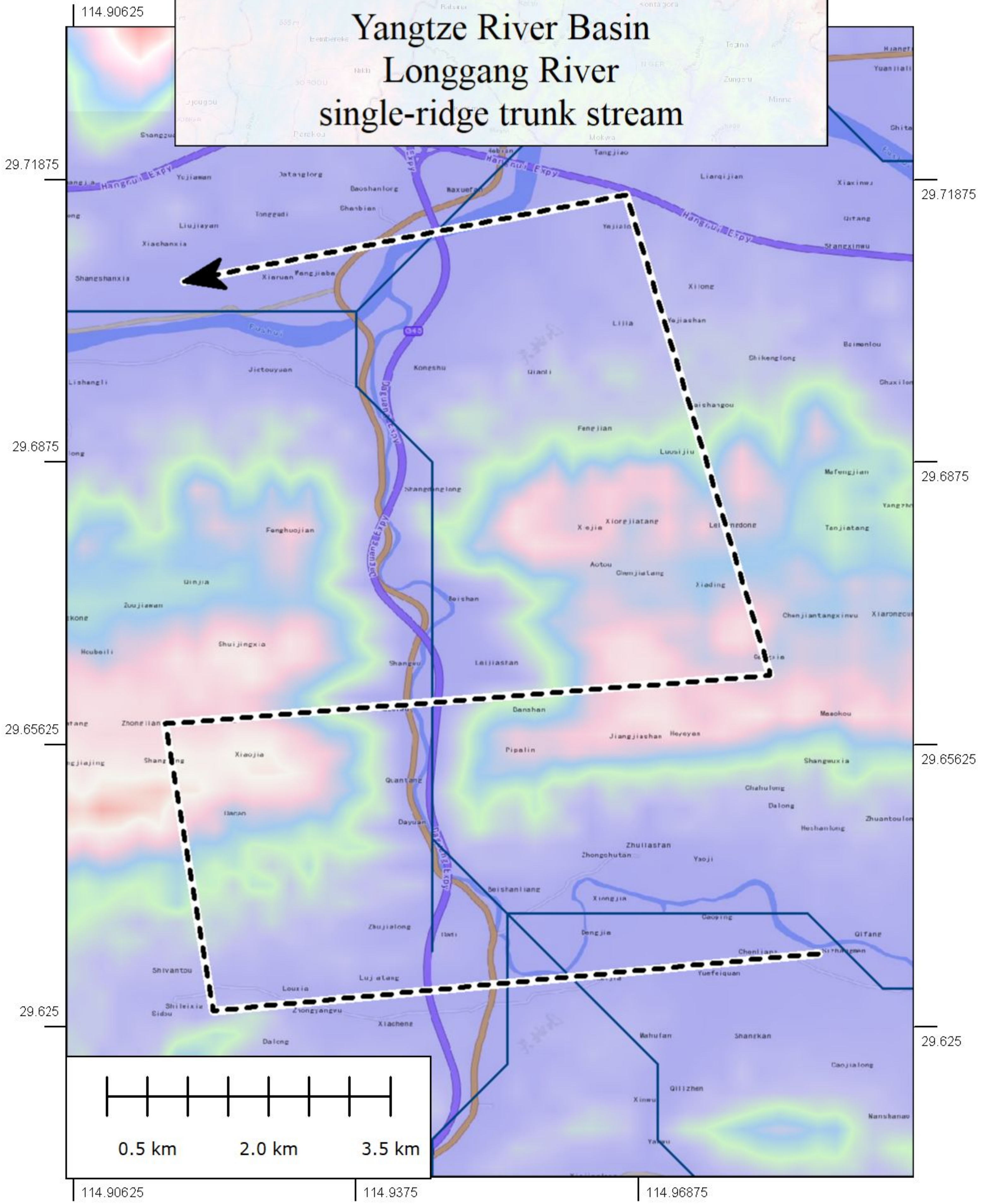

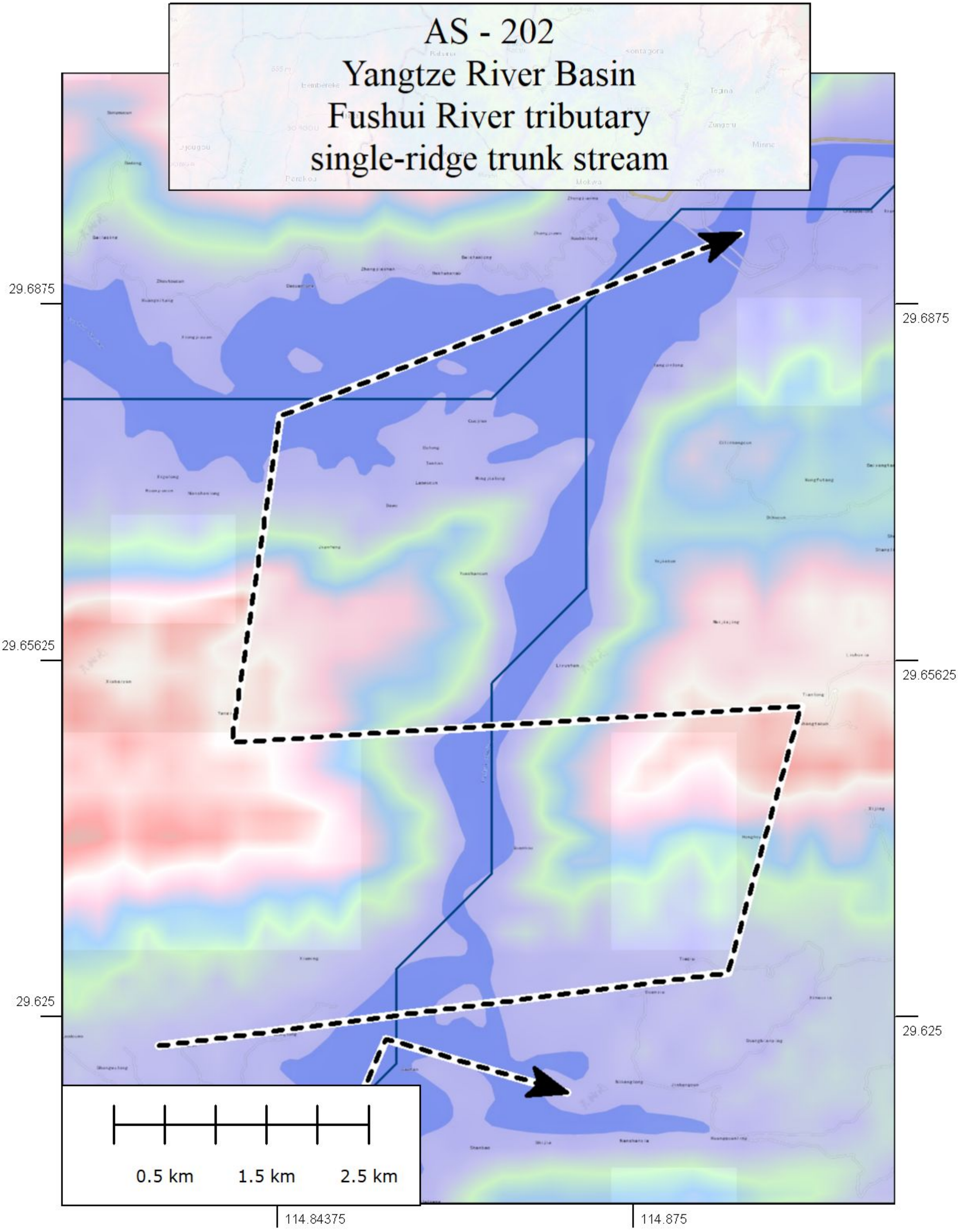
AS - 202
Yangtze River Basin
Fushui River tributary
single-ridge trunk stream
29.6875
29.65625
29.625
0.5 km
1.5 km
2.5 km
114.84375
114.875

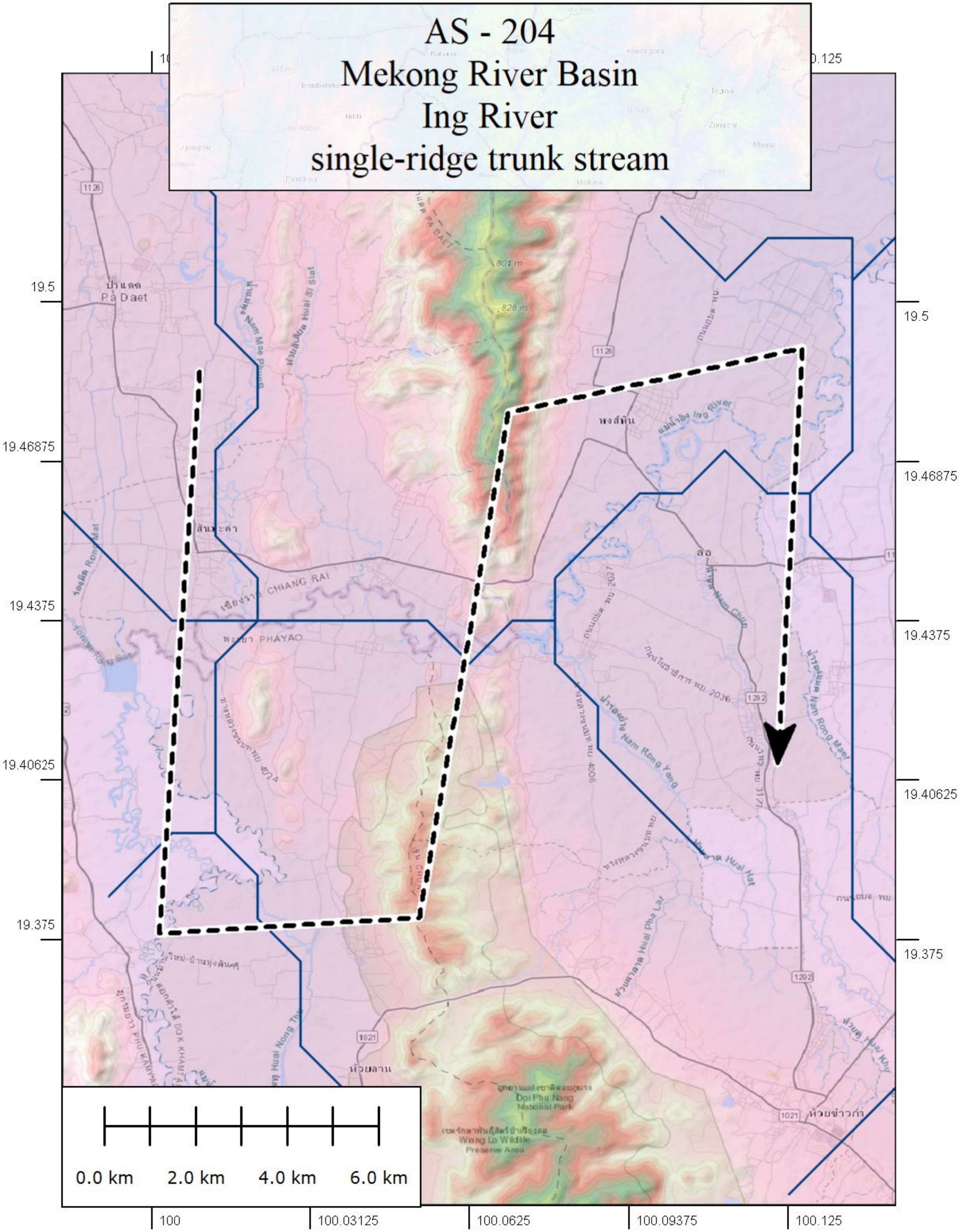
AS - 204
Mekong River Basin
Ing River
single-ridge trunk stream
19.5
19.46875
19.4375
19.40625
19.375
100
100.03125
100.0625
100.09375
100.125
0.0 km
2.0 km
4.0 km
6.0 km
Pa Daet
Nam Mae Phung
Huai Si Siat
801 m
828 m
Ing River
CHIANG RAI
PHAYAO
Nam Chun
Nam Rong Yang
Nam Rong Maet
Huai Hat
Huai Pha Lat
Huai Nong Thi
Doi Phu Nang National Park
Wiang Lo Wildlife Preserve Area
1126
1292
1021

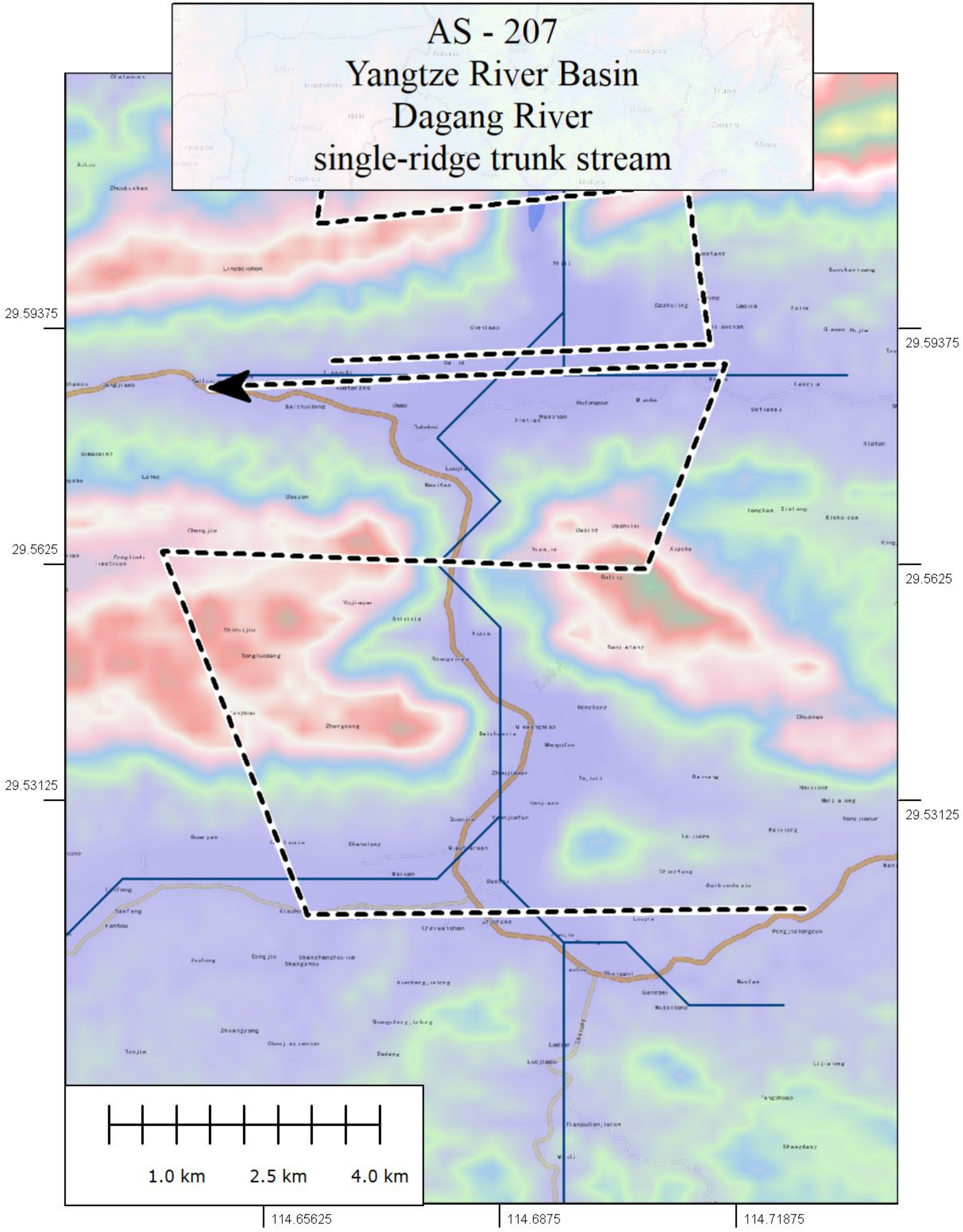
AS - 207
Yangtze River Basin
Dagang River
single-ridge trunk stream
29.59375
29.5625
29.53125
1.0 km
2.5 km
4.0 km
114.65625
114.6875
114.71875

AS - 208
Yangtze River Basin
Wangying Reservoir
single-ridge head stream

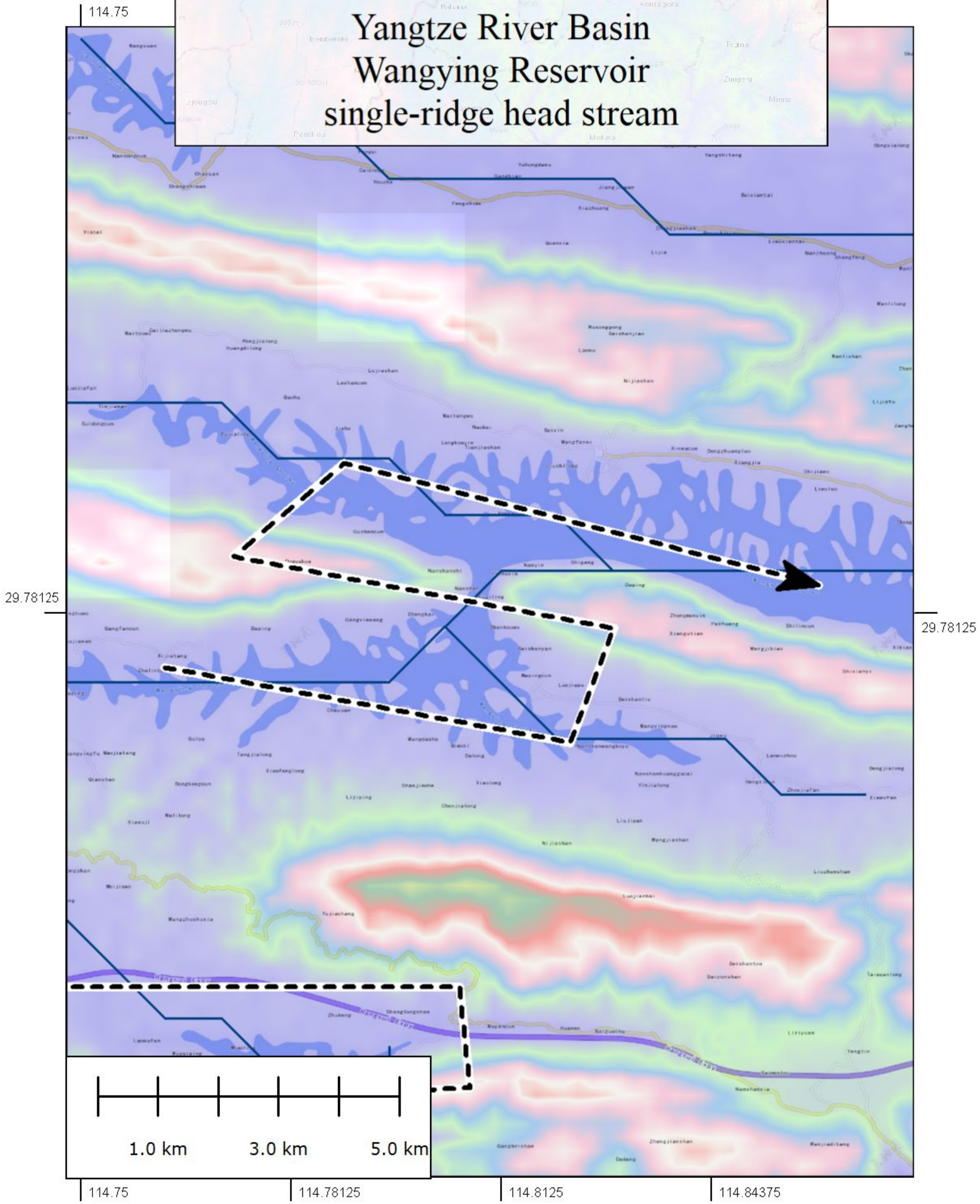

AS - 209
Yangtze River Basin
Fushui River tributary
single-ridge head stream

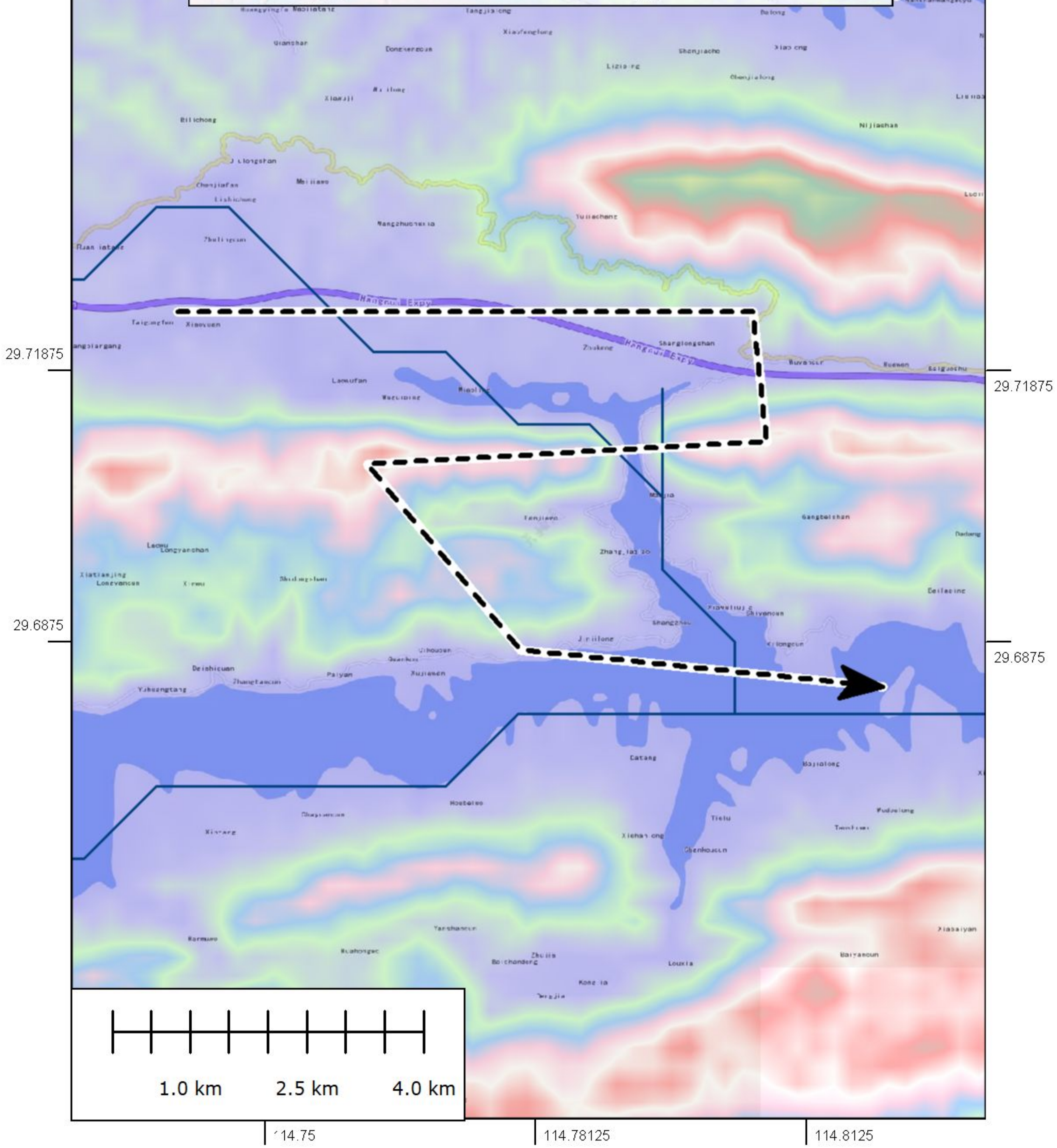

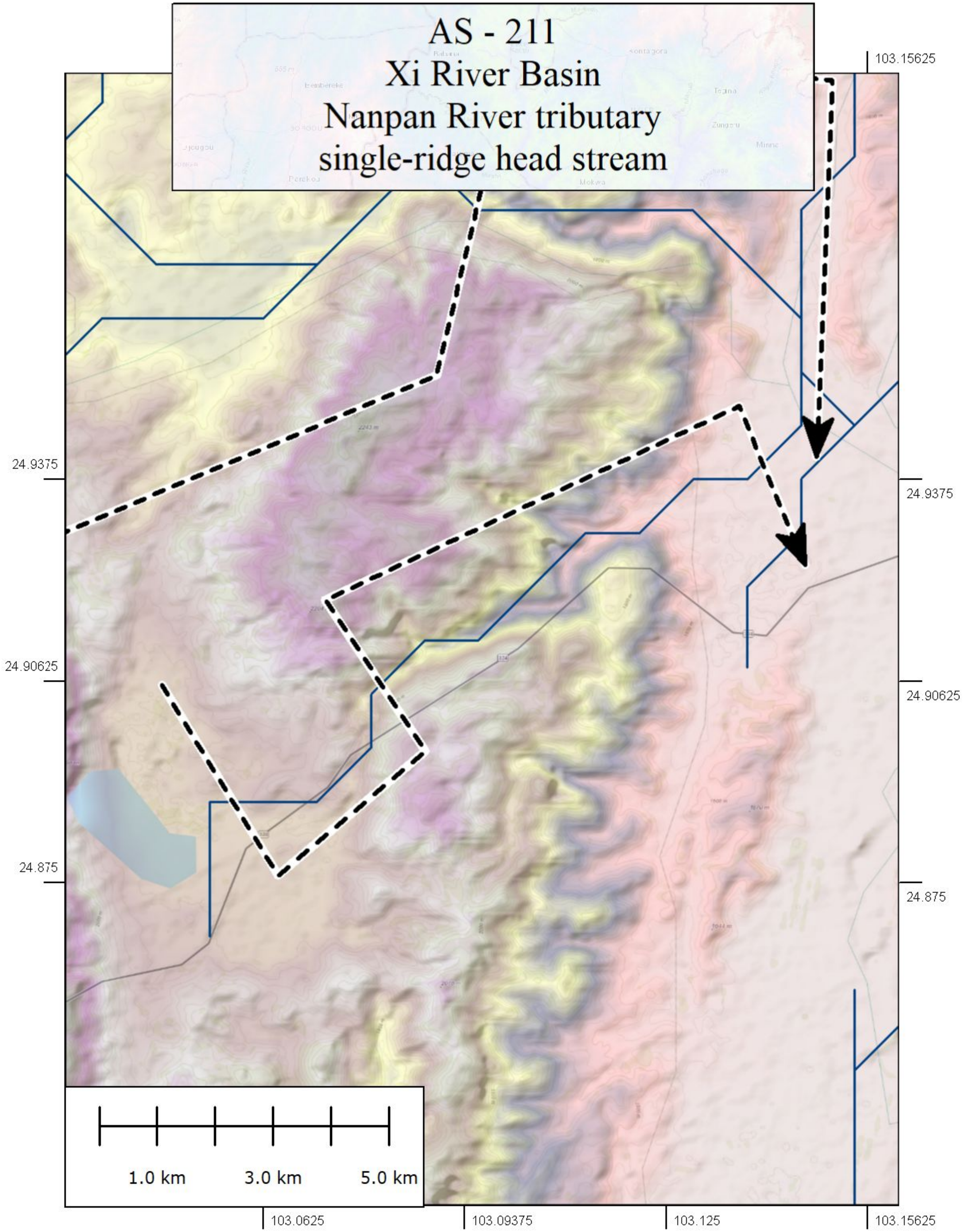

AS - 211
Xi River Basin
Nanpan River tributary
single-ridge head stream
103.15625
24.9375
24.9375
24.90625
24.90625
24.875
24.875
1.0 km
3.0 km
5.0 km
103.0625
103.09375
103.125
103.15625

AS - 213
Endorheic basin
Chu RIver
single-ridge trunk stream

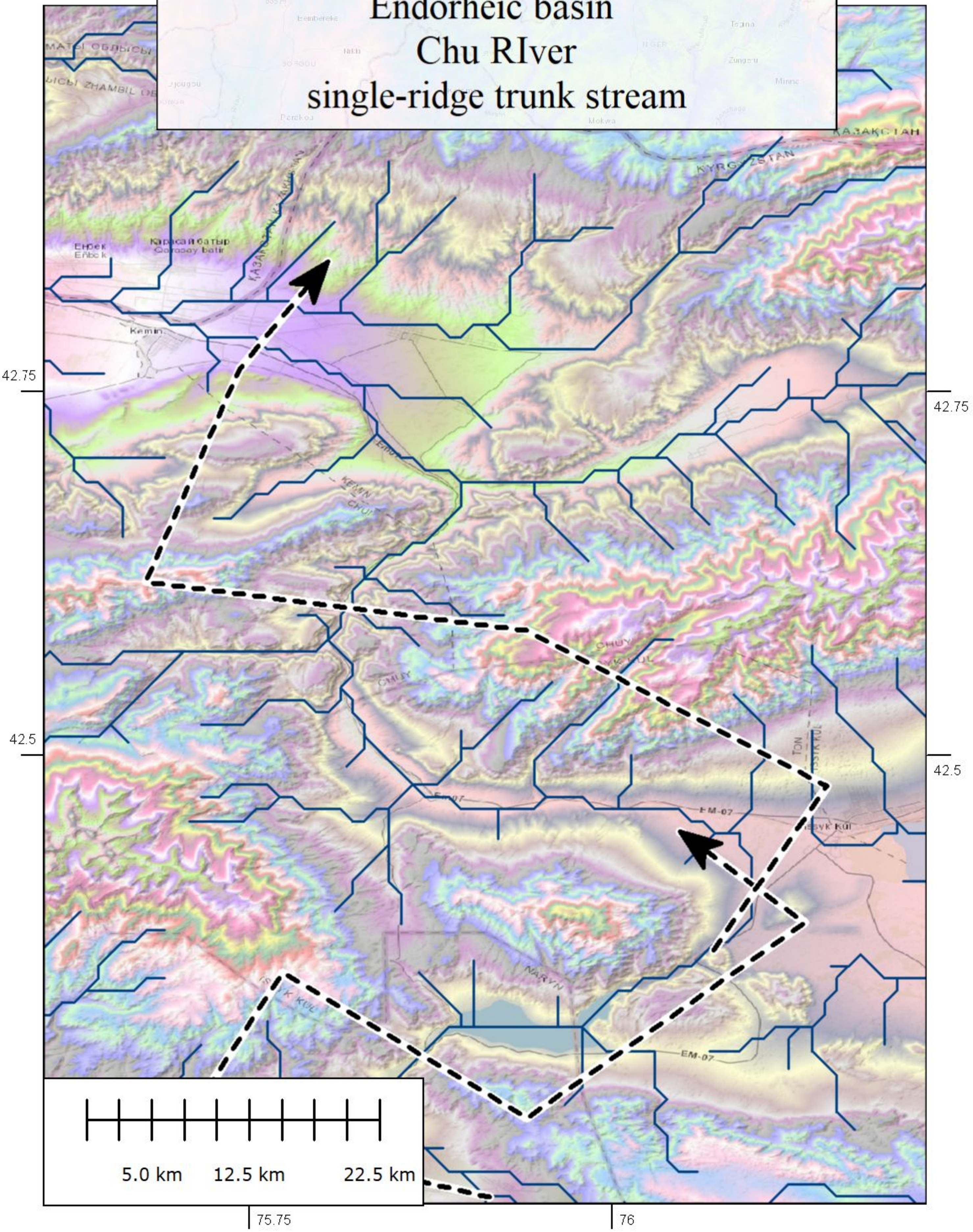

AS - 216
Ganges River Basin
Ram Ganga River
single-ridge trunk stream

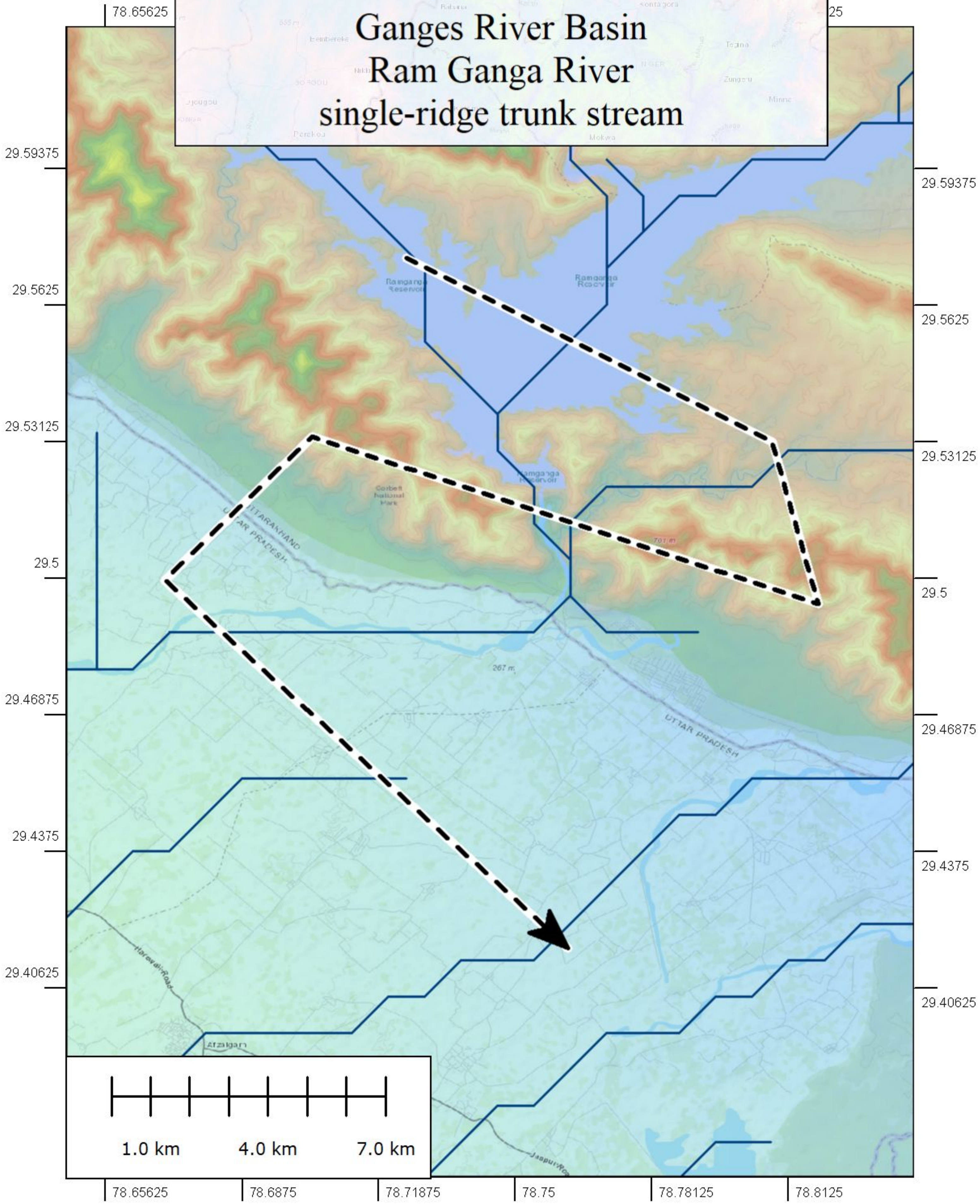

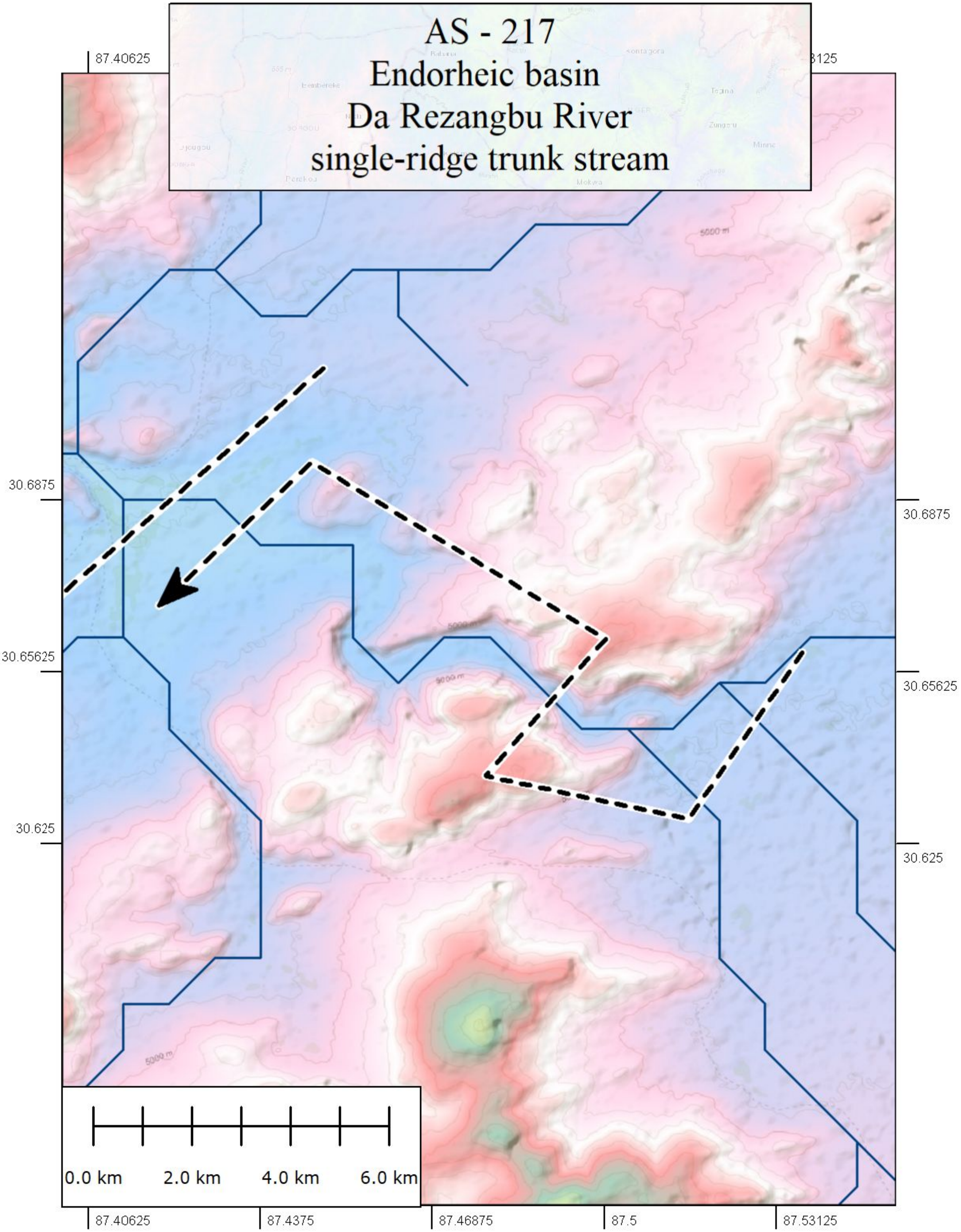

AS - 217
Endorheic basin
Da Rezangbu River
single-ridge trunk stream
87.40625
87.4375
87.46875
87.5
87.53125
30.6875
30.65625
30.625
5000 m
0.0 km
2.0 km
4.0 km
6.0 km

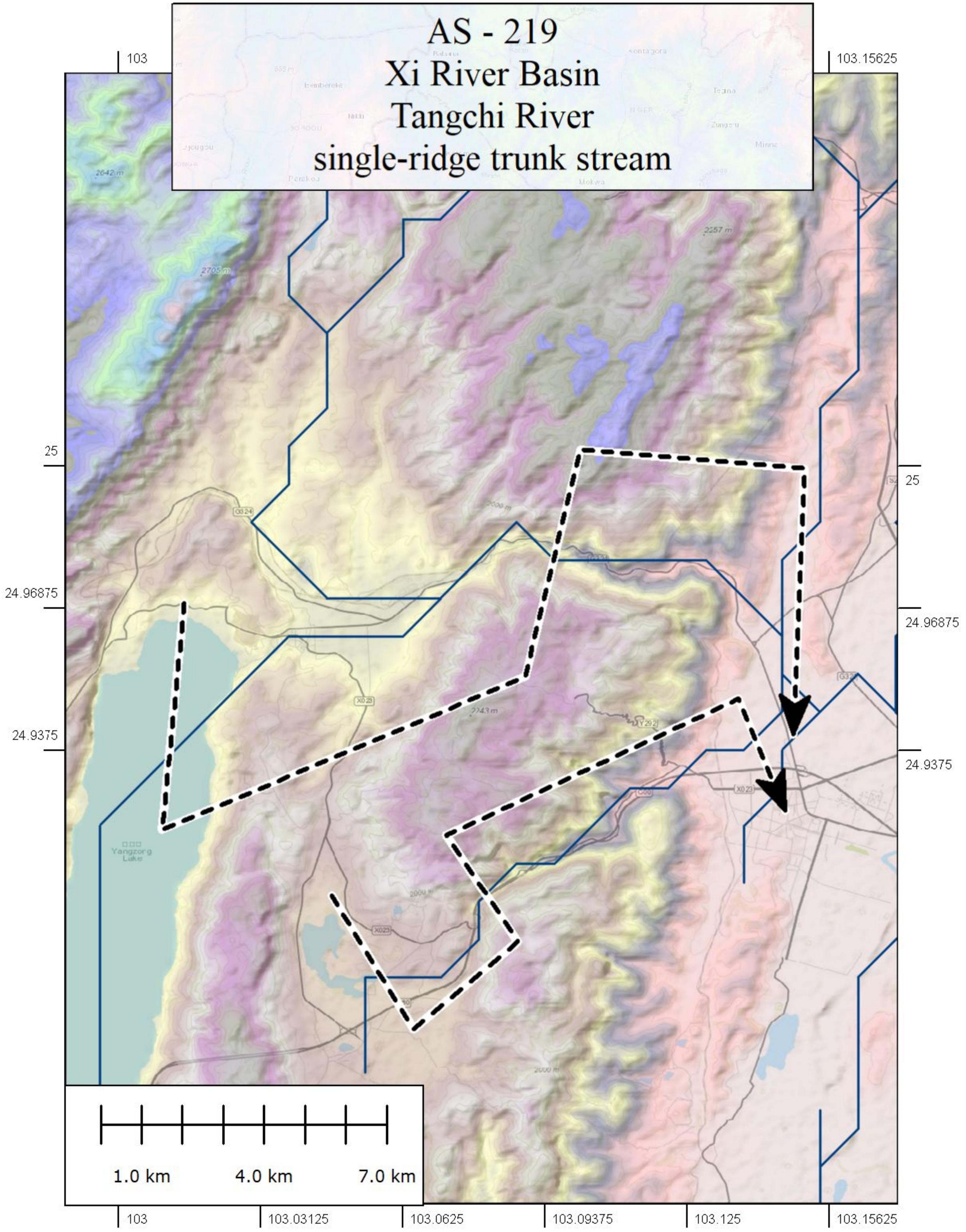

AS - 219
Xi River Basin
Tangchi River
single-ridge trunk stream
103
103.15625
25
24.96875
24.9375
103.03125
103.0625
103.09375
103.125
1.0 km
4.0 km
7.0 km
Yangzong Lake
2705 m
2642 m
2257 m
2000 m
2743 m
G324
X023
Y292

AS - 220
Mekong River Basin
Hueang River tributary
single-ridge trunk stream

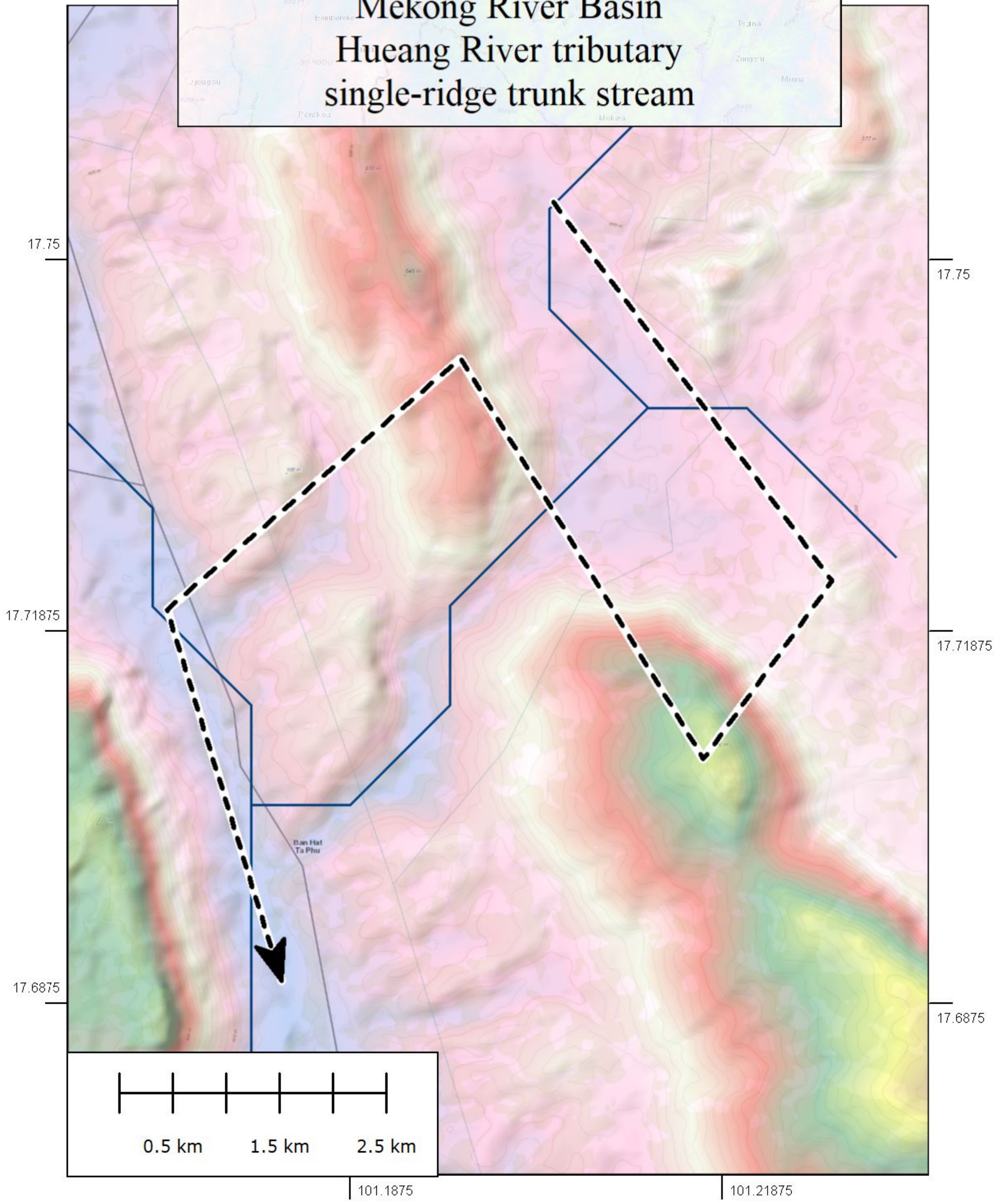

AS - 221
Mekong River Basin
Nam Houng River
single-ridge trunk stream

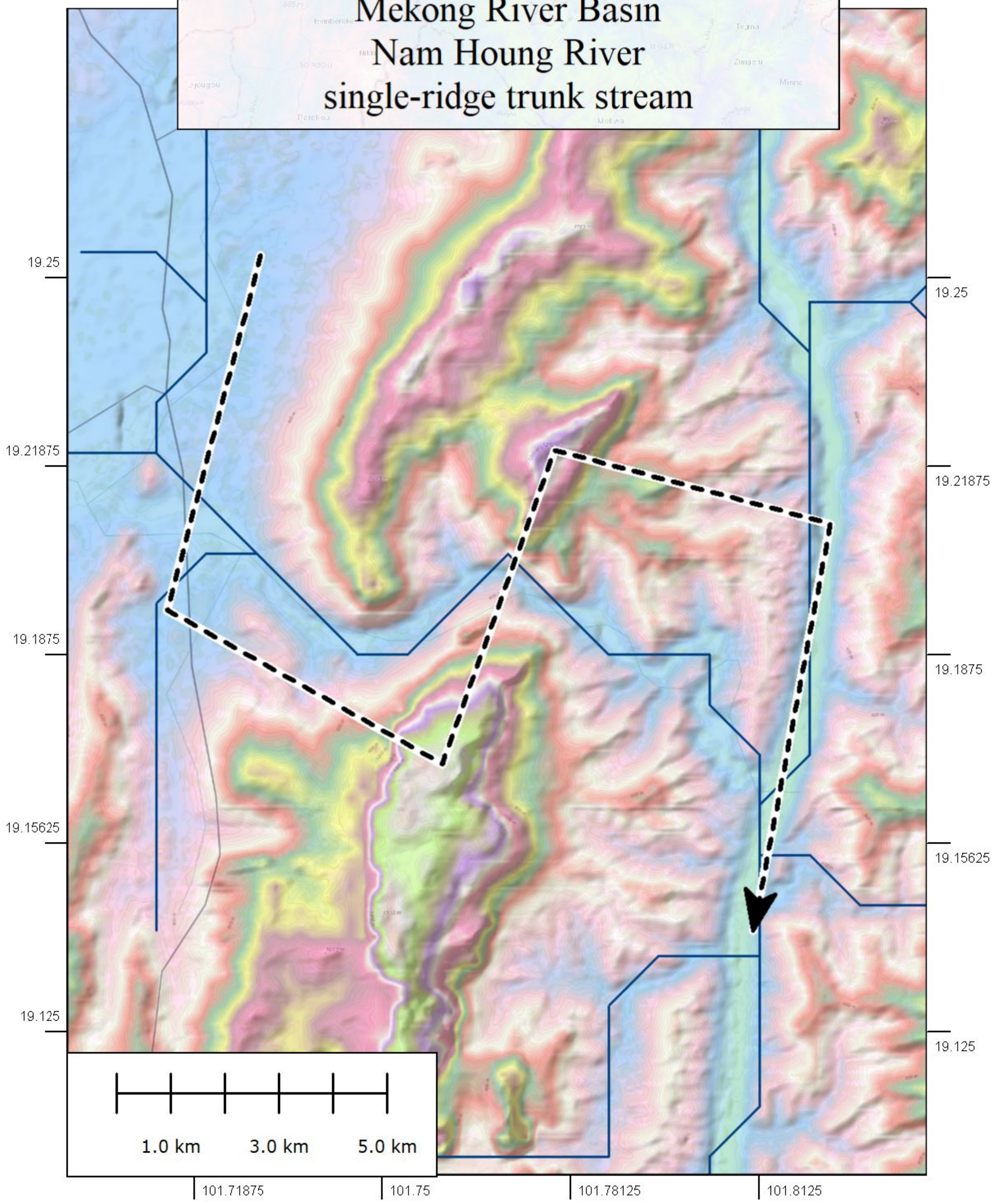

AS - 223
Ganges River Basin
Yamuna River
single-ridge trunk stream

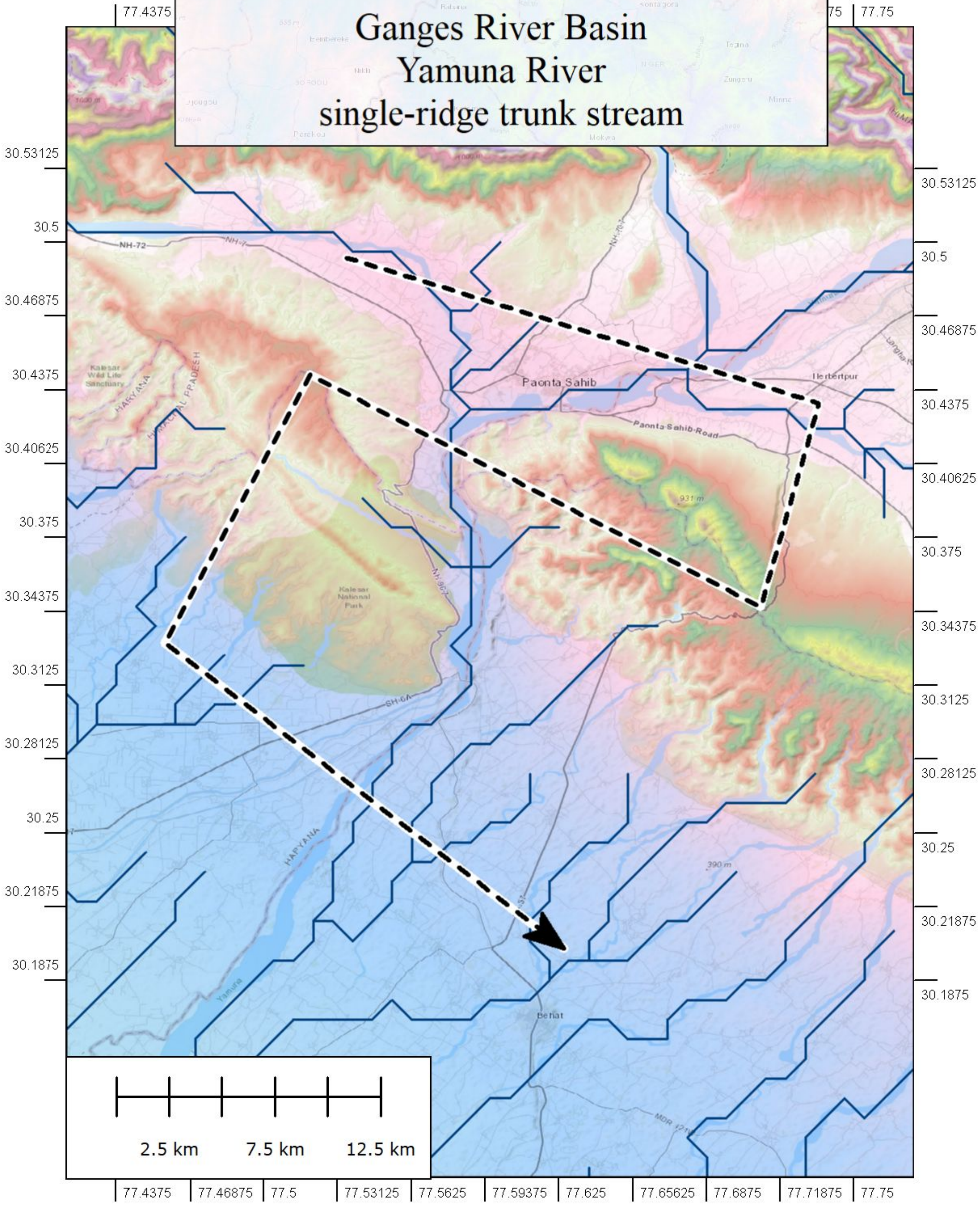

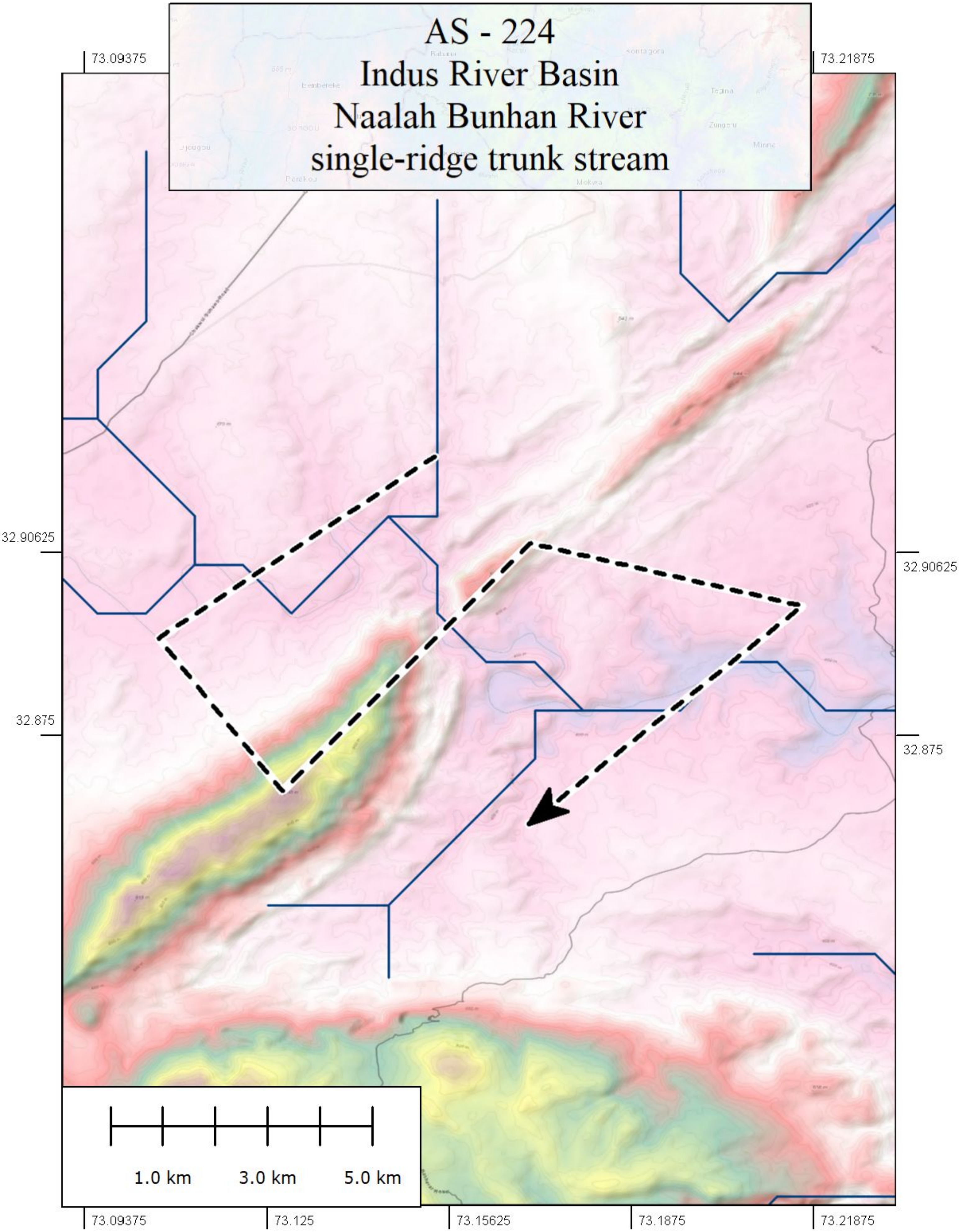
AS - 224
Indus River Basin
Naalah Bunhan River
single-ridge trunk stream
73.09375
73.21875
32.90625
32.90625
32.875
32.875
1.0 km
3.0 km
5.0 km
73.09375
73.125
73.15625
73.1875
73.21875

AS - 225
Yangtze River Basin
Fengxiang River
single-ridge head stream

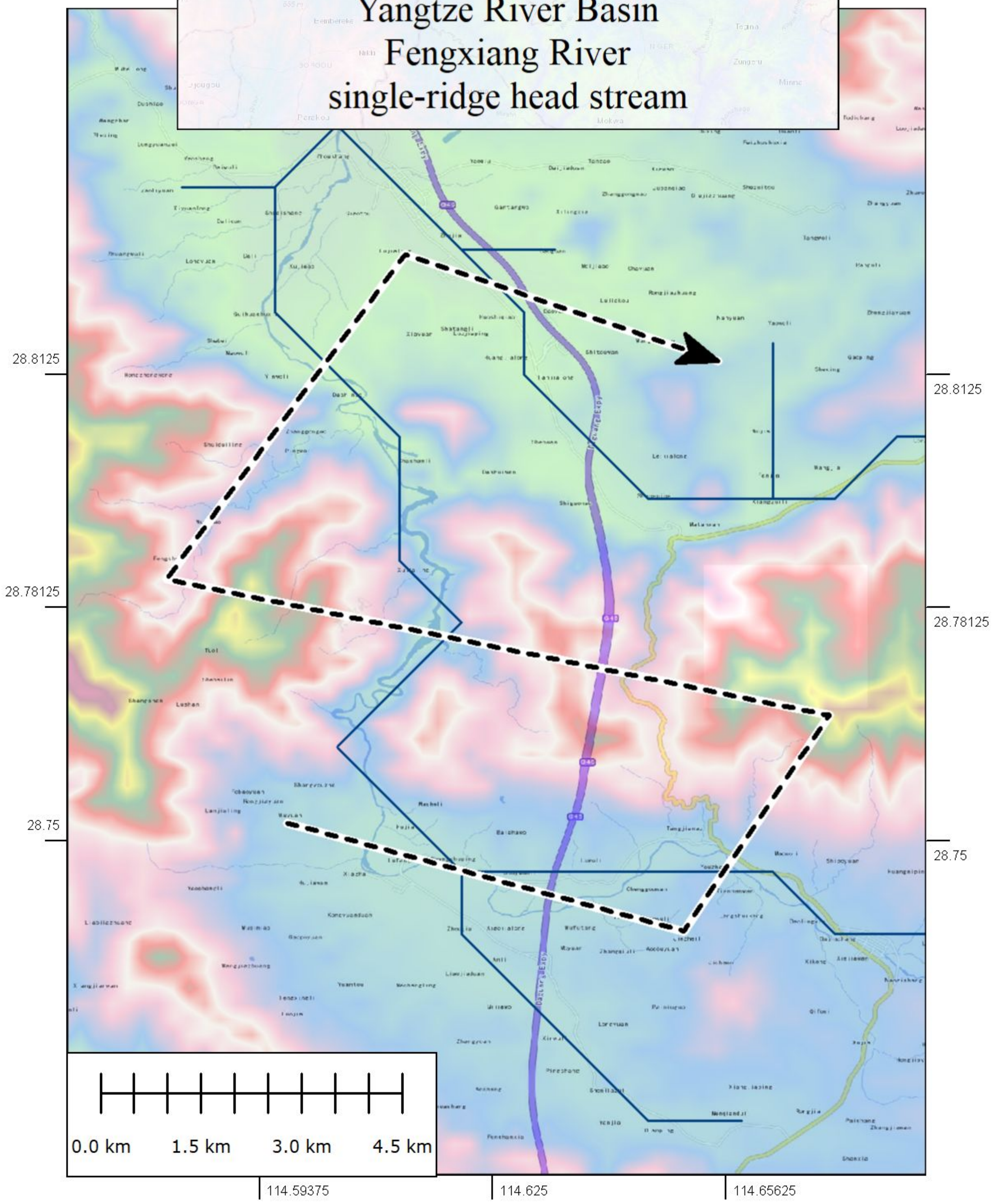

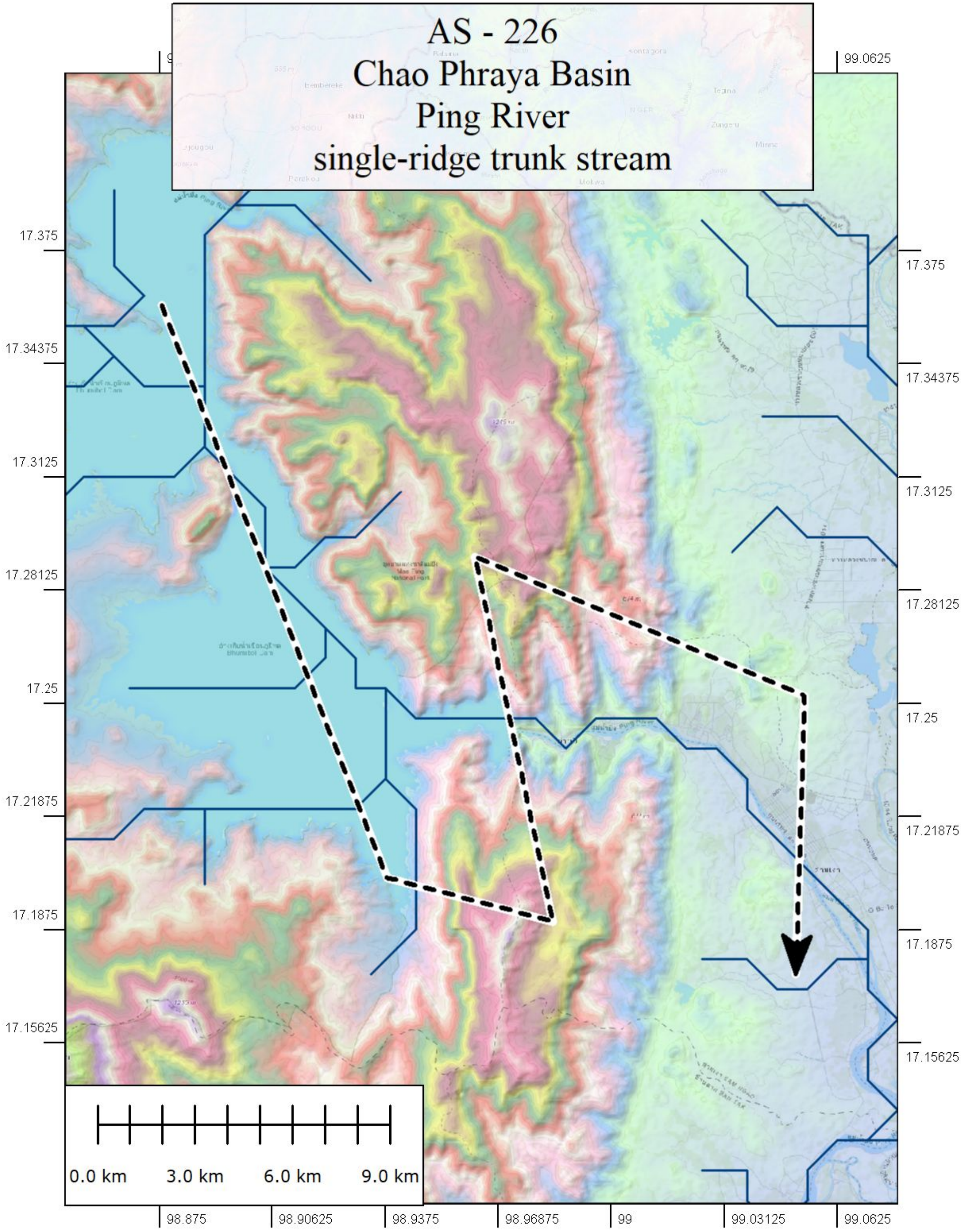

AS - 226
Chao Phraya Basin
Ping River
single-ridge trunk stream
17.375
17.34375
17.3125
17.28125
17.25
17.21875
17.1875
17.15625
98.875
98.90625
98.9375
98.96875
99
99.03125
99.0625
0.0 km
3.0 km
6.0 km
9.0 km

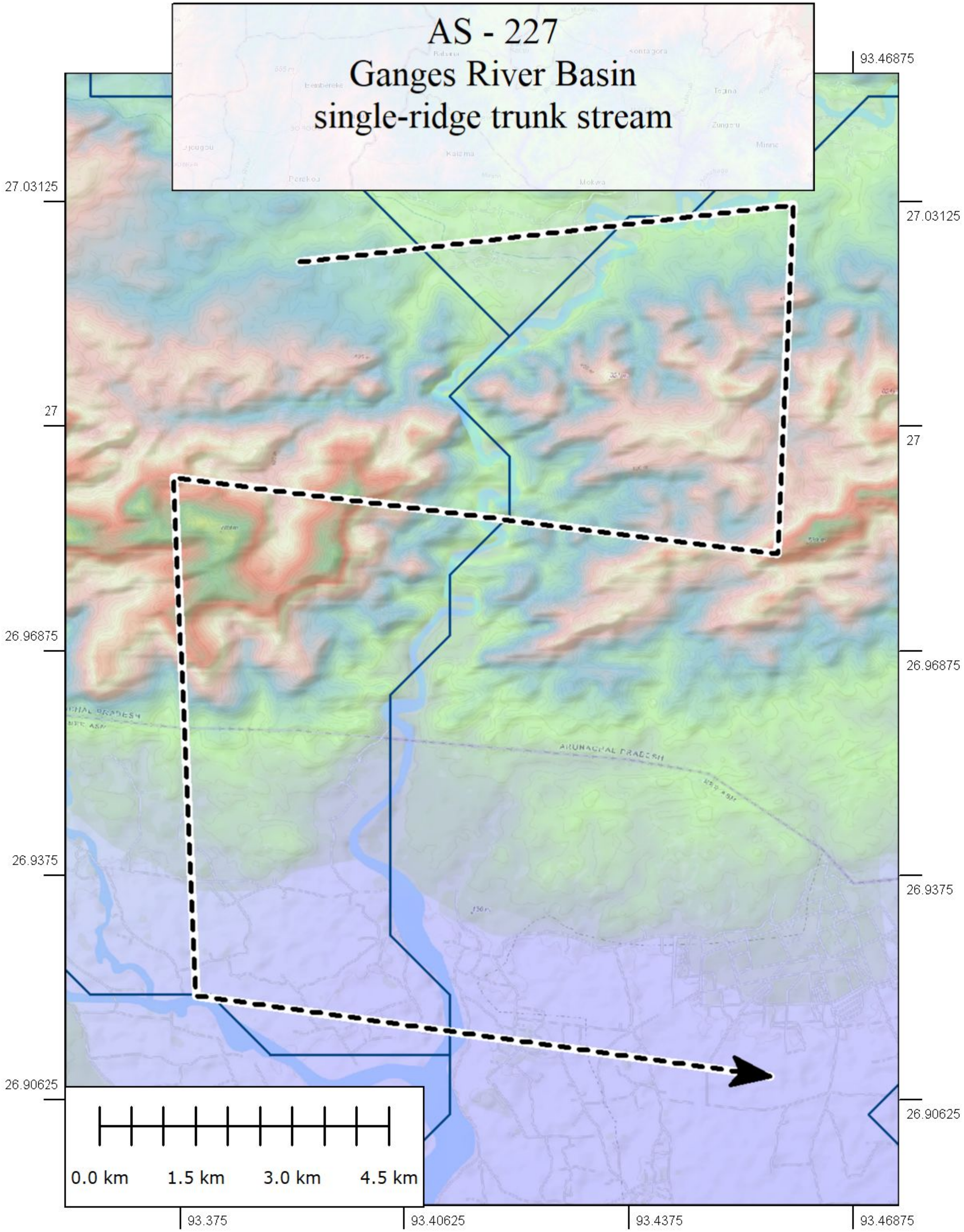
AS - 227
Ganges River Basin
single-ridge trunk stream
93.46875
27.03125
27
26.96875
26.9375
26.90625
93.375
93.40625
93.4375
0.0 km
1.5 km
3.0 km
4.5 km
ARUNACHAL PRADESH

AS - 229
Indus River Basin
Indus River
single-ridge trunk stream

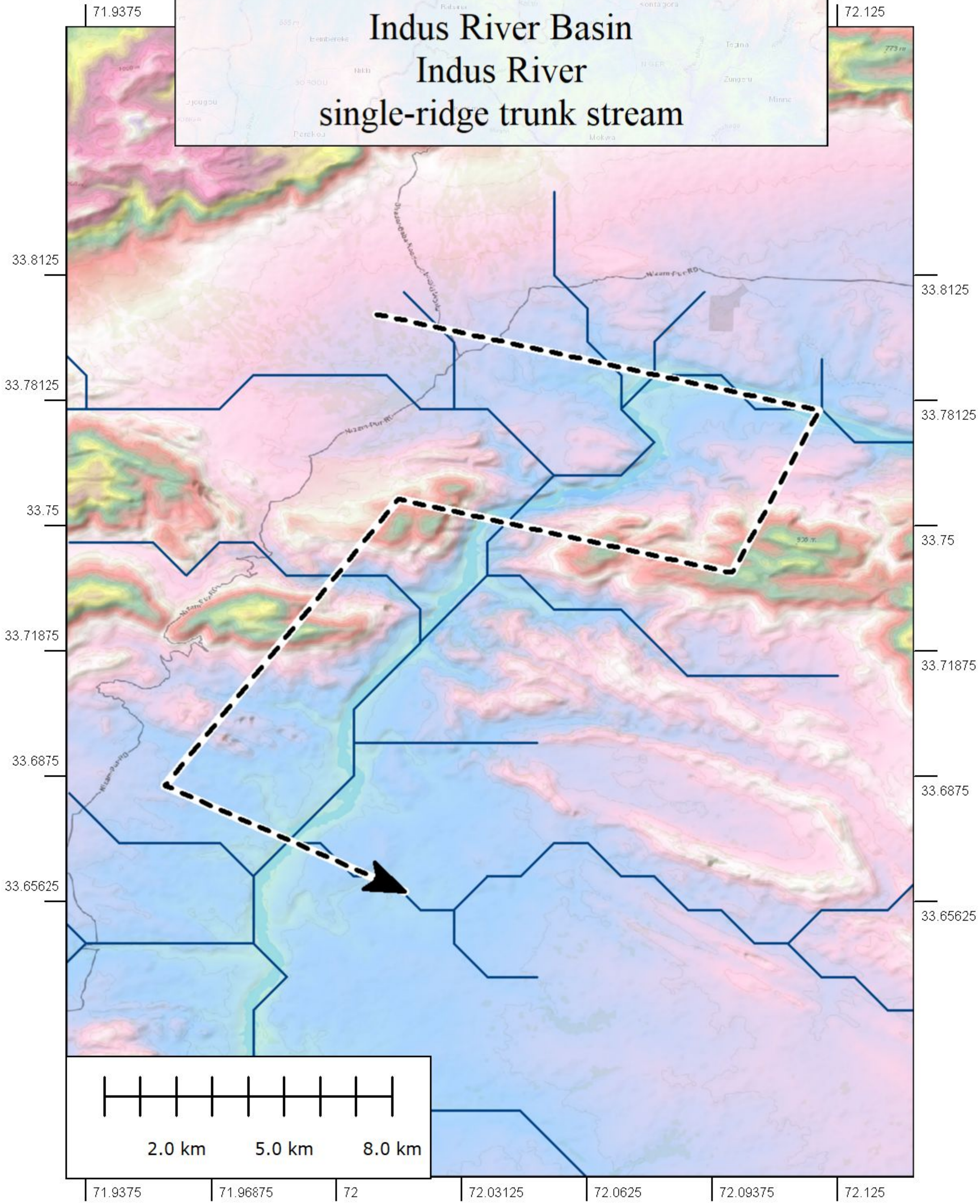

AS - 230
Yangtze River Basin
Ganshui River
single-ridge trunk stream

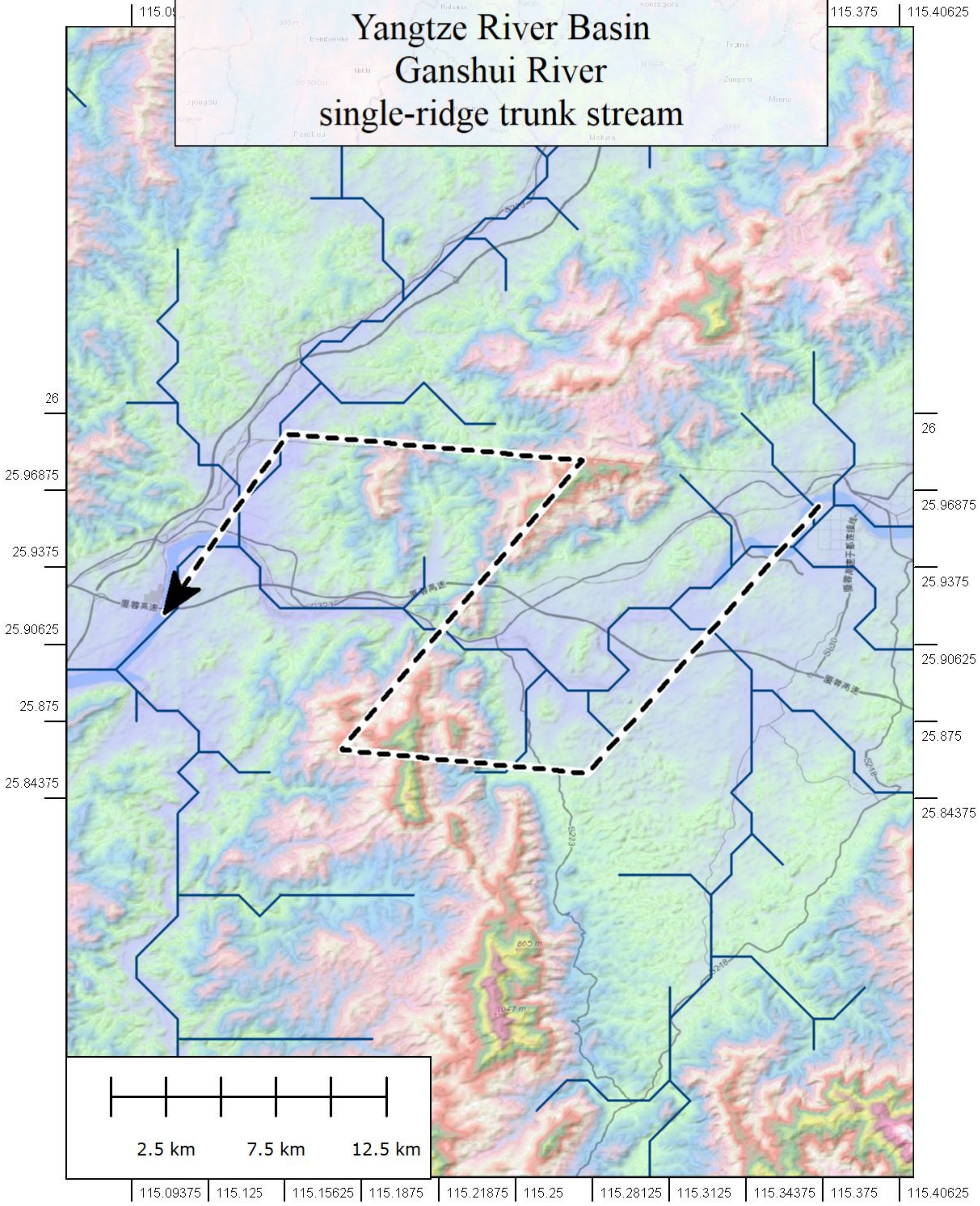

AS - 231
Dong River Basin
Xinfeng River Xinfengjiang Reservoir
single-ridge trunk stream

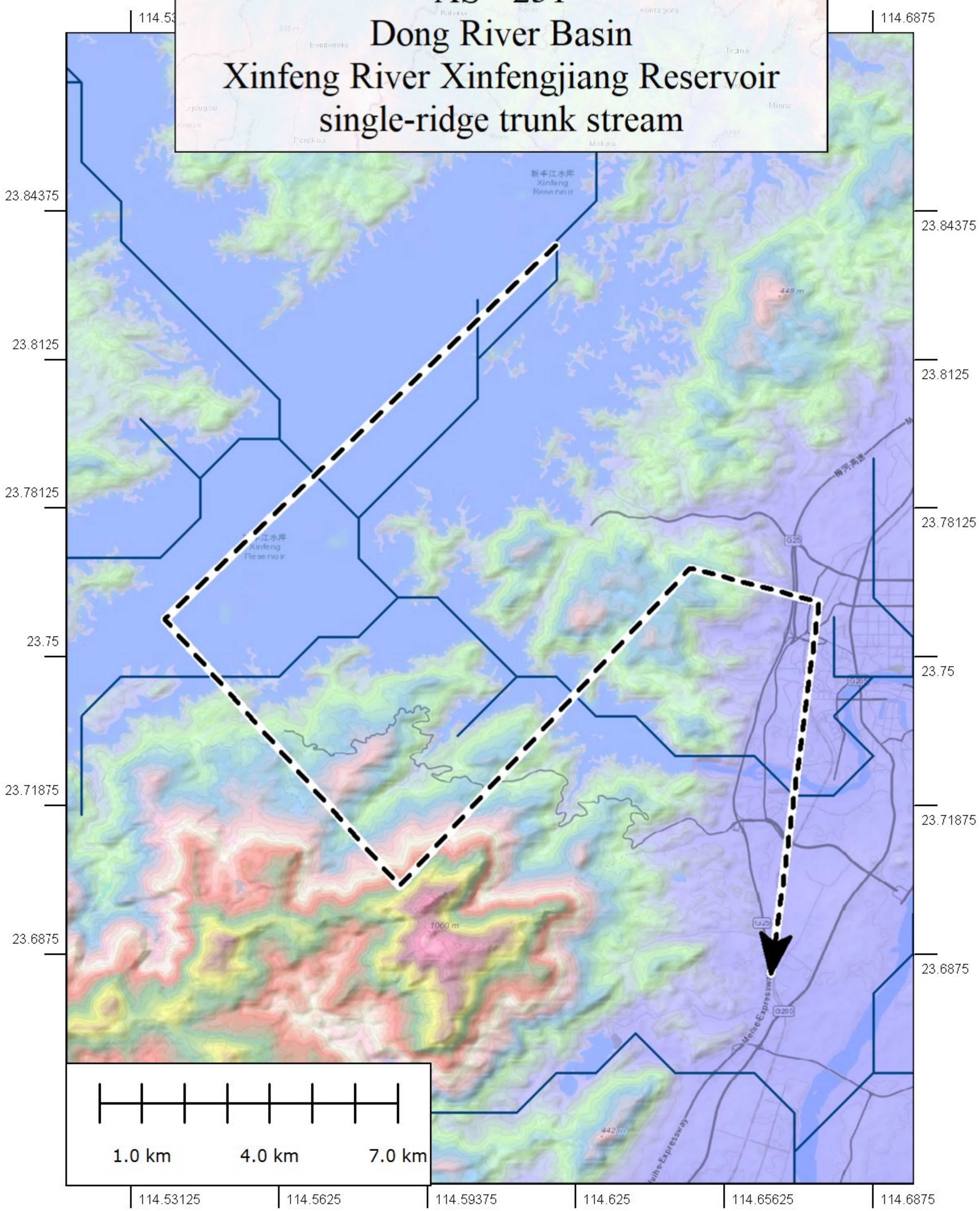

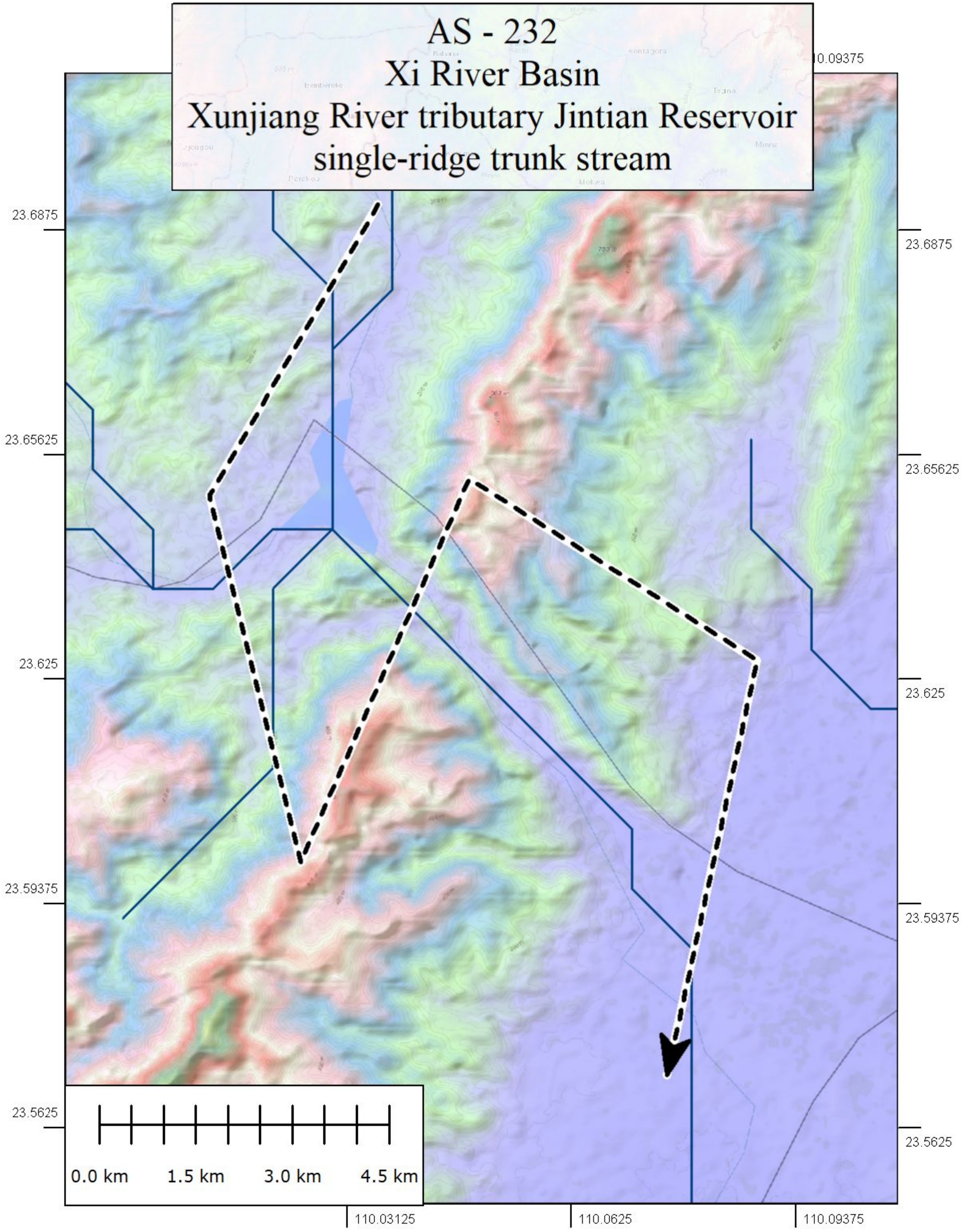
AS - 232
Xi River Basin
Xunjiang River tributary Jintian Reservoir
single-ridge trunk stream
23.6875
23.65625
23.625
23.59375
23.5625
110.03125
110.0625
110.09375
0.0 km
1.5 km
3.0 km
4.5 km

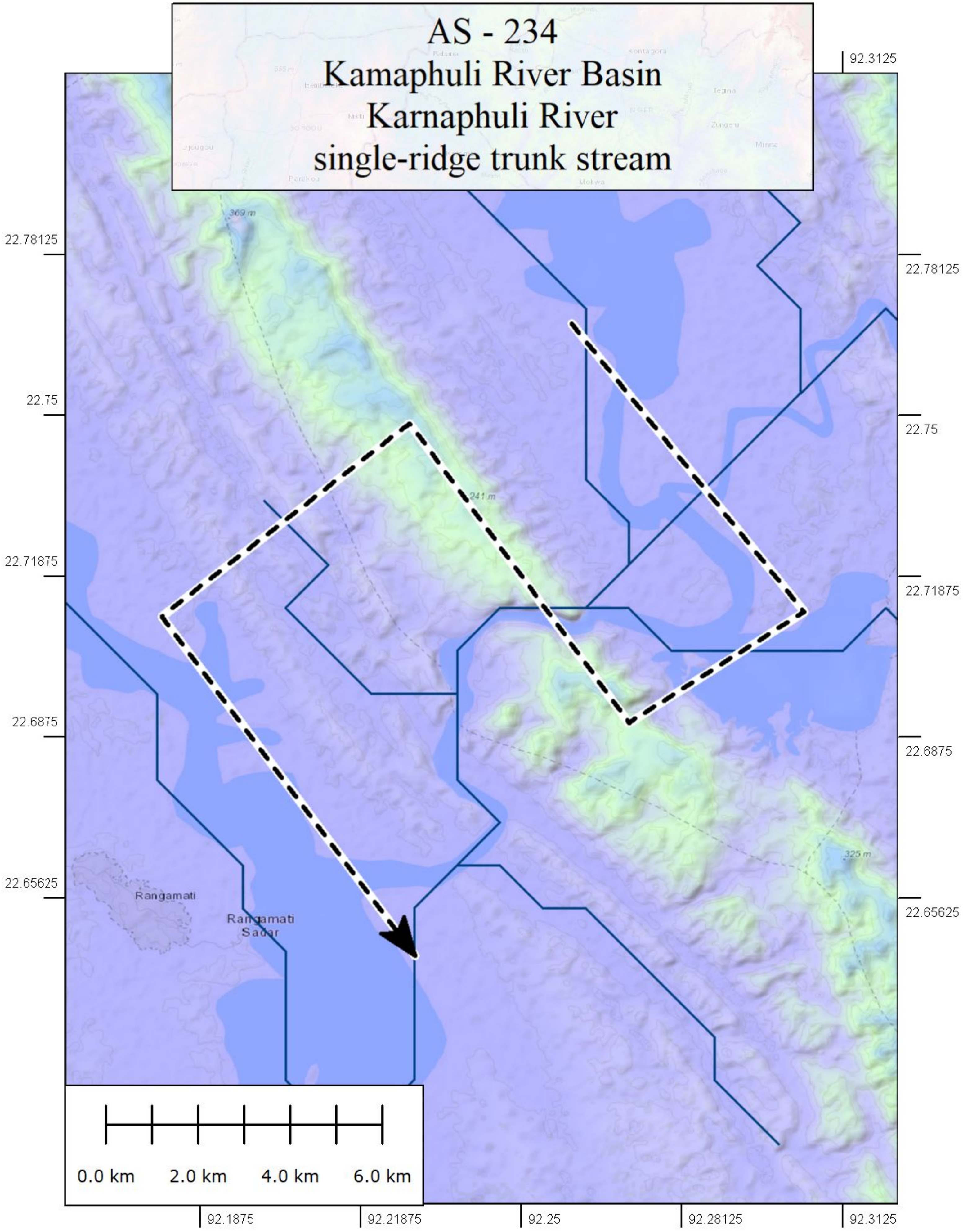
AS - 234
Kamaphuli River Basin
Karnaphuli River
single-ridge trunk stream
92.3125
22.78125
22.75
22.71875
22.6875
22.65625
369 m
241 m
325 m
Rangamati
Rangamati Sadar
0.0 km
2.0 km
4.0 km
6.0 km
92.1875
92.21875
92.25
92.28125

AS - 236
Irrawaddy River Basin
Yu River
single-ridge trunk stream

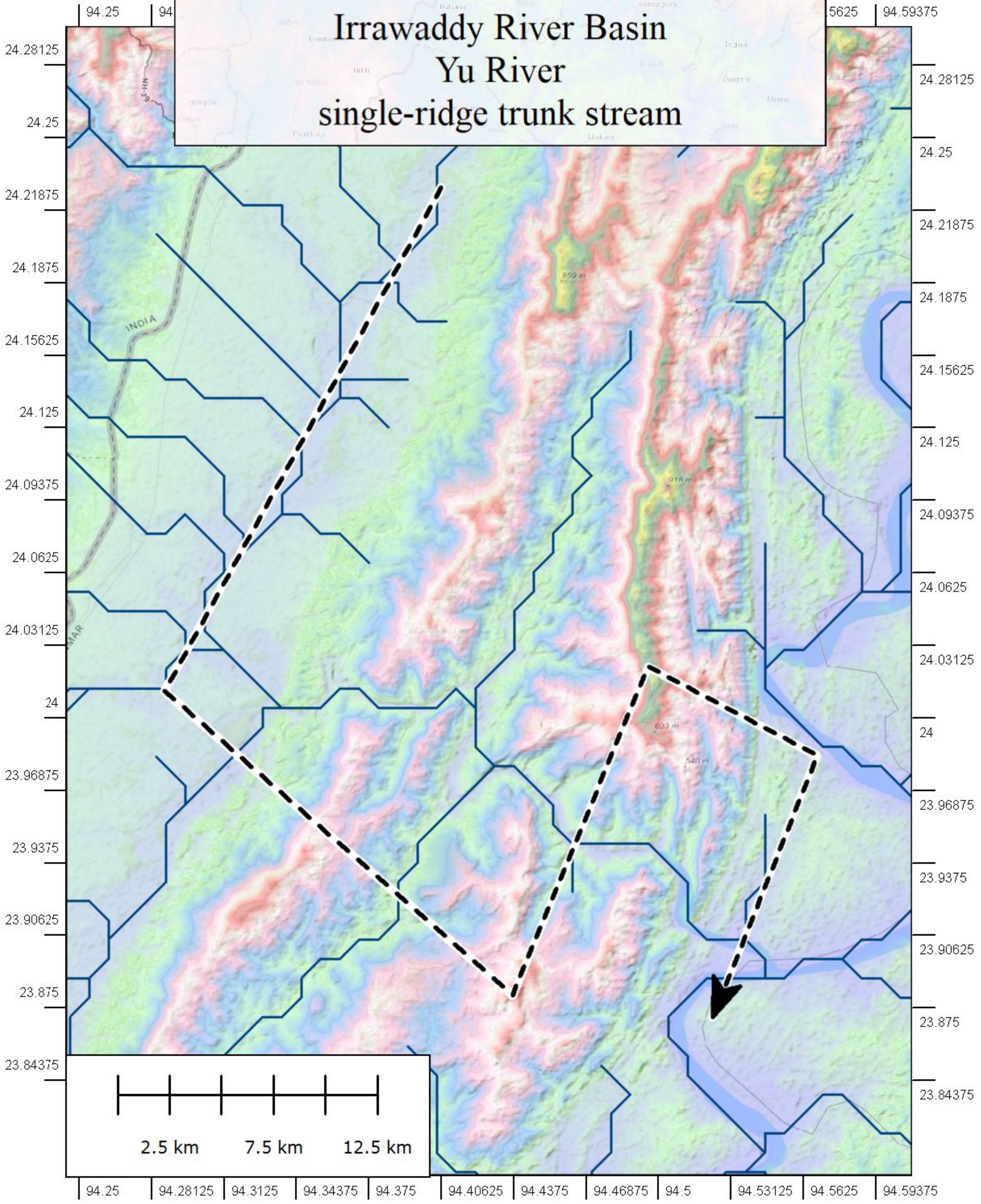

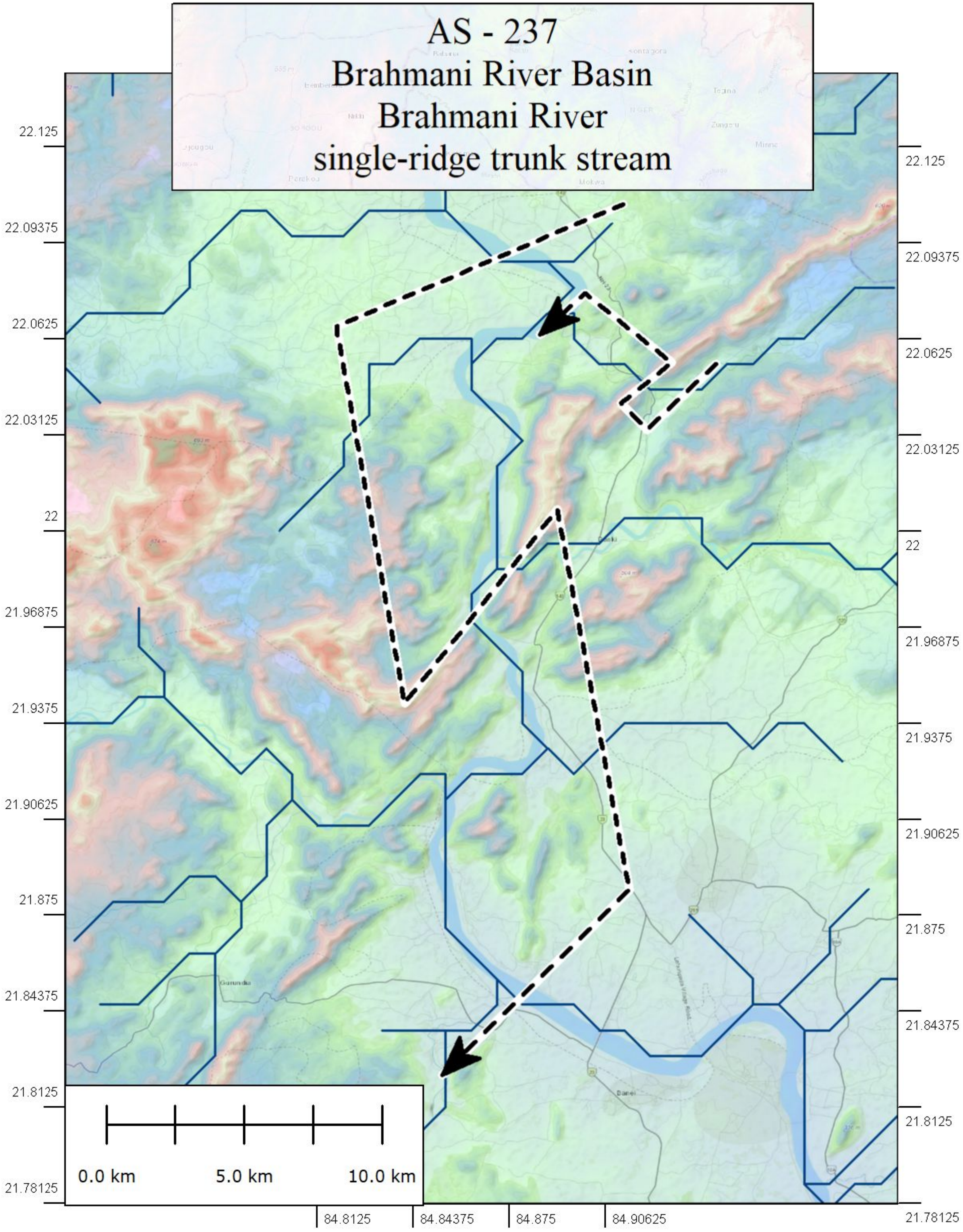
AS - 237
Brahmani River Basin
Brahmani River
single-ridge trunk stream
0.0 km
5.0 km
10.0 km
22.125
22.09375
22.0625
22.03125
22
21.96875
21.9375
21.90625
21.875
21.84375
21.8125
21.78125
84.8125
84.84375
84.875
84.90625

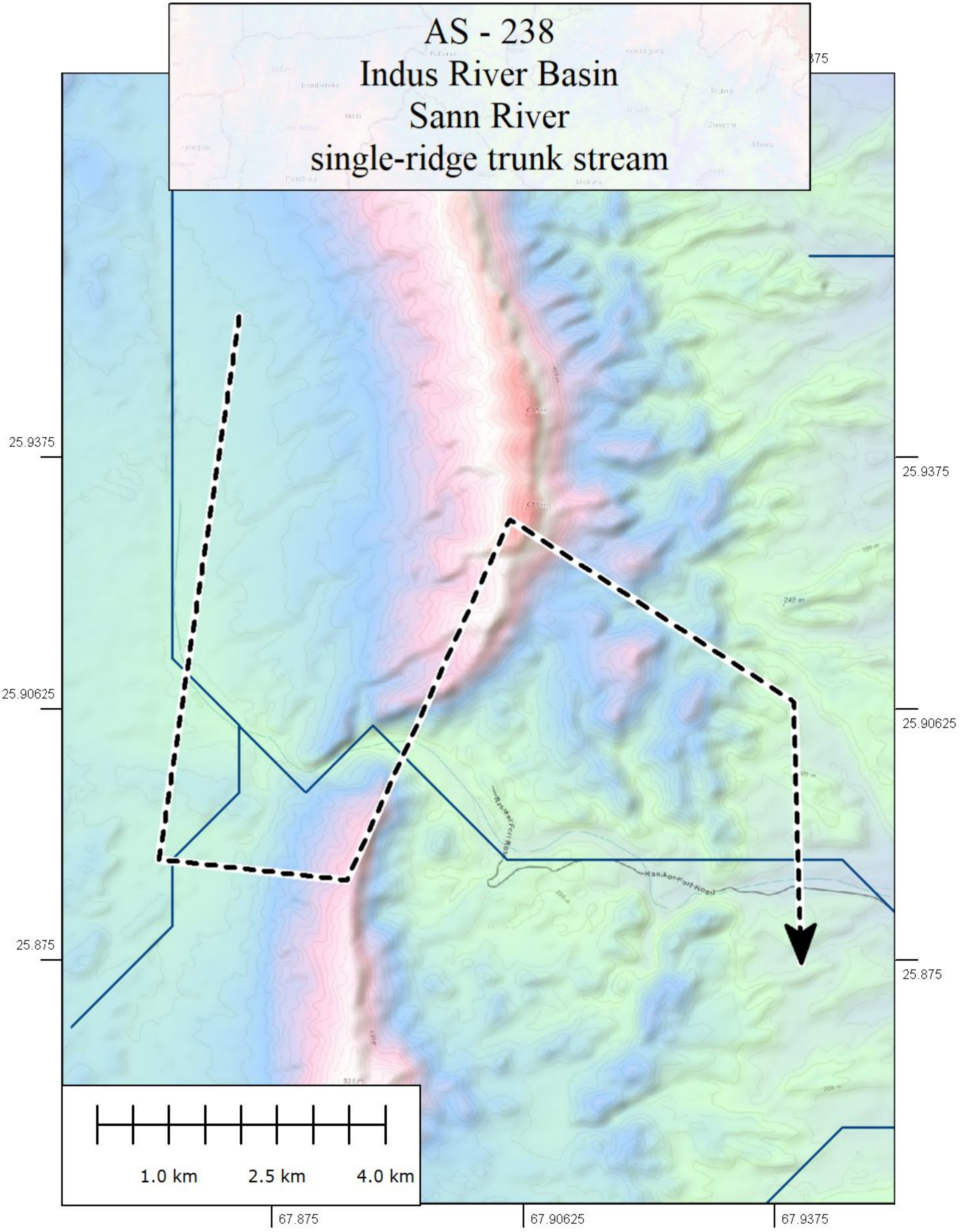
AS - 238
Indus River Basin
Sann River
single-ridge trunk stream
25.9375
25.90625
25.875
25.9375
25.90625
25.875
67.875
67.90625
67.9375
1.0 km
2.5 km
4.0 km

AS - 240
Irrawaddy River Basin
Chindwin River
single-ridge trunk stream

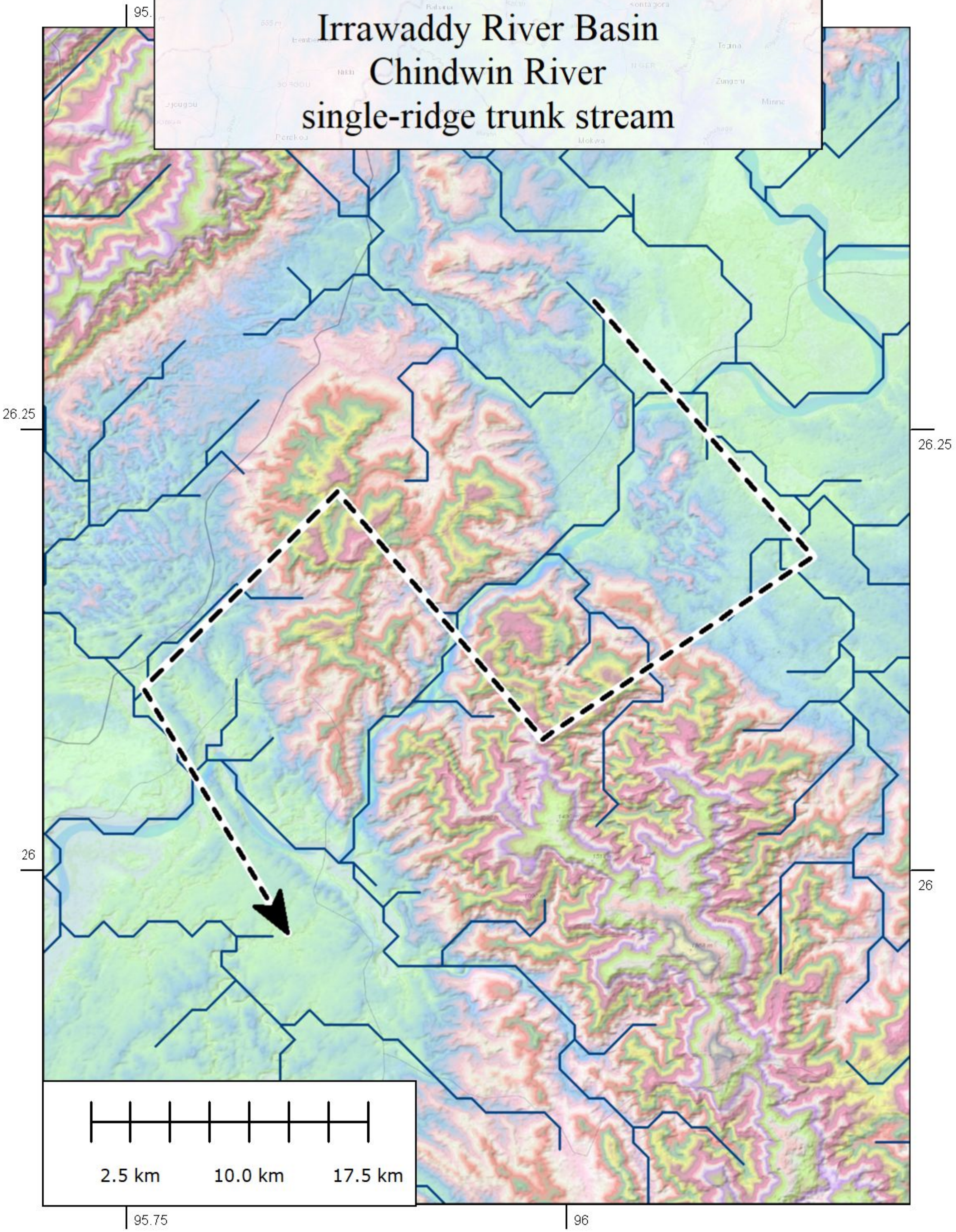

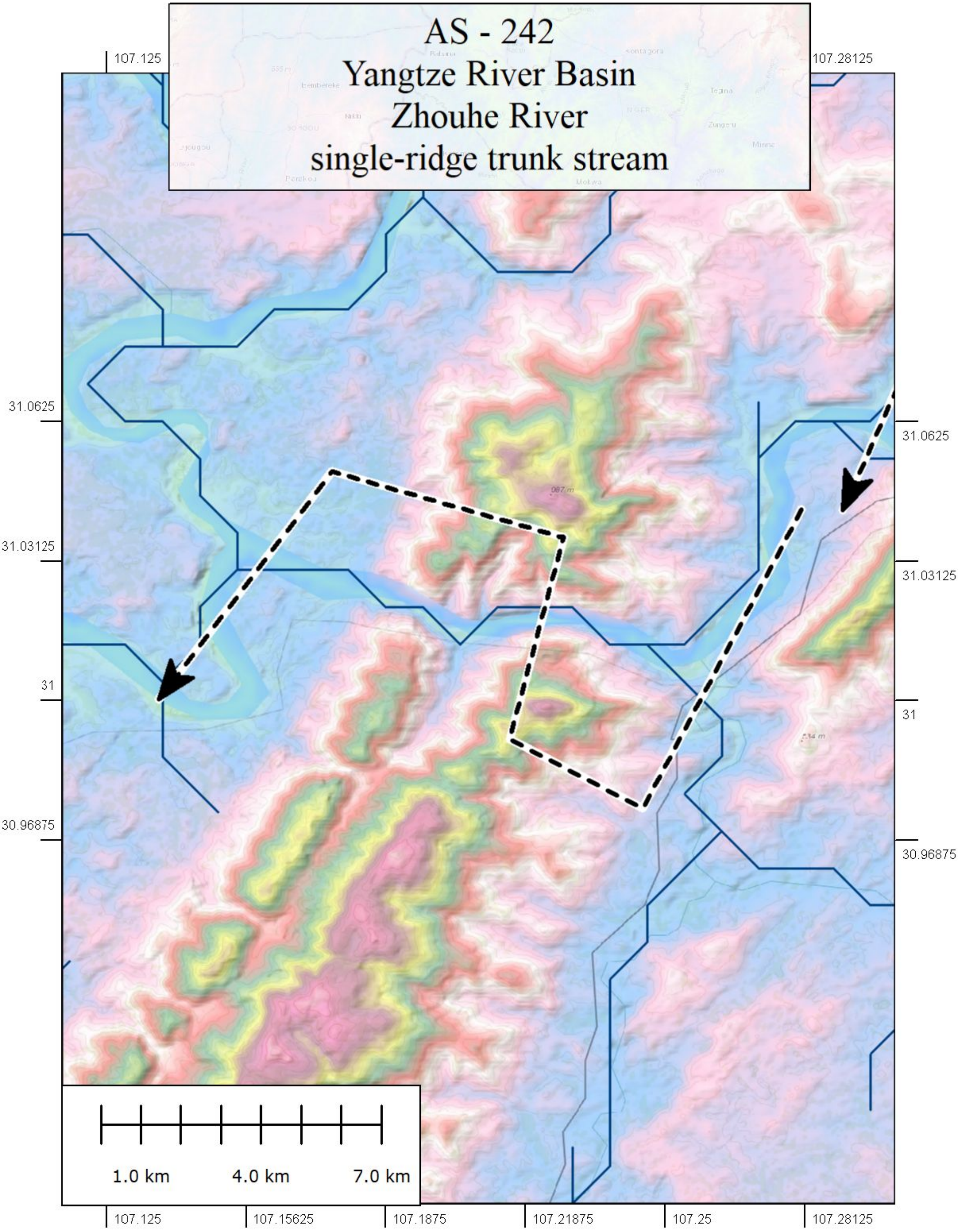
AS - 242
Yangtze River Basin
Zhouhe River
single-ridge trunk stream
107.125
107.15625
107.1875
107.21875
107.25
107.28125
31.0625
31.03125
31
30.96875
1.0 km
4.0 km
7.0 km

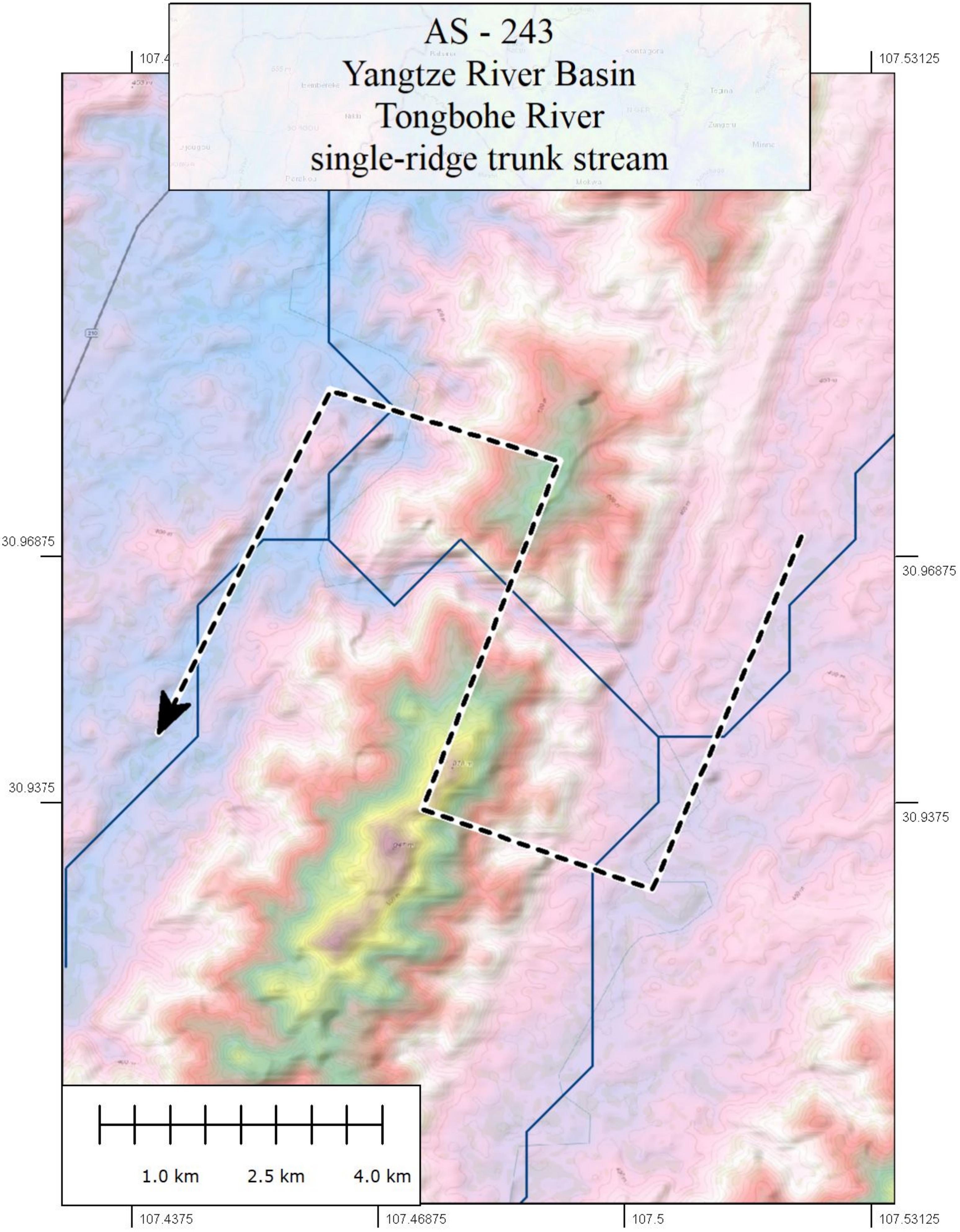
AS - 243
Yangtze River Basin
Tongbohe River
single-ridge trunk stream
107.53125
30.96875
30.96875
30.9375
30.9375
1.0 km
2.5 km
4.0 km
107.4375
107.46875
107.5
107.53125

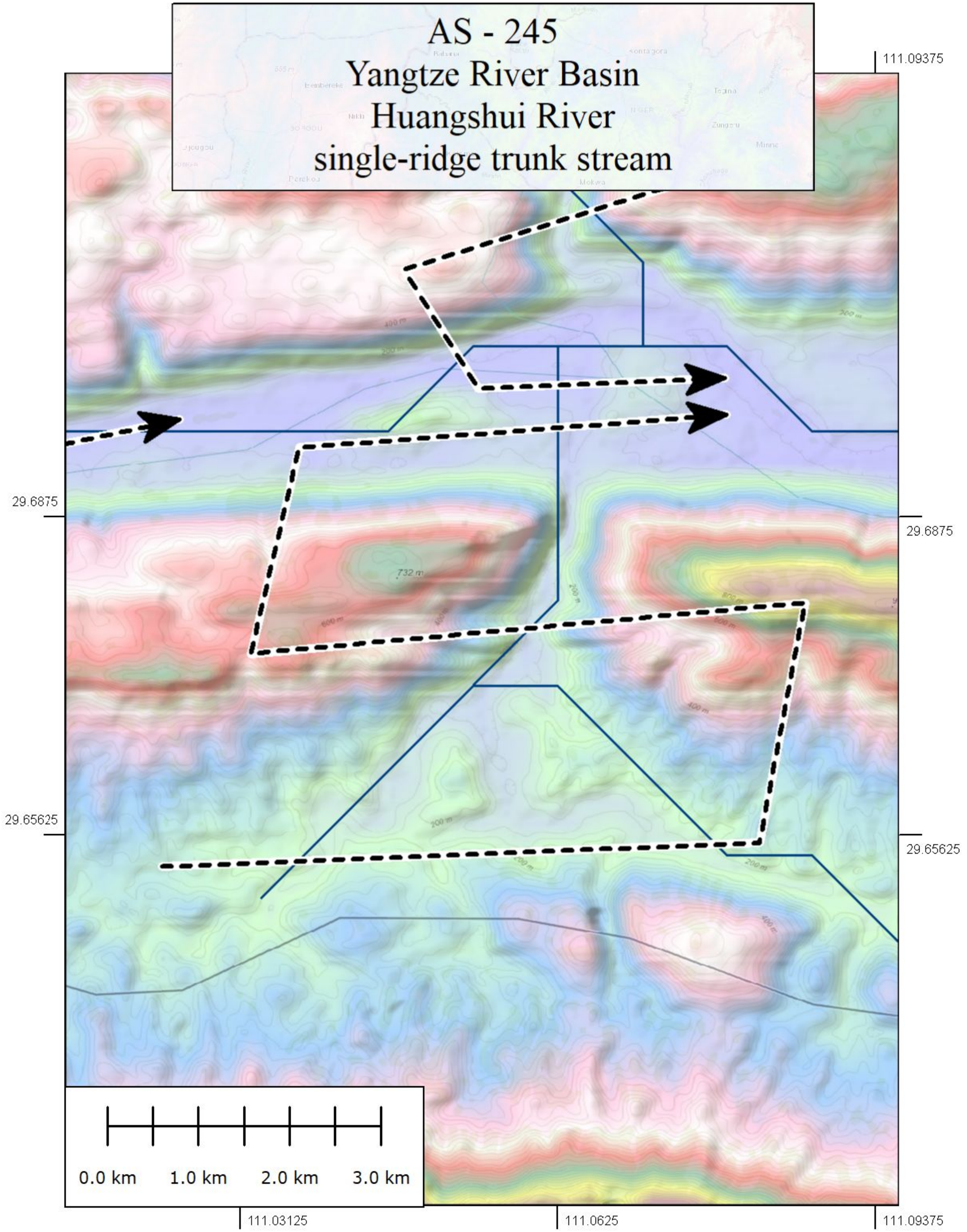

AS - 245
Yangtze River Basin
Huangshui River
single-ridge trunk stream
111.09375
29.6875
29.6875
29.65625
29.65625
732 m
0.0 km
1.0 km
2.0 km
3.0 km
111.03125
111.0625
111.09375

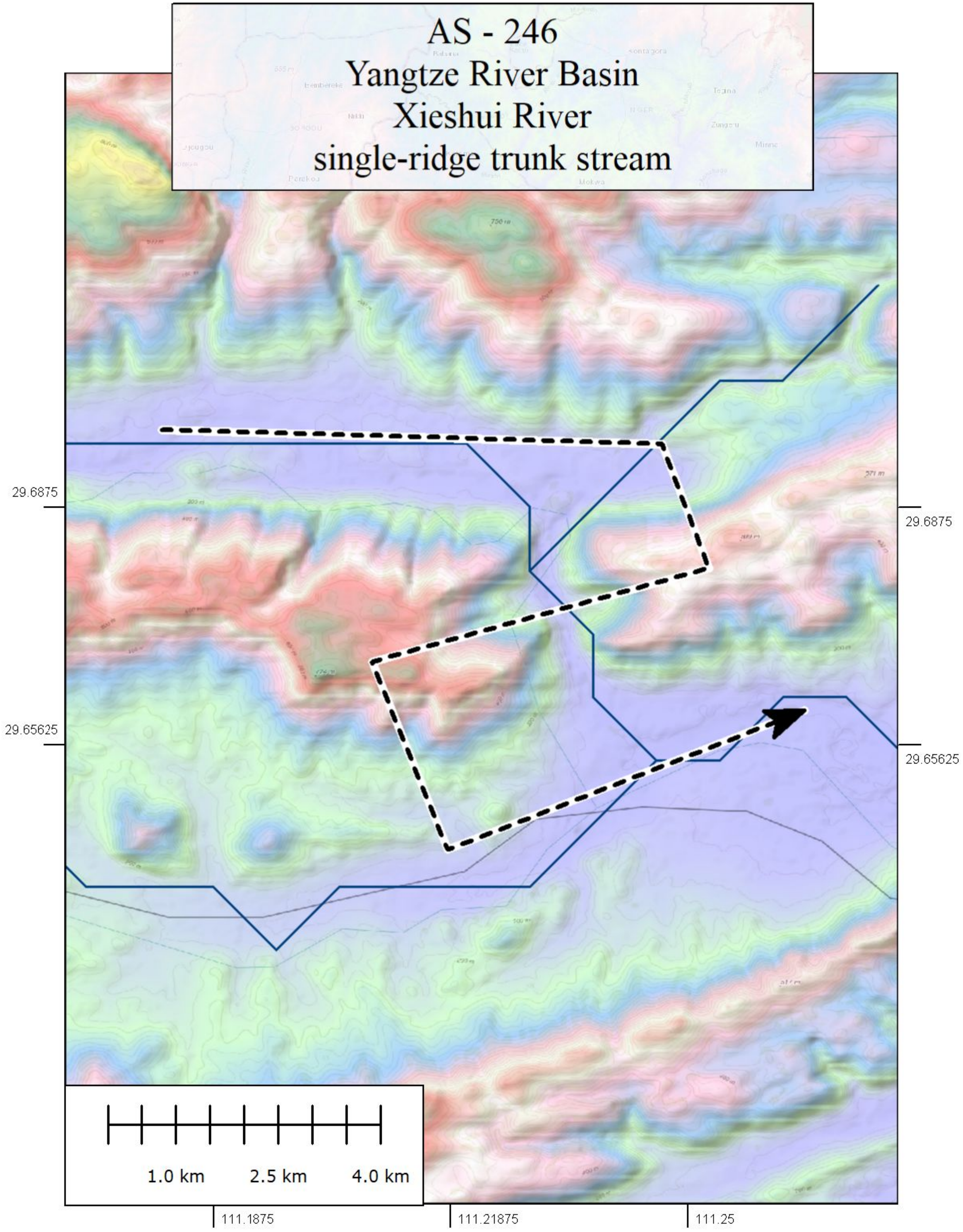
AS - 246
Yangtze River Basin
Xieshui River
single-ridge trunk stream
29.6875
29.65625
29.6875
29.65625
1.0 km
2.5 km
4.0 km
111.1875
111.21875
111.25

AS - 248
Ganges River Basin
Chathe River
single-ridge trunk stream

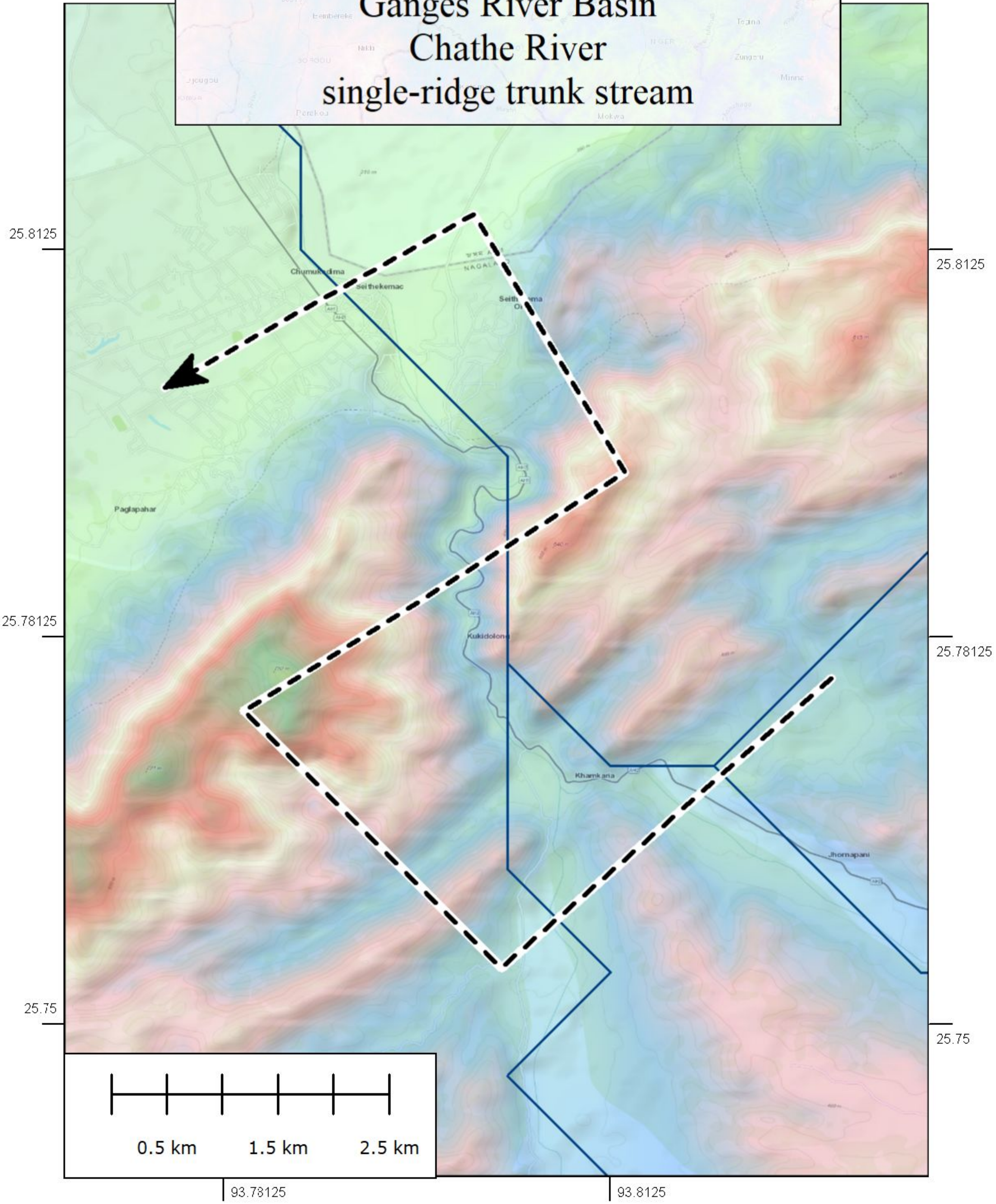

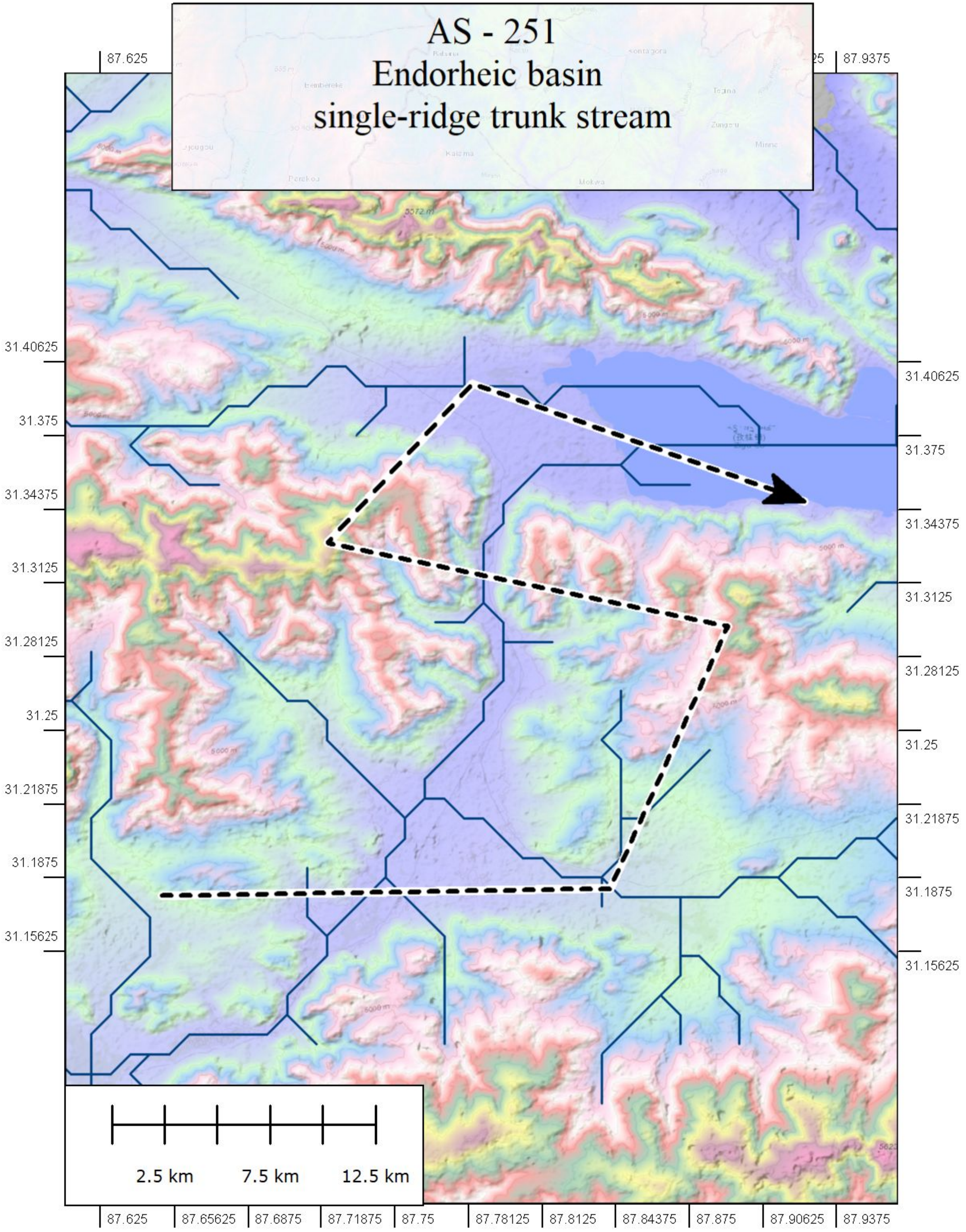
AS - 251
Endorheic basin
single-ridge trunk stream
87.625
87.65625
87.6875
87.71875
87.75
87.78125
87.8125
87.84375
87.875
87.90625
87.9375
31.40625
31.375
31.34375
31.3125
31.28125
31.25
31.21875
31.1875
31.15625
2.5 km
7.5 km
12.5 km

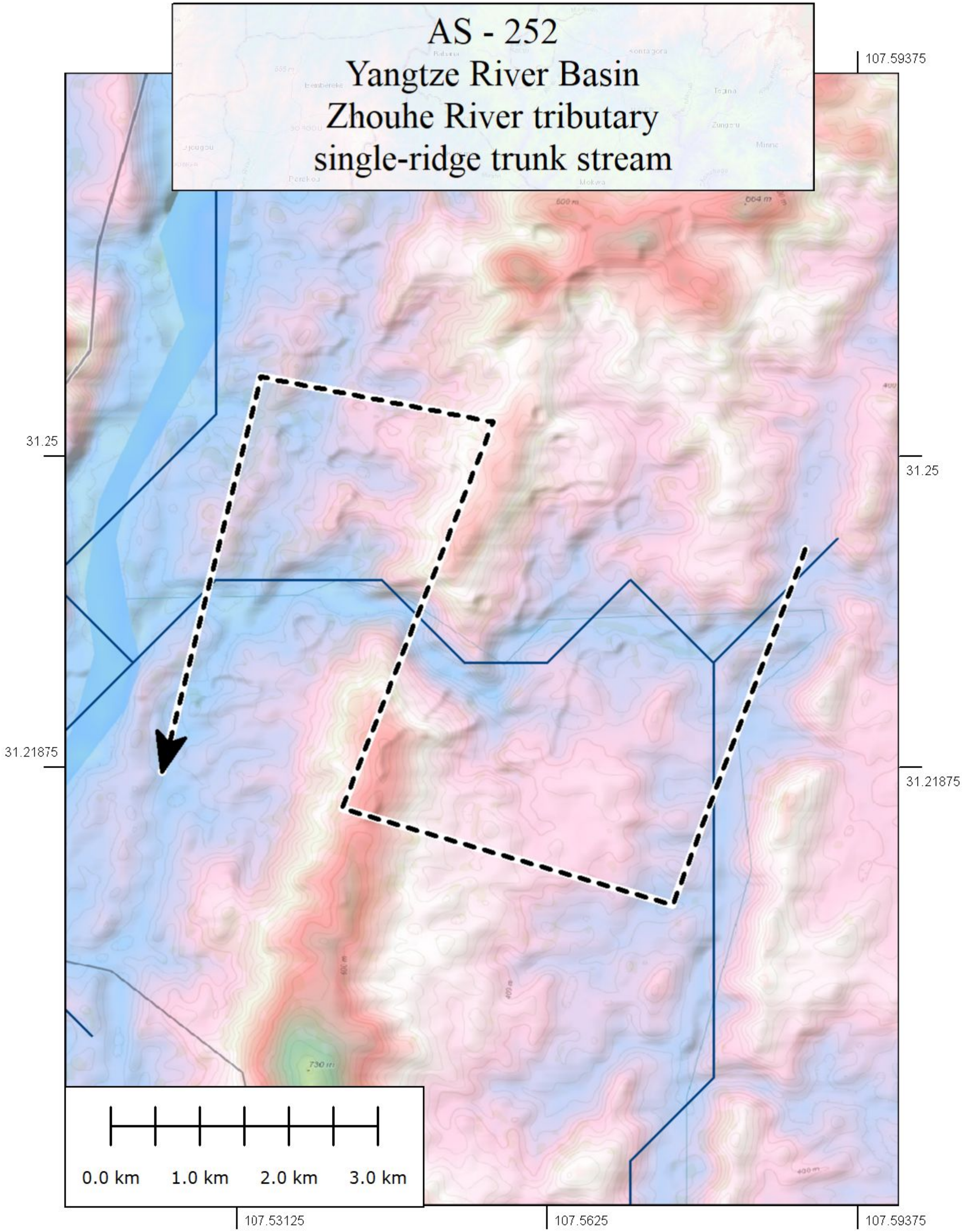
AS - 252
Yangtze River Basin
Zhouhe River tributary
single-ridge trunk stream
107.59375
31.25
31.25
31.21875
31.21875
107.53125
107.5625
107.59375
0.0 km
1.0 km
2.0 km
3.0 km

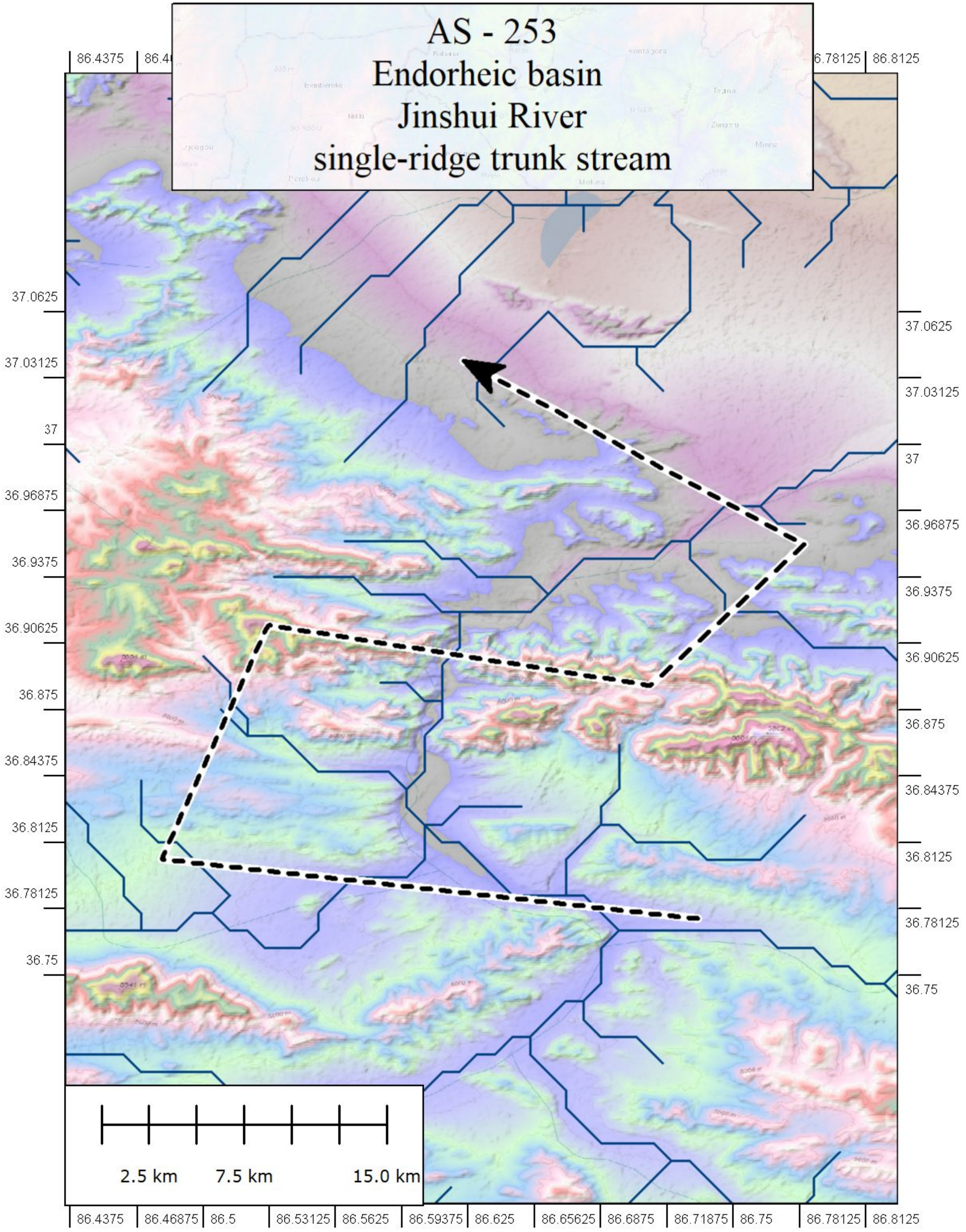
AS - 253
Endorheic basin
Jinshui River
single-ridge trunk stream
86.4375
86.46875
86.5
86.53125
86.5625
86.59375
86.625
86.65625
86.6875
86.71875
86.75
86.78125
86.8125
37.0625
37.03125
37
36.96875
36.9375
36.90625
36.875
36.84375
36.8125
36.78125
36.75
2.5 km
7.5 km
15.0 km

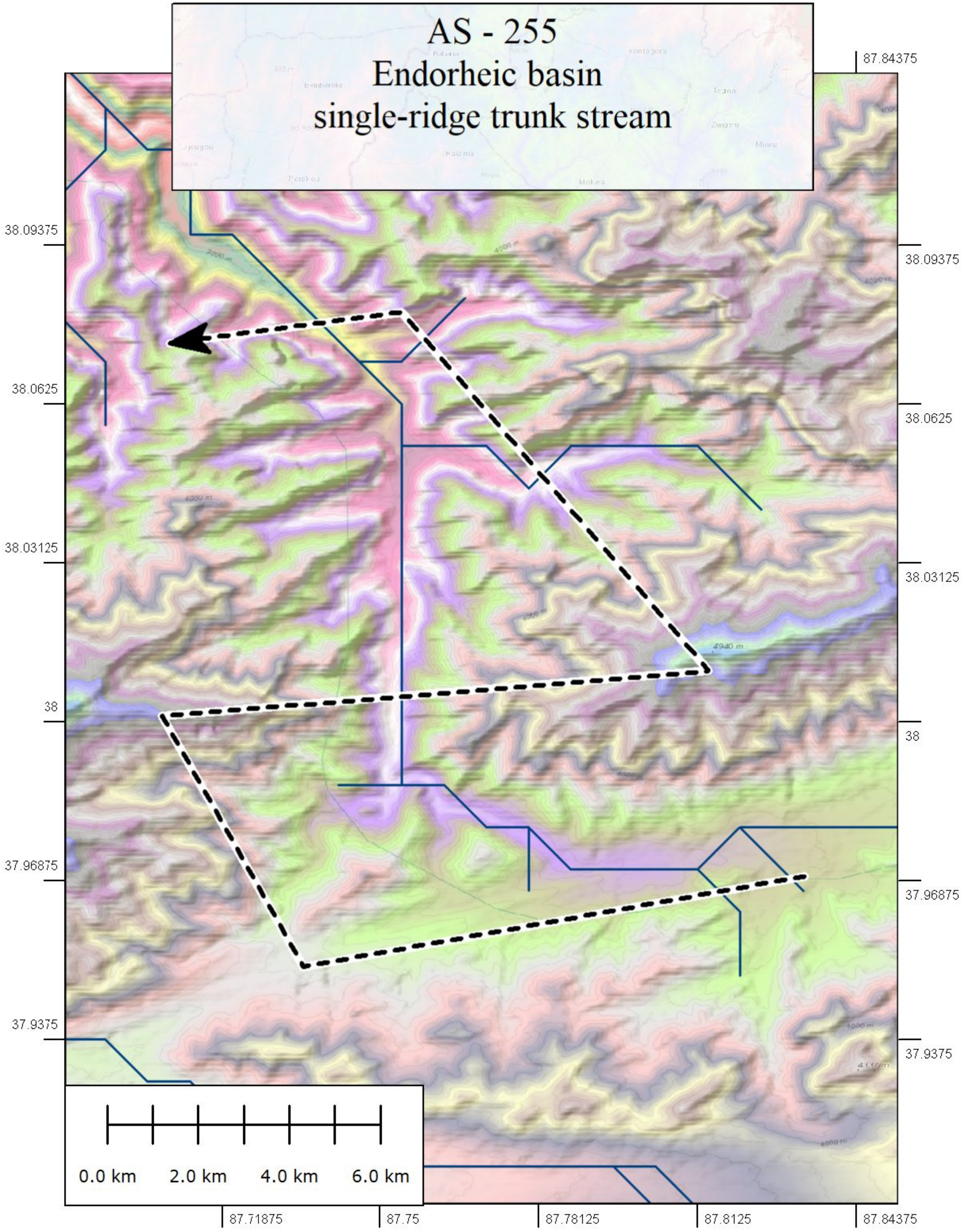
AS - 255
Endorheic basin
single-ridge trunk stream
38.09375
38.0625
38.03125
38
37.96875
37.9375
87.84375
87.71875
87.75
87.78125
87.8125
0.0 km
2.0 km
4.0 km
6.0 km
4940 m

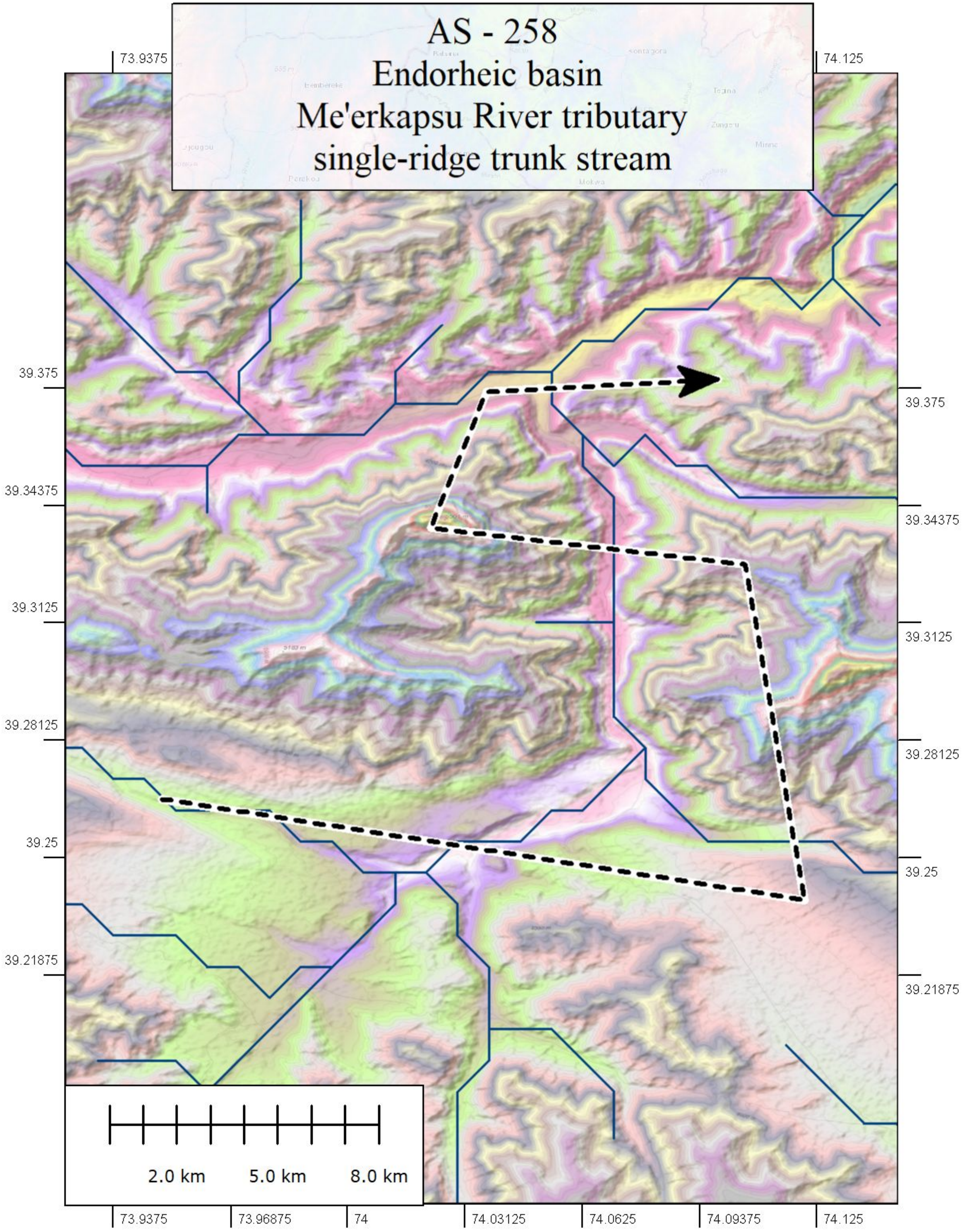
AS - 258
Endorheic basin
Me'erkapsu River tributary
single-ridge trunk stream
73.9375
74.125
73.96875
74
74.03125
74.0625
74.09375
39.375
39.34375
39.3125
39.28125
39.25
39.21875
2.0 km
5.0 km
8.0 km

AS - 259
Indus River Basin
Indus River
single-ridge trunk stream

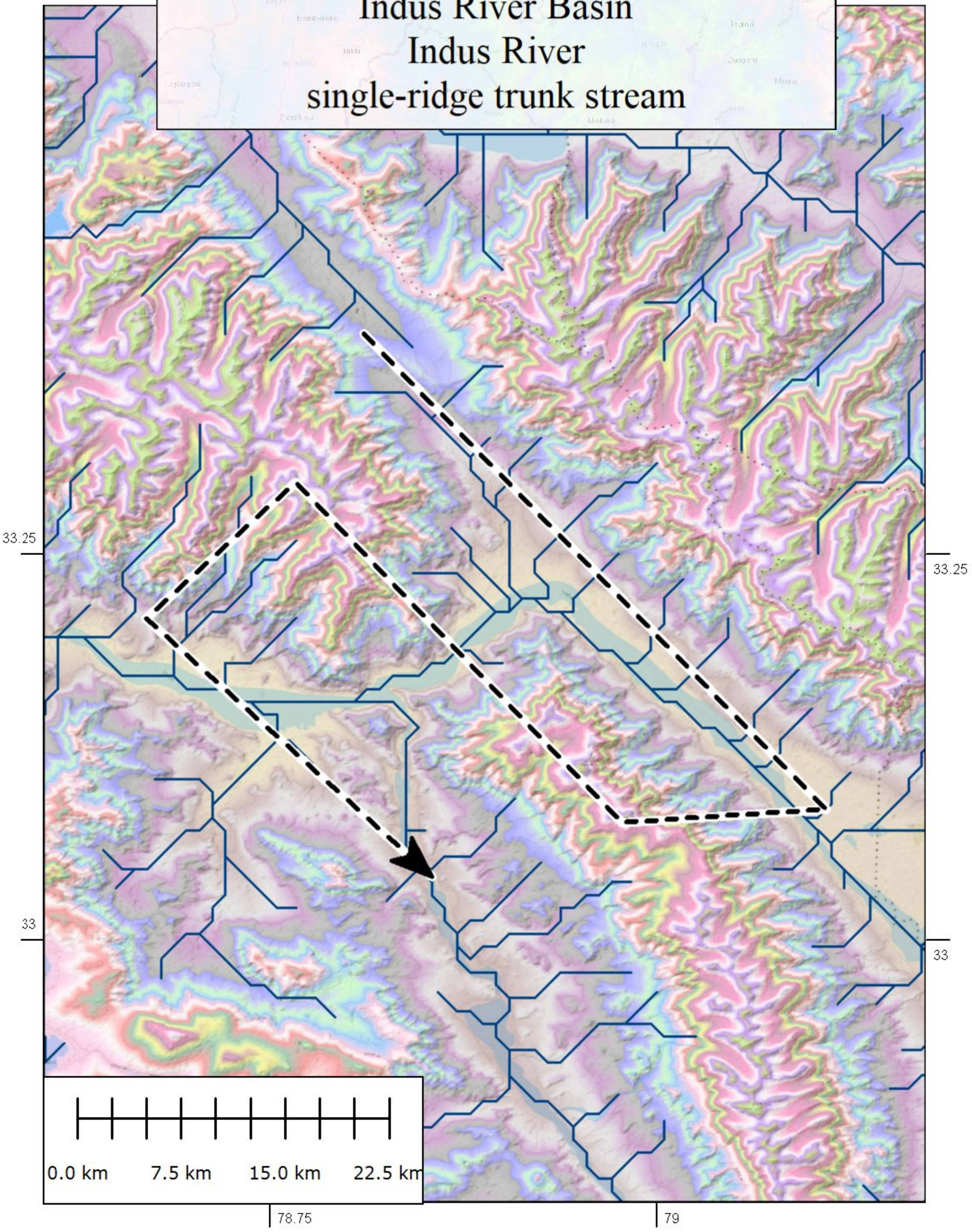

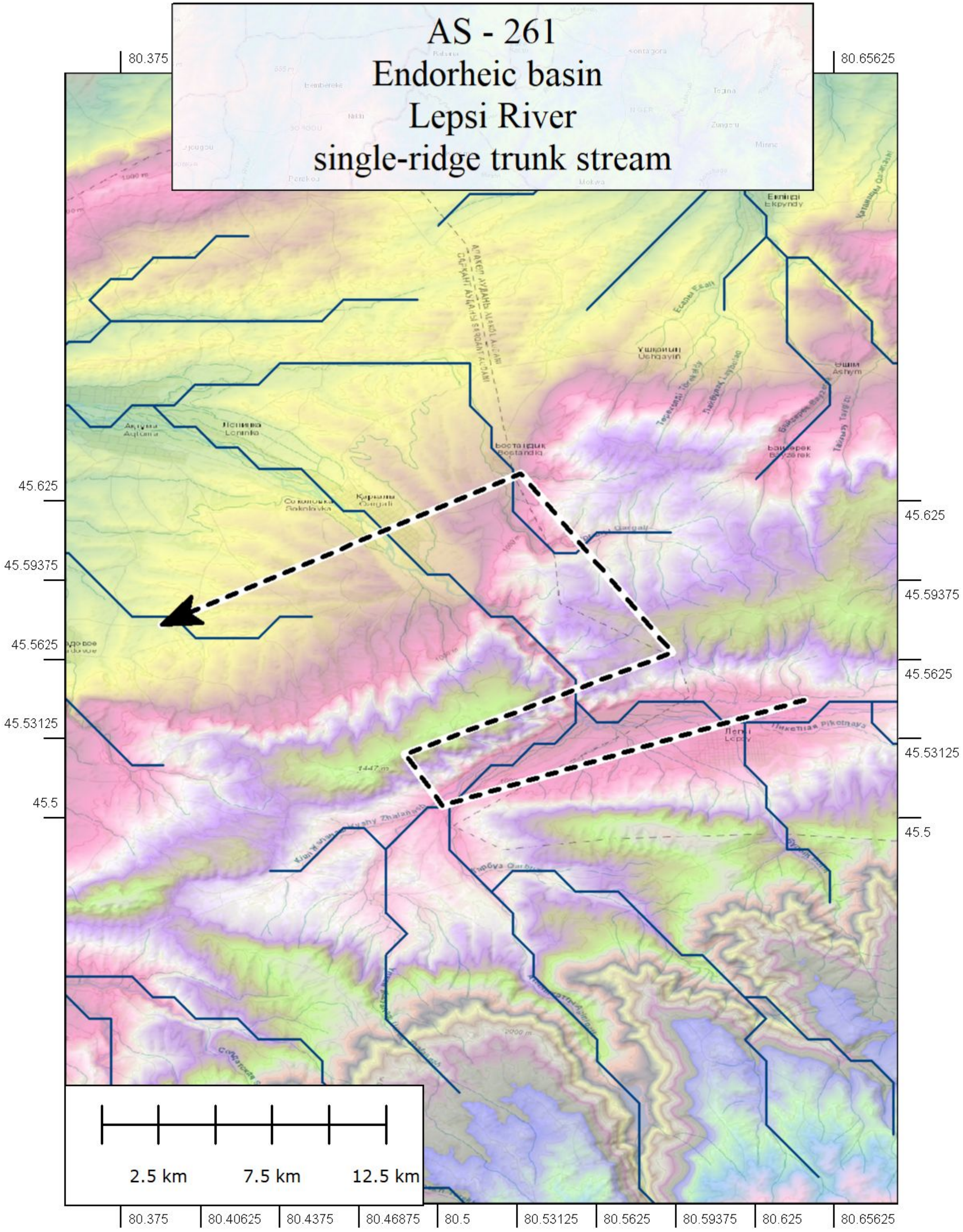
AS - 261
Endorheic basin
Lepsi River
single-ridge trunk stream
80.375
80.65625
45.625
45.59375
45.5625
45.53125
45.5
45.625
45.59375
45.5625
45.53125
45.5
2.5 km
7.5 km
12.5 km
80.375
80.40625
80.4375
80.46875
80.5
80.53125
80.5625
80.59375
80.625
80.65625

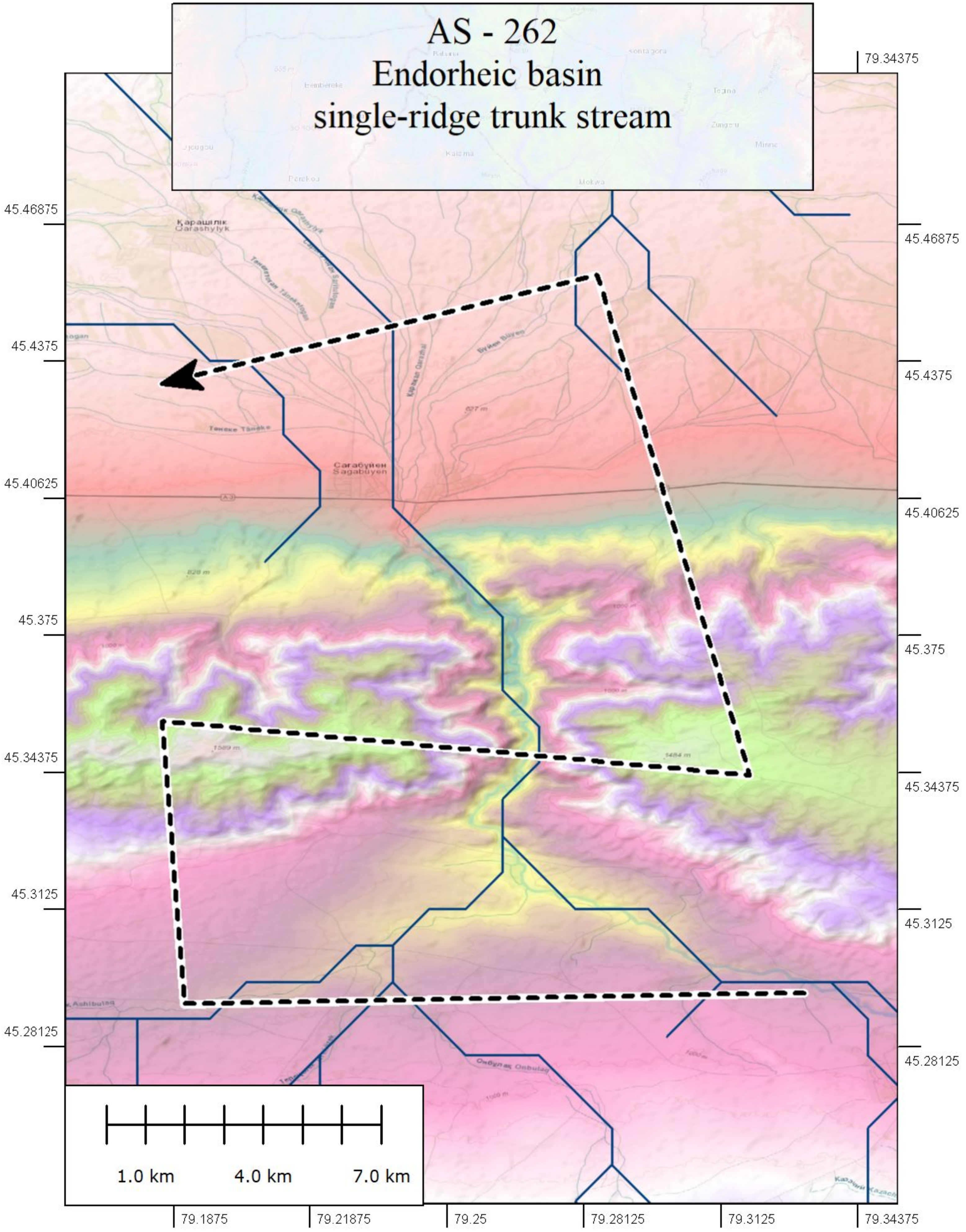

AS - 262
Endorheic basin
single-ridge trunk stream
45.46875
45.4375
45.40625
45.375
45.34375
45.3125
45.28125
79.34375
79.1875
79.21875
79.25
79.28125
79.3125
79.34375
Қарашилік
Qarashylyk
Сағабуйен
Sagabuyen
Теңеке Täneke
Бүйен Büyen
1.0 km
4.0 km
7.0 km

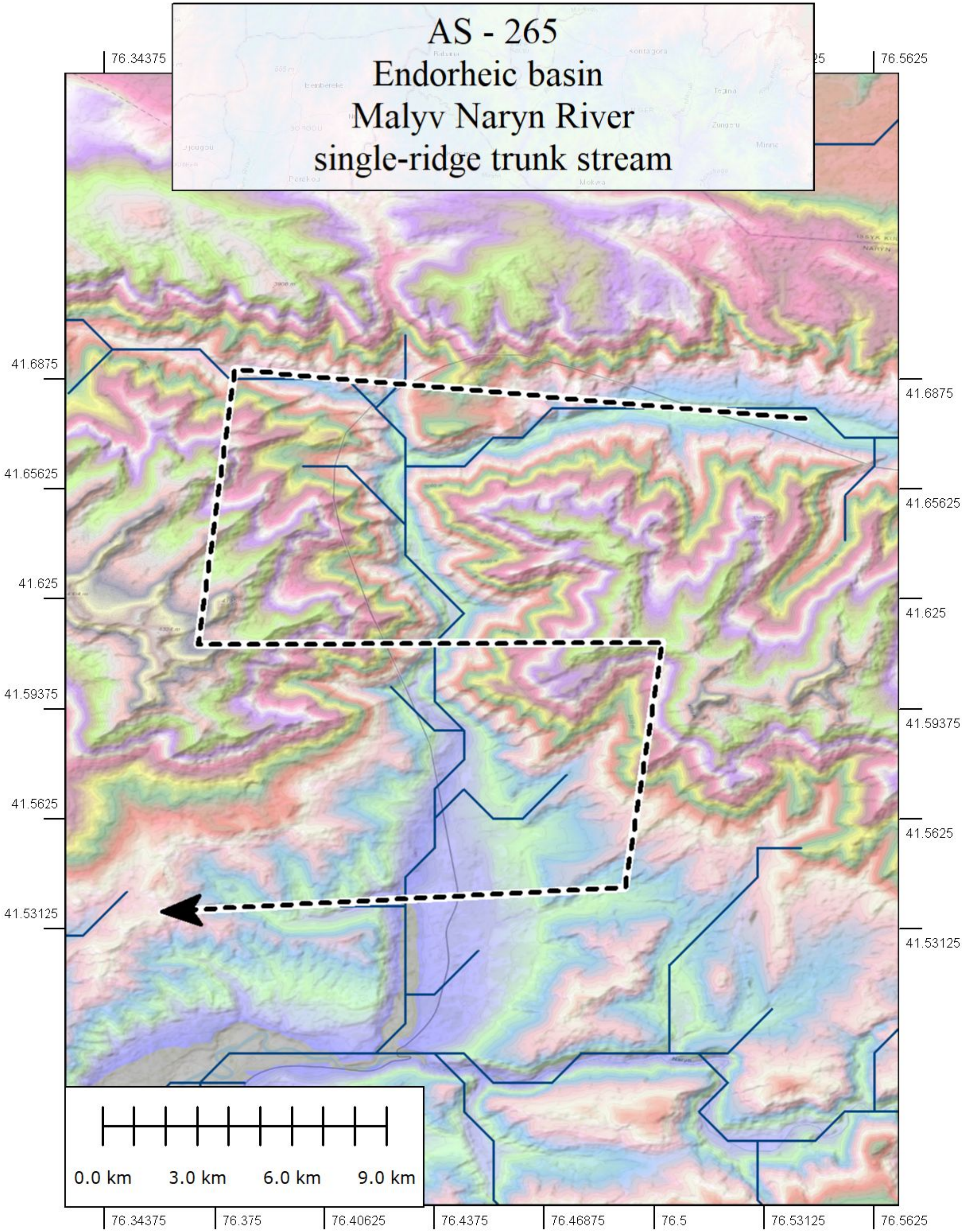
AS - 265
Endorheic basin
Malyv Naryn River
single-ridge trunk stream
76.34375
76.5625
41.6875
41.65625
41.625
41.59375
41.5625
41.53125
0.0 km
3.0 km
6.0 km
9.0 km
76.34375
76.375
76.40625
76.4375
76.46875
76.5
76.53125
76.5625

AS - 266
Endorheic basin
single-ridge head stream

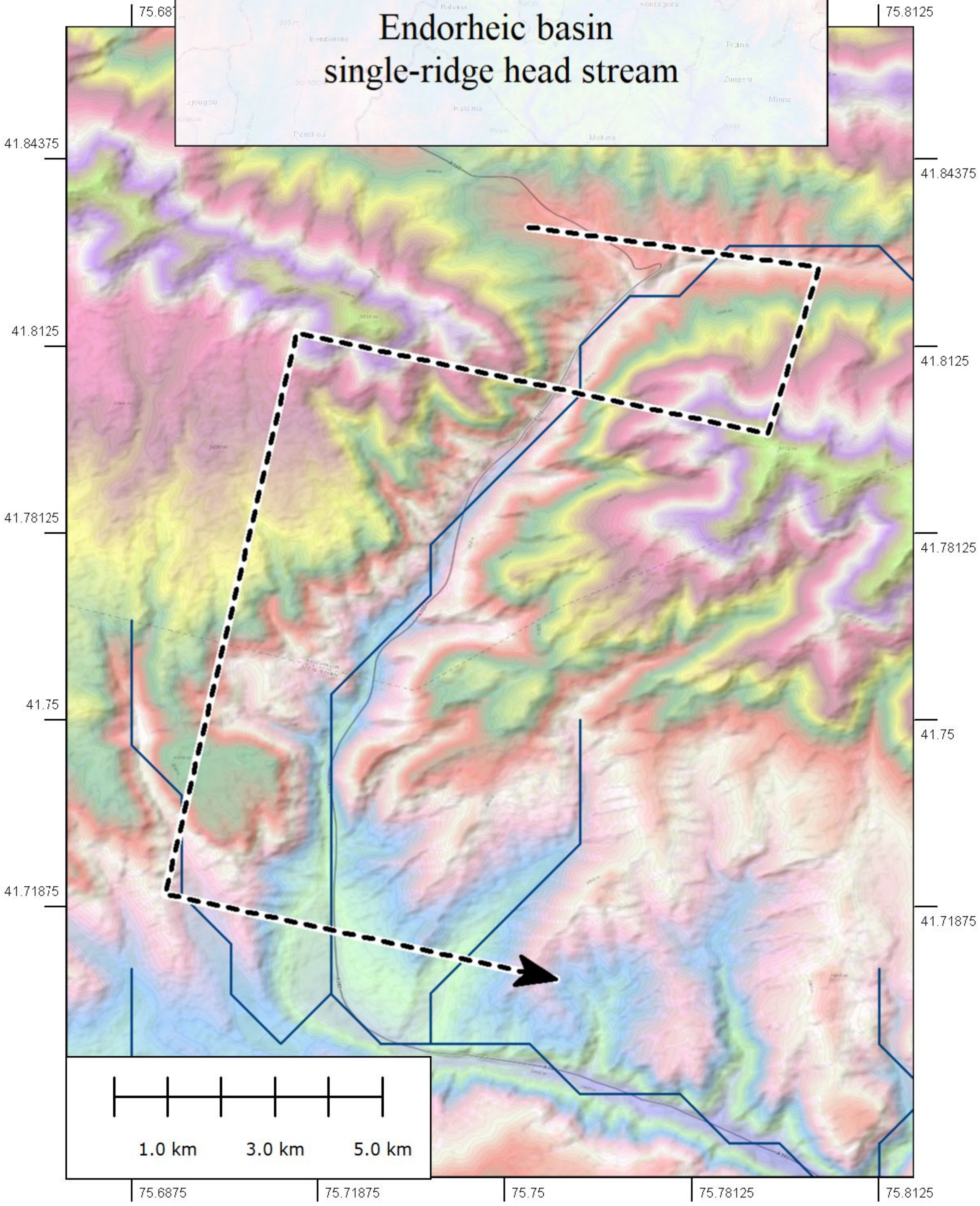

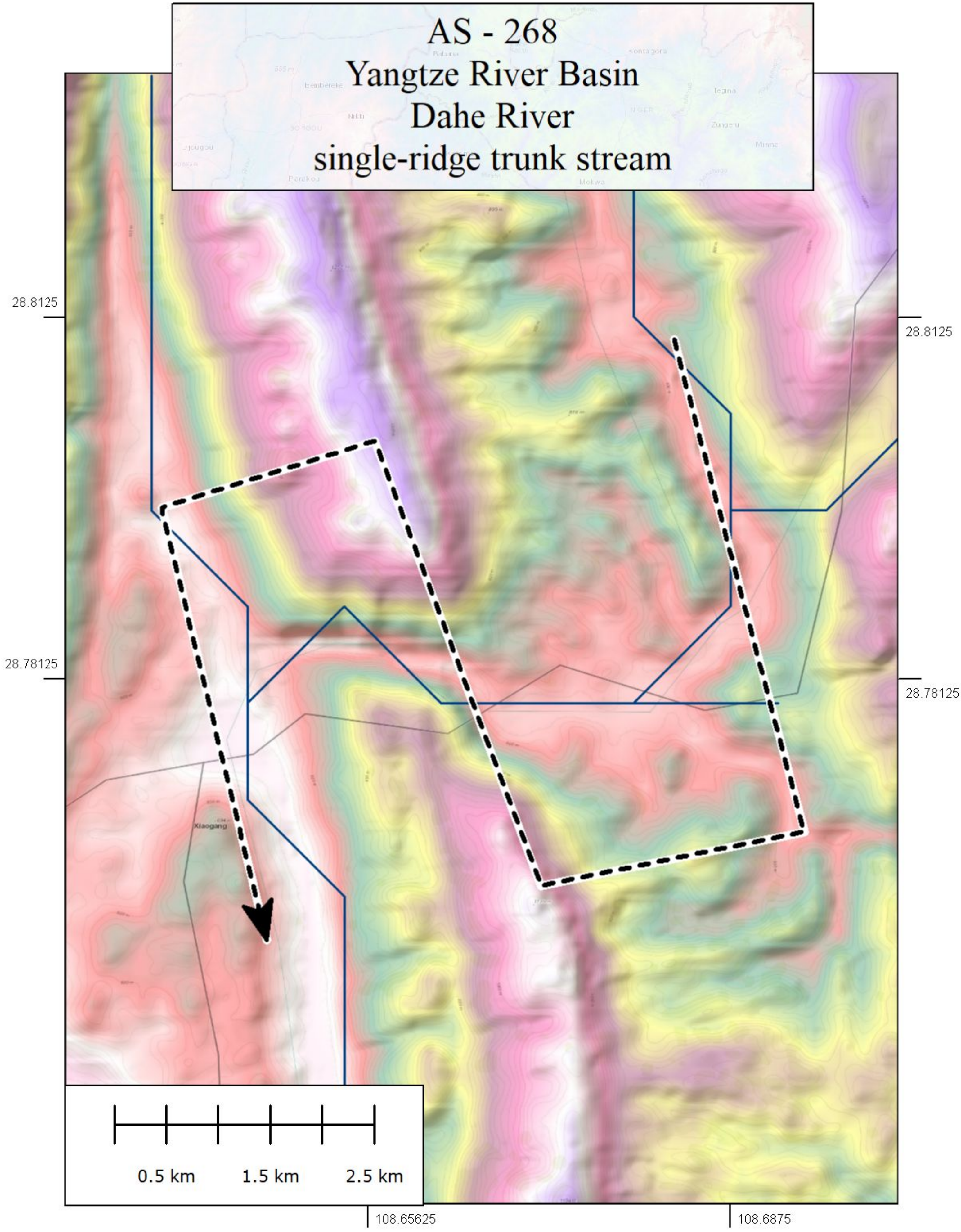
AS - 268
Yangtze River Basin
Dahe River
single-ridge trunk stream
28.8125
28.8125
28.78125
28.78125
Xiaogang
0.5 km
1.5 km
2.5 km
108.65625
108.6875

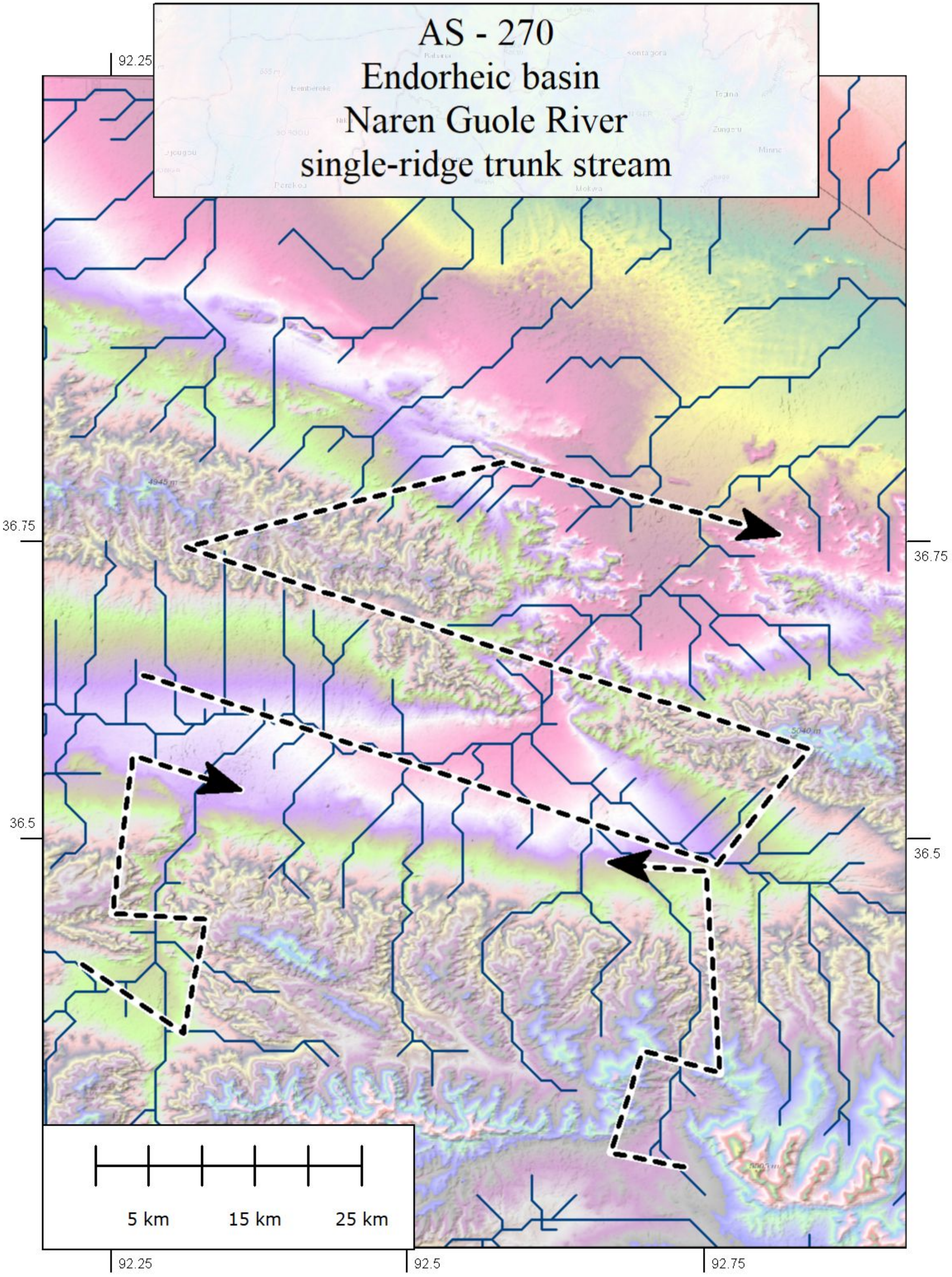
AS - 270
Endorheic basin
Naren Guole River
single-ridge trunk stream
92.25
92.5
92.75
36.75
36.5
5 km
15 km
25 km

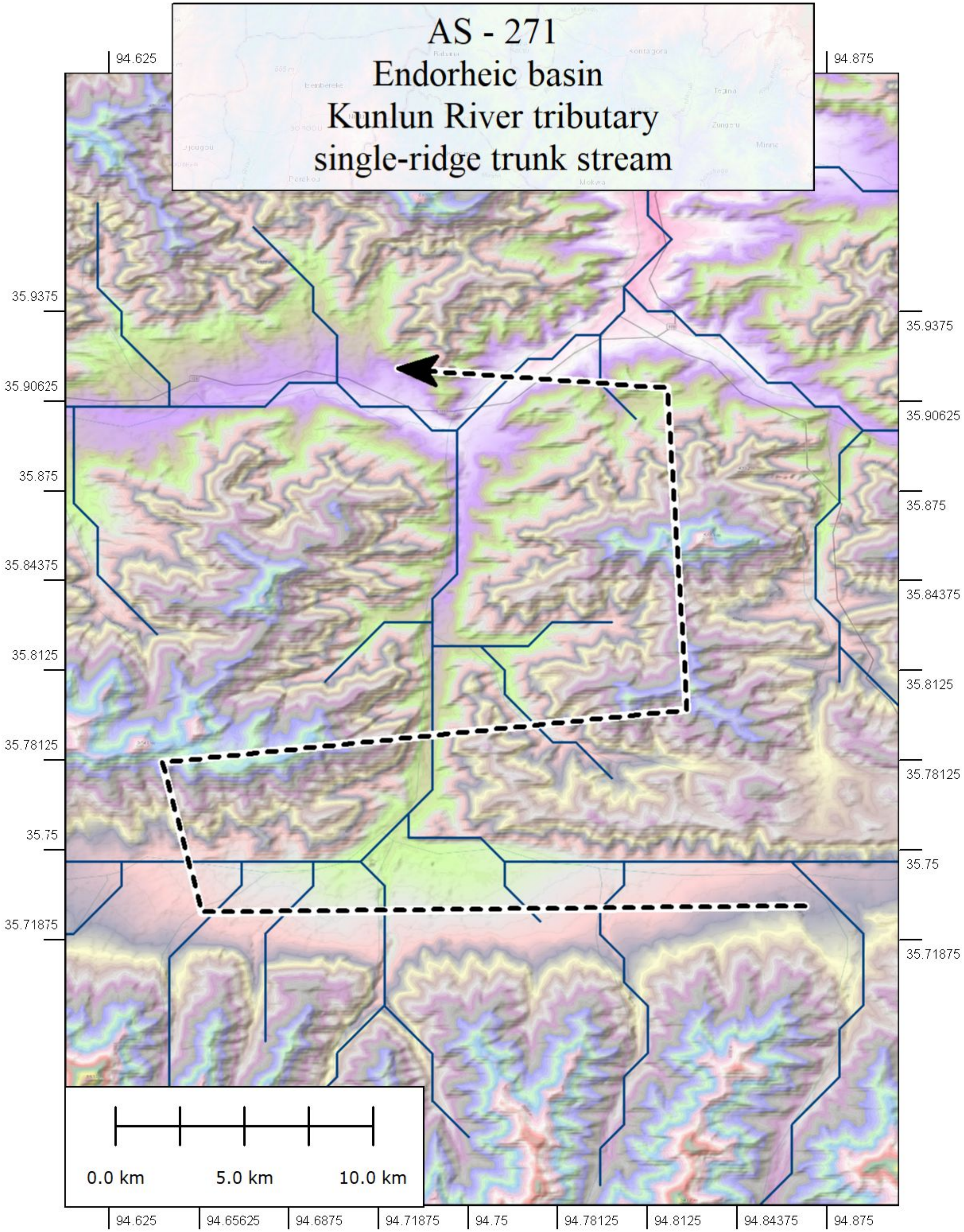
AS - 271
Endorheic basin
Kunlun River tributary
single-ridge trunk stream
94.625
94.875
35.9375
35.90625
35.875
35.84375
35.8125
35.78125
35.75
35.71875
94.625
94.65625
94.6875
94.71875
94.75
94.78125
94.8125
94.84375
94.875
0.0 km
5.0 km
10.0 km

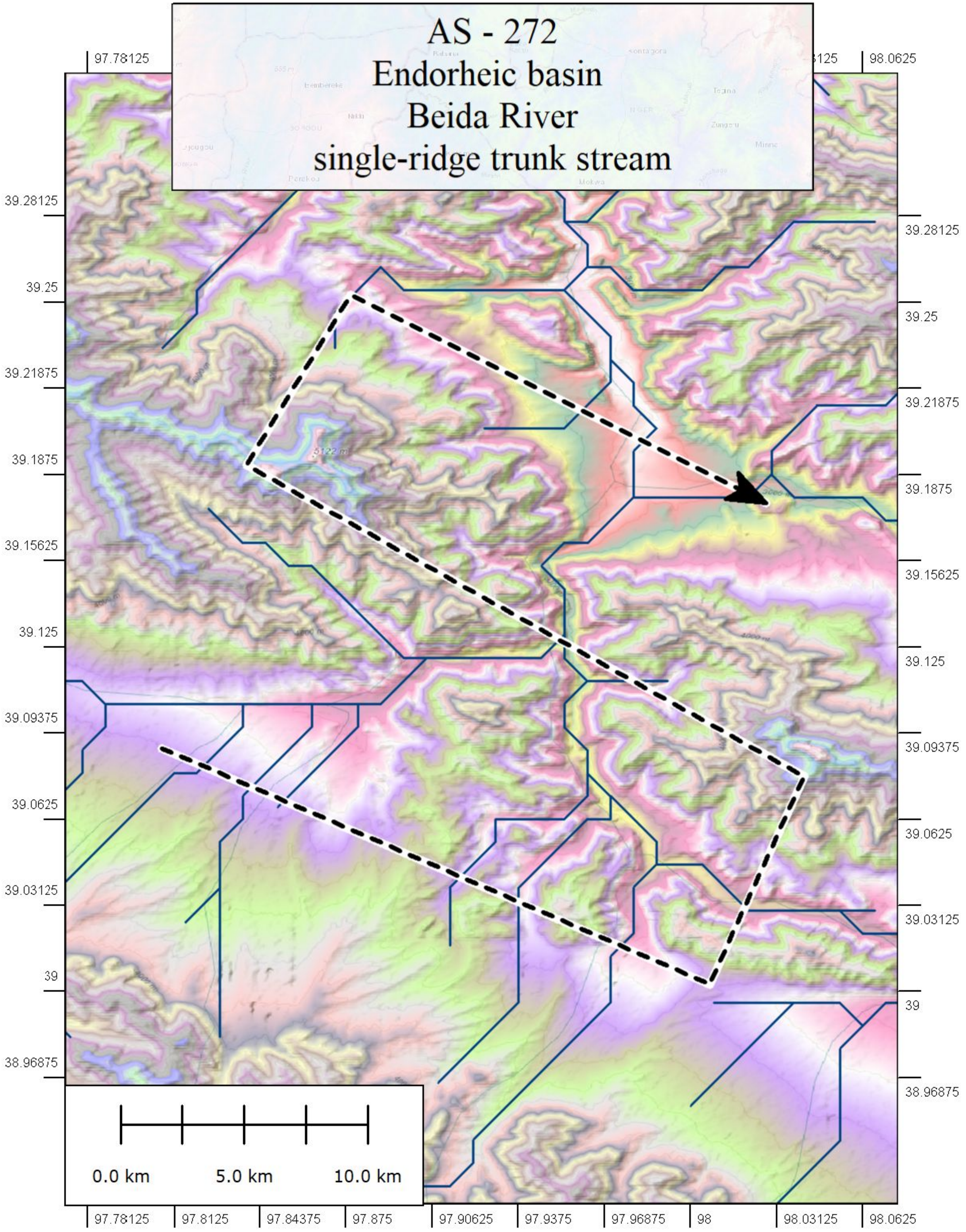
AS - 272
Endorheic basin
Beida River
single-ridge trunk stream
97.78125
98.0625
39.28125
39.25
39.21875
39.1875
39.15625
39.125
39.09375
39.0625
39.03125
39
38.96875
0.0 km
5.0 km
10.0 km
97.78125
97.8125
97.84375
97.875
97.90625
97.9375
97.96875
98
98.03125
98.0625

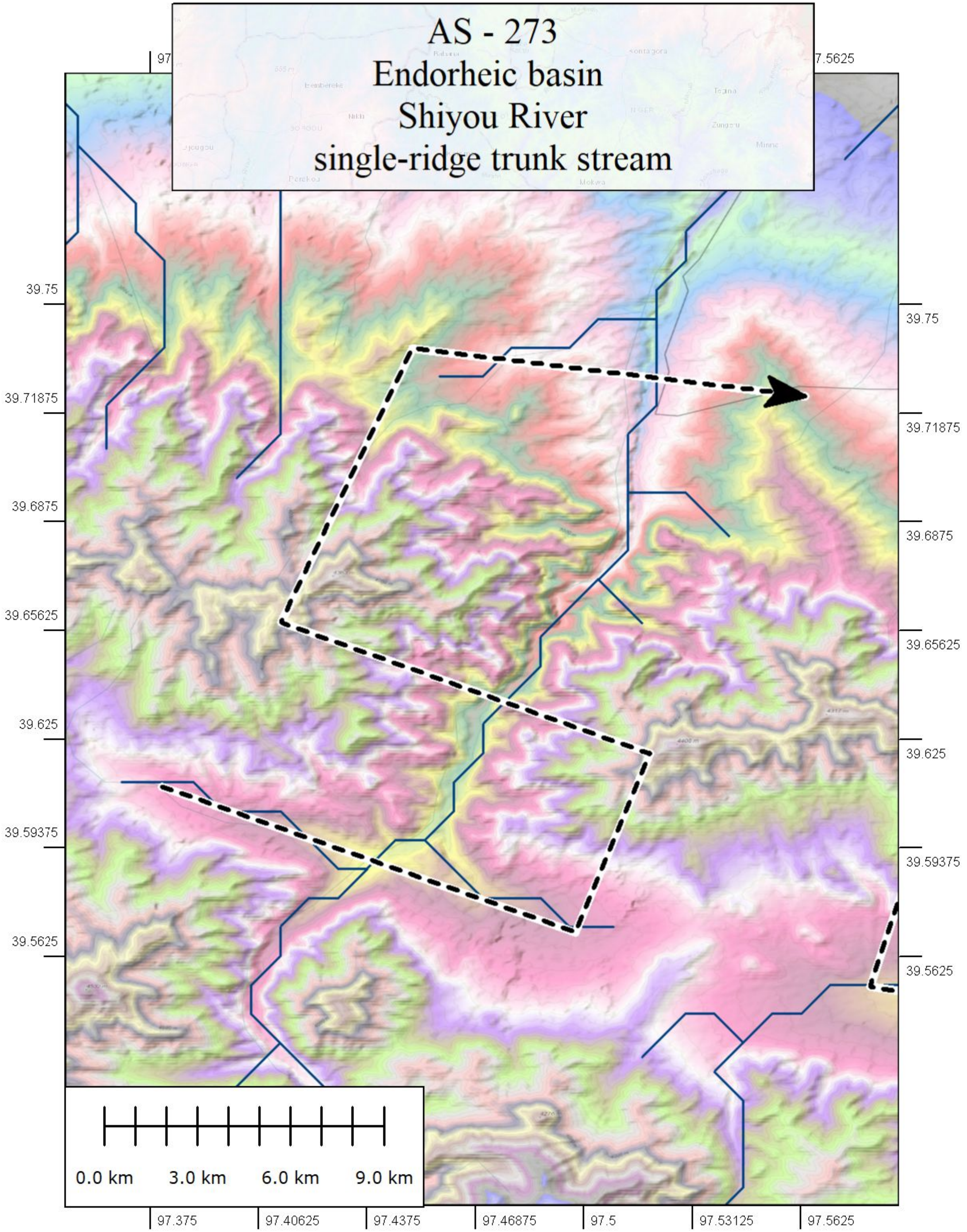

AS - 273
Endorheic basin
Shiyou River
single-ridge trunk stream
39.75
39.71875
39.6875
39.65625
39.625
39.59375
39.5625
97.375
97.40625
97.4375
97.46875
97.5
97.53125
97.5625
0.0 km
3.0 km
6.0 km
9.0 km

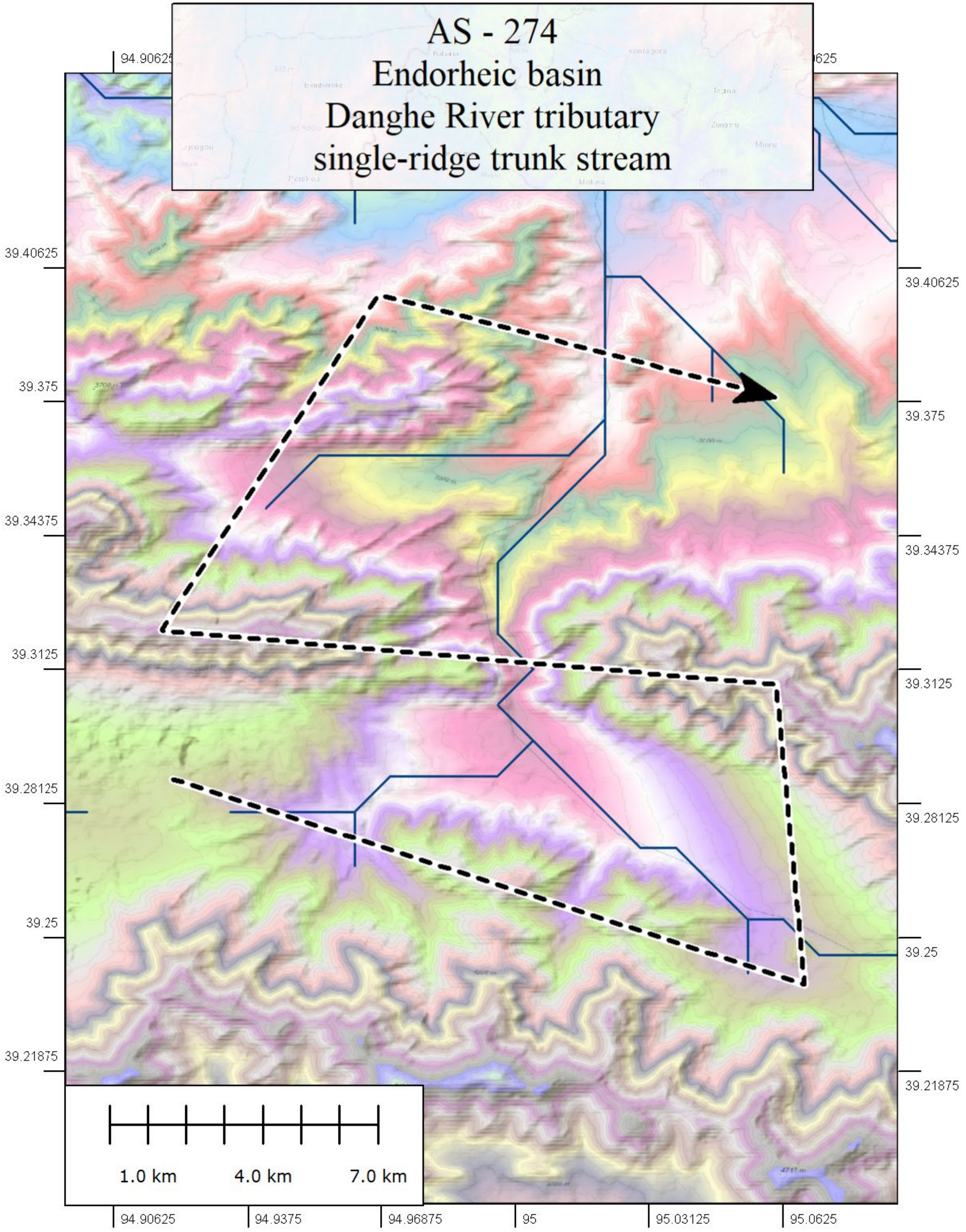
AS - 274
Endorheic basin
Danghe River tributary
single-ridge trunk stream
94.90625
94.9375
94.96875
95
95.03125
95.0625
39.40625
39.375
39.34375
39.3125
39.28125
39.25
39.21875
1.0 km
4.0 km
7.0 km

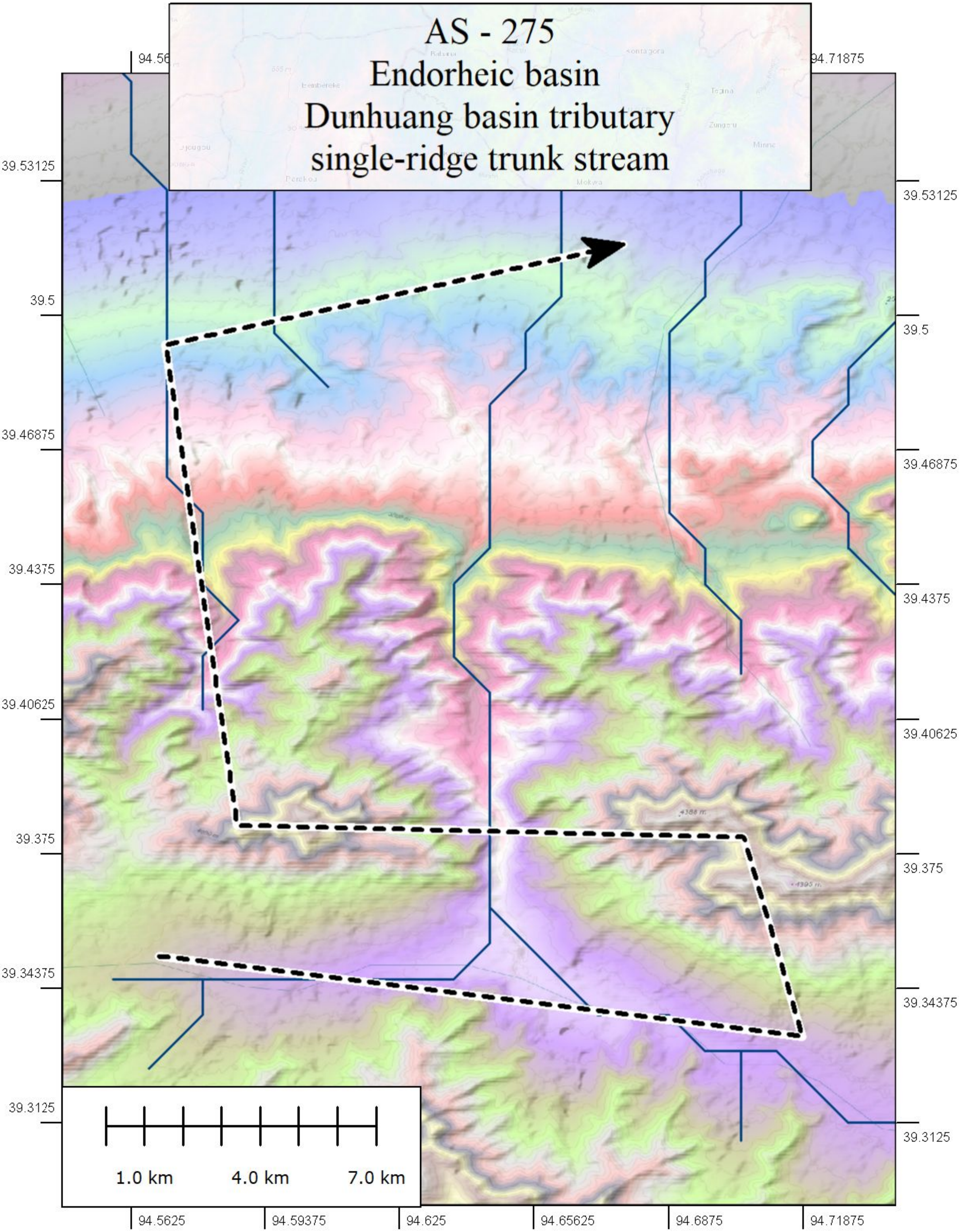
AS - 275
Endorheic basin
Dunhuang basin tributary
single-ridge trunk stream
94.56
94.71875
39.53125
39.5
39.46875
39.4375
39.40625
39.375
39.34375
39.3125
1.0 km
4.0 km
7.0 km
94.5625
94.59375
94.625
94.65625
94.6875
94.71875

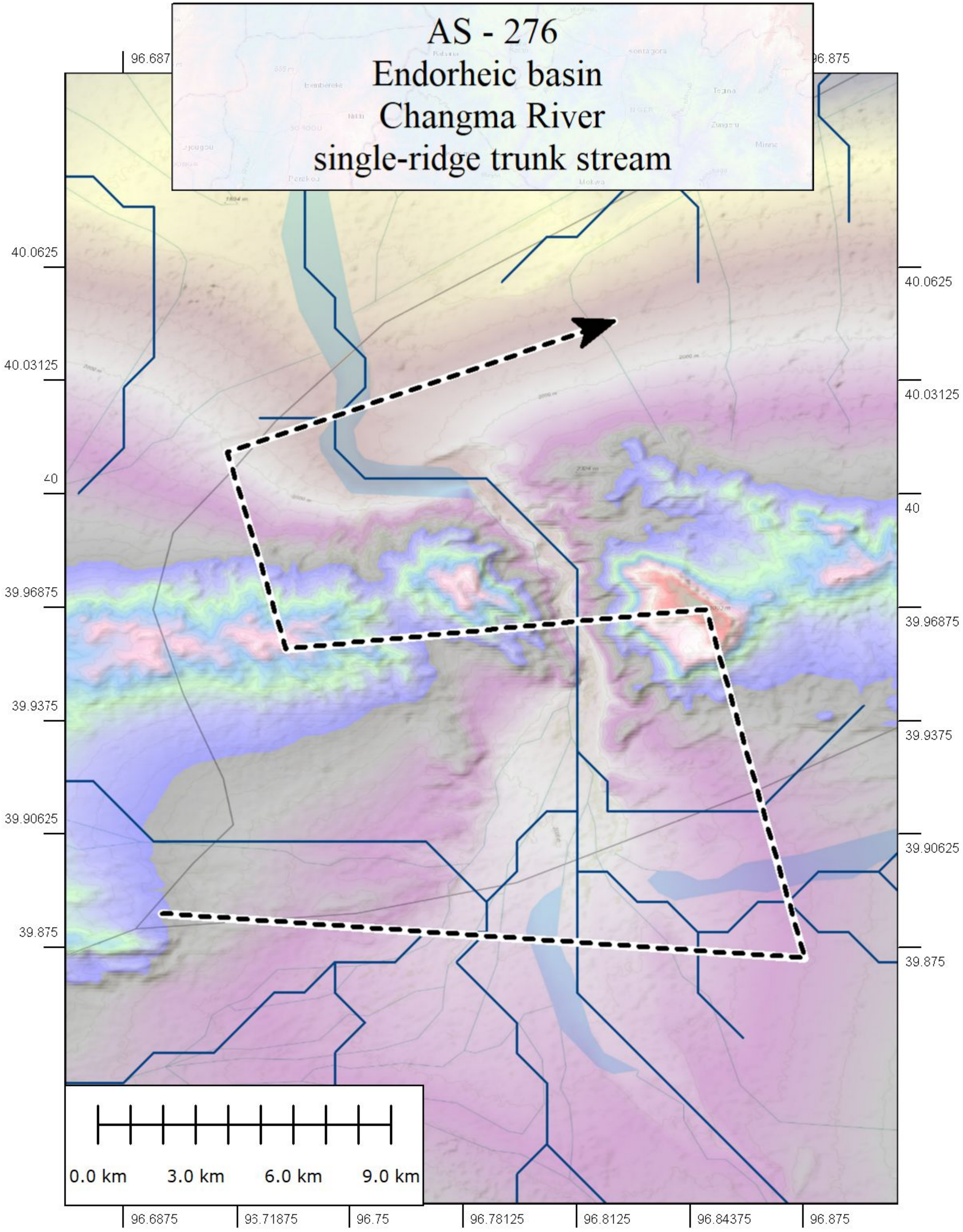
AS - 276
Endorheic basin
Changma River
single-ridge trunk stream
96.687
96.875
40.0625
40.03125
40
39.96875
39.9375
39.90625
39.875
40.0625
40.03125
40
39.96875
39.9375
39.90625
39.875
96.6875
93.71875
96.75
96.78125
96.8125
96.84375
96.875
0.0 km
3.0 km
6.0 km
9.0 km

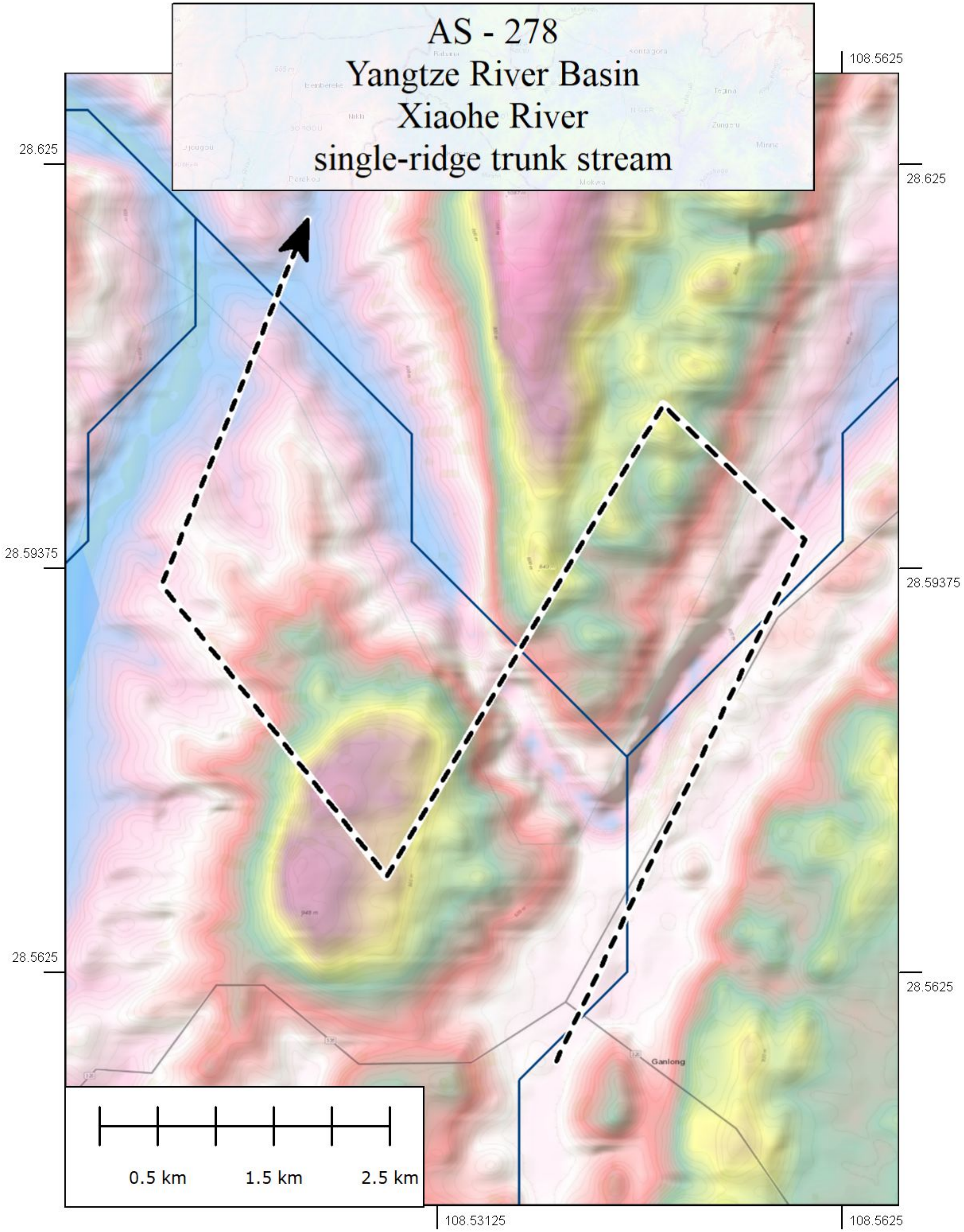

AS - 278
Yangtze River Basin
Xiaohe River
single-ridge trunk stream
108.5625
28.625
28.625
28.59375
28.59375
28.5625
28.5625
108.53125
108.5625
Ganlong
0.5 km
1.5 km
2.5 km

AS - 279
Endorheic basin
Susamyr River
single-ridge trunk stream

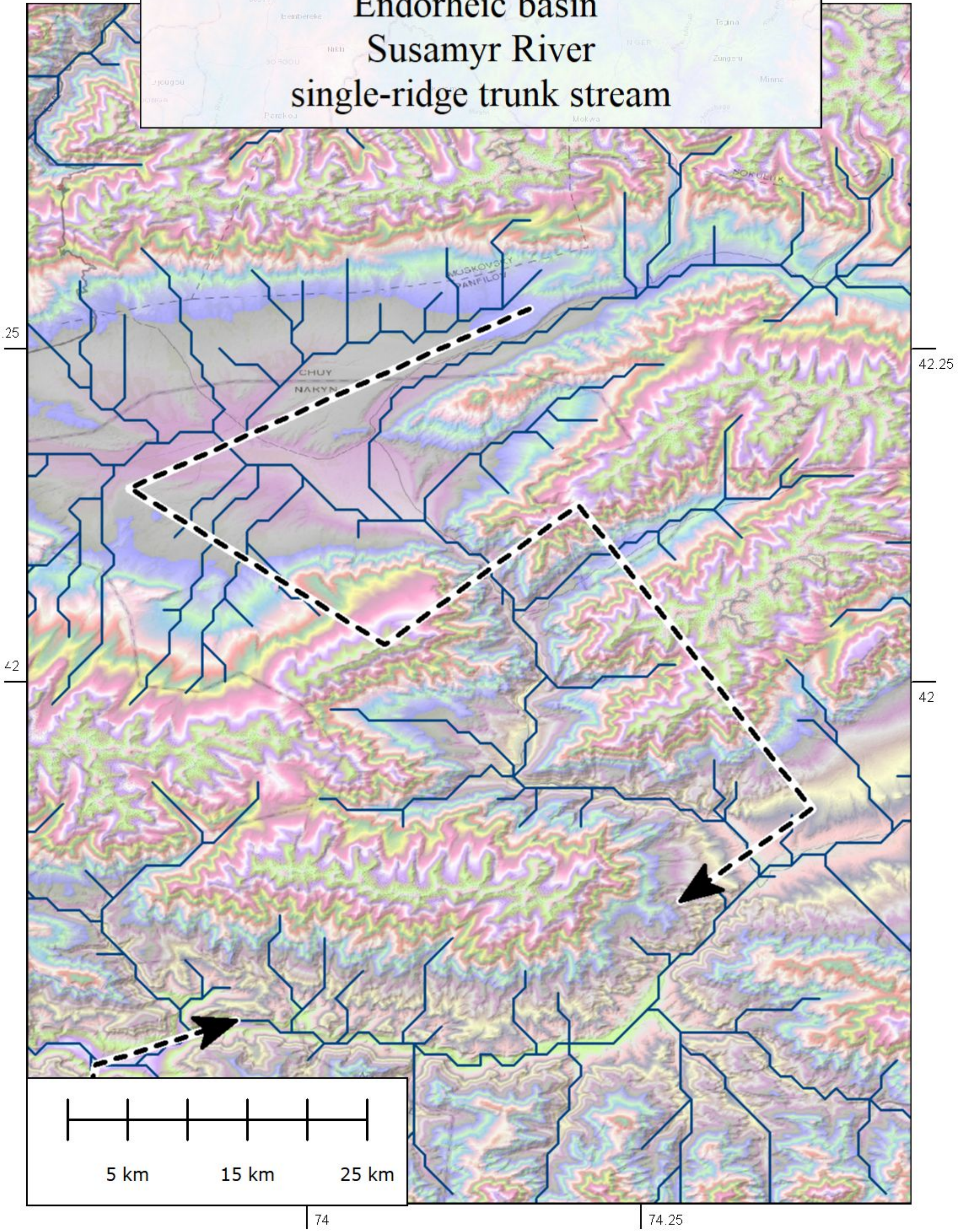

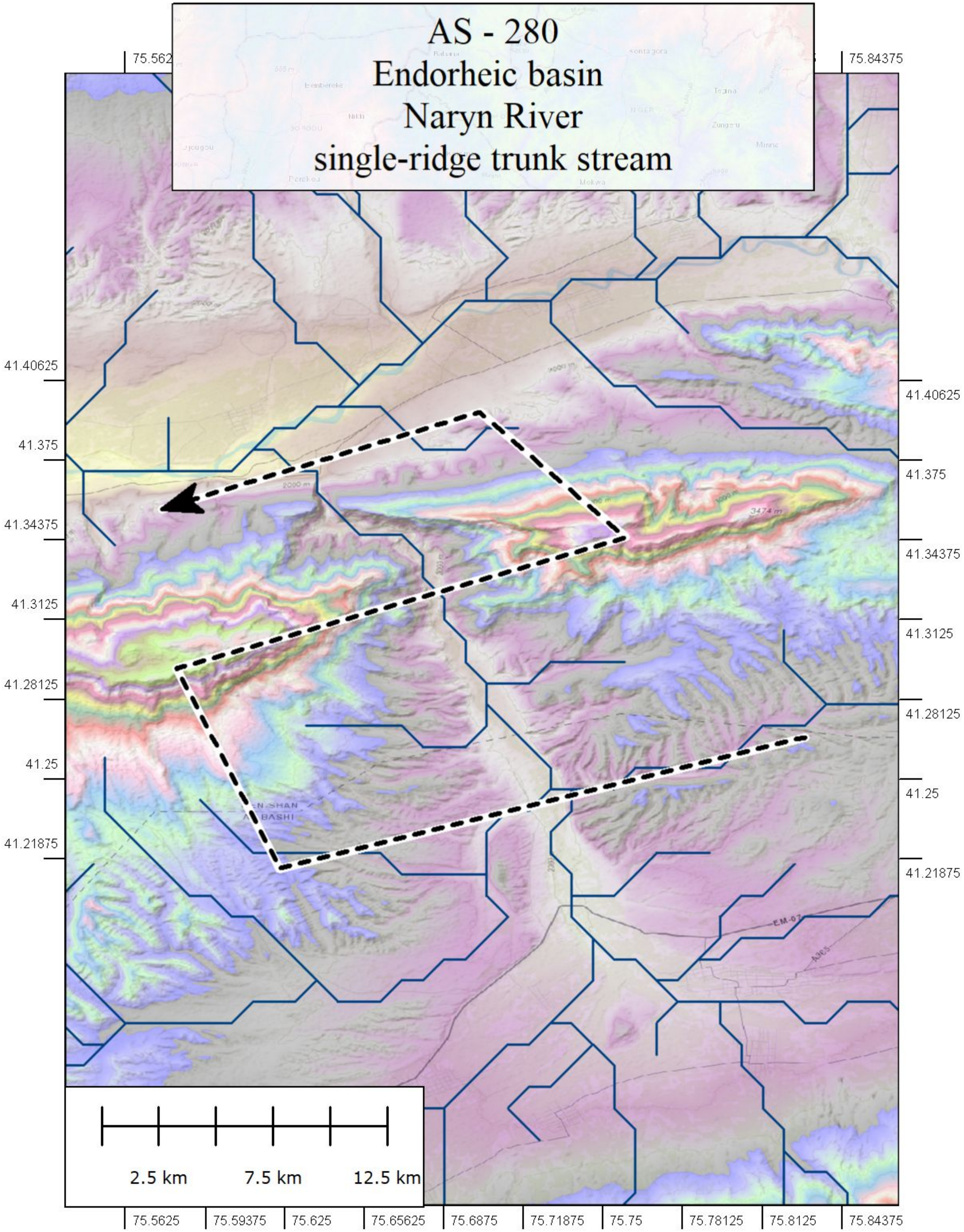
AS - 280
Endorheic basin
Naryn River
single-ridge trunk stream
75.5625
75.59375
75.625
75.65625
75.6875
75.71875
75.75
75.78125
75.8125
75.84375
41.40625
41.375
41.34375
41.3125
41.28125
41.25
41.21875
2.5 km
7.5 km
12.5 km

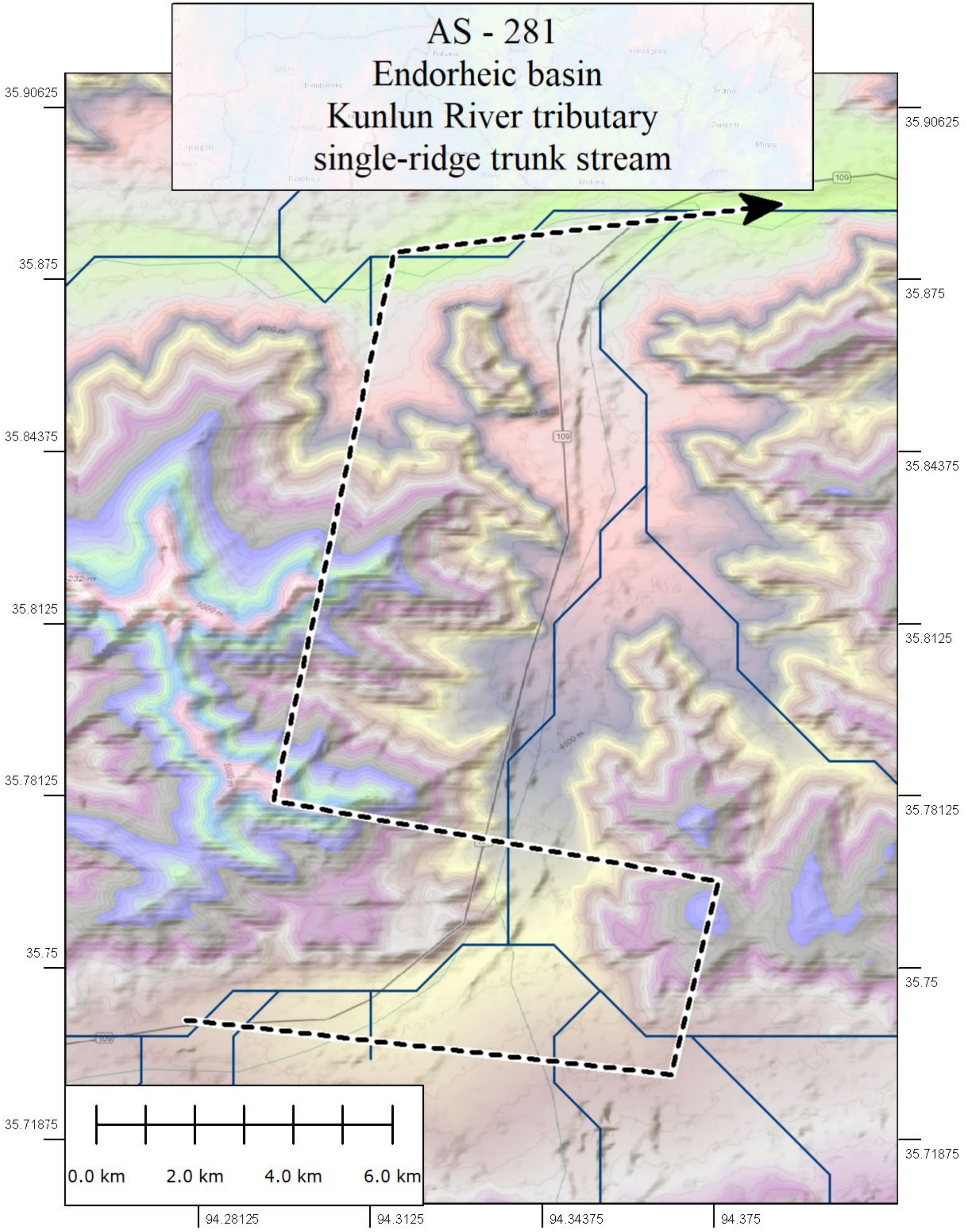

AS - 281
Endorheic basin
Kunlun River tributary
single-ridge trunk stream
35.90625
35.875
35.84375
35.8125
35.78125
35.75
35.71875
94.28125
94.3125
94.34375
94.375
4000 m
5000 m
109
0.0 km
2.0 km
4.0 km
6.0 km

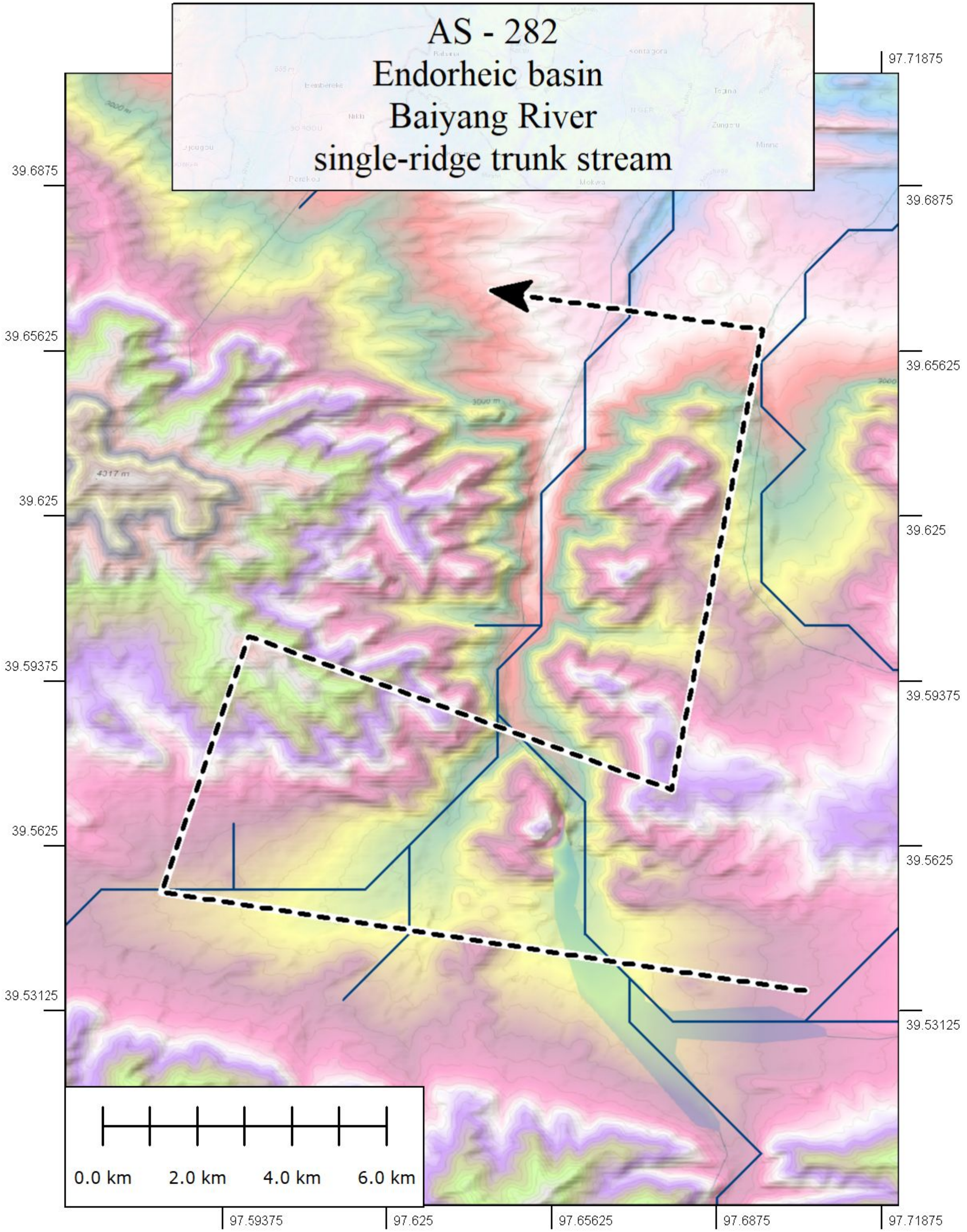
AS - 282
Endorheic basin
Baiyang River
single-ridge trunk stream
97.71875
39.6875
39.65625
39.625
39.59375
39.5625
39.53125
0.0 km
2.0 km
4.0 km
6.0 km
97.59375
97.625
97.65625
97.6875
4317 m
3000 m

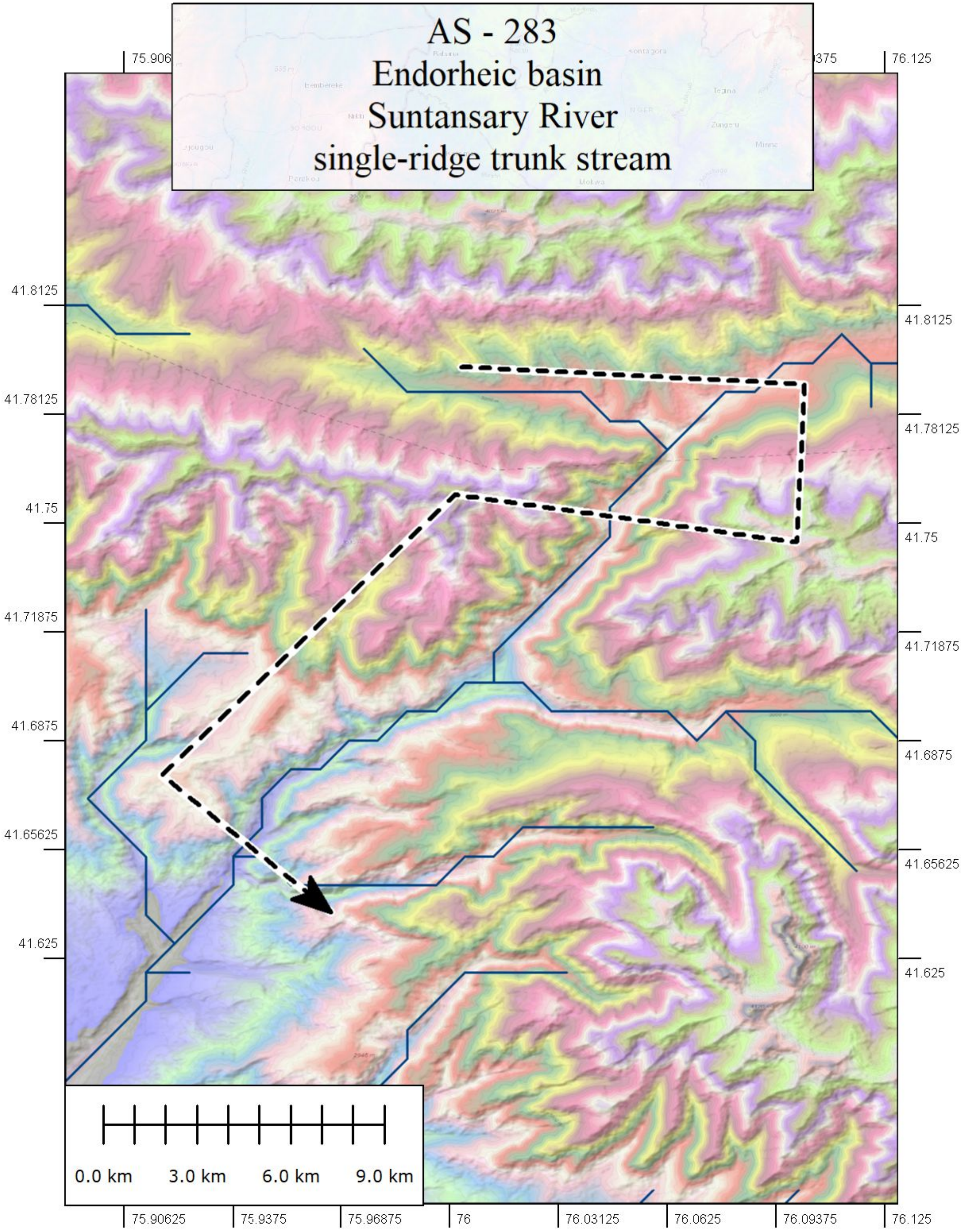
AS - 283
Endorheic basin
Suntansary River
single-ridge trunk stream
75.906
375
76.125
41.8125
41.78125
41.75
41.71875
41.6875
41.65625
41.625
0.0 km
3.0 km
6.0 km
9.0 km
75.90625
75.9375
75.96875
76
76.03125
76.0625
76.09375

AS - 287
Vitim Basin
Reka Vitim River
single-ridge trunk stream

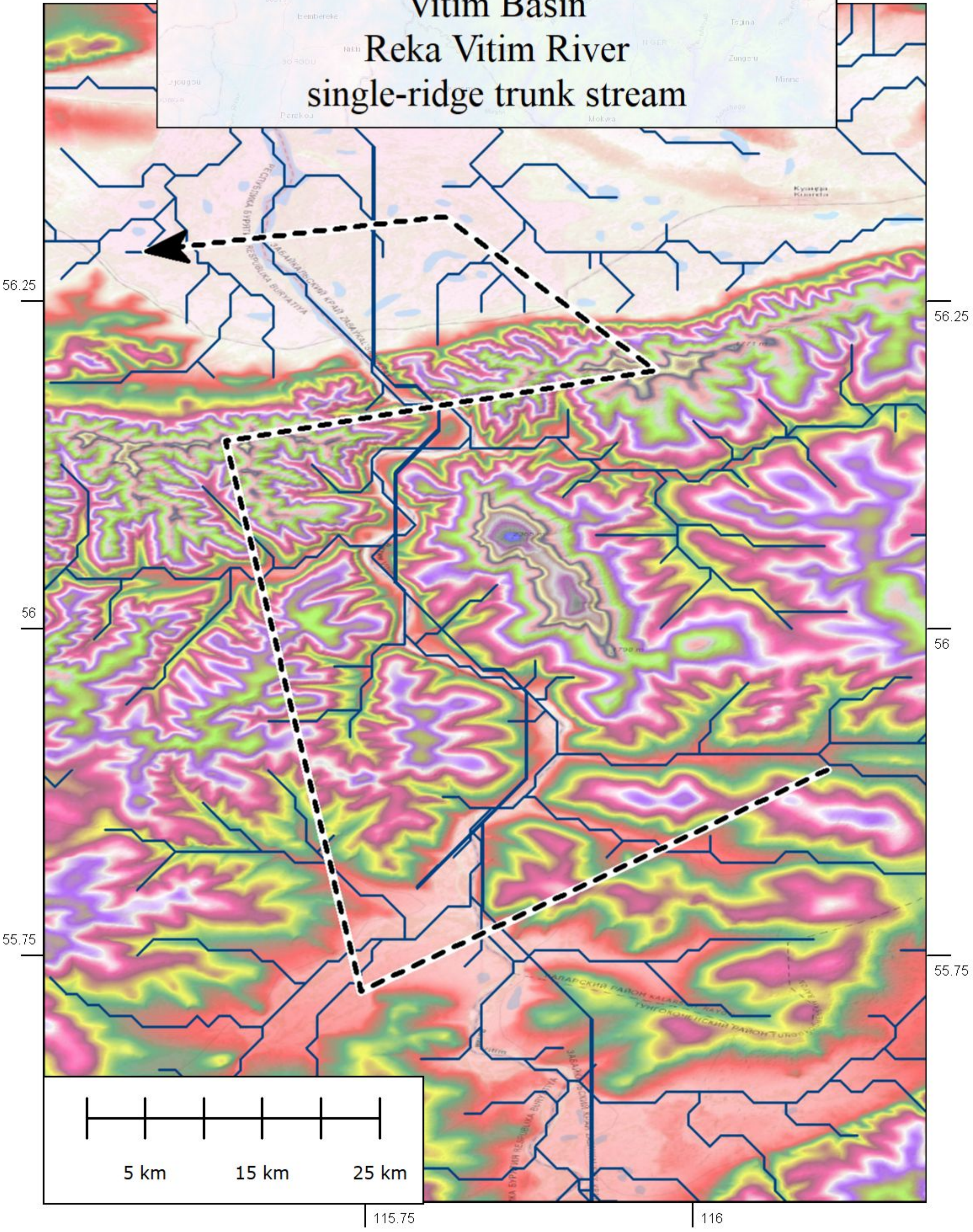

AS - 293
Mekong River Basin
Mekong River
single-ridge trunk stream

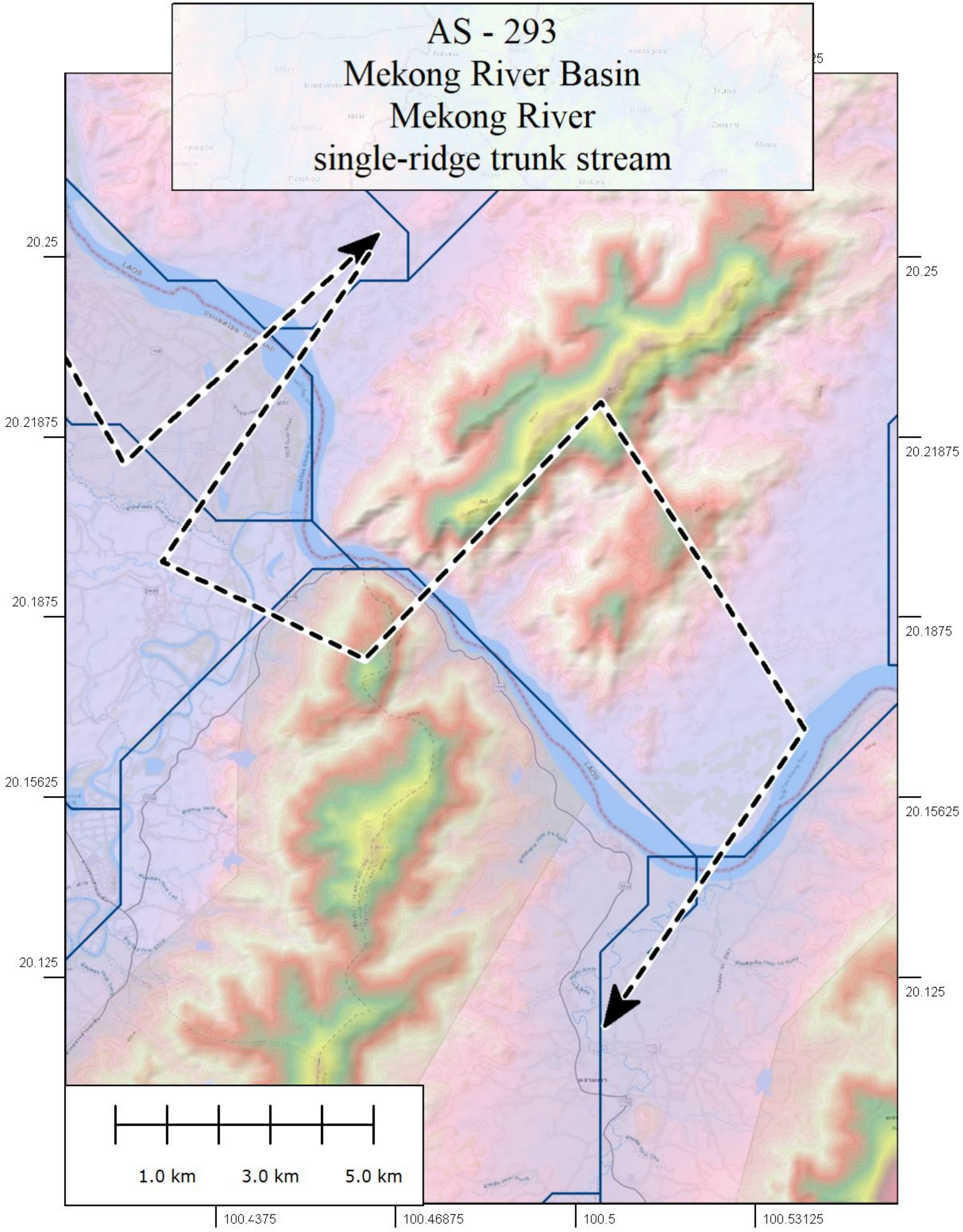

AS - 297
Mekong River Basin
Lancang River tributary
single-ridge trunk stream

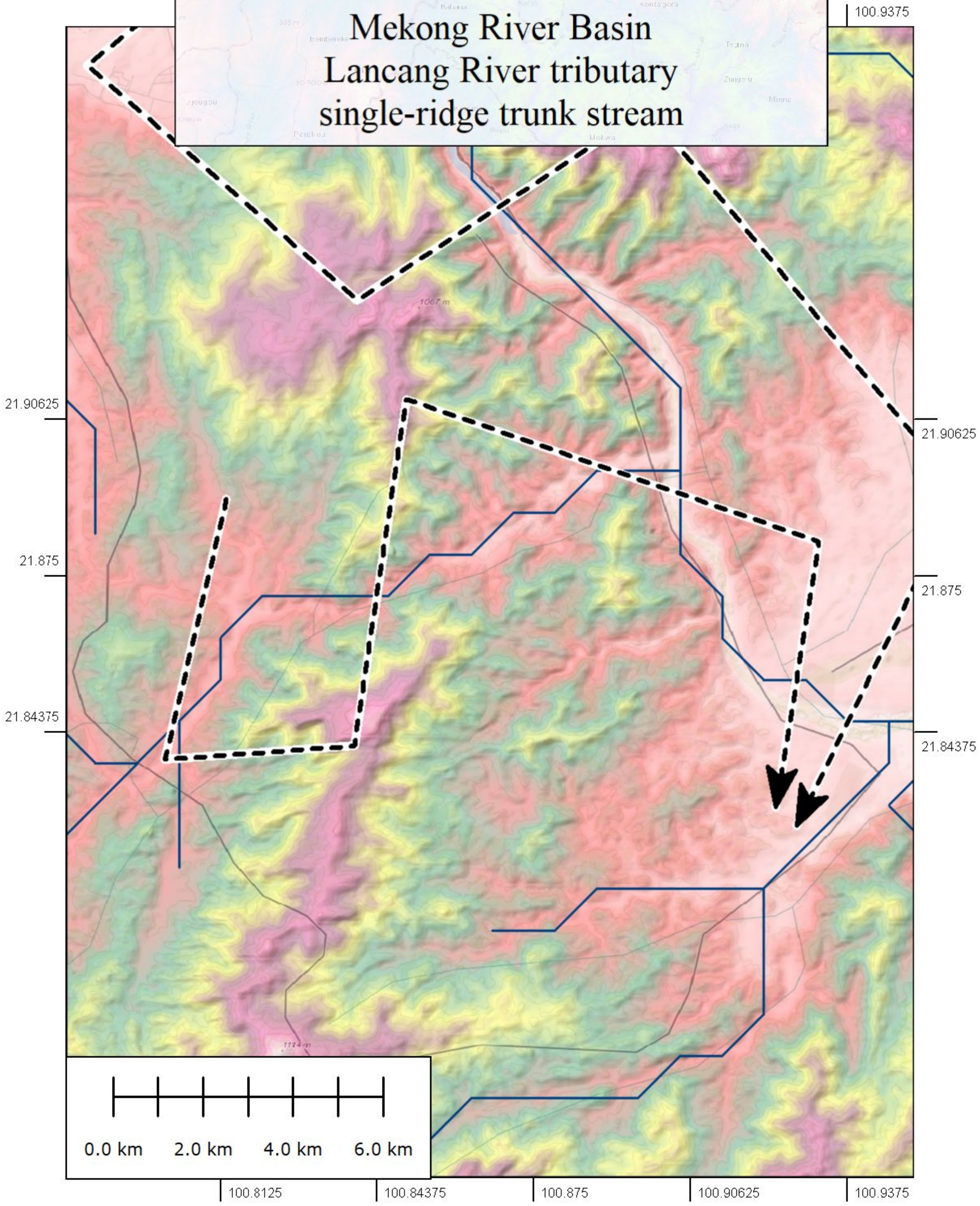

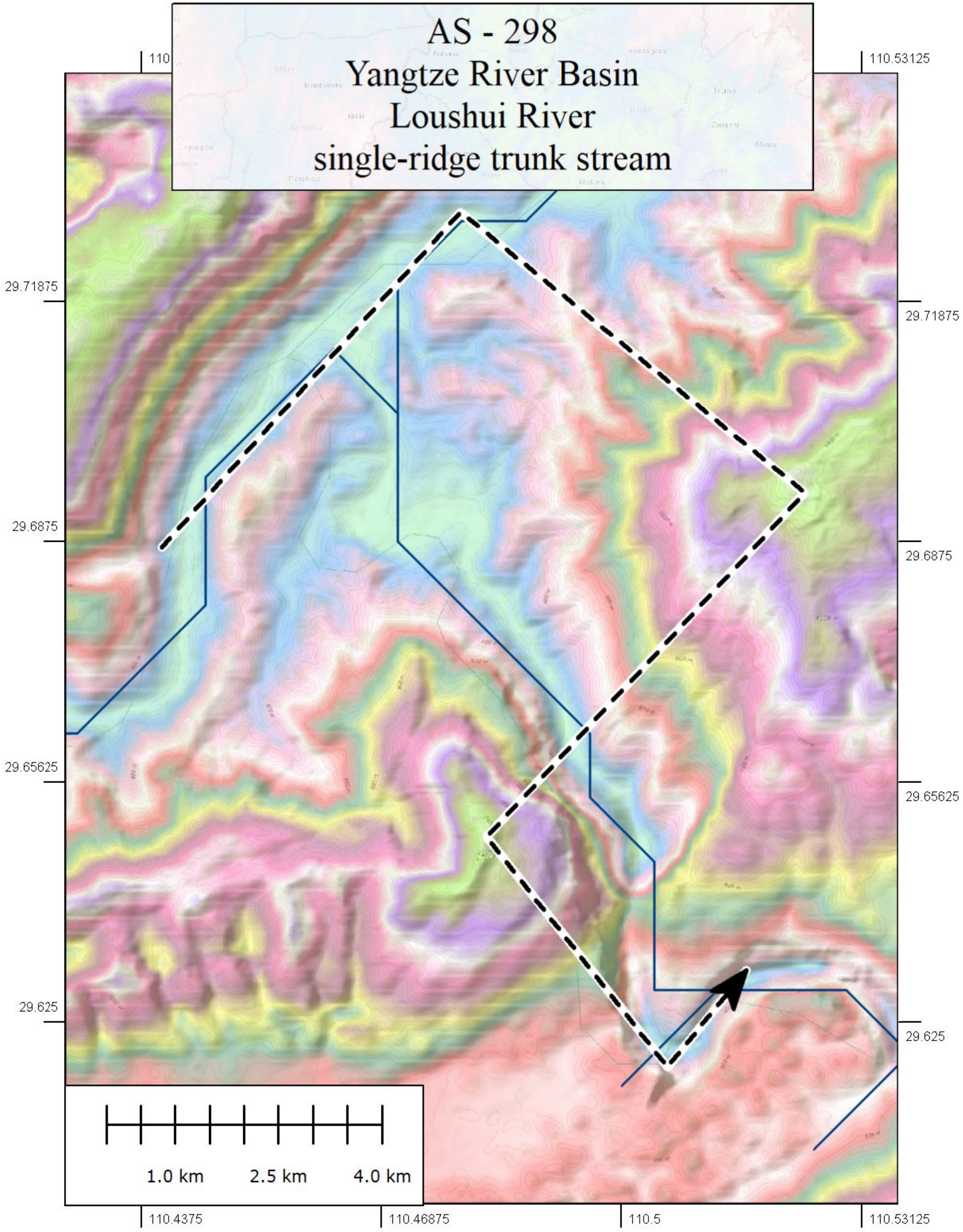
AS - 298
Yangtze River Basin
Loushui River
single-ridge trunk stream
29.71875
29.6875
29.65625
29.625
110.53125
110.4375
110.46875
110.5
1.0 km
2.5 km
4.0 km

AS - 299
Yangtze River Basin
Fengle River
single-ridge trunk stream

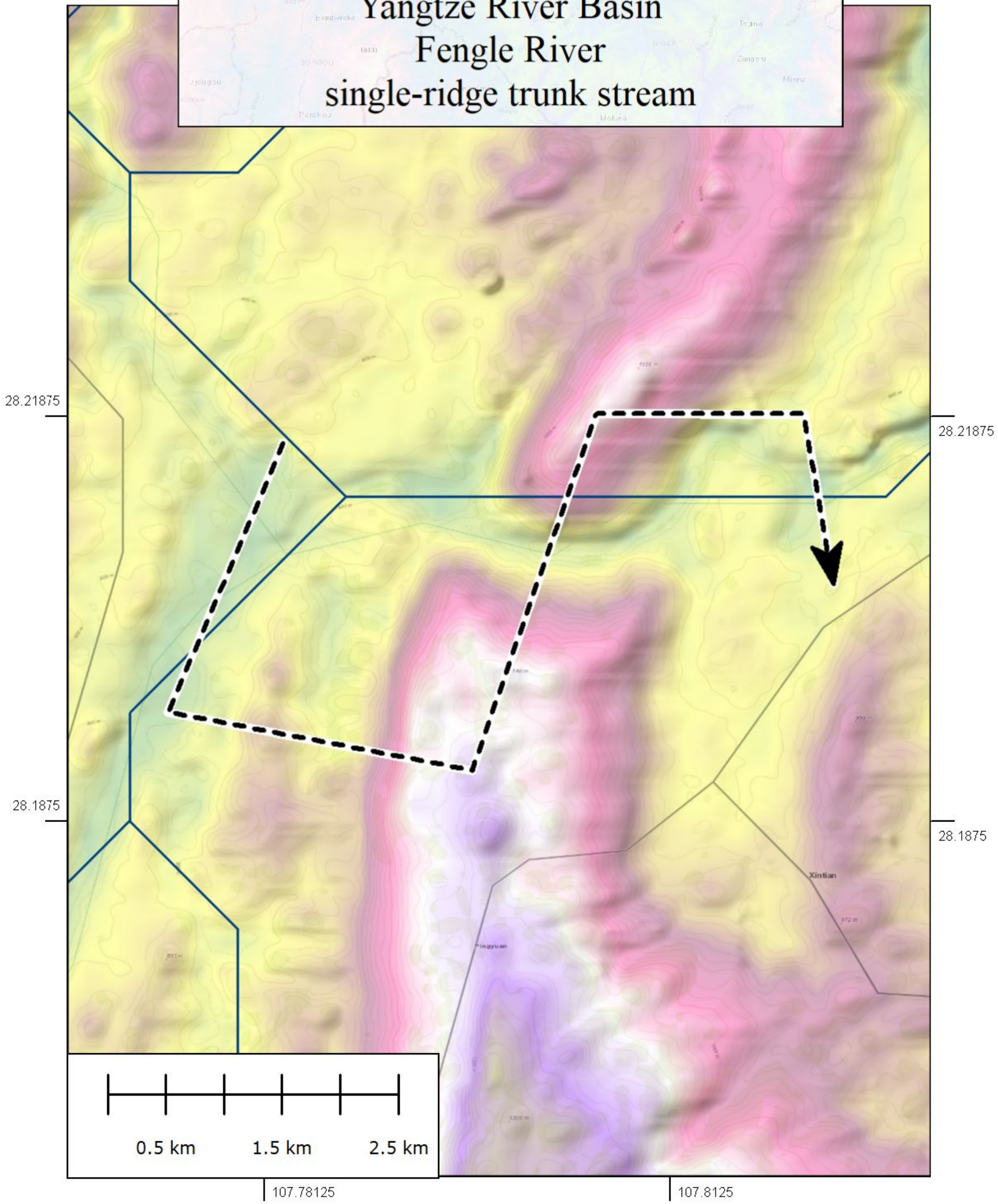

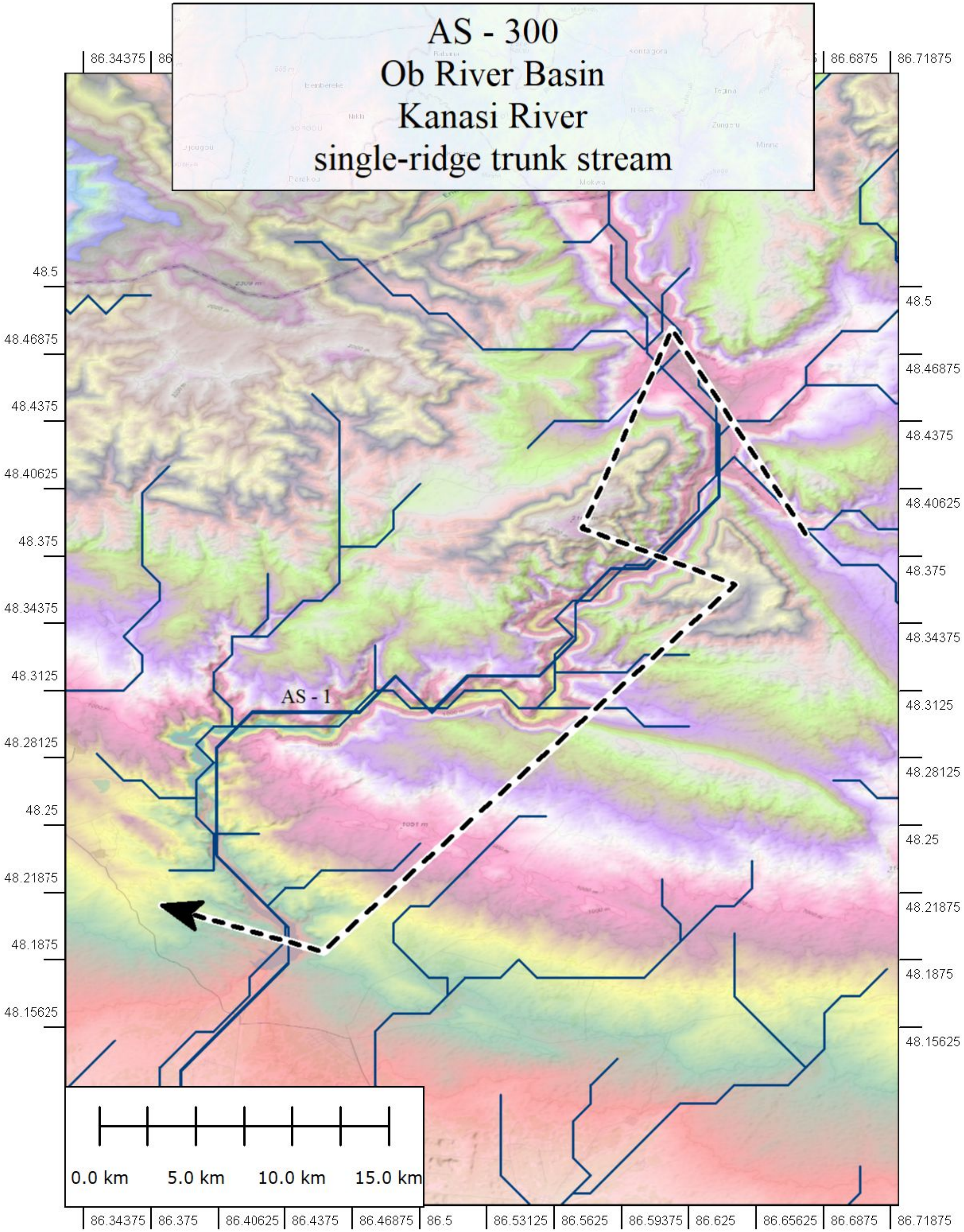
AS - 300
Ob River Basin
Kanasi River
single-ridge trunk stream
AS - 1
0.0 km
5.0 km
10.0 km
15.0 km
86.34375
86.375
86.40625
86.4375
86.46875
86.5
86.53125
86.5625
86.59375
86.625
86.65625
86.6875
86.71875
48.5
48.46875
48.4375
48.40625
48.375
48.34375
48.3125
48.28125
48.25
48.21875
48.1875
48.15625

AS - 301
Yangtze River Basin
Fushui River tributary
single-ridge trunk stream

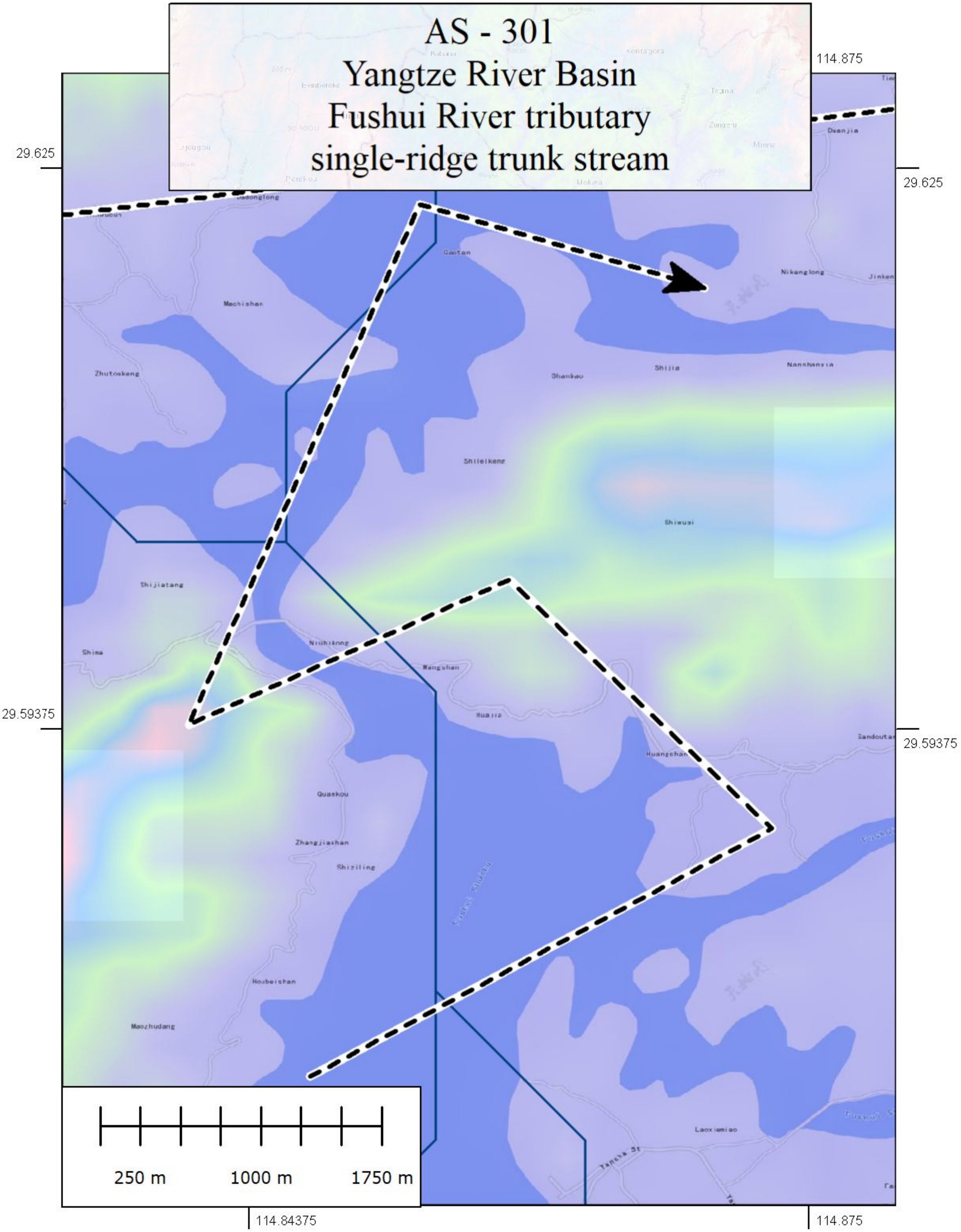

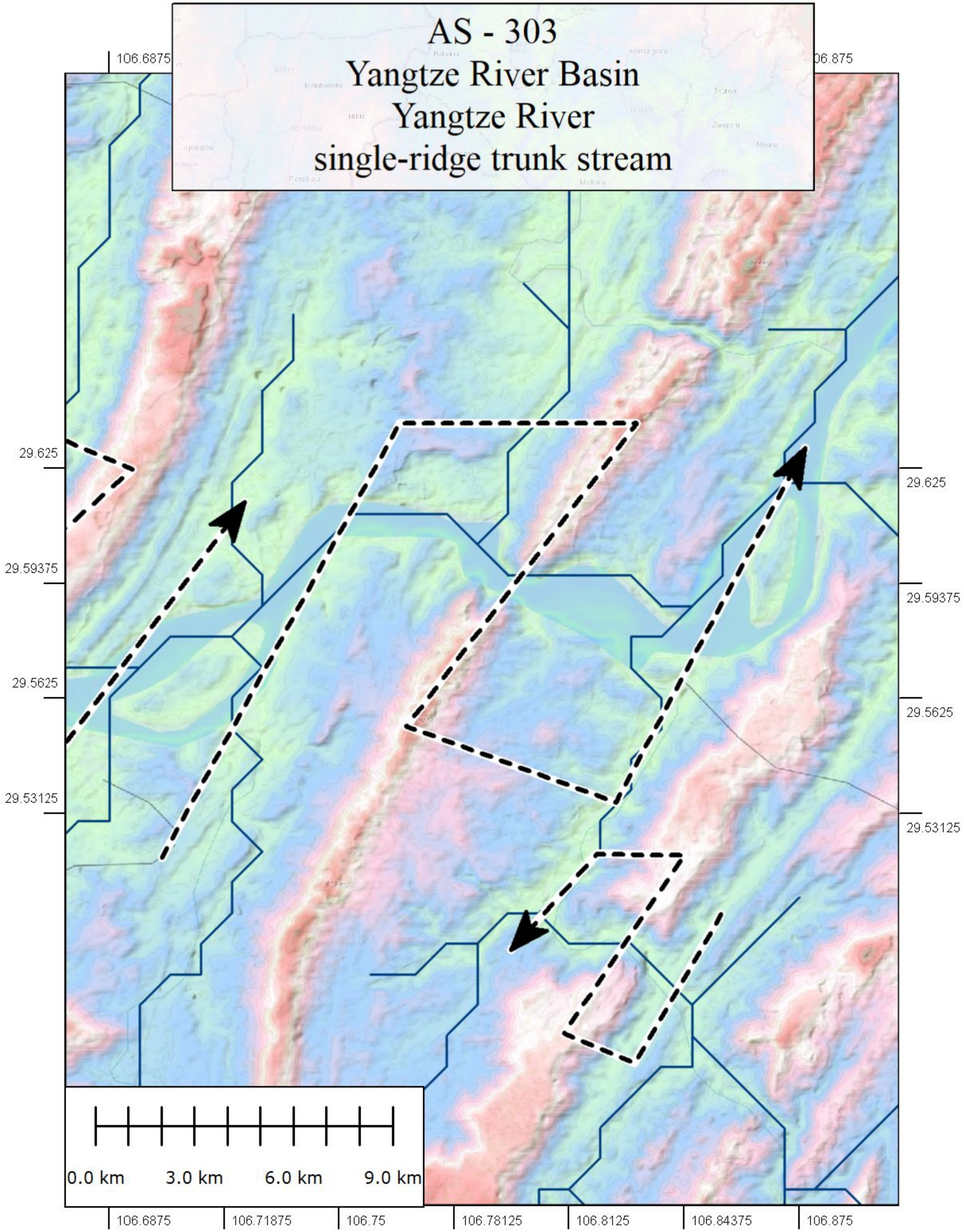
AS - 303
Yangtze River Basin
Yangtze River
single-ridge trunk stream
106.6875
06.875
29.625
29.59375
29.5625
29.53125
29.625
29.59375
29.5625
29.53125
0.0 km
3.0 km
6.0 km
9.0 km
106.6875
106.71875
106.75
106.78125
106.8125
106.84375
106.875

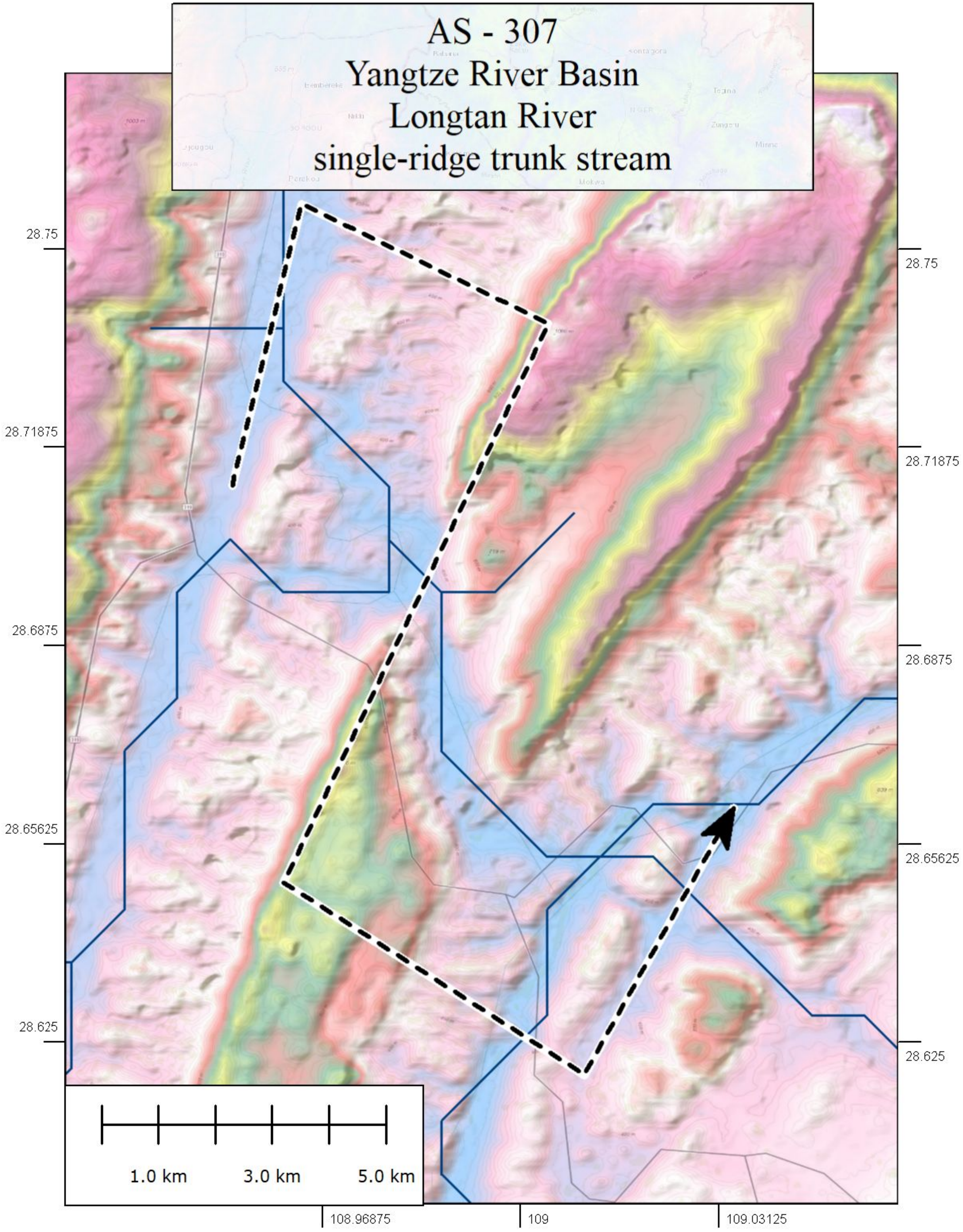
AS - 307
Yangtze River Basin
Longtan River
single-ridge trunk stream
28.75
28.71875
28.6875
28.65625
28.625
28.75
28.71875
28.6875
28.65625
28.625
108.96875
109
109.03125
1.0 km
3.0 km
5.0 km

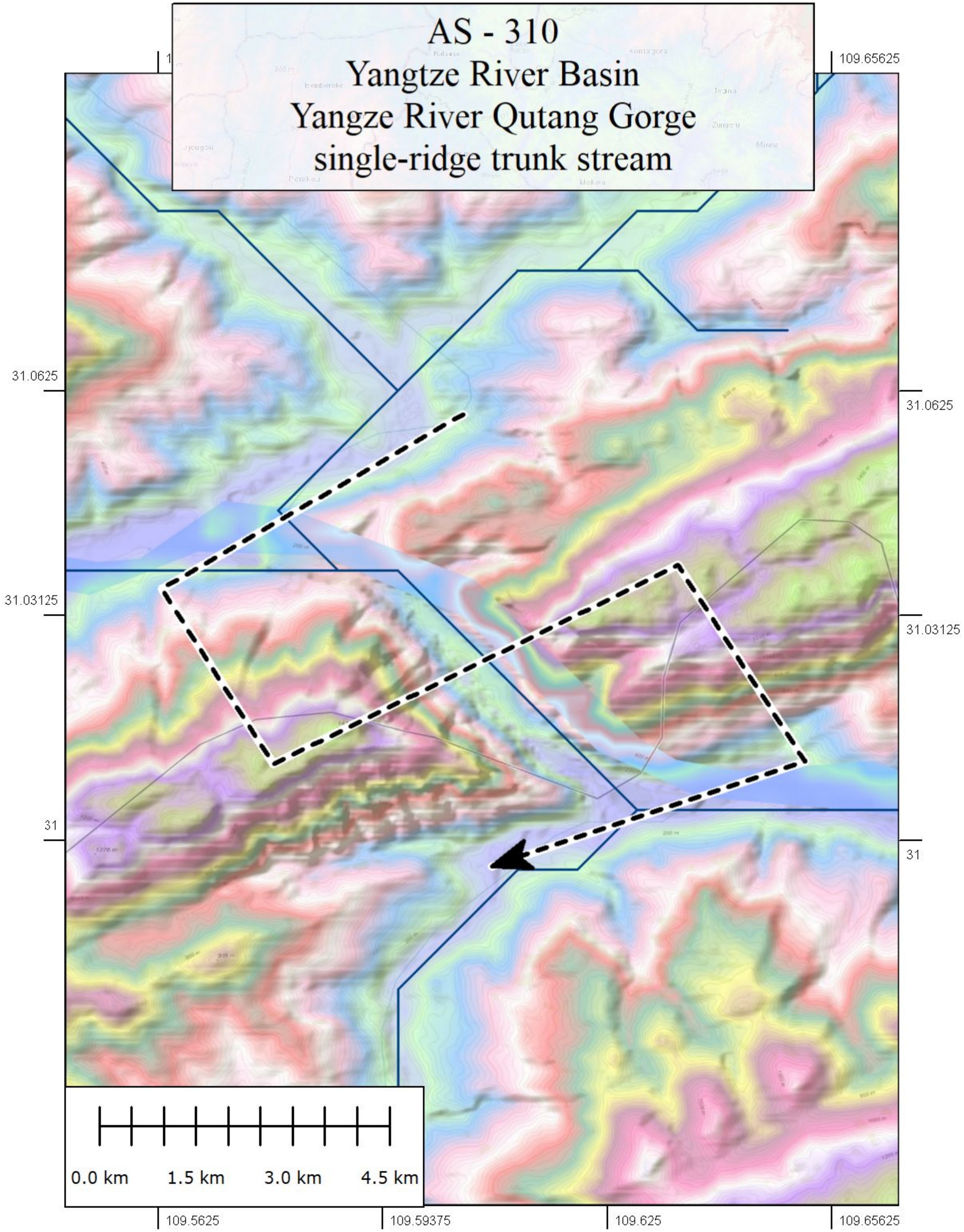
AS - 310
Yangtze River Basin
Yangze River Qutang Gorge
single-ridge trunk stream
109.65625
31.0625
31.0625
31.03125
31.03125
31
31
0.0 km
1.5 km
3.0 km
4.5 km
109.5625
109.59375
109.625
109.65625

AS - 311
Yangtze River Basin
Laoxia River
single-ridge trunk stream

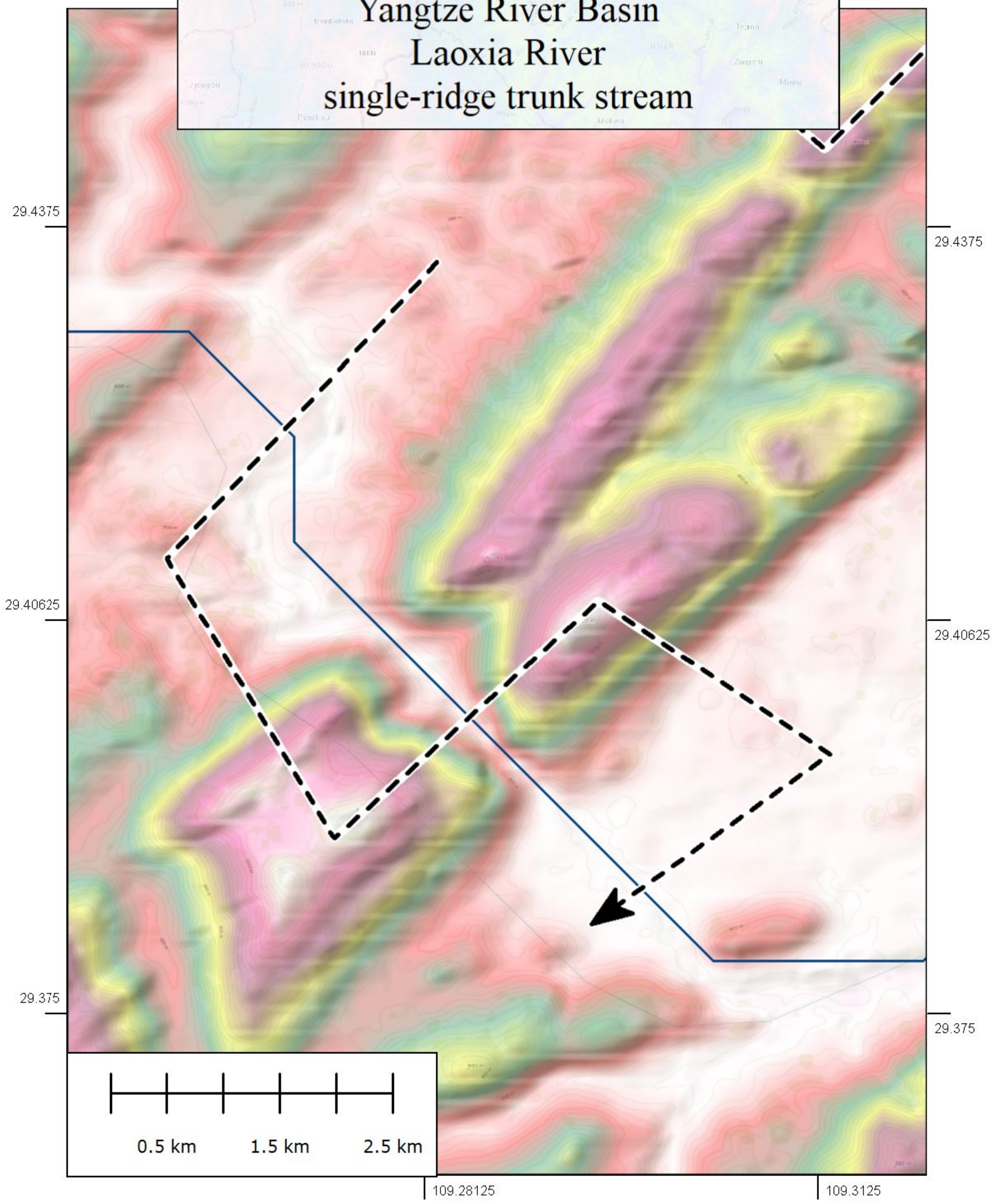

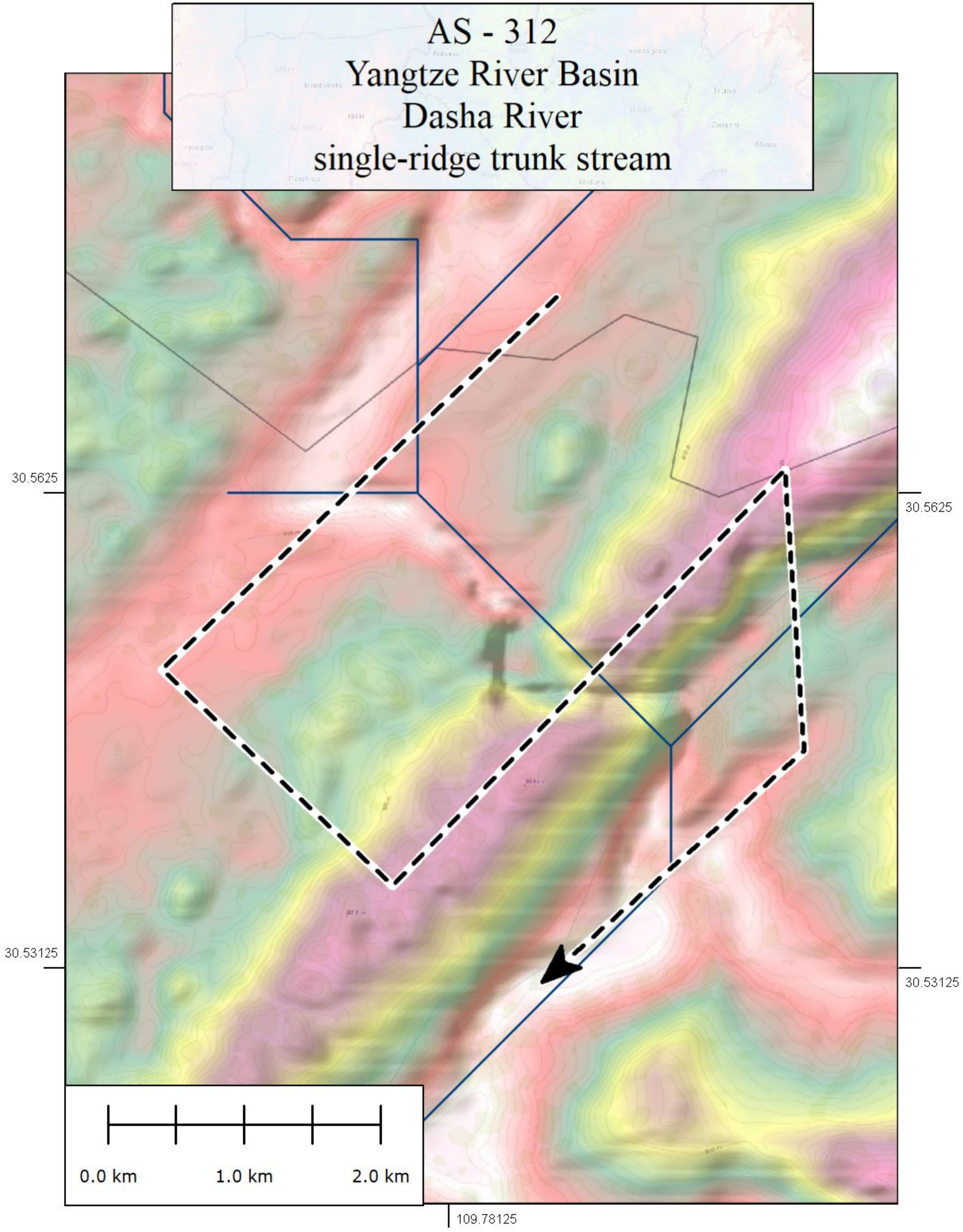
AS - 312
Yangtze River Basin
Dasha River
single-ridge trunk stream
30.5625
30.5625
30.53125
30.53125
109.78125
0.0 km
1.0 km
2.0 km

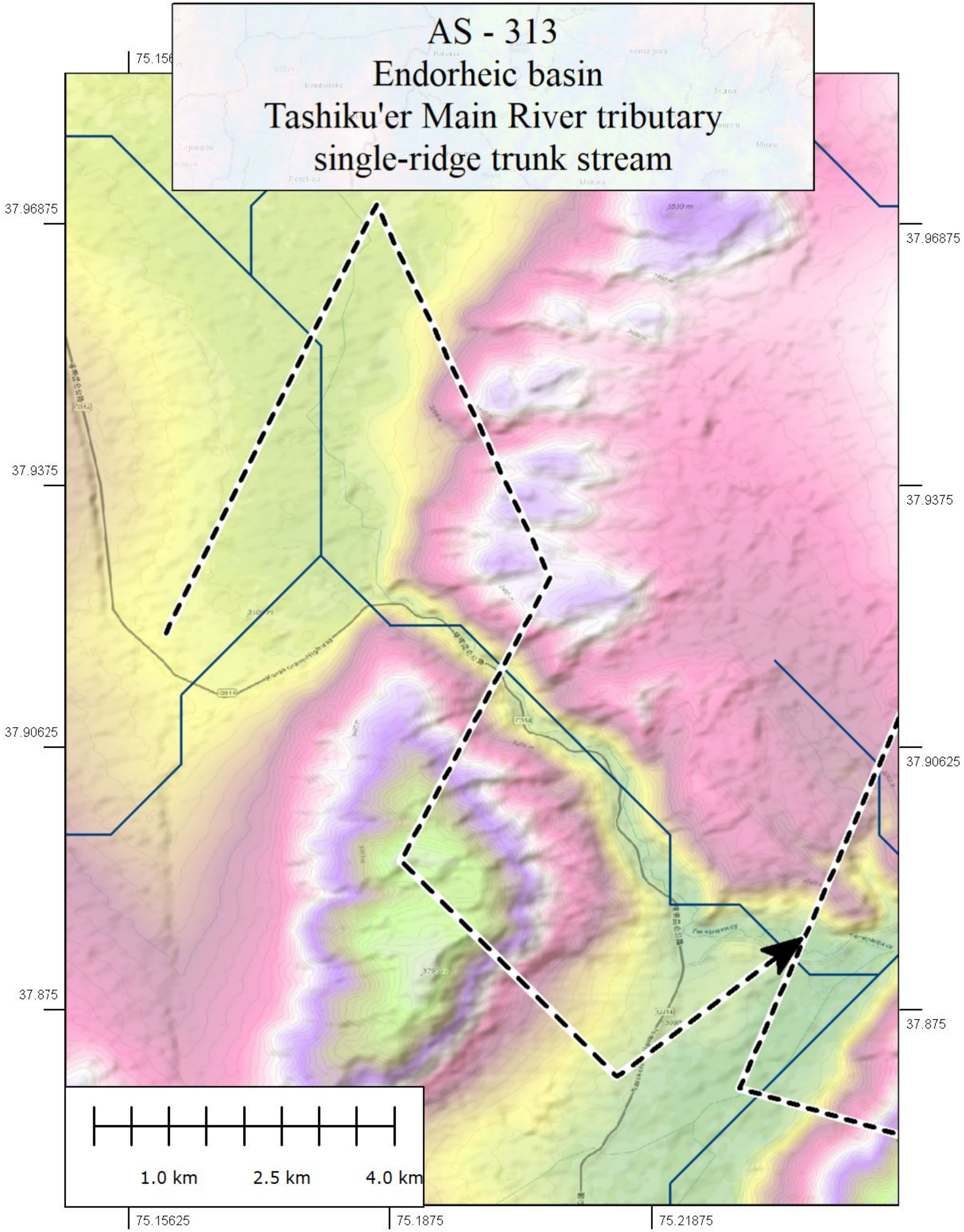
AS - 313
Endorheic basin
Tashiku'er Main River tributary
single-ridge trunk stream
75.15625
75.1875
75.21875
37.96875
37.9375
37.90625
37.875
1.0 km
2.5 km
4.0 km

AS - 314
Yangtze River Basin
Mengdont River
single-ridge trunk stream

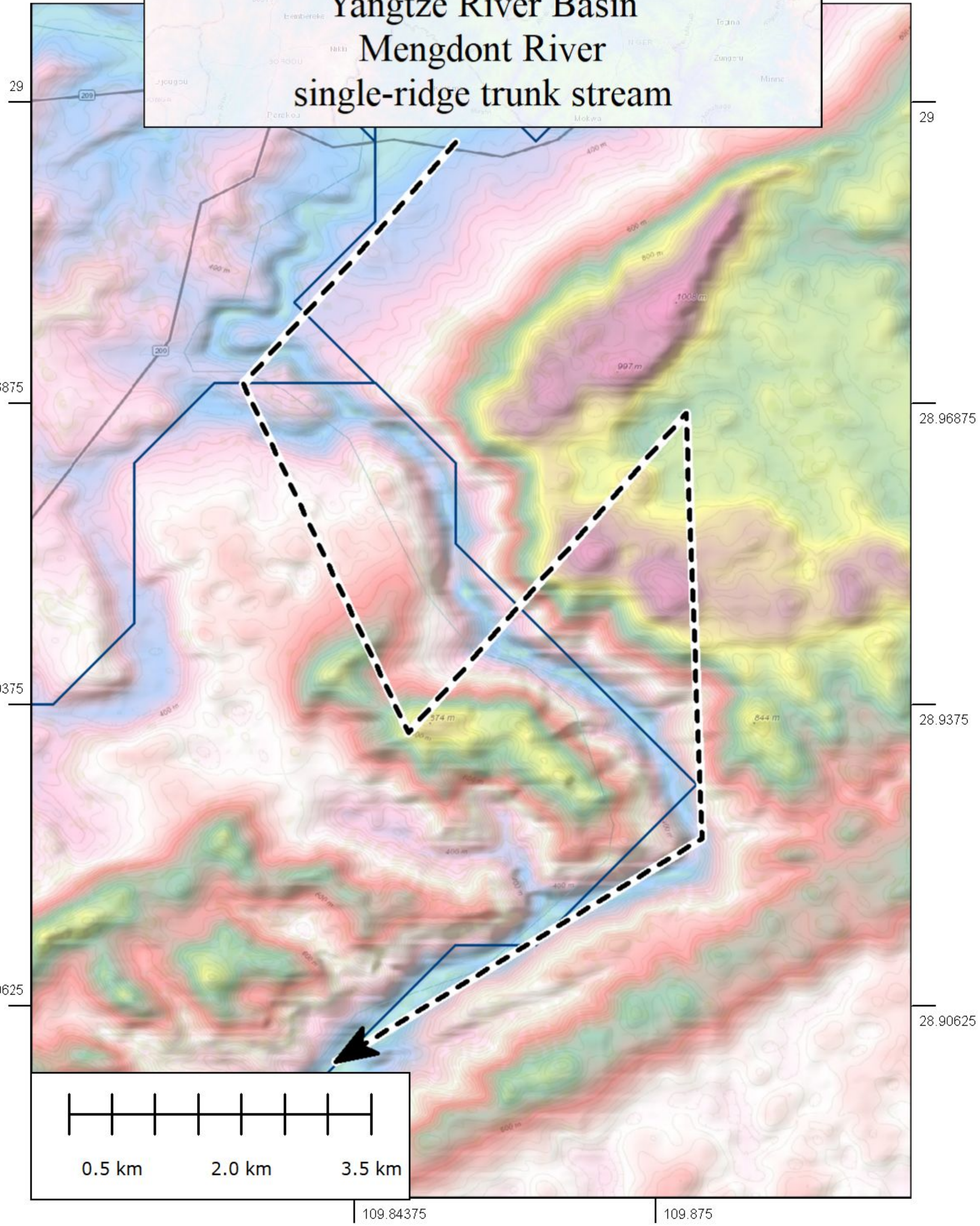

AS - 315
Yangtze River Basin
Daning River
single-ridge trunk stream

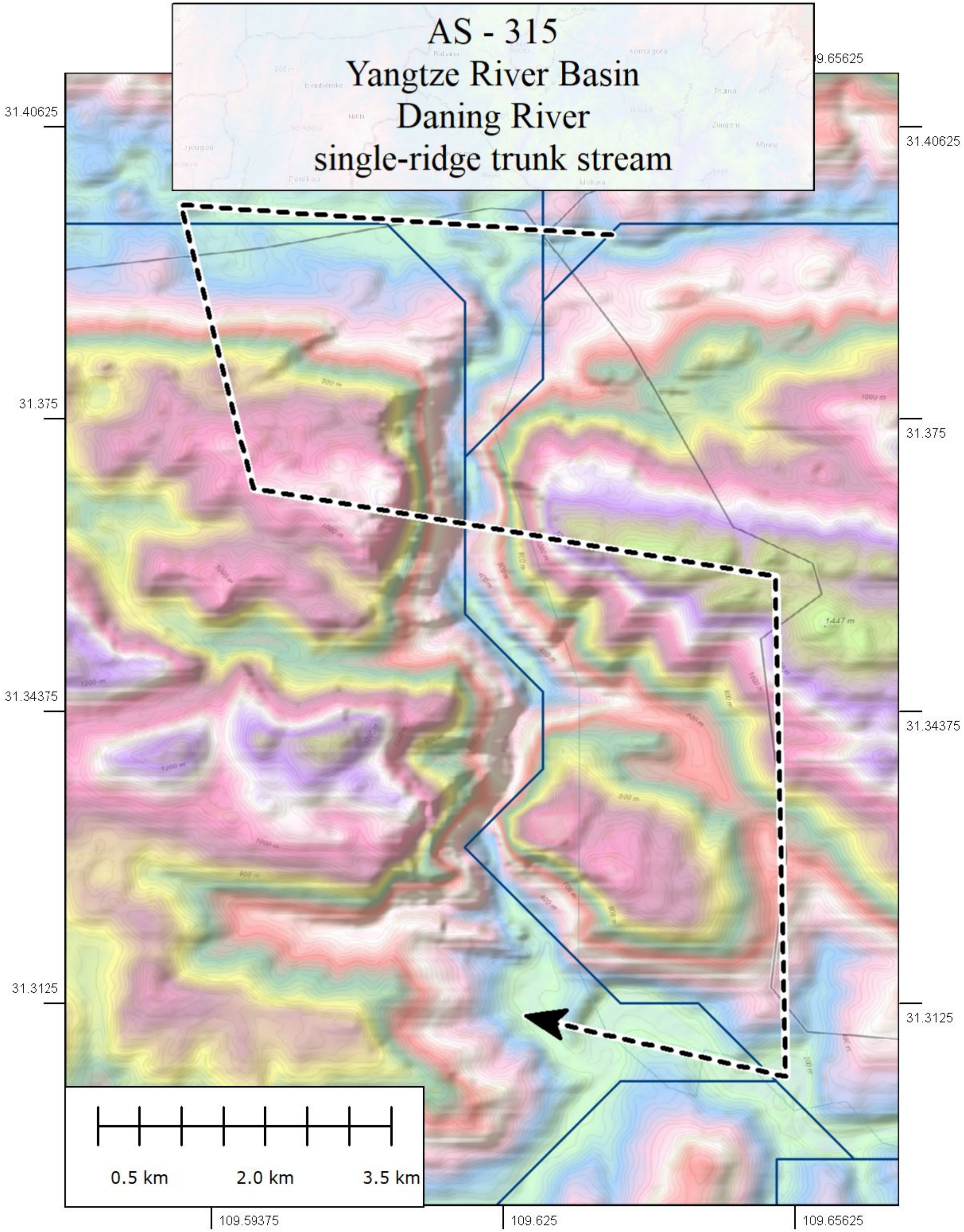

AS - 316
Yangtze River Basin
Shiqian River tributary
single-ridge head stream

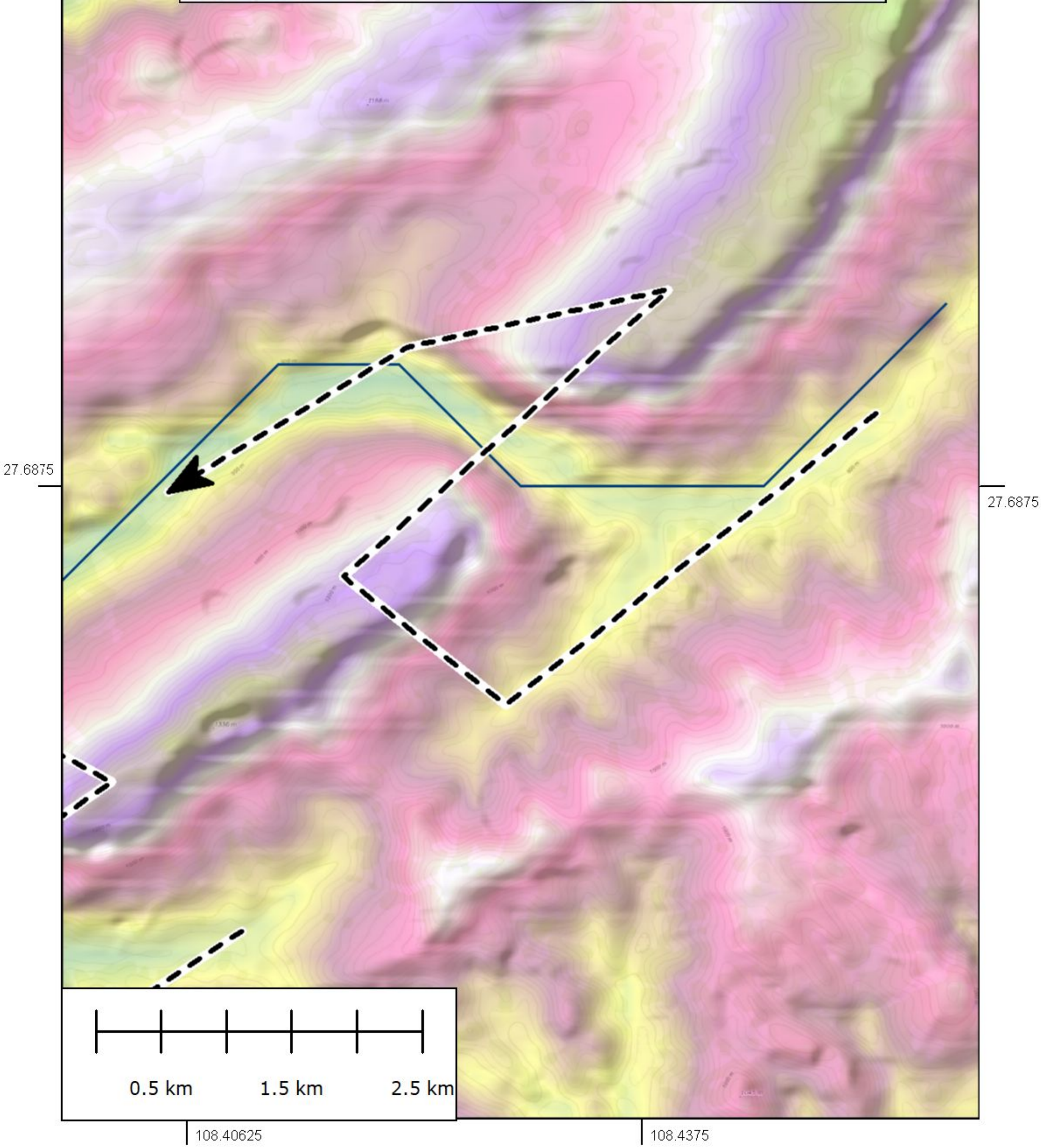

AS - 317
Yangtze River Basin
Qujiang River tributary
single-ridge head stream

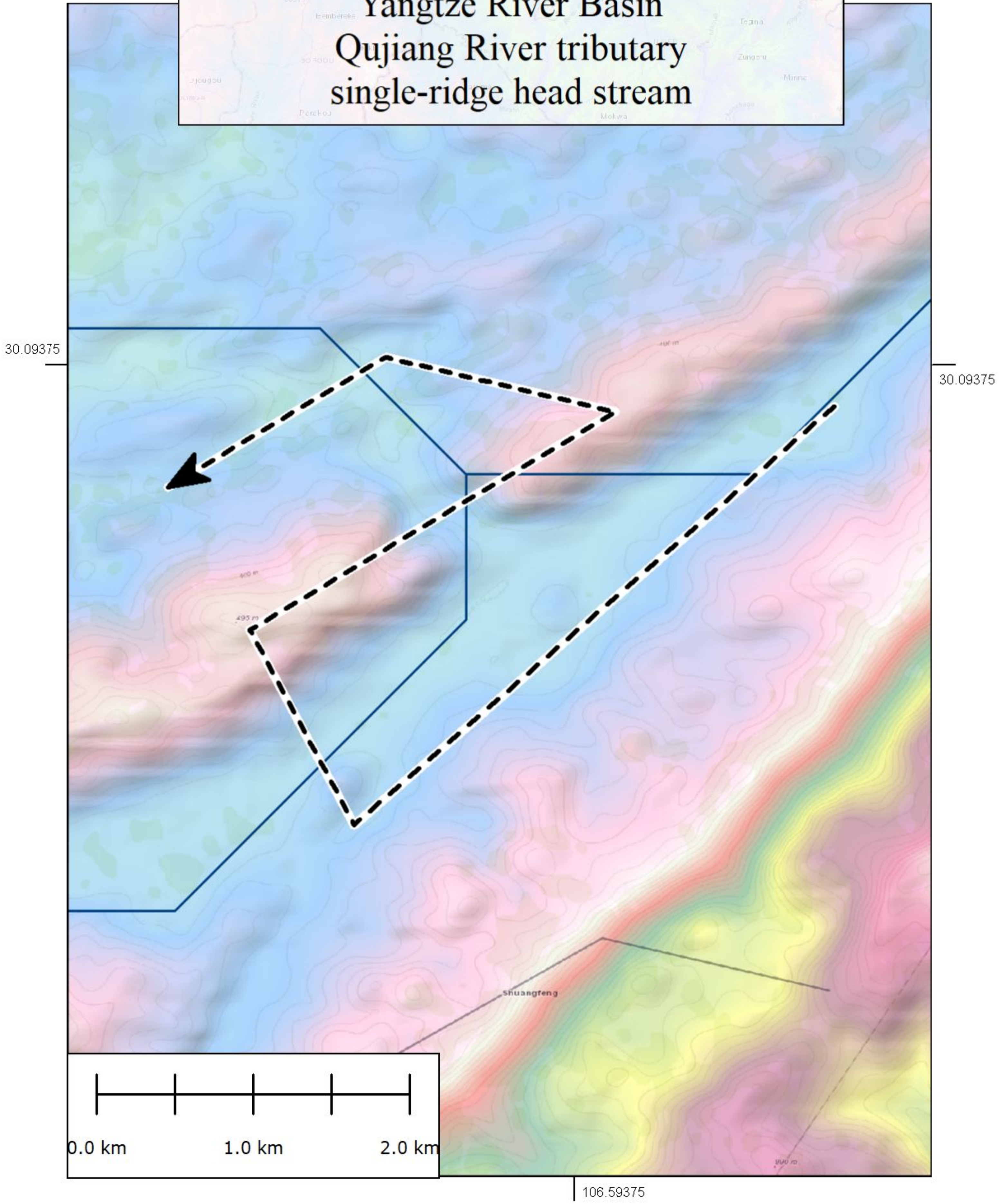

AS - 318
Yangtze River Basin
Jiashihe River
single-ridge trunk stream

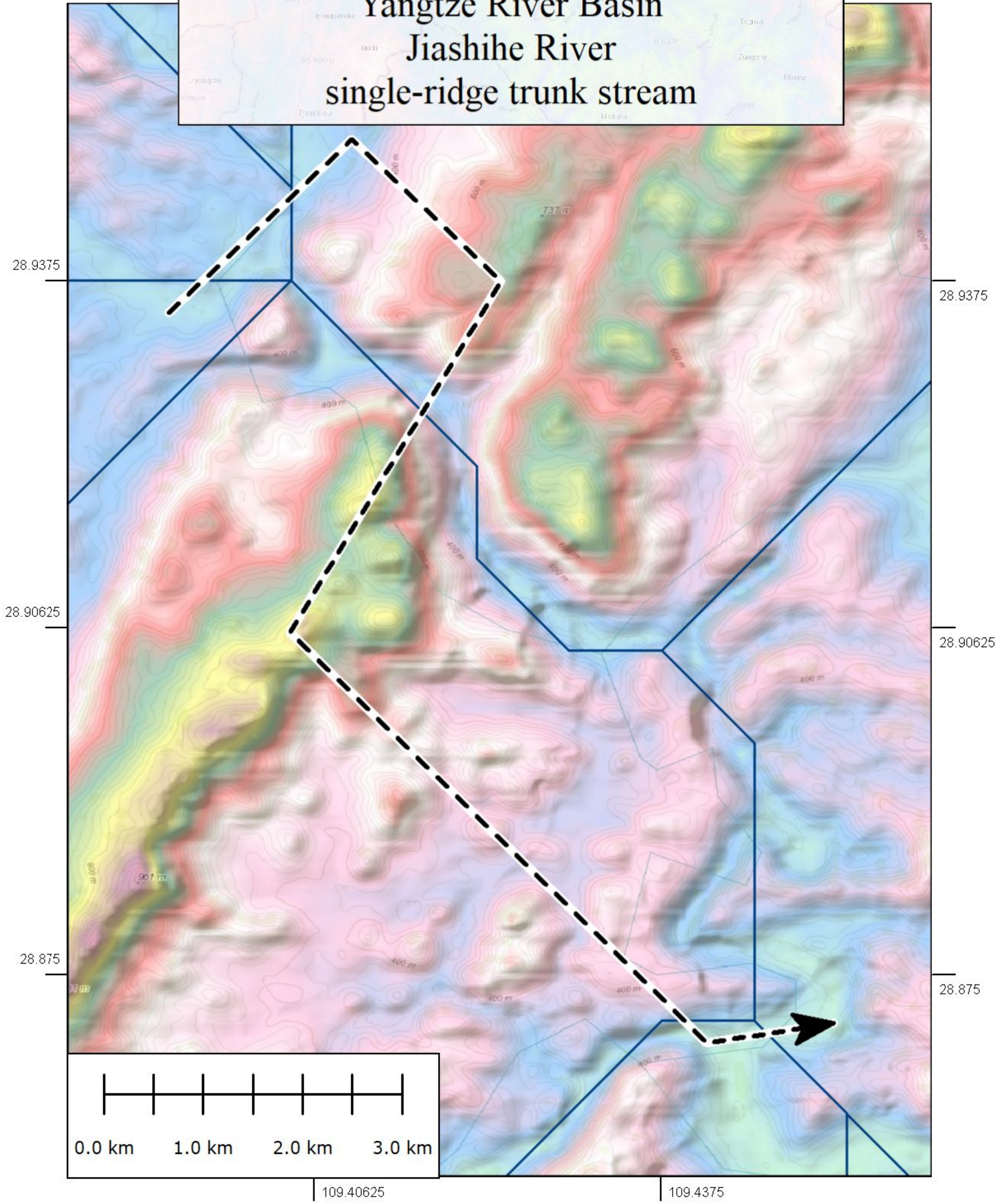

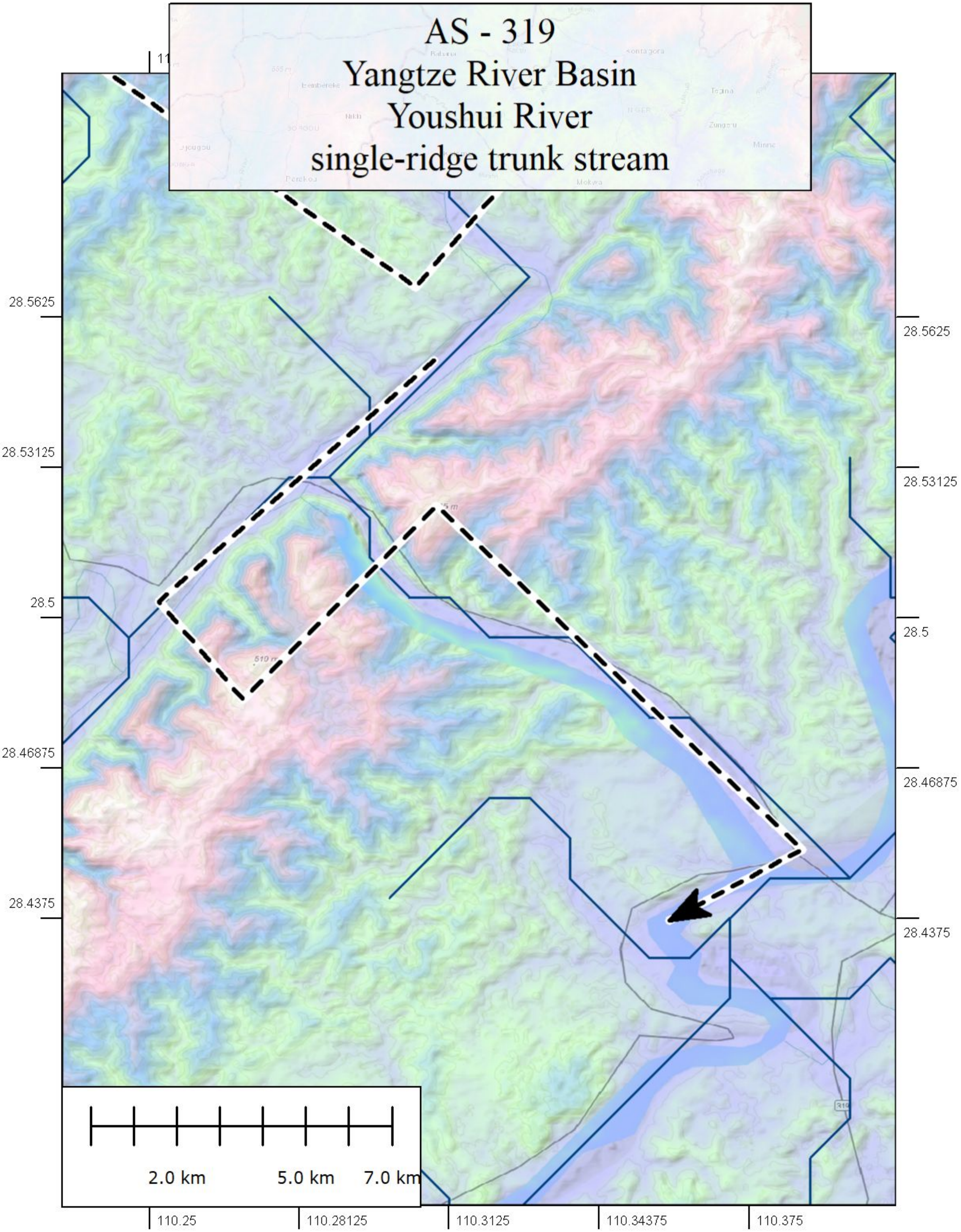
AS - 319
Yangtze River Basin
Youshui River
single-ridge trunk stream
28.5625
28.53125
28.5
28.46875
28.4375
110.25
110.28125
110.3125
110.34375
110.375
2.0 km
5.0 km
7.0 km

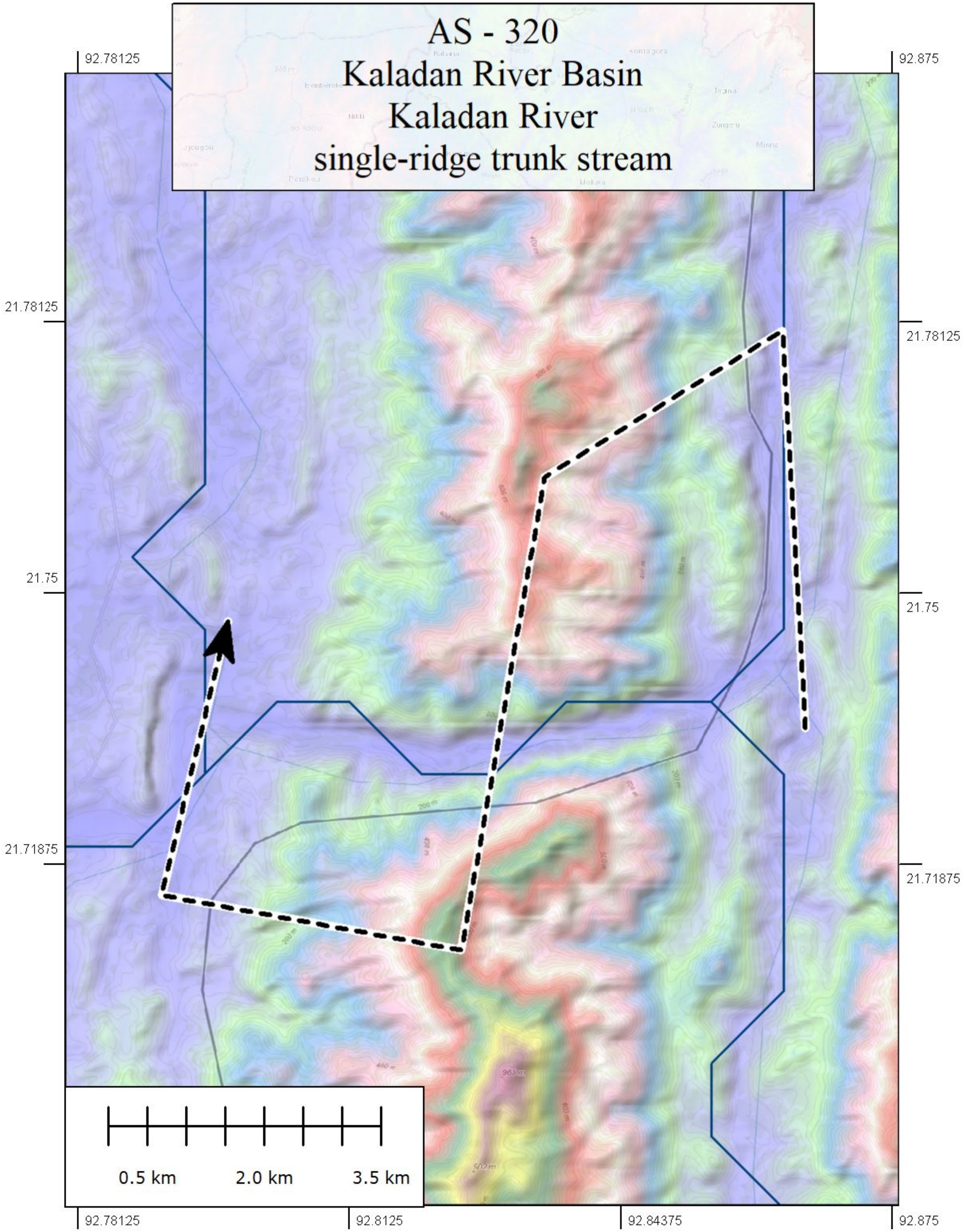
AS - 320
Kaladan River Basin
Kaladan River
single-ridge trunk stream
92.78125
92.875
21.78125
21.75
21.71875
21.78125
21.75
21.71875
0.5 km
2.0 km
3.5 km
92.78125
92.8125
92.84375
92.875

AS - 323
Huang (Yellow) River Basin
Yellow River
single-ridge trunk stream

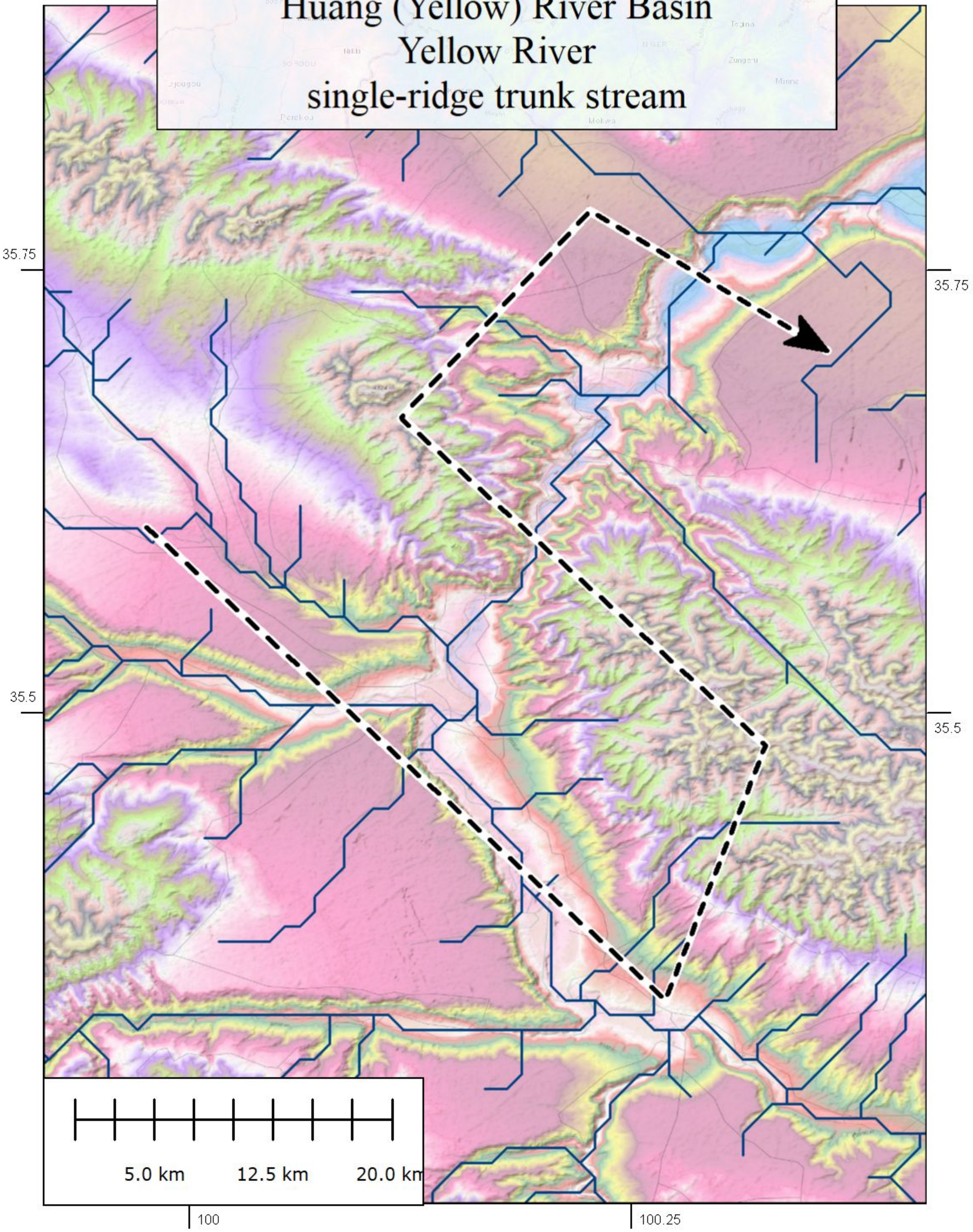

AS - 325
Indus River Basin
single-ridge trunk stream

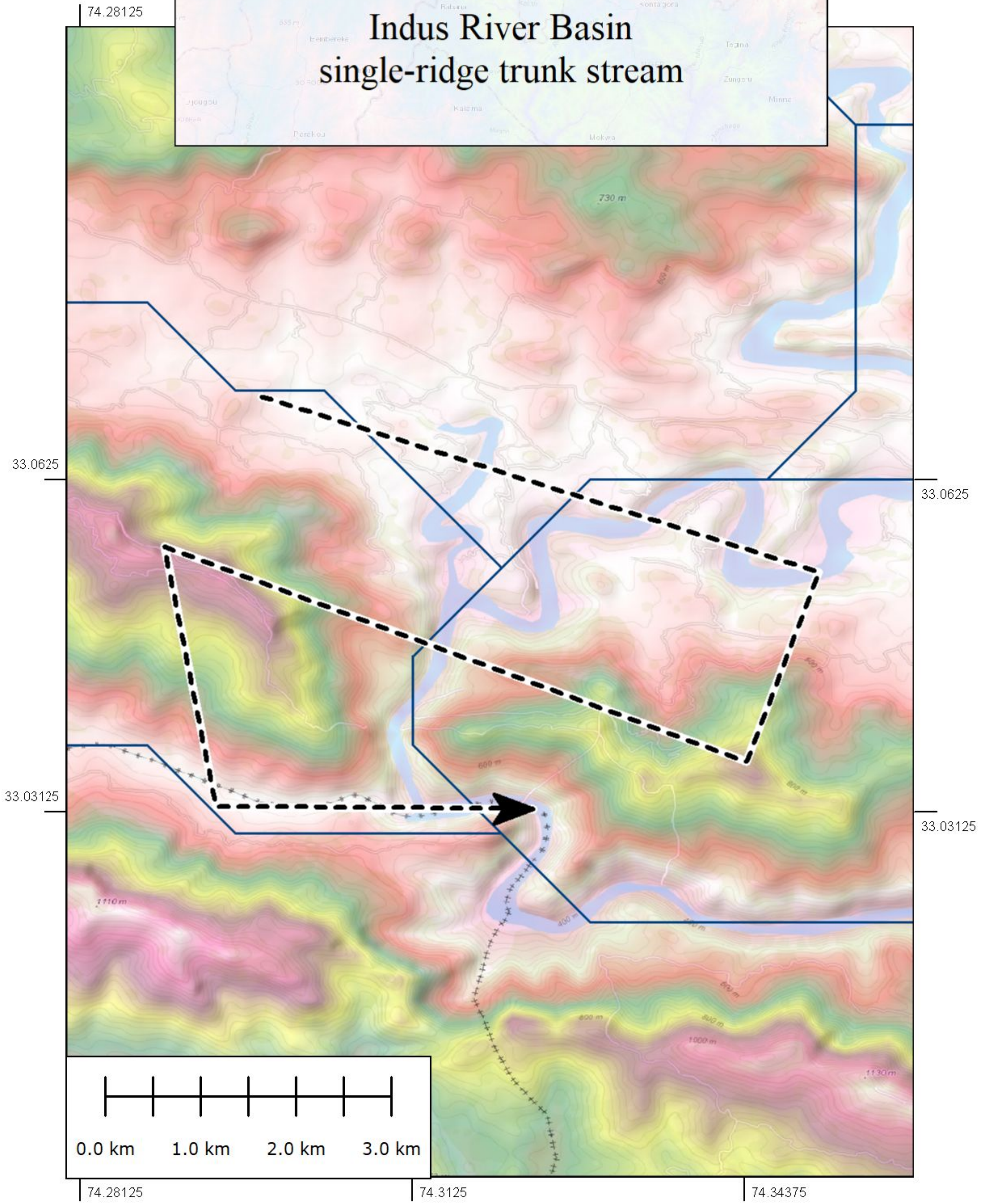

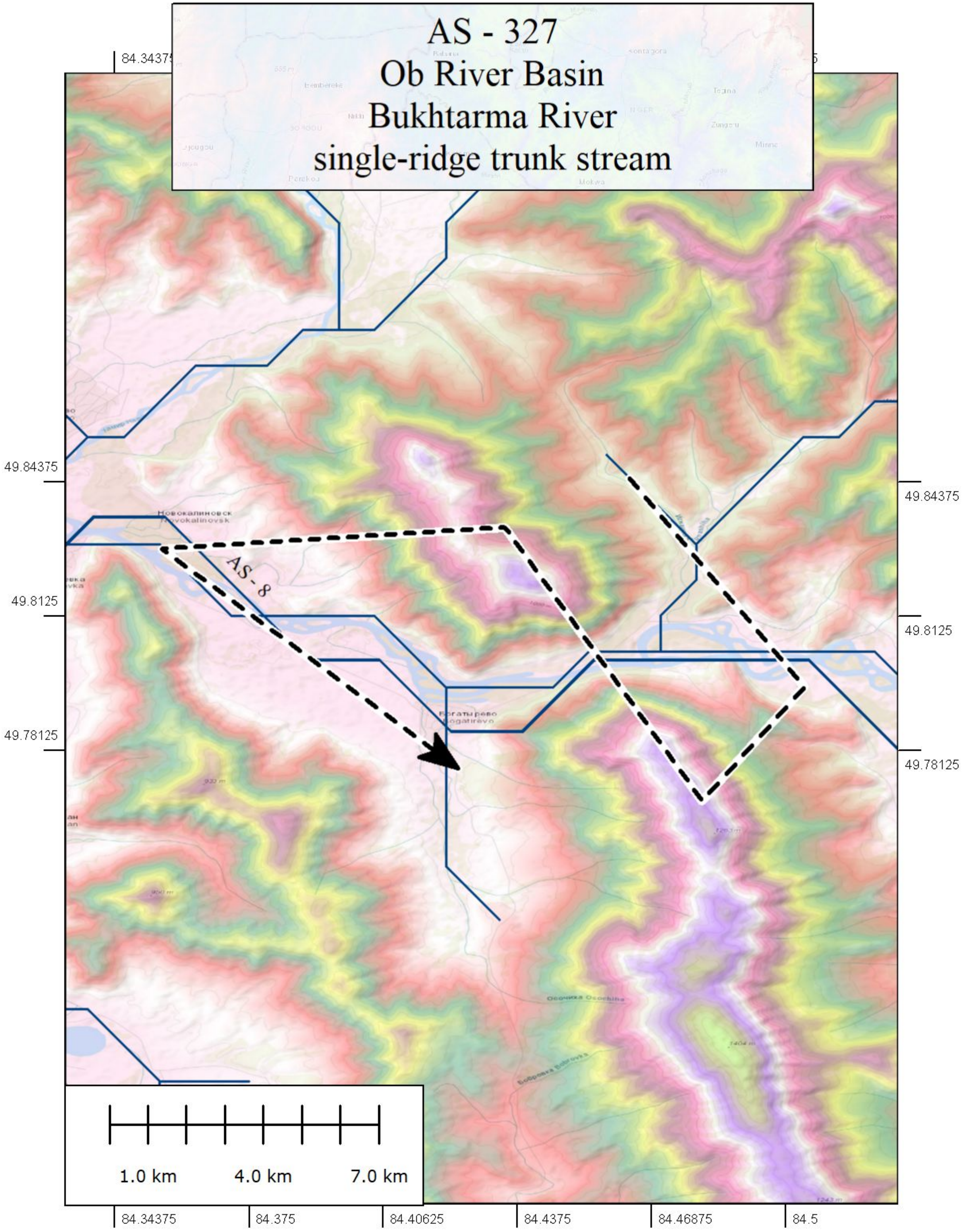
AS - 327
Ob River Basin
Bukhtarma River
single-ridge trunk stream
AS - 8
Новокалиновск
Novokalinovsk
Богатырево
Bogatirevo
Осочиха Osochiha
Бобровка Bobrovka
1.0 km
4.0 km
7.0 km
49.84375
49.8125
49.78125
84.34375
84.375
84.40625
84.4375
84.46875
84.5

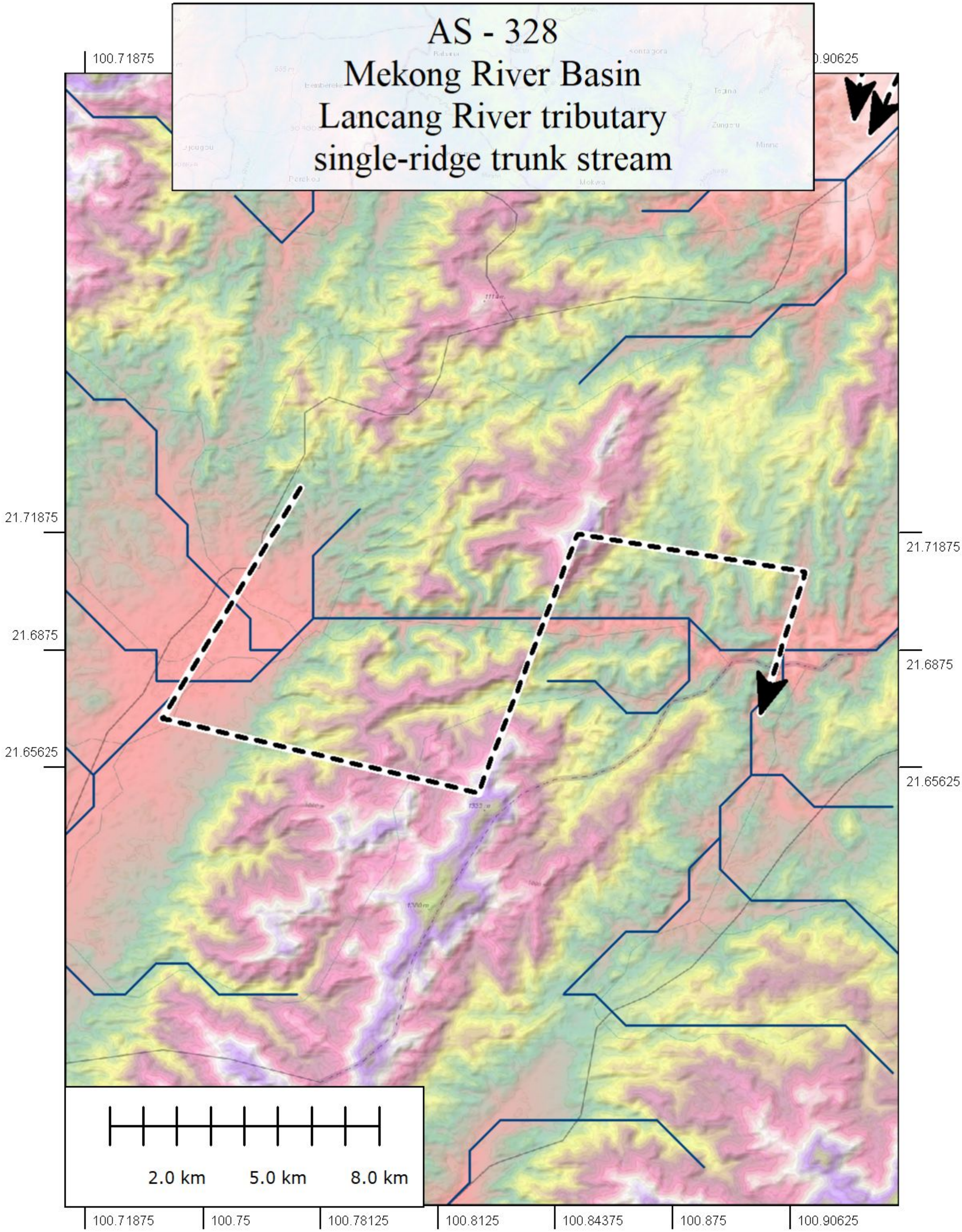
AS - 328
Mekong River Basin
Lancang River tributary
single-ridge trunk stream
100.71875
100.75
100.78125
100.8125
100.84375
100.875
100.90625
21.71875
21.6875
21.65625
2.0 km
5.0 km
8.0 km

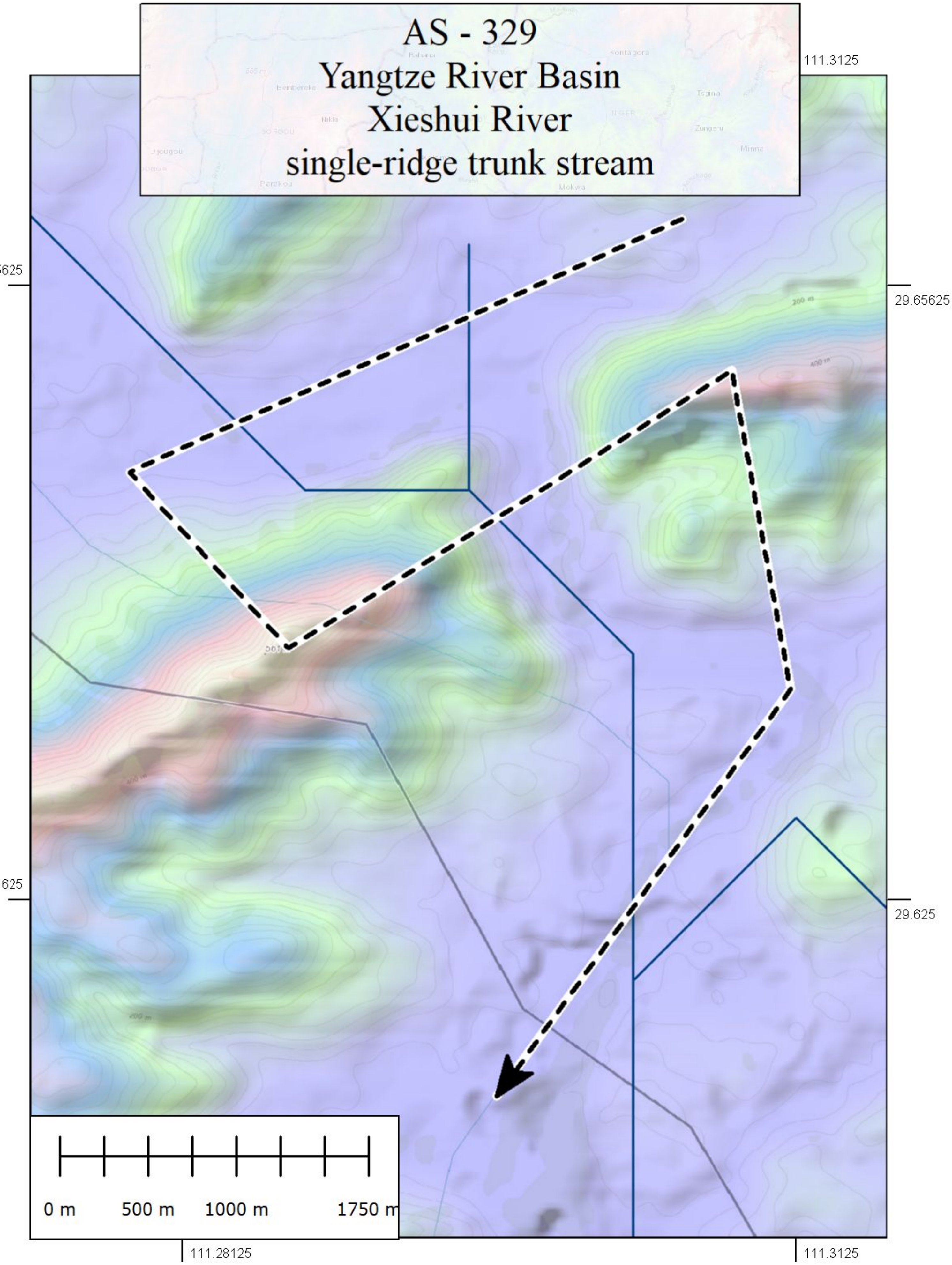
AS - 329
Yangtze River Basin
Xieshui River
single-ridge trunk stream
111.3125
29.65625
29.65625
29.625
29.625
0 m
500 m
1000 m
1750 m
111.28125
111.3125

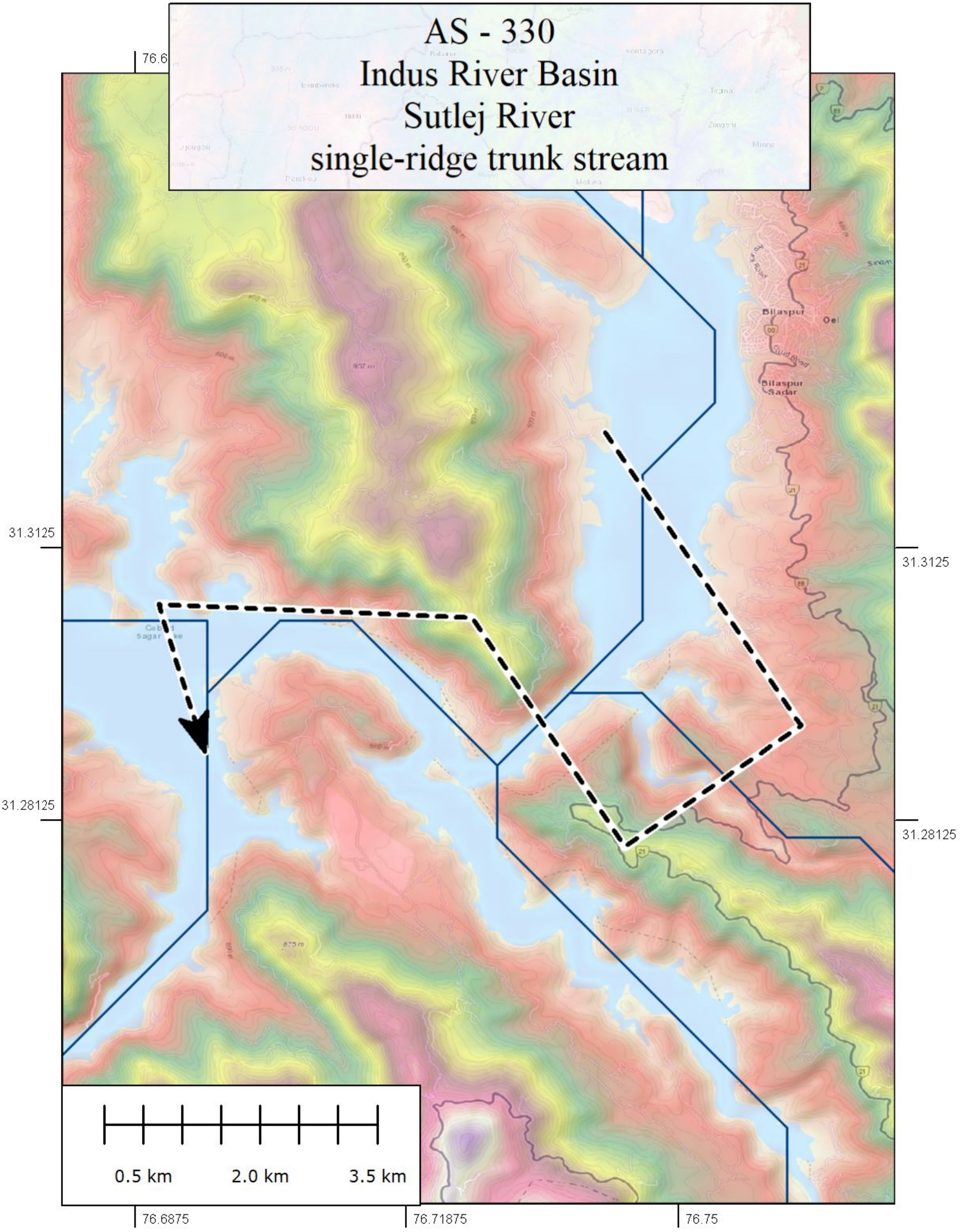
AS - 330
Indus River Basin
Sutlej River
single-ridge trunk stream
76.6875
76.71875
76.75
31.3125
31.28125
Bilaspur
Oel
Bilaspur Sadar
0.5 km
2.0 km
3.5 km

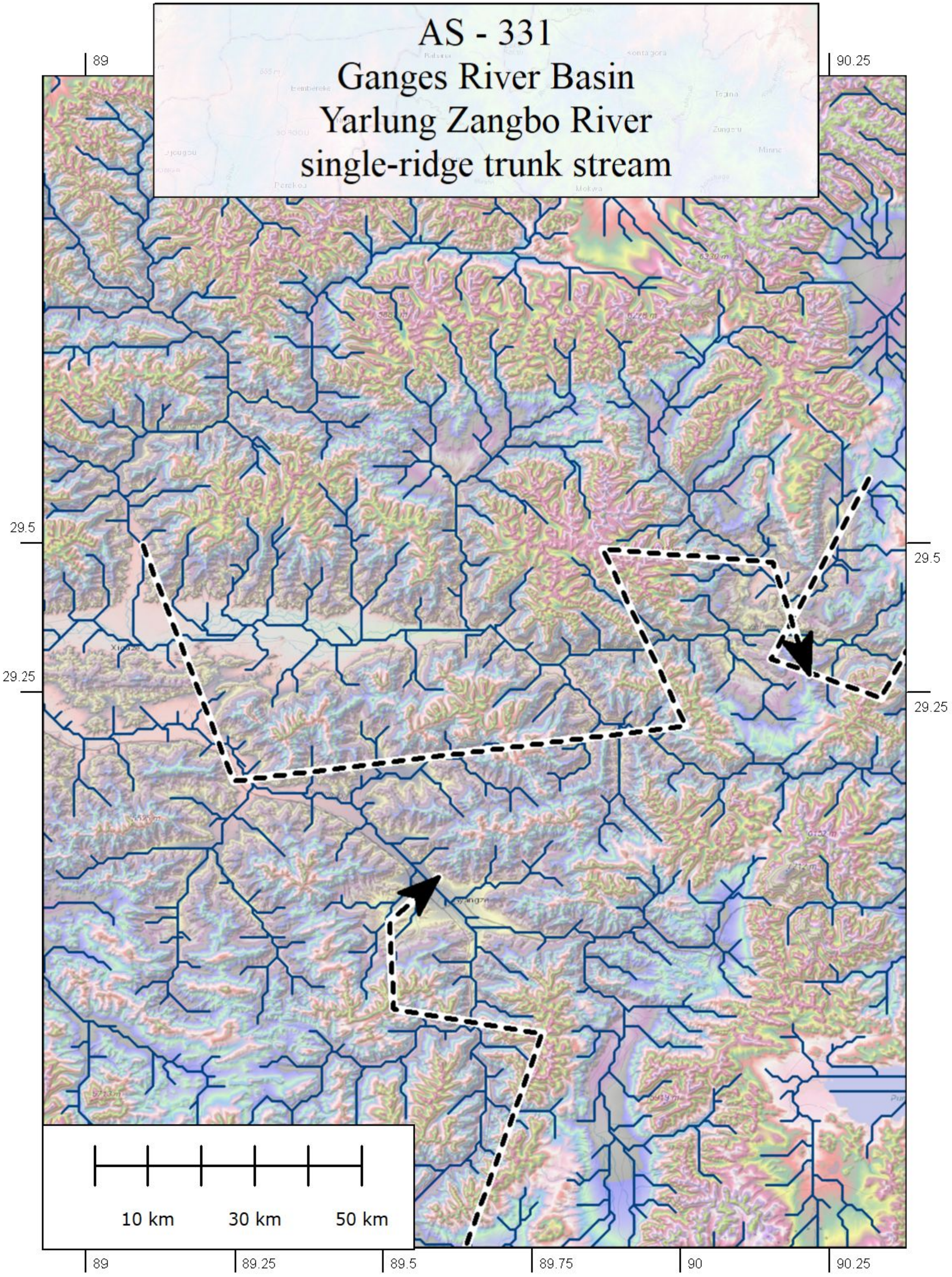
AS - 331
Ganges River Basin
Yarlung Zangbo River
single-ridge trunk stream
89
89.25
89.5
89.75
90
90.25
29.5
29.25
10 km
30 km
50 km

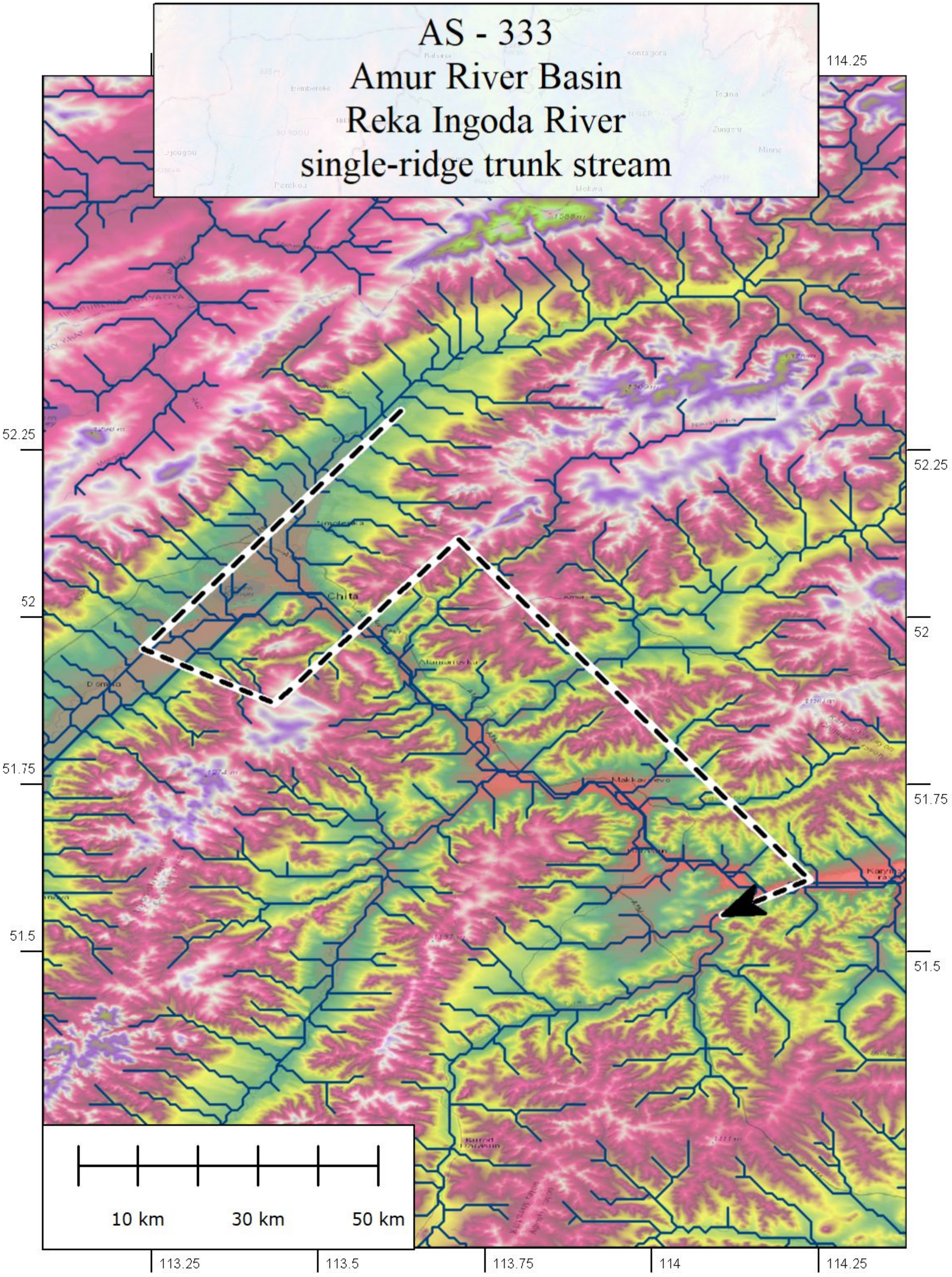
AS - 333
Amur River Basin
Reka Ingoda River
single-ridge trunk stream
52.25
52
51.75
51.5
113.25
113.5
113.75
114
114.25
Chita
10 km
30 km
50 km

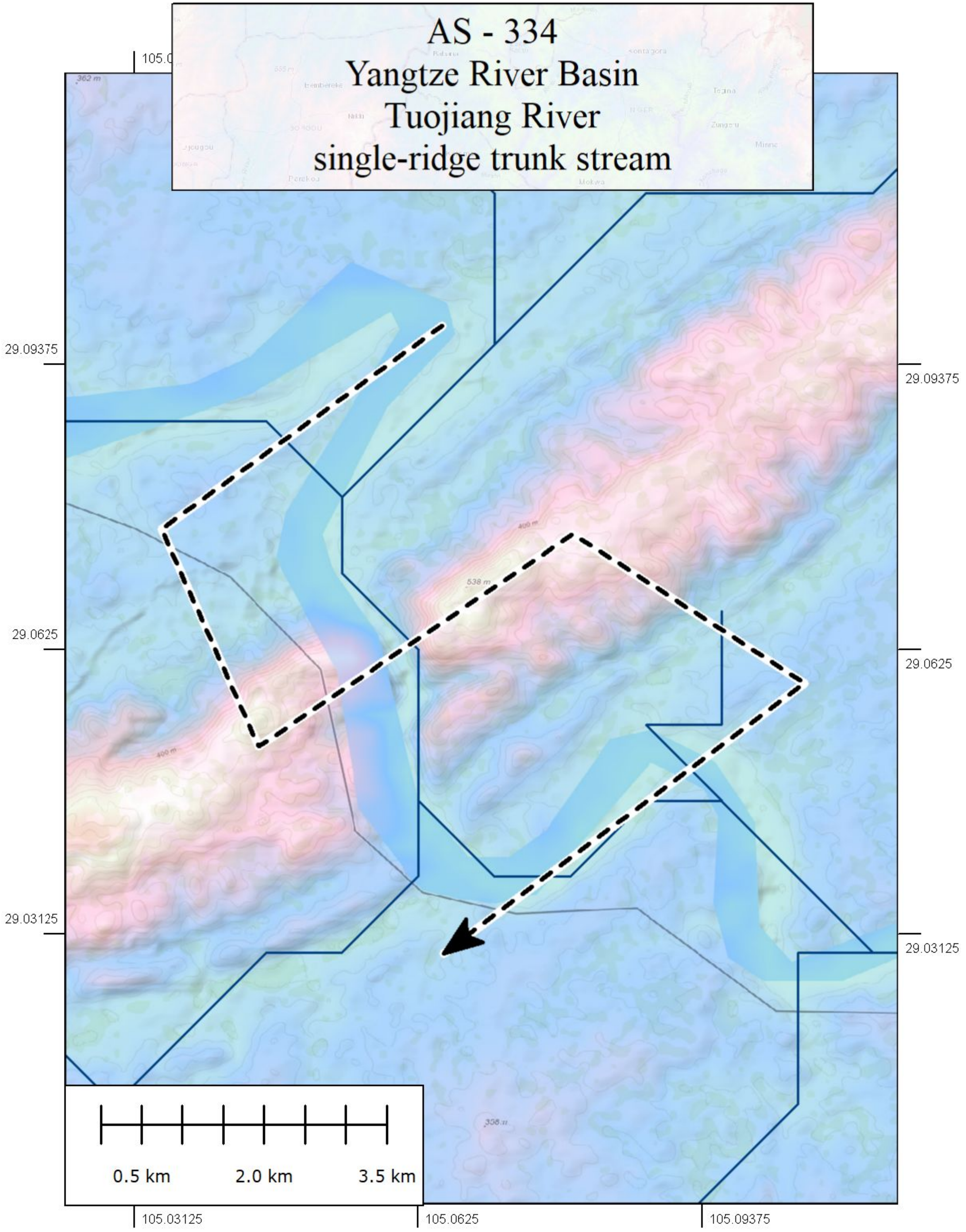
AS - 334
Yangtze River Basin
Tuojiang River
single-ridge trunk stream
362 m
400 m
538 m
400 m
358 m
29.09375
29.0625
29.03125
29.09375
29.0625
29.03125
105.03125
105.0625
105.09375
0.5 km
2.0 km
3.5 km
