# Supplementary material for: A global database of large-scale transverse drainages
Source: Data Brief. 2019 Jan 14;23:103650. doi: 10.1016/j.dib.2018.12.088 (PMC6369416; doi:10.1016/j.dib.2018.12.088)

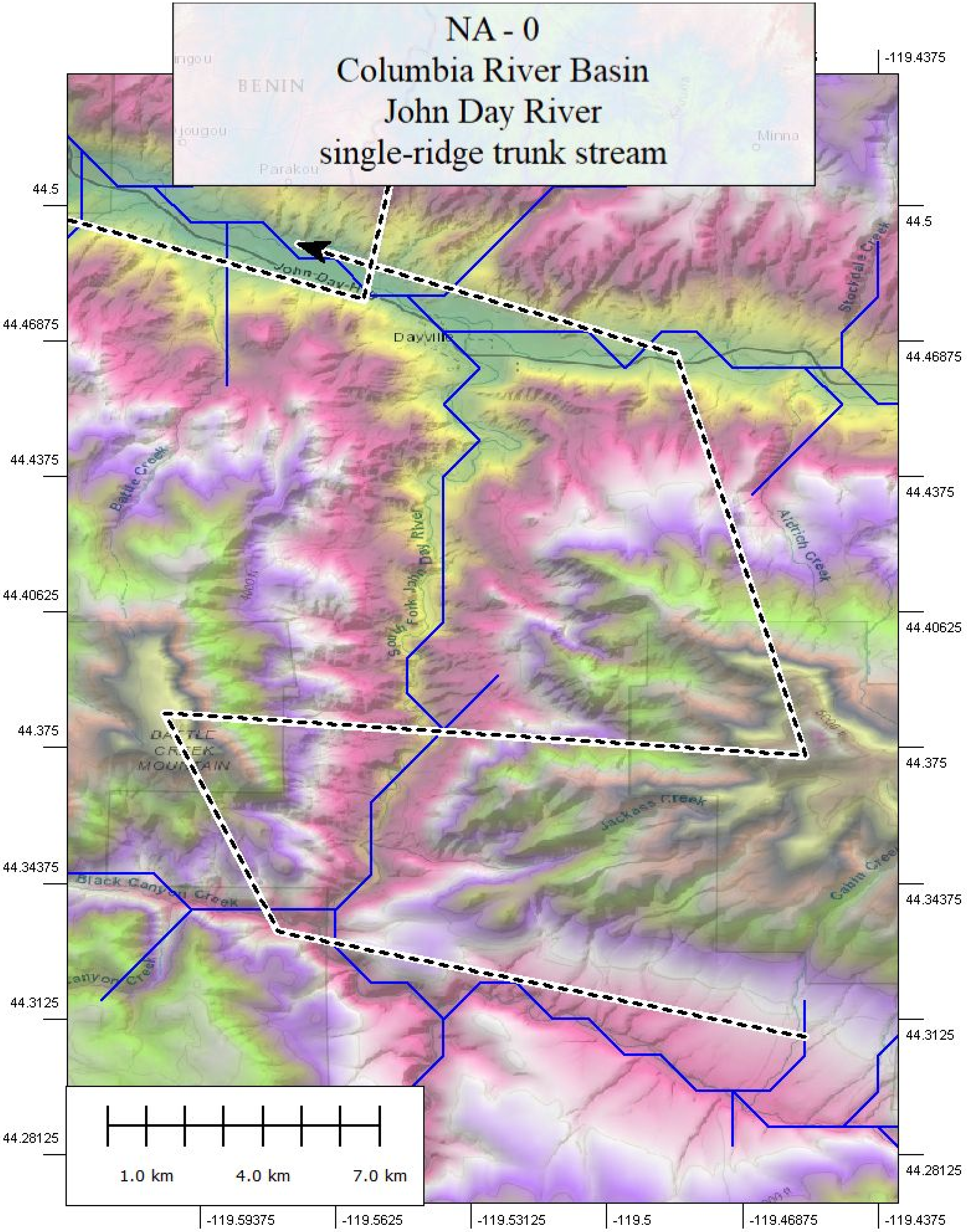

NA - 0
Columbia River Basin
John Day River
single-ridge trunk stream
John Day River
Dayville
Stockdale Creek
Battle Creek
South Fork John Day River
Aldrich Creek
BATTLE CREEK MOUNTAIN
Jackass Creek
Black Canyon Creek
Canyon Creek
Cabin Creek
1.0 km
4.0 km
7.0 km
44.5
44.46875
44.4375
44.40625
44.375
44.34375
44.3125
44.28125
-119.59375
-119.5625
-119.53125
-119.5
-119.46875
-119.4375

NA - 1
Fraser River Basin
West Road (Blackwater) River
single-ridge trunk stream

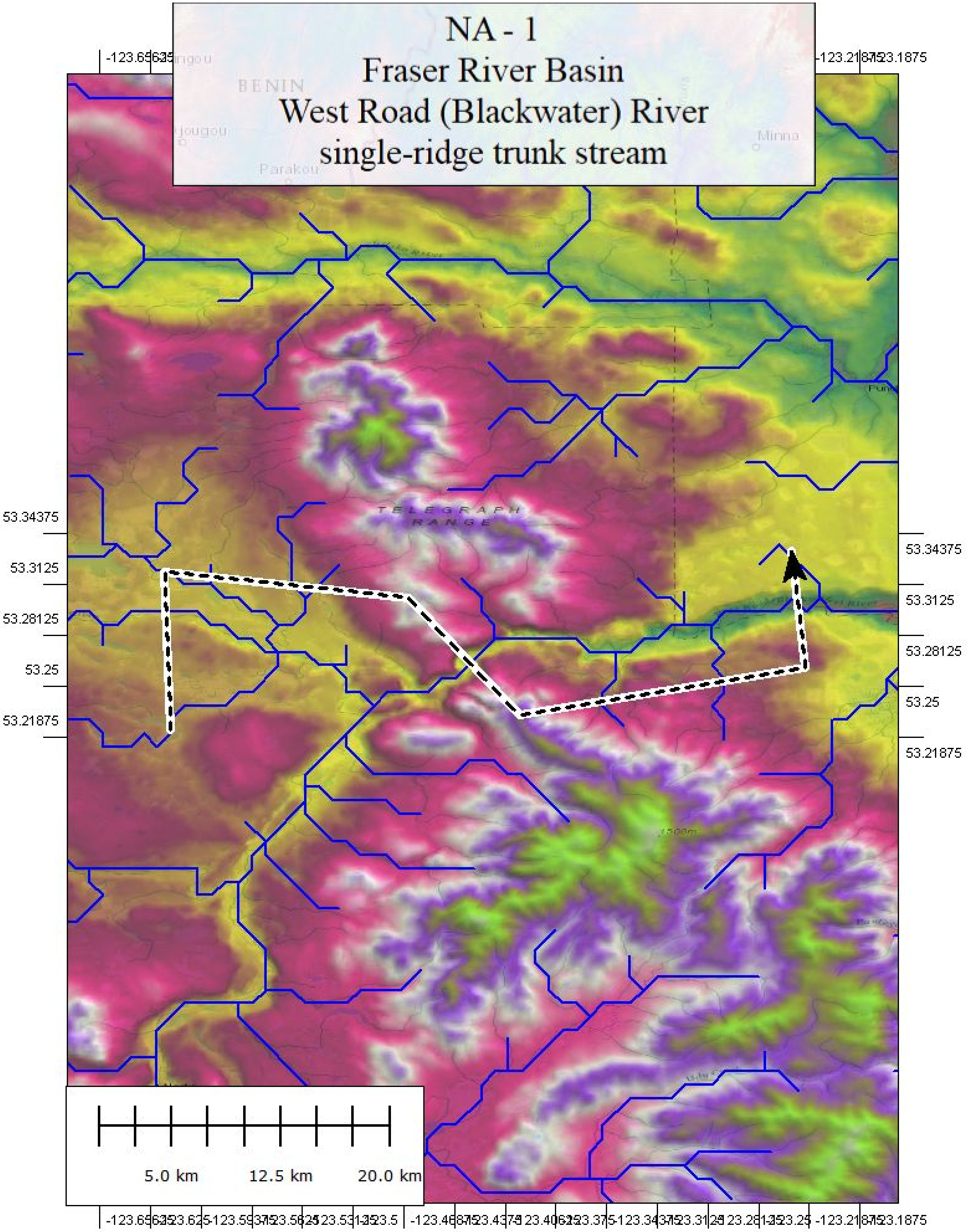

NA - 10
Rio Shutaque Basin
Rio Grande de Zacapa tributary
irregular high ground trunk stream

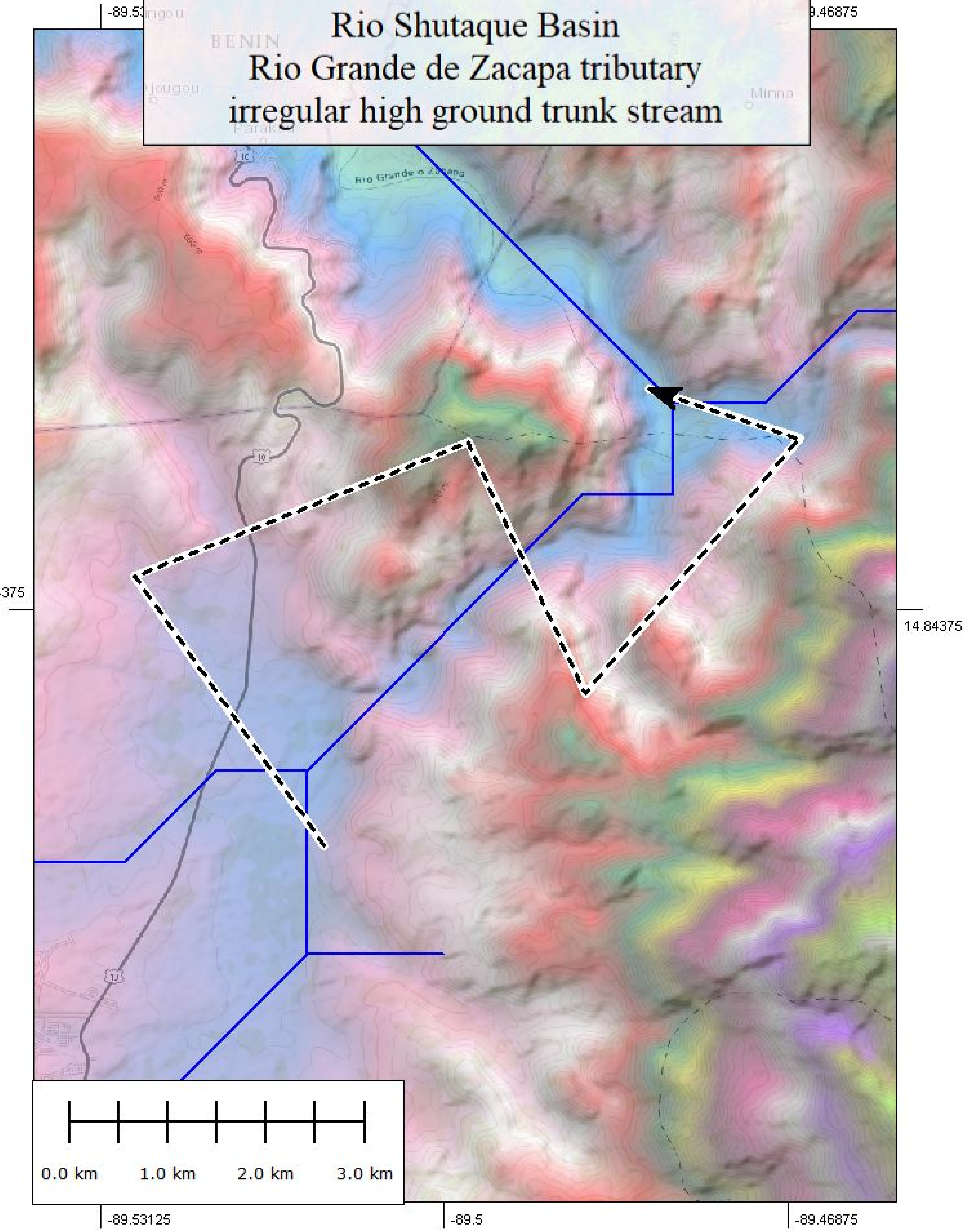

NA - 100
Mississippi River Basin
Dry Run
multi-ridge head stream

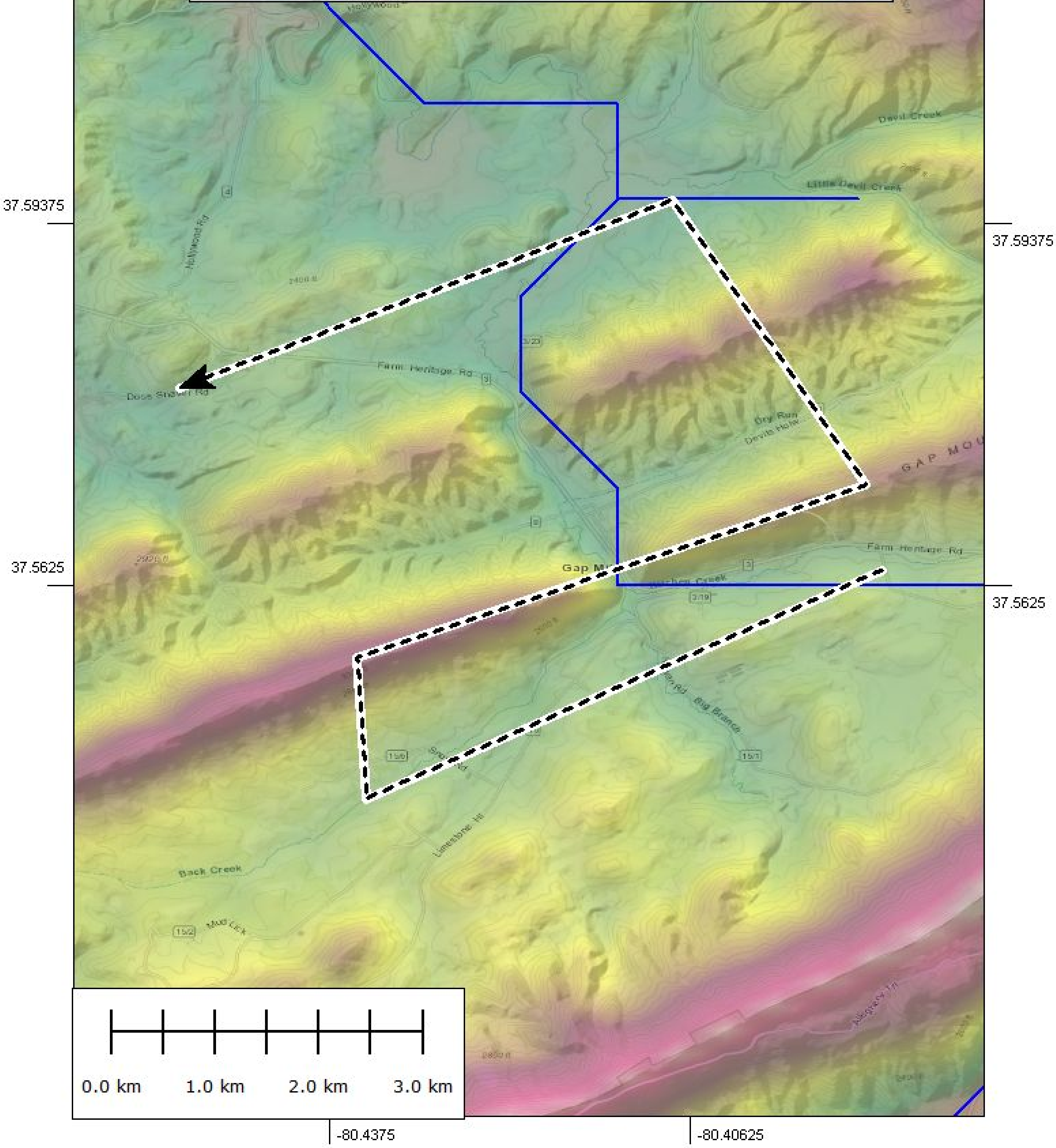

NA - 101
Sacramento River Basin
Carquinez Strait
irregular high ground trunk stream

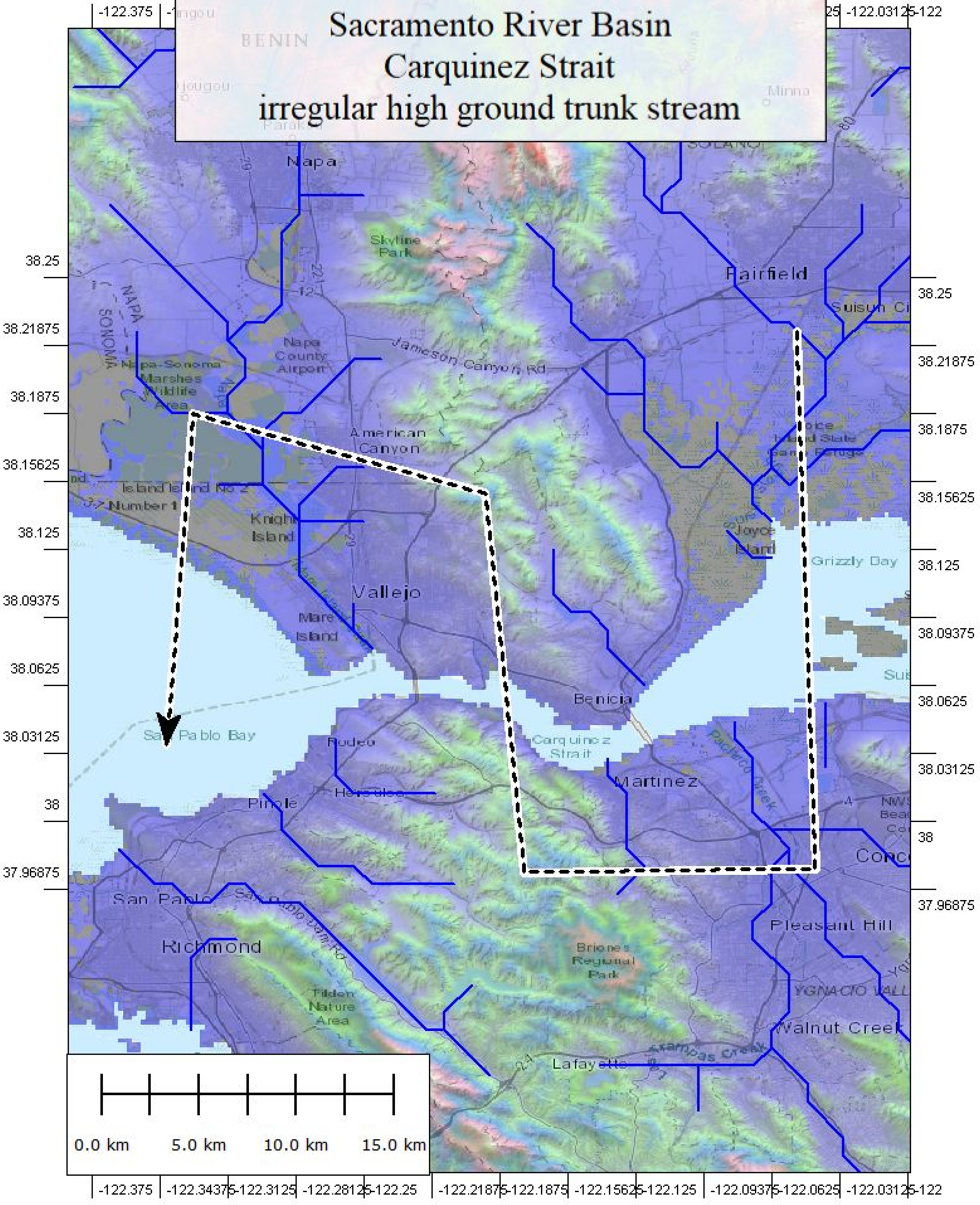

NA - 102
Susquehanna River Basin
Felix Run
single-ridge head stream

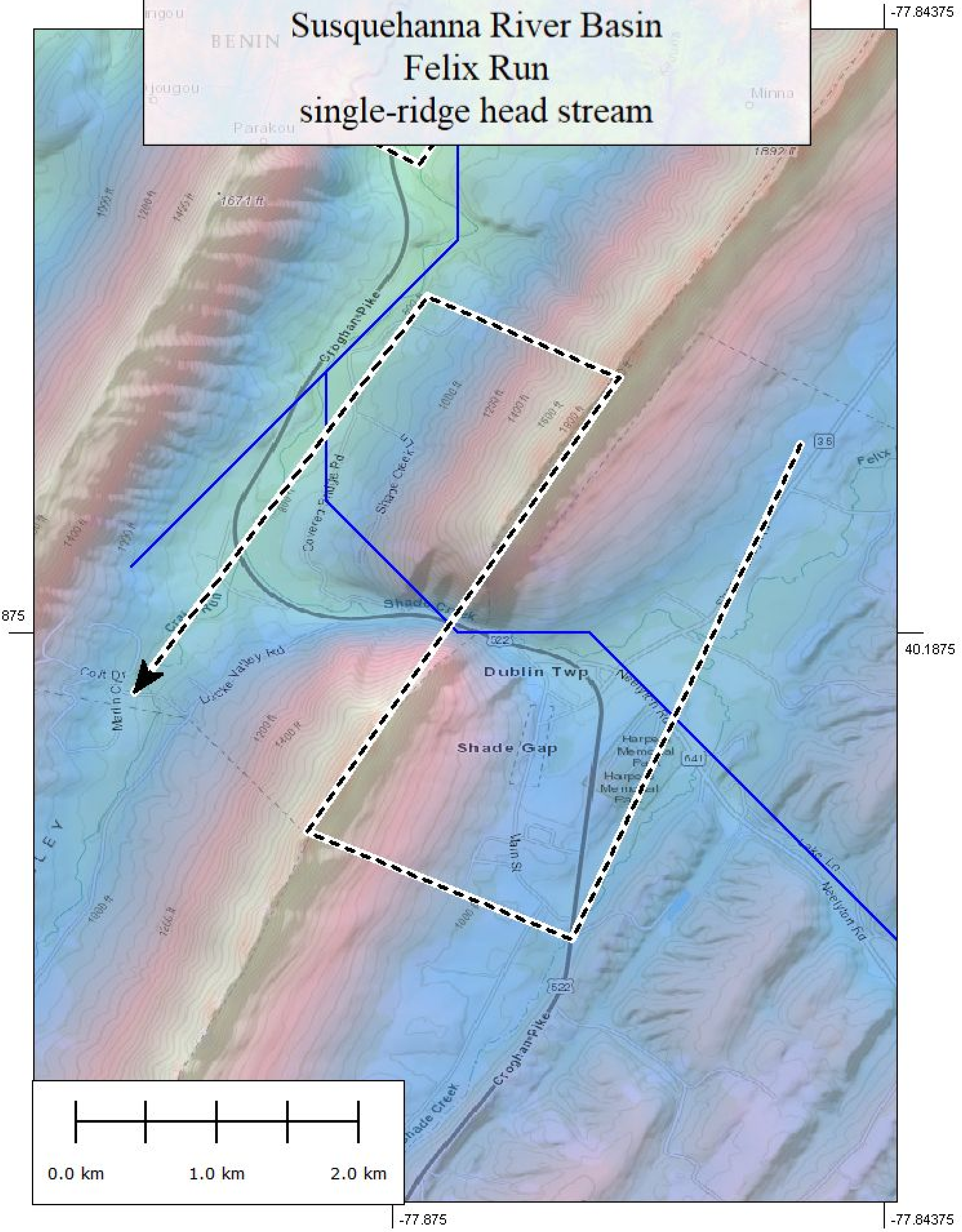

NA - 103
Mississippi River Basin
Carlock Creek
single-ridge head stream

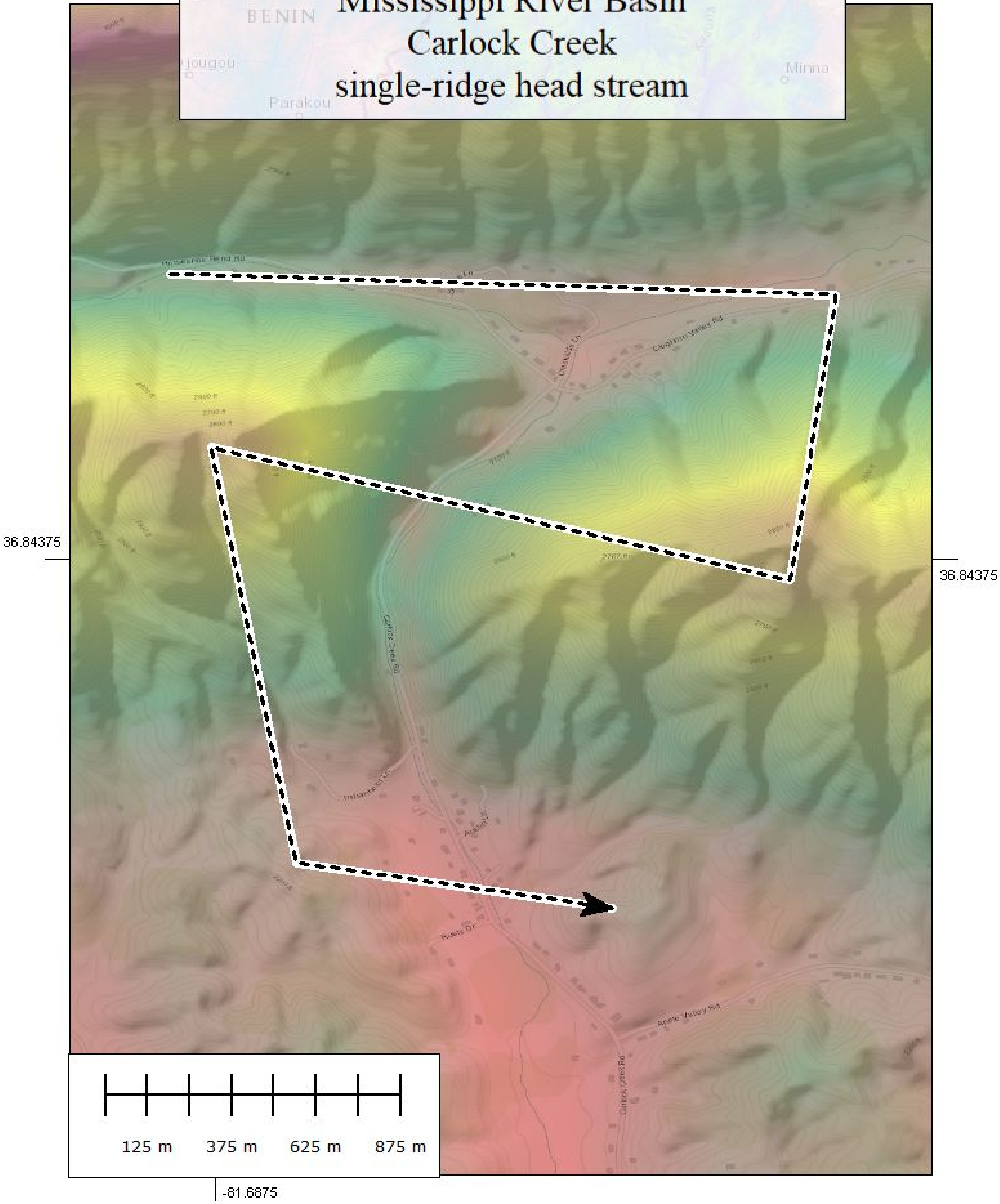

NA - 104
Susquehanna River Basin
Buffalo Run
single-ridge head stream

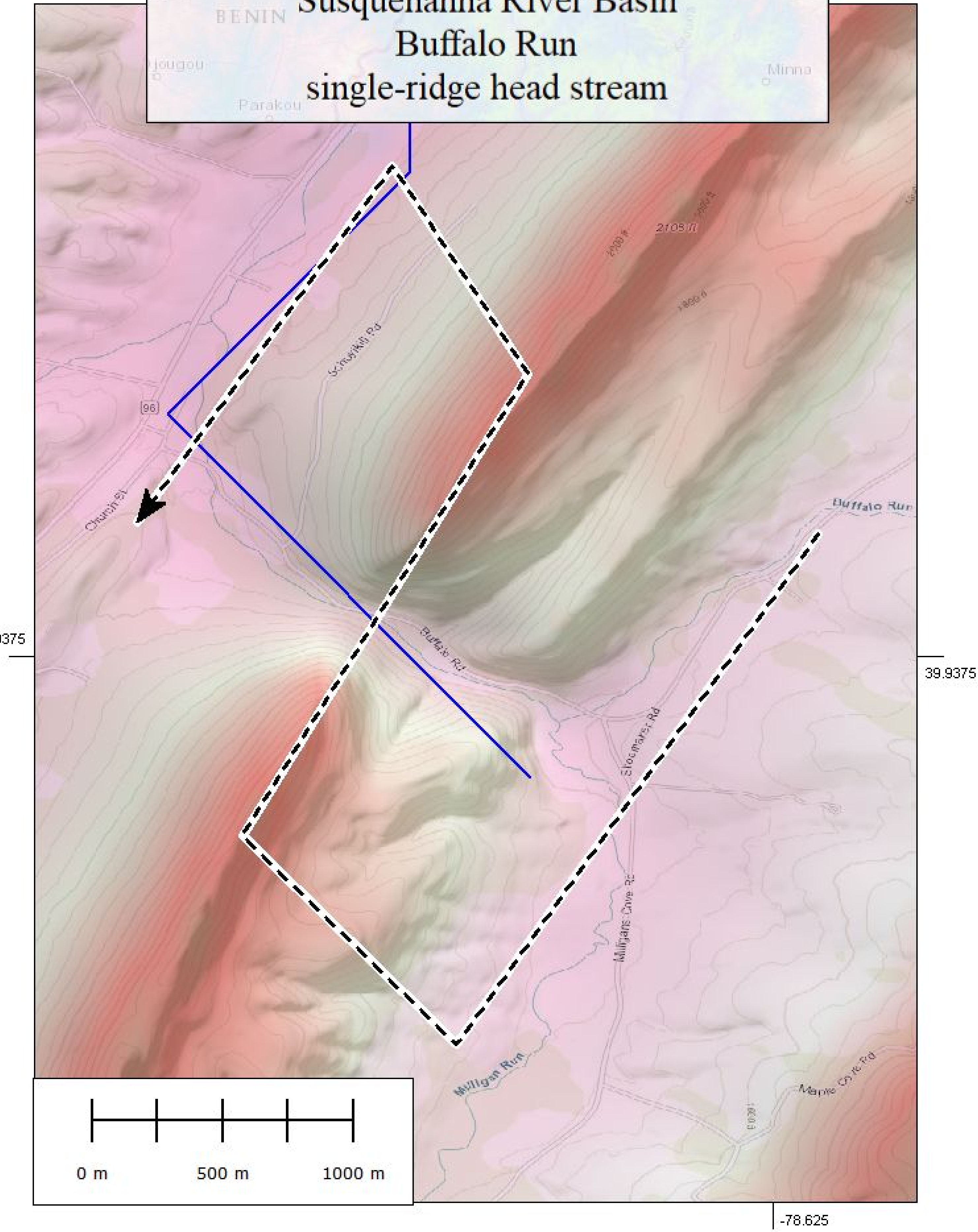

NA - 105
Sacramento River Basin
Stone Corral Creek
single-ridge head stream

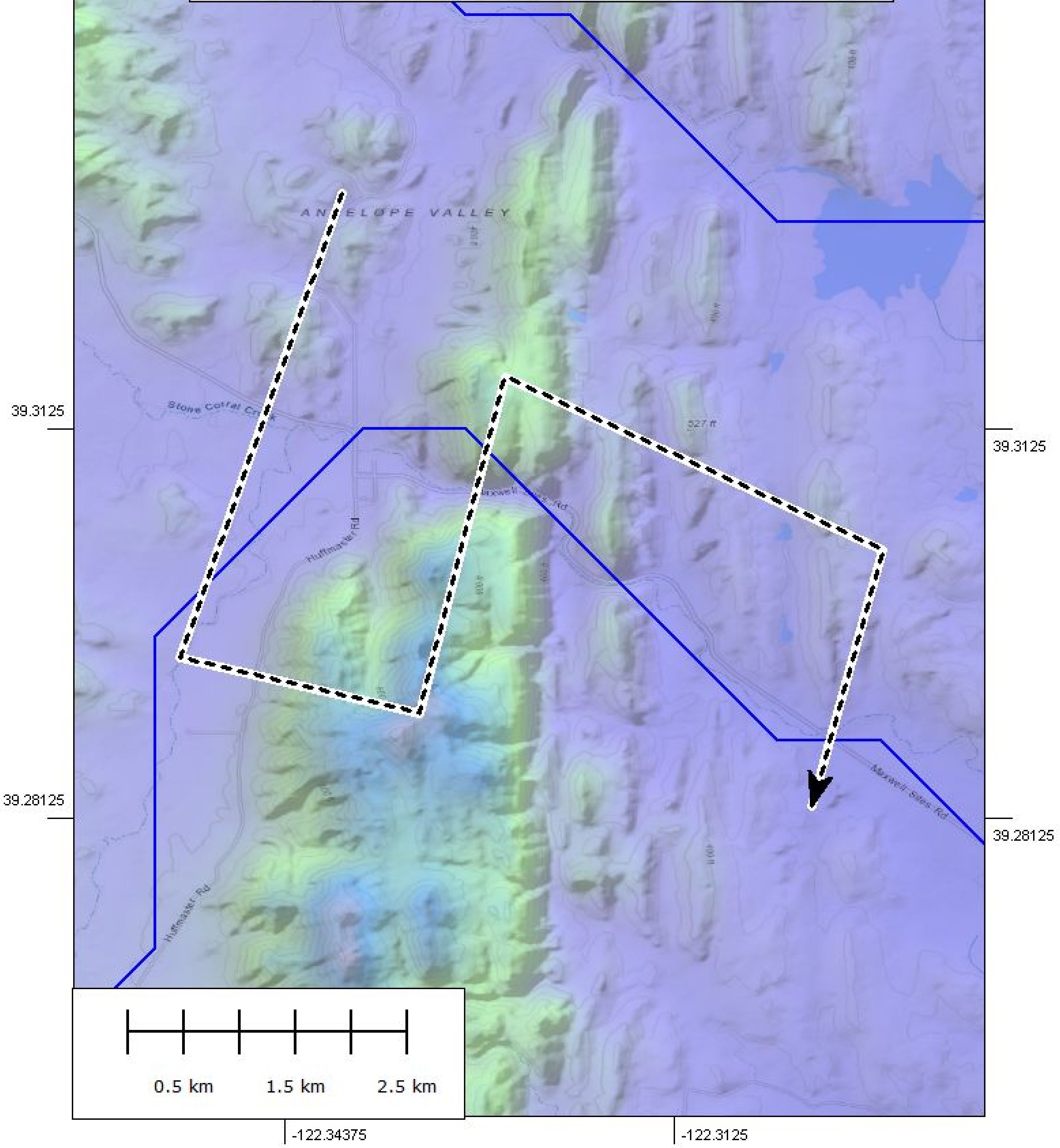

NA - 106
Mississippi River Basin
North Fork Holston River tributary
single-ridge head stream

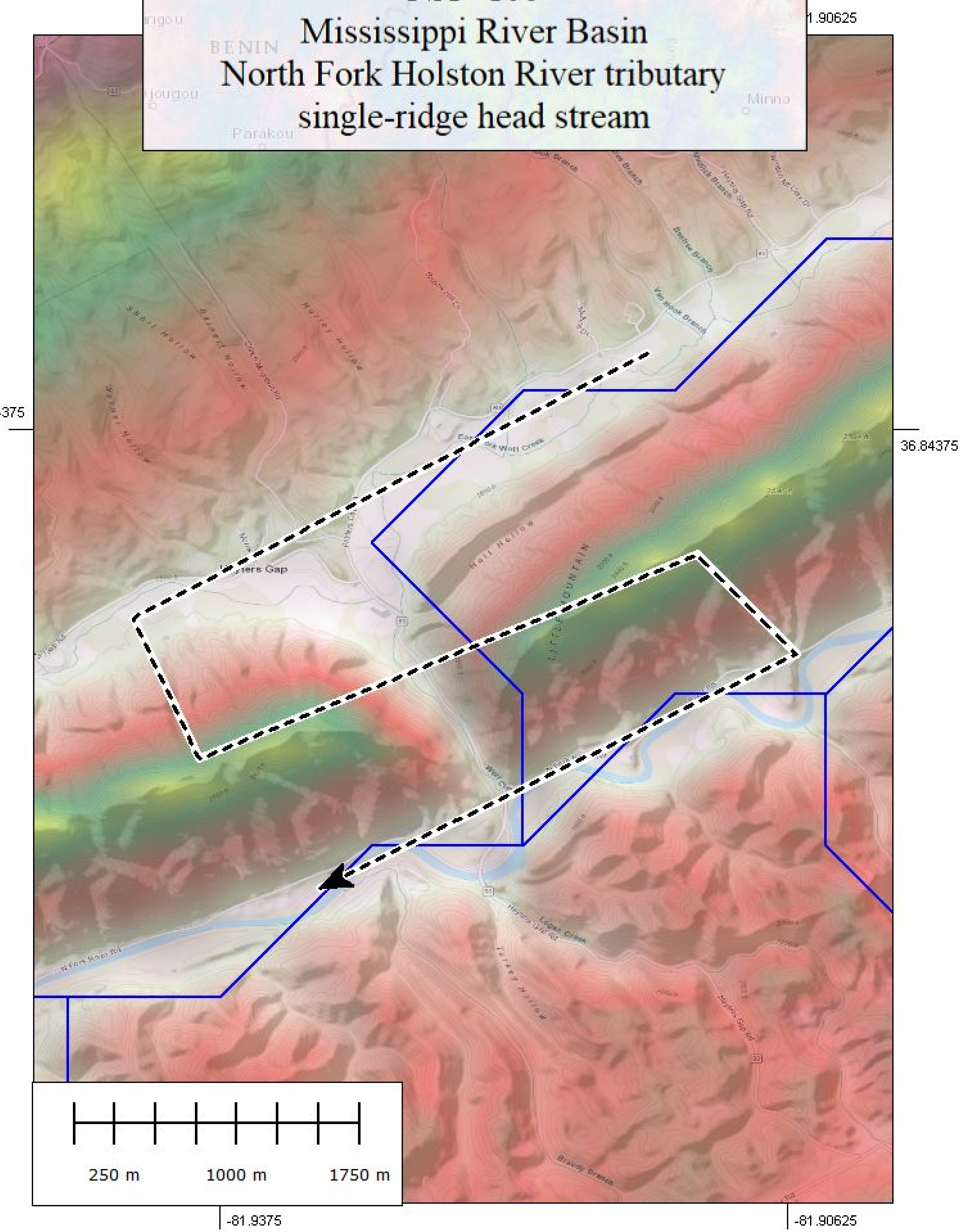

NA - 107
Columbia River Basin
Willow Creek
single-ridge head stream

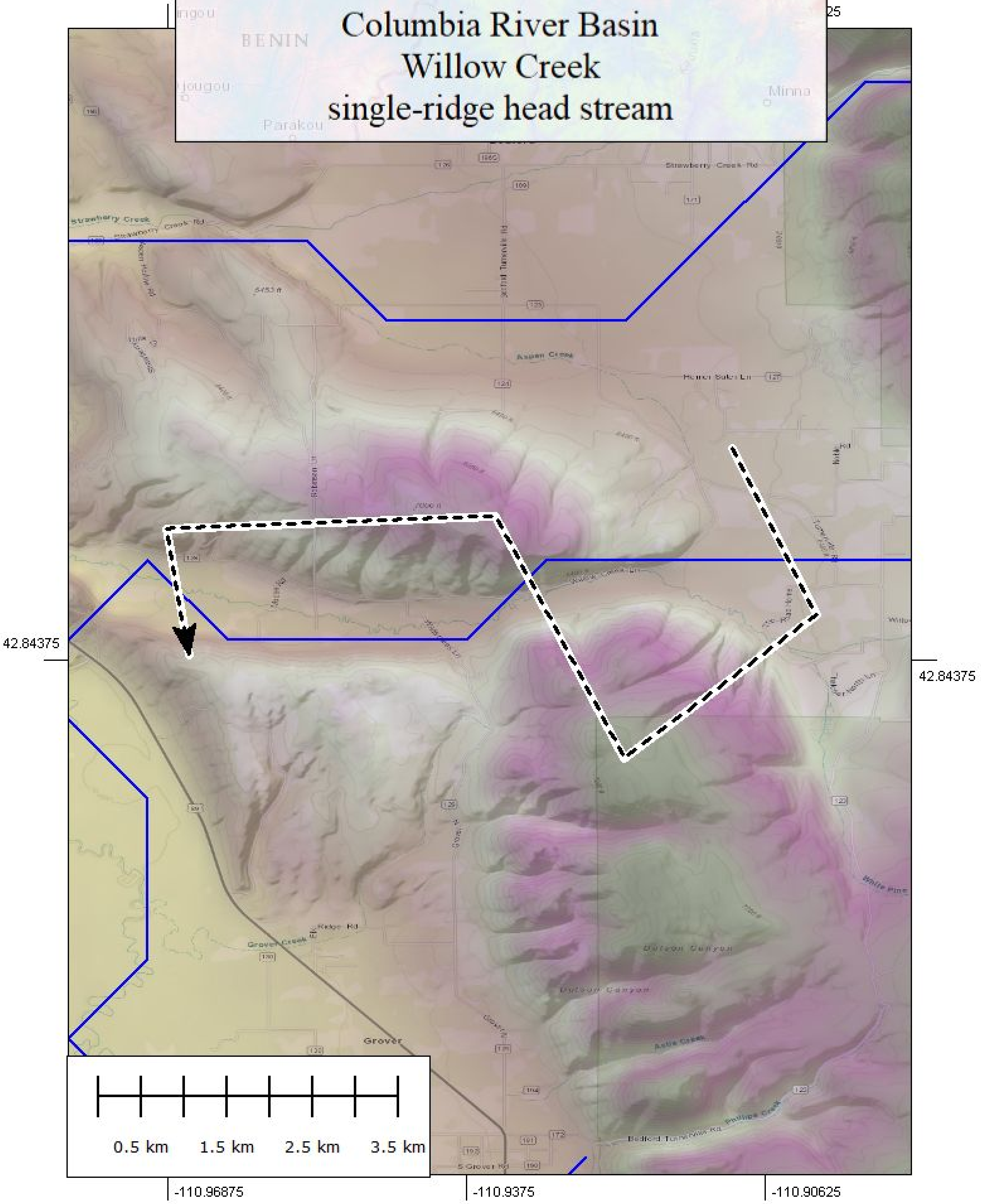

NA - 108
James River Basin
Barbours Creek
single-ridge head stream

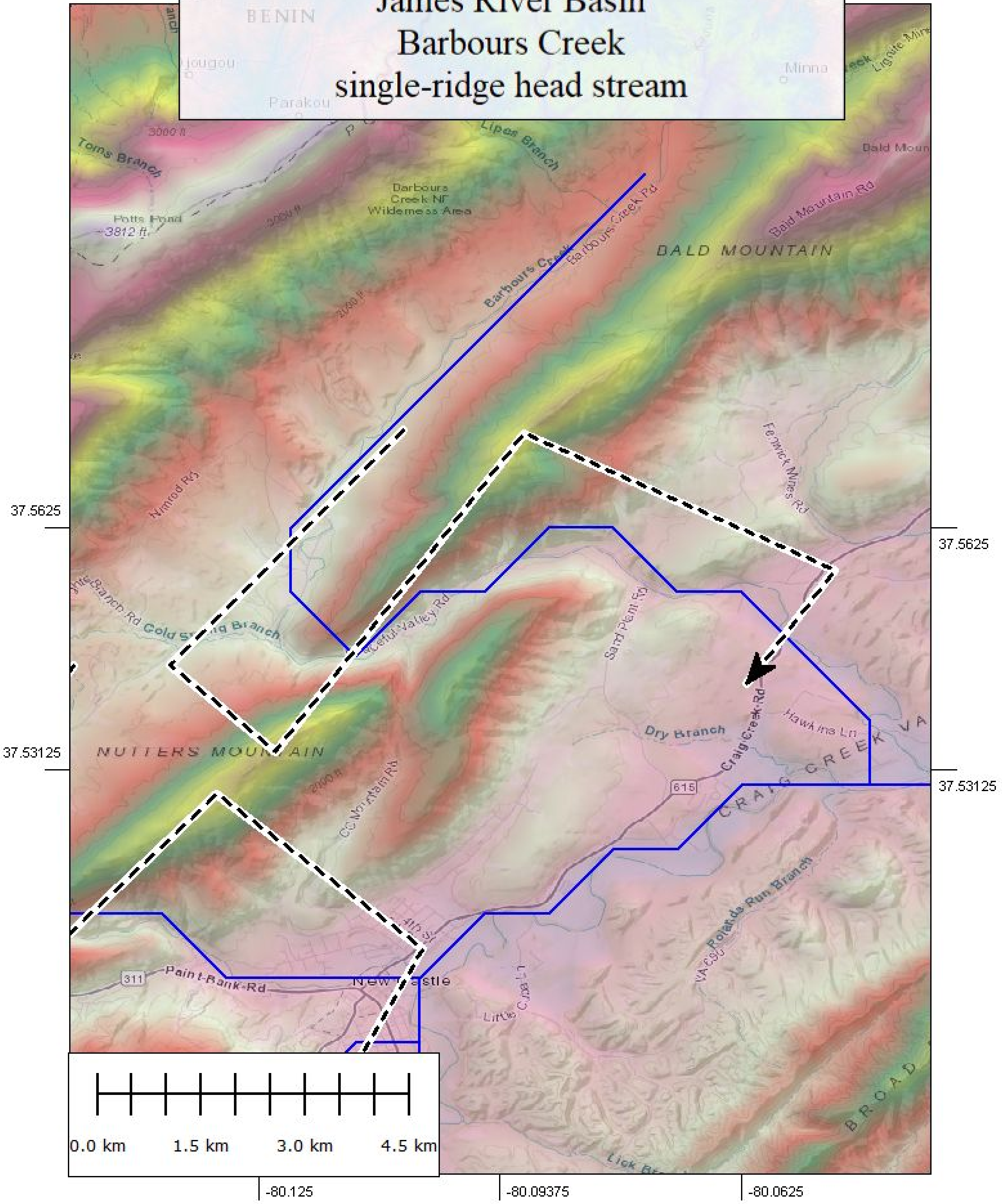

NA - 109
Colorado River (Arizona) Basin
Soldier Creek, 9 Mile Canyon
single-ridge head stream

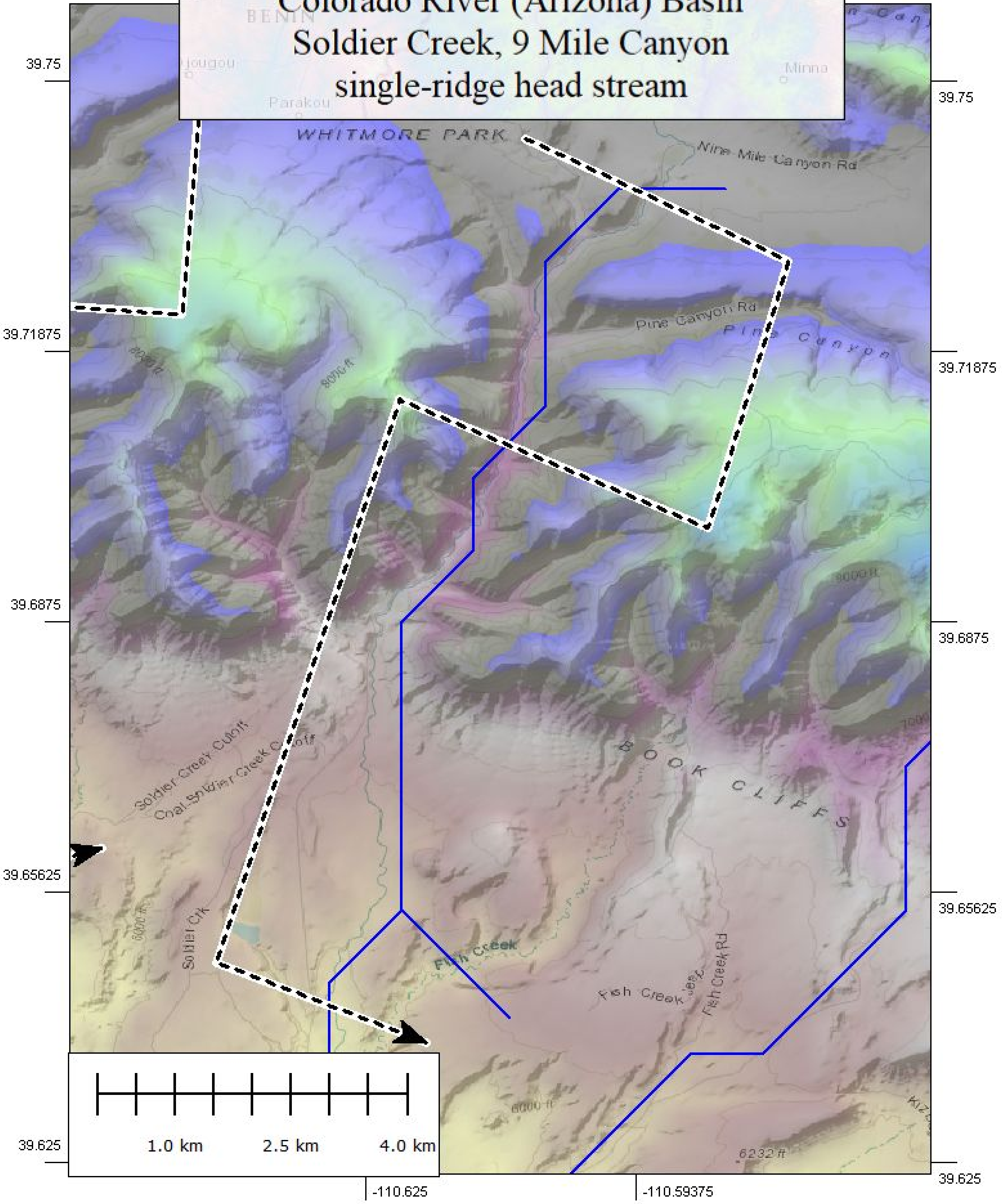

NA - 11
Colorado River (Arizona) Basin
Colorado River, Salt Wash
plateau trunk stream

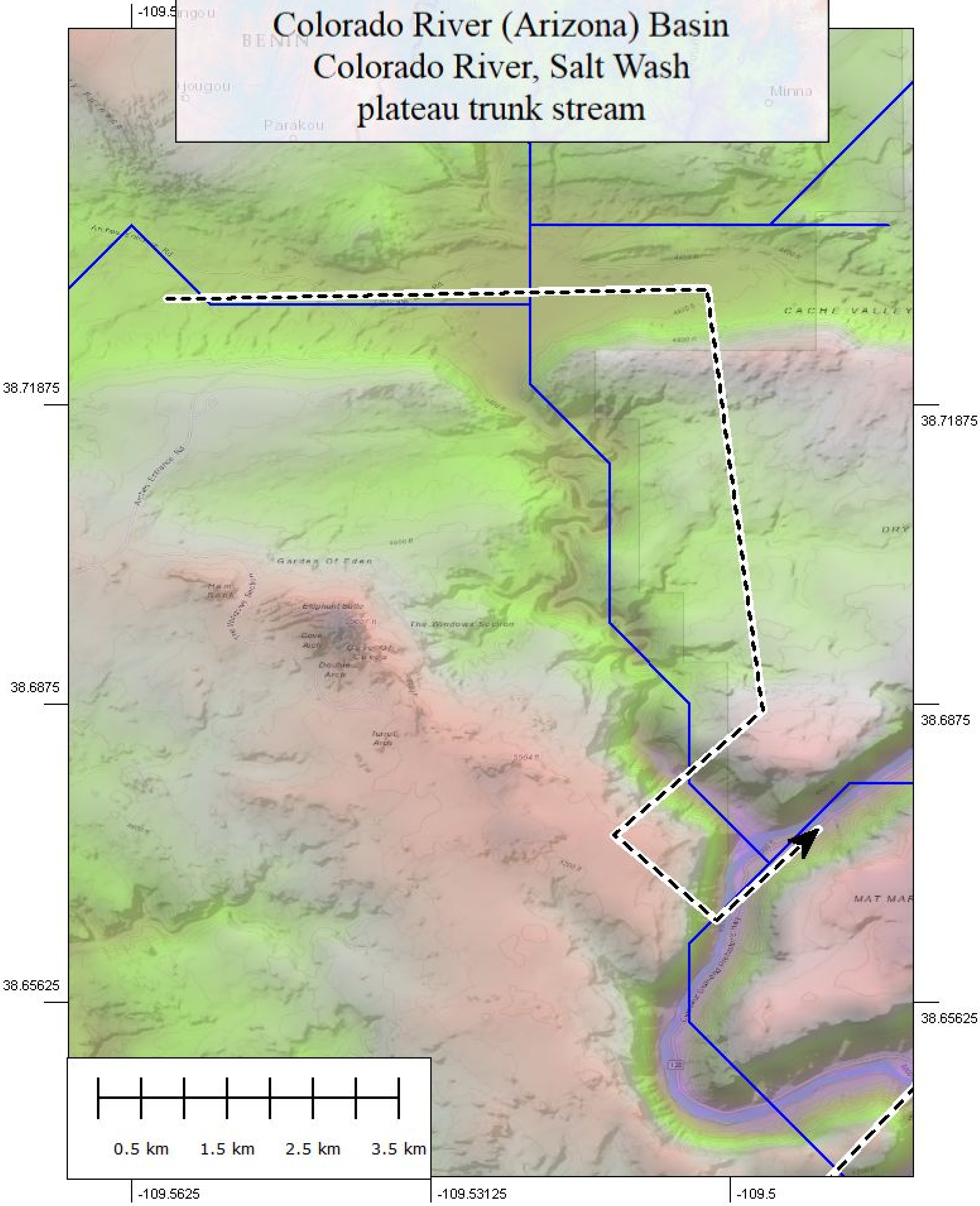

NA - 110
Colorado River (Arizona) Basin
Clearwater Creek
single-ridge head stream

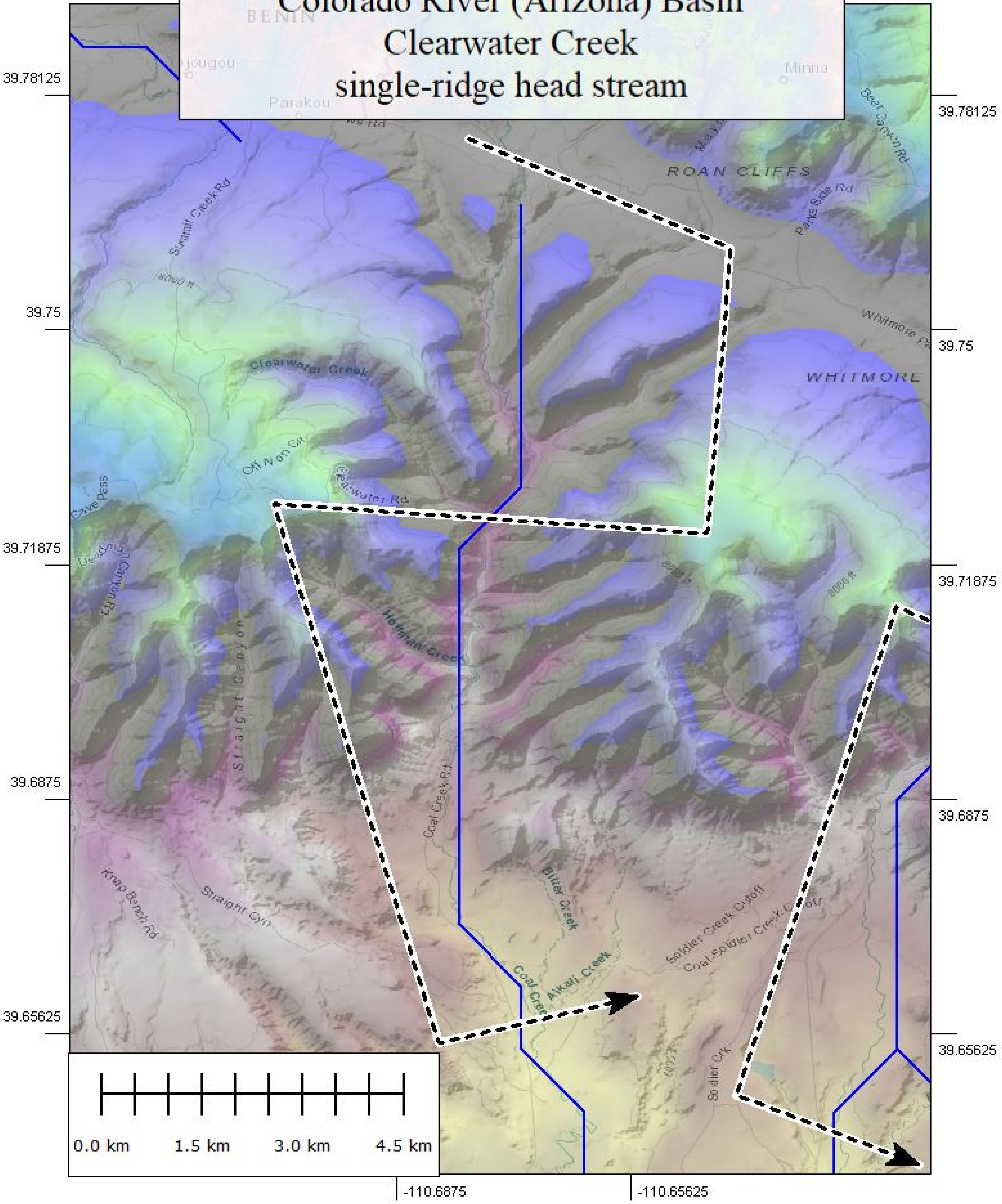

NA - 111
Susquehanna River Basin
Manada Creek
single-ridge head stream

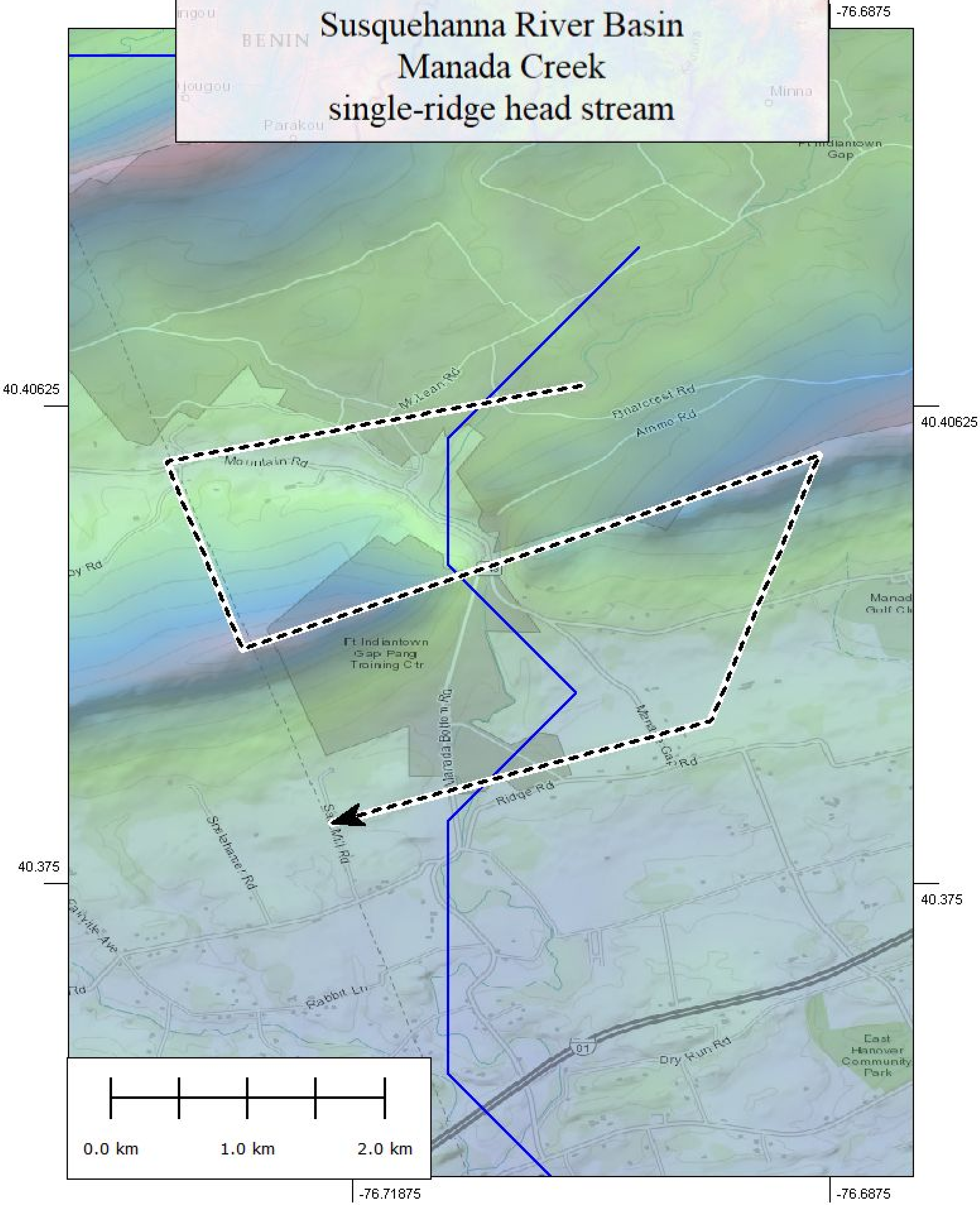

NA - 112
Pnuco River Basin
Las Canalitas
single-ridge head stream

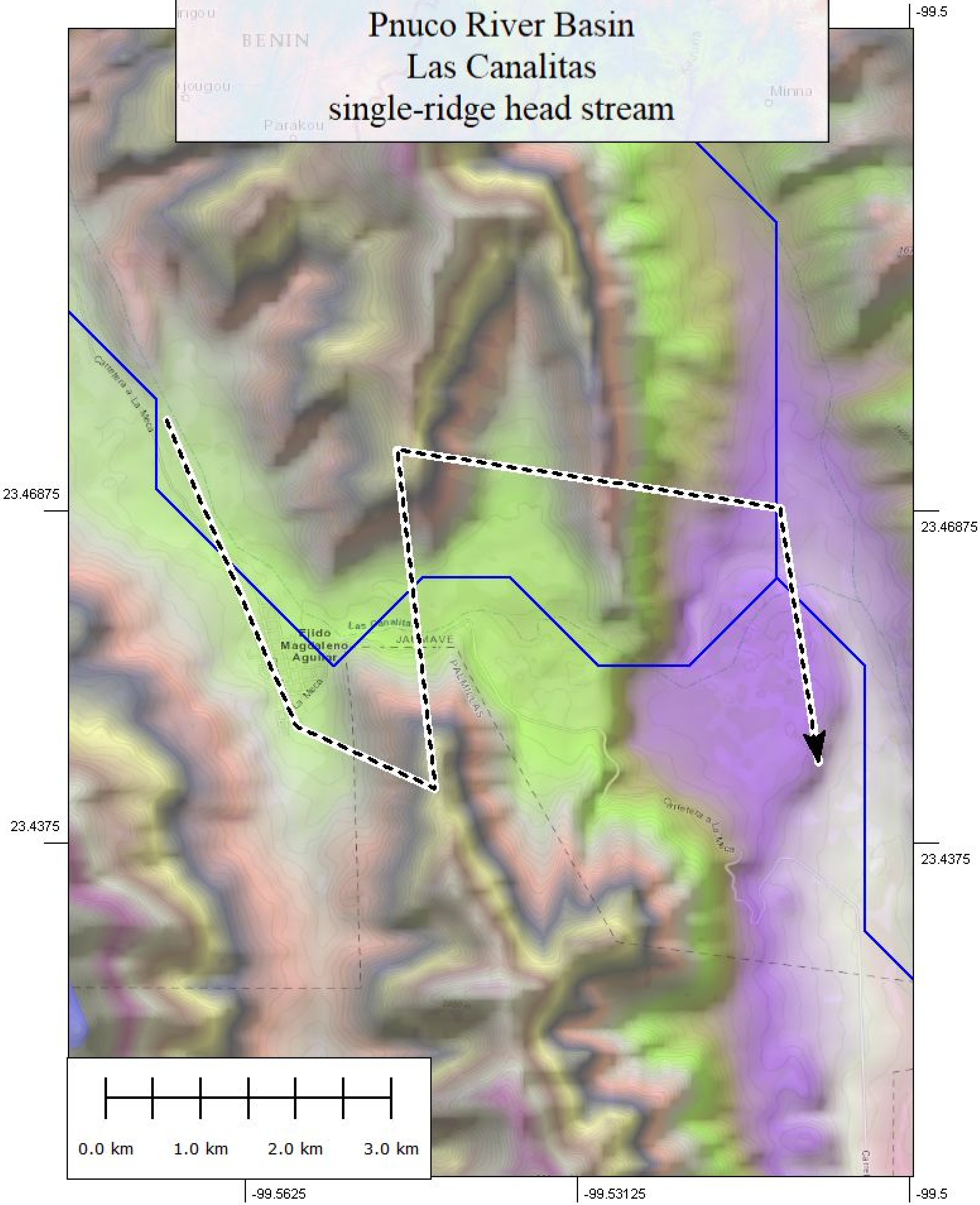

NA - 113
Rio Grande (Texas-Mexico) Basin
single-ridge head stream

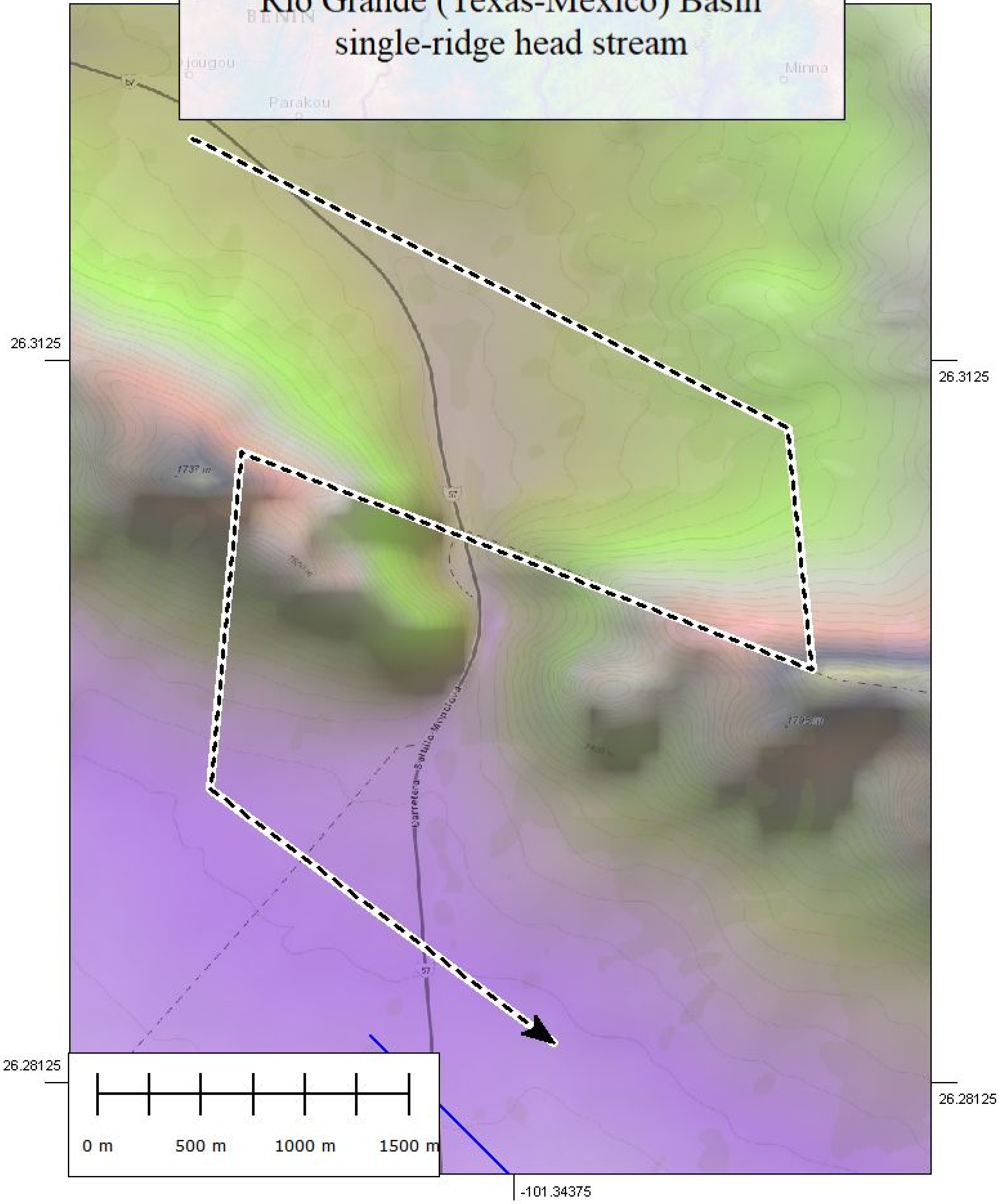

NA - 114
Rio Grande (Texas-Mexico) Basin
Gallina River
plateau trunk stream

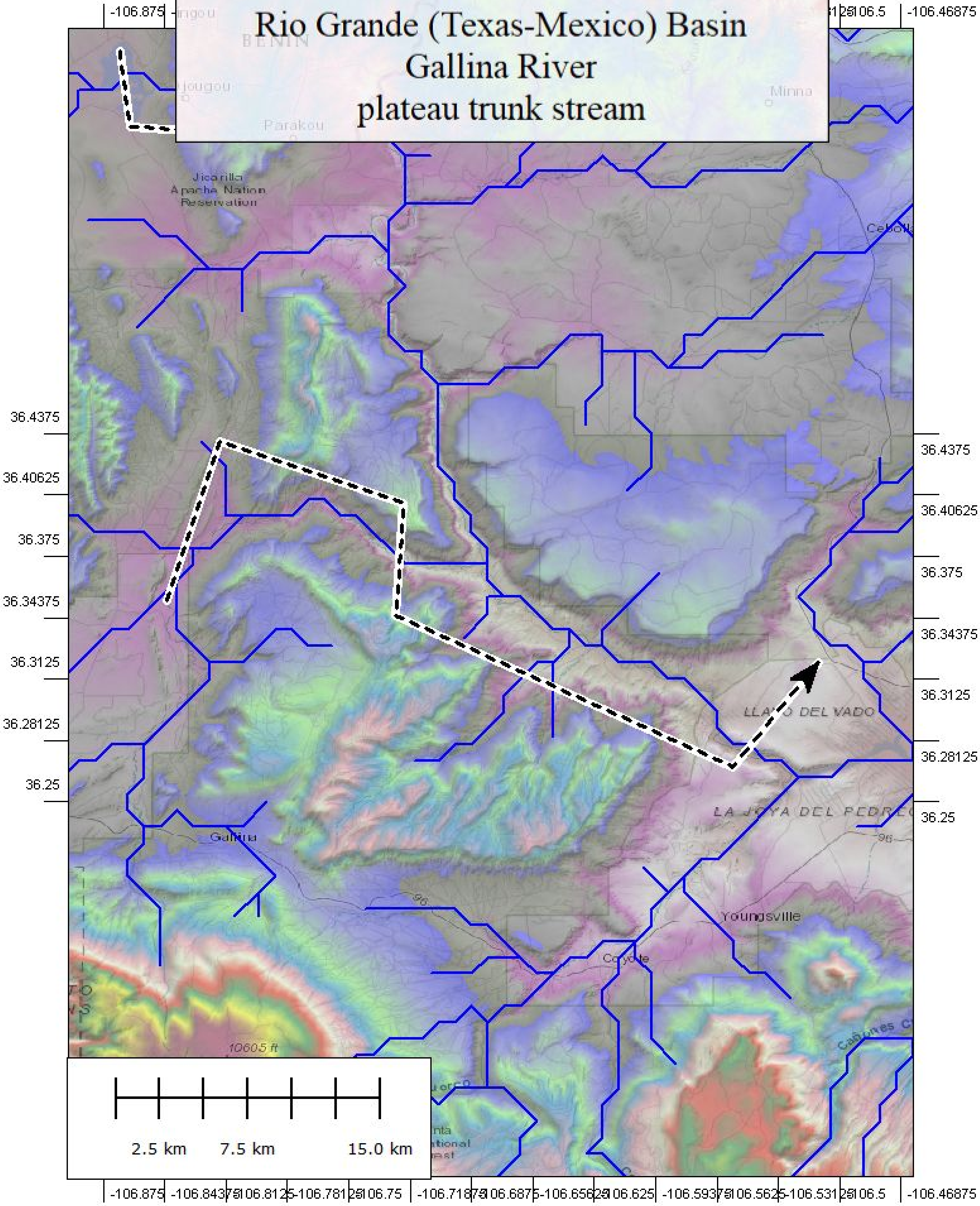

NA - 115
Colorado River (Arizona) Basin
Colorado River, Grand Canyon
plateau trunk stream

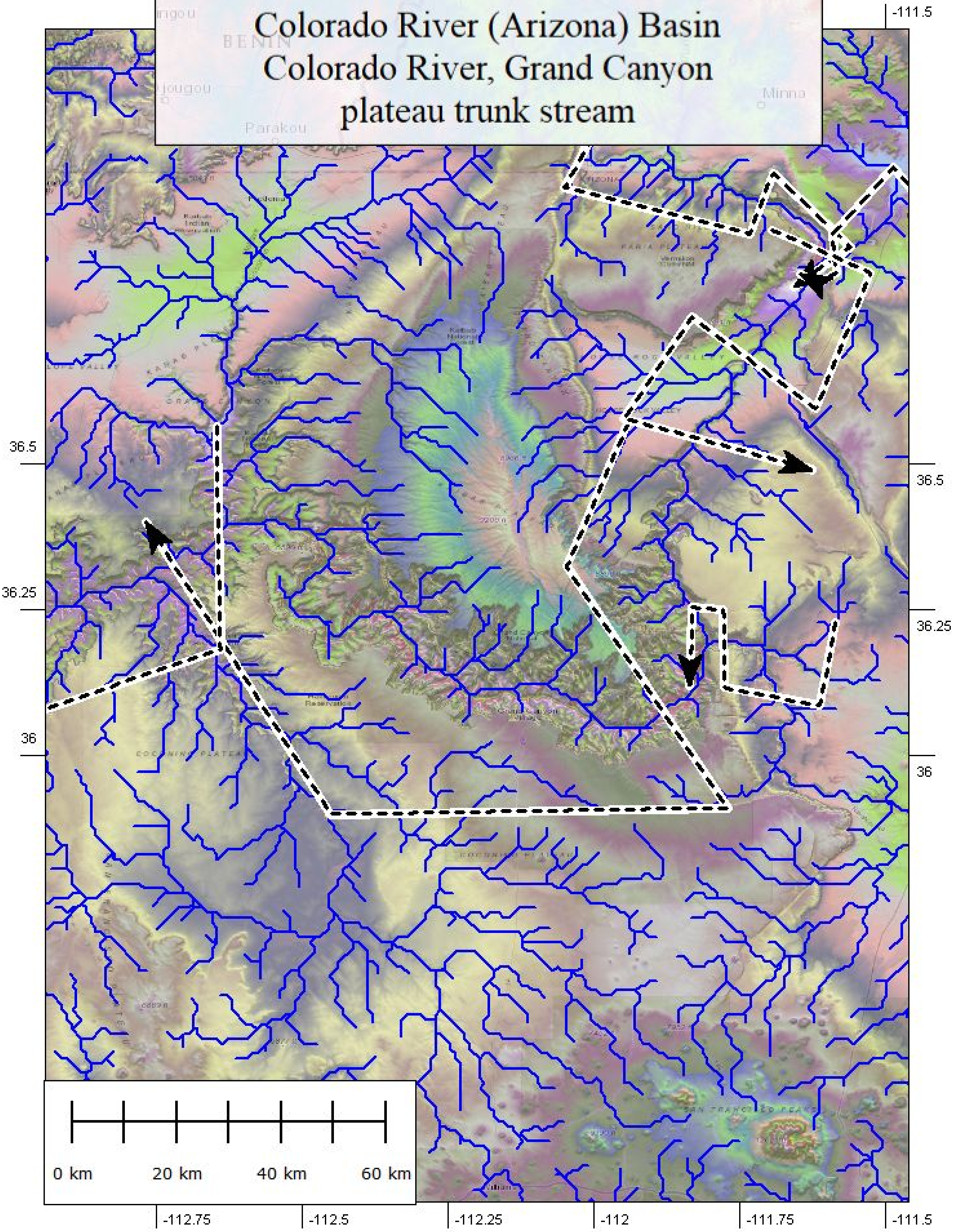

NA - 116
Colorado River (Arizona) Basin
Kaibab Gulch
plateau trunk stream

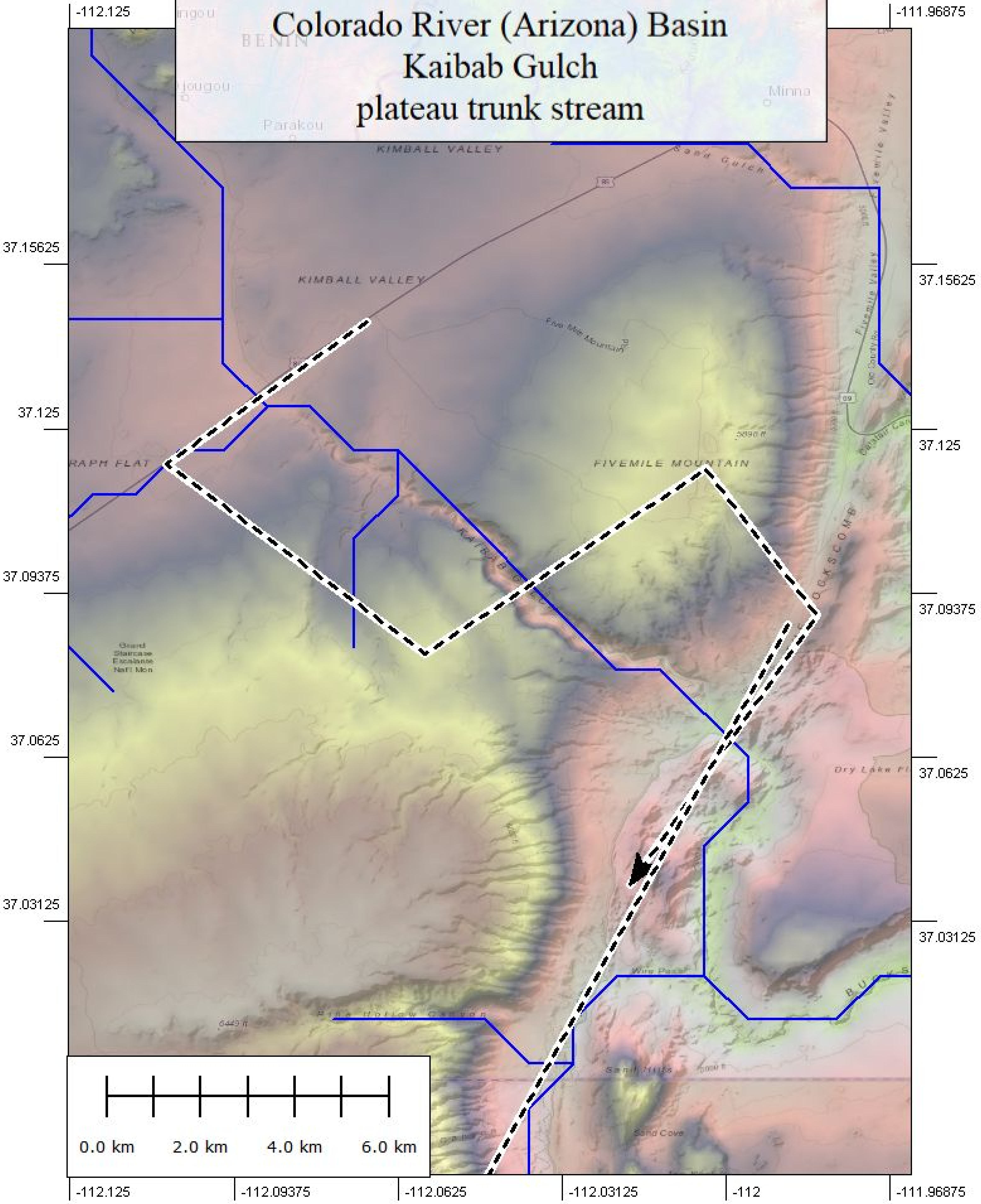

NA - 117
Mississippi River Basin
Doe River
single-ridge trunk stream

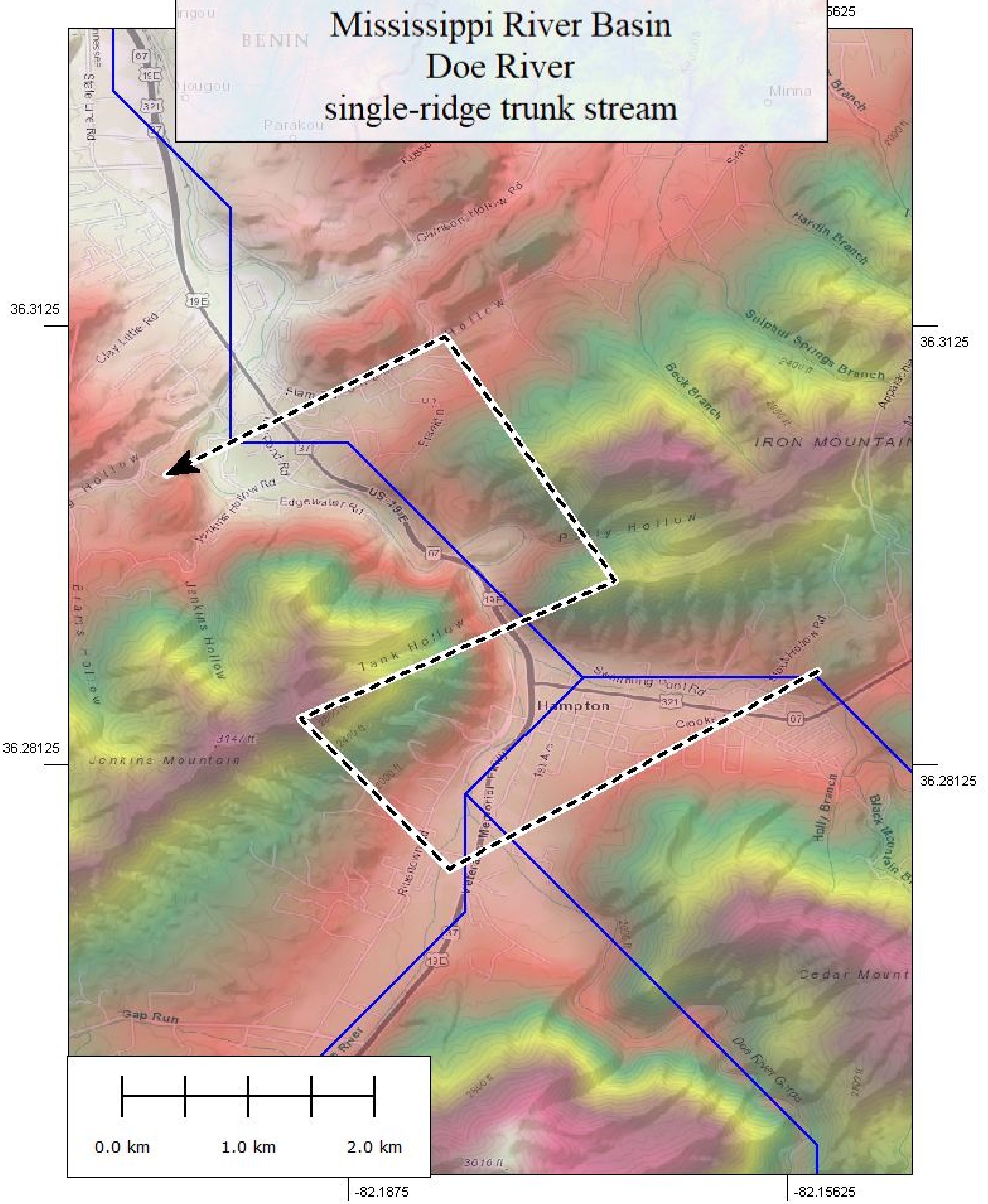

NA - 118
Mississippi River Basin
Youghiogheny River
single-ridge trunk stream

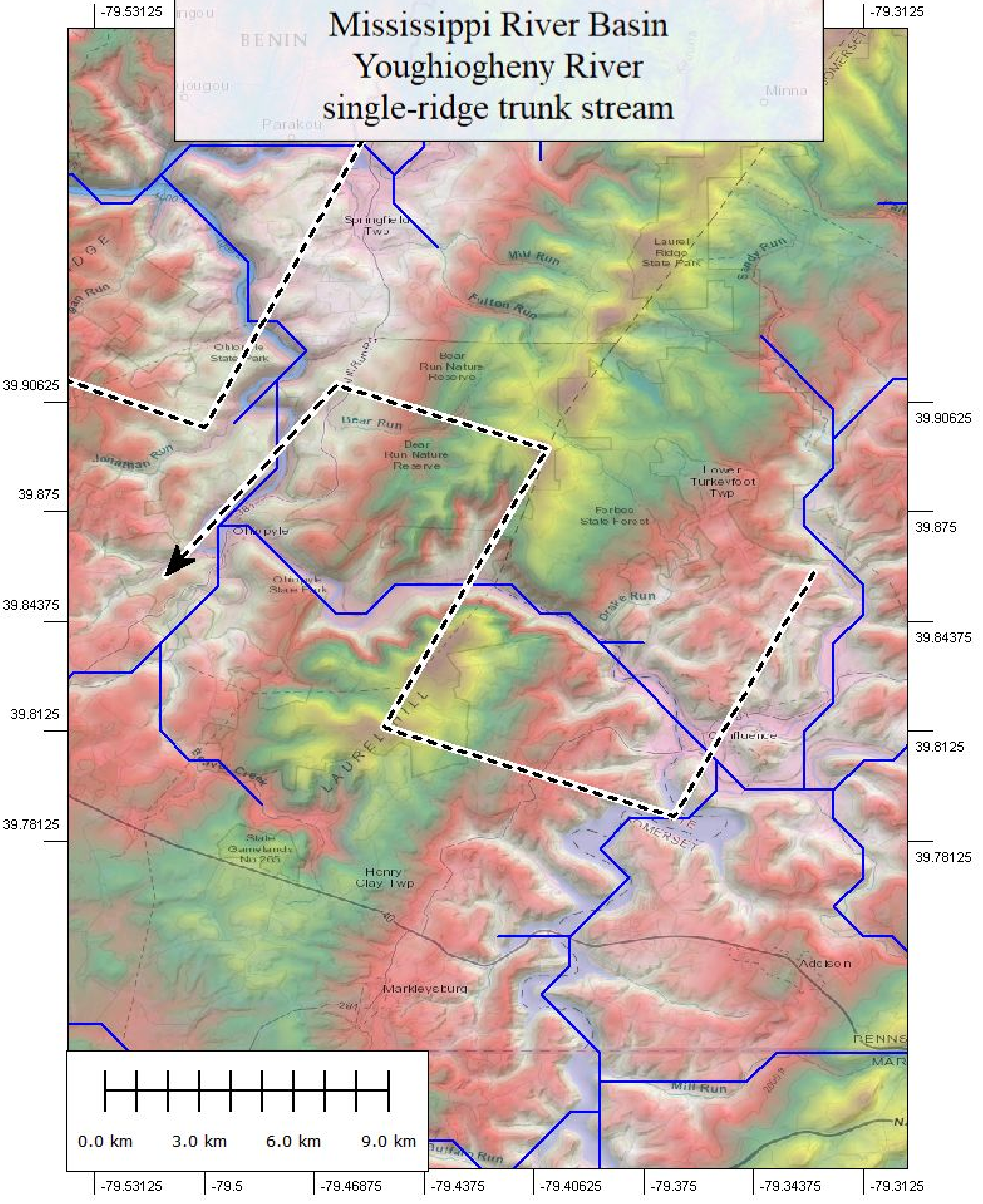

NA - 119
Colorado River (Arizona) Basin
Fremont RIver
plateau trunk stream

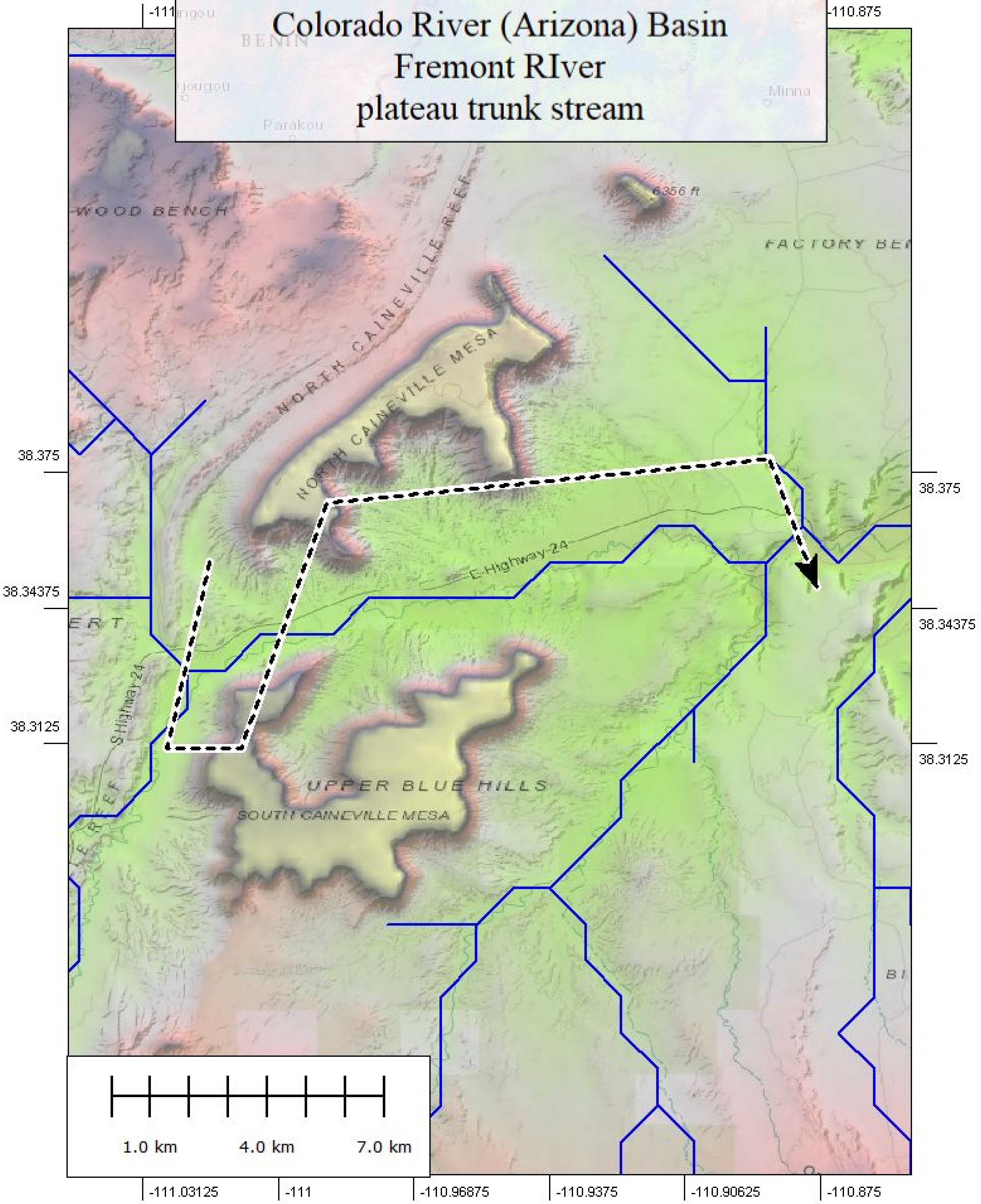

NA - 12
Mackenzie River (Canada) Basin
Crooked River tributary
irregular high ground trunk stream

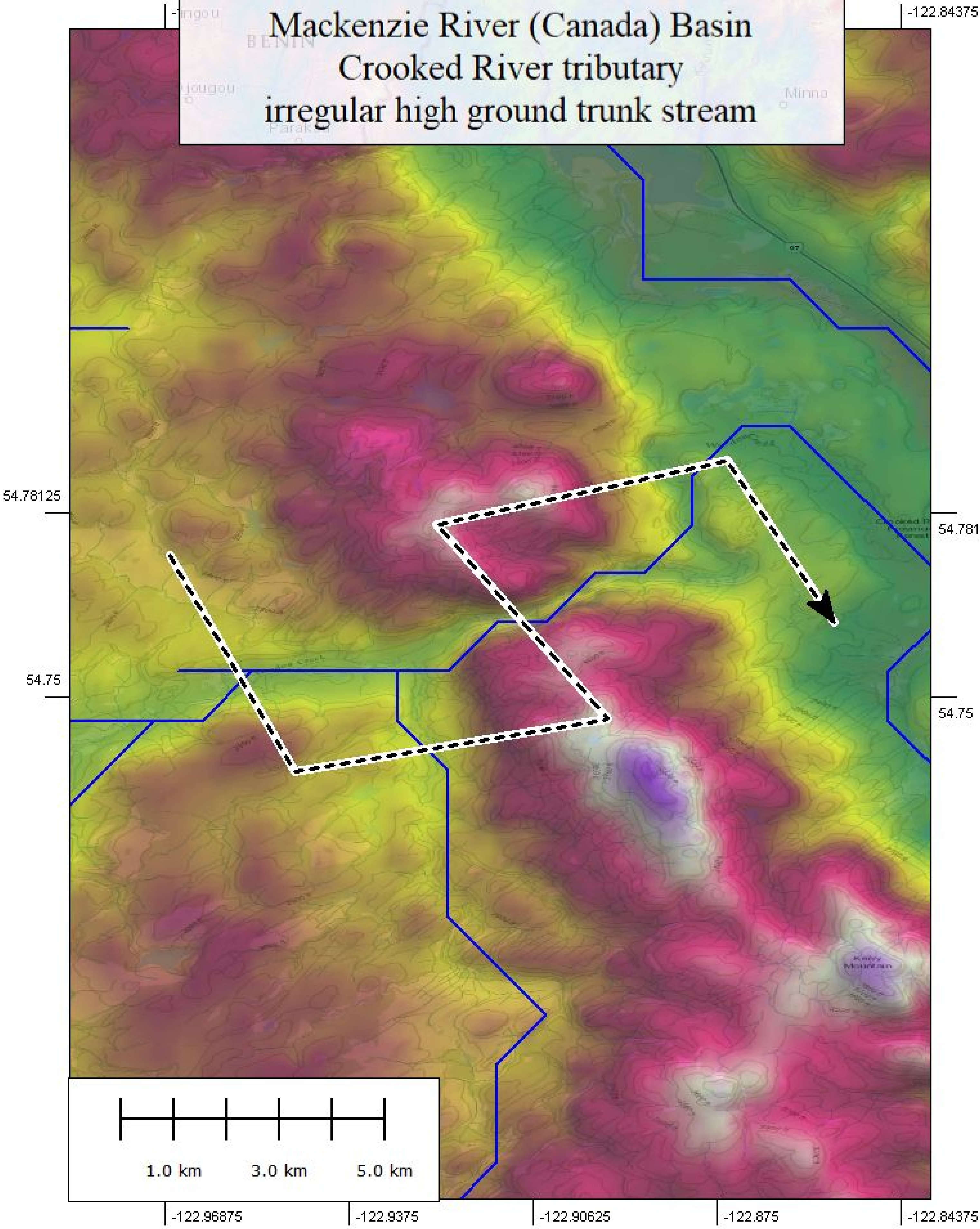

NA - 120
Columbia River Basin
Powder River
irregular high ground trunk stream

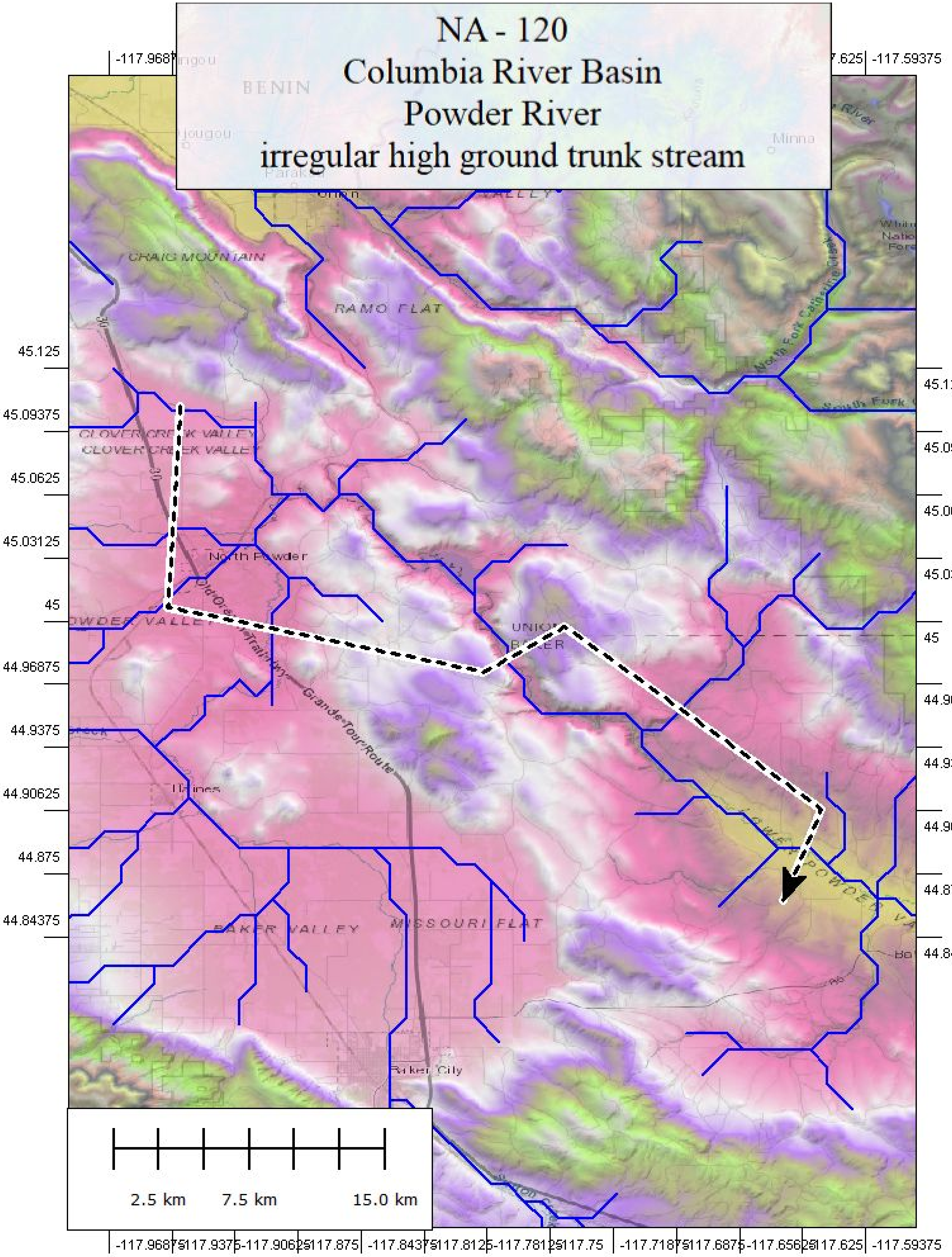

NA - 121
Colorado River (Arizona) Basin
Gila River
single-ridge trunk stream

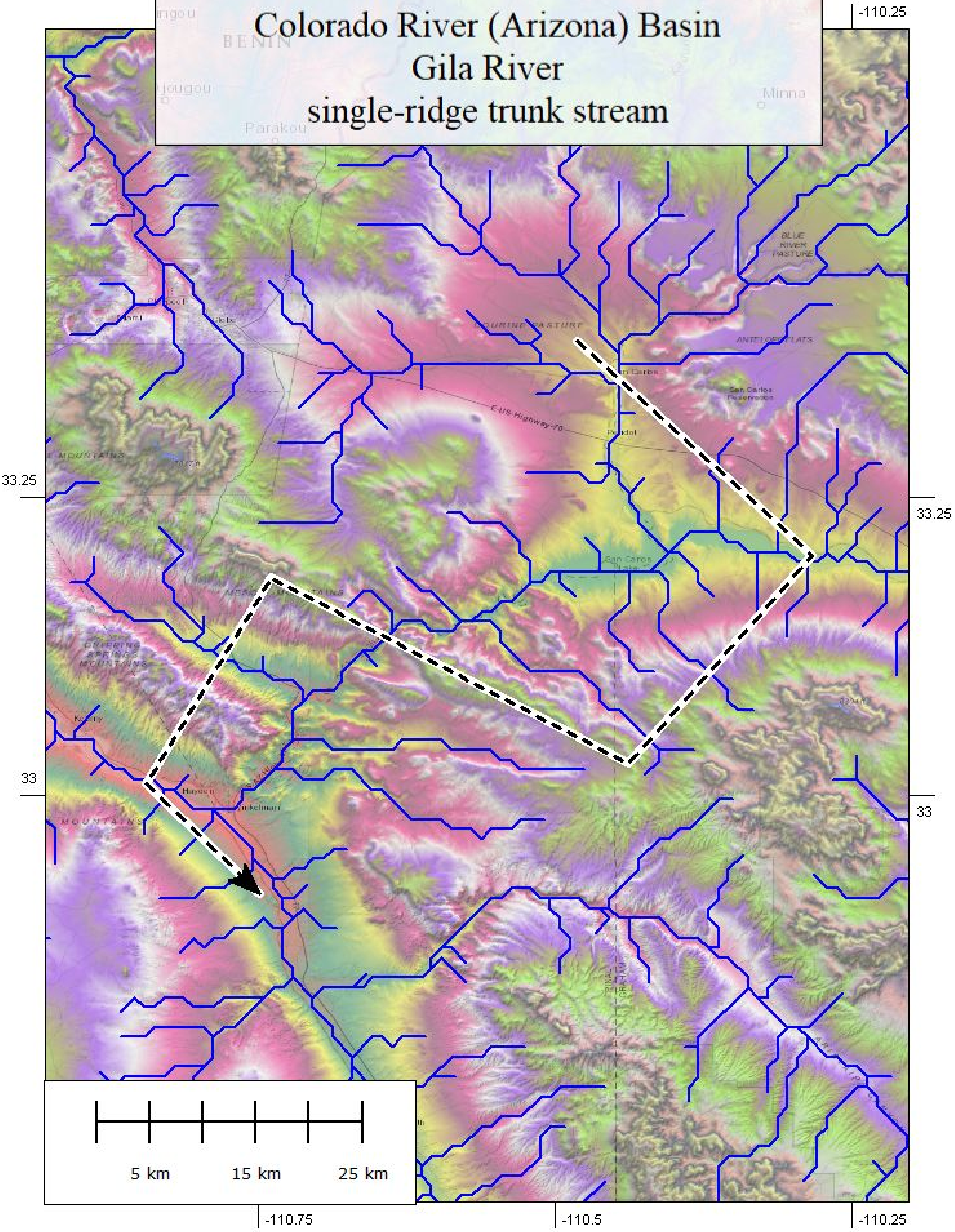

NA - 122
Rio Grande (Texas-Mexico) Basin
Chama River tributary
single-ridge trunk stream

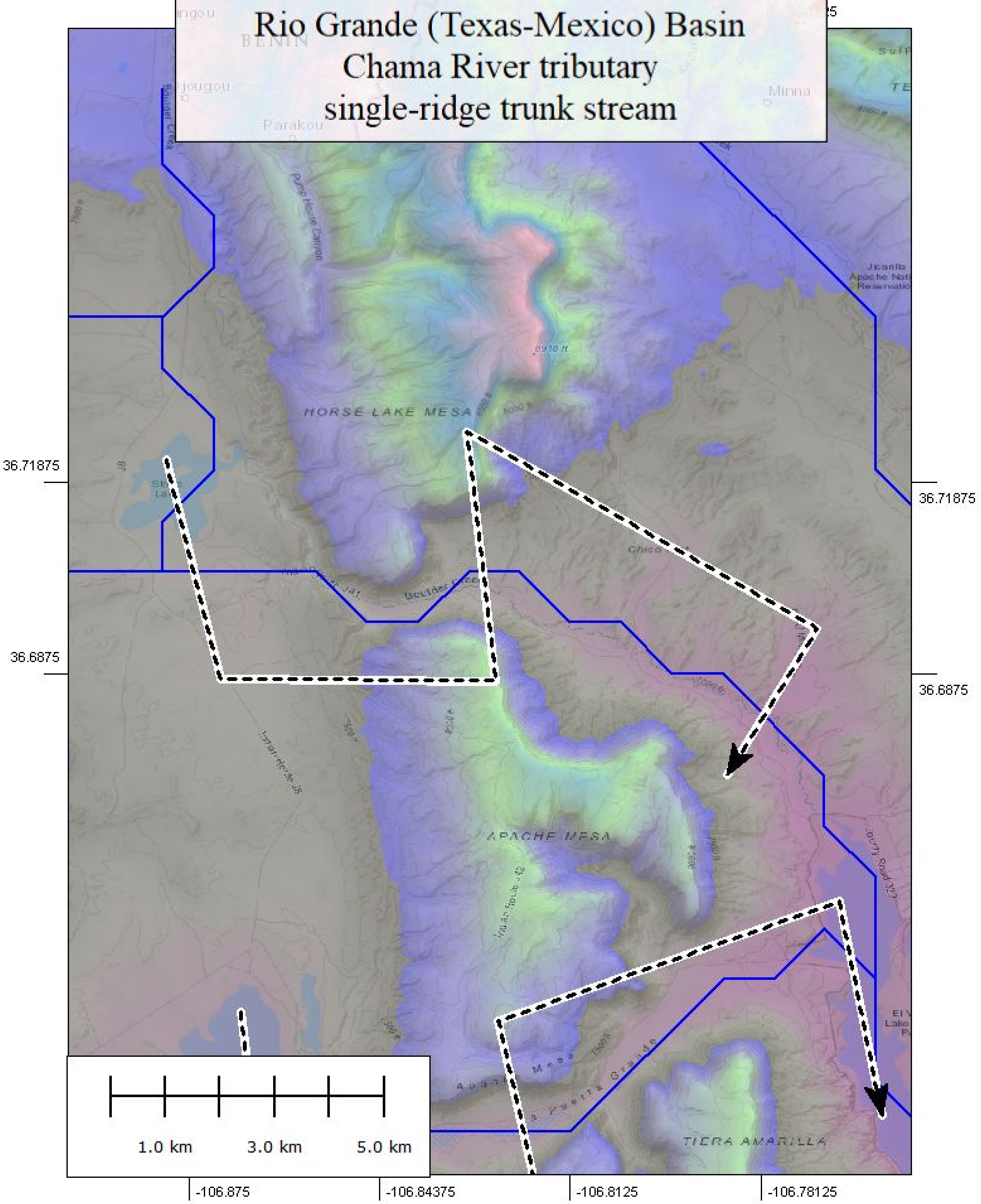

NA - 123
Mississippi River Basin
Grasshopper Creek
irregular high ground trunk stream

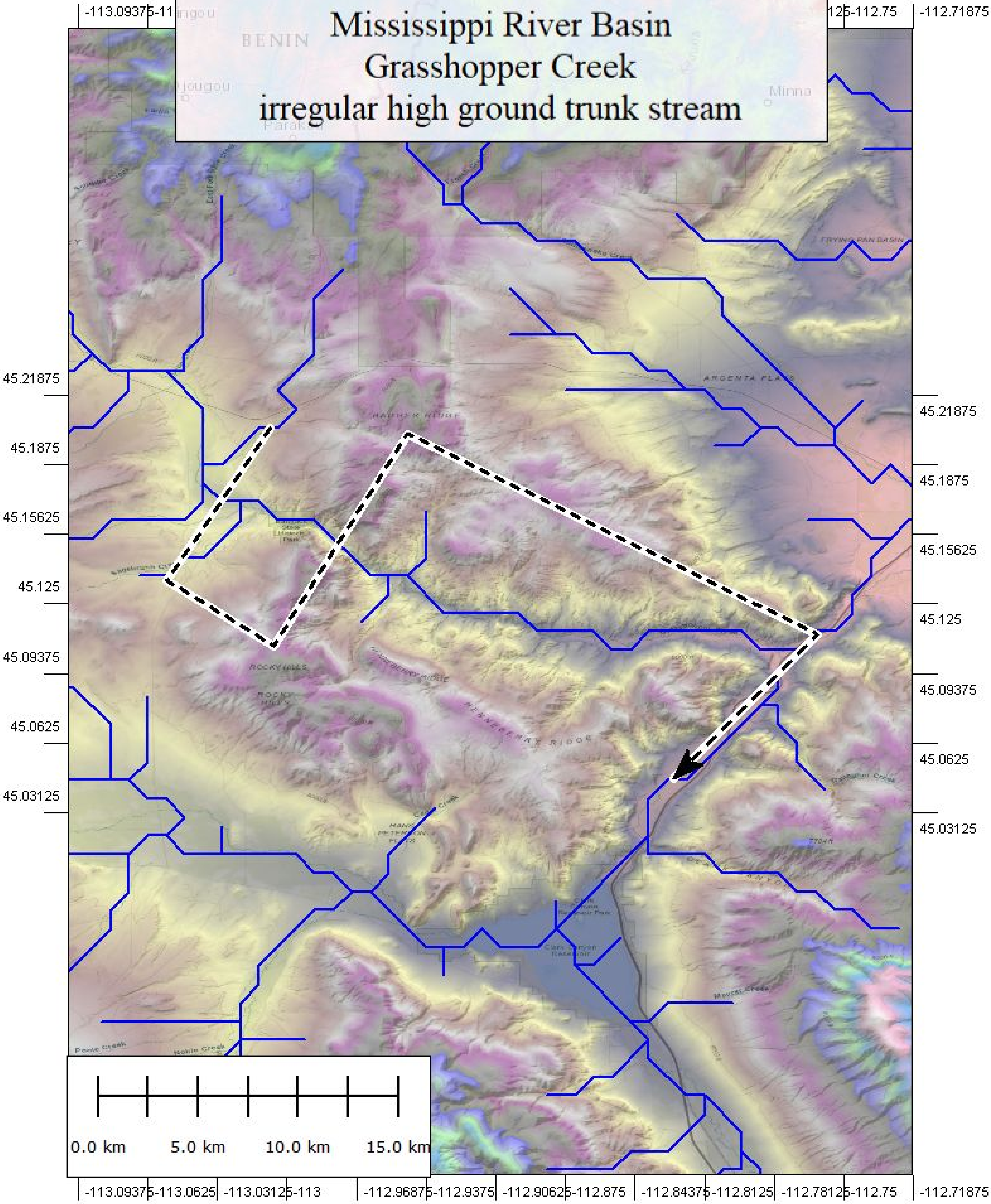

NA - 124
Colorado River (Arizona) Basin
Yampa River
irregular high ground trunk stream

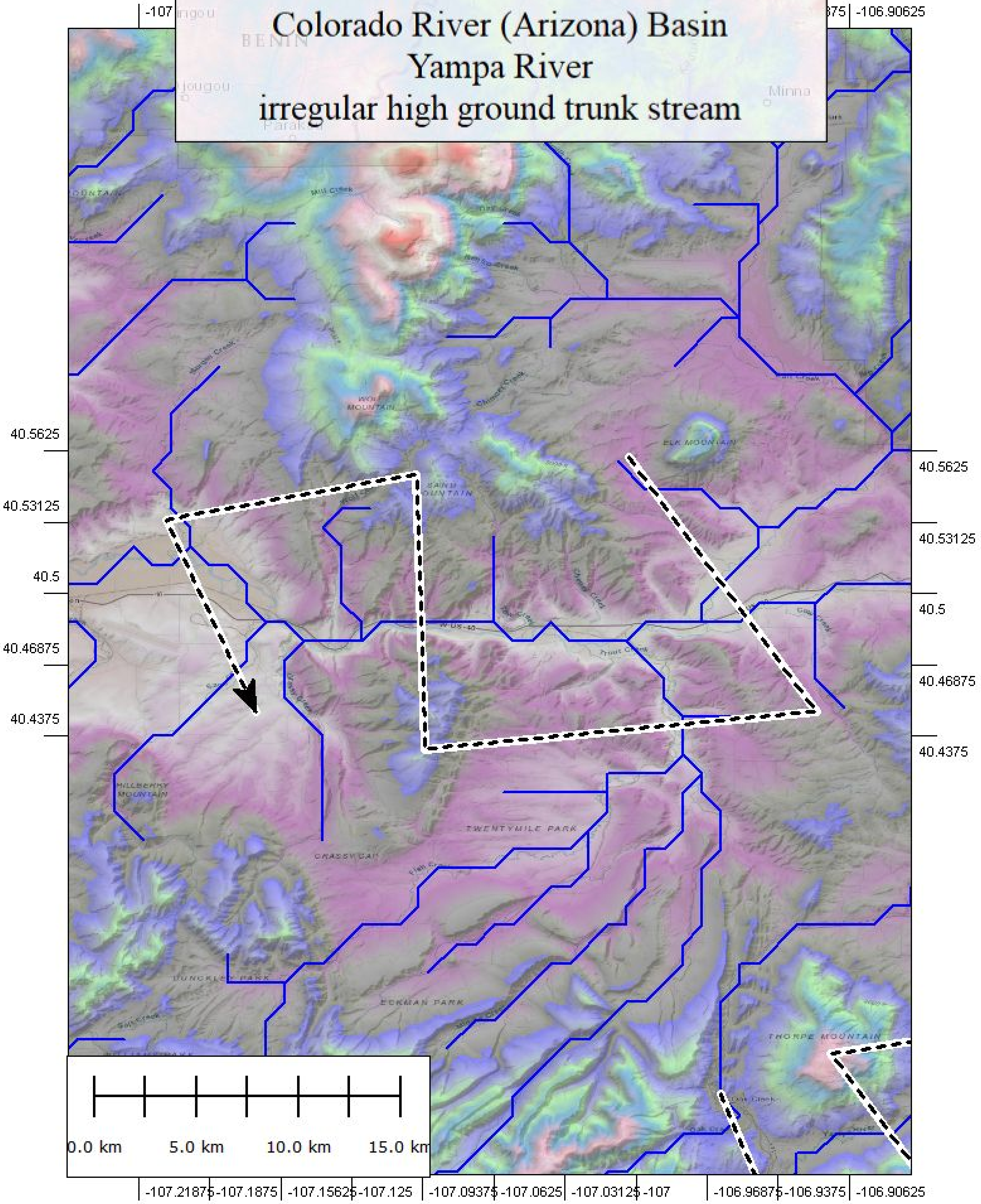

NA - 125
Rio Grande (Texas-Mexico) Basin
Sabinas River, Ojo de Agua Bustamante Canyon

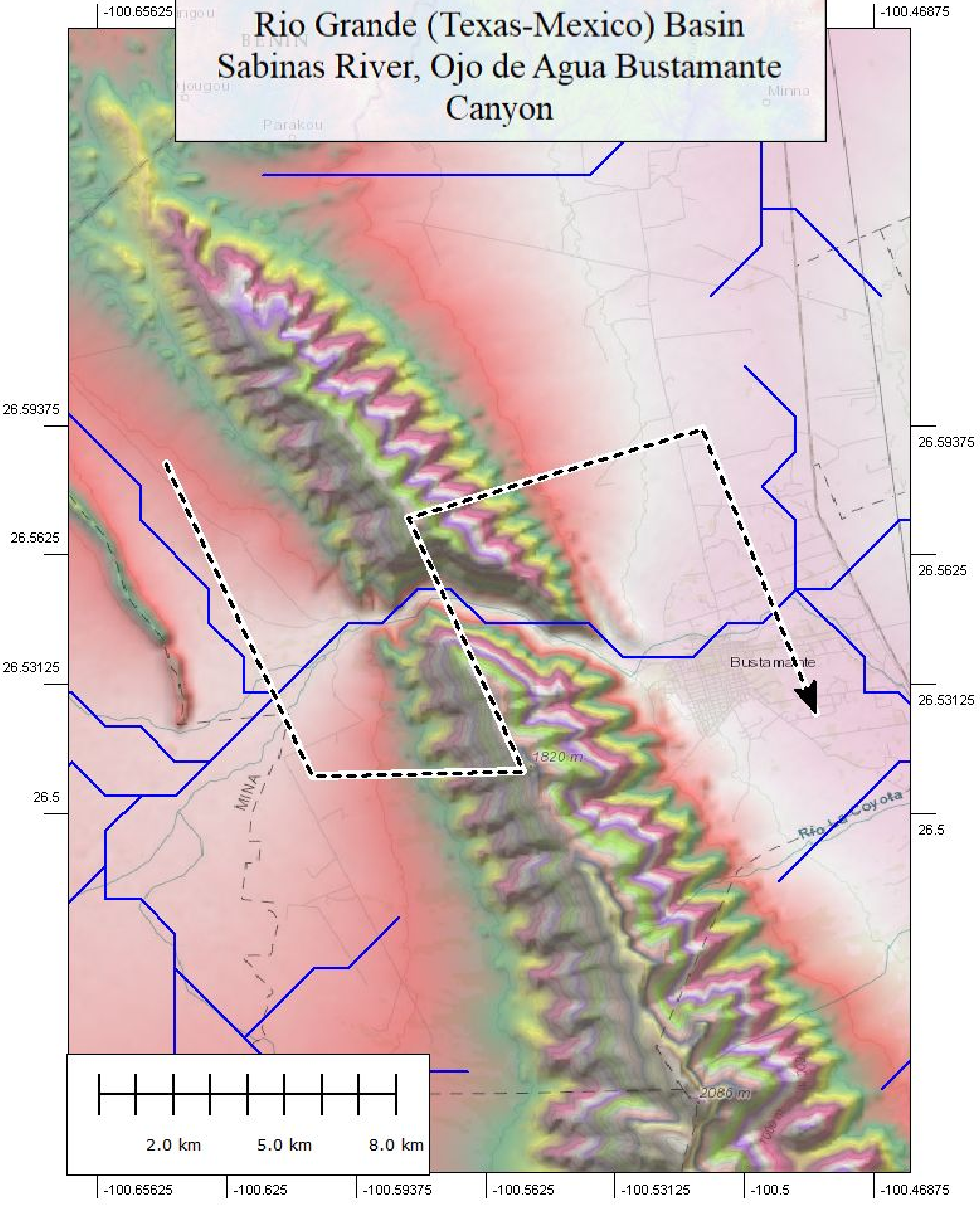

NA - 126
Rio Grande (Texas-Mexico) Basin
Arroyo La Chueca
single-ridge trunk stream

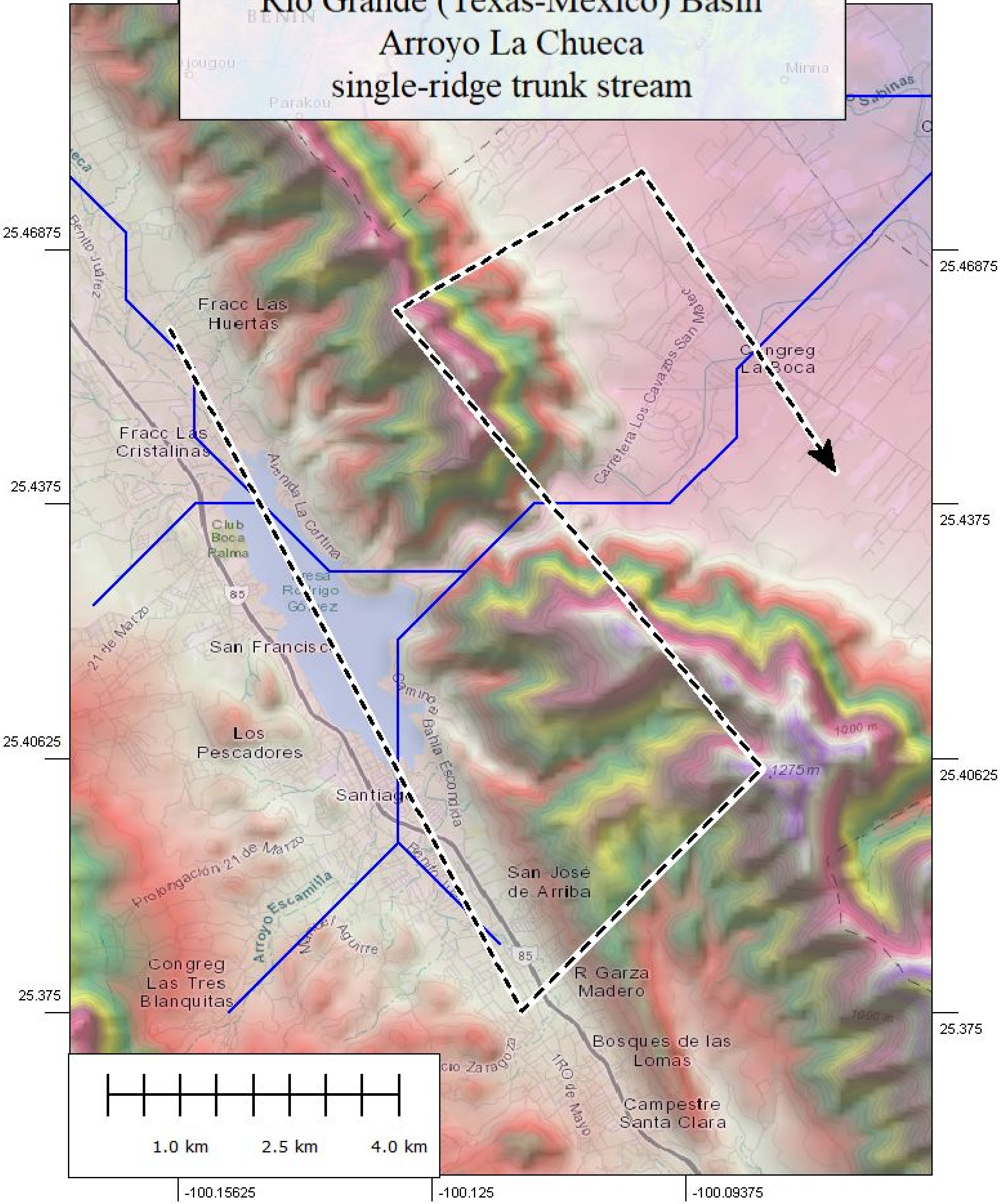

NA - 127
Columbia River Basin
Columbia River
single-ridge trunk stream

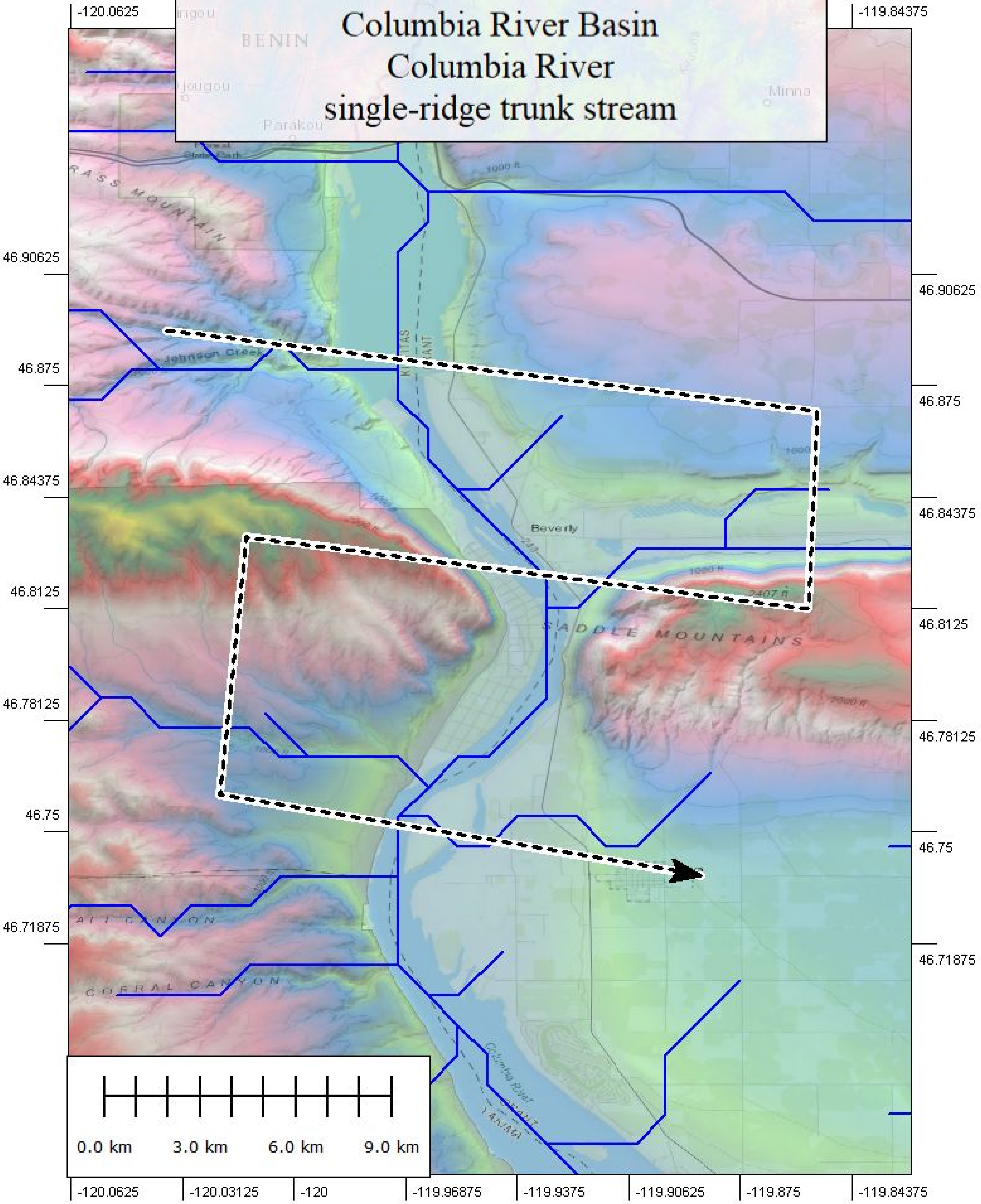

NA - 128
Columbia River Basin
Yakima River
single-ridge trunk stream

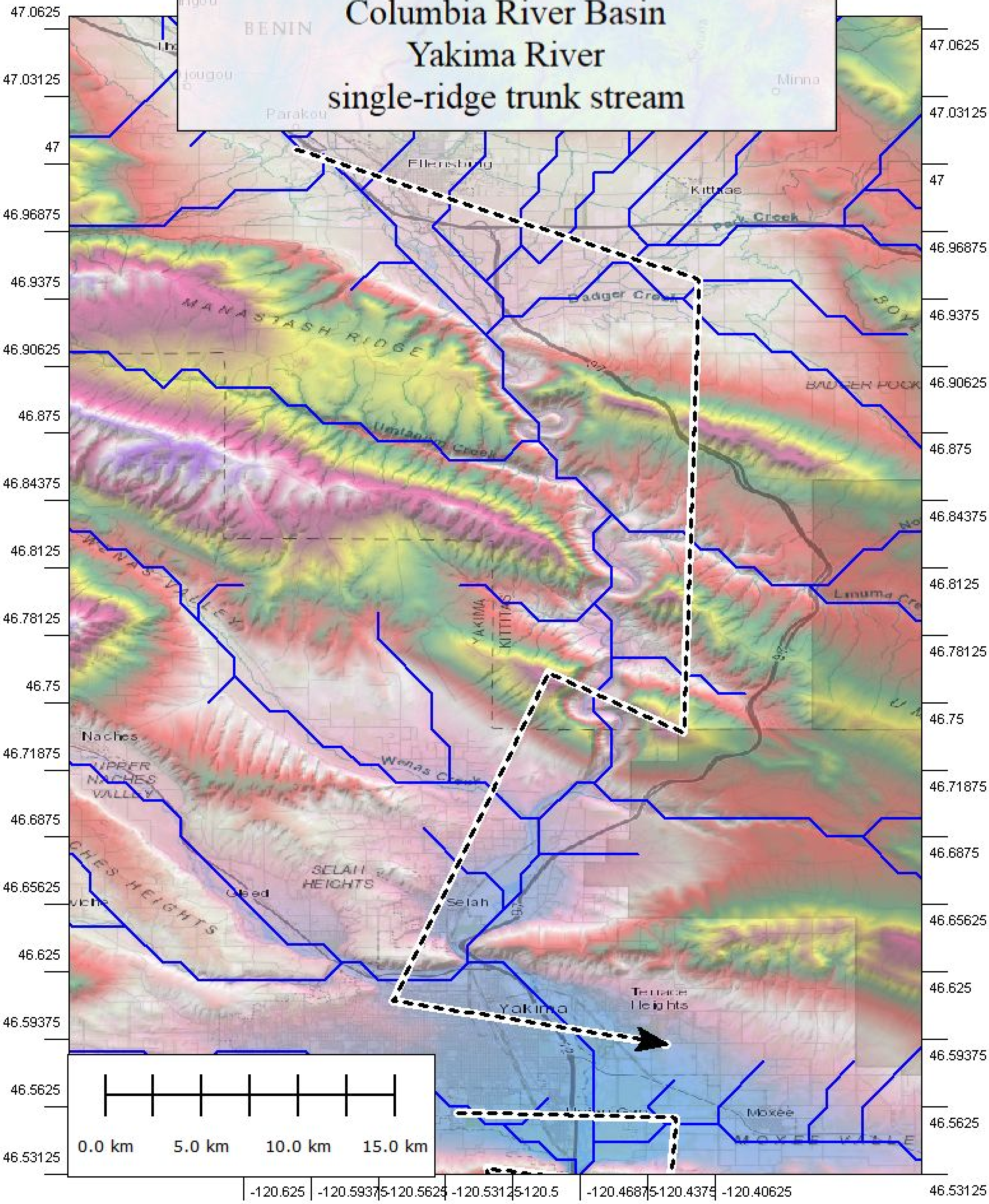

NA - 129
Columbia River Basin
Deschutes River
downdip plateau trunk stream

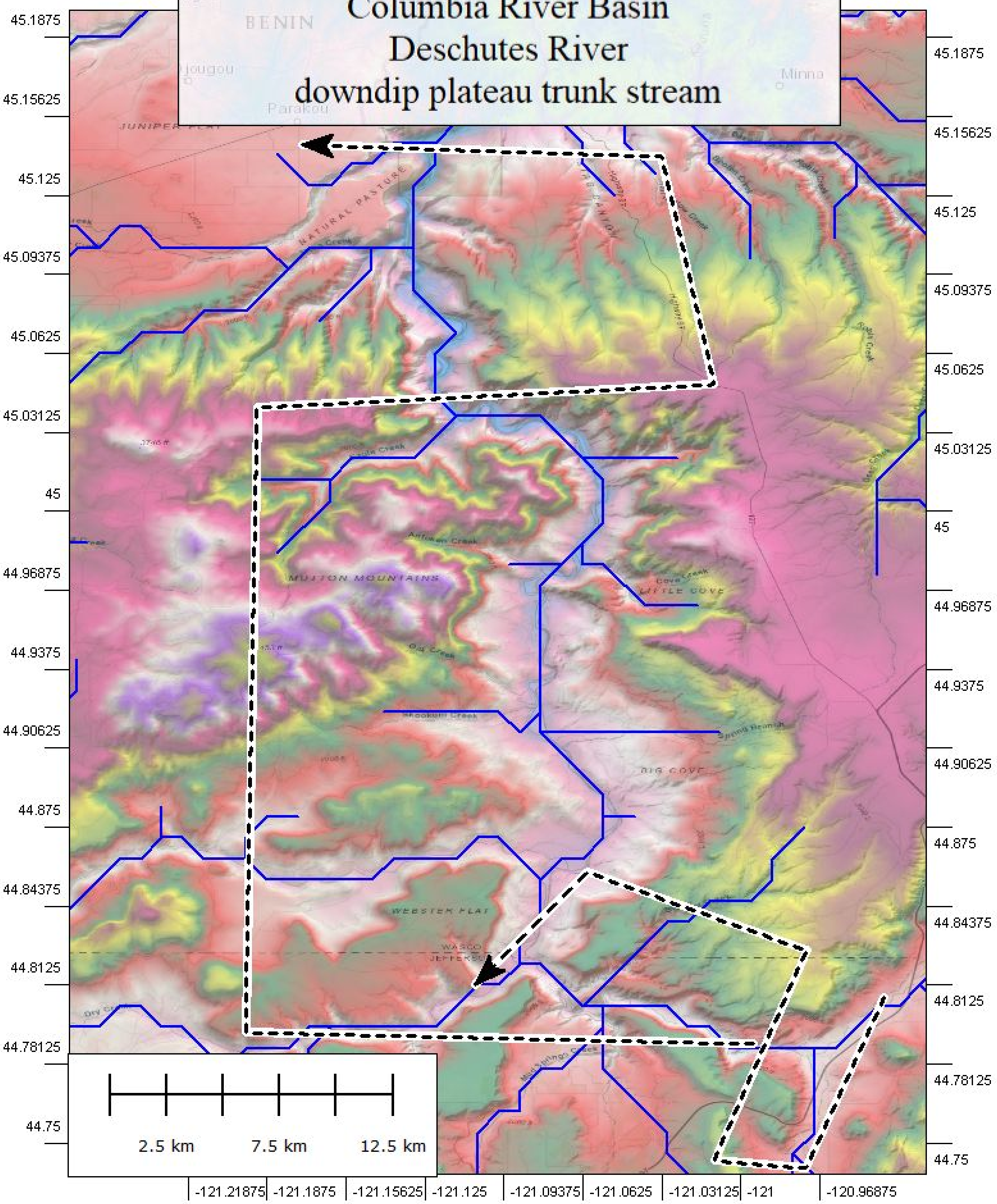

NA - 13
Rio Shutaque Basin
Rio Grande de Zacapa
irregular high ground trunk stream

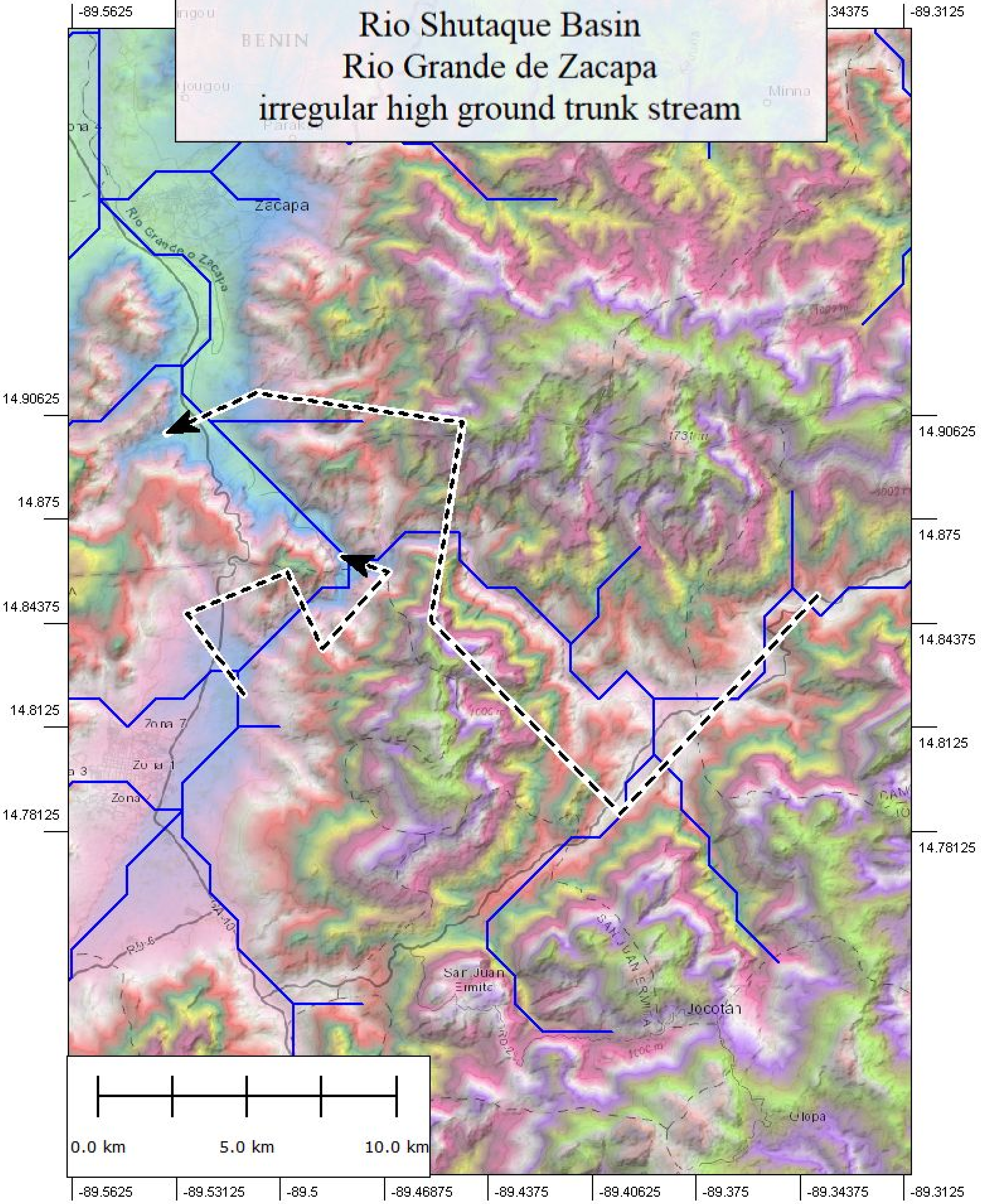

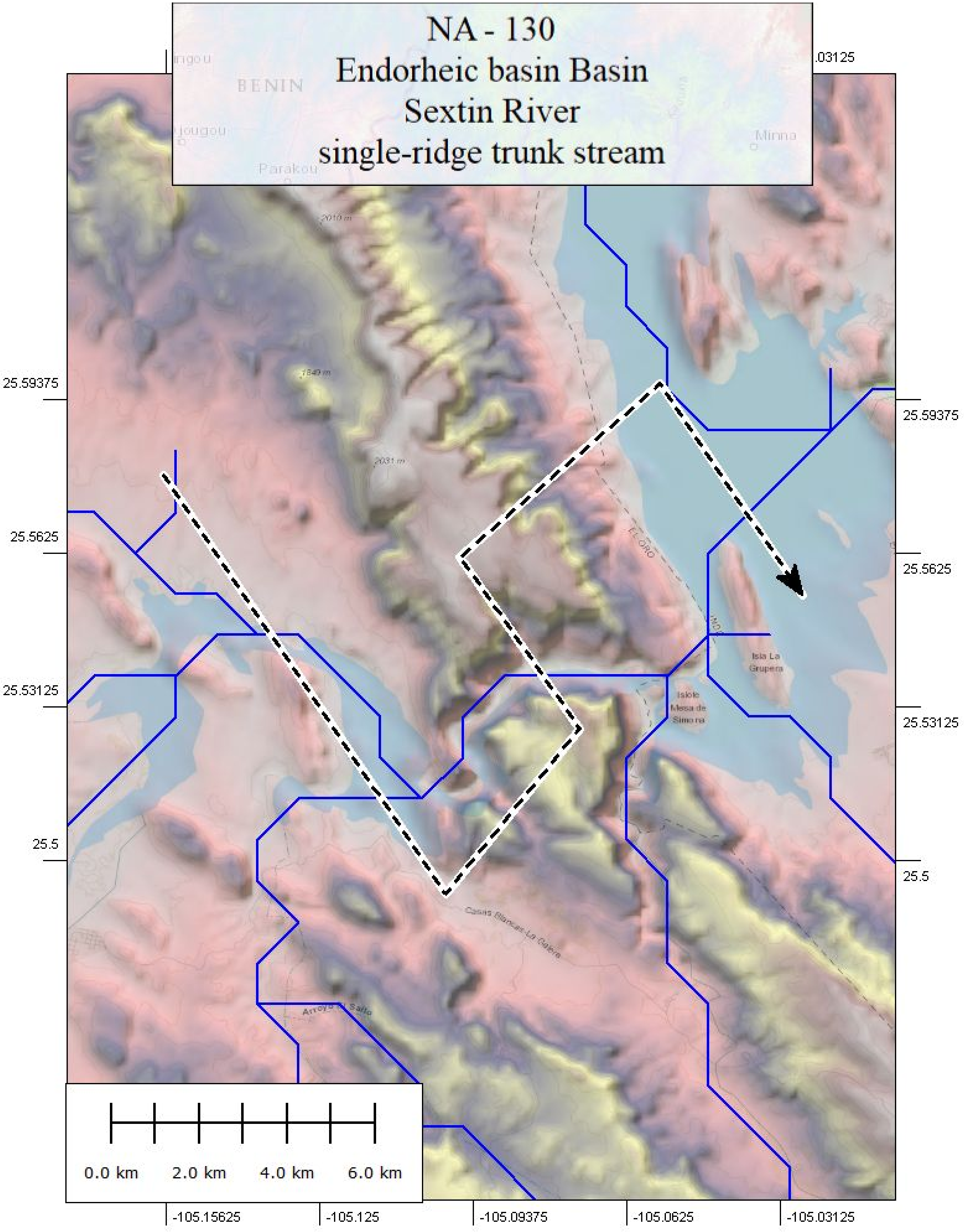
NA - 130
Endorheic basin Basin
Sextin River
single-ridge trunk stream
BENIN
Parakou
Minna
2010 m
1849 m
2031 m
Isla La Grupera
Islote Mesa de Simona
Casas Blancas La Galera
25.59375
25.5625
25.53125
25.5
-105.15625
-105.125
-105.09375
-105.0625
-105.03125
0.0 km
2.0 km
4.0 km
6.0 km

NA - 131
Mississippi River Basin
Madison River, Madison Slide
irregular high ground trunk stream

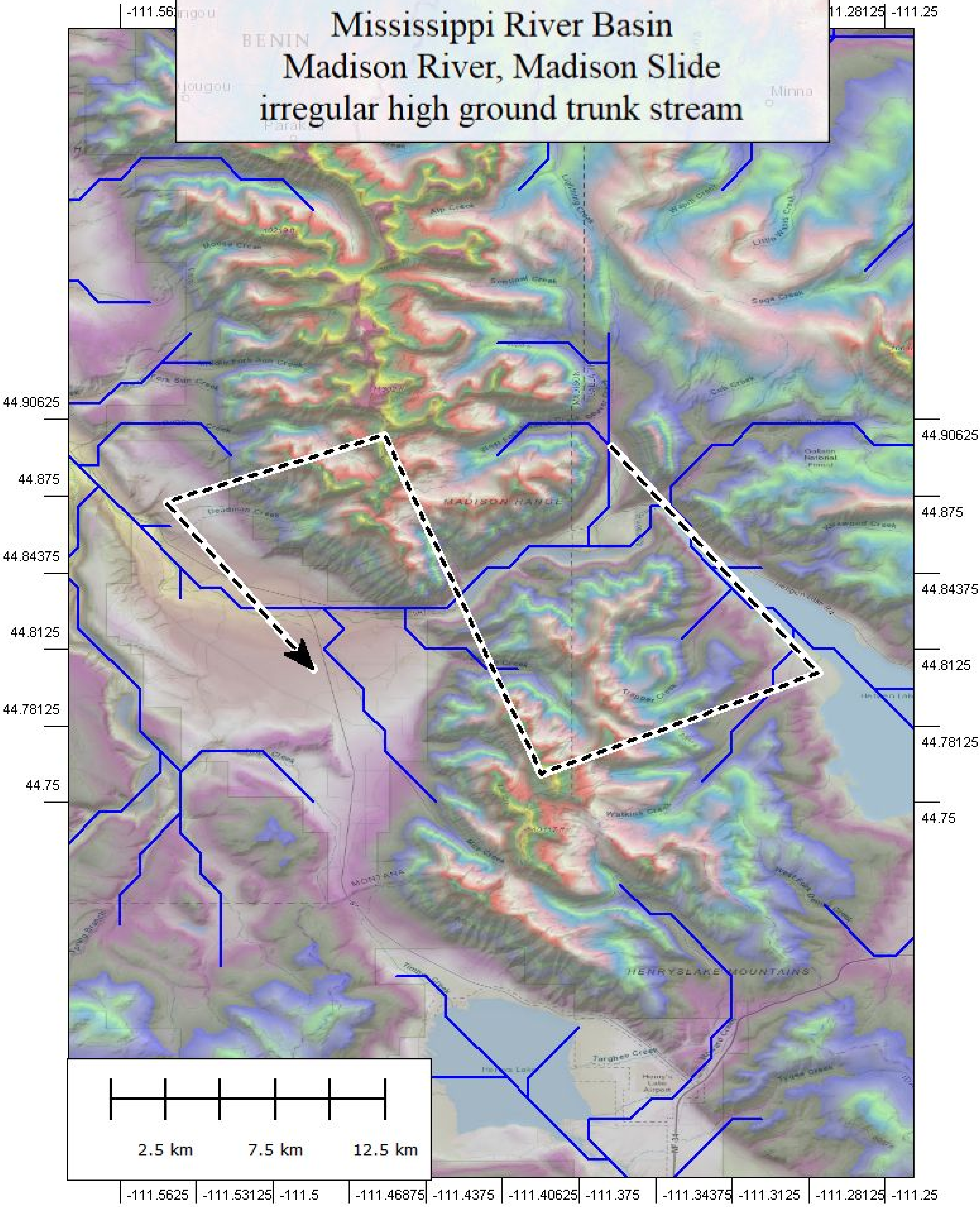

NA - 132
Rio Grande (Texas-Mexico) Basin
Canon del Chorro
single-ridge trunk stream

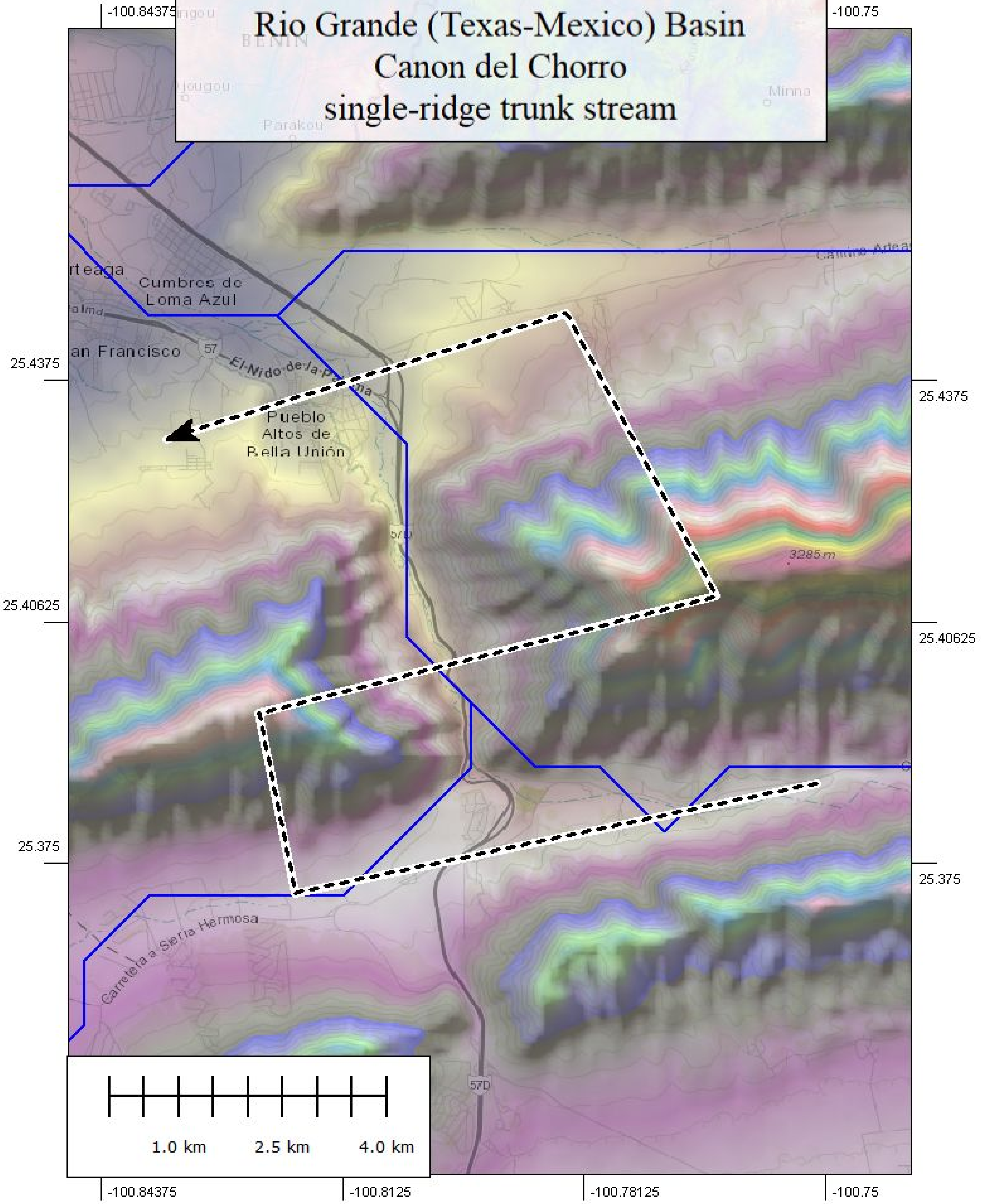

NA - 133
Colorado River (Arizona) Basin
Colorado River
downdip plateau trunk stream

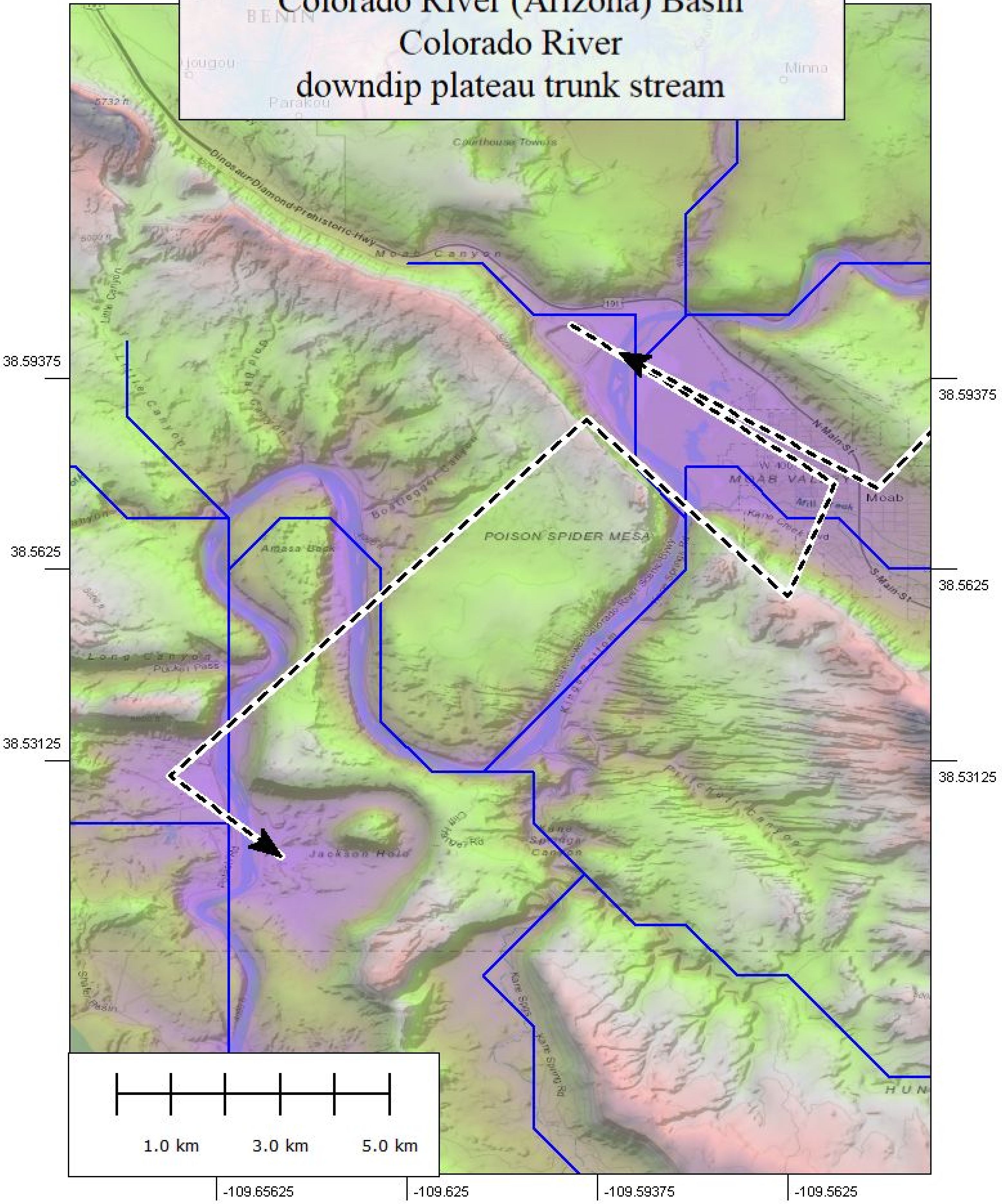

NA - 134
Colorado River (Arizona) Basin
Dolores River
plateau trunk stream

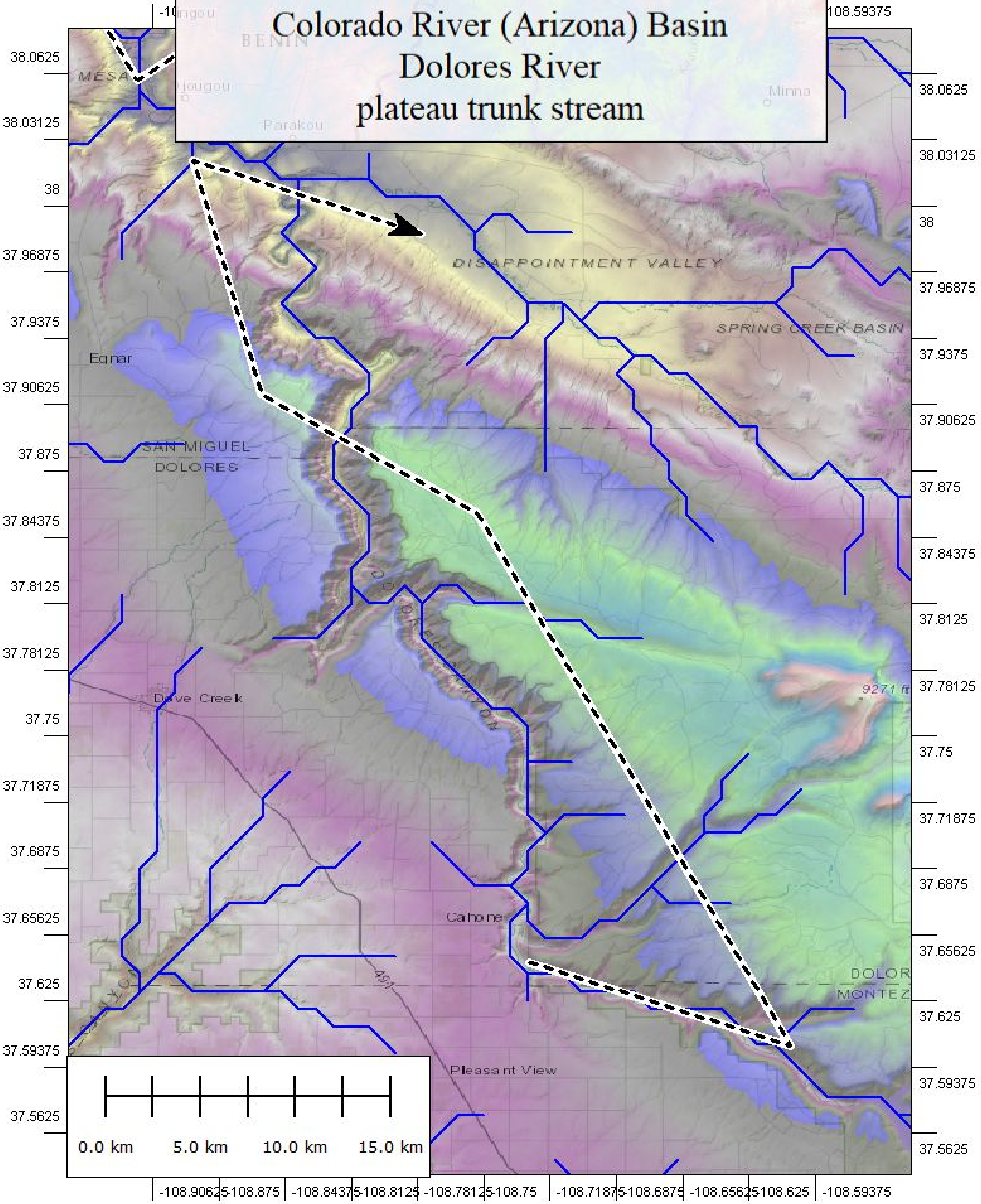

NA - 135
Endorheic basin Basin
East Fork Sevier River, Kingston Canyon
irregular high ground trunk stream

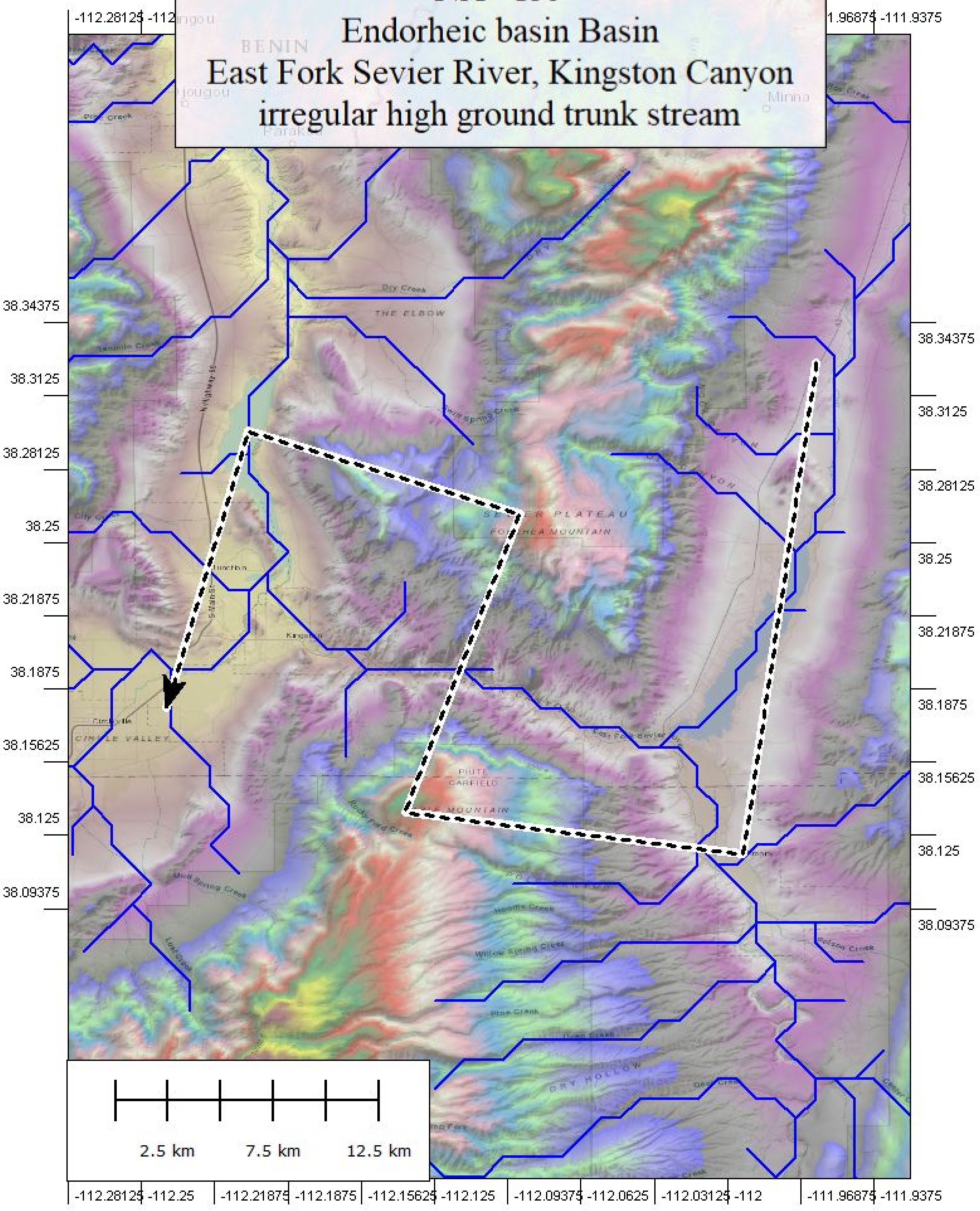

NA - 136
Endorheic basin Basin
Nazas River
irregular high ground trunk stream

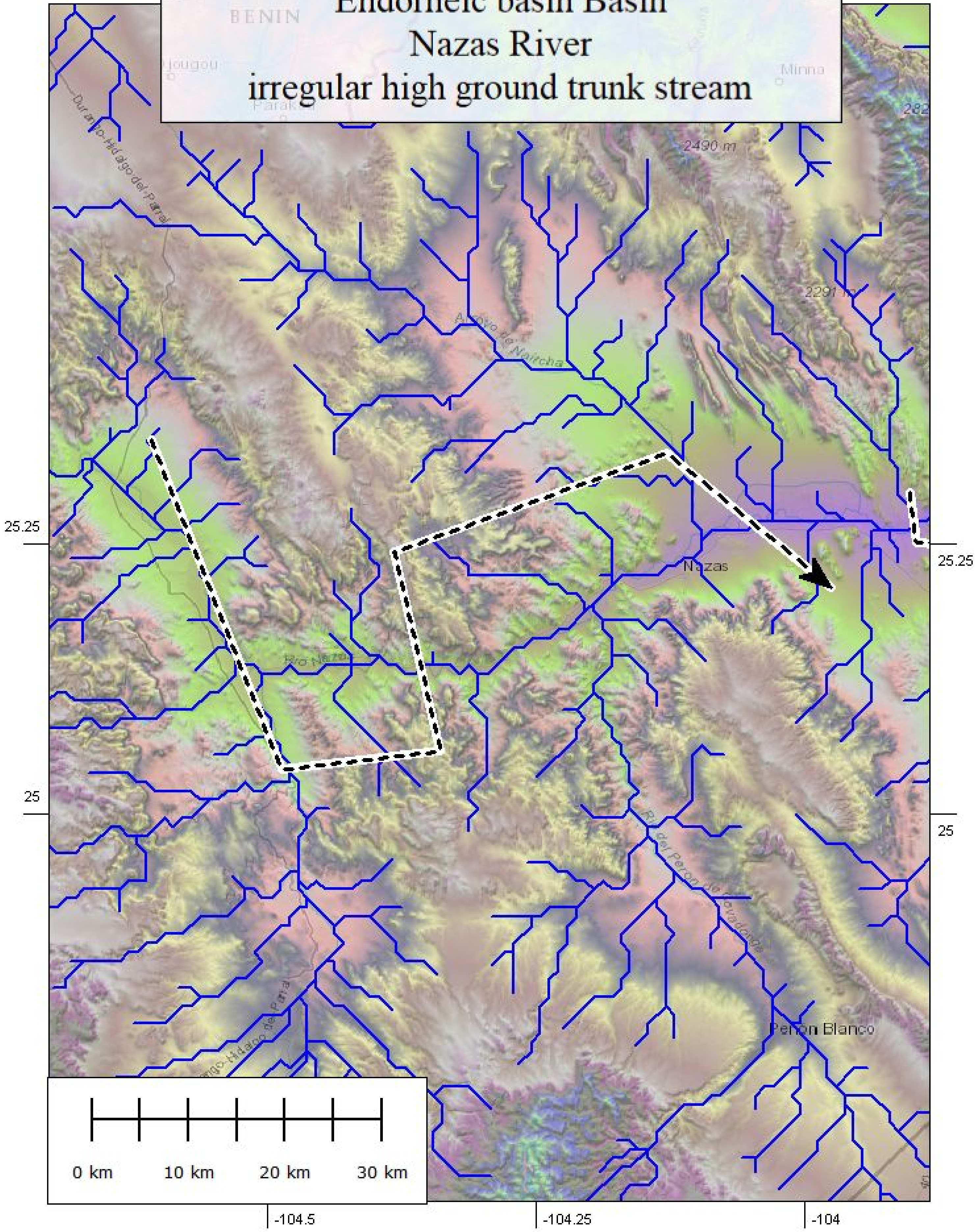

NA - 137
Colorado River (Arizona) Basin
Erickson Canyon
single-ridge trunk stream

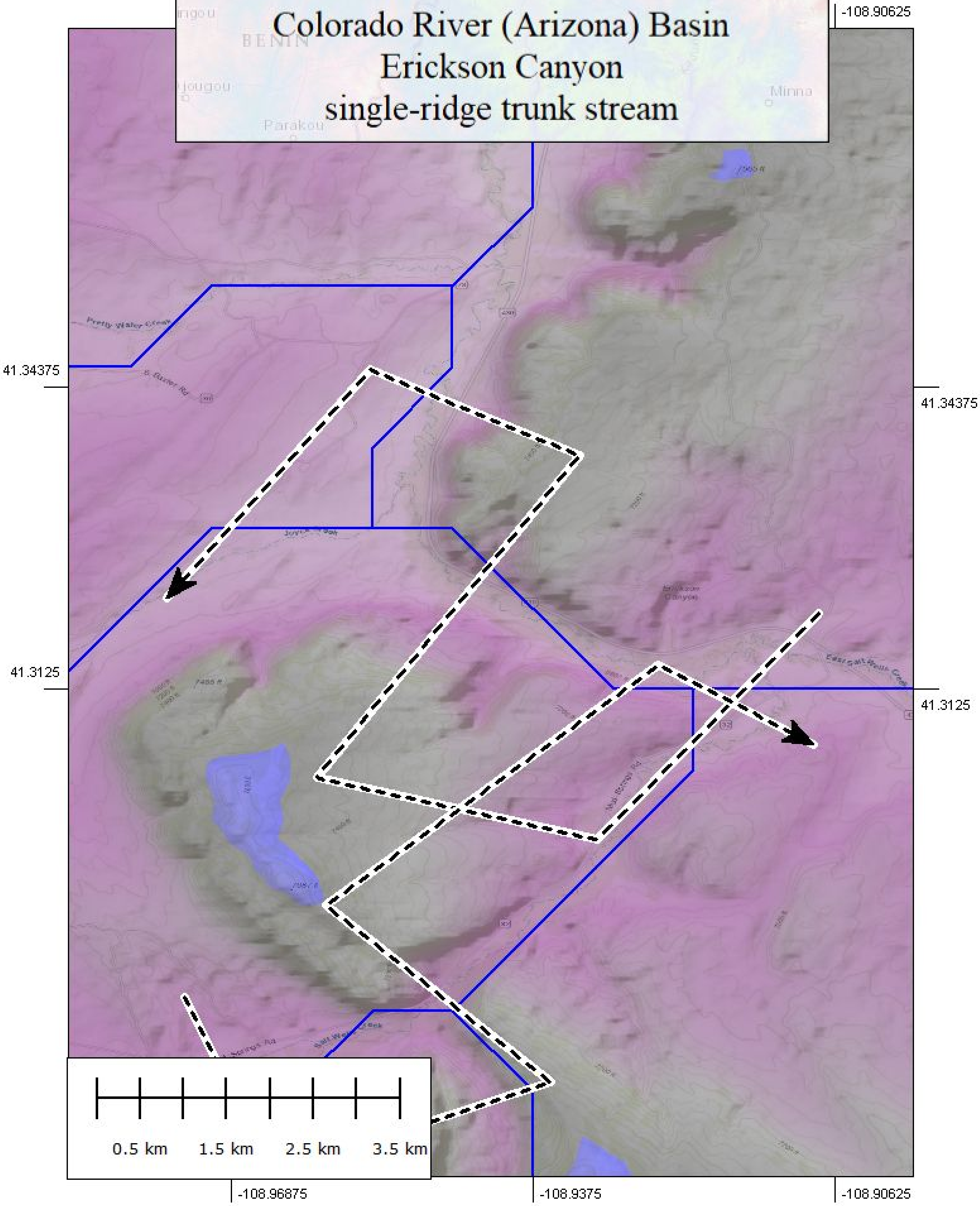

NA - 138
Colorado River (Arizona) Basin
Fontanelle Creek, Fontanelle Gap
single-ridge trunk stream

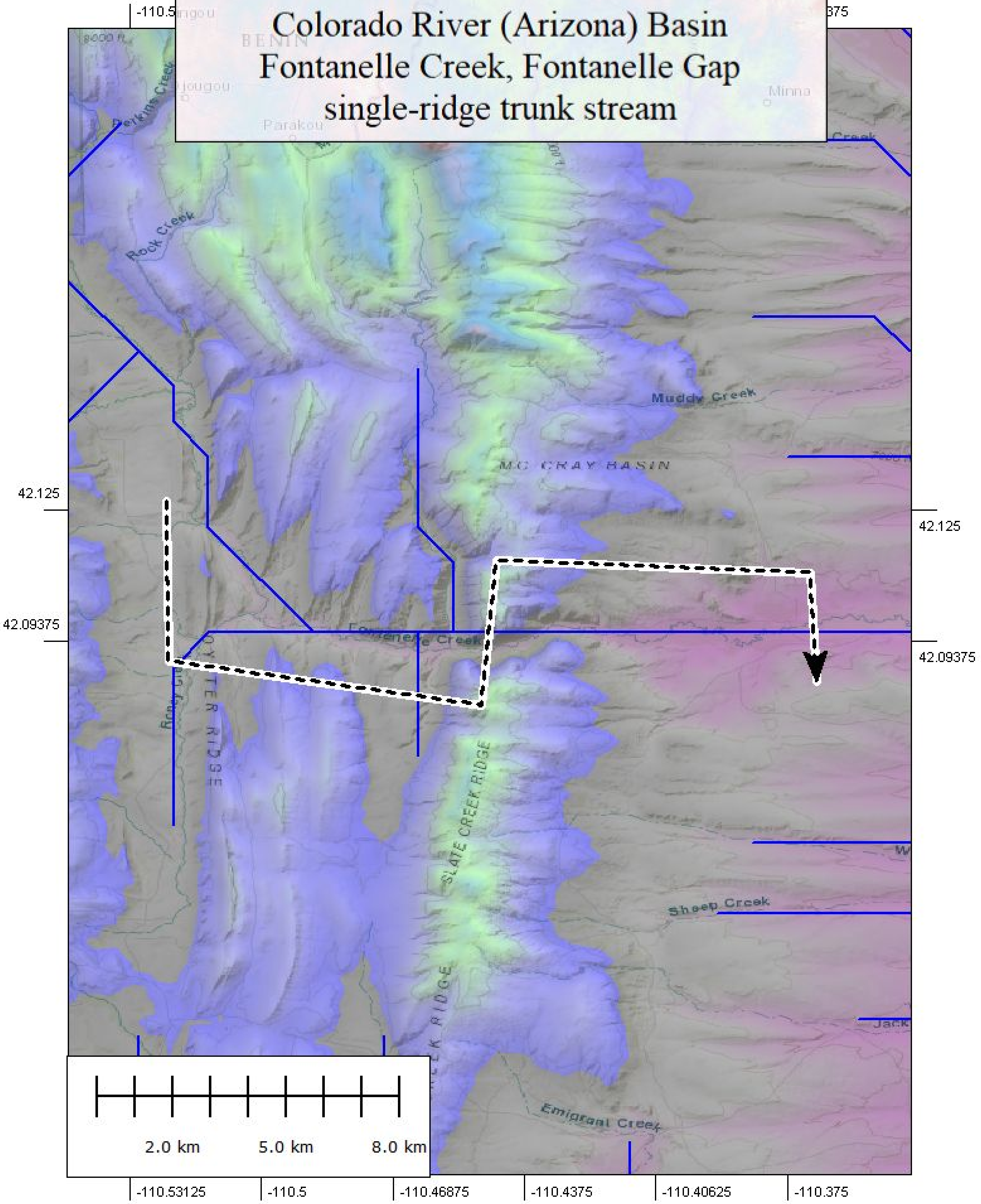

NA - 139
Colorado River (Arizona) Basin
Gunnison River
irregular high ground trunk stream

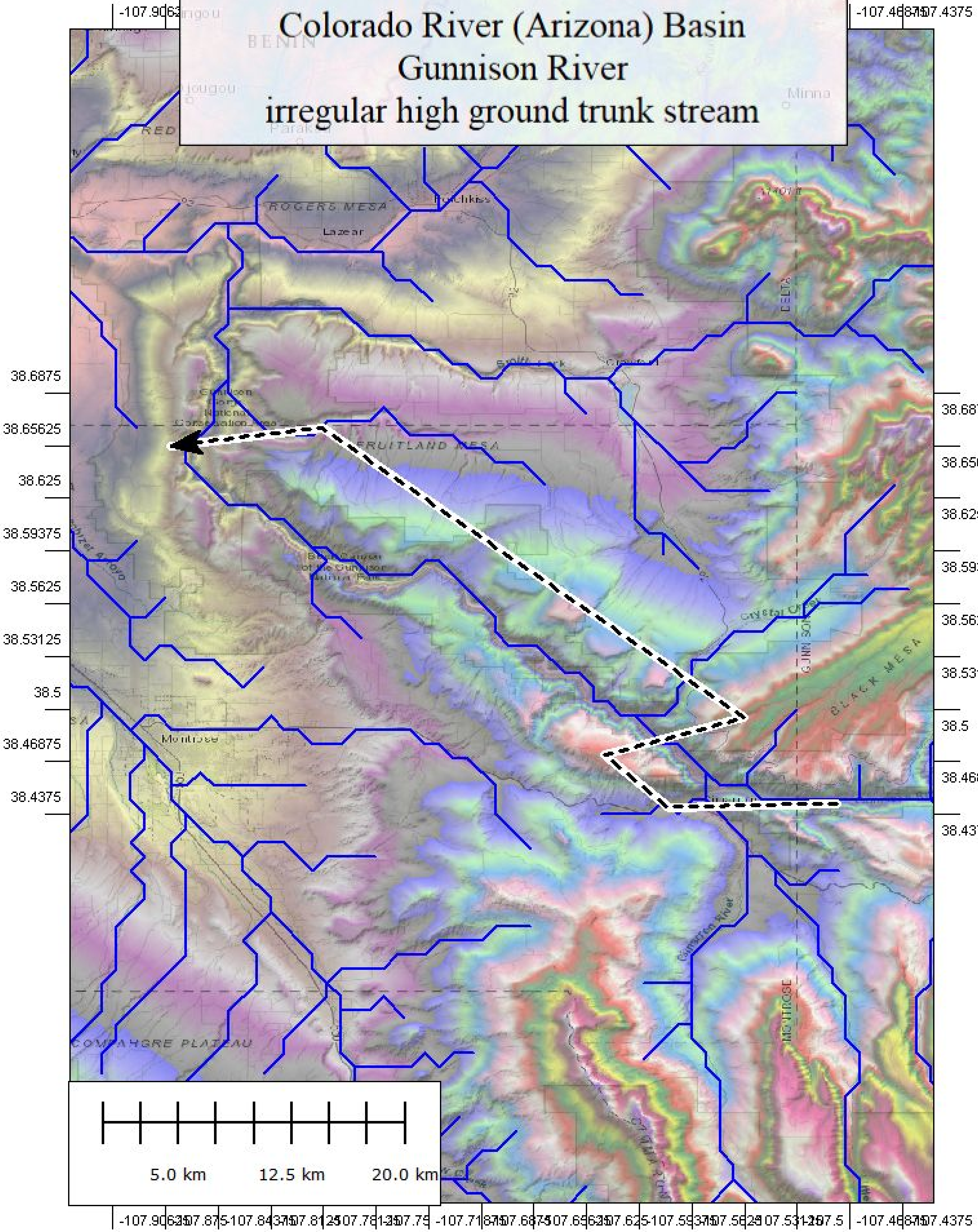

NA - 14
Colorado River (Arizona) Basin
Green River
updip plateau trunk stream

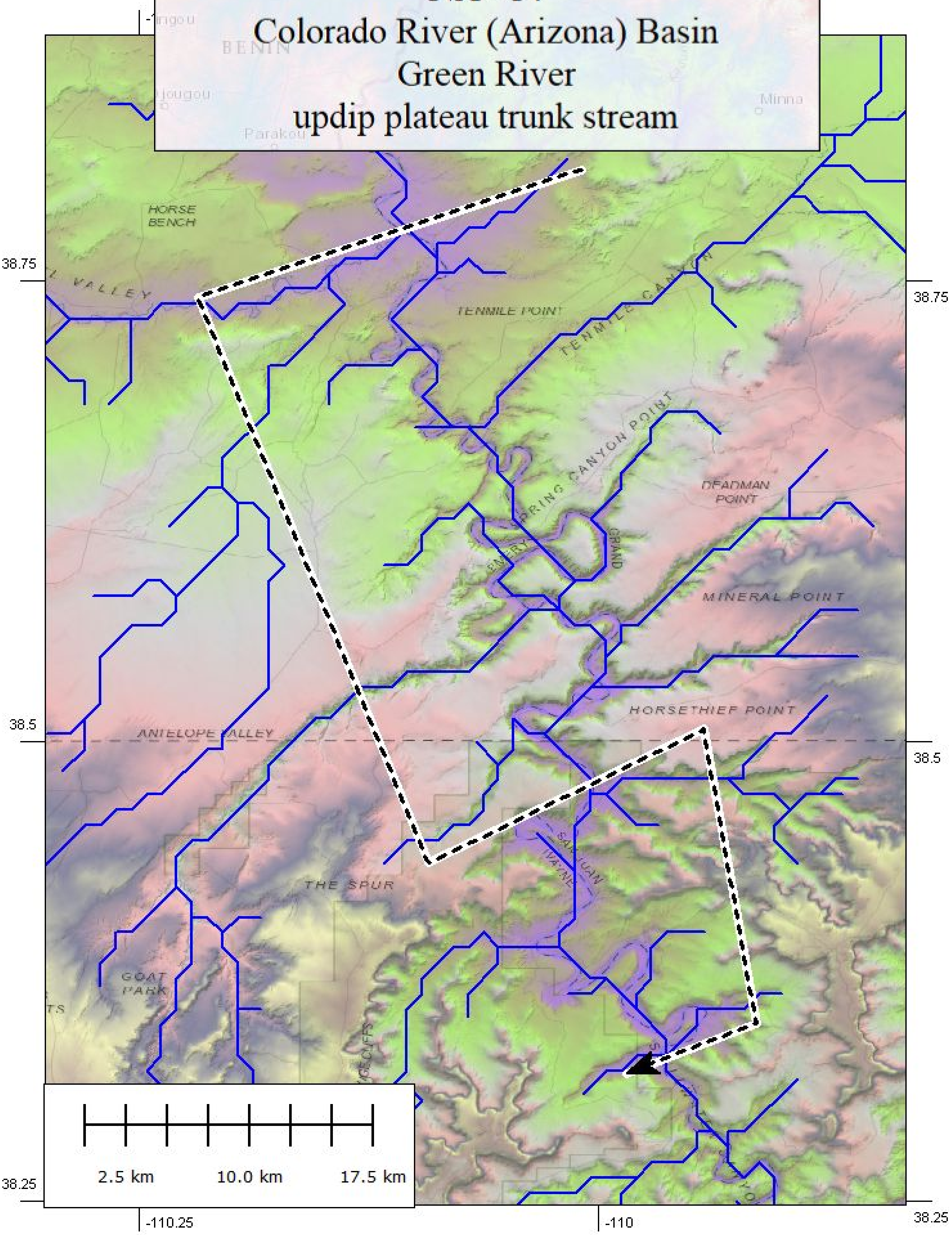

NA - 140
Columbia River Basin
Hoback River
irregular high ground trunk stream

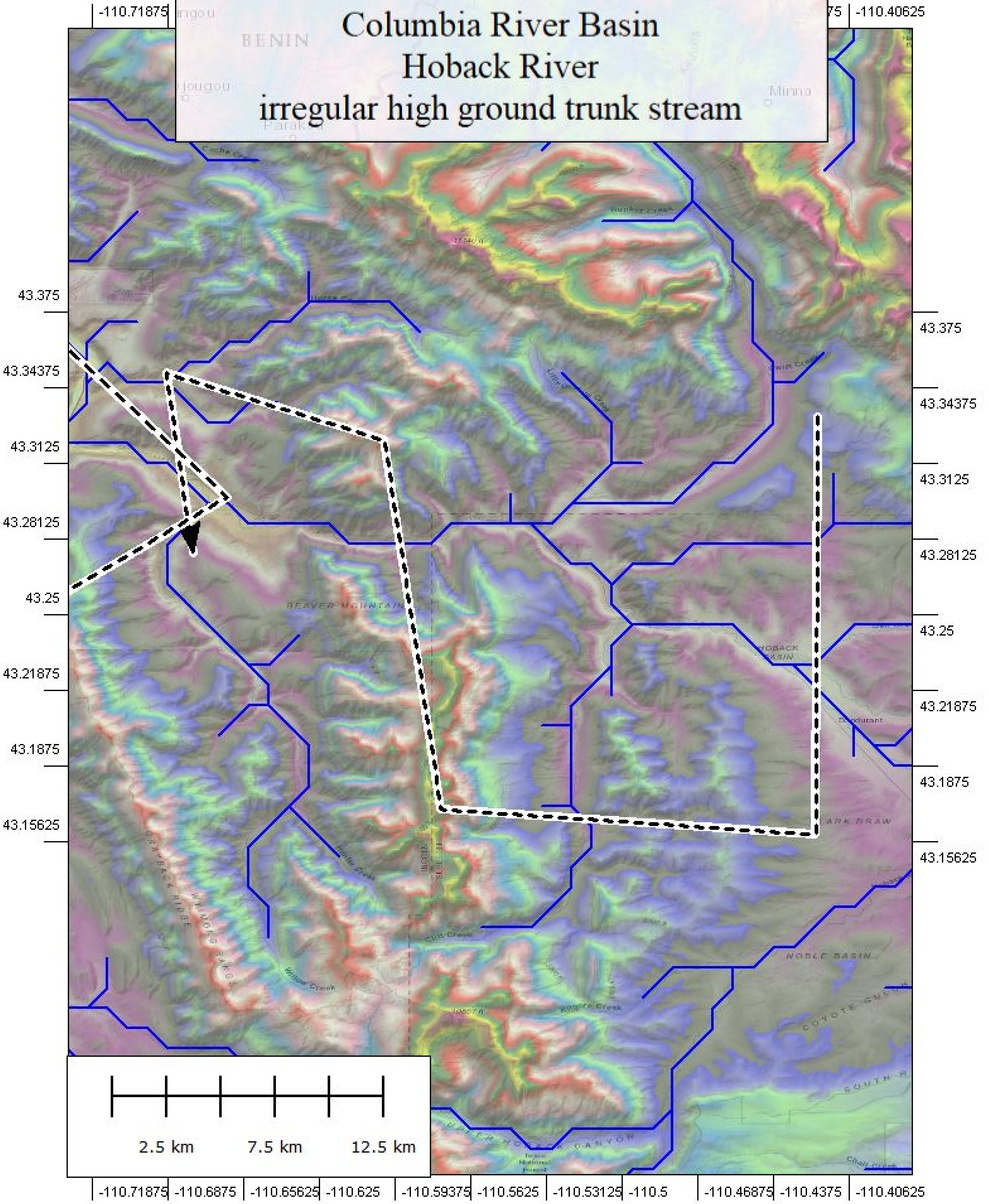

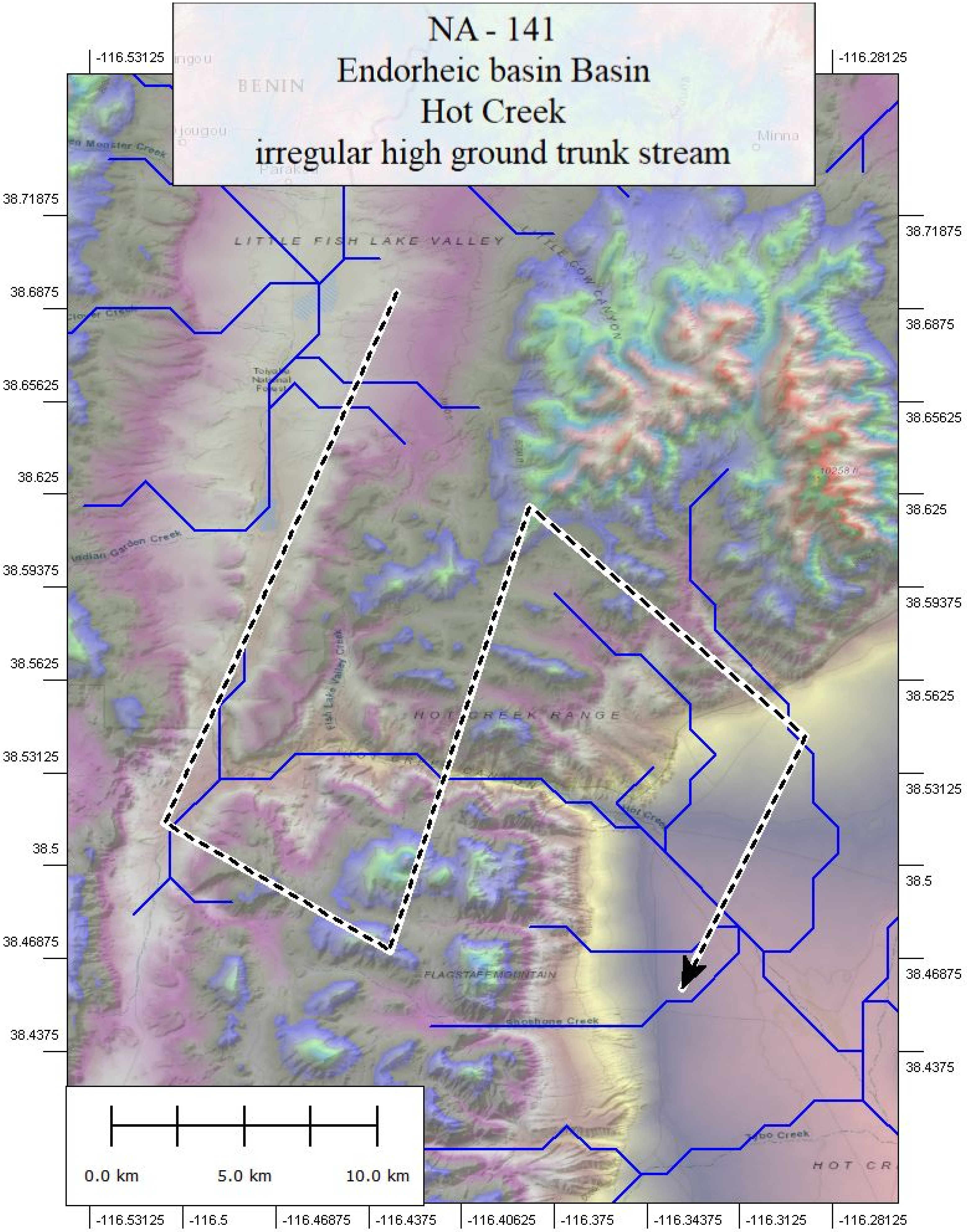
NA - 141
Endorheic basin Basin
Hot Creek
irregular high ground trunk stream
-116.53125
-116.28125
38.71875
38.6875
38.65625
38.625
38.59375
38.5625
38.53125
38.5
38.46875
38.4375
LITTLE FISH LAKE VALLEY
LITTLE COW CANYON
Toiyabe National Forest
Indian Garden Creek
Fish Lake Valley Creek
HOT CREEK RANGE
Hot Creek
FLAGSTAFF MOUNTAIN
Shoshone Creek
Tybo Creek
BENIN
Minna
0.0 km
5.0 km
10.0 km
-116.5
-116.46875
-116.4375
-116.40625
-116.375
-116.34375
-116.3125

NA - 142
Endorheic basin Basin
Aguanaval River
multi-ridge trunk stream

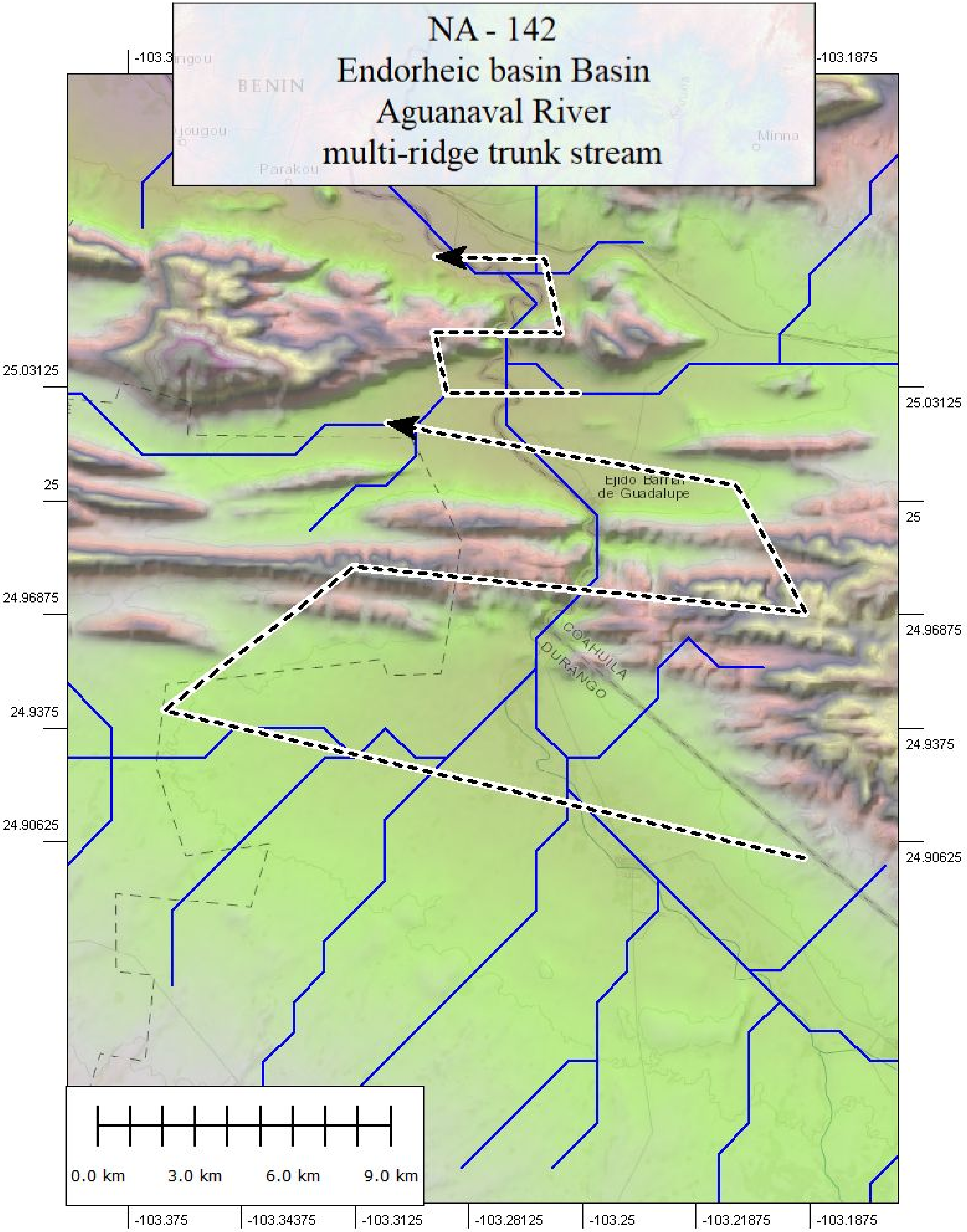

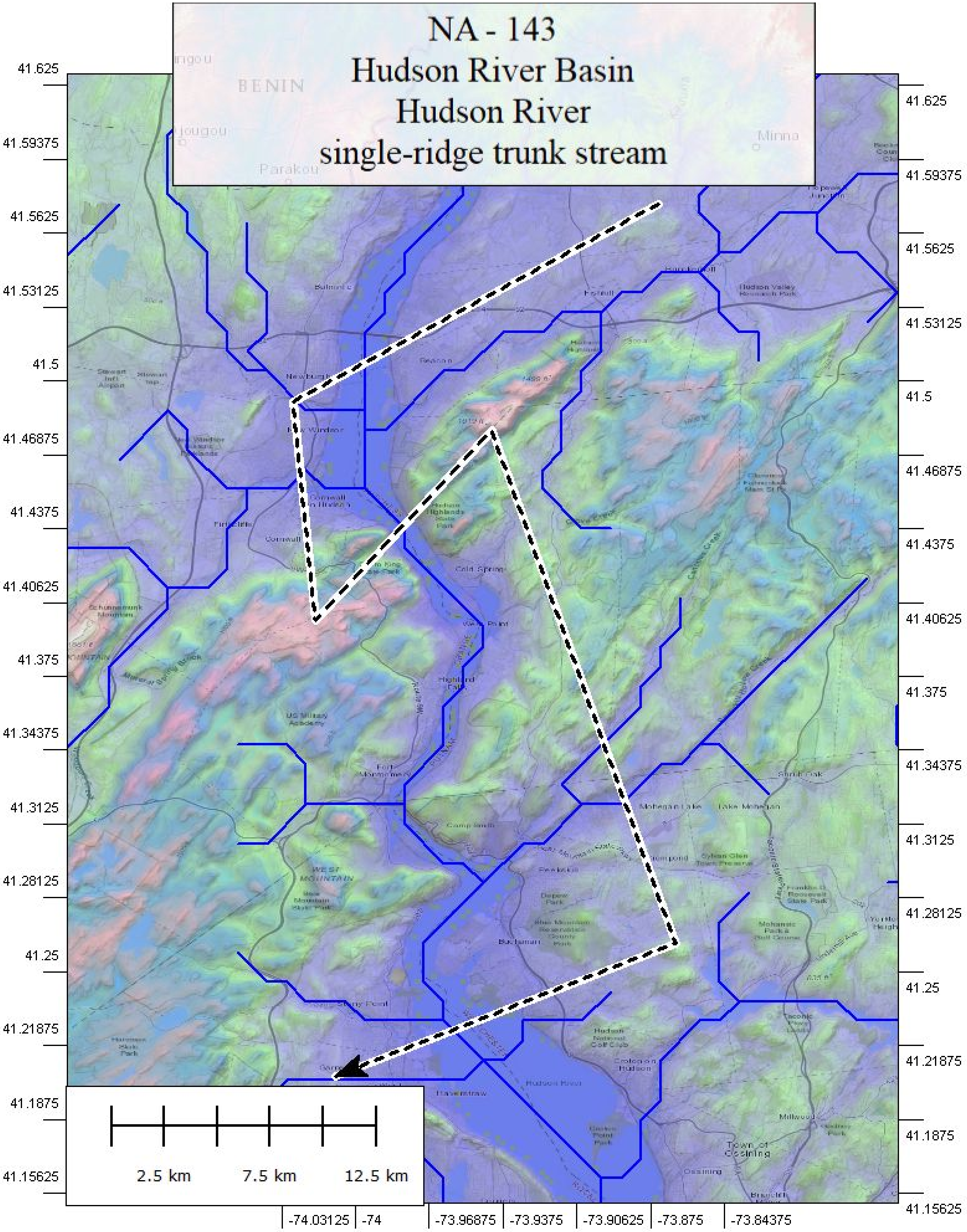

NA - 143
Hudson River Basin
Hudson River
single-ridge trunk stream
41.625
41.59375
41.5625
41.53125
41.5
41.46875
41.4375
41.40625
41.375
41.34375
41.3125
41.28125
41.25
41.21875
41.1875
41.15625
-74.03125
-74
-73.96875
-73.9375
-73.90625
-73.875
-73.84375
2.5 km
7.5 km
12.5 km
BENIN
Parakou
Minna
Newburgh
Beacon
Cornwall
Cold Spring
West Point
US Military Academy
Camp Smith
Mohegan Lake
Lake Mohegan
Peekskill
Buchanan
Haverstraw
Hudson River
Millwood
Ossining
Town of Ossining
WEST MOUNTAIN

NA - 144
James River Basin
James River
single-ridge trunk stream

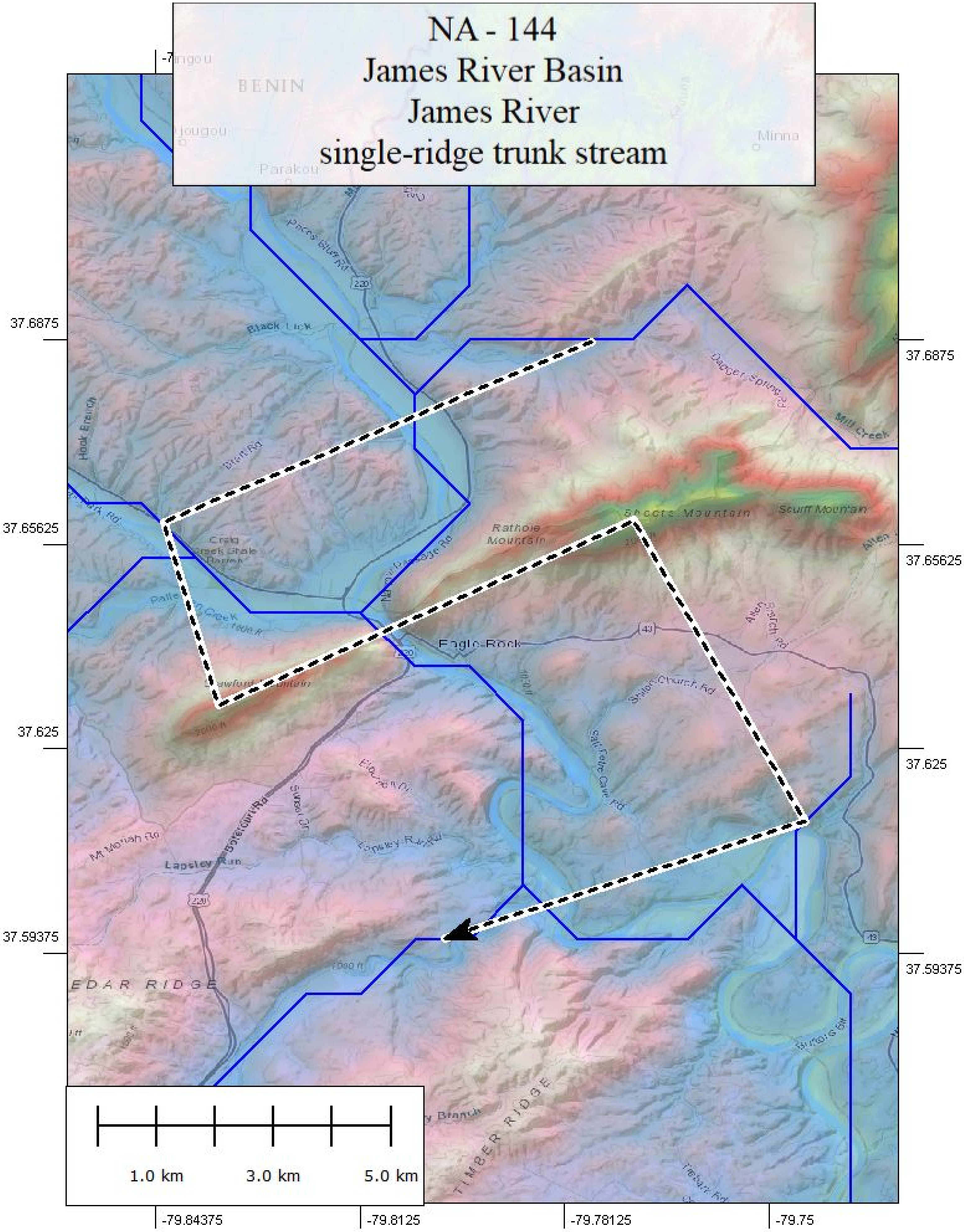

NA - 145
James River Basin
James River
single-ridge trunk stream

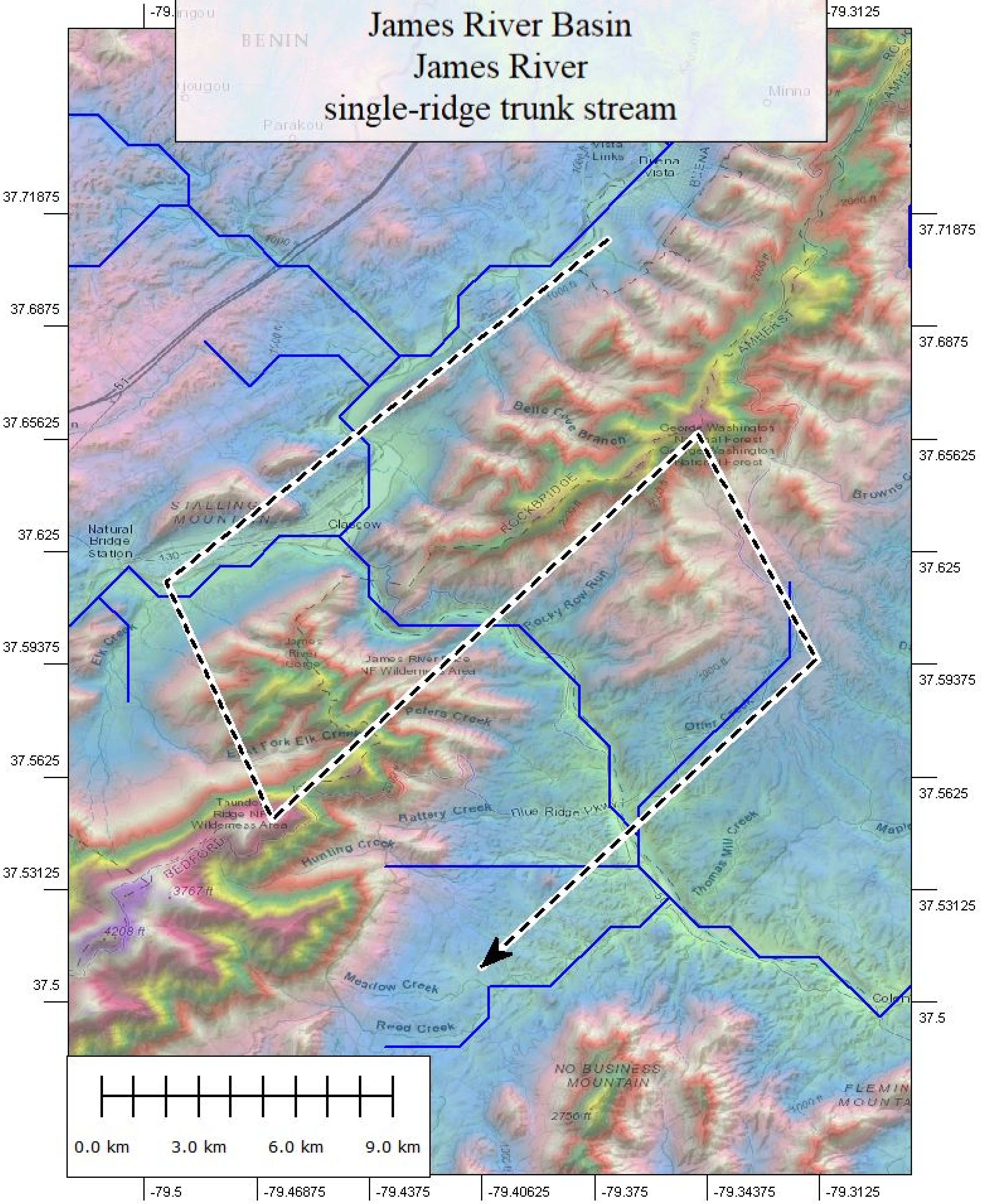

NA - 146
Mississippi River Basin
Jefferson River
irregular high ground trunk stream

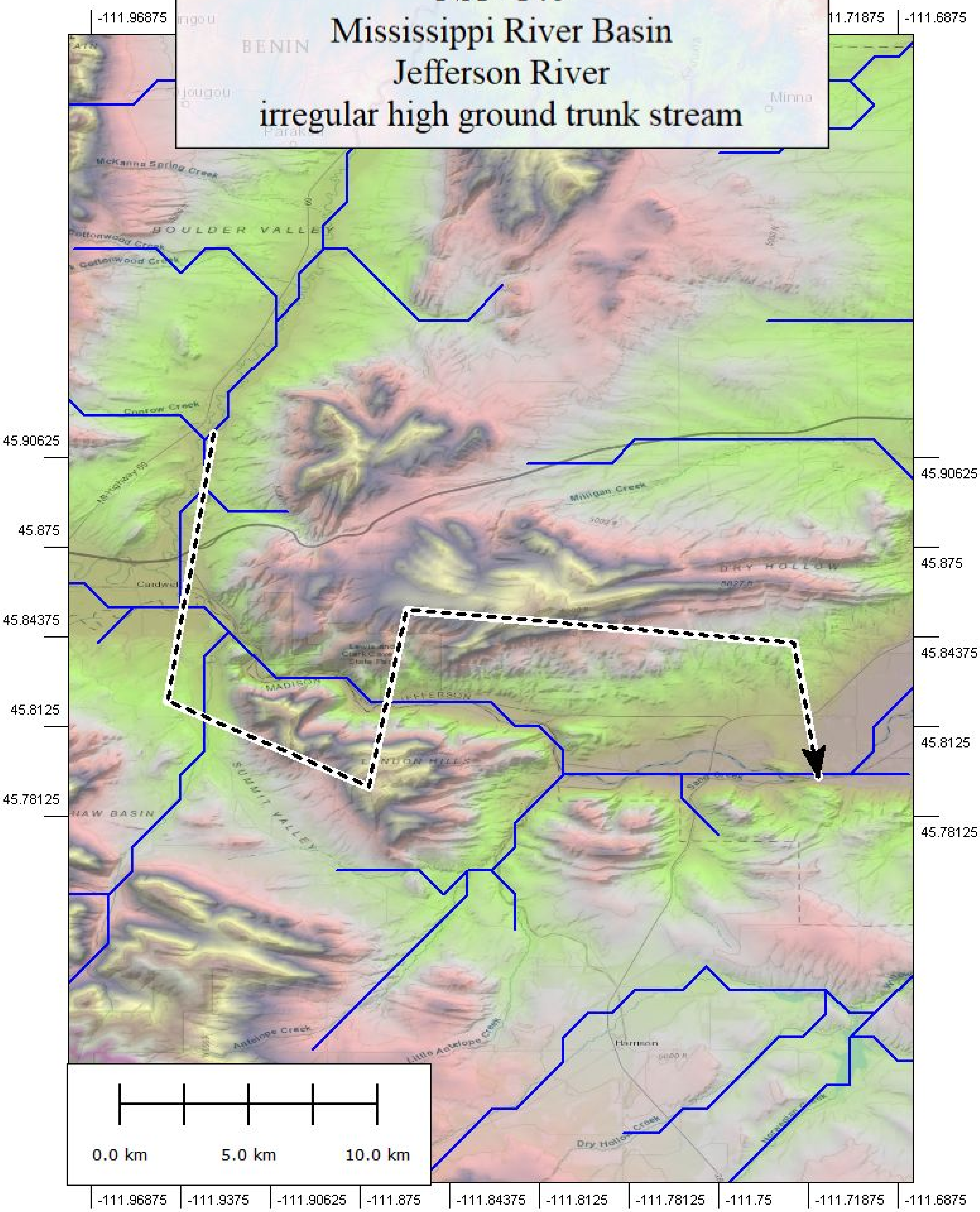

NA - 147
Susquehanna River Basin
Kishacoquillas Creek
single-ridge trunk stream

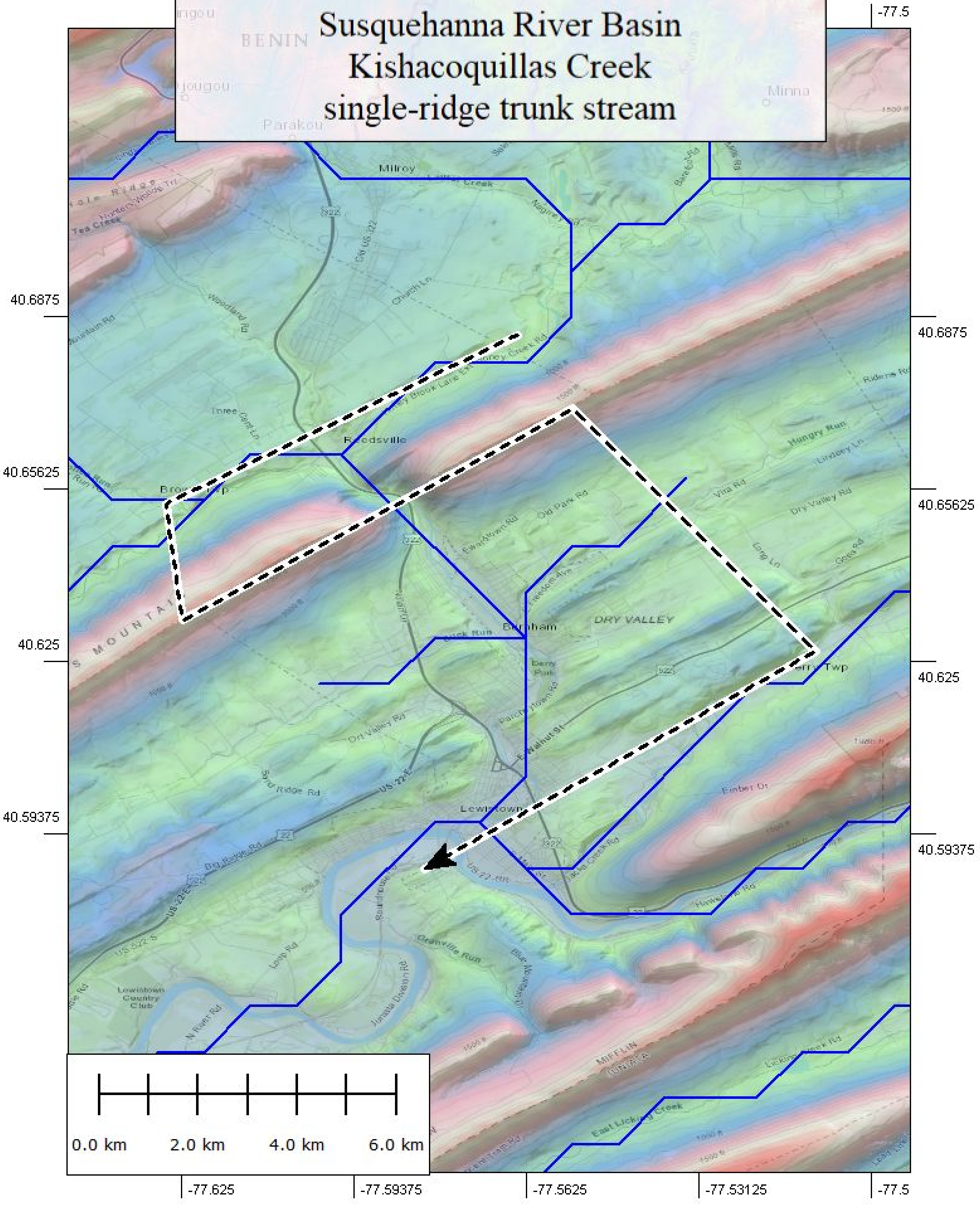

NA - 148
Columbia River Basin
Kootenay River
irregular high ground trunk stream

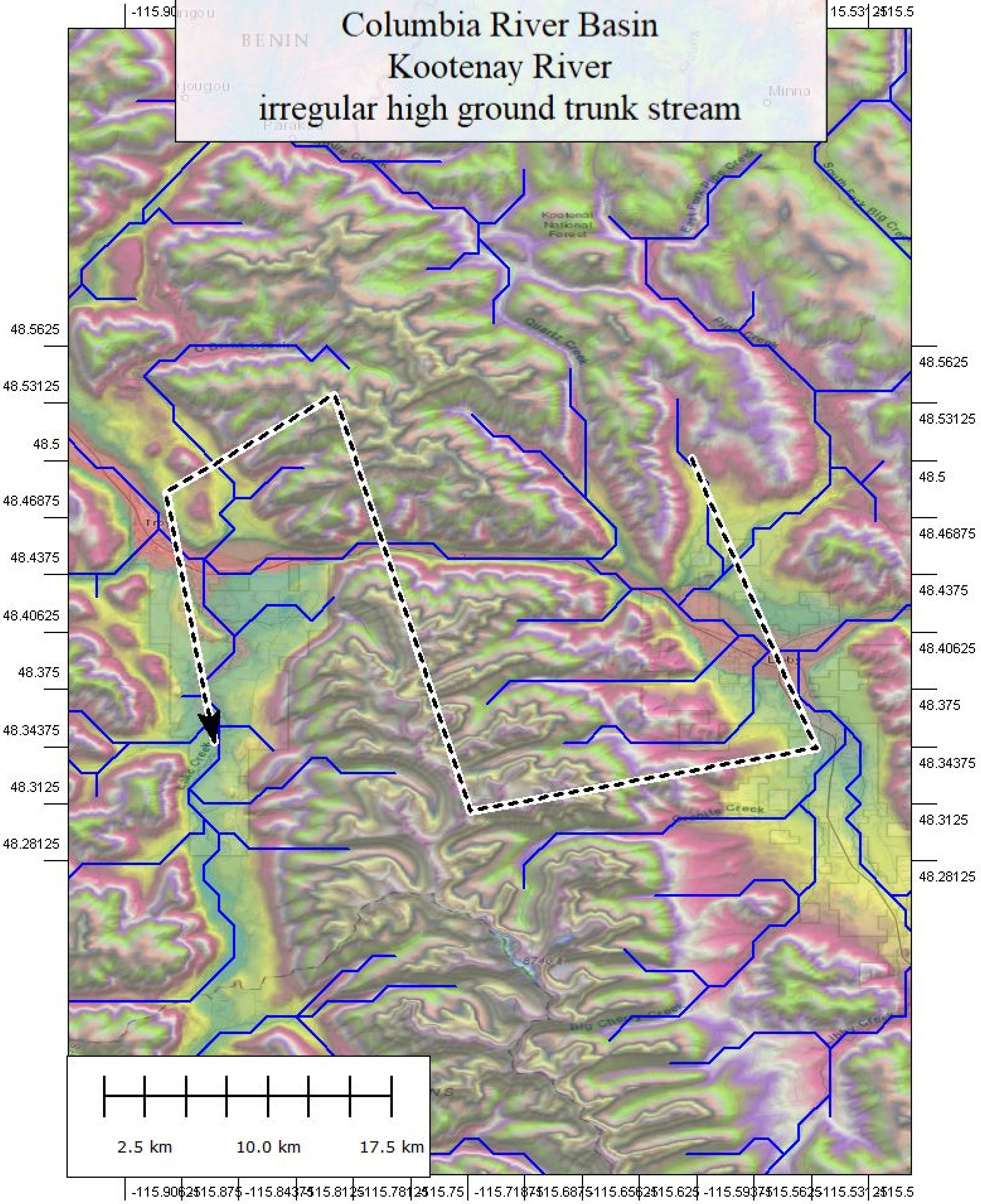

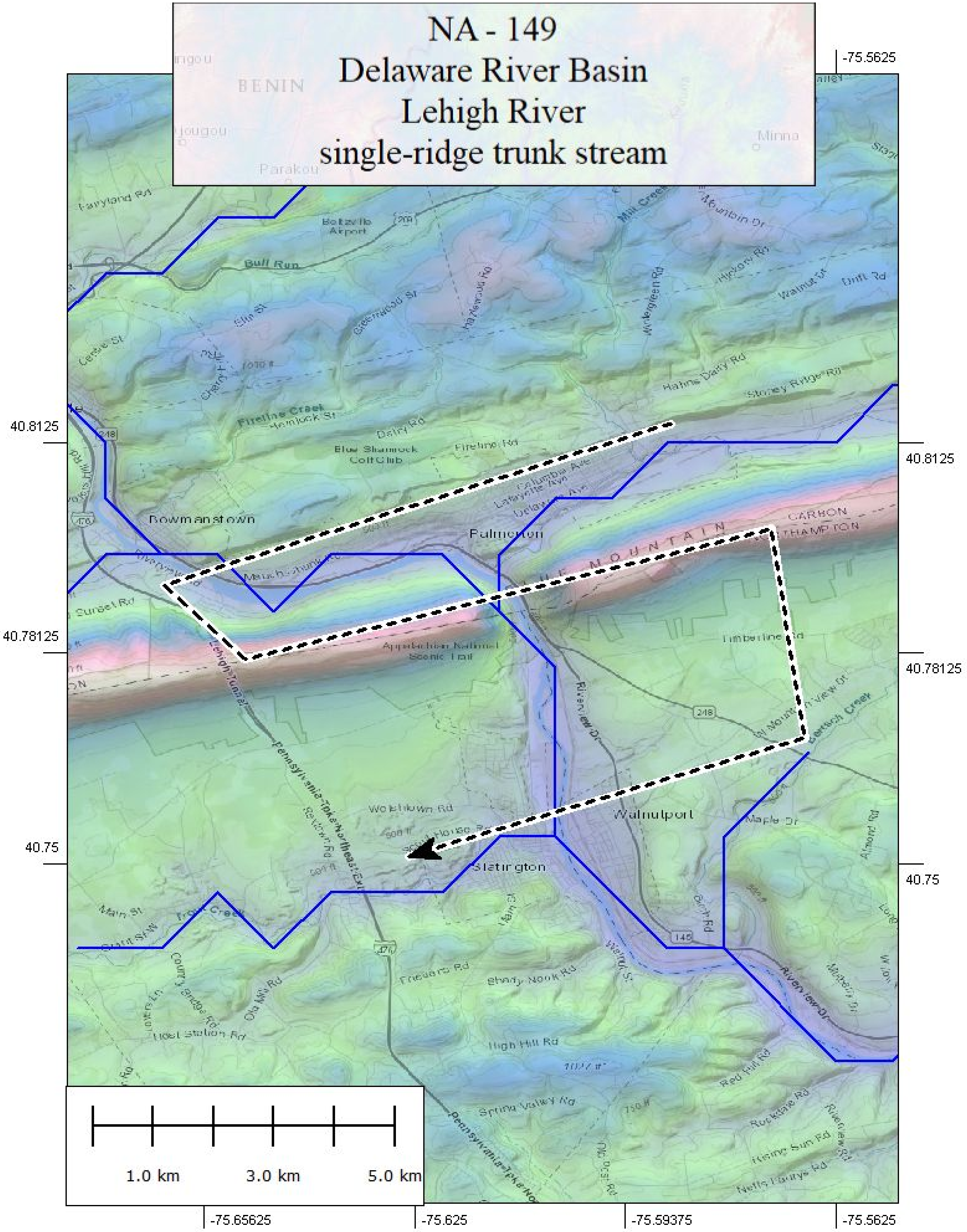
NA - 149
Delaware River Basin
Lehigh River
single-ridge trunk stream
-75.5625
40.8125
40.8125
40.78125
40.78125
40.75
40.75
-75.65625
-75.625
-75.59375
-75.5625
1.0 km
3.0 km
5.0 km
Bowmanstown
Palmerton
Walnutport
Slatington
Appalachian National Scenic Trail
Blue Shamrock Golf Club
Boltzville Airport
Bull Run
Fireline Creek
Mill Creek
Bertsch Creek
Trout Creek
CARBON
NORTHAMPTON
Pennsylvania Tpke Northeast Ext
Riverview Dr
Lehigh Tunnel

NA - 15
Mackenzie River (Canada) Basin
irregular high ground trunk stream

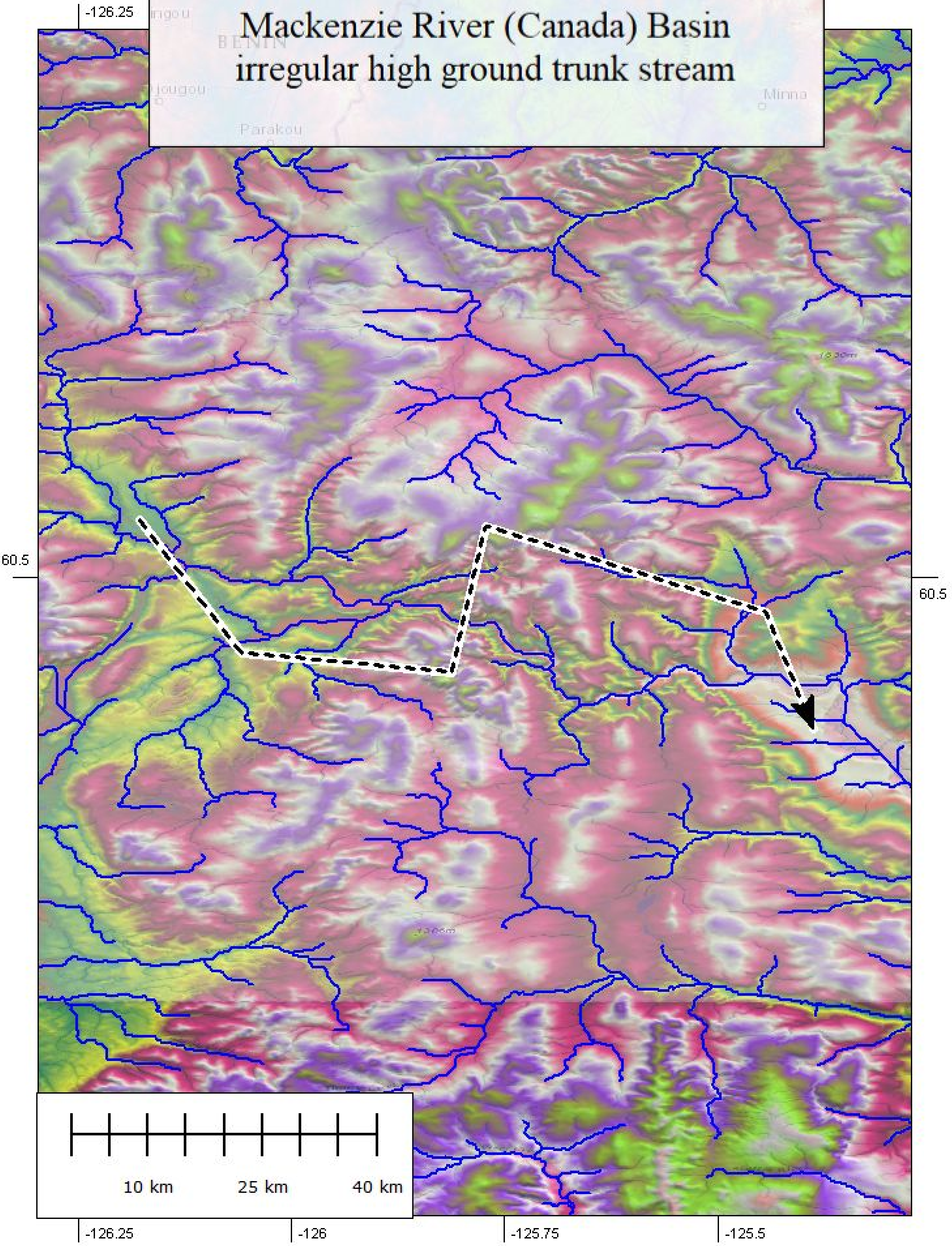

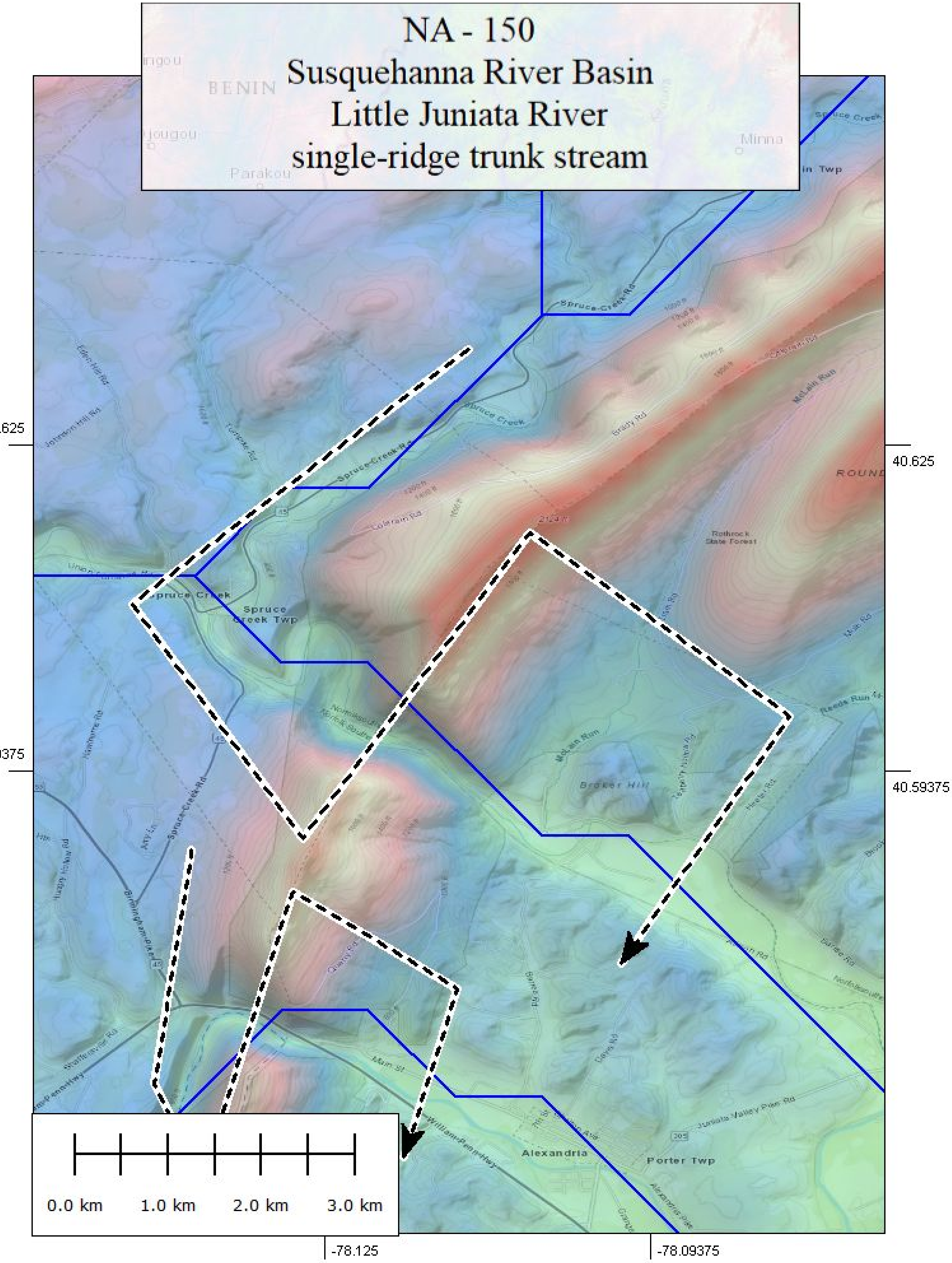
NA - 150
Susquehanna River Basin
Little Juniata River
single-ridge trunk stream
40.625
40.59375
-78.125
-78.09375
0.0 km
1.0 km
2.0 km
3.0 km
Spruce Creek
Spruce Creek Twp
Spruce Creek Rd
Brady Rd
McLain Run
Rothrock State Forest
Broker Hill
Alexandria
Porter Twp
Main St
William Penn Hwy
Juniata Valley Pike Rd
Reeds Run
Colerain Rd
Johnson Hill Rd
Eden Hill Rd
Turnpike Rd
Birmingham Pike
Shaffersville Rd
Quarry Rd
Barree Rd
Davis Rd

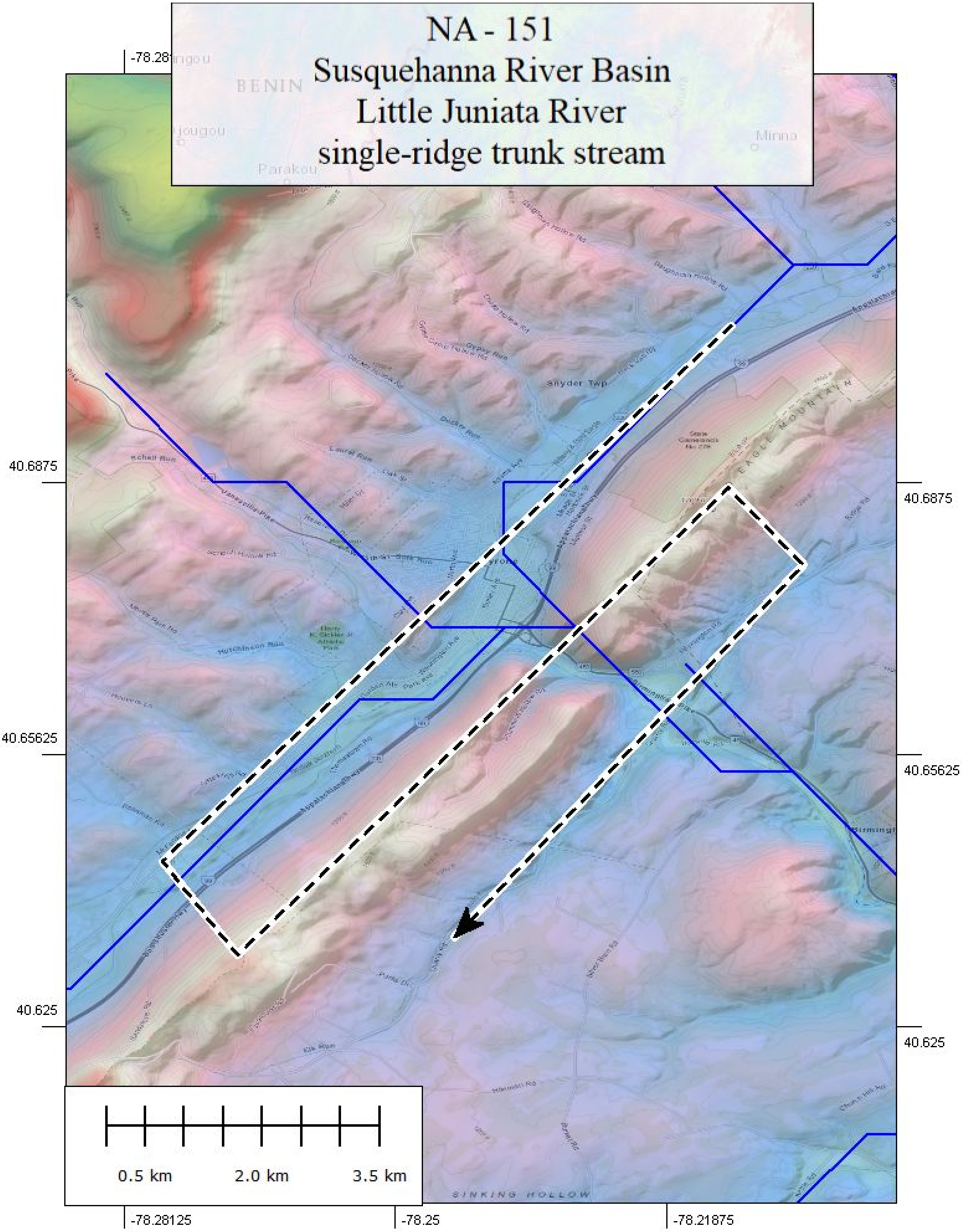
NA - 151
Susquehanna River Basin
Little Juniata River
single-ridge trunk stream
-78.28125
-78.25
-78.21875
40.6875
40.65625
40.625
0.5 km
2.0 km
3.5 km
Snyder Twp
EAGLE MOUNTAIN
SINKING HOLLOW

NA - 152
James River Basin
Maury River
single-ridge trunk stream

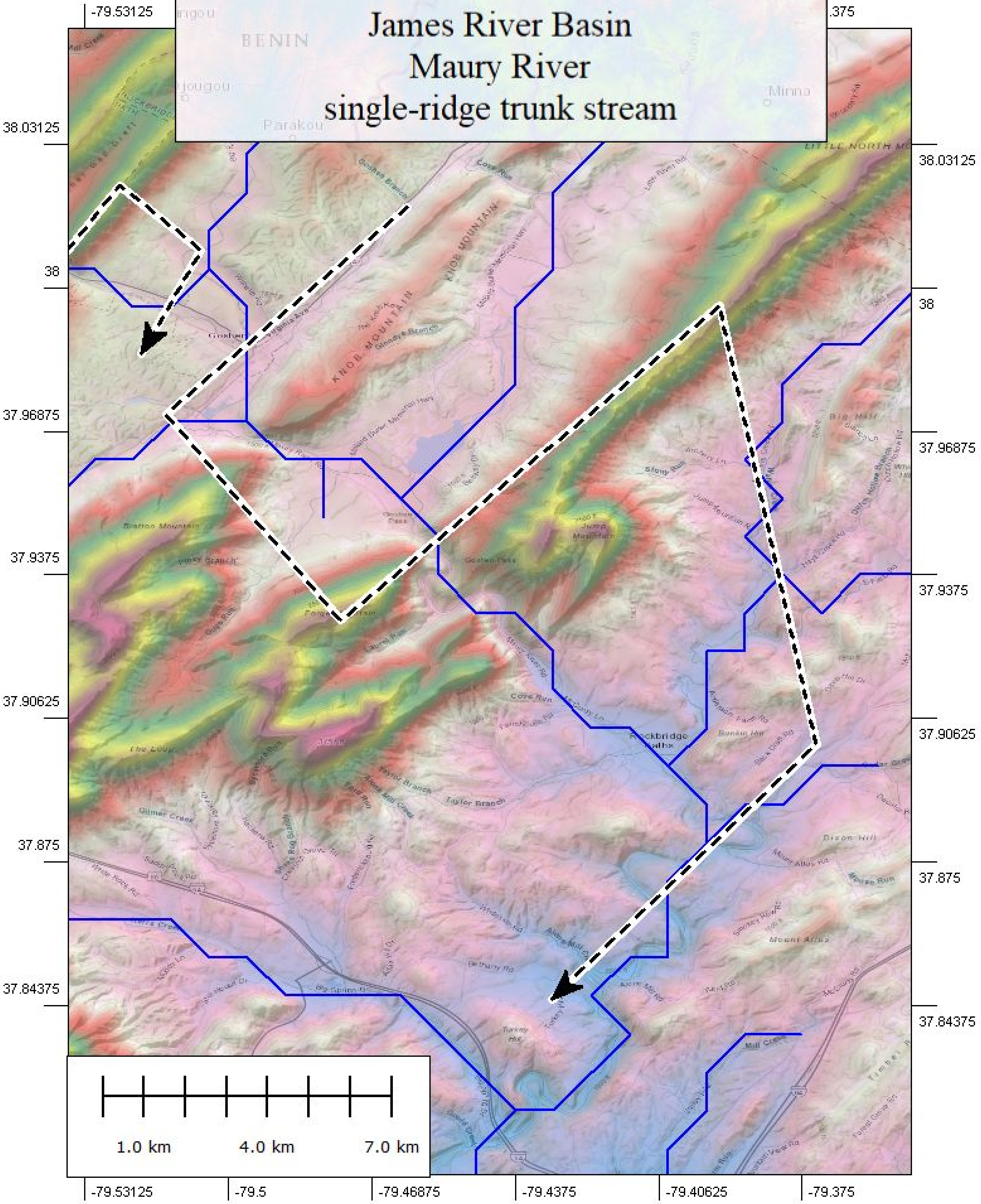

NA - 153
Colorado River (Arizona) Basin
Dolores River
plateau trunk stream

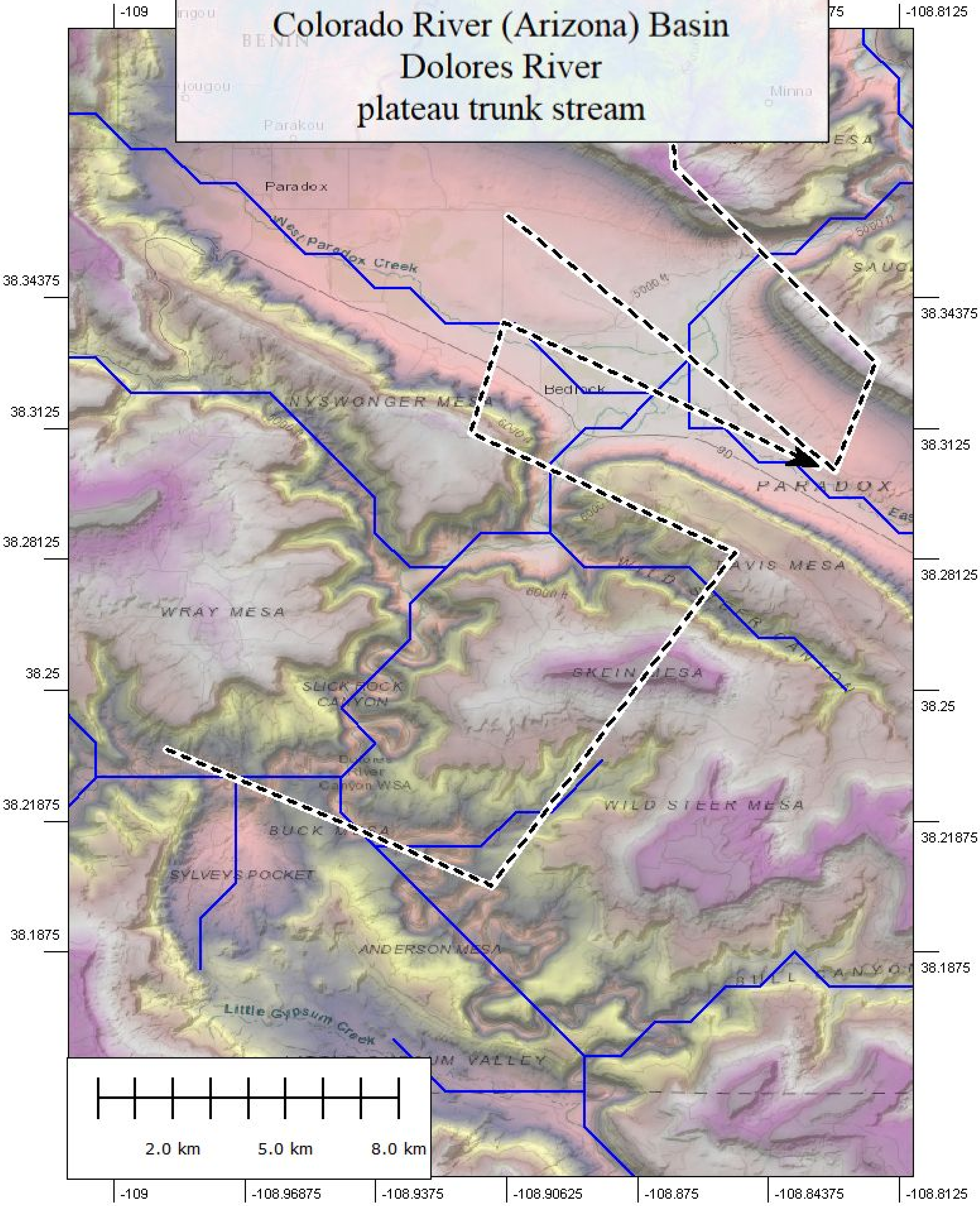

NA - 154
Colorado River (Arizona) Basin
Green River
irregular high ground trunk stream

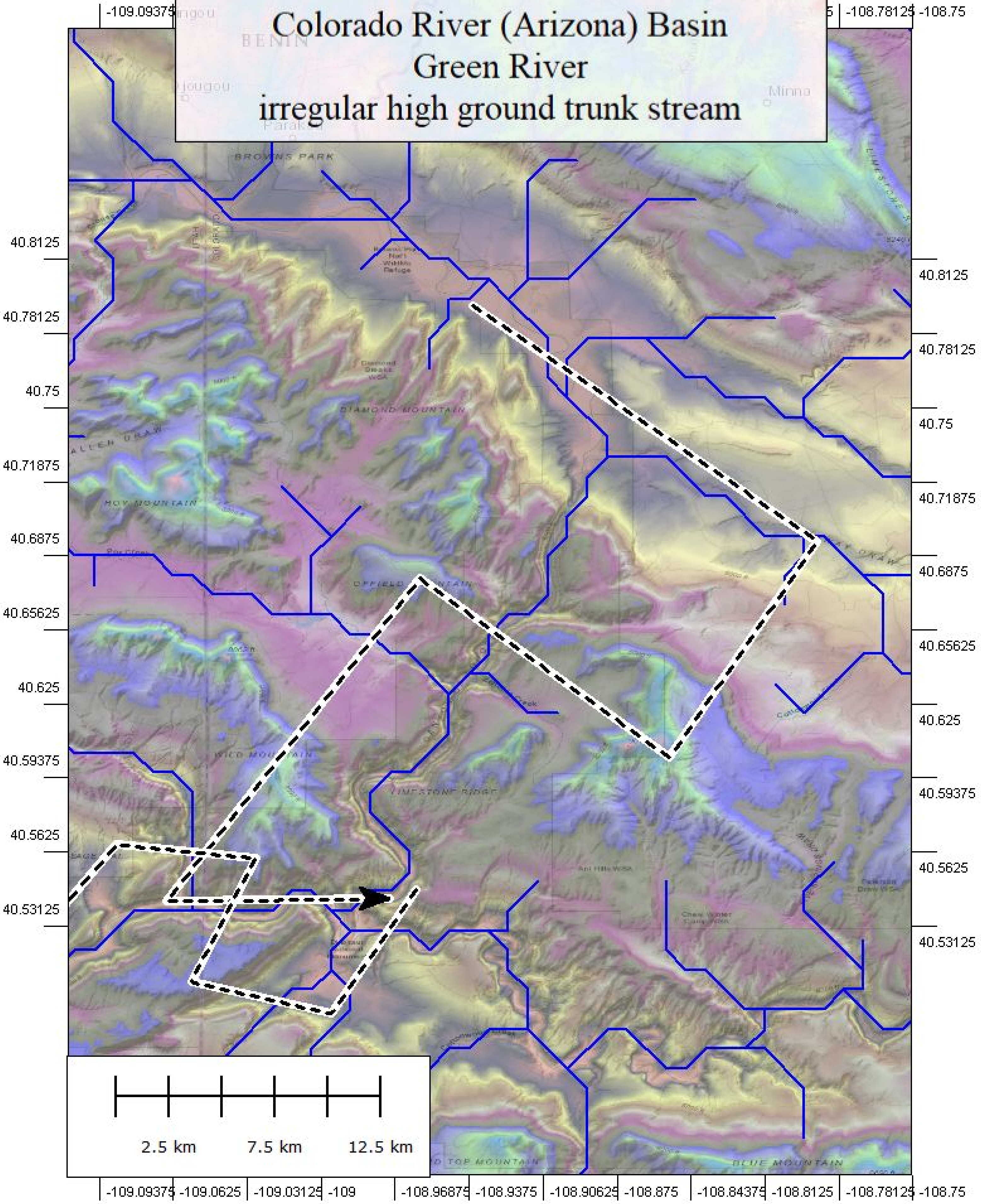

NA - 155
Colorado River (Arizona) Basin
Green River
irregular high ground trunk stream

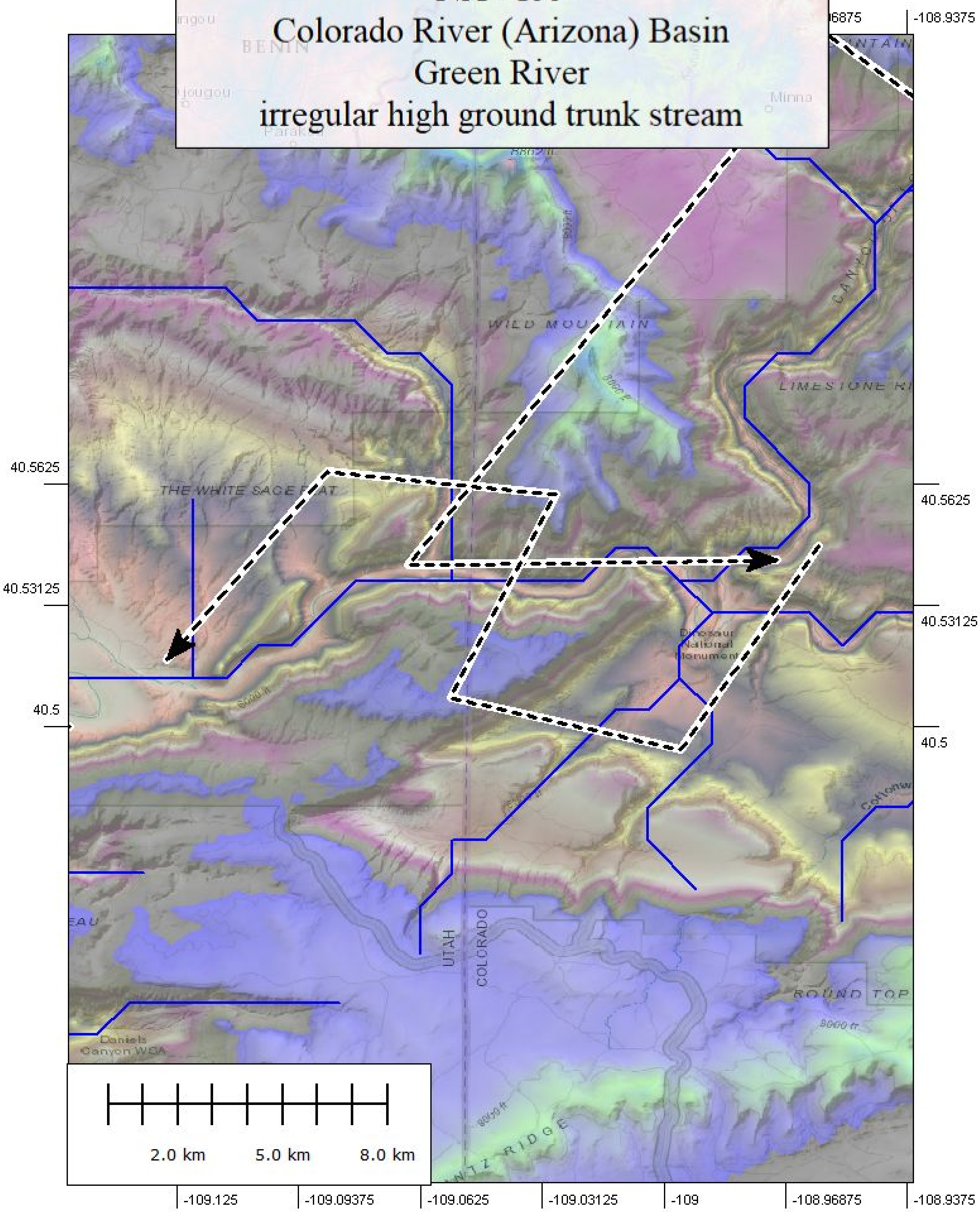

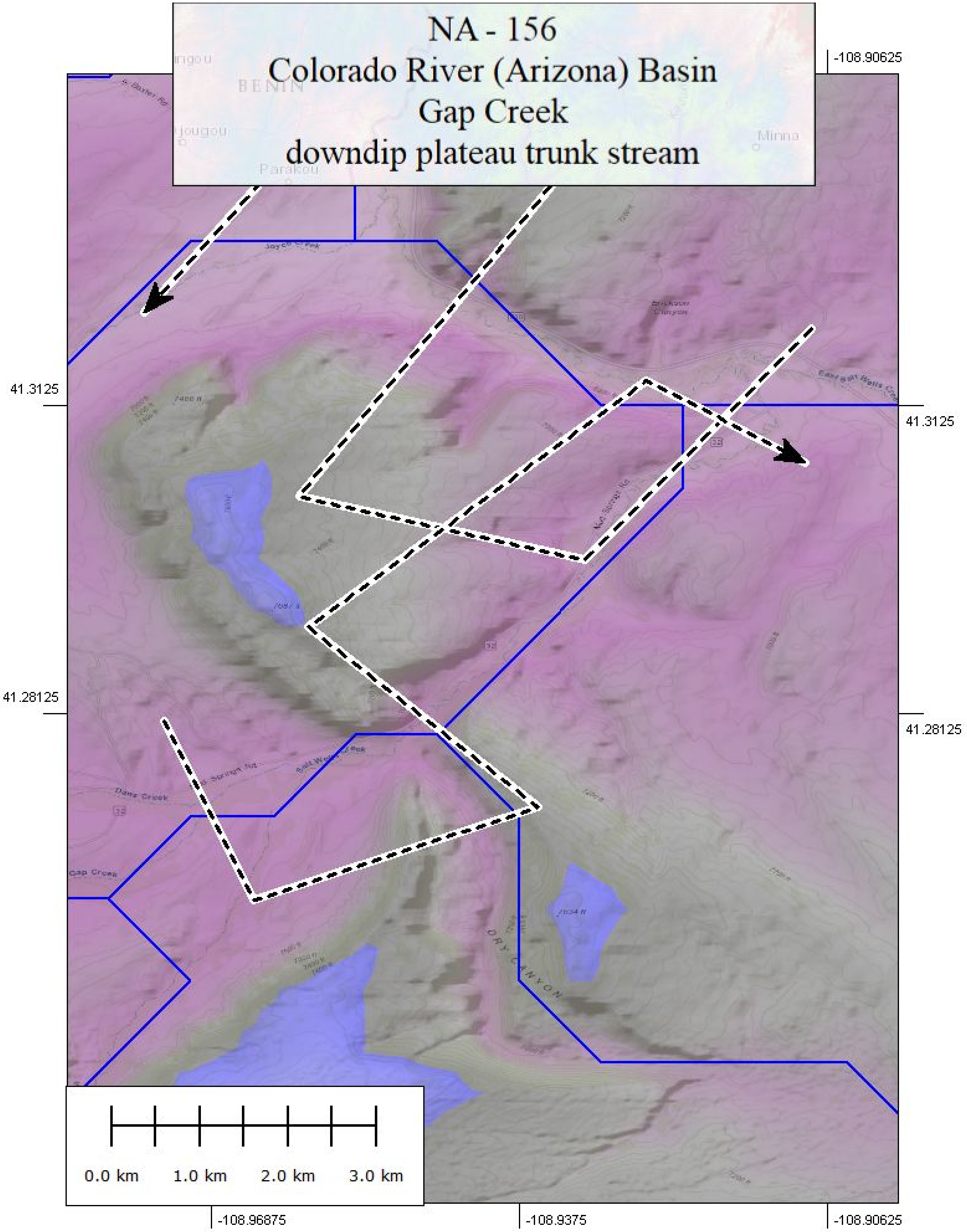
NA - 156
Colorado River (Arizona) Basin
Gap Creek
downdip plateau trunk stream
-108.90625
41.3125
41.3125
41.28125
41.28125
0.0 km
1.0 km
2.0 km
3.0 km
-108.96875
-108.9375
-108.90625

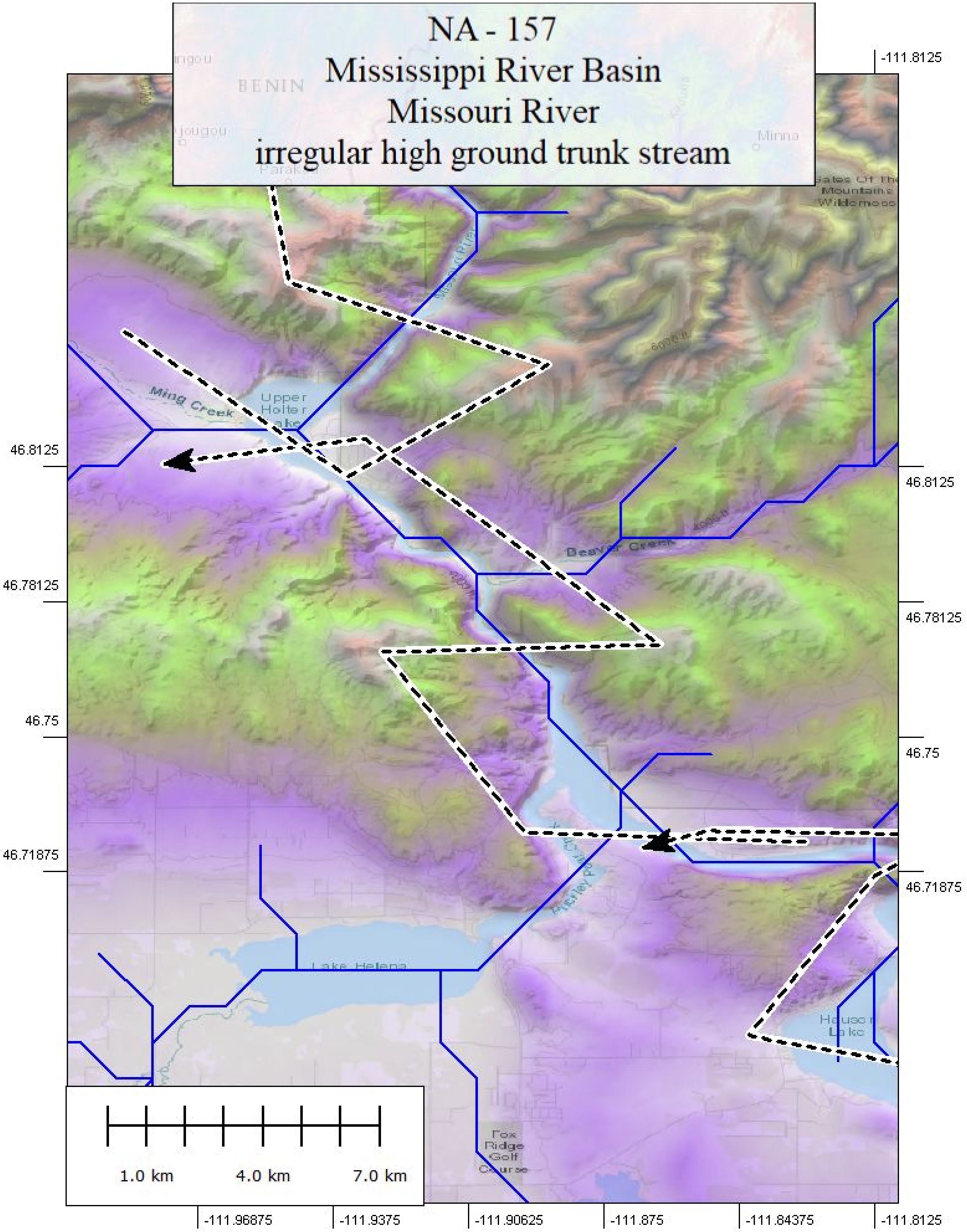
NA - 157
Mississippi River Basin
Missouri River
irregular high ground trunk stream
BENIN
Minna
Gates Of The
Mountains
Wilderness
Missouri River
Ming Creek
Upper
Holter
Lake
Beaver Creek
Lake Helena
Hauser
Lake
Fox
Ridge
Golf
Course
1.0 km
4.0 km
7.0 km
-111.8125
46.8125
46.78125
46.75
46.71875
46.8125
46.78125
46.75
46.71875
-111.96875
-111.9375
-111.90625
-111.875
-111.84375
-111.8125

NA - 158
Pnuco River Basin
Verde River tributary
single-ridge trunk stream

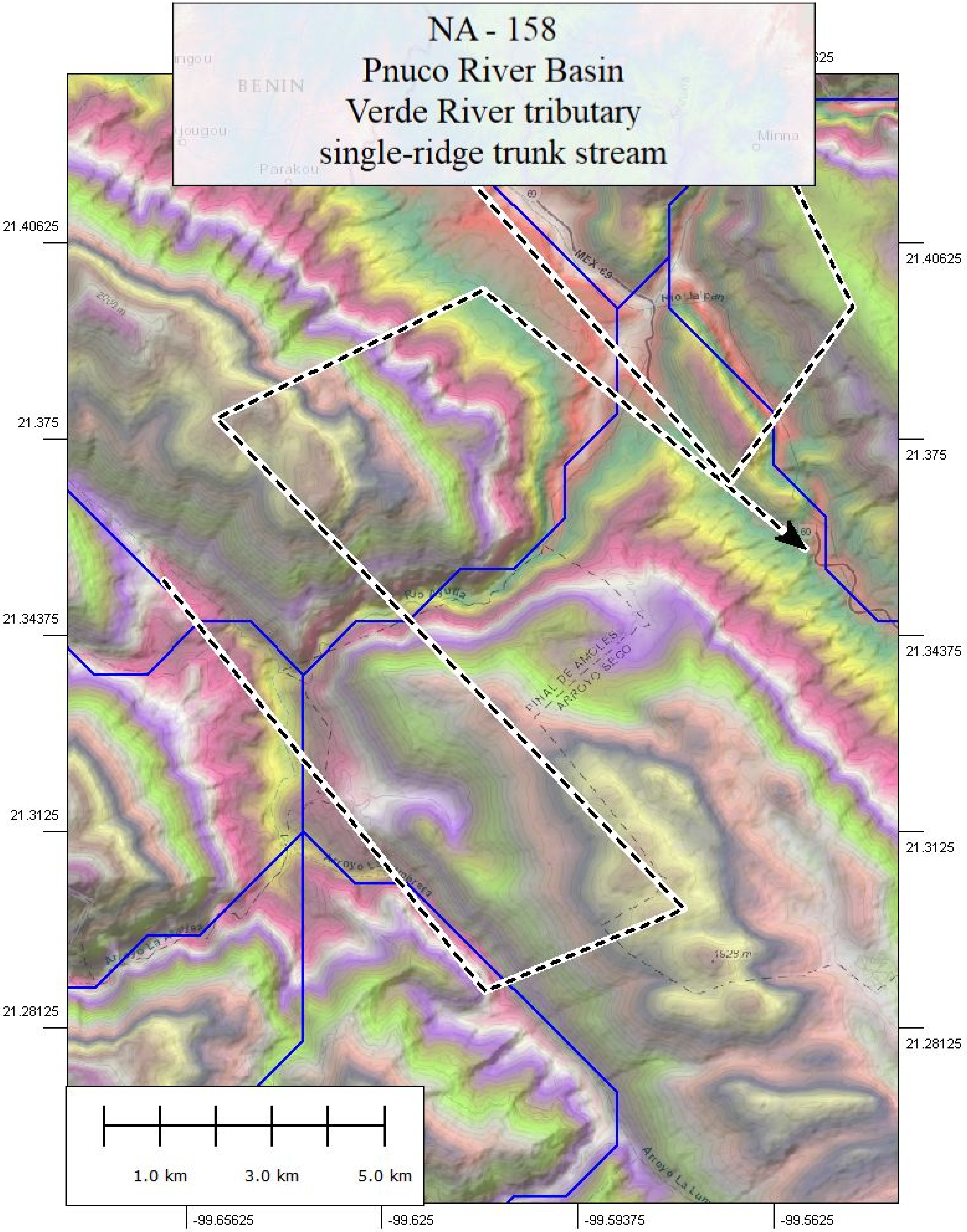

NA - 159
Mississippi River Basin
Badger Creek
irregular high ground trunk stream

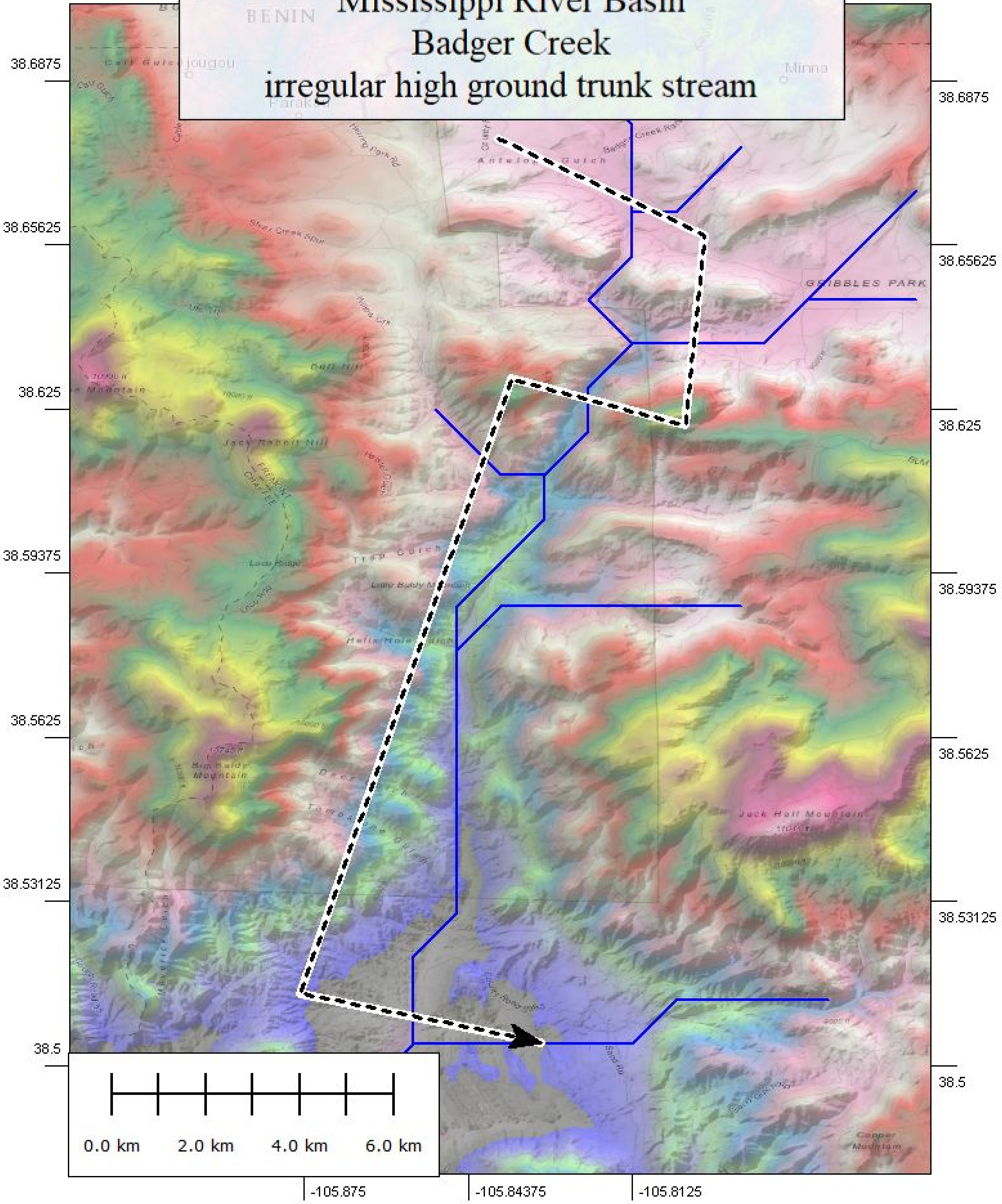

NA - 16
Mackenzie River (Canada) Basin
Coal River tributary
single-ridge trunk stream

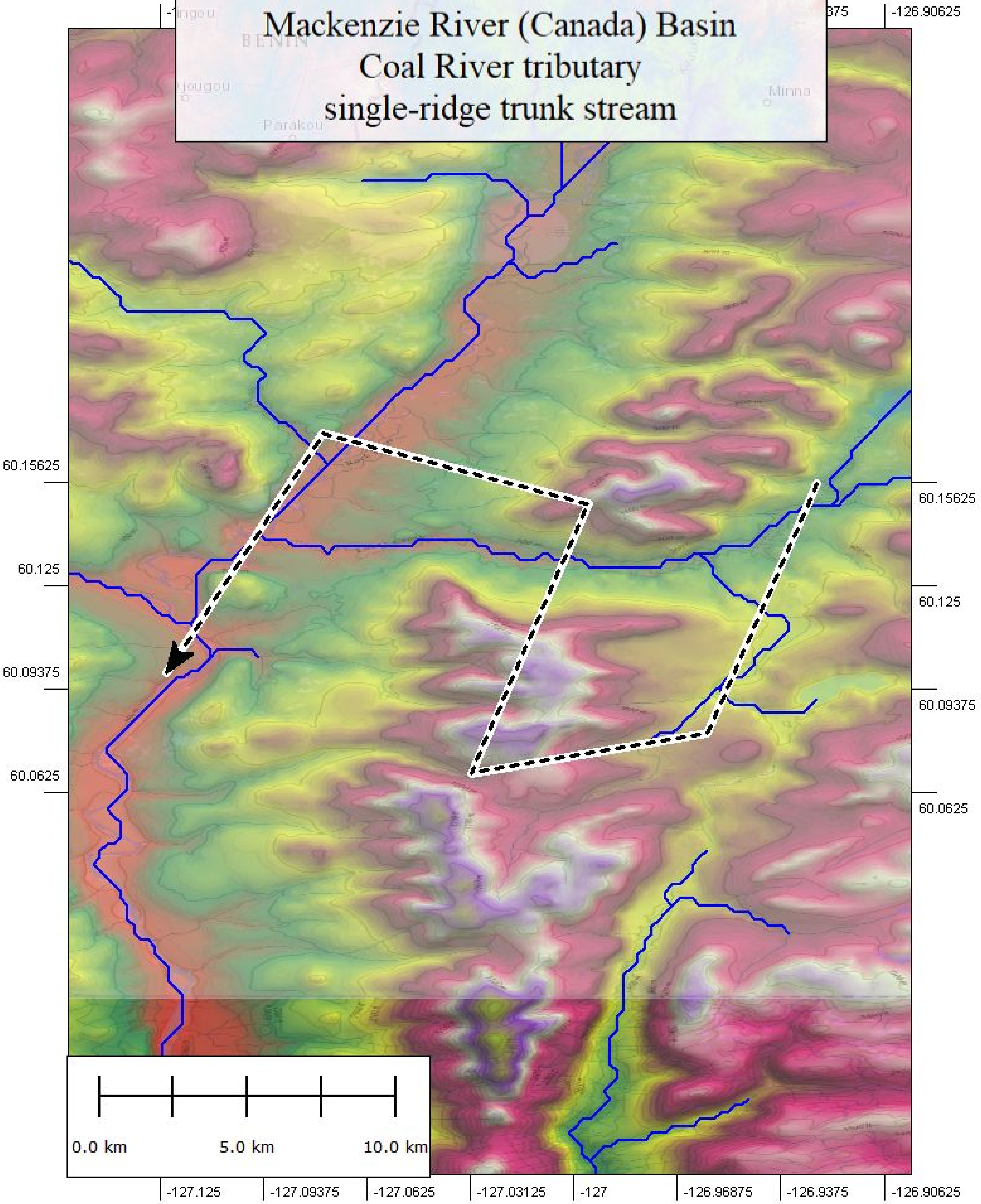

NA - 160
Mississippi River Basin
New River
irregular high ground trunk stream

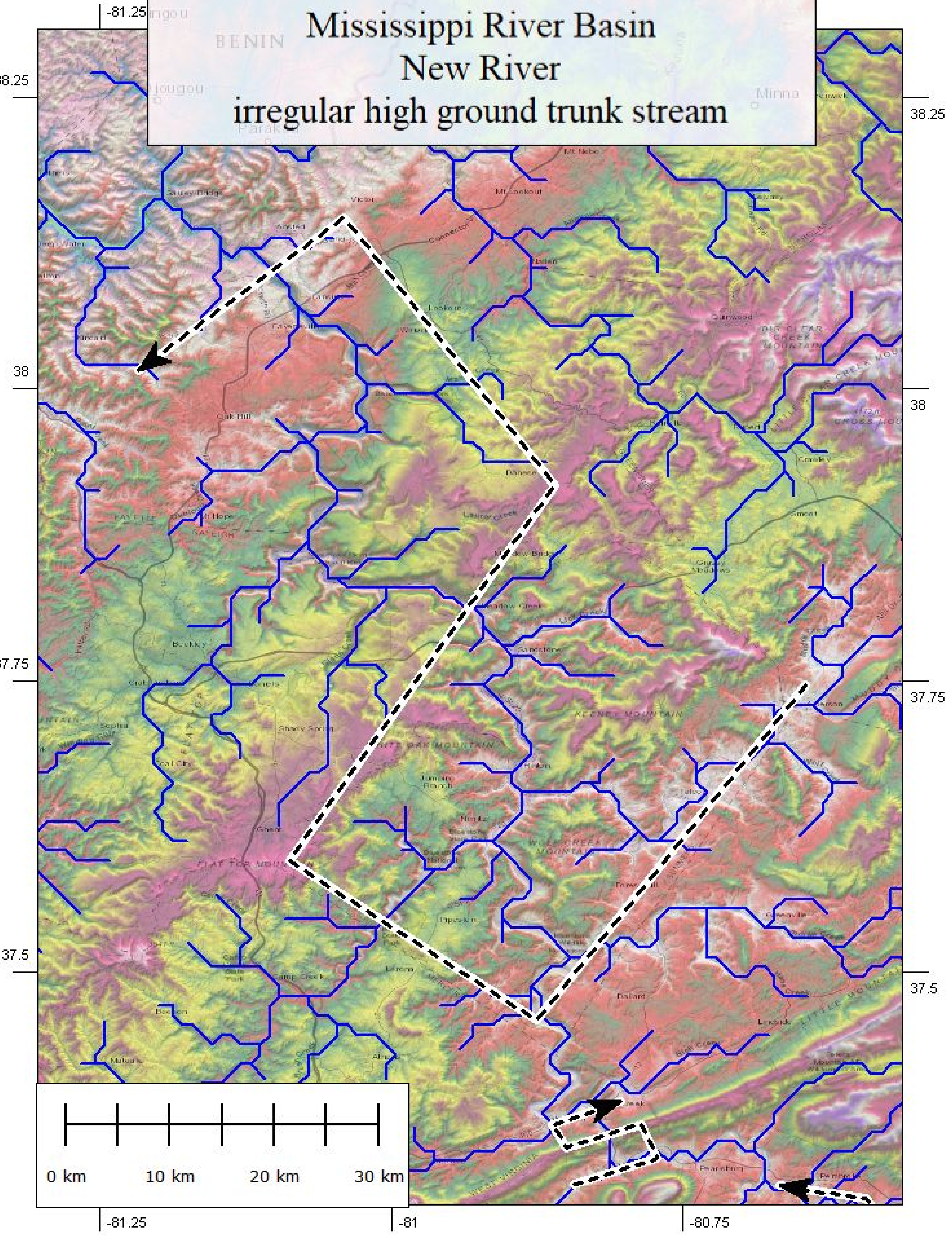

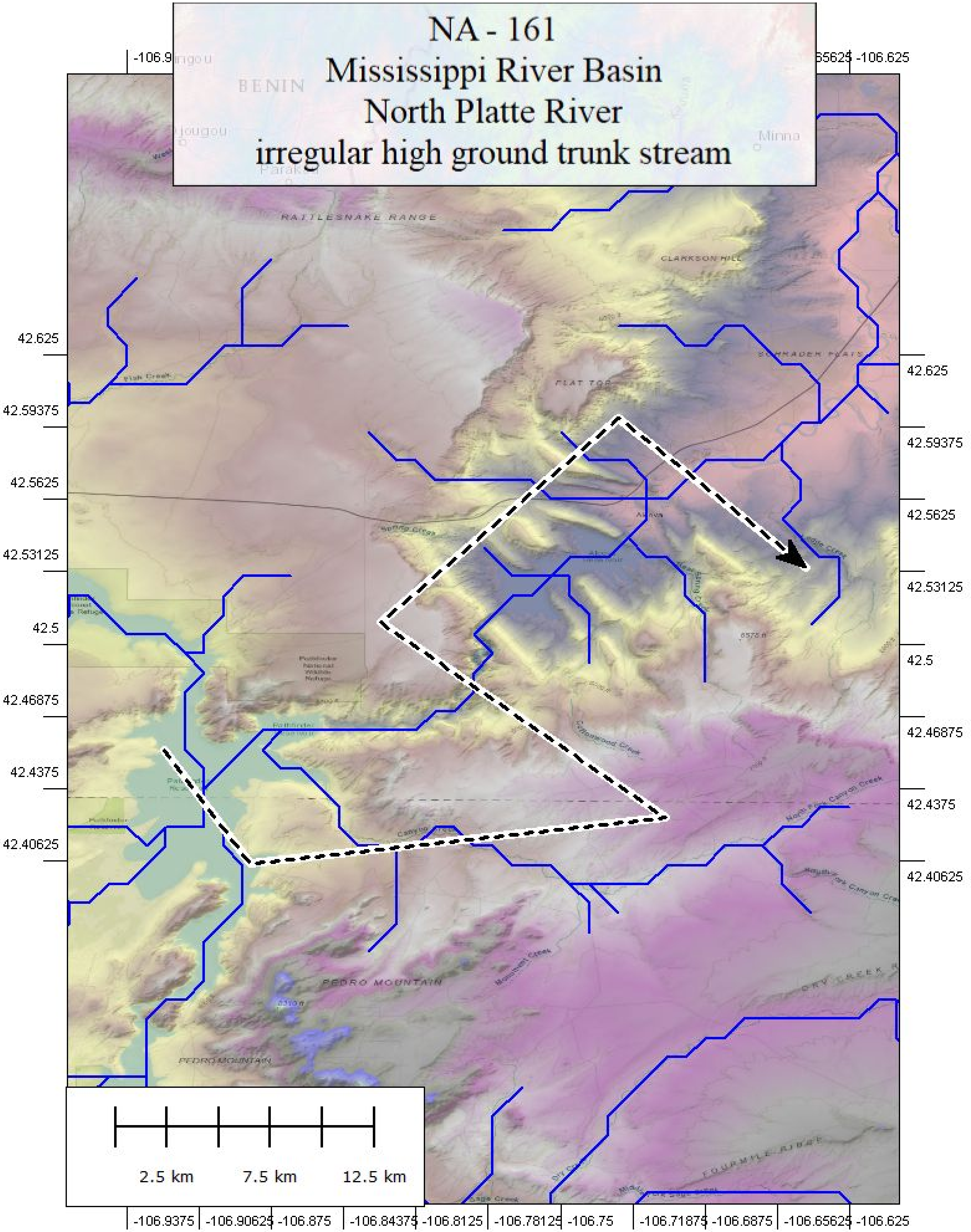
NA - 161
Mississippi River Basin
North Platte River
irregular high ground trunk stream
RATTLESNAKE RANGE
CLARKSON HILL
SCHRADER FLATS
FLAT TOP
Fish Creek
PEDRO MOUNTAIN
FOURMILE RIDGE
DRY CREEK
2.5 km
7.5 km
12.5 km
42.625
42.59375
42.5625
42.53125
42.5
42.46875
42.4375
42.40625
-106.9375
-106.90625
-106.875
-106.84375
-106.8125
-106.78125
-106.75
-106.71875
-106.6875
-106.65625
-106.625

NA - 162
Mississippi River Basin
Kortes Reservoir
single-ridge trunk stream

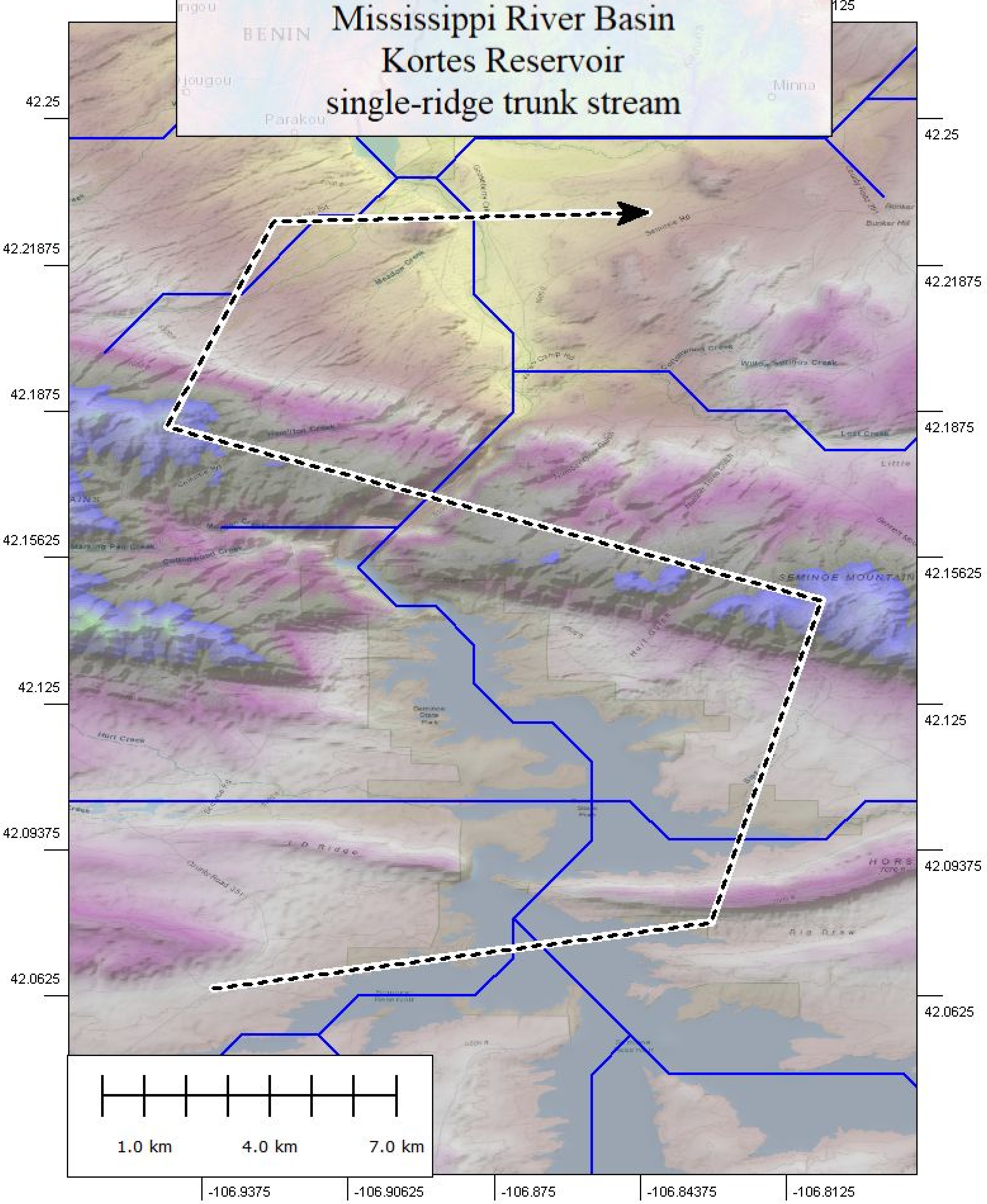

NA - 163
Columbia River Basin
Owyhee River
irregular high ground trunk stream

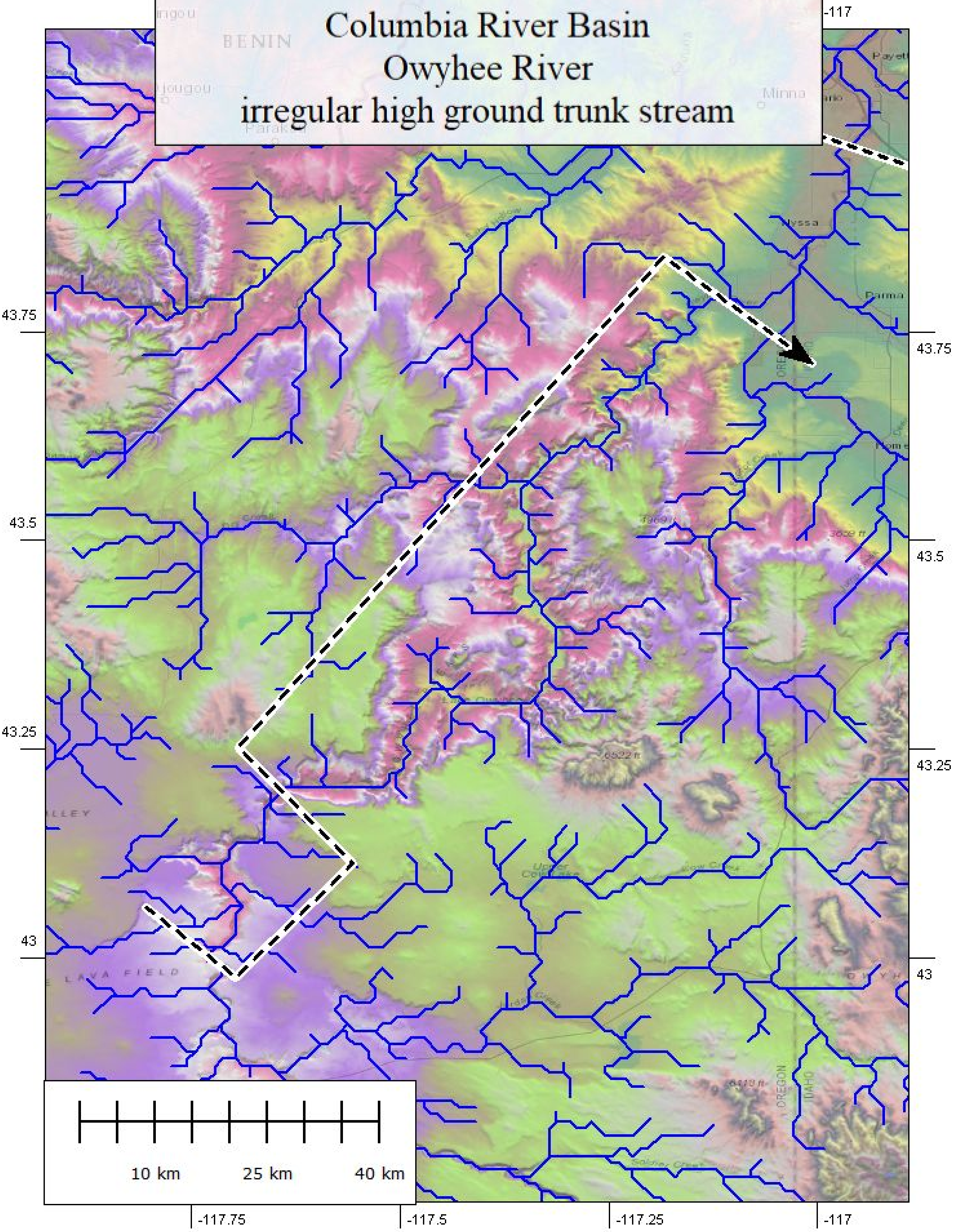

NA - 164
Columbia River Basin
Wild Horse Reservoir tributary
irregular high ground trunk stream

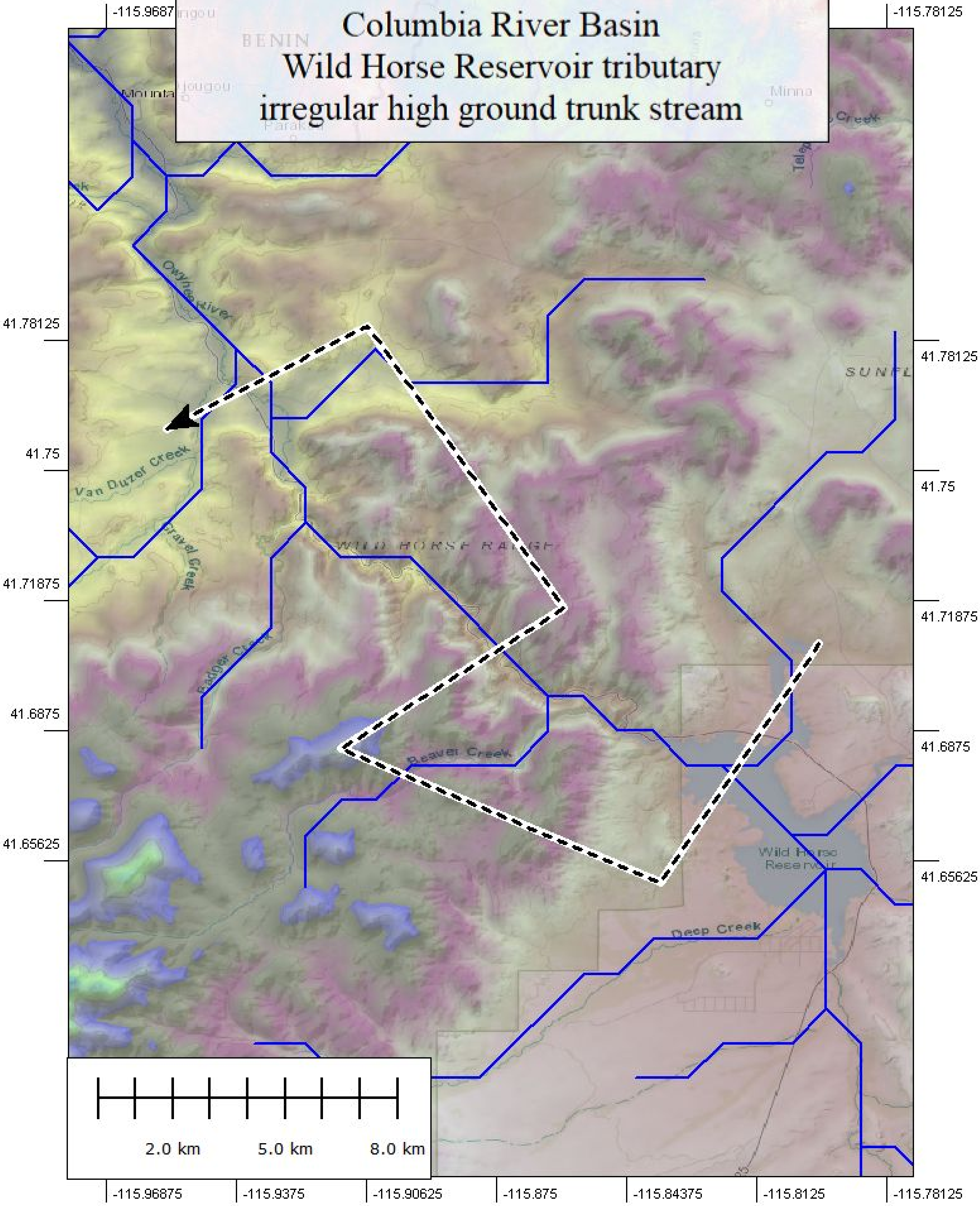

NA - 165
Columbia River Basin
Bull Run, NV
irregular high ground trunk stream

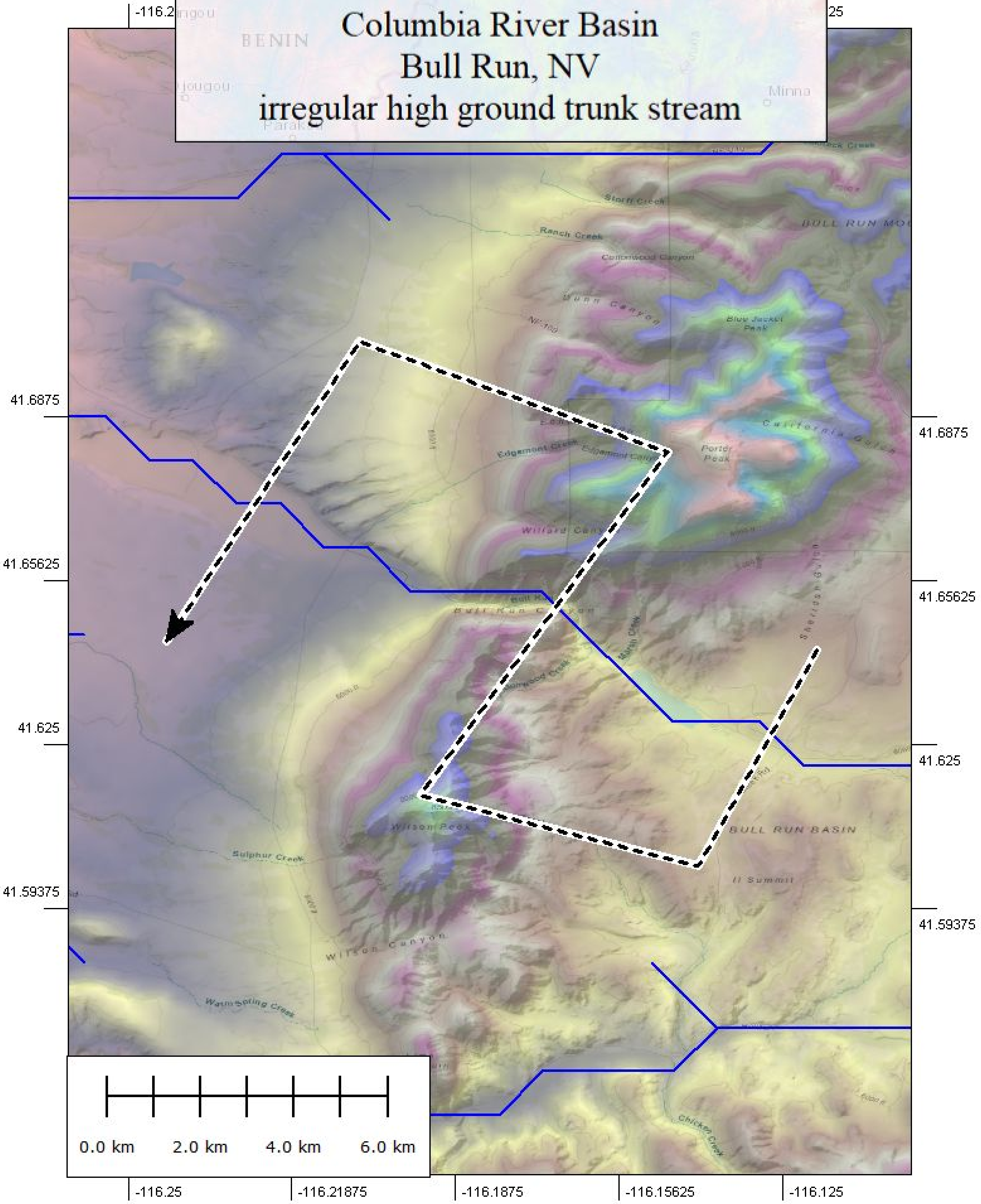

NA - 166
Columbia River Basin
Portneuf River
single-ridge trunk stream

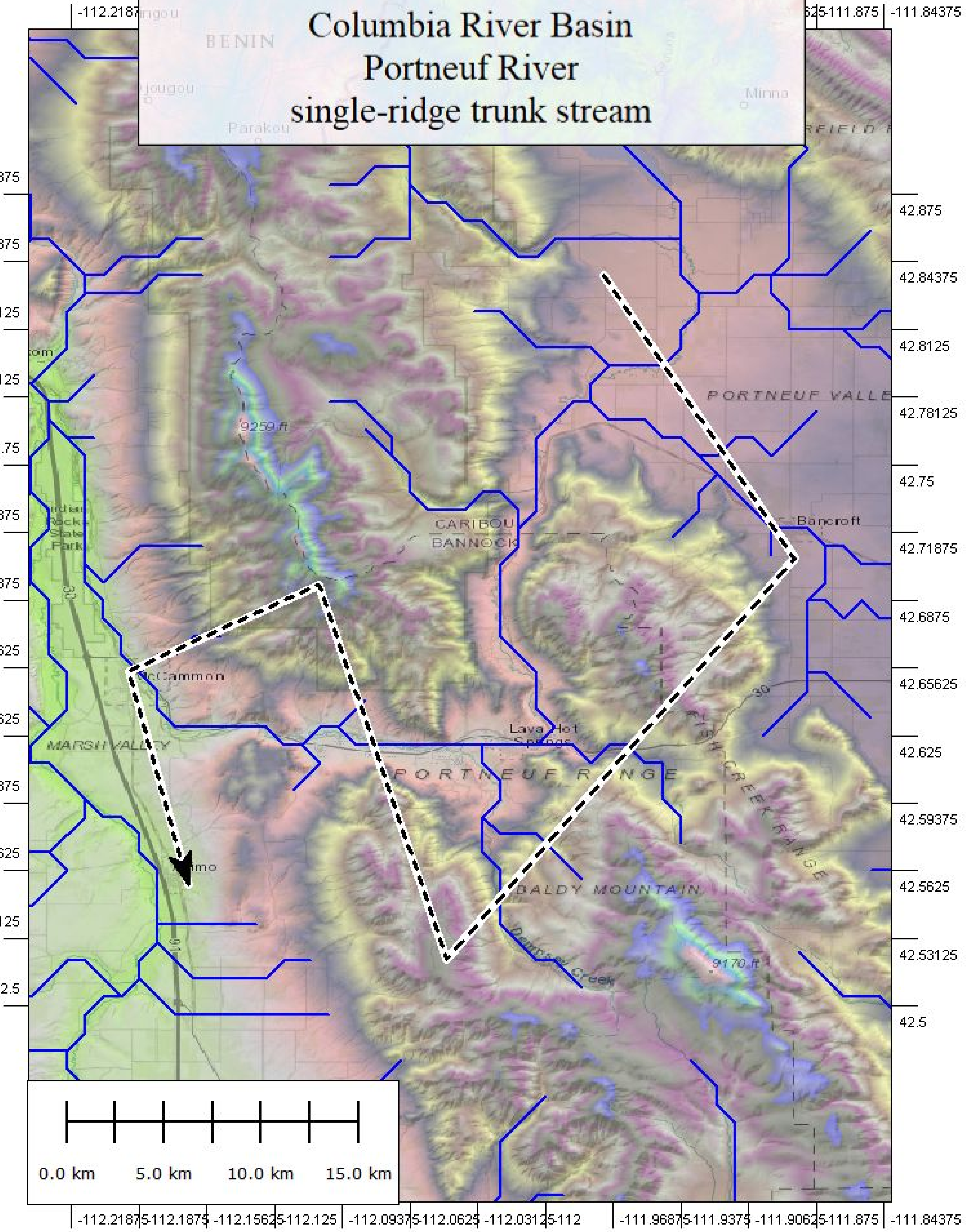

NA - 167
Potomac River Basin
Harper's Ferry
multi-ridge trunk stream

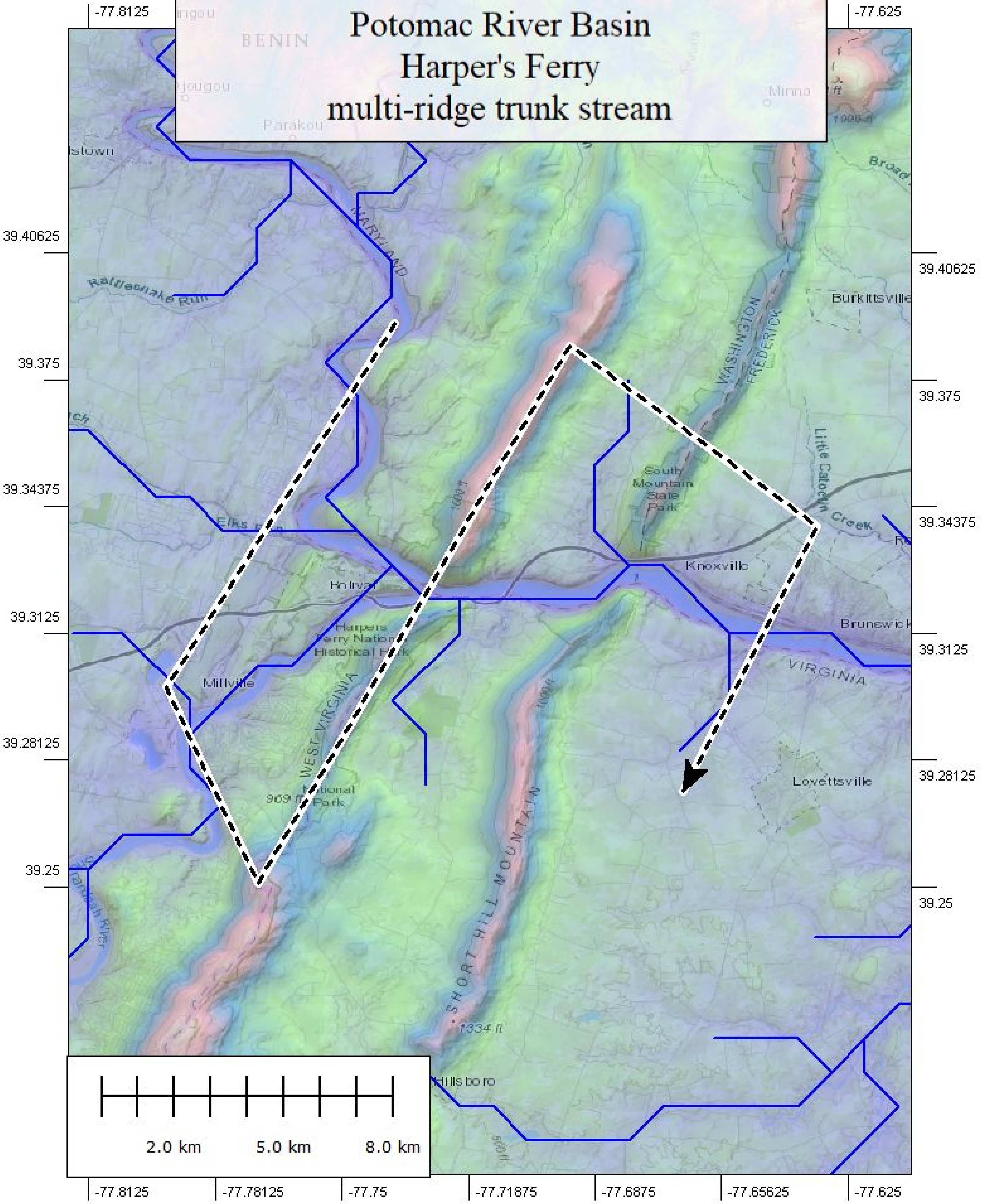

NA - 168
Potomac River Basin
Potomac River
single-ridge trunk stream

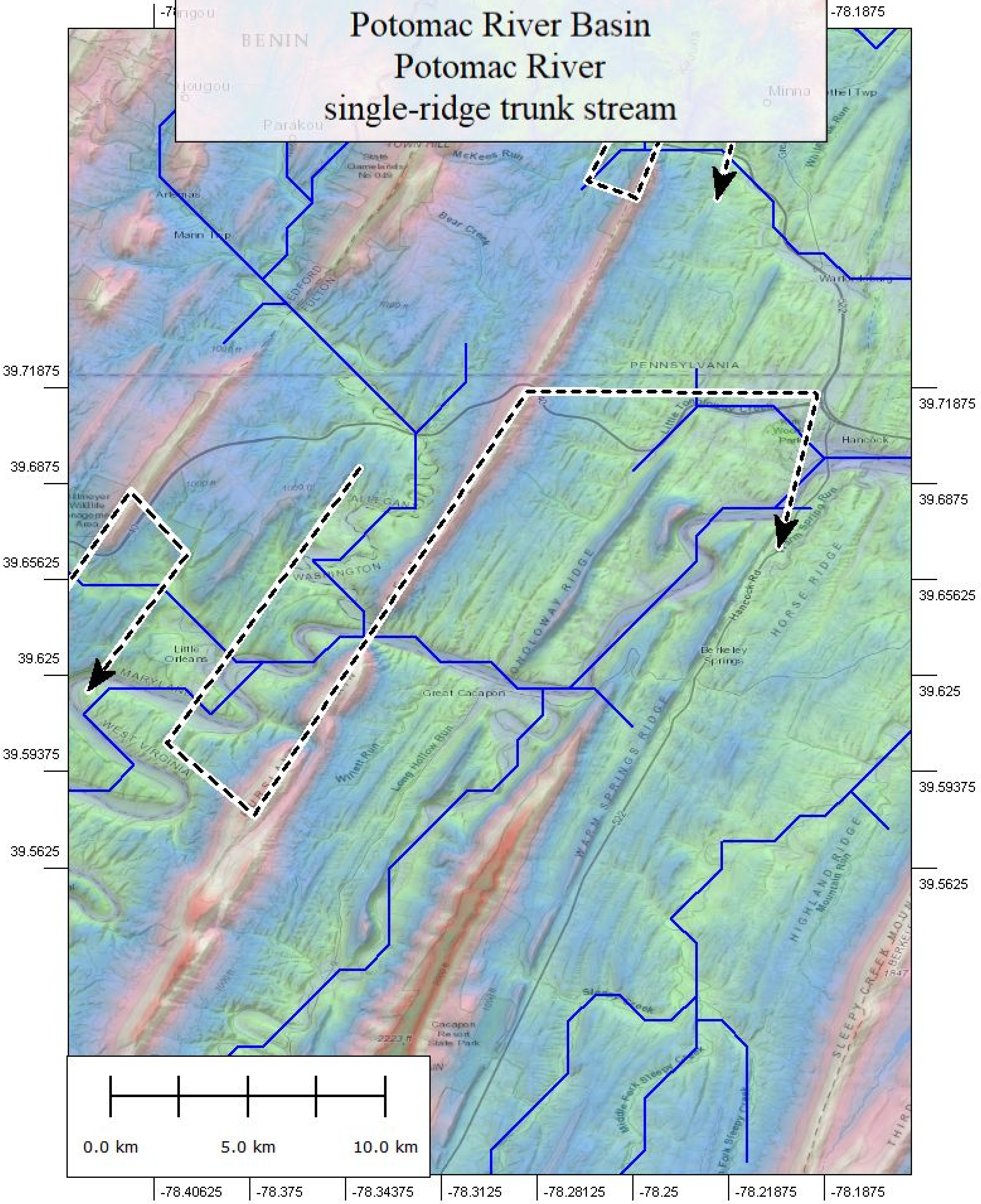

NA - 169
Endorheic basin Basin
Provo River
irregular high ground trunk stream

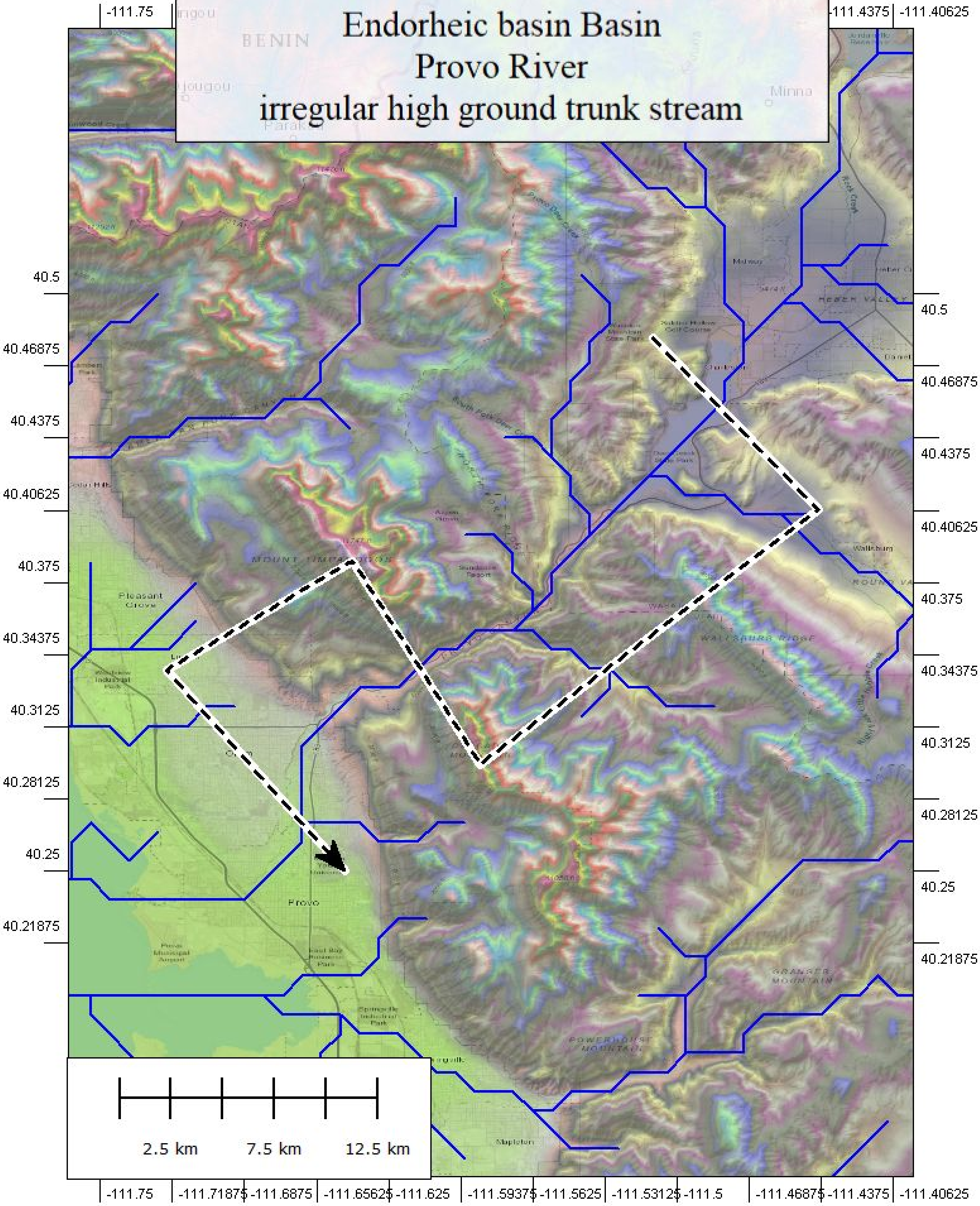

NA - 17
Colorado River (Arizona) Basin
Green River
irregular high ground trunk stream

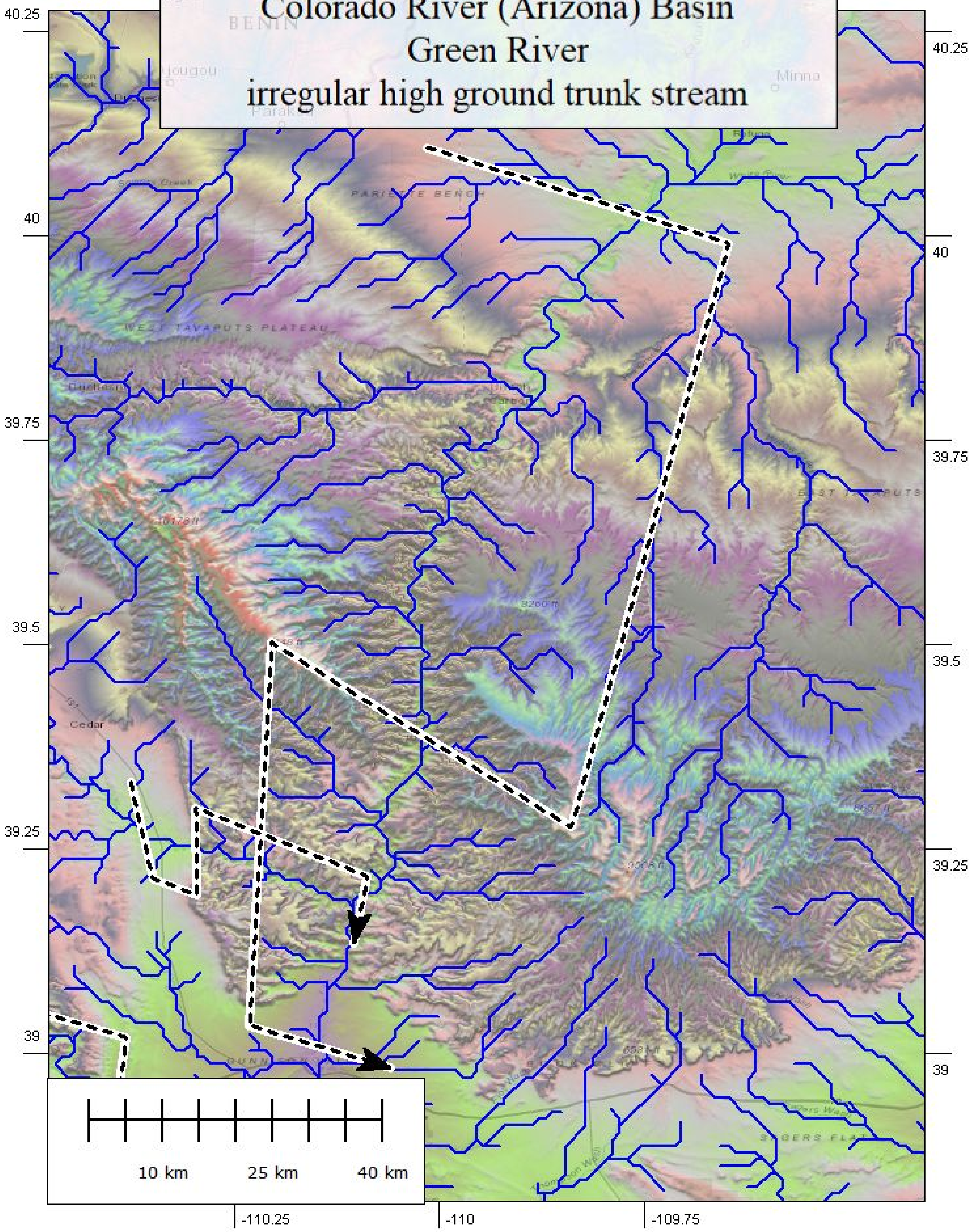

NA - 170
Mississippi River Basin
Nolichucky River
single-ridge trunk stream

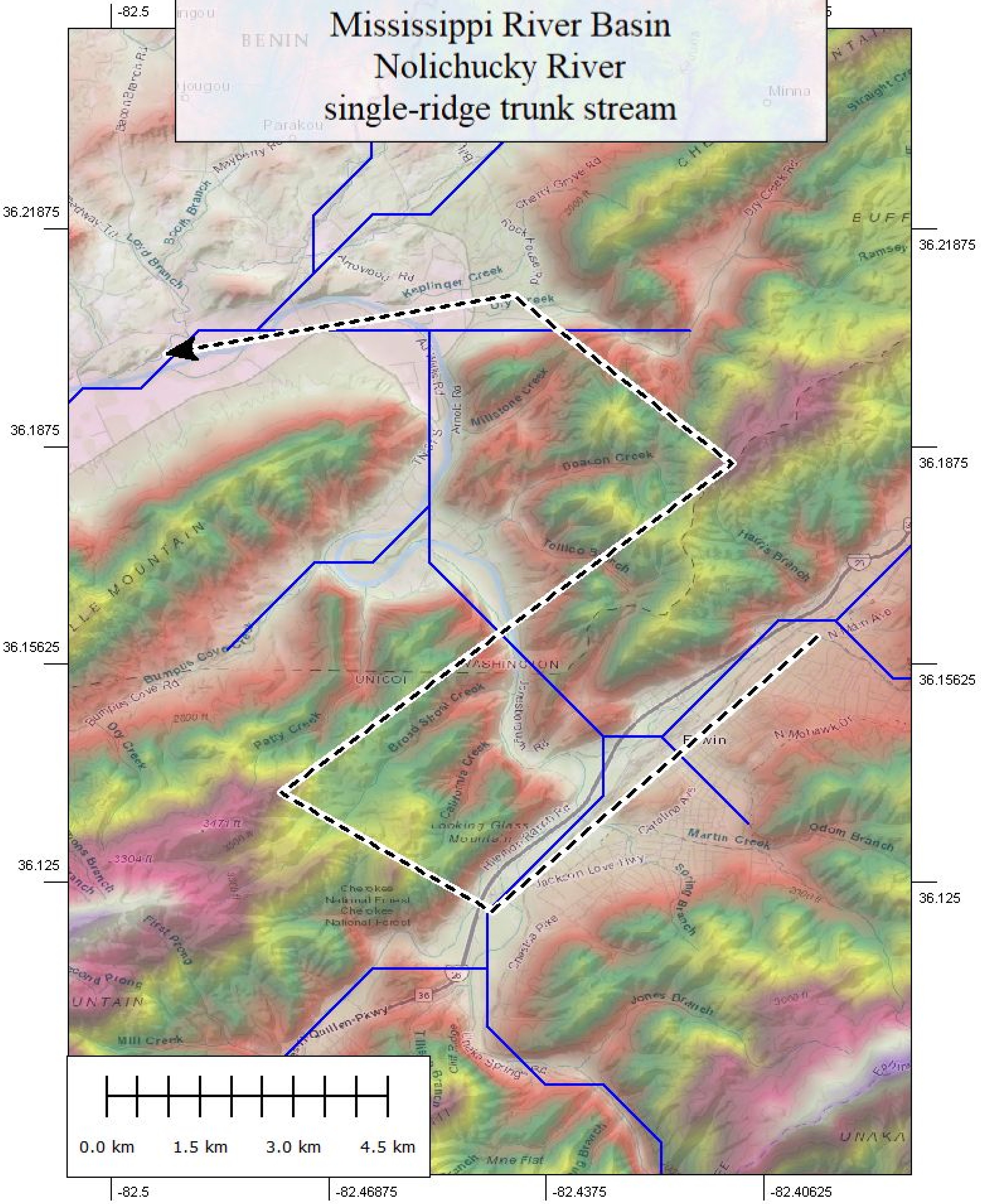

NA - 171
Coahuayana River Basin
Trojes Reservoir tributary
single-ridge trunk stream

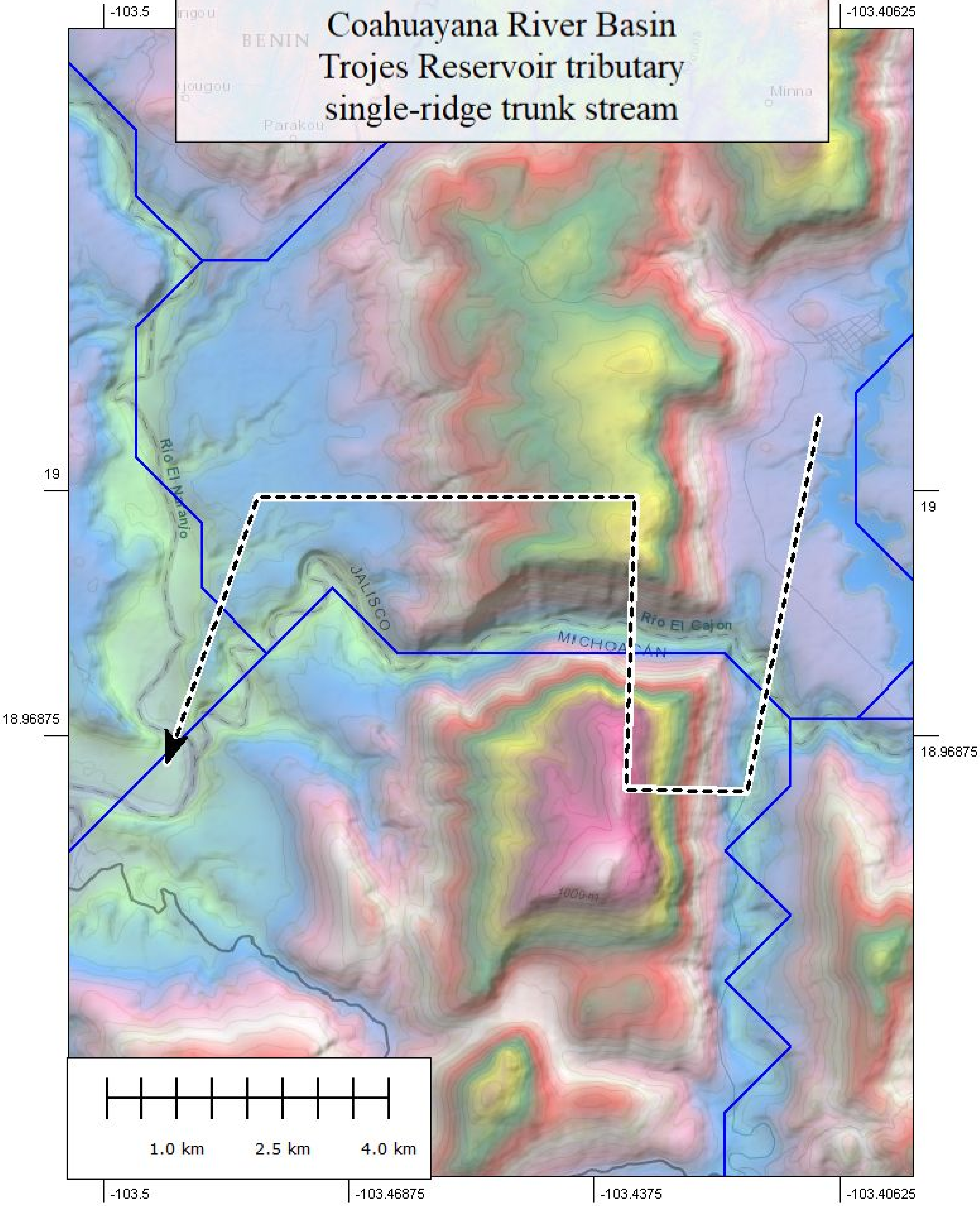

NA - 172
Endorheic basin Basin
Twin Creek
single-ridge trunk stream

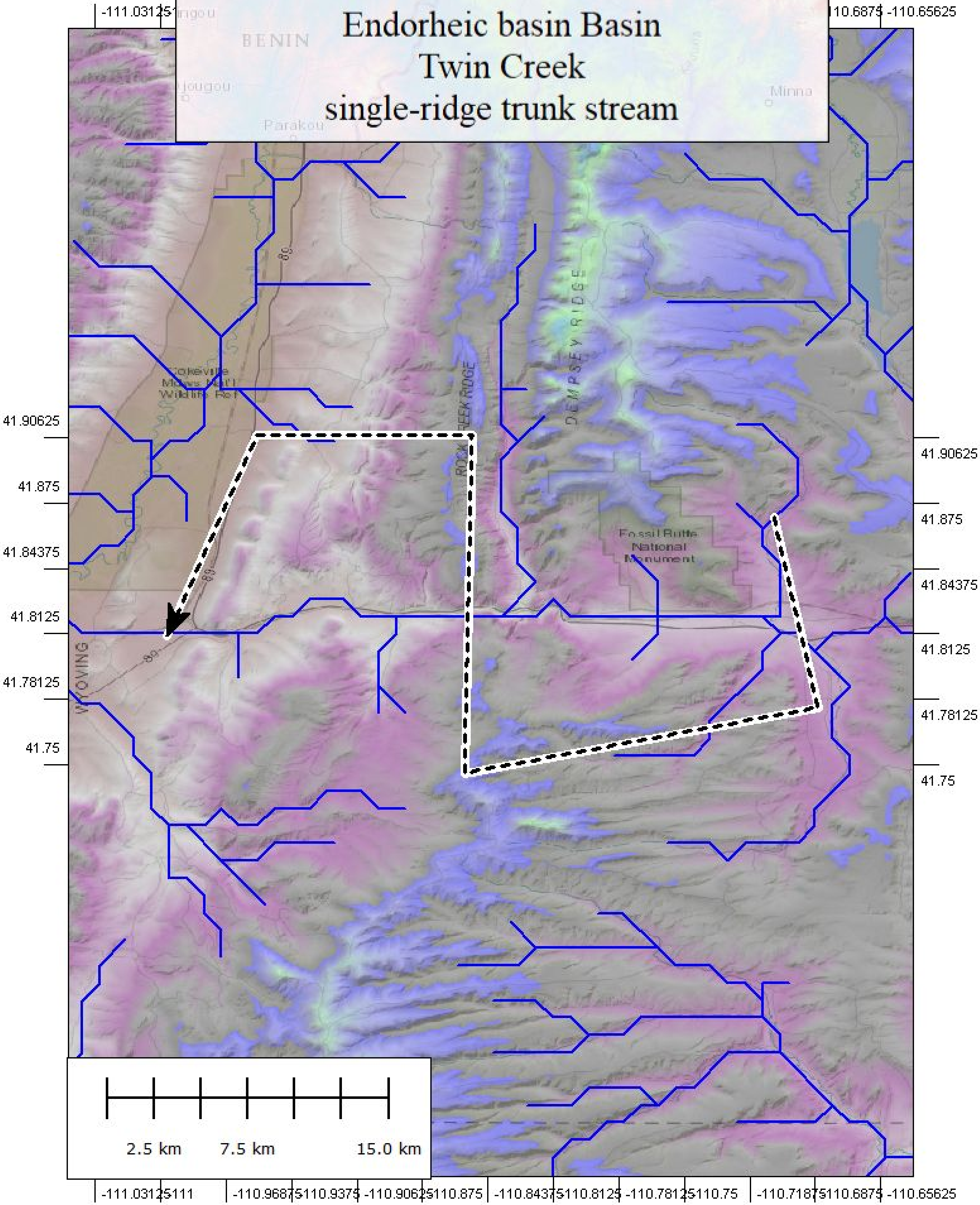

NA - 173
Endorheic basin Basin
Salina Creek
irregular high ground trunk stream

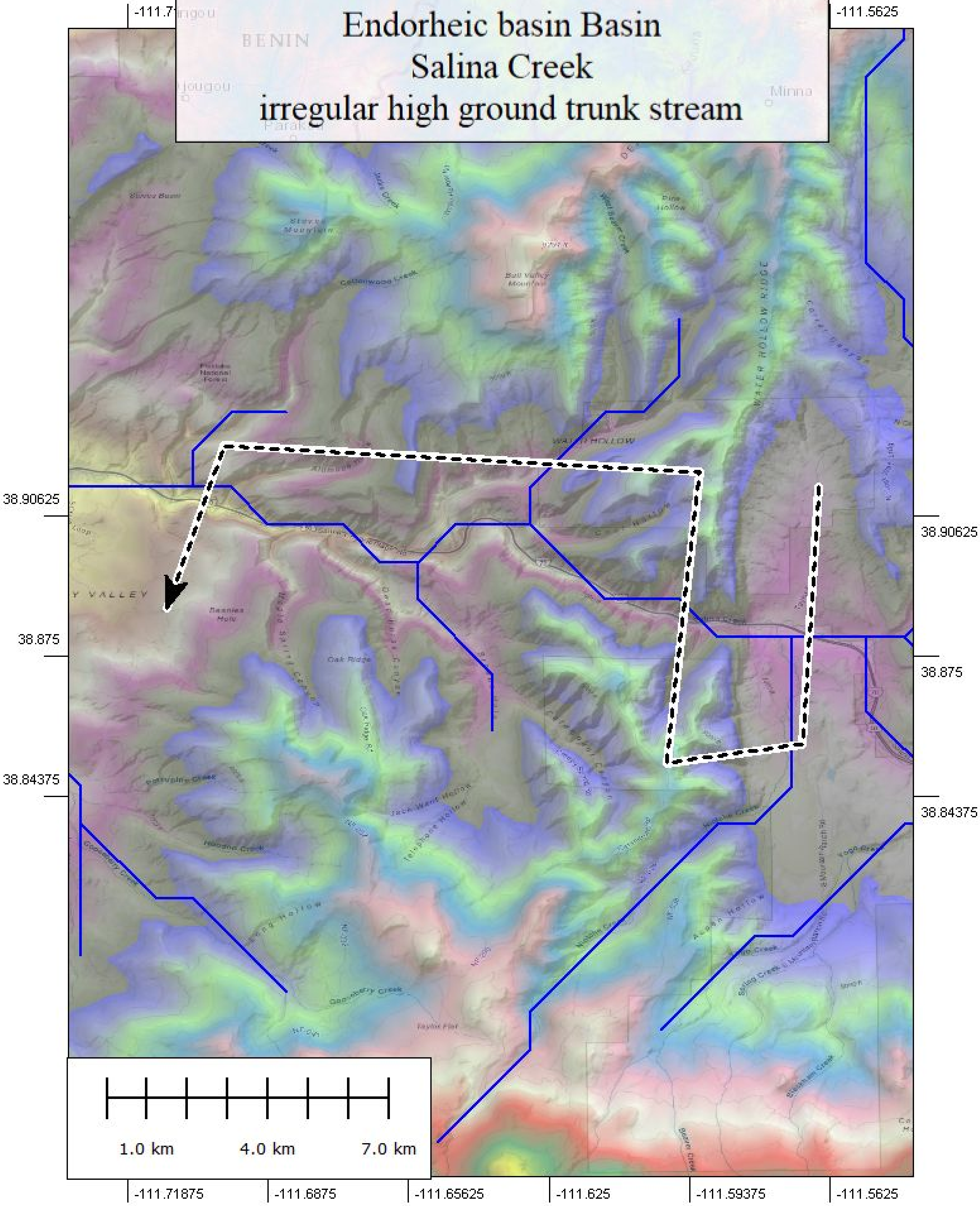

NA - 174
Columbia River Basin
Salmon Falls Creek
irregular high ground trunk stream

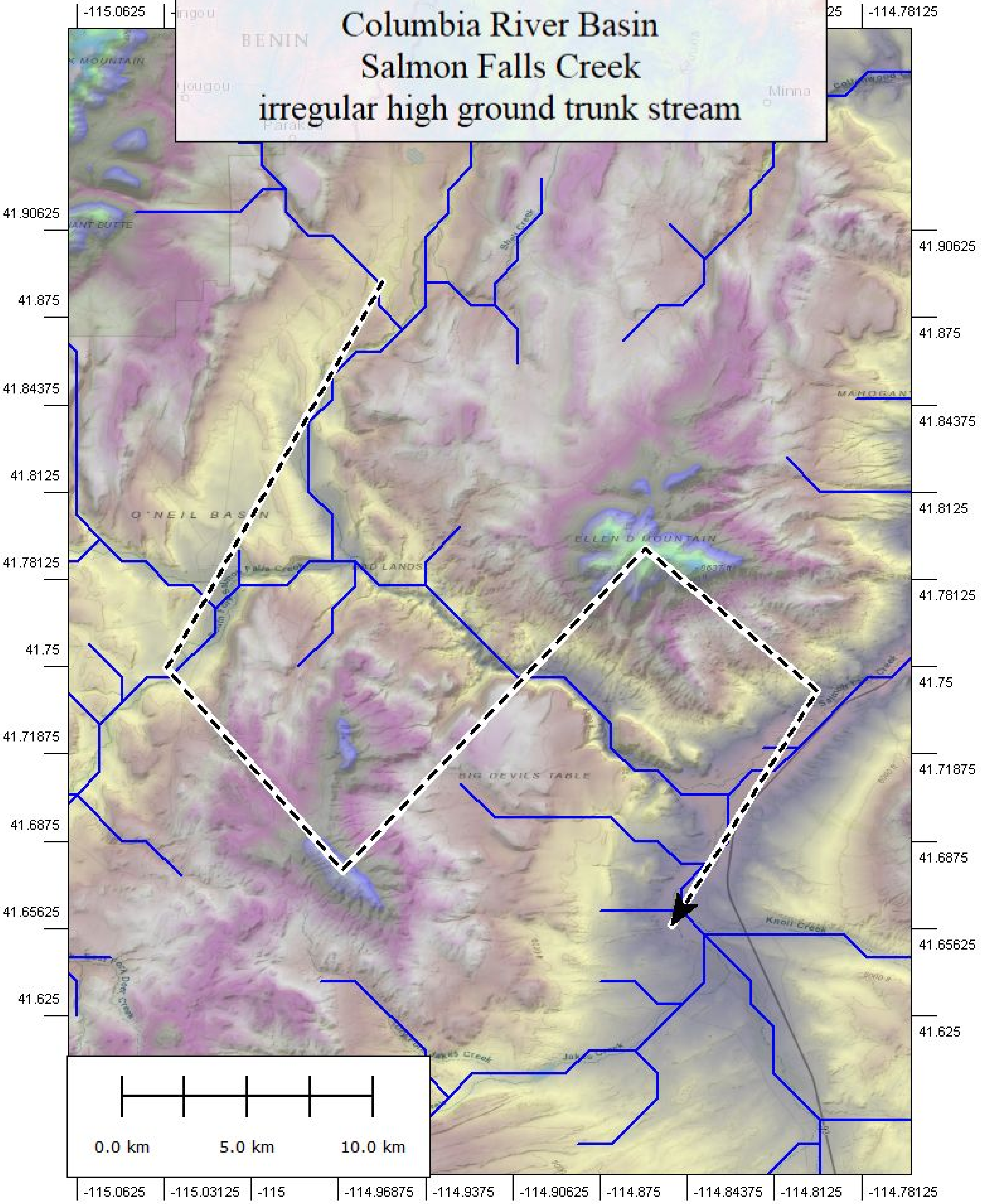

NA - 175
Colorado River (Arizona) Basin
Salt River
irregular high ground trunk stream

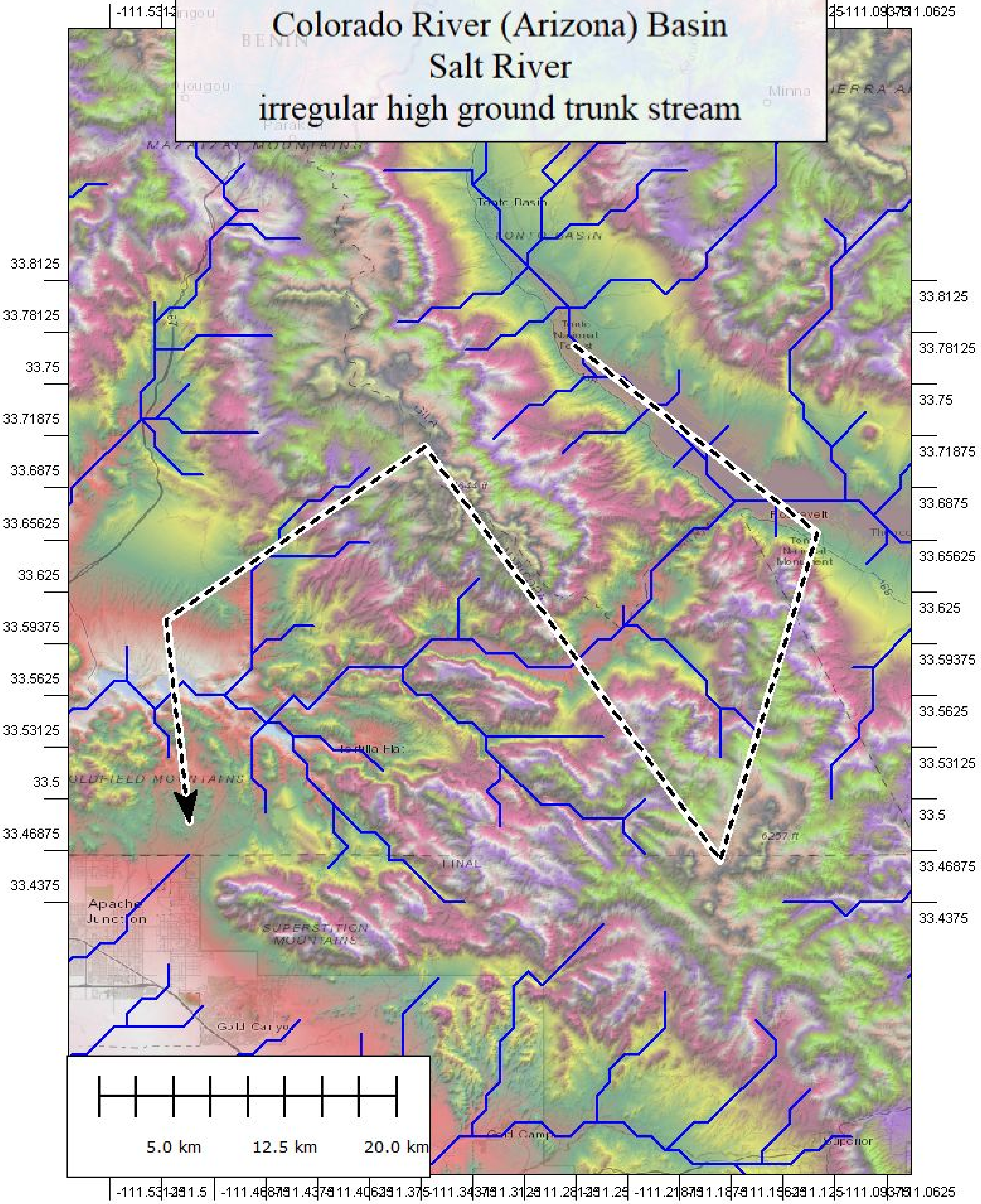

NA - 176
Colorado River (Arizona) Basin
San Francisco River
irregular high ground trunk stream

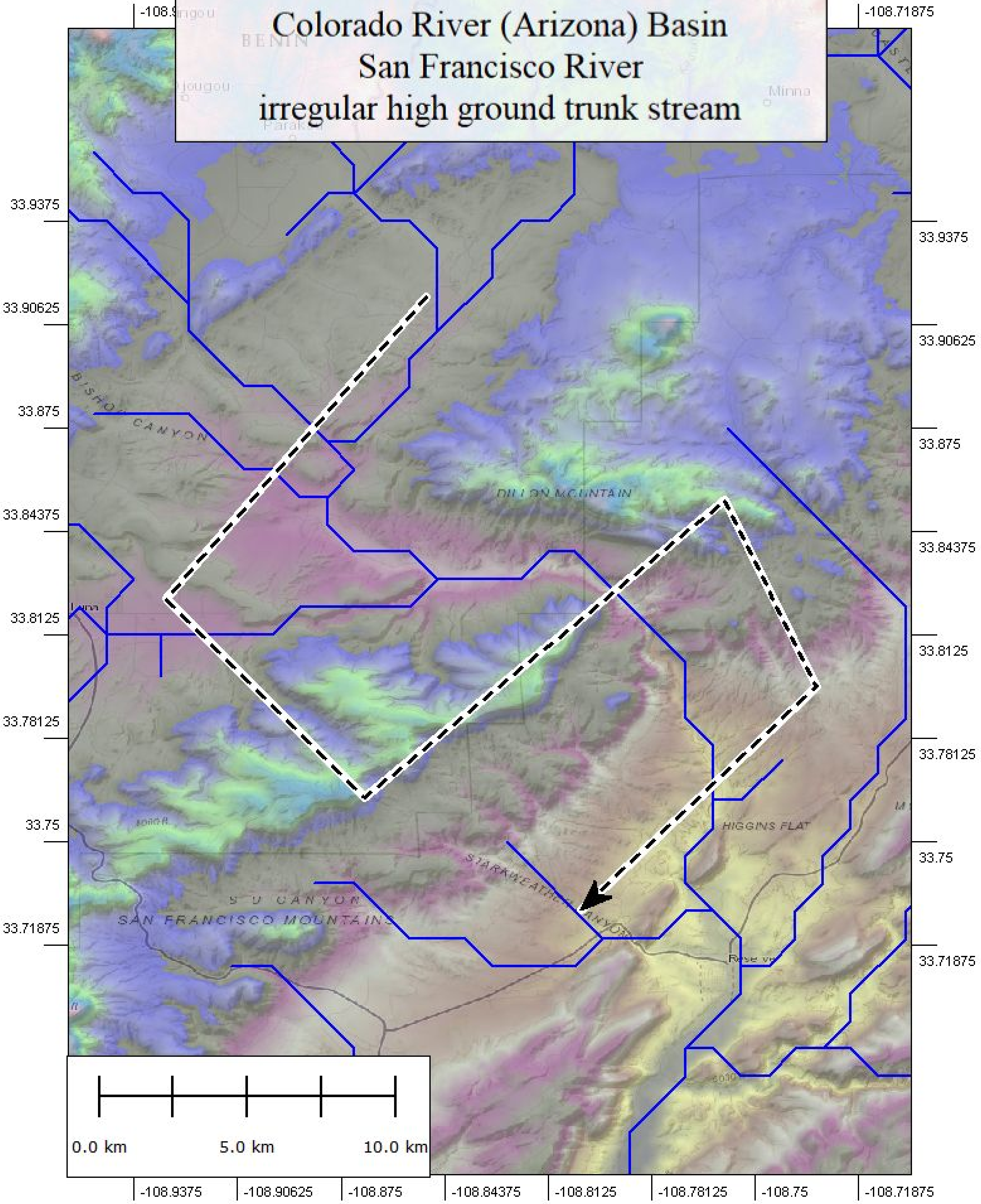

NA - 177
Potomac River Basin
North Fork Shenandoah River
single-ridge trunk stream

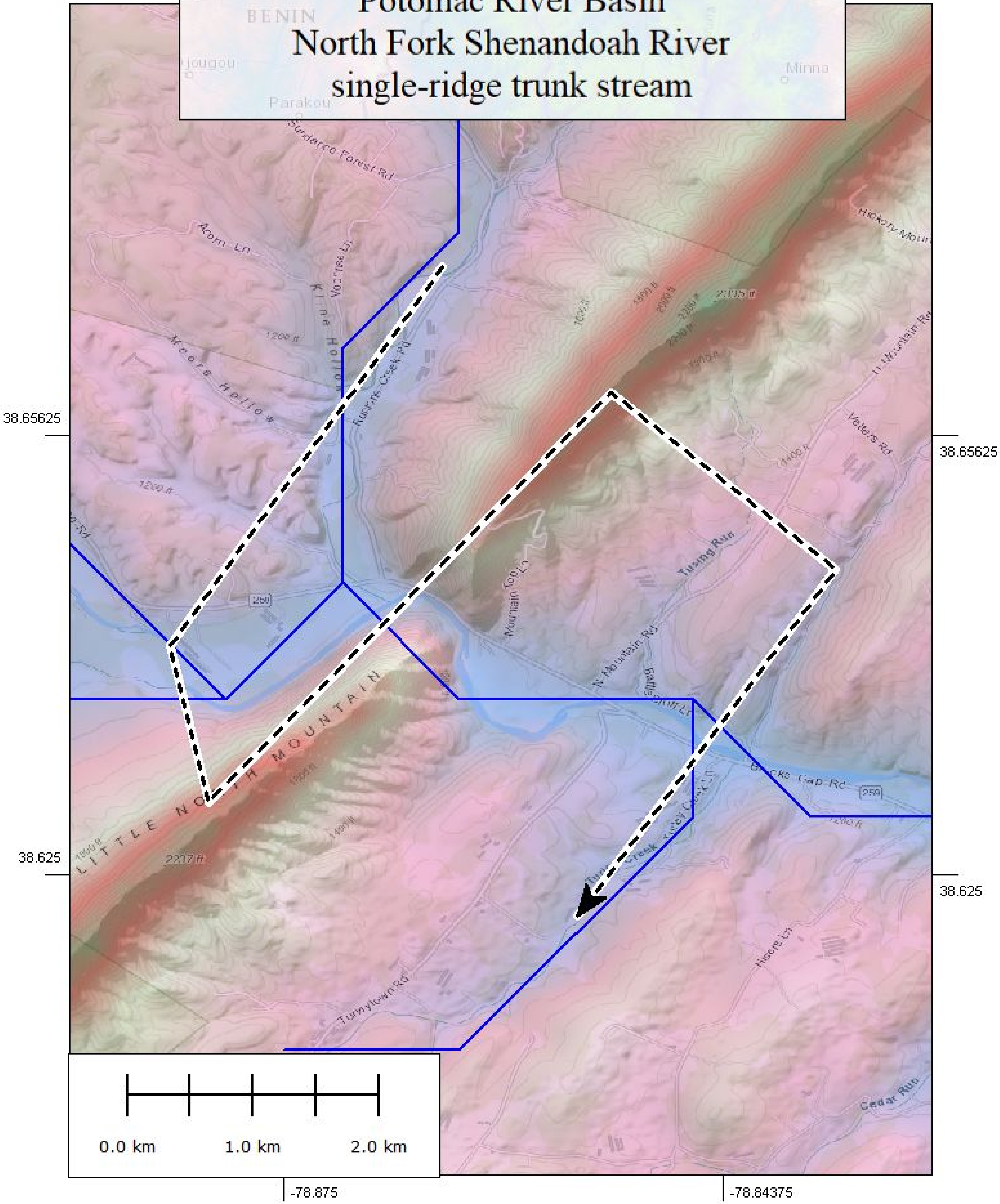

NA - 178
Colorado River (Arizona) Basin
San Rafael River
plateau trunk stream

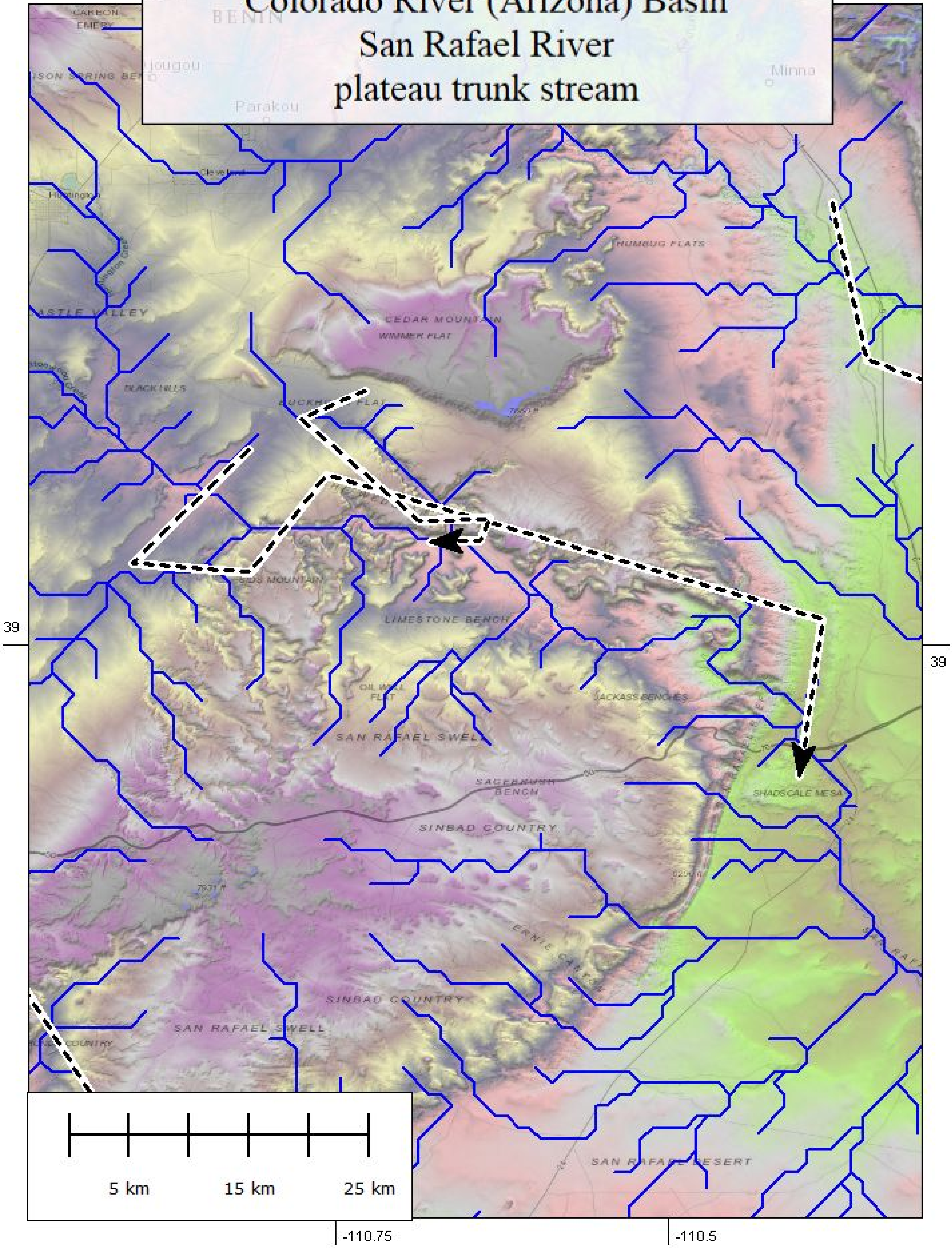

NA - 179
Santa Ana River Basin
Santa Ana River
irregular high ground trunk stream

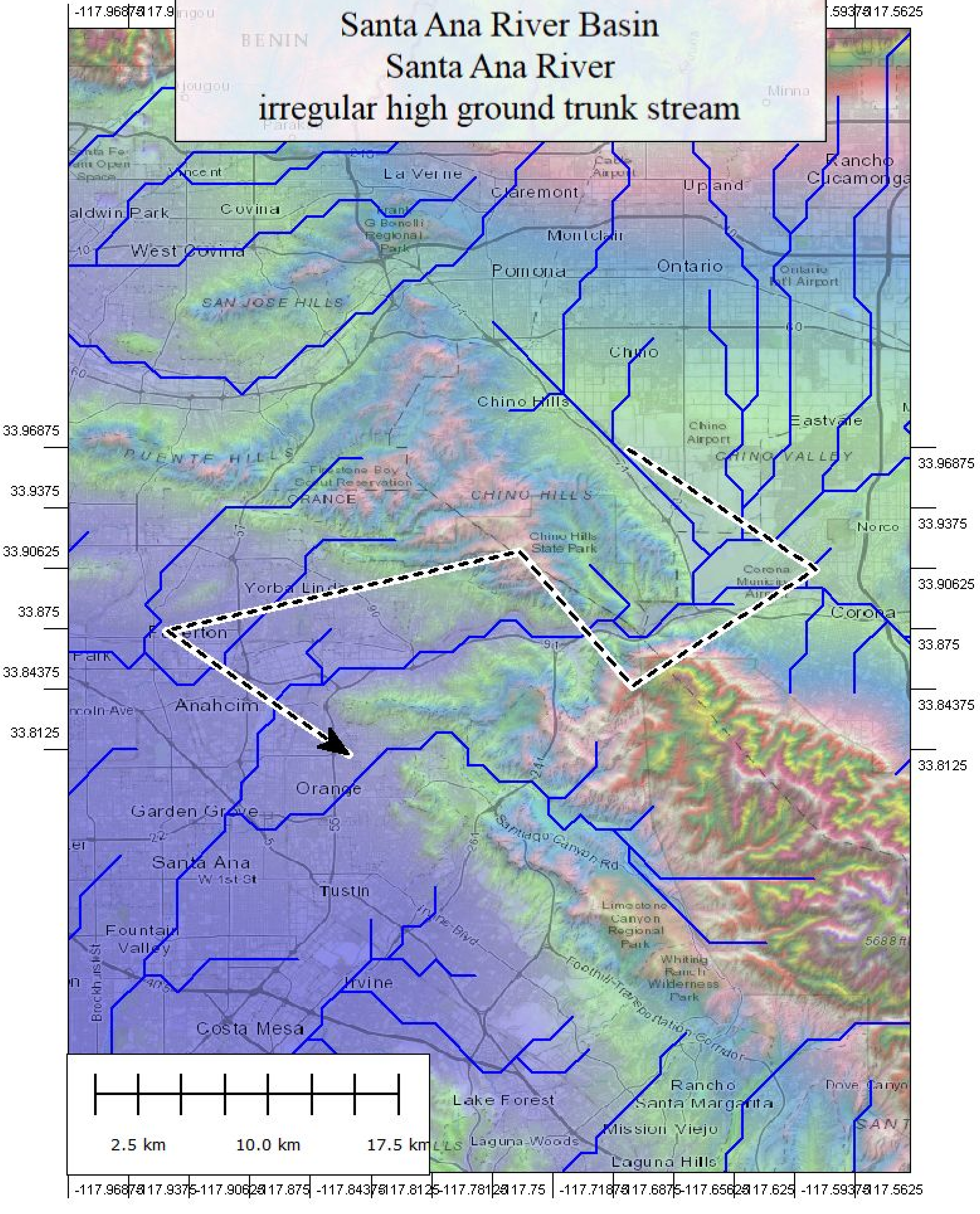

NA - 18
Pnuco River Basin
Verde River tributary
single-ridge trunk stream

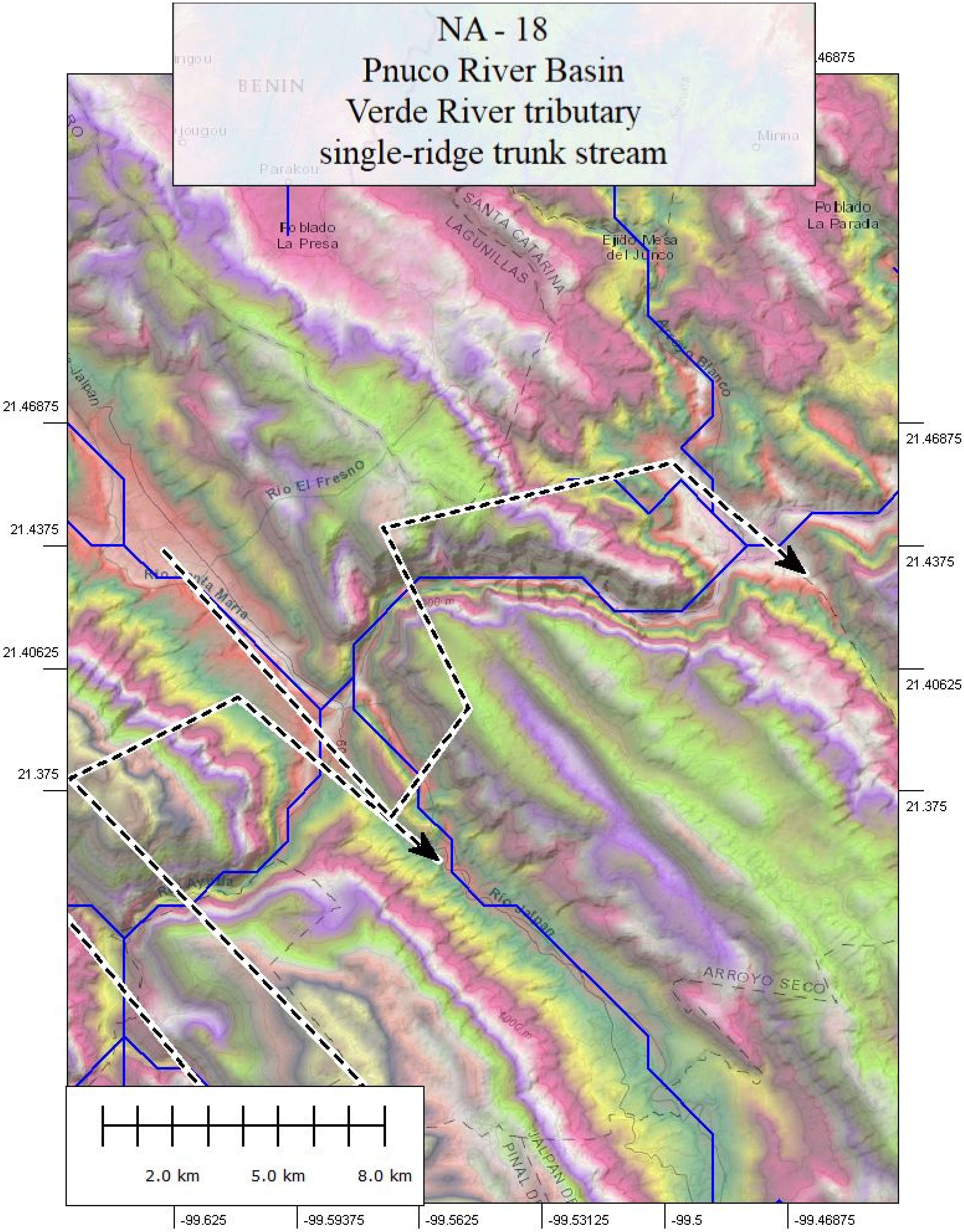

NA - 180
Delaware River Basin
Schuylkill River
single-ridge trunk stream

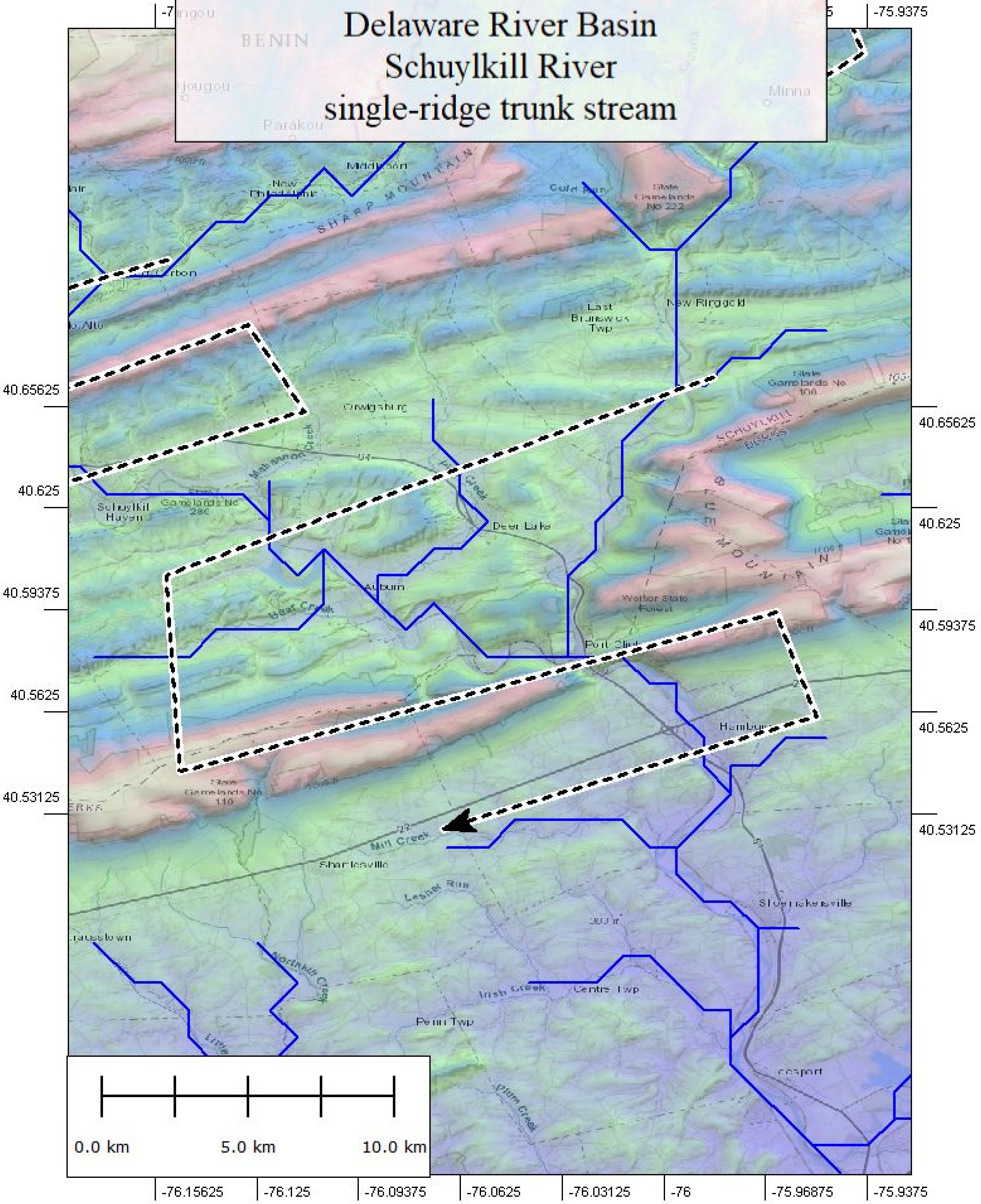

NA - 181
Endorheic basin Basin
Currant Creek, Goshen Canyon
single-ridge trunk stream

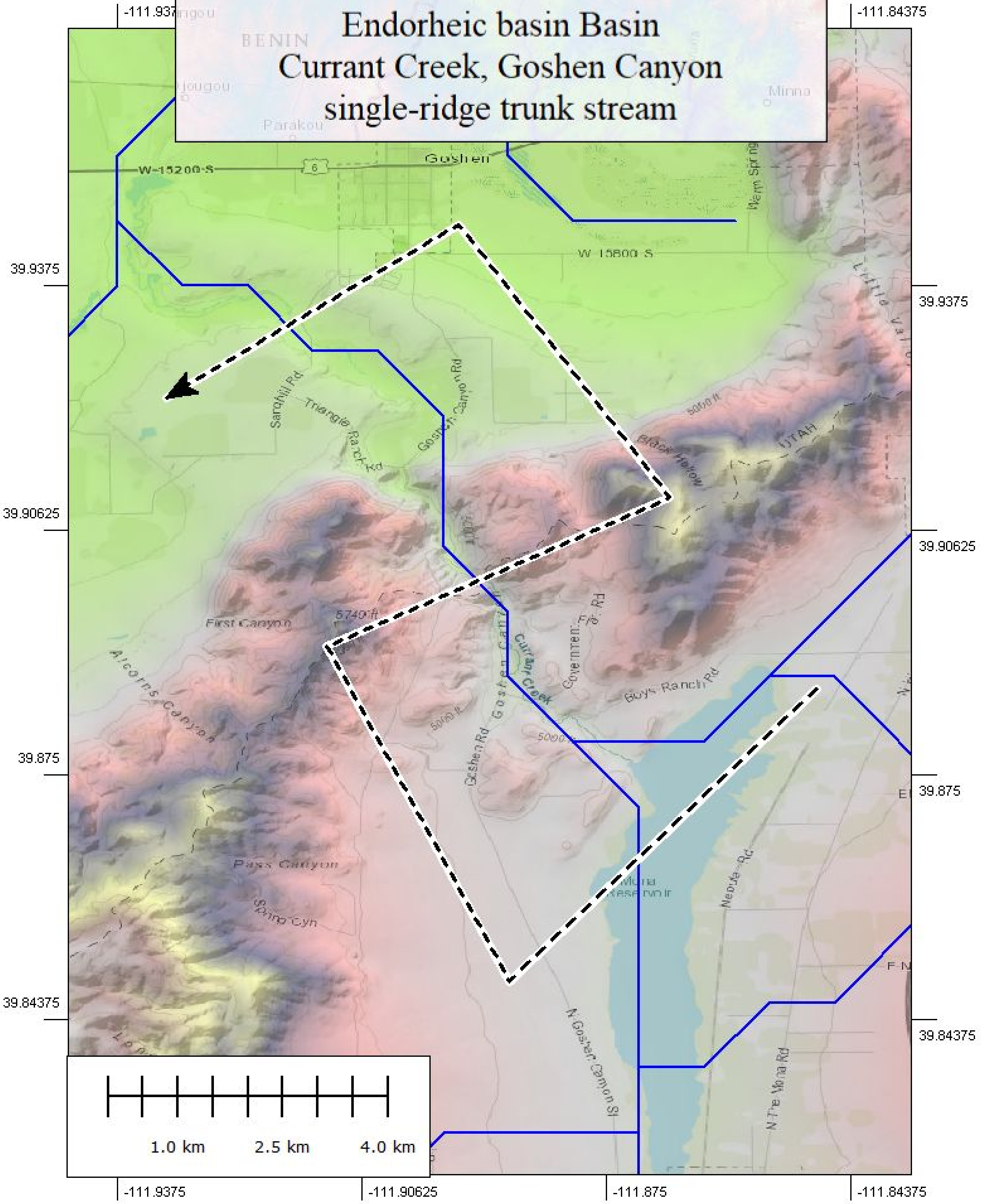

NA - 182
Columbia River Basin
Payette River
irregular high ground trunk stream

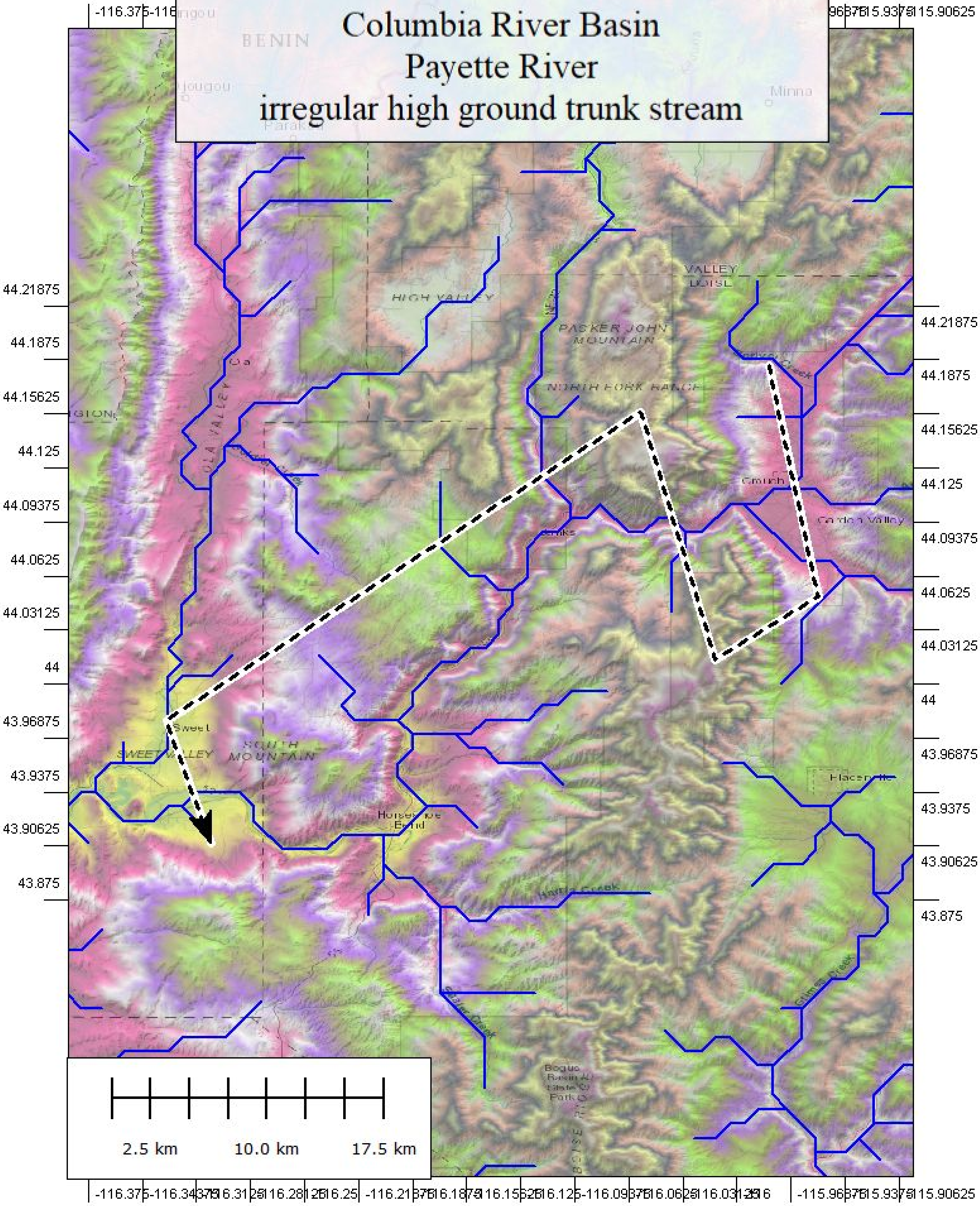

NA - 183
Colorado River (Arizona) Basin
Seely Creek, Joes Valley Reservoir
plateau trunk stream

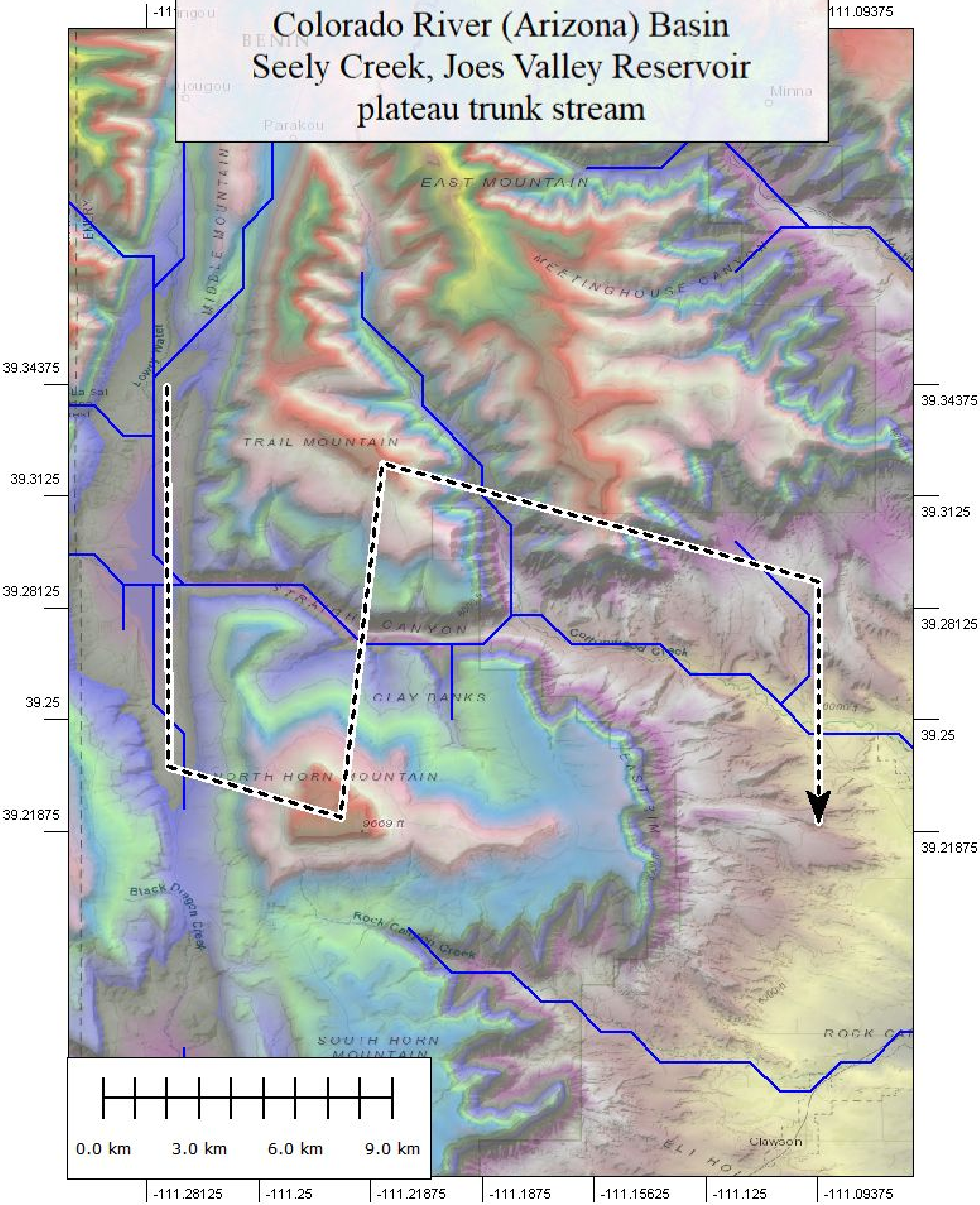

NA - 184
Colorado River (Arizona) Basin
Red Creek
updip plateau trunk stream

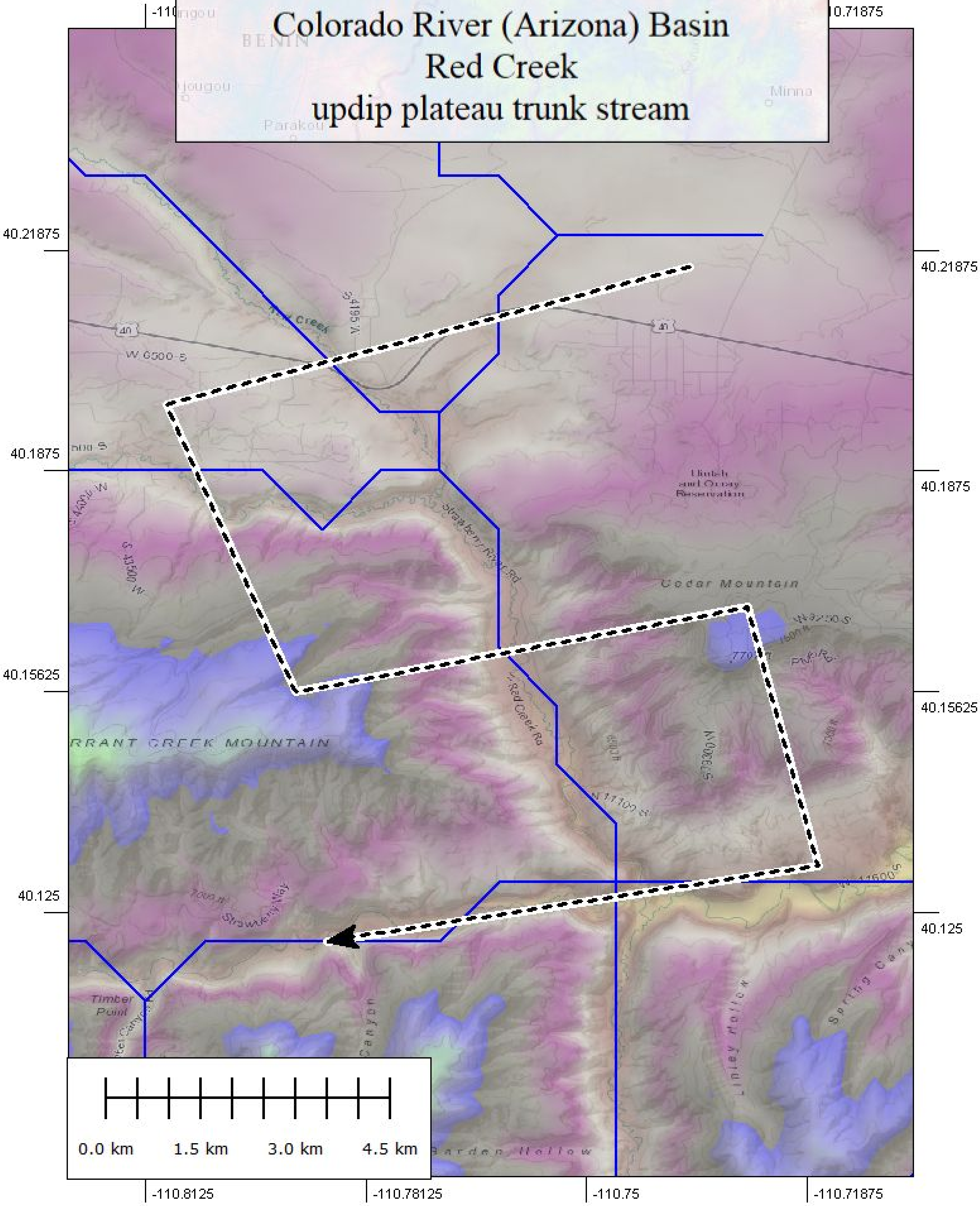

NA - 185
Mississippi River Basin
North Platte River
single-ridge trunk stream

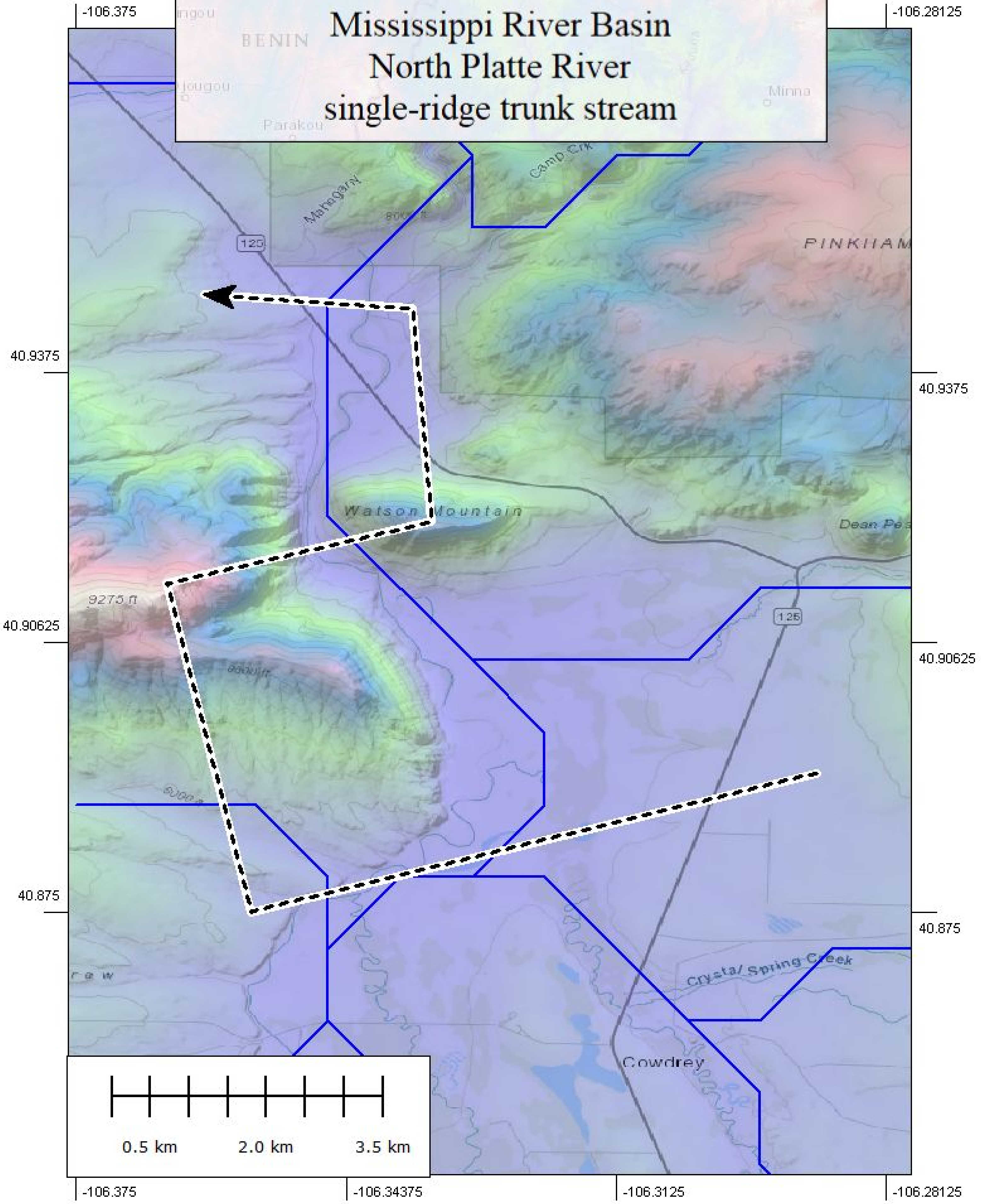

NA - 186
Mississippi River Basin
Sun River
irregular high ground trunk stream

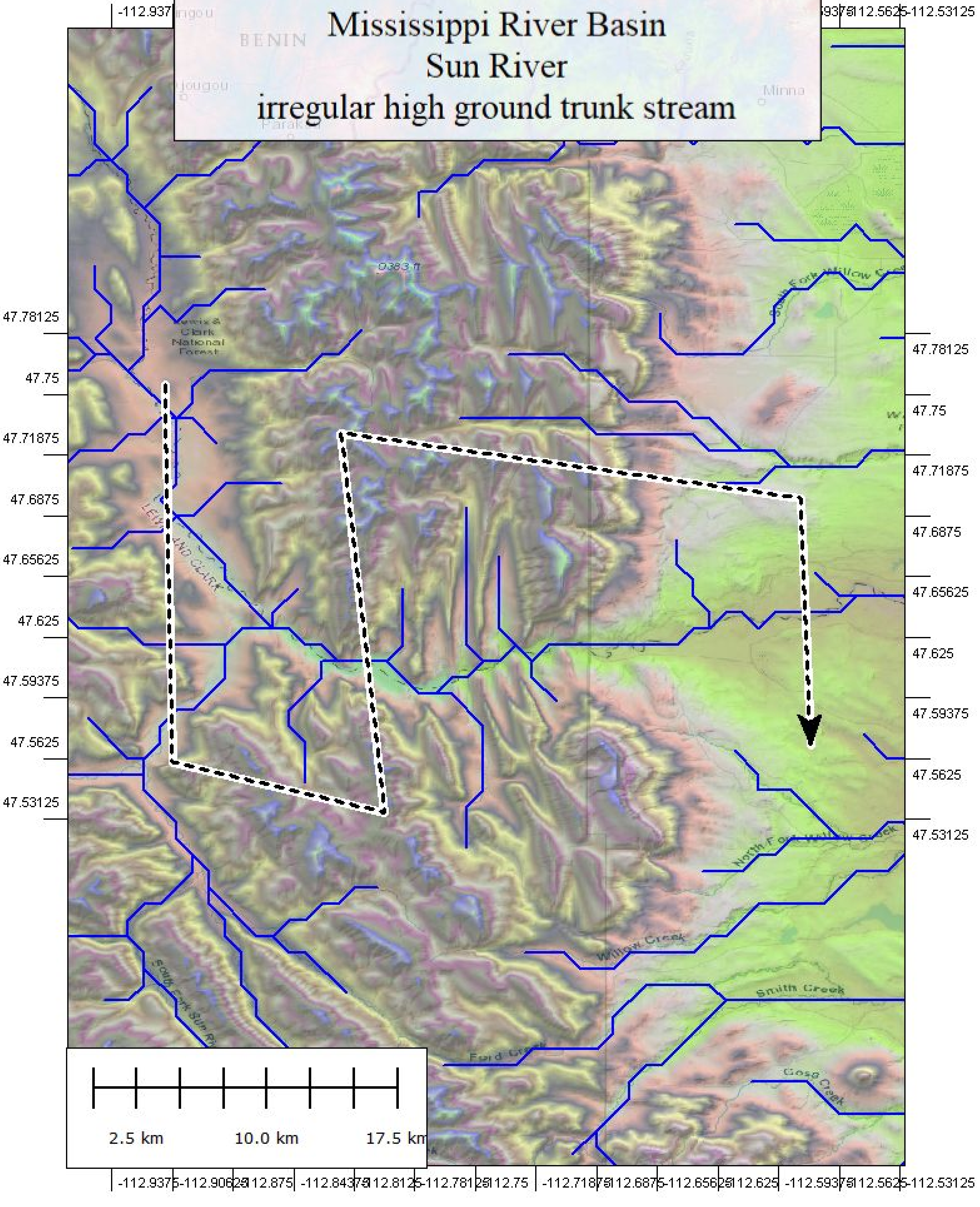

NA - 187
Endorheic basin Basin
Weber River, Weber Canyon
single-ridge trunk stream

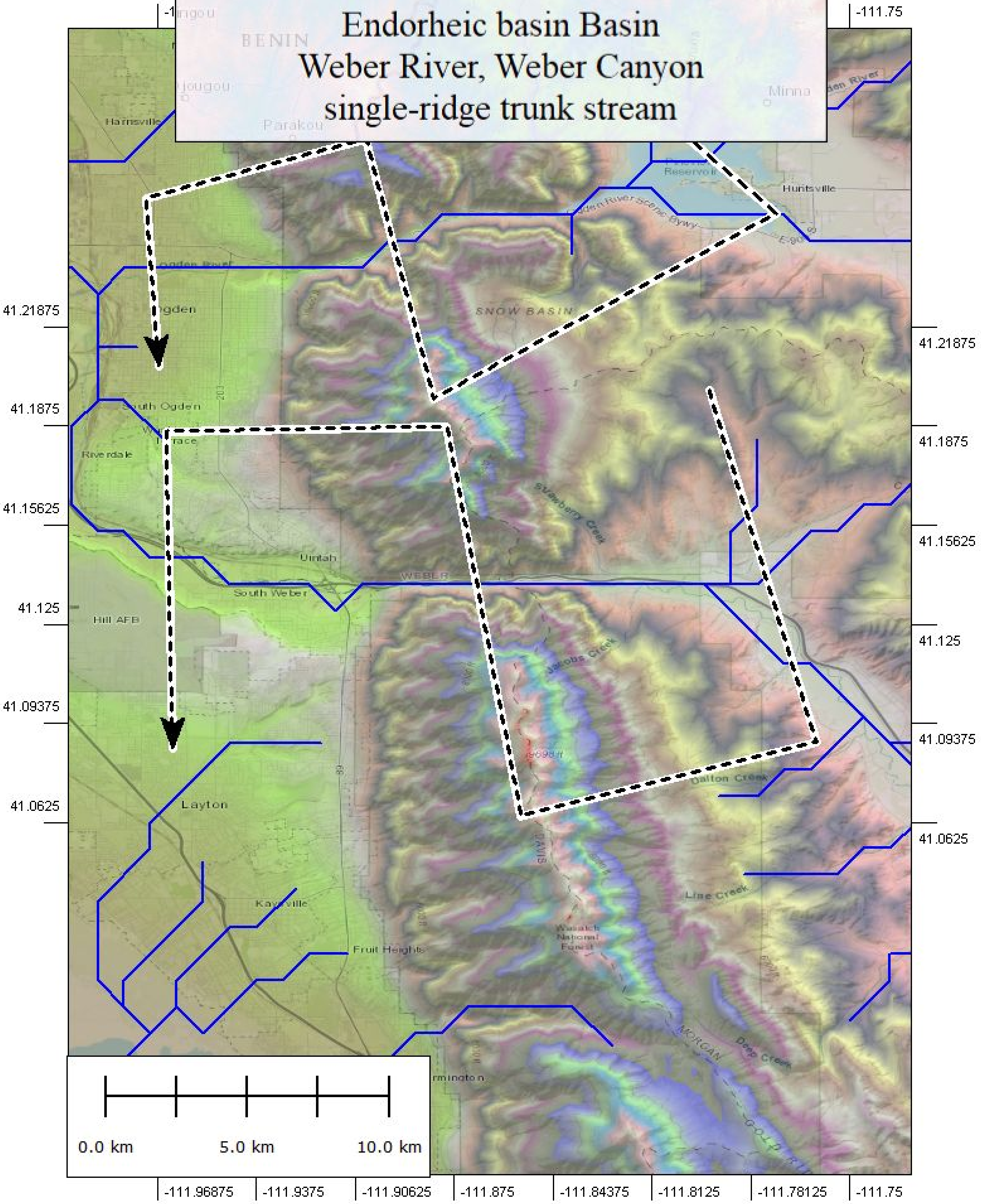

NA - 188
Endorheic basin Basin
Ogden River, Ogden Canyon
irregular high ground trunk stream

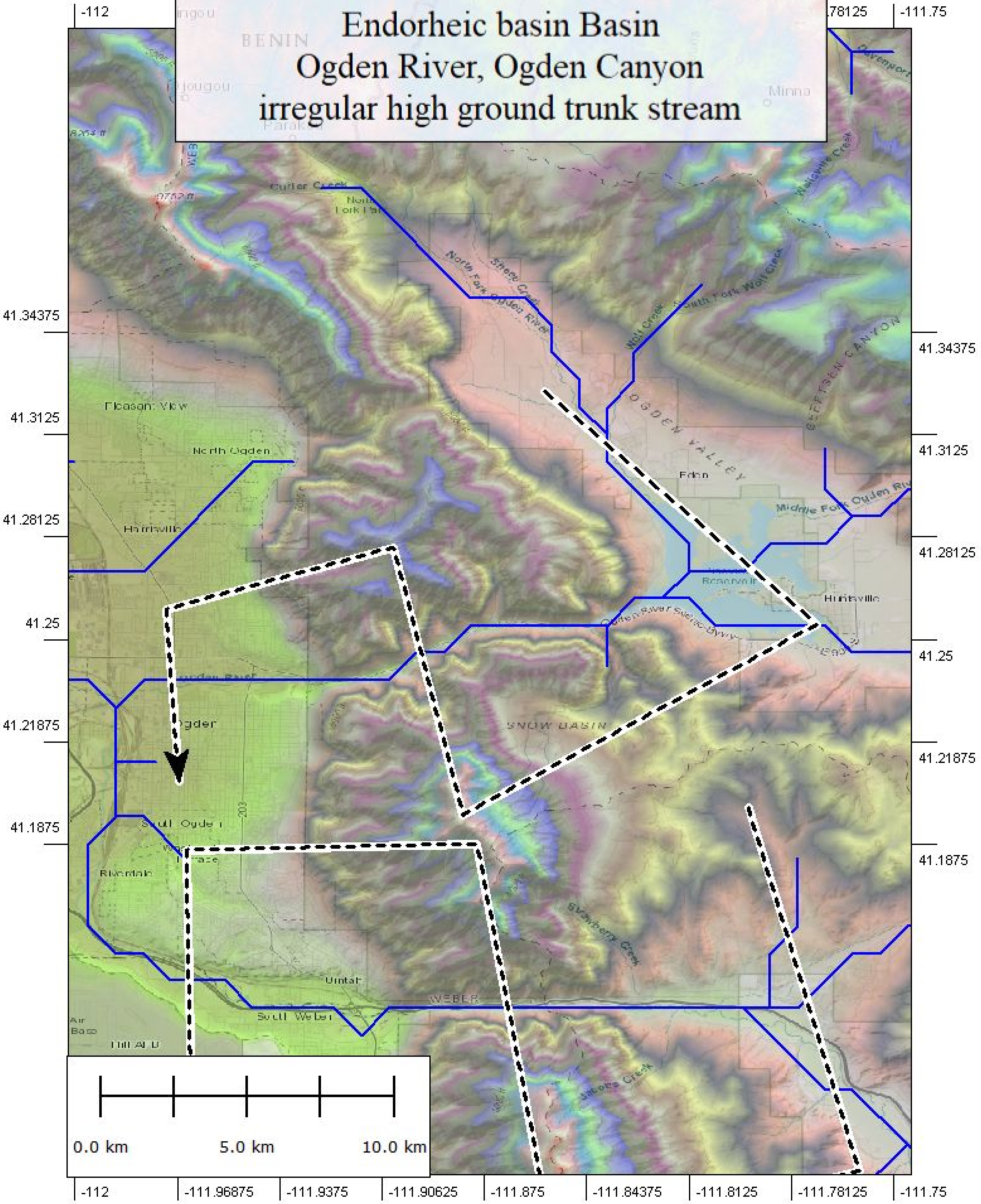

NA - 189
Delaware River Basin
Schuylkill River
multi-ridge trunk stream

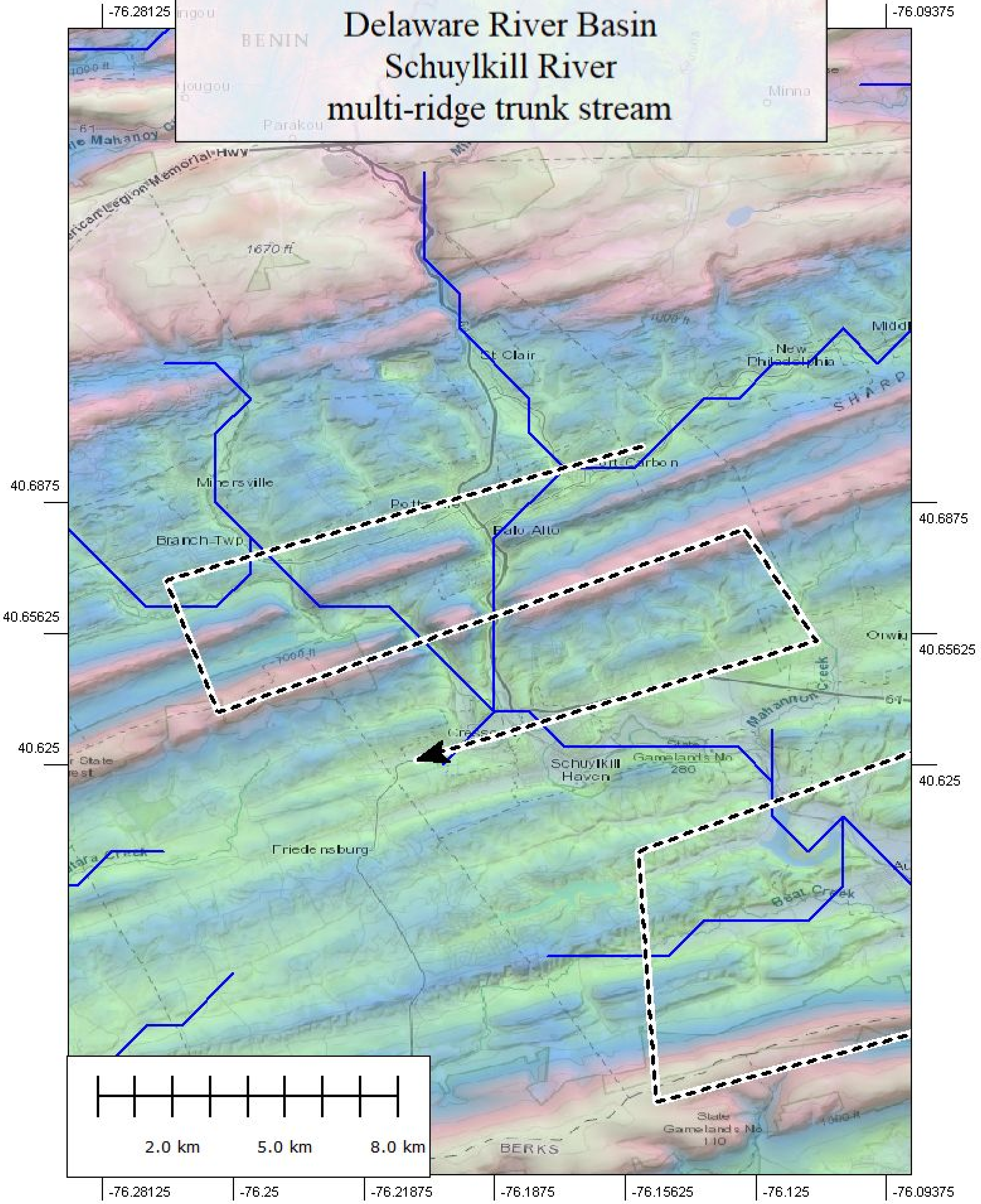

NA - 19
Mississippi River Basin
Missouri River
irregular high ground trunk stream

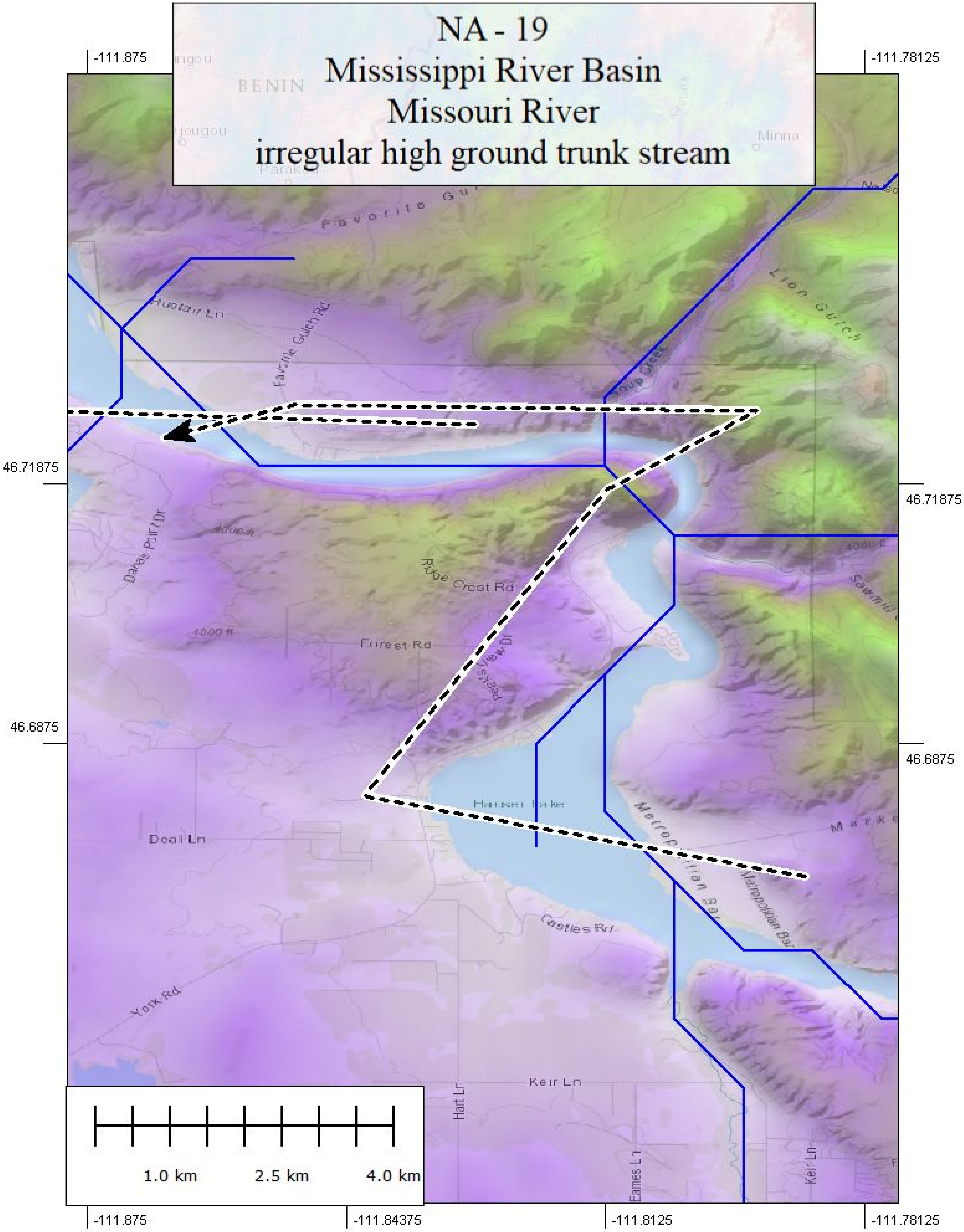

NA - 190
Susquehanna River Basin
West Branch Susquehanna River
single-ridge trunk stream

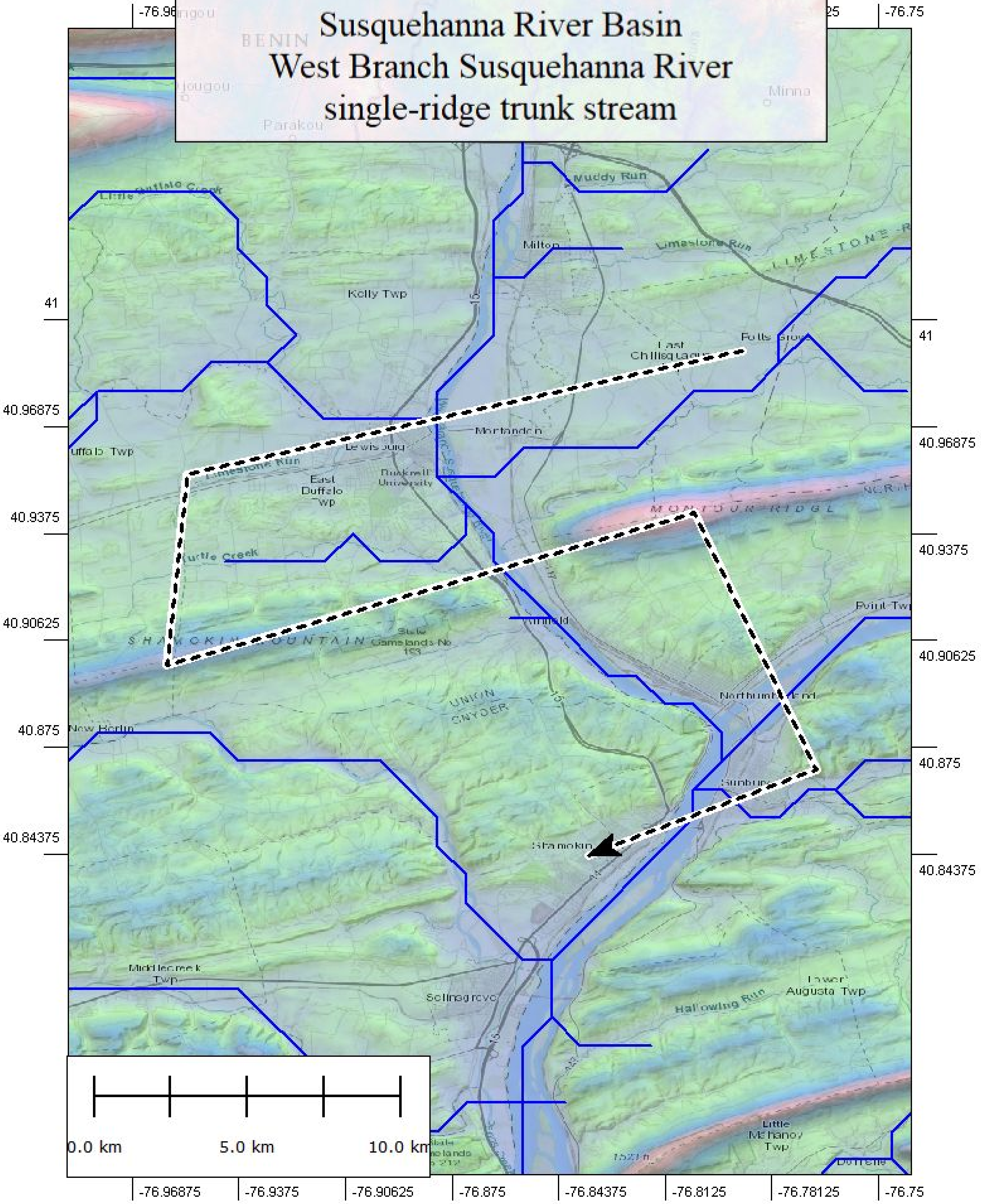

NA - 191
Columbia River Basin
Willamette River
plateau trunk stream

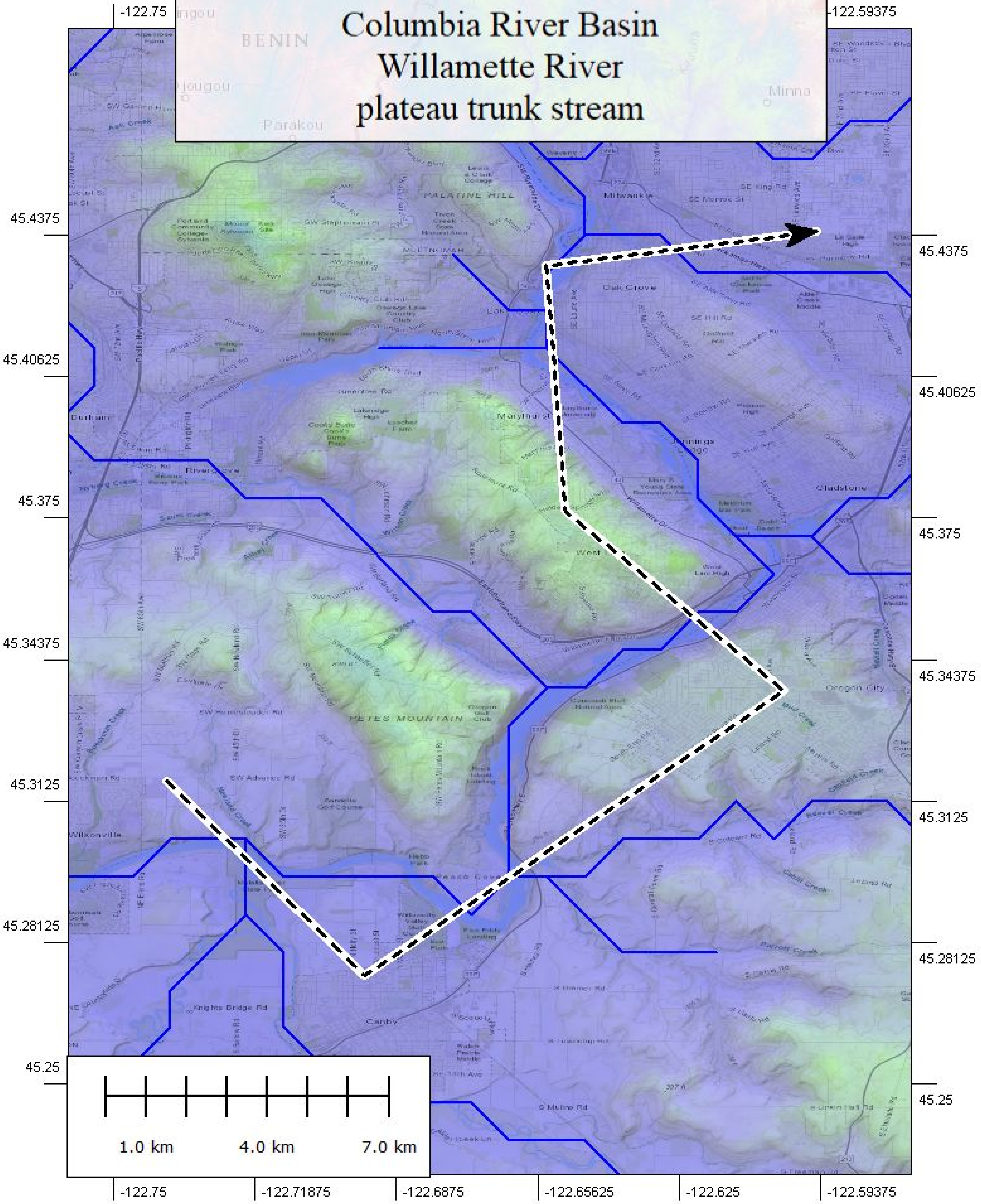

NA - 192
Columbia River Basin
Willamette River
single-ridge trunk stream

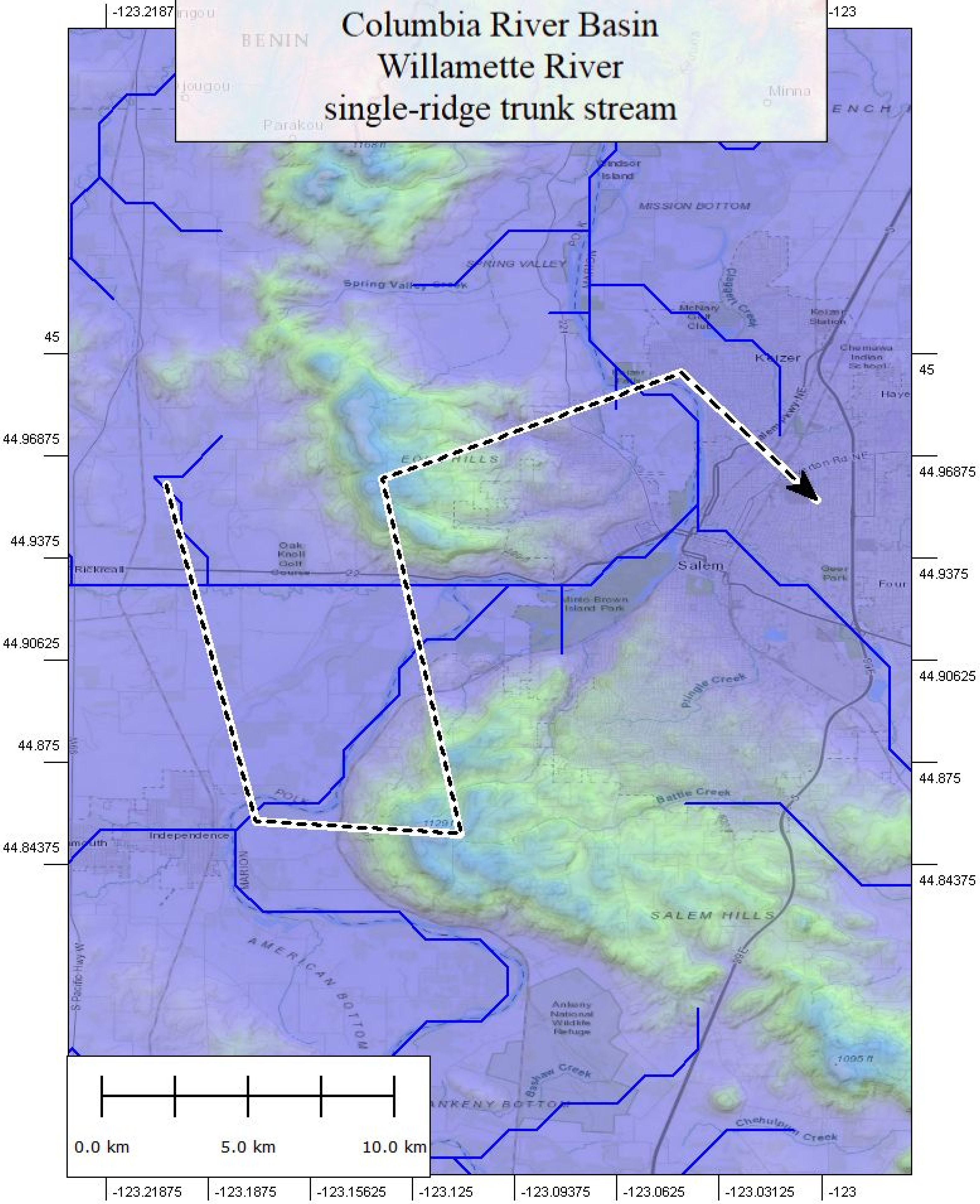

NA - 193
Potomac River Basin
North River
single-ridge trunk stream

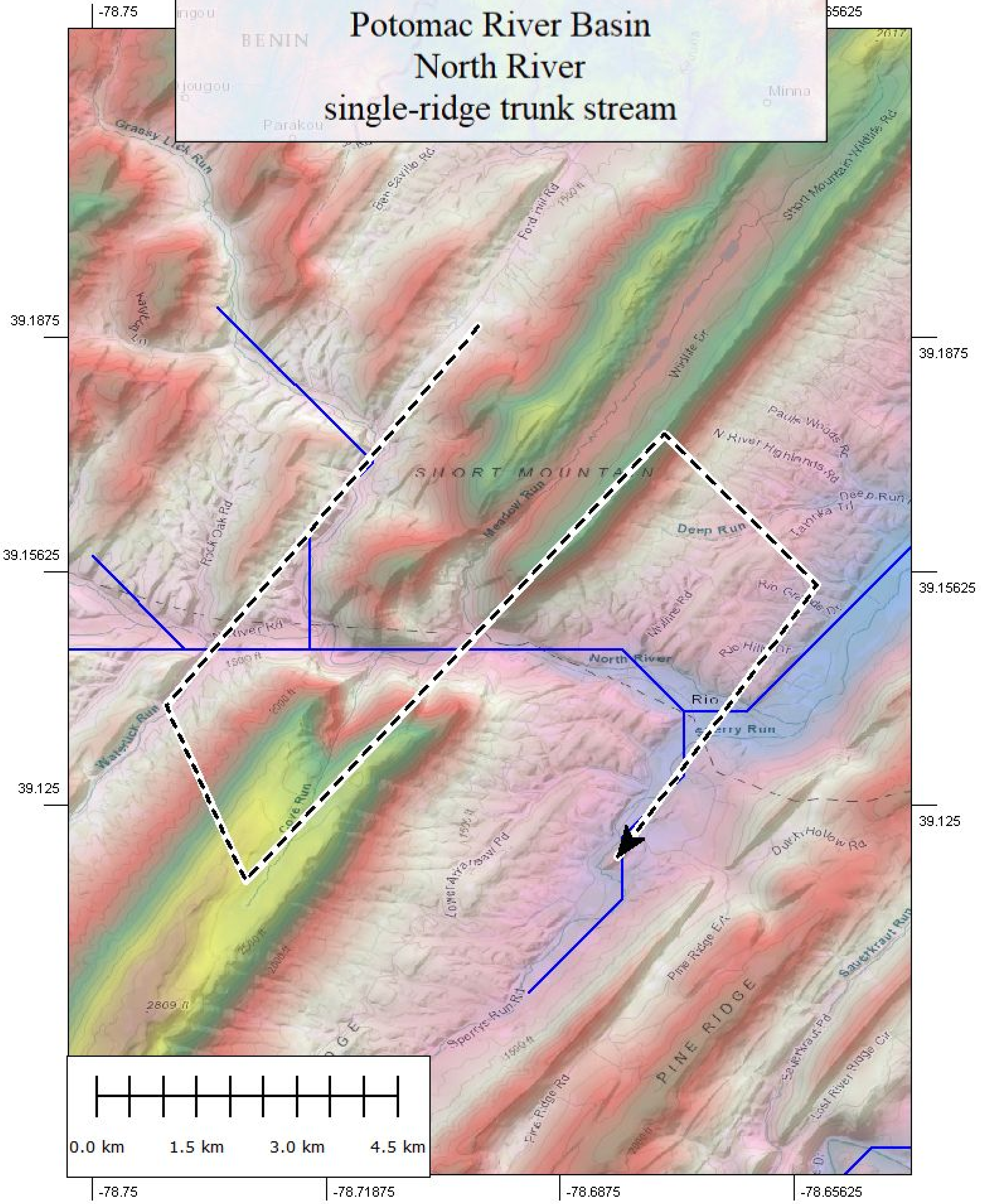

NA - 194
Mississippi River Basin
Wind River, Wind River Canyon
downdip plateau trunk stream

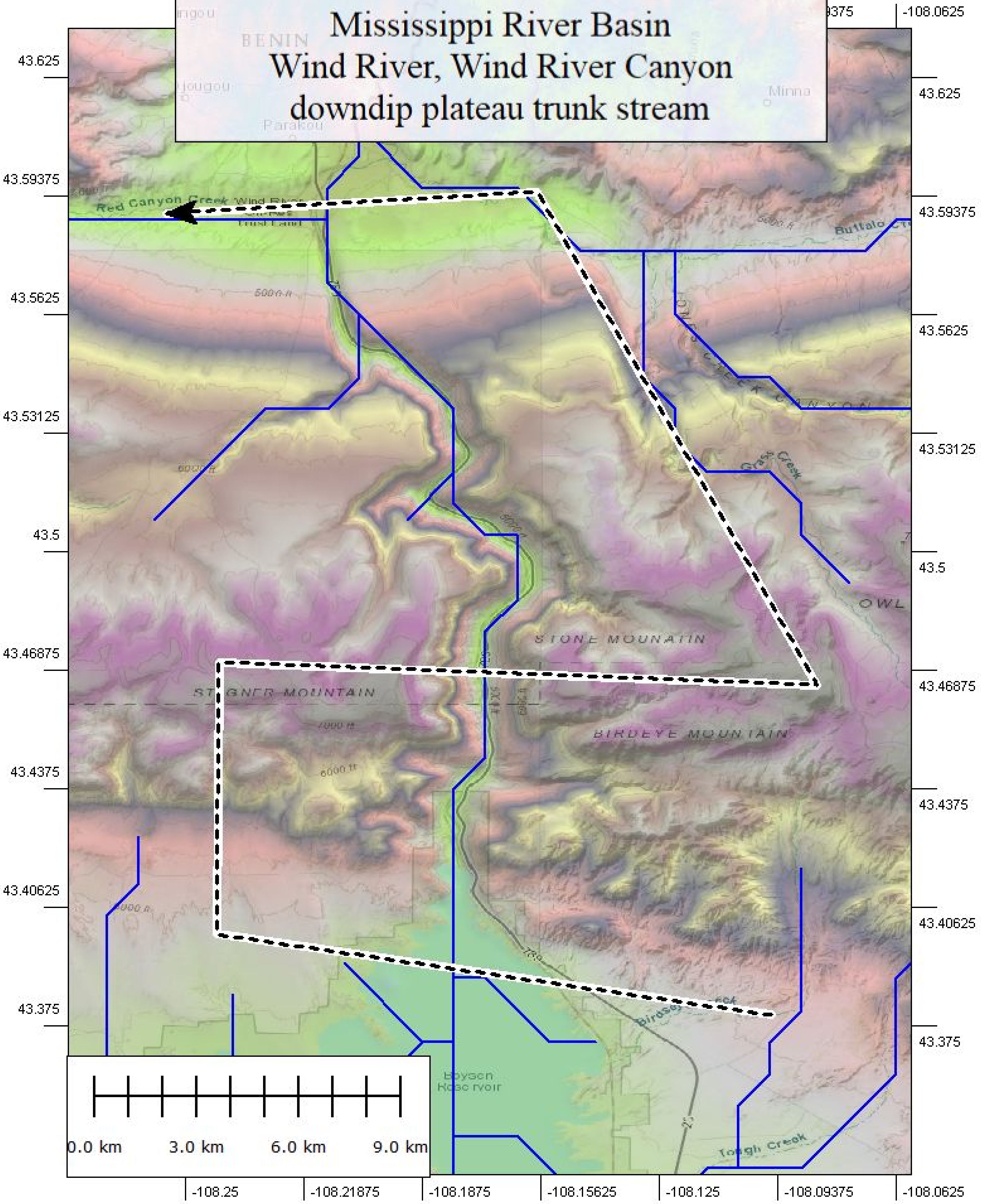

NA - 195
St. Lawrence River Basin
Missisquoi River
irregular high ground trunk stream

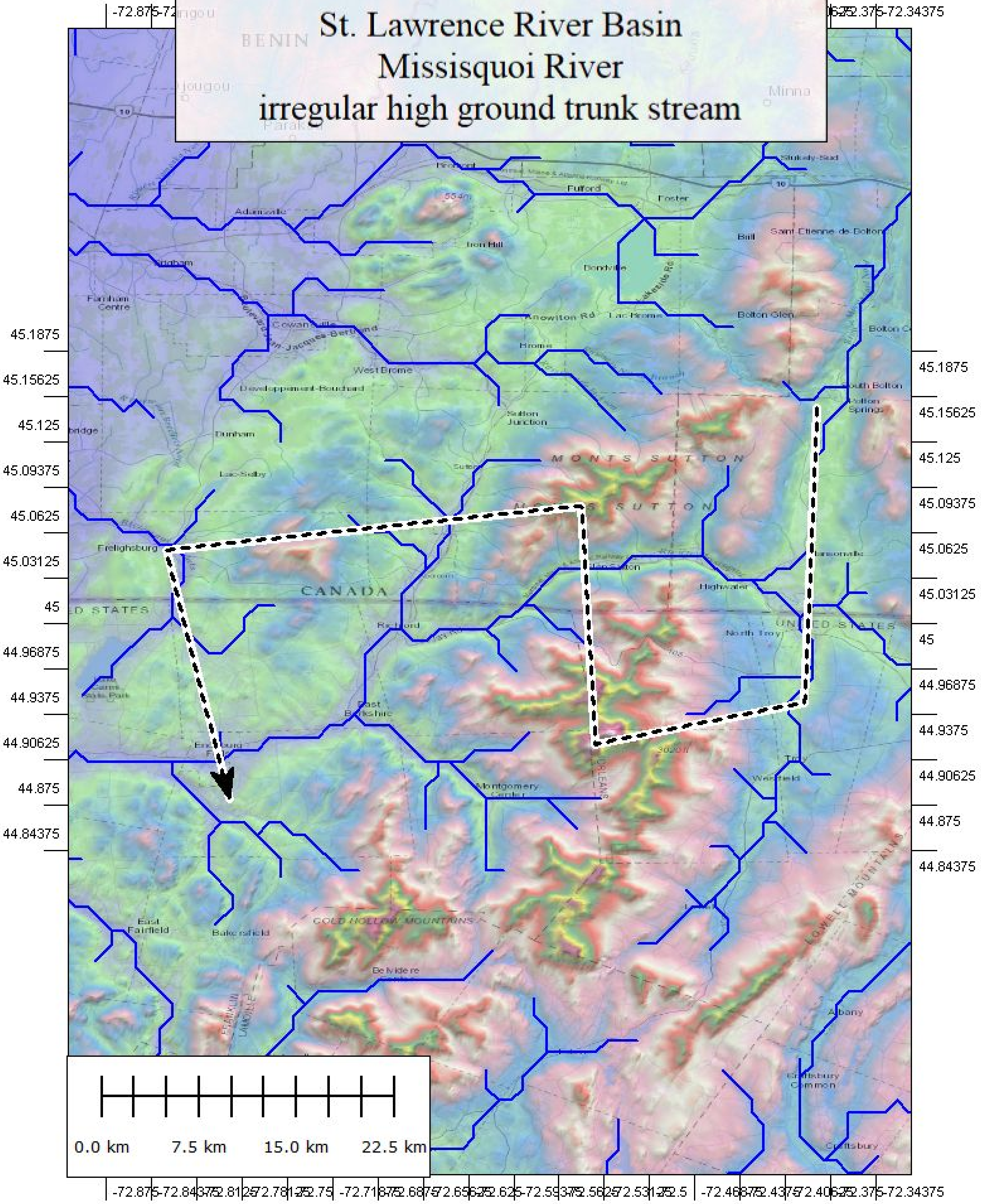

NA - 196
Rio Grande (Texas-Mexico) Basin
Conchos River, El Granero Reservoir
single-ridge trunk stream

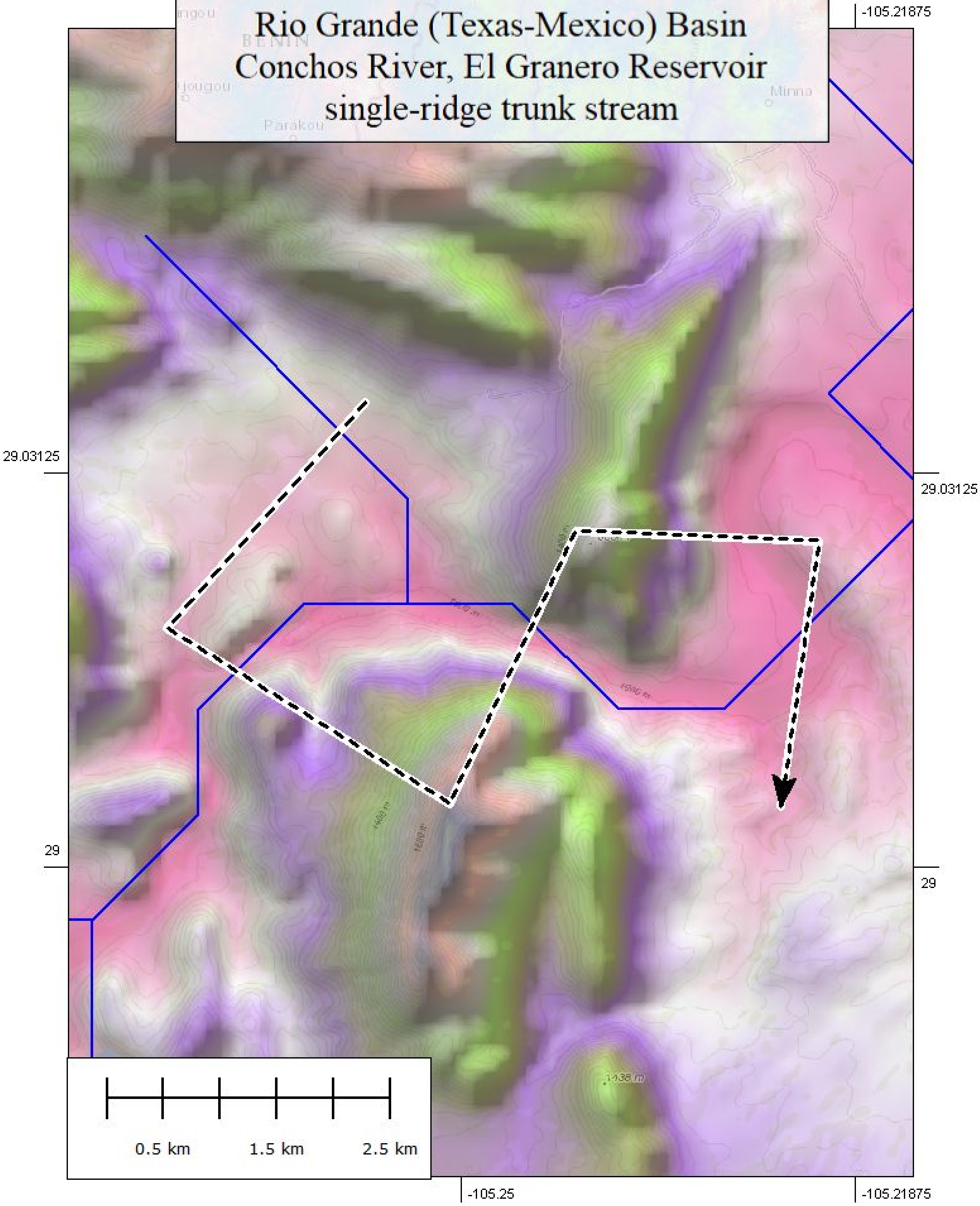

NA - 197
Endorheic basin Basin
South Fork River
irregular high ground trunk stream

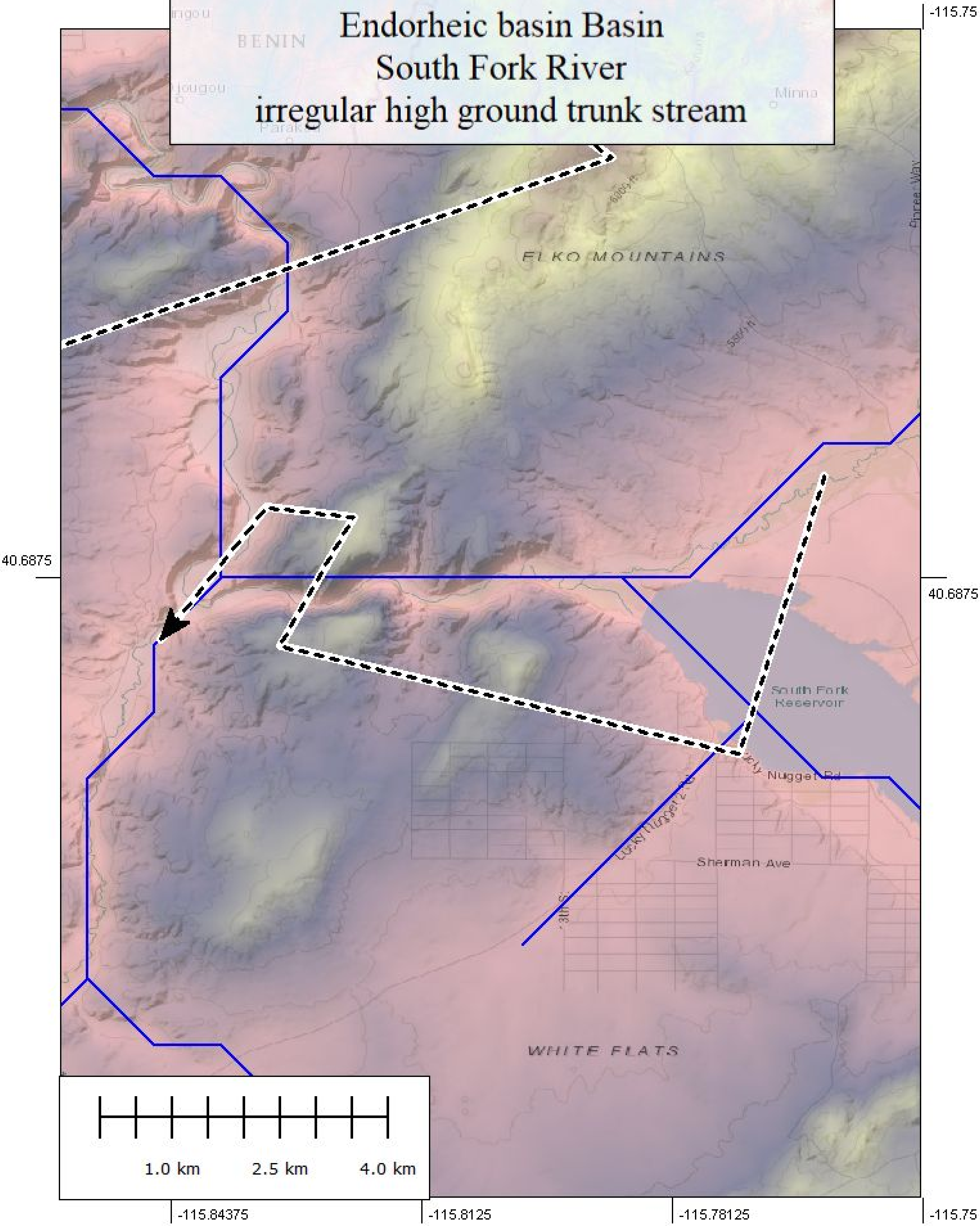

NA - 198
Endorheic basin Basin
Humboldt River
irregular high ground trunk stream

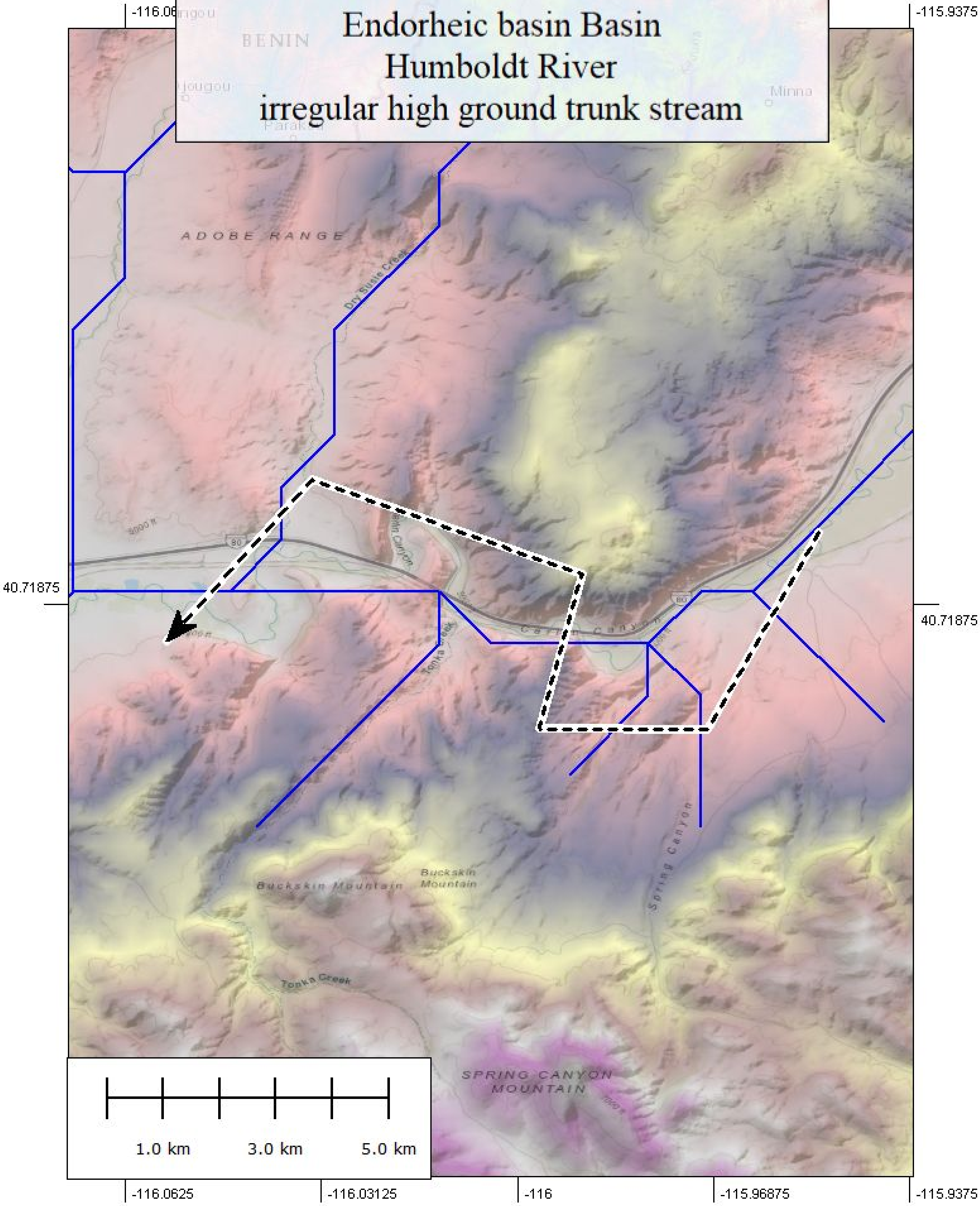

NA - 199
Colorado River (Arizona) Basin
Yampa River
single-ridge trunk stream

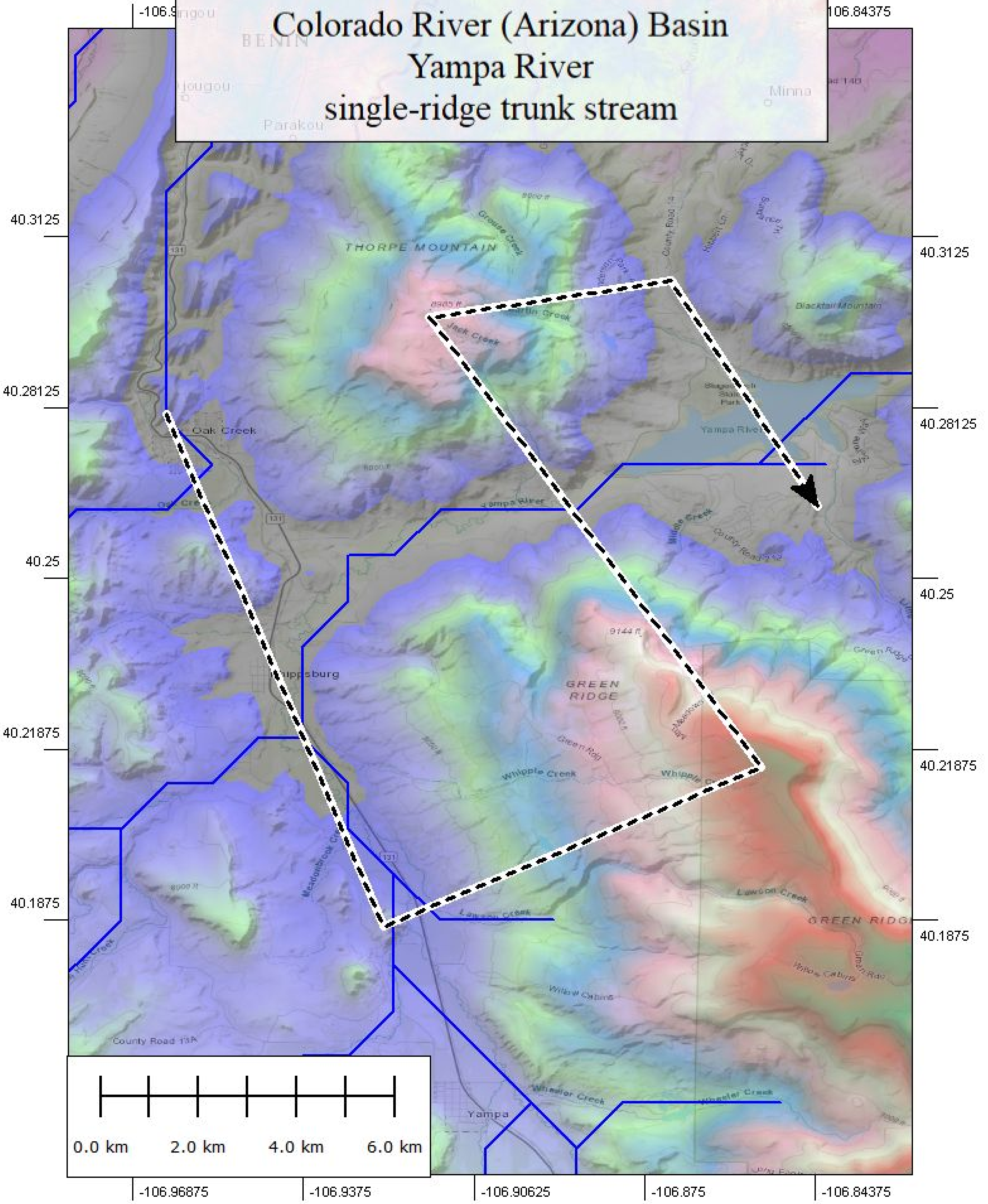

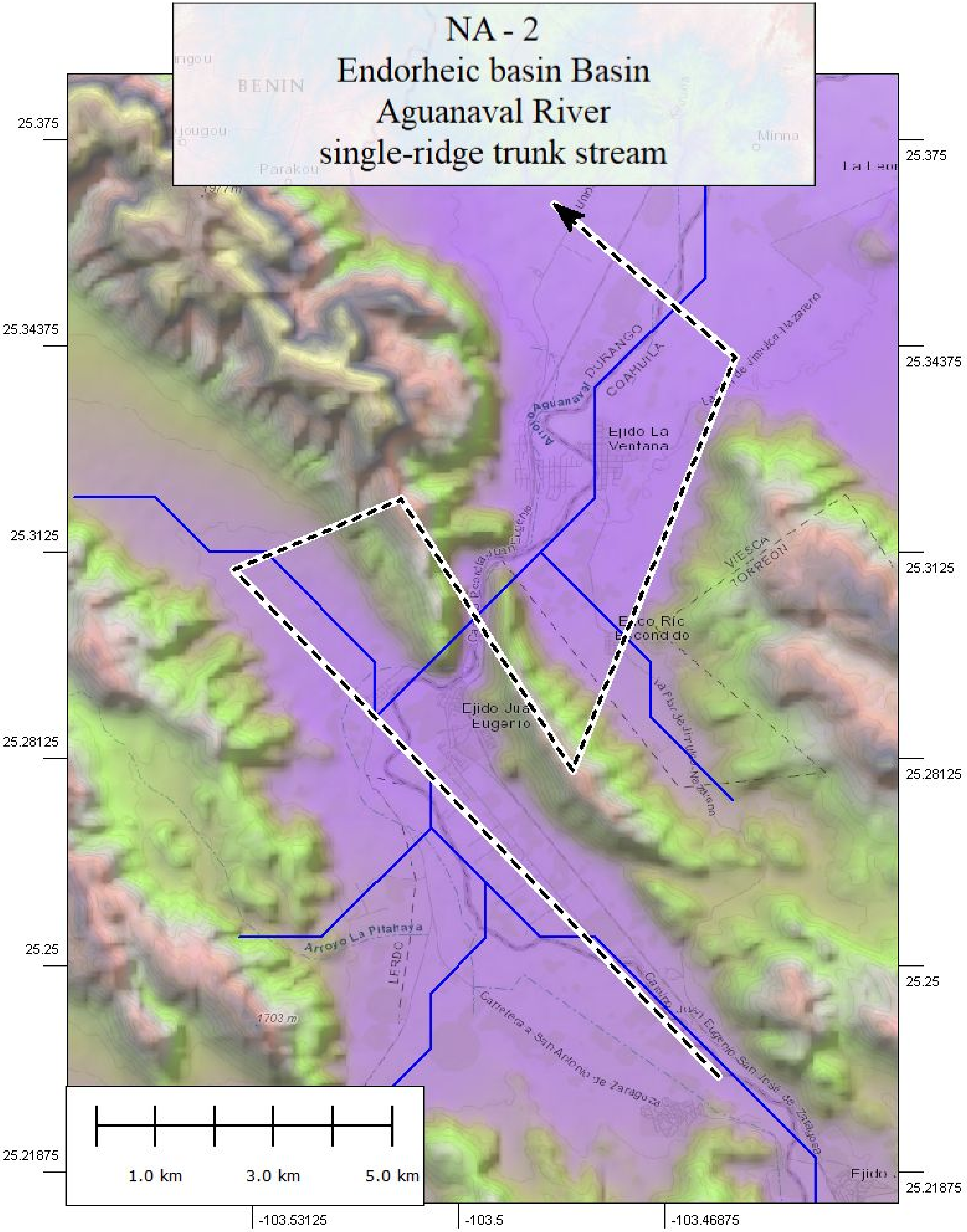

NA - 2
Endorheic basin Basin
Aguanaval River
single-ridge trunk stream

NA - 20
Rio Balsas Basin
Rio Nexapa
irregular high ground trunk stream

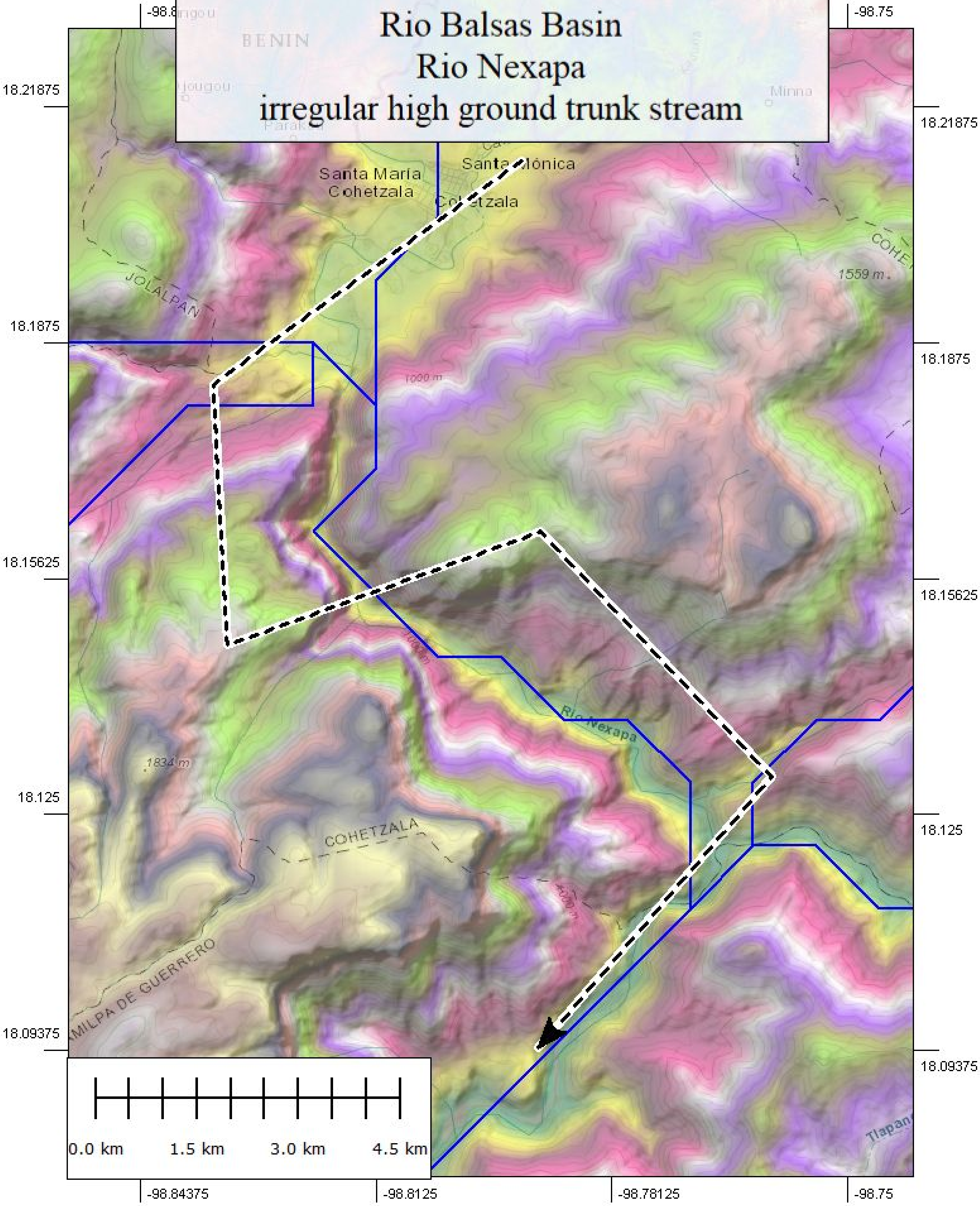

NA - 200
Mississippi River Basin
Little River, Chilhowee Gap
single-ridge trunk stream

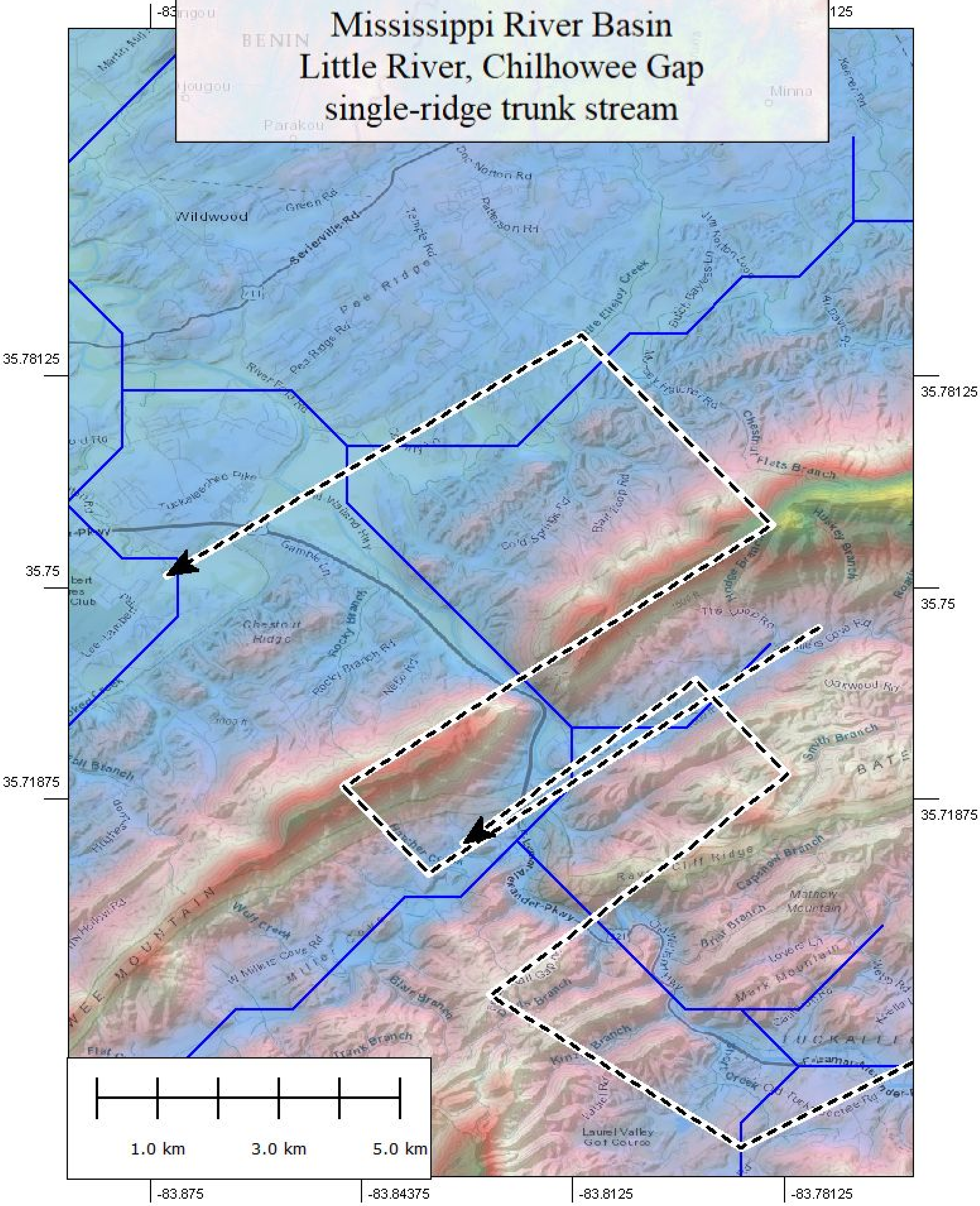

NA - 201
Rio Grande (Texas-Mexico) Basin
Arroyo Zanja
single-ridge trunk stream

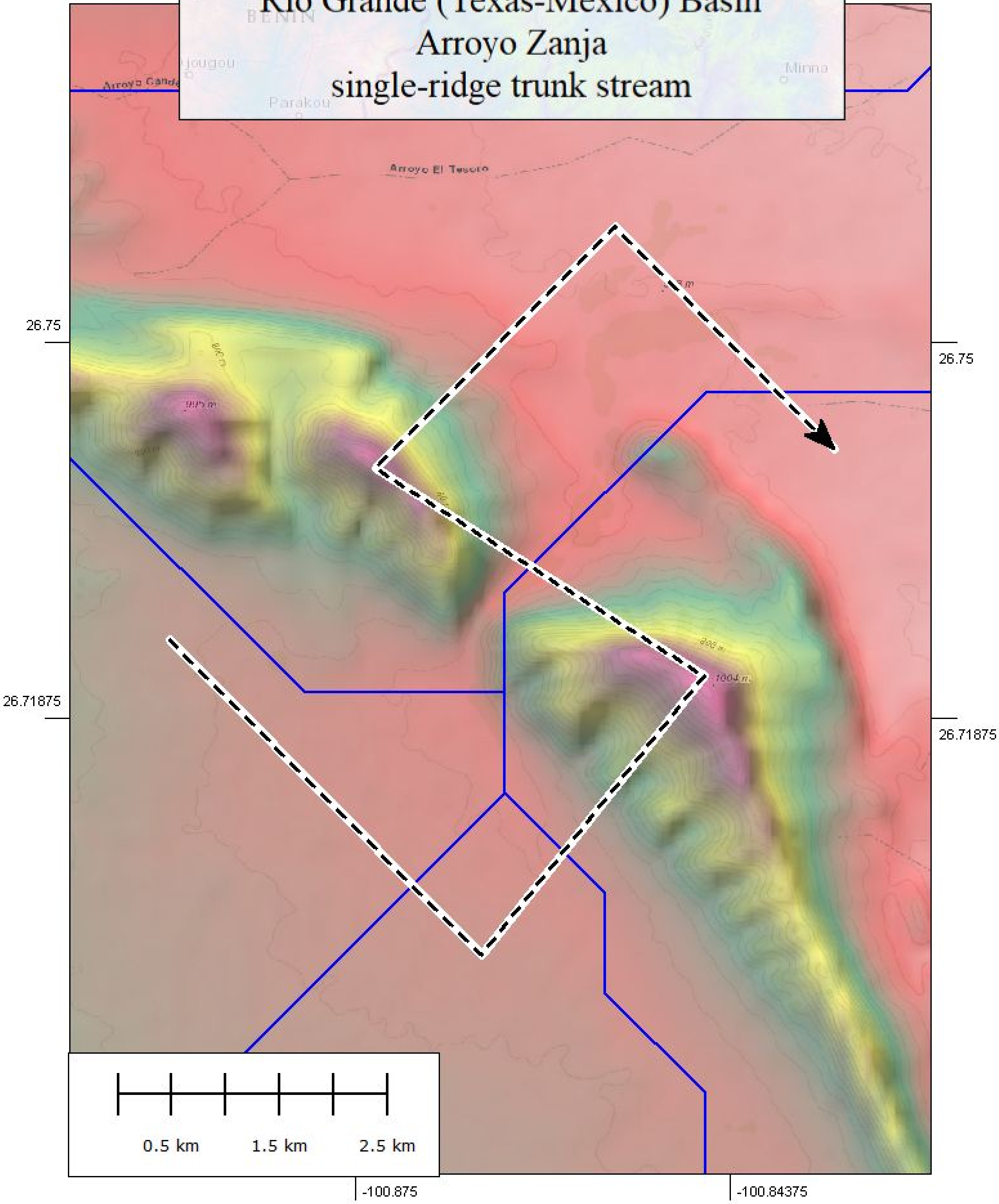

NA - 202
Santa Margarita River Basin
Santa Margarita River
irregular high ground trunk stream

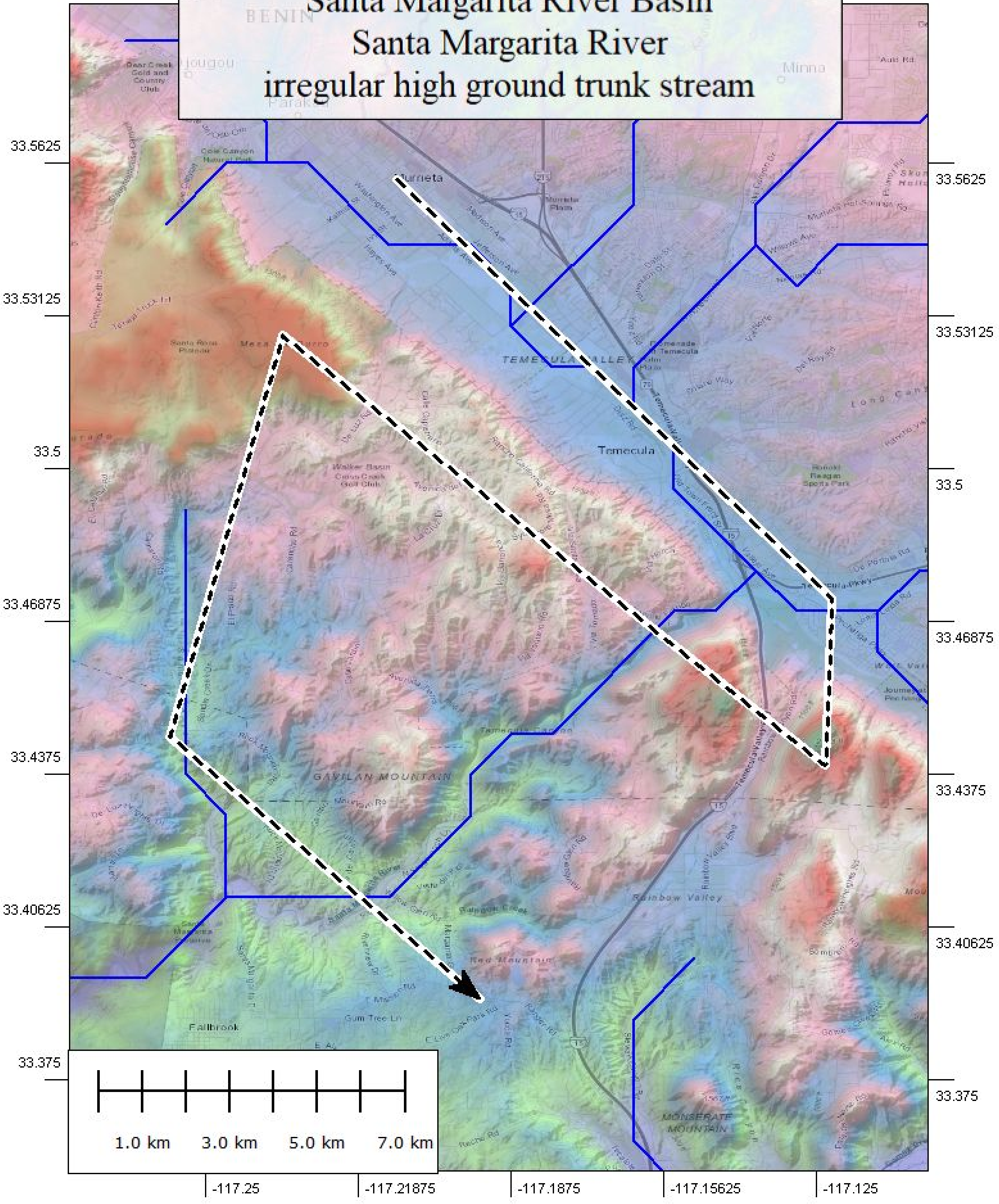

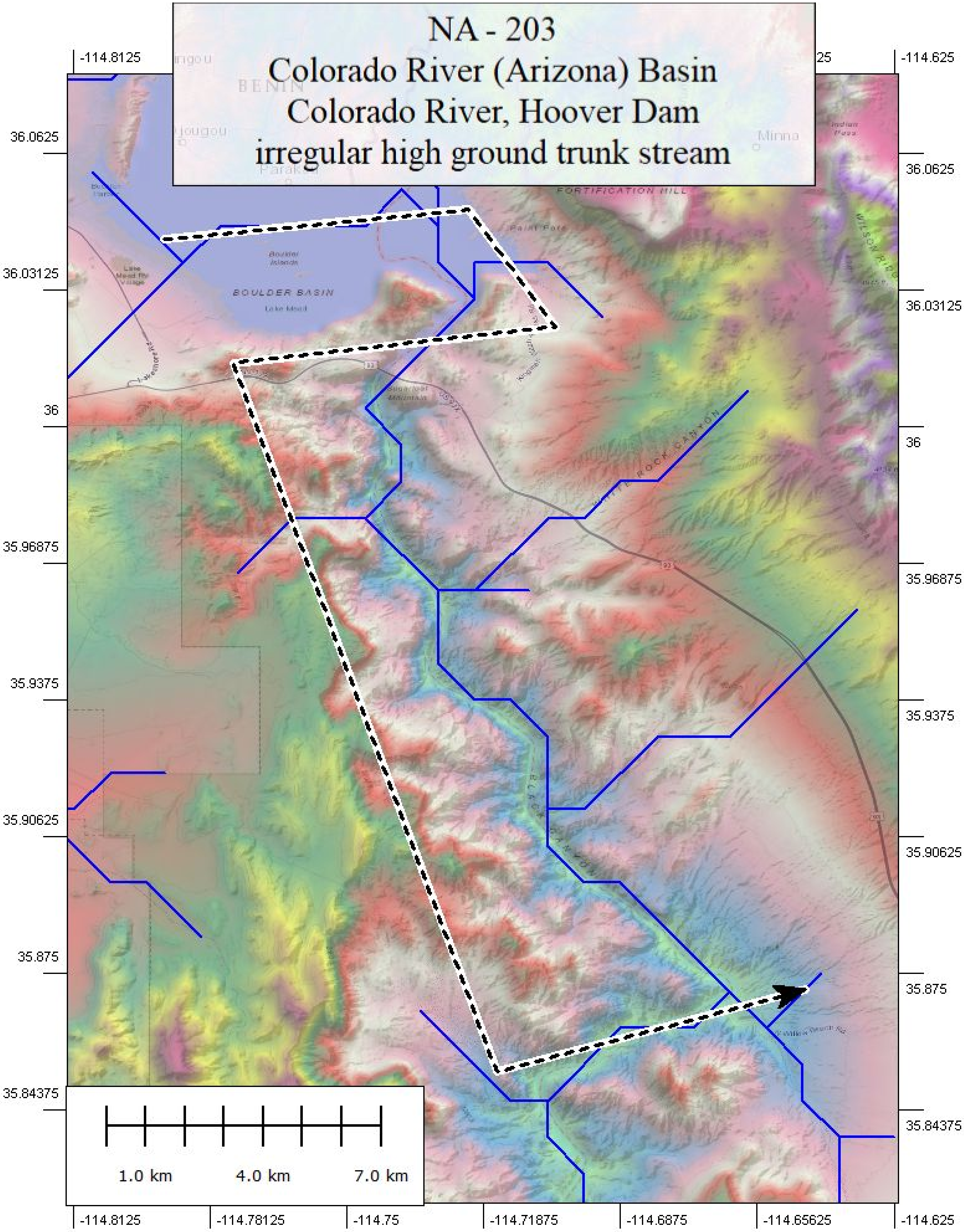
NA - 203
Colorado River (Arizona) Basin
Colorado River, Hoover Dam
irregular high ground trunk stream
-114.8125
-114.78125
-114.75
-114.71875
-114.6875
-114.65625
-114.625
36.0625
36.03125
36
35.96875
35.9375
35.90625
35.875
35.84375
BOULDER BASIN
Lake Mead
FORTIFICATION HILL
WHITE ROCK CANYON
BLACK CANYON
1.0 km
4.0 km
7.0 km

NA - 204
James River Basin
Rivanna River
single-ridge trunk stream

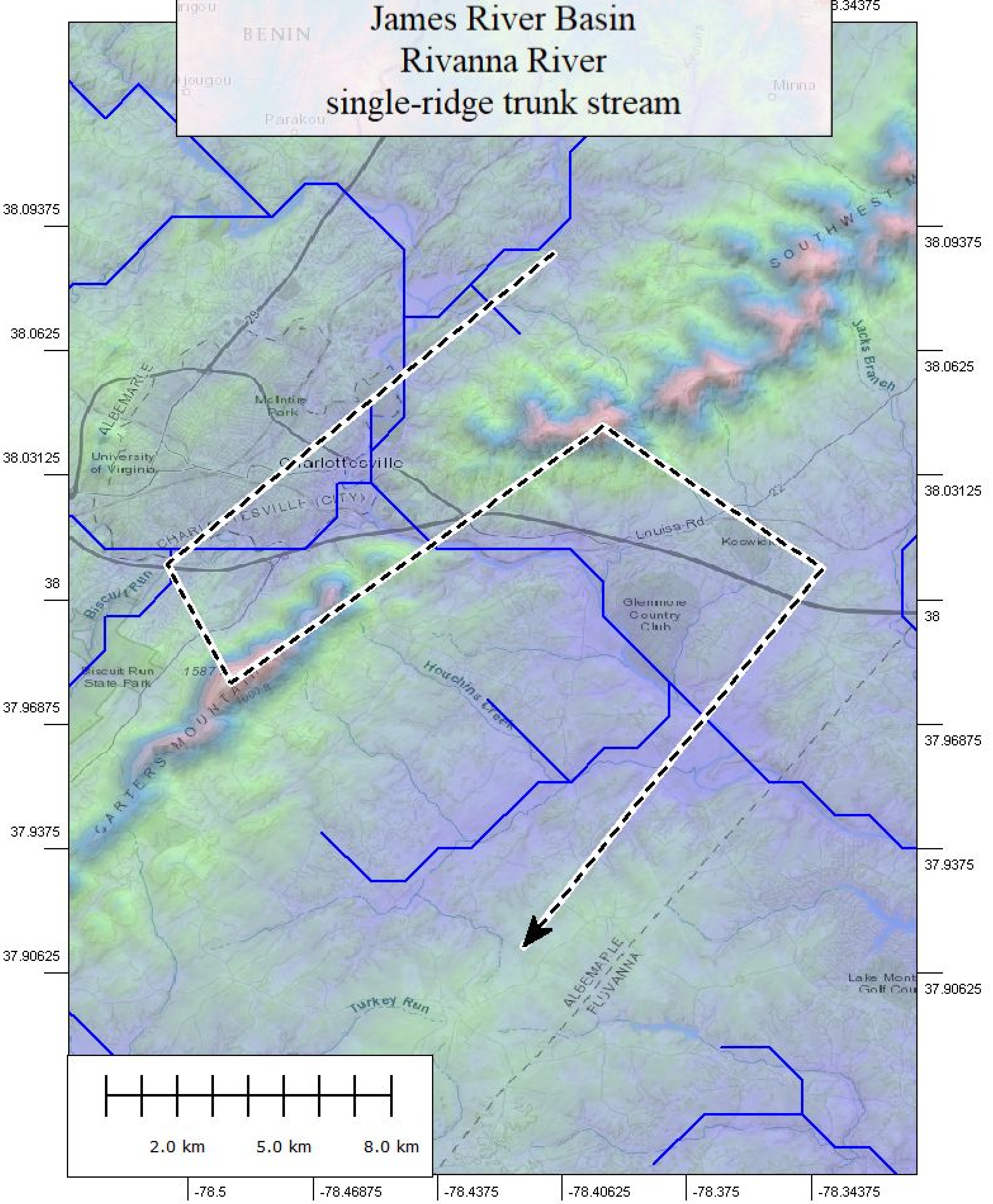

NA - 205
Colorado River (Arizona) Basin
Greem Rove, Split Mountain
single-ridge trunk stream

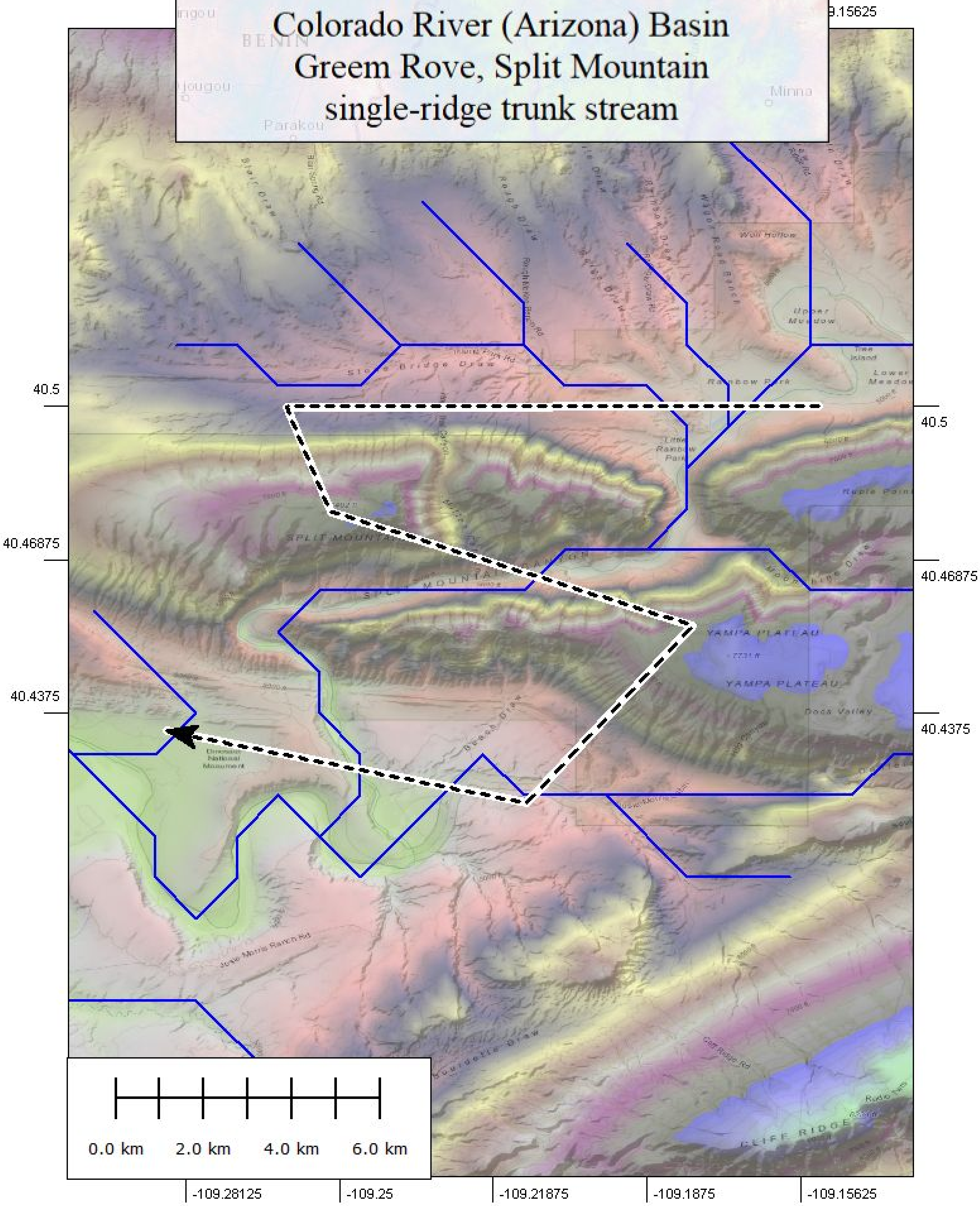

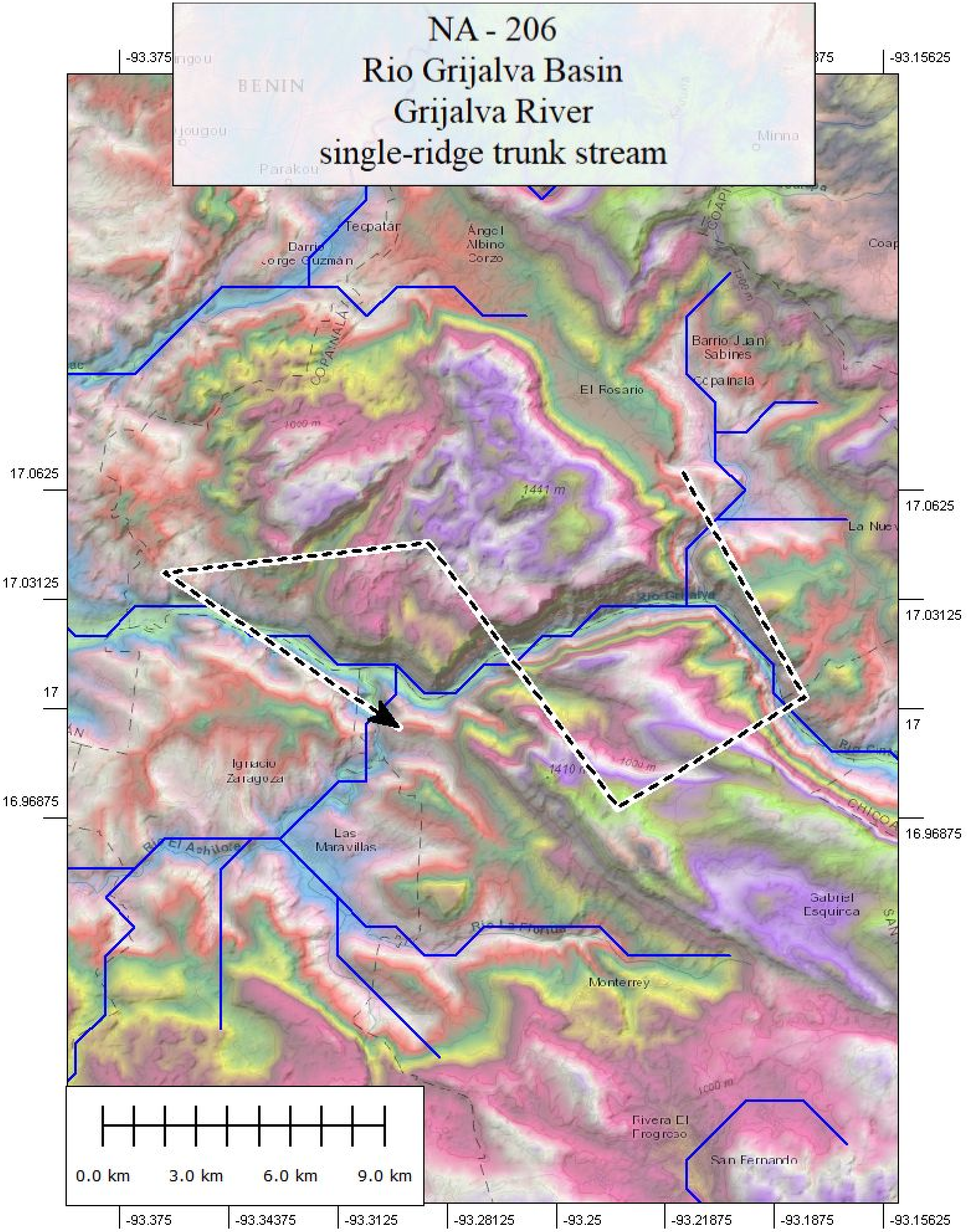

NA - 206
Rio Grijalva Basin
Grijalva River
single-ridge trunk stream
Tecpatán
Barrio Jorge Guzmán
Angel Albino Corzo
Barrio Juan Sabines
Copainalá
El Rosario
1441 m
1000 m
Rio Grijalva
La Nuev
Ignacio Zaragoza
Las Maravillas
1410 m
Gabriel Esquinca
Monterrey
Rivera El Progreso
San Fernando
0.0 km
3.0 km
6.0 km
9.0 km
-93.375
-93.34375
-93.3125
-93.28125
-93.25
-93.21875
-93.1875
-93.15625
17.0625
17.03125
17
16.96875

NA - 207
Mississippi River Basin
Watauga River
single-ridge trunk stream

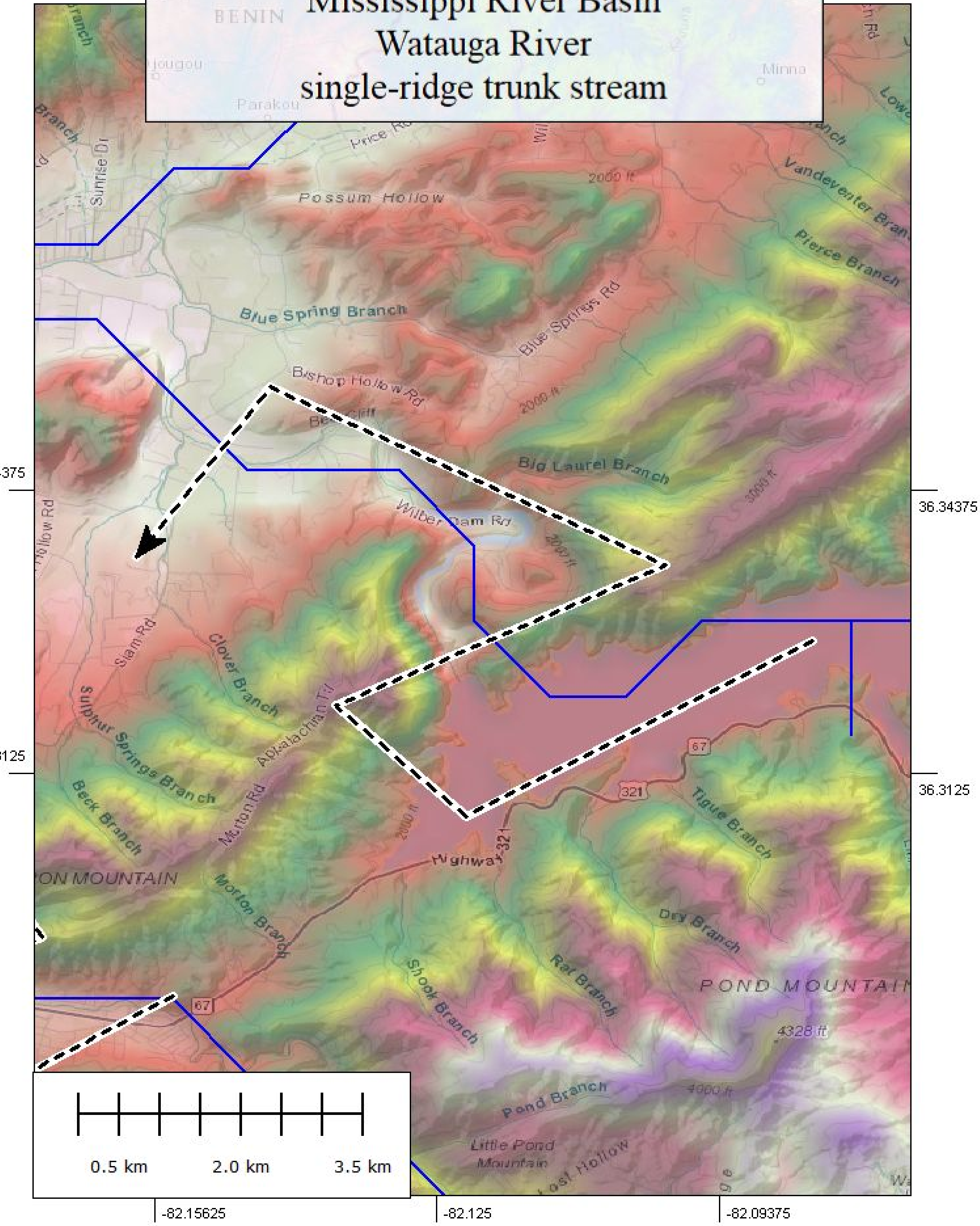

NA - 208
Colorado River (Arizona) Basin
White River, Fairfield Gulch
single-ridge trunk stream

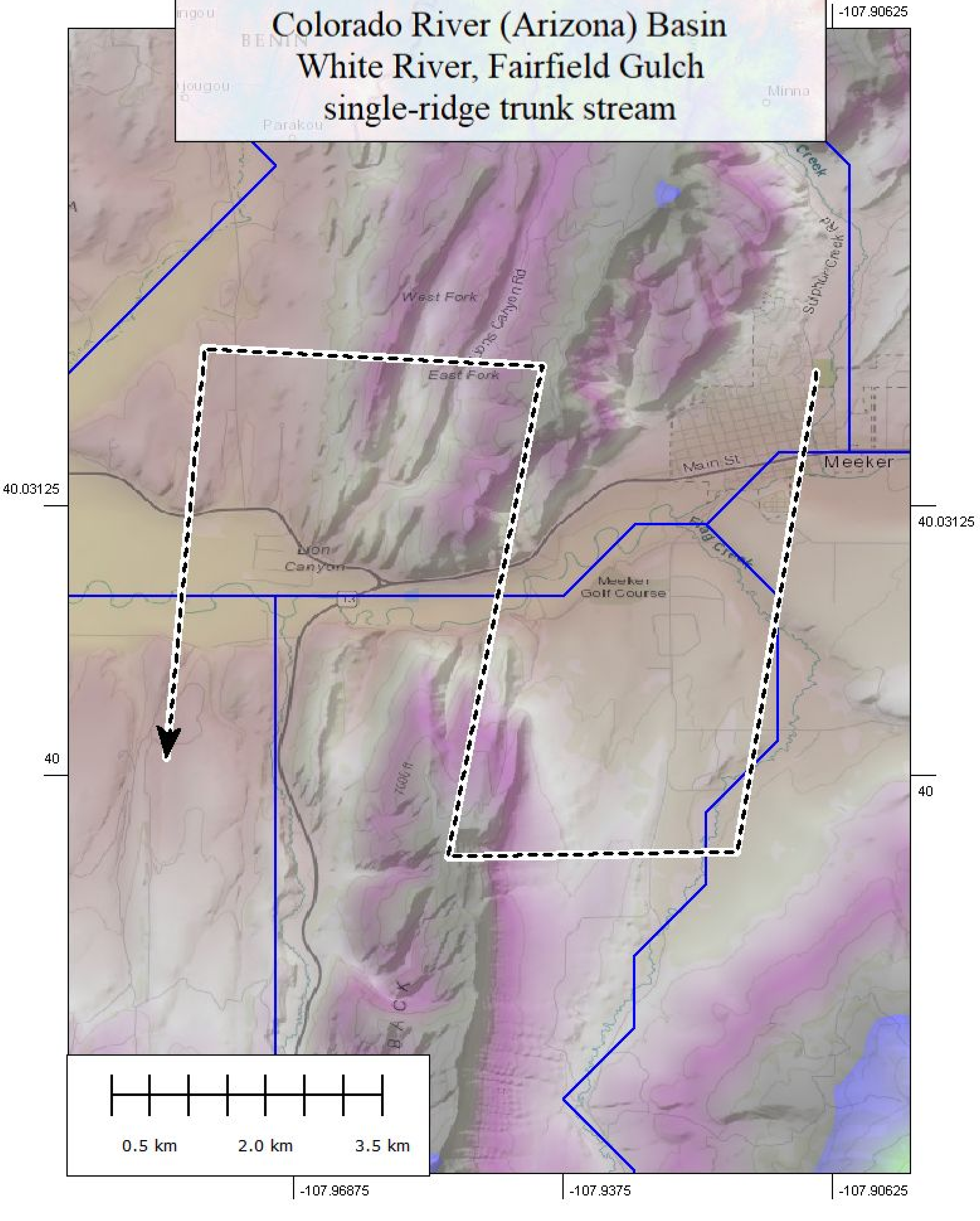

NA - 209
Colorado River (Arizona) Basin
Rifle Creek, Rifle Gap
single-ridge trunk stream

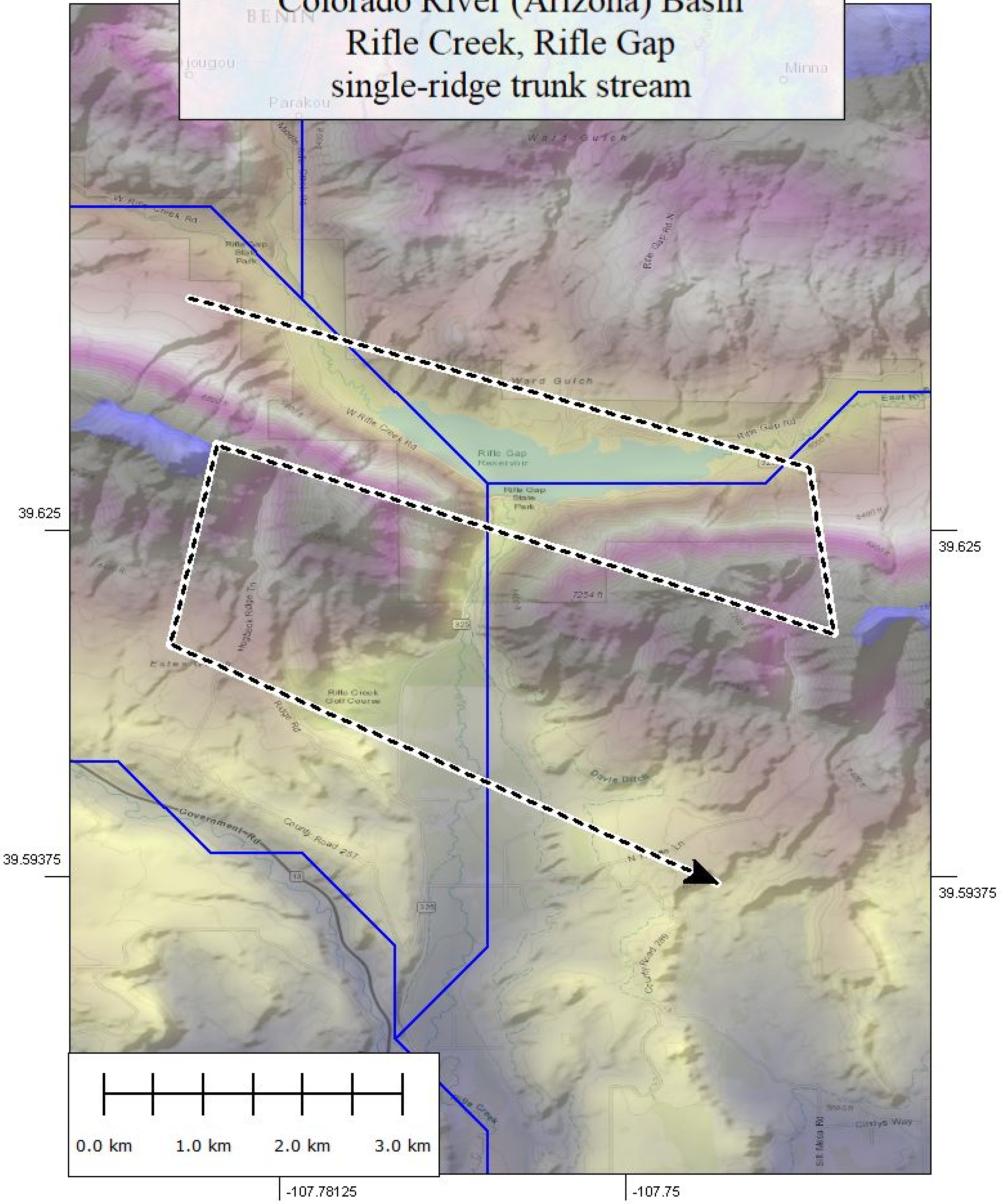

NA - 21
Colorado River (Arizona) Basin
Muddy Creek
updip plateau trunk stream

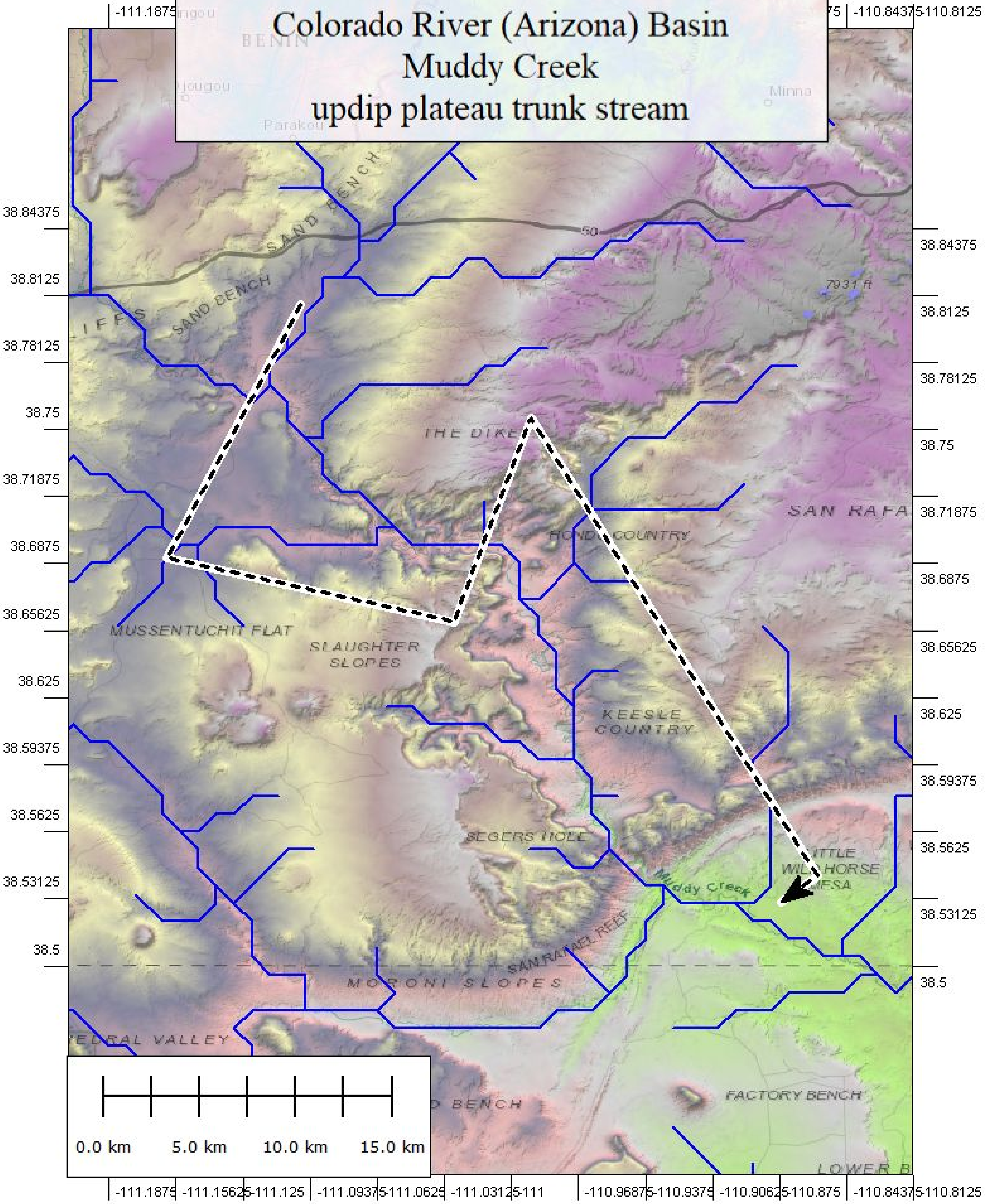

NA - 210
Mississippi River Basin
Youghiogheny River
single-ridge trunk stream

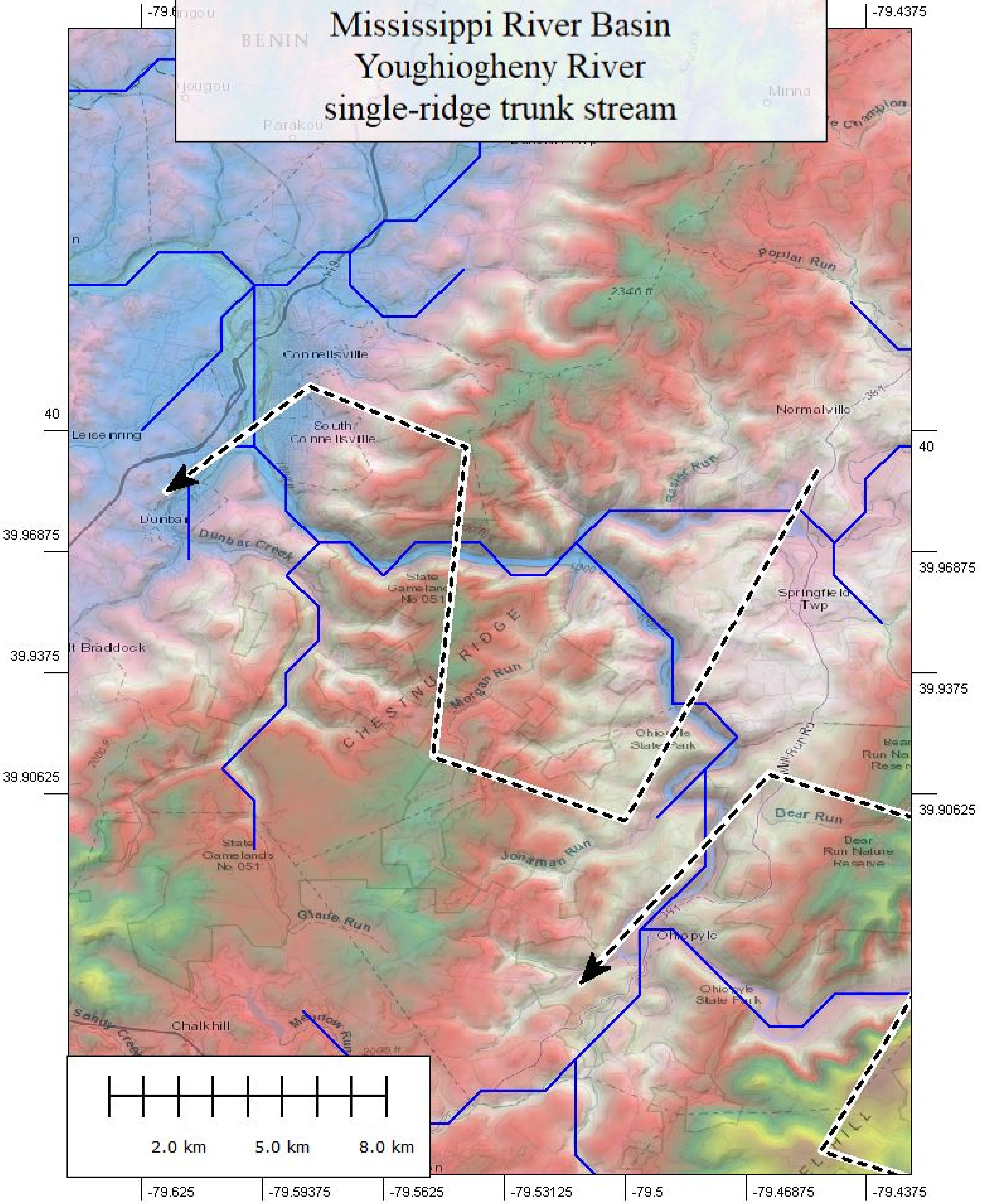

NA - 211
Rio Grijalva Basin
Petalcingo River tributary
single-ridge trunk stream

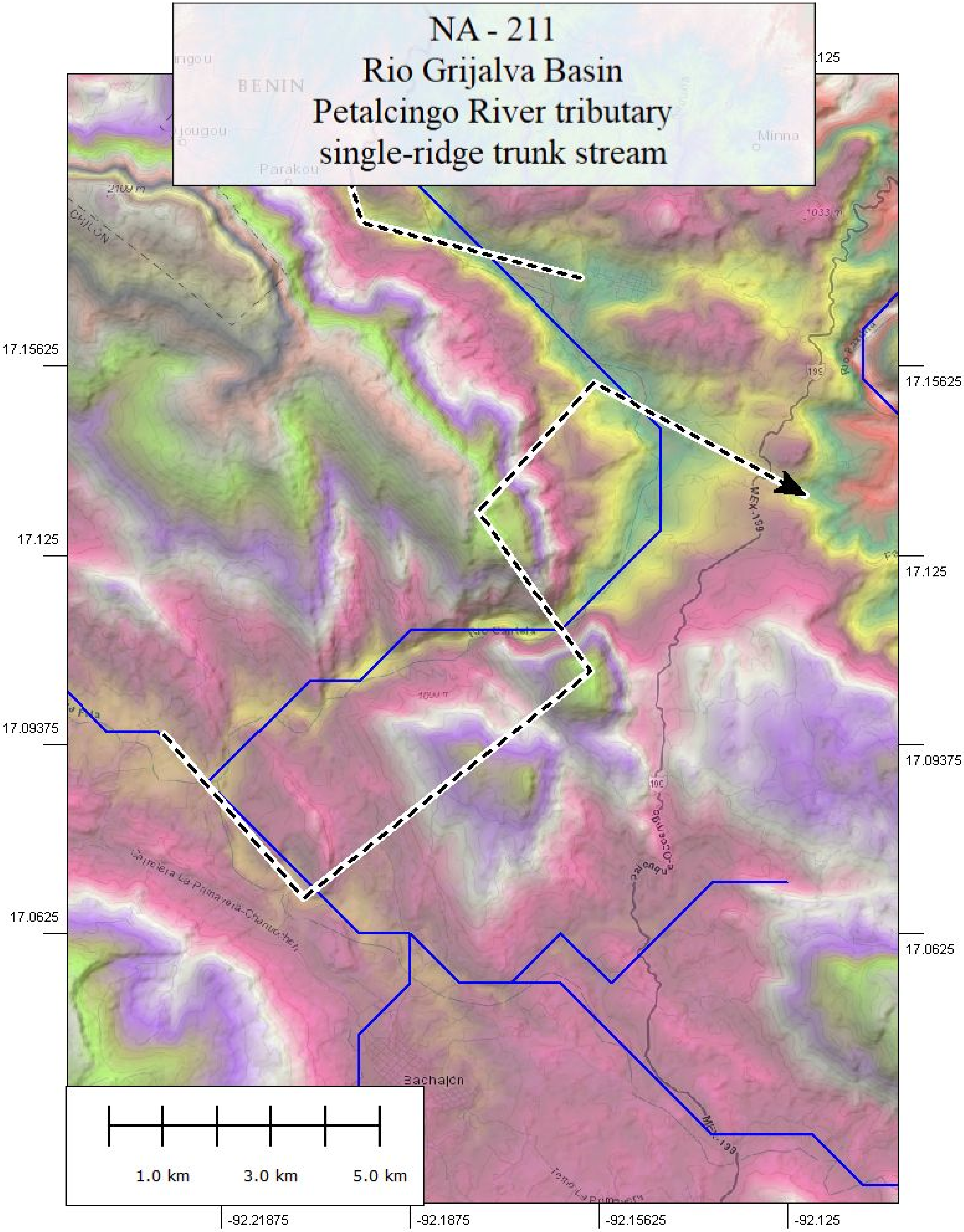

NA - 212
Rio Balsas Basin
Atoyac River
irregular high ground trunk stream

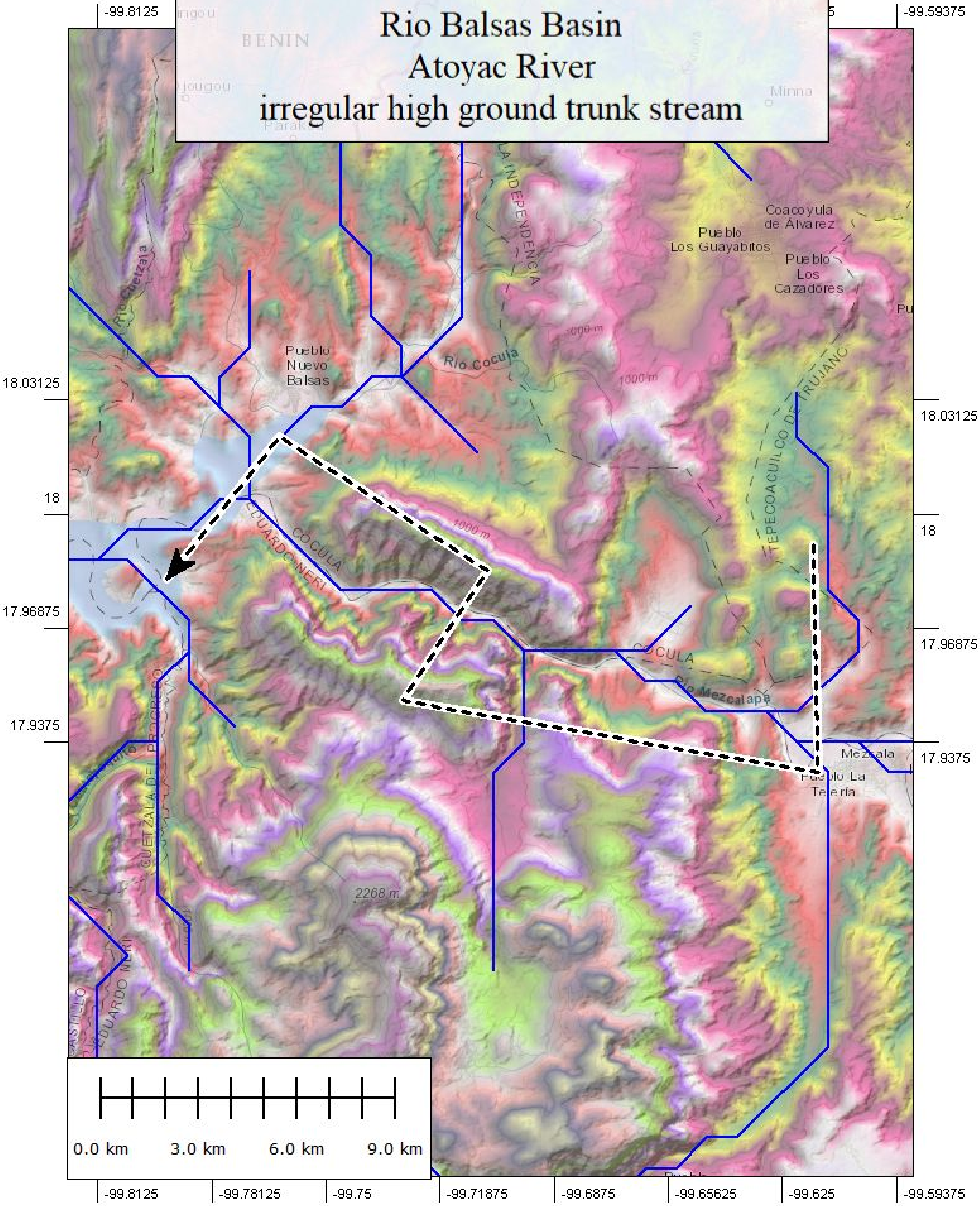

NA - 213
Pnuco River Basin
Escanela River, Angostura Canyon
single-ridge trunk stream

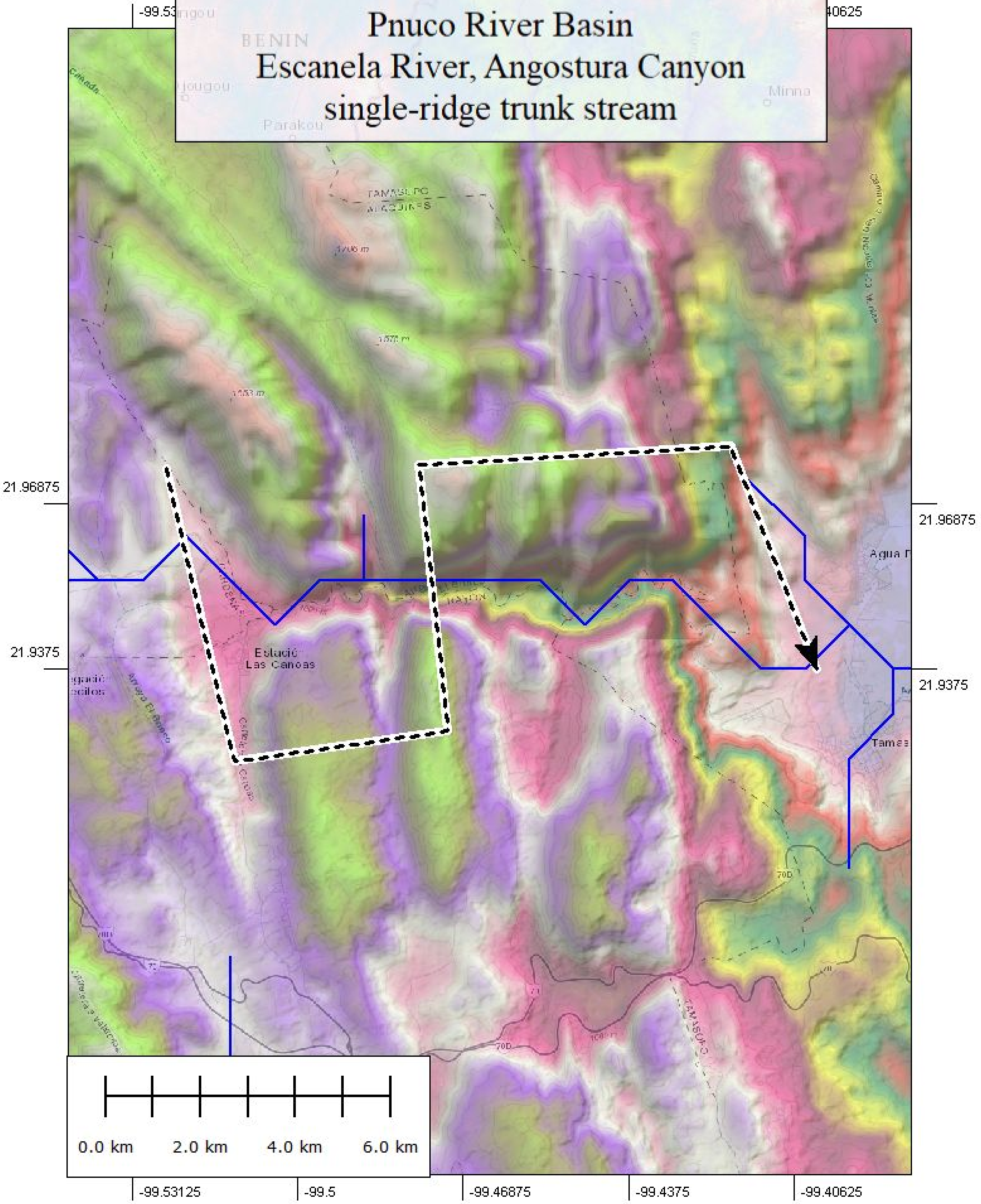

NA - 214
Rio Grijalva Basin
Petalcingo River tributary
irregular high ground trunk stream

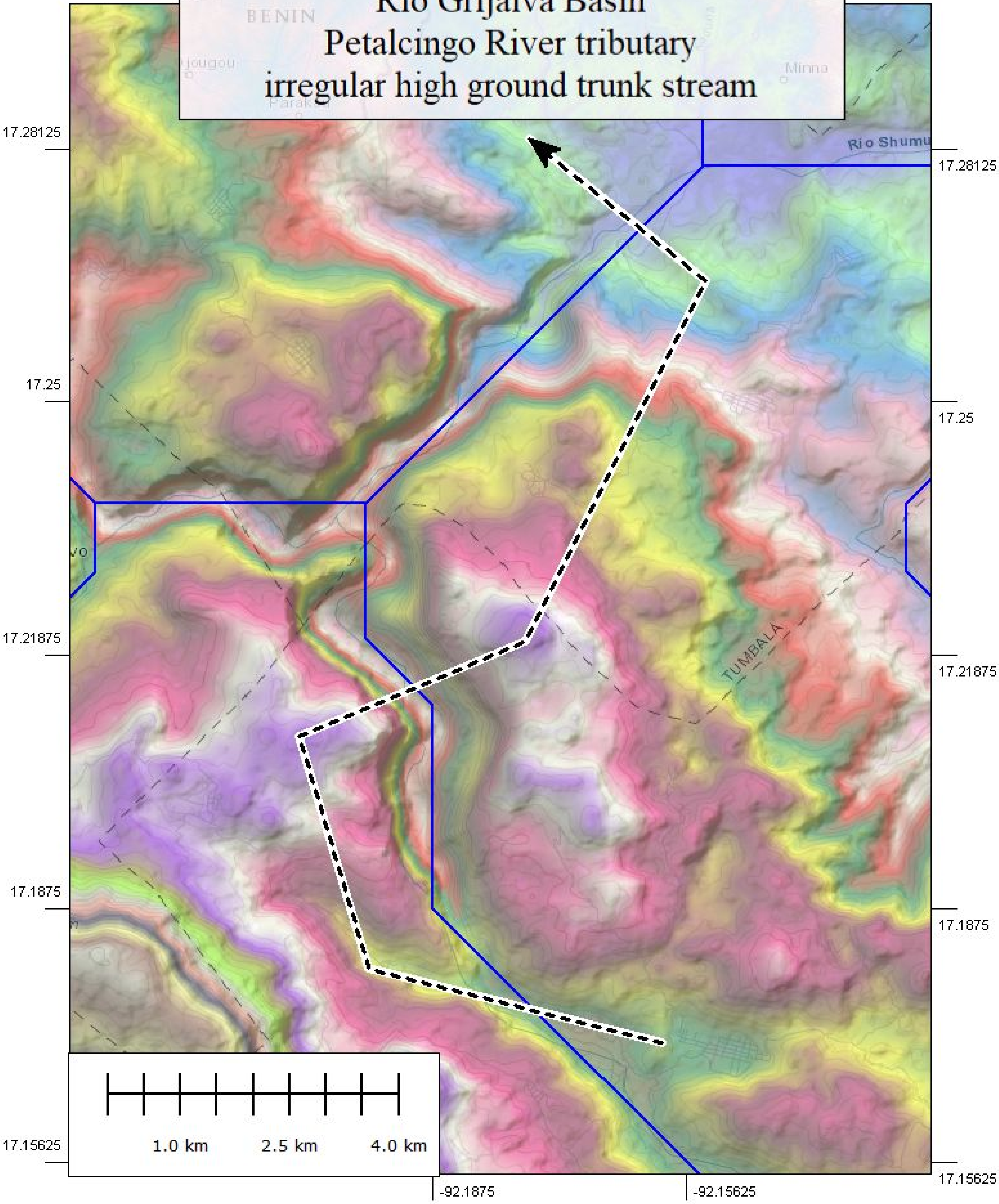

NA - 215
Rio Grande (Texas-Mexico) Basin
El Berrendo de Arriba Pass
single-ridge trunk stream

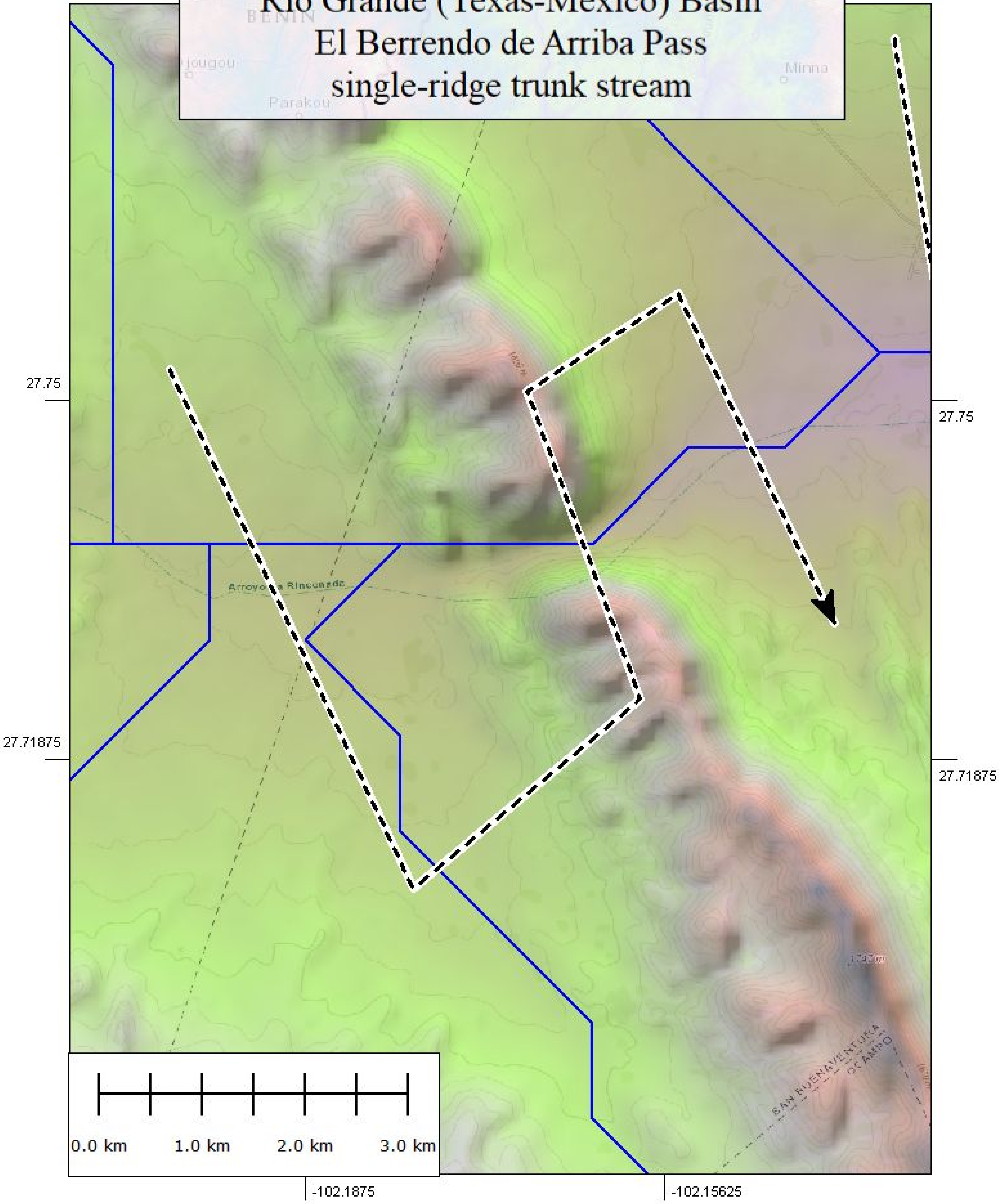

NA - 216
Rio Grande (Texas-Mexico) Basin
El Berrendo de Arriba Pass
single-ridge trunk stream

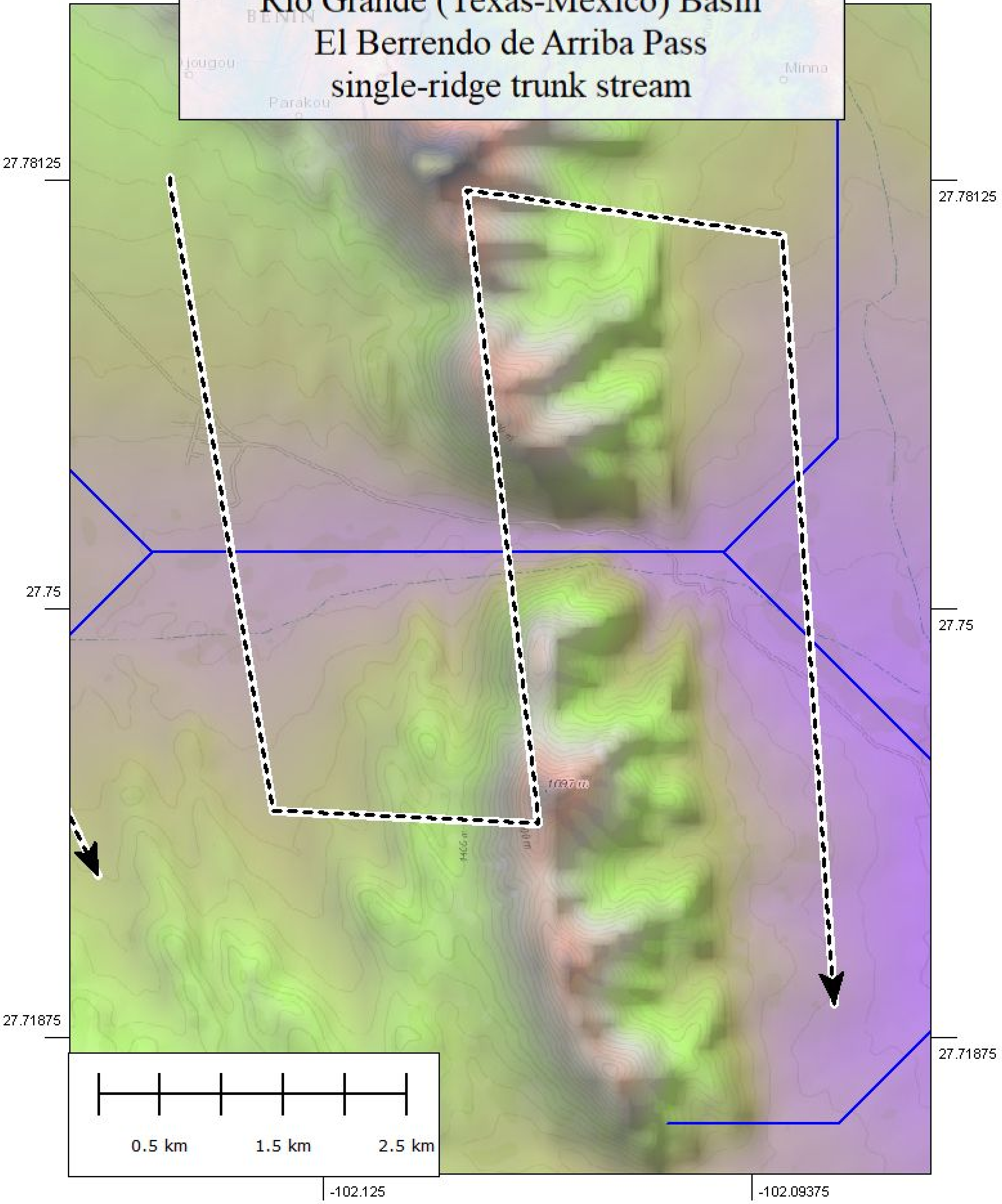

NA - 217
Rio Grande (Texas-Mexico) Basin
Santa Ana Pass
single-ridge trunk stream

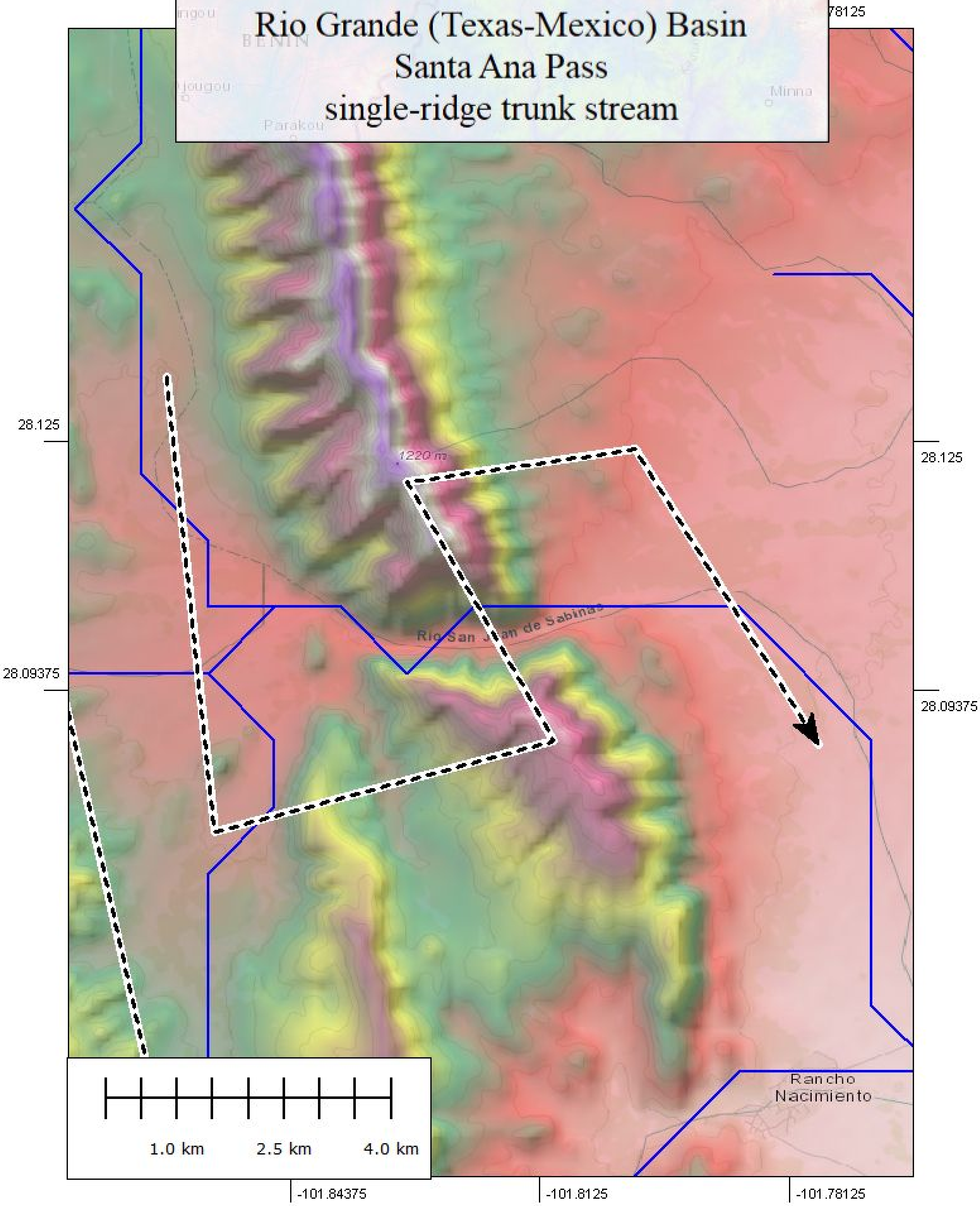

NA - 218
Rio Grande (Texas-Mexico) Basin
Sabinas Hidalgo River
single-ridge trunk stream

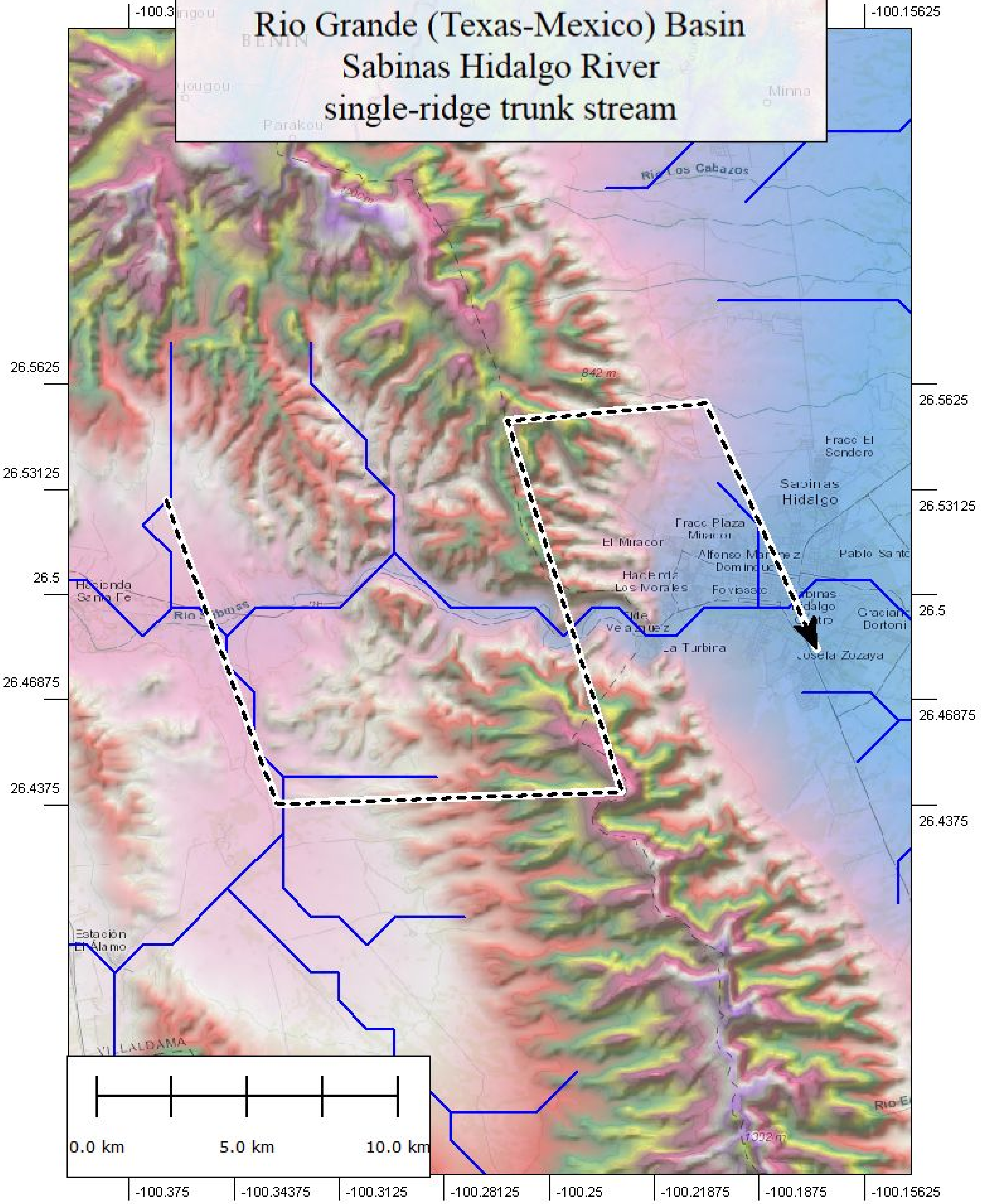

NA - 219
Rio Grande (Texas-Mexico) Basin
single-ridge trunk stream

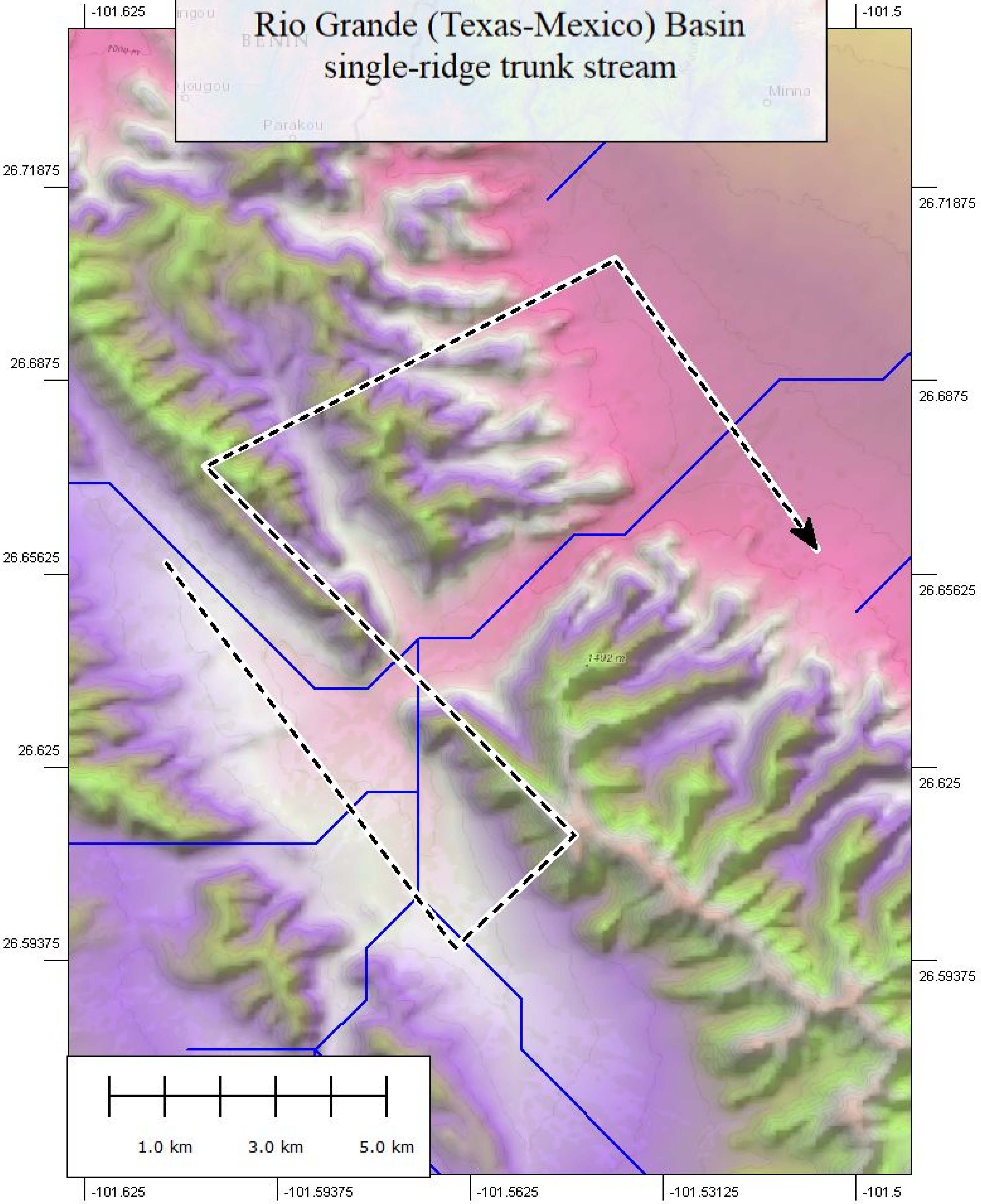

NA - 22
Sacramento River Basin
Golden Gate Narrows
irregular high ground trunk stream

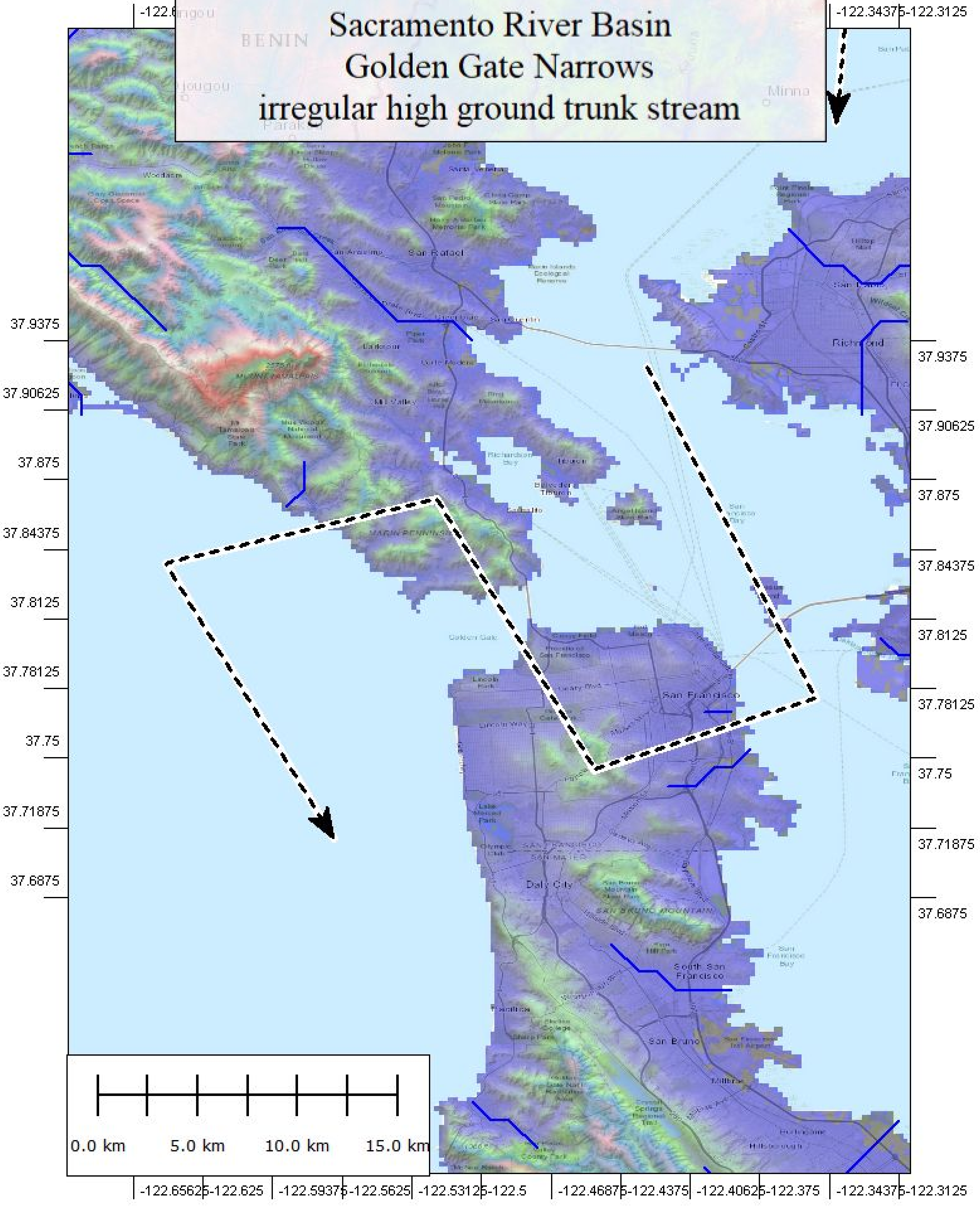

NA - 220
Terraba River Basin
Rio Grande de Terraba
single-ridge trunk stream

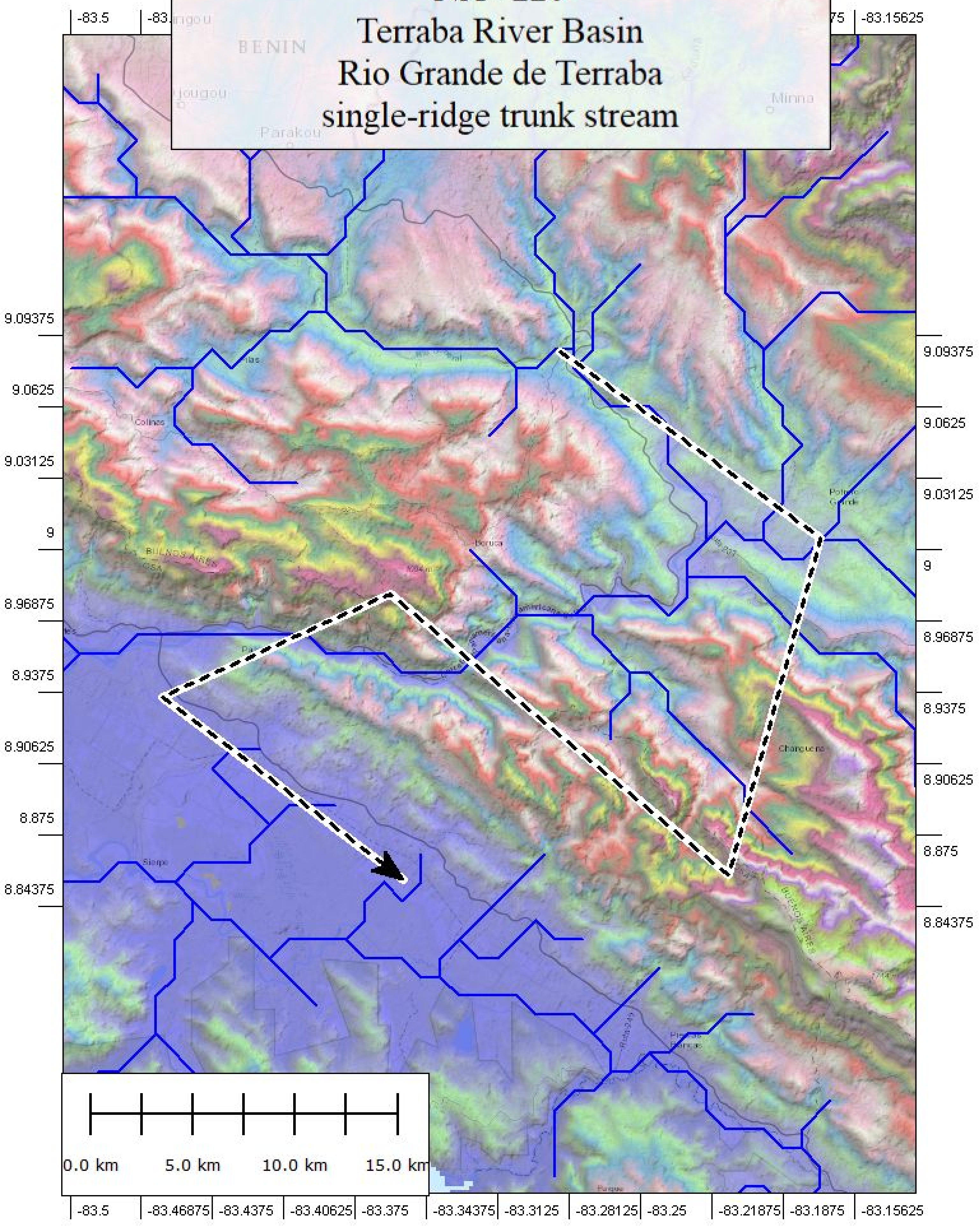

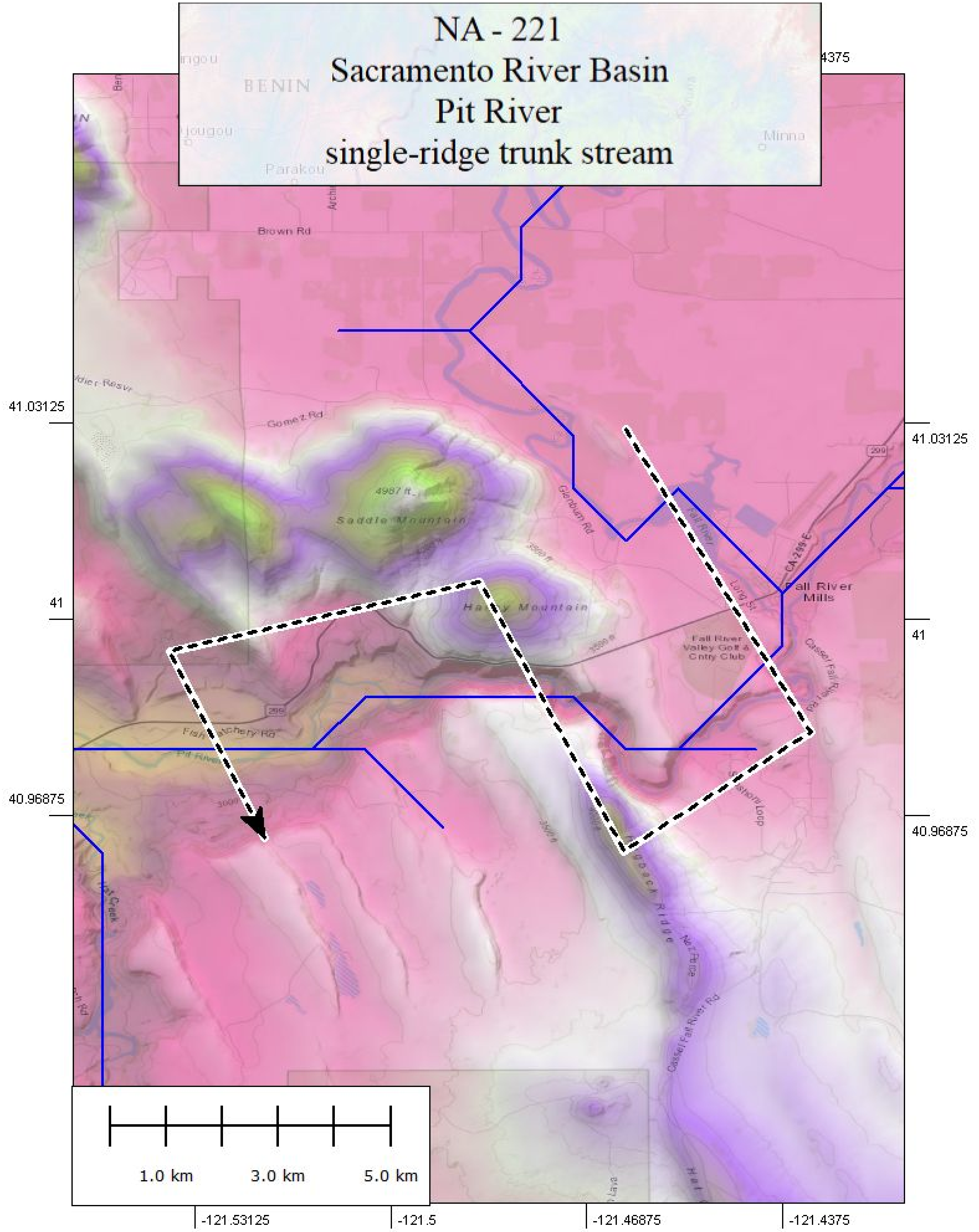
NA - 221
Sacramento River Basin
Pit River
single-ridge trunk stream
41.03125
41
40.96875
-121.53125
-121.5
-121.46875
-121.4375
1.0 km
3.0 km
5.0 km
Saddle Mountain
4987 ft
Hatchet Mountain
Fall River Mills
Fall River Valley Golf & Cntry Club
Brown Rd
Gomez Rd
Glenburn Rd
Fish Hatchery Rd
Pit River
Cassel Fall River Rd
CA-299-E
Long St
Hogback Ridge

NA - 222
Rio Grijalva Basin
Grijalva River tributary
downdip plateau trunk stream

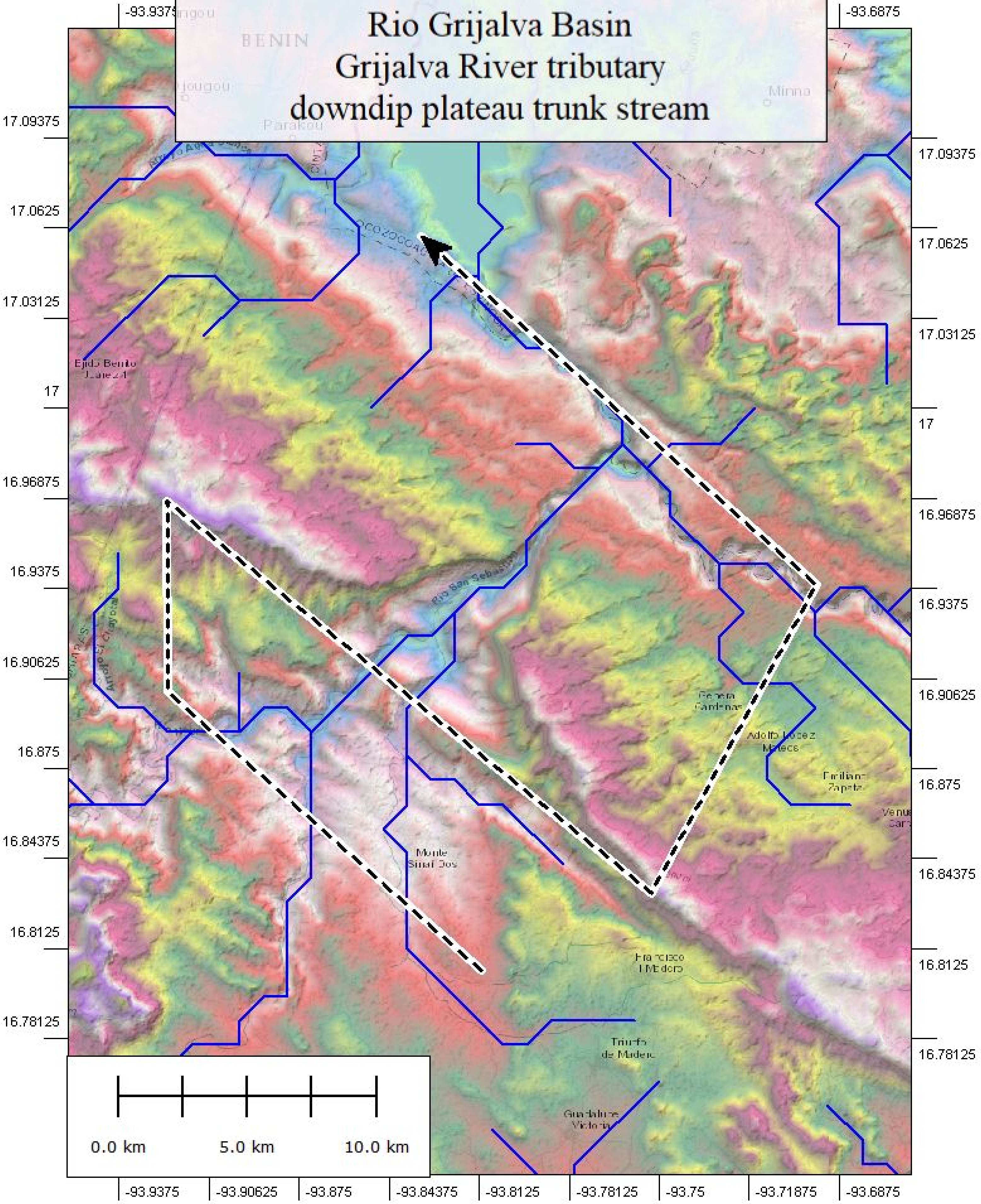

NA - 223
Rio Grijalva Basin
Grijalva River tributary
plateau trunk stream

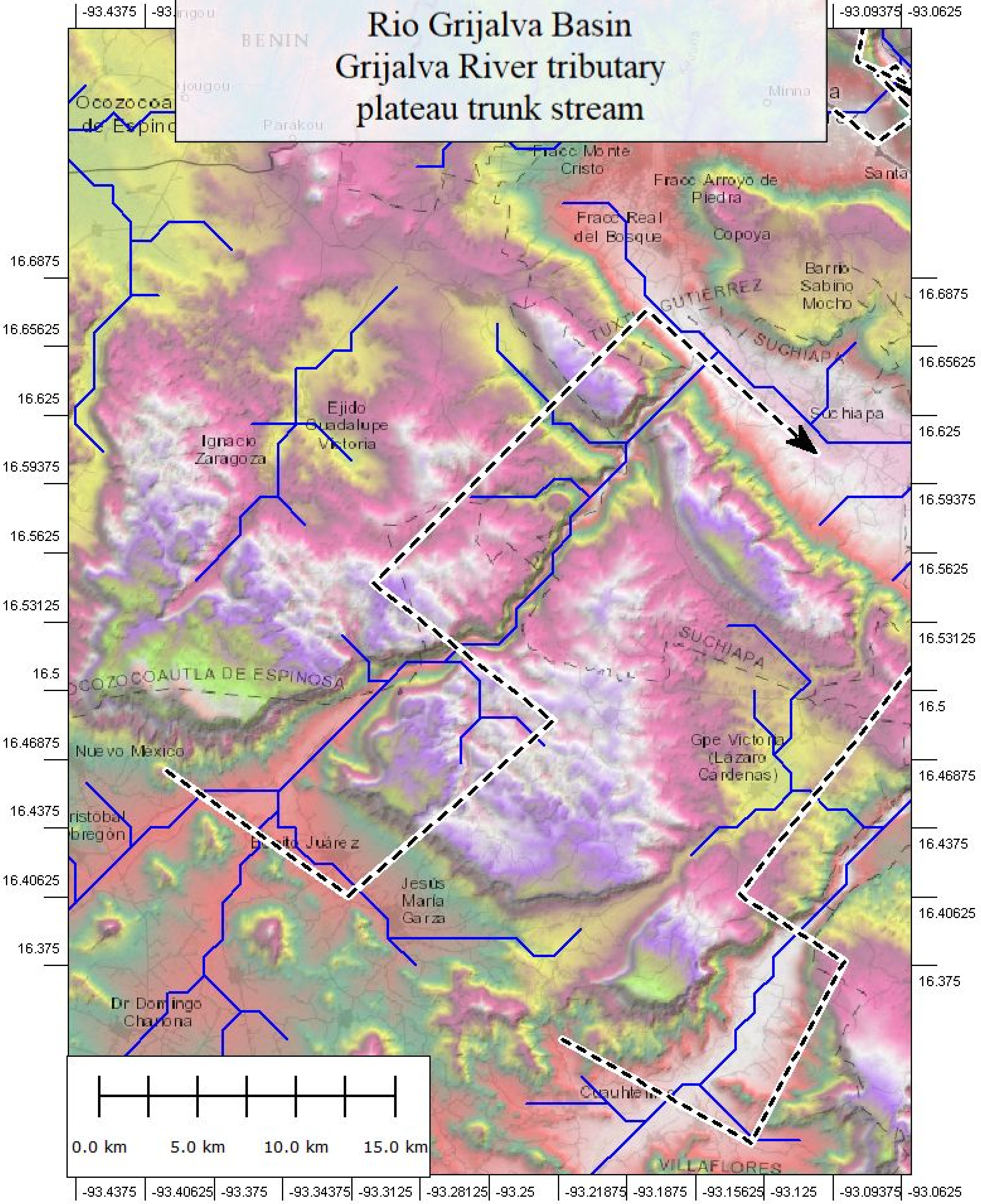

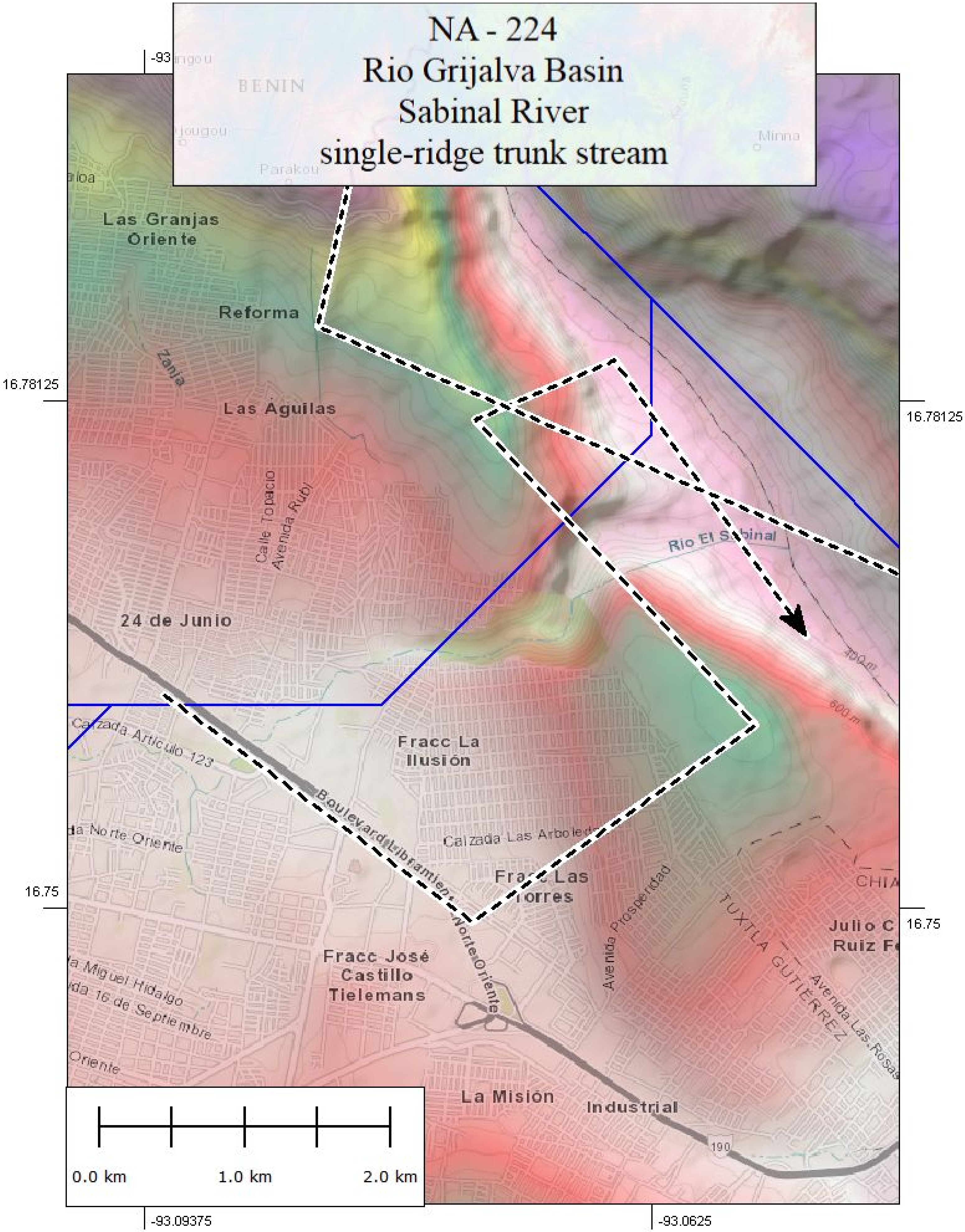

NA - 224
Rio Grijalva Basin
Sabinal River
single-ridge trunk stream
-93
16.78125
16.78125
16.75
16.75
-93.09375
-93.0625
Las Granjas Oriente
Reforma
Zanja
Las Aguilas
Calle Topacio
Avenida Ruby
Rio El Sabinal
24 de Junio
Calzada Articulo 123
Fracc La Ilusión
Boulevard Libramiento Norte Oriente
Calzada Las Arboledas
Fracc Las Torres
Avenida Prosperidad
TUXTLA GUTIÉRREZ
Avenida Las Rosas
Fracc José Castillo Tielemans
La Misión
Industrial
190
400 m
600 m
0.0 km
1.0 km
2.0 km

NA - 225
Rio Grijalva Basin
Grijalva Rivertributary
single-ridge trunk stream

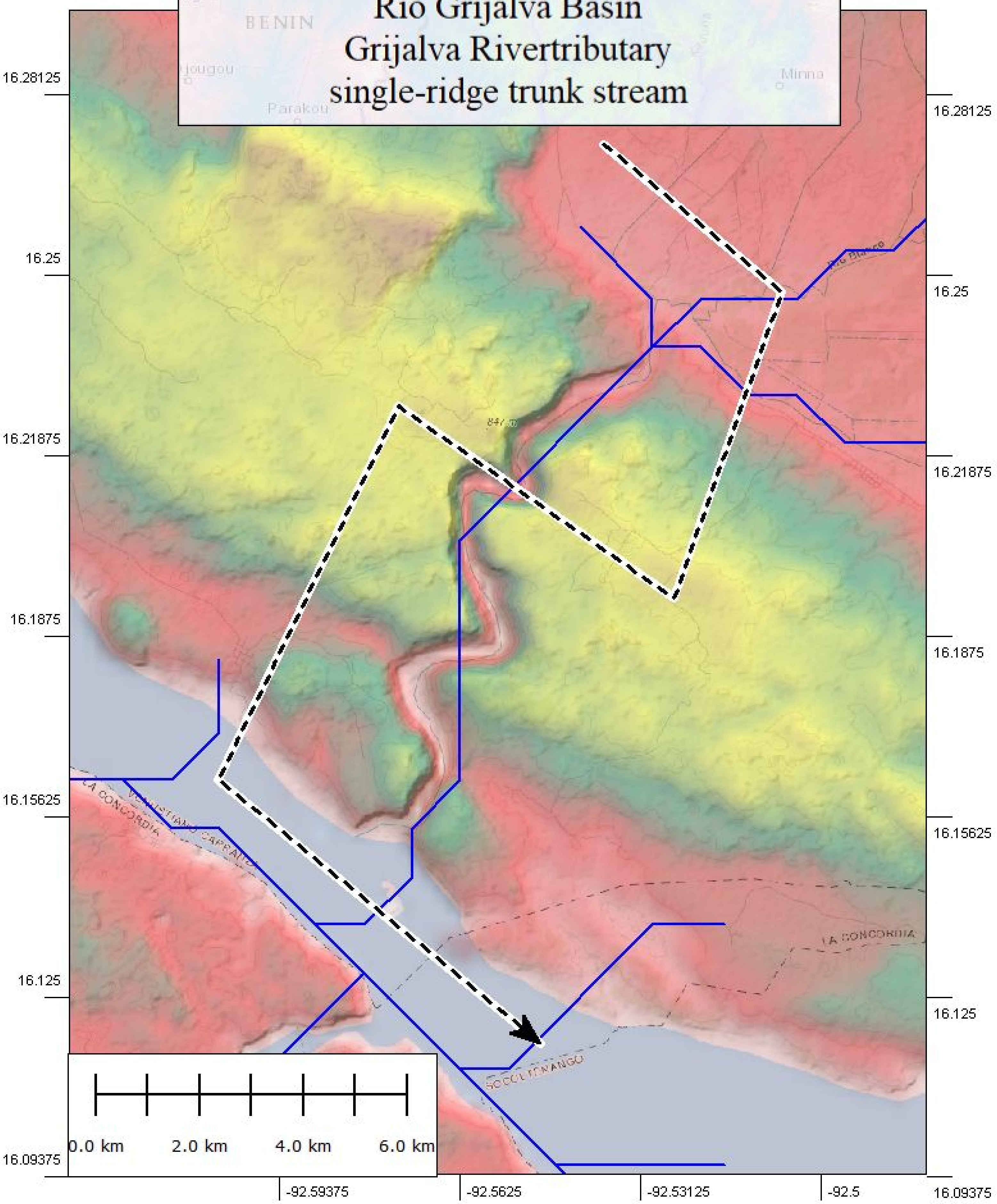

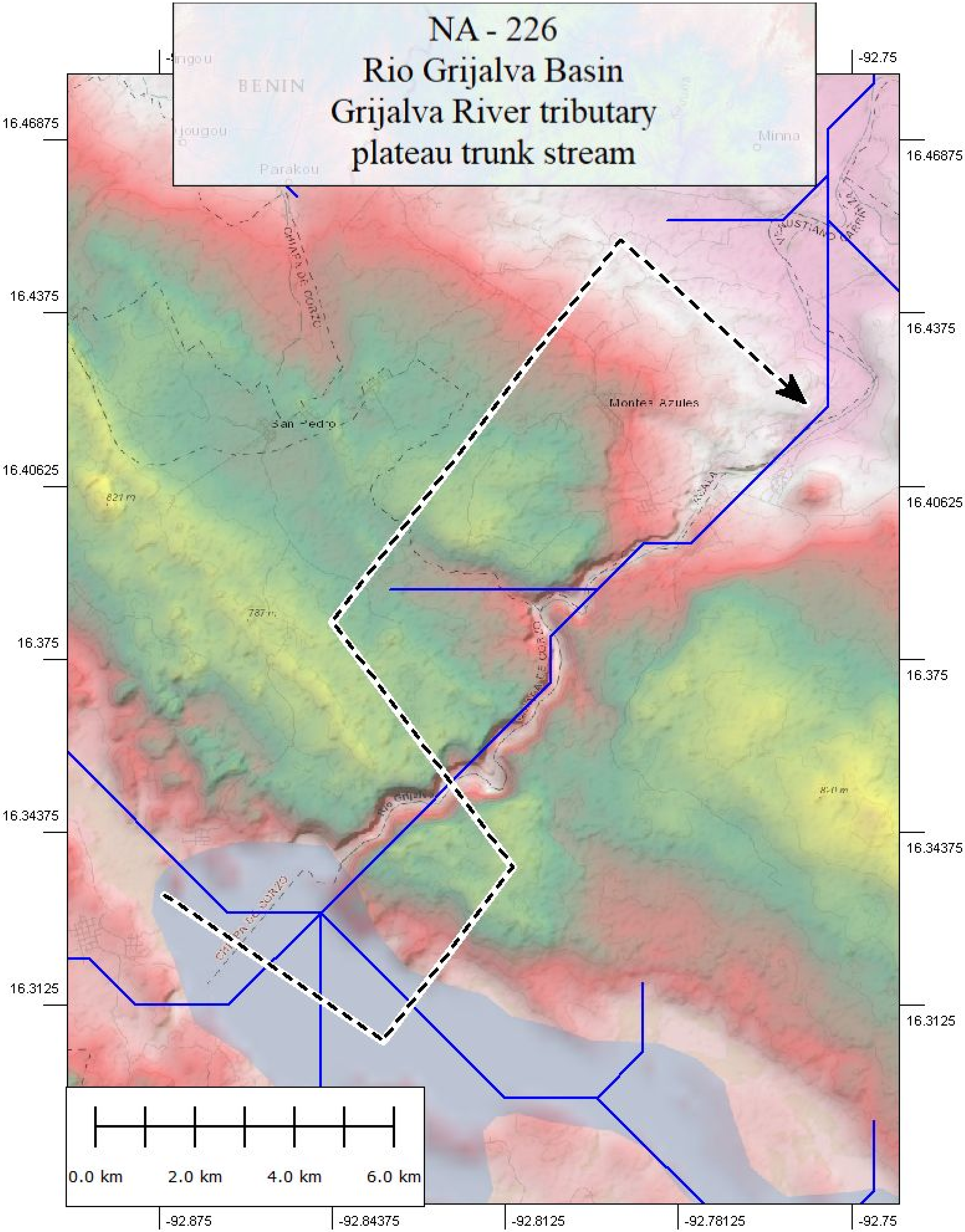
NA - 226
Rio Grijalva Basin
Grijalva River tributary
plateau trunk stream
Montes Azules
San Pedro
821 m
787 m
16.46875
16.4375
16.40625
16.375
16.34375
16.3125
-92.875
-92.84375
-92.8125
-92.78125
-92.75
0.0 km
2.0 km
4.0 km
6.0 km

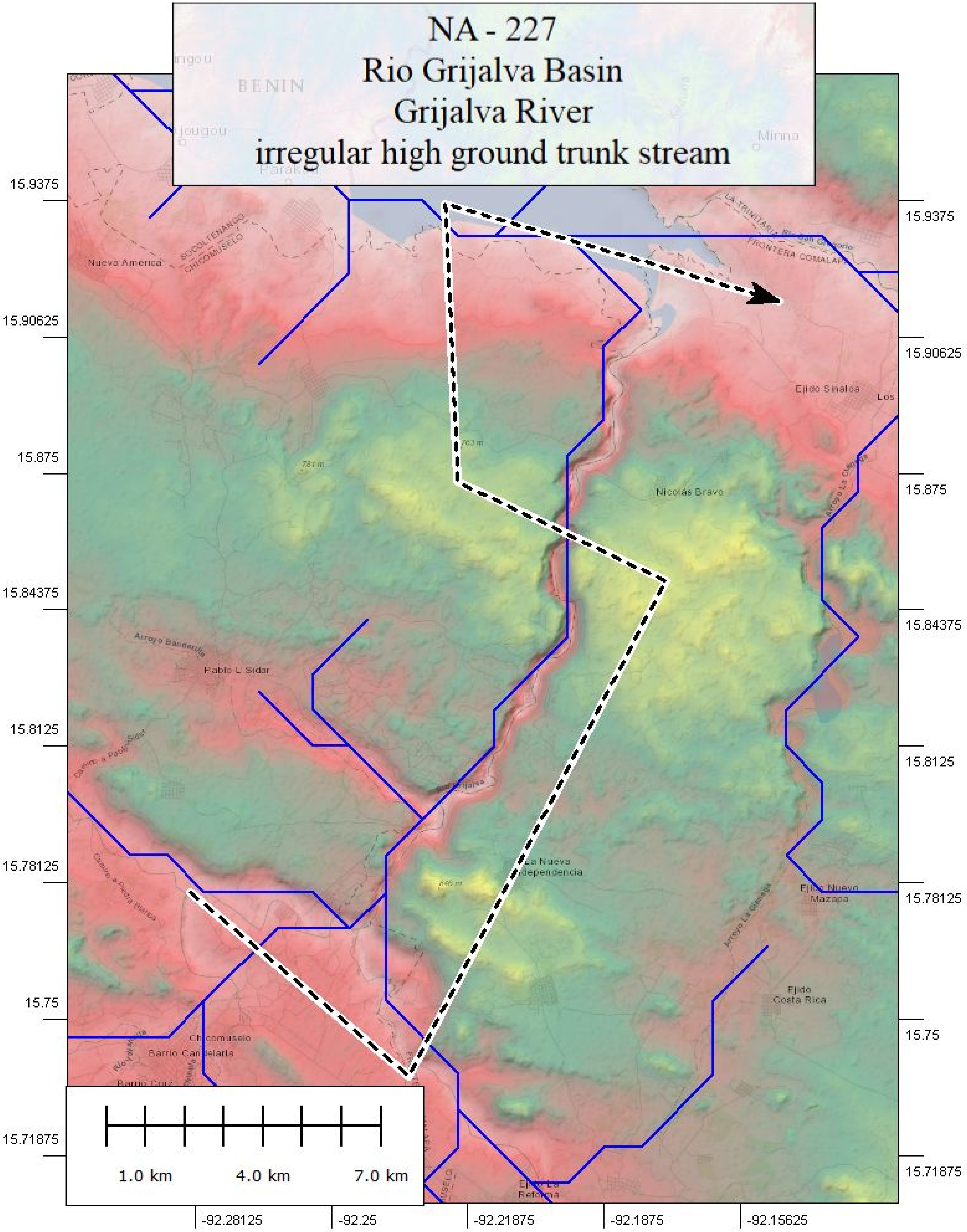
NA - 227
Rio Grijalva Basin
Grijalva River
irregular high ground trunk stream
15.9375
15.90625
15.875
15.84375
15.8125
15.78125
15.75
15.71875
-92.28125
-92.25
-92.21875
-92.1875
-92.15625
1.0 km
4.0 km
7.0 km
BENIN
Nueva America
SOCOLTENANGO
CHICOMUSELO
LA TRINITARIA
FRONTERA COMALAPA
Ejido Sinaloa
Los
763 m
781 m
Nicolás Bravo
Arroyo La Ciénega
Arroyo Banderilla
Pablo L Sidar
Rio Grijalva
La Nueva
Independencia
646 m
Ejido Nuevo
Mazapa
Ejido
Costa Rica
Chicomuselo
Barrio Candelaria
Barrio Cruz
Ejido La
Reforma

NA - 228
Yaqui River Basin
Rio Chico
single-ridge trunk stream

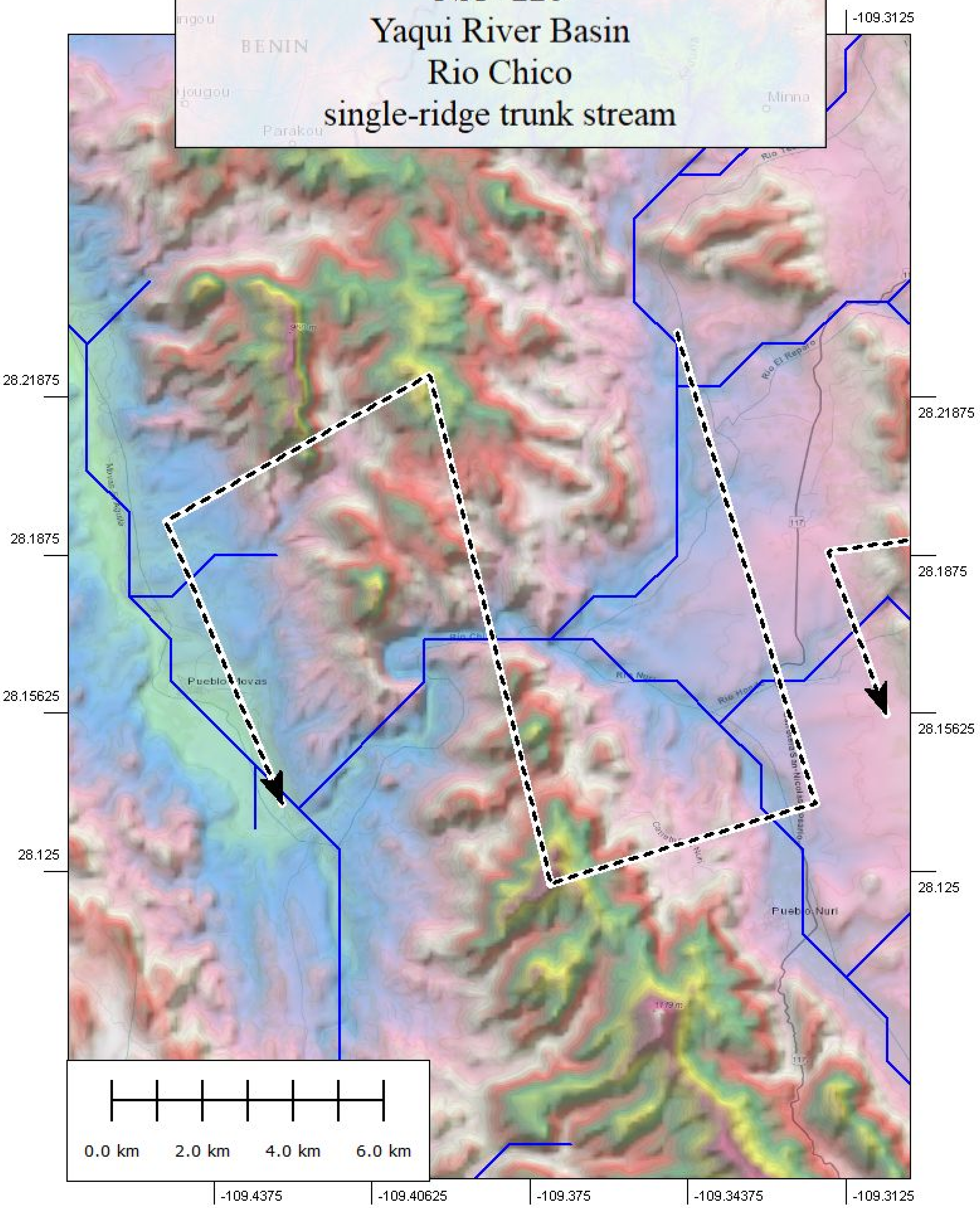

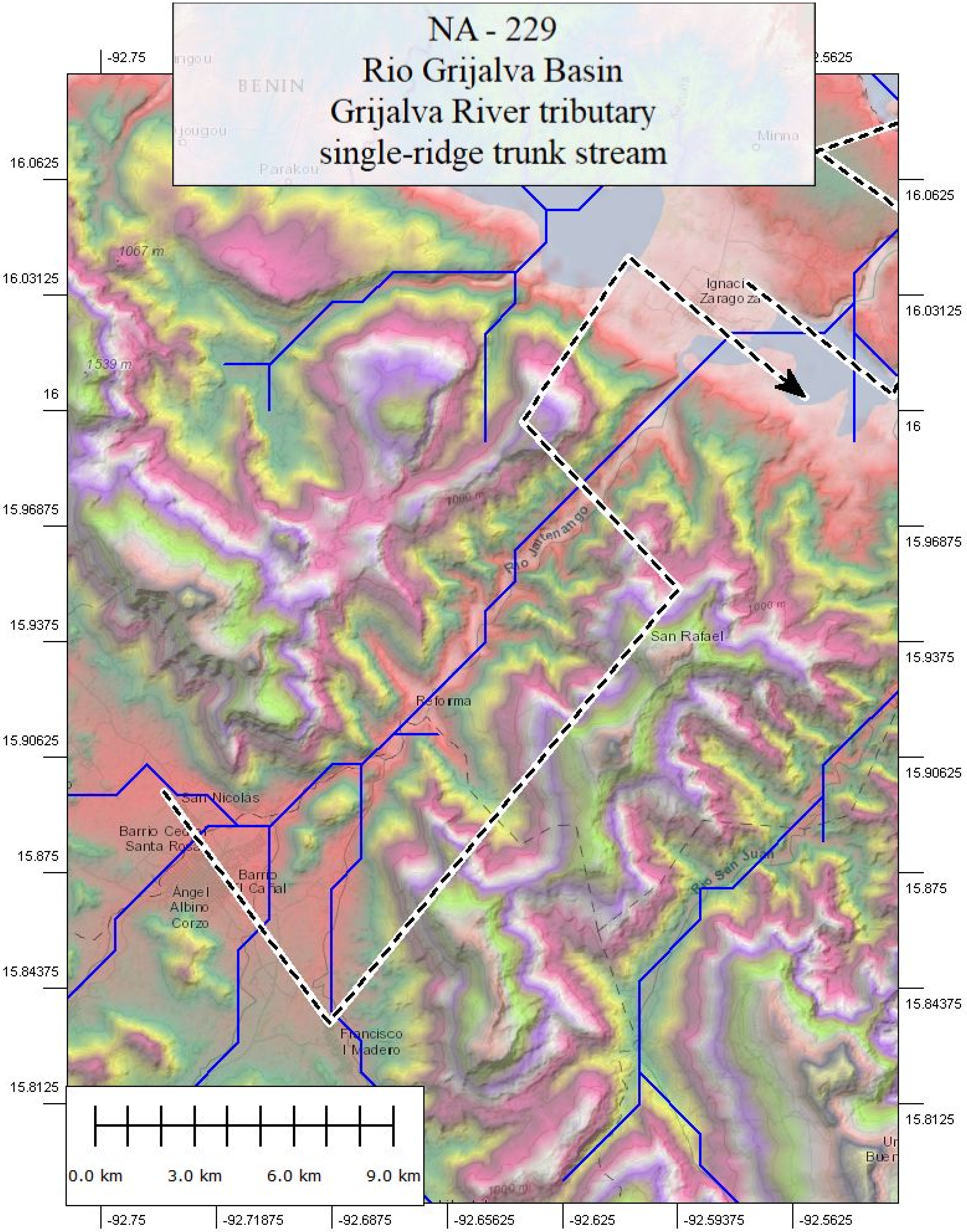
NA - 229
Rio Grijalva Basin
Grijalva River tributary
single-ridge trunk stream
-92.75
-92.71875
-92.6875
-92.65625
-92.625
-92.59375
-92.5625
16.0625
16.03125
16
15.96875
15.9375
15.90625
15.875
15.84375
15.8125
1067 m
1539 m
1000 m
Ignaci
Zaragoza
Rio Jaltenango
San Rafael
Reforma
San Nicolás
Barrio Cecil
Santa Rosa
Barrio
l Canal
Ángel
Albino
Corzo
Francisco
I Madero
Rio San Juan
0.0 km
3.0 km
6.0 km
9.0 km

NA - 23
Roanoke River Basin
Mason Creek
single-ridge head stream

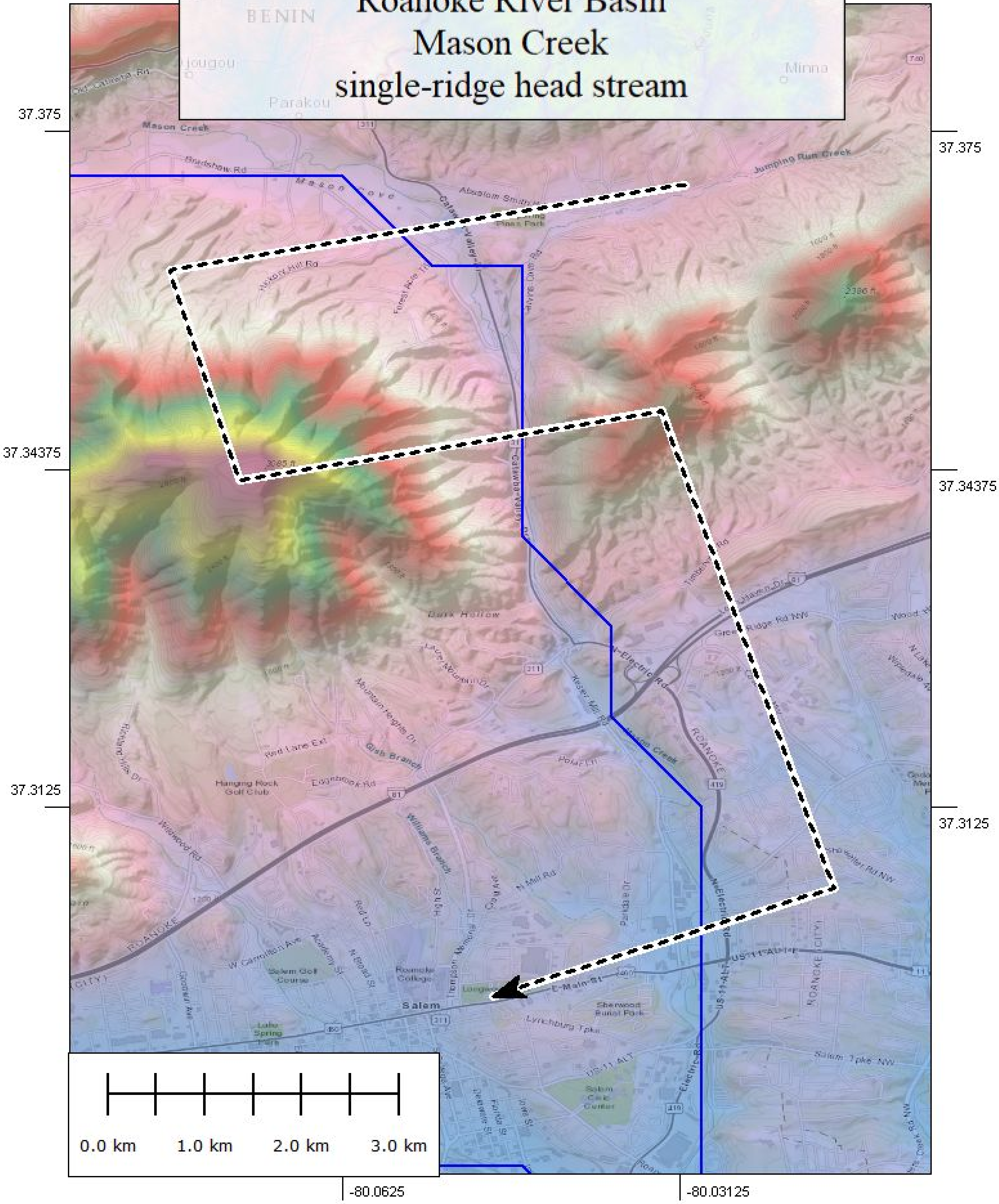

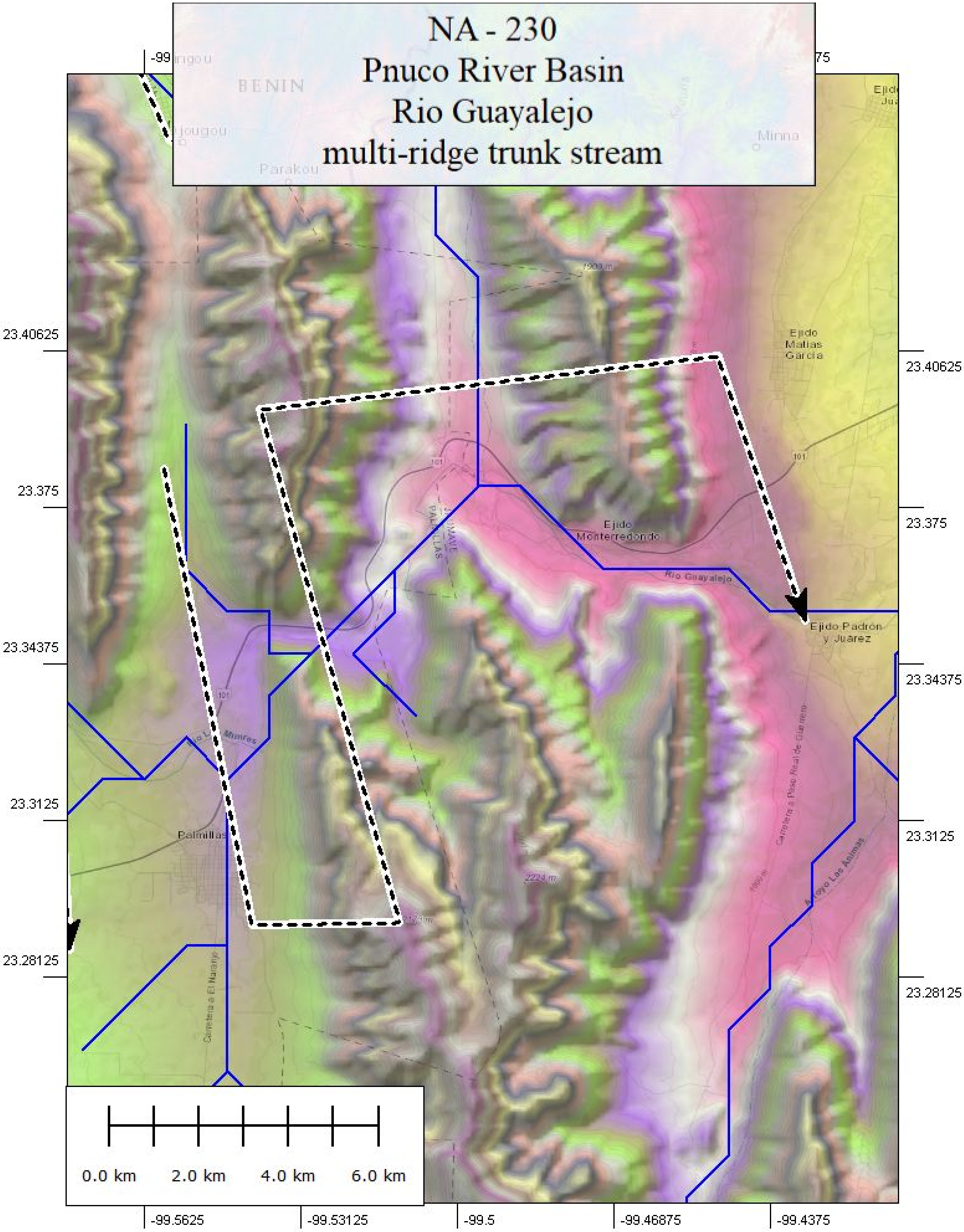
NA - 230
Pnuco River Basin
Rio Guayalejo
multi-ridge trunk stream
Ejido Matias Garcia
Ejido Monterredondo
Rio Guayalejo
Ejido Padrón y Juarez
Palmillas
Arroyo Las Animas
Carretera a Pisos Real de Guanamé
Carretera a El Naranjo
101
2224 m
23.40625
23.375
23.34375
23.3125
23.28125
-99.5625
-99.53125
-99.5
-99.46875
-99.4375
0.0 km
2.0 km
4.0 km
6.0 km

NA - 231
Tehuantepec River Basin
Tehuantepec River
irregular high ground trunk stream

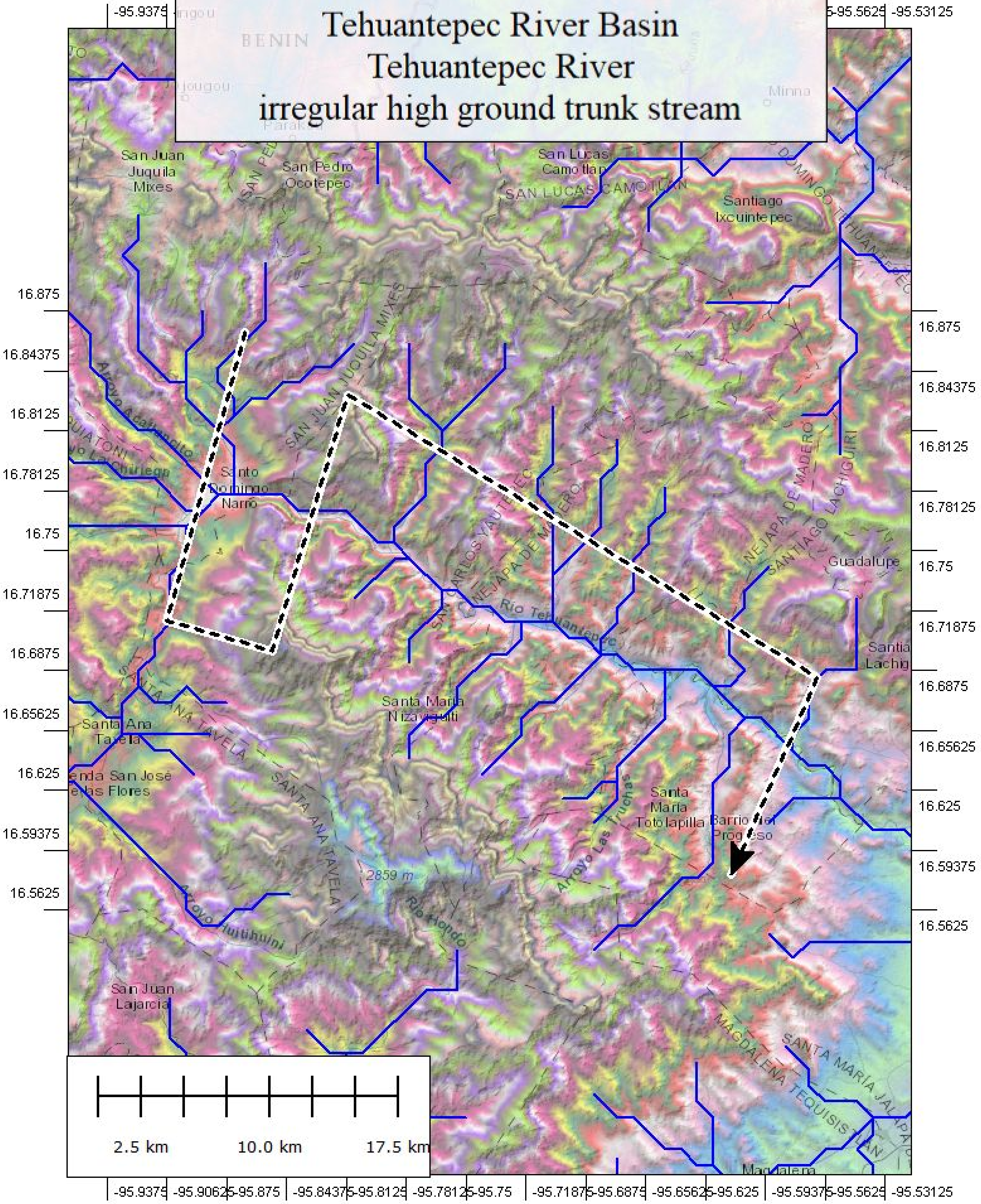

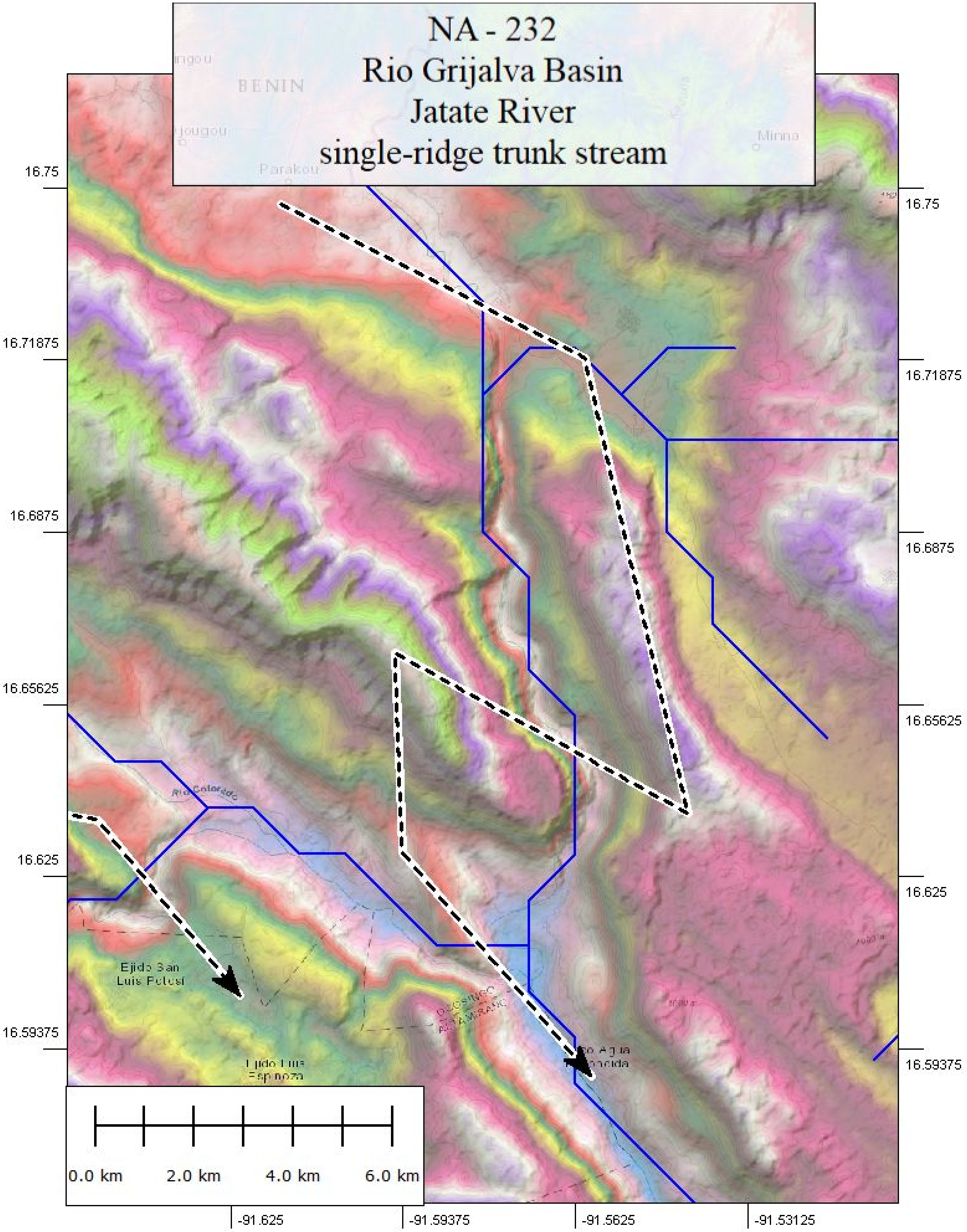

NA - 232
Rio Grijalva Basin
Jatate River
single-ridge trunk stream
BENIN
Parakou
Rio Colorado
Ejido San Luis Potosi
16.75
16.71875
16.6875
16.65625
16.625
16.59375
-91.625
-91.59375
-91.5625
-91.53125
0.0 km
2.0 km
4.0 km
6.0 km

NA - 233
Colorado River (Arizona) Basin
Price River
downdip plateau trunk stream

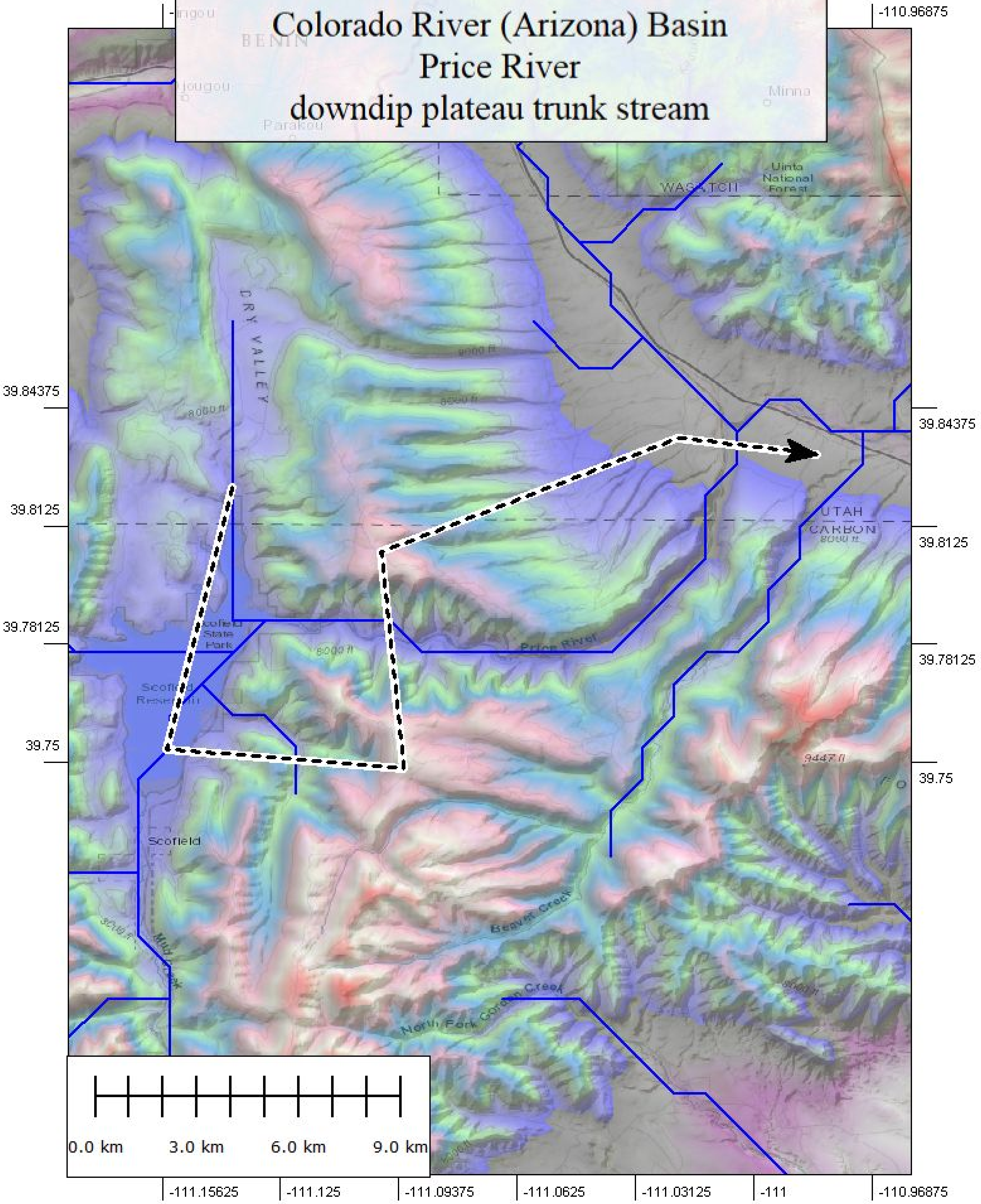

NA - 234
Rio Grijalva Basin
Jatate River tributary
single-ridge trunk stream

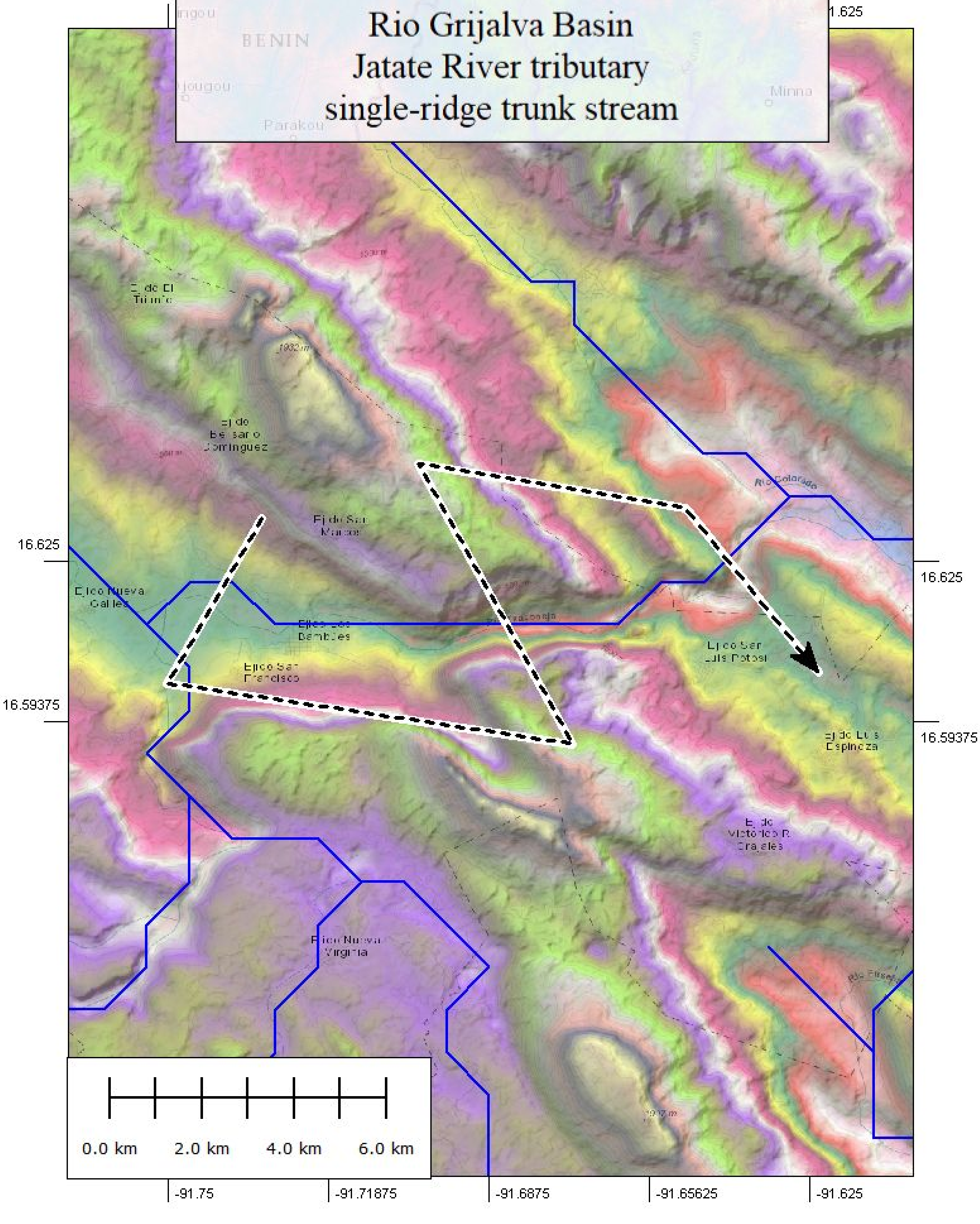

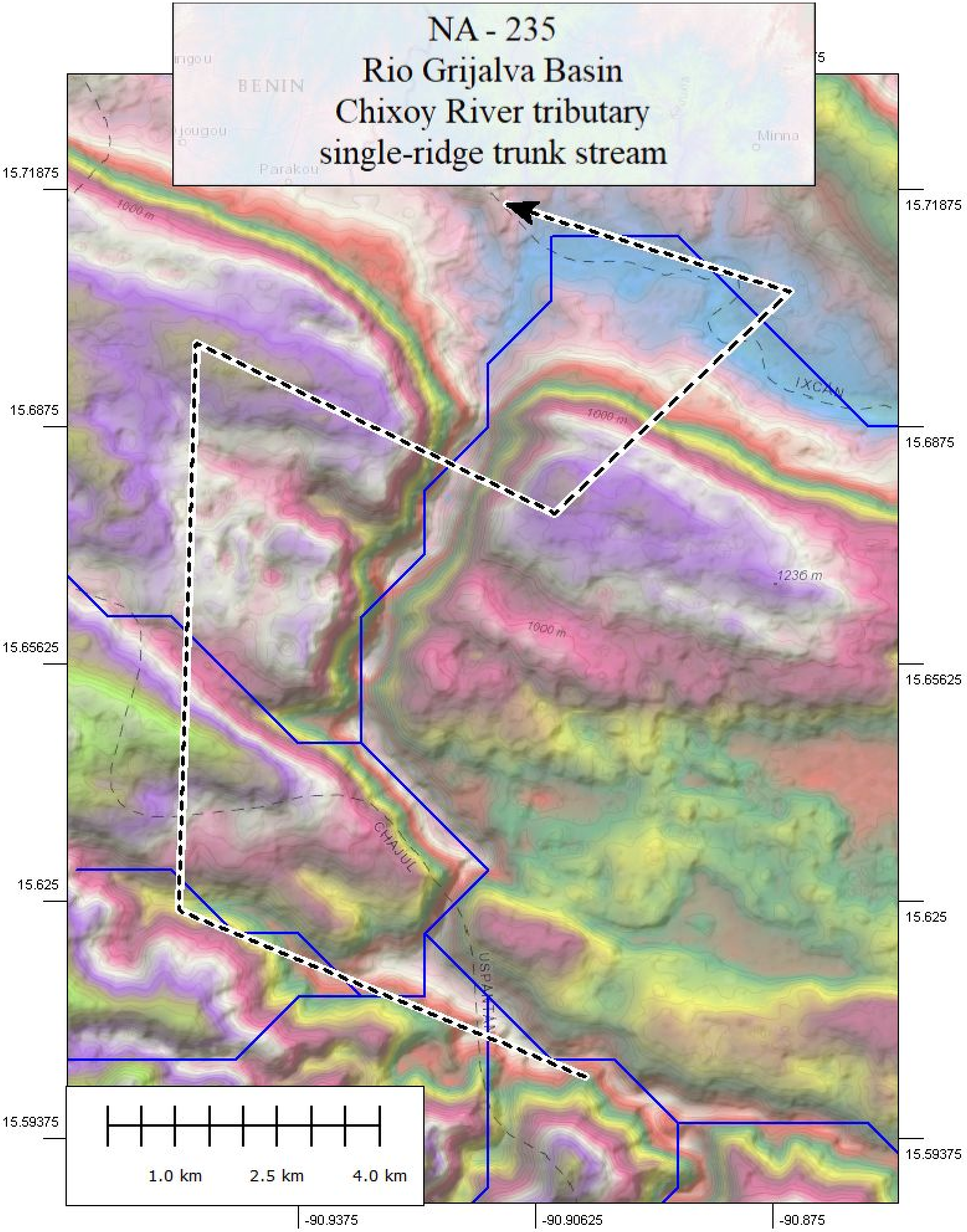
NA - 235
Rio Grijalva Basin
Chixoy River tributary
single-ridge trunk stream
BENIN
Djougou
Parakou
Minna
1000 m
1000 m
1000 m
1236 m
IXCAN
CHAJUL
USPANTAN
15.71875
15.6875
15.65625
15.625
15.59375
15.71875
15.6875
15.65625
15.625
15.59375
-90.9375
-90.90625
-90.875
1.0 km
2.5 km
4.0 km

NA - 236
Papaloapan River Basin
Papalapan River, Cerro de Oro dam
multi-ridge trunk stream

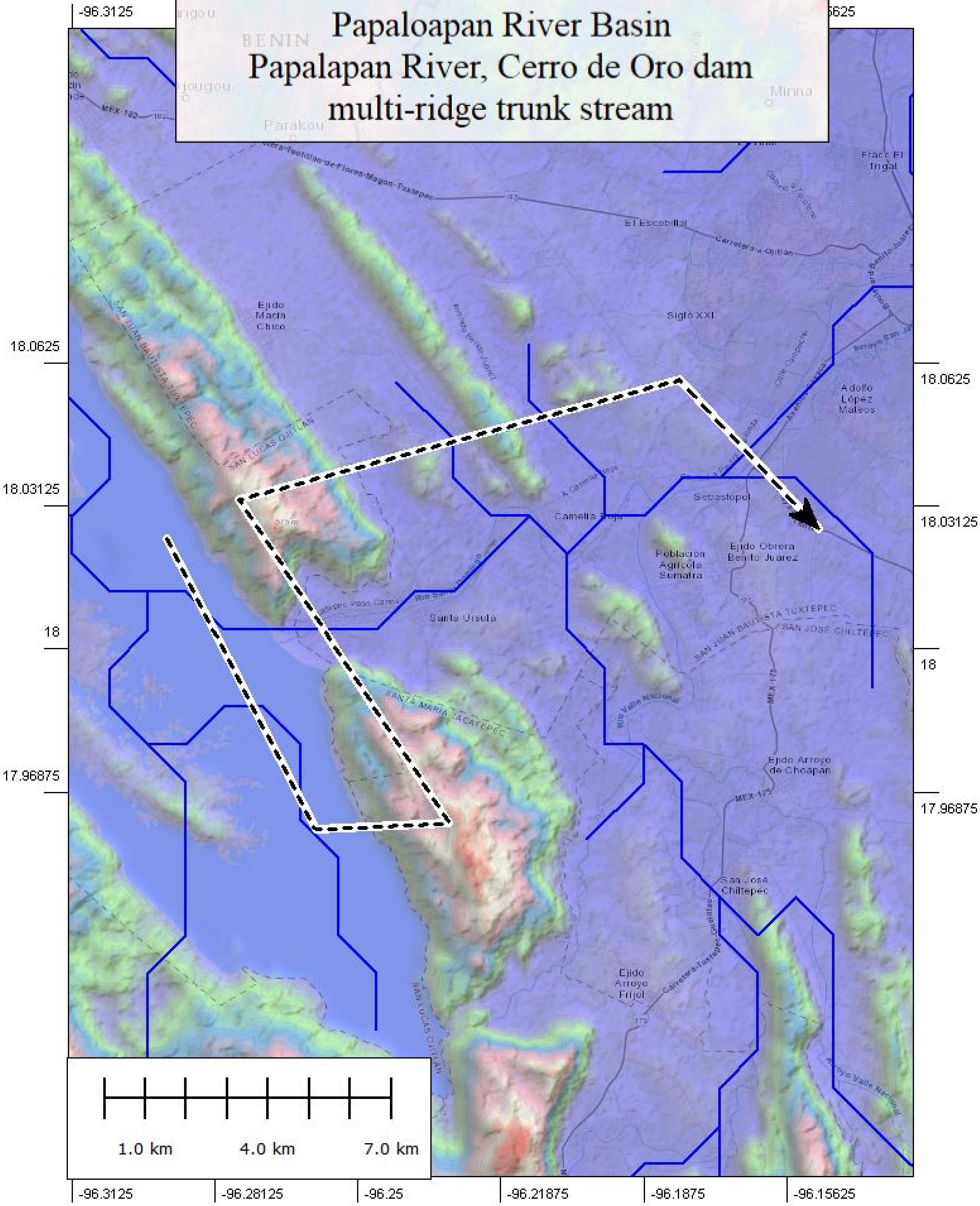

NA - 237
Rio Patuca Basin
Patuca River
single-ridge trunk stream

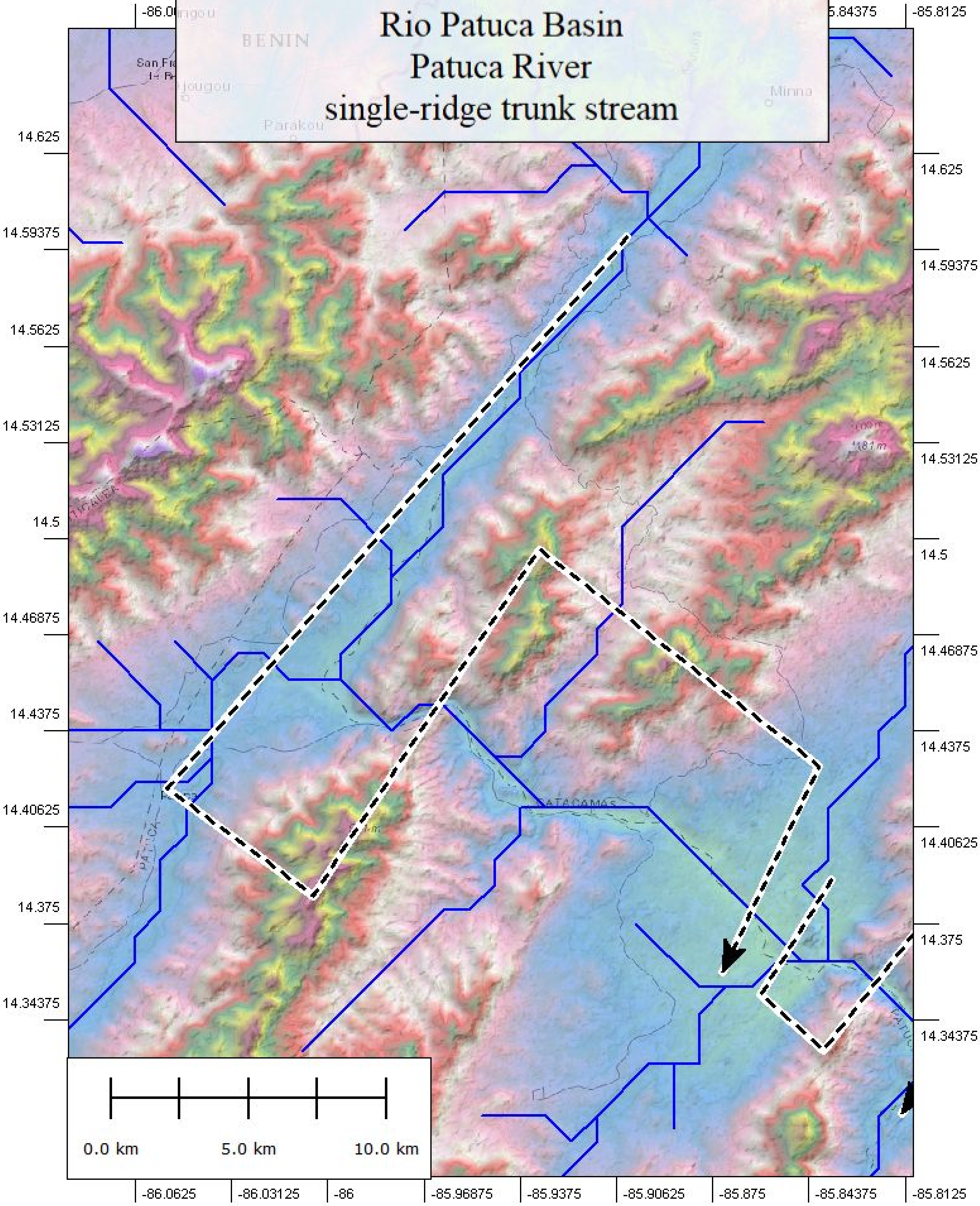

NA - 238
Rio Patuca Basin
Telica River
single-ridge trunk stream

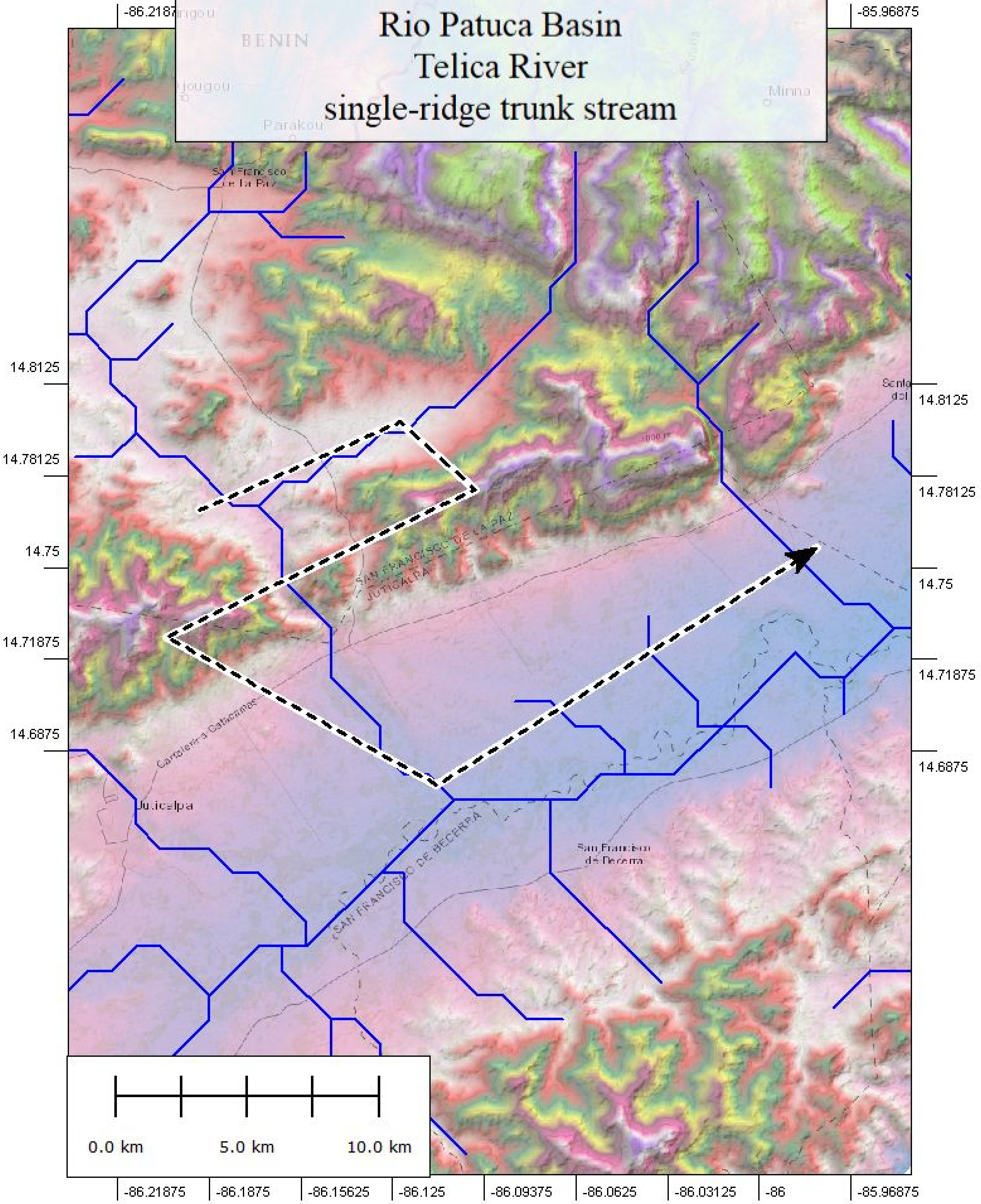

NA - 239
Rio Grande (Texas-Mexico) Basin
Obayos Pass
single-ridge trunk stream

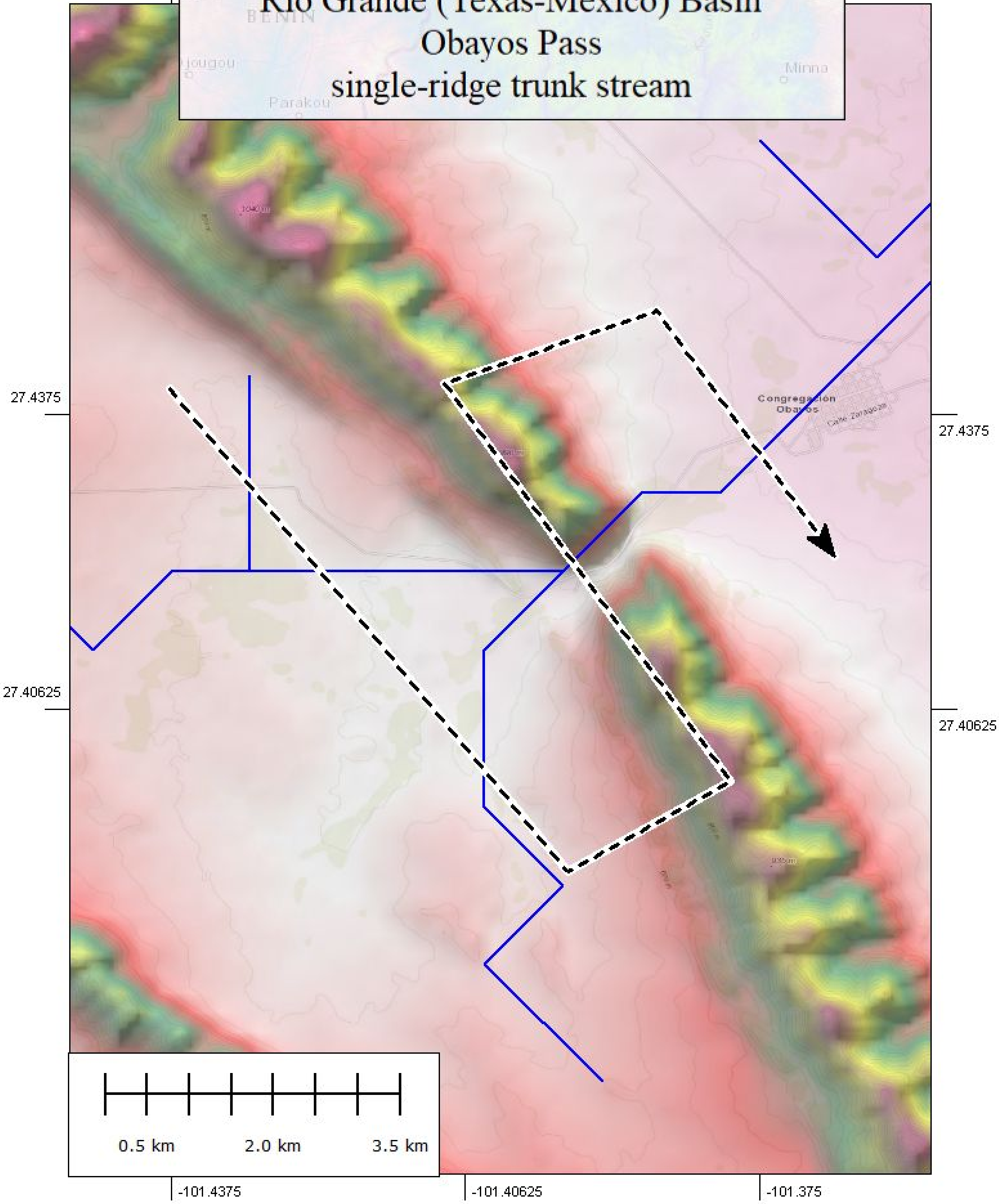

NA - 24
Alameda Creek Basin
Alameda Creek, Niles Canyon
irregular high ground trunk stream

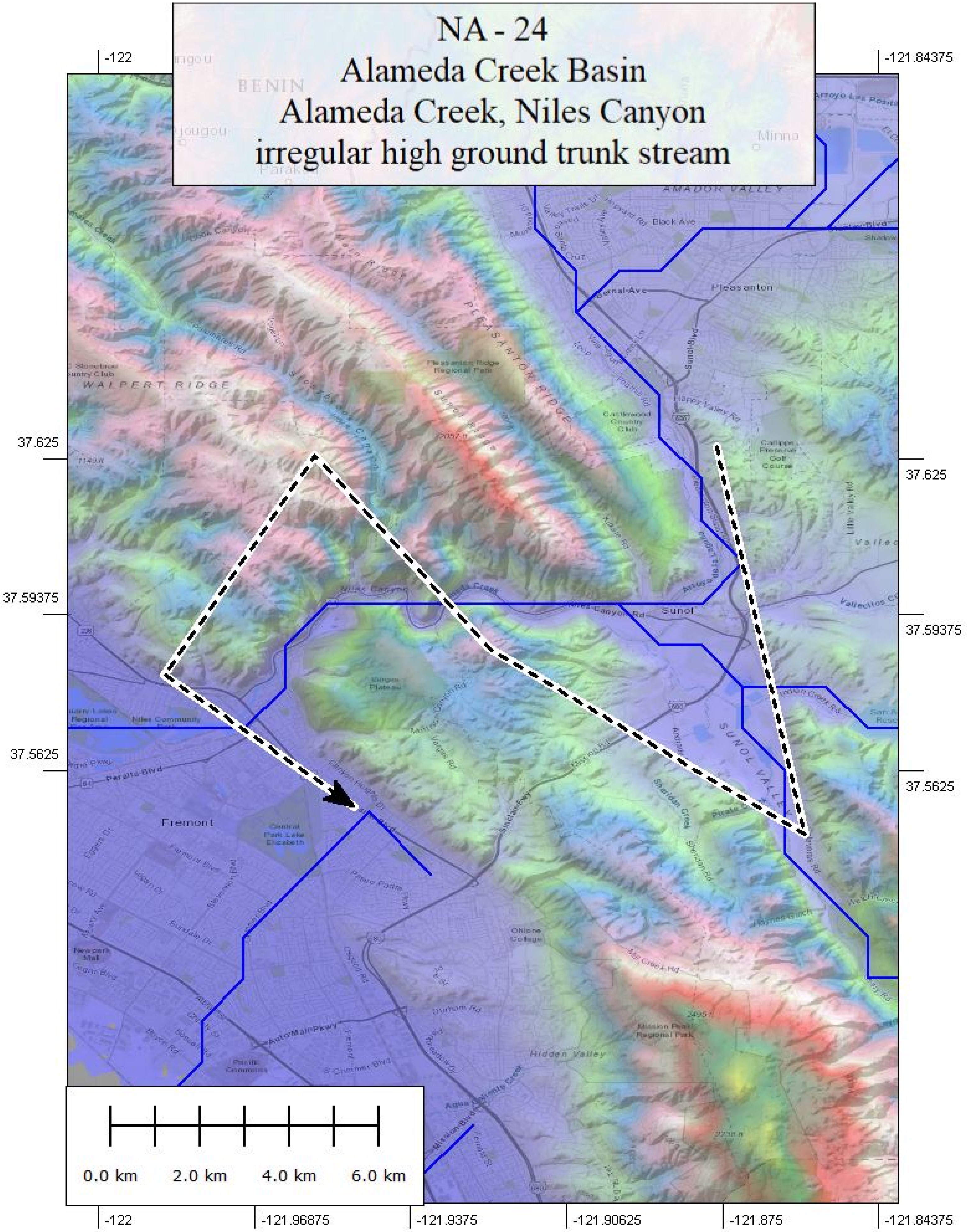

NA - 240
Rio San Lorenzo Basin
San Lorenzo River
irregular high ground trunk stream

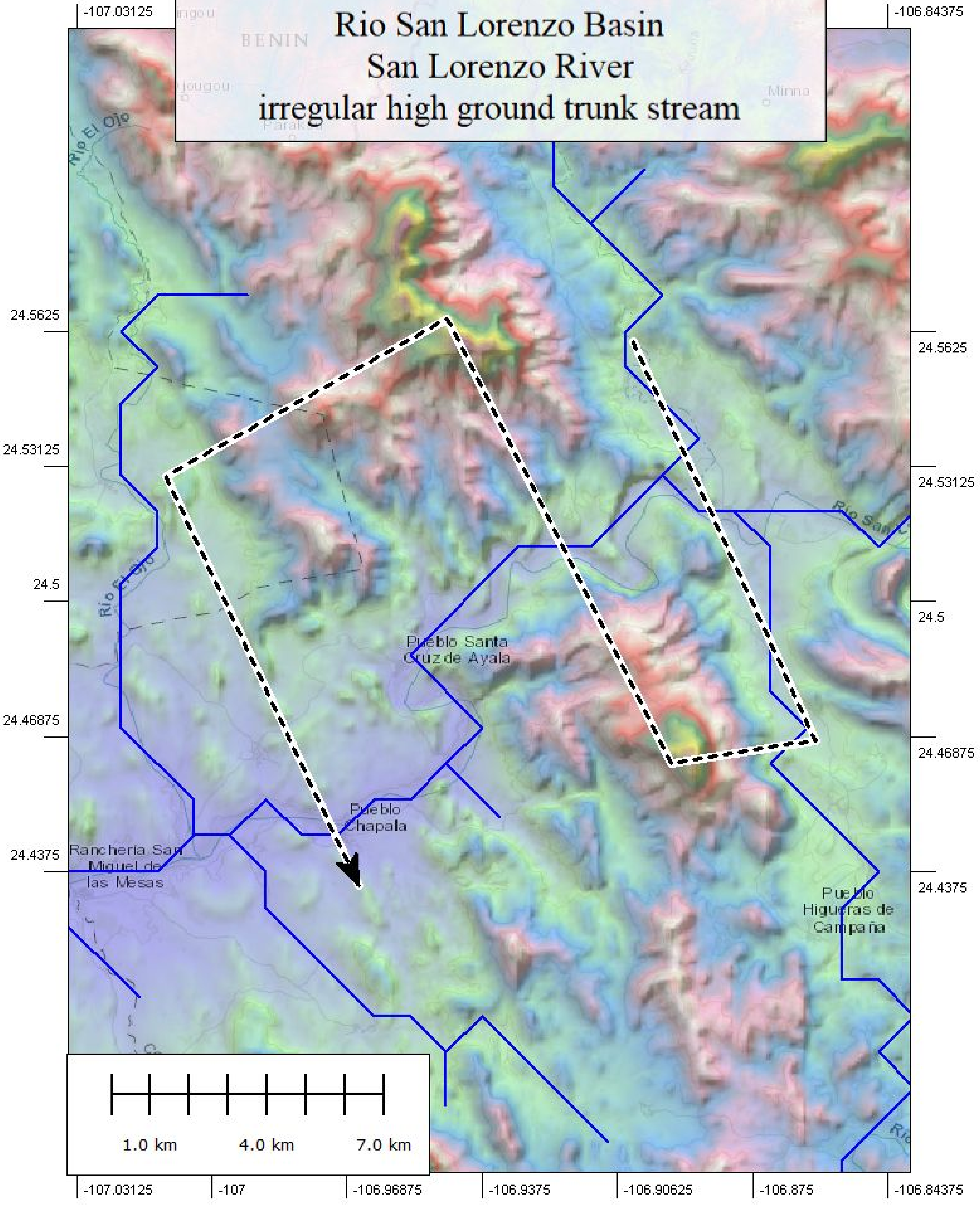

NA - 241
Colorado River (Arizona) Basin
Coloarado River
single-ridge trunk stream

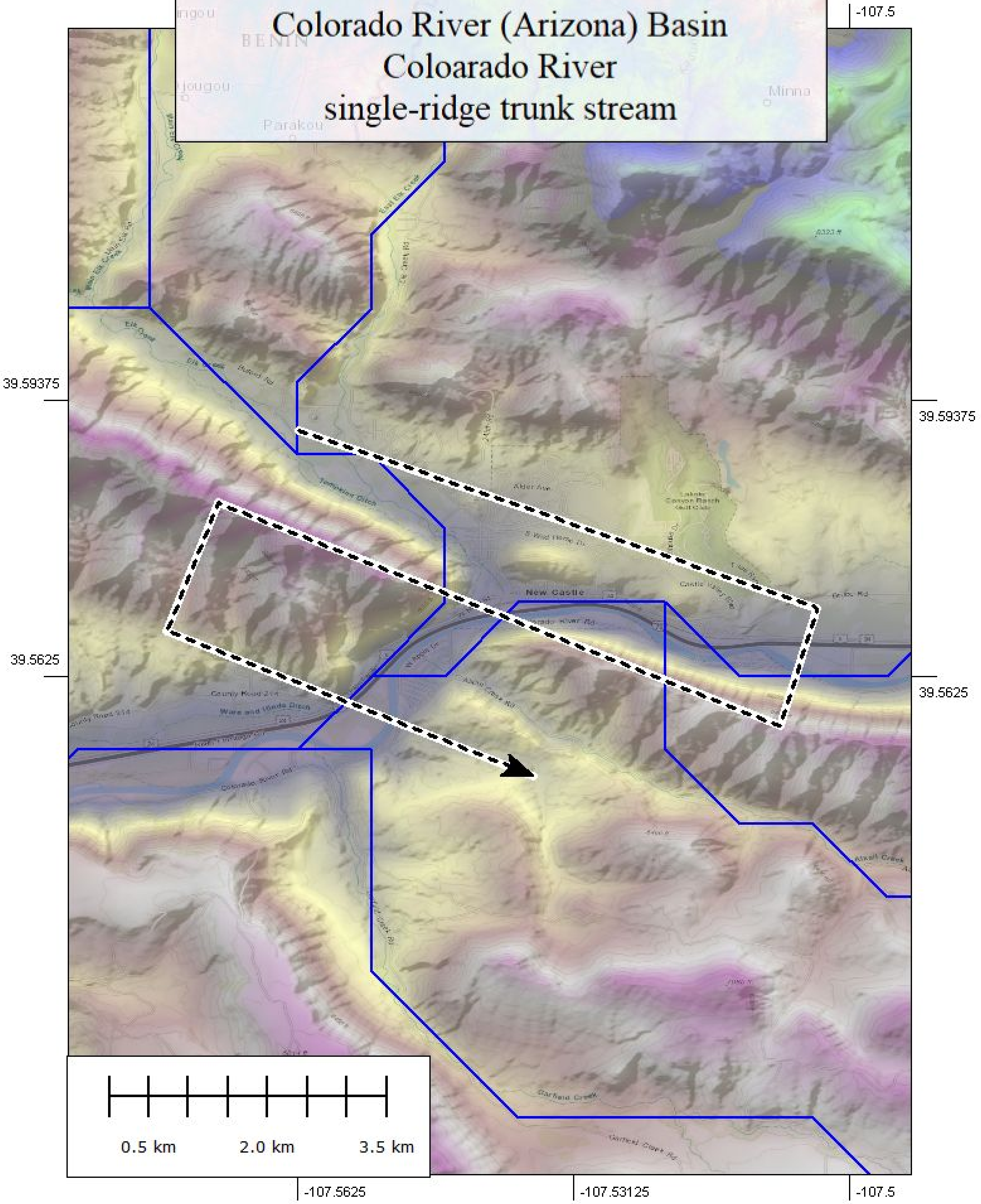

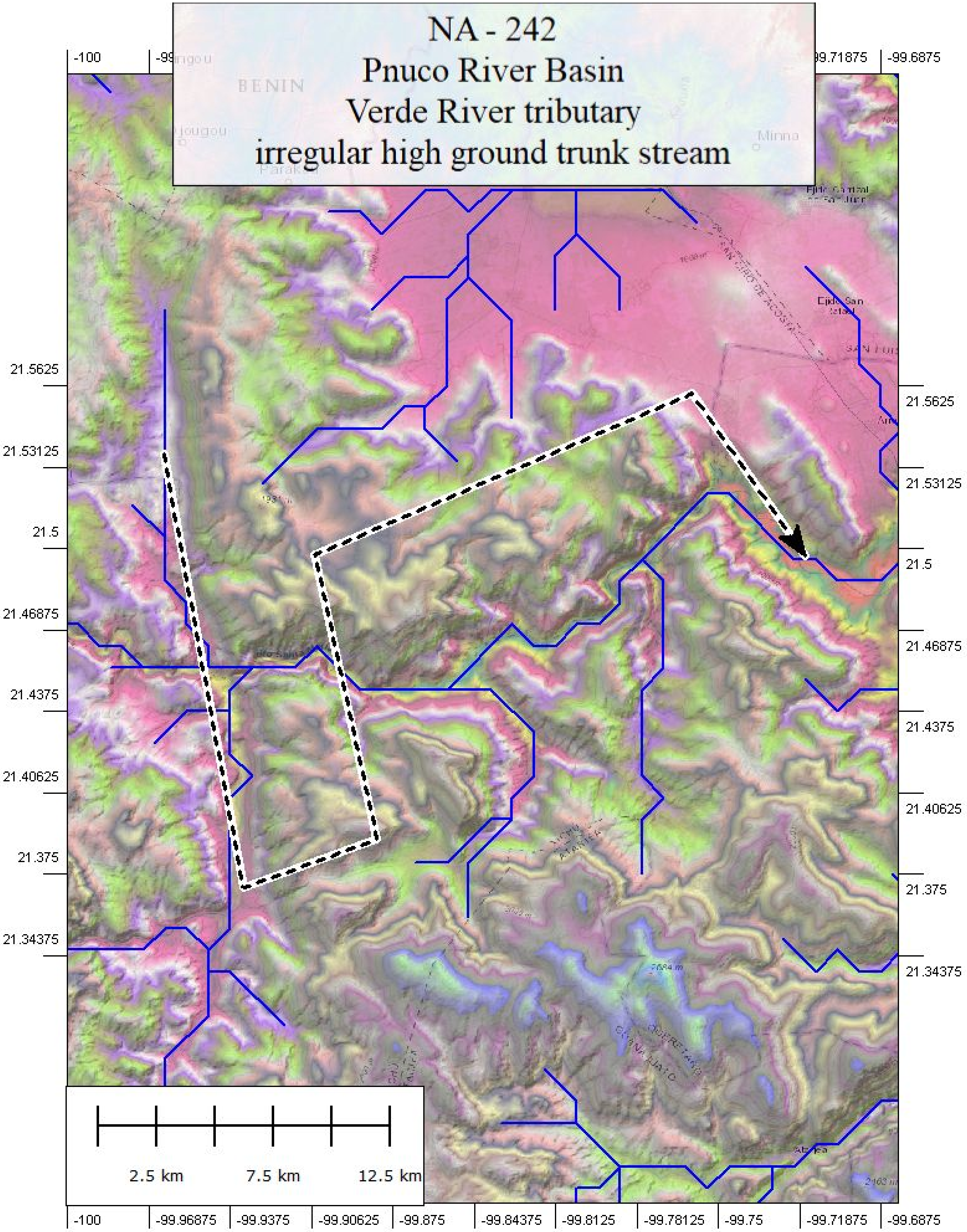
NA - 242
Pnuco River Basin
Verde River tributary
irregular high ground trunk stream
-100
-99.96875
-99.9375
-99.90625
-99.875
-99.84375
-99.8125
-99.78125
-99.75
-99.71875
-99.6875
21.5625
21.53125
21.5
21.46875
21.4375
21.40625
21.375
21.34375
2.5 km
7.5 km
12.5 km

NA - 243
Papaloapan River Basin
Tonto River
irregular high ground trunk stream

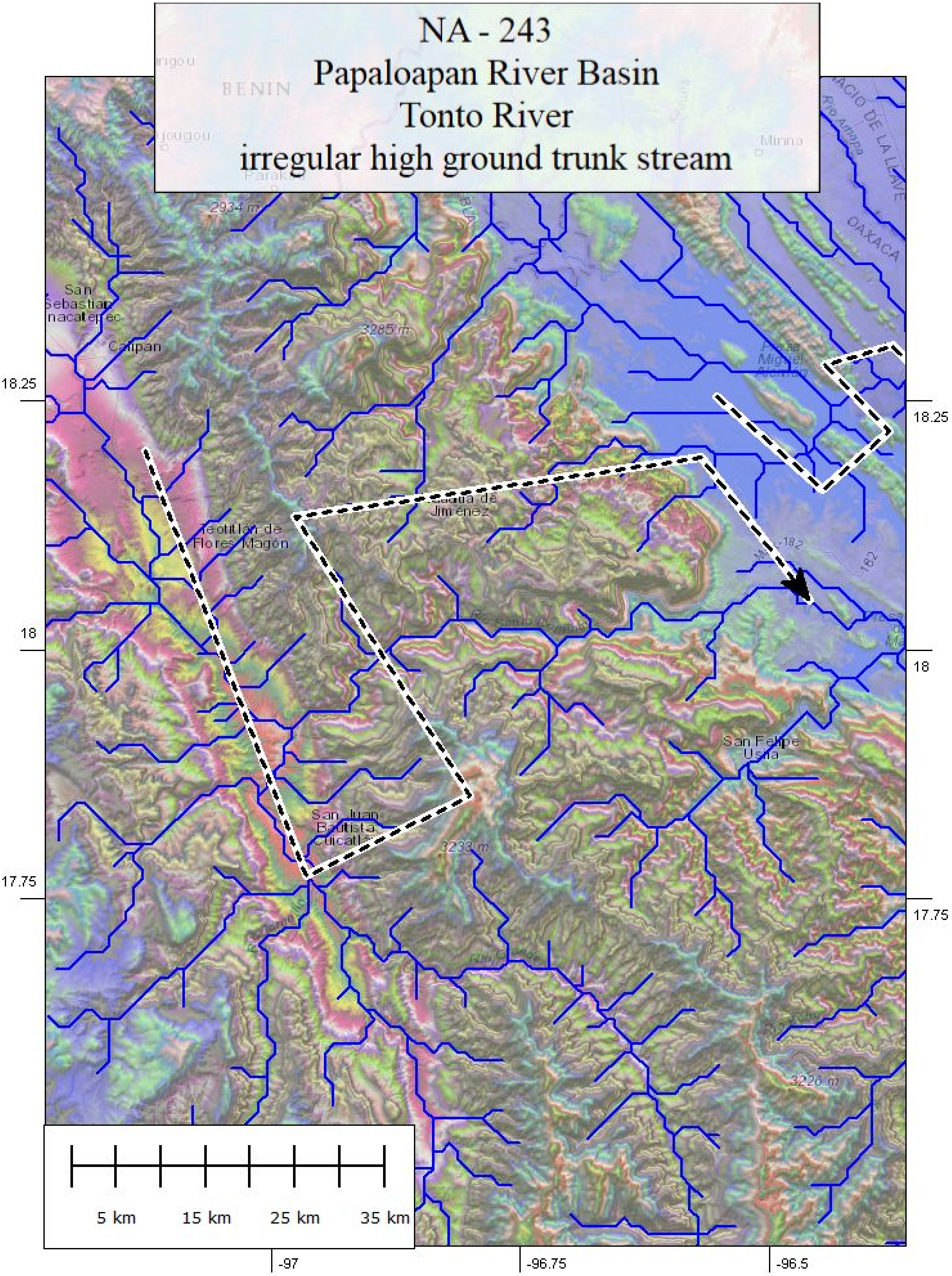

NA - 244
Papaloapan River Basin
Rio Barranca Seca
single-ridge trunk stream

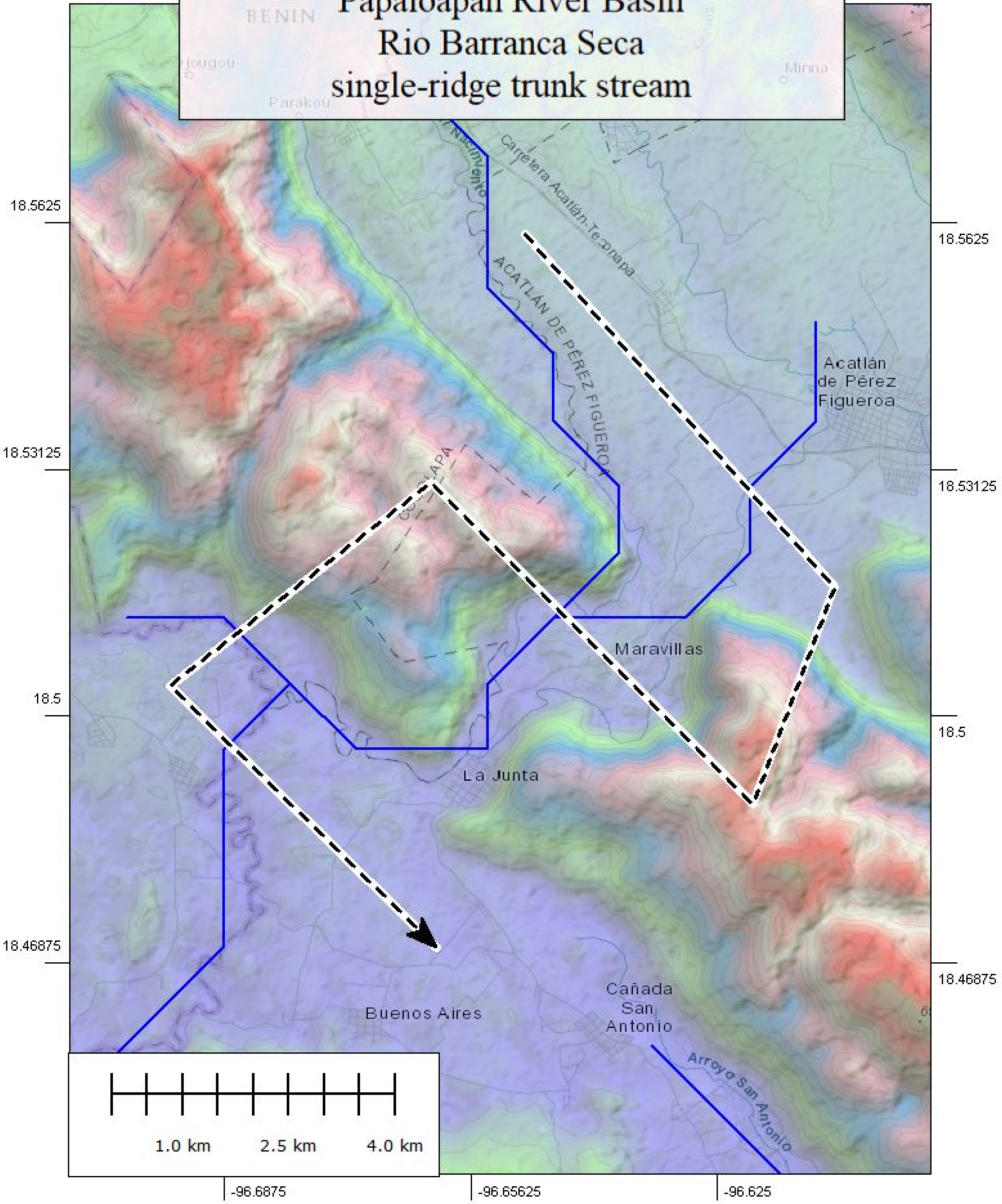

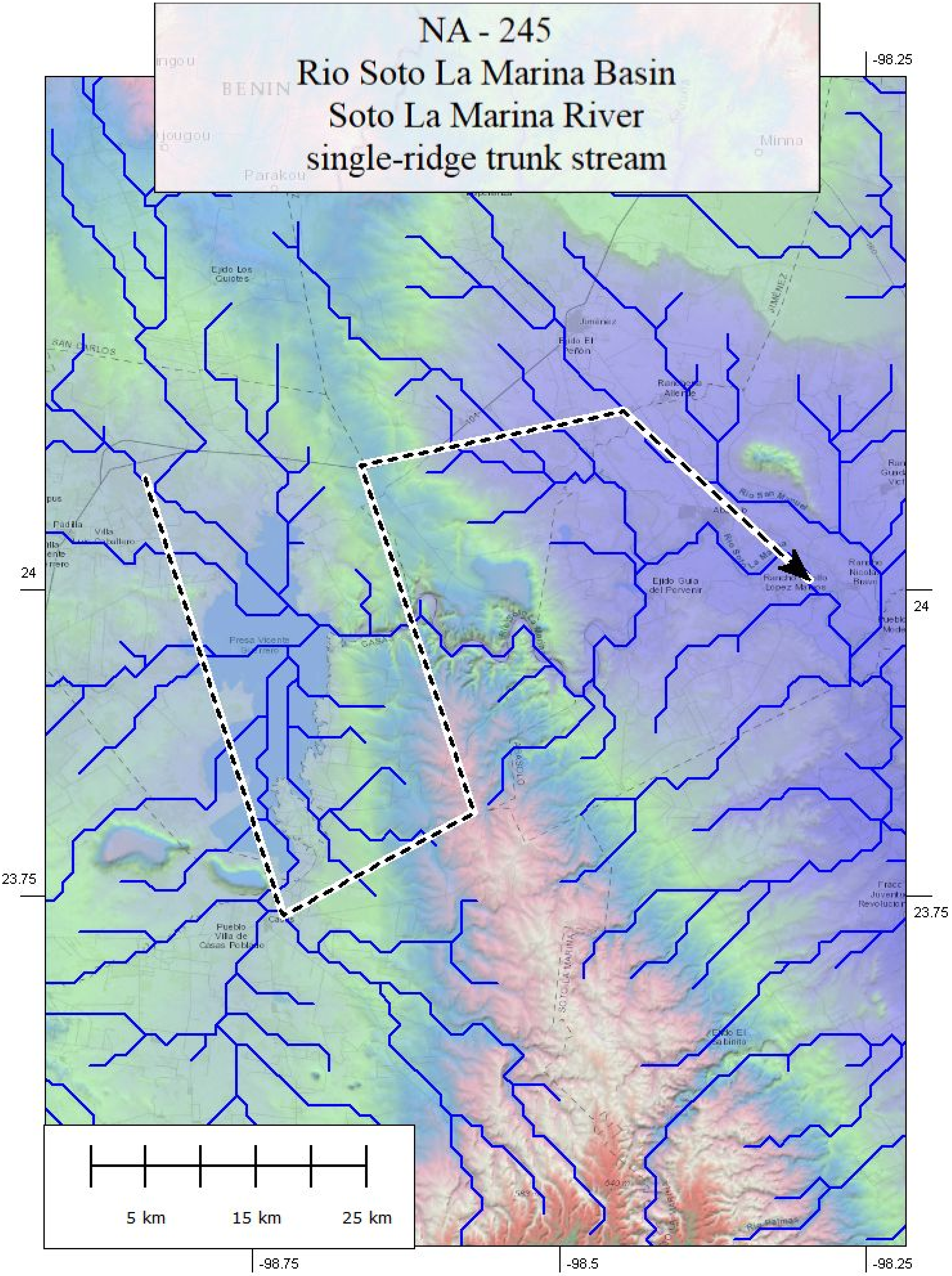
NA - 245
Rio Soto La Marina Basin
Soto La Marina River
single-ridge trunk stream
-98.25
24
23.75
-98.75
-98.5
5 km
15 km
25 km
Ejido Los Guiotes
Jiménez
Ejido El Peñón
SAN CARLOS
Padilla
Villa Caballero
Presa Vicente Guerrero
Ejido Guia del Porvenir
Rio San Marcos
Rancho Nicolas Bravo
Pueblo Villa de Casas Poblano
SOTO LA MARINA
Ejido El Gabinito
Parakou
BENIN

NA - 246
Papaloapan River Basin
Tesechoacan River
single-ridge trunk stream

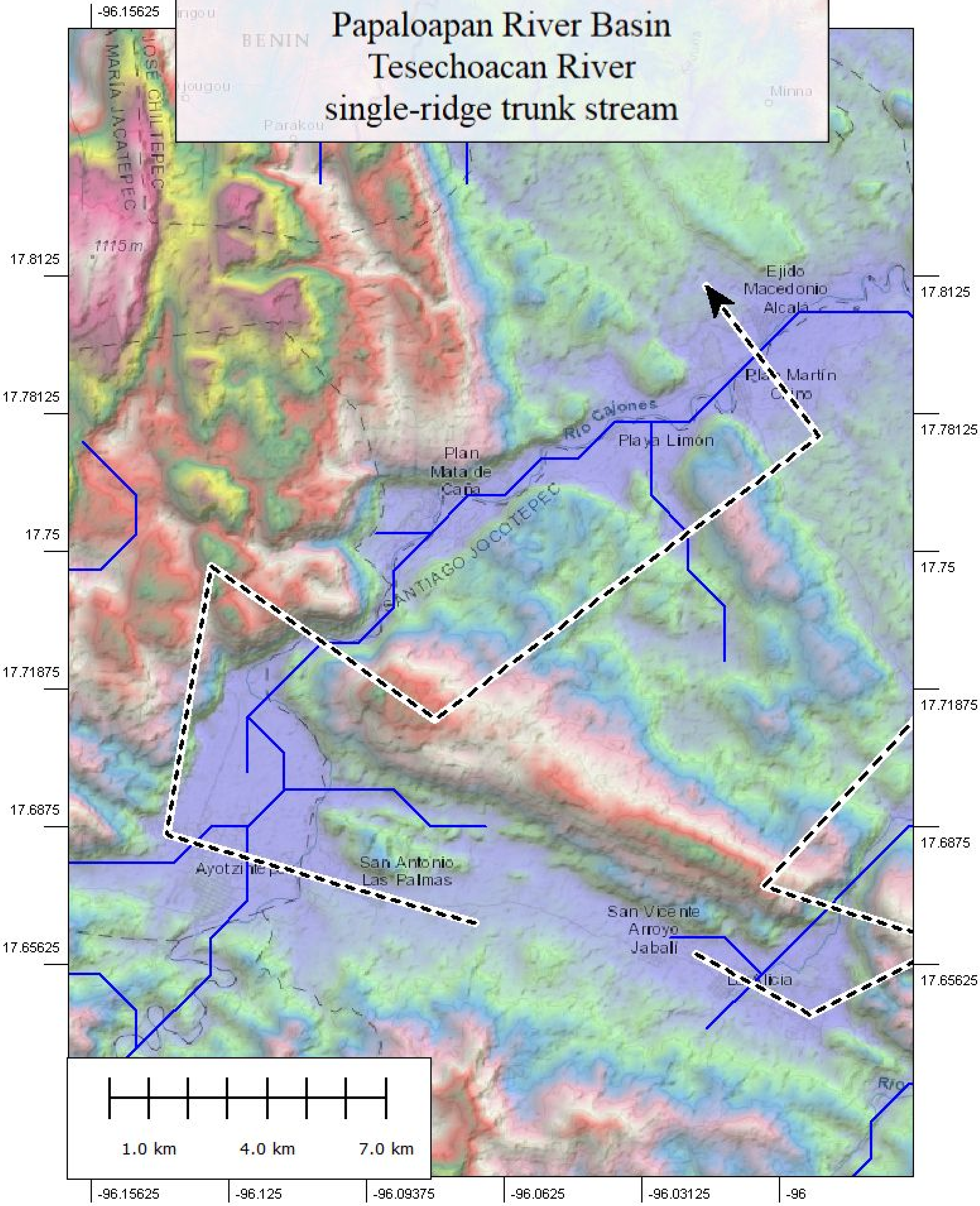

NA - 247
Papaloapan River Basin
Tonto River, Temascal Dam
multi-ridge trunk stream

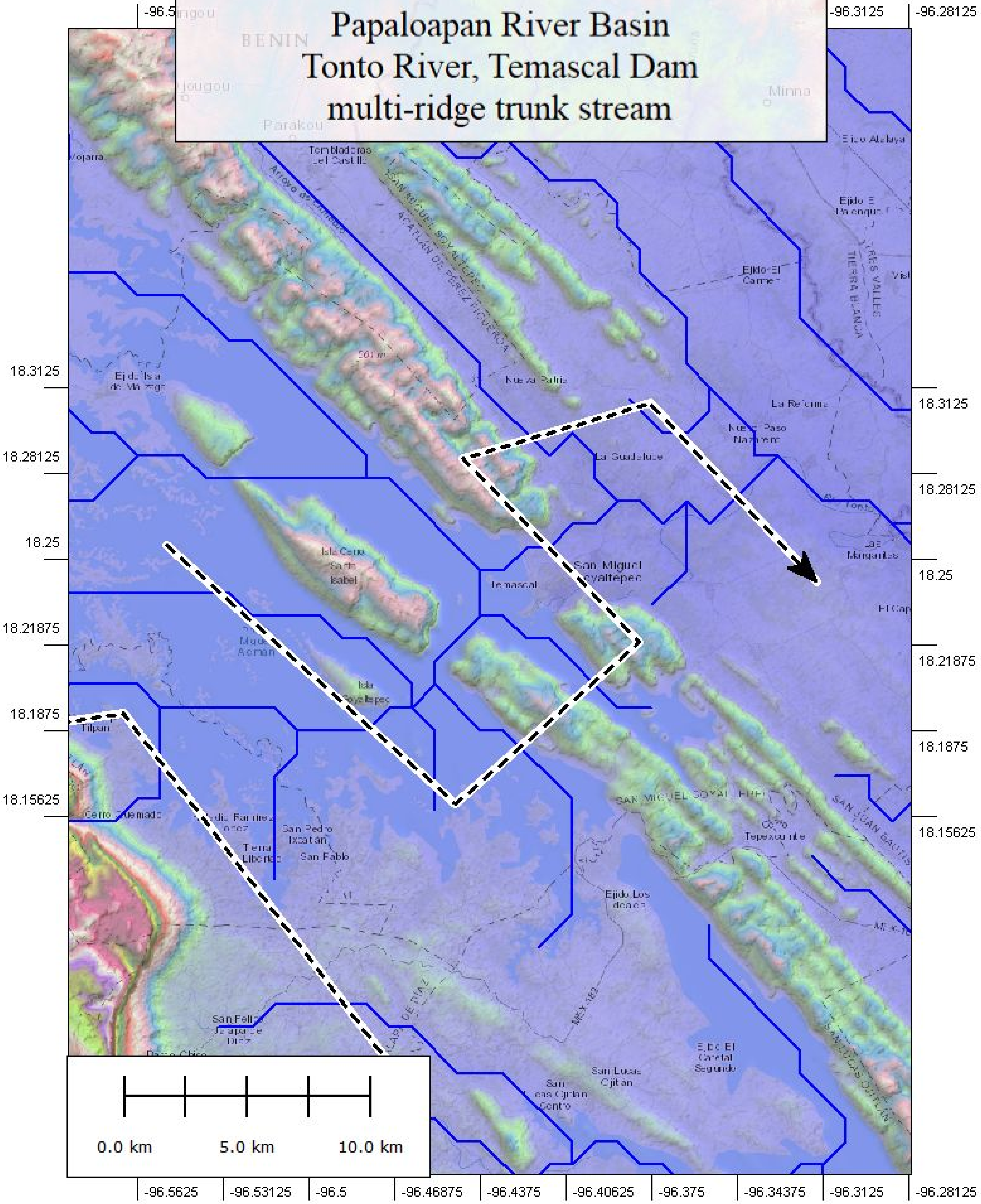

NA - 248
Mississippi River Basin
Conemaugh River
single-ridge trunk stream

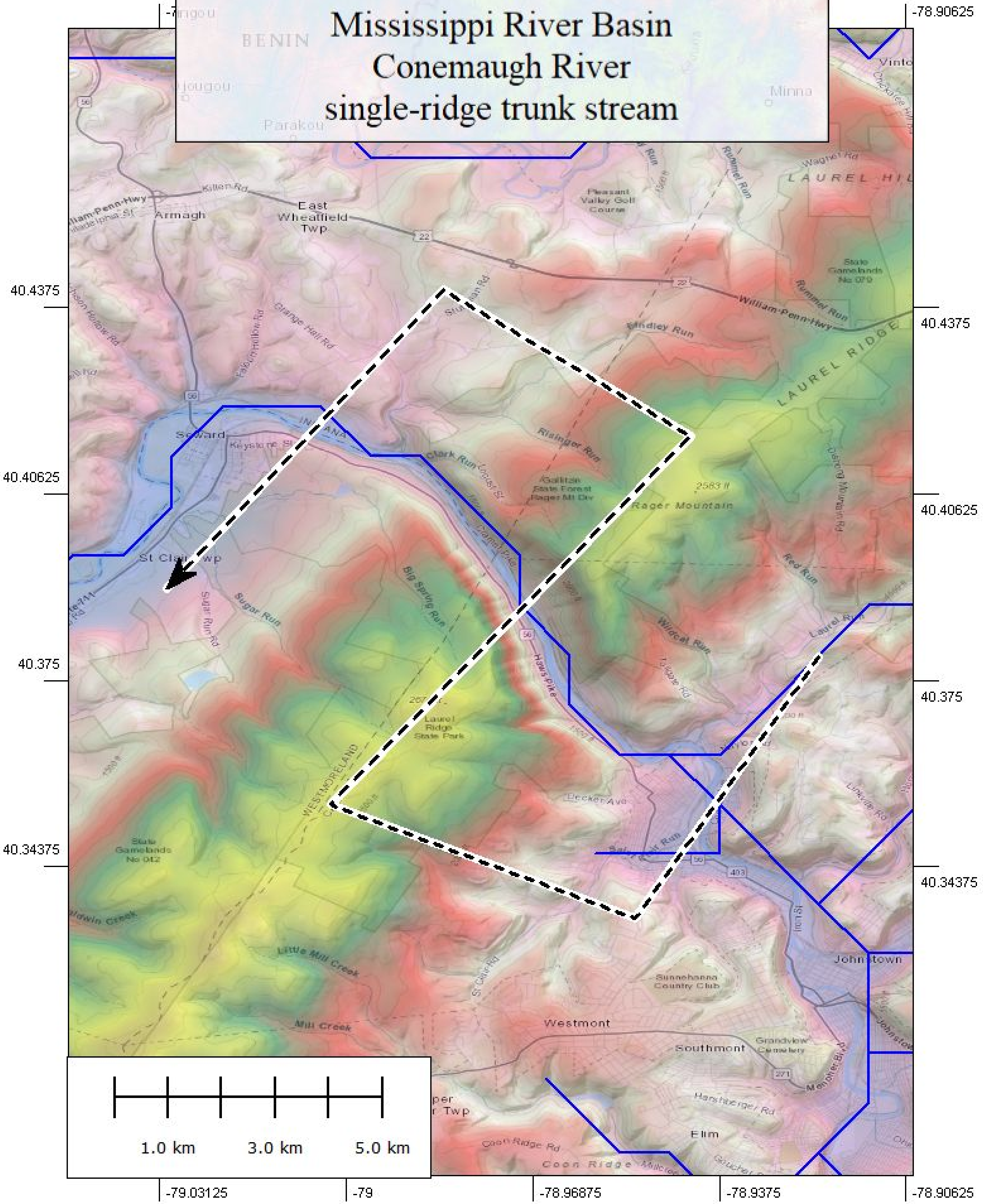

NA - 249
Rio Grande (Texas-Mexico) Basin
Salado Pass
multi-ridge trunk stream

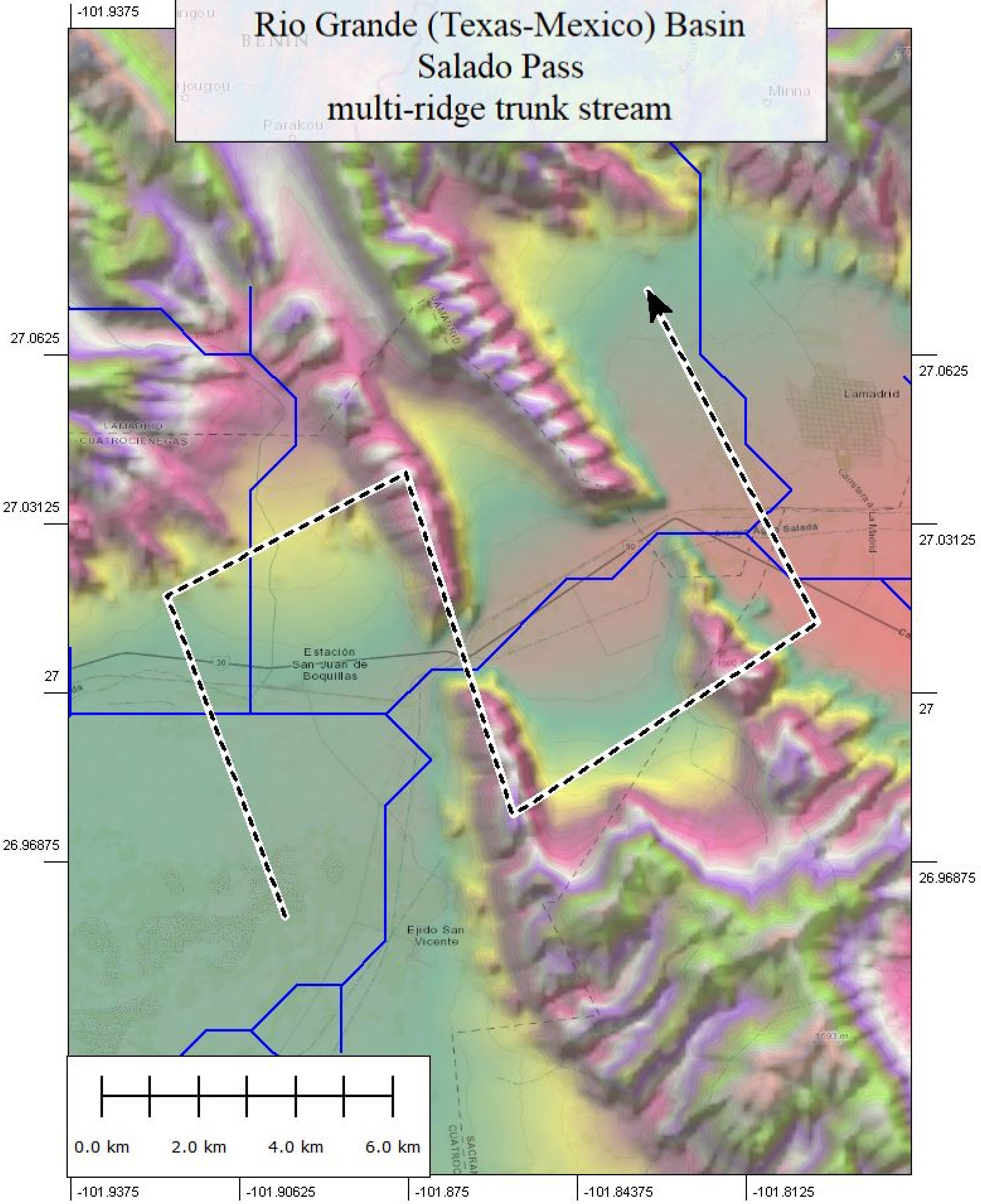

NA - 25
East Fork Russian River Basin
East Fork Russian River
single-ridge trunk stream

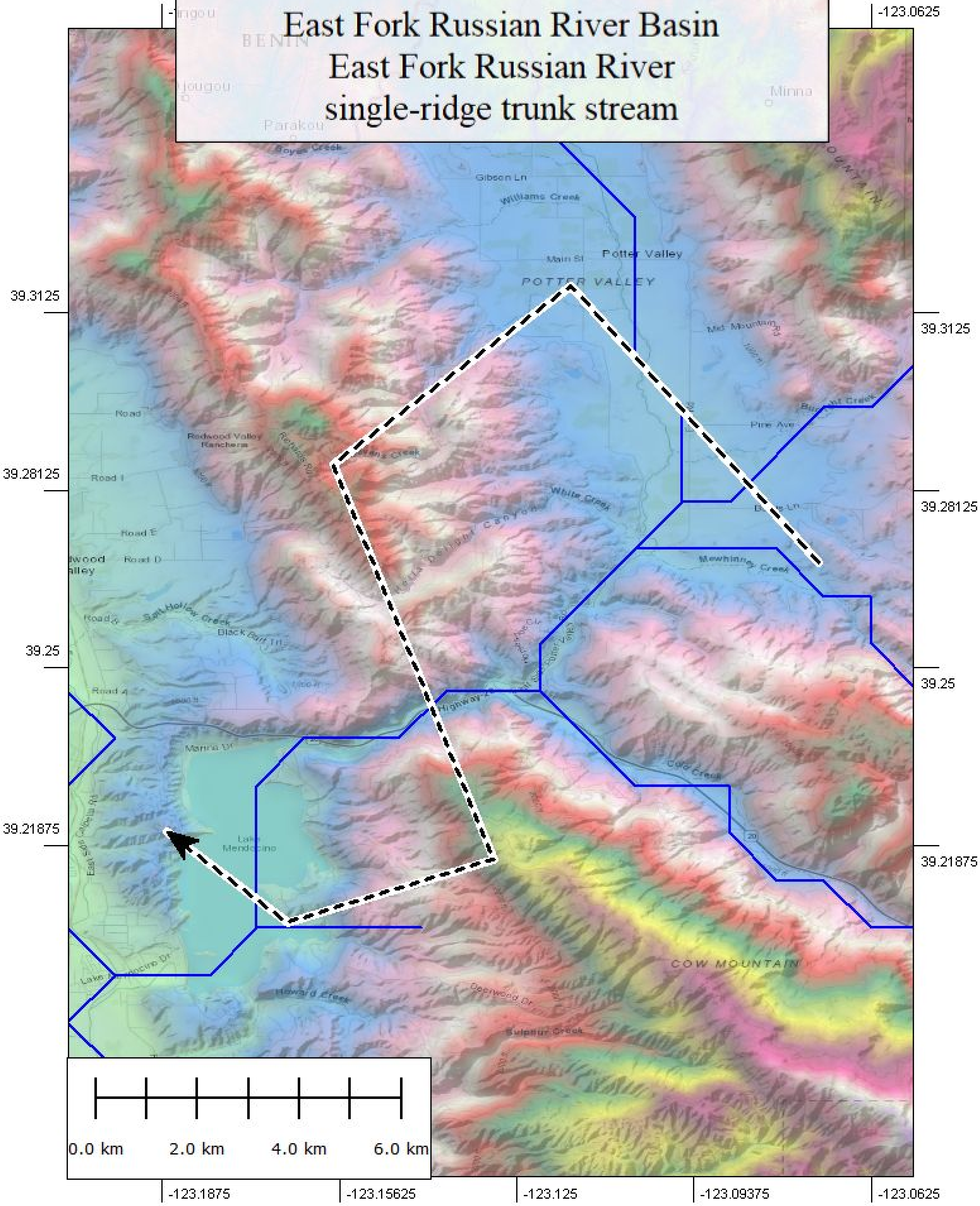

NA - 250
Rio Grande (Texas-Mexico) Basin
Arroyo Chupadero
single-ridge trunk stream

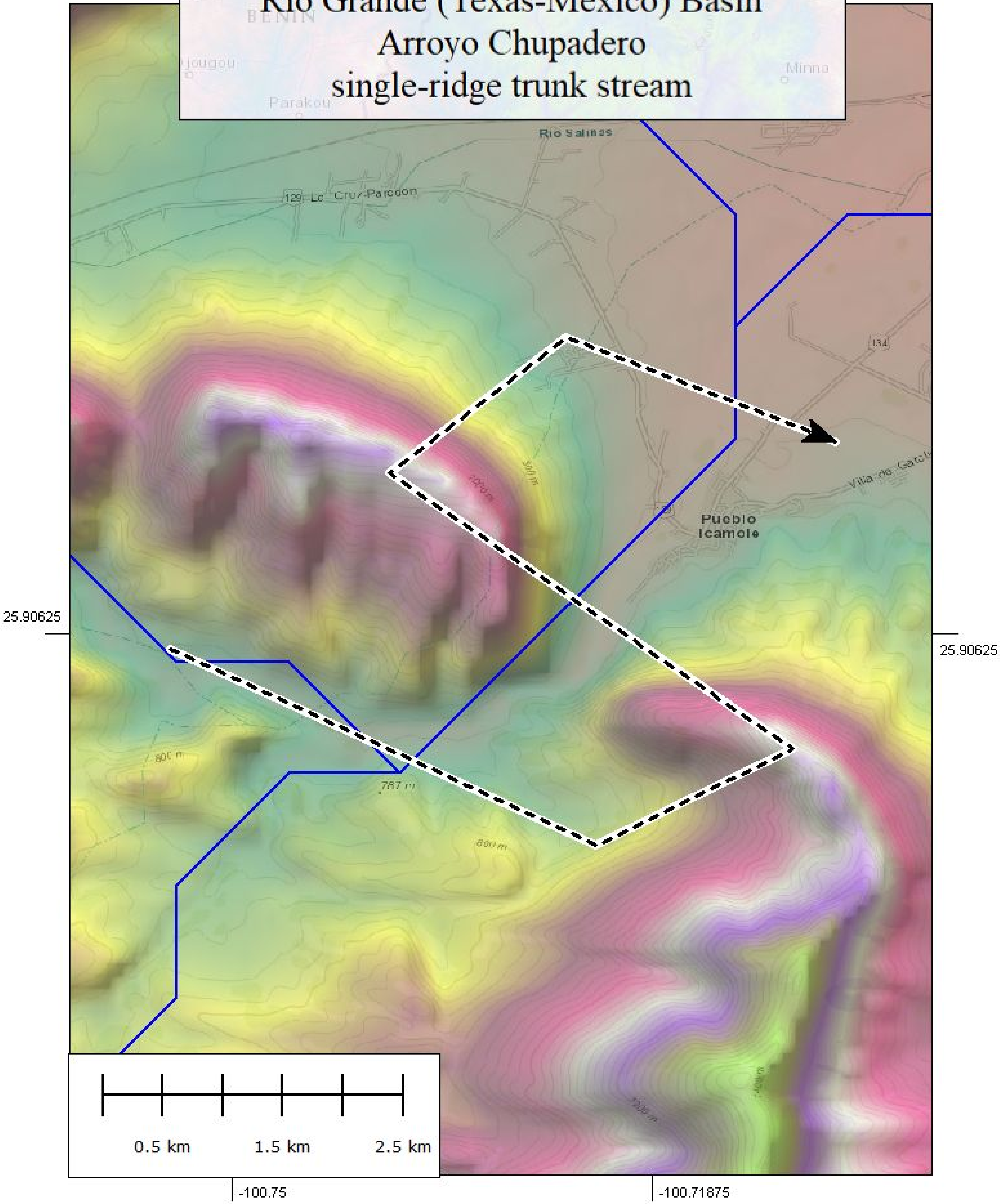

NA - 251
Rio Soto La Marina Basin
Soto la Marina River tributary
single-ridge trunk stream

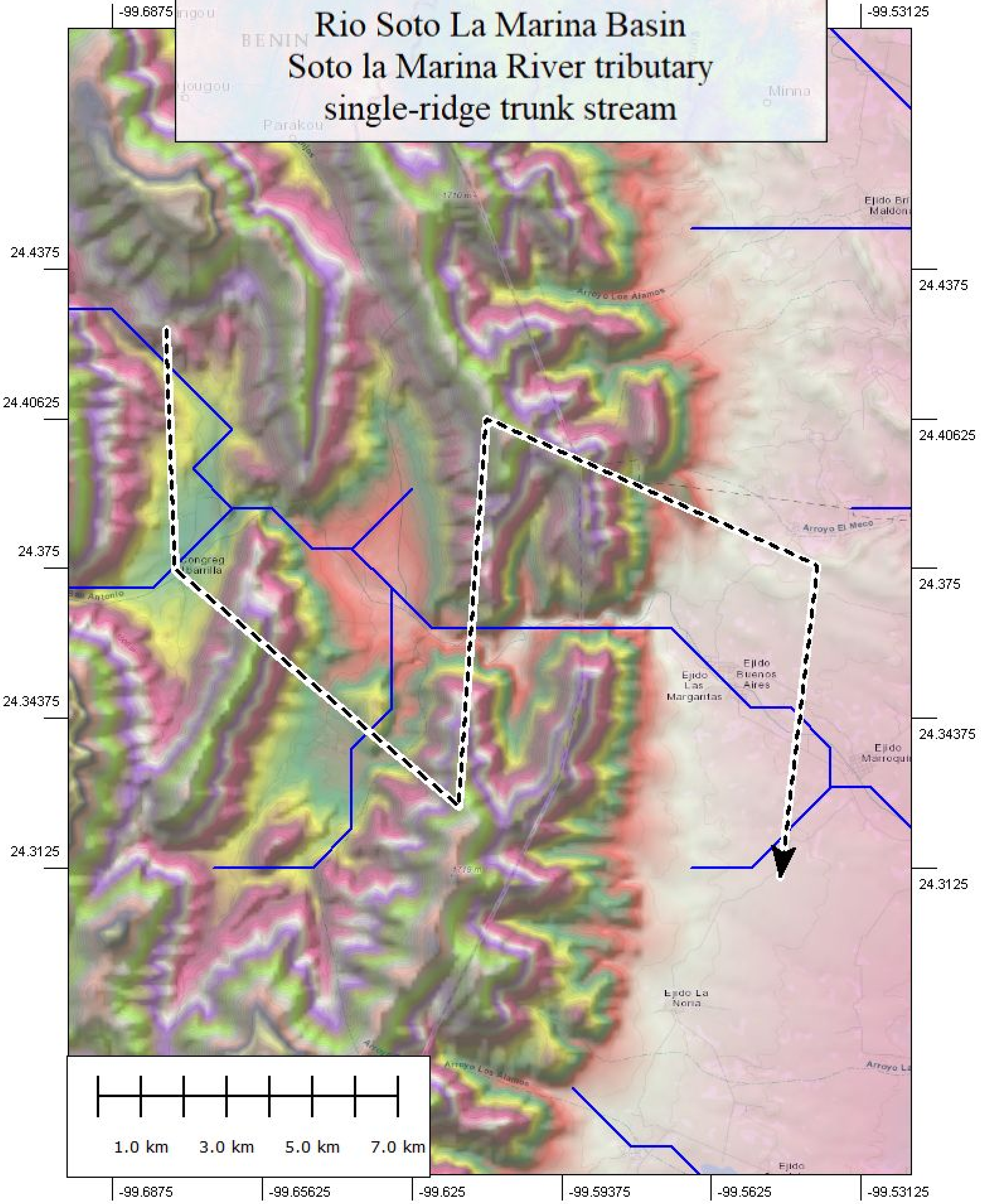

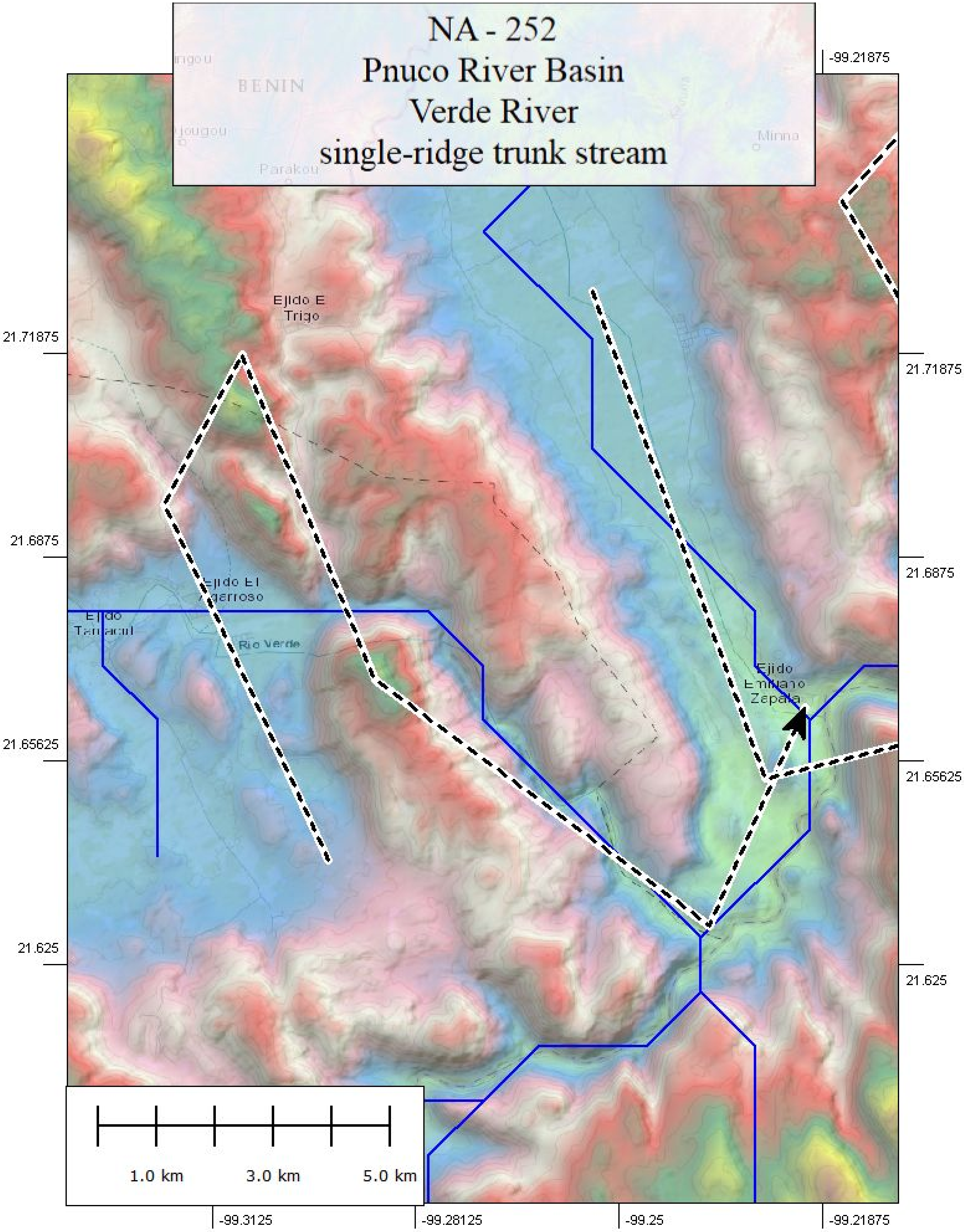
NA - 252
Pnuco River Basin
Verde River
single-ridge trunk stream
BENIN
Parakou
Minna
Ejido E
Trigo
Ejido El
Agarroso
Ejido
Tamacul
Rio Verde
Ejido
Emiliano
Zapata
21.71875
21.6875
21.65625
21.625
-99.3125
-99.28125
-99.25
-99.21875
1.0 km
3.0 km
5.0 km

NA - 253
James River Basin
Jackson River, Rainbow Gap
single-ridge trunk stream

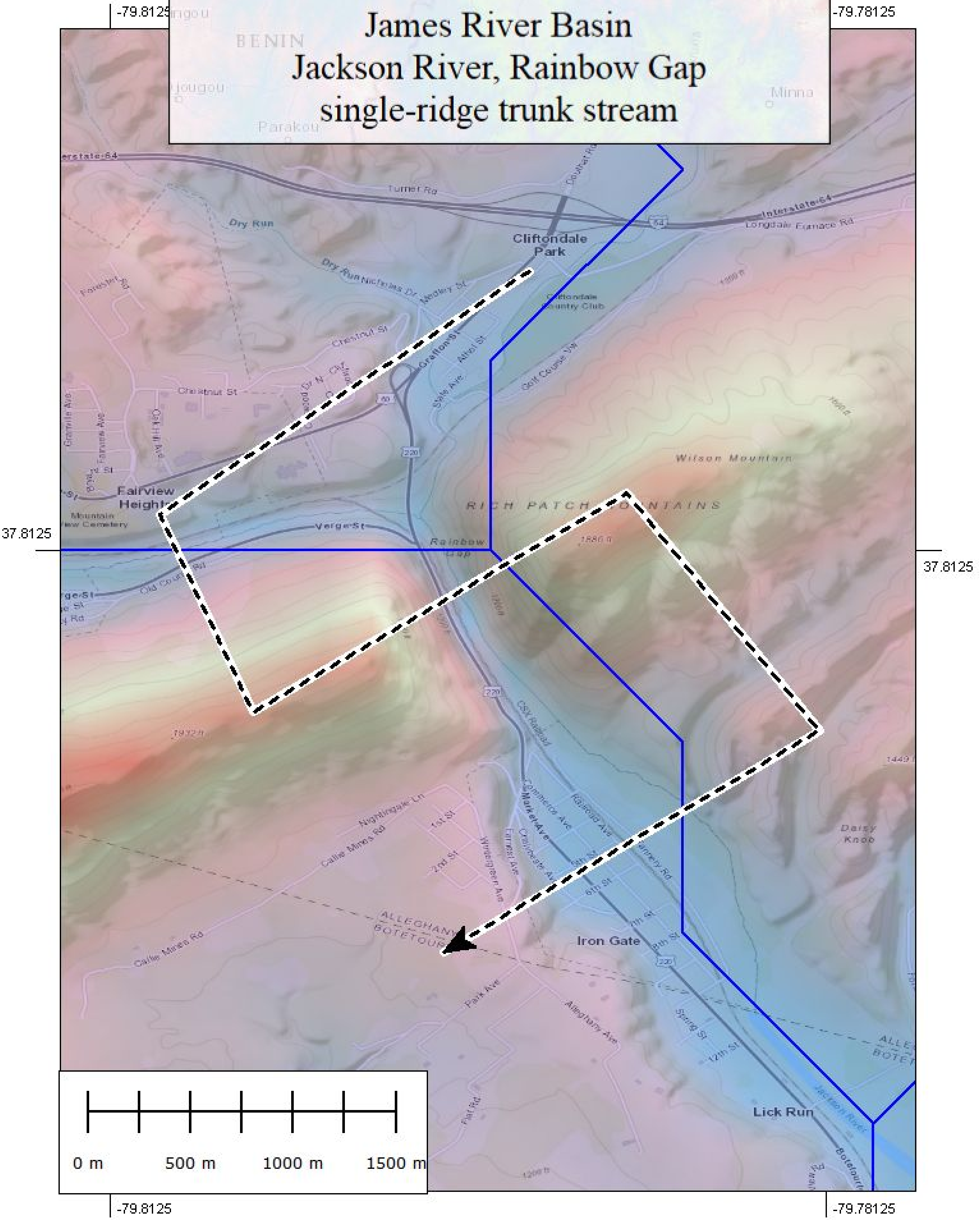

NA - 254
Rio Soto La Marina Basin
Rio Las Huertas
multi-ridge trunk stream

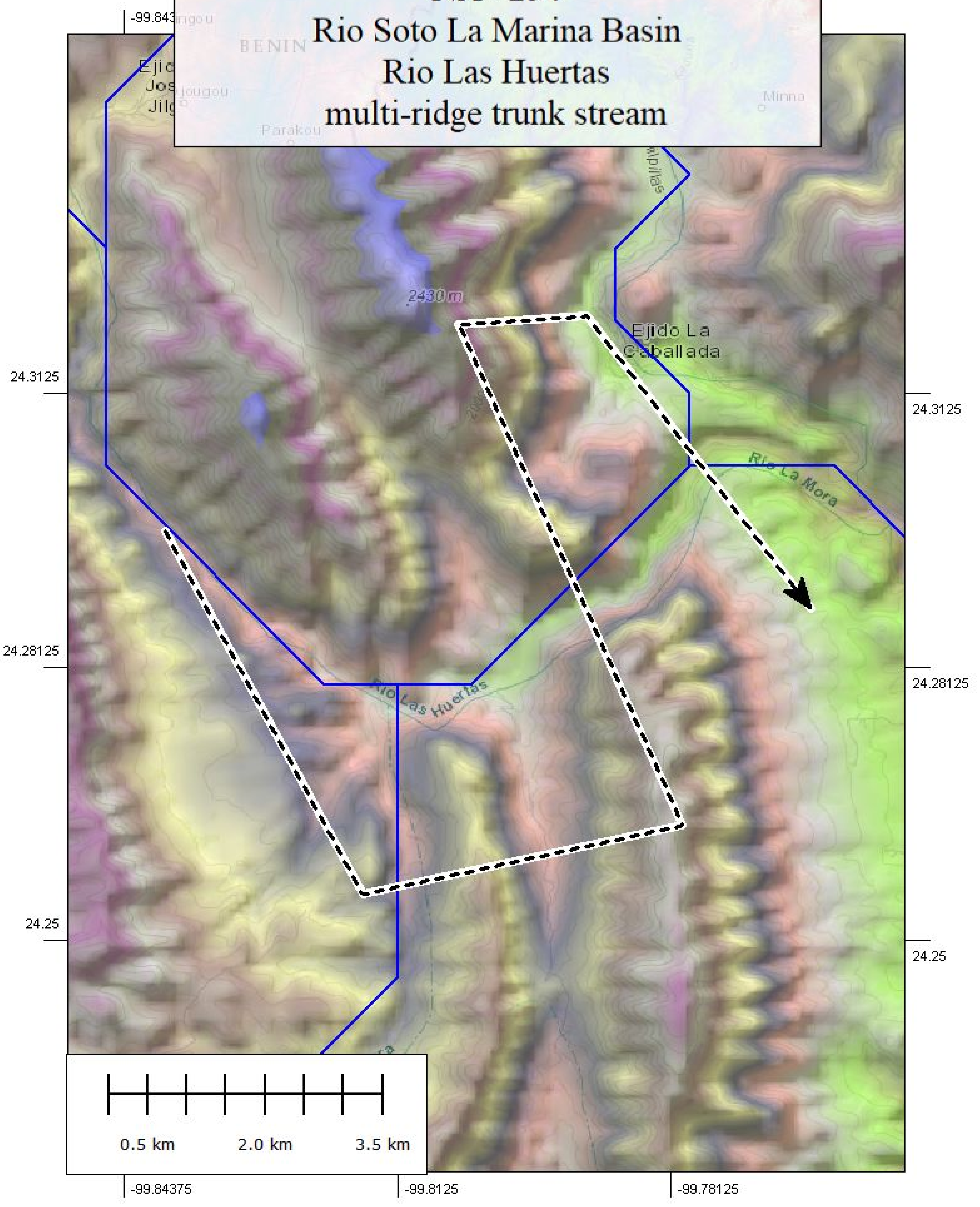

NA - 255
Rio Verde Basin
Rio Miahuatlan
single-ridge trunk stream

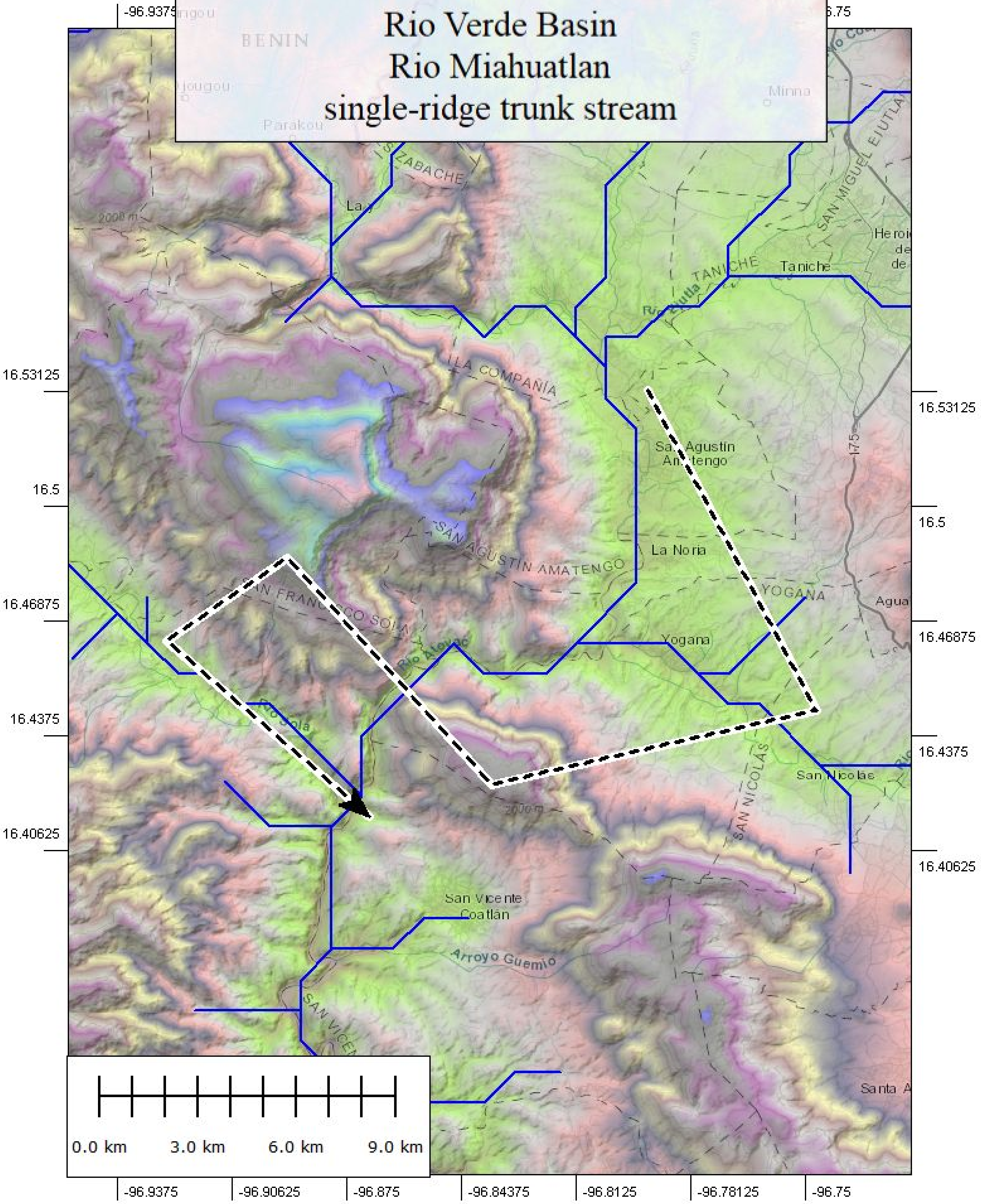

NA - 256
Papaloapan River Basin
Tonto River tributary
single-ridge trunk stream

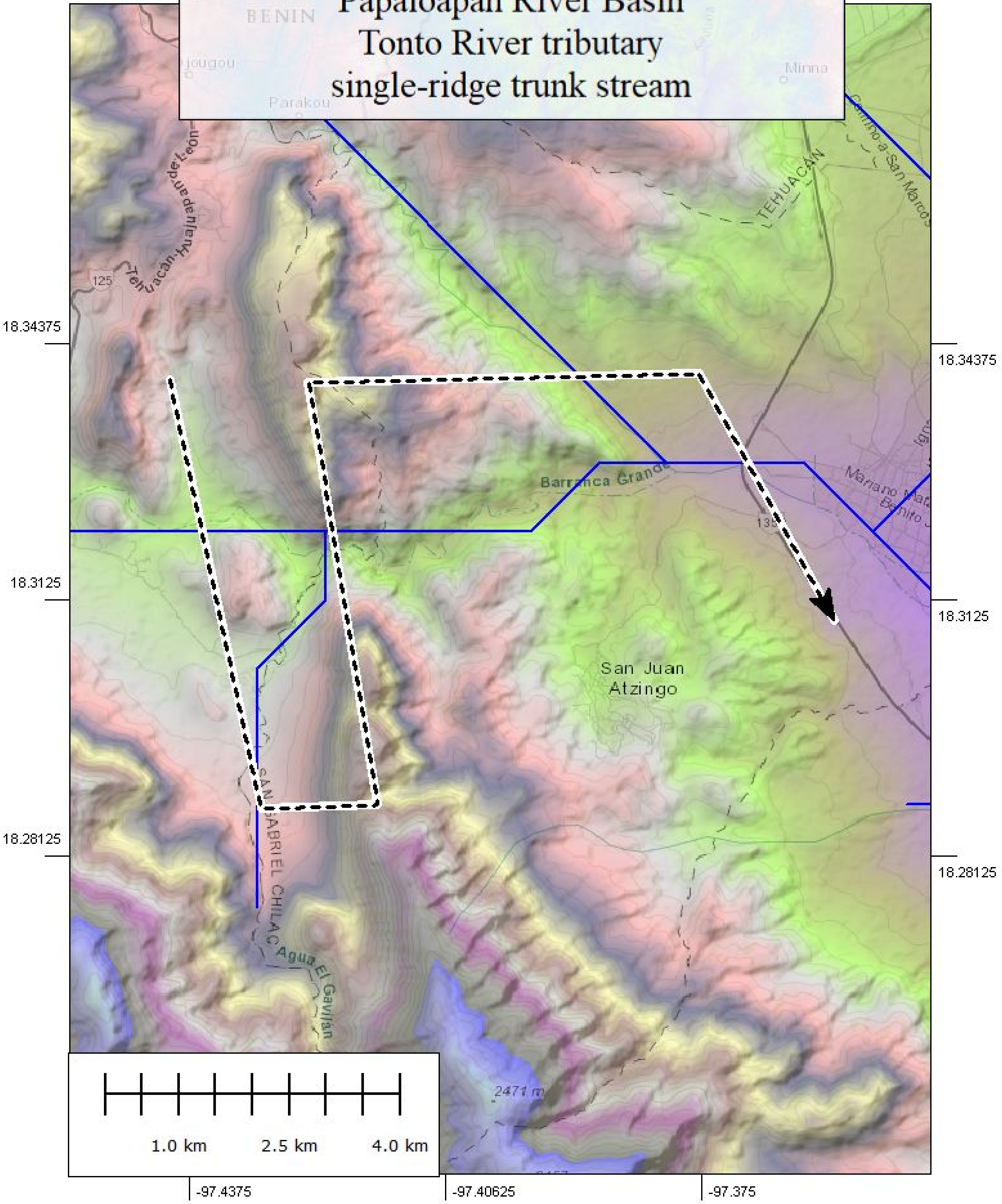

NA - 257
Rio Verde Basin
Rio Penoles
single-ridge trunk stream

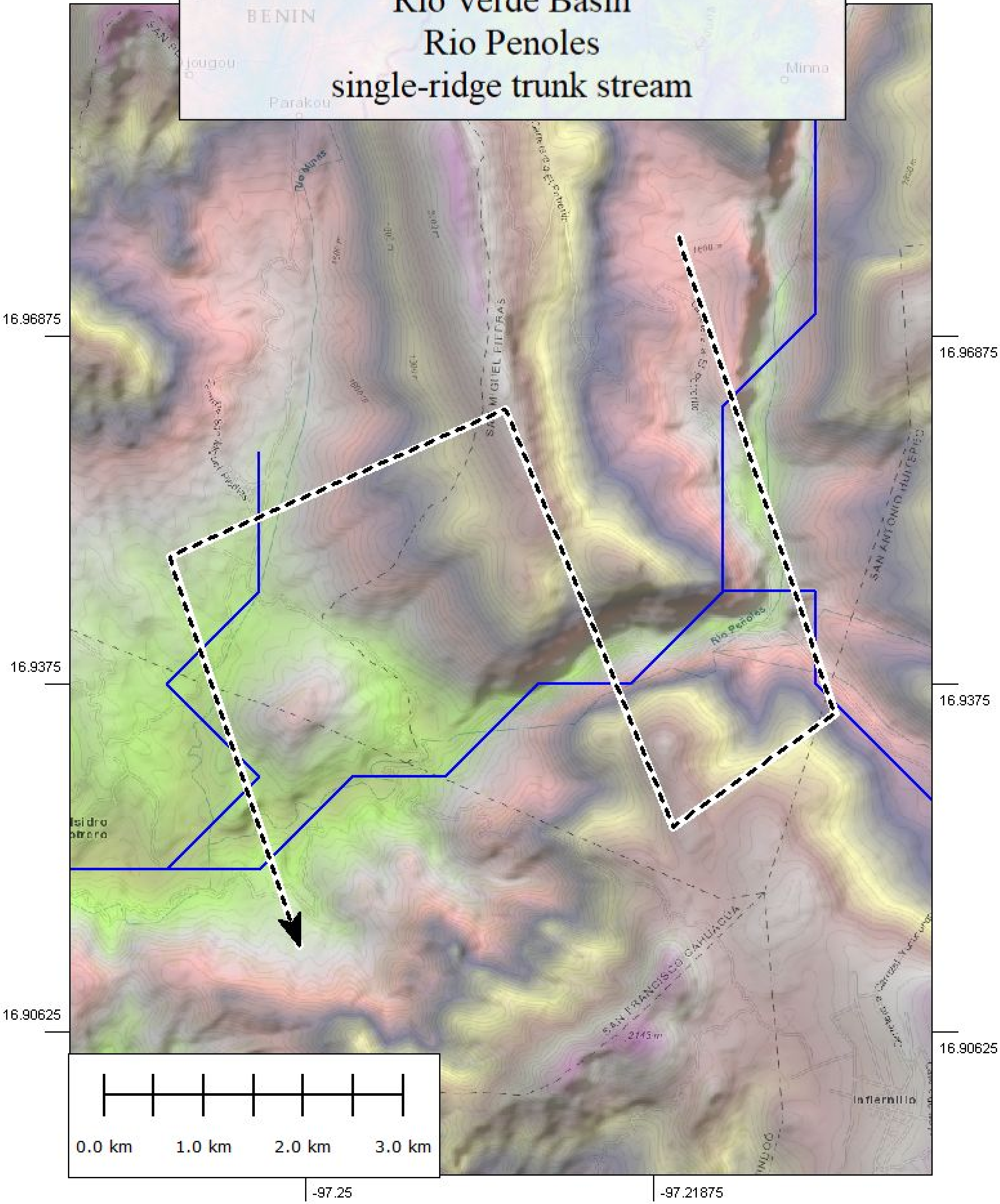

NA - 258
Columbia River Basin
Stump Creek
single-ridge trunk stream

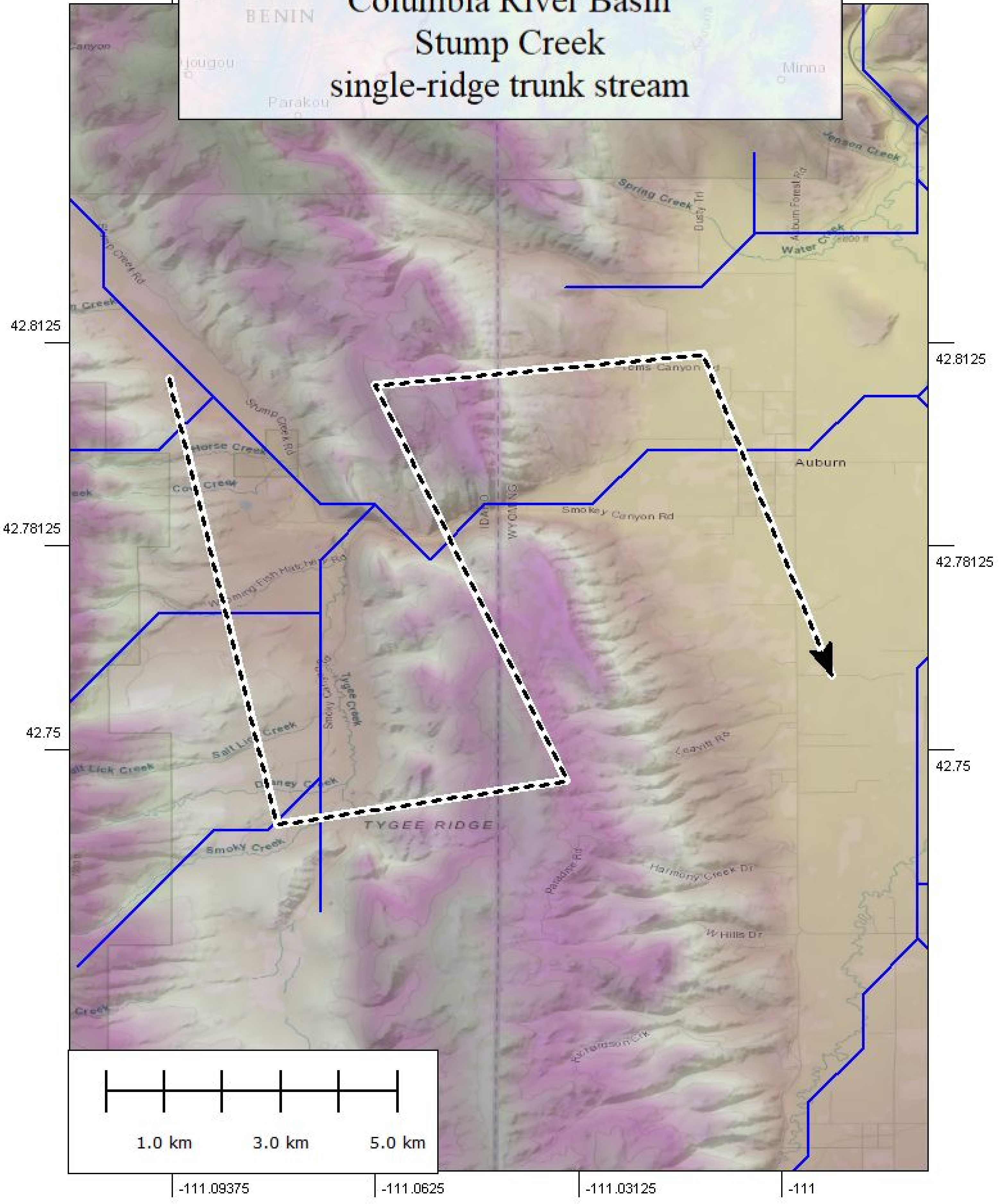

NA - 259
Endorheic basin Basin
Maggie Creek, Maggie Creek Canyon
single-ridge trunk stream

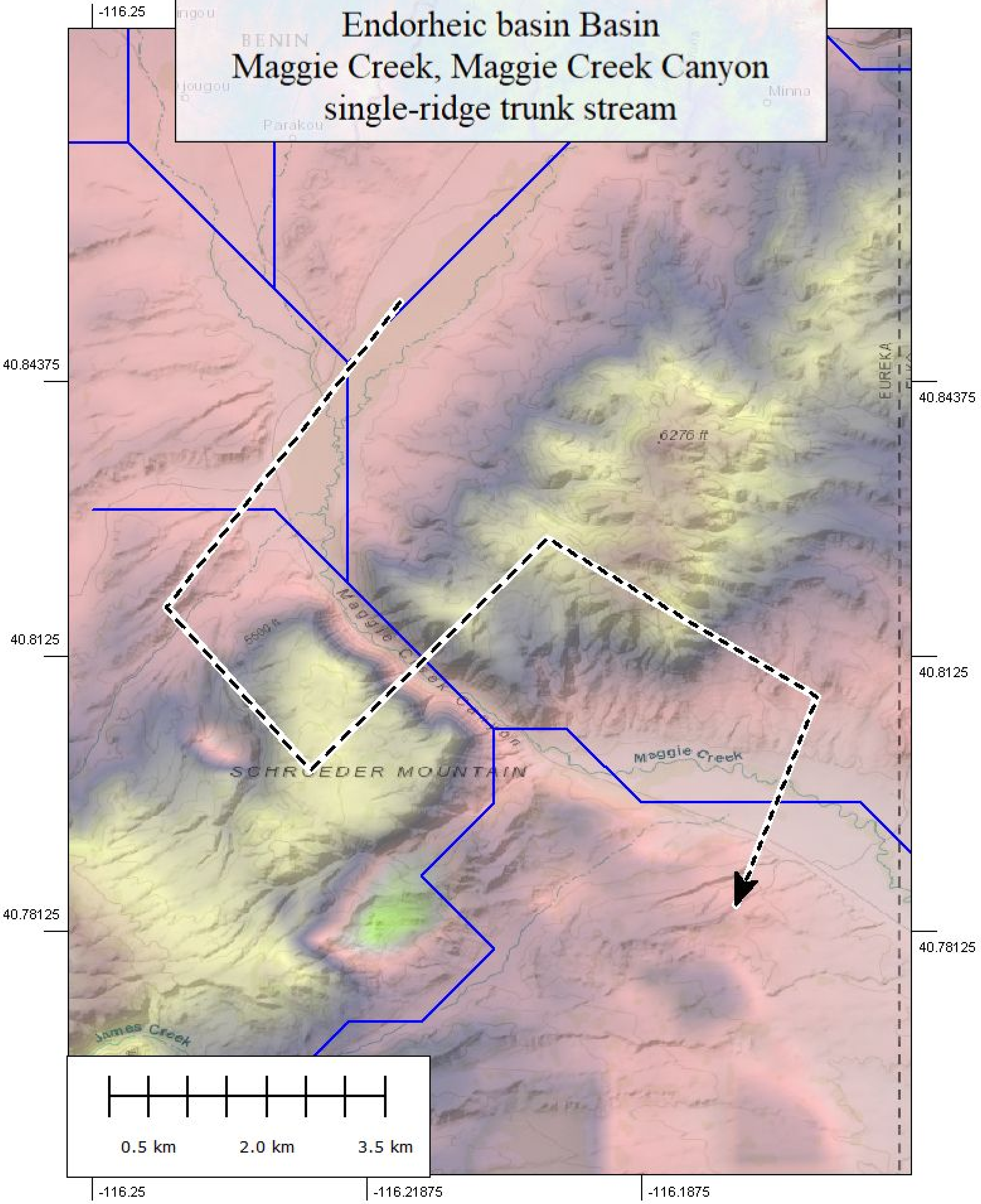

NA - 26
Malibu Creek Basin
Malibu Creek, Malibu Canyon
irregular high ground trunk stream

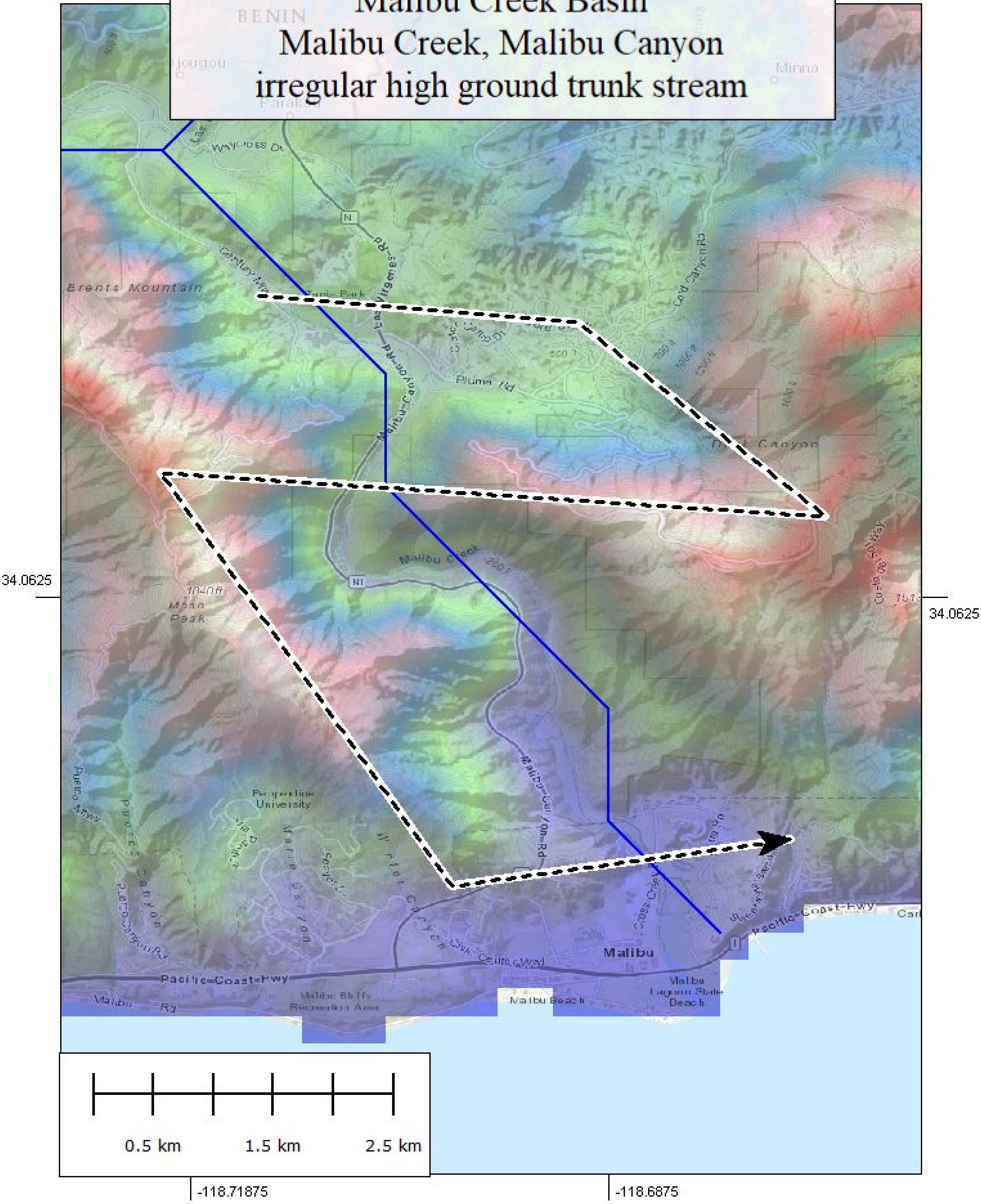

NA - 260
Rio Grande (Texas-Mexico) Basin
Rio Grande
irregular high ground trunk stream

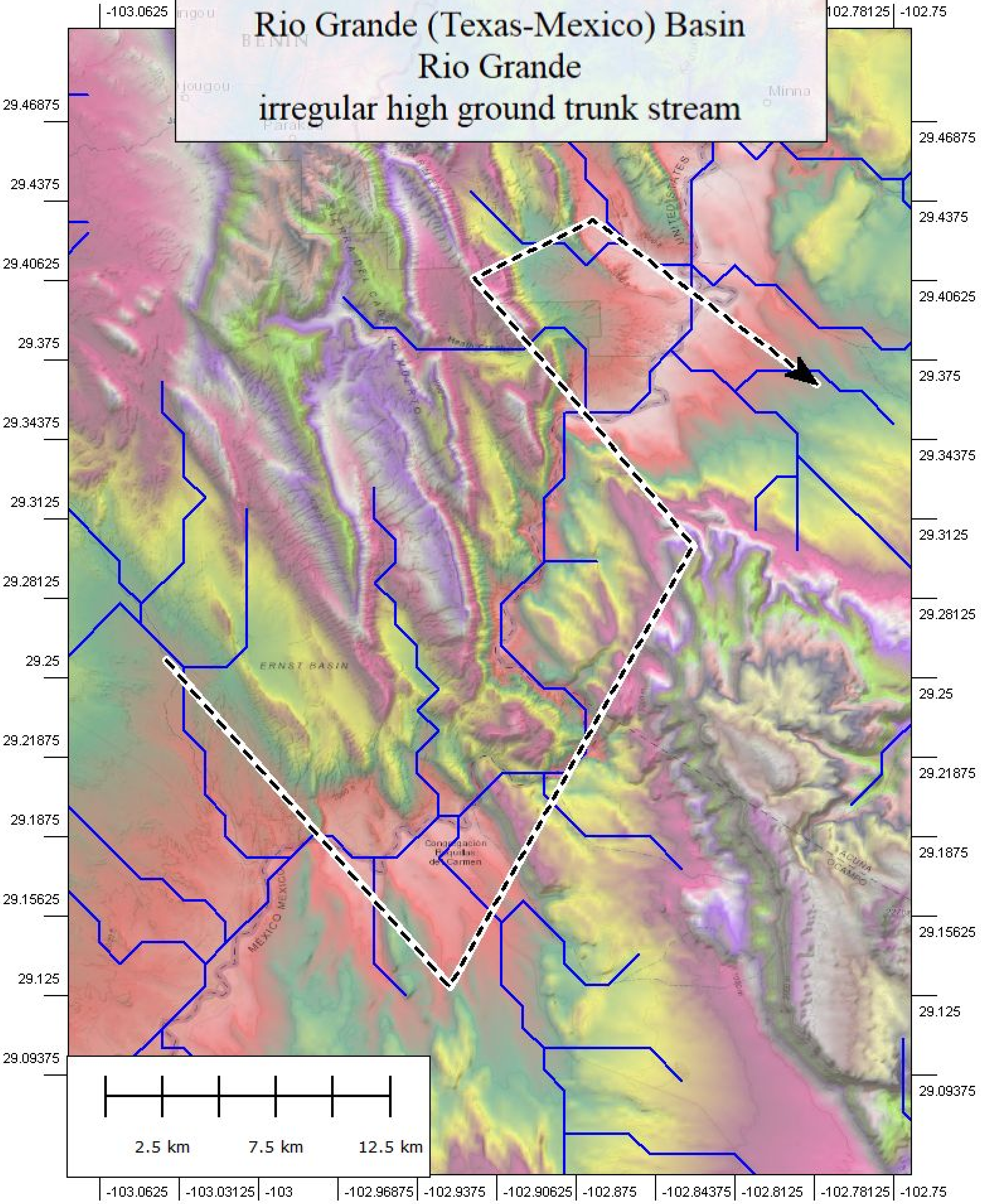

NA - 261
Columbia River Basin
John Day River, Devils Gulch
irregular high ground trunk stream

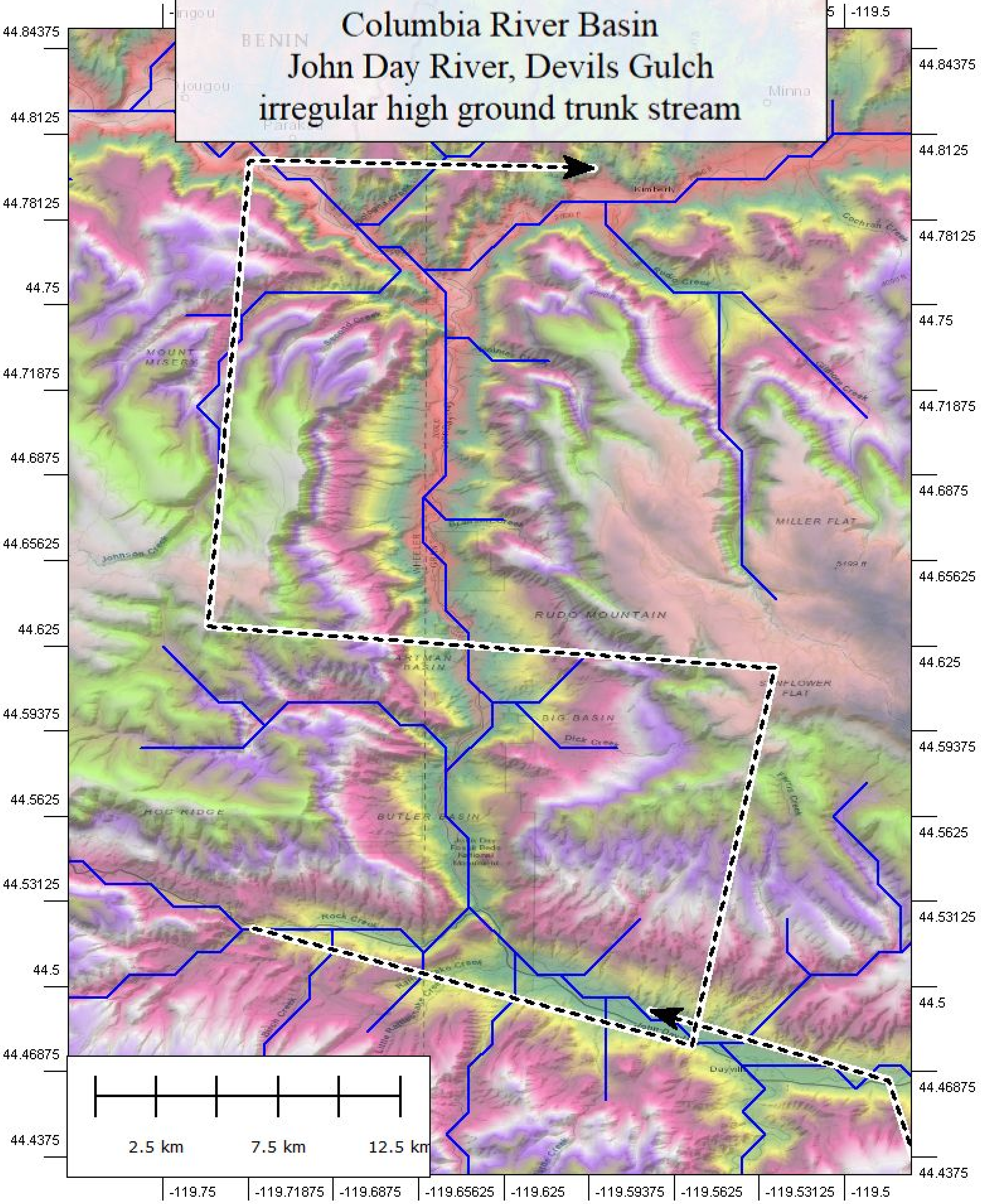

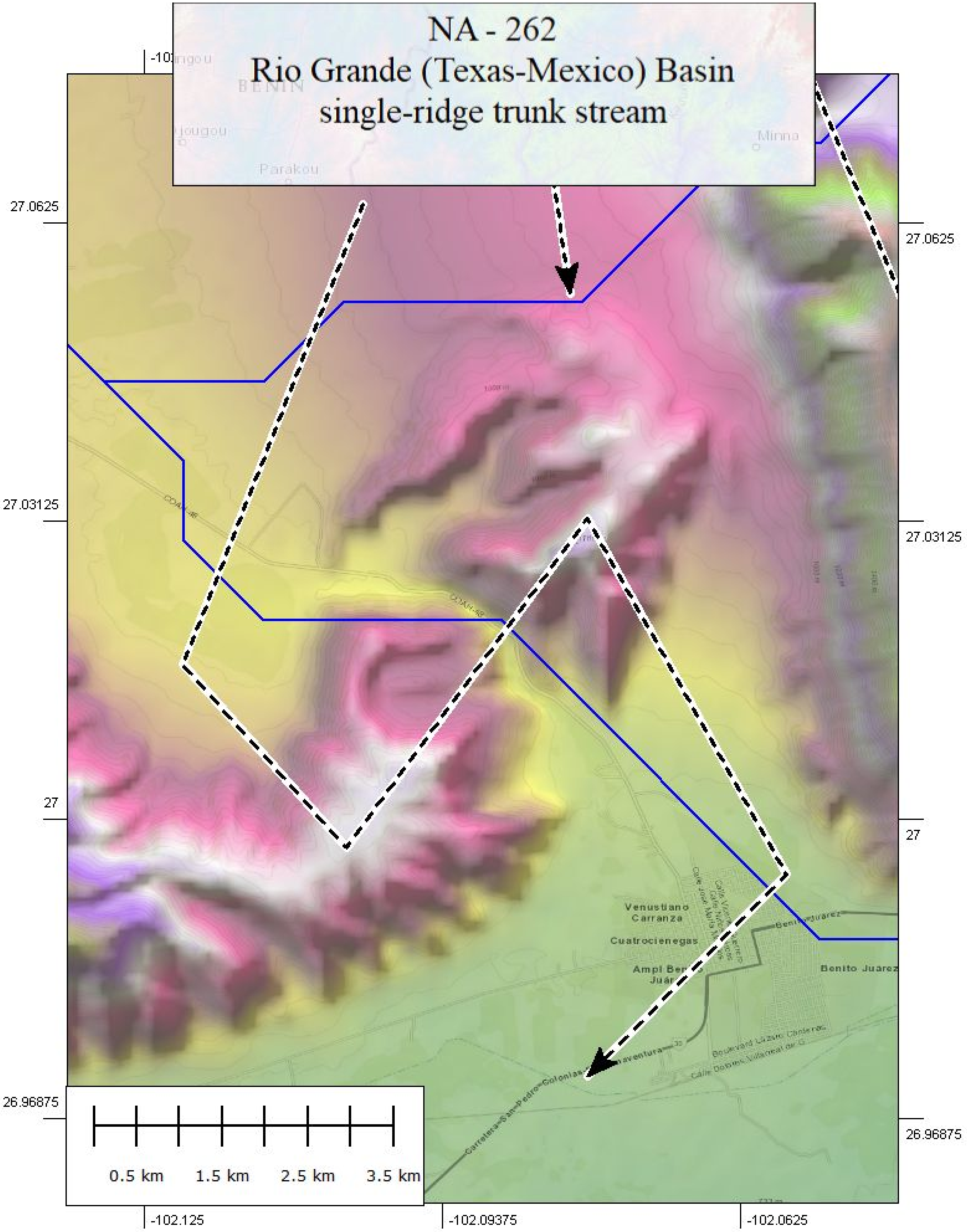
NA - 262
Rio Grande (Texas-Mexico) Basin
single-ridge trunk stream
27.0625
27.03125
27
26.96875
-102.125
-102.09375
-102.0625
Parakou
Venustiano Carranza
Cuatrocienegas
Benito Juarez
0.5 km
1.5 km
2.5 km
3.5 km

NA - 263
Sacramento River Basin
Cosumnes River
single-ridge trunk stream

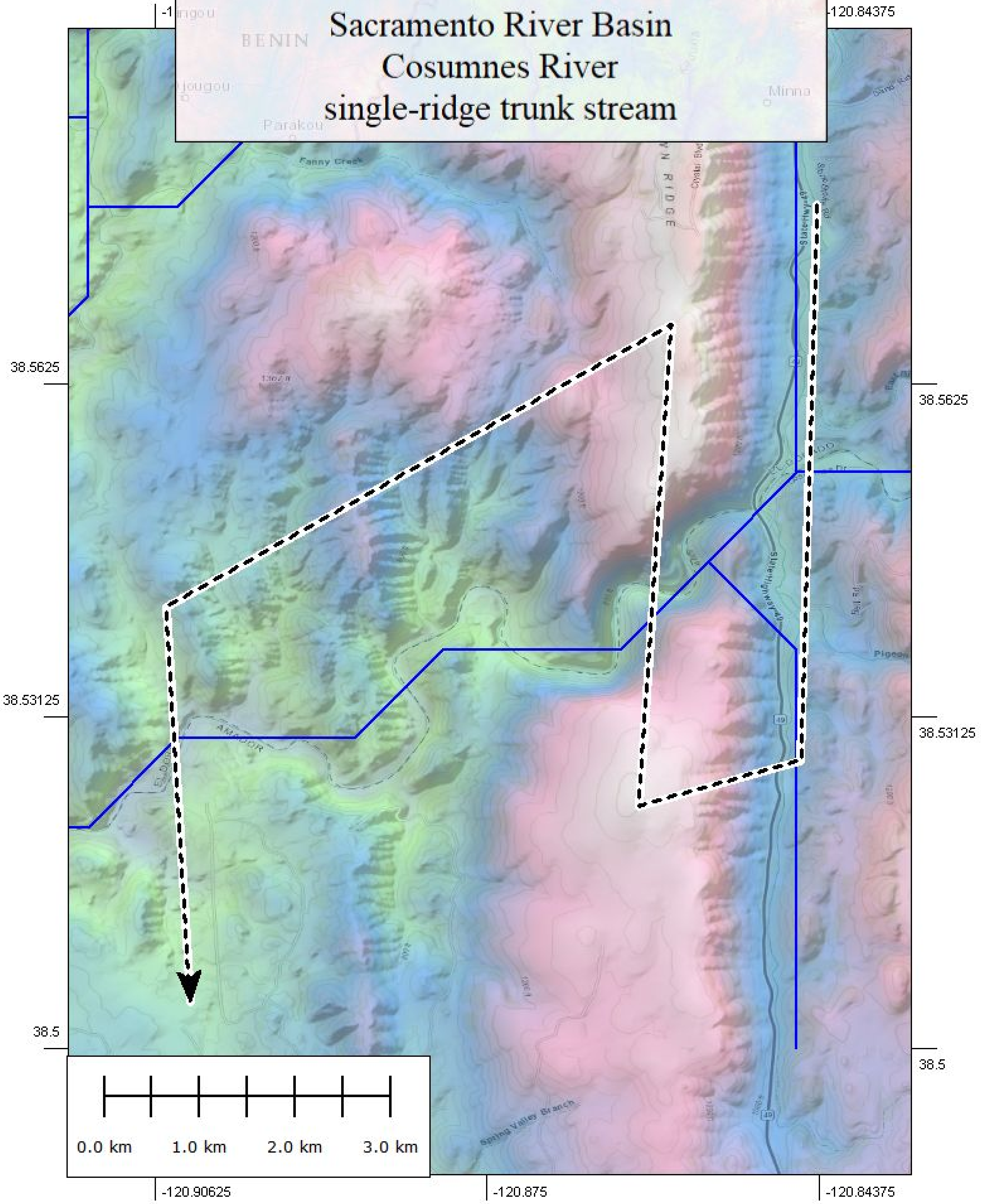

NA - 264
Mackenzie River (Canada) Basin
Peace River, Williston Lake
irregular high ground trunk stream

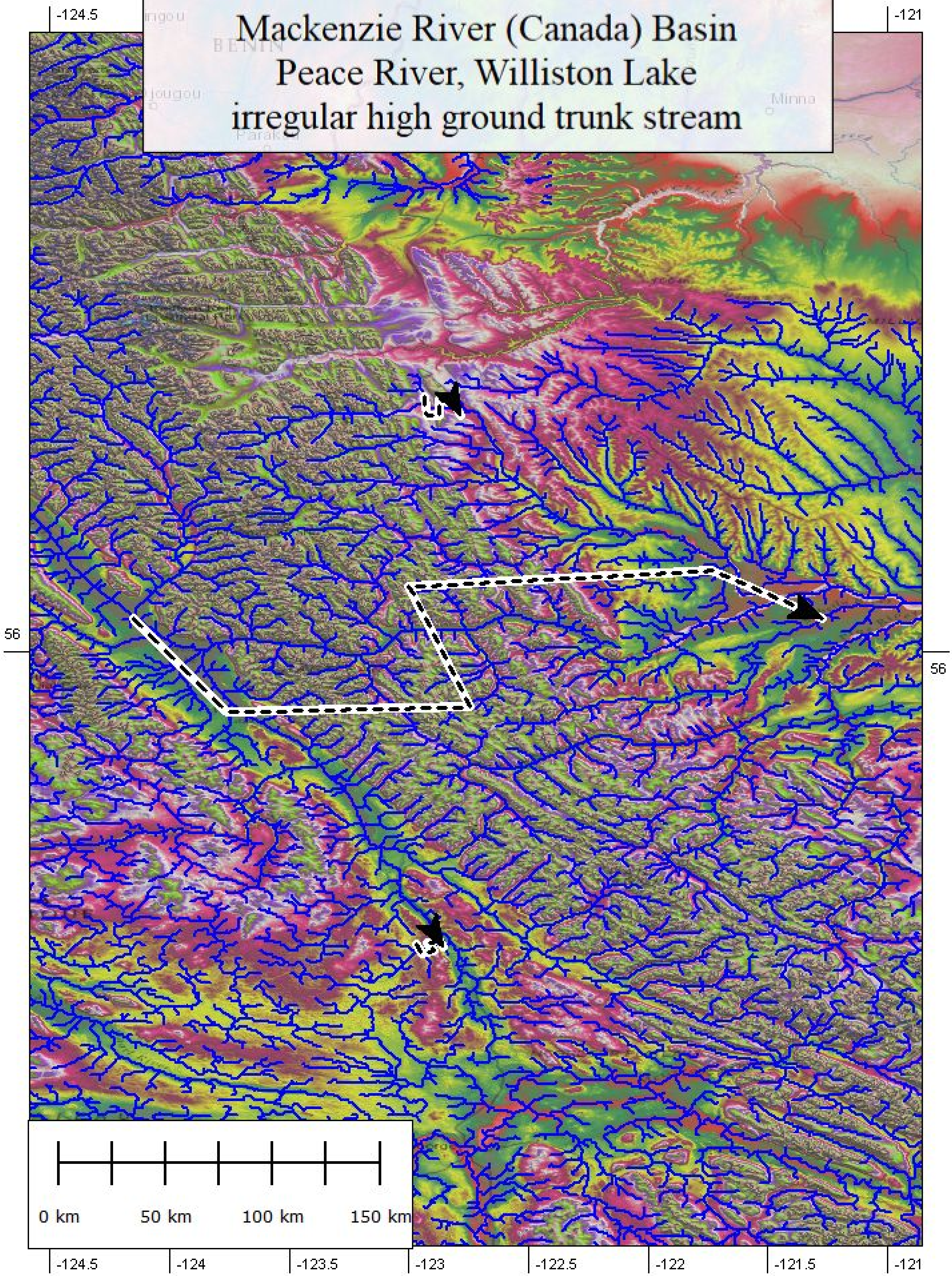

NA - 265
Kliniklini River Basin
Klinaklini River
irregular high ground trunk stream

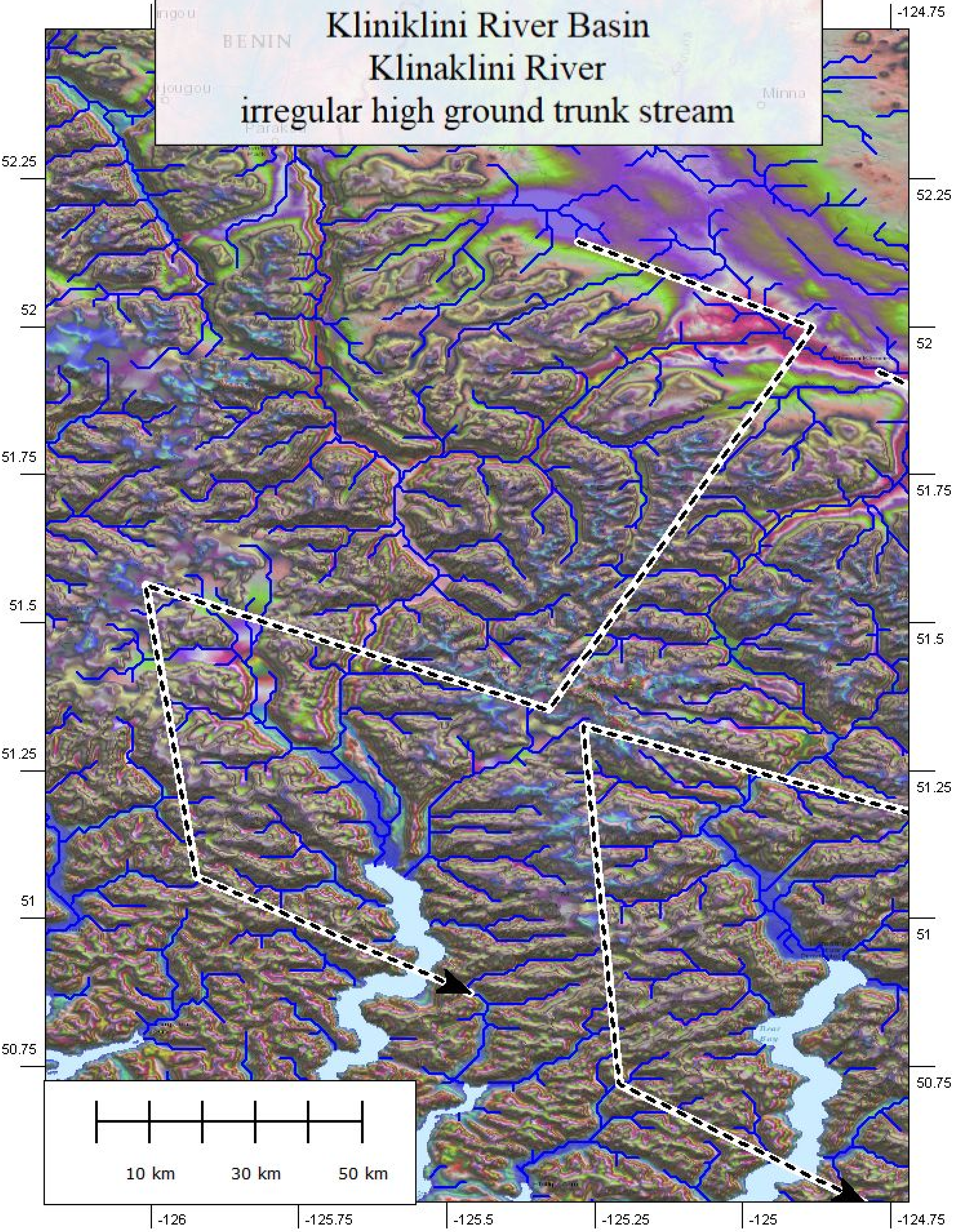

NA - 266
Rio Grande (Texas-Mexico) Basin
El Carmen Pass
single-ridge trunk stream

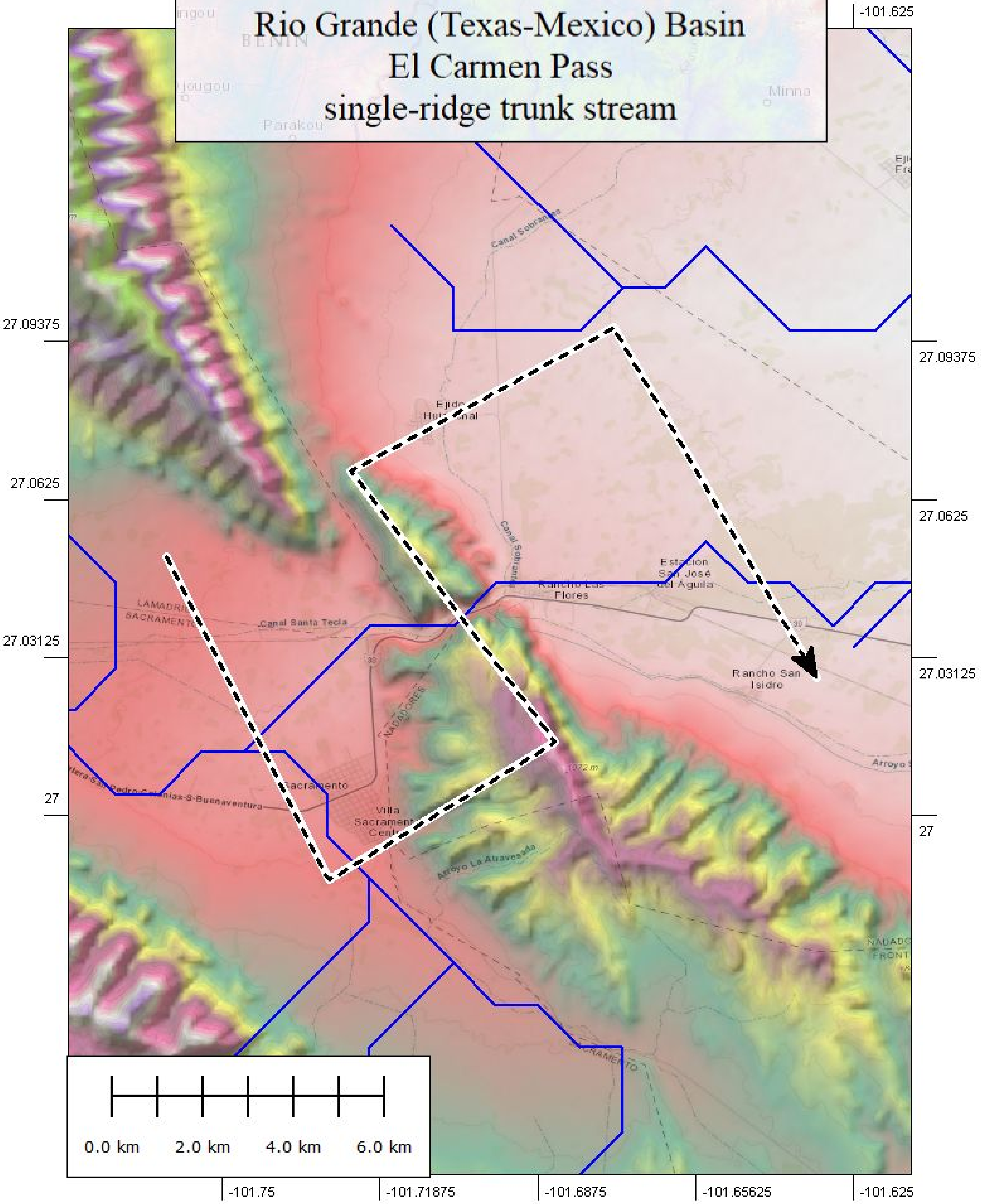

NA - 267
Rio Grande (Texas-Mexico) Basin
Arroyo El Colorado
single-ridge trunk stream

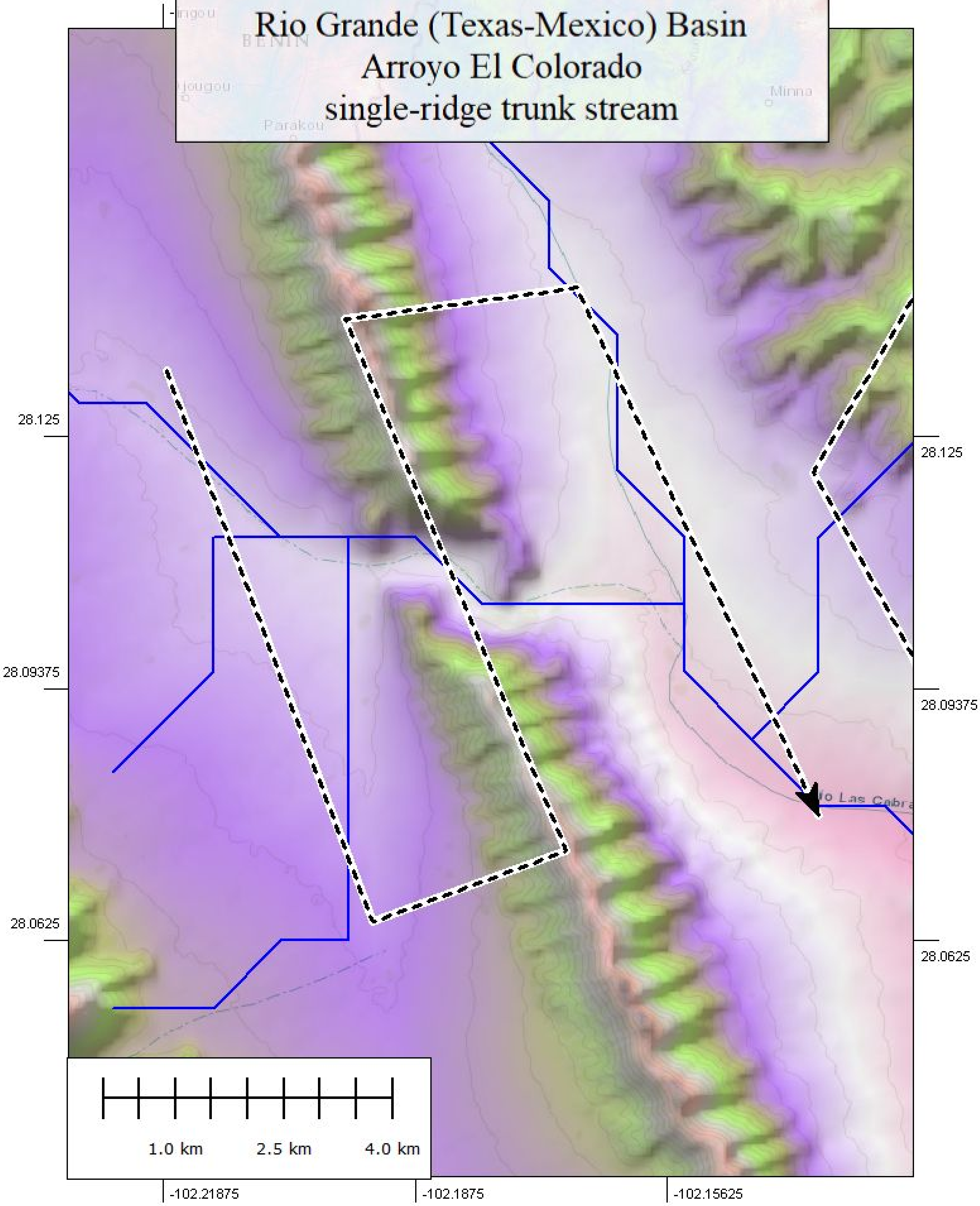

NA - 268
Rio Grande (Texas-Mexico) Basin
Pesqueria River
single-ridge trunk stream

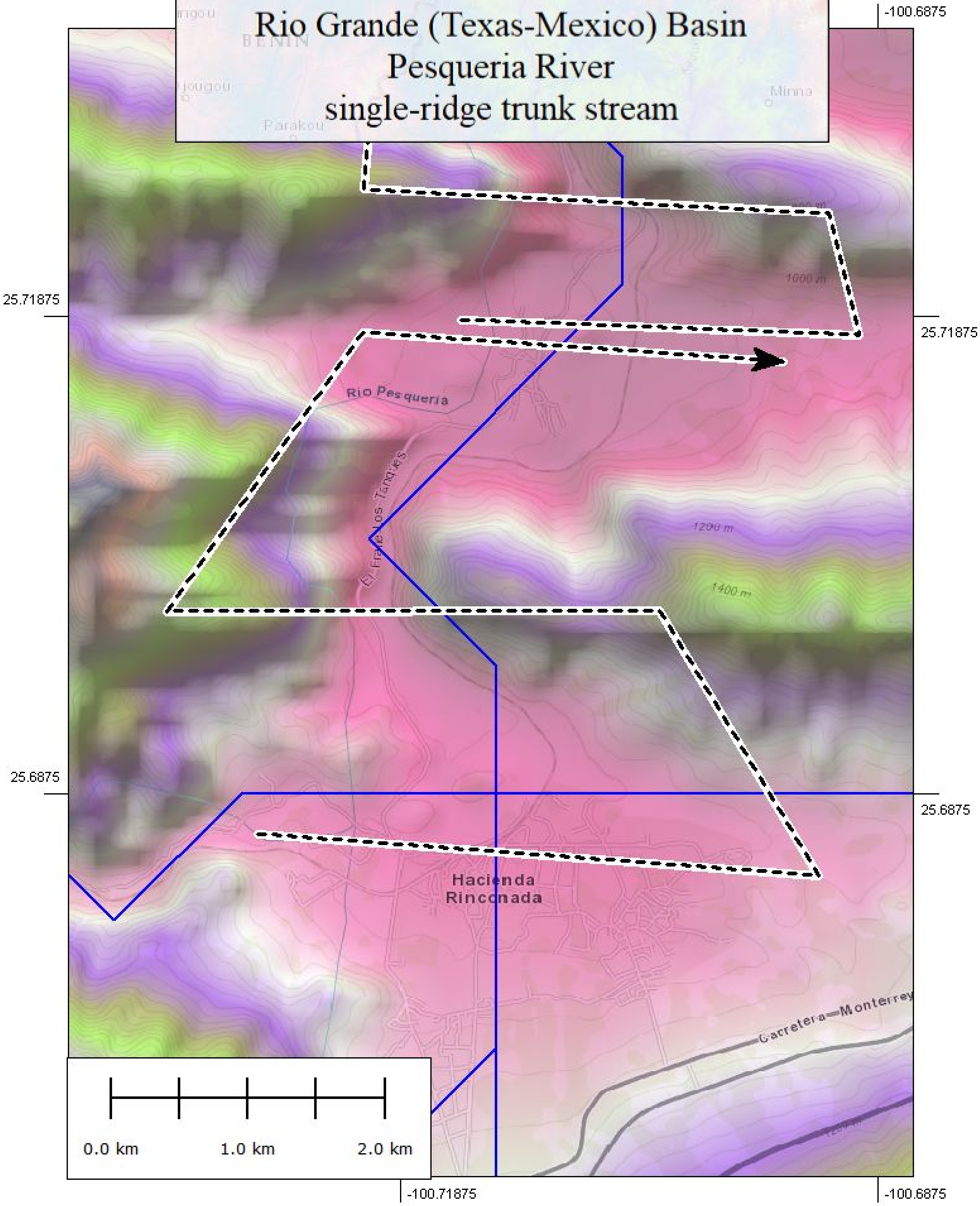

NA - 269
Rio Grijalva Basin
Rio Tabilicum
plateau trunk stream

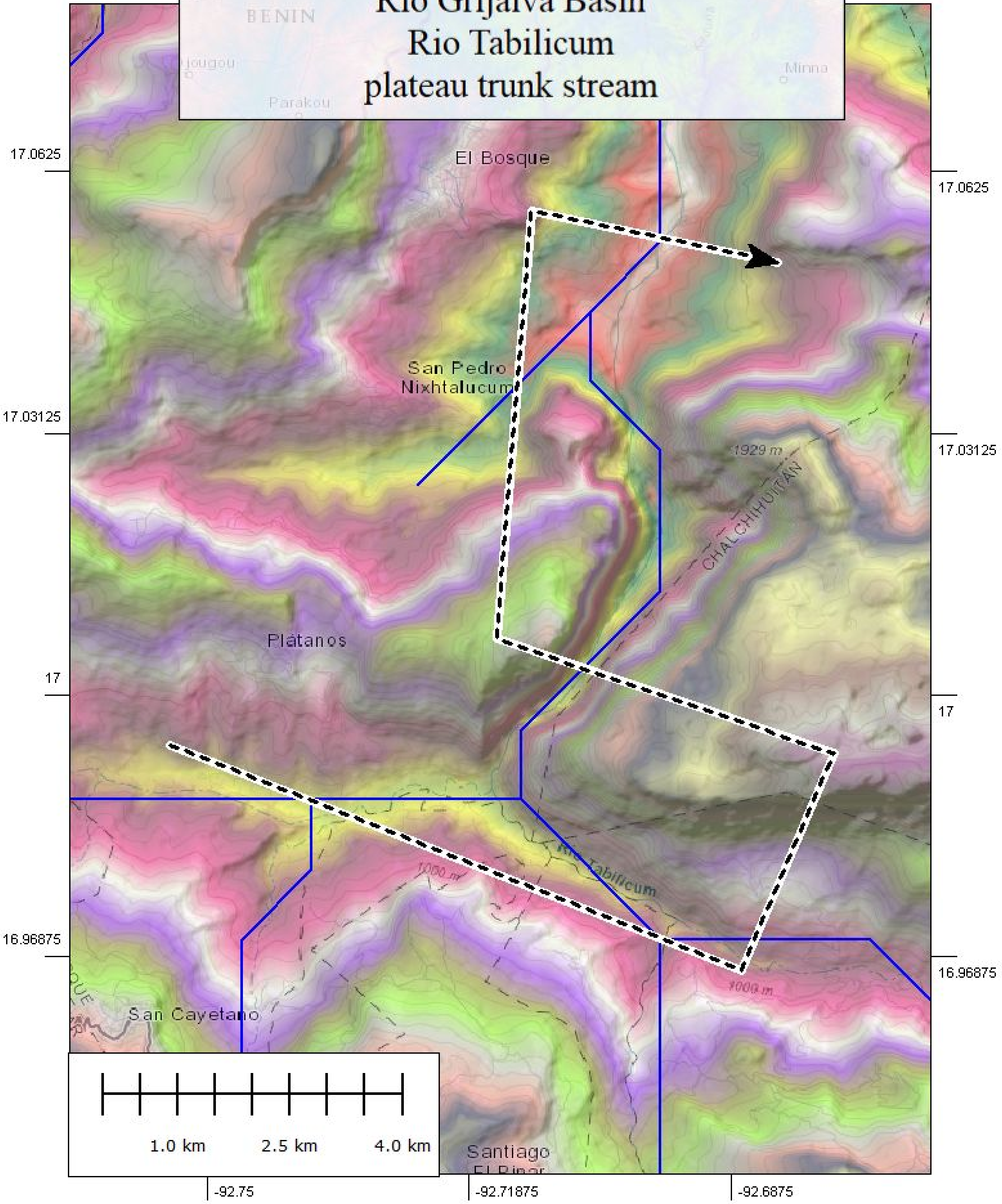

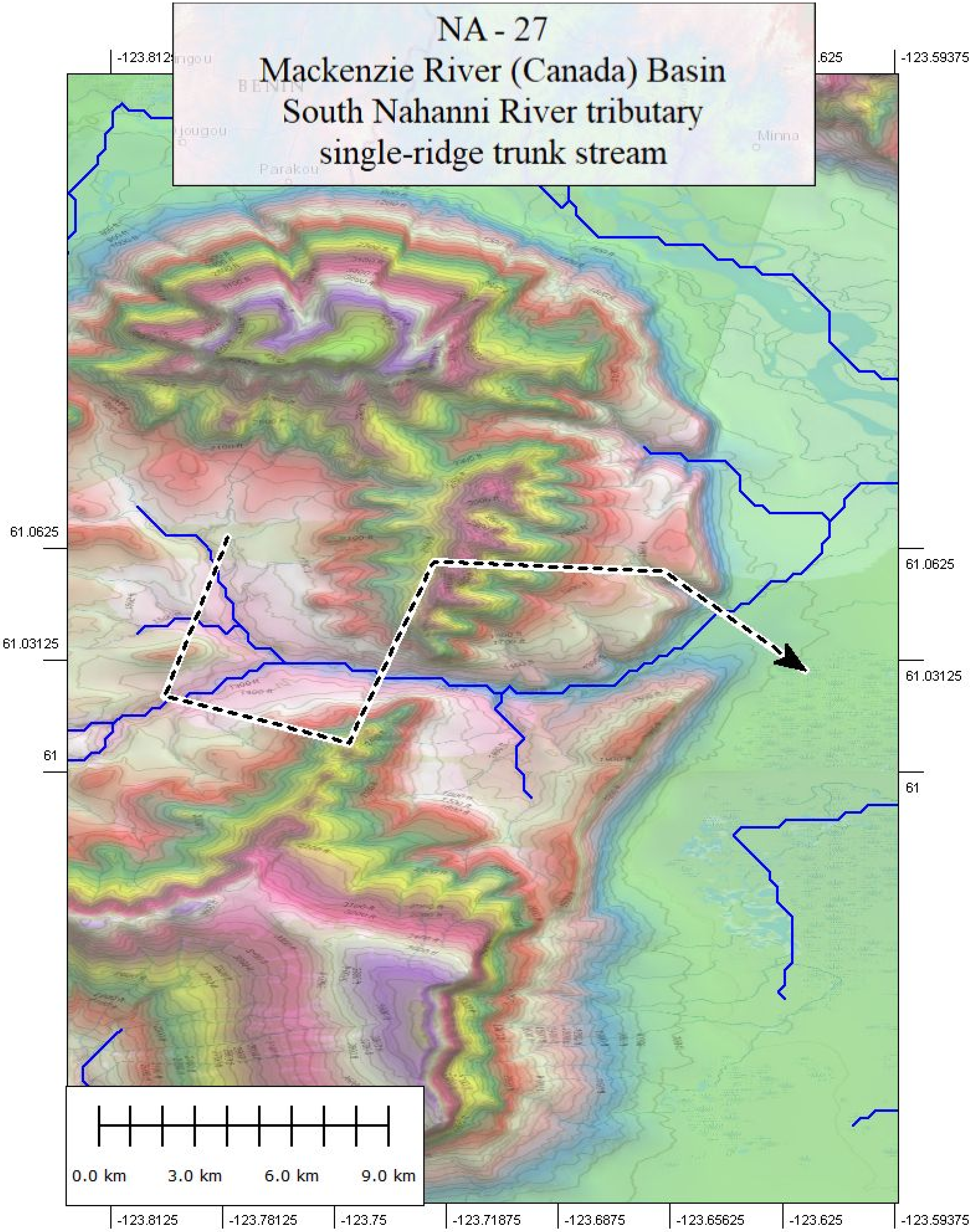
NA - 27
Mackenzie River (Canada) Basin
South Nahanni River tributary
single-ridge trunk stream
-123.8125
-123.78125
-123.75
-123.71875
-123.6875
-123.65625
-123.625
-123.59375
61.0625
61.03125
61
0.0 km
3.0 km
6.0 km
9.0 km

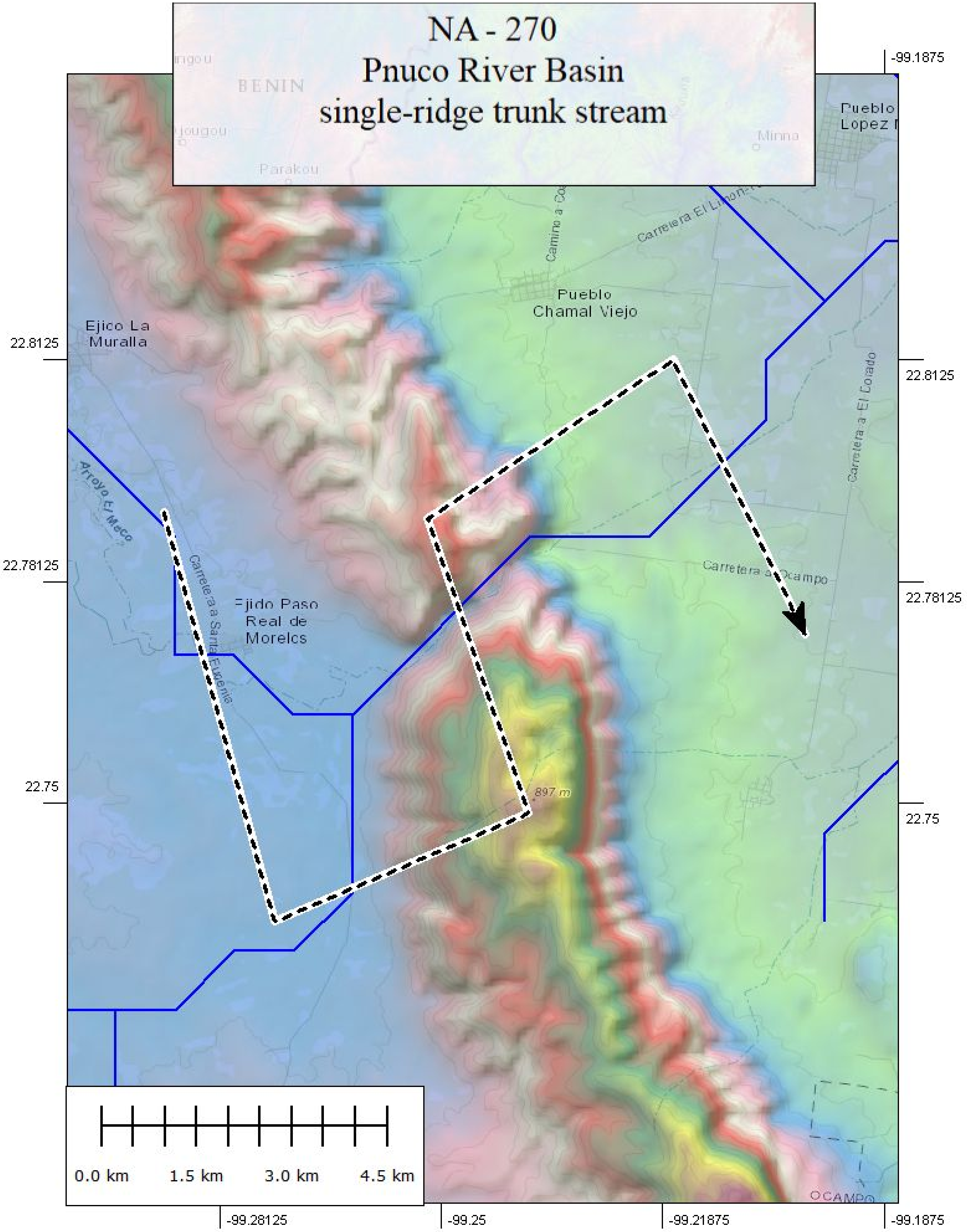

NA - 270
Pnuco River Basin
single-ridge trunk stream
BENIN
Parakou
Minno
Pueblo
Lopez
Carretera El Limon
Pueblo
Chamal Viejo
Ejico La
Muralla
Arroyo El Meco
Carretera a Santa Eugenia
Ejido Paso
Real de
Morelcs
Carretera a Ocampo
Carretera a El Corado
897 m
OCAMPO
22.8125
22.78125
22.75
-99.28125
-99.25
-99.21875
-99.1875
0.0 km
1.5 km
3.0 km
4.5 km

NA - 271
Susquehanna River Basin
Mahoning Creek
single-ridge trunk stream

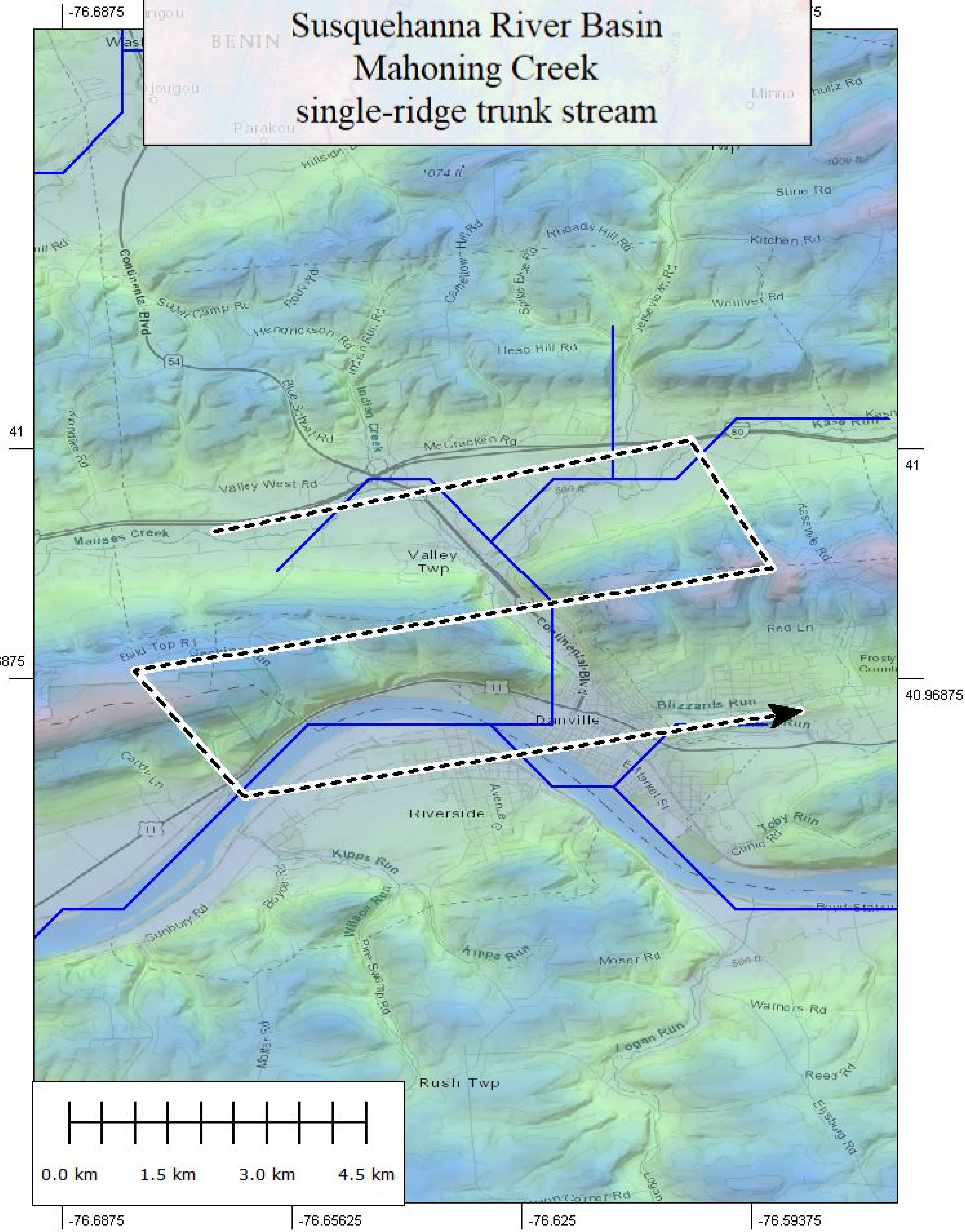

NA - 272
Pnuco River Basin
Valles River
single-ridge trunk stream

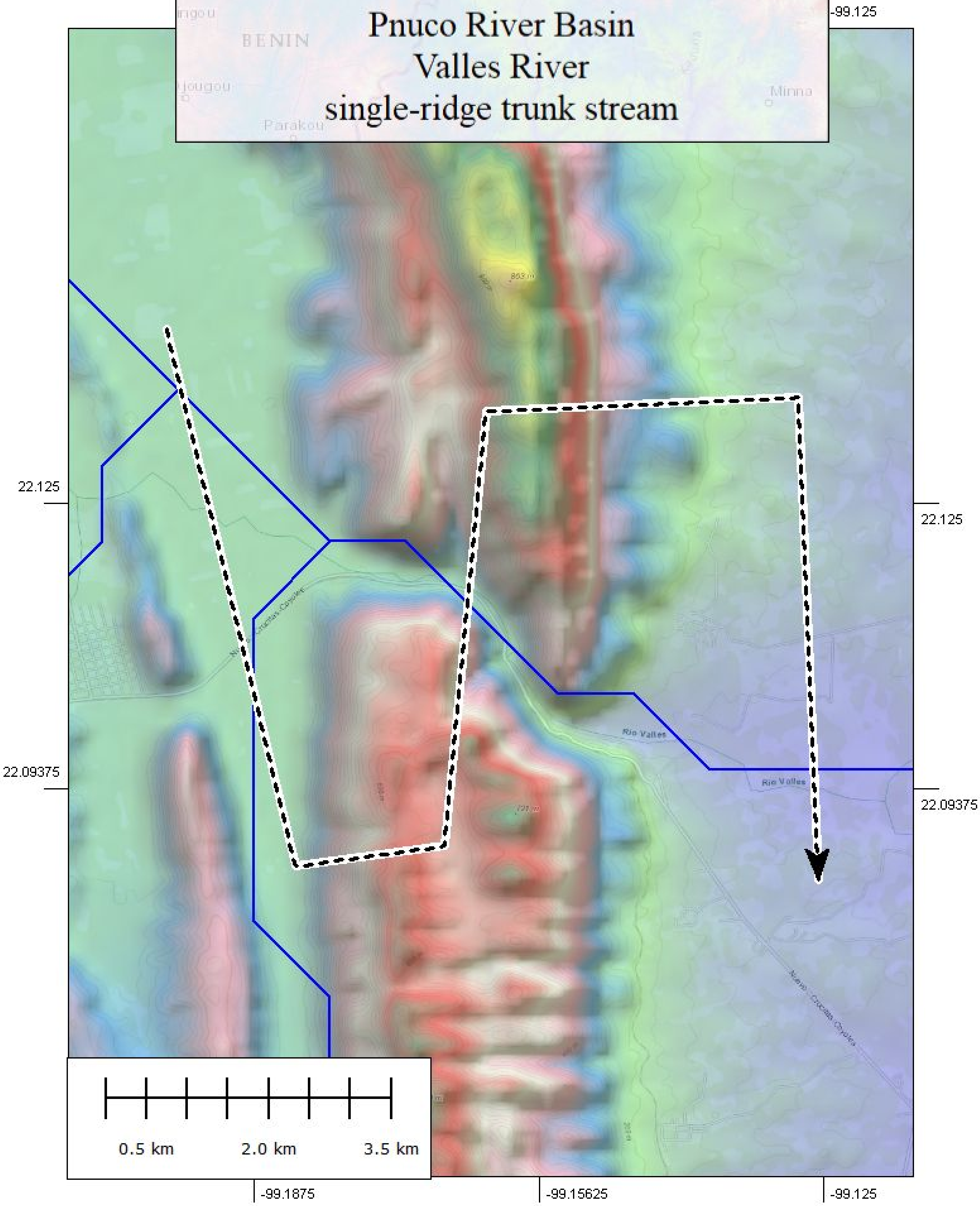

NA - 273
Pnuco River Basin
Rio Guayalejo
single-ridge trunk stream

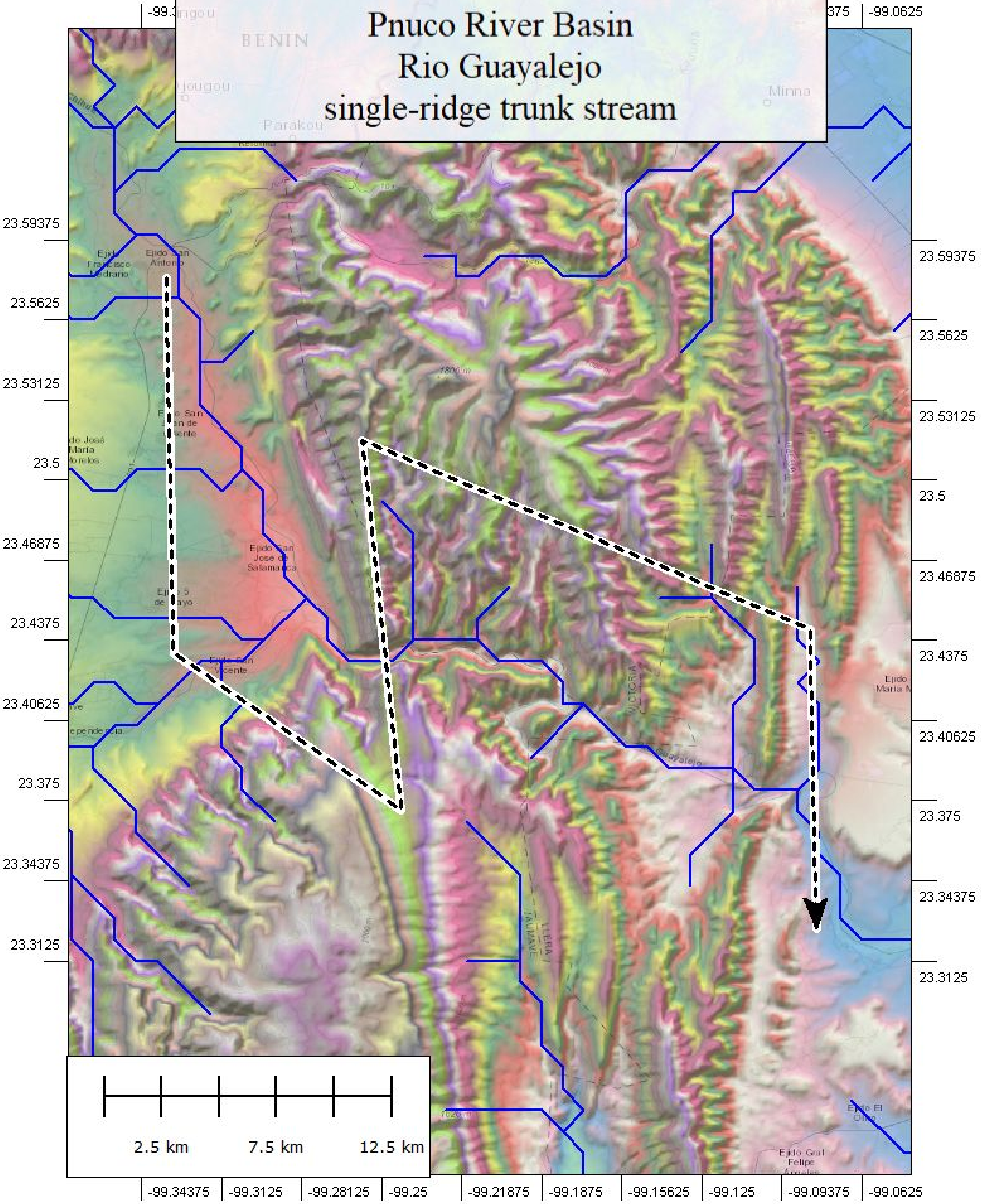

NA - 274
Mississippi River Basin
Anthony Creek
single-ridge trunk stream

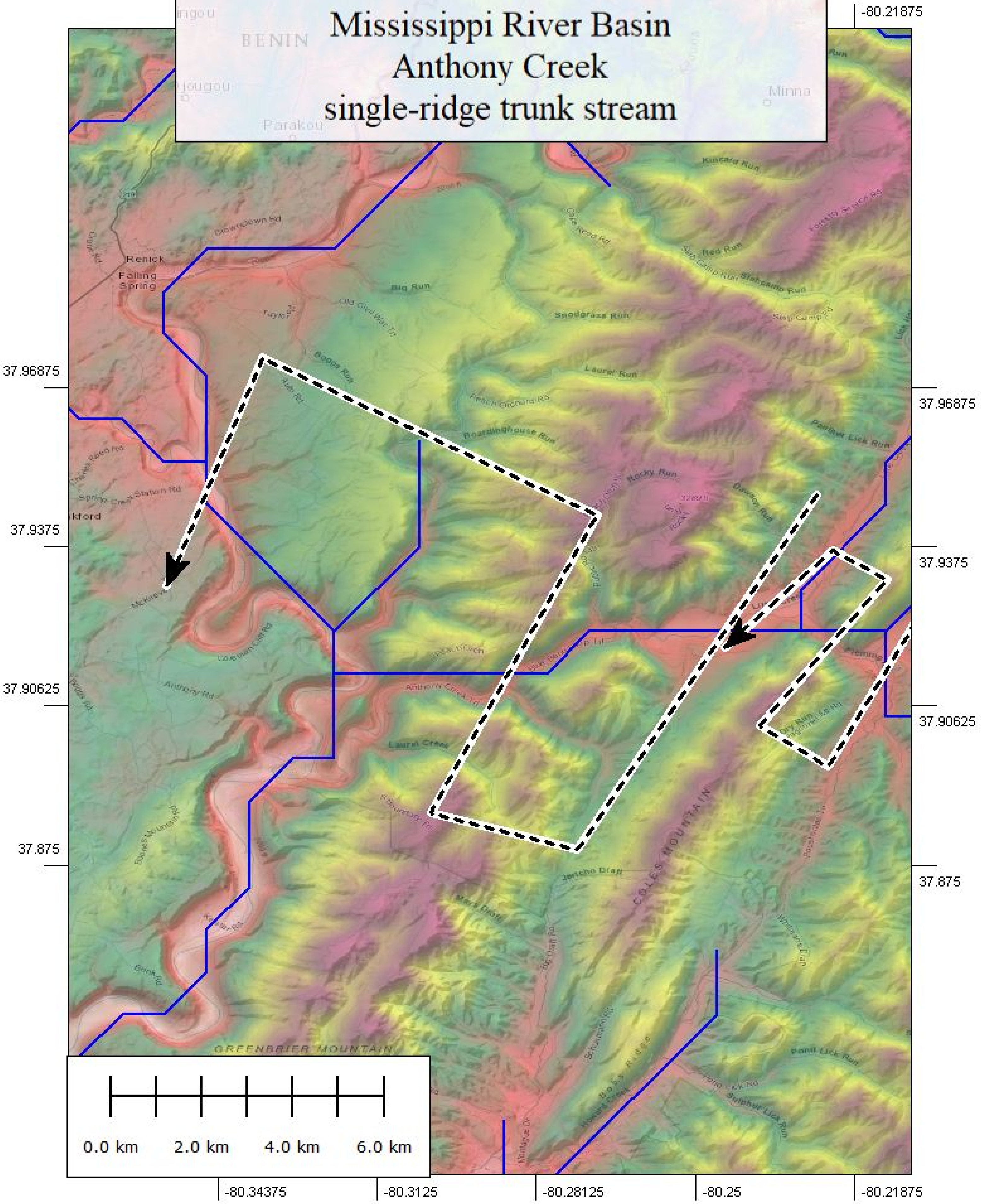

NA - 275
Pnuco River Basin
Rio Verde tributary
single-ridge trunk stream

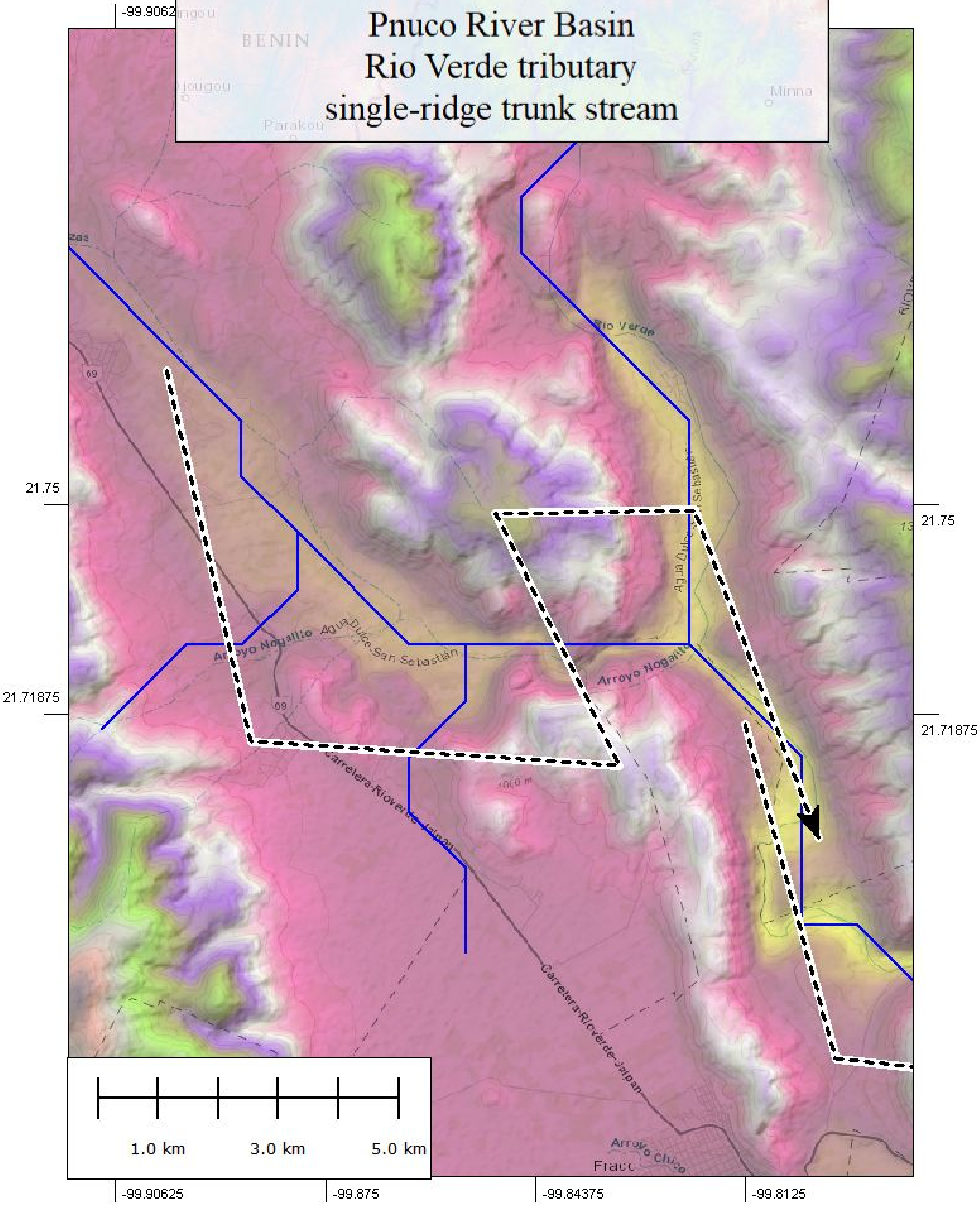

NA - 276
James River Basin
Jackson River
single-ridge trunk stream

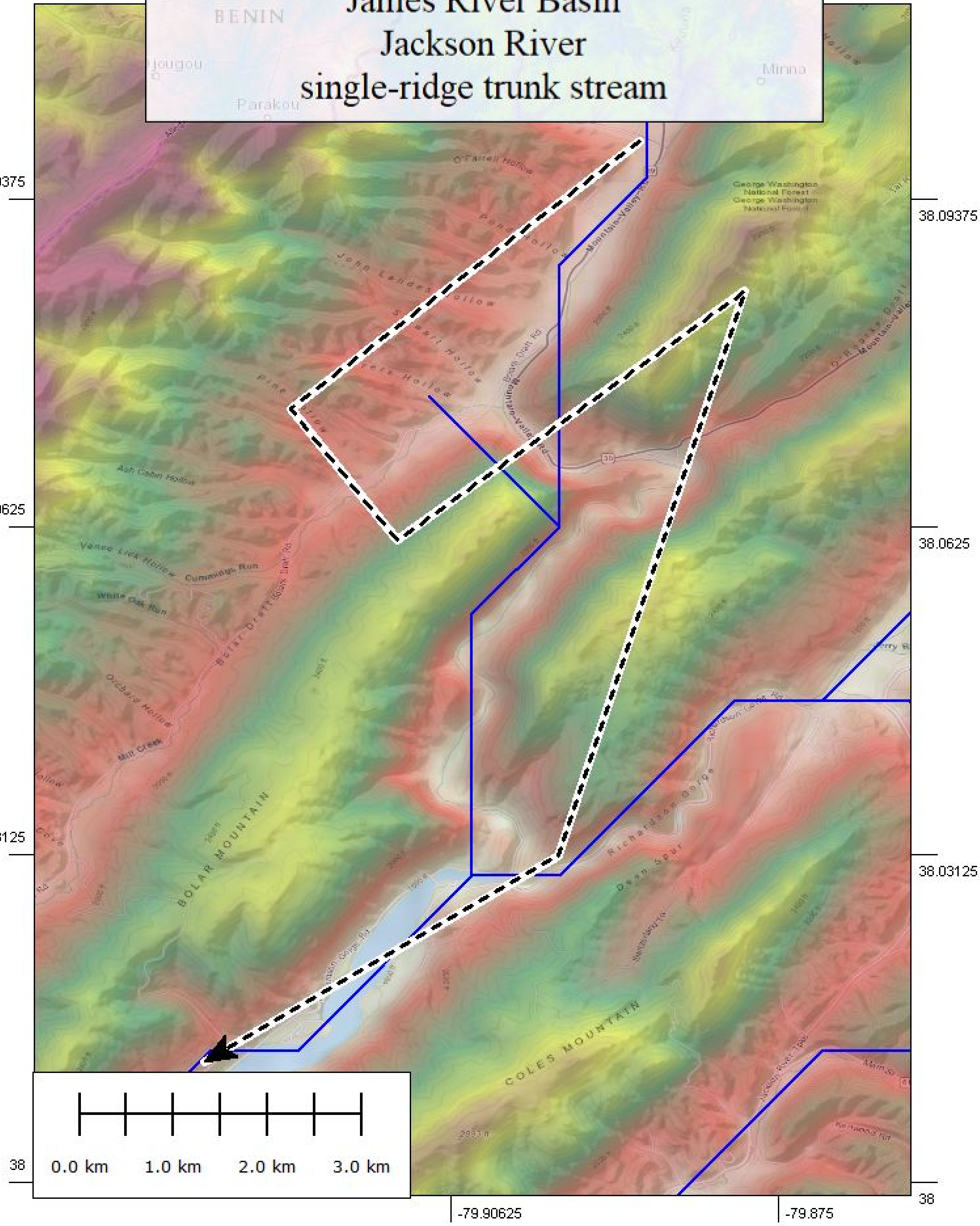

NA - 277
James River Basin
Jackson River
single-ridge trunk stream

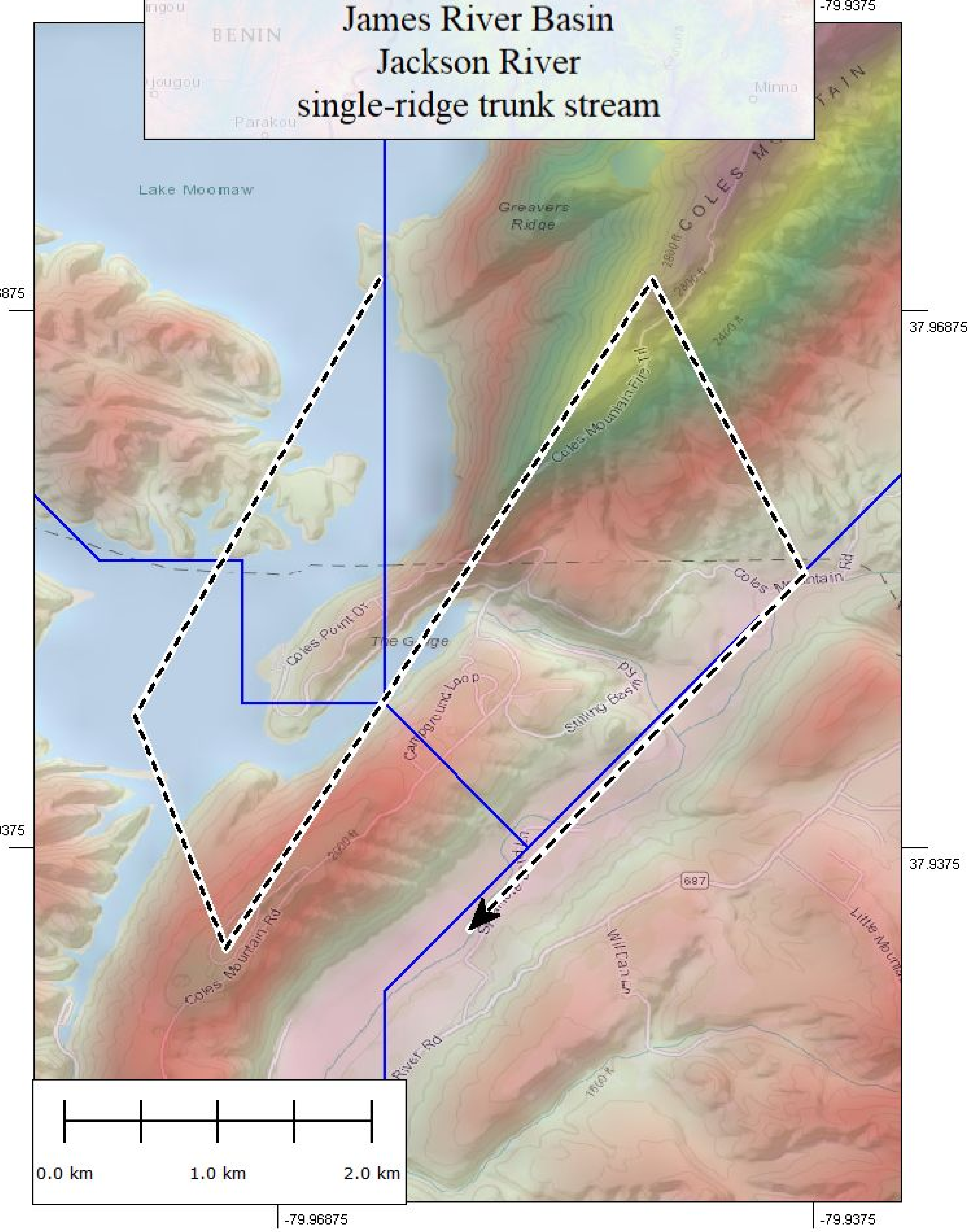

NA - 278
Mississippi River Basin
Sitlington Creek, Campbell Hollow
single-ridge trunk stream

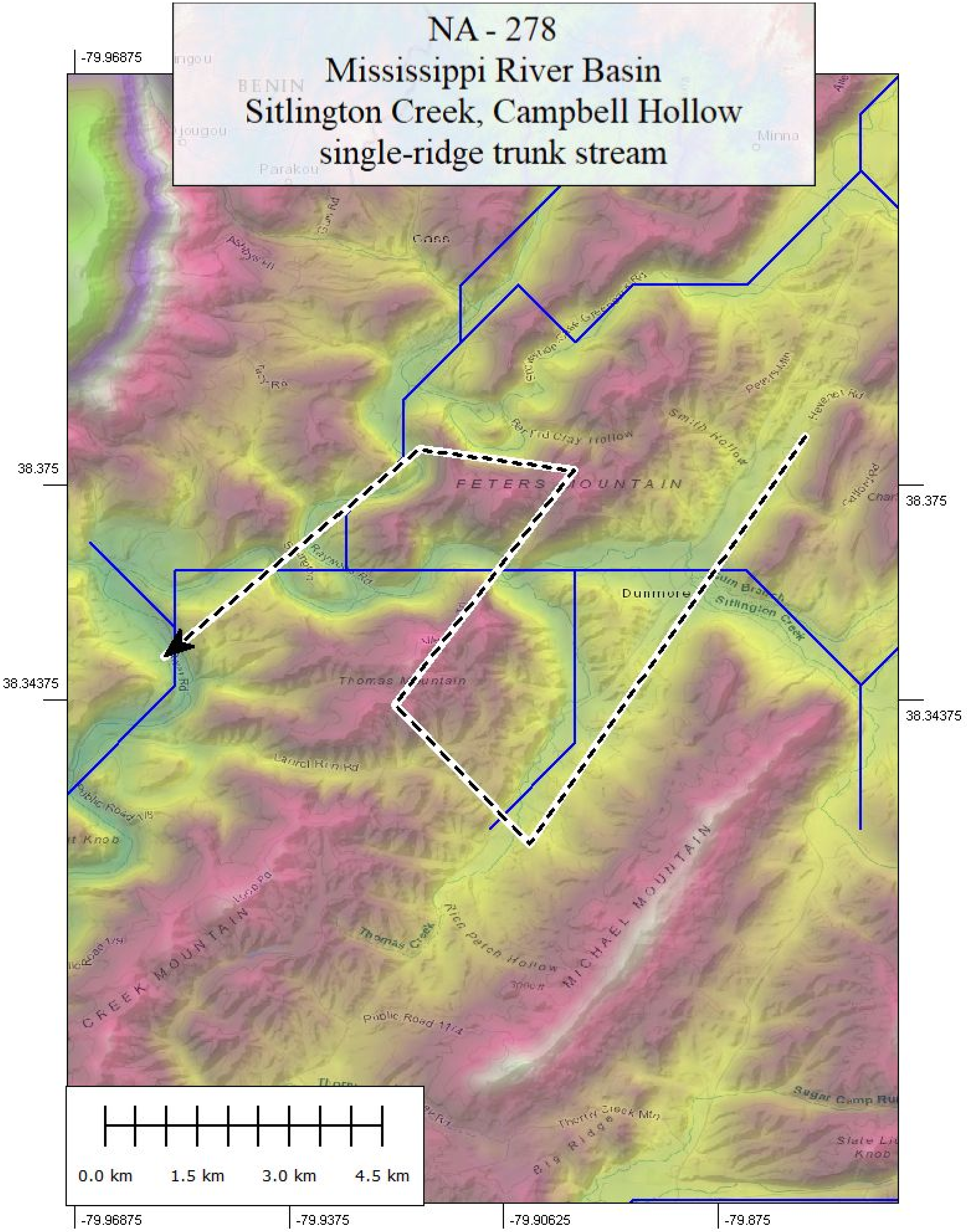

NA - 279
Mackenzie River (Canada) Basin
Halfway River
single-ridge trunk stream

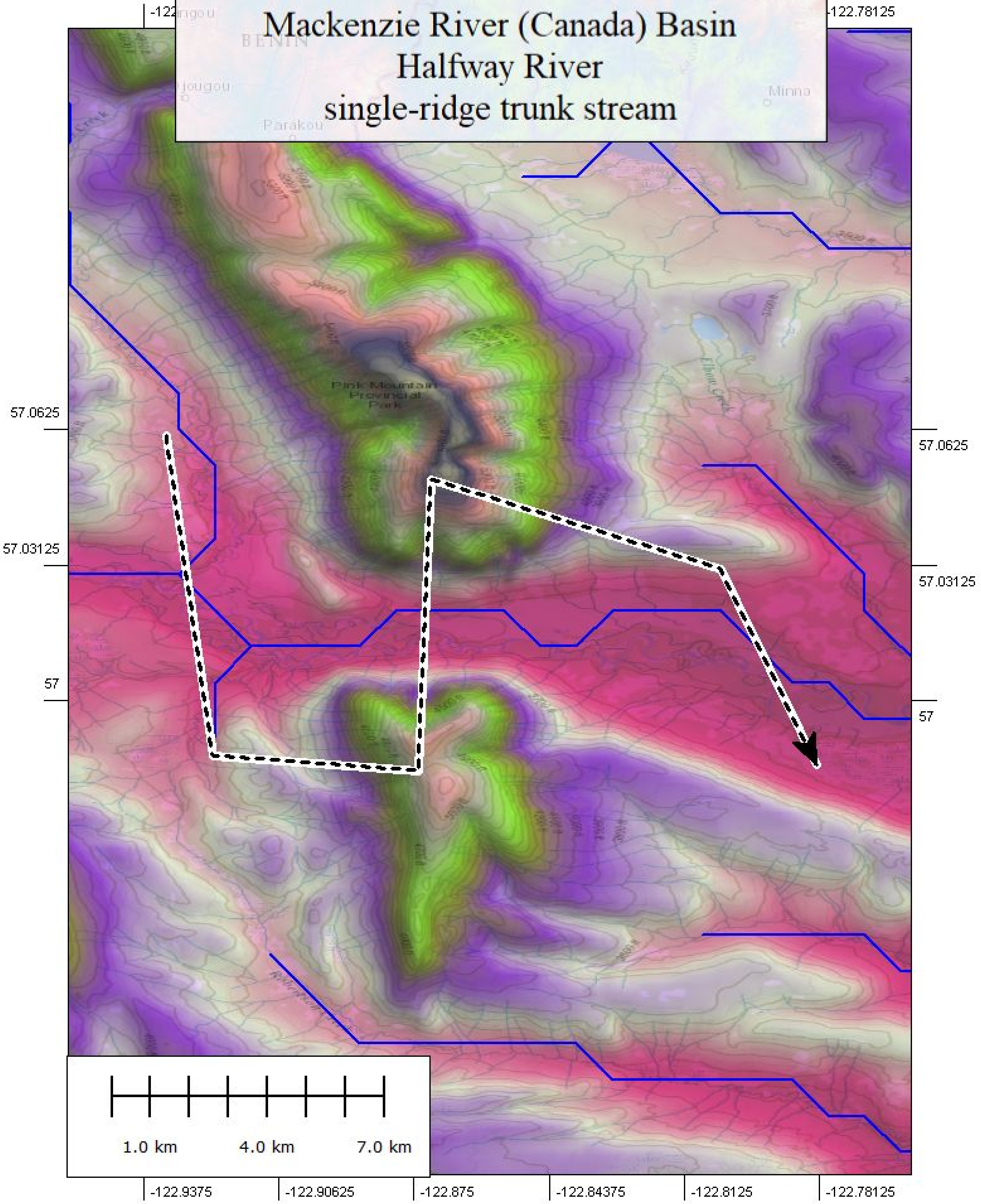

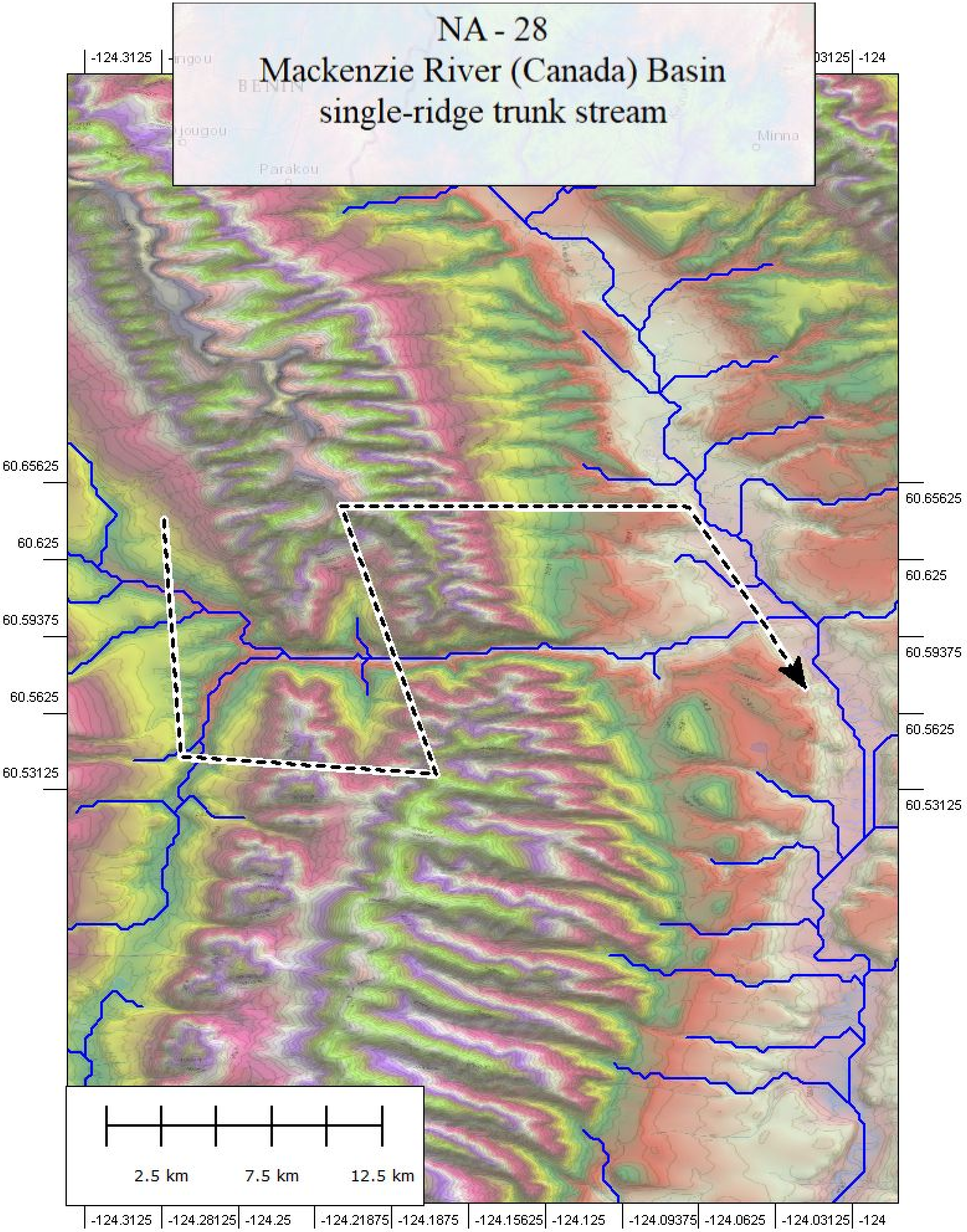
NA - 28
Mackenzie River (Canada) Basin
single-ridge trunk stream
-124.3125
-124.28125
-124.25
-124.21875
-124.1875
-124.15625
-124.125
-124.09375
-124.0625
-124.03125
-124
60.65625
60.625
60.59375
60.5625
60.53125
2.5 km
7.5 km
12.5 km

NA - 280
Potomac River Basin
Wills Creek, Cumberland Narrows
single-ridge trunk stream

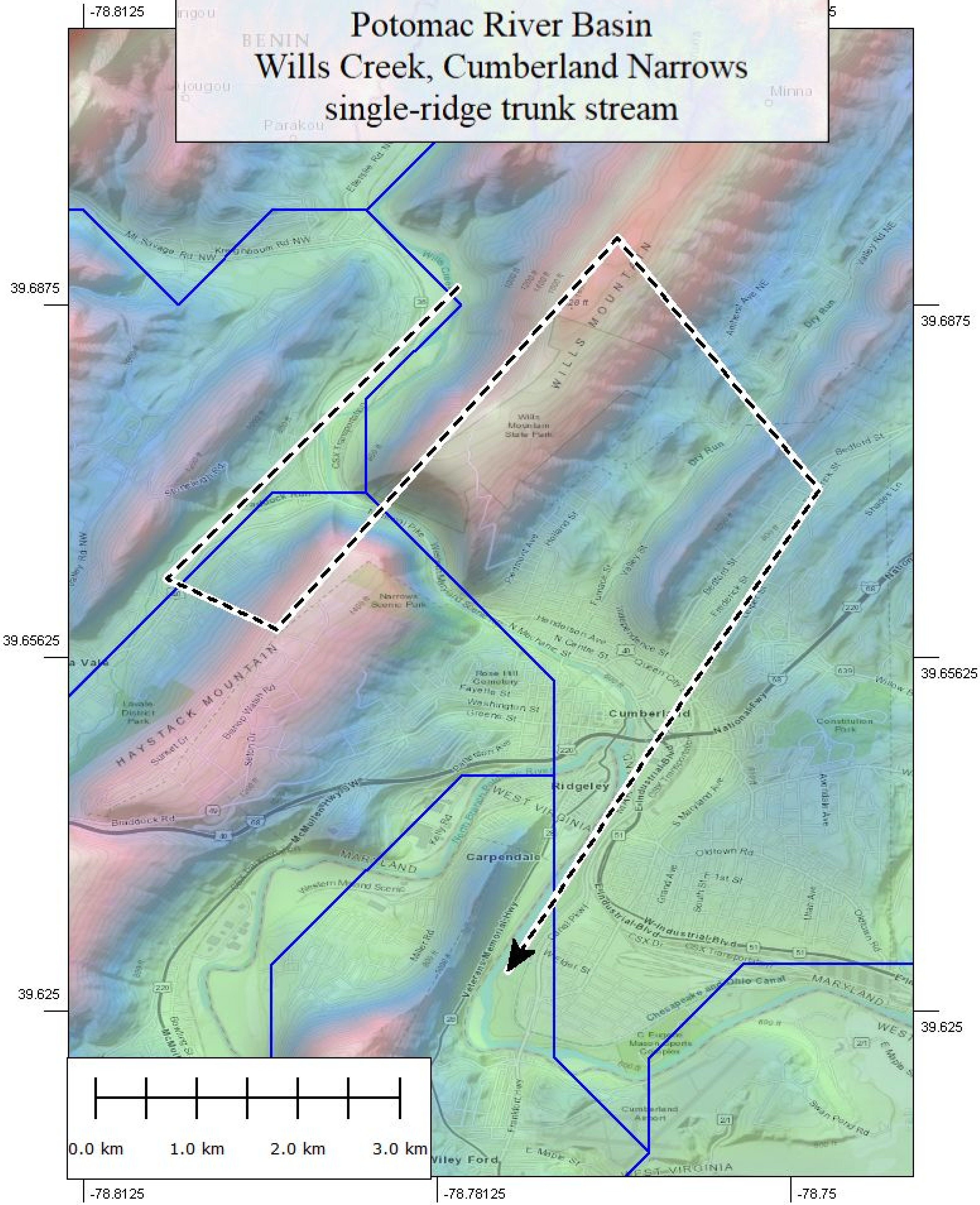

NA - 281
Potomac River Basin
Fifteenmile Creek
multi-ridge trunk stream

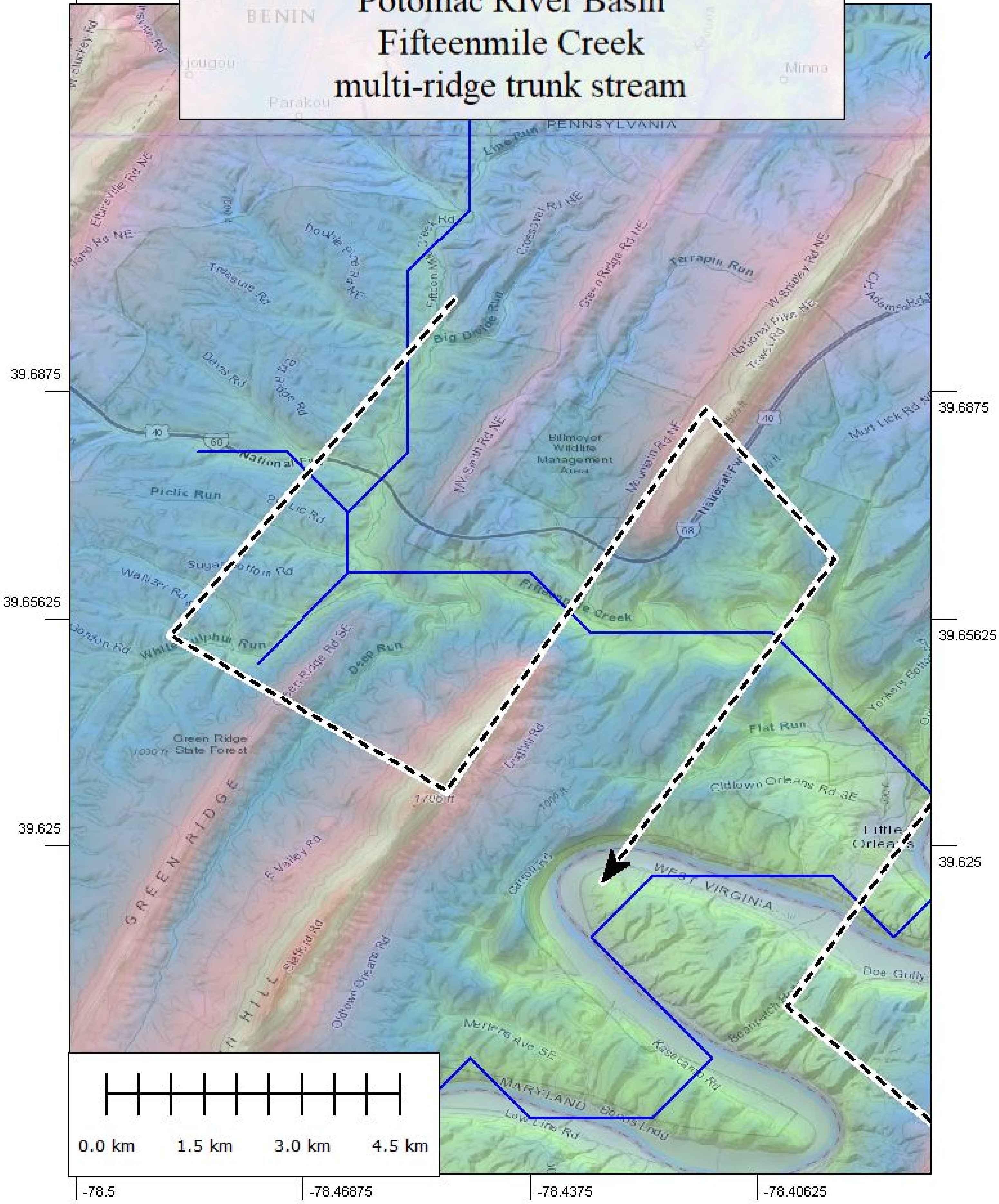

NA - 282
Susquehanna River Basin
Aughwick Creek tributary
single-ridge trunk stream

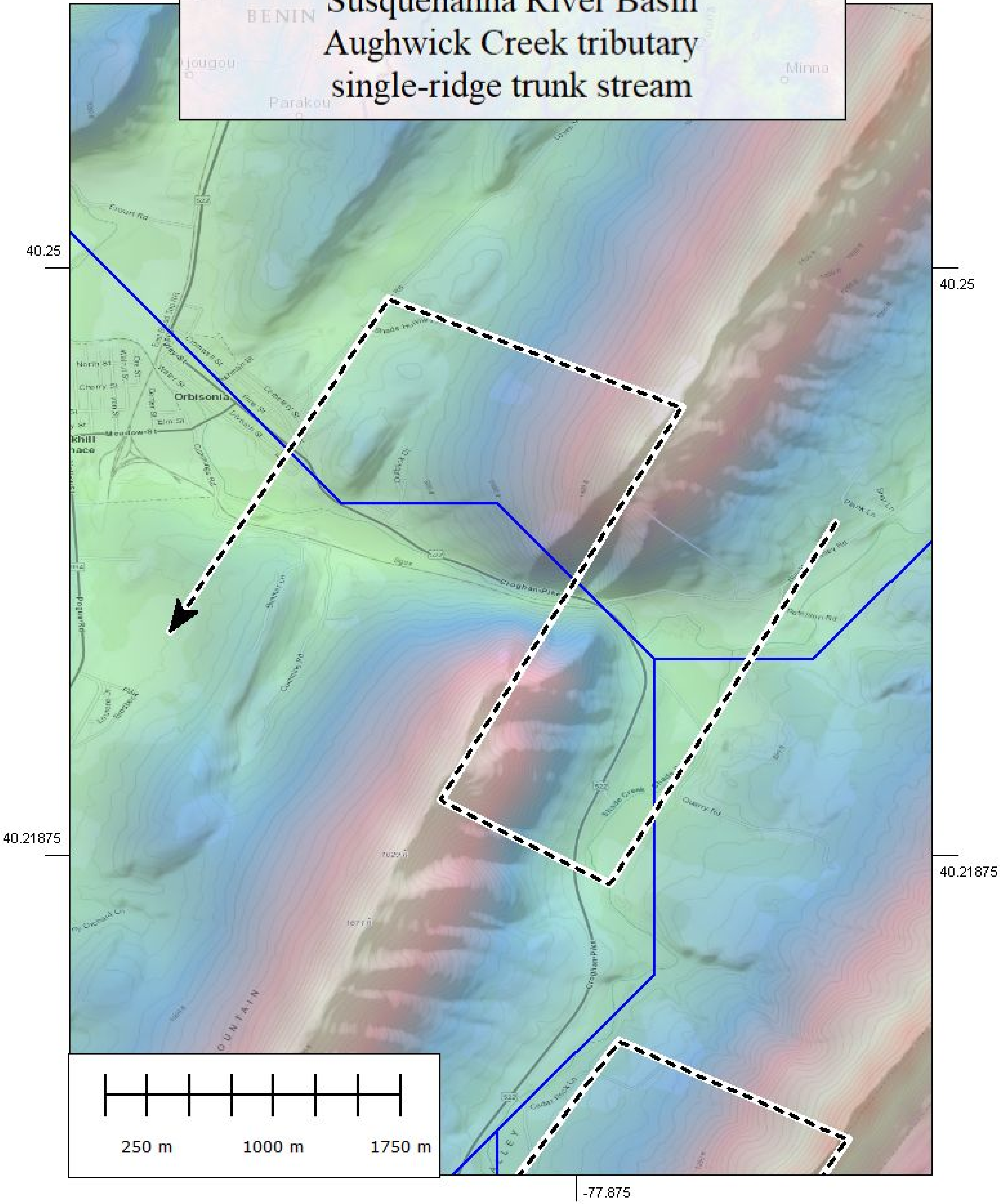

NA - 283
Susquehanna River Basin
Tusarora Creek tributary, Concord Narrows
single-ridge head stream

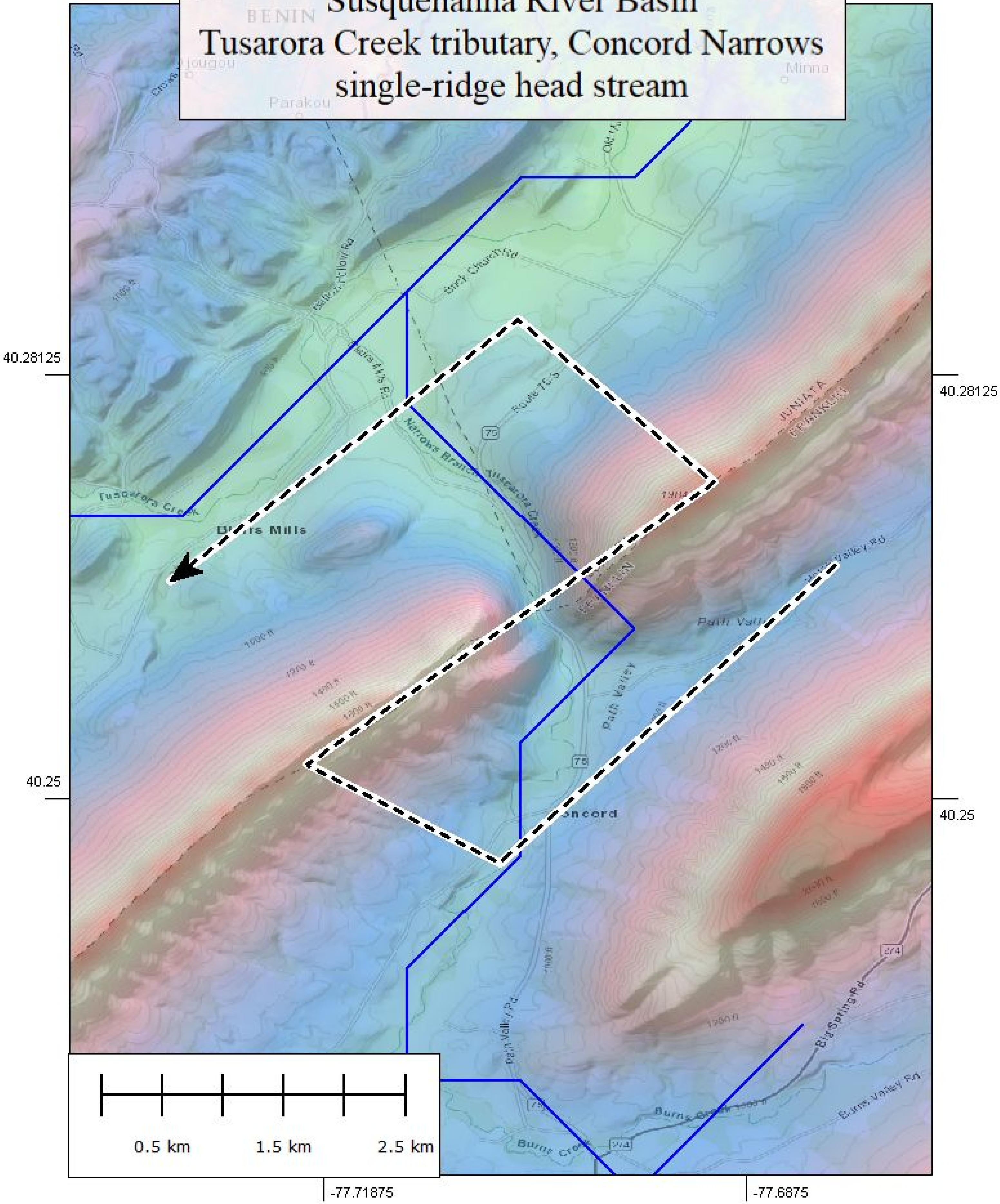

NA - 284
James River Basin
Dunlap Creek
single-ridge trunk stream

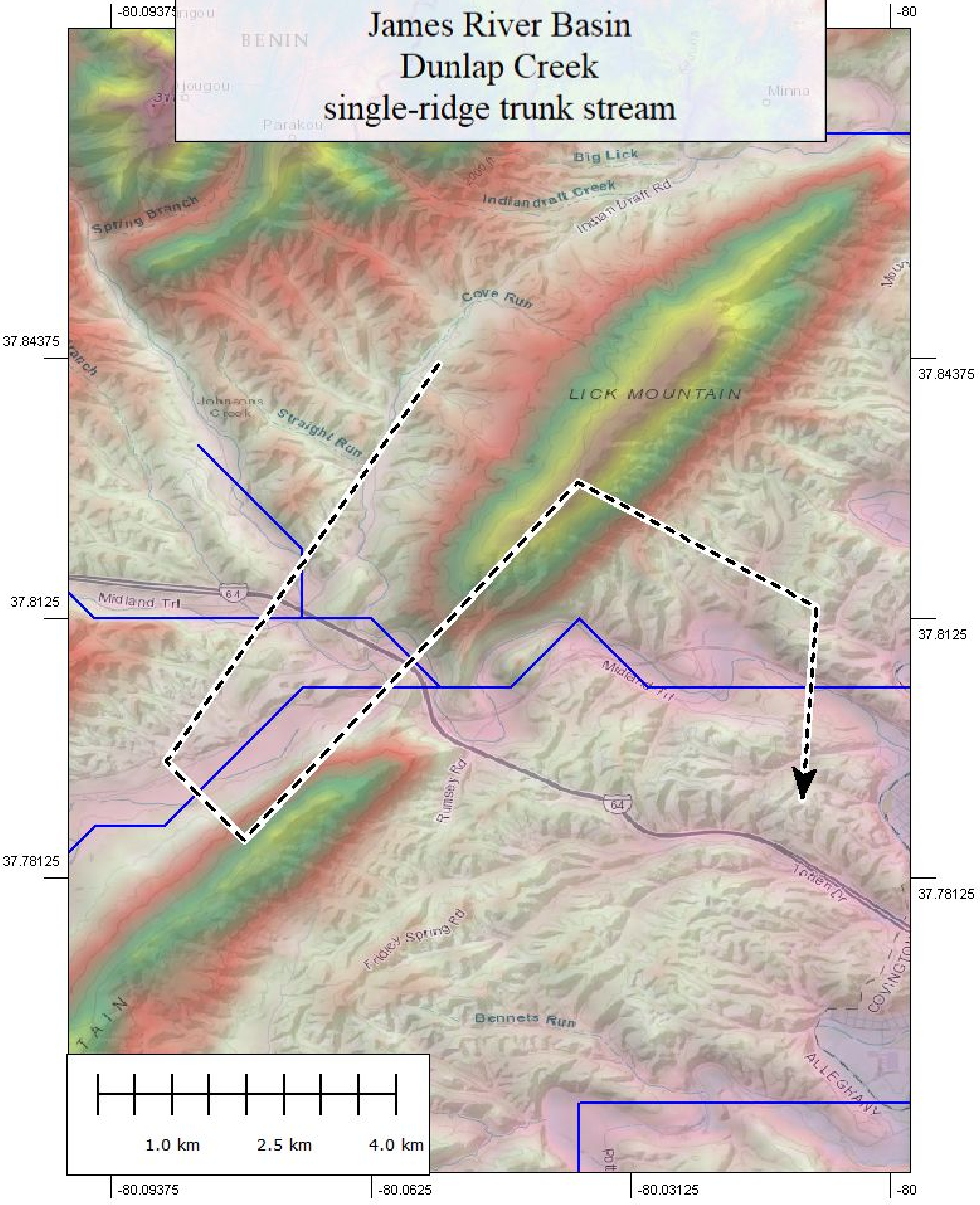

NA - 285
Susquehanna River Basin
Frankstown Branch Juniata River
single-ridge trunk stream

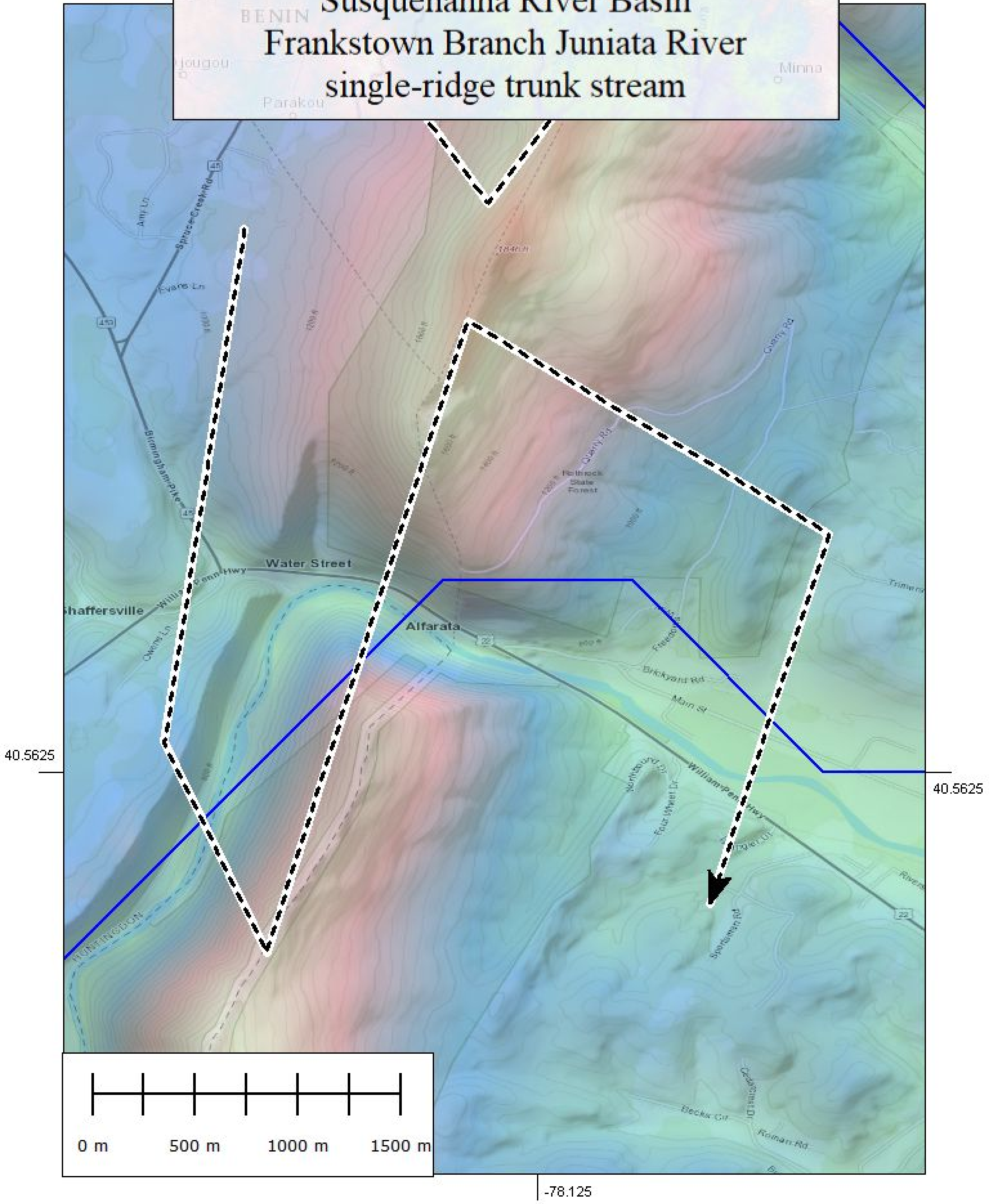

NA - 286
Mississippi River Basin
Howard Creek
single-ridge trunk stream

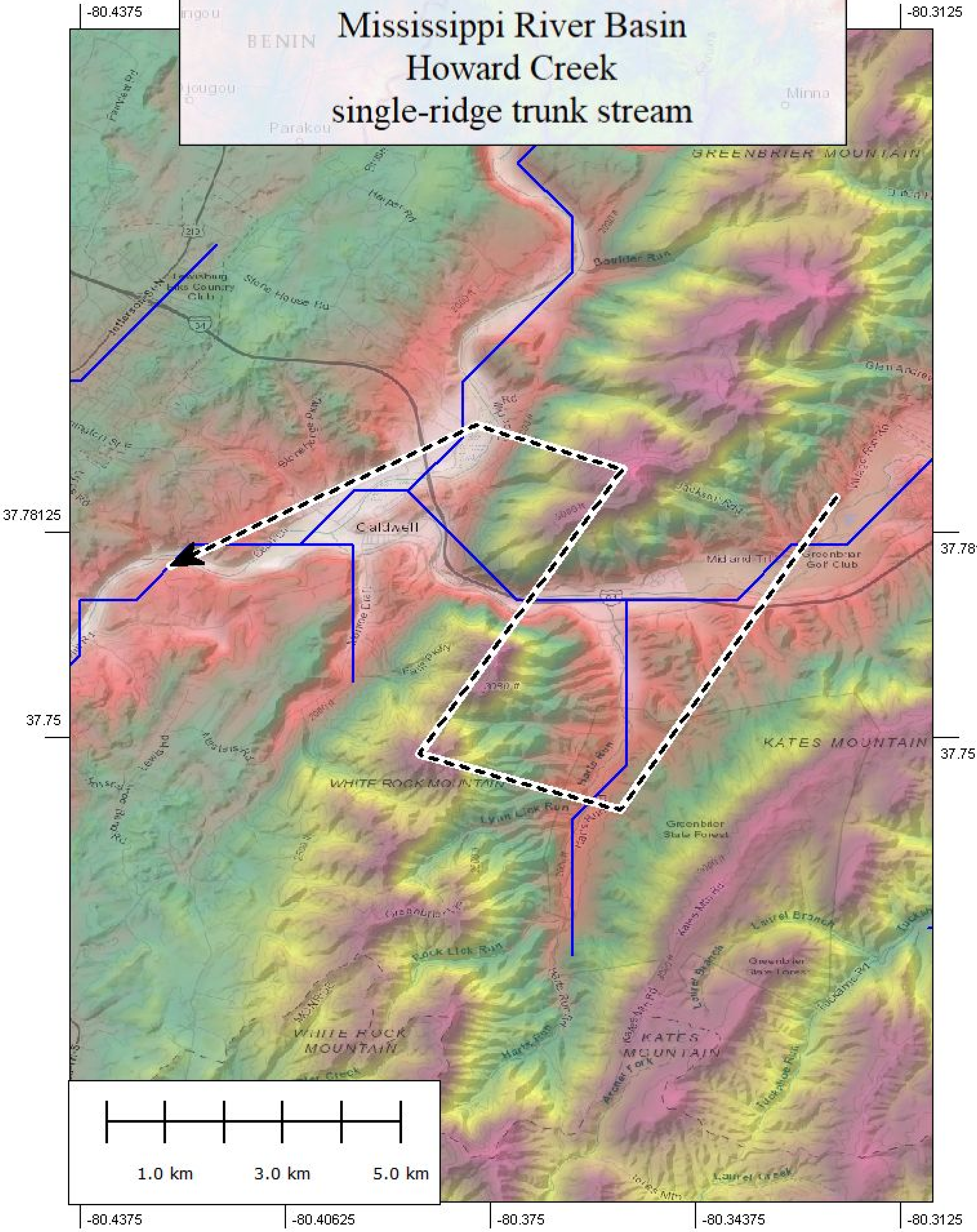

NA - 287
Susquehanna River Basin
Juniata River, Jacks Narrows
single-ridge trunk stream

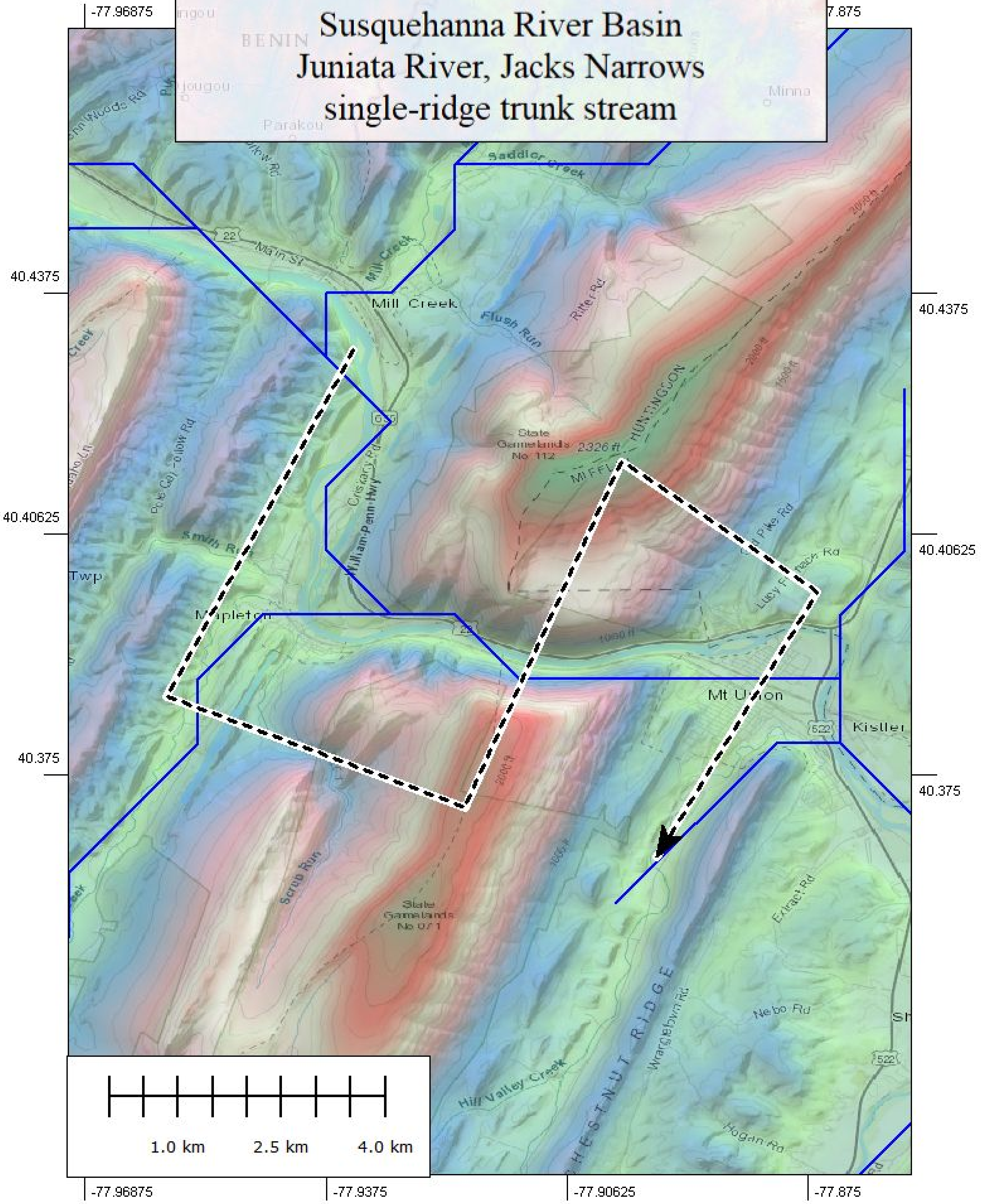

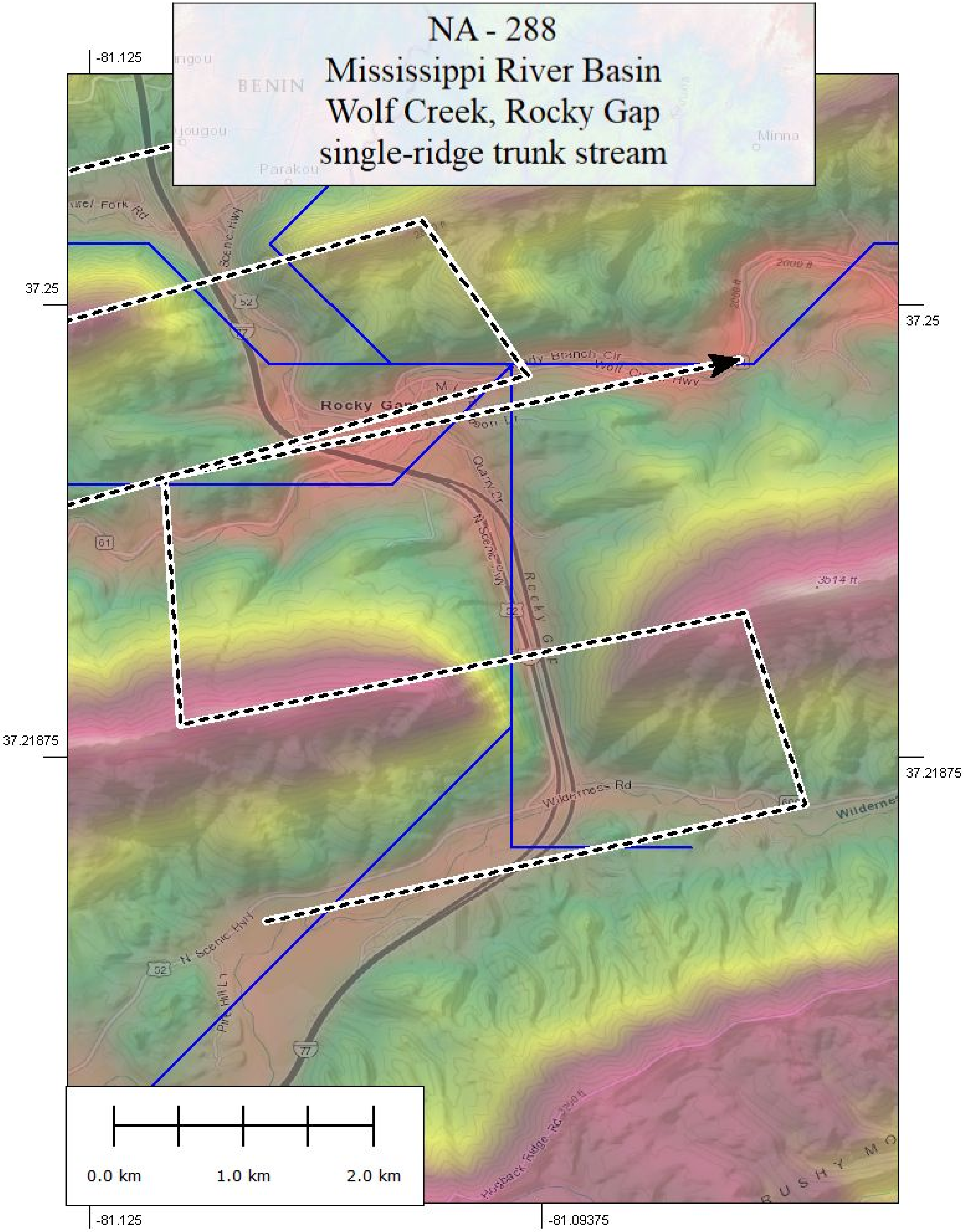
NA - 288
Mississippi River Basin
Wolf Creek, Rocky Gap
single-ridge trunk stream
-81.125
37.25
37.25
37.21875
37.21875
Rocky Gap
Wilderness Rd
N Scenic Hwy
Quarry Cir
0.0 km
1.0 km
2.0 km
-81.125
-81.09375

NA - 289
Rio Grande (Texas-Mexico) Basin
single-ridge trunk stream

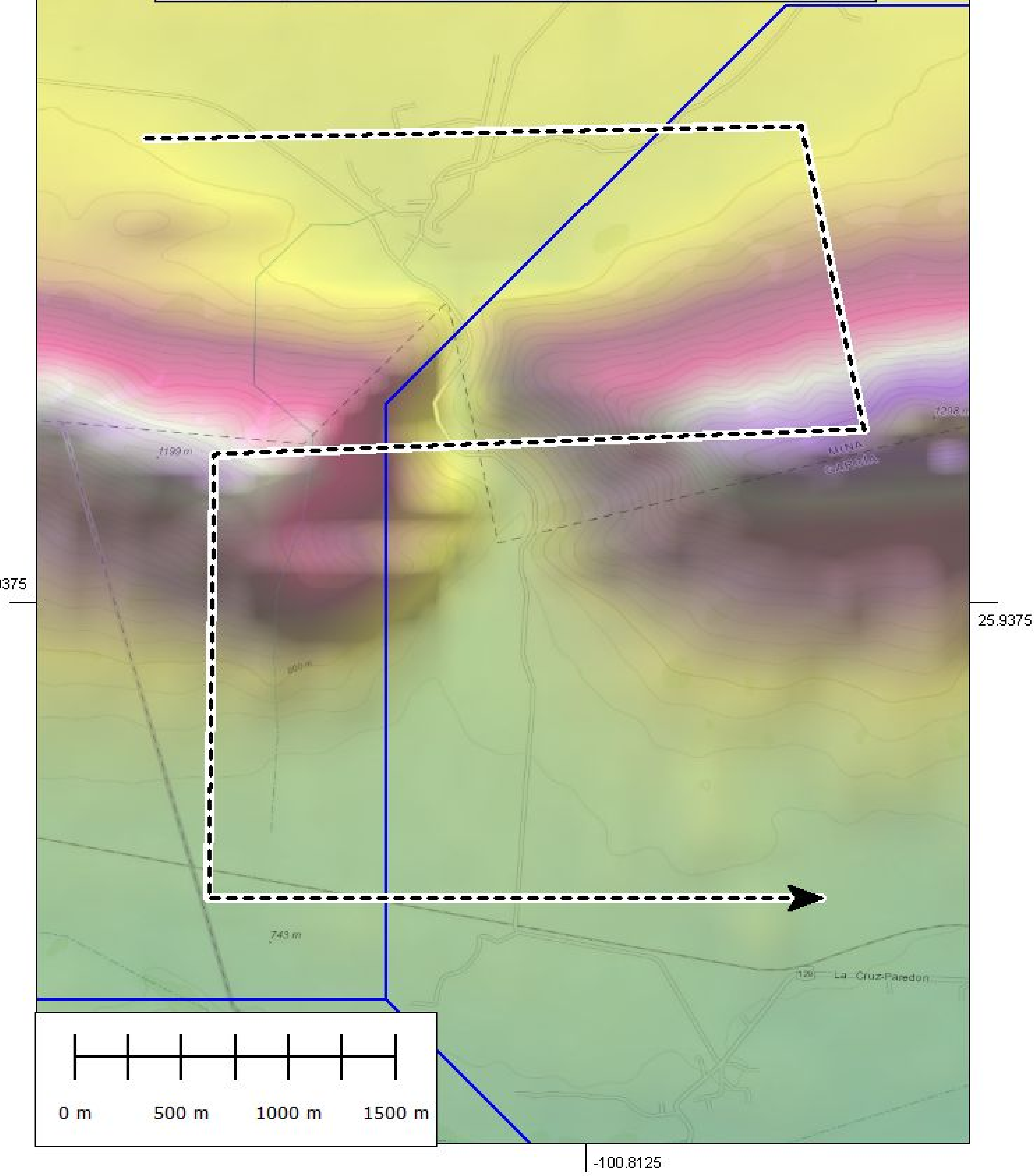

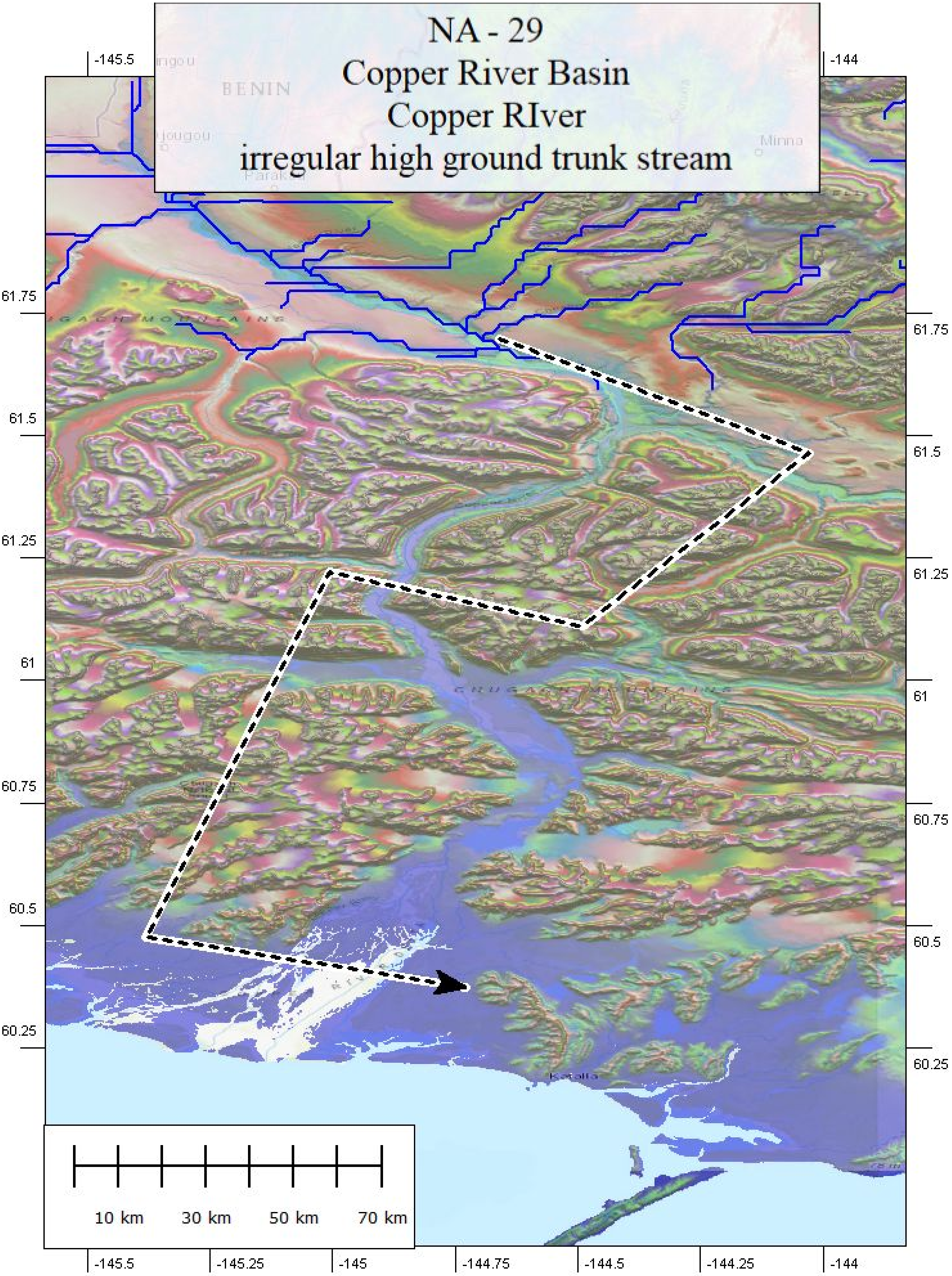
NA - 29
Copper River Basin
Copper RIver
irregular high ground trunk stream
-145.5
-144
61.75
61.5
61.25
61
60.75
60.5
60.25
-145.25
-145
-144.75
-144.5
-144.25
10 km
30 km
50 km
70 km

NA - 290
Kliniklini River Basin
Homathko& Southgate Rivers, Bute Inlet
irregular high ground trunk stream

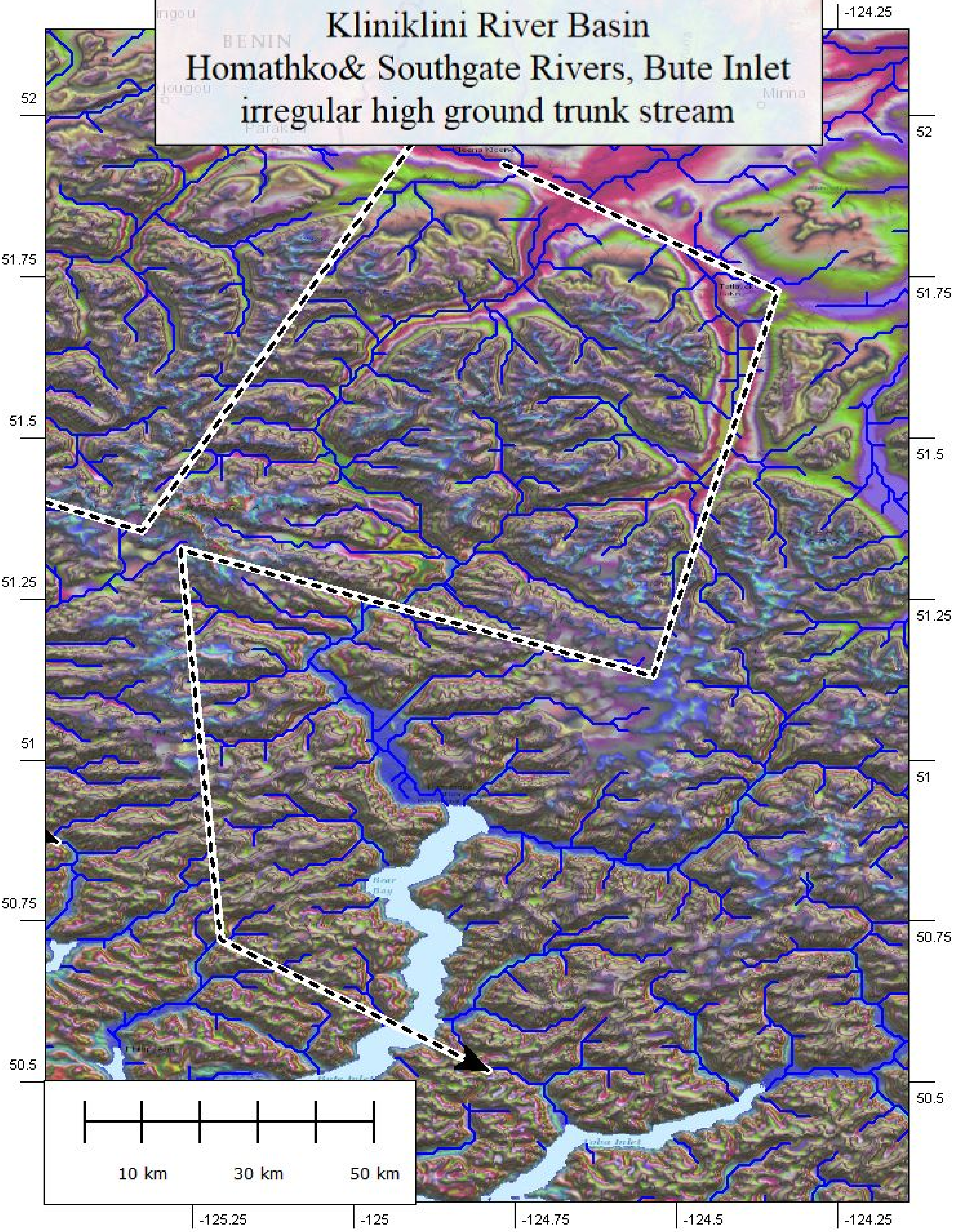

NA - 291
Mississippi River Basin
North Platte River
single-ridge trunk stream

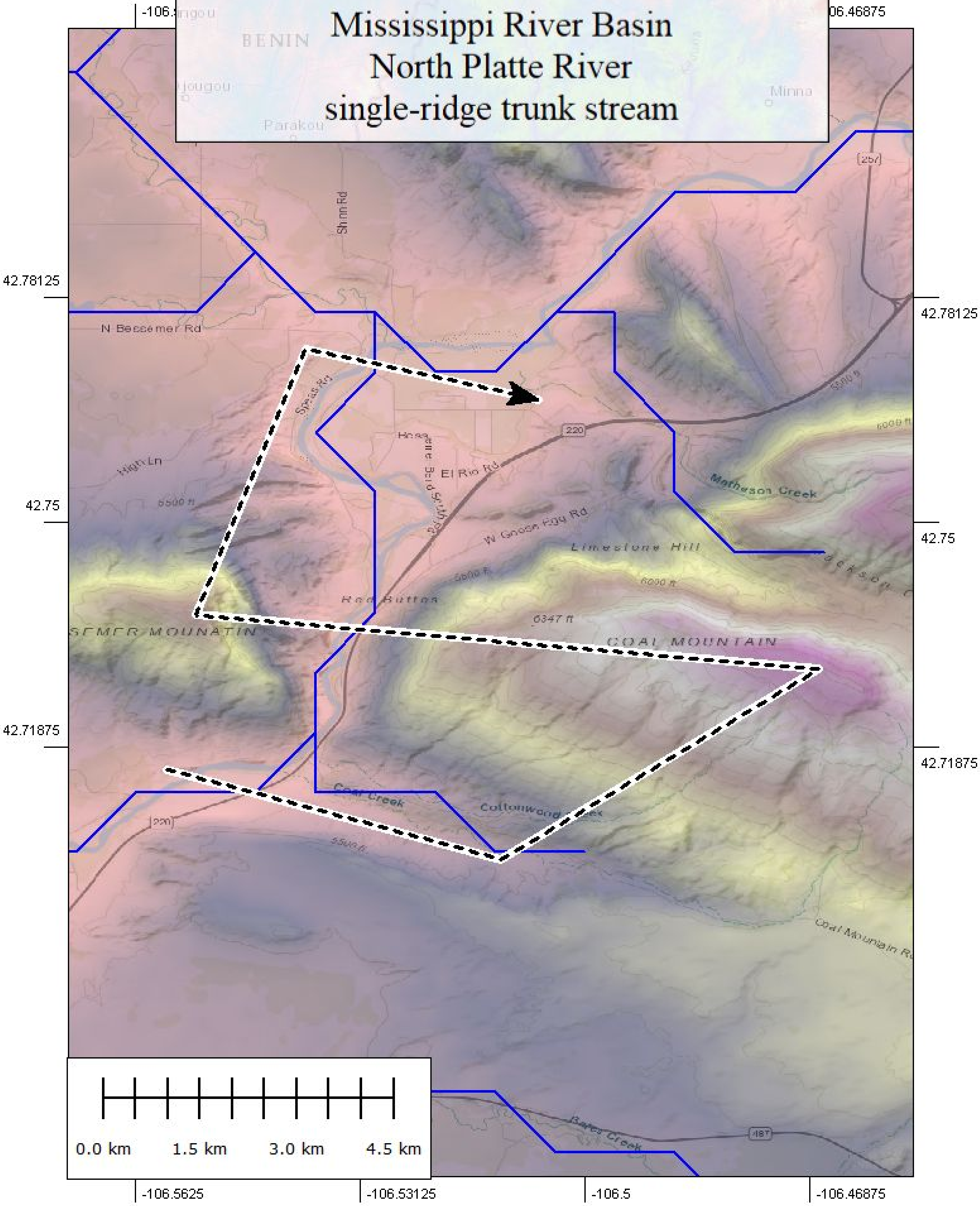

NA - 292
Mississippi River Basin
Big Creek
downdip plateau trunk stream

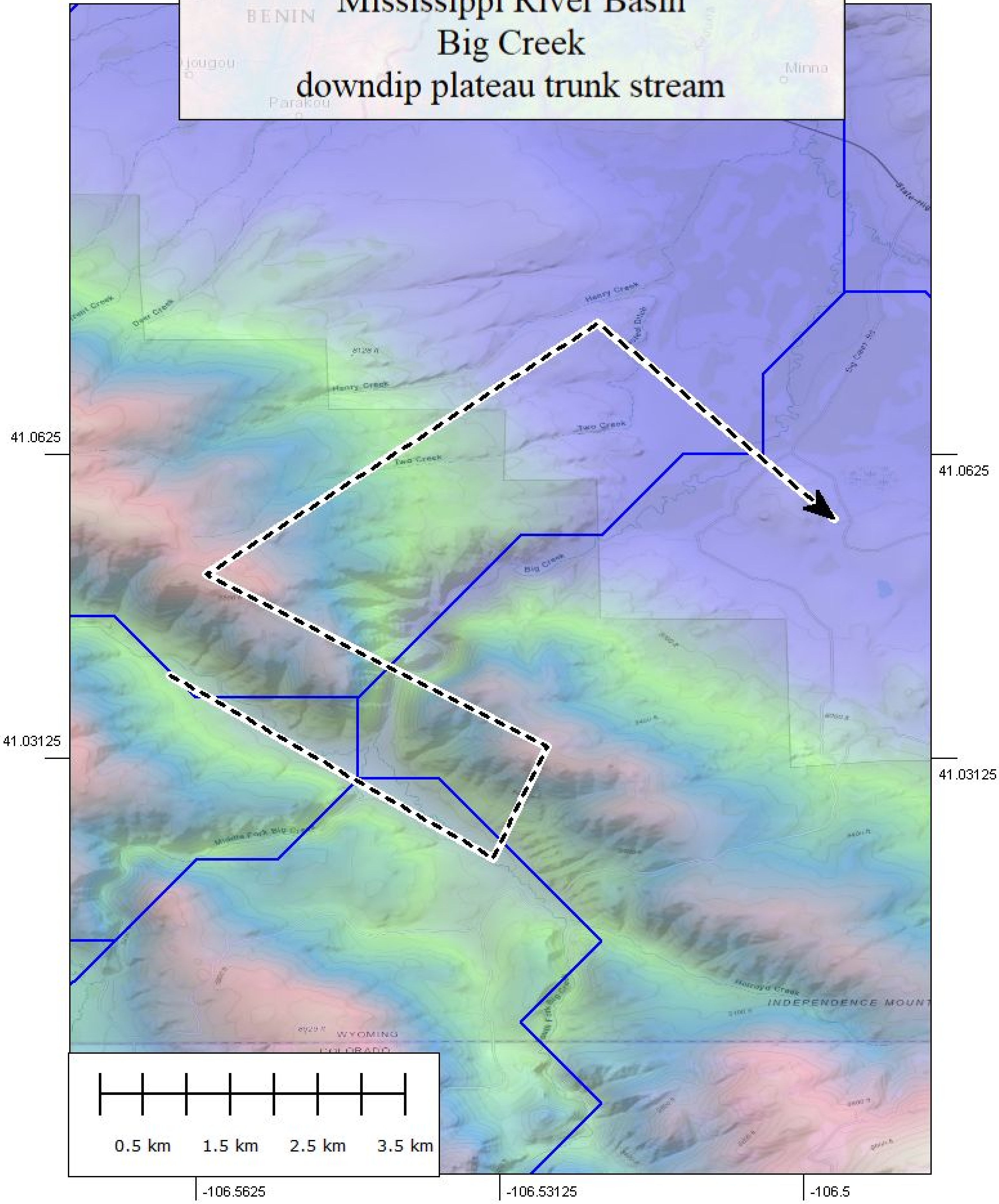

NA - 293
Endorheic basin Basin
Rio Ramos
irregular high ground trunk stream

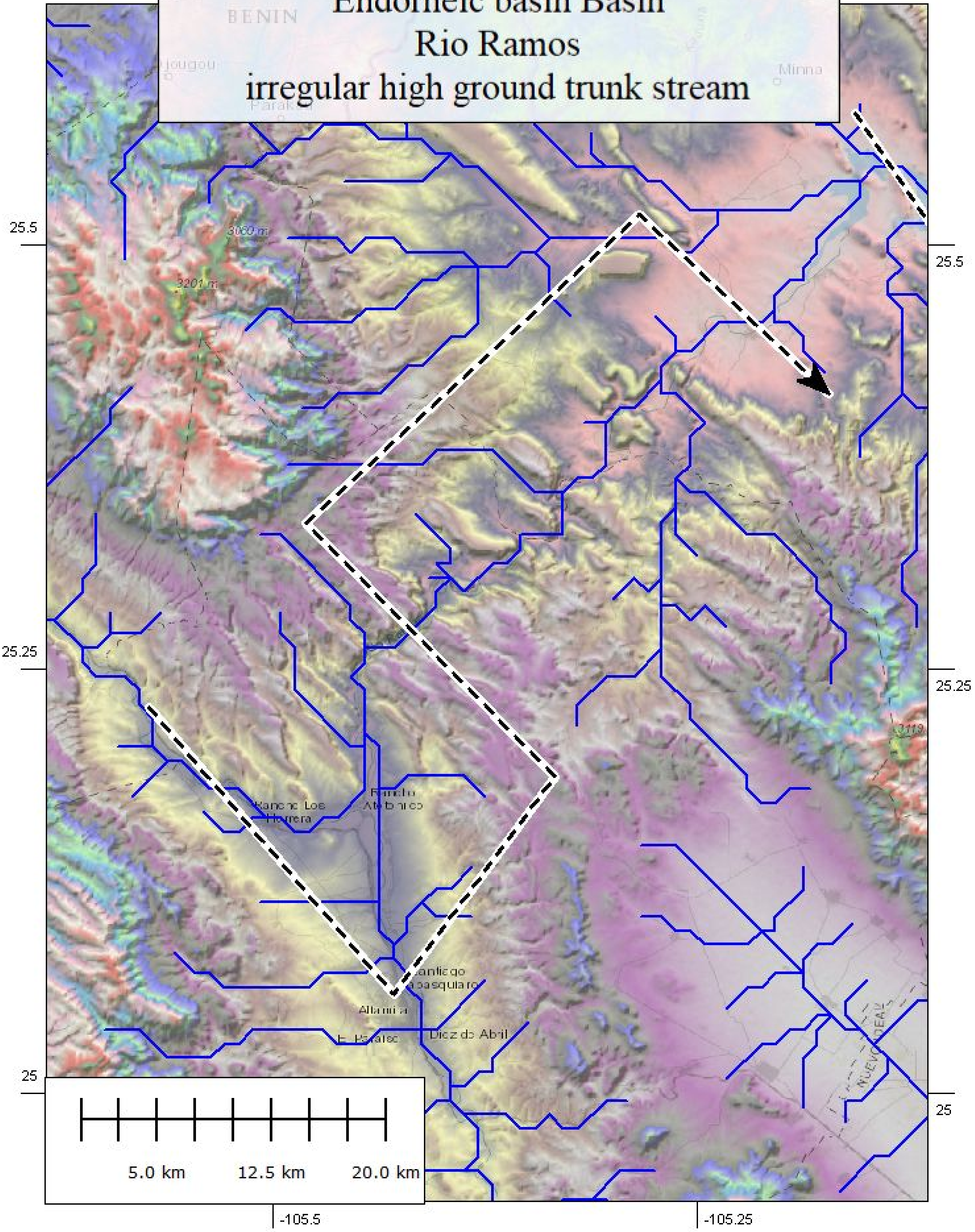

NA - 294
Mississippi River Basin
New River
single-ridge trunk stream

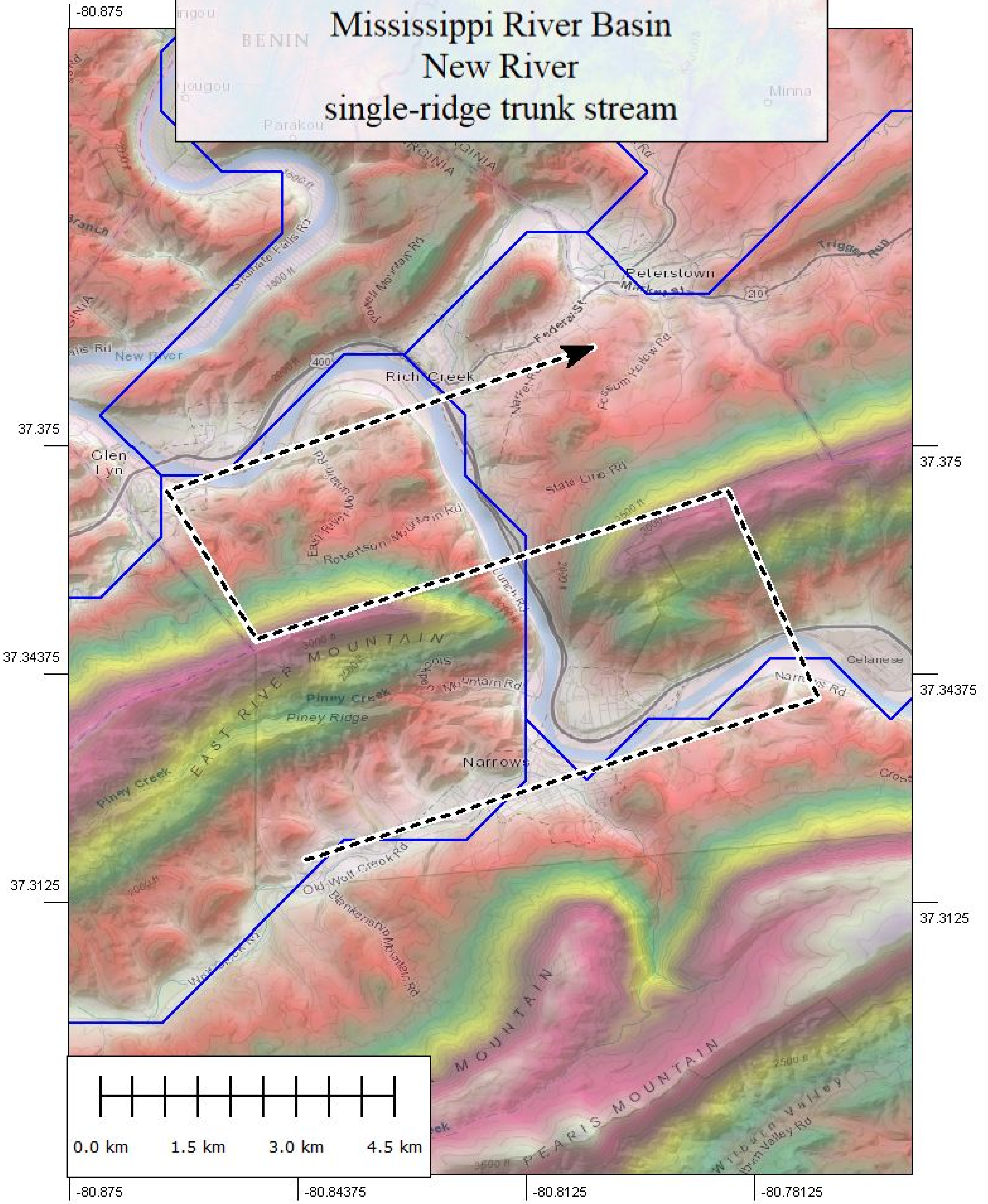

NA - 295
Susquehanna River Basin
Sidling Hill Creek
single-ridge trunk stream

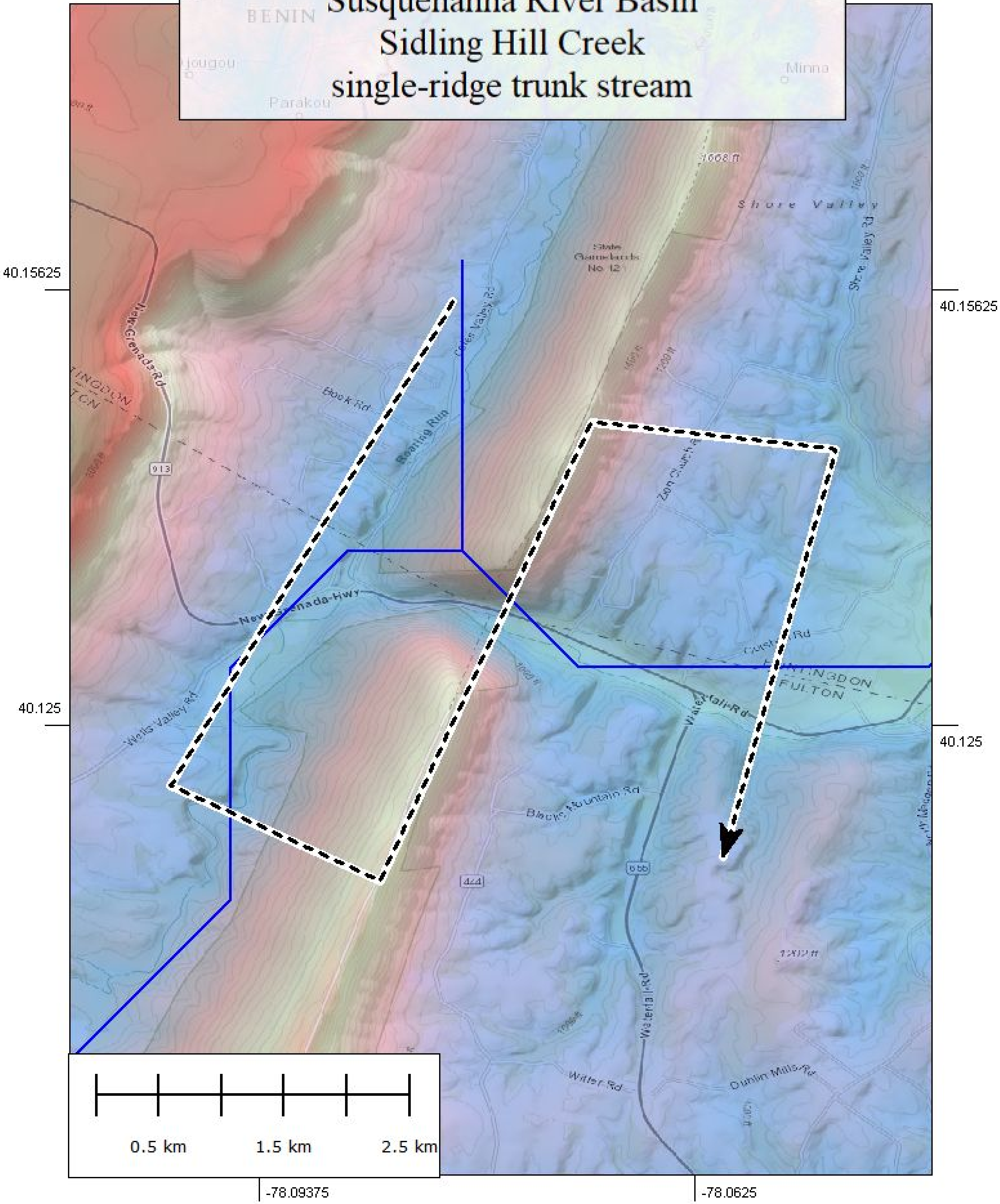

NA - 296
Colorado River (Arizona) Basin
Willow Creek
updip plateau trunk stream

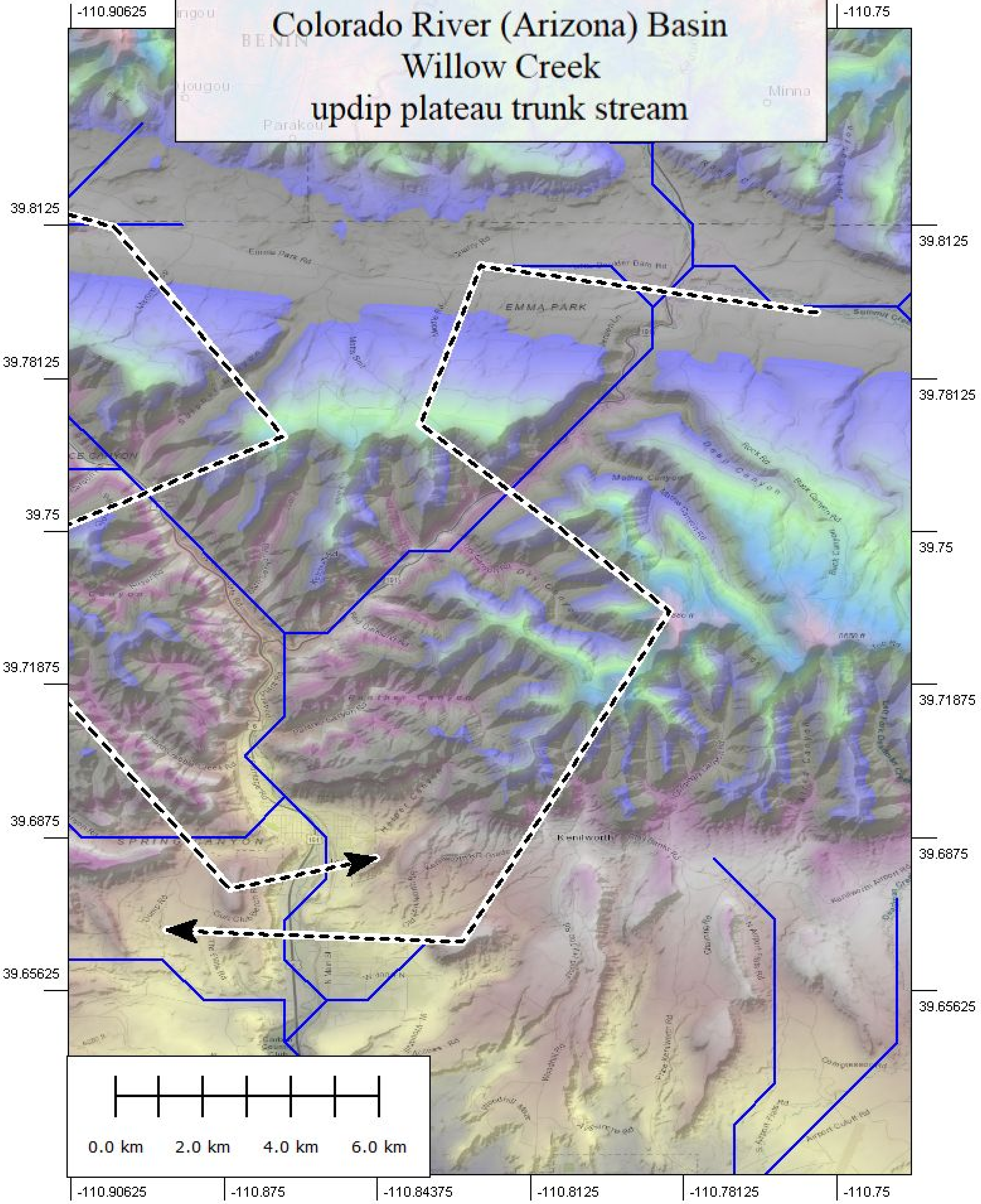

NA - 297
Colorado River (Arizona) Basin
Paria River
updip plateau trunk stream

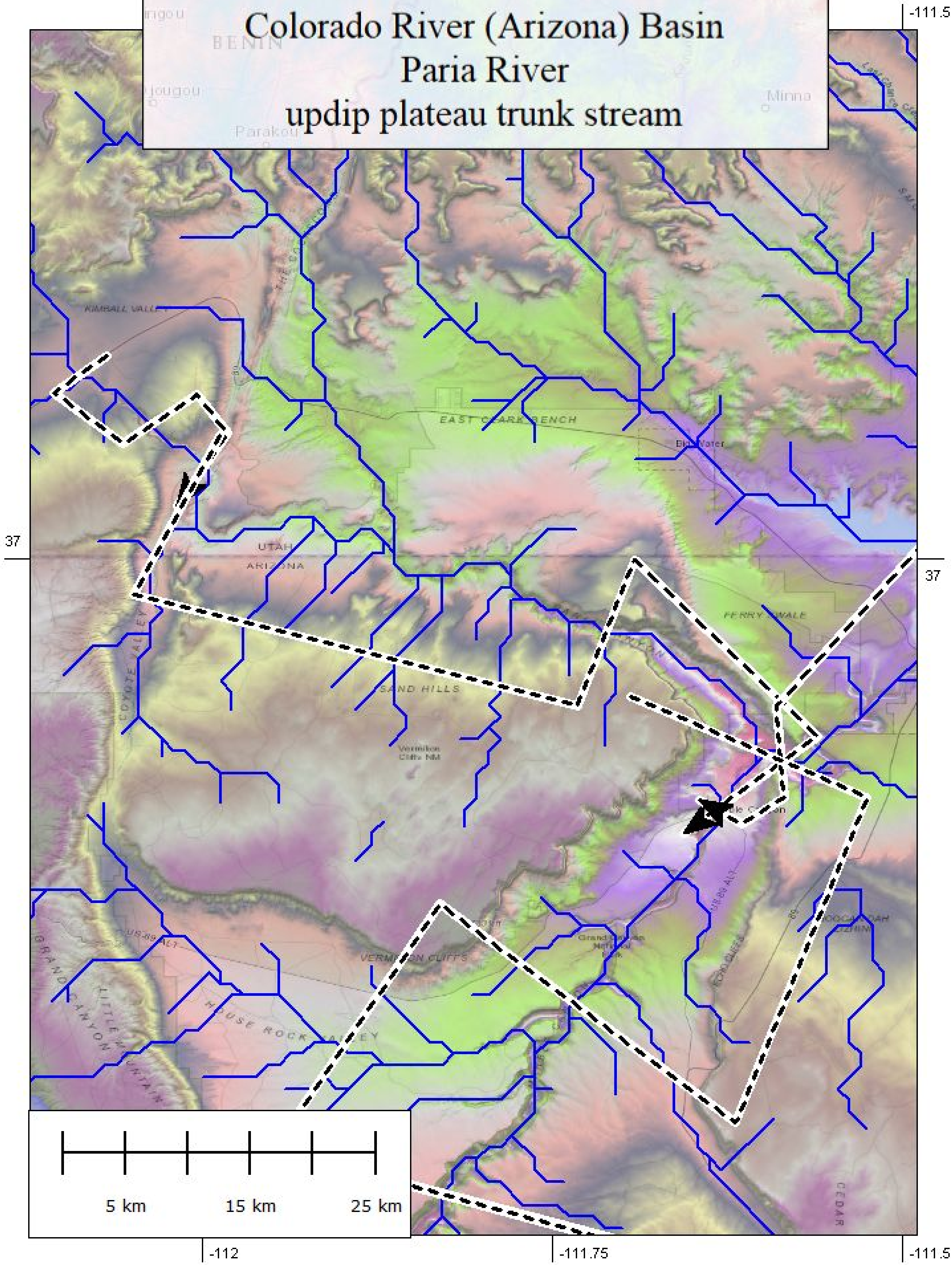

NA - 298
Colorado River (Arizona) Basin
Colorado River
irregular high ground trunk stream

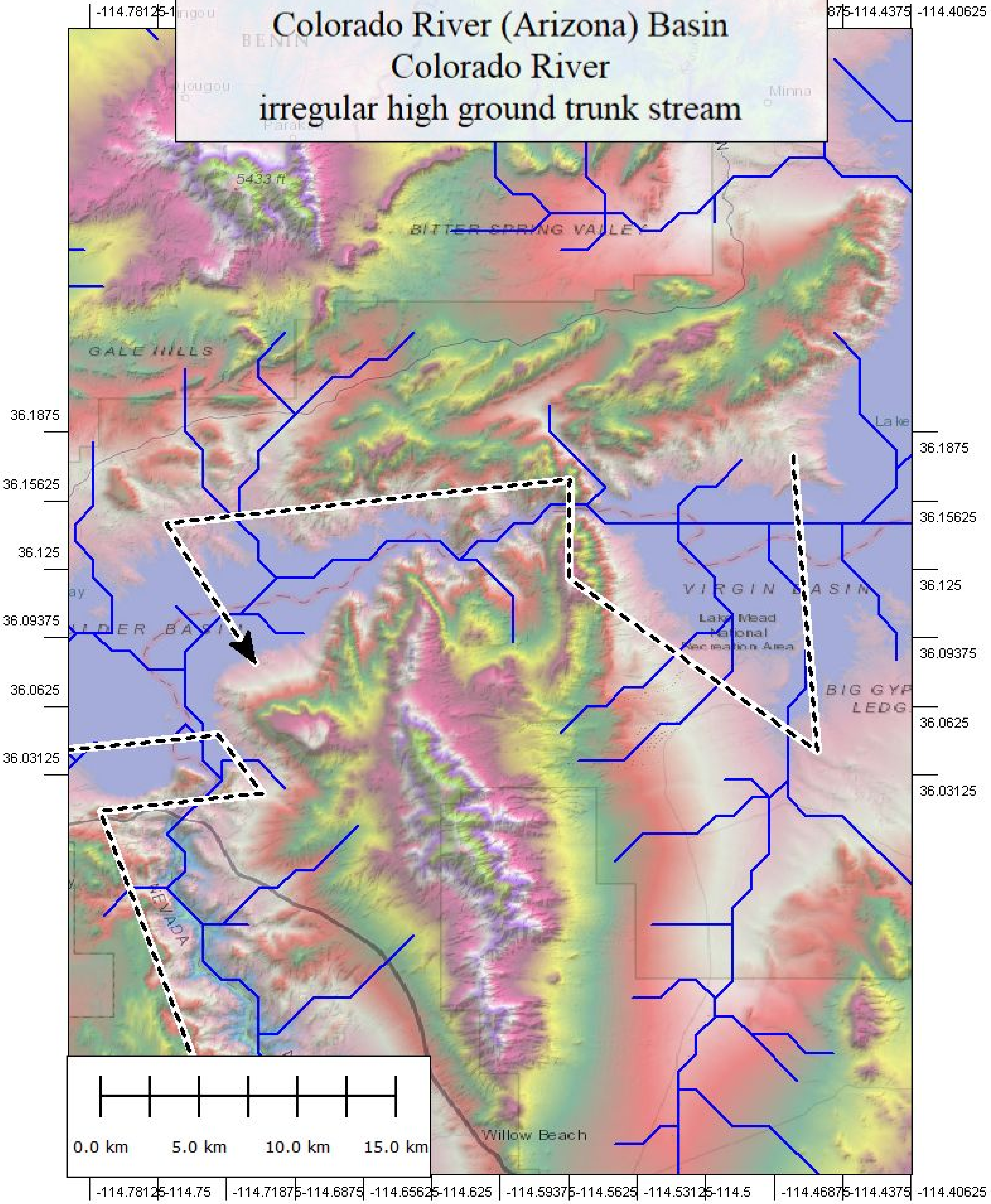

NA - 299
Colorado River (Arizona) Basin
Virgin River
updip plateau trunk stream

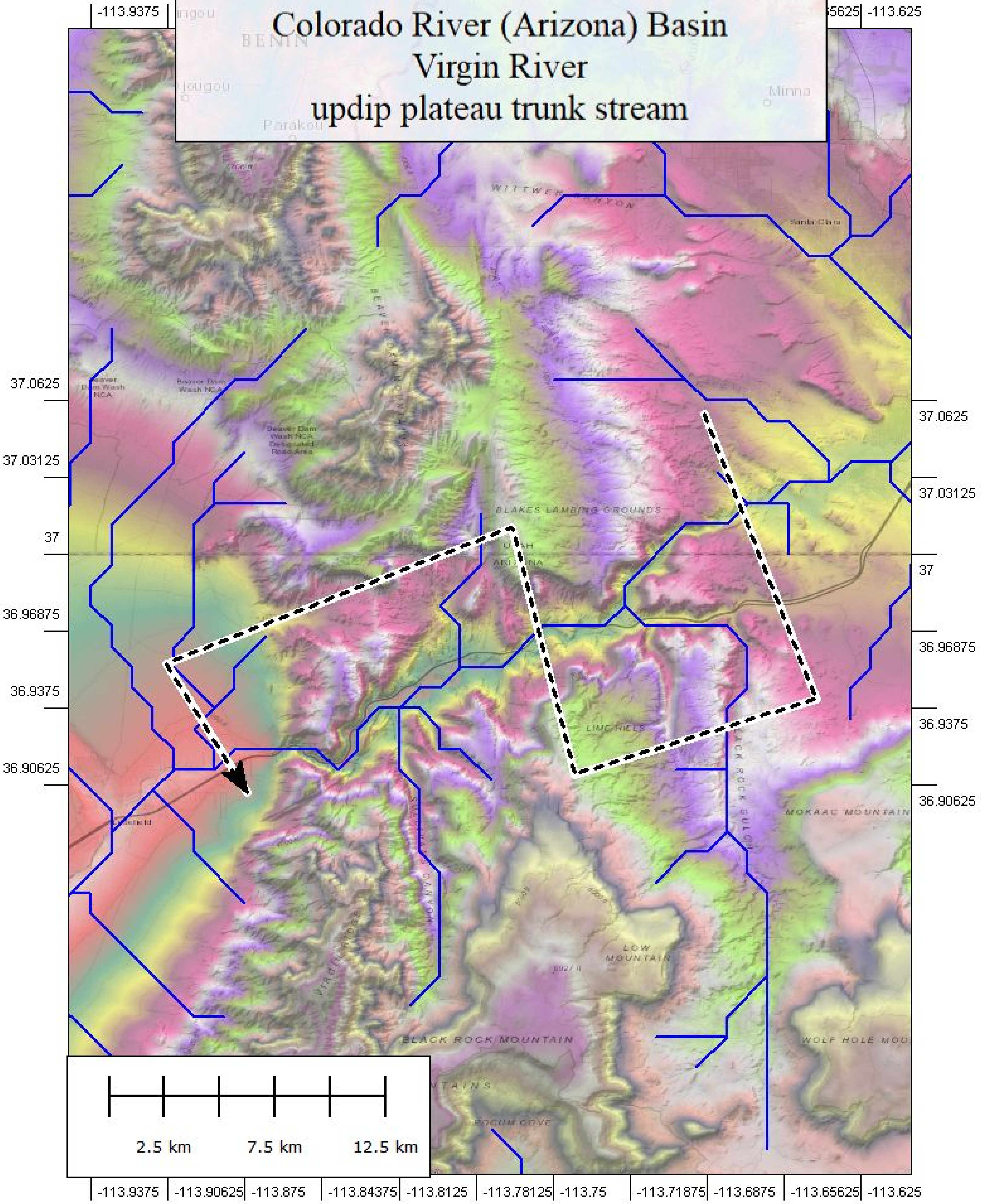

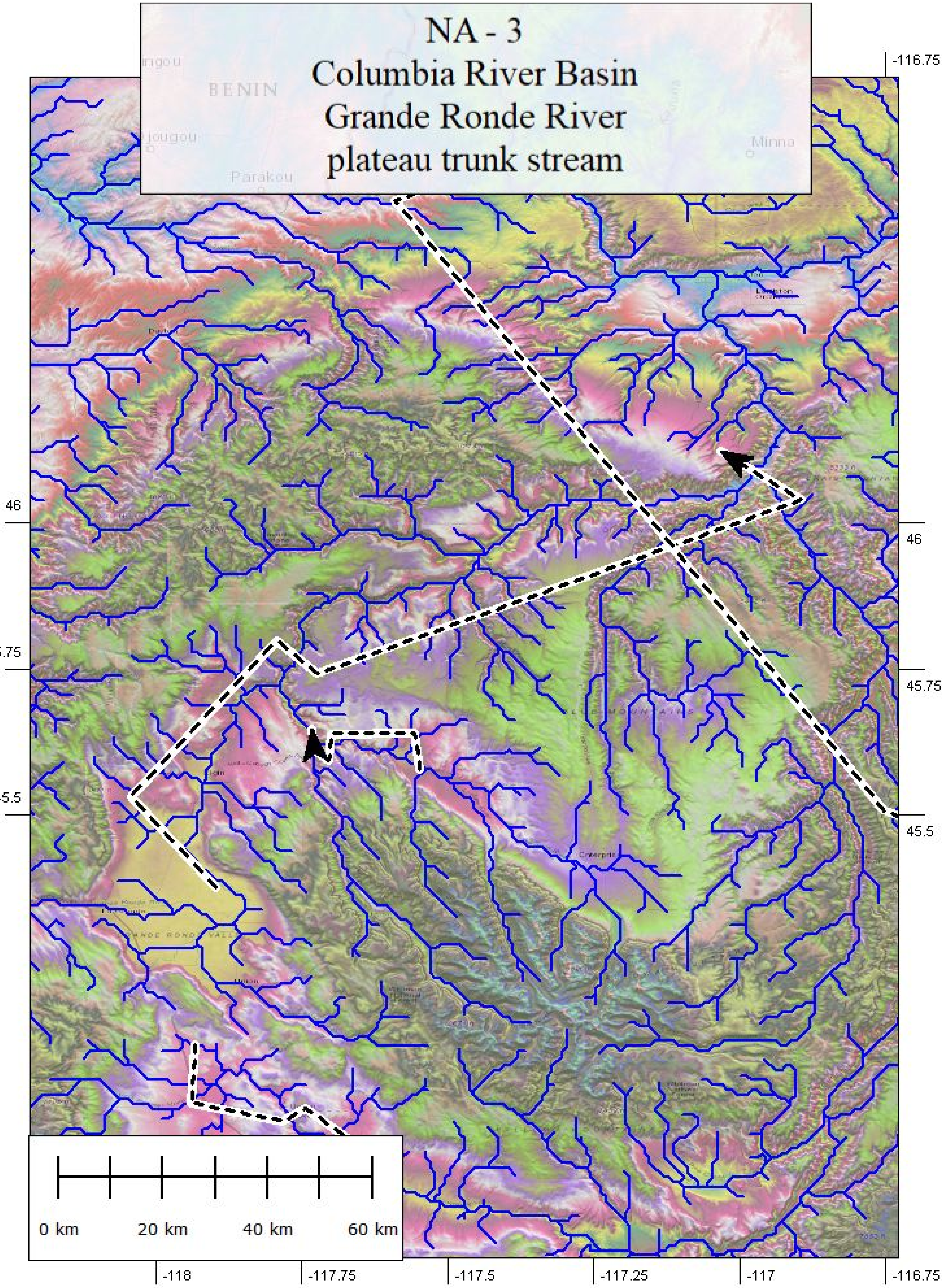
NA - 3
Columbia River Basin
Grande Ronde River
plateau trunk stream
-116.75
46
45.75
45.5
0 km
20 km
40 km
60 km
-118
-117.75
-117.5
-117.25
-117
-116.75

NA - 30
Mackenzie River (Canada) Basin
Mackenzie River tributary, Blackwater Lake outflow

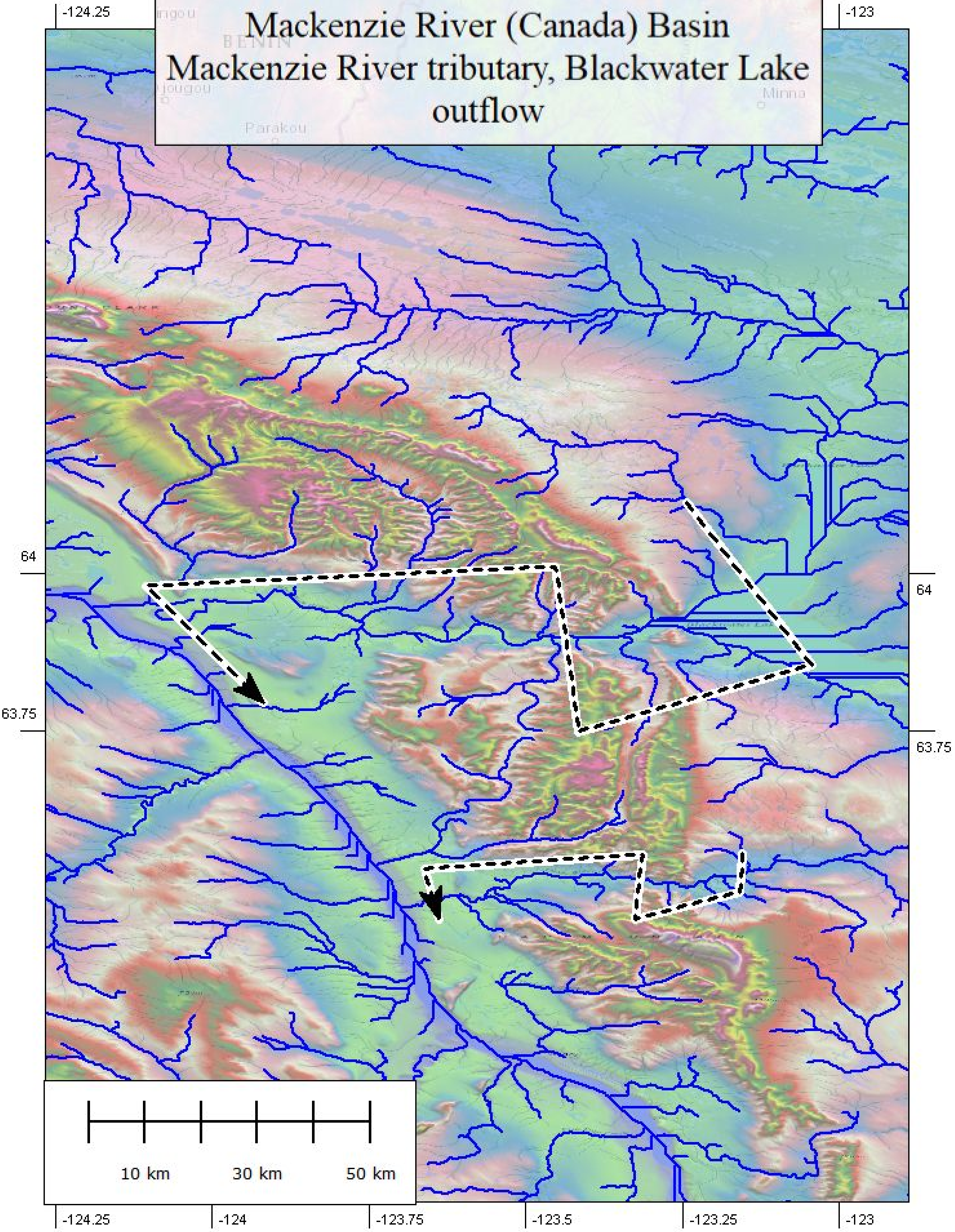

NA - 300
Columbia River Basin
Columbia River
irregular high ground trunk stream

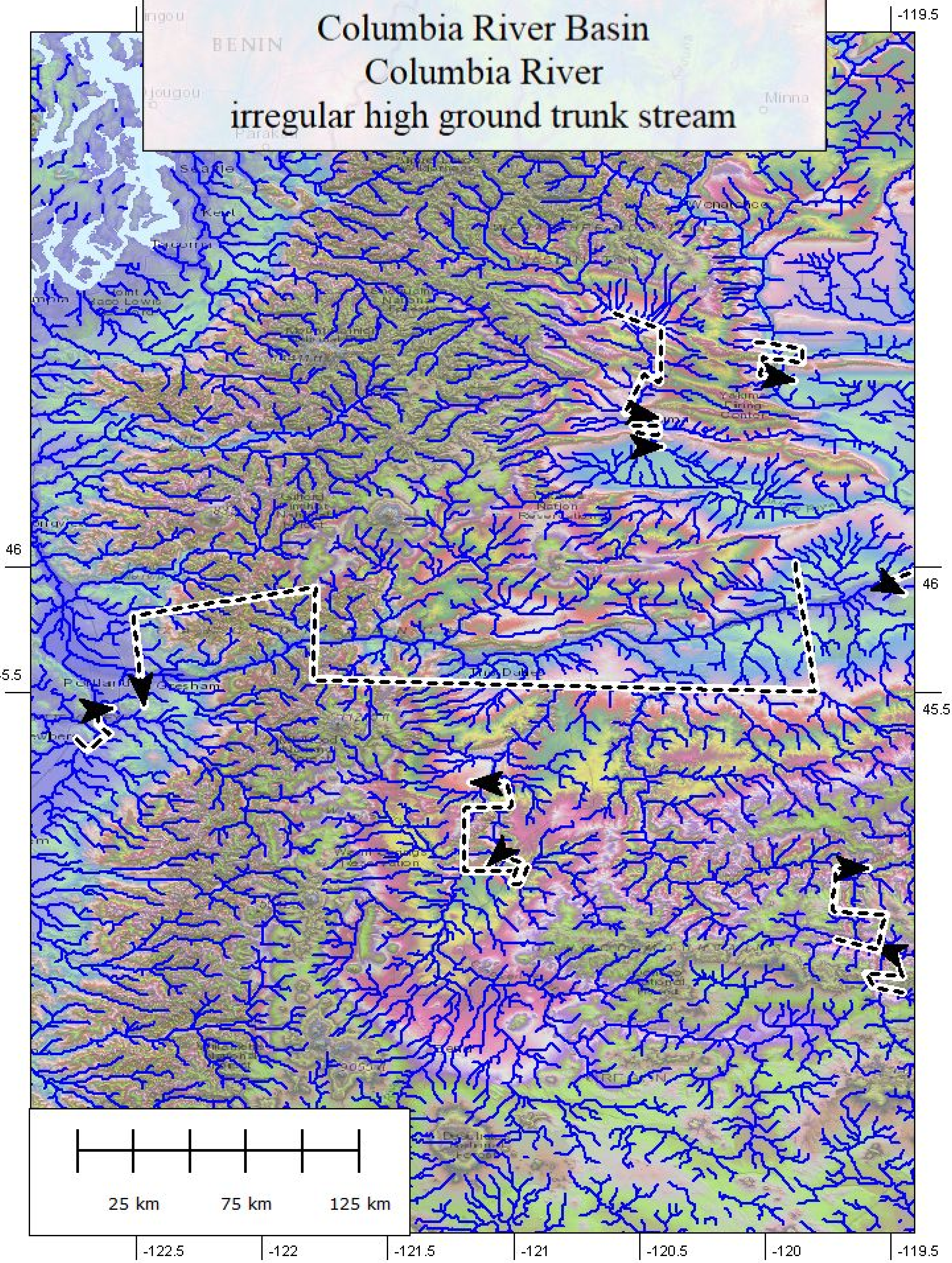

NA - 301
Columbia River Basin
Trout Creek
plateau trunk stream

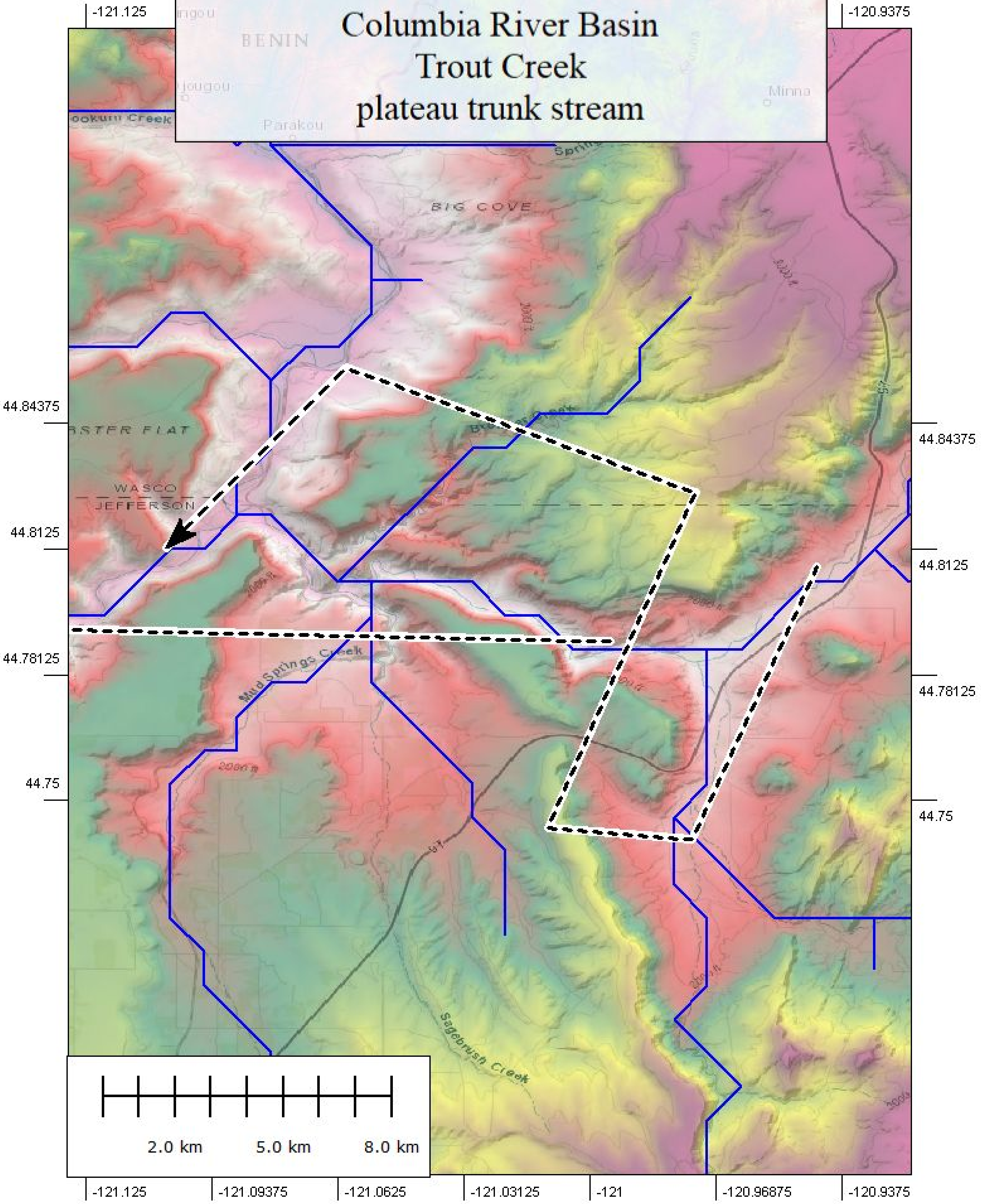

NA - 302
Colorado River (Arizona) Basin
Colorado River
updip plateau trunk stream

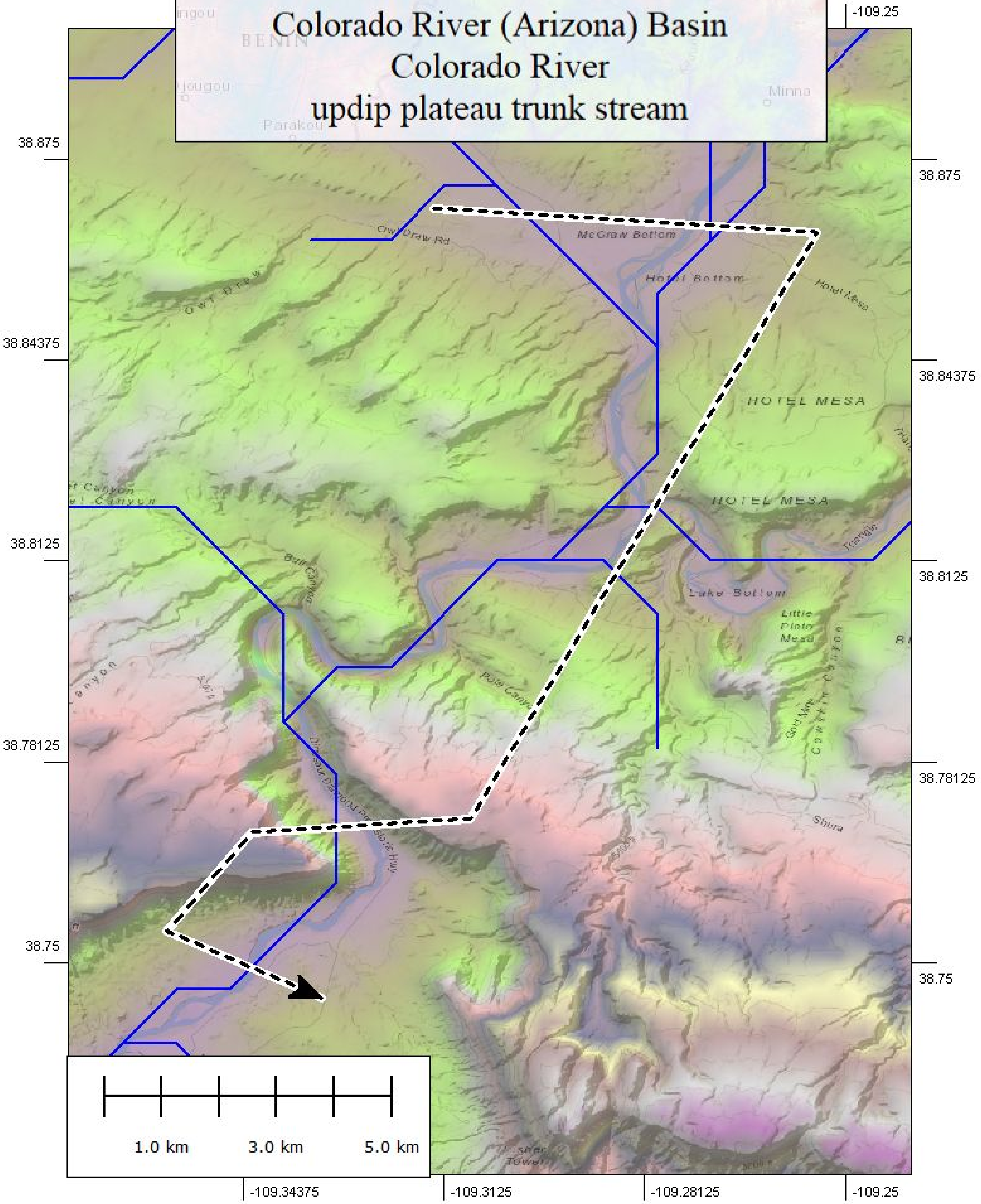

NA - 303
Colorado River (Arizona) Basin
Dolores River
single-ridge trunk stream

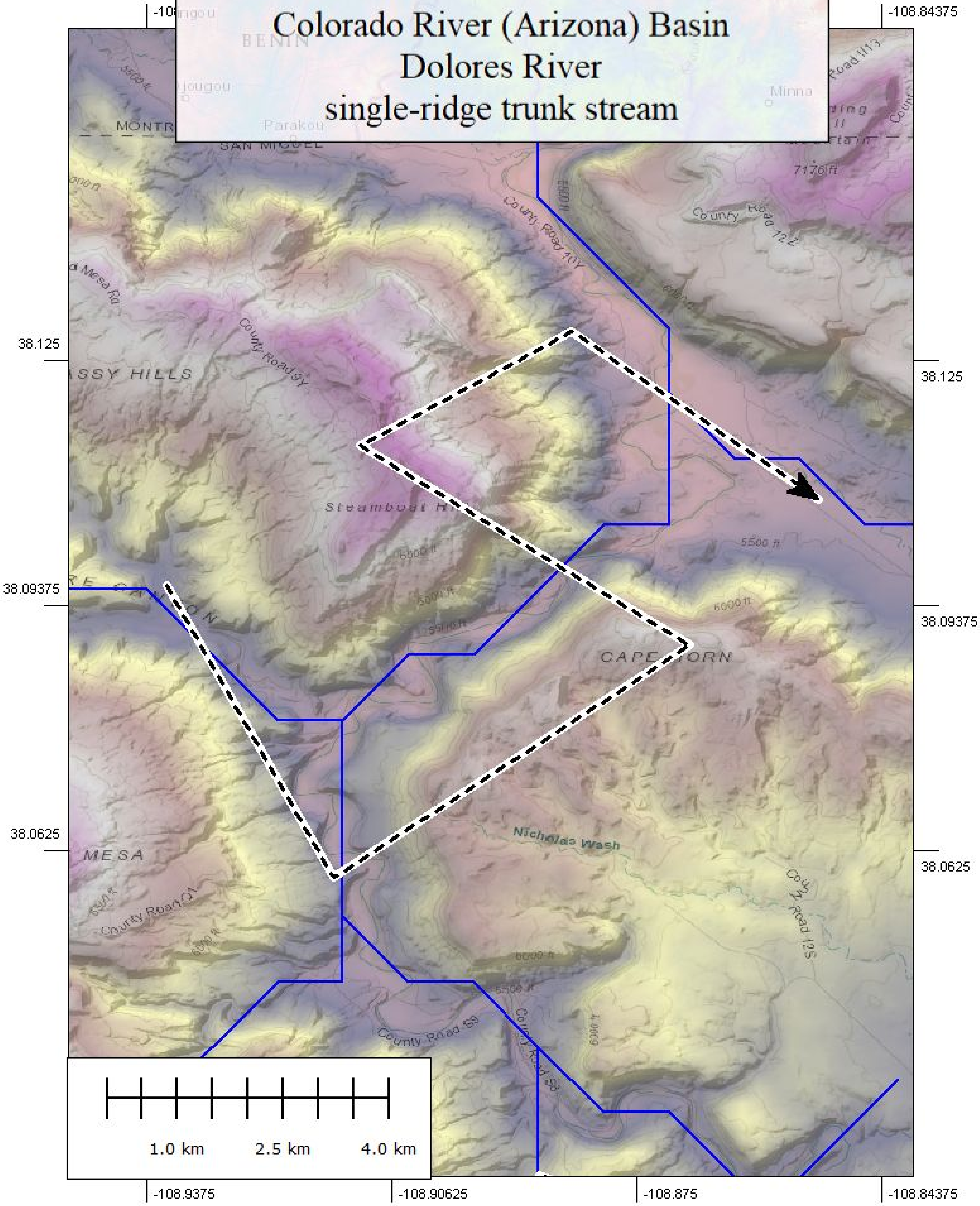

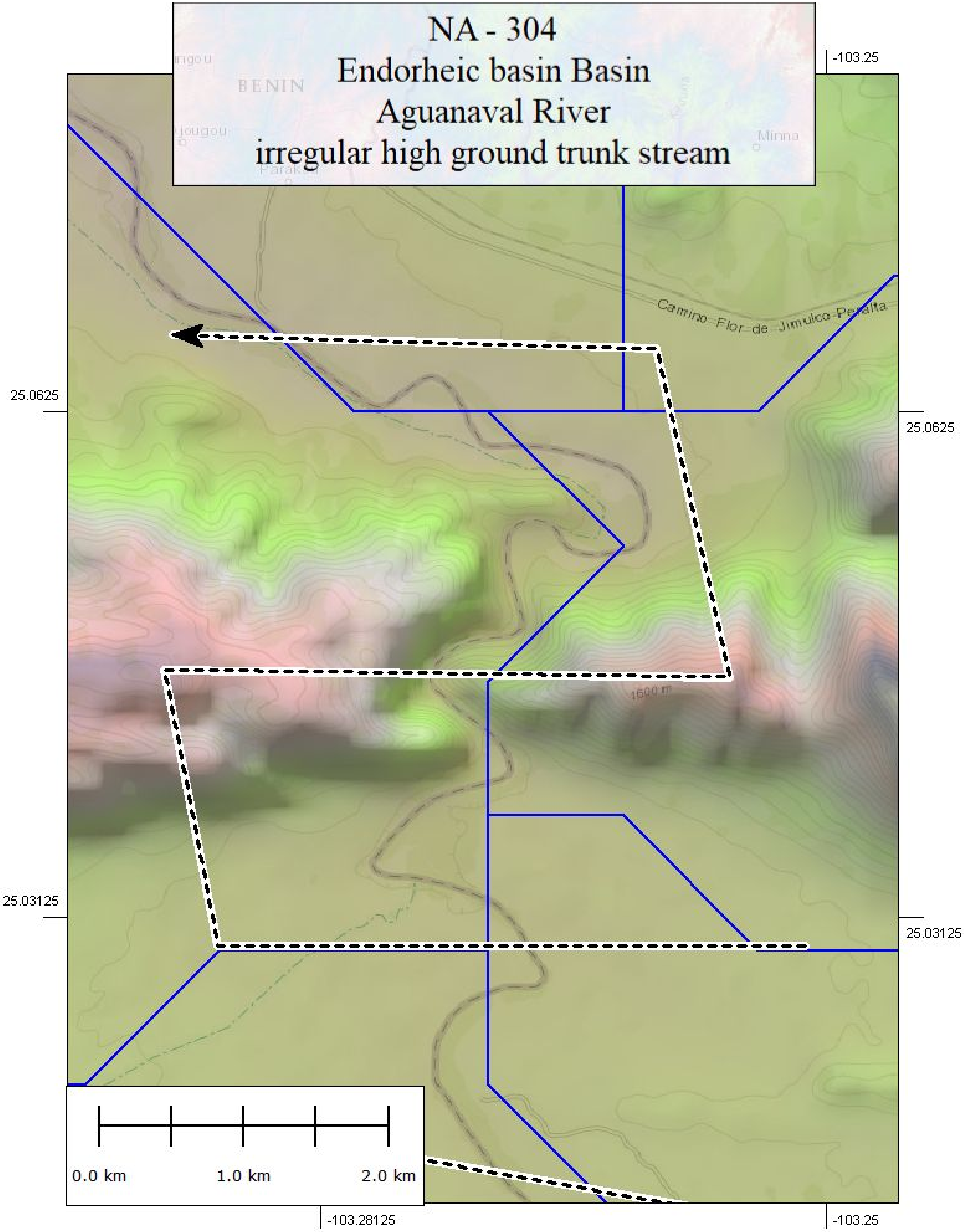
NA - 304
Endorheic basin Basin
Aguanaval River
irregular high ground trunk stream
-103.25
25.0625
25.0625
25.03125
25.03125
-103.28125
-103.25
Camino Flor de Jimulco Peralta
1600 m
0.0 km
1.0 km
2.0 km

NA - 305
Endorheic basin Basin
Box Elder Creek, Box Elder Canyon
single-ridge head stream

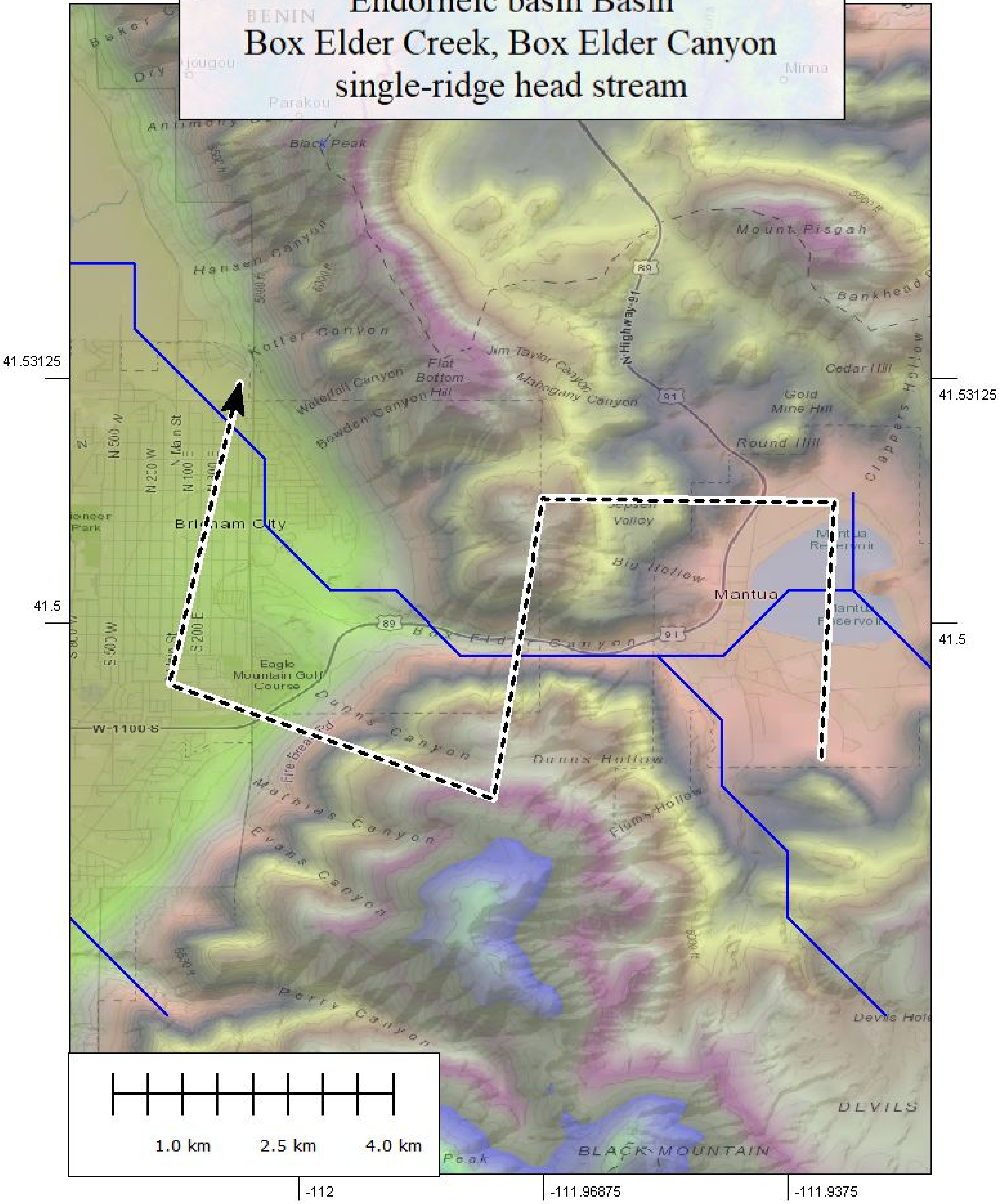

NA - 306
Sacramento River Basin
Pit River
single-ridge trunk stream

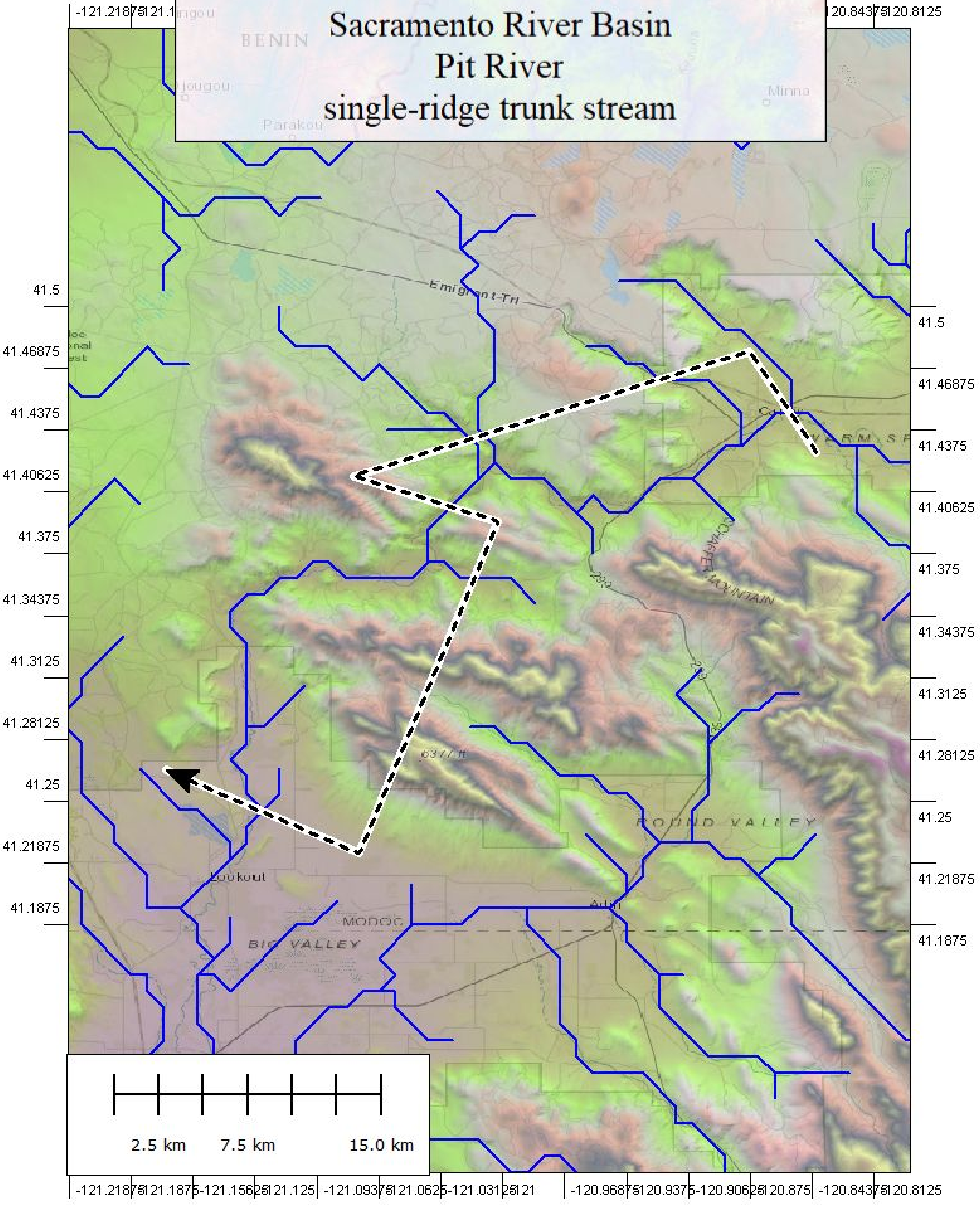

NA - 307
Colorado River (Arizona) Basin
San Rafael River tributary, Buckhorn Wash
updip plateau trunk stream

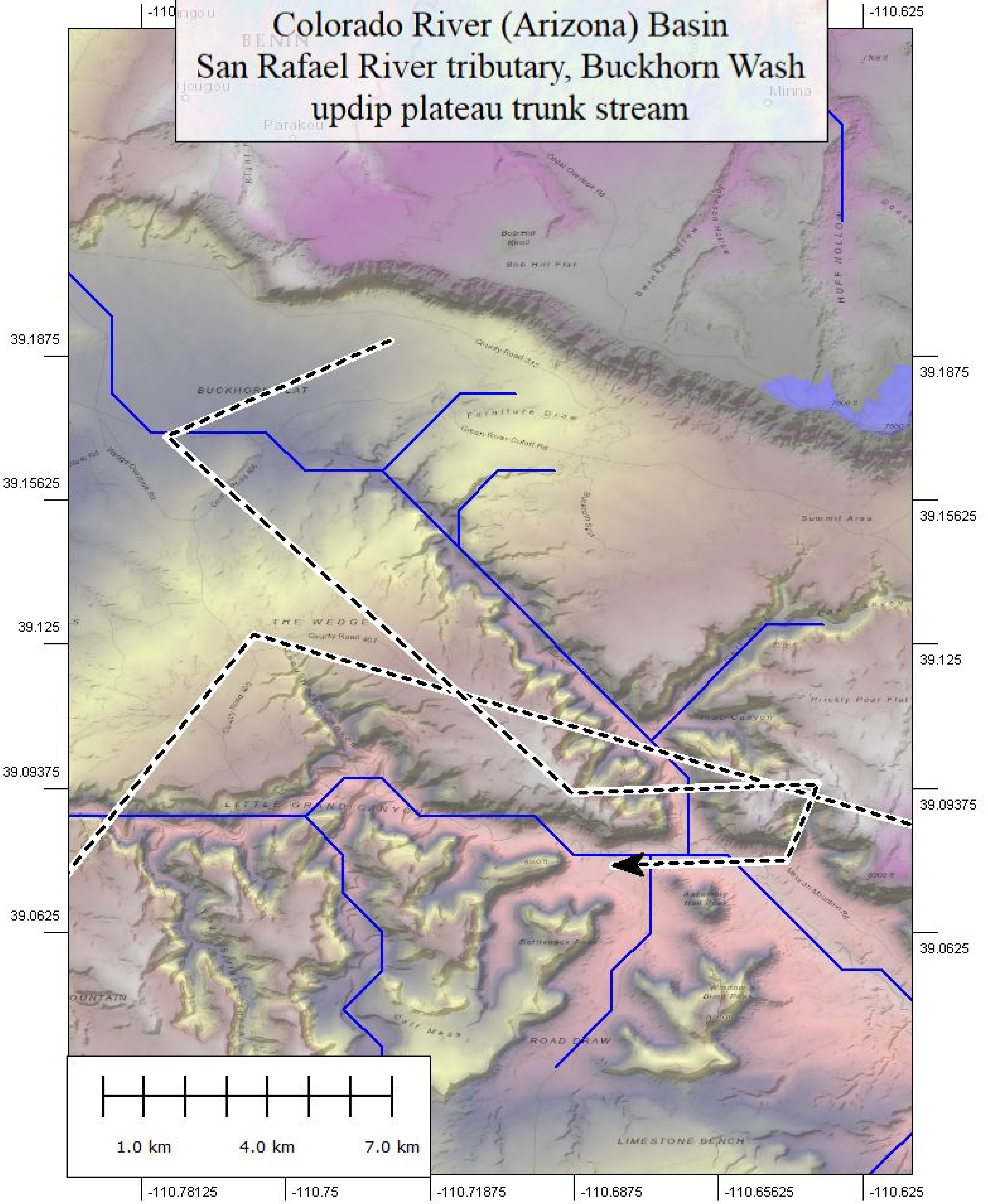

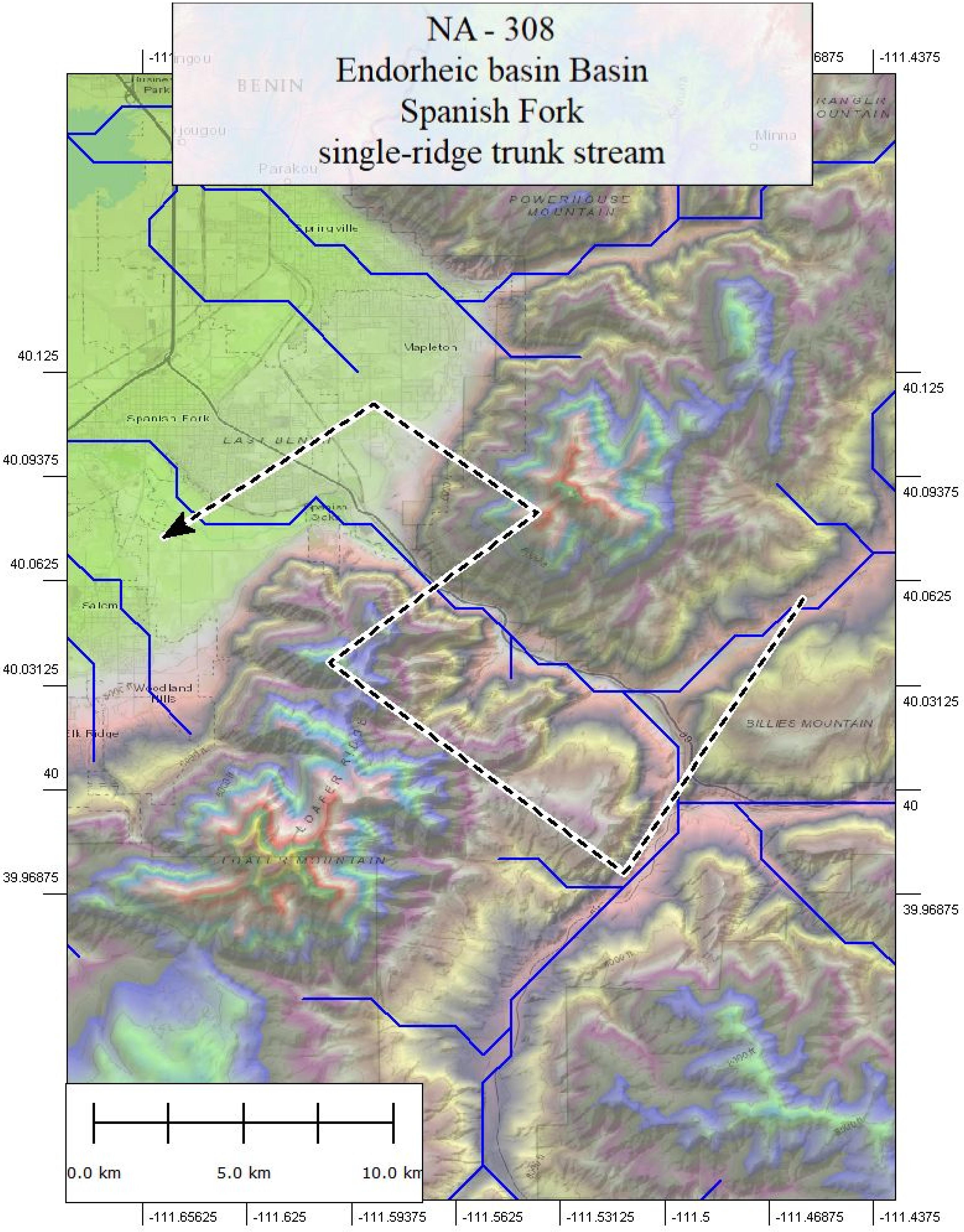
NA - 308
Endorheic basin Basin
Spanish Fork
single-ridge trunk stream
POWERHOUSE MOUNTAIN
Springville
Mapleton
Spanish Fork
Salem
Woodland Hills
Elk Ridge
LOAFER MOUNTAIN
BILLIES MOUNTAIN
40.125
40.09375
40.0625
40.03125
40
39.96875
-111.65625
-111.625
-111.59375
-111.5625
-111.53125
-111.5
-111.46875
-111.4375
0.0 km
5.0 km
10.0 km

NA - 309
Endorheic basin Basin
South Fork River
single-ridge trunk stream

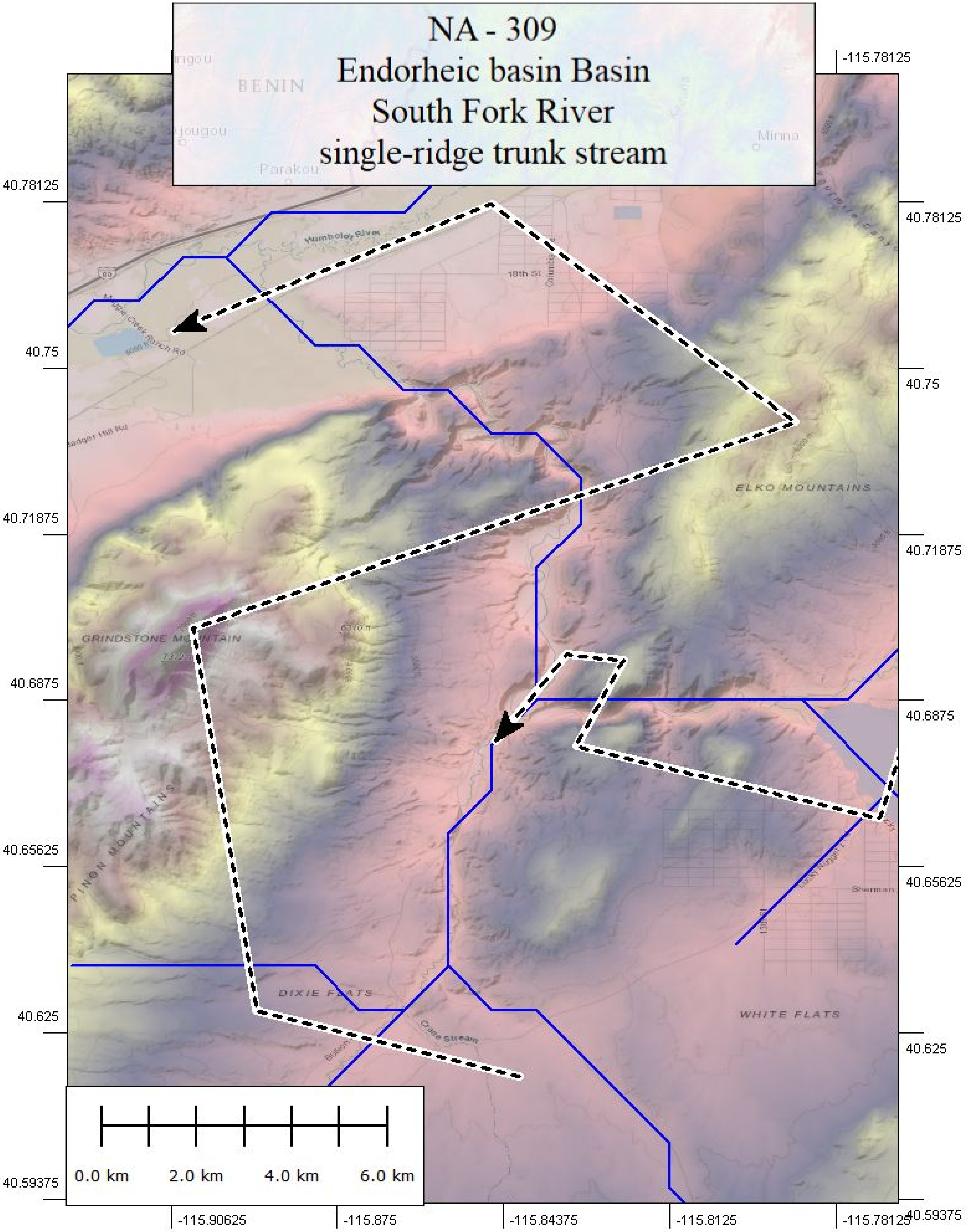

NA - 31
Mackenzie River (Canada) Basin
Liard River tributary
single-ridge trunk stream

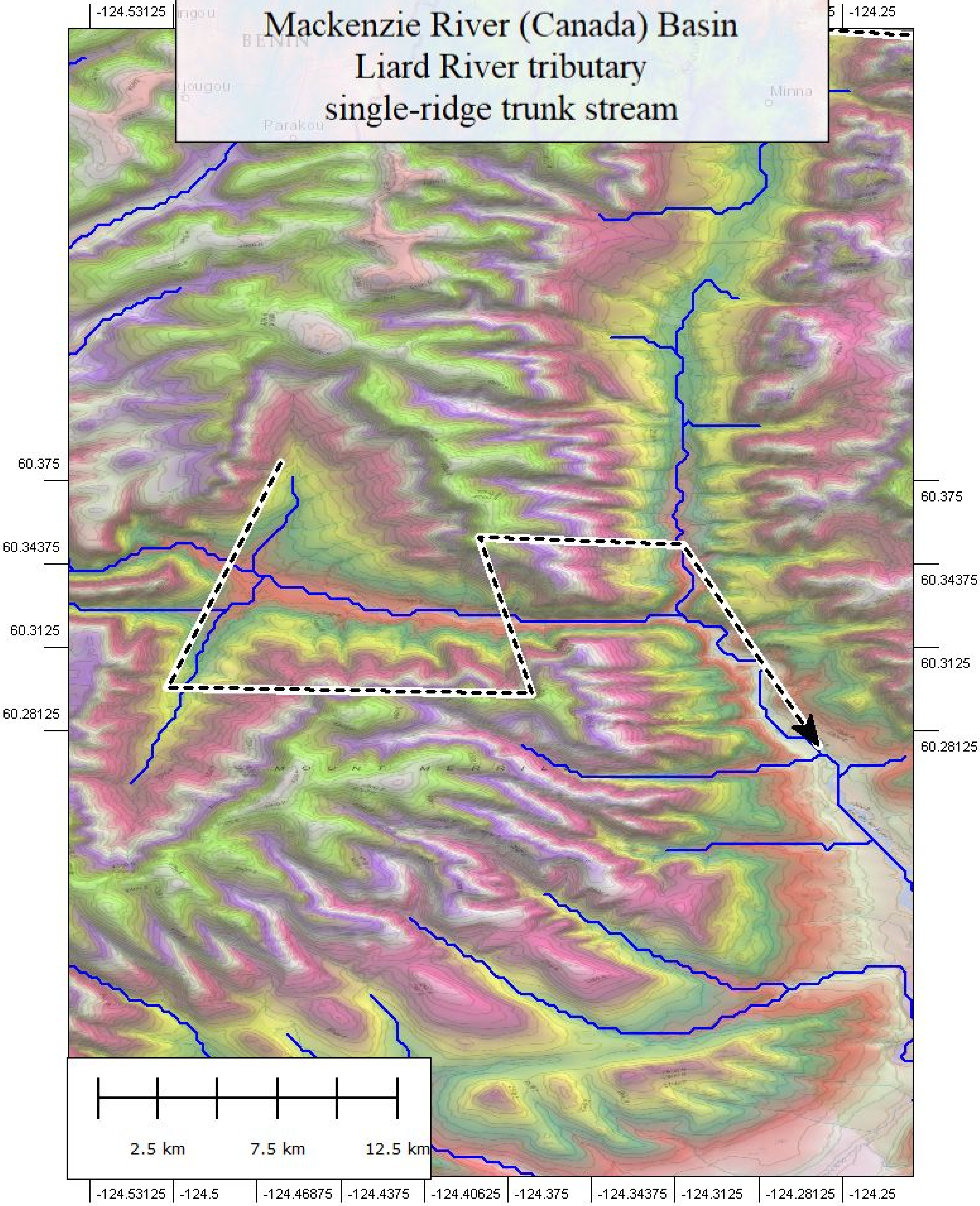

NA - 310
Susquehanna River Basin
Susquehanna River
single-ridge trunk stream

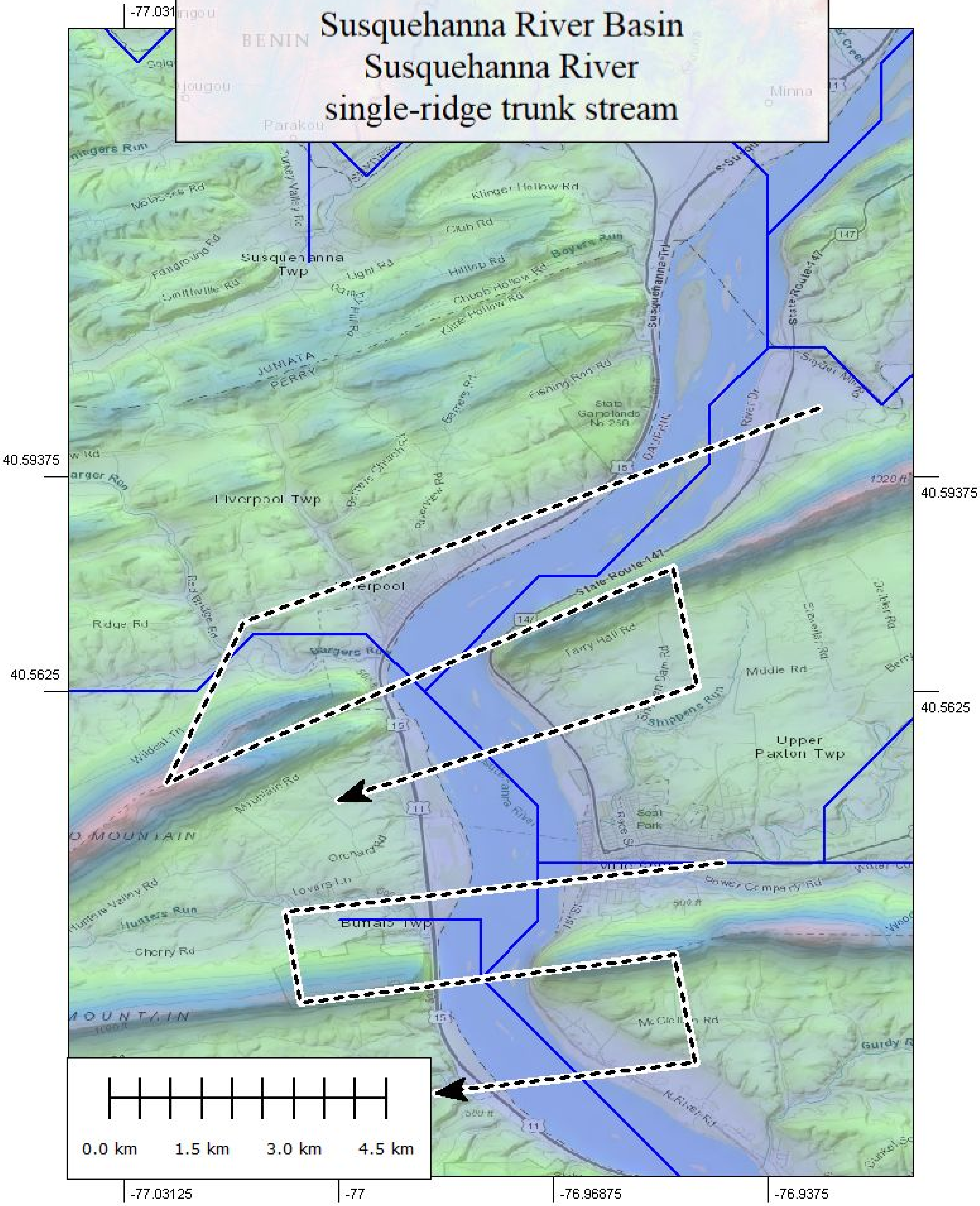

NA - 311
Mississippi River Basin
Big Sheep Creek
irregular high ground trunk stream

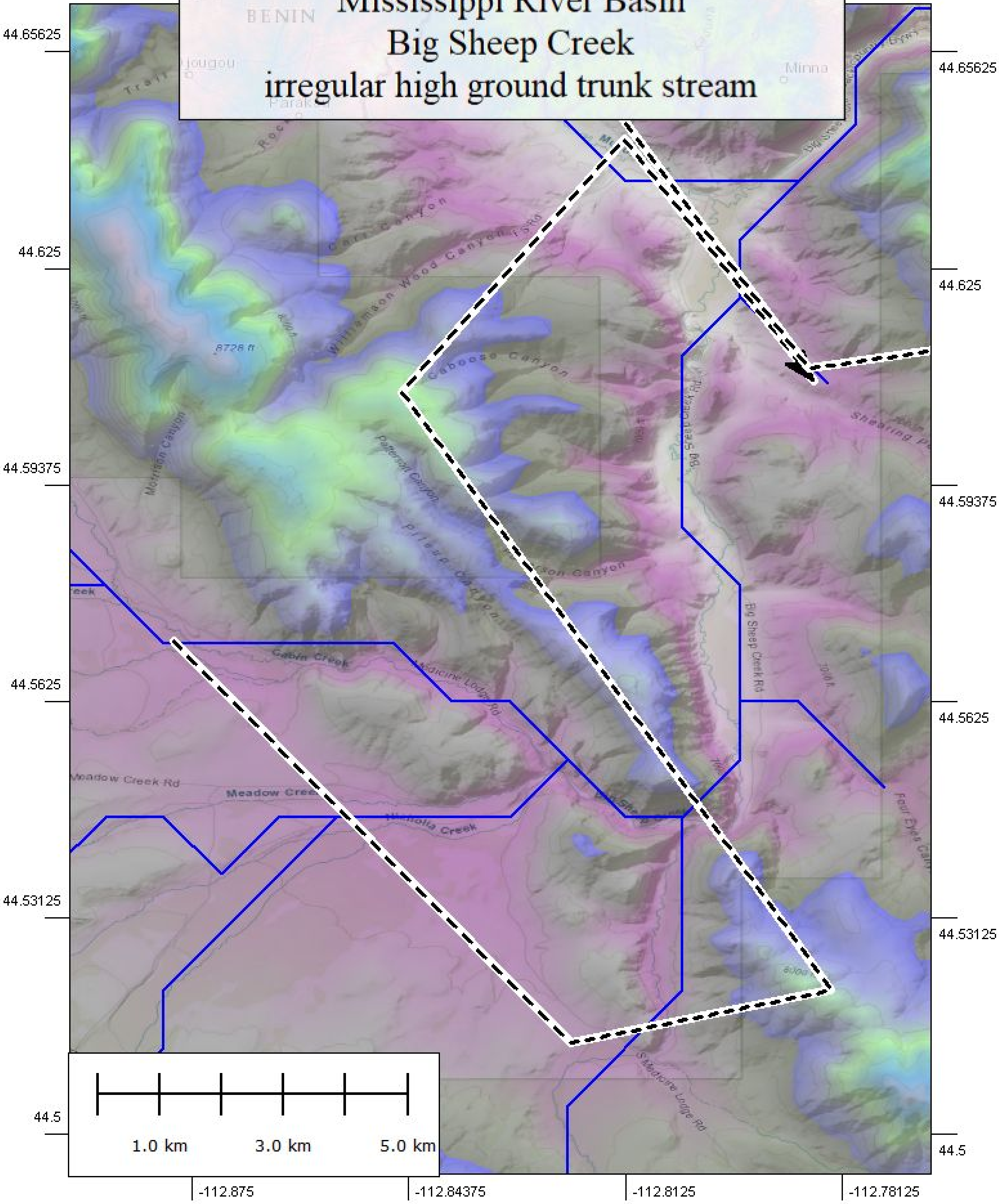

NA - 312
Rio Patuca Basin
Patuca River
single-ridge trunk stream

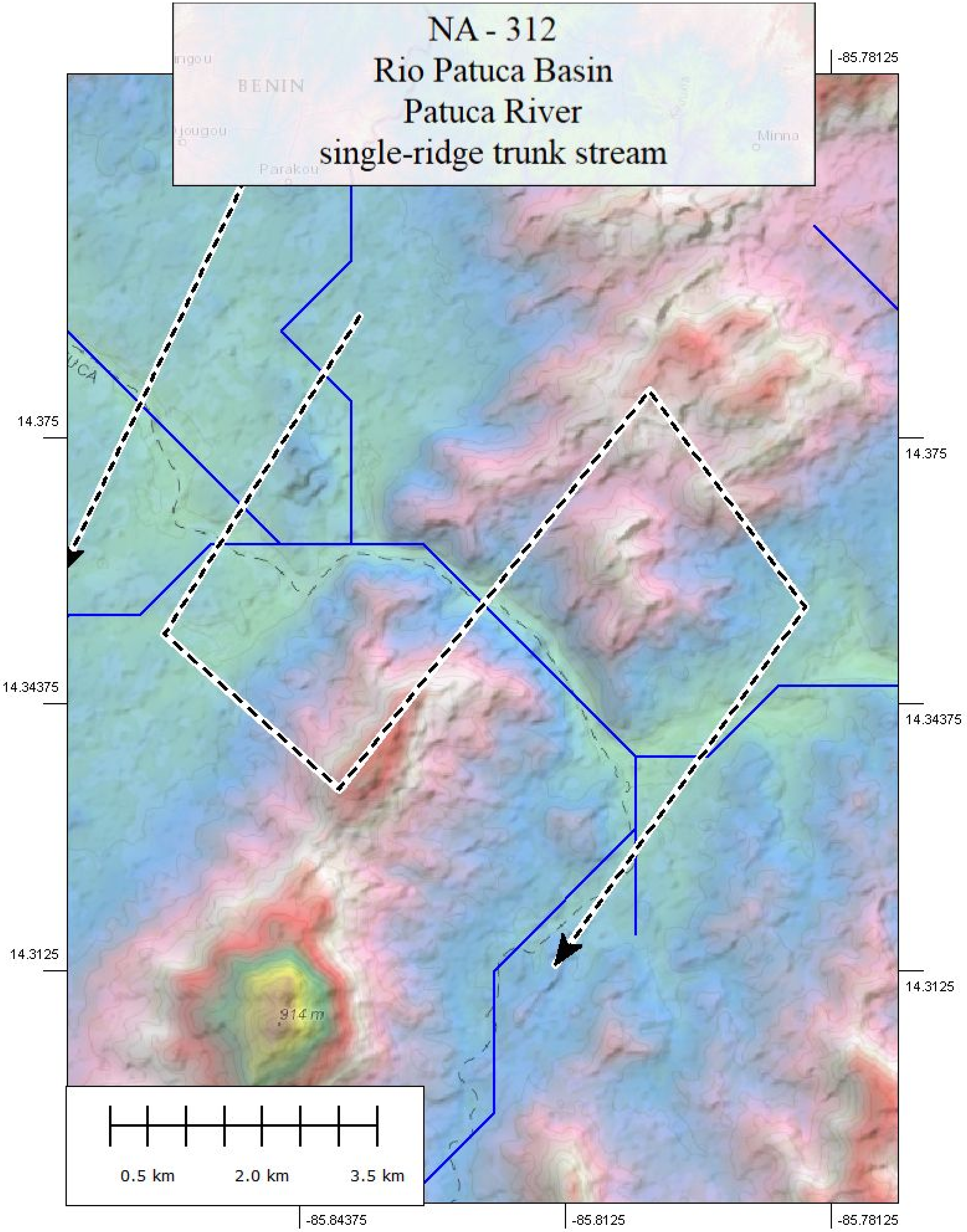

NA - 313
Delaware River Basin
Delaware River, Delaware Water Gap
single-ridge trunk stream

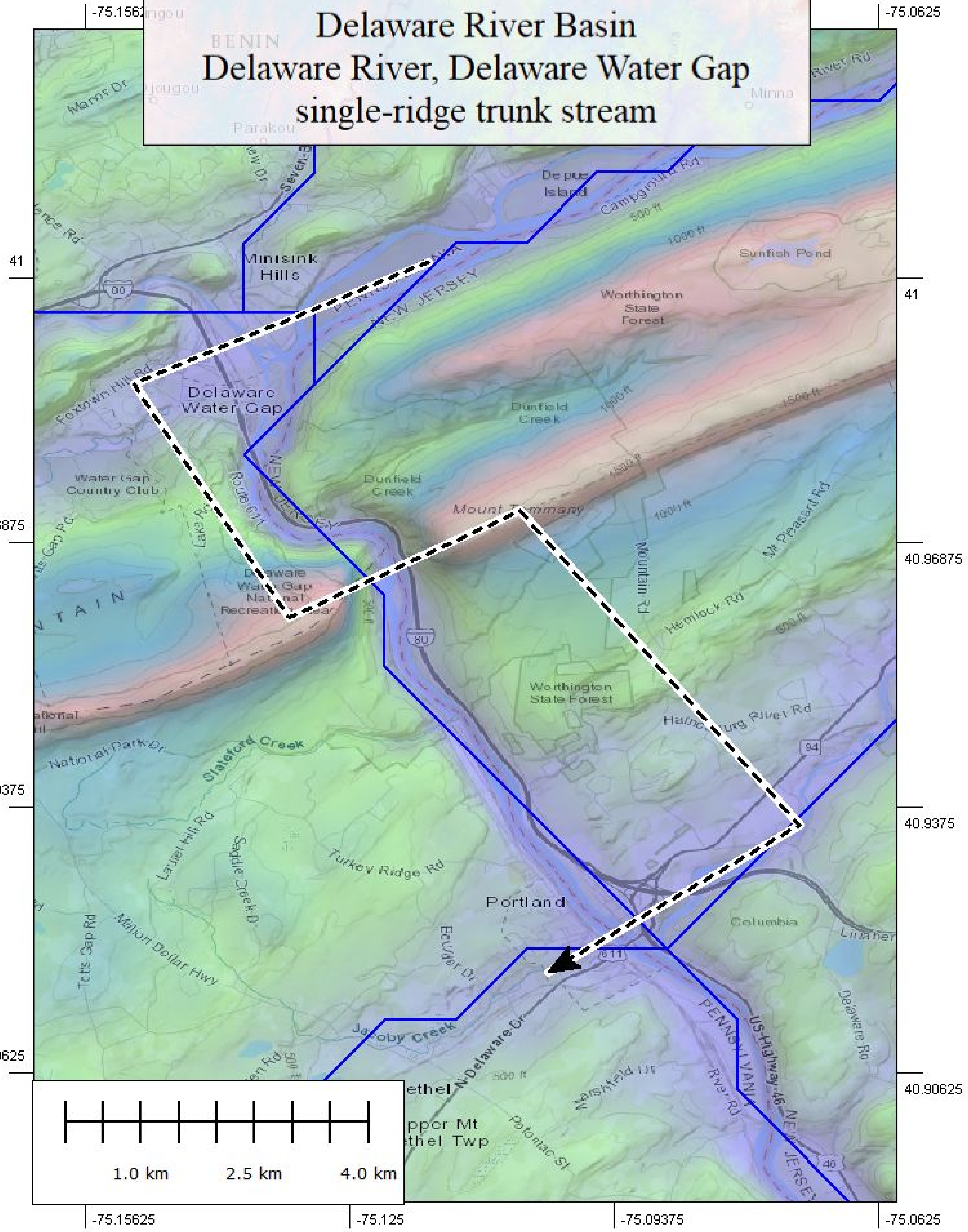

NA - 314
Susquehanna River Basin
Swatara Creek
single-ridge trunk stream

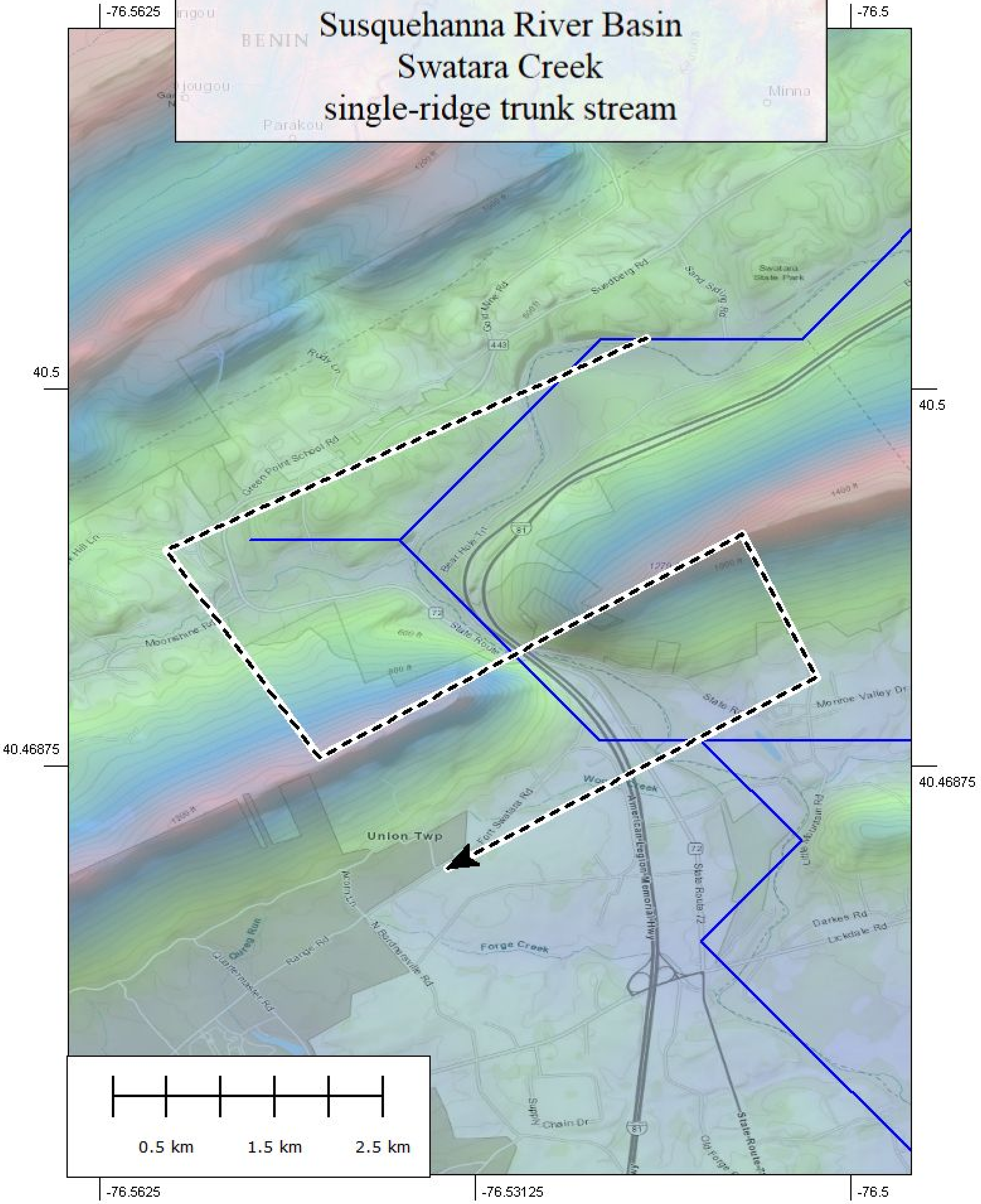

NA - 315
Susquehanna River Basin
Yellow Creek
single-ridge trunk stream

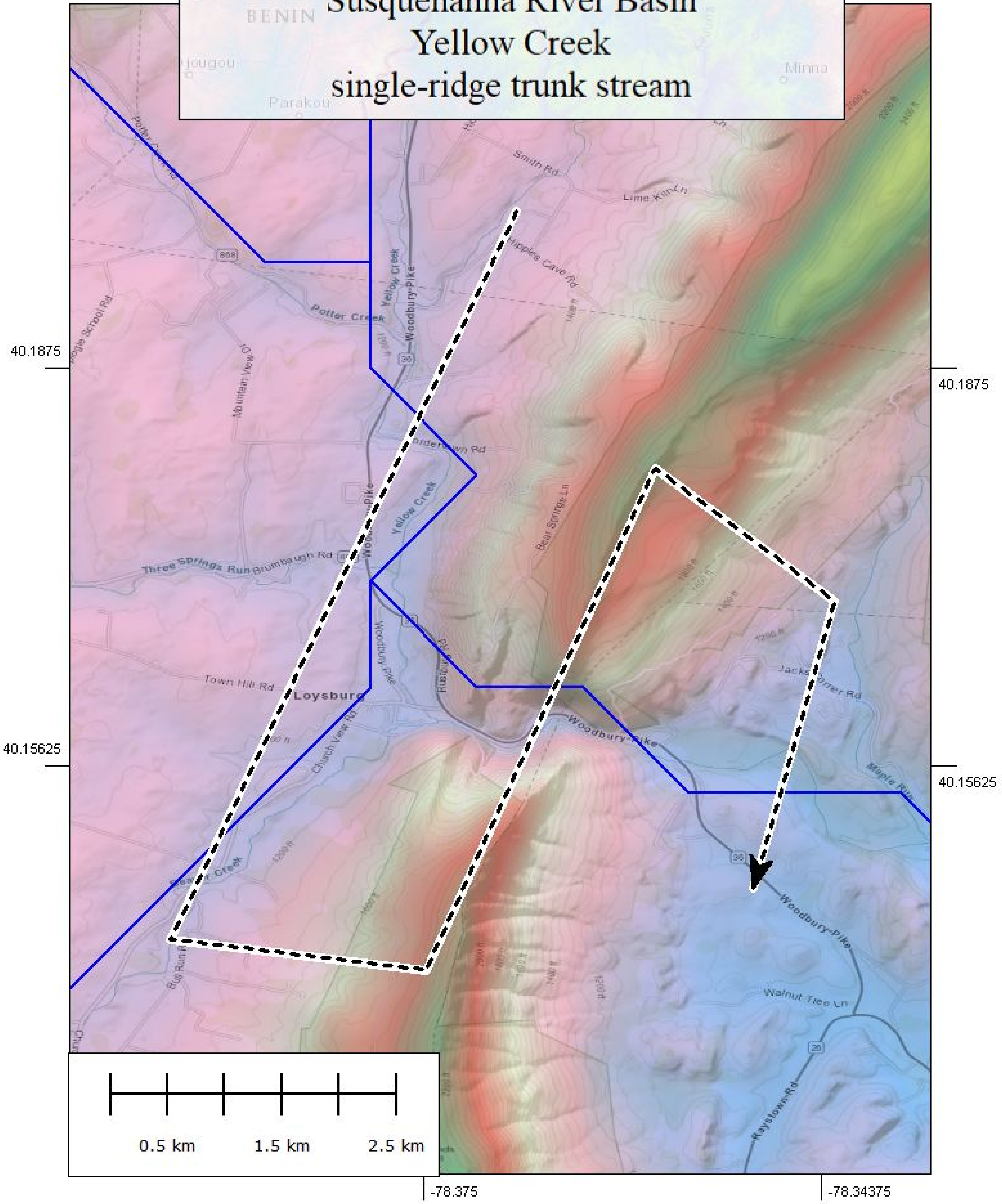

NA - 316
Susquehanna River Basin
Brush Creek
single-ridge head stream

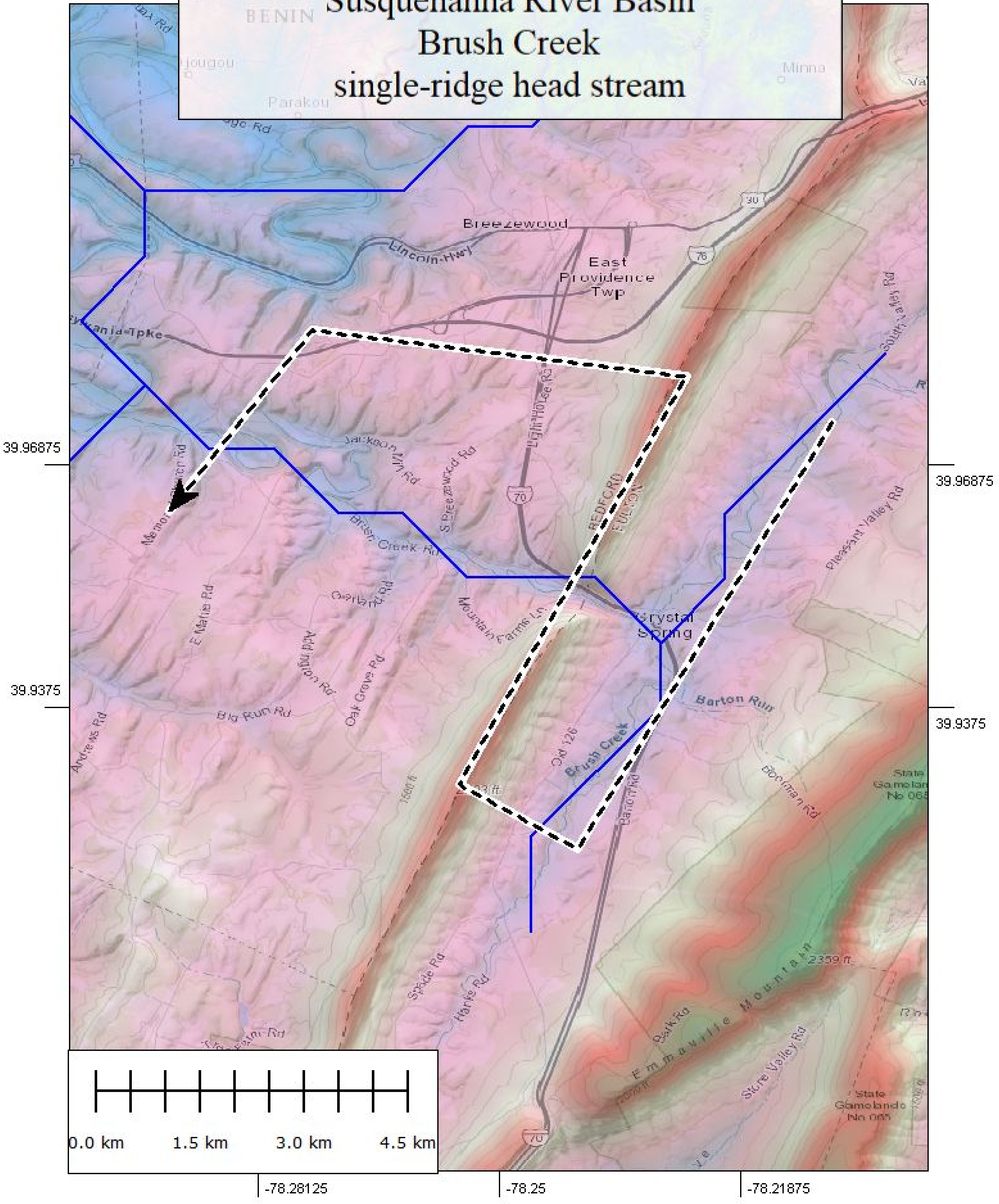

NA - 317
Potomac River Basin
Tonoloway Creek
single-ridge head stream

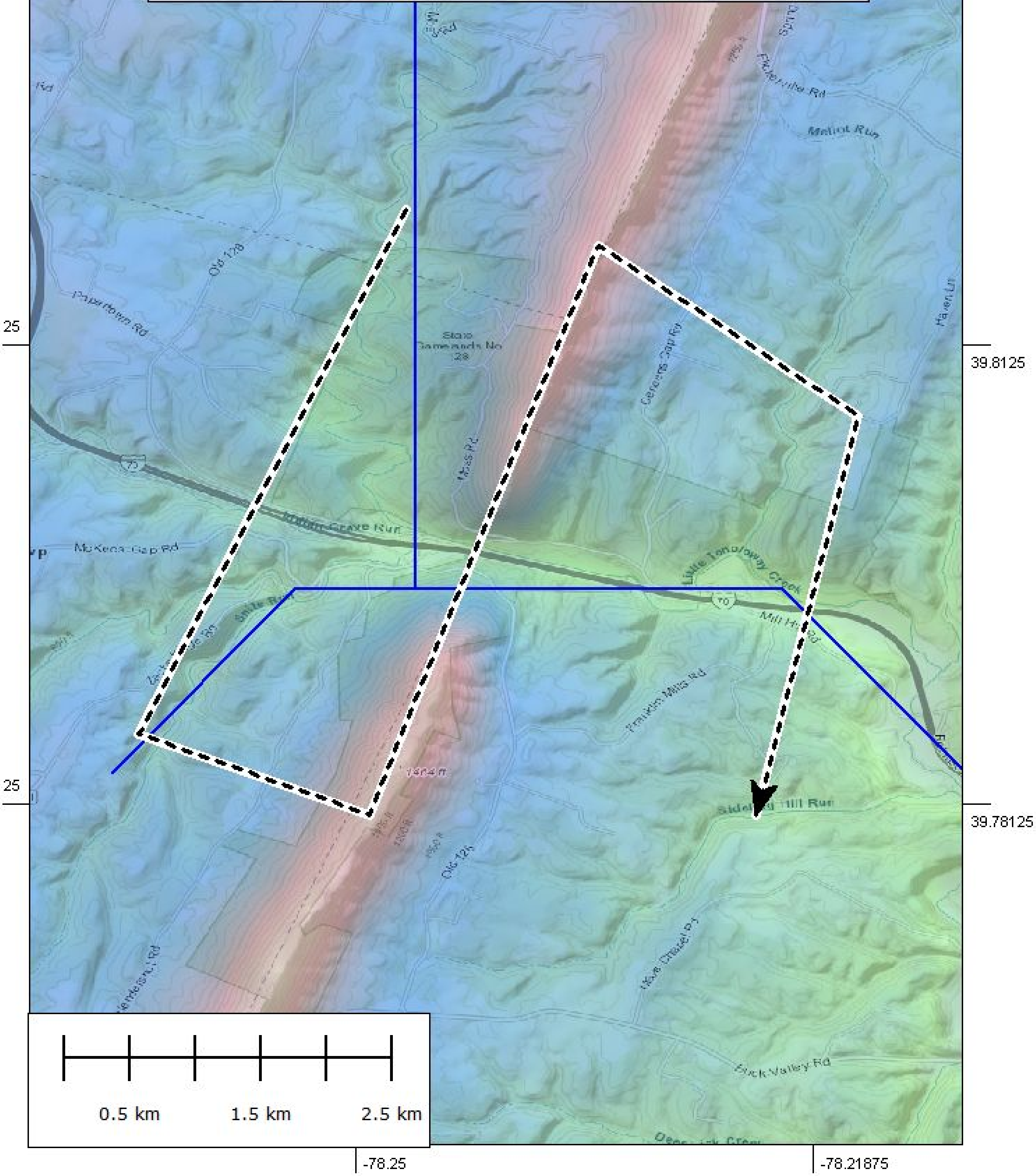

NA - 318
Susquehanna River Basin
Juniata River, Millerstown Gap
multi-ridge trunk stream

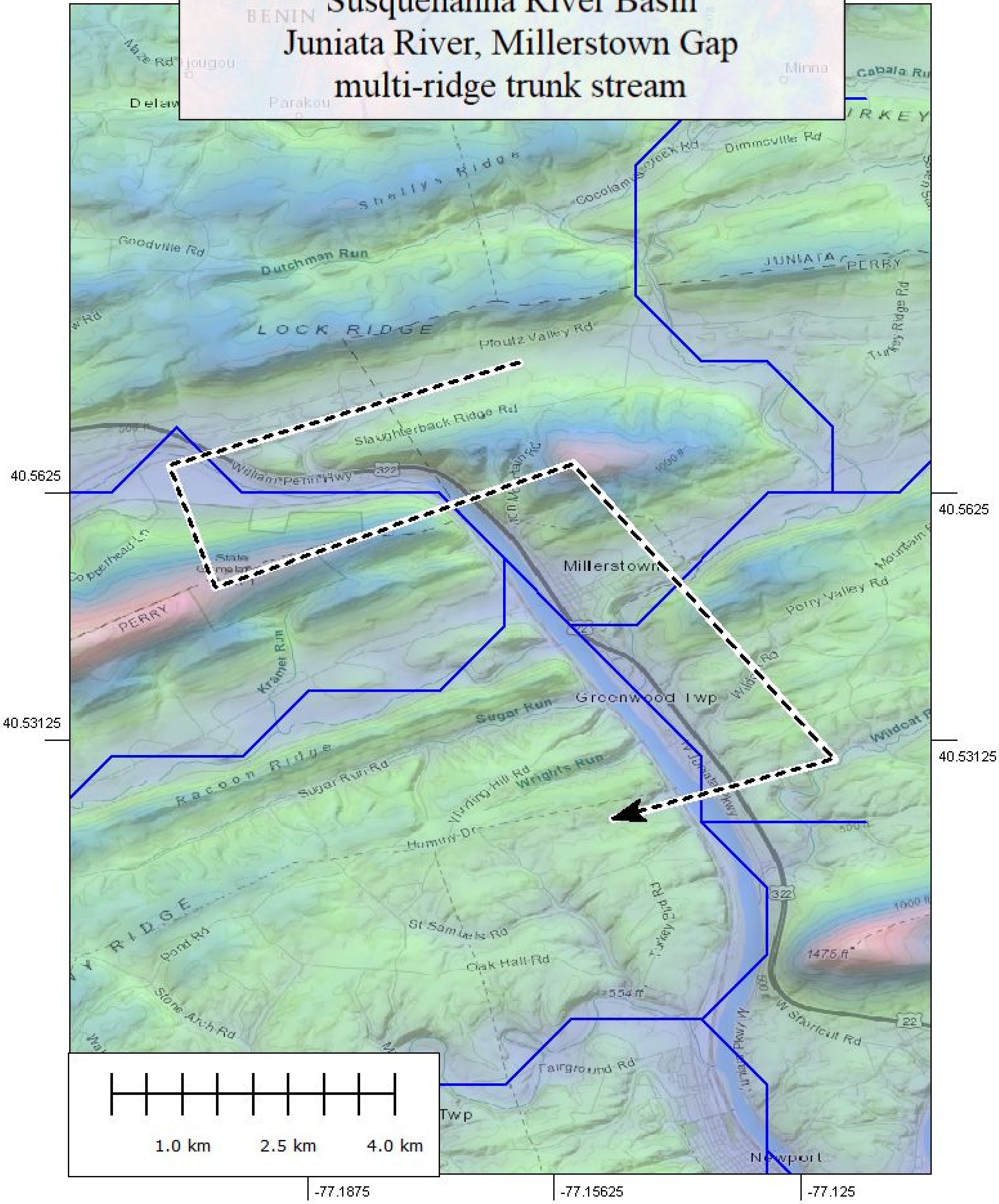

NA - 319
Sacramento River Basin
Putah Creek, Thompson Canyon& Monticello Dam

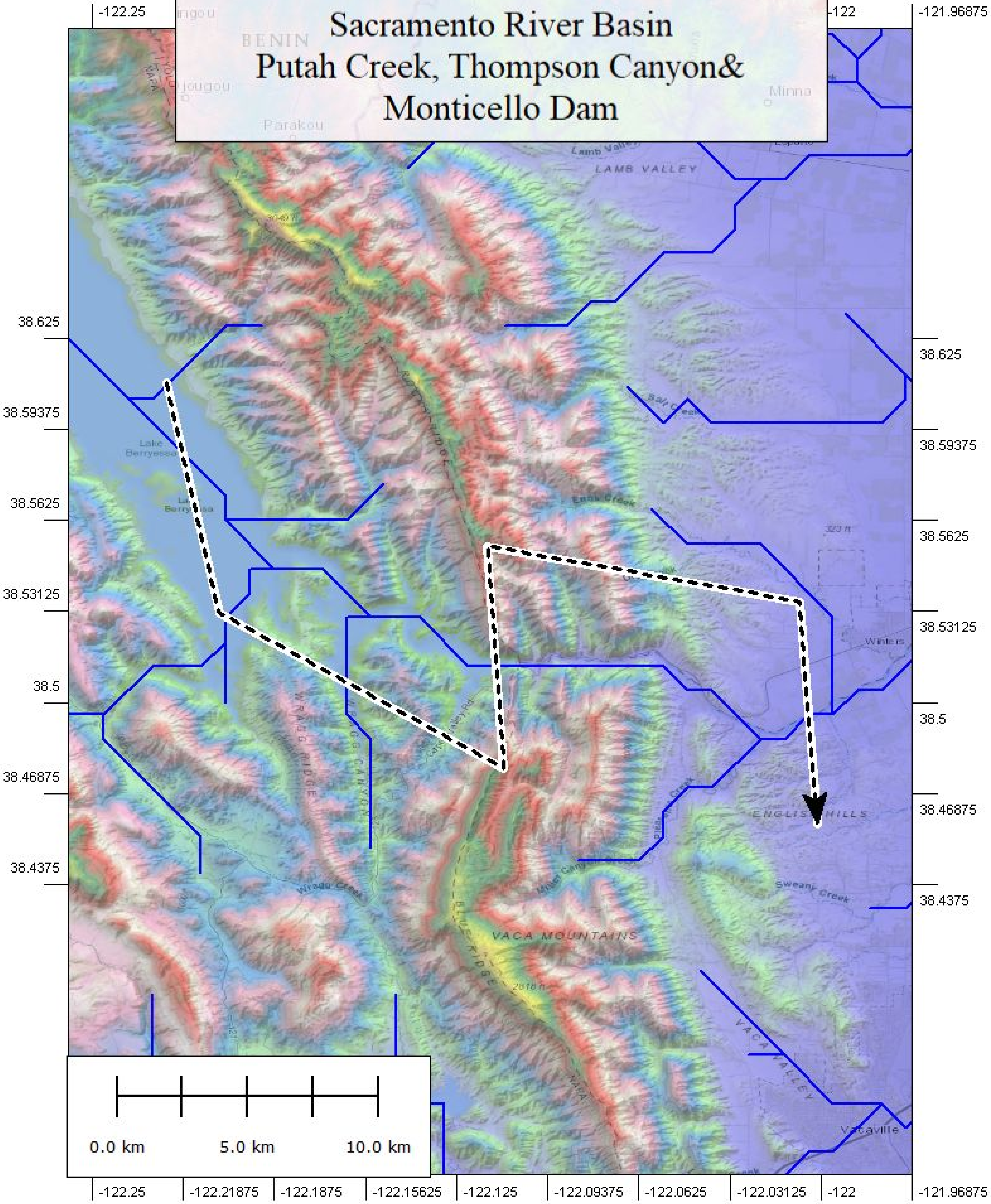

NA - 32
Mackenzie River (Canada) Basin
Little Doctor Lake
single-ridge trunk stream

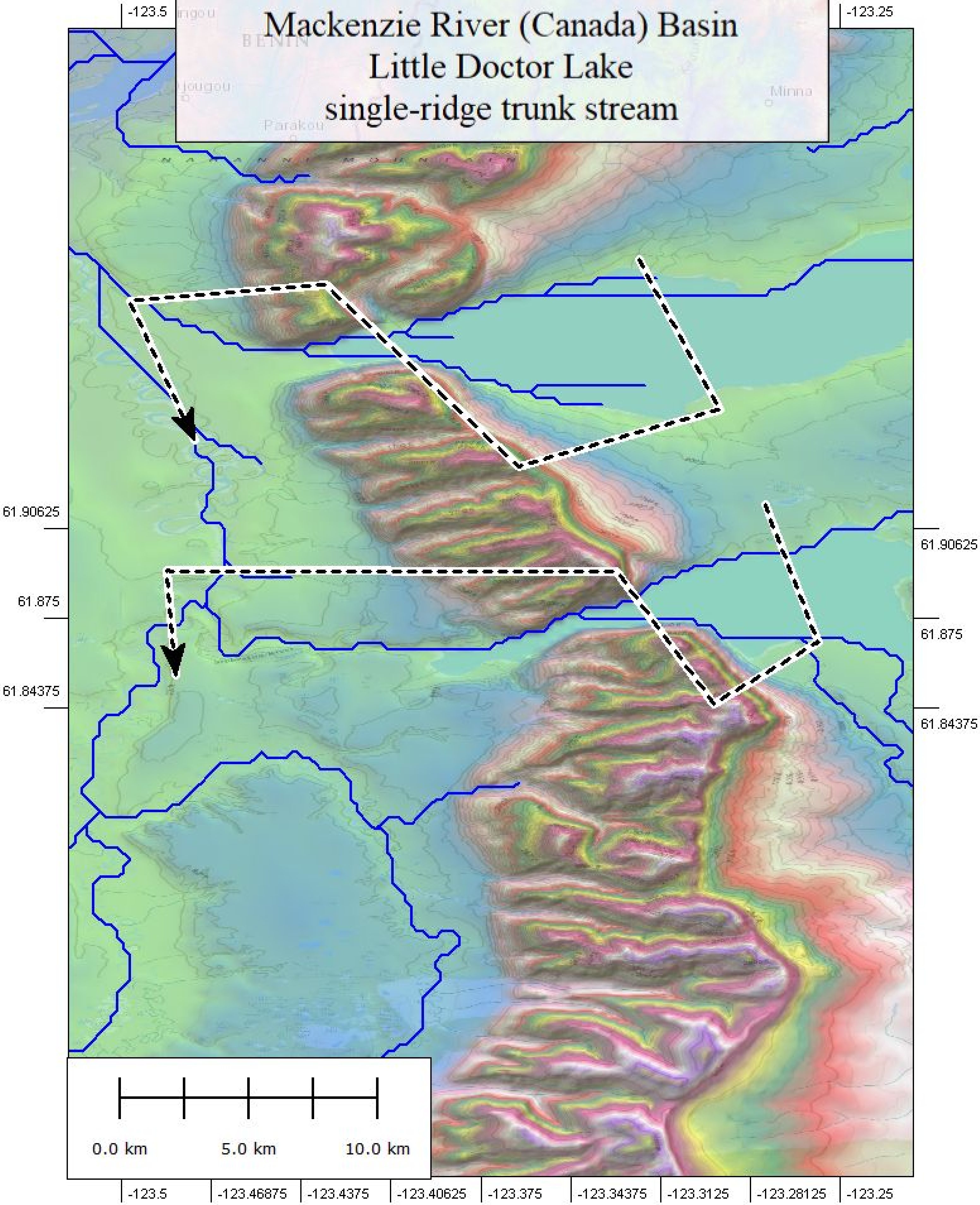

NA - 320
Sacramento River Basin
Calvins Creek
single-ridge head stream

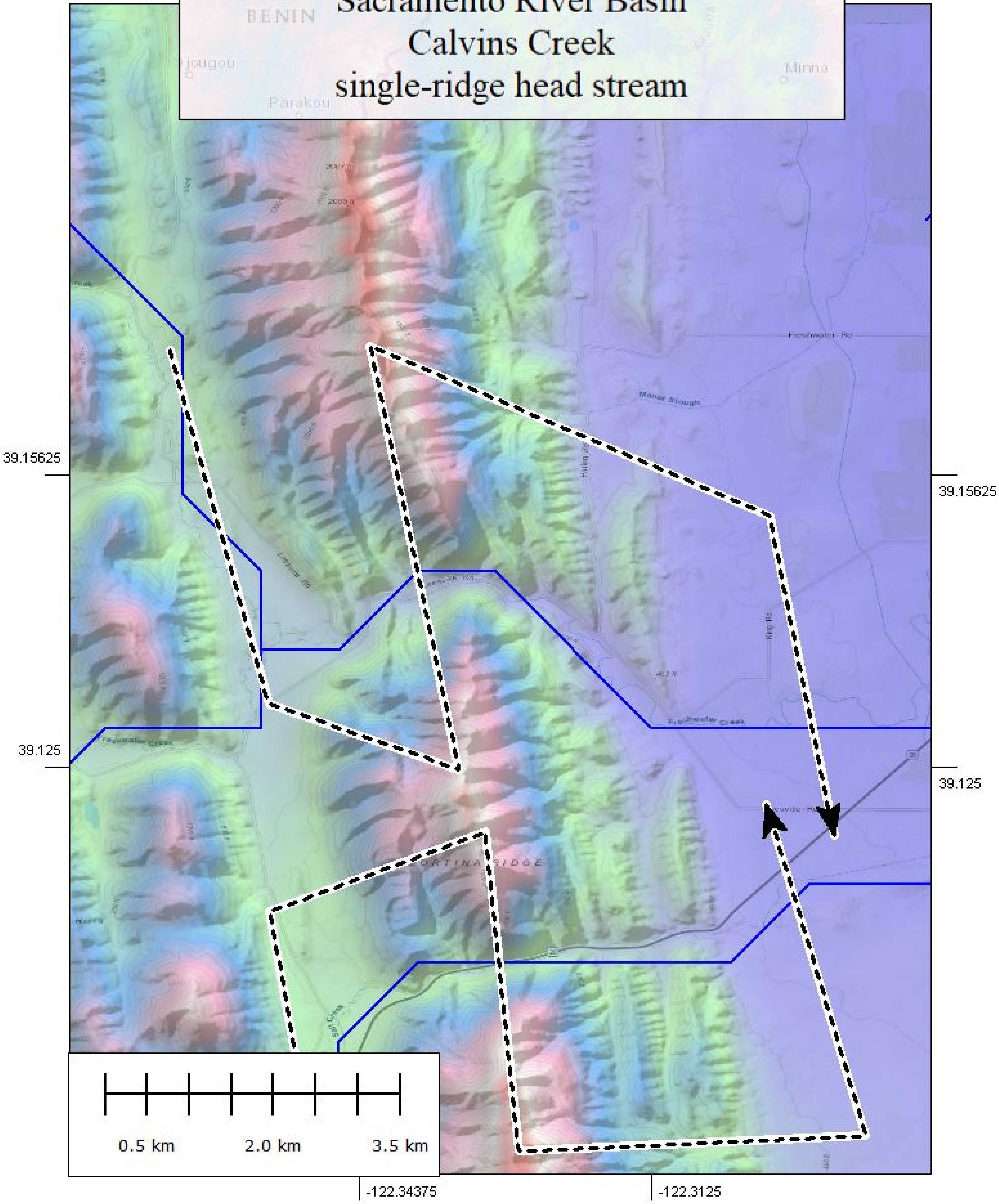

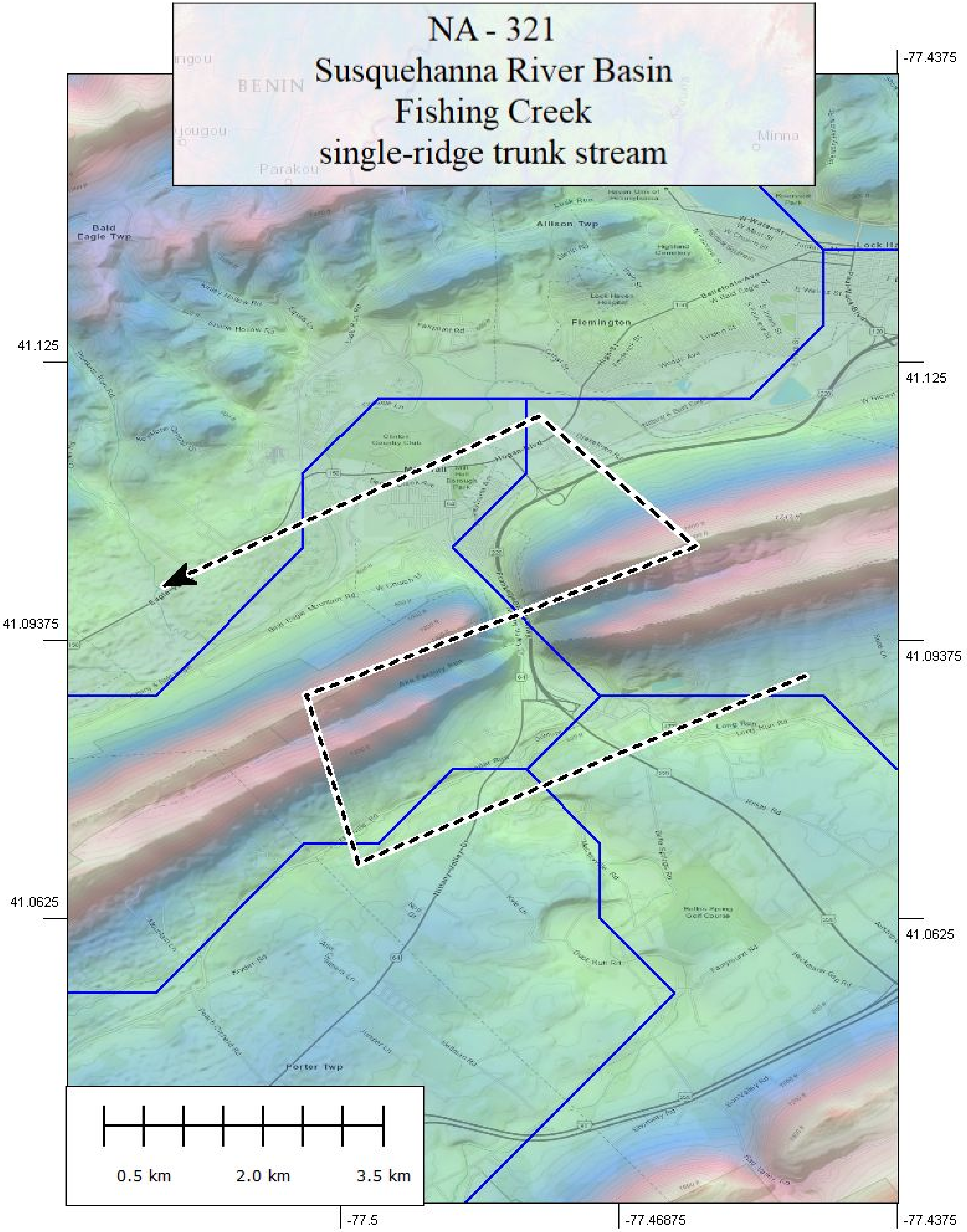
NA - 321
Susquehanna River Basin
Fishing Creek
single-ridge trunk stream
-77.4375
41.125
41.09375
41.0625
-77.5
-77.46875
0.5 km
2.0 km
3.5 km
Bald Eagle Twp
Allison Twp
Flemington
Porter Twp

NA - 322
Susquehanna River Basin
Spring Creek tributary
single-ridge trunk stream

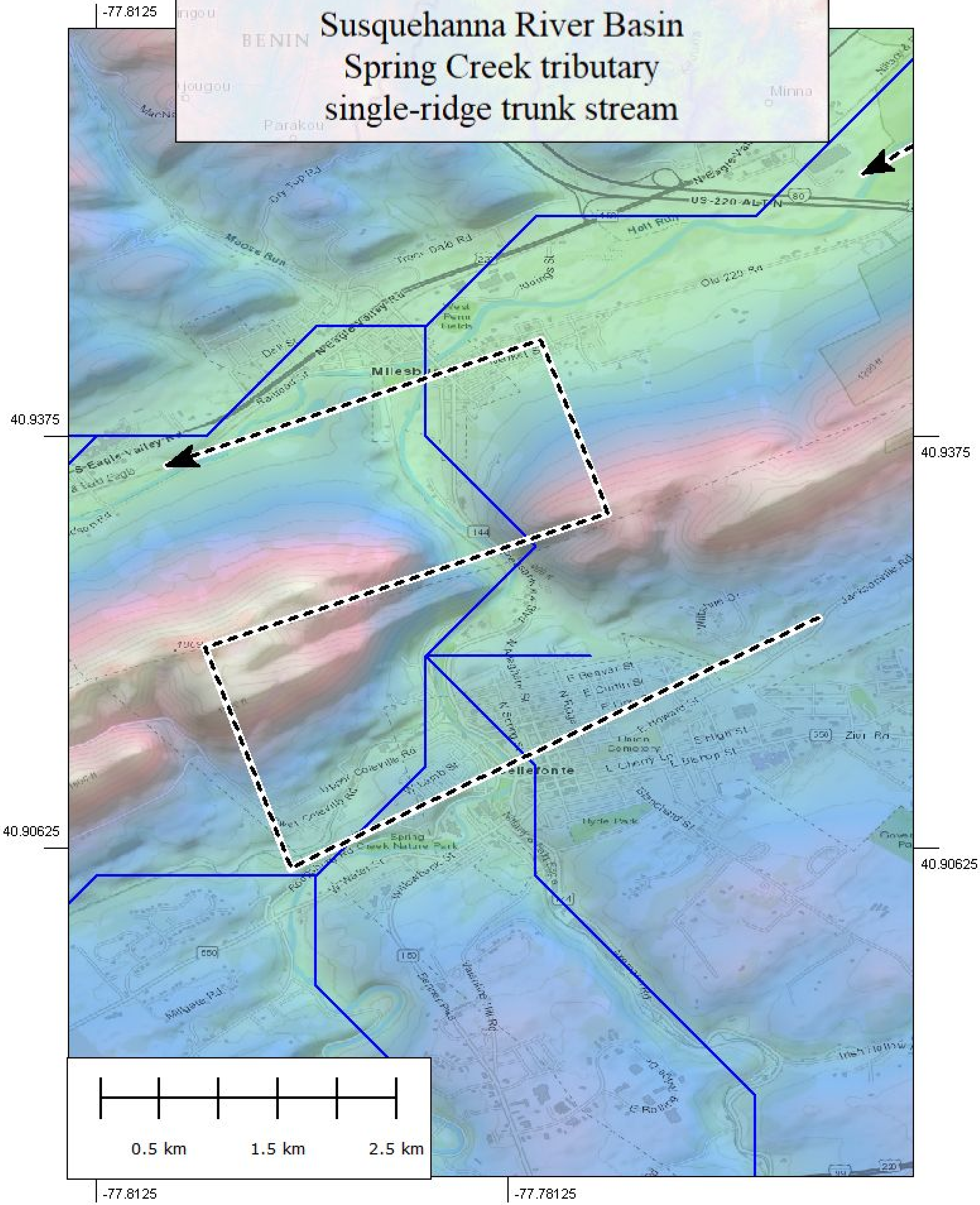

NA - 323
Susquehanna River Basin
Antes Creek
single-ridge trunk stream

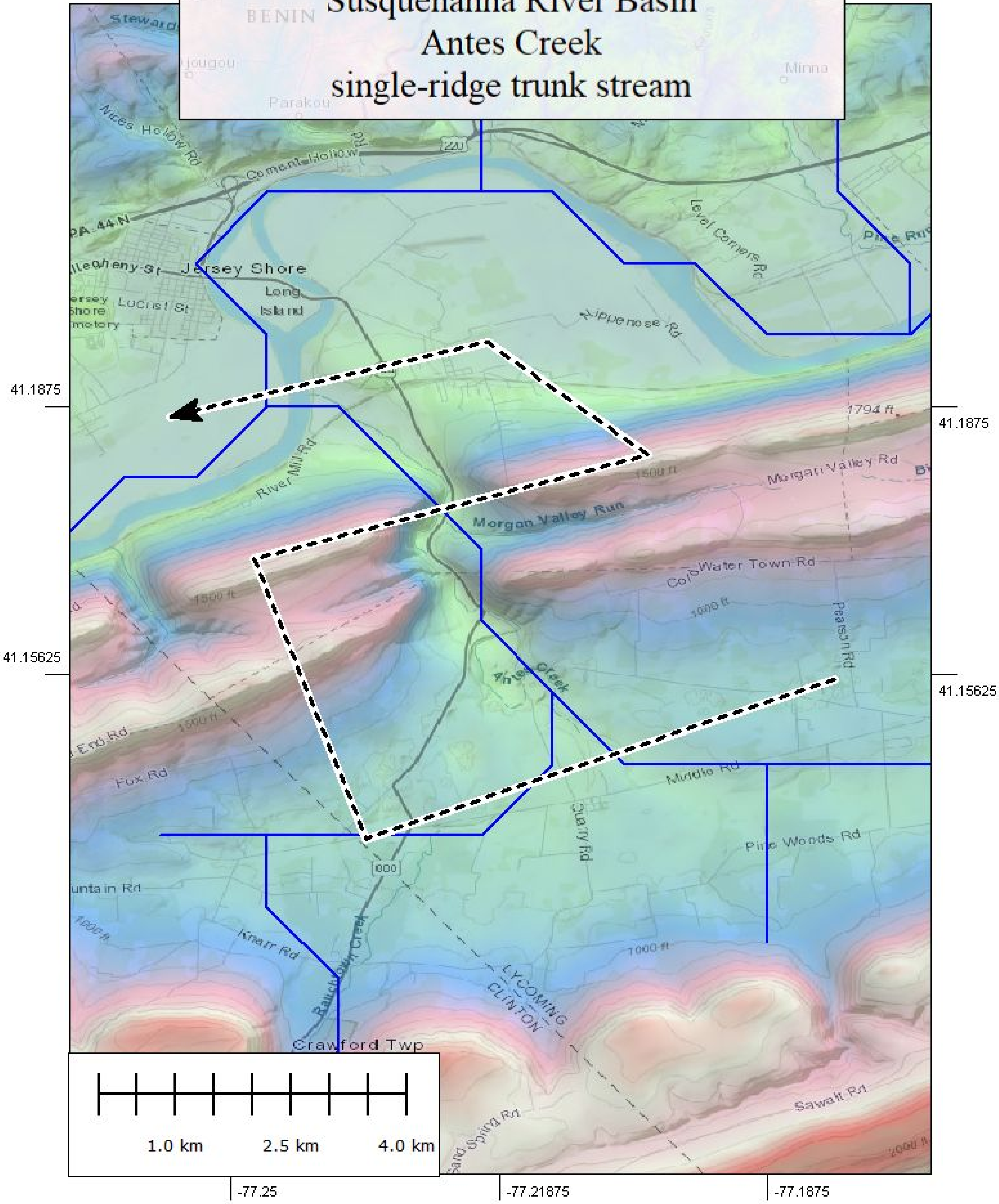

NA - 324
Susquehanna River Basin
Susquehanna River
single-ridge trunk stream

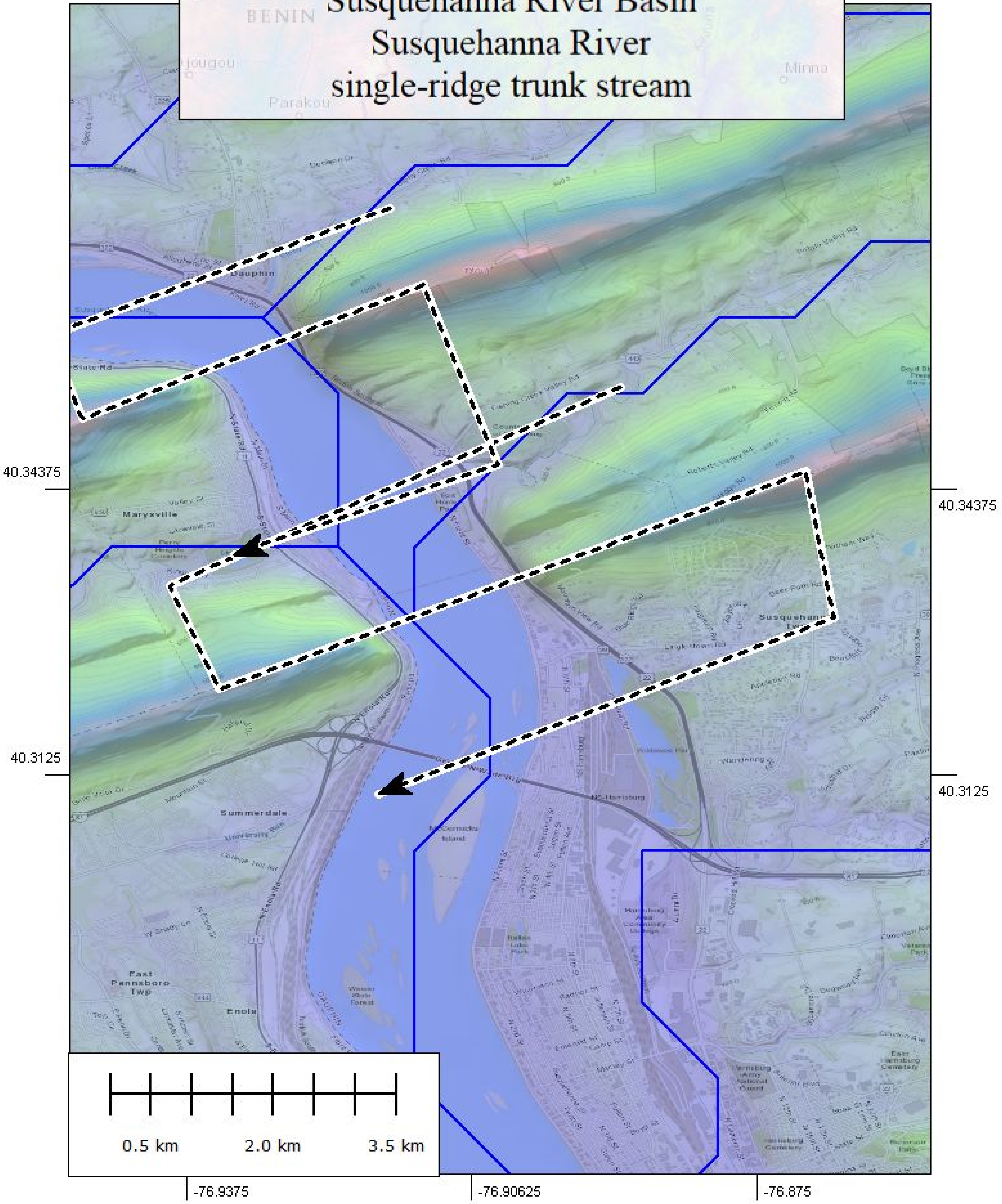

NA - 325
Susquehanna River Basin
Susquehanna River
single-ridge trunk stream

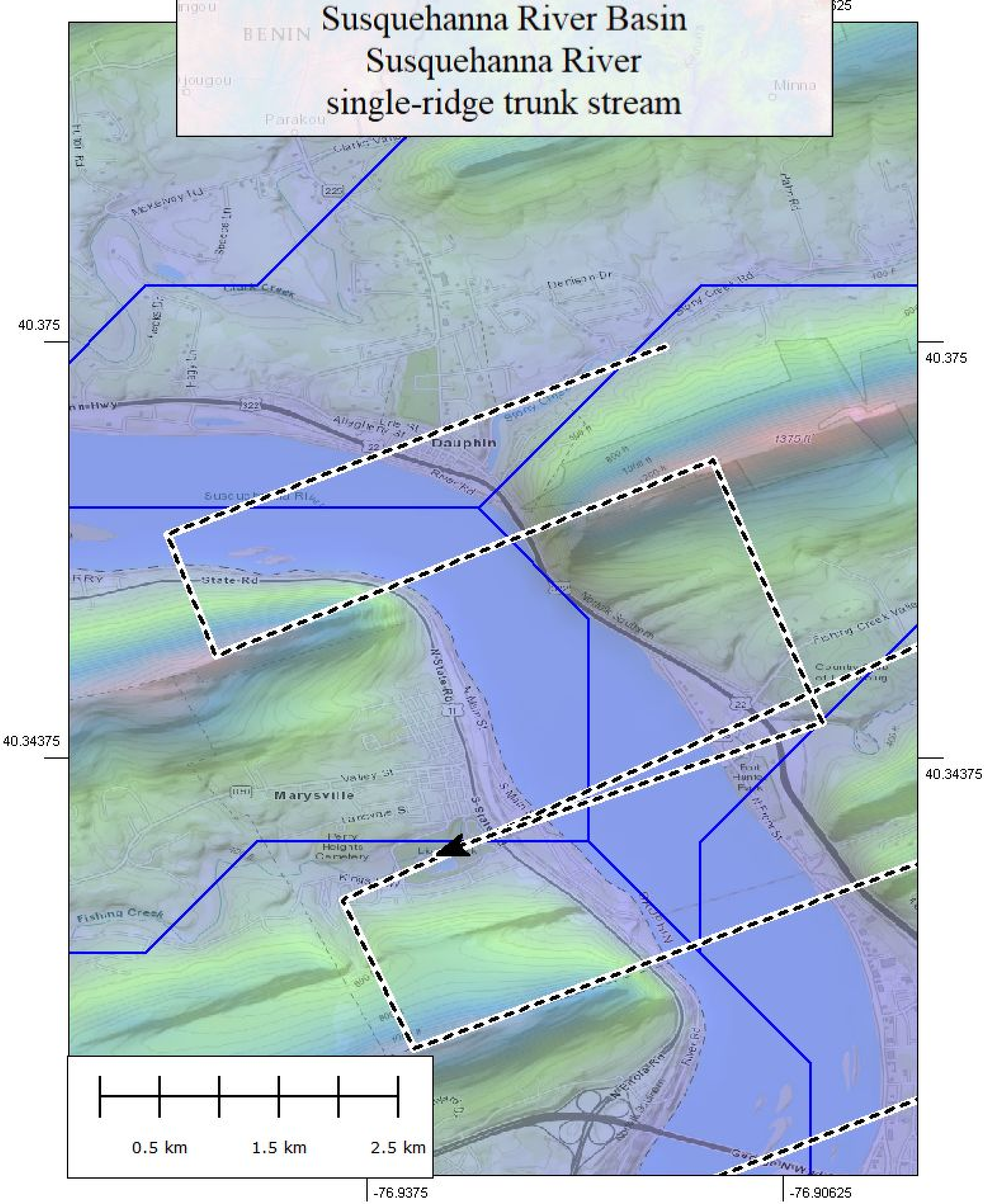

NA - 326
Susquehanna River Basin
Susquehanna River
single-ridge trunk stream

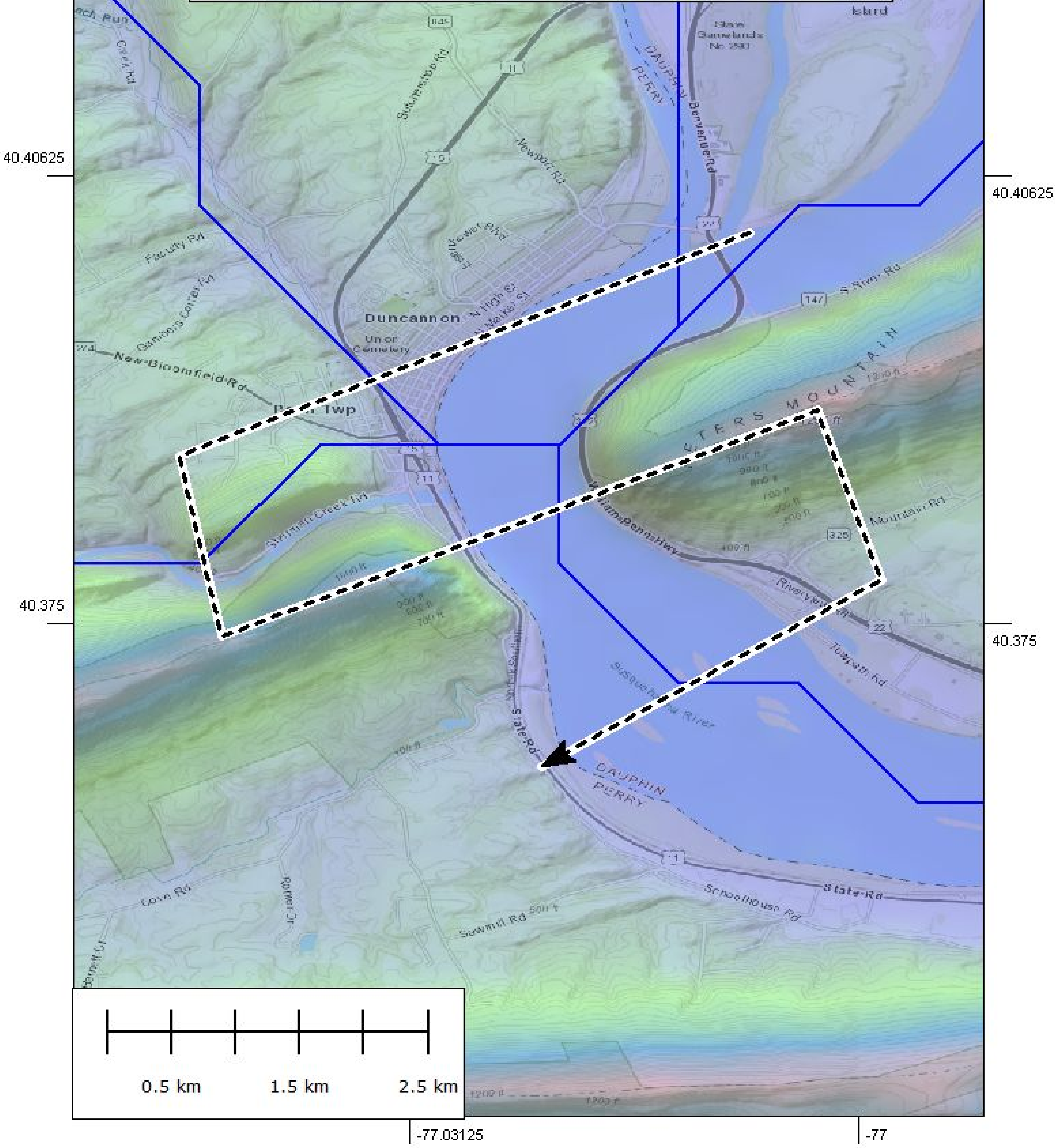

NA - 327
Susquehanna River Basin
Catawissa Creek
single-ridge trunk stream

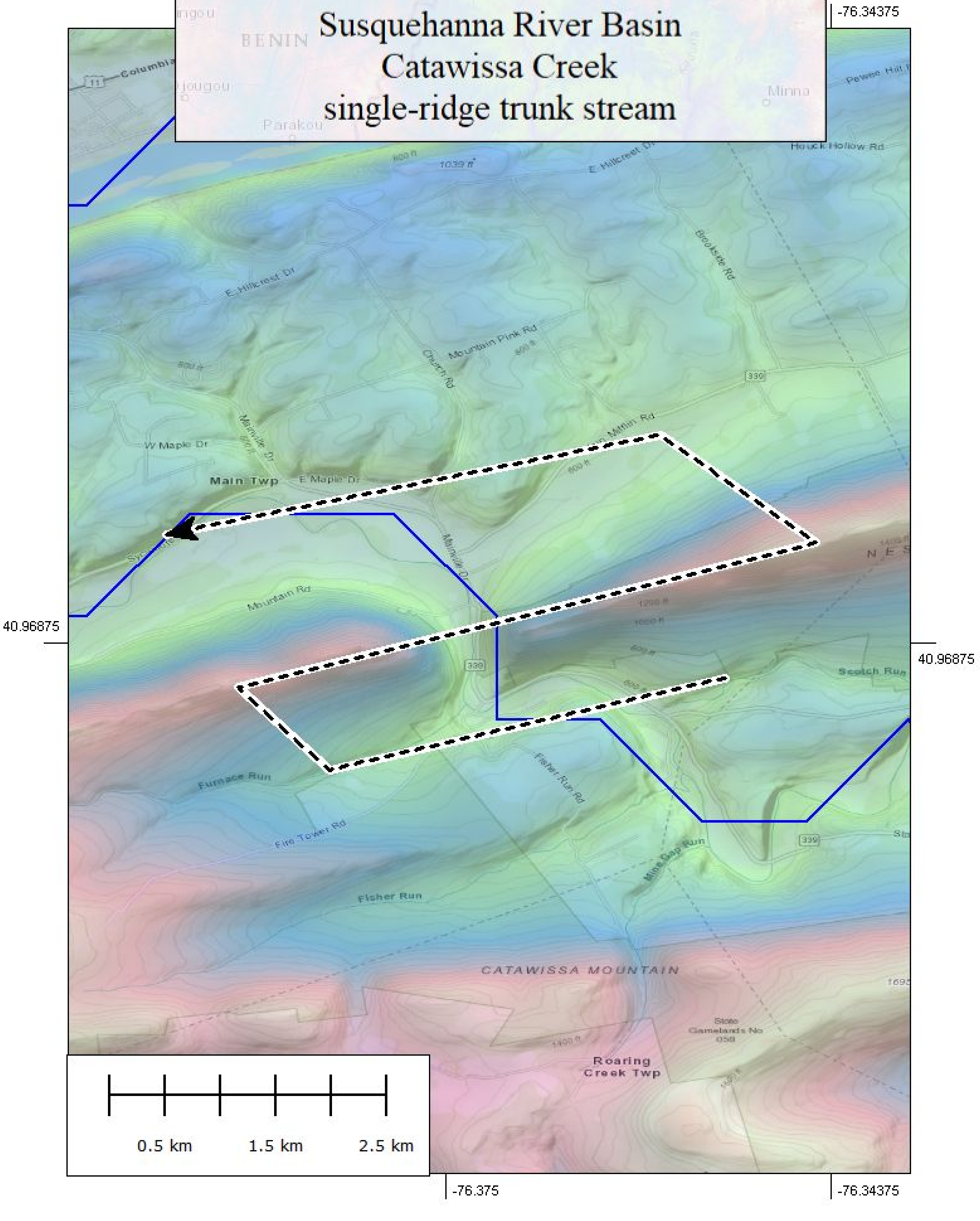

NA - 328
Mississippi River Basin
Pass Creek
irregular high ground trunk stream

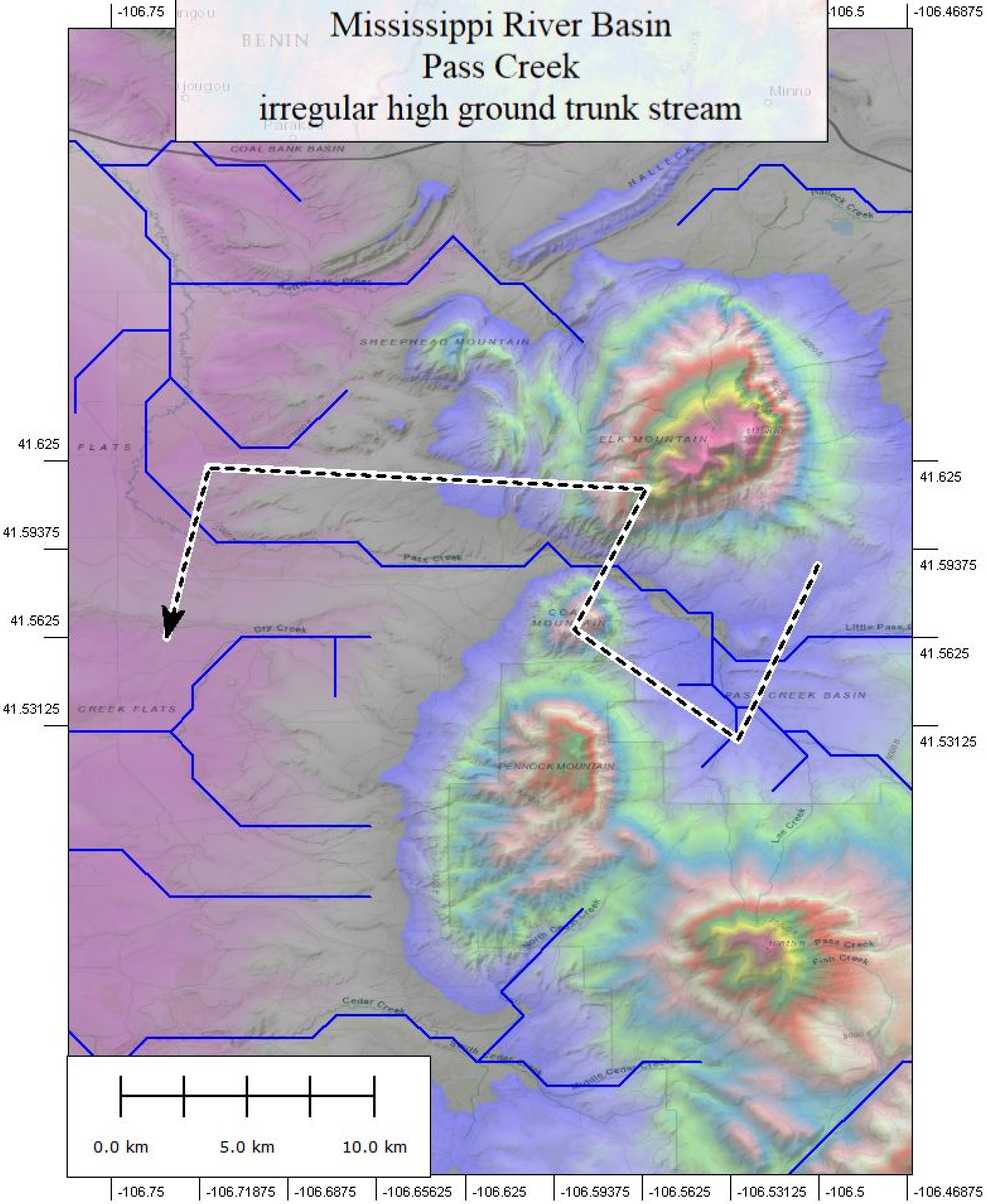

NA - 329
Colorado River (Arizona) Basin
Muddy Creek
single-ridge trunk stream

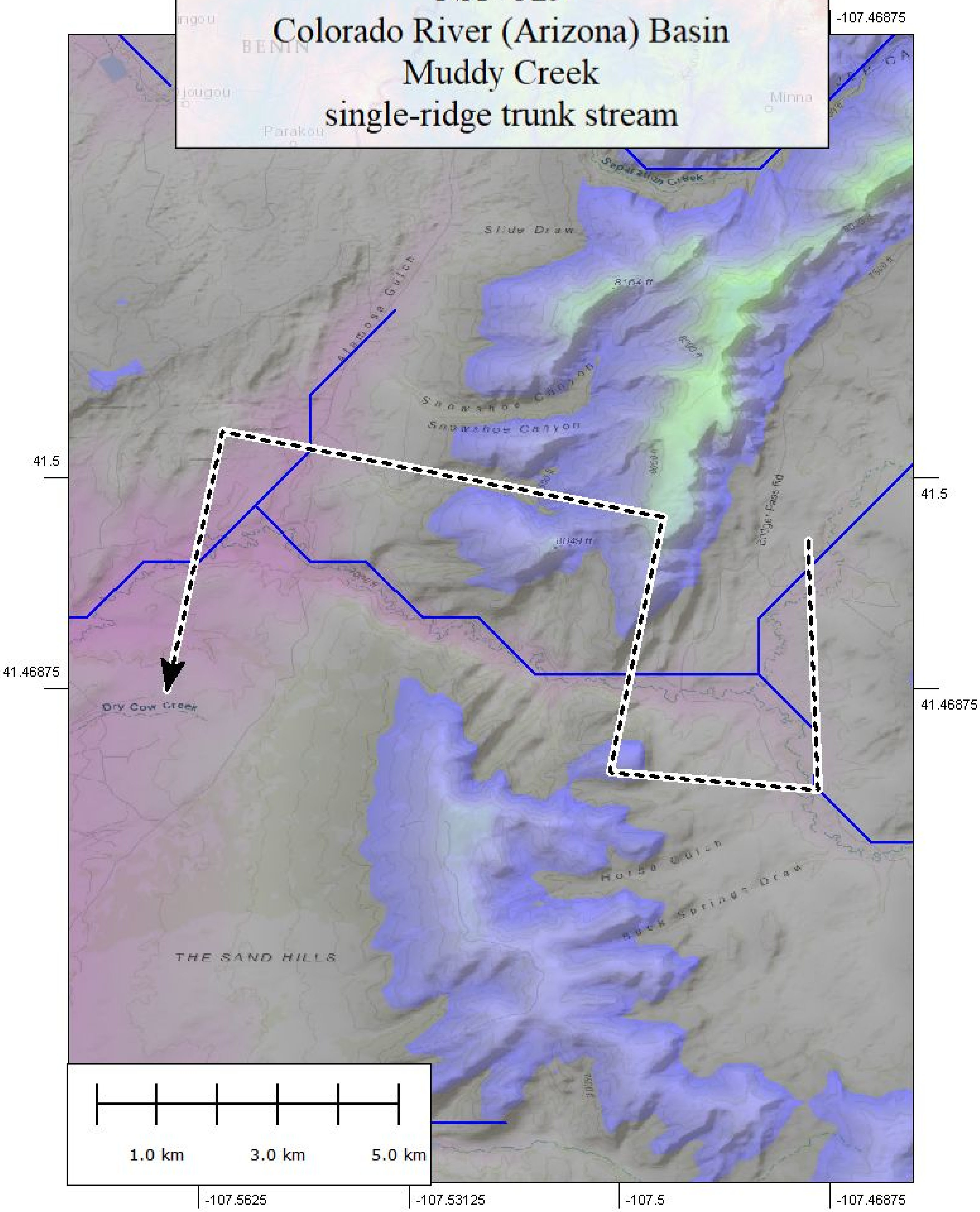

NA - 33
Mackenzie River (Canada) Basin
Cli Lake
single-ridge head stream

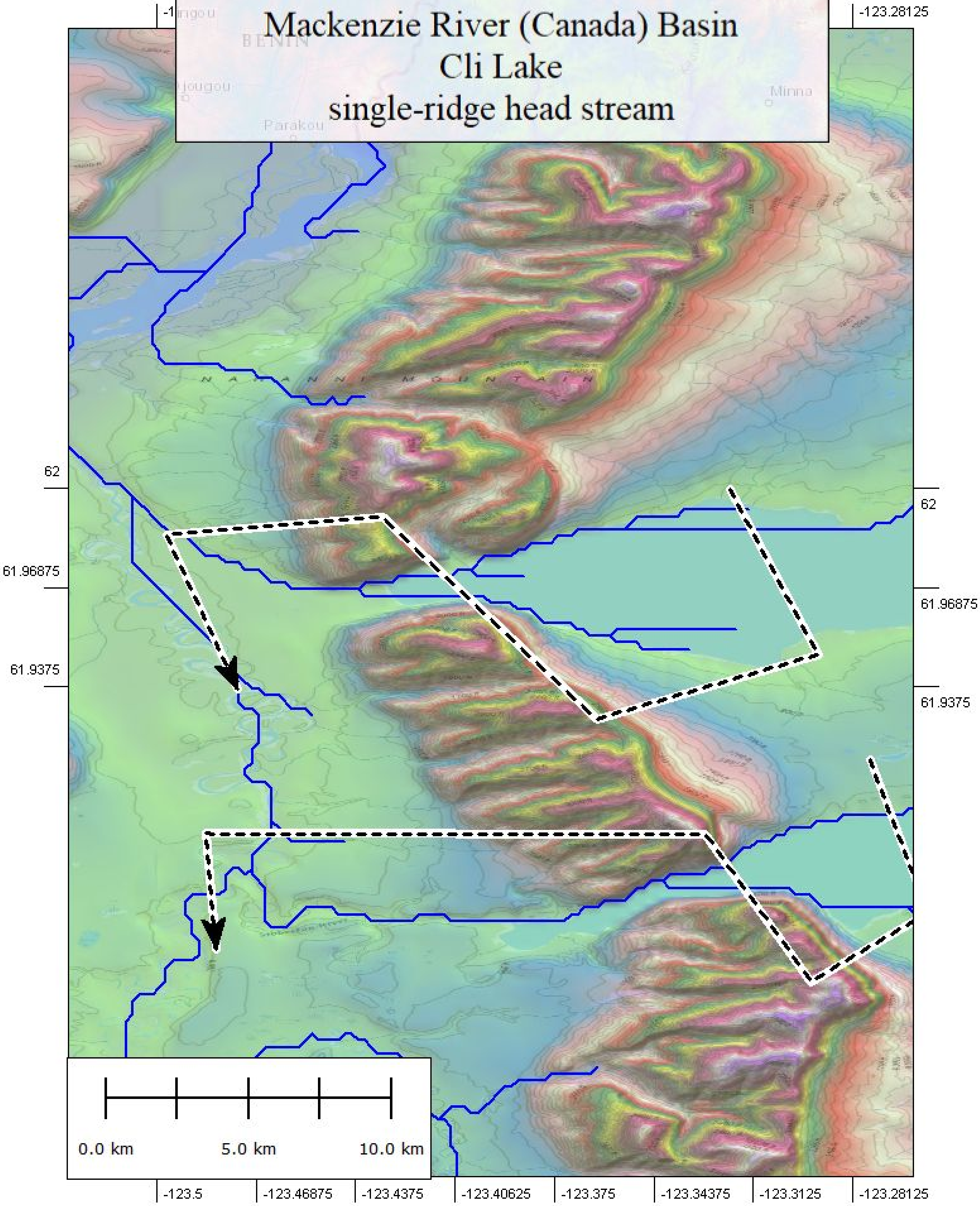

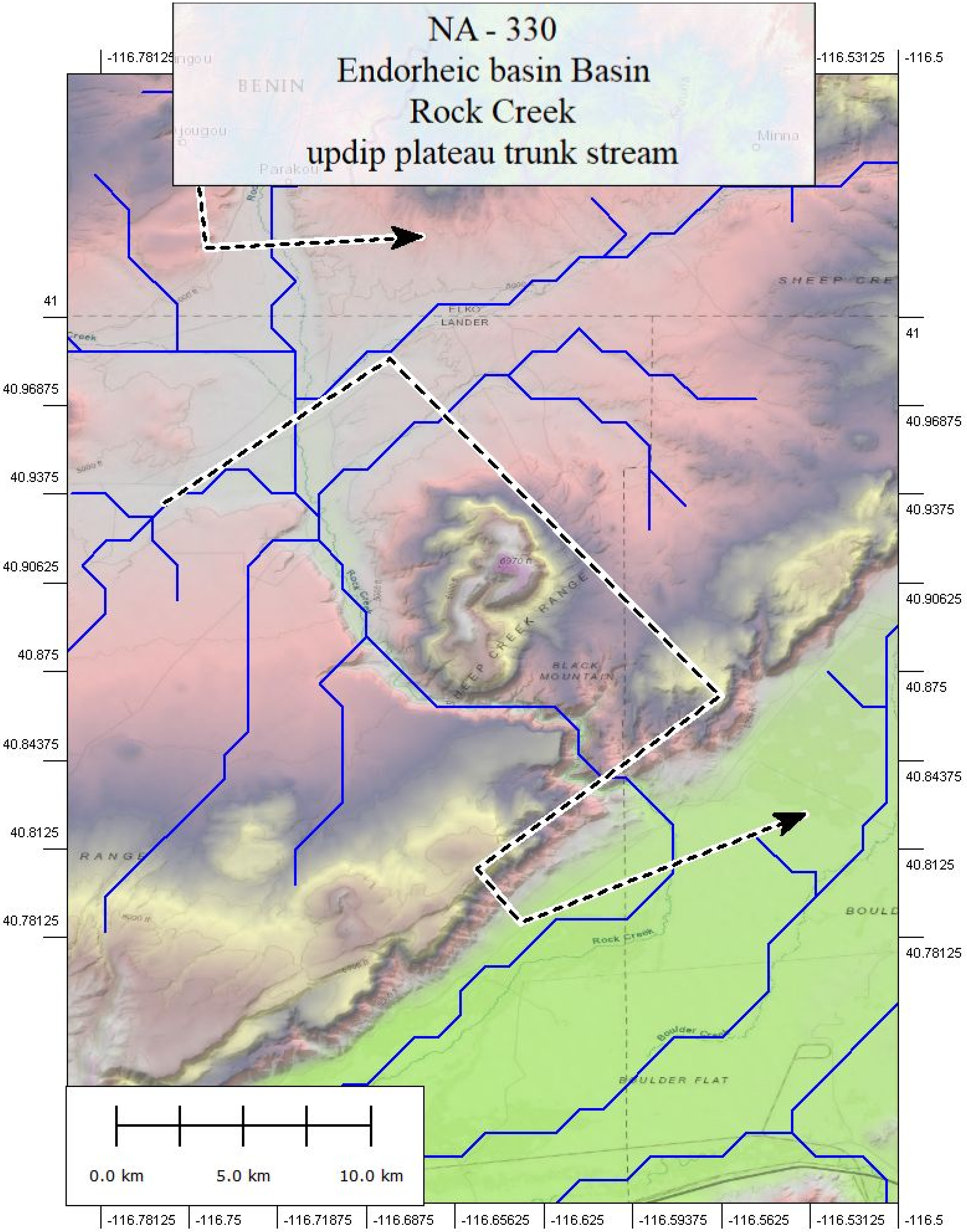
NA - 330
Endorheic basin Basin
Rock Creek
updip plateau trunk stream
-116.78125
-116.53125
-116.5
BENIN
Djougou
Parakou
Minna
41
40.96875
40.9375
40.90625
40.875
40.84375
40.8125
40.78125
SHEEP CRE
ELKO
LANDER
Creek
Rock Creek
6970 ft
SHEEP CREEK RANGE
BLACK MOUNTAIN
RANGE
BOULD
Rock Creek
Boulder Creek
BOULDER FLAT
0.0 km
5.0 km
10.0 km
-116.75
-116.71875
-116.6875
-116.65625
-116.625
-116.59375
-116.5625

NA - 331
Rio Grande (Texas-Mexico) Basin
Arroyo La Cueva
single-ridge trunk stream

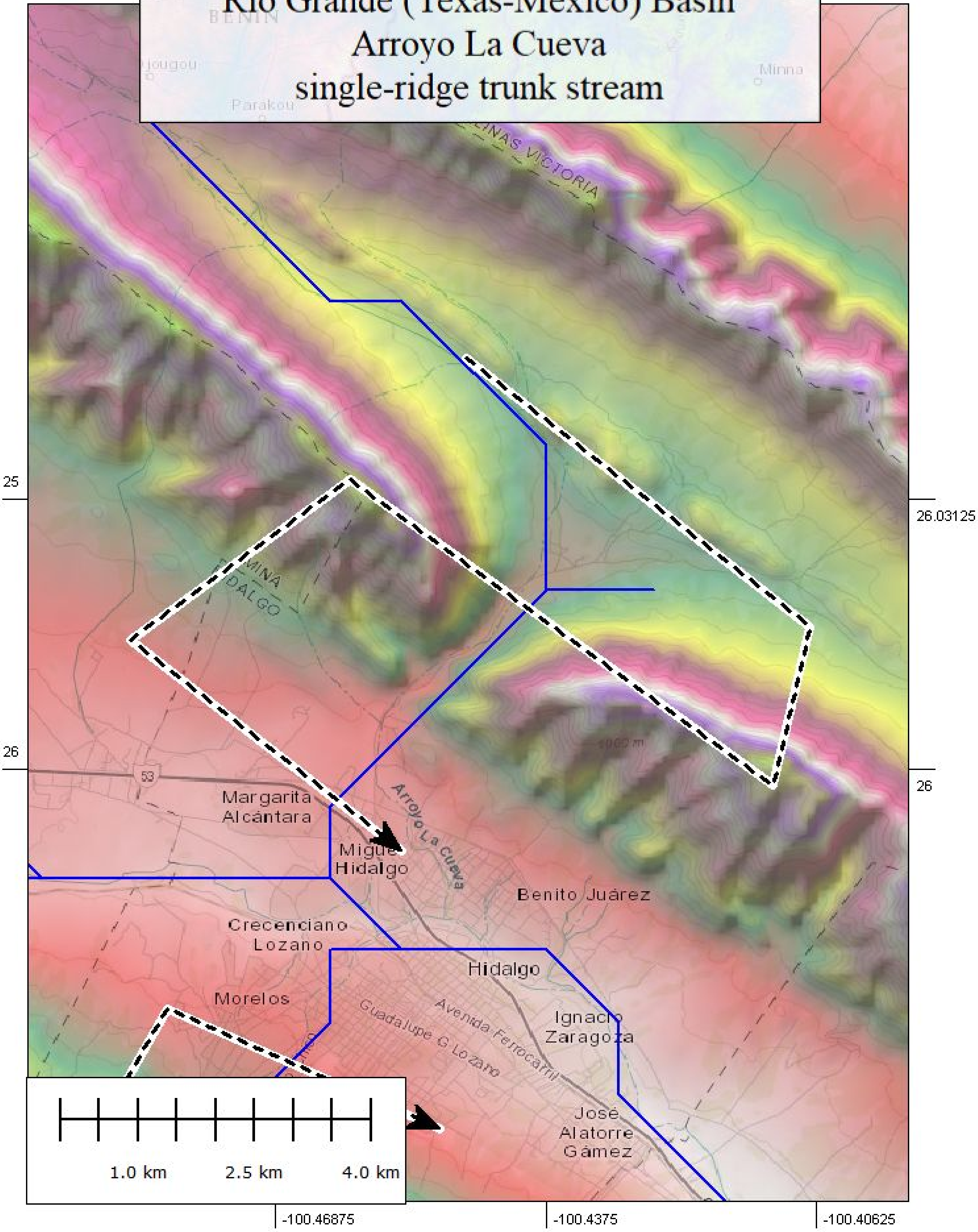

NA - 332
Endorheic basin Basin
Truckee River
irregular high ground trunk stream

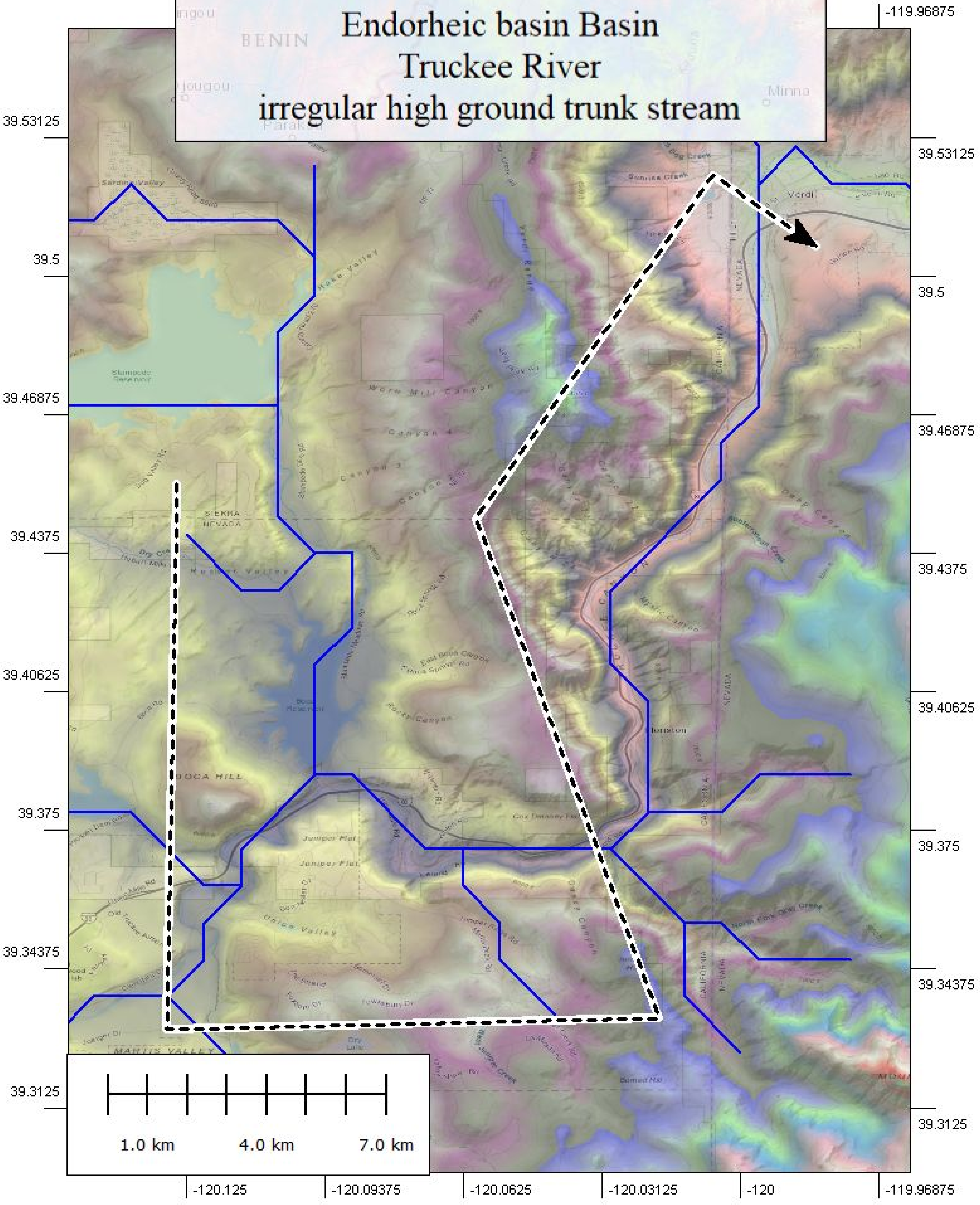

NA - 333
Potomac River Basin
Wills Creek tributary
single-ridge trunk stream

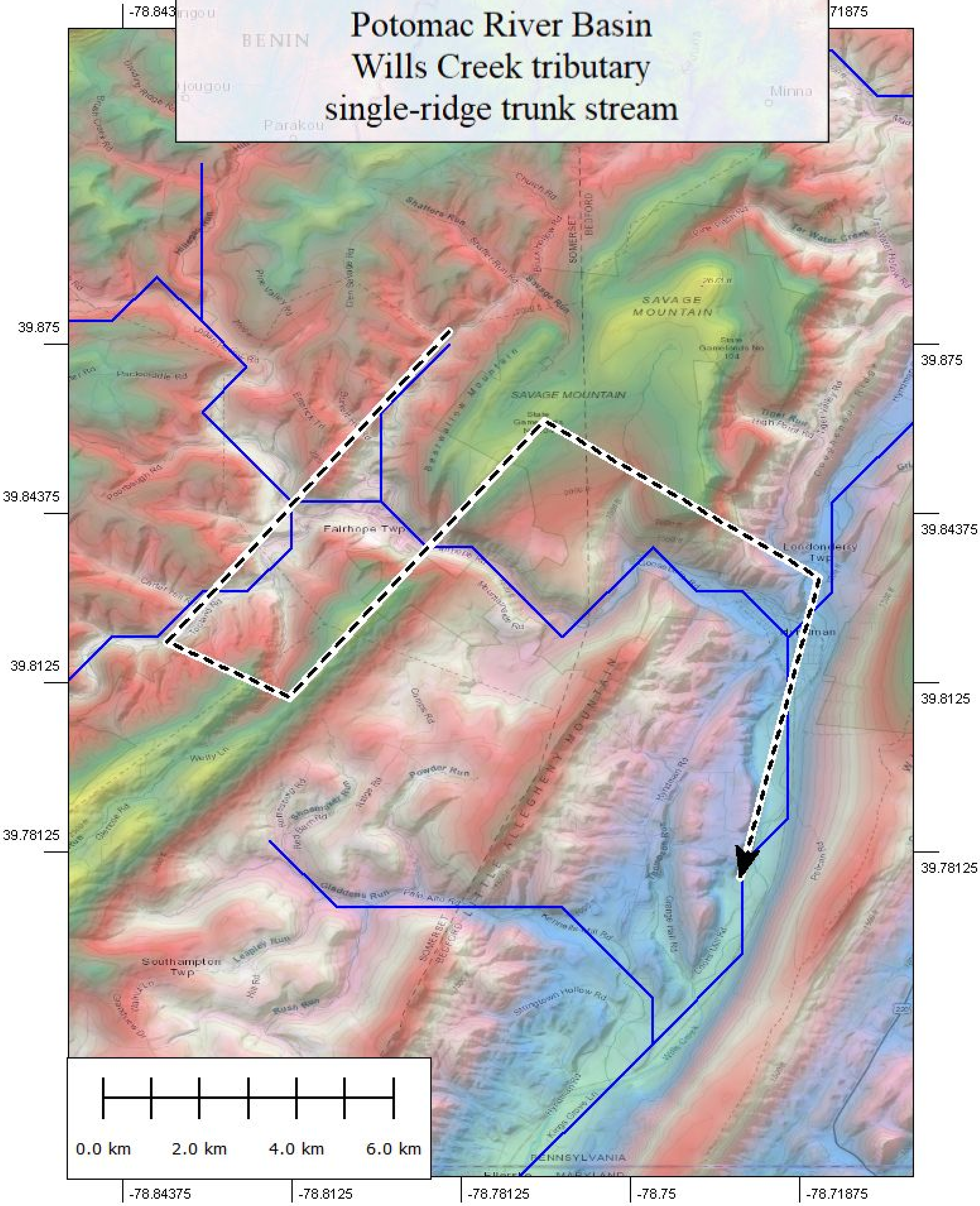

NA - 334
Colorado River (Arizona) Basin
Black Creek
plateau trunk stream

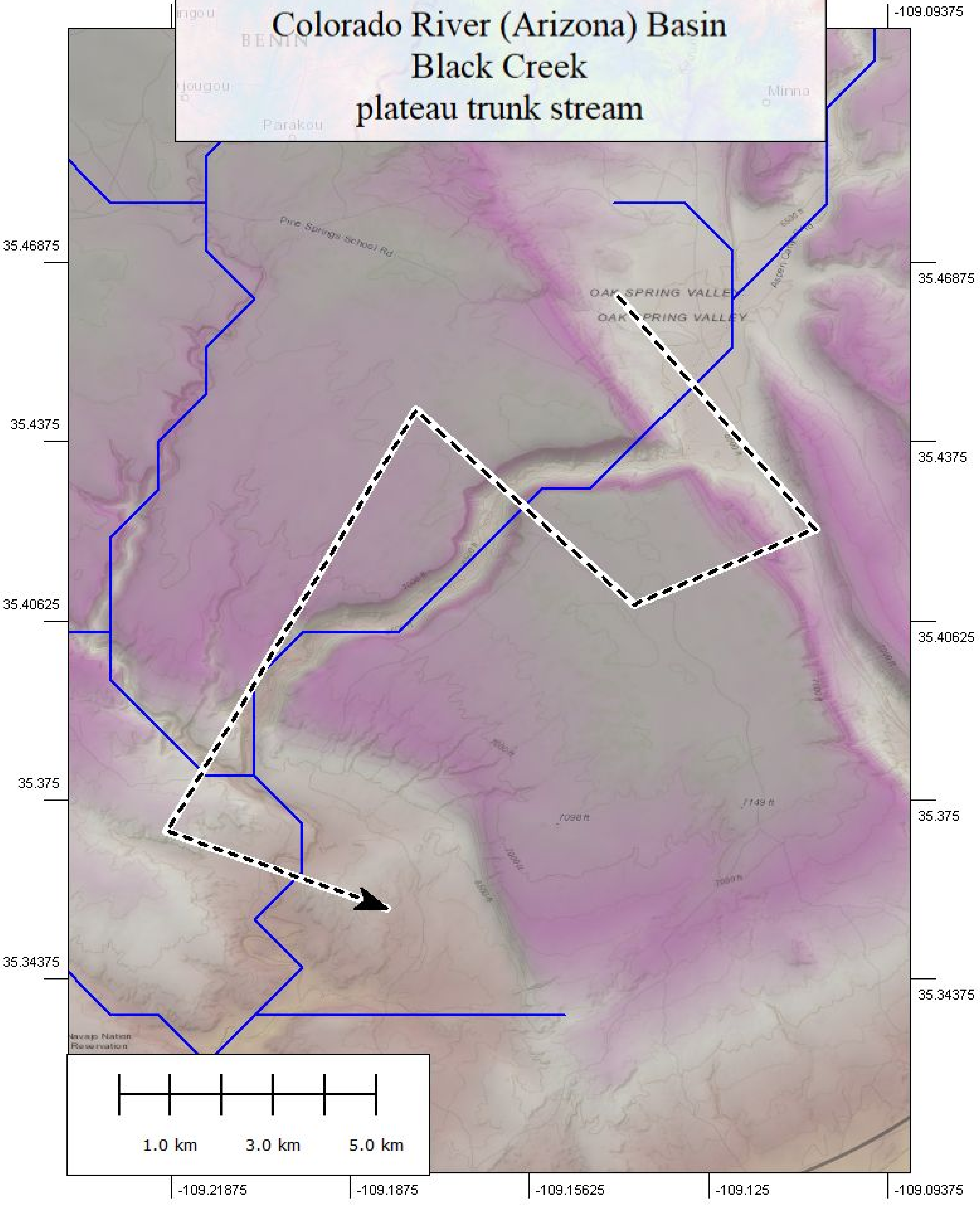

NA - 335
Endorheic basin Basin
Sheep Creek
single-ridge trunk stream

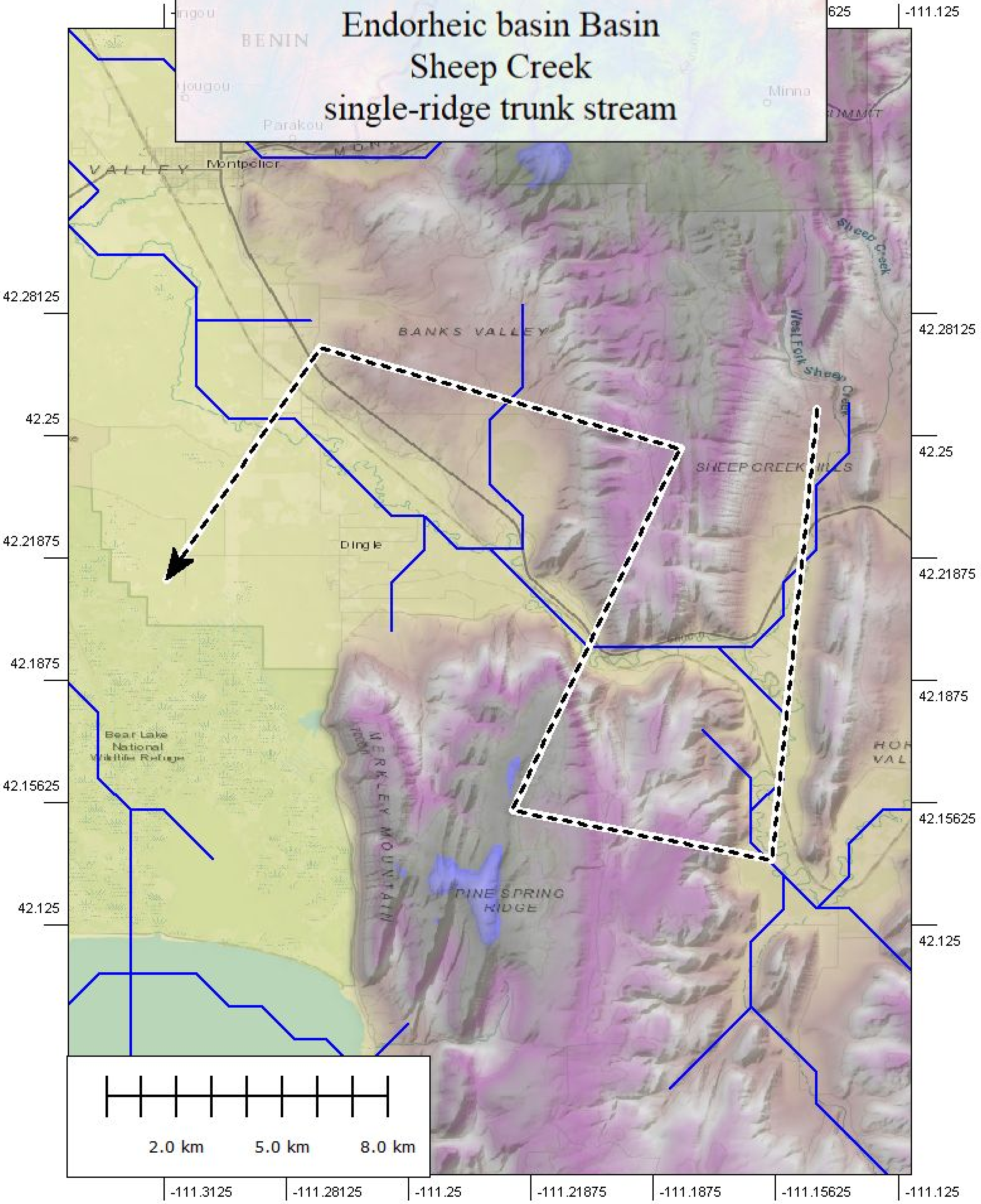

NA - 336
Endorheic basin Basin
Bear River
single-ridge trunk stream

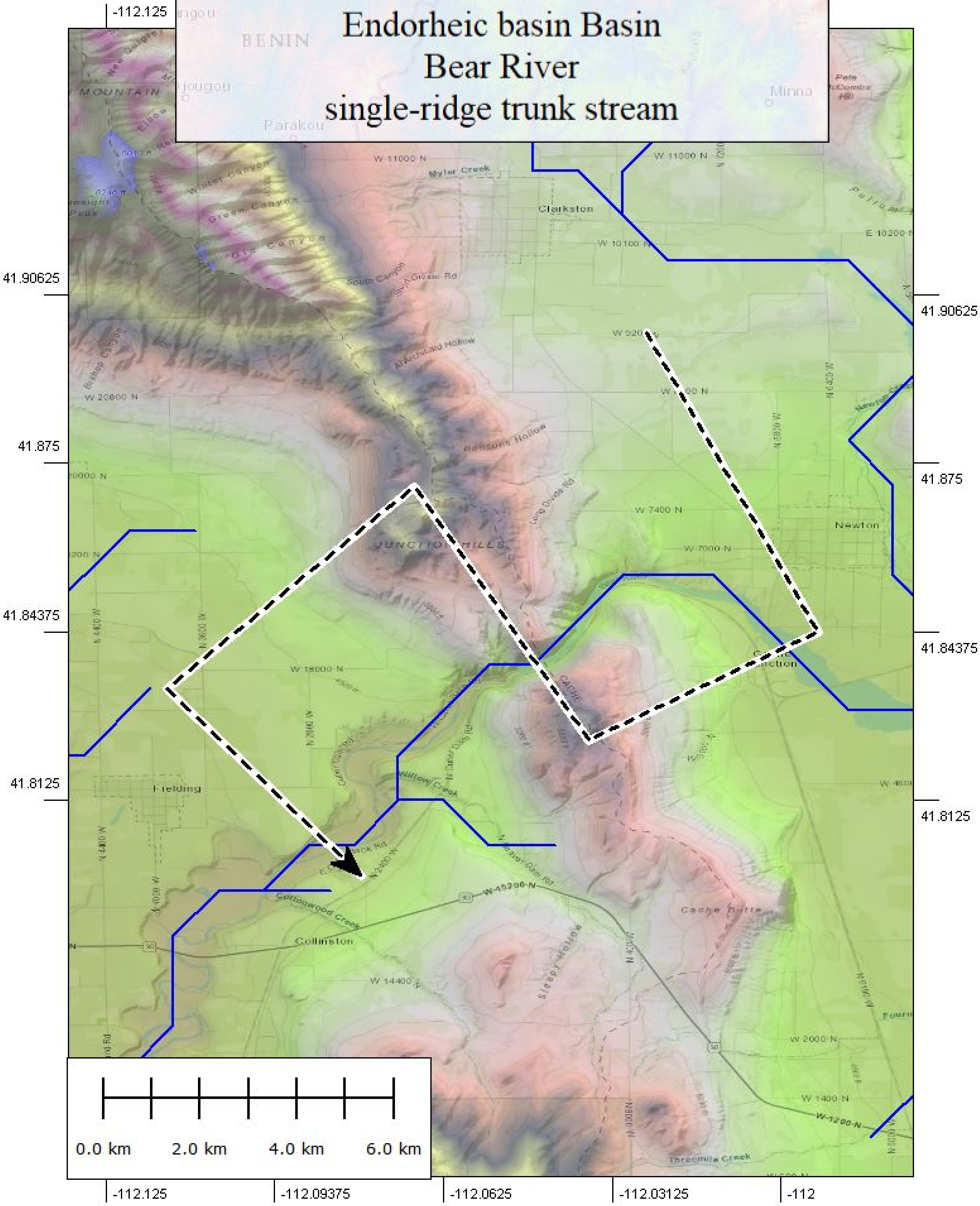

NA - 337
Mississippi River Basin
Big Hole River
irregular high ground trunk stream

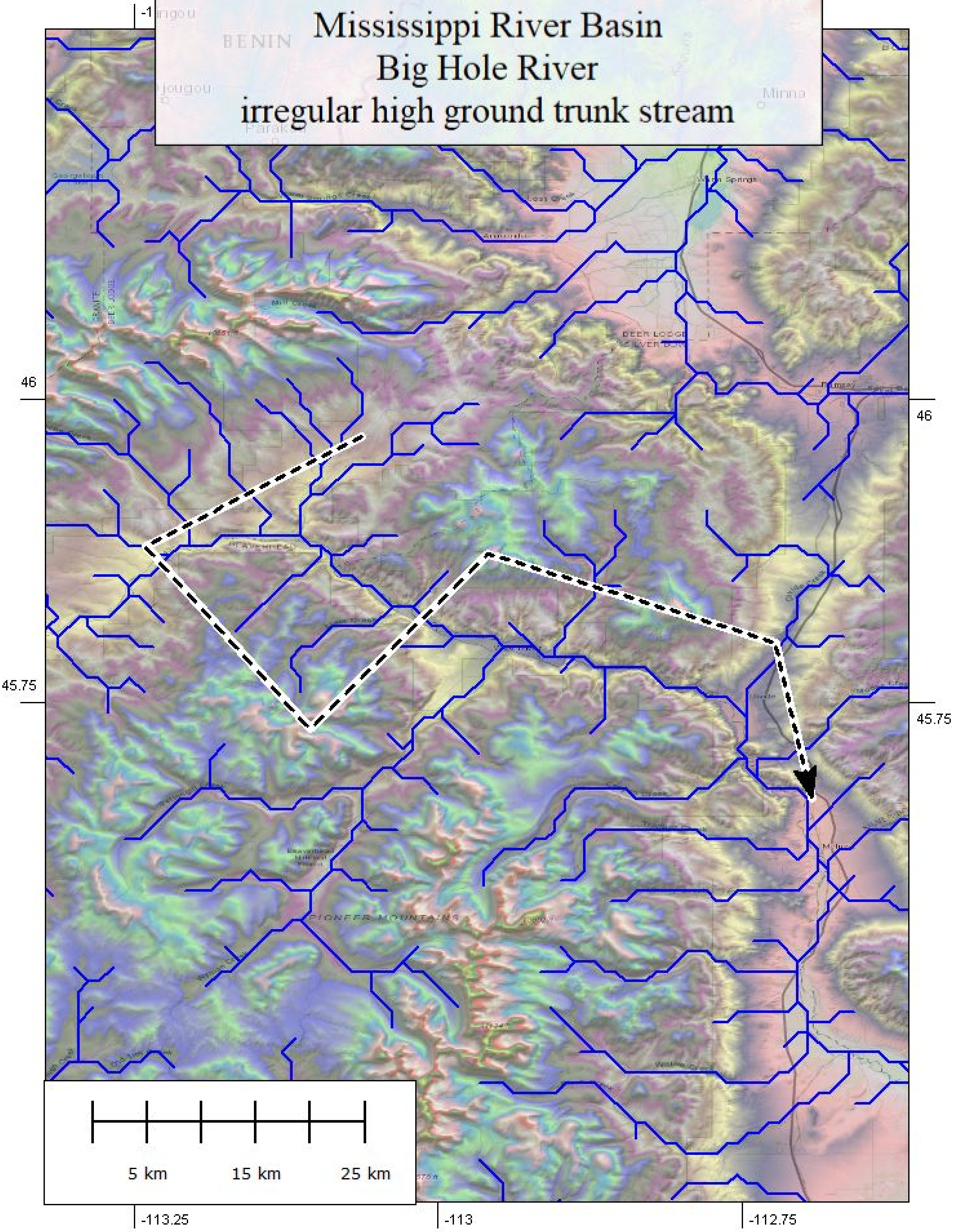

NA - 338
Mississippi River Basin
Big Sheep Creek
single-ridge trunk stream

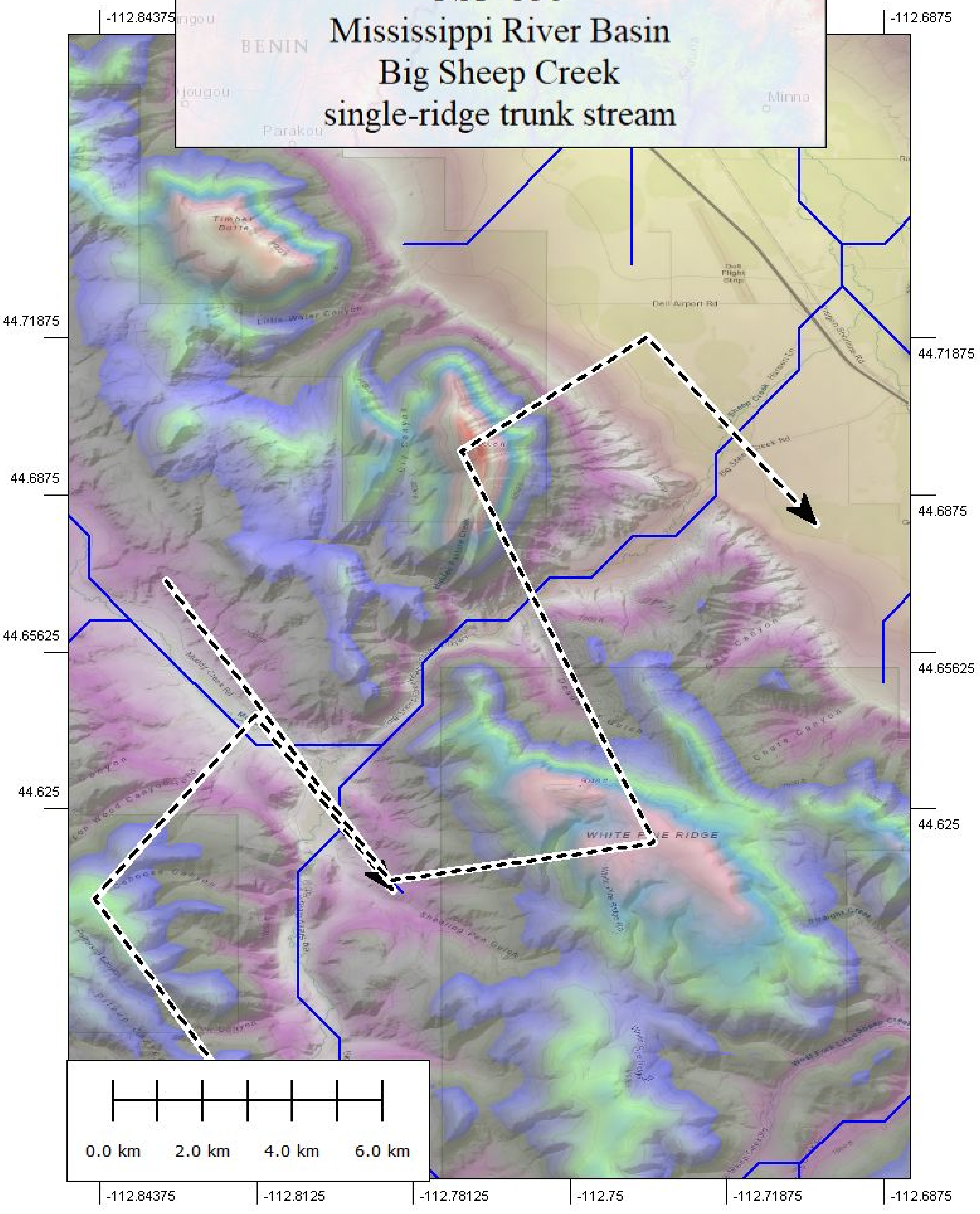

NA - 339
Mississippi River Basin
Bighorn River, Bighorn Canyon
irregular high ground trunk stream

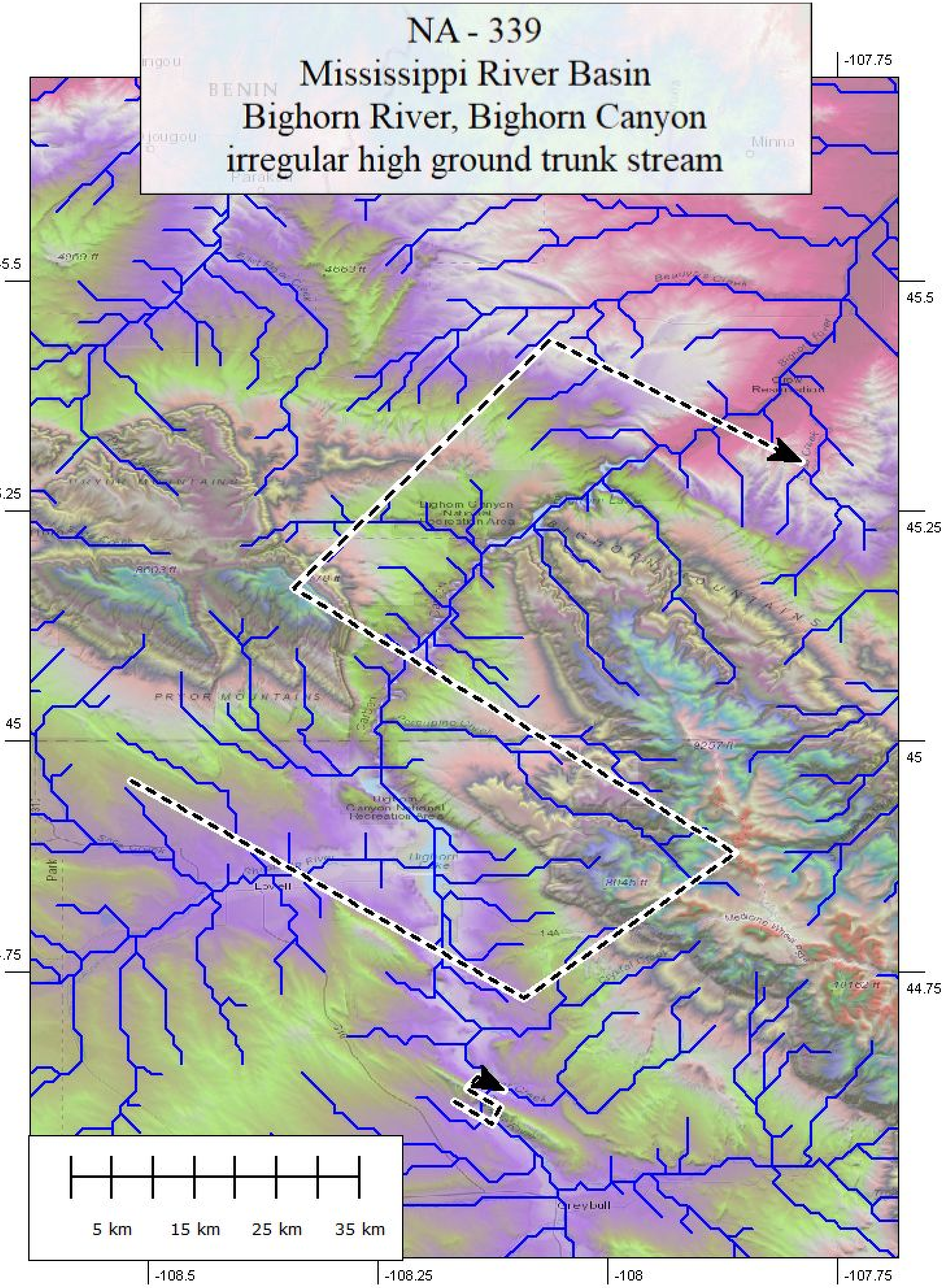

NA - 34
Mackenzie River (Canada) Basin
single-ridge head stream

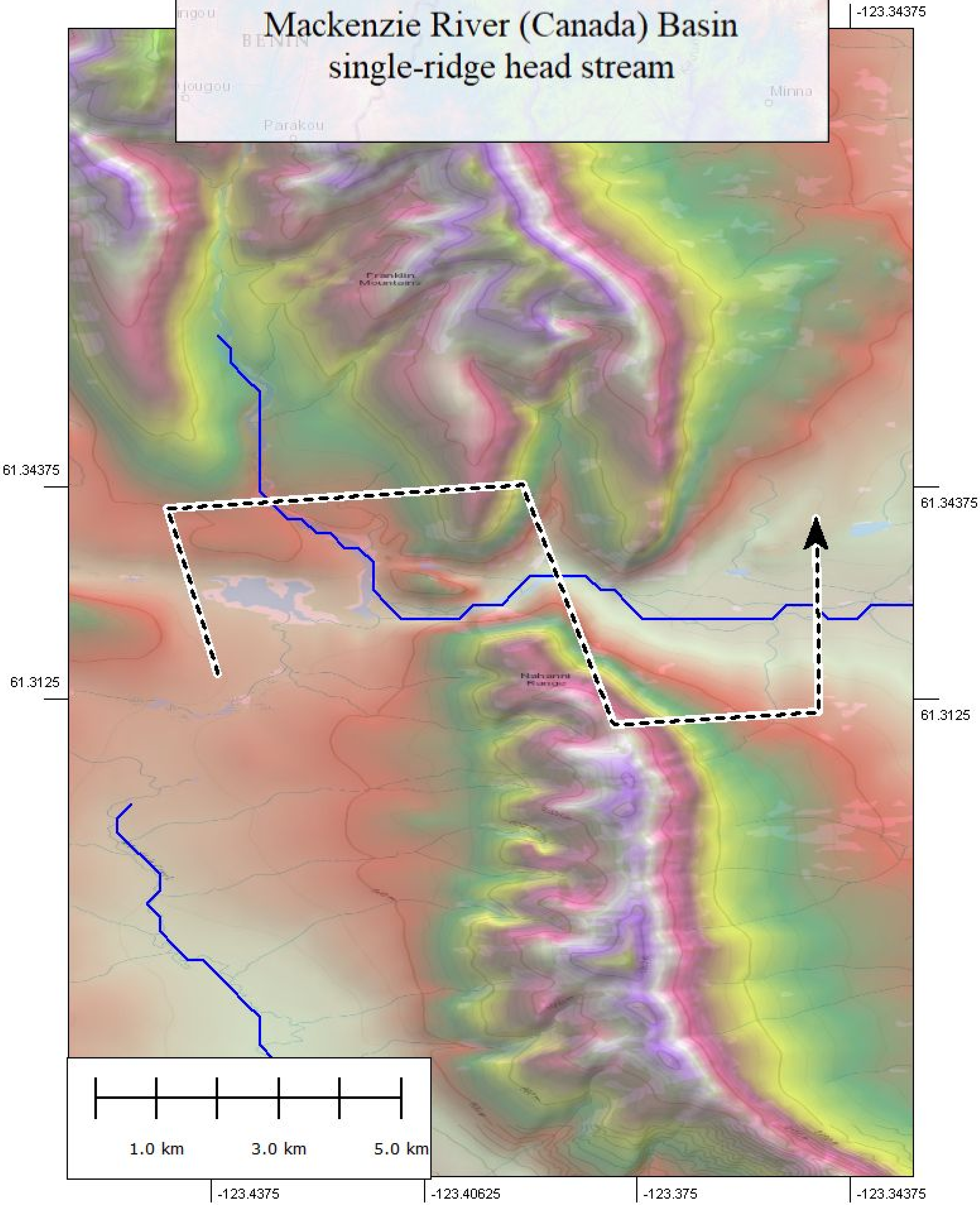

NA - 340
Columbia River Basin
Blackfoot River
irregular high ground trunk stream

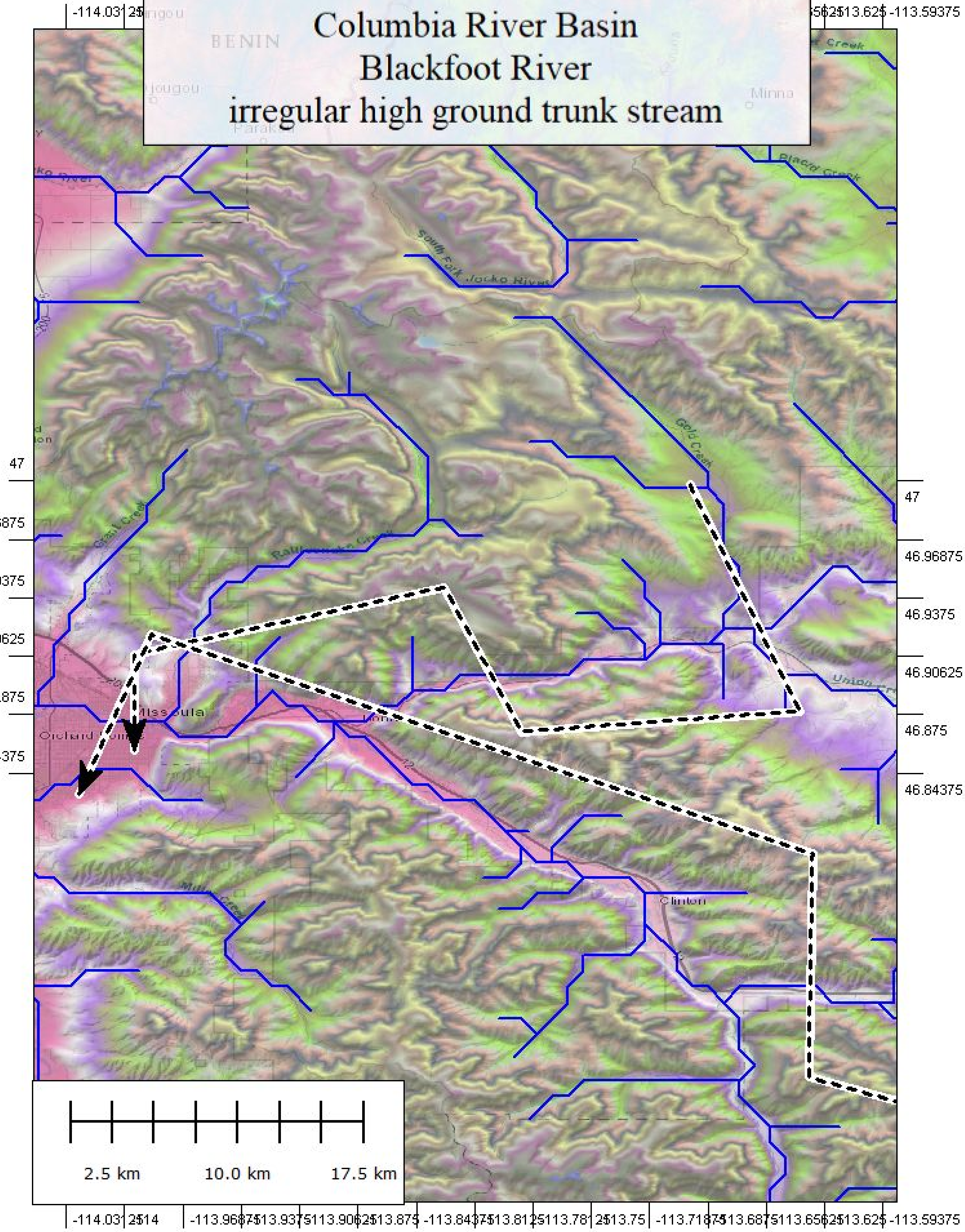

NA - 341
Mississippi River Basin
Casselman River
single-ridge trunk stream

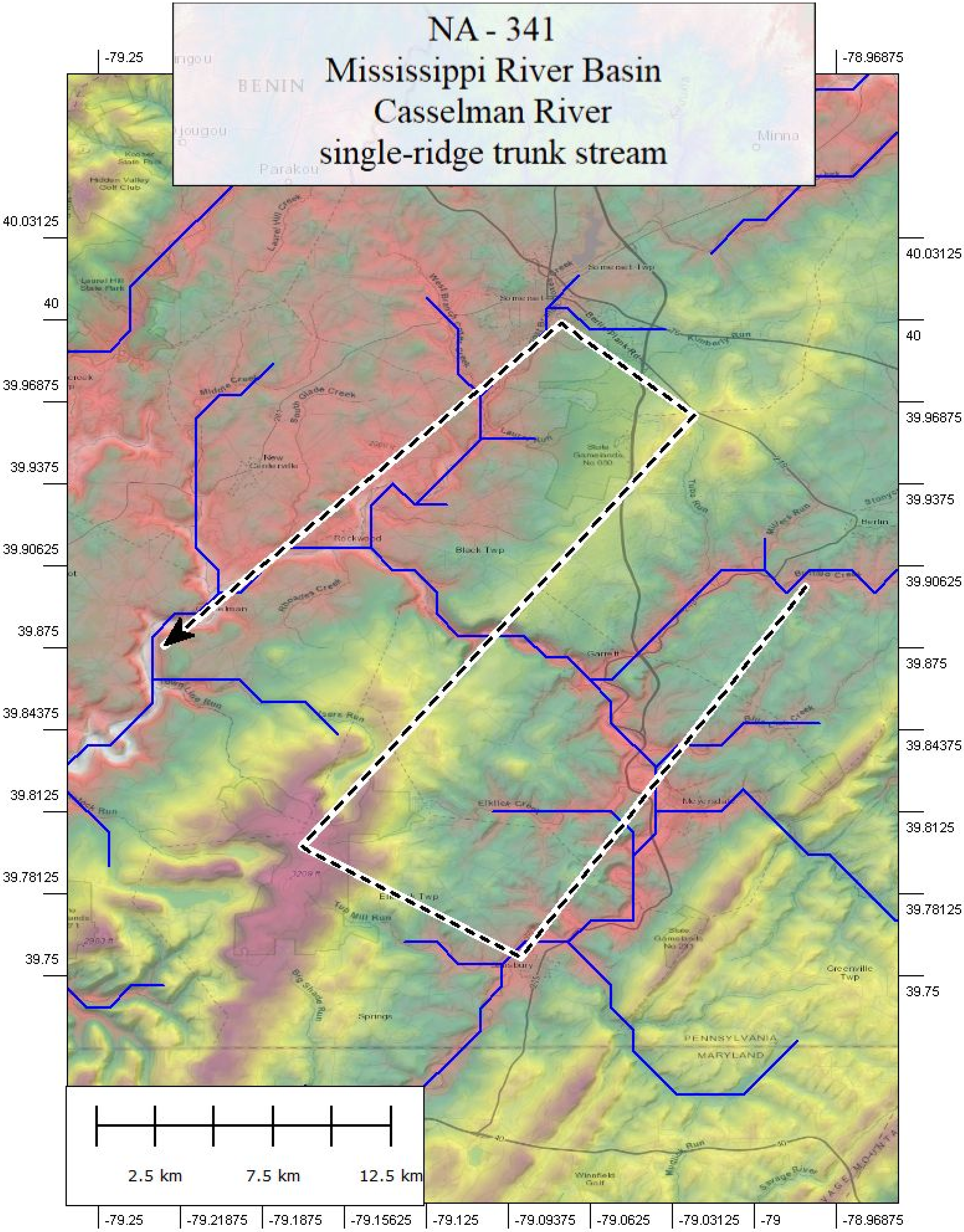

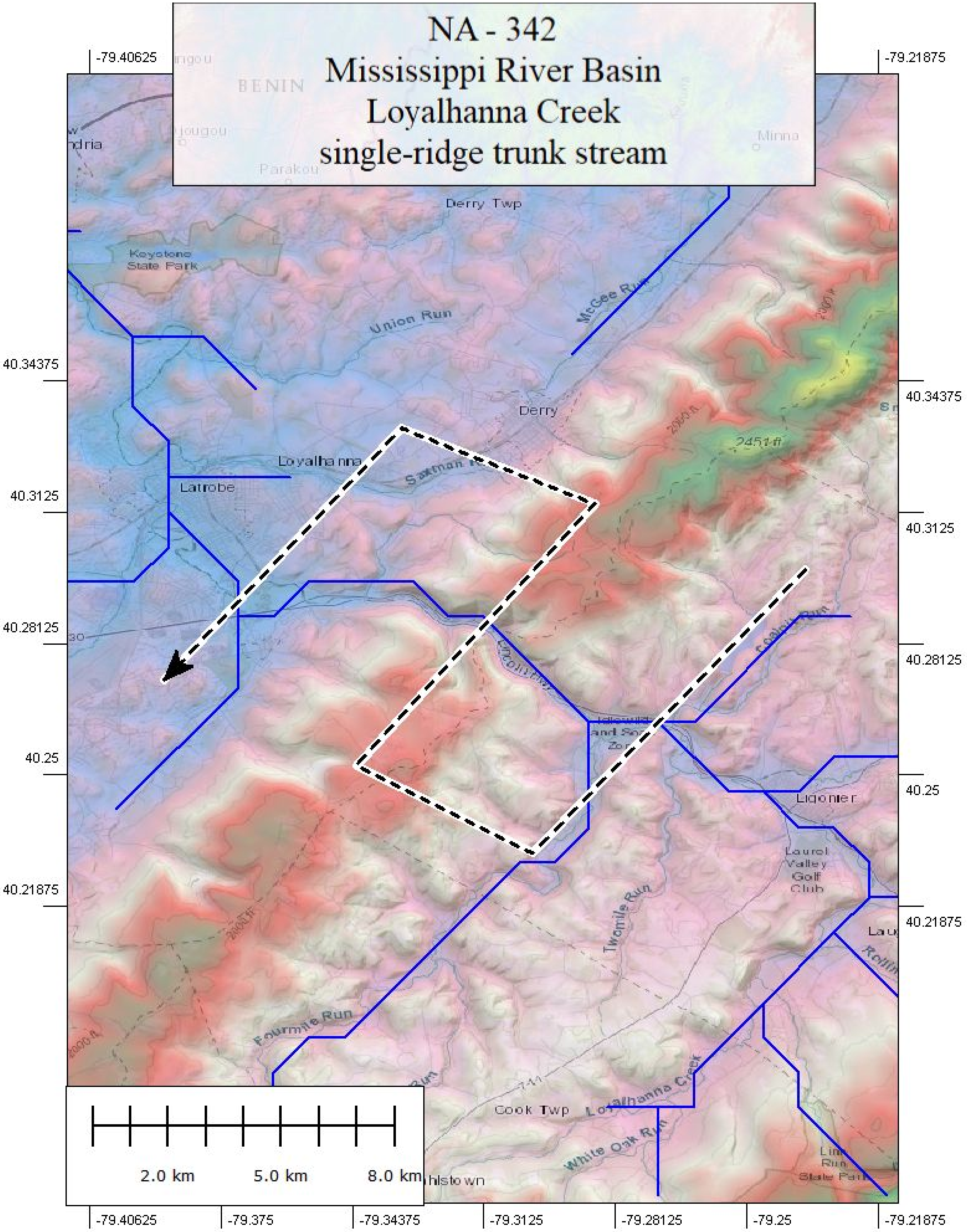
NA - 342
Mississippi River Basin
Loyalhanna Creek
single-ridge trunk stream
-79.40625
-79.21875
Derry Twp
Keystone State Park
Union Run
McGee Run
40.34375
Derry
Loyalhanna
Latrobe
40.3125
40.28125
40.25
Ligonier
Laurel Valley Golf Club
40.21875
Twomile Run
Fourmile Run
Cook Twp
Loyalhanna Creek
White Oak Run
2.0 km
5.0 km
8.0 km
-79.375
-79.34375
-79.3125
-79.28125
-79.25

NA - 343
Mississippi River Basin
Conemaugh River
single-ridge trunk stream

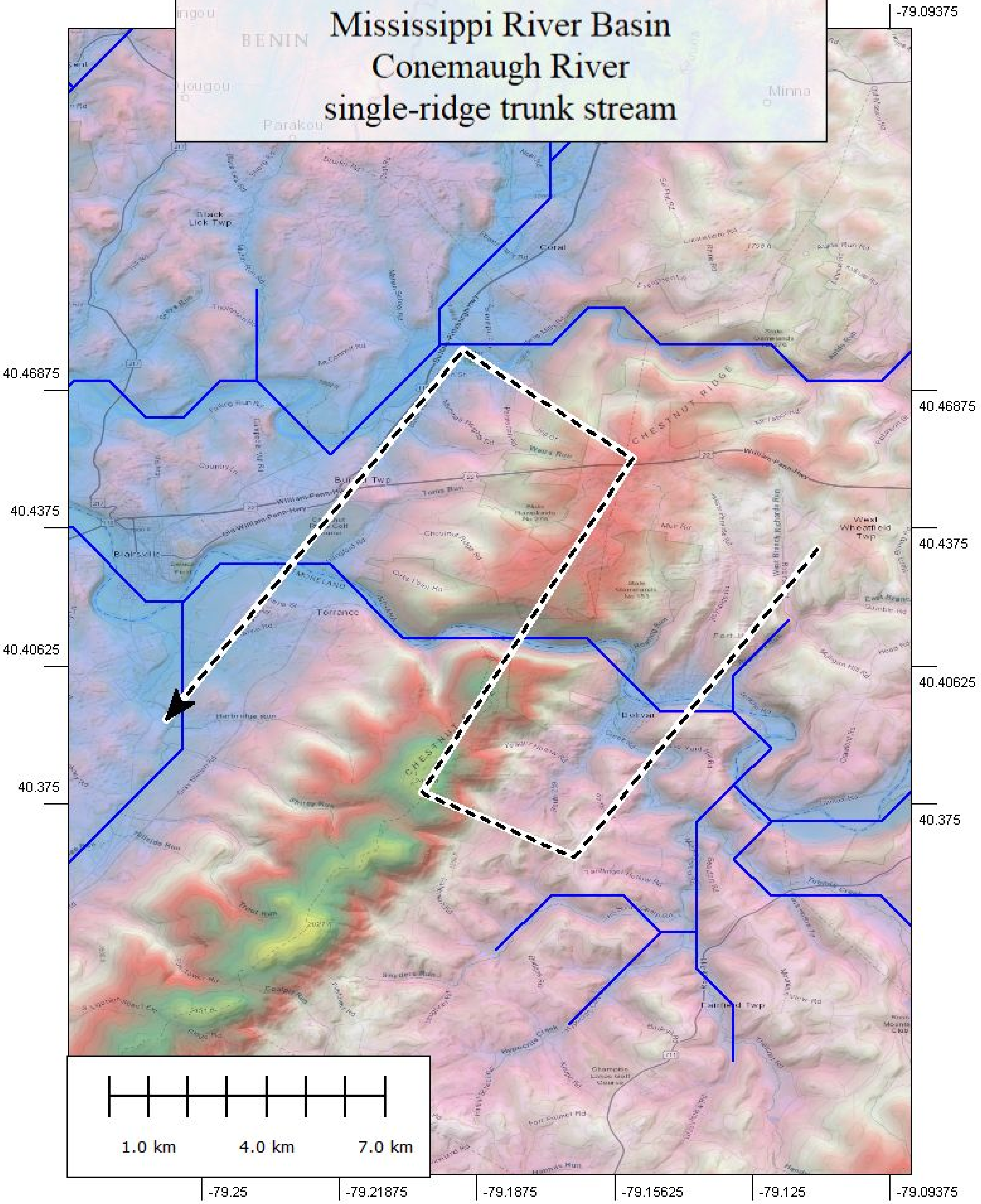

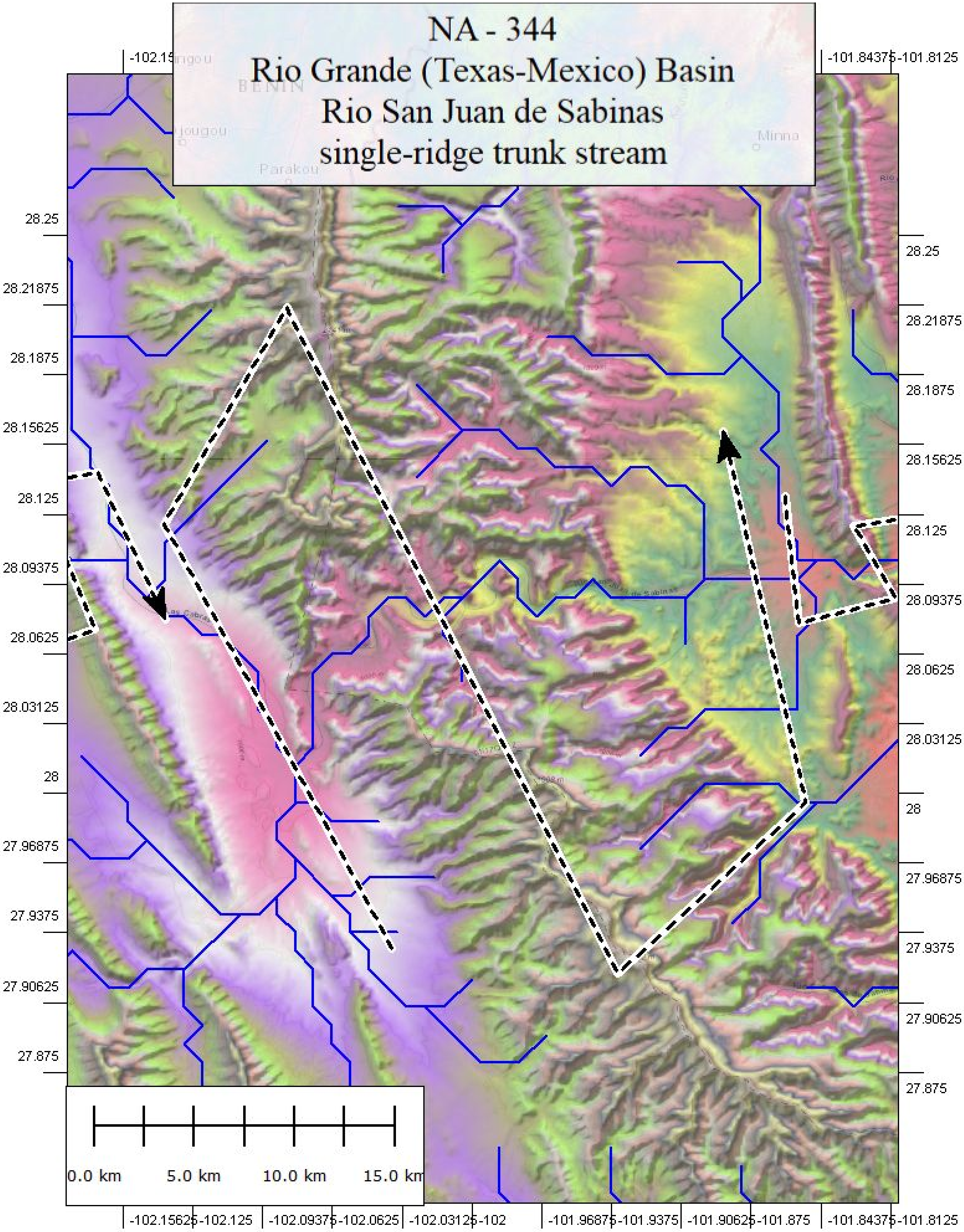
NA - 344
Rio Grande (Texas-Mexico) Basin
Rio San Juan de Sabinas
single-ridge trunk stream
-102.15625
-102.125
-102.09375
-102.0625
-102.03125
-102
-101.96875
-101.9375
-101.90625
-101.875
-101.84375
-101.8125
28.25
28.21875
28.1875
28.15625
28.125
28.09375
28.0625
28.03125
28
27.96875
27.9375
27.90625
27.875
0.0 km
5.0 km
10.0 km
15.0 km

NA - 345
Mississippi River Basin
Cheat River
irregular high ground trunk stream

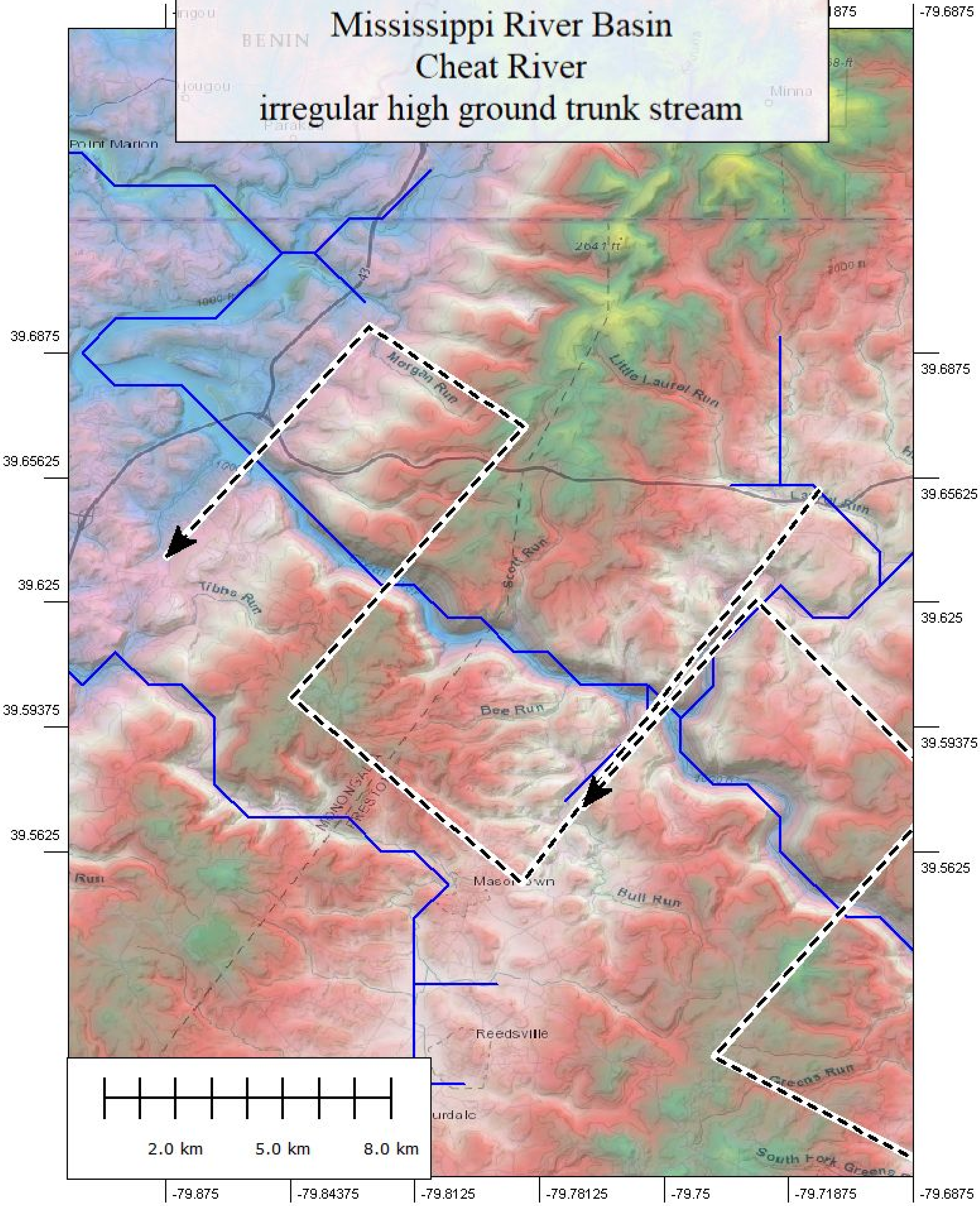

NA - 346
Mississippi River Basin
Cimarron River
irregular high ground trunk stream

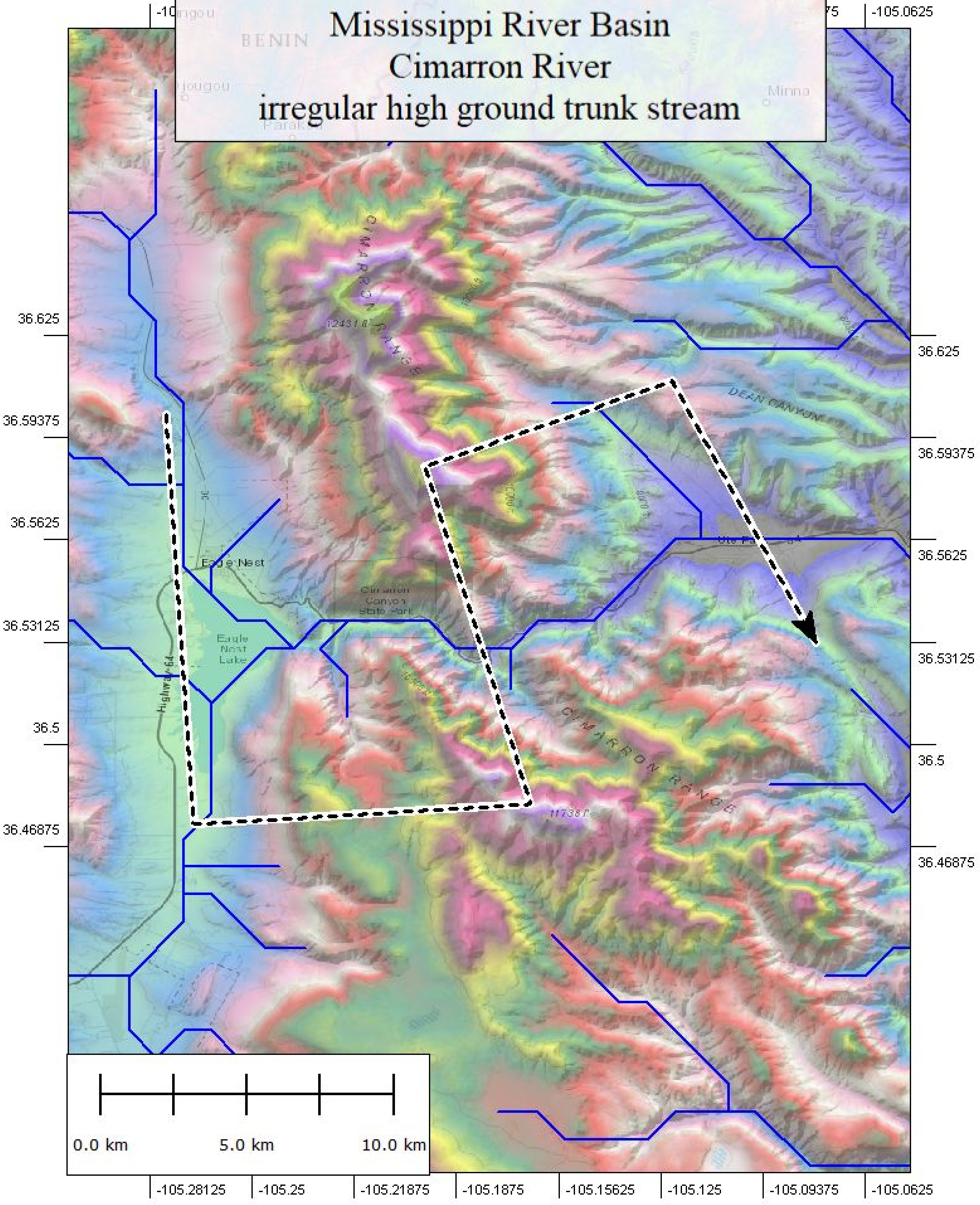

NA - 347
Rio Grande (Texas-Mexico) Basin
Pesqueria River
single-ridge trunk stream

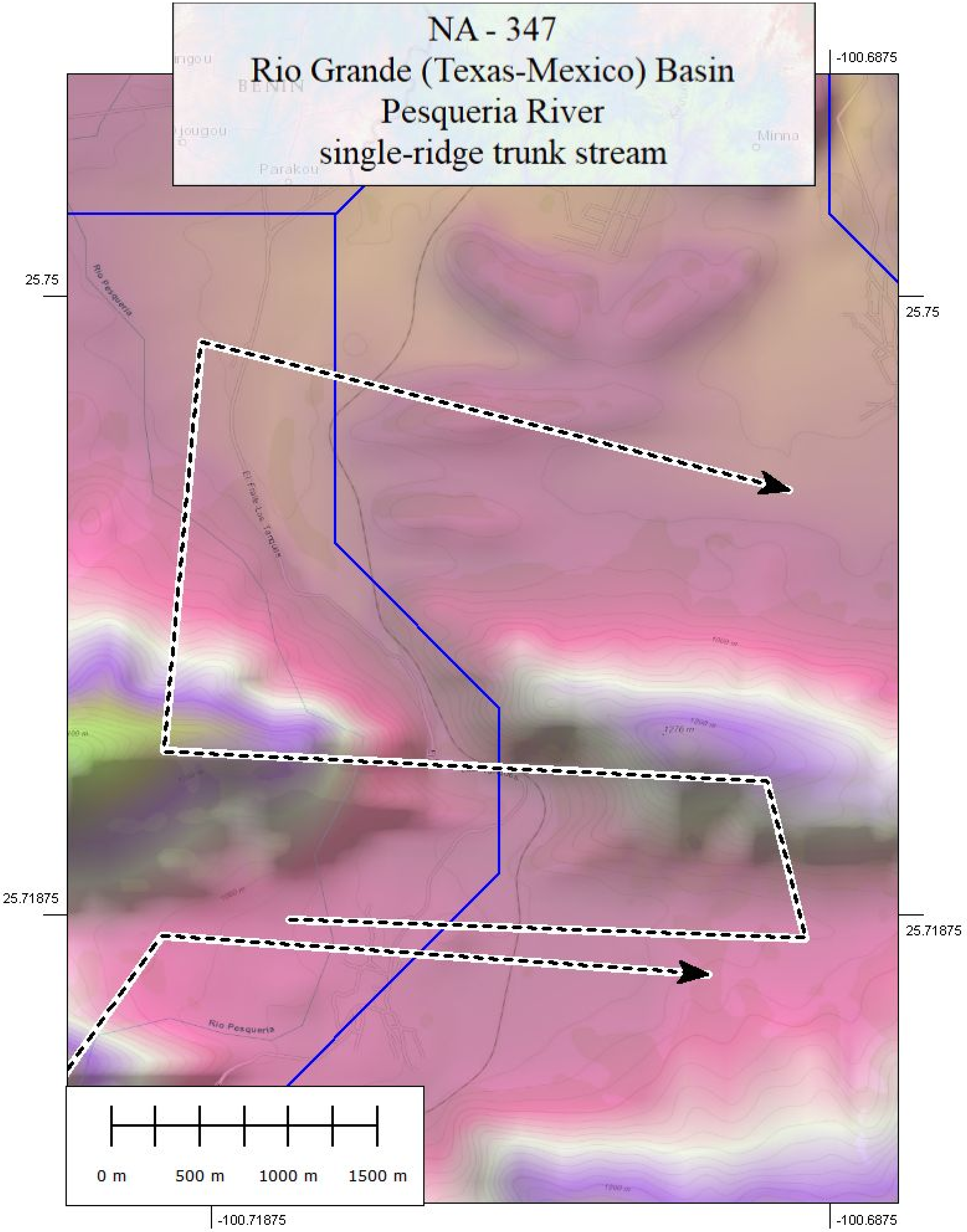

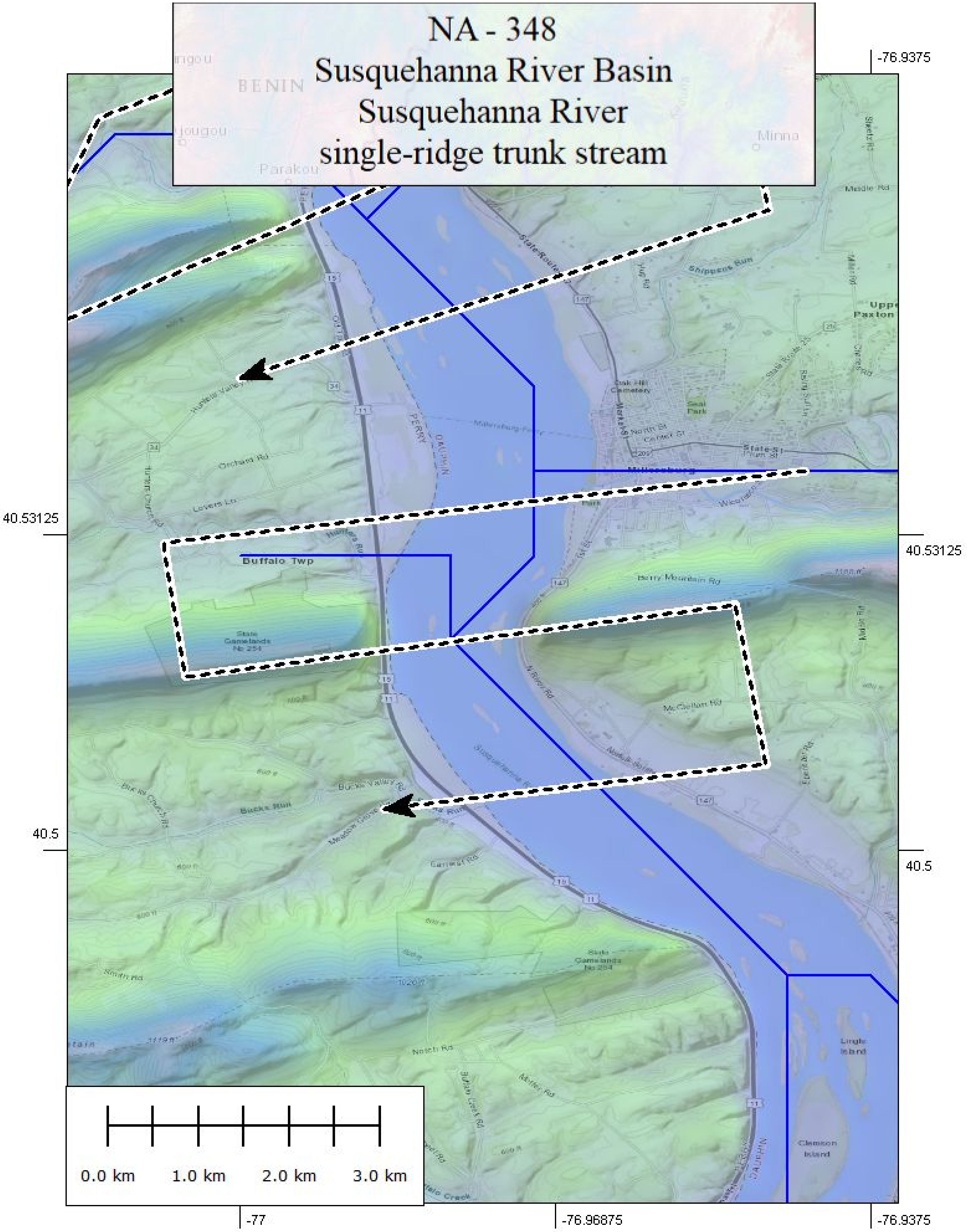
NA - 348
Susquehanna River Basin
Susquehanna River
single-ridge trunk stream
-76.9375
40.53125
40.53125
40.5
40.5
-77
-76.96875
-76.9375
0.0 km
1.0 km
2.0 km
3.0 km
Buffalo Twp
Berry Mountain Rd
Orchard Rd
Lovers Ln
Shippens Run
Oak Hill Cemetery
Seal Park
North St
Center St
State St
McClellan Rd
Bucks Valley Rd
Bucks Run
Susquehanna River
Smith Rd
Notch Rd
State Gamelands No 254
PERRY
DAUPHIN
Lingle Island
Clemson Island

NA - 349
Mississippi River Basin
Bighorn River, Sheep Canyon
single-ridge trunk stream

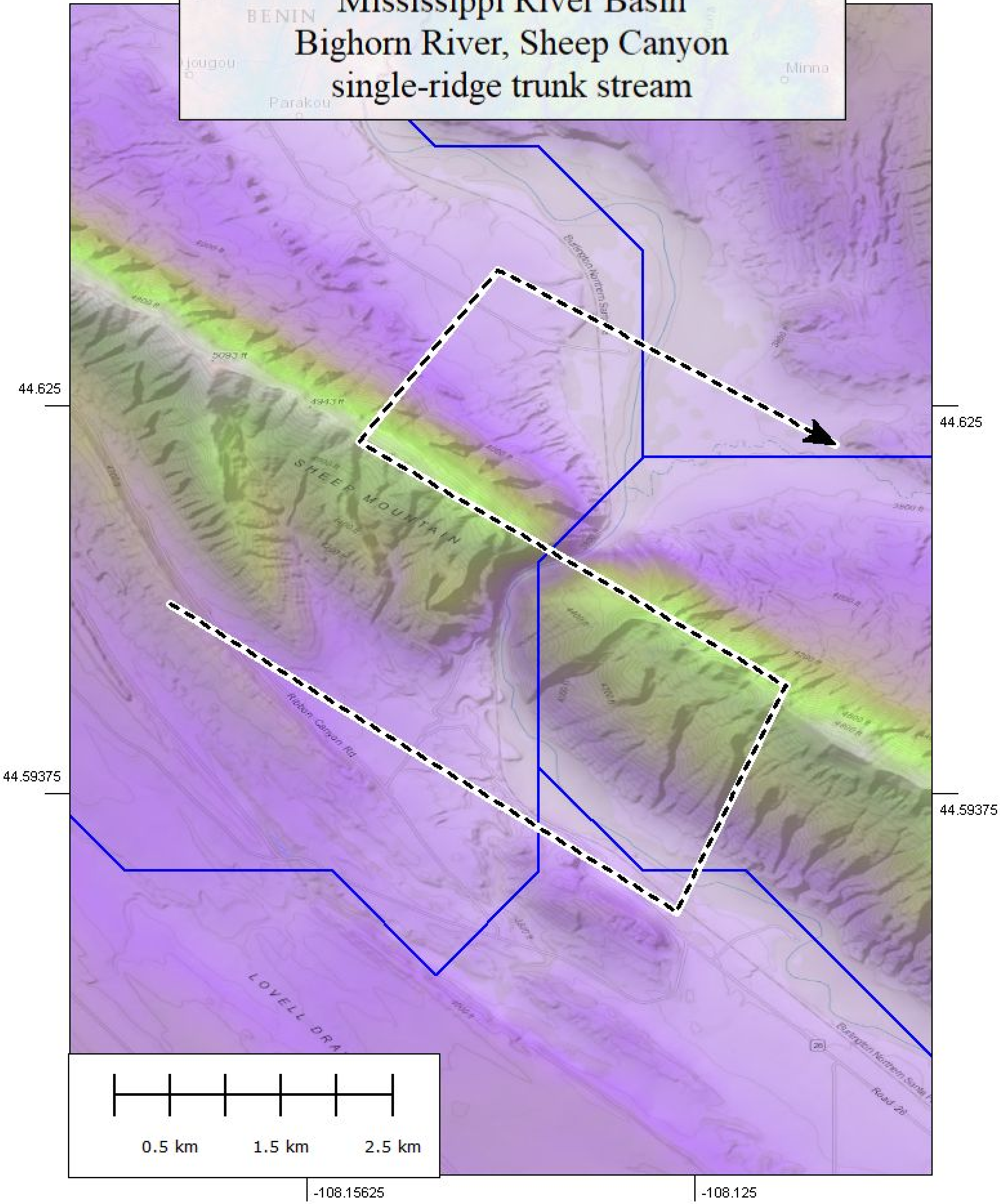

NA - 35
Mackenzie River (Canada) Basin
Liard River tributary
single-ridge trunk stream

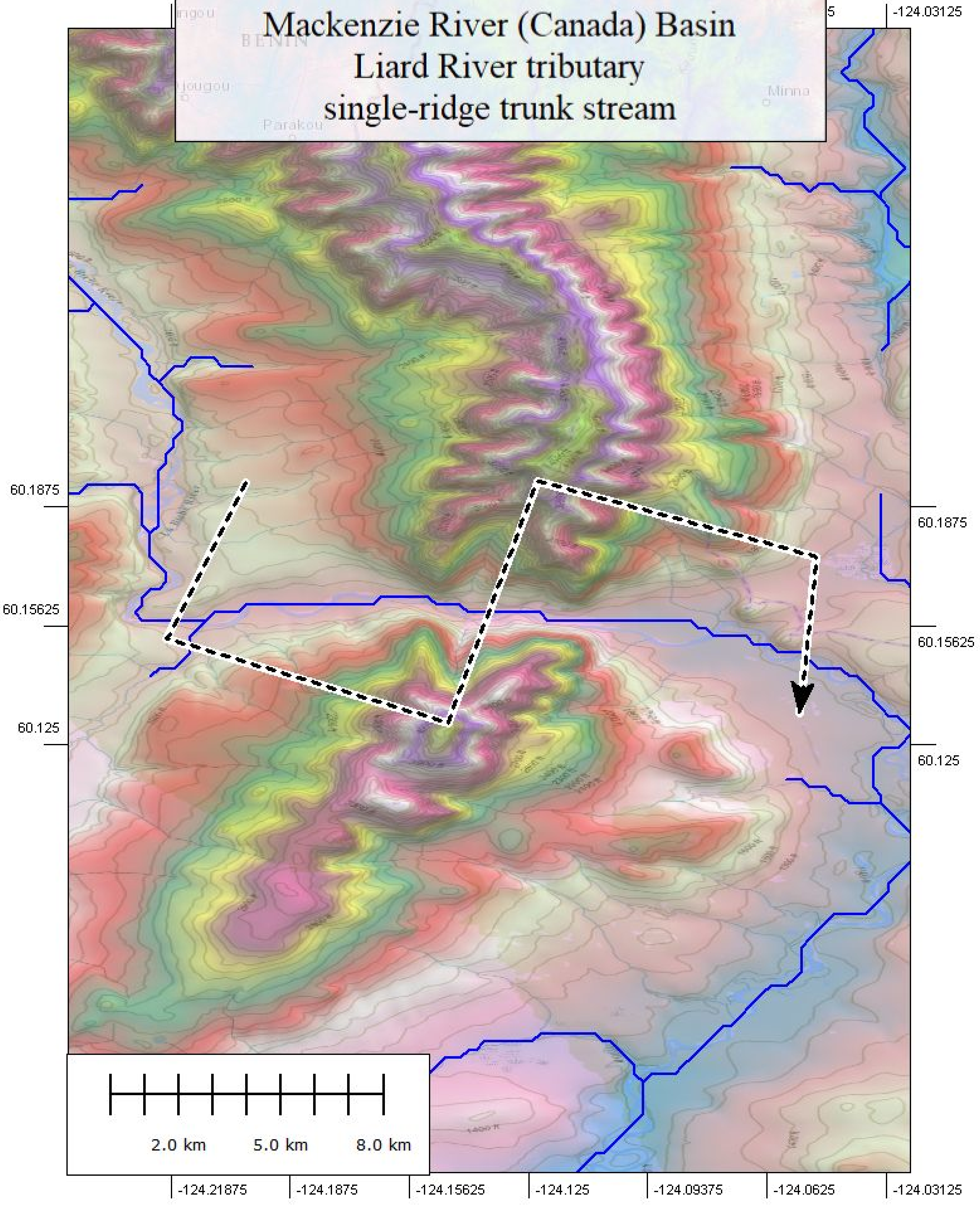

NA - 350
Mississippi River Basin
Cheat River
irregular high ground trunk stream

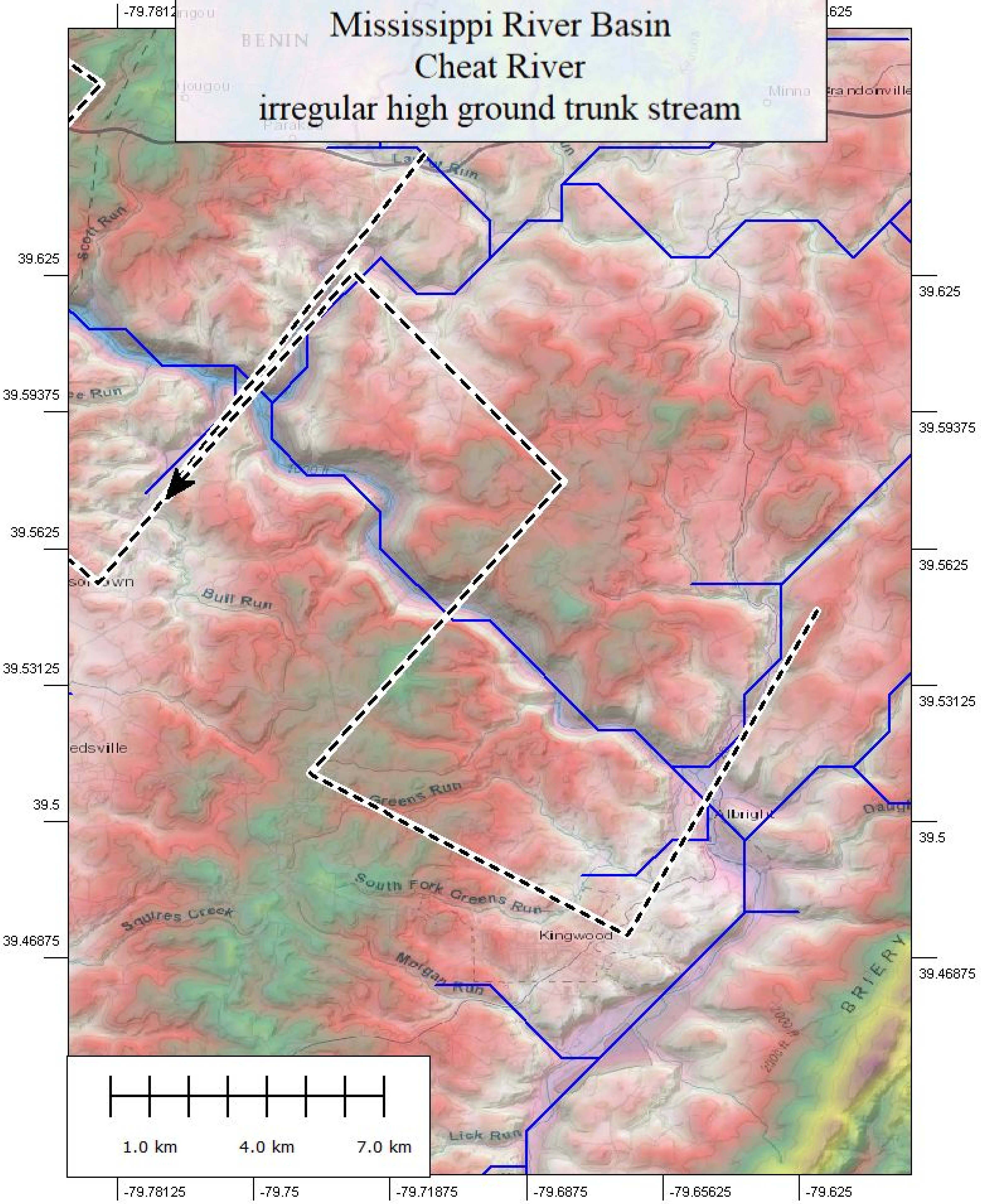

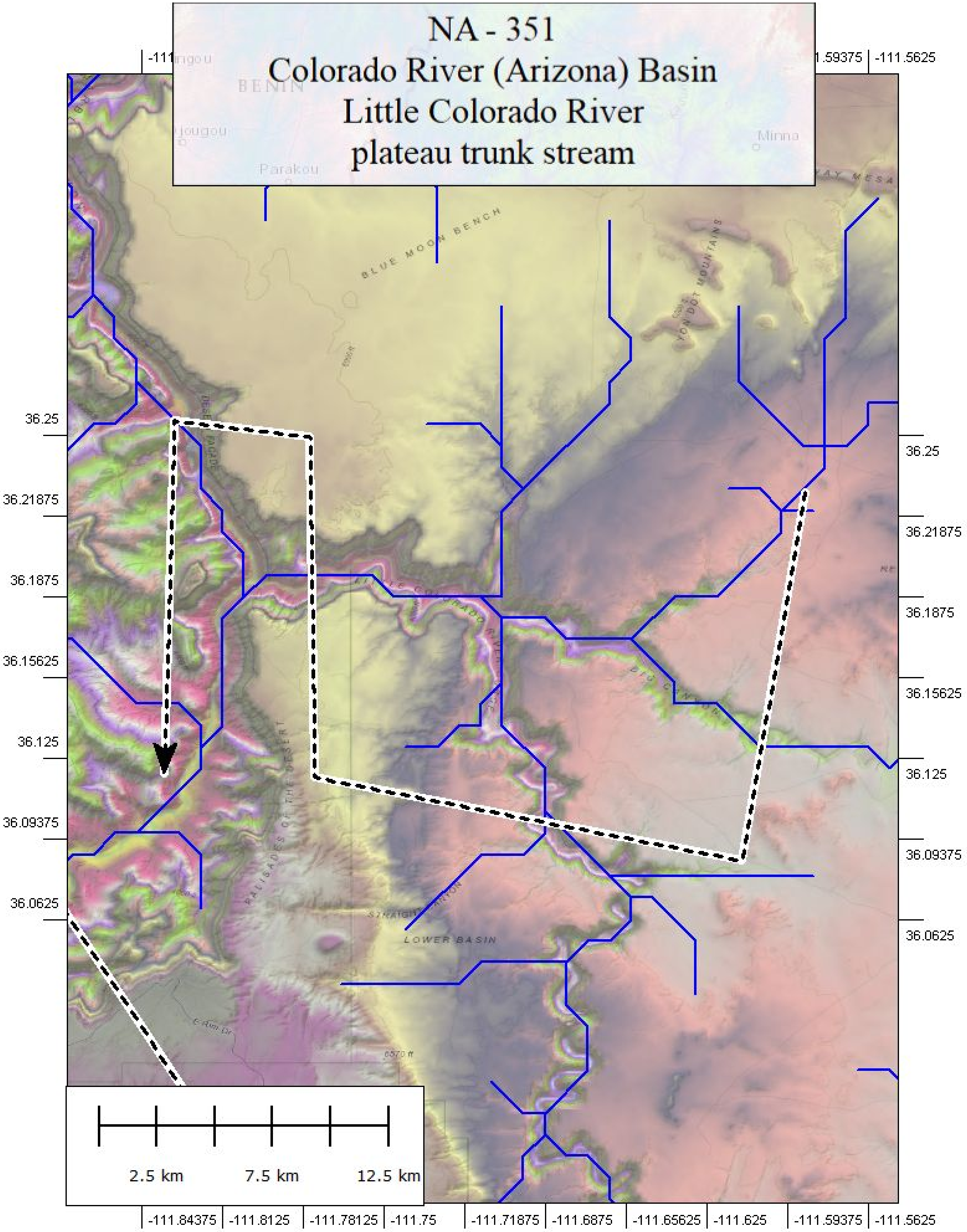
NA - 351
Colorado River (Arizona) Basin
Little Colorado River
plateau trunk stream
BLUE MOON BENCH
PALISADES OF THE DESERT
STRAIGHT CANYON
LOWER BASIN
36.25
36.21875
36.1875
36.15625
36.125
36.09375
36.0625
-111.84375
-111.8125
-111.78125
-111.75
-111.71875
-111.6875
-111.65625
-111.625
-111.59375
-111.5625
2.5 km
7.5 km
12.5 km

NA - 352
Rio Grande (Texas-Mexico) Basin
La Escondida Canyon
single-ridge trunk stream

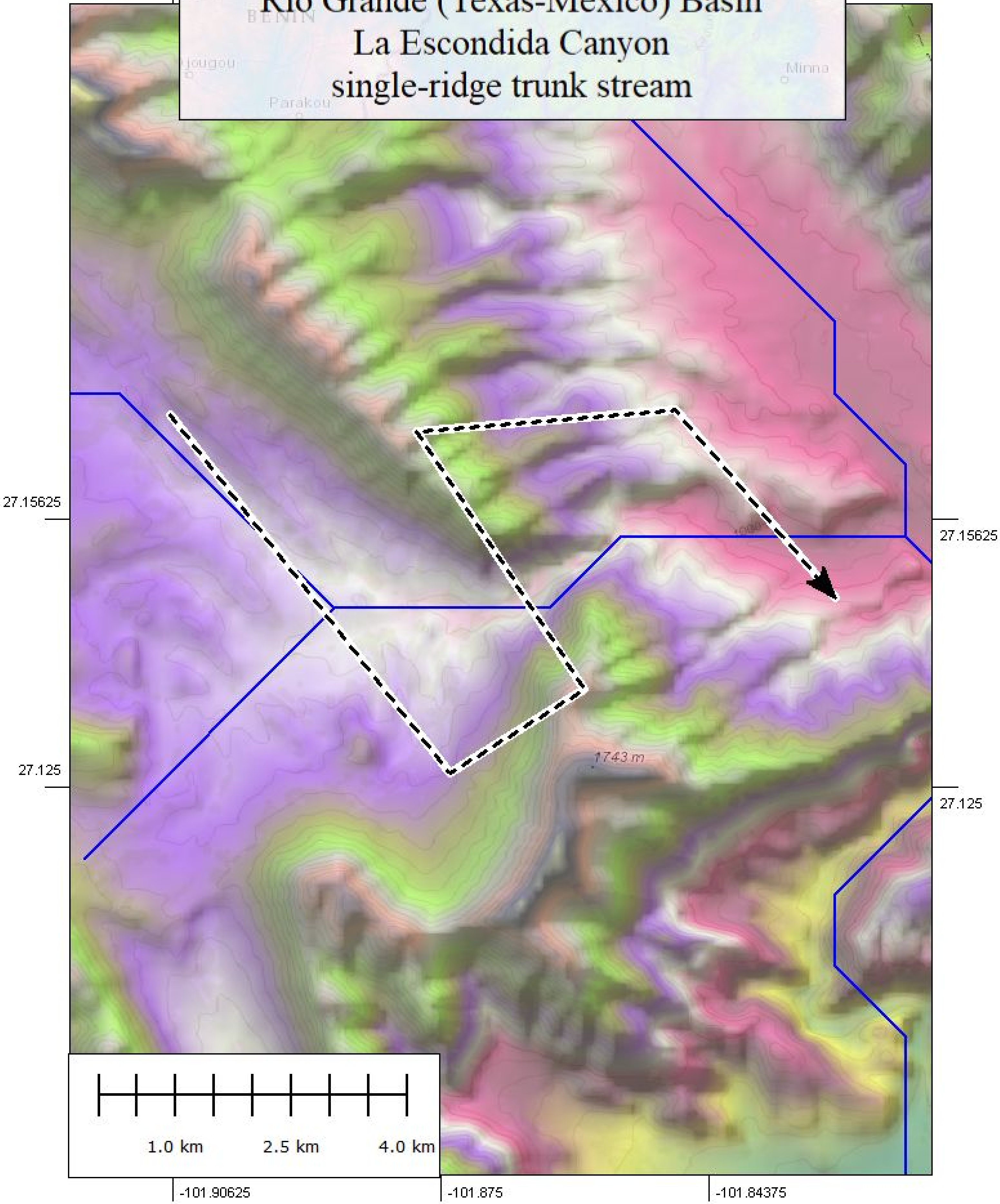

NA - 353
Colorado River (Arizona) Basin
Colorado River
irregular high ground trunk stream

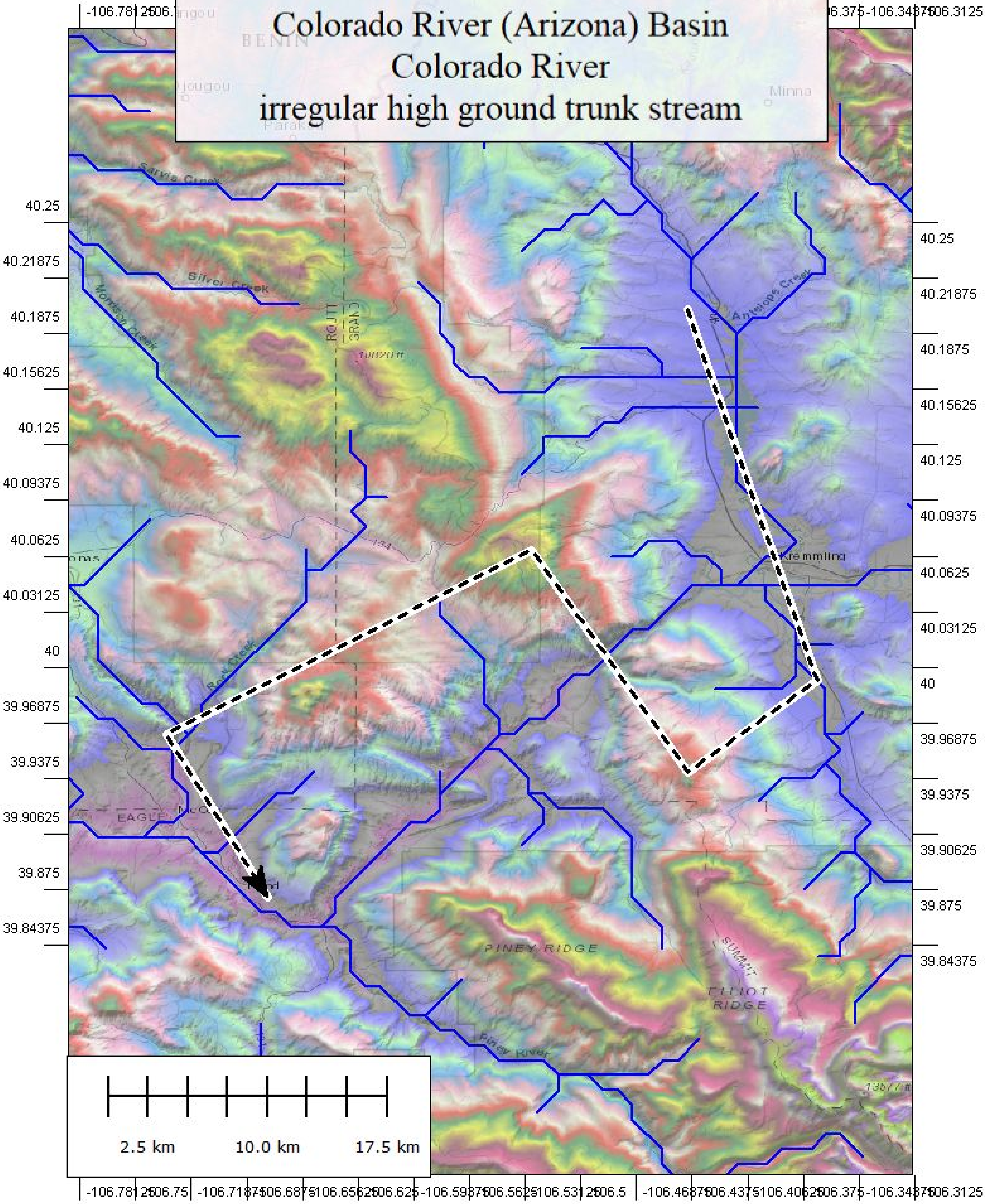

NA - 354
Rio Grande (Texas-Mexico) Basin
Santa Catarina River
multi-ridge trunk stream

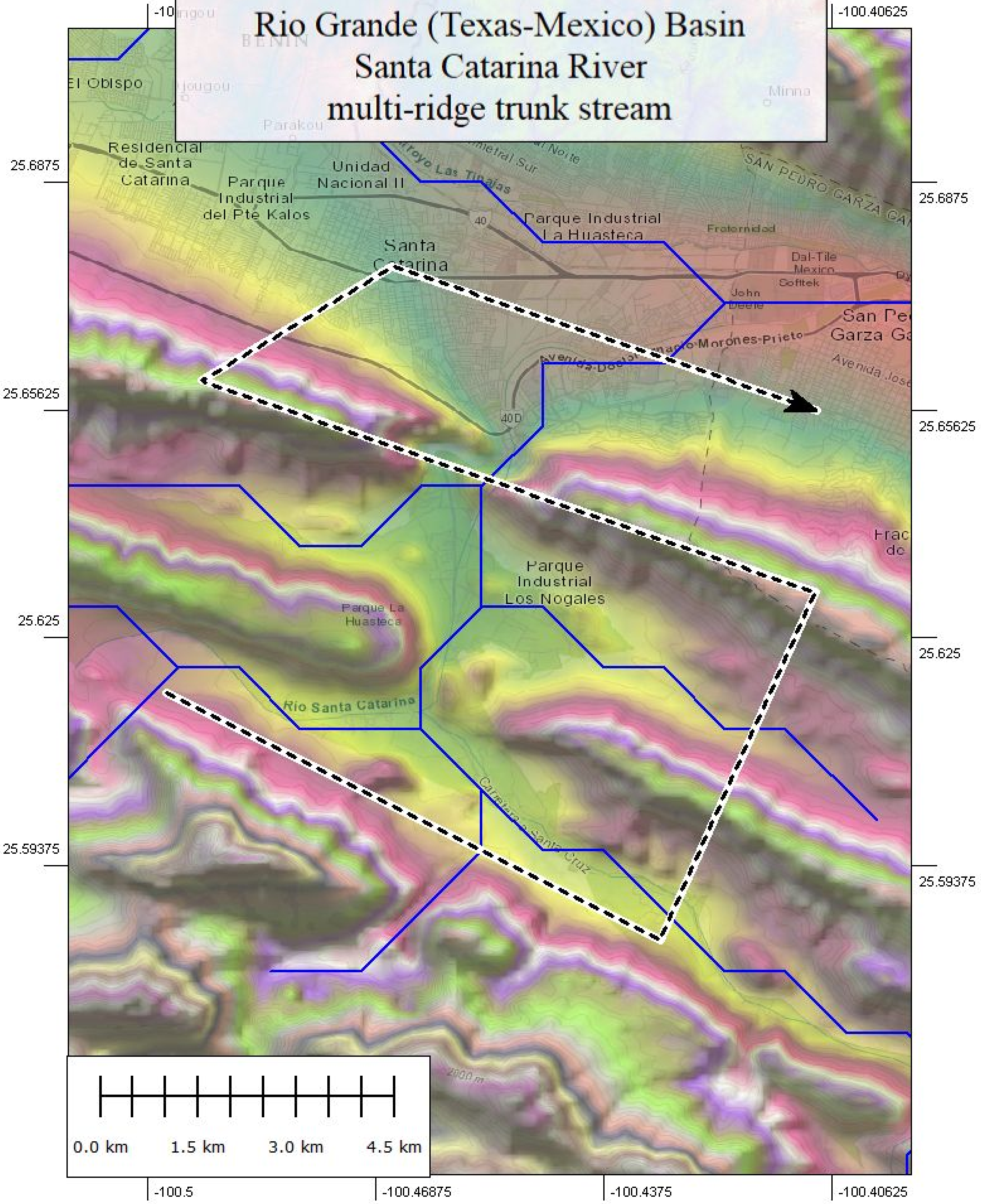

NA - 355
Colorado River (Arizona) Basin
Colorado River
updip plateau trunk stream

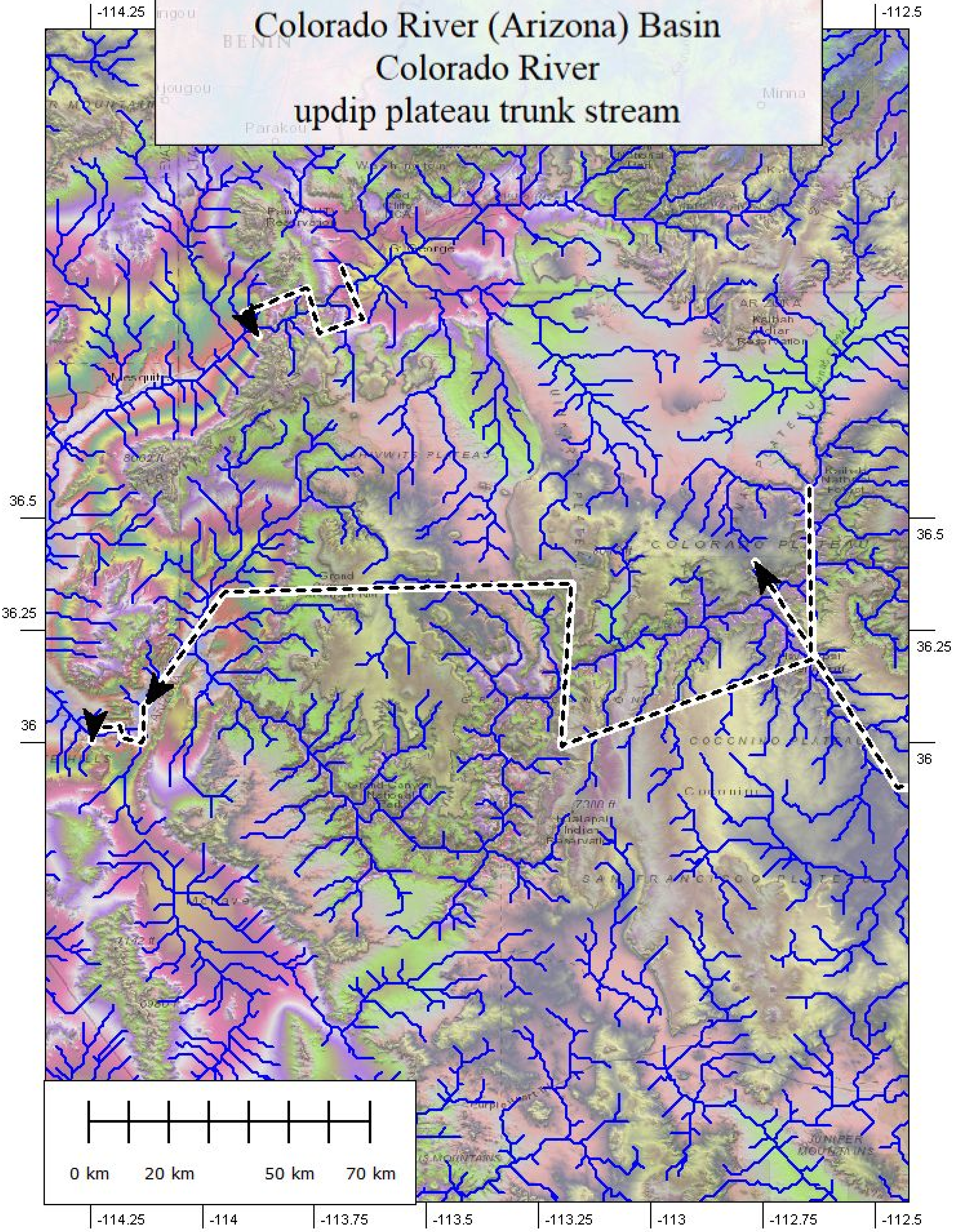

NA - 356
Colorado River (Arizona) Basin
Eagle River
irregular high ground trunk stream

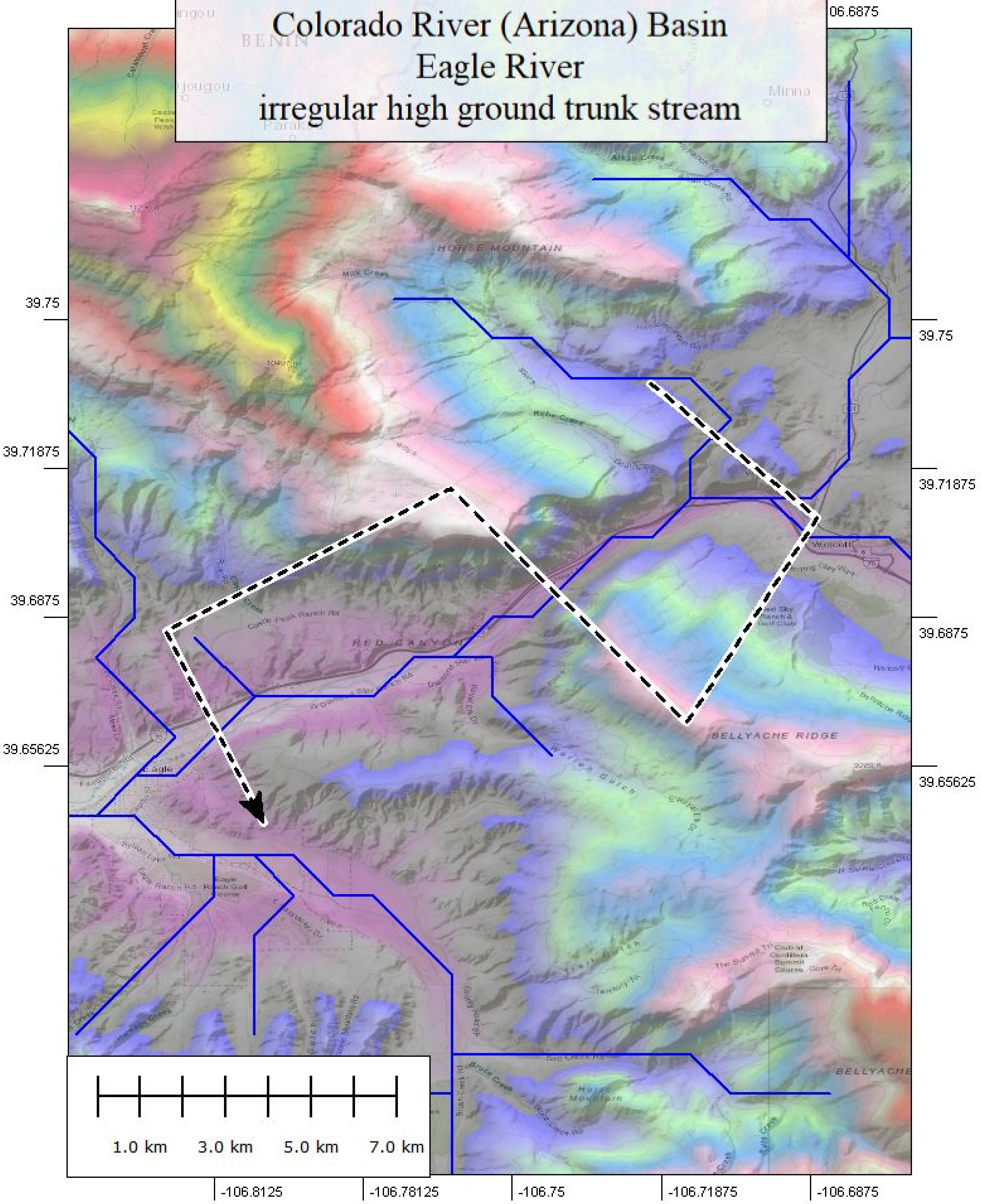

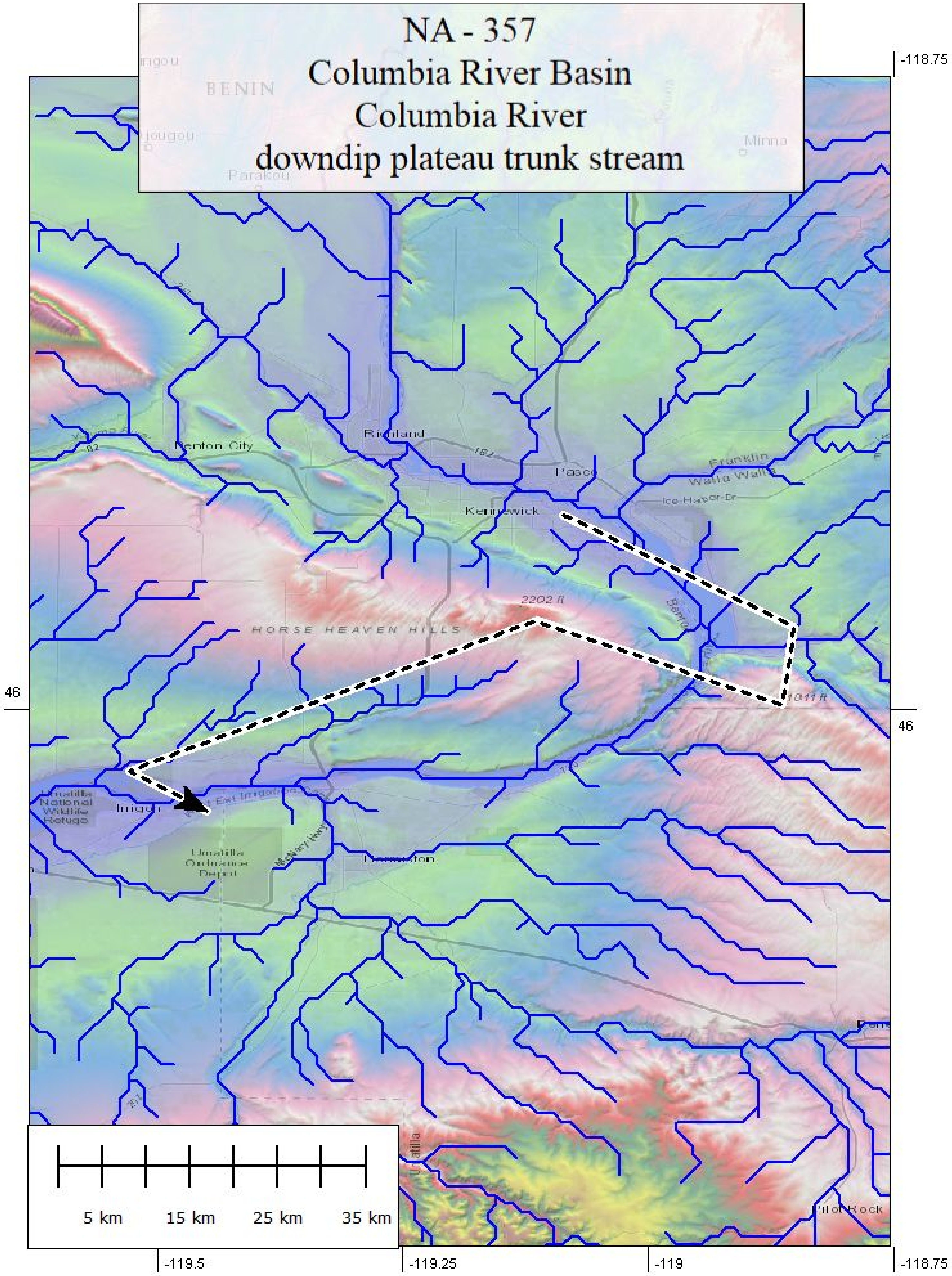
NA - 357
Columbia River Basin
Columbia River
downdip plateau trunk stream
-118.75
Richland
Benton City
Pasco
Kennewick
Franklin
Walla Walla
Ice Harbor Dr
2202 ft
HORSE HEAVEN HILLS
1811 ft
46
46
Umatilla National Wildlife Refuge
Irrigon
Umatilla Ordnance Depot
Pilot Rock
5 km
15 km
25 km
35 km
-119.5
-119.25
-119
-118.75

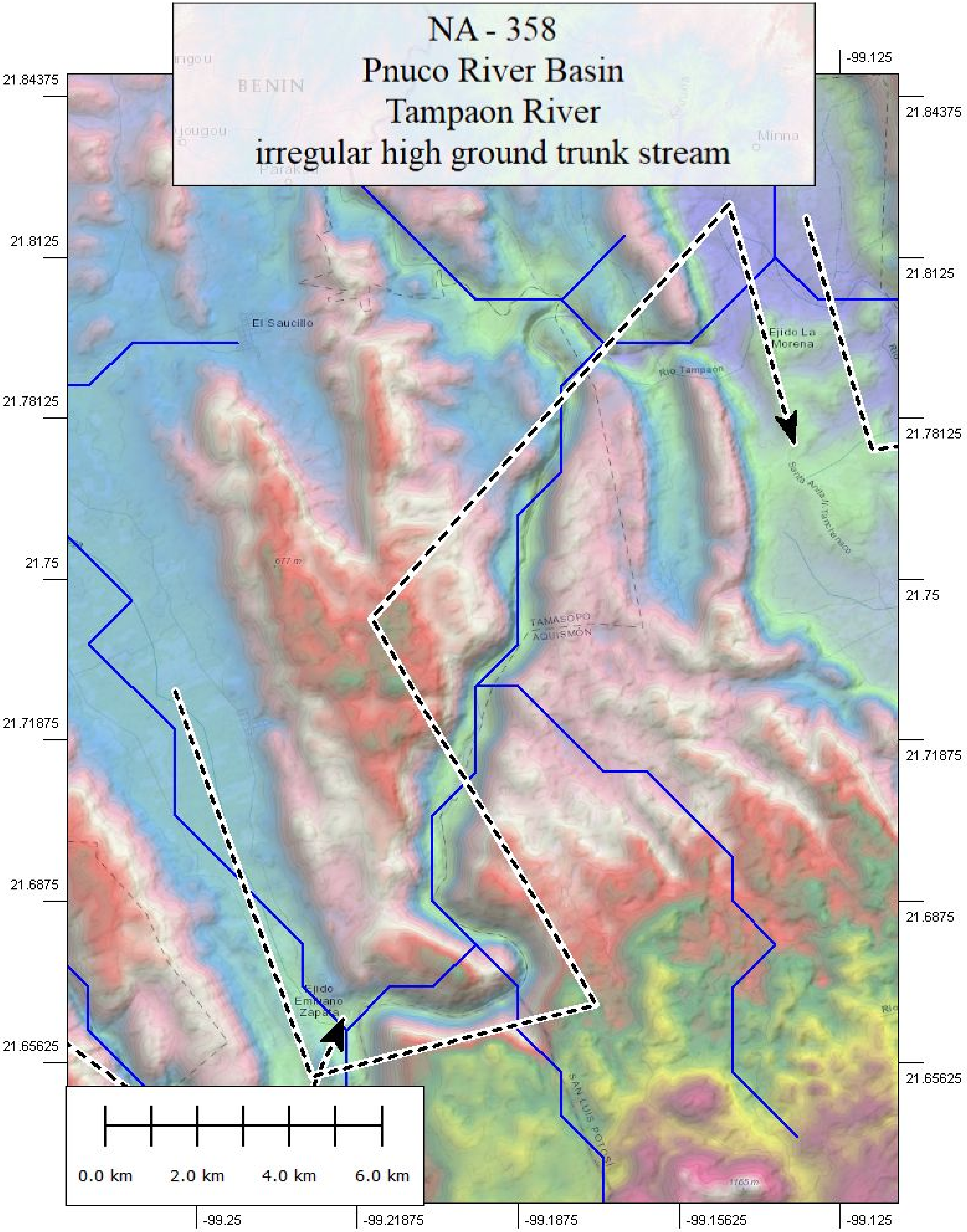
NA - 358
Pnuco River Basin
Tampaon River
irregular high ground trunk stream
21.84375
21.8125
21.78125
21.75
21.71875
21.6875
21.65625
-99.25
-99.21875
-99.1875
-99.15625
-99.125
El Saucillo
Ejido La Morena
Rio Tampaon
TAMASOPO
AQUISMON
SAN LUIS POTOSI
0.0 km
2.0 km
4.0 km
6.0 km

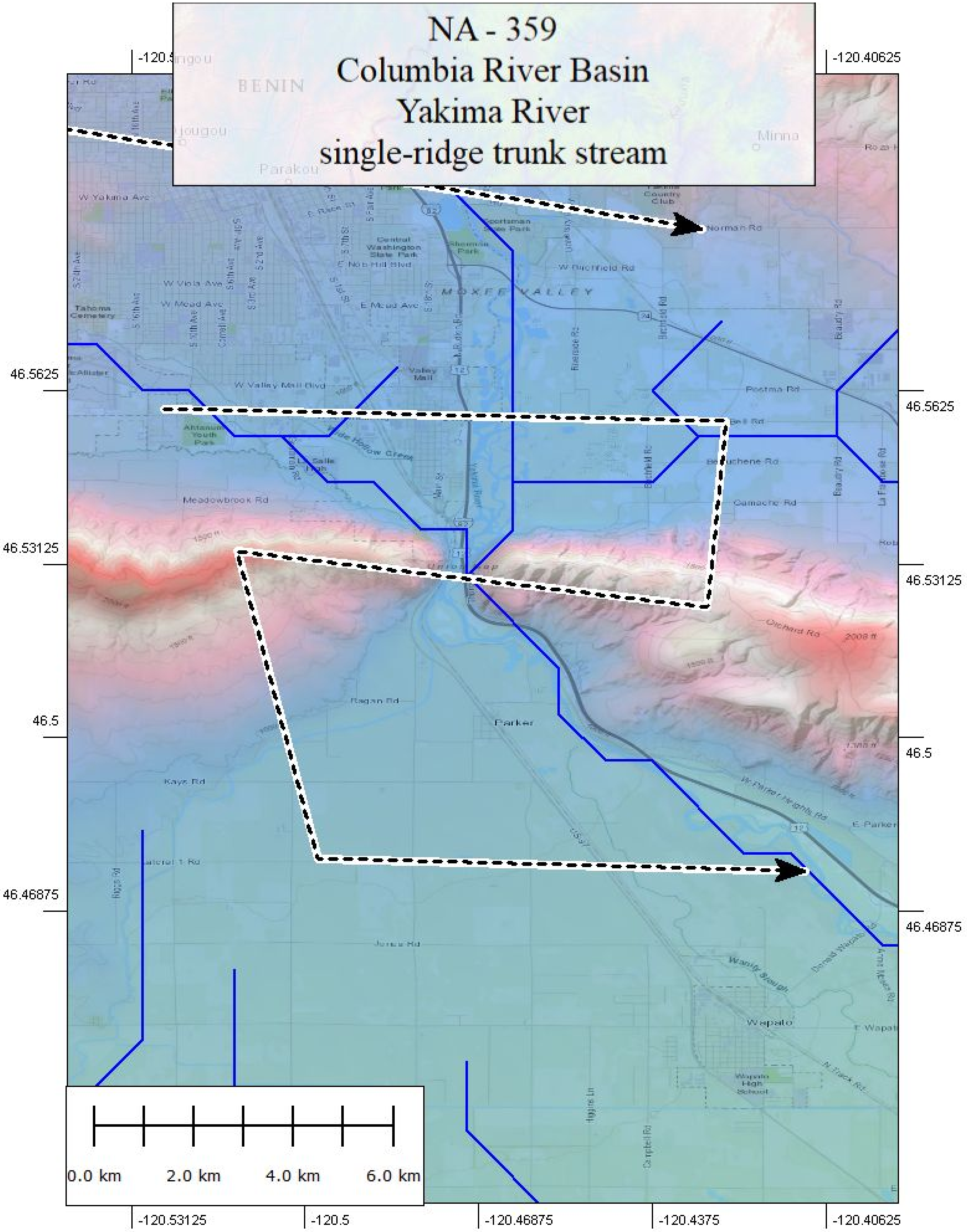

NA - 359
Columbia River Basin
Yakima River
single-ridge trunk stream
-120.53125
-120.5
-120.46875
-120.4375
-120.40625
46.5625
46.53125
46.5
46.46875
0.0 km
2.0 km
4.0 km
6.0 km
MOXEE VALLEY
Parker
Wapato
W Yakima Ave
W Valley Mall Blvd
Meadowbrook Rd
Ragan Rd
Kays Rd
Jones Rd
Norman Rd
Postma Rd
Beauchene Rd
Gamache Rd
Orchard Rd
Wanity Slough
Central Washington State Park
Sherman Park
Valley Mall

NA - 36
Mackenzie River (Canada) Basin
Mackenzie River tributary
single-ridge trunk stream

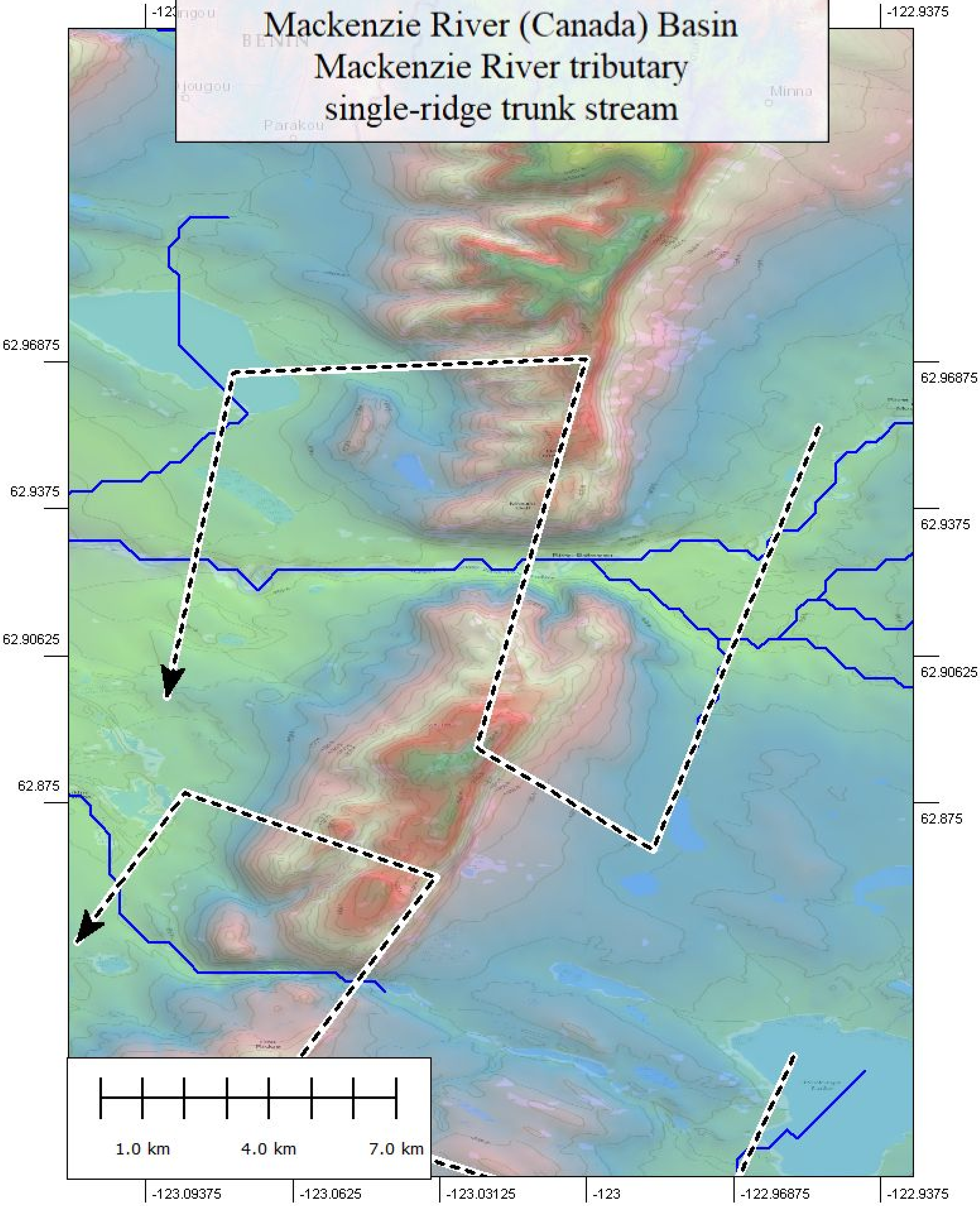

NA - 360
Potomac River Basin
Savage River
single-ridge trunk stream

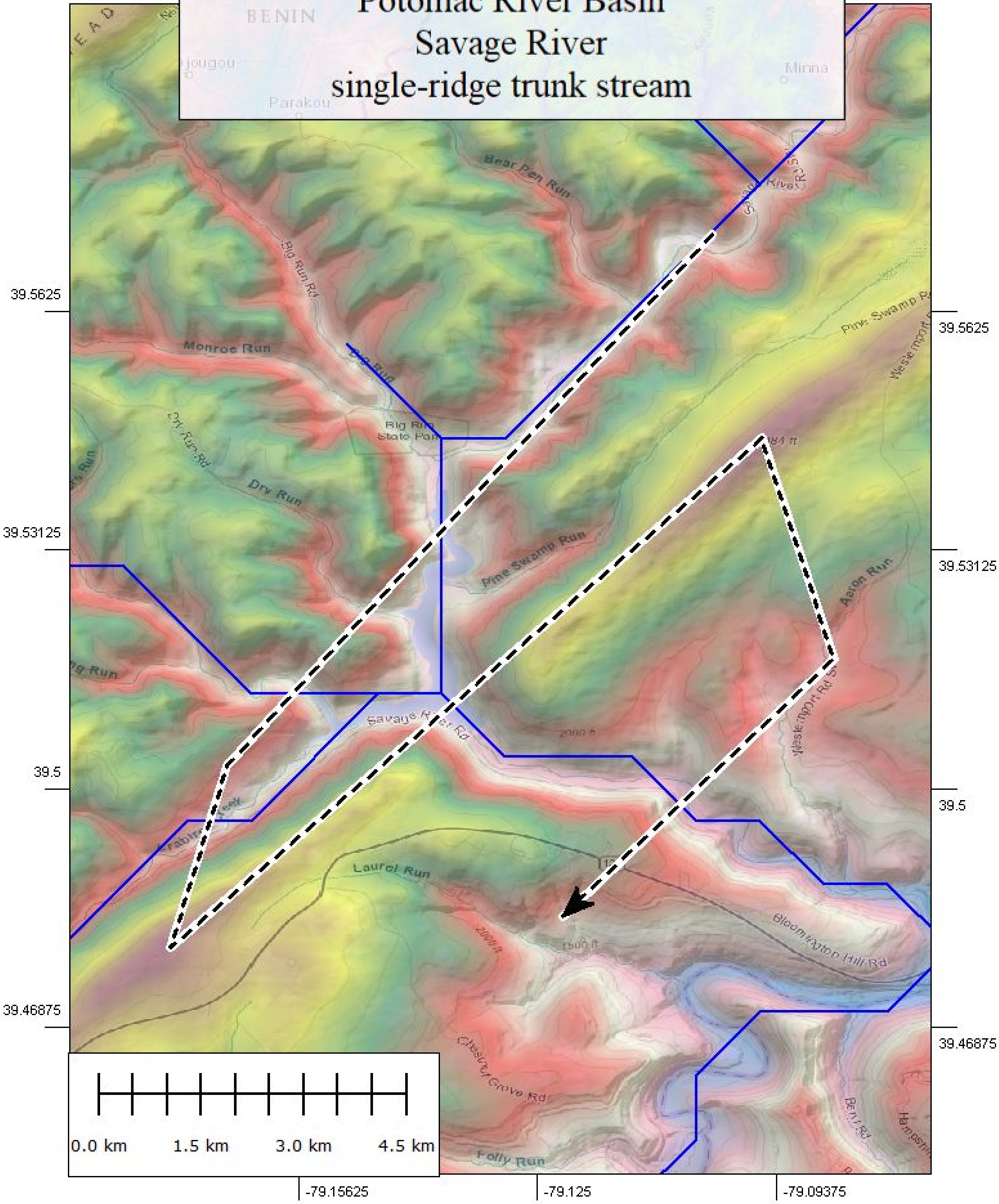

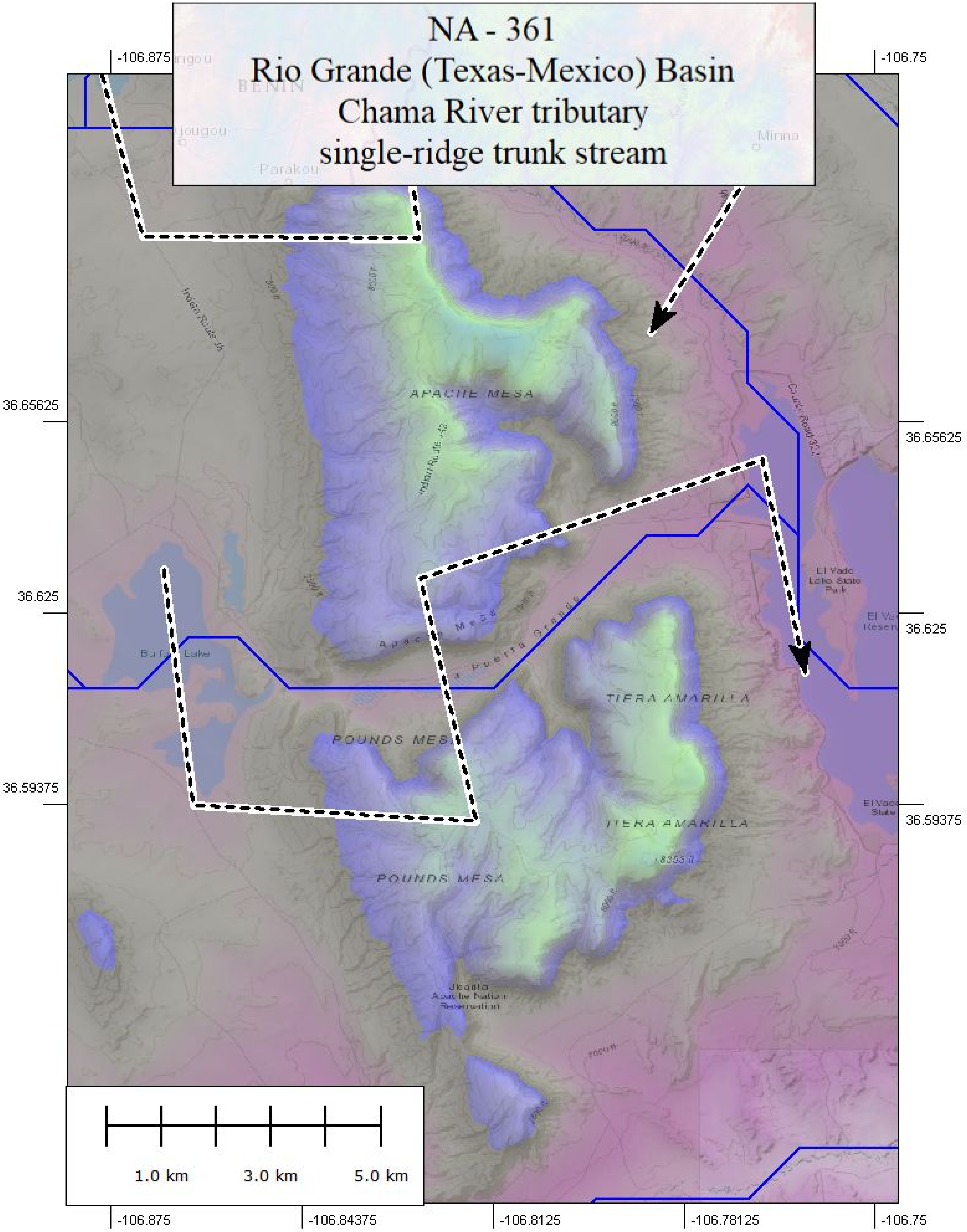
NA - 361
Rio Grande (Texas-Mexico) Basin
Chama River tributary
single-ridge trunk stream
-106.875
-106.75
36.65625
36.625
36.59375
-106.84375
-106.8125
-106.78125
APACHE MESA
Apache Mesa
POUNDS MESA
TIERA AMARILLA
El Vado Lake State Park
Jicarilla Apache Nation Reservation
1.0 km
3.0 km
5.0 km

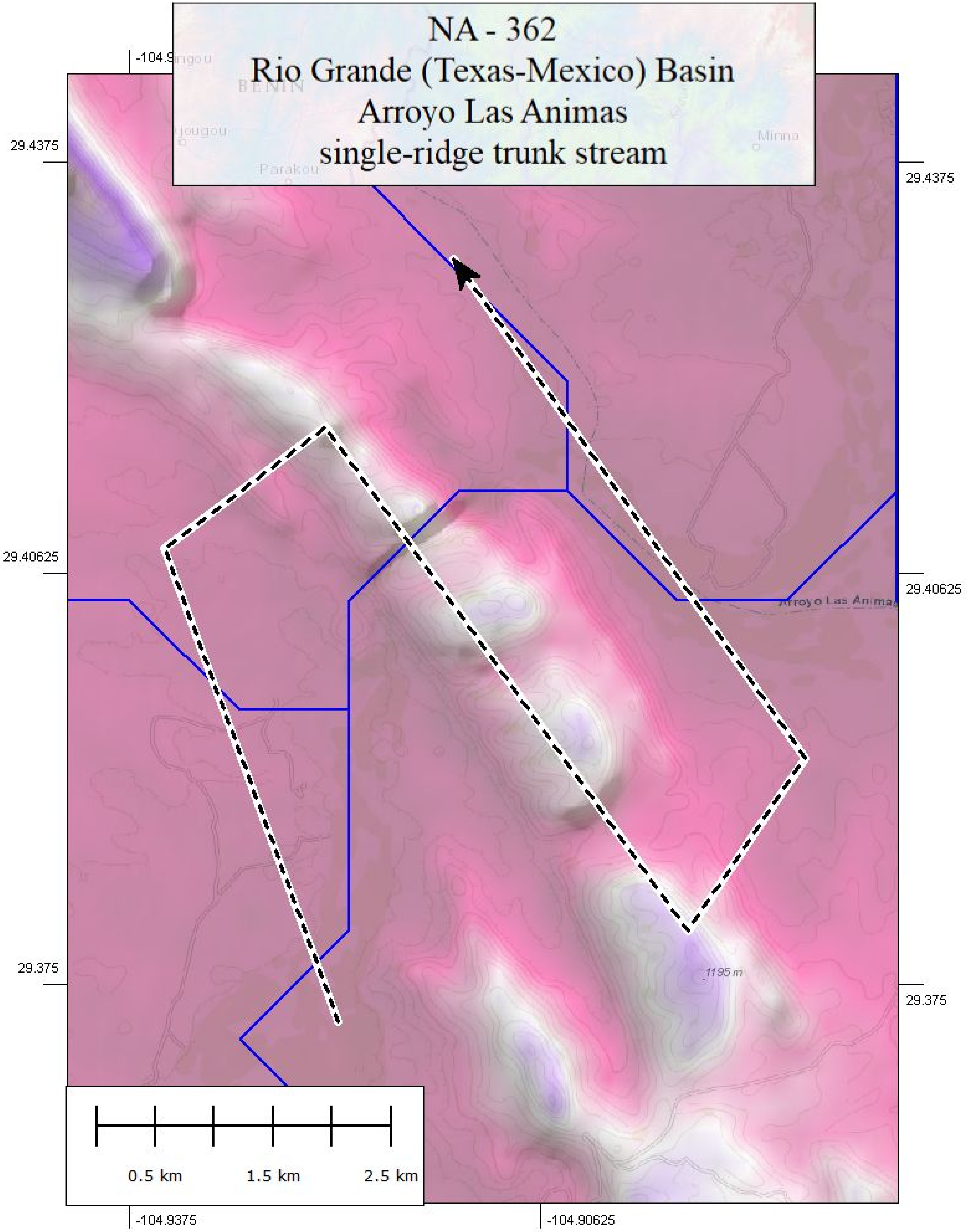

NA - 362
Rio Grande (Texas-Mexico) Basin
Arroyo Las Animas
single-ridge trunk stream
29.4375
29.40625
29.375
-104.9375
-104.90625
Arroyo Las Animas
1195 m
0.5 km
1.5 km
2.5 km

NA - 363
Rio Grande (Texas-Mexico) Basin
single-ridge trunk stream

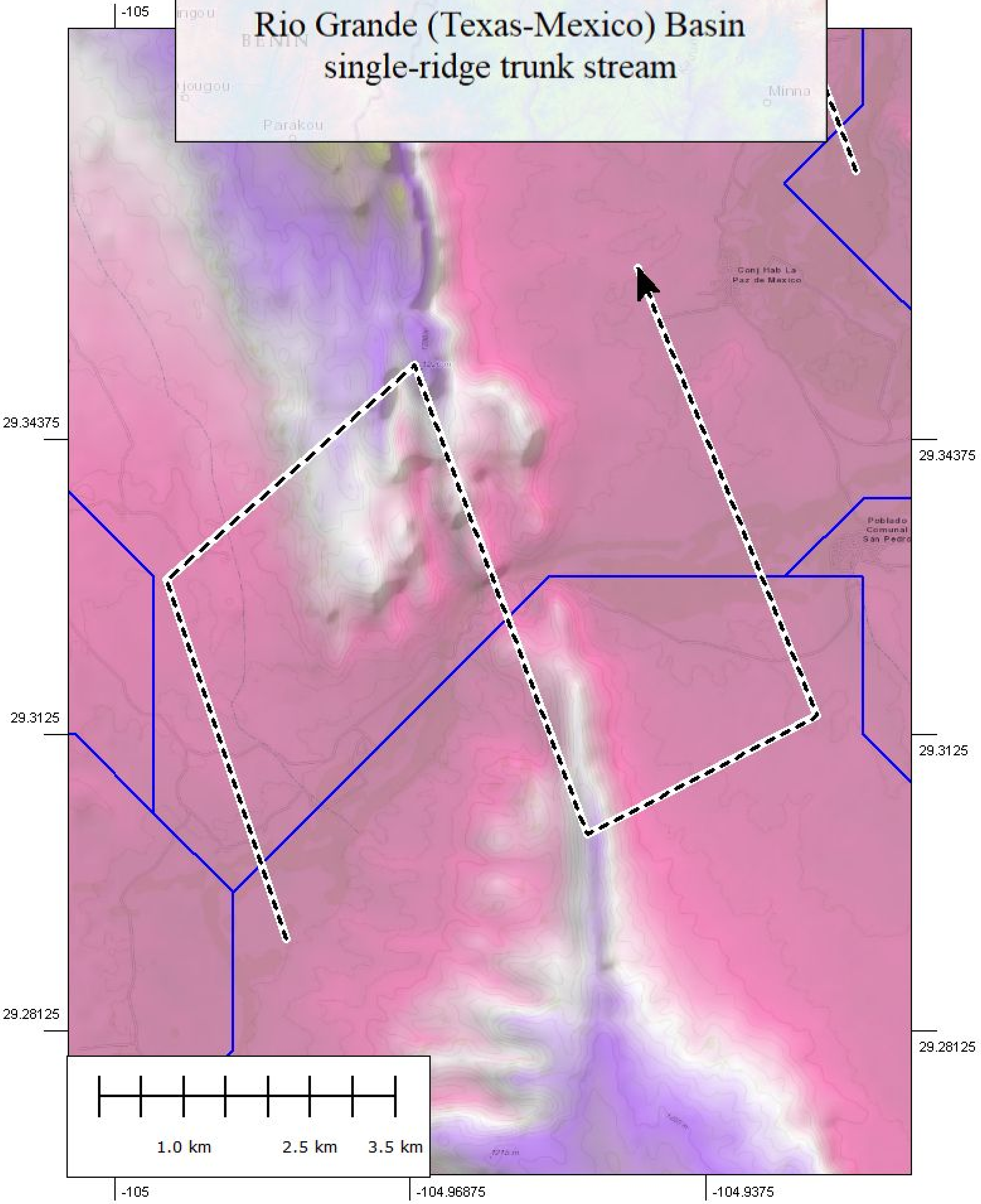

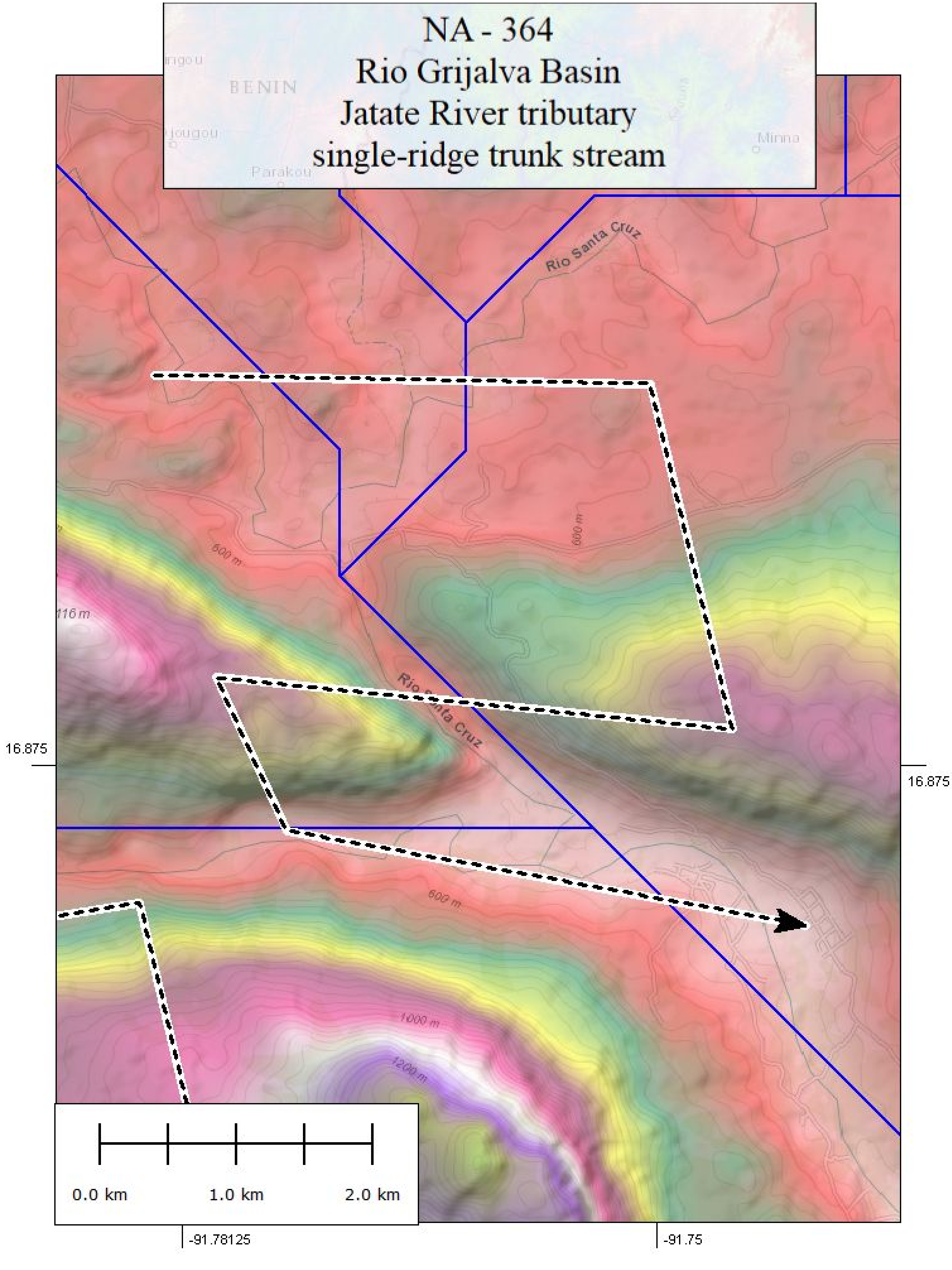
NA - 364
Rio Grijalva Basin
Jatate River tributary
single-ridge trunk stream
BENIN
Parakou
Minna
Rio Santa Cruz
600 m
1116 m
Rio Santa Cruz
600 m
1000 m
1200 m
16.875
16.875
0.0 km
1.0 km
2.0 km
-91.78125
-91.75

NA - 365
Mississippi River Basin
Tygart Valley River
single-ridge trunk stream

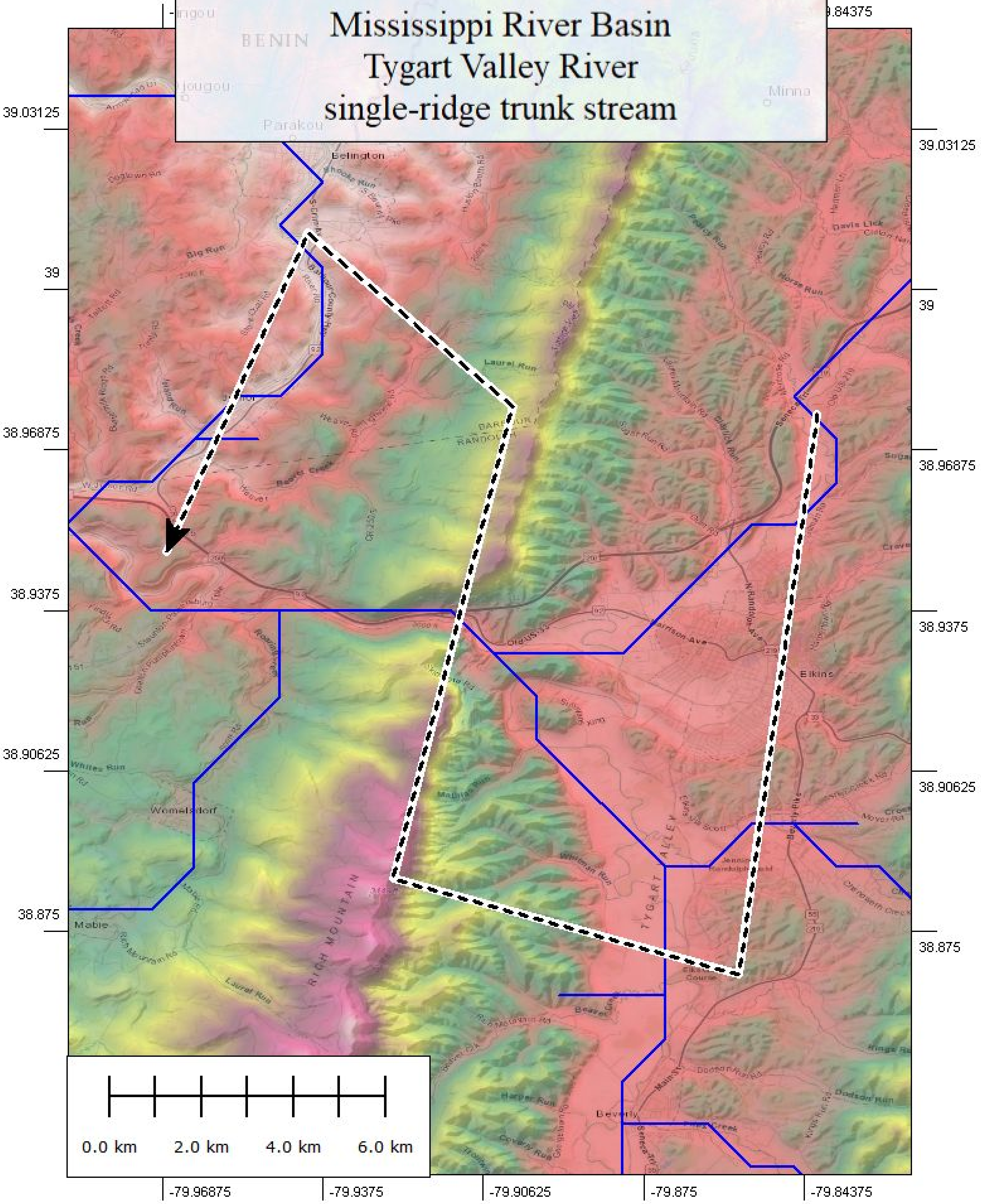

NA - 366
Rio Grijalva Basin
Jatate River, Jatate Canyon
irregular high ground trunk stream

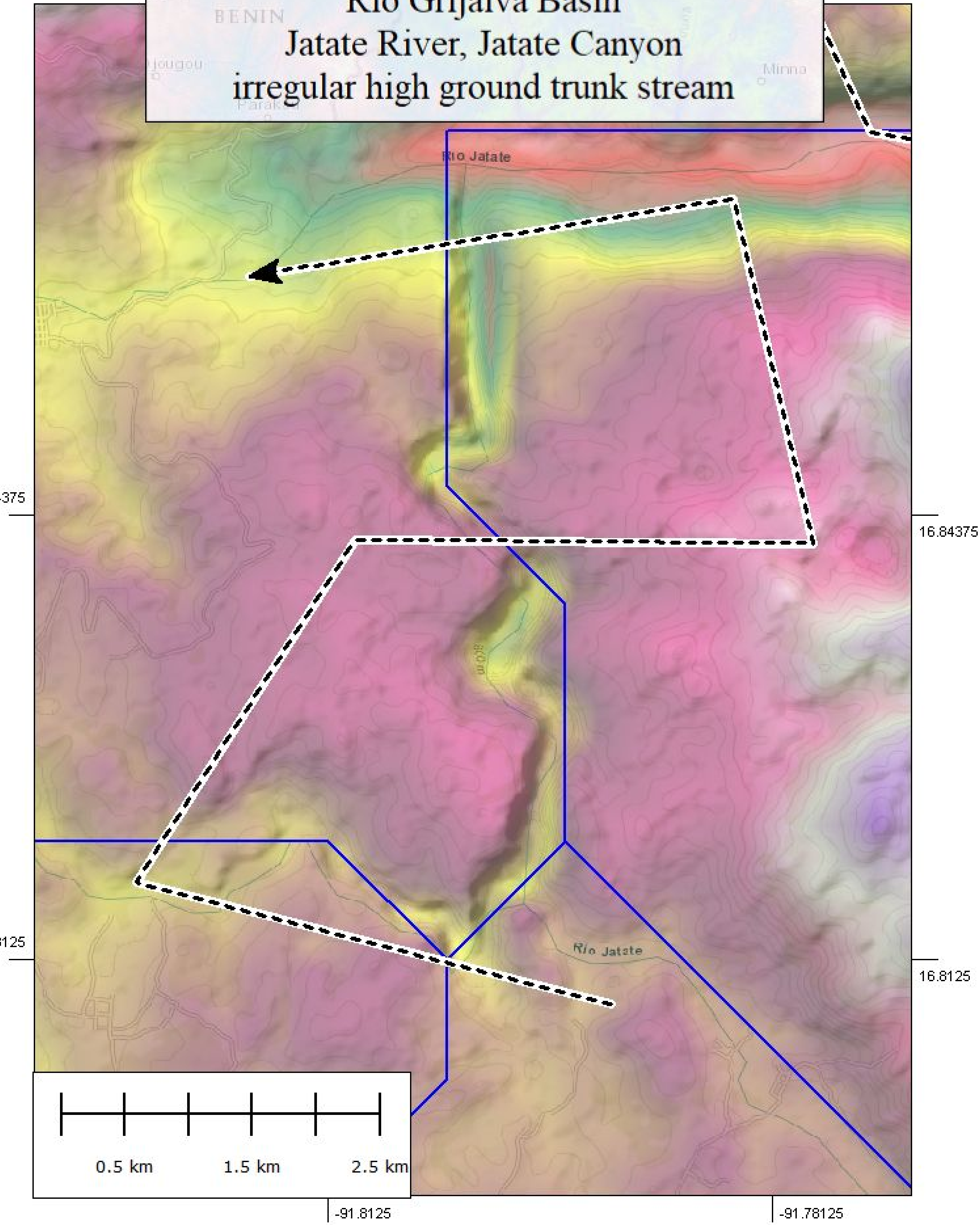

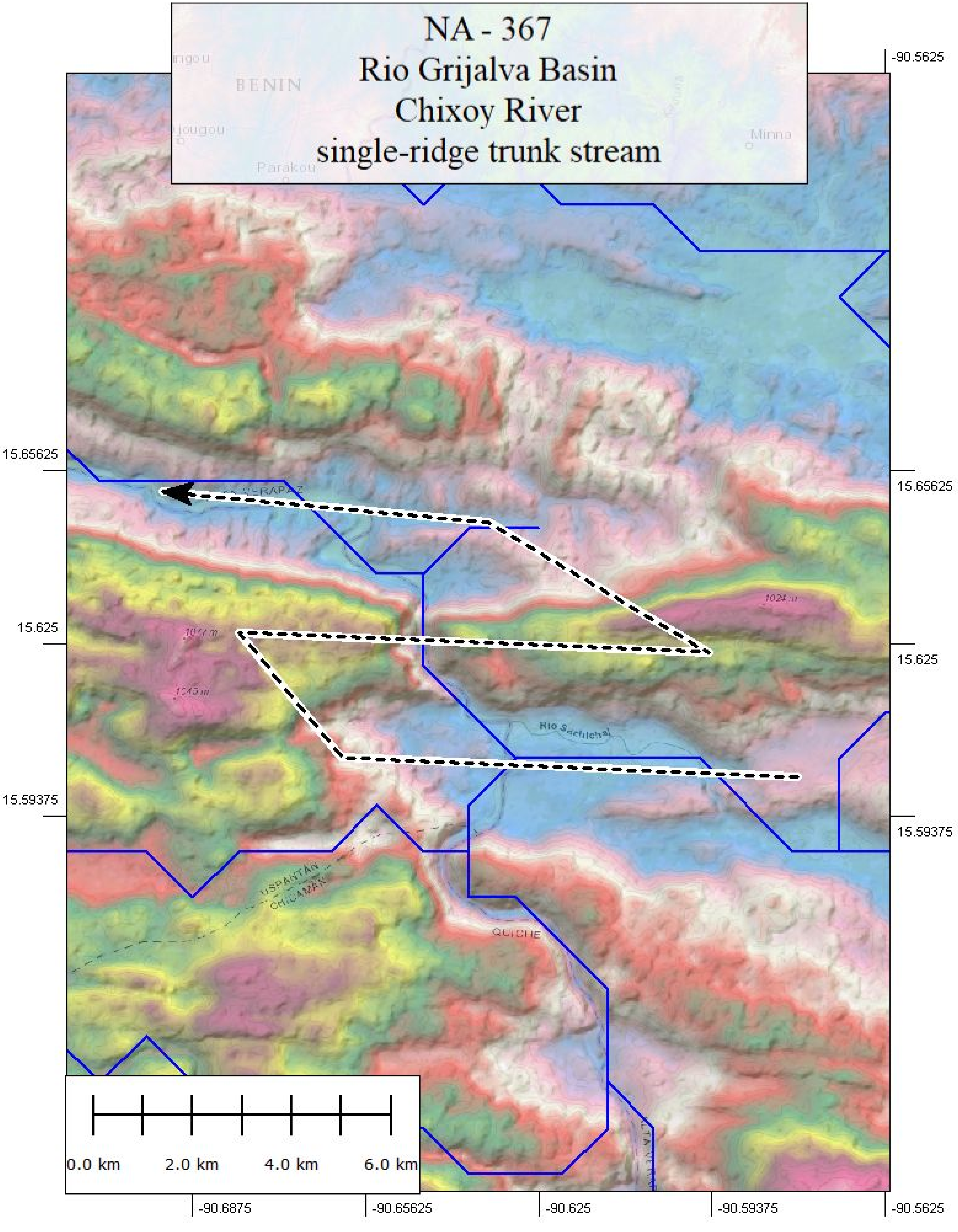
NA - 367
Rio Grijalva Basin
Chixoy River
single-ridge trunk stream
BENIN
Parakou
-90.5625
15.65625
15.625
15.59375
QUICHE
USPANTAN
CHICAMAN
0.0 km
2.0 km
4.0 km
6.0 km
-90.6875
-90.65625
-90.625
-90.59375

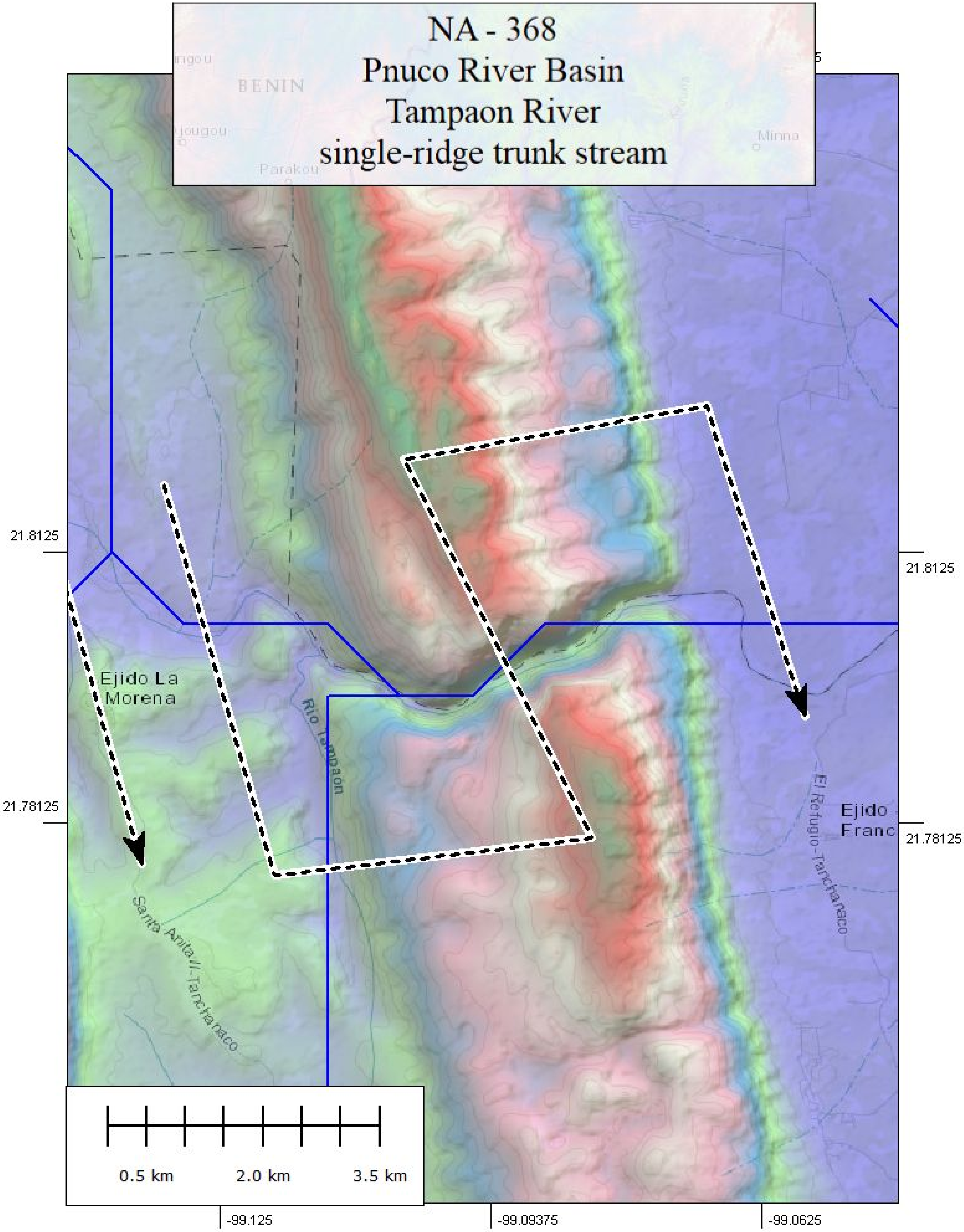
NA - 368
Pnuco River Basin
Tampaon River
single-ridge trunk stream
BENIN
Parakou
Minna
Ejido La Morena
Rio Tampaon
Ejido Franc
El Refugio-Tanchanaco
Santa Anita II-Tanchanaco
21.8125
21.8125
21.78125
21.78125
0.5 km
2.0 km
3.5 km
-99.125
-99.09375
-99.0625

NA - 369
Mississippi River Basin
Missouri River
irregular high ground trunk stream

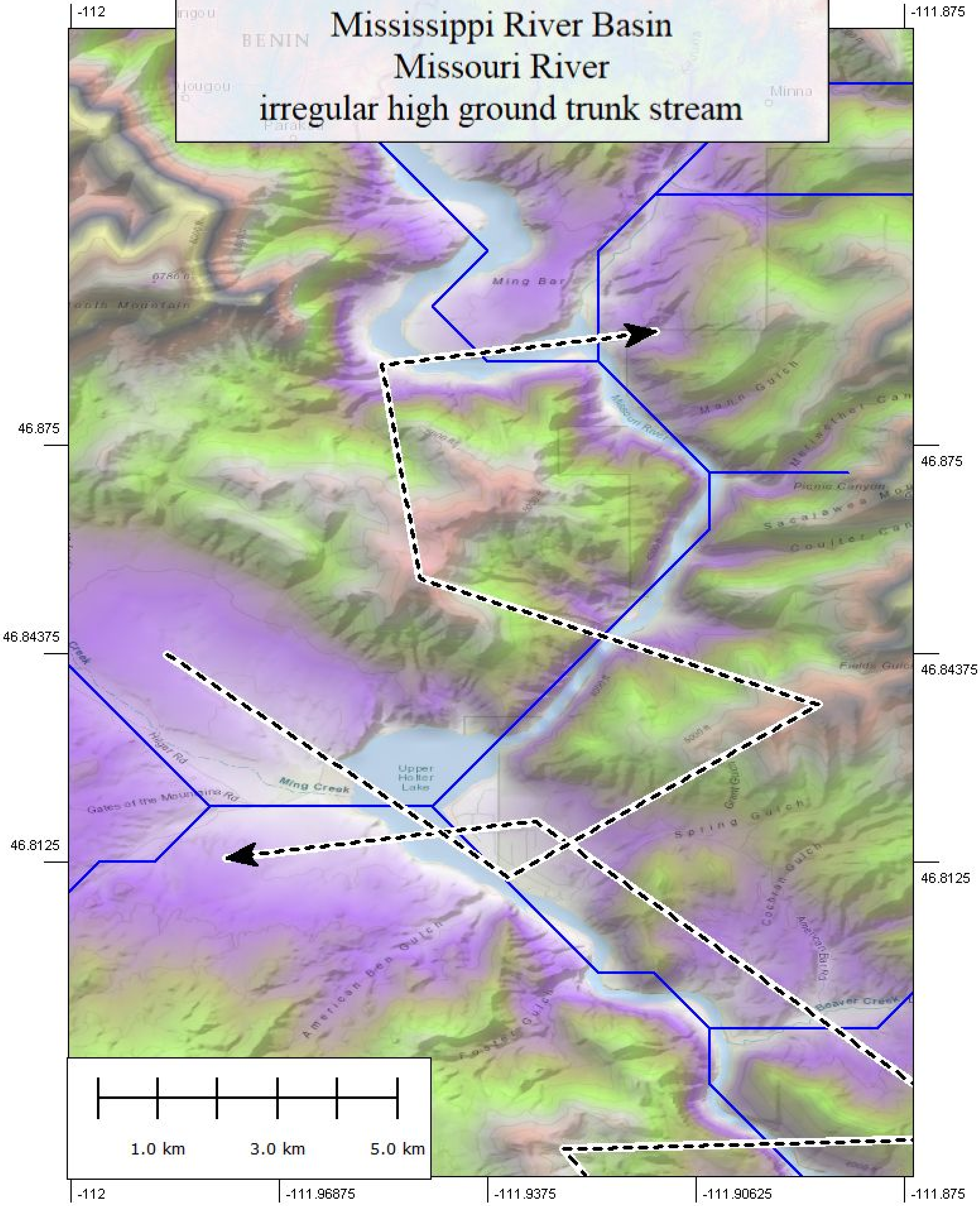

NA - 37

Mackenzie River (Canada) Basin

Beaver River

single-ridge trunk stream

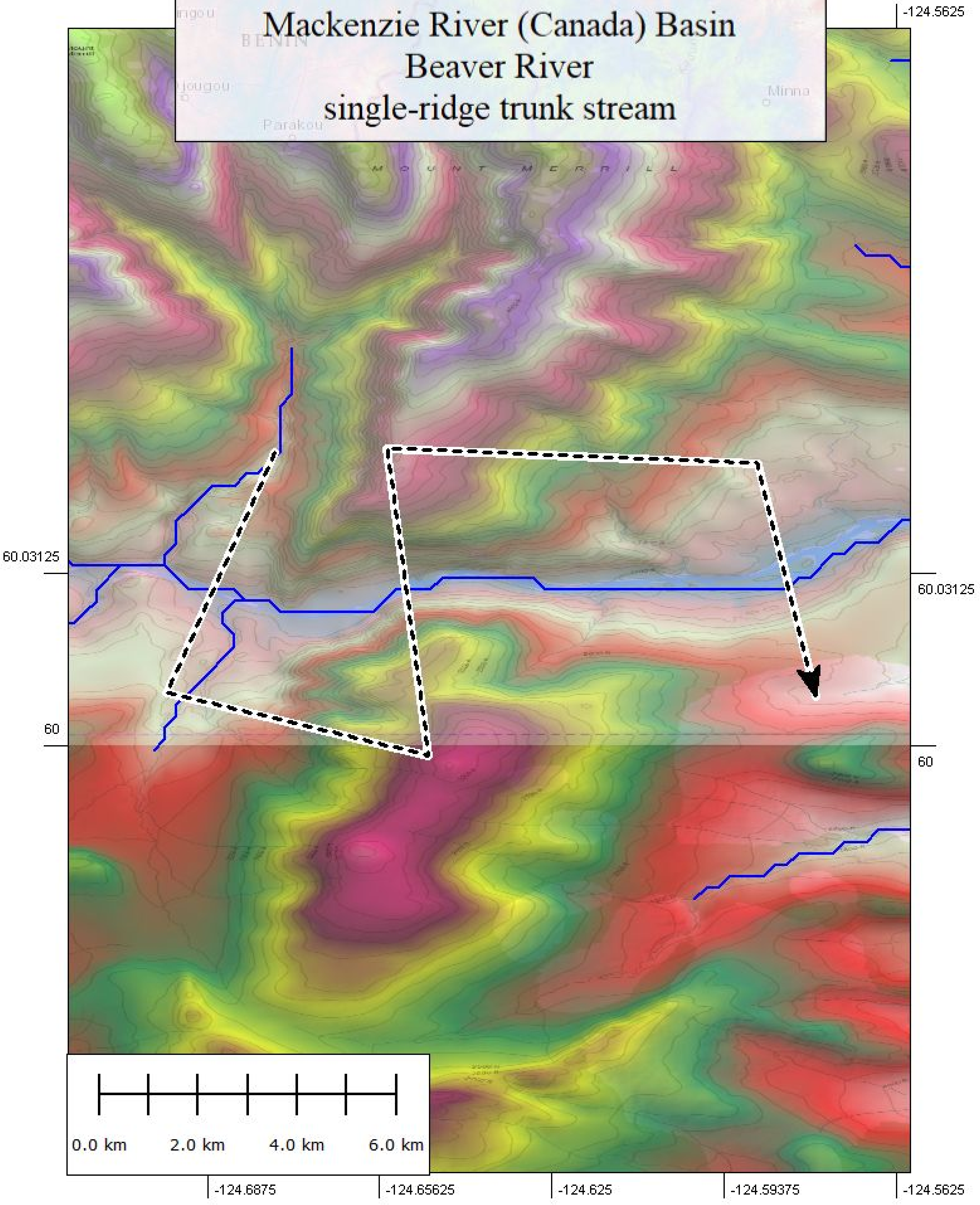

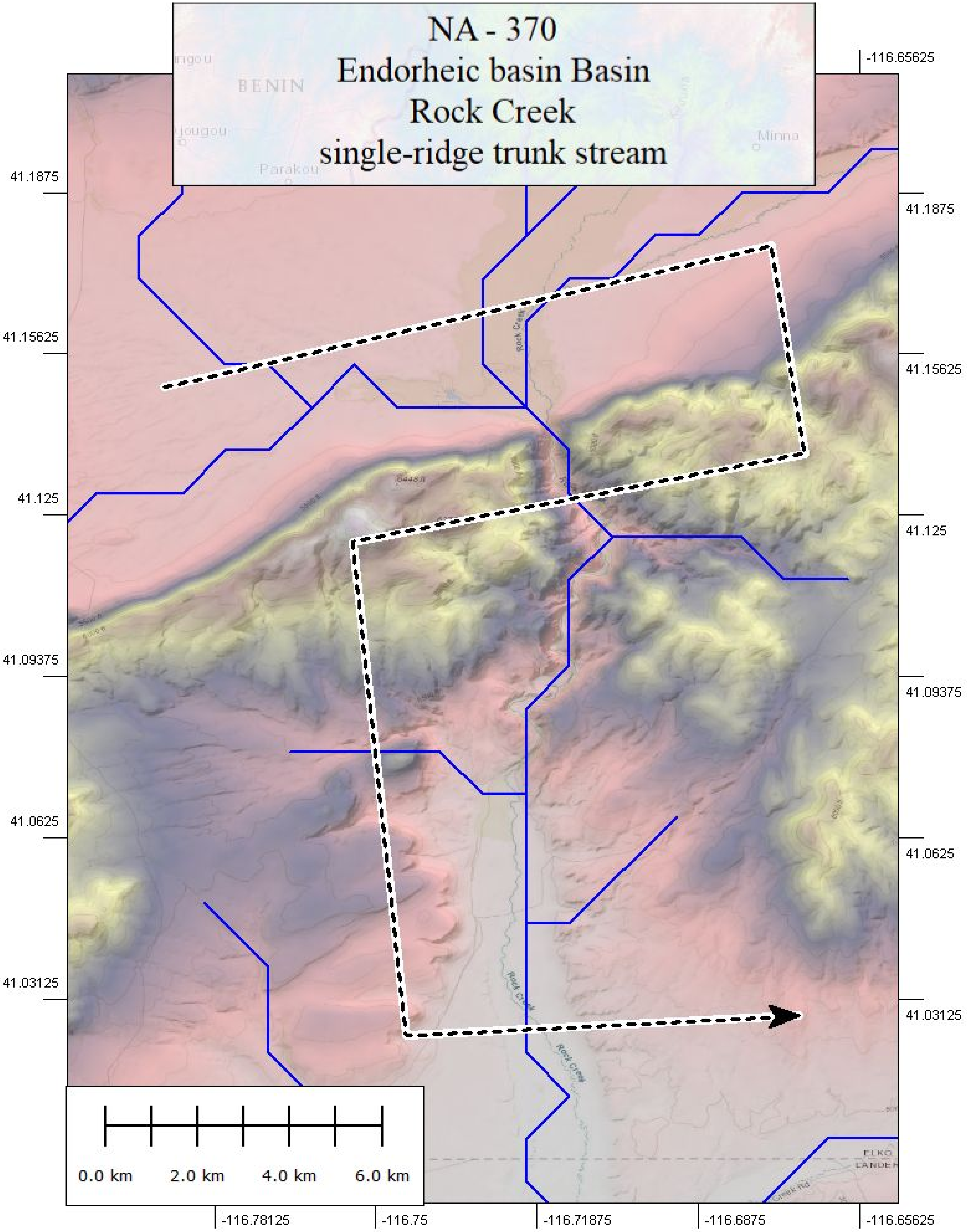
NA - 370
Endorheic basin Basin
Rock Creek
single-ridge trunk stream
-116.65625
41.1875
41.15625
41.125
41.09375
41.0625
41.03125
-116.78125
-116.75
-116.71875
-116.6875
0.0 km
2.0 km
4.0 km
6.0 km
Rock Creek
ELKO
BENIN
Parakou
Minna

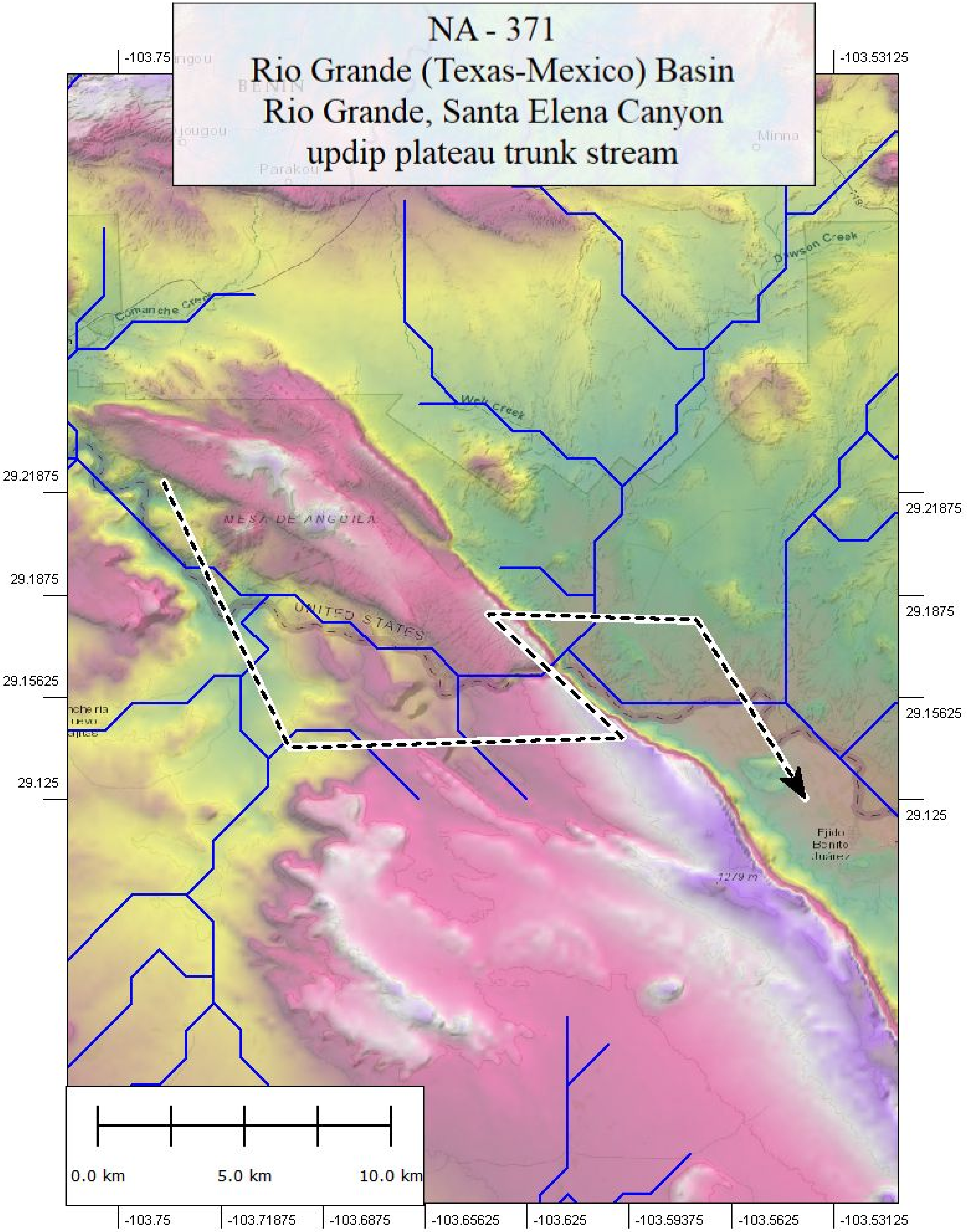

NA - 371
Rio Grande (Texas-Mexico) Basin
Rio Grande, Santa Elena Canyon
updip plateau trunk stream
-103.75
-103.53125
Comanche Creek
Dawson Creek
Well Creek
MESA DE ANGUILA
UNITED STATES
29.21875
29.1875
29.15625
29.125
Ejido Benito Juárez
1279 m
0.0 km
5.0 km
10.0 km
-103.71875
-103.6875
-103.65625
-103.625
-103.59375
-103.5625

NA - 372
Rio Grande (Texas-Mexico) Basin
Dog Canyon
single-ridge trunk stream

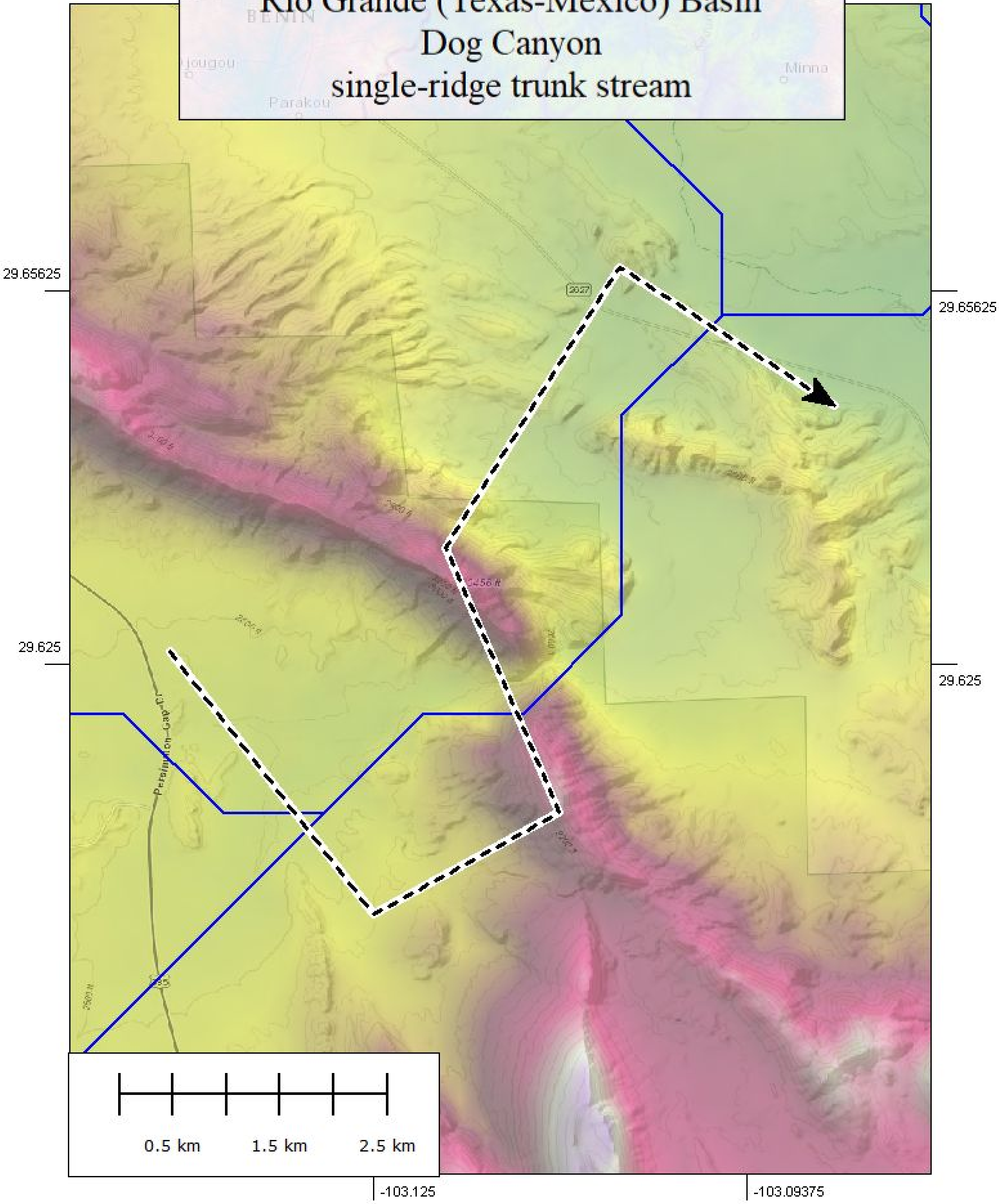

NA - 373
Rio Grande (Texas-Mexico) Basin
Rio Conchos tributary
single-ridge trunk stream

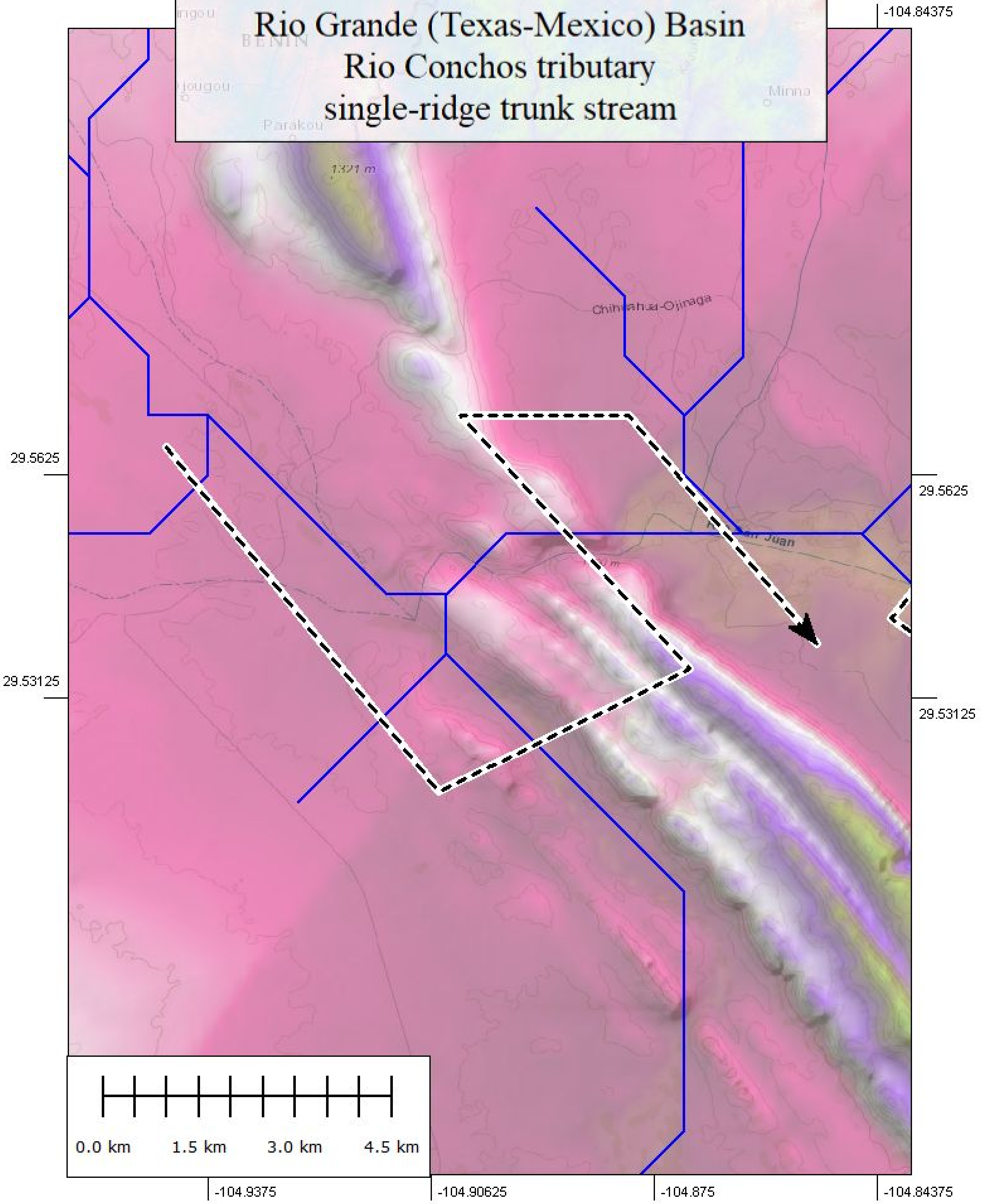

NA - 374
Rio Grande (Texas-Mexico) Basin
Rio Conchos, Peguis Canyon
irregular high ground trunk stream

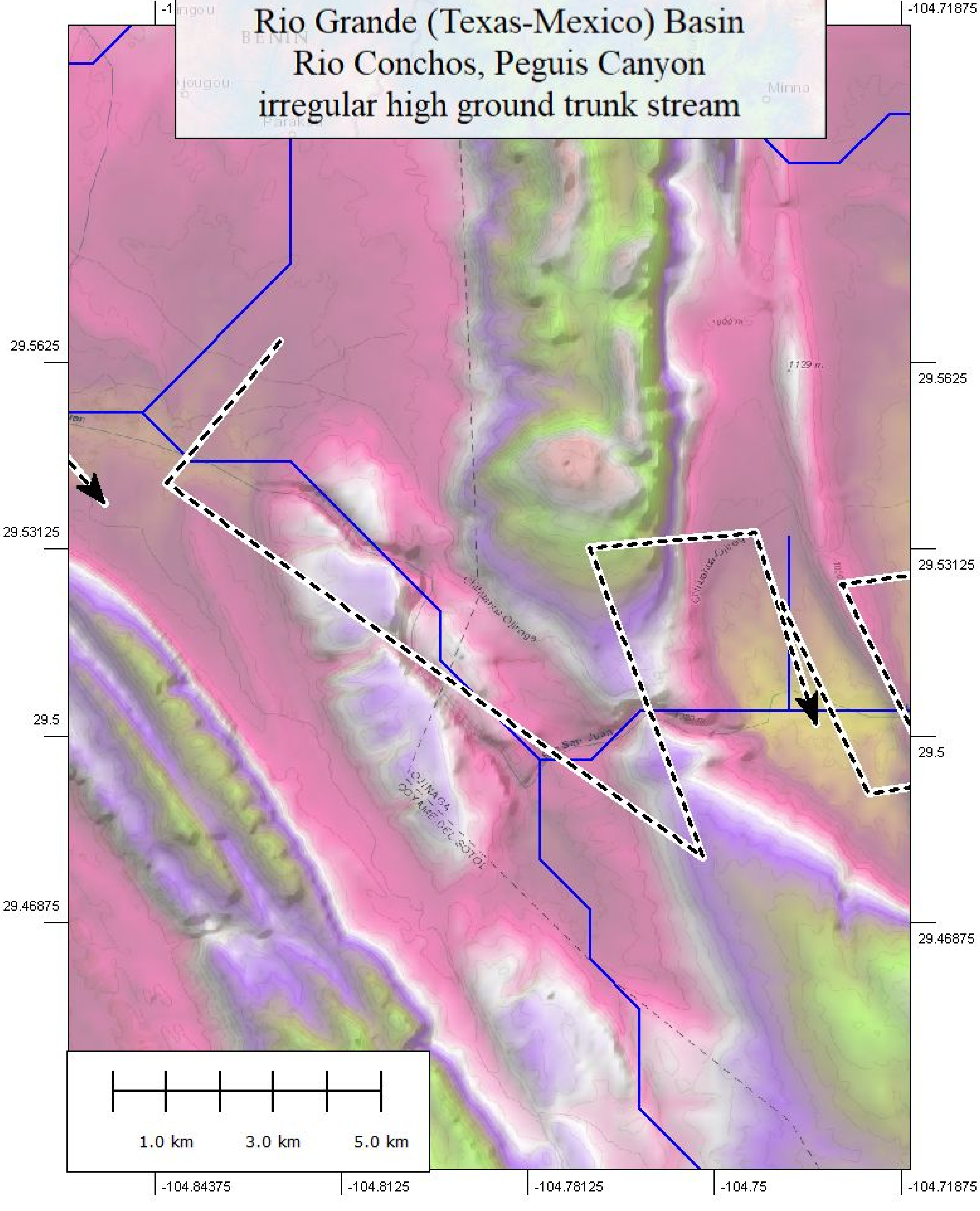

NA - 375
Mississippi River Basin
Kimberling Creek
single-ridge trunk stream

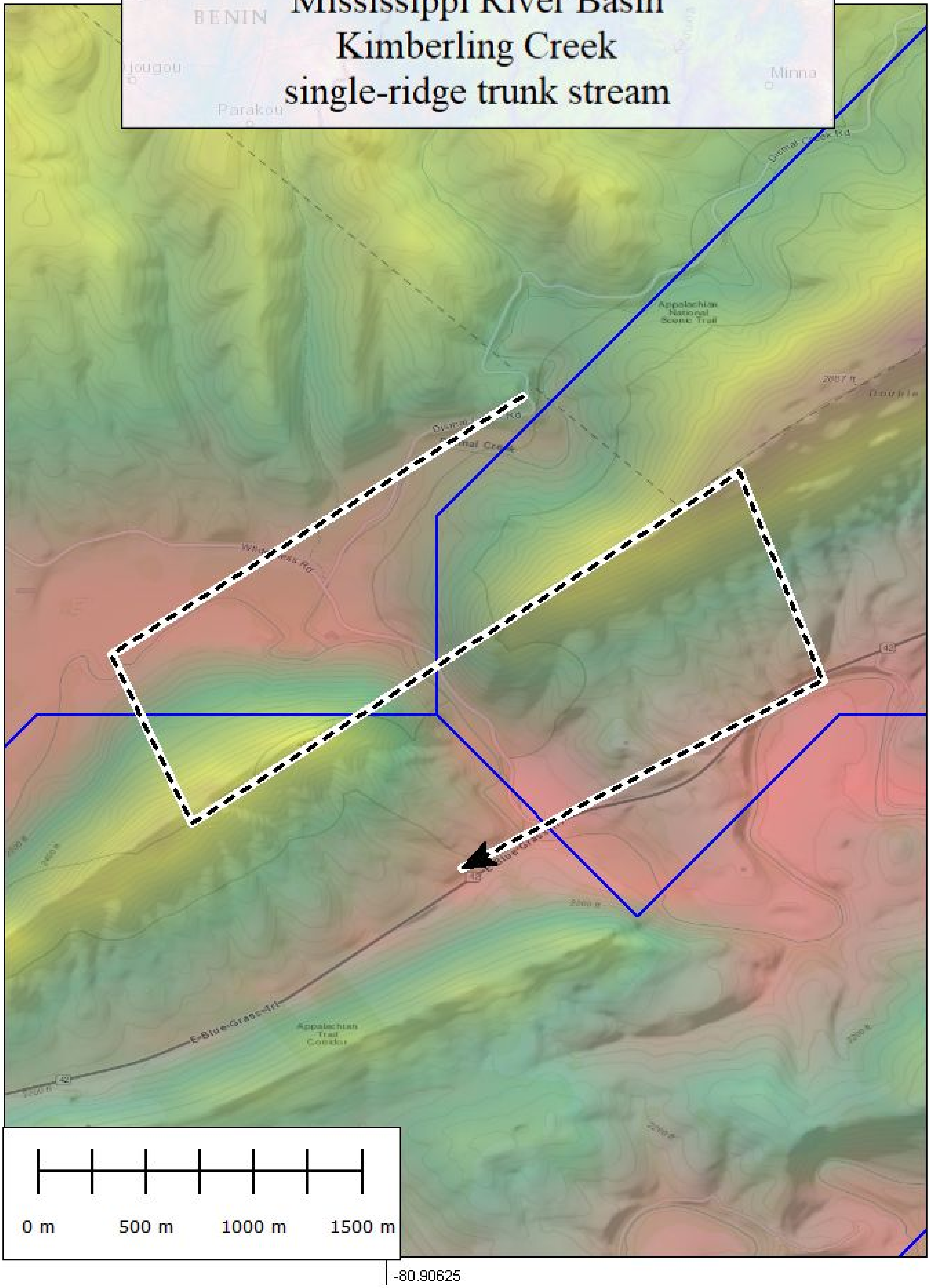

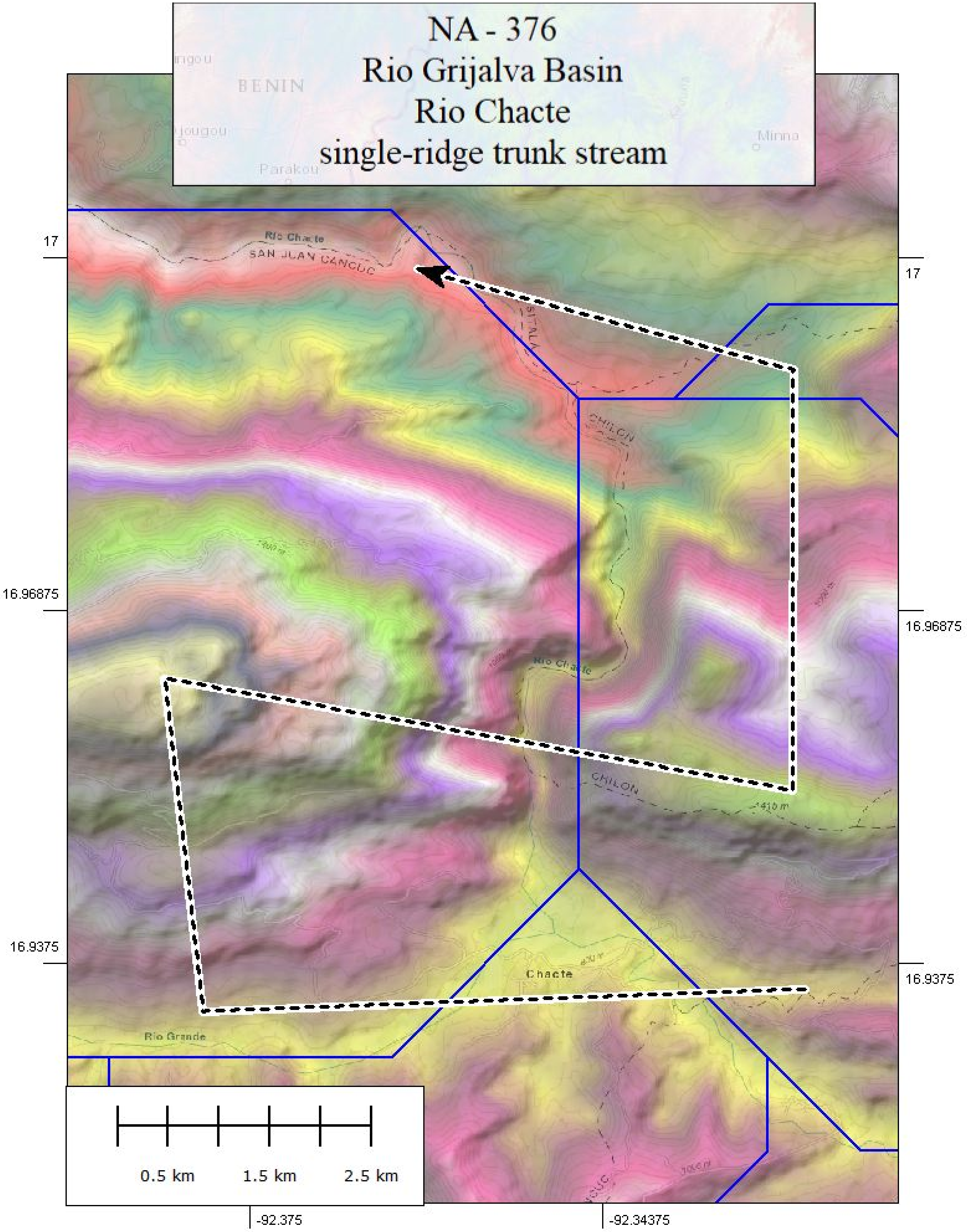
NA - 376
Rio Grijalva Basin
Rio Chacte
single-ridge trunk stream
BENIN
Parakou
Minna
Rio Chacte
SAN JUAN CANCUC
CHILON
Rio Chacte
CHILON
Chacte
Rio Grande
17
17
16.96875
16.96875
16.9375
16.9375
-92.375
-92.34375
0.5 km
1.5 km
2.5 km

NA - 377
Connecticut River Basin
Connecticut River, Mt Tom
single-ridge trunk stream

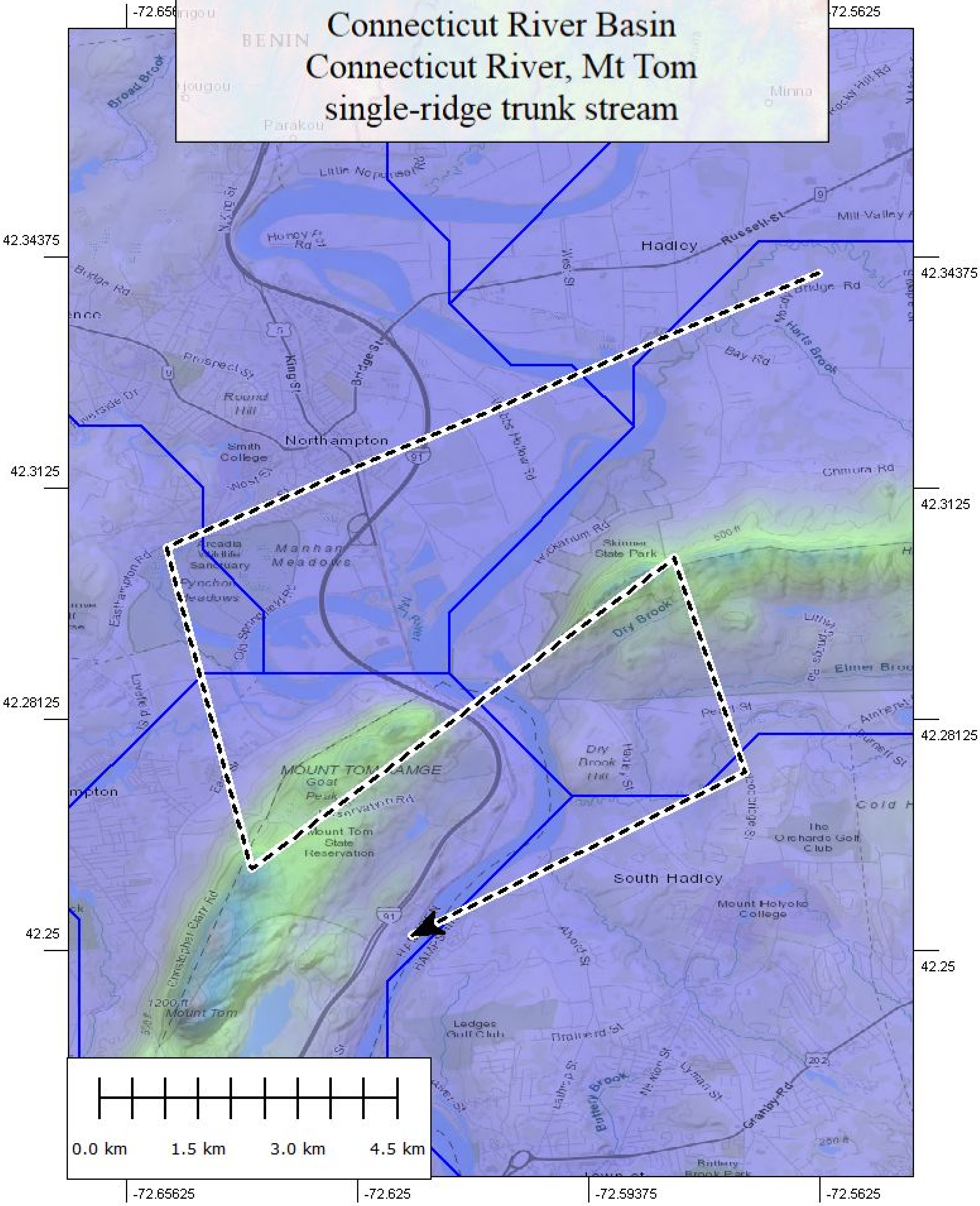

NA - 378
Mississippi River Basin
Anthony Creek
single-ridge trunk stream

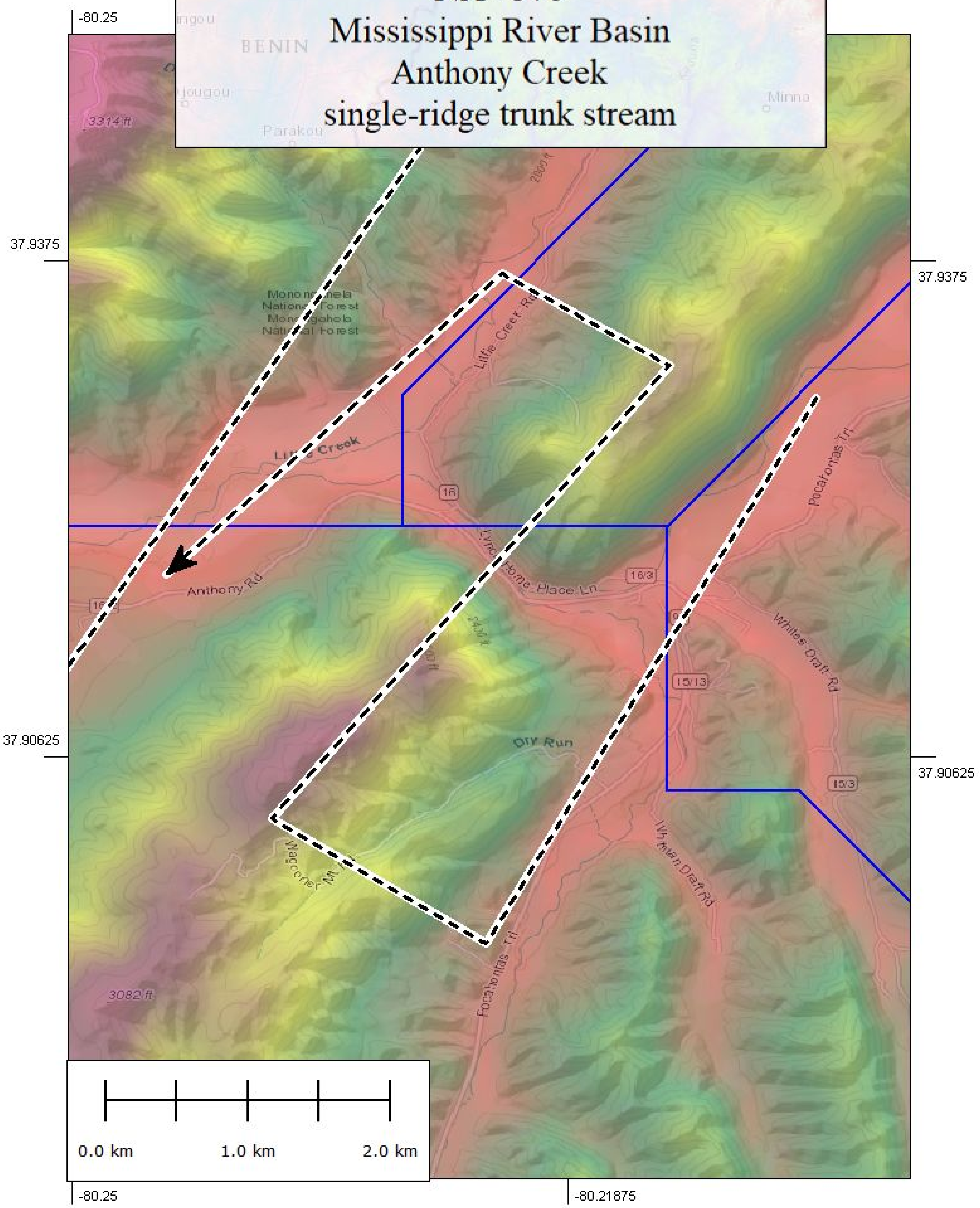

NA - 379
James River Basin
Johns Creek
single-ridge trunk stream

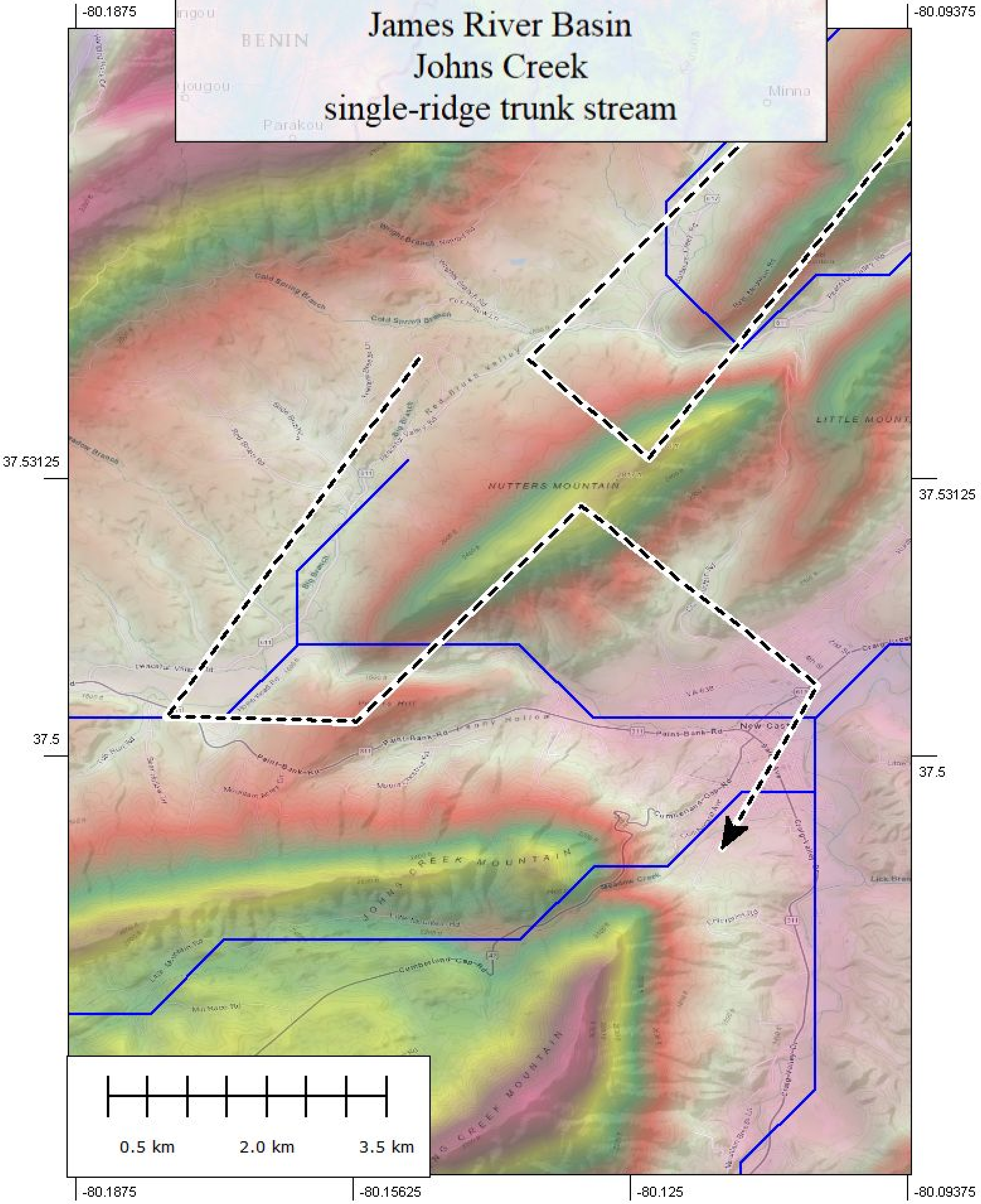

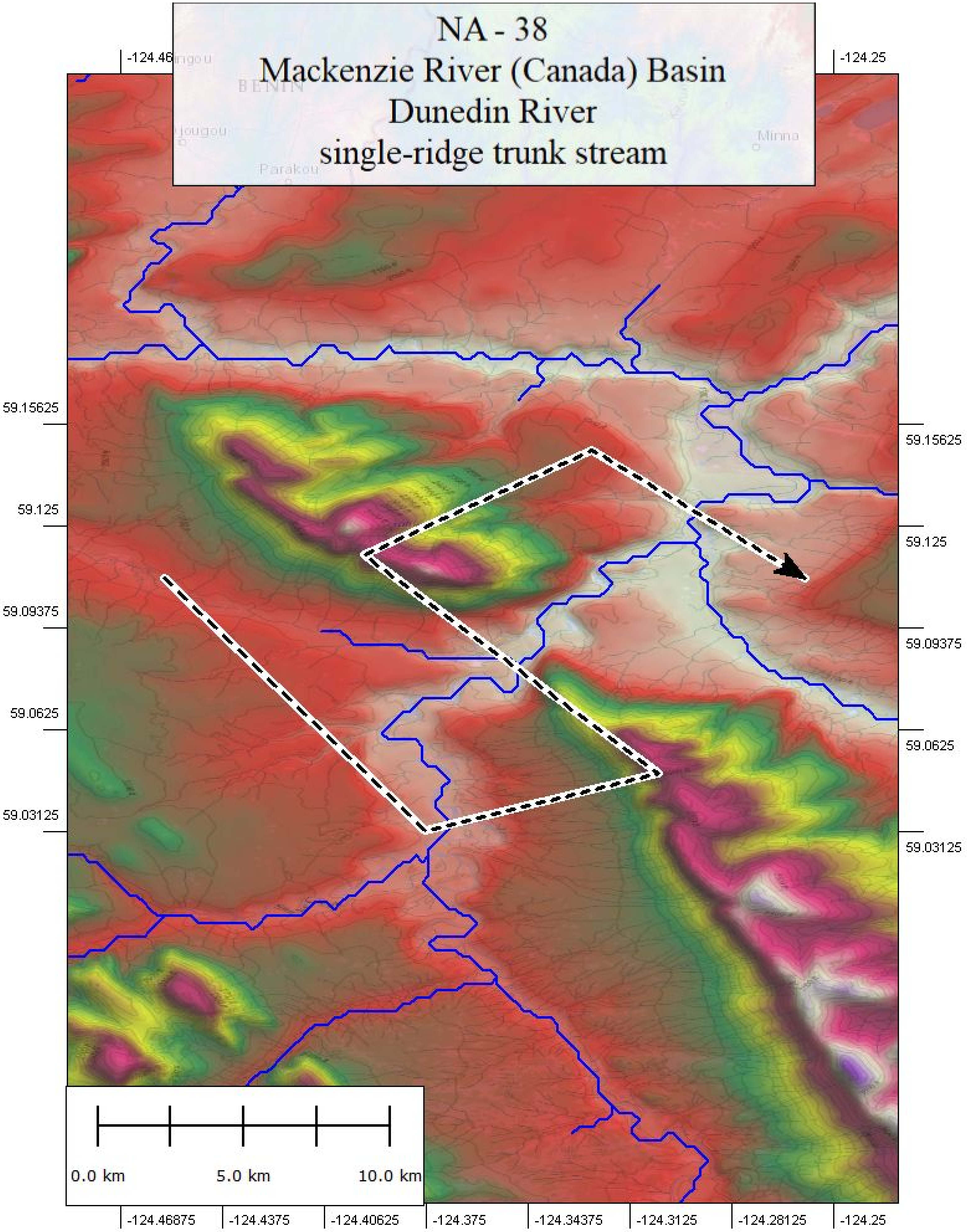
NA - 38
Mackenzie River (Canada) Basin
Dunedin River
single-ridge trunk stream
-124.46
-124.25
59.15625
59.125
59.09375
59.0625
59.03125
0.0 km
5.0 km
10.0 km
-124.46875
-124.4375
-124.40625
-124.375
-124.34375
-124.3125
-124.28125
-124.25

NA - 380
Colorado River (Arizona) Basin
Dolores River
downdip plateau trunk stream

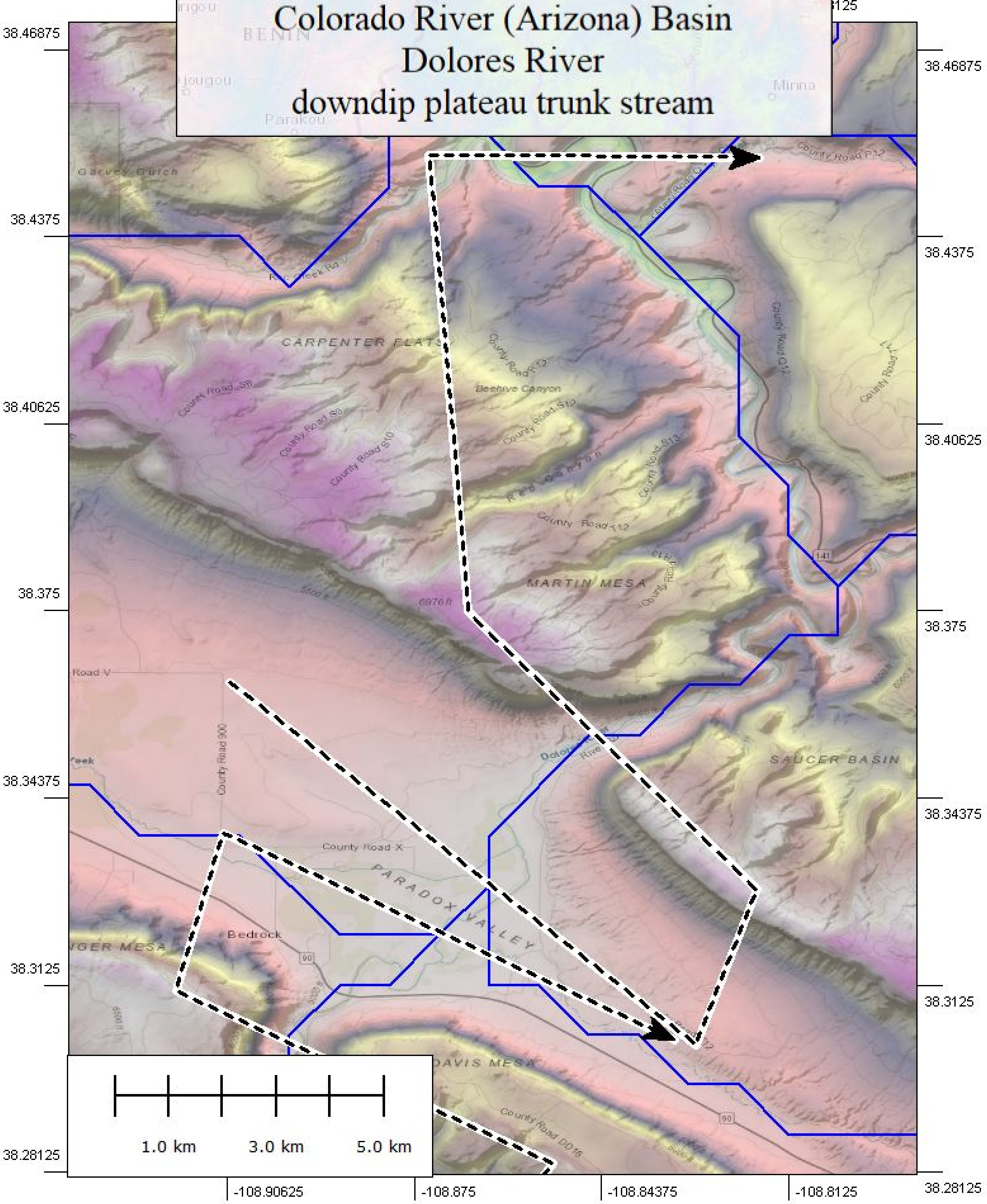

NA - 381
Sacramento River Basin
Feather River
irregular high ground trunk stream

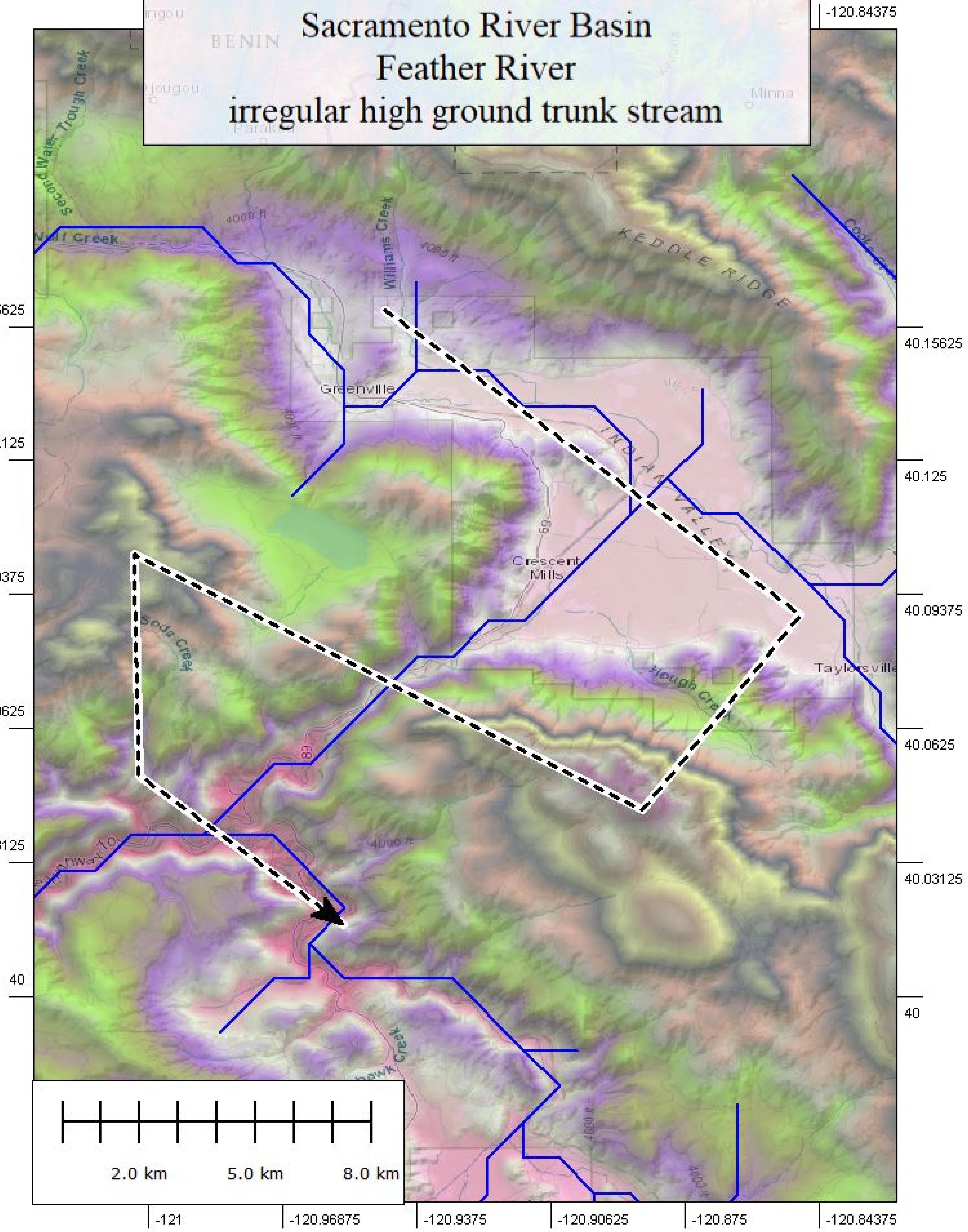

NA - 382
Potomac River Basin
South Branch Potomac River
single-ridge trunk stream

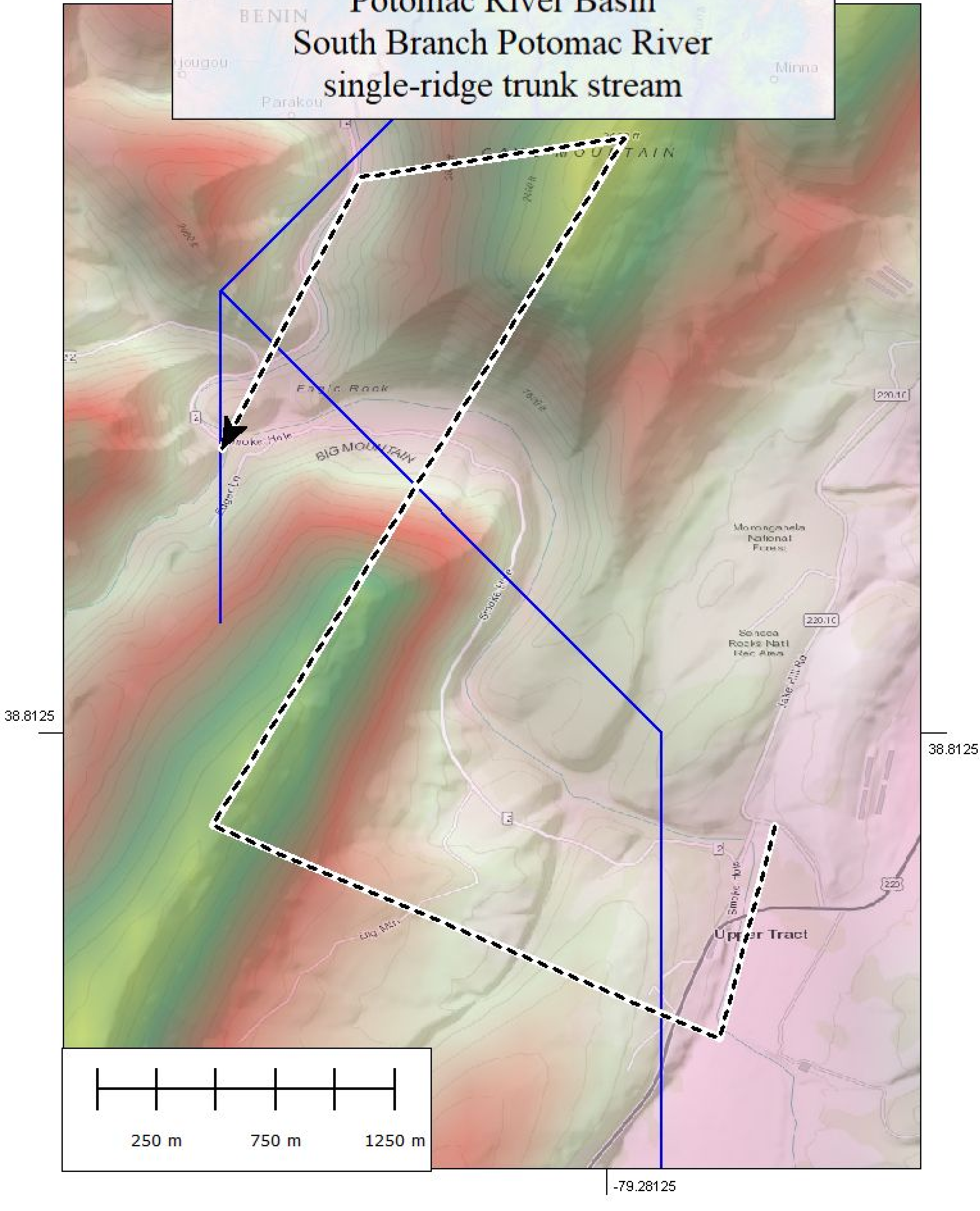

NA - 383
Susquehanna River Basin
Frankstown Branch Juniata River
single-ridge trunk stream

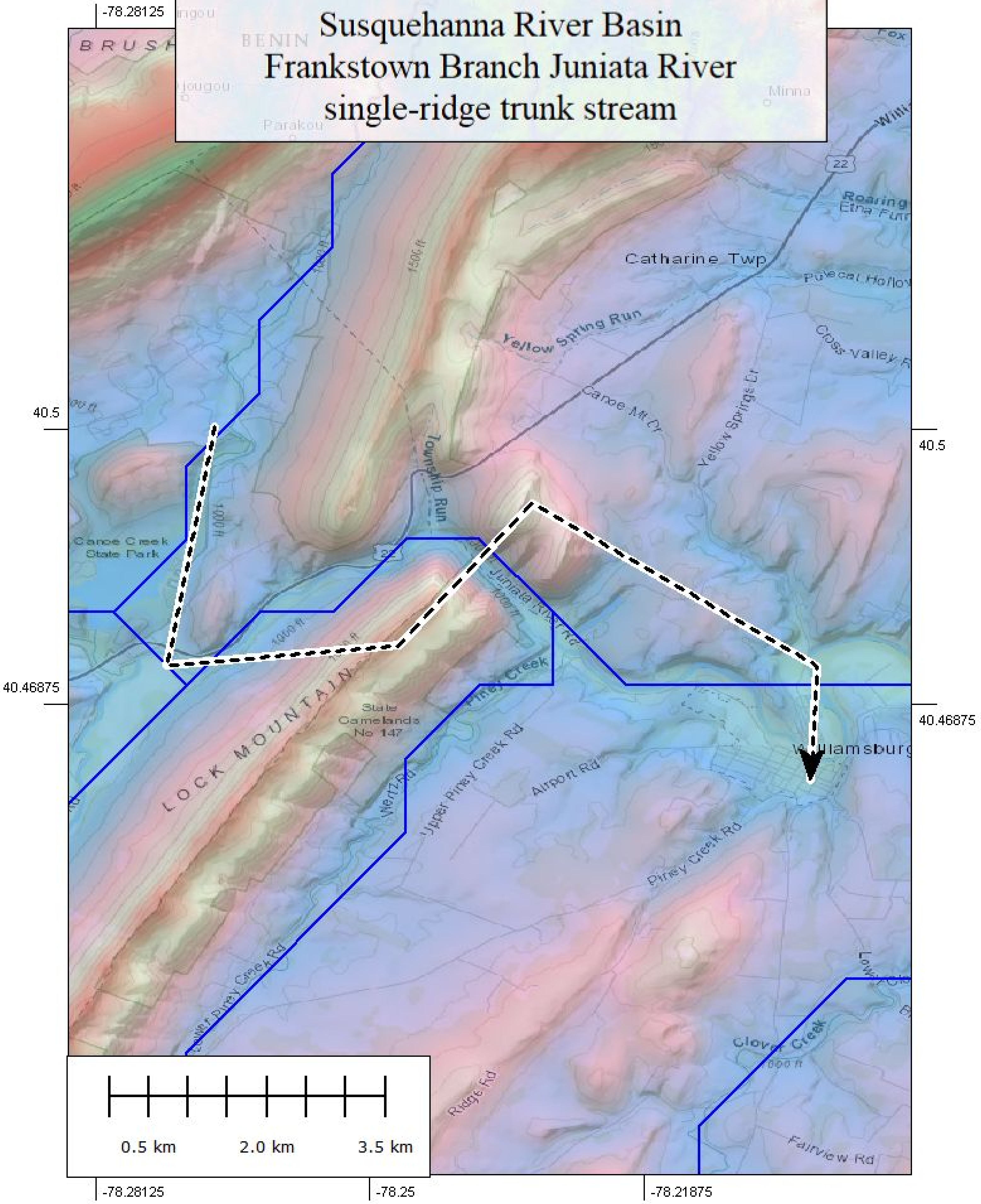

NA - 384
Susquehanna River Basin
Raystown Branch Juniata River
single-ridge trunk stream

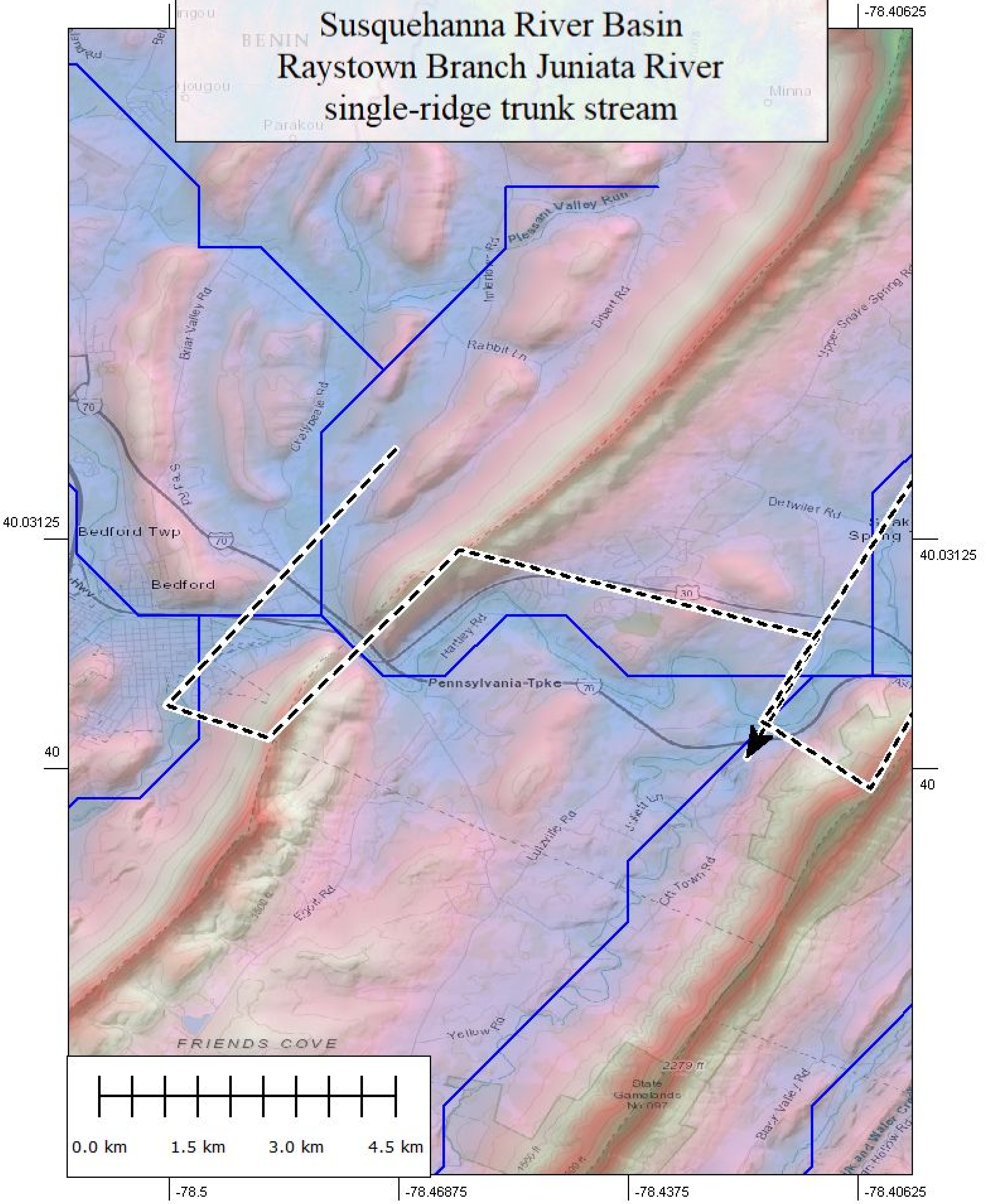

NA - 385
Susquehanna River Basin
Raystown Branch Juniata River
single-ridge trunk stream

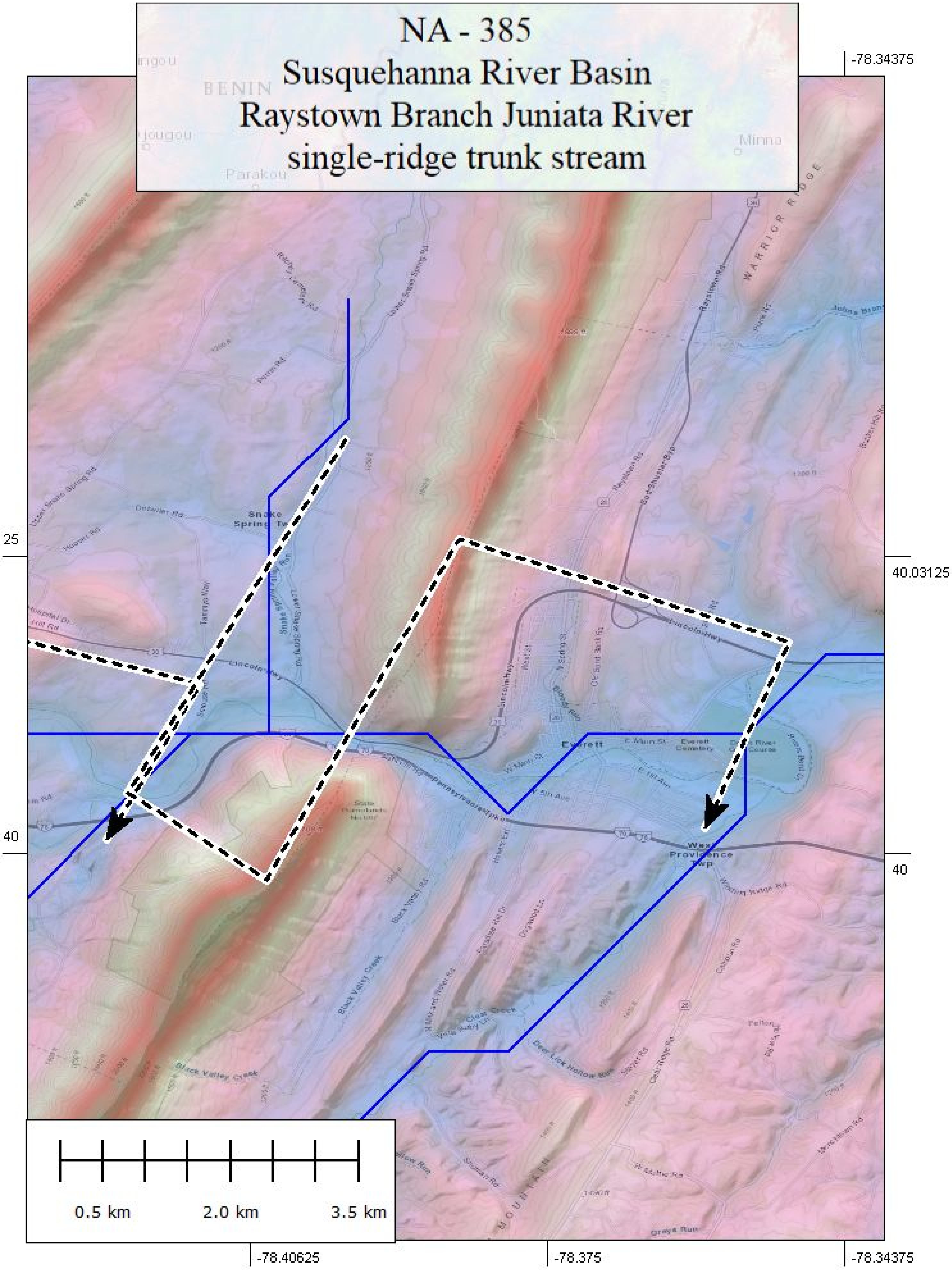

NA - 386
Mississippi River Basin
Tennesee River
single-ridge trunk stream

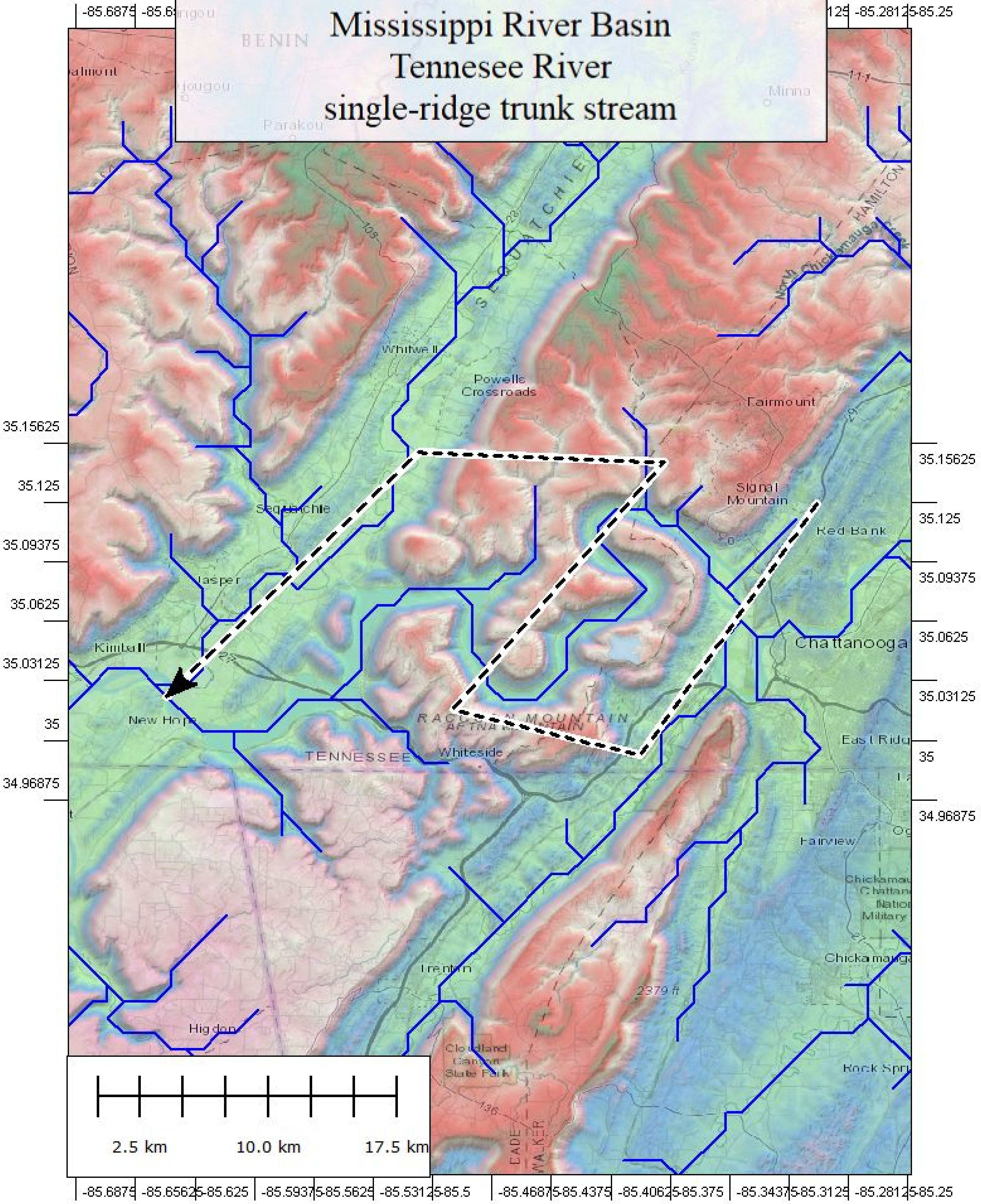

NA - 387
Mississippi River Basin
Hiwassee River, Hiwassee Gap
single-ridge trunk stream

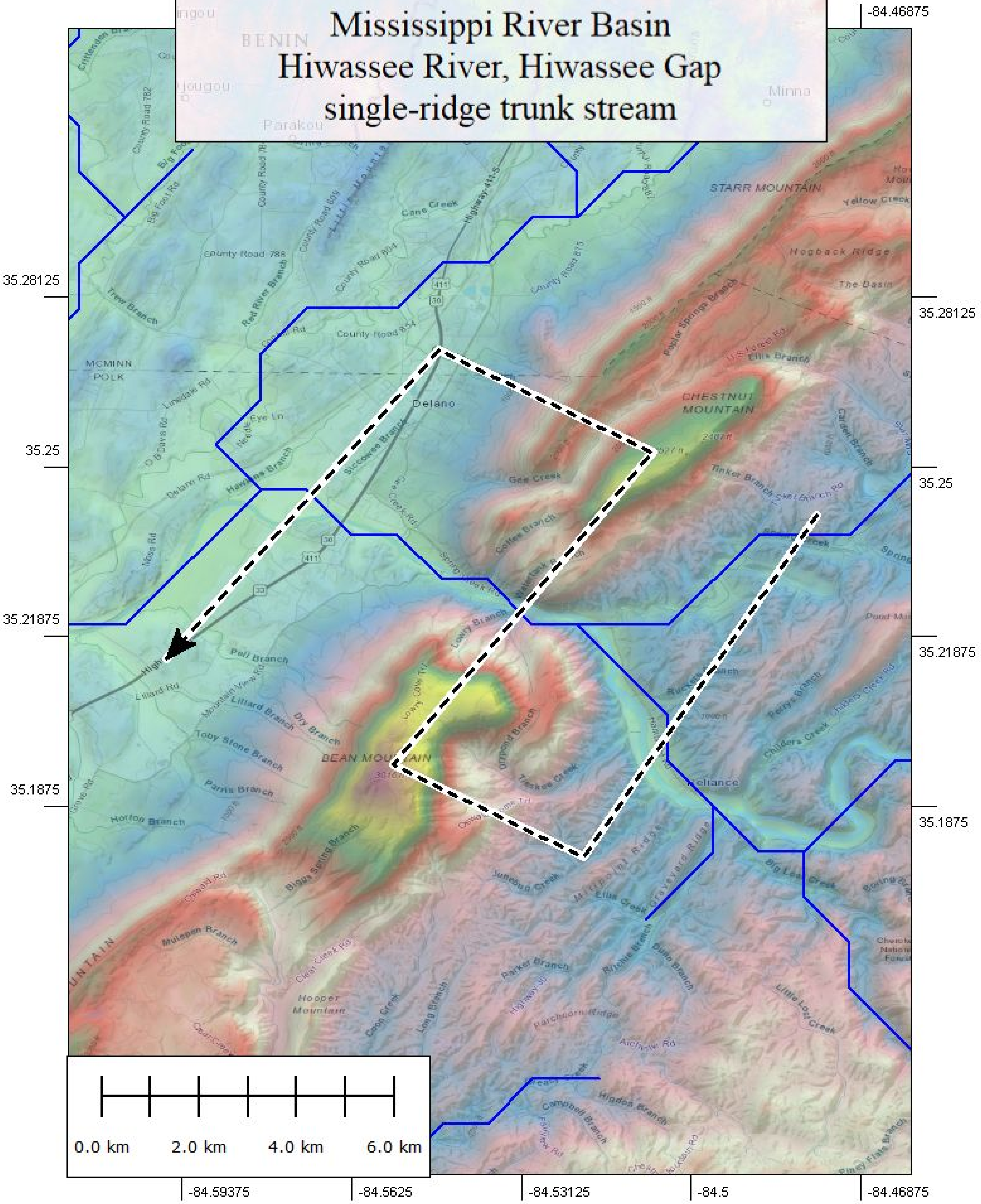

NA - 388
Susquehanna River Basin
Mahanoy Creek, Dorns Gap
single-ridge trunk stream

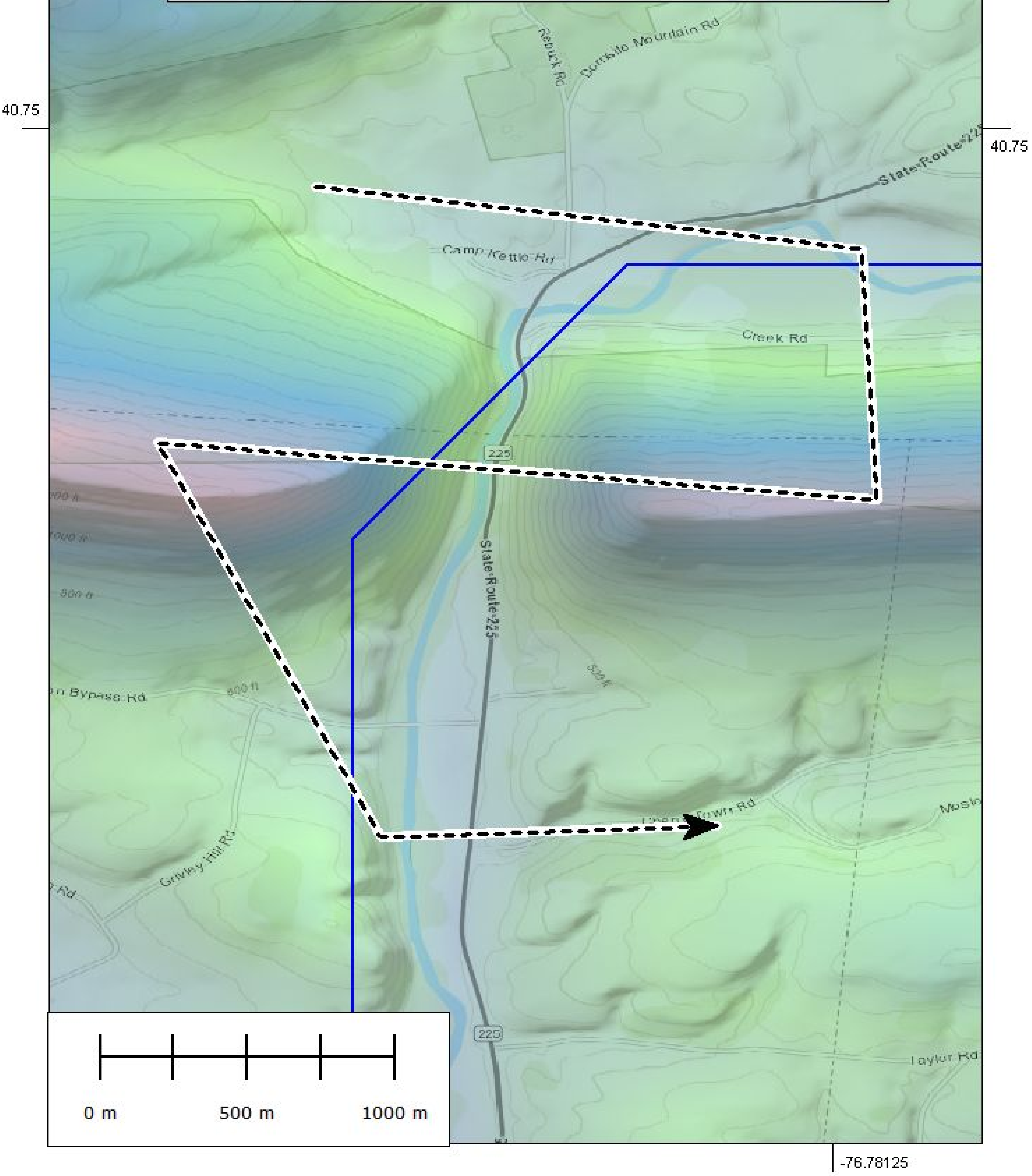

NA - 389
Colorado River (Arizona) Basin
Vermillion Creek
single-ridge trunk stream

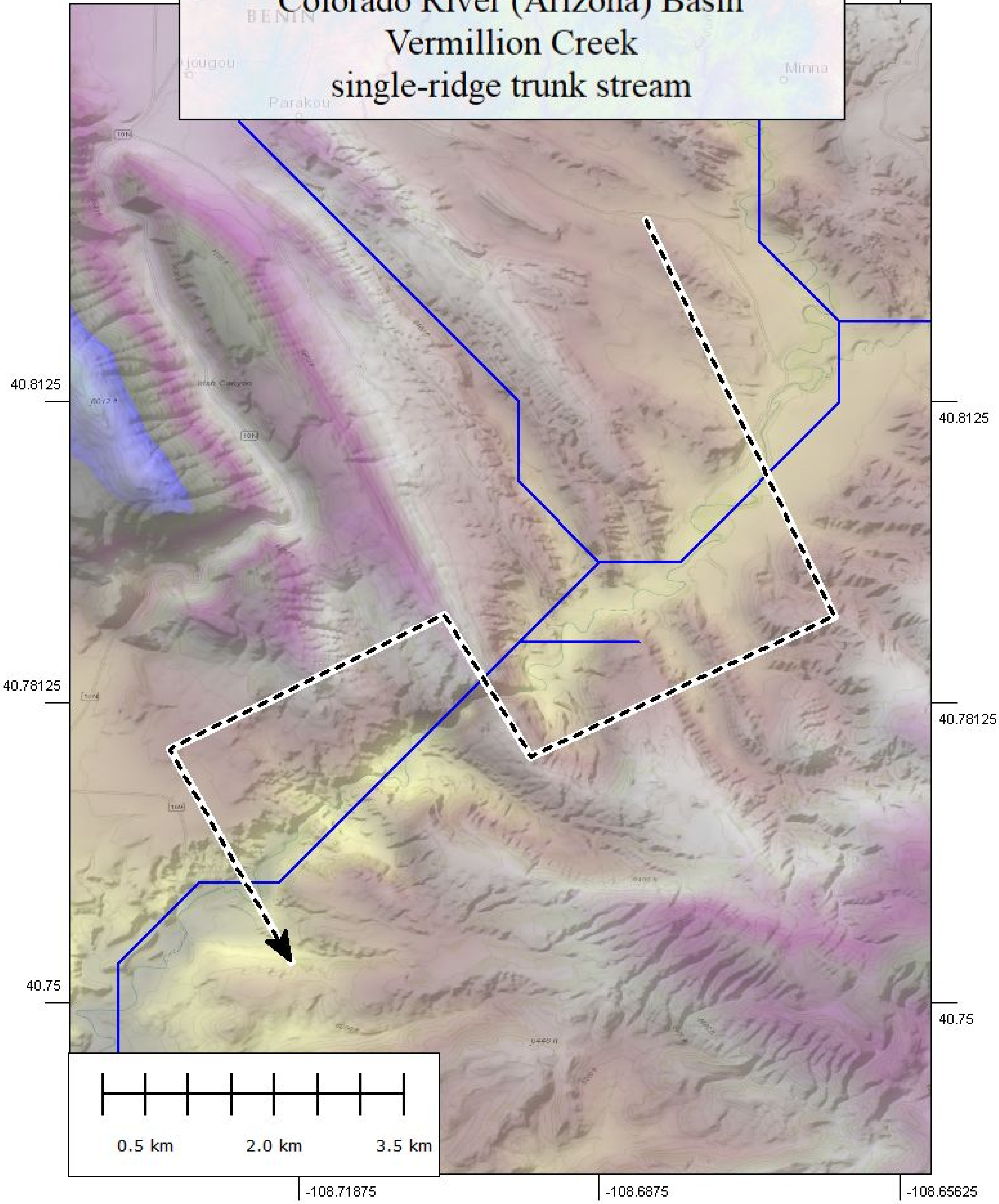

NA - 39
Mackenzie River (Canada) Basin
Dunedin River tributary
single-ridge head stream

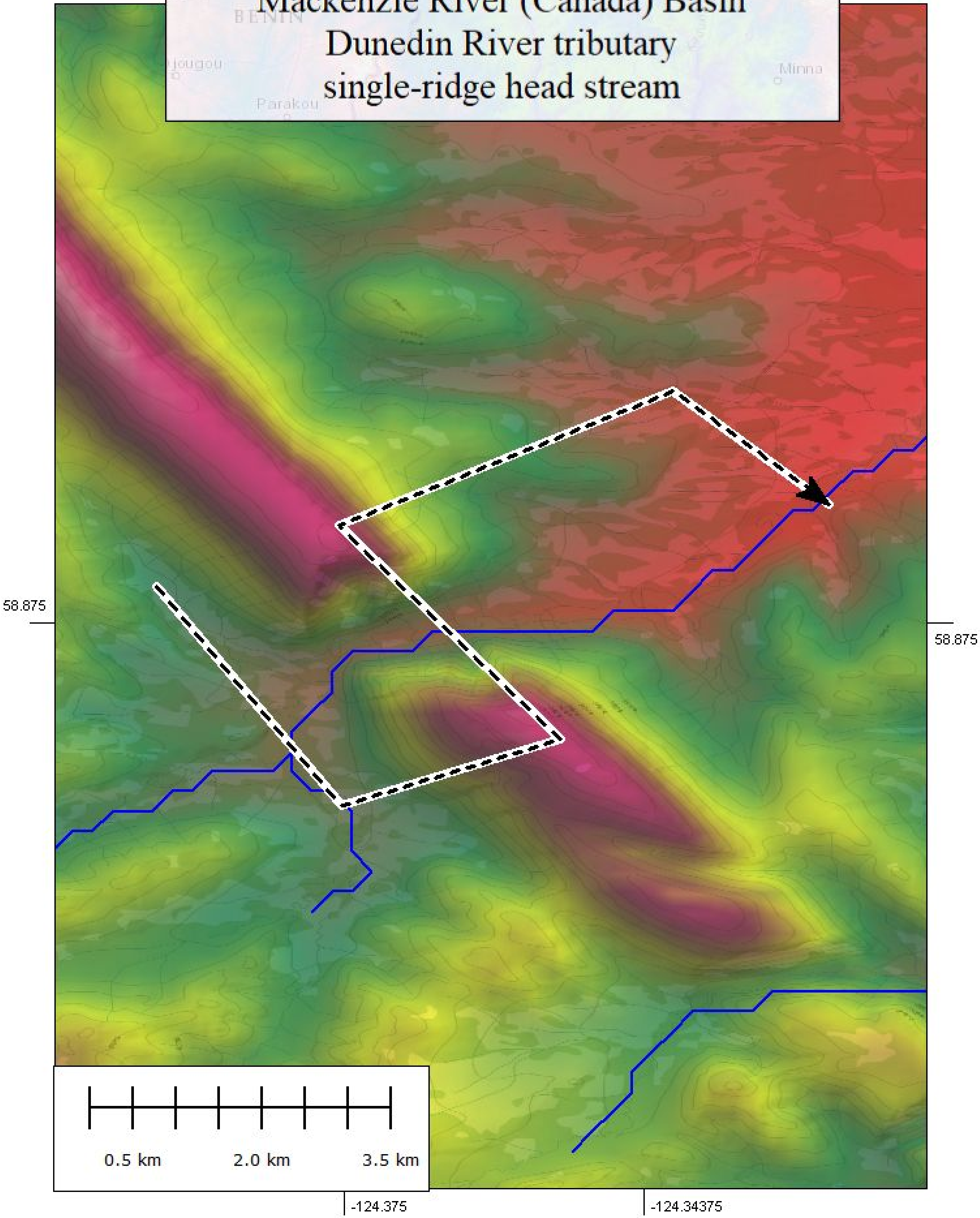

NA - 390
Susquehanna River Basin
Nescopeck Creek
single-ridge trunk stream

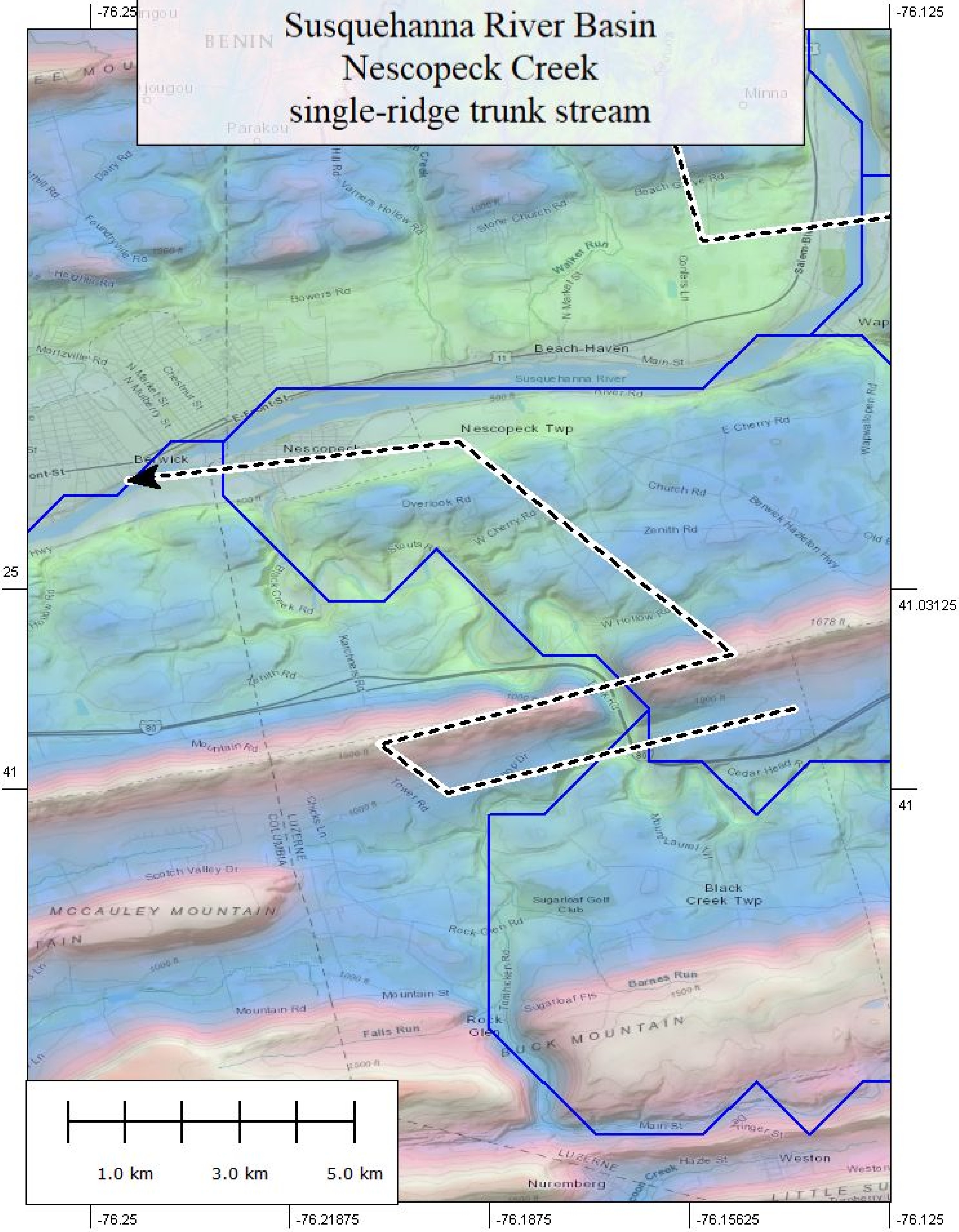

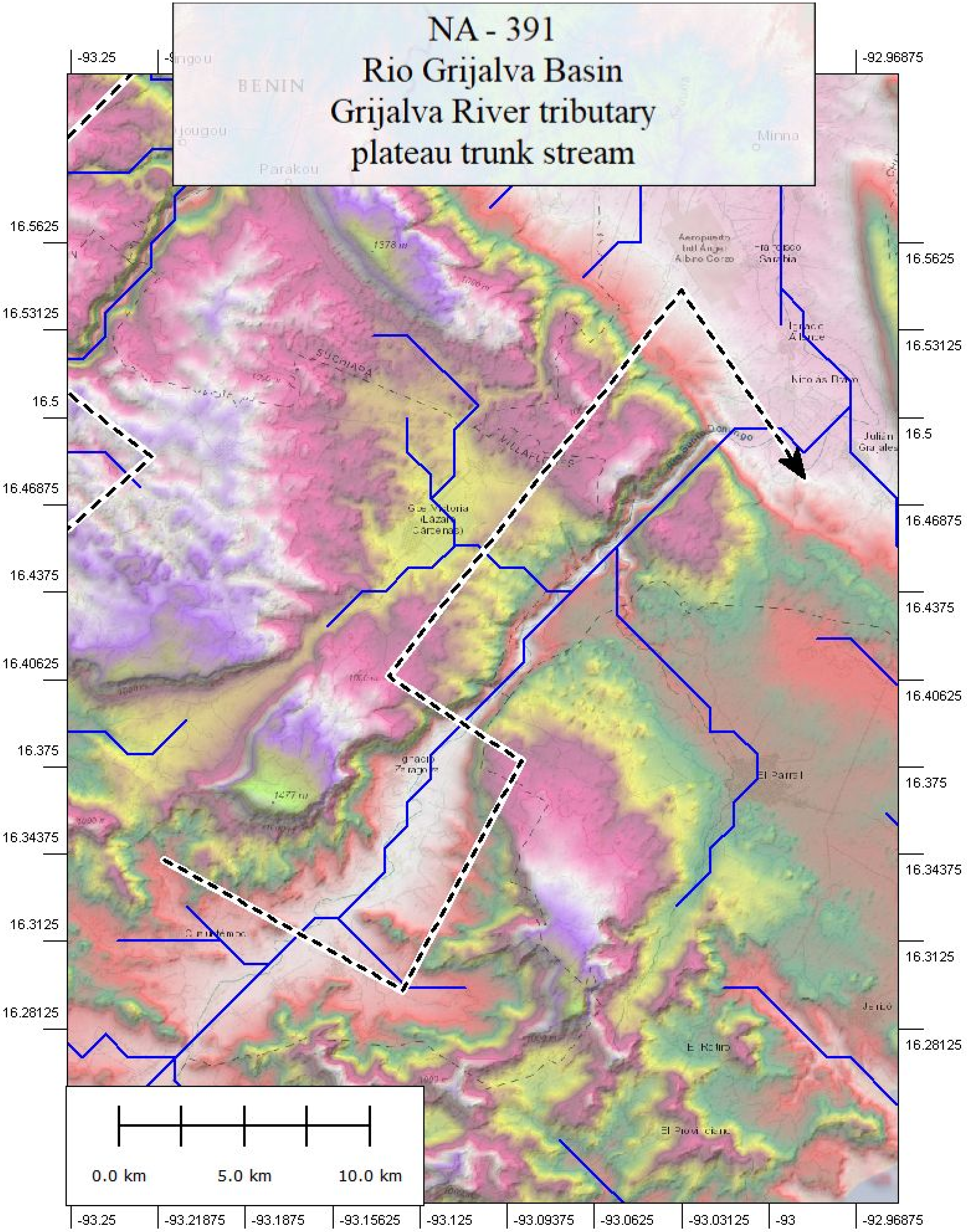
NA - 391
Rio Grijalva Basin
Grijalva River tributary
plateau trunk stream
-93.25
-93.21875
-93.1875
-93.15625
-93.125
-93.09375
-93.0625
-93.03125
-93
-92.96875
16.5625
16.53125
16.5
16.46875
16.4375
16.40625
16.375
16.34375
16.3125
16.28125
BENIN
Parakou
SUCHIAPA
VILLAFLORES
El Parral
El Retiro
0.0 km
5.0 km
10.0 km

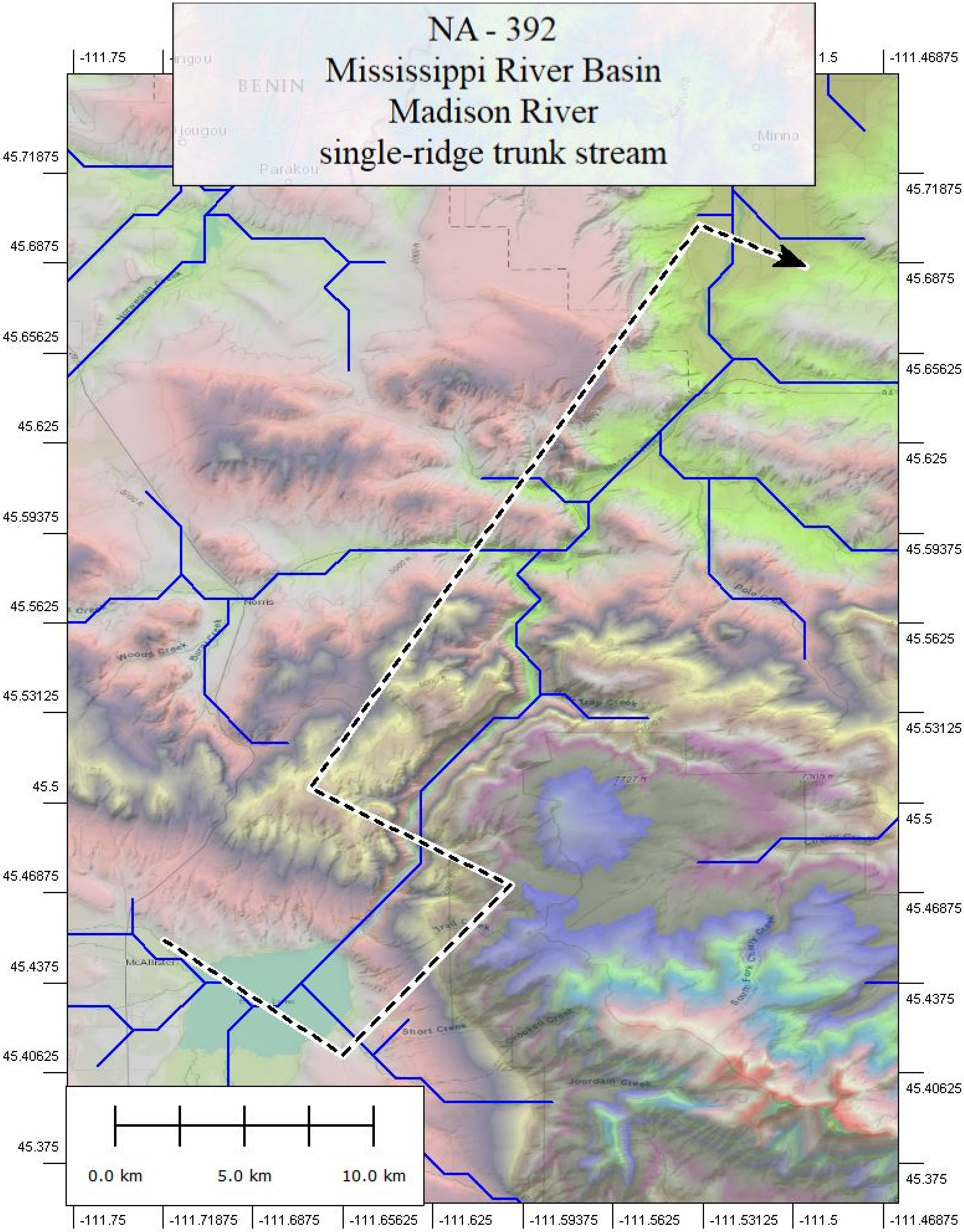
NA - 392
Mississippi River Basin
Madison River
single-ridge trunk stream
-111.75
-111.71875
-111.6875
-111.65625
-111.625
-111.59375
-111.5625
-111.53125
-111.5
-111.46875
45.71875
45.6875
45.65625
45.625
45.59375
45.5625
45.53125
45.5
45.46875
45.4375
45.40625
45.375
0.0 km
5.0 km
10.0 km
Norris
Woods Creek
McAllister
Short Creek
Trail Creek
Jourdain Creek
Crooked Creek
South Fork Cherry Creek
Trap Creek

NA - 393
Columbia River Basin
Snake River
single-ridge trunk stream

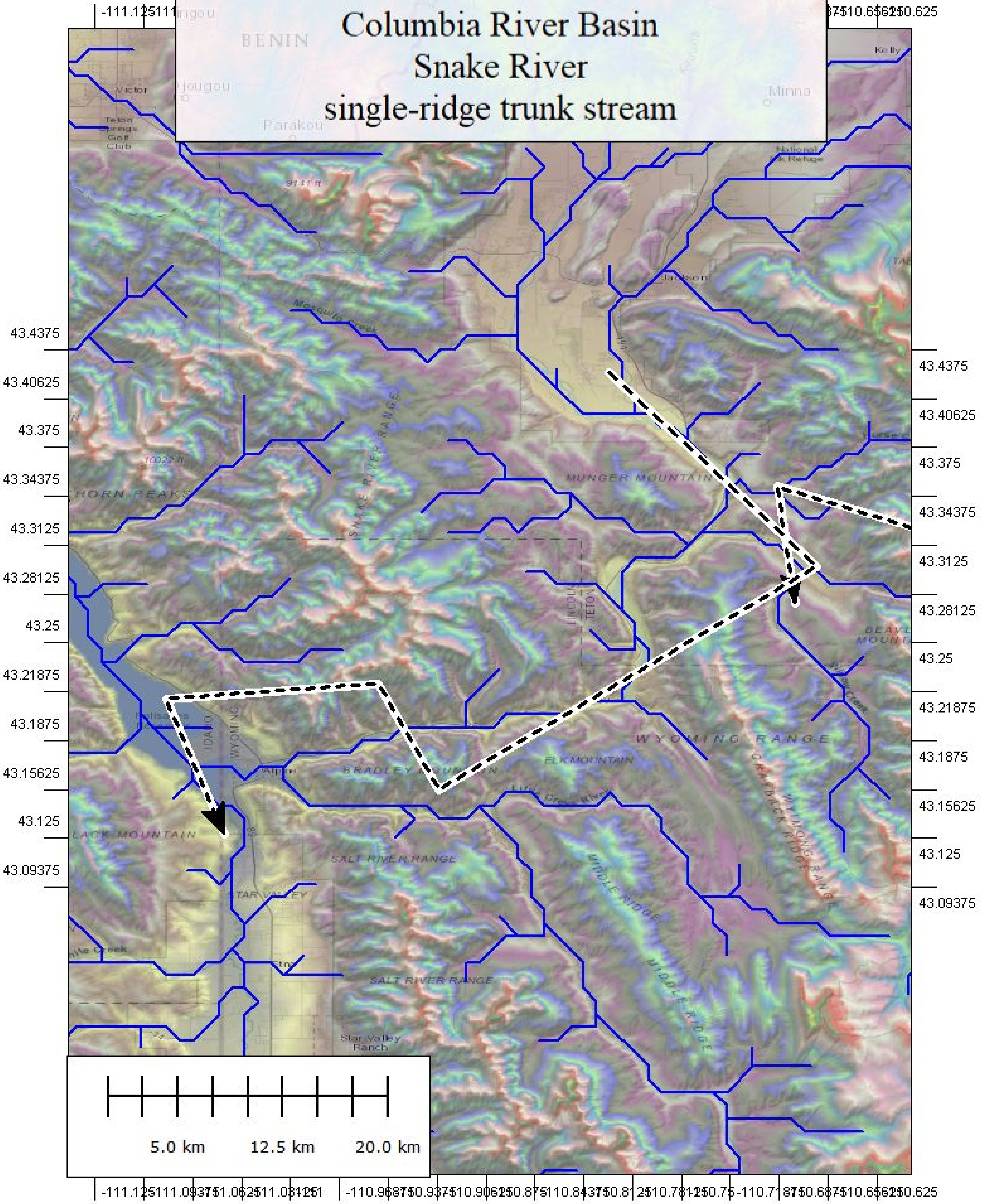

NA - 394
Colorado River (Arizona) Basin
Price River
irregular high ground trunk stream

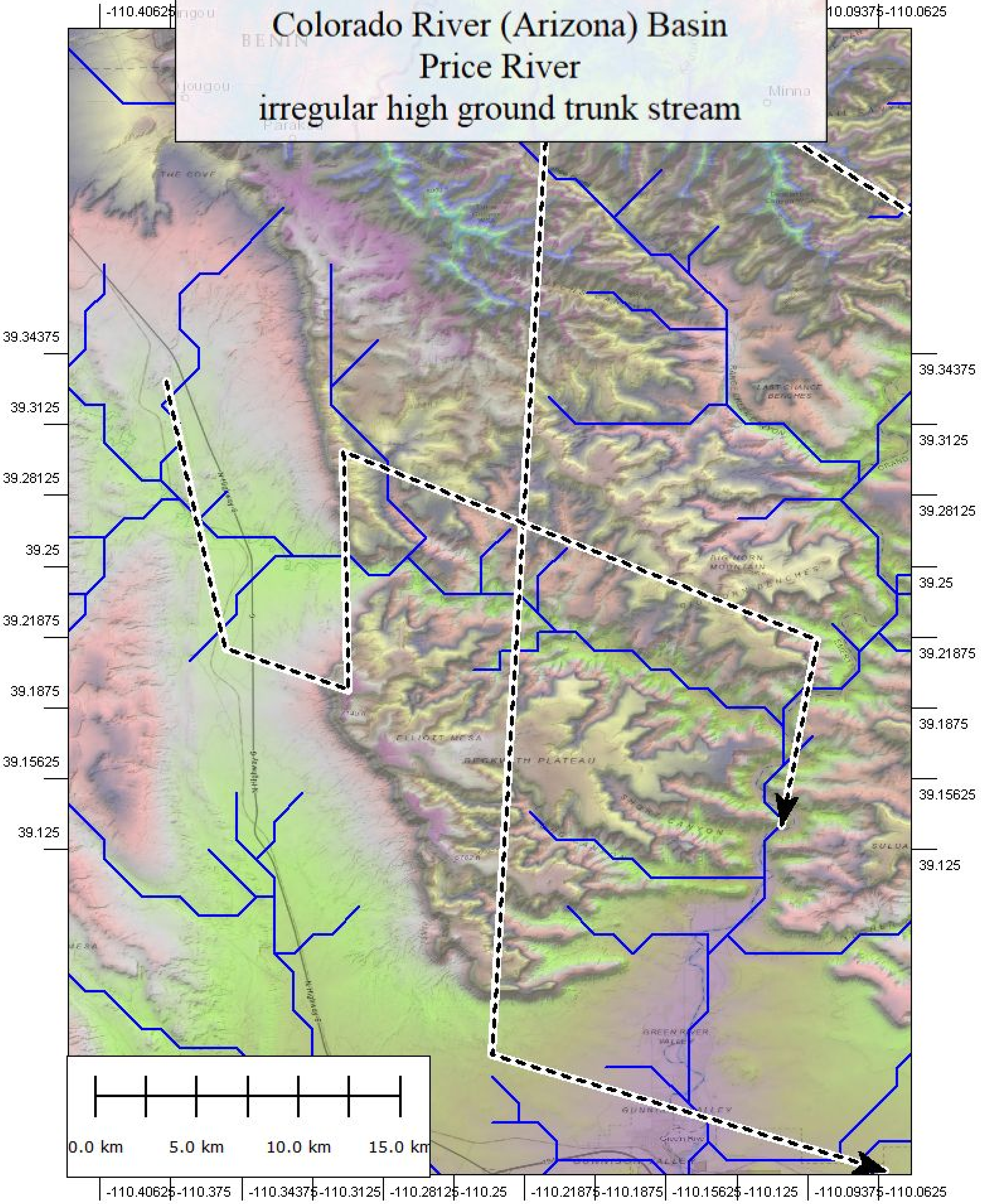

NA - 395
Pnuco River Basin
Rio Verde
single-ridge trunk stream

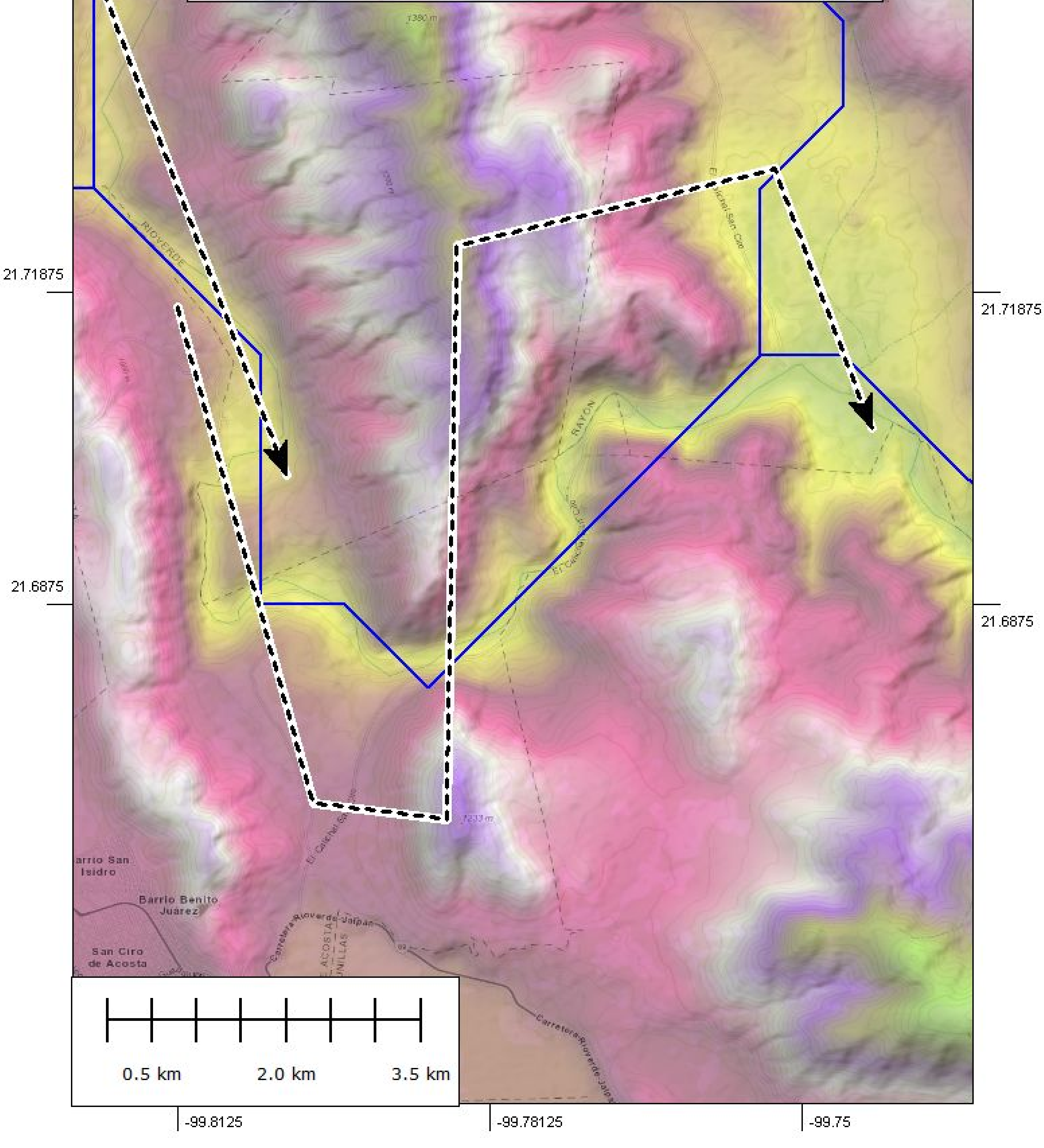

NA - 396
Colorado River (Arizona) Basin
Price River, Price Canyon
single-ridge trunk stream

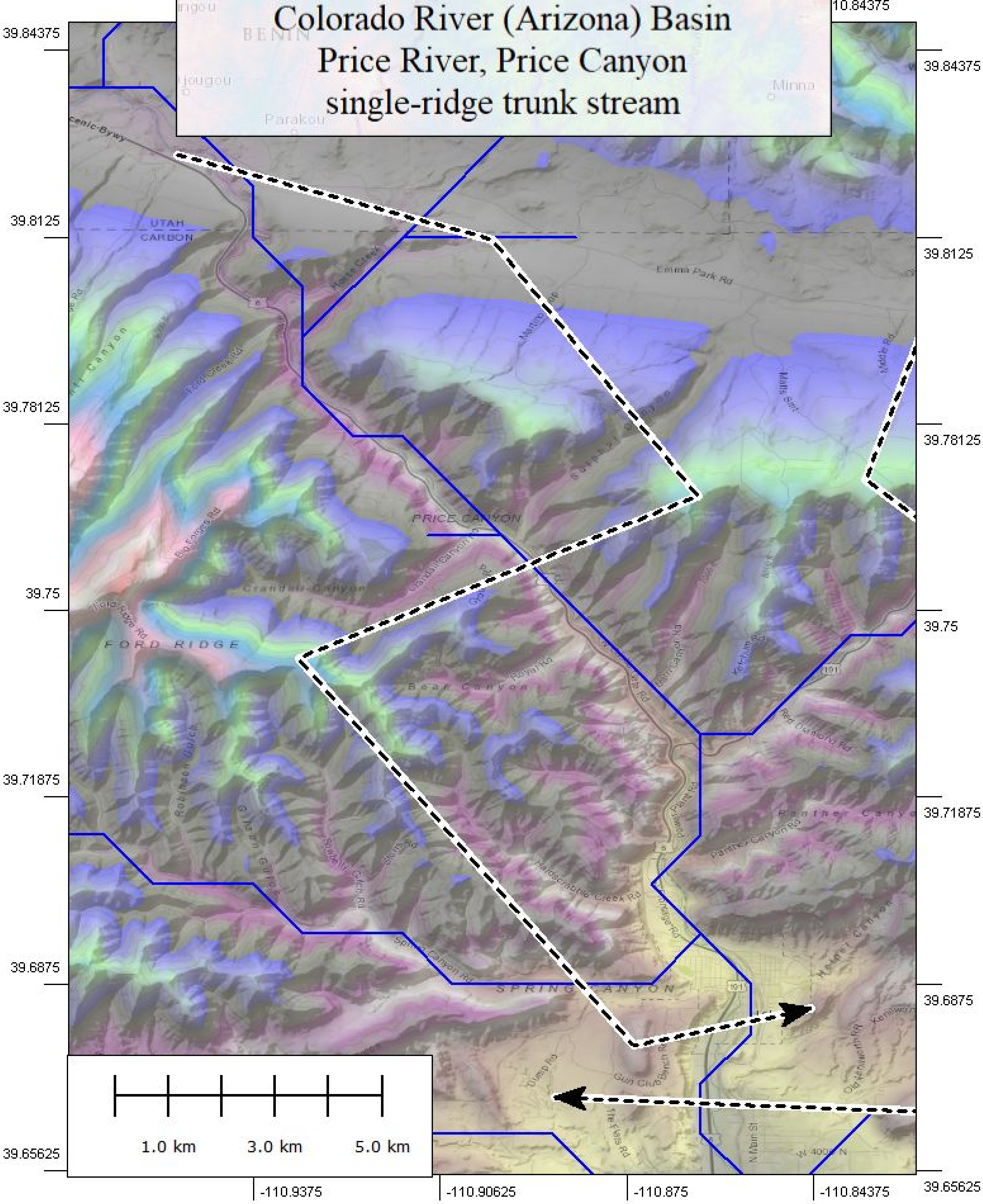

NA - 397
Endorheic basin Basin
Nazas River
multi-ridge trunk stream

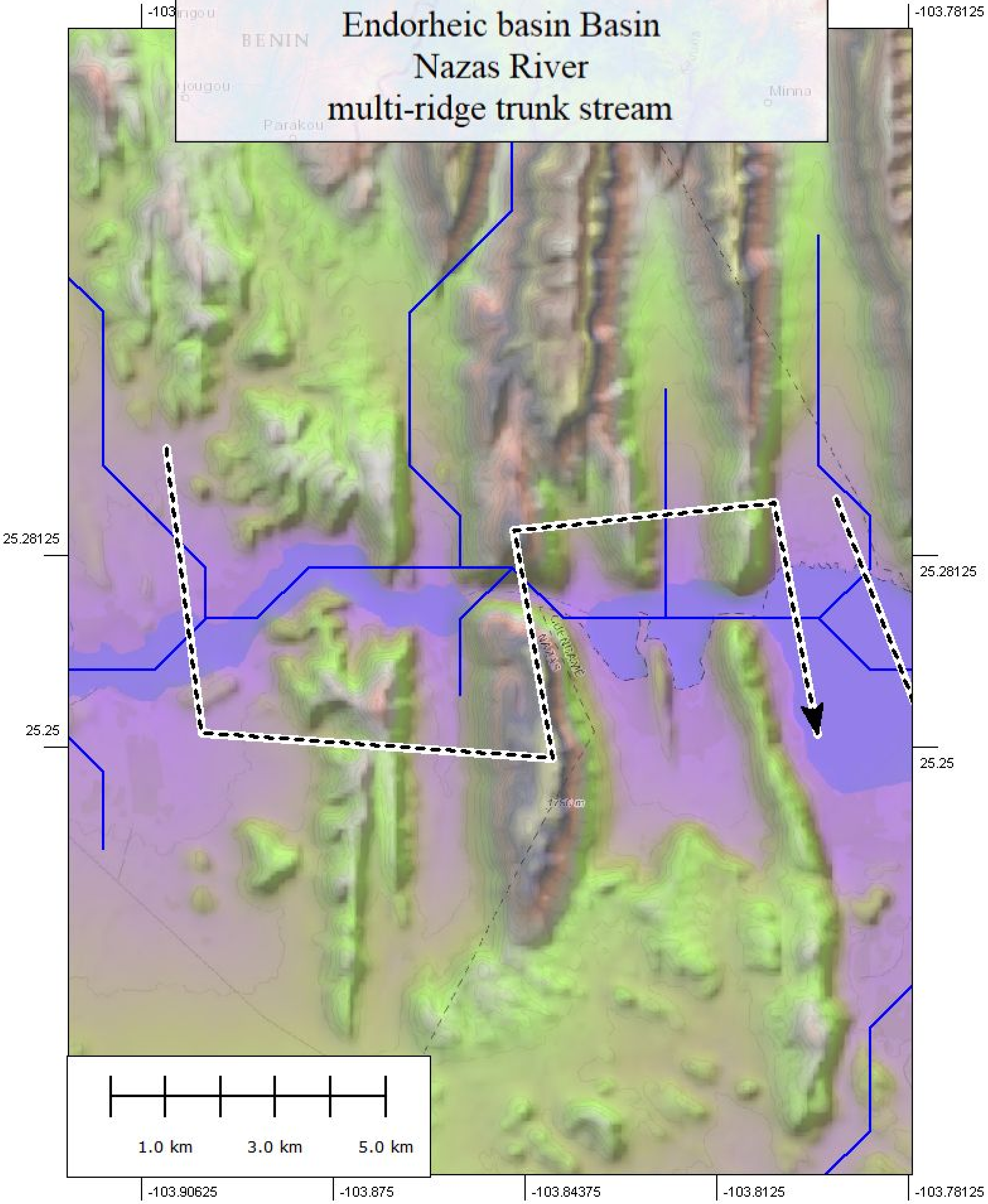

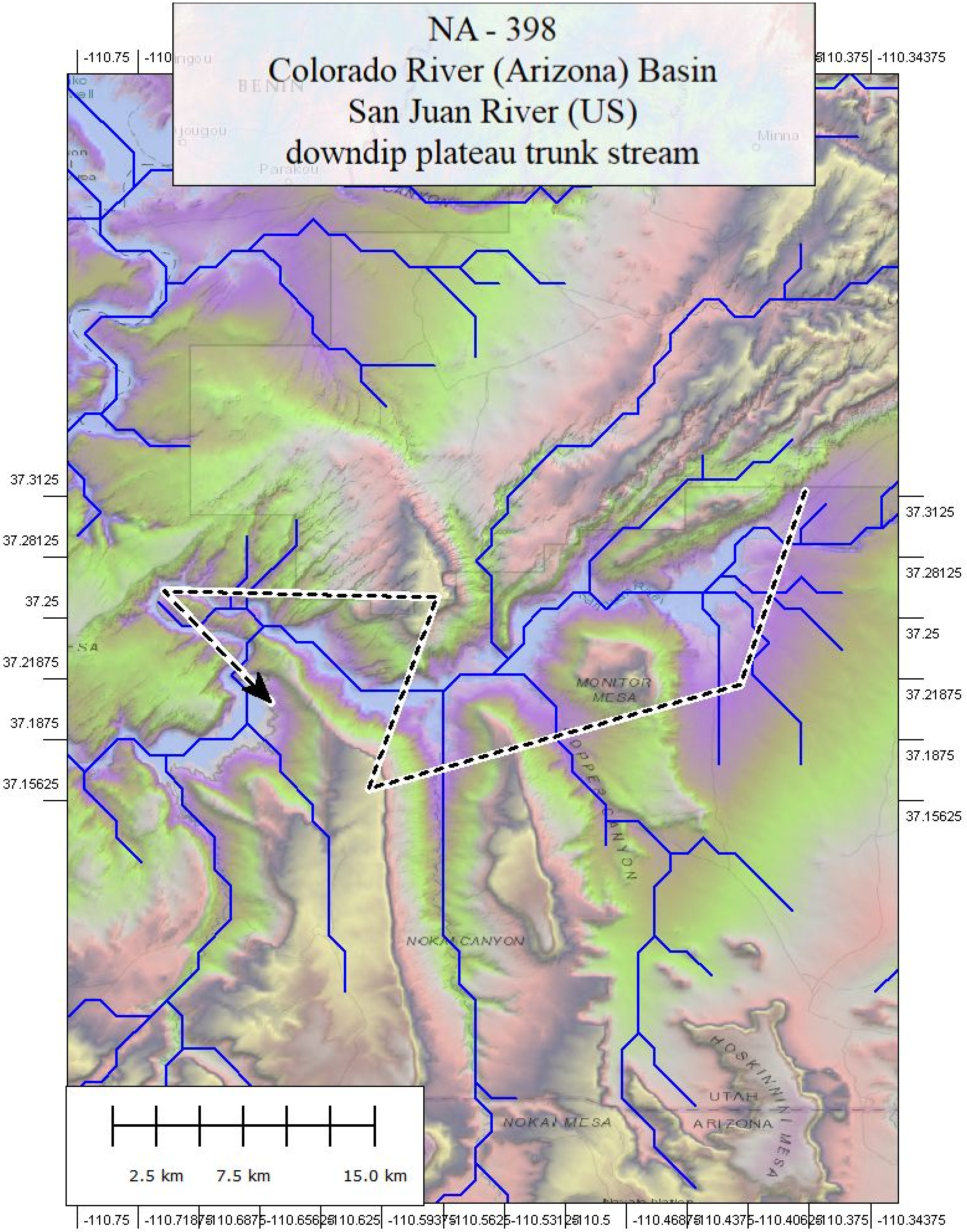
NA - 398
Colorado River (Arizona) Basin
San Juan River (US)
downdip plateau trunk stream
-110.75
-110.71875
-110.6875
-110.65625
-110.625
-110.59375
-110.5625
-110.53125
-110.5
-110.46875
-110.4375
-110.40625
-110.375
-110.34375
37.3125
37.28125
37.25
37.21875
37.1875
37.15625
MONITOR MESA
NOKAI CANYON
NOKAI MESA
HOSKINNINI MESA
UTAH
ARIZONA
2.5 km
7.5 km
15.0 km

NA - 399
Colorado River (Arizona) Basin
San Miguel River tributary
single-ridge trunk stream

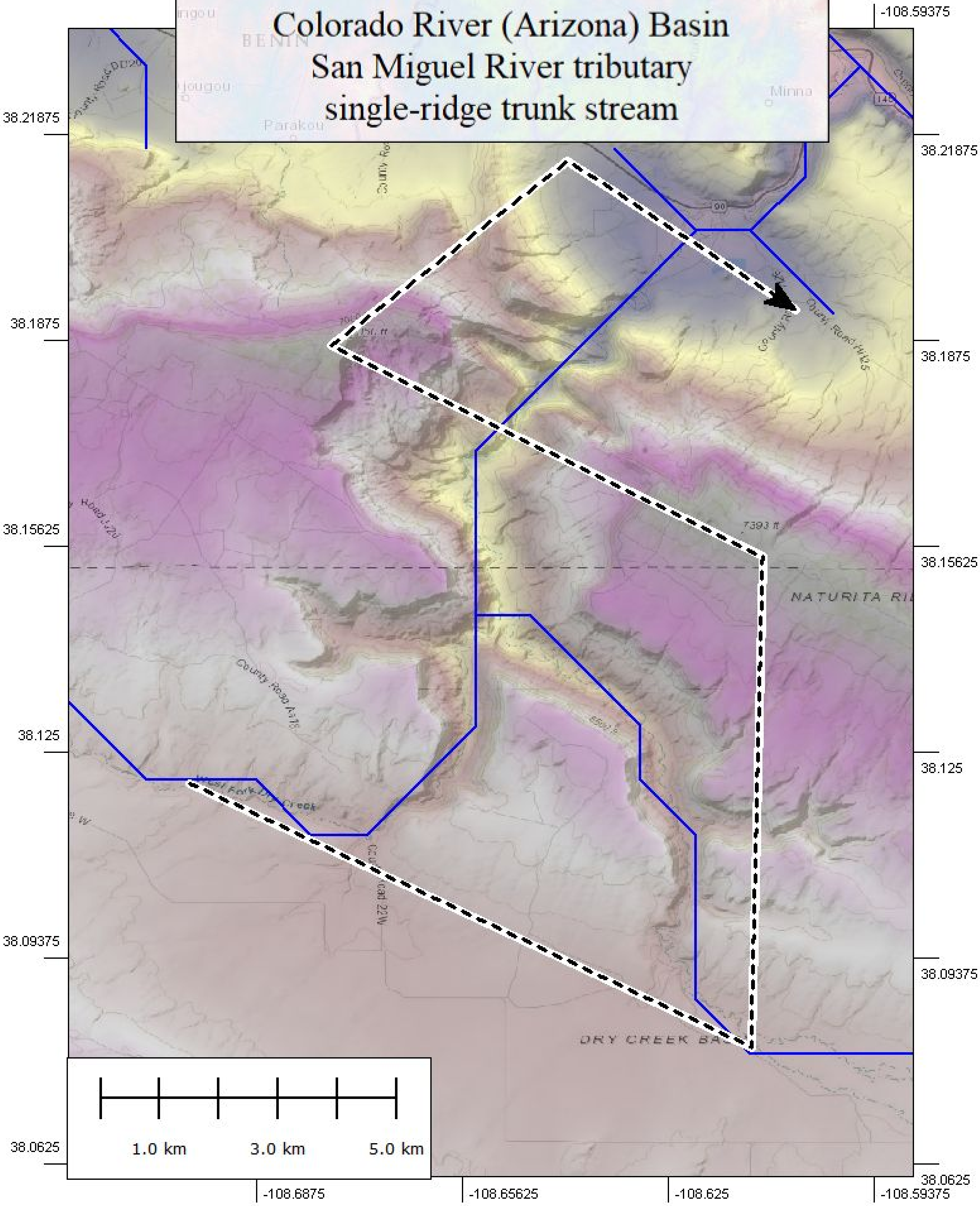

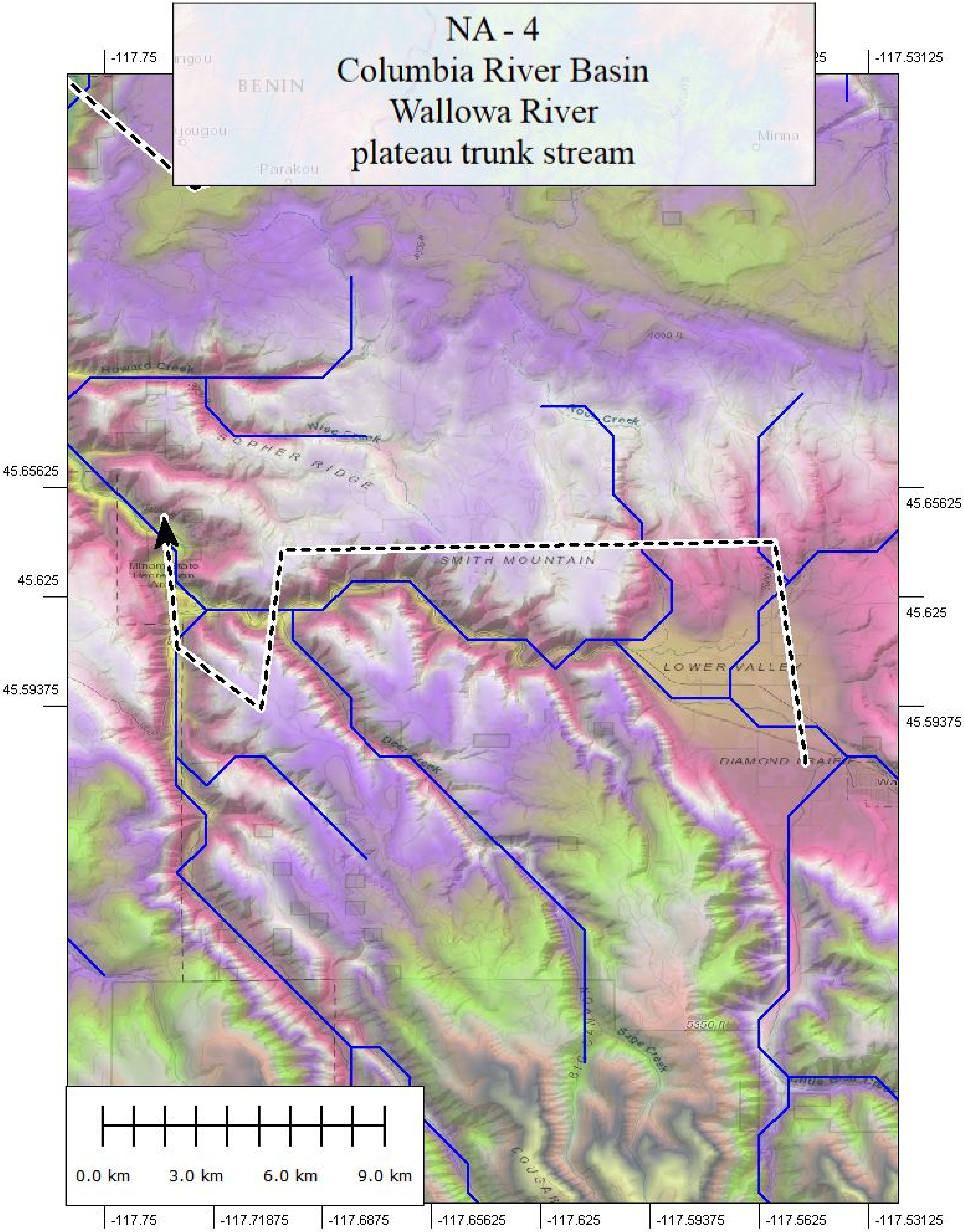
NA - 4
Columbia River Basin
Wallowa River
plateau trunk stream
-117.75
-117.53125
45.65625
45.625
45.59375
Howard Creek
Wise Creek
GOPHER RIDGE
SMITH MOUNTAIN
LOWER VALLEY
DIAMOND PRAIRIE
Deer Creek
Sage Creek
5350 ft
0.0 km
3.0 km
6.0 km
9.0 km
-117.71875
-117.6875
-117.65625
-117.625
-117.59375
-117.5625

NA - 40
Mackenzie River (Canada) Basin
Bluefish Lake
single-ridge head stream

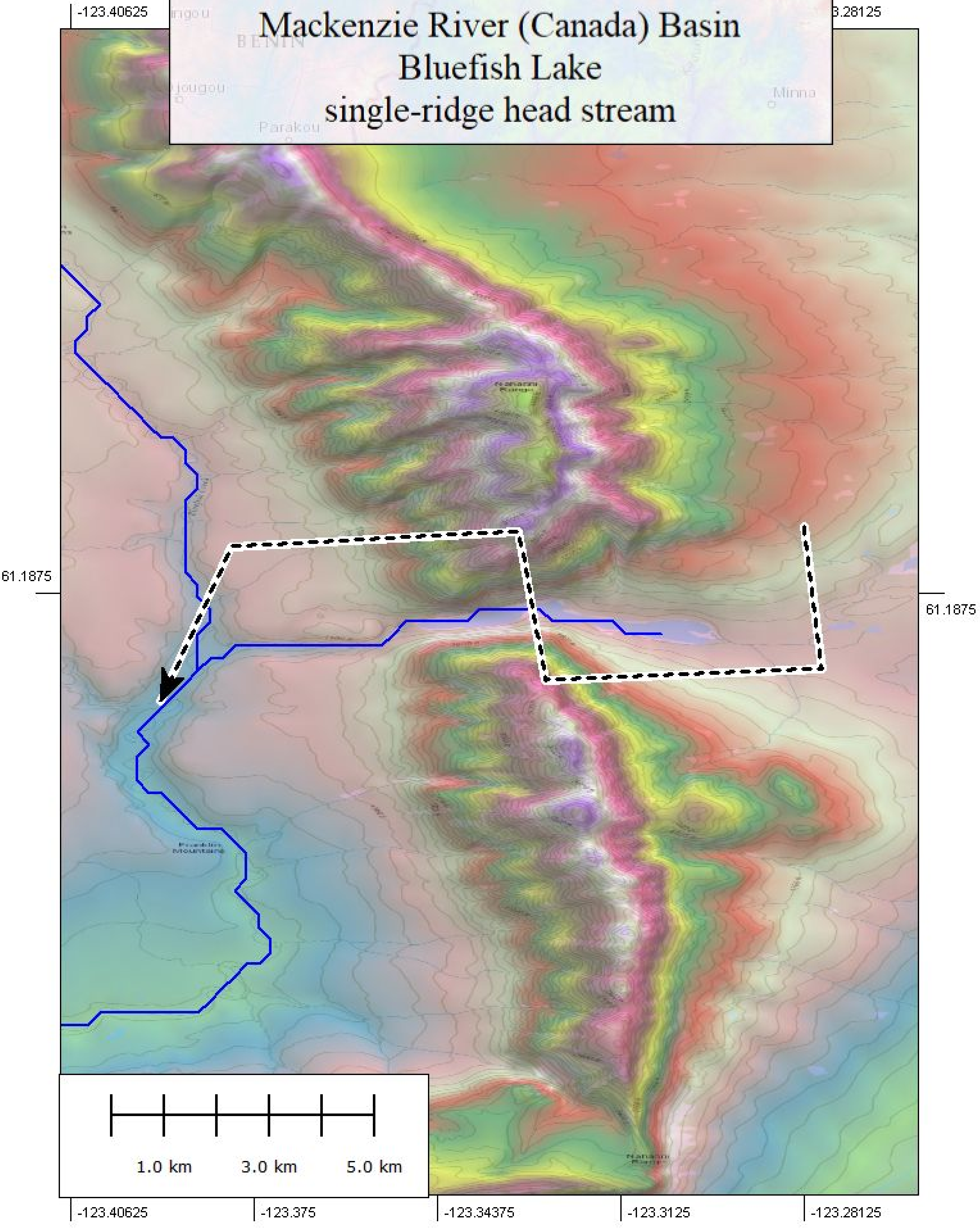

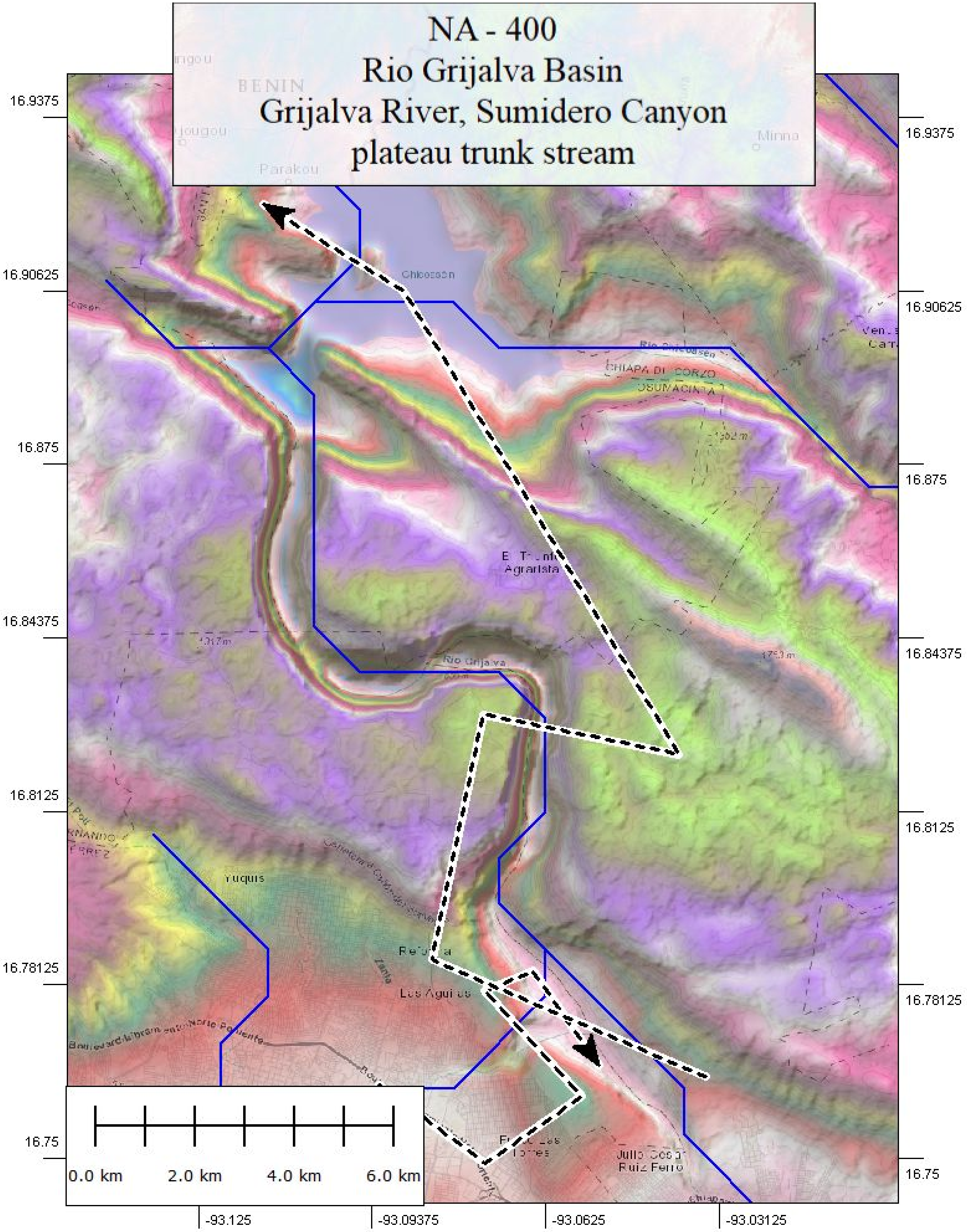
NA - 400
Rio Grijalva Basin
Grijalva River, Sumidero Canyon
plateau trunk stream
16.9375
16.90625
16.875
16.84375
16.8125
16.78125
16.75
-93.125
-93.09375
-93.0625
-93.03125
Parakou
BENIN
Minna
Chicoasén
CHIAPA DE CORZO
USUMACINTA
El Triunfo Agrarista
Rio Grijalva
Yuquis
Reforma
Los Aguilas
Julio Cesar Ruiz Ferro
0.0 km
2.0 km
4.0 km
6.0 km

NA - 401
Colorado River (Arizona) Basin
Green River, Flaming Gorge
downdip plateau trunk stream

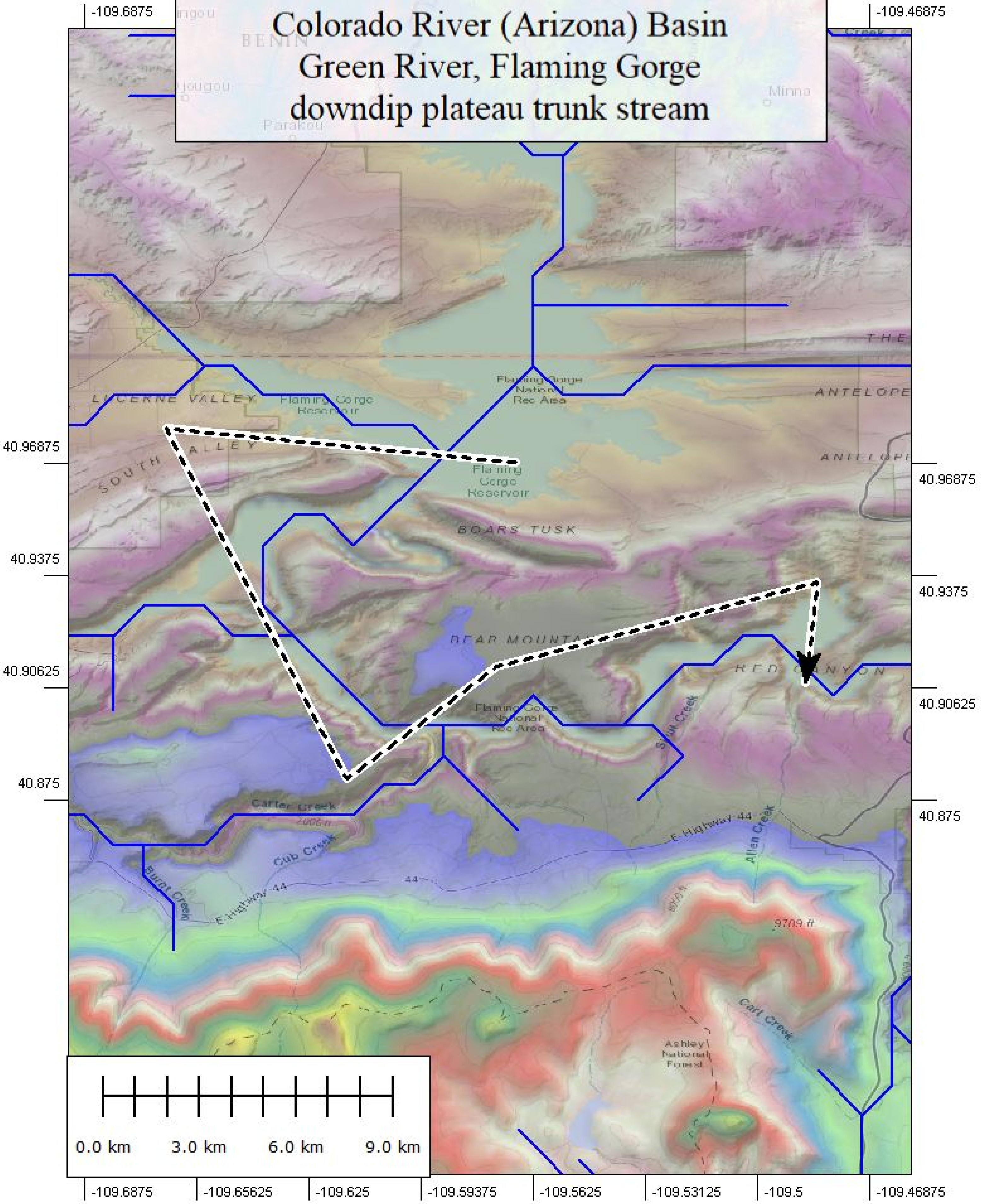

NA - 402
Delaware River Basin
Lehigh River
plateau trunk stream

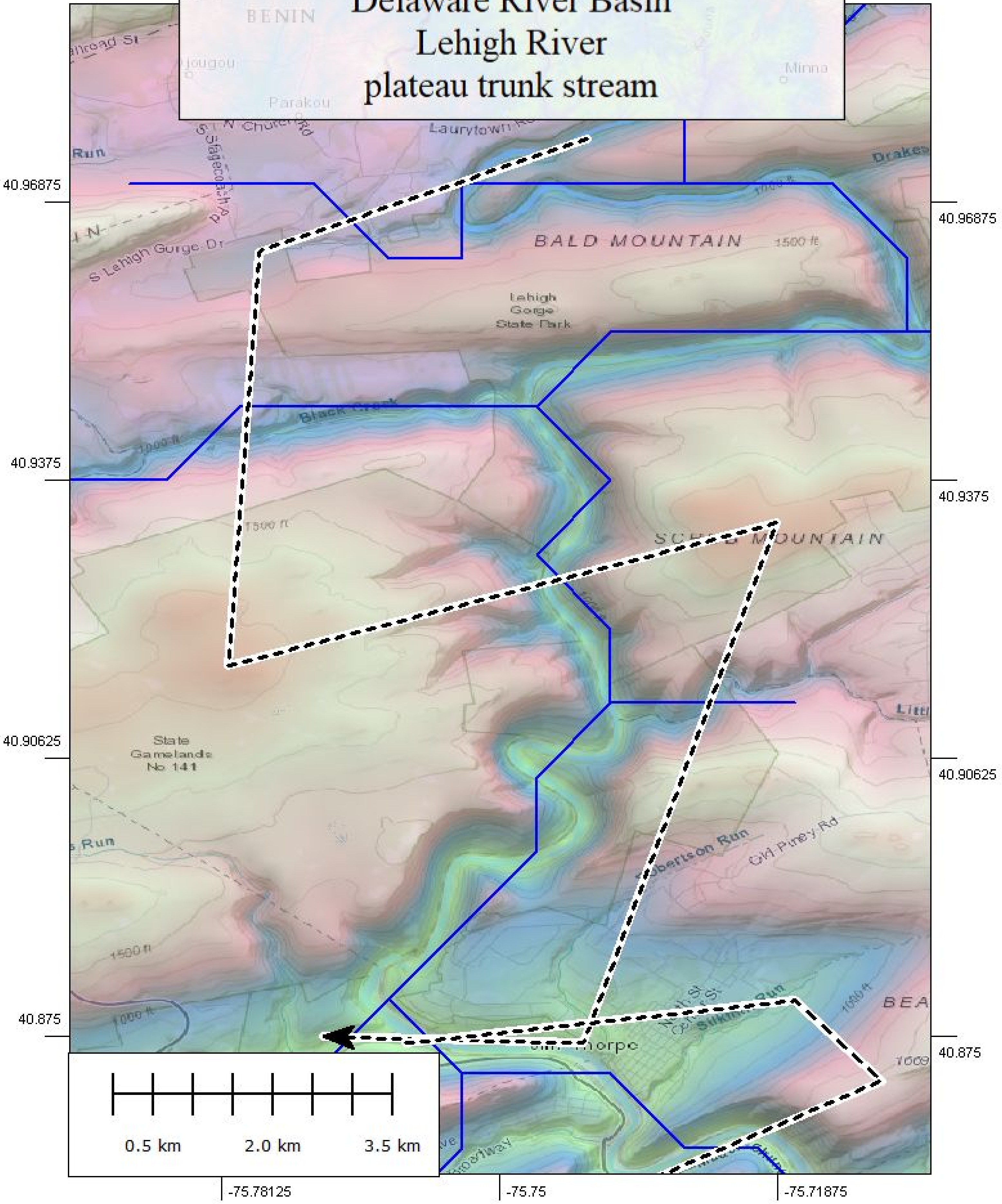

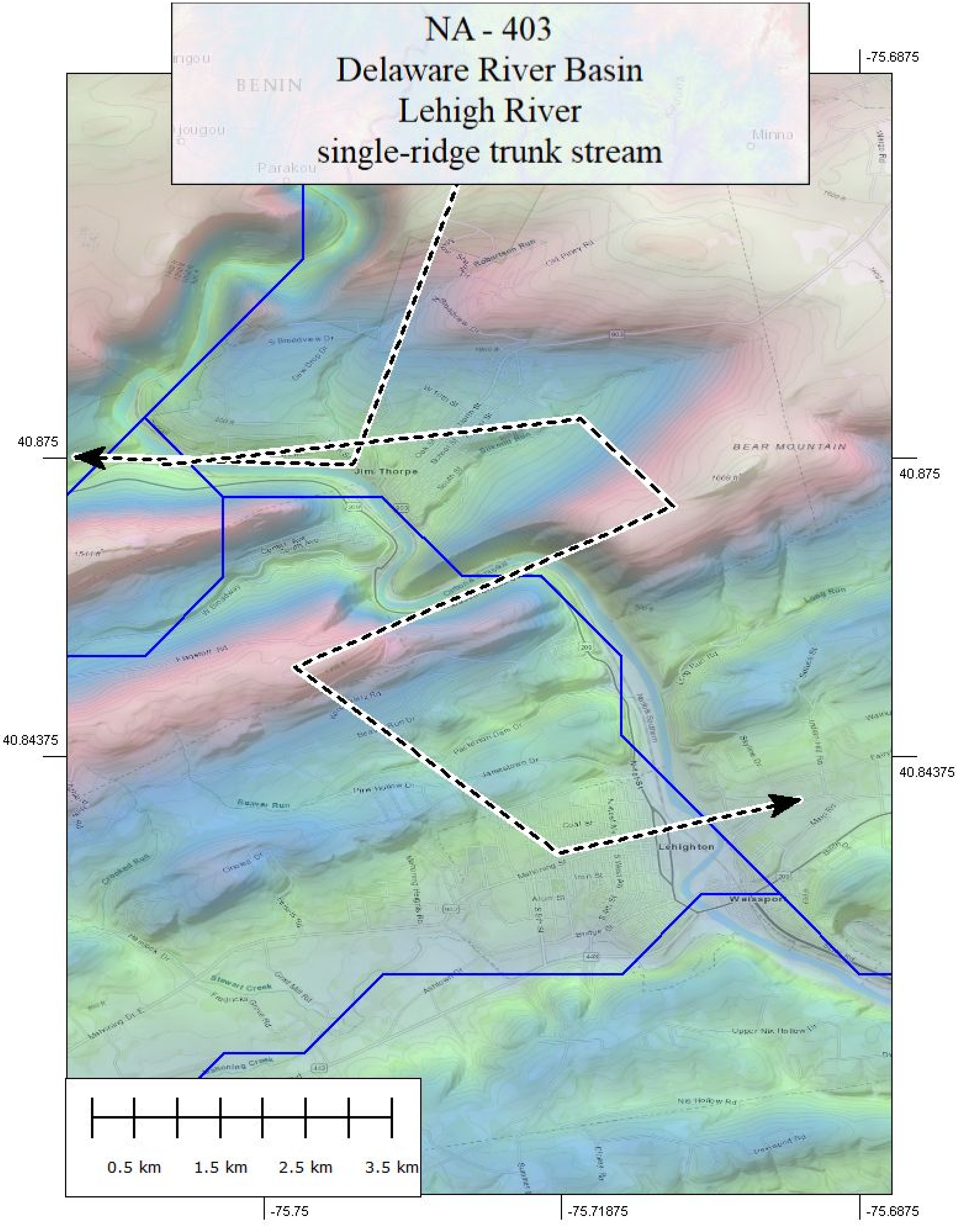
NA - 403
Delaware River Basin
Lehigh River
single-ridge trunk stream
-75.6875
40.875
40.875
40.84375
40.84375
-75.75
-75.71875
-75.6875
0.5 km
1.5 km
2.5 km
3.5 km
Jim Thorpe
Lehighton
BEAR MOUNTAIN
Robertson Run
Beaver Run
Crooked Run
Stewart Creek
Long Run
Mahoning Creek
Weissport

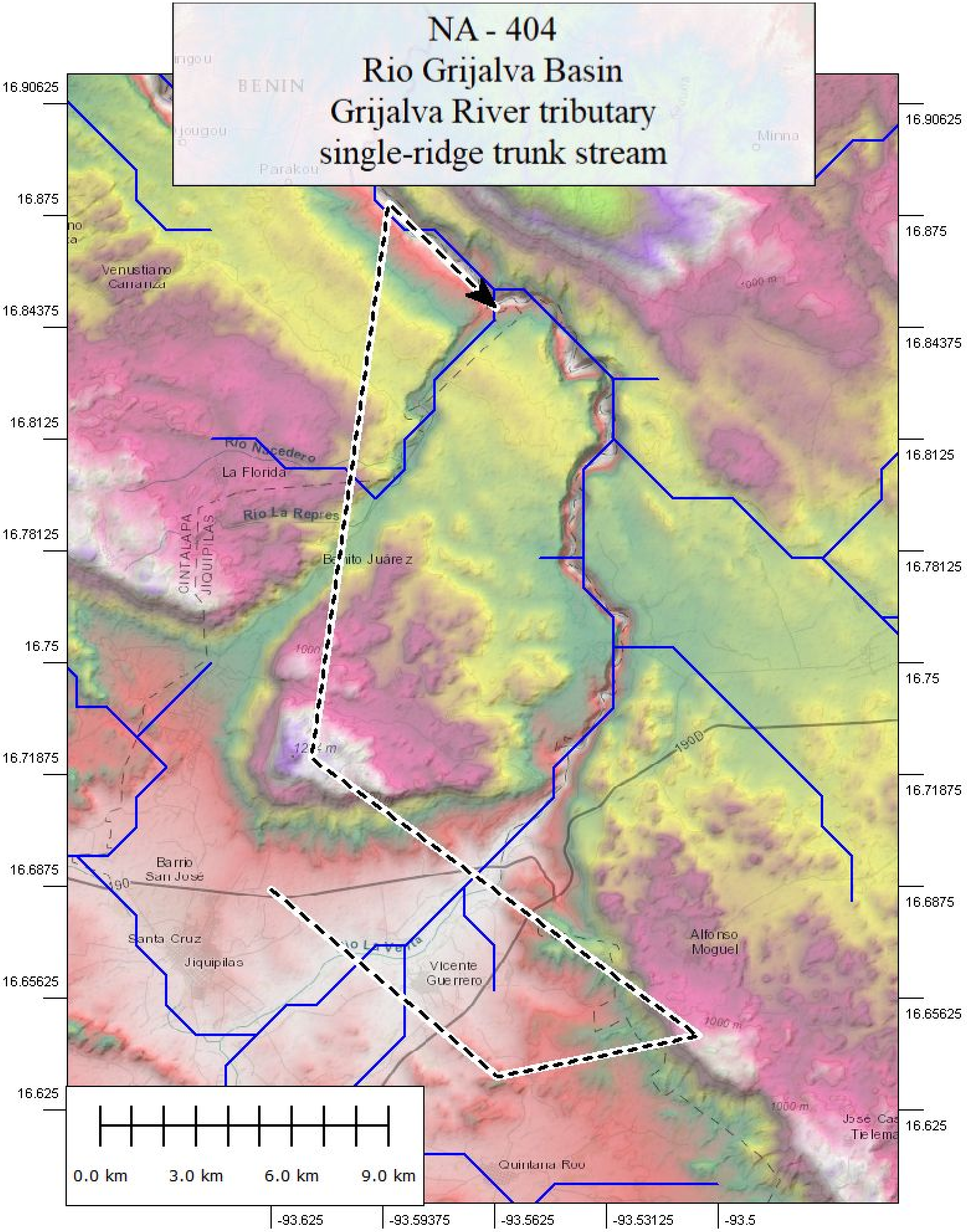
NA - 404
Rio Grijalva Basin
Grijalva River tributary
single-ridge trunk stream
Venustiano Carranza
Rio Nacedero
La Florida
Rio La Repres
CINTALAPA
JIQUIPILAS
Benito Juárez
1000 m
190D
Barrio San José
190
Santa Cruz
Jiquipilas
Vicente Guerrero
Alfonso Moguel
Quintana Roo
1000 m
1000 m
0.0 km
3.0 km
6.0 km
9.0 km
16.90625
16.875
16.84375
16.8125
16.78125
16.75
16.71875
16.6875
16.65625
16.625
-93.625
-93.59375
-93.5625
-93.53125
-93.5

NA - 405
Delaware River Basin
Little Schuylkill River
multi-ridge trunk stream

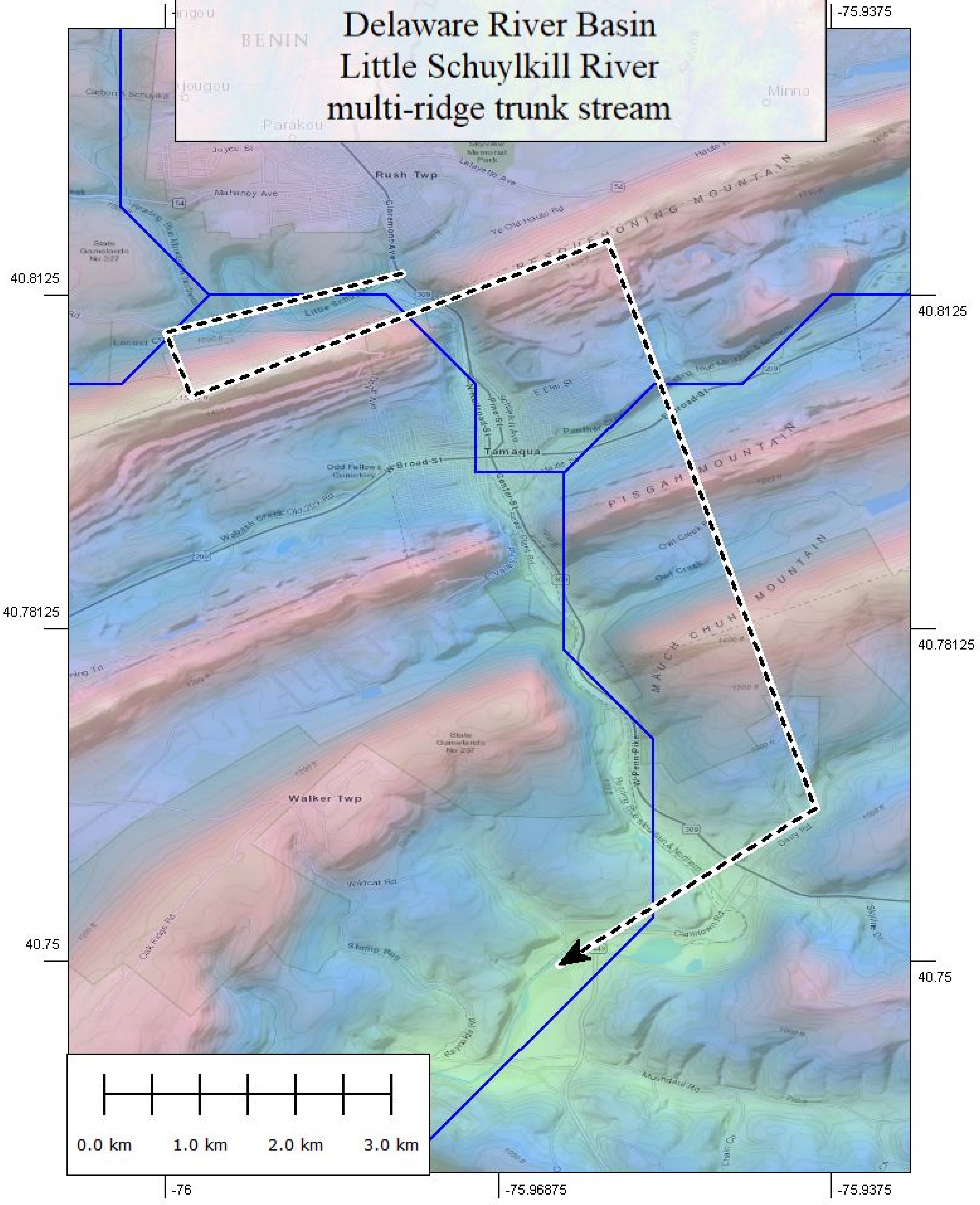

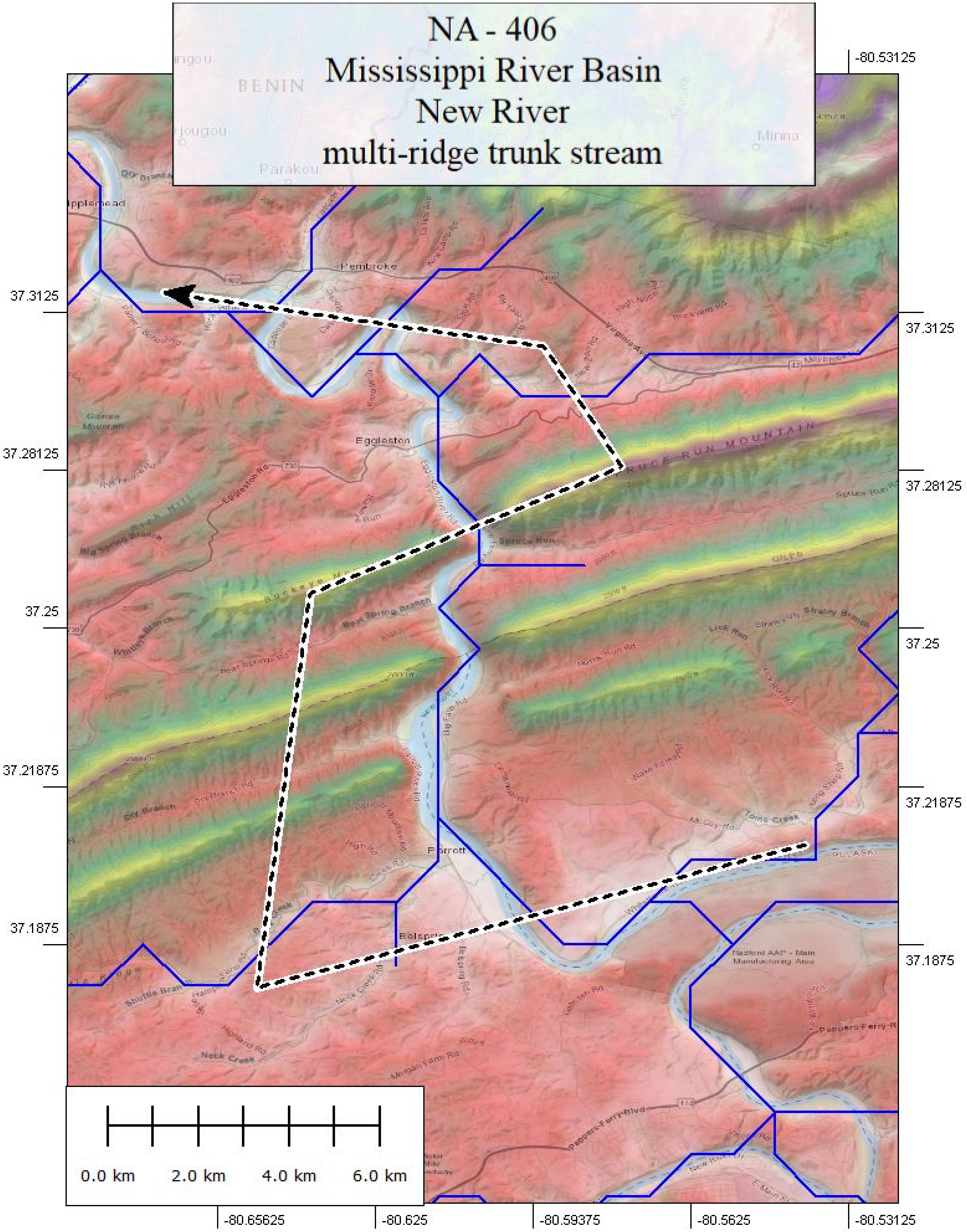
NA - 406
Mississippi River Basin
New River
multi-ridge trunk stream
-80.53125
37.3125
37.3125
37.28125
37.28125
37.25
37.25
37.21875
37.21875
37.1875
37.1875
-80.65625
-80.625
-80.59375
-80.5625
-80.53125
0.0 km
2.0 km
4.0 km
6.0 km
Pembroke
Eggleston
Spruce Run Mountain
Spruce Run
Pearisburg Rd
Parrott
Belspring
Radford AAP - Main Manufacturing Area
Peppers Ferry Rd
Peppers Ferry Blvd
Pulaski
Giles
Rich Hill
Big Spring Branch
Bear Spring Branch
Shuffle Branch
Neck Creek
Toms Creek
Stanley Branch
Lick Run

NA - 407
Colorado River (Arizona) Basin
Colorado River, Glen Canyon
updip plateau trunk stream

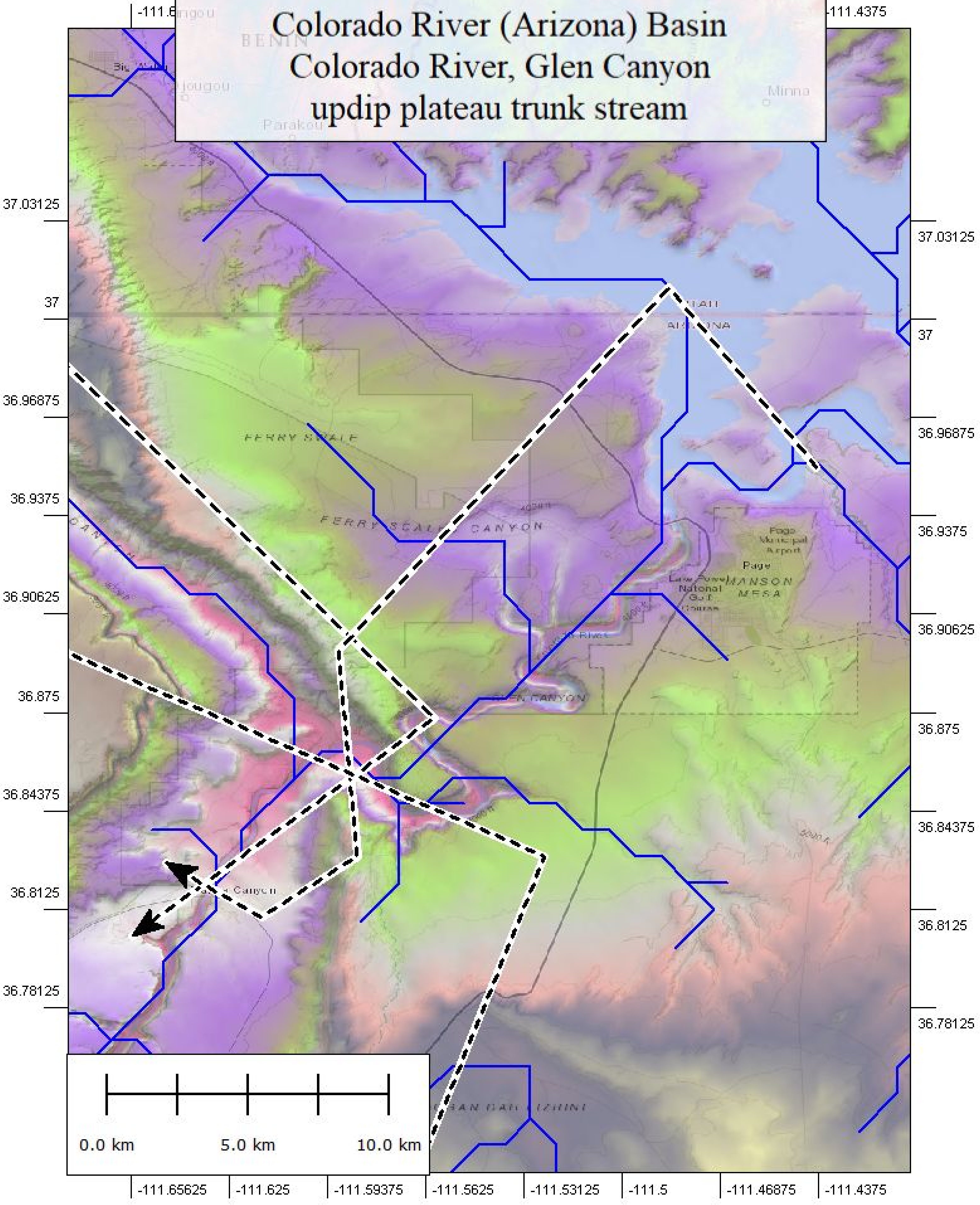

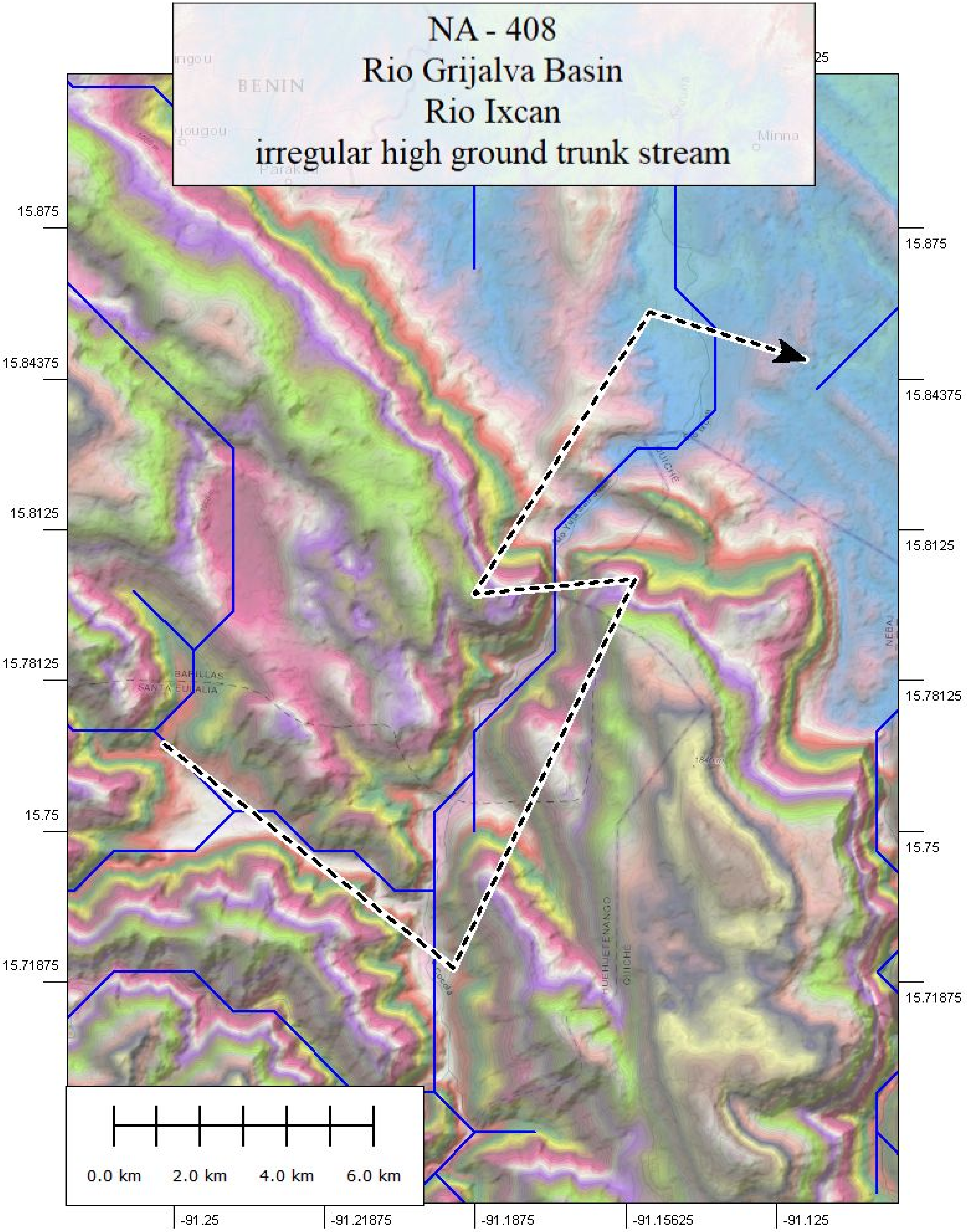
NA - 408
Rio Grijalva Basin
Rio Ixcan
irregular high ground trunk stream
15.875
15.84375
15.8125
15.78125
15.75
15.71875
-91.25
-91.21875
-91.1875
-91.15625
-91.125
0.0 km
2.0 km
4.0 km
6.0 km
BARILLAS
SANTA EULALIA
HUEHUETENANGO
QUICHE
NEBAJ

NA - 409
Endorheic basin Basin
Nazas River
multi-ridge trunk stream

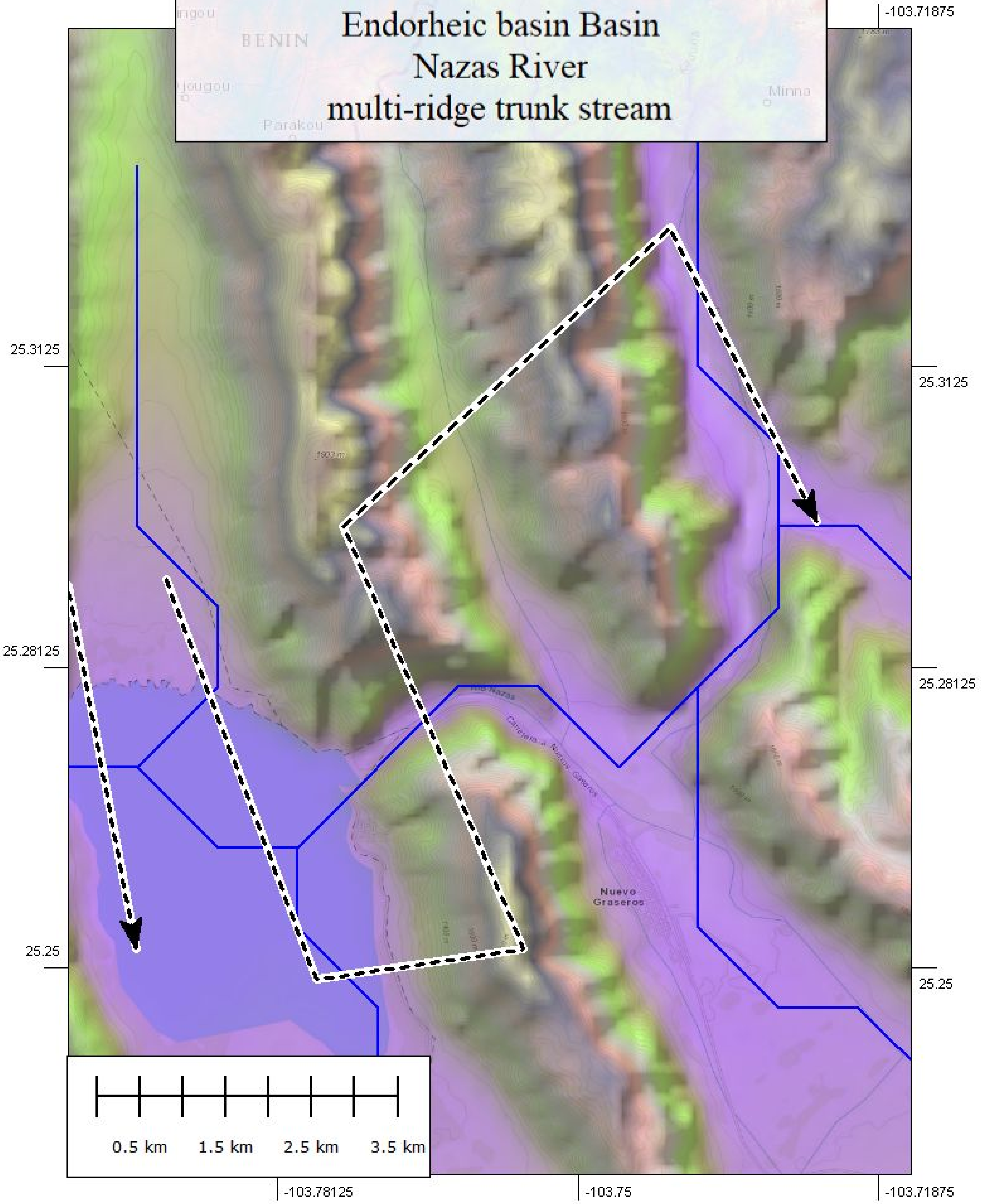

NA - 41
Mackenzie River (Canada) Basin
Root River
irregular high ground trunk stream

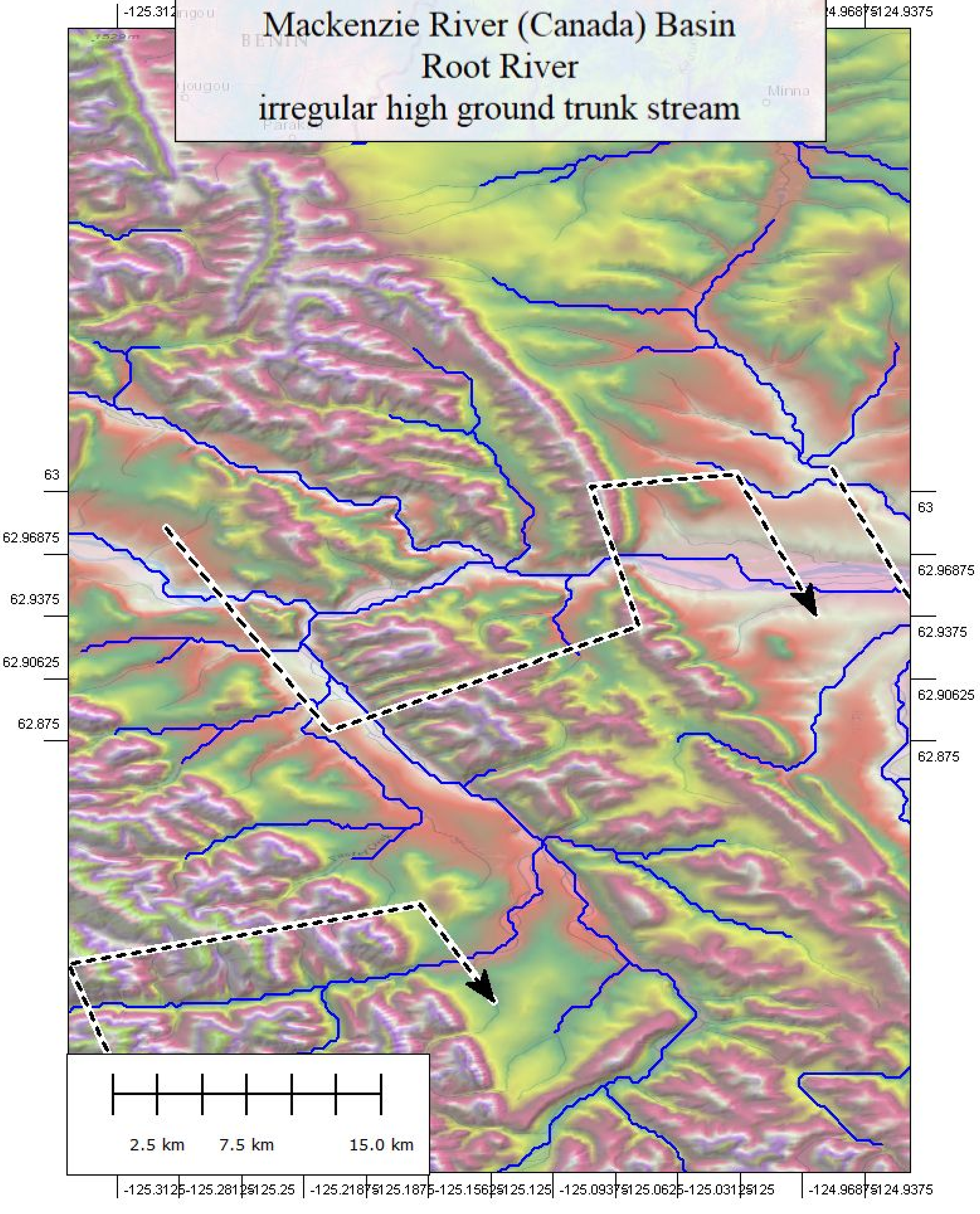

NA - 410
Colorado River (Arizona) Basin
Red Creek
single-ridge trunk stream

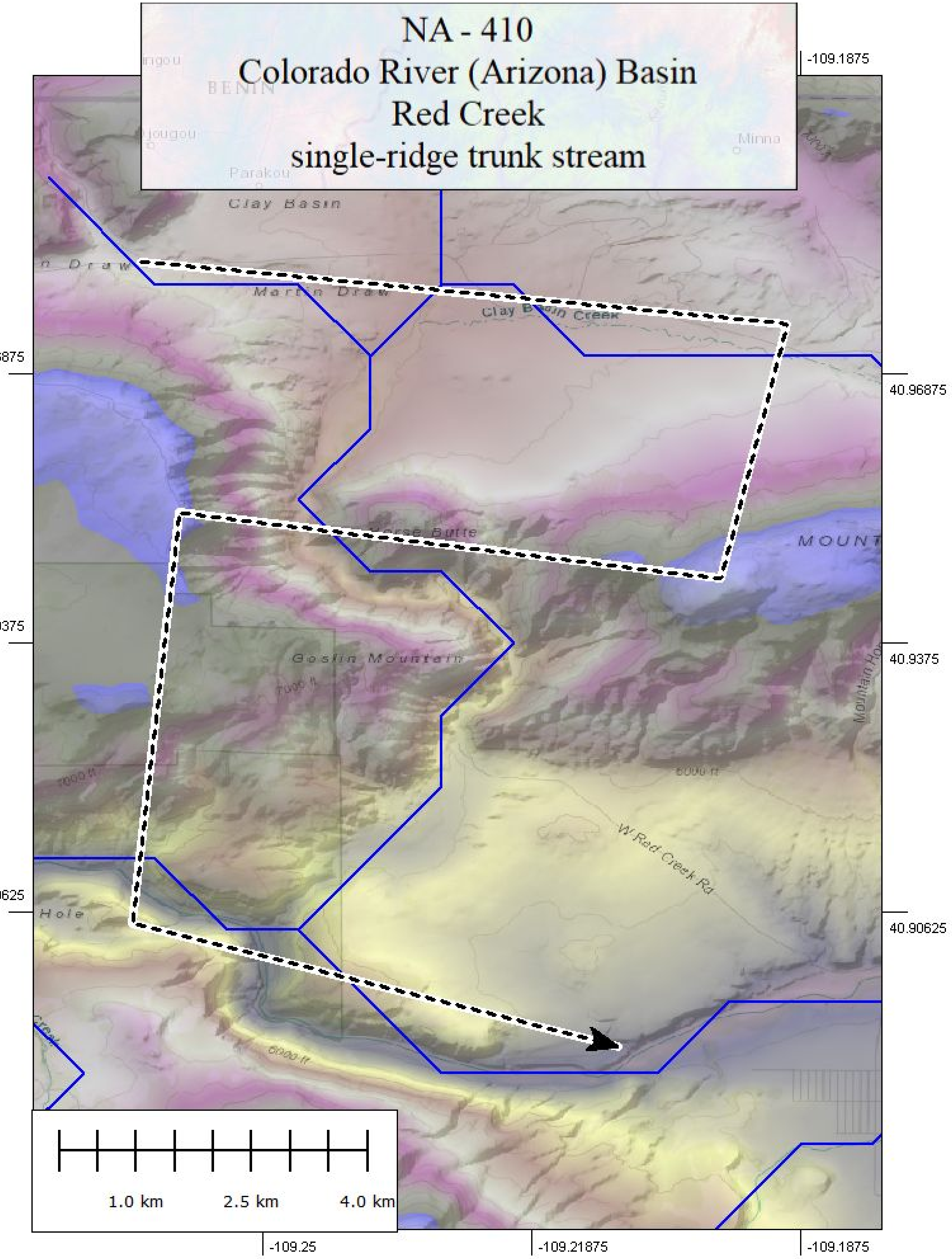

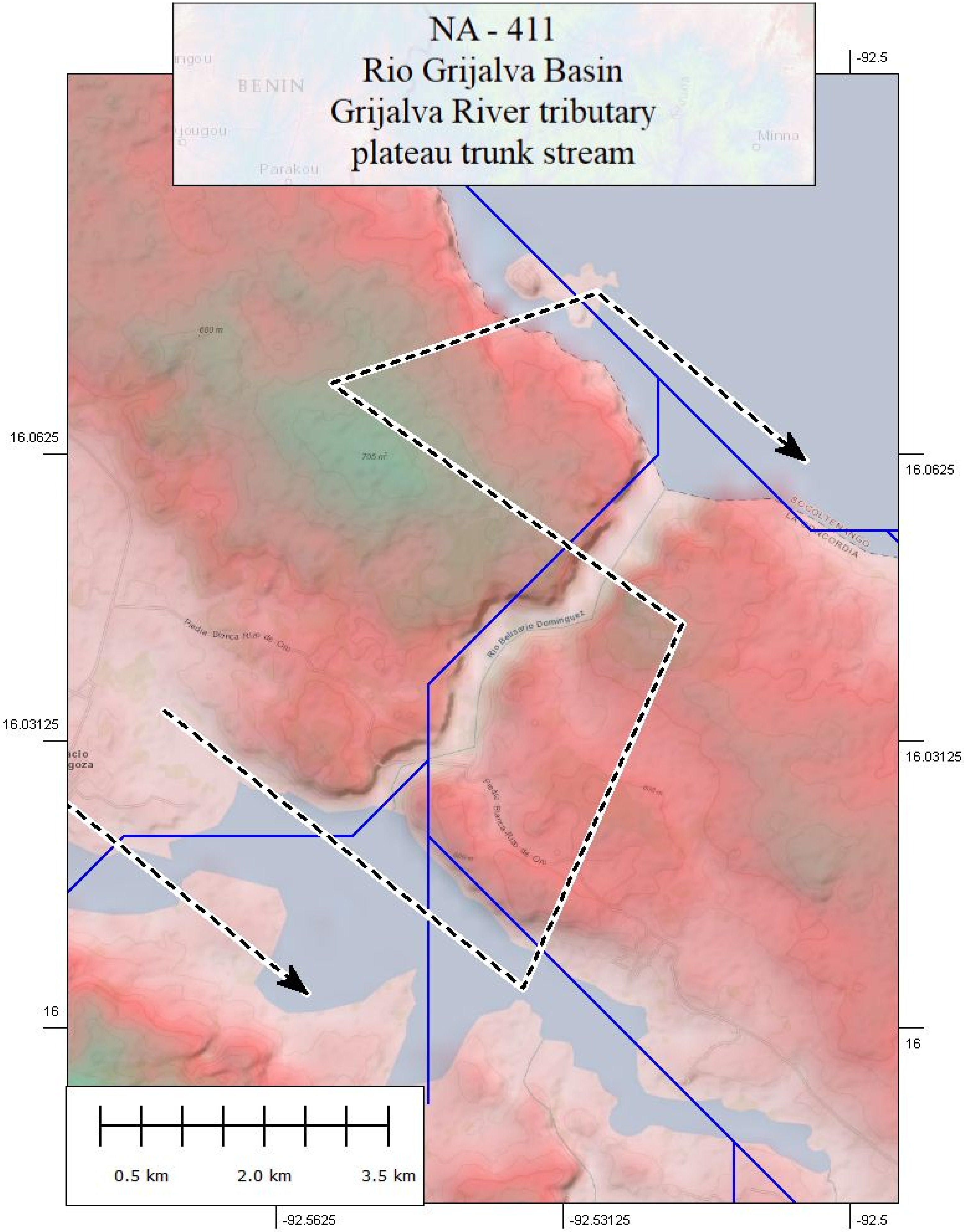
NA - 411
Rio Grijalva Basin
Grijalva River tributary
plateau trunk stream
BENIN
Parakou
Minna
680 m
705 m
Rio Belisario Dominguez
16.0625
16.03125
16
-92.5
-92.5625
-92.53125
0.5 km
2.0 km
3.5 km

NA - 412
Colorado River (Arizona) Basin
Colorado River
updip plateau trunk stream

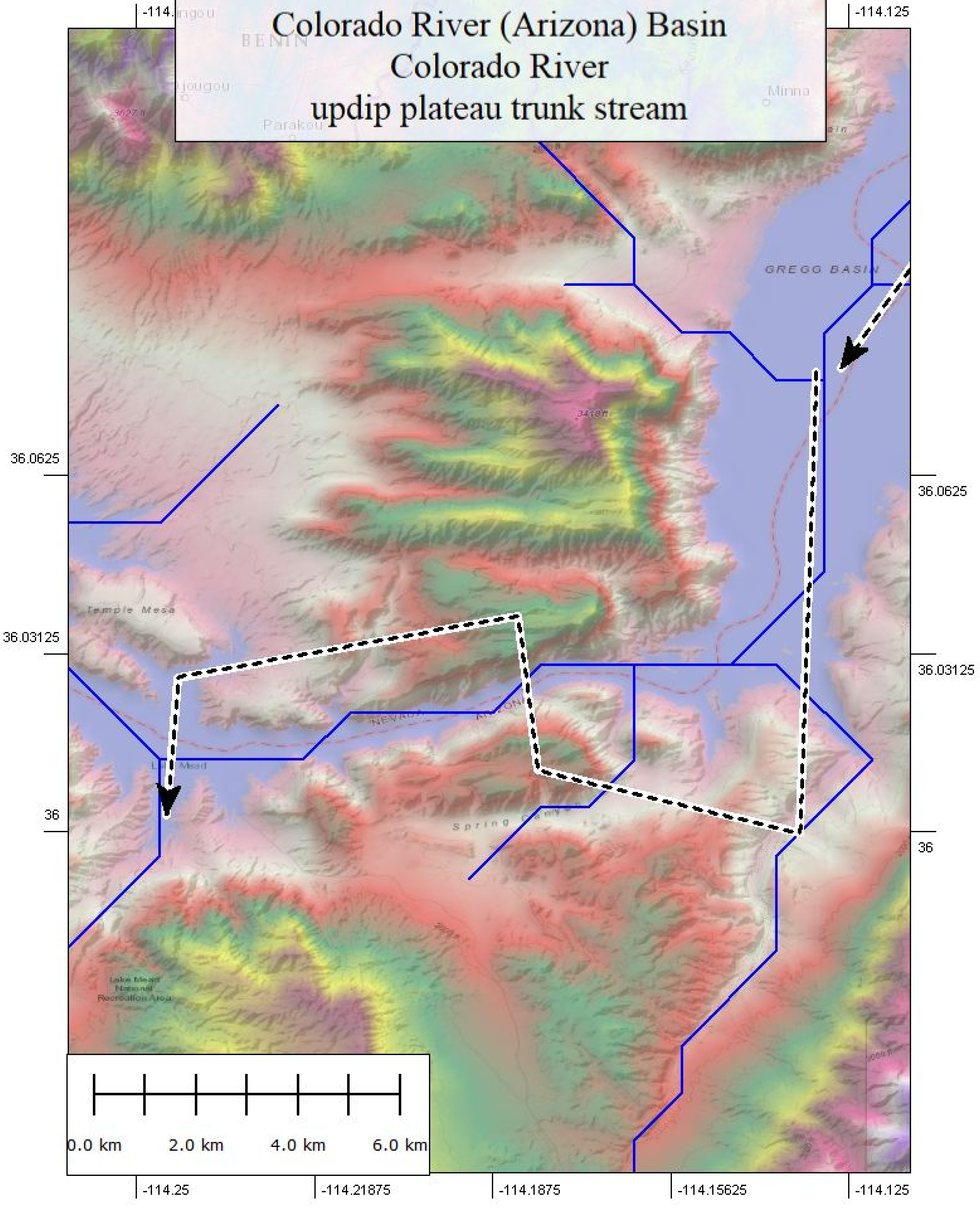

NA - 413
Sacramento River Basin
Spanish Creek
irregular high ground trunk stream

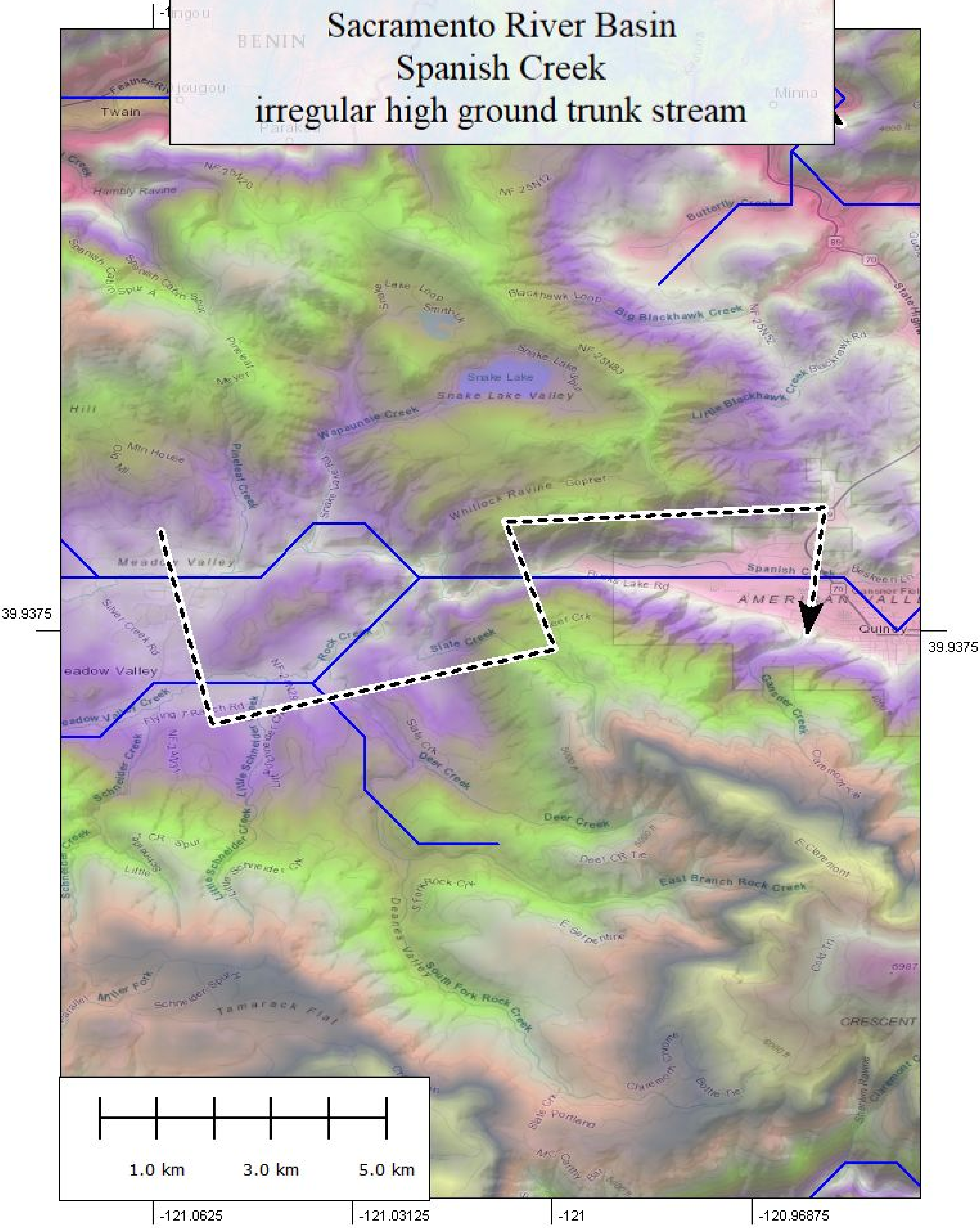

NA - 414
Columbia River Basin
Clearwater River
irregular high ground trunk stream

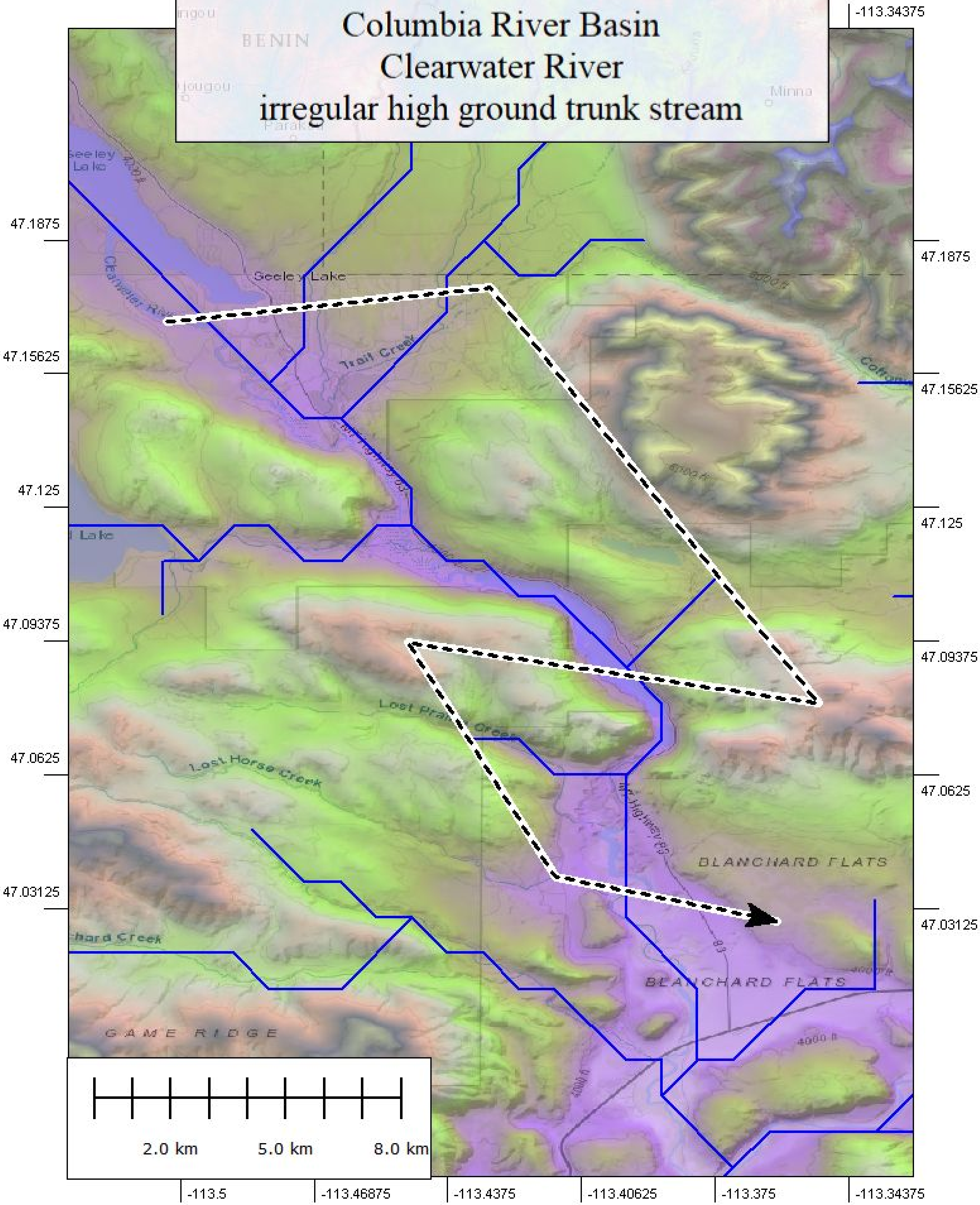

NA - 415
Sacramento River Basin
Pit River
irregular high ground trunk stream

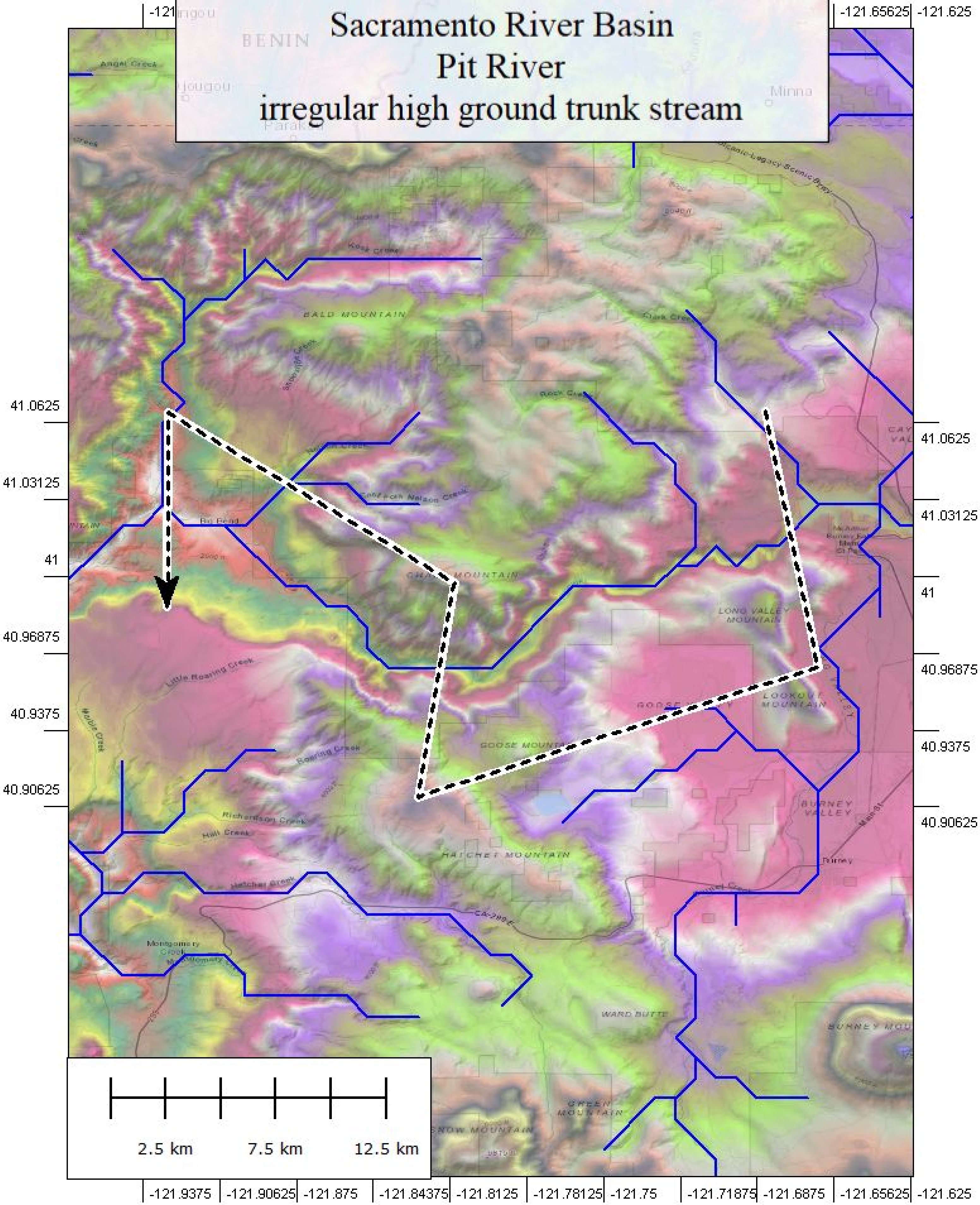

NA - 416
Columbia River Basin
Clark Fork River
irregular high ground trunk stream

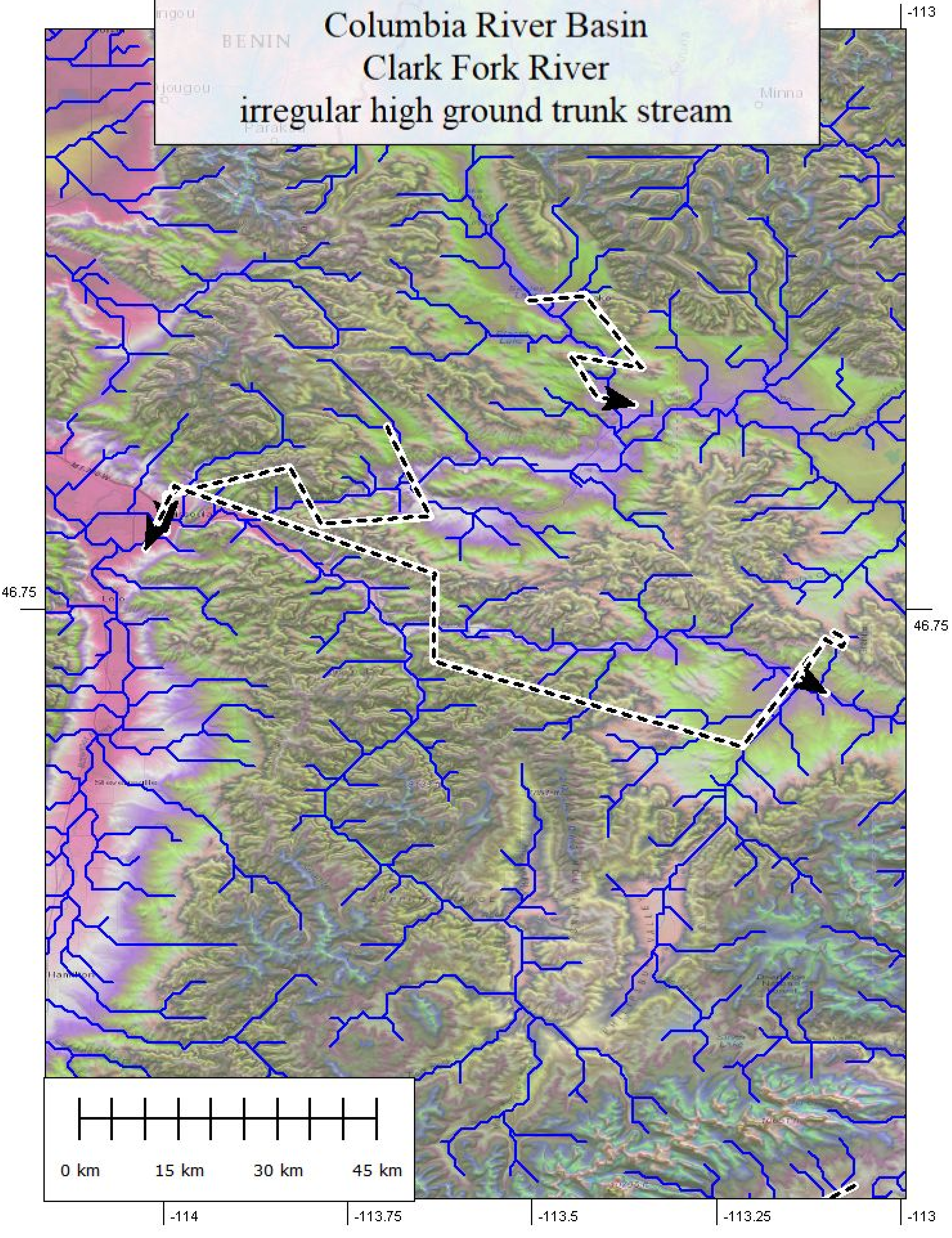

NA - 417
Eel River Basin
Eel River
irregular high ground trunk stream

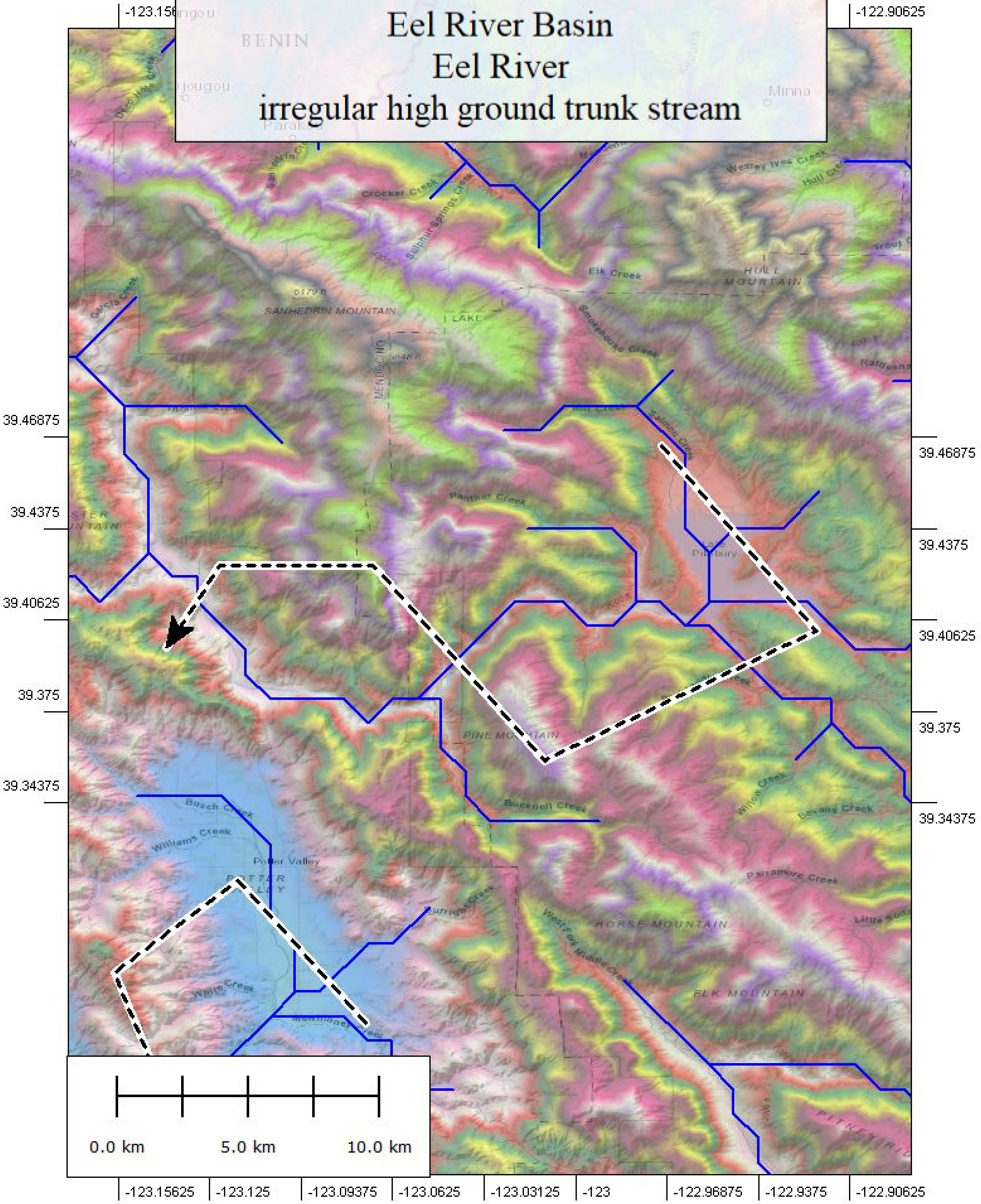

NA - 418
Colorado River (Arizona) Basin
Colorado River
updip plateau trunk stream

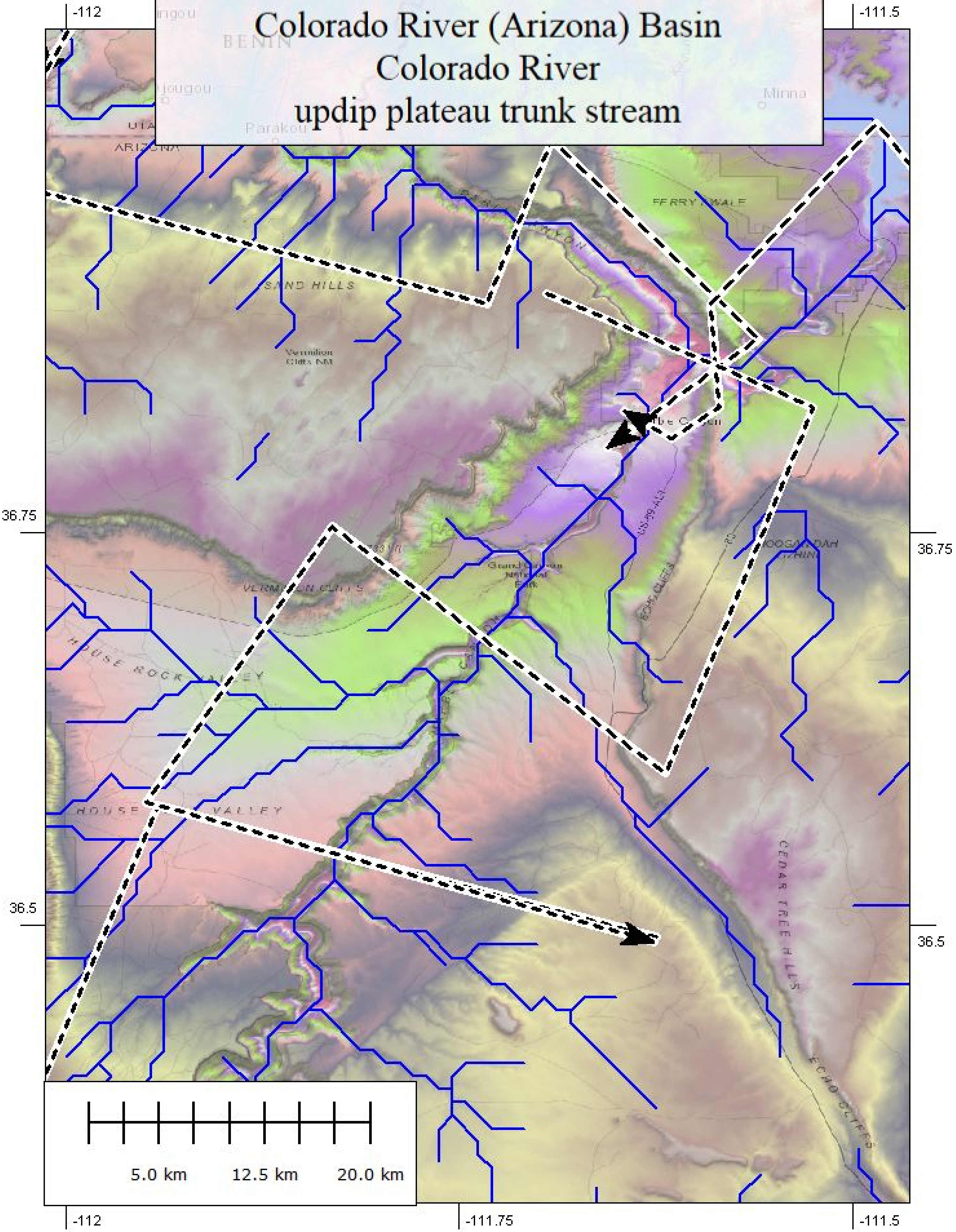

NA - 419
Colorado River (Arizona) Basin
San Juan River (US)
downdip plateau trunk stream

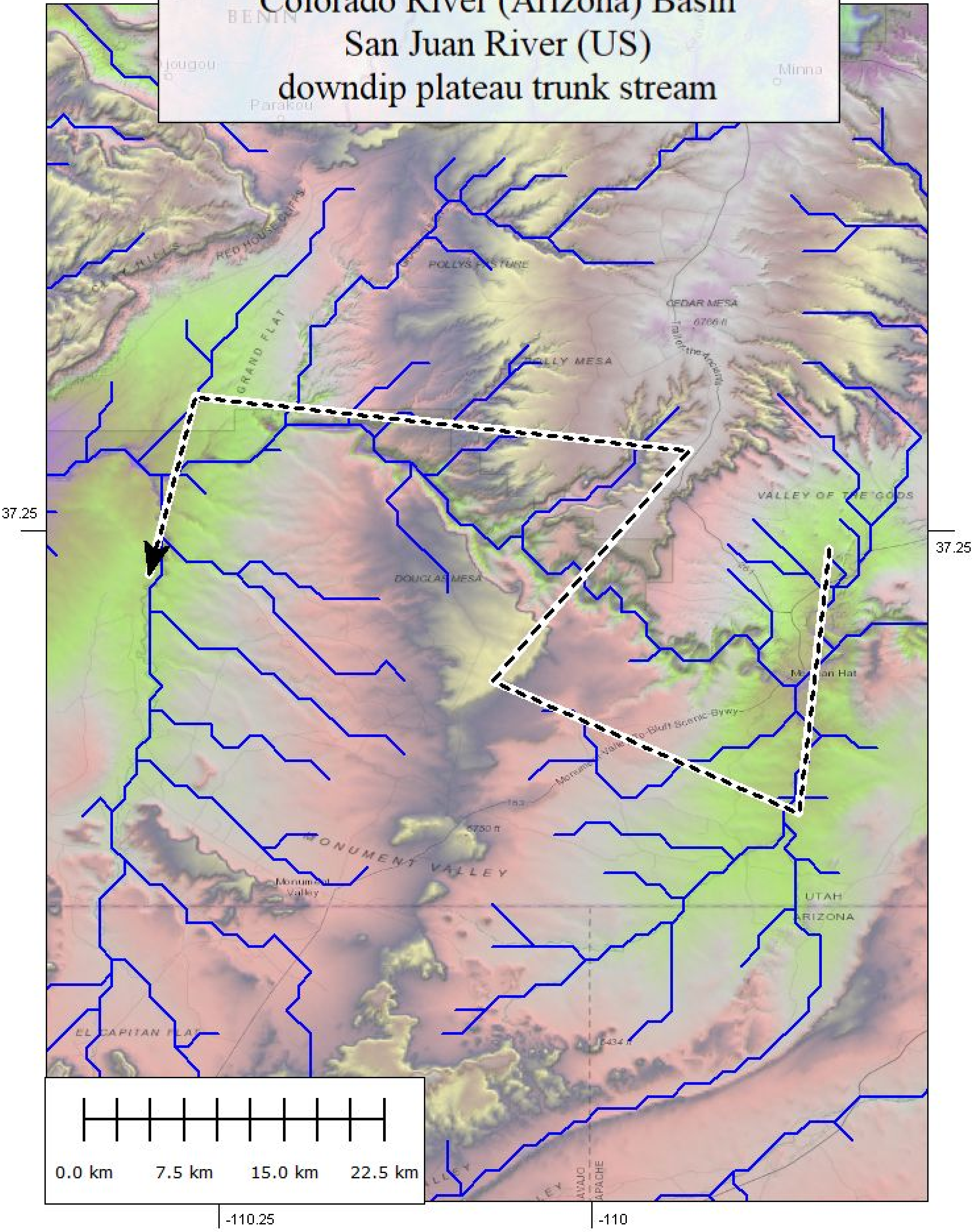

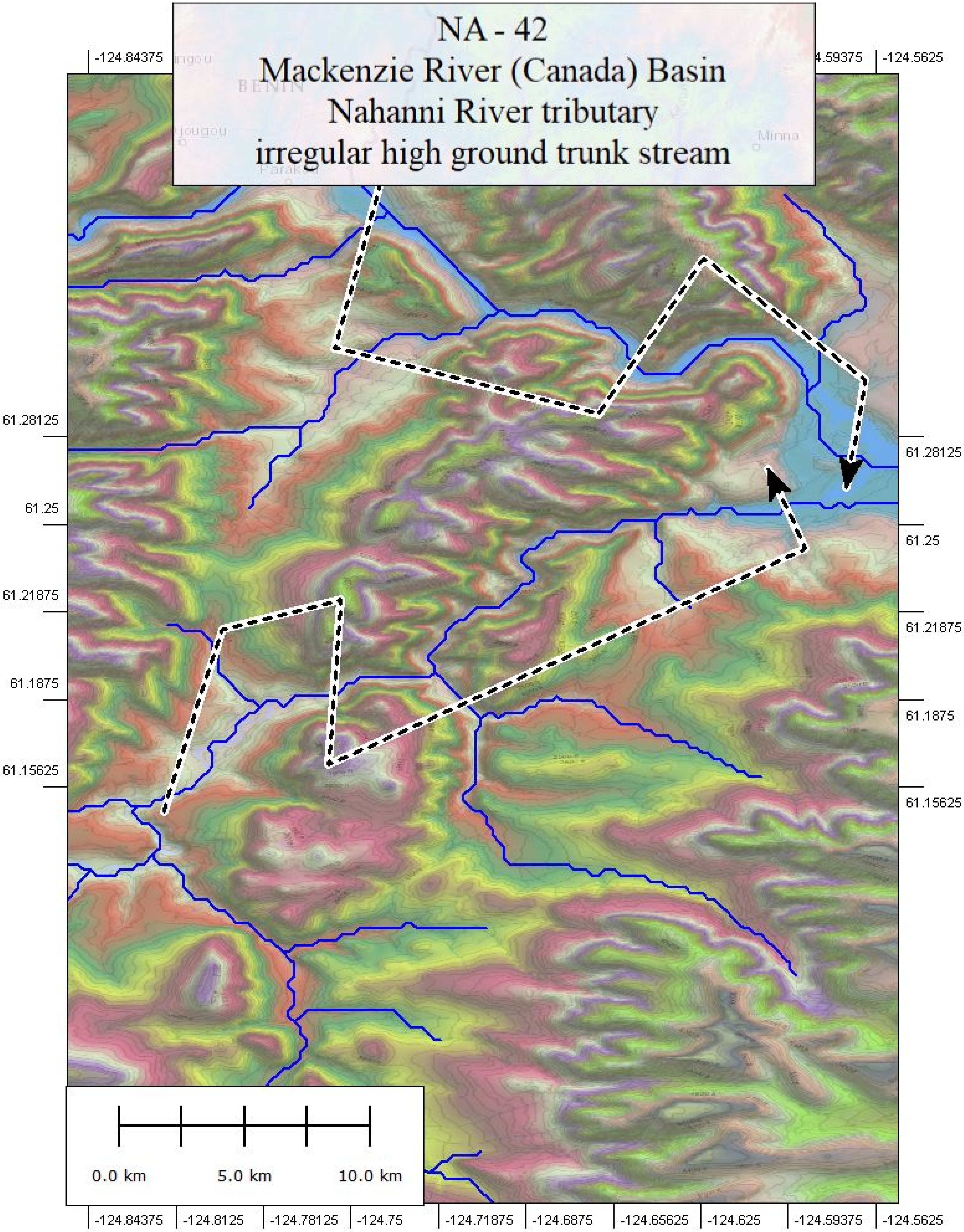

NA - 42
Mackenzie River (Canada) Basin
Nahanni River tributary
irregular high ground trunk stream

NA - 420
Rio Grande (Texas-Mexico) Basin
Rio Grande River
single-ridge trunk stream

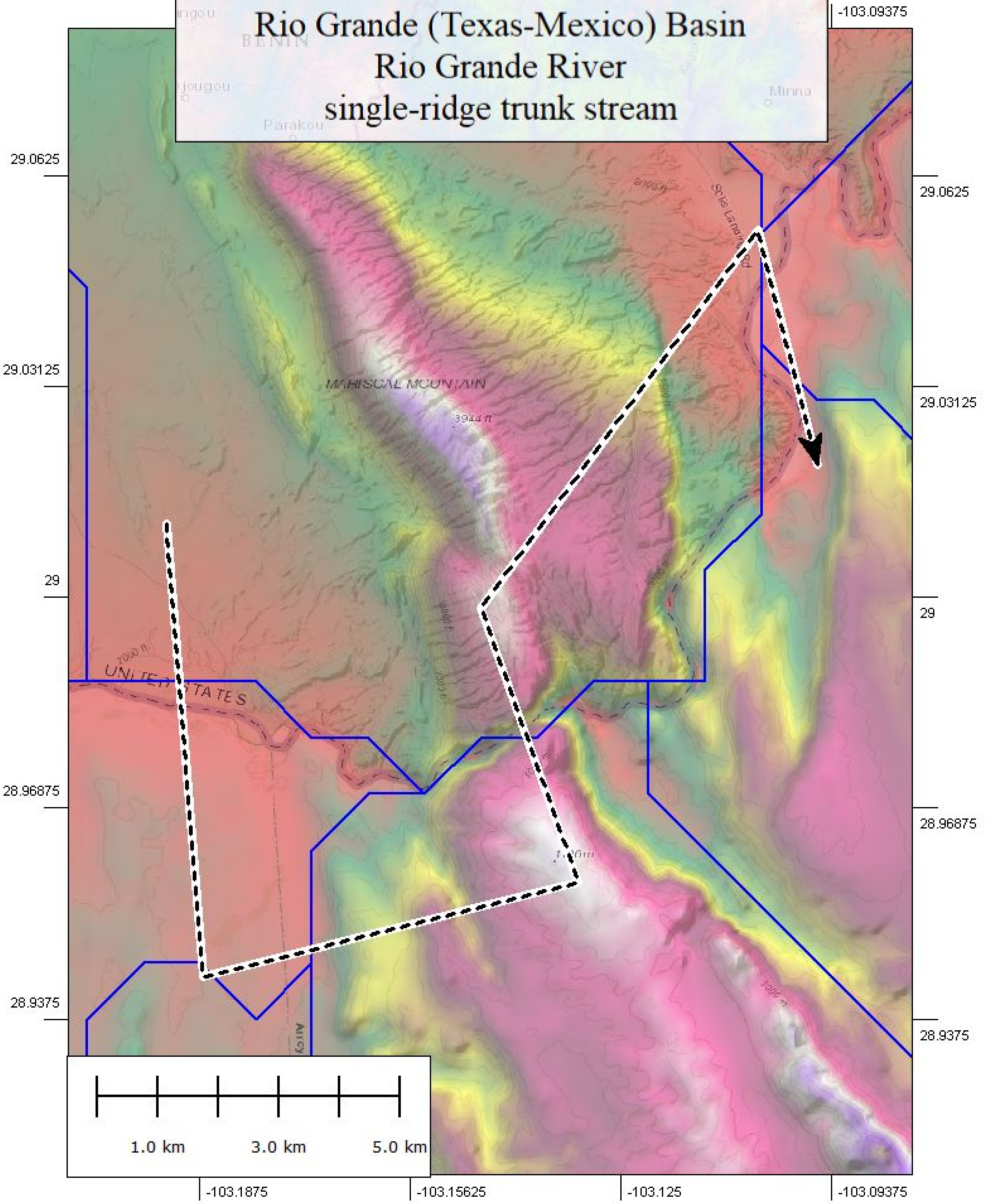

NA - 421
Columbia River Basin
Snake River
irregular high ground trunk stream

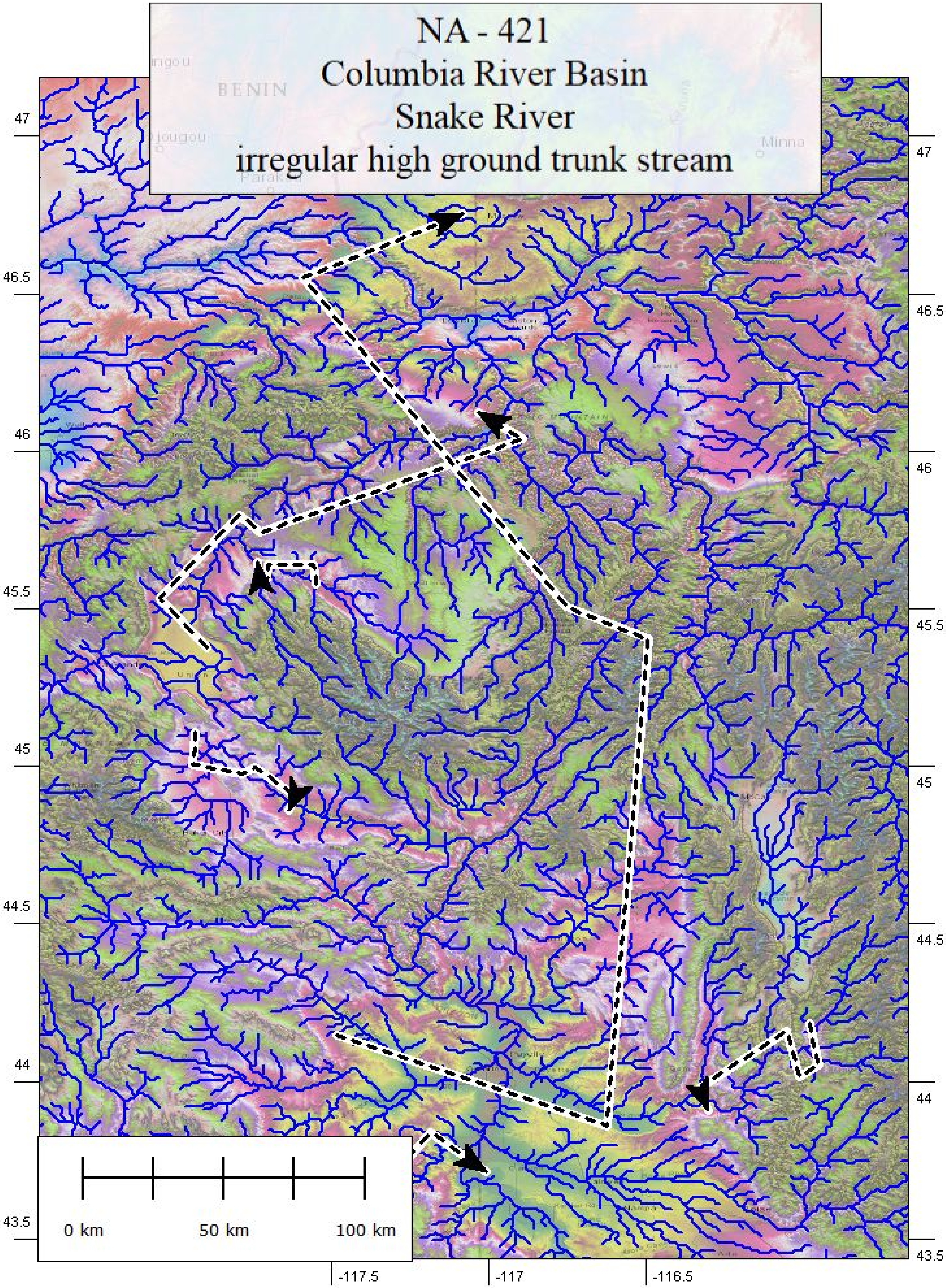

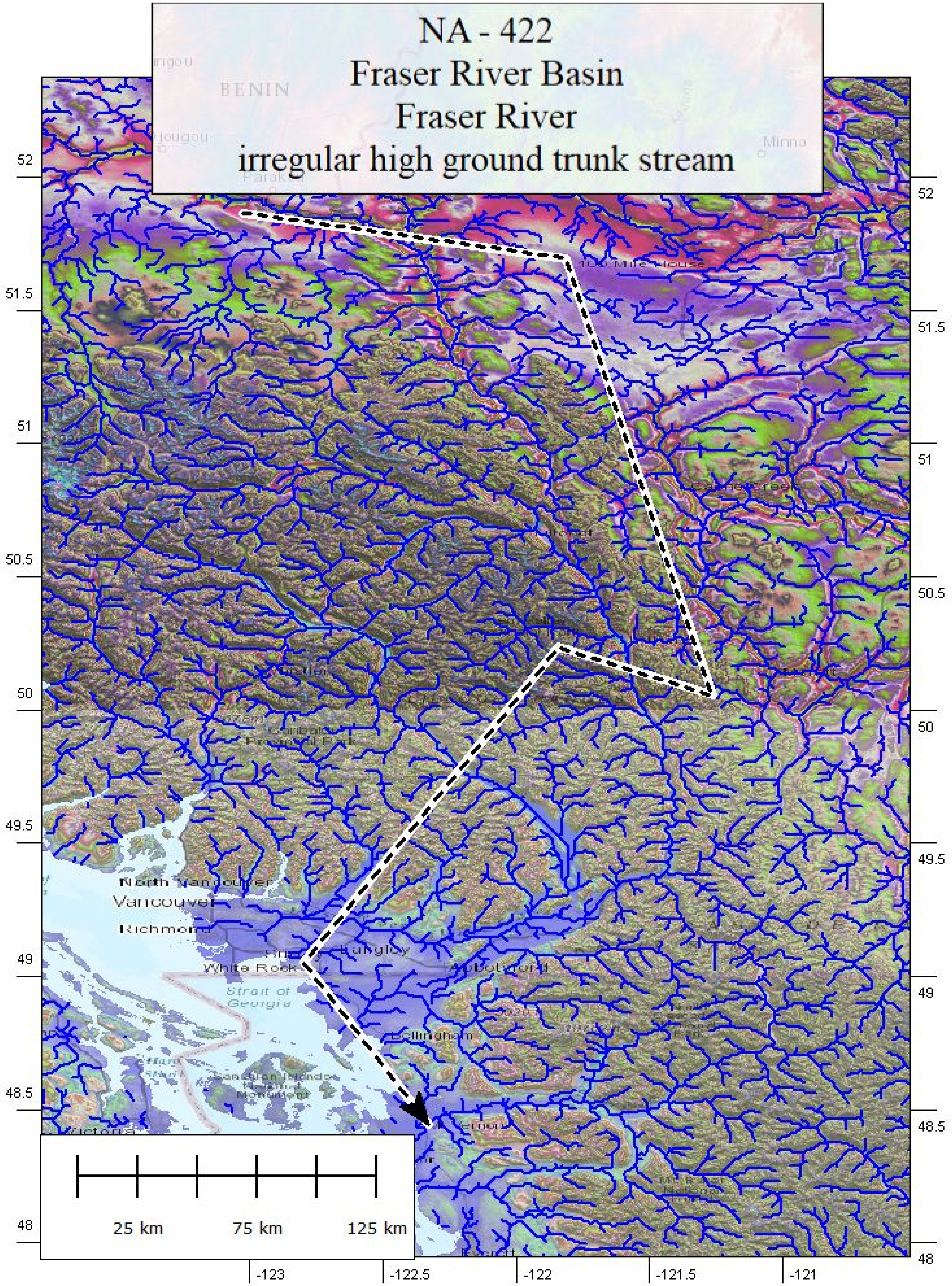
NA - 422
Fraser River Basin
Fraser River
irregular high ground trunk stream
52
51.5
51
50.5
50
49.5
49
48.5
48
-123
-122.5
-122
-121.5
-121
25 km
75 km
125 km
BENIN
Minna
100 Mile House
North Vancouver
Vancouver
Richmond
White Rock
Langley
Abbotsford
Strait of Georgia
Bellingham

NA - 423
Rogue RIver Basin
Rogue River
irregular high ground trunk stream

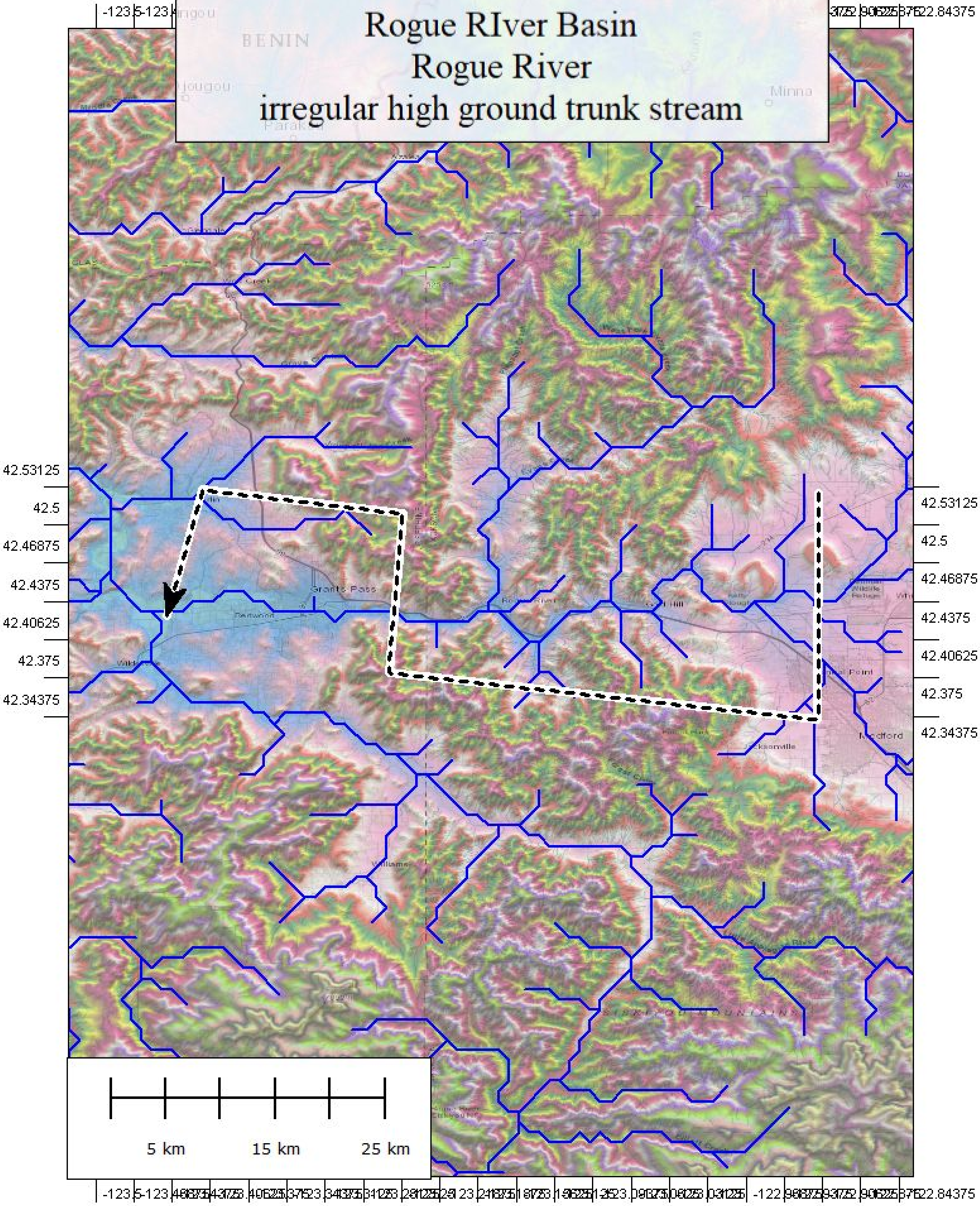

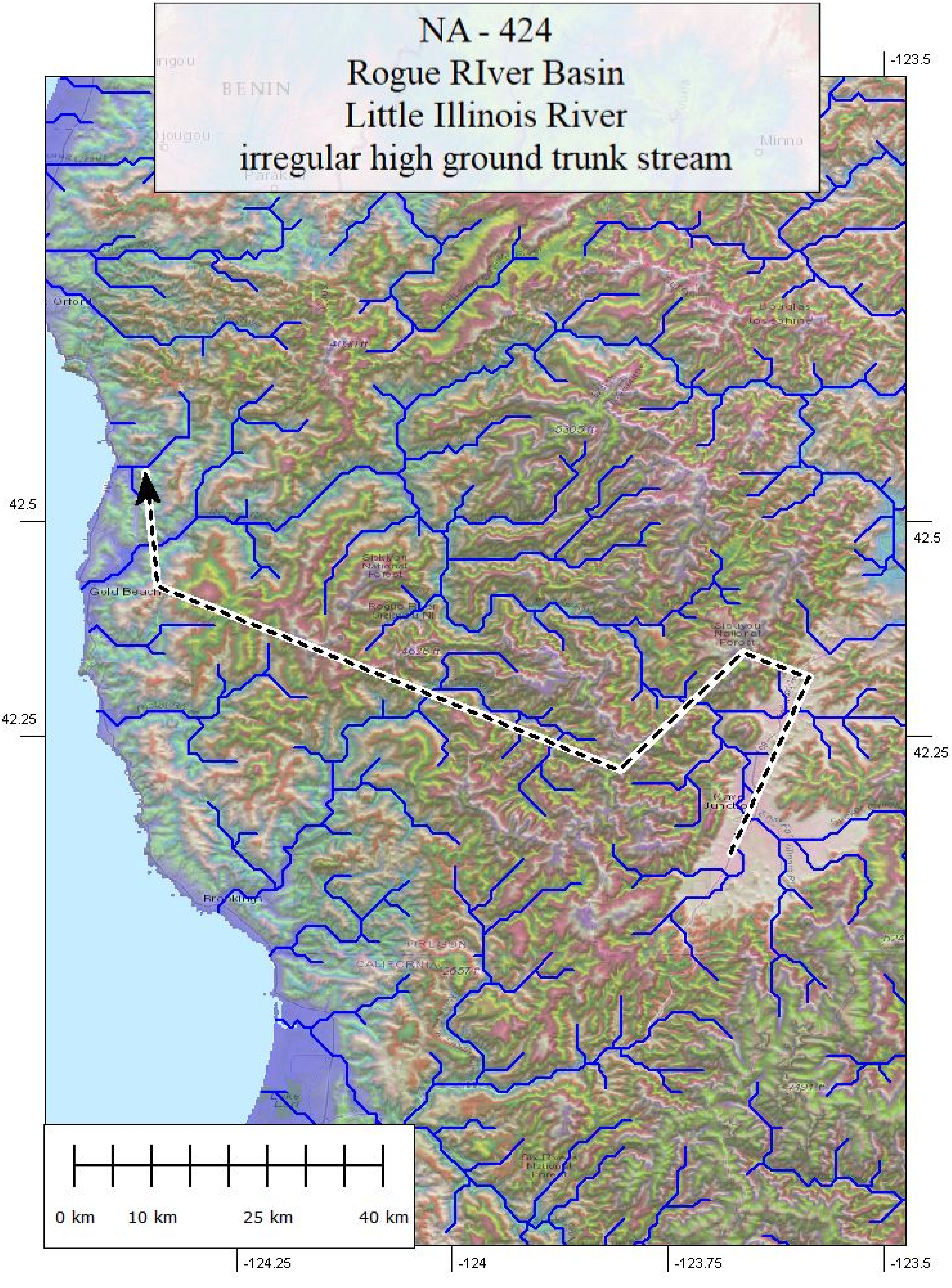
NA - 424
Rogue RIver Basin
Little Illinois River
irregular high ground trunk stream
Gold Beach
OREGON
CALIFORNIA
42.5
42.25
-123.5
-124.25
-124
-123.75
0 km
10 km
25 km
40 km

NA - 425
South Umpqua River Basin
Umpqua River
irregular high ground trunk stream

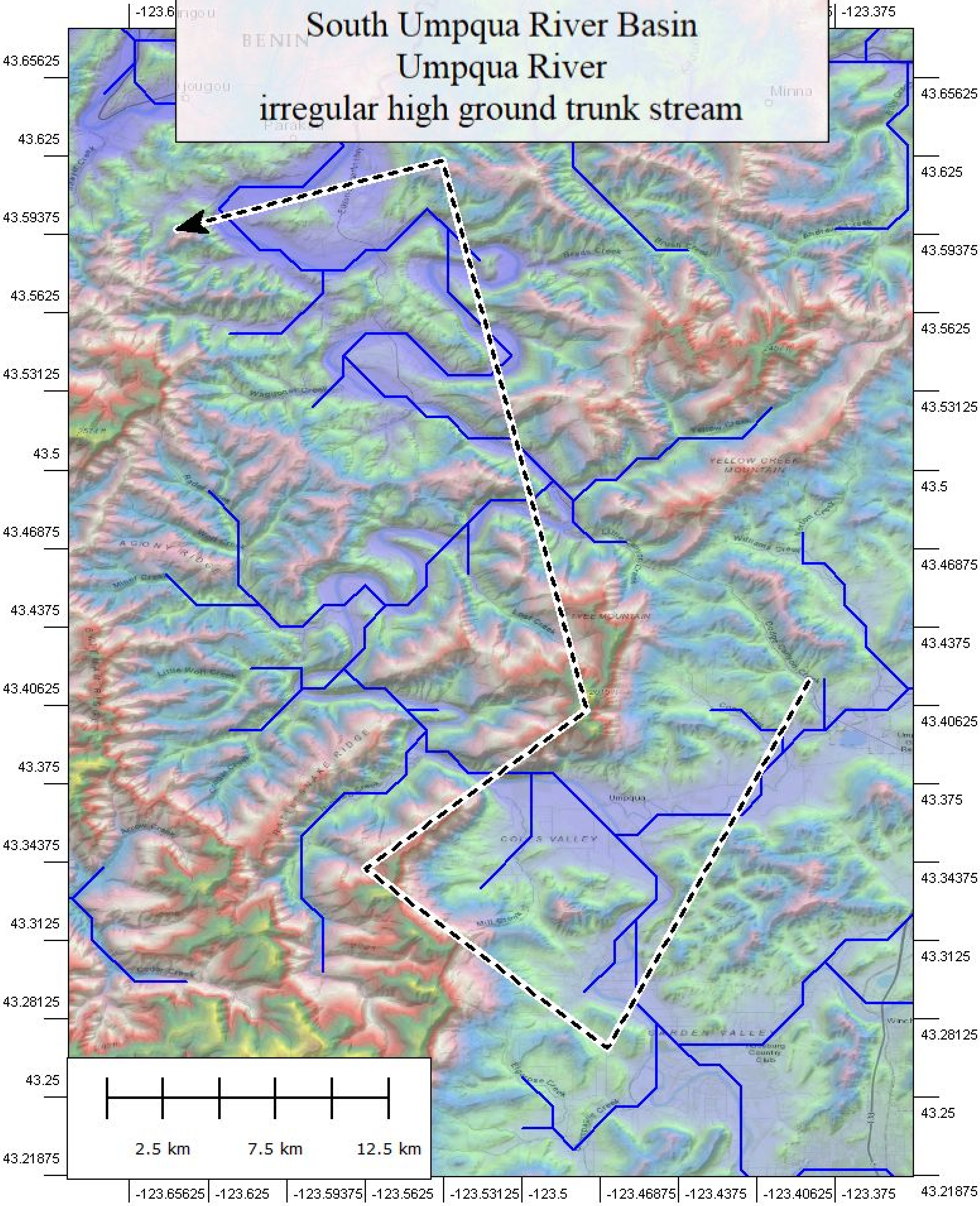

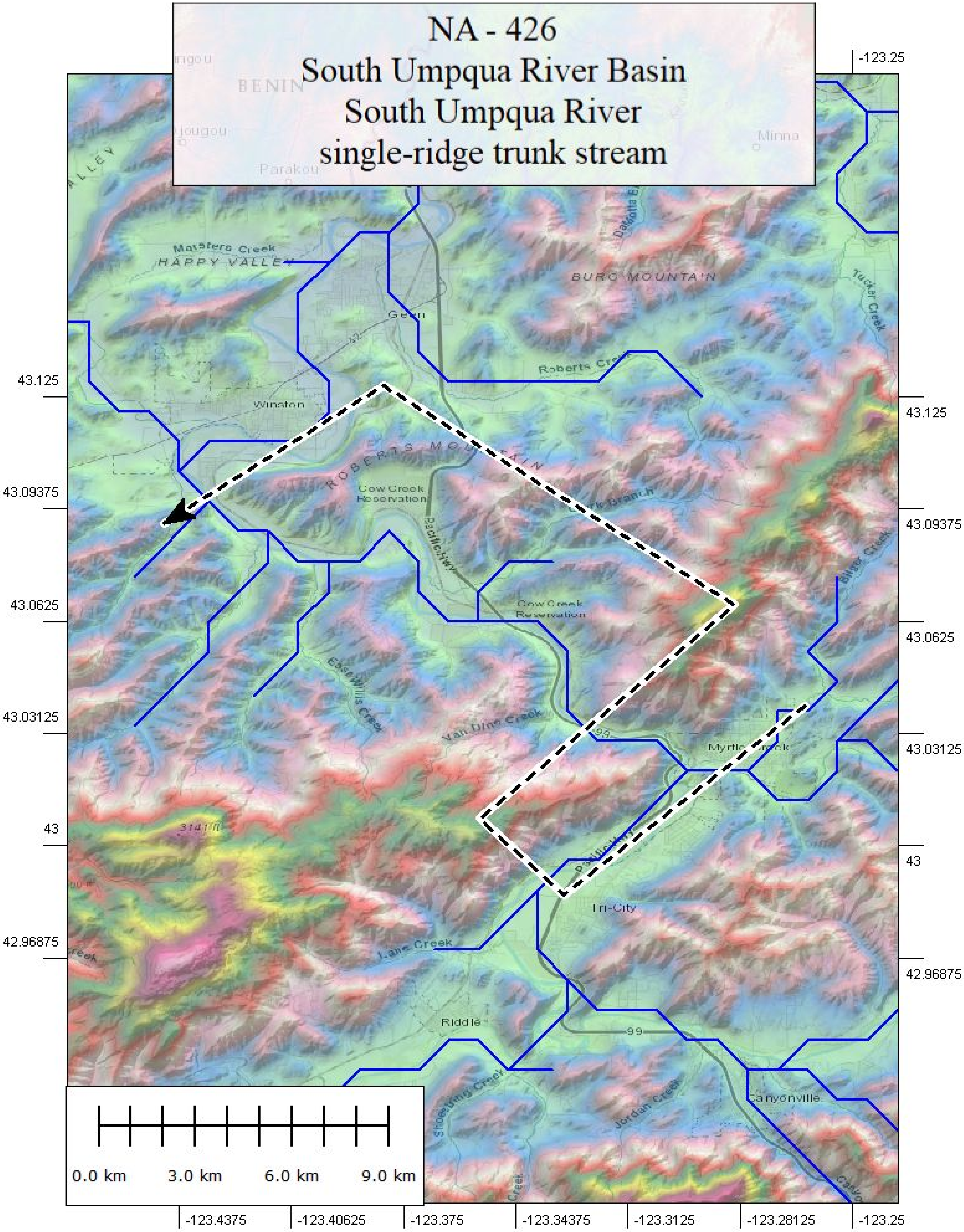

NA - 426
South Umpqua River Basin
South Umpqua River
single-ridge trunk stream
Masters Creek
HAPPY VALLEY
BURO MOUNTAIN
Tucker Creek
Green
Roberts Creek
Winston
ROBERTS MOUNTAIN
Cow Creek Reservation
Pacific Hwy
Cow Creek Reservation
Bilger Creek
Van Dine Creek
Myrtle Creek
99
3141 ft
Tri-City
Lane Creek
Riddle
99
Canyonville
Shoestring Creek
Jordan Creek
0.0 km
3.0 km
6.0 km
9.0 km
43.125
43.09375
43.0625
43.03125
43
42.96875
-123.4375
-123.40625
-123.375
-123.34375
-123.3125
-123.28125
-123.25

NA - 427
Mississippi River Basin
Little River
single-ridge trunk stream

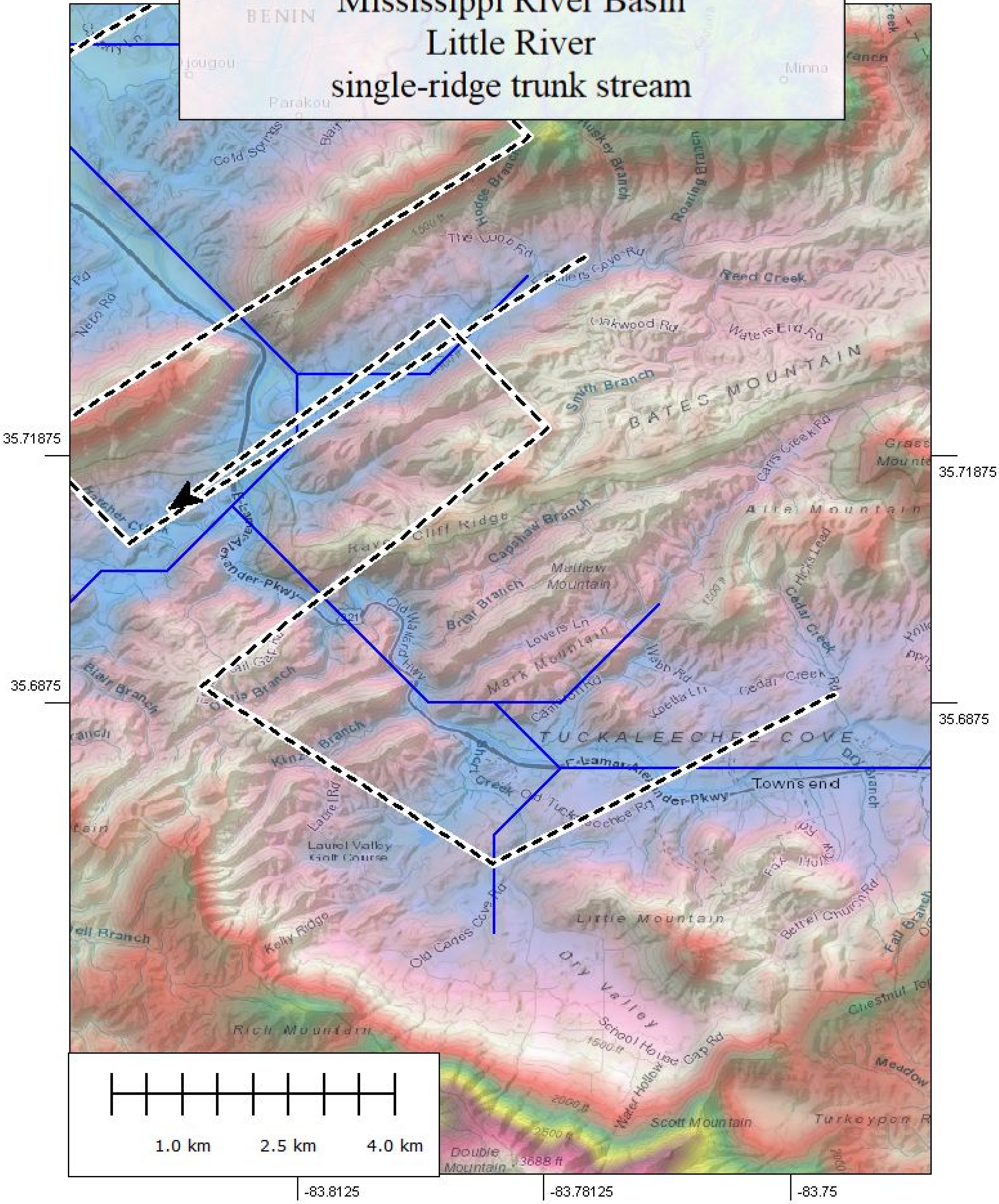

NA - 428
Mobile River Basin
Mill Creek
single-ridge head stream

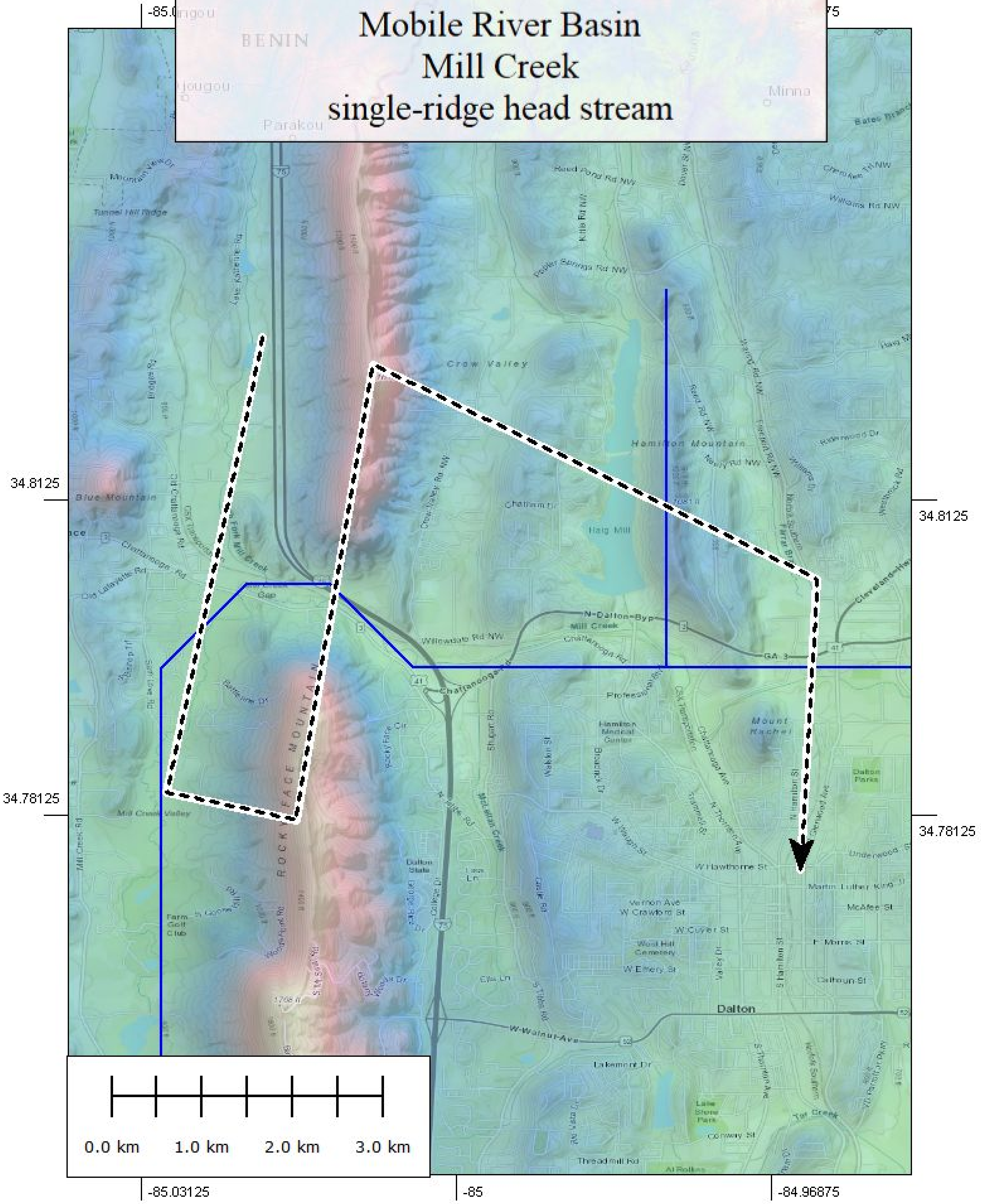

NA - 429
Mississippi River Basin
South Chickamauga Creek
single-ridge trunk stream

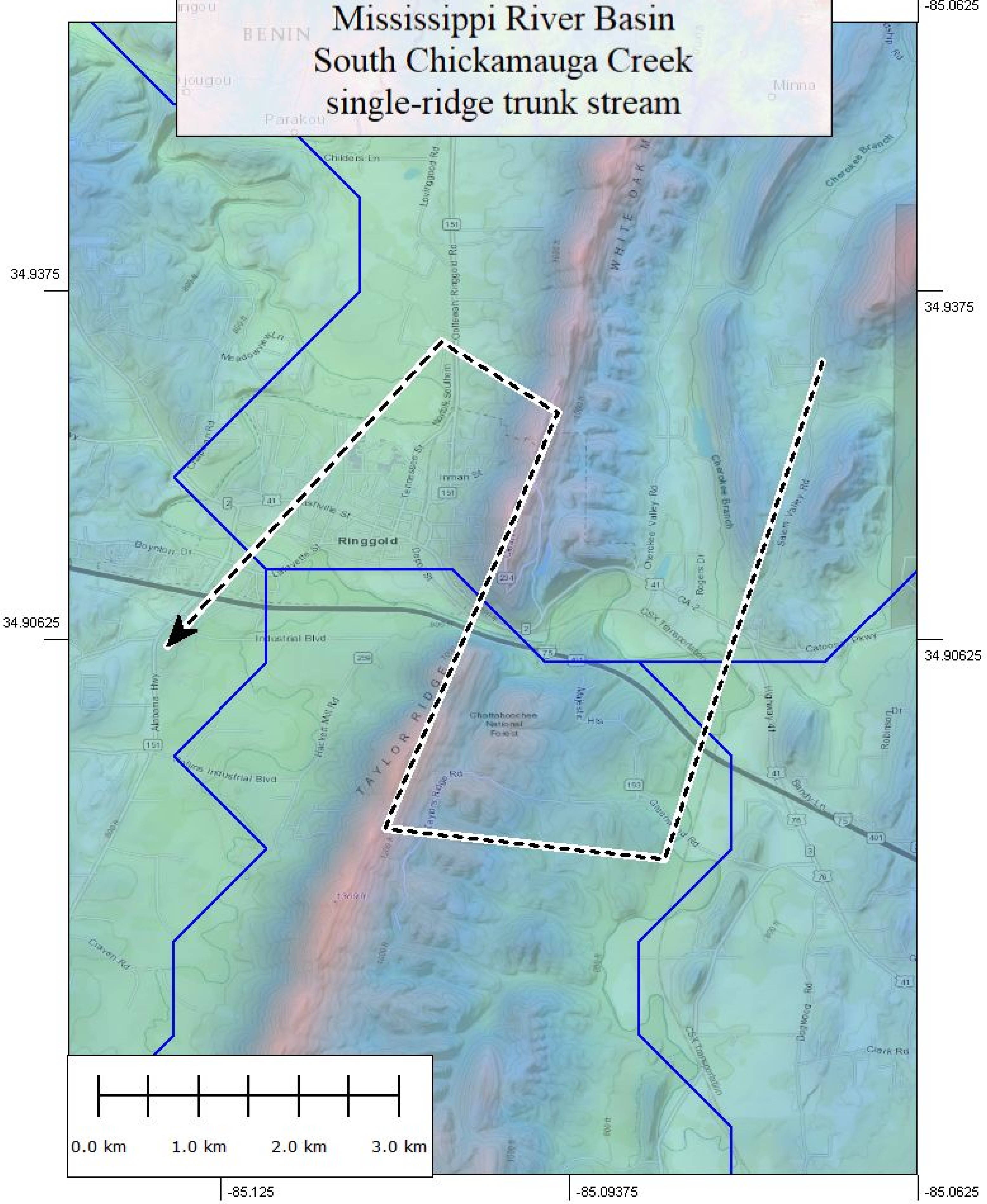

NA - 43
Mackenzie River (Canada) Basin
Root River tributary
irregular high ground trunk stream

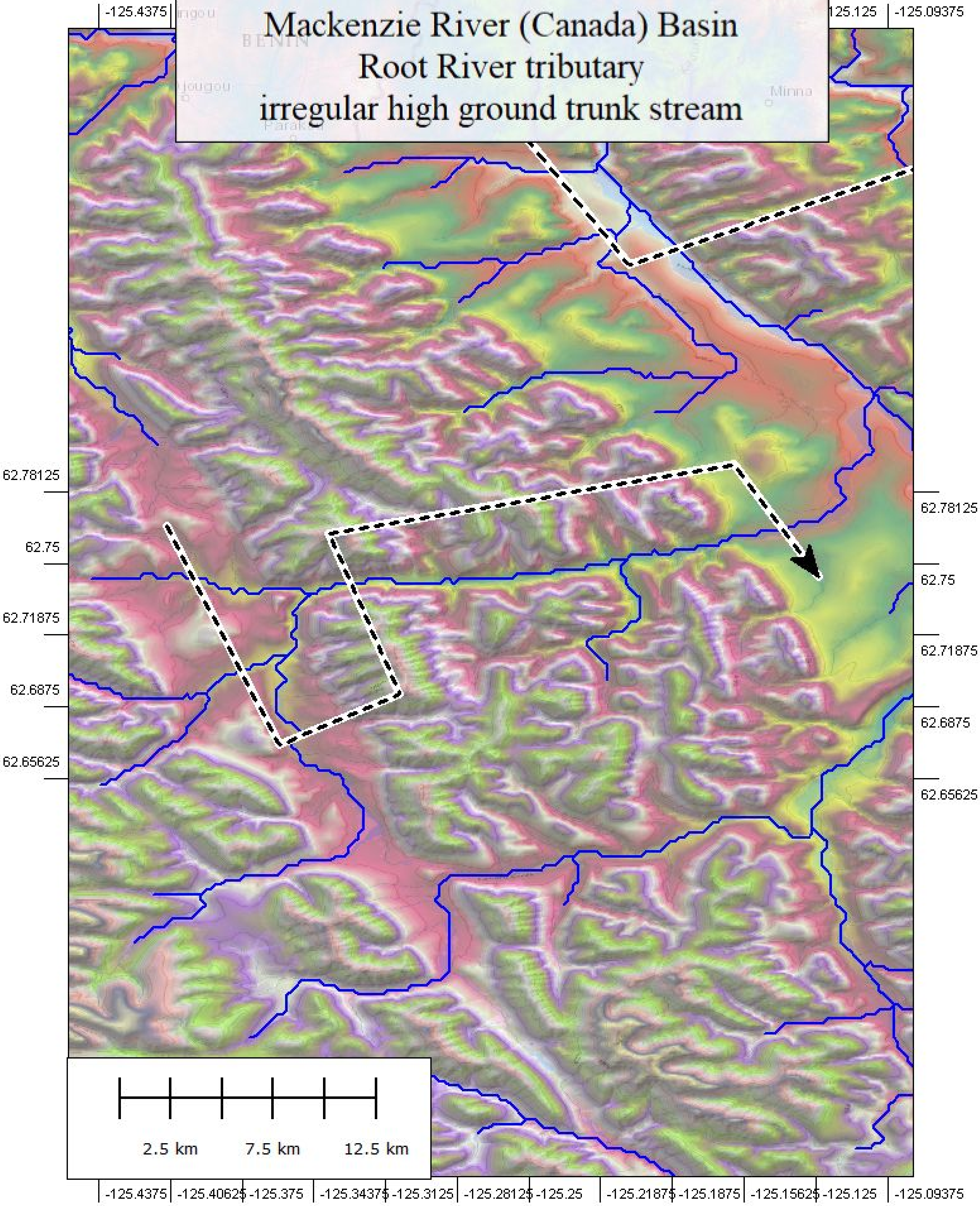

NA - 430
Mississippi River Basin
Cumberland River
single-ridge trunk stream

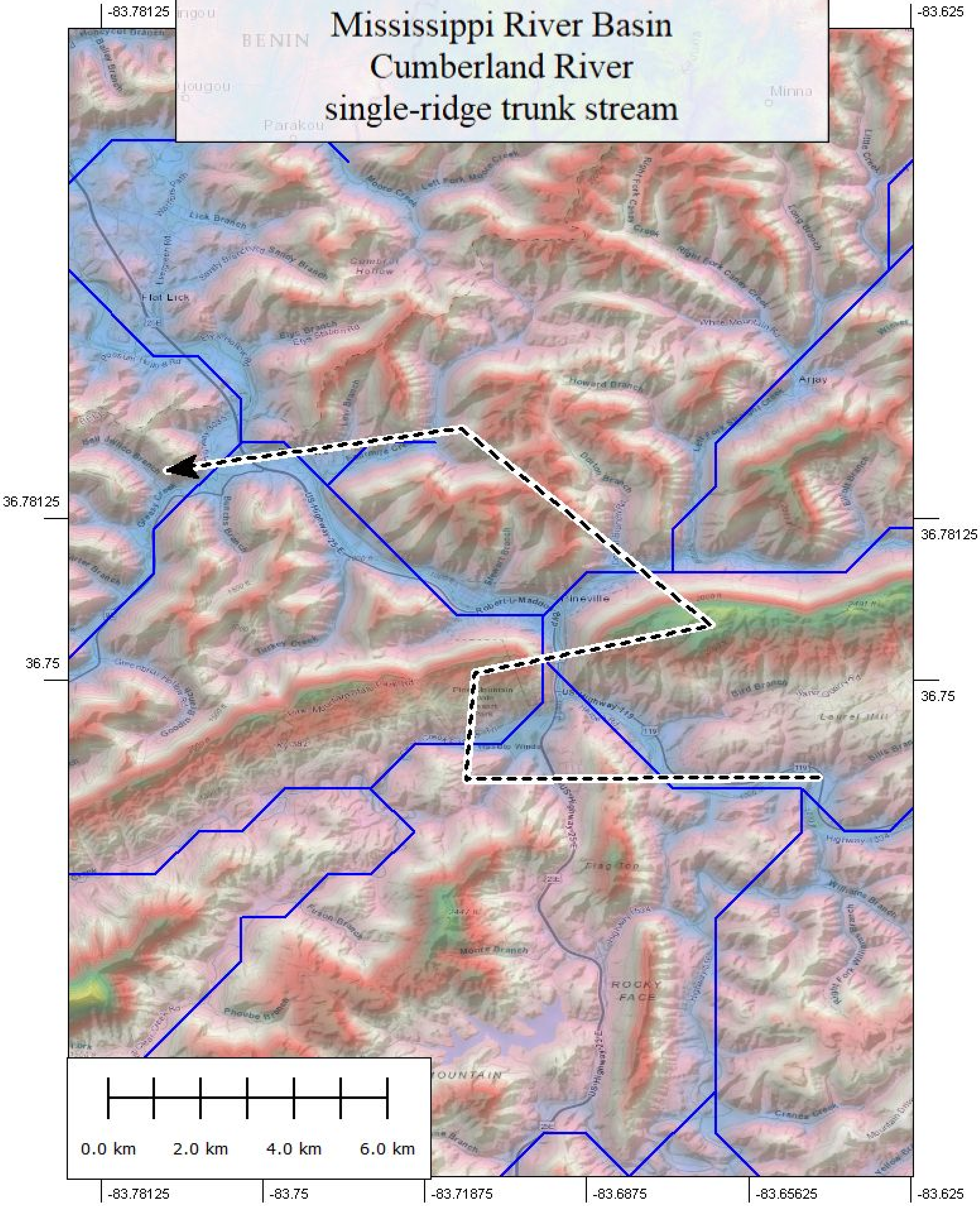

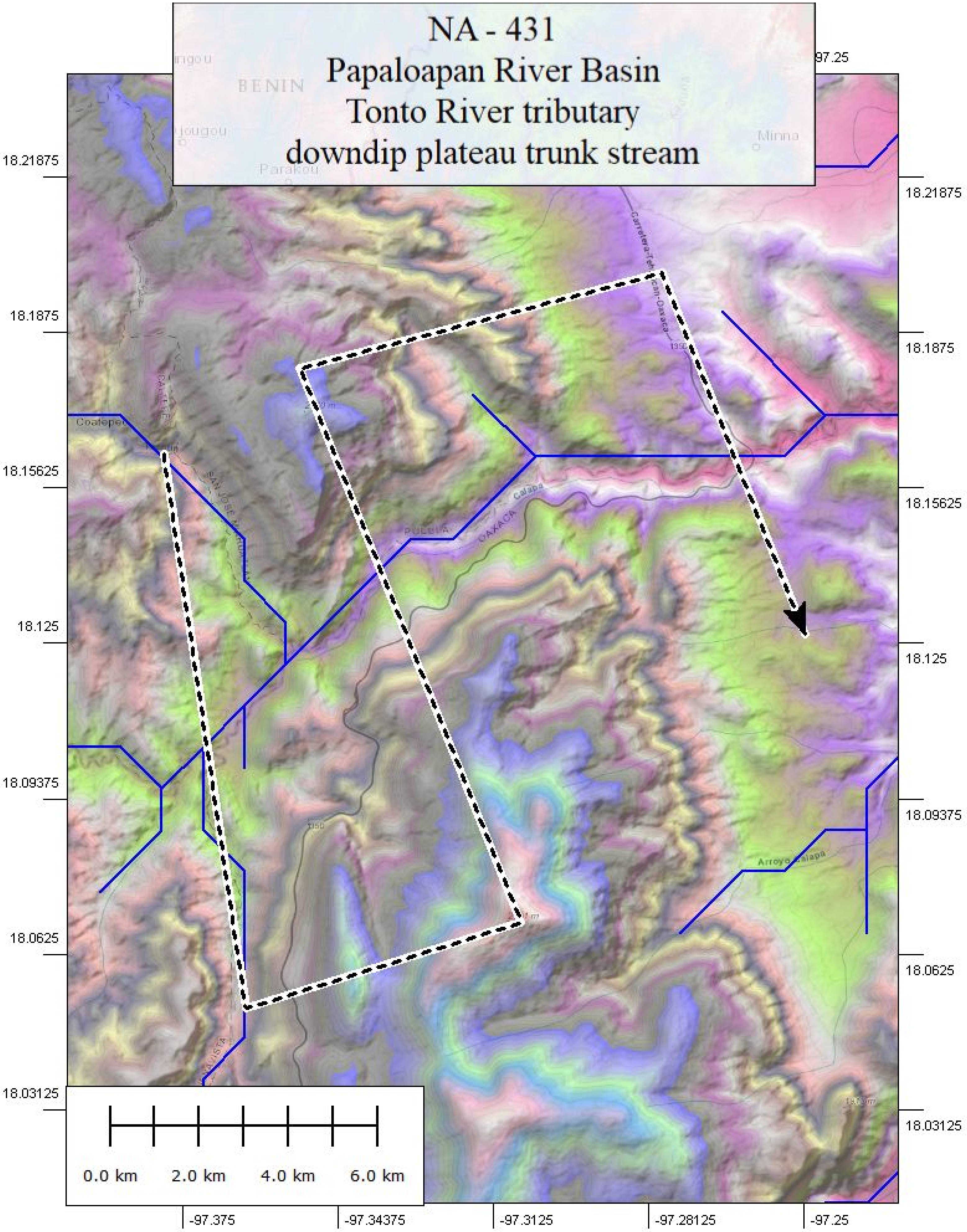

NA - 431
Papaloapan River Basin
Tonto River tributary
downdip plateau trunk stream
18.21875
18.1875
18.15625
18.125
18.09375
18.0625
18.03125
-97.375
-97.34375
-97.3125
-97.28125
-97.25
0.0 km
2.0 km
4.0 km
6.0 km
Coatepec
Calapa
OAXACA
Arroyo Calapa
BENIN
Parakou
Djougou
Minna

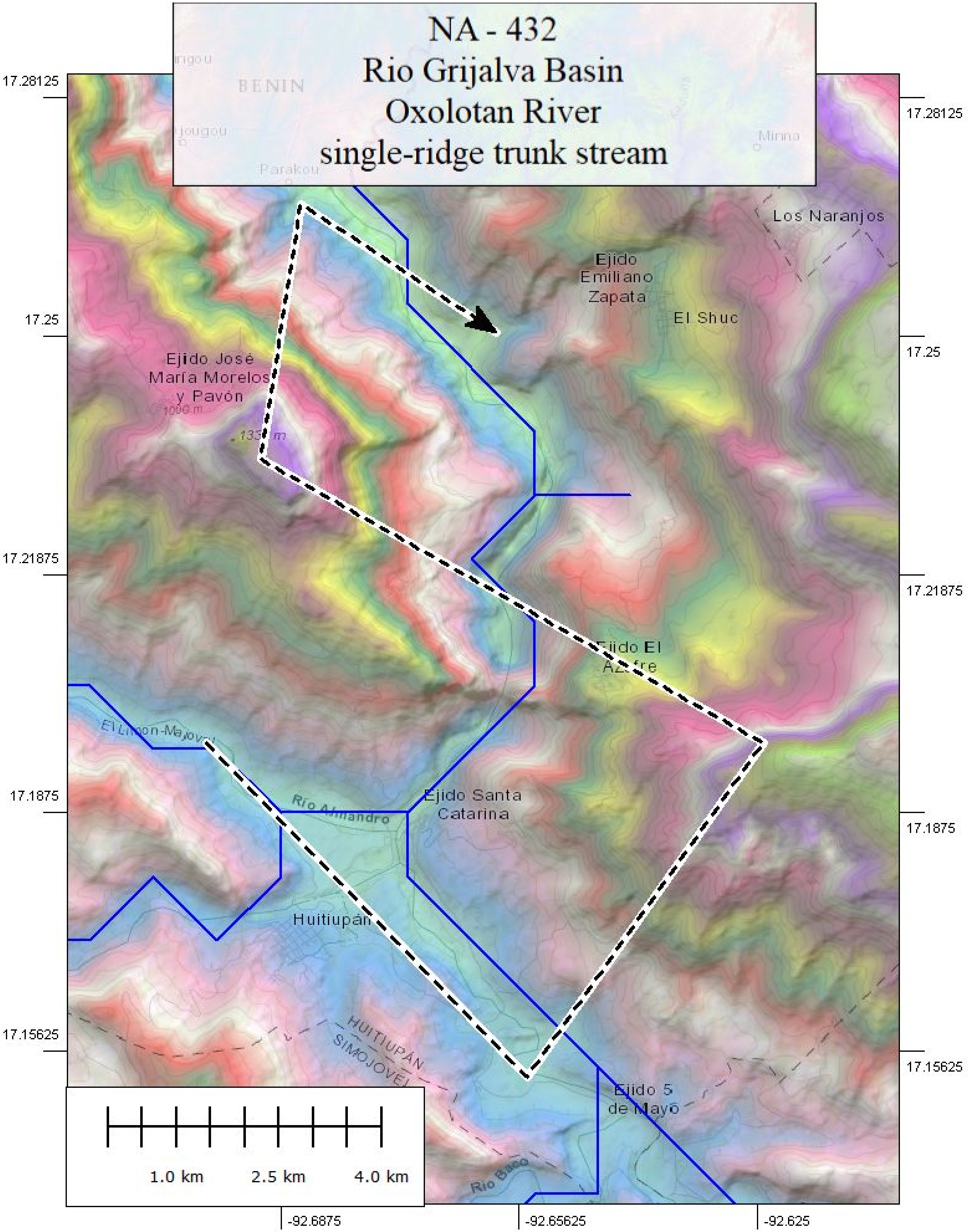
NA - 432
Rio Grijalva Basin
Oxolotan River
single-ridge trunk stream
Los Naranjos
Ejido Emiliano Zapata
El Shuc
Ejido José María Morelos y Pavón
1000 m
Ejido El Azufre
El Limon-Majoval
Río Almandro
Ejido Santa Catarina
Huitiupán
HUITIUPÁN SIMOJOVEL
Ejido 5 de Mayo
Río Baco
17.28125
17.25
17.21875
17.1875
17.15625
-92.6875
-92.65625
-92.625
1.0 km
2.5 km
4.0 km

NA - 433
Mississippi River Basin
Sixteenmile Creek
irregular high ground trunk stream

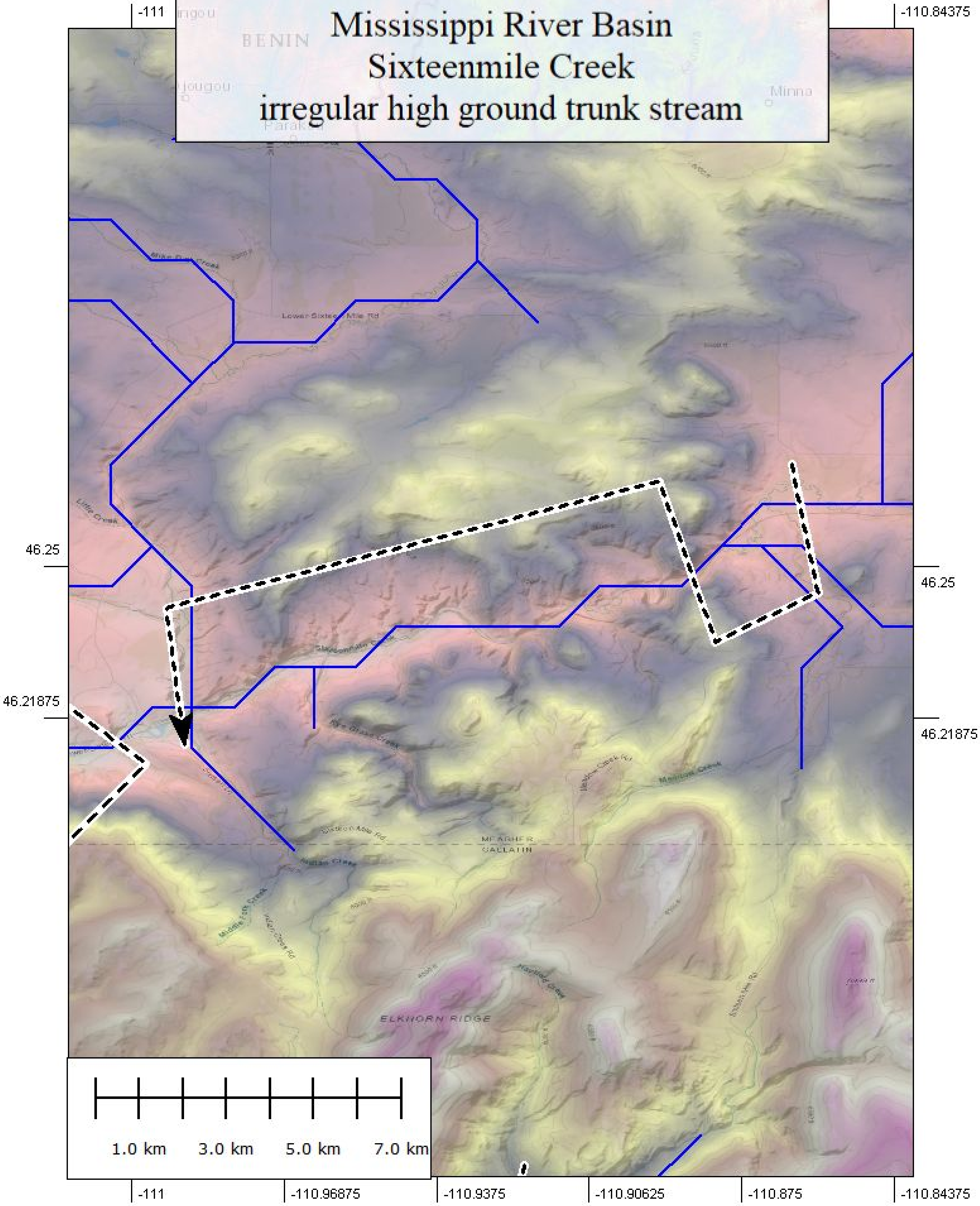

NA - 434
Mississippi River Basin
Sixteenmile Creek
single-ridge trunk stream

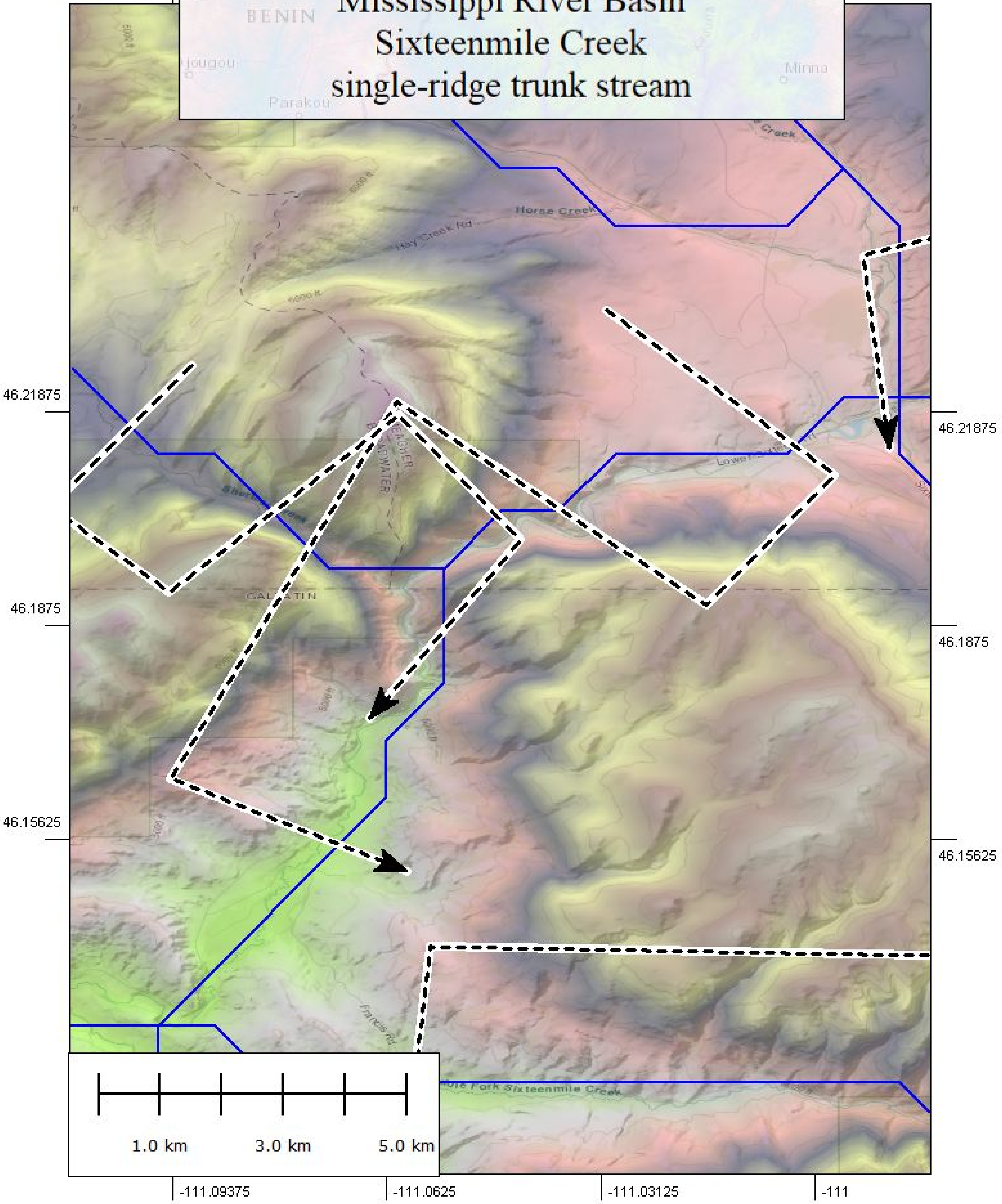

NA - 435
Mississippi River Basin
Shoshone River
single-ridge trunk stream

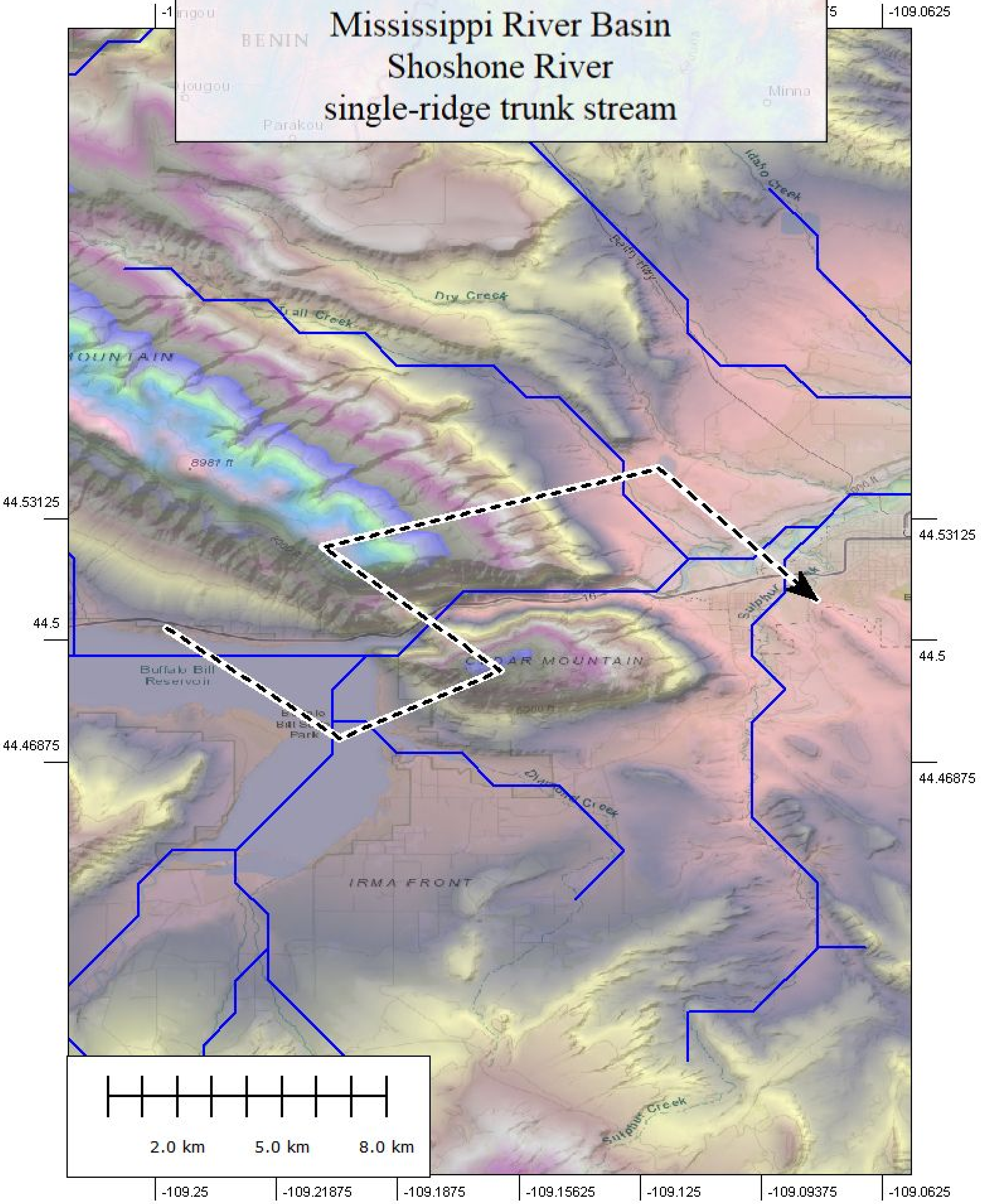

NA - 436
Mississippi River Basin
Missouri River
irregular high ground trunk stream

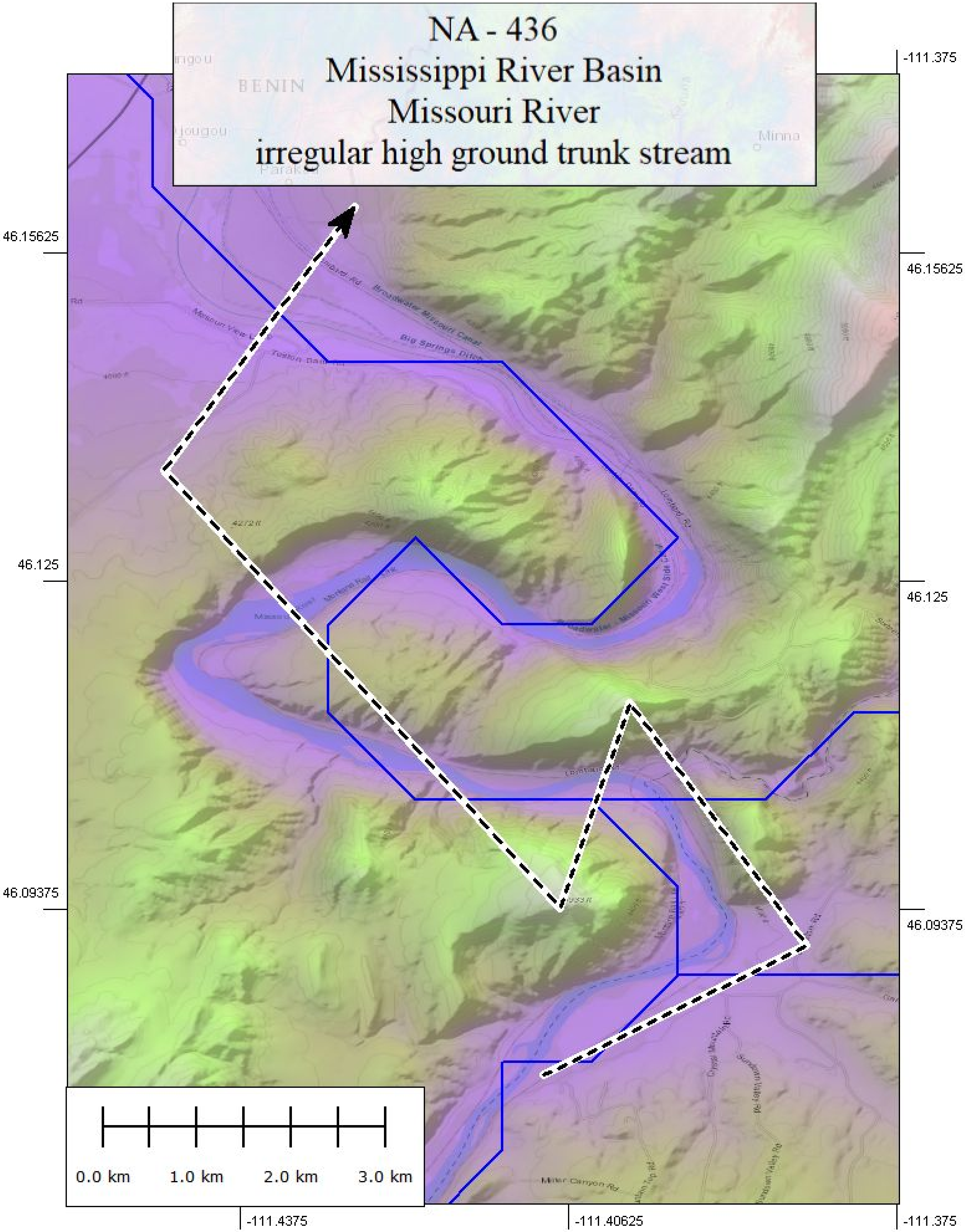

NA - 437
Mississippi River Basin
Yellowstone River
plateau trunk stream

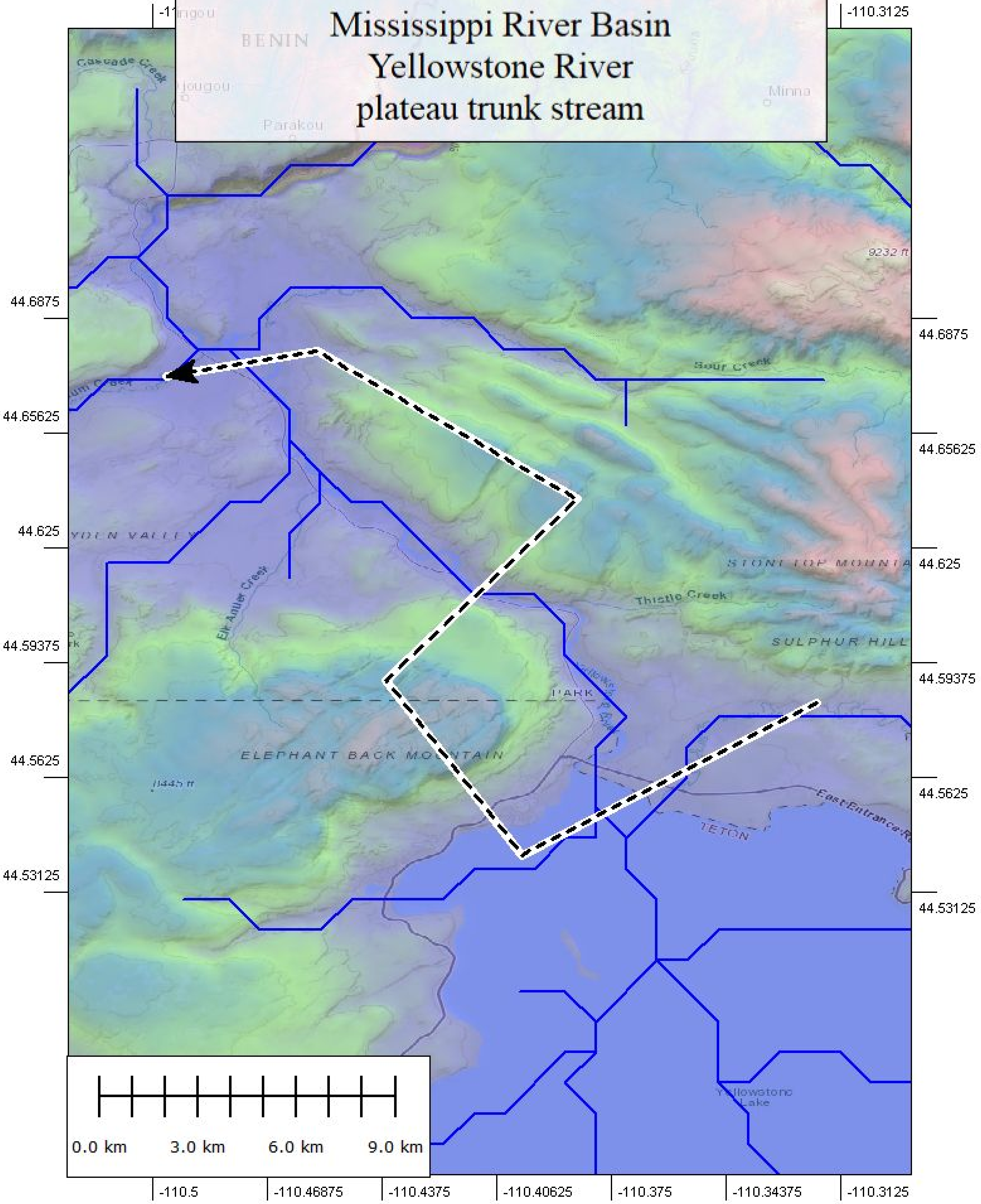

NA - 438
Mississippi River Basin
South Fork Sixteenmile Creek
single-ridge head stream

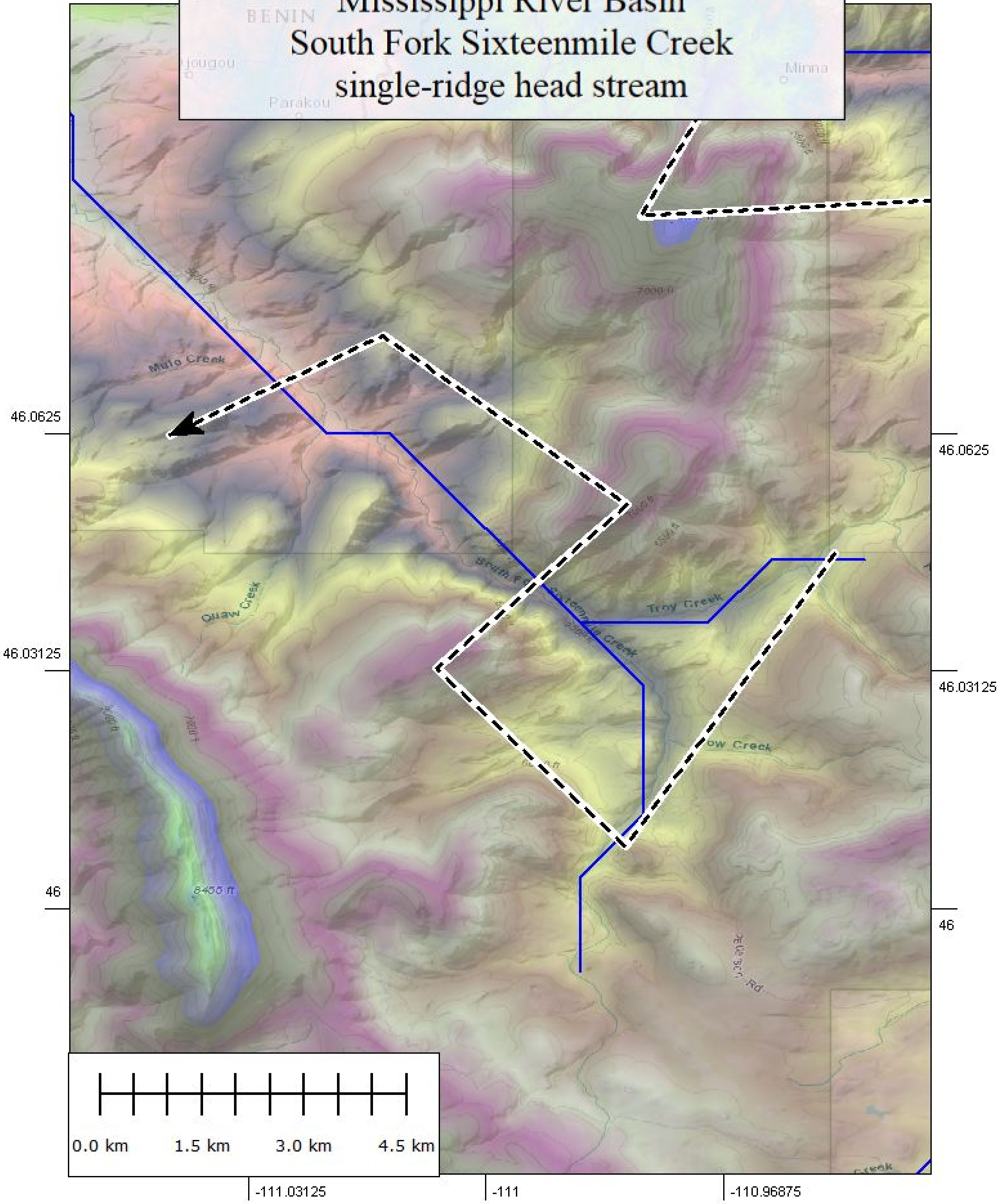

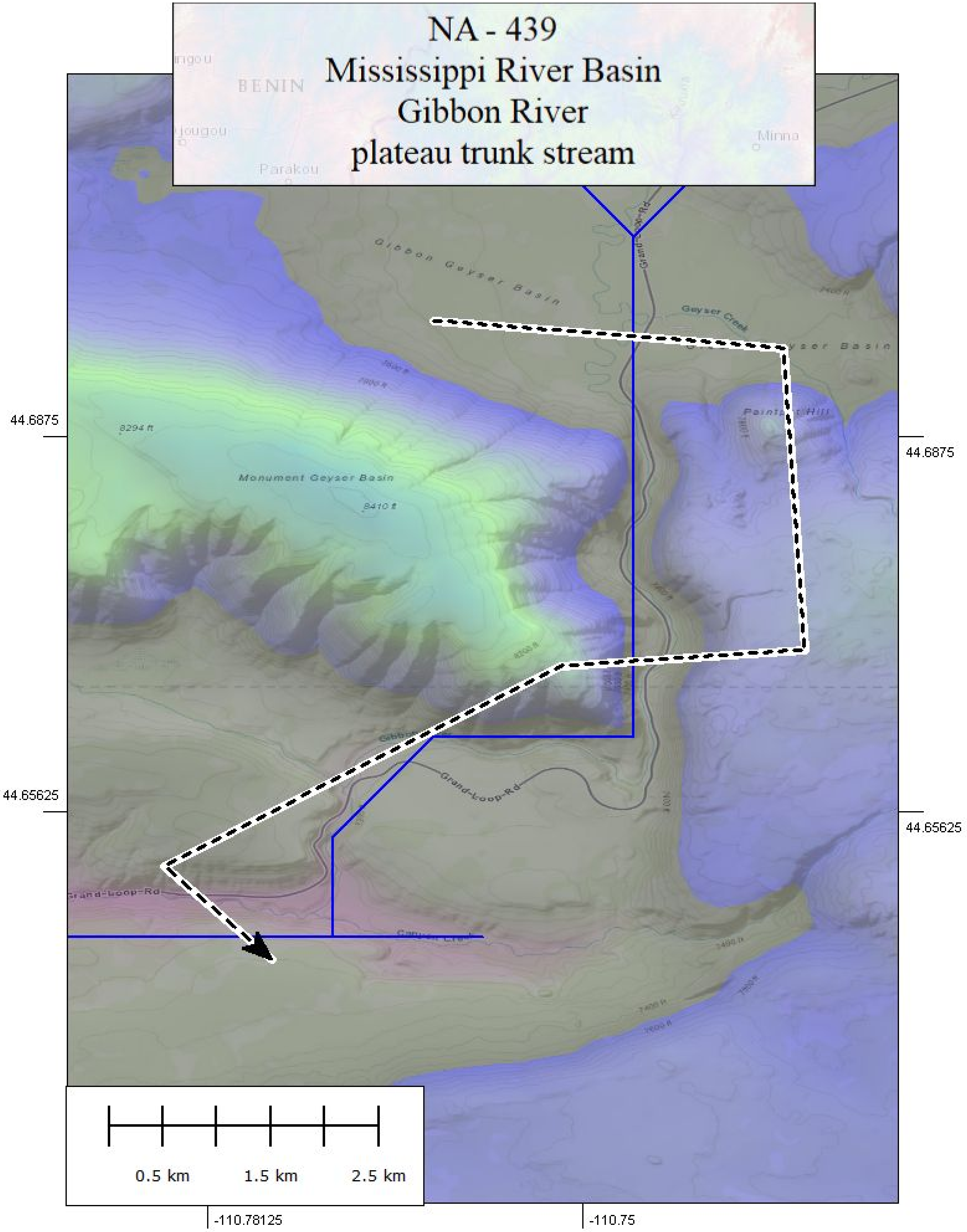

NA - 439
Mississippi River Basin
Gibbon River
plateau trunk stream
BENIN
Parakou
Minna
Gibbon Geyser Basin
Geyser Creek
Monument Geyser Basin
8294 ft
8410 ft
Paintpot Hill
Grand Loop Rd
Canyon Creek
44.6875
44.65625
-110.78125
-110.75
0.5 km
1.5 km
2.5 km

NA - 44
Mackenzie River (Canada) Basin
English Chief River
irregular high ground trunk stream

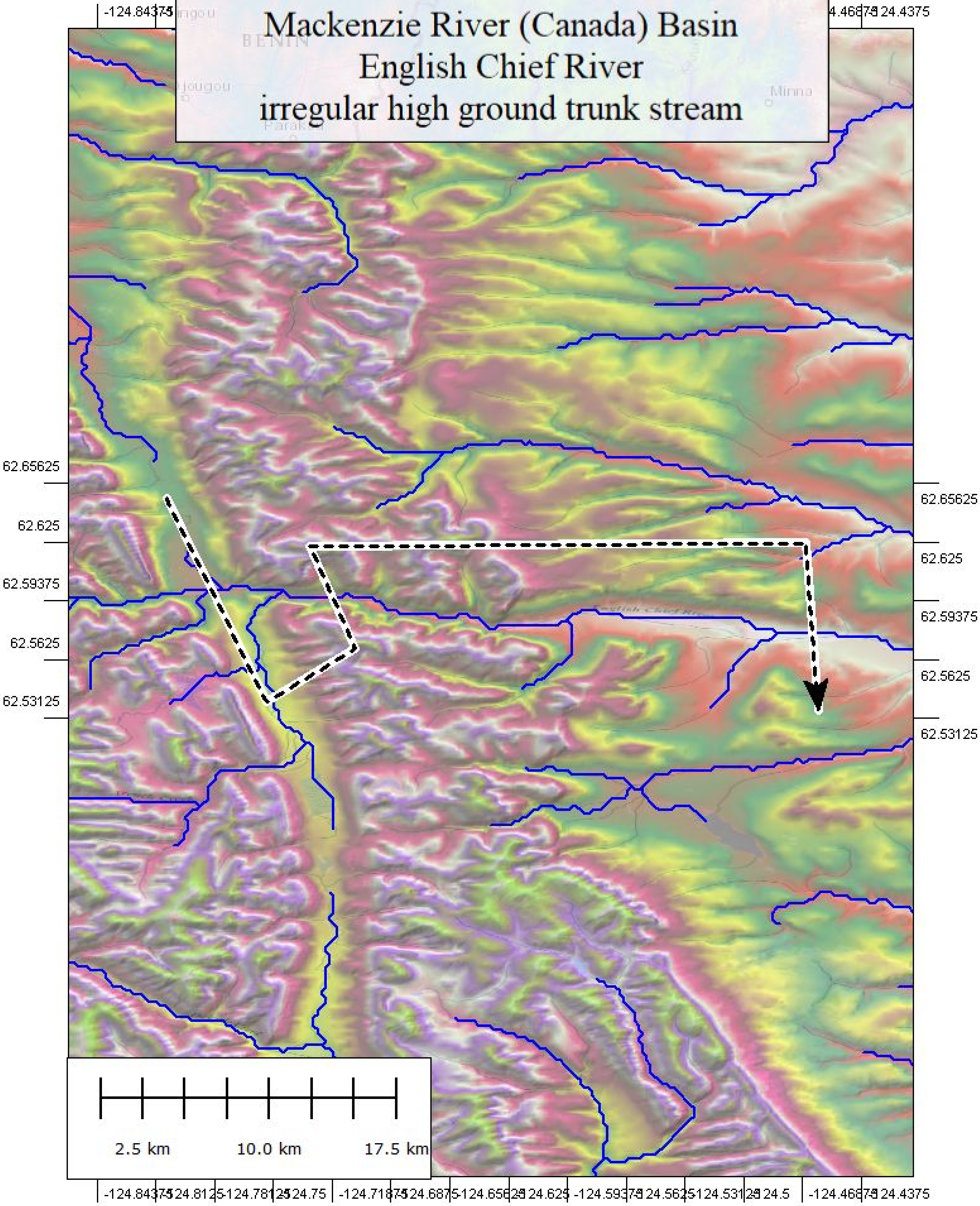

NA - 441
Colorado River (Arizona) Basin
South Piney Creek
irregular high ground trunk stream

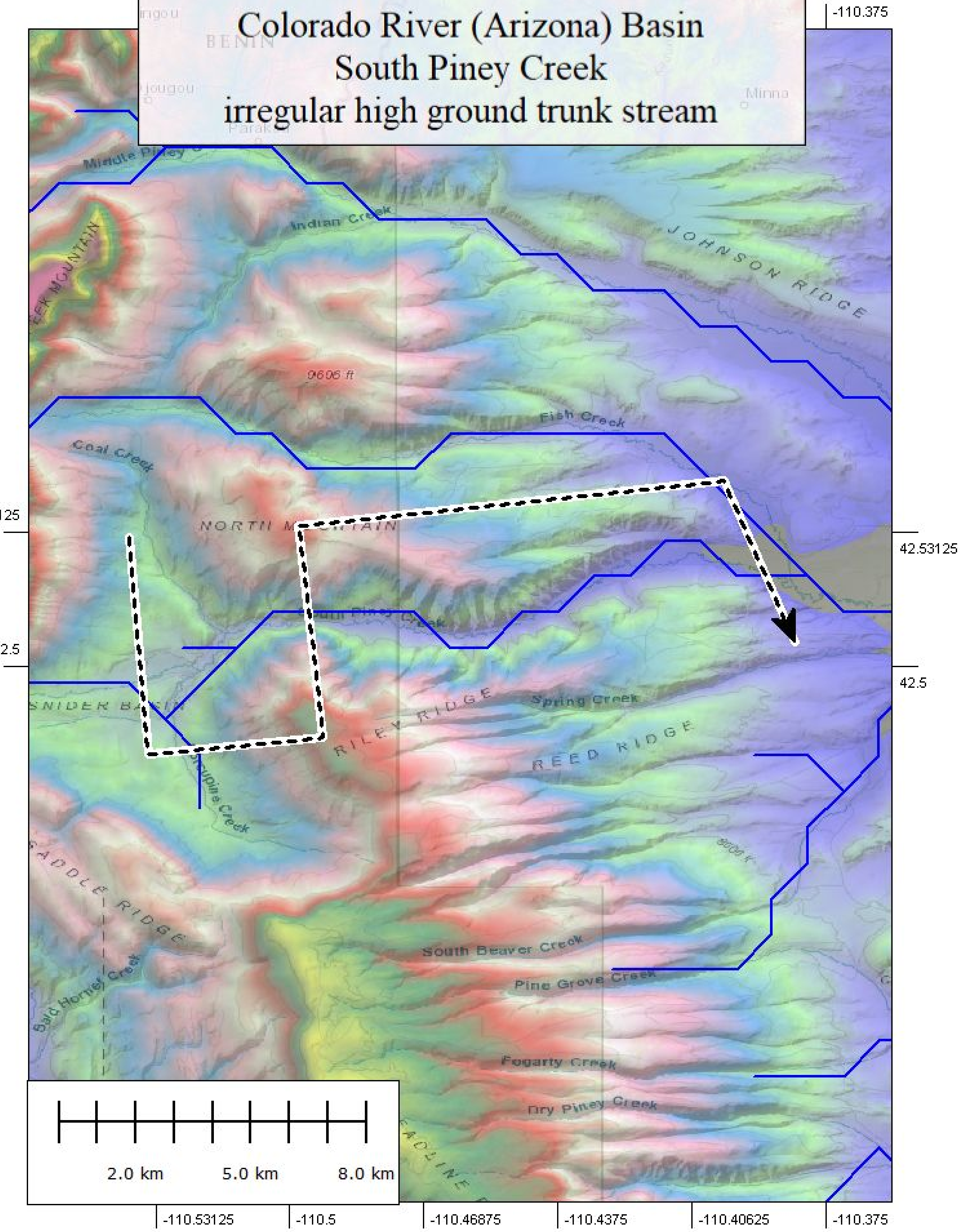

NA - 442
Mississippi River Basin
Sherlock Creek
single-ridge head stream

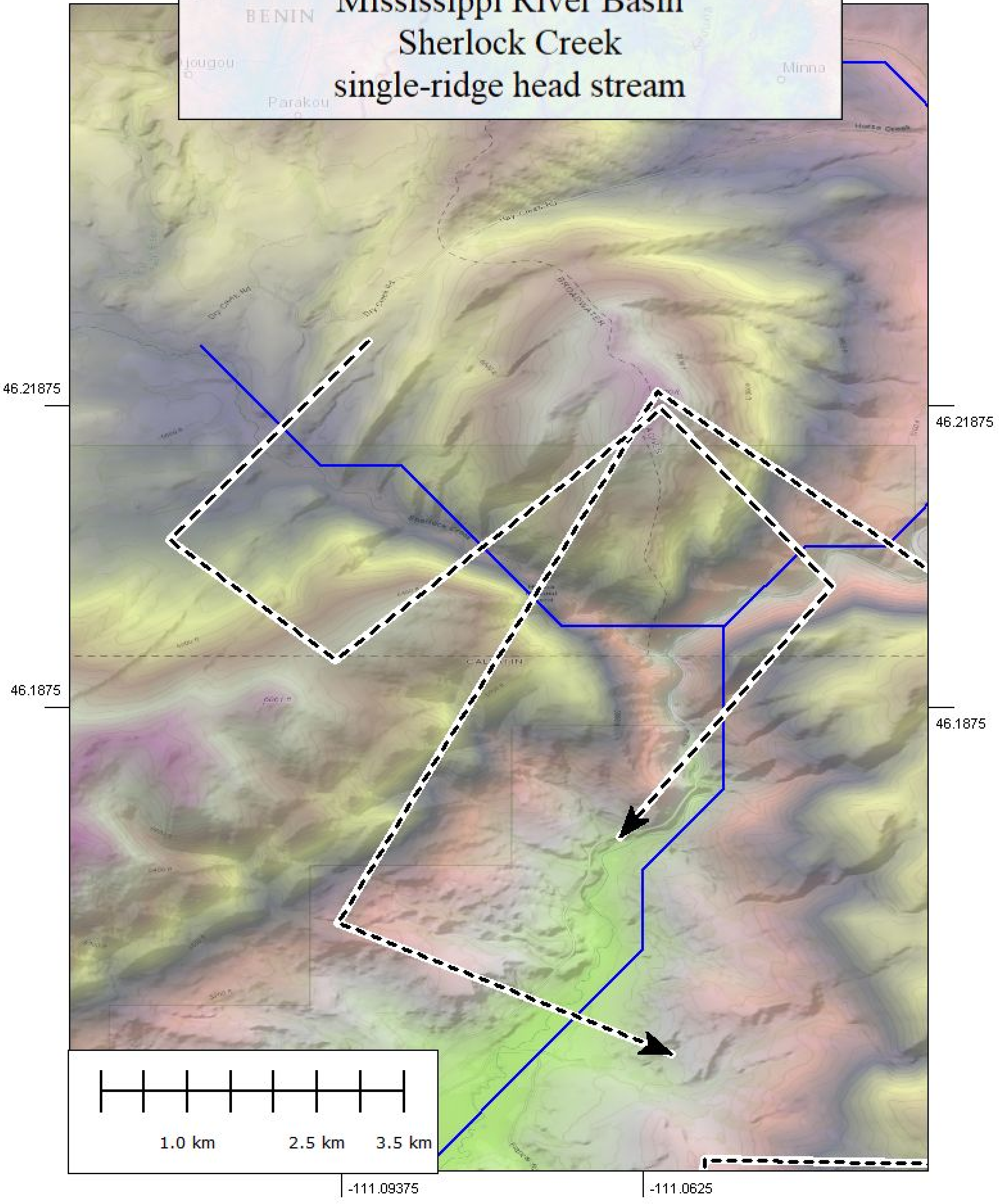

NA - 443
Mississippi River Basin
Middle Fork Sixteenmile Creek
single-ridge trunk stream

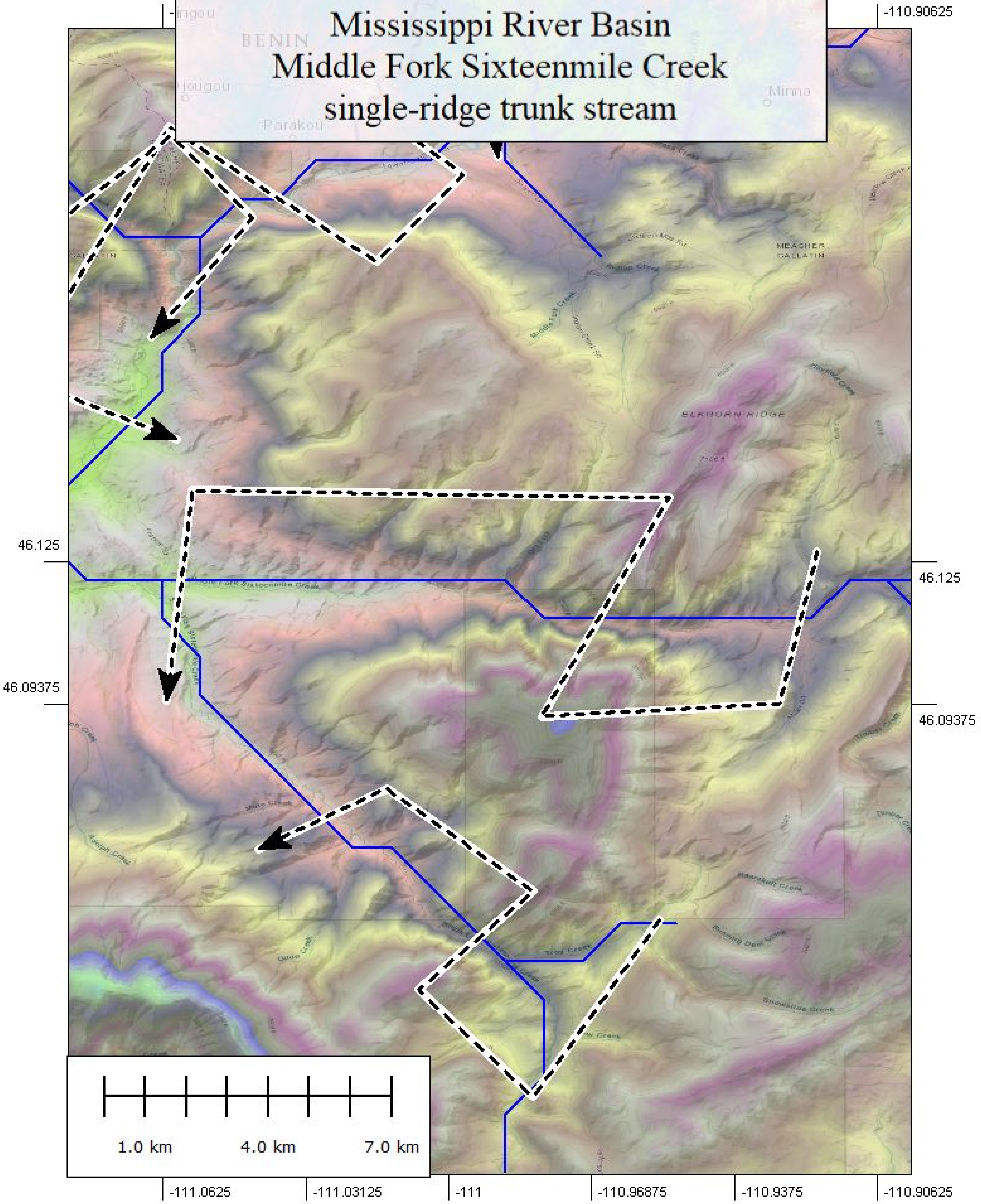

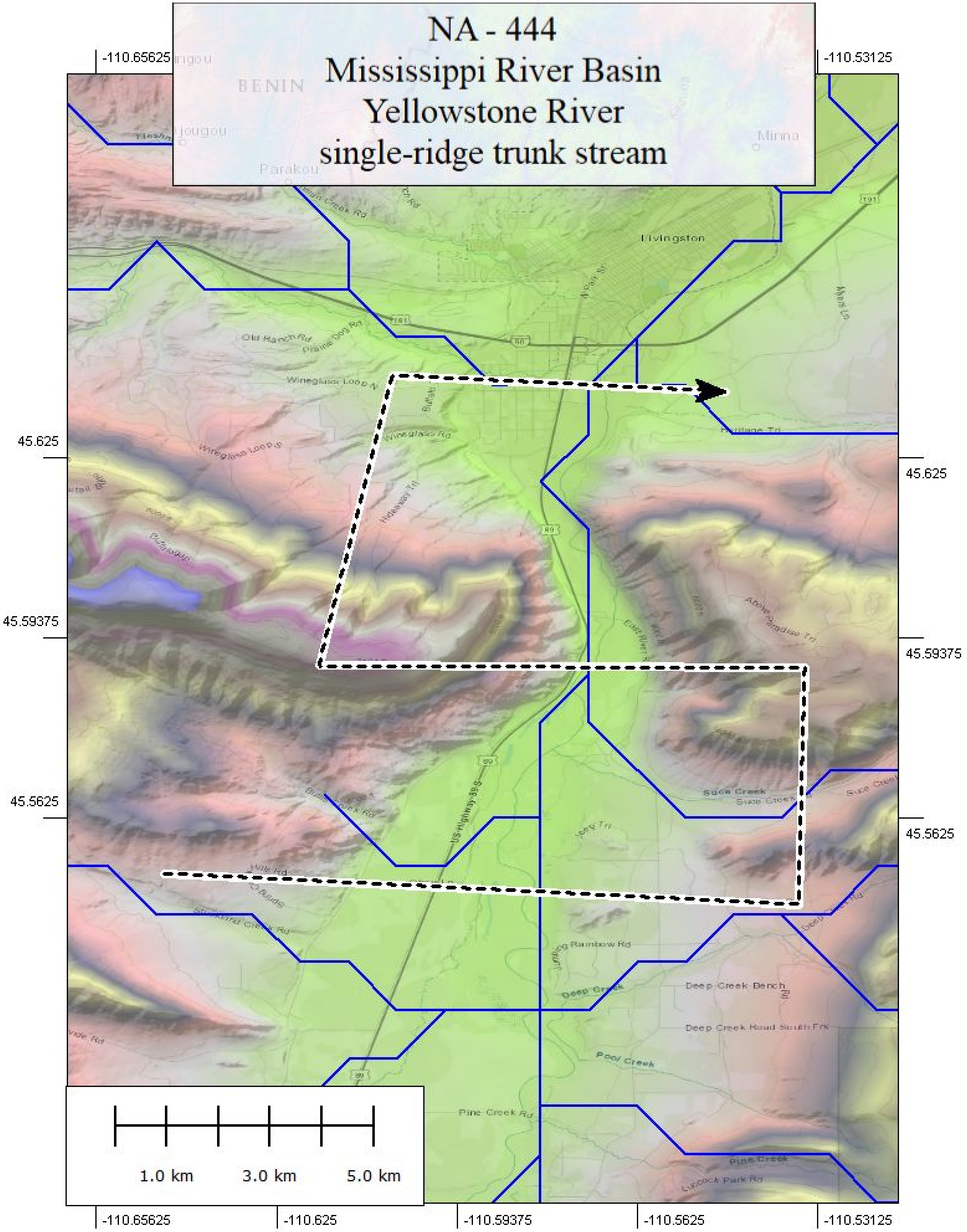
NA - 444
Mississippi River Basin
Yellowstone River
single-ridge trunk stream
-110.65625
-110.53125
-110.625
-110.59375
-110.5625
45.625
45.59375
45.5625
Livingston
Old Ranch Rd
Prairie Dog Rd
Wineglass Loop N
Wineglass Rd
Wineglass Loop S
East River Rd
Suce Creek
Rainbow Rd
Deep Creek
Deep Creek Bench
Deep Creek Road South Fork
Pool Creek
Pine Creek Rd
Pine Creek
US Highway 89 S
1.0 km
3.0 km
5.0 km

NA - 445
Mississippi River Basin
Sixteenmile Creek
single-ridge trunk stream

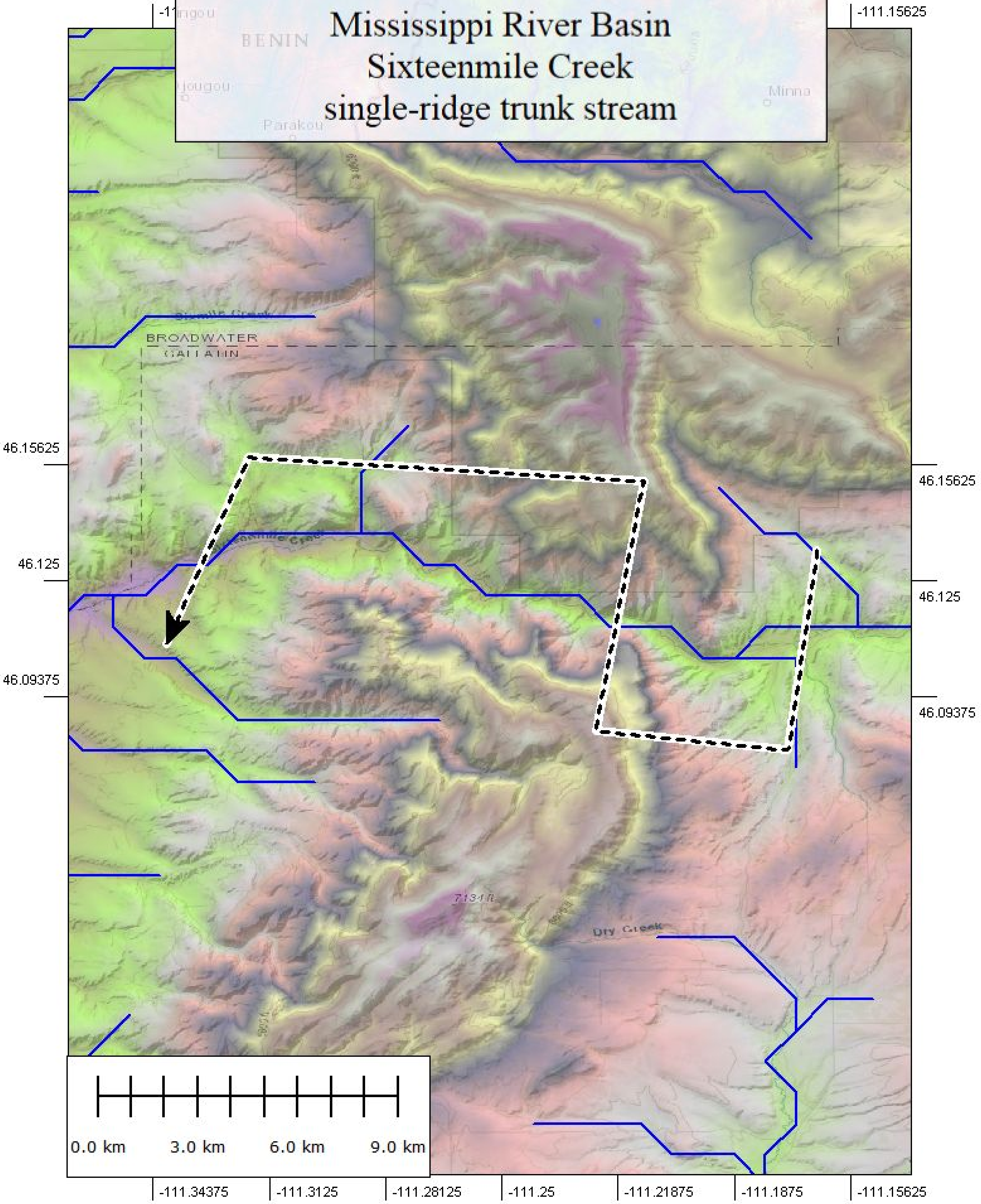

NA - 45
Mackenzie River (Canada) Basin
North Nahanni River
irregular high ground trunk stream

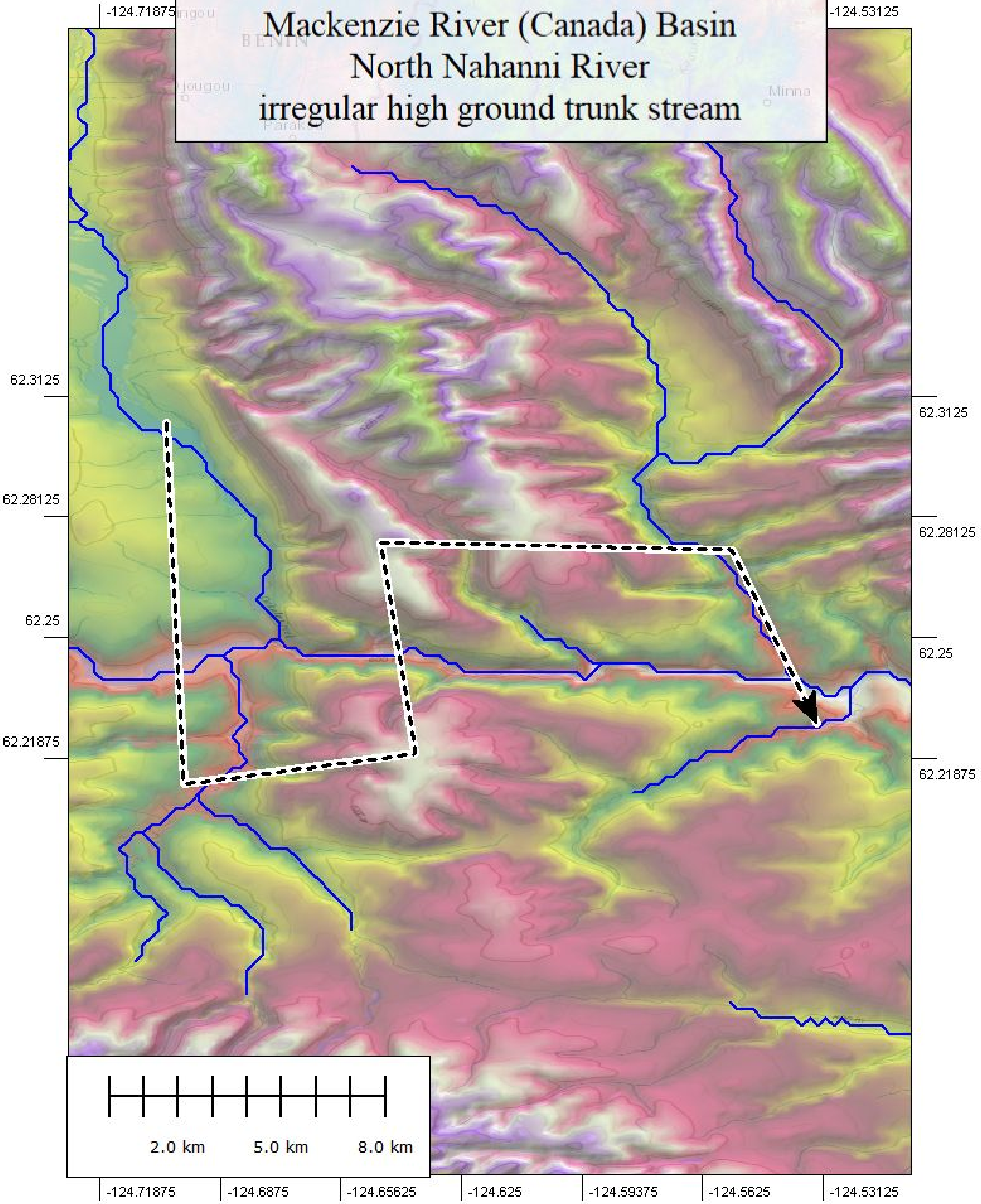

NA - 46
Mackenzie River (Canada) Basin
Root River
single-ridge trunk stream

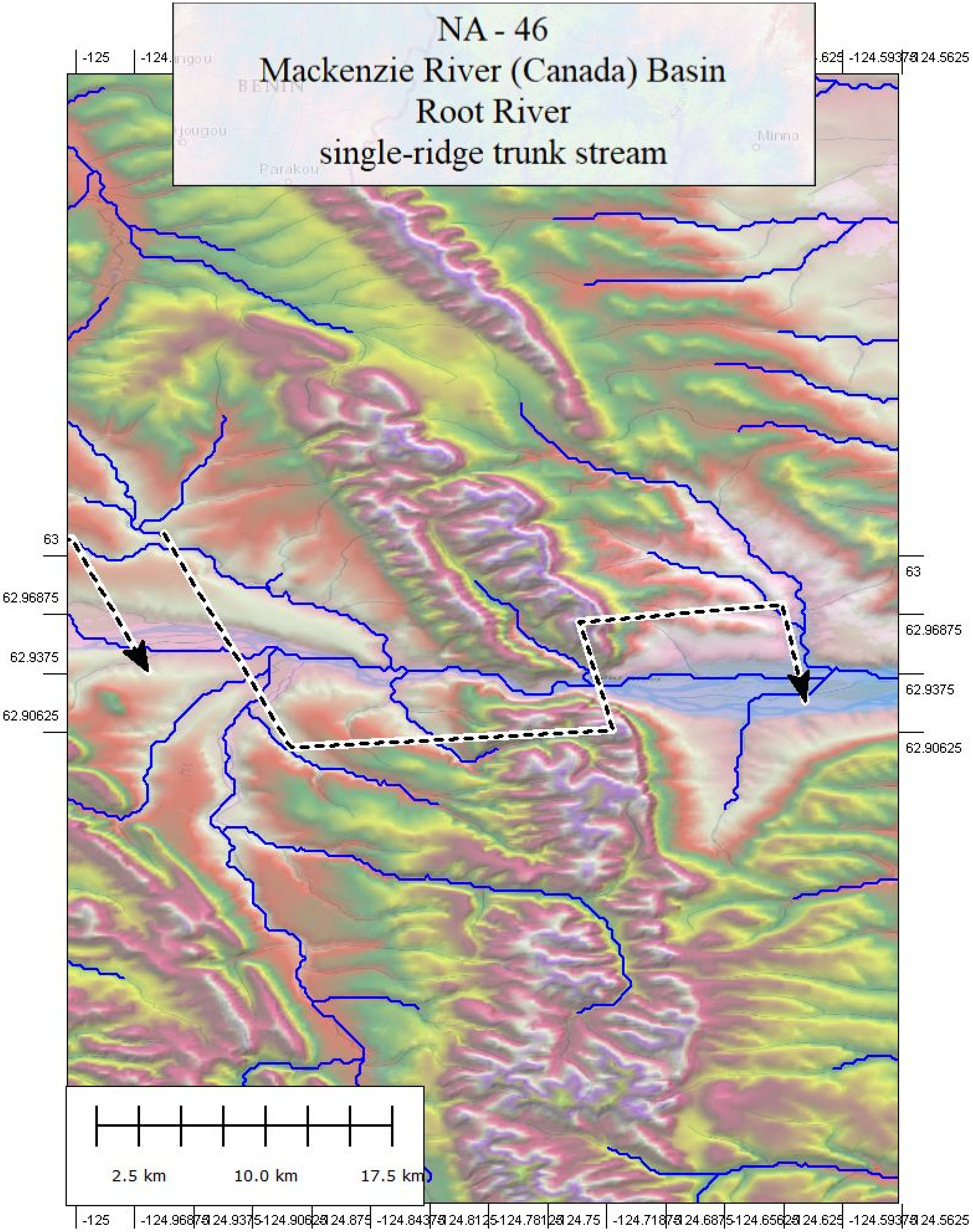

NA - 47
Yukon River Basin
Delta River
irregular high ground trunk stream

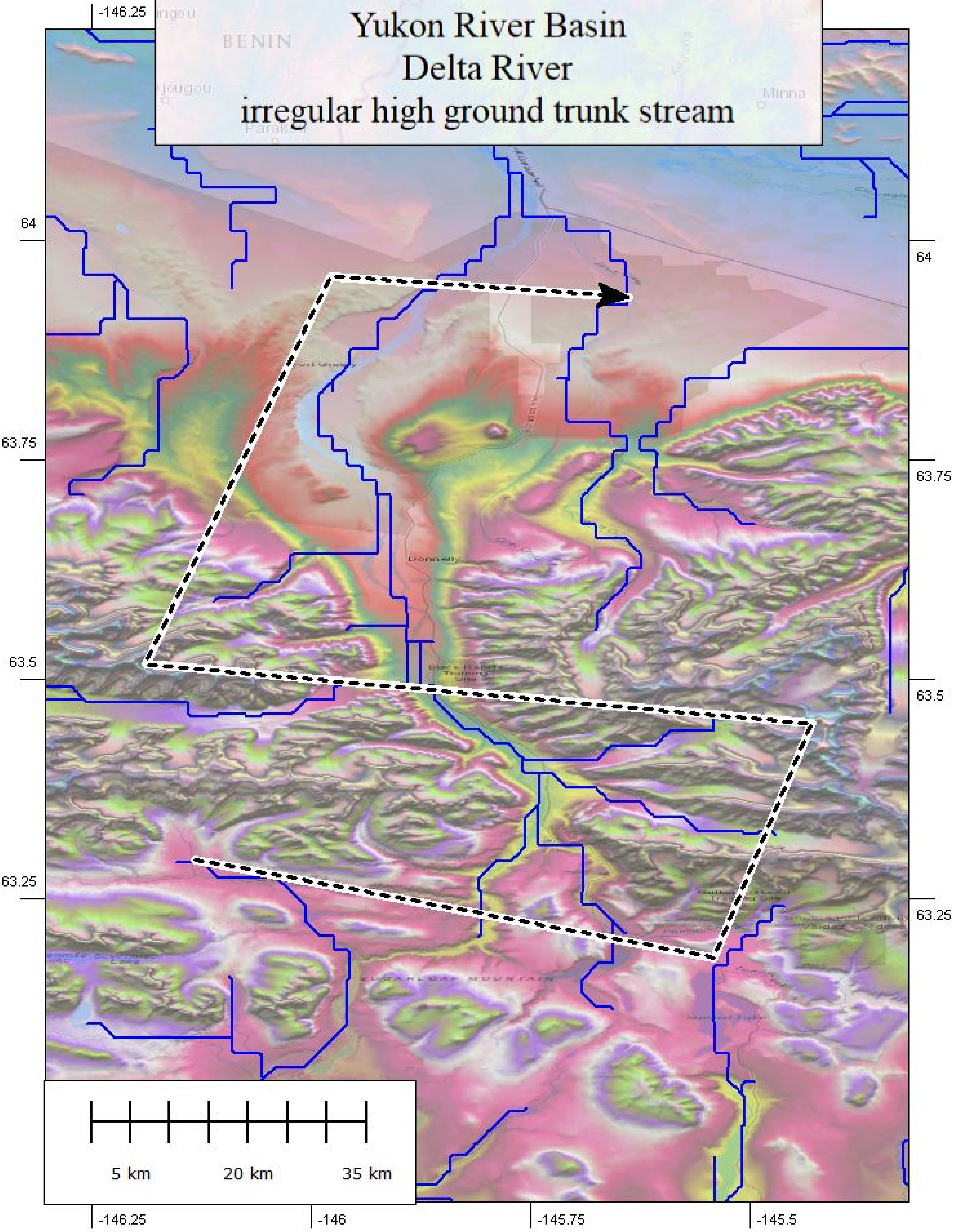

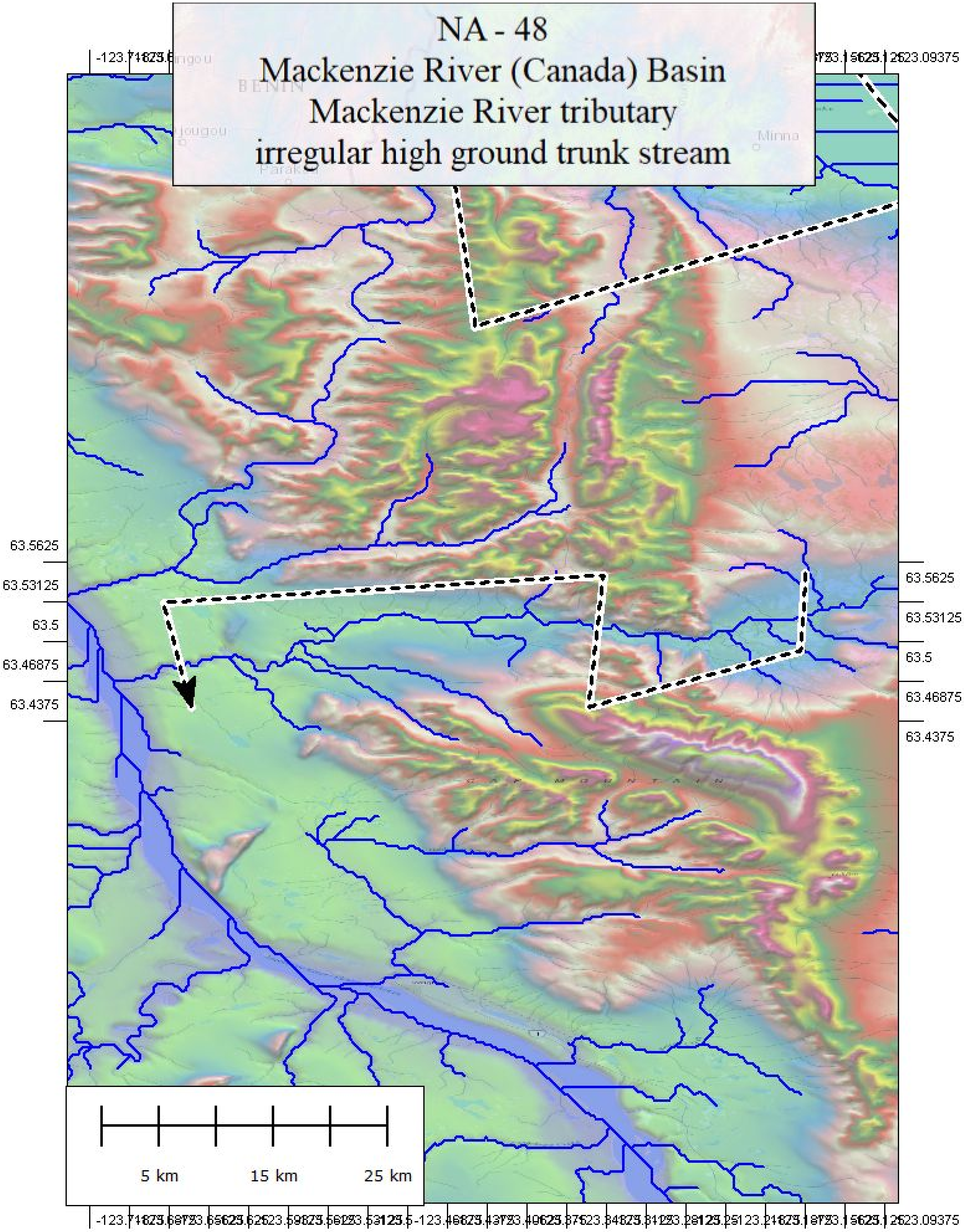
NA - 48
Mackenzie River (Canada) Basin
Mackenzie River tributary
irregular high ground trunk stream
63.5625
63.53125
63.5
63.46875
63.4375
5 km
15 km
25 km

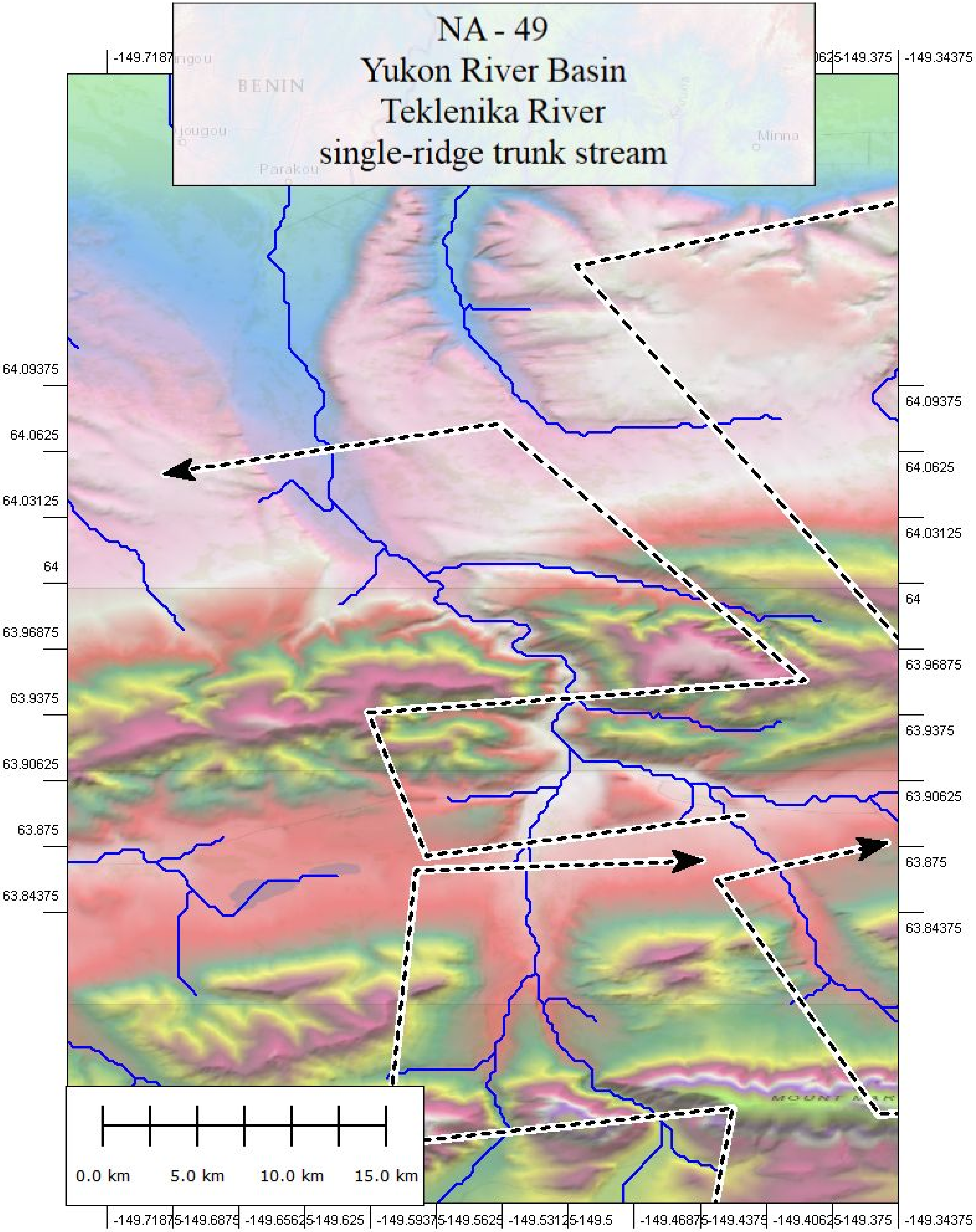
NA - 49
Yukon River Basin
Teklenika River
single-ridge trunk stream
-149.71875
-149.6875
-149.65625
-149.625
-149.59375
-149.5625
-149.53125
-149.5
-149.46875
-149.4375
-149.40625
-149.375
-149.34375
64.09375
64.0625
64.03125
64
63.96875
63.9375
63.90625
63.875
63.84375
0.0 km
5.0 km
10.0 km
15.0 km
BENIN
Djougou
Parakou

NA - 5
Endorheic basin Basin
Truckee River
irregular high ground trunk stream

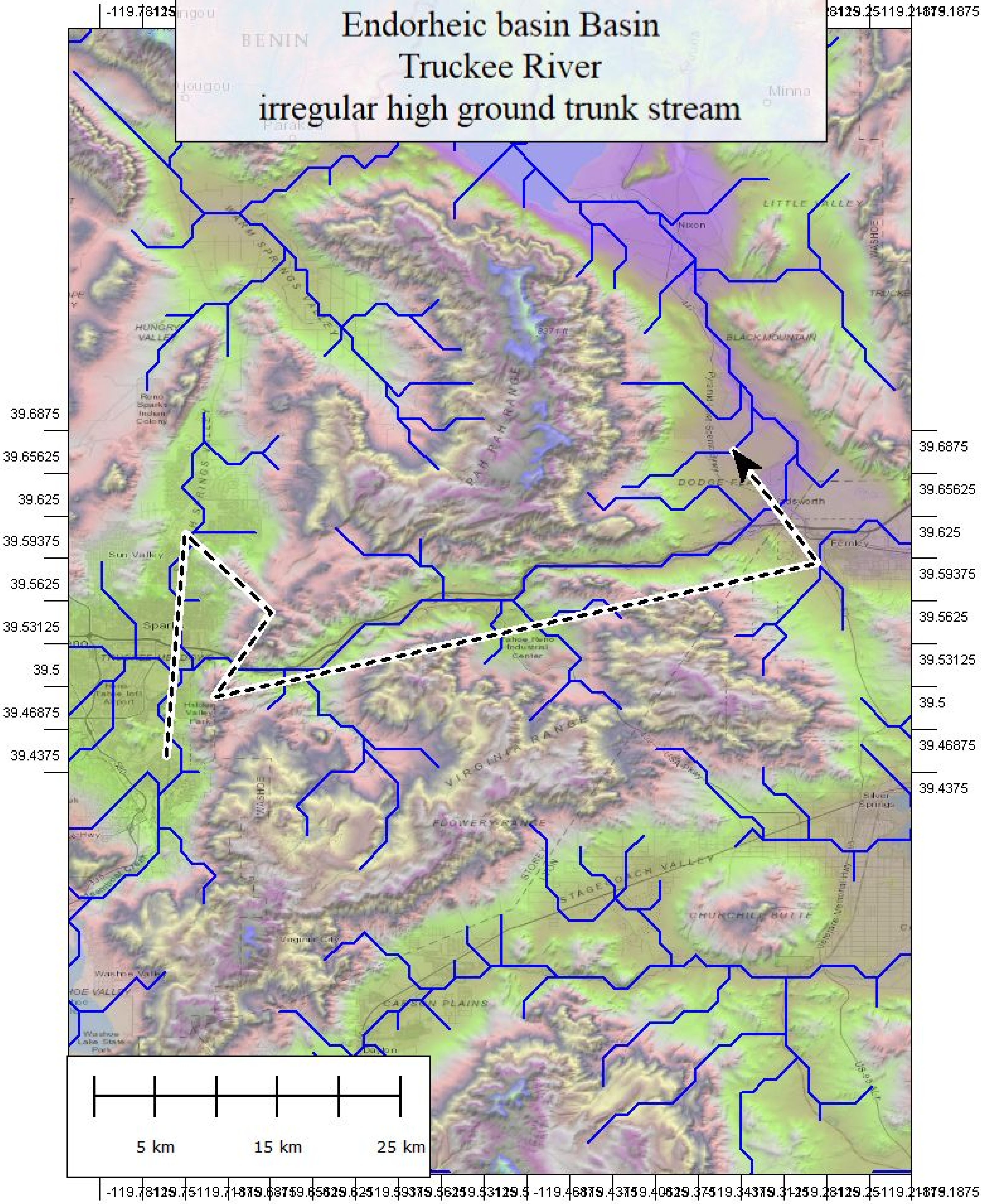

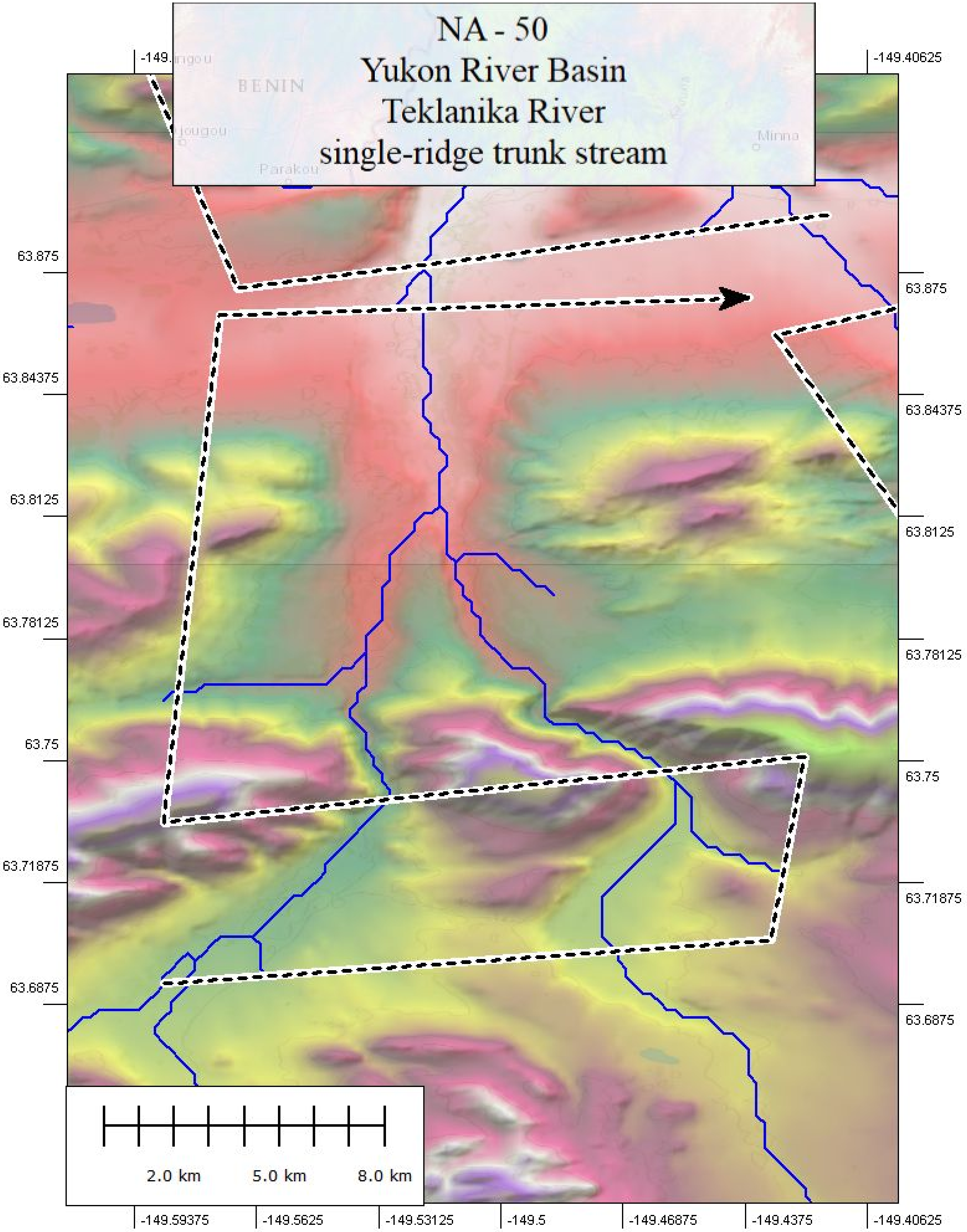
NA - 50
Yukon River Basin
Teklanika River
single-ridge trunk stream
63.875
63.84375
63.8125
63.78125
63.75
63.71875
63.6875
-149.59375
-149.5625
-149.53125
-149.5
-149.46875
-149.4375
-149.40625
2.0 km
5.0 km
8.0 km

NA - 51
Yukon River Basin
Savage River
multi-ridge trunk stream

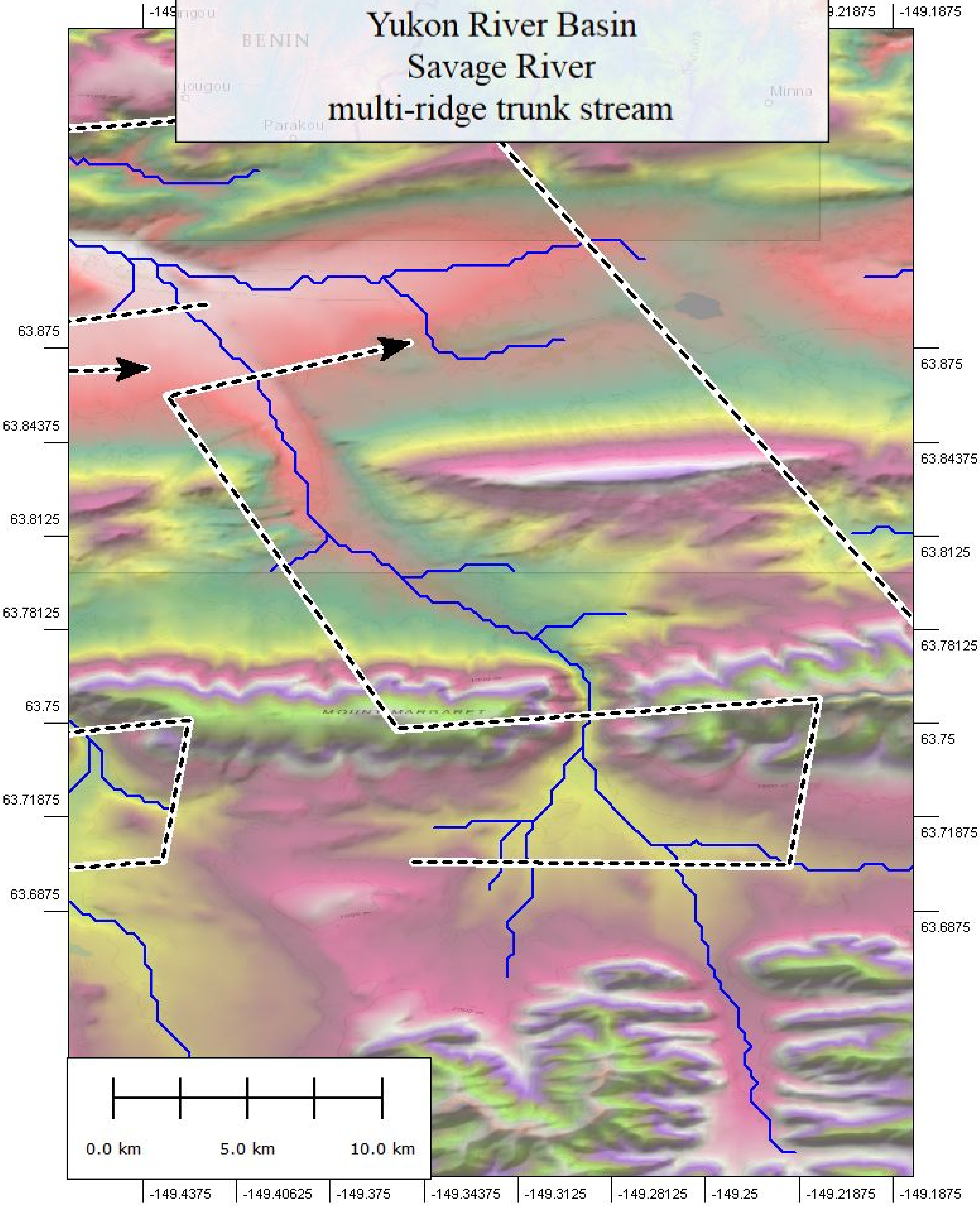

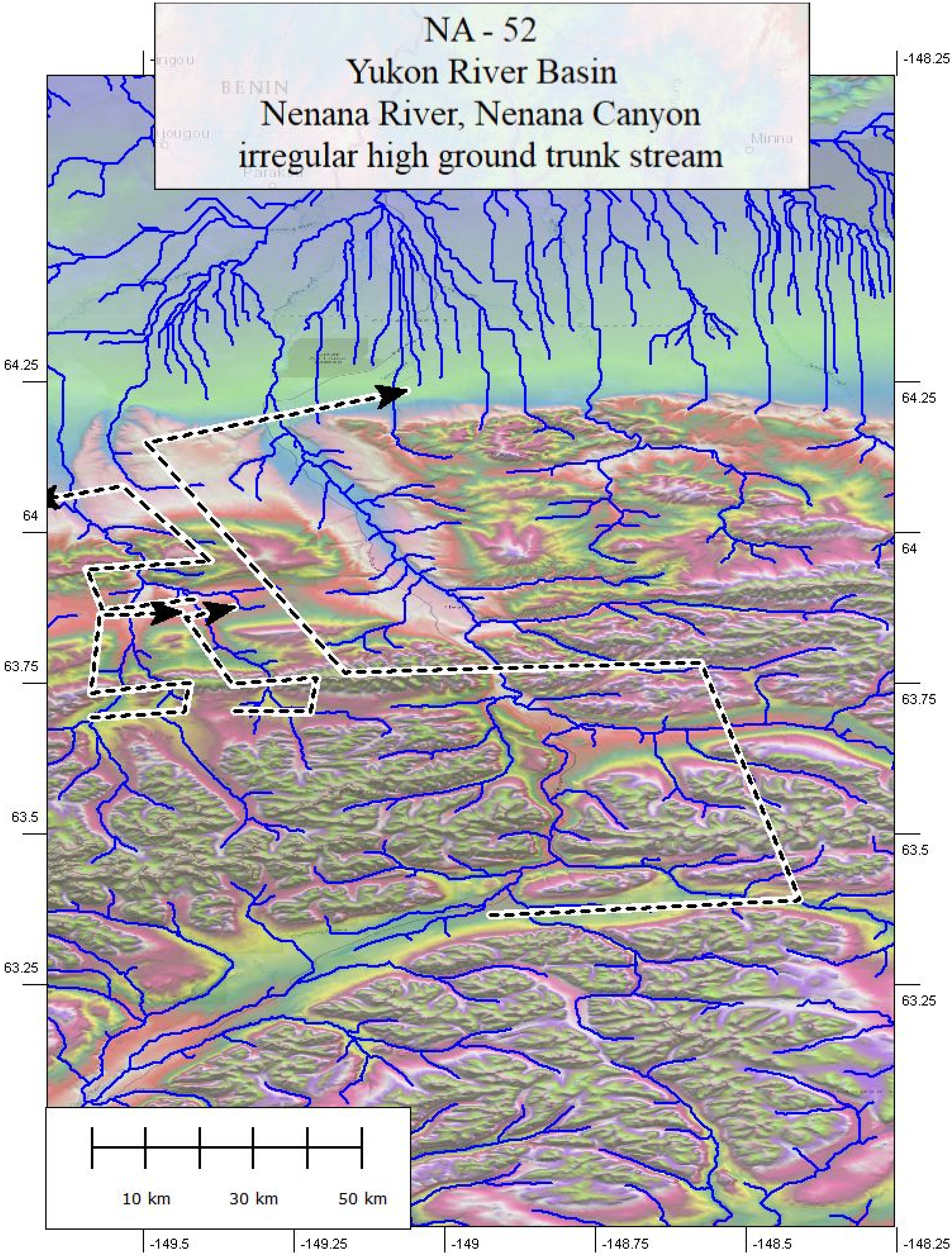
NA - 52
Yukon River Basin
Nenana River, Nenana Canyon
irregular high ground trunk stream
64.25
64
63.75
63.5
63.25
-148.25
-149.5
-149.25
-149
-148.75
-148.5
10 km
30 km
50 km

NA - 53
Mackenzie River (Canada) Basin
Mackenzie River tributary single-ridge head stream

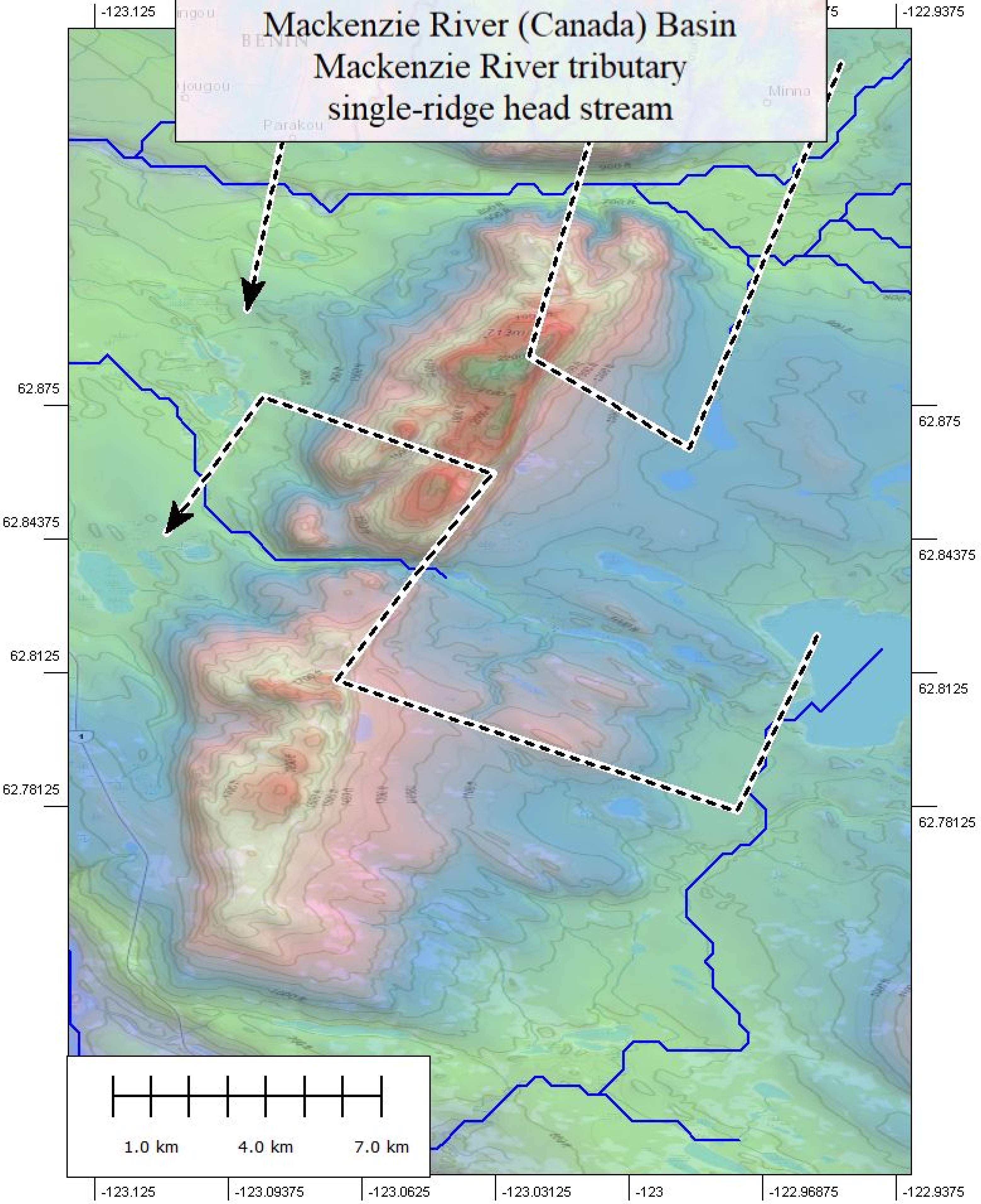

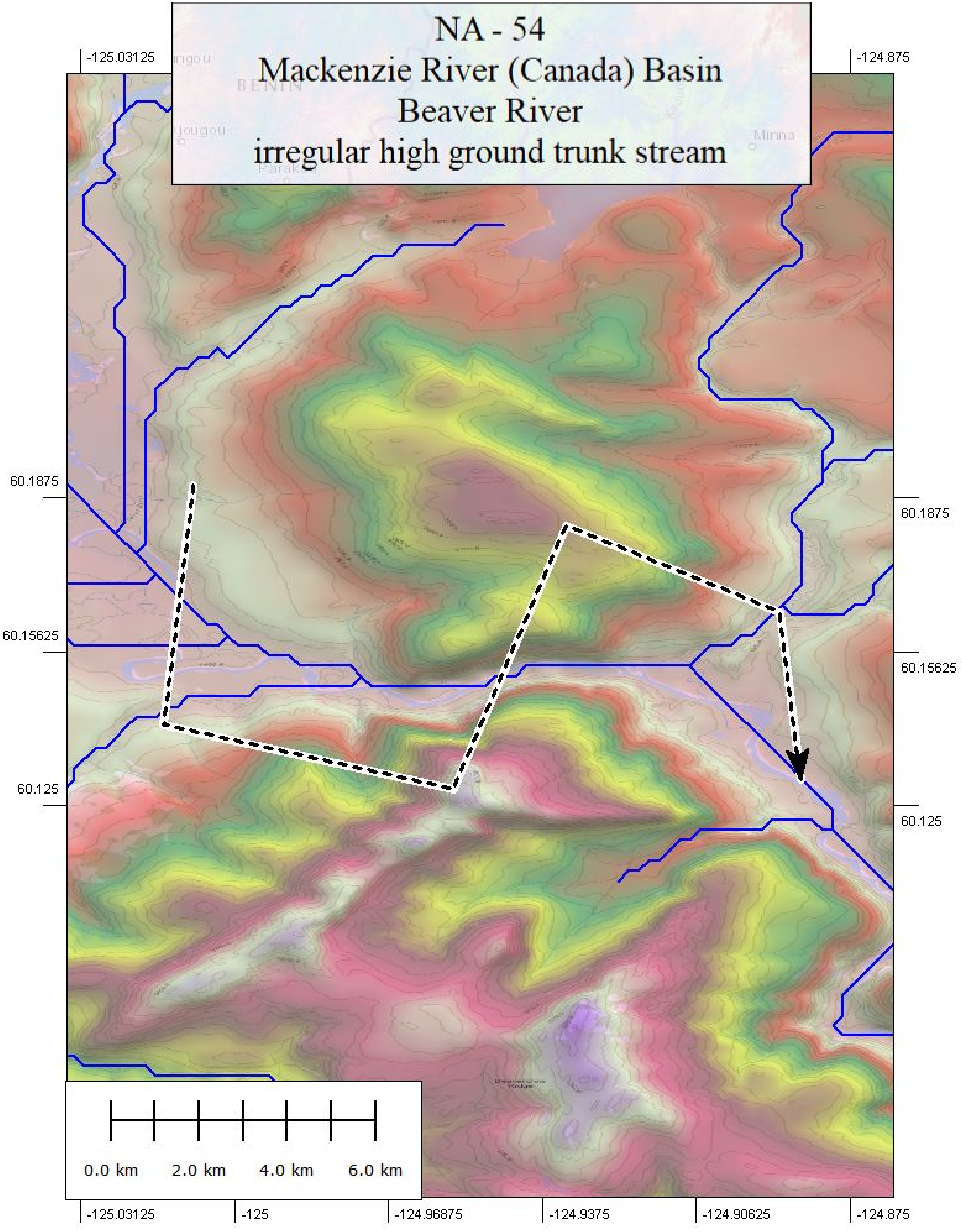

NA - 54
Mackenzie River (Canada) Basin
Beaver River
irregular high ground trunk stream
-125.03125
-124.875
60.1875
60.15625
60.125
-125
-124.96875
-124.9375
-124.90625
0.0 km
2.0 km
4.0 km
6.0 km

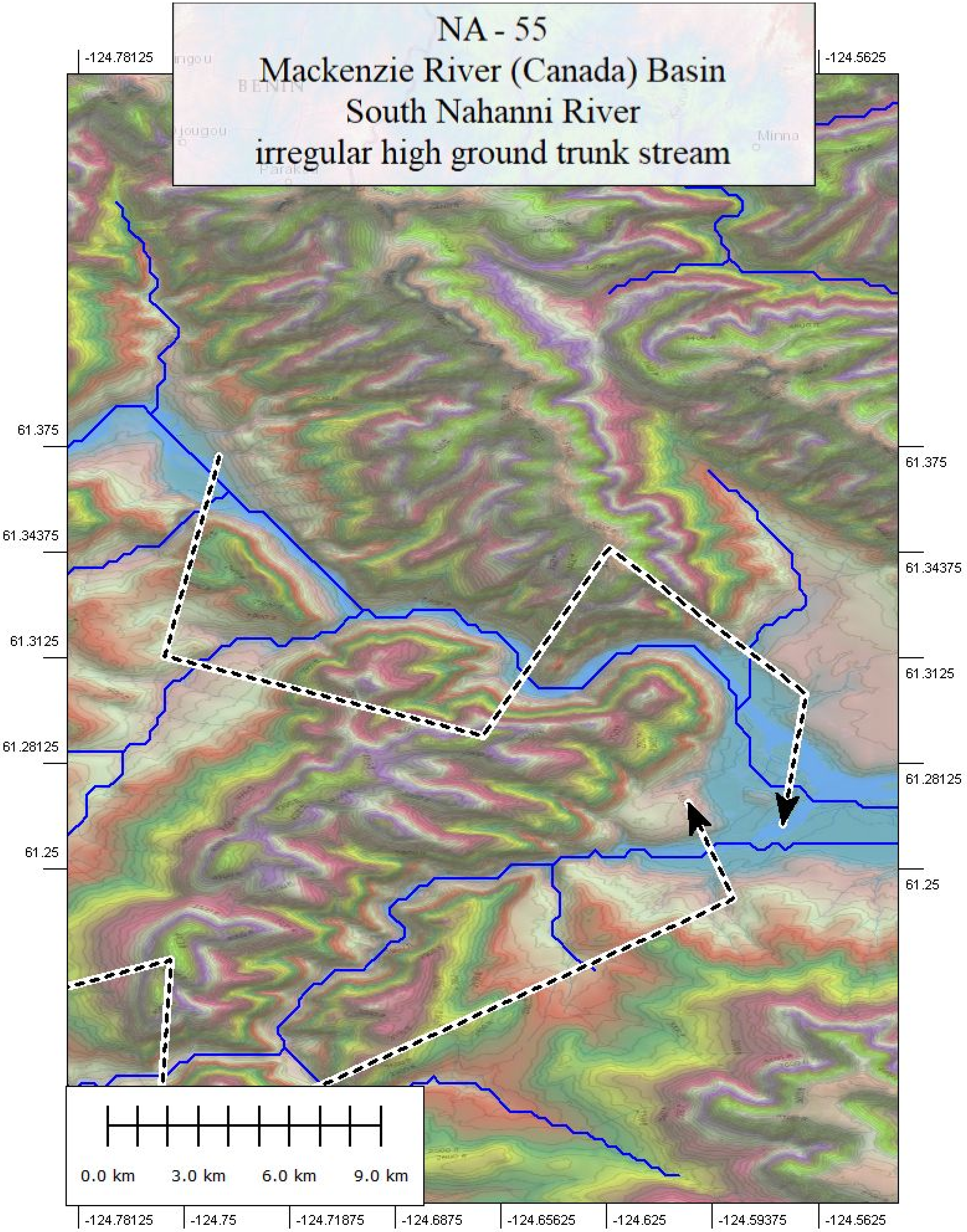
NA - 55
Mackenzie River (Canada) Basin
South Nahanni River
irregular high ground trunk stream
-124.78125
-124.5625
61.375
61.34375
61.3125
61.28125
61.25
0.0 km
3.0 km
6.0 km
9.0 km
-124.75
-124.71875
-124.6875
-124.65625
-124.625
-124.59375

NA - 56
Mackenzie River (Canada) Basin
Redstone River
single-ridge trunk stream

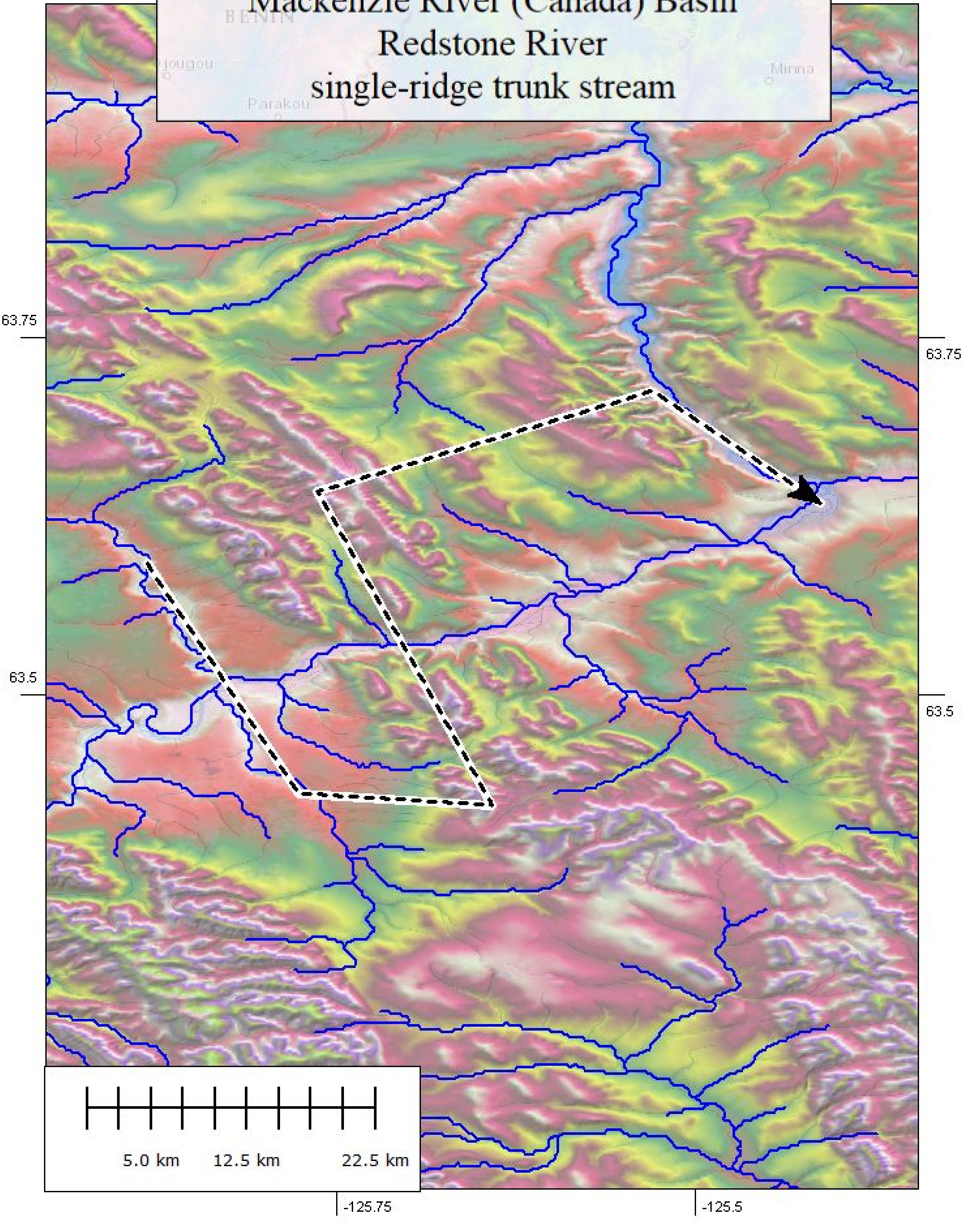

NA - 57
Susquehanna River Basin
Elk Creek, Milheim Narrows
single-ridge head stream

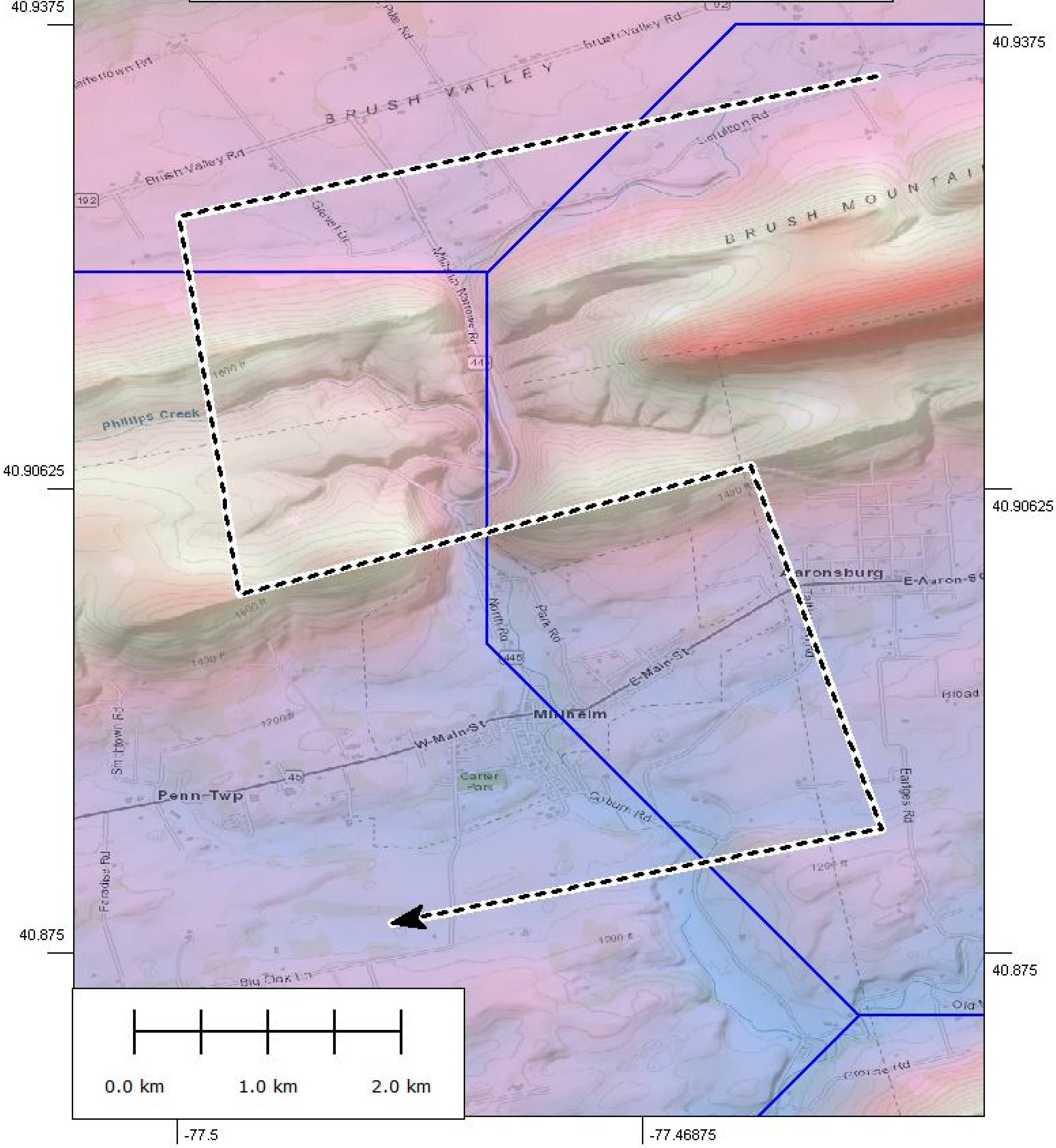

NA - 58
Colorado River (Arizona) Basin
Salt Creek
downdip plateau head stream

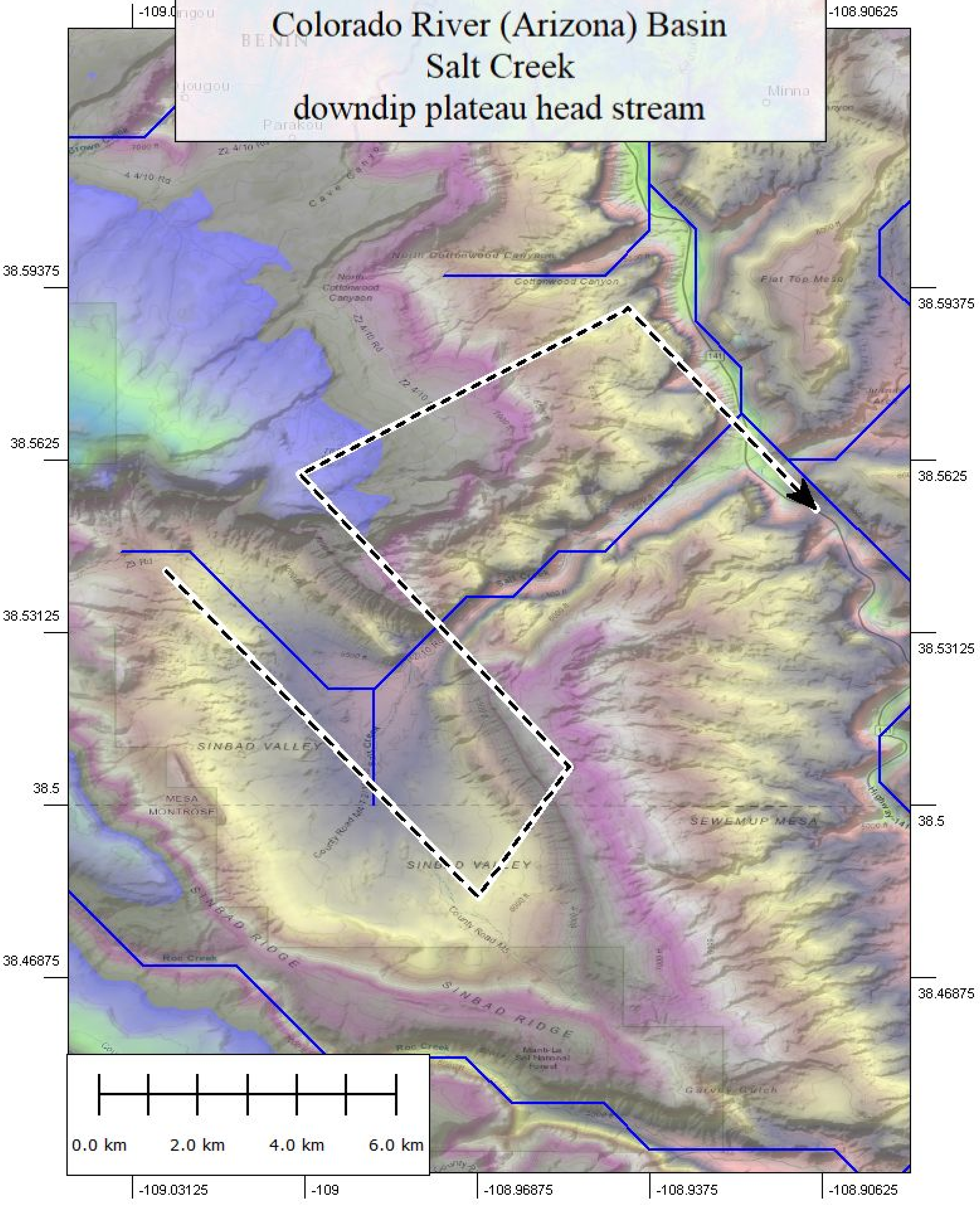

NA - 59
James River Basin
Mill Creek, Panther Gap
single-ridge head stream

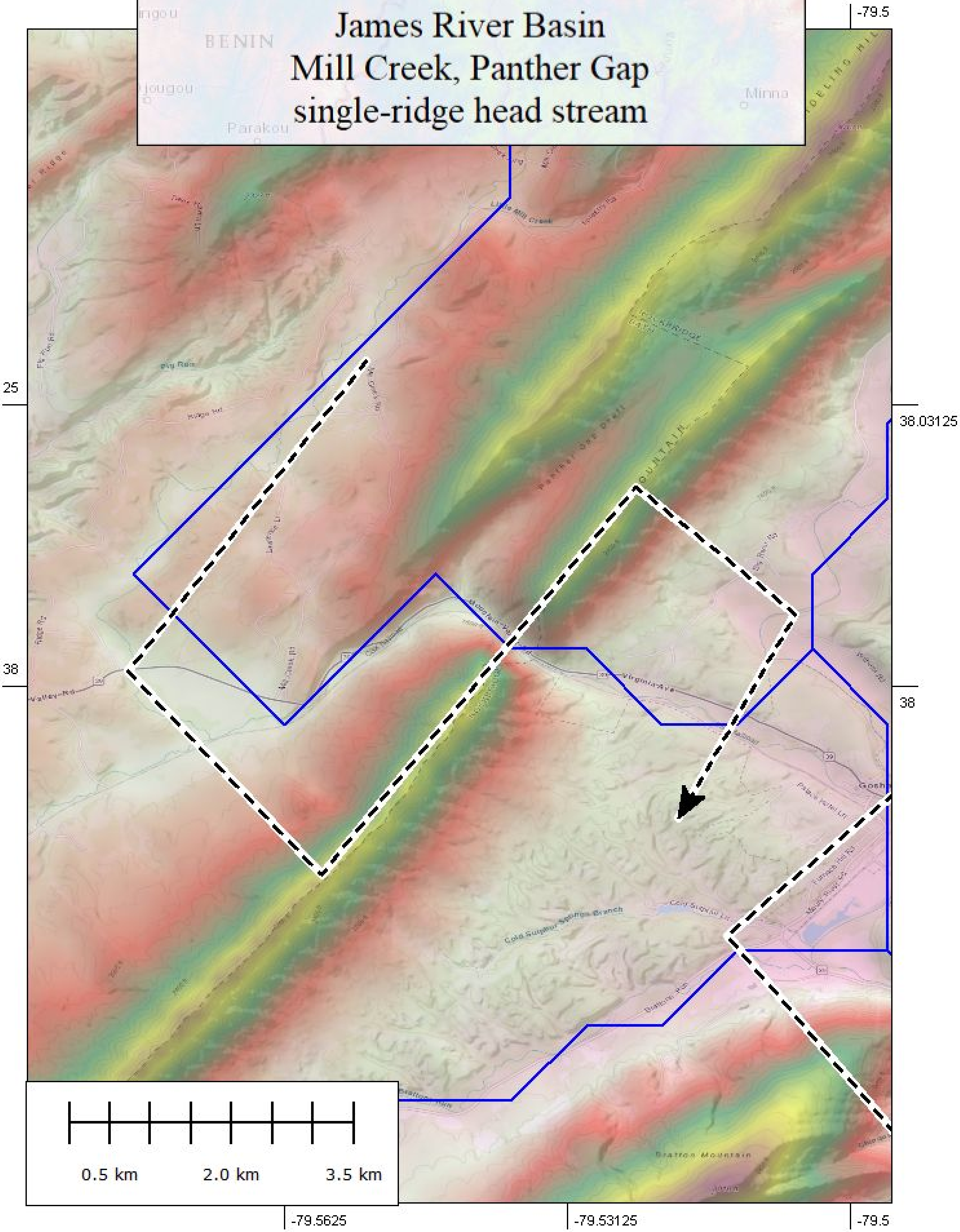

NA - 6
Rio Grande (Texas-Mexico) Basin
Rio Conchos
single-ridge trunk stream

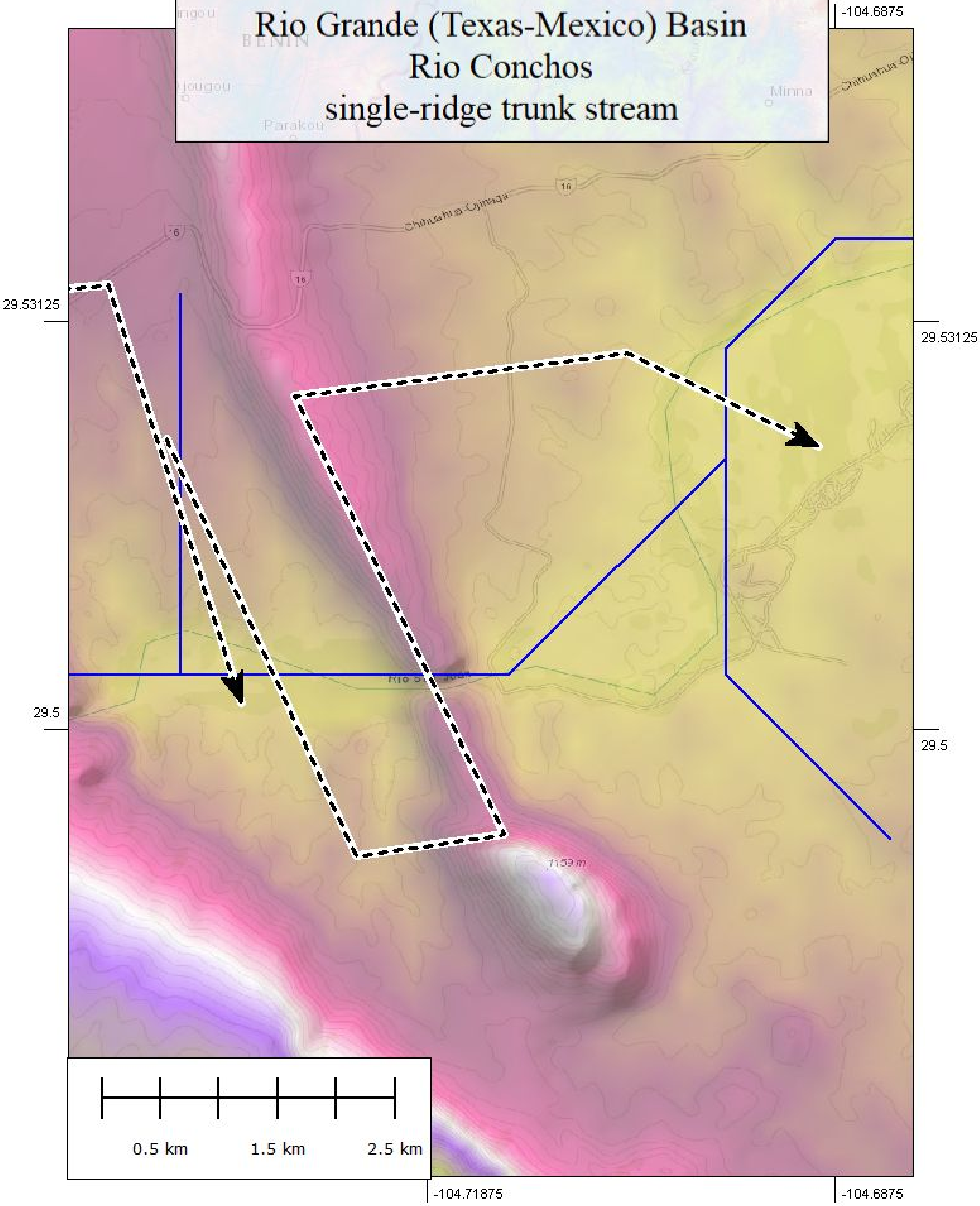

NA - 60
Rio Grande (Texas-Mexico) Basin
Padilla Pass
single-ridge head stream

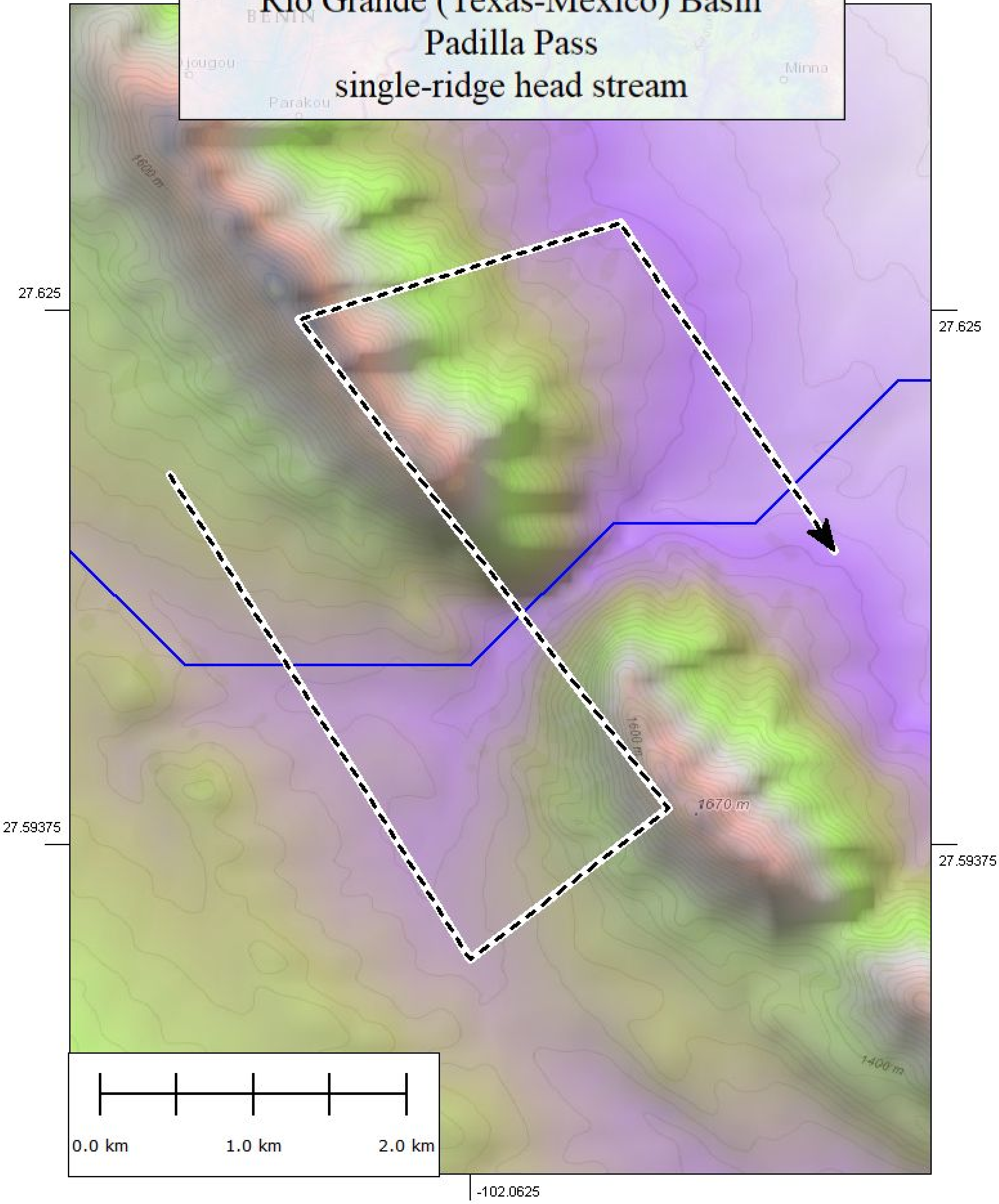

NA - 61
Rio Grande (Texas-Mexico) Basin
La Escondida Pass
single-ridge head stream

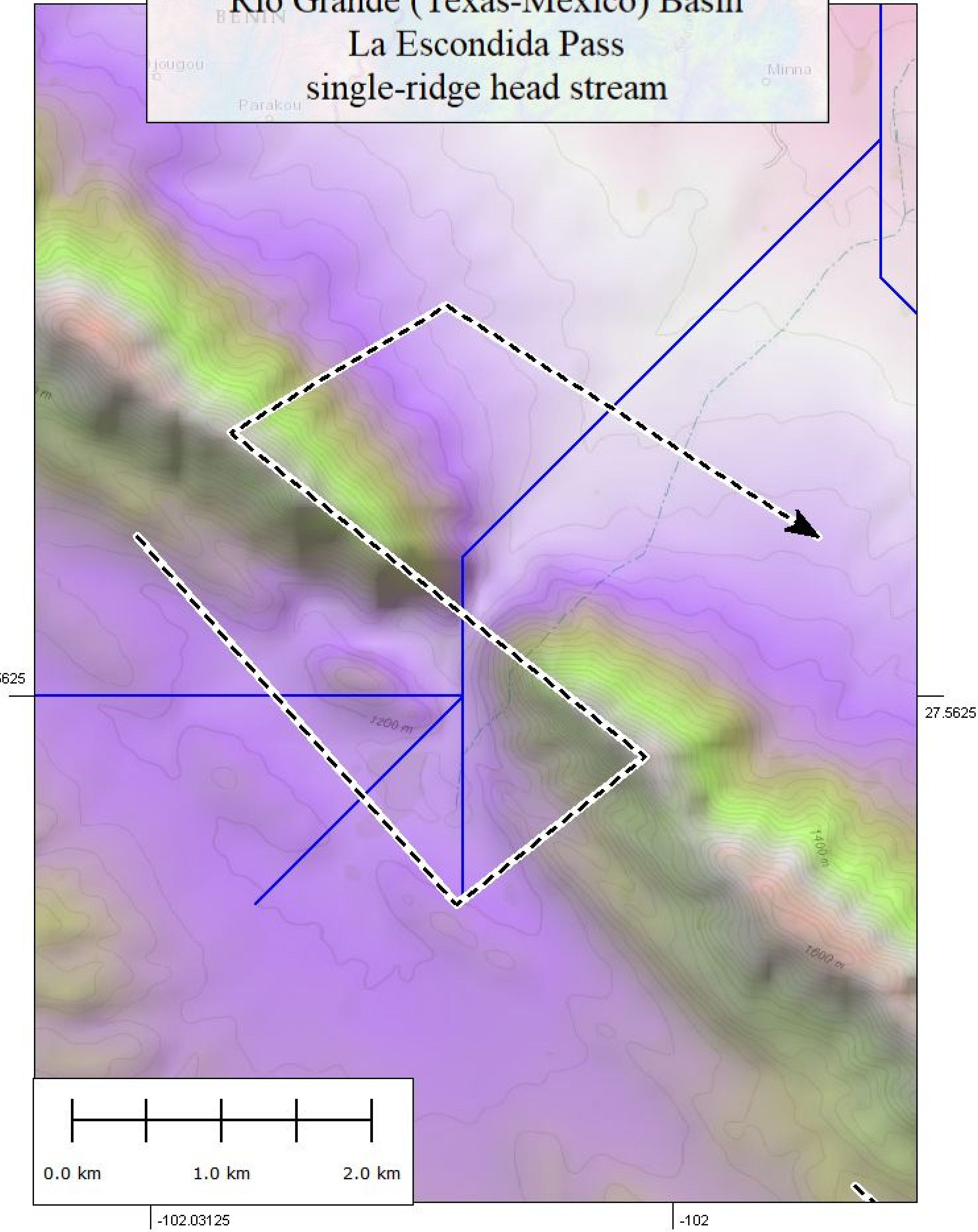

NA - 62
Colorado River (Arizona) Basin
Fourteenmile Creek
irregular high ground head stream

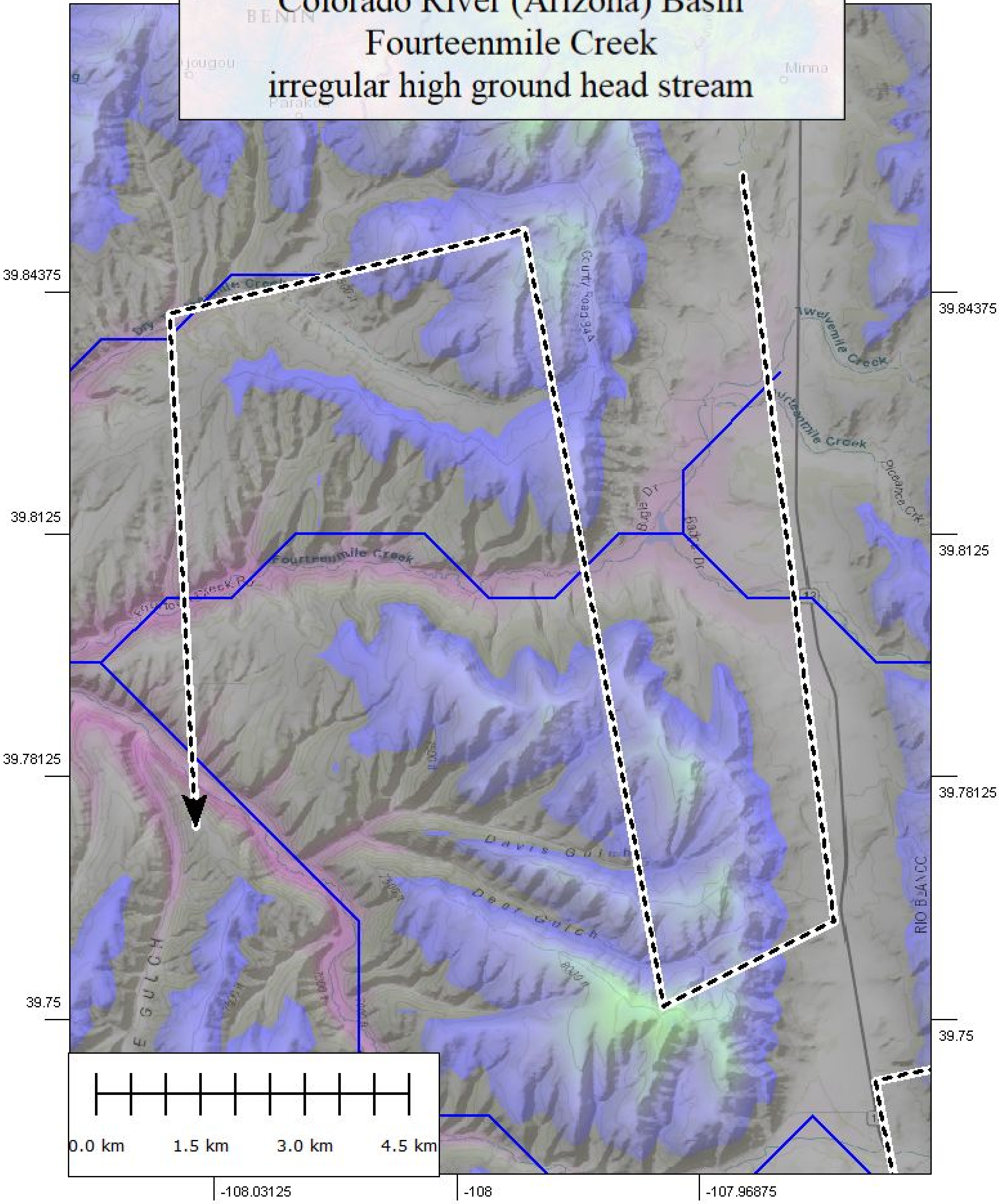

NA - 63
Rio Grande (Texas-Mexico) Basin
El Pino Pass
single-ridge head stream

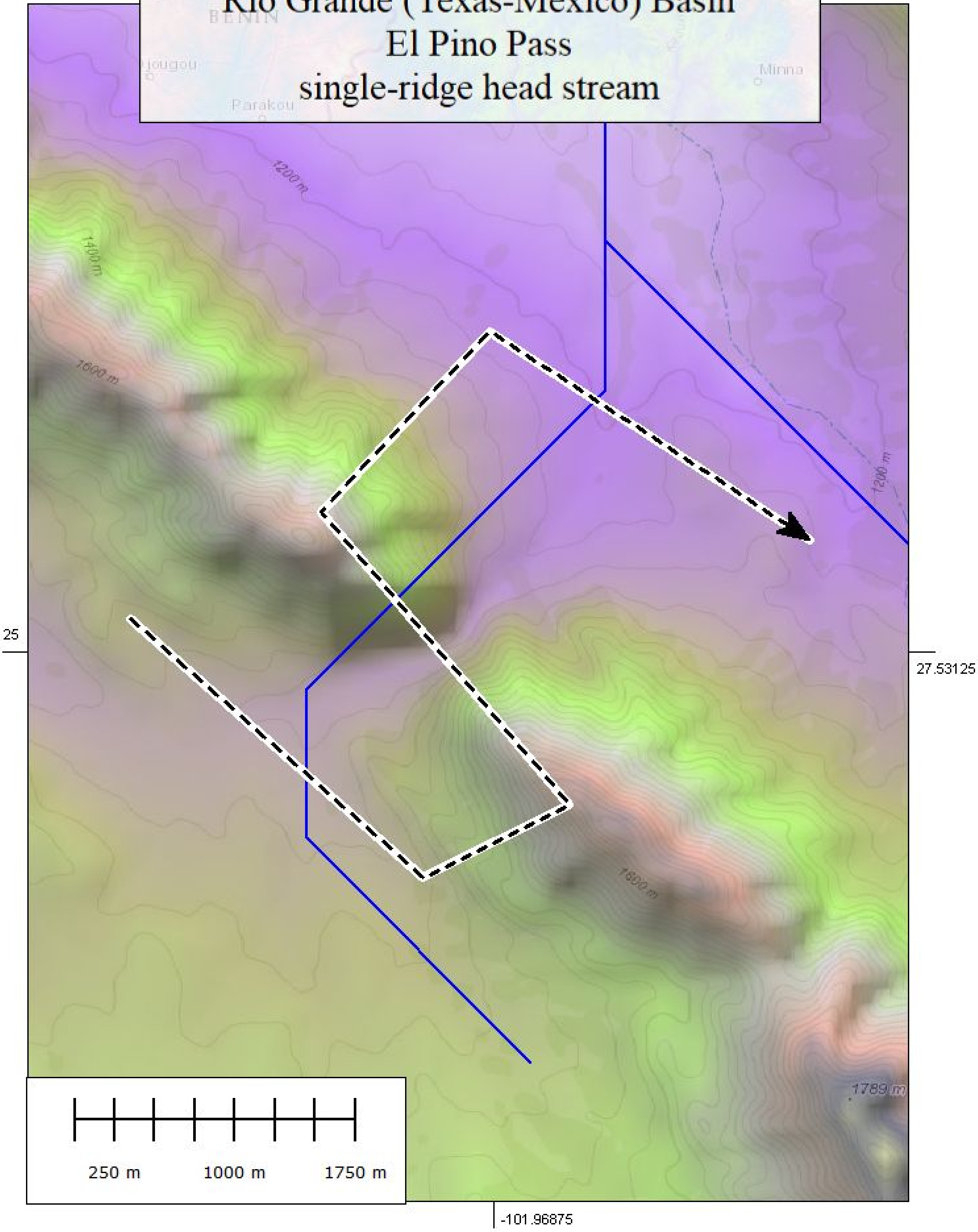

NA - 64
Rio Grande (Texas-Mexico) Basin
single-ridge head stream

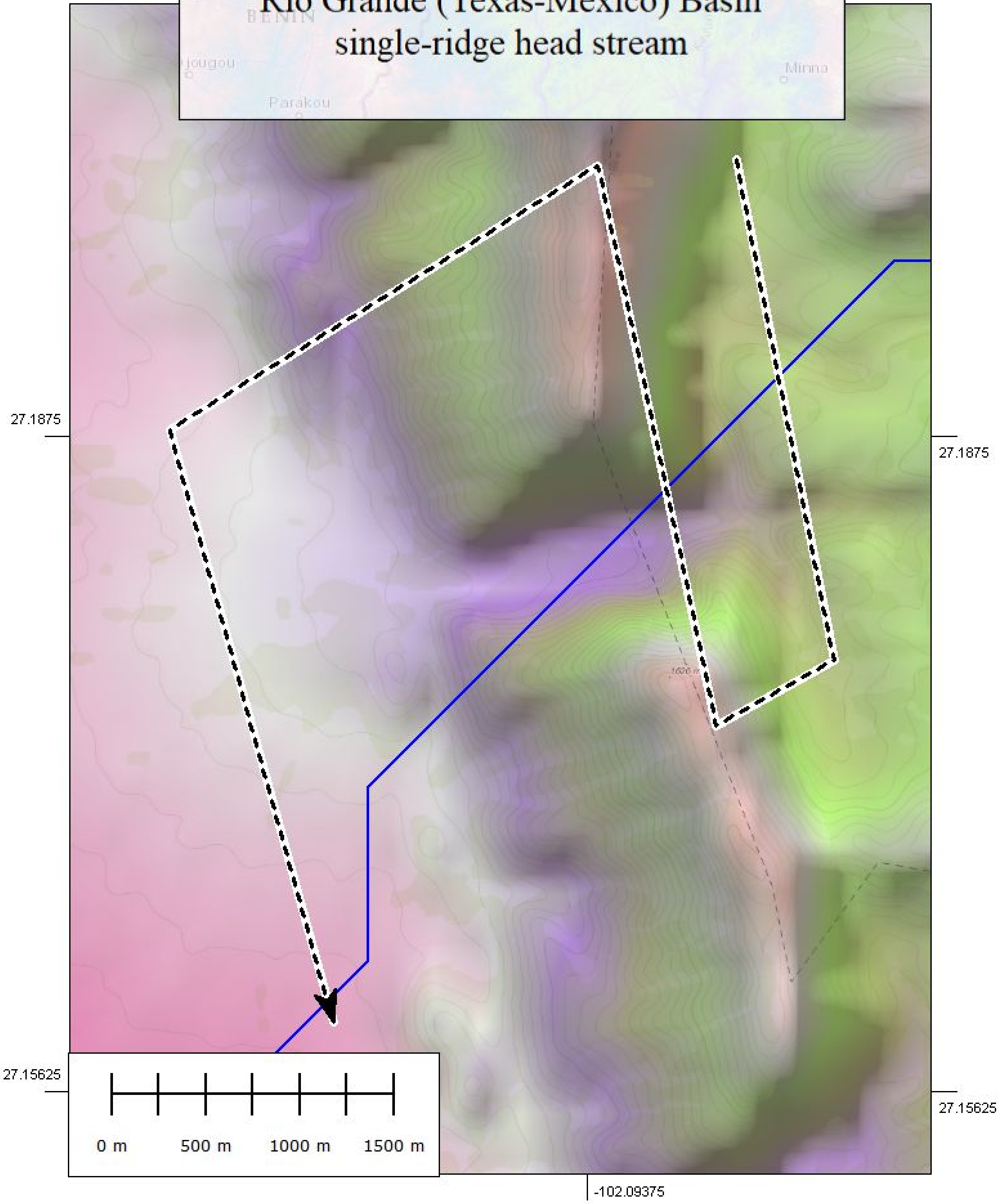

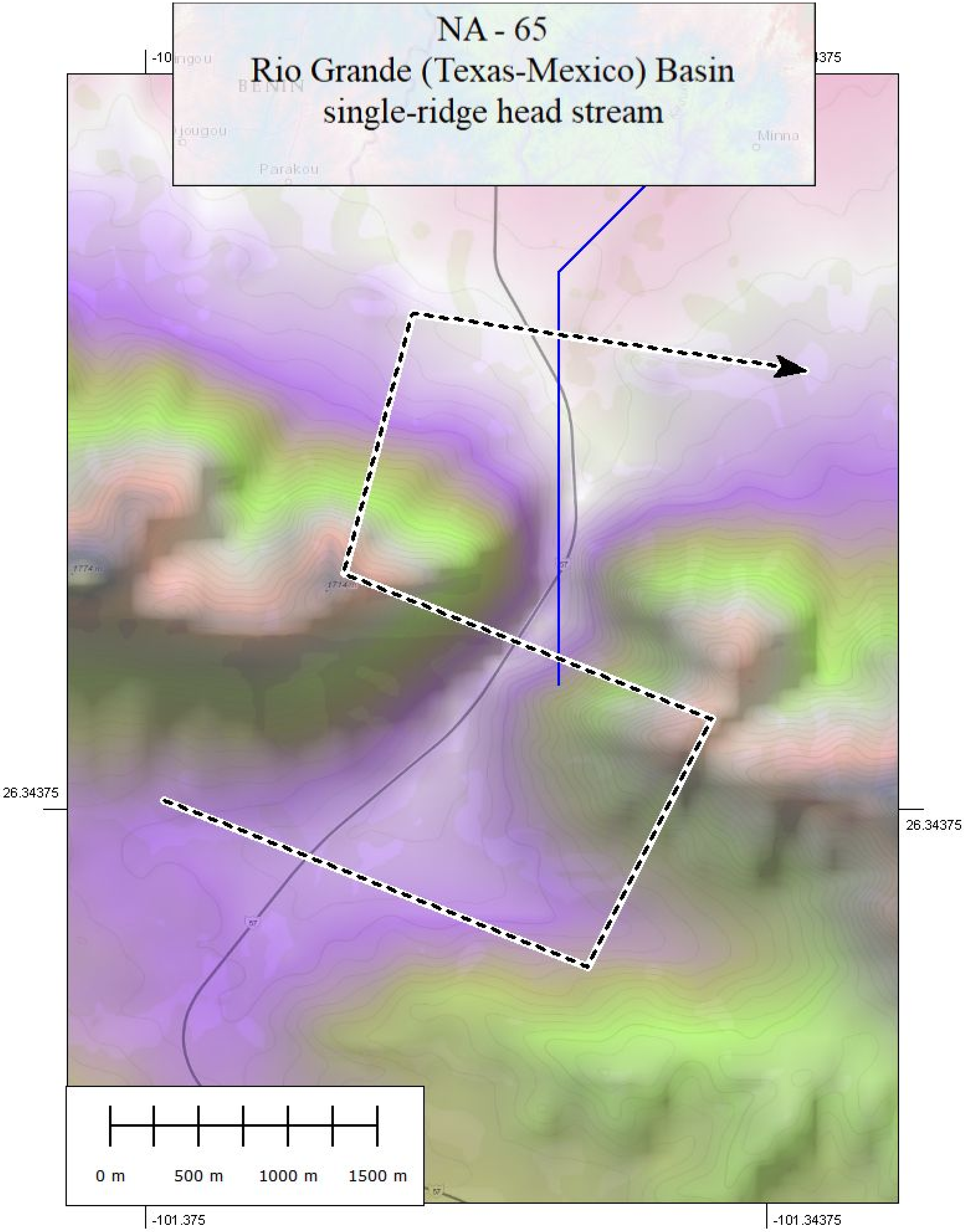
NA - 65
Rio Grande (Texas-Mexico) Basin
single-ridge head stream
BENIN
Parakou
Minna
26.34375
26.34375
0 m
500 m
1000 m
1500 m
-101.375
-101.34375

NA - 66
Rio Grande (Texas-Mexico) Basin
single-ridge head stream

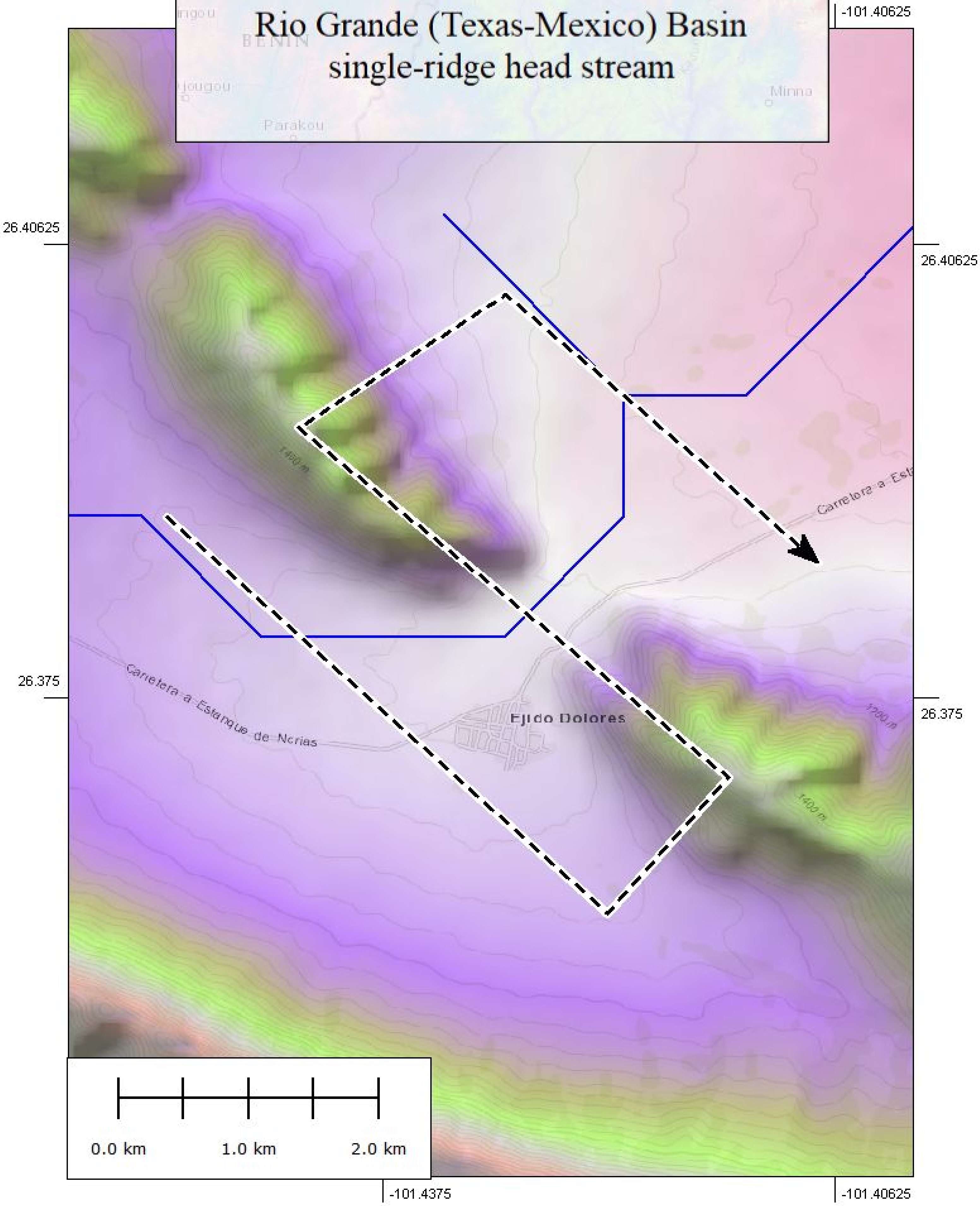

NA - 67
Pnuco River Basin
single-ridge head stream

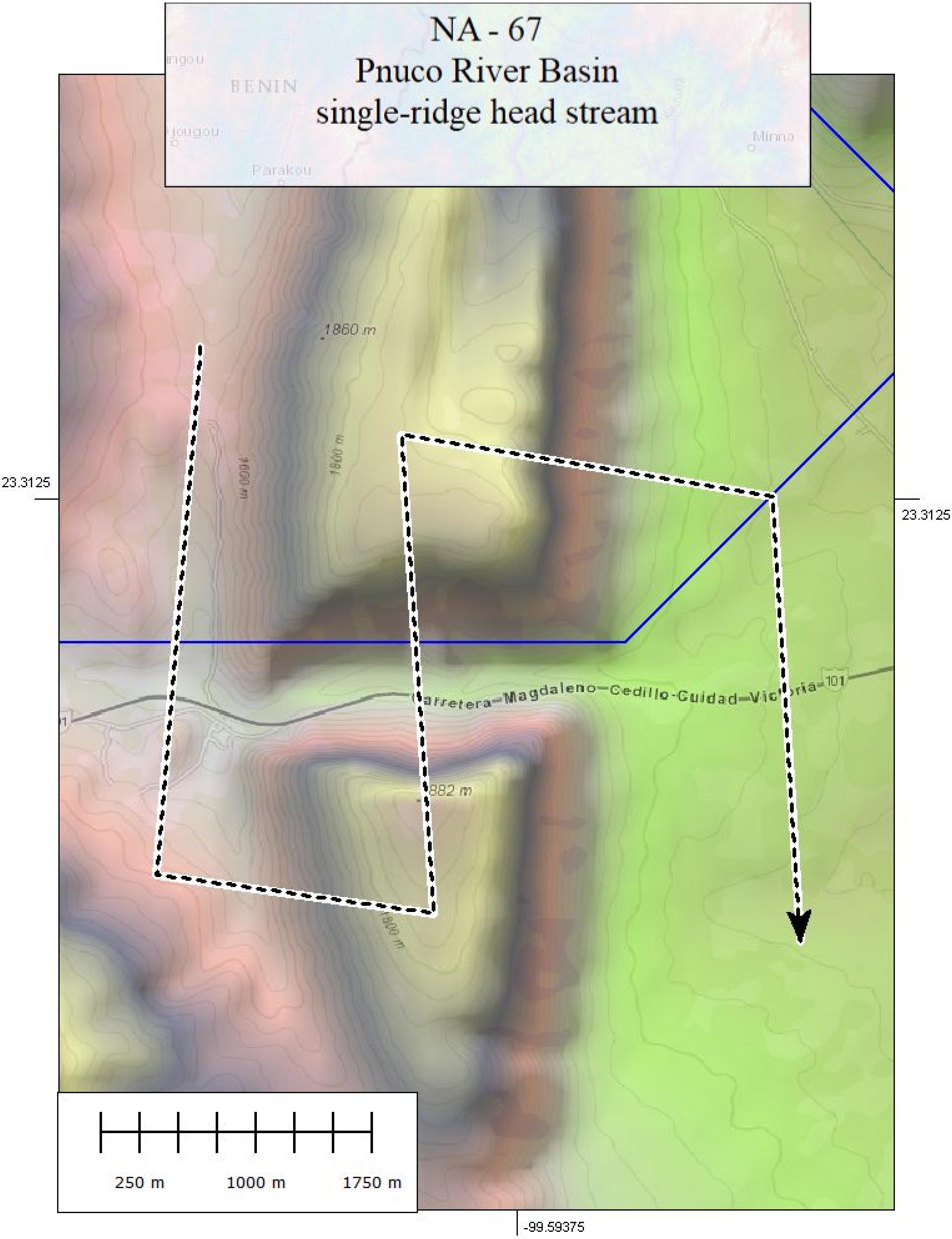

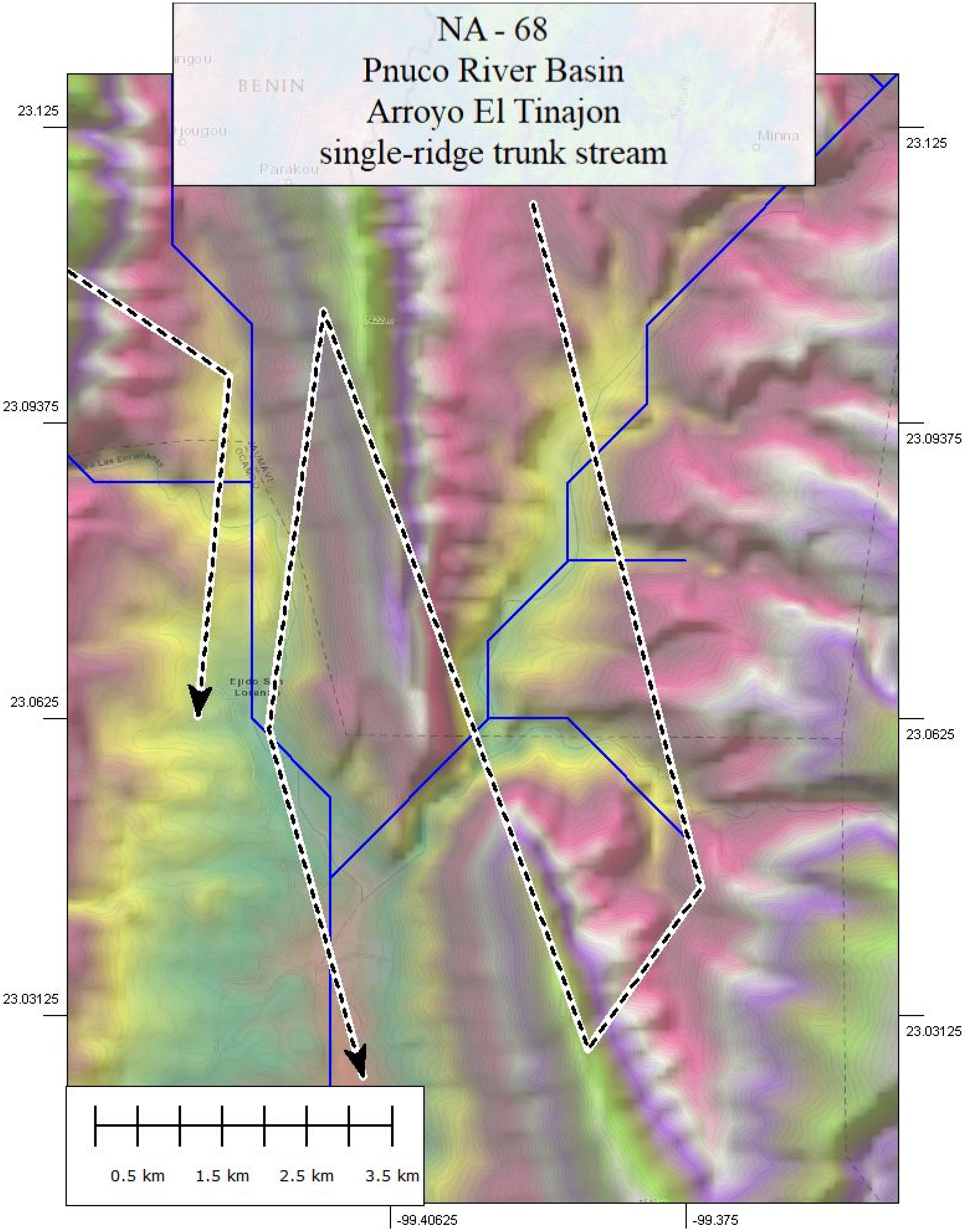
NA - 68
Pnuco River Basin
Arroyo El Tinajon
single-ridge trunk stream
23.125
23.125
23.09375
23.09375
23.0625
23.0625
23.03125
23.03125
-99.40625
-99.375
0.5 km
1.5 km
2.5 km
3.5 km

NA - 69
Pnuco River Basin
Arroyo Las Enramadas
single-ridge head stream

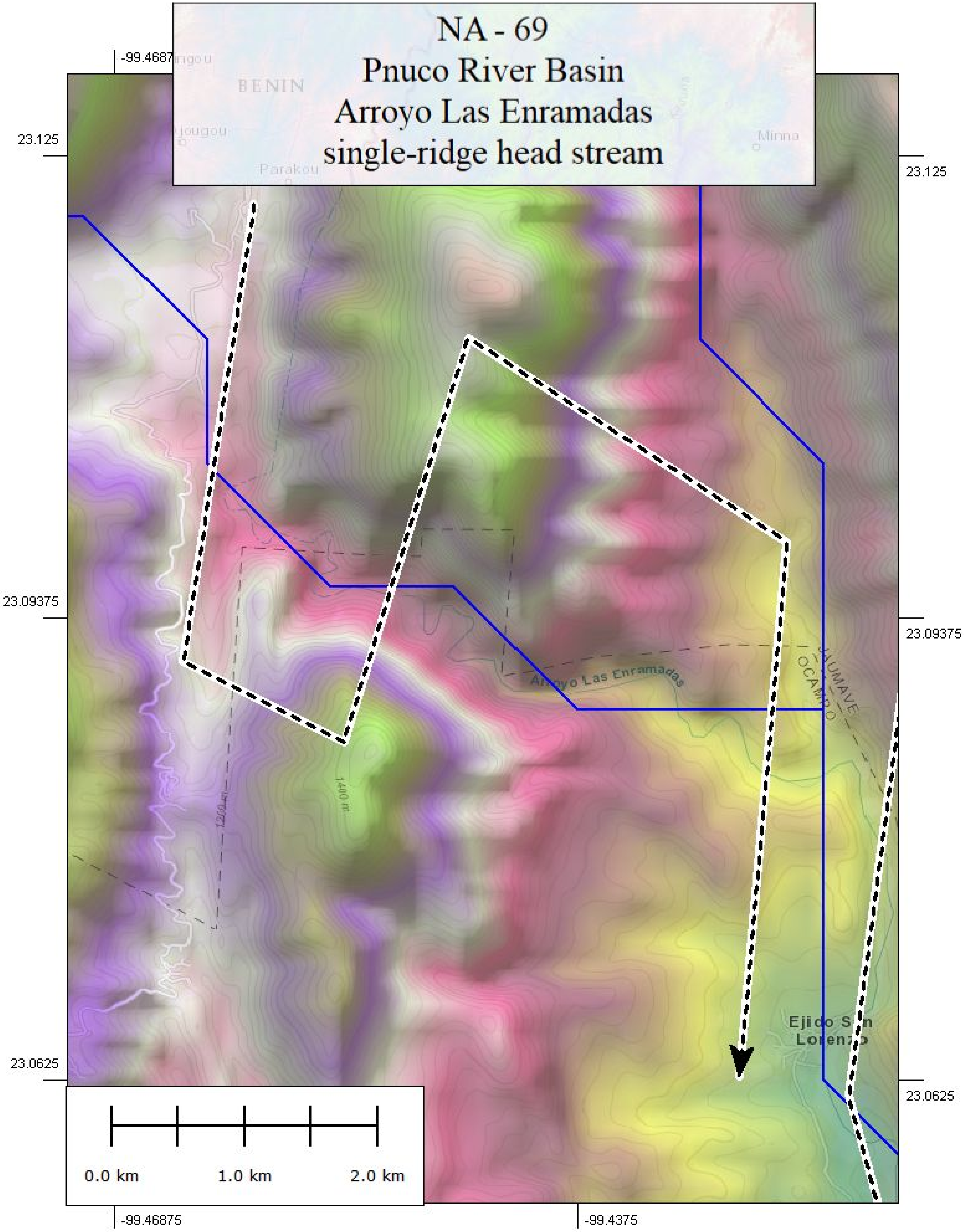

NA - 7
Columbia River Basin
Blackfoot River
irregular high ground trunk stream

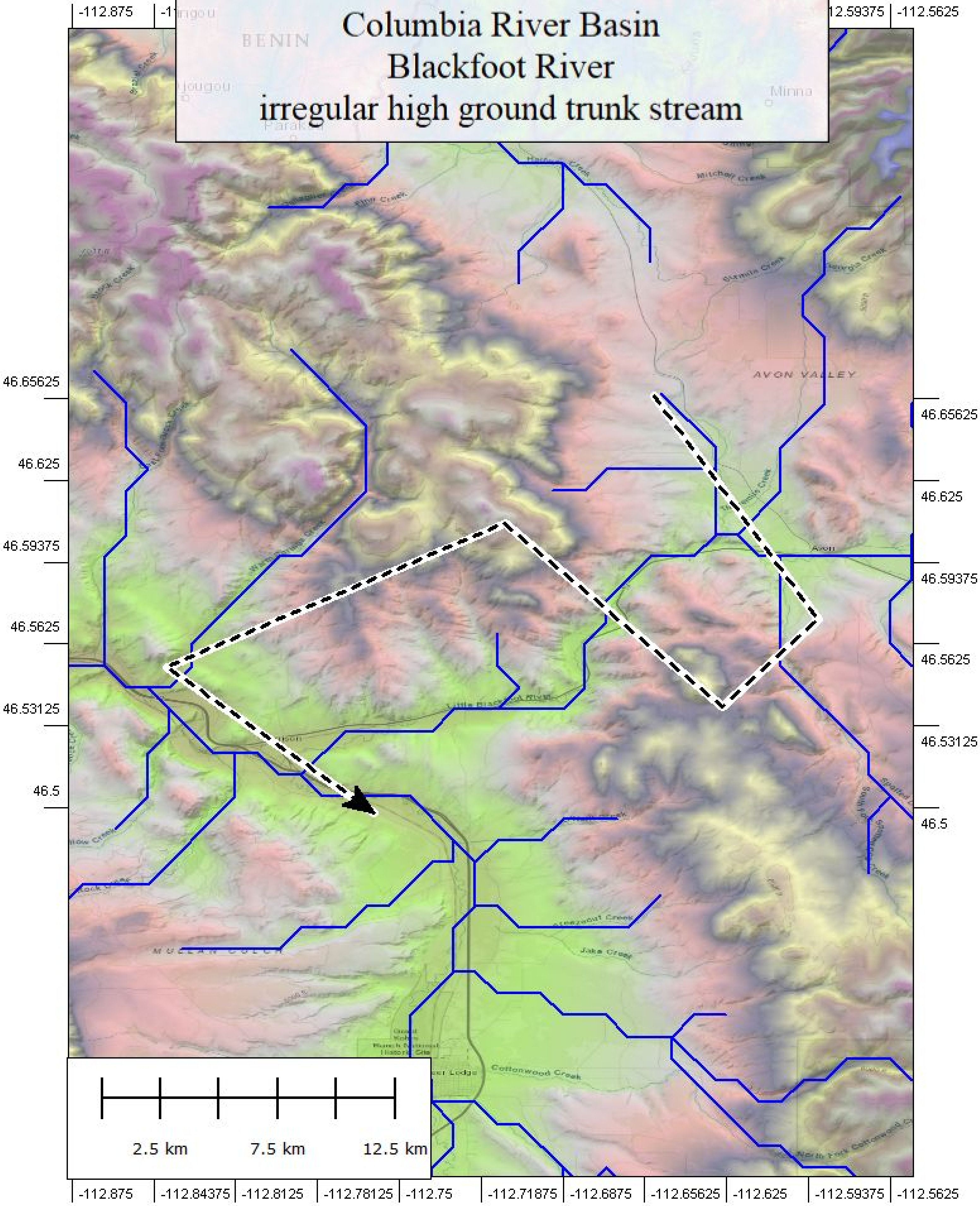

NA - 70
Rio Grande (Texas-Mexico) Basin
La Tortola Canyon
single-ridge head stream

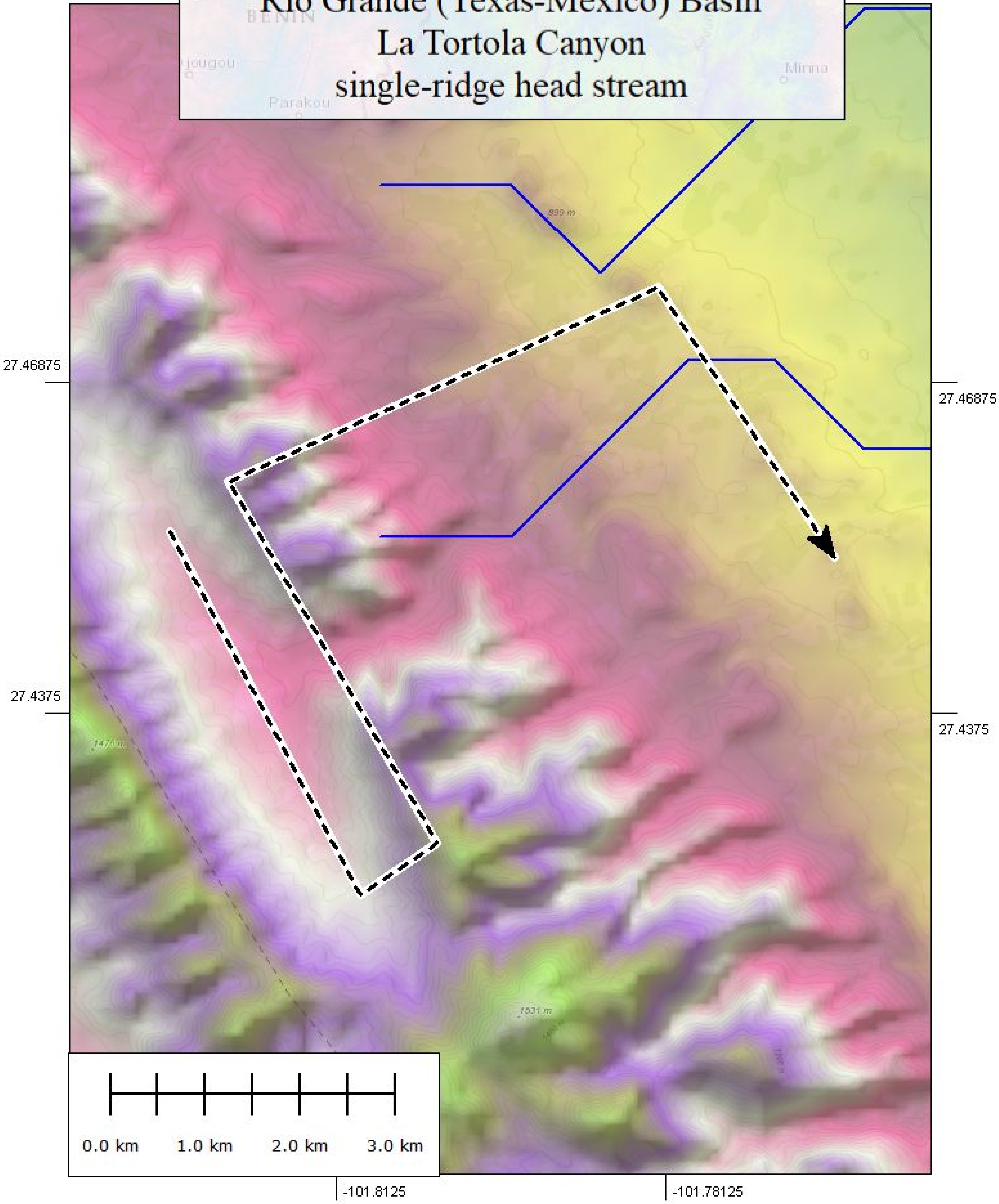

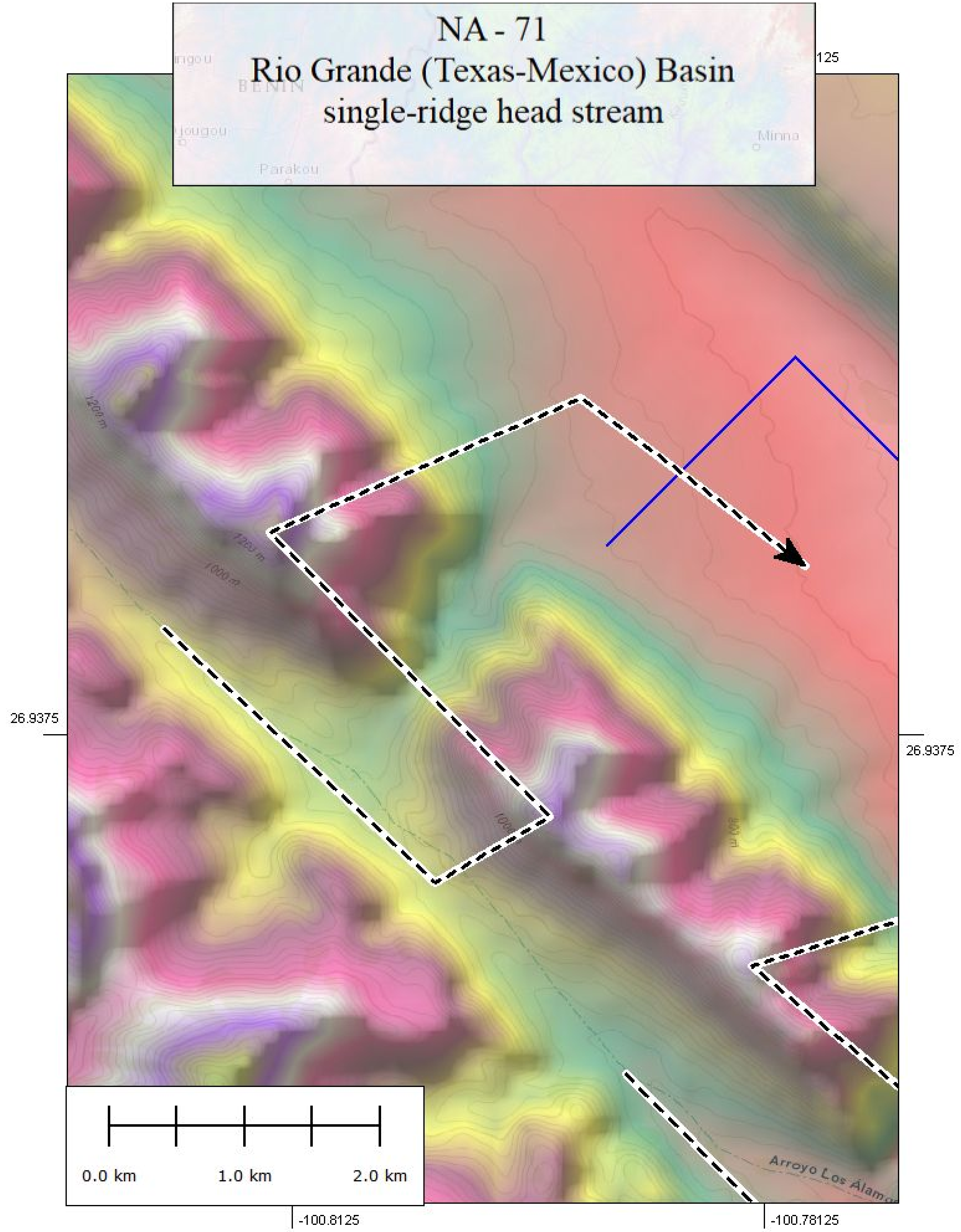
NA - 71
Rio Grande (Texas-Mexico) Basin
single-ridge head stream
Parakou
Minna
26.9375
26.9375
-100.8125
-100.78125
0.0 km
1.0 km
2.0 km
Arroyo Los Álamos
1200 m
1000 m

NA - 72
Rio Grande (Texas-Mexico) Basin
single-ridge head stream

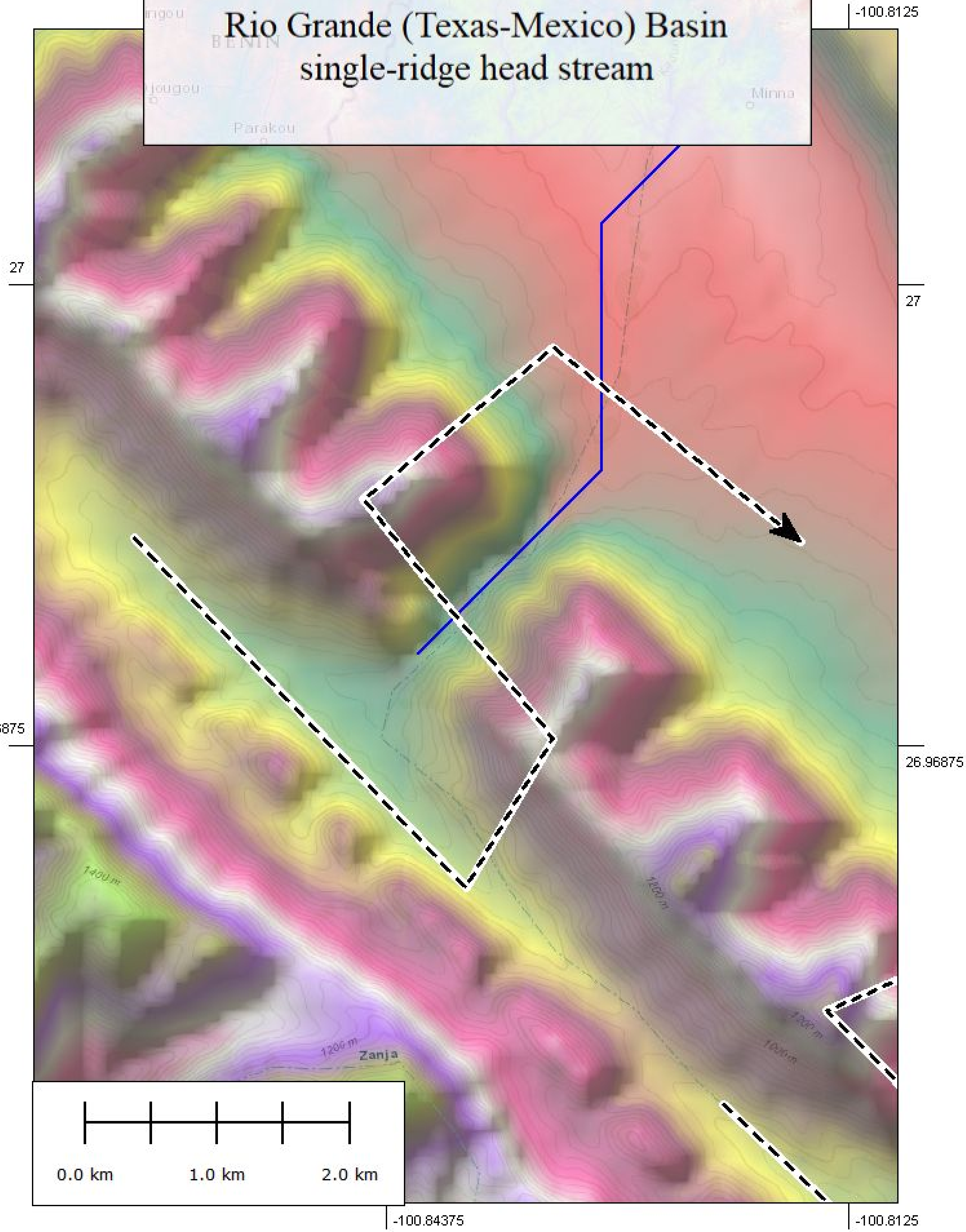

NA - 73
Rio Grande (Texas-Mexico) Basin
Arroyo Los Alamos
single-ridge head stream

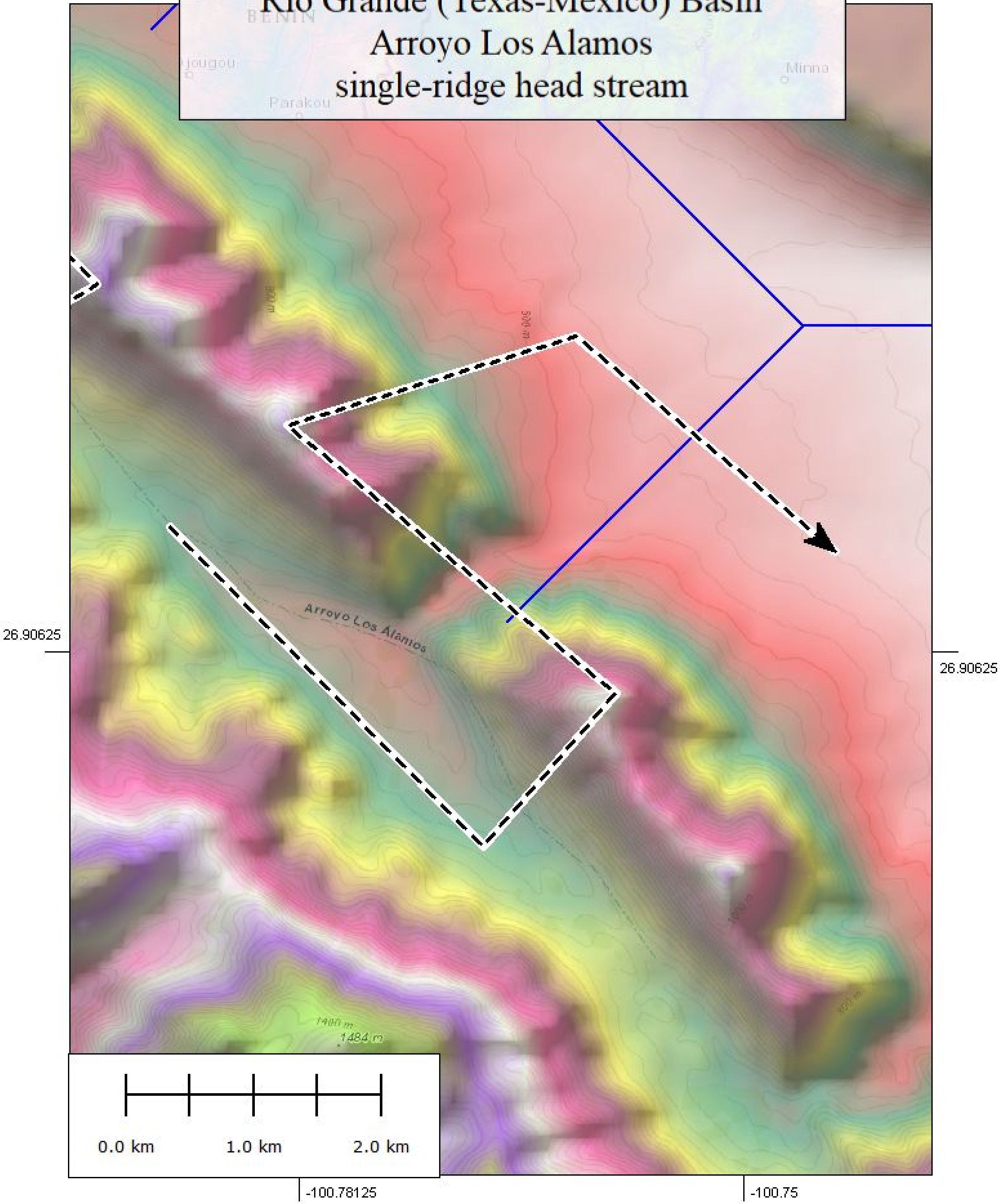

NA - 74
Rio Grande (Texas-Mexico) Basin
Santa Caterina River tributary
single-ridge head stream

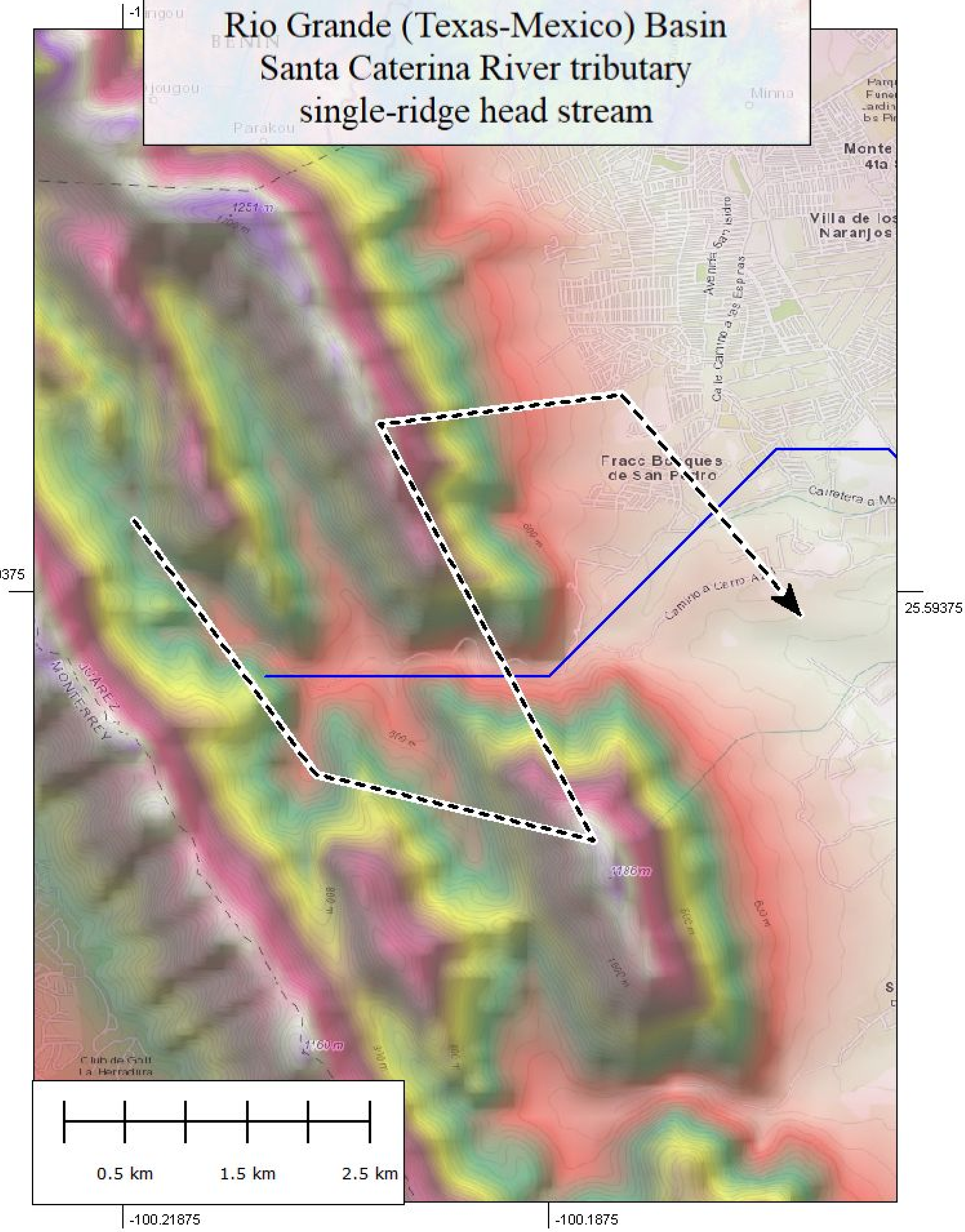

NA - 75
James River Basin
Bolar Run, Bolar Gap
single-ridge head stream

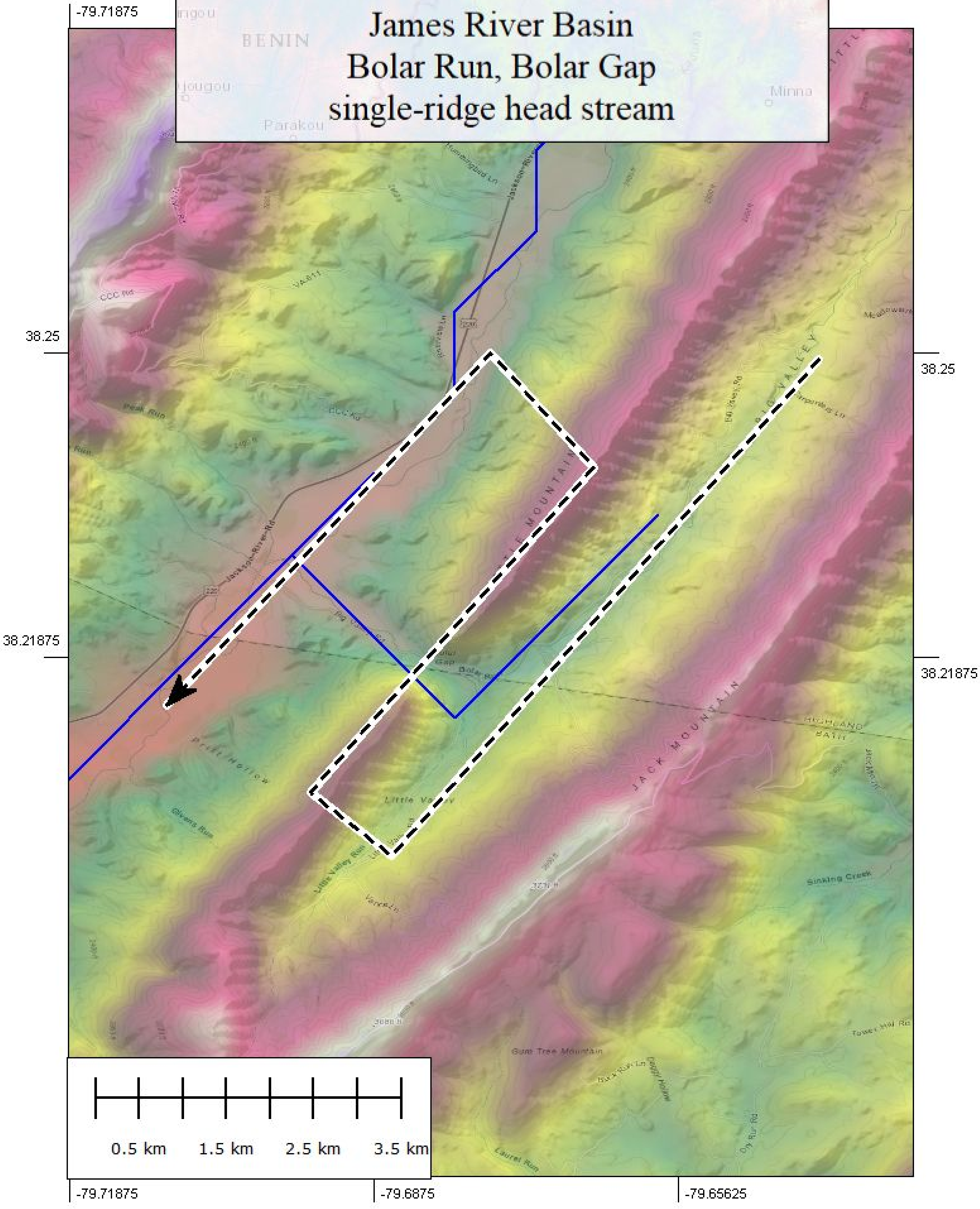

NA - 76
Rio Grande (Texas-Mexico) Basin
Canon de la Virgen
single-ridge head stream

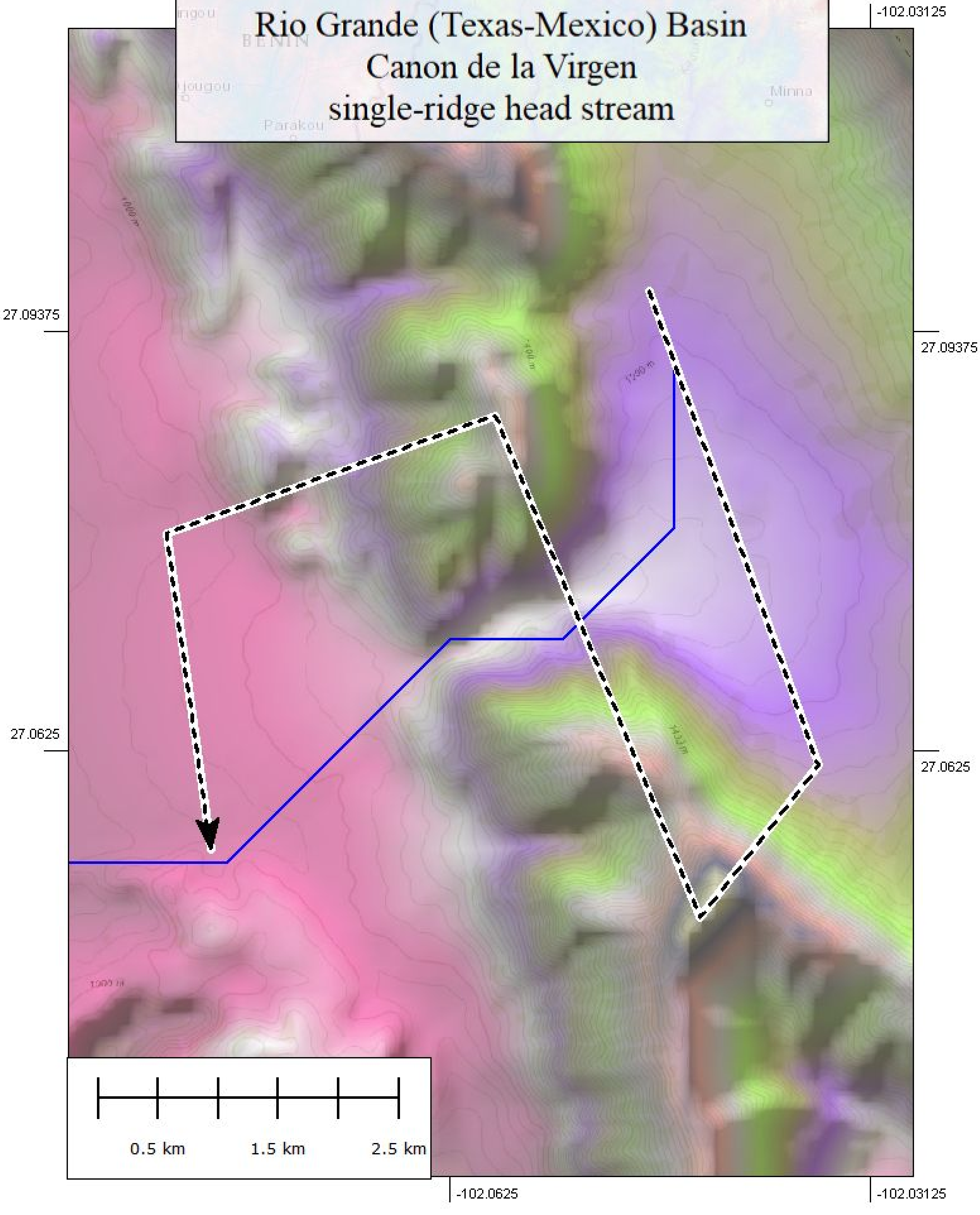

NA - 77
James River Basin
Piney Branch, McGraw Gap
single-ridge head stream

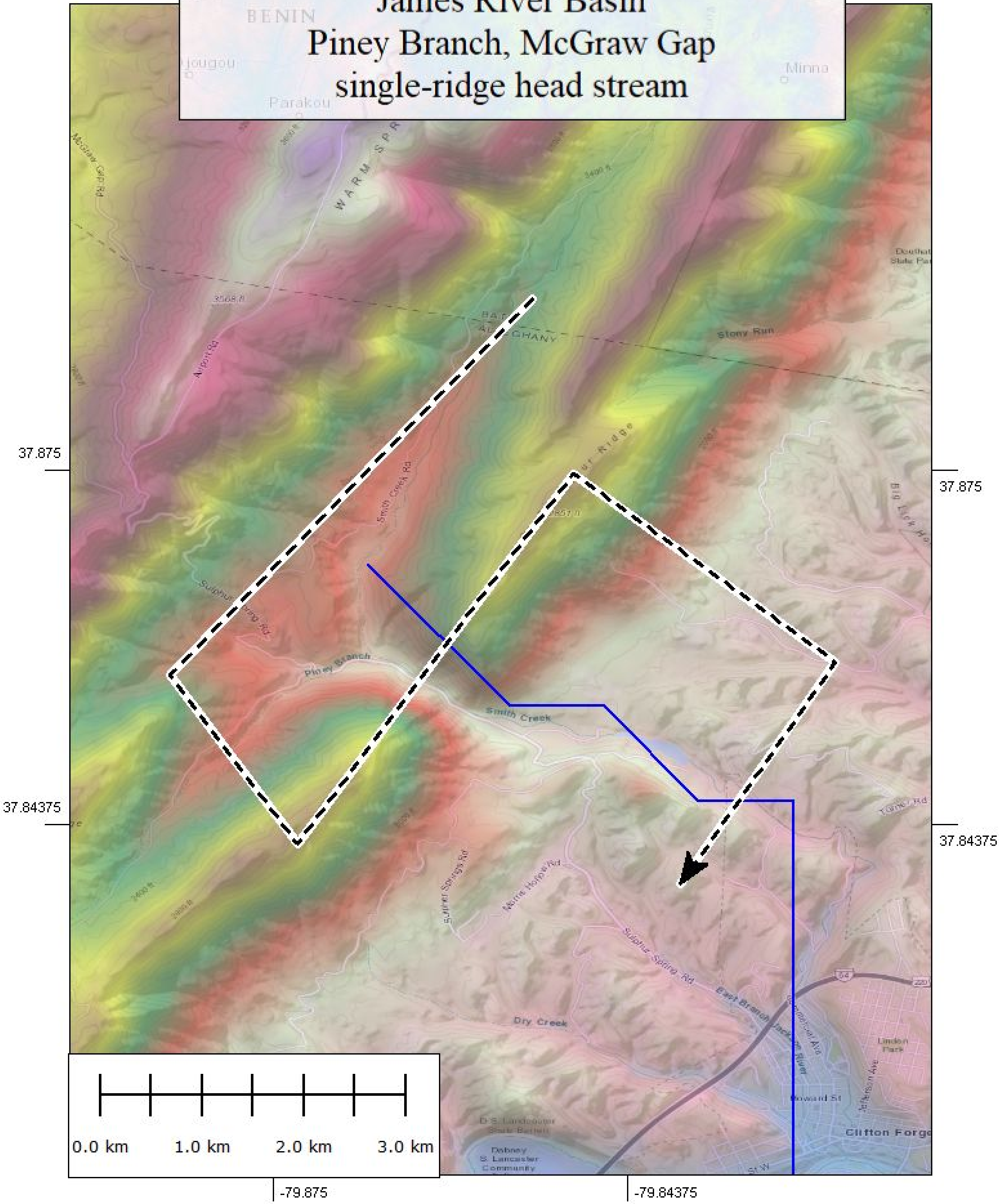

NA - 78
James River Basin
Jackson River, Vanderpool Gap
single-ridge head stream

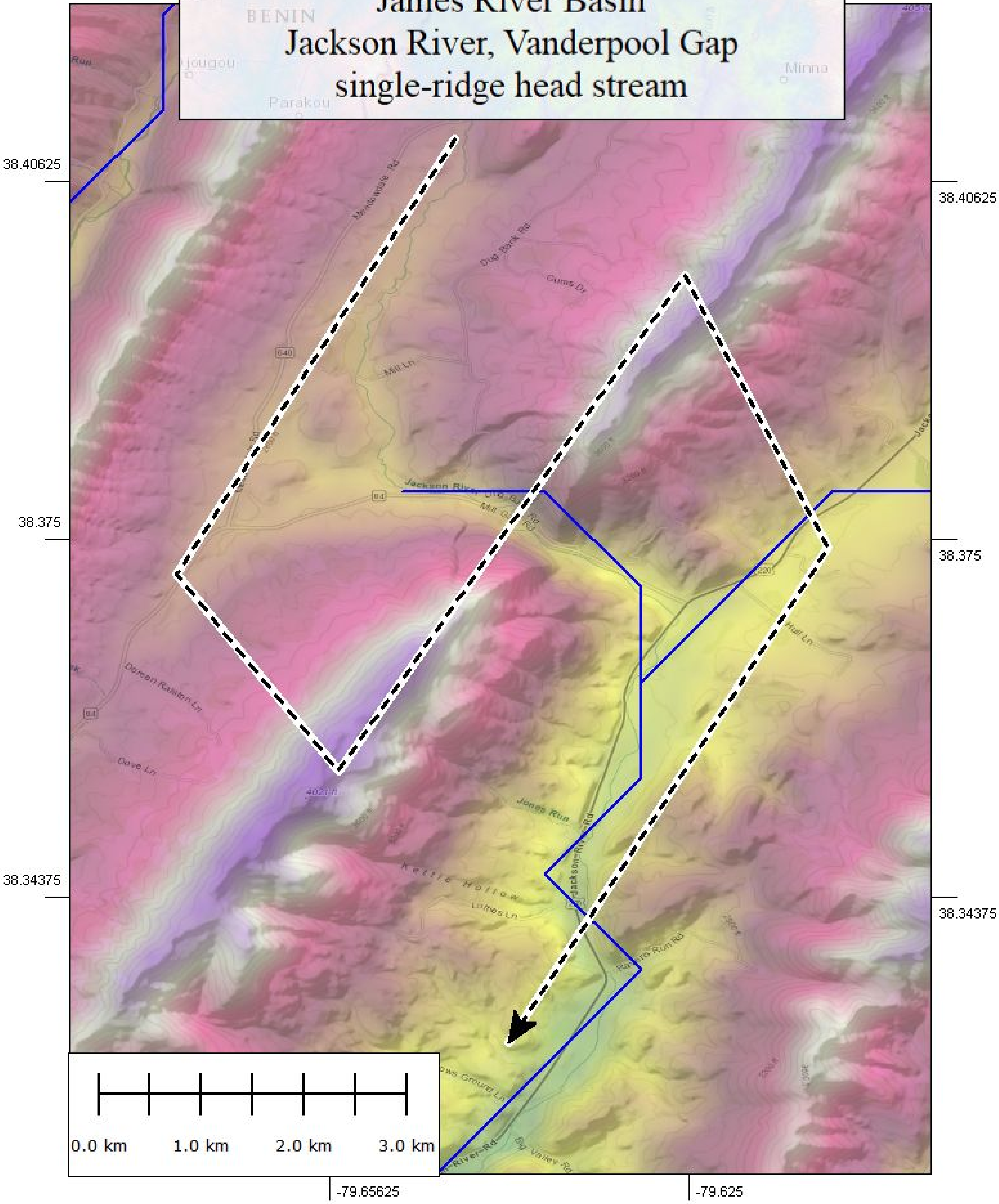

NA - 79
Potomac River Basin
South Branch Potomac River
single-ridge head stream

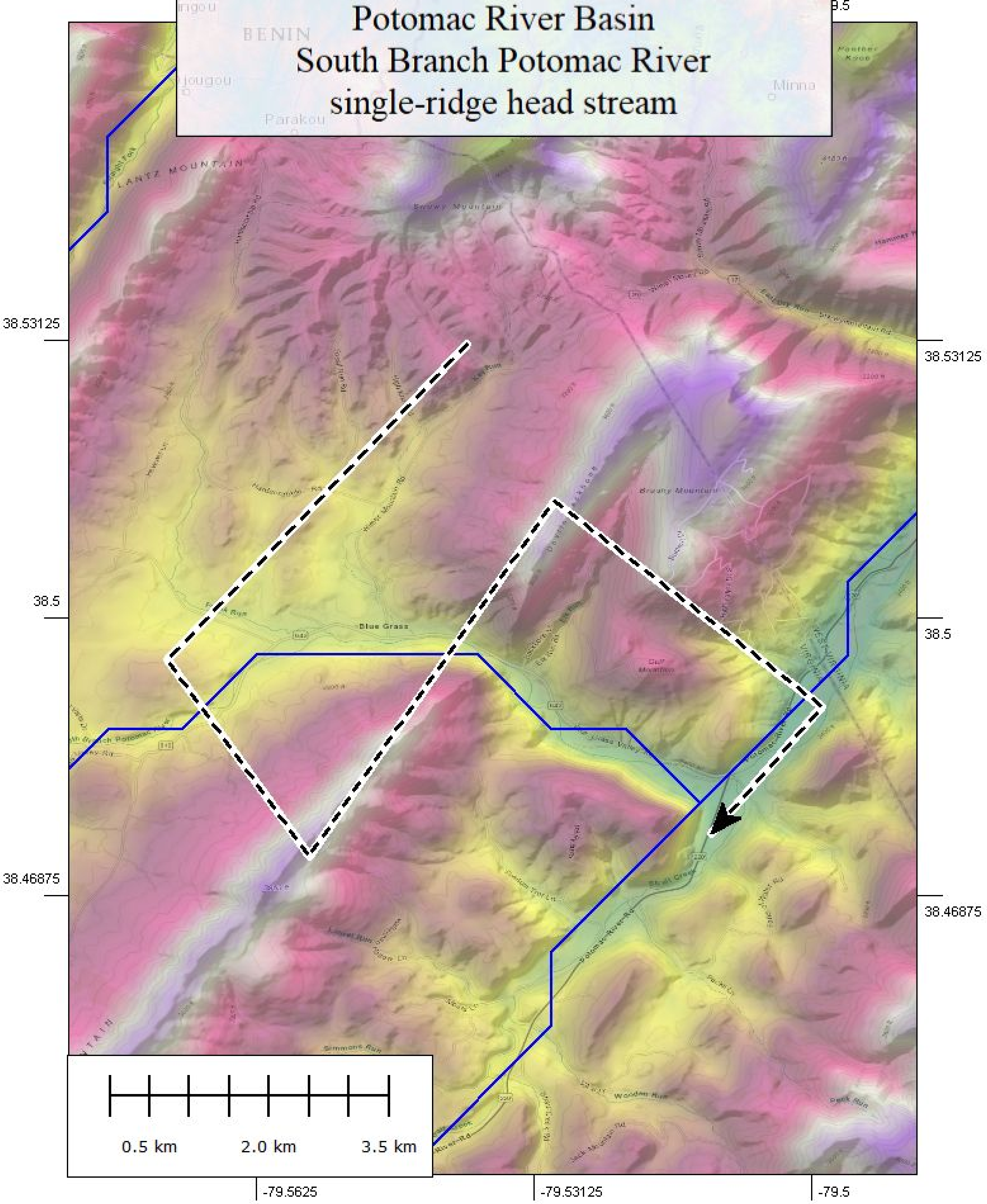

NA - 8
Colorado River (Arizona) Basin
Colorado River
downdip plateau trunk stream

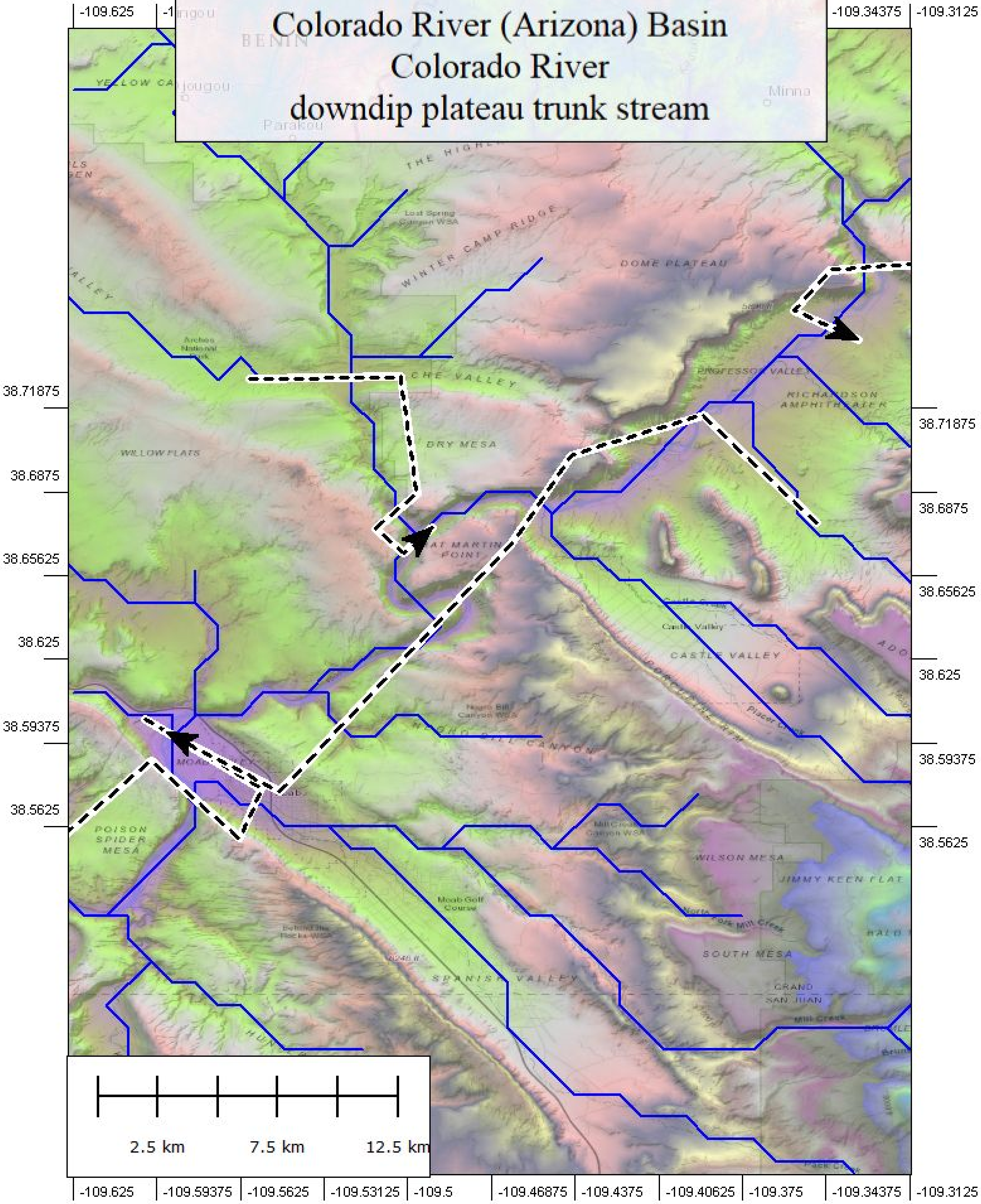

NA - 80
Potomac River Basin
Smith Creek
single-ridge head stream

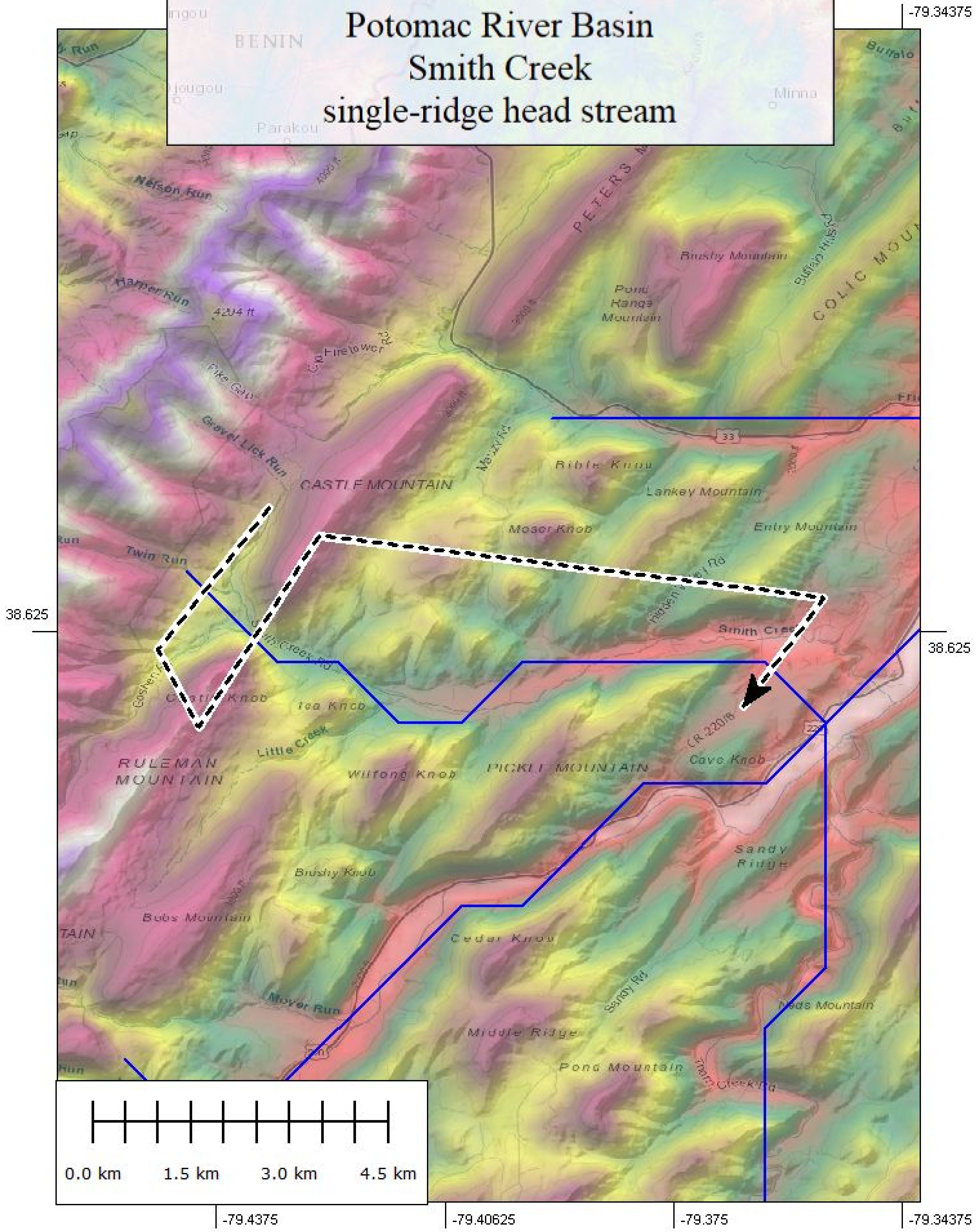

NA - 81
Potomac River Basin
Baker RUn
single-ridge head stream

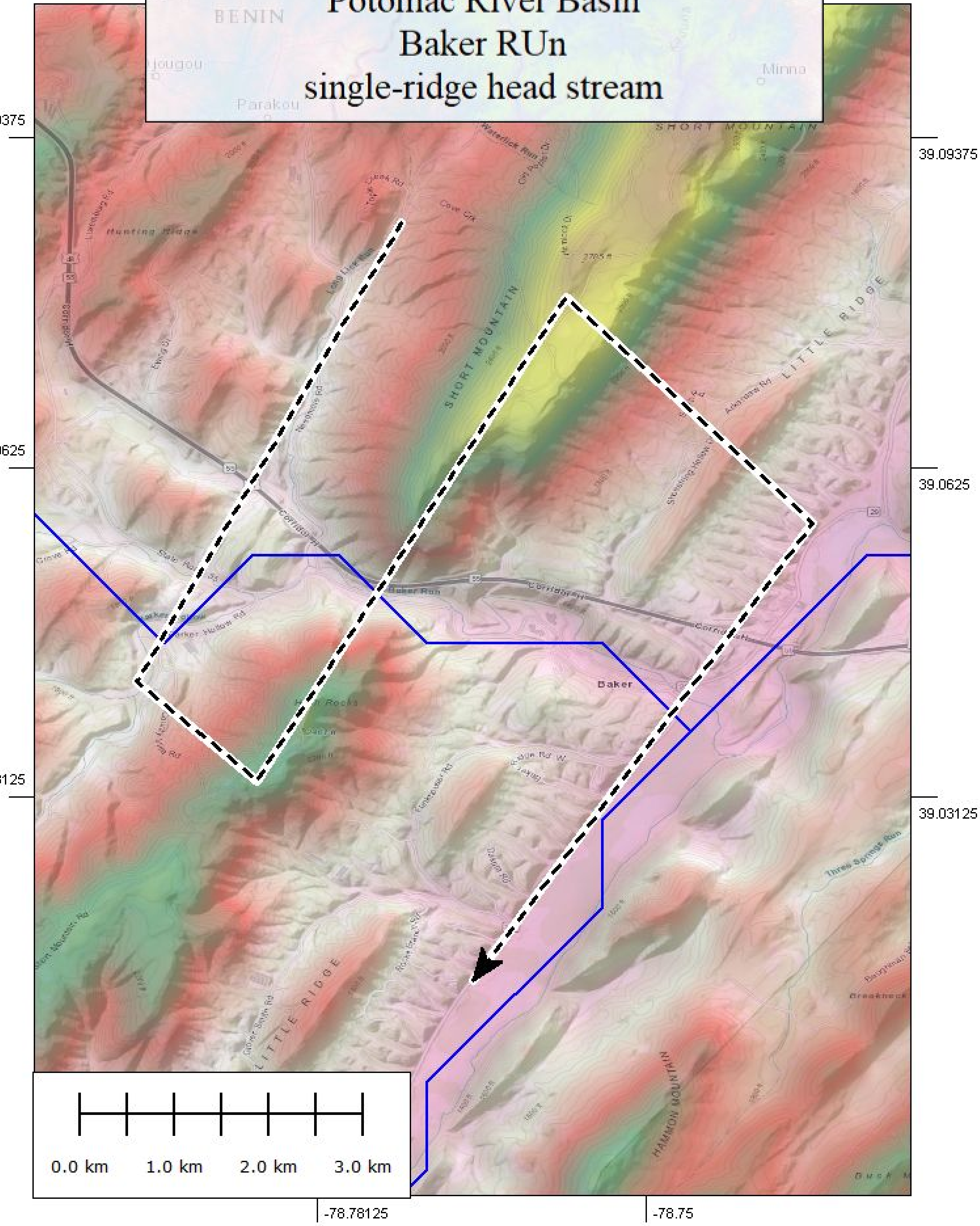

NA - 82
Mississippi River Basin
Cove Creek
single-ridge head stream

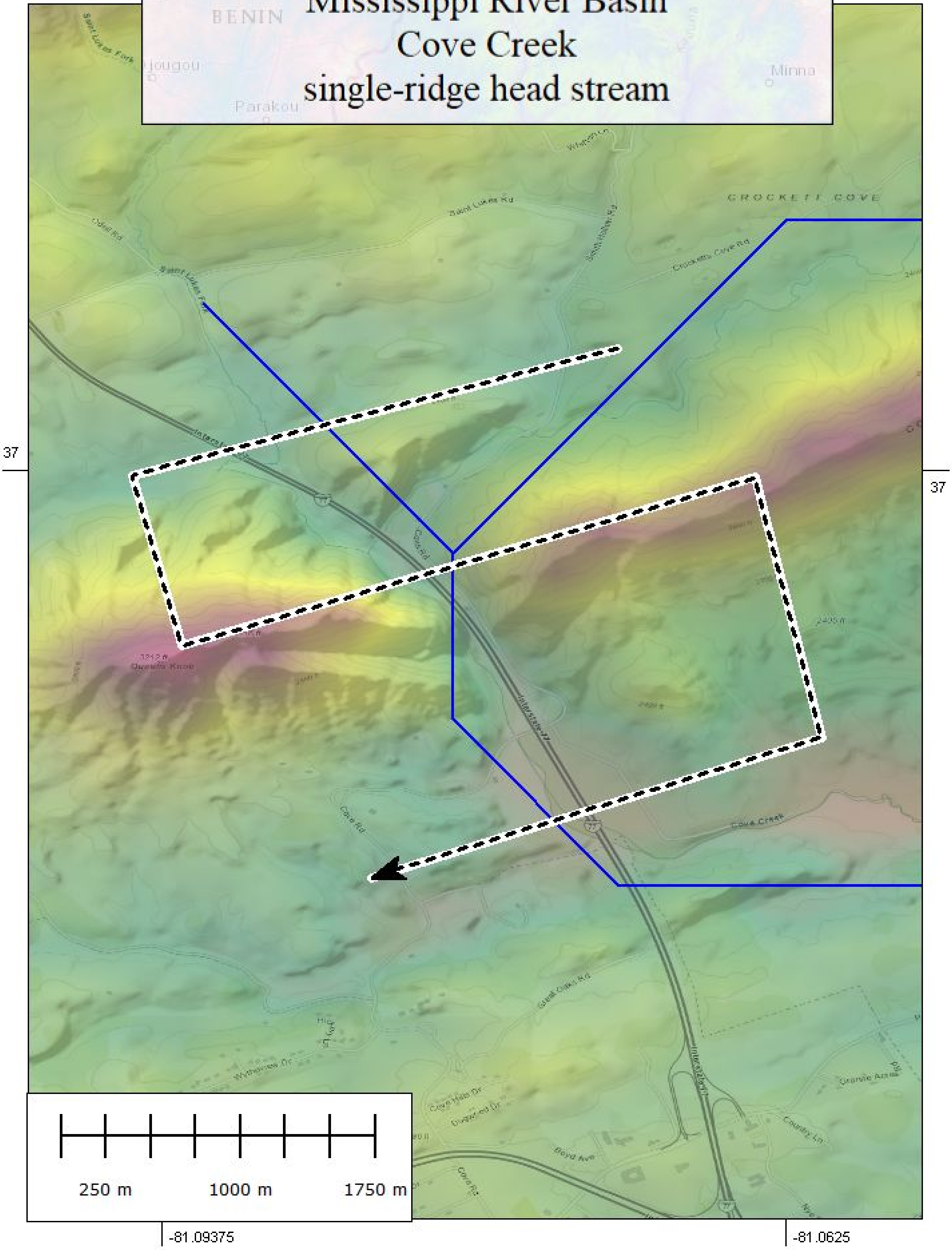

NA - 83
Mississippi River Basin
North Fork Holston River, Mocassin Gap
single-ridge head stream

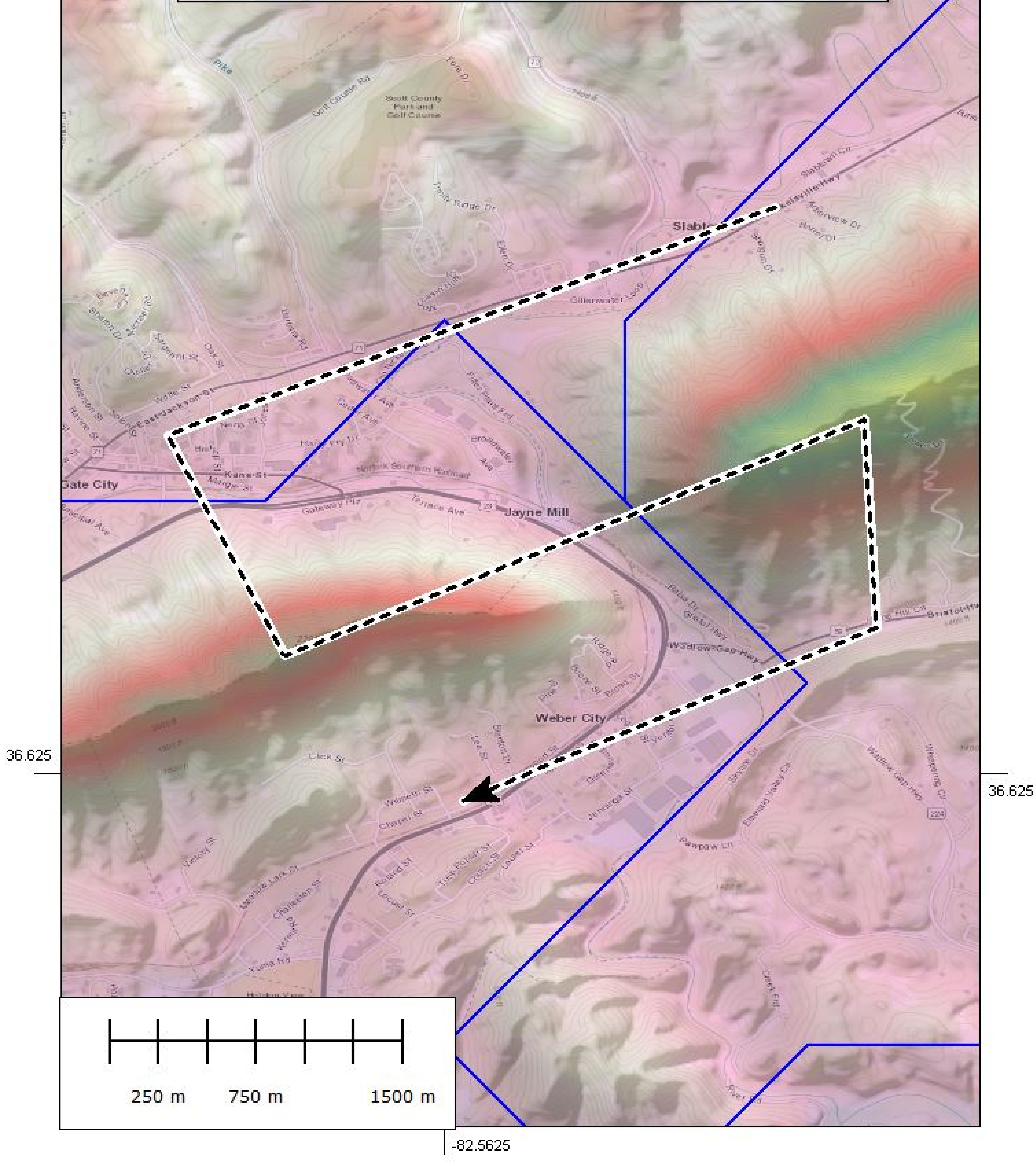

NA - 84
Mississippi River Basin
Laurel Creek
single-ridge head stream

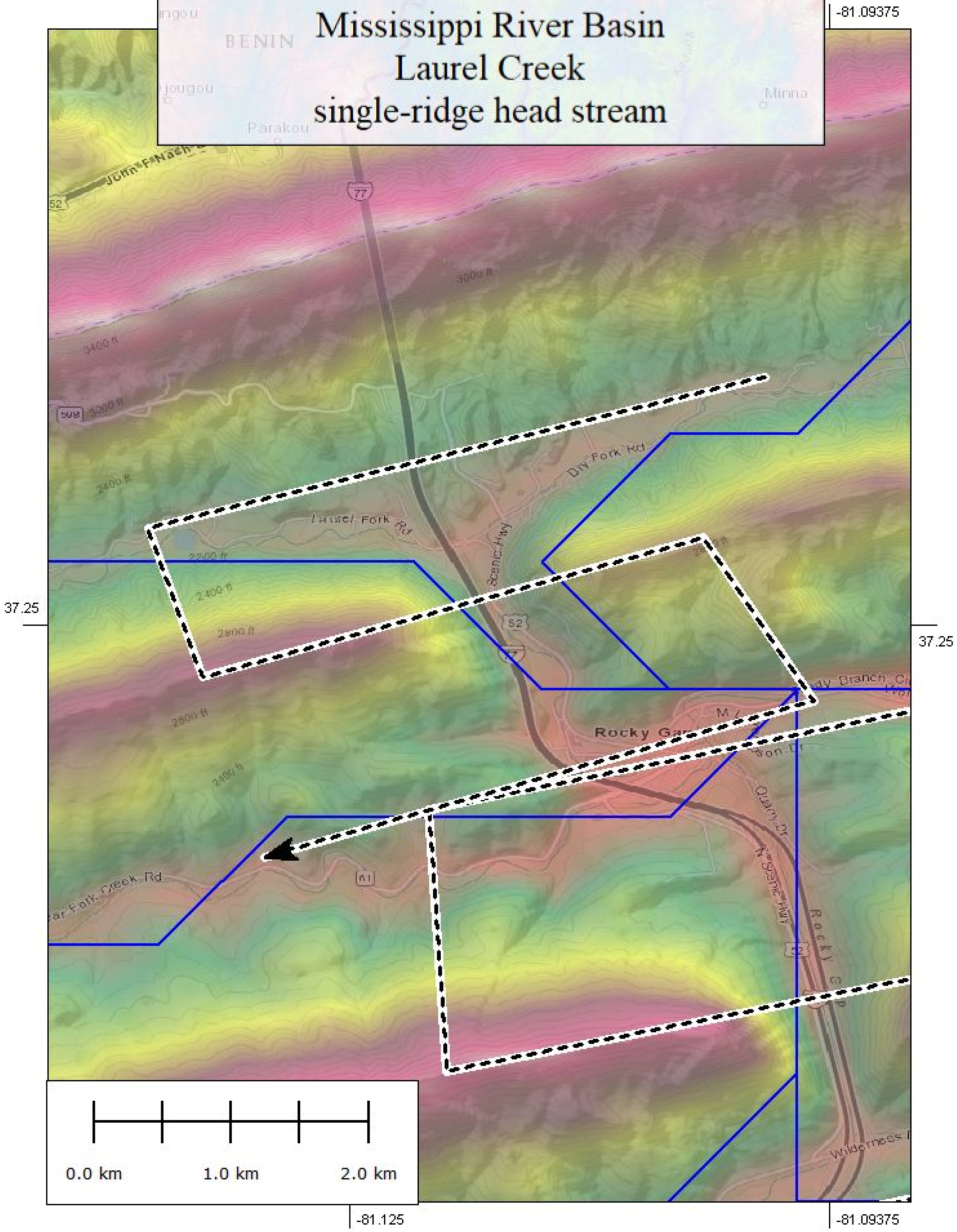

NA - 85
Susquehanna River Basin
Indiantown Run, Indiantown Gap
single-ridge head stream

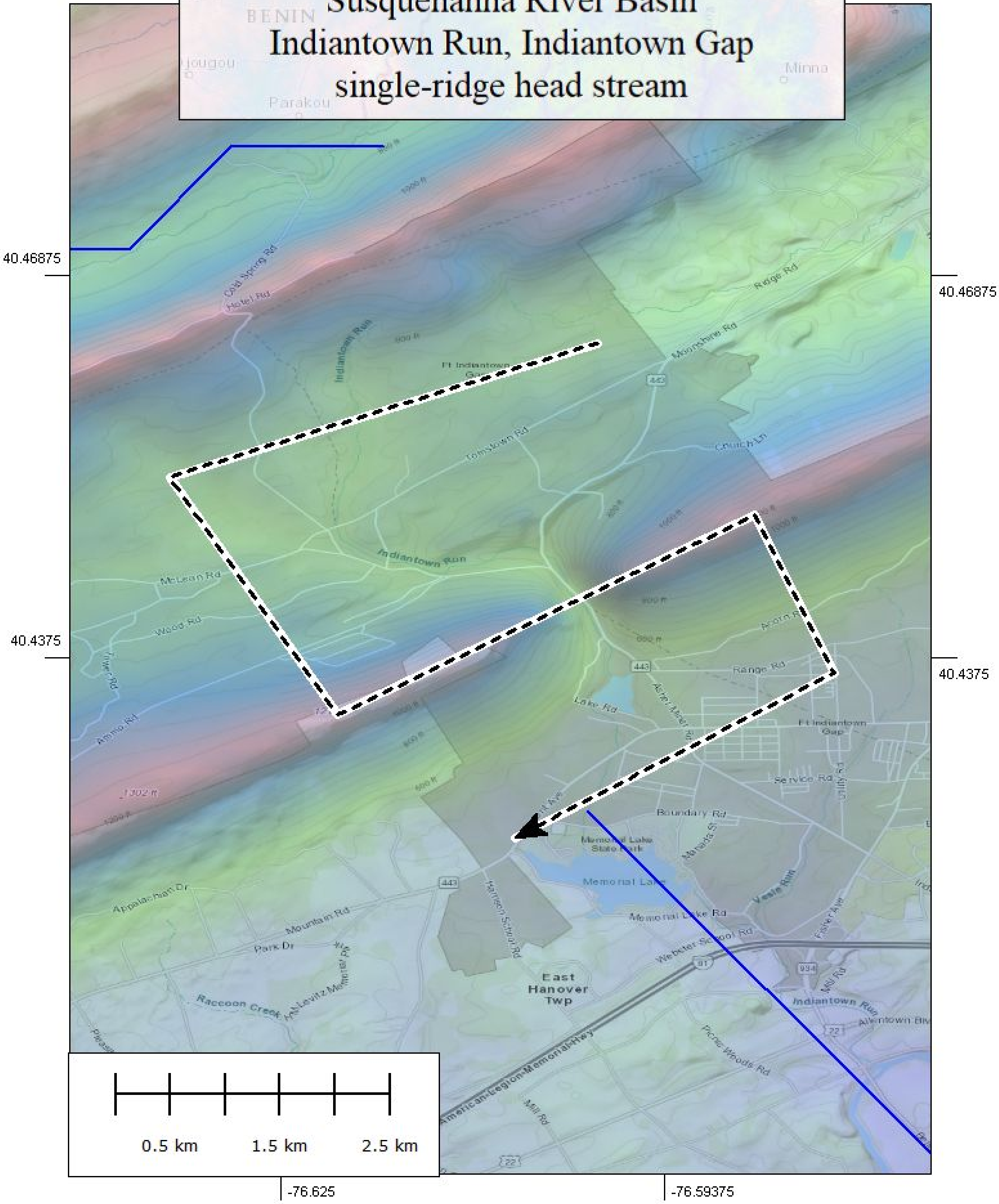

NA - 86
Susquehanna River Basin
Shamokin Creek
multi-ridge head stream

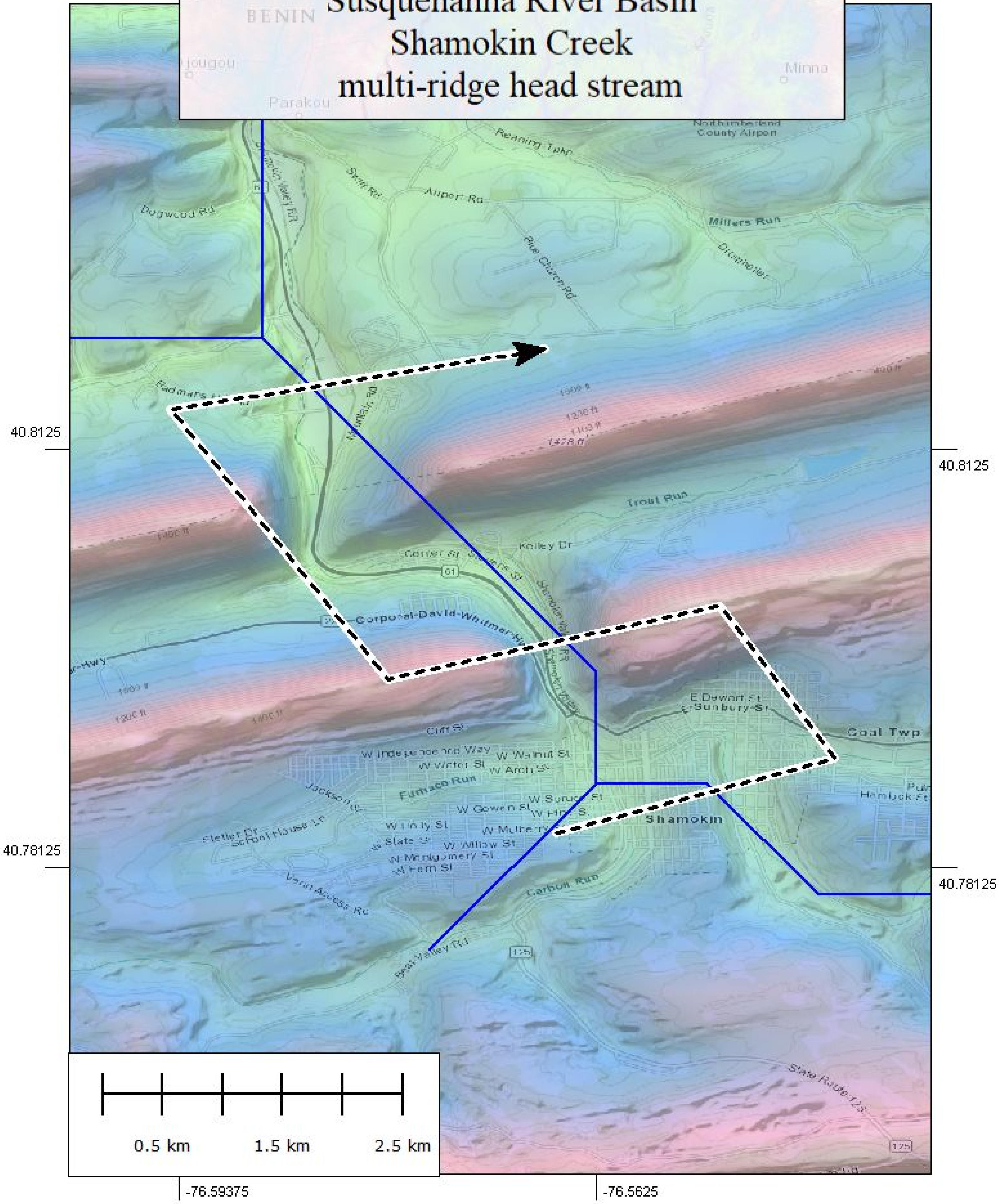

NA - 87
Susquehanna River Basin
Susquehanna River tributary
multi-ridge head stream

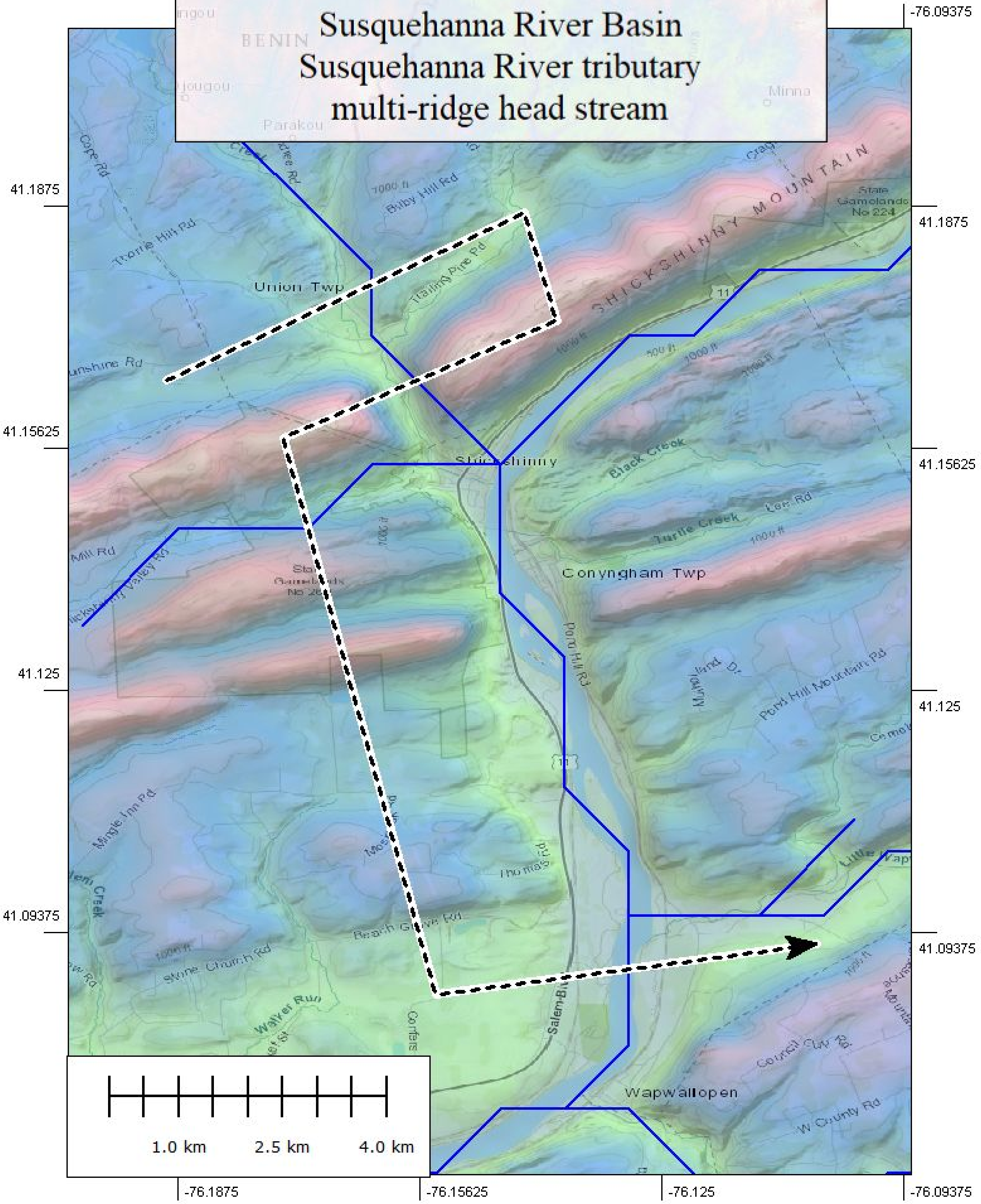

NA - 88
Potomac River Basin
Mill Creek
single-ridge head stream

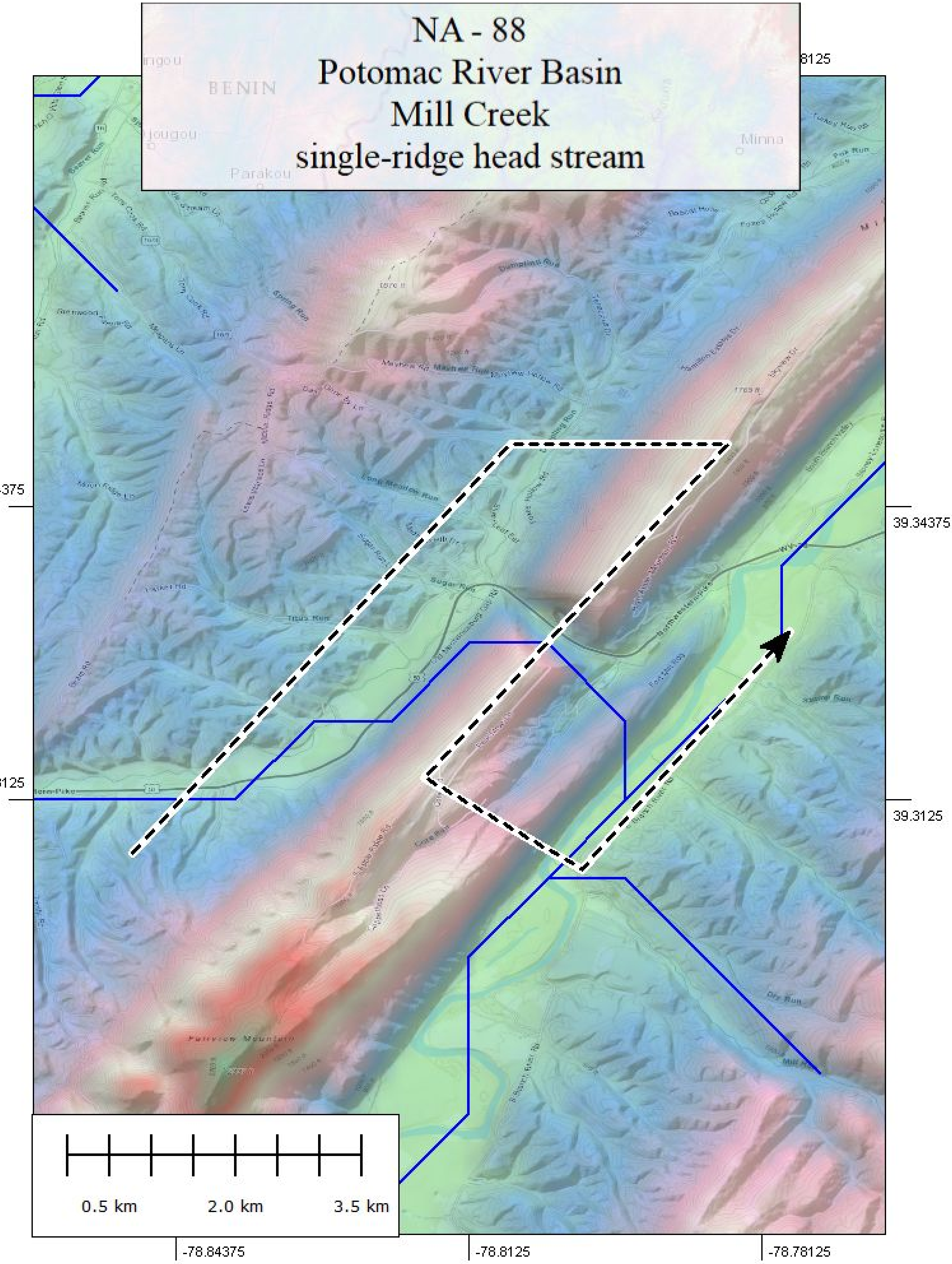

NA - 89
Susquehanna River Basin
Hatter Creek, McKee Gap
single-ridge head stream

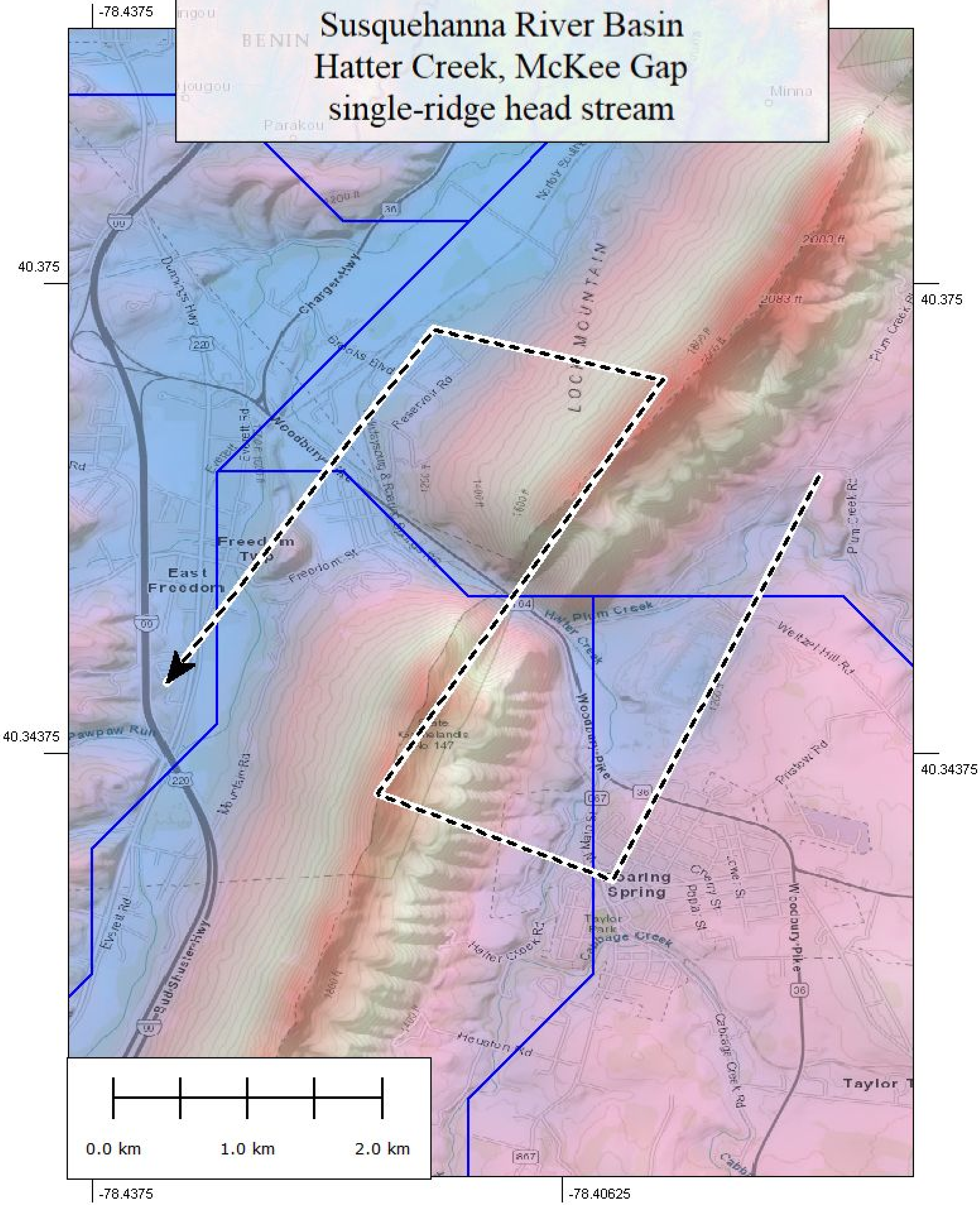

NA - 9
Columbia River Basin
Clark Fork River tributary
single-ridge head stream

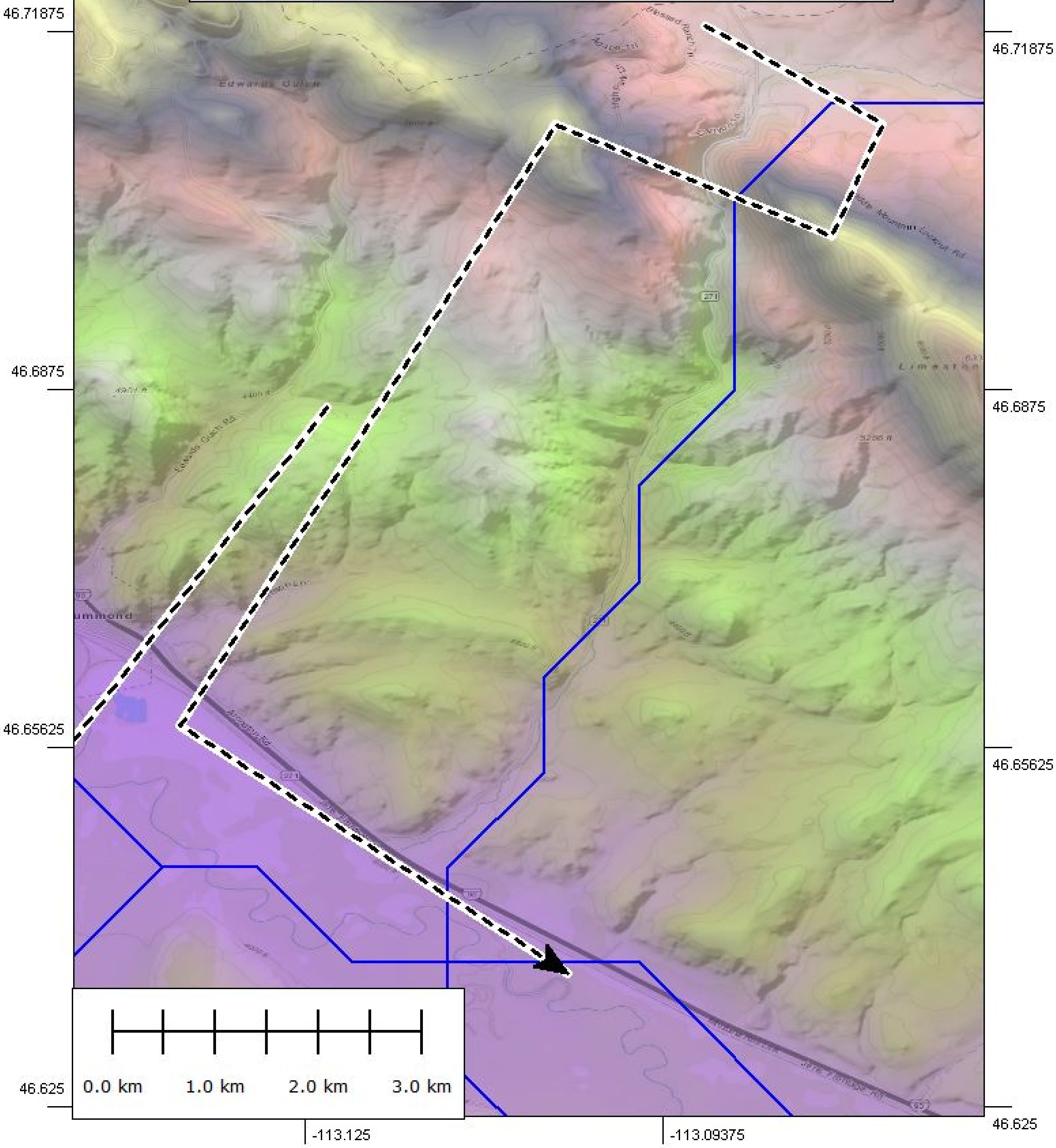

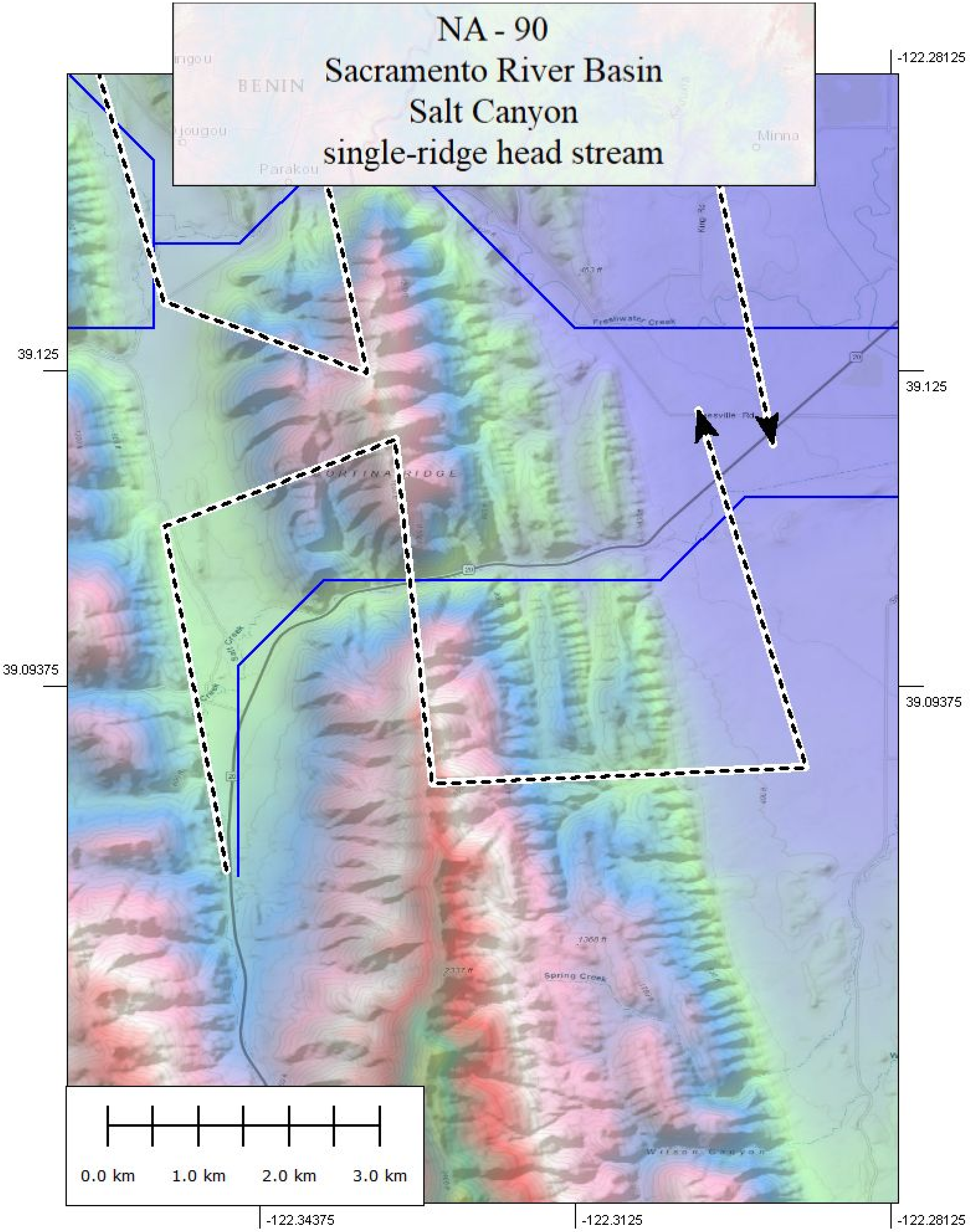

NA - 90
Sacramento River Basin
Salt Canyon
single-ridge head stream
-122.28125
39.125
39.125
39.09375
39.09375
-122.34375
-122.3125
-122.28125
0.0 km
1.0 km
2.0 km
3.0 km
Freshwater Creek
Salt Creek
Spring Creek
Wilson Canyon
BENIN
Parakou
Minna

NA - 91
Susquehanna River Basin
Lick Run, Howard Gap
single-ridge head stream

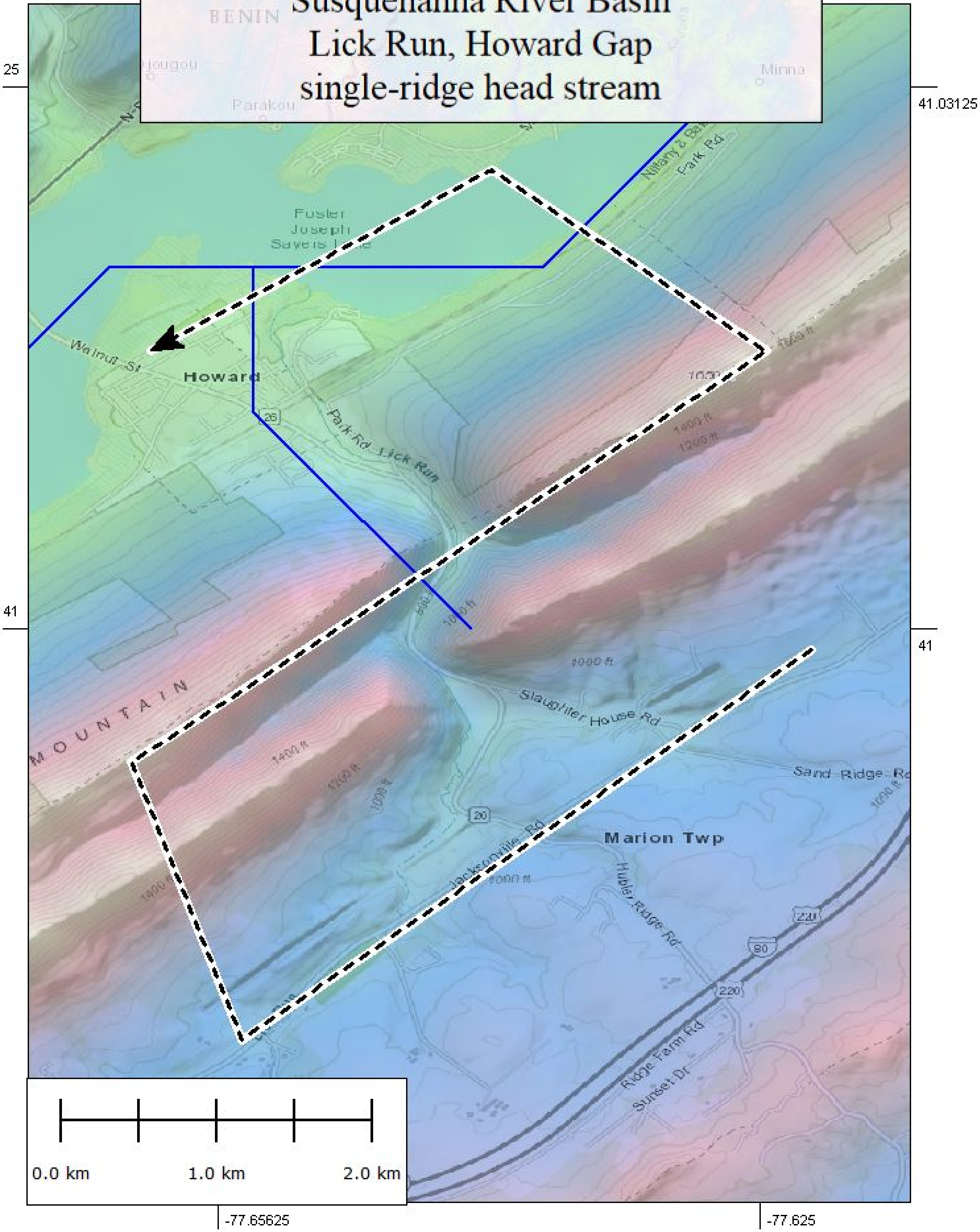

NA - 92
Susquehanna River Basin
Nittany Creek, Curtin Gap
single-ridge head stream

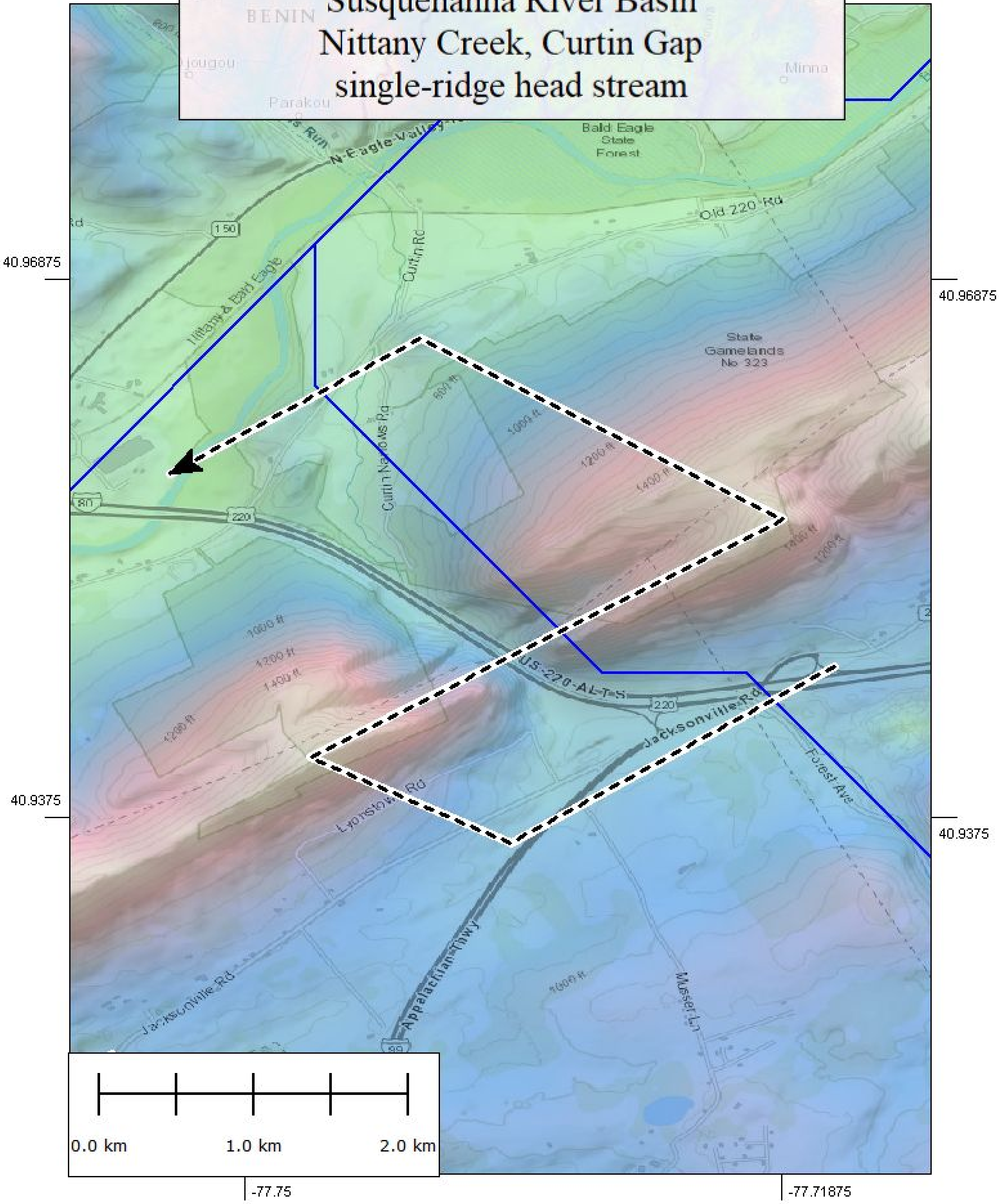

NA - 93
Susquehanna River Basin
Kansas Valley Run
single-ridge head stream

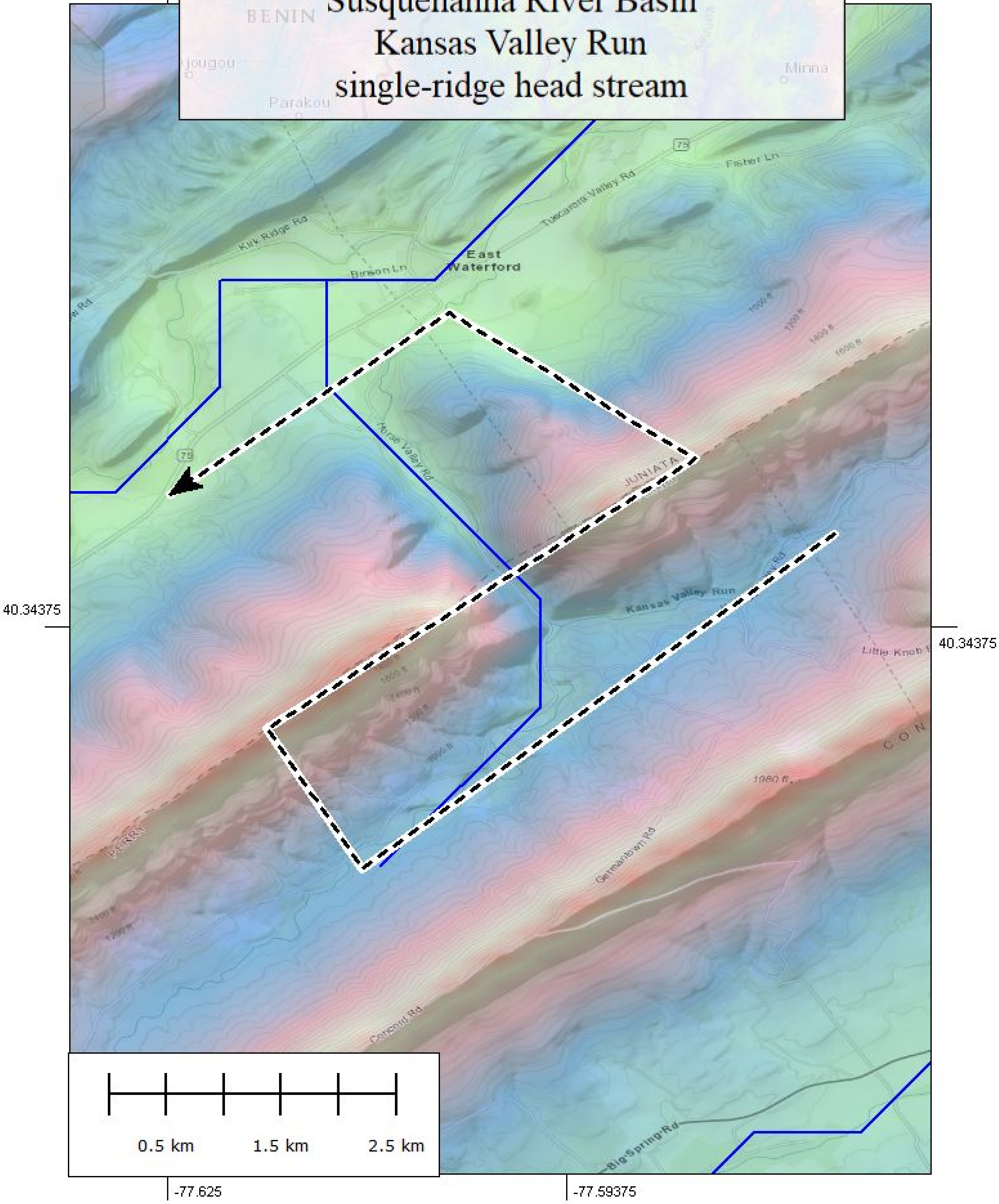

NA - 94
Rio Grande (Texas-Mexico) Basin
Potrero Chico
single-ridge head stream

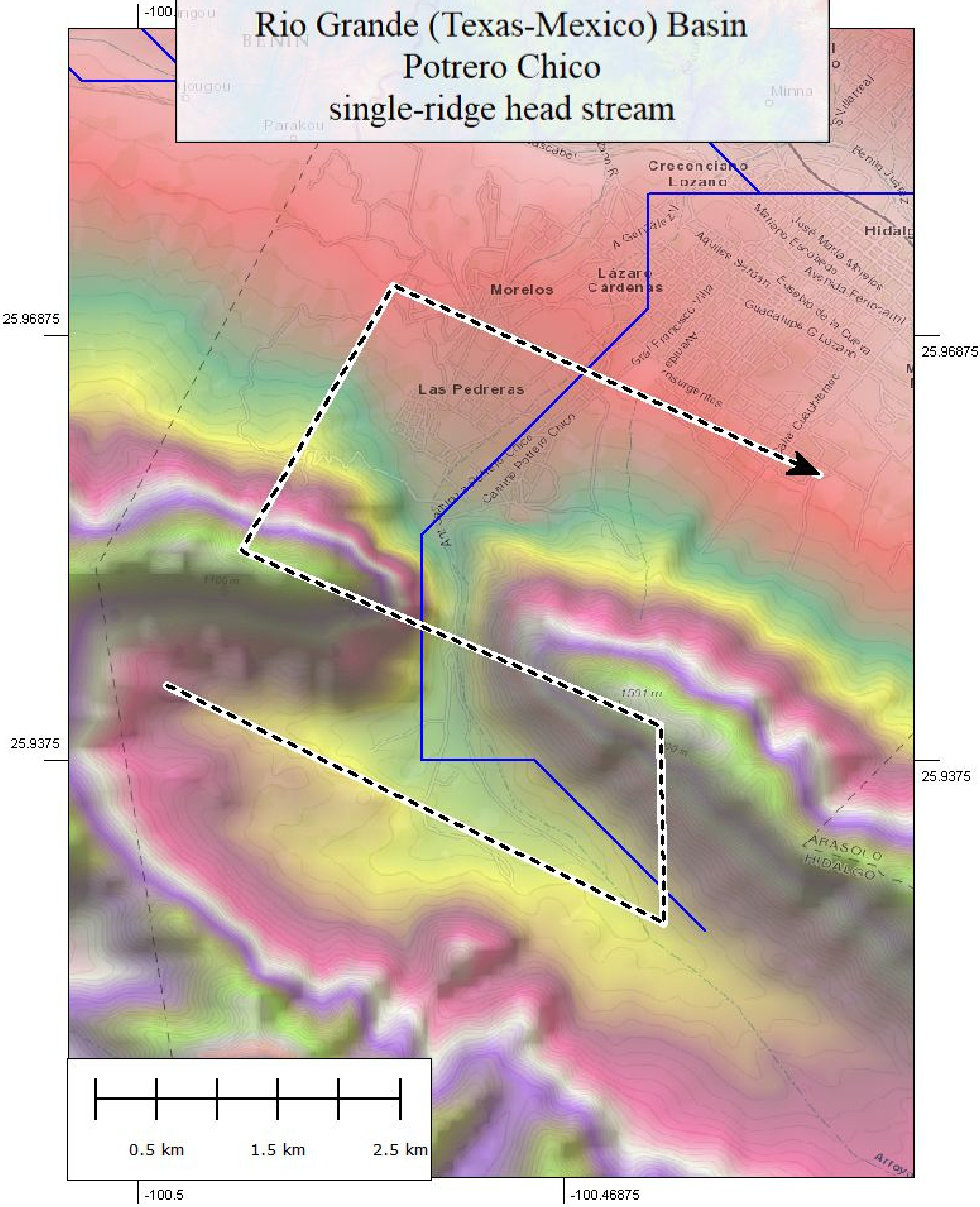

NA - 95
Mississippi River Basin
Little Walker Creek
single-ridge head stream

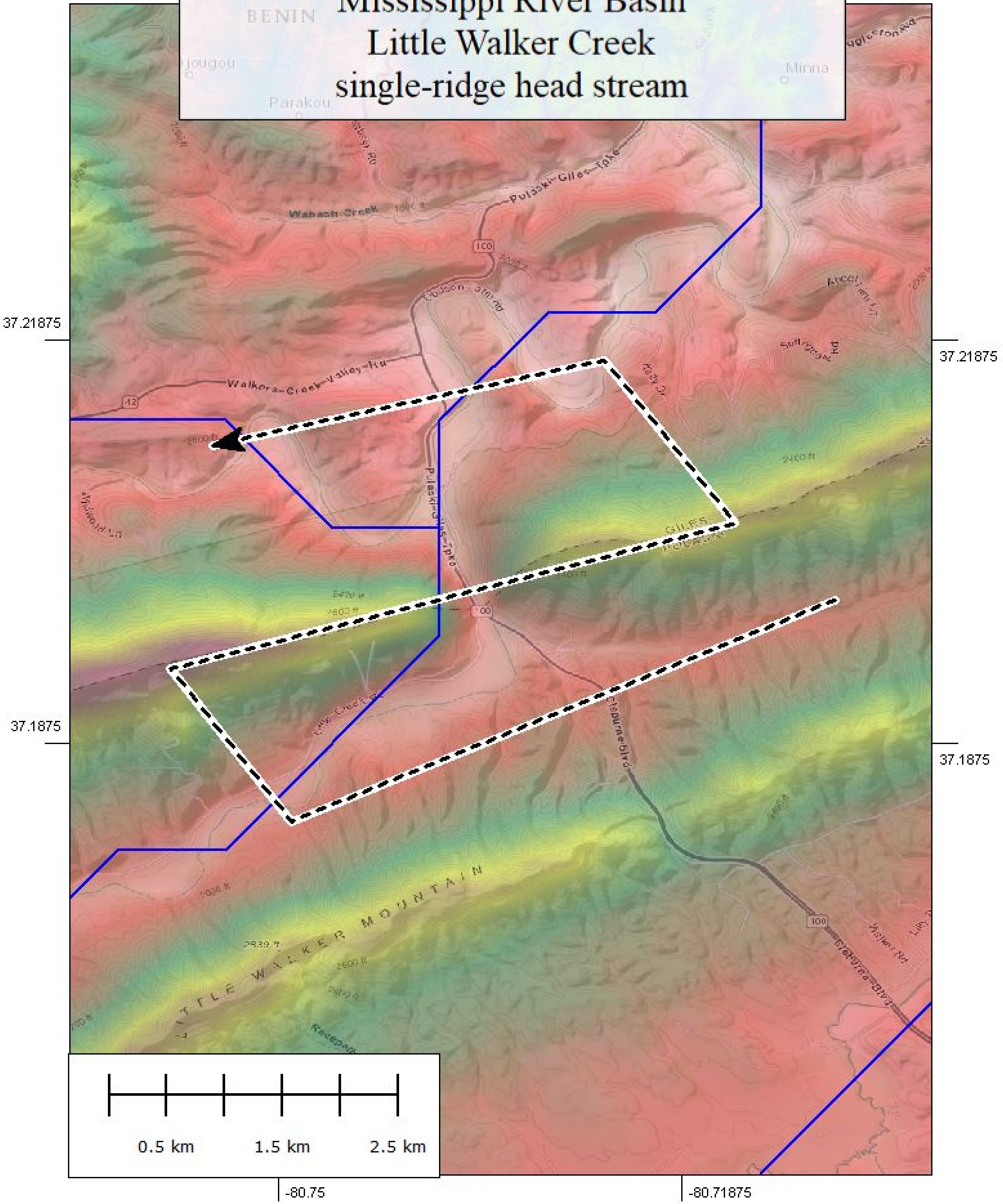

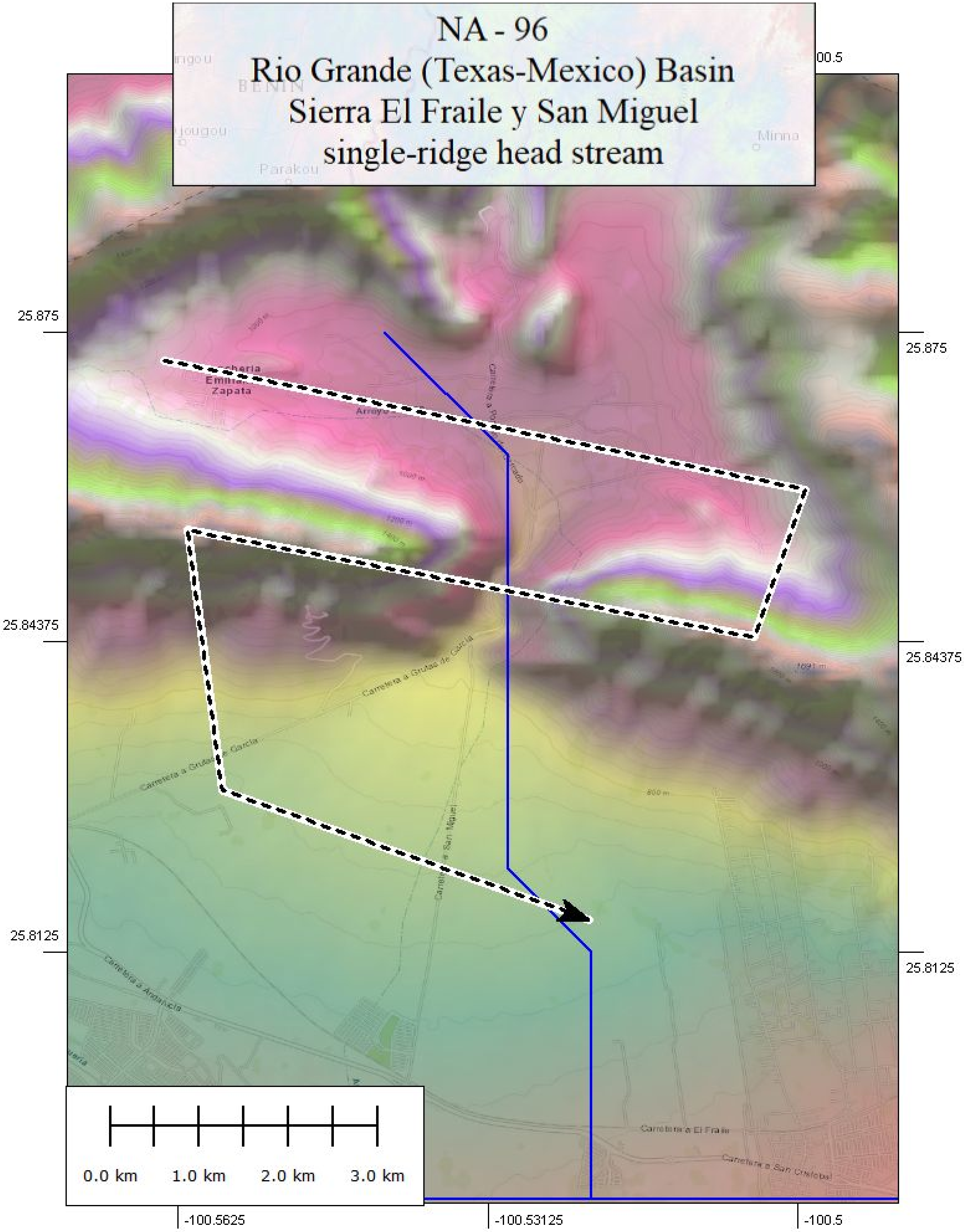

NA - 96
Rio Grande (Texas-Mexico) Basin
Sierra El Fraile y San Miguel
single-ridge head stream
25.875
25.875
25.84375
25.84375
25.8125
25.8125
-100.5
-100.5625
-100.53125
-100.5
0.0 km
1.0 km
2.0 km
3.0 km
Carretera a Grutas de García
Carretera a San Miguel
Carretera a El Fraile
Carretera a San Cristobal
Carretera a Andalucia

NA - 97
Yaqui River Basin
Rio Hondo
single-ridge head stream

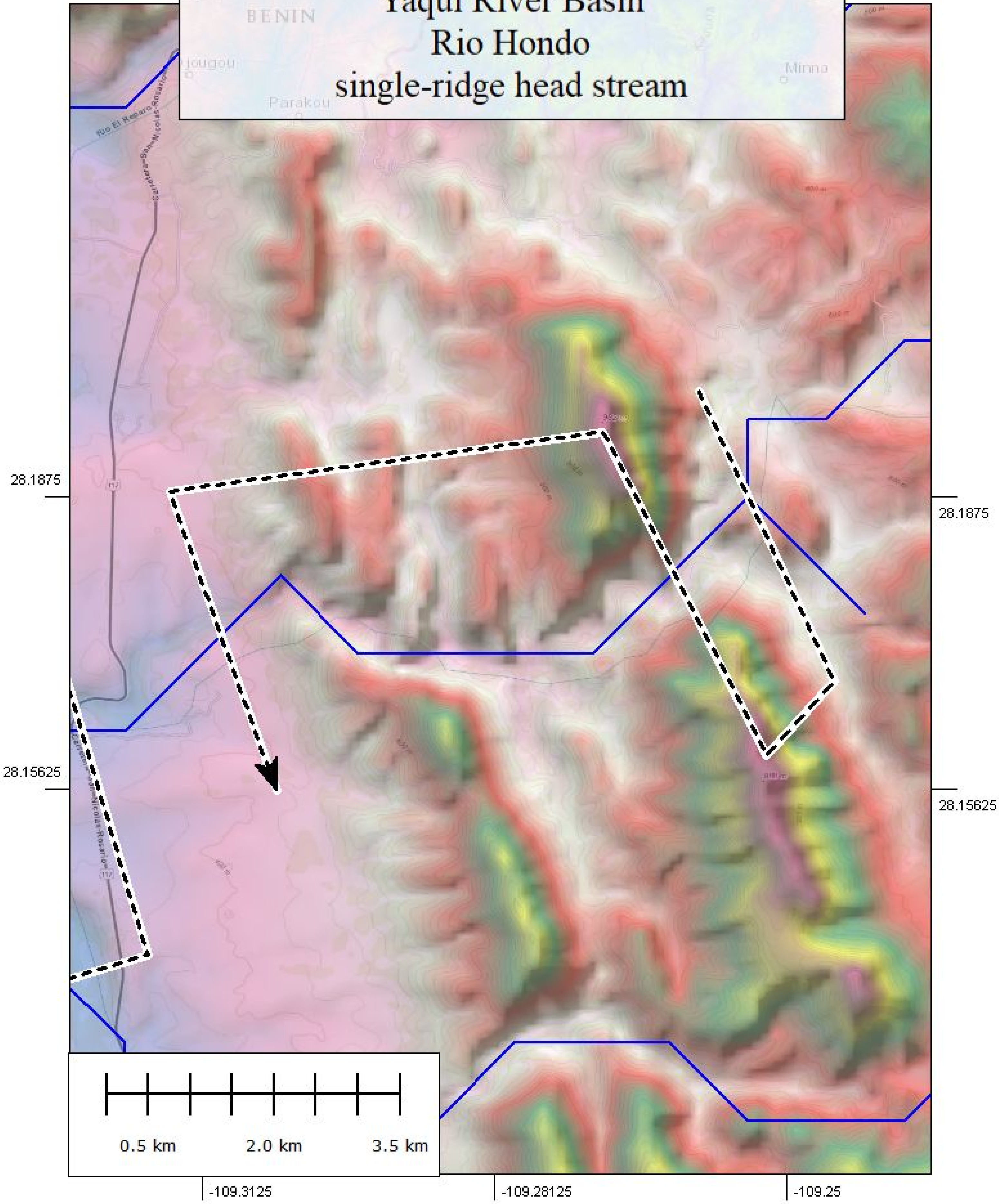

NA - 98
Colorado River (Arizona) Basin
Piceance Creek
single-ridge head stream

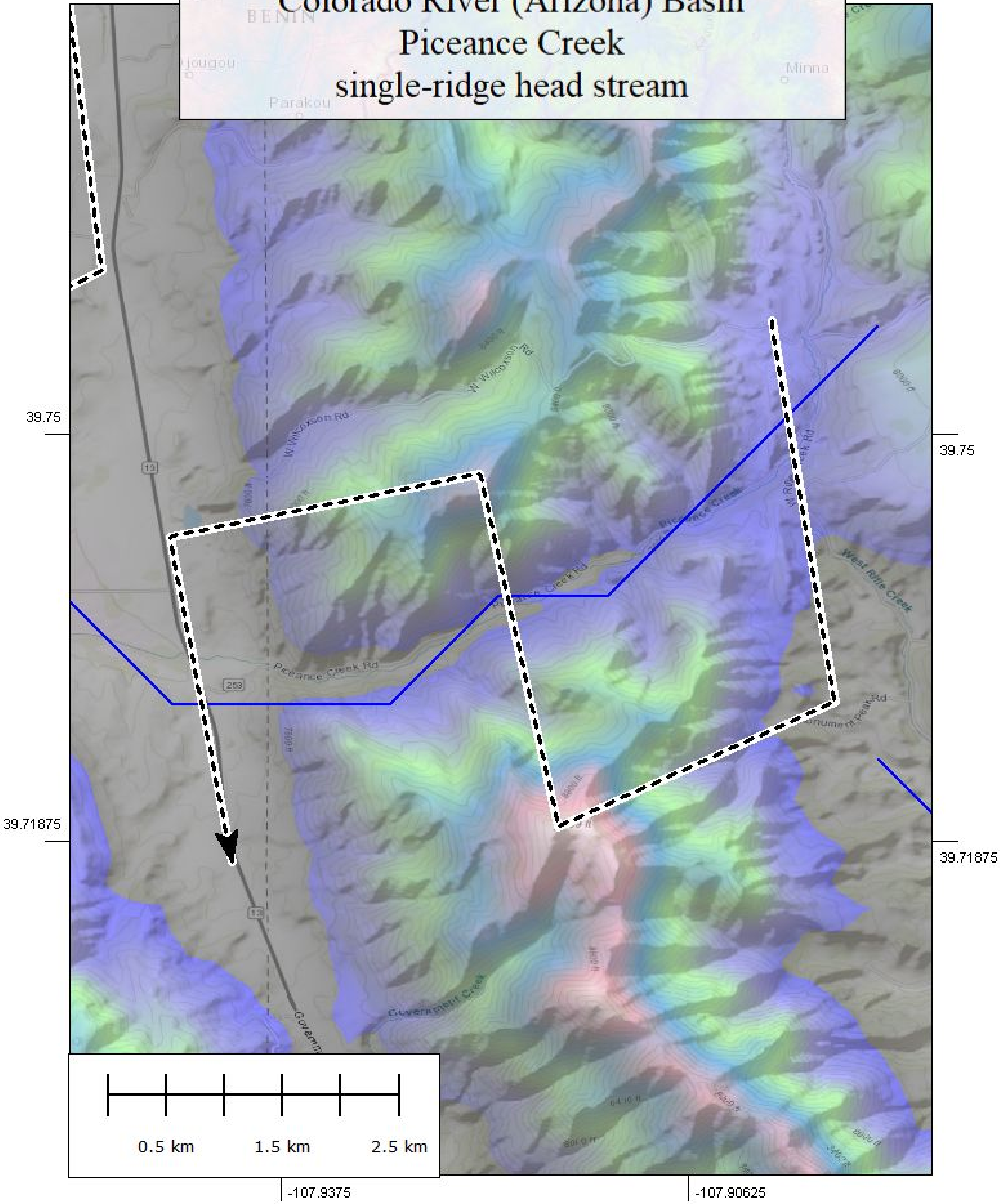

NA - 99
Papaloapan River Basin
Tesechoacan River tributary
single-ridge head stream

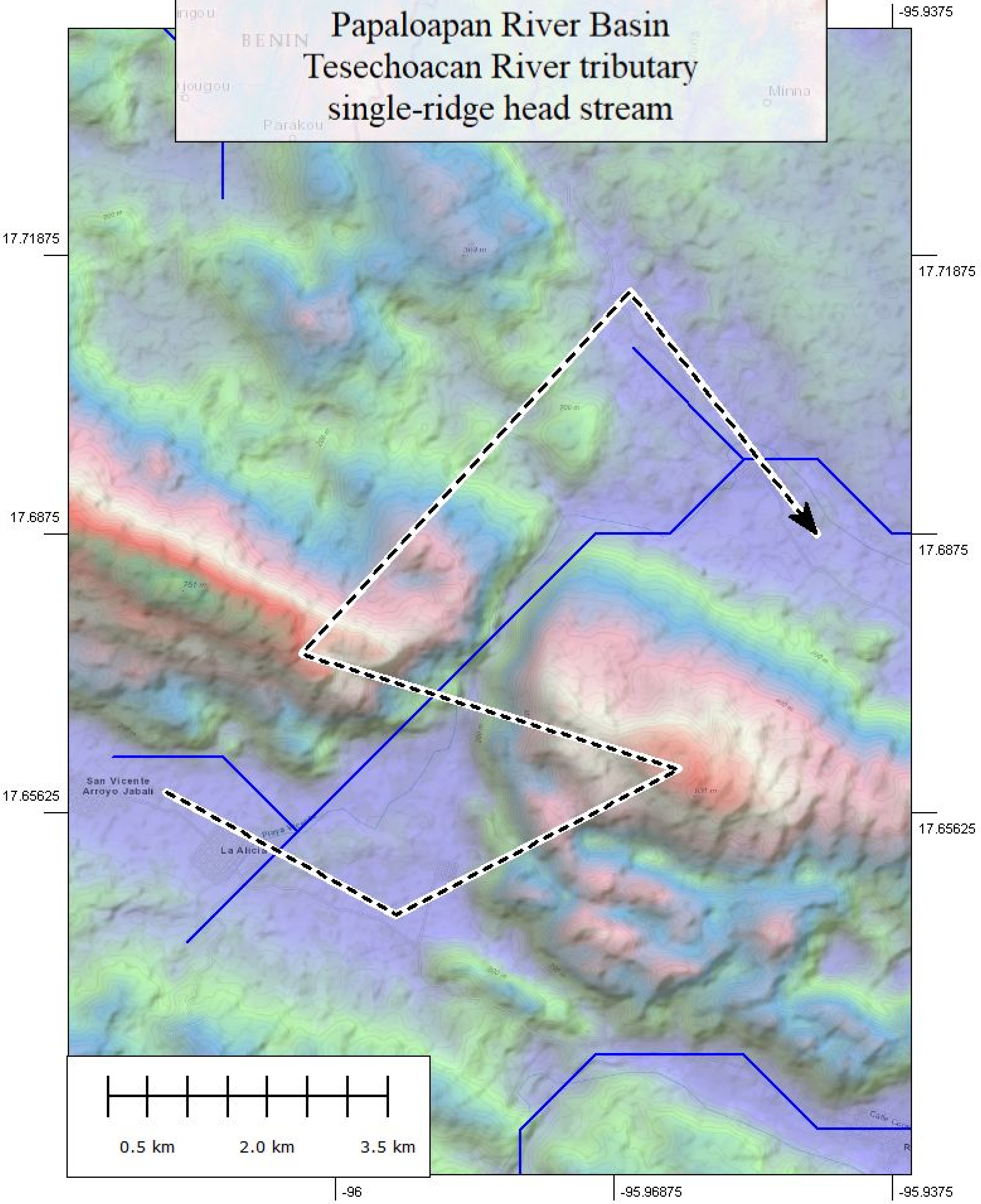

Supplement: Supplementary file 8 — Supplementary material [file mmc8.pdf]
